# KSHV MicroRNAs Mediate Cellular Transformation and Tumorigenesis by Redundantly Targeting Cell Growth and Survival Pathways

**DOI:** 10.1371/journal.ppat.1003857

**Published:** 2013-12-26

**Authors:** Rosalie Moody, Ying Zhu, Yufei Huang, Xiaodong Cui, Tiffany Jones, Roble Bedolla, Xiufen Lei, Zhiqiang Bai, Shou-Jiang Gao

**Affiliations:** 1 Department of Pediatrics, University of Texas Health Science Center at San Antonio, San Antonio, Texas, United States of America; 2 Department of Microbiology and Immunology, University of Texas Health Science Center at San Antonio, San Antonio, Texas, United States of America; 3 Department of Molecular Microbiology and Immunology, Keck School of Medicine, University of Southern California, Los Angeles, California, United States of America; 4 Department of Electrical and Computer Engineering, University of Texas at San Antonio, San Antonio, Texas, United States of America; Heinrich-Pette-Institute, Leibniz Institute for Experimental Virology, Germany

## Abstract

Kaposi's sarcoma-associated herpesvirus (KSHV) is causally linked to several human cancers, including Kaposi's sarcoma, primary effusion lymphoma and multicentric Castleman's disease, malignancies commonly found in HIV-infected patients. While KSHV encodes diverse functional products, its mechanism of oncogenesis remains unknown. In this study, we determined the roles KSHV microRNAs (miRs) in cellular transformation and tumorigenesis using a recently developed KSHV-induced cellular transformation system of primary rat mesenchymal precursor cells. A mutant with a cluster of 10 precursor miRs (pre-miRs) deleted failed to transform primary cells, and instead, caused cell cycle arrest and apoptosis. Remarkably, the oncogenicity of the mutant virus was fully restored by genetic complementation with the miR cluster or several individual pre-miRs, which rescued cell cycle progression and inhibited apoptosis in part by redundantly targeting IκBα and the NF-κB pathway. Genomic analysis identified common targets of KSHV miRs in diverse pathways with several cancer-related pathways preferentially targeted. These works define for the first time an essential viral determinant for KSHV-induced oncogenesis and identify NF-κB as a critical pathway targeted by the viral miRs. Our results illustrate a common theme of shared functions with hierarchical order among the KSHV miRs.

## Author Summary

Kaposi's sarcoma-associated herpesvirus (KSHV) is the causal agent of several human cancers. KSHV encodes over two dozen genes that regulate diverse cellular pathways. However, the molecular mechanism of KSHV-induced oncogenesis remains unknown. In this study, we determined the roles of KSHV microRNAs (miRs) in KSHV-induced oncogenesis using a recently developed KSHV cellular transformation system of primary rat mesenchymal precursor cells. A KSHV mutant with a cluster of 10 precursor miRs (pre-miRs) deleted failed to transform primary cells, and instead, caused cell cycle arrest and apoptosis. Expression of the miR cluster or several pre-miRs was sufficient to restore the oncogenicity of the mutant virus. KSHV miRs regulated cell cycle progression and inhibited apoptosis in part by redundantly targeting IκBα and the NF-κB pathway. By integrating gene expression profiling and target prediction, we identified common targets of KSHV miRs in diverse pathways. Importantly, several cancer-related pathways were preferentially targeted by KSHV miRs. These works have demonstrated for the first time the important roles of KSHV miRs in oncogenesis and identified NF-κB as a critical pathway targeted by the miRs. Our results reveal that shared function is a common theme of KSHV miRs, which manifest functional hierarchical order.

## Introduction

Infection by Kaposi's sarcoma-associated herpesvirus (KSHV) is associated with Kaposi's sarcoma (KS), the most common cancer in HIV-infected patients [1]. KSHV is also linked to the development of several other lymphoproliferative malignancies including primary effusion lymphoma (PEL) and a subset of multicentric Castleman's disease (MCD) [1].

KSHV encodes over 90 genes and more than two dozen microRNAs (miRs) derived from 12 precursor miRs (pre-miRs) [1], [2]. While diverse functions have been identified for these viral products, viral and cellular determinants required for KSHV-induced oncogenesis remain unknown primarily because of the lack of a trackable system for KSHV cellular transformation [1]. The recent development of a model of KSHV efficient infection and transformation of primary rat mesenchymal precursor cells (MM) provides for the first time a reliable system for identifying the viral and cellular determinants essential for KSHV-induced oncogenesis [3]. In this model, KSHV-induced tumors manifest the typical virological and pathological features of human KS tumors.

While KS has all the typical cancer hallmarks, unlike other cancers that depend on genome instability and mutation to enable the cancer features, no uniform genetic alteration has been identified in KS tumors so far [4], [5]. In fact, recent studies have shown that KSHV-induced cellular transformation and tumorigenesis depend on the viral genome [3], [6]. This unique feature indicates that the induction of KS tumors or at least early stage of KS tumors depends on the KSHV genome and the expression of KSHV genes. Thus, identification of KSHV genes required for cellular transformation and tumorigenesis can provide direct insights into the mechanism of KSHV-induced oncogenesis.

Similar to other herpesviruses, the life cycle of KSHV consists of latency and lytic replication phases [7]. Following acute infection, KSHV establishes latency in the immunocompetent hosts. Upon stimulation by specific signals, latent KSHV can be reactivated into lytic replication. During lytic replication, KSHV expresses almost all lytic proteins and produces infectious virions, which often results in cell death. In contrast, KSHV only expresses a limited number of viral proteins during latency. Thus, KSHV latent infection is an effective strategy for evading host immune detection [7]. In KS lesions, most of the tumor cells are latently infected by KSHV indicating that viral latency and latent products are likely essential for the development of KS tumors [7], [8].

MicroRNAs (miRs) are a class of ∼22 nt long non-coding small RNAs involved in diverse cellular functions and in all phases of cancer development [9]. MiRs primarily regulate gene expression at post-transcriptional level mainly through binding to the 3′ untranslated region (3′UTR) of the target mRNAs [9]. The identification of miRs encoded by KSHV implicates that this mode of gene regulation also exists for this herpesvirus [2].

KSHV miRs are highly expressed during latency and in KS tumors [10]–[15], implicating their essential functions in the viral life cycle and in the development of KS tumors. Indeed, several KSHV miRs regulate viral latency by directly targeting viral genes or indirectly targeting cellular pathways [16]–[21]. KSHV miRs also regulate diverse cellular pathways [17], [22]–[36], which might contribute to the development of KSHV-related malignancies. In this study, by using the newly developed cellular transformation system combined with a reverse genetics approach [3], [37], we have demonstrated that viral miRs are essential for KSHV-induced cellular transformation and tumorigenesis. Our results show that KSHV miRs redundantly target the NF-κB pathway to regulate cell cycle progression and apoptosis. By using a genomic approach, we have found that KSHV miRs redundantly regulate diverse cellular pathways, however, with several cancer-related pathways preferentially targeted, highlighting the intricacies of KSHV-cell interactions.

## Results

### KSHV miR cluster is essential for cellular transformation and tumorigenesis

**Figure 1:**
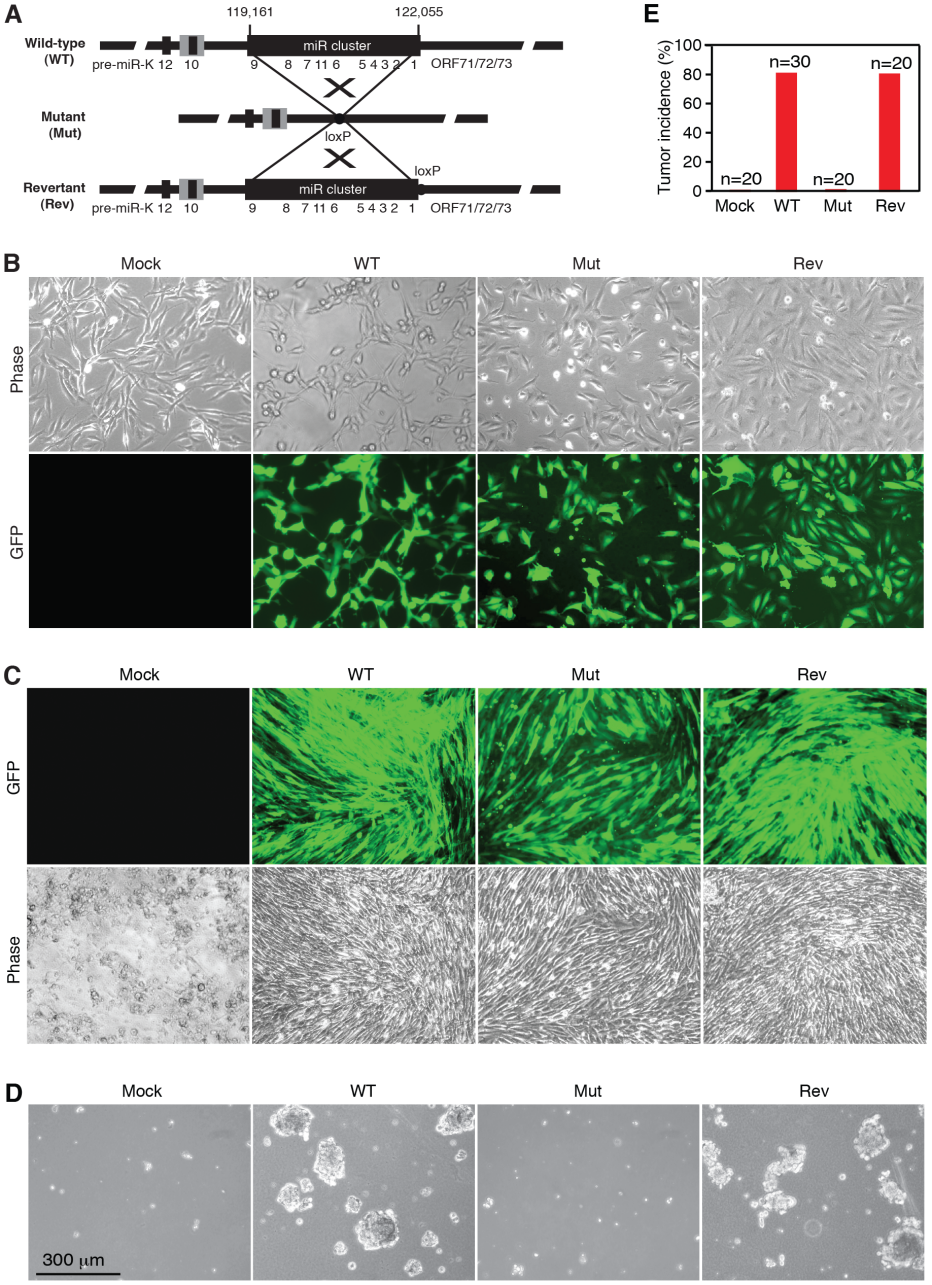
KSHV miR cluster is required for cellular transformation and tumorigenesis. (A) Schematic illustration of wild-type (WT) KSHV recombinant virus, and a mutant virus containing a cluster of 10 KSHV pre-miRs deleted (Mut) and its revertant (Rev). (B) Morphology and GFP expression of MM cells (Mock) and MM cells infected by the KSHV recombinant viruses. (C) MM cells infected by WT and Rev viruses formed foci in cultures while Mock cells and MM cells infected by the Mut virus did not. (D) Formation of colonies in semisolid softagar medium plated with MM cells and MM cells infected by the KSHV recombinant viruses. (E) Tumor incidences in nude mice inoculated with MM cells and MM cells infected by the KSHV recombinant viruses.

To determine the roles of KSHV miRs in cellular transformation, we infected MM cells with a mutant virus containing a deletion of a cluster of 10 pre-miRs including pre-miR-K1–9 and -K11 (Mut) together with its corresponding revertant (Rev) virus and the wild-type (WT) virus (Figure 1A). There is no obvious morphological difference between cells infected by the WT and Mut viruses (Figure 1B). Expression analysis of viral mRNAs and proteins showed that, similar to cells infected by WT and Rev viruses, most Mut cells were in the latent state (Figure S1 and S2), which were consistent with previous reports using 293 cells [17], [18]. In culture, Mut cells neither formed any foci nor grew any overlapping cultures while WT and Rev cells did (Figure 1C). While WT and Rev cells formed large colonies in semisolid softagar, Mut cells only formed small colonies of 3–5 cells (Figure 1D). These results indicated that, similar to uninfected cells (Mock), Mut cells were anchorage-dependent and contact-inhibited, and thus were not transformed. Accordingly, no tumor was induced by Mut cells in the nude mice while WT and Rev cells efficiently induced tumors with 80% incidence rates (Figure 1E). Therefore, the Mut virus was neither transforming nor tumorigenic.

**Figure 2:**
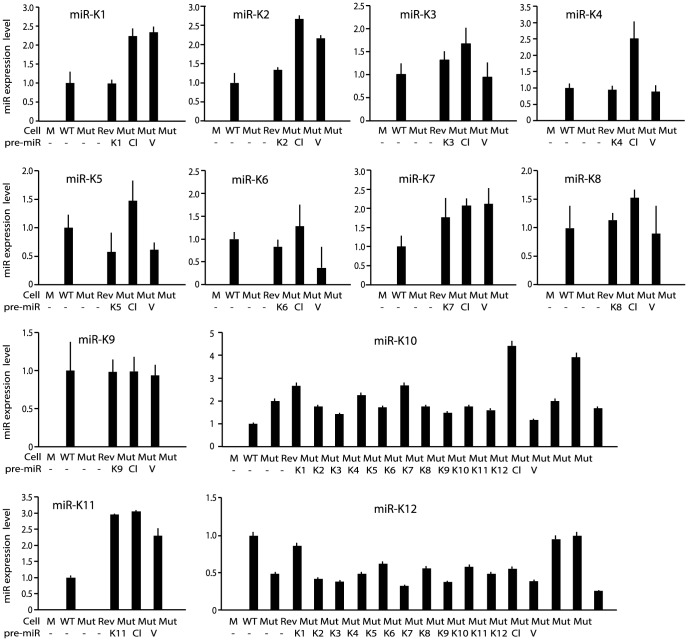
Expression of KSHV miRs in MM cells infected by WT, Mut and Rev viruses, as well as MM cells infected by Mut virus complemented by pre-miRs (Ki), miR cluster (Cl) or vector control (V). MiRs were detected by quantitative real-time reverse transcription PCR (RT-qPCR).

**Figure 3:**
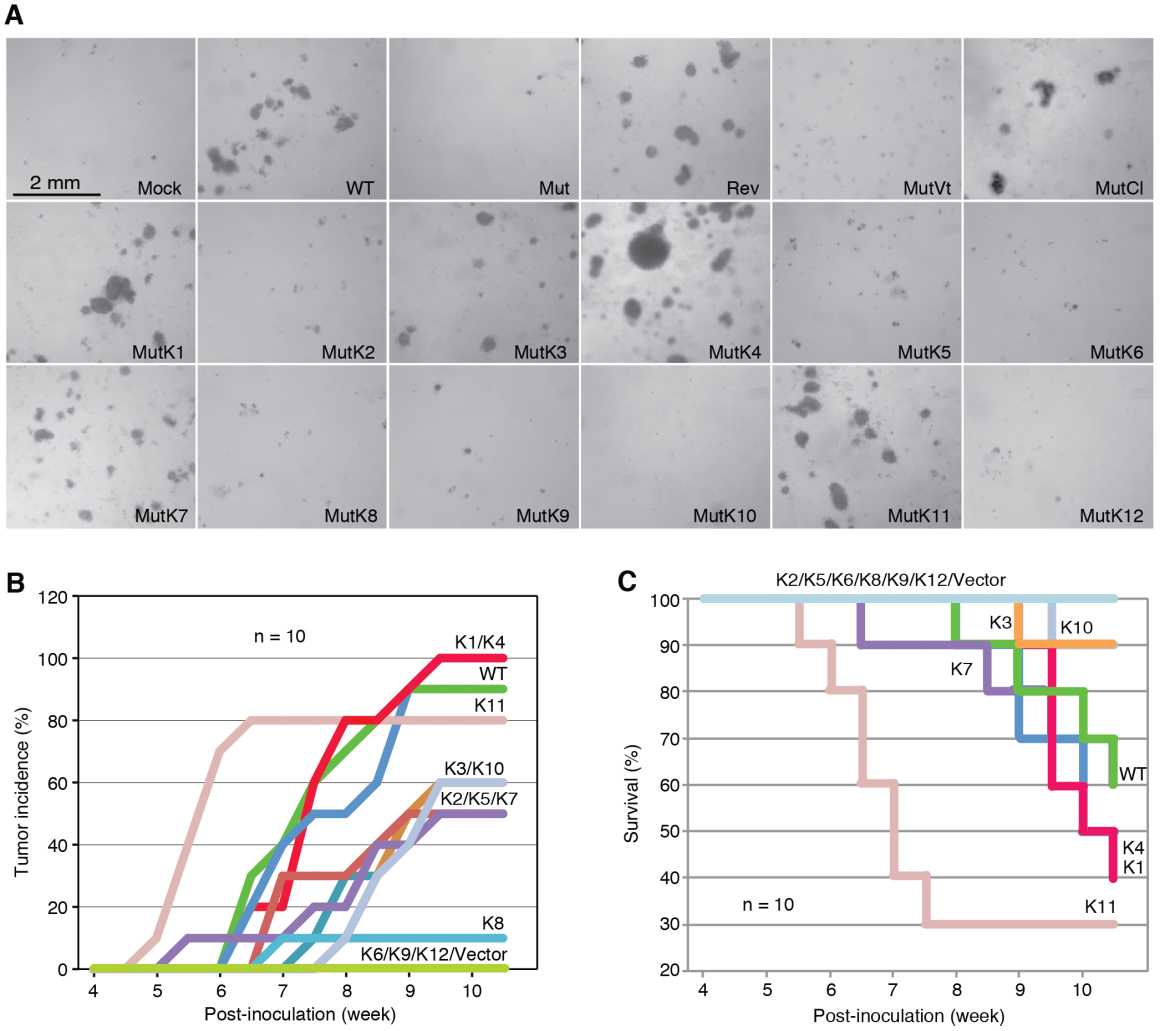
Multiple KSHV miRs rescue cellular transformation and tumorigenesis of the Mut virus. (A) Formation of colonies in softagar medium plated with MM cells infected by WT, REV and Mut viruses and Mut virus complemented by individual KSHV pre-miRs (MutKi), miR cluster (MutCl) or vector control (MutVt). (B–C) Tumor incidences over time (B) and Kaplan-Meier survival curves (C) of nude mice inoculated with MM cells infected by WT virus or Mut virus complemented by individual KSHV pre-miRs or vector control. Tumor volume of 0.2 cm^3^ was used as a threshold for tumor incidence. Tumor analyses were performed at 10 weeks following inoculation of the cells or when the volumes reached 1 cm^3^.

We performed genetic complementation by stably expressing the miR cluster in Mut cells (MutCl). The expression levels of miRs in MutCl cells were within 1- to 3-fold of those of WT cells (Figure 2). The miR cluster (MutCl) but not the vector control (MutVt) restored anchorage independence of Mut cells (Figure 3A), confirming its essential role in KSHV-induced cellular transformation.

To determine whether the Mut virus could immortalize MM cells, we serially passaged the Mut cells. Mock cells underwent crisis at around passage 27–30 as previously reported [3]. Similar to WT and Rev cells, Mut cells were continuously passaged for far beyond the crisis point, indicating that the Mut virus had immortalized the primary cells (Figure S3). Therefore, the miR cluster was not required for KSHV immortalization of MM cells.

### Multiple individual KSHV pre-miRs rescue the oncogenicity of the Mut virus

To identify specific miRs that mediate KSHV cellular transformation, we performed genetic complementation by stably expressing individual pre-miRs in Mut cells (MutKi). The expression levels of miRs in the respective MutKi cells were similar to those of MutCl cells (Figure 2). As expected, all MutKi cells were immortalized (Figure S3). MutK1, MutK4, and MutK11 cells formed large colonies in softagar and efficiently induced tumors in nude mice (Figure 3 and Figure S4). In fact, MutK11 cells induced faster tumor formation and progression than WT cells (Figure 3B–3C and Figure S4), which might reflect the 3-fold higher miR-K11 expression level in MutK11 cells than in WT cells (Figure 2), and hence a dose-dependent effect. These results are not surprising because miR-K11 is a functional ortholog of miR-155, a human oncogenic miR [26], [34]. Tumors induced by MutK1 and MutK4 cells had tumor formation and progression rates similar to those of WT cells (Figure 3B–3C and Figure S4). Tumors induced by MutK7 cells had a slower tumor formation rate than that of WT cells but the two cell types induced tumors with similar progression rates. MutK2, MutK3, and MutK5 cells had partial cellular transformation phenotype, forming smaller colonies in softagar and inducing slower rates of tumor formation and progression (Figure 3 and Figure S4). MutK10 cells had no visible colony in softagar but induced tumors with slower tumor formation and progression rates than those of WT cells (Figure 3 and Figure S4). While miR-K10 was not deleted in Mut virus, it was expressed 4.5-fold higher in MutK10 than WT cells, suggesting a dose-dependent effect, and that miR-K10 expression from Mut virus alone was insufficient to sustain cellular transformation. Together, these results indicated that multiple miRs mediated KSHV cellular transformation and tumorigenesis.

### KSHV miRs promote cell cycle progression and inhibit apoptosis

**Figure 4:**
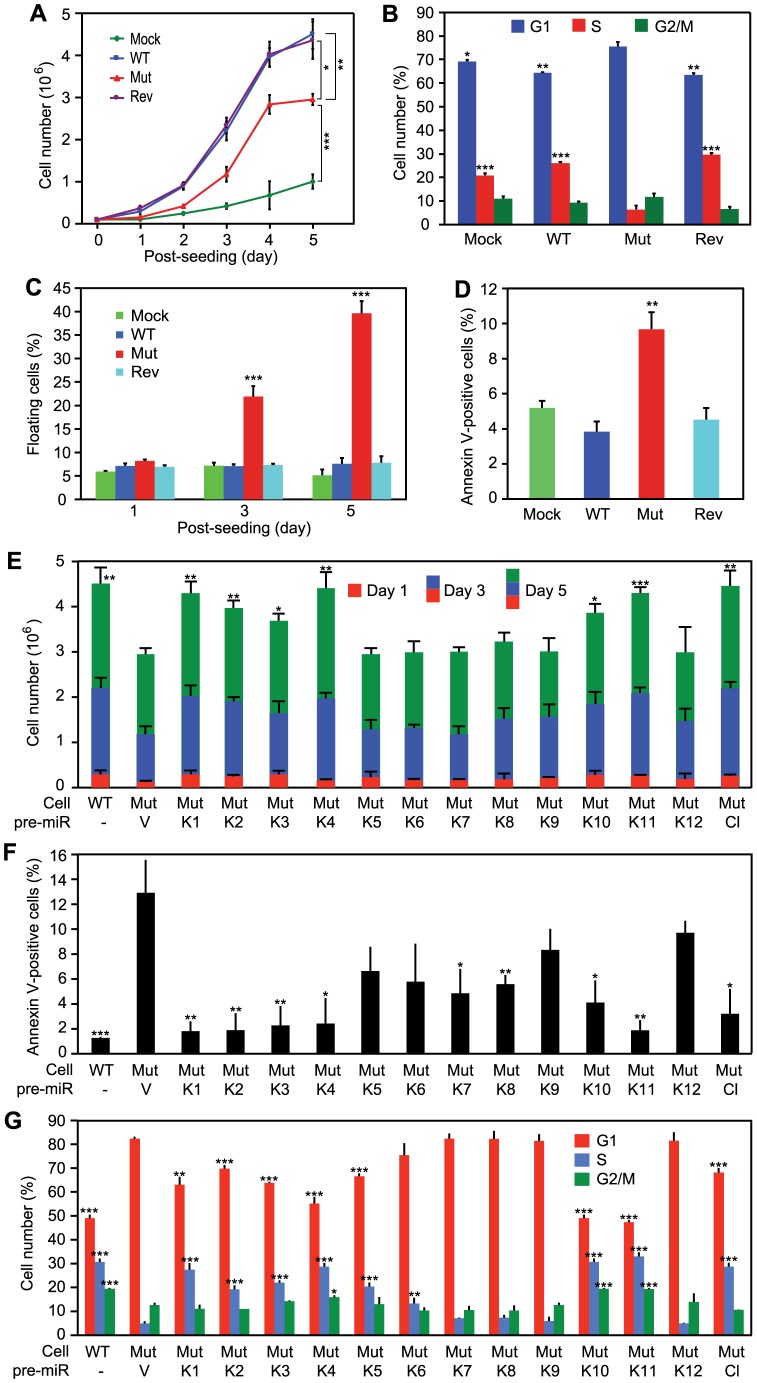
KSHV miRs promote cellular proliferation by regulating cell cycle and inhibiting apoptosis. (A–D) Growth curves (A), cell cycle profiles (B), floating cells (C), and annexin V-positive adherent cells (D) in cultures of MM cells infected by different KSHV recombinant viruses. (E–G) Cell growth (E), annexin V-positive adherent cells (F), and cell cycle profiles (G) of cultures of MM cells infected by the Mut virus complemented by individual KSHV pre-miRs (MutKi), miR cluster (MutCl) or vector control (MutVt). Cell cycle and apoptosis were analyzed at day 5 post-seeding. All statistical analyses were performed by comparing other cells with the MutVt cells.

WT virus increases cellular proliferation by promoting cell cycle progression [3]. While Mut cells retained growth advantage over Mock cells, their growth rates were significantly lower than those of WT and Rev cells, particularly after day 3 post-seeding when cultures reached confluency (Figure 4A), which were consistent with their anchorage-dependence. Cell cycle analysis at day 5 post-seeding showed that Mut cells had significantly more G1-phase cells and less S-phase cells than WT and Rev cells had (Figure 4B). In fact, there were more G1-phase cells and less S-phase cells in Mut cells than those of Mock cells. Furthermore, we observed significantly more floating cells in the Mut cultures than the WT and Rev cultures, particularly after they reached confluency (Figure 4C). Analysis of adherent cells showed that Mut cultures had more apoptotic cells than WT and Rev cultures had (Figure 4D). Complementation of Mut cells with the miR cluster rescued the growth rates, reduced the number of apoptotic cells, and increased the number of S-phase cells in cell cycle to levels similar to those of WT cells (Figure 4E–4G). These results indicated that the Mut virus induced cell cycle arrest and apoptosis, which was rescued by the miR cluster.

While the Mut cells had higher KSHV lytic activity, over 90% of them were latently infected by the Mut virus (Figure S1 and S2). Thus, the increased viral lytic activity in a small number of cells was unlikely the cause of the 40% floating cells observed in the Mut cell cultures. Furthermore, treatment with Ganciclovir, an inhibitor of herpesvirus DNA polymerase, did not reduce the number of floating cells in Mut and WT cell cultures (Figure S5). We therefore concluded that the miR cluster directly regulated cell cycle progression and apoptosis in the KSHV-transformed cells.

Because multiple miRs could independently rescue cellular transformation of the Mut virus, it suggested that they might have redundant functions. Indeed, compared to WT cells, the cell growth rates were fully rescued in MutK1, MutK4 and MutK11 cells, and partially in MutK2, MutK3 and MutK10 cells (Figure 4E). All MutKi cells had lower apoptosis rates than Mut cells (Figure 4F). In particular, MutK1, MutK2, MutK3, MutK4 and MutK11 cells had apoptosis rates as low as that of WT cells (Figure 4F). Similarly, cell cycle profiles of MutK1, MutK4, MutK10 and MutK11 cells were fully rescued to that of WT cells (Figure 4G). These results indicated that several miRs regulated either one or both cell cycle and apoptosis pathways. In agreement with their effects on cellular transformation and tumor induction, pre-miR-K1, -K4 and -K11 strongly regulated cell cycle and apoptosis.

### Regulation of cellular pathways by KSHV miRs

**Figure 5:**
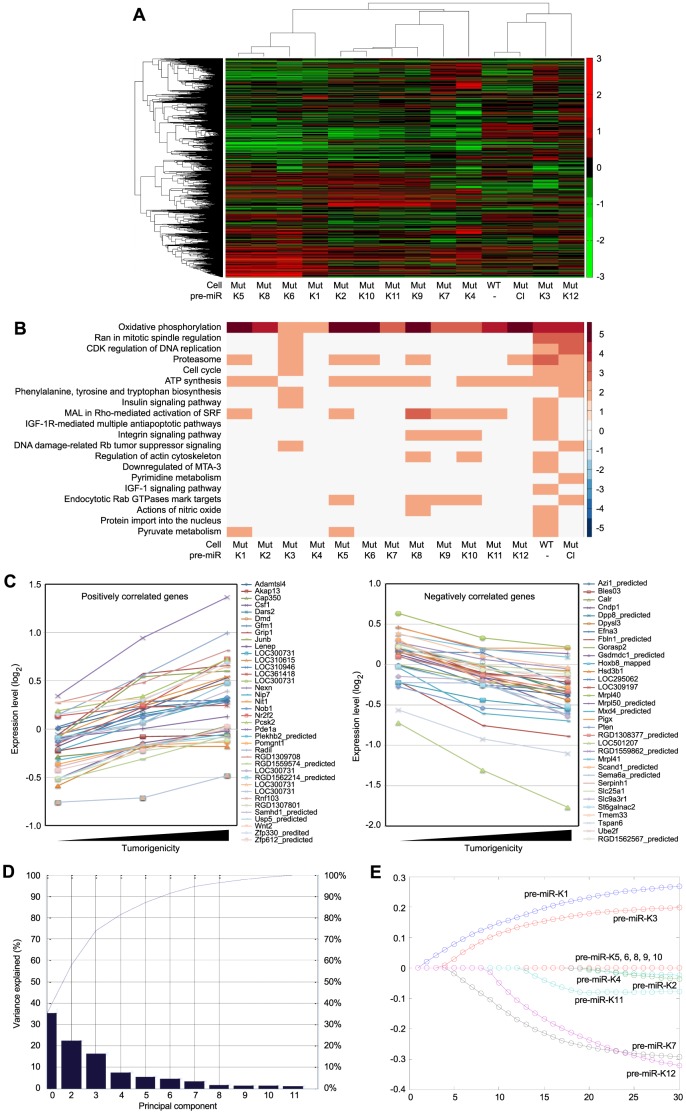
Gene expression profiling analysis of KSHV miRs. (A) Unsupervised clustering of gene expression profiles of WT cells, and Mut cells complemented with the miR cluster (Cl) or individual pre-miRs (Ki). Note that the Mut cells condition was subtracted from all the MutKi cells to eliminate the effect of other unrelated viral genes (see Materials and Methods for details). (B) Top 20 most enriched pathways in WT cells, and Mut cells complemented with the miR cluster or individual pre-miRs compared to Mut cells complemented with the vector control. The color scale represents the GSEA normalized enrichment score. The expression fold changes of all genes of these cells are in Table S5. (C) Signature genes that are positively or negatively correlated with tumorigenicity identified by Anova. (D) Principle components (PCs) obtained from the expression data of MutKi cells and the corresponding percentages of explained expression variances. Note that >95% of expression variances of the 16,501 genes in MutKi cells can be explained using only 8 PCs. (E) Lasso fitting to determine the linear combinatory effect of individual pre-miRs to the overall expression pattern of Mut cells complemented with the miR cluster. The x-axis denotes the Lasso iteration and the y-axis represents the coefficients or predicted effects for each MutKi. The Lasso reached the converged predictions at the 30th iteration.

To reveal the cellular pathways targeted by miRs that might be essential for KSHV cellular transformation, we compared gene expression profiles of WT and Mut cells. Consistent with the enhanced growth and survival phenotypes (Figure 4A–4D), Gene Set Enrichment Analysis (GSEA) confirmed that oxidative phosphorylation pathway and several other metabolic and energy consumption-related pathways, as well as a number of cell cycle- and apoptosis-related pathways were enriched in WT cells (Table 1). Unsupervised clustering with other MutKi cells showed similar profiles of MutCl and WT cells with both falling in the same subgroup, indicating that MutCl cells copied the gene expression profile of WT cells, thus validating the genetic rescue experimental approach (Figure 5A). In contrast, the profiles of MutKi cells fell into distinct subgroups implicating their functional divergences. Nevertheless, similar to WT and MutCl cells, GSEA showed that all MutKi cells were enriched for the oxidative phosphorylation pathway with some also enriched for cell cycle- and apoptosis-related pathways (Figure 5B and Table S1), which were consistent with the ability of the pre-miRs to rescue the cell cycle and apoptosis phenotypes of Mut cells (Figure 4F–4G).

**Table 1:** Top enriched pathways in WT cells compared to Mut cells.

NAME	PATHWAY NAME	SIZE	ES	NES	NOM p-val	FDR q-val	FWER p-val	RANK AT MAX	LEADING EDGE
HSA00190	Oxidative phosphorylation	72	0.38443	3.875	0	0	0	3603	tags = 58%, list = 20%, signal = 73%
H_RANMSPATHWAY	Role of Ran in mitotic spindle regulation	5	0.93719	2.6367	0	0.0068837	0.013	1133	tags = 100%, list = 6%, signal = 107%
HSA03050	Proteasome	27	0.4238	2.5803	0	0.0063235	0.018	5709	tags = 74%, list = 32%, signal = 108%
H_MCMPATHWAY	CDK Regulation of DNA Replication	13	0.56196	2.4812	0	0.0086194	0.033	2350	tags = 69%, list = 13%, signal = 80%
HSA04110	Cell cycle	64	0.2652	2.4768	0	0.0071126	0.034	2833	tags = 42%, list = 16%, signal = 50%
HSA04910	Insulin signaling pathway	76	0.20299	2.1107	0.0019569	0.074815	0.353	4645	tags = 46%, list = 26%, signal = 62%
H_MALPATHWAY	Role of MAL in Rho-Mediated Activation of SRF	13	0.47619	2.0336	0.0058939	0.10793	0.526	2508	tags = 62%, list = 14%, signal = 71%
H_IGF1RPATHWAY	Multiple antiapoptotic pathways from IGF-1R signaling lead to BAD phosphorylation	13	0.46411	2.0095	0.0059406	0.11021	0.576	4108	tags = 69%, list = 23%, signal = 90%
H_INTEGRINPATHWAY	Integrin Signaling Pathway	19	0.39047	1.9888	0.0019305	0.11261	0.616	2449	tags = 53%, list = 14%, signal = 61%
HSA04810	Regulation of actin cytoskeleton	127	0.14694	1.9245	0.0059289	0.15204	0.764	2614	tags = 29%, list = 15%, signal = 34%
H_MTA3PATHWAY	Downregulated of MTA-3 in ER-negative Breast Tumors	10	0.47574	1.9132	0.0039761	0.14796	0.789	2238	tags = 60%, list = 12%, signal = 68%
H_IGF1PATHWAY	IGF-1 Signaling Pathway	11	0.46332	1.8633	0.013752	0.18541	0.879	4750	tags = 73%, list = 26%, signal = 99%
H_NO1PATHWAY	Actions of Nitric Oxide in the Heart	14	0.41225	1.8495	0.011472	0.1855	0.9	4151	tags = 64%, list = 23%, signal = 84%
HSA00193	ATP synthesis	27	0.29377	1.8319	0.018367	0.19189	0.917	4048	tags = 52%, list = 23%, signal = 67%
H_NPCPATHWAY	Mechanism of Protein Import into the Nucleus	5	0.6701	1.8279	0.012821	0.18303	0.923	5934	tags = 100%, list = 33%, signal = 149%
HSA00620	Pyruvate metabolism	27	0.29628	1.8063	0.012245	0.19359	0.945	3337	tags = 48%, list = 19%, signal = 59%
HSA04510	Focal adhesion	92	0.15985	1.8026	0.015414	0.18512	0.95	3589	tags = 36%, list = 20%, signal = 45%
HSA00010	Glycolysis/Gluconeogenesis	33	0.25389	1.7651	0.017375	0.21432	0.977	4160	tags = 48%, list = 23%, signal = 63%

To identify the functional genes sets that might be correlated with the tumor phenotype, we divided the MutKi cells into three classes. Class 1 had high tumorigenicity, which included MutK1, MutK4 and MutK11 cells; Class 2 had medium tumorigenicity, which included MutK2, MutK3, MutK5, MutK7 and MutK10 cells; and Class 3 had low or no tumorigenicity, which included MutK6, MutK8, MutK9 and MutK12 cells (Figure 3). We then performed Analysis of Variance (ANOVA) on all genes to predict the subset of signature genes whose expression levels showed significant differences among these three classes. A total of 153 signature genes with significantly differential expression levels across the three classes (P-value<0.05) were obtained (Table S2). Pathway enrichment of the signature genes by Ingenuity Pathway Analysis (IPA) revealed that the top enriched network functions were cellular development, cellular growth and proliferation, and reproductive system development and function (Table 2). To understand how the signature genes were involved in regulating the enriched pathways, we calculated the average expression levels of all signature genes within each class and mapped them to the top enriched networks (Figure S6). As expected, differential expression of the signature genes among the three classes was evident. We further isolated the 38 signature genes whose expression levels exhibited positive correlation with tumorigenicity and 33 signature genes whose expression levels exhibited negative correlation with tumorigenicity (Figure 5C). Significantly, a number of genes with positive correlation with tumorigenicity such as Akap13, CSF1, Grip1, JunB, Nexn, Nob1, Nrf2f2, Pcsk2, Pde1a, Pomgnt1, Radil, Usp5 and Wnt2 had previously been shown to have oncogenic, growth-promoting or pro-angiogenic activities [38]–[54] while several genes with negative correlation with tumorigenicity such as Calr, Dpp8, Fbln1, Hsd3b1, Mxd4, Pten and Mrpl41 had previously been shown to have tumor suppressive, growth inhibitory, proapoptotic or immune regulatory activities [55]–[63]. The deregulation of these genes by KSHV miRs was likely to contribute to KSHV-induced tumorigenesis.

**Table 2:** Top networks of signature genes associated with tumorigenicity induced by KSHV miRs.

ID	Associated network functions	Score
1	Cellular Development, Cellular Growth and Proliferation, Reproductive System Development and Function	45
2	Small Molecule Biochemistry, Cellular Assembly and Organization, Developmental Disorder	32
3	Infectious Disease, Inflammatory Disease, Neurological Disease	29
4	Cell-To-Cell Signaling and Interaction, Hereditary Disorder, Nervous System Development and Function	26
5	Cardiovascular System Development and Function, Lipid Metabolism, Small Molecule Biochemistry	24

We further determined the linear combinatory effect of individual miRs to the overall expression pattern of MutCl cells by Lasso fitting [64]. We performed principal component analysis (PCA), which projected the expression of all analyzed genes to 8 most significant principle components (Figure 5D). Lasso was then applied to regress the principle components among samples to infer the combinatory impact from miRs and identified pre-miR-K1 as the largest contributor followed by pre-miR-K3 (Figure 5D and 5E).

### MiR-K1 targeting of IκBα but not p21 is essential for promoting cell cycle progression and inhibiting apoptosis

**Figure 6:**
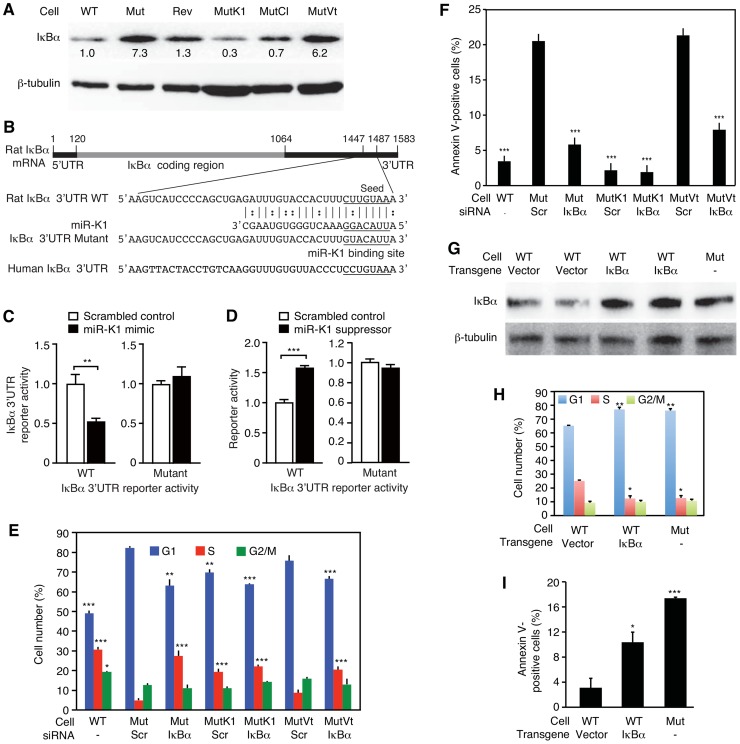
MiR-K1 targeting of IκBα is essential and sufficient for KSHV subversion of cell cycle and apoptosis pathways. (A) Expression of IκBα protein in cells with and without the expression of miR-K1 measured by Western-blotting. Cells analyzed were WT cells (WT), Mut cells (Mut), Rev cells (Rev), and Mut cells complemented with miR-K1 (MutK1), miR cluster (MutCl and vector control (MutVt). (B) Sequence alignment of miR-K1 with rat IκBα 3′UTR WT reporter and its mutant reporter containing a mutation in the putative miR-K1 targeting site, and the corresponding human IκBα 3′UTR sequence. (C) Suppression of IκBα 3′UTR WT reporter activity but not its mutant reporter activity by KSHV miR-K1. 293 cells were cotransfected with the IκBα 3′UTR WT reporter or its mutant reporter together with a miR-K1 mimic or a scrambled control and a β-galatosidase expression construct for 48 h and measured for relative luciferase activities. (D) Derepression of the inhibitory effect of miR-K1 on IκBα 3′UTR WT reporter but not its mutant reporter in WT cells by a miR-K1 suppressor. (E-F) Cell cycle profiles (E) and apoptosis(F) in Mut cells, Mut cells complemented with miR-K1 or vector control with knock down of IκBα using a specific siRNA or a scrambled control. (G–I) Expression of IκBα in WT cells(G) is sufficient to cause a shift in cell cycle profile (H) or apoptosis rate (I) to that resembling Mut cells. Statistical analyses were performed by comparing other cells with Mut cells transfected with scrambled siRNA (E–F) or WT cells transfected with control vector (H–I).

MiR-K1 targets IκBα, an inhibitor of the pro-survival NF-κB pathway, and cyclin-dependent protein kinase inhibitor p21/WAF1, a cell cycle regulator [17], [65]. Because of the robust phenotypes of MutK1 cells (Figure 3 and 4), we examined the consequence of targeting IκBα. Indeed, the expression level of IκBα protein was lower in cells expressing miR-K1, including WT, Rev, MutK1 and MutCl cells, than cells without miR-K1, including Mut cells, and cells complemented with vector control (MutVt) (Figure 6A and Figure S7). Examination of the 3′UTR of rat IκBα indeed identified a conserved miR-K1 targeting site with a single nucleotide difference (U to C) from the human site (Fig. 6B). Our previous study has shown that this site is a functional targeting site of miR-K1 in human cells [17]. In a reporter assay, miR-K1 suppressed the rat IκBα 3UTR reporter activity by 50% (Fig. 6C). Mutation of the putative targeting site abolished the repressive effect of miR-K1. In WT cells, a miR-K1 suppressor increased the IκBα 3UTR reporter activity by 1.6-fold but had no effect on the mutant 3′UTR reporter activity (Fig. 6D). Together, these results indicated that, similar to human IκBα, rat IκBα was also a target of KSHV miR-K1.

To investigate if miR-K1 regulated cell cycle and apoptosis by targeting IκBα, we performed siRNA knock down of IκBα in the Mut cells (Figure S8A and S8B). SiRNA knock down of IκBα in Mut and MutVt cells was sufficient to rescue cell cycle profiles to those of WT cells, reducing G1-phase cells from 82% and 78% to 63% and 65%, and increasing S-phase cells from 4% and 6% to 27% and 20%, respectively (Figure 6E, and Figure S8A and S8B). Similarly, knock down of IκBα was sufficient to reduce apoptotic cells from 20.5% and 22% to 6% and 7.5% in Mut and MutVt cells, respectively (Figure 6F). Significantly, expression of IκBα with a construct lacking its native 3′UTR, thus escaping miR-K1 targeting, in WT cells was sufficient to alter cell cycle profiles to those resembling Mut cells (Figure 6G and 6H), and increase apoptotic cells from 3% to 10.5% (Figure 6I). Together, these results indicated that IκBα targeting by miR-K1 was necessary and sufficient for cell cycle progression and inhibition of apoptosis in KSHV-transformed cells.

Similar to IκBα, the expression level of p21 was lower in cells expressing miR-K1 than cells without miR-K1 (Figure S9A and S9B). However, siRNA knock down of p21 in Mut and MutVt cells had no effect on cell cycle (Figure S8C, S8D and S9C), indicating that p21 targeting was not required for miR-K1 regulation of cell cycle. Interestingly, G1-phase cells was increased from 59% to 65%, and S-phase cells was reduced from 26% to 19% following knock down of p21 in MutK1 cells. This was accompanied with an increase in apoptotic cells from 4% to 23% (Figure S9D). Knock down of p21 increased apoptotic cells from 21% to 38% in Mut cells, and from 25% to 43% in MutVt cells. These results indicated that miR-K1 targeting of p21 was not required for miR-K1 inhibition of apoptosis. On the contrary, persistent low level of p21 was likely essential for maintaining the homeostasis and survival of KSHV-transformed cells.

### Multiple KSHV miRs redundantly regulate IκBα and the NF-κB pathway

**Figure 7:**
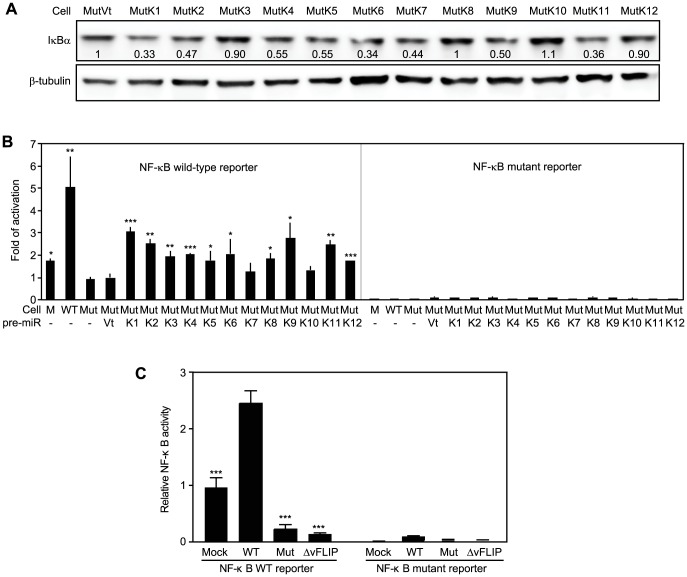
Multiple KSHV miRs target the IκBα and NF-κB pathway. (A) IκBα expression in MM cells infected by Mut virus complemented by individual KSHV pre-miRs (MutKi) or vector control (MutVt). Cell grown to confluency were examined for IκBα protein expression by Western-blotting. (B) NF-κB reporter activity in MM cells infected by Mut virus complemented by individual KSHV pre-miRs or vector control. Cells were transfected with a NF-κB reporter construct or it's a mutant reporter together with a β-galatosidase expression construct for 48 h and measured for relative luciferase activities. (C) Deletion of either the miR cluster or vFLIP from the KSHV genome is sufficient to abolish KSHV activation of the NF-κB pathway. Uninfected cells or cells infected by WT and mutants of miRNA cluster (Mut) or vFLIP (ΔvFLIP) were transfected with either NF-κB WT luciferase reporter or NF-κB mutant luciferase reporter together with a β-galactosidase construct for 48 h. Cells were collected and measured for relative luciferase activities. Statistical analyses were performed by comparing other cells with Mut cells (B) or WT cells (C).

The above results indicated that IκBα and its related pathways could be redundantly regulated by KSHV miRs. Bioinformatics and 3′UTR screenings have failed to identify IκBα as the direct target of other KSHV miRs [17]. Nevertheless, IκBα is regulated by as many as 60 cellular pathways (Table S3). Indeed, IκBα levels were reduced by more than 40% in MutK2, MutK4, MutK5, MutK6, MutK7, MutK9 and MutK11 cells besides MutK1 cells (Figure 7A). However, MutK3, MutK8, MutK10 and MutK12 cells had minimal changes in IκBα level, suggesting regulation of downstream pathways by these miRs.

Because NF-κB is the common effector pathway of IκBα inhibition, we examined its activation by KSHV miRs. All MutKi cells except MutK7 and MutK10 had 1.8- to 3-fold higher NF-κB activities than Mut cells had (Figure 7B). These miRs might synergistically or additively contribute to the 5-fold constitutive NF-κB activation in WT cells. Indeed, compared to Mock cells, many components and downstream targets of the NF-κB pathway were upregulated (Figure S10).

In addition to IκBα, several other identified cellular targets of KSHV miRs also regulate cell growth and survival (Table 3). MiR-K5, 9, 10a and 10b target Bcl2-associated factor BCLAF1 [36]; miR-K1, 3 and 4-3p target caspase 3 [35]; and miR-K10a targets tumor necrosis factor-like weak inducer of apoptosis receptor (TWEAKR) [23]. Furthermore, miR-K10a and its variants also target TGF-β type II receptor [28] while miR-K11 is an ortholog of cellular miR-155 [26], [34], which is implicated in cancer [66]. It has been shown that miR-K11 targets BACH1 and SMAD5 [26], [29], [31], [34]. Thus, these miRs could directly regulate cell cycle and apoptosis by targeting genes that are downstream of the NF-κB or other pathways.

**Table 3:** Experimentally confirmed cellular genes targeted by KSHV miRs.

Targeting gene	Pre-miR	Functional consequences	Reference
IκBα	K1	Cell growth and survival, inflammation	[17]
p21	K1	Cell cycle	[65]
Caspases 3	K1, 3, 4	Cell survival	[35]
BCLAF1	K5, 9, 10	Cell survival	[36]
TGF-βRII	K10	Cell growth and survival	[28]
TWEAKR	K10	Cell survival	[23]
BACH1	K11	Cell growth and viability	[26], [31], [34]
SMAD5	K11	Cell growth and survival	[29]

Previous studies have shown that overexpression of KSHV vFLIP activates the NF-κB pathway [67], [68]. To assess the relative contribution of KSHV miRs and vFLIP to the activated NF-κB activity in the KSHV-transformed cells, we examined the NF-κB reporter activity in cells infected with a KSHV mutant with vFLIP deleted (ΔvFLIP) [69]. Similar to the Mut cells, deletion of vFLIP abolished the activation of the NF-κB pathway (Figure 7C). In fact, both Mut and ΔvFLIP cells had lower NF-κB activity than the Mock cells. These results indicated that, in the context of KSHV infection, both miRs and vFLIP were required for the activation of the NF-κB pathway.

### KSHV-transformed cells are addicted to the NF-κB pathway

**Figure 8:**
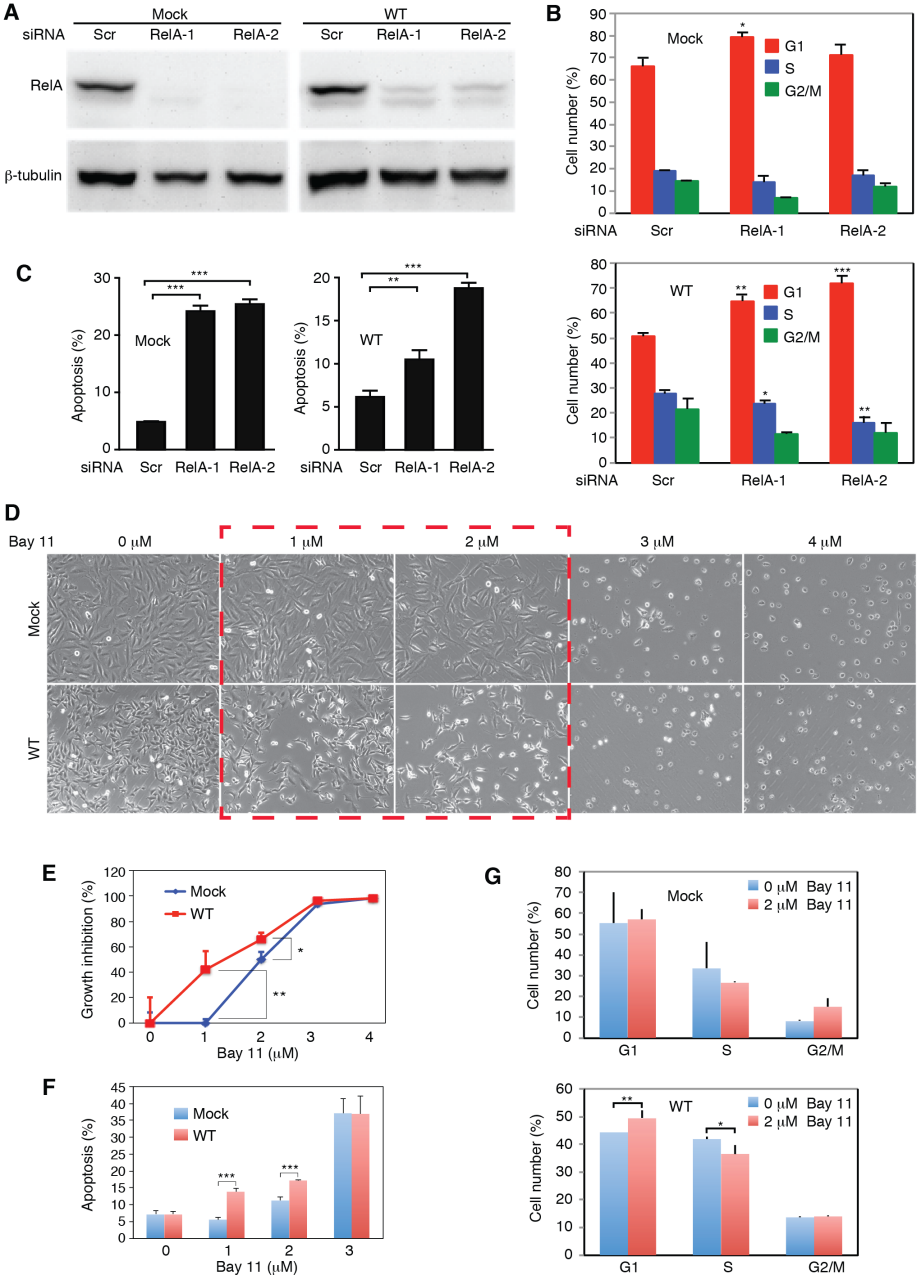
Inhibition of the NF-κB pathway prevents cell cycle progression and induces apoptosis in KSHV-transformed cells. (A) SiRNA knock down of RelA in Mock and WT cells. Cells were transfected with RelA siRNAs (RelA-1 or RelA-2) or a scrambled control (Scr) for 48 h and the expression of RelA protein was examined by Western-blotting. (B–C) Knock down of RelA inhibits cell cycle progression in WT cells with minimal effect on the Mock cells (B) but induces apoptosis in both Mock and WT cells (C). Cells were transfected with RelA siRNAs or a scrambled control (Scr) for 48 h and total apoptotic cells and cell cycle were examined. (D–G) Effects of the NF-κB pathway inhibitor Bay-11 on cell morphology (D), cell growth (E), apoptosis (F) and cell cycle progression (G) in Mock and WT cells. Statistical analyses were performed by comparing cells transfected with RelA siRNAs with scrambled control (B–C), and Mock cells with WT cells (E–G).

Because of the observed activation of the NF-κB pathway in WT cells, we explored if targeting of this pathway was sufficient to inhibit cell growth and survival of WT cells. We performed siRNA knock down of RelA, a key component of the NF-κB complexes (Figure 8A). Knock down of RelA significantly induced cell cycle arrest in WT cells (Figure 8B). The two RelA siRNAs had minimal effect on the cell cycle profiles of Mock cells. However, in WT cells, they increased the number of G1-phase cells from 51% to 65% and 72%, respectively (P<0.001 and P<0.001), and S-phase cells from 28% to 23% and 16%, respectively (P<0.05 and P<0.01). Nevertheless, knock down of RelA efficiently induced cell apoptosis in both WT and Mock cells, indicating that RelA and NF-κB activity were required for the survival of both types of cells (Figure 8C).

We further explored the use of specific NF-κB inhibitor Bay-11. Unlike the siRNA approach, the effect of Bay-11 can be more easily titrated. Significantly, inhibition of the NF-κB pathway in WT cells with low doses of Bay-11 was sufficient to induce cell growth arrest and apoptosis, and change cell cycle profile by increasing G1-phase cells and reducing S-phase cells (Figure 8D–8G). Under the same condition, Mock cells had no increase in apoptotic cells and only marginal change in cell cycle (Figure 8D–8G). However, at higher doses (>3 µM), Bay-11 was toxic to both Mock and WT cells. Thus, while the growth and survival of both Mock and WT cells required the NF-κB pathway, WT cells were more susceptible to the NF-κB inhibitor. These results further confirmed that multiple KSHV miRs might regulate cell cycle and apoptosis by activating the NF-κB pathway.

### Redundant functions are a common theme of KSHV miRs

**Figure 9:**
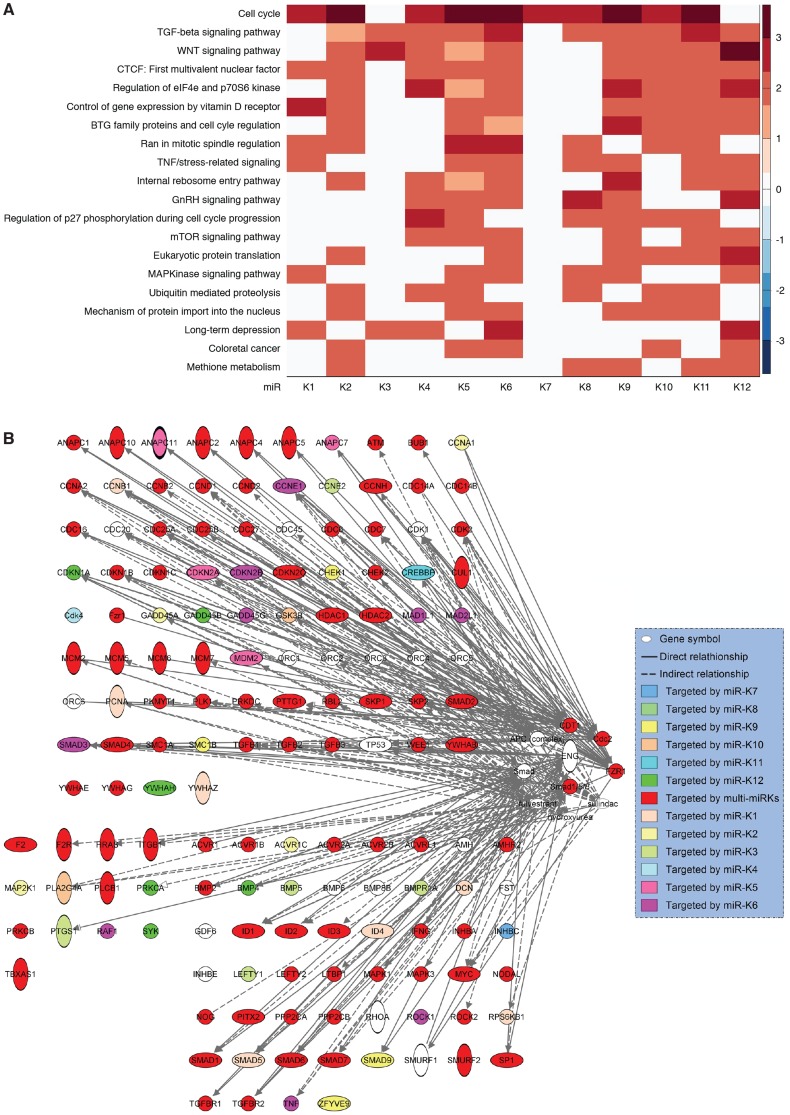
KSHV miRs preferentially target cancer-related pathways. (A) Top 20 most enriched pathways containing targets of KSHV miRs identified by combining SVMicrO predicted targets with gene expression results (Table S4). The color scale represents the GSEA normalized enrichment score. (B) Mapping of targets of KSHV miRs to the top three enriched pathways.

Our results and those from previous studies indicate that KSHV miRs have redundant functions and regulate several common cellular pathways. To examine the extent of these shared functions, we identified other potential targets of KSHV miRs by performing target prediction with SVMicrO [70], and integrating the identified targets with gene expression profiles using the Borda merging method to improve target prediction precision. Surprisingly, almost all the validated targets of KSHV miRs were identified to have high SVMicro scores by this approach (Table S4), confirming the effectiveness of this approach. Among those validated target genes, besides the identified miRs, a number of KSHV miRs were newly predicted to target these genes, confirming the theme of redundant functions among KSHV miRs. In fact, results of these systemic analyses showed that KSHV miRs redundantly target a large number of cellular genes in diverse pathways, further revealing the common functions of these miRs (Table S4). Significantly, a number of cellular pathways including cell cycle, TGF-β signaling, WNT signaling, Vitamin D receptor signaling, TNF/stress-related signaling, mTOR signaling and MAPK signaling were highly enriched (Figure 9A), indicating their top hierarchical positions among the pathways that were regulated by KSHV miRs. Many of these pathways have been implicated in the development of cancer and regulation of metabolic pathways [4]. We mapped the predicted targets of KSHV miRs of the top three pathways (Figure 9B). It was evident that over half of the genes (71 of 135) were the targets of more than one KSHV miRs.

## Discussion

KSHV encodes diverse genes and miRs with cellular regulatory functions [1], [2]. When tested by overexpression out of the context of KSHV infection, several KSHV genes manifest cellular transforming potentials [71]–[78]. Nevertheless, the roles of these viral genes in KSHV-induced tumorigenesis remain unclear because of the lack of a KSHV cellular transformation system. The recent development of a model of efficient KSHV infection and transformation of primary MM cells should facilitate the delineation of viral and cellular determinants required for KSHV-induced oncogenesis in the context viral infection [3]. Using this system combined with a reverse genetics approach, we have identified for the first time a viral determinant, the miR cluster, required for KSHV-induced cellular transformation and tumorigenesis.

While KSHV lytic replication in a small number of infected cells might promote KS tumor progression through an autocrine and paracrine mechanism as a result of de novo infection and expression of viral lytic gene products, most tumor cells in KS lesions are latently infected by KSHV [7]. Furthermore, results from the new cell model reveal that KSHV-induced cellular transformation depends on the viral genome [3]. Most KSHV-transformed cells as well as tumors derived from this model are latently infected by KSHV. These clinical and laboratory observations implicate that malignant proliferation of KSHV latent cells are the essential driving force behind the full growth of KS tumors. As a result, KSHV latent products are likely to have critical roles in the development of KSHV-induced tumors.

KSHV miRs are highly expressed during latency and in KS tumors [10]–[15]. The identification of KSHV miRs as the essential determinant for KSHV-induced tumorigenesis substantiates the critical role of latent infection in KSHV-induced oncogenesis. While several other viral latent genes including LANA, vFLIP and vCyclin possess oncogenic properties [74], [75], [77], [78], the requirement for KSHV miR cluster implicates that the combined effects of these viral genes are not sufficient to cause cellular transformation. Paradoxically, our results have shown that the miR cluster is not required for KSHV-induced cellular immortalization (Figure S3) indicating the involvement of other viral genes in KSHV-induced oncogenesis in addition to miRs. Indeed, the Mut virus induces cell cycle arrest and apoptosis in MM cells (Figure 4B–4D). These phenotypes are consistent with the outcomes of oncogenic insults, likely exerted by KSHV oncogenes, in primary cells. The ability of KSHV miRs to rescue cell cycle arrest and inhibit apoptosis (Figure 4) indicates that they primarily function in protective roles to rescue the KSHV-infected cells from oncogenic insults. Thus, a fine balance between uncontrolled cell growth elicited by oncogenic signals and cell homeostasis exerted by pro-survival signals as a result of the intricate interactions of KSHV miRs with other viral oncogenes are likely essential for successful KSHV-induced cellular transformation.

**Figure 10:**
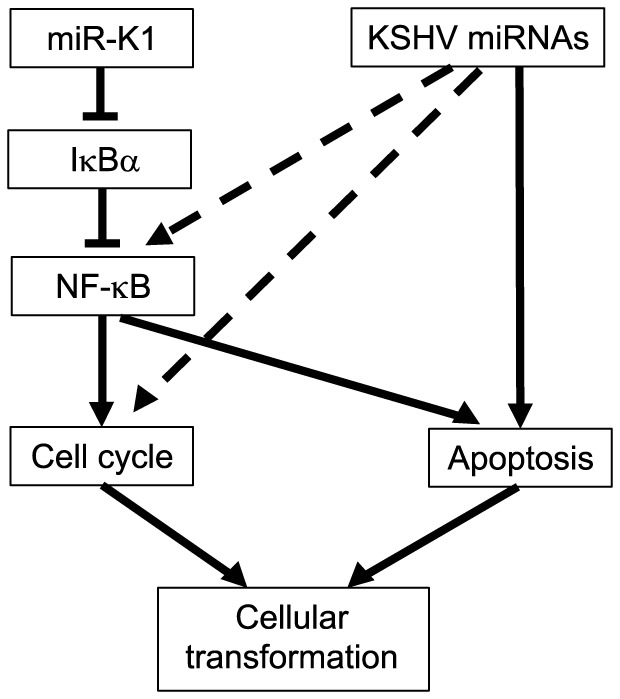
A model illustrating the regulation of cellular transformation by KSHV miRs.

Our results show that multiple individual KSHV miRs are capable of effectively rescuing the oncogenicity of the Mut virus (Figure 3). These observations point to the redundant functions of the miRs. Indeed, most KSHV miRs rescue cell cycle progression and inhibit apoptosis in the Mut cells (Figure 4E–4G). Significantly, most KSHV miRs in addition to miR-K1 exert these protective functions at least in part by targeting IκBα and the NF-κB pathway, which contribute to KSHV-induced cellular transformation (Figure 10). While it would be interesting in examining the role of targeting IκBα in KSHV-induced cellular transformation of human cells, unfortunately, there is currently no valid human cell model available for such studies. As a result of targeting IκBα and the NF-κB pathway, knock down of RelA is sufficient to cause cell cycle arrest in KSHV-transformed cells but has less effect in the uninfected cells (Figure 8B). While knock down of RelA or treatment of the cells with high doses of NF-κB inhibitor kill both uninfected and KSHV-transformed cells, lower doses of the NF-κB inhibitor differentially block cell growth by inducing apoptosis and inhibiting cell cycle progression of KSHV-transformed cells (Figure 8). Thus, KSHV-transformed cells are addicted to this essential pro-survival pathway, which is constitutively and redundantly activated by KSHV miRs (Figure 7B). Previous studies have shown that the NF-κB pathway is essential for the survival of PEL cells [79]. However, in this model, activation of the NF-κB pathway is primarily exerted by KSHV vFLIP protein [68], [80], [81]. In contrast, our results indicate that both KSHV vFLIP and miRs are required for the activation of the NF-κB pathway (Figure 7C). Furthermore, our results have shown that activation of the NF-κB pathway by KSHV miRs is essential for cellular transformation in the KS model (Figure 10). It can be speculated that vFLIP might also be required for KSHV-induced cellular transformation. It would be important to determine how vFLIP and miRs might concertedly regulate the NF-κB pathway and contribute to KSHV-induced oncogenesis in both PEL and KS models.

While our results have shown the important roles of KSHV miRs in cellular transformation, expression of these miRs individually or as a cluster alone out of the context of KSHV infection is not sufficient to induce cellular transformation (data not shown). These outcomes are expected as it is well-known that disruption of multiple checkpoints/tumor suppressor pathways is required for cellular transformation [82]. Similarly, while activation of the NF-κB pathway and inhibition of IκBα are required for KSHV-induced cellular transformation, they are unlikely to be sufficient to cause cellular transformation in primary cells out of the context of KSHV infection. Nevertheless, our observations implicate the essential role of activating the NF-κB pathway and inhibiting IκBα in the development of other cancers. In fact, the role of activated NF-κB pathway in cancer development has been well established [83]. It is also worth noted that knock out of IκBα has been shown to induce tumors in mice while overexpression of IκBα inhibits tumor formation [84], [85].

Our results suggest that it might be attractive to develop specific inhibitors or suppressors of KSHV miRs for targeting their essential functions in KSHV-induced oncogenesis. However, their redundant functions could make it challenging for therapeutic application. On the other hand, the NF-κB pathway might be a more feasible therapeutic target as it is identified as a common essential target of KSHV miRs. It would be interesting to test the preclinical application of targeting the NF-κB pathway in the KS animal model.

The redundant functions of KSHV miRs in activating the NF-κB pathway and regulating cell growth and survival implicate a common theme of shared functions among these miRs. Previous studies have also identified a number of common targets of KSHV miRs. For examples, miR-K1, 3 and 4-3p target caspase 3 while miR-K5, 9 and 10a/b target BCLAF1 [35], [36]. Our initial systemic genomic approach has led to the identification of potential targets in diverse cellular pathways (Table S4). These results are consistent with the diverse expression patterns of different MutKi cells revealed in the gene expression clustering analysis (Figure 5A). Nevertheless, results of GSEA show that all MutKi cells are enriched in oxidative phosphorylation and cell cycle pathways (Figure 5B), reflecting their enhanced proliferative rates promoted by the miRs. Indeed, among the diverse pathways targeted by KSHV miRs, a number of cancer-related pathways are highly enriched (Figure 9A). Among the top enriched pathways, most of the genes regulated by the miRs are targeted by multiple KSHV miRs (Figure 9B). These results indicate that despite the seemingly complexities, a hierarchal order of functions of KSHV miRs exists with a number of essential cellular pathways positioning at the top ranks. Confirmation of the essential roles of these pathways in KSHV cellular transformation and tumorigenesis should shed further light on the mechanism of KSHV-induced oncogenesis.

## Materials and Methods

### Cells, recombinant viruses, and growth of tumors

KSHV recombinant viruses were previously described [17], [37], [69]. Assays for cell growth and proliferation, and methods for foci formation, growth in softagar and tumor growth were previously described [3], [17], [28].

### Plasmids, siRNAs and miR suppressor

Constructs of KSHV pre-miRs were obtained by cloning fragments of the pri-miRs into retroviral vector pSUPER.retro.puro as previously described [86]. The rat IκBα 3′UTR WT luciferase reporter plasmid was obtained by inserting the full-length 3′UTR of IκBα (Genbank accession no. NM_001105720.2) into the Kpn I and Xho I sites downstream of the luciferase coding sequence in the pGL3 cm vector following PCR amplification. PCR primers used were 5′AGTGGTACCCCAAAGGAACGTGGACTTGT (forward) and 5′AGTCTCGAGCCAAAATAATTACCAACAAAATACACC (reverse) with the restriction enzyme sites underlined. Mutagenesis was carried out using the WT reporter as a template to generate the mutant reporter IκBα 3′UTR mutant containing a mutation in the putative site by PCR amplification. The modified primers were 5′AGTGGTACCCCAAAGGAACGTGGACTTGT (forward) and 5′AGTCTCGAGCCAAAATAATTACCAACAAAATACACCATATACAACATAATGTACAAAGT (reverse) with the restriction enzyme sites underlined. The rat IκBα expression construct lacking its native 3′UTR was obtained by PCR amplification of the IκBα cDNA using primers 5′GCGACCGCCACGACGGCGAC (forward) and 5′GTGGAGGCCGCTGTGCGGGTC (reverse). The fragment was cloned into the mammalian expression vector pCMV4 to derive plasmid pCMV4-IκBα (Addgene, Cambridge, MA).

SiRNAs to IκBα, p21, RelA and scrambled controls were obtained from Invitrogen (Carlsbad, CA). Lock-nucleic acid (LNA)-based miR-K1 suppresser and the scrambled control were previously described [17].

### Analysis of cell cycle and apoptosis

Cell cycle and apoptosis were analyzed as previously described [3], [28]. Cell cycle was analyzed by propidium iodide (PI) staining. Apoptotic cells were detected by PI staining and with a FITC Annexin V Apoptosis Detection kit (BD Biosciences, San Jose, CA).

### Real-time quantitative reverse transcription-PCR (RT-qPCR)

RT-qPCR for KSHV genes (vFLIP, LANA, ORF50, ORF57 and ORF25), KSHV miRs and their primers were previously described [17].

### Luciferase reporter assay

Reporter assays were performed as previously described [17], [28], [69]. To determine the NF-κB activity, a NF-κB luciferase reporter construct or a mutant construct and a β-galactosidase expression plasmid pSV-β-gal (Promega, Madison, WI) were cotransfected into cells cultured in 24-well plates using the F2 transfection reagent (Targeting Systems, El Cajon, CA). Reporter assays for the 3′UTR reporters were carried out by cotransfection of the luciferase reporter plasmid with pRL-TK (Promega, Madison, WI) and a miR mimic (Sigma, St. Louis, MO). The pRL-TK vector providing the constitutive expression of Renilla luciferase was used as an internal control. Other reporter assays were performed with the indicated expression plasmids. Transfection was performed in duplicate or triplicate, and all experiments were independently repeated at least three times. At the indicated time, cells were then lysed, and the luciferase and β-gal activities were measured using luciferase and β-galactosidase kits (Promega). Luciferase activity was normalized to β-galactosidase activity. For the 3′UTR reporter assays, the Dual-Luciferase Reporter Assay System was used as instructed by the manufacturer (Promega).

### Immunofluorescence assay

Cells were fixed for 30 min with 2% paraformaldehyde (Sigma-Aldrich) or 10 min with methanol, permeabilized with 1% saponin (Sigma-Aldrich) for 60 min and blocked with DMEM with 10% FBS for 1 h. The slide was then stained for 1 h with a primary antibody followed for 1 h with an Alex Fluor 568 secondary antibody (Invitrogen) and stained with 4′, 6′-diamidino-2-phenylindole (DAPI) (Sigma-Aldrich). A rat monoclonal antibody to LANA (Abcam, Cambridge, MA), a mouse monoclonal antibody to ORF65 [87], a mouse monoclonal antibody to p21 (Santa Cruz, Santa Cruz, CA), and a rabbit polyclonal antibody to IκBα (Abcam) were used for the experiments.

### Western-blotting

Western-blotting was performed as previously described [88]. Protein lysates were resolved in SDS-PAGE and transferred to Hybond-C extra membranes (GE Healthcare Bio-Sciences, Pittsburgh, PA). Following incubation with primary and secondary antibodies, the membranes were developed in chemiluminescence substrate (Pierce Chemical, Dallas, TX). Images were captured using a BioSpectrum 810 Advanced Imaging System (UVP, Upland, CA).

### Analysis of gene expression profiles

Cells were grown to 70–80% confluency. Total RNAs were isolated with Trizol reagent (Invitrogen). The RNA samples were labeled with Biotin using an Illumina TotalPrep RNA Amplification Kit (Illumina, San Diego, CA). Biotin-labeled cRNA samples were hybridized with Rat-Ref-12-V1 Beadchips using the standard protocol recommended by the manufacturer (Illumina). The Beadchips were scanned using a HiScanSQ scanner. The data were analyzed and quantile-normalized using the GenomeStudio software (Illumina). All the array data were submitted to GEO (http://www.ncbi.nlm.nih.gov/geo/query/acc.cgi?acc=GSE50381).

### Removal of vector effect

Vector effects were observed between MM cells (Mock) and MM cells expressing vector control (MockVt), and between Mut cells and MutVt (Figure S11A and S11B). To remove the vector effect, logarithmic gene expression fold changes in cells with *vs* without vector were calculated and denoted as MockVt/Mock and MutVt/Mut, respectively. Under the null hypothesis that fold changes of genes (MockVt/Mock and MutVt/Mut) were independent of vector effect and distributed jointly according to a zero mean bivariate Gaussian distribution (Figure S11C), 2,064 genes were identified (P<0.1) to depend on the vector, and thus removed (Figure S11D and S11E).

### Removal of genes with no differential expression

Genes show no differential expression in MutKi cells *vs* MutVt cells were removed. A gene is considered not differentially expressed if its expression fold change (MutKi/MutVt) can be explained by the vector effect in all MutKi cells, *i.e.* MutKi/MutVt<σ^2^_m_ for i = 1, …, or 12, where σ^2^_m_ is the variance of vector effect estimated in the step of removal of vector effect. A total of 4,236 genes were identified to have no differential expression in all 12 MutKi cells, and thus were removed. The subsequent analysis was carried out on the remaining 16,501 genes.

### Identification of cellular genes with correlation with tumorigenicity

We divided the MutKi cells into three classes based on the results of tumor formation and progression (Figure 3). Class 1 had high tumorigenicity, which included MutK1, MutK4 and MutK11 cells; Class 2 had medium tumorigenicity, which included MutK2, MutK3, MutK5, MutK7 and MutK10 cells; and Class 3 had low or no tumorigenicity, which included MutK6, MutK8, MutK9 and MutK12 cells. We then performed Analysis of Variance (ANOVA) on all genes to predict the subset of signature genes whose expressions showed significant differences among these three classes. ANOVA is a statistical test for testing if mean expression levels of a gene in multiple classes of samples are equal. It resembles a generalization of *t*-test for differential expression under multiple conditions. Genes with P-values<0.05 were considered to have significantly differential expression across the three classes (Table S2). Pathway enrichment of the signature genes by Ingenuity Pathway Analysis (IPA) was performed to reveal the top enriched networks (Table 2). To visualize the expression of signature genes in the networks, we calculated the average expression within each class for all signature genes and then mapped the average expression to the top enriched networks (Figure S6). To obtain cellular genes that were correlated with tumorigenicity, we isolated the signature genes whose expression levels exhibit strong positive and negative correlation with tumorigenicity (Figure 5C).

### The Lasso fitting

The linear combinatory effect of individual pre-miRs to the overall expression pattern of MutCl cells was determined by the Lasso fitting. Principle component analysis (PCA) was first applied to the expression data of all MutKi cells to reduce the high dimension of 16,501 genes in individual MutKi cells down to 8 principle components (PCs), which were sufficient to explain >95% of the variances in the gene expression (Figure 5D). The projection matrix obtained from the PCA was subsequently used to project the expression of MutCl cells, which were also reduced to 8 dimensions. The Lasso was then applied to the 8-dimensional projected expression data to infer the combinatory effect of the expression of individual MutKi cells to that of MutCl cells. Since the Lasso is designed to promote sparse models, it sets the effect (coefficients) to “0” if a pre-miR is predicted to be insignificant (Figure 5E).

### Prediction of miR targets

Genome-wide targets of miRs in rat were predicted using SVMicrO [70]. For each miR, target genes were ranked with the decreasing order of SVMicrO score, *i.e.*, the top ranked genes were more likely to be targets. At the same time, genes were also ranked according to their expression fold changes in the corresponding MutKi cells with more down-regulated genes ranking higher in the list. The ranked genes in the target list and down-regulated gene list were combined using the Borda merging method into a single gene list, where a higher ranked gene was likely to have a higher SVMicrO score and a larger fold change of down-regulated expression, and thus was likely a target of the miR.

### Gene set enrichment analysis of pathways

A total of 457 human pathways were downloaded from NCI Pathway Interaction Database (NCI-PID) (http://pid.nci.nih.gov/). A total of 356 corresponding rat pathways were obtained by mapping human genes in the pathways to their homologues of rat genes. Pathways with less than 5 genes were excluded. Two different GSEA implementations were carried out. To identify differential expressed pathways in each of MutKi cells, GESA was performed on the gene expression fold changes (Figure 5A and Table S5). To predict pathways that are directly targeted by miRs, GSEA was applied to the ranked list obtained by combining SVMicrO scores and the expression fold changes (Figure 9A and Table S4).

### Statistics

Data are shown as mean ± SD (standard deviations) where appropriate. The 1-tailed Student's test was used to compare data between the experimental groups. Statistical significance was assumed at P values less than 0.05, 0.01 or 0.001, which is represented by “*”, “**” or “***” respectively.

### Study approval

This study was carried out in strict accordance with the recommendations in the Guide for the Care and Use of Laboratory Animals of the National Institutes of Health. The animal protocol was approved by the Institutional Animal Care and Use Committee of the University of Texas Health Science Center at San Antonio (Animal Welfare Assurance no. A3345-01). All surgery was performed under sodium pentobarbital anesthesia, and all efforts were made to minimize suffering.

### Web site

A web site to facilitate the search of cellular gene targets of KSHV miRs, together with SVMicrO scores, expression levels, and enriched pathways has been established at: http://compgenomics.utsa.edu/kshv/.

## Supporting Information

Figure S1 **Expression of viral latent genes in MM cells infected by KSHV recombinant viruses.** (**A–B**) LANA protein expression revealed by immunofluorescence assay at low resolution (**A**) and high resolution showing the typical LANA nuclear speckle pattern (**B**). (**C**) Expression of KSHV latent vFLIP and LANA transcripts detected by quantitative real-time reverse transcription PCR.
(TIF)

Figure S2 **Expression of viral lytic genes in MM cells infected by KSHV recombinant viruses.** (**A**) ORF65 protein revealed by immunofluorescence assay. (**B**) Summary of the percentages of ORF65-positive cells. (**C**) Expression of KSHV lytic ORF50, ORF57 and ORF25 transcripts detected by quantitative real-time reverse transcription PCR. Similar to WT and Rev cells, most Mut cells were latently infected by KSHV albeit an increase of lytic activity in a small number of cells. Statistical analyses were performed by comparing other cells with WT cells (B–C).
(TIF)

Figure S3 **KSHV miRs are not required for KSHV immortalization of MM cells.** Uninfected MM cells (Mock) underwent crisis at around 27–30 passages. MM cells infected by WT and Rev viruses, and Mut virus with or without complementation with individual pre-miRs (MutKi), miR cluster (MutCl) or vector control (MutVt) grew continuously without any crisis.
(EPS)

Figure S4 **Tumor growth curves.** Tumor volumes were measured twice a week in nude mice subcutaneously inoculated with WT cells or Mut cells complemented with individual pre-miRs (MutKi) or vector control (MutVt) control. Tumor growth curves are shown for cells that induced tumors. Tumor volume of 0.2 cm^3^ was used as a threshold for tumor incidence. Tumor analyses were performed at 10 weeks following inoculation or when the volumes reached 1 cm^3^.
(EPS)

Figure S5 **Ganciclovir does not affect the number of floating cells and death cells in WT and Mut cells.** Cells were treated with different concentrations of ganciclovir for 24 h and 72 h and the status of the cells were examined.
(EPS)

Figure S6 **Networks of signature genes that are correlated with tumorigenicity identified by ANOVA.** Cells were divided into three classes based on their tumorigenicity: Class 1 had high tumorigenicity including MutK1, MutK4 and MutK11 cells (right panels); Class 2 had medium tumorigenicity including MutK2, MutK3, MutK5, MutK7 and MutK10 cells (middle panels); and Class 3 had low or no tumorigenicity including MutK6, MutK8, MutK9 and MutK12 cells (left panels). The average expression levels of the signature genes were mapped to the networks.
(TIF)

Figure S7 **Expression of IκBα protein in cells with and without the expression of miR-K1 measured by immunofluorescence assay.**
(EPS)

Figure S8 **SiRNA knock down of IκBα and p21 proteins in MM cells infected by Mut virus.** (**A–B**) Expression of IκBα protein following siRNA knock down examined by Western-blotting (**A**) and immunofluorescence assay (**B**). (**C–D**) Expression of p21 protein following siRNA knock down examined by Western-blotting (**C**) and immunofluorescence assay (**D**).
(TIF)

Figure S9 **MiR-K1 targeting of p21 is not required for KSHV subversion of cell cycle and apoptosis pathways.** (**A–B**) Expression of p21 protein in cells with and without the expression of miR-K1 measured by Western-blotting (**A**) and immunofluorescence assay (**B**). (**C–D**) Cell cycle profiles (**C**) and apoptosis(**D**) in Mut, MutK1 or MutVt cells with knock down of p21 using specific siRNAs or scrambled controls. All statistical analyses were performed by comparing other cells with Mut cells transfected with scrambled siRNA.
(TIF)

Figure S10 **Regulation of the NF-κB network by KSHV.** The network was constructed by Ingenuity Pathway Analysis.
(EPS)

Figure S11 **Removal of vector effect.** (**A–B**) Scatterplots of cellular gene expression in MM cells with *vs* without vector (MockVt *vs* Mock) (**A**), and Mut cells with *vs* without vector (MutVt *vs* Mut) (**B**). Expression outliers due to vector effect can be clearly seen. (**C**) Scatterplot of vector noise in MockVt *vs* MutVt, which was fitted by a bi-variant Gaussian distribution. A total of 2,064 genes (brown points) were identified (P<0.1) to be vector-dependent, and thus removed. (**D–E**) Gene expression scatterplots after removal of vector effect in MM cells with *vs* without vector (MockVt *vs* Mock) (**D**), and Mut cells with *vs* without vector (MutVt *vs* Mut) (**E**).
(EPS)

Table S1 **Expression levels of individual genes in the top enriched pathways in MutKi cells compared to Mut cells.**
(PDF)

Table S2 **Expression of signature genes associated with tumorigenicity mediated by KSHV miRs.**
(PDF)

Table S3 **Cellular pathways that regulate IκBα.**
(PDF)

Table S4 **Target prediction by combining SVMicrO predicted targets with gene expression results.**
(PDF)

Table S5 **Expression fold change (log2) of MTKi, MutCl and WT cells vs. MTVt cells.**
(PDF)
