# Correction: KSHV MicroRNAs Mediate Cellular Transformation and Tumorigenesis by Redundantly Targeting Cell Growth and Survival Pathways

**DOI:** 10.1371/annotation/582f0298-9999-46ea-be7e-6718608b11c5

**Published:** 2014-01-21

**Authors:** Rosalie Moody, Ying Zhu, Yufei Huang, Xiaodong Cui, Tiffany Jones, Roble Bedolla, Xiufen Lei, Zhiqiang Bai, Shou-Jiang Gao

There was an error in the Funding section. The corrected Funding section should appear as follows:

This study was supported by grants from National Institute of Health (CA096512, CA124332, CA132637 and CA177377) to SJC (www.nih.gov). The funders had no role in study design, data collection and analysis, decision to publish, or preparation of the manuscript.

