# Supplementary figures and images for: KSHV MicroRNAs Mediate Cellular Transformation and Tumorigenesis by Redundantly Targeting Cell Growth and Survival Pathways
Source: PLoS Pathog. 2013 Dec 26;9(12):e1003857. doi: 10.1371/journal.ppat.1003857 (PMC3873467; doi:10.1371/journal.ppat.1003857)

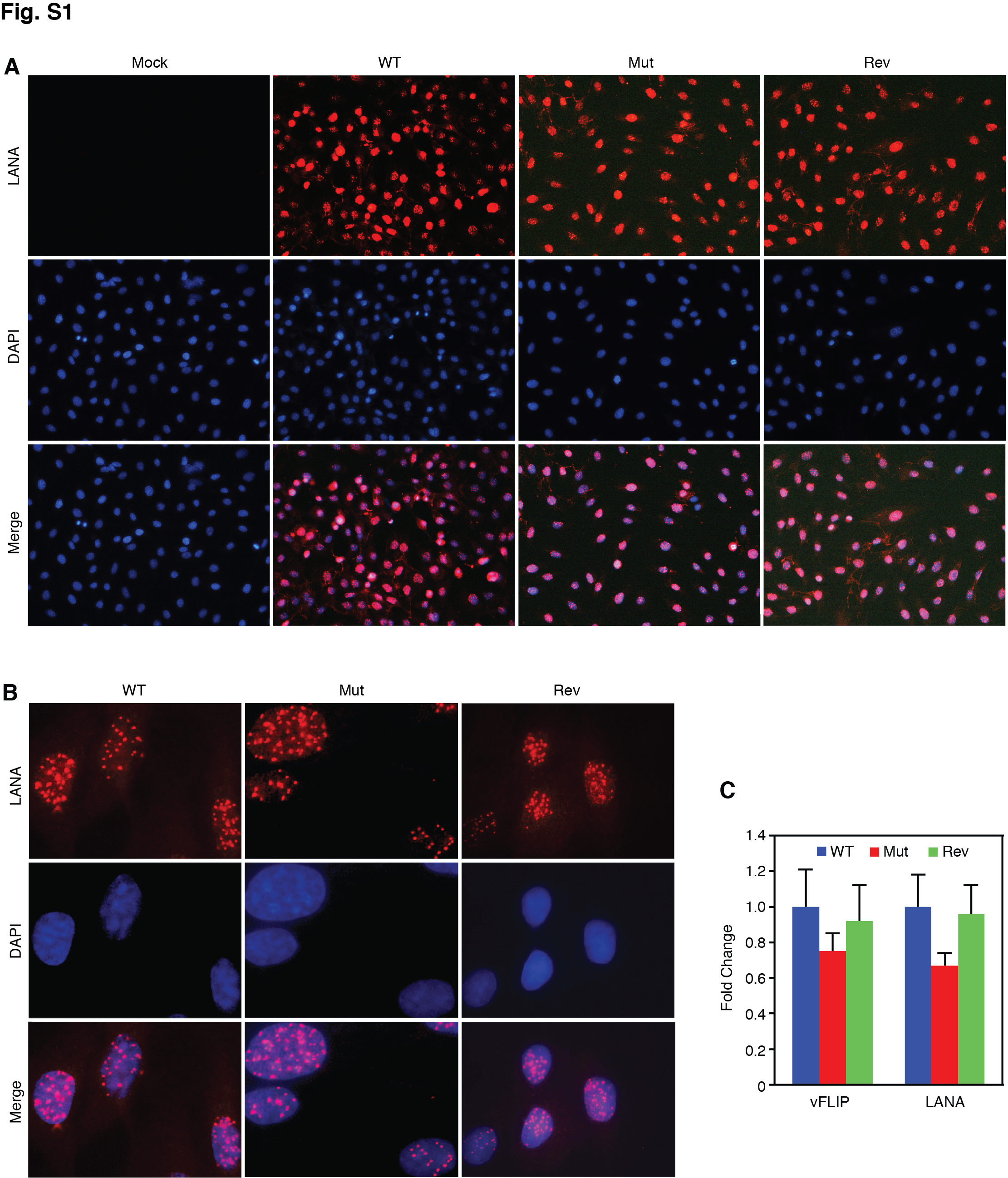

Supplement: Figure S1 — Expression of viral latent genes in MM cells infected by KSHV recombinant viruses. (A–B) LANA protein expression revealed by immunofluorescence assay at low resolution (A) and high resolution showing the typical LANA nuclear speckle pattern (B). (C) Expression of KSHV latent vFLIP and LANA transcripts detected by quantitative real-time reverse transcription PCR. (TIF) [file ppat.1003857.s001.tif]

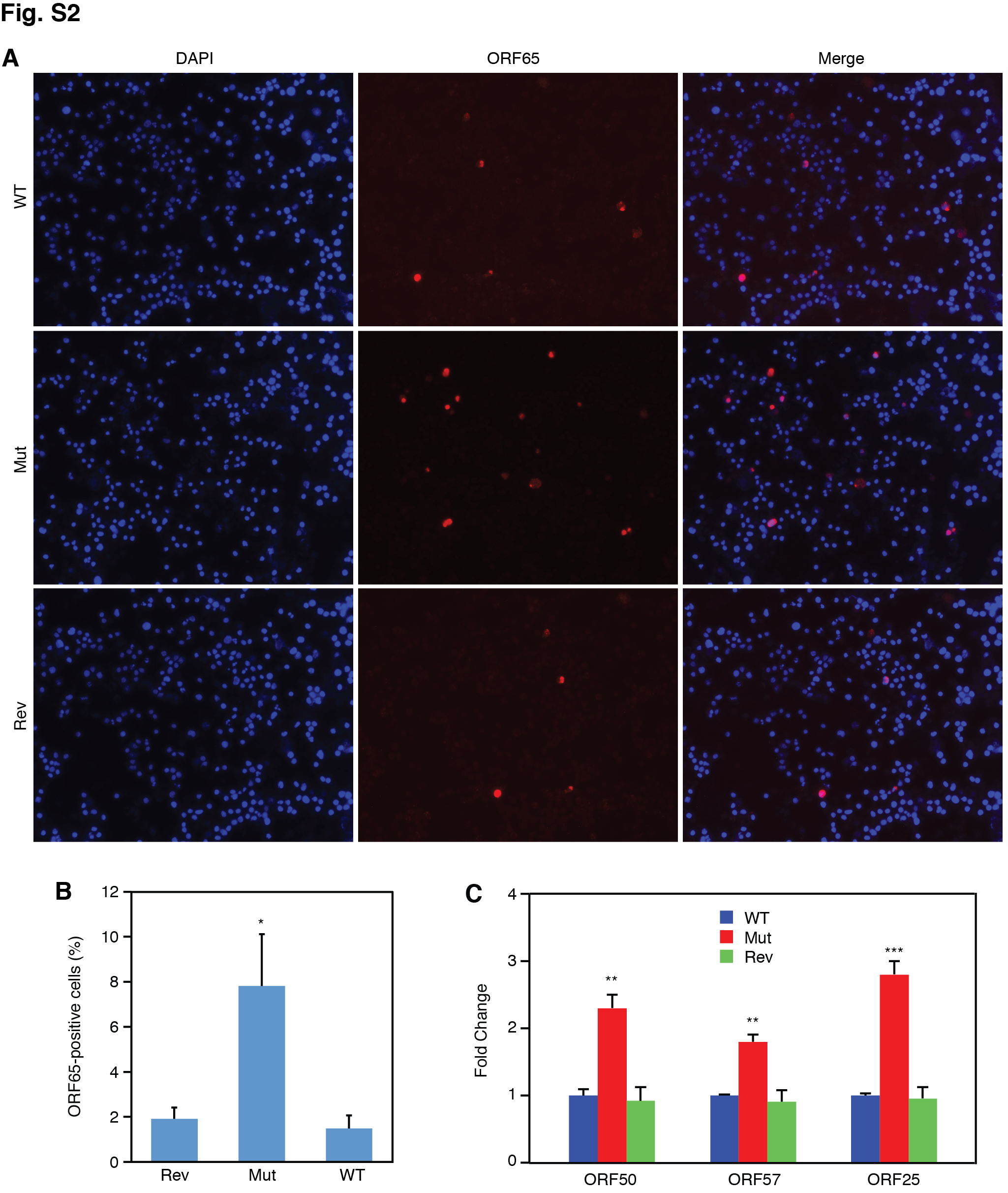

Supplement: Figure S2 — Expression of viral lytic genes in MM cells infected by KSHV recombinant viruses. (A) ORF65 protein revealed by immunofluorescence assay. (B) Summary of the percentages of ORF65-positive cells. (C) Expression of KSHV lytic ORF50, ORF57 and ORF25 transcripts detected by quantitative real-time reverse transcription PCR. Similar to WT and Rev cells, most Mut cells were latently infected by KSHV albeit an increase of lytic activity in a small number of cells. Statistical analyses were performed by comparing other cells with WT cells (B–C). (TIF) [file ppat.1003857.s002.tif]

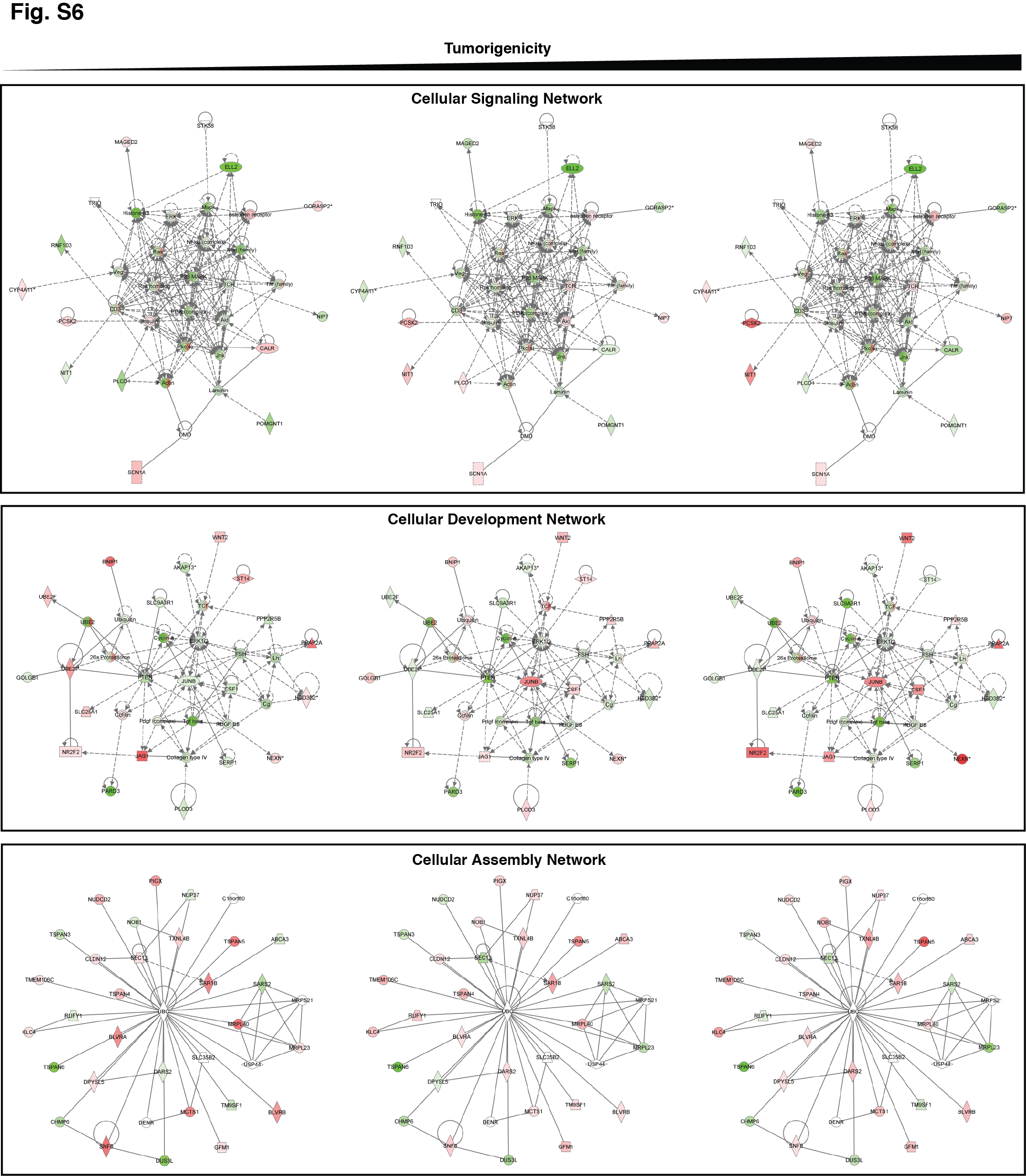

Supplement: Figure S6 — Networks of signature genes that are correlated with tumorigenicity identified by ANOVA. Cells were divided into three classes based on their tumorigenicity: Class 1 had high tumorigenicity including MutK1, MutK4 and MutK11 cells (right panels); Class 2 had medium tumorigenicity including MutK2, MutK3, MutK5, MutK7 and MutK10 cells (middle panels); and Class 3 had low or no tumorigenicity including MutK6, MutK8, MutK9 and MutK12 cells (left panels). The average expression levels of the signature genes were mapped to the networks. (TIF) [file ppat.1003857.s006.tif]

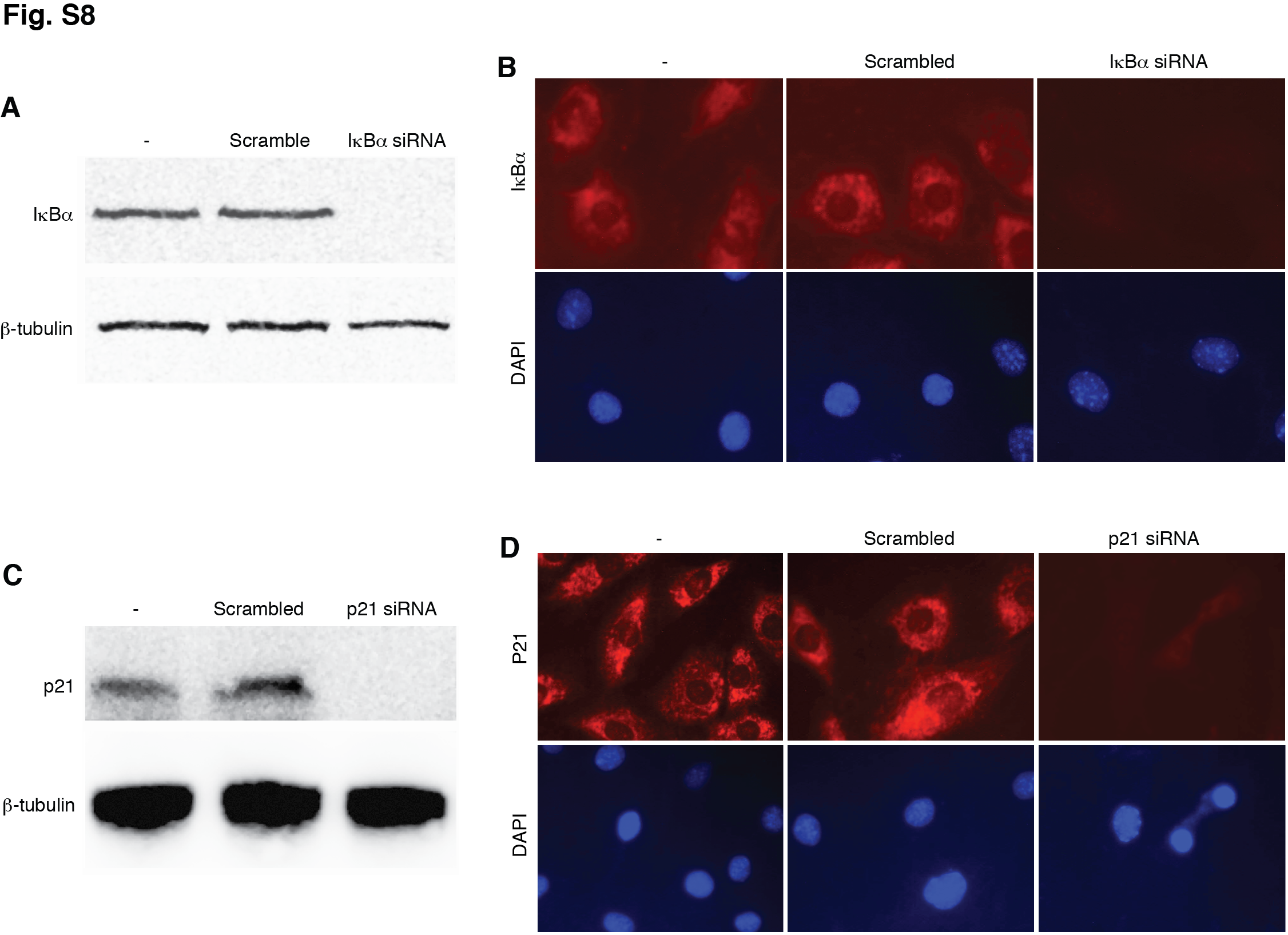

Supplement: Figure S8 — SiRNA knock down of IκBα and p21 proteins in MM cells infected by Mut virus. (A–B) Expression of IκBα protein following siRNA knock down examined by Western-blotting (A) and immunofluorescence assay (B). (C–D) Expression of p21 protein following siRNA knock down examined by Western-blotting (C) and immunofluorescence assay (D). (TIF) [file ppat.1003857.s008.tif]

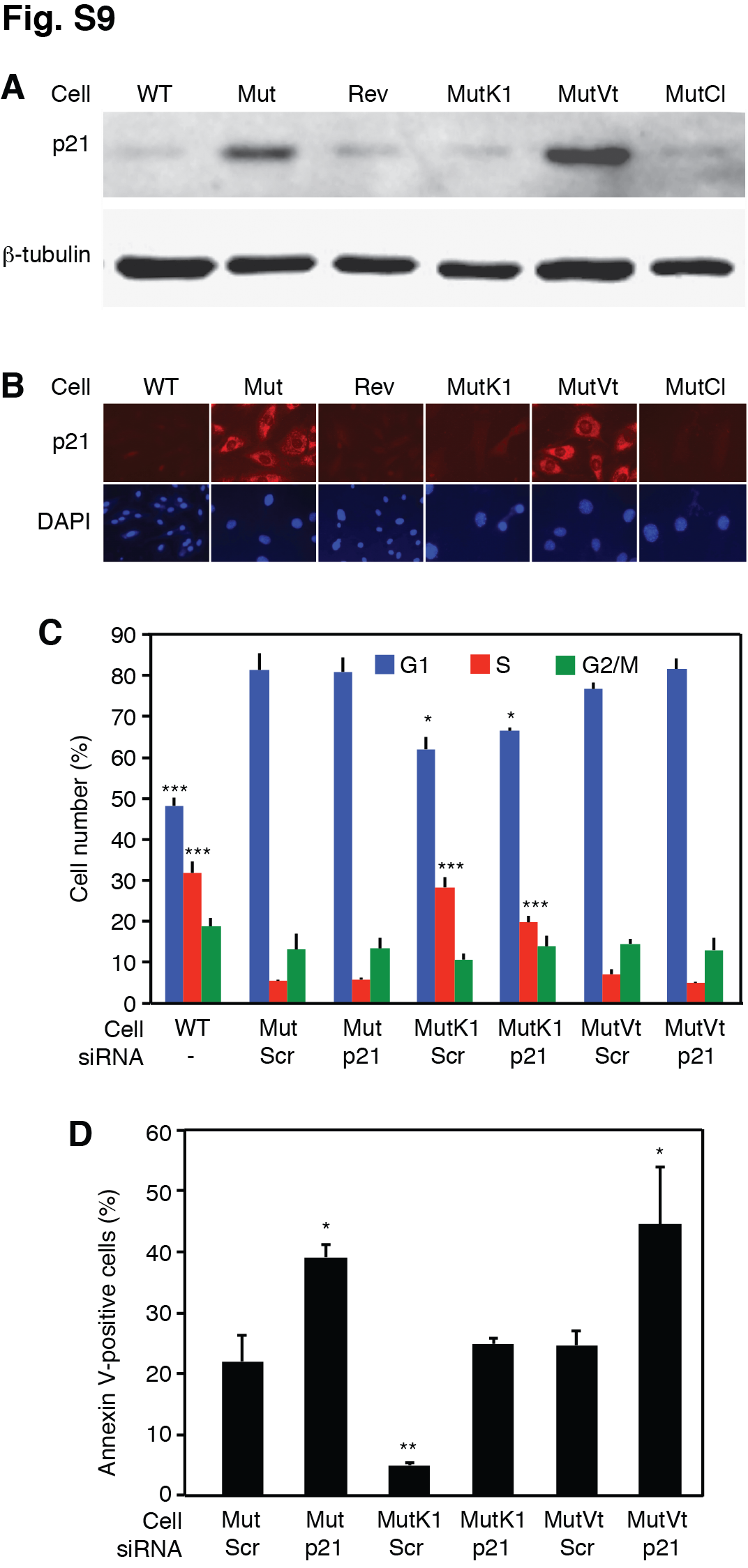

Supplement: Figure S9 — MiR-K1 targeting of p21 is not required for KSHV subversion of cell cycle and apoptosis pathways. (A–B) Expression of p21 protein in cells with and without the expression of miR-K1 measured by Western-blotting (A) and immunofluorescence assay (B). (C–D) Cell cycle profiles (C) and apoptosis(D) in Mut, MutK1 or MutVt cells with knock down of p21 using specific siRNAs or scrambled controls. All statistical analyses were performed by comparing other cells with Mut cells transfected with scrambled siRNA. (TIF) [file ppat.1003857.s009.tif]
