# Supplementary material for: KSHV MicroRNAs Mediate Cellular Transformation and Tumorigenesis by Redundantly Targeting Cell Growth and Survival Pathways
Source: PLoS Pathog. 2013 Dec 26;9(12):e1003857. doi: 10.1371/journal.ppat.1003857 (PMC3873467; doi:10.1371/journal.ppat.1003857)
Supplement: Table S1 — Expression levels of individual genes in the top enriched pathways in MutKi cells compared to Mut cells. (PDF) [file ppat.1003857.s012.pdf]

Table S1. Expression levels of individual genes in the top enriched pathways in MutKi cells compared to Mut cells

Oxidative phosphorylation

Gene symbol	Refseq	K1	K2	K3	K4	K5	K6	K7	K8	K9	K10	K11	K12	Cluster	WT
Atp6v1a1_predicted	NM_001108318	0.9181	1.5308	-0.358	0.096	0.7776	-0.233	0.3781	0.8492	0.803	1.1606	0.5643	0.0072	0.5305	0.8477
Atp6v1c1_predicted	NM_001011992	0.6871	0.8957	-0.617	0.1493	0.6104	0.1304	0.2409	0.7861	0.5337	0.7967	0.1133	0.1748	0.6288	0.7839
Ndufa8	NM_001047862	0.4826	0.3865	-0.096	-0.383	0.8326	0.8689	-0.071	0.3912	0.3341	0.5758	0.174	0.6572	0.5762	0.6631
Cyc1_predicted	NM_001130491	0.5094	0.3192	0.5286	0.6693	0.5428	0.7447	0.0732	0.6316	0.33	0.3564	0.4081	0.4136	0.4942	0.6598
Ndufb9_predicted	NM_001127294	0.3198	0.1751	0.4705	0.3282	0.4347	0.6953	0.1006	0.4479	0.4533	0.2463	0.2336	0.6224	0.6844	0.6524
RGD1563463_predicted	NM_017311	0.1484	-0.143	0.2162	0.3165	0.6224	1.5033	-0.131	0.3712	-0.044	0.086	0.0889	1.0468	0.5467	0.6121
Ndufb7_predicted	NM_001108442	1.2034	0.2311	0.6436	0.723	1.0223	1.3553	0.6312	0.7586	0.0856	0.1513	0.3273	0.5501	0.3462	0.6049
Ndufb6_predicted	NM_001106646	0.3589	0.4228	0.1825	-0.341	0.5537	0.7204	0.28	0.3339	0.3379	0.4445	0.4542	0.6942	0.3915	0.5944
Ndufab1_predicted	NM_001106294	0.703	0.1472	0.4627	-0.133	0.7712	0.9984	0.2848	0.6317	0.3552	0.3102	0.3319	0.5196	0.3595	0.5695
Cox8a	NM_134345	0.7407	0.6533	0.2795	0.2743	0.9176	0.9686	0.4899	0.9033	0.7221	0.7611	0.8789	0.2907	0.5008	0.5664
Atp5b	NM_134364	0.2516	0.0969	0.3704	-0.074	0.2568	-0.094	0.3167	0.3655	-0.045	0.0835	0.1873	0.0205	0.5939	0.5372
Atp6v1h	NM_001013929	0.7269	1.012	0.3433	1.0393	1.1	0.8961	0.6848	0.9842	0.7101	0.8466	0.8813	0.307	0.5553	0.4996
Ndufs3_predicted	NM_001106489	0.1062	0.2898	-0.06	0.112	0.6919	0.7293	0.3421	0.7134	0.4517	0.27	0.4666	0.4586	0.614	0.4919
Ndufs7	NM_001008525	0.291	0.2373	0.2503	0.1206	0.491	0.8886	0.2121	0.3928	0.4426	0.3586	0.4407	0.6514	0.4142	0.4775
Cox6a1	NM_012814	0.3282	0.1548	0.0906	0.4613	0.2755	0.6034	0.4164	0.4621	0.3849	0.2342	0.3816	0.3122	0.332	0.4579
Cox5b	NM_053586	0.3729	0.1129	0.1703	-0.105	0.4157	0.7094	-0.128	0.3444	0.1507	0.0155	-0.096	0.3033	0.3673	0.456
LOC316632	NM_182671	0.406	0.5309	0.1752	-0.155	0.399	0.4911	0.1265	0.47	0.582	0.6168	0.5342	0.337	0.3836	0.4092
Atp5d	NM_139106	0.4714	0.3074	-0.027	-0.42	0.5325	0.6157	-0.009	0.5541	0.374	0.2931	0.3388	0.3967	0.3685	0.3839
Ndufb5_predicted	NM_001106426	0.5618	0.0644	0.1593	-0.213	0.4651	0.5099	0.1766	0.4581	0.4102	0.0461	0.3402	0.3318	0.1765	0.3769
Ndufb8_predicted	NM_001106360	0.4196	0.332	0.0179	0.1204	0.5024	0.5126	-0.12	0.2329	0.155	0.2089	0.2354	0.2334	0.2706	0.3667
Cox5a	NM_145783	0.488	0.0586	0.0084	-0.191	0.6514	0.7458	0.2331	0.6017	0.1162	0.1353	0.1204	0.2357	0.2786	0.3508
Atp6v1b2	NM_057213	0.6269	0.3801	0.0451	0.1199	0.6093	0.374	0.2313	0.5246	0.101	0.3561	0.2511	-0.05	0.3348	0.3493
Uqcrfs1	NM_001008888	0.6217	0.3649	0.0308	0.4017	0.6885	0.7065	0.0771	0.8684	0.1717	0.2569	0.224	0.2524	0.3657	0.3448
Ndufa7_predicted	NM_001106772	-0.149	0.0947	0.0934	-0.205	-0.072	-0.043	0.1458	-0.044	-0.019	0.1514	0.3702	0.3986	0.422	0.3402
Atp6v0c	NM_130823	0.5884	0.1526	0.3434	-0.247	0.3026	0.0032	0.2098	0.3041	-0.024	0.1974	0.0599	-0.504	0.134	0.3301
Ndufa5	NM_012985	0.6917	0.2536	0.2119	0.8959	1.0061	0.9315	0.2842	0.7509	0.2505	0.4232	0.3633	0.0966	0.2674	0.3292
Cox7b	NM_182819	0.7107	-0.039	0.8256	0.3776	0.4387	0.4907	0.6297	0.2102	0.2883	0.3619	0.2098	0.5857	0.2797	0.3264
Atp6v0e	NM_053578	0.6262	0.5289	0.0458	0.2901	0.7129	0.8466	0.7337	0.7817	0.5967	0.5939	0.612	0.7831	0.474	0.3004
Atp5g1	NM_017311	-0.019	-0.31	0.1752	-0.073	0.3457	0.5832	-0.3	0.125	-0.169	-0.031	-0.057	0.6002	0.2248	0.2993
Cox4i1	NM_017202	0.4663	0.1138	0.2844	0.0637	0.4497	0.4124	0.4613	0.2364	0.0334	0.1524	-0.078	0.3335	0.3411	0.2912
Cox17	NM_053540	0.8637	0.2605	0.3006	0.8679	0.6905	1.3164	0.1524	0.4049	0.0688	0.1872	0.1476	0.3565	0.2461	0.2779
Atp6v1c2	NM_001014199	-0.103	0.2475	0.5951	-0.125	-0.28	-0.117	0.6104	-0.152	0.093	0.254	0.098	0.135	0.2442	0.2763
Ndufa10	NM_182671	0.4079	0.4479	0.1098	-0.279	0.3531	0.5067	0.1045	0.4132	0.4351	0.5471	0.4634	0.332	0.2949	0.269
Atp5o	NM_138883	0.142	-0.025	0.1467	-0.019	0.3559	0.3977	0.4267	0.3771	0.2212	0.1483	0.1087	0.3241	0.2916	0.2587
Ndufs5	NM_001030052	0.2927	0.03	0.494	0.3856	0.5966	0.9299	0.2172	0.3899	0.3843	0.2617	0.3437	0.5291	0.2147	0.2529
Atp5c1	NM_053825	0.1016	0.2656	0.2581	0.0436	0.2803	0.5008	-0.117	0.3313	0.2734	0.0637	0.3318	0.5051	0.241	0.2526
Atp6v1c1	NM_001011992	0.2411	0.3366	0.3551	0.6174	0.0616	-0.181	0.5351	0.2006	0.2439	0.1583	0.4683	-0.123	0.4589	0.2403
Sdhb_predicted	NM_001100539	0.4374	0.2498	-0.166	0.0818	0.8601	0.6332	-0.231	0.7211	0.2997	0.2541	0.3571	0.149	0.257	0.2367
Pyp_mapped	NM_001100834	-0.18	-0.735	0.4776	0.5216	-0.445	-0.366	-0.172	-0.3	-0.38	-0.431	-0.45	0.0418	0.3115	0.2302
Uqcrc1	NM_001004250	0.0373	-0.363	0.1066	-0.078	0.613	0.5431	-0.045	0.5459	-0.288	-0.205	-0.212	-0.262	-0.008	0.2205

Ndufa11	NM_212517	0.2476	-0.163	0.1974	-0.317	0.3761	0.652	-0.323	0.2882	-0.063	-0.071	-0.025	0.493	0.2802	0.2184
Atp5i	NM_080481	0.267	0.089	0.4474	-0.357	0.3913	0.3762	0.2103	0.2393	0.0401	-0.013	0.1474	0.3183	0.2283	0.2056
Ndufa12_predicted	NM_001106781	-0.188	-0.216	0.0784	-0.28	-0.004	0.1655	-0.671	-0.021	0.1962	-0.132	-0.162	0.3556	0.2926	0.2017
Atp5j	NM_053602	0.2722	0.2249	0.2168	0.2032	0.7883	0.6643	0.2408	0.5764	0.4126	0.2285	0.4423	0.2105	0.2477	0.1997
Ndufv1	NM_001006972	0.44	0.1459	0.1014	-0.003	0.7823	0.5448	0.0318	0.6427	0.0797	0.2769	0.0402	-0.012	0.1879	0.1979
MGC72942	NM_212516	0.368	0.1131	0.3051	0.1804	0.5976	0.5575	0.3459	0.3273	0.0824	0.2431	0.2884	0.3801	0.1657	0.1804
Ndufa1_predicted	NM_001108813	0.6506	0.2959	0.2555	0.1728	0.5558	0.6065	0.2496	0.4226	0.6035	0.4912	0.7308	0.5257	0.2377	0.174
Atp6v1d	NM_199386	0.553	0.2113	-0.157	0.9401	0.5003	0.3442	0.6952	0.4446	0.1116	0.164	0.1158	0.1266	0.1842	0.1683
Ndufb11_predicted	NM_001106756	0.2027	0.1043	-0.471	-0.267	0.4597	0.3356	-0.238	0.1958	-0.024	0.0794	0.0105	0.0691	0.0335	0.1662
Sdha	NM_130428	0.3308	0.7056	-0.092	0.7797	0.6931	0.6961	0.4505	0.6233	0.5312	0.7574	0.3493	-0.021	0.0638	0.1647
Atp6v0d1	NM_001011927	0.3668	0.3246	-0.15	-0.321	0.0151	0.1045	0.1092	0.0038	0.1283	0.0646	0.0978	0.3027	0.2686	0.1462
Atp6ap1	NM_031785	0.2541	0.3622	0.1034	-0.157	-0.327	-0.416	-0.16	-0.282	0.4226	0.4996	0.3428	0.088	0.4263	0.1454
LOC499529	NM_001025146	-0.046	-0.007	-0.018	0.1046	-0.016	0.0986	-0.054	0.1377	0.0734	0.0585	0.0241	-0.026	-0.024	0.1436
Cox6c1	NM_173303	-0.014	0.0235	0.0456	0.3683	0.6354	0.576	0.0088	0.4397	0.4367	-0.002	0.2661	0.5304	0.4597	0.1422
Ndufs8_predicted	NM_001106322	0.0833	-0.103	-0.096	-0.268	0.0333	0.3347	-0.549	-0.131	0.1351	-0.111	-0.098	0.2837	-0.132	0.1103
Atp6v1g1_predicted	NM_001106660	0.0605	-0.128	-0.029	0.3817	0.1892	0.2842	0.4459	0.1164	0.1695	-0.188	0.0689	0.4638	-0.09	0.1094
Atp6v1e1	NM_198745	0.6777	0.6213	0.0798	0.2115	0.2697	0.3794	0.4706	0.3335	0.5529	0.7012	0.7641	0.598	0.146	0.1044
Ndufb3_predicted	NM_001106912	0.5826	0.3257	0.0582	0.2129	0.5536	0.5454	0.4574	0.5328	0.5507	0.3845	0.4149	0.2061	0.1957	0.0928
Atp12a	NM_133517	-0.213	-0.13	-0.029	-0.007	-0.033	-0.126	-0.046	0.0563	-0.173	-0.123	-0.121	0.2638	0.3109	0.0765
Ndufs1	NM_001005550	0.3404	0.1271	-0.073	0.0884	0.3757	0.2901	-0.03	0.4664	-0.103	0.0022	-0.117	-0.308	0.0094	0.0639
Atp5g3	NM_053756	0.1004	0.2499	0.0019	-0.102	0.2048	0.3088	0.0832	0.0973	0.0526	-0.167	-0.026	0.2065	0.1543	0.0598
Atp6v0d2	NM_001011972	0.1345	0.0807	0.0482	0.2617	-0.035	0.1052	0.1665	0.1584	-0.057	0.0248	-0.051	0.0381	-0.023	0.0548
Sdhc	NM_001005534	0.475	0.4478	0.1845	0.3785	0.1936	0.3053	0.404	0.1511	0.2195	0.2952	0.3794	0.3044	-0.083	0.0399
Atp6v1f	NM_053884	0.8148	0.2021	-0.314	0.0756	0.5065	0.9232	0.017	0.3268	0.3529	0.0484	0.316	0.1121	-0.052	0.0296
Ndufc2	NM_001009290	0.2591	0.1314	-0.186	-0.15	-0.03	0.2918	0.0775	-0.339	0.0576	-0.179	-0.108	0.1512	-0.246	0.0261
Ndufa6_predicted	NM_001130505	0.2788	0.2322	0.1935	0.7996	0.4428	0.5641	-0.072	0.2666	0.1786	0.2497	0.4834	0.3957	0.0516	0.01
Uqcrb_predicted	NM_001127553	-0.288	0.1529	-0.221	0.011	-0.082	0.1112	0.1206	-0.029	0.0963	0.0758	0.2879	0.316	0.1264	0.0034
LOC500560	NM_053602	-0.04	0.0341	-0.044	0.0244	-0.038	0.0385	0.028	0.1304	0.1243	0.436	0.183	0.141	0.2255	0.0023
Atp6v0d1_predicted	NM_001011927	-0.222	0.039	-0.134	0.2221	0.0247	-0.071	-0.068	-0.021	0.0203	0.1558	-0.041	-0.004	0.1915	-0.007
Atp5a1	NM_023093	-0.073	0.0106	-0.017	-0.253	0.1654	-0.147	0.1207	0.238	-0.141	0.1293	-0.108	-0.228	-0.208	-0.012
Ndufv3l	NM_022607	0.0661	-0.049	-0.388	-0.235	0.2412	0.1592	-0.36	0.0912	-0.034	-0.085	-0.064	-0.159	-0.053	-0.062
Atp6v0a1	NM_031604	0.5059	0.321	1.0349	0.5287	-0.171	-0.023	0.4398	-0.159	0.2848	0.3797	0.5353	0.2367	0.1255	-0.062
Atp4b	NM_012510	-0.037	0.0903	-0.005	-0.006	0.1717	0.1849	0.0321	0.1906	0.0365	-0.044	0.1136	-0.026	0.0776	-0.063
Atp6v0a4_predicted	NM_001106591	0.0109	0.1069	-0.121	0.0156	0.0872	-0.072	-0.154	-0.074	0.0499	0.0577	-0.096	0.0322	-0.015	-0.071
Sdhd	NM_198788	0.3127	-0.163	-0.298	-0.023	0.5618	0.3766	-0.126	0.4651	-0.16	-0.375	-0.148	-0.183	-0.06	-0.114
Ndufv2	NM_031064	0.0552	0.2484	-0.15	-0.504	0.1173	0.3411	-0.104	0.2898	0.2046	0.2474	0.1751	0.327	0.1308	-0.141
Atp6v1e2_predicted	NM_001108979	-5E-04	-0.098	0.0077	-0.058	0.1299	0.2031	-0.088	0.0947	-0.024	-0.12	-0.14	-0.097	-0.097	-0.147
Ndufb2_predicted	NM_001108624	0.0936	-0.343	-0.272	-0.014	-0.037	0.1992	-0.451	-0.272	-0.171	-0.329	-0.161	-0.079	-0.258	-0.148
Atp6v1g3_predicted	NM_001105991	-0.04	0.1839	0.1341	0.1343	-0.006	0.1553	0.0159	-0.02	-0.013	-0.174	-0.121	0.3528	-0.084	-0.196
Cox15_predicted	NM_001033699	0.3851	0.3204	0.1174	0.6704	0.2382	0.2088	-0.078	0.2662	0.2086	0.3467	0.2955	-0.002	-0.27	-0.204
Cox7a2l_predicted	NM_001106704	0.4664	0.6956	-1.042	-0.188	0.3478	-0.015	-0.339	0.2182	0.6206	0.603	0.6255	-5E-04	-0.232	-0.264
Atp6v1b1_predicted	NM_001107867	-0.19	-0.167	-0.385	-0.222	-0.143	-0.141	0.0718	-0.335	-0.208	-0.171	-0.151	-0.202	-0.264	-0.273
Atp5g2	NM_133556	-0.176	0.1944	-0.521	-0.768	-0.25	-0.056	-0.54	-0.336	0.5529	0.1957	0.1824	0.3852	-0.364	-0.28
RGD1566212_predicted	NM_133556	-0.358	-0.134	-0.303	-0.842	-0.303	-0.005	-0.769	-0.397	-0.213	-0.35	-0.003	0.3338	-0.497	-0.337

Ppa2_predicted	NM_001135871	0.0284	-0.247	-0.483	-0.731	-0.348	-0.036	-0.336	-0.319	-0.441	-0.436	-0.4	0.0412	-0.455	-0.363
Cox6a2	NM_001109994	0.8163	-0.297	-0.602	-0.12	-0.272	-0.281	-0.275	-0.285	-0.381	-0.296	-0.159	-0.653	-0.453	-0.643
Atp6v1g2	NM_212490	-0.626	-0.455	-0.226	-0.305	-0.574	-0.196	-0.026	-0.522	-0.478	-0.553	-0.567	-0.574	-0.717	-0.685
Tcirg1	NM_199089	-0.341	0.2398	0.0964	0.8046	0.3497	0.5093	0.1918	0.3552	-0.281	0.0033	-0.041	-0.114	-0.539	-0.713

Role of Ran in mitotic spindle regulation

Refseq	Gene symbol	K1	K2	K3	K4	K5	K6	K7	K8	K9	K10	K11	K12	Cluster	WT
Ran	NM_053439	-0.055	-0.526	0.5668	-0.17	-0.213	-0.444	-0.043	-0.285	-0.321	-0.518	-0.537	0.1394	0.5209	0.8222
Stk6	NM_153296	-0.198	-0.83	0.5693	-0.142	-0.492	-0.574	-0.614	-0.4	0.2826	-0.727	-0.395	0.4752	0.619	0.8079
Tpx2_predicted	NM_001107790	-0.363	-1.097	0.9722	0.0812	-0.771	-0.565	-0.829	-0.893	-0.315	-0.824	-0.742	0.7682	0.4491	0.6959
Kpnb1	NM_017063	-0.279	-0.306	-0.381	-0.885	-0.196	-0.767	-0.479	0.0171	-0.272	-0.095	-0.466	-0.208	0.697	0.659
Rangap1	NM_001012199	-0.02	-0.611	0.6586	-0.15	-0.103	-0.17	-0.558	-0.265	-0.087	-0.39	-0.306	0.1442	0.388	0.5868
LOC681932	NM_053483	-0.645	-1.718	0.4367	-1.04	-1.219	-1.418	-1.477	-1.218	-0.838	-0.988	-1.15	0.1831	0.3514	0.4776
Rangap1_predicted	NM_001012199	-0.055	-0.698	0.7397	-0.104	-0.281	-0.448	-0.478	-0.626	-0.797	-0.8	-0.794	0.0167	0.3348	0.458
Kif15	NM_181635	-0.215	-0.247	0.5148	0.1976	-0.58	-0.751	-0.167	-0.575	0.0013	-0.072	-0.104	-0.037	0.4083	0.3557

Proteasome

Refseq	Gene symbol	K1	K2	K3	K4	K5	K6	K7	K8	K9	K10	K11	K12	Cluster	WT
Psmd13_predicted	NM_001108925	0.5403	0.1904	0.529	0.7681	0.6179	0.4783	0.4082	0.5975	0.4603	0.3791	0.2591	0.301	0.7348	0.8719
Psmc5	NM_031149	-0.074	-0.321	0.4192	-0.104	0.3015	0.3948	-0.061	0.3483	0.0343	0.1735	-0.157	0.3045	0.7799	0.8022
Psmd12	NM_001005875	0.4345	0.033	0.3257	0.1424	0.4833	0.5031	0.4041	0.3925	0.0207	0.1835	0.1313	0.2349	0.8016	0.598
Psmb3	NM_017285	0.3994	0.0249	0.2819	0.2283	0.5004	0.7912	-0.146	0.3431	0.1261	0.3458	0.1316	0.5881	0.5308	0.5621
Psmb7	NM_053532	0.4148	0.295	0.1751	0.2053	0.6302	0.841	0.3359	0.5393	0.1222	0.079	0.1316	0.3274	0.4881	0.5596
Psmb4	NM_031629	0.7132	0.5749	0.1272	0.3544	0.7306	0.5935	0.3532	0.618	0.4605	0.6715	0.6513	0.4916	0.7003	0.5591
Psmd1	NM_031978	0.0937	0.6648	0.8566	1.827	0.2447	0.5786	0.3884	0.0501	0.6695	0.6478	0.8846	0.4505	0.395	0.4626
Psmd4	NM_031331	0.51	-0.059	0.7135	0.7221	0.2728	0.4582	0.3805	0.0064	-0.246	-0.275	-0.083	0.2243	0.3343	0.4441
Psmd2	NM_001031639	0.1559	0.178	0.6227	0.4312	0.3656	0.43	0.157	0.276	0.153	0.2424	0.1407	0.2135	0.379	0.4357
Psmb6	NM_057099	0.5281	-0.139	0.2016	0.2755	0.4973	0.9336	-0.03	0.3933	0.0792	-0.182	0.1014	0.5107	0.1371	0.3902
Psmc1	NM_057123	0.0369	-0.098	0.4477	0.1798	0.0899	0.115	0.2985	0.2051	-0.045	-0.024	-0.012	0.3494	0.5111	0.3516
Psmd6	NM_198730	0.1828	0.399	0.2127	0.7729	0.4968	0.2885	0.5296	0.4546	0.1807	0.3955	0.2177	0.038	0.346	0.3137
Psmc3	NM_031595	-0.404	-0.27	0.3288	-1.031	-0.152	0.129	-0.343	-0.234	-0.085	-0.068	0.0366	0.5523	0.4166	0.3039
Psma4	NM_017281	0.2451	-0.177	0.0822	0.4	0.3242	0.3904	0.0171	0.2665	0.2348	-0.06	-0.049	0.0972	0.2474	0.2847
Psmd7_predicted	NM_001107426	0.1755	0.0262	0.2964	0.0076	-0.307	-0.17	-0.091	-0.287	0.0832	-0.146	0.0993	-0.028	0.109	0.2807
Psmc4	NM_057122	-0.051	-0.355	0.2986	0.2679	-0.054	0.0256	-0.029	-0.037	-0.383	-0.304	-0.245	0.1531	0.1615	0.2504
Psmb1	NM_053590	0.0863	0.1016	0.2198	0.2386	0.2802	0.3421	0.1265	0.309	0.1297	0.116	0.2597	0.4	0.0193	0.2182
Psmd8	NM_001100831	0.1487	-0.093	0.4365	0.5998	0.3239	0.7302	0.2324	0.5942	0.1985	-0.253	0.1308	0.2372	0.023	0.1863
Psmd11_predicted	NM_001107027	0.2329	0.1108	0.0117	0.2281	0.4242	0.0606	0.4833	0.4136	0.0852	-0.079	0.0814	-0.389	0.2279	0.1727
Psma1	NM_017278	0.2798	0.0286	0.1328	0.4884	0.2707	0.3121	-0.013	0.4592	0.2367	0.0995	0.2785	0.1559	0.206	0.1407
Psmc6	NM_001100509	-0.09	0.1137	0.041	-0.142	-0.121	-0.328	0.1613	-0.26	0.1066	-0.178	0.114	-0.278	0.0828	0.1366
Psma3	NM_017280	0.3843	0.1824	-0.003	-0.161	0.1488	0.3148	0.0023	0.2681	0.1431	0.1674	-0.053	0.2861	0.3419	0.1089
Psmc2	NM_033236	0.0155	-0.411	0.0261	-0.139	-0.188	-0.085	-0.102	-0.079	-0.241	-0.177	-0.166	-0.179	0.0709	0.0921
Psma6	NM_017283	0.2359	-0.026	-0.272	-0.034	0.5196	0.2988	-0.001	0.5894	0.0805	-0.225	-0.104	0.1761	0.1243	0.0878
LOC363748	NM_017285	-0.164	-0.183	-0.201	-0.214	-0.15	-0.216	0.0903	-0.173	-0.031	-0.03	-0.184	-0.122	-0.087	0.0323
Psma7	NM_001008217	0.024	-0.126	0.1964	-0.565	0.1393	-0.128	0.001	0.1243	0.1344	0.0372	-0.093	-0.195	0.1762	0.0191

LOC310988	NM_001100509	0.0683	-0.138	0.0382	-0.061	0.0222	0.0259	-0.028	-0.051	0.0328	0.0192	0.1437	-0.007	0.1607	0.017
Psma2	NM_017279	-0.16	-0.067	-0.033	0.0622	-0.204	-0.396	0.2827	-0.19	0.1729	0.0502	-0.054	-0.078	-0.134	-4E-04
Psmb5	NM_001105727	-0.155	-0.391	0.0167	-0.596	-0.383	-0.077	-0.304	-0.551	-0.158	-0.541	-0.34	0.3154	0.073	-0.005
Psmd3	NM_001008281	-0.335	-0.169	0.0685	-0.072	-0.185	-0.335	-0.393	-0.225	-0.089	0.092	-0.208	0.0145	-0.013	-0.008
Psmb2	NM_017284	0.0332	-0.234	-0.035	-0.185	0.1288	0.0992	0.054	-0.128	-0.147	-0.116	-0.238	0.0833	-0.177	-0.061
Psma5	NM_017282	0.2983	-0.126	0.0841	0.0427	0.1116	0.1124	-0.158	-0.261	-0.06	-0.048	-0.043	-0.063	-0.206	-0.097

CDK Regulation of DNA Replication

Refseq	Gene symbol	K1	K2	K3	K4	K5	K6	K7	K8	K9	K10	K11	K12	Cluster	WT
Cdc6_predicted	NM_001108298	-0.303	-0.457	1.1581	-0.469	-0.764	-0.605	-0.443	-0.823	-0.095	-0.313	-0.052	0.1718	0.907	0.9988
Orc6l	NM_001033690	0.3106	-0.358	0.8637	-0.022	-0.121	-0.007	-0.393	-0.136	0.116	-0.012	-0.046	0.5101	1.0214	0.9668
Ris2_predicted	NM_001106192	0.1896	-0.23	0.7239	-0.033	-0.506	-0.576	-0.12	-0.486	-0.132	-6E-04	-0.164	-0.02	0.9272	0.919
Mcm5_predicted	NM_001106170	-0.152	-0.526	0.1527	-0.602	-0.304	-0.531	-0.617	-0.62	-0.275	-0.36	-0.247	-0.133	0.6931	0.7404
Orc1l	NM_177931	-0.732	-0.917	0.9951	-0.511	-0.871	-0.893	-0.439	-0.848	-0.605	-0.576	-0.598	-0.118	0.5462	0.6643
Mcm6	NM_017287	-0.412	-0.629	0.7967	-0.733	-1	-1.165	-0.82	-1.04	-0.598	-0.541	-0.653	0.2378	0.6931	0.6309
Mcm5_predicted	NM_001106170	-0.409	-0.558	-0.002	-0.986	-0.872	-0.648	-0.818	-0.869	-0.405	-0.595	-0.291	0.2116	0.3624	0.5449
Ccne1	NM_001100821	0.2233	0.0373	0.305	-0.178	0.0396	0.3271	-0.53	-0.015	-0.134	-0.145	-0.076	0.2155	0.4606	0.4482
Mcm7	NM_001004203	-1.234	-1.628	0.6408	-0.999	-1.827	-1.663	-0.94	-1.919	-1.044	-1.276	-1.07	-0.321	0.2851	0.3199
Mcm2_predicted	NM_001107873	-0.374	-0.909	0.3283	-0.929	-0.871	-1.152	-0.967	-1.027	-0.727	-0.59	-0.928	-0.354	0.1203	0.2329
Orc2l_predicted	NM_001012003	-0.061	-0.253	0.0752	-0.116	-0.101	-0.099	0.0107	-0.22	-0.177	-0.156	-0.107	0.0091	-0.206	0.0619
Orc4l	NM_199092	0.1187	-0.135	0.1239	0.2237	0.1759	0.2101	0.3322	0.271	-0.117	-0.046	-0.045	0.0424	0.2761	0.0231
Kitl	NM_021844	0.128	-0.016	-0.119	0.1927	-0.044	-0.114	-0.163	-0.135	0.084	-0.095	-0.144	0.0093	-0.056	0.005
Orc5l	NM_001014186	0.0961	-0.14	0.119	0.2197	-0.421	-0.536	0.0619	-0.428	-0.118	-0.281	-0.146	-0.4	-0.258	-0.26
Orc2l	NM_001012003	-0.328	0.0302	0.1054	1.001	-0.351	-0.878	0.2683	-0.079	-0.496	-0.334	-0.353	-0.737	-0.241	-0.297
Cdkn1b	NM_031762	-0.387	-0.522	-0.608	-0.177	-0.985	-0.733	-0.471	-0.816	-0.366	-0.262	-0.313	-0.097	-0.726	-0.754

Cell cycle

Refseq	Gene symbol	K1	K2	K3	K4	K5	K6	K7	K8	K9	K10	K11	K12	Cluster	WT
Cdc6_predicted	NM_001108298	-0.303	-0.457	1.1581	-0.469	-0.764	-0.605	-0.443	-0.823	-0.095	-0.313	-0.052	0.1718	0.907	0.9988
Orc6l	NM_001033690	0.3106	-0.358	0.8637	-0.022	-0.121	-0.007	-0.393	-0.136	0.116	-0.012	-0.046	0.5101	1.0214	0.9668
Plk1	NM_017100	-0.812	-1.357	1.1488	-0.459	-0.97	-0.917	-1.005	-1.387	-0.461	-0.89	-0.718	0.6025	0.5198	0.8132
Ccna2	NM_053702	-0.487	-1.484	0.6987	-0.756	-1.324	-1.298	-1.079	-1.333	-0.513	-0.877	-0.918	0.2591	0.7294	0.8087
Mcm5_predicted	NM_001106170	-0.152	-0.526	0.1527	-0.602	-0.304	-0.531	-0.617	-0.62	-0.275	-0.36	-0.247	-0.133	0.6931	0.7404
Orc1l	NM_177931	-0.732	-0.917	0.9951	-0.511	-0.871	-0.893	-0.439	-0.848	-0.605	-0.576	-0.598	-0.118	0.5462	0.6643
Cdc20	NM_171993	-0.655	-1.546	0.9239	-0.539	-0.981	-0.789	-1.086	-1.196	-0.364	-0.766	-1.02	0.8313	0.5677	0.6463
Mcm6	NM_017287	-0.412	-0.629	0.7967	-0.733	-1	-1.165	-0.82	-1.04	-0.598	-0.541	-0.653	0.2378	0.6931	0.6309
Ywhag	NM_019376	0.3475	0.3446	0.0346	0.0854	0.0935	0.2706	0.2342	0.3399	-0.076	0.04	0.149	-0.21	0.3573	0.6236
Ywhaz	NM_013011	-0.065	0.1086	0.3973	-0.09	0.1446	-0.276	0.3439	0.2262	-0.105	-0.151	-0.075	-0.118	0.4368	0.597
Mcm5_predicted	NM_001106170	-0.409	-0.558	-0.002	-0.986	-0.872	-0.648	-0.818	-0.869	-0.405	-0.595	-0.291	0.2116	0.3624	0.5449
Bub1_predicted	NM_001106507	-0.576	-0.83	0.886	-0.028	-0.735	-0.933	-0.363	-0.862	-0.23	-0.636	-0.723	0.2018	0.541	0.5369
Ccnb2	NM_001009470	-0.141	-1.435	0.866	-0.462	-1.111	-0.981	-0.852	-1.217	-0.235	-1.117	-0.848	0.8013	0.4818	0.491
Pcna	NM_022381	-0.133	-0.69	0.4443	-0.869	-0.911	-0.964	-0.579	-0.909	-0.39	-0.581	-0.458	-0.116	0.4921	0.4511
Ccne1	NM_001100821	0.2233	0.0373	0.305	-0.178	0.0396	0.3271	-0.53	-0.015	-0.134	-0.145	-0.076	0.2155	0.4606	0.4482
Anapc10_predicted	NM_001108445	0.6868	0.4221	0.1726	-0.068	-0.009	0.0873	0.4157	-0.229	0.1836	0.2807	0.5466	0.3831	0.3855	0.4425

Mdm2_predicted	NM_001108099	0.4744	0.8391	0.2347	1.6785	0.7314	0.32	0.8229	0.9969	0.17	0.6039	0.4085	-0.371	0.462	0.4132
Ywhah	NM_013052	0.4296	-0.728	0.4673	-0.196	0.0234	0.1733	0.0595	0.2224	-0.386	-0.294	-0.614	-0.018	0.2599	0.401
Cdk4	NM_053593	-0.371	-0.369	0.1459	-0.795	-0.55	-0.539	-0.674	-0.735	-0.125	-0.326	-0.174	0.4945	0.3634	0.3866
Chek1	NM_080400	0.0609	0.2265	0.1012	0.2004	0.1285	0.2934	0.0875	0.1017	-0.278	-0.079	0.2802	0.0048	0.4345	0.3776
Pkmyt1_predicted	NM_001105766	-0.161	-1.095	0.5835	0.4747	-0.732	-0.674	-0.714	-0.645	-0.661	-0.979	-0.786	-0.156	0.3617	0.3444
Mcm7	NM_001004203	-1.234	-1.628	0.6408	-0.999	-1.827	-1.663	-0.94	-1.919	-1.044	-1.276	-1.07	-0.321	0.2851	0.3199
Ccnh	NM_052981	0.4538	0.4073	0.0842	1.3194	0.4034	0.4022	0.362	0.499	0.6173	0.5218	0.6232	0.1465	0.5273	0.3196
Ccne2_predicted	NM_001108656	-0.237	-0.258	-0.189	-0.721	-0.74	-1.066	-0.726	-0.48	-0.436	-0.235	-0.47	-0.235	0.4481	0.2981
Wee1	NM_001012742	0.1536	-0.303	0.2409	-0.109	-0.302	-0.489	-0.276	-0.215	-0.03	-0.223	-0.044	0.2739	0.1094	0.2717
Cdc27	NM_001024793	-0.068	0.3243	-0.322	0.0178	0.0152	-0.071	-0.238	-0.072	-0.063	0.0338	-0.149	-0.256	0.3287	0.2695
Gsk3b	NM_032080	0.0289	-0.087	0.1025	0.1719	0.2685	0.094	0.051	0.2256	0.1967	0.0974	-0.003	-0.132	0.2443	0.264
Cdc2a	NM_019296	-0.21	-1.116	0.1973	-0.674	-1.015	-1.105	-1.375	-1.087	-0.338	-0.909	-0.802	0.2107	0.1896	0.2501
RGD1561880_predicted	NM_001126082	0.0611	0.0876	0.0582	0.2524	0.3132	0.3236	0.2685	0.2067	0.0754	0.1148	0.1568	0.2964	0.1666	0.2434
Mcm2_predicted	NM_001107873	-0.374	-0.909	0.3283	-0.929	-0.871	-1.152	-0.967	-1.027	-0.727	-0.59	-0.928	-0.354	0.1203	0.2329
RGD1562456_predicted	NM_001106416	-0.053	0.0198	0.0352	-0.172	-0.082	0.5701	0.1865	0.3318	-0.007	0.4423	0.0172	-0.246	0.5334	0.2179
Cdkn1c	NM_182735	0.3203	0.2147	0.2883	0.0254	0.2927	0.3271	0.2429	0.1391	-0.044	-0.014	0.1779	0.0639	0.1322	0.2178
Smc1l2_predicted	NM_001130498	0.0382	0.0067	0.2584	-0.05	0.0313	0.0942	0.1397	0.2792	-0.037	-0.06	0.0092	0.0353	-0.095	0.2019
Anapc2	NM_001100532	0.3249	0.0722	0.43	0.0882	0.0601	-0.038	0.159	0.1801	0.0553	0.0786	0.2279	0.1003	0.1706	0.1541
Ywhae	NM_031603	0.2562	0.0621	-0.003	-0.126	0.2201	0.4413	0.0776	0.1229	-0.109	-0.119	-0.146	0.4384	0.1398	0.1345
Hdac1_predicted	NM_001025409	0.0319	0.0879	-0.185	-0.225	0.0652	0.3582	0.1009	0.1132	0.0179	0.086	0.2472	0.5122	-0.047	0.1204
Ywhab	NM_019377	-0.244	0.2053	-0.059	-0.665	-0.106	-0.471	-0.197	-0.151	0.109	0.1387	0.0596	-0.025	0.1096	0.1148
Cdc7_predicted	NM_001108352	-0.127	######	-0.165	-0.167	0.0839	-0.245	0.0052	0.0149	-0.038	-0.218	-0.161	-0.251	-0.08	0.1102
Anapc7_predicted	NM_001107142	0.5313	0.3976	-0.005	0.8907	0.7174	0.6948	0.3155	0.6092	0.354	0.1543	0.0382	0.0529	-0.043	0.1049
Anapc5_predicted	NM_001080147	-0.038	-0.202	-0.196	-0.546	-0.311	-0.489	-0.283	-0.372	-0.216	-0.195	-0.149	0.127	0.0993	0.0809
Cdc25b	NM_133572	-0.085	-0.214	0.3274	-0.057	-0.407	-0.451	-0.269	-0.473	-0.37	-0.476	-0.337	0.1498	0.084	0.0808
Mad2l1_predicted	NM_001106594	-0.282	-0.419	0.2223	-0.047	-0.283	-0.324	-0.173	-0.376	-0.452	-0.209	-0.362	0.3531	-0.058	0.074
Smad3	NM_013095	0.0732	0.0869	0.1658	0.029	0.0009	0.147	0.0799	0.1123	-0.058	0.0216	0.1028	0.1047	0.0458	0.0739
Smc1a	NM_031683	-0.427	-0.139	0.605	-0.195	-0.879	-1.019	-0.141	-0.854	0.0529	0.015	0.0215	0.0046	0.0384	0.0677
Cdc25a	NM_133571	-0.064	-0.377	0.1402	-0.308	-0.211	-0.45	-0.264	-0.322	0.1943	-0.169	-0.033	-0.114	0.0535	0.0653
Orc2l_predicted	NM_001012003	-0.061	-0.253	0.0752	-0.116	-0.101	-0.099	0.0107	-0.22	-0.177	-0.156	-0.107	0.0091	-0.206	0.0619
Cdkn1a	NM_080782	0.2927	-0.128	-0.061	1.0608	0.5435	1.1683	0.4702	0.293	-0.214	-0.005	-0.191	-7E-04	-0.169	0.0359
Hdac1	NM_001025409	0.0644	0.2457	-0.223	-0.04	0.2635	-0.069	0.0685	0.3033	-0.078	-0.068	0.2756	0.1281	-0.086	0.0251
Orc4l	NM_199092	0.1187	-0.135	0.1239	0.2237	0.1759	0.2101	0.3322	0.271	-0.117	-0.046	-0.045	0.0424	0.2761	0.0231
Atm_mapped	NM_001106821	-0.036	0.0281	-0.072	0.2959	-0.014	-0.01	0.1963	0.1224	0.0464	0.0916	0.0546	0.0806	0.1393	0.0026
Cdc14a_predicted	NM_001134856	0.0142	0.7654	-0.51	-0.494	-0.288	-0.633	0.0143	-0.053	0.4335	0.4291	0.3845	-0.196	-0.104	-0.006
Tp53	NM_030989	-0.55	-0.386	0.1187	-0.04	-0.466	0.0555	-1E-03	-0.631	-0.428	-0.529	-0.442	0.1478	0.0812	-0.015
Ccna1	NM_001011949	0.7244	0.3829	-0.065	0.3101	0.5256	0.6234	0.0568	0.5047	0.4418	0.589	0.4777	0.3696	-0.082	-0.024
Cdkn2b	NM_130812	-0.03	-0.015	0.0423	0.0154	0.1153	0.0815	-0.02	-0.047	-0.068	0.112	-0.05	-0.028	0.0712	-0.029
Cdkn2c	NM_131902	-0.53	-0.004	0.2347	-0.189	-0.733	-0.727	-0.474	-0.819	-0.183	-0.204	-0.408	0.3323	-0.139	-0.038
Fzr1_predicted	NM_001108074	-0.327	-0.604	0.2499	-0.347	-0.469	-0.738	-0.575	-0.736	-0.512	-0.728	-0.749	-0.072	-0.084	-0.042
Cdc25c_predicted	NM_001107396	-0.224	-0.322	-0.077	-0.185	-0.276	-0.444	-0.271	-0.178	-0.244	-0.43	-0.412	-0.137	-0.024	-0.043
Hdac2	NM_053447	-0.525	-0.629	-0.421	-0.289	-0.486	-0.567	-0.571	-0.391	-0.36	-0.714	-0.509	-0.424	-0.295	-0.121
Ccnd2	NM_022267	-0.32	0.1044	-0.287	-0.189	0.2221	0.1569	-0.068	-0.08	-0.305	-0.184	-0.084	-0.193	-0.364	-0.139
Smad2	NM_019191	-0.763	-0.942	-0.405	-1.401	-0.052	-0.637	-0.508	-0.311	-0.841	-0.985	-0.861	-0.61	-0.025	-0.182

Cdc16	NM_001024744	-0.343	-0.567	-0.121	-0.083	-0.497	-0.216	-0.352	-0.491	-0.385	-0.542	-0.4	0.3819	-0.317	-0.212
Skp1a	NM_001007608	-0.285	-0.067	-0.215	-0.34	-0.495	-0.612	-0.065	-0.451	-0.026	-0.252	-0.101	-0.2	-0.273	-0.227
Smad4	NM_019275	-0.242	0.0136	-0.21	-0.217	-0.323	-0.509	0.1376	-0.015	-0.127	-0.091	-0.205	-0.337	-0.241	-0.253
Orc5l	NM_001014186	0.0961	-0.14	0.119	0.2197	-0.421	-0.536	0.0619	-0.428	-0.118	-0.281	-0.146	-0.4	-0.258	-0.26
Cdc16	NM_001024744	-0.487	-0.603	-0.267	-0.258	-0.737	-0.574	-0.341	-0.721	-0.458	-0.704	-0.735	0.0126	-0.319	-0.27
Chek2	NM_053677	-0.576	-1.185	0.144	-0.302	-0.86	-0.757	-0.38	-0.687	-0.425	-1.076	-0.639	0.0776	-0.189	-0.286
Gadd45a	NM_024127	1.4057	0.7213	0.0861	2.0662	1.0515	0.8384	0.0972	0.9763	0.5986	0.7877	0.692	0.3807	-0.334	-0.286
Orc2l	NM_001012003	-0.328	0.0302	0.1054	1.001	-0.351	-0.878	0.2683	-0.079	-0.496	-0.334	-0.353	-0.737	-0.241	-0.297
Hdac1	NM_001025409	-0.444	-0.38	-0.26	-0.359	-0.339	-0.182	-0.364	-0.355	-0.146	-0.206	-0.351	-0.019	-0.496	-0.332
Tgfb3	NM_013174	-0.783	-0.514	-0.275	-0.749	-0.733	-0.655	-0.695	-0.731	-0.295	-0.499	-0.255	0.1508	-0.407	-0.362
Ccnd1	NM_171992	-0.255	-0.335	-0.38	-0.973	-0.7	-0.479	-0.699	-0.87	-0.26	-0.447	-0.377	-0.225	-0.441	-0.362
Anapc4	NM_001107220	-0.543	-0.676	0.2696	0.3861	-0.768	-0.863	0.2391	-0.769	-0.464	-0.479	-0.58	-0.303	-0.305	-0.364
Tgfb1	NM_021578	-0.644	-0.222	-0.412	-0.825	-0.64	-0.931	-0.476	-0.798	-0.43	-0.189	-0.404	-0.514	-0.298	-0.378
Gadd45g	NM_001077640	-0.401	-1.022	-0.084	1.5095	0.1861	0.868	-0.237	0.4537	-0.949	-0.424	-0.87	-0.611	-0.451	-0.378
Prkdc_predicted	NM_001108327	-0.847	-0.439	0.3296	0.5745	-0.44	-1.151	0.1197	-0.488	-0.331	-0.518	-0.18	-0.4	-0.479	-0.382
Cdc14b_predicted	NM_001108404	-0.439	-0.513	-0.182	-0.136	-0.285	-0.529	-0.158	-0.21	-0.379	-0.416	-0.437	-0.154	-0.412	-0.401
Anapc1_predicted	NM_001107771	-0.879	-0.648	-0.471	-0.291	-0.255	-0.099	-0.872	-0.449	-0.556	-0.668	-0.826	-0.012	-0.452	-0.503
Cul1_predicted	NM_001108627	-0.348	-0.461	-0.527	-1.096	-0.344	-0.288	-0.077	-0.317	-0.417	-0.348	-0.246	-0.334	-0.445	-0.558
Rbl2	NM_031094	-0.606	-0.231	-0.608	-0.843	-0.742	-0.359	-0.211	-0.867	-0.074	-0.167	-0.198	0.3601	-0.415	-0.681
Cdkn1b	NM_031762	-0.387	-0.522	-0.608	-0.177	-0.985	-0.733	-0.471	-0.816	-0.366	-0.262	-0.313	-0.097	-0.726	-0.754
Gadd45b	NM_001008321	-0.571	-0.551	-0.672	1.1825	-0.37	0.0768	0.0226	-0.389	-0.736	-0.425	-0.499	-0.243	-0.733	-0.759
Tgfb2	NM_031131	-2.061	-1.36	-2.587	-1.62	-0.36	-0.445	-1.762	-0.578	-1.812	-1.575	-1.411	-2.506	-2.091	-2.058

Insulin Signaling Pathway

Refseq	Gene symbol	K1	K2	K3	K4	K5	K6	K7	K8	K9	K10	K11	K12	Cluster	WT
Rasa1	NM_013135	0.3577	0.7515	0.6959	-0.179	-0.257	-0.992	1.3839	-0.257	0.5887	0.4704	0.6542	-0.016	0.2942	0.3738
Csnk2a1	NM_053824	0.2273	0.2902	-0.332	0.0135	0.4822	0.0976	-0.079	0.2249	0.0931	0.3945	0.146	0.0104	0.2452	0.3272
Sos1	NM_001100716	0.2397	0.139	-0.004	0.0468	-0.003	0.038	-0.061	-0.065	0.2328	0.0498	-0.039	-0.069	0.0004	0.3145
Map2k1	NM_031643	0.3113	0.3264	0.1669	-0.12	0.2482	-0.013	0.0275	0.1835	0.1637	0.1875	0.3065	-0.355	0.1177	0.2264
Elk1	NM_001108059	-0.072	0.0453	-0.413	-0.069	-0.229	-0.384	-0.152	0.2371	-0.154	0.0505	-0.126	-0.383	-0.022	0.1651
Grb2	NM_030846	-0.247	-0.049	0.0856	0.0804	-0.238	-0.117	0.0561	-0.194	0.1711	0.1036	-0.011	-0.201	-0.145	0.1431
RGD1559787_predicted	NM_001109302	-0.342	0.0996	-0.029	0.1352	-0.178	-0.219	-0.335	-0.041	0.0091	0.0193	0.1017	-0.192	-0.025	0.1207
Ptpn11	NM_013088	0.0567	-0.195	-0.328	-0.376	0.1738	0.2723	-0.102	0.182	-0.096	-0.198	-0.187	0.0339	0.1258	0.1195
Jun	NM_021835	-0.641	-0.647	0.1331	-0.725	-0.331	0.1199	-0.449	-0.381	-0.254	-0.531	-0.284	0.3566	0.0265	0.0485
Pik3cg_predicted	NM_001106723	0.0832	-0.019	0.1252	0.098	-0.169	0.1087	0.1463	0.005	0.29	0.0961	0.2092	0.1469	0.237	0.0007
Insr	NM_017071	0.1291	-0.027	-0.045	-0.185	-0.072	0.0476	-0.059	0.0879	0.2929	-0.006	-0.028	-0.038	0.0644	-0.01
Slc2a4	NM_012751	0.0044	0.1633	0.0796	-0.027	0.0048	0.1072	-0.044	-0.083	-0.002	0.0475	-0.003	-0.082	0.0828	-0.126
Fos	NM_022197	0.3026	0.231	-0.035	0.0949	-0.26	-0.178	0.1259	-0.061	-0.065	-0.088	0.1793	0.1172	-0.247	-0.173
Mapk3	NM_017347	0.3283	0.1773	-0.828	-0.429	0.4243	0.0725	-0.043	0.3563	-0.346	-0.064	-0.135	-0.558	-0.214	-0.188
Irs1	NM_012969	0.0722	0.0697	-0.048	-0.249	0.0496	-0.16	-0.11	-0.088	-0.202	-0.109	-0.241	-0.274	-0.393	-0.197
Raf1	NM_012639	-0.549	-0.579	-0.52	-1.026	-0.697	-0.636	-0.332	-0.764	-0.575	-0.753	-0.587	-0.438	-0.72	-0.684

Role of MAL in Rho-Mediated Activation of SRF

Refseq	Gene symbol	K1	K2	K3	K4	K5	K6	K7	K8	K9	K10	K11	K12	Cluster	WT

Limk1	NM_031727	0.7733	0.347	0.9583	0.6432	0.7727	1.0035	0.5921	0.4208	0.4245	0.6679	0.9059	0.8677	0.5357	0.8638
Rhoc_predicted	NM_057132	0.9976	0.5872	0.2094	0.3536	1.2204	1.063	0.4802	1.2089	0.6036	0.5459	0.6634	0.4648	0.4268	0.5849
Mapk1	NM_053842	0.1899	0.2365	-0.224	-0.254	0.1591	-0.095	0.2903	0.2148	-0.078	-0.077	-0.123	-0.284	0.3741	0.4622
Rac1	NM_134366	0.4318	-2E-04	-0.275	-0.456	0.5524	0.3213	0.0464	0.4818	0.202	-0.052	-0.196	-0.192	0.2267	0.3903
Rock1	NM_031098	0.1393	0.4089	-0.645	0.3067	-0.232	-0.9	0.2423	0.4314	0.1614	0.5512	0.0017	-0.24	0.2955	0.3169
Acta1	NM_019212	0.5333	0.0816	0.7952	1.4517	1.2983	1.9875	-0.074	1.6537	0.1019	0.1697	0.2598	0.0845	0.3393	0.2275
Hnf1a	NM_012669	-0.031	-0.011	-0.083	0.0568	0.1294	0.0497	-0.075	0.0512	0.0214	0.0663	0.2266	0.0572	0.2263	0.2272
Map2k1	NM_031643	0.3113	0.3264	0.1669	-0.12	0.2482	-0.013	0.0275	0.1835	0.1637	0.1875	0.3065	-0.355	0.1177	0.2264
Mal	NM_012798	1.3417	0.3199	0.2302	0.3768	1.8403	1.4952	0.2064	1.4655	0.487	0.6893	0.4388	-0.03	0.1163	0.2229
RGD1559787_predicted	NM_001109302	-0.342	0.0996	-0.029	0.1352	-0.178	-0.219	-0.335	-0.041	0.0091	0.0193	0.1017	-0.192	-0.025	0.1207
Map3k1	NM_053887	0.3083	0.0547	0.1056	-0.07	0.0823	-1E-03	0.0512	0.193	0.1275	0.0938	0.0094	0.1465	0.021	0.0456
Diap1_predicted	NM_001107393	0.2	0.0842	-0.223	-0.2	0.1684	-0.109	0.08	0.268	0.1788	0.1218	0.2141	0.1795	-0.078	0.0004
Rhoa	NM_057132	0.1223	0.1374	-0.093	0.3954	0.5676	0.4536	0.2125	0.5139	0.1944	0.3573	0.2653	0.2919	0.0461	-0.154
Map4k2_predicted	NM_001106329	-0.134	-0.14	-0.06	-0.103	-0.195	-0.062	-0.082	-0.149	-0.169	0.0904	-0.079	-0.158	0.0467	-0.161
Mapk3	NM_017347	0.3283	0.1773	-0.828	-0.429	0.4243	0.0725	-0.043	0.3563	-0.346	-0.064	-0.135	-0.558	-0.214	-0.188
Raf1	NM_012639	-0.549	-0.579	-0.52	-1.026	-0.697	-0.636	-0.332	-0.764	-0.575	-0.753	-0.587	-0.438	-0.72	-0.684
Map2k2	NM_133283	-1.234	-1.161	-0.847	-1.54	-1.145	-1.317	-1.084	-1.244	-1.202	-1.179	-1.136	-0.744	-0.969	-1.004

Multiple antiapoptotic pathways from IGF-1R signaling lead to BAD phosphorylation

Refseq	Gene symbol	K1	K2	K3	K4	K5	K6	K7	K8	K9	K10	K11	K12	Cluster	WT
Mapk1	NM_053842	0.1899	0.2365	-0.224	-0.254	0.1591	-0.095	0.2903	0.2148	-0.078	-0.077	-0.123	-0.284	0.3741	0.4622
Igf1r	NM_052807	-0.492	-0.066	0.6372	0.996	-0.027	0.2992	0.4027	-0.013	-0.273	-0.047	-0.043	0.0231	0.4745	0.4566
Ywhah	NM_013052	0.4296	-0.728	0.4673	-0.196	0.0234	0.1733	0.0595	0.2224	-0.386	-0.294	-0.614	-0.018	0.2599	0.401
Prkar2a	NM_019264	-0.049	0.1908	0.4218	0.0919	0.0874	0.168	0.0591	0.1264	0.1603	0.0141	0.3626	0.2132	0.3039	0.3171
Sos1	NM_001100716	0.2397	0.139	-0.004	0.0468	-0.003	0.038	-0.061	-0.065	0.2328	0.0498	-0.039	-0.069	0.0004	0.3145
Bad	NM_022698	-0.018	-0.224	0.0514	-0.61	-0.243	-0.058	-0.663	-0.376	0.0096	-0.123	0.0257	0.3288	0.2393	0.262
Map2k1	NM_031643	0.3113	0.3264	0.1669	-0.12	0.2482	-0.013	0.0275	0.1835	0.1637	0.1875	0.3065	-0.355	0.1177	0.2264
Akt1	NM_033230	0.0085	-0.07	0.3608	-0.631	0.2019	-0.172	-0.008	0.2784	-0.022	-0.162	-0.375	-0.352	-0.019	0.1763
Grb2	NM_030846	-0.247	-0.049	0.0856	0.0804	-0.238	-0.117	0.0561	-0.194	0.1711	0.1036	-0.011	-0.201	-0.145	0.1431
Adcy1_predicted	NM_001107239	-0.061	0.0391	0.0054	-0.036	-0.044	-0.034	0.122	0.054	0.0192	-0.028	0.0286	-0.003	-0.025	0.0193
Rps6ka1	NM_031107	0.001	-0.118	0.2065	0.0767	0.1657	0.4422	-0.037	-0.03	-0.236	-0.007	-0.106	-0.152	-0.186	-0.046
Mapk3	NM_017347	0.3283	0.1773	-0.828	-0.429	0.4243	0.0725	-0.043	0.3563	-0.346	-0.064	-0.135	-0.558	-0.214	-0.188
Irs1	NM_012969	0.0722	0.0697	-0.048	-0.249	0.0496	-0.16	-0.11	-0.088	-0.202	-0.109	-0.241	-0.274	-0.393	-0.197
Prkar2b	NM_001030020	0.3406	-0.204	-0.157	0.3313	-0.228	-0.312	-0.229	-0.328	-0.046	-0.333	-0.373	0.6673	-0.381	-0.338
Prkacb_predicted	NM_001077645	-0.25	-0.269	-1.219	-1.031	-0.114	-0.424	-0.323	-0.055	-0.227	-0.23	-0.379	-0.322	-0.452	-0.644
Raf1	NM_012639	-0.549	-0.579	-0.52	-1.026	-0.697	-0.636	-0.332	-0.764	-0.575	-0.753	-0.587	-0.438	-0.72	-0.684

Integrin Signaling Pathway

Refseq	Gene symbol	K1	K2	K3	K4	K5	K6	K7	K8	K9	K10	K11	K12	Cluster	WT
Cav1	NM_133651	0.9602	0.721	-1.167	-1.514	1.3532	-0.118	-0.397	1.7173	0.2314	0.5471	-0.017	-0.843	1.1984	1.0568
Cav1	NM_133651	0.876	1.1396	-1.256	-1.13	1.1003	-0.34	0.1111	1.5069	0.1574	0.8312	-0.099	-0.992	0.9348	0.8436
Bcar1	NM_012931	0.7385	0.6697	1.0586	0.9247	0.9258	0.7494	0.7055	0.8182	0.6028	0.719	0.5179	0.5281	0.5173	0.7716
Actn1	NM_031005	-0.334	-0.008	0.5405	0.3751	0.3205	-0.339	-0.333	0.2886	0.3462	0.102	0.0036	-0.563	0.4607	0.7322
Rhoc_predicted	NM_057132	0.9976	0.5872	0.2094	0.3536	1.2204	1.063	0.4802	1.2089	0.6036	0.5459	0.6634	0.4648	0.4268	0.5849

Capns1	NM_017118	0.5248	0.7534	-0.58	-0.754	0.3605	-0.146	-0.269	0.1809	0.6505	0.6146	0.4186	-0.185	0.312	0.4676
Mapk1	NM_053842	0.1899	0.2365	-0.224	-0.254	0.1591	-0.095	0.2903	0.2148	-0.078	-0.077	-0.123	-0.284	0.3741	0.4622
Rock1	NM_031098	0.1393	0.4089	-0.645	0.3067	-0.232	-0.9	0.2423	0.4314	0.1614	0.5512	0.0017	-0.24	0.2955	0.3169
Sos1	NM_001100716	0.2397	0.139	-0.004	0.0468	-0.003	0.038	-0.061	-0.065	0.2328	0.0498	-0.039	-0.069	0.0004	0.3145
Crkl	NM_001008284	-0.226	0.1178	0.0982	-0.363	-0.178	-0.218	-0.234	-0.152	0.025	0.1856	0.0809	0.1363	0.2659	0.2929
Vcl_predicted	NM_001107248	0.3328	0.1098	0.1915	0.2133	0.2164	0.293	0.0491	0.475	0.0497	0.1453	0.3223	0.2573	0.4344	0.2462
Actn3	NM_133424	0.1706	0.23	0.0273	-0.024	0.1492	0.1164	0.1259	0.092	0.2819	0.1398	0.2606	0.1454	0.0593	0.2287
Acta1	NM_019212	0.5333	0.0816	0.7952	1.4517	1.2983	1.9875	-0.074	1.6537	0.1019	0.1697	0.2598	0.0845	0.3393	0.2275
Map2k1	NM_031643	0.3113	0.3264	0.1669	-0.12	0.2482	-0.013	0.0275	0.1835	0.1637	0.1875	0.3065	-0.355	0.1177	0.2264
Grb2	NM_030846	-0.247	-0.049	0.0856	0.0804	-0.238	-0.117	0.0561	-0.194	0.1711	0.1036	-0.011	-0.201	-0.145	0.1431
Zyx	NM_053761	0.0497	0.3525	0.1718	0.0527	0.1254	0.5228	0.0224	0.0546	0.2935	-0.154	0.2403	0.2922	0.0664	0.0973
Jun	NM_021835	-0.641	-0.647	0.1331	-0.725	-0.331	0.1199	-0.449	-0.381	-0.254	-0.531	-0.284	0.3566	0.0265	0.0485
Fyn	NM_012755	0.278	-0.033	0.2423	-0.047	0.1119	0.3185	-0.017	0.3044	0.0514	0.1384	0.166	0.1324	0.2074	0.0362
Zyx	NM_053761	0.0574	-0.06	-0.082	0.0329	-0.047	0.058	0.0098	-0.039	0.0752	0.0627	-0.046	0.0094	-0.022	0.0084
Csk_predicted	NM_001030039	-0.087	-0.42	-0.078	-0.087	0.0436	0.4153	-0.292	0.1998	-0.453	-0.359	-0.332	-0.114	-0.034	-0.016
Tln1	NM_001039025	-0.071	0.3027	0.1156	-0.283	-0.056	-0.083	-0.294	-0.296	0.1485	0.1839	0.231	0.1391	-0.107	-0.124
Rhoa	NM_057132	0.1223	0.1374	-0.093	0.3954	0.5676	0.4536	0.2125	0.5139	0.1944	0.3573	0.2653	0.2919	0.0461	-0.154
Ppp1r12b_predicted	NM_001107178	0.0008	-0.028	-0.106	-0.02	0.1745	-0.009	0.0377	0.0819	-0.027	-0.071	0.1665	0.2017	0.0111	-0.167
Mapk3	NM_017347	0.3283	0.1773	-0.828	-0.429	0.4243	0.0725	-0.043	0.3563	-0.346	-0.064	-0.135	-0.558	-0.214	-0.188
Capn1	NM_019152	-0.131	0.045	0.0325	-0.412	-3E-04	0.0683	-0.208	-0.087	0.1379	-0.068	-0.076	0.0716	-0.015	-0.194
Itgb1	NM_017022	-0.372	0.5851	-1.086	-0.317	0.0142	-0.479	-0.349	-0.025	0.1225	0.2547	0.0189	-0.375	-0.078	-0.231
Raf1	NM_012639	-0.549	-0.579	-0.52	-1.026	-0.697	-0.636	-0.332	-0.764	-0.575	-0.753	-0.587	-0.438	-0.72	-0.684
Map2k2	NM_133283	-1.234	-1.161	-0.847	-1.54	-1.145	-1.317	-1.084	-1.244	-1.202	-1.179	-1.136	-0.744	-0.969	-1.004

Regulation of actin cytoskeleton

Refseq	Gene symbol	K1	K2	K3	K4	K5	K6	K7	K8	K9	K10	K11	K12	Cluster	WT
Rras2	NM_001013434	0.7975	0.2862	0.4787	0.9886	1.2151	1.0363	0.6676	1.3277	0.7401	0.609	0.3557	0.5264	0.9018	1.1172
Baiap2	NM_057196	0.3062	0.3094	1.255	0.5184	0.5013	0.5974	1.0448	0.2639	0.3881	0.741	0.8477	0.5031	0.9938	0.9798
Pak1	NM_017198	0.5735	1.1758	0.7418	1.6668	0.7971	0.9404	0.2769	0.8054	1.0242	1.1549	1.0189	0.7802	0.9218	0.9462
Limk1	NM_031727	0.7733	0.347	0.9583	0.6432	0.7727	1.0035	0.5921	0.4208	0.4245	0.6679	0.9059	0.8677	0.5357	0.8638
Bcar1	NM_012931	0.7385	0.6697	1.0586	0.9247	0.9258	0.7494	0.7055	0.8182	0.6028	0.719	0.5179	0.5281	0.5173	0.7716
Msn	NM_030863	0.6279	1.1187	-1.429	-0.782	0.6991	-0.611	-0.137	0.8921	0.5826	1.0791	0.2006	-0.664	0.9282	0.7698
Wasf1	NM_001025114	0.4858	0.3344	0.6513	0.3998	0.4766	0.2774	0.6493	0.4933	0.6082	0.5716	0.3553	0.6545	0.8511	0.767
Actn1	NM_031005	-0.334	-0.008	0.5405	0.3751	0.3205	-0.339	-0.333	0.2886	0.3462	0.102	0.0036	-0.563	0.4607	0.7322
Pdgfra	NM_012802	0.2186	1.871	-0.002	-0.103	0.1154	-0.022	1.2581	0.1133	1.4972	1.7244	1.4788	0.8427	0.6398	0.717
Rhoc_predicted	NM_057132	0.9976	0.5872	0.2094	0.3536	1.2204	1.063	0.4802	1.2089	0.6036	0.5459	0.6634	0.4648	0.4268	0.5849
Pfn2	NM_030873	1.2049	0.4024	-0.506	0.6479	0.9333	0.6943	0.2684	1.0652	0.3495	0.5649	0.0845	0.0306	0.6125	0.5824
Pfn1	NM_022511	-0.255	-0.279	-0.504	-1.215	-0.195	-0.697	-0.441	-0.229	0.1025	-0.305	-0.178	0.0614	0.4062	0.5694
Ppp1r12a	NM_053890	0.1017	0.5328	0.0991	0.6456	0.2123	0.231	0.314	0.2714	0.3991	0.4693	0.1608	0.2962	0.4708	0.555
Actb	NM_031144	0.0815	-0.035	-0.254	-0.673	-0.236	-1.146	-0.04	0.1146	0.1424	0.1382	-0.191	0.0164	0.491	0.5533
Itgav_predicted	NM_001106549	0.5155	0.803	0.2433	1.2994	1.0343	0.8493	0.5735	1.1846	0.5653	0.6854	0.8009	0.0702	0.6528	0.5222
Fgfr2	NM_001109892	0.0262	-0.047	-0.001	0.0627	0.0032	-0.033	0.1388	-0.019	0.1086	0.1751	0.0432	0.0463	-0.073	0.5197
Arhgef12	NM_001013246	0.2178	-0.108	0.157	0.1631	0.1308	-0.104	0.0463	0.2729	-0.023	0.1246	0.0364	-0.09	0.3523	0.5175
Cfl1	NM_017147	-0.019	-0.005	0.3049	-0.152	0.1876	-0.115	-0.073	0.0517	0.1142	0.1573	0.0584	0.1277	0.4106	0.5156

Mapk1	NM_053842	0.1899	0.2365	-0.224	-0.254	0.1591	-0.095	0.2903	0.2148	-0.078	-0.077	-0.123	-0.284	0.3741	0.4622
Itga5_mapped	NM_001108118	0.747	0.9083	1.1645	1.2258	1.2158	0.7962	0.823	0.9774	0.5807	0.5219	0.6025	0.1911	0.1032	0.4497
Nckap1	NM_031618	0.1342	0.3388	-0.341	0.0398	0.4396	-0.448	0.0198	0.5613	0.1684	0.4468	-0.023	-0.255	0.4196	0.4365
Fgfr4	NM_001109904	0.4353	0.4585	0.3149	0.2521	0.2906	0.2967	0.2902	0.4239	0.4666	0.4577	0.0942	0.5642	0.3155	0.4347
Crk	NM_019302	0.3082	0.0691	0.2988	0.0964	0.5378	0.6265	0.3411	0.3819	-0.086	0.3491	0.3081	0.3329	0.3625	0.4064
Rac1	NM_134366	0.4318	-2E-04	-0.275	-0.456	0.5524	0.3213	0.0464	0.4818	0.202	-0.052	-0.196	-0.192	0.2267	0.3903
Gna12	NM_031034	-0.065	0.1462	-0.115	-0.033	-0.043	-0.496	-0.048	0.3261	0.3973	0.0396	0.01	-0.12	0.0423	0.3437
Myl3	NM_012606	0.0126	0.176	0.1	0.0645	-0.036	0.3209	0.0318	0.1924	0.0161	-0.008	0.3125	0.3801	0.0854	0.3368
Rock1	NM_031098	0.1393	0.4089	-0.645	0.3067	-0.232	-0.9	0.2423	0.4314	0.1614	0.5512	0.0017	-0.24	0.2955	0.3169
Pak2	NM_053306	0.108	0.3677	-0.658	-0.542	0.044	-0.836	-0.277	0.2073	0.3431	0.3562	0.0564	-0.188	0.2376	0.316
Sos1	NM_001100716	0.2397	0.139	-0.004	0.0468	-0.003	0.038	-0.061	-0.065	0.2328	0.0498	-0.039	-0.069	0.0004	0.3145
Nckap1l_predicted	NM_001108119	2.217	0.7942	0.5247	0.7815	2.1981	2.4741	0.7869	2.0508	0.7256	0.995	1.0124	0.1817	0.3474	0.3107
Itga3_predicted	NM_001108292	0.5297	-0.056	0.264	0.2853	0.73	-0.018	0.1966	0.3364	0.1312	0.1163	-0.065	-0.294	0.1343	0.3098
Pak3	NM_019210	-0.086	-0.309	-0.433	-0.648	-0.314	-0.44	-0.151	-0.271	-0.535	-0.222	-0.38	-0.573	-0.135	0.3066
Myh9	NM_013194	-0.501	0.538	-0.34	-0.108	-0.144	-0.523	-0.505	-0.112	0.3375	0.6141	0.161	0.007	0.2953	0.305
Cfl2_predicted	NM_001108982	-0.135	0.0396	0.1671	0.4894	-0.106	-0.499	-0.086	-0.042	0.1419	0.0948	0.0651	0.0611	0.4059	0.3027
LOC360883	NM_001127449	0.0666	-0.103	-0.014	-0.086	0.1102	-0.018	-0.236	-0.187	-0.017	0.0652	-0.196	-0.182	0.5484	0.301
Crkl	NM_001008284	-0.226	0.1178	0.0982	-0.363	-0.178	-0.218	-0.234	-0.152	0.025	0.1856	0.0809	0.1363	0.2659	0.2929
Pik3r2	NM_022185	0.0799	0.2557	0.1992	-0.127	0.1345	-0.105	0.2451	0.108	0.3234	0.3592	0.252	-0.117	-0.219	0.29
Fgf5	NM_022211	0.0227	0.0231	0.2384	0.1159	0.2519	0.2015	0.1775	0.3227	0.0859	0.328	0.0721	0.0717	0.1602	0.2798
Mylk2	NM_057209	0.1619	-0.058	0.0492	0.0021	-2E-04	0.3018	-0.06	0.0644	-0.043	-0.019	0.4676	0.441	0.0082	0.2783
Pip4k2b	NM_053550	0.1023	0.0837	0.0136	-0.09	0.0004	-0.024	0.0012	0.1775	0.0987	0.0697	0.0541	-0.104	0.0988	0.2652
Bdkrb1	NM_030851	-0.056	0.3673	0.2491	0.1625	0.0324	-0.049	0.0847	-0.078	-0.113	-0.067	-0.182	0.0131	0.1091	0.2631
Pfn4	NM_001009503	-0.067	-0.014	0.2416	0.0616	0.0175	0.1327	-0.031	0.0049	0.3961	0.269	0.0044	0.0404	-0.008	0.2613
Pxn_predicted	NM_001012147	0.1372	-0.016	0.1146	-0.008	0.0213	0.1775	0.0336	0.0691	-0.024	0.1038	0.1046	0.1469	-0.018	0.2589
Vcl_predicted	NM_001107248	0.3328	0.1098	0.1915	0.2133	0.2164	0.293	0.0491	0.475	0.0497	0.1453	0.3223	0.2573	0.4344	0.2462
Chrm2	NM_031016	0.2365	0.1024	0.1582	0.0423	0.1888	0.1138	0.1453	0.0968	-0.022	0.0363	0.0773	0.0793	0.0408	0.2333
Actn3	NM_133424	0.1706	0.23	0.0273	-0.024	0.1492	0.1164	0.1259	0.092	0.2819	0.1398	0.2606	0.1454	0.0593	0.2287
Map2k1	NM_031643	0.3113	0.3264	0.1669	-0.12	0.2482	-0.013	0.0275	0.1835	0.1637	0.1875	0.3065	-0.355	0.1177	0.2264
Rdx	NM_001005889	-0.155	0.0162	-0.063	-0.446	-0.012	-0.013	-0.157	-0.1	0.1125	-0.022	-0.045	-0.137	0.4509	0.2238
Ppp1ca	NM_031527	0.1196	0.0143	0.2943	-0.846	0.0817	-0.013	-0.319	-0.013	-0.009	0.0134	-0.014	0.0692	0.1227	0.217
RGD1308470	NM_173100	-0.114	-0.051	-0.255	0.0474	0.0117	-0.066	0.2313	0.0151	0.1499	-0.187	0.0207	0.0715	0.1634	0.2071
Actb	NM_031144	-0.22	-0.126	-0.294	-0.635	-0.31	-0.904	-0.11	-0.351	-0.533	-0.357	-0.42	-0.144	0.0711	0.1894
Pip5k1c_predicted	NM_001009967	-0.086	-0.051	-0.068	0.2228	0.0633	0.0303	-0.007	0.1607	0.059	-0.002	-0.07	0.0251	0.1103	0.1798
Fgf20	NM_023961	-0.001	0.0244	0.0471	0.0102	-0.035	0.0754	0.089	0.0124	-0.064	0.1178	0.0244	-1E-04	-0.015	0.1768
Pik3cb	NM_053481	0.0846	-0.3	-0.363	0.1314	0.1796	-0.105	-0.171	0.4984	-0.348	-0.303	-0.465	-0.541	-0.23	0.174
Pxn	NM_001012147	-5E-04	0.3074	-0.053	-0.127	-0.066	-0.468	0.0947	-0.166	0.0228	0.0625	-0.011	-0.411	0.0546	0.1686
Ssh2_predicted	NM_001107024	-0.006	0.1102	0.0158	0.1473	0.1006	0.1392	0.0915	0.2094	0.2655	0.0011	-0.053	0.12	-0.007	0.1682
Fgf2	NM_019305	0.2135	0.0531	0.1787	0.2991	0.215	0.531	0.0297	0.2286	-0.039	0.0296	-0.069	0.2598	0.0445	0.1655
Fgd1	NM_001037546	0.0075	0.2854	0.0833	0.0847	0.008	0.0739	0.0599	0.006	-0.007	0.0573	0.1426	0.2963	-0.059	0.1615
Arpc3_predicted	NM_001105933	0.3368	0.2646	-0.244	0.3674	0.5522	0.45	0.5495	0.266	0.3289	0.3963	0.2824	0.0204	-0.089	0.1581
Actg_predicted	NM_001127449	-0.234	-0.139	0.2129	-0.367	-0.151	-0.461	-0.118	-0.098	-0.568	-0.227	-0.448	-0.172	0.0412	0.1498
Fgf6	NM_131908	0.043	0.0681	-0.03	0.1422	0.0184	-0.004	-0.051	0.0418	0.1285	0.042	0.0966	0.0211	-0.026	0.1434
Rras_predicted	NM_001108481	0.5164	0.5179	0.0566	0.8249	0.865	1.0369	0.3214	0.789	0.4356	0.4122	0.4213	0.1917	-0.124	0.1355

Apc	NM_012499	0.0162	-0.055	0.1087	0.0827	-0.065	-0.051	0.0368	-0.067	0.1656	-0.077	-0.031	0.2036	-0.002	0.1346
Nras	NM_080766	0.2289	0.0505	-0.163	-0.566	0.0018	-0.466	0.037	0.1195	-0.175	-0.202	-0.367	-0.551	0.1088	0.1292
Pak4_predicted	NM_001106238	-0.148	0.0521	0.3564	0.1822	-0.142	-0.092	0.0485	0.0422	-0.086	-0.191	0.0522	-0.002	0.2507	0.1252
Arpc4_predicted	NM_001106615	0.3434	0.1223	-0.061	-0.875	0.0851	-0.125	-0.415	-0.127	0.0699	0.0212	-0.031	-0.103	0.0732	0.1236
Mras	NM_012981	-0.122	0.2271	0.2612	-0.223	-0.312	-0.171	0.0398	-0.278	0.2537	0.1166	-0.007	0.1294	0.2013	0.1123
LOC303202	NM_001013434	0.0604	0.0354	0.0103	0.0123	0.1356	0.0558	-0.092	0.0753	0.0546	0.2005	0.2296	-0.022	0.2324	0.109
Pip5k2c	NM_080480	0.3073	0.1297	0.5423	0.5986	0.1617	-0.292	-0.076	0.3378	-0.082	0.3809	0.194	-0.054	0.1772	0.1076
Iqgap1_predicted	NM_001108489	-0.35	0.19	-0.927	-0.49	0.1171	-0.468	-0.389	0.3481	-0.123	0.0096	-0.414	-0.729	0.1154	0.0923
Git1	NM_031814	0.3293	-0.157	0.1766	0.0358	0.1278	0.296	0.0011	0.0284	-0.214	-0.125	-0.075	0.0826	0.1605	0.0918
Arhgef6	NM_001005565	0.0991	0.3926	-0.244	-0.062	-0.01	-0.085	0.1943	-0.189	0.0374	0.213	0.1885	-0.097	0.0084	0.0904
Apc2_predicted	NM_001106769	-0.092	-0.002	0.0187	-0.036	-0.004	-0.06	-0.034	-0.136	0.2163	-0.067	0.0716	0.052	-0.114	0.0882
Chrm3	NM_012527	0.175	-0.117	0.0945	0.2611	0.3028	0.0432	0.0142	-0.167	0.0329	0.118	0.0344	-0.036	0.0479	0.0867
Chrm1	NM_080773	0.1681	0.0894	0.0798	0.0773	0.0371	0.0161	0.0768	0.1648	0.0999	0.2278	0.2439	0.0513	0.0909	0.084
Fgf9	NM_012952	0.0044	-0.045	-0.055	-0.094	-0.017	-0.132	-0.07	-0.078	-0.144	0.2178	0.0453	-0.095	-0.141	0.0821
Kras	NM_031515	0.0074	-0.294	-0.295	-0.186	0.0713	-0.095	-0.171	0.2968	-0.003	-0.214	-0.138	-0.053	-0.003	0.0792
Fgfr3	NM_053429	-0.221	-0.131	0.052	-0.126	0.446	0.7827	-0.177	0.4398	0.0485	0.0506	0.0546	-0.209	-0.043	0.0767
F2	NM_022924	0.0032	0.196	0.1451	0.1653	0.098	0.1875	0.3726	0.4434	0.1445	0.1282	0.1022	0.2974	0.3385	0.0765
Matk	NM_021859	-0.381	-0.209	0.0086	-0.121	-0.27	-0.064	-0.208	-0.327	-0.298	-0.351	-0.449	0.1166	-0.165	0.0699
Itga10_predicted	NM_001107699	0.1327	0.1495	0.1072	0.0881	0.7962	0.7524	0.3246	0.5065	0.0788	0.2214	0.1145	0.3779	0.1567	0.0683
Mlc3	NM_020104	1.0921	0.1433	0.1919	0.2722	0.0128	0.184	0.4379	0.4449	-0.076	-0.034	0.0394	0.1297	0.1666	0.067
Fgf22	NM_130751	-0.039	-0.02	-0.058	-0.08	-0.033	-0.018	-0.11	-0.056	0.0255	0.0586	0.0017	0.0743	0.0482	0.0661
Fgf15	NM_130753	0.0544	-0.027	0.2302	0.1974	-0.008	0.095	0.0635	0.0374	0.0104	0.1143	0.0874	0.0358	0.0259	0.0535
Vav2_predicted	NM_001106563	0.1294	0.0066	0.2391	0.0809	0.1736	0.0368	0.225	0.1835	0.1001	0.2343	-0.038	0.019	0.0802	0.053
Gna13	NM_001013119	0.0504	0.2987	0.0997	0.1626	0.16	0.139	0.1398	0.1528	0.1719	0.1865	0.2124	0.0201	0.2272	0.0416
Fgf10	NM_012951	0.0936	-0.045	0.077	0.3513	0.1082	-0.006	0.0422	0.053	0.0627	0.0401	-0.04	-0.003	0.1591	0.0415
Itgal	NM_001033998	0.0184	-0.111	-0.015	-0.004	-0.045	0.0841	-0.044	-0.105	0.0435	-0.043	-0.041	0.0121	0.0704	0.0057
Arhgef7	NM_001113521	0.1779	0.0691	-0.07	0.1568	0.2367	0.1042	0.1346	0.2112	0.0182	0.1519	0.0388	-0.04	0.0243	0.0027
Pik3cg_predicted	NM_001106723	0.0832	-0.019	0.1252	0.098	-0.169	0.1087	0.1463	0.005	0.29	0.0961	0.2092	0.1469	0.237	0.0007
Diap1_predicted	NM_001107393	0.2	0.0842	-0.223	-0.2	0.1684	-0.109	0.08	0.268	0.1788	0.1218	0.2141	0.1795	-0.078	0.0004
Pip5k1b	NM_001012743	0.1684	0.0339	0.0702	0.1753	0.2066	0.1487	0.0147	0.0468	-0.006	-0.079	-0.035	-0.004	0.1098	-0.001
Itga7	NM_030842	0.0824	0.8883	1.4744	1.2978	-0.033	0.1627	1.7669	-0.069	0.3234	0.8206	0.5865	-0.239	-0.088	-0.013
Csk_predicted	NM_001030039	-0.087	-0.42	-0.078	-0.087	0.0436	0.4153	-0.292	0.1998	-0.453	-0.359	-0.332	-0.114	-0.034	-0.016
Fgf17	NM_019198	0.0878	-0.093	-0.051	0.0115	-0.077	0.0358	0.0172	-0.064	-0.027	-0.081	-0.061	0.0986	0.0009	-0.022
Itgad	NM_031691	-0.14	0.0064	-0.102	-0.081	0.0744	-0.073	0.0545	-0.069	-0.069	0.1647	-0.141	-0.005	-0.107	-0.022
Fgf3	NM_130817	0.1294	0.1202	0.0628	-0.093	0.1786	-0.127	-0.086	0.0451	-0.046	-0.077	-0.121	-0.12	0.2088	-0.023
Ptk2	NM_013081	-0.57	0.0402	-0.276	-0.572	-0.343	-0.304	-0.229	-0.33	-0.016	0.0105	-0.087	-0.036	-0.131	-0.029
Bdkrb2	NM_173100	-0.126	-0.115	-0.247	-0.108	0.0233	-0.083	-0.18	-0.091	-0.046	-0.237	-0.284	-0.057	-0.07	-0.038
Itgam	NM_012711	0.0201	0.0667	0.0281	0.1961	0.0963	-0.047	0.2404	0.0628	0.0225	0.1731	0.1686	0.051	-0.071	-0.051
Arpc2_predicted	NM_001106919	0.1863	-0.002	0.1765	0.2421	0.2526	-0.023	0.3127	0.2154	0.0293	-0.016	0.1378	0.027	0.1809	-0.053
Fgf21	NM_130752	-0.024	-0.078	-0.039	0.0781	-0.087	0.0277	-0.009	-0.037	0.1059	-0.017	-0.033	-0.078	-0.022	-0.054
Pip5k2a	NM_053926	-0.328	-0.191	-0.317	-0.651	-0.142	-0.278	-0.352	-0.134	-0.328	-0.156	-0.309	-0.029	0.0694	-0.055
Fgf13	NM_053428	-0.051	-0.092	0.0278	0.0201	-0.088	-0.053	0.0907	0.0417	-0.037	-0.005	-0.035	0.16	0.0505	-0.057
Chrm5	NM_017362	-0.126	0.0457	0.07	0.0654	-0.032	0.011	0.0335	-0.032	0.0842	0.1119	-0.076	0.2193	0.0932	-0.058
Ssh3	NM_001012217	0.0683	0.0997	-0.112	0.035	0.1322	0.3855	0.0323	0.3414	-0.248	-0.506	0.2071	-0.217	-0.05	-0.059

Chrm4	NM_031547	0.0202	-0.076	0.0417	0.0293	-0.085	-0.081	0.1272	-0.035	0.0528	0.1176	-0.134	0.471	0.1754	-0.066
Fgf14	NM_022223	-0.026	-0.095	-1E-04	-0.131	0.0222	-0.102	-0.034	0.1666	-0.054	-0.109	0.0102	0.0779	-0.046	-0.067
LOC291926	NM_001110810	0.0686	0.0152	0.013	-0.028	-0.04	0.0601	-0.092	-0.14	0.0195	-0.092	0.0325	0.0627	-0.051	-0.069
Pik3cd_predicted	NM_001108978	0.1475	0.0967	0.0062	0.1372	0.0844	-0.297	0.1596	0.3111	-0.046	-0.078	0.1223	-0.168	0.3211	-0.072
Itgb2	NM_001037780	0.1975	-0.217	0.1595	-0.142	-0.005	-0.215	-0.012	-0.087	-0.045	-0.191	-0.027	-0.203	0.0818	-0.073
Actg1	NM_001127449	-0.08	-0.085	0.0092	-0.101	-0.125	-0.101	0.084	-0.197	0.251	-0.085	-0.088	-0.172	-0.067	-0.082
Fgf12	NM_130814	-0.035	-0.111	-0.046	-0.17	-0.09	-0.04	-0.127	-0.154	-0.051	0.0469	-0.063	-0.087	-0.11	-0.094
Fgf11	NM_130816	0.0267	-0.139	-0.041	-0.072	-0.107	0.0116	-0.142	-0.076	-0.113	0.0263	0.0391	-0.131	-0.088	-0.097
LOC316717	NM_013065	-0.095	-0.104	-0.108	-0.14	-0.107	-0.125	-0.126	-0.023	-0.149	0.0963	-0.13	-0.152	-0.019	-0.101
Fgf7	NM_022182	0.0723	0.5446	-1.785	-0.541	-1.912	-2.141	-0.238	-1.634	0.1374	0.2302	0.1169	-0.619	-0.086	-0.106
Fgf8	NM_133286	-0.014	-0.211	0.0373	-0.072	-0.006	0.3088	-0.252	0.1153	0.0265	-0.221	0.0876	0.0465	-0.187	-0.108
Mylk_predicted	NM_001105874	-0.063	-0.186	-0.185	-0.108	-0.15	-0.111	-0.121	0.0085	-0.113	-0.185	-0.134	-0.152	-0.144	-0.113
Pdgfb	NM_031524	-0.079	-0.067	0.0038	-0.107	-0.127	-0.107	-0.11	-0.081	-0.106	-0.11	0.0862	-0.105	-0.089	-0.114
Itgb8_predicted	NM_001108726	0.1678	0.2558	0.0208	0.808	-0.025	0.0073	0.3564	0.0133	0.3082	0.0384	0.2887	-0.149	-0.1	-0.119
Egfr	NM_031507	-0.395	0.4898	-0.08	0.2402	-0.416	-0.322	######	-0.223	0.0938	0.27	0.3576	-0.154	-0.254	-0.121
Fgf23	NM_130754	-0.053	-0.101	-0.161	-0.053	-0.103	-0.068	0.1468	-0.092	-0.072	0.0107	0.0367	-0.086	-0.092	-0.127
Actn4	NM_031675	-0.384	-0.538	0.2457	0.2626	-0.298	-0.558	-0.516	-0.462	-0.667	-0.465	-0.562	-0.978	-0.275	-0.135
Vav1	NM_012759	-0.394	-0.067	-0.049	-0.139	-0.047	-0.018	-0.042	-0.206	0.0374	0.1275	0.1971	-0.227	0.0297	-0.146
Fn1	NM_019143	-0.169	-0.285	0.0019	-0.352	-0.088	-0.226	0.2217	-0.051	-0.367	-0.336	-0.549	0.1277	-0.133	-0.148
Rac2	NM_001008384	0.0764	-0.035	0.0845	0.0496	0.0751	0.0555	-0.143	-0.103	-0.088	0.1856	0.0144	-0.024	-0.225	-0.154
Rhoa	NM_057132	0.1223	0.1374	-0.093	0.3954	0.5676	0.4536	0.2125	0.5139	0.1944	0.3573	0.2653	0.2919	0.0461	-0.154
Ppp1r12b_predicted	NM_001107178	0.0008	-0.028	-0.106	-0.02	0.1745	-0.009	0.0377	0.0819	-0.027	-0.071	0.1665	0.2017	0.0111	-0.167
Cyfip1_predicted	NM_001107517	-0.624	-0.019	-0.181	-0.73	-0.434	-0.483	-0.059	-0.511	-0.168	-0.131	-0.375	-0.001	-0.232	-0.175
Arpc1a	NM_031146	-0.189	0.2512	-0.173	-0.574	-0.347	-0.568	-0.017	-0.412	-0.129	0.1647	-0.054	-0.327	-0.214	-0.184
Mapk3	NM_017347	0.3283	0.1773	-0.828	-0.429	0.4243	0.0725	-0.043	0.3563	-0.346	-0.064	-0.135	-0.558	-0.214	-0.188
Arpc5	NM_001025717	-0.202	-0.343	-0.344	-0.652	-0.727	-0.855	-0.201	-0.832	-0.19	-0.418	-0.404	-0.368	-0.22	-0.193
Pak7_predicted	NM_001107781	-0.174	-0.115	-0.034	-0.22	-0.047	-0.194	-0.124	-0.124	0.0265	-0.125	-0.027	-0.127	-0.182	-0.193
Itgb6	NM_001004263	-0.227	-0.211	-0.138	-0.2	-0.292	-0.252	-0.284	-0.268	-0.203	-0.079	-0.243	-0.142	-0.095	-0.195
Fgd3_predicted	NM_001108409	-0.24	-0.204	-0.206	-0.256	-0.211	0.0284	-0.106	-0.204	0.0319	-0.262	-0.235	-0.163	-0.204	-0.208
Coro1c_predicted	NM_001107154	-0.013	-0.075	0.5241	0.8599	-0.028	0.1924	0.1997	0.0336	0.1193	-0.057	0.0587	0.0272	-0.329	-0.219
Arhgef1	NM_021694	0.0852	0.1736	0.7274	0.8244	0.5232	0.7567	0.6652	0.5364	0.1279	0.2912	0.2721	-0.1	-0.222	-0.224
Itgb1	NM_017022	-0.372	0.5851	-1.086	-0.317	0.0142	-0.479	-0.349	-0.025	0.1225	0.2547	0.0189	-0.375	-0.078	-0.231
LOC500109	NM_024146	-0.03	0.0908	0.0786	-0.067	-0.155	0.1279	-0.161	-0.018	-0.165	-0.244	0.0074	0.0866	-0.199	-0.239
Itga4_mapped	NM_001107737	-0.252	-0.247	-0.173	-0.124	0.0247	-0.265	-0.127	-0.132	-0.273	0.0252	-0.041	-0.279	-0.152	-0.244
Myh14	NM_001100690	-0.409	-0.406	-0.323	-0.236	-0.286	-0.181	-0.218	-0.13	-0.372	-0.283	-0.024	-0.209	-0.279	-0.255
Pik3r3	NM_022213	-0.395	-0.192	-0.435	-0.26	-0.669	-0.721	-0.401	-0.422	-0.226	-0.061	-0.377	-0.183	-0.361	-0.264
Arpc1b	NM_019289	-0.066	0.1836	-0.075	-0.037	0.05	0.2889	0.7866	-0.054	0.1085	0.3748	0.0232	0.2952	-0.201	-0.266
Rock2	NM_013022	0.2801	0.1127	0.1471	1.3847	0.2383	-0.205	0.9631	0.3271	-0.221	-0.245	0.1036	-0.53	-0.175	-0.273
Fgf16	NM_021867	-0.095	-0.211	-0.494	-0.093	-0.271	-0.073	-0.089	-0.408	-0.18	-0.79	0.041	-0.203	-0.32	-0.287
Fgf4	NM_053809	-0.304	-0.217	-0.348	-0.192	-0.348	-0.245	-0.219	-0.121	-0.087	-0.29	-0.032	-0.187	-0.184	-0.301
Actb	NM_031144	-0.08	-0.037	0.0444	-0.032	-0.183	-0.214	0.0674	-0.022	-0.173	-0.083	-0.309	0.047	-0.081	-0.31
Slc9a1	NM_012652	-0.103	-0.165	0.361	-0.041	-0.287	-0.113	-0.311	-0.305	-0.355	-0.139	-0.123	0.1006	-0.377	-0.342
Egf	NM_012842	-0.039	-0.229	0.4491	0.2961	-0.342	-0.251	0.4999	-0.293	-0.133	-0.152	-0.038	-0.23	-0.174	-0.343
F2r	NM_012950	-0.577	-0.061	-0.628	-0.055	-0.054	-0.152	-0.782	-0.12	-0.257	-0.186	0.0459	-0.119	-0.269	-0.422

Vil2	NM_019357	-0.406	-0.936	-0.533	-0.475	0.2542	-0.056	-0.908	0.09	-1.072	-0.957	-0.977	-1.208	-0.665	-0.665
Raf1	NM_012639	-0.549	-0.579	-0.52	-1.026	-0.697	-0.636	-0.332	-0.764	-0.575	-0.753	-0.587	-0.438	-0.72	-0.684
Fgf1	NM_012846	-0.785	-0.758	-0.803	-0.879	-0.597	-0.757	-0.793	-0.784	-0.722	-0.913	-0.792	-0.57	-0.847	-0.705
Ppp1cb	NM_013065	-0.82	-0.484	-1.219	-0.91	-0.738	-1.29	-0.694	-0.689	-0.661	-0.501	-0.613	-1.056	-0.614	-0.895
Limk2	NM_024135	-1.498	-1.287	-0.697	-1.324	-1.2	-1.377	-1.149	-1.227	-1.178	-1.32	-1.428	-1.031	-0.932	-0.922
Map2k2	NM_133283	-1.234	-1.161	-0.847	-1.54	-1.145	-1.317	-1.084	-1.244	-1.202	-1.179	-1.136	-0.744	-0.969	-1.004
Gsn	NM_001004080	-0.33	0.1864	0.0068	-0.105	-0.344	-0.208	-0.135	-0.383	-0.045	-0.027	-0.075	-0.171	-0.965	-1.018
Cd14	NM_021744	-0.877	-0.393	-1.585	-2.306	-0.317	-0.376	-0.83	-0.305	-0.278	-0.5	-0.552	-0.729	-1.36	-1.258
Myh10	NM_031520	-1.513	-1.738	-1.624	-1.692	-1.459	-1.541	-2.098	-1.351	-0.874	-1.987	-1.886	-0.051	-1.627	-1.508
Itgb4	NM_013180	-1.435	-1.356	-2.061	-1.898	-1.763	-2.015	-1.414	-1.785	-1.269	-1.404	-1.52	-1.37	-2.737	-2.629
Itga11_predicted	NM_001108156	-2.195	-1.539	-2.872	-2.67	-2.945	-2.66	-3.969	-2.689	-0.823	-1.492	-1.856	-0.275	-3.276	-3.473

Downregulated of MTA-3 in ER-negative Breast Tumors

Refseq	Gene symbol	K1	K2	K3	K4	K5	K6	K7	K8	K9	K10	K11	K12	Cluster	WT
Tuba4a	NM_001007004	0.1616	-0.079	0.9773	-0.07	0.2423	0.3448	-0.356	0.0967	0.0651	-0.083	-0.011	0.3317	1.2277	1.0832
Hspb1	NM_031970	0.8203	0.9264	0.2019	1.1218	0.787	0.7705	0.6502	0.6694	0.7574	0.9508	0.7032	0.3831	0.2358	0.5038
Mta3_predicted	NM_001106705	0.3889	-0.173	-0.222	0.0784	1.0096	0.7966	-0.267	0.6802	0.0001	0.0151	-0.257	0.1589	0.4023	0.5002
Mbd3_predicted	NM_001108735	-0.041	-0.45	0.2911	-0.694	-0.401	-0.536	-0.611	-0.654	-0.017	-0.238	-0.454	0.3364	0.1649	0.4527
Snai1	NM_053805	0.2096	0.4673	0.4212	0.568	0.3572	0.2341	0.0844	0.3401	0.2534	0.6905	0.2853	-0.042	0.1795	0.3361
Aldoa	NM_012495	0.8342	0.9033	-0.243	-0.355	0.8624	0.2648	-0.332	1.0121	0.5665	0.807	0.5938	-0.32	0.401	0.2944
Aldoa	NM_012495	0.7227	0.7813	0.5222	0.3822	0.8001	0.6361	-0.203	0.6797	0.1274	0.7727	0.8144	-0.261	0.2335	0.2921
Cdh1	NM_031334	0.1759	0.1353	0.0938	0.2496	0.0699	-0.036	0.121	0.0744	0.0536	0.2699	0.0835	0.1504	0.0436	0.2405
LOC365889	NM_022298	-0.009	0.09	0.2778	0.0444	-0.015	0.0151	0.0556	-0.018	0.0784	-0.011	0.1435	0.0908	-0.003	0.161
Hspb2	NM_130431	0.2137	0.1888	0.0541	0.1291	0.1895	0.2226	-0.001	0.064	0.2204	0.0565	0.1754	0.4658	0.1744	0.1439
Hdac1_predicted	NM_001025409	0.0319	0.0879	-0.185	-0.225	0.0652	0.3582	0.1009	0.1132	0.0179	0.086	0.2472	0.5122	-0.047	0.1204
Ctsd	NM_134334	0.7987	0.8464	-0.389	0.6976	0.7391	0.6926	0.5098	0.7943	0.4258	0.4174	0.6185	-0.064	-0.019	0.0597
Hdac1	NM_001025409	0.0644	0.2457	-0.223	-0.04	0.2635	-0.069	0.0685	0.3033	-0.078	-0.068	0.2756	0.1281	-0.086	0.0251
Tuba1a	NM_022298	-0.878	-0.72	0.562	-0.951	-0.573	-0.995	-0.563	-0.545	-0.274	-0.666	-0.706	0.0835	0.093	-0.005
RGD1565155_predicted	NM_022298	0.0353	0.1979	-0.047	0.2457	-0.022	0.2037	-0.01	0.0619	-0.086	-0.088	0.0725	0.1311	-0.053	-0.012
LOC500319	NM_022298	0.0073	-0.005	-0.026	0.1115	0.0679	0.1242	0.0551	0.2035	0.1412	0.0176	0.047	0.041	0.0239	-0.02
Esr1	NM_012689	-0.072	-0.093	-0.105	-0.125	-0.032	0.1546	-0.061	0.0441	-0.11	-0.06	-0.077	-0.02	-0.028	-0.083
LOC680876	NM_022298	-0.15	0.0386	0.0133	-0.19	-0.26	-0.765	-0.326	-0.382	-0.094	0.136	-0.094	0.1666	-0.162	-0.235
Mta1	NM_022588	-0.533	-0.373	-0.275	-0.665	-0.581	-0.834	-0.675	-0.317	-0.319	-0.337	-0.475	-0.692	-0.32	-0.283
Hdac1	NM_001025409	-0.444	-0.38	-0.26	-0.359	-0.339	-0.182	-0.364	-0.355	-0.146	-0.206	-0.351	-0.019	-0.496	-0.332

IGF-1 Signaling Pathway

Refseq	Gene symbol	K1	K2	K3	K4	K5	K6	K7	K8	K9	K10	K11	K12	Cluster	WT
Igf1r	NM_052807	-0.492	-0.066	0.6372	0.996	-0.027	0.2992	0.4027	-0.013	-0.273	-0.047	-0.043	0.0231	0.4745	0.4566
Rasa1	NM_013135	0.3577	0.7515	0.6959	-0.179	-0.257	-0.992	1.3839	-0.257	0.5887	0.4704	0.6542	-0.016	0.2942	0.3738
Csnk2a1	NM_053824	0.2273	0.2902	-0.332	0.0135	0.4822	0.0976	-0.079	0.2249	0.0931	0.3945	0.146	0.0104	0.2452	0.3272
Sos1	NM_001100716	0.2397	0.139	-0.004	0.0468	-0.003	0.038	-0.061	-0.065	0.2328	0.0498	-0.039	-0.069	0.0004	0.3145
Map2k1	NM_031643	0.3113	0.3264	0.1669	-0.12	0.2482	-0.013	0.0275	0.1835	0.1637	0.1875	0.3065	-0.355	0.1177	0.2264
Elk1	NM_001108059	-0.072	0.0453	-0.413	-0.069	-0.229	-0.384	-0.152	0.2371	-0.154	0.0505	-0.126	-0.383	-0.022	0.1651
Grb2	NM_030846	-0.247	-0.049	0.0856	0.0804	-0.238	-0.117	0.0561	-0.194	0.1711	0.1036	-0.011	-0.201	-0.145	0.1431

RGD1559787_predicted	NM_001109302	-0.342	0.0996	-0.029	0.1352	-0.178	-0.219	-0.335	-0.041	0.0091	0.0193	0.1017	-0.192	-0.025	0.1207
Ptpn11	NM_013088	0.0567	-0.195	-0.328	-0.376	0.1738	0.2723	-0.102	0.182	-0.096	-0.198	-0.187	0.0339	0.1258	0.1195
Jun	NM_021835	-0.641	-0.647	0.1331	-0.725	-0.331	0.1199	-0.449	-0.381	-0.254	-0.531	-0.284	0.3566	0.0265	0.0485
Pik3cg_predicted	NM_001106723	0.0832	-0.019	0.1252	0.098	-0.169	0.1087	0.1463	0.005	0.29	0.0961	0.2092	0.1469	0.237	0.0007
Fos	NM_022197	0.3026	0.231	-0.035	0.0949	-0.26	-0.178	0.1259	-0.061	-0.065	-0.088	0.1793	0.1172	-0.247	-0.173
Mapk3	NM_017347	0.3283	0.1773	-0.828	-0.429	0.4243	0.0725	-0.043	0.3563	-0.346	-0.064	-0.135	-0.558	-0.214	-0.188
Irs1	NM_012969	0.0722	0.0697	-0.048	-0.249	0.0496	-0.16	-0.11	-0.088	-0.202	-0.109	-0.241	-0.274	-0.393	-0.197
Raf1	NM_012639	-0.549	-0.579	-0.52	-1.026	-0.697	-0.636	-0.332	-0.764	-0.575	-0.753	-0.587	-0.438	-0.72	-0.684

Actions of Nitric Oxide in the Heart

Refseq	Gene symbol	K1	K2	K3	K4	K5	K6	K7	K8	K9	K10	K11	K12	Cluster	WT
Cav1	NM_133651	0.9602	0.721	-1.167	-1.514	1.3532	-0.118	-0.397	1.7173	0.2314	0.5471	-0.017	-0.843	1.1984	1.0568
Cav1	NM_133651	0.876	1.1396	-1.256	-1.13	1.1003	-0.34	0.1111	1.5069	0.1574	0.8312	-0.099	-0.992	0.9348	0.8436
Slc7a1	NM_013111	0.2359	-0.307	0.6417	-0.023	0.2606	0.2699	0.1714	0.5038	-0.316	-0.405	-0.147	0.1042	0.3918	0.5726
Hsp90aa1	NM_175761	0.1626	-0.117	0.6893	0.9032	-0.007	-0.191	-0.236	0.4974	0.1389	-0.056	0.0811	-0.117	0.6404	0.4709
Tnni1	NM_017184	-0.028	0.1282	0.0634	0.0491	0.194	0.0669	0.3368	0.1545	-0.074	-0.062	0.0049	0.2341	0.0044	0.3698
Prkar2a	NM_019264	-0.049	0.1908	0.4218	0.0919	0.0874	0.168	0.0591	0.1264	0.1603	0.0141	0.3626	0.2132	0.3039	0.3171
Acta1	NM_019212	0.5333	0.0816	0.7952	1.4517	1.2983	1.9875	-0.074	1.6537	0.1019	0.1697	0.2598	0.0845	0.3393	0.2275
RGD1308470	NM_173100	-0.114	-0.051	-0.255	0.0474	0.0117	-0.066	0.2313	0.0151	0.1499	-0.187	0.0207	0.0715	0.1634	0.2071
Vegfa	NM_001110335	0.5841	0.29	-0.243	0.6237	0.4685	0.1591	-0.257	0.5515	0.128	0.3236	0.1458	-0.23	0.049	0.2018
Akt1	NM_033230	0.0085	-0.07	0.3608	-0.631	0.2019	-0.172	-0.008	0.2784	-0.022	-0.162	-0.375	-0.352	-0.019	0.1763
Nos3	NM_021838	0.9939	0.1642	0.7431	0.3343	0.3733	0.6297	0.6364	0.7229	0.4439	0.0985	0.0695	0.4569	-0.05	0.1644
Pde3b	NM_017229	0.0399	0.0866	0.1539	0.1344	-0.036	0.0054	0.2894	0.0817	0.0063	0.1481	0.2136	0.1129	-0.145	0.1441
Pde3a	NM_017337	0.0509	0.1281	0.1267	0.1297	0.1562	0.14	0.3367	0.357	0.2194	0.5202	0.2702	0.1477	0.2368	0.1415
Chrm1	NM_080773	0.1681	0.0894	0.0798	0.0773	0.0371	0.0161	0.0768	0.1648	0.0999	0.2278	0.2439	0.0513	0.0909	0.084
Bdkrb2	NM_173100	-0.126	-0.115	-0.247	-0.108	0.0233	-0.083	-0.18	-0.091	-0.046	-0.237	-0.284	-0.057	-0.07	-0.038
Chrna1	NM_024485	-0.012	-0.048	0.2118	-0.047	-0.073	0.0857	0.1366	0.0534	-0.018	-0.068	0.0116	0.1155	0.0784	-0.054
Flt1	NM_019306	0.3371	-0.177	-0.133	0.1924	0.2663	0.0484	0.0609	0.4272	-0.043	-0.22	0.0957	0.1294	0.2163	-0.23
Prkar2b	NM_001030020	0.3406	-0.204	-0.157	0.3313	-0.228	-0.312	-0.229	-0.328	-0.046	-0.333	-0.373	0.6673	-0.381	-0.338
Prkacb_predicted	NM_001077645	-0.25	-0.269	-1.219	-1.031	-0.114	-0.424	-0.323	-0.055	-0.227	-0.23	-0.379	-0.322	-0.452	-0.644

ATP synthesis

Refseq	Gene symbol	K1	K2	K3	K4	K5	K6	K7	K8	K9	K10	K11	K12	Cluster	WT
Atp6v1a1_predicted	NM_001108318	0.9181	1.5308	-0.358	0.096	0.7776	-0.233	0.3781	0.8492	0.803	1.1606	0.5643	0.0072	0.5305	0.8477
Atp6v1c1_predicted	NM_001011992	0.6871	0.8957	-0.617	0.1493	0.6104	0.1304	0.2409	0.7861	0.5337	0.7967	0.1133	0.1748	0.6288	0.7839
RGD1563463_predicted	NM_017311	0.1484	-0.143	0.2162	0.3165	0.6224	1.5033	-0.131	0.3712	-0.044	0.086	0.0889	1.0468	0.5467	0.6121
Atp5b	NM_134364	0.2516	0.0969	0.3704	-0.074	0.2568	-0.094	0.3167	0.3655	-0.045	0.0835	0.1873	0.0205	0.5939	0.5372
Atp6v1h	NM_001013929	0.7269	1.012	0.3433	1.0393	1.1	0.8961	0.6848	0.9842	0.7101	0.8466	0.8813	0.307	0.5553	0.4996
Atp5d	NM_139106	0.4714	0.3074	-0.027	-0.42	0.5325	0.6157	-0.009	0.5541	0.374	0.2931	0.3388	0.3967	0.3685	0.3839
Atp6v1b2	NM_057213	0.6269	0.3801	0.0451	0.1199	0.6093	0.374	0.2313	0.5246	0.101	0.3561	0.2511	-0.05	0.3348	0.3493
Atp6v0c	NM_130823	0.5884	0.1526	0.3434	-0.247	0.3026	0.0032	0.2098	0.3041	-0.024	0.1974	0.0599	-0.504	0.134	0.3301
Atp6v0e	NM_053578	0.6262	0.5289	0.0458	0.2901	0.7129	0.8466	0.7337	0.7817	0.5967	0.5939	0.612	0.7831	0.474	0.3004
Atp5g1	NM_017311	-0.019	-0.31	0.1752	-0.073	0.3457	0.5832	-0.3	0.125	-0.169	-0.031	-0.057	0.6002	0.2248	0.2993
Atp6v1c2	NM_001014199	-0.103	0.2475	0.5951	-0.125	-0.28	-0.117	0.6104	-0.152	0.093	0.254	0.098	0.135	0.2442	0.2763

Atp5o	NM_138883	0.142	-0.025	0.1467	-0.019	0.3559	0.3977	0.4267	0.3771	0.2212	0.1483	0.1087	0.3241	0.2916	0.2587
Atp5c1	NM_053825	0.1016	0.2656	0.2581	0.0436	0.2803	0.5008	-0.117	0.3313	0.2734	0.0637	0.3318	0.5051	0.241	0.2526
Atp6v1c1	NM_001011992	0.2411	0.3366	0.3551	0.6174	0.0616	-0.181	0.5351	0.2006	0.2439	0.1583	0.4683	-0.123	0.4589	0.2403
Atp5i	NM_080481	0.267	0.089	0.4474	-0.357	0.3913	0.3762	0.2103	0.2393	0.0401	-0.013	0.1474	0.3183	0.2283	0.2056
Atp5j	NM_053602	0.2722	0.2249	0.2168	0.2032	0.7883	0.6643	0.2408	0.5764	0.4126	0.2285	0.4423	0.2105	0.2477	0.1997
MGC72942	NM_212516	0.368	0.1131	0.3051	0.1804	0.5976	0.5575	0.3459	0.3273	0.0824	0.2431	0.2884	0.3801	0.1657	0.1804
Atp6v1d	NM_199386	0.553	0.2113	-0.157	0.9401	0.5003	0.3442	0.6952	0.4446	0.1116	0.164	0.1158	0.1266	0.1842	0.1683
Atp6v0d1	NM_001011927	0.3668	0.3246	-0.15	-0.321	0.0151	0.1045	0.1092	0.0038	0.1283	0.0646	0.0978	0.3027	0.2686	0.1462
Atp6ap1	NM_031785	0.2541	0.3622	0.1034	-0.157	-0.327	-0.416	-0.16	-0.282	0.4226	0.4996	0.3428	0.088	0.4263	0.1454
Atp6v1g1_predicted	NM_001106660	0.0605	-0.128	-0.029	0.3817	0.1892	0.2842	0.4459	0.1164	0.1695	-0.188	0.0689	0.4638	-0.09	0.1094
Atp6v1e1	NM_198745	0.6777	0.6213	0.0798	0.2115	0.2697	0.3794	0.4706	0.3335	0.5529	0.7012	0.7641	0.598	0.146	0.1044
Atp5g3	NM_053756	0.1004	0.2499	0.0019	-0.102	0.2048	0.3088	0.0832	0.0973	0.0526	-0.167	-0.026	0.2065	0.1543	0.0598
Atp6v0d2	NM_001011972	0.1345	0.0807	0.0482	0.2617	-0.035	0.1052	0.1665	0.1584	-0.057	0.0248	-0.051	0.0381	-0.023	0.0548
Atp6v1f	NM_053884	0.8148	0.2021	-0.314	0.0756	0.5065	0.9232	0.017	0.3268	0.3529	0.0484	0.316	0.1121	-0.052	0.0296
LOC500560	NM_053602	-0.04	0.0341	-0.044	0.0244	-0.038	0.0385	0.028	0.1304	0.1243	0.436	0.183	0.141	0.2255	0.0023
Atp6v0d1_predicted	NM_001011927	-0.222	0.039	-0.134	0.2221	0.0247	-0.071	-0.068	-0.021	0.0203	0.1558	-0.041	-0.004	0.1915	-0.007
Atp5a1	NM_023093	-0.073	0.0106	-0.017	-0.253	0.1654	-0.147	0.1207	0.238	-0.141	0.1293	-0.108	-0.228	-0.208	-0.012
Atp6v0a1	NM_031604	0.5059	0.321	1.0349	0.5287	-0.171	-0.023	0.4398	-0.159	0.2848	0.3797	0.5353	0.2367	0.1255	-0.062
Atp6v0a4_predicted	NM_001106591	0.0109	0.1069	-0.121	0.0156	0.0872	-0.072	-0.154	-0.074	0.0499	0.0577	-0.096	0.0322	-0.015	-0.071
Atp6v1e2_predicted	NM_001108979	-5E-04	-0.098	0.0077	-0.058	0.1299	0.2031	-0.088	0.0947	-0.024	-0.12	-0.14	-0.097	-0.097	-0.147
Atp6v1g3_predicted	NM_001105991	-0.04	0.1839	0.1341	0.1343	-0.006	0.1553	0.0159	-0.02	-0.013	-0.174	-0.121	0.3528	-0.084	-0.196
Atp6v1b1_predicted	NM_001107867	-0.19	-0.167	-0.385	-0.222	-0.143	-0.141	0.0718	-0.335	-0.208	-0.171	-0.151	-0.202	-0.264	-0.273
Atp5g2	NM_133556	-0.176	0.1944	-0.521	-0.768	-0.25	-0.056	-0.54	-0.336	0.5529	0.1957	0.1824	0.3852	-0.364	-0.28
RGD1566212_predicted	NM_133556	-0.358	-0.134	-0.303	-0.842	-0.303	-0.005	-0.769	-0.397	-0.213	-0.35	-0.003	0.3338	-0.497	-0.337
Atp6v1g2	NM_212490	-0.626	-0.455	-0.226	-0.305	-0.574	-0.196	-0.026	-0.522	-0.478	-0.553	-0.567	-0.574	-0.717	-0.685
Tcirg1	NM_199089	-0.341	0.2398	0.0964	0.8046	0.3497	0.5093	0.1918	0.3552	-0.281	0.0033	-0.041	-0.114	-0.539	-0.713

Mechanism of Protein Import into the Nucleus

Refseq	Gene symbol	K1	K2	K3	K4	K5	K6	K7	K8	K9	K10	K11	K12	Cluster	WT
Ran	NM_053439	-0.055	-0.526	0.5668	-0.17	-0.213	-0.444	-0.043	-0.285	-0.321	-0.518	-0.537	0.1394	0.5209	0.8222
Kpnb1	NM_017063	-0.279	-0.306	-0.381	-0.885	-0.196	-0.767	-0.479	0.0171	-0.272	-0.095	-0.466	-0.208	0.697	0.659
Rangap1	NM_001012199	-0.02	-0.611	0.6586	-0.15	-0.103	-0.17	-0.558	-0.265	-0.087	-0.39	-0.306	0.1442	0.388	0.5868
LOC681932	NM_053483	-0.645	-1.718	0.4367	-1.04	-1.219	-1.418	-1.477	-1.218	-0.838	-0.988	-1.15	0.1831	0.3514	0.4776
Rangap1_predicted	NM_001012199	-0.055	-0.698	0.7397	-0.104	-0.281	-0.448	-0.478	-0.626	-0.797	-0.8	-0.794	0.0167	0.3348	0.458
Nup62	NM_023098	0.0567	0.0646	0.2287	0.1177	-0.053	0.1308	0.0176	0.0177	0.0056	0.0235	-0.026	-0.038	-0.025	0.2437
Nup153	NM_001100470	-0.395	-0.29	0.2125	0.5072	-0.436	-0.794	0.0434	-0.336	-0.392	-0.239	-0.497	-0.369	0.0663	0.127
Nutf2	NM_001007629	0.105	-0.057	-0.056	0.275	-0.085	0.0588	0.1124	-0.111	-2E-04	-0.064	0.1615	-0.218	-0.028	0.0809
Nup210	NM_053322	-2.956	-2.253	-1.546	-2.327	-1.513	-1.558	-2.094	-1.553	-2.621	-2.325	-2.246	-3.021	-2.085	-1.847

Pyruvate metabolism

Refseq	Gene symbol	K1	K2	K3	K4	K5	K6	K7	K8	K9	K10	K11	K12	Cluster	WT
Pkm2	NM_053297	1.0932	0.9847	0.8593	0.1077	0.7902	0.4884	0.6378	0.8492	1.0282	1.0986	1.1897	0.0315	0.8456	0.9684
Aldh9a1	NM_022273	0.3003	0.5536	-0.247	0.0074	-0.051	-0.899	0.0975	-0.262	0.3116	0.3366	-0.012	-0.059	0.5243	0.816
Me2_predicted	NM_001107376	0.9285	0.6963	0.2496	-0.42	0.6221	0.293	0.0642	0.67	0.7551	0.8446	0.5727	0.1155	0.668	0.6944

Ldha	NM_017025	0.5762	0.6752	0.6336	0.0015	0.599	0.2661	0.1332	0.4393	0.3591	0.7912	0.4485	-0.143	0.6359	0.6769
Hagh	NM_033349	0.7763	0.3314	0.8517	1.0019	0.6963	1.2514	0.6131	0.6624	0.2672	0.5122	0.7071	0.567	0.5129	0.574
RGD1561179_predicted	NM_053297	0.4072	0.6073	0.9511	-0.238	0.3602	-0.158	0.1472	0.2359	-0.07	0.2319	-0.022	-0.197	0.3775	0.5592
Mdh2	NM_031151	0.2467	-0.255	0.3397	-0.286	0.2221	0.0292	0.219	0.2284	-0.039	0.3381	-0.105	0.057	0.1853	0.5341
RGD1561681_predicted	NM_053297	0.2467	0.5385	0.9962	-0.391	0.1569	-0.396	-0.08	0.1667	0.224	0.3532	0.3929	-0.602	0.4677	0.4126
Me1	NM_012600	0.4316	0.3469	0.0231	0.4867	0.4295	0.1912	-0.335	0.3934	0.3047	0.2398	-0.167	0.2062	0.5405	0.4104
Acaca	NM_022193	0.0942	0.0796	0.1202	0.2891	0.166	0.2309	0.3296	0.1702	0.4119	-0.043	0.1141	-0.025	0.1893	0.3826
Pklr	NM_012624	0.143	0.2716	0.3378	0.1915	0.4559	0.1017	0.1031	0.384	0.189	0.1232	0.4533	0.104	0.1116	0.2397
Dld	NM_199385	0.1959	0.4005	-0.14	0.1086	-0.06	-0.425	0.2057	0.3326	0.1452	0.2828	0.177	-0.288	0.2161	0.1882
Pdha2	NM_053994	-0.046	-0.009	-0.087	0.0569	0.1863	0.0751	0.1238	0.066	0.0277	0.1488	0.216	-0.121	-0.064	0.1854
Acyp1_predicted	NM_001106746	-0.105	0.033	0.6607	0.5438	-0.097	0.4128	0.0821	-0.163	0.2518	0.1858	0.2968	0.596	0.2327	0.1838
Aldh3a2	NM_031731	0.1682	0.1606	0.2803	0.3567	0.061	-0.086	0.3022	0.0659	0.1618	0.1637	-0.265	0.3969	0.1576	0.1806
LOC311254	NM_001044242	0.4255	0.2642	0.0624	0.5084	0.4478	0.4574	0.3045	0.3615	-0.063	0.2406	0.0872	0.0618	0.5251	0.1789
Ldhc	NM_017266	0.0945	0.1087	-0.148	0.2342	-0.041	0.0061	-0.067	0.0462	0.0769	-0.098	-0.146	0.0561	0.1422	0.1767
Akr1b1	NM_012498	0.7225	0.0427	0.0481	1.1708	0.6269	0.5918	0.9549	0.7207	-0.073	-0.13	0.1038	-0.128	0.0048	0.1364
Acat2	NM_001006995	-0.962	-1.129	1.8203	0.3109	-0.94	-0.923	-0.151	-1.2	-0.073	-0.749	-0.751	0.734	0.2332	0.1213
RGD1560120_predicted	NM_012498	-0.009	0.0718	0.1352	0.1852	0.0226	0.0868	0.2064	0.0141	0.4068	-0.151	-0.078	0.0091	0.0761	0.1138
LOC364149	NM_001006995	0.1236	0.0156	0.1496	0.1067	-0.036	-0.005	-0.009	-0.011	-0.035	-0.011	0.0786	0.1021	0.093	0.058
Mdh1	NM_033235	0.4619	0.2984	0.1033	-0.076	0.2898	0.2427	-0.027	0.1003	0.4141	0.1572	0.3266	0.1057	0.0948	0.0577
Me3_predicted	NM_001108491	0.1952	0.0448	0.0593	0.0752	0.1329	0.0008	-0.068	0.0175	-0.044	-0.046	0.0673	0.1633	0.1148	0.0325
Aldh1b1	NM_001011975	-0.213	-0.074	0.0326	-0.103	-0.206	0.2237	0.2528	-0.064	0.2952	-0.081	-0.152	-0.287	-0.186	0.0292
Pck1	NM_198780	-0.114	0.0419	-0.092	0.0519	0.0239	0.0133	0.1296	0.0894	0.0283	-0.166	-0.159	-0.057	0.0293	0.0006
Ldhd	NM_001008893	1.0157	0.9995	-0.085	0.7476	0.8177	1.1626	-0.141	0.8736	0.5048	0.8032	0.756	0.5066	-0.213	-0.002
Pdhb	NM_001007620	0.044	-0.226	-0.075	0.2926	0.06	-0.122	-0.103	0.2313	-0.036	-0.176	-0.119	-0.21	-0.083	-0.011
LOC683694	NM_001113754	0.1486	0.1529	-0.056	-0.207	-0.049	0.2881	-0.2	-0.027	0.2229	-0.121	0.1061	0.0522	-0.148	-0.018
Ldhb	NM_012595	-0.089	0.3	-1.17	-0.742	-0.823	-0.881	-0.233	-0.834	0.0206	0.2432	0.2639	-0.713	0.0029	-0.033
Aldh1a3	NM_153300	-0.149	-0.066	0.0868	-0.113	-0.155	-0.164	0.0278	-0.085	-0.194	0.0677	-0.152	-0.044	0.0854	-0.038
Acat1	NM_017075	-0.477	-0.145	-0.438	-0.68	-0.594	-0.837	-0.398	-0.507	-0.162	-0.327	-0.302	-0.154	-0.299	-0.065
Ldhal6b	NM_183334	-0.063	0.193	0.0408	-0.077	0.1326	0.0502	0.0681	-0.054	0.0508	-0.069	-0.118	-0.126	-0.006	-0.071
Acot12	NM_130747	0.1567	0.015	-2E-04	0.062	0.1934	0.1323	0.0946	-0.029	0.0086	0.0797	0.1527	0.1224	0.0651	-0.074
Aldh3a2	NM_031731	-0.119	-0.14	-0.054	-0.229	-0.138	-0.035	-0.021	-0.276	0.0086	-0.372	-0.081	-0.135	-0.118	-0.081
Acss2_predicted	NM_001107793	0.7603	0.9589	0.607	1.0057	0.9684	0.8484	0.3417	0.7545	0.2244	0.7025	0.3949	0.3241	-0.053	-0.157
Glo1	NM_207594	-0.42	-0.391	-0.883	-0.647	-0.282	-0.241	-1.047	-0.354	-0.27	-0.57	-0.508	-0.051	-0.274	-0.269
Dlat	NM_031025	-0.399	-0.279	-0.406	-0.747	-0.444	-0.266	-0.626	-0.403	-0.383	-0.233	-0.308	-0.733	-0.467	-0.568
Pcx	NM_012744	0.0542	0.0591	-0.606	-0.549	0.1999	-0.038	-0.435	-0.065	-0.199	-0.273	-0.146	-0.3	-0.844	-0.771
Pck2_predicted	NM_001108377	-0.908	-1.119	-0.393	-1.071	-1.085	-1.356	-1.076	-1.079	-0.908	-1.239	-1.237	-0.701	-1.014	-1.093
Aldh3a1	NM_031972	-1.546	-1.216	-1.34	-1.572	-1.47	-1.571	-1.18	-1.718	-0.604	-1.675	-1.321	0.4767	-1.753	-1.759
