# Supplementary material for: KSHV MicroRNAs Mediate Cellular Transformation and Tumorigenesis by Redundantly Targeting Cell Growth and Survival Pathways
Source: PLoS Pathog. 2013 Dec 26;9(12):e1003857. doi: 10.1371/journal.ppat.1003857 (PMC3873467; doi:10.1371/journal.ppat.1003857)
Supplement: Table S2 — Expression of signature genes associated with tumorigenicity mediated by KSHV miRs. (PDF) [file ppat.1003857.s013.pdf]

Table S2. Expression of signature genes associated with tumorigenicity mediated by KSHV miRs

Refseq	Gene symbol	K1	K2	K3	K4	K5	K6	K7	K8	K9	K10	K11	K12
XM_001054650.1	Abca3	0.132	0.250	0.448	0.259	0.208	-0.004	0.413	0.035	-0.243	0.038	0.243	-0.357
NM_001107776	Adam33_predicted	-0.281	-0.096	0.013	-0.339	-0.597	-0.670	0.281	-0.586	-0.327	-0.042	-0.205	-0.603
NM_001034012	Adamtsl4	0.332	0.305	0.248	0.268	-0.045	0.211	0.401	-0.097	-0.021	0.230	0.264	-0.067
NM_001106271	Akap13	-0.102	-0.032	0.030	0.031	-0.120	-0.404	-0.152	-0.168	-0.138	-0.110	-0.124	-0.202
NM_019383.1	Atp5h	0.300	0.019	-0.078	-0.336	0.234	0.805	-0.250	0.363	0.435	0.055	0.254	0.475
NM_001108310	Azi1_predicted	-0.477	-0.270	-0.159	-0.442	-0.288	0.007	-0.384	0.003	-0.164	-0.225	-0.232	0.118
NM_001024233	Bles03	-0.389	-0.160	-0.241	-0.130	0.010	0.298	-0.045	0.059	0.217	-0.058	-0.139	0.178
NM_053850	Blvra	0.391	0.274	0.188	-0.048	0.219	0.513	-0.153	0.251	0.584	0.126	0.283	0.742
NM_080897	Bnip1	0.566	0.167	0.221	0.347	0.494	1.007	0.112	0.590	0.391	0.202	0.356	0.612
NM_022399	Calr	-0.346	-0.076	-0.162	-0.423	0.278	0.382	-0.340	0.411	-0.011	0.105	-0.144	0.240
XM_341136.3	Cap350	-0.169	-0.329	-0.025	0.279	-0.159	-0.299	-0.197	-0.215	-0.344	-0.314	-0.018	-0.284
XM_340885.3	Cbx1_predicted	-0.191	-0.082	-0.480	-0.214	-0.054	0.260	-0.462	0.042	0.344	0.004	0.114	0.603
NM_001017470	Cep70	-0.111	-0.140	-0.186	-0.013	-0.260	-0.225	-0.208	-0.182	-0.007	-0.205	-0.040	-0.290
NM_001106509	Chchd5_predicted	0.390	0.050	-0.073	-0.048	0.218	0.684	-0.434	0.206	0.523	0.191	0.196	0.453
NM_001007687	Cndp1	-0.215	-0.225	-0.103	-0.558	0.220	0.114	-0.399	0.033	-0.063	-0.282	-0.327	0.415
NM_001033692	Commd9	0.390	0.083	0.080	0.403	0.317	0.522	0.098	0.217	0.256	-0.045	0.235	0.318
NM_001034925	Cpt1c	-0.293	0.284	0.394	-0.193	-0.279	-0.275	0.181	-0.336	-0.386	0.023	-0.061	-0.063
NM_023981	Csf1	0.456	0.435	-0.067	0.774	0.021	-0.342	0.142	0.094	0.101	0.434	0.372	-0.575
NM_175837	Cyp4a10	-0.126	-0.074	-0.213	0.063	-0.187	-0.033	-0.232	-0.036	-0.034	-0.103	-0.028	-0.043
NM_001034143	Dars2	0.398	0.244	0.099	0.229	0.132	0.036	0.040	-0.330	0.062	0.263	0.310	-0.197
NM_001005244	Dmd	0.229	0.462	0.091	0.627	0.114	0.104	0.220	0.166	0.170	0.286	0.596	0.100
NM_001108159	Dpp8_predicted	-0.109	-0.208	0.024	-0.806	0.032	0.223	0.086	0.080	0.172	0.059	-0.135	0.316
NM_012934	Dpysl3	-0.112	-0.215	-0.157	-0.748	0.362	0.093	-0.272	0.386	-0.042	-0.347	-0.461	0.333
XM_001072657.1	Efna3	-0.391	-0.321	-0.262	-0.266	0.000	0.370	-0.228	0.266	-0.087	-0.431	-0.318	-0.109
NM_001106693	Eif4g3_predicted	-0.457	-0.165	0.256	-0.325	-0.163	-0.490	0.204	-0.355	-0.489	-0.262	-0.060	-0.520
XM_001054852.1	Ell2	-0.853	-0.917	-1.149	-0.695	-0.913	-0.800	-0.952	-0.722	-0.507	-0.816	-0.987	-0.511
NM_001127547	Fbln1_predicted	-0.898	-1.018	-0.863	-0.975	-0.306	0.038	-0.955	-0.312	-0.536	-0.638	-0.796	-0.014
NM_001108434	Fech_predicted	0.130	-0.145	-0.514	-0.397	-0.244	-0.320	-0.272	0.033	0.084	-0.330	0.030	-0.019
NM_053625	Gfm1	0.267	0.271	0.251	0.406	0.189	0.097	0.375	0.067	0.052	0.339	0.219	-0.130
NM_138885	Golgb1	-0.051	0.235	0.042	-0.154	0.357	-0.091	0.318	-0.057	-0.006	0.203	0.103	-0.203
NM_001007720	Gorasp2	-0.334	-0.055	-0.335	-0.509	-0.113	-0.012	-0.126	-0.050	-0.096	-0.433	-0.179	0.146
NM_032069	Grip1	0.533	0.489	0.607	0.916	-0.014	-0.303	1.104	0.152	0.370	0.694	0.525	-0.463
NM_001130553	Gsdmdc1_predicted	-0.693	-0.019	-0.081	-0.789	-0.289	0.160	-0.448	-0.130	0.132	0.040	-0.195	0.477
NM_001037978	Gtl3	0.305	0.085	0.085	-0.009	-0.014	0.081	-0.021	0.066	0.136	0.138	0.102	0.127
XM_236362.3	Herc1_predicted	-0.297	-0.062	0.326	0.049	-0.188	-0.735	-0.042	-0.361	-0.628	-0.146	-0.175	-0.336

XM_220888.3	Hoxb8_mapped	-0.535	-0.514	-0.456	-0.665	-0.394	-0.359	-0.544	-0.383	-0.214	-0.280	-0.452	0.067
NM_017265	Hsd3b1	-0.046	0.062	-0.116	-0.002	0.038	-0.168	-0.235	0.090	-0.050	-0.005	-0.184	-0.094
NM_001108101	Irak3_predicted	0.036	0.664	0.355	0.244	0.090	-0.175	0.812	0.003	0.239	0.463	0.325	-0.141
NM_019147	Jag1	0.204	-0.176	0.104	0.768	0.438	1.048	-0.005	0.674	0.298	-0.172	0.482	0.993
NM_021836	Junb	0.637	0.824	0.161	0.339	0.269	0.008	0.800	0.037	0.164	0.645	0.825	-0.586
NM_053614	Lenep	-0.086	-0.336	-0.008	0.281	-0.394	-0.669	0.132	-0.331	-0.416	-0.109	-0.253	-0.644
NM_001024869	LOC295062	-0.150	0.402	-0.088	-0.583	0.146	-0.035	0.072	0.242	0.249	0.198	-0.039	0.173
NM_053330	LOC300731	-0.526	-0.532	-0.701	-0.510	-1.184	-1.116	-0.510	-1.148	-0.438	-0.639	-0.415	-0.330
XM_219560.3	LOC309197	-0.099	-0.116	-0.170	-0.193	0.186	0.692	-0.307	0.024	0.039	-0.201	-0.170	0.373
NM_001033895	LOC310615	-0.219	-0.149	-0.064	-0.015	-0.539	-0.803	0.132	-0.541	-0.330	-0.277	-0.303	-0.677
NM_001034926	LOC310946	0.439	0.117	0.230	0.340	0.186	-0.128	-0.037	0.166	-0.062	0.189	0.060	-0.200
NM_001024896	LOC361418	0.391	0.280	0.345	0.221	-0.054	-0.306	0.384	-0.092	-0.112	0.186	0.127	-0.093
NM_053330	LOC300731	-0.526	-0.532	-0.701	-0.510	-1.184	-1.116	-0.510	-1.148	-0.438	-0.639	-0.415	-0.330
NM_001108283	LOC367923	-0.154	-0.371	-0.453	0.019	-0.146	0.006	-0.549	-0.268	-0.234	-0.727	-0.133	-0.210
NM_183402	LOC498325	-0.220	-0.309	-0.663	-0.403	-0.195	-0.164	-0.145	-0.181	-0.329	-0.232	-0.246	-0.273
NM_130432	LOC684988	-0.317	-0.384	-0.394	-0.154	-0.257	0.033	-0.594	-0.243	-0.194	-0.473	-0.233	-0.157
NM_001108283	LOC367923	-0.154	-0.371	-0.453	0.019	-0.146	0.006	-0.549	-0.268	-0.234	-0.727	-0.133	-0.210
NM_001109331	LOC692000	0.450	0.149	0.283	0.201	0.249	0.832	0.145	0.335	0.433	0.393	0.278	0.706
NM_080479	Maged2	0.076	0.029	-0.239	0.369	-0.145	0.037	-0.353	-0.093	0.102	-0.370	-0.038	0.404
NM_001044237	Mcts1	0.446	0.005	0.263	0.375	0.421	0.658	0.080	0.620	0.316	-0.043	0.136	0.624
NM_001135813	Med31_predicted	0.122	-0.132	-0.257	0.241	-0.077	0.508	-0.482	0.169	0.134	-0.012	0.042	0.037
NM_001024865	Mrpl40	0.376	0.255	0.362	-0.015	0.489	0.934	0.194	0.377	0.519	0.349	0.287	0.716
NM_001108665	Mrpl50_predicted	0.195	0.161	0.181	0.007	0.423	0.689	-0.009	0.389	0.421	0.168	0.201	0.349
NM_001007696	mrpl9	0.547	0.167	-0.133	0.373	0.177	0.064	-0.159	0.262	0.034	-0.048	0.289	-0.231
NM_001108364	Mxd4_predicted	-0.725	-0.591	-0.849	-0.710	-0.200	0.013	-0.676	-0.318	-0.120	-0.700	-0.656	0.287
NM_031520	Myh10	-1.513	-1.738	-1.624	-1.692	-1.459	-1.541	-2.098	-1.351	-0.874	-1.987	-1.886	-0.051
NM_139230	Nexn	0.064	0.092	-0.084	0.152	-0.047	-0.152	-0.038	-0.144	-0.010	0.103	0.179	-0.062
NM_138847	Nip7	0.391	-0.090	0.246	0.389	-0.017	-0.309	0.362	-0.067	-0.109	-0.128	0.176	-0.414
NM_001082580	Nit1	0.702	0.372	0.151	0.753	-0.009	-0.243	0.564	0.052	0.192	0.280	0.173	-0.285
NM_199086	Nob1	0.145	0.054	0.058	0.636	-0.004	0.018	0.380	-0.239	-0.098	0.114	0.212	-0.206
NM_080778	Nr2f2	0.734	0.532	-0.252	0.782	0.054	0.088	0.313	0.373	0.207	0.537	0.661	-0.127
NM_001009621	Nudcd2	0.428	-0.166	-0.212	0.179	0.242	0.402	-0.335	0.318	0.162	-0.275	-0.083	0.387
NM_031235	Pard3	-0.927	-0.242	-0.593	-0.798	-0.593	-0.858	-0.234	-0.709	-0.659	-0.417	-0.443	-0.758
NM_012746	Pcsk2	1.031	0.552	0.195	0.452	0.020	0.326	0.660	0.126	0.307	0.274	0.685	-0.002
NM_030871	Pde1a	1.369	1.402	1.035	1.594	0.374	0.334	0.811	0.323	0.722	1.091	1.137	0.000
NM_001100651	Pigx	0.185	0.203	0.082	0.095	0.357	0.393	0.176	0.484	0.546	0.205	0.331	0.405
NM_017035	Plcd1	-0.297	0.129	0.428	-0.151	-0.353	-0.600	0.236	-0.433	-0.205	0.060	0.004	-0.220
NM_001106899	Plekhb2_predicted	0.102	-0.032	-0.241	-0.046	-0.160	-0.486	-0.191	-0.244	-0.250	-0.199	-0.205	-0.313

NM_178101	Plod3	0.052	0.391	0.081	-0.025	0.000	-0.169	0.276	0.037	-0.100	0.228	0.161	-0.413
NM_001007747	Pomgnt1	-0.166	-0.183	0.006	-0.004	-0.299	-0.400	-0.127	-0.413	-0.383	-0.220	-0.236	-0.261
NM_022538	Ppap2a	0.657	0.194	0.433	0.961	0.472	0.668	0.333	0.844	0.611	0.133	0.607	0.393
NM_001107911	Ppp1r8_predicted	0.289	0.312	-0.077	-0.174	0.148	-0.226	0.358	-0.063	-0.195	0.226	0.155	-0.138
NM_181379	Ppp2r5b	-0.145	0.185	0.423	0.322	0.110	-0.480	0.369	-0.063	-0.058	-0.011	-0.067	-0.182
NM_001038588	Prodh2	0.726	-0.126	-0.194	0.377	-0.038	0.228	0.049	0.206	-0.049	-0.261	0.124	-0.281
NM_031606	Pten	-0.550	-0.707	-0.503	-0.470	-0.333	-0.376	-0.584	-0.060	-0.465	-0.565	-0.794	-0.189
NM_001105883	Pvrl3_predicted	-0.153	-0.062	-0.005	-0.236	0.048	-0.142	-0.118	-0.018	-0.201	0.131	-0.394	0.014
NM_001037218	Radil	0.732	0.627	0.637	1.498	0.369	0.285	0.647	0.085	0.348	0.506	0.762	-0.125
NM_001135259	RGD1305283_predicted	0.847	0.211	-0.008	0.227	0.476	0.795	-0.145	0.659	0.637	0.427	0.422	0.559
NM_001044252	RGD1306809_predicted	-0.684	-0.495	-0.463	-0.495	-0.376	-0.909	-0.282	-0.620	-0.661	-0.374	-0.526	-0.757
XM_001075785.1	RGD1307772	0.613	0.465	0.673	0.423	0.439	0.142	0.549	0.444	0.346	0.627	0.431	-0.069
NM_001107334	RGD1308377_predicted	-0.170	-0.240	-0.239	-0.520	0.058	0.104	-0.105	0.289	-0.052	-0.161	-0.307	0.047
NM_001014131	RGD1309708	0.929	0.773	0.414	0.653	0.436	0.310	0.128	0.287	0.583	0.654	0.863	-0.082
NM_001014102	RGD1309930	-1.567	-1.767	-1.199	-1.958	-0.773	-0.795	-1.322	-0.828	-0.953	-1.474	-1.772	-0.316
NM_001012238	RGD1311980	0.895	0.825	0.783	0.161	0.773	0.338	0.917	0.376	0.573	0.886	0.855	0.108
NM_001100534	RGD1559574_predicted	-0.177	-0.014	-0.589	-0.017	-0.406	-0.739	-0.368	-0.471	-0.308	-0.182	-0.002	-0.633
XM_577351.2	RGD1559774_predicted	0.307	-0.264	0.006	0.050	0.469	0.738	0.003	0.392	0.390	0.097	0.031	0.478
NM_001024280	RGD1559795_predicted	0.031	-0.519	-0.244	-0.128	-0.048	-0.037	-0.713	-0.153	-0.063	-0.379	-0.002	-0.196
XM_574479.2	RGD1559862_predicted	-0.057	0.042	-0.084	-0.127	0.227	0.522	0.060	0.231	0.130	0.074	0.024	0.220
NM_001108678	RGD1559909_predicted	0.449	0.352	0.168	0.601	0.442	0.610	0.252	0.359	0.585	0.336	0.424	0.531
XM_226796.2	RGD1560076_predicted	0.095	-0.031	-0.470	-0.032	0.120	0.543	-0.349	0.293	0.231	-0.041	0.195	0.292
NM_001008309	RGD1560568_predicted	-0.287	-0.417	-0.505	-0.374	-0.206	-0.317	-0.283	-0.201	-0.268	-0.528	-0.316	-0.160
NM_031112	RGD1560729_predicted	0.094	-0.189	0.070	0.524	0.047	0.101	-0.355	0.144	-0.078	-0.085	-0.106	0.055
NM_001013426	RGD1560917_predicted	0.227	-0.083	0.427	-0.040	0.348	0.624	0.146	0.349	0.154	0.113	0.087	0.381
NM_001126088	RGD1560991_predicted	0.148	-0.088	-0.393	0.040	-0.197	-0.021	-0.633	-0.125	0.127	-0.042	0.006	0.113
NM_053330	LOC300731	-0.526	-0.532	-0.701	-0.510	-1.184	-1.116	-0.510	-1.148	-0.438	-0.639	-0.415	-0.330
XM_001074676.1	RGD1561789_predicted	-0.058	-0.399	-0.385	0.031	-0.104	0.139	-0.322	0.093	0.194	-0.060	0.088	-0.080
NM_001127497	RGD1562046_predicted	0.161	0.570	0.224	0.282	0.362	-0.344	0.391	0.000	0.175	0.567	-0.076	0.216
XM_575654.2	RGD1562214_predicted	0.559	0.164	-0.017	0.755	0.043	-0.173	0.196	-0.089	0.162	-0.051	0.115	-0.247
NM_001007603	RGD1562725_predicted	-0.157	-0.251	-0.309	-0.094	-0.255	0.091	-0.370	-0.117	-0.189	-0.434	-0.065	0.074
NM_001007603	RGD1562725_predicted	-0.157	-0.251	-0.309	-0.094	-0.255	0.091	-0.370	-0.117	-0.189	-0.434	-0.065	0.074
NM_053330	LOC300731	-0.526	-0.532	-0.701	-0.510	-1.184	-1.116	-0.510	-1.148	-0.438	-0.639	-0.415	-0.330
NM_001114391	RGD1562953_predicted	-0.333	-0.368	-1.442	-0.712	-0.253	-0.160	-1.166	-0.221	-0.202	-0.291	-0.223	-0.503
NM_001007603	RGD1562725_predicted	-0.157	-0.251	-0.309	-0.094	-0.255	0.091	-0.370	-0.117	-0.189	-0.434	-0.065	0.074
NM_001114391	RGD1562953_predicted	-0.333	-0.368	-1.442	-0.712	-0.253	-0.160	-1.166	-0.221	-0.202	-0.291	-0.223	-0.503
NM_053867	RGD1563739_predicted	0.082	0.038	-0.197	0.180	0.101	-0.199	-0.015	-0.097	0.398	-0.029	0.120	-0.036
XM_001056192.1	RGD1563958_predicted	0.212	-0.179	-0.189	-0.143	-0.003	0.558	-0.364	0.046	0.104	-0.192	-0.202	0.272

NM_024139	RGD1564956_predicted	0.139	0.281	0.150	-0.412	0.057	0.269	0.207	0.202	-0.096	0.269	-0.043	-0.062
NM_017150	RGD1565306_predicted	-0.180	-0.301	-1.005	-0.531	-0.176	-0.138	-0.933	-0.144	-0.153	-0.380	-0.232	-0.190
NM_053330	LOC300731	-0.526	-0.532	-0.701	-0.510	-1.184	-1.116	-0.510	-1.148	-0.438	-0.639	-0.415	-0.330
XM_576332.2	RGD1565798_predicted	0.042	-0.039	-0.281	-0.013	-0.239	-0.260	-0.180	-0.100	0.265	-0.100	0.174	0.011
XM_225053.3	RGD1565806_predicted	0.015	-0.100	-0.322	0.151	0.097	0.753	-0.360	0.129	0.147	-0.018	-0.001	0.169
NM_022593	RGD1565815_predicted	0.078	-0.265	0.099	0.118	-0.079	0.113	-0.039	-0.013	0.271	-0.045	-0.088	0.316
NM_001114391	RGD1562953_predicted	-0.333	-0.368	-1.442	-0.712	-0.253	-0.160	-1.166	-0.221	-0.202	-0.291	-0.223	-0.503
NM_001128065	RGD1566373_predicted	-0.101	-0.393	-0.191	-0.182	-0.179	-0.296	-0.605	-0.147	-0.192	-0.301	-0.112	-0.085
NM_053438	Rnf103	-0.095	-0.111	-0.270	0.056	0.038	-0.427	-0.181	-0.161	-0.530	-0.334	0.029	-0.454
NM_001025727	Rnf8	0.114	-0.022	-0.091	-0.074	-0.196	-0.664	-0.396	-0.433	-0.405	-0.422	-0.382	-0.535
NM_022515	Rpl24	0.164	-0.120	-0.352	0.339	0.235	0.883	-0.480	0.298	0.218	-0.182	0.133	0.454
NM_001100727	Rufy1	-0.107	0.424	0.331	-0.235	-0.093	-0.222	0.323	-0.032	-0.120	0.270	0.307	-0.163
XM_001067782.1	Samhd1_predicted	0.509	0.157	0.186	0.558	-0.036	-0.239	0.103	-0.129	-0.124	-0.122	0.110	0.045
NM_001108599	Scand1_predicted	-0.299	-0.315	-0.008	-0.659	-0.181	0.587	-0.271	-0.080	0.049	-0.243	0.122	0.684
NM_030875	Scn1a	0.047	-0.124	0.056	0.086	0.235	0.213	0.023	0.344	0.445	0.071	0.057	0.216
NM_001108430	Sema6a_predicted	-0.619	-0.264	-0.280	-0.766	-0.226	-0.596	-0.163	-0.066	0.088	-0.223	-0.484	-0.328
NM_030835	Serp1	-0.513	-0.590	-0.246	-0.337	-0.243	-0.145	-0.749	-0.069	-0.232	-0.331	-0.373	0.083
NM_017173	Serpinh1	-0.258	-0.107	-0.109	0.144	0.059	0.371	-0.400	0.156	0.179	-0.146	-0.320	0.038
NM_017307	Slc25a1	-0.038	0.064	-0.015	-0.149	0.295	0.393	-0.313	0.202	0.121	-0.059	-0.080	0.273
NM_001106098	Slc35d2_predicted	-0.367	0.200	0.006	-0.631	-0.178	-0.149	0.207	-0.404	-0.248	-0.193	-0.287	-0.230
NM_021594	Slc9a3r1	-0.474	-0.266	0.068	-0.973	-0.050	-0.217	-0.435	-0.138	-0.274	-0.307	-0.489	0.046
NM_001031652	St6galnac2	-0.671	-0.251	-0.219	-0.430	-0.049	0.038	-0.373	-0.030	-0.017	-0.380	-0.430	-0.083
NM_001105828	Supt4h2_predicted	0.118	0.190	-0.042	0.124	0.274	0.768	0.270	0.312	0.354	0.216	0.325	0.563
NM_001106451	Tbx15_predicted	-0.135	-0.446	-0.687	-0.723	-0.229	0.116	-0.510	0.011	-0.104	-0.282	-0.346	0.224
NM_021673	Tcam1	0.161	0.031	0.007	0.574	-0.022	-0.001	0.044	0.236	0.108	0.128	0.292	0.140
NM_001106111	Them2_predicted	0.910	0.253	0.438	0.649	0.605	1.009	0.451	0.744	0.584	0.282	0.483	0.690
NM_001012155	Tm9sf1	-0.298	0.116	0.056	-0.108	-0.019	-0.266	0.041	-0.042	-0.273	0.053	-0.198	-0.218
NM_021671	Tmem33	0.083	0.078	-0.123	-0.105	0.400	0.670	0.087	0.466	0.093	0.090	-0.080	0.315
NM_001107903	Tmem68_predicted	0.043	0.234	0.022	-0.148	0.116	-0.549	0.386	0.086	-0.083	0.099	-0.011	-0.554
NM_001107691	Trim46_predicted	-0.330	-0.420	0.202	-0.304	-0.302	-0.489	-0.127	-0.530	-0.587	-0.271	-0.329	-0.462
NM_001107658	Trio	-0.036	0.280	0.673	0.405	0.458	-0.154	0.396	0.228	0.079	0.307	-0.042	0.008
NM_001135876	Trmu_predicted	0.410	0.020	0.368	0.567	0.134	0.394	0.076	0.128	0.278	0.237	0.347	0.368
NM_001107750	Tspan18_predicted	0.170	0.124	0.111	0.107	-0.023	0.120	0.086	0.455	0.203	0.038	0.008	0.717
NM_001100672	Tspan6	-1.252	-0.755	-1.349	-1.192	-0.737	-0.824	-0.880	-0.544	-0.584	-0.872	-0.851	-0.293
NM_001008381	Ube2f	-0.068	0.044	-0.332	-0.037	0.255	0.359	-0.170	0.272	0.214	-0.028	-0.425	0.240
NM_013050	Ube2i	0.036	0.034	-0.263	-0.088	0.020	0.404	-0.185	-0.093	0.138	0.041	-0.308	0.431
NM_001106619	Usp5_predicted	0.065	-0.188	0.176	0.050	-0.270	-0.613	-0.181	-0.276	-0.441	-0.367	-0.360	-0.750
XM_575397.2	Wnt2	0.540	0.396	0.441	0.504	0.209	0.308	0.324	0.060	0.216	0.174	0.907	0.527

NM_172017	Yif1	0.053	-0.272	-0.003	0.029	0.128	0.699	-0.123	0.039	0.089	-0.161	-0.117	0.455
NM_001108443	Zfp330_predicted	0.626	0.151	0.318	0.940	0.118	0.045	0.593	0.294	0.170	0.311	0.497	0.088
NM_001107428	Zfp612_predicted	0.382	-0.043	-0.317	-0.122	-0.368	-0.378	-0.031	-0.409	-0.485	-0.352	-0.174	-0.427
NM_001106689	Znf593_predicted	0.466	0.051	0.156	0.155	0.579	1.042	-0.011	0.627	0.381	0.171	0.129	0.544
