# Supplementary material for: KSHV MicroRNAs Mediate Cellular Transformation and Tumorigenesis by Redundantly Targeting Cell Growth and Survival Pathways
Source: PLoS Pathog. 2013 Dec 26;9(12):e1003857. doi: 10.1371/journal.ppat.1003857 (PMC3873467; doi:10.1371/journal.ppat.1003857)
Supplement: Table S3 — Cellular pathways that regulate IκBα. (PDF) [file ppat.1003857.s014.pdf]

Table S3. Cellular pathways that regulate IκBα

4-1BB Signaling in T Lymphocytes;
Activation of IRF by Cytosolic Pattern Recognition Receptors;
Acute Phase Response Signaling;
Angiopoietin Signaling;
Apoptosis Signaling;
April Mediated Signaling;
ATM Signaling;
B Cell Activating Factor Signaling;
B Cell Receptor Signaling;
CD27 Signaling in Lymphocytes;
CD28 Signaling in T Helper Cells;
CD40 Signaling;
Death Receptor Signaling;
Dendritic Cell Maturation;
Erythropoietin Signaling;
fMLP Signaling in Neutrophils;
Glucocorticoid Receptor Signaling;
G-Protein Coupled Receptor Signaling;
Gα12/13 Signaling; Hepatic Cholestasis;
Hypoxia Signaling in the Cardiovascular System;
iCOS-iCOSL Signaling in T Helper Cells; IL-10 Signaling;
IL-17A Signaling in Airway Cells;
IL-17A Signaling in Fibroblasts;
IL-1 Signaling;
IL-6 Signaling;
Induction of Apoptosis by HIV1;
iNOS Signaling;
LPS-stimulated MAPK Signaling;
Lymphotoxin β Receptor Signaling;
MIF-mediated Glucocorticoid Regulation;

MIF Regulation of Innate Immunity;
Molecular Mechanisms of Cancer;
NF-κB Activation by Viruses;
NF-κB Signaling;
OX40 Signaling Pathway;
PI3K/AKT Signaling;
PI3K Signaling in B Lymphocytes;
PKCθ Signaling in T Lymphocytes;
PPAR Signaling; PPARα/RXRα Activation;
Production of Nitric Oxide and Reactive Oxygen Species in Macrophages;
Prostate Cancer Signaling;
Protein Kinase A Signaling;
RANK Signaling in Osteoclasts;
Regulation of IL-2 Expression in Activated and Anergic T Lymphocytes;
Relaxin Signaling;
Role of IL-17A in Arthritis;
Role of Macrophages, Fibroblasts and Endothelial Cells in Rheumatoid Arthritis;
Role of NFAT in Regulation of the Immune Response;
Role of Osteoblasts, Osteoclasts and Chondrocytes in Rheumatoid Arthritis;
Role of PI3K/AKT Signaling in the Pathogenesis of Influenza;
Role of PKR in Interferon Induction and Antiviral Response;
Role of RIG1-like Receptors in Antiviral Innate Immunity;
Small Cell Lung Cancer Signaling;
T Cell Receptor Signaling;
TNFR1 Signaling;
TNFR2 Signaling;
Toll-like Receptor Signaling;
TWEAK Signaling;
Type I Diabetes Mellitus Signaling;
Type II Diabetes Mellitus Signaling

Source of data: Ingenuity Systems, Redwood City, CA
