# Supplementary material for: KSHV MicroRNAs Mediate Cellular Transformation and Tumorigenesis by Redundantly Targeting Cell Growth and Survival Pathways
Source: PLoS Pathog. 2013 Dec 26;9(12):e1003857. doi: 10.1371/journal.ppat.1003857 (PMC3873467; doi:10.1371/journal.ppat.1003857)
Supplement: Table S4 — Target prediction by combining SVMicrO predicted targets with gene expression results. (PDF) [file ppat.1003857.s015.pdf]

Table S4 Target prediction by combining SVMicrO predicted targets with gene expression results

Notes: Scores based on combining expression fold change (MutKi vs MutVt) and SVMicrO target prediction score.

Ref_seq	gene_symbol	K1	K2	K3	K4	K5	K6	K7	K8	K9	K10	K11	K12
NM_001014048	Ctdspl2	0.0161	0.0818	0.2118	0.4036	0.0526	0.5562	0.1248	0.0118	0.2967	0.0395	0.4483	0.0664
NM_001008344	Etf1	0.5568	0.4439	0.1730	0.2233	0.1020	0.1762	0.9499	0.3300	0.6622	0.5428	0.8673	0.8281
NM_001008346	RGD1308302	0.8080	0.9493	0.1253	0.9184	0.8000	0.8951	0.9518	0.7482	0.9576	0.8437	0.7227	0.8070
NM_001008347	Fank1	0.7385	0.8068	0.6640	0.9053	0.6506	0.7269	0.8112	0.7930	0.7284	0.5458	0.8658	0.7350
NM_001008350	Taf11	0.8967	0.9599	0.5071	0.7981	0.8432	0.8857	0.2116	0.7730	0.6546	0.7800	0.7213	0.1798
NM_001012748	Cdh20	0.5768	0.3378	0.8273	0.5576	0.5599	0.1618	0.5815	0.4328	0.7500	0.4187	0.3598	0.6006
NM_001009344	Tbrg1	0.2450	0.7149	0.3488	0.0620	0.2856	0.1553	0.7714	0.4550	0.7910	0.8042	0.8365	0.1383
NM_001008354	Mrrf	0.6260	0.4432	0.9013	0.9623	0.8528	0.9817	0.5865	0.9704	0.6293	0.8316	0.5524	0.8864
NM_001008355	Tmem111	0.2287	0.0952	0.2236	0.0678	0.0082	0.1203	0.1592	0.2981	0.1489	0.1769	0.0067	0.4606
NM_001008356	Slc39a3	0.8349	0.9327	0.7557	0.7731	0.9072	0.8134	0.7770	0.9293	0.8228	0.8563	0.8718	0.9477
NM_001008357	Hcfc2	0.5090	0.7159	0.1933	0.0510	0.0441	0.1267	0.0878	0.0561	0.1461	0.4023	0.0912	0.0345
NM_001008360	RGD1310686	0.7356	0.2458	0.9481	0.8367	0.8803	0.9622	0.6208	0.7608	0.6444	0.1637	0.1531	0.9573
NM_001047893	Shroom2	0.8384	0.8926	0.8917	0.9328	0.5530	0.7407	0.9475	0.6590	0.5331	0.6071	0.8264	0.7584
NM_001008365	Tom1	0.1420	0.2594	0.0089	0.0610	0.2024	0.1877	0.4193	0.2548	0.1854	0.1166	0.3089	0.3884
NM_001008367	Isoc2b	0.6777	0.0522	0.2762	0.8113	0.4640	0.1338	0.7831	0.6762	0.8229	0.3247	0.8768	0.1013
NM_001008368	Sirt2	0.0322	0.0555	0.7985	0.6386	0.0250	0.0522	0.4136	0.2785	0.0463	0.0422	0.0210	0.6931
NM_001008369	Pitpnm1	0.5348	0.4323	0.2412	0.2864	0.6180	0.3329	0.1787	0.5070	0.6076	0.1127	0.4558	0.6008
NM_001011963	Asb6	0.5516	0.4105	0.6350	0.6002	0.0839	0.3848	0.5589	0.4506	0.4500	0.3685	0.7475	0.7857
NM_001008371	RGD1309216	0.3181	0.0907	0.9453	0.9118	0.2845	0.3219	0.5566	0.3709	0.1618	0.1071	0.2843	0.2251
NM_001012047	Btd	0.5709	0.5742	0.2988	0.6260	0.6023	0.5720	0.9035	0.7842	0.6254	0.8438	0.8380	0.3930
NM_001012464	Terf1	0.9352	0.6281	0.7022	0.5600	0.8838	0.6830	0.9060	0.9352	0.2453	0.8841	0.3814	0.4045
NM_001008374	Pgrmc2	0.4684	0.4411	0.5790	0.1515	0.6276	0.5887	0.4249	0.4449	0.5784	0.5589	0.5758	0.7449
NM_001008325	Tmem218	0.2714	0.0743	0.5992	0.0680	0.2118	0.1702	0.1209	0.0970	0.0056	0.0193	0.0152	0.1239
NM_001008376	Trappc3	0.2878	0.4218	0.7906	0.1586	0.4199	0.1931	0.1407	0.4233	0.8170	0.8107	0.0988	0.7955
NM_001008378	Tspan31	0.0621	0.0489	0.0152	0.1228	0.2107	0.0427	0.1434	0.1980	0.2467	0.2119	0.0068	0.3693
NM_001008382	Cnot8	0.9787	0.9261	0.8759	0.3751	0.9094	0.9194	0.5779	0.5530	0.8937	0.4323	0.8887	0.6764
NM_001012177	Mccc2	0.5310	0.2479	0.1562	0.4946	0.3656	0.4974	0.2881	0.4367	0.3229	0.4309	0.4765	0.3971
NM_001011972	Atp6v0d2	0.2106	0.1528	0.2638	0.0393	0.5255	0.2967	0.1304	0.0769	0.4281	0.3451	0.4944	0.1949
NM_001008375	Map2k1ip1	0.2983	0.0126	0.8154	0.8295	0.0777	0.0125	0.2201	0.3292	0.0054	0.1456	0.2018	0.1051
NM_001012167	Pld3	0.2030	0.0719	0.8741	0.6788	0.0367	0.5917	0.6232	0.1077	0.0133	0.1580	0.0653	0.5377
NM_001008511	Esrra	0.5175	0.5109	0.4248	0.7081	0.5116	0.6827	0.7007	0.6222	0.5915	0.7295	0.5063	0.7249
NM_001008514	Cldn19	0.0952	0.4086	0.2972	0.2697	0.1686	0.0851	0.3927	0.4368	0.5177	0.5590	0.0398	0.2407
NM_001008513	Ccl21b	0.2096	0.0955	0.2695	0.1651	0.5315	0.4955	0.0341	0.4359	0.3419	0.0675	0.0286	0.4171
NM_001012029	Ifi204	0.1975	0.1228	0.0831	0.1918	0.1618	0.1251	0.1773	0.1196	0.2047	0.3527	0.2916	0.2631
NM_001008515	C1qa	0.1047	0.0521	0.1309	0.5291	0.1851	0.3696	0.2257	0.1718	0.0926	0.1417	0.1424	0.2383
NM_001008519	Lrpprc	0.6233	0.2894	0.0863	0.0959	0.3852	0.8032	0.0864	0.9495	0.7270	0.5747	0.5937	0.7467
NM_013001	Pax6	0.6800	0.4980	0.3902	0.7585	0.4198	0.8401	0.5785	0.3312	0.5621	0.9075	0.4008	0.8190
NM_001012063	Sae1	0.7567	0.1985	0.6277	0.2318	0.6672	0.5008	0.4722	0.7176	0.3553	0.6125	0.1408	0.8120
NM_001012053	Zkscan3	0.5640	0.5397	0.8984	0.6390	0.8616	0.7659	0.7041	0.1734	0.7094	0.7339	0.6238	0.7504
NM_001012052	Cage1	0.0402	0.0973	0.0289	0.0105	0.0860	0.0909	0.0252	0.0256	0.3350	0.0581	0.0525	0.0438
NM_001008525	Ndufs7	0.4138	0.0274	0.1903	0.1038	0.0260	0.0620	0.3331	0.1809	0.1064	0.0102	0.0071	0.0144
NM_001008526	Bag5	0.8440	0.8975	0.9588	0.9032	0.7913	0.8102	0.8371	0.9582	0.9385	0.8645	0.9727	0.8340
NM_001008554	RGD1306446	0.7147	0.9295	0.8326	0.9003	0.8523	0.3873	0.8427	0.5112	0.9112	0.9485	0.9098	0.8241

NM_001012011	Lig3	0.8584	0.8147	0.3599	0.8505	0.9553	0.9254	0.9793	0.8668	0.9816	0.9885	0.9769	0.9944
NM_001012064	Pvrl2	0.2766	0.1469	0.1223	0.0987	0.4204	0.2586	0.3339	0.2236	0.6414	0.0715	0.1913	0.7853
NM_001008560	Prss35	0.9696	0.2512	0.9882	0.2725	0.9989	0.7413	0.9543	0.9904	0.0037	0.3149	0.3173	0.1282
NM_001008559	Hapln3	0.2628	0.8286	0.6517	0.0882	0.0169	0.0220	0.6559	0.0579	0.8630	0.5259	0.8789	0.7377
NM_001012017	Dirc2	0.4451	0.5426	0.8164	0.9985	0.3433	0.6168	0.5346	0.6981	0.3291	0.4794	0.8565	0.6972
NM_001008721	Ebna1bp2	0.7658	0.8102	0.1128	0.6720	0.7703	0.3554	0.7355	0.6145	0.8523	0.9192	0.9138	0.1341
NM_001012111	Lpin1	0.7041	0.3891	0.2967	0.2531	0.4332	0.5078	0.3085	0.3214	0.5816	0.3595	0.8757	0.6706
NM_001011971	Xkr4	0.6501	0.5630	0.3037	0.6988	0.4824	0.5757	0.6561	0.6244	0.7159	0.6516	0.6920	0.6683
NM_001012077	Mtmr12	0.5473	0.7599	0.5056	0.6181	0.3794	0.7718	0.7695	0.5067	0.6467	0.6001	0.5732	0.5282
NM_001011986	Sf3a2	0.4643	0.6242	0.5416	0.5614	0.7391	0.6668	0.8823	0.5942	0.6376	0.2232	0.7197	0.9431
NM_001011990	Eif3e	0.6435	0.0537	0.8582	0.0897	0.8321	0.6903	0.8059	0.8300	0.1043	0.1454	0.2509	0.5671
NM_001008767	Txnip	0.1662	0.0566	0.9946	0.2635	0.1460	0.2642	0.0140	0.1576	0.0371	0.1542	0.1013	0.9924
NM_001012094	Zbtb43	0.9242	0.9801	0.9966	0.5201	0.7491	0.8635	0.9018	0.8027	0.7874	0.9639	0.9933	0.9541
NM_001008768	Prim1	0.1084	0.8833	0.4043	0.7539	0.8117	0.7621	0.8068	0.9800	0.8027	0.8074	0.9456	0.1748
NM_001008773	Eif1a	0.5164	0.3563	0.5109	0.5347	0.3771	0.4701	0.6971	0.2272	0.6933	0.4538	0.5704	0.2129
NM_001012097	Atg7	0.0151	0.0810	0.1915	0.2141	0.1037	0.0120	0.3444	0.1745	0.2342	0.0672	0.0954	0.2124
NM_001011998	Fbxo9	0.8084	0.7843	0.4196	0.4631	0.9073	0.8806	0.3000	0.8758	0.7757	0.8681	0.7791	0.6235
NM_001012148	Ankrd13a	0.3397	0.3577	0.1938	0.2014	0.3725	0.5922	0.2988	0.1162	0.2819	0.1441	0.2794	0.4970
NM_001037360	Znf830	0.7431	0.7629	0.9047	0.9073	0.3386	0.4660	0.4275	0.7124	0.9085	0.9038	0.9204	0.6849
NM_001029925	Ostm1	0.0981	0.8620	0.8656	0.2059	0.0504	0.4573	0.0534	0.1049	0.0165	0.5718	0.0239	0.8111
NM_001012176	Rsph1	0.1453	0.3354	0.3003	0.3308	0.2140	0.2097	0.0720	0.1133	0.5865	0.2452	0.4842	0.8243
NM_001012182	Sike	0.0585	0.1075	0.1541	0.2578	0.3052	0.1148	0.0318	0.2367	0.5646	0.6266	0.4769	0.9660
NM_001025008	Klhl38	0.9047	0.8399	0.6495	0.8143	0.6825	0.8391	0.8082	0.6595	0.0918	0.8407	0.7923	0.6708
NM_001008827	RT1-A1	0.5364	0.4309	0.5986	0.5635	0.6760	0.2853	0.5371	0.5910	0.4335	0.3380	0.2362	0.2703
NM_001008831	RT1-Ba	0.3287	0.6164	0.5022	0.4909	0.8104	0.5137	0.7139	0.0961	0.1417	0.1253	0.5351	0.1554
NM_001012081	Polr3c	0.0545	0.7389	0.7943	0.7527	0.6608	0.7986	0.7106	0.5831	0.7475	0.7969	0.8003	0.7300
NM_001008848	RT1-Ha	0.4185	0.3452	0.4739	0.1560	0.1466	0.1985	0.2218	0.4611	0.0504	0.0440	0.1057	0.0950
NM_001012104	Ddost	0.5438	0.0227	0.7999	0.9164	0.0166	0.1715	0.8700	0.0572	0.0981	0.1905	0.1844	0.7418
NM_001011984	Asb2	0.3182	0.5861	0.4571	0.5760	0.2361	0.5286	0.5982	0.4184	0.0848	0.2407	0.2395	0.3260
NM_001008858	RT1-T24-1	0.0598	0.0367	0.7894	0.0271	0.0062	0.0434	0.1048	0.0710	0.2313	0.1785	0.0147	0.8092
NM_001012214	Serpinb6b	0.2079	0.2236	0.2693	0.4097	0.2928	0.2057	0.2692	0.6378	0.1077	0.3225	0.5536	0.4502
NM_001012210	Trim13	0.5008	0.4469	0.5935	0.6113	0.5175	0.3877	0.6568	0.5471	0.4944	0.6311	0.4170	0.4605
NM_001013170	Wars	0.2236	0.9095	0.2562	0.7853	0.9336	0.2023	0.8977	0.6292	0.6849	0.9591	0.8255	0.2201
NM_001012228	Xkr5	0.7710	0.9010	0.0240	0.7118	0.9853	0.9405	0.7739	0.9975	0.8533	0.8805	0.6828	0.1213
NM_001012230	Xkrx	0.5086	0.5034	0.4980	0.4284	0.7664	0.2236	0.4556	0.1034	0.1944	0.6038	0.2416	0.2327
NM_001012229	Xkr9	0.6819	0.4289	0.5588	0.6932	0.4123	0.5916	0.3883	0.7474	0.8961	0.9083	0.5598	0.0693
NM_001012348	Fam113a	0.4451	0.4234	0.4419	0.4068	0.2477	0.4353	0.3852	0.3313	0.2796	0.4320	0.3320	0.2047
NM_001008884	RT1-Db1	0.2339	0.2213	0.1023	0.1548	0.1028	0.2221	0.4007	0.2397	0.5315	0.4377	0.5404	0.5773
NM_001012098	Recql	0.0697	0.7524	0.0219	0.1558	0.3129	0.5450	0.9040	0.0737	0.5995	0.7244	0.4976	0.0735
NM_001008891	Ssr1	0.7518	0.5169	0.5849	0.5751	0.2752	0.9090	0.6228	0.3744	0.2930	0.8349	0.4735	0.5748
NM_001013050	Pold2	0.9944	0.7680	0.7003	0.7721	0.7940	0.8118	0.8921	0.7696	0.7526	0.9438	0.8563	0.7548
NM_001011954	Cybrd1	0.7052	0.5469	0.0810	0.4305	0.5294	0.4901	0.7093	0.4712	0.2351	0.2668	0.3929	0.6068
NM_001012355	Senp8	0.5743	0.3301	0.5933	0.4021	0.3210	0.3520	0.4321	0.3585	0.2213	0.7074	0.4492	0.3546
NM_001008893	Ldhd	0.0016	0.0029	0.4705	0.0076	0.0031	0.0037	0.5673	0.0080	0.0045	0.0017	0.0035	0.0064
NM_001012354	Lrrc57	0.9380	0.8471	0.9318	0.6094	0.7152	0.3449	0.8902	0.9506	0.6450	0.8951	0.5252	0.9082
NM_001014003	Got1l1	0.8006	0.1014	0.8041	0.2658	0.5362	0.5924	0.8449	0.8408	0.0460	0.8985	0.9215	0.5944
NM_001014218	Atg9a	0.9513	0.8656	0.8456	0.7710	0.9562	0.9479	0.8871	0.9752	0.9745	0.9756	0.7497	0.9796
NM_001012154	Tbrg4	0.7581	0.9347	0.8123	0.8570	0.7276	0.9207	0.5698	0.7423	0.9187	0.8223	0.8651	0.8283
NM_001009255	Sfrs9	0.6530	0.5402	0.4629	0.9101	0.1159	0.1654	0.4274	0.5621	0.8182	0.1298	0.1897	0.1878

NM_001009258	Chfr	0.4487	0.9649	0.8832	0.4571	0.2516	0.2958	0.2292	0.2917	0.7773	0.8731	0.8528	0.7745
NM_001033665	Bcan	0.7762	0.5534	0.6935	0.3343	0.8183	0.7796	0.6270	0.8524	0.8231	0.6721	0.9384	0.7869
NM_001009275	RGD1310224	0.0265	0.1042	0.0729	0.1088	0.0264	0.0803	0.0202	0.0304	0.0103	0.0311	0.1200	0.1740
NM_001009290	Ndufc2	0.4204	0.3322	0.8605	0.6877	0.4209	0.1317	0.3402	0.8045	0.3855	0.6906	0.7584	0.1333
NM_001009672	Sumo1	0.6100	0.7511	0.9535	0.3834	0.6402	0.7510	0.3498	0.1570	0.6910	0.3766	0.6218	0.1258
NM_001009967	Pip5k1c	0.8889	0.6760	0.6508	0.2445	0.6558	0.7128	0.7125	0.6571	0.6355	0.6194	0.4291	0.6889
NM_001009357	Rqcd1	0.9862	0.9923	0.5092	0.9536	0.9605	0.9767	0.8474	0.9953	0.9846	0.9870	0.9765	0.7929
NM_001012472	Dgcr14	0.0155	0.7268	0.0449	0.0409	0.0916	0.3669	0.5761	0.0899	0.3010	0.0629	0.6263	0.1843
NM_001009405	Arhgap29	0.0581	0.0195	0.7314	0.2436	0.2101	0.6760	0.3868	0.2681	0.0034	0.0032	0.0757	0.1195
NM_001012124	Rpp25	0.2169	0.6950	0.2359	0.7836	0.5565	0.7022	0.5917	0.5597	0.4671	0.3389	0.0441	0.8660
NM_001012122	Tymp	0.4561	0.3098	0.4847	0.5985	0.4264	0.4613	0.1207	0.4462	0.3712	0.3229	0.1887	0.4465
NM_001009422	Nipsnap3a	0.0661	0.0148	0.0341	0.6057	0.1569	0.0750	0.0897	0.0517	0.0929	0.0836	0.1441	0.2029
NM_001033862	Ceacam1	0.8506	0.6352	0.9150	0.7145	0.6614	0.8986	0.9526	0.9740	0.8316	0.8947	0.8951	0.8638
NM_001033861	Ceacam1	0.8506	0.6352	0.9150	0.7145	0.6614	0.8986	0.9526	0.9740	0.8316	0.8947	0.8951	0.8638
NM_012595	Ldhb	0.5393	0.1519	0.9998	0.9963	0.9097	0.8873	0.7573	0.9562	0.3189	0.0533	0.1084	0.8948
NM_001009482	Mycbpap	0.4623	0.4819	0.3780	0.3454	0.2569	0.2974	0.2312	0.2210	0.2931	0.1817	0.4638	0.4649
NM_001009448	Cdh19	0.5845	0.1227	0.1213	0.0329	0.2638	0.0344	0.3644	0.1956	0.0133	0.4587	0.5722	0.0315
NM_001009466	Gemin6	0.3731	0.9238	0.3009	0.1232	0.0448	0.0154	0.8340	0.2070	0.1931	0.7280	0.9383	0.0739
NM_001009470	Ccnb2	0.6318	0.8120	0.0737	0.9154	0.8328	0.8850	0.8751	0.8820	0.7997	0.8703	0.9315	0.0597
NM_001012140	Rab34	0.3381	0.0280	0.6338	0.7677	0.1014	0.0915	0.8411	0.7284	0.0887	0.0824	0.3485	0.3653
NM_001015016	Cd72	0.6799	0.4916	0.8056	0.6932	0.8646	0.8939	0.7691	0.4727	0.7094	0.6359	0.7388	0.6857
NM_001012476	Rnase11	0.2068	0.4514	0.3247	0.7249	0.4534	0.4222	0.4730	0.4228	0.5311	0.7232	0.6053	0.5389
NM_001009491	Oas1h	0.1459	0.2093	0.1647	0.1821	0.2869	0.5888	0.7269	0.1453	0.2560	0.3614	0.6551	0.3021
NM_001009501	Ly49i5	0.5549	0.5475	0.4656	0.5441	0.3394	0.4289	0.6638	0.2426	0.3131	0.1039	0.6588	0.5463
NM_001009496	Ly49i9	0.6490	0.2841	0.4999	0.2548	0.4690	0.2239	0.5674	0.3469	0.6975	0.2314	0.3583	0.3842
NM_001014032	Msl3l2	0.3884	0.2981	0.2092	0.2276	0.1983	0.4101	0.2312	0.2209	0.1872	0.1817	0.4107	0.2045
NM_001013033	Tspyl1	0.9820	0.6321	0.9736	0.7565	0.7099	0.8724	0.7816	0.4694	0.9838	0.8382	0.9201	0.8351
NM_001009540	Tacstd2	0.9007	0.5611	0.7011	0.6704	0.6421	0.8203	0.6212	0.3316	0.7947	0.4255	0.3077	0.3306
NM_001009541	Ier2	0.0526	0.0064	0.1805	0.1458	0.1790	0.2505	0.2065	0.7912	0.1010	0.0073	0.1451	0.1702
NM_001017457	Degs2	0.1069	0.6230	0.4854	0.6983	0.1239	0.1617	0.6594	0.2409	0.8270	0.5775	0.2369	0.2529
NM_001013142	Cmtm2a	0.7228	0.6472	0.7194	0.7244	0.5759	0.6028	0.4574	0.5162	0.9758	0.7690	0.7668	0.4325
NM_001013141	Zfp35	0.3411	0.5898	0.7799	0.6516	0.6814	0.6347	0.6426	0.2147	0.7027	0.5857	0.8136	0.4108
NM_001009600	Arhgdib	0.9882	0.8414	0.8649	0.9068	0.9205	0.9795	0.8147	0.9282	0.8949	0.9839	0.8535	0.9300
NM_001014057	Tsen2	0.0937	0.7957	0.1914	0.8910	0.8776	0.1129	0.4866	0.6712	0.8972	0.3379	0.9311	0.5860
NM_001012129	Satb1	0.9367	0.9155	0.7987	0.8575	0.7858	0.5318	0.7858	0.9123	0.9426	0.9000	0.9966	0.8462
NM_001014093	Parp16	0.9471	0.4899	0.9864	0.9577	0.9597	0.8773	0.9343	0.8818	0.6061	0.8928	0.4203	0.9801
NM_001009604	Bri3	0.8176	0.4142	0.8783	0.8056	0.6548	0.8777	0.0584	0.9729	0.3779	0.2159	0.5702	0.0435
NM_001009605	Brms1	0.0484	0.0241	0.5743	0.0516	0.3436	0.2683	0.5078	0.0524	0.2735	0.2852	0.3580	0.8020
NM_001009618	Sub1	0.1273	0.1244	0.3371	0.6335	0.0175	0.2838	0.1795	0.0980	0.2594	0.2074	0.3914	0.0489
NM_001009619	Nubp1	0.7937	0.7624	0.0201	0.1757	0.8475	0.8007	0.4760	0.8401	0.6308	0.4924	0.6024	0.0151
NM_001009623	Tnfsf13	0.5589	0.0085	0.1052	0.3603	0.9282	0.6886	0.2357	0.8508	0.1923	0.1114	0.1054	0.0302
NM_001009625	Ifi35	0.0902	0.0918	0.1217	0.0092	0.0120	0.1492	0.0460	0.0451	0.0210	0.0889	0.0027	0.0027
NM_001012057	Gpt2	0.3346	0.2588	0.2595	0.2272	0.2998	0.2632	0.4942	0.5392	0.5402	0.2730	0.1980	0.2668
NM_001034943	Slc22a12	0.3819	0.8113	0.6035	0.3259	0.2367	0.4627	0.7570	0.2743	0.8485	0.6095	0.6489	0.8377
NM_001035232	Slc19a1	0.7812	0.9292	0.4351	0.8631	0.3970	0.3572	0.9723	0.6609	0.9703	0.7109	0.7570	0.9313
NM_001012215	Pcdhgb7	0.8852	0.8992	0.2785	0.9222	0.9247	0.7874	0.2636	0.9573	0.9838	0.8913	0.3616	0.9595
NM_001009629	Rfc3	0.9270	0.7664	0.0197	0.7403	0.7795	0.7776	0.8406	0.8576	0.8415	0.9378	0.8438	0.0453
NM_001012068	Uros	0.1358	0.0249	0.4119	0.3866	0.4084	0.4729	0.3651	0.4594	0.2848	0.1814	0.2482	0.2051
NM_001011959	Ppgb	0.6776	0.6893	0.4368	0.6303	0.5510	0.5131	0.3866	0.2551	0.4802	0.5906	0.4152	0.3578

NM_001012164	Cd97	0.1116	0.8112	0.2691	0.5419	0.4841	0.4428	0.5919	0.5069	0.6158	0.6251	0.2642	0.7372
NM_001009645	Kif22	0.6933	0.9520	0.0243	0.4964	0.7316	0.6731	0.3792	0.7521	0.6704	0.8474	0.8325	0.0379
NM_001009647	Mrpl16	0.0344	0.4152	0.2886	0.3043	0.1148	0.0505	0.1792	0.5010	0.1054	0.3194	0.1195	0.1654
NM_001009649	Lpxn	0.7585	0.3529	0.9046	0.7136	0.6007	0.9106	0.9024	0.5949	0.4007	0.7667	0.6759	0.6800
NM_001009651	Clic2	0.0448	0.0064	0.0026	0.0282	0.1442	0.0278	0.0176	0.0459	0.0249	0.1526	0.0231	0.0133
NM_001012193	Rdh11	0.4899	0.3697	0.9547	0.8163	0.8287	0.5188	0.3974	0.8540	0.7738	0.5870	0.5867	0.3915
NM_001013118	Abi3	0.5594	0.6523	0.6779	0.7797	0.5552	0.6809	0.8310	0.6739	0.6030	0.3865	0.8929	0.1565
NM_001012131	Inpp1	0.2406	0.3193	0.6548	0.2916	0.3330	0.1548	0.3727	0.1249	0.2399	0.3320	0.5165	0.1669
NM_001012130	Pgk2	0.3549	0.8057	0.7126	0.6741	0.3488	0.6456	0.6368	0.0795	0.2304	0.6083	0.4900	0.6264
NM_001024309	LOC499749	0.8564	0.6985	0.0172	0.1442	0.0352	0.1584	0.0347	0.2527	0.9054	0.3117	0.1842	0.0675
NM_001012038	Mtmr3	0.3360	0.9585	0.9301	0.8178	0.6720	0.4550	0.5530	0.2057	0.8186	0.7938	0.4486	0.8762
NM_001009660	Spsb2	0.0744	0.6215	0.5946	0.9076	0.4989	0.1700	0.1657	0.5048	0.1479	0.5902	0.8714	0.0620
NM_001009661	Wbp11	0.8617	0.8290	0.1774	0.7727	0.5664	0.8973	0.7758	0.6379	0.7441	0.8369	0.8529	0.9242
NM_001009662	Car8	0.6395	0.6324	0.8351	0.5266	0.8133	0.3808	0.6197	0.4993	0.2964	0.5699	0.5009	0.0755
NM_001011976	Wdr31	0.4061	0.1991	0.2090	0.2271	0.1982	0.2085	0.4498	0.2208	0.1870	0.1815	0.4371	0.4024
NM_001011987	Glipr1	0.1218	0.0288	0.0333	0.0062	0.0404	0.0061	0.0401	0.1312	0.0020	0.3249	0.0867	0.0025
NM_001011933	Far1	0.3561	0.3071	0.4596	0.6607	0.3717	0.2084	0.2308	0.3544	0.3493	0.4349	0.7327	0.2043
NM_031544	Ampd3	0.1600	0.3720	0.6262	0.4706	0.0823	0.4797	0.5946	0.3663	0.7272	0.3873	0.1474	0.9269
NM_001009670	Abhd14a	0.5707	0.1370	0.2264	0.0238	0.0382	0.0761	0.4917	0.1841	0.5202	0.2280	0.1626	0.2382
NM_001011953	Pigk	0.3301	0.5724	0.9173	0.2464	0.7371	0.5528	0.6531	0.5135	0.5915	0.7940	0.8896	0.2454
NM_001009674	Itm2c	0.8115	0.0535	0.9562	0.3049	0.9372	0.8879	0.2507	0.8914	0.0710	0.1061	0.0096	0.5056
NM_001013228	Dynlt3	0.2792	0.2680	0.9996	0.2839	0.2997	0.3415	0.5658	0.2745	0.3571	0.1930	0.3686	0.7997
NM_001013037	Allc	0.0330	0.4974	0.0944	0.6878	0.0204	0.0401	0.1664	0.0078	0.4925	0.2035	0.0978	0.4422
NM_001013103	Cdc34	0.4846	0.3423	0.7050	0.8046	0.3834	0.3920	0.4548	0.2385	0.8280	0.4183	0.6213	0.5835
NM_001012174	Fkbp5	0.1631	0.2672	0.2913	0.2359	0.1106	0.2894	0.3563	0.2665	0.0867	0.0691	0.0834	0.3507
NM_001009682	Oasl2	0.0423	0.0027	0.0031	0.0407	0.0302	0.0179	0.2086	0.0767	0.1022	0.0204	0.0529	0.1420
NM_001009683	Tor3a	0.0369	0.5187	0.5948	0.6517	0.2696	0.0798	0.3978	0.2215	0.2014	0.3474	0.3351	0.4401
NM_001009684	Hsd17b13	0.8287	0.7597	0.2559	0.2010	0.7072	0.8095	0.2665	0.6608	0.7353	0.7692	0.6844	0.8059
NM_001009685	Drg1	0.8060	0.7896	0.7074	0.8738	0.9505	0.9660	0.8232	0.9659	0.7957	0.8906	0.8201	0.5384
NM_001009690	Rom1	0.7483	0.8653	0.1548	0.7445	0.6179	0.1049	0.9548	0.6950	0.8101	0.7292	0.5005	0.0176
NM_001009700	Imp4	0.3157	0.8289	0.1201	0.3179	0.5466	0.1923	0.2609	0.4031	0.4026	0.7745	0.6855	0.9050
NM_001009689	Cdc42ep2	0.0355	0.0227	0.2345	0.8085	0.4183	0.4723	0.1544	0.3803	0.0585	0.1038	0.0173	0.1015
NM_001013244	Tspan13	0.7181	0.4360	0.5897	0.6016	0.6136	0.5652	0.7119	0.5583	0.7018	0.5381	0.6278	0.3411
NM_001009692	Sh3glb2	0.1797	0.7503	0.7274	0.8281	0.4538	0.6956	0.8287	0.8627	0.6957	0.9442	0.9431	0.7875
NM_001009698	Lysmd3	0.0399	0.4455	0.7855	0.1525	0.0075	0.0069	0.1521	0.0184	0.2238	0.6403	0.2754	0.5830
NM_001009693	Thrap3	0.7563	0.0623	0.9653	0.3007	0.8944	0.9510	0.5805	0.4577	0.1140	0.6179	0.4015	0.7328
NM_001009703	Tmed9	0.9154	0.8246	0.8350	0.2021	0.2112	0.2389	0.8615	0.0968	0.6743	0.9653	0.9549	0.6103
NM_001009704	Sipa1l2	0.6518	0.7362	0.5448	0.5921	0.2076	0.6914	0.5637	0.6875	0.6287	0.7043	0.6927	0.5197
NM_001013041	Clk4	0.7386	0.6671	0.2316	0.0111	0.7270	0.7463	0.0351	0.7390	0.6859	0.5790	0.6493	0.7438
NM_001009708	Lmo4	0.9854	0.9265	0.9924	0.9259	0.8612	0.9377	0.9529	0.9860	0.9478	0.9987	0.9931	0.9988
NM_001009699	Mad2l1bp	0.6032	0.3449	0.2385	0.1452	0.0629	0.1385	0.5619	0.0699	0.0970	0.1192	0.5967	0.4684
NM_001009712	Yipf4	0.3916	0.2161	0.9874	0.3411	0.3099	0.6976	0.4976	0.1640	0.2874	0.4491	0.5763	0.8418
NM_001025144	Prmt2	0.9444	0.8554	0.9279	0.7576	0.8311	0.9726	0.7150	0.8307	0.9514	0.7507	0.9518	0.8871
NM_001009717	Lrg1	0.6394	0.3591	0.4457	0.5800	0.4301	0.5102	0.5302	0.4091	0.4402	0.4891	0.3699	0.4721
NM_001009825	Ppp1r7	0.8673	0.2831	0.0833	0.9553	0.5020	0.6761	0.1349	0.4180	0.2062	0.3218	0.0198	0.2356
NM_001014070	LOC313672	0.8285	0.8562	0.5830	0.7615	0.6665	0.7947	0.6124	0.8473	0.7091	0.7663	0.2697	0.8008
NM_001009953	Rtn3	0.4649	0.7028	0.6980	0.5273	0.6450	0.5498	0.7143	0.3673	0.4830	0.4491	0.4610	0.7242
NM_001034072	Tpm1	0.4367	0.7303	0.6371	0.6080	0.4992	0.4851	0.6099	0.4830	0.4713	0.7143	0.3045	0.7344
NM_001010956	Gmcl1	0.6438	0.6994	0.6255	0.5574	0.5777	0.4506	0.5678	0.5740	0.4633	0.4844	0.6198	0.6551

NM_001013074	Pknox1	0.5262	0.6406	0.2607	0.3501	0.2407	0.4228	0.3184	0.3989	0.2598	0.3054	0.4970	0.2515
NM_001012463	Taf9	0.7092	0.5150	0.5446	0.4458	0.3787	0.5867	0.4531	0.5445	0.3405	0.5574	0.2840	0.2471
NM_001013938	Vwa1	0.8837	0.8929	0.8568	0.6826	0.9111	0.7197	0.8590	0.9883	0.9197	0.9675	0.9551	0.9803
NM_001013224	Nmnat3	0.2003	0.6630	0.6765	0.6176	0.6340	0.5299	0.2628	0.6080	0.5274	0.6245	0.7121	0.6656
NM_001009964	Tbx5	0.0614	0.3959	0.0584	0.4288	0.5090	0.1334	0.2379	0.0846	0.5162	0.1199	0.0766	0.2013
NM_001012087	40,975.0000	0.4861	0.1471	0.7583	0.5967	0.0144	0.3476	0.3560	0.1575	0.1487	0.5487	0.3307	0.2173
NM_001009966	Pacsin3	0.8590	0.5279	0.8849	0.8370	0.3765	0.5531	0.6860	0.6757	0.5428	0.5550	0.8592	0.7518
NM_001013869	Cc2d1a	0.0633	0.5833	0.0224	0.0446	0.7400	0.8322	0.0539	0.9429	0.3937	0.8319	0.1279	0.9158
NM_001012275	Arih2	0.7281	0.6221	0.7158	0.6928	0.6288	0.5742	0.6767	0.6277	0.5021	0.5684	0.4538	0.5647
NM_001030039	Csk	0.7551	0.8706	0.7641	0.6645	0.6335	0.2083	0.9796	0.4313	0.9982	0.8689	0.9508	0.9186
NM_001024327	Znf467	0.3989	0.4802	0.2632	0.6532	0.4815	0.5018	0.7544	0.6004	0.6173	0.8348	0.7940	0.0989
NM_001024325	LOC500077	0.3429	0.8491	0.5461	0.0460	0.5272	0.3944	0.7572	0.5855	0.6902	0.4627	0.0495	0.1063
NM_001013896	Tmem135	0.9400	0.9784	0.9974	0.9347	0.7418	0.9290	0.9606	0.5056	0.8696	0.8797	0.8086	0.4809
NM_001013860	Ptrh2	0.9630	0.9601	0.1421	0.5942	0.9110	0.9393	0.4986	0.9198	0.3573	0.5866	0.7228	0.0795
NM_001014207	Taf1d	0.7568	0.7281	0.1413	0.0868	0.7813	0.7238	0.0475	0.8789	0.8445	0.7253	0.7657	0.1731
NM_001013082	Pon2	0.5330	0.8228	0.9718	0.9884	0.5338	0.7205	0.8354	0.7444	0.7633	0.9102	0.7926	0.7980
NM_017013	Gsta2	0.0029	0.7417	0.5214	0.4753	0.5242	0.6173	0.2646	0.3904	0.6487	0.4431	0.2272	0.4207
NM_001011889	Cldn9	0.3355	0.3742	0.1403	0.2585	0.4478	0.3251	0.1853	0.4635	0.3687	0.2652	0.1337	0.3324
NM_001011890	Rnps1	0.7366	0.8278	0.9270	0.7750	0.6534	0.7608	0.6664	0.6479	0.7087	0.7001	0.8970	0.7278
NM_001012000	Plscr4	0.5194	0.0657	0.9303	0.9395	0.6663	0.8854	0.5024	0.3360	0.1096	0.0868	0.5049	0.4133
NM_001011901	Hsph1	0.4784	0.5501	0.4883	0.5606	0.5258	0.5140	0.5664	0.3480	0.5152	0.4815	0.5592	0.5740
NM_001011891	Nubp2	0.2842	0.3056	0.0218	0.3854	0.1186	0.0471	0.0828	0.0744	0.2051	0.3310	0.0897	0.1252
NM_001012207	Pigc	0.5066	0.3418	0.5706	0.4062	0.2238	0.5357	0.5124	0.2850	0.3674	0.2353	0.6517	0.2280
NM_001011892	Serpinf2	0.6265	0.3967	0.6532	0.8204	0.8501	0.4929	0.7197	0.6887	0.5183	0.5066	0.3769	0.5939
NM_001012219	Lhx8	0.7268	0.5051	0.4782	0.5839	0.7013	0.2356	0.2500	0.6128	0.5952	0.6810	0.5997	0.4191
NM_001013878	Fam149b1	0.9654	0.7583	0.6409	0.8594	0.7454	0.8424	0.5901	0.9481	0.9516	0.8518	0.8387	0.7806
NM_001011893	41,156.0000	0.6609	0.6996	0.6957	0.5171	0.8156	0.5975	0.8076	0.9462	0.8678	0.7741	0.5198	0.8678
NM_001012004	Rnf25	0.6947	0.5204	0.2843	0.0231	0.4173	0.1984	0.0685	0.8965	0.6374	0.8186	0.8508	0.1304
NM_001011894	Psme3	0.6887	0.5577	0.5681	0.7139	0.6188	0.7103	0.5853	0.7012	0.6752	0.6116	0.5591	0.7223
NM_001011905	Stat2	0.3433	0.2954	0.1087	0.4094	0.4875	0.1915	0.1844	0.3539	0.4742	0.4467	0.2217	0.5477
NM_001012109	Galnt14	0.7335	0.4003	0.5394	0.4316	0.1429	0.4236	0.7510	0.1357	0.7105	0.3322	0.6404	0.5553
NM_012724	Fcer1a	0.9240	0.8234	0.7138	0.7453	0.5934	0.4953	0.6928	0.5393	0.7409	0.4605	0.3884	0.7405
NM_012720	Mobp	0.5106	0.4587	0.4548	0.5560	0.6616	0.6229	0.6916	0.6465	0.5974	0.6238	0.7102	0.6919
NM_001011896	Abcf3	0.3432	0.0959	0.1734	0.0599	0.3772	0.0995	0.0940	0.5418	0.4460	0.2278	0.0613	0.7538
NM_001012007	Irgm	0.2605	0.1572	0.3397	0.6998	0.2542	0.2187	0.2320	0.4777	0.1938	0.2420	0.6348	0.4569
NM_001012195	Rnf41	0.7974	0.1977	0.3205	0.3087	0.4203	0.8843	0.5072	0.5469	0.6727	0.0928	0.8584	0.7461
NM_001012194	Vrk1	0.6802	0.5766	0.2869	0.4196	0.9024	0.6529	0.5987	0.5409	0.4107	0.3528	0.5618	0.0955
NM_001012350	Fam174a	0.5325	0.4891	0.2223	0.2528	0.3388	0.5648	0.2936	0.5501	0.1988	0.4336	0.4966	0.2482
NM_001012345	Dgat2	0.8921	0.6109	0.7851	0.5530	0.8933	0.7833	0.7957	0.7280	0.7110	0.9023	0.5551	0.7809
NM_001105825	Ppm1d	0.9168	0.9173	0.7573	0.2938	0.9479	0.9708	0.3542	0.7805	0.9263	0.9505	0.9745	0.8441
NM_001013130	Sptbn1	0.1741	0.0492	0.5102	0.2558	0.3262	0.3247	0.1075	0.2692	0.0657	0.0776	0.0709	0.4492
NM_001011898	Hcls1	0.6260	0.6810	0.9096	0.9132	0.5015	0.9387	0.8183	0.7761	0.2902	0.4409	0.4475	0.4089
NM_001012010	Rnf135	0.7129	0.6240	0.4059	0.2488	0.4145	0.4535	0.6862	0.4950	0.6146	0.2093	0.7017	0.5129
NM_001013199	Sdccag10	0.1552	0.0082	0.1632	0.1006	0.1226	0.0113	0.0369	0.1224	0.0182	0.1041	0.0435	0.0544
NM_001012009	Pipox	0.4599	0.2600	0.7585	0.7034	0.6015	0.1197	0.7240	0.5146	0.4593	0.4994	0.5215	0.6182
NM_001012349	Ppapdc3	0.7494	0.7663	0.3916	0.8757	0.8237	0.8249	0.6827	0.5465	0.6971	0.6617	0.7183	0.6068
NM_001011914	Dr1	0.9082	0.9032	0.7333	0.9389	0.3011	0.8445	0.8972	0.3523	0.8967	0.8684	0.9174	0.7682
NM_001011913	Fignl1	0.8522	0.9139	0.8856	0.8400	0.7053	0.7041	0.9750	0.9241	0.7263	0.7393	0.9934	0.4270
NM_001013882	Dctd	0.4531	0.5417	0.5227	0.5373	0.6778	0.6264	0.4436	0.4999	0.5438	0.4964	0.4715	0.7095

NM_001014228	Cct6b	0.1928	0.4045	0.4458	0.2268	0.2644	0.2082	0.3374	0.2206	0.2724	0.3564	0.4329	0.2038
NM_001014244	Cyb5r2	0.4105	0.2513	0.1419	0.4417	0.6309	0.4909	0.2465	0.5856	0.7946	0.3029	0.1304	0.2081
NM_001012015	Lamp3	0.1549	0.8008	0.2022	0.6597	0.5667	0.7179	0.8547	0.1977	0.8296	0.7040	0.4730	0.3955
NM_001014014	Nip30	0.5659	0.5575	0.5695	0.6427	0.5987	0.4098	0.2460	0.2369	0.5286	0.3640	0.2149	0.5653
NM_001012016	Tbccd1	0.0865	0.0628	0.4400	0.0711	0.2583	0.2029	0.2734	0.0096	0.6433	0.0847	0.1215	0.3939
NM_001011918	Anxa11	0.6143	0.3976	0.9529	0.7852	0.3754	0.7970	0.6829	0.4878	0.7902	0.1548	0.2086	0.9839
NM_001013890	Ttc29	0.5805	0.3512	0.6095	0.1154	0.3750	0.0963	0.6472	0.5302	0.3721	0.3997	0.1721	0.1275
NM_001011920	Sf4	0.5277	0.1413	0.6090	0.5399	0.8449	0.6532	0.5386	0.8790	0.1377	0.3167	0.5893	0.6098
NM_001011921	Pdgfrl	0.9030	0.8526	0.9042	0.7813	0.8423	0.8367	0.9550	0.8478	0.8998	0.8239	0.9875	0.7794
NM_001017473	Rmnd5b	0.7548	0.3236	0.5452	0.8165	0.1418	0.7674	0.1069	0.3148	0.4814	0.3643	0.5295	0.4838
NM_001012226	Stat4	0.5400	0.2378	0.2585	0.3370	0.3146	0.4069	0.3461	0.4250	0.4321	0.3600	0.3831	0.3584
NM_001012023	Epb4.1l5	0.4975	0.6205	0.2366	0.6643	0.5320	0.5704	0.6139	0.3906	0.2032	0.5662	0.5959	0.3257
NM_001011924	Mbd1	0.9350	0.5373	0.5528	0.5772	0.1273	0.6162	0.8102	0.0559	0.8779	0.8049	0.8069	0.9715
NM_001013923	Ndrg3	0.6915	0.3726	0.6958	0.4934	0.3315	0.6153	0.6566	0.5395	0.3135	0.5786	0.4304	0.7248
NM_001012096	Tia1	0.6938	0.3198	0.7208	0.8234	0.7480	0.7492	0.3993	0.7685	0.6116	0.6842	0.7398	0.4339
NM_001013902	Pdilt	0.4493	0.3434	0.4004	0.2268	0.2849	0.3494	0.4122	0.3334	0.3038	0.2719	0.4375	0.2037
NM_001012027	Serpinc1	0.5696	0.4582	0.3811	0.6242	0.5402	0.4139	0.3920	0.4502	0.3545	0.6956	0.5031	0.4642
NM_001012742	Wee1	0.5033	0.9824	0.3377	0.9034	0.9386	0.9800	0.9568	0.9525	0.8376	0.9120	0.8515	0.2948
NM_001011930	Trip13	0.8431	0.8899	0.1131	0.7540	0.8345	0.9547	0.9144	0.7576	0.8390	0.6775	0.7712	0.3563
NM_001013894	Lilrb4	0.6463	0.8420	0.2648	0.5822	0.5492	0.5845	0.8740	0.6237	0.7451	0.9211	0.6922	0.7289
NM_001012030	Zfp281	0.5791	0.3711	0.6827	0.7282	0.5269	0.3991	0.2781	0.2755	0.4058	0.6233	0.4476	0.2726
NM_001011934	Hbs1l	0.1061	0.4042	0.5642	0.2050	0.1938	0.4591	0.1756	0.2157	0.4877	0.6990	0.6420	0.2389
NM_001011936	Bag3	0.3136	0.2080	0.6195	0.7911	0.1010	0.3636	0.7914	0.3394	0.0764	0.2719	0.2601	0.8394
NM_001013063	Raet1l	0.4176	0.7755	0.0742	0.8250	0.7529	0.8558	0.9955	0.7321	0.5981	0.7754	0.7926	0.0288
NM_001011931	Mill1	0.4929	0.7340	0.3644	0.6732	0.7656	0.8576	0.1080	0.5206	0.6628	0.5568	0.5065	0.3998
NM_001011932	Btbd1	0.2940	0.2985	0.9445	0.3146	0.2054	0.0304	0.5126	0.0849	0.1431	0.2345	0.1657	0.1180
NM_001012033	Paqr3	0.4924	0.5308	0.4600	0.7097	0.0580	0.2775	0.3659	0.2932	0.3039	0.5345	0.3612	0.5820
NM_001013974	Ccdc47	0.4311	0.2385	0.3982	0.3616	0.5821	0.9278	0.3200	0.5354	0.1534	0.3082	0.4782	0.6779
NM_001012460	41,153.0000	0.0592	0.1192	0.2532	0.3429	0.1984	0.0238	0.1230	0.2232	0.0893	0.0584	0.0513	0.2326
NM_001017386	Opalin	0.6522	0.4827	0.3552	0.6681	0.2034	0.5184	0.5004	0.6603	0.6602	0.4228	0.6852	0.4328
NM_001013194	Tssc4	0.7118	0.8392	0.7365	0.8169	0.3319	0.2116	0.6429	0.6596	0.7695	0.8505	0.7890	0.6376
NM_001012040	Samd8	0.3905	0.8830	0.6422	0.1493	0.0110	0.0186	0.5015	0.0286	0.2611	0.8220	0.1813	0.2425
NM_001024318	Asb4	0.2967	0.6096	0.3016	0.4037	0.4608	0.4753	0.5222	0.5209	0.2229	0.6063	0.6009	0.3131
NM_001013936	Slc25a34	0.7863	0.6830	0.8249	0.6874	0.6413	0.7553	0.8769	0.8783	0.6949	0.6986	0.7346	0.8104
NM_001011941	Cdc37l1	0.0401	0.1501	0.5066	0.9439	0.4752	0.7467	0.3709	0.6132	0.2215	0.3534	0.5824	0.8749
NM_001012043	Tdrd3	0.8175	0.7708	0.7497	0.6883	0.7550	0.8121	0.7836	0.7568	0.8458	0.8944	0.7397	0.7194
NM_001012044	Lcp1	0.4579	0.4362	0.3354	0.6910	0.1673	0.3485	0.4065	0.0810	0.7160	0.5873	0.7873	0.3293
NM_001011945	Pcbp3	0.5739	0.4564	0.5259	0.1279	0.2574	0.4468	0.6647	0.5449	0.3708	0.2965	0.1784	0.3962
NM_001013895	Prkd2	0.6364	0.0706	0.7572	0.3873	0.5939	0.3480	0.8125	0.2124	0.2344	0.2488	0.2990	0.7198
NM_001011947	Rai14	0.8045	0.8475	0.7938	0.9311	0.7919	0.7514	0.8398	0.7139	0.7480	0.9144	0.9422	0.8382
NM_001011948	Slc7a12	0.3773	0.3381	0.3103	0.2267	0.4580	0.2985	0.2305	0.5126	0.6582	0.4440	0.2868	0.4460
NM_001011951	Sf3b4	0.1203	0.5558	0.0144	0.7032	0.2271	0.6797	0.0507	0.3721	0.4156	0.3918	0.5933	0.8550
NM_001013036	Gmpr2	0.6846	0.2122	0.3323	0.6823	0.5851	0.2203	0.7318	0.6247	0.4750	0.7114	0.2090	0.5664
NM_001011956	Lcmt2	0.6890	0.6956	0.7062	0.7754	0.7211	0.7418	0.7521	0.7471	0.6812	0.9038	0.7103	0.6635
NM_001012059	Mcoln3	0.0264	0.1950	0.2265	0.0126	0.1640	0.3591	0.1083	0.2238	0.1700	0.4560	0.2883	0.0306
NM_001014064	Pars2	0.8772	0.7647	0.8172	0.4873	0.4778	0.8284	0.7825	0.4629	0.6079	0.9117	0.7871	0.8019
NM_001014040	Tars2	0.0461	0.0493	0.0288	0.0335	0.0132	0.2007	0.0912	0.1095	0.0446	0.0984	0.1756	0.0442
NM_001012062	Map3k7ip2	0.7948	0.2966	0.9836	0.2857	0.4137	0.4584	0.7880	0.6068	0.9017	0.7367	0.8715	0.8053
NM_001014145	Cdyl	0.0145	0.2268	0.7670	0.0806	0.0237	0.0089	0.0957	0.0326	0.0537	0.0932	0.0821	0.2583

NM_001013909	Wdr70	0.0988	0.5974	0.1831	0.1159	0.4469	0.0460	0.1559	0.3918	0.8001	0.8579	0.3958	0.4198
NM_001014049	Sel1l2	0.0883	0.0289	0.0371	0.1137	0.6167	0.1130	0.1828	0.0618	0.1768	0.3883	0.0090	0.3089
NM_001024320	Hyal6	0.3817	0.0581	0.3774	0.5061	0.1800	0.3688	0.3974	0.3824	0.4857	0.5651	0.8158	0.0811
NM_001011964	Mapkap1	0.1043	0.6403	0.3414	0.1511	0.5209	0.2260	0.7059	0.1073	0.2249	0.1979	0.2994	0.0922
NM_001012067	Grwd1	0.4938	0.7135	0.4736	0.4343	0.2151	0.4018	0.5048	0.3036	0.5954	0.6738	0.2326	0.2473
NM_001012743	Pip5k1b	0.4211	0.6129	0.6553	0.4732	0.2913	0.2898	0.3538	0.1989	0.3714	0.8067	0.3558	0.7789
NM_001012069	Mtl5	0.5738	0.2268	0.2993	0.6269	0.2265	0.3021	0.4466	0.3061	0.5471	0.7588	0.1206	0.2247
NM_001011969	Strap	0.4314	0.9752	0.8131	0.8878	0.1865	0.3030	0.9431	0.5208	0.8971	0.9596	0.5778	0.7965
NM_001013184	Hmgn1	0.5670	0.9900	0.7597	0.7602	0.9677	0.8930	0.9895	0.9182	0.8623	0.9434	0.9449	0.4144
NM_001011929	Sh3glb1	0.2554	0.3438	0.6073	0.7854	0.6931	0.4463	0.5368	0.4542	0.4030	0.7797	0.6292	0.3357
NM_001013870	Trmt1	0.5012	0.1118	0.0937	0.0606	0.8864	0.9214	0.2955	0.9449	0.4879	0.0751	0.2448	0.7064
NM_001025684	Pex11b	0.3495	0.9761	0.7749	0.7787	0.6495	0.6437	0.8314	0.7260	0.9026	0.5557	0.7092	0.8564
NM_001025122	Cyhr1	0.7653	0.8063	0.6324	0.7953	0.7989	0.7396	0.8942	0.7202	0.3995	0.7879	0.8049	0.2448
NM_001012143	Jmjd6	0.2445	0.2083	0.4572	0.4990	0.2915	0.4713	0.4647	0.3234	0.3150	0.4291	0.5123	0.2376
NM_001013945	Ighg	0.1425	0.1483	0.1176	0.0800	0.4837	0.0934	0.4189	0.3054	0.5675	0.5229	0.1753	0.2526
NM_001012042	Xkr6	0.3566	0.4419	0.1746	0.3603	0.1662	0.2359	0.4027	0.4011	0.5540	0.8848	0.5433	0.8689
NM_001011942	Cnnm2	0.4629	0.3033	0.8238	0.8980	0.9411	0.9212	0.3421	0.9846	0.1194	0.0604	0.2418	0.9629
NM_001012046	Spry2	0.4471	0.9900	0.9938	0.9568	0.8586	0.1424	0.0573	0.6303	0.9827	0.9743	0.9209	0.9821
NM_001012461	Dntt	0.7196	0.2362	0.7088	0.7641	0.5998	0.4405	0.9106	0.7186	0.7349	0.7598	0.7579	0.8275
NM_001012049	Mfap3l	0.3816	0.3085	0.0632	0.1114	0.4660	0.7056	0.1219	0.1523	0.6089	0.4666	0.5242	0.8810
NM_001013907	Dnajb12	0.7357	0.7399	0.8003	0.2741	0.3488	0.8150	0.6383	0.5323	0.8810	0.2304	0.3151	0.7982
NM_001011949	Ccna1	0.0127	0.3615	0.6622	0.0885	0.0360	0.0213	0.2710	0.0142	0.0916	0.0770	0.1278	0.0131
NM_001011950	Dap3	0.2230	0.7696	0.0515	0.7437	0.6534	0.6212	0.1866	0.6997	0.2330	0.7400	0.3373	0.1623
NM_001012051	Zfp367	0.4465	0.8518	0.6717	0.9761	0.9354	0.9689	0.9479	0.4656	0.9252	0.8943	0.9795	0.5497
NM_001011952	Slc39a8	0.6697	0.7954	0.9036	0.9315	0.9594	0.5805	0.9064	0.6686	0.5817	0.7907	0.5641	0.0089
NM_001012054	Calml3	0.5063	0.3444	0.4194	0.5346	0.5454	0.2225	0.5197	0.4422	0.6559	0.1980	0.2406	0.3659
NM_001012055	Cdh16	0.7482	0.0518	0.0216	0.1177	0.1188	0.1423	0.0953	0.1592	0.2709	0.2288	0.1118	0.5275
NM_001012746	Rcan3	0.5823	0.5393	0.6576	0.6203	0.4343	0.3502	0.5757	0.4044	0.4490	0.4651	0.5853	0.6884
NM_001012060	Rnf146	0.5029	0.2353	0.9980	0.9071	0.1394	0.9512	0.1274	0.1097	0.4933	0.2245	0.8842	0.8555
NM_001013990	Srd5a3	0.8044	0.7482	0.8768	0.7647	0.8743	0.8150	0.5461	0.8840	0.2937	0.8611	0.4347	0.9816
NM_001024323	Fam71f2	0.0973	0.0947	0.1167	0.4991	0.2897	0.1073	0.6781	0.0995	0.0546	0.2163	0.3676	0.1518
NM_001035007	Mbtps2	0.4867	0.4593	0.5712	0.0509	0.0603	0.5076	0.0820	0.1894	0.8143	0.0734	0.6788	0.4608
NM_001011965	Stom	0.5202	0.2314	0.4106	0.3996	0.6261	0.3938	0.7307	0.6654	0.6311	0.4623	0.2086	0.5902
NM_001011966	Smarcd3	0.6486	0.4927	0.9129	0.7208	0.8989	0.7130	0.8929	0.8382	0.9048	0.7765	0.7803	0.9631
NM_001013083	Cpa2	0.5704	0.6178	0.7678	0.5971	0.5994	0.5948	0.4949	0.4697	0.5169	0.6732	0.6208	0.4071
NM_001012070	Gpr44	0.3788	0.0939	0.6118	0.0358	0.2991	0.4309	0.3220	0.4028	0.1330	0.5211	0.1382	0.4420
NM_001011970	Tm7sf3	0.1713	0.4727	0.3111	0.3177	0.2746	0.6823	0.4517	0.5253	0.5256	0.4266	0.0584	0.1672
NM_001012072	Ppp1r3c	0.5478	0.5266	0.8842	0.9024	0.8532	0.9311	0.9491	0.7893	0.6207	0.6122	0.2129	0.5158
NM_001012075	Tspyl4	0.6644	0.1628	0.8269	0.6448	0.5935	0.6090	0.1410	0.4840	0.4468	0.3709	0.4300	0.5337
NM_001013034	Glrx2	0.0761	0.7538	0.5963	0.4200	0.9792	0.6612	0.3621	0.4709	0.2842	0.4647	0.7924	0.6491
NM_001011978	Dhdds	0.3002	0.0549	0.4635	0.0899	0.3428	0.0142	0.0691	0.1577	0.0190	0.1207	0.1470	0.1151
NM_001011973	Actl7a	0.5360	0.5438	0.7236	0.2810	0.2442	0.7152	0.7216	0.4846	0.4197	0.4639	0.8457	0.5957
NM_001011974	Akap2	0.0023	0.1500	0.0770	0.0078	0.0013	0.0863	0.0756	0.2334	0.0735	0.0742	0.1875	0.2286
NM_001012078	Sema4a	0.3646	0.3109	0.3505	0.5082	0.3811	0.7828	0.6543	0.2905	0.5125	0.4855	0.4817	0.6814
NM_001012079	Arhgef2	0.4119	0.7635	0.3190	0.9794	0.9344	0.9682	0.9869	0.9085	0.9809	0.8929	0.9278	0.9881
NM_001011980	Ppan	0.8171	0.9519	0.0237	0.0199	0.8178	0.7946	0.6078	0.8642	0.7474	0.8895	0.7386	0.4552
NM_001013089	Galt	0.0428	0.1351	0.1342	0.0561	0.0789	0.0332	0.0320	0.0566	0.0711	0.0554	0.1173	0.0020
NM_001013185	Cabc1	0.9822	0.7955	0.9411	0.7648	0.8060	0.9850	0.9824	0.7477	0.9147	0.8253	0.8311	0.9542
NM_001011981	Snx17	0.4385	0.4682	0.6479	0.8364	0.7291	0.3461	0.7887	0.9231	0.9610	0.6754	0.7967	0.5249

NM_001034074	Tpm1	0.4367	0.7303	0.6371	0.6080	0.4992	0.4851	0.6099	0.4830	0.4713	0.7143	0.3045	0.7344
NM_001025405	Ruvbl2	0.4226	0.5111	0.4804	0.2262	0.2544	0.2076	0.2302	0.2202	0.2671	0.5669	0.3909	0.2987
NM_001017459	Mdm1	0.2890	0.6539	0.1308	0.1145	0.9021	0.8736	0.1039	0.5420	0.9136	0.0468	0.4135	0.0546
NM_001011983	Coq6	0.2250	0.1142	0.1428	0.1131	0.0511	0.0195	0.1877	0.0469	0.0862	0.1734	0.1185	0.1549
NM_001014117	Ublcp1	0.4274	0.9249	0.3881	0.5020	0.2535	0.5011	0.1238	0.6463	0.1744	0.5459	0.6614	0.3744
NM_001025121	Cdkl1	0.1462	0.6356	0.1394	0.6554	0.1494	0.3298	0.1497	0.5297	0.3358	0.6051	0.3063	0.6793
NM_001013221	Rabl2b	0.5237	0.3640	0.5781	0.9472	0.7820	0.6550	0.9444	0.7427	0.9920	0.7525	0.6995	0.9259
NM_001013190	Rad23a	0.1080	0.0442	0.3771	0.6582	0.1565	0.0777	0.0355	0.2182	0.0163	0.0408	0.0167	0.2864
NM_001012085	Stam2	0.5273	0.1078	0.2101	0.0968	0.4241	0.4054	0.1173	0.5371	0.0410	0.2808	0.5494	0.1013
NM_001013109	Dapk2	0.5774	0.2312	0.4418	0.5164	0.5030	0.2656	0.5469	0.2468	0.2946	0.2857	0.6091	0.2344
NM_001013097	Rnaseh1	0.8338	0.8836	0.6524	0.9263	0.8465	0.9600	0.6422	0.9452	0.8896	0.8660	0.9606	0.7391
NM_001012086	Cytip	0.0798	0.0238	0.9802	0.0529	0.7657	0.8494	0.3579	0.7689	0.1207	0.0068	0.1877	0.7854
NM_001012161	Tbce	0.7678	0.7623	0.2931	0.0882	0.7310	0.8012	0.1817	0.7558	0.9563	0.7519	0.8973	0.5466
NM_001013960	Acot9	0.0424	0.2278	0.7060	0.2010	0.1614	0.0917	0.2745	0.3115	0.0955	0.2097	0.0660	0.0575
NM_001014104	Metrnl	0.3873	0.7532	0.6913	0.1641	0.0291	0.0324	0.8365	0.2822	0.2477	0.7644	0.7814	0.0365
NM_001011988	Cnot2	0.9182	0.5400	0.4822	0.0715	0.4702	0.7805	0.6169	0.3546	0.7071	0.3862	0.4843	0.2257
NM_001012100	Slc7a13	0.5140	0.2117	0.2896	0.3924	0.0648	0.3326	0.3578	0.3096	0.0365	0.2192	0.2673	0.2003
NM_001012089	Dusp2	0.7840	0.1735	0.1475	0.6351	0.4351	0.6062	0.2448	0.2480	0.5396	0.6455	0.1911	0.7667
NM_001012090	Zc3h8	0.8053	0.7646	0.6637	0.4949	0.8459	0.8340	0.1835	0.7891	0.7382	0.8915	0.8517	0.8064
NM_001014240	Akr1c13	0.8076	0.5068	0.7917	0.5733	0.8847	0.9017	0.8721	0.8769	0.3177	0.3607	0.5237	0.4920
NM_001014195	Tmem214	0.8948	0.8124	0.2377	0.7114	0.8950	0.2889	0.8543	0.8645	0.8548	0.9213	0.5515	0.6280
NM_001014021	Ccdc81	0.2744	0.8064	0.3869	0.5029	0.7361	0.0475	0.4225	0.3697	0.1379	0.3905	0.6552	0.4924
NM_001011989	Gns	0.7407	0.0962	0.5943	0.1180	0.7490	0.7491	0.6277	0.6013	0.0729	0.0030	0.0392	0.1453
NM_001012101	Coro2a	0.2579	0.3026	0.4899	0.2783	0.0706	0.1273	0.3555	0.2871	0.6751	0.8311	0.5173	0.6702
NM_001013207	Rbm39	0.7886	0.6888	0.7618	0.1126	0.7999	0.8396	0.1796	0.8982	0.7002	0.8529	0.8278	0.8620
NM_001009474	Pir	0.5802	0.0540	0.8252	0.6021	0.0083	0.1147	0.8553	0.1894	0.1513	0.0187	0.2178	0.7025
NM_001014204	Dnajc22	0.4306	0.5220	0.2680	0.2260	0.4147	0.5242	0.6521	0.3388	0.3334	0.3811	0.4380	0.4784
NM_001013226	Tesp1	0.2860	0.3767	0.2641	0.2259	0.3924	0.5549	0.5947	0.4043	0.2968	0.3577	0.3025	0.2499
NM_001014120	Fkbp10	0.1369	0.0969	0.0420	0.1472	0.1881	0.1477	0.1090	0.0110	0.1095	0.0013	0.1108	0.1883
NM_001013105	Fkbp11	0.3660	0.3745	0.4896	0.4616	0.6301	0.6763	0.7206	0.3911	0.6506	0.6310	0.2062	0.6293
NM_001012103	Trim32	0.3927	0.6614	0.4859	0.5881	0.5904	0.3289	0.6850	0.5307	0.2981	0.6895	0.7134	0.5332
NM_001011994	Gga1	0.6596	0.1888	0.0820	0.4220	0.8205	0.8119	0.2528	0.6463	0.5227	0.5837	0.1526	0.3338
NM_001001509	Hdmcp	0.1471	0.5906	0.4553	0.8500	0.6317	0.5182	0.3832	0.7314	0.3541	0.2244	0.4865	0.1981
NM_001012105	Zbtb17	0.1982	0.4164	0.1227	0.1702	0.2346	0.2176	0.3715	0.2126	0.0782	0.2287	0.1661	0.3006
NM_001001512	Gtf2i	0.9736	0.8861	0.9252	0.8914	0.9860	0.9921	0.4944	0.9652	0.9734	0.9582	0.8566	0.9613
NM_001012095	Rnf32	0.2974	0.3619	0.7454	0.6658	0.5889	0.6786	0.6695	0.6486	0.4991	0.8288	0.4122	0.6301
NM_001011995	Tuba1c	0.4115	0.6814	0.6784	0.7352	0.4947	0.7202	0.6010	0.6310	0.6031	0.6222	0.6047	0.3933
NM_001012107	Gpr157	0.4877	0.2475	0.0498	0.4114	0.6701	0.7118	0.2791	0.5706	0.4531	0.2052	0.3382	0.3613
NM_001012473	Polr2c	0.4148	0.2958	0.2359	0.7165	0.5077	0.4965	0.3370	0.5804	0.2136	0.7052	0.3849	0.2301
NM_001012106	Mad2l2	0.0362	0.8616	0.0988	0.6609	0.6957	0.2200	0.7233	0.6897	0.8409	0.9623	0.6355	0.1451
NM_001014026	Clrn3	0.8142	0.8379	0.8504	0.3139	0.7397	0.7120	0.8178	0.7243	0.5275	0.8513	0.2955	0.8038
NM_001013925	Nub1	0.0181	0.2322	0.9180	0.2014	0.0443	0.3461	0.1688	0.0292	0.1163	0.2067	0.2089	0.7477
NM_001011997	Tmod3	0.4895	0.6681	0.3830	0.5768	0.5397	0.2648	0.3845	0.2969	0.2867	0.4324	0.4390	0.2166
NM_001014002	Mak16	0.4362	0.7372	0.0834	0.1734	0.5654	0.1776	0.0850	0.7350	0.8357	0.7594	0.6533	0.1398
NM_001009413	Krcc1	0.7971	0.8921	0.9533	0.9597	0.9151	0.7831	0.9794	0.7980	0.7416	0.7864	0.9816	0.7700
NM_001013128	Dusp18	0.8108	0.6084	0.5132	0.4987	0.8214	0.8825	0.7750	0.6711	0.3897	0.5578	0.6818	0.8814
NM_001013126	Cyb5r1	0.6707	0.9201	0.8369	0.8802	0.8246	0.9110	0.5771	0.9809	0.9473	0.9941	0.7241	0.9729
NM_001079940	Habp4	0.4957	0.4216	0.5822	0.0427	0.0832	0.0398	0.6612	0.1457	0.3007	0.4138	0.4256	0.7712
NM_001013888	Klkbl4	0.3921	0.2596	0.6474	0.3041	0.4140	0.3487	0.3548	0.3819	0.3161	0.4139	0.1977	0.5401

NM_001001799	Tmem35	0.5245	0.2097	0.4863	0.5758	0.7914	0.6898	0.8148	0.4327	0.6916	0.6806	0.6291	0.4941
NM_001011999	Morf4l1	0.6513	0.4054	0.3219	0.6219	0.2566	0.2075	0.3414	0.2200	0.4523	0.4514	0.4075	0.2897
NM_001025711	Atg4b	0.0109	0.2151	0.1535	0.2471	0.2220	0.3206	0.1741	0.1748	0.2156	0.1957	0.2912	0.2020
NM_001024897	Ehd2	0.3881	0.4431	0.2560	0.2878	0.5260	0.5811	0.3111	0.6225	0.6003	0.3733	0.3424	0.3682
NM_001013139	Fars2	0.3370	0.3101	0.0789	0.4291	0.4273	0.1232	0.6452	0.0486	0.3129	0.3280	0.2197	0.0462
NM_001012118	Osr2	0.5639	0.0877	0.4514	0.0813	0.2495	0.0755	0.0574	0.6085	0.1638	0.2133	0.4264	0.3988
NM_001012116	Spag1	0.8823	0.0138	0.3058	0.2716	0.9160	0.9078	0.4474	0.7185	0.4683	0.5891	0.8784	0.0947
NM_001012113	Ptdss1	0.7534	0.2162	0.7052	0.4232	0.4488	0.7431	0.1603	0.7911	0.5436	0.3238	0.8624	0.3060
NM_001014029	Fam122a	0.5512	0.6490	0.6710	0.9078	0.7044	0.8811	0.8091	0.4916	0.7129	0.7925	0.8489	0.8994
NM_001013929	Atp6v1h	0.0177	0.0489	0.0430	0.0900	0.0186	0.0166	0.0302	0.0070	0.0106	0.0199	0.0342	0.0682
NM_001013150	Map3k11	0.7321	0.5564	0.0473	0.9631	0.6183	0.5305	0.6563	0.8332	0.9425	0.9643	0.3829	0.6716
NM_001012123	C1qtnf5	0.0186	0.0869	0.9277	0.8518	0.8369	0.9245	0.5680	0.9753	0.1946	0.1355	0.0462	0.0725
NM_001002279	Rnf166	0.0235	0.0362	0.9116	0.1039	0.7211	0.4608	0.3861	0.6102	0.3520	0.1673	0.2683	0.3490
NM_001012125	Loxl1	0.4964	0.5342	0.4476	0.8608	0.1244	0.0733	0.7630	0.2336	0.2730	0.1444	0.3445	0.3175
NM_001013158	B3galnt1	0.5247	0.2382	0.1512	0.9744	0.7360	0.6979	0.0385	0.6530	0.1353	0.1368	0.0381	0.0050
NM_001012128	Zfp105	0.4215	0.4920	0.3050	0.2257	0.2501	0.5009	0.2297	0.5164	0.3634	0.3830	0.4083	0.3796
NM_001013191	Cbfb	0.4564	0.3226	0.9991	0.9838	0.9969	0.9922	0.8918	0.9963	0.4175	0.0589	0.3027	0.0907
NM_001002290	Kprp	0.7570	0.7859	0.1319	0.1411	0.2305	0.0718	0.5591	0.1160	0.2263	0.6609	0.7613	0.0218
NM_001012146	Dgcr2	0.6201	0.5551	0.3790	0.6613	0.5696	0.4615	0.5358	0.3844	0.5684	0.4594	0.4696	0.5570
NM_001013164	Ccbl1	0.0202	0.0643	0.0811	0.8741	0.0100	0.1253	0.5992	0.0685	0.0551	0.1019	0.0129	0.1303
NM_001007752	Mns1	0.4143	0.7521	0.0911	0.0228	0.1107	0.1907	0.5115	0.1566	0.0790	0.7497	0.5984	0.0529
NM_001012133	Sp140	0.4872	0.1193	0.0090	0.0518	0.1587	0.0755	0.0414	0.4592	0.8337	0.4143	0.2522	0.1523
NM_001014033	41,162.0000	0.0191	0.4356	0.7625	0.7464	0.1567	0.4807	0.7434	0.0224	0.4296	0.0201	0.6535	0.7038
NM_001012135	Mlph	0.1213	0.1094	0.2833	0.4324	0.5896	0.6512	0.3461	0.2935	0.1473	0.3303	0.0365	0.6155
NM_001013910	Gnb4	0.4468	0.6104	0.1654	0.7643	0.1926	0.2221	0.4623	0.4134	0.1651	0.4094	0.0940	0.0796
NM_001012139	Plscr3	0.5932	0.8813	0.2956	0.8850	0.6117	0.6020	0.8845	0.4985	0.6275	0.5752	0.4550	0.3977
NM_001013197	Stk19	0.6158	0.7783	0.0560	0.1192	0.4353	0.4475	0.5837	0.1113	0.6214	0.8004	0.1453	0.1387
NM_001012144	Tfg	0.9204	0.6308	0.3829	0.8747	0.2873	0.3306	0.6371	0.4349	0.9036	0.6623	0.9875	0.9478
NM_001033914	Epn2	0.9682	0.9820	0.9962	0.7430	0.9552	0.9680	0.8495	0.8760	0.9496	0.9980	0.9750	0.9887
NM_012574	Grin2b	0.7572	0.3893	0.3437	0.7845	0.8882	0.7932	0.8510	0.5121	0.6382	0.4372	0.2772	0.5990
NM_001037441	Klrc1	0.4959	0.4931	0.3491	0.4912	0.4340	0.2374	0.3588	0.2501	0.4590	0.3560	0.3086	0.4053
NM_001030031	Lta4h	0.1065	0.0038	0.0780	0.3989	0.2709	0.4265	0.2909	0.1246	0.0018	0.0020	0.0380	0.0501
NM_001002818	Fkbpl	0.1034	0.3055	0.4933	0.5004	0.0044	0.0051	0.0744	0.0171	0.0064	0.0318	0.1388	0.0123
NM_001012151	Stx18	0.5412	0.2201	0.5801	0.6264	0.3363	0.4373	0.3327	0.6177	0.3717	0.6021	0.2375	0.3776
NM_001008349	Nfkb2	0.5397	0.1842	0.0071	0.0411	0.2577	0.2710	0.1203	0.3232	0.9547	0.2826	0.2155	0.5893
NM_001012156	Cdadc1	0.7456	0.4085	0.5964	0.9117	0.8276	0.8052	0.3205	0.8057	0.6993	0.4976	0.7740	0.4026
NM_001012155	Tm9sf1	0.8158	0.2166	0.3658	0.7429	0.3255	0.8151	0.2905	0.4511	0.7425	0.2814	0.8332	0.8235
NM_001012159	Upf3a	0.5949	0.2417	0.5813	0.6872	0.8853	0.0648	0.9473	0.7168	0.3163	0.6826	0.3425	0.0110
NM_001012160	Gkap1	0.7803	0.8913	0.9817	0.8268	0.6457	0.2782	0.1337	0.5851	0.8659	0.9339	0.8637	0.8378
NM_001024256	Med4	0.7404	0.7706	0.9091	0.8333	0.4907	0.8784	0.9489	0.3639	0.3019	0.5014	0.6148	0.2310
NM_001013110	Tf	0.3399	0.1981	0.2694	0.2257	0.2393	0.5553	0.4116	0.4095	0.4717	0.3091	0.2988	0.2619
NM_001012163	Lims2	0.5710	0.2246	0.1210	0.1999	0.2771	0.2160	0.5374	0.5044	0.9165	0.3253	0.5719	0.7944
NM_001012165	Cox4nb	0.2612	0.8525	0.2850	0.8409	0.9631	0.2452	0.4388	0.8034	0.5692	0.4099	0.6025	0.0089
NM_001013980	Cyyr1	0.6909	0.7805	0.8694	0.5186	0.5316	0.5810	0.5514	0.2203	0.7378	0.8773	0.4442	0.7492
NM_001014019	Hnrnph2	0.8442	0.7673	0.6957	0.7969	0.7932	0.8943	0.8407	0.9213	0.9263	0.9162	0.8442	0.7303
NM_001002855	Prm3	0.1996	0.0993	0.2767	0.0760	0.1045	0.5098	0.5171	0.1569	0.4012	0.3526	0.6867	0.5531
NM_001012166	Abcb10	0.9361	0.4948	0.5648	0.7807	0.5231	0.6466	0.1719	0.4045	0.5141	0.3951	0.3733	0.5828
NM_001003409	LOC298116	0.2341	0.3269	0.4752	0.4797	0.6545	0.2431	0.3510	0.3857	0.6329	0.3850	0.3701	0.2863
NM_001012168	Tulp2	0.0420	0.0577	0.1366	0.1545	0.3074	0.1239	0.0885	0.1427	0.1530	0.0962	0.1783	0.4177

NM_001009542	Pdcd10	0.8779	0.6961	0.9611	0.6194	0.6951	0.4486	0.8522	0.5790	0.4325	0.9609	0.9841	0.7306
NM_001003706	LOC360228	0.1224	0.0303	0.0720	0.3129	0.5706	0.1321	0.0511	0.2220	0.0238	0.0206	0.2980	0.0084
NM_001012169	Zfp143	0.6403	0.6932	0.7893	0.5891	0.5387	0.1562	0.6202	0.2579	0.5068	0.7285	0.9069	0.5956
NM_001012170	Nap1l4	0.5660	0.3609	0.4639	0.2998	0.4028	0.3945	0.4347	0.1072	0.4060	0.4455	0.2414	0.3565
NM_001012171	Bscl2	0.2984	0.3326	0.3800	0.3757	0.4230	0.4949	0.2293	0.5366	0.2597	0.3851	0.3878	0.4389
NM_001012172	Rfx3	0.3568	0.6143	0.8481	0.7755	0.7544	0.7172	0.7424	0.7616	0.9107	0.9060	0.9213	0.9792
NM_001013984	Npl	0.1537	0.2702	0.3315	0.2789	0.1959	0.1934	0.2858	0.2685	0.4077	0.7404	0.1816	0.0980
NM_001012178	Mynn	0.5210	0.5990	0.5017	0.5354	0.7173	0.4878	0.4667	0.2442	0.3467	0.5517	0.4456	0.4398
NM_001013114	Haghl	0.1705	0.1689	0.3280	0.1556	0.3776	0.2534	0.1104	0.9133	0.8066	0.9141	0.0848	0.8195
NM_001012201	Cadm1	0.6298	0.7990	0.7052	0.5501	0.7250	0.4583	0.8776	0.6110	0.6496	0.4906	0.4958	0.4549
NM_001012351	Cpsf4	0.4112	0.4804	0.7217	0.5372	0.6010	0.6892	0.5815	0.7255	0.4477	0.1927	0.4452	0.2275
NM_001034921	Nars2	0.8759	0.6077	0.7162	0.0660	0.6277	0.8198	0.6030	0.5466	0.8455	0.7510	0.7958	0.6471
NM_001003975	G4	0.1153	0.5973	0.5335	0.3075	0.5366	0.8586	0.2672	0.2006	0.9762	0.5277	0.3093	0.1148
NM_001014186	Orc5l	0.3460	0.8211	0.4364	0.3304	0.9569	0.9941	0.1686	0.9597	0.8633	0.9642	0.8460	0.9733
NM_001014051	Ttll9	0.3125	0.5171	0.5360	0.4179	0.4931	0.6081	0.7241	0.3121	0.1236	0.4421	0.3703	0.6285
NM_001013249	Dhx30	0.7765	0.6192	0.0624	0.1535	0.6731	0.6831	0.0366	0.8805	0.4591	0.5502	0.9031	0.4257
NM_001024340	LOC500392	0.9221	0.8190	0.6416	0.7302	0.9009	0.6611	0.0983	0.7213	0.3515	0.6866	0.9220	0.6019
NM_001004069	Ng23	0.2650	0.2596	0.3953	0.3505	0.5795	0.3705	0.5512	0.3343	0.2061	0.2206	0.2643	0.6859
NM_001014042	Mybphl	0.6942	0.4669	0.7449	0.8330	0.7216	0.6500	0.7897	0.8216	0.5708	0.8646	0.9863	0.6533
NM_001012184	Rbms1	0.0726	0.0333	0.2383	0.9548	0.1040	0.5992	0.3600	0.1359	0.1319	0.0704	0.0547	0.8702
NM_001012187	Klhl7	0.3264	0.1460	0.8181	0.7804	0.5215	0.3873	0.5267	0.5629	0.6269	0.5280	0.7192	0.5067
NM_001012191	Dtnb	0.5861	0.4122	0.2153	0.4249	0.7388	0.8623	0.2156	0.7043	0.6999	0.1543	0.3742	0.4950
NM_001013985	Sccpdh	0.0712	0.0803	0.9574	0.0142	0.3881	0.4397	0.1573	0.8161	0.1120	0.0486	0.2579	0.2074
NM_001013157	Nnt	0.0822	0.0416	0.3502	0.2161	0.3055	0.5528	0.4248	0.0702	0.0915	0.0107	0.2067	0.0694
NM_001017492	Apobec4	0.0588	0.0898	0.3032	0.0914	0.1003	0.0948	0.5597	0.0512	0.0822	0.2515	0.0551	0.0908
NM_001012199	Rangap1	0.6541	0.9765	0.1737	0.8841	0.8466	0.9642	0.9218	0.9558	0.8702	0.9249	0.7437	0.4916
NM_001012211	Phf7	0.7588	0.5579	0.9489	0.3542	0.6949	0.5851	0.7261	0.7794	0.7052	0.8140	0.7424	0.8804
NM_001012213	Sfxn1	0.8172	0.8596	0.9749	0.8877	0.7081	0.7718	0.7888	0.6303	0.8880	0.9748	0.7544	0.9258
NM_001013889	LOC291863	0.1607	0.2709	0.2545	0.3493	0.4039	0.1887	0.3843	0.2884	0.1389	0.6341	0.8179	0.2535
NM_001012221	Oxct2a	0.6311	0.5705	0.2921	0.4854	0.8084	0.7331	0.6012	0.3718	0.3920	0.4339	0.6703	0.3369
NM_001012220	Catsper2	0.3923	0.2880	0.3084	0.2255	0.3310	0.3455	0.2290	0.2194	0.3918	0.3123	0.3286	0.2033
NM_001013954	Lca5	0.7489	0.6718	0.7125	0.1960	0.6829	0.5170	0.4344	0.3138	0.5074	0.0603	0.6064	0.4656
NM_001004091	Il17re	0.5201	0.0673	0.1268	0.9155	0.5560	0.3331	0.8670	0.8669	0.2354	0.0100	0.0344	0.9396
NM_001004094	Psma3l	0.5402	0.2741	0.3717	0.4764	0.6593	0.2797	0.4302	0.5350	0.6611	0.3188	0.6443	0.2813
NM_001014050	Fam110a	0.0704	0.1281	0.0344	0.0763	0.2521	0.0829	0.1130	0.2049	0.0899	0.0555	0.0397	0.2769
NM_001014054	Tmem168	0.9032	0.8476	0.9941	0.9650	0.9830	0.9555	0.9455	0.9791	0.6225	0.9224	0.9609	0.8689
NM_001014055	Tmem209	0.2425	0.5916	0.0515	0.2843	0.1443	0.7716	0.1233	0.3817	0.2554	0.4407	0.1588	0.1362
NM_001014058	Usp18	0.0686	0.0537	0.3119	0.1189	0.0489	0.1337	0.2805	0.0371	0.1832	0.0746	0.2139	0.9166
NM_001015037	Kat3	0.2975	0.4666	0.2753	0.3160	0.2637	0.3459	0.4900	0.4933	0.5269	0.4397	0.3478	0.2438
NM_001004102	ST7	0.3686	0.9587	0.6210	0.6164	0.7870	0.7812	0.2324	0.7415	0.0166	0.1013	0.2242	0.0747
NM_001034130	Iqub	0.8797	0.7204	0.6164	0.1325	0.4617	0.7270	0.1867	0.3098	0.7228	0.2860	0.7992	0.6599
NM_001013211	Prp2	0.5326	0.3606	0.1661	0.7718	0.3994	0.1688	0.0995	0.1171	0.3108	0.0594	0.5610	0.6021
NM_001013969	Slc46a1	0.6012	0.2363	0.6936	0.7074	0.8077	0.8944	0.8452	0.8230	0.8099	0.5761	0.1535	0.3072
NM_001014053	Lrrc26	0.1760	0.1215	0.1177	0.4368	0.3190	0.2001	0.1999	0.3174	0.2110	0.1325	0.6125	0.0893
NM_001013953	Ccdc153	0.4498	0.4669	0.4245	0.7280	0.3398	0.4502	0.9293	0.4186	0.2437	0.6295	0.0773	0.6846
NM_001014136	Ngly1	0.9438	0.8938	0.7804	0.8409	0.9362	0.9982	0.6333	0.7296	0.6549	0.8600	0.6505	0.6885
NM_001012462	Supv3l1	0.8961	0.7331	0.2820	0.2442	0.7951	0.6307	0.5848	0.7448	0.6177	0.6816	0.8293	0.6769
NM_001004201	Lrrc46	0.6025	0.5304	0.4726	0.2396	0.3576	0.1518	0.7597	0.4438	0.4586	0.5607	0.5369	0.6169
NM_001013987	Rpap2	0.6407	0.6607	0.8281	0.5540	0.6293	0.2823	0.7372	0.5413	0.6572	0.8039	0.2271	0.4197

NM_001004204	MGC94190	0.6034	0.0486	0.6754	0.0353	0.0103	0.3176	0.1888	0.2392	0.3936	0.1116	0.2137	0.1427
NM_001009620	Tmem204	0.7188	0.8257	0.7479	0.8797	0.9526	0.5211	0.8452	0.9310	0.8672	0.8378	0.6671	0.3127
NM_001012357	Ccl9	0.6299	0.6218	0.2134	0.3697	0.4256	0.3672	0.4673	0.7409	0.3753	0.4415	0.3906	0.7391
NM_001014161	Idh2	0.4167	0.1547	0.6295	0.8285	0.2507	0.2318	0.5459	0.1351	0.5158	0.3400	0.3217	0.3749
NM_001012459	Clec11a	0.2443	0.7446	0.0857	0.1397	0.6176	0.4258	0.4516	0.1863	0.6221	0.4662	0.2140	0.7731
NM_001012465	Lman1l	0.5950	0.5392	0.5764	0.2925	0.4356	0.5670	0.7946	0.0768	0.1308	0.5622	0.5694	0.6471
NM_001012468	Rab18	0.9257	0.0860	0.9391	0.7418	0.8197	0.9730	0.6892	0.7262	0.3192	0.3287	0.1589	0.6939
NM_001012469	Il21r	0.7079	0.5947	0.6983	0.5721	0.4081	0.7756	0.3913	0.5717	0.8733	0.4941	0.3773	0.4692
NM_001012471	Zbtb38	0.4480	0.0515	0.3956	0.8207	0.3619	0.6499	0.3880	0.5771	0.1676	0.0363	0.4791	0.7088
NM_001013971	Cuedc1	0.7851	0.3836	0.3161	0.7687	0.6794	0.7627	0.4820	0.9771	0.2070	0.5601	0.0704	0.2343
NM_001004219	RGD1303117	0.5804	0.2694	0.1584	0.0827	0.7969	0.3044	0.0126	0.6708	0.7785	0.7082	0.2192	0.0632
NM_001004221	MGC93975	0.9645	0.4858	0.7905	0.8647	0.9047	0.9588	0.3605	0.9356	0.2915	0.8593	0.1635	0.4196
NM_001039686	Gkn2	0.5990	0.5332	0.2501	0.5154	0.4747	0.5101	0.3570	0.4498	0.2306	0.6150	0.8397	0.6318
NM_212510	Rpl18a	0.2073	0.4346	0.4295	0.3247	0.4704	0.1279	0.5548	0.3029	0.8375	0.8085	0.5878	0.5798
NM_212494	Cuta	0.4609	0.1978	0.2077	0.2253	0.4042	0.2866	0.2287	0.4879	0.1868	0.4681	0.1973	0.2635
NM_199498	Krt19	0.0450	0.8204	0.4489	0.3278	0.0525	0.0117	0.4127	0.0278	0.6989	0.0662	0.7504	0.7087
NM_001024252	Pcaf	0.4465	0.0604	0.9814	0.9556	0.3720	0.9205	0.4444	0.3267	0.3492	0.3145	0.3597	0.9108
NM_001014162	RGD1309578	0.4131	0.7765	0.6773	0.3578	0.5460	0.5419	0.8165	0.2909	0.5209	0.6111	0.4960	0.2420
NM_001012737	Cdh7	0.0405	0.0911	0.1536	0.0253	0.1919	0.0793	0.0658	0.2814	0.0556	0.0456	0.0305	0.0601
NM_001012739	Fau	0.5958	0.0383	0.7272	0.0786	0.3877	0.2567	0.4236	0.4205	0.1636	0.1437	0.0306	0.2020
NM_001014006	F12	0.3154	0.1561	0.1346	0.2326	0.1108	0.1780	0.0736	0.4960	0.1402	0.0738	0.0383	0.0652
NM_001008766	Ciao1	0.0872	0.0848	0.1457	0.5570	0.3159	0.0421	0.0292	0.1535	0.2617	0.5582	0.3179	0.0370
NM_001013919	Galk2	0.3260	0.5572	0.4505	0.4422	0.6946	0.3949	0.2800	0.4856	0.2480	0.3744	0.2678	0.6472
NM_001004237	Aurkaip1	0.4914	0.8507	0.6933	0.8744	0.6439	0.1355	0.7327	0.8156	0.3414	0.8632	0.5455	0.0311
NM_001012744	Enpp5	0.9320	0.9271	0.7865	0.8351	0.7691	0.8589	0.8656	0.8213	0.9099	0.9763	0.9155	0.7871
NM_001014256	Bhmt2	0.4865	0.6628	0.2170	0.2381	0.5306	0.5566	0.2740	0.5960	0.4819	0.6070	0.6782	0.5184
NM_001014078	Wdr89	0.4096	0.1433	0.0703	0.4271	0.2014	0.4602	0.3383	0.5632	0.1463	0.2590	0.2138	0.9060
NM_001012745	Zfp422	0.6809	0.8955	0.9540	0.9927	0.9758	0.9139	0.7445	0.7514	0.9645	0.8334	0.9165	0.9018
NM_001017383	Ppil2	0.2707	0.3757	0.4077	0.3251	0.3067	0.4556	0.6921	0.7274	0.3748	0.1810	0.1971	0.3145
NM_001014073	Fam98a	0.3179	0.7960	0.1947	0.0980	0.1994	0.1906	0.4289	0.2779	0.4624	0.3933	0.3484	0.3741
NM_001004242	Arhgap8	0.0125	0.8107	0.0374	0.0866	0.0379	0.0167	0.1210	0.0698	0.8033	0.7307	0.7409	0.1138
NM_001012648	Klri2	0.4643	0.3155	0.2073	0.2250	0.4292	0.2068	0.4537	0.2190	0.3449	0.1810	0.1970	0.2032
NM_001008770	Cmbl	0.8417	0.7129	0.8914	0.7197	0.3929	0.2311	0.5059	0.8252	0.6072	0.4178	0.8622	0.4249
NM_001004244	RGD1303127	0.3809	0.3316	0.1203	0.0380	0.2432	0.1029	0.0304	0.4034	0.0140	0.0267	0.3593	0.0994
NM_001025633	Hnrnpc	0.6546	0.4429	0.2380	0.4207	0.5895	0.2573	0.4366	0.7303	0.5085	0.4566	0.4137	0.7331
NM_001004246	Ttc12	0.7963	0.6935	0.6737	0.9275	0.3033	0.6648	0.7992	0.7739	0.6618	0.5121	0.7909	0.5118
NM_001014046	Fam82a2	0.4469	0.1712	0.5691	0.2322	0.1187	0.2317	0.1806	0.0543	0.5522	0.1695	0.0633	0.7899
NM_001004248	Tmem30a	0.2125	0.3756	0.9991	0.9885	0.3943	0.8246	0.9555	0.5556	0.0236	0.2715	0.4303	0.3634
NM_001024292	LOC499330	0.1586	0.9151	0.9591	0.7099	0.8630	0.7678	0.6575	0.7590	0.4104	0.9893	0.7849	0.6465
NM_001013035	Phb2	0.7359	0.9226	0.8573	0.8171	0.9017	0.8407	0.8387	0.9074	0.9014	0.9291	0.9628	0.9293
NM_001004251	MGC94335	0.2662	0.2592	0.8928	0.9748	0.6684	0.6203	0.7955	0.5850	0.8750	0.7693	0.9732	0.7302
NM_001013038	Gdf6	0.4183	0.1989	0.2116	0.1245	0.0966	0.6532	0.0678	0.3009	0.2007	0.4141	0.3540	0.6185
NM_001014020	Tarsl2	0.6958	0.6969	0.5739	0.6375	0.7356	0.7571	0.8186	0.3429	0.7407	0.7224	0.7707	0.4144
NM_001034188	Ppil1	0.7810	0.7874	0.9885	0.7923	0.9100	0.8878	0.9127	0.7569	0.8247	0.7003	0.9802	0.4540
NM_001033926	Fhl1	0.6911	0.7219	0.9625	0.5510	0.9715	0.9663	0.2366	0.9986	0.3474	0.6532	0.7460	0.9802
NM_001024259	Bmpr1b	0.3516	0.2187	0.0682	0.1730	0.7371	0.7658	0.8329	0.1061	0.3176	0.0275	0.1418	0.0671
NM_001013042	Slc39a11	0.1238	0.5727	0.3481	0.7179	0.2575	0.4136	0.3850	0.3583	0.3363	0.1385	0.7471	0.2642
NM_001013043	Sectm1a	0.2884	0.6979	0.2094	0.5078	0.5253	0.1217	0.3245	0.4265	0.6463	0.2444	0.5544	0.2329
NM_001013045	Ccl24	0.6202	0.5044	0.4331	0.6454	0.5963	0.5266	0.6697	0.6692	0.2218	0.5720	0.5613	0.6513

NM_001013044	Cryzl1	0.6928	0.8041	0.8823	0.9711	0.5863	0.5775	0.7956	0.8867	0.8421	0.8775	0.8265	0.9370
NM_001004261	RGD1303232	0.5793	0.1201	0.3969	0.1058	0.1370	0.0262	0.7285	0.4646	0.8322	0.5077	0.4172	0.6551
NM_001004262	Cog6	0.1227	0.0770	0.7083	0.1392	0.0722	0.6217	0.1606	0.2160	0.0502	0.1088	0.7800	0.5508
NM_001024742	Ard1b	0.6304	0.4038	0.8906	0.5604	0.6913	0.8996	0.5460	0.8564	0.6182	0.7992	0.7538	0.4728
NM_001013046	Rab35	0.6817	0.6354	0.4924	0.6012	0.6618	0.6385	0.5884	0.6834	0.6515	0.6442	0.7164	0.6768
NM_001004264	Traf4af1	0.8047	0.8040	0.0213	0.8421	0.9347	0.9199	0.8223	0.8027	0.7440	0.9107	0.7921	0.1419
NM_001013048	Igfbp7	0.5874	0.0323	0.3393	0.0765	0.7924	0.8129	0.0777	0.9051	0.1679	0.2459	0.0941	0.0605
NM_001004267	Tmem106b	0.1028	0.1300	0.9083	0.7194	0.0746	0.1082	0.0177	0.0069	0.2434	0.2231	0.1770	0.9227
NM_001004268	RGD1303271	0.8644	0.4529	0.7323	0.6273	0.8088	0.8735	0.6357	0.7334	0.8816	0.5764	0.9231	0.6981
NM_001013055	Rpp40	0.0128	0.2255	0.0784	0.0481	0.0026	0.0038	0.0157	0.1404	0.0177	0.0219	0.1962	0.0054
NM_001004272	MGC94192	0.9384	0.3683	0.4644	0.4079	0.6525	0.8932	0.3529	0.7670	0.5105	0.8592	0.4786	0.3893
NM_001013060	Rit2	0.5756	0.7353	0.4621	0.7830	0.8161	0.5149	0.4706	0.7398	0.8984	0.5478	0.6093	0.2939
NM_001013058	Rbm17	0.7859	0.8635	0.8471	0.7356	0.9985	0.9939	0.8842	0.8278	0.7172	0.7846	0.7976	0.8740
NM_001106616	Csgalnact2	0.1207	0.1179	0.9322	0.5886	0.0807	0.3368	0.0293	0.0082	0.4881	0.3102	0.9550	0.8533
NM_001013059	Ndfip1	0.4658	0.1533	0.9005	0.8893	0.2548	0.6193	0.1109	0.0498	0.0596	0.0856	0.3185	0.7053
NM_001004276	Commd10	0.8220	0.9181	0.7903	0.8106	0.8983	0.8574	0.9758	0.7994	0.5720	0.9311	0.4230	0.3879
NM_001109065	RGD1563482	0.6042	0.7470	0.6974	0.9978	0.7942	0.7572	0.4280	0.8354	0.5028	0.9244	0.8076	0.2002
NM_001004278	Tsga10ip	0.2130	0.3344	0.1019	0.4235	0.1688	0.3404	0.1810	0.2317	0.1389	0.1835	0.2173	0.2401
NM_001108788	Foxp4	0.8891	0.9671	0.9910	0.9795	0.9954	0.9806	0.9790	0.9802	0.9805	0.8789	0.9154	0.9944
NM_001004281	Tbc1d20	0.5262	0.1638	0.2930	0.6580	0.0182	0.0672	0.6456	0.0201	0.6405	0.5097	0.8382	0.3107
NM_012520	Cat	0.0057	0.3196	0.7582	0.9343	0.0042	0.1917	0.2597	0.2382	0.1429	0.2519	0.1132	0.0772
NM_012530	Ckm	0.1543	0.1701	0.1546	0.1521	0.1103	0.4520	0.1097	0.5952	0.0928	0.5536	0.1397	0.4296
NM_012695	Smp2a	0.2693	0.2854	0.7292	0.3407	0.2708	0.2787	0.4852	0.2188	0.4486	0.2943	0.6038	0.2031
NM_001013070	Tspan4	0.0736	0.0615	0.7740	0.8916	0.1745	0.7037	0.3677	0.3092	0.1211	0.0550	0.0162	0.3214
NM_001013073	Btbd9	0.1105	0.0891	0.0313	0.5131	0.0318	0.0897	0.0334	0.1074	0.0266	0.0202	0.1221	0.0070
NM_001013072	Sfxn2	0.4761	0.6552	0.5989	0.5184	0.6072	0.2514	0.6003	0.6637	0.5336	0.5649	0.6323	0.5293
NM_001013076	Dnajb4	0.0007	0.0164	0.7122	0.0046	0.0155	0.0748	0.1262	0.0060	0.0187	0.0338	0.0038	0.3070
NM_001031647	Dnali1	0.8813	0.9485	0.8161	0.8044	0.9117	0.7887	0.6698	0.6900	0.5901	0.9683	0.6329	0.7281
NM_001013077	Plekha3	0.2689	0.2523	0.7612	0.0074	0.2665	0.2509	0.1464	0.0325	0.1137	0.1711	0.2158	0.9695
NM_001013080	Clic3	0.1333	0.4531	0.0236	0.1075	0.3462	0.0194	0.4678	0.3352	0.1214	0.1899	0.0885	0.1468
NM_001013079	Osbpl2	0.6303	0.6166	0.6165	0.5779	0.5237	0.5483	0.2836	0.6373	0.3227	0.6639	0.6440	0.6145
NM_001024779	Cyp2u1	0.4449	0.6579	0.6853	0.3576	0.2435	0.4707	0.3865	0.6051	0.1971	0.5320	0.1724	0.3047
NM_001013085	Snx10	0.1779	0.5001	0.9341	0.5699	0.1520	0.0693	0.0441	0.0343	0.7440	0.3372	0.0269	0.9853
NM_001013084	Akr1b10	0.3377	0.1970	0.5797	0.3987	0.5833	0.3192	0.3223	0.4204	0.2230	0.6137	0.6769	0.3719
NM_001014044	Tifa	0.0566	0.1290	0.6081	0.9624	0.8394	0.9093	0.0237	0.9285	0.0124	0.1844	0.2324	0.0695
NM_001079942	Sema3b	0.0737	0.0021	0.1053	0.0293	0.0011	0.0664	0.0324	0.0050	0.0396	0.0507	0.0023	0.0472
NM_001004447	Spata21	0.2138	0.4898	0.0853	0.1172	0.1425	0.4222	0.5819	0.2631	0.1254	0.1653	0.5688	0.1104
NM_001013088	Fhl5	0.3159	0.0078	0.0191	0.0551	0.1109	0.0386	0.3403	0.0521	0.0362	0.2829	0.1367	0.2812
NM_001013086	Capg	0.1003	0.3883	0.8118	0.7528	0.0558	0.2548	0.7479	0.0609	0.4184	0.7831	0.2902	0.8419
NM_001033757	Cdkn1c	0.5953	0.4758	0.5969	0.4767	0.4504	0.6506	0.5312	0.2441	0.8198	0.7427	0.5118	0.6519
NM_001013101	Moap1	0.2832	0.4615	0.3951	0.5144	0.4775	0.2930	0.2546	0.3247	0.2020	0.2022	0.2687	0.3733
NM_001013090	Rg9mtd3	0.8076	0.8547	0.7397	0.7662	0.9290	0.5490	0.8169	0.7453	0.4149	0.5702	0.7394	0.5169
NM_001013102	Theg	0.3382	0.1974	0.2068	0.2245	0.3219	0.3535	0.2284	0.5739	0.3287	0.3037	0.3795	0.2649
NM_001013094	Ccnl2	0.9198	0.9148	0.3741	0.1412	0.9355	0.9853	0.1826	0.9608	0.9919	0.9558	0.8266	0.9865
NM_001013104	Eef1d	0.7450	0.7265	0.4129	0.2126	0.3682	0.3428	0.8547	0.3346	0.6191	0.6561	0.7625	0.0300
NM_001024255	Txk	0.1331	0.1498	0.2285	0.2867	0.1789	0.2044	0.0484	0.0642	0.0785	0.1290	0.0128	0.0524
NM_001013096	Mycn	0.8761	0.5335	0.9865	0.8350	0.2052	0.1416	0.3262	0.2032	0.8077	0.4729	0.8836	0.8621
NM_001013963	RGD1306568	0.8710	0.9149	0.6379	0.7735	0.9755	0.9871	0.6944	0.8871	0.3907	0.5862	0.1368	0.2431
NM_001013107	Dazap2	0.5991	0.5980	0.6907	0.7179	0.6551	0.5543	0.6746	0.6370	0.5828	0.6480	0.7226	0.4968

NM_001014105	Apool	0.2571	0.6319	0.9983	0.0875	0.0845	0.0357	0.8433	0.0533	0.5550	0.5923	0.6919	0.4199
NM_001014260	LOC366431	0.3260	0.5487	0.5393	0.5898	0.2179	0.4938	0.2429	0.5757	0.2171	0.4744	0.5009	0.6844
NM_001005531	RGD1359156	0.4210	0.1926	0.2430	0.1004	0.0193	0.4172	0.0626	0.1904	0.0799	0.0569	0.1754	0.1262
NM_001005532	MGC95210	0.4669	0.2012	0.4324	0.0577	0.2048	0.6262	0.0531	0.3668	0.5305	0.0415	0.0594	0.5875
NM_001013991	Tmem175	0.0184	0.0739	0.1393	0.4572	0.0803	0.0457	0.0173	0.1177	0.0126	0.0791	0.4636	0.1435
NM_001013964	Mgrn1	0.6729	0.8492	0.6867	0.6957	0.8724	0.7918	0.9144	0.7968	0.9614	0.9274	0.3549	0.9732
NM_001013115	Guk1	0.1498	0.0126	0.3017	0.0738	0.5826	0.3075	0.0719	0.1573	0.6963	0.1508	0.0377	0.0785
NM_001014148	Paip2	0.2503	0.5703	0.9456	0.2593	0.6057	0.6343	0.3589	0.5308	0.2300	0.9657	0.3388	0.7535
NM_001013122	Kdelr2	0.2876	0.4398	0.3603	0.4158	0.7316	0.2065	0.3404	0.2187	0.3446	0.4035	0.4831	0.2030
NM_001013121	Snapc2	0.5598	0.3712	0.5257	0.5055	0.5600	0.4024	0.6416	0.4229	0.4249	0.5606	0.2451	0.5070
NM_001005542	Wdr34	0.6878	0.4847	0.8125	0.8561	0.5866	0.8547	0.7911	0.8160	0.0399	0.1435	0.5716	0.0157
NM_001013125	Bfar	0.3999	0.5340	0.6520	0.2328	0.4195	0.6436	0.2561	0.5853	0.4146	0.4189	0.6616	0.4775
NM_001013124	Ung	0.4731	0.9325	0.1827	0.5179	0.6886	0.7248	0.9696	0.9289	0.9151	0.6328	0.9195	0.3075
NM_001005544	Morn1	0.6940	0.1865	0.8158	0.5295	0.5204	0.4648	0.5911	0.4440	0.7392	0.7808	0.7555	0.6557
NM_001012080	Hfe2	0.0013	0.7572	0.8388	0.9475	0.9297	0.7530	0.9427	0.9225	0.9064	0.9144	0.8111	0.8562
NM_001014125	Pdia5	0.7887	0.8976	0.6723	0.0482	0.3288	0.1491	0.7780	0.8537	0.8742	0.8166	0.8108	0.0389
NM_001005548	Leo1	0.8374	0.7839	0.7777	0.8722	0.8859	0.7336	0.7171	0.8622	0.7241	0.8096	0.8878	0.7104
NM_001025655	Cmpk1	0.9183	0.7103	0.9425	0.9520	0.9209	0.9261	0.9675	0.7270	0.5594	0.5142	0.6904	0.5469
NM_001013127	Tagln2	0.2123	0.8638	0.7812	0.9940	0.3009	0.9264	0.9344	0.8837	0.1445	0.2456	0.3049	0.9467
NM_001033913	Gimap5	0.3020	0.2873	0.4325	0.3926	0.2371	0.3301	0.3881	0.3581	0.4372	0.4626	0.6596	0.2832
NM_001033716	Cast	0.6583	0.3763	0.9610	0.9512	0.3357	0.6714	0.5842	0.5342	0.6643	0.4498	0.6294	0.6838
NM_001014142	Ufsp2	0.1415	0.0128	0.0680	0.0093	0.0049	0.1296	0.1030	0.0134	0.1727	0.1269	0.2717	0.0649
NM_001014121	Amz2	0.8302	0.9083	0.5971	0.8509	0.8436	0.5047	0.4310	0.8080	0.1186	0.5628	0.6247	0.6003
NM_001013137	Cxcl14	0.5902	0.6311	0.4902	0.5466	0.6299	0.3309	0.4512	0.7205	0.5776	0.4390	0.4453	0.6886
NM_001013136	Rfxank	0.1071	0.5117	0.2832	0.2265	0.4229	0.4079	0.4188	0.8924	0.1901	0.5339	0.4076	0.4402
NM_001013138	Fbxo23	0.1924	0.1972	0.2067	0.2243	0.1975	0.2065	0.2279	0.2185	0.1864	0.1808	0.1968	0.2025
NM_001013143	Terf2ip	0.5109	0.3363	0.8132	0.3261	0.3714	0.9134	0.5575	0.0735	0.0311	0.3518	0.4962	0.2013
NM_001013145	Ccr6	0.0783	0.1416	0.7263	0.5048	0.4222	0.0806	0.6215	0.3354	0.1025	0.4242	0.2733	0.5175
NM_001013151	Gna14	0.4108	0.5767	0.6022	0.4233	0.4335	0.5155	0.4458	0.6600	0.6184	0.3763	0.6054	0.1561
NM_001013148	Plekhf1	0.0033	0.1341	0.1504	0.0622	0.1135	0.1026	0.1078	0.1582	0.0278	0.0190	0.3163	0.0822
NM_001025735	Tcea1	0.7207	0.6232	0.9275	0.8815	0.6705	0.7212	0.3776	0.7505	0.5537	0.6016	0.7937	0.8116
NM_001013152	Rcl1	0.3812	0.5833	0.5054	0.6062	0.4387	0.4734	0.2626	0.2464	0.3284	0.5782	0.3127	0.4601
NM_001013154	Pcgf6	0.7825	0.6936	0.8814	0.2713	0.9850	0.9544	0.6539	0.9736	0.5556	0.8572	0.8991	0.8154
NM_001017378	Myst1	0.7108	0.4096	0.4486	0.3524	0.2698	0.5715	0.3305	0.5218	0.3205	0.4808	0.3840	0.2023
NM_001013156	Zcchc9	0.9209	0.9653	0.4110	0.4799	0.6858	0.3612	0.4981	0.9044	0.8102	0.9057	0.9220	0.1097
NM_001025006	Dohh	0.4984	0.2445	0.2191	0.2649	0.5180	0.6048	0.4144	0.4499	0.5144	0.4751	0.6737	0.6975
NM_001014080	Uba1	0.2732	0.2595	0.4258	0.5276	0.3136	0.2760	0.2279	0.5496	0.3914	0.2701	0.1967	0.5253
NM_001013163	Bmyc	0.3864	0.1618	0.4727	0.4808	0.4184	0.6039	0.4411	0.1565	0.4542	0.4622	0.3791	0.4293
NM_001013161	Cstf1	0.5423	0.6212	0.6895	0.6286	0.6623	0.5400	0.6019	0.5924	0.6258	0.4746	0.6843	0.5727
NM_001030028	Elmo3	0.9218	0.9889	0.0042	0.0355	0.7980	0.8618	0.0501	0.7520	0.9111	0.8137	0.8864	0.4768
NM_001024303	Lix1l	0.8448	0.3927	0.9595	0.6786	0.9958	0.9629	0.3876	0.9419	0.5295	0.6959	0.4598	0.3321
NM_001014772	Adam9	0.6974	0.6625	0.7193	0.4624	0.2159	0.4696	0.3505	0.4231	0.3448	0.4895	0.6686	0.6230
NM_001033680	Syt1	0.4533	0.0308	0.0942	0.1464	0.6832	0.7001	0.0203	0.3862	0.1074	0.0624	0.0951	0.2185
NM_001013166	Gsg1	0.4006	0.5738	0.7463	0.6914	0.5974	0.5023	0.6889	0.2657	0.4176	0.7878	0.2573	0.3035
NM_001013168	Dnajc8	0.9002	0.5508	0.8265	0.5756	0.5576	0.3824	0.8580	0.4409	0.5434	0.1395	0.5364	0.8957
NM_001014106	Rfk	0.1627	0.2965	0.9791	0.3502	0.1422	0.2086	0.1474	0.0214	0.2478	0.1101	0.2660	0.0654
NM_001014187	Lancl2	0.6330	0.2466	0.2521	0.2243	0.4961	0.2536	0.4433	0.5421	0.2301	0.2463	0.4229	0.3702
NM_001013171	Gulp1	0.8932	0.1091	0.4310	0.4004	0.7900	0.5516	0.2188	0.4157	0.1849	0.1162	0.2404	0.0773
NM_001009478	Saa4	0.8757	0.3426	0.5402	0.7583	0.8857	0.2086	0.8260	0.9235	0.7515	0.5776	0.8668	0.7239

NM_001013175	Apol3	0.0601	0.0801	0.6176	0.0879	0.5548	0.5147	0.1628	0.5264	0.0245	0.1587	0.1473	0.5307
NM_001009668	Etfa	0.1305	0.4082	0.8794	0.7767	0.2021	0.0709	0.8684	0.1606	0.0390	0.1461	0.3609	0.4586
NM_001008510	Isg20	0.8385	0.8236	0.7973	0.1379	0.8506	0.8427	0.8286	0.8331	0.7860	0.7614	0.8900	0.9096
NM_001008336	Tnk2	0.9781	0.8022	0.3731	0.1390	0.8550	0.9746	0.9471	0.8387	0.8015	0.9567	0.7132	0.9379
NM_001005880	Dcir2	0.4056	0.1449	0.5852	0.2622	0.4315	0.3569	0.2852	0.3133	0.6421	0.5600	0.5450	0.4777
NM_001005882	RGD1359713	0.8801	0.9231	0.8185	0.9570	0.8172	0.6655	0.7587	0.6992	0.9453	0.9513	0.7647	0.7390
NM_001013176	Btg4	0.4227	0.7043	0.7173	0.7098	0.5240	0.6427	0.6447	0.2413	0.1830	0.3155	0.5064	0.1249
NM_001005884	Letm1	0.4809	0.6032	0.4691	0.6492	0.2062	0.4605	0.4076	0.4804	0.8237	0.5622	0.5348	0.5149
NM_001013180	Scgb3a1	0.2019	0.3517	0.4936	0.6160	0.3141	0.1289	0.6361	0.5320	0.4151	0.4039	0.1635	0.5998
NM_001014099	Paqr8	0.4595	0.4372	0.3523	0.2240	0.1971	0.2986	0.3815	0.3796	0.2589	0.3046	0.1965	0.3139
NM_001013181	Zbtb16	0.3395	0.2538	0.5212	0.0783	0.1246	0.1787	0.2535	0.6521	0.6020	0.3670	0.3646	0.7610
NM_001013179	Hes6	0.9472	0.9193	0.9274	0.4925	0.8474	0.8703	0.9113	0.9732	0.8498	0.9899	0.9030	0.7428
NM_001013186	Bin3	0.4142	0.2542	0.6722	0.5051	0.7210	0.6941	0.4318	0.5813	0.4643	0.5247	0.4548	0.5684
NM_001013188	Leprotl1	0.0982	0.0602	0.9899	0.6014	0.3161	0.2755	0.3338	0.2101	0.0139	0.1185	0.3279	0.1395
NM_001005890	Clec4a1	0.1975	0.2500	0.0603	0.2241	0.2934	0.4168	0.5262	0.1462	0.1631	0.2495	0.1115	0.4791
NM_001013200	Anp32e	0.0257	0.4204	0.2655	0.9709	0.1012	0.1010	0.8753	0.2015	0.5213	0.5139	0.9151	0.2456
NM_001005893	Repin1	0.0337	0.2634	0.2310	0.1290	0.0442	0.3273	0.2564	0.4324	0.1500	0.1264	0.1680	0.2855
NM_001014100	Lincr	0.4605	0.4807	0.1164	0.5773	0.3642	0.3974	0.1487	0.4619	0.2681	0.5652	0.4409	0.5835
NM_001013999	Lrrc18	0.2263	0.6586	0.2794	0.4589	0.5651	0.3470	0.4367	0.2710	0.2013	0.3031	0.2508	0.4093
NM_001013989	G3bp2	0.4981	0.3413	0.9438	0.9535	0.8129	0.5780	0.7579	0.6386	0.4733	0.4498	0.4398	0.3660
NM_001013196	Dnajc4	0.3000	0.3340	0.4174	0.0948	0.0591	0.1983	0.3938	0.0802	0.4136	0.2594	0.2906	0.3347
NM_001013195	Pold4	0.6421	0.6631	0.7805	0.8057	0.4717	0.1280	0.9617	0.4575	0.5566	0.8433	0.5268	0.8160
NM_001005904	Ccin	0.6875	0.6432	0.5806	0.7506	0.7907	0.5548	0.3760	0.5883	0.0874	0.5307	0.5195	0.2623
NM_001017537	Tex261	0.0388	0.1355	0.5520	0.5843	0.3661	0.4287	0.2752	0.7816	0.4324	0.8256	0.2716	0.2923
NM_001014089	Tbcel	0.7668	0.5148	0.6426	0.0577	0.4675	0.4581	0.4913	0.0733	0.4325	0.1012	0.7020	0.3147
NM_001013204	Ccndbp1	0.2121	0.1355	0.8121	0.5597	0.0885	0.0967	0.1504	0.0672	0.1397	0.0298	0.0367	0.0594
NM_001014254	Clk2	0.9509	0.9590	0.6746	0.1155	0.8740	0.8649	0.3833	0.8726	0.9316	0.8625	0.7973	0.9148
NM_001014232	Cnrip1	0.3596	0.4709	0.1521	0.4002	0.2487	0.1220	0.5075	0.2716	0.6737	0.1171	0.4577	0.3905
NM_001013209	Dnajb6	0.5925	0.9606	0.9992	0.9827	0.9802	0.8891	0.8118	0.8742	0.9560	0.8354	0.9601	0.9482
NM_001014031	Ftsjd2	0.5955	0.5794	0.5833	0.4100	0.5528	0.5817	0.5583	0.5359	0.4853	0.5841	0.4594	0.5206
NM_001014226	Nat15	0.3627	0.3804	0.6477	0.7299	0.9277	0.6297	0.7922	0.4989	0.8743	0.6600	0.6487	0.8215
NM_001013229	Eral1	0.1657	0.3017	0.7958	0.6306	0.5682	0.7408	0.7655	0.0457	0.1269	0.2018	0.5220	0.8839
NM_001013233	Gypc	0.0209	0.4129	0.4645	0.3577	0.1600	0.2508	0.0959	0.3246	0.3354	0.3061	0.4395	0.3534
NM_001014091	Ccdc33	0.6220	0.3594	0.5767	0.6368	0.5218	0.4004	0.5859	0.3077	0.8190	0.6498	0.3799	0.3371
NM_001009379	Oas1d	0.5162	0.5379	0.3923	0.3692	0.6448	0.5998	0.6136	0.4684	0.8419	0.8596	0.7521	0.2897
NM_001014253	Selt	0.5985	0.6317	0.6136	0.5485	0.5788	0.4607	0.5182	0.4495	0.1972	0.6893	0.6766	0.6375
NM_001013238	Pttg1ip	0.8914	0.3772	0.7576	0.9655	0.9598	0.8155	0.9339	0.9801	0.4457	0.6482	0.6768	0.3361
NM_001006964	MGC94542	0.6915	0.7568	0.1984	0.7307	0.0940	0.0944	0.5429	0.2487	0.7375	0.7580	0.5225	0.1629
NM_001013240	Cdc26	0.2109	0.4073	0.8302	0.9289	0.3728	0.3284	0.5360	0.2186	0.3747	0.5005	0.5795	0.1590
NM_001013239	Zdhhc12	0.0076	0.4903	0.1045	0.6254	0.1462	0.0435	0.3738	0.3152	0.5442	0.0858	0.2097	0.1228
NM_001013243	Slc30a3	0.1209	0.1162	0.1015	0.3105	0.1136	0.5594	0.4163	0.3338	0.1505	0.1286	0.2175	0.6823
NM_001014229	Stard3	0.6959	0.1264	0.1592	0.4528	0.4877	0.6776	0.6528	0.6496	0.1469	0.1002	0.5706	0.6820
NM_001008323	Pmm1	0.0755	0.3366	0.2120	0.0694	0.3521	0.2268	0.9000	0.3109	0.2262	0.3699	0.5670	0.1166
NM_001013247	Sln	0.4550	0.2869	0.4152	0.3380	0.2946	0.3811	0.4447	0.2180	0.2519	0.2959	0.1963	0.5176
NM_001013246	Arhgef12	0.1658	0.6390	0.2668	0.0725	0.1641	0.5509	0.3813	0.2747	0.3054	0.0670	0.1733	0.4693
NM_001013250	Mageh1	0.4937	0.4203	0.2219	0.4484	0.3603	0.2269	0.3654	0.5142	0.1942	0.5459	0.7025	0.2156
NM_001013427	Rarres2	0.3180	0.6234	0.3640	0.6058	0.4124	0.2819	0.3161	0.4393	0.2822	0.0955	0.3511	0.5470
NM_001013430	Rhoh	0.6167	0.7199	0.3528	0.5529	0.9452	0.7848	0.5077	0.4464	0.7536	0.5808	0.0646	0.1548
NM_001013429	Cldn14	0.2751	0.3952	0.5680	0.4094	0.2572	0.4000	0.3130	0.3656	0.3517	0.2460	0.2893	0.4222

NM_001025670	Agpat9	0.7602	0.2274	0.0640	0.7580	0.0732	0.3450	0.0704	0.1842	0.4817	0.1352	0.3270	0.0574
NM_001013433	Arl11	0.7337	0.6510	0.7945	0.6167	0.6298	0.5786	0.9083	0.9250	0.6294	0.5270	0.7599	0.8940
NM_001079943	Zfp426	0.6769	0.4754	0.6938	0.2329	0.5817	0.5684	0.4612	0.5933	0.5089	0.4344	0.4277	0.4795
NM_001024275	Rassf4	0.2025	0.2029	0.6403	0.5883	0.6330	0.5761	0.2756	0.6339	0.4947	0.6026	0.3018	0.5206
NM_001025705	Azi2	0.9764	0.9695	0.7808	0.8104	0.8750	0.9095	0.5038	0.8100	0.9198	0.8481	0.9835	0.7193
NM_001014118	Slc47a1	0.3151	0.5218	0.5884	0.2498	0.0717	0.1631	0.1228	0.3571	0.4692	0.1133	0.7757	0.4675
NM_001006982	Uxt	0.1285	0.4108	0.7551	0.6975	0.1346	0.0230	0.7618	0.2994	0.1532	0.2050	0.0802	0.1191
NM_001014133	Abcg3l2	0.7228	0.5957	0.3092	0.1453	0.4037	0.5510	0.5910	0.7211	0.1536	0.6012	0.4944	0.6679
NM_001014110	Prickle3	0.2313	0.2864	0.0650	0.2887	0.7549	0.6834	0.1196	0.6815	0.2948	0.4115	0.5604	0.5358
NM_001031662	Coq4	0.0508	0.2312	0.5982	0.5099	0.3113	0.2503	0.7754	0.1788	0.2190	0.5427	0.6531	0.2118
NM_001014141	Tmem192	0.1116	0.3931	0.2390	0.2261	0.0992	0.0740	0.4120	0.4531	0.1832	0.3727	0.2466	0.3848
NM_001006989	MGC94600	0.1660	0.3985	0.9049	0.9602	0.2815	0.4509	0.6116	0.2095	0.2473	0.0097	0.1617	0.0834
NM_001013874	Ociad1	0.4696	0.7169	0.7610	0.0645	0.3778	0.2164	0.2548	0.5983	0.6000	0.5223	0.5253	0.1769
NM_001014149	Rell2	0.0027	0.2687	0.1126	0.0200	0.1600	0.2440	0.3454	0.1002	0.1143	0.2461	0.4352	0.4718
NM_001033699	Cox15	0.2259	0.3811	0.3294	0.2884	0.1664	0.2079	0.7644	0.3471	0.1771	0.0167	0.1767	0.6617
NM_001013887	Dnajc18	0.0263	0.0379	0.0049	0.0069	0.0180	0.0224	0.0700	0.0315	0.1167	0.0209	0.0300	0.0170
NM_001024886	Dcun1d3	0.4026	0.3922	0.5827	0.4687	0.2602	0.1736	0.7947	0.3461	0.2079	0.5232	0.7485	0.1661
NM_001013891	Txnl4b	0.1228	0.0493	0.7874	0.1792	0.2410	0.2850	0.2807	0.1323	0.3071	0.3028	0.4253	0.8824
NM_001006996	Ddx18	0.6452	0.7875	0.0079	0.1192	0.7621	0.8796	0.2530	0.8485	0.7387	0.6659	0.6631	0.9602
NM_001009920	Yc2	0.7430	0.4755	0.7311	0.6096	0.8056	0.5772	0.8217	0.4396	0.8265	0.7569	0.6661	0.4723
NM_001014166	Il33	0.8338	0.0740	0.6295	0.5408	0.7438	0.5950	0.9125	0.7301	0.2903	0.3335	0.5522	0.7199
NM_001007000	Pcgf1	0.7908	0.9438	0.9494	0.1229	0.7323	0.6892	0.5666	0.7522	0.8207	0.7383	0.9606	0.9211
NM_001013212	Snapc3	0.3280	0.0884	0.7325	0.1972	0.0438	0.0700	0.3705	0.0376	0.2221	0.0810	0.2807	0.0331
NM_001013217	Trim54	0.7174	0.2428	0.5110	0.2448	0.0747	0.4561	0.5282	0.5119	0.5326	0.1929	0.0311	0.3936
NM_001007003	Cnot10	0.7755	0.8752	0.2104	0.0908	0.6334	0.7715	0.2262	0.9117	0.7494	0.7457	0.8043	0.7246
NM_001013218	Reep6	0.1573	0.9209	0.6175	0.3511	0.7637	0.7223	0.7706	0.7161	0.9707	0.4433	0.3231	0.7636
NM_001011911	Lyar	0.7629	0.8876	0.0049	0.0313	0.3329	0.0778	0.0974	0.1398	0.7557	0.8564	0.8217	0.4518
NM_001007009	MGC94915	0.1762	0.5072	0.5966	0.4066	0.1478	0.4718	0.3375	0.2723	0.7711	0.5564	0.2167	0.3789
NM_001007010	Dyx1c1	0.4853	0.4349	0.2675	0.3786	0.2351	0.4081	0.7125	0.4254	0.0751	0.5579	0.4056	0.2358
NM_001007011	Zpbp2	0.9381	0.8448	0.7539	0.7203	0.7872	0.5713	0.6175	0.7601	0.5134	0.7266	0.5189	0.5356
NM_001024258	Znf574	0.6430	0.3030	0.0246	0.2447	0.7347	0.6287	0.3537	0.2803	0.8677	0.3858	0.4378	0.5045
NM_001013047	Mtm1	0.6267	0.4435	0.6418	0.5010	0.2346	0.5335	0.2647	0.5559	0.5948	0.4531	0.2573	0.5507
NM_001008880	Scn4b	0.3866	0.6506	0.5787	0.7317	0.1576	0.1966	0.8338	0.2901	0.5841	0.4102	0.1957	0.3871
NM_001008876	Rsl1d1	0.5798	0.8645	0.9824	0.6437	0.5819	0.3119	0.9905	0.3812	0.8627	0.7554	0.5264	0.5127
NM_001011991	Ndrg1	0.2903	0.1513	0.3546	0.1230	0.3760	0.4745	0.4865	0.4136	0.3741	0.3229	0.3734	0.3523
NM_001013222	Rnd1	0.0206	0.7113	0.2766	0.1200	0.0325	0.1216	0.3372	0.0437	0.7972	0.1754	0.2801	0.9927
NM_001014185	Sfrs6	0.9460	0.9637	0.7521	0.9976	0.9172	0.9681	0.6583	0.9909	0.9558	0.9749	0.9557	0.9624
NM_001014200	Sppl2b	0.2004	0.4241	0.6512	0.2370	0.4136	0.5739	0.5030	0.5566	0.4533	0.4553	0.3113	0.5849
NM_001024763	Nif3l1	0.3503	0.1768	0.0824	0.3214	0.2956	0.5328	0.3121	0.4914	0.2011	0.2090	0.4120	0.2969
NM_001014156	Dbndd1	0.8681	0.6498	0.7241	0.9311	0.8626	0.7059	0.7765	0.8327	0.5898	0.8060	0.8770	0.7752
NM_001033975	Lhb	0.4603	0.2036	0.4406	0.2692	0.4816	0.2874	0.2186	0.4718	0.8255	0.3133	0.6179	0.4391
NM_001034028	Hyou1	0.9714	0.8776	0.6214	0.8074	0.9299	0.7807	0.9519	0.5859	0.9481	0.4057	0.6329	0.9377
NM_001013916	Cd302	0.2497	0.6789	0.5404	0.3164	0.4825	0.5042	0.0875	0.3912	0.0944	0.0820	0.0984	0.4654
NM_001014015	Rbm34	0.7445	0.3718	0.9259	0.0164	0.1596	0.1261	0.5551	0.0098	0.2493	0.2209	0.3142	0.3670
NM_001014177	Spata1	0.7021	0.2551	0.3027	0.2882	0.3939	0.3778	0.2767	0.6041	0.6123	0.3146	0.6253	0.3318
NM_001024307	Lrriq3	0.2201	0.0950	0.2890	0.1134	0.4289	0.1457	0.6474	0.1898	0.1469	0.1073	0.0647	0.2697
NM_001037658	LOC652955	0.4495	0.4159	0.2065	0.4241	0.2553	0.3847	0.3367	0.4380	0.1862	0.6869	0.7264	0.2017
NM_001015035	B3gntl1	0.1220	0.4767	0.3736	0.0784	0.4988	0.9300	0.4662	0.5979	0.2210	0.6048	0.4273	0.6287
NM_001013922	RGD1307752	0.4236	0.8892	0.3294	0.1450	0.3281	0.9194	0.6280	0.5249	0.6359	0.8771	0.4307	0.5514

NM_001014025	RGD1305014	0.7206	0.4263	0.5388	0.9555	0.5496	0.8117	0.4553	0.7529	0.7929	0.7774	0.3705	0.6540
NM_001034088	Carm1	0.4734	0.3336	0.5459	0.2944	0.6659	0.6579	0.6721	0.7265	0.5366	0.5982	0.4628	0.6362
NM_001024996	RGD1306932	0.8316	0.8542	0.8488	0.7999	0.9752	0.6230	0.8118	0.8463	0.8224	0.7526	0.6758	0.5257
NM_001014027	Slc25a22	0.7381	0.3174	0.7558	0.6819	0.4432	0.9452	0.8075	0.7289	0.6206	0.7958	0.7404	0.6253
NM_001014039	Tnfaip8l2	0.4377	0.1585	0.1141	0.0523	0.5049	0.2545	0.3344	0.2821	0.5446	0.3759	0.5580	0.7769
NM_001014222	Dmrtc1c	0.7841	0.6641	0.4648	0.3215	0.8352	0.4686	0.5742	0.6719	0.3000	0.5830	0.8618	0.8446
NM_001013927	RGD1308734	0.2913	0.3625	0.1889	0.2250	0.2807	0.3005	0.2643	0.0774	0.3769	0.0973	0.1567	0.0722
NM_001007605	Svs3	0.3457	0.4938	0.1481	0.4200	0.3356	0.1247	0.1329	0.1279	0.2194	0.3281	0.0568	0.2210
NM_001033758	Cdkn1c	0.5953	0.4758	0.5969	0.4767	0.4504	0.6506	0.5312	0.2441	0.8198	0.7427	0.5118	0.6519
NM_001025563	Pus1	0.6849	0.5706	0.1639	0.2426	0.7474	0.6606	0.1565	0.8791	0.8946	0.8743	0.9013	0.5035
NM_001013940	Dync2li1	0.3046	0.3123	0.9988	0.3176	0.6185	0.2990	0.4693	0.8115	0.2367	0.0510	0.2551	0.8660
NM_001013946	Akap8l	0.8229	0.1531	0.2745	0.0897	0.6774	0.6206	0.0783	0.9024	0.7531	0.6862	0.0207	0.0536
NM_001013948	Ankrd46	0.4663	0.1817	0.8948	0.4698	0.1764	0.5666	0.5778	0.2821	0.3070	0.3359	0.1612	0.4992
NM_001013950	Lmbr1l	0.8223	0.5233	0.0815	0.0835	0.6933	0.3129	0.2179	0.8137	0.3874	0.7509	0.3641	0.0516
NM_001014227	Glod4	0.2092	0.0965	0.8135	0.8000	0.4367	0.5676	0.9541	0.1355	0.0911	0.7299	0.3032	0.9955
NM_001014239	Fbxo25	0.5061	0.4702	0.5960	0.2767	0.5451	0.6004	0.4662	0.4306	0.3590	0.6073	0.2203	0.5082
NM_001014231	Wdr42a	0.2411	0.2942	0.2043	0.8692	0.6234	0.8971	0.4850	0.8500	0.4420	0.5535	0.7015	0.8390
NM_001013957	Pqbp1	0.9283	0.7861	0.4044	0.8081	0.8192	0.3478	0.5984	0.7149	0.3346	0.9288	0.5493	0.0508
NM_001015025	Stk38	0.8857	0.3009	0.3246	0.9048	0.9786	0.9934	0.3124	0.9433	0.3183	0.8578	0.7947	0.9345
NM_001014249	Pdss2	0.5521	0.6291	0.6207	0.5717	0.3976	0.4130	0.2527	0.6090	0.4761	0.6430	0.2215	0.7230
NM_001014062	RGD1306195	0.8662	0.5458	0.8695	0.7191	0.4045	0.7153	0.4772	0.9343	0.0205	0.3898	0.7916	0.8568
NM_001013965	Tekt4	0.2037	0.5061	0.6691	0.4584	0.3594	0.1012	0.5031	0.7630	0.5664	0.4395	0.2934	0.5413
NM_001014065	Zcchc12	0.3674	0.6469	0.4236	0.4131	0.2096	0.0547	0.1267	0.3898	0.6698	0.1825	0.3380	0.2954
NM_001014067	Ccdc17	0.6590	0.6087	0.5365	0.6991	0.7759	0.5709	0.8986	0.6678	0.5653	0.6629	0.3206	0.5064
NM_001012092	Xkr7	0.9502	0.9443	0.6644	0.7383	0.3225	0.8004	0.9351	0.5645	0.2654	0.4901	0.8275	0.3911
NM_001014069	Snip1	0.6132	0.5210	0.2322	0.3208	0.4232	0.2372	0.4230	0.7580	0.2717	0.9303	0.2038	0.1418
NM_001007632	Ascc1	0.8654	0.9165	0.9251	0.8095	0.9279	0.4571	0.7949	0.9520	0.7293	0.9500	0.7411	0.7257
NM_001025282	Orc3l	0.1921	0.1969	0.4146	0.2236	0.1968	0.2059	0.2273	0.2177	0.1862	0.1806	0.1959	0.2016
NM_001017445	Faf2	0.0977	0.3339	0.6252	0.0223	0.3147	0.1609	0.4383	0.1213	0.2837	0.1523	0.4147	0.1169
NM_001014071	Errfi1	0.7637	0.9418	0.8629	0.3318	0.6024	0.5527	0.9269	0.1332	0.9396	0.5153	0.8526	0.9577
NM_001007635	tGap1	0.5204	0.5638	0.3654	0.6310	0.7730	0.3394	0.5670	0.7617	0.2591	0.5534	0.5003	0.3518
NM_001007638	RGD1359334	0.3923	0.6695	0.0917	0.1313	0.0955	0.3610	0.4706	0.3187	0.6809	0.5489	0.5389	0.4726
NM_001007639	St7l	0.5476	0.5115	0.5980	0.8725	0.5036	0.4687	0.0927	0.6052	0.0884	0.3987	0.0991	0.2064
NM_001014075	RGD1311648	0.3499	0.1649	0.3080	0.9125	0.4378	0.1879	0.5448	0.1949	0.2418	0.3367	0.0481	0.1428
NM_001014076	Nol10	0.0292	0.8227	0.0923	0.6703	0.2902	0.3593	0.1868	0.0965	0.7588	0.3521	0.4553	0.3154
NM_001007645	MGC95152	0.8232	0.9027	0.9796	0.5477	0.3481	0.2625	0.9811	0.1759	0.7136	0.9460	0.9898	0.5955
NM_001014077	Clec14a	0.6539	0.6090	0.5892	0.1235	0.9034	0.0980	0.6542	0.4760	0.6632	0.7030	0.9467	0.2608
NM_001013976	RGD1305007	0.4780	0.6701	0.9402	0.7691	0.8166	0.8441	0.8372	0.7659	0.5983	0.6424	0.7895	0.6575
NM_001009654	Spc25	0.9713	0.9882	0.1368	0.8949	0.8852	0.7757	0.9588	0.9424	0.7219	0.8372	0.9546	0.1480
NM_001007649	MGC94282	0.4137	0.9896	0.6557	0.9398	0.7654	0.9253	0.8301	0.8023	0.6409	0.9707	0.9382	0.6747
NM_001014082	Ncln	0.7555	0.9673	0.8040	0.8514	0.7666	0.9991	0.8035	0.9861	0.9735	0.7557	0.8095	0.9704
NM_001024767	Dyrk3	0.0108	0.0307	0.0998	0.0467	0.0730	0.0140	0.0130	0.0662	0.1505	0.1070	0.2569	0.2300
NM_001013983	RGD1307736	0.5015	0.5219	0.3086	0.2358	0.1850	0.2704	0.5159	0.3858	0.5254	0.3418	0.3481	0.2351
NM_001024776	Hipk4	0.4870	0.2436	0.3041	0.1966	0.6137	0.1788	0.2672	0.6470	0.5359	0.3905	0.2701	0.4013
NM_001009663	Serpina6	0.1926	0.4909	0.2704	0.3158	0.0931	0.1312	0.1843	0.4425	0.4005	0.1281	0.4873	0.3860
NM_001038597	Oprm1	0.5747	0.5691	0.6230	0.6104	0.5073	0.7519	0.4963	0.8155	0.6760	0.6699	0.7131	0.3974
NM_001007657	RGD1359127	0.5690	0.8694	0.7617	0.9804	0.9091	0.7687	0.4039	0.9157	0.2184	0.6077	0.1358	0.1805
NM_001007658	RGD1359378	0.0590	0.1043	0.2895	0.2830	0.0464	0.1370	0.2891	0.1035	0.3834	0.0328	0.0208	0.8056
NM_001007659	RGD1359310	0.7881	0.8860	0.8382	0.1197	0.3681	0.1243	0.9463	0.7969	0.0708	0.4303	0.7609	0.0249

NM_001009664	Zfp414	0.9522	0.7178	0.1062	0.6376	0.8414	0.7737	0.7561	0.8187	0.7145	0.6378	0.8504	0.0293
NM_001007663	RGD1359616	0.6830	0.9105	0.1650	0.7619	0.7789	0.6389	0.7305	0.8296	0.7406	0.9497	0.7094	0.0566
NM_001025641	Psg29	0.4705	0.6712	0.4686	0.8277	0.7690	0.5522	0.5830	0.4745	0.5325	0.1651	0.0251	0.4802
NM_001013992	LOC305691	0.2379	0.5688	0.4276	0.5113	0.6970	0.2054	0.2981	0.2175	0.2758	0.5313	0.7065	0.3646
NM_001024891	Rhbdd1	0.2678	0.4463	0.3201	0.4973	0.1100	0.3646	0.1362	0.4575	0.1032	0.2249	0.4472	0.1732
NM_001014843	Faim3	0.4494	0.3719	0.3104	0.3342	0.2811	0.2945	0.3284	0.6074	0.1861	0.2781	0.1958	0.4551
NM_001007671	Cyb5d2	0.0547	0.0135	0.4598	0.5513	0.0391	0.2185	0.1119	0.3200	0.6577	0.4786	0.1094	0.5729
NM_001007672	Tmem98	0.3963	0.8034	0.2694	0.9500	0.6045	0.6644	0.6652	0.9344	0.1894	0.6201	0.3880	0.1679
NM_001007673	Mmd	0.2017	0.2693	0.1218	0.1809	0.0930	0.3211	0.1788	0.2663	0.2701	0.2412	0.2589	0.1127
NM_001017467	Ly6e	0.4217	0.3021	0.4148	0.0220	0.2420	0.6139	0.0922	0.5033	0.6127	0.4948	0.1217	0.6518
NM_001013994	Rcor2	0.6668	0.6299	0.1312	0.7846	0.6630	0.8092	0.8185	0.8762	0.9205	0.5829	0.2065	0.7727
NM_001017452	Nsun7	0.3466	0.1252	0.1795	0.6015	0.0979	0.1628	0.0842	0.0772	0.0193	0.0963	0.3080	0.0335
NM_001014108	Gpc4	0.7902	0.7424	0.6556	0.3329	0.6004	0.4574	0.8386	0.5008	0.1276	0.7740	0.4904	0.2606
NM_001017477	Gps2	0.5290	0.0409	0.5482	0.5923	0.4554	0.0558	0.6090	0.6422	0.3407	0.7288	0.2258	0.2490
NM_001007683	Glt8d1	0.9108	0.8332	0.8730	0.9973	0.7463	0.9976	0.8861	0.8177	0.7931	0.8244	0.8109	0.6790
NM_001014113	Utp14a	0.1294	0.5097	0.0495	0.0915	0.0301	0.3701	0.0549	0.3285	0.7151	0.3570	0.6450	0.2543
NM_001014114	RGD1305733	0.5487	0.6942	0.5568	0.5524	0.7129	0.6290	0.4757	0.5451	0.7058	0.6025	0.4820	0.6470
NM_001007688	RGD1359600	0.8885	0.9614	0.8352	0.9861	0.1590	0.8867	0.6942	0.2865	0.9238	0.9024	0.8475	0.3193
NM_001007689	Ciapin1	0.2386	0.1917	0.0948	0.0162	0.2201	0.0825	0.0882	0.0404	0.0581	0.0342	0.1202	0.2627
NM_001017471	Atf7ip2	0.6045	0.1941	0.5585	0.2641	0.6874	0.6372	0.7187	0.1421	0.4951	0.5049	0.5383	0.6340
NM_001017478	Cxcl16	0.3275	0.3502	0.3249	0.4258	0.2289	0.1391	0.5345	0.0888	0.4189	0.1642	0.3877	0.2449
NM_001017466	Chmp4c	0.7930	0.2421	0.8024	0.7114	0.9525	0.8140	0.0137	0.8672	0.1366	0.1383	0.1471	0.0224
NM_001007693	Ttc9c	0.6813	0.7019	0.3754	0.6910	0.5981	0.5353	0.5258	0.5030	0.4844	0.6319	0.6082	0.5681
NM_001017464	Btbd16	0.2303	0.0525	0.5937	0.0337	0.1265	0.0951	0.1997	0.1813	0.2494	0.0990	0.1806	0.5079
NM_012844	Ephx1	0.7451	0.2651	0.8034	0.6930	0.6395	0.4953	0.4872	0.7430	0.4718	0.5098	0.3991	0.3980
NM_001014129	Noc4l	0.8151	0.7725	0.3378	0.1746	0.2025	0.6031	0.1185	0.7267	0.5897	0.7761	0.6583	0.5981
NM_001014144	RGD1305679	0.0110	0.6220	0.6288	0.3574	0.2022	0.0500	0.9391	0.5552	0.7100	0.4478	0.6679	0.0711
NM_001017504	Zrsr1	0.5692	0.3734	0.0523	0.3209	0.1319	0.0232	0.0523	0.3137	0.1075	0.2137	0.3560	0.4164
NM_001014146	RGD1308114	0.7966	0.0118	0.8432	0.6653	0.5474	0.4682	0.0547	0.5561	0.0189	0.0211	0.0851	0.3657
NM_001007702	RGD1359108	0.2664	0.5953	0.6720	0.3962	0.3379	0.2940	0.1435	0.1686	0.0204	0.2733	0.2813	0.7035
NM_001012145	Hgd	0.5075	0.4944	0.6855	0.0846	0.3016	0.1108	0.5259	0.5942	0.0529	0.0846	0.1815	0.1566
NM_001014143	Thex1	0.2413	0.5131	0.6626	0.2425	0.5214	0.4789	0.6882	0.2240	0.5943	0.2676	0.6973	0.6177
NM_001007707	Brp16	0.9304	0.9057	0.2325	0.7321	0.8870	0.8386	0.7674	0.9781	0.9102	0.9289	0.4574	0.8649
NM_001007708	RGD1359634	0.1577	0.1828	0.3054	0.2356	0.2779	0.0296	0.7656	0.3389	0.2831	0.1191	0.0235	0.2006
NM_001012747	Nol12	0.5496	0.4017	0.0830	0.6328	0.6243	0.4433	0.4146	0.7847	0.1814	0.7114	0.0107	0.0445
NM_001009702	Rrp15	0.6142	0.8089	0.0802	0.4608	0.8137	0.7139	0.6106	0.8815	0.6497	0.8444	0.9783	0.0896
NM_001017488	Slc24a6	0.9003	0.6832	0.1736	0.0697	0.6228	0.8613	0.2403	0.7348	0.8643	0.5102	0.6209	0.9812
NM_001007713	Tmbim1	0.2094	0.0094	0.9378	0.1917	0.0810	0.2898	0.1332	0.1102	0.2229	0.1268	0.0071	0.9754
NM_001007715	Ribc1	0.0678	0.5911	0.1126	0.5579	0.2586	0.2706	0.3477	0.1321	0.2182	0.2193	0.1711	0.6974
NM_001007718	ste2	0.4166	0.3796	0.7391	0.5619	0.7479	0.5939	0.5840	0.4508	0.5551	0.2071	0.7444	0.4957
NM_001014172	Rsrc1	0.0446	0.1539	0.0756	0.1095	0.0054	0.0817	0.0843	0.0342	0.0206	0.4178	0.5400	0.1017
NM_001014167	RGD1309804	0.5685	0.3982	0.7675	0.6307	0.5937	0.5829	0.1915	0.6655	0.2528	0.3385	0.2478	0.1446
NM_001007722	LOC360504	0.2774	0.4778	0.2063	0.2232	0.4361	0.3941	0.2271	0.2173	0.4074	0.1806	0.1958	0.2985
NM_001011961	Srms	0.5271	0.7639	0.7180	0.6100	0.2145	0.6397	0.4051	0.5976	0.4447	0.5465	0.5632	0.7188
NM_001007724	Dcakd	0.9530	0.0940	0.8885	0.7538	0.9831	0.9079	0.9251	0.8321	0.2404	0.3869	0.0975	0.9568
NM_001017512	Zfp61	0.7380	0.6923	0.0521	0.4098	0.7320	0.7296	0.1255	0.7371	0.7104	0.6334	0.7240	0.7254
NM_001014180	Alkbh3	0.0625	0.0788	0.4982	0.0464	0.0852	0.2471	0.0221	0.0218	0.1000	0.3429	0.0326	0.0659
NM_001007727	Lypd1	0.9955	0.9482	0.8997	0.9569	0.9586	0.9949	0.9778	0.9010	0.9407	0.9585	0.9860	0.9741
NM_001079939	Lmf2	0.9651	0.7416	0.1598	0.3171	0.9423	0.9231	0.9159	0.9394	0.5220	0.5273	0.6237	0.2275

NM_001025667	Rnf2	0.5366	0.2677	0.8870	0.5521	0.0889	0.4666	0.3179	0.1060	0.3019	0.1502	0.3447	0.7365
NM_001014190	RGD1307218	0.8855	0.6652	0.7047	0.2424	0.3823	0.5906	0.5357	0.4957	0.7240	0.7465	0.2864	0.6057
NM_001024234	Wscd1	0.4778	0.4687	0.6164	0.3857	0.7266	0.5968	0.6732	0.5240	0.5151	0.5503	0.3411	0.3653
NM_001024290	Gal3st3	0.1837	0.4040	0.1098	0.6972	0.7613	0.3260	0.6786	0.7948	0.8817	0.3606	0.2700	0.8229
NM_001024286	Spib	0.7630	0.8092	0.8994	0.8120	0.7890	0.6726	0.6967	0.6416	0.7431	0.8631	0.7414	0.8077
NM_001007734	Echdc1	0.7115	0.7053	0.8665	0.4794	0.8306	0.7836	0.3971	0.5461	0.6243	0.8485	0.6118	0.4977
NM_001007735	Sertad1	0.7809	0.8669	0.6911	0.8129	0.9971	0.8905	0.4009	0.8718	0.2454	0.8836	0.7231	0.0460
NM_001007737	RGD1359158	0.4898	0.0417	0.8973	0.8161	0.3153	0.1219	0.1188	0.0349	0.4154	0.1364	0.3154	0.1230
NM_001024274	Gtdc1	0.7366	0.7979	0.8540	0.6625	0.6447	0.9021	0.8442	0.9253	0.3808	0.9148	0.9252	0.1666
NM_001017456	Klhdc10	0.1229	0.4146	0.7714	0.0746	0.3432	0.5658	0.5193	0.1384	0.8670	0.2148	0.5030	0.8144
NM_001014205	Tmem123	0.3744	0.2748	0.7807	0.2376	0.1426	0.2628	0.9468	0.2239	0.3060	0.6644	0.4420	0.5745
NM_001024257	Cenpt	0.8156	0.7648	0.0022	0.1249	0.9496	0.8173	0.8469	0.9045	0.7376	0.8562	0.8937	0.0689
NM_001024975	Morn4	0.3722	0.1713	0.8713	0.2457	0.6516	0.0579	0.8383	0.6110	0.2284	0.5709	0.2816	0.5628
NM_001025019	Mettl11a	0.1328	0.1369	0.5077	0.1115	0.0407	0.1035	0.2167	0.3090	0.5195	0.8079	0.1162	0.5267
NM_001024748	Rshl1	0.4415	0.3972	0.3374	0.3514	0.2169	0.2303	0.1373	0.4717	0.0641	0.1404	0.0776	0.2599
NM_001007745	Tmem43	0.7669	0.9927	0.9729	0.9782	0.8667	0.9772	0.8149	0.7881	0.9678	0.9516	0.9912	0.8882
NM_001007746	MGC94199	0.9562	0.5524	0.8026	0.4515	0.8957	0.6662	0.5419	0.7145	0.3065	0.6264	0.6193	0.8301
NM_001014208	Yipf2	0.8903	0.8987	0.7405	0.7350	0.1903	0.4562	0.9454	0.2961	0.8300	0.9333	0.9249	0.4096
NM_001007748	Tmem18	0.4654	0.2676	0.7856	0.3968	0.1632	0.4503	0.3279	0.1523	0.7870	0.0343	0.3489	0.0555
NM_001024314	Abhd12	0.4393	0.4071	0.2883	0.2618	0.3479	0.4526	0.3607	0.5034	0.7001	0.4357	0.6982	0.2845
NM_001014212	RGD1309784	0.3278	0.2839	0.6332	0.1643	0.3207	0.1452	0.0772	0.0230	0.1255	0.0699	0.1563	0.1845
NM_001024306	Nola1	0.7304	0.9955	0.3504	0.8009	0.9228	0.9296	0.8054	0.9105	0.8085	0.9678	0.8226	0.6806
NM_001007751	MGC94207	0.7476	0.3556	0.0527	0.3206	0.5930	0.4962	0.7116	0.9602	0.2881	0.5619	0.0760	0.1598
NM_001011922	Nedd9	0.9930	0.9952	0.8874	0.8475	0.9556	0.9213	0.9646	0.9993	0.9120	0.9990	0.9937	0.4965
NM_001012008	Znf18	0.1540	0.1406	0.1943	0.6433	0.1402	0.0857	0.3493	0.1441	0.0850	0.1516	0.3502	0.1560
NM_001024305	Prpf38b	0.7462	0.8045	0.8809	0.3927	0.4817	0.8732	0.2636	0.4249	0.4602	0.5836	0.8025	0.9555
NM_001025663	Rbmx	0.6835	0.5755	0.4884	0.5473	0.3427	0.5069	0.6142	0.3173	0.7283	0.6277	0.5142	0.7220
NM_001024338	Bcl2l14	0.0634	0.6779	0.9187	0.1112	0.4809	0.5059	0.8020	0.6729	0.7352	0.8252	0.6753	0.5778
NM_001024333	Cidec	0.3577	0.7583	0.6219	0.3173	0.7568	0.7125	0.7961	0.8628	0.8568	0.4121	0.7493	0.1872
NM_001024332	Arl8b	0.5815	0.6014	0.7042	0.7132	0.7165	0.7086	0.7181	0.7244	0.7119	0.7171	0.6532	0.5897
NM_001024990	Amdhd2	0.3544	0.8864	0.3482	0.8522	0.6680	0.8081	0.8426	0.7868	0.8351	0.8078	0.6713	0.7788
NM_001014245	RGD1305441	0.9274	0.9388	0.2979	0.8937	0.9802	0.7266	0.2728	0.9635	0.9944	0.8935	0.9356	0.9720
NM_001024347	Slfnl1	0.2234	0.1057	0.0643	0.1569	0.0216	0.1712	0.3214	0.3487	0.1267	0.1255	0.4437	0.5892
NM_001014246	RGD1309482	0.5976	0.2388	0.6776	0.4870	0.2189	0.2432	0.5648	0.3923	0.2268	0.2600	0.3348	0.3113
NM_012868	Npr3	0.5191	0.0661	0.2479	0.3261	0.0479	0.0187	0.2232	0.0063	0.0202	0.1177	0.0666	0.1026
NM_001024351	Apof	0.3631	0.3903	0.5303	0.3645	0.4465	0.1835	0.2422	0.3252	0.1023	0.1761	0.1858	0.2989
NM_001007802	Cmtm6	0.5342	0.4105	0.6579	0.5466	0.5934	0.5753	0.7148	0.5287	0.5314	0.3900	0.6593	0.5914
NM_001014241	RGD1308454	0.0836	0.2643	0.2304	0.5362	0.5550	0.4720	0.4234	0.4236	0.2790	0.6296	0.3489	0.1209
NM_001025424	Usp3	0.5174	0.1633	0.8249	0.2879	0.1648	0.6466	0.3292	0.1544	0.6891	0.4547	0.5367	0.4438
NM_001014242	Isoc1	0.2856	0.2447	0.6089	0.3327	0.3321	0.3446	0.8862	0.2824	0.2605	0.7816	0.3765	0.1211
NM_001024356	H1fnt	0.1886	0.2100	0.1410	0.2211	0.4030	0.3400	0.5834	0.1376	0.0137	0.1013	0.6630	0.2207
NM_001024354	Lrrc14	0.6532	0.8809	0.4604	0.7541	0.9170	0.9798	0.7142	0.8830	0.6913	0.5924	0.8231	0.5774
NM_001014250	LOC365601	0.9602	0.9963	0.9729	0.7524	0.9704	0.9648	0.9394	0.9130	0.9446	0.9827	0.9318	0.9849
NM_001014269	Lrrfip1	0.9978	0.0855	0.5775	0.5456	0.9256	0.9649	0.5630	0.7757	0.7804	0.2407	0.3355	0.7938
NM_001014773	Pcdhga7	0.5242	0.6526	0.1268	0.4688	0.3365	0.8005	0.3803	0.5681	0.8062	0.5655	0.4131	0.6935
NM_001014274	Armcx2	0.4360	0.2754	0.2772	0.0170	0.8174	0.8747	0.0248	0.9187	0.2328	0.0744	0.3075	0.0383
NM_001024370	Pigy	0.4878	0.8447	0.8449	0.9986	0.7670	0.8638	0.9022	0.9426	0.3803	0.2898	0.8931	0.9348
NM_001008283	RGD1306284	0.4499	0.6823	0.8307	0.5885	0.2164	0.0653	0.5803	0.2639	0.7364	0.8311	0.7450	0.7180
NM_001008285	Ccdc52	0.5866	0.7172	0.1232	0.3536	0.9554	0.8271	0.6828	0.5859	0.6865	0.7293	0.5895	0.8479

NM_001008287	Donson	0.6341	0.9785	0.1680	0.2112	0.7597	0.7739	0.8458	0.5289	0.6310	0.8464	0.9969	0.0951
NM_001008288	RGD1306954	0.0252	0.2287	0.2205	0.8048	0.3596	0.3029	0.3711	0.1170	0.1558	0.3410	0.4302	0.0982
NM_001008289	Sbds	0.8207	0.4223	0.5499	0.2363	0.8698	0.9109	0.0529	0.8140	0.8596	0.3979	0.8078	0.8223
NM_001008290	RGD1310861	0.2762	0.0575	0.7974	0.2555	0.0741	0.2547	0.2204	0.2795	0.0999	0.1509	0.0642	0.0216
NM_001014785	Ncbp1	0.9940	0.9331	0.4289	0.6169	0.8898	0.9029	0.4626	0.8201	0.7364	0.7896	0.8578	0.8100
NM_001014787	Igsf8	0.4295	0.3061	0.7057	0.2909	0.3801	0.3734	0.4280	0.4665	0.5182	0.2425	0.3784	0.7312
NM_001024879	Dnpep	0.8167	0.8743	0.7669	0.9999	0.7517	0.9576	0.8745	0.9367	0.8323	0.9002	0.9385	0.8359
NM_001014792	Kpna3	0.7176	0.3720	0.8480	0.9648	0.5008	0.9729	0.9235	0.1547	0.8461	0.6477	0.9078	0.9679
NM_001015006	Dmap1	0.2901	0.3134	0.3782	0.3733	0.1966	0.2938	0.4322	0.2170	0.1859	0.1805	0.4293	0.2012
NM_001014810	Yif1b	0.2848	0.3273	0.3825	0.4351	0.2419	0.3447	0.5202	0.4569	0.3208	0.4649	0.3030	0.5430
NM_001013871	Tmem183a	0.3814	0.8803	0.6608	0.3447	0.2860	0.4946	0.6044	0.1303	0.6669	0.6878	0.8933	0.4380
NM_001014790	Rarres1	0.2368	0.5160	0.3170	0.4113	0.2247	0.3641	0.3509	0.4520	0.3290	0.5908	0.4892	0.7326
NM_001012082	Slc35a3	0.3968	0.7932	0.8272	0.8355	0.6746	0.4116	0.6909	0.8539	0.7629	0.1765	0.7551	0.3051
NM_001010964	Klrb1a	0.7294	0.6113	0.1395	0.3647	0.4801	0.3601	0.6133	0.2639	0.3233	0.2122	0.3091	0.4355
NM_001011665	Trim26	0.6815	0.8698	0.9135	0.9589	0.9086	0.7615	0.9938	0.6215	0.9784	0.8213	0.9388	0.9813
NM_001008307	RGD1311257	0.9891	0.9857	0.9576	0.1251	0.8196	0.7656	0.9913	0.9933	0.9930	0.9178	0.7068	0.9693
NM_001015010	Cib2	0.5846	0.5181	0.8016	0.2186	0.4527	0.2996	0.5608	0.0577	0.6388	0.8586	0.6820	0.3447
NM_001015012	Rab30	0.1816	0.1611	0.1234	0.0519	0.0890	0.0923	0.1544	0.0087	0.1181	0.1173	0.3177	0.0358
NM_001015011	Il17f	0.5697	0.3929	0.2391	0.3292	0.4840	0.2287	0.2530	0.2410	0.5850	0.2902	0.4706	0.4678
NM_001008313	Cbx3	0.8642	0.9791	0.9949	0.9842	0.9663	0.9940	0.9457	0.7487	0.8768	0.8987	0.9776	0.7744
NM_001015019	Chchd2	0.4762	0.3170	0.3463	0.4965	0.4626	0.3731	0.6238	0.3969	0.1858	0.4708	0.2995	0.3700
NM_001008318	RGD1311578	0.3664	0.2045	0.1692	0.2571	0.1941	0.1697	0.3076	0.0896	0.0802	0.1141	0.1980	0.0187
NM_001008319	Maea	0.9167	0.9818	0.7168	0.2772	0.2251	0.3210	0.2443	0.3376	0.8995	0.8967	0.8876	0.9130
NM_001015021	Dnajb11	0.6745	0.4541	0.4153	0.6982	0.3090	0.2272	0.6996	0.2083	0.4204	0.4546	0.8009	0.6637
NM_001025049	Acrbp	0.0861	0.1980	0.3996	0.7611	0.5311	0.4146	0.6946	0.5149	0.5279	0.3090	0.0829	0.2497
NM_001025634	A2ld1	0.7550	0.9580	0.7661	0.7979	0.8368	0.7332	0.9533	0.7798	0.8904	0.9638	0.7243	0.9557
NM_001015027	Crebl2	0.5448	0.2793	0.8060	0.7007	0.6474	0.8513	0.7899	0.8697	0.9091	0.7739	0.8912	0.5268
NM_001015034	Tnfrsf14	0.6326	0.6860	0.7811	0.7660	0.8366	0.9004	0.9245	0.6673	0.9100	0.6650	0.8318	0.7084
NM_001025623	Vnn1	0.2430	0.7431	0.3877	0.1735	0.7234	0.1133	0.3336	0.4485	0.6104	0.1367	0.7496	0.1259
NM_001008329	RGD1311745	0.0841	0.0293	0.0200	0.0671	0.0950	0.0168	0.0990	0.0462	0.0198	0.2423	0.0168	0.1020
NM_001008330	Polr1c	0.9741	0.8687	0.3466	0.8352	0.7083	0.6945	0.7850	0.7914	0.1677	0.2687	0.2577	0.1511
NM_001008331	Wdfy1	0.1680	0.7774	0.2860	0.0260	0.1782	0.1399	0.1938	0.3856	0.2957	0.7336	0.7357	0.4719
NM_001017376	Phyhip	0.6860	0.6967	0.3681	0.4418	0.4692	0.7429	0.6704	0.6839	0.6065	0.4989	0.6986	0.5534
NM_001017377	Paqr4	0.8723	0.9156	0.9110	0.9273	0.9194	0.9875	0.9485	0.8619	0.8849	0.6989	0.9100	0.8629
NM_001025635	Nars	0.4354	0.6711	0.2537	0.5637	0.2199	0.2359	0.5349	0.4570	0.4959	0.2359	0.3651	0.6125
NM_001025275	Rad23b	0.3224	0.2758	0.2963	0.0471	0.0962	0.2579	0.3447	0.2618	0.2017	0.3363	0.2566	0.0715
NM_001008337	Rg9mtd1	0.2986	0.0749	0.3379	0.1799	0.1451	0.0297	0.6123	0.0357	0.2943	0.4124	0.0151	0.7554
NM_001033997	Ubox5	0.2321	0.7965	0.5088	0.4989	0.2362	0.8454	0.9277	0.7174	0.7824	0.8405	0.3602	0.8496
NM_001017382	Frs3	0.7121	0.3474	0.3678	0.6616	0.5805	0.9018	0.4834	0.6894	0.6760	0.4192	0.4396	0.7386
NM_001008341	Prl7a4	0.3715	0.4001	0.3425	0.4742	0.1574	0.2425	0.3185	0.1614	0.0933	0.4350	0.0722	0.0480
NM_001017444	Tm2d2	0.0118	0.2320	0.1324	0.6662	0.0402	0.2177	0.0240	0.0255	0.1453	0.0095	0.1369	0.0325
NM_001017385	Kdelr1	0.9231	0.8536	0.9812	0.9686	0.7620	0.6955	0.8418	0.8846	0.8419	0.9047	0.5744	0.6613
NM_001034111	Prlr	0.3917	0.8980	0.8191	0.6832	0.6154	0.4844	0.5082	0.7328	0.7527	0.8900	0.8007	0.6541
NM_001024872	Nkap	0.1056	0.2696	0.1144	0.0252	0.0547	0.0127	0.4200	0.2129	0.0243	0.1937	0.1651	0.1021
NM_001033867	Crkrs	0.8825	0.9533	0.6086	0.6430	0.7702	0.7264	0.4901	0.8125	0.8688	0.9369	0.7244	0.5860
NM_001008351	RGD1309594	0.9969	0.7163	0.9256	0.8031	0.9569	0.9986	0.9136	0.7692	0.9899	0.9488	0.7706	0.9126
NM_001024236	Gtf2h3	0.8148	0.9459	0.8780	0.9674	0.7375	0.8844	0.2540	0.9080	0.9138	0.8368	0.9524	0.9118
NM_001024908	Mfsd3	0.7625	0.8323	0.7558	0.9409	0.7202	0.7538	0.7635	0.8283	0.8558	0.8866	0.4391	0.5097
NM_001008358	Tmem106c	0.0573	0.0373	0.4362	0.2116	0.1925	0.8522	0.0452	0.6579	0.2946	0.0377	0.0628	0.4209

NM_001024240	RGD1310251	0.7061	0.9080	0.3141	0.8074	0.5561	0.4826	0.5988	0.6504	0.6830	0.3087	0.6266	0.4335
NM_001008361	Rnf167	0.6135	0.8168	0.8429	0.0584	0.0995	0.0782	0.4697	0.3636	0.8161	0.8425	0.5485	0.6797
NM_001011960	Zswim2	0.3675	0.3582	0.2061	0.2229	0.2848	0.2050	0.2267	0.2169	0.2965	0.2786	0.3552	0.2752
NM_001008363	Zfand2a	0.4960	0.8660	0.2345	0.1767	0.8289	0.8758	0.0825	0.9189	0.9041	0.5103	0.6702	0.8710
NM_001025722	Cdk10	0.5981	0.8468	0.5746	0.3550	0.7466	0.6650	0.2624	0.5029	0.9358	0.5633	0.8005	0.5568
NM_001024242	Ctsw	0.8741	0.7385	0.7198	0.8332	0.9126	0.7690	0.7146	0.8165	0.6730	0.7991	0.6544	0.6775
NM_001014152	Phkb	0.6801	0.2354	0.6162	0.4594	0.2294	0.2434	0.3302	0.2980	0.7118	0.3553	0.6315	0.2264
NM_001011919	Gtpbp3	0.2613	0.4476	0.2554	0.4400	0.3866	0.2495	0.6855	0.5266	0.3451	0.3778	0.4019	0.7003
NM_001024247	Lactb2	0.5904	0.1375	0.1093	0.9251	0.2883	0.7750	0.0481	0.2504	0.0295	0.1328	0.0948	0.0451
NM_001024244	Vtcn1	0.8801	0.9071	0.7927	0.8190	0.9018	0.7626	0.9557	0.6524	0.8510	0.8023	0.9590	0.6982
NM_001024250	Bop1	0.6534	0.8895	0.0622	0.1167	0.2840	0.2959	0.5359	0.1041	0.7771	0.7078	0.5846	0.1529
NM_001024253	Upk1b	0.3008	0.2374	0.7451	0.5379	0.4485	0.4975	0.1342	0.5079	0.3441	0.2684	0.5780	0.6337
NM_001025059	Tbc1d22b	0.9272	0.5620	0.7143	0.6587	0.9230	0.7809	0.6506	0.8728	0.8171	0.4997	0.9457	0.9604
NM_001025045	Ttc26	0.1961	0.4282	0.2059	0.7586	0.2391	0.4184	0.8357	0.3130	0.3762	0.6439	0.5048	0.7793
NM_001024899	Sufu	0.8898	0.4920	0.5186	0.3800	0.2888	0.8086	0.6614	0.8679	0.2645	0.2995	0.2382	0.1032
NM_001031655	Manba	0.3400	0.7088	0.6063	0.2621	0.5653	0.4982	0.6175	0.7239	0.5369	0.7547	0.2973	0.1348
NM_001033967	Ak2	0.5921	0.6105	0.4642	0.1839	0.2624	0.4950	0.6734	0.6315	0.3237	0.5626	0.4802	0.2963
NM_001008377	RGD1307315	0.1146	0.3723	0.6133	0.7040	0.0975	0.1664	0.0835	0.1706	0.2532	0.1457	0.1175	0.3903
NM_001012028	Nuf2	0.8407	0.8704	0.1288	0.6013	0.8468	0.7853	0.8220	0.8882	0.7575	0.9545	0.9390	0.0351
NM_001048044	Cdc42ep3	0.7448	0.9676	0.9057	0.8478	0.9432	0.9390	0.9978	0.9380	0.8204	0.9753	0.9589	0.9101
NM_001111127	Hist3h2ba	0.2188	0.4055	0.1814	0.5233	0.0861	0.6685	0.4824	0.3965	0.4955	0.4935	0.2991	0.3643
NM_001113543	Wtap	0.9811	0.9628	0.9886	0.7917	0.8805	0.6479	0.9574	0.7922	0.9117	0.9436	0.9845	0.6997
NM_001113542	Wtap	0.9811	0.9628	0.9886	0.7917	0.8805	0.6479	0.9574	0.7922	0.9117	0.9436	0.9845	0.6997
NM_001025016	Chac2	0.6136	0.0746	0.0711	0.1672	0.6610	0.5620	0.0569	0.6186	0.0136	0.1436	0.0614	0.4631
NM_001024983	RGD1559532	0.2539	0.7045	0.5201	0.3471	0.6494	0.6555	0.6601	0.4487	0.3596	0.5499	0.3996	0.3768
NM_001024267	MGC109340	0.9345	0.9523	0.8152	0.9172	0.8783	0.9225	0.7340	0.6484	0.8717	0.2795	0.6874	0.7801
NM_001008509	Brd8	0.9531	0.9359	0.4928	0.9082	0.9153	0.8877	0.7782	0.9263	0.9444	0.9391	0.9539	0.7679
NM_001013112	Hibch	0.3395	0.2944	0.9787	0.7637	0.2167	0.0674	0.4887	0.1941	0.3221	0.2112	0.2087	0.3488
NM_001025740	Rrm2	0.3490	0.7059	0.5507	0.9252	0.6403	0.9120	0.9948	0.0551	0.7685	0.7322	0.9636	0.9360
NM_001024269	Luc7l	0.8915	0.7563	0.0591	0.0967	0.8081	0.3109	0.1621	0.3301	0.7750	0.7572	0.7714	0.1539
NM_001008517	RGD1310553	0.9264	0.9755	0.6398	0.8157	0.3382	0.2978	0.0810	0.2229	0.9633	0.9368	0.9859	0.7815
NM_001008518	MGC105649	0.2767	0.2834	0.2527	0.3582	0.4299	0.1738	0.0103	0.3247	0.0878	0.2505	0.0777	0.9877
NM_001024276	Mettl7b	0.5881	0.7317	0.5855	0.6557	0.8401	0.5933	0.4821	0.4611	0.8321	0.8335	0.9311	0.8328
NM_001025696	Yars	0.6301	0.9453	0.1803	0.7228	0.6456	0.7905	0.9927	0.9649	0.7322	0.7305	0.8697	0.9202
NM_001030025	Upp1	0.0572	0.0030	0.0025	0.3117	0.1296	0.0339	0.1130	0.0613	0.0016	0.0966	0.1176	0.0053
NM_001008520	Abhd1	0.8105	0.4319	0.3964	0.3334	0.6152	0.7749	0.8502	0.7810	0.4103	0.7947	0.6918	0.8231
NM_001025761	Mapkapk5	0.8190	0.8505	0.7592	0.6564	0.7105	0.6621	0.7257	0.7272	0.7346	0.8955	0.8473	0.6828
NM_001025754	Neil1	0.2800	0.4906	0.4266	0.1512	0.7043	0.5251	0.4549	0.5715	0.3963	0.2475	0.1690	0.3177
NM_001012050	Fbxo8	0.1587	0.1302	0.8206	0.8569	0.2632	0.0258	0.1250	0.0373	0.3599	0.3520	0.3660	0.1960
NM_001012041	Irf9	0.8553	0.5248	0.4113	0.3631	0.4568	0.1232	0.0908	0.0675	0.5034	0.7310	0.4222	0.7291
NM_001011940	Best1	0.1168	0.6705	0.0725	0.4340	0.1255	0.1171	0.1777	0.1804	0.4234	0.4193	0.2611	0.7187
NM_001025029	Tifab	0.0755	0.2987	0.8037	0.5222	0.7403	0.3152	0.9105	0.7640	0.5005	0.8502	0.4945	0.5379
NM_001025028	Fam35a	0.2871	0.8478	0.8107	0.0833	0.0504	0.5277	0.1990	0.0286	0.5552	0.5616	0.0679	0.7519
NM_001025066	Apol9a	0.0469	0.2845	0.1242	0.0876	0.0020	0.0991	0.1049	0.0109	0.1326	0.0638	0.0085	0.1163
NM_001008527	Aox3	0.6598	0.3371	0.3341	0.7045	0.1057	0.5678	0.1354	0.5321	0.2219	0.3819	0.1623	0.1205
NM_001024998	Txndc15	0.3846	0.0190	0.8314	0.9051	0.0312	0.2074	0.1039	0.4127	0.0952	0.0818	0.3529	0.0365
NM_001024997	Ccdc93	0.4888	0.3220	0.1017	0.4265	0.7741	0.8521	0.7056	0.6221	0.2513	0.1112	0.0702	0.0510
NM_001025119	Plekho1	0.1920	0.4794	0.2829	0.4302	0.1965	0.3784	0.3406	0.2168	0.4466	0.1804	0.1956	0.2828
NM_001008561	Rnase9	0.5408	0.7244	0.1833	0.5939	0.3552	0.7763	0.7126	0.7992	0.2466	0.9559	0.8680	0.1722

NM_023969	Lpar3	0.6014	0.5473	0.5343	0.6674	0.6097	0.6007	0.6268	0.5968	0.2316	0.7100	0.3664	0.6727
NM_022959	Rassf9	0.8349	0.8343	0.8308	0.9373	0.7546	0.8685	0.7747	0.7767	0.7381	0.8604	0.8847	0.6271
NM_001024740	Ccdc116	0.3118	0.3400	0.2764	0.5066	0.2376	0.3958	0.6974	0.6251	0.2471	0.2671	0.4448	0.4063
NM_001024741	Chaf1b	0.6991	0.7590	0.0900	0.7568	0.8370	0.7953	0.9220	0.7475	0.4291	0.7617	0.9426	0.0936
NM_001033704	Xylb	0.4118	0.4135	0.3214	0.1297	0.8172	0.4671	0.3465	0.1435	0.4769	0.3467	0.0700	0.4628
NM_001014168	Poll	0.1773	0.1814	0.7739	0.3665	0.0875	0.4451	0.7981	0.2510	0.0391	0.0973	0.0373	0.9053
NM_001012073	Rrp1	0.7592	0.9624	0.3079	0.5775	0.6521	0.4983	0.3439	0.8050	0.8352	0.4862	0.8251	0.9006
NM_001024744	Cdc16	0.8310	0.8957	0.8436	0.6667	0.9593	0.6758	0.9214	0.8609	0.7651	0.8514	0.9627	0.2176
NM_001024745	Slc39a6	0.4589	0.2787	0.1643	0.3374	0.0840	0.1959	0.0364	0.2062	0.1072	0.1173	0.3050	0.8041
NM_001008764	Psenen	0.0835	0.0847	0.6983	0.5926	0.3516	0.1982	0.1437	0.4433	0.1707	0.0176	0.0233	0.3646
NM_001037795	Fmc1	0.2247	0.6146	0.1540	0.4229	0.1196	0.2119	0.4223	0.3637	0.3067	0.4447	0.1509	0.0529
NM_001037218	Radil	0.1119	0.1719	0.0277	0.0152	0.1803	0.0690	0.0072	0.2468	0.2461	0.2185	0.0120	0.7666
NM_001034147	Pex7	0.2752	0.0522	0.3124	0.1639	0.2884	0.7261	0.2795	0.1017	0.0380	0.2221	0.1973	0.0521
NM_001024747	Phf10	0.7022	0.6101	0.2372	0.7437	0.4820	0.2556	0.7776	0.5473	0.4915	0.8427	0.4163	0.1440
NM_001008771	Wdr77	0.1652	0.5774	0.8869	0.6476	0.9381	0.7499	0.9318	0.5055	0.6803	0.1277	0.7684	0.9043
NM_001024750	Pold3	0.7171	0.6481	0.6161	0.3615	0.6622	0.0969	0.7013	0.8285	0.8532	0.7870	0.8624	0.5493
NM_001025731	Gle1	0.0169	0.2665	0.0769	0.1963	0.0173	0.0602	0.0900	0.0150	0.0293	0.1103	0.2077	0.3218
NM_001024752	Snx15	0.5457	0.0744	0.1372	0.1296	0.1595	0.1948	0.3709	0.4967	0.1456	0.0558	0.1478	0.8567
NM_001025727	Rnf8	0.1026	0.6604	0.4913	0.7512	0.5472	0.9558	0.4696	0.5641	0.3491	0.5477	0.4816	0.7309
NM_001012132	Hsfy2	0.2467	0.6328	0.3403	0.4224	0.2515	0.2535	0.5111	0.3638	0.3537	0.0234	0.4761	0.3512
NM_001012136	Gtf2a1l	0.4843	0.2759	0.2830	0.3248	0.2396	0.2050	0.3224	0.5226	0.3190	0.4807	0.3183	0.2464
NM_001024757	Wwp1	0.6548	0.7705	0.6579	0.4049	0.3538	0.7116	0.4657	0.4764	0.7265	0.7828	0.8052	0.5915
NM_001024759	Tmem5	0.6578	0.0330	0.6903	0.0072	0.5752	0.4978	0.2464	0.7742	0.3591	0.4225	0.0499	0.0103
NM_001033671	Bcl2l1	0.6446	0.5666	0.6885	0.6086	0.5832	0.7071	0.6674	0.6460	0.6880	0.6262	0.4395	0.7091
NM_001033670	Bcl2l1	0.6446	0.5666	0.6885	0.6086	0.5832	0.7071	0.6674	0.6460	0.6880	0.6262	0.4395	0.7091
NM_001033955	Calca	0.5146	0.2222	0.4223	0.7027	0.6452	0.3174	0.2607	0.4353	0.3327	0.4308	0.4268	0.5937
NM_001014101	Als2cr12	0.0567	0.1144	0.0849	0.0646	0.0388	0.1560	0.5620	0.1293	0.3405	0.3207	0.2905	0.5666
NM_001024761	Lrrfip2	0.0298	0.5505	0.0686	0.0418	0.0136	0.0706	0.3148	0.0144	0.7411	0.7603	0.4038	0.5028
NM_001024762	Prim2	0.0166	0.3895	0.0311	0.0546	0.1437	0.0733	0.4800	0.0694	0.2680	0.0563	0.2028	0.0353
NM_001025730	Fbxw5	0.1268	0.1590	0.5179	0.3151	0.3103	0.3292	0.0776	0.1965	0.0903	0.2146	0.2231	0.3608
NM_001014024	Orai3	0.9629	0.9616	0.2916	0.5674	0.8945	0.5343	0.7819	0.7368	0.9733	0.8889	0.8391	0.5562
NM_001011977	Tekt2	0.5320	0.3881	0.1364	0.0992	0.1161	0.5800	0.6352	0.1429	0.5081	0.0879	0.4063	0.5614
NM_001024765	Nbr1	0.3580	0.3185	0.9951	0.2310	0.2246	0.7951	0.2289	0.1392	0.0251	0.3094	0.3124	0.5683
NM_001008830	RT1-A3	0.5362	0.4320	0.5983	0.5704	0.6792	0.2885	0.5387	0.5902	0.4308	0.3408	0.2397	0.2705
NM_001008833	RT1-CE10	0.3177	0.4530	0.3062	0.2894	0.2531	0.2307	0.3713	0.1460	0.6181	0.3854	0.0626	0.2892
NM_001008841	RT1-CE3	0.0607	0.1079	0.1367	0.1380	0.1371	0.1126	0.0296	0.0631	0.4241	0.0632	0.0334	0.1109
NM_001008845	RT1-CE7	0.3335	0.1963	0.2058	0.2224	0.3653	0.2048	0.2265	0.2165	0.3866	0.4757	0.1954	0.3038
NM_001024768	Elf3	0.5976	0.6559	0.2625	0.4458	0.5172	0.7174	0.4791	0.3032	0.8055	0.6724	0.3574	0.2672
NM_001024769	Cdc73	0.4396	0.8179	0.5557	0.8685	0.8915	0.3829	0.0588	0.8007	0.6489	0.5127	0.3960	0.4704
NM_001033672	Bcl2l1	0.6446	0.5666	0.6885	0.6086	0.5832	0.7071	0.6674	0.6460	0.6880	0.6262	0.4395	0.7091
NM_201272	Plekhg5	0.7012	0.0834	0.3200	0.5699	0.9485	0.9591	0.8722	0.9566	0.4059	0.3569	0.5664	0.1057
NM_001024771	Tnip2	0.1732	0.4256	0.2614	0.1510	0.4904	0.6066	0.0715	0.2098	0.9351	0.3334	0.3456	0.8246
NM_001025751	Blk	0.7650	0.8578	0.7934	0.9211	0.3699	0.5380	0.3050	0.9705	0.4863	0.1196	0.6031	0.7260
NM_001025746	Rhox2	0.1280	0.0815	0.0263	0.4190	0.2997	0.0744	0.0538	0.1972	0.1082	0.1471	0.2408	0.6475
NM_001024775	Zfp110	0.6955	0.6605	0.3487	0.1752	0.6924	0.5859	0.3413	0.5233	0.8438	0.8047	0.3920	0.2632
NM_001012222	Fbxo7	0.0877	0.0691	0.0264	0.0921	0.0025	0.2701	0.1200	0.0071	0.0482	0.0536	0.0525	0.0561
NM_001012218	Trim55	0.5365	0.0656	0.5349	0.3663	0.4929	0.5022	0.5085	0.8410	0.8590	0.2040	0.7711	0.8636
NM_001108379	RGD1307201	0.1450	0.4782	0.1030	0.5611	0.1929	0.2293	0.7058	0.2674	0.3683	0.1319	0.0127	0.0184
NM_001108499	Cyp2r1	0.4290	0.8288	0.5182	0.7314	0.6217	0.0514	0.4644	0.2793	0.2236	0.5413	0.6500	0.0312

NM_001108383	Ebf2	0.8882	0.8732	0.9011	0.7707	0.8560	0.8820	0.8913	0.6458	0.4816	0.8344	0.9048	0.6278
NM_001033063	Rpp38	0.5853	0.2581	0.2349	0.5685	0.1705	0.1204	0.0273	0.3329	0.7633	0.7830	0.2351	0.2933
NM_001025637	Psmb10	0.0037	0.4591	0.0270	0.2152	0.6472	0.0129	0.0464	0.8229	0.5417	0.3992	0.1486	0.0039
NM_001024778	Rad17	0.5625	0.3141	0.4244	0.1761	0.4893	0.8455	0.1067	0.4805	0.2066	0.2480	0.4366	0.2034
NM_001033693	Slc31a2	0.2820	0.0678	0.4333	0.1559	0.3224	0.3769	0.2574	0.1375	0.6839	0.3203	0.1210	0.9620
NM_001024780	Prkra	0.4454	0.4739	0.8558	0.9326	0.8103	0.8067	0.9099	0.5043	0.3284	0.1403	0.2712	0.3743
NM_001013428	Pla2g2d	0.8368	0.0957	0.8017	0.5050	0.9260	0.6125	0.8991	0.3429	0.8856	0.6900	0.8249	0.6161
NM_001034146	Usp10	0.3340	0.8714	0.1247	0.7897	0.6382	0.9882	0.2565	0.7729	0.9737	0.8570	0.5700	0.9000
NM_001024783	Creld1	0.9147	0.9271	0.1158	0.1726	0.8131	0.9624	0.6022	0.8175	0.9591	0.8622	0.9710	0.9955
NM_001025146	Ndufs4	0.5703	0.7222	0.7919	0.5060	0.7086	0.5051	0.8616	0.5416	0.4171	0.5011	0.6620	0.5732
NM_001024788	RGD1310036	0.2795	0.4421	0.5033	0.5149	0.3397	0.3698	0.3648	0.4014	0.4241	0.0653	0.3191	0.3675
NM_001025695	Cops5	0.2947	0.1534	0.2209	0.1042	0.1987	0.3874	0.0213	0.0410	0.2490	0.6336	0.6502	0.3188
NM_001025707	Tfeb	0.4667	0.7005	0.1265	0.5705	0.5538	0.3684	0.1279	0.8448	0.2220	0.8412	0.2269	0.7544
NM_001025142	Trim35	0.1402	0.2695	0.4877	0.3728	0.2146	0.2277	0.6783	0.3265	0.2647	0.2824	0.5321	0.2406
NM_001024800	Txndc1	0.0053	0.2149	0.9228	0.2436	0.1898	0.7663	0.4186	0.2344	0.0506	0.1257	0.2563	0.5620
NM_001014000	Cdkn2aip	0.8426	0.2345	0.0664	0.1428	0.6583	0.4659	0.0424	0.4568	0.0882	0.2877	0.2335	0.0678
NM_001014259	Polr3k	0.3701	0.5880	0.3963	0.6579	0.2439	0.3600	0.4196	0.6601	0.3745	0.6298	0.3366	0.3562
NM_001014255	Aph1a	0.2365	0.6697	0.9732	0.9797	0.9503	0.9662	0.9285	0.8718	0.8555	0.7810	0.3514	0.9642
NM_001012162	Mllt10	0.6155	0.7617	0.7171	0.6523	0.7694	0.5371	0.7590	0.7349	0.3532	0.7062	0.7431	0.3020
NM_001012358	Tmem41b	0.6544	0.7256	0.6446	0.6400	0.6093	0.2460	0.4480	0.5832	0.1896	0.6979	0.6540	0.4842
NM_001025640	Vta1	0.6638	0.4388	0.5632	0.3714	0.3919	0.2925	0.2479	0.3418	0.3135	0.2552	0.3642	0.2714
NM_001024793	Cdc27	0.8437	0.1727	0.9244	0.7332	0.4673	0.8190	0.8794	0.6369	0.4049	0.3858	0.8082	0.9456
NM_001009180	Capza2	0.3129	0.3600	0.9677	0.9867	0.3144	0.9707	0.8970	0.5699	0.0940	0.3734	0.8798	0.9224
NM_001033964	Pfkfb2	0.5721	0.5921	0.7296	0.5564	0.6767	0.7084	0.8268	0.8376	0.7158	0.6081	0.7405	0.5834
NM_001012056	Ces7	0.3511	0.4316	0.6239	0.4865	0.4153	0.6234	0.7874	0.2392	0.6045	0.2753	0.5445	0.3331
NM_001025287	Tipin	0.4689	0.7585	0.4623	0.4160	0.6297	0.3982	0.3633	0.3189	0.4081	0.5230	0.6740	0.2642
NM_001024881	Tns4	0.7468	0.5334	0.6022	0.6272	0.6956	0.6944	0.5808	0.1180	0.1014	0.5125	0.6175	0.3476
NM_001024884	RGD1309362	0.2623	0.2116	0.0460	0.1061	0.3556	0.5623	0.1010	0.0414	0.0009	0.1344	0.1317	0.0357
NM_001009271	Nt5dc2	0.9123	0.9024	0.6042	0.8638	0.7527	0.8507	0.7865	0.8787	0.7455	0.8653	0.7637	0.0040
NM_001025665	Ccdc63	0.5397	0.0686	0.4887	0.1188	0.4483	0.1866	0.2603	0.4178	0.0466	0.0651	0.0748	0.4745
NM_001024887	Sigirr	0.0457	0.6173	0.7532	0.4421	0.8020	0.4546	0.6653	0.2289	0.4503	0.6775	0.4320	0.7359
NM_001009283	RGD1304580	0.8276	0.8911	0.7433	0.4527	0.5719	0.5473	0.4600	0.6919	0.6814	0.4576	0.9926	0.7695
NM_001009292	Giyd2	0.9078	0.3405	0.2135	0.3901	0.9192	0.9135	0.6048	0.8586	0.9744	0.9955	0.8108	0.5276
NM_001009316	RGD1310660	0.3087	0.4926	0.7248	0.7183	0.0310	0.0433	0.8170	0.0628	0.2757	0.3216	0.1663	0.0571
NM_001024896	Hsdl1	0.1950	0.0662	0.0656	0.0627	0.7033	0.6659	0.1752	0.7513	0.4865	0.3899	0.1196	0.5663
NM_001025014	Tmem50b	0.7821	0.3731	0.9176	0.8876	0.2356	0.4560	0.2639	0.3257	0.3492	0.2934	0.2711	0.1025
NM_001025703	Fam152b	0.2488	0.7627	0.2678	0.3498	0.3819	0.5228	0.8172	0.3266	0.7169	0.7990	0.6153	0.9419
NM_001025036	LOC499339	0.5773	0.5704	0.5979	0.7907	0.3655	0.6331	0.3880	0.4200	0.6228	0.5231	0.6958	0.7379
NM_001009369	Mrpl38	0.3314	0.4938	0.3729	0.5098	0.4132	0.3409	0.2260	0.2163	0.3561	0.4601	0.1953	0.4686
NM_001024964	Exoc3	0.0346	0.0905	0.3651	0.0895	0.0485	0.0111	0.0184	0.1047	0.0086	0.1985	0.0546	0.6407
NM_001025065	Angptl3	0.8777	0.5306	0.6953	0.1419	0.4743	0.4040	0.6476	0.7103	0.9200	0.8216	0.7788	0.8671
NM_001024970	RGD1305572	0.5772	0.6817	0.4709	0.6676	0.6838	0.2126	0.5184	0.5237	0.6791	0.5640	0.4940	0.5916
NM_001025765	Zfp90	0.3803	0.0409	0.6505	0.7220	0.2043	0.4170	0.2419	0.5098	0.7116	0.4278	0.0785	0.2618
NM_001024967	Tmem106a	0.8790	0.4598	0.7689	0.6480	0.8385	0.5645	0.7840	0.5062	0.6777	0.2025	0.3973	0.3658
NM_001034110	Ctsf	0.3489	0.5000	0.2232	0.4369	0.3984	0.3664	0.3614	0.2658	0.2293	0.7314	0.4624	0.5520
NM_001025773	Tnfrsf9	0.4159	0.3498	0.1403	0.6477	0.7295	0.2834	0.7200	0.6962	0.3583	0.1873	0.1235	0.2046
NM_001024978	RGD1305844	0.6898	0.8116	0.9085	0.8562	0.8659	0.7585	0.7139	0.5407	0.8682	0.3349	0.8228	0.5710
NM_001024986	RGD1306186	0.4675	0.5992	0.8976	0.5084	0.5439	0.3123	0.5518	0.7685	0.5308	0.7118	0.7221	0.7103
NM_012621	Pfkfb1	0.8715	0.9324	0.0529	0.7756	0.8766	0.9698	0.7296	0.9715	0.6995	0.9028	0.6815	0.2547

NM_001009455	Armc5	0.9618	0.9093	0.8563	0.8831	0.8791	0.8643	0.9107	0.9267	0.7763	0.9384	0.9205	0.7916
NM_001024995	Lrrc33	0.5453	0.2377	0.1031	0.2962	0.6052	0.4472	0.2330	0.5361	0.0451	0.4633	0.8435	0.1609
NM_001025111	Cd300lf	0.3184	0.5586	0.4818	0.2307	0.5656	0.1469	0.2711	0.3916	0.5211	0.4951	0.5246	0.2933
NM_012495	Aldoa	0.0958	0.1436	0.4259	0.2804	0.0089	0.0450	0.7817	0.0895	0.3043	0.1249	0.1000	0.9008
NM_001029914	Cmtm1	0.2321	0.8420	0.6601	0.7257	0.4638	0.8804	0.9073	0.4161	0.9485	0.9603	0.7896	0.5959
NM_001009487	Ly49s4	0.0406	0.5335	0.2991	0.2892	0.3179	0.4178	0.0811	0.5091	0.2408	0.1183	0.3053	0.2198
NM_001009488	Ly49s6	0.2948	0.5830	0.4064	0.1564	0.5843	0.0928	0.1785	0.3210	0.1758	0.2549	0.3965	0.2100
NM_001009489	Oas1k	0.1598	0.7779	0.6340	0.4898	0.8284	0.7051	0.8855	0.4165	0.7794	0.6179	0.6271	0.5714
NM_001009490	Oas1f	0.4516	0.2328	0.3865	0.3499	0.5359	0.6439	0.4279	0.2990	0.3193	0.8412	0.5201	0.5582
NM_001029913	Nkapl	0.2322	0.3480	0.2414	0.4520	0.4473	0.3375	0.3005	0.2826	0.4786	0.3797	0.3573	0.2264
NM_001009494	Ly49s7	0.2545	0.0605	0.3569	0.3465	0.1988	0.1033	0.4889	0.3632	0.1922	0.5251	0.3250	0.4011
NM_001009495	Ly49i4	0.0658	0.1912	0.4927	0.2858	0.2724	0.5077	0.0945	0.4626	0.4609	0.2203	0.2202	0.6140
NM_001025271	Sfpq	0.9293	0.7572	0.7065	0.9620	0.9798	0.8721	0.7510	0.8361	0.7309	0.7392	0.9454	0.2489
NM_001025147	Gpr160	0.3799	0.3279	0.2908	0.2219	0.3848	0.5213	0.3457	0.2162	0.2750	0.3760	0.3775	0.3667
NM_001025132	Cnih2	0.0427	0.0084	0.1410	0.4619	0.1639	0.3530	0.1497	0.3589	0.4246	0.2328	0.0093	0.3199
NM_001029921	Eps15l1	0.6467	0.5370	0.6932	0.3367	0.8106	0.6885	0.6897	0.8644	0.2420	0.5754	0.7078	0.2725
NM_001009503	Pfn4	0.7137	0.3040	0.0773	0.1860	0.5906	0.1421	0.4650	0.5236	0.0982	0.1663	0.7006	0.3843
NM_001009505	LOC494224	0.1373	0.5909	0.5095	0.5742	0.0235	0.1002	0.1143	0.0552	0.3263	0.0714	0.6018	0.3406
NM_001013227	Cops8	0.0620	0.2612	0.6266	0.0708	0.4482	0.3018	0.4846	0.7979	0.0328	0.1629	0.3341	0.0979
NM_001013053	Defcr24	0.4682	0.5256	0.5884	0.7432	0.6157	0.3186	0.3506	0.7313	0.7710	0.2352	0.3739	0.3002
NM_001013064	Fbxo17	0.6674	0.6662	0.2452	0.6309	0.7974	0.8489	0.8359	0.4882	0.3801	0.6231	0.7799	0.5503
NM_001009524	Bin2a	0.4136	0.2568	0.4464	0.6385	0.2917	0.1173	0.3029	0.0726	0.7723	0.1039	0.7965	0.4898
NM_022546	Dapk3	0.1917	0.1962	0.2057	0.4355	0.4307	0.3912	0.2259	0.2161	0.2872	0.3465	0.1951	0.3122
NM_001025272	Pgam5	0.3233	0.3007	0.9293	0.6749	0.1483	0.8838	0.6105	0.2230	0.3881	0.1536	0.4065	0.9373
NM_001013095	Eif3g	0.4727	0.3182	0.2055	0.2217	0.2761	0.6025	0.2258	0.3957	0.2618	0.1799	0.1950	0.3792
NM_001025279	Reep4	0.3339	0.4053	0.4162	0.2675	0.3047	0.2416	0.2962	0.5793	0.5169	0.2264	0.4168	0.4689
NM_001009538	MGC72612	0.7283	0.5257	0.1423	0.5535	0.5253	0.6477	0.4101	0.8451	0.7201	0.8531	0.2405	0.0484
NM_001033703	Apobec3f	0.8919	0.9449	0.5332	0.1019	0.9430	0.6168	0.8409	0.7965	0.6969	0.8793	0.8785	0.7662
NM_001033690	Orc6l	0.0253	0.7321	0.0318	0.5044	0.5230	0.4346	0.9511	0.5824	0.2524	0.4746	0.4217	0.0464
NM_001033070	Cip29	0.8123	0.6383	0.8889	0.9140	0.7542	0.4127	0.7590	0.8791	0.8294	0.8620	0.9202	0.1196
NM_001025400	Smndc1	0.4839	0.7341	0.9112	0.9260	0.4878	0.5910	0.6509	0.3589	0.1958	0.4423	0.2718	0.4533
NM_001025294	Mbp	0.3217	0.2552	0.5839	0.6952	0.7808	0.2483	0.4483	0.5076	0.5358	0.5647	0.5904	0.5811
NM_001009601	Klc4	0.4485	0.0893	0.5808	0.0439	0.1931	0.4273	0.2219	0.4307	0.0631	0.1334	0.1257	0.9322
NM_001025289	Mbp	0.3217	0.2552	0.5839	0.6952	0.7808	0.2483	0.4483	0.5076	0.5358	0.5647	0.5904	0.5811
NM_001025291	Mbp	0.3217	0.2552	0.5839	0.6952	0.7808	0.2483	0.4483	0.5076	0.5358	0.5647	0.5904	0.5811
NM_001025292	Mbp	0.3217	0.2552	0.5839	0.6952	0.7808	0.2483	0.4483	0.5076	0.5358	0.5647	0.5904	0.5811
NM_001025293	Mbp	0.3217	0.2552	0.5839	0.6952	0.7808	0.2483	0.4483	0.5076	0.5358	0.5647	0.5904	0.5811
NM_001031651	Tmub2	0.8905	0.8718	0.8075	0.1657	0.4129	0.5878	0.9387	0.5886	0.7800	0.6203	0.4524	0.8569
NM_001009621	Nudcd2	0.0203	0.9485	0.9468	0.2738	0.3816	0.2723	0.9204	0.2472	0.1078	0.9060	0.8954	0.0138
NM_001013859	Inpp5k	0.3671	0.5135	0.2516	0.3066	0.2423	0.4722	0.3397	0.3097	0.4263	0.3630	0.3098	0.3867
NM_001025402	Umps	0.1194	0.0675	0.1108	0.6871	0.0140	0.0507	0.0257	0.0079	0.0224	0.0822	0.0743	0.0196
NM_001033061	Cenpl	0.1329	0.5117	0.0275	0.1002	0.7595	0.8588	0.5964	0.7747	0.4880	0.5895	0.5631	0.0260
NM_001025403	Rbms2	0.1506	0.0277	0.9844	0.0195	0.4022	0.0659	0.9448	0.3698	0.7926	0.2599	0.2798	0.7584
NM_001033060	Rpain	0.0406	0.0562	0.7878	0.1173	0.0289	0.0058	0.0735	0.1250	0.1298	0.0884	0.1552	0.3689
NM_001025406	Exosc9	0.1141	0.0224	0.0933	0.1044	0.0201	0.0189	0.0046	0.0068	0.0228	0.0980	0.1414	0.0644
NM_001009630	RGD1311899	0.8936	0.8897	0.7481	0.3660	0.8709	0.4943	0.0955	0.6420	0.5996	0.9009	0.2396	0.8650
NM_001009631	Tmco1	0.3988	0.0753	0.0920	0.0769	0.8341	0.6252	0.1324	0.5524	0.0989	0.1704	0.4218	0.0862
NM_001013178	Med20	0.2725	0.1135	0.9694	0.4560	0.4496	0.7096	0.5793	0.3794	0.4332	0.4263	0.5762	0.6165
NM_001013160	Trim69	0.5667	0.4029	0.0744	0.2873	0.7557	0.3515	0.4547	0.5984	0.5497	0.3443	0.4326	0.3864

NM_001009635	RGD1306437	0.5974	0.7548	0.8120	0.1046	0.8768	0.7893	0.8821	0.8676	0.5421	0.3512	0.9282	0.8511
NM_001013182	Hn1l	0.6620	0.6259	0.4658	0.6993	0.5741	0.6608	0.6481	0.6053	0.5108	0.6494	0.6376	0.3529
NM_001025408	Mrps25	0.7849	0.2276	0.1067	0.3366	0.0830	0.2581	0.7386	0.8427	0.3101	0.0609	0.0639	0.1193
NM_001009638	RGD1311805	0.7985	0.8722	0.2112	0.5374	0.8906	0.7325	0.1473	0.7440	0.7298	0.8695	0.9777	0.7835
NM_001009640	Cirh1a	0.0541	0.0587	0.0585	0.0503	0.1061	0.1514	0.0663	0.1247	0.0497	0.0760	0.0333	0.0602
NM_001024371	Ncald	0.9852	0.9609	0.9741	0.9926	0.9856	0.9969	0.9669		0.9989	0.9991	0.9984	0.9623
NM_001024336	Tuba3b	0.4580	0.2940	0.2054	0.3586	0.2540	0.3862	0.3407	0.5036	0.4177	0.2917	0.3299	0.5036
NM_001033694	Srebf2	0.6405	0.8155	0.3198	0.0301	0.9286	0.9456	0.8926	0.9726	0.6550	0.5380	0.9322	0.9491
NM_001025410	Chmp5	0.2971	0.0716	0.6968	0.0194	0.1241	0.0385	0.0667	0.1120	0.4460	0.3567	0.2649	0.7530
NM_001025416	Dok1	0.0053	0.0072	0.0721	0.1469	0.0060	0.1646	0.0183	0.1093	0.0075	0.0436	0.0892	0.0402
NM_001025417	Actl7b	0.7479	0.7641	0.4524	0.5588	0.5101	0.7710	0.6276	0.6577	0.4833	0.7882	0.6795	0.8566
NM_001025418	Ppp2r1b	0.4565	0.7009	0.3398	0.3953	0.2467	0.4144	0.3721	0.4102	0.4581	0.3141	0.3358	0.4088
NM_001025419	Tax1bp3	0.6715	0.5801	0.4979	0.9881	0.8801	0.9587	0.9788	0.9643	0.3300	0.7220	0.2270	0.1708
NM_001025422	Irak2	0.3947	0.2193	0.0284	0.2153	0.1398	0.2751	0.2596	0.3083	0.1632	0.5278	0.0621	0.8314
NM_001025421	Cugbp1	0.9292	0.8012	0.5229	0.9757	0.5231	0.9038	0.9379	0.3771	0.5002	0.8114	0.8555	0.8791
NM_001009655	RGD1307465	0.7950	0.8937	0.1577	0.9285	0.6290	0.9052	0.9963	0.9566	0.9132	0.8005	0.9126	0.3620
NM_001009656	Zgpat	0.6039	0.7798	0.0751	0.2398	0.4559	0.7363	0.3226	0.6539	0.5701	0.4509	0.6158	0.5599
NM_001009659	RGD1305225	0.0518	0.6008	0.0183	0.0151	0.0384	0.0219	0.0236	0.0732	0.1839	0.2607	0.1151	0.1577
NM_001034143	Dars2	0.1560	0.0420	0.3360	0.2134	0.2191	0.2488	0.6140	0.6781	0.1655	0.1124	0.2229	0.6094
NM_001033697	Akr1c1	0.3294	0.3662	0.3232	0.2383	0.4180	0.2581	0.3788	0.3594	0.2406	0.3270	0.4377	0.2358
NM_001034032	Dnajc12	0.4978	0.6617	0.4327	0.4901	0.4002	0.4669	0.4457	0.3504	0.5032	0.5964	0.2476	0.6649
NM_013057	F3	0.7338	0.9413	0.8464	0.1669	0.9124	0.9807	0.9824	0.9749	0.9269	0.9797	0.8077	0.7697
NM_001029927	Cpvl	0.1940	0.3669	0.2046	0.1482	0.4352	0.2330	0.4682	0.3655	0.4282	0.1090	0.0971	0.5198
NM_001012066	Sphk2	0.4294	0.7554	0.6631	0.7439	0.5922	0.6006	0.9011	0.6015	0.5561	0.5306	0.6762	0.8249
NM_001025624	Hmgn2	0.9532	0.9999	0.7704	0.9918	0.9864	0.9627	0.9886	0.9417	0.9549	0.9920	0.9886	0.2936
NM_001025629	Mospd3	0.1422	0.1012	0.8294	0.8198	0.6916	0.5079	0.5953	0.5271	0.2173	0.0548	0.1096	0.7268
NM_001034006	Acap2	0.6306	0.7695	0.8950	0.6004	0.7751	0.2239	0.6954	0.4451	0.7939	0.7881	0.7801	0.6693
NM_001037362	MGC125086	0.3904	0.1504	0.4332	0.8681	0.0665	0.0947	0.6039	0.5676	0.4452	0.5563	0.1620	0.7515
NM_001009675	Tceal1	0.5635	0.4622	0.7635	0.2438	0.2626	0.0974	0.5435	0.0547	0.5763	0.8795	0.6938	0.3352
NM_001037768	Nagk	0.8775	0.7246	0.4003	0.7525	0.7964	0.8467	0.4672	0.7586	0.9417	0.9904	0.8184	0.9448
NM_001037316	Nat1	0.4680	0.4861	0.3385	0.4069	0.4889	0.2163	0.7030	0.3367	0.5104	0.3886	0.5554	0.3886
NM_001009677	Plekhm1	0.5374	0.3450	0.1866	0.3785	0.1592	0.2309	0.0279	0.2630	0.4839	0.2504	0.4186	0.4000
NM_001009678	RGD1311458	0.8454	0.7860	0.0880	0.2013	0.8571	0.9039	0.5959	0.9315	0.7604	0.8342	0.8312	0.6202
NM_001025638	Stx11	0.2168	0.7686	0.8736	0.7482	0.8226	0.2985	0.4924	0.6799	0.8485	0.8170	0.5190	0.7950
NM_012570	Glud1	0.9729	0.8207	0.6781	0.9896	0.6875	0.9701	0.6877	0.9821	0.8013	0.6171	0.6758	0.9318
NM_001025643	Fbl	0.8983	0.9062	0.2989	0.9285	0.7392	0.8409	0.9395	0.9498	0.7473	0.8845	0.9259	0.2641
NM_001025644	Syngr4	0.4065	0.5959	0.1440	0.4521	0.5715	0.5891	0.9147	0.2995	0.8988	0.4953	0.4904	0.3446
NM_001035238	Csnk2b	0.3330	0.3661	0.5590	0.3503	0.3109	0.3475	0.3291	0.4094	0.2633	0.4797	0.4271	0.3404
NM_001025647	RGD1310597	0.4796	0.1961	0.3527	0.3658	0.4117	0.2750	0.4342	0.2156	0.4594	0.3807	0.7097	0.5089
NM_001009686	Wdr23	0.3900	0.8958	0.3469	0.7700	0.8004	0.9231	0.3322	0.6100	0.9506	0.8284	0.4078	0.7866
NM_001012738	Ckmt1	0.4080	0.4342	0.2792	0.2214	0.2979	0.2747	0.3819	0.4964	0.3777	0.2757	0.5062	0.2945
NM_001009688	Thumpd1	0.9831	0.8614	0.9134	0.3174	0.9675	0.9762	0.7051	0.9368	0.6845	0.9495	0.8894	0.8501
NM_001025650	Dusp11	0.7650	0.7409	0.6695	0.7627	0.8780	0.6750	0.9087	0.6706	0.7391	0.8309	0.9470	0.6482
NM_001025649	Tm9sf4	0.5295	0.1112	0.2745	0.5942	0.1661	0.6201	0.4616	0.1715	0.0888	0.5590	0.2538	0.7971
NM_001025651	Sytl1	0.8870	0.9016	0.8667	0.8573	0.8609	0.8380	0.9787	0.8432	0.9058	0.9470	0.9177	0.8671
NM_001009694	LOC314140	0.3910	0.2633	0.3680	0.7288	0.3719	0.3047	0.5529	0.6735	0.2053	0.4683	0.4908	0.4467
NM_001025672	Pspc1	0.0254	0.1507	0.4737	0.7716	0.6119	0.1606	0.2439	0.2279	0.2484	0.0996	0.4189	0.0336
NM_012489	Acaa1	0.4013	0.1705	0.1359	0.5342	0.3685	0.3546	0.5440	0.2650	0.5966	0.3248	0.0835	0.3841
NM_001009696	Dcun1d5	0.4948	0.9111	0.1307	0.6540	0.6362	0.5605	0.7189	0.5486	0.4001	0.6521	0.9597	0.2008

NM_001025677	RGD1308782	0.3552	0.2618	0.0662	0.4658	0.0781	0.3102	0.0710	0.0481	0.2949	0.0311	0.0497	0.4667
NM_001025676	Rbm22	0.3089	0.6009	0.6392	0.6334	0.6873	0.4417	0.6106	0.6192	0.2739	0.2236	0.4838	0.3858
NM_001009701	Itfg3	0.1309	0.1704	0.2334	0.0862	0.1149	0.1527	0.1719	0.0794	0.4044	0.0251	0.0106	0.7708
NM_001013855	Timd2	0.6656	0.8137	0.8290	0.7477	0.5575	0.6815	0.7081	0.5337	0.6093	0.7652	0.7775	0.7445
NM_001025666	RGD1310572	0.2933	0.1648	0.0479	0.0083	0.4774	0.3133	0.1559	0.1093	0.1783	0.1584	0.0693	0.2209
NM_001025660	Ei24	0.3231	0.0871	0.3015	0.4378	0.6916	0.9253	0.1701	0.6640	0.1472	0.1554	0.0067	0.4794
NM_001013856	Cnot6	0.3435	0.1931	0.7581	0.8922	0.0872	0.8849	0.4502	0.3602	0.0459	0.0223	0.0947	0.6233
NM_001009707	RGD1359508	0.0176	0.0069	0.0314	0.4768	0.0655	0.0242	0.0728	0.0350	0.0218	0.0085	0.0331	0.0195
NM_001025675	Tubb6	0.0482	0.9864	0.0174	0.6714	0.6342	0.7456	0.6564	0.7540	0.9169	0.9656	0.7598	0.7111
NM_001009710	Lrrc41	0.6007	0.1177	0.5912	0.5794	0.6368	0.5853	0.0599	0.4129	0.9035	0.5153	0.4862	0.9149
NM_001009711	RGD1304567	0.2583	0.6543	0.0138	0.4611	0.8566	0.7208	0.7075	0.7394	0.0464	0.0432	0.0473	0.1404
NM_001025678	Fkrp	0.2530	0.3552	0.4337	0.8180	0.6698	0.8734	0.7390	0.8838	0.4696	0.1395	0.5766	0.7200
NM_001025682	Cdr2	0.1593	0.1825	0.3233	0.0166	0.0623	0.3762	0.7208	0.0962	0.8459	0.9260	0.8947	0.4893
NM_001025680	Gpr4	0.1178	0.3149	0.1225	0.0677	0.5562	0.1109	0.3087	0.3382	0.2715	0.4759	0.2381	0.6268
NM_001025043	Itpripl1	0.6737	0.1041	0.5633	0.5146	0.2528	0.2323	0.4999	0.0729	0.2845	0.4416	0.8031	0.4115
NM_001024304	Gstm4	0.6480	0.4160	0.4818	0.6735	0.4500	0.6814	0.5572	0.5948	0.5285	0.3821	0.2182	0.2722
NM_001025683	RGD1311946	0.0901	0.4622	0.0215	0.8693	0.1134	0.0972	0.6117	0.3710	0.0719	0.6857	0.0363	0.2814
NM_001009716	Asphd2	0.2021	0.5029	0.0577	0.5302	0.6508	0.1557	0.2916	0.5568	0.0300	0.3523	0.1558	0.3098
NM_001034913	Gpbp1l1	0.8976	0.3088	0.9117	0.8776	0.8168	0.9604	0.6165	0.6845	0.9040	0.7743	0.8830	0.8024
NM_001009718	Ly49i6	0.6689	0.7551	0.8660	0.8081	0.1486	0.7794	0.7215	0.5302	0.4050	0.3887	0.8864	0.4887
NM_001025700	Fbxl12	0.4208	0.0932	0.6516	0.1382	0.4725	0.9256	0.1868	0.2141	0.0772	0.2200	0.0907	0.2363
NM_001025692	Sdcbp2	0.2550	0.3676	0.3984	0.3712	0.5871	0.7102	0.6872	0.3160	0.4530	0.2084	0.3951	0.3024
NM_001034127	Alg8	0.9893	0.8081	0.1095	0.8858	0.8852	0.9895	0.8809	0.9946	0.9519	0.9836	0.9823	0.7226
NM_001025726	Srpk1	0.8619	0.8478	0.7145	0.9009	0.9806	0.6077	0.9672	0.7777	0.8211	0.7663	0.8126	0.9826
NM_012490	Acr	0.1757	0.1147	0.5296	0.2501	0.1308	0.1734	0.1950	0.1377	0.1589	0.0609	0.0635	0.1929
NM_001009962	Metrn	0.1127	0.3647	0.7576	0.6966	0.3412	0.0665	0.8391	0.6212	0.5380	0.5710	0.1134	0.0347
NM_001025709	Pdcl3	0.8587	0.9031	0.8119	0.9180	0.8462	0.7928	0.7881	0.6597	0.8490	0.4193	0.6073	0.6689
NM_001025717	Arpc5	0.9640	0.9533	0.9645	0.9351	0.9636	0.9620	0.9496	0.9425	0.8953	0.9718	0.7640	0.9774
NM_001025725	Hirip3	0.8068	0.8774	0.0350	0.1302	0.7720	0.6812	0.6689	0.7387	0.7739	0.9209	0.9489	0.1360
NM_001033709	Alg6	0.6858	0.8834	0.9209	0.8358	0.8692	0.6783	0.9336	0.5691	0.8283	0.8493	0.9386	0.8535
NM_001009972	Ggnbp1	0.0905	0.0767	0.0734	0.3556	0.6963	0.1988	0.3320	0.1838	0.5461	0.3919	0.0693	0.2148
NM_001009976	Spetex-2A	0.3420	0.1959	0.3792	0.2210	0.2802	0.2043	0.2255	0.2151	0.1852	0.1797	0.1947	0.1997
NM_001014211	Gcom1	0.2126	0.4490	0.6175	0.4236	0.2119	0.2213	0.4472	0.5483	0.6876	0.6249	0.7322	0.5395
NM_001013040	Ptpn9	0.0431	0.1113	0.7354	0.0591	0.1584	0.5273	0.2156	0.7205	0.4187	0.2355	0.6915	0.5991
NM_001010921	LOC494499	0.0674	0.8737	0.6533	0.8474	0.6206	0.5299	0.4992	0.8001	0.7964	0.8418	0.7365	0.6276
NM_001013862	Ccdc45	0.8965	0.6558	0.2681	0.0064	0.6258	0.4762	0.0111	0.8575	0.8914	0.7707	0.6382	0.4966
NM_001010946	Ficd	0.0983	0.6738	0.6506	0.7497	0.0600	0.3743	0.4989	0.0665	0.6570	0.8391	0.6947	0.7183
NM_001034104	Tmc4	0.8227	0.5720	0.6033	0.0537	0.1444	0.8463	0.7475	0.7645	0.6183	0.7863	0.3773	0.8539
NM_001033887	Prrc1	0.5757	0.8362	0.6875	0.1899	0.0589	0.2222	0.8943	0.0148	0.3993	0.8116	0.7936	0.1141
NM_001010961	Tmem17	0.1629	0.0579	0.2137	0.0604	0.0506	0.1154	0.1184	0.1297	0.0957	0.0078	0.1693	0.0497
NM_001025766	Il34	0.8918	0.6092	0.7790	0.5604	0.2027	0.4480	0.5939	0.1076	0.4301	0.1326	0.3657	0.2409
NM_001010963	Zc3h15	0.8906	0.9420	0.8089	0.9454	0.8457	0.7353	0.7446	0.6907	0.7335	0.7000	0.9083	0.8786
NM_001025698	Sf3a3	0.4610	0.4614	0.3480	0.8926	0.4713	0.5491	0.7160	0.4319	0.3933	0.5796	0.4668	0.4068
NM_001025714	Pldn	0.4312	0.6124	0.6068	0.5896	0.7042	0.6798	0.6475	0.5750	0.5893	0.5456	0.6373	0.6401
NM_001034144	Nkx3-1	0.2995	0.2978	0.0334	0.1406	0.1727	0.1346	0.3182	0.3106	0.3256	0.1057	0.6636	0.3142
NM_001025721	Colec12	0.2395	0.3200	0.3105	0.3001	0.1678	0.0724	0.1395	0.6808	0.7065	0.5208	0.6993	0.5592
NM_001033901	Tut1	0.3094	0.6608	0.0163	0.0913	0.1312	0.1464	0.1504	0.3547	0.5440	0.5335	0.4609	0.3392
NM_001031650	Mrpl44	0.0329	0.1122	0.4381	0.1740	0.1006	0.0310	0.0583	0.1028	0.4502	0.1829	0.1526	0.1409
NM_012642	Ren	0.3191	0.4244	0.1395	0.5205	0.6411	0.1703	0.6138	0.3787	0.2563	0.4018	0.1282	0.4263

NM_001033891	Mobkl1b	0.6962	0.5635	0.3909	0.6684	0.6752	0.6215	0.6986	0.4847	0.6155	0.4113	0.7049	0.6497
NM_001034135	Rbx1	0.3759	0.5459	0.9374	0.4873	0.2933	0.1521	0.9624	0.2188	0.2034	0.3186	0.0247	0.0530
NM_001031645	Acp6	0.1185	0.2887	0.7954	0.1391	0.1381	0.0239	0.5806	0.1458	0.0980	0.0140	0.1627	0.5864
NM_001014008	Aspn	0.8962	0.0510	0.8612	0.0097	0.8084	0.7587	0.7525	0.9379	0.2823	0.0839	0.0387	0.0085
NM_001013873	Fam152a	0.8569	0.9512	0.6808	0.9013	0.9642	0.7708	0.9169	0.9150	0.7082	0.8957	0.8897	0.5043
NM_001025732	Nol5a	0.6311	0.7077	0.6019	0.3971	0.5130	0.5309	0.4848	0.5970	0.4980	0.7942	0.5489	0.7544
NM_001014028	Fam108b1	0.3420	0.0654	0.9158	0.7784	0.1421	0.3179	0.0147	0.0153	0.3884	0.0713	0.0293	0.8526
NM_001025733	Procr	0.2069	0.0249	0.0995	0.0330	0.0421	0.1145	0.5468	0.0136	0.0148	0.1559	0.1543	0.6384
NM_012613	Npr1	0.8507	0.8563	0.7726	0.4792	0.4133	0.7549	0.1960	0.5481	0.3378	0.6353	0.6431	0.1866
NM_001025737	Smpdl3b	0.2167	0.2165	0.2454	0.2653	0.2144	0.2412	0.4635	0.3153	0.6720	0.5884	0.6202	0.4519
NM_001025739	Rpl11	0.5679	0.4570	0.1369	0.2198	0.5133	0.4730	0.5180	0.4531	0.3496	0.5151	0.7831	0.4414
NM_001025749	Grap	0.5282	0.7852	0.1895	0.2043	0.5126	0.7669	0.6538	0.7571	0.4097	0.2725	0.4286	0.4154
NM_001033987	Map2k5	0.6005	0.7676	0.5538	0.7732	0.8463	0.7961	0.9525	0.6141	0.9125	0.6528	0.8566	0.5215
NM_001025767	Blnk	0.0493	0.0641	0.1193	0.2247	0.0550	0.0162	0.1234	0.0632	0.0465	0.0946	0.0309	0.0275
NM_012782	Bckdha	0.1618	0.4571	0.7714	0.6324	0.0471	0.2610	0.6032	0.2333	0.1978	0.0692	0.0464	0.9413
NM_001033903	Rpe	0.6160	0.6696	0.6215	0.6509	0.5965	0.6079	0.6722	0.6674	0.6301	0.6978	0.4709	0.2082
NM_001030035	Rpgrip1	0.8614	0.7643	0.4915	0.8410	0.5927	0.8802	0.6069	0.8911	0.7662	0.2700	0.5277	0.4480
NM_001038600	Oprm1	0.5747	0.5691	0.6230	0.6104	0.5073	0.7519	0.4963	0.8155	0.6760	0.6699	0.7131	0.3974
NM_001030030	Cpsf3	0.4863	0.4704	0.0518	0.8329	0.4394	0.2854	0.8343	0.2900	0.8593	0.4355	0.4418	0.5509
NM_001012741	Lipg	0.3762	0.1514	0.5719	0.8275	0.6445	0.9000	0.6320	0.4700	0.4815	0.5773	0.4409	0.3664
NM_001011904	Rnf10	0.9765	0.8798	0.5567	0.7959	0.5001	0.9818	0.7368	0.8827	0.7835	0.9163	0.9771	0.8983
NM_001037180	Fkbp8	0.0741	0.1339	0.0384	0.4437	0.2178	0.2696	0.3651	0.4814	0.0616	0.2727	0.0320	0.8106
NM_001034998	Ctps2	0.5103	0.2504	0.2763	0.2965	0.2864	0.3061	0.3326	0.3475	0.3487	0.2388	0.3817	0.2326
NM_001025750	Plek	0.2354	0.4902	0.1430	0.4789	0.5467	0.2588	0.3077	0.7830	0.2615	0.4556	0.5799	0.3828
NM_001011906	Acbd6	0.8803	0.8143	0.7546	0.9452	0.8744	0.8148	0.8238	0.8452	0.8607	0.8258	0.8779	0.7944
NM_001037979	Adipor2	0.0054	0.0073	0.0211	0.3713	0.1177	0.0228	0.0369	0.3277	0.6286	0.0649	0.0522	0.7499
NM_001030021	Rplp2	0.6886	0.7072	0.4602	0.7171	0.5980	0.4011	0.7161	0.6791	0.6002	0.3394	0.5571	0.4193
NM_001030026	Ifi30	0.9682	0.7801	0.9328	0.9862	0.9343	0.9820	0.9371	0.8416	0.9586	0.7747	0.8012	0.8358
NM_001038601	Oprm1	0.5747	0.5691	0.6230	0.6104	0.5073	0.7519	0.4963	0.8155	0.6760	0.6699	0.7131	0.3974
NM_001011909	Gpatc2	0.2410	0.1486	0.1890	0.3480	0.0570	0.1213	0.1142	0.2459	0.3201	0.0673	0.1637	0.3785
NM_012705	Cd4	0.2216	0.6189	0.7244	0.6481	0.5529	0.0907	0.7423	0.4436	0.8743	0.7484	0.3140	0.6436
NM_001034936	Mare	0.8502	0.9660	0.3712	0.9133	0.9775	0.9303	0.9697	0.8867	0.9103	0.6864	0.7072	0.8536
NM_181636	Col23a1	0.5963	0.1458	0.5928	0.4326	0.2907	0.7068	0.7766	0.7995	0.5029	0.0067	0.0747	0.3560
NM_057205	Uba3	0.1971	0.4220	0.9229	0.3185	0.1620	0.2352	0.0139	0.1476	0.3172	0.5203	0.5793	0.6059
NM_001011917	Cab39l	0.4640	0.5045	0.5225	0.0412	0.3212	0.1211	0.5967	0.1057	0.3513	0.3050	0.6534	0.2562
NM_001109393	Timp4	0.4876	0.2442	0.5342	0.5203	0.4886	0.5360	0.4589	0.5765	0.7007	0.2499	0.4494	0.4560
NM_133560	Trak2	0.9096	0.9726	0.9773	0.9514	0.9078	0.9497	0.9729	0.9232	0.9901	0.9264	0.9957	0.9562
NM_001012356	Nono	0.3828	0.6379	0.2176	0.5913	0.6425	0.5890	0.3039	0.6445	0.5751	0.5077	0.6016	0.7028
NM_001029909	Syne1	0.4601	0.6513	0.6096	0.5735	0.6160	0.6714	0.8220	0.8938	0.6913	0.5075	0.6584	0.6577
NM_001013876	Fam161a	0.4793	0.6059	0.4462	0.4396	0.5612	0.4821	0.3733	0.5069	0.4888	0.3196	0.1540	0.3570
NM_001013210	Fkbp14	0.5718	0.5713	0.4687	0.6270	0.5078	0.4284	0.4000	0.6045	0.3727	0.4509	0.6406	0.4889
NM_001011926	Fts	0.5528	0.3613	0.8456	0.5725	0.5375	0.7344	0.6376	0.5851	0.0370	0.1803	0.0769	0.5240
NM_001034199	Nptx2	0.4817	0.3147	0.4706	0.3287	0.6078	0.5782	0.3368	0.2456	0.5357	0.4832	0.4319	0.1093
NM_012798	Mal	0.0012	0.0514	0.1872	0.1788	0.1447	0.1274	0.1580	0.1497	0.2753	0.1082	0.1943	0.6137
NM_012498	Akr1b1	0.1057	0.3010	0.5668	0.0436	0.0804	0.1241	0.2008	0.3352	0.1474	0.6302	0.4467	0.6177
NM_001034145	Elp2	0.7688	0.5444	0.2428	0.1799	0.7782	0.8085	0.2012	0.7620	0.5733	0.4630	0.7686	0.5184
NM_001033893	N4bp3	0.4750	0.6517	0.4977	0.1667	0.6469	0.8101	0.8742	0.9023	0.9576	0.8015	0.6416	0.9427
NM_001029920	RGD1310423	0.6413	0.7653	0.3074	0.6364	0.1413	0.5699	0.7697	0.4835	0.8598	0.4982	0.3374	0.1481
NM_001012740	Cbln2	0.6535	0.4520	0.4153	0.5188	0.6727	0.7496	0.6766	0.1698	0.0423	0.4590	0.6116	0.6483

NM_001034924	Sfrs8	0.5335	0.6683	0.6335	0.7341	0.6054	0.3561	0.9344	0.7229	0.4038	0.5782	0.7975	0.2796
NM_001011937	Doc2g	0.4506	0.6574	0.5231	0.5320	0.7029	0.7190	0.5169	0.8842	0.4521	0.2546	0.4771	0.5391
NM_001031642	Serpinb1a	0.0138	0.0121	0.0253	0.0029	0.0129	0.0014	0.0035	0.0628	0.0100	0.0162	0.0246	0.5822
NM_001031646	Stoml2	0.8713	0.8867	0.1806	0.9954	0.7065	0.7483	0.5994	0.7932	0.7076	0.5475	0.6185	0.0426
NM_001034117	Becn1	0.9401	0.9260	0.9801	0.9987	0.9693	0.7572	0.7621	0.7806	0.7484	0.9791	0.9774	0.9297
NM_001011943	Obfc1	0.4224	0.1813	0.5921	0.7388	0.4089	0.7593	0.5600	0.5190	0.3389	0.3447	0.3588	0.1479
NM_001011944	Slc37a1	0.6259	0.8759	0.6254	0.8610	0.7916	0.4148	0.3687	0.7878	0.7343	0.5354	0.5297	0.8319
NM_001031658	Slc11a1	0.5217	0.7763	0.1413	0.6150	0.1026	0.1773	0.6990	0.8201	0.2699	0.4063	0.1908	0.3733
NM_001031660	Msrb2	0.6523	0.8357	0.1555	0.3162	0.4582	0.6350	0.4427	0.3423	0.2718	0.6279	0.4620	0.0098
NM_001037096	Rassf2	0.3930	0.7266	0.6550	0.6703	0.4383	0.4603	0.5071	0.0574	0.2692	0.6321	0.4181	0.7009
NM_001031661	B4galt7	0.0099	0.0412	0.0570	0.0157	0.0701	0.1958	0.0480	0.1135	0.1345	0.3563	0.0921	0.1106
NM_001032397	Tcf21	0.3193	0.4867	0.8376	0.2550	0.0999	0.3107	0.5605	0.8879	0.8238	0.2228	0.3408	0.1890
NM_001033062	Cldn23	0.4284	0.6667	0.5751	0.6210	0.4816	0.4967	0.2695	0.4371	0.4511	0.1971	0.3537	0.5569
NM_001011957	Ell3	0.6922	0.5719	0.9085	0.9521	0.6301	0.6619	0.7405	0.7059	0.5453	0.6292	0.7765	0.7835
NM_001033069	Eif4ebp2	0.5094	0.7803	0.8006	0.8491	0.4925	0.8078	0.1748	0.3937	0.2813	0.9001	0.5513	0.8959
NM_012527	Chrm3	0.2874	0.8519	0.5494	0.2041	0.4063	0.6368	0.7477	0.6022	0.6959	0.4289	0.6785	0.7237
NM_001034961	Sohlh2	0.4841	0.3467	0.5408	0.6397	0.5700	0.6671	0.4970	0.2473	0.6254	0.3803	0.5650	0.5306
NM_001025415	Ch25h	0.4112	0.4526	0.5424	0.4847	0.4347	0.7293	0.3369	0.3128	0.2848	0.1213	0.4103	0.5283
NM_001013087	Elmod3	0.4174	0.3875	0.7517	0.9435	0.3899	0.4998	0.6657	0.2420	0.9325	0.5760	0.4582	0.4929
NM_001035251	Slc38a9	0.3573	0.0964	0.2578	0.0602	0.1430	0.3130	0.0361	0.0260	0.1781	0.4709	0.0318	0.0952
NM_001079892	RGD1565983	0.6509	0.6420	0.0070	0.0822	0.4678	0.8220	0.0856	0.1284	0.1984	0.0722	0.4507	0.1758
NM_001079895	Rbm9	0.8720	0.6560	0.3380	0.8007	0.6059	0.5622	0.7517	0.7199	0.6238	0.8936	0.7997	0.0910
NM_001011968	Gimap6	0.0789	0.5961	0.3952	0.1274	0.1642	0.1228	0.2594	0.2565	0.2954	0.4311	0.2532	0.1773
NM_001033679	Prkar1b	0.6790	0.6299	0.6476	0.6172	0.4089	0.6830	0.2367	0.7087	0.4933	0.1964	0.2446	0.6900
NM_012841	Dcc	0.8144	0.6204	0.5621	0.6818	0.8912	0.5575	0.8473	0.7858	0.5130	0.6689	0.5275	0.1356
NM_012523	Cd53	0.9114	0.7886	0.8018	0.9223	0.9185	0.8957	0.5476	0.5127	0.9392	0.7166	0.9079	0.7581
NM_001033683	Vat1	0.1444	0.1454	0.1796	0.0277	0.3000	0.2250	0.2971	0.1239	0.1962	0.2872	0.0155	0.2961
NM_001037367	Asb12	0.2692	0.1955	0.2041	0.2203	0.2957	0.2740	0.2251	0.3841	0.5525	0.4107	0.1995	0.4454
NM_012563	Gad2	0.8016	0.7174	0.7264	0.7298	0.3308	0.4967	0.7481	0.5801	0.3360	0.3967	0.4895	0.5911
NM_001033687	Ushbp1	0.4392	0.2372	0.2857	0.4156	0.3028	0.3471	0.4100	0.6748	0.4121	0.4275	0.7275	0.2500
NM_012497	Aldoc	0.0758	0.8946	0.9418	0.6888	0.5587	0.6483	0.9875	0.8372	0.8045	0.4879	0.8701	0.6864
NM_012515	Tspo	0.2857	0.1367	0.6771	0.9334	0.7257	0.4455	0.2676	0.8215	0.0175	0.2162	0.0605	0.0431
NM_001033999	Rpap1	0.5143	0.4574	0.1374	0.5482	0.6326	0.5353	0.2834	0.3763	0.6142	0.6299	0.7333	0.4408
NM_001033710	Prss16	0.6725	0.3578	0.5256	0.1903	0.5744	0.1764	0.1421	0.1664	0.4262	0.5409	0.7253	0.5224
NM_001034069	Tpm1	0.4367	0.7303	0.6371	0.6080	0.4992	0.4851	0.6099	0.4830	0.4713	0.7143	0.3045	0.7344
NM_001034068	Tpm1	0.4367	0.7303	0.6371	0.6080	0.4992	0.4851	0.6099	0.4830	0.4713	0.7143	0.3045	0.7344
NM_012576	Nr3c1	0.6096	0.8189	0.9518	0.8520	0.2466	0.2938	0.3411	0.8864	0.5635	0.6232	0.6631	0.9392
NM_001033852	Cdw92	0.7218	0.5101	0.8357	0.9485	0.6167	0.6931	0.6239	0.5232	0.8863	0.7039	0.7500	0.3854
NM_001034071	Tpm1	0.4367	0.7303	0.6371	0.6080	0.4992	0.4851	0.6099	0.4830	0.4713	0.7143	0.3045	0.7344
NM_001011996	Tmem24	0.0492	0.2547	0.1317	0.0172	0.1434	0.3436	0.2607	0.5508	0.3271	0.3217	0.2064	0.5500
NM_001034002	Yap1	0.3007	0.4087	0.8574	0.4855	0.3520	0.4649	0.5218	0.8019	0.7623	0.4652	0.0664	0.7155
NM_001012001	Lrrc2	0.6070	0.5571	0.5734	0.3088	0.2038	0.6320	0.7347	0.6833	0.5176	0.5781	0.5593	0.5492
NM_001025736	Gnl2	0.6515	0.4301	0.1183	0.1414	0.0584	0.4457	0.1385	0.0210	0.4122	0.5299	0.3409	0.7441
NM_001025738	Fusip1	0.8542	0.6073	0.5872	0.8813	0.8356	0.8998	0.8024	0.8724	0.5990	0.2091	0.9358	0.4248
NM_001025742	Dazap1	0.8900	0.7481	0.0926	0.4211	0.8837	0.8636	0.7986	0.9778	0.9121	0.9010	0.8522	0.6410
NM_001030024	Slc19a2	0.6068	0.1032	0.8303	0.2491	0.2990	0.8664	0.7169	0.3048	0.3864	0.6335	0.7426	0.6377
NM_001033892	Cpsf3l	0.0611	0.0565	0.0663	0.1090	0.0292	0.0402	0.1375	0.0562	0.0559	0.0069	0.1097	0.0549
NM_001033707	Arfgap2	0.7776	0.8510	0.6388	0.9580	0.9666	0.9969	0.9831	0.9027	0.9143	0.9111	0.6537	0.4825
NM_001012012	Snx11	0.6129	0.8248	0.9608	0.9300	0.6222	0.4102	0.6260	0.6592	0.1428	0.5050	0.5133	0.9577

NM_001012013	Acbd4	0.7425	0.4717	0.7185	0.7016	0.7116	0.4203	0.4381	0.8552	0.3802	0.8779	0.4424	0.5952
NM_012502	Ar	0.8819	0.9298	0.8720	0.8572	0.7489	0.5835	0.6225	0.4525	0.9884	0.6289	0.5206	0.7857
NM_012582	Hp	0.1033	0.7371	0.0234	0.0121	0.5659	0.7310	0.2263	0.8939	0.7253	0.8904	0.5745	0.7774
NM_012579	Hist1h1t	0.1032	0.3349	0.2025	0.4313	0.0282	0.3277	0.0876	0.0155	0.2623	0.1650	0.4793	0.1871
NM_001034935	Ppef1	0.4980	0.7468	0.7129	0.8130	0.6687	0.7019	0.7509	0.7172	0.7407	0.5859	0.8756	0.8252
NM_001013884	Uimc1	0.8848	0.8160	0.7589	0.0834	0.7225	0.6518	0.6881	0.7555	0.6879	0.6322	0.9337	0.9026
NM_001013903	Tmem171	0.1913	0.4534	0.2039	0.2201	0.1954	0.2034	0.2247	0.2145	0.3829	0.1794	0.1940	0.1994
NM_001024363	Als2cr11	0.7487	0.7550	0.6497	0.7254	0.5881	0.6323	0.6237	0.4618	0.7145	0.8388	0.8388	0.6025
NM_001035233	Slc1a2	0.4918	0.2181	0.6152	0.3460	0.6767	0.4787	0.6113	0.2641	0.4120	0.5095	0.2439	0.6315
NM_001034951	Acsf2	0.4512	0.2038	0.2196	0.5288	0.5446	0.6127	0.7324	0.4797	0.6186	0.1897	0.6718	0.6936
NM_001012032	Arhgap24	0.3331	0.4722	0.5536	0.4538	0.3080	0.2946	0.5769	0.0912	0.2751	0.6348	0.3066	0.7521
NM_012836	Cpd	0.4721	0.1664	0.5368	0.2032	0.3671	0.8576	0.3919	0.4427	0.5538	0.3629	0.0611	0.8726
NM_001031640	Eif3s9	0.3955	0.4123	0.4375	0.4194	0.5313	0.6107	0.3979	0.3695	0.4766	0.6684	0.4788	0.3704
NM_001031639	Psmd2	0.1557	0.1642	0.0269	0.3138	0.0923	0.1953	0.0647	0.0741	0.1649	0.1856	0.1344	0.2125
NM_012531	Comt	0.4756	0.1035	0.2123	0.7190	0.0738	0.0316	0.5018	0.0856	0.0885	0.2582	0.0213	0.4301
NM_001035257	Coq9	0.3876	0.5217	0.2086	0.3889	0.1973	0.0382	0.1740	0.0875	0.1360	0.0395	0.0889	0.1107
NM_001033655	Dnah1	0.5855	0.5074	0.4784	0.3611	0.2598	0.5232	0.2605	0.4710	0.8379	0.4033	0.0684	0.1192
NM_001039010	Ppcs	0.0444	0.3626	0.1983	0.3817	0.0124	0.2420	0.1427	0.2736	0.1498	0.1247	0.0136	0.0951
NM_001037556	Nmnat1	0.7780	0.4389	0.4193	0.5295	0.3776	0.3035	0.6870	0.4752	0.1621	0.4778	0.8925	0.3729
NM_001031656	Serinc2	0.5296	0.4446	0.5582	0.3260	0.3496	0.6437	0.6669	0.8732	0.9403	0.8612	0.6206	0.9673
NM_001012048	Sh2d4a	0.1692	0.1102	0.3360	0.2604	0.2699	0.1661	0.3563	0.0064	0.0921	0.0332	0.0144	0.0802
NM_001031659	Trip12	0.5147	0.3559	0.9034	0.8882	0.1588	0.8017	0.1789	0.1194	0.4193	0.3977	0.3791	0.8513
NM_001037217	Mmd2	0.8004	0.6454	0.4856	0.5822	0.8281	0.3261	0.9733	0.3181	0.7741	0.5698	0.7305	0.7578
NM_001013899	Ubqlnl	0.4671	0.1925	0.1371	0.2700	0.1910	0.2729	0.5975	0.2227	0.3656	0.2723	0.3558	0.4964
NM_001033067	Tmem45b	0.9895	0.9409	0.7903	0.9638	0.9521	0.9511	0.8455	0.9615	0.9700	0.9813	0.9770	0.8662
NM_133584	Pde5a	0.5124	0.5805	0.9393	0.6316	0.9466	0.5899	0.8928	0.6466	0.9587	0.3071	0.6749	0.5347
NM_001008340	Thap1	0.3984	0.2985	0.3076	0.5135	0.3222	0.2033	0.2245	0.3790	0.2581	0.3364	0.4148	0.1993
NM_012493	Afp	0.0578	0.0915	0.3978	0.4500	0.0125	0.0148	0.3742	0.0590	0.3637	0.1726	0.3202	0.4241
NM_001033652	Tmprss11d	0.2652	0.5004	0.4242	0.7253	0.3170	0.3070	0.4111	0.9026	0.8928	0.4065	0.5689	0.2831
NM_001033884	Rexo4	0.1970	0.3565	0.6103	0.6461	0.3327	0.3527	0.6486	0.2541	0.2741	0.6811	0.1447	0.3344
NM_001034960	Spag6	0.5128	0.3763	0.7843	0.5759	0.5202	0.8056	0.6293	0.8426	0.7788	0.5422	0.7114	0.4536
NM_001034953	Cdca7l	0.5979	0.6250	0.4680	0.6584	0.5770	0.6710	0.6798	0.2514	0.4383	0.6042	0.2923	0.5538
NM_001012065	Adck4	0.6054	0.5058	0.5713	0.6124	0.9291	0.7114	0.6841	0.9311	0.7751	0.6535	0.6798	0.7099
NM_001037975	LOC297568	0.4054	0.4862	0.5592	0.4465	0.6383	0.5677	0.5833	0.4944	0.7632	0.5410	0.6136	0.5353
NM_001034125	Per1	0.1174	0.0474	0.0030	0.0035	0.0708	0.0958	0.0499	0.0788	0.0379	0.1908	0.0237	0.4766
NM_001033674	Bicd2	0.6744	0.9285	0.7174	0.7163	0.6923	0.5990	0.8005	0.6721	0.4751	0.5206	0.8042	0.6427
NM_012560	Foxg1	0.7683	0.6269	0.7552	0.5871	0.6512	0.4800	0.4929	0.1086	0.6678	0.7551	0.8162	0.3435
NM_001013071	Tm7sf2	0.8116	0.8545	0.2711	0.7609	0.7269	0.7611	0.1708	0.6353	0.8776	0.7983	0.7304	0.7346
NM_001033681	Eif5a	0.7552	0.7871	0.9500	0.9128	0.4719	0.8484	0.9547	0.9748	0.6512	0.9348	0.7177	0.9327
NM_001014017	Zfp819	0.4873	0.7138	0.1071	0.0900	0.3312	0.5465	0.2851	0.3400	0.4347	0.4823	0.5622	0.2991
NM_001013914	Slc44a3	0.6084	0.6496	0.3314	0.4238	0.2734	0.3683	0.6888	0.4083	0.4049	0.4565	0.4806	0.3398
NM_001012074	Herc4	0.9403	0.5810	0.8420	0.3754	0.6517	0.7457	0.3587	0.8173	0.5856	0.5368	0.8642	0.7174
NM_001013917	Arhgap15	0.7024	0.1047	0.2417	0.5211	0.5573	0.5169	0.5298	0.8185	0.3614	0.5613	0.7396	0.7188
NM_001033688	Dsc2	0.7483	0.7306	0.8532	0.8845	0.7683	0.2479	0.7803	0.1795	0.8169	0.5339	0.9442	0.5954
NM_001038495	Atg12	0.3497	0.0812	0.9847	0.2144	0.0749	0.2128	0.3356	0.2959	0.6141	0.3709	0.2942	0.2962
NM_001037135	Pcdhga10	0.5850	0.8964	0.4894	0.7790	0.8718	0.7804	0.8780	0.4615	0.8918	0.8018	0.7664	0.7782
NM_001033691	Irf7	0.5368	0.6648	0.1205	0.7631	0.5462	0.2734	0.1576	0.4941	0.6017	0.7777	0.8919	0.7288
NM_001012083	Snx7	0.2052	0.1613	0.0592	0.0177	0.5989	0.7066	0.1314	0.8502	0.0642	0.0135	0.0831	0.0061
NM_012577	Gstp1	0.0190	0.0467	0.6987	0.0389	0.0784	0.0276	0.7632	0.1122	0.0393	0.0231	0.1203	0.0035

NM_001033696	Hnrpdl	0.9329	0.7526	0.2814	0.0334	0.8116	0.7709	0.0887	0.8318	0.9897	0.8887	0.9358	0.8707
NM_001033695	L3mbtl2	0.9262	0.8543	0.1461	0.0860	0.9576	0.9511	0.2762	0.6340	0.9878	0.5560	0.5446	0.8282
NM_012620	Serpine1	0.2179	0.9747	0.6892	0.2602	0.9861	0.3826	0.5856	0.9260	0.9872	0.7421	0.7660	0.9781
NM_001033708	Rae1	0.5999	0.5296	0.4753	0.1311	0.3414	0.3796	0.9441	0.3510	0.7101	0.5815	0.6234	0.3304
NM_001039013	Tle2	0.8474	0.7547	0.5973	0.8933	0.8807	0.8354	0.7347	0.9551	0.9184	0.9557	0.9475	0.8321
NM_001037285	Hnrnpf	0.8875	0.9597	0.9698	0.7130	0.8443	0.9277	0.9244	0.8831	0.8546	0.9349	0.9676	0.7430
NM_001012093	Zfp64	0.7053	0.6478	0.2527	0.5273	0.1989	0.3057	0.3781	0.2143	0.2195	0.2469	0.3224	0.5324
NM_001013924	Gtpbp5	0.5515	0.4068	0.4440	0.1147	0.6058	0.2774	0.4343	0.2283	0.7600	0.6860	0.5020	0.6792
NM_001038588	Prodh2	0.0961	0.5178	0.6950	0.1115	0.5141	0.0448	0.1715	0.0537	0.3602	0.8617	0.2813	0.7398
NM_001039014	Zdhhc3	0.6605	0.5282	0.7121	0.9899	0.8630	0.8088	0.7393	0.7114	0.2793	0.7246	0.4548	0.4420
NM_001033898	Calu	0.7254	0.7912	0.9966	0.8714	0.9853	0.9966	0.9904	0.9843	0.6645	0.7510	0.9369	0.9932
NM_001037348	Jtv1	0.4558	0.4016	0.2039	0.3683	0.3786	0.3949	0.3982	0.2141	0.4505	0.4206	0.3263	0.1991
NM_001012099	Xkr8	0.9365	0.8016	0.8640	0.6184	0.9356	0.7223	0.7192	0.9529	0.9482	0.6903	0.9813	0.9540
NM_001037287	Hnrnpf	0.8875	0.9597	0.9698	0.7130	0.8443	0.9277	0.9244	0.8831	0.8546	0.9349	0.9676	0.7430
NM_001037286	Hnrnpf	0.8875	0.9597	0.9698	0.7130	0.8443	0.9277	0.9244	0.8831	0.8546	0.9349	0.9676	0.7430
NM_001034070	Tpm1	0.4367	0.7303	0.6371	0.6080	0.4992	0.4851	0.6099	0.4830	0.4713	0.7143	0.3045	0.7344
NM_012819	Acadl	0.1235	0.6819	0.8823	0.8662	0.9071	0.8074	0.8364	0.7862	0.4995	0.7026	0.7617	0.6855
NM_001039031	Dak	0.5619	0.2360	0.3862	0.5448	0.5639	0.3883	0.3954	0.3089	0.6763	0.4851	0.2518	0.6429
NM_001012108	Thumpd2	0.4946	0.5040	0.4160	0.3905	0.5845	0.2199	0.7040	0.2404	0.5925	0.2010	0.4748	0.5310
NM_001034009	Mcam	0.6482	0.5048	0.6329	0.4866	0.4411	0.5425	0.5177	0.6059	0.4942	0.3772	0.8326	0.6181
NM_001013906	Lhfpl5	0.3752	0.5821	0.5075	0.1384	0.3559	0.6257	0.4600	0.1537	0.0503	0.1263	0.2168	0.5419
NM_001034015	Calcr	0.6574	0.6123	0.7032	0.7262	0.7249	0.4590	0.7658	0.4708	0.8241	0.6804	0.5363	0.6839
NM_001012112	Ankrd9	0.7088	0.8353	0.0562	0.3949	0.7667	0.8241	0.9163	0.7474	0.8286	0.8601	0.9791	0.8225
NM_001037338	Ndufb4	0.1880	0.0721	0.2880	0.0376	0.1049	0.0467	0.5130	0.0955	0.2280	0.0315	0.1373	0.0088
NM_001037336	Lrrc4	0.7232	0.6991	0.6891	0.6558	0.7139	0.6774	0.6481	0.5191	0.6465	0.6586	0.6572	0.6785
NM_001034020	Syn2	0.6827	0.7593	0.6898	0.6252	0.6666	0.8793	0.4728	0.4291	0.4994	0.4229	0.5407	0.6417
NM_001012115	Creb3l3	0.0639	0.5244	0.5051	0.4556	0.1734	0.2937	0.4565	0.3724	0.4762	0.6266	0.1025	0.0875
NM_012558	Fbp1	0.3211	0.0908	0.2210	0.1946	0.0454	0.4248	0.2119	0.8857	0.5123	0.0601	0.0773	0.0362
NM_001039016	Zdhhc9	0.5854	0.7008	0.6203	0.5742	0.6747	0.6939	0.5554	0.6561	0.5595	0.7357	0.7038	0.7243
NM_001012119	Cbx6	0.9546	0.6159	0.8735	0.8246	0.5213	0.9589	0.4364	0.7025	0.6552	0.4538	0.0736	0.8777
NM_001012121	Prr5	0.5996	0.3123	0.5793	0.3699	0.1867	0.1031	0.3442	0.5697	0.4600	0.4325	0.6726	0.7489
NM_012547	Drd2	0.7769	0.4653	0.8195	0.1190	0.5790	0.5217	0.9293	0.4929	0.6945	0.7375	0.5675	0.1802
NM_001034831	Zfp384	0.9937	0.9787	0.5288	0.9209	0.9855	0.8887	0.7694	0.9318	0.9602	0.9908	0.9435	0.9496
NM_001034830	Zfp384	0.9937	0.9787	0.5288	0.9209	0.9855	0.8887	0.7694	0.9318	0.9602	0.9908	0.9435	0.9496
NM_001012126	Tmem22	0.8427	0.5434	0.8052	0.9423	0.8597	0.4645	0.7853	0.3477	0.7295	0.8751	0.9537	0.6664
NM_001033931	Gmcl1	0.6438	0.6994	0.6255	0.5574	0.5777	0.4506	0.5678	0.5740	0.4633	0.4844	0.6198	0.6551
NM_001033957	Kcng3	0.4754	0.4350	0.6794	0.3052	0.5923	0.3807	0.4596	0.3185	0.5347	0.5799	0.4136	0.6758
NM_019361	Arc	0.2144	0.4437	0.1763	0.4262	0.3534	0.5138	0.1395	0.6197	0.2386	0.6932	0.3219	0.4140
NM_001033961	Kcnip2	0.5152	0.3958	0.2930	0.6152	0.4791	0.6706	0.5488	0.5655	0.7193	0.4522	0.6099	0.5872
NM_012554	Eno1	0.4781	0.2509	0.2522	0.8765	0.3471	0.5819	0.9360	0.4218	0.5500	0.2949	0.3751	0.5884
NM_001012134	B3gnt7	0.4457	0.1002	0.3652	0.2626	0.2197	0.5112	0.7637	0.4496	0.3434	0.7809	0.3326	0.4587
NM_012639	Raf1	0.9344	0.9881	0.7681	0.9732	0.9681	0.9878	0.9707	0.9309	0.9156	0.9659	0.9305	0.9386
NM_012725	Klkb1	0.5940	0.1579	0.4021	0.3003	0.4950	0.5690	0.6546	0.3890	0.3984	0.5853	0.2827	0.2357
NM_001012137	Fus	0.9116	0.7657	0.0988	0.7106	0.8787	0.7721	0.4665	0.7679	0.9978	0.9745	0.9103	0.4081
NM_012662	Svs4	0.4668	0.1522	0.3083	0.1293	0.7524	0.4922	0.5609	0.7920	0.5515	0.6000	0.4954	0.4576
NM_001012141	Tada2l	0.2940	0.1263	0.1049	0.1170	0.1534	0.2523	0.2130	0.0333	0.2128	0.0253	0.5723	0.5302
NM_001012142	Scrn2	0.5313	0.7360	0.4624	0.8480	0.8220	0.8206	0.5455	0.8434	0.8387	0.5296	0.6840	0.2766
NM_001037346	Rps19	0.2786	0.5585	0.6881	0.2244	0.5031	0.3838	0.7530	0.3661	0.4699	0.2395	0.3009	0.2585
NM_012663	Vamp2	0.9545	0.7092	0.4179	0.6149	0.9289	0.9636	0.5292	0.9333	0.6458	0.8030	0.6351	0.8290

NM_012687	Tbxas1	0.2343	0.2455	0.3485	0.1030	0.5985	0.3942	0.1759	0.2251	0.3175	0.3890	0.4910	0.0900
NM_012667	Tacr1	0.9122	0.7491	0.4702	0.4683	0.9653	0.6739	0.5274	0.7113	0.9241	0.8994	0.7026	0.8997
NM_012655	Sp1	0.2944	0.5526	0.3772	0.3381	0.4944	0.7351	0.3869	0.1024	0.5614	0.7070	0.3492	0.3958
NM_001013935	Fam54b	0.1832	0.6947	0.9449	0.7834	0.0689	0.3753	0.8039	0.0973	0.7070	0.5419	0.7083	0.4951
NM_001033971	RGD708545	0.2128	0.3889	0.4235	0.6647	0.2260	0.5157	0.7230	0.2867	0.3875	0.2374	0.2568	0.8062
NM_001012152	Tbc1d14	0.8925	0.9342	0.8395	0.9266	0.7814	0.7977	0.9370	0.5525	0.7524	0.9558	0.8576	0.8631
NM_001013069	Stard10	0.4421	0.1949	0.2599	0.2199	0.2403	0.2639	0.3330	0.3879	0.4238	0.2680	0.1937	0.6055
NM_012508	Atp2b2	0.8391	0.6340	0.9318	0.9165	0.9785	0.8776	0.9590	0.9631	0.9724	0.9076	0.8958	0.9391
NM_001012158	Letm2	0.7239	0.8490	0.5117	0.7520	0.8555	0.7149	0.8294	0.8878	0.7562	0.7988	0.9373	0.7646
NM_001037646	Fuz	0.8877	0.7343	0.3616	0.6833	0.4959	0.5644	0.3996	0.9214	0.7659	0.5347	0.8892	0.8490
NM_001034083	Snn	0.7158	0.5382	0.9105	0.6569	0.8969	0.8920	0.5754	0.6017	0.8929	0.6558	0.8209	0.7698
NM_001037644	Ccdc130	0.1457	0.0464	0.1502	0.1153	0.0414	0.2741	0.0158	0.1374	0.2511	0.0427	0.1705	0.3897
NM_001037554	Bex4	0.9164	0.8425	0.3713	0.3431	0.2623	0.0430	0.2308	0.6313	0.1949	0.0442	0.6037	0.1067
NM_001037365	Bex1	0.6625	0.3256	0.4063	0.2197	0.3212	0.3538	0.3923	0.4274	0.2855	0.2901	0.4911	0.5208
NM_012500	Apeh	0.0621	0.2332	0.0293	0.7556	0.1967	0.5383	0.8253	0.0840	0.3658	0.0796	0.0955	0.6300
NM_001033984	Ank3	0.7210	0.7056	0.4647	0.6855	0.5948	0.5547	0.5921	0.6848	0.7186	0.7348	0.6500	0.6718
NM_012529	Ckb	0.8010	0.9627	0.8461	0.9970	0.9973	0.9105	0.7685	0.8962	0.9302	0.8163	0.8873	0.8307
NM_012544	Ace	0.1348	0.0440	0.2987	0.2782	0.2833	0.4942	0.5277	0.5907	0.6789	0.0626	0.4851	0.1208
NM_001034107	Ing3	0.5299	0.6820	0.4402	0.8196	0.9564	0.8908	0.8267	0.5241	0.8346	0.7757	0.9214	0.7474
NM_001033998	Itgal	0.5119	0.9005	0.4869	0.3357	0.5988	0.5886	0.5739	0.8643	0.4014	0.7088	0.6098	0.7252
NM_001012173	Trub1	0.9893	0.8450	0.8756	0.0982	0.8670	0.9227	0.8013	0.7725	0.6927	0.9305	0.7587	0.7543
NM_001034108	40,970.0000	0.5444	0.2366	0.3727	0.6681	0.5868	0.6693	0.6299	0.3832	0.1965	0.2169	0.6017	0.3037
NM_001012175	Zfand3	0.2015	0.4422	0.5135	0.9139	0.2762	0.3671	0.4960	0.3780	0.5471	0.2503	0.5078	0.9198
NM_001013939	Raver1	0.8146	0.7983	0.1941	0.0235	0.8914	0.8820	0.5491	0.9760	0.8996	0.8205	0.4243	0.3865
NM_012496	Aldob	0.0571	0.2148	0.0623	0.1024	0.3710	0.5187	0.0299	0.1590	0.0752	0.0795	0.0548	0.2339
NM_001037651	Prtg	0.3318	0.4423	0.8078	0.7734	0.4984	0.9182	0.9311	0.5283	0.2561	0.8159	0.3717	0.6536
NM_001034112	Mlx	0.0945	0.7809	0.2809	0.1501	0.9005	0.3600	0.3995	0.9337	0.9835	0.9522	0.5986	0.3880
NM_001012180	Bbs7	0.1334	0.0459	0.0355	0.0635	0.1625	0.0064	0.0175	0.0129	0.1331	0.1050	0.1516	0.1877
NM_001110838	Toag1	0.4408	0.7716	0.5540	0.2152	0.4693	0.8014	0.4622	0.5031	0.6190	0.6688	0.6870	0.3788
NM_001107498	Samd4b	0.0998	0.8283	0.1646	0.1588	0.2399	0.2715	0.3050	0.3572	0.4576	0.3704	0.3760	0.9165
NM_001013942	Clip4	0.0073	0.0436	0.2579	0.0236	0.7610	0.6870	0.0034	0.6622	0.0686	0.0777	0.1182	0.0392
NM_012589	Il6	0.1835	0.1335	0.2185	0.1023	0.0388	0.0689	0.0614	0.0915	0.0384	0.4781	0.2887	0.0820
NM_001034124	Mfap4	0.5561	0.2935	0.0609	0.0711	0.1089	0.3717	0.5396	0.6190	0.7177	0.6060	0.3328	0.2040
NM_012603	Myc	0.9731	0.9122	0.8793	0.9695	0.9427	0.9449	0.8224	0.8986	0.9605	0.8147	0.8228	0.8409
NM_001034126	Nit2	0.9597	0.7850	0.6647	0.7605	0.7787	0.7219	0.8500	0.8568	0.7991	0.8293	0.8207	0.7714
NM_001012188	Creb3l2	0.8793	0.9749	0.4309	0.8194	0.8998	0.7802	0.9603	0.5149	0.8991	0.7783	0.6915	0.2940
NM_001024243	Nudt3	0.8135	0.3064	0.9145	0.3545	0.9103	0.8553	0.6761	0.9063	0.9253	0.5793	0.9024	0.9088
NM_001012190	Ubap1	0.2991	0.2929	0.6768	0.3492	0.1490	0.8234	0.1537	0.0442	0.2470	0.3272	0.1242	0.9351
NM_012505	Atp1a2	0.2432	0.4242	0.5392	0.6705	0.3135	0.4012	0.2516	0.5735	0.6493	0.3635	0.1541	0.2274
NM_012504	Atp1a1	0.2577	0.3587	0.4583	0.7595	0.1323	0.0087	0.2031	0.1831	0.8834	0.7230	0.7351	0.6810
NM_001012192	Tssc1	0.3821	0.3189	0.5200	0.5266	0.4805	0.5728	0.3178	0.2472	0.4790	0.2800	0.3844	0.4232
NM_001037787	Rpo1-3	0.7591	0.6879	0.7230	0.8419	0.8717	0.7420	0.4859	0.9378	0.3485	0.8639	0.4128	0.7453
NM_012597	Lipc	0.9284	0.7298	0.6981	0.7918	0.7887	0.6383	0.8445	0.6827	0.7420	0.6997	0.7299	0.4711
NM_001034075	Tpm1	0.4367	0.7303	0.6371	0.6080	0.4992	0.4851	0.6099	0.4830	0.4713	0.7143	0.3045	0.7344
NM_001034008	Ncoa4	0.6166	0.6882	0.6331	0.7221	0.7292	0.5121	0.4832	0.6044	0.4116	0.6737	0.6950	0.5853
NM_001012198	Arhgap9	0.6423	0.5667	0.6832	0.5419	0.3440	0.3999	0.5965	0.2535	0.5711	0.5864	0.4973	0.5093
NM_001012203	Klhdc3	0.4992	0.2125	0.4425	0.3388	0.5009	0.6509	0.4191	0.3725	0.5830	0.6958	0.2257	0.7259
NM_001012204	Traf3ip1	0.5722	0.5089	0.1541	0.2618	0.8903	0.6636	0.0518	0.7437	0.7528	0.4476	0.0905	0.1438
NM_001034131	Foxp1	0.7255	0.7588	0.7408	0.7630	0.7196	0.6906	0.8747	0.7248	0.7460	0.7524	0.7589	0.8575

NM_001012206	Phlda3	0.4432	0.9470	0.5090	0.5053	0.5646	0.3905	0.7966	0.7629	0.9176	0.9822	0.7542	0.6755
NM_001034133	Klhdc2	0.5560	0.4660	0.4652	0.5565	0.5476	0.4086	0.3154	0.6070	0.3259	0.2704	0.4191	0.3795
NM_001034134	Eif3s6ip	0.4114	0.2591	0.3721	0.2194	0.4113	0.4708	0.3796	0.4311	0.4973	0.3440	0.2985	0.1986
NM_001034136	Mrpl2	0.1911	0.4868	0.2952	0.2193	0.1951	0.3825	0.2242	0.2139	0.1847	0.4977	0.1934	0.2666
NM_001034137	Sp110	0.0926	0.0196	0.0208	0.0995	0.0097	0.1217	0.0045	0.0332	0.0411	0.1341	0.0228	0.0292
NM_001012212	Calr3	0.0908	0.0530	0.3328	0.3127	0.1036	0.2247	0.0768	0.0569	0.1747	0.1702	0.5028	0.0410
NM_001017449	Dph5	0.0549	0.3407	0.2421	0.0887	0.0739	0.0682	0.2090	0.2564	0.5638	0.0477	0.0911	0.3057
NM_001037770	Fbxo22	0.8186	0.8470	0.3572	0.9788	0.6830	0.6965	0.5510	0.7737	0.4434	0.6045	0.7446	0.6090
NM_001017497	Smr3a	0.8073	0.3210	0.2853	0.8118	0.8250	0.2390	0.7547	0.5801	0.7306	0.7647	0.8329	0.6692
NM_001034150	Srpr	0.9933	0.9734	0.6912	0.0919	0.3954	0.8656	0.3358	0.4124	0.6499	0.9298	0.6810	0.8673
NM_001012223	Bin2	0.7636	0.0996	0.0380	0.2533	0.0357	0.0355	0.0763	0.5335	0.3153	0.5033	0.0486	0.6154
NM_012596	Lepr	0.3519	0.2290	0.1708	0.0933	0.0859	0.2769	0.2496	0.3206	0.4064	0.3414	0.0558	0.5438
NM_001014111	Msl3l1	0.5903	0.6914	0.5854	0.6443	0.2040	0.4137	0.3622	0.4762	0.3088	0.6479	0.6940	0.3714
NM_001014061	Ccdc91	0.1822	0.7590	0.8767	0.7821	0.4787	0.8949	0.5389	0.8929	0.4427	0.7661	0.5750	0.7875
NM_001037353	Etv6	0.6787	0.3614	0.2283	0.5602	0.2122	0.5200	0.3532	0.4556	0.5456	0.1847	0.2810	0.3000
NM_001034198	Tmem33	0.6122	0.6732	0.9130	0.8593	0.6695	0.3082	0.5347	0.5539	0.6685	0.7249	0.8604	0.2774
NM_001100967	Nyx	0.1844	0.1068	0.4724	0.1744	0.3852	0.0554	0.6898	0.4358	0.2162	0.3480	0.1478	0.0168
NM_001106303	Hmx2	0.8855	0.8795	0.7796	0.7227	0.8713	0.7599	0.7508	0.8023	0.9101	0.5565	0.7288	0.7383
NM_001105868	Ephb3	0.9696	0.7665	0.8976	0.7562	0.5351	0.7958	0.9212	0.9538	0.1614	0.3331	0.7963	0.7777
NM_012708	Psmb9	0.0115	0.0791	0.1459	0.1975	0.0653	0.0030	0.1115	0.0077	0.0671	0.0917	0.0190	0.0808
NM_001035002	Atad1	0.6087	0.2059	0.9658	0.9656	0.0257	0.9387	0.6214	0.2296	0.6804	0.4288	0.9370	0.8026
NM_001012347	RGD1309360	0.4391	0.2536	0.2919	0.2190	0.4154	0.2959	0.4551	0.3607	0.2324	0.5004	0.5896	0.3200
NM_184048	Taf9	0.7092	0.5150	0.5446	0.4458	0.3787	0.5867	0.4531	0.5445	0.3405	0.5574	0.2840	0.2471
NM_001012353	MGC108823	0.7101	0.2482	0.5940	0.7363	0.4937	0.4214	0.9333	0.5557	0.5475	0.6565	0.6814	0.6645
NM_001017446	Ino80c	0.6397	0.4097	0.6835	0.4348	0.7255	0.9737	0.5182	0.9676	0.2973	0.2028	0.0264	0.5672
NM_001024743	Ugp2	0.2976	0.3526	0.7707	0.0242	0.0515	0.7619	0.7569	0.4067	0.1708	0.4697	0.4593	0.6522
NM_001034916	Rpl8	0.7429	0.5092	0.2621	0.1471	0.5034	0.4403	0.5342	0.8787	0.5094	0.6799	0.6144	0.0921
NM_001034918	Iws1	0.5883	0.8823	0.2752	0.1508	0.3503	0.1613	0.0819	0.1018	0.0587	0.0560	0.2850	0.2804
NM_001034922	Atad3a	0.8313	0.8693	0.0668	0.7696	0.8811	0.8598	0.8914	0.9667	0.9137	0.8423	0.8748	0.8238
NM_001034934	Arid5a	0.9290	0.8237	0.7770	0.8327	0.4269	0.9431	0.3626	0.6824	0.8845	0.6967	0.7469	0.9248
NM_001034938	Endog	0.1910	0.1948	0.2036	0.2189	0.1950	0.2032	0.4409	0.2137	0.1844	0.5002	0.1932	0.3198
NM_012761	Sp4	0.6859	0.3395	0.7859	0.8329	0.8072	0.7904	0.4040	0.8822	0.3465	0.7470	0.8684	0.8911
NM_012604	Myh3	0.0515	0.0740	0.1113	0.0033	0.1519	0.0664	0.0042	0.0054	0.0469	0.0013	0.1067	0.1041
NM_001013913	Slc16a4	0.9160	0.2945	0.6997	0.8463	0.7148	0.6394	0.6953	0.6205	0.2732	0.4312	0.5984	0.1757
NM_001014090	Oaf	0.0512	0.2016	0.0137	0.6364	0.4731	0.6434	0.1355	0.5423	0.1057	0.0072	0.2132	0.1155
NM_001014068	Hpdl	0.6092	0.7153	0.4187	0.7766	0.3840	0.8894	0.9514	0.6638	0.8745	0.6741	0.7859	0.7004
NM_001013966	Thg1l	0.5353	0.9370	0.0379	0.8336	0.8386	0.1976	0.8345	0.7099	0.7966	0.7943	0.6452	0.0638
NM_001034944	Grap2	0.5126	0.3381	0.4125	0.6891	0.3351	0.4321	0.8197	0.7897	0.5245	0.3409	0.7081	0.6162
NM_001035234	Smndc1	0.4839	0.7341	0.9112	0.9260	0.4878	0.5910	0.6509	0.3589	0.1958	0.4423	0.2718	0.4533
NM_012652	Slc9a1	0.8187	0.8987	0.3564	0.7667	0.9810	0.8045	0.9440	0.9325	0.8429	0.8019	0.6693	0.4907
NM_012648	Scnn1b	0.1942	0.3781	0.4762	0.2960	0.2356	0.1662	0.3451	0.0936	0.4016	0.2821	0.3594	0.6373
NM_001035236	Tagln3	0.5755	0.4251	0.6597	0.4033	0.5526	0.6298	0.5136	0.6396	0.2246	0.2347	0.5409	0.3522
NM_001035237	Tcfe2a	0.8033	0.7013	0.4874	0.4137	0.5227	0.3850	0.5069	0.9718	0.9249	0.6417	0.8848	0.5399
NM_012654	Slc9a3	0.6547	0.5639	0.5258	0.5724	0.6847	0.4196	0.4741	0.5053	0.5970	0.3574	0.5880	0.5177
NM_012601	Mpg	0.1066	0.0273	0.0077	0.0839	0.0377	0.0538	0.0153	0.4072	0.4463	0.4995	0.2961	0.0096
NM_012721	P2rx6	0.6241	0.4711	0.1276	0.2799	0.4234	0.9153	0.4568	0.6376	0.0952	0.4385	0.1835	0.3227
NM_012643	Ret	0.9914	0.6441	0.9497	0.8543	0.5628	0.5902	0.9333	0.8131	0.9162	0.6801	0.7398	0.9820
NM_017265	Hsd3b6	0.4811	0.1549	0.5571	0.5965	0.5402	0.5724	0.8936	0.1624	0.7088	0.7384	0.6835	0.5815
NM_012982	Msx2	0.9805	0.9300	0.9072	0.6392	0.8723	0.5404	0.9224	0.5888	0.9382	0.9287	0.9653	0.5735

NM_001037137	Pcdhga5	0.4177	0.7258	0.3013	0.7799	0.5337	0.9209	0.3104	0.4035	0.4955	0.5027	0.4710	0.9221
NM_001037156	Pcdhga8	0.5357	0.6757	0.2443	0.6040	0.5919	0.6877	0.6256	0.7121	0.7026	0.5373	0.5041	0.7214
NM_001037154	Pcdhga3	0.6232	0.3292	0.2006	0.5782	0.4985	0.5454	0.7910	0.8222	0.4318	0.4293	0.3992	0.7874
NM_012592	Ivd	0.7454	0.7460	0.9408	0.8144	0.7424	0.9397	0.8806	0.8911	0.9915	0.7680	0.8881	0.8118
NM_001037158	Pcdhga9	0.5356	0.6756	0.2441	0.6038	0.5918	0.6877	0.6255	0.7119	0.7025	0.5371	0.5040	0.7213
NM_001012749	Ly49s5	0.2539	0.3874	0.3764	0.3778	0.2383	0.5968	0.3994	0.2134	0.2050	0.2366	0.5459	0.2182
NM_001008339	Whsc2	0.9518	0.9890	0.9696	0.9739	0.6840	0.9905	0.8037	0.9082	0.7932	0.7906	0.9993	0.4307
NM_001037179	Lrrc8c	0.9087	0.7578	0.6985	0.8220	0.7394	0.5557	0.8221	0.8005	0.7370	0.7936	0.8984	0.1563
NM_012562	Fuca1	0.0579	0.1112	0.8384	0.1908	0.0590	0.2443	0.3778	0.0165	0.1241	0.0043	0.1239	0.4475
NM_012744	Pc	0.3826	0.5875	0.7860	0.9114	0.1828	0.5146	0.8636	0.6216	0.6170	0.9361	0.7897	0.9644
NM_012715	Adm	0.0942	0.0927	0.9489	0.1790	0.1290	0.0064	0.8922	0.1821	0.1982	0.0122	0.1435	0.2159
NM_001037192	RGD1307890	0.2816	0.4120	0.5895	0.6388	0.2422	0.3391	0.4861	0.2133	0.2662	0.2966	0.7123	0.4118
NM_001037204	Taf1a	0.2803	0.8643	0.5100	0.0366	0.5763	0.6366	0.1910	0.7079	0.3892	0.7008	0.5699	0.4364
NM_001037198	RGD1304644	0.4431	0.6245	0.4367	0.4287	0.7553	0.5446	0.5641	0.4234	0.5808	0.0526	0.3744	0.7594
NM_001037208	Creld2	0.8956	0.7730	0.0047	0.0744	0.0748	0.0246	0.7664	0.0323	0.8233	0.0911	0.2931	0.0216
NM_012636	Pthlh	0.9982	0.9868	0.9493	0.7939	0.8764	0.9901	0.2952	0.8703	0.8532	0.8583	0.9413	0.9533
NM_001037199	Dhrs3	0.5499	0.4880	0.2237	0.2814	0.4453	0.6410	0.2738	0.6707	0.4905	0.2992	0.2540	0.6158
NM_012981	Mras	0.8600	0.3367	0.2403	0.9408	0.9349	0.9128	0.6714	0.9532	0.3800	0.5392	0.2781	0.5202
NM_012669	Hnf1a	0.6499	0.6511	0.8240	0.3169	0.4757	0.6697	0.8045	0.5211	0.5571	0.3365	0.0288	0.5540
NM_012735	Hk2	0.2748	0.4390	0.9304	0.8580	0.9376	0.9376	0.9732	0.5644	0.5861	0.4528	0.0778	0.9968
NM_012736	Gpd2	0.7238	0.7519	0.3865	0.8685	0.4933	0.7340	0.7317	0.8603	0.8463	0.9954	0.8801	0.7238
NM_001013054	Adprhl1	0.8852	0.8251	0.8921	0.8315	0.9304	0.8678	0.9067	0.9736	0.9813	0.9277	0.7943	0.9560
NM_001013057	Akr1c21	0.5006	0.4936	0.0608	0.1610	0.2437	0.5182	0.0773	0.3054	0.1450	0.1986	0.1628	0.3132
NM_001025657	Ssu72	0.0336	0.2943	0.3755	0.7925	0.0435	0.1078	0.7242	0.1632	0.0959	0.1006	0.3860	0.0246
NM_001013975	RGD1307119	0.1272	0.0101	0.1033	0.0700	0.0445	0.0056	0.0216	0.1395	0.1865	0.0257	0.0970	0.6489
NM_001013065	Ggn	0.5864	0.5277	0.2788	0.0212	0.5166	0.4593	0.2024	0.3767	0.3445	0.3427	0.0725	0.3286
NM_001013066	Sipa1l3	0.8649	0.6349	0.7299	0.2706	0.6118	0.2275	0.6491	0.2647	0.4434	0.5608	0.5957	0.4258
NM_001013068	Mrpl46	0.1005	0.4145	0.0839	0.0696	0.0850	0.0703	0.4456	0.5485	0.0966	0.7628	0.4701	0.0452
NM_001013245	Tbca	0.5186	0.2604	0.2102	0.5048	0.7116	0.5138	0.7280	0.4363	0.5953	0.3996	0.7271	0.1983
NM_012686	Vsnl1	0.2437	0.2040	0.2858	0.0955	0.1601	0.1348	0.0078	0.1373	0.1619	0.2871	0.1798	0.5522
NM_001037327	Csmd1	0.4655	0.3854	0.5312	0.4447	0.6389	0.6554	0.8187	0.6371	0.6801	0.6335	0.3198	0.2686
NM_001037358	Lmo2	0.2893	0.7296	0.4273	0.2686	0.5086	0.3722	0.4734	0.3898	0.4464	0.3261	0.5448	0.4778
NM_012826	Azgp1	0.5610	0.4881	0.3907	0.4372	0.1092	0.1101	0.5394	0.0817	0.3354	0.1610	0.1493	0.1392
NM_001013078	Fsip1	0.5838	0.6726	0.8631	0.7936	0.8421	0.7496	0.6932	0.7994	0.8576	0.7529	0.8011	0.7292
NM_001013081	Phyhd1	0.7315	0.0463	0.0945	0.6236	0.7652	0.8725	0.0125	0.8030	0.2725	0.0735	0.0533	0.2886
NM_001037544	Slc6a14	0.0965	0.0798	0.6233	0.0715	0.1792	0.0174	0.3989	0.3813	0.0696	0.3149	0.5842	0.0619
NM_012651	Slc4a1	0.1658	0.7981	0.5597	0.5642	0.7772	0.8923	0.8577	0.4771	0.4188	0.7059	0.5209	0.8882
NM_012808	Tst	0.4800	0.7786	0.8871	0.8529	0.6883	0.7469	0.8100	0.8820	0.7361	0.7769	0.8097	0.5721
NM_012806	Mapk10	0.1462	0.0421	0.5098	0.1456	0.0668	0.3860	0.1414	0.5898	0.6595	0.0737	0.1086	0.6525
NM_001037774	Cldn8	0.6627	0.1229	0.5671	0.6050	0.5862	0.1801	0.7749	0.3194	0.8342	0.5915	0.8677	0.5464
NM_001017461	Ogdh	0.0619	0.4280	0.1050	0.0770	0.0782	0.3670	0.0899	0.1503	0.7128	0.4954	0.6969	0.7823
NM_001017381	Fli1	0.6705	0.5363	0.4767	0.7510	0.7335	0.3432	0.5494	0.5346	0.5896	0.6365	0.4617	0.8397
NM_012537	Cyp11b1	0.7697	0.3707	0.1573	0.5302	0.8956	0.9706	0.2160	0.9851	0.1999	0.3810	0.0051	0.0916
NM_001037777	Lrp10	0.0178	0.0671	0.0529	0.1168	0.0533	0.1067	0.0966	0.1763	0.0981	0.0555	0.1090	0.7335
NM_001013092	Creb3	0.1492	0.5850	0.2131	0.2460	0.1147	0.1403	0.1851	0.1255	0.7979	0.2525	0.2768	0.1777
NM_001013093	Ubxd3	0.6763	0.5739	0.8187	0.1368	0.3504	0.7627	0.9178	0.3046	0.5883	0.5617	0.5012	0.9715
NM_001037788	Styxl1	0.1069	0.1625	0.8136	0.2767	0.4077	0.2594	0.1948	0.0919	0.1660	0.1407	0.0516	0.6848
NM_001037974	Jph2	0.2039	0.0376	0.0894	0.6460	0.0115	0.2064	0.0675	0.0623	0.0583	0.4239	0.0962	0.5391
NM_001037792	RGD1311122	0.8155	0.8611	0.9140	0.8465	0.9168	0.9490	0.7117	0.7135	0.5780	0.5355	0.6792	0.3378

NM_001013098	Dhrs7	0.4911	0.5163	0.5314	0.6525	0.6025	0.3058	0.5453	0.3146	0.5623	0.4051	0.5032	0.8486
NM_001013099	Slc38a6	0.6061	0.4187	0.5032	0.1270	0.8971	0.8630	0.3829	0.9296	0.5067	0.3375	0.0787	0.5999
NM_207605	Sh2d2a	0.1452	0.6294	0.5411	0.1578	0.3925	0.1895	0.6492	0.5447	0.3863	0.5091	0.3526	0.5219
NM_207604	Tlr6	0.0975	0.0049	0.5796	0.0926	0.0063	0.0184	0.0739	0.0075	0.0333	0.0894	0.0338	0.1289
NM_207593	Psbpc2	0.5463	0.5728	0.7935	0.5698	0.4009	0.5265	0.5299	0.5273	0.7318	0.7547	0.7842	0.6625
NM_207603	Fcgr3a	0.4018	0.4366	0.4887	0.6560	0.0951	0.6266	0.8222	0.8006	0.5122	0.4657	0.5233	0.8123
NM_207602	St3gal6	0.4521	0.7085	0.6351	0.7803	0.9425	0.5197	0.2644	0.6457	0.6339	0.6050	0.7012	0.0290
NM_199385	Dld	0.1668	0.1049	0.6057	0.1357	0.4376	0.7099	0.0638	0.1252	0.0645	0.1215	0.4697	0.8430
NM_198738	Psat1	0.5498	0.3767	0.3592	0.5487	0.5759	0.5567	0.3913	0.5518	0.4744	0.4760	0.6217	0.5222
NM_198727	Tmem132d	0.4326	0.5949	0.4905	0.7065	0.3229	0.0518	0.6807	0.5142	0.7917	0.7044	0.3978	0.8244
NM_212463	Ehmt2	0.8273	0.7828	0.5811	0.7513	0.8358	0.9008	0.8276	0.8919	0.6944	0.8479	0.8917	0.9918
NM_012750	Gfra2	0.2785	0.4706	0.3372	0.2806	0.2833	0.1040	0.4156	0.1829	0.8676	0.6712	0.6586	0.4860
NM_001014079	Fam164c	0.4965	0.6542	0.6624	0.6808	0.2639	0.8420	0.8586	0.2829	0.2533	0.7985	0.9241	0.4312
NM_001013120	Igsf11	0.2759	0.6864	0.7394	0.6393	0.8034	0.7466	0.6823	0.5363	0.1836	0.5945	0.2535	0.7343
NM_001038494	Tmem147	0.6164	0.7714	0.7394	0.8602	0.7473	0.5369	0.8646	0.7001	0.8927	0.7008	0.6670	0.7987
NM_012583	Hprt1	0.1823	0.0644	0.4265	0.2411	0.0545	0.2904	0.1760	0.2361	0.0635	0.1242	0.1646	0.2745
NM_013116	Tshb	0.8513	0.3451	0.8077	0.6365	0.5426	0.4662	0.8573	0.5305	0.1358	0.6367	0.6092	0.1575
NM_001038591	Ing1	0.1236	0.9337	0.8070	0.9005	0.7879	0.7878	0.7828	0.5182	0.5899	0.0740	0.1866	0.2289
NM_001038596	Dnaja3	0.3165	0.5369	0.3465	0.4704	0.3485	0.4695	0.4702	0.3516	0.2426	0.7153	0.2529	0.4037
NM_001013131	Ttc5	0.8147	0.4407	0.5904	0.3599	0.4509	0.7505	0.1168	0.5971	0.0447	0.0849	0.8206	0.3358
NM_001013132	Fbxo16	0.1495	0.1643	0.0353	0.2844	0.0905	0.0654	0.0668	0.1879	0.1569	0.2785	0.2458	0.2154
NM_001013133	Rhobtb2	0.1869	0.6927	0.1780	0.7922	0.7443	0.3733	0.9034	0.3752	0.5999	0.0955	0.3913	0.3718
NM_001013135	Sdccag3	0.8545	0.8871	0.4262	0.6944	0.8874	0.9743	0.4159	0.9624	0.7875	0.9201	0.7813	0.8752
NM_001039001	Trap1	0.0222	0.0391	0.1767	0.0444	0.0870	0.1538	0.5454	0.1627	0.1543	0.4732	0.3866	0.5617
NM_001039004	Cpsf5	0.5022	0.3549	0.4611	0.4551	0.3358	0.3673	0.2235	0.3040	0.3055	0.4922	0.5939	0.3613
NM_012993	Nrd1	0.0464	0.2068	0.0111	0.0612	0.0333	0.0438	0.0437	0.0585	0.0145	0.0958	0.0134	0.0231
NM_001039005	Mcoln2	0.4907	0.6473	0.5200	0.6114	0.1676	0.2051	0.4054	0.5248	0.3502	0.5688	0.6791	0.5489
NM_001014094	Plscr2	0.0288	0.0557	0.2799	0.2468	0.0948	0.1293	0.0652	0.0268	0.0523	0.0276	0.1662	0.0165
NM_012785	Coq7	0.0972	0.7076	0.2828	0.7928	0.2549	0.0424	0.7326	0.2801	0.5093	0.3669	0.6191	0.0162
NM_012700	Stx1b	0.6500	0.2540	0.8627	0.4647	0.5333	0.3503	0.7097	0.3603	0.4875	0.2734	0.4138	0.4230
NM_001039011	Cnksr1	0.3759	0.0737	0.6841	0.8912	0.7275	0.6008	0.6194	0.4753	0.5570	0.2033	0.9417	0.7570
NM_001013149	Mesdc1	0.8608	0.3156	0.8117	0.8423	0.7906	0.9222	0.9975	0.8256	0.8861	0.3213	0.8035	0.9235
NM_013190	Pfkl	0.0457	0.1468	0.0569	0.7620	0.7578	0.7976	0.9056	0.8877	0.1110	0.1035	0.0412	0.8857
NM_012791	Dyrk1a	0.1817	0.9210	0.5062	0.2527	0.7533	0.8132	0.1440	0.1945	0.9555	0.9778	0.8499	0.5544
NM_001013153	Ubtd1	0.1025	0.1439	0.0489	0.0408	0.0202	0.1753	0.0847	0.1450	0.1084	0.1649	0.0475	0.2891
NM_001039259	Zdhhc19	0.1106	0.1523	0.1119	0.0655	0.2204	0.1438	0.1880	0.1511	0.1648	0.3030	0.3806	0.3452
NM_012822	Alox5	0.7302	0.6672	0.7340	0.3446	0.7177	0.6374	0.6921	0.8265	0.0931	0.6768	0.5314	0.5787
NM_001039020	Zfp207	0.7781	0.8463	0.2254	0.8687	0.7825	0.8047	0.7812	0.9129	0.5255	0.8059	0.9480	0.2491
NM_001013988	Stbd1	0.8689	0.7950	0.6671	0.8460	0.7803	0.7852	0.9555	0.8673	0.7358	0.9304	0.7903	0.8149
NM_001014088	Eepd1	0.5731	0.4941	0.3766	0.6850	0.2204	0.5276	0.7047	0.3852	0.2088	0.6074	0.3431	0.2248
NM_012710	Semg1	0.8619	0.6241	0.2534	0.6483	0.3295	0.4385	0.6148	0.6418	0.3835	0.6536	0.6145	0.7800
NM_001013162	Scrn3	0.2742	0.0606	0.7257	0.5488	0.1409	0.1905	0.1802	0.4224	0.3186	0.7839	0.3297	0.8206
NM_001039024	RGD1311267	0.5139	0.2116	0.2114	0.5146	0.5197	0.6586	0.7317	0.7075	0.6570	0.4455	0.6873	0.6876
NM_001007008	Chchd10	0.0776	0.2206	0.6039	0.4871	0.0681	0.0236	0.1099	0.0835	0.4063	0.2776	0.0732	0.8551
NM_001039028	Actr1b	0.6585	0.7229	0.5727	0.6728	0.6321	0.6273	0.5531	0.6955	0.5857	0.6557	0.5334	0.5855
NM_001039033	Actl6a	0.8823	0.9503	0.1362	0.2362	0.6743	0.8774	0.4829	0.6404	0.9020	0.9025	0.9712	0.8518
NM_001039035	Sfrs7	0.9653	0.9011	0.1612	0.4397	0.8823	0.7314	0.4718	0.9798	0.7994	0.5759	0.7518	0.4424
NM_001039034	Wdr5	0.6169	0.6368	0.3438	0.2865	0.6407	0.7286	0.6756	0.3913	0.7977	0.4644	0.3738	0.4285
NM_001013172	Fhl4	0.9807	0.5290	0.9392	0.7250	0.9613	0.3829	0.8787	0.9647	0.4595	0.8038	0.9668	0.6669

NM_001039037	Zdhhc13	0.9671	0.7496	0.8862	0.7172	0.1785	0.4029	0.3926	0.0714	0.7859	0.8460	0.9169	0.9840
NM_013156	Ctsl1	0.0087	0.1164	0.2610	0.3069	0.0086	0.0830	0.1293	0.0057	0.0169	0.1732	0.1323	0.1715
NM_001039197	Tor1b	0.7148	0.6815	0.8431	0.6866	0.8248	0.9518	0.1727	0.8825	0.8314	0.9160	0.7194	0.8217
NM_001039208	RGD1305613	0.9824	0.7512	0.2434	0.3192	0.9919	0.8829	0.5585	0.9679	0.8925	0.9940	0.9257	0.4812
NM_001013183	Narfl	0.0691	0.2119	0.0610	0.1630	0.1820	0.2605	0.3618	0.4182	0.4740	0.1351	0.1500	0.4998
NM_001013187	Slc25a30	0.1509	0.4185	0.3567	0.4140	0.2483	0.4305	0.1479	0.3362	0.4726	0.3748	0.2736	0.6253
NM_001013189	Pqlc1	0.3683	0.1352	0.6792	0.8137	0.2074	0.4062	0.4786	0.4260	0.7669	0.1220	0.1685	0.3058
NM_001013192	Olfml1	0.6210	0.6539	0.4305	0.5599	0.5431	0.4873	0.5259	0.6072	0.6007	0.7282	0.4401	0.6309
NM_012609	Nf1	0.4150	0.4872	0.6026	0.7924	0.3324	0.8974	0.5807	0.5479	0.3826	0.8071	0.5966	0.9464
NM_001014153	Prmt7	0.1907	0.3698	0.2030	0.2185	0.1949	0.4407	0.2234	0.2126	0.3781	0.1788	0.1929	0.1978
NM_012600	Me1	0.1027	0.3771	0.5321	0.3018	0.0937	0.4345	0.8991	0.3491	0.1973	0.4142	0.8788	0.1379
NM_019334	Pitx2	0.4986	0.5690	0.7241	0.9550	0.7959	0.8541	0.2889	0.8691	0.5648	0.6746	0.8601	0.2922
NM_012698	Dmd	0.4951	0.5137	0.5400	0.6191	0.4648	0.3607	0.3657	0.6325	0.1998	0.5577	0.5026	0.3961
NM_001013201	Gdap2	0.3391	0.3178	0.2716	0.0847	0.1005	0.1447	0.3985	0.0104	0.3042	0.1857	0.3449	0.3640
NM_001013202	Clca2	0.3027	0.5418	0.2813	0.3054	0.4180	0.3579	0.3214	0.2306	0.2737	0.5123	0.5078	0.2838
NM_012488	A2m	0.8313	0.8481	0.8805	0.7186	0.7429	0.6476	0.8347	0.7110	0.7806	0.7131	0.8798	0.8521
NM_001013205	Myef2	0.6804	0.7096	0.6943	0.5246	0.6783	0.5229	0.5197	0.5463	0.6091	0.7033	0.6254	0.8124
NM_133620	Zhx1	0.9343	0.3054	0.9255	0.9196	0.7258	0.9553	0.2516	0.7499	0.4508	0.2613	0.1695	0.4739
NM_012924	Cd44	0.1382	0.1062	0.2334	0.2726	0.1607	0.1686	0.2185	0.0089	0.2264	0.1080	0.2316	0.7249
NM_012668	Tat	0.7711	0.3120	0.4399	0.0269	0.7051	0.5463	0.5659	0.1729	0.5374	0.1725	0.6751	0.5637
NM_012503	Asgr1	0.5141	0.1098	0.7234	0.7135	0.5583	0.4120	0.5920	0.7481	0.0649	0.0104	0.0476	0.0073
NM_001014116	Jmjd8	0.6569	0.7434	0.1105	0.1340	0.7939	0.8830	0.8005	0.2713	0.4707	0.8356	0.7163	0.8344
NM_001025627	Leprel1	0.0157	0.0212	0.0048	0.0035	0.0119	0.0015	0.4276	0.1174	0.0834	0.0684	0.0509	0.0371
NM_001017489	Lgtn	0.6993	0.3869	0.2028	0.4793	0.4599	0.5306	0.3360	0.2125	0.4162	0.4852	0.4587	0.3935
NM_001013213	Itgb3bp	0.0927	0.4498	0.0867	0.4363	0.7245	0.8031	0.2304	0.8443	0.0168	0.1052	0.0115	0.0029
NM_001013214	Ttc4	0.4366	0.7994	0.1874	0.2264	0.3189	0.7764	0.5579	0.6314	0.9047	0.4641	0.7202	0.9565
NM_012625	Pmch	0.4725	0.4676	0.6463	0.5695	0.8985	0.5027	0.4508	0.4412	0.4358	0.0940	0.4596	0.6388
NM_012573	Grin2a	0.6759	0.5758	0.5055	0.0402	0.5495	0.5019	0.3566	0.4168	0.6539	0.5826	0.2691	0.4103
NM_012521	S100g	0.3010	0.1857	0.2765	0.1664	0.2591	0.1126	0.5499	0.1563	0.0537	0.2336	0.2637	0.3006
NM_012608	Mme	0.9956	0.2850	0.8246	0.9292	0.8549	0.9918	0.9979	0.9769	0.0617	0.1180	0.2537	0.0423
NM_001013223	Pcbp2	0.9703	0.2984	0.7798	0.8790	0.8128	0.8160	0.7838	0.7722	0.3930	0.1988	0.1172	0.3752
NM_001015015	Usp1	0.9627	0.9203	0.5397	0.9902	0.9068	0.7896	0.9445	0.9709	0.8669	0.9362	0.9498	0.2949
NM_012747	Stat3	0.6051	0.2610	0.5000	0.0165	0.3284	0.1617	0.2420	0.2530	0.7844	0.2316	0.1532	0.6592
NM_012627	Pkib	0.7377	0.3768	0.6857	0.7063	0.4593	0.2181	0.9464	0.7141	0.7287	0.5407	0.5869	0.3041
NM_013069	Cd74	0.4774	0.1457	0.2108	0.5275	0.2329	0.5122	0.7448	0.4353	0.6479	0.2454	0.0900	0.6868
NM_012694	Slc6a3	0.4624	0.2778	0.3432	0.2753	0.3372	0.3344	0.4086	0.4080	0.2702	0.3243	0.1659	0.0978
NM_001013235	Scoc	0.3277	0.3298	0.2888	0.4389	0.6336	0.8303	0.5048	0.2979	0.4117	0.1708	0.1697	0.1946
NM_013126	Dgkg	0.3286	0.4090	0.4586	0.3531	0.5165	0.6792	0.2480	0.5915	0.6688	0.6175	0.2340	0.5709
NM_012619	Pah	0.7501	0.7434	0.1221	0.3945	0.2491	0.2186	0.1599	0.3262	0.5377	0.3711	0.8803	0.2056
NM_001013241	Lzic	0.9010	0.9089	0.7702	0.8398	0.8240	0.9421	0.9533	0.8485	0.5587	0.8893	0.9412	0.2659
NM_001013242	Dnajc5g	0.6686	0.4026	0.6381	0.4065	0.6014	0.5324	0.6399	0.5576	0.3212	0.7814	0.1433	0.2657
NM_012629	Prl	0.7940	0.8066	0.7278	0.8412	0.8363	0.5630	0.7248	0.8633	0.7221	0.8830	0.7428	0.7895
NM_012615	Odc1	0.9841	0.9483	0.2941	0.9991	0.9292	0.9771	0.9646	0.8483	0.9711	0.9562	0.9730	0.7995
NM_012939	Ctsh	0.5637	0.5142	0.8633	0.6504	0.6067	0.6037	0.5896	0.9522	0.6738	0.7763	0.7191	0.8522
NM_012512	B2m	0.0100	0.0286	0.1568	0.0690	0.0104	0.0119	0.0373	0.0346	0.1149	0.0168	0.1603	0.0097
NM_012607	Nefh	0.5513	0.5029	0.2218	0.3711	0.1312	0.2334	0.3682	0.2108	0.4031	0.1977	0.1692	0.5120
NM_012624	Pklr	0.0795	0.3454	0.1825	0.3625	0.1784	0.5826	0.3309	0.2735	0.3566	0.1499	0.0886	0.5345
NM_001013248	Foxb1	0.5916	0.5481	0.7186	0.7176	0.5356	0.5210	0.4778	0.5854	0.4414	0.6692	0.2109	0.5583
NM_001025401	Nup85	0.8137	0.9377	0.0472	0.8289	0.7104	0.7870	0.7634	0.9528	0.7180	0.9347	0.9257	0.1454

NM_001025032	Cd96	0.5491	0.5997	0.3527	0.3143	0.2295	0.2572	0.4102	0.2123	0.4448	0.3389	0.3955	0.3370
NM_001013252	Srprb	0.0850	0.1383	0.3889	0.0162	0.1013	0.0955	0.4609	0.1206	0.6581	0.1993	0.5666	0.0741
NM_213562	Trim39	0.7461	0.9587	0.8932	0.8158	0.9729	0.6911	0.9774	0.9820	0.6301	0.8722	0.9950	0.9308
NM_212545	Krt17	0.9535	0.7556	0.6359	0.7600	0.8378	0.1352	0.6571	0.8304	0.8592	0.5154	0.6544	0.8677
NM_001013421	Lsg1	0.7428	0.9619	0.0346	0.0351	0.6887	0.5029	0.0525	0.8343	0.4871	0.4577	0.6449	0.2101
NM_001013426	Mrpl41	0.0553	0.5571	0.0809	0.3790	0.0416	0.0756	0.1154	0.0738	0.0995	0.3400	0.1487	0.0749
NM_001013431	Chchd4	0.2448	0.7223	0.2608	0.2217	0.1698	0.2570	0.6207	0.3027	0.6872	0.4778	0.1545	0.6107
NM_012516	C4bpa	0.2799	0.1889	0.1096	0.4999	0.2058	0.0334	0.0824	0.1674	0.2575	0.3184	0.1100	0.2151
NM_012926	Cd80	0.0293	0.4052	0.3051	0.0404	0.4205	0.2699	0.2617	0.2749	0.4098	0.2214	0.2803	0.4822
NM_012518	Calm3	0.2624	0.1945	0.7154	0.7266	0.3343	0.4291	0.2232	0.4554	0.4974	0.7168	0.1928	0.7326
NM_001013857	Lrrc48	0.4693	0.8321	0.4398	0.3211	0.1213	0.1011	0.4782	0.2701	0.5388	0.0718	0.4206	0.1171
NM_001013858	Tlcd1	0.5370	0.3174	0.3575	0.3345	0.2123	0.2355	0.2685	0.2682	0.6088	0.7222	0.2443	0.3886
NM_001024754	Gmps	0.6432	0.6619	0.0309	0.6865	0.6005	0.5597	0.5776	0.6685	0.6749	0.4895	0.5402	0.5473
NM_001024361	LOC501110	0.8550	0.8183	0.3384	0.6041	0.5189	0.6182	0.8219	0.4736	0.1316	0.4955	0.7417	0.1447
NM_001013861	RGD1307851	0.2536	0.0584	0.1143	0.3475	0.6006	0.5611	0.4630	0.5618	0.1106	0.3487	0.7517	0.5181
NM_001014096	Cldn18	0.4869	0.2137	0.4276	0.5011	0.5310	0.5741	0.2843	0.3260	0.2752	0.3141	0.2025	0.4710
NM_001013996	Slc25a37	0.3653	0.3793	0.5487	0.4763	0.5672	0.3840	0.2759	0.3994	0.6626	0.4575	0.2420	0.5046
NM_001013864	Lpp	0.6995	0.3816	0.4335	0.2717	0.3630	0.8612	0.3192	0.7528	0.5882	0.6834	0.5533	0.5904
NM_001013865	Tmem39a	0.7464	0.7997	0.7863	0.0748	0.1643	0.1190	0.2113	0.1054	0.2438	0.5070	0.9641	0.1014
NM_001013866	RGD1309437	0.1939	0.7587	0.7644	0.8121	0.5170	0.3239	0.8124	0.1593	0.5643	0.4451	0.8527	0.0848
NM_001013867	RGD1311893	0.6118	0.5106	0.7005	0.8987	0.3812	0.6378	0.8724	0.4872	0.7116	0.6717	0.5773	0.5278
NM_001013868	LOC288521	0.1349	0.2533	0.3464	0.6317	0.4566	0.5039	0.3716	0.4680	0.3311	0.1136	0.5615	0.3486
NM_001014109	Magix	0.4571	0.6039	0.3118	0.7168	0.2725	0.3511	0.2586	0.4407	0.5149	0.4091	0.4449	0.1522
NM_001014107	Mospd1	0.2859	0.3002	0.9769	0.8811	0.3675	0.9620	0.8907	0.5360	0.8441	0.8151	0.8199	0.7894
NM_001024794	Mettl3	0.8091	0.9487	0.5232	0.6137	0.8219	0.8731	0.0288	0.8152	0.5669	0.7212	0.9110	0.9775
NM_012908	Faslg	0.6664	0.6358	0.3301	0.8303	0.7128	0.8503	0.6768	0.4986	0.7891	0.3192	0.5962	0.4155
NM_001013872	LOC289035	0.5931	0.1944	0.3922	0.2181	0.1947	0.2895	0.2231	0.4716	0.3971	0.4764	0.1926	0.1977
NM_012882	Sstr5	0.7946	0.4265	0.3707	0.8382	0.1232	0.0522	0.9112	0.5484	0.6920	0.7934	0.8744	0.6130
NM_012876	Rps29	0.8582	0.7396	0.2793	0.7534	0.8009	0.2070	0.7421	0.8129	0.9740	0.8843	0.6209	0.0380
NM_001013877	RGD1307937	0.7107	0.7764	0.6354	0.9558	0.6455	0.7216	0.4124	0.8360	0.7437	0.9598	0.7178	0.2686
NM_001024365	Cesl1	0.5535	0.6366	0.4280	0.3567	0.2341	0.3533	0.4877	0.2120	0.2309	0.4156	0.4261	0.2406
NM_001013879	RGD1311847	0.2174	0.4458	0.2290	0.4818	0.4794	0.6480	0.4400	0.2778	0.5576	0.2370	0.4506	0.3197
NM_001008343	RGD1307279	0.2261	0.6658	0.3012	0.1429	0.5271	0.7200	0.1651	0.1542	0.4471	0.7090	0.8300	0.1031
NM_012681	Ttr	0.1021	0.3114	0.3202	0.3532	0.1181	0.2294	0.5001	0.0403	0.3110	0.0622	0.1711	0.1376
NM_001013883	RGD1310414	0.3719	0.1168	0.0522	0.0355	0.1168	0.0403	0.1158	0.1344	0.0472	0.0883	0.1820	0.0337
NM_001015009	Ptgr2	0.0062	0.3738	0.2483	0.0292	0.1611	0.2349	0.3126	0.2542	0.0400	0.2157	0.1330	0.3007
NM_001014263	Sypl1	0.0410	0.0554	0.9503	0.5963	0.0104	0.0054	0.0372	0.1232	0.0308	0.0249	0.3096	0.7060
NM_001013885	RGD1307537	0.4749	0.1152	0.4646	0.4651	0.5995	0.6292	0.5375	0.4088	0.5516	0.0435	0.8697	0.1853
NM_012623	Abcb1b	0.0009	0.0879	0.0633	0.0027	0.0013	0.0031	0.0991	0.0060	0.0032	0.1466	0.0959	0.0030
NM_001024277	Gcat	0.3944	0.3333	0.3905	0.5953	0.2266	0.2545	0.3156	0.6265	0.5243	0.3800	0.3706	0.2373
NM_001024268	Lig1	0.8540	0.8296	0.3873	0.6033	0.8759	0.8787	0.8900	0.8583	0.8404	0.8988	0.7904	0.3850
NM_001014159	Rbm42	0.7348	0.7376	0.2683	0.7048	0.0231	0.4365	0.4912	0.4840	0.5198	0.1994	0.1707	0.8725
NM_001014158	Zfp52	0.0646	0.2402	0.1179	0.1916	0.0261	0.3522	0.0610	0.0584	0.0071	0.1182	0.0900	0.4820
NM_012767	Gnrh1	0.9983	0.8603	0.1040	0.0497	0.8979	0.8714	0.1770	0.9482	0.9897	0.8495	0.8717	0.7113
NM_001013893	LOC292516	0.0342	0.0312	0.1675	0.2249	0.0682	0.0215	0.3622	0.0200	0.0283	0.0777	0.0312	0.0151
NM_012921	Alx1	0.3453	0.6724	0.5138	0.5260	0.0942	0.4691	0.4804	0.5340	0.0853	0.2665	0.4521	0.4556
NM_017291	Gabrr1	0.8470	0.6583	0.1690	0.5270	0.8754	0.5822	0.7524	0.8519	0.5200	0.7918	0.8191	0.5782
NM_012683	Ugt1a1	0.3599	0.6904	0.6653	0.6416	0.4874	0.4900	0.4268	0.2863	0.3794	0.4167	0.5323	0.6246
NM_012677	Ton	0.7498	0.2010	0.6708	0.2853	0.3935	0.2103	0.5131	0.7598	0.0441	0.2675	0.0877	0.5115

NM_001013897	LOC293103	0.1524	0.2190	0.1553	0.8684	0.5464	0.3383	0.8958	0.7550	0.7614	0.4283	0.3115	0.3419
NM_001013898	RGD1311703	0.8979	0.8993	0.7920	0.4822	0.9483	0.6596	0.5593	0.9357	0.4023	0.4122	0.7817	0.4224
NM_001014202	Derl1	0.6816	0.6426	0.7332	0.6478	0.6236	0.6967	0.5874	0.2889	0.4809	0.6188	0.4444	0.6940
NM_001014191	Tmem38b	0.5750	0.4294	0.6914	0.6305	0.6878	0.6140	0.7201	0.6260	0.7137	0.5903	0.4297	0.2067
NM_001004214	Nqo2	0.1749	0.0717	0.5800	0.8354	0.1065	0.0483	0.6313	0.1242	0.0422	0.0126	0.0395	0.0217
NM_013073	Pcmt1	0.2401	0.2300	0.8609	0.3861	0.2340	0.4524	0.1530	0.0368	0.2733	0.3054	0.3504	0.4038
NM_001013904	LOC293989	0.1906	0.1943	0.2024	0.6572	0.4181	0.3223	0.3306	0.7300	0.5885	0.1785	0.3509	0.2500
NM_001013905	RGD1307648	0.5450	0.5693	0.4264	0.9012	0.4419	0.2028	0.4097	0.6066	0.4635	0.0124	0.0307	0.4761
NM_001014188	Isy1	0.5672	0.8329	0.9980	0.7093	0.7806	0.7734	0.9837	0.7314	0.6682	0.7009	0.8595	0.8439
NM_001025762	Smyd3	0.7599	0.4349	0.6224	0.5511	0.3609	0.5599	0.5944	0.0705	0.6423	0.3731	0.7400	0.7790
NM_001013908	RGD1306583	0.7047	0.0943	0.1483	0.0709	0.1971	0.4763	0.0738	0.3229	0.0177	0.1372	0.2019	0.1042
NM_012670	Tcp1	0.8994	0.8014	0.4673	0.9095	0.8184	0.8535	0.7507	0.6653	0.6870	0.8271	0.9428	0.7228
NM_013199	Dnm2	0.2481	0.3214	0.2429	0.5025	0.1540	0.1426	0.1892	0.2587	0.2486	0.0340	0.1540	0.4404
NM_001013911	Ift80	0.2829	0.1566	0.1815	0.1162	0.5668	0.2746	0.0202	0.7381	0.2102	0.1115	0.0706	0.0111
NM_001013912	Mllt11	0.1219	0.8176	0.7912	0.6889	0.6612	0.6644	0.7735	0.1319	0.8620	0.6225	0.6794	0.9270
NM_012535	Prl3b1	0.3337	0.2333	0.4844	0.7092	0.3146	0.4138	0.5935	0.7732	0.0824	0.4148	0.5510	0.5194
NM_013105	Cyp3a23/3a1	0.5474	0.4895	0.3943	0.4501	0.5473	0.8133	0.7642	0.6917	0.5545	0.2559	0.7344	0.4351
NM_013021	Prph2	0.5624	0.4197	0.8786	0.6026	0.4675	0.4429	0.5489	0.8022	0.6222	0.6411	0.3528	0.8526
NM_001014181	Atpbd4	0.7201	0.3503	0.4058	0.5836	0.8714	0.6646	0.6500	0.1377	0.8150	0.5891	0.1461	0.3513
NM_001013918	RGD1309540	0.3067	0.2398	0.3611	0.4948	0.3870	0.6138	0.3698	0.3169	0.5161	0.2003	0.2266	0.2953
NM_001025752	Ccrk	0.7299	0.9266	0.5248	0.6318	0.7882	0.3315	0.8798	0.4375	0.9373	0.5100	0.6441	0.9529
NM_001013920	RGD1359452	0.4636	0.1941	0.2023	0.2175	0.1945	0.2026	0.2228	0.2116	0.4658	0.3536	0.1926	0.1973
NM_001013921	Dtwd1	0.3355	0.8460	0.1570	0.0190	0.0809	0.0712	0.0467	0.0537	0.0564	0.6791	0.1881	0.0909
NM_012661	Sts	0.4214	0.7499	0.7354	0.8518	0.8099	0.4318	0.4562	0.7518	0.9439	0.8347	0.8763	0.7844
NM_001025053	Galnt4	0.5686	0.3730	0.6424	0.3918	0.2051	0.6143	0.6520	0.4384	0.3979	0.6364	0.2237	0.3228
NM_001024342	Dnai1	0.5449	0.1453	0.1785	0.1648	0.1250	0.1302	0.0366	0.4911	0.2618	0.7060	0.3419	0.6188
NM_001014196	Ppp2r3c	0.1101	0.0635	0.8401	0.3796	0.2633	0.1053	0.1770	0.0962	0.1574	0.2894	0.0946	0.1407
NM_001013926	Tprkb	0.9052	0.9489	0.7561	0.8700	0.7995	0.7316	0.9363	0.9304	0.7933	0.8097	0.8584	0.8797
NM_012666	Tac1	0.8379	0.5443	0.8929	0.8376	0.9096	0.8212	0.4845	0.6012	0.3701	0.7514	0.6959	0.4666
NM_001014214	Dalrd3	0.3781	0.6181	0.2741	0.3771	0.2009	0.3009	0.4294	0.2114	0.2755	0.3972	0.5082	0.1972
NM_001013930	LOC298139	0.8170	0.8434	0.6989	0.7372	0.8299	0.1900	0.2236	0.4940	0.5337	0.7446	0.3624	0.3998
NM_001013931	RGD1311249	0.9171	0.7684	0.9143	0.8894	0.9519	0.9038	0.9685	0.8020	0.9347	0.8254	0.9717	0.9410
NM_001013932	LOC298250	0.3893	0.2682	0.2021	0.4096	0.2881	0.2025	0.3863	0.5291	0.2531	0.3001	0.4249	0.3148
NM_012821	Adcy6	0.3405	0.2349	0.6499	0.6840	0.6171	0.8674	0.3025	0.6262	0.6099	0.4415	0.5914	0.9161
NM_001013934	RGD1310425	0.0613	0.3518	0.2106	0.1069	0.4369	0.2606	0.4837	0.0944	0.2160	0.8409	0.4468	0.3725
NM_001014194	Dnajc16	0.4053	0.2888	0.3036	0.3221	0.5672	0.1293	0.3264	0.8209	0.2202	0.5506	0.4968	0.2102
NM_001014215	Cenpq	0.5113	0.4596	0.5080	0.2174	0.3842	0.3087	0.2847	0.3931	0.3624	0.2476	0.6264	0.4509
NM_001013937	Actrt2	0.3784	0.6529	0.4339	0.7927	0.6643	0.6208	0.4688	0.2613	0.4838	0.7112	0.8625	0.7206
NM_012549	Edn2	0.3406	0.1791	0.3789	0.2722	0.1438	0.4408	0.4187	0.2915	0.1599	0.1586	0.1738	0.3105
NM_001025414	Hars	0.4239	0.4355	0.3240	0.2874	0.3721	0.2405	0.6264	0.4704	0.2040	0.5055	0.4537	0.3274
NM_001024782	Lrrc8a	0.6504	0.7744	0.7744	0.9672	0.9445	0.8833	0.7581	0.4308	0.8153	0.5957	0.8202	0.8657
NM_001013941	LOC298795	0.3992	0.5039	0.4384	0.5001	0.5220	0.2101	0.2300	0.1363	0.3025	0.1473	0.5391	0.3839
NM_001024781	Sox18	0.7197	0.2993	0.4944	0.2729	0.7552	0.1264	0.1732	0.5178	0.1039	0.3116	0.2064	0.3294
NM_001013943	Aldoal1	0.5208	0.3885	0.3694	0.6273	0.3858	0.5002	0.2842	0.2912	0.3247	0.4223	0.4098	0.3709
NM_001013944	RGD1309051	0.1331	0.0617	0.8312	0.2166	0.2768	0.5157	0.0553	0.3978	0.0364	0.1015	0.0630	0.3160
NM_013133	Glra1	0.9783	0.7614	0.6582	0.5643	0.6414	0.1487	0.8674	0.7883	0.4851	0.8786	0.9537	0.2596
NM_001013947	RGD1304605	0.3872	0.5870	0.4199	0.5771	0.6664	0.6243	0.8740	0.8997	0.5237	0.6572	0.7058	0.7063
NM_001013949	Ribc2	0.0595	0.2350	0.0366	0.3916	0.1006	0.3545	0.2442	0.2695	0.2222	0.0640	0.0763	0.1676
NM_012556	Fabp1	0.4437	0.8482	0.6140	0.5074	0.7138	0.3777	0.4987	0.6446	0.7073	0.6333	0.7361	0.2508

NM_001013952	LOC300308	0.8086	0.7433	0.7518	0.4243	0.7836	0.4051	0.6747	0.3784	0.6165	0.7621	0.7861	0.7085
NM_001014793	Kpna4	0.2127	0.2082	0.9581	0.2549	0.0457	0.5381	0.4335	0.2523	0.2686	0.0090	0.3971	0.3762
NM_001014236	Ssbp4	0.7610	0.8593	0.5299	0.8280	0.7041	0.9282	0.7527	0.8917	0.7688	0.9074	0.3947	0.1657
NM_001013956	RGD1309049	0.2110	0.6932	0.2199	0.3974	0.7775	0.4492	0.5713	0.7880	0.9163	0.2717	0.7990	0.9378
NM_012874	Ros1	0.7732	0.9448	0.8345	0.7933	0.8961	0.7617	0.6317	0.7783	0.6808	0.8884	0.7512	0.6045
NM_001013958	Wdr45	0.6227	0.8171	0.3976	0.7056	0.0147	0.0913	0.1502	0.1450	0.8450	0.3726	0.7120	0.7602
NM_001013959	LOC302576	0.4345	0.1999	0.2393	0.3265	0.4165	0.3322	0.2892	0.2662	0.5654	0.2351	0.2635	0.2540
NM_001033900	Ngrn	0.0143	0.0709	0.7312	0.0291	0.1676	0.0518	0.0404	0.1687	0.0338	0.2690	0.0506	0.1815
NM_001013961	Actrt1	0.2120	0.0638	0.0765	0.1000	0.0933	0.0541	0.1837	0.2906	0.0196	0.0931	0.1840	0.2086
NM_001013962	LOC302845	0.1549	0.1250	0.0072	0.1717	0.0034	0.1199	0.0111	0.0130	0.0042	0.0108	0.1530	0.0013
NM_012637	Ptpn1	0.1447	0.0294	0.0614	0.0886	0.0507	0.0716	0.1346	0.1293	0.0543	0.2279	0.0795	0.1755
NM_012564	Gc	0.1287	0.2160	0.1267	0.2385	0.1105	0.0754	0.1177	0.6902	0.2224	0.0429	0.1062	0.0865
NM_012532	Cp	0.3780	0.2859	0.2721	0.3034	0.2047	0.5142	0.6455	0.4366	0.3078	0.3691	0.2653	0.2435
NM_001013967	RGD1311260	0.6218	0.7070	0.6270	0.6498	0.4987	0.4426	0.5524	0.6980	0.5933	0.6603	0.4095	0.5960
NM_001013970	Slfn8	0.9197	0.7263	0.0052	0.8581	0.9313	0.7419	0.1453	0.8977	0.6402	0.6975	0.7729	0.1493
NM_001014266	Ccdc41	0.7973	0.3091	0.0778	0.0124	0.9761	0.8482	0.0278	0.7406	0.5289	0.9084	0.3002	0.0448
NM_001105797	Slc16a11	0.8274	0.7528	0.7487	0.7633	0.7905	0.6730	0.7660	0.7416	0.7444	0.7494	0.9099	0.8856
NM_001013973	Rnf190	0.2311	0.7925	0.7261	0.4756	0.4812	0.8357	0.3395	0.0746	0.1374	0.1377	0.7155	0.4820
NM_012851	Hsd17b1	0.9025	0.7561	0.8151	0.9480	0.7336	0.7343	0.8123	0.8049	0.7842	0.5672	0.8514	0.7589
NM_001013979	LOC304131	0.4376	0.9685	0.9897	0.9975	0.9962	0.7376	0.9899	0.9977	0.9482	0.7627	0.9399	0.8868
NM_012572	Grik4	0.5235	0.6642	0.0558	0.1570	0.2106	0.6343	0.0602	0.5575	0.4940	0.2183	0.5368	0.3780
NM_001013981	LOC304396	0.6853	0.7999	0.3896	0.5666	0.8796	0.8529	0.4880	0.8865	0.7713	0.4484	0.8579	0.8326
NM_012801	Pdgfa	0.9023	0.9454	0.9808	0.8538	0.8266	0.8822	0.0392	0.6559	0.7859	0.9281	0.7336	
NM_001024344	Sit1	0.9183	0.6520	0.6277	0.7934	0.3531	0.8964	0.8683	0.3066	0.7973	0.0775	0.6709	0.6696
NM_001017450	Iip45	0.5270	0.9565	0.0651	0.2922	0.6396	0.5307	0.8172	0.7950	0.6436	0.6655	0.3886	0.0693
NM_013138	Itpr3	0.8410	0.3856	0.3559	0.5902	0.4966	0.3858	0.1566	0.6788	0.2022	0.2384	0.3471	0.4646
NM_001013986	LOC305076	0.8607	0.9299	0.0908	0.5841	0.8422	0.7619	0.1041	0.8083	0.8430	0.9904	0.8617	0.6504
NM_001025130	Bag4	0.5404	0.6102	0.5842	0.3792	0.4224	0.6598	0.2869	0.2852	0.4873	0.5932	0.6305	0.2333
NM_001024991	Fahd1	0.9285	0.8387	0.5903	0.8683	0.6415	0.6681	0.3916	0.6488	0.1522	0.2126	0.7678	0.1008
NM_001024289	Ptprcap	0.4319	0.0935	0.3733	0.1117	0.3687	0.3811	0.1718	0.6614	0.4234	0.2608	0.6027	0.3778
NM_001014273	Armcx3	0.9155	0.8703	0.9566	0.9489	0.3413	0.6796	0.8835	0.1865	0.9224	0.9507	0.9758	0.8681
NM_001014791	Rnf113a1	0.0951	0.0096	0.0042	0.1830	0.0594	0.0238	0.0337	0.0310	0.0337	0.0047	0.0503	0.0180
NM_012728	Glp1r	0.6939	0.7499	0.1819	0.1136	0.4971	0.8403	0.8011	0.8145	0.8577	0.8799	0.1989	0.9111
NM_001013993	LOC305806	0.6910	0.5049	0.9359	0.8793	0.8973	0.6824	0.3320	0.5916	0.4496	0.6034	0.8040	0.6894
NM_001013995	RGD1305288	0.8589	0.9886	0.8515	0.8168	0.8164	0.8278	0.8903	0.8252	0.8401	0.9241	0.8087	0.6031
NM_001017443	Lrp2bp	0.5577	0.4005	0.3107	0.4600	0.2748	0.6779	0.3903	0.0855	0.1892	0.0255	0.3890	0.1581
NM_001013997	RGD1309522	0.9056	0.8558	0.3417	0.1791	0.8644	0.7792	0.2914	0.9273	0.7501	0.8558	0.9028	0.5720
NM_001033870	Csnk1g2	0.2137	0.4698	0.7759	0.9779	0.2307	0.9085	0.9209	0.7747	0.2000	0.6057	0.7109	0.6359
NM_001025716	Vps4b	0.2900	0.2949	0.9609	0.5319	0.3259	0.8271	0.2748	0.8183	0.2433	0.0926	0.2646	0.8925
NM_001014001	RGD1307325	0.1017	0.1356	0.5016	0.9186	0.5292	0.4178	0.1237	0.5513	0.2149	0.1533	0.0622	0.6773
NM_001024310	Arl6ip6	0.7980	0.0723	0.9018	0.8969	0.8233	0.7333	0.5918	0.7389	0.1506	0.1519	0.7369	0.5713
NM_001024273	Cmah	0.5222	0.2326	0.1709	0.0795	0.5256	0.4804	0.0633	0.6670	0.4950	0.2072	0.5315	0.2398
NM_001033965	Pfkfb2	0.5721	0.5921	0.7296	0.5564	0.6767	0.7084	0.8268	0.8376	0.7158	0.6081	0.7405	0.5834
NM_001029898	Mrpl19	0.0301	0.5902	0.7081	0.5093	0.1359	0.0147	0.7463	0.0413	0.6198	0.5030	0.7986	0.1940
NM_012730	Cyp2d2	0.0881	0.0951	0.0323	0.0908	0.0654	0.1093	0.0146	0.0121	0.0683	0.1982	0.0092	0.2644
NM_001014007	LOC306766	0.5045	0.2174	0.6044	0.7420	0.1039	0.1032	0.4996	0.2668	0.1454	0.6817	0.0682	0.3976
NM_001014163	Cend1	0.6431	0.9435	0.6111	0.9001	0.6980	0.7826	0.8967	0.8212	0.9551	0.9533	0.8868	0.9357
NM_001014010	RGD1308147	0.4163	0.5158	0.2293	0.3069	0.4100	0.4818	0.2724	0.5615	0.2811	0.2682	0.4215	0.6444
NM_001014011	Gramd3	0.8984	0.8588	0.5170	0.2216	0.8263	0.8838	0.4375	0.6988	0.3149	0.7582	0.7169	0.7045

NM_012673	Thy1	0.6691	0.3812	0.7590	0.9448	0.6020	0.9618	0.7579	0.6294	0.8583	0.6674	0.7153	0.9274
NM_001014013	RGD1305215	0.2280	0.6555	0.3144	0.2455	0.7254	0.7225	0.5224	0.5998	0.6616	0.6301	0.5609	0.7273
NM_001014176	Alg14	0.6077	0.5668	0.5995	0.7324	0.5062	0.4778	0.5226	0.4252	0.3737	0.6902	0.6282	0.3699
NM_001014170	Fam48a	0.7051	0.6317	0.5647	0.5764	0.6311	0.6848	0.2822	0.6844	0.5805	0.4975	0.6497	0.7134
NM_012938	Ctse	0.5253	0.6592	0.7545	0.6629	0.5326	0.3831	0.8238	0.7695	0.6955	0.6390	0.2608	0.1735
NM_001014016	RGD1311107	0.3082	0.0162	0.5287	0.0849	0.2502	0.1396	0.1443	0.2953	0.8652	0.7094	0.3097	0.2651
NM_001014018	RGD1309326	0.9838	0.7645	0.4618	0.8395	0.8661	0.6635	0.9502	0.8556	0.9598	0.9965	0.6839	0.5480
NM_001014203	Ccdc65	0.2882	0.0811	0.5415	0.6212	0.5788	0.1057	0.0255	0.3941	0.0412	0.0184	0.1841	0.0192
NM_001014184	Lpin3	0.3362	0.5542	0.2174	0.3378	0.4512	0.3180	0.5242	0.3116	0.6949	0.2464	0.6075	0.5685
NM_001014022	Btbd10	0.3213	0.7128	0.3183	0.3019	0.3133	0.4263	0.2363	0.0277	0.9423	0.1428	0.4295	0.8088
NM_001014023	RGD1304579	0.1968	0.2639	0.5608	0.4784	0.5009	0.3725	0.2421	0.6191	0.4043	0.1992	0.5357	0.4996
NM_012795	Gp5	0.4389	0.2374	0.1246	0.3124	0.1759	0.3548	0.3030	0.4670	0.5741	0.4098	0.2098	0.6697
NM_012740	Th	0.0857	0.3496	0.1042	0.3780	0.2482	0.0244	0.4522	0.0360	0.5052	0.7096	0.2838	0.3689
NM_001014030	RGD1309313	0.6264	0.8925	0.8980	0.7867	0.8194	0.3972	0.9164	0.5346	0.9700	0.8922	0.9910	0.9917
NM_012789	Dpp4	0.6555	0.4629	0.4449	0.4230	0.4475	0.7525	0.0564	0.4214	0.5375	0.3592	0.5273	0.5490
NM_012731	Ntrk2	0.5842	0.6013	0.6791	0.6679	0.5626	0.7207	0.6434	0.6584	0.5582	0.6552	0.6303	0.6479
NM_012659	Sst	0.3531	0.1812	0.1291	0.0858	0.3005	0.0540	0.5495	0.2429	0.2988	0.1855	0.1915	0.2188
NM_013023	Sag	0.8343	0.8273	0.7907	0.8286	0.8936	0.7612	0.8887	0.9557	0.8292	0.8524	0.9062	0.1775
NM_001014034	Qrsl1	0.6566	0.9127	0.1076	0.0037	0.1400	0.0624	0.3458	0.3109	0.3903	0.4123	0.0993	0.2313
NM_001014036	RGD1307509	0.4207	0.3007	0.7124	0.4414	0.6732	0.6913	0.1919	0.3385	0.8009	0.7414	0.3490	0.8416
NM_001014037	RGD1306227	0.8821	0.7380	0.0745	0.1064	0.7263	0.6473	0.7931	0.8248	0.8990	0.8329	0.9518	0.0914
NM_012660	Eef1a2	0.3977	0.4880	0.0409	0.0526	0.0327	0.3154	0.1924	0.0922	0.5317	0.2811	0.1694	0.6962
NM_001025134	Uqcrq	0.5500	0.7165	0.7131	0.6076	0.5041	0.4308	0.5145	0.5426	0.4611	0.6059	0.5373	0.4359
NM_001014041	LOC310721	0.6726	0.8378	0.8942	0.9444	0.7607	0.3051	0.6359	0.1373	0.7204	0.8677	0.8027	0.6765
NM_001024801	Zw10	0.2428	0.3654	0.1621	0.0485	0.1225	0.2661	0.1156	0.6124	0.1103	0.5353	0.1697	0.3810
NM_001024774	Def8	0.5753	0.8890	0.6430	0.9250	0.9227	0.9869	0.9151	0.8041	0.9530	0.4902	0.8660	0.9959
NM_001014217	Nhej1	0.7013	0.7292	0.2202	0.8126	0.9216	0.6924	0.7743	0.7874	0.9626	0.7921	0.8939	0.6941
NM_001014216	Obfc2a	0.1520	0.0870	0.1861	0.2798	0.3209	0.3527	0.3268	0.1124	0.2869	0.2436	0.2209	0.5571
NM_001014045	RGD1311463	0.2924	0.0182	0.1835	0.6198	0.0288	0.4131	0.1466	0.1209	0.0286	0.1416	0.2274	0.1542
NM_001014225	Cluap1	0.1945	0.2879	0.2962	0.2992	0.2244	0.3906	0.6915	0.2970	0.5766	0.2421	0.4453	0.2960
NM_001014047	LOC311352	0.7785	0.8335	0.2286	0.9185	0.9161	0.7841	0.4829	0.8795	0.5452	0.3880	0.5712	0.5044
NM_001014219	Ankzf1	0.8451	0.6561	0.3716	0.1996	0.7589	0.0846	0.0795	0.3765	0.5693	0.9240	0.8338	0.8205
NM_001014233	Tmem55b	0.8091	0.9760	0.2377	0.2910	0.4744	0.2559	0.3546	0.3881	0.9726	0.8214	0.8922	0.6024
NM_001014235	Phf11l	0.3210	0.5562	0.2371	0.5667	0.4319	0.3849	0.4082	0.4907	0.5558	0.2774	0.2442	0.4498
NM_001014052	RGD1309726	0.4439	0.4006	0.0769	0.6618	0.4005	0.4688	0.3464	0.1393	0.4747	0.4375	0.1742	0.3881
NM_001015020	Tgif1	0.9180	0.8823	0.9678	0.0275	0.8564	0.7838	0.2509	0.9061	0.9384	0.9123	0.9483	0.9197
NM_001014056	RGD1304879	0.7983	0.1413	0.4734	0.2151	0.0395	0.4123	0.5606	0.0380	0.0675	0.6415	0.5126	0.4729
NM_013063	Parp1	0.6335	0.6206	0.5579	0.7793	0.8485	0.7750	0.8673	0.9758	0.5739	0.6433	0.9621	0.7101
NM_012557	Fancc	0.3358	0.2061	0.3753	0.5582	0.6208	0.0838	0.7307	0.7895	0.3090	0.3878	0.7069	0.1464
NM_012770	Gucy1b2	0.6317	0.8574	0.5602	0.1133	0.4412	0.5132	0.8725	0.8271	0.4660	0.4396	0.3802	0.5606
NM_001014059	RGD1304952	0.8809	0.7917	0.6079	0.1272	0.9639	0.7320	0.9255	0.8900	0.8802	0.7619	0.7822	0.9167
NM_001014060	LOC312831	0.2477	0.2797	0.4280	0.6357	0.2000	0.2675	0.4526	0.5846	0.2498	0.7191	0.6302	0.5561
NM_017191	Adra1a	0.7032	0.4505	0.5848	0.2158	0.4881	0.6496	0.8379	0.5499	0.4843	0.4902	0.5025	0.0714
NM_013066	Map2	0.2292	0.1219	0.0957	0.1020	0.1754	0.1064	0.3851	0.3687	0.0417	0.1508	0.1118	0.5031
NM_012612	Nppa	0.2315	0.4476	0.5217	0.4488	0.2478	0.2028	0.0910	0.5146	0.6806	0.4668	0.3835	0.7197
NM_012631	Prnp	0.1520	0.5608	0.9188	0.2218	0.0981	0.2090	0.1112	0.1445	0.8924	0.2772	0.8168	0.9895
NM_012611	Nos2	0.0394	0.6925	0.5473	0.5950	0.5063	0.6937	0.0793	0.7681	0.5595	0.3453	0.4918	0.8227
NM_001024751	Sox6	0.6873	0.6546	0.8757	0.6688	0.6083	0.6968	0.6543	0.5415	0.6411	0.8323	0.8457	0.9510
NM_001014072	RGD1311517	0.6616	0.4012	0.3731	0.2967	0.3615	0.5539	0.6357	0.2287	0.6010	0.4202	0.4077	0.3651

NM_001014268	Lrrc1	0.8162	0.7710	0.8422	0.5807	0.8573	0.9176	0.6403	0.7536	0.7890	0.9510	0.9016	0.9908
NM_001014074	LOC313936	0.3720	0.5518	0.1002	0.1803	0.4597	0.1122	0.9169	0.2225	0.1162	0.4837	0.5242	0.1780
NM_013060	Id2	0.1036	0.8618	0.9793	0.9696	0.8430	0.9285	0.9139	0.9562	0.8614	0.8798	0.9255	0.4183
NM_001034090	Ephx1	0.7451	0.2651	0.8034	0.6930	0.6395	0.4953	0.4872	0.7430	0.4718	0.5098	0.3991	0.3980
NM_001031649	Sirt6	0.7290	0.7598	0.9798	0.3835	0.8627	0.9395	0.7554	0.9646	0.9593	0.7563	0.8567	0.9515
NM_001031643	Trdmt1	0.8732	0.3135	0.4849	0.8452	0.1671	0.5618	0.1309	0.0858	0.1116	0.1582	0.3542	0.5859
NM_013221	Hbp1	0.8544	0.3489	0.8839	0.6495	0.3662	0.7890	0.2747	0.6223	0.8503	0.4132	0.4657	0.8825
NM_012682	Ucp1	0.6285	0.1734	0.6261	0.4016	0.9020	0.5966	0.8020	0.9060	0.6412	0.3537	0.8153	0.3114
NM_001014083	RGD1307947	0.2203	0.4901	0.4169	0.3131	0.5017	0.2455	0.2225	0.3240	0.0786	0.3593	0.2769	0.1918
NM_001014084	RGD1359449	0.3174	0.5455	0.2407	0.0720	0.5836	0.0676	0.3947	0.4887	0.3254	0.1390	0.0734	0.6189
NM_001014085	Maf1	0.8966	0.4302	0.7579	0.7500	0.7115	0.8733	0.8769	0.9646	0.7747	0.3088	0.1464	0.7244
NM_001014087	Ccdc67	0.4429	0.3749	0.5750	0.8255	0.6071	0.3682	0.8079	0.4967	0.4259	0.2970	0.4373	0.5475
NM_013058	Id3	0.3089	0.7376	0.5506	0.9200	0.8330	0.5514	0.8866	0.7823	0.2187	0.6288	0.2497	0.1463
NM_012956	Gabrb1	0.7920	0.8959	0.2013	0.2210	0.7458	0.2817	0.5065	0.3125	0.5184	0.4818	0.5526	0.4439
NM_001014092	Paqr5	0.8515	0.8540	0.4567	0.9045	0.8834	0.3695	0.5949	0.6944	0.1842	0.3697	0.5505	0.4398
NM_001025056	Gmppa	0.3806	0.0611	0.3810	0.4153	0.1047	0.4702	0.9645	0.1428	0.4404	0.5408	0.5224	0.7995
NM_012581	Hoxa2	0.2408	0.7937	0.7176	0.9162	0.7967	0.6513	0.8864	0.7317	0.9142	0.8540	0.8268	0.7496
NM_001014095	Dzip1l	0.1273	0.7805	0.1523	0.1816	0.2958	0.4003	0.7938	0.8096	0.5203	0.6501	0.4714	0.1372
NM_001014097	LOC315970	0.2779	0.2934	0.3428	0.2165	0.7255	0.2827	0.3679	0.5408	0.4507	0.3109	0.3966	0.1969
NM_001014098	Ccdc51	0.0453	0.0342	0.1365	0.0273	0.0191	0.0568	0.0541	0.0669	0.1119	0.0628	0.3578	0.6377
NM_001017453	Pik3ip1	0.8799	0.9709	0.9967	0.7816	0.9212	0.9763	0.8711	0.9928	0.9711	0.9867	0.9991	0.8793
NM_012578	H1f0	0.9712	0.3858	0.5146	0.2634	0.8055	0.4069	0.1404	0.8393	0.1961	0.3682	0.1391	0.1137
NM_012959	Gfra1	0.5225	0.7867	0.6272	0.8238	0.5223	0.6126	0.4094	0.5848	0.8142	0.9548	0.6324	0.3802
NM_001014102	RGD1309930	0.8646	0.7924	0.9471	0.8959	0.8269	0.8069	0.9534	0.8933	0.8515	0.9173	0.5496	0.5780
NM_001015026	Tspan12	0.9658	0.9926	0.9898	0.7995	0.9757	0.9491	0.8988	0.9585	0.9922	0.9905	0.9930	0.9437
NM_001025131	Sult2a2	0.3372	0.3886	0.4772	0.2992	0.1978	0.4262	0.3155	0.3265	0.4379	0.2905	0.3870	0.2249
NM_001024755	Ube2l6	0.0990	0.0313	0.0173	0.0108	0.1051	0.1690	0.0268	0.1364	0.0321	0.0355	0.0474	0.1300
NM_012633	Prph	0.0657	0.2272	0.3916	0.0619	0.1428	0.4767	0.3482	0.0317	0.2402	0.1122	0.1214	0.3091
NM_001025404	Ceacam11	0.2918	0.5227	0.4256	0.4651	0.2920	0.2613	0.3020	0.0529	0.1540	0.2468	0.3238	0.5351
NM_001014112	LOC317471	0.4440	0.5987	0.5039	0.5446	0.3448	0.5856	0.5252	0.2945	0.1086	0.4699	0.5682	0.2482
NM_012588	Igfbp3	0.8628	0.3803	0.7380	0.8291	0.8848	0.5564	0.9013	0.8445	0.4492	0.5801	0.5619	0.6239
NM_013157	Ass1	0.1389	0.4309	0.3855	0.3558	0.0332	0.1815	0.3772	0.0388	0.4606	0.5852	0.4930	0.5947
NM_001014115	LOC360479	0.2453	0.7152	0.2246	0.3385	0.4882	0.3140	0.3701	0.4443	0.5218	0.2268	0.3177	0.5504
NM_001025409	Hdac1	0.6948	0.6075	0.6877	0.8718	0.5827	0.3691	0.6432	0.4661	0.5268	0.3839	0.1804	0.4486
NM_012955	Cenpi	0.0993	0.8360	0.0452	0.3816	0.8845	0.8867	0.5691	0.7627	0.2186	0.8976	0.3815	0.0401
NM_001014119	RGD1307935	0.4658	0.4004	0.3544	0.7596	0.7079	0.6096	0.1508	0.7545	0.8573	0.5143	0.0685	0.9495
NM_012591	Irf1	0.9140	0.4907	0.6135	0.0879	0.5177	0.8397	0.1430	0.6131	0.6439	0.5302	0.1808	0.7728
NM_001014122	Mif4gd	0.9548	0.9594	0.3425	0.8844	0.7633	0.8028	0.9615	0.9991	0.8383	0.8266	0.7013	0.8074
NM_012846	Fgf1	0.8588	0.9392	0.9067	0.9837	0.9345	0.8637	0.8111	0.8896	0.9469	0.9978	0.9068	0.9654
NM_001014126	RGD1306410	0.8471	0.8988	0.5183	0.9720	0.9028	0.9192	0.2887	0.9192	0.1050	0.8757	0.7693	0.3260
NM_001014128	LOC360807	0.9585	0.9635	0.7423	0.6488	0.7669	0.8317	0.3936	0.7046	0.6498	0.9381	0.9134	0.9480
NM_012859	Lipe	0.3996	0.1008	0.0598	0.2721	0.2579	0.5123	0.4521	0.2601	0.4753	0.6469	0.1723	0.3275
NM_001014130	RGD1309106	0.3524	0.4070	0.4852	0.2956	0.5379	0.5178	0.3696	0.1008	0.4270	0.4760	0.2745	0.6263
NM_001014131	RGD1309708	0.0887	0.0191	0.0648	0.0464	0.0368	0.0834	0.3662	0.3876	0.0209	0.0476	0.0025	0.5205
NM_001014132	Traf3ip3	0.3781	0.3073	0.0747	0.6530	0.0433	0.0596	0.0802	0.2742	0.0691	0.0512	0.1506	0.0762
NM_001017469	Fam71d	0.1903	0.1935	0.2015	0.2163	0.1942	0.2018	0.2220	0.2105	0.1834	0.3232	0.1922	0.3090
NM_001014135	Wdr1	0.6932	0.9809	0.2403	0.1853	0.3916	0.7576	0.9249	0.2809	0.4980	0.9183	0.9129	0.9058
NM_001014137	LOC361016	0.6159	0.5846	0.3570	0.3321	0.8699	0.7267	0.4354	0.4331	0.3891	0.5314	0.6276	0.6453
NM_001106876	Mllt1	0.9369	0.9867	0.7981	0.8671	0.9385	0.9520	0.9953	0.9831	0.9584	0.9927	0.9876	0.9696

NM_001014140	RGD1309676	0.2617	0.7363	0.4314	0.0965	0.0961	0.1310	0.0789	0.2268	0.4563	0.5425	0.0957	0.4615
NM_001017480	Hoxb7	0.9613	0.4890	0.8103	0.8737	0.8121	0.9856	0.3770	0.8342	0.6087	0.2644	0.1315	0.2557
NM_001017481	Ddc8	0.6632	0.5197	0.0065	0.2794	0.5648	0.6003	0.3120	0.6859	0.7506	0.5272	0.5006	0.7953
NM_001017479	Tmem100	0.8492	0.6195	0.6869	0.2994	0.6938	0.5015	0.2384	0.1015	0.8089	0.7929	0.9464	0.4688
NM_012628	Prkcg	0.8036	0.8441	0.4211	0.0731	0.6915	0.8371	0.6985	0.2284	0.9245	0.9363	0.9477	0.9778
NM_001014147	RGD1310571	0.1611	0.2829	0.2168	0.6093	0.7559	0.6015	0.9922	0.3081	0.3059	0.3845	0.1578	0.1964
NM_001017484	Lca5l	0.3879	0.2530	0.1013	0.3613	0.1216	0.4375	0.1870	0.1795	0.2137	0.2236	0.3100	0.0124
NM_001017487	Bri3bp	0.5023	0.8769	0.7626	0.8791	0.5267	0.1788	0.8006	0.6028	0.8897	0.7724	0.7329	0.8715
NM_001017486	Gbas	0.4036	0.1261	0.7816	0.4080	0.1283	0.5519	0.3573	0.6779	0.2308	0.1557	0.5257	0.6772
NM_001014150	Yipf5	0.3966	0.5339	0.9118	0.1611	0.3081	0.3067	0.2101	0.3282	0.0794	0.5659	0.7391	0.0805
NM_001017490	Tmem81	0.7110	0.2232	0.5841	0.0941	0.7623	0.2798	0.2888	0.5617	0.4627	0.3942	0.3584	0.9489
NM_001024787	Osgepl1	0.9757	0.7918	0.3700	0.3375	0.7773	0.9209	0.2884	0.6499	0.9250	0.8218	0.6275	0.7468
NM_001024295	Sumo3	0.8679	0.4649	0.5193	0.3763	0.9319	0.8193	0.8277	0.8883	0.8651	0.9380	0.8628	0.3824
NM_001024278	Map4	0.7847	0.8400	0.6182	0.5219	0.7913	0.5972	0.8971	0.8104	0.7805	0.7142	0.7705	0.8590
NM_001017494	Blzf1	0.8985	0.4094	0.6124	0.1144	0.9738	0.7383	0.0362	0.8682	0.0789	0.0587	0.4278	0.9412
NM_001017482	Tex19	0.1332	0.0203	0.1249	0.3345	0.2358	0.1603	0.3495	0.0285	0.3817	0.2228	0.3125	0.7411
NM_001017503	Purb	0.2488	0.9442	0.6852	0.8033	0.3499	0.7168	0.5746	0.4733	0.9663	0.8983	0.8357	0.7296
NM_001017491	Lax1	0.4084	0.0225	0.1463	0.1857	0.2970	0.2672	0.6295	0.0796	0.1808	0.1229	0.0108	0.3261
NM_001014154	Lrrc50	0.3823	0.7721	0.0853	0.8380	0.4974	0.8059	0.1359	0.8146	0.8873	0.5195	0.6364	0.9151
NM_001014155	Taf1c	0.7423	0.8663	0.0055	0.0656	0.8605	0.9358	0.1737	0.8776	0.7752	0.7349	0.8143	0.5744
NM_001014157	Ltv1	0.4875	0.5757	0.1556	0.0320	0.0772	0.0984	0.0968	0.5385	0.6523	0.6499	0.5081	0.1003
NM_012732	Lipa	0.8791	0.2503	0.9709	0.7601	0.4259	0.9007	0.9512	0.9365	0.5431	0.8692	0.6048	0.9388
NM_001017505	Itgbl1	0.0977	0.0529	0.0537	0.2421	0.6268	0.1113	0.2280	0.5169	0.0427	0.1759	0.0176	0.0778
NM_001014160	Gramd1a	0.9437	0.9259	0.7133	0.1834	0.7184	0.9696	0.8954	0.9897	0.8909	0.8934	0.7441	0.9537
NM_012763	Ptpra	0.5840	0.7126	0.6199	0.5118	0.4255	0.2017	0.6037	0.4770	0.7195	0.5386	0.5741	0.2371
NM_012693	Cyp2a2	0.6871	0.2156	0.8086	0.8119	0.4758	0.7559	0.4233	0.8813	0.4100	0.6355	0.8551	0.5827
NM_001014164	Cybasc3	0.9146	0.7035	0.9253	0.9518	0.7888	0.9138	0.9562	0.9776	0.4680	0.5733	0.9580	0.9533
NM_001014165	RGD1310039	0.8380	0.6361	0.7921	0.2996	0.6714	0.8520	0.5974	0.4724	0.8923	0.7754	0.8243	0.7427
NM_012605	Mylpf	0.0960	0.5950	0.0129	0.7289	0.3451	0.3922	0.2614	0.2767	0.1188	0.6677	0.4124	0.7460
NM_001025423	Adhfe1	0.1666	0.0195	0.9664	0.0534	0.1738	0.0254	0.1918	0.1220	0.0242	0.0475	0.2657	0.4466
NM_001014169	LOC361776	0.1737	0.0285	0.2861	0.6172	0.2076	0.1723	0.5051	0.0785	0.1595	0.0369	0.0375	0.4900
NM_001017507	Mef2b	0.0764	0.7525	0.5624	0.1057	0.1088	0.0922	0.7453	0.4033	0.6862	0.7360	0.0973	0.3984
NM_001017502	Ccdc117	0.6545	0.7546	0.6357	0.7579	0.4939	0.3587	0.5855	0.5861	0.5667	0.5279	0.8053	0.8593
NM_001017496	Cxcl13	0.4656	0.4610	0.1153	0.3788	0.6538	0.1874	0.2807	0.4216	0.5829	0.6451	0.1554	0.3525
NM_001014171	Veph1	0.7068	0.6661	0.2969	0.7868	0.4562	0.7929	0.5773	0.9372	0.5300	0.6242	0.7457	0.5962
NM_012920	Camk2a	0.4810	0.4775	0.3845	0.3265	0.5421	0.7146	0.3197	0.5531	0.6505	0.0591	0.1035	0.2660
NM_012925	Cd59	0.0932	0.1161	0.6160	0.3405	0.0555	0.0489	0.2761	0.0053	0.1509	0.2739	0.1083	0.0328
NM_001014173	RGD1311265	0.4019	0.2472	0.3063	0.0991	0.3441	0.4415	0.1614	0.3995	0.3234	0.2153	0.4933	0.6156
NM_001014174	LOC361985	0.4079	0.3229	0.5277	0.7060	0.5211	0.5030	0.5051	0.2833	0.6383	0.5064	0.3566	0.5693
NM_001014175	LOC361990	0.7708	0.5312	0.8476	0.5835	0.8342	0.7066	0.7227	0.6662	0.9211	0.6720	0.7399	0.6213
NM_001025694	Fam166a	0.7846	0.7100	0.7088	0.7794	0.7137	0.5877	0.7628	0.7070	0.5962	0.1868	0.8971	0.3696
NM_001025648	Snapap	0.6314	0.7310	0.5687	0.3582	0.4106	0.4666	0.7293	0.5918	0.5111	0.4295	0.6710	0.4638
NM_001017514	Izumo1	0.3130	0.3470	0.5531	0.3183	0.3035	0.0996	0.6070	0.1066	0.2888	0.2197	0.2021	0.3958
NM_001014178	Fam69b	0.6981	0.6157	0.8492	0.7554	0.0638	0.0694	0.8733	0.6385	0.5674	0.2005	0.4354	0.4935
NM_001014179	Wdsub1	0.6727	0.4763	0.3483	0.3620	0.3542	0.2015	0.2939	0.3112	0.2836	0.4405	0.3688	0.2442
NM_012664	Syp	0.7927	0.5292	0.9201	0.7405	0.9442	0.8526	0.8423	0.8418	0.7132	0.7652	0.0335	0.7186
NM_001014182	Mall	0.2872	0.5586	0.6019	0.5537	0.6613	0.5904	0.4817	0.5118	0.5047	0.4518	0.5362	0.6172
NM_001014183	RGD1306991	0.5160	0.8849	0.7469	0.2985	0.9092	0.7024	0.8044	0.6215	0.6699	0.0829	0.3720	0.7433
NM_001024262	Tspan11	0.8468	0.8736	0.8254	0.5357	0.7731	0.8882	0.6932	0.1738	0.8037	0.8761	0.5738	0.8413

NM_001024271	Rnf185	0.4374	0.9471	0.9028	0.9298	0.4964	0.4494	0.8881	0.7579	0.3597	0.9320	0.3569	0.9329
NM_012692	Cyp2a1	0.0865	0.4958	0.0549	0.4470	0.1774	0.3516	0.6354	0.1506	0.2244	0.6661	0.3744	0.2786
NM_001017932	Wdr55	0.3191	0.2450	0.2011	0.5240	0.3528	0.3676	0.2900	0.2638	0.3293	0.3714	0.3108	0.2308
NM_012690	Abcb4	0.3613	0.5832	0.3138	0.4110	0.2936	0.0543	0.6558	0.1959	0.4704	0.1686	0.7184	0.1951
NM_001014192	Tmem39b	0.7767	0.6393	0.2370	0.5327	0.6855	0.7012	0.1337	0.8940	0.8102	0.3457	0.2044	0.5055
NM_001014193	RGD1359529	0.9976	0.8300	0.9526	0.8820	0.7728	0.8479	0.2872	0.9645	0.7636	0.9208	0.8788	0.8720
NM_013087	Cd81	0.7179	0.8547	0.8982	0.9496	0.0418	0.1912	0.9260	0.0989	0.8900	0.9615	0.7541	0.9084
NM_012820	Acsl1	0.1515	0.4367	0.2649	0.1392	0.0827	0.3839	0.9813	0.1512	0.7665	0.4905	0.8468	0.3956
NM_022502	Ppt1	0.0635	0.0591	0.7937	0.8854	0.7400	0.7657	0.0547	0.9698	0.1776	0.0413	0.0798	0.3777
NM_022400	Bcat2	0.3602	0.0751	0.6850	0.5957	0.1301	0.3135	0.6770	0.0626	0.3033	0.2257	0.3740	0.3144
NM_001029911	Cit	0.3071	0.0720	0.5360	0.2126	0.2999	0.3312	0.3559	0.2769	0.0264	0.0062	0.3296	0.5228
NM_001024297	Spz1	0.5050	0.3192	0.1210	0.4760	0.0489	0.1261	0.8201	0.3613	0.3204	0.5710	0.6497	0.7429
NM_001014197	RGD1310311	0.8638	0.4014	0.5234	0.0943	0.1286	0.0647	0.2680	0.3326	0.2495	0.0999	0.2391	0.2911
NM_001014198	RGD1308470	0.5291	0.7311	0.6961	0.5600	0.6046	0.7165	0.2445	0.7252	0.5986	0.6256	0.6211	0.4915
NM_012779	Aqp5	0.1625	0.1243	0.2755	0.0542	0.0819	0.6979	0.4666	0.3414	0.2422	0.1852	0.2507	0.4479
NM_012713	Prkcb	0.6022	0.6313	0.6293	0.6369	0.5157	0.4208	0.0524	0.6342	0.8859	0.7799	0.4343	0.7047
NM_001014201	Cd320	0.6429	0.9802	0.2003	0.1330	0.6482	0.6925	0.8285	0.8093	0.3557	0.8561	0.8927	0.1055
NM_017200	Tfpi	0.0326	0.0206	0.0606	0.0259	0.0370	0.0089	0.0536	0.0482	0.1224	0.0916	0.1337	0.0318
NM_001014206	RGD1309534	0.1693	0.1284	0.9725	0.4293	0.3772	0.5455	0.0576	0.0722	0.2879	0.2419	0.2409	0.0183
NM_001014209	LOC363060	0.8727	0.8513	0.7711	0.7952	0.8044	0.2172	0.8271	0.7373	0.9511	0.7818	0.7852	0.9468
NM_001014210	Tbc1d21	0.1902	0.2004	0.4412	0.2159	0.2678	0.4287	0.6417	0.5290	0.3856	0.6528	0.1918	0.2578
NM_001024283	Scgb3a2	0.0202	0.0329	0.3927	0.4919	0.0406	0.3484	0.0190	0.1861	0.1557	0.0755	0.4641	0.0284
NM_001024281	Retnlb	0.3136	0.3441	0.4636	0.4884	0.7909	0.2170	0.5150	0.3460	0.4216	0.5517	0.3803	0.4639
NM_001024298	Tmem174	0.8599	0.4186	0.2761	0.5420	0.5113	0.4313	0.7619	0.3150	0.0698	0.6921	0.7729	0.6360
NM_012809	Cnp	0.2776	0.2900	0.5906	0.1152	0.1057	0.0480	0.0413	0.2112	0.2899	0.1268	0.1068	0.3423
NM_001024321	Hyal5	0.0351	0.4914	0.3632	0.7667	0.4479	0.0743	0.0853	0.3728	0.0657	0.5731	0.3599	0.3500
NM_201423	Ugt1a2	0.3597	0.6903	0.6652	0.6415	0.4872	0.4899	0.4267	0.2862	0.3793	0.4167	0.5322	0.6246
NM_001024326	Rab19	0.6657	0.6939	0.0373	0.2390	0.4507	0.2339	0.6596	0.1433	0.8819	0.5588	0.4962	0.0278
NM_001014221	LOC363337	0.1054	0.3820	0.2864	0.1312	0.2382	0.3614	0.1281	0.2012	0.3298	0.4830	0.4264	0.2351
NM_001025646	Cep55	0.1434	0.9014	0.3174	0.6111	0.9053	0.8757	0.9327	0.8756	0.9559	0.9355	0.9484	0.1942
NM_001033663	Araf	0.8196	0.4581	0.5266	0.6122	0.5844	0.5822	0.6369	0.7391	0.4322	0.3968	0.4016	0.9104
NM_001024299	Zfp458	0.1542	0.5077	0.9648	0.1347	0.6092	0.8536	0.4731	0.8832	0.5084	0.5746	0.8145	0.6826
NM_012816	Amacr	0.7832	0.8121	0.9761	0.6898	0.7304	0.7682	0.2164	0.7357	0.9160	0.7634	0.8172	0.4092
NM_012769	Gucy1b3	0.5421	0.4509	0.7800	0.6521	0.3412	0.5545	0.1403	0.5273	0.6658	0.4626	0.7161	0.7300
NM_001024324	Fam71f1	0.2489	0.2964	0.5269	0.2703	0.6201	0.2319	0.4946	0.7063	0.2754	0.5200	0.5929	0.3149
NM_001033963	Prkx	0.7565	0.4876	0.8079	0.5948	0.2967	0.3712	0.5565	0.3690	0.5439	0.4279	0.7734	0.6166
NM_001025659	Lrrc6	0.2440	0.2468	0.2666	0.0717	0.3287	0.1752	0.0967	0.2465	0.1242	0.2818	0.0727	0.2896
NM_001014230	Iqcg	0.5280	0.5170	0.6744	0.3032	0.5642	0.3770	0.2603	0.3462	0.6345	0.5666	0.8791	0.4804
NM_001025685	Cd5l	0.5486	0.3624	0.7195	0.5897	0.4945	0.5671	0.2668	0.4921	0.7139	0.6616	0.5889	0.6213
NM_001024331	Rab43	0.0175	0.3028	0.1384	0.2840	0.0500	0.0256	0.2743	0.0405	0.0591	0.1617	0.5966	0.1028
NM_013022	Rock2	0.1076	0.1282	0.4805	0.0565	0.1392	0.6663	0.0822	0.0564	0.9130	0.8621	0.1324	0.9872
NM_001024335	Cd27	0.6389	0.3963	0.3383	0.1721	0.4802	0.3308	0.1081	0.4548	0.3969	0.4284	0.2558	0.6501
NM_001024341	Fam110b	0.8205	0.0904	0.5233	0.6434	0.6910	0.3038	0.0373	0.6304	0.0602	0.2809	0.0649	0.1352
NM_001024345	Tpm2	0.4620	0.3753	0.2960	0.2157	0.3983	0.3833	0.2217	0.2099	0.3277	0.3842	0.5174	0.3576
NM_001014237	LOC364558	0.2584	0.2460	0.5740	0.2156	0.2245	0.2539	0.3248	0.3012	0.3736	0.6064	0.7091	0.1963
NM_001014238	LOC364620	0.2009	0.5190	0.3117	0.6863	0.5353	0.6786	0.5052	0.3289	0.4825	0.5713	0.5588	0.3989
NM_012618	S100a4	0.8363	0.8941	0.7528	0.5639	0.7381	0.5081	0.6235	0.7397	0.8833	0.8591	0.9105	0.5340
NM_001014243	RGD1309888	0.7138	0.7600	0.4749	0.5355	0.7354	0.9030	0.8576	0.8700	0.8709	0.7914	0.7743	0.9115
NM_012704	Ptger3	0.4414	0.5993	0.1065	0.6106	0.1683	0.2024	0.4810	0.2777	0.4660	0.5348	0.2996	0.5524

NM_012762	Casp1	0.0311	0.0016	0.0258	0.0061	0.8052	0.8507	0.0431	0.7111	0.0278	0.0181	0.0252	0.0387
NM_001014247	Lzts2	0.6670	0.4445	0.5644	0.5824	0.4962	0.5484	0.6150	0.6019	0.4135	0.5894	0.6086	0.6520
NM_001014248	RGD1308127	0.8888	0.9683	0.9551	0.9515	0.1066	0.5954	0.9220	0.3195	0.9070	0.9564	0.9799	0.7558
NM_012784	Cnr1	0.1567	0.4051	0.6213	0.1864	0.4633	0.6624	0.2214	0.0684	0.1908	0.5172	0.1712	0.5264
NM_012781	Arnt2	0.6829	0.6621	0.9198	0.9614	0.5166	0.8128	0.9183	0.0423	0.2775	0.6381	0.1434	0.8921
NM_001014252	LOC365791	0.8320	0.5205	0.5403	0.6298	0.5268	0.5395	0.3387	0.5044	0.3861	0.3567	0.5358	0.3538
NM_012756	Igf2r	0.9973	0.9642	0.9321	0.5199	0.9084	0.9639	0.7252	0.9958	0.9786	0.9300	0.9711	0.7522
NM_001024366	Arl13a	0.2160	0.2392	0.1862	0.3978	0.0672	0.2679	0.1137	0.1855	0.0759	0.2380	0.4158	0.1980
NM_001024758	Ttll10	0.5669	0.2916	0.6093	0.0373	0.2259	0.1577	0.5098	0.7171	0.6657	0.1195	0.2946	0.4075
NM_145670	Bcas1	0.9890	0.8557	0.9390	0.8932	0.9283	0.8881	0.8609	0.9563	0.9001	0.9744	0.9406	0.5232
NM_001014265	Mterfd3	0.0460	0.4101	0.1378	0.3042	0.2236	0.1824	0.3429	0.1412	0.1448	0.1506	0.1351	0.1169
NM_001024355	Ccdc134	0.8717	0.8572	0.0173	0.6397	0.9426	0.7279	0.9242	0.9427	0.7209	0.9121	0.5344	0.2911
NM_001024367	Armcx1	0.9152	0.7677	0.8964	0.2657	0.6412	0.4212	0.9835	0.4309	0.7532	0.9821	0.9612	0.9030
NM_001014267	RGD1305207	0.8502	0.8028	0.7070	0.4811	0.6575	0.6263	0.6570	0.6172	0.8673	0.4444	0.5484	0.9811
NM_001024368	Slc10a3	0.2852	0.2238	0.0255	0.6085	0.0928	0.4914	0.3939	0.6660	0.1654	0.2859	0.0820	0.7786
NM_012722	Eln	0.9157	0.0781	0.2632	0.2413	0.9251	0.7753	0.1647	0.9430	0.2886	0.2621	0.0101	0.8460
NM_001014270	LOC367321	0.3237	0.3475	0.3324	0.3386	0.2607	0.3197	0.4735	0.5193	0.3185	0.4579	0.4496	0.2528
NM_001014271	LOC367515	0.2699	0.4696	0.2489	0.3942	0.3055	0.5320	0.3062	0.2097	0.1831	0.4980	0.3472	0.2678
NM_001014272	LOC367808	0.5084	0.6290	0.5275	0.6562	0.5637	0.4013	0.5877	0.4386	0.4366	0.5924	0.6541	0.3182
NM_012626	Ppy	0.5712	0.5876	0.1192	0.3932	0.1309	0.0849	0.0315	0.1258	0.0381	0.0857	0.0915	0.1296
NM_012890	Slc30a2	0.9496	0.7608	0.8452	0.6549	0.9791	0.8194	0.3814	0.8514	0.2689	0.8070	0.7341	0.7112
NM_001014275	Tceal8	0.8675	0.9966	0.9840	0.9966	0.9352	0.9955	0.9713	0.7851	0.9611	0.9816	0.9797	0.7562
NM_001014762	Pdrg1	0.1827	0.2405	0.3495	0.1686	0.2526	0.2119	0.0300	0.2184	0.1510	0.1159	0.1122	0.2498
NM_001036626	Zfp36l2	0.9902	0.9806	0.9153	0.9938	0.9940	0.9625	0.9137	0.9998	0.4132	0.9525	0.7648	0.9059
NM_001024358	Glb1l3	0.4759	0.2229	0.8826	0.2984	0.1195	0.6346	0.6385	0.3889	0.9802	0.4758	0.8019	0.7169
NM_001014780	Pcdhb6	0.4775	0.4396	0.5831	0.5656	0.2940	0.2147	0.0713	0.7570	0.7547	0.0590	0.4964	0.6543
NM_001113783	Golt1b	0.9649	0.7812	0.8282	0.1184	0.6808	0.2388	0.7546	0.7648	0.8417	0.9333	0.9558	0.1154
NM_012922	Casp3	0.1131	0.1847	0.1468	0.5070	0.8002	0.7514	0.0166	0.4702	0.0817	0.0016	0.1161	0.0677
NM_012804	Abcd3	0.6891	0.9401	0.6568	0.9864	0.7758	0.7727	0.8609	0.9384	0.8746	0.9575	0.8833	0.2711
NM_017017	Hgf	0.5260	0.8006	0.3248	0.2456	0.2085	0.2332	0.1381	0.5637	0.4585	0.7583	0.3697	0.0637
NM_013103	Hnf1b	0.8633	0.7833	0.5398	0.0723	0.6469	0.6927	0.3436	0.2414	0.5639	0.5026	0.5341	0.1799
NM_012792	Fmo1	0.0255	0.0076	0.1466	0.0737	0.7851	0.4877	0.0095	0.3511	0.0213	0.0026	0.1496	0.1606
NM_012800	P2ry1	0.7195	0.8544	0.6996	0.7571	0.3767	0.7861	0.7176	0.7811	0.8838	0.6655	0.5950	0.6612
NM_012788	Dlg1	0.1610	0.0929	0.7337	0.6783	0.0203	0.8615	0.4920	0.0111	0.2626	0.0339	0.5817	0.6327
NM_012640	Rbp2	0.0173	0.2607	0.5674	0.1686	0.1175	0.0586	0.1519	0.0478	0.6438	0.2106	0.3322	0.5051
NM_001024772	Naf1	0.6376	0.6649	0.5976	0.2799	0.4048	0.5719	0.2467	0.2376	0.5409	0.6907	0.4155	0.5341
NM_001015003	Crbn	0.1101	0.2396	0.8943	0.2691	0.0248	0.1777	0.2240	0.1966	0.1667	0.3353	0.4780	0.5695
NM_001015004	Vgll4	0.0019	0.3681	0.1803	0.2724	0.4580	0.0824	0.2449	0.6736	0.2102	0.2865	0.0869	0.1580
NM_001015005	Ddx47	0.9961	0.8304	0.7045	0.2694	0.8180	0.8458	0.4798	0.7394	0.8465	0.8834	0.7457	0.7151
NM_012857	Lamp1	0.2108	0.0419	0.3610	0.3242	0.8211	0.8239	0.0798	0.7523	0.5332	0.3178	0.4686	0.8091
NM_001015007	Dph2	0.7480	0.8457	0.5436	0.4134	0.6587	0.7049	0.7427	0.7613	0.7924	0.5135	0.6075	0.8726
NM_001015008	Tcea3	0.0072	0.1066	0.7490	0.6199	0.0752	0.0082	0.7564	0.0817	0.6396	0.4916	0.3515	0.1236
NM_012909	Aqp2	0.4481	0.6287	0.4498	0.5992	0.6465	0.4200	0.3021	0.8167	0.4711	0.1108	0.5071	0.4961
NM_013002	Pcp4	0.7855	0.5272	0.5826	0.4434	0.8445	0.6443	0.1322	0.8392	0.8641	0.7681	0.4606	0.4076
NM_012780	Arnt	0.0828	0.1007	0.1616	0.0080	0.0210	0.4881	0.1172	0.0758	0.2275	0.3954	0.5837	0.6590
NM_001015013	Mtvr2	0.9133	0.8819	0.1314	0.8253	0.9186	0.8569	0.9833	0.9875	0.9207	0.9631	0.4111	0.2200
NM_133317	Tob1	0.8677	0.7587	0.9540	0.9482	0.5712	0.5999	0.8949	0.7787	0.9715	0.5820	0.9815	0.0766
NM_080770	Scgb2a1	0.0832	0.4503	0.4764	0.4396	0.8111	0.3476	0.2257	0.2656	0.3361	0.3175	0.1757	0.0967
NM_053930	Gp1bb	0.8539	0.8159	0.9437	0.7195	0.9240	0.9799	0.9898	0.9307	0.9306	0.7365	0.8721	0.8901

NM_001015017	Olfm2	0.0979	0.0097	0.5710	0.1407	0.2950	0.0144	0.1596	0.0504	0.2108	0.0215	0.0135	0.2673
NM_013092	Cma1	0.3484	0.2047	0.3094	0.3884	0.1079	0.4852	0.5909	0.3488	0.4114	0.4740	0.3828	0.4495
NM_001015023	Rab24	0.9661	0.9122	0.3401	0.2031	0.8349	0.8219	0.1403	0.7497	0.8284	0.9504	0.9600	0.7497
NM_013089	Gys2	0.4989	0.7854	0.6892	0.5018	0.5704	0.3822	0.3100	0.6121	0.4911	0.7190	0.3439	0.3494
NM_012839	Cycs	0.2567	0.9049	0.2618	0.9190	0.2749	0.2394	0.7403	0.1182	0.4274	0.6428	0.5535	0.1178
NM_013083	Hspa5	0.9828	0.8212	0.4097	0.1682	0.0158	0.1141	0.9987	0.1324	0.9977	0.9133	0.9807	0.6549
NM_012855	Jak3	0.8634	0.8678	0.8507	0.7867	0.8585	0.9412	0.9179	0.8606	0.8848	0.7761	0.9525	0.9527
NM_012778	Aqp1	0.2709	0.1003	0.2958	0.2367	0.0458	0.1711	0.2305	0.2065	0.1995	0.1881	0.0573	0.1610
NM_001015028	Anks6	0.8067	0.2527	0.1723	0.2343	0.1858	0.9114	0.5748	0.9312	0.9670	0.1658	0.7366	0.5516
NM_012649	Sdc4	0.3006	0.2723	0.3472	0.7654	0.6454	0.3156	0.4382	0.4189	0.3167	0.0517	0.0044	0.1593
NM_001015030	Ubl3	0.8906	0.9945	0.9875	0.9195	0.2153	0.8911	0.9606	0.4213	0.9529	0.9740	0.9779	0.9784
NM_001015031	Arl5b	0.6179	0.7184	0.8239	0.4371	0.4900	0.8591	0.7788	0.7808	0.4069	0.8709	0.8838	0.4980
NM_001108453	Cbfa2t3	0.9229	0.9113	0.5117	0.9489	0.7351	0.8609	0.6997	0.5665	0.9189	0.1397	0.6340	0.9729
NM_012650	Shbg	0.1973	0.6296	0.1441	0.4491	0.3401	0.2185	0.4251	0.2568	0.4449	0.4149	0.6002	0.0642
NM_199268	Pde8b	0.6066	0.7767	0.3515	0.3657	0.3915	0.1686	0.7605	0.1337	0.6816	0.6268	0.7312	0.4534
NM_212516	Atp5l	0.1131	0.2170	0.1585	0.1359	0.0763	0.1139	0.0815	0.0244	0.3338	0.0680	0.0643	0.0256
NM_212466	Cfb	0.0278	0.2330	0.3168	0.1699	0.0576	0.2658	0.2017	0.1544	0.0327	0.4570	0.5487	0.3028
NM_013186	Kcnb1	0.6748	0.6723	0.6910	0.7666	0.7324	0.8131	0.8600	0.5364	0.8650	0.5833	0.8321	0.6289
NM_012653	Slc9a2	0.7634	0.6191	0.8769	0.2473	0.6244	0.2779	0.6790	0.6652	0.7114	0.7379	0.6363	0.8012
NM_001024796	Spin1	0.4333	0.1233	0.9144	0.2596	0.0868	0.0961	0.8322	0.0449	0.0983	0.2533	0.5931	0.2202
NM_001024369	Ypel4	0.6056	0.1517	0.7643	0.5353	0.4926	0.5737	0.9065	0.8274	0.3057	0.5212	0.2488	0.9068
NM_001017447	RGD1305235	0.2139	0.4204	0.1170	0.0460	0.1294	0.1209	0.0188	0.0158	0.0270	0.0695	0.3338	0.1072
NM_001017448	RGD1311358	0.0699	0.1432	0.1939	0.1082	0.3012	0.2320	0.3160	0.0544	0.0540	0.1356	0.3460	0.0129
NM_001024792	Slc25a39	0.0511	0.3537	0.5635	0.6805	0.0295	0.1548	0.7797	0.2615	0.0771	0.1496	0.1966	0.5584
NM_001017451	RGD1309228	0.1626	0.5191	0.2981	0.2617	0.3088	0.3623	0.3164	0.1009	0.0892	0.3237	0.1726	0.3955
NM_001024876	Nsmce2	0.0609	0.3699	0.1539	0.0772	0.2080	0.0157	0.1885	0.2377	0.1630	0.1923	0.3075	0.0824
NM_001017454	RGD1307799	0.3860	0.2654	0.1436	0.8083	0.9285	0.9188	0.2598	0.6560	0.4211	0.3636	0.0991	0.5198
NM_001017455	Tmem80	0.6165	0.5255	0.1461	0.9552	0.7197	0.1779	0.8467	0.5847	0.9072	0.8787	0.4850	0.3441
NM_001025645	Mus81	0.3530	0.2914	0.4091	0.8216	0.3605	0.0702	0.2600	0.2422	0.7112	0.4313	0.7920	0.5751
NM_001017458	Tcp11l2	0.3040	0.0930	0.8180	0.5977	0.2721	0.4371	0.2266	0.1723	0.2828	0.6397	0.2500	0.0603
NM_001033686	Cyp4f18	0.5869	0.3502	0.4229	0.7016	0.7706	0.2248	0.7235	0.7825	0.8452	0.6640	0.7520	0.9191
NM_001017462	LOC361346	0.0556	0.0142	0.0044	0.0030	0.6629	0.7030	0.0056	0.6798	0.1570	0.0060	0.0102	0.0973
NM_001017463	LOC361635	0.6346	0.1754	0.4817	0.9201	0.3239	0.5778	0.1034	0.1584	0.2894	0.1923	0.0755	0.6991
NM_001025753	Rem1	0.1097	0.0111	0.1101	0.5786	0.3532	0.4369	0.1828	0.2507	0.0111	0.0922	0.0126	0.4015
NM_001017465	LOC361914	0.6161	0.7288	0.3858	0.2774	0.6800	0.5866	0.4004	0.8091	0.8712	0.7657	0.7449	0.5326
NM_013170	Gucy2c	0.3042	0.1558	0.3737	0.4634	0.3940	0.3840	0.2378	0.4259	0.1179	0.2129	0.0883	0.1317
NM_001017468	Mill2	0.1669	0.5885	0.3614	0.5297	0.6753	0.3584	0.4533	0.2748	0.7903	0.4322	0.5788	0.6751
NM_001024877	Ncaph2	0.7797	0.8524	0.0425	0.6912	0.8158	0.9101	0.6930	0.9804	0.5535	0.9182	0.8122	0.0234
NM_001024802	Tmem165	0.7742	0.8874	0.8723	0.9471	0.5103	0.8692	0.8686	0.1329	0.9063	0.7351	0.9918	0.7253
NM_001024799	Fam133b	0.4292	0.9579	0.5062	0.5142	0.9504	0.7527	0.3607	0.9441	0.8721	0.9410	0.8728	0.6420
NM_001017472	LOC497899	0.0558	0.3361	0.4186	0.4469	0.1511	0.0468	0.6019	0.0344	0.0693	0.0291	0.2771	0.0861
NM_001033956	Calca	0.5146	0.2222	0.4223	0.7027	0.6452	0.3174	0.2607	0.4353	0.3327	0.4308	0.4268	0.5937
NM_001017474	LOC497934	0.4218	0.4353	0.8271	0.4640	0.2458	0.6597	0.5186	0.1205	0.2027	0.1555	0.3995	0.1504
NM_001017475	LOC497938	0.1997	0.5201	0.7736	0.7452	0.3208	0.3106	0.4080	0.2135	0.2926	0.2656	0.6772	0.1700
NM_001017476	LOC497940	0.6281	0.3065	0.1574	0.4988	0.5711	0.4672	0.7514	0.7601	0.7140	0.7976	0.6798	0.7057
NM_001024864	Rogdi	0.4194	0.0362	0.3503	0.2338	0.1931	0.0198	0.2661	0.2505	0.1261	0.1417	0.0692	0.2792
NM_001024866	Ccdc104	0.5049	0.0491	0.6625	0.5852	0.8637	0.3930	0.8603	0.8074	0.0643	0.2957	0.0680	0.0112
NM_001017483	LOC498063	0.3172	0.6231	0.5579	0.7103	0.5686	0.7456	0.8509	0.2232	0.7550	0.8533	0.5482	0.6798
NM_001024867	Vps37a	0.1226	0.6451	0.8765	0.7862	0.0318	0.1192	0.2853	0.1451	0.4919	0.3273	0.5017	0.4652

NM_001017485	LOC498145	0.3591	0.2643	0.7201	0.4438	0.3354	0.2375	0.1032	0.2323	0.4937	0.3934	0.4024	0.2750
NM_001024869	Wwtr1	0.6376	0.0380	0.7263	0.8271	0.0869	0.4906	0.3910	0.1968	0.2335	0.0898	0.6094	0.1038
NM_001024868	Pih1d1	0.1500	0.8604	0.0690	0.6193	0.6562	0.3683	0.6650	0.5670	0.4783	0.8238	0.6422	0.0610
NM_012813	St8sia1	0.3621	0.7042	0.8809	0.5651	0.4074	0.1368	0.5716	0.8678	0.2289	0.7787	0.7836	0.5252
NM_012966	Hspe1	0.1136	0.6383	0.0700	0.7659	0.4415	0.3007	0.8281	0.5969	0.7726	0.3342	0.7033	0.0707
NM_012946	Sparcl1	0.0753	0.1241	0.0641	0.2221	0.1414	0.3028	0.2987	0.0624	0.1322	0.5072	0.2437	0.4855
NM_001024980	Ankrd34a	0.3668	0.6015	0.5862	0.6244	0.4620	0.5839	0.7198	0.6899	0.5568	0.5552	0.2089	0.5997
NM_001024870	Ctnnbl1	0.4221	0.8551	0.1830	0.4914	0.1283	0.1170	0.1744	0.0477	0.5359	0.4718	0.8146	0.4762
NM_012696	Kng1	0.8118	0.9082	0.8557	0.9455	0.7752	0.8145	0.9782	0.9059	0.8408	0.8940	0.9381	0.7613
NM_001017493	LOC498265	0.8346	0.9079	0.1428	0.2414	0.7192	0.8629	0.7077	0.7296	0.7797	0.8891	0.9111	0.5963
NM_001017495	LOC498330	0.8764	0.6659	0.7833	0.6722	0.4284	0.2538	0.6879	0.6055	0.0839	0.5591	0.2299	0.7256
NM_001025010	Nlrx1	0.3348	0.6922	0.5510	0.4509	0.4902	0.6362	0.6597	0.3283	0.4928	0.2102	0.4026	0.4299
NM_001017498	LOC498350	0.7837	0.3713	0.5748	0.4074	0.3614	0.1886	0.5339	0.7321	0.3053	0.3251	0.5216	0.2533
NM_001017499	LOC498353	0.1013	0.0158	0.0564	0.7537	0.1082	0.1151	0.1074	0.2465	0.3115	0.0322	0.0772	0.0289
NM_001017500	LOC498368	0.9295	0.9642	0.9476	0.8134	0.9100	0.9502	0.9675	0.9537	0.9938	0.9771	0.9617	0.9064
NM_001017501	LOC498400	0.5772	0.2691	0.4709	0.0374	0.3219	0.5814	0.2926	0.5797	0.3238	0.0624	0.2746	0.4946
NM_001024874	Rhox9	0.0772	0.6309	0.1216	0.0539	0.1215	0.0458	0.1308	0.0972	0.4341	0.2474	0.0965	0.3872
NM_001025000	Iyd	0.1740	0.6132	0.1329	0.3611	0.0628	0.4516	0.2386	0.7533	0.2216	0.0741	0.1081	0.1293
NM_001024901	Rhox12	0.5078	0.0518	0.6113	0.1031	0.4074	0.5499	0.1871	0.5438	0.1696	0.1611	0.5871	0.1626
NM_001024889	Rhox4g	0.0940	0.6518	0.3223	0.1359	0.0580	0.0128	0.1026	0.0419	0.4514	0.2520	0.1919	0.4271
NM_001024885	Ccdc90b	0.7200	0.4269	0.9850	0.7998	0.6031	0.9541	0.0682	0.4558	0.0132	0.0210	0.1734	0.4862
NM_001025012	Gin1	0.3316	0.2808	0.3181	0.4089	0.1936	0.2008	0.2214	0.3449	0.2600	0.1779	0.3253	0.1955
NM_001017508	LOC498664	0.9598	0.6391	0.7769	0.7590	0.5804	0.5552	0.8707	0.9077	0.4248	0.8762	0.6562	0.9099
NM_001017509	MGC114246	0.4568	0.5368	0.6149	0.4904	0.4143	0.3421	0.3960	0.1286	0.1330	0.2562	0.4121	0.6273
NM_001017510	LOC498750	0.7038	0.2867	0.6312	0.7591	0.7340	0.7503	0.2978	0.7543	0.6978	0.6608	0.5678	0.7533
NM_001024756	Ythdf1	0.9869	0.8503	0.8472	0.6145	0.8536	0.5373	0.6617	0.9155	0.7959	0.7454	0.9248	0.1367
NM_001017513	MGC108974	0.6726	0.3892	0.6071	0.4926	0.1535	0.6318	0.8195	0.7153	0.4180	0.7897	0.5454	0.3951
NM_001025013	Mageb16	0.4708	0.5397	0.4507	0.6022	0.2196	0.2297	0.3777	0.2666	0.1974	0.2088	0.6005	0.2172
NM_012753	Cyp17a1	0.8435	0.9499	0.7372	0.7223	0.6703	0.6954	0.5861	0.5022	0.7218	0.6290	0.7104	0.8695
NM_001025004	Vsig4	0.4476	0.1529	0.2643	0.4852	0.2158	0.4680	0.4044	0.4571	0.3036	0.3790	0.3479	0.1009
NM_012864	Mmp7	0.5921	0.3247	0.8393	0.4246	0.9303	0.6665	0.7436	0.5196	0.6057	0.7637	0.7257	0.2811
NM_012818	Aanat	0.2150	0.1811	0.5064	0.0958	0.3157	0.4504	0.4133	0.4756	0.3124	0.5972	0.2929	0.5642
NM_001017988	LOC303566	0.8339	0.6825	0.0928	0.8216	0.9875	0.8715	0.7360	0.9087	0.8319	0.7331	0.8755	0.6389
NM_012796	Gstt2	0.0130	0.3291	0.8489	0.0615	0.0853	0.0071	0.7263	0.2290	0.0097	0.0613	0.2053	0.1125
NM_012665	Syt2	0.7143	0.5778	0.4535	0.4949	0.8066	0.6987	0.6459	0.3325	0.3351	0.3300	0.8240	0.7018
NM_013159	Ide	0.2629	0.2248	0.9247	0.9008	0.6274	0.9958	0.7809	0.4690	0.3730	0.2253	0.4737	0.9374
NM_012803	Proc	0.8270	0.5927	0.6471	0.6003	0.3446	0.6839	0.7363	0.8064	0.6938	0.6774	0.4642	0.6032
NM_012889	Vcam1	0.1160	0.2424	0.1349	0.1248	0.9844	0.8125	0.0075	0.8528	0.0260	0.0940	0.0966	0.0888
NM_001124768	Tac1	0.8379	0.5443	0.8929	0.8376	0.9096	0.8212	0.4845	0.6012	0.3701	0.7514	0.6959	0.4666
NM_012676	Tnnt2	0.2566	0.0333	0.7908	0.2836	0.7543	0.7323	0.7676	0.8176	0.1606	0.0433	0.0103	0.0191
NM_012671	Tgfa	0.2548	0.0044	0.2243	0.1683	0.9602	0.9574	0.0136	0.9467	0.2403	0.2285	0.2857	0.1531
NM_001024245	Ccdc32	0.6268	0.1477	0.9548	0.7456	0.1963	0.5284	0.7276	0.5080	0.4842	0.0817	0.6426	0.9487
NM_001024246	RGD1310794	0.5655	0.1452	0.5786	0.0521	0.3689	0.5640	0.3803	0.0395	0.3200	0.6914	0.4571	0.8597
NM_013034	Slc6a4	0.2999	0.0981	0.1891	0.2366	0.4140	0.8451	0.0841	0.5546	0.3132	0.2413	0.2003	0.3491
NM_013196	Ppara	0.3892	0.6535	0.7445	0.8474	0.7873	0.3661	0.4938	0.6329	0.6567	0.4734	0.6918	0.9631
NM_013010	Prkag1	0.7883	0.3747	0.1316	0.2896	0.9187	0.8835	0.4291	0.9976	0.0739	0.3386	0.6409	0.3883
NM_012746	Pcsk2	0.1108	0.1899	0.0443	0.0651	0.5389	0.0874	0.0391	0.1717	0.0637	0.1026	0.0494	0.4023
NM_012678	Tpm4	0.8432	0.0994	0.8361	0.1770	0.7142	0.8251	0.7688	0.7357	0.8757	0.7515	0.7150	0.8553
NM_001024965	Trappc2	0.6934	0.7032	0.5728	0.8807	0.6055	0.3919	0.0779	0.6903	0.2771	0.4947	0.2326	0.4607

NM_001025630	Arl6ip4	0.3199	0.3633	0.3714	0.3253	0.6366	0.4058	0.3424	0.3212	0.2393	0.2654	0.3300	0.3349
NM_001024260	Nostrin	0.9151	0.5786	0.7056	0.6522	0.6312	0.5914	0.7008	0.7903	0.7032	0.2128	0.7349	0.6061
NM_012751	Slc2a4	0.3284	0.0526	0.3443	0.5564	0.3419	0.4969	0.7962	0.7059	0.5976	0.6321	0.5908	0.8434
NM_001024265	RGD1311251	0.0279	0.5414	0.4149	0.2239	0.4762	0.4949	0.3353	0.0660	0.0808	0.0485	0.0895	0.2041
NM_001024266	Lztfl1	0.3262	0.2967	0.8048	0.2202	0.0199	0.0291	0.0280	0.1161	0.0485	0.0262	0.2300	0.4027
NM_012684	Vcsa1	0.5395	0.5839	0.3416	0.3704	0.5942	0.1784	0.7979	0.4844	0.6054	0.3902	0.7722	0.4369
NM_017005	Fh1	0.1385	0.5678	0.0066	0.0342	0.4023	0.6104	0.1825	0.5334	0.4686	0.2107	0.8336	0.1839
NM_001024270	Pelp1	0.0879	0.0556	0.0032	0.4222	0.1589	0.0211	0.0875	0.0164	0.7211	0.0859	0.2227	0.7655
NM_001024272	Phf11	0.0895	0.2569	0.0528	0.5847	0.2499	0.1188	0.1579	0.2130	0.3905	0.0802	0.1969	0.4268
NM_017064	Stat5a	0.8582	0.8195	0.8420	0.0299	0.8717	0.8977	0.6503	0.6615	0.4807	0.4964	0.2102	0.3356
NM_013096	Hba-a2	0.6245	0.2861	0.5975	0.4537	0.5080	0.5339	0.5811	0.7770	0.5963	0.5099	0.5692	0.7958
NM_013179	Hcrt	0.7984	0.6641	0.1839	0.3086	0.8047	0.4221	0.4395	0.6548	0.2386	0.6189	0.7169	0.4049
NM_012771	Lyz2	0.4805	0.5382	0.3271	0.6027	0.4216	0.3705	0.6419	0.2593	0.4732	0.2476	0.3390	0.3029
NM_012758	Syk	0.5308	0.3312	0.6643	0.4872	0.7256	0.4801	0.4161	0.7400	0.4229	0.2625	0.0279	0.8259
NM_001025026	Stap2	0.4312	0.9467	0.7862	0.8318	0.9962	0.7844	0.8429	0.8286	0.7910	0.8160	0.7555	0.9038
NM_001024284	MGC105567	0.7586	0.4771	0.6807	0.3809	0.4053	0.7008	0.5313	0.7338	0.1754	0.4195	0.0560	0.2423
NM_001024285	Ahrr	0.3592	0.9879	0.9095	0.2176	0.2389	0.3272	0.7592	0.2074	0.9762	0.9889	0.9713	0.9433
NM_001024287	LOC499240	0.4305	0.1920	0.3289	0.1973	0.1367	0.3791	0.5912	0.4476	0.5454	0.3934	0.1720	0.1777
NM_001024288	LOC499279	0.4050	0.4399	0.6674	0.5385	0.4654	0.2905	0.3025	0.1645	0.3499	0.4842	0.3810	0.2014
NM_001025283	Fam118b	0.4180	0.7330	0.6448	0.4325	0.6807	0.5680	0.2572	0.7078	0.7294	0.3180	0.3398	0.3110
NM_001025034	Fam107b	0.9315	0.9119	0.8840	0.9199	0.8261	0.7347	0.8679	0.6792	0.9034	0.9742	0.9580	0.8783
NM_001024291	LOC499306	0.3721	0.2480	0.7579	0.6248	0.0813	0.2107	0.2796	0.4711	0.6824	0.7235	0.0194	0.3315
NM_012850	Ghrhr	0.6677	0.3945	0.3680	0.5054	0.7822	0.1390	0.1498	0.6104	0.5719	0.4673	0.5898	0.4202
NM_001024293	LOC499331	0.3387	0.2744	0.3672	0.2952	0.1330	0.0311	0.4314	0.1908	0.0475	0.3712	0.4852	0.4289
NM_001025042	Fibin	0.9614	0.1353	0.8648	0.1710	0.9996	0.8700	0.0083	0.9941	0.0960	0.1264	0.1558	0.0042
NM_001024296	LOC499418	0.0478	0.0550	0.2810	0.3977	0.2927	0.7284	0.2341	0.1163	0.0830	0.1285	0.1052	0.2951
NM_001024987	Cwc15	0.3366	0.5430	0.1849	0.2104	0.0468	0.1013	0.2525	0.0622	0.3064	0.2976	0.3162	0.0579
NM_001024300	LOC499626	0.1893	0.1927	0.3890	0.2148	0.1934	0.2004	0.2207	0.2086	0.1826	0.1777	0.1914	0.1953
NM_001024301	LOC499643	0.5508	0.7419	0.7512	0.5461	0.6911	0.5679	0.2723	0.8206	0.5752	0.5524	0.6366	0.8891
NM_001024302	Lysmd1	0.3538	0.3483	0.3556	0.3877	0.0976	0.1405	0.8565	0.2628	0.4768	0.4004	0.8100	0.6013
NM_001033656	Man1a1	0.8000	0.8710	0.6663	0.8012	0.8332	0.6038	0.7813	0.7958	0.9315	0.8549	0.8476	0.5178
NM_001025046	Fam131b	0.3221	0.5966	0.9066	0.9525	0.7881	0.9136	0.7640	0.1362	0.7637	0.4624	0.8682	0.4787
NM_001025051	Fam110c	0.3607	0.4412	0.4428	0.5551	0.6706	0.6097	0.4681	0.5986	0.0594	0.8725	0.4020	0.5075
NM_001024311	LOC499806	0.0930	0.3520	0.3619	0.4014	0.1559	0.2242	0.2181	0.0558	0.5351	0.1512	0.4878	0.2506
NM_001024312	LOC499886	0.5631	0.1184	0.2361	0.5619	0.6554	0.8368	0.9294	0.7496	0.0912	0.1842	0.6283	0.7213
NM_001024313	LOC499900	0.8911	0.5988	0.9512	0.1863	0.6347	0.1694	0.8117	0.4873	0.6851	0.0689	0.7553	0.3424
NM_001024971	Ny-sar-48	0.0422	0.7825	0.0221	0.0079	0.1061	0.0561	0.3763	0.1563	0.8760	0.6052	0.5594	0.2206
NM_001024315	LOC499949	0.6296	0.5073	0.7773	0.2450	0.8003	0.5293	0.7550	0.0832	0.2387	0.5913	0.1691	0.5723
NM_001024317	LOC499971	0.7800	0.8220	0.9233	0.8338	0.7793	0.7298	0.8795	0.1248	0.8248	0.4342	0.7210	0.5973
NM_001024993	Smarce1	0.6610	0.5011	0.8300	0.8240	0.5392	0.2214	0.9247	0.9167	0.7872	0.6291	0.6068	0.1244
NM_001024988	Herpud2	0.6573	0.1377	0.9631	0.2444	0.4390	0.8225	0.6118	0.1749	0.4813	0.4250	0.5379	0.8468
NM_001025129	Fam107a	0.4342	0.8033	0.7434	0.8331	0.7537	0.5274	0.1606	0.2739	0.4062	0.7179	0.3052	0.6115
NM_001024322	LOC500054	0.1315	0.4675	0.4379	0.1617	0.5912	0.9216	0.2127	0.5050	0.3138	0.3259	0.1333	0.7236
NM_001024968	Slc46a3	0.0636	0.0246	0.2795	0.0455	0.1720	0.7311	0.0919	0.0954	0.0260	0.1226	0.1092	0.7644
NM_013037	Il1rl1	0.4577	0.4260	0.3171	0.1662	0.4241	0.3903	0.5096	0.3478	0.4136	0.3074	0.4198	0.3671
NM_001024976	Fam26f	0.3808	0.2848	0.3704	0.2146	0.2943	0.3960	0.2206	0.2085	0.1825	0.1777	0.5173	0.3209
NM_001024977	Fam26e	0.3680	0.6068	0.4292	0.7053	0.5835	0.5799	0.4300	0.5746	0.6217	0.5334	0.5976	0.3049
NM_001024328	Gimap7	0.3263	0.4986	0.3163	0.4319	0.2292	0.5860	0.5831	0.2999	0.2665	0.5312	0.4931	0.3682
NM_001024329	LOC500124	0.0381	0.3793	0.8505	0.5820	0.3722	0.5626	0.4058	0.1189	0.8970	0.3344	0.4003	0.7471

NM_001024330	LOC500227	0.5725	0.2185	0.3343	0.5586	0.5131	0.4764	0.4618	0.2094	0.0883	0.1142	0.5586	0.6063
NM_001033860	Ceacam1	0.8506	0.6352	0.9150	0.7145	0.6614	0.8986	0.9526	0.9740	0.8316	0.8947	0.8951	0.8638
NM_001024979	Gpatch4	0.2555	0.7463	0.0340	0.7202	0.8539	0.8805	0.2545	0.9032	0.9442	0.8001	0.8524	0.8363
NM_001024999	Snx20	0.6664	0.1269	0.8711	0.5964	0.8303	0.8040	0.5074	0.7647	0.5162	0.6124	0.5381	0.7006
NM_001024334	LOC500300	0.0486	0.9283	0.8577	0.8839	0.0632	0.0404	0.9506	0.0582	0.6978	0.6844	0.6789	0.9253
NM_001025124	Ntan1	0.1891	0.3994	0.2005	0.6150	0.6008	0.4169	0.4125	0.2081	0.1823	0.3647	0.4221	0.4514
NM_001024337	LOC500331	0.1246	0.6030	0.1658	0.5183	0.3466	0.1283	0.5630	0.4263	0.2892	0.2265	0.3248	0.2391
NM_012775	Tgfbr1	0.9121	0.8560	0.4975	0.8323	0.7535	0.4724	0.8754	0.5686	0.8808	0.9501	0.9156	0.8582
NM_001025138	Tmem156	0.5193	0.6620	0.8918	0.6066	0.7901	0.5634	0.9871	0.9694	0.7700	0.8818	0.1620	0.5386
NM_001030041	Carm1	0.4734	0.3336	0.5459	0.2944	0.6659	0.6579	0.6721	0.7265	0.5366	0.5982	0.4628	0.6362
NM_001024343	LOC500445	0.2740	0.8309	0.3326	0.5104	0.4858	0.2094	0.4896	0.7330	0.6589	0.6822	0.6820	0.4456
NM_012776	Adrbk1	0.7919	0.1984	0.5961	0.8902	0.3765	0.8884	0.1054	0.6161	0.8793	0.9194	0.9227	0.5462
NM_001024346	LOC500475	0.0436	0.2432	0.2424	0.6715	0.2471	0.4642	0.5492	0.6182	0.6381	0.5162	0.2944	0.4453
NM_001024348	LOC500567	0.2093	0.1614	0.1043	0.0830	0.3669	0.3016	0.6336	0.2756	0.3909	0.0715	0.2078	0.4097
NM_001024349	LOC500598	0.1495	0.4687	0.6092	0.5662	0.7420	0.2174	0.3163	0.8339	0.5765	0.5851	0.1830	0.7407
NM_001024350	LOC500705	0.8295	0.3634	0.1380	0.4745	0.9462	0.3291	0.4934	0.6695	0.0915	0.9297	0.8449	0.0764
NM_001024352	LOC500827	0.4316	0.4456	0.5757	0.5788	0.2790	0.2516	0.7214	0.4947	0.4129	0.5053	0.3433	0.2304
NM_001033684	Cct6a	0.9430	0.9714	0.4485	0.6113	0.6565	0.8674	0.4893	0.9489	0.8540	0.6983	0.9935	0.7443
NM_001024357	MGC105560	0.0714	0.2899	0.9496	0.8501	0.1222	0.6485	0.2568	0.1621	0.2932	0.3475	0.2640	0.8045
NM_001024359	Pmp22cd	0.1387	0.2890	0.0402	0.0317	0.3926	0.5538	0.1305	0.2285	0.1571	0.2161	0.1628	0.2266
NM_001024360	nod3l	0.2731	0.0453	0.0544	0.5094	0.0358	0.1800	0.1569	0.2192	0.1184	0.0344	0.1297	0.2742
NM_001025118	Fam63a	0.4611	0.0642	0.3355	0.7998	0.4829	0.6260	0.6343	0.7163	0.3226	0.5090	0.0909	0.8133
NM_001025115	Stap1	0.6297	0.6284	0.6324	0.5818	0.2986	0.6259	0.6292	0.8494	0.4754	0.6742	0.6044	0.7485
NM_001024364	LOC501180	0.2339	0.6277	0.4560	0.0350	0.3119	0.2593	0.1096	0.6852	0.5846	0.4812	0.5420	0.4880
NM_019301	Cr1l	0.3494	0.2003	0.4946	0.6610	0.6631	0.8096	0.4065	0.6672	0.2443	0.3532	0.4544	0.2036
NM_012757	Mas1	0.5506	0.8931	0.9405	0.7780	0.9673	0.8103	0.6813	0.9216	0.7064	0.5559	0.8302	0.7287
NM_001025120	Gtf3c2	0.7210	0.5242	0.4320	0.7102	0.6662	0.4247	0.4818	0.4546	0.5133	0.6682	0.4532	0.5922
NM_001025127	Rufy3	0.0363	0.6775	0.1797	0.0192	0.0954	0.4048	0.3278	0.0975	0.0805	0.0297	0.2713	0.4893
NM_001037095	Cpox	0.3088	0.1312	0.6439	0.2026	0.1539	0.3369	0.9156	0.0283	0.4828	0.0915	0.3671	0.7137
NM_012706	Grpr	0.6636	0.2654	0.1432	0.5862	0.1993	0.1939	0.1006	0.2722	0.0937	0.1786	0.3395	0.1126
NM_012847	Fnta	0.4172	0.1392	0.6244	0.1497	0.1087	0.5632	0.0617	0.3291	0.2925	0.4313	0.2721	0.5645
NM_012942	Cyp7a1	0.5658	0.2209	0.1921	0.7154	0.5266	0.5040	0.6196	0.8532	0.6385	0.8252	0.4294	0.7206
NM_012940	Cyp1b1	0.0077	0.0576	0.0845	0.0328	0.5932	0.8189	0.1391	0.5113	0.0081	0.0195	0.0598	0.1729
NM_001024739	Tekt3	0.3752	0.4153	0.2154	0.3537	0.4293	0.1836	0.0809	0.4105	0.3906	0.5029	0.3031	0.1900
NM_012823	Anxa3	0.8112	0.8372	0.8751	0.9467	0.0417	0.0606	0.9071	0.1555	0.7686	0.8403	0.8213	0.0913
NM_001025770	Tsga14	0.8535	0.9892	0.8166	0.9965	0.9176	0.9985	0.9234	0.8338	0.6108	0.9758	0.9946	0.9340
NM_012786	Cox8b	0.5937	0.7754	0.5871	0.5895	0.6148	0.3611	0.3716	0.3029	0.5827	0.8381	0.6435	0.7278
NM_013078	Otc	0.7219	0.5753	0.4595	0.8929	0.8594	0.1244	0.5735	0.5834	0.3627	0.9139	0.7561	0.5182
NM_012699	Dnajb9	0.3741	0.2286	0.9794	0.3636	0.1064	0.0975	0.0397	0.0809	0.3151	0.3603	0.3562	0.9371
NM_001024749	RGD1305713	0.9735	0.9202	0.9195	0.8629	0.7005	0.8136	0.9595	0.8107	0.7650	0.8397	0.6824	0.6122
NM_013113	Atp1b1	0.9861	0.9990	0.9798	0.1715	0.9394	0.9813	0.9484	0.9744	0.9823	0.9788	0.9744	0.9844
NM_012714	Gipr	0.8124	0.8437	0.8122	0.7695	0.1118	0.4683	0.9658	0.7997	0.7740	0.7013	0.5457	0.7633
NM_001024753	Ifit2	0.3846	0.6115	0.1363	0.5389	0.3800	0.3364	0.5703	0.4949	0.5154	0.5187	0.5785	0.6699
NM_012748	Stx2	0.6738	0.6694	0.5157	0.6064	0.1170	0.8337	0.6012	0.7374	0.4354	0.6681	0.6520	0.9725
NM_001025035	Thnsl1	0.0395	0.0411	0.0385	0.1640	0.0009	0.0024	0.0089	0.0038	0.0481	0.1079	0.1475	0.5009
NM_012718	Andpro	0.0966	0.0400	0.0453	0.3430	0.1509	0.2230	0.5088	0.2974	0.5378	0.6490	0.0733	0.3096
NM_012717	Calcrl	0.3802	0.1319	0.5730	0.4849	0.5861	0.6779	0.4485	0.7631	0.1900	0.2392	0.4386	0.4860
NM_001024760	RGD1307682	0.6223	0.5477	0.8700	0.8407	0.4718	0.2198	0.6458	0.6009	0.1025	0.2930	0.0797	0.0459
NM_012815	Gclc	0.9986	0.9981	0.8035	0.2280	0.9883	0.9738	0.9438	0.9773	0.9893	0.9926	0.9659	0.9454

NM_021265	Mpp4	0.0768	0.4513	0.6313	0.2570	0.4623	0.1892	0.1377	0.5102	0.4494	0.7561	0.2206	0.4625
NM_019357	Ezr	0.9261	0.9243	0.9835	0.9176	0.2023	0.4938	0.9834	0.1587	0.9146	0.9969	0.9973	0.9827
NM_012860	Mat1a	0.9540	0.8805	0.9467	0.6619	0.4583	0.8398	0.9804	0.7455	0.8936	0.9705	0.8907	0.7709
NM_012765	Htr2c	0.6040	0.9190	0.7514	0.5859	0.4134	0.8510	0.2517	0.4678	0.9467	0.8430	0.6410	0.5511
NM_001024764	Slc36a3	0.2914	0.3554	0.2054	0.4087	0.5302	0.1722	0.0803	0.1319	0.4212	0.3040	0.3048	0.3243
NM_001024766	Wdr5b	0.9028	0.3219	0.5509	0.6586	0.7953	0.6057	0.6711	0.7009	0.2130	0.2989	0.3675	0.6458
NM_012897	Adrbk2	0.3098	0.0358	0.2503	0.1777	0.4632	0.2459	0.1529	0.2342	0.3390	0.2836	0.0454	0.2157
NM_012726	Atxn1	0.4327	0.4732	0.2977	0.2620	0.7004	0.5740	0.6835	0.6960	0.7570	0.6929	0.0745	0.4281
NM_001024770	Sec3l1	0.5230	0.2541	0.0274	0.0613	0.8377	0.8706	0.0961	0.4451	0.2487	0.0749	0.2198	0.6425
NM_012996	Oxt	0.2801	0.1440	0.2192	0.5073	0.1661	0.1600	0.1710	0.3295	0.1636	0.3956	0.5325	0.0794
NM_001025136	Hexim1	0.6449	0.5888	0.6964	0.6510	0.5840	0.4999	0.3389	0.5459	0.2520	0.4855	0.5520	0.4192
NM_001024773	Brf2	0.6842	0.3104	0.1591	0.0969	0.5894	0.6601	0.0951	0.2897	0.5322	0.3006	0.1595	0.4044
NM_013109	Otx1	0.2371	0.1184	0.1474	0.1766	0.4905	0.4441	0.0649	0.5514	0.3314	0.2274	0.2661	0.6423
NM_001030037	Capn7	0.8011	0.6704	0.9536	0.9802	0.7988	0.8400	0.6532	0.5963	0.9358	0.9338	0.8854	0.6887
NM_013108	Adrb3	0.8159	0.9474	0.8085	0.6343	0.8958	0.7733	0.8950	0.8375	0.8466	0.7376	0.8899	0.1505
NM_013125	Scn5a	0.6063	0.8528	0.7075	0.0988	0.3110	0.3675	0.6849	0.8447	0.8557	0.4196	0.5740	0.2773
NM_012733	Rbp1	0.8636	0.6721	0.8497	0.8376	0.7965	0.7966	0.8415	0.8106	0.7649	0.7302	0.7820	0.7717
NM_001024785	Mcart1	0.8952	0.6416	0.9477	0.9956	0.9031	0.8969	0.9258	0.9725	0.7889	0.8281	0.9023	0.9526
NM_001024786	Wdr78	0.4665	0.5488	0.3574	0.3669	0.6431	0.6517	0.4952	0.6567	0.4075	0.5716	0.5617	0.5118
NM_012783	Bsg	0.2183	0.6099	0.6185	0.5263	0.6509	0.5722	0.4784	0.5120	0.4160	0.3032	0.2504	0.2851
NM_001024789	Nap1l2	0.5950	0.6466	0.6815	0.5081	0.2059	0.2914	0.2432	0.5752	0.5592	0.6090	0.6816	0.2090
NM_012716	Slc16a1	0.0119	0.2524	0.0395	0.1900	0.1399	0.3194	0.8493	0.1951	0.0894	0.2315	0.3194	0.4747
NM_013007	Pnoc	0.9194	0.5454	0.8890	0.8502	0.8890	0.6181	0.7346	0.6681	0.8520	0.6700	0.8688	0.9177
NM_001024791	Epn3	0.1708	0.7248	0.7328	0.8725	0.5477	0.3896	0.8718	0.4411	0.8371	0.7643	0.9007	0.8452
NM_001025413	Tmem184a	0.0064	0.4305	0.4491	0.8489	0.1221	0.0557	0.9263	0.1869	0.4423	0.2259	0.7701	0.7035
NM_001025152	Tc2n	0.9359	0.2026	0.4801	0.3264	0.6555	0.5694	0.5761	0.4724	0.4198	0.0808	0.6438	0.4166
NM_017141	Polb	0.5205	0.0941	0.6279	0.1332	0.6597	0.0814	0.2136	0.8635	0.5390	0.6605	0.1252	0.5355
NM_001024795	Rnf44	0.7851	0.8205	0.6499	0.6721	0.7271	0.5945	0.5942	0.1792	0.8491	0.6651	0.1806	0.8204
NM_012745	Klrd1	0.8098	0.5291	0.4950	0.4556	0.1748	0.3616	0.3517	0.3733	0.5113	0.6924	0.2789	0.2848
NM_001024797	LOC499653	0.0801	0.3602	0.2050	0.4590	0.3590	0.5920	0.1930	0.5499	0.2905	0.7289	0.3566	0.5422
NM_001025285	Ankrd54	0.5874	0.5471	0.6119	0.7207	0.4344	0.7208	0.5991	0.5835	0.5987	0.7147	0.5184	0.4698
NM_012912	Atf3	0.8640	0.8070	0.4065	0.4947	0.3105	0.3499	0.5854	0.3305	0.4139	0.3916	0.1945	0.3906
NM_012742	Foxa1	0.8075	0.7913	0.5305	0.6776	0.5707	0.7820	0.5990	0.6355	0.3758	0.8624	0.9024	0.5910
NM_012950	F2r	0.9493	0.6888	0.9667	0.6484	0.6064	0.8166	0.9741	0.5267	0.9620	0.8889	0.5148	0.7931
NM_013032	Slc1a1	0.2135	0.2063	0.4703	0.4738	0.4966	0.2830	0.3688	0.7953	0.5596	0.5599	0.8544	0.4836
NM_012743	Foxa2	0.8489	0.7870	0.9545	0.9660	0.8897	0.8638	0.7332	0.9318	0.7268	0.9091	0.9307	0.7820
NM_001024803	LOC364427	0.3288	0.4277	0.3230	0.3732	0.3777	0.7583	0.4116	0.2145	0.3242	0.2610	0.3069	0.7395
NM_013061	Si	0.6828	0.3710	0.3989	0.2638	0.7502	0.2738	0.1222	0.3531	0.1821	0.1309	0.2473	0.1113
NM_001025654	Rhox7	0.1824	0.2105	0.0694	0.2744	0.0720	0.1303	0.1412	0.2415	0.0991	0.0614	0.2246	0.4150
NM_001025407	Alg5	0.7627	0.5129	0.1972	0.9874	0.6740	0.5952	0.0380	0.7116	0.0889	0.0430	0.0661	0.1611
NM_001033897	Noc2l	0.7865	0.8789	0.0752	0.6205	0.7548	0.7947	0.1075	0.8827	0.6526	0.9105	0.8119	0.7605
NM_001033068	Edc4	0.3272	0.4644	0.1377	0.3393	0.4116	0.5262	0.2628	0.2545	0.5082	0.4749	0.3116	0.6072
NM_001034941	Tarbp2	0.6864	0.8376	0.0781	0.1177	0.0664	0.7145	0.6262	0.9159	0.6362	0.5163	0.5503	0.2753
NM_001025658	Tex21	0.5391	0.4473	0.2480	0.3973	0.3980	0.4542	0.1556	0.7856	0.5563	0.2505	0.2353	0.5024
NM_001025412	Fam58b	0.1927	0.0442	0.5162	0.1529	0.1963	0.4328	0.3088	0.2603	0.0756	0.3183	0.1355	0.1578
NM_001030054	Akirin1	0.6908	0.6877	0.7006	0.6936	0.7076	0.6652	0.5491	0.6601	0.6038	0.6576	0.5886	0.6773
NM_001024871	Rabepk	0.0513	0.0733	0.3793	0.0808	0.2496	0.0452	0.8760	0.0679	0.1107	0.0882	0.2793	0.0871
NM_001033883	Cxcl12	0.9132	0.6546	0.9875	0.9736	0.9935	0.8409	0.9510	0.9723	0.6400	0.6419	0.4038	0.7140
NM_001025631	Aytl1b	0.6724	0.2750	0.0864	0.2417	0.5290	0.6076	0.1517	0.2314	0.1328	0.4523	0.1676	0.2370

NM_001033882	Cxcl12	0.9132	0.6546	0.9875	0.9736	0.9935	0.8409	0.9510	0.9723	0.6400	0.6419	0.4038	0.7140
NM_001024875	RGD1307254	0.0606	0.4487	0.0678	0.2737	0.1452	0.0192	0.0797	0.0222	0.1643	0.3283	0.1933	0.0920
NM_001025673	Mlf1ip	0.1324	0.4153	0.1508	0.1753	0.3188	0.2614	0.1444	0.2170	0.2911	0.2058	0.1332	0.3003
NM_001037206	Bphl	0.8570	0.7190	0.8855	0.9569	0.7582	0.8805	0.8157	0.9478	0.5617	0.9293	0.8995	0.8013
NM_001025671	Rassf6	0.5070	0.7966	0.7755	0.3494	0.8746	0.9365	0.3665	0.8436	0.5313	0.4970	0.4364	0.3727
NM_001024878	Znrf4	0.4676	0.1516	0.3993	0.1854	0.1427	0.4390	0.2550	0.3274	0.4569	0.4817	0.0482	0.2760
NM_001024880	MGC114483	0.4231	0.4291	0.1174	0.0799	0.3434	0.6063	0.2107	0.5816	0.5610	0.0300	0.2384	0.2026
NM_001024882	Ccdc19	0.6733	0.6329	0.5532	0.7762	0.8618	0.3913	0.2844	0.6175	0.7688	0.4612	0.6399	0.4235
NM_001024883	RGD1310810	0.5437	0.2927	0.1584	0.4019	0.3036	0.3634	0.1406	0.5814	0.4478	0.3448	0.0461	0.7371
NM_001033958	Obp3	0.2498	0.4680	0.2506	0.2140	0.6626	0.4298	0.4033	0.3716	0.6553	0.3945	0.7159	0.2910
NM_001033924	Fcer2	0.5605	0.3392	0.4759	0.6288	0.4996	0.5183	0.5670	0.7015	0.8022	0.3666	0.5420	0.6212
NM_001024888	Gatad2b	0.2405	0.9322	0.8038	0.9683	0.4240	0.5621	0.9434	0.3953	0.9387	0.5674	0.9762	0.7872
NM_001024890	MGC114520	0.6908	0.5645	0.7477	0.8882	0.5830	0.5472	0.8374	0.3763	0.3364	0.6594	0.3690	0.5657
NM_012910	Arrb1	0.2204	0.1246	0.4616	0.1852	0.2966	0.2956	0.4495	0.4285	0.3426	0.0744	0.0191	0.7659
NM_001024892	Rnf12	0.1546	0.0802	0.9807	0.2283	0.0317	0.0932	0.4909	0.2002	0.0535	0.1067	0.7552	0.1029
NM_001024893	MGC114427	0.2097	0.2393	0.2493	0.2712	0.2218	0.4647	0.3363	0.2585	0.2455	0.5149	0.6748	0.2224
NM_001024894	Plac1	0.1140	0.1651	0.1993	0.4393	0.0841	0.1802	0.4455	0.5007	0.1909	0.3745	0.0644	0.0875
NM_001024895	MGC114492	0.3479	0.4810	0.3851	0.3853	0.6273	0.3041	0.4561	0.5488	0.5421	0.4180	0.4479	0.3397
NM_013046	Trh	0.4341	0.5730	0.6112	0.6614	0.6989	0.6275	0.6549	0.6014	0.7335	0.6345	0.6966	0.6576
NM_001024898	Paf1	0.0974	0.1945	0.0164	0.4899	0.0646	0.0247	0.1348	0.1019	0.1308	0.3725	0.0325	0.0156
NM_001025756	Tmem186	0.3162	0.2454	0.3770	0.2046	0.4403	0.8012	0.3272	0.3901	0.8068	0.3507	0.4337	0.4082
NM_001025734	Tada3l	0.6308	0.9002	0.8416	0.9515	0.7705	0.9936	0.6651	0.9835	0.8012	0.9216	0.6121	0.9657
NM_001025686	Sec22b	0.2088	0.2415	0.4008	0.0325	0.3588	0.1116	0.4377	0.3185	0.4088	0.2217	0.6227	0.1547
NM_001024900	Tmem55a	0.9620	0.9146	0.9438	0.9994	0.9910	0.9768	0.3135	0.9686	0.6455	0.9671	0.8795	0.4054
NM_001025701	Rad21	0.9511	0.9509	0.5434	0.8838	0.9873	0.9755	0.9494	0.9835	0.5501	0.7081	0.9478	0.3573
NM_001024902	Lrrc56	0.8029	0.9086	0.4960	0.9493	0.8332	0.7577	0.7934	0.8394	0.7171	0.9282	0.7300	0.6794
NM_001024903	Sertad2	0.9625	0.8576	0.8821	0.6913	0.7707	0.6403	0.8430	0.9442	0.8103	0.9391	0.8208	0.9314
NM_001024905	MGC116121	0.4679	0.2010	0.0806	0.0451	0.2287	0.3287	0.0519	0.2957	0.0728	0.2838	0.3516	0.0628
NM_001024906	Arl2bp	0.4348	0.1303	0.4671	0.7498	0.5295	0.8846	0.5332	0.5414	0.9144	0.7461	0.7837	0.2205
NM_001024907	MGC114499	0.7093	0.2795	0.5728	0.5851	0.4067	0.5115	0.7558	0.3344	0.6245	0.5159	0.7248	0.7574
NM_001024909	MGC114464	0.3384	0.4800	0.5506	0.4718	0.5241	0.0632	0.5478	0.5509	0.5223	0.5630	0.7957	0.4575
NM_012752	Cd24	0.9921	0.9997	0.3683	0.9369	0.0499	0.2446	0.9661	0.1517	0.3166	0.9994	0.9413	0.2648
NM_019239	Mgat3	0.9663	0.7115	0.7157	0.5972	0.9083	0.9067	0.9552	0.8969	0.4647	0.3636	0.9638	0.9061
NM_001024966	Samd14	0.0455	0.1912	0.2580	0.1351	0.3108	0.1415	0.5318	0.2651	0.0436	0.2127	0.3222	0.6553
NM_001037195	Rhog	0.8806	0.6809	0.9521	0.9099	0.6131	0.5234	0.7620	0.7564	0.5078	0.4061	0.3003	0.3984
NM_001024972	RGD1310061	0.9815	0.9664	0.9136	0.9436	0.8873	0.9987	0.8370	0.7790	0.9771	0.9272	0.9380	0.4548
NM_001024974	RGD1311863	0.3173	0.4580	0.4346	0.1774	0.2884	0.6692	0.7381	0.1424	0.5046	0.3868	0.4446	0.4214
NM_012998	P4hb	0.7842	0.7172	0.8356	0.7466	0.9685	0.9460	0.8428	0.9347	0.8237	0.8429	0.8776	0.9102
NM_012911	Arrb2	0.3734	0.2531	0.1483	0.5216	0.5606	0.3230	0.6527	0.1423	0.4373	0.6481	0.6491	0.1582
NM_001024981	RGD1304978	0.9248	0.8125	0.9253	0.7641	0.8942	0.8339	0.7904	0.7739	0.8514	0.7330	0.9502	0.9627
NM_001024982	Cpne9	0.4318	0.3748	0.3933	0.5595	0.4247	0.6064	0.7471	0.5293	0.7563	0.3035	0.3984	0.5792
NM_001024984	RGD1310606	0.3369	0.8832	0.8773	0.8391	0.4169	0.7125	0.6675	0.2492	0.9661	0.6559	0.9004	0.9017
NM_012862	Mgp	0.8491	0.7758	0.7681	0.8678	0.8001	0.8285	0.0031	0.9313	0.7761	0.4361	0.9338	0.9027
NM_001034164	Stat1	0.7508	0.7091	0.7007	0.6717	0.6358	0.5474	0.5955	0.5909	0.6813	0.5798	0.6060	0.5438
NM_001025774	Ccdc105	0.0903	0.1000	0.2385	0.2730	0.0417	0.1072	0.1275	0.1800	0.3579	0.5214	0.1146	0.3039
NM_001034849	Gimap1	0.4598	0.1923	0.2002	0.4601	0.3314	0.1996	0.2199	0.5113	0.1819	0.3431	0.5156	0.1948
NM_001030027	Gins4	0.3977	0.9843	0.0369	0.3312	0.6869	0.6442	0.7334	0.8623	0.9852	0.8000	0.9230	0.0310
NM_013040	Abcc9	0.8753	0.7426	0.7025	0.0202	0.4558	0.8328	0.8153	0.7967	0.7935	0.8543	0.7669	0.2526
NM_001079937	Pbk	0.8668	0.9888	0.0617	0.9790	0.7582	0.9955	0.7876	0.9388	0.7453	0.9746	0.8272	0.2437

NM_001029904	Rnase1	0.7817	0.6777	0.7648	0.8786	0.3361	0.2973	0.8983	0.5526	0.7316	0.7676	0.7209	0.2306
NM_001030052	Ndufs5	0.0920	0.1854	0.1472	0.0726	0.0611	0.1081	0.0387	0.0804	0.0751	0.0842	0.0555	0.0441
NM_012506	Atp1a3	0.4083	0.3320	0.2301	0.0904	0.5236	0.4290	0.2959	0.3184	0.1086	0.0914	0.1016	0.3246
NM_012634	Prps2	0.2113	0.0023	0.1196	0.3080	0.3268	0.8851	0.4049	0.4794	0.0011	0.1832	0.2002	0.6091
NM_001025001	LOC308990	0.9649	0.9979	0.9163	0.9691	0.8798	0.7743	0.8349	0.7838	0.9381	0.7928	0.8664	0.9808
NM_001025003	RGD1305178	0.1470	0.8275	0.1634	0.1395	0.2080	0.1855	0.0228	0.3670	0.2567	0.8673	0.1061	0.2539
NM_001025723	Mtrf1l	0.3029	0.3487	0.1730	0.0238	0.0135	0.0540	0.0879	0.1276	0.2005	0.4138	0.2318	0.0511
NM_001025007	RGD1310852	0.1133	0.2548	0.3581	0.3426	0.1621	0.2906	0.2296	0.0316	0.1193	0.0786	0.2580	0.0527
NM_001025009	Josd1	0.6327	0.4084	0.9348	0.7628	0.5791	0.8555	0.4293	0.7294	0.6305	0.4880	0.5168	0.8955
NM_001031644	Ercc3	0.7816	0.4342	0.2370	0.1077	0.6187	0.2560	0.3469	0.6935	0.5803	0.1915	0.1880	0.0636
NM_012749	Ncl	0.9441	0.9477	0.8567	0.7708	0.9664	0.8680	0.7685	0.8641	0.8624	0.8318	0.8966	0.8348
NM_001025015	Gtpbp8	0.0353	0.3843	0.9076	0.8297	0.1388	0.7384	0.0386	0.4425	0.0367	0.1426	0.6135	0.4497
NM_001025017	Them4	0.2559	0.4287	0.6477	0.2811	0.1062	0.3935	0.4012	0.3781	0.0599	0.2199	0.5881	0.4829
NM_001025018	Tmem77	0.2107	0.3176	0.8829	0.8402	0.0855	0.2079	0.3786	0.0820	0.0601	0.1739	0.7418	0.2097
NM_001025020	RGD1310753	0.7821	0.8076	0.8725	0.8694	0.8040	0.8584	0.3307	0.3215	0.6689	0.5970	0.7721	0.5091
NM_001025021	LOC362526	0.3555	0.6784	0.6318	0.0384	0.5507	0.3523	0.1181	0.2570	0.7047	0.9669	0.6403	0.6569
NM_001025022	RGD1308059	0.9355	0.3160	0.9609	0.7605	0.9923	0.9780	0.3028	0.9915	0.0120	0.2609	0.2101	0.0481
NM_001025024	RGD1311362	0.9046	0.8690	0.4061	0.3934	0.5776	0.8605	0.0794	0.7850	0.8644	0.9532	0.8792	0.4643
NM_001025025	LOC363267	0.6371	0.0114	0.6453	0.0307	0.5280	0.3411	0.0947	0.6197	0.2059	0.0716	0.0958	0.8308
NM_001025768	Dsn1	0.9681	0.8729	0.1404	0.5623	0.9587	0.9006	0.9017	0.8805	0.8147	0.9781	0.3722	0.7339
NM_001025710	Fam126b	0.3078	0.3083	0.7357	0.2750	0.5951	0.8389	0.0813	0.4283	0.6180	0.0441	0.5828	0.4765
NM_001025027	Fundc1	0.3324	0.1622	0.8366	0.4183	0.1964	0.2095	0.3853	0.2082	0.0355	0.2767	0.4614	0.1998
NM_001037775	Chst12	0.2232	0.0169	0.3843	0.1152	0.1090	0.0820	0.0340	0.0621	0.0750	0.0389	0.1431	0.0638
NM_001079936	Rab5b	0.9798	0.9162	0.9926	0.9846	0.9980	0.9917	0.9983	0.9888	0.3820	0.9111	0.9384	0.9097
NM_001025031	LOC497888	0.5238	0.4672	0.3358	0.5274	0.1945	0.0443	0.4365	0.3229	0.3831	0.5531	0.4836	0.6933
NM_001031653	Polr3d	0.1208	0.7612	0.1743	0.2185	0.3677	0.7754	0.6099	0.6636	0.9212	0.3812	0.6886	0.7797
NM_001031638	Cd68	0.1180	0.0580	0.3974	0.0361	0.2135	0.0043	0.3141	0.0511	0.0357	0.0607	0.0250	0.5337
NM_001025033	LOC498154	0.7403	0.9595	0.1517	0.8899	0.8629	0.5870	0.4345	0.8194	0.8773	0.9293	0.7974	0.3082
NM_001025776	Rhox8	0.2441	0.2599	0.2756	0.3396	0.5829	0.2789	0.3664	0.6025	0.4513	0.2539	0.3122	0.5965
NM_013031	Slc18a2	0.5212	0.2355	0.3322	0.7267	0.2017	0.3710	0.2621	0.4385	0.4165	0.6195	0.2543	0.3030
NM_012856	Kcnc1	0.5305	0.6918	0.6927	0.9133	0.8177	0.3887	0.3585	0.8036	0.5209	0.7146	0.8402	0.6290
NM_001025038	LOC499465	0.6382	0.3400	0.2776	0.1679	0.1190	0.1970	0.7392	0.1484	0.8073	0.1217	0.8882	0.8337
NM_001025039	LOC499602	0.4527	0.2262	0.8334	0.3464	0.3746	0.7732	0.1047	0.6526	0.7266	0.4280	0.3391	0.6596
NM_001025041	Ier5l	0.8703	0.1190	0.2036	0.8703	0.8818	0.7405	0.0491	0.6812	0.7917	0.8806	0.6388	0.2244
NM_001025044	LOC499980	0.6344	0.9872	0.8441	0.1705	0.5537	0.8750	0.4904	0.4896	0.2317	0.7512	0.5638	0.3478
NM_001025748	Taz	0.9190	0.5386	0.8613	0.8385	0.8300	0.9581	0.9691	0.8692	0.8857	0.8303	0.8526	0.9349
NM_001030032	Slc25a38	0.7649	0.8731	0.8893	0.8927	0.8355	0.9758	0.7775	0.9509	0.9995	0.8420	0.7881	0.8474
NM_001025047	LOC500251	0.0591	0.2947	0.9022	0.2341	0.1265	0.1996	0.2287	0.4655	0.3046	0.0132	0.2659	0.5923
NM_001025050	Cdca8	0.6941	0.9498	0.0217	0.8833	0.6356	0.6586	0.9581	0.8689	0.7848	0.8875	0.8038	0.0649
NM_001025052	LOC500825	0.5527	0.5113	0.4057	0.1619	0.2210	0.4564	0.3017	0.6179	0.5178	0.6767	0.5968	0.4407
NM_001025757	Trpv3	0.6905	0.6854	0.4107	0.0480	0.3095	0.2847	0.6513	0.3513	0.6934	0.4996	0.1642	0.1939
NM_001025054	LOC500956	0.2171	0.4049	0.3302	0.2605	0.5453	0.6562	0.6441	0.7503	0.5541	0.3952	0.5478	0.7065
NM_001034163	Blmh	0.2831	0.5107	0.2898	0.9321	0.8226	0.4651	0.5799	0.6283	0.3718	0.4353	0.2464	0.1915
NM_001025058	LOC502372	0.1930	0.4313	0.1670	0.5495	0.4206	0.5891	0.1783	0.4818	0.4661	0.0487	0.2914	0.1544
NM_001025760	Zkscan1	0.4108	0.0693	0.9231	0.4562	0.3663	0.7390	0.6618	0.2072	0.3857	0.5506	0.1799	0.9046
NM_001025060	LOC502684	0.5089	0.7144	0.6473	0.7796	0.5657	0.3955	0.7133	0.2930	0.8047	0.5038	0.5969	0.7583
NM_001025063	Scrn1	0.0335	0.7942	0.0286	0.9397	0.0724	0.0478	0.6606	0.0751	0.0950	0.0358	0.7722	0.1532
NM_001025064	LOC502894	0.3526	0.8034	0.4512	0.2049	0.6170	0.7289	0.5424	0.1016	0.9319	0.5732	0.8174	0.8401
NM_017074	Cth	0.2726	0.2061	0.0158	0.0903	0.4334	0.6363	0.2970	0.0854	0.2555	0.0143	0.3253	0.0657

NM_012635	Prss1	0.1011	0.3818	0.1152	0.2050	0.3391	0.1341	0.3734	0.0375	0.7463	0.6285	0.1059	0.2974
NM_001029917	Memo1	0.9889	0.9215	0.3382	0.9360	0.9014	0.8418	0.8731	0.9836	0.9676	0.9801	0.9847	0.7342
NM_001025113	Kpna5	0.0296	0.3374	0.0414	0.2936	0.3832	0.4604	0.2942	0.4491	0.3783	0.0515	0.2822	0.4114
NM_001105729	Dpf1	0.3578	0.4139	0.1240	0.7916	0.8514	0.7583	0.3238	0.6973	0.7202	0.7265	0.1598	0.3353
NM_001106297	Cd2bp2	0.7903	0.9626	0.2578	0.2119	0.8636	0.8548	0.6436	0.8588	0.9311	0.1338	0.5691	0.2546
NM_001105805	Txndc17	0.7421	0.8784	0.5915	0.7085	0.6987	0.7956	0.3315	0.7490	0.0129	0.6783	0.5576	0.0271
NM_001105775	Pdia2	0.3821	0.1921	0.3212	0.2138	0.3204	0.3278	0.3487	0.3398	0.2579	0.3162	0.1909	0.3129
NM_001105710	Cad	0.9820	0.9987	0.0038	0.7009	0.9532	0.7595	0.8252	0.9998	0.8658	0.9376	0.8259	0.7847
NM_012759	Vav1	0.9161	0.5652	0.4349	0.9283	0.3883	0.4353	0.4634	0.7085	0.2608	0.1246	0.2071	0.7284
NM_001025123	Kdelc2	0.1267	0.1520	0.6157	0.9538	0.3034	0.3376	0.9784	0.1644	0.4992	0.4024	0.2486	0.6512
NM_017022	Itgb1	0.9885	0.1111	0.9703	0.9176	0.5959	0.9846	0.9886	0.8038	0.3262	0.3862	0.7068	0.8722
NM_012760	Plagl1	0.4425	0.8554	0.7496	0.2958	0.7632	0.5414	0.5325	0.9067	0.8588	0.3360	0.8104	0.8309
NM_001025125	Sumf2	0.0036	0.0762	0.3902	0.7169	0.1207	0.2062	0.2773	0.0955	0.3241	0.2556	0.0550	0.0354
NM_001025126	RGD1304773	0.2569	0.8201	0.7936	0.6751	0.8809	0.7700	0.6782	0.9039	0.5128	0.2752	0.1314	0.7720
NM_001029916	Depdc7	0.1330	0.5919	0.2673	0.0555	0.4890	0.5178	0.4989	0.7467	0.1897	0.0978	0.2388	0.0245
NM_001025128	RGD1304774	0.2084	0.5495	0.2747	0.5476	0.2125	0.2267	0.4658	0.3805	0.3858	0.6198	0.2421	0.6653
NM_012727	Camk4	0.1481	0.6750	0.2663	0.0860	0.4043	0.0753	0.3611	0.5528	0.7511	0.7622	0.7275	0.1060
NM_013175	Scg5	0.9594	0.5695	0.8247	0.7378	0.7910	0.9145	0.5466	0.5588	0.8495	0.8358	0.5678	0.7122
NM_001025133	Capn13	0.7236	0.5717	0.3447	0.8231	0.5283	0.7406	0.6344	0.9011	0.2200	0.6003	0.6509	0.1490
NM_001029918	Ttc30a1	0.7621	0.6626	0.8499	0.9371	0.9095	0.9695	0.7518	0.9042	0.8586	0.7108	0.3655	0.7182
NM_001025137	Ier5	0.1917	0.4058	0.5965	0.2321	0.4227	0.7485	0.0673	0.7179	0.1152	0.1492	0.4035	0.8027
NM_001025139	Zpbp	0.2482	0.4264	0.0660	0.4165	0.0390	0.0251	0.2433	0.2527	0.6379	0.4890	0.0491	0.4919
NM_001025141	Ccnb1ip1	0.7079	0.7226	0.6829	0.3136	0.5812	0.6546	0.4007	0.6523	0.5958	0.6662	0.8786	0.6266
NM_001029919	Necap1	0.6872	0.6155	0.2206	0.7147	0.5588	0.6258	0.6042	0.3747	0.6274	0.6717	0.6587	0.5591
NM_001025143	LOC498606	0.1239	0.5509	0.2368	0.7041	0.7495	0.9314	0.7044	0.8504	0.7814	0.3280	0.1696	0.6069
NM_001025145	Zfp365	0.7993	0.5775	0.9505	0.2205	0.8218	0.5979	0.4670	0.8828	0.6024	0.6897	0.2522	0.8005
NM_012870	Tnfrsf11b	0.3396	0.2849	0.1956	0.2015	0.8789	0.9309	0.2041	0.9633	0.2635	0.2379	0.1450	0.2822
NM_001025148	Galntl5	0.7377	0.5627	0.8229	0.5849	0.5235	0.3603	0.6255	0.3960	0.4569	0.5106	0.6482	0.5497
NM_001025151	LOC500625	0.8107	0.9293	0.8901	0.9354	0.6942	0.8916	0.4484	0.9316	0.8587	0.8204	0.6126	0.8792
NM_001029922	Zbbx	0.7199	0.7390	0.7176	0.7732	0.3865	0.3635	0.5057	0.5494	0.6408	0.1609	0.5859	0.6139
NM_001025153	Iqcf3	0.2101	0.2992	0.5501	0.2816	0.3286	0.6353	0.4914	0.6128	0.2877	0.4554	0.3221	0.4878
NM_001025155	Lrrc17	0.1118	0.1208	0.1397	0.5194	0.1351	0.2653	0.2101	0.0704	0.2447	0.2323	0.3677	0.0212
NM_013187	Plcg1	0.9995	0.9267	0.9923	0.3437	0.9325	0.9881	0.9939	0.9510	0.9911	0.9242	0.9889	0.9409
NM_019190	Cd46	0.6773	0.8129	0.3320	0.6262	0.7611	0.3557	0.6397	0.5040	0.7698	0.7718	0.0965	0.2219
NM_001033653	Akap12	0.8854	0.5725	0.7863	0.3727	0.7106	0.6773	0.8847	0.7066	0.6321	0.6376	0.7996	0.7327
NM_001025276	RGD1305807	0.0068	0.6282	0.7776	0.5740	0.0605	0.0600	0.8863	0.1566	0.4491	0.8174	0.6508	0.4311
NM_001025278	RGD1306402	0.6796	0.7612	0.7409	0.2231	0.9474	0.7689	0.5355	0.9286	0.7305	0.8456	0.9416	0.7742
NM_012879	Slc2a2	0.6307	0.7377	0.1027	0.8918	0.2853	0.3564	0.6713	0.3016	0.8805	0.3043	0.6082	0.4426
NM_012764	Gata1	0.7081	0.5986	0.3941	0.5380	0.5828	0.7274	0.7069	0.3512	0.3756	0.4044	0.4974	0.9304
NM_012565	Gck	0.4385	0.9570	0.5669	0.6664	0.5126	0.8684	0.9058	0.5648	0.8108	0.9805	0.6190	0.8817
NM_001029924	Stra6	0.8528	0.7332	0.7537	0.2957	0.7640	0.6940	0.8808	0.3207	0.5013	0.5973	0.5377	0.8289
NM_001025284	Rpusd4	0.6573	0.1521	0.4625	0.2876	0.2136	0.4587	0.3968	0.4689	0.3359	0.4287	0.0645	0.2937
NM_001031663	Fam82b	0.2103	0.1176	0.8666	0.4661	0.0862	0.0486	0.0189	0.0305	0.3295	0.1286	0.0084	0.0551
NM_012880	Sod3	0.0059	0.0340	0.1954	0.0945	0.2314	0.2108	0.1589	0.2667	0.0997	0.0998	0.1377	0.1036
NM_013059	Alpl	0.2540	0.8889	0.8406	0.8476	0.9498	0.7070	0.8788	0.8412	0.6996	0.9397	0.7295	0.2607
NM_012898	Ahsg	0.3203	0.2096	0.4142	0.1562	0.4696	0.2542	0.5174	0.1552	0.7333	0.8583	0.0370	0.3055
NM_012774	Gpc3	0.4967	0.5728	0.5338	0.2348	0.3482	0.5725	0.1197	0.0759	0.1227	0.1734	0.0980	0.1461
NM_022386	Mafg	0.9699	0.9870	0.9016	0.9478	0.9782	0.9307	0.9900	0.9839	0.9984	0.9672	0.9878	0.9547
NM_001037349	Bloc1s2	0.0074	0.2819	0.0910	0.1534	0.1393	0.2228	0.0144	0.1218	0.4231	0.2391	0.1066	0.2913

NM_012997	P2rx1	0.6216	0.7215	0.9384	0.8958	0.7826	0.5662	0.8924	0.6578	0.6536	0.9492	0.8123	0.9497
NM_013100	Ptger1	0.1212	0.5729	0.3192	0.0922	0.4602	0.1969	0.1175	0.3078	0.1410	0.4265	0.2220	0.1820
NM_013085	Plau	0.8209	0.7191	0.9416	0.6644	0.7618	0.8477	0.5487	0.7459	0.3295	0.7535	0.7809	0.6795
NM_012842	Egf	0.6708	0.9145	0.2898	0.2897	0.8033	0.9444	0.0704	0.6790	0.6571	0.7805	0.3733	0.9092
NM_012963	Hmgb1	0.7315	0.7347	0.6412	0.6670	0.6016	0.5698	0.7201	0.6553	0.5955	0.5252	0.6638	0.6346
NM_013084	Acadsb	0.0472	0.0406	0.4005	0.4707	0.2182	0.4924	0.2755	0.4799	0.0499	0.2002	0.3903	0.1216
NM_001025411	Dnaja4	0.3743	0.6670	0.8364	0.8383	0.4244	0.6528	0.9231	0.1269	0.1891	0.8161	0.9017	0.6196
NM_001080096	Acsbg2	0.0501	0.1210	0.0290	0.2752	0.4198	0.1635	0.3669	0.1701	0.0591	0.0427	0.2490	0.2287
NM_001033706	Aldh16a1	0.1885	0.1918	0.2934	0.2137	0.4501	0.1993	0.2193	0.2071	0.3764	0.4116	0.1908	0.2682
NM_012953	Fosl1	0.5473	0.5977	0.5199	0.6650	0.4545	0.5127	0.5532	0.5222	0.6734	0.6768	0.3064	0.4031
NM_013050	Ube2i	0.5056	0.6392	0.8331	0.7347	0.6072	0.2884	0.7648	0.5959	0.2107	0.3915	0.7375	0.0963
NM_017153	Rps3a	0.0548	0.0372	0.0588	0.0183	0.1829	0.5055	0.0646	0.0420	0.5489	0.5951	0.2020	0.3575
NM_017152	Rps17	0.1699	0.2972	0.4022	0.0242	0.1420	0.0835	0.1767	0.3349	0.2458	0.1181	0.1002	0.4979
NM_012990	Nog	0.3315	0.5993	0.5941	0.8176	0.7205	0.0878	0.5708	0.4811	0.9225	0.5308	0.6779	0.9224
NM_001109986	Ddit3	0.3942	0.9500	0.5607	0.1854	0.4323	0.3427	0.5441	0.5147	0.9985	0.9605	0.8893	0.5135
NM_012933	Dpysl4	0.4807	0.8105	0.8758	0.5031	0.3744	0.7303	0.8826	0.8511	0.4244	0.8250	0.7733	0.8922
NM_001039007	Hrasls5	0.2556	0.3321	0.3300	0.6105	0.3031	0.3563	0.3287	0.1075	0.3380	0.2436	0.4118	0.3355
NM_013013	Psap	0.0916	0.0236	0.8951	0.6601	0.4430	0.7415	0.7603	0.5565	0.0084	0.0082	0.2657	0.9511
NM_001025625	Stub1	0.3919	0.1717	0.4931	0.3720	0.2524	0.3269	0.4446	0.8118	0.4568	0.1336	0.5282	0.1146
NM_001025626	RGD1306595	0.1392	0.2205	0.3784	0.8303	0.0524	0.1060	0.0144	0.2181	0.6744	0.4247	0.0683	0.1129
NM_001037292	Ube2d2	0.5685	0.7312	0.5161	0.4324	0.5424	0.4257	0.2188	0.6230	0.6119	0.6813	0.4366	0.6496
NM_001025628	Ropn1	0.3573	0.2416	0.1384	0.3630	0.5423	0.1520	0.0721	0.1697	0.1491	0.0902	0.1256	0.0853
NM_012978	Lhcgr	0.7301	0.4639	0.8517	0.8701	0.8110	0.5932	0.8784	0.8105	0.7919	0.9230	0.6600	0.1305
NM_012962	Gss	0.0686	0.3663	0.0354	0.5907	0.1450	0.0831	0.0844	0.0196	0.7238	0.7625	0.6146	0.3061
NM_012929	Col2a1	0.4874	0.5175	0.2121	0.1782	0.3836	0.1563	0.4962	0.5604	0.5148	0.7323	0.2200	0.8105
NM_013015	Ptgds	0.0327	0.0413	0.0666	0.2032	0.0500	0.0028	0.0613	0.0431	0.0639	0.0213	0.0045	0.3581
NM_001025636	RGD1311742	0.3446	0.7610	0.8584	0.6366	0.8444	0.2347	0.7336	0.6563	0.6188	0.7939	0.8220	0.6162
NM_012930	Cpt2	0.4158	0.1213	0.7175	0.8217	0.0674	0.1981	0.8607	0.0960	0.3711	0.0372	0.0815	0.4334
NM_001025639	LOC292543	0.2273	0.1167	0.2672	0.1661	0.2173	0.3436	0.4037	0.4240	0.0461	0.1577	0.7568	0.0599
NM_001034078	Cbx8	0.8332	0.6962	0.7787	0.7843	0.5430	0.3101	0.2087	0.6171	0.2530	0.4337	0.5328	0.2700
NM_001025642	Fbxo46	0.5965	0.0320	0.3492	0.5213	0.4192	0.4997	0.6014	0.5097	0.9410	0.2368	0.4868	0.7991
NM_013101	Pde4a	0.2904	0.3887	0.3940	0.1099	0.2946	0.1360	0.2108	0.1793	0.1552	0.0849	0.0284	0.6900
NM_012975	Lgals4	0.8656	0.8339	0.8663	0.7736	0.8642	0.8711	0.7819	0.8925	0.9980	0.7579	0.7635	0.8493
NM_012858	Lhb	0.4603	0.2036	0.4406	0.2692	0.4816	0.2874	0.2186	0.4718	0.8255	0.3133	0.6179	0.4391
NM_012899	Alad	0.0031	0.0187	0.1999	0.2795	0.0812	0.0302	0.4356	0.1368	0.0024	0.0509	0.0448	0.0301
NM_012892	Accn1	0.4310	0.4189	0.2804	0.3756	0.2264	0.4054	0.7649	0.3018	0.7473	0.5791	0.3381	0.5724
NM_012854	Il10	0.0227	0.4707	0.4454	0.0483	0.0079	0.0136	0.0356	0.0374	0.4705	0.6652	0.7036	0.5662
NM_012794	Glycam1	0.5566	0.4837	0.3760	0.1055	0.3424	0.2508	0.4112	0.8417	0.4900	0.5938	0.5791	0.1801
NM_001025652	RGD1305420	0.5249	0.7543	0.8816	0.6791	0.5449	0.6654	0.6961	0.3297	0.3033	0.4939	0.6413	0.4352
NM_001025653	Lrrc42	0.3795	0.7344	0.1509	0.1133	0.0112	0.2233	0.1674	0.0499	0.1591	0.2581	0.5091	0.2390
NM_001039009	Zdhhc21	0.7113	0.7252	0.7336	0.8801	0.1582	0.8412	0.8712	0.1821	0.7143	0.7541	0.4413	0.9665
NM_012797	Id1	0.7831	0.8055	0.8439	0.8456	0.7781	0.9129	0.9493	0.8329	0.8731	0.9602	0.8189	0.8784
NM_001025656	LOC298442	0.9944	0.8161	0.0649	0.0442	0.8376	0.7635	0.8017	0.8136	0.8667	0.9096	0.8141	0.5105
NM_001034920	Cblc	0.2608	0.4341	0.5823	0.2668	0.1781	0.4611	0.4580	0.2431	0.4405	0.7913	0.3638	0.7858
NM_001025661	Crb3	0.7317	0.3629	0.4360	0.5728	0.5694	0.6859	0.0493	0.8531	0.8448	0.2610	0.7157	0.0932
NM_001025662	LOC301165	0.4492	0.1298	0.3508	0.0740	0.1715	0.5083	0.3891	0.1868	0.4604	0.4827	0.4573	0.4544
NM_001033685	Sap18	0.6622	0.2881	0.2235	0.4273	0.6542	0.1478	0.2854	0.4918	0.1763	0.1033	0.4338	0.5034
NM_001033682	Shpk	0.7685	0.4360	0.4088	0.3350	0.2513	0.5523	0.4059	0.3339	0.3612	0.4835	0.3257	0.1520
NM_001025664	Wsb1	0.6893	0.9008	0.7150	0.6168	0.7564	0.9465	0.6313	0.2352	0.9498	0.9943	0.9955	0.7572

NM_001130988	Slbp	0.9455	0.9602	0.9573	0.7860	0.4638	0.8315	0.9231	0.6769	0.8790	0.5621	0.9020	0.7711
NM_012886	Timp3	0.3765	0.1010	0.2206	0.4320	0.6454	0.1455	0.9050	0.7863	0.8797	0.3298	0.5677	0.2663
NM_012799	Nmbr	0.6091	0.4393	0.1151	0.4156	0.6105	0.3123	0.6514	0.4103	0.2392	0.0895	0.2714	0.0507
NM_001025668	RGD1306783	0.7036	0.6116	0.2427	0.6740	0.6698	0.6823	0.5562	0.7264	0.6883	0.8776	0.8676	0.8543
NM_001025669	RGD1307161	0.2654	0.3183	0.9122	0.9693	0.3072	0.7401	0.0400	0.4456	0.1805	0.3047	0.3735	0.8080
NM_001031641	Aga	0.0519	0.0297	0.0696	0.0144	0.0342	0.1985	0.0286	0.0085	0.0007	0.0500	0.0386	0.0562
NM_017012	Grm5	0.6188	0.3831	0.6805	0.2612	0.0401	0.4162	0.0706	0.2302	0.0180	0.0595	0.4372	0.0615
NM_019330	Pcca	0.4776	0.7106	0.1997	0.4171	0.6996	0.6239	0.3341	0.4430	0.3026	0.6182	0.7070	0.6566
NM_001025679	LOC308394	0.5042	0.4780	0.4049	0.5181	0.4261	0.6177	0.4529	0.4133	0.5686	0.4802	0.6560	0.5124
NM_001025681	Ttc23	0.6860	0.0763	0.8330	0.8605	0.7963	0.6745	0.3388	0.7323	0.3671	0.1515	0.4058	0.0488
NM_012810	Sycp1	0.2866	0.2494	0.2502	0.4945	0.6701	0.3493	0.5022	0.6077	0.3526	0.0804	0.4927	0.4188
NM_012811	Mfge8	0.7576	0.8967	0.7834	0.8751	0.9390	0.9980	0.9317	0.9184	0.9959	0.8574	0.7540	0.9430
NM_012812	Cox6a2	0.4544	0.9142	0.8347	0.4790	0.7349	0.7889	0.6391	0.8466	0.7987	0.7779	0.8378	0.8140
NM_013102	Fkbp1a	0.8742	0.6033	0.5873	0.8828	0.9831	0.6412	0.9841	0.7432	0.9336	0.6584	0.9012	0.3806
NM_012814	Cox6a1	0.0570	0.1960	0.2907	0.0922	0.0599	0.0495	0.0406	0.0138	0.0733	0.0543	0.0540	0.1333
NM_001025687	Dclre1b	0.8294	0.5152	0.3214	0.2969	0.7892	0.1451	0.4699	0.4349	0.7269	0.5595	0.3324	0.3846
NM_001025688	Palmd	0.4956	0.7869	0.3221	0.2989	0.4271	0.0776	0.6739	0.3040	0.6456	0.5961	0.8817	0.3631
NM_001025691	RGD1311739	0.2937	0.8303	0.8172	0.6289	0.5304	0.7968	0.8267	0.5500	0.1390	0.6180	0.1566	0.6488
NM_012913	Atp1b3	0.0428	0.1211	0.4076	0.2899	0.1631	0.2006	0.1063	0.0329	0.1492	0.2275	0.0042	0.2514
NM_001025693	Cdca7	0.9928	0.9966	0.8940	0.9617	0.9600	0.9262	0.9745	0.9895	0.9972	0.9376	0.8442	0.8933
NM_013079	Asns	0.7383	0.8192	0.9025	0.8213	0.7171	0.9126	0.9198	0.8459	0.9230	0.8225	0.8884	0.8580
NM_013161	Pnlip	0.7352	0.1915	0.1816	0.3928	0.0946	0.5655	0.3750	0.4946	0.3014	0.3152	0.0394	0.3410
NM_001034000	Ncapd3	0.4596	0.7125	0.2326	0.1962	0.5257	0.5022	0.6936	0.5842	0.7248	0.7653	0.7955	0.2325
NM_013016	Sirpa	0.9998	0.8674	0.9964	0.9668	0.9922	0.7394	0.9650	0.9689	0.8852	0.9807	0.9904	0.9998
NM_001034022	Mprip	0.7589	0.4098	0.9364	0.9706	0.9929	0.9551	0.8811	0.9467	0.9849	0.8714	0.8974	0.8484
NM_012817	Igfbp5	0.3168	0.1278	0.1140	0.1406	0.5486	0.2166	0.0558	0.5928	0.6711	0.3075	0.0436	0.5134
NM_001025706	Fem1a	0.5936	0.3834	0.5819	0.8635	0.2543	0.5848	0.5995	0.2476	0.7808	0.7499	0.6381	0.9288
NM_001025708	Ogfrl1	0.9875	0.7545	0.8398	0.8869	0.7724	0.7488	0.8738	0.9358	0.7866	0.9487	0.8683	0.8656
NM_001034080	Pyy	0.5291	0.7171	0.7965	0.8121	0.7299	0.7936	0.6986	0.6991	0.6367	0.7435	0.9026	0.7617
NM_001025712	Itm2a	0.8695	0.8409	0.8865	0.9377	0.3328	0.7379	0.9406	0.2844	0.7950	0.8034	0.8704	0.7960
NM_001025713	MGC114529	0.8972	0.5785	0.7614	0.7986	0.6958	0.2752	0.0109	0.7783	0.9413	0.5175	0.8818	0.4153
NM_001025715	Limd2	0.1338	0.3189	0.5701	0.8671	0.7252	0.2127	0.4777	0.5262	0.6137	0.1316	0.1103	0.3099
NM_013080	Ptprz1	0.6782	0.7096	0.4730	0.6575	0.6037	0.3392	0.3723	0.5826	0.4328	0.5299	0.5312	0.4368
NM_001025718	MGC112830	0.9954	0.9989	0.9078	0.9471	0.9751	0.9943	0.7835	0.9938	0.7505	0.8925	0.9455	0.8740
NM_001025719	RGD1311307	0.8923	0.9393	0.8800	0.9618	0.3385	0.3951	0.9958	0.8240	0.8966	0.9860	0.9520	0.8406
NM_012511	Atp7b	0.8105	0.8314	0.6683	0.4132	0.7442	0.7560	0.9043	0.8677	0.5236	0.6390	0.4898	0.5326
NM_001031657	Tdp1	0.3438	0.0675	0.1381	0.1553	0.2820	0.7534	0.1243	0.0964	0.7513	0.4991	0.2398	0.1391
NM_013088	Ptpn11	0.2627	0.7374	0.8468	0.9296	0.2604	0.0492	0.5877	0.4831	0.4866	0.8747	0.6470	0.4017
NM_012916	Bcan	0.7762	0.5534	0.6935	0.3343	0.8183	0.7796	0.6270	0.8524	0.8231	0.6721	0.9384	0.7869
NM_012824	Apoc1	0.4334	0.1913	0.6094	0.2132	0.3529	0.6462	0.3789	0.4441	0.3804	0.2913	0.1904	0.4319
NM_001025724	Pnldc1	0.4336	0.5684	0.4070	0.0517	0.6589	0.3878	0.2954	0.7459	0.2366	0.5474	0.7505	0.3574
NM_012827	Bmp4	0.6903	0.4964	0.6198	0.7488	0.6668	0.7126	0.7029	0.7352	0.3650	0.8248	0.4640	0.8966
NM_138825	Serpina12	0.4075	0.1911	0.1996	0.3885	0.1927	0.3069	0.2180	0.2065	0.3637	0.1773	0.1904	0.3224
NM_012828	Cacnb3	0.8275	0.2470	0.0773	0.2836	0.8696	0.7958	0.7606	0.6394	0.2642	0.6513	0.1773	0.3588
NM_012830	Cd2	0.7609	0.5732	0.8575	0.5941	0.4477	0.7324	0.5596	0.4898	0.1731	0.4898	0.5847	0.7755
NM_001025729	Zbed3	0.7257	0.6790	0.8391	0.7216	0.5323	0.3665	0.6146	0.5829	0.6469	0.8792	0.9342	0.9677
NM_012992	Npm1	0.8409	0.6046	0.9907	0.9562	0.6214	0.7907	0.9136	0.8891	0.6360	0.5032	0.8843	0.9367
NM_012835	Cort	0.5609	0.6572	0.5702	0.2297	0.0683	0.0622	0.5948	0.1538	0.4948	0.6581	0.6031	0.6393
NM_013076	Lep	0.0860	0.3918	0.1563	0.1871	0.0901	0.0896	0.3029	0.2496	0.3936	0.1256	0.3666	0.3213

NM_013000	Pam	0.9521	0.9678	0.9674	0.9997	0.9463	0.9505	0.9622	0.9104	0.9746	0.9995	0.9697	0.9580
NM_012837	Cst3	0.0355	0.0946	0.0182	0.0237	0.0884	0.3118	0.0239	0.1662	0.0235	0.0187	0.0123	0.0313
NM_012840	Cyct	0.6031	0.8912	0.8727	0.5298	0.3532	0.0415	0.6132	0.8214	0.5601	0.5477	0.8384	0.0812
NM_013098	G6pc	0.5177	0.3802	0.1571	0.3270	0.3276	0.3448	0.6282	0.2659	0.4028	0.3294	0.6008	0.1437
NM_013026	Sdc1	0.6447	0.9940	0.5598	0.9015	0.2988	0.2674	0.9997	0.5051	0.9751	0.9812	0.9529	0.8361
NM_031597	Kcnq3	0.3409	0.7139	0.2617	0.2695	0.2369	0.5957	0.5190	0.7266	0.6390	0.6766	0.8707	0.3385
NM_021668	Cml1	0.8684	0.6997	0.2872	0.8289	0.9504	0.9131	0.7344	0.7965	0.7189	0.4235	0.9225	0.7265
NM_001025743	Wdr61	0.3985	0.4522	0.8836	0.1862	0.5504	0.4323	0.0656	0.3050	0.2348	0.2079	0.4370	0.3698
NM_001025744	Tusc4	0.0991	0.0184	0.9867	0.1072	0.0041	0.0660	0.6615	0.1382	0.0528	0.0452	0.0289	0.0814
NM_001025745	Zfand2b	0.6451	0.7868	0.1672	0.0653	0.6210	0.0449	0.1741	0.8040	0.2819	0.7208	0.8417	0.3459
NM_001025747	Yipf6	0.3035	0.1202	0.8126	0.7290	0.1623	0.0049	0.1683	0.2028	0.0530	0.2561	0.7124	0.1202
NM_001033896	Tmem79	0.4603	0.1833	0.2523	0.4764	0.6505	0.2170	0.7751	0.0479	0.7298	0.2985	0.5802	0.8607
NM_013042	Tff3	0.1750	0.2316	0.0466	0.1086	0.3656	0.5447	0.0222	0.4666	0.2912	0.3728	0.2294	0.5254
NM_013127	Cd38	0.0418	0.1743	0.3893	0.3384	0.7596	0.2207	0.5165	0.4365	0.6982	0.4691	0.4923	0.5581
NM_012843	Emp1	0.2810	0.6640	0.9439	0.9977	0.5604	0.9428	0.2329	0.3464	0.9831	0.8229	0.9684	0.9594
NM_001037643	Ube2z	0.1505	0.2508	0.8412	0.8300	0.2433	0.8456	0.5710	0.2628	0.2774	0.2301	0.0658	0.2883
NM_012941	Cyp51	0.9800	0.8933	0.1599	0.1395	0.9836	0.9359	0.7068	0.9747	0.7552	0.9555	0.9719	0.0900
NM_001025755	MGC116197	0.8070	0.7549	0.3325	0.7874	0.6851	0.8293	0.6855	0.2908	0.7207	0.4541	0.6913	0.7459
NM_001033894	Jakmip1	0.5073	0.5597	0.5228	0.5279	0.2821	0.0689	0.2663	0.1648	0.4812	0.7849	0.0665	0.1369
NM_012575	Grin2c	0.0919	0.0189	0.2388	0.1032	0.4711	0.4952	0.5161	0.2526	0.2612	0.2440	0.1014	0.1184
NM_001034109	Aarsd1	0.5175	0.5556	0.5673	0.2527	0.6264	0.7240	0.5825	0.6055	0.5764	0.6786	0.4177	0.6391
NM_001025758	LOC497959	0.8439	0.5636	0.6127	0.5999	0.5832	0.5149	0.8815	0.7122	0.5433	0.9661	0.8308	0.6701
NM_001025759	MGC112692	0.0667	0.5495	0.1085	0.3554	0.5697	0.7047	0.8382	0.4085	0.2862	0.7903	0.8666	0.8207
NM_013055	Map3k12	0.9680	0.0475	0.8560	0.8209	0.7440	0.8874	0.7295	0.9798	0.7520	0.2698	0.2724	0.3310
NM_013045	Tnr	0.7155	0.7518	0.5512	0.9212	0.7952	0.7892	0.9380	0.9016	0.9631	0.9402	0.9135	0.9326
NM_001034011	Sft2d2	0.6151	0.8340	0.8383	0.9396	0.9169	0.8779	0.4192	0.9071	0.5018	0.2931	0.5463	0.8691
NM_012514	Brca1	0.5009	0.5506	0.5063	0.3051	0.7265	0.5647	0.5447	0.5150	0.1920	0.4636	0.6026	0.6330
NM_001025764	LOC498933	0.8808	0.6870	0.8572	0.5753	0.4243	0.7901	0.8698	0.7744	0.6996	0.6470	0.8267	0.9795
NM_001037357	Lilrb3l	0.4763	0.4462	0.4350	0.6275	0.4266	0.2737	0.2461	0.3822	0.3215	0.3556	0.5457	0.3450
NM_001033927	Ola1	0.2587	0.9310	0.9269	0.7792	0.0977	0.1141	0.7645	0.3791	0.8858	0.7408	0.6567	0.3388
NM_001025769	C20orf165	0.5079	0.4111	0.5522	0.1957	0.2099	0.2759	0.3360	0.2898	0.0672	0.6380	0.2969	0.5928
NM_001025771	LOC500118	0.5619	0.9856	0.8960	0.8183	0.7268	0.8115	0.8752	0.9141	0.7124	0.8025	0.8491	0.8504
NM_001025772	MGC114440	0.1913	0.1715	0.0607	0.1597	0.2735	0.1443	0.4724	0.1661	0.6805	0.0166	0.2665	0.4806
NM_001033951	Zc3h14	0.6351	0.7010	0.4858	0.3332	0.6027	0.6917	0.6102	0.6278	0.5611	0.6765	0.5475	0.3679
NM_001025775	LOC501194	0.7606	0.3536	0.0388	0.1130	0.8273	0.8681	0.0344	0.8863	0.7743	0.4768	0.1249	0.8638
NM_001106465	Ntng1	0.6078	0.5524	0.5160	0.5457	0.6352	0.2254	0.5526	0.3972	0.3320	0.3530	0.4226	0.4743
NM_013160	Mxi1	0.6906	0.6433	0.6231	0.5719	0.7131	0.5028	0.7111	0.7136	0.6457	0.6337	0.7035	0.7229
NM_012866	Nfyc	0.3242	0.8568	0.3895	0.9445	0.9770	0.8216	0.1577	0.9657	0.1130	0.4378	0.4776	0.2740
NM_017031	Pde4b	0.1046	0.2108	0.1506	0.1694	0.3627	0.7563	0.0750	0.7637	0.1697	0.1133	0.0471	0.6002
NM_012867	Ninj1	0.1957	0.2110	0.2698	0.6440	0.1732	0.2289	0.2770	0.0586	0.2425	0.0140	0.0613	0.1286
NM_012869	Npy5r	0.4233	0.8394	0.9479	0.8630	0.8575	0.6690	0.8857	0.6382	0.3461	0.7343	0.6285	0.6315
NM_001130536	Fzd6	0.4913	0.5939	0.6358	0.6258	0.5767	0.4681	0.5894	0.6307	0.5748	0.6578	0.6132	0.4780
NM_017039	Ppp2ca	0.5058	0.4506	0.6965	0.7405	0.3716	0.8244	0.4613	0.1176	0.6560	0.4886	0.5962	0.7451
NM_017033	Pgm1	0.5468	0.3127	0.5180	0.8312	0.1217	0.7321	0.8375	0.3094	0.2038	0.0882	0.4478	0.9807
NM_013111	Slc7a1	0.3461	0.9743	0.3209	0.6512	0.3597	0.3231	0.4307	0.0377	0.9842	0.9842	0.5568	0.5297
NM_012877	Scn2b	0.6850	0.3368	0.6058	0.4247	0.6109	0.3932	0.4826	0.3934	0.0156	0.1306	0.3540	0.1446
NM_012928	Cnga2	0.1582	0.5090	0.8296	0.5379	0.7054	0.3880	0.9021	0.7197	0.7109	0.6711	0.6898	0.7184
NM_012875	Rpl39	0.4873	0.6644	0.0941	0.0984	0.4845	0.4418	0.2298	0.7887	0.5883	0.7411	0.2320	0.4117
NM_016990	Add1	0.7050	0.5445	0.8916	0.9563	0.8373	0.9888	0.8696	0.4637	0.9394	0.7849	0.5024	0.9303

NM_017048	Slc4a2	0.8145	0.8853	0.0386	0.9031	0.9303	0.7989	0.2074	0.9484	0.7957	0.9282	0.8169	0.9945
NM_012872	Pdc	0.2052	0.5672	0.1704	0.8191	0.4045	0.3534	0.4469	0.1777	0.1415	0.7556	0.6548	0.6281
NM_012888	Tshr	0.8051	0.3347	0.5700	0.6892	0.2257	0.7258	0.7225	0.5823	0.7871	0.6465	0.7478	0.3151
NM_012887	Tmpo	0.9722	0.9988	0.2980	0.6349	0.9827	0.9825	0.9882	0.9933	0.9789	0.9665	0.9926	0.1725
NM_017049	Slc4a3	0.8193	0.9419	0.5041	0.7046	0.7843	0.8554	0.6497	0.9323	0.9451	0.9566	0.9995	0.8791
NM_001029915	Bxdc2	0.6615	0.4494	0.8743	0.5073	0.4711	0.8701	0.1088	0.3738	0.6410	0.3498	0.2190	0.8560
NM_001034138	Ccdc99	0.5754	0.6168	0.0589	0.0832	0.2131	0.4269	0.3363	0.7798	0.3244	0.4975	0.7697	0.0374
NM_012915	Atpif1	0.3639	0.6827	0.2216	0.5627	0.6860	0.4183	0.1682	0.3587	0.5424	0.5039	0.8351	0.6951
NM_001034946	Itln1	0.2838	0.0868	0.0939	0.1171	0.3698	0.0697	0.3547	0.4473	0.1243	0.1330	0.2154	0.2432
NM_001034912	Fam134b	0.5545	0.6828	0.4638	0.5341	0.7020	0.2148	0.0962	0.5367	0.8254	0.8506	0.8513	0.7818
NM_001034911	Ubfd1	0.2035	0.9123	0.4023	0.7915	0.7464	0.3731	0.8435	0.5269	0.6869	0.2265	0.7659	0.2253
NM_012935	Cryab	0.0273	0.0139	0.0155	0.0333	0.1252	0.0904	0.0396	0.0427	0.0361	0.0147	0.0460	0.1321
NM_012895	Adk	0.9570	0.8963	0.7474	0.9587	0.9002	0.8080	0.7889	0.9171	0.9507	0.9206	0.9364	0.8552
NM_001034947	Ttrap	0.4746	0.0731	0.9103	0.1534	0.1295	0.2156	0.0672	0.0356	0.2556	0.1781	0.0902	0.7906
NM_001029926	LOC500893	0.5607	0.5560	0.6686	0.9006	0.7149	0.8780	0.7609	0.8223	0.8187	0.1504	0.5978	0.6475
NM_001034942	Zbtb44	0.9450	0.6684	0.7746	0.6511	0.9314	0.7994	0.8236	0.2254	0.7369	0.6327	0.5486	0.4427
NM_001029928	Lair1	0.4702	0.1905	0.1996	0.2127	0.6319	0.4422	0.3560	0.4062	0.1817	0.1772	0.1902	0.1945
NM_001107897	Gdap1	0.9500	0.8597	0.9867	0.5902	0.9758	0.6910	0.8369	0.6626	0.6640	0.9709	0.5684	0.6331
NM_012849	Gast	0.4327	0.4978	0.2807	0.2720	0.5920	0.1398	0.2343	0.1275	0.1589	0.0585	0.1306	0.6516
NM_013141	Ppard	0.2546	0.1274	0.3303	0.1820	0.7841	0.4299	0.1659	0.2730	0.2518	0.4418	0.6362	0.3607
NM_013052	Ywhah	0.2147	0.9287	0.2887	0.8245	0.5763	0.4409	0.5660	0.0682	0.9802	0.9787	0.9819	0.7732
NM_001030029	Commd7	0.3428	0.7619	0.4232	0.1243	0.4888	0.7135	0.8282	0.8664	0.8335	0.6646	0.7331	0.4481
NM_017342	Sftpc	0.6386	0.6900	0.7594	0.6296	0.8439	0.8475	0.4689	0.8172	0.6236	0.4997	0.7789	0.7144
NM_013095	Smad3	0.5506	0.4797	0.4134	0.4663	0.7320	0.5382	0.1613	0.4660	0.7942	0.6054	0.5410	0.5140
NM_013143	Mep1a	0.2091	0.4762	0.3961	0.2247	0.5765	0.1528	0.4243	0.4382	0.6783	0.4456	0.6619	0.3777
NM_001034079	Med24	0.6543	0.3830	0.3629	0.3970	0.3561	0.2502	0.4605	0.5424	0.5552	0.2292	0.5107	0.5802
NM_001030033	C1galt1c1	0.0685	0.0586	0.1005	0.0311	0.0255	0.0343	0.0497	0.0630	0.0134	0.0886	0.0933	0.0117
NM_001030034	Rhbdf1	0.8999	0.2999	0.4229	0.9540	0.7757	0.9389	0.0573	0.8189	0.2142	0.8421	0.5415	0.9417
NM_001030036	RGD1308087	0.8783	0.6411	0.8470	0.7955	0.9444	0.7372	0.7254	0.7241	0.9759	0.4853	0.8780	0.9085
NM_001030038	RGD1305314	0.9801	0.9948	0.9711	0.9875	0.8965	0.9136	0.9818	0.9947	0.9755	0.9982	0.9948	0.9572
NM_013158	Dbh	0.4743	0.6270	0.4838	0.5180	0.2239	0.3341	0.6128	0.3742	0.7385	0.9493	0.0813	0.7482
NM_012863	Bhlhb8	0.2351	0.8395	0.7524	0.4920	0.7626	0.7060	0.4241	0.7896	0.7840	0.3461	0.4276	0.5316
NM_001030042	Rad9b	0.5248	0.9289	0.7246	0.2736	0.7946	0.6058	0.5307	0.3872	0.5428	0.8693	0.8186	0.6847
NM_001034093	Skiv2l2	0.1961	0.4746	0.4207	0.1297	0.4504	0.3085	0.4056	0.3903	0.2852	0.4713	0.3842	0.5824
NM_001033866	Surf2	0.6798	0.2825	0.4815	0.3903	0.7169	0.5747	0.4622	0.5083	0.5932	0.4640	0.4951	0.3271
NM_001031627	LOC606294	0.9583	0.7105	0.7598	0.7932	0.9234	0.8705	0.8587	0.8714	0.8072	0.6456	0.9093	0.8428
NM_013200	Cpt1b	0.8526	0.6842	0.2639	0.1747	0.7262	0.6524	0.2306	0.6952	0.6265	0.6750	0.7076	0.8304
NM_012945	Hbegf	0.4103	0.3596	0.6952	0.4936	0.0212	0.1999	0.9875	0.3221	0.8839	0.1714	0.7880	0.3827
NM_012891	Acadvl	0.1134	0.0794	0.8849	0.7441	0.1004	0.0425	0.7793	0.3549	0.0483	0.0330	0.3021	0.2191
NM_013145	Gnai1	0.4783	0.7188	0.8212	0.4607	0.5931	0.4633	0.8307	0.1507	0.7470	0.8081	0.7830	0.8175
NM_013149	Ahr	0.4169	0.0014	0.7421	0.0147	0.0675	0.6942	0.0032	0.0409	0.0006	0.0009	0.0020	0.6928
NM_019167	Sptbn2	0.8141	0.2331	0.4627	0.6249	0.3953	0.8227	0.7268	0.6550	0.3401	0.5061	0.4929	0.6382
NM_012903	Anp32a	0.8022	0.9212	0.9656	0.9800	0.8679	0.9871	0.8737	0.8683	0.7517	0.9600	0.8908	0.9903
NM_013004	Phex	0.6890	0.5866	0.3021	0.1413	0.7120	0.6425	0.0774	0.5810	0.7072	0.1537	0.7023	0.1869
NM_019298	Chrnd	0.7216	0.4912	0.5833	0.3116	0.3743	0.2512	0.2778	0.7094	0.0699	0.0278	0.5286	0.0787
NM_001031648	Efhd2	0.2412	0.8932	0.3954	0.9148	0.3247	0.2736	0.9048	0.0369	0.7317	0.9067	0.7431	0.9145
NM_001031652	St6galnac2	0.8487	0.9164	0.8027	0.8283	0.6595	0.6005	0.7582	0.6696	0.5348	0.8856	0.8512	0.8010
NM_001031654	Anxa8	0.9376	0.8666	0.8599	0.7903	0.9992	0.7731	0.8181	0.8287	0.9460	0.7978	0.9958	0.9843
NM_013166	Cntf	0.4681	0.2168	0.0259	0.0650	0.6994	0.3229	0.0473	0.5145	0.6223	0.1720	0.5708	0.1459

NM_012904	Anxa1	0.2260	0.0124	0.0751	0.0993	0.0226	0.0966	0.0848	0.0939	0.0687	0.0115	0.0169	0.3337
NM_013099	Mc4r	0.3633	0.8167	0.7310	0.8450	0.5849	0.6310	0.6737	0.7037	0.6441	0.1828	0.6937	0.4766
NM_017101	Ppia	0.8557	0.9256	0.3323	0.8711	0.7695	0.5731	0.7855	0.7597	0.8230	0.7371	0.8344	0.4852
NM_012584	Hsd3b5	0.0491	0.6128	0.5277	0.7318	0.2659	0.1817	0.5469	0.3778	0.1905	0.7922	0.4589	0.5567
NM_013006	Lypla1	0.9798	0.9665	0.9387	0.9747	0.8396	0.9616	0.8114	0.9469	0.5017	0.8535	0.7631	0.6994
NM_013119	Scn3a	0.4443	0.6052	0.3650	0.4955	0.3936	0.9034	0.5139	0.4995	0.8328	0.4869	0.7057	0.8665
NM_017034	Pim1	0.1630	0.1635	0.3975	0.2030	0.4666	0.2370	0.1921	0.2934	0.4677	0.2855	0.3700	0.2621
NM_013177	Got2	0.1437	0.1132	0.2344	0.7144	0.2777	0.0926	0.7824	0.1424	0.0737	0.2150	0.3110	0.9786
NM_017298	Cacna1d	0.9331	0.8095	0.5880	0.8588	0.8849	0.8392	0.7727	0.8694	0.6914	0.6459	0.7133	0.9529
NM_013008	Pou1f1	0.6381	0.8403	0.9220	0.6666	0.6119	0.2963	0.6695	0.7611	0.7663	0.7875	0.8276	0.4697
NM_001034948	Zfyve19	0.8597	0.7528	0.8642	0.7237	0.9769	0.6459	0.7488	0.8592	0.9082	0.6281	0.7720	0.7479
NM_012548	Edn1	0.1696	0.3479	0.4815	0.0813	0.1647	0.0856	0.7555	0.2949	0.1833	0.7124	0.2536	0.0535
NM_001033064	Kazald1	0.8422	0.9778	0.9217	0.9329	0.9183	0.9718	0.9522	0.9850	0.7360	0.9219	0.8929	0.1355
NM_017060	Pla2g16	0.0137	0.0176	0.0486	0.1400	0.0183	0.0467	0.0027	0.1228	0.0271	0.0399	0.0796	0.0427
NM_013097	Dnase1	0.1007	0.1086	0.3814	0.2756	0.4434	0.1117	0.0156	0.2745	0.2891	0.1085	0.2371	0.3295
NM_013011	Ywhaz	0.8721	0.4569	0.2983	0.8836	0.4804	0.9071	0.3491	0.2196	0.7611	0.8879	0.8348	0.7577
NM_001033066	Ddhd1	0.4612	0.5563	0.2143	0.6087	0.5624	0.3321	0.7045	0.5808	0.2675	0.4448	0.5300	0.2216
NM_001012088	Pex16	0.2847	0.2153	0.2482	0.3481	0.0912	0.2912	0.1057	0.2127	0.1015	0.0688	0.0249	0.5654
NM_017218	Erbb3	0.1110	0.0245	0.1056	0.0453	0.0883	0.3134	0.2875	0.1803	0.1041	0.1654	0.4398	0.1002
NM_001033071	Clul1	0.0730	0.1472	0.0983	0.3306	0.1596	0.2333	0.3381	0.0933	0.1840	0.1006	0.2639	0.1153
NM_001033072	LOC500959	0.1877	0.1902	0.1994	0.2123	0.3583	0.3851	0.2173	0.2059	0.1815	0.1770	0.1899	0.2809
NM_017173	Serpinh1	0.7796	0.7930	0.6730	0.0929	0.5271	0.1856	0.7925	0.2090	0.3182	0.8032	0.9808	0.4097
NM_012914	Atp2a3	0.7459	0.9362	0.9897	0.8935	0.9829	0.9933	0.9946	0.9845	0.9917	0.9887	0.9642	0.9985
NM_017125	Cd63	0.0245	0.1525	0.1199	0.3112	0.3620	0.3188	0.0431	0.2549	0.0715	0.0111	0.1682	0.0396
NM_017179	Uncx	0.4445	0.2279	0.6525	0.5163	0.7145	0.7855	0.9103	0.8030	0.4856	0.7862	0.6313	0.6277
NM_013018	Rab3a	0.1276	0.4281	0.3632	0.3814	0.2306	0.3218	0.7430	0.2949	0.5819	0.5056	0.8291	0.6665
NM_001033666	Dstn	0.3599	0.7577	0.7871	0.4921	0.2311	0.4767	0.5455	0.3142	0.7303	0.6618	0.6232	0.8329
NM_012919	Cacna2d1	0.8602	0.5084	0.9762	0.1904	0.8779	0.9975	0.8678	0.8061	0.9493	0.5128	0.7652	0.9930
NM_017029	Nefm	0.5742	0.5427	0.6685	0.4329	0.8336	0.8291	0.1534	0.7259	0.8163	0.5495	0.7460	0.5482
NM_017199	Ssr4	0.6989	0.7017	0.0417	0.3947	0.4033	0.0166	0.7234	0.8611	0.1139	0.6602	0.2786	0.0344
NM_013019	Rab4a	0.8983	0.1283	0.9380	0.2833	0.0286	0.0149	0.8843	0.0238	0.2435	0.3108	0.1356	0.1076
NM_019180	Tpsb2	0.2746	0.5026	0.6926	0.1970	0.2194	0.1088	0.3140	0.4699	0.4473	0.2339	0.2478	0.3288
NM_012923	Ccng1	0.2707	0.2266	0.9131	0.1894	0.0340	0.6197	0.4047	0.2934	0.3932	0.4251	0.3031	0.9308
NM_017131	Casq2	0.1859	0.5232	0.1228	0.3759	0.8751	0.3092	0.8205	0.2255	0.9089	0.0252	0.1849	0.7233
NM_013414	Bglap	0.8286	0.6801	0.1582	0.5197	0.6784	0.7249	0.8580	0.4860	0.7005	0.7945	0.6402	0.7452
NM_013219	Cadps	0.9705	0.9242	0.9355	0.9913	0.9099	0.8712	0.9573	0.8501	0.9453	0.9252	0.7660	0.9791
NM_182667	Myocd	0.1456	0.1099	0.3261	0.2582	0.2421	0.1342	0.2146	0.1323	0.3126	0.2472	0.3344	0.1695
NM_001033692	Commd9	0.0480	0.1503	0.1389	0.0366	0.2581	0.3175	0.1302	0.3167	0.4039	0.5919	0.1219	0.1615
NM_017078	Chrna5	0.4425	0.6981	0.5374	0.4520	0.4565	0.1170	0.2992	0.4964	0.5637	0.1178	0.4668	0.5794
NM_013041	Sycp3	0.1503	0.4575	0.2481	0.5764	0.6693	0.6303	0.5484	0.2489	0.1907	0.5305	0.5848	0.5996
NM_013027	Sepw1	0.1649	0.6772	0.9542	0.9286	0.6978	0.5861	0.7474	0.4828	0.7190	0.6669	0.6573	0.9070
NM_013028	Shox2	0.5352	0.7413	0.4662	0.2745	0.6155	0.3729	0.3183	0.4738	0.1730	0.8949	0.3862	0.1956
NM_013030	Slc34a1	0.5200	0.5615	0.7881	0.6095	0.8144	0.5847	0.7282	0.7049	0.8608	0.6148	0.6424	0.7380
NM_001033698	Ankrd16	0.7519	0.9040	0.4976	0.4195	0.5344	0.4523	0.3083	0.8574	0.7371	0.0977	0.4204	0.5837
NM_017248	Hnrnpa1	0.8886	0.9397	0.7976	0.9193	0.8703	0.8342	0.8804	0.9751	0.8412	0.8780	0.9570	0.3205
NM_019134	Slc12a1	0.2272	0.4887	0.5631	0.4174	0.2982	0.2196	0.2489	0.6258	0.7691	0.6314	0.4460	0.1859
NM_020077	Adam2	0.3389	0.3731	0.5761	0.4328	0.6601	0.4134	0.1213	0.3858	0.5370	0.5484	0.7289	0.5979
NM_001033702	Rnf126	0.2536	0.2451	0.4738	0.5757	0.3723	0.3434	0.2170	0.5503	0.3603	0.3127	0.2572	0.6631
NM_013025	Ccl3	0.0885	0.3995	0.2010	0.2570	0.3495	0.1328	0.0904	0.1025	0.0230	0.3199	0.6626	0.5349

NM_013024	Sbp	0.0867	0.4000	0.4029	0.0821	0.1541	0.0809	0.0337	0.0088	0.4015	0.1117	0.2037	0.3085
NM_012927	Cdh6	0.6037	0.5871	0.5713	0.5578	0.6389	0.5884	0.7299	0.6059	0.5919	0.6106	0.5217	0.2890
NM_001033705	Zfp451	0.5862	0.5671	0.3476	0.6061	0.2184	0.5233	0.3854	0.4015	0.4107	0.6092	0.4902	0.6275
NM_001035255	Sfrs11	0.8652	0.9875	0.8043	0.2352	0.7540	0.9441	0.1526	0.8796	0.9678	0.9027	0.9632	0.9003
NM_012641	Reg1a	0.6229	0.3850	0.5189	0.2907	0.3134	0.3843	0.4054	0.3851	0.4981	0.3927	0.4088	0.5196
NM_019179	Tyms	0.8262	0.8169	0.4913	0.9702	0.9838	0.8307	0.8261	0.7774	0.7674	0.8394	0.9809	0.0660
NM_013029	St8sia3	0.2686	0.2894	0.5630	0.3061	0.3927	0.4824	0.4849	0.5063	0.2250	0.3458	0.5818	0.3407
NM_012932	Crmp1	0.5292	0.6135	0.5615	0.6142	0.4960	0.4083	0.7168	0.0820	0.7345	0.5540	0.6503	0.4030
NM_012931	Bcar1	0.0474	0.1191	0.0091	0.0580	0.0038	0.0285	0.3055	0.0293	0.1714	0.1879	0.0181	0.1123
NM_013051	Scgb1a1	0.0956	0.1292	0.0839	0.0372	0.0554	0.0695	0.0184	0.0666	0.3497	0.4156	0.1962	0.2504
NM_013106	Gnai3	0.6812	0.6984	0.6470	0.6852	0.6861	0.7132	0.7106	0.6740	0.3012	0.6388	0.6569	0.3074
NM_012737	Apoa4	0.2558	0.1900	0.2679	0.2118	0.3280	0.3207	0.2168	0.4763	0.4240	0.2951	0.3097	0.3943
NM_013151	Plat	0.0102	0.0363	0.9638	0.2123	0.2126	0.0470	0.0296	0.0093	0.1458	0.0971	0.1538	0.6660
NM_017000	Nqo1	0.0756	0.0119	0.0995	0.3468	0.2671	0.0707	0.0445	0.1168	0.0515	0.1252	0.0180	0.1326
NM_031545	Nppb	0.0078	0.0407	0.0121	0.0766	0.0369	0.1166	0.2020	0.0211	0.0094	0.0239	0.1093	0.1253
NM_013036	Sstr4	0.0722	0.5793	0.0966	0.1446	0.5436	0.5535	0.3096	0.1799	0.7811	0.2763	0.4424	0.3728
NM_182671	Ndufa10l1	0.0884	0.0134	0.3505	0.7543	0.0177	0.2457	0.4128	0.2106	0.1034	0.0136	0.0756	0.0350
NM_001037140	Pcdhga1	0.5355	0.6754	0.2439	0.6037	0.5917	0.6876	0.6254	0.7117	0.7023	0.5370	0.5039	0.7212
NM_017135	Ak3l1	0.0032	0.0053	0.5465	0.4044	0.0096	0.1006	0.7470	0.0287	0.0073	0.0045	0.0064	0.7456
NM_013038	Stxbp1	0.4719	0.1968	0.1206	0.1460	0.3907	0.9287	0.3196	0.2698	0.8115	0.1205	0.4064	0.9473
NM_001033868	Surf4_predictec	0.5258	0.7338	0.5382	0.6695	0.6061	0.7110	0.5689	0.6120	0.6054	0.1933	0.5779	0.6028
NM_016993	Bcl2	0.3965	0.3013	0.6099	0.7903	0.5000	0.6527	0.7692	0.1774	0.2064	0.8929	0.2979	0.5584
NM_012969	Irs1	0.6419	0.6185	0.8550	0.9261	0.6483	0.7983	0.7950	0.6920	0.9475	0.8482	0.9357	0.9588
NM_013068	Fabp2	0.5401	0.6748	0.4095	0.5702	0.1505	0.5661	0.5937	0.6317	0.5624	0.1170	0.6897	0.0765
NM_019280	Gja5	0.9749	0.5615	0.9336	0.7514	0.9951	0.9579	0.0680	0.8810	0.8752	0.5706	0.7639	0.9517
NM_020093	Park2	0.1145	0.0206	0.0835	0.1908	0.0286	0.1275	0.6800	0.1672	0.1090	0.3004	0.3624	0.6733
NM_013064	Hcrtr1	0.6529	0.9606	0.5675	0.8288	0.6547	0.8620	0.8831	0.8051	0.8033	0.9249	0.7165	0.8748
NM_001037159	Pcdhgb8	0.7539	0.8559	0.0646	0.8249	0.8153	0.5371	0.5128	0.5439	0.6327	0.6935	0.5910	0.7001
NM_001037139	Pcdhga2	0.8524	0.5119	0.7018	0.9526	0.9511	0.9748	0.9117	0.8139	0.8663	0.2177	0.2141	0.8228
NM_017016	Hdc	0.5998	0.4967	0.6693	0.8061	0.8914	0.4709	0.6525	0.7383	0.6625	0.6681	0.3347	0.4156
NM_001033889	Cog7	0.3728	0.4076	0.7725	0.8752	0.9284	0.4610	0.9305	0.8497	0.8965	0.0413	0.1649	0.0871
NM_001033890	RGD1311072	0.3737	0.6938	0.6846	0.7031	0.3248	0.3181	0.7681	0.5633	0.3470	0.3944	0.2835	0.2952
NM_001037200	Fam82a	0.4753	0.5480	0.0759	0.6157	0.7319	0.7009	0.1021	0.7559	0.2781	0.7184	0.1171	0.2083
NM_001037216	Nxf7	0.4798	0.7124	0.4024	0.6692	0.4820	0.4685	0.4170	0.2389	0.8801	0.8686	0.6972	0.5169
NM_001037201	Ddx21	0.6438	0.6452	0.3254	0.3731	0.9766	0.3357	0.4853	0.6122	0.9408	0.7016	0.9139	0.8248
NM_013224	Rps26	0.6577	0.8239	0.7457	0.8215	0.7078	0.7045	0.7183	0.7292	0.6090	0.6807	0.5015	0.6097
NM_001037656	Lace1	0.3101	0.3674	0.6377	0.3902	0.4318	0.6372	0.4212	0.5116	0.3187	0.7169	0.1897	0.4050
NM_001037356	Pak1ip1	0.3264	0.8831	0.0609	0.1118	0.2005	0.0486	0.0163	0.3373	0.7718	0.6797	0.9624	0.2767
NM_012587	Ibsp	0.2922	0.6917	0.7676	0.8835	0.7052	0.9330	0.8918	0.1915	0.2662	0.6710	0.5241	0.8765
NM_012586	Iapp	0.6601	0.0771	0.6032	0.0792	0.8607	0.6840	0.2538	0.1733	0.4019	0.4212	0.7804	0.0250
NM_001039345	Afaf	0.2424	0.3139	0.6535	0.3289	0.7625	0.4654	0.0264	0.3694	0.5989	0.0577	0.6751	0.7533
NM_012510	Atp4b	0.3756	0.4725	0.3123	0.4569	0.0630	0.2203	0.2443	0.2294	0.4141	0.7970	0.4228	0.5522
NM_012567	Gja1	0.3776	0.2313	0.6804	0.7427	0.9863	0.9534	0.1763	0.9951	0.3077	0.3025	0.3450	0.2943
NM_012555	Ets1	0.8881	0.9624	0.9764	0.7782	0.4763	0.9238	0.9595	0.9493	0.9809	0.9874	0.9755	0.9499
NM_001037196	Jmjd5	0.5117	0.7397	0.2287	0.4984	0.4452	0.7474	0.3753	0.4361	0.6717	0.3549	0.6550	0.3408
NM_001033923	Gimap8	0.5097	0.1465	0.0792	0.5862	0.4758	0.3735	0.8084	0.5311	0.8499	0.8370	0.4914	0.6041
NM_017015	Gusb	0.6267	0.1138	0.7969	0.7598	0.7728	0.9578	0.0725	0.6783	0.0476	0.1592	0.0314	0.2879
NM_017052	Sord	0.9125	0.9620	0.8533	0.6426	0.8296	0.9256	0.7608	0.7071	0.6795	0.9110	0.9453	0.9083
NM_001039023	Net1	0.2383	0.2635	0.9975	0.9750	0.5020	0.6418	0.2377	0.1719	0.2450	0.0266	0.2446	0.9722

NM_001037776	Tmem177	0.0488	0.1650	0.9015	0.7725	0.7006	0.7506	0.5899	0.6956	0.1676	0.0553	0.5417	0.8806
NM_013136	Mak	0.1681	0.2796	0.3352	0.4839	0.3165	0.4601	0.5619	0.3317	0.6126	0.2695	0.5488	0.3390
NM_021774	Fhit	0.1873	0.1897	0.1992	0.2117	0.1923	0.1979	0.2165	0.2057	0.1812	0.1768	0.1896	0.1943
NM_001039012	Rer1	0.2251	0.7536	0.9048	0.8726	0.4460	0.7634	0.8083	0.8689	0.8347	0.6402	0.7951	0.4988
NM_012948	Emd	0.7647	0.6110	0.3818	0.0239	0.4418	0.2259	0.6211	0.5531	0.5650	0.6072	0.7819	0.3257
NM_013048	Ttpa	0.9323	0.8543	0.5168	0.9040	0.6929	0.7602	0.9265	0.9120	0.9114	0.8260	0.3497	0.5980
NM_017346	Cacnb1	0.8676	0.9136	0.8213	0.6732	0.9365	0.9804	0.9280	0.9334	0.8388	0.9056	0.3318	0.9799
NM_012569	Gls	0.6785	0.5378	0.5970	0.7216	0.9848	0.9911	0.6159	0.9137	0.6822	0.5351	0.7014	0.3715
NM_017035	Plcd1	0.6900	0.2101	0.0226	0.6379	0.8667	0.7537	0.1261	0.8806	0.7337	0.6547	0.5268	0.8686
NM_013049	Tnfrsf4	0.4607	0.0687	0.0507	0.0166	0.5378	0.8423	0.2316	0.6264	0.1185	0.2971	0.1237	0.4816
NM_013131	Nr3c2	0.1743	0.2306	0.1966	0.2305	0.1891	0.1634	0.1878	0.3091	0.1992	0.3103	0.2604	0.2282
NM_017025	Ldha	0.1780	0.2835	0.1652	0.4933	0.1651	0.2396	0.0965	0.2924	0.1250	0.1981	0.0780	0.7475
NM_012952	Fgf9	0.5461	0.6050	0.5238	0.7941	0.7845	0.7414	0.6155	0.7664	0.8297	0.2548	0.3953	0.5973
NM_019276	Ugt8	0.4450	0.3704	0.4114	0.5241	0.3616	0.3694	0.0640	0.6021	0.3512	0.5165	0.3255	0.3977
NM_019253	Ptpn5	0.0286	0.4621	0.4643	0.2386	0.3099	0.1297	0.6401	0.1381	0.7635	0.5085	0.6847	0.3773
NM_199267	Rela	0.6288	0.7296	0.7748	0.9402	0.8610	0.6069	0.0732	0.9079	0.8435	0.2984	0.8682	0.5510
NM_198777	Tmem138	0.6375	0.9667	0.0689	0.8751	0.7580	0.4093	0.7663	0.8902	0.8859	0.7405	0.7285	0.1977
NM_001039691	Ugt1a6	0.3595	0.6902	0.6651	0.6415	0.4871	0.4898	0.4265	0.2860	0.3791	0.4166	0.5320	0.6245
NM_182955	Zfp191	0.0659	0.5176	0.2303	0.4658	0.5290	0.7211	0.4246	0.4171	0.5330	0.7585	0.7320	0.1226
NM_212529	Hsd17b8	0.0529	0.2341	0.9002	0.0796	0.3248	0.2543	0.3229	0.2163	0.2465	0.1561	0.0165	0.9912
NM_001024235	Znf294	0.6666	0.9026	0.8360	0.1306	0.3316	0.3694	0.5244	0.0271	0.6567	0.8470	0.8675	0.8625
NM_198742	Etfdh	0.2478	0.0456	0.7460	0.7396	0.0537	0.0378	0.1148	0.0506	0.0186	0.0091	0.0273	0.2397
NM_001033985	RT1-CE14	0.5413	0.5573	0.4054	0.3900	0.7189	0.2518	0.3771	0.5051	0.2747	0.2494	0.2260	0.3082
NM_001033986	RT1-CE5	0.2824	0.1013	0.5641	0.2739	0.5475	0.3995	0.3389	0.3120	0.3731	0.4938	0.0160	0.1071
NM_019383	Atp5h	0.2276	0.5174	0.5639	0.2548	0.4529	0.1200	0.4641	0.0765	0.5994	0.4784	0.2586	0.4082
NM_013174	Tgfb3	0.9289	0.9654	0.9165	0.9947	0.9130	0.9419	0.9600	0.7919	0.9458	0.9198	0.9579	0.3526
NM_017057	Tnp2	0.1837	0.6604	0.1764	0.5021	0.0169	0.3672	0.6914	0.6295	0.0442	0.1494	0.5518	0.1165
NM_012568	Glra2	0.8215	0.6655	0.7668	0.7886	0.4624	0.5963	0.8078	0.6428	0.7798	0.5543	0.5965	0.5931
NM_012566	Gfi1	0.1128	0.1536	0.5792	0.7625	0.2116	0.6458	0.3611	0.1543	0.3879	0.6868	0.1605	0.5296
NM_012559	Fgg	0.0242	0.0103	0.0158	0.0849	0.1072	0.1616	0.2873	0.0709	0.0373	0.1232	0.1432	0.0078
NM_001037186	Slc25a40	0.2114	0.6428	0.6757	0.5178	0.5478	0.6889	0.7191	0.6977	0.2763	0.4203	0.7111	0.4245
NM_001037189	Ccdc55	0.4574	0.4471	0.5050	0.3746	0.3782	0.2428	0.4726	0.3490	0.2119	0.2953	0.5093	0.2882
NM_013178	Scn4a	0.8411	0.4979	0.6550	0.7532	0.6432	0.5146	0.6984	0.5542	0.4780	0.1788	0.2376	0.5729
NM_001034003	RGD1308874	0.9311	0.4625	0.9618	0.9958	0.8281	0.9338	0.9644	0.7414	0.6856	0.1968	0.6929	0.8623
NM_001034004	Jam2	0.6621	0.7332	0.3718	0.2351	0.6387	0.6165	0.2563	0.6162	0.7295	0.6882	0.4066	0.5042
NM_001039022	Coq5	0.2681	0.7761	0.3920	0.2482	0.0279	0.3036	0.2346	0.1337	0.7913	0.6473	0.7728	0.4915
NM_001035221	Ypel5	0.3518	0.2820	0.9990	0.2237	0.2465	0.1363	0.6954	0.3202	0.2807	0.1556	0.2221	0.8333
NM_012524	Cebpa	0.5195	0.6466	0.2418	0.7885	0.4555	0.6269	0.5549	0.6180	0.5655	0.8720	0.4592	0.7523
NM_001038599	Oprm1	0.5747	0.5691	0.6230	0.6104	0.5073	0.7519	0.4963	0.8155	0.6760	0.6699	0.7131	0.3974
NM_017067	Chm	0.4290	0.3548	0.4513	0.3610	0.1731	0.0562	0.3800	0.0853	0.1670	0.1701	0.2975	0.0446
NM_013082	Sdc2	0.3307	0.2753	0.9120	0.3302	0.7389	0.9615	0.2932	0.9663	0.2651	0.2250	0.1117	0.5950
NM_001034010	RGD1310827	0.5157	0.3826	0.8996	0.8837	0.6806	0.6559	0.8578	0.6054	0.5908	0.4077	0.2075	0.4267
NM_012943	Dlx5	0.7813	0.9995	0.8085	0.9955	0.8663	0.8330	0.8994	0.9007	0.7620	0.8292	0.9698	0.9431
NM_001034021	Tbc1d14	0.8925	0.9342	0.8395	0.9266	0.7814	0.7977	0.9370	0.5525	0.7524	0.9558	0.8576	0.8631
NM_012944	Drd4	0.6126	0.6259	0.7485	0.6780	0.2011	0.4538	0.8420	0.5672	0.6568	0.5623	0.1737	0.5612
NM_013044	Tmod1	0.9440	0.8385	0.7547	0.9787	0.4362	0.6515	0.9271	0.9583	0.9038	0.9371	0.8751	0.9633
NM_013047	Trhr	0.4510	0.0699	0.5696	0.0427	0.3957	0.2768	0.3231	0.3205	0.4238	0.1464	0.3598	0.0235
NM_017159	Hal	0.4052	0.2668	0.5476	0.1679	0.3683	0.4480	0.4680	0.5648	0.3233	0.2115	0.1915	0.3975
NM_022930	Chrna9	0.3167	0.7167	0.1915	0.3787	0.6316	0.7778	0.3260	0.8640	0.7340	0.3618	0.0545	0.1782

NM_013081	Ptk2	0.9918	0.6222	0.9376	0.9627	0.9846	0.9469	0.9301	0.6921	0.6014	0.6140	0.8933	0.6234
NM_017010	Grin1	0.3560	0.5071	0.6486	0.1247	0.7738	0.4050	0.1471	0.4320	0.2637	0.2393	0.0267	0.7304
NM_019248	Ntrk3	0.1367	0.1009	0.4447	0.1591	0.5810	0.2305	0.5232	0.8313	0.0329	0.4338	0.4552	0.5265
NM_017011	Grm1	0.7193	0.5535	0.6226	0.6718	0.7964	0.6303	0.2344	0.8374	0.6530	0.6983	0.5260	0.5095
NM_013091	Tnfrsf1a	0.2853	0.0582	0.1511	0.7399	0.2221	0.1515	0.1376	0.0640	0.9257	0.2620	0.6337	0.8369
NM_012958	Galr1	0.4654	0.1982	0.4033	0.2540	0.2826	0.7281	0.3274	0.6273	0.2299	0.3794	0.5290	0.3900
NM_031046	Itpr2	0.2089	0.2264	0.1442	0.5382	0.2262	0.3351	0.1583	0.3465	0.1260	0.2524	0.2415	0.8678
NM_020074	Srgn	0.1405	0.1836	0.9176	0.9216	0.3022	0.0726	0.0817	0.4214	0.5379	0.1645	0.0703	0.0709
NM_012960	Ggh	0.7889	0.7892	0.8758	0.8649	0.7746	0.8354	0.8840	0.8509	0.7746	0.7485	0.8121	0.8084
NM_013167	Ucp3	0.9310	0.4436	0.7942	0.8298	0.6453	0.8563	0.2730	0.8914	0.6386	0.4693	0.5185	0.2272
NM_019175	Klk6	0.1578	0.4991	0.1802	0.5937	0.1267	0.3880	0.0540	0.3270	0.1081	0.0474	0.6880	0.5380
NM_019246	Pcsk7	0.1100	0.7785	0.0396	0.4597	0.5351	0.3371	0.4400	0.4244	0.8947	0.7317	0.6442	0.8001
NM_019126	Psg19	0.5120	0.3737	0.6754	0.0979	0.6217	0.5925	0.4091	0.1877	0.8290	0.7056	0.1941	0.3616
NM_019207	Neurog1	0.5424	0.4775	0.4751	0.3693	0.2185	0.1661	0.4927	0.4837	0.4092	0.5509	0.4909	0.8558
NM_012492	Adrb2	0.9276	0.9720	0.9517	0.8818	0.7864	0.9852	0.6757	0.7412	0.8878	0.9620	0.9900	0.8611
NM_017097	Ctsc	0.9088	0.2115	0.6419	0.7888	0.8152	0.5880	0.5200	0.5480	0.1170	0.5006	0.6296	0.5661
NM_022273	Aldh9a1	0.1472	0.1463	0.9519	0.3021	0.5031	0.7475	0.3609	0.9226	0.2820	0.3172	0.5031	0.8492
NM_021672	Gdf9	0.4641	0.2243	0.4056	0.1708	0.3595	0.5584	0.6731	0.3431	0.4689	0.7172	0.5229	0.1232
NM_001037649	Rbm43	0.4246	0.4612	0.7002	0.7131	0.1938	0.8617	0.6380	0.6796	0.0460	0.2087	0.7875	0.4758
NM_013134	Hmgcr	0.9616	0.9983	0.1352	0.3847	0.9892	0.9379	0.6663	0.9994	0.9718	0.9645	0.9838	0.3456
NM_012964	Hmmr	0.0615	0.7821	0.0179	0.0802	0.7534	0.7403	0.6011	0.1071	0.6606	0.6709	0.8183	0.1242
NM_017004	Es1	0.4691	0.4270	0.0964	0.1305	0.1958	0.0402	0.0964	0.5860	0.4001	0.1622	0.1442	0.0696
NM_017003	Erbb2	0.7027	0.7602	0.1945	0.4126	0.5557	0.7873	0.2991	0.8453	0.5384	0.5080	0.3693	0.5210
NM_012968	Il1rap	0.1871	0.1895	0.4536	0.2114	0.1922	0.1978	0.4182	0.2056	0.1810	0.1766	0.1894	0.1941
NM_017006	G6pd	0.0218	0.1843	0.0092	0.1337	0.0713	0.0260	0.3851	0.3441	0.5680	0.2294	0.2626	0.9263
NM_001034132	RGD1559786	0.9213	0.8364	0.6699	0.7180	0.8244	0.6449	0.6586	0.7718	0.7796	0.2593	0.4941	0.1688
NM_013070	Utrn	0.3273	0.5288	0.4400	0.0209	0.5531	0.0871	0.3521	0.0969	0.1284	0.2903	0.1322	0.8846
NM_012973	Kcne1	0.4025	0.3046	0.3664	0.4586	0.1270	0.1608	0.2567	0.0864	0.0770	0.7714	0.6842	0.1050
NM_012971	Kcna4	0.4919	0.8894	0.2003	0.4035	0.4471	0.2614	0.3671	0.3665	0.6454	0.4433	0.6666	0.0267
NM_001037765	Spryd4	0.4237	0.1875	0.6321	0.9420	0.8924	0.6612	0.4370	0.3451	0.8561	0.7838	0.7600	0.9100
NM_001037657	Rbm12	0.5430	0.7321	0.5753	0.5442	0.5615	0.4907	0.6379	0.2583	0.4346	0.6375	0.6261	0.3101
NM_001034139	Lrrc8e	0.4672	0.5543	0.5239	0.4239	0.1862	0.3562	0.5784	0.4247	0.5521	0.8565	0.8248	0.4013
NM_001034140	Baiap2l1	0.5579	0.5186	0.2187	0.6024	0.3557	0.4558	0.2559	0.5987	0.1950	0.2066	0.4570	0.2164
NM_012553	Ela2a	0.1546	0.3964	0.6573	0.3767	0.4179	0.5785	0.3393	0.1985	0.0512	0.3243	0.7828	0.1207
NM_016997	Cel	0.2850	0.3332	0.1032	0.1519	0.1779	0.4958	0.1994	0.5155	0.2353	0.1450	0.5559	0.3386
NM_016986	Acadm	0.1719	0.2854	0.9016	0.3758	0.3533	0.6874	0.2766	0.5360	0.1480	0.1486	0.2180	0.7702
NM_001039002	Fam43a	0.9205	0.8949	0.9272	0.7137	0.8529	0.8499	0.8888	0.8513	0.8952	0.8256	0.6605	0.8291
NM_001037981	Trat1	0.1728	0.3752	0.4975	0.3174	0.3044	0.4545	0.5822	0.1048	0.7014	0.0260	0.8729	0.3181
NM_001037789	Ccdc28a	0.6791	0.5834	0.5834	0.2597	0.3581	0.5063	0.5732	0.4402	0.4965	0.2081	0.2402	0.4824
NM_016999	Cyp4b1	0.0804	0.4640	0.3867	0.1847	0.1036	0.0630	0.1668	0.1207	0.4279	0.7534	0.4384	0.2457
NM_001034149	Ddi2	0.3767	0.1706	0.6285	0.3993	0.6423	0.4202	0.3779	0.4063	0.5061	0.6520	0.4436	0.5080
NM_013075	Hoxa1	0.1716	0.3171	0.3331	0.6602	0.5225	0.3897	0.2373	0.5756	0.7735	0.3852	0.2621	0.4105
NM_013074	Hcrtr2	0.3733	0.2454	0.7044	0.4091	0.6591	0.1376	0.3425	0.2909	0.2028	0.1239	0.6448	0.2737
NM_001034151	Tmem54	0.1451	0.2446	0.1261	0.2883	0.0835	0.5263	0.4028	0.6022	0.7786	0.2496	0.3846	0.1957
NM_013071	Oprm1	0.5747	0.5691	0.6230	0.6104	0.5073	0.7519	0.4963	0.8155	0.6760	0.6699	0.7131	0.3974
NM_001034152	Nsg2	0.6584	0.1805	0.6944	0.1045	0.5064	0.4021	0.3449	0.6659	0.5404	0.6645	0.4224	0.8007
NM_001034153	MGC125213	0.4823	0.1236	0.1497	0.0454	0.3726	0.4534	0.2810	0.2552	0.0721	0.0593	0.5800	0.1198
NM_001034154	MGC125002	0.3648	0.0221	0.4403	0.2394	0.2370	0.0971	0.3221	0.3172	0.0068	0.3027	0.3308	0.0791
NM_012976	Lgals5	0.0555	0.0377	0.1611	0.0587	0.0535	0.0751	0.3295	0.1442	0.1166	0.0641	0.0511	0.5065

NM_017138	Rpsa	0.4710	0.7165	0.5213	0.6858	0.6788	0.3286	0.7085	0.7441	0.1527	0.4480	0.5739	0.3304
NM_017040	Ppp2cb	0.5911	0.4862	0.4575	0.8624	0.7031	0.8240	0.2650	0.6684	0.3116	0.4142	0.6100	0.8157
NM_001034187	Sh3rf2	0.6653	0.6394	0.8596	0.5239	0.1990	0.5495	0.5502	0.5520	0.1604	0.6329	0.8747	0.8095
NM_012865	Nfya	0.7702	0.6963	0.8671	0.8865	0.5750	0.7143	0.4499	0.3619	0.6581	0.7463	0.9664	0.3253
NM_017268	Hmgcs1	0.9999	0.9971	0.2076	0.1292	0.9545	0.9997	0.9466	0.9947	0.9489	0.9607	0.9994	0.1010
NM_012980	Mmp11	0.9374	0.6563	0.7192	0.7337	0.5183	0.8394	0.7821	0.8600	0.8639	0.3831	0.4158	0.9177
NM_017041	Ppp3ca	0.1916	0.8906	0.1843	0.2777	0.1424	0.6091	0.1441	0.3640	0.6894	0.7829	0.1565	0.4857
NM_001034835	RGD1309220	0.9001	0.6684	0.4429	0.1068	0.8367	0.9354	0.6980	0.0354	0.8943	0.9806	0.8706	0.5733
NM_017130	Neu2	0.1298	0.3282	0.6455	0.3722	0.5381	0.4646	0.5718	0.5702	0.4166	0.4833	0.1111	0.5642
NM_017061	Lox	0.3435	0.1152	0.1733	0.8423	0.1137	0.4548	0.5862	0.2038	0.2648	0.4105	0.7913	0.0995
NM_001034854	Ppapdc2	0.6365	0.5447	0.5820	0.6831	0.6170	0.6002	0.5979	0.6387	0.5333	0.5570	0.6274	0.7178
NM_001034855	Gpr153_predict	0.5230	0.6483	0.7352	0.4477	0.4642	0.6021	0.2712	0.6363	0.5656	0.5952	0.4034	0.5512
NM_001039008	Tmem176a	0.7626	0.8302	0.8115	0.5739	0.8604	0.6396	0.7542	0.7346	0.8084	0.9169	0.7353	0.8293
NM_012550	Ednra	0.6016	0.6732	0.7089	0.6568	0.7297	0.5596	0.2337	0.6961	0.5372	0.6879	0.6387	0.5793
NM_013222	Gfer	0.3859	0.4939	0.3977	0.3766	0.5394	0.2461	0.2895	0.6138	0.6681	0.5024	0.2468	0.4195
NM_001034917	Mks1	0.1626	0.6833	0.4882	0.8279	0.3025	0.1111	0.8531	0.4364	0.8744	0.3632	0.3118	0.6947
NM_017014	Gstm1	0.0749	0.2030	0.8000	0.8041	0.3845	0.0202	0.8732	0.1562	0.1053	0.1664	0.0330	0.0467
NM_013194	Myh9	0.7084	0.0264	0.8655	0.7740	0.8108	0.9373	0.7413	0.4900	0.3681	0.0333	0.3681	0.7438
NM_001034919	RGD1562218	0.9608	0.4748	0.8068	0.9731	0.7878	0.6043	0.0100	0.1524	0.8377	0.4839	0.7650	0.6833
NM_001039344	Tes	0.8560	0.9991	0.9347	0.8477	0.9984	0.7319	0.7969	0.9683	0.9902	0.8838	0.9046	0.9181
NM_001039017	Specc1	0.1591	0.0252	0.0450	0.2633	0.4517	0.3222	0.1170	0.1875	0.0372	0.0232	0.2334	0.2756
NM_001034923	Dus3l	0.8732	0.7197	0.0278	0.0112	0.7359	0.7345	0.3051	0.7568	0.5565	0.7219	0.8456	0.4665
NM_001098724	Necab3	0.2033	0.6208	0.1639	0.0402	0.6856	0.7342	0.5336	0.8786	0.8194	0.0915	0.6152	0.9642
NM_181635	Kif15	0.5997	0.8624	0.0064	0.0496	0.8918	0.8450	0.5849	0.8660	0.4012	0.3923	0.4683	0.4271
NM_001034929	Slc39a9	0.6789	0.6792	0.5437	0.7765	0.7771	0.6899	0.2124	0.3273	0.7280	0.8778	0.8340	0.4842
NM_001034930	LOC314655	0.2972	0.4822	0.4233	0.5028	0.2395	0.2623	0.3145	0.3682	0.4727	0.2639	0.4621	0.2649
NM_001108541	Wdr41	0.8133	0.9409	0.3000	0.9598	0.9724	0.9250	0.8962	0.9082	0.9528	0.7070	0.9402	0.2591
NM_001100475	Hip1	0.9462	0.8390	0.2879	0.0227	0.6352	0.5818	0.2154	0.6778	0.9152	0.8802	0.8581	0.9669
NM_019365	Rassf5	0.7857	0.9860	0.9758	0.9456	0.7966	0.9517	0.9936	0.9001	0.9615	0.8590	0.9908	0.8831
NM_001108539	Hace1	0.9853	0.9031	0.9472	0.9791	0.9365	0.8722	0.8925	0.9533	0.8892	0.9406	0.9286	0.9409
NM_001108710	Coch	0.4270	0.3754	0.6051	0.5729	0.2517	0.1586	0.3065	0.7577	0.3111	0.5955	0.4937	0.1161
NM_001108597	Cst9l	0.2392	0.1119	0.2508	0.0689	0.2660	0.1327	0.3171	0.1157	0.1237	0.0615	0.0904	0.1543
NM_001108909	Cnfn	0.2842	0.1483	0.0440	0.0563	0.2747	0.0289	0.4253	0.4571	0.5572	0.3756	0.0945	0.2259
NM_001108899	Clec3a	0.0386	0.0955	0.4326	0.4662	0.4849	0.4310	0.4022	0.1495	0.3929	0.0421	0.2199	0.3018
NM_001034932	C1qtnf6	0.5340	0.1470	0.9082	0.7616	0.3176	0.8648	0.4062	0.1493	0.2262	0.0027	0.3337	0.6759
NM_012983	Myo1d	0.4087	0.7756	0.5547	0.7212	0.9845	0.9867	0.6918	0.9733	0.6524	0.7139	0.6693	0.9935
NM_017050	Sod1	0.2162	0.1262	0.0558	0.0507	0.0363	0.0827	0.1084	0.2008	0.1669	0.0416	0.0937	0.0207
NM_017043	Ptgs1	0.4928	0.3585	0.7037	0.2476	0.5825	0.5141	0.6493	0.5981	0.5547	0.5400	0.5254	0.4110
NM_001039085	Cox6b2	0.1867	0.3284	0.1988	0.3323	0.2457	0.2835	0.5764	0.3370	0.2549	0.3381	0.3387	0.6949
NM_001034937	Phgdhl1	0.3398	0.4271	0.3166	0.3104	0.7911	0.3380	0.5782	0.5331	0.4831	0.6313	0.8765	0.2534
NM_017358	Cdon	0.4617	0.4245	0.7205	0.2539	0.4234	0.4064	0.7286	0.5228	0.4957	0.4645	0.6110	0.5847
NM_001034939	Obfc2b	0.3745	0.4400	0.0871	0.4187	0.6914	0.4051	0.8366	0.1264	0.7327	0.7028	0.7762	0.0824
NM_001034940	Zcrb1	0.3716	0.9826	0.2731	0.8676	0.7701	0.5812	0.8099	0.7561	0.0497	0.9577	0.5866	0.0372
NM_001039099	Zdhhc1	0.1083	0.0461	0.6883	0.5174	0.8208	0.5520	0.6234	0.9234	0.6632	0.0769	0.2355	0.4664
NM_001039196	Slc39a13		0.9846	0.3720	0.8290	0.9977		0.9185	0.9637	0.9864	0.9857	0.7933	0.9399
NM_012599	Mbl1	0.6628	0.3389	0.5967	0.6955	0.4735	0.4913	0.4276	0.5105	0.6836	0.4305	0.7283	0.4064
NM_012606	Myl3	0.2685	0.0417	0.4573	0.2938	0.5501	0.1254	0.6263	0.1395	0.4401	0.4234	0.0536	0.1644
NM_001039340	Zdhhc17	0.5011	0.6015	0.5934	0.4910	0.6159	0.6423	0.6976	0.6649	0.5511	0.6163	0.5822	0.4035
NM_001039342	Zdhhc11	0.0786	0.3592	0.8673	0.1166	0.4880	0.7252	0.5450	0.5137	0.1450	0.2411	0.7929	0.2947

NM_001034952	Pqlc3	0.1582	0.2963	0.9053	0.3157	0.9248	0.9086	0.1917	0.8765	0.4347	0.1403	0.3288	0.9660
NM_001034959	LOC619574	0.6646	0.5647	0.6396	0.5806	0.6407	0.6667	0.2515	0.6972	0.7255	0.7137	0.2028	0.6562
NM_001039455	Specc1l	0.1222	0.1046	0.2426	0.4433	0.3546	0.0912	0.7584	0.4290	0.2769	0.2528	0.2146	0.0177
NM_012542	Cyp2a3a	0.1854	0.2261	0.0749	0.6381	0.2090	0.6484	0.5162	0.1546	0.4440	0.3651	0.6022	0.2161
NM_001039454	Fam113b	0.3351	0.8269	0.3887	0.0912	0.5926	0.1292	0.2967	0.0567	0.4767	0.1143	0.5983	0.8086
NM_001034994	Rwdd4a	0.2550	0.8831	0.8196	0.2437	0.3887	0.8583	0.9340	0.6097	0.6268	0.5519	0.4789	0.1583
NM_012793	Gamt	0.6437	0.3197	0.3178	0.7909	0.5898	0.0336	0.7802	0.3411	0.0206	0.0445	0.0074	0.0645
NM_012907	Apobec1	0.0269	0.5666	0.5330	0.1124	0.0452	0.1795	0.3526	0.3943	0.1236	0.3269	0.4355	0.1551
NM_001034950	Rup2	0.4788	0.3042	0.4797	0.5928	0.4958	0.2815	0.4384	0.5895	0.1076	0.5742	0.4959	0.6304
NM_001038598	Oprm1	0.5747	0.5691	0.6230	0.6104	0.5073	0.7519	0.4963	0.8155	0.6760	0.6699	0.7131	0.3974
NM_012580	Hmox1	0.2812	0.1490	0.1386	0.8524	0.5521	0.0992	0.2558	0.4156	0.0059	0.0658	0.0086	0.2452
NM_013197	Alas2	0.6316	0.3208	0.3129	0.4806	0.0361	0.4119	0.3255	0.6477	0.0740	0.2819	0.2823	0.3746
NM_012984	Myo9b	0.9536	0.9196	0.8712	0.2650	0.8814	0.9572	0.9188	0.9084	0.9925	0.9185	0.8471	0.8652
NM_013121	Cd28	0.2474	0.4258	0.1286	0.4942	0.1363	0.1299	0.0606	0.2142	0.4570	0.2108	0.0242	0.1260
NM_012985	Ndufa5	0.0684	0.0999	0.3441	0.0044	0.0014	0.0840	0.1087	0.1003	0.0199	0.0055	0.1028	0.1086
NM_016988	Acp2	0.7876	0.0970	0.5395	0.9871	0.8660	0.7078	0.3040	0.6101	0.1658	0.2181	0.2271	0.4683
NM_001044295	Tpd52l1	0.2375	0.4221	0.2348	0.4830	0.5114	0.6623	0.7016	0.4322	0.2183	0.2270	0.6117	0.4555
NM_001044301	Muc15	0.2285	0.6150	0.4920	0.3837	0.4336	0.2602	0.2992	0.4954	0.4359	0.2457	0.3315	0.3195
NM_001044293	Nap1l5	0.7286	0.4065	0.6511	0.7295	0.2032	0.3559	0.2996	0.7139	0.7044	0.5086	0.6326	0.4995
NM_001044302	Psrc1	0.3619	0.3906	0.6702	0.3367	0.5366	0.2551	0.4129	0.5754	0.7236	0.3499	0.2359	0.6085
NM_001035252	Myl2	0.7296	0.6237	0.6844	0.7374	0.2877	0.5128	0.0714	0.6077	0.0924	0.4767	0.4101	0.3717
NM_001035253	LOC500148	0.6658	0.4402	0.7001	0.8005	0.5027	0.2073	0.6978	0.5292	0.6881	0.8827	0.7730	0.6096
NM_012617	Oprd1	0.4942	0.4971	0.3901	0.5552	0.5289	0.6073	0.2569	0.4974	0.4364	0.2434	0.6109	0.3422
NM_012614	Npy	0.3919	0.3095	0.2151	0.5533	0.1476	0.3226	0.4351	0.2776	0.2604	0.3279	0.3413	0.6971
NM_012610	Ngfr	0.1709	0.9102	0.2367	0.3160	0.2451	0.2485	0.0661	0.2261	0.4632	0.9423	0.2694	0.7580
NM_001035517	RGD1310127	0.8065	0.7894	0.7292	0.8951	0.4598	0.6687	0.8944	0.8845	0.8887	0.9165	0.9545	0.9698
NM_183052	Ube2v2	0.6104	0.3542	0.9389	0.3896	0.8085	0.9082	0.3676	0.2588	0.7152	0.5287	0.3537	0.5348
NM_182952	Cxcl11	0.1618	0.0814	0.1926	0.4444	0.0691	0.0705	0.0129	0.0127	0.2391	0.5001	0.5942	0.0390
NM_001037094	LOC292069	0.7421	0.7227	0.0601	0.2370	0.7028	0.6852	0.5314	0.6879	0.8140	0.7412	0.8991	0.1948
NM_022277	Casp8	0.4125	0.8968	0.8985	0.8678	0.7062	0.6863	0.1341	0.7477	0.5607	0.7834	0.6996	0.7861
NM_012988	Nfia	0.9832	0.9810	0.9017	0.9391	0.9182	0.9439	0.9435	0.9290	0.9104	0.9779	0.8715	0.5398
NM_013090	Vamp1	0.7277	0.9044	0.9345	0.4330	0.9102	0.9484	0.9357	0.5277	0.8284	0.5012	0.0758	0.8425
NM_017080	Hsd11b1	0.1637	0.0681	0.0067	0.0911	0.7678	0.7829	0.0389	0.8282	0.0058	0.2251	0.0147	0.0397
NM_012598	Lpl	0.3009	0.0056	0.5559	0.5768	0.1182	0.0118	0.1193	0.3167	0.0973	0.0281	0.2705	0.5961
NM_012526	Chgb	0.0886	0.1326	0.0731	0.0290	0.0122	0.0216	0.0031	0.2378	0.0142	0.1318	0.1283	0.0060
NM_175597	Ssx2ip	0.2653	0.3247	0.2530	0.1344	0.0668	0.1482	0.0318	0.1151	0.8038	0.2632	0.8538	0.5435
NM_012901	Ambp	0.3553	0.0954	0.5677	0.3666	0.2462	0.2701	0.2269	0.0713	0.1600	0.1874	0.5775	0.4126
NM_001037181	RGD1308517	0.0787	0.2295	0.0940	0.7910	0.6600	0.4786	0.0122	0.4146	0.3349	0.4457	0.0638	0.1502
NM_001037182	LOC290876	0.5688	0.8630	0.9313	0.3156	0.8324	0.8493	0.7907	0.3766	0.9304	0.9335	0.4351	0.8778
NM_001037183	Mrpl47	0.3472	0.3869	0.3880	0.1261	0.1905	0.0527	0.1107	0.0758	0.4673	0.4857	0.2493	0.5180
NM_001037184	RGD1307100	0.8928	0.9859	0.4771	0.1840	0.8572	0.9887	0.0415	0.9609	0.9774	0.9747	0.9310	0.7308
NM_017169	Prdx2	0.1038	0.4684	0.4363	0.4615	0.1482	0.0960	0.7473	0.4000	0.1611	0.0929	0.0567	0.1718
NM_013165	Cckbr	0.6320	0.7428	0.8478	0.7293	0.7943	0.7935	0.6815	0.8580	0.8492	0.9306	0.9486	0.6560
NM_001037187	LOC302680	0.6351	0.9187	0.7933	0.8356	0.8425	0.7815	0.8999	0.6343	0.8073	0.1047	0.4369	0.8127
NM_001037188	RGD1307155	0.0174	0.1624	0.9788	0.9142	0.0871	0.3243	0.9176	0.2787	0.3688	0.1802	0.0142	0.8693
NM_012719	Sstr1	0.7048	0.4374	0.7692	0.7914	0.7407	0.8324	0.7926	0.7485	0.8688	0.5387	0.5186	0.7988
NM_001037190	LOC303448	0.0464	0.5162	0.3818	0.8904	0.3990	0.4750	0.8174	0.4040	0.5912	0.5819	0.4726	0.5455
NM_001037191	RGD1306873	0.1753	0.2327	0.2425	0.4065	0.3667	0.0736	0.1605	0.0756	0.3412	0.1313	0.0590	0.2256
NM_019238	Fdft1	0.8226	0.9104	0.2139	0.2778	0.8498	0.8187	0.9648	0.3468	0.9544	0.9728	0.7955	0.2212

NM_001037194	Det1	0.6624	0.9534	0.8099	0.9302	0.6636	0.7990	0.8383	0.7024	0.5533	0.5603	0.9868	0.7582
NM_012829	Cck	0.5576	0.3027	0.9643	0.7579	0.0114	0.0085	0.7350	0.0315	0.6299	0.5845	0.1857	0.8115
NM_019275	Smad4	0.6926	0.2231	0.7066	0.8595	0.7339	0.8009	0.0716	0.3005	0.6549	0.5640	0.7810	0.7922
NM_019266	Scn8a	0.4711	0.2692	0.2165	0.6967	0.8701	0.5797	0.3254	0.1828	0.2996	0.5110	0.2842	0.6591
NM_012991	Npap60	0.6098	0.6235	0.5358	0.5383	0.6981	0.4663	0.6688	0.3025	0.6338	0.1924	0.4268	0.6992
NM_001037202	Plekhh3	0.7873	0.8027	0.2498	0.8335	0.9307	0.7268	0.6744	0.9298	0.8855	0.7503	0.7338	0.8618
NM_022259	Cd244	0.3832	0.3229	0.5984	0.7779	0.5755	0.4207	0.3884	0.1909	0.7983	0.5556	0.2526	0.3697
NM_001037205	LOC360997	0.6277	0.6703	0.3804	0.2679	0.3282	0.5308	0.6482	0.6058	0.2309	0.6497	0.5657	0.6449
NM_012543	Dbp	0.7589	0.6198	0.7686	0.6649	0.7698	0.7052	0.5231	0.5284	0.1891	0.7398	0.0301	0.9452
NM_012528	Chrnb1	0.4222	0.7488	0.1564	0.3017	0.1479	0.1517	0.4121	0.4196	0.6918	0.6710	0.0133	0.8167
NM_001037209	Mterfd2	0.2950	0.1476	0.5420	0.0459	0.3567	0.1643	0.0845	0.0929	0.0651	0.3000	0.1120	0.5388
NM_001037211	LOC367830	0.2886	0.2749	0.3169	0.9062	0.1199	0.4361	0.3251	0.4518	0.5941	0.7606	0.7137	0.8222
NM_001037212	Zfp94	0.5288	0.3082	0.3386	0.0085	0.0838	0.3749	0.1353	0.4116	0.8074	0.1690	0.1184	0.1315
NM_001037214	Cdca4	0.9989	0.9640	0.6716	0.9157	0.9858	0.9640	0.9587	0.9887	0.9850	0.9711	0.9529	0.9875
NM_001037215	Dsm-1	0.2512	0.3076	0.1578	0.6768	0.3597	0.2541	0.8527	0.4567	0.2333	0.1453	0.1169	0.2235
NM_012754	Esr2	0.1082	0.1323	0.5116	0.1436	0.3468	0.1123	0.1279	0.0300	0.1860	0.3154	0.0627	0.1313
NM_012845	Ms4a2	0.4855	0.8036	0.5475	0.5436	0.7949	0.5457	0.9256	0.8052	0.8667	0.7315	0.6946	0.5530
NM_012838	Cstb	0.0739	0.5023	0.2542	0.1053	0.5602	0.4260	0.0438	0.6940	0.5828	0.3549	0.0801	0.7136
NM_012703	Thrsp	0.0803	0.8434	0.5661	0.6125	0.8584	0.8420	0.8900	0.5222	0.8447	0.3509	0.5249	0.6829
NM_012680	Tsc2	0.7166	0.5612	0.1848	0.6651	0.7393	0.7818	0.4965	0.8089	0.9333	0.7423	0.8678	0.8189
NM_020301	Adam7	0.9317	0.9030	0.8449	0.5251	0.7174	0.7201	0.8420	0.9541	0.9154	0.8905	0.8675	0.6017
NM_013104	Igfbp6	0.8085	0.0364	0.0577	0.0990	0.8583	0.9413	0.0269	0.7704	0.0403	0.0483	0.0175	0.0171
NM_012994	Nxph1	0.8353	0.6074	0.8394	0.8183	0.8155	0.7698	0.1564	0.6441	0.3644	0.9214	0.7489	0.0584
NM_001114180	Plek2	0.7666	0.9661	0.9933	0.9504	0.9749	0.8937	0.9765	0.8042	0.9890	0.9964	0.8128	0.8717
NM_001037220	Kiaa0415	0.9300	0.6382	0.1044	0.1893	0.5129	0.5168	0.2664	0.1937	0.9153	0.0862	0.5378	0.8714
NM_013094	Plin	0.2083	0.2498	0.8492	0.6353	0.7714	0.3172	0.3298	0.5619	0.2986	0.5634	0.4233	0.4137
NM_017066	Ptn	0.8710	0.2317	0.6247	0.5900	0.1258	0.1349	0.3116	0.5071	0.7451	0.6830	0.4815	0.5588
NM_012995	Ocm	0.1302	0.0454	0.0213	0.0788	0.0055	0.0613	0.0726	0.0463	0.0566	0.0127	0.0371	0.5375
NM_139326	Pomc	0.1841	0.1427	0.1712	0.0280	0.5886	0.5281	0.1723	0.2512	0.0795	0.0923	0.1505	0.4674
NM_139254	Tubb3	0.1663	0.1548	0.1283	0.6154	0.1254	0.6261	0.3031	0.2708	0.3889	0.7223	0.5934	0.9028
NM_017193	Aadat	0.0748	0.0220	0.0175	0.0186	0.0038	0.0314	0.0172	0.0836	0.0350	0.0144	0.2791	0.0513
NM_017083	Myo5b	0.5645	0.5797	0.5328	0.1745	0.0424	0.2061	0.8132	0.4003	0.7304	0.7858	0.5281	0.6744
NM_017084	Gnmt	0.0410	0.1337	0.2328	0.1072	0.0409	0.0093	0.5400	0.1304	0.1778	0.2455	0.1533	0.0444
NM_139194	Fas	0.6424	0.5389	0.9962	0.9693	0.9399	0.7283	0.7972	0.9138	0.9081	0.4861	0.5899	0.7559
NM_001037337	Pcdhga12	0.4060	0.6928	0.2094	0.4938	0.5828	0.3884	0.4960	0.6204	0.3474	0.6849	0.2552	0.8801
NM_013189	Gnaz	0.6512	0.4645	0.5347	0.7161	0.7101	0.8176	0.7353	0.1599	0.7290	0.8769	0.1430	0.6037
NM_001044770	Cyp4a2	0.6107	0.4044	0.2664	0.2973	0.4102	0.4479	0.2998	0.2719	0.4998	0.2205	0.2521	0.3823
NM_001080380	Tmem30b	0.7177	0.4567	0.7056	0.6665	0.5152	0.3132	0.2808	0.2213	0.4045	0.2381	0.7875	0.5488
NM_013185	Hck	0.7774	0.7047	0.5508	0.6106	0.8283	0.8459	0.6297	0.3472	0.6212	0.4913	0.6803	0.8708
NM_012884	Cntn2	0.7205	0.9017	0.7832	0.6073	0.7494	0.8698	0.9217	0.7612	0.7487	0.9648	0.7686	0.6272
NM_001037354	Tcfcp2l2	0.3147	0.3260	0.7154	0.3648	0.2269	0.2484	0.2092	0.1539	0.4045	0.1025	0.7487	0.4727
NM_012848	Fth1	0.1514	0.0482	0.1335	0.2660	0.0578	0.0367	0.0098	0.0111	0.0726	0.0860	0.0304	0.0435
NM_017320	Ctss	0.4955	0.5598	0.4757	0.2399	0.3734	0.3421	0.0478	0.2763	0.1837	0.3781	0.1758	0.1587
NM_017155	Adora1	0.4628	0.1965	0.2825	0.3605	0.0121	0.0342	0.3423	0.1148	0.2266	0.3707	0.2189	0.2245
NM_012522	Cbs	0.7959	0.7034	0.2644	0.9042	0.7049	0.4630	0.9616	0.5247	0.2708	0.2237	0.6015	0.6009
NM_001037363	Lrrn1	0.4212	0.4240	0.4237	0.3201	0.4158	0.4165	0.4744	0.3386	0.2271	0.5728	0.4333	0.1410
NM_001037364	LOC500990	0.8130	0.6647	0.8092	0.4199	0.4554	0.8362	0.5097	0.2417	0.3784	0.5898	0.6308	0.6915
NM_012571	Got1	0.1878	0.6304	0.7081	0.9795	0.1192	0.2179	0.3649	0.0318	0.6591	0.3268	0.8745	0.7548
NM_012545	Ddc	0.1398	0.3270	0.2961	0.3414	0.0644	0.6972	0.1532	0.3640	0.5974	0.4157	0.7042	0.2458

NM_001037368	Defb29	0.3842	0.4708	0.4579	0.4753	0.3799	0.3836	0.2157	0.3403	0.3522	0.4326	0.1889	0.5915
NM_053788	Stx1a	0.8917	0.7210	0.4909	0.4664	0.4474	0.7235	0.5687	0.7441	0.8274	0.8387	0.7883	0.8857
NM_053677	Chek2	0.7181	0.7571	0.0693	0.6579	0.7350	0.8547	0.7170	0.7386	0.7017	0.7500	0.8730	0.1265
NM_001037508	Defb24	0.1866	0.3887	0.3178	0.4106	0.3399	0.2900	0.2155	0.6580	0.3669	0.3298	0.3271	0.2988
NM_001037515	Defb36	0.3150	0.1890	0.2124	0.5189	0.2503	0.4331	0.2153	0.3878	0.3998	0.1762	0.5104	0.2560
NM_001037519	Defb27	0.4567	0.2961	0.4463	0.4266	0.4233	0.3460	0.2152	0.4066	0.4596	0.4866	0.4791	0.3951
NM_001037523	Defb33	0.2881	0.3218	0.3567	0.2105	0.1918	0.4036	0.3795	0.2051	0.1807	0.4659	0.4340	0.1937
NM_001037524	Defb52	0.5510	0.2977	0.2929	0.0946	0.6039	0.5428	0.2879	0.0652	0.2738	0.1045	0.1835	0.0890
NM_012999	Pcsk6	0.9900	0.9858	0.9245	0.8133	0.9886	0.9583	0.9809	0.9960	0.9389	0.8969	0.8918	0.9784
NM_021753	Pga5	0.1139	0.1231	0.4696	0.1824	0.0820	0.2848	0.4632	0.3156	0.1117	0.5704	0.0852	0.0954
NM_001037546	Fgd1	0.2449	0.3266	0.3724	0.3301	0.5150	0.3709	0.4720	0.6250	0.6367	0.5451	0.3776	0.2191
NM_001037547	Defb51	0.2253	0.4429	0.2407	0.4807	0.4949	0.2123	0.2934	0.5382	0.2153	0.5532	0.4340	0.1935
NM_012647	Scn2a1	0.9297	0.9690	0.9572	0.9418	0.8970	0.9761	0.9901	0.8555	0.9754	0.9738	0.9471	0.9406
NM_013110	Il7	0.4389	0.6397	0.2336	0.2512	0.4856	0.4282	0.2531	0.4007	0.1914	0.3317	0.4938	0.2115
NM_001037645	Rab2b	0.3502	0.0714	0.8528	0.9685	0.8169	0.8376	0.7048	0.8221	0.3373	0.1781	0.2301	0.5064
NM_013173	Slc11a2	0.9851	0.9996	0.0835	0.9836	0.9751	0.9641	0.8708	0.8481	0.9325	0.9937	0.9989	0.3065
NM_001037647	Unc45a	0.8653	0.4659	0.3660	0.1793	0.7977	0.6270	0.3955	0.6062	0.4557	0.8411	0.6306	0.4028
NM_012883	Sult1e1	0.3032	0.2559	0.4517	0.2935	0.4751	0.3309	0.3836	0.3891	0.2770	0.2428	0.3934	0.3755
NM_012893	Actg2	0.5329	0.6114	0.6575	0.6724	0.0696	0.0269	0.7541	0.4842	0.8552	0.7214	0.4820	0.9007
NM_017171	Prkce	0.6373	0.6453	0.7273	0.6997	0.7659	0.6221	0.7769	0.6455	0.6847	0.3316	0.6930	0.9203
NM_001037652	Zdhhc6	0.4190	0.1896	0.7013	0.6083	0.1767	0.5166	0.6294	0.2435	0.6335	0.2039	0.7089	0.8546
NM_001037654	Dixdc1	0.8455	0.6557	0.8715	0.8034	0.6043	0.7979	0.4925	0.9256	0.9739	0.9147	0.9554	0.9301
NM_001037655	RGD1306230	0.6916	0.3930	0.6200	0.6746	0.5930	0.2167	0.5990	0.6501	0.5744	0.3548	0.1940	0.2824
NM_017145	Mcpt1	0.4192	0.0942	0.0506	0.3677	0.2848	0.2645	0.5255	0.2839	0.6587	0.1911	0.1238	0.1273
NM_013112	Apoa2	0.6527	0.6549	0.5260	0.2851	0.7303	0.3865	0.7665	0.8209	0.5490	0.5848	0.5890	0.7579
NM_001037767	Arpc5l	0.9189	0.8100	0.8401	0.8541	0.9459	0.8447	0.7558	0.7058	0.9414	0.9266	0.6628	0.4206
NM_001037769	RGD1304719	0.0525	0.1331	0.8509	0.7502	0.0508	0.1413	0.5183	0.1254	0.4956	0.3960	0.2082	0.0275
NM_001037772	LOC300963	0.9117	0.8157	0.8674	0.8887	0.7264	0.7272	0.8599	0.8074	0.7699	0.8816	0.8465	0.8239
NM_001100564	Lin54	0.4420	0.9964	0.1588	0.4164	0.8518	0.8197	0.7796	0.8225	0.9376	0.9569	0.9668	0.1595
NM_013114	Selp	0.0537	0.7305	0.7896	0.4463	0.1165	0.5153	0.1160	0.0977	0.3292	0.0661	0.1093	0.5003
NM_001037778	Dctn5	0.1498	0.0860	0.9197	0.7546	0.0842	0.8591	0.3305	0.0963	0.0406	0.1337	0.1802	0.0815
NM_001106343	Zfp275	0.5037	0.5046	0.5020	0.4880	0.3168	0.4623	0.2567	0.6046	0.6885	0.4085	0.3268	0.6888
NM_001107344	Mylip	0.7329	0.2204	0.9267	0.2150	0.6563	0.8575	0.8074	0.3811	0.1592	0.3575	0.5794	0.9898
NM_001107324	Atp11a	0.6684	0.9455	0.1048	0.8976	0.4766	0.6837	0.8300	0.3664	0.7829	0.5553	0.5875	0.3908
NM_001037784	Vsig1	0.0762	0.1103	0.2736	0.0322	0.2686	0.4482	0.3037	0.1253	0.5777	0.0431	0.2422	0.3068
NM_013118	Guca2a	0.5995	0.3974	0.4220	0.1277	0.6087	0.6421	0.5837	0.7256	0.5271	0.6529	0.1371	0.5454
NM_017020	Il6ra	0.4825	0.3620	0.3618	0.8261	0.3408	0.5825	0.2756	0.8158	0.3384	0.1159	0.1302	0.9426
NM_017023	Kcnj1	0.1007	0.5802	0.4445	0.4206	0.1760	0.5136	0.2707	0.2203	0.1008	0.3257	0.7009	0.0503
NM_001037794	MGC125233	0.0282	0.0506	0.2701	0.1883	0.2303	0.3037	0.1021	0.0231	0.1537	0.1507	0.5053	0.0154
NM_001039021	Zdhhc8	0.4552	0.5147	0.5155	0.4427	0.1722	0.6961	0.0529	0.3867	0.6756	0.4642	0.2587	0.9135
NM_001037797	LOC500354	0.8528	0.7427	0.6296	0.6902	0.8735	0.5724	0.2817	0.5001	0.5584	0.5573	0.6624	0.5791
NM_001037973	Dusp9	0.7880	0.5229	0.4039	0.3099	0.2627	0.3491	0.6831	0.3152	0.5027	0.2797	0.1425	0.7877
NM_013122	Igfbp2	0.4068	0.3090	0.3600	0.3273	0.2751	0.1184	0.0865	0.3949	0.4598	0.9051	0.3059	0.4485
NM_012546	Drd1a	0.9338	0.5905	0.8012	0.8598	0.7444	0.8696	0.7203	0.1808	0.5142	0.6623	0.6515	0.2163
NM_012517	Cacna1c	0.9674	0.9678	0.4919	0.6401	0.9015	0.9773	0.9402	0.9874	0.9995	0.9635	0.9915	0.9625
NM_001037978	Gtl3	0.1633	0.5165	0.1613	0.3255	0.5755	0.2199	0.3647	0.1046	0.1458	0.1224	0.2933	0.1160
NM_019333	Pfkfb4	0.2594	0.1846	0.1898	0.1858	0.0362	0.8388	0.0611	0.3538	0.0891	0.5794	0.4779	0.4435
NM_017100	Plk1	0.9635	0.9205	0.0665	0.9508	0.9746	0.9111	0.9424	0.9172	0.8749	0.7973	0.8795	0.0359
NM_021859	Matk	0.8062	0.6025	0.4921	0.7883	0.6215	0.5223	0.9572	0.8288	0.8353	0.8813	0.8986	0.1524

NM_021838	Nos3	0.0238	0.0744	0.0080	0.2652	0.0938	0.1311	0.0609	0.2356	0.1643	0.1086	0.2031	0.0999
NM_017024	Lcat	0.7348	0.9357	0.0043	0.0649	0.7456	0.8762	0.0084	0.7651	0.7426	0.7508	0.9042	0.7115
NM_001038992	Ccdc23	0.4443	0.3982	0.5095	0.4022	0.3156	0.4713	0.5602	0.2916	0.2768	0.2516	0.3969	0.2293
NM_001038994	Ccdc23	0.4443	0.3982	0.5095	0.4022	0.3156	0.4713	0.5602	0.2916	0.2768	0.2516	0.3969	0.2293
NM_017070	Srd5a1	0.7247	0.0465	0.9035	0.6857	0.5259	0.4612	0.9409	0.5779	0.8005	0.7395	0.5685	0.7097
NM_017051	Sod2	0.2181	0.2185	0.1077	0.2379	0.0762	0.1202	0.0695	0.0467	0.1239	0.0154	0.2295	0.1680
NM_017001	Epo	0.8473	0.6538	0.6806	0.7299	0.5293	0.7350	0.7717	0.7194	0.9012	0.6392	0.2002	0.7506
NM_001108011	Asxl2	0.8904	0.4573	0.9524	0.2228	0.8266	0.9053	0.3877	0.6994	0.7313	0.2974	0.4475	0.9213
NM_001107863	Ppm1k	0.4296	0.2421	0.4816	0.5132	0.1669	0.2643	0.5550	0.5335	0.5506	0.3161	0.3329	0.1120
NM_001039006	Klhl25	0.5083	0.3465	0.3978	0.5724	0.2207	0.5779	0.3989	0.6323	0.1159	0.0288	0.6265	0.5852
NM_001039100	Zdhhc24	0.3376	0.8467	0.7153	0.7297	0.7033	0.5928	0.8223	0.4798	0.6210	0.0853	0.4070	0.7034
NM_013132	Anxa5	0.0141	0.0678	0.7697	0.9023	0.6068	0.6129	0.0407	0.9291	0.7696	0.0552	0.4728	0.0803
NM_001039015	RGD1310230	0.9714	0.7848	0.9142	0.1697	0.9416	0.7882	0.1464	0.9828	0.5967	0.9172	0.9714	0.9440
NM_001039336	Zdhhc20	0.7798	0.5337	0.9175	0.6344	0.0322	0.8353	0.0675	0.0734	0.3840	0.8285	0.5435	0.7657
NM_183326	Gabra1	0.9132	0.5425	0.7103	0.8500	0.8622	0.6918	0.5179	0.9374	0.4239	0.3421	0.6007	0.7017
NM_017091	Pcsk1	0.5693	0.5223	0.5043	0.4323	0.5483	0.6897	0.6854	0.4388	0.6016	0.7468	0.7634	0.6453
NM_001039338	Zdhhc5	0.2235	0.2305	0.3018	0.2102	0.5381	0.2376	0.5433	0.4172	0.3665	0.3974	0.3802	0.7006
NM_017136	Sqle	0.8004	0.8612	0.0526	0.1146	0.8455	0.8874	0.9100	0.8671	0.7660	0.7594	0.9631	0.0493
NM_001039026	Rbaf600	0.8026	0.4820	0.3252	0.1328	0.9302	0.6888	0.7566	0.8963	0.7447	0.8740	0.9190	0.9574
NM_001039027	Wdr18	0.8495	0.6470	0.5844	0.5869	0.8310	0.6251	0.2828	0.5224	0.3619	0.8168	0.7826	0.4151
NM_017290	Atp2a2	0.7318	0.7335	0.6491	0.7287	0.6940	0.7190	0.5783	0.6974	0.6391	0.7068	0.7151	0.7777
NM_017277	Ap1b1	0.6324	0.9871	0.1491	0.8603	0.8350	0.5582	0.9397	0.9593	0.9170	0.4868	0.6421	0.2295
NM_016991	Adra1b	0.0599	0.2142	0.3406	0.6394	0.3197	0.2795	0.2455	0.3448	0.1617	0.6395	0.2384	0.9528
NM_012861	Mgmt	0.2659	0.6492	0.2158	0.0831	0.3610	0.5885	0.4060	0.2986	0.0453	0.4176	0.1641	0.4696
NM_001039032	Lemd2	0.3049	0.4830	0.8311	0.1367	0.1266	0.8041	0.6247	0.4232	0.6741	0.3021	0.8533	0.9791
NM_017071	Insr	0.3282	0.8049	0.7237	0.8745	0.6948	0.5938	0.7563	0.3578	0.3314	0.3950	0.7231	0.5665
NM_013139	Clps	0.0582	0.6664	0.2492	0.5428	0.7153	0.2533	0.5449	0.0553	0.5033	0.5916	0.6204	0.2441
NM_017044	Pth	0.8030	0.7395	0.6863	0.7499	0.5432	0.7941	0.3829	0.7317	0.5186	0.8707	0.9658	0.3215
NM_001039036	Gabpb1	0.7295	0.6958	0.7100	0.7310	0.4027	0.5431	0.5050	0.6583	0.6414	0.6975	0.5304	0.6945
NM_001039044	Cdc42se1	0.2870	0.4401	0.1893	0.9628	0.3787	0.3419	0.7486	0.4513	0.3776	0.4495	0.5998	0.1894
NM_012853	Htr4	0.5806	0.5153	0.4984	0.5882	0.5530	0.5164	0.5123	0.8099	0.5299	0.5430	0.3166	0.3969
NM_012900	Ambn	0.3074	0.3932	0.8781	0.2786	0.1333	0.2709	0.7983	0.5768	0.6728	0.7022	0.7892	0.6725
NM_016998	Cpa1	0.2787	0.3844	0.4302	0.3421	0.3213	0.0384	0.1577	0.1392	0.5014	0.0728	0.4999	0.7908
NM_012519	Camk2d	0.8461	0.3384	0.7049	0.7331	0.9778	0.9346	0.6186	0.7936	0.3690	0.2702	0.2122	0.8242
NM_001039101	Zdhhc15	0.7945	0.4233	0.7418	0.4757	0.3810	0.8631	0.5010	0.5946	0.5689	0.3720	0.6085	0.9260
NM_001039163	Tusc5	0.1942	0.1260	0.2789	0.2192	0.6764	0.4396	0.6411	0.3925	0.6825	0.1636	0.5565	0.5857
NM_001039174	LOC654482	0.6329	0.2780	0.3821	0.6265	0.4440	0.3599	0.3921	0.4284	0.2044	0.3323	0.2776	0.2149
NM_013144	Igfbp1	0.3506	0.8092	0.6814	0.4255	0.2937	0.0901	0.6077	0.3574	0.9877	0.7353	0.6965	0.3626
NM_017226	Padi2	0.7651	0.9276	0.3351	0.6815	0.5203	0.8346	0.8638	0.8462	0.6246	0.9466	0.6149	0.8962
NM_001039207	Narf	0.5591	0.4249	0.9583	0.8416	0.2961	0.9565	0.4439	0.1670	0.2990	0.3585	0.6886	0.5650
NM_001039325	Zdhhc22	0.7604	0.4933	0.4729	0.4788	0.4175	0.2676	0.3759	0.7715	0.6205	0.4828	0.5627	0.5261
NM_001039337	Fubp3	0.7934	0.7564	0.8737	0.3959	0.7979	0.7030	0.6406	0.6589	0.8446	0.9842	0.8967	0.1389
NM_001039339	Zdhhc18	0.8745	0.8032	0.7036	0.4256	0.8772	0.3898	0.8641	0.7107	0.8780	0.7428	0.4743	0.8453
NM_013130	Smad1	0.6374	0.1644	0.7762	0.3161	0.2621	0.6236	0.6523	0.7713	0.8572	0.8460	0.2680	0.6404
NM_001039341	LOC497978	0.8615	0.9282	0.9753	0.7816	0.8479	0.9211	0.9140	0.9373	0.8594	0.9686	0.9340	0.8698
NM_012949	Eno3	0.7368	0.7488	0.0045	0.7638	0.7433	0.8085	0.8714	0.7663	0.7758	0.7538	0.7449	0.1187
NM_012987	Nes	0.3954	0.2035	0.7972	0.6297	0.6490	0.7013	0.1711	0.9073	0.4176	0.0414	0.6444	0.5974
NM_013146	Cald1	0.0733	0.1428	0.9743	0.9017	0.2470	0.9100	0.9442	0.2452	0.3203	0.2668	0.2006	0.7885
NM_001039378	Trappc1	0.0089	0.0255	0.6276	0.8762	0.5770	0.0787	0.7236	0.8359	0.3610	0.3965	0.1152	0.1334

NM_001039393	Crisp4	0.4047	0.3538	0.3971	0.2611	0.5006	0.3918	0.3515	0.2870	0.1986	0.2113	0.7268	0.4269
NM_017008	Gapdh	0.4370	0.2523	0.4255	0.2101	0.2974	0.5378	0.4433	0.5515	0.4212	0.6680	0.3510	0.5495
NM_012894	Adarb1	0.2532	0.9282	0.7303	0.2920	0.4561	0.6341	0.6847	0.5770	0.6461	0.9299	0.3428	0.7781
NM_175758	Slc1a5	0.1184	0.7182	0.2084	0.2701	0.3809	0.1259	0.2980	0.1411	0.8997	0.8554	0.7938	0.6735
NM_001044288	Kti12	0.3390	0.5298	0.3305	0.4081	0.2055	0.2650	0.4298	0.7070	0.4278	0.5875	0.2708	0.4355
NM_198745	Atp6v1e1	0.2915	0.1779	0.5964	0.2308	0.1203	0.0722	0.1792	0.2221	0.0143	0.2479	0.0172	0.1489
NM_001039607	LOC294154	0.3723	0.7074	0.6613	0.7327	0.6370	0.6605	0.5822	0.7223	0.6326	0.6371	0.4934	0.7046
NM_001039608	RGD1306730	0.9179	0.8793	0.7562	0.6360	0.8770	0.7950	0.8779	0.8732	0.9794	0.8305	0.8395	0.7635
NM_198735	Art2b	0.4461	0.6999	0.4080	0.3040	0.4934	0.3929	0.5023	0.3421	0.3516	0.4000	0.3595	0.3093
NM_175754	Agrn	0.9867	0.2431	0.5714	0.0352	0.9353	0.9495	0.9500	0.9598	0.4236	0.4561	0.2686	0.8343
NM_001039612	RGD1306626	0.1345	0.3666	0.1215	0.0845	0.1636	0.4270	0.6805	0.4976	0.3713	0.4880	0.2818	0.4553
NM_001034926	Lrrc40	0.0433	0.5000	0.3381	0.2734	0.1021	0.6067	0.4025	0.0782	0.3912	0.0533	0.3284	0.6809
NM_001044231	Sbsn	0.7554	0.5781	0.8379	0.8228	0.8466	0.8776	0.7057	0.8958	0.8536	0.8842	0.2027	0.7355
NM_001044230	Lass1	0.2191	0.5751	0.5857	0.2308	0.7302	0.6828	0.7089	0.7305	0.6292	0.6086	0.5740	0.6505
NM_001039722	Slc6a19	0.6562	0.5018	0.6274	0.5336	0.3492	0.1972	0.2730	0.2550	0.5056	0.2042	0.2646	0.1932
NM_001047101	Adamts7	0.0285	0.0464	0.0019	0.0450	0.0939	0.0737	0.0099	0.0433	0.0933	0.0529	0.0930	0.5061
NM_001039587	Wdr45l	0.9394	0.0841	0.7947	0.0955	0.2863	0.7945	0.3811	0.0742	0.2105	0.0686	0.1049	0.8711
NM_001080207	Znf652	0.4297	0.6412	0.7607	0.6012	0.4978	0.6348	0.7979	0.6780	0.4306	0.6252	0.7384	0.4733
NM_001047878	F5	0.0326	0.0820	0.0842	0.0458	0.0007	0.0022	0.0637	0.0039	0.0263	0.0582	0.0255	0.0700
NM_001044300	MGC112715	0.4706	0.2041	0.7178	0.3422	0.4270	0.4204	0.5699	0.5332	0.4298	0.1928	0.2156	0.4734
NM_178096	Nrep	0.5403	0.5564	0.6282	0.8125	0.4762	0.2270	0.9766	0.5269	0.5945	0.6330	0.8363	0.6048
NM_001040156	Aste1	0.8980	0.6972	0.4249	0.2454	0.0821	0.1155	0.1236	0.8309	0.6278	0.8380	0.9096	0.6148
NM_181388	Spg7	0.0452	0.5238	0.0921	0.1377	0.0693	0.0479	0.2835	0.0185	0.1111	0.1254	0.2567	0.5311
NM_001045843	P2ry5	0.2951	0.2798	0.4495	0.3336	0.2522	0.4844	0.5563	0.4669	0.3524	0.3789	0.6148	0.2415
NM_181550	Sqstm1	0.6568	0.4590	0.4052	0.5100	0.6676	0.5003	0.3327	0.5608	0.4362	0.2988	0.5647	0.2566
NM_181479	Kir3dl1	0.6401	0.3425	0.6738	0.6534	0.6377	0.6026	0.6370	0.4128	0.3299	0.6055	0.5977	0.5073
NM_181628	Tsg101	0.5975	0.6594	0.3421	0.4118	0.8278	0.7057	0.1002	0.0744	0.3094	0.4087	0.6823	0.6102
NM_001077674	Hlx	0.3706	0.7645	0.6356	0.5783	0.0527	0.0558	0.2943	0.1554	0.2910	0.3836	0.1755	0.3412
NM_001042561	Wsb1	0.6893	0.9008	0.7150	0.6168	0.7564	0.9465	0.6313	0.2352	0.9498	0.9943	0.9955	0.7572
NM_181633	Gpr151	0.6880	0.6765	0.5448	0.8552	0.5842	0.9456	0.5493	0.6885	0.6922	0.7645	0.7350	0.6414
NM_001047858	Srxn1	0.0985	0.1381	0.1700	0.6185	0.4085	0.4531	0.2699	0.4316	0.4100	0.1934	0.0368	0.2213
NM_199404	Man2b1	0.8491	0.0536	0.0098	0.0100	0.5835	0.5622	0.0198	0.8823	0.8867	0.3612	0.3817	0.5388
NM_199394	Entpd5	0.1421	0.0977	0.2257	0.1882	0.0067	0.0387	0.1811	0.1114	0.0302	0.0937	0.0364	0.1385
NM_183332	Myadm	0.9926	0.9582	0.7349	0.9966	0.9839	0.9963	0.9576	0.9505	0.9997	0.9724	0.9825	0.9864
NM_198773	Ppm1e	0.7243	0.0900	0.4741	0.1309	0.1896	0.2161	0.1992	0.6153	0.1577	0.4660	0.4032	0.3949
NM_198757	Srr	0.3870	0.9365	0.9064	0.9214	0.9122	0.8229	0.7159	0.8231	0.9044	0.9301	0.9475	0.9220
NM_001044228	LOC289740	0.1090	0.5837	0.8383	0.8605	0.2237	0.7468	0.7408	0.0624	0.3552	0.8272	0.5907	0.8093
NM_001044232	Rg9mtd2	0.0507	0.7793	0.3465	0.0848	0.0403	0.1229	0.5929	0.0473	0.5071	0.5854	0.8463	0.2018
NM_001044235	RGD1310899	0.0596	0.1763	0.2214	0.1061	0.0708	0.0112	0.1003	0.0625	0.1245	0.0368	0.0736	0.0736
NM_001044237	Mcts1	0.1292	0.4870	0.4079	0.0944	0.0497	0.0477	0.1215	0.0508	0.0115	0.5316	0.1527	0.0031
NM_184047	Il18rap	0.4699	0.8462	0.5582	0.7587	0.5300	0.4191	0.6975	0.5339	0.3082	0.2895	0.7406	0.6621
NM_184046	Rtkn	0.8344	0.7256	0.6501	0.8643	0.7608	0.7045	0.8536	0.8893	0.8647	0.9723	0.8546	0.9247
NM_001044238	Gdpd1	0.0466	0.0438	0.8202	0.9467	0.8546	0.9147	0.2057	0.9464	0.1704	0.0151	0.1867	0.9344
NM_001037790	Leng8	0.7654	0.7373	0.7671	0.7670	0.8255	0.8292	0.8208	0.9553	0.8190	0.7869	0.7584	0.7384
NM_001044240	Gdf1	0.4713	0.1761	0.1366	0.4025	0.5519	0.3417	0.1247	0.4639	0.3633	0.1488	0.3893	0.1316
NM_001081751	Pou5f2	0.6476	0.4837	0.7249	0.5435	0.2207	0.0399	0.1637	0.7367	0.4067	0.7784	0.9242	0.6163
NM_184051	Prkag2	0.1686	0.9301	0.2627	0.7165	0.3058	0.2728	0.2283	0.0697	0.9764	0.9804	0.9486	0.9471
NM_199208	Rdh2	0.4368	0.4117	0.4830	0.5508	0.1724	0.6211	0.4073	0.4931	0.0870	0.6851	0.2698	0.2213
NM_198771	Fam3c	0.8517	0.9878	0.9454	0.9893	0.9151	0.8011	0.8844	0.7786	0.9151	0.8910	0.8190	0.9793

NM_199119	Ddx24	0.4687	0.4074	0.4138	0.6798	0.0473	0.2852	0.2848	0.1385	0.6440	0.6044	0.4196	0.8408
NM_001048042	Nmnat2	0.1465	0.3045	0.4491	0.1194	0.3162	0.4805	0.2726	0.1143	0.5526	0.1601	0.1548	0.3311
NM_001077434	Lrrc52	0.5897	0.4826	0.1651	0.8263	0.6960	0.1696	0.3818	0.6746	0.4437	0.7709	0.1851	0.7921
NM_001048243	Ubl5	0.8667	0.7879	0.6968	0.6389	0.7815	0.9114	0.6806	0.6861	0.6008	0.8104	0.9112	0.9155
NM_001044258	LOC498940	0.1127	0.2926	0.6010	0.4554	0.2533	0.2742	0.2543	0.6423	0.2382	0.4307	0.2023	0.1200
NM_001085369	Kif2c	0.6964	0.8685	0.2184	0.7753	0.8635	0.9981	0.8439	0.9330	0.7084	0.9122	0.7554	0.4902
NM_001077435	Bex2	0.4887	0.4120	0.6536	0.6624	0.3375	0.0682	0.1481	0.5160	0.4758	0.4934	0.1275	0.1699
NM_001044266	Trim24	0.4236	0.5741	0.2684	0.6313	0.2431	0.4026	0.5833	0.0289	0.2515	0.3469	0.3015	0.6313
NM_001044252	RGD1306809	0.9599	0.9667	0.9431	0.9829	0.9616	0.9658	0.9846	0.7670	0.9947	0.9332	0.9693	0.8897
NM_001100970	Aebp1	0.0426	0.0132	0.0242	0.1035	0.0165	0.1149	0.0923	0.1418	0.1246	0.0375	0.0427	0.0544
NM_001044270	Tuba1b	0.4849	0.3079	0.3011	0.4735	0.5708	0.3311	0.4582	0.5215	0.3944	0.4189	0.3500	0.3502
NM_201989	Xirp2	0.5442	0.7081	0.6930	0.5621	0.6367	0.5313	0.7338	0.4778	0.6462	0.6449	0.6383	0.6740
NM_001044275	LOC679532	0.1866	0.1884	0.4542	0.2099	0.2912	0.1968	0.2145	0.5661	0.4634	0.4799	0.1883	0.3137
NM_001044277	MGC94891	0.2409	0.2426	0.4931	0.3029	0.5936	0.2767	0.3345	0.4659	0.2746	0.3072	0.2895	0.2613
NM_001044278	LOC681395	0.3822	0.1883	0.4090	0.2097	0.3015	0.1966	0.4152	0.2046	0.1802	0.1759	0.3923	0.1928
NM_001044281	MGC93861	0.3126	0.4840	0.2485	0.3101	0.2778	0.2662	0.2145	0.2813	0.4889	0.4633	0.4738	0.6602
NM_001044282	MGC94725	0.5674	0.3905	0.8134	0.5249	0.4871	0.4186	0.3562	0.1286	0.7400	0.4607	0.6866	0.4377
NM_001044283	LOC684097	0.7168	0.3251	0.7219	0.7037	0.7059	0.5262	0.5273	0.4695	0.1941	0.6735	0.3816	0.4066
NM_001082574	Fastkd3	0.8579	0.4905	0.2576	0.0875	0.7007	0.8156	0.5280	0.8450	0.1207	0.3713	0.4314	0.4468
NM_001044289	LOC686841	0.4639	0.1881	0.3955	0.2096	0.1916	0.1965	0.2143	0.2045	0.4580	0.3328	0.5191	0.1927
NM_001044292	MGC116202	0.1865	0.3540	0.1982	0.4359	0.1914	0.1963	0.3842	0.5417	0.1800	0.1757	0.4660	0.1926
NM_001082549	Spint2	0.3717	0.2814	0.6606	0.4478	0.3185	0.4341	0.2820	0.4393	0.2426	0.2649	0.2805	0.6526
NM_001044297	LOC689907	0.4477	0.4306	0.3585	0.2094	0.3012	0.3443	0.2142	0.4155	0.2384	0.5479	0.3019	0.6861
NM_001044299	LOC690784	0.5095	0.4760	0.2166	0.3203	0.6338	0.5390	0.2513	0.4838	0.5006	0.5824	0.2435	0.3802
NM_001044303	LOC691416	0.1864	0.3199	0.1981	0.2094	0.2648	0.1963	0.2141	0.2044	0.3630	0.1756	0.4841	0.2781
NM_001083624	Pds5a	0.1863	0.1879	0.1981	0.2091	0.1913	0.1962	0.2140	0.2043	0.4654	0.1755	0.1882	0.1926
NM_001044268	RGD1563444	0.3402	0.2488	0.5523	0.4265	0.5485	0.3827	0.3858	0.4367	0.3219	0.3101	0.4345	0.2678
NM_001044702	Prl5a2	0.3066	0.4658	0.3469	0.3557	0.7011	0.1961	0.5426	0.2043	0.2361	0.3060	0.3349	0.3045
NM_001097582	Tlr7	0.6803	0.7915	0.8455	0.7709	0.7568	0.6282	0.5684	0.8130	0.6617	0.6244	0.8411	0.7017
NM_001098667	Zbtb24	0.7705	0.6801	0.7028	0.8601	0.8168	0.7518	0.8468	0.4890	0.6785	0.9413	0.3351	0.8113
NM_001108569	Gbp5	0.7235	0.6654	0.3591	0.8819	0.8851	0.5161	0.3176	0.5130	0.8925	0.6338	0.4600	0.5547
NM_001039610	Ube2cbp	0.7509	0.2452	0.8093	0.7517	0.6560	0.6128	0.6560	0.6244	0.2125	0.4753	0.6391	0.2155
NM_199237	Fshr	0.6191	0.7650	0.9055	0.4152	0.0839	0.6150	0.8391	0.7673	0.6348	0.5575	0.4403	0.4982
NM_199207	Fam21c	0.4028	0.4022	0.2609	0.3669	0.3470	0.4555	0.4061	0.4681	0.6670	0.5765	0.4686	0.4476
NM_199110	Mfng	0.7872	0.7769	0.4666	0.8300	0.7398	0.7768	0.8348	0.7354	0.5645	0.7511	0.6733	0.5986
NM_001047093	Klhl5	0.8697	0.7102	0.9487	0.3348	0.8190	0.8385	0.7560	0.2146	0.8980	0.4640	0.8114	0.8780
NM_201415	Rpl17	0.2011	0.6326	0.4050	0.2440	0.1504	0.1674	0.5537	0.1499	0.1435	0.3063	0.3956	0.3847
NM_001080783	Hisppd2a	0.9943	0.9115	0.9553	0.2492	0.3660	0.8770	0.8016	0.7173	0.9332	0.9875	0.8129	0.9871
NM_001047088	Gpr120	0.1968	0.4695	0.6619	0.2953	0.0748	0.3894	0.2195	0.4300	0.1531	0.4159	0.3289	0.2422
NM_001100922	Prkaca	0.1580	0.4486	0.2778	0.8803	0.6634	0.6521	0.1218	0.6306	0.6563	0.4926	0.2984	0.8046
NM_201426	Adm2	0.8981	0.0711	0.0935	0.1467	0.3788	0.3984	0.1269	0.9341	0.0329	0.0602	0.0807	0.0732
NM_199111	Nfkbie	0.4266	0.3743	0.4754	0.3842	0.5268	0.7758	0.8344	0.4469	0.6729	0.5596	0.3454	0.3771
NM_199109	Flvcr2	0.4253	0.9262	0.8242	0.6680	0.9263	0.6291	0.8537	0.9492	0.7954	0.8959	0.6795	0.8661
NM_201420	Tcfap2c	0.0345	0.3938	0.1810	0.5743	0.1101	0.1330	0.5639	0.5495	0.2295	0.0378	0.3926	0.5292
NM_001039665	Sult2b1	0.5315	0.1095	0.0746	0.1214	0.2556	0.1663	0.2039	0.2738	0.1036	0.2676	0.2554	0.1362
NM_001047104	LOC361128	0.0675	0.2288	0.3457	0.8722	0.7076	0.9638	0.6702	0.6288	0.2656	0.2023	0.1225	0.9458
NM_199114	Fgfrl1	0.2306	0.0054	0.8038	0.1463	0.1734	0.1119	0.0169	0.2982	0.2135	0.0636	0.0195	0.0892
NM_201560	RGD1303142	0.1934	0.6021	0.7252	0.2985	0.1352	0.0908	0.2655	0.2010	0.2633	0.7298	0.5306	0.1275
NM_001047108	Lbx1	0.0284	0.1146	0.0351	0.0999	0.1796	0.1046	0.0882	0.3018	0.1219	0.0123	0.0832	0.4716

NM_001047110	LOC499770	0.9107	0.7598	0.1635	0.9387	0.9347	0.8162	0.8755	0.9791	0.8934	0.9918	0.6143	0.8584
NM_001047111	RGD1311642	0.4404	0.6585	0.4131	0.8847	0.5305	0.1402	0.0443	0.2991	0.6153	0.6082	0.1417	0.8530
NM_001047116	MGC124740	0.3953	0.6995	0.6250	0.7099	0.5864	0.6790	0.5268	0.6521	0.5569	0.6703	0.6402	0.6924
NM_001047743	MGC112883	0.9441	0.9264	0.6488	0.9143	0.9176	0.7870	0.9808	0.6432	0.9871	0.9444	0.9836	0.6402
NM_001044241	Atl3	0.5433	0.5533	0.4370	0.3272	0.3580	0.2703	0.3824	0.4162	0.2564	0.4773	0.4257	0.3857
NM_001077666	RGD1308065	0.1755	0.3790	0.3836	0.6177	0.0222	0.3076	0.3931	0.1353	0.6648	0.2338	0.1882	0.5903
NM_199375	Fam125a	0.0267	0.1123	0.7575	0.7692	0.0456	0.2789	0.1632	0.2723	0.0965	0.3078	0.0609	0.0800
NM_201655	Tepp	0.6353	0.5575	0.6170	0.1885	0.6934	0.5215	0.5181	0.7072	0.5411	0.2324	0.0212	0.5660
NM_001044394	Ablim1	0.4651	0.5258	0.5404	0.4465	0.1163	0.4229	0.2678	0.1610	0.0753	0.2890	0.2892	0.7556
NM_001047860	RGD1306344	0.0231	0.0041	0.0497	0.1253	0.0530	0.5513	0.0158	0.0389	0.0223	0.0256	0.0069	0.5270
NM_001105726	Ptpmt1	0.6305	0.5982	0.9216	0.7486	0.3192	0.4044	0.7074	0.5405	0.0988	0.4989	0.3022	0.7908
NM_001102408	Uck2	0.3471	0.9088	0.8128	0.7895	0.6798	0.8314	0.9791	0.9581	0.9677	0.9418	0.9572	0.8944
NM_001104528	Ephb1	0.8755	0.8872	0.9723	0.4424	0.8322	0.8336	0.9757	0.8871	0.8511	0.3753	0.2265	0.6405
NM_201992	Fcar	0.9172	0.8856	0.8216	0.7015	0.6716	0.5267	0.8489	0.5540	0.6378	0.6432	0.5010	0.7607
NM_001106045	Sox7	0.6695	0.8039	0.5865	0.3138	0.4249	0.5452	0.8557	0.7007	0.3990	0.8327	0.8540	0.6865
NM_001083122	Slc30a2	0.9496	0.7608	0.8452	0.6549	0.9791	0.8194	0.3814	0.8514	0.2689	0.8070	0.7341	0.7112
NM_001105727	Psmb5	0.6649	0.8136	0.3392	0.7260	0.8648	0.4160	0.6877	0.8957	0.8610	0.7197	0.6846	0.0158
NM_001100944	RGD1562638	0.3192	0.2506	0.7452	0.2487	0.7158	0.6536	0.6934	0.8486	0.6250	0.3647	0.3285	0.8429
NM_203325	Mup5	0.3062	0.4273	0.2714	0.2089	0.6504	0.2599	0.3250	0.3753	0.5693	0.3468	0.3836	0.2352
NM_203332	LOC360395	0.6988	0.7368	0.6069	0.7545	0.2899	0.2025	0.8550	0.6318	0.2193	0.7759	0.3785	0.6528
NM_203333	Scgb2a2	0.5547	0.3934	0.5637	0.5193	0.7735	0.2682	0.4536	0.6737	0.6105	0.8758	0.5528	0.4545
NM_001105713	Prkca	0.9144	0.8652	0.6245	0.9402	0.6086	0.5828	0.9610	0.9018	0.7774	0.7385	0.9308	0.9208
NM_207592	Gpi	0.1342	0.0738	0.0465	0.8784	0.0702	0.8717	0.9475	0.1278	0.4969	0.0526	0.0037	0.9701
NM_198130	Myd88	0.3980	0.2679	0.7387	0.9631	0.6954	0.2603	0.1913	0.8395	0.2767	0.2144	0.2688	0.9897
NM_203336	Esrrg	0.7909	0.6455	0.8285	0.7697	0.7746	0.6816	0.8933	0.8858	0.5606	0.9045	0.8630	0.7615
NM_001081980	Tbc1d10c	0.2758	0.0567	0.2319	0.4917	0.4612	0.7318	0.4941	0.5199	0.1737	0.1110	0.2911	0.4994
NM_203338	Vkorc1l1	0.9896	0.9849	0.9282	0.9787	0.8619	0.6575	0.8067	0.9734	0.8850	0.9972	0.9782	0.9457
NM_001126294	RGD1559690	0.8879	0.9436	0.1104	0.4996	0.8841	0.8860	0.5693	0.7267	0.8983	0.8932	0.9469	0.3535
NM_001047895	LOC317588	0.1861	0.1876	0.1980	0.2088	0.1908	0.1957	0.2138	0.2042	0.1799	0.1752	0.1879	0.1922
NM_001099758	Pigp	0.0709	0.6170	0.7480	0.8443	0.5405	0.0274	0.4492	0.1027	0.2093	0.6037	0.7991	0.2738
NM_138856	Dnajc21	0.4939	0.7776	0.2040	0.4251	0.3230	0.0573	0.3502	0.0447	0.3638	0.6068	0.5690	0.7037
NM_001047903	LOC360933	0.0481	0.1384	0.0593	0.1029	0.0861	0.4155	0.2381	0.4909	0.0217	0.0200	0.2999	0.3683
NM_001047908	RGD1305413	0.1860	0.1874	0.1979	0.2087	0.1907	0.3429	0.2137	0.4153	0.1797	0.4090	0.1878	0.1921
NM_001100630	Ints2	0.2906	0.1396	0.0909	0.0781	0.2275	0.5020	0.0937	0.0474	0.0122	0.0050	0.3129	0.3648
NM_001010947	RGD1307789	0.7723	0.7829	0.6311	0.7116	0.5406	0.7590	0.7506	0.6765	0.5240	0.6570	0.7446	0.8494
NM_203369	Zmynd11	0.5750	0.7208	0.6194	0.7141	0.5337	0.6688	0.6691	0.6721	0.6819	0.5593	0.6878	0.6749
NM_203409	Ncam2	0.5351	0.6998	0.6372	0.6092	0.5872	0.2101	0.4341	0.2293	0.1876	0.7303	0.7060	0.6428
NM_001105719	Mip	0.6019	0.4606	0.7788	0.2871	0.6106	0.4473	0.3865	0.6978	0.3494	0.8547	0.7802	0.4371
NM_001103361	Adat3	0.3515	0.3785	0.5280	0.4275	0.3861	0.4863	0.5796	0.3646	0.2164	0.4931	0.3517	0.3071
NM_001081447	RGD1563634	0.7781	0.9251	0.5733	0.7699	0.7609	0.8399	0.9715	0.8620	0.7537	0.9541	0.8391	0.6910
NM_001047936	LOC498971	0.3448	0.5110	0.5629	0.2086	0.2783	0.2822	0.6518	0.3276	0.3466	0.2915	0.3893	0.1919
NM_001047940	LOC499469	0.1256	0.3740	0.0977	0.0243	0.0160	0.3008	0.1421	0.0272	0.1174	0.2396	0.1755	0.1228
NM_001047961	LOC500712	0.1858	0.1873	0.1978	0.2086	0.1905	0.1955	0.2134	0.2040	0.1795	0.1749	0.1876	0.1918
NM_001047963	LOC500877	0.2157	0.2788	0.0227	0.1833	0.3561	0.4427	0.3723	0.1069	0.1499	0.0353	0.1999	0.2226
NM_001047871	Ifrd2	0.9451	0.8287	0.5380	0.7728	0.8233	0.8210	0.9952	0.9931	0.9531	0.7809	0.9938	0.9854
NM_001048043	RGD1307399	0.3733	0.4118	0.3529	0.7242	0.7094	0.5619	0.4869	0.5348	0.5222	0.6088	0.5399	0.4567
NM_013188	Pygb	0.2620	0.2236	0.1592	0.0136	0.3143	0.5105	0.5329	0.2345	0.3834	0.3089	0.0220	0.6225
NM_183329	Sra1	0.0663	0.2436	0.6830	0.0420	0.0337	0.1178	0.3054	0.0395	0.2897	0.6104	0.2617	0.2288
NM_001077656	Myl1	0.2325	0.3863	0.1977	0.2084	0.4453	0.4065	0.2927	0.2873	0.5060	0.5950	0.2862	0.2622

NM_001079709	Acot5	0.4534	0.6210	0.9133	0.6713	0.8157	0.5173	0.4480	0.3124	0.4897	0.3373	0.3883	0.7120
NM_001076553	Pkib	0.7377	0.3768	0.6857	0.7063	0.4593	0.2181	0.9464	0.7141	0.7287	0.5407	0.5869	0.3041
NM_001076793	Lilrc1	0.2161	0.5262	0.3333	0.4555	0.3952	0.2466	0.5066	0.4532	0.2445	0.3571	0.4802	0.2504
NM_001048215	Kirrel3	0.2473	0.5069	0.4684	0.4853	0.5417	0.5037	0.2210	0.5677	0.4805	0.7001	0.4383	0.4574
NM_001048046	RGD1564534	0.5741	0.7098	0.5433	0.5026	0.7312	0.6263	0.2950	0.2311	0.6818	0.7189	0.5517	0.4557
NM_001105730	Rps28	0.0738	0.0745	0.1738	0.5750	0.2906	0.3423	0.4943	0.3821	0.3408	0.6567	0.2429	0.3313
NM_001077590	Bves	0.6322	0.8587	0.7663	0.4298	0.6885	0.5068	0.4018	0.7412	0.7791	0.6947	0.1522	0.9032
NM_001077592	Rpl38	0.3009	0.2112	0.6879	0.1875	0.2381	0.0892	0.1666	0.1957	0.0130	0.3671	0.6228	0.1024
NM_001077635	Acbd5	0.1856	0.1872	0.1977	0.2082	0.1902	0.7256	0.2132	0.2039	0.4803	0.3234	0.1874	0.1917
NM_212515	Nhp2l1	0.4248	0.4007	0.4800	0.5316	0.5326	0.3178	0.3956	0.3987	0.2568	0.4846	0.3423	0.4868
NM_182951	Serinc1	0.9391	0.9235	0.9838	0.8308	0.5388	0.9584	0.0246	0.5019	0.4662	0.9116	0.4332	0.8733
NM_182949	Prss27	0.7006	0.3378	0.4377	0.7174	0.5340	0.4911	0.4624	0.4988	0.0522	0.7639	0.4572	0.4039
NM_201990	Pgap1	0.0981	0.2221	0.3785	0.2982	0.0685	0.0330	0.0464	0.1431	0.0247	0.0093	0.1595	0.0958
NM_181090	Slc38a2	0.8827	0.9953	0.9947	0.9365	0.9711	0.9686	0.9998	0.9989	0.9939	0.9952	0.9962	0.9831
NM_001077648	Prdm2	0.2207	0.7151	0.3740	0.1963	0.3686	0.1401	0.7779	0.2503	0.9223	0.6156	0.4697	0.9329
NM_178847	Cyp27a1	0.4279	0.0231	0.8226	0.7148	0.3131	0.6448	0.7940	0.6917	0.1068	0.0457	0.1898	0.6629
NM_175598	Ric8b	0.6637	0.5942	0.6478	0.7489	0.6427	0.8490	0.8323	0.6906	0.2827	0.6255	0.7330	0.8588
NM_001077651	Elavl4	0.0763	0.0737	0.0701	0.0733	0.1321	0.0715	0.5090	0.5259	0.0875	0.4155	0.7296	0.1486
NM_153470	Lzts1	0.3004	0.3709	0.2713	0.6396	0.3166	0.2690	0.6799	0.0231	0.2429	0.3926	0.4150	0.2243
NM_001079700	Cdc42ep1	0.9466	0.8965	0.1992	0.6672	0.8753	0.9455	0.9273	0.8132	0.3997	0.7394	0.9977	0.3268
NM_001134603	RGD1560846	0.1145	0.1508	0.4575	0.3175	0.4303	0.2954	0.3280	0.1732	0.7250	0.1008	0.1121	0.5601
NM_001044267	Mknk1	0.1809	0.1718	0.2948	0.0217	0.2873	0.6246	0.1138	0.4442	0.0708	0.2958	0.1396	0.3571
NM_199386	Atp6v1d	0.1604	0.0399	0.8275	0.1300	0.2909	0.0396	0.0165	0.0280	0.4722	0.3137	0.3704	0.2624
NM_153467	Gsbs	0.6396	0.7056	0.5229	0.8445	0.7844	0.8231	0.8266	0.8499	0.5042	0.5529	0.5833	0.4119
NM_001044304	Eid3	0.5033	0.2833	0.2821	0.4025	0.5100	0.1951	0.6820	0.3465	0.1793	0.3550	0.4064	0.1916
NM_153317	Rab8b	0.1855	0.2976	0.1975	0.2081	0.2899	0.3328	0.2130	0.3933	0.7309	0.3070	0.1873	0.5151
NM_001100945	Rspry1	0.6987	0.2252	0.1829	0.2610	0.3274	0.6172	0.1210	0.4448	0.2084	0.2437	0.0148	0.5998
NM_199370	Krt8	0.8327	0.8264	0.1679	0.9270	0.8512	0.9252	0.8504	0.8032	0.9094	0.7860	0.9292	0.1374
NM_001077668	Drap1	0.1771	0.2327	0.2600	0.6067	0.2418	0.2003	0.5081	0.0194	0.1118	0.0628	0.2737	0.1312
NM_199269	Mdga2	0.0039	0.0045	0.0547	0.0549	0.7865	0.7317	0.0145	0.8802	0.0389	0.2560	0.0799	0.2169
NM_001044243	Tasp1	0.8213	0.6217	0.7884	0.9370	0.9220	0.9435	0.7354	0.7376	0.6607	0.5927	0.5028	0.6806
NM_001107641	Sim1	0.8421	0.9247	0.9448	0.7485	0.8145	0.5809	0.7936	0.0225	0.9793	0.9114	0.9737	0.9285
NM_181389	Nsmaf	0.1855	0.1871	0.1974	0.2080	0.2809	0.1949	0.2128	0.4000	0.1792	0.1748	0.1872	0.3496
NM_181432	Hps6	0.6289	0.2410	0.6335	0.7146	0.1858	0.3387	0.5792	0.0792	0.4958	0.7989	0.6591	0.5843
NM_181440	Grpca	0.1707	0.1798	0.4813	0.1808	0.1605	0.0999	0.2673	0.2325	0.2131	0.1589	0.0504	0.1567
NM_181475	Cd2ap	0.2957	0.1870	0.3353	0.2079	0.4310	0.1948	0.2126	0.3493	0.4710	0.4949	0.3983	0.3173
NM_001100976	Pbxip1	0.0577	0.0173	0.8827	0.7003	0.3100	0.6920	0.0498	0.0173	0.1346	0.1497	0.0741	0.8537
NM_001079701	RGD1310641	0.3034	0.2308	0.6977	0.2199	0.0989	0.3969	0.2261	0.2737	0.4294	0.0493	0.4225	0.0289
NM_001044285	Sepx1	0.4941	0.2254	0.4032	0.2076	0.3255	0.2385	0.5774	0.2621	0.3179	0.2308	0.4863	0.5889
NM_001079708	RGD1564149	0.2615	0.7438	0.3953	0.2901	0.6145	0.3142	0.6227	0.3420	0.0610	0.0825	0.6316	0.7143
NM_001079706	RGD1305412	0.1413	0.0893	0.3486	0.2508	0.1520	0.6893	0.6008	0.2417	0.5782	0.5473	0.5054	0.7642
NM_001042621	Pip5k1a	0.6436	0.7193	0.6992	0.6156	0.7014	0.5066	0.6307	0.7155	0.5180	0.5219	0.6598	0.6574
NM_001079710	Gpr18	0.4422	0.3254	0.4436	0.2076	0.2938	0.2808	0.2125	0.2037	0.4630	0.3145	0.3922	0.2817
NM_001079712	LOC689582	0.2077	0.5422	0.5953	0.2981	0.4191	0.4565	0.4392	0.6209	0.3772	0.3888	0.5465	0.4036
NM_001047847	Morg1	0.0706	0.0156	0.0166	0.0149	0.1185	0.3563	0.1126	0.2119	0.3431	0.2980	0.1243	0.0120
NM_001106268	Chsy1	0.9933	0.9821	0.9976	0.9473	0.9808	0.9513	0.9893	0.8990	0.9627	0.9987	0.8956	0.9240
NM_207595	Ankra2	0.9357	0.7897	0.8069	0.5761	0.4461	0.3754	0.4038	0.3085	0.3289	0.8302	0.8903	0.5226
NM_001044226	RGD1306772	0.5705	0.6884	0.8244	0.7012	0.5945	0.3169	0.7971	0.8170	0.8471	0.9018	0.6889	0.5141
NM_001048184	Rragc	0.3241	0.4552	0.7725	0.9002	0.2400	0.3901	0.9004	0.4241	0.9024	0.5579	0.8690	0.7936

NM_199095	Svs1	0.7871	0.8825	0.8685	0.7972	0.7924	0.7510	0.7273	0.9599	0.8195	0.8375	0.6891	0.8112
NM_148889	Aptx	0.0071	0.0817	0.0348	0.7446	0.0537	0.0136	0.2635	0.0815	0.1684	0.5414	0.2413	0.0917
NM_001079890	Gprc5a	0.8098	0.7649	0.9485	0.7410	0.3416	0.2076	0.6830	0.1508	0.8864	0.9583	0.7477	0.9974
NM_001077676	RGD1560212	0.7475	0.9406	0.4779	0.9926	0.8120	0.8209	0.5310	0.9093	0.9493	0.8190	0.6602	0.7976
NM_001079699	Rrs1	0.6806	0.9094	0.0793	0.3364	0.6009	0.9292	0.7601	0.8544	0.7775	0.7645	0.9226	0.9421
NM_001077678	RGD1562433	0.7512	0.6080	0.8451	0.6759	0.6859	0.5462	0.0959	0.3895	0.2439	0.3022	0.4511	0.7022
NM_171996	Decr2	0.6565	0.6639	0.4833	0.2900	0.5848	0.4113	0.6947	0.5957	0.4867	0.2333	0.5630	0.5515
NM_147214	LOC259246	0.2420	0.4168	0.4698	0.4857	0.6546	0.2432	0.3603	0.3920	0.6333	0.3894	0.3730	0.2858
NM_001079896	LOC367975	0.7894	0.6130	0.6448	0.6656	0.0722	0.4813	0.5270	0.5734	0.4960	0.4978	0.4173	0.4653
NM_199086	Nob1	0.1157	0.3221	0.1834	0.0791	0.3267	0.7315	0.0387	0.6972	0.4954	0.1617	0.1833	0.7564
NM_199083	Pno1	0.3632	0.0356	0.0962	0.0642	0.0523	0.0598	0.1226	0.0187	0.0659	0.2392	0.0155	0.1231
NM_198787	Sgsm3	0.8771	0.0701	0.8004	0.1579	0.1767	0.5837	0.7630	0.3982	0.1409	0.1439	0.1348	0.9508
NM_012970	Kcna2	0.5779	0.9116	0.5198	0.5113	0.4391	0.8055	0.3538	0.1281	0.3744	0.5888	0.5395	0.5190
NM_013184	Ntf4	0.6694	0.2308	0.7745	0.7369	0.1282	0.5258	0.1734	0.2321	0.2411	0.0077	0.1238	0.8584
NM_013150	Nrcam	0.6085	0.6761	0.6801	0.6421	0.7097	0.4687	0.5235	0.7154	0.6507	0.6546	0.7239	0.5788
NM_012790	Dspp	0.8457	0.3672	0.1480	0.5299	0.4715	0.6552	0.7224	0.5615	0.6047	0.7283	0.6969	0.7253
NM_012630	Prlr	0.3917	0.8980	0.8191	0.6832	0.6154	0.4844	0.5082	0.7328	0.7527	0.8900	0.8007	0.6541
NM_012675	Tnf	0.9322	0.5923	0.8222	0.6054	0.9187	0.8542	0.8734	0.7665	0.9424	0.7243	0.5396	0.8951
NM_012590	Inha	0.8968	0.9117	0.9049	0.9297	0.9732	0.7906	0.9562	0.8548	0.9738	0.8093	0.7531	0.9296
NM_013191	S100b	0.3797	0.6253	0.7817	0.4480	0.4136	0.4629	0.3047	0.5929	0.5704	0.5803	0.8166	0.2496
NM_012697	Slc22a1	0.3401	0.3477	0.3536	0.0595	0.2408	0.1684	0.1068	0.2697	0.0351	0.1042	0.3099	0.3784
NM_013218	Ak3	0.0297	0.0188	0.8656	0.9883	0.2474	0.2290	0.1371	0.8804	0.0249	0.1150	0.2757	0.9297
NM_012967	Icam1	0.1411	0.0058	0.1150	0.2381	0.1545	0.1822	0.1896	0.2401	0.0976	0.1973	0.0522	0.1457
NM_053794	Wnk1	0.0246	0.5037	0.9460	0.7808	0.6159	0.2476	0.8104	0.3562	0.9191	0.9833	0.9914	0.8799
NM_053556	Bud31	0.6956	0.8382	0.2966	0.6127	0.3510	0.1434	0.0684	0.3139	0.7225	0.6167	0.7718	0.0475
NM_031541	Scarb1	0.9775	0.9947	0.8722	0.9352	0.8314	0.9760	0.8980	0.9754	0.7891	0.9894	0.7409	0.8651
NM_031824	Ctcf	0.9947	0.9595	0.4619	0.8822	0.9911	0.9796	0.5711	0.8598	0.9404	0.9370	0.9487	0.8888
NM_001080150	RGD1309969	0.8346	0.8280	0.9155	0.4549	0.9106	0.7036	0.8481	0.4561	0.2666	0.2325	0.6335	0.8266
NM_001080151	Ccdc21	0.7670	0.4481	0.2717	0.2774	0.9657	0.9816	0.2774	0.8831	0.8151	0.9636	0.4067	0.6394
NM_001100885	Myl9	0.5328	0.6633	0.4835	0.7992	0.7120	0.6078	0.4611	0.5865	0.6052	0.8485	0.9268	0.5371
NM_001100815	Gtpbp10	0.0628	0.2846	0.0554	0.1736	0.0439	0.0412	0.1358	0.0670	0.4285	0.1638	0.8195	0.0493
NM_001103351	Parp9	0.3642	0.2560	0.6565	0.2728	0.5144	0.2447	0.5208	0.2693	0.2153	0.3482	0.2462	0.2260
NM_001080782	Tect2	0.3144	0.1867	0.3931	0.4406	0.2413	0.3720	0.5933	0.5817	0.2918	0.1987	0.3508	0.4107
NM_198746	Klra5	0.4193	0.2902	0.4080	0.7201	0.4513	0.3810	0.6962	0.5828	0.4016	0.5378	0.4580	0.7622
NM_199495	Ndufa10	0.5092	0.5343	0.5431	0.3474	0.2073	0.6100	0.5960	0.5784	0.5391	0.3167	0.4613	0.4927
NM_198744	Tpd52l2	0.0184	0.2836	0.9728	0.8155	0.3006	0.6894	0.1906	0.1217	0.3939	0.6153	0.8017	0.7808
NM_001080789	Arhgap9	0.6423	0.5667	0.6832	0.5419	0.3440	0.3999	0.5965	0.2535	0.5711	0.5864	0.4973	0.5093
NM_022505	Rhd	0.2320	0.0944	0.3442	0.0590	0.2656	0.3426	0.1297	0.6267	0.2448	0.3470	0.2163	0.3779
NM_001102417	Svs3b	0.6564	0.6630	0.1028	0.0103	0.3732	0.6131	0.7707	0.1853	0.8053	0.7528	0.5374	0.5042
NM_001105757	Atrx	0.7202	0.3913	0.4937	0.6010	0.4034	0.6158	0.4244	0.5080	0.4582	0.8073	0.3672	0.7235
NM_001077429	Lnp	0.1563	0.3499	0.0464	0.0180	0.0217	0.0502	0.1570	0.1786	0.1607	0.0406	0.1141	0.1254
NM_212528	Col11a2	0.9012	0.6153	0.2484	0.4660	0.5398	0.4215	0.1920	0.4047	0.8070	0.3581	0.5733	0.4591
NM_001081958	Gnl3l	0.1852	0.1864	0.1974	0.2075	0.1901	0.3250	0.2123	0.2036	0.1791	0.1746	0.1869	0.1913
NM_201350	Shank2	0.8295	0.8358	0.8412	0.8812	0.6399	0.8180	0.7678	0.6648	0.8974	0.7852	0.4408	0.7264
NM_198736	Kctd13	0.0040	0.0086	0.1272	0.0533	0.2576	0.2374	0.0506	0.0126	0.0654	0.0118	0.3136	0.4689
NM_212538	Phf1	0.7700	0.9318	0.5289	0.1387	0.7832	0.9034	0.3999	0.8081	0.9190	0.8736	0.7590	0.9312
NM_198734	Yif1b	0.2848	0.3273	0.3825	0.4351	0.2419	0.3447	0.5202	0.4569	0.3208	0.4649	0.3030	0.5430
NM_198733	Cyp2b21	0.5859	0.5146	0.6884	0.2397	0.3630	0.1798	0.5721	0.4215	0.4456	0.2317	0.2399	0.3624
NM_001100992	Etv3l	0.1908	0.1482	0.4893	0.2832	0.3663	0.0661	0.7114	0.4938	0.3726	0.2210	0.5489	0.5449

NM_001100990	Rspo3	0.8672	0.0588	0.6743	0.4428	0.4942	0.2745	0.6141	0.7316	0.0967	0.4492	0.6138	0.3128
NM_198726	Kpna1	0.1445	0.1467	0.0635	0.0140	0.0760	0.0973	0.1483	0.0180	0.3011	0.3170	0.6320	0.5460
NM_199408	Gpr177	0.6380	0.2196	0.7472	0.7340	0.3932	0.5417	0.5820	0.6087	0.6800	0.3540	0.5599	0.4843
NM_001100966	Scap	0.7759	0.5813	0.7867	0.9131	0.6428	0.7588	0.6020	0.4901	0.2327	0.5593	0.5137	0.9609
NM_198133	Uts2d	0.2696	0.4447	0.3076	0.1748	0.2091	0.1424	0.3058	0.1468	0.2459	0.3633	0.1632	0.4158
NM_198050	Kif27	0.3358	0.2781	0.2688	0.5117	0.3652	0.4218	0.3960	0.3269	0.3543	0.4208	0.3781	0.1909
NM_001083313	Chmp1a	0.9784	0.1641	0.9360	0.9518	0.4431	0.9959	0.9068	0.4782	0.1200	0.0185	0.3740	0.9206
NM_001100978	RGD1309744	0.9859	0.8466	0.9903	0.7685	0.9206	0.9860	0.9921	0.7772	0.9905	0.9426	0.8009	0.9498
NM_001100982	Siva1	0.9603	0.9350	0.1760	0.9817	0.9363	0.7026	0.9929	0.7539	0.3503	0.9598	0.7952	0.1291
NM_001101003	Kcne1l	0.1851	0.1860	0.4165	0.3351	0.3087	0.2984	0.2120	0.3372	0.2802	0.1744	0.4146	0.2925
NM_199395	Geft	0.3815	0.0110	0.1525	0.1990	0.8819	0.7562	0.0244	0.7792	0.0675	0.0633	0.1282	0.0885
NM_198134	Bst2	0.2090	0.6803	0.7560	0.5124	0.2389	0.2036	0.6935	0.4302	0.4736	0.8969	0.4477	0.0915
NM_001101001	Ptpla	0.2778	0.2824	0.3967	0.2072	0.3543	0.1946	0.3416	0.6270	0.4529	0.4003	0.3636	0.3577
NM_001105718	Vps35	0.1991	0.0102	0.2934	0.1062	0.0099	0.2677	0.2212	0.0179	0.3322	0.1665	0.2517	0.3817
NM_001102413	Irx6	0.1196	0.3048	0.0618	0.2304	0.1780	0.3207	0.2210	0.1434	0.3350	0.1037	0.1275	0.2595
NM_199099	Impdh2	0.7753	0.7574	0.5830	0.6954	0.7167	0.7183	0.7589	0.7419	0.8506	0.4877	0.6055	0.6751
NM_207615	Clcf1	0.2116	0.2701	0.1197	0.2532	0.8357	0.8519	0.2593	0.7502	0.1919	0.5353	0.8593	0.9326
NM_001083898	Ccdc92	0.2065	0.5499	0.6755	0.2565	0.4852	0.4519	0.4881	0.6569	0.5781	0.2120	0.2103	0.5764
NM_207613	Cdh15	0.6760	0.4384	0.3078	0.2513	0.3143	0.2763	0.6415	0.4189	0.4835	0.1154	0.0340	0.1110
NM_001077436	Hmox3	0.4353	0.4274	0.6481	0.7510	0.6491	0.0286	0.5411	0.5012	0.2762	0.6255	0.1226	0.5007
NM_001039713	Fto	0.3862	0.0846	0.8707	0.4657	0.0482	0.0059	0.7016	0.2805	0.1888	0.0290	0.0871	0.0197
NM_198741	Hla-dma	0.0111	0.4913	0.1099	0.1097	0.3303	0.0441	0.3736	0.2483	0.0526	0.1340	0.0997	0.0944
NM_198739	Ly6g5c	0.0173	0.3753	0.0919	0.5961	0.5986	0.5657	0.5195	0.3105	0.5832	0.1096	0.0292	0.5533
NM_198740	Hla-dmb	0.8434	0.5651	0.3369	0.2465	0.2280	0.5707	0.6908	0.7077	0.7560	0.5999	0.7286	0.5668
NM_001085352	Apoc2	0.5717	0.2302	0.3288	0.1498	0.4252	0.6361	0.1650	0.1803	0.4782	0.2038	0.4149	0.7096
NM_199384	Laptm4a	0.7968	0.0208	0.5666	0.8901	0.5067	0.4038	0.0840	0.4108	0.0181	0.4128	0.3637	0.0936
NM_201562	Rtn2	0.3482	0.3524	0.0517	0.2755	0.0437	0.5607	0.3162	0.6570	0.2742	0.1227	0.5351	0.1170
NM_001105881	Nat13	0.3843	0.9484	0.2984	0.3107	0.2968	0.1842	0.6448	0.2936	0.4776	0.1464	0.9411	0.3073
NM_001077681	RGD1564345	0.7845	0.3969	0.5913	0.4832	0.4798	0.4204	0.1000	0.7460	0.7780	0.5537	0.7594	0.4555
NM_001044272	Jagn1	0.5529	0.6044	0.3338	0.5718	0.0919	0.6794	0.1077	0.2144	0.2076	0.3879	0.1860	0.3332
NM_001044269	Tmem196	0.7235	0.1506	0.3172	0.4104	0.3417	0.4634	0.2501	0.0874	0.0588	0.1391	0.1708	0.0901
NM_198755	Vwa5a	0.8807	0.7741	0.6473	0.3995	0.6716	0.7889	0.7718	0.7465	0.8616	0.6832	0.8708	0.4742
NM_177419	Xrcc5	0.8415	0.1139	0.5906	0.9129	0.9912	0.9164	0.5324	0.9208	0.0272	0.1603	0.3038	0.0679
NM_001105902	Pex11g	0.3690	0.7348	0.6141	0.6876	0.5489	0.6296	0.3221	0.9726	0.9113	0.4574	0.7718	0.7859
NM_001127297	Wipi1	0.8268	0.8374	0.9394	0.9654	0.9559	0.7085	0.7483	0.7732	0.9912	0.9783	0.9088	0.7941
NM_175762	Ldlr	0.8202	0.8811	0.2923	0.1729	0.6865	0.8001	0.7845	0.6343	0.9335	0.7827	0.6837	0.6029
NM_001098803	Zfhx2	0.7312	0.9213	0.5491	0.5873	0.7363	0.7372	0.7086	0.9031	0.8486	0.7954	0.7749	0.9598
NM_001044263	Rfxap	0.8812	0.8750	0.7063	0.4758	0.7520	0.8176	0.4746	0.9160	0.3379	0.3645	0.7710	0.8980
NM_001098239	Asph	0.8957	0.4028	0.9571	0.8680	0.6461	0.8227	0.8558	0.8651	0.5542	0.4927	0.9677	0.9395
NM_001047090	Aph1b	0.0971	0.1090	0.7652	0.7823	0.2477	0.5996	0.1202	0.1984	0.0056	0.0212	0.0137	0.2509
NM_001097581	Sav1	0.7276	0.5511	0.8951	0.0574	0.9330	0.9165	0.4331	0.7133	0.0963	0.7063	0.5781	0.1596
NM_001044265	Steap4	0.3570	0.7038	0.5862	0.3236	0.5210	0.1155	0.4833	0.7434	0.2717	0.8965	0.6674	0.6203
NM_001104560	Kcnmb3	0.2370	0.4240	0.2780	0.5626	0.5982	0.8358	0.3531	0.5958	0.4105	0.7439	0.1801	0.3684
NM_001100972	Epc1	0.9985	0.9933	0.3506	0.9310	0.7903	0.9518	0.3094	0.8103	0.8343	0.8307	0.6877	0.4598
NM_001100971	RGD1305110	0.1938	0.1526	0.0491	0.0058	0.0298	0.0560	0.0893	0.0788	0.0946	0.0869	0.3620	0.0416
NM_001100974	RGD1560471	0.2834	0.7795	0.8234	0.6178	0.3341	0.9286	0.4756	0.4728	0.6072	0.9158	0.6831	0.8472
NM_001134546	RGD1307235	0.9882	0.9312	0.6076	0.5250	0.9908	0.9886	0.3503	0.9774	0.9445	0.6574	0.9466	0.9219
NM_001105721	Notch1	0.9968	0.8850	0.5197	0.2142	0.4474	0.8415	0.6310	0.4965	0.9815	0.7910	0.9330	0.9271
NM_001100977	Mtfr1	0.0597	0.0761	0.4030	0.8464	0.0714	0.1630	0.0142	0.0560	0.2302	0.0384	0.4031	0.4152

NM_001100983	Myl6l	0.5440	0.1859	0.3722	0.3873	0.2422	0.2872	0.2119	0.3223	0.3270	0.2872	0.3062	0.3078
NM_001100979	RGD1311084	0.1898	0.1250	0.3823	0.1327	0.1728	0.1301	0.2276	0.3052	0.6050	0.2712	0.3675	0.1715
NM_001105781	Mrpl22	0.0672	0.4563	0.0477	0.1548	0.1343	0.0864	0.0287	0.0152	0.1561	0.0402	0.1260	0.0091
NM_198778	Mpped2	0.7108	0.6000	0.8529	0.4054	0.5212	0.3706	0.2611	0.4208	0.7363	0.8027	0.2602	0.4527
NM_198776	MGC72973	0.3769	0.5841	0.2301	0.3994	0.6211	0.5176	0.0526	0.5568	0.4173	0.2271	0.5302	0.2980
NM_001105829	Lpo	0.4714	0.3847	0.4138	0.0710	0.5097	0.2098	0.3083	0.4329	0.6980	0.9205	0.4824	0.8570
NM_001130537	Zbtb39	0.6049	0.7456	0.4459	0.8762	0.6422	0.9206	0.7313	0.3720	0.5787	0.8950	0.9490	0.2522
NM_001100497	Hoxb9	0.7358	0.6550	0.8065	0.6557	0.6967	0.9516	0.5828	0.4671	0.9637	0.2635	0.6922	0.8873
NM_001100576	Akr1c19	0.2481	0.0856	0.0112	0.0493	0.7732	0.8627	0.0029	0.8479	0.1004	0.0148	0.2364	0.0118
NM_001106464	Gstm6l	0.6415	0.5124	0.5513	0.6227	0.4012	0.9344	0.6108	0.5944	0.9811	0.5843	0.5184	0.7547
NM_001106409	Actbl2	0.2209	0.2046	0.5889	0.5630	0.6122	0.3073	0.2199	0.2323	0.3988	0.5975	0.6177	0.2364
NM_001099647	LOC685045	0.5055	0.2465	0.2295	0.2843	0.2209	0.3204	0.2727	0.2029	0.2040	0.4149	0.4634	0.3002
NM_001100123	Lilrc2	0.3364	0.2557	0.3596	0.2070	0.3318	0.1944	0.3431	0.2027	0.6747	0.2983	0.4348	0.3782
NM_199082	Sectm1b	0.3299	0.3542	0.7078	0.1531	0.2834	0.2385	0.7531	0.4556	0.1686	0.4102	0.1555	0.2713
NM_199081	Slc35b1	0.1377	0.7444	0.0375	0.4845	0.0574	0.0459	0.0489	0.0149	0.2280	0.6941	0.5136	0.3033
NM_001105769	Tbc1d24	0.8459	0.9176	0.5193	0.2822	0.4865	0.3815	0.7491	0.8268	0.6225	0.4625	0.5182	0.5041
NM_001108335	Rasal1	0.4347	0.2610	0.6088	0.4814	0.3199	0.5650	0.7335	0.9281	0.2871	0.1234	0.4689	0.7760
NM_001108487	Aen	0.9424	0.9904	0.7306	0.1264	0.8531	0.8174	0.9224	0.7473	0.7606	0.9702	0.9939	0.9209
NM_001108349	Rcsd1	0.3332	0.7753	0.7128	0.3376	0.4449	0.3709	0.5571	0.7108	0.1490	0.3352	0.6453	0.5537
NM_001108345	Kif14	0.4229	0.8094	0.5004	0.6570	0.8024	0.8973	0.8678	0.7449	0.5345	0.3811	0.8909	0.4976
NM_001108643	Tmem72	0.4021	0.4990	0.6145	0.8740	0.8509	0.8899	0.2632	0.6484	0.8464	0.8242	0.6108	0.8939
NM_001113779	Pabpnl1	0.3757	0.4877	0.3087	0.5211	0.3889	0.6732	0.5795	0.5950	0.4547	0.1875	0.1674	0.2460
NM_001113780	Tmem222	0.7192	0.7408	0.0635	0.5876	0.9081	0.5980	0.8098	0.9376	0.8910	0.3129	0.1231	0.2602
NM_001134602	RGD1565374	0.2733	0.8769	0.8671	0.6033	0.4653	0.5302	0.0697	0.7511	0.3593	0.2185	0.5140	0.4526
NM_001105790	Rangrf	0.2708	0.5726	0.6567	0.2574	0.0794	0.3227	0.0846	0.0948	0.3827	0.5331	0.2452	0.0788
NM_001105786	B9d1	0.6602	0.7652	0.7969	0.9147	0.6997	0.2223	0.8393	0.6888	0.3869	0.8147	0.8329	0.4224
NM_001108013	Matn3	0.7625	0.3553	0.2665	0.1036	0.3553	0.2078	0.4182	0.3489	0.1699	0.5096	0.5547	0.0589
NM_001108584	Rapsn	0.8160	0.6273	0.0965	0.9009	0.7027	0.9271	0.9111	0.7554	0.1527	0.7427	0.5385	0.1958
NM_001098793	Prkrip1	0.8217	0.9405	0.5664	0.2443	0.9646	0.6915	0.0931	0.9673	0.9474	0.6575	0.7612	0.8424
NM_199085	Serpinb6a	0.1510	0.7685	0.0672	0.7730	0.1115	0.0688	0.6572	0.1245	0.8657	0.5631	0.8279	0.8596
NM_199084	Rcbtb2	0.7345	0.0381	0.2955	0.8872	0.7335	0.7368	0.7329	0.7448	0.0049	0.1163	0.4658	0.0208
NM_001100986	Hipk1	0.5608	0.8126	0.4880	0.8964	0.7619	0.7508	0.5946	0.6452	0.0424	0.4258	0.1069	0.6720
NM_021741	Cep78	0.4629	0.1857	0.1968	0.2069	0.3250	0.1943	0.2116	0.3920	0.1789	0.1743	0.4840	0.1906
NM_001100748	Trpc4ap	0.1207	0.1279	0.1559	0.6535	0.2600	0.4036	0.4046	0.1011	0.4656	0.0481	0.1065	0.7759
NM_001108463	Rshl2	0.1045	0.4320	0.4764	0.5166	0.1487	0.0345	0.1333	0.4735	0.0868	0.2020	0.2151	0.0555
NM_001100813	Cldn12	0.6586	0.3094	0.5153	0.3077	0.0776	0.2980	0.4211	0.3669	0.6428	0.9336	0.8868	0.7069
NM_001098838	Lyg1	0.6766	0.7249	0.5784	0.7230	0.4427	0.4830	0.8100	0.8129	0.5387	0.6492	0.8296	0.1800
NM_001108556	RGD1304953	0.2588	0.4352	0.4910	0.3337	0.5373	0.4964	0.1543	0.1202	0.4574	0.5451	0.2722	0.0213
NM_001108550	Postn	0.9753	0.9536	0.9854	0.9187	0.8648	0.7304	0.9279	0.6756	0.8582	0.9701	0.7526	0.7761
NM_001108658	Topors	0.5818	0.1109	0.2763	0.3472	0.0907	0.3526	0.0119	0.2494	0.2048	0.0967	0.1829	0.0376
NM_001108737	Mier2	0.4316	0.1553	0.3935	0.0457	0.7785	0.2939	0.3625	0.3309	0.5792	0.4946	0.3206	0.3820
NM_001004209	Hsd17b11	0.9016	0.4594	0.9495	0.9824	0.9507	0.8523	0.7997	0.9590	0.5352	0.4841	0.5480	0.8500
NM_001108729	Silv	0.8782	0.2257	0.0398	0.5878	0.8157	0.8232	0.7801	0.8000	0.0548	0.0174	0.1068	0.0386
NM_001108616	Agap3	0.9523	0.9170	0.3668	0.9247	0.8916	0.9864	0.8869	0.9680	0.9271	0.9962	0.9424	0.9544
NM_001098782	Serf2	0.9920	0.9214	0.6290	0.9491	0.9700	0.9547	0.9561	0.9762	0.9970	0.8718	0.9419	0.9914
NM_199088	Stk22s1	0.7211	0.7136	0.1279	0.1573	0.7402	0.9313	0.0588	0.7527	0.7066	0.6899	0.6780	0.6135
NM_001109194	RGD1560880	0.2523	0.3491	0.0492	0.3096	0.4823	0.8024	0.1761	0.5459	0.1957	0.2352	0.1791	0.9428
NM_001109188	Lce1f	0.1847	0.1856	0.4906	0.2068	0.4983	0.2563	0.4515	0.2021	0.3125	0.4387	0.5071	0.3328
NM_001109187	RGD1562234	0.3268	0.4811	0.6539	0.3002	0.1825	0.1324	0.3533	0.3170	0.2693	0.2104	0.8464	0.3666

NM_001109185	Rit1	0.0592	0.0974	0.3363	0.2677	0.0105	0.2734	0.0446	0.0584	0.2559	0.1072	0.0358	0.8128
NM_001098788	Dhx58	0.0235	0.0890	0.4204	0.1557	0.0123	0.0199	0.0226	0.0924	0.0917	0.0635	0.0866	0.1140
NM_001105210	Mrvi1	0.5838	0.7245	0.6054	0.4668	0.7061	0.7073	0.7175	0.5543	0.7148	0.2964	0.4649	0.5891
NM_199101	Plekha4	0.7752	0.6161	0.2363	0.7969	0.7342	0.6718	0.3669	0.8469	0.7498	0.5859	0.4977	0.7590
NM_001100980	Efnb3	0.8866	0.7844	0.1513	0.7503	0.9301	0.9678	0.5104	0.8801	0.7437	0.8012	0.1666	0.8751
NM_001100175	Tmem38a	0.3036	0.4011	0.4880	0.2854	0.5016	0.2371	0.5827	0.3131	0.6144	0.2529	0.2630	0.5978
NM_199093	Serping1	0.5074	0.3493	0.0503	0.3372	0.4832	0.5674	0.0466	0.4658	0.1789	0.1807	0.0819	0.6948
NM_199104	Zfyve27	0.4857	0.3764	0.3244	0.4647	0.8314	0.5017	0.4607	0.6602	0.9594	0.6423	0.7511	0.8978
NM_001040190	Atcay	0.7786	0.7217	0.8978	0.4113	0.8248	0.8026	0.5125	0.5448	0.5944	0.8860	0.5930	0.8892
NM_001104527	RGD1311589	0.9067	0.8477	0.9470	0.9227	0.8948	0.8642	0.8073	0.9629	0.7971	0.9868	0.9988	0.9597
NM_001134413	Gltp	0.7035	0.9253	0.8918	0.9598	0.9880	0.7347	0.9907	0.9991	0.9295	0.8404	0.9576	0.5715
NM_199096	Necap2	0.1875	0.0129	0.1627	0.2306	0.0995	0.2575	0.1912	0.1483	0.0315	0.2188	0.3060	0.4162
NM_199107	Gyltl1b	0.4410	0.3693	0.4172	0.6648	0.5042	0.0740	0.2618	0.5888	0.1264	0.0815	0.1819	0.3692
NM_001108544	Adamts6	0.9085	0.4634	0.4299	0.2640	0.7839	0.7685	0.3415	0.9108	0.9420	0.9664	0.8793	0.9858
NM_001108441	Dnajb1	0.2416	0.7324	0.4313	0.8114	0.8141	0.8230	0.7634	0.9085	0.5788	0.7672	0.6122	0.5785
NM_001105768	Kctd5	0.2146	0.2983	0.5393	0.7234	0.3076	0.4868	0.8681	0.6761	0.2371	0.3432	0.2662	0.8182
NM_001105833	Phospho1	0.0022	0.3022	0.3720	0.4262	0.1236	0.2678	0.0306	0.2555	0.0424	0.0677	0.0163	0.3933
NM_001105764	Znf213	0.5080	0.3307	0.1641	0.4866	0.5535	0.5807	0.1086	0.4980	0.2608	0.5331	0.2152	0.7154
NM_001105839	Nkiras2	0.1169	0.3136	0.6167	0.8089	0.4158	0.2504	0.1225	0.2952	0.5009	0.1791	0.2239	0.6601
NM_001100965	RGD1563440	0.2793	0.3253	0.4397	0.3526	0.5135	0.3630	0.6728	0.6233	0.5600	0.1371	0.4178	0.3891
NM_199097	Adi1	0.5839	0.7518	0.6352	0.8890	0.0480	0.5432	0.6866	0.7013	0.8737	0.2655	0.8346	0.1241
NM_199098	Tmem19	0.4092	0.1125	0.5149	0.5325	0.8070	0.8058	0.9920	0.4651	0.0342	0.0188	0.0445	0.6305
NM_001100993	LOC499719	0.3060	0.4909	0.4528	0.4353	0.2375	0.3238	0.3694	0.2021	0.2352	0.3583	0.3210	0.2708
NM_001106006	Ube2k	0.9803	0.8590	0.9429	0.9843	0.9763	0.9891	0.9474	0.7095	0.9466	0.9109	0.9812	0.9678
NM_001100968	Usp43	0.4993	0.6146	0.5699	0.4844	0.6812	0.5291	0.6594	0.6175	0.8549	0.5310	0.5387	0.3929
NM_199108	Hp1bp3	0.9708	0.9068	0.7496	0.9511	0.9710	0.9933	0.9016	0.8776	0.9061	0.8671	0.9318	0.8672
NM_001101002	LOC681092	0.5583	0.4641	0.5153	0.5258	0.2887	0.5946	0.2468	0.3717	0.4681	0.2080	0.5418	0.4720
NM_001101005	LOC689852	0.2568	0.3640	0.5292	0.2068	0.2143	0.6863	0.4991	0.5966	0.6106	0.7005	0.5852	0.7308
NM_001101008	LOC683722	0.3890	0.4344	0.4694	0.3488	0.5252	0.6815	0.3254	0.4123	0.2962	0.4282	0.4133	0.2777
NM_001101012	LOC686288	0.2930	0.4506	0.2667	0.3312	0.2666	0.1942	0.3720	0.3542	0.3296	0.1742	0.5607	0.1904
NM_199105	MGC72614	0.1845	0.1479	0.2068	0.1592	0.8014	0.3691	0.2456	0.0749	0.1814	0.0304	0.2393	0.0469
NM_001101018	Oaz3	0.2980	0.1855	0.5617	0.2067	0.6552	0.2903	0.3712	0.4644	0.3118	0.4358	0.3104	0.3431
NM_001039516	Kcnk5	0.3442	0.2472	0.5569	0.2162	0.7145	0.4502	0.2967	0.2861	0.4869	0.4379	0.0806	0.5734
NM_001105714	Wnt1	0.4062	0.8675	0.6748	0.6195	0.7581	0.2868	0.8041	0.4301	0.6057	0.3620	0.7504	0.4213
NM_001105936	Iscu	0.6076	0.0551	0.9223	0.9808	0.1457	0.0455	0.1179	0.4810	0.5642	0.0400	0.1795	0.1143
NM_001105855	Dnah17	0.2326	0.4300	0.2514	0.2065	0.6899	0.5091	0.2948	0.3696	0.3558	0.3603	0.2715	0.3305
NM_001122677	Zcchc2	0.6660	0.6431	0.5091	0.7061	0.7520	0.5238	0.6055	0.6817	0.3900	0.7649	0.2935	0.4807
NM_001042356	Camk2b	0.6197	0.6376	0.7087	0.5906	0.7241	0.7017	0.6473	0.7210	0.6742	0.5040	0.6041	0.7338
NM_001105738	Gpx5	0.2223	0.1389	0.4349	0.6019	0.2787	0.3474	0.2749	0.2360	0.4860	0.0233	0.1023	0.1186
NM_001105858	Dysfip1	0.4063	0.7709	0.4287	0.5513	0.2775	0.1656	0.5804	0.6619	0.9127	0.8289	0.8218	0.1989
NM_001105860	Fam128b	0.6358	0.3403	0.8616	0.7566	0.7604	0.7216	0.5305	0.7845	0.5495	0.0768	0.4871	0.8284
NM_001127294	Ndufb9	0.1316	0.1803	0.0976	0.0795	0.0964	0.0052	0.0985	0.1655	0.0046	0.0888	0.1494	0.0276
NM_001107721	Abca4	0.4887	0.0979	0.1130	0.1869	0.0963	0.0466	0.1035	0.1724	0.0796	0.0453	0.3777	0.1397
NM_001103353	LOC691995	0.6687	0.5128	0.5339	0.2387	0.5498	0.6167	0.3749	0.4853	0.5515	0.2077	0.5473	0.4612
NM_001103355	Fbxl13	0.2423	0.5130	0.5135	0.4205	0.2256	0.2877	0.4413	0.4309	0.3413	0.5404	0.2800	0.2329
NM_001103356	LOC10012536[illegible]	0.5344	0.7299	0.4537	0.6548	0.4685	0.6209	0.2538	0.6928	0.4836	0.7119	0.4246	0.5554
NM_001103357	LOC10012536[illegible]	0.4966	0.4348	0.2177	0.5469	0.3923	0.2841	0.2719	0.6412	0.3535	0.6168	0.4311	0.4693
NM_001103358	Ceacam6	0.5218	0.4287	0.4286	0.4167	0.4495	0.4698	0.4232	0.2393	0.2127	0.6684	0.4527	0.5274
NM_001105755	Pkn2	0.7582	0.3159	0.8889	0.9686	0.5774	0.8208	0.7230	0.4586	0.5757	0.5542	0.9147	0.9192

NM_001105766	Pkmyt1	0.7548	0.7539	0.0876	0.0114	0.8045	0.9108	0.8688	0.9393	0.8830	0.8261	0.8409	0.8890
NM_001103360	Pnrc2	0.5972	0.6188	0.6241	0.6102	0.6479	0.4582	0.5986	0.5956	0.5114	0.6577	0.6150	0.3366
NM_001103363	LOC10012538	0.2352	0.2495	0.2625	0.3619	0.2228	0.3790	0.3882	0.5020	0.5413	0.3677	0.5853	0.2849
NM_001103364	LOC10012538	0.3245	0.3665	0.3160	0.4802	0.2244	0.2505	0.2115	0.4698	0.3274	0.3236	0.1864	0.2380
NM_199117	Cbx7	0.8738	0.7681	0.9538	0.7558	0.4869	0.8311	0.6219	0.5553	0.9767	0.6940	0.1551	0.8872
NM_213563	Vars2	0.3824	0.4413	0.5055	0.3334	0.4523	0.6866	0.4509	0.4723	0.2921	0.4249	0.2247	0.5742
NM_199113	Popdc2	0.3985	0.3865	0.4558	0.0184	0.0864	0.6051	0.2714	0.3294	0.5348	0.8074	0.2875	0.3042
NM_199112	RGD735175	0.8125	0.0971	0.1287	0.8596	0.5105	0.3899	0.1505	0.3261	0.7356	0.9471	0.3718	0.9169
NM_001085475	Ddi1	0.2212	0.4131	0.7109	0.4994	0.6468	0.4105	0.6026	0.8017	0.0700	0.6324	0.8362	0.4883
NM_001085485	Selo	0.8134	0.7916	0.3141	0.8646	0.4615	0.9002	0.3507	0.2477	0.2065	0.3866	0.6507	0.3524
NM_199270	Bre	0.0459	0.0200	0.7972	0.7204	0.0463	0.3146	0.0395	0.1625	0.0792	0.0122	0.0157	0.3155
NM_001078647	Tmem134	0.2348	0.4335	0.8543	0.7785	0.3476	0.2528	0.8667	0.5325	0.2941	0.2863	0.3442	0.5239
NM_199291	Doxl2	0.2609	0.4560	0.1694	0.3761	0.2902	0.2020	0.5980	0.5758	0.2085	0.4512	0.3114	0.1840
NM_001105889	Tmem30c	0.3020	0.2686	0.3283	0.2064	0.4342	0.3397	0.3617	0.2019	0.2803	0.3438	0.1864	0.2700
NM_001105932	Vps29	0.9066	0.8599	0.3584	0.2616	0.9157	0.6817	0.1379	0.7048	0.4882	0.9124	0.6682	0.3786
NM_001105928	Vps37b	0.9626	0.8634	0.3659	0.9431	0.9719	0.9224	0.6179	0.9900	0.9147	0.9310	0.6539	0.9278
NM_199371	Tmprss8	0.5011	0.7427	0.6676	0.6691	0.9456	0.7547	0.2318	0.3428	0.1989	0.7725	0.8400	0.4009
NM_199372	Eif4a1	0.8626	0.9430	0.5451	0.9205	0.9949	0.9386	0.9447	0.9612	0.9534	0.9985	0.8820	0.9646
NM_199373	Wrb	0.2704	0.2359	0.2119	0.4240	0.1053	0.3078	0.3614	0.1773	0.2416	0.1137	0.2450	0.2225
NM_199376	Sil1	0.0530	0.0787	0.7950	0.0962	0.0440	0.1223	0.8568	0.0806	0.0677	0.0678	0.3807	0.0831
NM_199374	Spata18	0.4335	0.3794	0.2317	0.2055	0.6278	0.0590	0.0533	0.3410	0.1560	0.1381	0.6286	0.1088
NM_199377	Abcc12	0.4729	0.1875	0.1990	0.4404	0.2045	0.6286	0.7920	0.2567	0.4584	0.5936	0.5787	0.4987
NM_199382	Bpgm	0.3666	0.8640	0.9127	0.3263	0.2060	0.0544	0.0106	0.0232	0.8135	0.9949	0.7249	0.9988
NM_001078648	Tmem134	0.2348	0.4335	0.8543	0.7785	0.3476	0.2528	0.8667	0.5325	0.2941	0.2863	0.3442	0.5239
NM_199380	Eif2s2	0.3888	0.8875	0.6097	0.6612	0.1939	0.4624	0.7843	0.4084	0.8252	0.9562	0.8326	0.6860
NM_199378	Cfdp1	0.1553	0.6172	0.6899	0.3280	0.6538	0.7379	0.3081	0.5491	0.2506	0.5862	0.2909	0.1132
NM_001104612	Jrk	0.3628	0.2885	0.3460	0.8307	0.4318	0.4497	0.1370	0.4769	0.7373	0.3654	0.3591	0.6548
NM_001098240	Mpv17l	0.8361	0.8843	0.8689	0.6255	0.6477	0.7361	0.9192	0.8349	0.5817	0.8675	0.7668	0.6763
NM_199383	Yipf1	0.1431	0.2549	0.7527	0.6453	0.5190	0.4351	0.6977	0.7092	0.0704	0.0507	0.4972	0.0729
NM_001105821	RGD1560058	0.6508	0.5954	0.4145	0.6377	0.1608	0.2142	0.4764	0.3868	0.5585	0.5744	0.3988	0.3188
NM_001105814	Abr	0.1994	0.2647	0.1702	0.9346	0.8420	0.9429	0.2014	0.9336	0.4685	0.1831	0.0343	0.3866
NM_199401	Cyp20a1	0.5868	0.0937	0.9958	0.9386	0.8433	0.8313	0.9556	0.7238	0.4295	0.3267	0.3497	0.0887
NM_199402	Spata20	0.4083	0.5648	0.3298	0.1890	0.4325	0.4535	0.1875	0.5357	0.3983	0.5801	0.3180	0.8431
NM_001079766	Prpf6	0.3682	0.3385	0.1967	0.2062	0.2640	0.1941	0.3867	0.2017	0.1787	0.5054	0.3663	0.1903
NM_199392	Gcx1	0.6901	0.5188	0.4260	0.4225	0.4251	0.5093	0.5788	0.2967	0.3964	0.4439	0.6142	0.6428
NM_001106022	RGD1308179	0.0842	0.7672	0.8910	0.3529	0.5100	0.1523	0.8583	0.5904	0.7833	0.6056	0.4871	0.0949
NM_001077649	Ctrc	0.0777	0.6229	0.1783	0.3665	0.1073	0.1221	0.0607	0.2352	0.2971	0.3894	0.3695	0.3868
NM_001040019	RGD1562373	0.2823	0.4036	0.1965	0.2061	0.2498	0.2851	0.3887	0.2017	0.7185	0.5022	0.5050	0.3035
NM_001105742	Klf3	0.4101	0.6762	0.9294	0.3762	0.3371	0.3783	0.8867	0.8656	0.5577	0.6790	0.5324	0.5260
NM_199405	Lcmt1	0.6097	0.4175	0.4227	0.5662	0.5123	0.2265	0.7932	0.4781	0.1504	0.2367	0.6590	0.0896
NM_199407	Unc5c	0.7300	0.2583	0.7435	0.6971	0.9772	0.8027	0.8206	0.7366	0.2242	0.0044	0.0061	0.0893
NM_001105749	Il16	0.6458	0.4502	0.4669	0.7661	0.8190	0.7288	0.6581	0.5887	0.4588	0.5343	0.5226	0.6998
NM_199398	Panx3	0.7360	0.7241	0.7159	0.5267	0.6391	0.5120	0.4558	0.4747	0.4874	0.6006	0.4350	0.6261
NM_199410	Wdr12	0.0748	0.5590	0.2910	0.0370	0.0242	0.5331	0.0969	0.0120	0.0716	0.0183	0.4617	0.4148
NM_001079705	RGD1311558	0.9796	0.8726	0.8562	0.9405	0.8930	0.8564	0.9051	0.6794	0.8042	0.8130	0.3409	0.4624
NM_001039849	Gpx4	0.5142	0.4859	0.6208	0.3050	0.4602	0.4936	0.3046	0.6268	0.2971	0.4050	0.1863	0.5100
NM_199399	Gpr108	0.4594	0.6616	0.2253	0.6266	0.6953	0.1069	0.0540	0.9264	0.7876	0.1775	0.5016	0.8400
NM_199500	Map1lc3a	0.1094	0.0558	0.9905	0.9291	0.1289	0.3745	0.0937	0.0754	0.2541	0.0042	0.1693	0.9436
NM_001105758	Tmed7	0.7233	0.7342	0.7243	0.6822	0.5743	0.5323	0.3915	0.3436	0.6695	0.6197	0.6222	0.7090

NM_199503	Pcdha1	0.9036	0.5096	0.7253	0.8578	0.8879	0.4928	0.8408	0.9136	0.9158	0.7155	0.7777	0.9074
NM_199502	Chrdl1	0.8225	0.5801	0.8212	0.6346	0.8557	0.8652	0.9292	0.4139	0.7386	0.6572	0.5560	0.6906
NM_001106210	Rnaset2	0.2019	0.0302	0.7382	0.5851	0.1280	0.0096	0.0816	0.1339	0.1270	0.0326	0.1865	0.0319
NM_001105760	Ptar1	0.9110	0.9413	0.7288	0.9002	0.8756	0.0766	0.7241	0.8232	0.9682	0.8981	0.9296	0.8061
NM_199493	RGD735029	0.9774	0.9442	0.9415	0.8397	0.8777	0.9185	0.9498	0.9764	0.3819	0.8649	0.6934	0.0786
NM_001105668	Mdfic	0.6147	0.5837	0.6142	0.9585	0.9612	0.8501	0.8957	0.9364	0.7037	0.6841	0.9097	0.3138
NM_199504	Pcdha2	0.7658	0.5318	0.9248	0.9609	0.9294	0.7011	0.9390	0.9797	0.9300	0.4953	0.7374	0.4201
NM_199505	Pcdha5	0.6690	0.6171	0.6184	0.6797	0.7282	0.5103	0.7152	0.7285	0.6426	0.6853	0.6347	0.7075
NM_001017375	Mphosph8	0.3818	0.2239	0.6954	0.3454	0.0877	0.4556	0.1159	0.1921	0.4202	0.0460	0.2085	0.3932
NM_199506	Pcdha6	0.6696	0.6177	0.6190	0.6805	0.7286	0.5109	0.7158	0.7291	0.6432	0.6858	0.6354	0.7082
NM_001134570	RGD1560258	0.0708	0.3481	0.0511	0.1887	0.1737	0.0565	0.2758	0.0967	0.0285	0.2243	0.3522	0.0068
NM_199496	Zbtb25	0.5929	0.9029	0.7199	0.8562	0.8955	0.3299	0.8298	0.9161	0.8907	0.5787	0.8219	0.7628
NM_001105901	Car15	0.1179	0.7787	0.6885	0.3126	0.2947	0.2487	0.5251	0.1858	0.6393	0.0969	0.2432	0.7324
NM_001105739	Prrx2	0.7477	0.7347	0.8896	0.8903	0.0991	0.2302	0.8656	0.6969	0.5337	0.8283	0.9491	0.1744
NM_001105734	Dusp10	0.3077	0.3949	0.2662	0.1181	0.3215	0.2762	0.2752	0.2806	0.3072	0.1414	0.0488	0.3957
NM_199497	Slc4a8	0.5145	0.7283	0.0275	0.7038	0.3061	0.4127	0.7939	0.6874	0.6904	0.9394	0.8945	0.9162
NM_199507	Pcdha7	0.6693	0.6176	0.6190	0.6802	0.7285	0.5107	0.7157	0.7290	0.6432	0.6856	0.6353	0.7080
NM_001105927	Ogfod2	0.6495	0.5233	0.6487	0.3011	0.4263	0.8179	0.6716	0.2571	0.5993	0.3638	0.2624	0.3671
NM_199509	Pcdhac1	0.6693	0.6175	0.6188	0.6801	0.7284	0.5106	0.7156	0.7288	0.6429	0.6855	0.6352	0.7079
NM_001099340	Wdr35l	0.6526	0.0596	0.5723	0.6927	0.3097	0.5565	0.6649	0.4312	0.1061	0.1503	0.5223	0.6778
NM_001105750	Stim2	0.5183	0.9898	0.6724	0.8220	0.7468	0.7926	0.8906	0.5667	0.8155	0.9651	0.9575	0.2754
NM_001077645	Prkacb	0.6242	0.6429	0.7570	0.7558	0.4707	0.6597	0.6943	0.3818	0.6075	0.6049	0.6944	0.6837
NM_001024989	Trex1	0.4686	0.1853	0.1964	0.2058	0.1897	0.1938	0.2111	0.2014	0.4616	0.3134	0.3429	0.1901
NM_001105765	Zfp13	0.4356	0.2489	0.2912	0.4618	0.2680	0.4467	0.4420	0.5336	0.3747	0.3200	0.3966	0.2403
NM_212491	Wdr46	0.2968	0.5015	0.3184	0.4193	0.2360	0.2360	0.3349	0.3294	0.5406	0.4440	0.7217	0.3551
NM_212504	Hspa1b	0.6058	0.7405	0.8804	0.7652	0.9445	0.9201	0.9041	0.9669	0.7044	0.8674	0.6612	0.9009
NM_001082410	Rnf144a	0.1311	0.4844	0.6380	0.0435	0.4001	0.9127	0.3745	0.4904	0.0552	0.2242	0.1437	0.6060
NM_001105934	Unc119b	0.3063	0.6151	0.9151	0.8537	0.1652	0.3876	0.2938	0.3160	0.2682	0.4009	0.3209	0.1849
NM_001105774	Rpusd1	0.1360	0.0874	0.1186	0.1268	0.2908	0.0290	0.4807	0.3708	0.4221	0.4376	0.5179	0.3185
NM_001107318	Gtf2e2	0.0352	0.3641	0.7470	0.5090	0.1956	0.3298	0.2958	0.0402	0.1407	0.1005	0.5412	0.1784
NM_001115022	Commd1	0.2593	0.2587	0.2999	0.2057	0.2328	0.2607	0.2109	0.2014	0.5132	0.3007	0.3209	0.2457
NM_001127553	Uqcrb	0.7657	0.1542	0.7419	0.4826	0.7008	0.2995	0.2651	0.6490	0.5204	0.2597	0.1319	0.0205
NM_212496	Dhx16	0.7978	0.8416	0.8169	0.6637	0.8398	0.8582	0.8530	0.8716	0.8398	0.8330	0.8871	0.8014
NM_001105763	Zfp263	0.8937	0.9853	0.9717	0.8544	0.9621	0.9133	0.7878	0.5705	0.9159	0.8336	0.7115	0.7595
NM_001105762	RGD1565166	0.0289	0.0071	0.1892	0.1076	0.0413	0.2887	0.2461	0.3508	0.0676	0.0417	0.0541	0.3863
NM_212500	Gnl1	0.8673	0.7176	0.9710	0.7383	0.7561	0.4567	0.7076	0.7201	0.6473	0.6839	0.8542	0.7491
NM_212489	Btnl8	0.5808	0.2639	0.7207	0.3268	0.2463	0.3149	0.3804	0.3880	0.2397	0.2912	0.3190	0.1900
NM_212501	Gtf2h4	0.0610	0.4940	0.1865	0.7802	0.0453	0.6314	0.6590	0.6241	0.8086	0.6663	0.5863	0.7882
NM_001105773	Chtf18	0.3807	0.3265	0.2903	0.5159	0.3321	0.3658	0.3544	0.3534	0.4018	0.3029	0.4362	0.2602
NM_212505	Ier3	0.6743	0.9580	0.8930	0.0349	0.7123	0.3214	0.9133	0.8944	0.9608	0.9956	0.9262	0.8274
NM_212495	Brd2	0.9449	0.9876	0.1606	0.8169	0.8455	0.8943	0.9567	0.9745	0.8889	0.9404	0.9045	0.9187
NM_001105937	Sgsm1	0.4904	0.3744	0.4348	0.4807	0.3937	0.7546	0.4131	0.7595	0.6825	0.7832	0.2310	0.7751
NM_001105871	Hrasls	0.7843	0.6095	0.8696	0.2283	0.8219	0.5953	0.6186	0.1536	0.2592	0.6771	0.3273	0.8648
NM_001105787	RGD1307767	0.2077	0.4457	0.0594	0.1978	0.2594	0.4171	0.0757	0.0660	0.1386	0.3561	0.3315	0.0745
NM_212509	Nfkbil1	0.6465	0.6774	0.7393	0.8256	0.6883	0.1268	0.9761	0.7760	0.7462	0.8292	0.6141	0.0724
NM_212508	Nrm	0.8550	0.9773	0.1221	0.9565	0.6854	0.2291	0.9335	0.7090	0.8460	0.8704	0.9654	0.8326
NM_212511	Rpl35	0.0416	0.2311	0.0791	0.0718	0.2149	0.1168	0.1690	0.2654	0.2355	0.1084	0.0438	0.4086
NM_212512	Slc4a5	0.1403	0.4425	0.2243	0.7621	0.4267	0.0847	0.9042	0.1115	0.3340	0.6762	0.3176	0.1035
NM_001105966	Creg1	0.4249	0.0601	0.7539	0.0391	0.3914	0.3953	0.5019	0.7754	0.9062	0.1406	0.9281	0.7563

NM_001105954	Dennd1b	0.5429	0.2973	0.4676	0.6029	0.4507	0.6154	0.5172	0.6805	0.6929	0.7326	0.4469	0.4853
NM_212513	Lag3	0.8228	0.3876	0.2803	0.3956	0.7439	0.5931	0.5695	0.5535	0.7661	0.5078	0.0802	0.8002
NM_001105890	Gpr15	0.6398	0.3649	0.5583	0.5889	0.3027	0.4716	0.7798	0.2715	0.3274	0.4373	0.6887	0.3720
NM_212518	Abcb7	0.1291	0.2130	0.0639	0.6757	0.0614	0.0081	0.1383	0.0126	0.1010	0.5369	0.7641	0.1882
NM_212519	Tomm70a	0.3119	0.7852	0.6038	0.7947	0.5028	0.6878	0.8188	0.4899	0.7379	0.8724	0.9547	0.8701
NM_212523	Kif5a	0.8873	0.8356	0.6979	0.2734	0.8876	0.6874	0.4766	0.7769	0.9282	0.4184	0.7439	0.9331
NM_001106017	Myl7	0.2998	0.2055	0.1290	0.4499	0.3509	0.4957	0.7611	0.6227	0.3319	0.3625	0.4376	0.5688
NM_001105803	Sdf2	0.1675	0.0658	0.7525	0.8473	0.3922	0.3730	0.3218	0.1526	0.0857	0.0700	0.0616	0.1253
NM_001105793	Aloxe3	0.2308	0.5483	0.0852	0.5400	0.8364	0.0858	0.6068	0.4167	0.9079	0.8264	0.6003	0.7788
NM_212524	Abhd5	0.0006	0.0013	0.0278	0.0021	0.0009	0.0035	0.0040	0.0033	0.0008	0.0010	0.0033	0.0278
NM_212525	Tyrobp	0.1015	0.3128	0.1624	0.0652	0.3257	0.4604	0.7526	0.3123	0.8176	0.5467	0.4195	0.6616
NM_001105845	Plcd3	0.8756	0.7121	0.6903	0.4484	0.8982	0.7453	0.4837	0.8671	0.9733	0.8802	0.7614	0.9089
NM_031506	Cftr	0.8444	0.3998	0.7499	0.5286	0.2097	0.3971	0.3911	0.6386	0.4997	0.4014	0.6389	0.3395
NM_001127295	Polr3h	0.1513	0.0437	0.1765	0.8254	0.2619	0.2224	0.9349	0.2067	0.0398	0.0697	0.0751	0.1477
NM_001102403	Svs6	0.7002	0.6986	0.1097	0.4771	0.1432	0.1990	0.2933	0.3395	0.1829	0.6668	0.7166	0.5692
NM_001105982	Susd4	0.7159	0.3909	0.4993	0.4902	0.7710	0.3981	0.3735	0.6998	0.4929	0.3749	0.2704	0.5703
NM_212531	Bat5	0.8154	0.9986	0.8110	0.8264	0.9984	0.9449	0.8165	0.9870	0.7816	0.8462	0.9544	0.9172
NM_001106000	Cox18	0.4094	0.0214	0.2802	0.4000	0.1148	0.2246	0.3283	0.1406	0.6674	0.2192	0.5475	0.3791
NM_212534	Mrps18b	0.6965	0.9244	0.8518	0.0777	0.6739	0.1515	0.8362	0.7942	0.7403	0.6881	0.8129	0.6178
NM_001105807	Mnt	0.8742	0.9373	0.2076	0.7806	0.9632	0.9858	0.5176	0.9772	0.9173	0.9220	0.8139	0.9434
NM_001105808	Smg6	0.7177	0.6442	0.3047	0.5678	0.9314	0.8258	0.6023	0.9243	0.9374	0.9768	0.6079	0.8828
NM_001105812	Slc43a2	0.9454	0.9681	0.9285	0.7189	0.9664	0.9805	0.9347	0.9741	0.9431	0.9485	0.8520	0.8813
NM_001105810	Smyd4	0.1751	0.1543	0.5061	0.3297	0.3753	0.6204	0.5188	0.4813	0.5393	0.4568	0.8386	0.9014
NM_001106013	Nol14	0.7266	0.9468	0.1671	0.0197	0.1240	0.2252	0.0670	0.3406	0.8329	0.4924	0.8113	0.3693
NM_212536	Msh5	0.6521	0.9275	0.2021	0.8729	0.7599	0.8460	0.9443	0.8988	0.4302	0.3252	0.8689	0.8877
NM_001105820	Spaca3	0.0584	0.2490	0.3673	0.2530	0.0587	0.3934	0.0625	0.1237	0.3972	0.1546	0.5247	0.4640
NM_001105817	Sarm1	0.7898	0.6957	0.3556	0.5506	0.9179	0.7558	0.7976	0.8168	0.3452	0.7937	0.4485	0.5256
NM_001105816	Poldip2	0.7461	0.9572	0.9032	0.9992	0.9674	0.9896	0.8140	0.9710	0.8943	0.9599	0.9526	0.8387
NM_001105798	Alox12	0.7632	0.8953	0.9165	0.9590	0.9421	0.9762	0.7485	0.7226	0.9828	0.9759	0.8355	0.7019
NM_001106037	Fit1	0.3018	0.3621	0.3269	0.0084	0.4227	0.4325	0.1706	0.3910	0.3666	0.0726	0.3671	0.3885
NM_001106034	Cmtm5	0.5287	0.2348	0.5684	0.3401	0.7778	0.3782	0.4917	0.5589	0.5959	0.5349	0.0689	0.3398
NM_001106248	Znf507	0.4184	0.7366	0.6179	0.0758	0.6254	0.4256	0.3890	0.6048	0.5049	0.7123	0.7181	0.4231
NM_001105874	Mylk	0.7767	0.9135	0.9516	0.8871	0.9412	0.8893	0.9037	0.6626	0.9199	0.9565	0.9373	0.8210
NM_001105822	Ccl12	0.0959	0.4438	0.4291	0.1273	0.1035	0.2988	0.1638	0.3166	0.1646	0.5292	0.3035	0.1346
NM_212544	Prr3	0.9922	0.9231	0.9871	0.8953	0.9244	0.9494	0.9584	0.9090	0.7462	0.9984	0.9457	0.9841
NM_212542	Ppp1r11	0.9871	0.9788	0.9171	0.8823	0.9361	0.9693	0.9882	0.9653	0.9582	0.6918	0.9458	0.9744
NM_212541	Slc44a4	0.0623	0.2766	0.0524	0.0392	0.1367	0.0940	0.3194	0.2675	0.2651	0.4742	0.2297	0.1902
NM_022543	Ccdc80	0.8535	0.9385	0.9616	0.7645	0.8835	0.9899	0.9814	0.8975	0.9435	0.9923	0.8878	0.8949
NM_001105859	St6galnac1	0.1014	0.6527	0.1820	0.5786	0.6625	0.6212	0.5746	0.7332	0.3107	0.8871	0.8735	0.7585
NM_053018	Cd9	0.3991	0.2611	0.2311	0.5038	0.4073	0.2636	0.2367	0.2738	0.2523	0.5661	0.4866	0.3161
NM_001105863	Thap7	0.3449	0.2208	0.3237	0.7193	0.3450	0.3029	0.3203	0.6984	0.1066	0.4688	0.1154	0.4384
NM_001040189	Nkr-p1c	0.1969	0.4742	0.2637	0.4168	0.6416	0.3490	0.5952	0.3759	0.3435	0.4798	0.5752	0.5890
NM_212549	Ring1	0.6627	0.7302	0.4839	0.7967	0.6471	0.2009	0.5978	0.7548	0.7656	0.7252	0.6076	0.4517
NM_001106675	Echdc2	0.5389	0.5825	0.2743	0.2996	0.1924	0.1704	0.6009	0.1897	0.5001	0.2658	0.1424	0.1278
NM_001108781	Gmppb	0.0837	0.7304	0.0140	0.1574	0.1360	0.2631	0.6883	0.1851	0.6753	0.8178	0.9221	0.7687
NM_001047114	Pomt2	0.5875	0.3915	0.5788	0.3810	0.2996	0.2217	0.4527	0.5397	0.5798	0.6905	0.2649	0.5047
NM_030866	Nfix	0.0731	0.3574	0.0020	0.0286	0.1547	0.4747	0.1151	0.1736	0.1046	0.1091	0.1072	0.0627
NM_001106031	Np	0.7819	0.7554	0.7793	0.8122	0.9413	0.7919	0.8096	0.9606	0.8275	0.7533	0.8416	0.7450
NM_001048185	Agmat	0.8594	0.0201	0.6725	0.3374	0.8410	0.6465	0.8147	0.5982	0.0376	0.0424	0.2751	0.0874

NM_001104630	Sephs1	0.9873	0.9776	0.4355	0.9700	0.9922	0.9915	0.7462	0.9899	0.9894	0.9471	0.9533	0.9852
NM_001017374	Eif4g2	0.7221	0.5420	0.8539	0.8967	0.6437	0.7811	0.8471	0.5375	0.8874	0.5912	0.9089	0.7686
NM_213567	Znrd1	0.6712	0.8494	0.7666	0.8892	0.8545	0.3541	0.6335	0.7332	0.8209	0.6863	0.7177	0.7567
NM_213565	Zfp57	0.7970	0.6286	0.1328	0.1652	0.8868	0.6772	0.4811	0.8315	0.8958	0.5841	0.9339	0.7384
NM_213564	Zbtb9	0.8870	0.8002	0.8169	0.7946	0.7188	0.9445	0.8916	0.6541	0.9377	0.7477	0.8639	0.9905
NM_001105956	F13b	0.6474	0.2508	0.3558	0.1092	0.3443	0.0919	0.6408	0.7167	0.5589	0.2565	0.1576	0.2763
NM_001105897	Cct8	0.3338	0.7282	0.3454	0.6121	0.6694	0.6001	0.2510	0.2334	0.3623	0.5986	0.6023	0.3806
NM_001105923	Mrps17	0.4836	0.4957	0.2440	0.6641	0.9321	0.2893	0.2450	0.7192	0.1786	0.0592	0.0820	0.1099
NM_001077640	Gadd45g	0.7929	0.9471	0.4898	0.0101	0.0512	0.2915	0.9369	0.1666	0.9043	0.9317	0.7858	0.9236
NM_001106041	Ctsg	0.2131	0.5066	0.3367	0.1010	0.2814	0.6147	0.1475	0.4332	0.2474	0.7179	0.4773	0.3750
NM_001047095	Nop16	0.7952	0.9346	0.7468	0.4902	0.3014	0.1659	0.9728	0.1090	0.4577	0.4134	0.7182	0.4382
NM_213629	Arhgap20	0.7011	0.3427	0.2456	0.3989	0.2222	0.4667	0.3632	0.5580	0.3910	0.4454	0.3718	0.3680
NM_001044294	Gabarapl1	0.4567	0.6216	0.5785	0.3381	0.6130	0.5434	0.5250	0.7168	0.7251	0.6098	0.6297	0.6901
NM_001035249	Parl	0.6706	0.2456	0.6404	0.6186	0.7094	0.0807	0.1901	0.0903	0.0420	0.2812	0.1900	0.0938
NM_001106056	Trim52	0.6354	0.8069	0.2083	0.3643	0.3275	0.6727	0.6135	0.4632	0.0344	0.6671	0.3289	0.6648
NM_001106055	Mycbp2	0.5604	0.1322	0.6383	0.0134	0.5552	0.6854	0.1319	0.4313	0.3677	0.3847	0.6729	0.3815
NM_214458	Phactr2	0.4664	0.0512	0.9484	0.4352	0.3515	0.0918	0.7994	0.2153	0.0853	0.1519	0.7258	0.4985
NM_001106786	Tbk1	0.9387	0.3318	0.4596	0.7335	0.9536	0.9898	0.2403	0.9169	0.3600	0.5141	0.6868	0.3711
NM_001105895	Prss7	0.5676	0.8935	0.7979	0.7607	0.7060	0.6353	0.5536	0.8930	0.5264	0.5703	0.6829	0.5241
NM_001105851	Otop2	0.7103	0.6666	0.6783	0.6848	0.8048	0.5065	0.4373	0.6274	0.8589	0.6248	0.6139	0.8347
NM_001105856	Chmp6	0.1175	0.8150	0.6382	0.8748	0.8487	0.8925	0.9130	0.8816	0.9783	0.8122	0.7179	0.8251
NM_001083966	Rev3l	0.8942	0.5928	0.9227	0.7088	0.6839	0.8463	0.3951	0.6166	0.8600	0.3136	0.7915	0.5944
NM_001044251	Fzd8	0.9970	0.7634	0.8612	0.9960	0.9964	0.7973	0.8596	0.9914	0.9704	0.9353	0.9895	0.9333
NM_001106158	Fam13b1	0.5910	0.7111	0.6611	0.7237	0.6141	0.6332	0.6663	0.7237	0.7170	0.7205	0.5605	0.7333
NM_001105945	Nanos3	0.0468	0.3092	0.2075	0.0281	0.1248	0.1265	0.0915	0.1044	0.0349	0.3240	0.0606	0.4031
NM_001047098	Cops7a	0.2366	0.3482	0.2963	0.3490	0.2296	0.3440	0.4520	0.5082	0.2571	0.3956	0.2797	0.4170
NM_001047085	Ivns1abp	0.7418	0.7521	0.0192	0.7485	0.7453	0.7449	0.7402	0.7619	0.8208	0.8507	0.7502	0.0998
NM_001127303	Itpripl2	0.1929	0.8114	0.6379	0.7394	0.5679	0.5113	0.7623	0.7451	0.7306	0.7523	0.6689	0.8680
NM_001037153	Pcdhga11	0.5337	0.8338	0.0785	0.6423	0.5736	0.8723	0.7617	0.8352	0.6815	0.4054	0.6584	0.5759
NM_001083115	Trpc4	0.5285	0.3200	0.2890	0.6126	0.4836	0.4345	0.1795	0.3909	0.2679	0.4261	0.6151	0.6737
NM_001039549	Ugt1a5	0.3594	0.6899	0.6650	0.6413	0.4870	0.4897	0.4265	0.2858	0.3790	0.4165	0.5319	0.6244
NM_001040008	Tuba3a	0.4034	0.3877	0.1960	0.4085	0.1894	0.3463	0.2106	0.3804	0.4524	0.4593	0.4263	0.5439
NM_001109056	RGD1559643	0.4670	0.3162	0.2411	0.2196	0.2513	0.1945	0.3119	0.1541	0.3128	0.4383	0.2877	0.2053
NM_001106083	Ing2	0.6749	0.5198	0.6402	0.9825	0.9329	0.9819	0.7519	0.8510	0.8475	0.8660	0.9253	0.2368
NM_001106071	Ccdc124	0.0503	0.6495	0.4171	0.6108	0.3377	0.0391	0.8155	0.0882	0.2029	0.0337	0.2252	0.1077
NM_001044248	Traf3ip2	0.5725	0.6386	0.4456	0.6445	0.5199	0.7246	0.5961	0.1936	0.3084	0.6347	0.2223	0.6317
NM_001044257	Epsti1	0.1297	0.1316	0.5164	0.6994	0.2336	0.1813	0.6007	0.3600	0.2314	0.1635	0.4416	0.2684
NM_001105909	Usp42	0.7640	0.3299	0.3618	0.3058	0.3945	0.7470	0.0764	0.5437	0.6164	0.6118	0.3755	0.7153
NM_001008886	H2-T23	0.1842	0.1848	0.1959	0.2054	0.1892	0.1934	0.2105	0.2007	0.1784	0.1738	0.1860	0.1898
NM_001077641	Plcb1	0.9552	0.9951	0.9929	0.9780	0.6768	0.7111	0.9769	0.8719	0.9834	0.9912	0.9893	0.9446
NM_031566	Nfib	0.7995	0.8344	0.3666	0.9613	0.8819	0.8003	0.8080	0.5822	0.9718	0.9180	0.9076	0.3921
NM_001044276	Rprm	0.6780	0.4575	0.5987	0.6299	0.4941	0.5287	0.5830	0.6785	0.5726	0.5267	0.4864	0.4329
NM_001105913	Elfn1	0.9637	0.9128	0.8165	0.8396	0.8375	0.7925	0.7827	0.8816	0.8616	0.9325	0.6173	0.8761
NM_001106791	Irak4	0.5317	0.4180	0.3685	0.8632	0.3897	0.5046	0.2824	0.7998	0.2516	0.3444	0.3758	0.2995
NM_001106036	Nrl	0.8212	0.8996	0.7448	0.8109	0.7579	0.4924	0.8546	0.7843	0.6445	0.9104	0.6235	0.4891
NM_001106065	Ocel1	0.0632	0.1308	0.0668	0.0776	0.0486	0.0726	0.3252	0.1136	0.0553	0.1531	0.1251	0.0995
NM_031567	Nfic	0.2703	0.5662	0.0860	0.3799	0.4723	0.6497	0.6776	0.3179	0.6071	0.2856	0.7529	0.2429
NM_001108264	Alg1	0.0679	0.4109	0.1854	0.5401	0.4285	0.9655	0.3496	0.8562	0.7062	0.6123	0.0350	0.1751
NM_001108176	Zic4	0.5318	0.2084	0.2274	0.3726	0.3113	0.2590	0.2354	0.5612	0.1283	0.4513	0.2652	0.2544

NM_001105872	Mfi2	0.3053	0.3461	0.5313	0.1921	0.4223	0.3786	0.8534	0.8198	0.4695	0.3090	0.1951	0.5060
NM_001106221	Aurkc	0.7396	0.7273	0.0724	0.5440	0.8738	0.7998	0.1023	0.8860	0.7018	0.9026	0.7588	0.6584
NM_001098216	Tead3	0.5016	0.4255	0.5564	0.1399	0.5206	0.2998	0.8077	0.4623	0.8722	0.7648	0.9068	0.9692
NM_012831	Cebpg	0.9857	0.9006	0.9893	0.8870	0.9849	0.7952	0.9515	0.7688	0.9397	0.9856	0.9998	0.9866
NM_001105870	Rtp1	0.3550	0.2457	0.4978	0.3155	0.1395	0.0631	0.3952	0.0536	0.0409	0.4610	0.1241	0.3293
NM_001044255	Coq2	0.5779	0.6293	0.6706	0.8914	0.2331	0.5801	0.7172	0.6494	0.4439	0.0401	0.4242	0.9066
NM_001104633	Sema3d	0.9212	0.3012	0.9170	0.2803	0.9223	0.8191	0.1680	0.8439	0.1544	0.1275	0.5280	0.2464
NM_001044280	Slc25a18	0.6455	0.5421	0.1689	0.5517	0.0790	0.3517	0.4673	0.5186	0.1845	0.1541	0.4798	0.1453
NM_001044287	Evi2a	0.2772	0.2090	0.3220	0.7246	0.4238	0.6883	0.4553	0.4759	0.1932	0.4385	0.4731	0.2175
NM_001044286	Mea1	0.5675	0.5453	0.3760	0.5171	0.5693	0.4864	0.4020	0.2006	0.4455	0.4609	0.4326	0.3414
NM_001044284	Tsc22d4	0.4110	0.2134	0.6417	0.6266	0.3870	0.5410	0.5237	0.4370	0.6105	0.2834	0.5100	0.7277
NM_001106144	RGD1312005	0.5288	0.0836	0.1512	0.0340	0.6162	0.6153	0.3435	0.5695	0.0910	0.0496	0.1249	0.1061
NM_001077647	Ddx4	0.4862	0.2501	0.8113	0.2273	0.0941	0.1834	0.2076	0.5257	0.0872	0.4894	0.5989	0.5097
NM_001105879	Cdgap	0.4622	0.7034	0.4930	0.7872	0.7731	0.6749	0.7015	0.5990	0.6043	0.8669	0.8078	0.5756
NM_001127293	Zc3hc1	0.0766	0.9107	0.7673	0.1283	0.1851	0.1164	0.2013	0.0331	0.6940	0.7472	0.7509	0.7119
NM_001105896	Krtap15-1	0.5477	0.1472	0.3095	0.3087	0.3715	0.1325	0.4636	0.5099	0.2527	0.5895	0.5875	0.3739
NM_001105877	Stfa3	0.7468	0.3390	0.6001	0.4833	0.2916	0.3925	0.6965	0.3959	0.5166	0.1089	0.4140	0.4477
NM_001042354	Camk2b	0.6197	0.6376	0.7087	0.5906	0.7241	0.7017	0.6473	0.7210	0.6742	0.5040	0.6041	0.7338
NM_001079689	Chit1	0.0494	0.0826	0.6146	0.3523	0.5916	0.4233	0.5504	0.6402	0.2167	0.0953	0.0623	0.7497
NM_001105941	Bloc1s1	0.5841	0.6212	0.6036	0.8800	0.8029	0.7688	0.7114	0.7530	0.1070	0.6837	0.3698	0.0557
NM_001101022	Olr1867	0.3798	0.3473	0.1959	0.4429	0.2828	0.3164	0.3886	0.5058	0.3332	0.3341	0.4329	0.3333
NM_001105888	Senp7	0.6092	0.4884	0.9857	0.3559	0.7451	0.8808	0.7099	0.2588	0.2953	0.2778	0.5490	0.8101
NM_001105892	Dscr6	0.2095	0.2690	0.1352	0.1107	0.2003	0.3071	0.3427	0.4350	0.2090	0.7475	0.2544	0.4609
NM_001039018	Fbxo39	0.8398	0.8927	0.6481	0.4516	0.5644	0.0708	0.7988	0.8602	0.9172	0.7415	0.6879	0.7125
NM_001047863	Mrps33	0.2372	0.4783	0.6493	0.7268	0.0679	0.0816	0.7162	0.7234	0.0437	0.1483	0.0600	0.2072
NM_001106746	Acyp1	0.5927	0.5084	0.0057	0.1617	0.5367	0.0337	0.2281	0.5511	0.0374	0.0657	0.2972	0.0209
NM_001106460	Ptpn22	0.6838	0.9727	0.2849	0.7907	0.4416	0.5192	0.6779	0.2476	0.0653	0.2534	0.3682	0.4397
NM_001040154	Rab3gap2	0.3582	0.7604	0.0227	0.0067	0.9107	0.6388	0.1284	0.8926	0.8092	0.8921	0.7074	0.3242
NM_001105783	Wnt9a	0.8757	0.9536	0.8636	0.8486	0.9090	0.9350	0.8540	0.8474	0.9533	0.9631	0.6494	0.9469
NM_001047086	Irf2	0.3956	0.6139	0.6524	0.8707	0.7293	0.2273	0.5964	0.7672	0.8213	0.7176	0.7149	0.8271
NM_001044274	Odam	0.2383	0.2631	0.3705	0.3769	0.3958	0.1932	0.4516	0.4949	0.2151	0.6114	0.2856	0.2374
NM_001106014	RGD1560394	0.3605	0.4507	0.4870	0.2885	0.4634	0.2112	0.1457	0.6333	0.6389	0.2793	0.5802	0.0456
NM_001008332	Col4a4	0.5064	0.7623	0.7172	0.7576	0.6681	0.3440	0.7669	0.5922	0.8507	0.8511	0.7977	0.6728
NM_001044296	Amtn	0.4232	0.2376	0.3913	0.4033	0.4859	0.2829	0.5729	0.2003	0.2832	0.3928	0.2882	0.2631
NM_001083811	Gria2	0.7190	0.6768	0.8462	0.3530	0.7390	0.8537	0.8198	0.7832	0.7492	0.7364	0.8185	0.8528
NM_001040186	Mfge8	0.7576	0.8967	0.7834	0.8751	0.9390	0.9980	0.9317	0.9184	0.9959	0.8574	0.7540	0.9430
NM_001047096	Fig4	0.1257	0.0242	0.2551	0.2537	0.2724	0.0391	0.0693	0.0943	0.1329	0.1845	0.2731	0.0992
NM_001113750	Tesl	0.3137	0.3603	0.3593	0.1928	0.4494	0.1057	0.0694	0.2996	0.1887	0.0149	0.1976	0.4179
NM_001113778	Tmem11	0.4456	0.4922	0.3512	0.7308	0.1891	0.2869	0.4334	0.3456	0.1782	0.3369	0.5162	0.1897
NM_001109345	RGD1563349	0.6970	0.4891	0.6059	0.7208	0.3290	0.4488	0.3417	0.3107	0.6239	0.5738	0.6868	0.4073
NM_001108941	Tbl1xr1	0.9203	0.8512	0.9831	0.9453	0.6610	0.9781	0.8070	0.8529	0.8525	0.8865	0.3346	0.9800
NM_001105910	Grid2ip	0.5215	0.4113	0.5553	0.5101	0.8041	0.5288	0.8255	0.8063	0.7992	0.6743	0.5544	0.3920
NM_001082478	Igf1	0.5304	0.4676	0.6053	0.7230	0.5689	0.6631	0.4305	0.6835	0.2139	0.6217	0.6019	0.5162
NM_206846	isg12(b)	0.0002	0.0689	0.0392	0.0386	0.0187	0.0478	0.0729	0.0024	0.0806	0.0954	0.1265	0.0305
NM_001079698	Cxxc1	0.7563	0.7616	0.1905	0.7346	0.4396	0.8135	0.0900	0.8293	0.9311	0.7192	0.6778	0.9847
NM_001105999	Ankrd17	0.9301	0.9696	0.9714	0.9438	0.9254	0.8880	0.8981	0.6689	0.9954	0.9838	0.9778	0.8696
NM_199508	Pcdha9	0.8836	0.7477	0.8073	0.8707	0.9169	0.6736	0.8396	0.8721	0.8369	0.8447	0.8665	0.9023
NM_001042579	Unc13b	0.5390	0.6263	0.6008	0.4997	0.4780	0.5902	0.6404	0.6910	0.2414	0.6955	0.7752	0.5693
NM_001105745	Pou2f3	0.6813	0.8203	0.8998	0.6986	0.9315	0.9653	0.9545	0.6806	0.9561	0.9680	0.9280	0.8700

NM_001105737	Tek	0.8215	0.4403	0.5561	0.4364	0.7259	0.4539	0.0325	0.8054	0.3743	0.4317	0.2878	0.1536
NM_001012238	Fam20c	0.1487	0.0926	0.1532	0.2188	0.0704	0.1573	0.2056	0.1439	0.1323	0.1463	0.1359	0.4533
NM_001105922	Fkbp6	0.5554	0.1333	0.1798	0.7100	0.5348	0.6856	0.2771	0.6941	0.5742	0.4045	0.2893	0.5115
NM_001106008	Tbc1d19	0.3092	0.5399	0.8112	0.8857	0.1476	0.8205	0.6706	0.1469	0.9940	0.8061	0.9206	0.8275
NM_001106004	Commd8	0.2494	0.7489	0.8100	0.6634	0.3898	0.3036	0.3909	0.5979	0.2564	0.8626	0.7768	0.2088
NM_001082479	Igf1	0.5304	0.4676	0.6053	0.7230	0.5689	0.6631	0.4305	0.6835	0.2139	0.6217	0.6019	0.5162
NM_001105993	Glmn	0.8699	0.8078	0.3596	0.6158	0.7812	0.6150	0.7085	0.6723	0.8522	0.8346	0.8054	0.6105
NM_001047862	Ndufa8	0.0049	0.0088	0.4724	0.6779	0.0019	0.0036	0.4337	0.0166	0.0096	0.0024	0.0405	0.0021
NM_001105952	Sox13	0.4051	0.2242	0.3752	0.5470	0.6409	0.4314	0.3200	0.3931	0.3611	0.5515	0.4864	0.7678
NM_001106245	Lin37	0.1018	0.1117	0.0060	0.0041	0.0584	0.0351	0.0093	0.0073	0.0986	0.1146	0.1258	0.4518
NM_001106242	Eif3k	0.6711	0.5429	0.7928	0.6838	0.5801	0.0850	0.5866	0.6232	0.6875	0.6095	0.9249	0.3678
NM_001106552	Sp5	0.9398	0.7856	0.5207	0.5611	0.4910	0.5420	0.6064	0.2411	0.9215	0.5615	0.3362	0.3628
NM_001106427	Spry1	0.8309	0.5294	0.5123	0.6438	0.6514	0.4174	0.5419	0.4278	0.6381	0.3084	0.4603	0.2681
NM_001106237	Med29	0.0058	0.5028	0.2876	0.5752	0.1810	0.2425	0.8095	0.3192	0.0426	0.3315	0.4240	0.0624
NM_001047913	Sqrdl	0.0078	0.0431	0.1699	0.1116	0.0400	0.0161	0.0864	0.0240	0.0110	0.1294	0.0376	0.4053
NM_001037193	Acd	0.0536	0.3241	0.3607	0.4918	0.4610	0.6789	0.0077	0.1597	0.2961	0.3511	0.1137	0.7889
NM_001034927	Sulf2	0.1616	0.0517	0.9889	0.1804	0.1502	0.2647	0.4396	0.3806	0.8961	0.4033	0.0632	0.9749
NM_001106095	Lig4	0.8536	0.7950	0.8121	0.3921	0.6704	0.6775	0.6753	0.5448	0.7237	0.8536	0.6678	0.6433
NM_001105715	Wnt3	0.9192	0.7022	0.8110	0.5109	0.4849	0.5735	0.4844	0.4024	0.9131	0.7895	0.5043	0.8386
NM_022176	Prl6a1	0.1723	0.0799	0.0814	0.3718	0.0420	0.0039	0.0022	0.0456	0.0144	0.0008	0.0636	0.1002
NM_001004282	Tmem178	0.2165	0.0572	0.2250	0.6721	0.9718	0.5393	0.7732	0.4091	0.4307	0.4284	0.9207	0.2538
NM_001004243	Rpap3	0.9756	0.6783	0.0310	0.4800	0.7715	0.7633	0.1019	0.8955	0.1088	0.3902	0.5251	0.1573
NM_001106563	Vav2	0.2745	0.7666	0.2998	0.5591	0.3834	0.7033	0.3592	0.4460	0.4729	0.3984	0.3573	0.6819
NM_001106048	Dok2	0.0082	0.1108	0.5542	0.6969	0.3350	0.7292	0.0938	0.9114	0.0172	0.1685	0.6977	0.5221
NM_001044229	RGD1564996	0.0821	0.3686	0.4092	0.3036	0.4155	0.4909	0.2490	0.4092	0.1177	0.5461	0.4322	0.1266
NM_001037093	Akap9	0.5000	0.4206	0.4966	0.1127	0.1689	0.1865	0.2497	0.1419	0.3652	0.8176	0.4849	0.3539
NM_001106142	Afap1l1	0.0124	0.4455	0.3388	0.1196	0.2397	0.1356	0.0988	0.1055	0.1538	0.8129	0.8318	0.1979
NM_001044224	Spag5	0.9791	0.8516	0.0149	0.1844	0.7426	0.9915	0.8729	0.9990	0.7666	0.9106	0.9769	0.0404
NM_001077585	Cnpy2	0.1838	0.1845	0.1955	0.2047	0.1889	0.1930	0.2100	0.1999	0.3733	0.2854	0.6191	0.5104
NM_001105847	Imp5	0.1943	0.4680	0.2461	0.1994	0.5996	0.2012	0.1417	0.3738	0.3962	0.2568	0.7096	0.1585
NM_001105935	Foxn4	0.7523	0.2516	0.7307	0.1912	0.7894	0.6813	0.7779	0.7854	0.7069	0.8102	0.3384	0.7710
NM_001105940	Ormdl2	0.9720	0.4655	0.6423	0.9340	0.8340	0.5846	0.9518	0.7492	0.5643	0.6598	0.8018	0.9638
NM_001044225	Taf6	0.8100	0.9958	0.4832	0.4725	0.4907	0.7103	0.2268	0.8781	0.7769	0.7095	0.6711	0.8155
NM_001044227	Cfhl1	0.7633	0.7739	0.5578	0.8849	0.6146	0.7334	0.5742	0.5611	0.5952	0.6620	0.6334	0.5775
NM_001105948	Serpinb8	0.2712	0.0822	0.2523	0.3705	0.2963	0.1857	0.1808	0.1649	0.1449	0.0128	0.2338	0.4261
NM_001106051	Esd	0.1138	0.2202	0.4522	0.0285	0.0898	0.0606	0.0791	0.0289	0.0171	0.0785	0.1818	0.0707
NM_001105946	RGD1564093	0.2317	0.5180	0.4173	0.6766	0.5482	0.2399	0.2956	0.4563	0.5650	0.3407	0.3448	0.4561
NM_001105944	Rfx1	0.9048	0.8158	0.2650	0.8827	0.8826	0.9993	0.4780	0.9514	0.9305	0.8179	0.7977	0.9979
NM_001071776	Bola1	0.2958	0.2095	0.1488	0.3891	0.2733	0.1759	0.3107	0.3177	0.1145	0.0534	0.3669	0.0420
NM_001048233	Ino80b	0.1191	0.0131	0.0678	0.7277	0.1055	0.3574	0.2400	0.2558	0.5704	0.3378	0.1082	0.2129
NM_001106146	Hdhd1a	0.4174	0.3805	0.3521	0.5008	0.1760	0.1500	0.4216	0.2224	0.3767	0.1891	0.4556	0.0776
NM_001106147	Trim36	0.1597	0.7484	0.6255	0.5987	0.1144	0.0598	0.8915	0.0950	0.9656	0.7960	0.9151	0.0412
NM_001105950	Slc35f5	0.9955	0.9853	0.9712	0.9789	0.9728	0.9664	0.9614	0.9984	0.7525	0.9606	0.9820	0.9438
NM_001044260	LOC498973	0.1932	0.5932	0.2095	0.2514	0.5082	0.4476	0.2340	0.6184	0.6661	0.5157	0.3371	0.5636
NM_001044246	Mageb18	0.6453	0.1545	0.3494	0.4680	0.2740	0.5175	0.4513	0.1175	0.1820	0.7611	0.5722	0.1133
NM_001044244	Tomm34	0.1182	0.2043	0.7543	0.6958	0.1120	0.7830	0.4266	0.3679	0.4821	0.0491	0.6333	0.9455
NM_001106154	Tmco6	0.0846	0.0566	0.3286	0.5686	0.0797	0.0534	0.2436	0.1700	0.0431	0.0848	0.1524	0.1297
NM_001106079	Atp13a1	0.8023	0.7717	0.0406	0.3120	0.9245	0.9085	0.6599	0.8522	0.9601	0.9322	0.8860	0.7924
NM_001106085	Dctn6	0.2992	0.4237	0.3864	0.2766	0.2652	0.1875	0.2580	0.0995	0.2833	0.0803	0.4397	0.1678

NM_023985	Trib1	0.5387	0.6595	0.6652	0.3333	0.0164	0.7361	0.2803	0.1126	0.1294	0.3097	0.1366	0.9521
NM_001106103	Ogn	0.9929	0.1904	0.9640	0.9546	0.9995	0.8968	0.8849	0.8904	0.1612	0.2493	0.3028	0.0856
NM_001105953	Tmem9	0.0604	0.0453	0.3196	0.0500	0.8115	0.7237	0.1457	0.7356	0.0930	0.0078	0.2852	0.1641
NM_001106053	RGD1306441	0.0683	0.0439	0.6805	0.4598	0.4339	0.1735	0.1836	0.4553	0.0515	0.0207	0.0625	0.2299
NM_013065	Ppp1cb	0.9679	0.9124	0.9623	0.9716	0.9551	0.8954	0.8237	0.8766	0.9750	0.8207	0.9722	0.7163
NM_013053	Ywhaq	0.5946	0.9686	0.9406	0.9649	0.7433	0.9586	0.9897	0.4291	0.5902	0.7626	0.9695	0.8785
NM_012507	Atp1b2	0.8388	0.7878	0.9302	0.9000	0.9879	0.6507	0.9589	0.8296	0.8886	0.6881	0.7024	0.8744
NM_001113775	RGD1304982	0.5223	0.3799	0.3966	0.4091	0.2826	0.7141	0.8832	0.7514	0.6213	0.5445	0.5055	0.3467
NM_001130013	Cdc42bpg	0.1698	0.9525	0.7692	0.4517	0.7462	0.8144	0.9276	0.8571	0.9032	0.8500	0.8645	0.3276
NM_001113781	Fam64a	0.5060	0.8672	0.0432	0.7052	0.9868	0.5639	0.8368	0.7325	0.9753	0.9015	0.4755	0.2762
NM_001037219	Foxk1	0.2265	0.2603	0.3131	0.5026	0.4911	0.2454	0.2908	0.4433	0.2133	0.2286	0.4265	0.4947
NM_001113776	Rell1	0.3344	0.1242	0.7806	0.3355	0.1473	0.7144	0.1945	0.1470	0.1367	0.1405	0.7415	0.8373
NM_001106016	Morc2	0.9609	0.9505	0.7326	0.8180	0.9635	0.9893	0.7503	0.9400	0.9427	0.9568	0.9522	0.8253
NM_001134590	RGD1559942	0.2880	0.2228	0.5087	0.2770	0.3349	0.3339	0.3136	0.2950	0.6149	0.2287	0.3979	0.3428
NM_001105957	Rgl1	0.7341	0.8481	0.0974	0.1416	0.3518	0.6135	0.8529	0.4304	0.2960	0.0910	0.8257	0.4094
NM_001106063	LOC290595	0.2053	0.0228	0.0184	0.0420	0.1772	0.0697	0.0753	0.0327	0.0091	0.0238	0.0245	0.1509
NM_001044250	Stat6	0.0310	0.1908	0.1021	0.0446	0.0281	0.2306	0.1515	0.0920	0.1873	0.6685	0.0446	0.0382
NM_001106108	Irf4	0.6538	0.4718	0.5456	0.5805	0.7274	0.5026	0.4746	0.1271	0.6810	0.7947	0.6417	0.7241
NM_001044696	RGD1565052	0.1718	0.1804	0.5192	0.0631	0.1864	0.1384	0.1936	0.2498	0.0104	0.2513	0.2752	0.0531
NM_001082977	Rasgrp2	0.2765	0.4465	0.7005	0.6515	0.0557	0.1099	0.5143	0.0615	0.3459	0.1721	0.6437	0.7980
NM_001105970	Nhlh1	0.9409	0.8914	0.9631	0.8868	0.8423	0.9602	0.9421	0.7573	0.7657	0.9199	0.7966	0.8646
NM_001105969	Vangl2	0.8632	0.7750	0.1599	0.1476	0.5925	0.5673	0.6578	0.3592	0.7706	0.6808	0.1080	0.9522
NM_001105965	Dpt	0.9533	0.1661	0.3098	0.0472	0.9143	0.7458	0.6926	0.8169	0.1291	0.1060	0.0031	0.9476
NM_001105964	Kifap3	0.1107	0.1822	0.7402	0.1625	0.5251	0.8665	0.3208	0.7583	0.2521	0.2548	0.0782	0.3611
NM_001106179	Tmem208	0.2430	0.5604	0.2060	0.2406	0.7908	0.9306	0.0593	0.9826	0.3841	0.2094	0.2282	0.0265
NM_001106299	Eraf	0.9150	0.7576	0.7596	0.8960	0.8200	0.7313	0.7746	0.9351	0.8827	0.8405	0.9065	0.9286
NM_001100963	Aass	0.5051	0.3360	0.5533	0.6529	0.5858	0.6661	0.3101	0.6245	0.4314	0.8295	0.7758	0.1298
NM_001105974	Grem2	0.8035	0.4853	0.6954	0.5287	0.3808	0.5043	0.5288	0.6481	0.5137	0.7655	0.6165	0.8423
NM_001105973	Slamf8	0.6295	0.7334	0.6142	0.8817	0.7500	0.8457	0.4157	0.5880	0.9034	0.4133	0.7381	0.9214
NM_001008334	Tmem97	0.3986	0.9012	0.0862	0.5288	0.6481	0.9515	0.6951	0.5821	0.7702	0.6821	0.4399	0.0965
NM_001047097	Zhx3	0.4015	0.5239	0.1622	0.3472	0.4413	0.3615	0.7222	0.2828	0.9386	0.7254	0.1284	0.5010
NM_001047109	LOC499531	0.1931	0.4793	0.6615	0.2290	0.5837	0.5226	0.6923	0.3369	0.6781	0.1843	0.2148	0.4969
NM_001004213	Tmem66	0.0676	0.0438	0.2515	0.1989	0.0428	0.2894	0.1182	0.0648	0.1757	0.2846	0.2747	0.3156
NM_001106122	RGD1309605	0.8506	0.2843	0.8710	0.4755	0.6111	0.9435	0.0646	0.2050	0.0479	0.4214	0.3216	0.7821
NM_001106304	Gprc5b	0.3065	0.1551	0.5077	0.1478	0.3438	0.2299	0.2763	0.0167	0.4225	0.3545	0.3178	0.6324
NM_001082477	Igf1	0.5304	0.4676	0.6053	0.7230	0.5689	0.6631	0.4305	0.6835	0.2139	0.6217	0.6019	0.5162
NM_001106204	Rnf217	0.9482	0.9700	0.9969	0.9348	0.4109	0.2806	0.9268	0.4195	0.9602	0.8245	0.8369	0.7730
NM_001106193	Trappc2l	0.0131	0.0022	0.5268	0.0066	0.0109	0.0180	0.0223	0.0048	0.1709	0.0471	0.0022	0.0333
NM_001106192	Cdt1	0.3813	0.9309	0.1101	0.7507	0.7167	0.8912	0.5694	0.8460	0.8356	0.2756	0.7796	0.4365
NM_001106208	Pabpc3	0.9420	0.8030	0.8219	0.6921	0.8816	0.3090	0.7505	0.6399	0.7362	0.8825	0.7872	0.6369
NM_001106220	Nlrp4a	0.2199	0.4782	0.3767	0.6417	0.6993	0.8165	0.6648	0.4310	0.4311	0.6653	0.7479	0.3180
NM_001106223	Fiz1	0.6018	0.6209	0.6363	0.6685	0.6976	0.4443	0.6574	0.4859	0.5282	0.7199	0.4573	0.7121
NM_001106227	Tmem160	0.0208	0.2152	0.0681	0.5748	0.0449	0.0182	0.2162	0.0051	0.0082	0.1140	0.4363	0.0343
NM_001106010	Gba3	0.1936	0.3823	0.1100	0.3223	0.3101	0.4032	0.1862	0.2474	0.0336	0.0748	0.2311	0.0476
NM_001047880	Slc25a15	0.0091	0.2408	0.0196	0.4681	0.0815	0.3171	0.5041	0.1274	0.0184	0.0562	0.1033	0.0280
NM_001013972	Efcab3	0.0703	0.1297	0.0764	0.5153	0.4438	0.2438	0.4203	0.0777	0.2923	0.2707	0.2419	0.1569
NM_001106058	Cldn10	0.9259	0.3567	0.8587	0.6217	0.4701	0.6534	0.2323	0.6183	0.4932	0.6382	0.3684	0.9348
NM_001047864	Dppa3	0.2485	0.1067	0.5209	0.2238	0.4604	0.3285	0.4507	0.3123	0.4142	0.3422	0.3496	0.3100
NM_001106107	RGD1309020	0.8240	0.7114	0.7529	0.0020	0.0650	0.1578	0.2495	0.0158	0.9051	0.8161	0.9891	0.8456

NM_001105752	Pop5	0.0009	0.1724	0.0036	0.0122	0.0024	0.0032	0.0128	0.0075	0.0030	0.0130	0.0034	0.0759
NM_001105746	Pou6f1	0.6968	0.6315	0.2087	0.6089	0.0736	0.7198	0.4857	0.2690	0.8436	0.7023	0.6828	0.3934
NM_001025117	Ehbp1l1	0.2657	0.4589	0.3812	0.3385	0.2393	0.2562	0.3581	0.4333	0.5659	0.3012	0.4711	0.6395
NM_001106162	Abhd3	0.2586	0.2369	0.2405	0.1654	0.0508	0.0700	0.0126	0.1024	0.1176	0.1825	0.2958	0.2122
NM_001106359	Wnt8b	0.6120	0.9039	0.9515	0.7657	0.8374	0.8429	0.6544	0.2528	0.5697	0.9187	0.7043	0.6904
NM_001106151	Pabpc2	0.8832	0.8042	0.3874	0.4552	0.7630	0.0370	0.6836	0.0396	0.8076	0.1144	0.9941	0.4713
NM_001106033	Efs	0.1872	0.1905	0.0229	0.3760	0.5739	0.2567	0.3897	0.3560	0.3248	0.5396	0.2467	0.6766
NM_001106035	RGD1564324	0.5896	0.4047	0.4102	0.5532	0.4533	0.3215	0.4404	0.4419	0.2855	0.3971	0.5643	0.5138
NM_001106168	Mmp15	0.7286	0.8738	0.8579	0.7652	0.8649	0.7113	0.3041	0.9322	0.5217	0.9086	0.8350	0.7689
NM_001047915	Rapgef5	0.8400	0.6102	0.7345	0.8713	0.7172	0.7081	0.4567	0.8026	0.7477	0.7752	0.7380	0.5955
NM_001106070	Map1s	0.3444	0.2570	0.0707	0.9465	0.1575	0.7477	0.3343	0.3387	0.1593	0.4702	0.1925	0.8213
NM_001106062	Ogdhl	0.6108	0.3031	0.4704	0.4479	0.6065	0.2796	0.5836	0.6208	0.1002	0.2982	0.0750	0.1452
NM_001106046	Adamdec1	0.6547	0.2821	0.5534	0.3935	0.2694	0.1159	0.2094	0.4516	0.6563	0.6437	0.2918	0.6296
NM_001106252	Klk11	0.6302	0.6141	0.7008	0.3486	0.7362	0.5311	0.8589	0.6052	0.3476	0.6827	0.5158	0.7833
NM_001106368	Pik3ap1	0.0639	0.4925	0.7212	0.2847	0.8230	0.8375	0.8081	0.9040	0.1683	0.2056	0.7622	0.5760
NM_001047087	Eif3s10	0.9903	0.9567	0.8336	0.9407	0.8298	0.8816	0.4797	0.9074	0.8612	0.9389	0.9700	0.7462
NM_001106057	Slitrk6	0.6840	0.3258	0.7763	0.4076	0.7329	0.6070	0.1201	0.8273	0.2875	0.7781	0.8758	0.2568
NM_001106052	Olfm4	0.6245	0.3032	0.5749	0.6157	0.5093	0.3148	0.3822	0.2958	0.6137	0.4182	0.5199	0.4449
NM_001106059	Thoc3	0.5361	0.7899	0.6817	0.7642	0.1564	0.2243	0.6588	0.1352	0.7881	0.6280	0.2017	0.4646
NM_001106060	Slmap	0.1047	0.6042	0.5673	0.4907	0.0515	0.6110	0.0522	0.3284	0.6671	0.3007	0.1401	0.8003
NM_001106183	Tmed6	0.1818	0.1290	0.0462	0.4189	0.2978	0.4087	0.7761	0.1823	0.0826	0.0259	0.5783	0.1142
NM_001105744	Eif2ak4	0.7075	0.7866	0.3932	0.0302	0.8637	0.6757	0.0624	0.6564	0.5879	0.6079	0.3589	0.7498
NM_001105740	Ndst2	0.9064	0.8140	0.2190	0.2095	0.9813	0.8118	0.8485	0.9320	0.9691	0.8565	0.6788	0.2565
NM_001103359	LOC10012537	0.5696	0.4093	0.5006	0.4473	0.3342	0.4359	0.4165	0.1534	0.4134	0.5250	0.4719	0.0842
NM_001047907	Sfrs3	0.9918	0.8763	0.7839	0.9952	0.9937	0.9919	0.9900	0.9109	0.8081	0.8313	0.8220	0.7401
NM_001106064	Cherp	0.5452	0.4798	0.1190	0.4762	0.8677	0.4272	0.6608	0.7827	0.6094	0.5421	0.6738	0.6198
NM_001005538	Fam160a2	0.6554	0.8015	0.3359	0.8326	0.7164	0.6790	0.1399	0.6364	0.6786	0.7423	0.8102	0.8366
NM_001106277	Fam154b	0.9022	0.9294	0.8971	0.9358	0.6442	0.6397	0.8454	0.8097	0.9049	0.8945	0.8205	0.8072
NM_001106253	Klk9	0.0624	0.0773	0.0659	0.0329	0.0824	0.0943	0.2736	0.4807	0.2876	0.1588	0.5178	0.6695
NM_001142363	Alg3	0.1834	0.1843	0.7156	0.4366	0.5333	0.4631	0.2093	0.5714	0.2655	0.4891	0.1850	0.2921
NM_001106186	Ftsjd1	0.7682	0.3298	0.3967	0.3858	0.8317	0.7074	0.0271	0.9203	0.1294	0.9027	0.3311	0.6875
NM_001106067	Glt25d1	0.6660	0.3314	0.9482	0.9724	0.3770	0.8512	0.9237	0.1341	0.6082	0.6030	0.6545	0.9439
NM_001106068	B3gnt3	0.9263	0.8997	0.7304	0.1570	0.8096	0.8798	0.8863	0.9348	0.8385	0.8567	0.4966	0.5702
NM_001100499	Pkp2	0.8264	0.8242	0.3873	0.1250	0.9402	0.9064	0.9352	0.9521	0.0391	0.8628	0.8046	0.1988
NM_001106189	Cdyl2	0.2675	0.4773	0.4415	0.1797	0.2766	0.0941	0.4233	0.3653	0.1755	0.3808	0.3369	0.3119
NM_001106185	Dhx38	0.6379	0.5069	0.0422	0.2113	0.9085	0.8325	0.6330	0.8236	0.8998	0.6444	0.3860	0.4547
NM_001080153	Tmub1	0.4697	0.8760	0.7648	0.9197	0.6990	0.9477	0.9071	0.8780	0.8983	0.7337	0.5651	0.8047
NM_001105678	Prps1l1	0.3403	0.1766	0.3436	0.5046	0.6553	0.2220	0.3346	0.2947	0.2134	0.2479	0.1497	0.4848
NM_001108450	Nudt7	0.0913	0.0476	0.1026	0.0060	0.2886	0.1566	0.2252	0.0981	0.1523	0.3010	0.2487	0.0846
NM_001106166	Tk2	0.0380	0.1308	0.8947	0.3456	0.2198	0.4927	0.4716	0.1977	0.1451	0.2783	0.2922	0.7724
NM_001106130	Atp9b	0.9858	0.9457	0.9945	0.7690	0.8055	0.9990	0.9762	0.9558	0.9903	0.9180	0.9504	0.9997
NM_001106076	Cope	0.0210	0.0091	0.1308	0.0547	0.0788	0.0306	0.2741	0.0833	0.0305	0.1330	0.0104	0.0518
NM_001083911	Sepp1	0.5034	0.7325	0.7425	0.6884	0.4850	0.4640	0.2519	0.7605	0.5686	0.1956	0.5549	0.5071
NM_001105779	Nola2	0.6957	0.8647	0.2276	0.8306	0.8419	0.6404	0.8969	0.9254	0.8512	0.7915	0.8195	0.4115
NM_001105777	Rars	0.0475	0.1742	0.0478	0.0115	0.0030	0.0810	0.1467	0.0668	0.0086	0.2105	0.0805	0.2134
NM_001047849	Agpat6	0.4982	0.9106	0.3306	0.0685	0.8576	0.8834	0.7525	0.5412	0.7418	0.3904	0.7995	0.5193
NM_001106288	Swap70	0.2973	0.2761	0.3075	0.3152	0.3921	0.6849	0.3807	0.0436	0.0067	0.0029	0.1703	0.2591
NM_001106286	Lyve1	0.7877	0.8703	0.8049	0.5706	0.5317	0.8785	0.5044	0.8700	0.5519	0.9616	0.7011	0.8639
NM_001106219	Prpf31	0.3149	0.6895	0.9268	0.7790	0.7560	0.5085	0.9085	0.8932	0.9350	0.8260	0.7577	0.8849

NM_001005330	Cr1l	0.3494	0.2003	0.4946	0.6610	0.6631	0.8096	0.4065	0.6672	0.2443	0.3532	0.4544	0.2036
NM_001005265	Cr1l	0.3494	0.2003	0.4946	0.6610	0.6631	0.8096	0.4065	0.6672	0.2443	0.3532	0.4544	0.2036
NM_001082580	Nit1	0.2531	0.5182	0.3091	0.3879	0.6050	0.8272	0.3629	0.3897	0.2248	0.5944	0.7041	0.7202
NM_001082541	Hnrpd	0.7105	0.7880	0.8604	0.8332	0.6938	0.3275	0.7459	0.6406	0.7968	0.4598	0.7435	0.6802
NM_001082540	Hnrpd	0.7105	0.7880	0.8604	0.8332	0.6938	0.3275	0.7459	0.6406	0.7968	0.4598	0.7435	0.6802
NM_001082539	Hnrpd	0.7105	0.7880	0.8604	0.8332	0.6938	0.3275	0.7459	0.6406	0.7968	0.4598	0.7435	0.6802
NM_001105857	Pycr1	0.5163	0.3494	0.2162	0.2742	0.4807	0.7137	0.3608	0.0520	0.5975	0.3546	0.1277	0.5136
NM_001083586	Cugbp2	0.3936	0.6237	0.3750	0.7325	0.4218	0.3604	0.4895	0.3658	0.6322	0.3887	0.7018	0.3520
NM_001106292	Eif3f	0.8241	0.1183	0.9856	0.4274	0.1116	0.9032	0.7696	0.3757	0.4562	0.1031	0.0274	0.5838
NM_001106507	Bub1	0.8797	0.8960	0.1055	0.7703	0.9784	0.9247	0.9587	0.9756	0.8543	0.9909	0.9798	0.2461
NM_001107005	Wnt3a	0.5790	0.4666	0.4516	0.5946	0.3442	0.6946	0.6859	0.4633	0.6284	0.5844	0.2024	0.5766
NM_001106308	Mmp21	0.7111	0.1688	0.7430	0.5147	0.3313	0.4111	0.3953	0.6199	0.8634	0.3789	0.1500	0.5905
NM_001105799	Med11	0.7510	0.7415	0.1187	0.9173	0.7437	0.0819	0.4789	0.6758	0.6449	0.7274	0.6356	0.1350
NM_001081974	Klc1	0.5384	0.5792	0.7357	0.4931	0.8012	0.8440	0.5928	0.9560	0.6811	0.5689	0.5988	0.9933
NM_001081973	Klc1	0.5384	0.5792	0.7357	0.4931	0.8012	0.8440	0.5928	0.9560	0.6811	0.5689	0.5988	0.9933
NM_001106309	Znf511	0.0243	0.8918	0.2292	0.0996	0.0152	0.0347	0.3906	0.0279	0.1680	0.7019	0.1617	0.0255
NM_019381	Tmbim6	0.6072	0.0017	0.7845	0.4932	0.7411	0.6791	0.8245	0.8311	0.1238	0.0248	0.2007	0.1630
NM_001081972	Klc1	0.5384	0.5792	0.7357	0.4931	0.8012	0.8440	0.5928	0.9560	0.6811	0.5689	0.5988	0.9933
NM_001100994	Rpl37a_predict	0.8382	0.7329	0.1948	0.9089	0.4540	0.3980	0.2581	0.7864	0.2947	0.3668	0.7655	0.1888
NM_001100506	Pctk3	0.4072	0.6544	0.2152	0.3552	0.4955	0.4571	0.7416	0.1548	0.8919	0.4270	0.3417	0.6435
NM_001106106	Eef1e1	0.2476	0.6783	0.3368	0.3436	0.4268	0.0742	0.5826	0.8109	0.5724	0.3044	0.8787	0.0825
NM_001004223	Eef1g	0.4463	0.0621	0.8616	0.8655	0.1323	0.6712	0.9809	0.3942	0.2427	0.8282	0.7577	0.7886
NM_001106250	Siglecg	0.0504	0.7692	0.2904	0.0867	0.0305	0.0871	0.6788	0.0273	0.1181	0.6815	0.8918	0.2817
NM_001105994	Abhd7	0.0962	0.4497	0.2352	0.0739	0.0992	0.2002	0.0696	0.2752	0.7166	0.1087	0.0943	0.7110
NM_001106284	Lrrc51	0.5415	0.0153	0.7096	0.5573	0.6019	0.3272	0.6757	0.1912	0.0837	0.0324	0.0910	0.0017
NM_001081660	Crygc	0.3957	0.3709	0.3691	0.5092	0.3762	0.3923	0.4320	0.5204	0.1673	0.6922	0.7443	0.1741
NM_001105712	Grm3	0.3504	0.4112	0.5035	0.2147	0.3380	0.1583	0.4807	0.5193	0.7892	0.4973	0.6926	0.6250
NM_001102383	Pds5b	0.9572	0.9871	0.6544	0.9336	0.9803	0.9610	0.8251	0.9397	0.7328	0.8477	0.9271	0.6791
NM_001105722	Rps5	0.8204	0.8536	0.8292	0.8096	0.9476	0.9356	0.8126	0.9939	0.7899	0.6205	0.9617	0.8970
NM_001102381	Nts	0.4323	0.5817	0.6572	0.3823	0.8612	0.4823	0.4527	0.4566	0.0291	0.4461	0.3545	0.1234
NM_001100136	Magmas	0.1260	0.0093	0.1339	0.2580	0.0256	0.1454	0.0245	0.1525	0.1131	0.0568	0.1149	0.0671
NM_001122975	Lrrc55	0.5393	0.2214	0.6750	0.5019	0.8062	0.7673	0.0846	0.5698	0.4205	0.2721	0.2998	0.6478
NM_053491	Plg	0.3274	0.3945	0.4123	0.4007	0.3854	0.6190	0.5668	0.5811	0.4801	0.6267	0.6675	0.4431
NM_001106127	Prtfdc1	0.7370	0.5123	0.9191	0.8205	0.8499	0.7661	0.9059	0.7812	0.4518	0.7827	0.7215	0.3421
NM_001101805	Pds5b	0.9572	0.9871	0.6544	0.9336	0.9803	0.9610	0.8251	0.9397	0.7328	0.8477	0.9271	0.6791
NM_001106327	Slc22a20	0.1791	0.1939	0.4615	0.3893	0.7956	0.6233	0.7296	0.7408	0.1405	0.7458	0.3704	0.4738
NM_001106105	Gcm2	0.3206	0.1173	0.2332	0.4051	0.4733	0.4431	0.2831	0.3635	0.5729	0.5128	0.3274	0.5493
NM_198737	Arl6ip1	0.6009	0.7231	0.5599	0.7281	0.5636	0.7136	0.7134	0.2534	0.7198	0.6207	0.4751	0.6336
NM_001105995	Klhl8	0.9619	0.4444	0.3423	0.4379	0.8747	0.9671	0.5768	0.8889	0.3335	0.4465	0.6367	0.0727
NM_001015018	Ddx17	0.8682	0.7670	0.7987	0.9404	0.6283	0.9611	0.2054	0.7426	0.6471	0.4190	0.3567	0.3650
NM_001106099	Cts7	0.5542	0.1532	0.5029	0.5917	0.7157	0.5959	0.4775	0.4524	0.6475	0.3990	0.4558	0.5648
NM_001082409	Fbxw17	0.0259	0.3490	0.7988	0.1362	0.0085	0.0899	0.3373	0.0236	0.5792	0.1201	0.5462	0.7162
NM_001106112	Gmnn	0.0359	0.0100	0.1432	0.0617	0.7941	0.2022	0.1071	0.6470	0.0061	0.0393	0.0164	0.0062
NM_001105756	Pbp2	0.1346	0.6917	0.4076	0.5586	0.4786	0.7565	0.3859	0.6497	0.8791	0.4780	0.2796	0.8911
NM_001106262	Otog	0.4632	0.5053	0.6166	0.4077	0.3392	0.5336	0.3123	0.6473	0.8244	0.7359	0.5122	0.5485
NM_001106116	Mrpl32	0.8173	0.3589	0.1754	0.0841	0.4321	0.0812	0.1070	0.2315	0.0536	0.4345	0.2695	0.0166
NM_001106265	Perp	0.7665	0.5195	0.4385	0.1468	0.2741	0.1765	0.7820	0.3299	0.4799	0.3529	0.3951	0.3180
NM_001100987	Aqr	0.4134	0.0868	0.0836	0.0873	0.1492	0.3469	0.0982	0.1656	0.8516	0.0949	0.8590	0.7664
NM_001106345	Lage3	0.7840	0.5099	0.3715	0.8107	0.6927	0.7745	0.8930	0.8219	0.4498	0.2880	0.4075	0.0473

NM_001100174	Arx	0.8102	0.5855	0.7732	0.7306	0.4647	0.1290	0.2059	0.4601	0.6395	0.7744	0.6012	0.5156
NM_001105818	Tp53i13	0.2483	0.0504	0.1361	0.0633	0.0687	0.0633	0.1821	0.4325	0.0198	0.0753	0.3224	0.5606
NM_001105939	Naca	0.6009	0.3343	0.6917	0.4738	0.6733	0.8114	0.0679	0.7612	0.0519	0.2134	0.4555	0.7221
NM_001100648	Foxn1	0.3476	0.8918	0.7441	0.6961	0.7816	0.6415	0.8635	0.9096	0.9178	0.9032	0.6646	0.9751
NM_001105794	Lsmd1	0.9023	0.7859	0.2181	0.1865	0.7422	0.3866	0.9701	0.8890	0.2814	0.9295	0.5696	0.3176
NM_001106356	Zfand5	0.3271	0.2198	0.9530	0.9347	0.2002	0.4657	0.0755	0.3236	0.3504	0.2155	0.2720	0.9846
NM_001100680	Ralgps2	0.8859	0.7883	0.8598	0.9244	0.7121	0.7441	0.9521	0.9813	0.8684	0.8092	0.8274	0.7846
NM_001105813	Vps53	0.1055	0.6484	0.8343	0.9123	0.1410	0.4828	0.7177	0.1651	0.5412	0.7961	0.7791	0.8317
NM_001106170	Mcm5	0.7571	0.7817	0.5124	0.8016	0.7465	0.8868	0.8758	0.9569	0.6953	0.7467	0.6474	0.4544
NM_001106163	Snrpd1	0.5520	0.5441	0.3071	0.6873	0.5687	0.4425	0.6621	0.1304	0.6650	0.2334	0.3186	0.1595
NM_001105804	Nek8	0.9054	0.9553	0.6030	0.5509	0.3527	0.0397	0.7532	0.1021	0.5728	0.8083	0.8746	0.1974
NM_001105924	Scand3	0.8692	0.6022	0.8237	0.3996	0.7230	0.5900	0.1166	0.3642	0.3576	0.9330	0.5471	0.2671
NM_001105921	Polr2j	0.8483	0.8221	0.6510	0.8325	0.5139	0.2537	0.0717	0.7815	0.2033	0.1937	0.0459	0.2475
NM_001105947	RGD1564058	0.0297	0.0551	0.7878	0.5584	0.0565	0.0571	0.3873	0.0864	0.0155	0.0156	0.0321	0.1340
NM_001105815	Ift20	0.9818	0.8128	0.1697	0.1403	0.6572	0.7925	0.7428	0.7705	0.1244	0.7546	0.4679	0.4388
NM_001106337	Ms4a12	0.1089	0.1619	0.2148	0.4582	0.3589	0.4625	0.4331	0.1602	0.1336	0.4240	0.7214	0.2967
NM_001106361	Pax2	0.3471	0.2792	0.4085	0.4731	0.3902	0.3310	0.0657	0.5484	0.3073	0.3755	0.1310	0.5148
NM_001106132	Loxhd1	0.5726	0.5247	0.5851	0.6088	0.5945	0.6683	0.7074	0.4788	0.5360	0.7111	0.6231	0.6408
NM_001105843	Lsm12	0.5430	0.9427	0.7720	0.9056	0.9359	0.4269	0.9802	0.9275	0.8386	0.9005	0.6553	0.6905
NM_001100573	Zfp184	0.3050	0.7267	0.1861	0.6322	0.4358	0.5134	0.8173	0.1868	0.1874	0.8569	0.3845	0.4572
NM_001109182	RGD1565989	0.1231	0.3728	0.2720	0.5342	0.3137	0.0889	0.2363	0.1340	0.0944	0.5835	0.2172	0.1097
NM_001106023	RGD1306508	0.5890	0.2385	0.5677	0.6222	0.5658	0.4706	0.2084	0.5551	0.2212	0.5221	0.4089	0.3133
NM_001105835	Cisd3	0.0803	0.1799	0.1107	0.9121	0.0833	0.0175	0.5860	0.0411	0.2812	0.2233	0.1165	0.1267
NM_001105919	Fis1	0.8699	0.3453	0.8252	0.7380	0.6116	0.0576	0.0849	0.7443	0.0864	0.0438	0.0896	0.1182
NM_001101011	Ndufv3l	0.5765	0.5346	0.8900	0.5970	0.1885	0.1922	0.6841	0.5415	0.4226	0.4036	0.3759	0.7807
NM_001105849	Ccdc46	0.3302	0.6455	0.2335	0.0929	0.4047	0.2474	0.5928	0.1581	0.7315	0.4248	0.5654	0.2877
NM_001105211	Mrvi1	0.5838	0.7245	0.6054	0.4668	0.7061	0.7073	0.7175	0.5543	0.7148	0.2964	0.4649	0.5891
NM_001106411	Gpx8	0.0082	0.2158	0.0518	0.0137	0.0626	0.0107	0.0197	0.0135	0.1637	0.0704	0.0088	0.0180
NM_001106296	RGD1308215	0.5615	0.4051	0.2785	0.1449	0.7618	0.7426	0.5594	0.5089	0.6854	0.3224	0.3278	0.5817
NM_001105736	Chic2	0.6817	0.3350	0.9511	0.4374	0.0544	0.3048	0.1140	0.2596	0.2204	0.6994	0.7122	0.1984
NM_001105728	Nthl1	0.8663	0.7910	0.8391	0.1821	0.1342	0.0564	0.8227	0.5251	0.9002	0.7263	0.8996	0.7536
NM_001100991	LOC499124	0.4634	0.2974	0.3800	0.5840	0.7712	0.4201	0.1298	0.6129	0.4338	0.7091	0.7040	0.8538
NM_001106140	Atp8b1	0.4050	0.2938	0.5918	0.4859	0.5055	0.3047	0.5739	0.5550	0.1127	0.8333	0.1458	0.3688
NM_001106366	Col17a1	0.2798	0.1776	0.2618	0.3369	0.1677	0.2255	0.3471	0.3249	0.1439	0.1521	0.1233	0.4886
NM_001106317	Rassf7	0.4717	0.4823	0.2048	0.5622	0.4636	0.4834	0.7488	0.3494	0.4330	0.3185	0.3875	0.0857
NM_001106310	RGD1311186	0.2518	0.7906	0.1025	0.1185	0.8846	0.6359	0.1850	0.6159	0.5315	0.4236	0.0434	0.4635
NM_001004205	Rilpl2	0.0909	0.0248	0.8367	0.1053	0.0394	0.0333	0.1964	0.0607	0.0111	0.2029	0.1133	0.0624
NM_001106371	Hells	0.9620	0.9435	0.6969	0.9395	0.9180	0.8567	0.9787	0.7550	0.9191	0.8700	0.9238	0.9947
NM_001105735	Litaf	0.3662	0.3224	0.2028	0.7672	0.1643	0.3771	0.0388	0.1727	0.2658	0.2791	0.2411	0.4358
NM_001105785	Nt5m	0.5584	0.1222	0.6759	0.6964	0.8810	0.1180	0.0227	0.6575	0.5923	0.4710	0.6088	0.3970
NM_001103352	Kifc3	0.9059	0.9741	0.7059	0.8492	0.6424	0.5824	0.8904	0.6448	0.8536	0.7312	0.9011	0.9425
NM_001106153	Ndufa2	0.3465	0.1996	0.7206	0.3207	0.3986	0.4775	0.5261	0.3104	0.2961	0.5753	0.4559	0.3266
NM_001106152	Slc25a2	0.0559	0.3382	0.3187	0.0552	0.1778	0.3045	0.4787	0.1488	0.0597	0.0455	0.1311	0.2050
NM_001106150	Spry4	0.6291	0.9450	0.9174	0.8843	0.8987	0.9221	0.7701	0.6546	0.6538	0.9130	0.6464	0.5424
NM_001106149	RGD1563060	0.3479	0.0933	0.1254	0.3476	0.3783	0.1636	0.2485	0.6390	0.2994	0.4120	0.1870	0.1923
NM_001113785	Ttc35	0.3467	0.0897	0.9555	0.0048	0.7554	0.0997	0.7132	0.5349	0.0769	0.0842	0.3040	0.3004
NM_001106181	Dus2l	0.0188	0.0477	0.2222	0.0309	0.0615	0.2068	0.0435	0.2398	0.0656	0.0709	0.1104	0.1243
NM_001106450	Etv3	0.4264	0.4178	0.4538	0.4073	0.1883	0.7247	0.4978	0.1992	0.4616	0.4371	0.4339	0.1886
NM_001105861	Top3b	0.1214	0.0956	0.0855	0.2860	0.4060	0.6399	0.1677	0.0925	0.1788	0.2650	0.6773	0.1042

NM_001105802	Dhx33	0.7556	0.4158	0.6339	0.3030	0.7602	0.5349	0.6059	0.9049	0.8069	0.3266	0.2159	0.4715
NM_001100985	RGD1563667	0.7323	0.7979	0.1547	0.4795	0.5368	0.7738	0.5951	0.4703	0.5381	0.4511	0.7309	0.2970
NM_001044249	Antxr1	0.1258	0.4236	0.5120	0.4524	0.6922	0.7885	0.8347	0.6832	0.5009	0.4870	0.3131	0.5992
NM_001106512	Ddrgk1	0.6735	0.5760	0.5859	0.4075	0.8092	0.6215	0.1748	0.7393	0.5592	0.1373	0.8944	0.7348
NM_214457	Phactr1	0.5692	0.2719	0.2739	0.2039	0.1882	0.3501	0.6468	0.1991	0.2972	0.4096	0.4642	0.5038
NM_001106340	Mphosph10	0.5003	0.5070	0.0372	0.1235	0.0925	0.1262	0.0152	0.0198	0.7111	0.1062	0.6672	0.5627
NM_001106398	Ccdc109a	0.8007	0.7465	0.8263	0.9958	0.9201	0.8084	0.9129	0.4751	0.6136	0.5697	0.9035	0.7588
NM_001106603	Cct7	0.1647	0.8591	0.5291	0.8823	0.7374	0.6095	0.9300	0.8996	0.7544	0.8150	0.8856	0.7688
NM_001106311	LOC293589	0.0792	0.2752	0.5070	0.2028	0.4041	0.4542	0.2466	0.4505	0.5646	0.2919	0.3081	0.3751
NM_001106410	Gpbp1	0.7289	0.7440	0.9504	0.6717	0.6141	0.9094	0.8533	0.5548	0.2544	0.7450	0.8244	0.7480
NM_001100989	RGD1562533	0.0587	0.2716	0.4218	0.5556	0.7888	0.6055	0.2544	0.8151	0.7329	0.7703	0.8016	0.8893
NM_001105811	Rilp	0.0984	0.0346	0.0856	0.3233	0.0690	0.2150	0.1951	0.1835	0.1652	0.1652	0.1159	0.1751
NM_001105711	Grm2	0.6225	0.5302	0.4184	0.7448	0.6492	0.3945	0.7387	0.4695	0.8253	0.8109	0.4993	0.4227
NM_001106363	Pprc1	0.8171	0.7972	0.1177	0.8037	0.7679	0.8518	0.9177	0.7961	0.7618	0.9318	0.8875	0.9899
NM_001105720	Nfkbia	0.4883	0.1519	0.6197	0.3487	0.6198	0.9223	0.0384	0.9571	0.7593	0.4786	0.5355	0.5991
NM_001105717	Dpysl2	0.8455	0.6487	0.7925	0.4445	0.7948	0.7019	0.6517	0.6243	0.7519	0.3964	0.5659	0.6012
NM_001105906	Uspl1	0.5699	0.6422	0.9722	0.6938	0.6271	0.9219	0.5983	0.9280	0.6826	0.8281	0.8549	0.6914
NM_001106087	Tex15	0.4674	0.3770	0.1946	0.2038	0.2742	0.1919	0.2082	0.1987	0.1774	0.3619	0.1846	0.4012
NM_001105716	Crabp1	0.1099	0.2069	0.3859	0.3317	0.5941	0.4699	0.0944	0.6646	0.3219	0.6427	0.8092	0.3135
NM_001134496	Tmem63a	0.8357	0.9058	0.9371	0.9619	0.9654	0.9661	0.9636	0.9036	0.9613	0.9416	0.8458	0.7779
NM_001100995	RGD1563224	0.1647	0.1175	0.8646	0.4533	0.2215	0.3488	0.8717	0.3770	0.4854	0.3016	0.5713	0.1085
NM_001106414	Ttc33	0.1892	0.2380	0.8934	0.7303	0.1407	0.1721	0.1447	0.1817	0.2548	0.1999	0.3685	0.2459
NM_001105723	Ubtf	0.3140	0.8115	0.4424	0.7240	0.8561	0.8382	0.4358	0.8779	0.4826	0.9666	0.2195	0.8236
NM_001105770	Znf598	0.9392	0.8844	0.3991	0.1544	0.9510	0.9554	0.5356	0.9815	0.7704	0.7258	0.7525	0.8178
NM_001106080	Tktl2	0.5471	0.5540	0.3348	0.6110	0.5617	0.2588	0.8627	0.4775	0.5000	0.2297	0.6968	0.3457
NM_001106078	Tssk6	0.1623	0.1157	0.0878	0.2428	0.3489	0.3820	0.1816	0.3208	0.1305	0.0235	0.6192	0.5765
NM_001106176	RGD1307418	0.0653	0.1170	0.4926	0.3931	0.4562	0.4098	0.5726	0.0992	0.1460	0.3496	0.3475	0.3586
NM_001105819	Rhbdl3	0.6057	0.5888	0.6116	0.5429	0.5882	0.6634	0.6215	0.7137	0.6761	0.6772	0.6854	0.7140
NM_001105724	Rasa2	0.3185	0.2166	0.0222	0.3999	0.3884	0.4892	0.0494	0.1681	0.6646	0.5269	0.5115	0.6438
NM_001106429	Pabpc4l	0.4388	0.3860	0.0925	0.0635	0.3243	0.4315	0.4926	0.1384	0.0398	0.3774	0.6564	0.4558
NM_001106448	Ube2q1	0.6258	0.9704	0.2398	0.9816	0.9816	0.9670	0.3647	0.8325	0.5376	0.9762	0.8304	0.6897
NM_001105733	Cacnb4	0.7820	0.7403	0.7827	0.4115	0.5461	0.5393	0.8260	0.7807	0.7516	0.5949	0.8678	0.2529
NM_013153	Has2	0.4827	0.8531	0.5657	0.8340	0.8193	0.4527	0.5584	0.6918	0.7975	0.6841	0.8014	0.6651
NM_001106437	Fhdc1	0.1286	0.8785	0.0691	0.2479	0.1746	0.2130	0.2931	0.0309	0.5167	0.3333	0.5056	0.1177
NM_001127296	Pwwp2a	0.5544	0.6208	0.6921	0.5891	0.5261	0.7106	0.5555	0.6688	0.6985	0.6274	0.4652	0.3558
NM_001106416	Skp2	0.5910	0.6564	0.6904	0.9468	0.8699	0.1082	0.1387	0.3814	0.6244	0.0863	0.7406	0.9497
NM_001106187	Sf3b3	0.7009	0.6776	0.4553	0.4228	0.4969	0.3755	0.2459	0.6062	0.3190	0.5010	0.2352	0.6023
NM_001105920	Alkbh4	0.5993	0.0796	0.6751	0.4388	0.1876	0.7586	0.3345	0.7249	0.7615	0.0469	0.1714	0.6122
NM_001105840	Rab5c	0.1440	0.3179	0.7970	0.7518	0.2986	0.4049	0.7492	0.0379	0.1152	0.1667	0.3769	0.3308
NM_001106190	RGD1311055	0.2427	0.7693	0.2696	0.6848	0.4348	0.7455	0.2712	0.6716	0.4288	0.8833	0.2061	0.2573
NM_001105837	Eif1	0.9093	0.8667	0.8489	0.5620	0.8313	0.6386	0.9772	0.9220	0.3484	0.4520	0.7144	0.3831
NM_001105824	Taf15	0.9347	0.8245	0.8972	0.7794	0.7342	0.8220	0.7366	0.7561	0.8894	0.8353	0.9692	0.6497
NM_001106447	Pygo2	0.6275	0.6548	0.1617	0.8091	0.5853	0.9609	0.2591	0.9329	0.8848	0.3435	0.4173	0.9188
NM_001106333	Lgals12	0.4812	0.5560	0.7034	0.0398	0.4253	0.6124	0.4396	0.4785	0.7262	0.5028	0.4733	0.1255
NM_001106713	Klhl29	0.9547	0.9707	0.8536	0.8067	0.9972	0.9467	0.9536	0.9005	0.5482	0.8958	0.9224	0.1402
NM_001107083	Rtp2	0.4745	0.1657	0.2582	0.0938	0.3075	0.0134	0.3522	0.0609	0.2699	0.1101	0.4845	0.2833
NM_001106840	Gsta4	0.4829	0.4468	0.5565	0.0969	0.7373	0.6416	0.2585	0.8761	0.1987	0.4124	0.5876	0.3104
NM_001106453	Trim45	0.7470	0.8471	0.4582	0.8369	0.7646	0.6048	0.9055	0.8217	0.8389	0.7329	0.8568	0.0364
NM_001105743	Shc3	0.0331	0.0705	0.4580	0.2498	0.0375	0.1233	0.2587	0.1755	0.5588	0.1166	0.0064	0.0658

NM_001101681	Ampd2	0.2740	0.4322	0.1786	0.7047	0.1056	0.8107	0.4213	0.1946	0.3034	0.1378	0.2410	0.8073
NM_001106202	Tdrd1	0.9808	0.9191	0.9052	0.8197	0.8067	0.6994	0.9838	0.1472	0.1745	0.9424	0.8997	0.3559
NM_001105753	Rasgrf1	0.4454	0.8085	0.4181	0.3409	0.5768	0.4876	0.3920	0.3892	0.5284	0.1551	0.6435	0.3642
NM_001105914	Dhrsx	0.7785	0.9093	0.0911	0.8027	0.8326	0.9234	0.7321	0.8909	0.7921	0.8918	0.8006	0.1173
NM_001105831	Mrpl27	0.0198	0.1266	0.0205	0.2101	0.0348	0.2855	0.1277	0.1165	0.0927	0.1482	0.0580	0.0199
NM_001105751	Hivep1	0.8541	0.9172	0.8477	0.2505	0.6003	0.0326	0.9032	0.8427	0.8448	0.8010	0.5121	0.8969
NM_001102364	Cldn6	0.8510	0.9150	0.7638	0.8708	0.8964	0.6231	0.9665	0.7907	0.6984	0.8175	0.6515	0.9689
NM_001105748	Kcnd1	0.6875	0.1901	0.1923	0.2378	0.0985	0.2619	0.2430	0.1915	0.2780	0.2889	0.1040	0.2217
NM_001105747	Il9	0.0946	0.4207	0.0276	0.5306	0.0272	0.0143	0.0238	0.1349	0.3529	0.7027	0.2285	0.0609
NM_001108377	Pck2	0.9037	0.9245	0.9561	0.9359	0.7790	0.8078	0.8674	0.8534	0.8920	0.8228	0.9666	0.9939
NM_001105876	Csta	0.6209	0.6826	0.8045	0.4198	0.1327	0.0379	0.7863	0.0394	0.7327	0.7424	0.6936	0.7315
NM_001105955	Aspm	0.9186	0.9934	0.0825	0.0856	0.7688	0.7704	0.8838	0.8558	0.6590	0.9688	0.9314	0.1622
NM_001106480	Prpf40a	0.4301	0.7437	0.8180	0.5343	0.3751	0.5960	0.3460	0.4222	0.1300	0.7437	0.8151	0.0271
NM_001106212	RGD1562747	0.0886	0.3317	0.0552	0.0548	0.0289	0.2114	0.1601	0.1199	0.0242	0.0794	0.0482	0.0356
NM_001106344	FAM50A	0.1829	0.2855	0.1944	0.5156	0.1880	0.2919	0.5045	0.4316	0.1772	0.3192	0.5577	0.2617
NM_001106498	Pak6	0.0717	0.3372	0.1239	0.0525	0.1257	0.2857	0.0709	0.1164	0.0795	0.0242	0.1827	0.1264
NM_001106396	Sesn1	0.9249	0.7794	0.5611	0.8639	0.9932	0.9971	0.9666	0.7387	0.0253	0.6173	0.2799	0.3046
NM_001105759	Dock9	0.9684	0.5346	0.9869	0.8986	0.5914	0.9130	0.9839	0.9396	0.9045	0.9383	0.7526	0.9579
NM_001105852	Otop3	0.5543	0.4584	0.4521	0.8736	0.3524	0.3174	0.5878	0.5362	0.6861	0.7760	0.5602	0.8711
NM_001134537	RGD1310159	0.8796	0.7209	0.8050	0.8011	0.8808	0.9459	0.9879	0.7844	0.8159	0.7917	0.8234	0.7868
NM_001106495	Tmem85	0.0683	0.1194	0.3122	0.0812	0.1050	0.1112	0.0862	0.0209	0.1461	0.0799	0.0521	0.2099
NM_001106504	Astl	0.4247	0.4285	0.5888	0.3412	0.6944	0.5502	0.4648	0.7903	0.2706	0.4269	0.7302	0.5747
NM_001106228	Ercc1	0.0020	0.0179	0.0046	0.7177	0.0650	0.0099	0.0038	0.0184	0.5420	0.8703	0.2750	0.1240
NM_001106225	Ttyh1	0.4542	0.1708	0.3158	0.0531	0.8586	0.3789	0.6684	0.2206	0.1216	0.8125	0.0557	0.4484
NM_001106378	Agpat3	0.9390	0.9336	0.9922	0.6449	0.9408	0.9118	0.8471	0.6117	0.9854	0.5911	0.9840	0.9649
NM_001106230	Qpctl	0.6994	0.9278	0.7436	0.7034	0.2320	0.6310	0.8512	0.4984	0.5536	0.8665	0.5293	0.1217
NM_001106424	RGD1562890	0.7800	0.1235	0.2200	0.4821	0.6384	0.7556	0.3789	0.5593	0.1496	0.0115	0.1855	0.0723
NM_001106423	Evi1	0.7091	0.5348	0.6763	0.7722	0.8090	0.8098	0.8472	0.5786	0.4609	0.6305	0.6951	0.3622
NM_001106443	Msto1	0.7574	0.6921	0.0337	0.3647	0.7509	0.8405	0.3894	0.7987	0.8062	0.5587	0.7607	0.7381
NM_001106432	Exosc8	0.8575	0.8326	0.1237	0.0039	0.7411	0.6117	0.0301	0.8457	0.8568	0.9104	0.7934	0.2121
NM_001106259	Akt1s1	0.8916	0.6030	0.9287	0.7509	0.8715	0.9177	0.4087	0.7976	0.9830	0.5497	0.9705	0.9892
NM_001106257	Mybpc2	0.6930	0.7388	0.4943	0.1141	0.7575	0.6930	0.4629	0.6105	0.1756	0.7237	0.6229	0.7403
NM_001105853	Recql5	0.5553	0.2962	0.2196	0.1500	0.4071	0.6523	0.2184	0.7619	0.1374	0.4834	0.2682	0.1534
NM_001106241	RGD1310133	0.6941	0.3519	0.2126	0.5320	0.9420	0.4297	0.8630	0.9586	0.7465	0.2803	0.5393	0.2859
NM_001106239	Mrps12	0.7735	0.9158	0.1649	0.6263	0.7367	0.6990	0.9480	0.8070	0.7204	0.8745	0.7664	0.0726
NM_001106238	Pak4	0.7523	0.3700	0.1004	0.2429	0.8767	0.7486	0.5725	0.6565	0.7401	0.9569	0.5569	0.7318
NM_001106233	RGD1311676	0.1425	0.3702	0.7422	0.5077	0.1974	0.1524	0.7738	0.4276	0.5708	0.5694	0.3746	0.6564
NM_001106689	Znf593	0.3546	0.1653	0.4948	0.1881	0.2124	0.1666	0.5340	0.1215	0.3128	0.2563	0.3214	0.2871
NM_001106550	Nkain4	0.2578	0.2279	0.1989	0.2666	0.3196	0.2083	0.4298	0.4281	0.1967	0.2438	0.1042	0.1048
NM_001106540	Pigt	0.7020	0.2025	0.7386	0.8080	0.4700	0.8687	0.8561	0.8401	0.3460	0.3164	0.1112	0.5573
NM_001105761	Tnfrsf17	0.4119	0.3527	0.5768	0.2918	0.2104	0.4052	0.3973	0.1496	0.3052	0.5087	0.4106	0.4018
NM_001105931	Cux2	0.1379	0.1021	0.2661	0.1760	0.2858	0.1090	0.0762	0.1331	0.3816	0.3036	0.0450	0.1665
NM_001105788	Rpl26	0.1188	0.2616	0.3783	0.0825	0.1659	0.0935	0.5215	0.1166	0.2259	0.5379	0.2485	0.2739
NM_001105767	Kremen2	0.3747	0.7928	0.6909	0.3724	0.8954	0.7459	0.5801	0.7696	0.4969	0.5597	0.7421	0.3987
NM_001106264	Reps1	0.9131	0.8994	0.9773	0.9858	0.9824	0.9930	0.9923	0.9398	0.9763	0.9861	0.9569	0.9501
NM_001106263	Htatip2	0.4562	0.5883	0.5724	0.0224	0.0657	0.0766	0.7902	0.1363	0.1694	0.7194	0.7893	0.1486
NM_001110143	Cldn22	0.7126	0.3664	0.9778	0.5833	0.3177	0.4684	0.9768	0.5239	0.7175	0.6946	0.7627	0.9299
NM_001106566	Fam73b	0.6427	0.5260	0.4065	0.7699	0.6184	0.3656	0.2348	0.4185	0.4918	0.8953	0.3634	0.4278
NM_001106451	Tbx15	0.7217	0.7391	0.9033	0.8988	0.8996	0.2783	0.9205	0.5837	0.7098	0.9398	0.7591	0.1738

NM_001106449	Tmod4	0.8429	0.0684	0.3526	0.1396	0.6843	0.6478	0.4717	0.2181	0.0144	0.4712	0.0229	0.6053
NM_001109359	RGD1562622	0.8452	0.9522	0.9307	0.8862	0.9262	0.8878	0.5371	0.9361	0.7676	0.6633	0.5842	0.8853
NM_001109356	RGD1562060	0.3113	0.2341	0.0948	0.0977	0.2407	0.4071	0.1694	0.3948	0.0638	0.1098	0.3780	0.0205
NM_001100572	Iars	0.8447	0.9479	0.7164	0.7447	0.8929	0.2924	0.8212	0.2381	0.8508	0.9071	0.9439	0.3486
NM_001109349	RGD1561816	0.6053	0.1442	0.6273	0.6214	0.3150	0.4580	0.4283	0.4360	0.4256	0.2484	0.5701	0.5595
NM_001109344	RGD1562846	0.8910	0.9001	0.9386	0.8172	0.8417	0.5508	0.6463	0.7589	0.9180	0.8157	0.6929	0.9339
NM_001109340	RGD1565469	0.6505	0.6006	0.7845	0.6659	0.9199	0.8169	0.8571	0.7596	0.6573	0.9173	0.4817	0.5893
NM_001106568	Ciz1	0.0456	0.0944	0.1260	0.0889	0.2291	0.1763	0.0458	0.0526	0.1173	0.0284	0.0432	0.3202
NM_001106462	Hbxip	0.1250	0.0360	0.9578	0.2983	0.1019	0.0144	0.2579	0.1483	0.0258	0.0448	0.0662	0.0198
NM_001106459	RGD1562344	0.3473	0.4993	0.4981	0.0550	0.5264	0.2876	0.5168	0.4289	0.3961	0.6555	0.5816	0.0705
NM_001106576	Armc10	0.0890	0.2296	0.9475	0.9336	0.8123	0.8743	0.8212	0.7566	0.1072	0.1231	0.4406	0.8487
NM_001105975	Adss	0.5419	0.2865	0.4255	0.2191	0.3738	0.8832	0.1066	0.2505	0.6373	0.3024	0.3438	0.9867
NM_001105771	Mrps34	0.7826	0.8824	0.1058	0.2183	0.0743	0.0420	0.2899	0.4795	0.6080	0.8461	0.7527	0.4658
NM_001105772	Prss34	0.4943	0.7976	0.7983	0.5870	0.2771	0.8689	0.8529	0.7056	0.7757	0.7347	0.7318	0.7464
NM_001106582	Gpr165	0.2956	0.7106	0.5628	0.2545	0.7717	0.4080	0.7121	0.2612	0.2804	0.1438	0.4922	0.4894
NM_001105968	Ppox	0.2229	0.3001	0.0209	0.2334	0.3073	0.1529	0.0454	0.6433	0.3808	0.6985	0.1078	0.0899
NM_001105865	Dgcr8	0.7884	0.4677	0.5196	0.7655	0.6112	0.8756	0.2912	0.7831	0.3645	0.4360	0.5197	0.8380
NM_001134499	RGD1311784	0.3952	0.5637	0.3159	0.0638	0.3457	0.7136	0.8325	0.9231	0.9055	0.7568	0.5326	0.8551
NM_001105782	Mrpl55	0.1262	0.2923	0.3327	0.1204	0.1299	0.0287	0.8358	0.0338	0.2298	0.1850	0.0890	0.2979
NM_001106474	Sec24b	0.9860	0.9664	0.8692	0.9555	0.9703	0.9395	0.9671	0.9093	0.9683	0.9213	0.9830	0.9352
NM_001105780	Sec24a	0.2658	0.7361	0.7503	0.1755	0.1345	0.3537	0.9478	0.0108	0.2627	0.9093	0.8530	0.3561
NM_001105886	Hoxd13	0.8743	0.0271	0.9675	0.8787	0.9038	0.9099	0.8536	0.9410	0.4780	0.8885	0.9793	0.9734
NM_001105885	Hoxd4	0.5346	0.4685	0.2254	0.7086	0.9074	0.8070	0.4435	0.5102	0.6799	0.8286	0.9801	0.6896
NM_001105880	Zbtb20	0.6285	0.6400	0.6674	0.6985	0.7332	0.7226	0.6755	0.7150	0.5127	0.7047	0.6882	0.6158
NM_001106487	Prg3	0.0505	0.0838	0.0741	0.0591	0.0141	0.3274	0.1435	0.1221	0.0240	0.1203	0.0040	0.1028
NM_001105800	Zmynd15	0.5062	0.6484	0.3309	0.1496	0.5964	0.1740	0.8225	0.5606	0.1400	0.7751	0.7372	0.4993
NM_001105981	Cnih4	0.2128	0.1491	0.1020	0.1385	0.9410	0.8546	0.1707	0.8752	0.3175	0.5521	0.2502	0.3111
NM_001109325	RGD1559597	0.5754	0.4578	0.4230	0.5368	0.7777	0.3185	0.4783	0.3324	0.3996	0.1064	0.4458	0.3722
NM_001109318	RGD1565785	0.5131	0.1399	0.4014	0.3830	0.2580	0.5763	0.1894	0.7558	0.0954	0.8274	0.3494	0.2378
NM_001106488	Mtch2	0.8927	0.9478	0.7098	0.9078	0.9924	0.9987	0.9282	0.9136	0.6354	0.7212	0.9671	0.3537
NM_001105976	Fam36a	0.2294	0.4404	0.1522	0.3950	0.8617	0.6566	0.3418	0.5573	0.2742	0.1055	0.1037	0.4531
NM_001105789	Arhgef15	0.5189	0.1752	0.4563	0.3789	0.3570	0.3160	0.6237	0.3168	0.4376	0.5488	0.6115	0.4006
NM_001106549	Itgav	0.2933	0.2648	0.3152	0.1818	0.2781	0.3124	0.2981	0.3002	0.1305	0.2940	0.2246	0.6132
NM_001106496	RGD1305045	0.2529	0.2579	0.8097	0.0893	0.3483	0.0195	0.0211	0.0334	0.0947	0.0635	0.0253	0.3001
NM_001077670	Eif3j	0.5377	0.9933	0.4318	0.9661	0.9063	0.8667	0.6773	0.8942	0.9625	0.9326	0.9883	0.7849
NM_001107081	Dvl3	0.6161	0.7474	0.6871	0.7871	0.6228	0.7159	0.0751	0.6276	0.8665	0.7536	0.7821	0.8697
NM_001106782	Mrpl42	0.0670	0.0310	0.0673	0.0621	0.2703	0.0411	0.2254	0.0083	0.0491	0.1250	0.0361	0.0381
NM_001106703	Glis1	0.3967	0.0267	0.4015	0.2242	0.1454	0.1498	0.1148	0.1121	0.2012	0.1184	0.7745	0.0391
NM_001106604	Alms1	0.9318	0.9643	0.8856	0.9904	0.9895	0.9736	0.9868	0.7894	0.9371	0.9995	0.9501	0.7792
NM_001113748	Rnf26	0.4421	0.6565	0.7470	0.8017	0.2446	0.5117	0.6990	0.0910	0.9667	0.9113	0.8638	0.2761
NM_001106619	Usp5	0.2962	0.6610	0.2088	0.4405	0.6464	0.8245	0.8736	0.7344	0.9322	0.7128	0.9086	0.9971
NM_001106615	Arpc4	0.2378	0.4688	0.6163	0.8883	0.4740	0.8075	0.9935	0.8981	0.5840	0.3263	0.7878	0.8371
NM_001106606	Nfu1	0.2219	0.5864	0.3179	0.5684	0.3478	0.1667	0.1374	0.3010	0.0945	0.0625	0.1191	0.1716
NM_001105791	Pfas	0.3307	0.4850	0.0344	0.6300	0.6561	0.8669	0.7195	0.8162	0.6948	0.5889	0.4632	0.7891
NM_001105792	Hes7	0.2706	0.2678	0.3863	0.5089	0.0647	0.6293	0.2693	0.3810	0.3377	0.3083	0.4279	0.6297
NM_001105801	Camta2	0.8765	0.8802	0.4735	0.8343	0.9608	0.7391	0.2096	0.9649	0.7869	0.9078	0.8547	0.9424
NM_001106315	Pkp3	0.5392	0.3934	0.7037	0.6099	0.1645	0.5034	0.8514	0.3712	0.4286	0.7715	0.6390	0.2604
NM_001106005	Gnpda2	0.9719	0.3849	0.9264	0.3349	0.3793	0.2081	0.0566	0.2843	0.1079	0.8040	0.2297	0.2206
NM_001106612	Eif4e3	0.3334	0.9173	0.9442	0.8712	0.2786	0.1235	0.9827	0.2481	0.9262	0.9308	0.5004	0.9824

NM_001105806	Tmem93	0.4583	0.3075	0.1132	0.3397	0.0816	0.1395	0.1462	0.3079	0.0053	0.1094	0.3617	0.0805
NM_001105795	RGD1563438	0.2735	0.2521	0.2749	0.4831	0.2325	0.6371	0.3142	0.2839	0.5941	0.6080	0.6093	0.2268
NM_001109207	Gins1	0.0305	0.9274	0.0024	0.8259	0.7149	0.7007	0.7320	0.9308	0.0340	0.6859	0.7882	0.0024
NM_001109213	RGD1561282	0.0434	0.2224	0.5216	0.0625	0.5522	0.0271	0.4905	0.4207	0.4970	0.5719	0.2388	0.0222
NM_001109206	RGD1565616	0.7600	0.1557	0.8129	0.7001	0.3670	0.8690	0.7130	0.5460	0.6557	0.8036	0.9343	0.5335
NM_001105893	Ifnar1	0.5665	0.6751	0.4322	0.2374	0.5148	0.6557	0.7671	0.7060	0.6044	0.5758	0.3196	0.7457
NM_001105826	Tubd1	0.3751	0.0159	0.1451	0.2898	0.0322	0.0886	0.3953	0.3861	0.0733	0.3205	0.1001	0.0591
NM_001106007	Pgm2	0.0829	0.0780	0.1388	0.0613	0.0355	0.0313	0.6758	0.1369	0.0261	0.0323	0.1809	0.0287
NM_001105918	Pop7	0.5929	0.7420	0.0287	0.8582	0.7344	0.6284	0.3218	0.9242	0.2385	0.2924	0.4837	0.0626
NM_001109677	LOC10012536[illegible]	0.4905	0.6571	0.7196	0.5535	0.6853	0.3662	0.4833	0.4286	0.2240	0.6460	0.5370	0.3446
NM_001106081	Tll1	0.5536	0.3362	0.7892	0.2536	0.6080	0.7620	0.5758	0.3111	0.7863	0.4151	0.6718	0.8432
NM_001106021	C1d	0.8077	0.7688	0.8994	0.5989	0.9697	0.9365	0.9205	0.6454	0.8969	0.7058	0.7603	0.0985
NM_001106729	Eapp	0.1826	0.7827	0.3937	0.7367	0.1878	0.3643	0.2079	0.1978	0.4233	0.1723	0.1842	0.4180
NM_001106380	Ube2g2	0.8778	0.6197	0.3337	0.0282	0.9416	0.9027	0.7863	0.9636	0.7060	0.7656	0.9932	0.9251
NM_001106338	Ms4a7	0.4922	0.9046	0.3838	0.3808	0.5966	0.6265	0.3240	0.3474	0.1005	0.1262	0.6496	0.6035
NM_001106336	Ms4a10	0.2063	0.3873	0.6397	0.3685	0.5371	0.5449	0.5058	0.4041	0.1678	0.1854	0.4086	0.2341
NM_001106737	Nin	0.6428	0.6065	0.1144	0.6444	0.5012	0.9608	0.8762	0.3461	0.1476	0.7811	0.4052	0.5441
NM_001106732	Nkx2-8	0.4120	0.0824	0.3633	0.7594	0.3041	0.6391	0.1206	0.7035	0.1391	0.1359	0.2017	0.0558
NM_001105834	Mrpl45	0.0702	0.1202	0.1198	0.2586	0.2360	0.0584	0.3888	0.0661	0.3976	0.0987	0.1511	0.2057
NM_001106351	RGD1564599	0.7420	0.1430	0.5986	0.5690	0.5809	0.4884	0.7568	0.5542	0.6262	0.5266	0.3061	0.6853
NM_001105938	B3gnt4	0.3926	0.4873	0.2308	0.4960	0.0703	0.4367	0.5124	0.7959	0.2883	0.4998	0.2491	0.0668
NM_001105846	Fmnl1	0.8805	0.7734	0.3674	0.7226	0.8412	0.7196	0.7547	0.6574	0.4914	0.7758	0.8842	0.7147
NM_001106082	Nek1	0.8090	0.5035	0.4669	0.4734	0.4338	0.5244	0.7368	0.5160	0.4163	0.4555	0.7606	0.5191
NM_001106335	Incenp	0.9292	0.9919	0.0115	0.6742	0.8496	0.4376	0.8204	0.7838	0.8500	0.9405	0.7817	0.0915
NM_001004077	Gk2	0.3673	0.7001	0.5605	0.5506	0.5960	0.4267	0.7919	0.2134	0.2179	0.1679	0.8474	0.5264
NM_001106430	Mgst2	0.4509	0.0146	0.5807	0.1801	0.8206	0.7703	0.0090	0.6961	0.0185	0.0519	0.5648	0.1184
NM_001105848	Helz	0.5399	0.1546	0.3451	0.4429	0.1104	0.4007	0.1133	0.4580	0.0882	0.0951	0.1542	0.5926
NM_001106388	Rfx6	0.8198	0.7542	0.8791	0.9057	0.7933	0.8602	0.7330	0.9340	0.3264	0.8954	0.7027	0.8687
NM_001106028	Cdkn3	0.0580	0.0519	0.1115	0.2788	0.1758	0.1282	0.3720	0.4318	0.6692	0.3045	0.5992	0.8051
NM_001105838	Klhl11	0.1923	0.2663	0.3723	0.2980	0.2549	0.4223	0.5898	0.3056	0.0627	0.4703	0.2459	0.0757
NM_001105836	Pcgf2	0.7597	0.8347	0.7467	0.6000	0.9937	0.7080	0.7734	0.7320	0.8285	0.7843	0.7971	0.8391
NM_001105832	Dlx3	0.0831	0.1151	0.1929	0.1016	0.1615	0.2403	0.2809	0.1306	0.2993	0.1241	0.2715	0.2868
NM_001105844	Higd1b	0.6099	0.7230	0.1244	0.1725	0.1787	0.0831	0.1549	0.7483	0.3575	0.8149	0.3633	0.4609
NM_001105842	Rdm1	0.7232	0.7831	0.9352	0.1291	0.8254	0.4849	0.8012	0.9459	0.7647	0.8125	0.9319	0.7077
NM_001105841	Ptrf	0.7858	0.6257	0.7528	0.7348	0.9490	0.8036	0.6822	0.7585	0.8141	0.7969	0.8364	0.7630
NM_001106463	Eps8l3	0.1382	0.2632	0.4600	0.6283	0.6100	0.7697	0.5036	0.5601	0.6178	0.7134	0.2400	0.5962
NM_001106358	Dmrt3	0.9073	0.9180	0.9602	0.9621	0.9867	0.8361	0.9122	0.8707	0.9437	0.9789	0.8968	0.9689
NM_001106355	RGD1310475	0.9000	0.3955	0.3708	0.2681	0.6090	0.7899	0.2836	0.5166	0.0533	0.7441	0.4985	0.8653
NM_001106354	Arhgap18	0.2934	0.3160	0.5109	0.3216	0.2754	0.3116	0.4601	0.4953	0.7189	0.2637	0.4566	0.4983
NM_001106352	Pkd2l1	0.9942	0.7278	0.9696	0.6834	0.9750	0.8260	0.9158	0.8949	0.9338	0.7229	0.9228	0.8286
NM_001106521	Nxt1	0.7981	0.9053	0.0439	0.9264	0.7272	0.8035	0.9084	0.7976	0.7551	0.9969	0.7944	0.0378
NM_001106364	Actr1a	0.4608	0.7166	0.5909	0.6856	0.5346	0.5791	0.9230	0.6954	0.8299	0.5827	0.6447	0.6457
NM_001004263	Itgb6	0.7435	0.8506	0.6800	0.8563	0.8694	0.6594	0.8877	0.7143	0.7821	0.7172	0.9687	0.5754
NM_001106365	Taf5	0.9318	0.9131	0.3232	0.3953	0.8113	0.5363	0.1307	0.8925	0.8328	0.8692	0.5762	0.0556
NM_001105963	Mrps14	0.1327	0.0778	0.8405	0.9213	0.9369	0.6503	0.0433	0.7978	0.1653	0.2148	0.0791	0.0304
NM_001105959	RGD1309104	0.1637	0.4161	0.6724	0.6710	0.3449	0.7747	0.0851	0.7709	0.4989	0.8123	0.7726	0.6836
NM_001134534	Btbd17	0.1501	0.3367	0.8319	0.6601	0.8175	0.6121	0.6612	0.6316	0.6844	0.8488	0.5180	0.6015
NM_001106372	40,973.0000	0.3341	0.2188	0.8049	0.9451	0.3126	0.1880	0.2810	0.3934	0.0109	0.1270	0.3238	0.0467
NM_001106369	Ccnj	0.5781	0.8842	0.8387	0.6292	0.1944	0.8256	0.5445	0.3626	0.3261	0.8911	0.5340	0.7129

NM_001134498	RGD1560070	0.8740	0.9799	0.9206	0.6589	0.9440	0.9344	0.9864	0.8069	0.8860	0.9053	0.9289	0.5372
NM_001106403	Bdp1	0.6544	0.9229	0.8752	0.9082	0.6832	0.4703	0.8138	0.7581	0.8025	0.5486	0.8596	0.4130
NM_001105971	Slamf9	0.6052	0.4138	0.5803	0.2195	0.5632	0.4566	0.6086	0.4891	0.5122	0.4958	0.5134	0.1380
NM_001105850	Ush1g	0.4738	0.5819	0.4290	0.5022	0.3332	0.4316	0.6006	0.5404	0.5871	0.3337	0.5777	0.4861
NM_001106155	Wnt8a	0.9017	0.8758	0.4906	0.6418	0.6121	0.2557	0.9663	0.7552	0.4152	0.7373	0.6188	0.7840
NM_001106066	Pgls	0.7730	0.8896	0.8284	0.7523	0.7926	0.7975	0.8451	0.9332	0.7000	0.9017	0.9297	0.6663
NM_001106032	Psmb11	0.8968	0.9347	0.8460	0.9663	0.6793	0.2052	0.9566	0.8430	0.9730	0.7154	0.8649	0.9463
NM_001105854	Fbf1	0.6041	0.9197	0.0123	0.7231	0.6986	0.4752	0.7503	0.9824	0.9804	0.7383	0.6838	0.8738
NM_001106580	Hepacam2	0.3409	0.4904	0.6877	0.1768	0.2015	0.2982	0.2963	0.3662	0.5294	0.2771	0.3555	0.2839
NM_001106537	Ttpal	0.9034	0.9833	0.7561	0.9989	0.9795	0.9865	0.9410	0.9613	0.7480	0.7436	0.9915	0.9617
NM_001106383	Slc5a4a	0.4252	0.3313	0.1615	0.1184	0.0526	0.1092	0.4494	0.0504	0.1785	0.3289	0.2838	0.1862
NM_001106461	Rhoc	0.3477	0.6974	0.2586	0.5013	0.5779	0.5337	0.4098	0.2617	0.2142	0.5948	0.2471	0.6944
NM_001106393	Nt5dc1	0.1327	0.2986	0.6394	0.1815	0.2921	0.0253	0.0187	0.1192	0.1939	0.0038	0.1547	0.3251
NM_001106402	Lhfpl2	0.9247	0.2866	0.8818	0.6464	0.8874	0.8509	0.6989	0.8895	0.9474	0.6965	0.3476	0.9141
NM_001106391	Bxdc1	0.8975	0.7270	0.5514	0.7496	0.7634	0.7213	0.8474	0.8637	0.8422	0.7674	0.8264	0.6311
NM_001142304	Clec2d4	0.0271	0.3440	0.0652	0.1123	0.0662	0.0619	0.0787	0.0664	0.0012	0.1138	0.1095	0.0143
NM_001106627	Casc1	0.5540	0.4331	0.6006	0.2678	0.4100	0.7772	0.5520	0.6419	0.4230	0.5350	0.7945	0.4737
NM_001106421	Tpd52	0.0107	0.0327	0.9607	0.6138	0.0802	0.0319	0.4869	0.1109	0.2398	0.1882	0.0831	0.7361
NM_001106418	Il7r	0.7538	0.9224	0.9434	0.8521	0.9617	0.8897	0.8444	0.6296	0.9666	0.9294	0.9403	0.7729
NM_001106444	Krtcap2	0.0824	0.9360	0.9060	0.9170	0.6811	0.2338	0.8202	0.9017	0.4380	0.8732	0.6309	0.0518
NM_001106440	Apoa1bp	0.0894	0.0035	0.7854	0.0125	0.0258	0.0843	0.0141	0.0544	0.0195	0.0758	0.0032	0.1489
NM_001108278	Sat2	0.3805	0.2064	0.2636	0.1402	0.3636	0.3963	0.1595	0.2256	0.1701	0.2301	0.1843	0.2997
NM_001106473	Bdh2	0.3125	0.0813	0.3882	0.8613	0.7296	0.3728	0.1056	0.7979	0.0246	0.0165	0.0749	0.0316
NM_001106404	Slc30a5	0.9532	0.2543	0.4691	0.6605	0.7080	0.7101	0.5000	0.8972	0.4749	0.0561	0.8097	0.5294
NM_001106413	Card6	0.1374	0.7748	0.8576	0.9937	0.1080	0.8828	0.9601	0.0438	0.8766	0.4578	0.9354	0.8515
NM_001106412	Mrps30	0.6603	0.5685	0.0812	0.0052	0.1276	0.1080	0.0542	0.0762	0.7457	0.1893	0.5238	0.6115
NM_001106417	Capsl	0.0968	0.0983	0.4244	0.0698	0.0211	0.0299	0.3650	0.0377	0.6115	0.3404	0.2417	0.5726
NM_001105864	Scarf2	0.8945	0.6954	0.7456	0.8047	0.9888	0.7677	0.9184	0.9711	0.9345	0.7052	0.9662	0.9928
NM_001105862	Hic2	0.4957	0.5398	0.5301	0.5121	0.6485	0.4084	0.7653	0.5440	0.6375	0.5463	0.5980	0.7275
NM_001106431	Stoml3	0.5758	0.1224	0.3962	0.8691	0.3537	0.0764	0.6741	0.5598	0.1633	0.5897	0.6337	0.2217
NM_001106438	S100a5	0.1117	0.1280	0.1656	0.0470	0.2386	0.0620	0.0114	0.1050	0.0368	0.1324	0.6129	0.4592
NM_001106436	RGD1560324	0.4733	0.1450	0.2299	0.3444	0.3430	0.5494	0.2559	0.5791	0.0902	0.0968	0.2739	0.1789
NM_001106634	Rdhe2	0.2008	0.2089	0.0757	0.2485	0.1672	0.0881	0.5541	0.1844	0.1231	0.2679	0.1228	0.1082
NM_001108561	Ahcyl1	0.5903	0.5149	0.9937	0.3084	0.9467	0.9808	0.9795	0.6266	0.9609	0.9655	0.9980	0.9670
NM_001109385	LOC679958	0.3249	0.4141	0.2741	0.4297	0.4426	0.2480	0.2896	0.4614	0.2424	0.3178	0.7052	0.2335
NM_001105984	Rps6kc1	0.5375	0.0315	0.4218	0.0133	0.9745	0.8883	0.1515	0.9460	0.8703	0.5181	0.7281	0.6891
NM_001105867	Klhl6	0.8291	0.6970	0.8806	0.6608	0.9044	0.9133	0.8932	0.8565	0.9494	0.8951	0.7292	0.7289
NM_001106446	Zbtb7b	0.0093	0.1058	0.8639	0.0089	0.0646	0.0719	0.3612	0.0311	0.0332	0.2502	0.0798	0.6144
NM_001105978	Gpr137b	0.4876	0.0660	0.1191	0.1159	0.5876	0.6674	0.5942	0.5718	0.4445	0.2861	0.7824	0.4475
NM_001108138	Vps11	0.6315	0.0931	0.7383	0.1541	0.2255	0.3353	0.2863	0.1619	0.2475	0.2196	0.1638	0.1423
NM_001106613	Ppp4r2	0.9991	0.8079	0.9889	0.9150	0.9961	0.9687	0.6665	0.9956	0.9500	0.9177	0.9497	0.8830
NM_001106652	Slc46a2	0.1586	0.1686	0.4947	0.7355	0.5262	0.7119	0.3929	0.6675	0.7584	0.0418	0.6888	0.3807
NM_001105979	Mixl1	0.6358	0.3749	0.4477	0.5701	0.5490	0.5289	0.7378	0.5046	0.5091	0.5395	0.4762	0.2598
NM_001105875	Ccdc58	0.3624	0.4799	0.0775	0.0721	0.0606	0.0255	0.1968	0.1490	0.1102	0.7543	0.5336	0.0699
NM_001106084	Tnks	0.8935	0.9526	0.3548	0.9018	0.6151	0.4908	0.6630	0.8549	0.8623	0.7288	0.6468	0.6753
NM_001106859	Atrip	0.7472	0.3268	0.0467	0.0909	0.7645	0.9169	0.0383	0.8184	0.7173	0.5357	0.5092	0.7435
NM_001106608	Chchd6	0.0323	0.9539	0.7348	0.8928	0.5814	0.1428	0.8365	0.2113	0.7346	0.8867	0.8413	0.1322
NM_001106509	Chchd5	0.0094	0.1388	0.4302	0.3749	0.0341	0.0050	0.7234	0.0466	0.0322	0.0896	0.0308	0.0491
NM_001108723	Cyp46a1	0.6228	0.5106	0.9296	0.2337	0.7476	0.7199	0.3962	0.8793	0.9220	0.8631	0.2377	0.9481

NM_001106690	Grhl3	0.0274	0.8479	0.8645	0.4386	0.2716	0.0071	0.1401	0.0159	0.4209	0.8340	0.2788	0.1860
NM_001106001	Enam	0.3170	0.3545	0.3342	0.3415	0.1383	0.1629	0.0729	0.1944	0.0955	0.5542	0.3721	0.4221
NM_001106135	Snx2	0.5259	0.3960	0.9381	0.1316	0.4683	0.7480	0.1723	0.2498	0.4083	0.7092	0.3457	0.5552
NM_001105878	Polq	0.0839	0.9552	0.4855	0.1628	0.8013	0.9460	0.8965	0.4404	0.7285	0.9191	0.7995	0.4595
NM_001106244	Polr2i	0.1941	0.2469	0.0260	0.2301	0.4115	0.0074	0.5832	0.1007	0.4207	0.6596	0.4051	0.0296
NM_001106469	Myoz2	0.0697	0.0497	0.0271	0.6468	0.1985	0.4240	0.1042	0.3446	0.1113	0.0844	0.0358	0.5235
NM_001105883	Pvrl3	0.7134	0.6692	0.4293	0.7744	0.3232	0.4993	0.6632	0.4447	0.6707	0.1941	0.8135	0.3194
NM_001105882	Tmprss7	0.8841	0.7224	0.8454	0.6684	0.8503	0.6983	0.9322	0.9424	0.7065	0.9351	0.9021	0.7318
NM_001106197	Arv1	0.9168	0.8100	0.2275	0.0178	0.8482	0.4501	0.0489	0.5470	0.7286	0.6221	0.8525	0.9420
NM_001105884	Hoxd1	0.3686	0.5979	0.2230	0.6733	0.3346	0.7312	0.1111	0.4124	0.6782	0.7801	0.8588	0.6118
NM_001105904	Cd209a	0.5799	0.7794	0.7640	0.3427	0.4694	0.4650	0.6534	0.8018	0.4095	0.8183	0.7563	0.2277
NM_001105903	Mcoln1	0.4147	0.3939	0.5460	0.2541	0.4755	0.4039	0.1572	0.4470	0.6545	0.7241	0.6300	0.4247
NM_001106479	Clcn6	0.1873	0.8893	0.7059	0.2159	0.4518	0.6338	0.6598	0.5477	0.4411	0.6915	0.7845	0.6873
NM_001105907	Trrap	0.8564	0.9396	0.3583	0.3980	0.8710	0.9407	0.1533	0.9556	0.8537	0.8220	0.6147	0.8238
NM_001105887	B3galt5	0.4683	0.2658	0.2296	0.4210	0.4693	0.7995	0.6387	0.5991	0.7216	0.4510	0.1024	0.8111
NM_001105891	Psmg1	0.5441	0.7819	0.0963	0.8034	0.7214	0.2443	0.7610	0.9258	0.4561	0.8108	0.8222	0.0107
NM_001106092	Vps36	0.3824	0.8331	0.1304	0.0155	0.5745	0.4579	0.6132	0.5722	0.7583	0.6412	0.8379	0.2704
NM_001105905	Dppa1	0.3373	0.4441	0.3147	0.6813	0.2662	0.3472	0.2342	0.4467	0.2785	0.2482	0.2308	0.1642
NM_001105894	Chodl	0.4840	0.6485	0.5286	0.5477	0.4448	0.7568	0.7131	0.4013	0.1055	0.6015	0.6241	0.4450
NM_001077646	Cd3g	0.2783	0.5934	0.3215	0.5303	0.5588	0.5919	0.6464	0.4679	0.7029	0.5792	0.5061	0.6655
NM_001106097	Med10	0.0911	0.2179	0.3763	0.5293	0.3748	0.0587	0.0681	0.6219	0.0636	0.1195	0.0723	0.0713
NM_001100559	Rwdd2b	0.5764	0.1408	0.2570	0.4560	0.3987	0.3370	0.4748	0.4560	0.2957	0.3287	0.1892	0.3747
NM_001106704	Cox7a2l	0.1192	0.2660	0.9668	0.9460	0.0168	0.4994	0.7450	0.0591	0.0360	0.0316	0.1678	0.7380
NM_001105898	Rbm11	0.6500	0.5782	0.9189	0.1377	0.7877	0.3385	0.4647	0.3527	0.3222	0.7980	0.5839	0.6312
NM_001106419	Rad1	0.9522	0.7737	0.7026	0.3092	0.4667	0.4053	0.8588	0.0421	0.5101	0.1936	0.5940	0.5063
NM_001106281	Prcp	0.0696	0.1344	0.9835	0.0482	0.1880	0.3163	0.3443	0.2802	0.2982	0.0369	0.0073	0.4875
NM_001106714	Slc7a15	0.7914	0.8576	0.7137	0.6896	0.9020	0.7387	0.7136	0.8536	0.5947	0.4755	0.8533	0.7527
NM_001106718	Fam49a	0.6048	0.5027	0.5191	0.6081	0.7352	0.3429	0.5652	0.4148	0.5384	0.6085	0.4554	0.4591
NM_001105899	Lipi	0.0376	0.0157	0.1601	0.2990	0.5265	0.1835	0.0500	0.3818	0.3681	0.0197	0.1528	0.0747
NM_001106717	Gen1	0.9896	0.7117	0.3710	0.9502	0.9718	0.7329	0.9627	0.7286	0.9379	0.7399	0.9442	0.8889
NM_001106501	Tp53bp1	0.5646	0.0945	0.0507	0.1096	0.2176	0.1076	0.1361	0.3001	0.2853	0.0472	0.4039	0.6344
NM_001106490	Mybpc3	0.2059	0.4716	0.2473	0.5640	0.9038	0.3605	0.6220	0.6429	0.3475	0.3779	0.1198	0.2381
NM_001106735	Klhl28	0.8466	0.9023	0.9779	0.9059	0.8309	0.9071	0.4811	0.9535	0.7925	0.5415	0.8493	0.1118
NM_001106734	Ctage5	0.5852	0.5743	0.3983	0.3862	0.5727	0.7387	0.6666	0.5333	0.5010	0.5977	0.6330	0.4363
NM_001106649	Ctnnal1	0.9208	0.9829	0.7670	0.7900	0.9493	0.8689	0.7431	0.7869	0.9422	0.9864	0.8928	0.9716
NM_001105912	Snx8	0.2501	0.8761	0.8188	0.5811	0.4596	0.4476	0.7711	0.6637	0.3765	0.7107	0.9196	0.1768
NM_001105911	Slc29a4	0.7309	0.5512	0.5612	0.4131	0.5685	0.1960	0.3902	0.2447	0.6991	0.3600	0.5980	0.7996
NM_001105916	Tfr2	0.0279	0.0448	0.3535	0.0824	0.0759	0.0111	0.4003	0.0570	0.6491	0.3642	0.4602	0.1521
NM_001105915	Asmtl	0.5623	0.7003	0.6494	0.4264	0.5619	0.0122	0.1186	0.4336	0.7233	0.8095	0.7575	0.3674
NM_001106039	Mdp-1	0.2317	0.3690	0.6128	0.7694	0.4409	0.0638	0.8150	0.8280	0.0749	0.6155	0.2202	0.1047
NM_001106974	A2bp1	0.3410	0.3010	0.2633	0.4469	0.3266	0.3333	0.3334	0.0443	0.2908	0.1945	0.2264	0.7013
NM_001106030	Parp2	0.8651	0.3760	0.0929	0.8917	0.7786	0.5935	0.1438	0.6332	0.2768	0.3095	0.5064	0.2246
NM_001106027	Fam3d	0.4935	0.5594	0.4374	0.4759	0.1496	0.2366	0.5349	0.8161	0.0693	0.1639	0.1895	0.8295
NM_001106024	Pde6b	0.3456	0.2419	0.1136	0.3570	0.1783	0.1332	0.1363	0.2156	0.1062	0.4428	0.1046	0.2593
NM_001106002	Polr2b	0.9576	0.3914	0.1473	0.2496	0.7515	0.7587	0.2330	0.5773	0.1970	0.7629	0.2732	0.1875
NM_001105917	Actl6b	0.4196	0.0976	0.4238	0.0740	0.0701	0.0729	0.1547	0.4726	0.4784	0.1349	0.0884	0.3762
NM_001106029	Cnih	0.7125	0.8933	0.8608	0.9175	0.9285	0.9868	0.9817	0.9366	0.8736	0.8925	0.9787	0.9925
NM_001106042	Xpo4	0.9795	0.9878	0.9265	0.9725	0.9837	0.9824	0.9236	0.7543	0.7435	0.9907	0.7472	0.7723
NM_001106585	Lsm8	0.9924	0.9378	0.6775	0.8490	0.9008	0.7632	0.7906	0.9236	0.9588	0.9521	0.9028	0.8079

NM_001106510	Tmc2	0.0901	0.2463	0.1266	0.1125	0.3670	0.2282	0.4479	0.1818	0.0690	0.2001	0.2951	0.2193
NM_001106131	Ctdp1	0.9120	0.9133	0.3670	0.9217	0.9213	0.4919	0.7535	0.3636	0.9206	0.8888	0.9166	0.7870
NM_001106680	Toe1	0.7834	0.7461	0.2550	0.4575	0.7660	0.8434	0.4895	0.9009	0.7075	0.3524	0.7187	0.0388
NM_001106742	Med6	0.0303	0.0853	0.5457	0.0118	0.0384	0.1734	0.0896	0.1241	0.0387	0.3021	0.0583	0.2867
NM_001106708	Slc30a6	0.2969	0.1405	0.4480	0.8021	0.6530	0.8132	0.4324	0.7779	0.1840	0.2921	0.2546	0.2505
NM_001106514	Mcm8	0.2973	0.7816	0.1250	0.8467	0.7873	0.6232	0.6368	0.9485	0.7377	0.9063	0.9761	0.2579
NM_001106655	Nans	0.1165	0.3316	0.0977	0.6714	0.0162	0.1410	0.1623	0.0889	0.1563	0.2589	0.3260	0.1318
NM_001106544	Dpm1	0.1986	0.1089	0.9422	0.3500	0.2486	0.2814	0.1893	0.0575	0.1742	0.2690	0.4129	0.3497
NM_001106688	Sh3bgrl3	0.1065	0.0051	0.3329	0.8504	0.3340	0.0076	0.2975	0.0493	0.3147	0.0527	0.1026	0.2857
NM_001106682	Lao1	0.3971	0.4367	0.7258	0.8494	0.1219	0.4942	0.0569	0.3551	0.5795	0.1077	0.6270	0.7075
NM_001105926	Dhx37	0.9752	0.9762	0.2938	0.1843	0.9678	0.9335	0.6922	0.9876	0.9217	0.9547	0.9680	0.4504
NM_001105929	Zcchc8	0.9560	0.5790	0.8620	0.1983	0.9001	0.9557	0.3357	0.9411	0.8309	0.5708	0.9391	0.9198
NM_001105930	Atxn2	0.6035	0.7359	0.5049	0.6514	0.7313	0.5451	0.6652	0.6971	0.7126	0.6484	0.6391	0.7341
NM_001106073	Lsm4	0.5213	0.8934	0.0356	0.9060	0.6320	0.1475	0.8610	0.8378	0.8626	0.7837	0.8949	0.1020
NM_001106575	Srpk2	0.7141	0.9750	0.9904	0.7772	0.7605	0.8890	0.9620	0.8255	0.9723	0.8559	0.9805	0.9740
NM_001106061	Arhgef3	0.7882	0.8514	0.9013	0.7492	0.3344	0.6202	0.1875	0.8782	0.8814	0.3702	0.4760	0.4629
NM_001106102	Higd2a	0.5944	0.2769	0.4345	0.3913	0.1512	0.1245	0.3002	0.0940	0.0299	0.3233	0.0467	0.0903
NM_001105992	Xpr1	0.2278	0.3868	0.5855	0.1806	0.5718	0.6959	0.1847	0.8599	0.2025	0.7496	0.1675	0.5889
NM_001106050	Dnajc15	0.8218	0.9163	0.9979	0.9361	0.7680	0.7954	0.9365	0.8684	0.7815	0.8414	0.8815	0.8300
NM_001106516	Ankrd5	0.1487	0.8721	0.4443	0.7415	0.1819	0.4367	0.4983	0.5263	0.3934	0.5746	0.4089	0.3532
NM_001106515	RGD1306816	0.5884	0.6556	0.3926	0.2911	0.6813	0.7536	0.6735	0.8809	0.5712	0.3204	0.6310	0.2087
NM_001105943	Il27ra	0.1283	0.6365	0.2141	0.2845	0.4021	0.0484	0.6172	0.2288	0.3508	0.5597	0.2049	0.0547
NM_001105942	Neurod4	0.8746	0.8336	0.8556	0.7170	0.8201	0.4451	0.5976	0.8616	0.5961	0.4391	0.7748	0.7798
NM_001106236	Blvrb	0.1001	0.3817	0.5646	0.0511	0.0060	0.0295	0.9287	0.0929	0.0309	0.0336	0.0759	0.3340
NM_001106530	Spag4l	0.3183	0.2413	0.0195	0.4782	0.3139	0.5345	0.2541	0.1388	0.4645	0.2634	0.3678	0.0285
NM_001106705	Mta3	0.0509	0.6678	0.7790	0.3243	0.0308	0.0533	0.8171	0.0511	0.3750	0.3405	0.8447	0.2242
NM_001106693	Eif4g3	0.9365	0.8655	0.0475	0.9646	0.5590	0.7140	0.2187	0.6687	0.8594	0.8300	0.6962	0.9829
NM_001106531	Bpil1	0.3425	0.6491	0.3427	0.5386	0.5228	0.7092	0.3165	0.4356	0.3680	0.2043	0.5639	0.7489
NM_001106077	RGD1308759	0.9833	0.9691	0.4067	0.6481	0.9571	0.8918	0.8687	0.9864	0.9851	0.9828	0.8176	0.9316
NM_001122976	Trmt12	0.5118	0.6926	0.8029	0.8390	0.6221	0.3141	0.6111	0.8135	0.4047	0.5469	0.1300	0.1792
NM_001106768	Med16	0.9947	0.9187	0.3065	0.7072	0.9696	0.7937	0.8803	0.9552	0.1447	0.5135	0.2068	0.1347
NM_001106541	Wfdc3	0.5098	0.2217	0.0832	0.4989	0.4641	0.8289	0.1448	0.3434	0.2254	0.0904	0.0515	0.6632
NM_001106538	Wfdc5	0.4244	0.2090	0.5473	0.2461	0.0969	0.3804	0.3266	0.4239	0.3400	0.4872	0.0673	0.2692
NM_001106723	Pik3cg	0.4371	0.7576	0.5363	0.4456	0.9392	0.5869	0.4878	0.6493	0.2525	0.5164	0.3818	0.5057
NM_001106548	RGD1307805	0.1593	0.0387	0.0096	0.1037	0.3978	0.0773	0.3807	0.6770	0.2723	0.2896	0.3848	0.0188
NM_001106546	Zfp93	0.5220	0.1127	0.1775	0.1687	0.3444	0.0580	0.2276	0.5785	0.0711	0.6522	0.1832	0.7593
NM_001106699	B3galt6	0.4673	0.7735	0.8907	0.3585	0.3501	0.8871	0.8711	0.5403	0.3559	0.1511	0.4346	0.7333
NM_001106673	Gpx7	0.9711	0.7743	0.0655	0.9784	0.7405	0.9667	0.7901	0.9805	0.6256	0.8812	0.9162	0.1183
NM_001106254	Klk7	0.4301	0.2396	0.5015	0.3898	0.2030	0.4585	0.3826	0.6164	0.7224	0.7274	0.6613	0.5084
NM_001106847	Tfdp2	0.6832	0.9849	0.9398	0.9846	0.9809	0.9453	0.7515	0.8397	0.9672	0.8325	0.9960	0.7889
NM_001106792	Znf641	0.3276	0.3486	0.8565	0.6475	0.5703	0.3816	0.4194	0.3526	0.5090	0.4201	0.1460	0.3661
NM_001106561	RGD1311501	0.7727	0.4166	0.7776	0.7664	0.2426	0.9291	0.9340	0.2136	0.5799	0.2567	0.4451	0.8835
NM_001106089	Ash2l	0.8932	0.1418	0.4272	0.3716	0.4035	0.4546	0.2993	0.4115	0.1009	0.2985	0.2526	0.1329
NM_001106196	Galnt2	0.2912	0.2810	0.3081	0.1806	0.0023	0.1755	0.2426	0.1123	0.1884	0.2166	0.2238	0.6737
NM_001106174	Mmaa	0.6212	0.9764	0.1446	0.1841	0.7961	0.6487	0.9569	0.8256	0.8534	0.7005	0.9850	0.1620
NM_001106801	Med17	0.9916	0.9639	0.1922	0.1399	0.5617	0.8743	0.1785	0.9700	0.7448	0.7806	0.6056	0.8507
NM_001106167	Setd6	0.5040	0.0595	0.0359	0.0701	0.0800	0.0668	0.1306	0.0097	0.8315	0.6908	0.3812	0.0442
NM_001106015	Pla2g3	0.8034	0.3117	0.6610	0.4726	0.7576	0.4462	0.2097	0.7627	0.5880	0.5023	0.8166	0.4748
NM_001106178	Exoc3l	0.0668	0.0027	0.0827	0.0449	0.0156	0.0115	0.0025	0.0180	0.0613	0.0021	0.0021	0.0044

NM_001106789	Saps2	0.2286	0.0653	0.4630	0.6310	0.7109	0.5196	0.5265	0.7319	0.8963	0.5702	0.6791	0.3126
NM_001106788	Trabd	0.8949	0.9307	0.1923	0.8860	0.9401	0.9690	0.9274	0.9508	0.9657	0.8785	0.8399	0.9185
NM_001106821	Atm	0.3981	0.3539	0.5692	0.0664	0.3089	0.4774	0.0769	0.3149	0.3562	0.1699	0.2957	0.1493
NM_001106605	Fam136a	0.1868	0.2650	0.9413	0.2239	0.1774	0.1925	0.9311	0.0814	0.0548	0.0264	0.4438	0.7619
NM_001106173	Tmem188	0.6111	0.1744	0.9684	0.9894	0.1503	0.8809	0.1059	0.1848	0.3623	0.4014	0.5660	0.9697
NM_001106171	Tox3	0.5168	0.8919	0.8538	0.7746	0.8867	0.7914	0.7433	0.4803	0.7378	0.9453	0.8598	0.1468
NM_001106804	Tmem205	0.1205	0.0180	0.7789	0.0244	0.0886	0.0019	0.2297	0.0114	0.0027	0.0131	0.0082	0.0027
NM_001106025	Zmynd17	0.7833	0.8670	0.0628	0.0971	0.8943	0.8594	0.5360	0.8744	0.9579	0.9145	0.8300	0.5977
NM_001106793	Ddx23	0.1950	0.0873	0.3193	0.1027	0.3263	0.8922	0.2674	0.4709	0.3123	0.9150	0.7447	0.8147
NM_001106767	Ela2	0.1562	0.0751	0.1477	0.2673	0.1374	0.1792	0.4271	0.1624	0.4966	0.1097	0.0827	0.5171
NM_001105960	RGD1308695	0.7519	0.8445	0.9651	0.2083	0.8786	0.9288	0.7129	0.9908	0.9154	0.8028	0.9849	0.7512
NM_001106747	Nek9	0.9634	0.5411	0.9846	0.7979	0.9144	0.9067	0.9558	0.4862	0.6771	0.6094	0.9753	0.9389
NM_001106808	Npsr1	0.4880	0.7565	0.5411	0.6339	0.8702	0.4680	0.6101	0.9278	0.3694	0.4459	0.4160	0.7773
NM_001106818	Mpzl2	0.5794	0.4478	0.7092	0.4682	0.2695	0.5086	0.6274	0.2984	0.7534	0.4516	0.3242	0.5863
NM_001106799	Mmp27	0.5446	0.3854	0.4848	0.2073	0.0173	0.1490	0.0514	0.2783	0.2320	0.1809	0.1650	0.3557
NM_001105958	RGD1308584	0.7646	0.6725	0.4120	0.3188	0.9114	0.8421	0.3542	0.9743	0.4365	0.7452	0.7118	0.1856
NM_001106573	Crygn	0.1822	0.1829	0.4609	0.2024	0.1872	0.3947	0.2070	0.1969	0.3699	0.3622	0.5212	0.1875
NM_001106570	Phf19	0.3491	0.5202	0.4413	0.4338	0.4237	0.8614	0.6567	0.7197	0.6985	0.3301	0.6939	0.4641
NM_022674	H2afz	0.9537	0.9467	0.1331	0.9854	0.9377	0.9168	0.9104	0.8467	0.8822	0.8525	0.9809	0.2640
NM_001106455	Igsf3	0.9373	0.9404	0.9740	0.9766	0.5463	0.9647	0.9798	0.2764	0.8469	0.9846	0.9699	0.4506
NM_001126120	Adh5	0.5445	0.4824	0.2475	0.4922	0.5254	0.2548	0.4564	0.4524	0.2256	0.2421	0.2737	0.3377
NM_031836	Vegfa	0.7331	0.7016	0.8891	0.5415	0.5108	0.5606	0.9797	0.4868	0.5533	0.6890	0.7006	0.9767
NM_001114939	RGD1563348	0.3880	0.8944	0.1092	0.1784	0.6188	0.1564	0.9020	0.6755	0.6951	0.5508	0.9144	0.0773
NM_001107086	Ubxn7	0.0180	0.2728	0.6820	0.9020	0.2011	0.8064	0.5480	0.2959	0.3347	0.3687	0.7759	0.3464
NM_001106843	Irak1bp1	0.2433	0.7627	0.9169	0.3373	0.6789	0.7476	0.4245	0.7972	0.1475	0.7793	0.5878	0.4324
NM_001105961	RGD1305609	0.3064	0.2732	0.7106	0.3807	0.2618	0.2833	0.2069	0.1967	0.2804	0.3799	0.3139	0.1873
NM_001106111	Them2	0.0862	0.1247	0.0076	0.1370	0.0040	0.0791	0.0471	0.0055	0.0280	0.0921	0.0039	0.0482
NM_001107014	Alox12e	0.0144	0.5957	0.1347	0.7325	0.1459	0.8250	0.1327	0.1557	0.2451	0.0540	0.3483	0.0961
NM_001106589	RGD1308226	0.1039	0.3541	0.1012	0.4511	0.3369	0.1344	0.5140	0.5101	0.0871	0.0723	0.2243	0.0355
NM_001106725	Agr2	0.7804	0.9445	0.7563	0.7577	0.5865	0.5864	0.5950	0.8906	0.8007	0.8383	0.7492	0.7986
NM_001105972	RGD1562464	0.0421	0.9363	0.4324	0.8138	0.6880	0.6026	0.9450	0.5688	0.6901	0.6916	0.5888	0.4595
NM_001106830	Iqch	0.1804	0.1213	0.2487	0.1462	0.5281	0.1534	0.2645	0.2291	0.1871	0.1793	0.4646	0.2572
NM_001106276	Cpeb1	0.2158	0.9022	0.0947	0.8361	0.7700	0.8437	0.6438	0.9413	0.6267	0.6926	0.9233	0.7994
NM_001105983	Disp1	0.1644	0.0133	0.0206	0.1308	0.3091	0.0390	0.0368	0.0944	0.6943	0.1709	0.3012	0.0321
NM_001105980	Nvl	0.4392	0.6739	0.2533	0.3135	0.5410	0.7665	0.2331	0.8625	0.3823	0.3802	0.9603	0.5722
NM_001105977	Efcab2	0.7707	0.8224	0.8154	0.9228	0.9645	0.6510	0.3941	0.9738	0.3720	0.5259	0.9806	0.6033
NM_001106607	Podxl2	0.4614	0.3938	0.2819	0.4000	0.4957	0.1937	0.0391	0.4141	0.2705	0.3140	0.0958	0.5515
NM_001106772	Ndufa7	0.7316	0.2687	0.5911	0.6890	0.7030	0.7242	0.1118	0.5990	0.3236	0.1972	0.1442	0.0449
NM_001106761	Brf1	0.6587	0.6288	0.0982	0.7966	0.7000	0.4973	0.5084	0.6895	0.7004	0.5385	0.8203	0.6228
NM_001106043	Dleu7	0.7224	0.8827	0.7478	0.5247	0.7604	0.7288	0.8511	0.5184	0.9067	0.6694	0.9070	0.6817
NM_001105985	Slc30a10	0.4726	0.2357	0.2284	0.2943	0.2564	0.7035	0.3816	0.0780	0.1033	0.2171	0.0787	0.0440
NM_001106800	Mmp20	0.8272	0.5184	0.8438	0.8195	0.9025	0.7098	0.4571	0.2117	0.7989	0.9107	0.8375	0.8346
NM_001106883	Apobec2	0.1050	0.1090	0.1478	0.5641	0.5950	0.7395	0.3170	0.8941	0.4382	0.2858	0.0533	0.2930
NM_001106878	Cd70	0.5698	0.3345	0.2702	0.7475	0.2065	0.3959	0.0322	0.0941	0.4285	0.8656	0.0913	0.1675
NM_001106755	Clmn	0.2066	0.2239	0.4518	0.2628	0.3739	0.4444	0.1208	0.6284	0.4952	0.0402	0.5680	0.5278
NM_001106622	Tapbpl	0.5763	0.4440	0.7733	0.8503	0.7593	0.6522	0.7765	0.7855	0.1317	0.7681	0.3696	0.2569
NM_001106621	Mrpl51	0.2438	0.8765	0.1544	0.2557	0.5004	0.1853	0.9439	0.0959	0.8455	0.6953	0.8710	0.3134
NM_001106003	Npal1	0.1155	0.2538	0.0980	0.2065	0.3182	0.4104	0.3481	0.1032	0.1465	0.4777	0.1972	0.3845
NM_001105988	Plxna2	0.6489	0.8709	0.3919	0.4127	0.2649	0.7522	0.4575	0.3383	0.6882	0.4127	0.5129	0.8709

NM_001106623	RGD1311164	0.0661	0.0961	0.3454	0.1992	0.0656	0.3114	0.1164	0.1112	0.1855	0.1849	0.2362	0.0885
NM_001106835	Bnip2	0.8605	0.2367	0.0265	0.3217	0.6042	0.6865	0.0056	0.7022	0.6187	0.5491	0.0697	0.7638
NM_001005891	Clec4a3	0.4531	0.3898	0.7739	0.0104	0.3123	0.5862	0.1035	0.1550	0.2596	0.1654	0.6201	0.2359
NM_001106125	Spag6l	0.2439	0.5665	0.7220	0.4327	0.2662	0.1904	0.3163	0.1966	0.6790	0.5202	0.2910	0.3740
NM_001106009	Ldb2	0.7883	0.8714	0.3477	0.7336	0.7883	0.6561	0.3228	0.6481	0.3275	0.2426	0.4017	0.1813
NM_001106631	Rbpsuh	0.2729	0.3590	0.3501	0.3046	0.2537	0.3547	0.0946	0.4445	0.3156	0.3651	0.4145	0.2980
NM_001106630	Jph1	0.5521	0.5466	0.7338	0.7971	0.3849	0.4253	0.4695	0.3829	0.5351	0.4015	0.7370	0.8977
NM_001106629	Steap1	0.0177	0.0500	0.0013	0.0016	0.0176	0.0374	0.0642	0.0021	0.0599	0.0539	0.0591	0.0016
NM_001106628	Mrps35	0.4058	0.9798	0.9152	0.5277	0.8968	0.5422	0.9619	0.9704	0.9369	0.9511	0.9663	0.9938
NM_001106129	Zadh2	0.5578	0.7727	0.9771	0.7764	0.8958	0.8965	0.8213	0.5936	0.9554	0.9314	0.7624	0.9282
NM_001106012	Evc2	0.7701	0.8284	0.5653	0.5746	0.7671	0.5644	0.6327	0.6537	0.9906	0.7596	0.9871	0.5228
NM_001106044	Tdh	0.0863	0.7642	0.7488	0.3824	0.2491	0.5211	0.2787	0.3580	0.0234	0.1871	0.5742	0.1173
NM_001106128	Ly86	0.0629	0.1773	0.3534	0.1523	0.2932	0.2280	0.3844	0.0518	0.0762	0.3189	0.3571	0.5870
NM_001106019	H2afv	0.9354	0.9800	0.8900	0.8825	0.7716	0.9398	0.9046	0.8126	0.9438	0.7630	0.9642	0.1083
NM_001106018	RGD1309870	0.5505	0.6098	0.6421	0.3626	0.1862	0.2087	0.1643	0.1959	0.5343	0.7369	0.7794	0.4238
NM_001106211	B3gnt6	0.5030	0.4593	0.5477	0.4306	0.5013	0.3497	0.7151	0.6529	0.3223	0.1718	0.0823	0.2270
NM_001106602	Dguok	0.2964	0.4408	0.3127	0.4259	0.4013	0.2801	0.2067	0.3390	0.4316	0.3436	0.5034	0.3144
NM_001106160	RGD1309102	0.9948	0.9381	0.6244	0.7387	0.7755	0.9747	0.0555	0.9213	0.9780	0.9263	0.8308	0.9512
NM_001105986	Lyplal1	0.8781	0.9356	0.8306	0.7961	0.9030	0.9118	0.8058	0.9420	0.9447	0.7739	0.9531	0.8055
NM_001106913	Clk1	0.9449	0.8103	0.1741	0.3000	0.7779	0.7281	0.0545	0.9081	0.7975	0.7758	0.8725	0.7274
NM_001106783	Myf5	0.4418	0.7008	0.3854	0.5375	0.5712	0.6060	0.8161	0.3709	0.7164	0.5438	0.7489	0.5789
NM_001106839	Lysmd2	0.5236	0.9092	0.9650	0.7791	0.7722	0.5773	0.8576	0.8662	0.8874	0.8952	0.5675	0.0490
NM_001106845	Ankrd34c	0.0533	0.3080	0.5638	0.7101	0.2750	0.4147	0.6779	0.1843	0.3251	0.5365	0.4950	0.9246
NM_001106648	RGD1306576	0.8251	0.5516	0.4967	0.7256	0.7407	0.3686	0.2803	0.8503	0.0570	0.5547	0.8304	0.0940
NM_001106645	Ddx58	0.4446	0.5456	0.8667	0.5049	0.3063	0.3669	0.5872	0.3065	0.3594	0.7292	0.0826	0.4425
NM_001106302	Hmx3	0.3669	0.0251	0.3683	0.2967	0.4041	0.2367	0.4538	0.7170	0.0688	0.1859	0.7234	0.2868
NM_001105933	Arpc3	0.0295	0.0606	0.8742	0.0597	0.0386	0.1512	0.0307	0.0801	0.0207	0.0687	0.1055	0.2169
NM_001106502	Usp8	0.1163	0.0352	0.0973	0.1289	0.2720	0.4609	0.0511	0.3011	0.1130	0.2039	0.1580	0.9381
NM_001110151	Snx27	0.9260	0.9824	0.8945	0.6851	0.7928	0.6837	0.9705	0.6538	0.9532	0.9220	0.9661	0.9090
NM_001106863	Tessp2	0.3575	0.4107	0.2497	0.5421	0.3577	0.1050	0.5395	0.1750	0.5758	0.2554	0.0670	0.3377
NM_001106837	Ecat1	0.0852	0.7968	0.5900	0.4272	0.4952	0.6000	0.2418	0.8492	0.5181	0.2796	0.3311	0.1101
NM_001106658	Zcchc7	0.6457	0.3807	0.7902	0.9549	0.1110	0.9025	0.4732	0.3623	0.6895	0.7321	0.8980	0.6492
NM_001106657	Zbtb5	0.9438	0.9636	0.9404	0.6175	0.9627	0.9593	0.7427	0.6261	0.9296	0.9694	0.9608	0.9522
NM_001106644	RGD1305158	0.0126	0.6160	0.3080	0.9466	0.2960	0.0629	0.7485	0.1031	0.0486	0.1431	0.4893	0.0560
NM_001106860	Spink8	0.1376	0.3303	0.5899	0.3383	0.1720	0.5204	0.6084	0.4142	0.7589	0.3094	0.6108	0.3861
NM_001106868	Hhatl	0.0568	0.5087	0.0739	0.1140	0.1423	0.2652	0.4262	0.1474	0.6043	0.2955	0.0555	0.0680
NM_001106874	Slc25a41	0.6331	0.7437	0.5210	0.1622	0.2775	0.1530	0.9373	0.5677	0.8384	0.1727	0.1029	0.5881
NM_001106873	Slc25a23	0.3626	0.4964	0.6484	0.6983	0.8295	0.7462	0.8594	0.4674	0.6151	0.7957	0.2307	0.8706
NM_001106091	Mrps31	0.1776	0.0269	0.3924	0.6225	0.3039	0.3081	0.1454	0.0938	0.3760	0.3910	0.0856	0.1763
NM_001106148	Myot	0.7332	0.7823	0.8643	0.5650	0.4324	0.7172	0.8166	0.8441	0.5148	0.9722	0.8921	0.8884
NM_001106235	Ccdc97	0.9402	0.8004	0.1299	0.2430	0.6123	0.9005	0.6874	0.6641	0.5713	0.6789	0.0804	0.7551
NM_001106246	Gpatch1	0.5766	0.8412	0.4884	0.0901	0.3654	0.0666	0.3001	0.3269	0.5073	0.0567	0.4389	0.3895
NM_001106661	RGD1304595	0.5514	0.4663	0.4672	0.1590	0.6800	0.6419	0.4844	0.5452	0.9004	0.7604	0.2050	0.8845
NM_001107008	Znf287	0.3091	0.1826	0.4562	0.5861	0.1197	0.2019	0.3935	0.4578	0.8096	0.1113	0.4751	0.6402
NM_001106856	Uba7	0.7390	0.1630	0.7779	0.7855	0.4936	0.7018	0.6184	0.5062	0.1134	0.8735	0.7666	0.8024
NM_001106180	RGD1307357	0.0419	0.9024	0.1894	0.7958	0.8238	0.8817	0.2901	0.9056	0.5812	0.8120	0.7825	0.9235
NM_001106049	Nudt15	0.0262	0.0095	0.0320	0.3954	0.0069	0.0055	0.0052	0.0868	0.0036	0.0071	0.0031	0.0049
NM_001107328	Efnb2	0.0923	0.5753	0.6619	0.4010	0.7754	0.3452	0.3683	0.6393	0.8215	0.5403	0.4740	0.6943
NM_001106916	Ikzf2	0.3079	0.7596	0.2803	0.4506	0.4056	0.3189	0.1027	0.1610	0.5874	0.4028	0.5046	0.5435

NM_001134535	Vwa5b2	0.4123	0.5088	0.0889	0.2279	0.2273	0.4285	0.2371	0.2539	0.0395	0.1850	0.3241	0.1678
NM_001106272	Znf592	0.9559	0.9742	0.0579	0.0428	0.9567	0.5142	0.4139	0.3327	0.9801	0.4506	0.9472	0.3231
NM_001106100	Agtpbp1	0.9390	0.8518	0.4634	0.8461	0.9893	0.9020	0.6556	0.7318	0.9965	0.9489	0.9308	0.8337
NM_001106040	RGD1308396	0.6740	0.6288	0.0202	0.2690	0.1592	0.1172	0.6231	0.1365	0.7556	0.2491	0.4588	0.5255
NM_001106038	Ipo4	0.6921	0.8438	0.0159	0.1879	0.8778	0.1871	0.8477	0.4457	0.8401	0.8456	0.7195	0.7969
NM_001106666	Bnc2	0.5634	0.6306	0.4574	0.6182	0.4510	0.4904	0.3672	0.7150	0.7227	0.6366	0.6039	0.5467
NM_001106169	RGD1565601	0.3841	0.4668	0.1450	0.2392	0.2885	0.1034	0.2760	0.2977	0.3395	0.3138	0.1253	0.2078
NM_001106334	Ganab	0.8982	0.2443	0.1961	0.1969	0.9544	0.7164	0.1468	0.6410	0.9819	0.8634	0.3922	0.8790
NM_001106324	B3gnt1	0.3107	0.3049	0.9955	0.8774	0.2342	0.1192	0.5251	0.0197	0.1059	0.1900	0.2954	0.3609
NM_001106848	Rnf7	0.0763	0.3941	0.2836	0.3965	0.5541	0.1483	0.1319	0.5016	0.2417	0.0762	0.3057	0.1537
NM_001127503	LOC100151767	0.5005	0.6307	0.5844	0.6719	0.4732	0.5754	0.6015	0.5833	0.7089	0.6554	0.6460	0.5635
NM_001109614	Polr2e	0.5632	0.0540	0.6463	0.3527	0.1233	0.1378	0.2294	0.4732	0.0715	0.0287	0.0572	0.1292
NM_001109599	Pou2af1	0.4011	0.3916	0.7143	0.5464	0.6865	0.2612	0.6819	0.2427	0.5881	0.6828	0.4144	0.4978
NM_001109500	Lce1m	0.2454	0.2603	0.4108	0.4339	0.4920	0.2945	0.4666	0.2894	0.3506	0.3742	0.5432	0.5813
NM_001109553	Crygs	0.0354	0.7679	0.0514	0.0331	0.3889	0.8543	0.1369	0.0973	0.1271	0.5646	0.8631	0.1369
NM_001109365	RGD1563528	0.3362	0.7991	0.3101	0.4416	0.5897	0.6517	0.8658	0.8403	0.6848	0.8438	0.7086	0.8666
NM_001109360	Cyp4f40	0.1321	0.0755	0.0821	0.1222	0.0821	0.0437	0.1359	0.1830	0.1515	0.0376	0.1716	0.0841
NM_001106920	Aamp	0.2649	0.1329	0.3487	0.3303	0.1916	0.3863	0.6163	0.4089	0.7447	0.4567	0.4897	0.3555
NM_001106285	Insc	0.3333	0.1057	0.3887	0.1457	0.3571	0.1146	0.2482	0.3335	0.4723	0.1257	0.0881	0.7358
NM_001106136	Ftmt	0.6907	0.4683	0.8762	0.7384	0.5855	0.2406	0.4909	0.9350	0.8854	0.8853	0.7497	0.7649
NM_001106074	Crlf1	0.5775	0.0246	0.1482	0.4749	0.1570	0.4780	0.1016	0.0522	0.0282	0.0225	0.0456	0.0424
NM_001106072	RGD1308064	0.0224	0.3476	0.0087	0.0751	0.0057	0.0582	0.1772	0.1257	0.5026	0.4150	0.0922	0.0322
NM_001106069	Fcho1	0.3523	0.4932	0.2470	0.3149	0.4515	0.6302	0.2944	0.6213	0.6673	0.2201	0.4557	0.6311
NM_001106684	Rhbdl2	0.9306	0.4959	0.5010	0.4402	0.5846	0.7356	0.7385	0.6115	0.5699	0.7532	0.7061	0.4817
NM_001106686	Eif2c4	0.6757	0.5874	0.5593	0.6372	0.7044	0.7000	0.6348	0.7110	0.6696	0.6714	0.7139	0.4735
NM_001106090	Whsc1l1	0.1656	0.8056	0.8374	0.5348	0.4249	0.5397	0.8297	0.6111	0.0428	0.6352	0.7560	0.6104
NM_001107020	Sgsm2	0.2357	0.4299	0.8475	0.2825	0.3155	0.3267	0.4001	0.3630	0.5219	0.4067	0.0211	0.6817
NM_001106205	Mrpl18	0.2682	0.8355	0.7936	0.3856	0.6894	0.2767	0.6485	0.0578	0.3488	0.6453	0.7291	0.4805
NM_001106200	Tectb	0.7748	0.7475	0.8224	0.5957	0.4379	0.7195	0.8620	0.0958	0.2863	0.3597	0.8155	0.7352
NM_001107116	Pcp2	0.6239	0.7179	0.5274	0.6110	0.7563	0.6345	0.5230	0.5379	0.6956	0.5104	0.8212	0.5498
NM_001106926	D2hgdh	0.4534	0.2956	0.0788	0.3882	0.0598	0.4846	0.3196	0.5905	0.5602	0.2409	0.0490	0.1555
NM_001106914	Kctd18	0.9255	0.3463	0.8566	0.3745	0.5884	0.7260	0.2129	0.9065	0.5220	0.5377	0.7064	0.4447
NM_001106199	RGD1559496	0.4247	0.5513	0.0100	0.4913	0.5526	0.7326	0.2493	0.1985	0.3401	0.5496	0.5403	0.6782
NM_001106306	Cpxm2	0.1359	0.0989	0.1809	0.0515	0.2108	0.1816	0.0973	0.2297	0.1105	0.2508	0.0439	0.3469
NM_001106494	Lpcat4	0.2490	0.1552	0.2443	0.3546	0.1141	0.1507	0.2585	0.1530	0.1538	0.1912	0.4998	0.6607
NM_001107030	Fndc8	0.3498	0.4156	0.2421	0.7344	0.5151	0.7392	0.4898	0.7385	0.5920	0.7565	0.8150	0.6065
NM_001106709	Slc4a1ap	0.3854	0.6599	0.2507	0.3221	0.4200	0.0691	0.0250	0.6003	0.7413	0.7390	0.4740	0.3187
NM_001106698	H6pd	0.6752	0.5752	0.7801	0.7560	0.7598	0.9526	0.9256	0.9158	0.9214	0.1733	0.1266	0.8187
NM_001106301	Nsmce4a	0.5681	0.4280	0.3029	0.3963	0.1868	0.1901	0.2064	0.1962	0.3684	0.4840	0.4387	0.1868
NM_001106291	Nlrp10	0.6742	0.1165	0.2280	0.5702	0.4677	0.6102	0.1621	0.1211	0.0453	0.3395	0.2158	0.6585
NM_001106110	Dcdc2	0.8630	0.9325	0.8807	0.6285	0.1985	0.8558	0.6288	0.6107	0.9577	0.7098	0.8886	0.4671
NM_001106165	Car7	0.4702	0.5060	0.4556	0.2319	0.8469	0.6367	0.7277	0.6419	0.8883	0.5942	0.9377	0.7596
NM_031035	Gnai2	0.2743	0.5803	0.8962	0.9777	0.3334	0.4854	0.8042	0.5223	0.9418	0.7575	0.9478	0.4157
NM_001115013	Selm	0.1951	0.6952	0.6896	0.8439	0.2153	0.0900	0.9138	0.3953	0.0550	0.8401	0.8082	0.0567
NM_001114599	RGD1560286	0.5851	0.5707	0.6206	0.7743	0.6263	0.7245	0.6743	0.6753	0.7999	0.7209	0.6137	0.7105
NM_001114604	Pcdhb21	0.2905	0.1511	0.5302	0.4801	0.5741	0.3334	0.3325	0.5610	0.4266	0.4446	0.5795	0.4254
NM_001114601	Pcdhb4	0.1527	0.1720	0.4166	0.3029	0.4576	0.4022	0.1098	0.0566	0.3894	0.0125	0.7562	0.1808
NM_001014783	Pcdhb3	0.5102	0.1858	0.0169	0.8457	0.5471	0.5344	0.1171	0.3778	0.4219	0.1078	0.0140	0.0528
NM_001106226	Gltscr1	0.1002	0.0418	0.2450	0.5330	0.0847	0.1392	0.3843	0.1101	0.2577	0.0586	0.0458	0.1442

NM_001106086	Ubxd6	0.5000	0.8724	0.5168	0.4298	0.7084	0.5143	0.4986	0.8743	0.6134	0.1030	0.8754	0.4009
NM_001106137	Poli	0.3562	0.3653	0.2465	0.6239	0.5475	0.1488	0.6712	0.0789	0.8288	0.8070	0.6163	0.7402
NM_001106255	RGD1309036	0.0496	0.4577	0.1053	0.5406	0.1139	0.0982	0.3225	0.1990	0.2063	0.8006	0.0287	0.3316
NM_001106251	Atpbd3	0.8971	0.8609	0.5990	0.8233	0.8576	0.7653	0.9817	0.8615	0.8215	0.8638	0.6350	0.8863
NM_001106373	Pank1	0.2194	0.6263	0.2288	0.9820	0.2765	0.2804	0.0639	0.3192	0.9492	0.9445	0.9511	0.9750
NM_001106231	RGD1562091	0.0122	0.0154	0.5309	0.1876	0.1263	0.0227	0.0510	0.2258	0.1661	0.0075	0.0145	0.2946
NM_001106101	Catsper3	0.6005	0.4020	0.4677	0.1966	0.6404	0.6339	0.1585	0.5535	0.3738	0.4140	0.5771	0.1285
NM_001105989	Cr2	0.2347	0.2435	0.3809	0.4020	0.0755	0.0646	0.3620	0.2664	0.2009	0.5811	0.2390	0.2620
NM_001106312	Cd163l1	0.1821	0.1825	0.1930	0.2016	0.1866	0.1897	0.2063	0.1961	0.1764	0.1715	0.1831	0.1868
NM_001106434	Tm4sf1	0.9481	0.9813	0.9984	0.8824	0.9077	0.7638	0.9551	0.7819	0.9909	0.9936	0.9817	0.8975
NM_001106273	Mesp2	0.2362	0.2645	0.4684	0.0871	0.0529	0.2136	0.3961	0.3239	0.1016	0.0850	0.0489	0.5201
NM_001106209	T	0.5466	0.5122	0.3443	0.3841	0.3271	0.5114	0.5863	0.4975	0.2360	0.5130	0.2394	0.3629
NM_001106203	Znhit6	0.7730	0.7134	0.7204	0.8446	0.8967	0.8029	0.8137	0.9387	0.7054	0.9050	0.9786	0.6879
NM_001106258	Tbc1d17	0.4141	0.3632	0.3229	0.6830	0.1183	0.8184	0.3983	0.5849	0.5518	0.3579	0.5519	0.3368
NM_001105990	Kif21b	0.3825	0.4790	0.4663	0.3150	0.5376	0.6741	0.2061	0.3886	0.3785	0.4692	0.2939	0.3894
NM_001106134	RGD1310784	0.7424	0.8633	0.1239	0.4630	0.8558	0.9532	0.9671	0.7917	0.7679	0.7730	0.7748	0.0268
NM_001106104	Ptpdc1	0.0086	0.1646	0.4269	0.1202	0.2664	0.0503	0.1624	0.2093	0.0077	0.2694	0.3660	0.6144
NM_001107046	Plxdc1	0.9689	0.9863	0.7994	0.8766	0.9125	0.9375	0.8765	0.8775	0.8199	0.9491	0.9420	0.9098
NM_001105996	Mrps18c	0.1087	0.6943	0.1293	0.6044	0.0198	0.0471	0.3814	0.2123	0.0490	0.1076	0.3714	0.0136
NM_001106740	Ppp2r5e	0.9573	0.8372	0.5790	0.5631	0.7015	0.7714	0.2754	0.3657	0.9776	0.6110	0.6219	0.9283
NM_001105998	Ccni	0.8575	0.3120	0.9355	0.9242	0.9638	0.9646	0.9827	0.9642	0.7764	0.2523	0.8813	0.9229
NM_001106749	RGD1310769	0.8990	0.7539	0.1109	0.9159	0.9682	0.5833	0.4571	0.9311	0.5737	0.1118	0.8893	0.3130
NM_001106656	Xpa	0.2113	0.0867	0.8409	0.3990	0.1514	0.1329	0.7969	0.1062	0.8345	0.5433	0.1031	0.5164
NM_001106744	RGD1307597	0.6220	0.3760	0.8332	0.6047	0.8892	0.9326	0.4629	0.5893	0.7976	0.2986	0.5591	0.9020
NM_001106953	Ppp1r2p9	0.4361	0.4705	0.3304	0.4567	0.2277	0.4996	0.4302	0.3705	0.2225	0.2569	0.3107	0.1867
NM_001106943	Foxo4	0.8591	0.9351	0.8137	0.9146	0.9560	0.9507	0.9591	0.9395	0.9234	0.9147	0.9130	0.8151
NM_001106957	Glod5	0.5785	0.5502	0.6395	0.4848	0.5352	0.0885	0.5677	0.4469	0.4904	0.6361	0.3894	0.3849
NM_198782	Dgki	0.9275	0.8328	0.2975	0.3737	0.8249	0.7603	0.6277	0.4134	0.6639	0.8728	0.6591	0.6082
NM_001107049	Ttc25	0.7294	0.6037	0.7453	0.5675	0.1435	0.7354	0.7397	0.8056	0.8542	0.6884	0.7024	0.9076
NM_001106750	Flrt2	0.4645	0.4416	0.8045	0.3120	0.1775	0.0704	0.2750	0.1762	0.4908	0.6044	0.4584	0.4515
NM_001106114	Hist1h2bn	0.8698	0.6183	0.4855	0.4062	0.5224	0.7183	0.7143	0.5025	0.3568	0.6108	0.5158	0.1247
NM_001106113	Hist1h1a	0.5860	0.1703	0.5205	0.5189	0.0849	0.0637	0.3166	0.2513	0.7210	0.4728	0.2617	0.8664
NM_001106756	Ndufb11	0.0735	0.1324	0.7165	0.6317	0.0424	0.1127	0.6426	0.2519	0.4220	0.1984	0.7578	0.2028
NM_001106754	Serpina9	0.3540	0.2249	0.7024	0.3545	0.2352	0.4167	0.5804	0.4804	0.4962	0.6363	0.5413	0.4932
NM_001106753	Cpsf2	0.3014	0.5498	0.4668	0.5633	0.3335	0.8601	0.0457	0.3629	0.4756	0.1962	0.2469	0.3979
NM_001107267	Lats2	0.4250	0.3670	0.3819	0.4358	0.4557	0.9576	0.4023	0.3703	0.2614	0.4724	0.7626	0.5069
NM_001106119	Fbxo18	0.8970	0.4075	0.1259	0.8567	0.6596	0.6997	0.0909	0.7746	0.4594	0.6919	0.7138	0.3654
NM_001106121	RGD1308977	0.4578	0.1323	0.6430	0.4357	0.1279	0.7034	0.3185	0.2383	0.0831	0.5472	0.4033	0.3225
NM_001106126	RGD1309839	0.1764	0.8621	0.5611	0.5994	0.1271	0.2934	0.6763	0.5120	0.4159	0.7380	0.7601	0.4799
NM_001106452	Man1a2	0.2175	0.9707	0.9919	0.9692	0.5740	0.8903	0.8870	0.4858	0.9421	0.7590	0.9599	0.7810
NM_001106897	LOC301289	0.4101	0.4953	0.5144	0.6301	0.5570	0.2237	0.2588	0.4905	0.2118	0.2822	0.5460	0.2345
NM_001106766	Polrmt	0.5885	0.4219	0.0384	0.1233	0.7346	0.5072	0.0080	0.7105	0.7986	0.7573	0.7112	0.5868
NM_001106353	Erlin1	0.2223	0.2566	0.3629	0.4817	0.3679	0.3667	0.5685	0.3979	0.5936	0.6150	0.8281	0.6329
NM_001005556	Visa	0.7498	0.1802	0.4902	0.9773	0.9550	0.7481	0.7867	0.9013	0.8824	0.8107	0.4551	0.9241
NM_001106124	Slc39a12	0.4434	0.2905	0.5894	0.3797	0.5445	0.3023	0.1513	0.3049	0.1003	0.2367	0.5815	0.2222
NM_001106357	Smc5	0.5783	0.3851	0.8740	0.3453	0.3712	0.6847	0.3744	0.1889	0.4995	0.8973	0.6499	0.9516
NM_001107153	Usp30	0.2977	0.4196	0.8922	0.0246	0.5391	0.4161	0.0635	0.5455	0.4310	0.2362	0.3853	0.7711
NM_001107042	Hoxb3	0.6420	0.7508	0.1224	0.5654	0.7830	0.7625	0.5704	0.8872	0.5573	0.6452	0.7697	0.5719
NM_001106933	Ap3s1	0.8846	0.4510	0.9486	0.9664	0.9804	0.9345	0.9253	0.8353	0.6769	0.4971	0.7583	0.7117

NM_001106963	Eif1ay	0.5623	0.6601	0.7695	0.5337	0.5327	0.6102	0.5018	0.2770	0.4868	0.5877	0.4766	0.7561
NM_001106961	Ofd1	0.2175	0.6811	0.6692	0.8727	0.6494	0.7034	0.4043	0.7678	0.0707	0.1160	0.4336	0.3329
NM_001106133	Dym	0.6551	0.8087	0.2259	0.5739	0.5374	0.1048	0.8088	0.7701	0.8372	0.9085	0.7944	0.1966
NM_001106234	Ethe1	0.7536	0.2619	0.8051	0.1974	0.0567	0.0529	0.9070	0.1091	0.7287	0.4047	0.8428	0.9724
NM_001108072	Plekhj1	0.2172	0.1196	0.3029	0.2017	0.1065	0.9056	0.6279	0.7088	0.0151	0.1619	0.0428	0.5144
NM_001009602	Actr10	0.1025	0.0193	0.1104	0.3876	0.0844	0.3443	0.0669	0.1268	0.0493	0.1297	0.0964	0.2367
NM_001007145	Ctnna1	0.9867	0.8005	0.9513	0.9362	0.5553	0.9342	0.9186	0.9237	0.9631	0.8345	0.9198	0.4895
NM_001106401	Fam172a	0.6753	0.7271	0.7132	0.6920	0.6621	0.5651	0.2332	0.6356	0.6037	0.7103	0.6242	0.5479
NM_001106862	Ngp	0.4461	0.2519	0.3928	0.6591	0.7536	0.3414	0.1908	0.3280	0.5897	0.5931	0.4505	0.7494
NM_001106138	Psmg2	0.1383	0.6977	0.1748	0.3007	0.3008	0.0657	0.6109	0.2063	0.1528	0.7069	0.4074	0.2366
NM_001106813	Crtam	0.5662	0.9373	0.8150	0.7752	0.7450	0.4920	0.3731	0.9071	0.8691	0.7438	0.7087	0.7238
NM_017146	Mcpt10	0.8386	0.4172	0.2719	0.4721	0.8839	0.5997	0.3692	0.6309	0.3943	0.1392	0.3737	0.4494
NM_001113751	Cdk2ap1	0.9233	0.9909	0.7700	0.8895	0.9487	0.6801	0.8925	0.6864	0.7611	0.8828	0.9056	0.2646
NM_001113390	Ptprr	0.6773	0.5142	0.9688	0.6484	0.5854	0.5375	0.5284	0.6098	0.4551	0.7071	0.5431	0.9502
NM_001113371	Synj2	0.9289	0.8790	0.9575	0.8633	0.7276	0.9402	0.9832	0.8164	0.9118	0.7186	0.8839	0.8685
NM_001107103	Ripk4	0.5569	0.6805	0.8146	0.9208	0.8212	0.5295	0.9240	0.9431	0.4682	0.4809	0.9492	0.8471
NM_001107040	Dlx4	0.2214	0.5467	0.2161	0.2408	0.2353	0.5167	0.5867	0.6275	0.6794	0.1914	0.2074	0.7133
NM_001106940	Lpar4	0.6845	0.3974	0.6114	0.5838	0.6639	0.5266	0.5598	0.1169	0.4453	0.7088	0.5827	0.2746
NM_001107138	Sbno1	0.7023	0.5161	0.9660	0.6278	0.2891	0.7473	0.5011	0.3797	0.7799	0.7297	0.9678	0.9188
NM_001106829	Pias1	0.9266	0.8439	0.4589	0.2345	0.7444	0.5587	0.2557	0.6495	0.1368	0.6374	0.5874	0.8838
NM_001106385	Cisd1	0.1436	0.1583	0.4284	0.0479	0.1127	0.0515	0.7783	0.2226	0.2558	0.2899	0.3036	0.8798
NM_001106376	Trim31	0.3896	0.1279	0.2712	0.2636	0.6713	0.7226	0.6311	0.6728	0.2276	0.6696	0.4055	0.6173
NM_001106832	Snx22	0.5629	0.5178	0.6291	0.7456	0.6081	0.4462	0.6021	0.5408	0.5071	0.3487	0.4988	0.5987
NM_001106831	RGD1565496	0.2562	0.7976	0.9440	0.5610	0.1454	0.8253	0.6661	0.0314	0.2116	0.9255	0.0122	0.9593
NM_001106834	Rora	0.6772	0.9782	0.9961	0.0764	0.2628	0.1148	0.9634	0.1631	0.9998	0.9953	0.7611	0.9832
NM_001106841	Mto1	0.5943	0.9100	0.1388	0.0333	0.0409	0.0631	0.2076	0.0398	0.6356	0.1841	0.5141	0.5017
NM_001134360	Wdr81	0.6792	0.6877	0.1653	0.0585	0.7481	0.8600	0.3193	0.9038	0.9507	0.4957	0.6757	0.8811
NM_001106846	Cldn2	0.9100	0.4229	0.8104	0.7567	0.7156	0.8236	0.9345	0.8907	0.6268	0.7907	0.8948	0.8723
NM_001106842	Senp6	0.9934	0.8749	0.9507	0.5693	0.9706	0.7327	0.6740	0.7890	0.8917	0.9791	0.9844	0.8727
NM_001107257	Fbxo34	0.9802	0.8063	0.8684	0.0368	0.6386	0.3000	0.8127	0.9162	0.9119	0.9862	0.8564	0.9315
NM_001106947	LOC302473	0.6461	0.5943	0.6990	0.5058	0.6778	0.5356	0.5569	0.6779	0.7220	0.6670	0.7224	0.4252
NM_001106852	Mrpl3	0.1493	0.1814	0.2137	0.5660	0.0142	0.1601	0.3026	0.3165	0.0635	0.3221	0.7798	0.1371
NM_001107105	Znf295	0.6599	0.7407	0.7747	0.8968	0.5083	0.7728	0.1979	0.8784	0.8786	0.3776	0.7929	0.8887
NM_001106950	LOC302495	0.6424	0.6812	0.6302	0.7184	0.6905	0.6616	0.6539	0.6939	0.6403	0.6088	0.6638	0.5429
NM_001106861	Smarcc1	0.6888	0.8765	0.5968	0.2699	0.5122	0.8320	0.6345	0.3748	0.5491	0.6843	0.7593	0.4237
NM_001106865	Ctdspl	0.7835	0.9907	0.6887	0.7450	0.9003	0.9563	0.9947	0.9964	0.0509	0.9869	0.9427	0.3686
NM_001106994	Ranbp17	0.3295	0.0268	0.3719	0.5039	0.4277	0.5402	0.3764	0.3947	0.1479	0.6342	0.3500	0.1836
NM_001107102	FAM3B	0.2851	0.0150	0.0572	0.0602	0.0005	0.0757	0.0660	0.0432	0.0183	0.0315	0.0690	0.0340
NM_001106986	Noxo1	0.6645	0.7187	0.5587	0.1888	0.5610	0.7233	0.4971	0.7521	0.9000	0.7143	0.4797	0.5795
NM_001106864	Ltf	0.4969	0.2298	0.2936	0.1880	0.0684	0.3347	0.3459	0.2159	0.7074	0.0912	0.2029	0.0970
NM_001107107	Ets2	0.9529	0.5064	0.4520	0.9382	0.7663	0.8276	0.5750	0.6867	0.8497	0.8072	0.7597	0.9902
NM_001106870	Fyco1	0.5739	0.6632	0.7428	0.4052	0.2489	0.8788	0.5263	0.7954	0.7642	0.5763	0.9200	0.8547
NM_001107108	Sim2	0.5562	0.8458	0.6745	0.6683	0.7921	0.6256	0.0605	0.4378	0.6532	0.7012	0.4577	0.5457
NM_001107002	41,160.0000	0.1720	0.2464	0.9522	0.2407	0.2530	0.1354	0.3691	0.2419	0.2082	0.0976	0.2588	0.1937
NM_001107141	Pptc7	0.8087	0.7668	0.6703	0.5142	0.4107	0.8938	0.2301	0.0554	0.7928	0.8543	0.6639	0.7626
NM_001106476	Metap1	0.8109	0.3416	0.8883	0.3231	0.7661	0.7493	0.5083	0.7934	0.8109	0.8357	0.8075	0.5624
NM_001106850	Sox14	0.6409	0.6717	0.9372	0.8001	0.8669	0.8245	0.3256	0.9070	0.9400	0.5769	0.3864	0.8716
NM_001106811	Adamts8	0.2142	0.3694	0.2705	0.0338	0.2925	0.2006	0.2850	0.1746	0.2332	0.4120	0.3133	0.8310
NM_001106535	RGD1311066	0.0970	0.1028	0.3980	0.0691	0.1366	0.4210	0.1117	0.3676	0.8012	0.0074	0.2759	0.2250

NM_001106320	Ppfia1	0.8469	0.2774	0.7485	0.8604	0.1574	0.7860	0.2399	0.4902	0.8565	0.3665	0.7503	0.8100
NM_001106115	Trim27	0.9736	0.8569	0.4524	0.2170	0.8638	0.9657	0.5474	0.9475	0.6974	0.4931	0.4053	0.7215
NM_001107173	Elk4	0.5899	0.3315	0.6070	0.7893	0.6129	0.7155	0.2224	0.4654	0.6780	0.1112	0.6983	0.0858
NM_001107130	Fbxo24	0.5358	0.0197	0.2735	0.7425	0.1805	0.3916	0.4945	0.7485	0.3664	0.5268	0.0518	0.2944
NM_001106445	Rag1ap1	0.2981	0.6975	0.9914	0.0504	0.7260	0.2061	0.9129	0.8116	0.6989	0.7838	0.5482	0.7013
NM_001106332	Otub1	0.6172	0.8226	0.7654	0.1540	0.6926	0.9092	0.3673	0.7649	0.8130	0.0827	0.2480	0.5879
NM_001106195	RGD1561507	0.6469	0.6831	0.7658	0.8526	0.8360	0.7303	0.8668	0.6671	0.5823	0.6776	0.6956	0.5394
NM_001106194	Sult5a1	0.8547	0.0013	0.7114	0.9210	0.7983	0.7775	0.9002	0.9129	0.0297	0.1111	0.1143	0.0395
NM_001106478	Hltf	0.2820	0.1429	0.9305	0.2071	0.5566	0.4917	0.8378	0.5895	0.5934	0.5768	0.4111	0.5739
NM_001106472	Dkk2	0.2581	0.3080	0.6971	0.1677	0.1324	0.0773	0.5924	0.0392	0.0624	0.4068	0.4869	0.2428
NM_001106387	Lrrtm3	0.7738	0.9440	0.8431	0.6531	0.4289	0.8766	0.8501	0.8290	0.9331	0.8851	0.7733	0.9429
NM_001106382	Mcm3ap	0.8520	0.7863	0.3748	0.0042	0.8750	0.8408	0.2818	0.8227	0.6857	0.8918	0.7761	0.2467
NM_001106207	Myct1	0.2182	0.6677	0.5189	0.7382	0.8845	0.3491	0.9577	0.7029	0.8472	0.3656	0.3976	0.2935
NM_001107016	Spag7	0.9210	0.9191	0.9427	0.5761	0.7222	0.3384	0.7623	0.9546	0.9636	0.8053	0.8724	0.6615
NM_001107144	Rnft2	0.5820	0.5614	0.4601	0.1358	0.7804	0.5195	0.8963	0.7662	0.6691	0.4502	0.2963	0.7848
NM_001107150	Ddx51	0.7405	0.5375	0.0151	0.2530	0.6197	0.8517	0.2051	0.5138	0.1725	0.0446	0.4882	0.8851
NM_001106218	Leng1	0.1148	0.7994	0.0958	0.0085	0.2031	0.1039	0.7983	0.2679	0.7564	0.9184	0.4392	0.3059
NM_001107835	Fbxw2	0.9005	0.6646	0.4353	0.9105	0.7181	0.3261	0.8867	0.7683	0.7301	0.4129	0.4718	0.2052
NM_053722	Clasp2	0.9445	0.9722	0.7812	0.3635	0.2007	0.3549	0.9110	0.3130	0.9487	0.9486	0.9577	0.8879
NM_001108249	Timm17b	0.0769	0.7103	0.4493	0.7696	0.1554	0.1633	0.8432	0.2799	0.3956	0.0660	0.0838	0.2854
NM_017187	Hmgb2	0.9410	0.9904	0.3635	0.9809	0.9074	0.8287	0.9222	0.9825	0.7046	0.9915	0.9577	0.1274
NM_001106927	Pdcd1	0.8056	0.8954	0.6362	0.7587	0.6879	0.8365	0.3396	0.6738	0.7879	0.7434	0.9044	0.2523
NM_001107026	Rhot1	0.7305	0.5596	0.5702	0.4342	0.7833	0.4234	0.2398	0.5090	0.5185	0.7579	0.6446	0.7672
NM_001106999	RGD1310352	0.8869	0.9578	0.9896	0.9923	0.9016	0.9125	0.9703	0.9589	0.8689	0.9684	0.9530	0.6398
NM_001106765	RGD1310212	0.9234	0.6685	0.5007	0.8553	0.6012	0.2348	0.8545	0.5393	0.3700	0.3631	0.9331	0.6443
NM_001106762	RGD1304563	0.4547	0.8177	0.9212	0.8732	0.9231	0.9270	0.2970	0.9276	0.8714	0.9398	0.6519	0.1659
NM_001106267	Tm2d3	0.1416	0.2979	0.5566	0.3485	0.0597	0.0888	0.0176	0.2341	0.4423	0.3891	0.0814	0.5922
NM_001106771	Ankrd24	0.5435	0.9584	0.1243	0.9922	0.9378	0.7497	0.9769	0.9910	0.8595	0.7951	0.7576	0.2828
NM_001106770	Mrpl54	0.7155	0.8725	0.6150	0.7285	0.9511	0.6834	0.7487	0.8404	0.8772	0.8756	0.9298	0.3918
NM_001106828	Parp6	0.7559	0.7233	0.4364	0.0311	0.6313	0.9696	0.1911	0.6684	0.9025	0.8608	0.8395	0.9864
NM_001106123	Mrc1	0.6483	0.8274	0.9518	0.2045	0.6355	0.4006	0.1824	0.3749	0.5382	0.3551	0.9453	0.5295
NM_001106781	Ndufa12	0.5721	0.7000	0.2690	0.8554	0.3459	0.1350	0.7496	0.4642	0.1671	0.6636	0.7084	0.0864
NM_001107074	Cd7	0.5259	0.5938	0.5331	0.4070	0.3625	0.3993	0.4385	0.1400	0.3876	0.4706	0.0794	0.2779
NM_001106745	Rps6kl1	0.7557	0.4072	0.4542	0.5209	0.5955	0.5163	0.5035	0.9304	0.6251	0.6608	0.6181	0.4562
NM_001106798	Aasdhppt	0.8185	0.6794	0.7328	0.7036	0.8729	0.6444	0.8805	0.6292	0.7231	0.6845	0.8104	0.7158
NM_001106806	Prkcsh	0.1986	0.1195	0.7048	0.4524	0.2236	0.5859	0.6258	0.5318	0.2509	0.3624	0.0584	0.6314
NM_001106962	Asb11	0.0242	0.4759	0.1258	0.4695	0.2878	0.5975	0.3489	0.1172	0.3232	0.6707	0.2844	0.4831
NM_001106295	Tufm	0.1105	0.7713	0.4868	0.7901	0.2103	0.4145	0.6858	0.3565	0.8725	0.6489	0.9177	0.8082
NM_001106282	Mrpl48	0.6373	0.6608	0.8510	0.8572	0.8485	0.8350	0.9786	0.9240	0.0233	0.6190	0.0424	0.3947
NM_001106331	Trpt1	0.5229	0.4477	0.5044	0.6201	0.9134	0.8800	0.8616	0.9523	0.8979	0.8172	0.7232	0.7771
NM_001106341	Mcee	0.0551	0.0295	0.7630	0.0894	0.0080	0.0041	0.2768	0.0627	0.1319	0.0334	0.0564	0.0348
NM_001005533	N4bp2l2	0.0594	0.0777	0.4427	0.5272	0.0592	0.0551	0.0113	0.2684	0.1091	0.5201	0.0447	0.0897
NM_001106232	Clptm1	0.6283	0.3058	0.1253	0.0401	0.7122	0.4739	0.3613	0.7791	0.8240	0.3354	0.1010	0.9035
NM_001110810	Mylk3	0.1826	0.3716	0.6910	0.6341	0.5057	0.2274	0.7149	0.7915	0.3882	0.5368	0.2908	0.1409
NM_001110138	Flad1	0.2010	0.5886	0.4566	0.6546	0.6322	0.7048	0.6296	0.7014	0.4527	0.2518	0.6280	0.5157
NM_053292	Vars	0.4473	0.8754	0.2573	0.6874	0.5595	0.3998	0.6460	0.6933	0.8441	0.6686	0.8588	0.7851
NM_001110488	Uchl5ip	0.8657	0.9938	0.5211	0.6544	0.8472	0.9732	0.5954	0.9587	0.9971	0.9787	0.9982	0.9368
NM_001110808	Capn12	0.2397	0.0821	0.4440	0.6868	0.4715	0.3746	0.3186	0.5192	0.3924	0.4536	0.2413	0.3641
NM_001110491	RGD1563982	0.8027	0.4558	0.6350	0.3836	0.5686	0.7739	0.0621	0.7710	0.2201	0.5129	0.8614	0.4911

NM_001107110	Cbr3	0.6617	0.0875	0.9077	0.8829	0.1431	0.3296	0.7722	0.7437	0.0828	0.2032	0.1292	0.7508
NM_001107067	Rhbdf2	0.1954	0.0892	0.0315	0.2484	0.1069	0.6254	0.3351	0.5971	0.2562	0.3065	0.1253	0.2038
NM_001106815	Trim29	0.0120	0.2389	0.1904	0.1212	0.0817	0.1610	0.1458	0.3003	0.1334	0.2736	0.0072	0.3303
NM_001106814	Tecta	0.7862	0.8798	0.6727	0.8379	0.3649	0.7825	0.7248	0.7676	0.8853	0.7259	0.5329	0.7629
NM_001106956	Suv39h1	0.7328	0.6230	0.5537	0.5083	0.5903	0.6460	0.4545	0.8220	0.7753	0.8391	0.8635	0.3517
NM_001106139	RGD1304731	0.6855	0.8318	0.9138	0.3100	0.7045	0.4180	0.5281	0.8959	0.6919	0.3754	0.8583	0.4218
NM_001106141	RGD1562699	0.3642	0.2921	0.0873	0.1777	0.0983	0.4626	0.0852	0.0491	0.0341	0.0657	0.0871	0.1428
NM_001106827	Brunol6	0.7152	0.7671	0.9208	0.7942	0.4660	0.6820	0.5462	0.6413	0.5762	0.8358	0.4227	0.5969
NM_001106825	RGD1311874	0.6362	0.6855	0.8057	0.6532	0.0053	0.0016	0.6100	0.0173	0.5493	0.7598	0.9613	0.2875
NM_001106946	Mageb5	0.5801	0.7697	0.6132	0.5469	0.4658	0.5235	0.3782	0.5994	0.7930	0.1883	0.2785	0.1628
NM_001107044	Osbpl7	0.6467	0.7005	0.7302	0.3387	0.5059	0.7511	0.3324	0.4975	0.7765	0.7847	0.5819	0.9257
NM_001106374	Lipk	0.3320	0.1566	0.6895	0.4730	0.7236	0.4295	0.3297	0.6426	0.3398	0.8819	0.8435	0.8809
NM_001106143	Tcof1	0.8011	0.9790	0.2136	0.8764	0.7399	0.2487	0.8132	0.7343	0.8136	0.7528	0.9340	0.3116
NM_001106386	Reep3	0.4356	0.7678	0.2419	0.7536	0.8889	0.6191	0.7309	0.9380	0.7626	0.4236	0.7925	0.4374
NM_001106145	Slc27a6	0.1518	0.4215	0.4991	0.4401	0.5018	0.1715	0.3367	0.1394	0.6150	0.1321	0.6923	0.2085
NM_001106407	Cenpk	0.0937	0.8272	0.0124	0.2097	0.7204	0.5858	0.6708	0.2890	0.8453	0.7801	0.8977	0.0314
NM_001106157	Srp19	0.6148	0.9623	0.7849	0.2975	0.7764	0.3739	0.7033	0.4600	0.7255	0.5175	0.8256	0.3825
NM_001106156	Pkd2l2	0.6435	0.4842	0.2307	0.3097	0.6657	0.2643	0.5968	0.2497	0.3686	0.6930	0.4118	0.4438
NM_001106326	Sf3b2	0.7680	0.5320	0.5298	0.9639	0.5717	0.6963	0.1091	0.7972	0.7083	0.8136	0.8270	0.3270
NM_001106206	Fbxo5	0.9760	0.8742	0.1496	0.7881	0.8365	0.7857	0.7965	0.9838	0.7415	0.7713	0.9726	0.0429
NM_001106360	Ndufb8	0.0385	0.0819	0.6070	0.2785	0.0262	0.0678	0.8031	0.1280	0.0835	0.0274	0.0949	0.0533
NM_001106222	Rfpl4a	0.2360	0.6459	0.5445	0.6734	0.2355	0.2436	0.7585	0.6181	0.7812	0.3820	0.8482	0.3282
NM_001106958	Gpr143	0.4413	0.5892	0.7622	0.3389	0.7427	0.3803	0.3241	0.6838	0.6232	0.7632	0.6744	0.3832
NM_001107045	Sp2	0.5888	0.7632	0.6558	0.7870	0.7821	0.7354	0.4856	0.8320	0.9644	0.4917	0.2104	0.9394
NM_001107085	Cpn2	0.9299	0.6179	0.7317	0.8360	0.8907	0.5583	0.8982	0.4383	0.7514	0.9476	0.7978	0.4048
NM_001106982	Ccdc64b	0.0718	0.1837	0.0256	0.1056	0.1304	0.0677	0.0628	0.6704	0.4715	0.2340	0.0452	0.0736
NM_001107101	Arl13b	0.9571	0.8256	0.8850	0.5472	0.9628	0.8076	0.1061	0.7182	0.9991	0.9225	0.9087	0.8821
NM_001108451	Dynlrb2	0.4317	0.4091	0.7568	0.7301	0.2268	0.2531	0.4423	0.1790	0.7941	0.7470	0.5360	0.2551
NM_001108448	Terf2	0.3486	0.4127	0.0933	0.1658	0.4569	0.2849	0.2362	0.0711	0.2824	0.0502	0.1491	0.8286
NM_001106164	Cmtm3	0.8543	0.5870	0.8055	0.8704	0.9843	0.9866	0.8090	0.9518	0.4450	0.1021	0.2424	0.1290
NM_001106161	Dsc1	0.5148	0.7855	0.7091	0.3572	0.6458	0.3736	0.7641	0.2634	0.3554	0.3756	0.7152	0.1310
NM_001106159	Stard4	0.8618	0.9680	0.2505	0.5393	0.0374	0.4307	0.1135	0.4069	0.3462	0.9331	0.6893	0.2670
NM_001106256	Josd2	0.7720	0.4560	0.4640	0.1737	0.5675	0.5884	0.6911	0.4296	0.7109	0.5088	0.1383	0.6120
NM_001106249	Siglec5	0.4845	0.8682	0.8253	0.6821	0.7526	0.3264	0.3520	0.4444	0.8711	0.7798	0.7129	0.8049
NM_001106247	Pdcd5	0.5301	0.8190	0.3833	0.0568	0.0758	0.0164	0.0624	0.1673	0.4655	0.6519	0.5122	0.0605
NM_001106175	RGD1308358	0.2596	0.5632	0.5931	0.4741	0.3853	0.4737	0.4966	0.0649	0.5996	0.4485	0.9082	0.4350
NM_001106891	Aars2	0.7059	0.9180	0.2816	0.3031	0.7024	0.7528	0.4910	0.2614	0.4194	0.6426	0.6072	0.2680
NM_001106880	Plcl2	0.4130	0.5633	0.7343	0.3044	0.3647	0.5040	0.6154	0.5581	0.6888	0.4177	0.4122	0.4969
NM_001106871	Xcr1	0.3672	0.0805	0.5651	0.5784	0.6281	0.4538	0.5702	0.5744	0.6562	0.8274	0.0822	0.6220
NM_001106869	Cdcp1	0.8280	0.9143	0.4626	0.8079	0.4216	0.4905	0.5875	0.5035	0.7643	0.9531	0.6442	0.2541
NM_001106182	Cog8	0.7063	0.5386	0.4578	0.1272	0.7318	0.0714	0.4918	0.7147	0.7289	0.2512	0.4563	0.0477
NM_001106177	Hsf4	0.3289	0.5193	0.0727	0.6237	0.4354	0.2796	0.6885	0.2966	0.4587	0.3469	0.4808	0.4400
NM_001107053	Nags	0.8446	0.2301	0.3742	0.2008	0.1654	0.6469	0.4029	0.5826	0.5834	0.0927	0.2319	0.0362
NM_001107037	Epx	0.6836	0.6690	0.5814	0.5868	0.7248	0.6752	0.8081	0.4482	0.5069	0.7685	0.7736	0.9039
NM_001106888	RGD1305680	0.3838	0.7161	0.6438	0.3323	0.6523	0.7679	0.8460	0.4573	0.4918	0.8696	0.3801	0.8395
NM_001106406	Ppwd1	0.3073	0.2995	0.0889	0.2924	0.4516	0.2370	0.0243	0.0551	0.1470	0.3655	0.2216	0.4228
NM_001106370	Kcnv2	0.4373	0.7422	0.7023	0.8609	0.8022	0.2168	0.7449	0.5217	0.8426	0.9020	0.4533	0.8854
NM_001106367	Sorcs3	0.5349	0.5736	0.9007	0.7143	0.8229	0.7432	0.9142	0.3816	0.3310	0.4571	0.7642	0.5751
NM_001107000	Fstl4	0.1043	0.1658	0.1035	0.2768	0.1607	0.0457	0.0490	0.2053	0.2237	0.1288	0.0322	0.0570

NM_001107125	Ftsj2	0.5969	0.1457	0.7566	0.6208	0.5952	0.6415	0.8498	0.5489	0.3370	0.2945	0.2240	0.4541
NM_001107164	Klf1	0.2669	0.3170	0.2098	0.1616	0.0705	0.0752	0.1678	0.2552	0.3907	0.0800	0.1533	0.2031
NM_001106307	Fam175b	0.3945	0.2814	0.7869	0.9249	0.2165	0.1926	0.3074	0.2574	0.3174	0.2307	0.2748	0.3102
NM_001106184	Wwp2	0.7341	0.7655	0.3774	0.7023	0.8073	0.7707	0.9140	0.8405	0.7428	0.7770	0.5419	0.5100
NM_001106894	Gpr110	0.9042	0.9621	0.7482	0.3993	0.8008	0.6252	0.6676	0.2799	0.8994	0.2247	0.9212	0.8628
NM_001106188	Wwox	0.5588	0.4377	0.7998	0.5215	0.3121	0.1805	0.4835	0.5884	0.5089	0.7129	0.6089	0.2004
NM_001106201	Dclre1a	0.7145	0.8035	0.1604	0.7105	0.9518	0.7467	0.2654	0.9268	0.9840	0.6834	0.9199	0.8963
NM_001106191	Banp	0.8249	0.7849	0.2474	0.0522	0.9927	0.9802	0.8555	0.9072	0.7582	0.7908	0.8071	0.9618
NM_001106497	Srp14	0.3608	0.2283	0.3020	0.1731	0.2717	0.1319	0.1076	0.1391	0.0203	0.0103	0.0178	0.4508
NM_001106442	Ssr2	0.6294	0.6757	0.8601	0.9701	0.8903	0.4840	0.7937	0.9735	0.8940	0.6757	0.5737	0.0702
NM_001107006	Atpaf2	0.8304	0.6795	0.5843	0.9010	0.9764	0.8942	0.7713	0.6866	0.8250	0.8671	0.8407	0.8796
NM_001107009	Ccdc42	0.4736	0.0470	0.1879	0.2212	0.0577	0.5026	0.6529	0.0596	0.2624	0.0922	0.0969	0.2049
NM_001106908	RGD1561425	0.4803	0.1730	0.4247	0.3527	0.1017	0.2818	0.0834	0.3537	0.7184	0.0312	0.6417	0.1730
NM_001106468	Usp53	0.4479	0.3115	0.9591	0.6769	0.8497	0.9023	0.0351	0.7945	0.9775	0.6185	0.8200	0.8449
NM_001106592	Znf282	0.9748	0.9830	0.9311	0.8096	0.9963	0.9423	0.9876	0.9823	0.9866	0.9934	0.7565	0.9523
NM_001106915	Carf	0.6368	0.6623	0.9678	0.8782	0.7886	0.6688	0.8377	0.7494	0.8354	0.8387	0.7735	0.3856
NM_001106405	Cd180	0.5179	0.4553	0.2080	0.5075	0.4395	0.5750	0.2667	0.6554	0.6399	0.2894	0.5481	0.5763
NM_001106198	Pgbd5	0.4613	0.3879	0.1818	0.0657	0.4320	0.4961	0.5595	0.1026	0.1365	0.4756	0.2519	0.2480
NM_001106214	Lix1	0.4201	0.5056	0.3959	0.4603	0.2928	0.6085	0.5208	0.1754	0.3223	0.4400	0.2289	0.1609
NM_001106217	Lrp11	0.8020	0.8266	0.8725	0.8424	0.3586	0.5041	0.2657	0.3570	0.4964	0.5093	0.0493	0.1152
NM_001106216	Fpr1	0.7379	0.8908	0.5917	0.8664	0.4940	0.5466	0.6114	0.6901	0.4305	0.4114	0.3614	0.1263
NM_001107124	Ttyh3	0.6593	0.8558	0.5240	0.8381	0.3674	0.7526	0.5893	0.9831	0.4620	0.8048	0.3234	0.9069
NM_001107133	Gigyf1	0.6504	0.4915	0.3898	0.0728	0.7696	0.7297	0.1832	0.8276	0.7826	0.7954	0.7324	0.6434
NM_001106350	Dkk1	0.6093	0.7724	0.7691	0.5378	0.6528	0.5702	0.4155	0.7312	0.3530	0.7310	0.7632	0.7635
NM_001134481	Plcxd2	0.4181	0.6687	0.7609	0.8511	0.7975	0.6244	0.3396	0.7067	0.5853	0.5966	0.0712	0.6279
NM_001106319	Cars	0.6714	0.9584	0.0538	0.8848	0.6458	0.5148	0.9507	0.9342	0.8976	0.9770	0.8741	0.6863
NM_001106810	Adamts15	0.6146	0.9722	0.5957	0.5931	0.9515	0.7728	0.9554	0.6594	0.9816	0.9356	0.8479	0.7804
NM_001106397	Mical1	0.7607	0.7832	0.0362	0.3324	0.2445	0.2759	0.8487	0.8799	0.6277	0.5899	0.6226	0.3878
NM_001107028	Unc45b	0.2468	0.2017	0.3130	0.2870	0.2320	0.4375	0.4273	0.3993	0.3329	0.3663	0.0873	0.3298
NM_001107027	Psmd11	0.3810	0.4134	0.7694	0.4026	0.2330	0.6695	0.2812	0.3375	0.4871	0.8443	0.6067	0.9931
NM_001134645	Cntrob	0.7066	0.6164	0.0028	0.2364	0.7837	0.7124	0.6499	0.7162	0.8597	0.6103	0.6195	0.1134
NM_001106470	RGD1304822	0.9083	0.8493	0.7782	0.6174	0.8179	0.8347	0.5291	0.9451	0.7482	0.7419	0.5821	0.9361
NM_001106229	RGD1307390	0.0744	0.3412	0.0551	0.0023	0.1411	0.1159	0.0395	0.1341	0.2235	0.0645	0.2633	0.4395
NM_001106316	Ptdss2	0.5013	0.3655	0.8187	0.7758	0.4613	0.7416	0.9331	0.3564	0.9638	0.5742	0.5512	0.8867
NM_001106797	Cbx5	0.9819	0.8484	0.7259	0.7820	0.9886	0.9081	0.9488	0.8661	0.0845	0.2573	0.8105	0.0474
NM_001106172	Nod2	0.9103	0.5937	0.5083	0.6561	0.2062	0.0348	0.8440	0.2720	0.1363	0.7161	0.3369	0.9505
NM_001106426	Ndufb5	0.2895	0.3323	0.1887	0.7896	0.0610	0.0566	0.2819	0.0748	0.0492	0.3144	0.2337	0.0229
NM_001106519	Ovol2	0.3887	0.7683	0.5068	0.4689	0.3932	0.1291	0.5858	0.6150	0.3723	0.3681	0.1871	0.0455
NM_001107195	Olfml2b	0.9409	0.8467	0.8853	0.8735	0.9202	0.9054	0.8985	0.8060	0.8699	0.9304	0.9733	0.3644
NM_001107134	Zan	0.6129	0.6450	0.4084	0.8202	0.4795	0.9466	0.9700	0.8148	0.9088	0.9451	0.9352	0.9245
NM_001107617	Fgd2	0.3205	0.3290	0.2203	0.2468	0.3711	0.2392	0.3294	0.3719	0.3728	0.6690	0.5491	0.4182
NM_001107187	Fam20b	0.5482	0.4851	0.6294	0.2319	0.5956	0.5014	0.6833	0.6612	0.6903	0.7279	0.7062	0.7148
NM_001134569	RGD1561157	0.6779	0.0666	0.0355	0.5953	0.1108	0.4668	0.6337	0.2865	0.4118	0.2851	0.1439	0.8508
NM_001107639	Prdm1	0.7938	0.6458	0.5560	0.6577	0.2983	0.5398	0.6413	0.4608	0.3047	0.4144	0.2518	0.6673
NM_001106565	Med27	0.8815	0.6150	0.3942	0.6639	0.3001	0.1865	0.5663	0.3293	0.5730	0.5696	0.7642	0.3297
NM_001107034	Tbx4	0.9372	0.9439	0.6538	0.8408	0.8488	0.8016	0.9075	0.6008	0.8758	0.7946	0.7528	0.2981
NM_001106934	Pof1b	0.8685	0.9744	0.6292	0.8620	0.8631	0.5666	0.8256	0.6480	0.4916	0.9626	0.8419	0.5544
NM_001106938	Gpr174	0.6702	0.6845	0.7316	0.6725	0.5533	0.4946	0.2345	0.6951	0.6880	0.6588	0.7196	0.2062
NM_001106936	RGD1559589	0.5466	0.0784	0.4189	0.6486	0.3792	0.2204	0.3524	0.1711	0.2748	0.3058	0.4246	0.4231

NM_001106935	RGD1562871	0.3047	0.5449	0.2236	0.3719	0.1619	0.6900	0.5973	0.1567	0.3922	0.6412	0.5282	0.2958
NM_001106456	RGD1308694	0.7905	0.6903	0.5441	0.8587	0.7098	0.7236	0.9487	0.3558	0.7455	0.9053	0.8674	0.8605
NM_001106394	Nodal	0.7452	0.6767	0.2852	0.2481	0.2666	0.6490	0.3371	0.6869	0.4213	0.4793	0.5442	0.4756
NM_001106269	Olig3	0.5958	0.2563	0.1755	0.3765	0.3435	0.7452	0.3147	0.4016	0.5435	0.3770	0.1854	0.7173
NM_001106266	Tjp1	0.6806	0.7608	0.9734	0.7305	0.7196	0.6724	0.7885	0.4116	0.6453	0.2920	0.3465	0.9355
NM_001106379	Aire	0.1124	0.0688	0.3121	0.1022	0.0347	0.3027	0.2737	0.1106	0.1429	0.1137	0.5718	0.1384
NM_001107486	Opa3	0.5606	0.8660	0.8626	0.8721	0.8840	0.8321	0.1777	0.9199	0.8003	0.5619	0.1805	0.9311
NM_001106275	Abhd2	0.8601	0.9969	0.6066	0.9605	0.8563	0.3209	0.8997	0.8472	0.9060	0.9900	0.8269	0.8433
NM_001106274	Rlbp1	0.7090	0.8601	0.9358	0.7535	0.9267	0.8391	0.9033	0.9787	0.9240	0.7321	0.8094	0.8850
NM_001106433	Spg20	0.5415	0.3235	0.9271	0.2789	0.2377	0.1627	0.1103	0.0426	0.2625	0.0175	0.3604	0.2472
NM_001106300	Ate1	0.3190	0.5017	0.3320	0.2682	0.3283	0.1719	0.4394	0.3306	0.3954	0.0088	0.4755	0.2009
NM_001106290	Rpl27a	0.7350	0.8282	0.5435	0.4564	0.8076	0.4532	0.8258	0.4520	0.7602	0.5084	0.5642	0.5621
NM_001106289	Tmem9b	0.1249	0.1366	0.7493	0.1348	0.0959	0.0510	0.0251	0.0878	0.0064	0.0133	0.1035	0.5013
NM_001106287	RGD1310717	0.1964	0.1070	0.4961	0.4856	0.2455	0.3363	0.1776	0.2567	0.2646	0.1999	0.1744	0.1809
NM_001106283	Folr2	0.1088	0.1128	0.1235	0.5481	0.0619	0.0129	0.1919	0.0480	0.2175	0.3008	0.0119	0.1530
NM_001106279	RGD1565584	0.9244	0.9656	0.9817	0.3368	0.9327	0.9078	0.7213	0.4093	0.9209	0.6014	0.9006	0.4819
NM_001106601	Bola3	0.3766	0.7800	0.6819	0.9271	0.9918	0.9096	0.9637	0.8642	0.9541	0.8924	0.9178	0.4956
NM_001107568	Fam18b2	0.1122	0.2522	0.9361	0.1722	0.0469	0.2883	0.1139	0.1134	0.1777	0.2165	0.3144	0.1904
NM_001106481	Upp2	0.6746	0.7090	0.7772	0.4444	0.7432	0.6545	0.1695	0.2172	0.6163	0.1795	0.8182	0.4764
NM_001106591	Atp6v0a4	0.4048	0.0991	0.6901	0.5954	0.4049	0.5316	0.6504	0.4797	0.1518	0.4417	0.7854	0.3504
NM_001107113	Bach1	0.8433	0.8075	0.8332	0.7037	0.8059	0.6172	0.6533	0.7576	0.6074	0.9540	0.6469	0.9366
NM_001106983	Prss32	0.4782	0.5261	0.7350	0.6467	0.2805	0.0610	0.0330	0.0166	0.3170	0.7701	0.0407	0.6690
NM_001106981	Zscan10	0.2507	0.4125	0.1927	0.4935	0.3010	0.1890	0.2053	0.4335	0.4837	0.3362	0.4949	0.5706
NM_001106988	Spsb3	0.1589	0.3786	0.1850	0.4307	0.1556	0.2897	0.1726	0.2699	0.5224	0.3771	0.5831	0.1460
NM_001106987	Rnf151	0.3508	0.7958	0.2347	0.7263	0.1679	0.2429	0.4867	0.7061	0.6721	0.8674	0.8351	0.6111
NM_001106985	Syngr3	0.9586	0.7771	0.8886	0.8247	0.9207	0.9060	0.9702	0.6084	0.9105	0.5935	0.6454	0.9055
NM_001107149	Ep400	0.9624	0.9362	0.5449	0.1922	0.9695	0.8383	0.3277	0.9852	0.9292	0.9937	0.9333	0.8031
NM_001107175	Ppp1r15b	0.6313	0.6262	0.8215	0.7519	0.4217	0.7740	0.8463	0.6050	0.8041	0.2916	0.5453	0.5486
NM_001107172	Slc26a9	0.1564	0.1197	0.0713	0.2544	0.2739	0.2513	0.2866	0.3723	0.3985	0.4526	0.1070	0.3601
NM_001107152	Pole	0.9826	0.9995	0.1727	0.9830	0.9683	0.9597	0.9735	0.9997	0.9558	0.9570	0.9611	0.7878
NM_001106294	Ndufab1	0.1884	0.3968	0.1217	0.9281	0.0753	0.2124	0.1566	0.3069	0.2506	0.3143	0.2276	0.1940
NM_001106293	Abca15	0.6911	0.7923	0.7699	0.6436	0.8848	0.5868	0.7430	0.8954	0.9028	0.9337	0.9562	0.8904
NM_001107245	Pcgf3	0.1983	0.5145	0.2978	0.6894	0.2160	0.0985	0.8516	0.1983	0.7185	0.0344	0.6673	0.0375
NM_001106511	Cpxm1	0.6511	0.1301	0.5195	0.3902	0.4024	0.3258	0.7154	0.7954	0.9656	0.4535	0.4665	0.6113
NM_001106305	Rnf152	0.7261	0.6557	0.7020	0.6001	0.5192	0.5795	0.6903	0.7019	0.6654	0.6637	0.6453	0.6007
NM_001107157	Dtx2	0.7699	0.7885	0.7881	0.8092	0.8704	0.7950	0.9966	0.9315	0.9291	0.7821	0.8000	0.8641
NM_001106313	Sirt3	0.0540	0.2131	0.6626	0.0423	0.2816	0.2234	0.0265	0.0475	0.1930	0.0809	0.0118	0.7129
NM_001106314	Ifitm1	0.0403	0.0311	0.0194	0.0359	0.0359	0.0151	0.0028	0.0791	0.1198	0.0119	0.0278	0.0075
NM_001107050	Gcn5l2	0.8753	0.9445	0.2476	0.3767	0.9455	0.8478	0.9358	0.9632	0.8106	0.8496	0.9925	0.9416
NM_001106949	Mbnl3	0.6347	0.8121	0.6287	0.8556	0.5570	0.3954	0.5528	0.1140	0.2373	0.7338	0.7949	0.6804
NM_001106951	Cul4b	0.9084	0.9426	0.9453	0.6590	0.9599	0.9042	0.9121	0.9472	0.9825	0.9409	0.9646	0.7955
NM_001106508	Acoxl	0.5279	0.1849	0.7165	0.4327	0.5470	0.7177	0.4625	0.7900	0.7695	0.4072	0.5582	0.5058
NM_001107224	Stk32b	0.6074	0.9638	0.6204	0.8077	0.9609	0.9322	0.7932	0.5088	0.6123	0.6149	0.7737	0.8322
NM_001107352	Hist1h2bh	0.8695	0.8990	0.5924	0.8452	0.8994	0.6022	0.5295	0.8226	0.4922	0.2932	0.6074	0.2630
NM_001106930	Efcab1	0.6153	0.5941	0.8076	0.3829	0.4493	0.2839	0.2642	0.5208	0.2570	0.6722	0.5466	0.5242
NM_001107058	Map3k3	0.5283	0.4297	0.3549	0.7542	0.4864	0.7702	0.3908	0.6782	0.3980	0.4007	0.3743	0.8342
NM_001100674	Map2k3	0.8509	0.7445	0.7875	0.9464	0.9140	0.9749	0.8249	0.9829	0.7131	0.7788	0.4529	0.4118
NM_001106955	Tbc1d25	0.8196	0.7903	0.6507	0.8198	0.8387	0.5521	0.4716	0.4855	0.6774	0.7106	0.8239	0.7825
NM_001106954	RGD1561661	0.7344	0.8497	0.7728	0.5233	0.6603	0.6743	0.9544	0.2825	0.9343	0.3838	0.5897	0.6870

NM_001107054	Hexim2	0.3698	0.1010	0.1000	0.1785	0.0411	0.2781	0.0657	0.3209	0.0813	0.1100	0.1183	0.0109
NM_001106614	Setd5	0.9901	0.9920	0.9614	0.9034	0.9762	0.9562	0.9889	0.9881	0.9875	0.9854	0.9818	0.9989
NM_001106567	Dolpp1	0.8329	0.8456	0.5246	0.8399	0.4698	0.9569	0.9679	0.9140	0.9550	0.2376	0.9681	0.8298
NM_001107234	Rnf215	0.9880	0.9416	0.4876	0.6931	0.9656	0.9301	0.9690	0.9038	0.9267	0.7479	0.7833	0.8998
NM_001107230	Eif4enif1	0.8204	0.9269	0.9621	0.8634	0.9616	0.8198	0.9375	0.8266	0.8609	0.8746	0.9591	0.9107
NM_001110823	Atp2a2	0.7318	0.7335	0.6491	0.7287	0.6940	0.7190	0.5783	0.6974	0.6391	0.7068	0.7151	0.7777
NM_172085	Pou3f2	0.4349	0.1740	0.5769	0.3690	0.8653	0.1550	0.1061	0.2448	0.3770	0.4685	0.1559	0.7357
NM_001110137	Prom1	0.7712	0.8783	0.8992	0.9584	0.8411	0.7961	0.8026	0.9302	0.8903	0.9374	0.8737	0.4731
NM_001107842	Golga1	0.9329	0.9873	0.7651	0.8669	0.9613	0.9904	0.7037	0.9329	0.8242	0.9448	0.7206	0.9124
NM_001106330	RGD1309710	0.5001	0.4789	0.2778	0.3082	0.2036	0.1191	0.5534	0.4539	0.1044	0.0747	0.3610	0.2807
NM_001106321	Lrp5	0.9795	0.0205	0.9603	0.5237	0.0773	0.2722	0.9115	0.0296	0.4251	0.1402	0.2477	0.8082
NM_001106553	Alx4	0.7848	0.6341	0.7027	0.6425	0.8415	0.8869	0.6124	0.8729	0.8095	0.9441	0.6932	0.7856
NM_001106499	Vps18	0.0461	0.1675	0.2965	0.1448	0.3506	0.4986	0.6273	0.1380	0.0129	0.1020	0.6102	0.7043
NM_001127451	Fam103a1	0.0655	0.0980	0.3112	0.2566	0.0909	0.0305	0.2622	0.0393	0.0215	0.0780	0.1813	0.0588
NM_001106471	Papss1	0.8957	0.8649	0.9420	0.9584	0.5933	0.6295	0.8837	0.6153	0.9722	0.9718	0.9250	0.9132
NM_001134601	RGD1565533	0.2029	0.4142	0.7390	0.4981	0.8146	0.1086	0.4171	0.3196	0.1782	0.1214	0.3056	0.3241
NM_001106390	Ptk9l	0.2574	0.4212	0.3349	0.2707	0.2327	0.2378	0.4669	0.4280	0.2639	0.5528	0.3865	0.4482
NM_001106395	Foxo3	0.5561	0.5224	0.6339	0.8365	0.4951	0.5553	0.7498	0.7454	0.8659	0.4435	0.2385	0.7780
NM_001106381	Susd2	0.8975	0.9414	0.6695	0.7307	0.9039	0.8037	0.9482	0.7096	0.9434	0.9871	0.7260	0.7939
NM_001106558	Phpt1	0.4716	0.4633	0.4305	0.2012	0.2359	0.3989	0.4712	0.3834	0.5482	0.3534	0.3049	0.4423
NM_001108489	Iqgap1	0.8969	0.2963	0.9963	0.9813	0.2384	0.9715	0.8976	0.0282	0.6970	0.7623	0.9858	0.9760
NM_001108490	Tm6sf1	0.0819	0.2194	0.8270	0.4975	0.1311	0.2923	0.2687	0.3235	0.1517	0.2845	0.3618	0.2747
NM_001108486	Lrrc28	0.3120	0.9882	0.9653	0.9376	0.7745	0.9652	0.9799	0.6751	0.5291	0.9517	0.9000	0.2279
NM_001107241	Ahsa2	0.6296	0.7277	0.6281	0.6277	0.7309	0.7151	0.6304	0.6295	0.5887	0.6294	0.6813	0.6537
NM_001106322	Ndufs8	0.3068	0.6912	0.6988	0.8585	0.2882	0.1322	0.9369	0.8103	0.3117	0.7717	0.4504	0.0457
NM_001106329	Map4k2	0.7533	0.9353	0.6811	0.6652	0.9140	0.7629	0.8074	0.9249	0.8840	0.5274	0.5052	0.8781
NM_001107246	Mfsd7	0.5261	0.5622	0.4871	0.5525	0.5581	0.6914	0.7291	0.5903	0.1723	0.9159	0.6213	0.4249
NM_001106325	Cd248	0.8199	0.6588	0.5654	0.3038	0.7404	0.8438	0.9858	0.8376	0.9237	0.7041	0.9303	0.8022
NM_001106323	Rhod	0.7356	0.3951	0.1310	0.5150	0.3293	0.0857	0.7513	0.3286	0.3210	0.5453	0.4245	0.1783
NM_001106342	Pnma3	0.1390	0.7851	0.4001	0.3852	0.6953	0.4432	0.5066	0.4801	0.9354	0.9041	0.9333	0.9179
NM_001106392	Hs3st5	0.8508	0.3510	0.5269	0.6435	0.6214	0.2952	0.6299	0.6106	0.5288	0.8566	0.5746	0.4678
NM_001108574	Snapc4	0.8335	0.7594	0.0990	0.3831	0.7530	0.8609	0.3177	0.7361	0.9289	0.8366	0.7486	0.7744
NM_001108674	RGD1305347	0.6897	0.5364	0.2217	0.4191	0.7600	0.6059	0.6484	0.3440	0.8903	0.1510	0.8317	0.2046
NM_001108573	RGD1307615	0.1727	0.2008	0.4274	0.0836	0.0158	0.3283	0.2206	0.3087	0.3015	0.5917	0.4688	0.3103
NM_001108670	Tm2d1	0.0152	0.1338	0.9041	0.1520	0.4091	0.2404	0.2310	0.1267	0.1413	0.2853	0.1933	0.6249
NM_001108571	Il1f10	0.0220	0.2307	0.2128	0.2584	0.2013	0.0473	0.5501	0.0845	0.0966	0.3338	0.1463	0.3017
NM_001108570	Il1f8	0.0752	0.1155	0.1606	0.0082	0.0329	0.0626	0.0866	0.0082	0.0573	0.2381	0.1468	0.1594
NM_001115046	LOC687565	0.5159	0.7186	0.5640	0.3331	0.4797	0.2901	0.2048	0.1955	0.2744	0.2914	0.1824	0.1858
NM_001108400	Ankrd37	0.4436	0.1077	0.7415	0.9349	0.1960	0.0669	0.9060	0.6240	0.1926	0.0671	0.1051	0.2962
NM_001107251	Fezf2	0.1275	0.1839	0.2700	0.1967	0.0472	0.0837	0.3434	0.2876	0.4510	0.0767	0.1476	0.5271
NM_001106972	RGD1309748	0.9269	0.7787	0.9629	0.4621	0.9246	0.9107	0.4586	0.8593	0.9490	0.4466	0.5812	0.9040
NM_001107232	Osbp2	0.5488	0.0776	0.6226	0.6708	0.5377	0.3149	0.6317	0.8607	0.7090	0.4340	0.6562	0.6176
NM_001106722	Gpr22	0.7156	0.6405	0.7205	0.5443	0.4062	0.4329	0.7740	0.6694	0.7108	0.7080	0.6734	0.5482
NM_001106701	Pin1	0.6496	0.7732	0.2547	0.5404	0.3353	0.0544	0.2432	0.5640	0.0969	0.1634	0.3313	0.2691
NM_001107096	Cep97	0.4307	0.3840	0.0898	0.4064	0.6651	0.6592	0.8052	0.3865	0.8222	0.2794	0.5657	0.1896
NM_001106993	Fbxw11	0.6761	0.5782	0.8824	0.7312	0.7790	0.5486	0.7545	0.6916	0.8411	0.5549	0.4795	0.7979
NM_001107080	Dgcr6	0.0209	0.0622	0.4812	0.1012	0.0562	0.0109	0.2272	0.1139	0.0414	0.0406	0.2570	0.0651
NM_001107079	Klhl22	0.1980	0.2172	0.6868	0.3519	0.1455	0.0581	0.8732	0.0345	0.6442	0.4240	0.1564	0.6766
NM_001106539	Matn4	0.6466	0.6409	0.5691	0.6778	0.4860	0.6531	0.7998	0.7660	0.4885	0.7474	0.5791	0.4808

NM_001106506	Nphp1	0.2572	0.6948	0.7921	0.8265	0.6803	0.7154	0.7419	0.7235	0.5268	0.7198	0.6049	0.5646
NM_001106349	Trpd52l3	0.7526	0.2574	0.5329	0.3298	0.2891	0.5392	0.3478	0.3414	0.4760	0.3893	0.4224	0.2889
NM_001106348	Mlana	0.0924	0.4200	0.4303	0.1210	0.0266	0.0301	0.0198	0.1899	0.0486	0.2156	0.1158	0.1331
NM_001106347	RGD1306839	0.0843	0.9507	0.4386	0.7702	0.4787	0.2396	0.8911	0.7129	0.6664	0.8733	0.8760	0.2400
NM_001107334	Hiatl1	0.8914	0.9464	0.9144	0.9551	0.5813	0.5364	0.9213	0.0351	0.8550	0.8672	0.9649	0.4104
NM_001107358	Zfp438	0.8152	0.7303	0.3612	0.6495	0.6969	0.6746	0.4884	0.7232	0.6050	0.7912	0.2993	0.4953
NM_001107636	Adamts14	0.1666	0.4484	0.7285	0.4642	0.4965	0.5280	0.7178	0.3433	0.2703	0.4479	0.6978	0.4383
NM_001134600	RGD1563273	0.8611	0.8421	0.8340	0.5722	0.6282	0.6504	0.5327	0.8143	0.2629	0.2281	0.7755	0.5817
NM_001107088	Lrch3	0.2302	0.5775	0.2513	0.4464	0.3220	0.4966	0.7615	0.3803	0.1511	0.0719	0.4916	0.1863
NM_001107495	Cyp2s1	0.4005	0.1993	0.0837	0.0609	0.1566	0.1446	0.4349	0.2767	0.4747	0.1074	0.0391	0.2537
NM_001106599	Htra2	0.2544	0.4838	0.2603	0.3365	0.5810	0.3614	0.5239	0.4572	0.5468	0.3543	0.4541	0.6781
NM_001106625	Erp27	0.3487	0.4148	0.4845	0.1977	0.3564	0.0615	0.7990	0.7342	0.4125	0.6852	0.4907	0.0911
NM_001106536	Mybl2	0.7104	0.9949	0.1976	0.8796	0.7857	0.9328	0.9270	0.9224	0.3654	0.3056	0.7981	0.2042
NM_001107244	Papolg	0.8973	0.4428	0.7706	0.9729	0.9536	0.6080	0.5686	0.8755	0.4183	0.4549	0.8149	0.2446
NM_001107093	Ift57	0.3196	0.5887	0.9324	0.4093	0.2697	0.2916	0.7361	0.2930	0.8794	0.7565	0.9723	0.5268
NM_001107095	Nfkbiz	0.9094	0.1672	0.9491	0.7476	0.6568	0.3803	0.3174	0.2836	0.8368	0.2623	0.6644	0.8145
NM_001107104	Prdm15	0.7269	0.4975	0.0435	0.4153	0.3784	0.5913	0.4542	0.8989	0.6701	0.6614	0.4619	0.6824
NM_001107371	Neto1	0.8571	0.9415	0.9184	0.8445	0.8595	0.8099	0.9417	0.7634	0.7485	0.9146	0.9061	0.9470
NM_001108042	Zfp410	0.9006	0.9323	0.9336	0.9245	0.9443	0.7352	0.3412	0.8994	0.9164	0.8642	0.8984	0.6430
NM_001107367	Nsun6	0.7165	0.8654	0.3472	0.0745	0.6487	0.3244	0.1105	0.6846	0.6309	0.8734	0.7704	0.0064
NM_001106571	Lhx2	0.5948	0.6885	0.5595	0.7070	0.2980	0.5282	0.5590	0.6690	0.3757	0.5764	0.4315	0.6224
NM_001109589	Rab36	0.1704	0.0496	0.1315	0.4757	0.2120	0.4596	0.6818	0.2698	0.5746	0.6303	0.3329	0.3345
NM_001106557	Edf1	0.0565	0.1592	0.9482	0.1600	0.0867	0.1271	0.2658	0.0128	0.0431	0.1222	0.0118	0.9519
NM_001106547	RGD1311378	0.7275	0.4092	0.3226	0.2733	0.6303	0.4733	0.6066	0.4755	0.4070	0.6153	0.5698	0.2908
NM_001106995	RGD1309452	0.6460	0.5284	0.7721	0.8060	0.5824	0.7719	0.5109	0.0715	0.6443	0.5346	0.2282	0.3662
NM_001106610	Hdac11	0.5113	0.8808	0.8136	0.9287	0.6294	0.7520	0.8066	0.6535	0.6706	0.8111	0.7445	0.6531
NM_001106564	RGD1306233	0.4006	0.2927	0.7034	0.4592	0.7117	0.8257	0.5561	0.4194	0.4150	0.0515	0.4402	0.0842
NM_001106560	Lcn9	0.6708	0.6424	0.8335	0.3251	0.8832	0.7168	0.5590	0.3909	0.6613	0.1735	0.3619	0.8086
NM_001106555	C8g	0.0485	0.3698	0.8260	0.8670	0.7788	0.0410	0.7211	0.6830	0.7459	0.1218	0.7969	0.6395
NM_001107250	Znf503	0.7503	0.9456	0.9632	0.9576	0.8641	0.7929	0.9980	0.9605	0.9477	0.9729	0.9944	0.9175
NM_001107482	Lrrc68	0.8138	0.8148	0.7664	0.6893	0.6902	0.9691	0.7502	0.9405	0.8930	0.6725	0.7700	0.8926
NM_001106574	RGD1306404	0.2634	0.8880	0.6407	0.0153	0.1253	0.9170	0.7523	0.4517	0.7915	0.6630	0.8692	0.8267
NM_001106425	Armc1	0.4145	0.3089	0.3946	0.2416	0.3903	0.3143	0.2408	0.3224	0.2619	0.1401	0.1386	0.0184
NM_001106400	Ankrd32	0.4952	0.3341	0.8194	0.2978	0.5621	0.2955	0.5490	0.1094	0.4081	0.3578	0.8028	0.2802
NM_031607	Hspb7	0.1073	0.0547	0.0679	0.1781	0.2601	0.2384	0.1254	0.1314	0.2896	0.1476	0.0719	0.2806
NM_001100636	Scamp1	0.3140	0.3152	0.9330	0.3876	0.2874	0.3651	0.2578	0.3188	0.2669	0.2516	0.4129	0.6734
NM_019267	Bckdhb	0.6946	0.7949	0.7163	0.8442	0.1238	0.1439	0.9596	0.3651	0.6569	0.6092	0.8049	0.1817
NM_001106572	Olfml2a	0.6852	0.1910	0.7900	0.7002	0.4556	0.7769	0.8000	0.8856	0.1953	0.2130	0.1327	0.3438
NM_001106577	Phtf2	0.5888	0.3848	0.7132	0.7550	0.7987	0.4652	0.6707	0.7208	0.8396	0.6660	0.5707	0.5083
NM_001106877	Rfx2	0.4111	0.2505	0.2992	0.3938	0.5606	0.6981	0.4406	0.3397	0.5257	0.4089	0.1981	0.7098
NM_001106636	Bsdc1	0.3258	0.2254	0.5178	0.7288	0.3053	0.7967	0.6084	0.3355	0.5043	0.3558	0.3662	0.4744
NM_001106620	Leprel2	0.7689	0.0118	0.9215	0.5460	0.7244	0.9764	0.0449	0.8563	0.0318	0.0535	0.0366	0.1500
NM_001134361	Med1	0.8940	0.6285	0.9020	0.7373	0.8114	0.6970	0.5146	0.9209	0.7113	0.6318	0.7629	0.7351
NM_001107109	Morc3	0.9157	0.8234	0.8993	0.4652	0.9898	0.9178	0.3069	0.4297	0.9367	0.9254	0.9652	0.9659
NM_001107396	Cdc25c	0.6846	0.7905	0.5993	0.8514	0.7449	0.8373	0.8231	0.7140	0.7302	0.9447	0.7878	0.5730
NM_001107347	Muted	0.7512	0.6401	0.8674	0.6216	0.6268	0.8323	0.8306	0.6018	0.8223	0.6220	0.7308	0.7729
NM_001107408	Gins3	0.7660	0.4315	0.8183	0.8085	0.6712	0.8773	0.4843	0.7714	0.4693	0.8777	0.7373	0.7086
NM_001106545	Kcng1	0.1601	0.0482	0.0318	0.3189	0.1397	0.1936	0.2523	0.2569	0.1249	0.2629	0.4417	0.3732
NM_001106399	Spata9	0.5484	0.1204	0.7841	0.7648	0.5929	0.7008	0.7435	0.8420	0.7750	0.8227	0.7051	0.7620

NM_001107115	Arhgef18	0.6113	0.4585	0.3956	0.2042	0.7026	0.3265	0.5233	0.7164	0.5738	0.3358	0.8162	0.3136
NM_001107112	Cldn17	0.4429	0.1887	0.5084	0.5802	0.4349	0.0711	0.7726	0.6179	0.7841	0.6384	0.6196	0.2080
NM_001106626	Aebp2	0.7033	0.6460	0.3908	0.5633	0.4532	0.9475	0.2117	0.7492	0.5381	0.2810	0.9064	0.8802
NM_001106696	Pla2g2e	0.7605	0.8147	0.6552	0.8449	0.7921	0.7028	0.5015	0.8419	0.8528	0.9437	0.7347	0.8110
NM_001106439	Slc27a3	0.9745	0.8514	0.3142	0.8236	0.7204	0.7524	0.3428	0.7725	0.5469	0.8259	0.8075	0.3499
NM_001106642	Ube2j1	0.1418	0.0911	0.8896	0.7822	0.6520	0.8795	0.8115	0.0523	0.1362	0.4810	0.8373	0.6241
NM_001107135	Cldn15	0.8450	0.7934	0.8372	0.5262	0.6434	0.6782	0.8643	0.8522	0.6992	0.5837	0.5231	0.6893
NM_001107132	RGD1310964	0.8252	0.6005	0.7544	0.7921	0.5146	0.8170	0.7437	0.8431	0.8846	0.0454	0.6708	0.9112
NM_001106594	Mad2l1	0.9574	0.9700	0.2814	0.8313	0.8105	0.9602	0.8868	0.9391	0.8809	0.7783	0.8070	0.1339
NM_001106774	Nuak1	0.6710	0.5226	0.7387	0.3285	0.8035	0.7600	0.8002	0.4488	0.6157	0.6699	0.9504	0.6707
NM_001107273	Cdca2	0.8089	0.9482	0.1537	0.7742	0.9916	0.7197	0.8321	0.7718	0.7644	0.7492	0.8475	0.0791
NM_001107278	Fndc3a	0.3070	0.1257	0.8919	0.3920	0.1527	0.2494	0.2365	0.3312	0.0013	0.2643	0.2497	0.9031
NM_001107140	Kntc1	0.8429	0.9042	0.0279	0.8590	0.8736	0.9289	0.9420	0.9494	0.8408	0.8158	0.8740	0.0845
NM_001107142	Anapc7	0.1356	0.1694	0.4150	0.0115	0.3082	0.1238	0.0784	0.1141	0.0773	0.1583	0.1948	0.3231
NM_001001505	Habp2	0.2532	0.0647	0.1147	0.0344	0.2830	0.1549	0.3080	0.0917	0.4700	0.1379	0.2533	0.6795
NM_001107331	Irx1	0.7895	0.5112	0.4811	0.6444	0.7112	0.3881	0.8051	0.7052	0.5640	0.6621	0.8432	0.8296
NM_001107392	Fchsd1_predict	0.4007	0.4647	0.4426	0.3624	0.3453	0.2626	0.3483	0.4480	0.4657	0.2614	0.2983	0.4286
NM_001106760	Nudt14	0.7291	0.6665	0.8761	0.7561	0.1330	0.0530	0.7672	0.4909	0.4882	0.8265	0.7737	0.1643
NM_001107411	Ogfod1	0.3853	0.4994	0.0951	0.5296	0.3781	0.2014	0.3201	0.0919	0.3356	0.4539	0.0066	0.4286
NM_001106457	Nhlh2	0.6247	0.8852	0.9445	0.4365	0.7036	0.5783	0.8139	0.9275	0.7207	0.9080	0.9033	0.6455
NM_001106454	Ttf2	0.0561	0.2502	0.0134	0.0435	0.6964	0.2672	0.1705	0.9042	0.3316	0.4001	0.3932	0.0042
NM_001134638	RGD1560593	0.2877	0.3940	0.3394	0.2111	0.6982	0.6570	0.3458	0.7278	0.0779	0.2319	0.3940	0.6631
NM_001035001	Tmem69	0.6867	0.6554	0.4178	0.7323	0.5860	0.5067	0.6052	0.5639	0.4977	0.5771	0.2080	0.4854
NM_001134497	RGD1559578	0.0270	0.0917	0.0300	0.2403	0.0843	0.1188	0.2883	0.2534	0.1418	0.0501	0.0191	0.0959
NM_001106467	Hiat1	0.6955	0.4551	0.2466	0.4623	0.3707	0.3218	0.2902	0.2963	0.5080	0.5838	0.5213	0.5257
NM_001106683	Mfsd2	0.3093	0.4536	0.1330	0.2166	0.1578	0.2393	0.4491	0.6441	0.3212	0.4552	0.4216	0.0436
NM_001108694	Dnajc11	0.3152	0.1502	0.3292	0.9649	0.0192	0.1665	0.9423	0.2563	0.5722	0.8454	0.2136	0.2898
NM_001106466	Prmt6	0.8874	0.8983	0.8614	0.8809	0.7247	0.4855	0.8318	0.8194	0.8885	0.7503	0.5686	0.2198
NM_001106542	Ube2c	0.8043	0.8602	0.1257	0.8451	0.9069	0.8226	0.7708	0.7578	0.8916	0.9343	0.8845	0.0181
NM_001106475	Adh6a	0.4260	0.3169	0.4581	0.4508	0.2297	0.4988	0.0715	0.1748	0.2191	0.4783	0.2504	0.2758
NM_001106587	Tnpo3	0.8178	0.9753	0.5703	0.8756	0.9565	0.8845	0.6814	0.9116	0.9604	0.9268	0.9234	0.9531
NM_001107166	Tnpo2	0.1628	0.2352	0.6834	0.5974	0.6869	0.5253	0.3000	0.6440	0.5400	0.7711	0.4054	0.4283
NM_001106600	Wbp1	0.6699	0.9616	0.8423	0.6259	0.4351	0.0988	0.9197	0.8442	0.7165	0.9311	0.9598	0.7549
NM_001106695	Mul1	0.1406	0.0224	0.4387	0.0409	0.2083	0.1588	0.3572	0.2210	0.2795	0.2651	0.2930	0.4948
NM_001106692	Ela3b	0.4152	0.2562	0.2861	0.2687	0.3475	0.3507	0.0134	0.2167	0.4565	0.6018	0.4426	0.3760
NM_001107174	Rbbp5	0.9125	0.9043	0.5707	0.3967	0.9744	0.8935	0.5728	0.9540	0.4572	0.1134	0.5449	0.4065
NM_001107390	Tcerg1	0.9170	0.8307	0.8315	0.4058	0.9423	0.7624	0.1833	0.6910	0.9617	0.9369	0.9460	0.5092
NM_001107171	Ccnt2	0.9929	0.9850	0.9316	0.4677	0.9931	0.9824	0.5891	0.9918	0.9973	0.9844	0.9902	0.8109
NM_001107303	Arrdc2	0.5900	0.6004	0.7325	0.2371	0.7163	0.6746	0.6950	0.6474	0.6349	0.5750	0.6953	0.7089
NM_001106524	Acss1	0.5067	0.7108	0.6837	0.4141	0.7155	0.3637	0.5102	0.6855	0.6401	0.7151	0.4341	0.4958
NM_001106517	Sptlc3	0.6075	0.6939	0.7507	0.8416	0.7603	0.4067	0.8522	0.8741	0.8754	0.4305	0.8580	0.4380
NM_001107170	Tcfcp2l1	0.3354	0.2428	0.4612	0.6473	0.3795	0.7803	0.8908	0.1372	0.8443	0.3421	0.5270	0.6624
NM_001107179	Lmod1	0.1355	0.1816	0.1390	0.3748	0.2134	0.4582	0.3409	0.4774	0.2463	0.3246	0.2499	0.4444
NM_001107176	Optc	0.3298	0.2218	0.2875	0.1489	0.0676	0.3200	0.3551	0.1446	0.4351	0.1963	0.4707	0.4209
NM_001106529	Kif3b	0.1253	0.4972	0.7150	0.9645	0.3838	0.6401	0.3075	0.6346	0.8197	0.6052	0.5456	0.7514
NM_001107089	Muc20	0.2945	0.2009	0.1184	0.1246	0.1599	0.6295	0.3301	0.5355	0.0428	0.6503	0.1510	0.5577
NM_001106971	Dusp21	0.6300	0.7049	0.4168	0.7614	0.4720	0.1065	0.7849	0.0793	0.6055	0.2829	0.5861	0.6092
NM_001107068	Mgat5b	0.2357	0.4970	0.7459	0.4571	0.6678	0.6637	0.7772	0.5095	0.3966	0.5382	0.0781	0.8174
NM_001107421	Gfod2	0.2103	0.0769	0.2111	0.2818	0.1146	0.5180	0.0249	0.5473	0.1547	0.0981	0.0356	0.4813

NM_001107369	Mastl	0.9072	0.8982	0.1812	0.4655	0.7658	0.5045	0.7941	0.4764	0.3308	0.9359	0.9001	0.1844
NM_001107338	Zfp346	0.7549	0.9910	0.9627	0.9344	0.7942	0.9851	0.9893	0.5748	0.9300	0.8762	0.9857	0.9609
NM_001106707	Nlrc4	0.6154	0.6765	0.6591	0.5775	0.5784	0.6219	0.7918	0.7198	0.4608	0.7696	0.8343	0.4415
NM_001106706	Ttc27	0.7231	0.4857	0.3263	0.4291	0.1863	0.4158	0.3689	0.1953	0.3941	0.2843	0.4236	0.3382
NM_001106646	Ndufb6	0.0328	0.1509	0.1476	0.6627	0.1143	0.0220	0.0195	0.0813	0.0758	0.0424	0.0843	0.0015
NM_001106483	Gca	0.9846	0.9686	0.9786	0.8363	0.6549	0.7618	0.8592	0.8903	0.9052	0.9733	0.9366	0.9264
NM_001106482	Pkp4	0.9636	0.9711	0.9994	0.9844	0.7470	0.9908	0.9982	0.8964	0.8935	0.8805	0.7912	0.9025
NM_001107430	Pdpr	0.1895	0.6706	0.0766	0.7006	0.4382	0.4356	0.2126	0.5897	0.8172	0.0986	0.4373	0.7550
NM_001106484	Fign	0.5250	0.8936	0.3620	0.3489	0.7944	0.6220	0.0398	0.5624	0.4413	0.5035	0.6340	0.7347
NM_001033968	Bat3	0.7611	0.9839	0.1926	0.2006	0.3627	0.2881	0.2859	0.1952	0.6199	0.4471	0.6595	0.6869
NM_001107240	B3gnt2	0.1862	0.2036	0.8478	0.1577	0.1497	0.3101	0.2338	0.2911	0.0031	0.0329	0.1209	0.9806
NM_001107353	Slc17a2	0.8448	0.7272	0.5085	0.3986	0.2411	0.5035	0.3765	0.7328	0.4993	0.5171	0.5087	0.3669
NM_001107349	RGD1563458	0.8493	0.8301	0.5863	0.7565	0.6851	0.5231	0.8528	0.4714	0.7827	0.8398	0.8797	0.1905
NM_001107201	Prox1	0.9478	0.9656	0.7698	0.8922	0.9369	0.9226	0.8319	0.9500	0.9148	0.9324	0.3358	0.8631
NM_001107200	Ptpn14	0.6266	0.8212	0.0768	0.7601	0.7517	0.6157	0.8848	0.9009	0.7422	0.8840	0.8802	0.4849
NM_001106711	Cenpa	0.8441	0.7190	0.8227	0.8631	0.8939	0.2733	0.6153	0.5456	0.7666	0.3982	0.8726	0.7046
NM_001106778	Ano4	0.6711	0.6278	0.6107	0.7467	0.5519	0.4856	0.5112	0.3540	0.2458	0.4713	0.5623	0.4064
NM_001106500	Ndufaf1	0.0260	0.0396	0.5817	0.8943	0.0945	0.0273	0.4836	0.0637	0.0609	0.0242	0.1031	0.1344
NM_001106489	Ndufs3	0.4949	0.1828	0.5429	0.0937	0.1331	0.0893	0.0133	0.0548	0.0224	0.1184	0.0638	0.0347
NM_001107139	Pitpnm2	0.5600	0.9020	0.7476	0.2930	0.4621	0.3360	0.6008	0.5364	0.8755	0.7889	0.5219	0.8083
NM_001106724	Agr3	0.0701	0.8920	0.8502	0.6854	0.4700	0.7077	0.4426	0.8078	0.2950	0.3076	0.6655	0.5329
NM_001107370	Cd226	0.3858	0.6306	0.0261	0.4332	0.2266	0.3052	0.4353	0.3000	0.5173	0.1730	0.6765	0.1349
NM_001107212	Adamts3	0.8895	0.8515	0.8486	0.7899	0.8388	0.7819	0.6623	0.8249	0.5490	0.7476	0.4282	0.8358
NM_001107194	Pogk	0.8690	0.7618	0.8952	0.6401	0.9563	0.8053	0.5888	0.6233	0.8741	0.4084	0.4057	0.7029
NM_001107193	Tbx19	0.8574	0.6226	0.9154	0.9119	0.9869	0.6337	0.9216	0.9830	0.9125	0.7605	0.7422	0.9398
NM_001106492	Apip	0.0921	0.0038	0.1154	0.0793	0.0787	0.0246	0.0235	0.2987	0.1403	0.0385	0.0898	0.0227
NM_001106503	Stard7	0.9443	0.0824	0.2581	0.0397	0.7452	0.9430	0.1152	0.5319	0.3862	0.0711	0.0922	0.7257
NM_001106493	Ehf	0.8318	0.4935	0.7372	0.2610	0.6781	0.4319	0.3012	0.2036	0.4664	0.6667	0.6162	0.1809
NM_001106635	Arid1a	0.9889	0.9793	0.7761	0.9833	0.9687	0.9966	0.9082	0.9112	0.9934	0.9652	0.9187	0.9911
NM_001106569	Psmd5	0.0516	0.3338	0.1122	0.0603	0.1340	0.1206	0.1450	0.1510	0.0169	0.2204	0.3155	0.6500
NM_001106505	Mrps5	0.1561	0.8346	0.1435	0.3944	0.2702	0.2563	0.4785	0.7541	0.4300	0.2977	0.5620	0.1516
NM_001106662	Frmd3	0.1177	0.5528	0.5425	0.2179	0.2578	0.1380	0.4789	0.5202	0.4329	0.7890	0.7898	0.3148
NM_001106653	Tex10	0.7265	0.3161	0.2840	0.0973	0.0949	0.0257	0.1452	0.0613	0.7183	0.7346	0.9371	0.0742
NM_001107210	Ppef2	0.2535	0.7292	0.7844	0.8140	0.7601	0.6012	0.5884	0.9227	0.6028	0.4687	0.4734	0.5967
NM_001107198	Exo1	0.7133	0.9238	0.2331	0.9641	0.8788	0.9884	0.9838	0.9415	0.9659	0.6379	0.9741	0.2558
NM_001107211	Mthfd2l	0.4135	0.6503	0.8046	0.2654	0.5948	0.9062	0.8241	0.5404	0.7714	0.1930	0.8766	0.6184
NM_001107209	Thap6	0.6318	0.5118	0.6415	0.4909	0.5457	0.5477	0.3904	0.2343	0.5464	0.5207	0.2574	0.6211
NM_001107213	Uba6	0.8871	0.7841	0.8658	0.5317	0.8525	0.5881	0.3392	0.3955	0.5239	0.8880	0.8602	0.8914
NM_001126495	Ctf8	0.3329	0.5826	0.6637	0.4607	0.5491	0.5624	0.5056	0.6372	0.4536	0.5218	0.4484	0.5513
NM_001106581	Pdk3	0.3041	0.1726	0.6571	0.2090	0.0540	0.1519	0.9252	0.1397	0.4336	0.2713	0.3581	0.8944
NM_001107389	Arhgap26	0.9121	0.2050	0.7023	0.4641	0.1221	0.7026	0.5037	0.7073	0.7617	0.5275	0.3331	0.3271
NM_001107294	Galntl2	0.4139	0.6461	0.6468	0.5844	0.5173	0.5504	0.7128	0.6732	0.4893	0.5714	0.5709	0.5613
NM_001134505	RGD1308333	0.2215	0.5328	0.1682	0.1363	0.4417	0.4674	0.4295	0.3689	0.5046	0.5881	0.6995	0.2820
NM_001107406	Elac1	0.4010	0.4451	0.1570	0.5091	0.3244	0.4815	0.1488	0.3745	0.1427	0.1719	0.4635	0.4653
NM_001109525	LOC689116	0.5611	0.4797	0.7294	0.3854	0.1811	0.6070	0.1290	0.3975	0.4265	0.3171	0.0987	0.4003
NM_001106758	Cinp	0.0628	0.0747	0.4120	0.8066	0.1879	0.2250	0.2792	0.1672	0.1846	0.0071	0.0724	0.2276
NM_001106757	Cfp	0.8770	0.6989	0.0021	0.0025	0.8658	0.5978	0.0104	0.8794	0.7958	0.6192	0.6857	0.0839
NM_001107432	Cntnap4	0.2898	0.1360	0.3139	0.4387	0.4741	0.4235	0.3087	0.0631	0.1375	0.1155	0.3141	0.3533
NM_001107131	Agfg2	0.4134	0.5419	0.4968	0.6219	0.7961	0.5555	0.7639	0.4987	0.8442	0.6423	0.3464	0.3346

NM_001107225	Sorcs2	0.8954	0.8241	0.3934	0.3538	0.9271	0.8351	0.8880	0.4693	0.7904	0.7037	0.5371	0.3715
NM_001106674	RGD1305274	0.7796	0.9034	0.1721	0.5210	0.3211	0.3214	0.4211	0.4604	0.5888	0.7226	0.2289	0.1608
NM_001106671	Ttc22	0.8168	0.8925	0.8875	0.9909	0.9905	0.9788	0.8380	0.9932	0.8681	0.8472	0.7067	0.2555
NM_001107227	Zfyve28	0.5437	0.9219	0.6699	0.7008	0.6810	0.6033	0.3512	0.7324	0.3186	0.7402	0.8446	0.6038
NM_001107450	Clca5	0.5752	0.6743	0.7957	0.4869	0.3693	0.2499	0.7297	0.4521	0.8403	0.4049	0.7242	0.6777
NM_001107238	Tmed4	0.1589	0.0835	0.4707	0.3006	0.3265	0.1025	0.1702	0.0169	0.8139	0.0412	0.3656	0.9561
NM_001107237	RGD1562979	0.0649	0.2120	0.8132	0.2184	0.4616	0.6948	0.4156	0.6040	0.9124	0.5416	0.6349	0.7628
NM_001107310	Spock3	0.6685	0.5628	0.6484	0.6299	0.4631	0.3861	0.6810	0.5762	0.4648	0.7164	0.6513	0.2071
NM_001106586	Irf5	0.1735	0.1133	0.0630	0.4931	0.2689	0.1144	0.0582	0.3035	0.3710	0.1944	0.3588	0.1082
NM_001106659	Prpf4	0.9418	0.7445	0.3511	0.4540	0.9448	0.8736	0.5102	0.4972	0.8496	0.9046	0.9409	0.4618
NM_001106520	RGD1566320	0.0300	0.0960	0.2127	0.1770	0.8842	0.4338	0.0064	0.6810	0.0620	0.0793	0.2663	0.0079
NM_001106802	Naalad2	0.7220	0.4813	0.5573	0.8433	0.7665	0.9160	0.6236	0.2581	0.9212	0.7318	0.5294	0.5057
NM_134401	Crtac1	0.4915	0.1865	0.5278	0.5037	0.3843	0.8577	0.1406	0.7179	0.2890	0.4782	0.2602	0.1745
NM_001107466	Fam120b	0.8899	0.0577	0.8301	0.9356	0.6241	0.7427	0.5789	0.4138	0.0979	0.2190	0.1684	0.5867
NM_001107511	Ap2a1	0.8052	0.8313	0.6889	0.6095	0.9033	0.9927	0.9828	0.9166	0.8736	0.6400	0.9302	0.9883
NM_001107414	Rpgrip1l	0.8933	0.6012	0.3010	0.7819	0.8082	0.7810	0.9253	0.9048	0.5163	0.4636	0.6578	0.7653
NM_001106721	Lamb1	0.1909	0.1745	0.3656	0.0075	0.3077	0.3305	0.1855	0.3327	0.2837	0.2925	0.2131	0.4362
NM_001106528	Plagl2	0.4148	0.3435	0.4300	0.5195	0.5508	0.6431	0.5640	0.5176	0.2829	0.3955	0.3544	0.2628
NM_001106526	Angpt4	0.0111	0.1097	0.2630	0.1375	0.0981	0.3513	0.7492	0.2623	0.1615	0.2538	0.0456	0.2729
NM_001106523	Cst7	0.5343	0.5891	0.8954	0.4820	0.2626	0.2490	0.7361	0.3281	0.4398	0.2862	0.2356	0.8612
NM_001106522	Cstl1	0.2862	0.3667	0.2818	0.1503	0.3138	0.5236	0.1338	0.3665	0.6583	0.4260	0.3436	0.2133
NM_001106534	Fer1l4	0.4379	0.0589	0.3866	0.0588	0.2677	0.1102	0.6037	0.7788	0.4659	0.4354	0.4422	0.0734
NM_001106533	Ergic3	0.7545	0.0645	0.9641	0.6870	0.8763	0.4489	0.2556	0.7614	0.1148	0.0198	0.4828	0.4453
NM_001106543	Ncoa5	0.5743	0.5965	0.0763	0.7423	0.8861	0.7987	0.8169	0.6795	0.4846	0.4990	0.5430	0.8948
NM_001106716	Osr1	0.1435	0.0986	0.8662	0.0387	0.1101	0.2014	0.2412	0.2847	0.1556	0.1250	0.2802	0.1947
NM_001106710	Emilin1	0.8585	0.1380	0.8561	0.8559	0.7970	0.8958	0.8184	0.9642	0.0755	0.0814	0.0878	0.8384
NM_001107535	Tyr	0.1081	0.7951	0.2758	0.5454	0.5555	0.5852	0.0195	0.6010	0.3714	0.0285	0.4888	0.1046
NM_001107420	Rbmxrtl	0.4276	0.1447	0.2339	0.2912	0.2813	0.6740	0.2675	0.1133	0.1398	0.3459	0.0767	0.1206
NM_001107416	Papd5	0.6342	0.9851	0.7693	0.9786	0.4337	0.4886	0.9104	0.3217	0.7731	0.6895	0.9361	0.3685
NM_001106554	Il1f6	0.1530	0.2028	0.4116	0.3836	0.4006	0.5361	0.4021	0.5663	0.3207	0.0405	0.4049	0.3277
NM_001106681	Atp6v0b	0.5131	0.3672	0.2760	0.3191	0.3274	0.4440	0.6204	0.4489	0.2685	0.2318	0.4342	0.5346
NM_001106647	Bag1	0.5641	0.7781	0.1024	0.8164	0.5890	0.4971	0.8799	0.2919	0.8715	0.8294	0.6665	0.0587
NM_001107478	Zbtb45	0.8463	0.8781	0.1326	0.7983	0.8747	0.8716	0.8941	0.9165	0.5129	0.8227	0.8125	0.9656
NM_001107469	Zc3h12d	0.8440	0.6408	0.7637	0.8045	0.7985	0.7940	0.9223	0.5783	0.8796	0.5522	0.7901	0.7446
NM_001107322	Ank1	0.1781	0.3525	0.2658	0.4260	0.1873	0.2600	0.3045	0.2916	0.4017	0.3573	0.3044	0.4857
NM_001107248	Vcl	0.1541	0.4179	0.2591	0.3192	0.4544	0.4077	0.5164	0.1843	0.6656	0.4589	0.3515	0.4007
NM_001106518	Snx5	0.4433	0.9096	0.8496	0.3403	0.8402	0.8273	0.8995	0.5662	0.4252	0.9136	0.4443	0.8133
NM_001107489	Znf575	0.1583	0.0122	0.0510	0.0111	0.1550	0.1499	0.0206	0.0104	0.4252	0.0065	0.0418	0.0494
NM_001106764	Morc2b	0.4455	0.3036	0.4628	0.7954	0.4263	0.6717	0.5095	0.3432	0.4703	0.8075	0.5765	0.7177
NM_001106763	Asnsd1	0.7068	0.9344	0.5073	0.0456	0.5129	0.9318	0.7490	0.3190	0.6032	0.4226	0.8443	0.7471
NM_001134509	RGD1311783	0.9984	0.9822	0.8764	0.9990	0.7484	0.9758	0.9970	0.9958	0.9234	0.7584	0.7778	0.9320
NM_001106551	RGD1306208	0.2611	0.5440	0.3545	0.2214	0.5443	0.7161	0.0999	0.7697	0.5493	0.4919	0.3406	0.6151
NM_001107491	Cxcl17	0.4582	0.1797	0.3400	0.2396	0.7736	0.5071	0.6171	0.3481	0.8637	0.6992	0.4104	0.2610
NM_001107558	Nkx6-2	0.7410	0.5626	0.7962	0.1299	0.3934	0.8045	0.7910	0.7515	0.8015	0.8527	0.7474	0.6371
NM_001106556	RGD1306215	0.0233	0.1253	0.4341	0.0246	0.0309	0.2063	0.6139	0.0468	0.0567	0.4453	0.0041	0.5768
NM_001108662	Melk	0.9642	0.9547	0.3842	0.9851	0.9475	0.9258	0.9726	0.9968	0.9383	0.9638	0.8962	0.3985
NM_001113372	Synj2	0.9289	0.8790	0.9575	0.8633	0.7276	0.9402	0.9832	0.8164	0.9118	0.7186	0.8839	0.8685
NM_001107433	Mon1b	0.3829	0.3464	0.4969	0.4467	0.5391	0.3649	0.3058	0.4698	0.2171	0.2112	0.1820	0.5402
NM_001107501	Clip3	0.1243	0.1018	0.0304	0.6426	0.4508	0.6600	0.0759	0.8460	0.3780	0.0807	0.2693	0.7842

NM_001106743	Zfyve1	0.1922	0.3829	0.4145	0.2479	0.7938	0.8765	0.2501	0.6492	0.3375	0.7641	0.4859	0.5805
NM_001106562	Slc2a6	0.2946	0.4095	0.0045	0.7362	0.1344	0.1580	0.0979	0.0966	0.9822	0.5900	0.4956	0.8620
NM_001106738	RGD1308917	0.2787	0.2960	0.2941	0.5498	0.4544	0.4211	0.4611	0.5168	0.3168	0.1707	0.3283	0.5976
NM_001106779	Nedd1	0.7144	0.3014	0.4140	0.4072	0.4921	0.8856	0.5712	0.6214	0.5940	0.8926	0.9325	0.4750
NM_001106593	RGD1306936	0.0373	0.5784	0.7238	0.6758	0.5287	0.1737	0.7209	0.4938	0.4209	0.6688	0.5331	0.7487
NM_001106679	Ipp	0.6377	0.7404	0.2837	0.3875	0.0960	0.6207	0.3823	0.1726	0.0576	0.1568	0.3477	0.6529
NM_001106617	Rad52	0.7060	0.3986	0.8732	0.8855	0.9238	0.8901	0.6726	0.8874	0.7235	0.9751	0.9179	0.8837
NM_001106664	Tyrp1	0.0201	0.2883	0.5766	0.0893	0.2840	0.1690	0.6055	0.1224	0.4528	0.4298	0.4254	0.0369
NM_001106590	Slc35b4	0.9008	0.9959	0.7632	0.9615	0.9851	0.9624	0.9766	0.9838	0.9402	0.9472	0.9421	0.9556
NM_001107508	Klk12	0.0985	0.2414	0.4985	0.3960	0.4090	0.4430	0.5337	0.7635	0.5486	0.1068	0.6127	0.3162
NM_001106597	Usp39	0.6442	0.8441	0.3903	0.0857	0.3648	0.1718	0.7716	0.5996	0.7888	0.5860	0.7384	0.7131
NM_001106643	Rars2	0.3650	0.7018	0.0899	0.5687	0.6204	0.5701	0.1511	0.4200	0.7576	0.4600	0.5850	0.8009
NM_001107272	Pnma2	0.5702	0.6524	0.4296	0.5911	0.7488	0.5261	0.6765	0.5280	0.6350	0.7480	0.6253	0.6606
NM_001107271	Zfp395	0.5715	0.7738	0.5801	0.5127	0.8435	0.7043	0.8448	0.8204	0.6283	0.9695	0.8271	0.8398
NM_001107275	Slc39a14	0.3200	0.3840	0.4425	0.6361	0.1396	0.5488	0.2869	0.2242	0.2394	0.3613	0.3521	0.4145
NM_001106715	Pum2	0.9766	0.9771	0.9602	0.9768	0.9705	0.8837	0.8809	0.9477	0.9941	0.8962	0.8944	0.9121
NM_001106748	Batf	0.8317	0.9085	0.8445	0.9126	0.2323	0.0173	0.8078	0.0779	0.9096	0.8205	0.7841	0.8718
NM_001106584	Rpa3	0.4250	0.8671	0.4451	0.6248	0.4722	0.3098	0.8404	0.6561	0.6220	0.7007	0.7395	0.5878
NM_001106588	Chchd3	0.2075	0.8608	0.9824	0.9547	0.3742	0.1675	0.7304	0.1926	0.4190	0.9422	0.8883	0.8351
NM_001107285	Gpc5	0.6398	0.8230	0.6008	0.6288	0.6913	0.4415	0.6038	0.8016	0.2887	0.7469	0.5380	0.6159
NM_001107284	Slitrk5	0.4320	0.6541	0.5034	0.5526	0.5054	0.1809	0.2270	0.4062	0.2652	0.6241	0.4202	0.6262
NM_001111310	LOC497796	0.2442	0.2439	0.5324	0.2950	0.5730	0.3363	0.3381	0.1950	0.3250	0.3835	0.2984	0.2462
NM_001107306	Armc6	0.6440	0.2578	0.3049	0.3779	0.3389	0.2825	0.6390	0.5694	0.3534	0.2641	0.3533	0.2348
NM_001107291	Itih1	0.1672	0.6798	0.8632	0.8016	0.5683	0.6949	0.6339	0.5967	0.2648	0.9101	0.9380	0.6762
NM_001106727	Npas3	0.4727	0.3283	0.3136	0.1444	0.2170	0.3090	0.2409	0.3556	0.1487	0.4194	0.5408	0.0911
NM_001107289	Lsm5	0.2469	0.8029	0.6344	0.8897	0.8312	0.6861	0.9118	0.8132	0.8570	0.9777	0.8293	0.7313
NM_001107290	Il17rb	0.7579	0.3990	0.5902	0.5949	0.8600	0.3942	0.3810	0.5764	0.9632	0.7805	0.7180	0.3219
NM_001107288	Flnb	0.8279	0.7837	0.6796	0.8636	0.8451	0.9413	0.7699	0.7207	0.8392	0.8299	0.4860	0.8509
NM_001107553	Znf668	0.8859	0.9182	0.2535	0.9191	0.9917	0.8560	0.9374	0.9796	0.8977	0.6258	0.8147	0.8132
NM_001107462	Kif13a	0.6036	0.6717	0.5124	0.4308	0.5411	0.7710	0.4055	0.4434	0.3969	0.0759	0.7268	0.5908
NM_001106595	Smyd1	0.1605	0.4205	0.1620	0.1266	0.6330	0.4394	0.6193	0.7658	0.1694	0.0870	0.0561	0.8228
NM_001106697	Mrto4	0.9086	0.8130	0.0408	0.5371	0.4724	0.2096	0.8935	0.6300	0.8197	0.7785	0.7186	0.0329
NM_001106596	Mrpl35	0.2218	0.5538	0.3415	0.0398	0.1293	0.0720	0.2447	0.1257	0.4539	0.6293	0.4244	0.2770
NM_001107621	Pofut2	0.9267	0.5884	0.9620	0.1509	0.5508	0.9289	0.9501	0.6161	0.9543	0.6910	0.7049	0.9576
NM_001107756	RGD1306717	0.2257	0.5523	0.2221	0.2779	0.3796	0.6869	0.5444	0.4396	0.3903	0.4811	0.2882	0.5242
NM_001107477	Zfp329	0.5452	0.0973	0.6327	0.2025	0.5973	0.3065	0.3681	0.0189	0.0330	0.6995	0.3628	0.3986
NM_001107304	Ell	0.1558	0.2704	0.2552	0.2334	0.3376	0.7090	0.3261	0.2388	0.2568	0.0852	0.3010	0.9548
NM_001106660	Atp6v1g1	0.6101	0.7630	0.5353	0.1345	0.3944	0.0405	0.3112	0.3935	0.2854	0.7499	0.4787	0.0147
NM_001106598	Ctnna2	0.6988	0.6865	0.6564	0.6284	0.4627	0.3883	0.4172	0.1305	0.7964	0.5775	0.3301	0.3355
NM_001106676	RGD1310376	0.7608	0.9202	0.4996	0.6880	0.8613	0.4933	0.6495	0.7944	0.8065	0.8192	0.8515	0.7716
NM_001106611	Lsm3	0.6314	0.9452	0.1353	0.7717	0.6239	0.0493	0.8247	0.7727	0.8056	0.9278	0.9584	0.0666
NM_001107472	LOC308320	0.0160	0.3202	0.5581	0.2340	0.0202	0.2011	0.5917	0.0677	0.0098	0.1588	0.0618	0.3048
NM_001107336	Dok3	0.7182	0.6946	0.6540	0.6960	0.4456	0.5255	0.5655	0.9176	0.7819	0.7963	0.6325	0.7737
NM_001107515	Hebp2	0.3038	0.3604	0.4633	0.5833	0.0151	0.2411	0.3638	0.2903	0.1763	0.1598	0.3300	0.0981
NM_001107549	Tnrc6a	0.9336	0.9446	0.6855	0.7779	0.9442	0.9724	0.8961	0.9854	0.9813	0.9866	0.9922	0.9894
NM_001106632	Mybl1	0.0117	0.9974	0.1761	0.1544	0.1942	0.1558	0.0323	0.2578	0.8804	0.4051	0.8249	0.3112
NM_001106836	Rnf111	0.8731	0.9183	0.7719	0.8455	0.8871	0.9703	0.3629	0.3761	0.9707	0.9006	0.9669	0.7860
NM_001107399	Ammecr1l	0.9657	0.5457	0.8491	0.9606	0.5647	0.9903	0.7292	0.4796	0.9442	0.5906	0.8180	0.9809
NM_001106633	Mrpl15	0.3583	0.8459	0.6400	0.9721	0.3368	0.1442	0.6749	0.8383	0.8093	0.9460	0.6042	0.2824

NM_001107555	Ikzf5	0.9321	0.9653	0.9759	0.9637	0.9289	0.6735	0.9539	0.9418	0.9098	0.9278	0.9832	0.8965
NM_001106638	Slc26a7	0.9496	0.9429	0.7784	0.9859	0.9942	0.7434	0.8040	0.7204	0.0547	0.8874	0.8914	0.0063
NM_001107562	B4galnt4	0.4523	0.5804	0.4878	0.7175	0.9064	0.7533	0.8248	0.6641	0.5229	0.4865	0.2475	0.7867
NM_001106650	Susd1	0.7013	0.4749	0.7696	0.9005	0.7884	0.5181	0.8856	0.6717	0.7258	0.7064	0.7334	0.4831
NM_001107055	Wnt9b	0.6496	0.7667	0.4857	0.8878	0.4664	0.5659	0.9565	0.1489	0.3266	0.1950	0.3029	0.8064
NM_001106654	Sec61b	0.0933	0.0788	0.0540	0.6677	0.0591	0.0376	0.3316	0.0886	0.2203	0.0293	0.0768	0.0051
NM_001107497	Supt5h	0.7805	0.3454	0.2524	0.1002	0.6999	0.6585	0.0160	0.6985	0.5426	0.0930	0.7586	0.7954
NM_001110797	Pclo	0.4338	0.7079	0.8178	0.7616	0.5674	0.7020	0.6952	0.4767	0.8324	0.8936	0.5798	0.6351
NM_001106415	NIPBL	0.8104	0.9817	0.9795	0.7692	0.9701	0.8987	0.8725	0.9812	0.9294	0.9983	0.9834	0.8420
NM_001106844	Fam46a	0.8877	0.8991	0.9940	0.2465	0.9105	0.8453	0.9830	0.8852	0.9188	0.9442	0.9167	0.9354
NM_001107070	RGD1310868	0.2663	0.6244	0.7623	0.2758	0.2888	0.4814	0.7050	0.0740	0.8501	0.3837	0.8044	0.9033
NM_001108362	Tmem128	0.7002	0.9461	0.4533	0.0678	0.0947	0.1730	0.1378	0.0956	0.5612	0.8540	0.3694	0.1914
NM_001106637	Gem	0.5198	0.6165	0.5359	0.5426	0.6792	0.7616	0.6183	0.6892	0.3386	0.4476	0.6200	0.8388
NM_001106640	Gpr63	0.4424	0.6117	0.3849	0.5071	0.0351	0.5733	0.4218	0.5767	0.1554	0.5269	0.7633	0.6020
NM_001106639	Otud6b	0.5581	0.1148	0.9423	0.7968	0.1552	0.7461	0.8129	0.0748	0.6745	0.4966	0.9240	0.7026
NM_001106790	Kif21a	0.4299	0.3353	0.4585	0.2422	0.3977	0.6670	0.4793	0.7355	0.6855	0.6284	0.1921	0.2396
NM_001106641	Rragd	0.7592	0.8191	0.8795	0.8495	0.0725	0.8103	0.7063	0.1122	0.8166	0.8796	0.8796	0.8657
NM_001106651	Snx30	0.7444	0.3511	0.5848	0.6744	0.6053	0.6145	0.8110	0.2580	0.7967	0.4595	0.9094	0.4177
NM_001107339	Ror2	0.7326	0.1898	0.3776	0.7149	0.5106	0.4727	0.5779	0.4225	0.4102	0.6593	0.3372	0.0864
NM_001106875	Asah3	0.4502	0.1373	0.2399	0.0755	0.5151	0.4882	0.2469	0.4959	0.6023	0.1059	0.5001	0.1726
NM_001106784	Lgr5	0.1621	0.4942	0.1426	0.4705	0.0331	0.4692	0.1654	0.2326	0.1121	0.0158	0.2066	0.0336
NM_001106833	Lactb	0.3328	0.3723	0.0151	0.0403	0.1744	0.0791	0.0345	0.1931	0.0188	0.2135	0.1529	0.0141
NM_001106826	Bbs4	0.6766	0.3888	0.6997	0.7309	0.8100	0.7579	0.6356	0.6099	0.5519	0.5136	0.8496	0.9400
NM_001106817	Bcl9l	0.8266	0.2534	0.1487	0.6005	0.6329	0.0970	0.1286	0.6510	0.2375	0.5004	0.0466	0.4413
NM_001106900	Lman2l	0.2255	0.5326	0.4527	0.5666	0.4547	0.5358	0.6040	0.2741	0.1809	0.3676	0.2558	0.5019
NM_001106879	Efhb	0.1265	0.4116	0.4389	0.6037	0.2495	0.0920	0.7092	0.2896	0.1822	0.3525	0.6796	0.1294
NM_001106669	Oma1	0.0726	0.0548	0.0670	0.6950	0.0806	0.0927	0.5634	0.2644	0.4418	0.2312	0.4470	0.1340
NM_001106668	Slc35d1	0.3649	0.2462	0.2959	0.5096	0.4291	0.3040	0.3958	0.1728	0.3521	0.0897	0.3316	0.5160
NM_001106672	Prpf38a	0.7466	0.6088	0.2877	0.2289	0.1665	0.4058	0.1861	0.1741	0.1863	0.0340	0.3126	0.2241
NM_001106670	C8a	0.2794	0.6849	0.6521	0.6303	0.3310	0.8344	0.6428	0.5397	0.8717	0.5665	0.5974	0.8489
NM_001106677	RGD1310351	0.9193	0.9631	0.9455	0.4858	0.8977	0.8861	0.9682	0.4345	0.9276	0.9435	0.8420	0.8476
NM_001106854	RGD1307461	0.6540	0.5470	0.6043	0.6615	0.4162	0.6962	0.6600	0.6426	0.5487	0.2246	0.7204	0.5177
NM_001106075	Klhl26	0.2858	0.5568	0.2734	0.7205	0.4030	0.7637	0.7776	0.7870	0.5349	0.7357	0.3401	0.8023
NM_001108524	Pycs	0.8677	0.9944	0.8325	0.4476	0.8264	0.5186	0.9770	0.3515	0.6398	0.7616	0.9476	0.8776
NM_001107024	Ssh2	0.6325	0.5517	0.7225	0.0934	0.4645	0.4225	0.5186	0.3119	0.3394	0.7152	0.4211	0.4815
NM_001107360	RGD1560155	0.9490	0.4056	0.5262	0.9634	0.9055	0.8752	0.6532	0.8785	0.6175	0.6729	0.8973	0.9199
NM_001107357	Arhgap12	0.7801	0.2071	0.3019	0.1720	0.3573	0.4917	0.3072	0.2115	0.1419	0.3729	0.2832	0.3125
NM_001106816	Abcg4	0.8062	0.9738	0.5726	0.2228	0.9407	0.8514	0.9416	0.8759	0.9796	0.3892	0.6705	0.6883
NM_001106896	Tcfap2b	0.4958	0.1753	0.6259	0.6186	0.5044	0.8514	0.6149	0.8005	0.7374	0.4643	0.7434	0.7873
NM_001107087	Ostalpha	0.8571	0.9513	0.9331	0.8921	0.9133	0.7678	0.8394	0.7134	0.8233	0.8136	0.8573	0.7444
NM_001106917	Pi15	0.7570	0.6837	0.8005	0.6068	0.4848	0.4438	0.6251	0.1335	0.3083	0.4596	0.2259	0.4867
NM_001106678	Nsun4	0.3365	0.7071	0.4213	0.9581	0.5773	0.8200	0.6226	0.8632	0.2331	0.2342	0.8978	0.3432
NM_001106928	Fert2	0.9286	0.7929	0.8857	0.7474	0.9030	0.6432	0.4898	0.3650	0.9364	0.8285	0.8859	0.8134
NM_001107409	Csnk2a2	0.2582	0.6249	0.5138	0.7223	0.1284	0.2909	0.6087	0.0260	0.1813	0.2586	0.6173	0.6682
NM_001106904	Map4k4	0.9361	0.8848	0.4320	0.3125	0.2904	0.6201	0.8998	0.9436	0.8423	0.9143	0.8392	0.6546
NM_001107374	RGD1308601	0.9296	0.7319	0.5208	0.7627	0.7611	0.5848	0.4505	0.9847	0.8806	0.8663	0.8359	0.8265
NM_001107373	RGD1311910	0.4822	0.6029	0.7492	0.6765	0.3323	0.2153	0.7452	0.3974	0.6705	0.2683	0.5222	0.1198
NM_001107534	Eftud1	0.1061	0.0922	0.4791	0.5426	0.1145	0.4271	0.5260	0.1345	0.3947	0.4965	0.5105	0.5942
NM_001107695	Vps72	0.1340	0.0511	0.1327	0.1393	0.0911	0.0275	0.0049	0.0458	0.0340	0.0911	0.0389	0.1210

NM_001107382	Znf532	0.7086	0.9328	0.6433	0.9769	0.9585	0.9925	0.2769	0.9654	0.9784	0.9584	0.8461	0.8769
NM_001107381	Spire1	0.8392	0.5394	0.8651	0.5542	0.4521	0.8573	0.6884	0.8834	0.7795	0.5998	0.8140	0.5708
NM_001106685	Csf3r	0.2389	0.2577	0.6125	0.3738	0.4962	0.4364	0.3263	0.7802	0.4181	0.2382	0.0463	0.6757
NM_001106700	G1p2	0.6520	0.2666	0.2495	0.6259	0.8251	0.3779	0.1587	0.7106	0.7509	0.8597	0.8008	0.6732
NM_001106687	Clspn	0.9309	0.9236	0.7101	0.6677	0.9167	0.8456	0.7059	0.7525	0.9169	0.8920	0.9447	0.9615
NM_001106702	Angptl6	0.1963	0.0220	0.1674	0.0773	0.2375	0.2910	0.0634	0.5609	0.5039	0.0428	0.0302	0.5033
NM_001106691	Zfp46	0.5456	0.8958	0.3530	0.6234	0.5111	0.5561	0.6585	0.4339	0.6051	0.6502	0.6412	0.5883
NM_001106752	Ttc8	0.5379	0.0962	0.8140	0.5918	0.6099	0.8175	0.1520	0.6885	0.5463	0.1284	0.5500	0.0629
NM_001107556	RGD1310799	0.4401	0.9618	0.8830	0.7840	0.7677	0.8137	0.9689	0.6692	0.9126	0.5349	0.8643	0.7654
NM_001107613	Anks1a	0.1307	0.5895	0.7508	0.4626	0.7302	0.7542	0.2986	0.5910	0.2429	0.7729	0.4878	0.6915
NM_001107547	St5	0.9583	0.7245	0.9238	0.8686	0.8716	0.9616	0.9515	0.8241	0.6598	0.8064	0.6812	0.6671
NM_001107632	Nepn	0.0627	0.0357	0.9051	0.5692	0.5843	0.1439	0.6041	0.4793	0.3578	0.4960	0.5843	0.4827
NM_001107599	Peo1	0.5332	0.3514	0.2605	0.8428	0.2382	0.9573	0.6938	0.6157	0.1363	0.4429	0.4569	0.4217
NM_001029899	Adam23	0.8273	0.5553	0.8072	0.5450	0.7366	0.8700	0.8875	0.7379	0.4762	0.5795	0.3605	0.5613
NM_001107643	Tet1	0.8413	0.6380	0.6998	0.5346	0.5186	0.5135	0.4757	0.4876	0.7081	0.5317	0.8235	0.3871
NM_001107464	Dact2	0.5093	0.9213	0.7682	0.7472	0.9629	0.5833	0.4375	0.8561	0.6887	0.4986	0.6743	0.8179
NM_001107463	Vps37c	0.8670	0.9204	0.7638	0.3821	0.7793	0.8987	0.9260	0.3977	0.9803	0.9103	0.2770	0.2690
NM_001107458	Znf541	0.8963	0.8058	0.9056	0.8720	0.8813	0.6674	0.7484	0.6813	0.8866	0.8594	0.8015	0.8824
NM_001106712	Gpr113	0.4232	0.4040	0.3429	0.7018	0.3331	0.0846	0.6070	0.3421	0.3805	0.1293	0.1501	0.4124
NM_001106866	Endogl1	0.5055	0.8138	0.5323	0.5964	0.8688	0.9321	0.9436	0.7659	0.8783	0.4944	0.8911	0.9929
NM_001134410	Tmem132e	0.5988	0.5200	0.7397	0.1550	0.7242	0.6698	0.7794	0.8118	0.5562	0.8058	0.6575	0.6323
NM_001106720	Sntg2	0.4752	0.8485	0.7684	0.6355	0.6470	0.6299	0.4842	0.6668	0.5383	0.3745	0.6807	0.7627
NM_001107395	Psd2	0.5602	0.6862	0.6040	0.6663	0.7842	0.7053	0.5645	0.5965	0.3417	0.7813	0.3471	0.5854
NM_001107672	Fbxo4	0.3950	0.0143	0.7594	0.8314	0.1258	0.2258	0.8332	0.0301	0.0479	0.2991	0.1725	0.6921
NM_001107652	Snx18	0.3526	0.3561	0.9579	0.6792	0.2913	0.8473	0.4197	0.2887	0.3208	0.3703	0.6838	0.8323
NM_001107035	Med13	0.8616	0.8185	0.6445	0.8503	0.2994	0.2283	0.6979	0.6709	0.8078	0.9409	0.5564	0.1455
NM_001107398	Wdr33	0.8325	0.4855	0.4650	0.6743	0.8073	0.5695	0.4691	0.6477	0.2800	0.0879	0.5784	0.7172
NM_001107407	Cdh5	0.8218	0.4375	0.5426	0.5375	0.3132	0.6553	0.1778	0.4591	0.3099	0.2767	0.0552	0.3118
NM_001107410	Gpr114	0.7336	0.5079	0.7630	0.7543	0.8765	0.7638	0.8956	0.7835	0.8264	0.8230	0.6308	0.8330
NM_001107675	Maml3	0.2879	0.2199	0.2394	0.3640	0.0792	0.1832	0.1396	0.1857	0.1519	0.6248	0.0765	0.1959
NM_001106109	Nrsn1	0.7728	0.6590	0.3341	0.1594	0.3961	0.0511	0.1534	0.1184	0.2851	0.0282	0.3910	0.5526
NM_001106694	Pink1	0.0814	0.0806	0.6023	0.4411	0.2865	0.1920	0.0934	0.1927	0.1883	0.1408	0.0201	0.6857
NM_001106726	RGD1310263	0.9495	0.9698	0.3645	0.9318	0.9777	0.9627	0.8955	0.9499	0.9882	0.8270	0.9748	0.5122
NM_001107419	Smarca5	0.2353	0.8777	0.8864	0.9130	0.9278	0.8343	0.8682	0.9222	0.4228	0.6631	0.4205	0.0223
NM_001107581	Plxna3	0.8531	0.8012	0.8256	0.6546	0.9570	0.8158	0.9202	0.9607	0.8921	0.9328	0.7610	0.7726
NM_001013117	Phf12	0.7598	0.6934	0.5175	0.1794	0.8673	0.6769	0.7470	0.8881	0.9539	0.9034	0.9091	0.8999
NM_001106728	RGD1565657	0.2543	0.0828	0.0642	0.1698	0.1556	0.0555	0.4578	0.4455	0.1893	0.1028	0.2853	0.4194
NM_001107423	Rbm35b	0.9375	0.7368	0.4546	0.5204	0.7899	0.9103	0.7518	0.3022	0.6242	0.6713	0.8067	0.8392
NM_001107592	Loxl4	0.3638	0.2457	0.2065	0.1239	0.3026	0.2481	0.3265	0.4235	0.3387	0.0988	0.5058	0.3391
NM_001106733	Trappc6b	0.8700	0.9447	0.8624	0.9202	0.8921	0.8205	0.8876	0.7557	0.7844	0.8759	0.9042	0.7525
NM_001106731	Brms1l	0.4032	0.4576	0.9707	0.8369	0.8623	0.9560	0.9313	0.9711	0.7782	0.8340	0.6746	0.9860
NM_001106906	Gpr45	0.6765	0.3919	0.6151	0.4891	0.7549	0.5746	0.4663	0.8281	0.4179	0.4315	0.5077	0.2533
NM_001106895	Tcfap2d	0.6065	0.8181	0.6886	0.4487	0.6592	0.7179	0.2002	0.7563	0.2098	0.3913	0.2278	0.5104
NM_001107697	Otud7b	0.7268	0.3396	0.8503	0.7481	0.7357	0.5024	0.9382	0.9148	0.8826	0.8947	0.8561	0.9104
NM_001106890	Mrpl14	0.2081	0.1994	0.1121	0.4447	0.1955	0.0842	0.4595	0.6959	0.5401	0.2053	0.4447	0.0220
NM_001106886	RGD1565959	0.4038	0.8294	0.6186	0.5244	0.8143	0.5942	0.1422	0.4256	0.5743	0.5037	0.4671	0.2928
NM_001106885	Trem1	0.2865	0.5222	0.4698	0.3305	0.1694	0.0383	0.1235	0.0865	0.3302	0.5007	0.1907	0.4333
NM_001106867	Eif1b	0.0651	0.9147	0.1443	0.9156	0.6776	0.2403	0.4436	0.8423	0.0323	0.4213	0.2235	0.1225
NM_001107451	Slc2a12	0.8307	0.6201	0.6712	0.8623	0.8381	0.5819	0.6754	0.9211	0.2578	0.4716	0.8957	0.6610

NM_001107834	Pbx3	0.9816	0.8679	0.8228	0.9968	0.3917	0.8199	0.7852	0.4248	0.9110	0.2170	0.8353	0.9337
NM_001107598	Mrpl43	0.1347	0.6196	0.9074	0.5500	0.2316	0.0903	0.4287	0.1420	0.6661	0.9070	0.7853	0.4178
NM_001107596	Tmem2	0.7933	0.6339	0.6237	0.9091	0.6849	0.5038	0.9200	0.6582	0.8718	0.9780	0.6133	0.7762
NM_001106739	Six4	0.7667	0.6348	0.9205	0.8167	0.4372	0.7558	0.5998	0.4730	0.9662	0.6316	0.8354	0.8226
NM_001107004	Zfp39	0.1290	0.1154	0.0126	0.3095	0.0213	0.0693	0.0520	0.1827	0.0617	0.1238	0.0803	0.0768
NM_001106892	Enpp4	0.8324	0.9718	0.5552	0.8672	0.8731	0.5191	0.4760	0.9351	0.8754	0.9264	0.8850	0.8878
NM_001107001	Aff4	0.9587	0.8352	0.9937	0.4007	0.9165	0.9765	0.6774	0.4106	0.9888	0.9704	0.8764	0.7388
NM_001107444	Nhlrc2	0.3775	0.6079	0.0157	0.8853	0.3406	0.2999	0.3924	0.5422	0.0781	0.0480	0.8671	0.1497
NM_001106858	Col7a1	0.6417	0.7704	0.5527	0.4031	0.8808	0.7417	0.5574	0.7361	0.8041	0.5638	0.6252	0.4530
NM_001107196	Atf6	0.6839	0.7625	0.2985	0.5157	0.3214	0.5988	0.7088	0.7674	0.5367	0.1541	0.8295	0.6538
NM_001106787	Mov10l1	0.3229	0.9408	0.8805	0.8529	0.5868	0.8673	0.8210	0.9019	0.9266	0.7949	0.4262	0.8037
NM_001107036	Mpo	0.3224	0.1119	0.5623	0.2819	0.2276	0.7274	0.4219	0.3403	0.3237	0.2450	0.8064	0.2893
NM_001107551	Znf629	0.7030	0.5006	0.5821	0.6009	0.8179	0.1873	0.1999	0.2201	0.1263	0.2439	0.1012	0.3527
NM_001107502	Lrfn3	0.2284	0.8741	0.8083	0.3763	0.8390	0.5473	0.7467	0.3675	0.7898	0.9044	0.8406	0.7692
NM_001107047	Ikzf3	0.6202	0.6652	0.4858	0.3777	0.3226	0.4749	0.5285	0.5712	0.5978	0.6517	0.5100	0.5180
NM_001106819	Nnmt	0.0236	0.0035	0.5685	0.0118	0.0017	0.1268	0.0562	0.0053	0.0301	0.0046	0.0325	0.0221
NM_001107468	Zfp53	0.3317	0.5362	0.3525	0.1999	0.2154	0.3481	0.3061	0.4815	0.2094	0.2224	0.2571	0.2799
NM_001107473	Zim1	0.7326	0.8047	0.3125	0.3640	0.7769	0.5437	0.2625	0.4945	0.5969	0.6861	0.4687	0.4059
NM_001107471	Cnot3	0.7154	0.1443	0.0904	0.3700	0.9208	0.8646	0.5813	0.5139	0.8148	0.4460	0.1903	0.7531
NM_001107470	Shprh	0.4195	0.4564	0.3687	0.4431	0.7688	0.7681	0.4402	0.3842	0.7533	0.3320	0.5936	0.2634
NM_001107483	Irf2bp1	0.8453	0.3132	0.8211	0.9255	0.9598	0.9826	0.8554	0.9601	0.6273	0.6957	0.5446	0.9941
NM_001107479	Npas1	0.4528	0.1221	0.0750	0.0630	0.1489	0.2992	0.1734	0.0435	0.5599	0.1172	0.4156	0.4685
NM_001107476	RGD1306181	0.5532	0.2257	0.1530	0.1135	0.1594	0.0535	0.3498	0.2774	0.3510	0.2490	0.1969	0.7940
NM_001107548	Usp31	0.5749	0.7492	0.9021	0.9129	0.9154	0.7162	0.6613	0.6874	0.6179	0.1834	0.8042	0.8686
NM_001106795	Aaas	0.1438	0.8627	0.0016	0.0206	0.5701	0.5874	0.6717	0.6828	0.7627	0.5364	0.6987	0.0843
NM_001108666	Smc2	0.9969	0.9917	0.3945	0.9818	0.9953	0.7591	0.9969	0.9695	0.9271	0.9733	0.9899	0.0324
NM_001107487	Zfp112	0.7760	0.8311	0.5208	0.9309	0.6103	0.8627	0.8823	0.7496	0.9891	0.8063	0.9842	0.6166
NM_001106759	RGD1307749	0.3946	0.4458	0.4036	0.5112	0.1272	0.0320	0.5467	0.0482	0.5267	0.7277	0.7478	0.1697
NM_001106911	RGD1562317	0.9339	0.9253	0.8494	0.9220	0.9658	0.7993	0.6793	0.9871	0.9108	0.8542	0.9744	0.6482
NM_001107493	Exosc5	0.7584	0.9666	0.5177	0.8153	0.7891	0.3765	0.7357	0.9857	0.8004	0.8544	0.8621	0.3965
NM_001107504	Chst8	0.6728	0.6139	0.8907	0.6903	0.7228	0.8209	0.8582	0.7551	0.9078	0.5941	0.6065	0.7529
NM_001106898	Bai3	0.7924	0.8896	0.6148	0.7985	0.4884	0.6419	0.2839	0.6271	0.6148	0.6564	0.8390	0.8413
NM_001106769	Apc2	0.9030	0.7867	0.5244	0.3798	0.5996	0.8413	0.7572	0.8369	0.2537	0.3922	0.3874	0.4864
NM_001107902	Sox17	0.7090	0.3862	0.3064	0.3872	0.0793	0.0279	0.1725	0.6345	0.0682	0.0813	0.6377	0.0968
NM_001107776	Adam33	0.9004	0.8533	0.7299	0.9330	0.9928	0.9811	0.3757	0.9855	0.9689	0.8054	0.7263	0.9596
NM_001107628	Mypn	0.4821	0.1114	0.1928	0.4513	0.5513	0.4436	0.0543	0.5380	0.5184	0.0557	0.4200	0.3853
NM_001107605	Neurl	0.2325	0.0185	0.3195	0.0346	0.0413	0.0638	0.0215	0.0606	0.0140	0.0503	0.0792	0.0622
NM_001107600	Fbxw4	0.8214	0.9184	0.8915	0.3214	0.8817	0.9415	0.7605	0.9882	0.8562	0.8897	0.7840	0.9173
NM_001106780	RGD1565947	0.1178	0.0902	0.1418	0.3621	0.1327	0.1556	0.1409	0.5570	0.3746	0.1200	0.4133	0.6000
NM_001106776	Ccdc53	0.4322	0.6572	0.5290	0.6917	0.3426	0.0792	0.4589	0.1611	0.1203	0.1148	0.1664	0.0206
NM_001107514	Heca	0.3452	0.8299	0.7442	0.8380	0.9194	0.8583	0.6091	0.7921	0.9029	0.4285	0.8773	0.6948
NM_001107574	Znhit2	0.2496	0.6076	0.6514	0.6963	0.3859	0.0813	0.8060	0.2974	0.8008	0.7072	0.8595	0.2346
NM_001107572	Ovol1	0.5369	0.3776	0.7963	0.0475	0.4808	0.7766	0.6046	0.7691	0.4131	0.5842	0.1058	0.3964
NM_001107725	Gstcd	0.3464	0.9004	0.4988	0.9689	0.9174	0.9339	0.9751	0.7663	0.8277	0.9529	0.9035	0.5117
NM_001106775	Nup37	0.6810	0.1810	0.0100	0.2230	0.7606	0.8580	0.1377	0.7762	0.0909	0.2578	0.1384	0.0855
NM_001106773	Bpil2	0.4168	0.3506	0.4911	0.5915	0.3208	0.2287	0.2473	0.2716	0.3087	0.2261	0.6900	0.3492
NM_001107516	Tubgcp5	0.8399	0.6353	0.3806	0.4900	0.7935	0.7322	0.0631	0.8511	0.9365	0.6254	0.2034	0.7413
NM_001107650	Ercc8	0.0272	0.9198	0.8087	0.3560	0.3402	0.1490	0.6597	0.6081	0.9653	0.8003	0.7175	0.8598
NM_001107520	Herc2	0.8934	0.9827	0.2902	0.8618	0.9651	0.9519	0.1555	0.7717	0.9712	0.8804	0.8619	0.9294

NM_001107518	Nipa2	0.1554	0.2850	0.8278	0.7974	0.0999	0.8411	0.1912	0.1403	0.0571	0.2628	0.3262	0.6278
NM_001107517	Cyfip1	0.7989	0.3187	0.8381	0.9438	0.6947	0.7041	0.6611	0.7574	0.8902	0.6340	0.8820	0.2982
NM_001107732	Ccdc148	0.1281	0.1841	0.1748	0.2163	0.5789	0.1859	0.2721	0.7423	0.3564	0.0860	0.1483	0.1450
NM_001107749	Tp53i11	0.6336	0.7914	0.4657	0.1343	0.7477	0.4116	0.9354	0.6164	0.6325	0.7099	0.8283	0.8177
NM_001107022	Scarf1	0.5961	0.5789	0.5930	0.6620	0.6041	0.3386	0.4149	0.3964	0.5321	0.3073	0.1490	0.3071
NM_001106893	Cyp39a1	0.1428	0.5480	0.5273	0.4502	0.8215	0.2132	0.5326	0.4081	0.5661	0.0630	0.2853	0.5772
NM_001106925	Dtymk	0.7130	0.6023	0.4127	0.7688	0.8428	0.7691	0.8146	0.9031	0.4092	0.9780	0.7311	0.2919
NM_001106918	Fsd1	0.0015	0.1403	0.0932	0.1218	0.0064	0.0188	0.1570	0.1406	0.0292	0.0823	0.1363	0.0299
NM_001107015	Tm4sf5	0.6704	0.5772	0.8168	0.3473	0.5362	0.2184	0.5680	0.1526	0.0254	0.5767	0.3181	0.9417
NM_001107755	Ramp	0.5892	0.3575	0.8248	0.8086	0.8409	0.7663	0.5306	0.7413	0.6060	0.0522	0.1060	0.6515
NM_001107761	Exdl1	0.5552	0.5567	0.3050	0.8201	0.5276	0.6106	0.5244	0.5619	0.7877	0.4480	0.7420	0.8514
NM_001107656	Cdh18	0.0482	0.8817	0.8157	0.0707	0.3317	0.1108	0.1295	0.0276	0.6724	0.8338	0.8660	0.4835
NM_001107654	RGD1306502	0.7384	0.5866	0.4214	0.4720	0.1506	0.5249	0.7322	0.1158	0.7888	0.6087	0.4748	0.5202
NM_001107667	Ccdc39	0.9566	0.4485	0.7222	0.7739	0.6635	0.6756	0.7173	0.6038	0.6708	0.7764	0.7914	0.8602
NM_001107655	Rnasen	0.9553	0.9687	0.6928	0.9158	0.9903	0.9437	0.9887	0.9965	0.9562	0.8933	0.8013	0.9488
NM_001106794	Pfdn5	0.1342	0.1563	0.2463	0.8471	0.4056	0.0780	0.2038	0.6927	0.0577	0.0075	0.0304	0.0028
NM_001106803	Mbd3l1	0.2710	0.3243	0.4262	0.2866	0.4404	0.6678	0.2551	0.0979	0.3239	0.1705	0.3821	0.5984
NM_001106910	Ercc5	0.9746	0.9787	0.9501	0.9290	0.9578	0.9900	0.8903	0.9824	0.9984	0.8392	0.9972	0.9586
NM_053312	Dbt	0.2813	0.5899	0.3738	0.2263	0.3081	0.4055	0.5768	0.2963	0.2188	0.3110	0.1719	0.7157
NM_053305	Tas1r1	0.1195	0.1023	0.2647	0.6204	0.1086	0.4735	0.1320	0.2508	0.3339	0.1926	0.1345	0.4236
NM_001110139	Atp2a2	0.7318	0.7335	0.6491	0.7287	0.6940	0.7190	0.5783	0.6974	0.6391	0.7068	0.7151	0.7777
NM_001100836	Fcgr1a	0.0339	0.3265	0.5178	0.4543	0.3667	0.4095	0.5647	0.4417	0.6912	0.5691	0.4606	0.1373
NM_001107526	Blm	0.9467	0.9702	0.2043	0.4980	0.9715	0.9656	0.9722	0.9929	0.8704	0.9276	0.9544	0.3167
NM_001107011	Mpdu1	0.3964	0.4226	0.9221	0.5720	0.9433	0.4782	0.9822	0.7688	0.3266	0.4109	0.3095	0.3084
NM_001107136	Auts2l	0.5156	0.2370	0.5185	0.4130	0.1822	0.2943	0.2979	0.3582	0.1698	0.4012	0.2690	0.3509
NM_001107309	Csgalnact1	0.0883	0.0444	0.0556	0.0421	0.0775	0.0431	0.0073	0.0093	0.0443	0.1312	0.2697	0.0895
NM_001107013	Gps2l	0.7585	0.9809	0.0748	0.5161	0.6729	0.7793	0.1490	0.7966	0.9846	0.9515	0.8178	0.9676
NM_001107680	Mlf1	0.4862	0.9518	0.7923	0.9951	0.7287	0.7117	0.8627	0.8722	0.8481	0.8484	0.9588	0.6926
NM_001107677	Gpr87	0.3107	0.4647	0.1544	0.2797	0.5369	0.2749	0.6353	0.1272	0.1119	0.0209	0.2438	0.5220
NM_001107670	Phf17	0.4273	0.5640	0.5938	0.3571	0.5301	0.5156	0.5147	0.5659	0.5891	0.5089	0.4855	0.8421
NM_001107528	Hddc3	0.0249	0.1181	0.6466	0.9124	0.9061	0.4484	0.8882	0.5727	0.3622	0.5144	0.2860	0.2158
NM_001107527	Man2a2	0.2034	0.0366	0.8411	0.1195	0.7062	0.8572	0.3426	0.6747	0.1339	0.1304	0.6317	0.8148
NM_001107270	Neil2	0.2377	0.2982	0.1097	0.1032	0.1357	0.1489	0.1632	0.3625	0.1282	0.0337	0.3462	0.0104
NM_001107687	Rab25	0.5918	0.6360	0.4742	0.7761	0.5867	0.1384	0.4847	0.4262	0.5498	0.3589	0.5588	0.1692
NM_001107683	Slitrk3	0.5243	0.3789	0.4028	0.7401	0.5355	0.3977	0.5643	0.4364	0.5573	0.5811	0.4489	0.3564
NM_001107533	Adamtsl3	0.7166	0.5396	0.7096	0.2332	0.6017	0.7001	0.6920	0.5207	0.6419	0.5417	0.4002	0.5192
NM_001106805	Rgl3	0.4785	0.9555	0.4756	0.0478	0.5874	0.8172	0.3056	0.5358	0.9065	0.9049	0.7790	0.7158
NM_001107856	LOC312273	0.3793	0.7669	0.6456	0.3632	0.5354	0.3553	0.5246	0.6663	0.4901	0.6350	0.9751	0.6223
NM_001107539	Fchsd2	0.5942	0.8406	0.9721	0.9857	0.9336	0.9650	0.9571	0.9904	0.7045	0.4393	0.9348	0.9900
NM_001107540	Rnf121	0.3303	0.6267	0.3156	0.7630	0.8969	0.7193	0.6031	0.8690	0.2868	0.3554	0.4113	0.3400
NM_001106989	Sox8	0.3357	0.3078	0.7398	0.6182	0.3233	0.4275	0.4295	0.4757	0.5688	0.5031	0.3501	0.8035
NM_001107071	Cbx2	0.8422	0.3675	0.5211	0.2007	0.4491	0.4141	0.9296	0.5690	0.8324	0.6288	0.2428	0.3721
NM_001107545	Ipo7	0.3128	0.4491	0.4800	0.2474	0.3284	0.1619	0.4301	0.2758	0.6427	0.8667	0.7066	0.8542
NM_001107064	Fads6	0.8559	0.4089	0.7314	0.9633	0.8305	0.8882	0.9746	0.8334	0.7953	0.7087	0.1271	0.9231
NM_001106964	Tbl1x	0.8893	0.4013	0.9210	0.7385	0.3130	0.9142	0.9010	0.8709	0.9066	0.9457	0.9283	0.9819
NM_001106807	Zfp653	0.7353	0.6368	0.2435	0.8558	0.8527	0.9614	0.7612	0.9439	0.9353	0.8683	0.9309	0.6454
NM_001107557	RGD1306820	0.6060	0.4993	0.2724	0.0312	0.5571	0.7424	0.1141	0.0341	0.2144	0.7466	0.4633	0.7991
NM_001106822	Slc35f2	0.3727	0.7605	0.7882	0.6604	0.4686	0.6383	0.7441	0.7345	0.6847	0.9523	0.4699	0.7369
NM_001106820	Ddx10	0.0377	0.3625	0.2614	0.0181	0.1899	0.4381	0.2397	0.0478	0.5075	0.1963	0.1073	0.9266

NM_001106824	Pstpip1	0.4395	0.4818	0.4757	0.2909	0.2443	0.4291	0.6331	0.1693	0.1533	0.3580	0.1871	0.0611
NM_001106823	RGD1308677	0.2013	0.0712	0.4096	0.3090	0.1847	0.1588	0.0790	0.4416	0.4793	0.3945	0.1504	0.6136
NM_001107563	Pddc1	0.0140	0.3500	0.6838	0.5628	0.2823	0.2206	0.1926	0.1845	0.1247	0.0864	0.1293	0.1000
NM_001108020	Pnpla8	0.2204	0.9188	0.7729	0.2627	0.5143	0.7332	0.6824	0.3884	0.5985	0.8223	0.7758	0.7758
NM_001107765	Cdan1	0.7369	0.9055	0.4549	0.7143	0.9781	0.9612	0.9453	0.8461	0.9145	0.7916	0.6128	0.4940
NM_001107567	Mrpl21	0.1393	0.9000	0.2945	0.7865	0.2155	0.0990	0.8256	0.1318	0.6817	0.8086	0.9188	0.0230
NM_001107699	Itga10	0.5022	0.4918	0.3910	0.3147	0.1884	0.2870	0.3102	0.1988	0.4535	0.3853	0.5234	0.1624
NM_001107907	Fam46b	0.4188	0.9692	0.3571	0.6670	0.0366	0.0240	0.9259	0.0497	0.6385	0.9127	0.9009	0.2385
NM_001109374	LOC679668	0.5627	0.4997	0.6085	0.6911	0.5567	0.3583	0.5550	0.4420	0.6168	0.4310	0.6533	0.6388
NM_001107711	Mov10	0.3764	0.8527	0.3044	0.9427	0.1589	0.1279	0.8410	0.7425	0.6923	0.5505	0.5555	0.9064
NM_001107661	Fam164a	0.5069	0.1832	0.4502	0.4127	0.4944	0.5908	0.7636	0.6677	0.3372	0.5797	0.8112	0.2304
NM_001107425	Nfat5	0.6489	0.9791	0.3631	0.3628	0.6285	0.9838	0.3276	0.9893	0.9841	0.3720	0.8805	0.7591
NM_001107069	Usp36	0.8928	0.6121	0.3164	0.0791	0.8793	0.9318	0.6162	0.7008	0.7861	0.8576	0.8510	0.9280
NM_001107051	Ezh1	0.8792	0.7948	0.4404	0.5921	0.8397	0.9403	0.6582	0.9052	0.9849	0.7967	0.2460	0.9624
NM_001107566	Tpcn2	0.9075	0.9200	0.2945	0.6250	0.9974	0.8797	0.3662	0.9569	0.9862	0.9803	0.8644	0.8936
NM_001107565	Oraov1	0.4840	0.7004	0.8776	0.8723	0.7349	0.3801	0.3665	0.9536	0.7938	0.5455	0.8211	0.1910
NM_001107564	Tmem16a	0.3106	0.5293	0.5794	0.4640	0.6782	0.1741	0.4072	0.2816	0.7882	0.6227	0.6678	0.4845
NM_001107082	Etv5	0.5448	0.6781	0.6972	0.7125	0.6230	0.6446	0.7184	0.7156	0.7160	0.6236	0.5985	0.6611
NM_001107077	Fn3krp	0.9610	0.3310	0.7152	0.7051	0.7697	0.8108	0.9467	0.7464	0.2528	0.3513	0.4428	0.6486
NM_001106975	Fam86a	0.4945	0.6193	0.8866	0.9306	0.7243	0.7706	0.7720	0.4406	0.5102	0.7848	0.9516	0.8435
NM_001107660	Car1	0.8390	0.8452	0.7379	0.7421	0.3419	0.4748	0.4740	0.1996	0.5082	0.3738	0.7724	0.2255
NM_001107585	Uhrf2	0.8548	0.8838	0.8020	0.8063	0.7953	0.8847	0.8494	0.9259	0.7837	0.8504	0.7806	0.8805
NM_001107582	Pdcd1lg2	0.5187	0.5246	0.2313	0.4629	0.6463	0.0464	0.0886	0.4076	0.4340	0.1983	0.3151	0.3247
NM_001109663	Armc9	0.5708	0.2028	0.2327	0.3166	0.8552	0.8275	0.2032	0.9089	0.1352	0.0390	0.2849	0.1854
NM_001107770	Sppl2a	0.7741	0.7132	0.6713	0.6965	0.6101	0.7685	0.5365	0.5872	0.6477	0.8106	0.3924	0.4125
NM_001107767	Duoxa1	0.1336	0.4805	0.4466	0.6545	0.5850	0.4364	0.6261	0.6395	0.8914	0.7823	0.7533	0.6220
NM_001106838	RGD1310552	0.8881	0.1209	0.8452	0.0855	0.9218	0.8631	0.0284	0.5467	0.3732	0.2806	0.4692	0.7566
NM_001107588	Chuk	0.5044	0.5208	0.8207	0.5283	0.4097	0.3335	0.6838	0.2400	0.2113	0.4849	0.8236	0.2994
NM_001108144	Usp28	0.9081	0.7313	0.8482	0.4466	0.9114	0.9913	0.1973	0.8680	0.5862	0.8315	0.9796	0.9231
NM_001107057	Wdr68	0.3663	0.9588	0.4422	0.9144	0.3645	0.4014	0.8996	0.4389	0.9504	0.6622	0.8995	0.7928
NM_001107778	Hspa12b	0.3508	0.5403	0.5220	0.6615	0.6375	0.7421	0.5908	0.4746	0.5559	0.5896	0.7948	0.6365
NM_001107784	Polr3f	0.8622	0.6415	0.9114	0.3694	0.6589	0.7574	0.4312	0.7507	0.8730	0.6697	0.8071	0.8819
NM_001107709	Ap4b1	0.8818	0.7125	0.5279	0.7247	0.7093	0.8640	0.7020	0.6702	0.9522	0.8016	0.7060	0.7481
NM_001106853	Hemk1	0.3500	0.4358	0.1678	0.2796	0.1472	0.7972	0.2708	0.6520	0.1235	0.6823	0.1085	0.1147
NM_001107145	Fbxw8	0.5728	0.1624	0.7197	0.9707	0.0711	0.9841	0.1675	0.3621	0.7653	0.6538	0.7037	0.8376
NM_001107147	Sirt4	0.7578	0.5856	0.6168	0.0210	0.4368	0.5965	0.0955	0.4802	0.8272	0.8304	0.7988	0.2570
NM_001107119	Zkscan5	0.5617	0.5613	0.2163	0.5172	0.4922	0.2752	0.5328	0.4715	0.6538	0.4722	0.6504	0.3387
NM_001106968	RGD1561230	0.4410	0.0594	0.0783	0.4775	0.4267	0.2453	0.2688	0.2654	0.4842	0.4041	0.4207	0.0866
NM_001136229	Trpm4	0.9408	0.7944	0.9182	0.5841	0.8127	0.8065	0.8372	0.6634	0.8868	0.7434	0.8495	0.9869
NM_001106857	Nckipsd	0.1018	0.2299	0.2221	0.1723	0.3605	0.3823	0.0117	0.2942	0.3477	0.2449	0.4013	0.8807
NM_001108757	Svs7	0.8597	0.2497	0.4688	0.4103	0.6156	0.6326	0.3310	0.5982	0.6771	0.2240	0.4053	0.3864
NM_001108509	Pnpla2	0.0715	0.0462	0.4115	0.0925	0.0973	0.3655	0.1264	0.1865	0.5249	0.0903	0.4275	0.9779
NM_001108615	Myt1	0.4742	0.4236	0.7940	0.8856	0.8081	0.5995	0.4964	0.7801	0.7521	0.8680	0.4774	0.6308
NM_001108613	RGD1311940	0.0890	0.1042	0.6334	0.0284	0.2412	0.1830	0.0780	0.2387	0.2177	0.3152	0.1285	0.2190
NM_001108696	Tp73	0.8786	0.0660	0.6259	0.5943	0.8899	0.9337	0.6079	0.8886	0.8835	0.7706	0.6573	0.9086
NM_001109127	Cbln1	0.5592	0.2018	0.7334	0.6241	0.5024	0.4655	0.6178	0.6932	0.6260	0.3734	0.4575	0.6774
NM_001108863	Fbxo48	0.4330	0.1683	0.3625	0.4697	0.4464	0.2913	0.1258	0.4450	0.0289	0.2547	0.0619	0.2308
NM_001108841	Gabpa	0.2887	0.5756	0.4118	0.9955	0.1666	0.6768	0.5626	0.3149	0.7056	0.6714	0.8062	0.6956
NM_001109968	Gls	0.6785	0.5378	0.5970	0.7216	0.9848	0.9911	0.6159	0.9137	0.6822	0.5351	0.7014	0.3715

NM_001108680	Adprhl2	0.8643	0.9041	0.7190	0.5827	0.9364	0.8294	0.7862	0.9816	0.7102	0.9604	0.7667	0.9062
NM_001108617	Pftk1	0.9728	0.9815	0.9791	0.9822	0.7100	0.9440	0.9925	0.3756	0.9525	0.9939	0.9908	0.9949
NM_001108593	Sec23b	0.7893	0.9300	0.3815	0.2093	0.3837	0.3700	0.4332	0.3651	0.5736	0.5784	0.7514	0.8806
NM_001108600	Actr5	0.6671	0.9931	0.5869	0.2090	0.9312	0.9849	0.9532	0.9406	0.9933	0.9566	0.9792	0.9461
NM_001108959	Tgm3	0.3261	0.7773	0.7718	0.6524	0.8960	0.4244	0.5462	0.7537	0.7528	0.8133	0.6689	0.8670
NM_001107779	Trmt6	0.7733	0.9784	0.3448	0.1795	0.9054	0.8816	0.8622	0.7840	0.8509	0.8781	0.4997	0.5678
NM_001107597	Dmrt2	0.8096	0.7984	0.0744	0.0204	0.8925	0.8671	0.8326	0.7816	0.0864	0.8450	0.9058	0.0067
NM_001107665	Usp13	0.1918	0.3294	0.2312	0.8089	0.5838	0.7263	0.7397	0.9375	0.7895	0.5685	0.0981	0.7562
NM_212521	Ush1c	0.7729	0.5377	0.6308	0.7684	0.7047	0.3774	0.7414	0.5244	0.8470	0.6360	0.6407	0.7743
NM_001108039	Papln	0.2743	0.6292	0.6482	0.3470	0.6736	0.6571	0.3787	0.8150	0.5476	0.4967	0.6786	0.3757
NM_001108402	Carkd	0.0216	0.2102	0.7910	0.7465	0.0271	0.0282	0.1992	0.3371	0.0489	0.4788	0.0576	0.3451
NM_001108070	Dos	0.5048	0.6950	0.1039	0.5899	0.8065	0.7604	0.7846	0.3463	0.8256	0.7195	0.6418	0.8529
NM_001107612	L3mbtl3	0.5819	0.6561	0.6546	0.6668	0.6563	0.4662	0.6830	0.4261	0.6693	0.6833	0.4082	0.2076
NM_001107611	Rbm20	0.6473	0.8683	0.6241	0.4345	0.5857	0.8177	0.7441	0.8358	0.5333	0.7836	0.6542	0.8895
NM_001108021	Klhl15	0.7590	0.8725	0.7431	0.6593	0.6436	0.5546	0.2102	0.5270	0.5926	0.8171	0.9143	0.6899
NM_001106990	Solh	0.8211	0.7248	0.7370	0.7186	0.2647	0.4448	0.4440	0.5980	0.6635	0.6404	0.5700	0.7588
NM_001109645	LOC691543	0.7271	0.6808	0.7231	0.6537	0.6247	0.6484	0.6110	0.2236	0.6255	0.5171	0.5177	0.5737
NM_001106887	Guca1a	0.8124	0.5702	0.8198	0.5938	0.1113	0.7430	0.5160	0.5389	0.3113	0.6610	0.7337	0.6257
NM_001106884	Trem2	0.0577	0.0800	0.2072	0.3477	0.0249	0.7262	0.3977	0.5671	0.4610	0.2036	0.2106	0.6642
NM_001107117	Lass4	0.3391	0.9098	0.5747	0.4419	0.4674	0.9123	0.9853	0.9613	0.1467	0.7666	0.2347	0.3803
NM_001107084	Bcl6	0.2780	0.2068	0.9718	0.3269	0.3086	0.3013	0.0879	0.2766	0.3576	0.2300	0.3083	0.9874
NM_001106881	Mocs1	0.4626	0.0425	0.1263	0.0100	0.2113	0.0926	0.2498	0.0278	0.2557	0.1648	0.4935	0.1576
NM_001107620	Ubash3a	0.0727	0.4604	0.1261	0.1388	0.3279	0.1285	0.1879	0.1760	0.4682	0.0936	0.4093	0.1938
NM_001107111	Il10rb	0.4963	0.0434	0.0345	0.6171	0.1471	0.4313	0.1062	0.3767	0.0582	0.2679	0.0138	0.6372
NM_001107892	Lrp6	0.9291	0.9332	0.9777	0.7296	0.9794	0.9897	0.8334	0.9780	0.9863	0.9663	0.9272	0.9828
NM_001106901	Cnnm3	0.8752	0.9329	0.2309	0.3073	0.7648	0.7748	0.7576	0.7856	0.8517	0.6306	0.7595	0.8151
NM_001107752	Ttc17	0.1480	0.3564	0.6932	0.3539	0.1487	0.7859	0.3072	0.3074	0.2951	0.4351	0.2125	0.8829
NM_001107094	Hoxd10	0.9905	0.9581	0.9858	0.8733	0.9840	0.8540	0.7679	0.9550	0.1698	0.6215	0.9213	0.1458
NM_001107802	Neurl2	0.0844	0.2410	0.4465	0.8512	0.5142	0.4465	0.1908	0.5941	0.0650	0.2541	0.0209	0.2324
NM_001107762	Nusap1	0.6503	0.9220	0.5772	0.9722	0.9889	0.9507	0.7854	0.9722	0.6128	0.9817	0.8922	0.7373
NM_001107759	Disp2	0.5634	0.9449	0.6925	0.2313	0.8859	0.6486	0.8270	0.7964	0.8732	0.7609	0.6797	0.6684
NM_001106903	Mrpl30	0.7647	0.3043	0.7540	0.9151	0.4095	0.0820	0.8831	0.5091	0.5721	0.7612	0.7700	0.7770
NM_001107003	Rapgef6	0.7004	0.6494	0.3311	0.6768	0.4669	0.5703	0.4684	0.6092	0.2273	0.3774	0.5428	0.6705
NM_001106948	Usp26	0.4381	0.3021	0.1725	0.5856	0.6819	0.7435	0.5553	0.4597	0.9246	0.6886	0.0672	0.7058
NM_001107663	RGD1307225	0.2078	0.1674	0.5418	0.8456	0.4727	0.4976	0.2139	0.6512	0.8700	0.5564	0.3858	0.7495
NM_001107060	Polg2	0.7764	0.8551	0.1122	0.0737	0.5297	0.3650	0.1315	0.3014	0.8035	0.3066	0.6924	0.5871
NM_001107032	Usp32	0.7639	0.0764	0.9186	0.6362	0.5919	0.9551	0.9081	0.2218	0.6330	0.4684	0.7326	0.9337
NM_001107097	Zbtb11	0.9115	0.2909	0.9789	0.9045	0.3484	0.9275	0.6812	0.8033	0.8133	0.8730	0.7827	0.3424
NM_001107824	Ddx31	0.9504	0.9313	0.3293	0.8158	0.9709	0.9813	0.7659	0.8758	0.9958	0.8858	0.6524	0.7449
NM_001107629	Rshl3	0.2822	0.0257	0.5106	0.1475	0.6354	0.1062	0.2749	0.0737	0.2185	0.6175	0.2036	0.2035
NM_001107787	Pax1	0.1335	0.4496	0.1402	0.0508	0.5639	0.1770	0.3452	0.4666	0.8032	0.1083	0.1888	0.5856
NM_001107625	Plekhk1	0.4709	0.9268	0.9005	0.7769	0.7578	0.8672	0.8773	0.4789	0.9272	0.8807	0.9720	0.9794
NM_001107796	Fam83d	0.7399	0.8641	0.2932	0.7142	0.8383	0.9540	0.7834	0.8406	0.7818	0.9981	0.9495	0.1857
NM_001107662	Phc3	0.8950	0.4137	0.4793	0.5108	0.6144	0.7902	0.3086	0.5836	0.7477	0.8164	0.8320	0.9600
NM_001107851	Tsga13	0.2772	0.3180	0.4116	0.3563	0.3846	0.3712	0.2034	0.3393	0.3303	0.4544	0.3946	0.2881
NM_001107826	Dolk	0.2599	0.0413	0.1543	0.9707	0.1788	0.0304	0.3340	0.0441	0.0221	0.0260	0.0469	0.1288
NM_001107817	Cobra1	0.7731	0.9284	0.4774	0.9545	0.8579	0.9630	0.7725	0.9377	0.9089	0.7077	0.7459	0.9148
NM_001107631	Gopc	0.6904	0.8263	0.9751	0.4927	0.7600	0.8530	0.8486	0.9246	0.8645	0.9061	0.6991	0.7340
NM_001106907	Tgfbrap1	0.8182	0.7761	0.7564	0.6846	0.7569	0.5808	0.9135	0.7254	0.6068	0.5157	0.6478	0.6064

NM_001107126	Cox19	0.6613	0.7956	0.9086	0.6215	0.9229	0.1868	0.6677	0.8302	0.7852	0.7812	0.3076	0.1031
NM_001106905	Il18r1	0.5084	0.9230	0.4465	0.4462	0.2893	0.1381	0.8685	0.5932	0.8124	0.3147	0.7689	0.6787
NM_001107627	Sirt1	0.3998	0.5196	0.5218	0.0801	0.1962	0.1473	0.0577	0.8547	0.6195	0.9164	0.7322	0.1100
NM_001107608	Hectd2	0.2590	0.2487	0.8692	0.1203	0.0863	0.2188	0.2406	0.1125	0.3297	0.1918	0.5715	0.0076
NM_001107583	Gldc	0.7428	0.8659	0.9042	0.8189	0.9661	0.8130	0.6981	0.7539	0.7142	0.7845	0.9868	0.6484
NM_001107282	Irg1	0.1430	0.6624	0.7432	0.7858	0.3233	0.6492	0.3597	0.3932	0.7990	0.2256	0.4235	0.6173
NM_001107827	Usp20	0.3679	0.2263	0.3613	0.3287	0.2195	0.5429	0.4761	0.3692	0.3514	0.7442	0.4569	0.8541
NM_001106909	RGD1309095	0.4054	0.9379	0.1264	0.1244	0.3372	0.1212	0.1134	0.1342	0.2323	0.2334	0.3930	0.1250
NM_001107123	Zfp469	0.9605	0.8845	0.8571	0.8065	0.7215	0.7694	0.4254	0.9107	0.2622	0.4696	0.2802	0.8121
NM_001106899	Plekhb2	0.4618	0.7050	0.9333	0.7943	0.8418	0.9843	0.6521	0.9054	0.9720	0.9445	0.9563	0.9346
NM_001107010	RGD1563106	0.2185	0.1051	0.1479	0.1465	0.0321	0.3069	0.3134	0.3222	0.1539	0.4753	0.0676	0.4217
NM_001005872	Kat5	0.9842	0.8607	0.1348	0.7005	0.9417	0.8827	0.0842	0.7973	0.9194	0.7518	0.7544	0.9471
NM_001107315	Pragmin	0.5324	0.4333	0.2942	0.7334	0.2190	0.1305	0.7688	0.2428	0.0136	0.1723	0.6358	0.4630
NM_001106912	Ndufb3	0.0872	0.0458	0.3258	0.1475	0.3306	0.0344	0.0799	0.1030	0.0022	0.0307	0.0405	0.1871
NM_001108009	Rasgrp3	0.1134	0.1741	0.1966	0.3505	0.1663	0.1239	0.0085	0.2895	0.2483	0.1484	0.0121	0.3548
NM_001106919	Arpc2	0.2585	0.6874	0.1800	0.2419	0.2990	0.7636	0.3636	0.0930	0.3119	0.6506	0.3631	0.6550
NM_001107017	Traf4	0.9465	0.9527	0.9752	0.7390	0.7487	0.9166	0.8278	0.9731	0.9999	0.7702	0.7899	0.9875
NM_001135600	Cyp4v3	0.2502	0.1265	0.5757	0.5499	0.4492	0.1135	0.1604	0.4164	0.2444	0.2558	0.3626	0.1118
NM_001106921	Prkag3	0.6234	0.7939	0.1171	0.3756	0.5329	0.6408	0.5714	0.6081	0.7955	0.8634	0.6478	0.5357
NM_001107019	Garnl4	0.4532	0.6337	0.8779	0.5747	0.7516	0.5850	0.3198	0.7650	0.7038	0.5191	0.4394	0.8540
NM_001108000	Fam132a	0.5736	0.5404	0.2866	0.4082	0.0972	0.5699	0.7039	0.4527	0.7942	0.3356	0.5764	0.3604
NM_001107859	Znf775	0.5780	0.8840	0.6003	0.4294	0.9280	0.7744	0.8385	0.9002	0.7466	0.6962	0.1918	0.9038
NM_001107766	Ttbk2	0.8397	0.7622	0.5098	0.7926	0.0721	0.7023	0.8663	0.1910	0.1168	0.4191	0.7985	0.4128
NM_001107689	Ash1l	0.7874	0.4715	0.4206	0.8112	0.7802	0.8607	0.3530	0.8449	0.9517	0.9334	0.4360	0.8889
NM_001107839	Rc3h2	0.8613	0.2210	0.1916	0.2785	0.4439	0.6470	0.1090	0.1770	0.1548	0.5671	0.7490	0.0784
NM_001106924	Cab39	0.9722	0.8661	0.9161	0.8592	0.9308	0.9829	0.2985	0.9853	0.5199	0.9601	0.9175	0.5248
NM_001107023	Rab11fip4	0.7415	0.1450	0.2509	0.4531	0.1308	0.4622	0.6130	0.2164	0.5485	0.2545	0.5013	0.7049
NM_001013116	Senp3	0.5684	0.4228	0.3356	0.3300	0.1125	0.7139	0.1345	0.1682	0.8557	0.2904	0.7522	0.2924
NM_173302	Slc22a9	0.3932	0.1122	0.5066	0.4833	0.4066	0.1799	0.4206	0.3883	0.4475	0.4928	0.4754	0.4890
NM_001107137	Tyw1	0.2087	0.3094	0.0842	0.4924	0.1131	0.1096	0.0831	0.0430	0.1931	0.0256	0.0565	0.0643
NM_001107659	Sema5a	0.4100	0.0024	0.6616	0.1828	0.9894	0.9686	0.0914	0.7605	0.0028	0.0016	0.2549	0.2194
NM_001107021	Hic1	0.8260	0.5539	0.3947	0.4029	0.3972	0.5518	0.4851	0.6726	0.7466	0.4427	0.5246	0.3551
NM_001107786	Rin2	0.1808	0.2444	0.3622	0.1541	0.2746	0.1544	0.0888	0.1923	0.2547	0.2141	0.1293	0.6035
NM_001107841	Rabgap1	0.7644	0.9586	0.9958	0.5368	0.8121	0.9595	0.5656	0.1325	0.5107	0.8702	0.5228	0.9671
NM_001107780	Hao1	0.5913	0.7176	0.7914	0.5928	0.8026	0.2554	0.9171	0.7212	0.6486	0.8741	0.9184	0.9016
NM_001107386	Pde6a	0.8912	0.9175	0.9074	0.9169	0.9716	0.6972	0.9376	0.8578	0.9667	0.8598	0.3493	0.7569
NM_001107025	RGD1304598	0.1839	0.5747	0.7359	0.7461	0.4889	0.7412	0.5841	0.7056	0.8270	0.6394	0.3982	0.7340
NM_001107846	Steap2	0.1508	0.2181	0.4964	0.0205	0.1335	0.1445	0.2428	0.1764	0.1903	0.1867	0.2423	0.7258
NM_001107129	Cops6	0.1600	0.4931	0.6305	0.7094	0.0666	0.0685	0.4830	0.1173	0.1203	0.6507	0.3291	0.1302
NM_001107127	RGD1566386	0.9139	0.6868	0.6590	0.8162	0.7644	0.0765	0.7348	0.8814	0.5512	0.7539	0.6104	0.9128
NM_001107822	RGD1307355	0.5794	0.4266	0.4783	0.5214	0.7096	0.4156	0.6245	0.5450	0.6687	0.6157	0.1722	0.5938
NM_001106932	Fem1c	0.9277	0.5361	0.8902	0.9163	0.7178	0.8267	0.2945	0.4663	0.3607	0.3929	0.2104	0.8043
NM_001107159	Mmp19	0.0114	0.1195	0.2081	0.1191	0.2114	0.1021	0.4327	0.2394	0.0788	0.1513	0.0130	0.0237
NM_001107155	Tmem119	0.6818	0.1778	0.9751	0.9307	0.9265	0.9607	0.3577	0.7674	0.1361	0.9168	0.3317	0.7454
NM_001107154	Ssh1	0.6682	0.7584	0.2035	0.1803	0.8260	0.4836	0.4098	0.7195	0.5059	0.8057	0.1404	0.6289
NM_001107031	Slfn2	0.6362	0.5240	0.2179	0.5455	0.2610	0.4251	0.6322	0.6114	0.3466	0.4292	0.2383	0.4823
NM_001107948	Atg4c	0.5564	0.2453	0.7182	0.7413	0.7305	0.7746	0.5575	0.4722	0.5143	0.5020	0.6990	0.6226
NM_001107384	Napg	0.3283	0.3036	0.5955	0.3641	0.2231	0.2346	0.3109	0.0076	0.5890	0.0957	0.3783	0.3395
NM_001106931	RGD1564712	0.5489	0.5978	0.7486	0.2872	0.6279	0.5467	0.8122	0.6825	0.6252	0.3174	0.7362	0.6835

NM_001107816	RGD1308019	0.2000	0.5623	0.4571	0.3560	0.2618	0.3819	0.4316	0.4121	0.0433	0.1261	0.1138	0.5393
NM_001100585	Rce1	0.8473	0.6769	0.5905	0.5717	0.7382	0.3689	0.9310	0.6862	0.8748	0.8978	0.6358	0.5573
NM_001107678	Dhx36	0.7566	0.4744	0.9485	0.3403	0.4970	0.8853	0.0917	0.0571	0.6908	0.5302	0.5152	0.5502
NM_001107033	Tbx2	0.6276	0.3163	0.3684	0.5264	0.3590	0.3305	0.1382	0.2453	0.0383	0.1701	0.2600	0.6260
NM_001107773	Stk35	0.0413	0.8453	0.5606	0.1666	0.2459	0.3167	0.7601	0.0291	0.4034	0.7429	0.9275	0.2616
NM_001107252	Nkiras1	0.2098	0.1224	0.4048	0.2209	0.0487	0.2218	0.1522	0.2447	0.0354	0.0035	0.1760	0.1970
NM_001107216	Arap2	0.4245	0.5841	0.5422	0.7079	0.0278	0.4231	0.4851	0.1617	0.1524	0.3299	0.1315	0.0504
NM_001107165	Rtbdn	0.2414	0.3567	0.3156	0.5731	0.7073	0.5909	0.1563	0.0659	0.2735	0.3816	0.1195	0.5740
NM_001108002	Fdx1l	0.1149	0.7436	0.0975	0.6765	0.0551	0.0866	0.0929	0.3033	0.2117	0.3414	0.0853	0.1606
NM_001108061	Amn	0.3770	0.4597	0.2220	0.7919	0.4482	0.6309	0.8235	0.4773	0.1798	0.3768	0.7863	0.4875
NM_001108057	Bcl11b	0.2072	0.7815	0.4697	0.5703	0.3104	0.5756	0.2694	0.7763	0.5242	0.6472	0.6607	0.7366
NM_001107883	Il17ra	0.3657	0.3459	0.0128	0.0634	0.3017	0.5752	0.2283	0.5096	0.1220	0.2063	0.0717	0.8918
NM_001107685	RGD1560010	0.6442	0.2170	0.8427	0.9081	0.8302	0.7623	0.9049	0.8140	0.1573	0.3787	0.7911	0.9255
NM_001107684	Rapgef2	0.9693	0.9980	0.8810	0.9796	0.9110	0.9756	0.9866	0.9715	0.9976	0.9622	0.9867	0.9922
NM_001107208	41,163.0000	0.9020	0.9530	0.8103	0.9889	0.9496	0.8955	0.9566	0.8363	0.7865	0.9388	0.9527	0.9668
NM_001100550	Slu7	0.2659	0.3275	0.1290	0.0222	0.1536	0.4220	0.0906	0.1934	0.0317	0.1266	0.3235	0.8832
NM_001111114	Grik1	0.9337	0.7699	0.8844	0.6345	0.6973	0.6754	0.7080	0.5252	0.4387	0.8605	0.7533	0.5539
NM_001107254	Samd4a	0.2015	0.5237	0.0705	0.0920	0.2310	0.1733	0.5088	0.0695	0.2728	0.6657	0.0369	0.4580
NM_001107484	Dmpk	0.1102	0.0007	0.0325	0.0761	0.4722	0.6771	0.0270	0.2025	0.0217	0.0136	0.0012	0.1375
NM_001107475	Suv420h2	0.7956	0.8924	0.1209	0.0249	0.8510	0.9471	0.2725	0.7671	0.9924	0.9035	0.8529	0.9369
NM_001107038	Stxbp4	0.7902	0.8900	0.8130	0.8212	0.7693	0.9478	0.8305	0.6720	0.7490	0.9095	0.6566	0.8747
NM_001107204	Lrrc8b	0.6863	0.7559	0.8225	0.3506	0.6223	0.9084	0.3907	0.3268	0.7276	0.5138	0.7638	0.7864
NM_001107190	Rabgap1l	0.1814	0.3259	0.5469	0.6886	0.2691	0.5099	0.3658	0.4160	0.2629	0.3476	0.4180	0.2696
NM_001107043	Tbx21	0.6272	0.7532	0.7504	0.7379	0.4420	0.6405	0.7482	0.3827	0.1938	0.4790	0.2022	0.5421
NM_001107151	Galnt9	0.4175	0.7148	0.5091	0.2418	0.7622	0.5131	0.7109	0.3144	0.5923	0.2054	0.5240	0.6466
NM_001107845	Pion	0.3943	0.6699	0.5033	0.6707	0.3947	0.4372	0.0627	0.6293	0.3457	0.3851	0.3352	0.3373
NM_001106942	Cdx4	0.7426	0.7921	0.0548	0.5854	0.5726	0.1276	0.1195	0.1142	0.4820	0.1621	0.3828	0.1471
NM_001107041	Hoxb13	0.4082	0.1750	0.5235	0.5200	0.1821	0.1726	0.3862	0.6235	0.4084	0.2906	0.6166	0.4111
NM_001106941	Magee2	0.7086	0.5791	0.6016	0.3674	0.6567	0.1883	0.8129	0.8364	0.4493	0.6076	0.1717	0.1982
NM_001107703	Bcl9	0.7891	0.6128	0.9362	0.9457	0.7014	0.7835	0.9307	0.4848	0.8048	0.4026	0.9277	0.8758
NM_001107693	Pogz	0.9919	0.9369	0.9029	0.9711	0.8947	0.9814	0.9580	0.9526	0.5174	0.6638	0.9561	0.9896
NM_001107694	Rfx5	0.9787	0.7213	0.9010	0.9688	0.9049	0.8487	0.7157	0.9416	0.9268	0.9689	0.9395	0.8950
NM_001107828	Gpr107	0.2516	0.2879	0.4012	0.0976	0.1208	0.2327	0.3624	0.2645	0.6036	0.1204	0.3539	0.3515
NM_001100581	RGD1310371	0.6383	0.9786	0.4986	0.6820	0.8481	0.2489	0.8952	0.9358	0.5040	0.3424	0.8726	0.8446
NM_001107039	Sgca	0.9252	0.9051	0.9203	0.7908	0.9340	0.9945	0.7959	0.8463	0.8305	0.8341	0.1357	0.8272
NM_001106939	RGD1560455	0.5621	0.6444	0.8245	0.9299	0.9234	0.7368	0.4752	0.9174	0.3181	0.7316	0.5427	0.9115
NM_001107704	Chd1l	0.3371	0.2652	0.3958	0.8481	0.2071	0.0389	0.6725	0.8845	0.5190	0.6533	0.3339	0.1889
NM_001107850	Rbm28	0.8705	0.9372	0.5367	0.5475	0.8069	0.9013	0.7455	0.9369	0.9081	0.6549	0.6817	0.8577
NM_001107375	Pex19	0.8978	0.8383	0.7705	0.9049	0.9698	0.9926	0.8476	0.9656	0.9567	0.8996	0.9288	0.8943
NM_001107214	Slain2	0.9726	0.8265	0.9673	0.8218	0.7811	0.9759	0.9842	0.9014	0.7445	0.9446	0.9362	0.7275
NM_001106944	Gdpd2	0.8687	0.0240	0.8672	0.8078	0.8872	0.9241	0.0628	0.8016	0.1065	0.0145	0.0818	0.8159
NM_001107156	Sart3	0.6021	0.8210	0.2858	0.0626	0.3001	0.8947	0.0645	0.6311	0.8075	0.9245	0.3779	0.3289
NM_001107707	Slc22a15	0.6618	0.7478	0.6906	0.6441	0.7266	0.7541	0.8380	0.5186	0.7273	0.0726	0.1825	0.2069
NM_001107855	Adck2	0.4159	0.4600	0.1726	0.7729	0.5205	0.4518	0.7718	0.8216	0.8156	0.5248	0.6780	0.1495
NM_001107253	Kctd6	0.9225	0.9962	0.8389	0.9879	0.9333	0.9189	0.8233	0.9658	0.7662	0.9506	0.9279	0.2730
NM_001108017	Cmpk2	0.8150	0.1988	0.5882	0.4170	0.5562	0.8962	0.1405	0.6703	0.3874	0.4488	0.0453	0.8050
NM_001107542	Usp47	0.9888	0.6786	0.8867	0.9665	0.9671	0.9317	0.8030	0.9177	0.9328	0.8491	0.9459	0.8577
NM_001107215	Guf1	0.5053	0.8755	0.9158	0.9657	0.6816	0.9154	0.6142	0.1737	0.5070	0.5755	0.9168	0.8015
NM_001012181	Elf2	0.9791	0.6134	0.8808	0.9481	0.9881	0.8390	0.9326	0.9765	0.8699	0.8078	0.7524	0.5549

NM_001033909	Elf2	0.9791	0.6134	0.8808	0.9481	0.9881	0.8390	0.9326	0.9765	0.8699	0.8078	0.7524	0.5549
NM_001106952	Efhc2	0.2254	0.4272	0.1605	0.6358	0.5742	0.1551	0.7784	0.7998	0.7334	0.9135	0.8369	0.4832
NM_001107052	Arf4l	0.7347	0.3188	0.2901	0.3181	0.1752	0.0198	0.8488	0.3221	0.4607	0.5887	0.3522	0.0935
NM_001107180	Ipo9	0.2553	0.9125	0.6000	0.6044	0.6848	0.4366	0.4940	0.6006	0.5886	0.7624	0.5053	0.3973
NM_001107048	RGD1564778	0.9724	0.9777	0.4956	0.7041	0.9897	0.9174	0.8783	0.9444	0.9413	0.9455	0.9655	0.6864
NM_001106984	Prss22	0.8393	0.5928	0.4608	0.5027	0.3939	0.6289	0.7947	0.5220	0.7895	0.5257	0.8117	0.7106
NM_001109024	Dnajc30	0.4906	0.6094	0.7802	0.7488	0.4595	0.1413	0.5618	0.5876	0.6189	0.3876	0.1985	0.7689
NM_001106959	RGD1563947	0.4592	0.3501	0.6383	0.2272	0.2288	0.4266	0.6445	0.5061	0.0435	0.4125	0.0928	0.2177
NM_001107092	Iqcb1	0.7352	0.9398	0.8873	0.2561	0.4981	0.9144	0.2383	0.6456	0.8612	0.5584	0.9304	0.7715
NM_001107061	Smurf2	0.3025	0.5618	0.6139	0.2993	0.4051	0.7148	0.4984	0.6527	0.4210	0.7162	0.7337	0.3417
NM_001106973	Pmm2	0.9817	0.9065	0.2409	0.1690	0.9823	0.9867	0.6230	0.9937	0.9741	0.9931	0.7000	0.9923
NM_001107063	Cdc42ep4	0.9103	0.9488	0.8707	0.8875	0.7874	0.8551	0.9266	0.9515	0.9980	0.9860	0.7339	0.8630
NM_001107861	Kbtbd2	0.4647	0.3108	0.9308	0.7913	0.7936	0.9840	0.2910	0.3218	0.4415	0.3648	0.3904	0.9834
NM_001107186	Abl2	0.1928	0.4387	0.7841	0.7866	0.7876	0.3117	0.4511	0.7646	0.7912	0.5963	0.6119	0.8081
NM_001107181	Pkp1	0.9667	0.9544	0.9397	0.9304	0.9682	0.8623	0.9972	0.9955	0.9868	0.9154	0.6594	0.9588
NM_001107120	Daglb	0.6581	0.1697	0.0908	0.4015	0.3040	0.3800	0.3232	0.1653	0.9513	0.7610	0.3921	0.8396
NM_001107073	Sirt7	0.4258	0.4453	0.0587	0.2932	0.2900	0.0252	0.1323	0.2069	0.8999	0.2648	0.0438	0.5173
NM_001107065	Caskin2	0.1880	0.7887	0.1698	0.4041	0.6596	0.4825	0.5564	0.3601	0.8439	0.2909	0.3995	0.4797
NM_001107189	Tnn	0.9457	0.5358	0.9792	0.9566	0.9918	0.9995		0.9987	0.8168	0.7753	0.8877	0.4515
NM_001107192	Klhl20	0.3963	0.8651	0.9563	0.3649	0.8984	0.8168	0.3643	0.8272	0.8090	0.5528	0.6249	0.9936
NM_001107736	Zfp385b	0.8277	0.5822	0.8149	0.5255	0.8608	0.5327	0.5640	0.7928	0.7720	0.7441	0.2186	0.8049
NM_001107735	Osbpl6	0.9596	0.6120	0.9122	0.1941	0.7686	0.1902	0.9336	0.5843	0.9705	0.8581	0.7476	0.6686
NM_001107733	Cobll1	0.0584	0.0404	0.1301	0.0729	0.0434	0.0432	0.0380	0.2384	0.0592	0.0606	0.3184	0.6912
NM_001107742	Slc43a1	0.8420	0.7448	0.6710	0.7104	0.8978	0.6273	0.9829	0.8062	0.7648	0.8471	0.9300	0.4366
NM_001107218	Gpr125	0.7772	0.6634	0.7494	0.0424	0.5875	0.6784	0.4581	0.6093	0.1135	0.3127	0.3362	0.0210
NM_001107207	Prdm8	0.3665	0.7589	0.7627	0.7521	0.5096	0.1593	0.4224	0.7054	0.9016	0.5677	0.7787	0.7645
NM_001107075	Foxk2	0.5994	0.5662	0.3827	0.9571	0.4519	0.8693	0.8004	0.3424	0.6834	0.8855	0.8695	0.6964
NM_001107771	Anapc1	0.9860	0.9916	0.9596	0.9682	0.9562	0.8116	0.9785	0.8385	0.9990	0.9073	0.9985	0.7653
NM_001107259	Peli2	0.4542	0.0198	0.9107	0.8067	0.8290	0.8783	0.9097	0.9381	0.9551	0.1130	0.2638	0.4060
NM_001106978	Glis2	0.8248	0.4549	0.3936	0.3054	0.5563	0.9827	0.3032	0.7623	0.3704	0.2744	0.4100	0.8802
NM_001107199	Kctd3	0.8106	0.8686	0.8747	0.6842	0.8197	0.9325	0.9861	0.9674	0.8129	0.9772	0.9493	0.8688
NM_001107903	Tmem68	0.6328	0.2250	0.5497	0.8436	0.5021	0.9552	0.2247	0.1350	0.5937	0.4081	0.3506	0.9690
NM_001107885	Bcl2l13	0.2949	0.7507	0.8015	0.7052	0.1521	0.0942	0.8598	0.5469	0.8968	0.1243	0.4794	0.5475
NM_001107222	Fbxl5	0.1545	0.2281	0.1157	0.2945	0.0027	0.0364	0.0924	0.0092	0.0227	0.1051	0.1978	0.1484
NM_001107059	Ddx42	0.5181	0.3589	0.9649	0.7649	0.8391	0.5351	0.8030	0.8482	0.3342	0.0419	0.2331	0.7049
NM_001107226	Sh3tc1	0.6894	0.0707	0.1619	0.3451	0.5847	0.5872	0.3390	0.2068	0.1190	0.4698	0.5811	0.5904
NM_001106970	Tmem32	0.4486	0.5185	0.7932	0.3247	0.8068	0.7865	0.6291	0.3939	0.8361	0.6666	0.8341	0.7108
NM_001134575	RGD1308106	0.6672	0.8747	0.8009	0.8278	0.9191	0.4088	0.8309	0.2866	0.4453	0.8399	0.5230	0.3203
NM_001107769	Slc24a5	0.4106	0.5216	0.2228	0.3696	0.4898	0.4433	0.3703	0.2687	0.2222	0.6863	0.5303	0.4798
NM_001107768	Sema6d	0.3909	0.5931	0.8634	0.5589	0.6833	0.3617	0.5691	0.2796	0.7735	0.3535	0.7264	0.9049
NM_001107940	Megf9	0.8751	0.5816	0.9166	0.8915	0.9184	0.8782	0.9026	0.5304	0.5213	0.5397	0.6813	0.9057
NM_001107882	40,976.0000	0.4198	0.6343	0.5503	0.9500	0.5418	0.9059	0.8462	0.7710	0.8235	0.5455	0.1326	0.3761
NM_001107879	Rybp	0.9567	0.9930	0.9984	0.9882	0.6050	0.7984	0.9891	0.8301	0.9452	0.9960	0.9911	0.9980
NM_001107330	Irx4	0.7425	0.6310	0.6542	0.4317	0.1814	0.2772	0.8175	0.1979	0.8218	0.8449	0.4400	0.7392
NM_001107263	Ngdn	0.1362	0.7824	0.1238	0.0362	0.0329	0.1118	0.0409	0.0100	0.0286	0.1366	0.2358	0.0596
NM_001106977	Ubn1	0.9650	0.6330	0.2467	0.5106	0.9204	0.9268	0.2951	0.8412	0.8628	0.6911	0.6884	0.7703
NM_001107329	Zfp828	0.3590	0.9321	0.4200	0.1950	0.7114	0.9514	0.6720	0.8227	0.2018	0.7066	0.5150	0.5325
NM_001107327	Abhd13	0.5808	0.2730	0.3238	0.4244	0.3766	0.5815	0.5384	0.3628	0.6890	0.2956	0.6184	0.5249
NM_001107326	Kbtbd11	0.2517	0.5121	0.8148	0.7020	0.3867	0.7355	0.4368	0.5493	0.3455	0.1864	0.4716	0.4818

NM_001107913	Zbtb8a	0.5599	0.5604	0.5188	0.6689	0.5777	0.5317	0.7204	0.5011	0.6655	0.3486	0.2648	0.2562
NM_001106992	Cpeb4	0.1404	0.1484	0.2570	0.0473	0.0624	0.1326	0.0096	0.0117	0.0265	0.2866	0.1507	0.3902
NM_001106991	Tmem8	0.4816	0.6433	0.7339	0.9437	0.6669	0.7676	0.7252	0.6543	0.6185	0.6545	0.8406	0.6499
NM_001107804	Mocs3	0.8451	0.2021	0.1489	0.7723	0.7048	0.5655	0.3798	0.4302	0.6097	0.4501	0.2817	0.4001
NM_001107983	Zmym1	0.8939	0.8306	0.8358	0.8224	0.7709	0.6639	0.8288	0.6725	0.7131	0.8869	0.8427	0.7455
NM_001107947	Kank4	0.7080	0.9724	0.7224	0.8387	0.7917	0.6785	0.9763	0.7699	0.9547	0.7259	0.7907	0.7426
NM_001108047	Foxn3	0.7215	0.2492	0.3356	0.8689	0.7240	0.6198	0.2997	0.5906	0.6399	0.4160	0.4868	0.8172
NM_001107249	Comtd1	0.3581	0.8086	0.7955	0.8243	0.3059	0.3290	0.3280	0.1850	0.2688	0.3332	0.8599	0.7747
NM_001004200	Cops3	0.8397	0.8851	0.2026	0.4673	0.5089	0.0879	0.4459	0.1502	0.3366	0.6089	0.9722	0.3794
NM_001004132	Pctk1	0.7290	0.8855	0.7128	0.9315	0.6123	0.9024	0.4340	0.5940	0.9395	0.8963	0.7865	0.9375
NM_001107106	Brwd1	0.7807	0.9130	0.9106	0.0422	0.7629	0.8425	0.1905	0.7154	0.5676	0.9775	0.8731	0.6049
NM_001108054	Serpina1f	0.6067	0.3891	0.4603	0.4044	0.5437	0.4838	0.4506	0.5437	0.5588	0.4993	0.7573	0.4704
NM_001106997	Maml1	0.8378	0.9053	0.9659	0.9635	0.6406	0.8927	0.9315	0.8114	0.9318	0.9325	0.7047	0.9748
NM_001107427	Chst4	0.9063	0.9288	0.1857	0.8982	0.7997	0.5004	0.6454	0.7365	0.6637	0.5391	0.8468	0.6100
NM_001107265	Cenpj	0.2043	0.7630	0.1817	0.0369	0.5169	0.8223	0.1033	0.4650	0.5682	0.8077	0.6949	0.7493
NM_001107098	Gpr128	0.7203	0.8080	0.8067	0.7937	0.1745	0.6351	0.7816	0.4942	0.6418	0.6039	0.6762	0.7417
NM_001107821	Agpat2	0.1947	0.3052	0.0343	0.8674	0.1818	0.1422	0.7729	0.5674	0.7259	0.2258	0.6651	0.7175
NM_001107951	Dmrta2	0.8180	0.5330	0.2912	0.3742	0.5200	0.7786	0.5871	0.4140	0.8117	0.3495	0.7670	0.3067
NM_001107818	Ndor1	0.4512	0.8986	0.2967	0.8515	0.8645	0.9146	0.6623	0.8380	0.6899	0.9580	0.9573	0.2566
NM_001107831	Uck1	0.2818	0.5173	0.4907	0.5057	0.5641	0.5433	0.5539	0.6357	0.1911	0.6566	0.5970	0.4388
NM_001107830	Lamc3	0.4256	0.4924	0.3114	0.1238	0.4991	0.4975	0.1195	0.3946	0.5391	0.4237	0.3171	0.7061
NM_001107391	Jakmip2	0.1979	0.1650	0.5345	0.7569	0.4481	0.1974	0.0179	0.5691	0.4718	0.2870	0.4753	0.1588
NM_001108022	RGD1304624	0.2754	0.7926	0.3909	0.6654	0.8554	0.8639	0.2718	0.8943	0.8526	0.7742	0.7433	0.8169
NM_001107840	Zbtb26	0.8511	0.8444	0.8399	0.9206	0.8544	0.9845	0.8964	0.7128	0.9540	0.9863	0.9689	0.8773
NM_001108068	Sbno2	0.9245	0.1928	0.0457	0.0047	0.7535	0.7523	0.0550	0.9144	0.8578	0.0360	0.1311	0.9762
NM_001108088	Epyc	0.8892	0.4069	0.9384	0.7741	0.2084	0.5391	0.9123	0.2197	0.2377	0.5834	0.9122	0.3418
NM_001107434	Gan	0.7272	0.7846	0.4117	0.8432	0.8471	0.1989	0.8569	0.2518	0.7395	0.4676	0.5580	0.3014
NM_001107128	Zfp68	0.5896	0.2345	0.5462	0.2738	0.2939	0.3905	0.6051	0.1938	0.3049	0.6698	0.4099	0.2362
NM_001107457	Kptn	0.7953	0.3703	0.2408	0.6314	0.6923	0.4194	0.3707	0.5579	0.7641	0.6339	0.7377	0.8249
NM_001107363	Pitrm1	0.7540	0.8182	0.0726	0.6042	0.7699	0.7827	0.6431	0.8141	0.7454	0.7699	0.9833	0.1040
NM_001107853	Luc7l2	0.9697	0.9222	0.9606	0.3130	0.9800	0.9672	0.4174	0.9930	0.9800	0.9769	0.9496	0.8046
NM_001107287	Farp1	0.8348	0.8413	0.3413	0.6812	0.8685	0.5855	0.7420	0.6257	0.8312	0.5605	0.8722	0.5895
NM_001108118	Itga5	0.1944	0.1936	0.0895	0.0662	0.2596	0.1448	0.1882	0.2388	0.2728	0.2745	0.0879	0.4005
NM_001108113	Csrnp2	0.8568	0.8770	0.9767	0.8208	0.9269	0.9603	0.9522	0.8861	0.9876	0.6024	0.8591	0.9846
NM_001107860	RGD1559747	0.5465	0.5439	0.5808	0.6404	0.6308	0.6369	0.6205	0.6693	0.6923	0.4048	0.6618	0.6118
NM_001107854	Slc37a3	0.7246	0.7336	0.7730	0.7624	0.4157	0.8710	0.8508	0.4495	0.1909	0.3913	0.9085	0.7946
NM_001005244	Dmd	0.4951	0.5137	0.5400	0.6191	0.4648	0.3607	0.3657	0.6325	0.1998	0.5577	0.5026	0.3961
NM_001107506	Tshz3	0.9758	0.9759	0.9986	0.9835	0.9967	0.9488	0.9932	0.9922	0.9943	0.9176	0.9489	0.9897
NM_001107297	Arhgap22	0.8423	0.8620	0.1595	0.9053	0.7466	0.7867	0.5452	0.9097	0.4605	0.7462	0.7053	0.2735
NM_001107162	RGD1304881	0.1415	0.7658	0.1035	0.0869	0.7019	0.8140	0.0413	0.9139	0.7671	0.9203	0.8560	0.7864
NM_001107161	RGD1310262	0.5706	0.7769	0.3435	0.7663	0.4514	0.1270	0.6360	0.2694	0.8386	0.8696	0.2863	0.8431
NM_001107158	Baz2a	0.6187	0.1005	0.4797	0.3587	0.6512	0.8485	0.5044	0.7990	0.6842	0.7725	0.2773	0.6275
NM_001107292	Bap1	0.9145	0.9038	0.6121	0.8257	0.7269	0.9725	0.7686	0.8913	0.6915	0.8831	0.5002	0.9123
NM_001107320	Prosc	0.0067	0.1160	0.0212	0.3395	0.1084	0.2725	0.1373	0.0740	0.0369	0.2606	0.1673	0.0704
NM_001108159	Dpp8	0.7400	0.9650	0.6780	0.9933	0.6472	0.4498	0.5599	0.5633	0.4356	0.5624	0.9146	0.2664
NM_001107871	RGD1566130	0.4065	0.5780	0.2671	0.3016	0.6552	0.4004	0.8158	0.8149	0.8619	0.3684	0.6035	0.6009
NM_001107870	Smyd5	0.4064	0.5141	0.6483	0.3561	0.4049	0.6590	0.7454	0.7456	0.6560	0.4897	0.7954	0.8209
NM_001107888	Emg1	0.4712	0.9607	0.8147	0.9602	0.7119	0.8696	0.8092	0.9461	0.9709	0.7673	0.7991	0.4703
NM_001107872	RGD1306746	0.9827	0.9780	0.2108	0.9320	0.9166	0.9722	0.7294	0.7652	0.9587	0.9285	0.8602	0.7314

NM_001107380	Znf474	0.1517	0.0685	0.3880	0.9042	0.4407	0.2319	0.6592	0.6878	0.0446	0.2151	0.3415	0.1857
NM_001107348	Rreb1	0.6734	0.8733	0.8897	0.8311	0.9644	0.7996	0.8873	0.9069	0.8358	0.9851	0.9900	0.9759
NM_001107203	Fbxo28	0.3496	0.1395	0.3491	0.1464	0.5415	0.3457	0.3631	0.1531	0.7687	0.2494	0.5356	0.4052
NM_001107923	Rngtt	0.8567	0.9064	0.9069	0.9616	0.7930	0.6390	0.8203	0.4058	0.5854	0.1322	0.8823	0.6334
NM_001107891	Ppp2r5a	0.2739	0.7652	0.2976	0.0997	0.8149	0.8551	0.3818	0.5379	0.7970	0.8736	0.5278	0.4839
NM_001107890	Tspan9	0.8863	0.9575	0.9276	0.9892	0.4295	0.9159	0.9322	0.3762	0.8803	0.9459	0.8562	0.9599
NM_001108190	Rtp3	0.3166	0.2343	0.4840	0.2656	0.1000	0.0407	0.1189	0.2727	0.0262	0.4791	0.2606	0.3795
NM_001108067	Hmha1	0.1187	0.7607	0.3888	0.0704	0.1749	0.0627	0.7675	0.2121	0.3325	0.3239	0.0355	0.3429
NM_001108193	Slc22a14	0.5062	0.8071	0.7916	0.6954	0.7808	0.7847	0.6733	0.5753	0.8437	0.2130	0.4539	0.7504
NM_001107640	Ascc3	0.5724	0.6072	0.7084	0.5280	0.2087	0.5225	0.6593	0.3956	0.4751	0.1868	0.5467	0.2145
NM_001107361	Larp5	0.9351	0.8187	0.9627	0.6686	0.8784	0.9590	0.9296	0.8792	0.9471	0.8991	0.9169	0.9986
NM_001107509	Klk8	0.7378	0.4714	0.2004	0.0388	0.2650	0.6954	0.4120	0.4229	0.4422	0.2789	0.8072	0.1315
NM_001107496	Dyrk1b	0.8711	0.2697	0.6131	0.8310	0.7513	0.9060	0.9019	0.2107	0.9621	0.6491	0.3533	0.7082
NM_001107364	Sfmbt2	0.7334	0.7400	0.8177	0.8260	0.8562	0.6525	0.9401	0.3197	0.7689	0.6269	0.8791	0.5460
NM_001107206	Aff1	0.2874	0.4094	0.5982	0.4754	0.4549	0.3625	0.3123	0.6332	0.4603	0.1179	0.2566	0.3763
NM_001107217	Med28	0.2721	0.8078	0.8791	0.3439	0.1735	0.1297	0.0742	0.0195	0.4326	0.6728	0.9293	0.1344
NM_001107971	Foxj3	0.3844	0.5066	0.5173	0.5709	0.5628	0.5578	0.5017	0.8915	0.3896	0.7983	0.5111	0.8746
NM_001108006	Vps13d	0.7723	0.2957	0.1776	0.5182	0.7764	0.7203	0.5481	0.3338	0.2379	0.5987	0.3621	0.4461
NM_001108164	Cgnl1	0.6094	0.7918	0.4945	0.2320	0.7017	0.0833	0.8969	0.8526	0.5810	0.9140	0.4720	0.7185
NM_001108087	Kera	0.7947	0.7455	0.4378	0.7612	0.2188	0.5438	0.8038	0.3854	0.7781	0.7554	0.7454	0.4288
NM_001107980	Rspo1	0.8253	0.8586	0.5659	0.8579	0.7298	0.7805	0.6355	0.5065	0.7669	0.8849	0.3431	0.2362
NM_001107546	Dennd5a	0.0247	0.1537	0.0489	0.0438	0.0470	0.0804	0.0346	0.1338	0.0684	0.1470	0.1274	0.0507
NM_001107898	Slco5a1	0.2910	0.0914	0.0484	0.1516	0.2941	0.7132	0.2112	0.2894	0.1895	0.1165	0.2367	0.4074
NM_001107428	Znf23	0.1506	0.6931	0.8833	0.7455	0.9741	0.6812	0.8023	0.9355	0.9219	0.9798	0.9326	0.9904
NM_001107401	Nol4	0.6971	0.8489	0.7929	0.9187	0.6644	0.4595	0.7616	0.8416	0.8050	0.8772	0.7321	0.6247
NM_001107242	Pex13	0.3897	0.2665	0.8751	0.6361	0.4919	0.4644	0.4073	0.1708	0.0024	0.2512	0.2304	0.6654
NM_001108001	RGD1304931	0.4747	0.5319	0.4273	0.6327	0.6364	0.7191	0.5121	0.4415	0.3430	0.5899	0.2193	0.6441
NM_001108229	Slc16a14	0.8629	0.5872	0.4787	0.7889	0.9737	0.6018	0.1986	0.9806	0.2862	0.5953	0.8421	0.6752
NM_001107937	Frmpd1	0.7076	0.8999	0.9239	0.6678	0.8124	0.8164	0.8552	0.9056	0.9238	0.8682	0.8163	0.5144
NM_001107938	Polr1e	0.7960	0.7687	0.1913	0.8161	0.8534	0.7879	0.6041	0.6905	0.8161	0.9262	0.6662	0.8052
NM_001107072	Fscn2	0.0462	0.3387	0.2508	0.4214	0.3420	0.1366	0.7467	0.0297	0.7571	0.0811	0.7675	0.1096
NM_001107076	Rab40b	0.4472	0.6142	0.9830	0.9682	0.4567	0.7185	0.9680	0.5560	0.4798	0.4815	0.9158	0.9044
NM_001106976	Ppl	0.6595	0.0775	0.6859	0.3252	0.1249	0.4879	0.5516	0.5015	0.1452	0.2689	0.0791	0.4242
NM_001107764	Pla2g4b	0.6325	0.8942	0.1283	0.0806	0.3378	0.5580	0.2237	0.5743	0.8019	0.1329	0.7188	0.8341
NM_001107760	Dll4	0.5671	0.5275	0.5763	0.5062	0.6095	0.6637	0.4773	0.5377	0.3742	0.5356	0.3708	0.3577
NM_001107758	Meis2	0.4946	0.2481	0.9549	0.8336	0.4471	0.6082	0.6420	0.5670	0.0962	0.5766	0.3187	0.2166
NM_001107757	Aven	0.6086	0.9433	0.1646	0.8285	0.8856	0.9523	0.7896	0.7764	0.7899	0.9174	0.8414	0.2821
NM_001107941	Wdr40b	0.7694	0.4027	0.6322	0.3432	0.5596	0.6311	0.6271	0.4221	0.4678	0.3087	0.5532	0.6743
NM_001107956	Car9	0.1304	0.1421	0.8813	0.5826	0.7937	0.7933	0.9821	0.4590	0.0044	0.0505	0.0192	0.8137
NM_001107790	Tpx2	0.9271	0.8354	0.0166	0.1775	0.8634	0.9673	0.8434	0.9244	0.8274	0.9829	0.7771	0.1668
NM_001107091	Sema5b	0.6920	0.7626	0.5620	0.6519	0.0959	0.2253	0.7288	0.3944	0.7417	0.6679	0.6966	0.3470
NM_001107220	Anapc4	0.8478	0.9345	0.2472	0.1090	0.7503	0.8149	0.0678	0.7971	0.7310	0.8307	0.8028	0.8017
NM_001107286	Tgds	0.8352	0.4568	0.8700	0.7271	0.8628	0.8157	0.9024	0.7401	0.4194	0.3476	0.6907	0.7201
NM_001107235	Sf3a1	0.9267	0.9524	0.2278	0.3604	0.3704	0.3392	0.5214	0.9306	0.9391	0.7248	0.8111	0.2731
NM_001107899	Depdc2	0.0159	0.6401	0.0504	0.6231	0.1937	0.1272	0.0516	0.1435	0.4208	0.7196	0.4278	0.5755
NM_001108069	Stk11	0.2716	0.5299	0.9630	0.1751	0.2770	0.6327	0.7586	0.5613	0.3605	0.5384	0.6885	0.1675
NM_001107231	Zfp278	0.9428	0.9421	0.9670	0.9319	0.9325	0.9791	0.9027	0.8836	0.9795	0.9928	0.9917	0.6640
NM_001107255	Wdhd1	0.2757	0.1882	0.0097	0.1100	0.4094	0.7341	0.1453	0.2132	0.2127	0.0826	0.1394	0.1683
NM_001107911	Ppp1r8	0.2729	0.2048	0.7897	0.8918	0.2606	0.9324	0.2370	0.5751	0.9612	0.4029	0.2955	0.8548

NM_001107957	Ccdc107	0.2421	0.7223	0.7229	0.5859	0.2164	0.4002	0.4822	0.2668	0.4030	0.2322	0.5492	0.4412
NM_001107795	RGD1305020	0.3515	0.2375	0.1936	0.0501	0.2578	0.4858	0.2722	0.0307	0.3424	0.2881	0.2076	0.3928
NM_001107260	Slc39a2	0.5276	0.5712	0.3040	0.7544	0.6935	0.6189	0.7096	0.6035	0.1493	0.7591	0.1264	0.5622
NM_001107811	Gpr155	0.7558	0.6881	0.7267	0.8816	0.6246	0.7963	0.6466	0.8085	0.7135	0.4952	0.6275	0.5615
NM_001107919	Fbxl4	0.4272	0.4542	0.9744	0.8938	0.7519	0.6853	0.3391	0.3476	0.6196	0.4047	0.7185	0.8988
NM_001107160	Asf1b	0.1123	0.9358	0.1396	0.3534	0.9132	0.6982	0.8690	0.9500	0.9820	0.7416	0.8086	0.2197
NM_001107099	Tbc1d23	0.9849	0.9836	0.9755	0.9342	0.9575	0.6957	0.9756	0.9885	0.9686	0.9991	0.9991	0.9151
NM_001106998	Phf15	0.7828	0.7214	0.7760	0.7358	0.6062	0.8341	0.5552	0.7656	0.6863	0.8960	0.8380	0.2885
NM_001107829	Fibcd1	0.8369	0.5806	0.7041	0.8550	0.9341	0.8962	0.9526	0.8711	0.8308	0.9100	0.3191	0.8497
NM_001107825	Ntng2	0.3475	0.2197	0.6564	0.3936	0.4360	0.3723	0.4795	0.5943	0.6967	0.0875	0.0999	0.1571
NM_001108019	Ankmy2	0.8930	0.6908	0.9774	0.9529	0.4736	0.9122	0.6678	0.7309	0.2797	0.8916	0.1181	0.9057
NM_001107268	Mtmr6	0.5574	0.3566	0.9935	0.9754	0.8441	0.9249	0.8421	0.3218	0.4354	0.2523	0.7348	0.9406
NM_001107118	Rnf6	0.3771	0.3232	0.7616	0.5804	0.3172	0.1714	0.3070	0.2907	0.5437	0.8532	0.3293	0.2650
NM_001107274	Dock5	0.7388	0.7046	0.7608	0.6001	0.6202	0.5536	0.8959	0.8321	0.9183	0.9561	0.9278	0.7642
NM_001107264	Nfatc4	0.6642	0.0545	0.1134	0.7656	0.5413	0.9080	0.0806	0.4038	0.0977	0.2068	0.0634	0.0458
NM_001107121	Zfp316	0.6439	0.6462	0.6361	0.2665	0.5469	0.5651	0.5557	0.6088	0.1421	0.3207	0.5127	0.2442
NM_001108031	RGD1305721	0.5689	0.7776	0.2953	0.9160	0.4913	0.1046	0.3655	0.4280	0.6517	0.5266	0.6479	0.3552
NM_001108341	Ulk1	0.8750	0.7958	0.8990	0.1103	0.8543	0.9783	0.9278	0.8540	0.9832	0.9367	0.7262	0.9309
NM_001107992	Ptchd2	0.3033	0.3658	0.1638	0.2145	0.4408	0.5917	0.4797	0.5731	0.4201	0.1046	0.4327	0.4657
NM_001108058	Spaca5	0.5080	0.6322	0.3840	0.5661	0.1218	0.2921	0.5335	0.0657	0.1581	0.3516	0.4810	0.2321
NM_001108056	Papola	0.9497	0.9507	0.9129	0.9758	0.9540	0.8394	0.8660	0.8624	0.7147	0.5604	0.9182	0.8982
NM_001107848	Ophn1	0.5525	0.3304	0.3931	0.8674	0.4594	0.5769	0.5064	0.4640	0.7297	0.2239	0.6167	0.6297
NM_001107143	Rbm19	0.4635	0.4929	0.5401	0.7679	0.8672	0.8940	0.6963	0.8734	0.7670	0.7671	0.5476	0.8029
NM_001107865	Tcf3	0.5226	0.5463	0.7926	0.8816	0.8519	0.1427	0.7042	0.8520	0.4145	0.3998	0.3796	0.7033
NM_001108136	Tmem136	0.2118	0.2407	0.2826	0.1883	0.1692	0.2408	0.6620	0.2904	0.2372	0.2124	0.3268	0.1388
NM_001107221	C1qtnf7	0.5100	0.7870	0.5822	0.7087	0.2217	0.6441	0.5013	0.3401	0.5734	0.7430	0.6380	0.1761
NM_001107168	Serpinb13	0.3539	0.5754	0.3409	0.4460	0.0799	0.0976	0.0354	0.5911	0.6952	0.4600	0.1136	0.2818
NM_001107167	Serpinb11	0.2290	0.5798	0.8784	0.7588	0.3833	0.1249	0.6635	0.4245	0.4192	0.6556	0.6842	0.5900
NM_001107169	Gli2	0.7829	0.9296	0.8507	0.9643	0.9647	0.8556	0.9656	0.9349	0.9033	0.6213	0.9202	0.5062
NM_001107183	Trove2	0.7192	0.9726	0.9190	0.9338	0.8734	0.5360	0.0778	0.8705	0.6503	0.9580	0.6264	0.9621
NM_001107182	Crb1	0.0690	0.0773	0.4637	0.2443	0.3869	0.1632	0.0984	0.2701	0.4949	0.2358	0.4001	0.2372
NM_001107378	Znf608	0.4835	0.9625	0.9198	0.9413	0.7855	0.9349	0.6286	0.7726	0.9769	0.9450	0.4623	0.9842
NM_001107343	Aof1	0.1242	0.5650	0.2447	0.6084	0.0354	0.7614	0.4826	0.0766	0.7762	0.5091	0.8605	0.5744
NM_001127504	Gas2	0.5786	0.3993	0.4613	0.5224	0.5360	0.5151	0.7682	0.8346	0.6750	0.8585	0.4894	0.7271
NM_001107877	Adamts9	0.9813	0.9609	0.9870	0.9947	0.9729	0.9596	0.9687	0.8834	0.9405	0.9911	0.9976	0.1846
NM_001107323	Tubgcp3	0.8488	0.9038	0.3210	0.8628	0.9580	0.9515	0.2719	0.8004	0.8979	0.9307	0.7358	0.6958
NM_001108938	Egflam	0.4691	0.2556	0.1152	0.2589	0.3258	0.1695	0.0224	0.2885	0.2811	0.5009	0.5893	0.1144
NM_001107188	Rasal2	0.9379	0.8835	0.4515	0.8881	0.5439	0.7798	0.1691	0.6513	0.7222	0.9133	0.4037	0.5705
NM_001107431	Chst5	0.4666	0.4447	0.1271	0.5568	0.3253	0.6607	0.8060	0.8819	0.2609	0.0410	0.0678	0.9509
NM_001108004	Dnajb5	0.8462	0.9500	0.4938	0.9318	0.4796	0.1506	0.9825	0.3584	0.9218	0.9009	0.5937	0.4846
NM_001107887	Cd163	0.3500	0.3978	0.5407	0.3592	0.3044	0.5786	0.4118	0.2324	0.6272	0.2886	0.2825	0.1053
NM_001107886	Phc1	0.9580	0.9118	0.6452	0.7492	0.9474	0.8826	0.8772	0.4478	0.9363	0.9380	0.7410	0.8946
NM_001107418	Usp38	0.8309	0.9101	0.5306	0.3562	0.8375	0.9635	0.7693	0.8478	0.7493	0.4225	0.6311	0.9486
NM_001107356	RGD1306613	0.4901	0.7568	0.7063	0.6816	0.4745	0.4890	0.1626	0.5728	0.4617	0.8507	0.6767	0.7191
NM_001107805	Nfatc2	0.3908	0.7656	0.4389	0.5185	0.3164	0.8013	0.7495	0.6743	0.6039	0.5204	0.5057	0.5632
NM_001107184	Dhx9	0.9349	0.9957	0.8426	0.9316	0.9093	0.9171	0.9642	0.7983	0.8084	0.8222	0.9660	0.0169
NM_001107974	Zmpste24	0.2184	0.0264	0.7624	0.9174	0.0539	0.7464	0.1428	0.4061	0.0284	0.3173	0.1294	0.8591
NM_001107276	Piwil2	0.8340	0.7725	0.8133	0.5932	0.8664	0.8516	0.4015	0.8802	0.3928	0.9088	0.3557	0.7478
NM_001107452	Pdcd6	0.7811	0.8017	0.9452	0.7714	0.7699	0.9642	0.2941	0.9468	0.8366	0.8536	0.4581	0.9300

NM_001107372	Kcng2	0.0950	0.2667	0.5357	0.0584	0.1142	0.1581	0.6501	0.0937	0.0995	0.5745	0.4084	0.9119
NM_001107480	Strn4	0.8802	0.8983	0.1534	0.5344	0.9538	0.8112	0.4828	0.9875	0.7887	0.9883	0.9378	0.8915
NM_001107332	Adamts16	0.5967	0.6957	0.7266	0.5124	0.5137	0.2816	0.9285	0.6837	0.2295	0.5400	0.8066	0.5876
NM_001134613	RGD1559884	0.0910	0.3261	0.1806	0.4270	0.5663	0.4960	0.3857	0.7484	0.5030	0.1848	0.2515	0.2929
NM_001107404	Cables1	0.5114	0.6446	0.7373	0.4796	0.4781	0.6901	0.7287	0.7841	0.8830	0.8727	0.1562	0.8292
NM_001108012	Ncoa1	0.9076	0.7496	0.7355	0.9463	0.9720	0.6316	0.4788	0.8188	0.4217	0.6396	0.9019	0.7649
NM_001107896	Ppfibp1	0.0223	0.0766	0.9199	0.4698	0.0092	0.6604	0.4742	0.0493	0.0093	0.0109	0.2629	0.7668
NM_001134612	Fam178a	0.4225	0.3611	0.7113	0.4151	0.4530	0.3165	0.7052	0.0245	0.3947	0.4887	0.3643	0.4949
NM_001107233	RGD1308775	0.5227	0.3461	0.3489	0.3749	0.3876	0.5615	0.3021	0.4077	0.4292	0.4006	0.3223	0.1779
NM_001107229	Depdc5	0.7593	0.7796	0.6986	0.5905	0.8884	0.8713	0.8412	0.7008	0.8728	0.1939	0.5518	0.8886
NM_001108008	Eml4	0.4755	0.4009	0.4467	0.3509	0.4080	0.7696	0.3476	0.8701	0.3589	0.3976	0.1148	0.5700
NM_001107933	Tbc1d2	0.1003	0.3101	0.1527	0.3309	0.1086	0.7400	0.0435	0.3145	0.2979	0.4553	0.3620	0.8741
NM_001107910	Eya3	0.5373	0.7423	0.5114	0.7608	0.7212	0.8565	0.1542	0.4497	0.4721	0.1086	0.4274	0.6678
NM_001107909	Map3k6	0.8258	0.8165	0.0840	0.5887	0.8666	0.6880	0.4858	0.8711	0.8397	0.8473	0.8689	0.7876
NM_001108180	Ky	0.1512	0.3509	0.3757	0.5503	0.4159	0.4100	0.3354	0.4667	0.3538	0.4262	0.4013	0.6973
NM_001107968	Znf691	0.7665	0.3902	0.4270	0.8625	0.2205	0.2735	0.4956	0.7794	0.2833	0.8882	0.3308	0.1962
NM_001108023	Fbxo33	0.3979	0.9391	0.8760	0.9774	0.2142	0.3068	0.3527	0.3776	0.5100	0.7193	0.8543	0.1673
NM_001108228	Slc19a3	0.4095	0.1411	0.0449	0.1237	0.0139	0.0553	0.1781	0.0502	0.1934	0.2196	0.0510	0.0164
NM_001107920	Map3k7	0.4556	0.9336	0.9405	0.9527	0.8988	0.9116	0.9795	0.8773	0.5166	0.7987	0.8389	0.7598
NM_001107918	Usp45	0.7213	0.4535	0.2968	0.3033	0.8316	0.8896	0.2857	0.5717	0.6968	0.4256	0.9743	0.7255
NM_001107917	Cpne3	0.2900	0.3312	0.8553	0.8251	0.0412	0.1319	0.4998	0.1360	0.3367	0.2522	0.0455	0.0569
NM_001107915	RGD1559904	0.9202	0.2729	0.7752	0.6940	0.5584	0.9304	0.0972	0.5852	0.9571	0.2978	0.2940	0.5286
NM_001107914	Bai2	0.0752	0.1892	0.6168	0.1867	0.1403	0.1399	0.6405	0.4354	0.7330	0.5643	0.1803	0.5864
NM_001107481	Pnmal2	0.4926	0.5349	0.6527	0.6222	0.5611	0.6936	0.7387	0.9287	0.6796	0.5840	0.1718	0.2404
NM_001107258	RGD1304610	0.7085	0.3238	0.5744	0.3661	0.5365	0.6142	0.7574	0.4500	0.0948	0.2578	0.5263	0.5414
NM_001108026	Prpf39	0.9993	0.9682	0.4540	0.1565	0.9878	0.8902	0.2334	0.8778	0.9544	0.9187	0.9122	0.9876
NM_001107429	Fuk	0.0530	0.4032	0.0177	0.0558	0.3172	0.0264	0.0442	0.0530	0.0730	0.4542	0.2232	0.0413
NM_001107262	Sall2	0.9200	0.8194	0.6506	0.9069	0.8946	0.8810	0.9715	0.7641	0.5806	0.5817	0.9025	0.9549
NM_001107261	Supt16h	0.4500	0.6928	0.4121	0.4928	0.6782	0.6594	0.5527	0.5985	0.3616	0.5433	0.5828	0.6413
NM_001108027	Klhdc1	0.1730	0.1365	0.4831	0.2303	0.1553	0.2373	0.0771	0.0911	0.4076	0.4445	0.8206	0.7219
NM_001107492	B3gnt8	0.4090	0.4255	0.3283	0.3195	0.2973	0.5448	0.2380	0.1403	0.1261	0.4730	0.0520	0.4136
NM_001107437	Jph3	0.4305	0.7200	0.7187	0.6051	0.6632	0.5021	0.2402	0.7166	0.7154	0.5330	0.3204	0.6582
NM_001108029	Arid4a	0.9278	0.3348	0.6797	0.9622	0.7058	0.9482	0.7929	0.9464	0.2499	0.5388	0.2891	0.7923
NM_001108032	Six6	0.6551	0.9077	0.8956	0.6539	0.8280	0.7550	0.2552	0.8015	0.8365	0.9313	0.6581	0.2238
NM_001108082	Pctk2	0.8166	0.9240	0.8895	0.8611	0.3864	0.8383	0.9045	0.8536	0.5813	0.8976	0.8620	0.7845
NM_001107929	Rnf20	0.4189	0.6955	0.1470	0.1543	0.3503	0.0955	0.1261	0.0217	0.3540	0.2057	0.2298	0.5142
NM_001108030	Daam1	0.8018	0.9756	0.7758	0.4523	0.7278	0.6054	0.9012	0.8235	0.9010	0.8700	0.6771	0.3946
NM_001005551	Car5b	0.9707	0.3582	0.9225	0.9856	0.9101	0.8478	0.9612	0.7336	0.7168	0.8463	0.7795	0.8878
NM_001107266	Ift88	0.3681	0.4960	0.8027	0.0932	0.6287	0.1911	0.4771	0.7532	0.4195	0.7993	0.3399	0.2258
NM_001107269	Wdfy2	0.6597	0.6737	0.6059	0.2295	0.3976	0.6882	0.5759	0.4244	0.7212	0.1822	0.1991	0.5969
NM_001107441	RGD1563235	0.6183	0.6863	0.7375	0.7718	0.9497	0.8628	0.8652	0.7215	0.7640	0.8196	0.6681	0.8146
NM_001107536	Uvrag	0.9631	0.4376	0.9023	0.7831	0.4635	0.8073	0.8103	0.8384	0.4867	0.4814	0.6599	0.5334
NM_001107513	Tmem143	0.3792	0.4109	0.7901	0.7366	0.6098	0.8641	0.3734	0.7675	0.5445	0.7202	0.4429	0.9226
NM_001107277	Lgi3	0.6356	0.4167	0.3235	0.4209	0.5377	0.6161	0.4398	0.7405	0.4532	0.2327	0.4655	0.2449
NM_001107443	Nrap	0.3789	0.4738	0.6225	0.5547	0.5734	0.6166	0.2941	0.6069	0.3068	0.3254	0.7388	0.6420
NM_001108040	RGD1307704	0.3031	0.1733	0.0363	0.7069	0.5775	0.2608	0.2413	0.7084	0.4416	0.4687	0.5059	0.2872
NM_001108038	Zfyve26	0.8580	0.5113	0.2096	0.2760	0.4687	0.5385	0.5723	0.4776	0.5035	0.3069	0.4162	0.4316
NM_001107442	Taf5l	0.8207	0.5241	0.2657	0.2060	0.5806	0.4075	0.3178	0.5148	0.6939	0.4586	0.5925	0.5641
NM_001108043	Mlh3	0.3480	0.0922	0.0483	0.2309	0.4542	0.2483	0.5433	0.3117	0.0154	0.4438	0.4775	0.5806

NM_001108044	RGD1309492	0.5357	0.6445	0.6345	0.5000	0.5471	0.8333	0.5341	0.5730	0.7946	0.8023	0.5653	0.8143
NM_001107944	Klhl9	0.1653	0.2621	0.7068	0.9948	0.2480	0.4700	0.5615	0.0181	0.0810	0.0778	0.2351	0.1308
NM_001107945	Dmrta1	0.4882	0.4713	0.6337	0.5370	0.4463	0.2515	0.2890	0.4202	0.4381	0.7678	0.5346	0.5783
NM_001107823	Gfi1b	0.4961	0.3778	0.3789	0.8871	0.5949	0.7648	0.7554	0.8573	0.8100	0.6773	0.5960	0.5798
NM_001107635	Lama4	0.4806	0.4231	0.4829	0.2723	0.2110	0.3413	0.0213	0.6634	0.4876	0.1422	0.2340	0.6197
NM_001107449	Clca3	0.5502	0.3102	0.3621	0.5865	0.3826	0.4379	0.2867	0.5776	0.3303	0.5126	0.4280	0.5940
NM_001107283	Slitrk1	0.3504	0.3679	0.5778	0.3141	0.3256	0.4510	0.7504	0.5799	0.3579	0.1954	0.2484	0.3364
NM_001107281	Klf12	0.6034	0.6941	0.7534	0.4109	0.8593	0.5108	0.2385	0.7955	0.5665	0.8303	0.3333	0.3406
NM_001107280	Pcdh20	0.8416	0.7002	0.8283	0.3399	0.3019	0.8283	0.4468	0.5081	0.7742	0.6665	0.6161	0.2996
NM_001107455	Tagap	0.4947	0.5177	0.9598	0.8441	0.5839	0.7142	0.7730	0.5760	0.6371	0.7067	0.3798	0.6442
NM_001108055	Ak7	0.7654	0.2726	0.1526	0.0807	0.8656	0.5218	0.5485	0.6075	0.0800	0.7835	0.8635	0.8894
NM_001108202	Dlk2	0.2989	0.1806	0.7061	0.3625	0.5043	0.3492	0.3207	0.5189	0.2826	0.5160	0.3635	0.3164
NM_001108197	Taf8	0.8516	0.4723	0.3173	0.4112	0.6130	0.8515	0.8976	0.4727	0.6698	0.9239	0.2333	0.8600
NM_001107952	Zfyve9	0.3705	0.5080	0.7771	0.9888	0.5724	0.8789	0.6157	0.3604	0.3870	0.5881	0.1497	0.8654
NM_001108215	Mfsd9	0.7517	0.3811	0.3938	0.7777	0.1325	0.5354	0.7736	0.1371	0.3952	0.0724	0.3254	0.5839
NM_001107301	Abhd8	0.0662	0.1609	0.9700	0.0935	0.0552	0.1250	0.7673	0.2025	0.0575	0.0573	0.0518	0.0948
NM_001107311	Enpp6	0.1440	0.6872	0.2939	0.6542	0.5577	0.5331	0.1523	0.6168	0.7355	0.3981	0.3323	0.1755
NM_001107293	Eaf1	0.8637	0.6437	0.3182	0.6442	0.3193	0.6917	0.7900	0.1695	0.9317	0.4669	0.1370	0.7191
NM_001107305	RGD1566239	0.0642	0.0231	0.1755	0.6350	0.8426	0.1536	0.1526	0.8812	0.0775	0.0333	0.1347	0.1417
NM_001108175	Tbc1d2b	0.6400	0.9273	0.6790	0.7551	0.7946	0.5854	0.2957	0.4423	0.8781	0.9703	0.5652	0.8858
NM_001107296	Ercc6	0.7224	0.8154	0.8682	0.4782	0.8572	0.7143	0.2751	0.8934	0.5921	0.1767	0.4821	0.4395
NM_001107379	Sncaip	0.5179	0.3359	0.5120	0.5950	0.3458	0.1611	0.2229	0.2945	0.4741	0.3686	0.6647	0.2115
NM_001107308	Gmip	0.4071	0.3584	0.4916	0.4133	0.6898	0.6468	0.6153	0.5957	0.5524	0.6464	0.2126	0.5096
NM_001107298	Mmrn2	0.5441	0.9304	0.0940	0.7375	0.1042	0.8332	0.5005	0.1454	0.4182	0.0542	0.0651	0.0690
NM_001107307	Cilp2	0.6808	0.4826	0.7818	0.5842	0.3962	0.6156	0.6735	0.2616	0.3765	0.6819	0.1321	0.7218
NM_001108084	Tmcc3	0.1833	0.0174	0.4025	0.3258	0.5138	0.4592	0.0281	0.6757	0.4292	0.3848	0.3766	0.2806
NM_001107299	Rgr	0.1612	0.5942	0.5372	0.6797	0.5085	0.8592	0.6510	0.0997	0.8043	0.8750	0.8060	0.5704
NM_001107488	RGD1565787	0.8258	0.6554	0.7342	0.4440	0.3961	0.1893	0.2054	0.4596	0.7367	0.4299	0.4198	0.1384
NM_001107316	Mfhas1	0.0153	0.4908	0.0331	0.0087	0.0056	0.0456	0.0242	0.0353	0.0971	0.0022	0.2035	0.1838
NM_001107314	RGD1304810	0.1721	0.2178	0.0822	0.1132	0.4187	0.3894	0.2548	0.3680	0.1301	0.1425	0.4690	0.4029
NM_001108240	Clptm1l	0.0689	0.6863	0.2397	0.2489	0.1054	0.2865	0.4389	0.0347	0.6798	0.1535	0.6087	0.8374
NM_001108239	Gtf3c3	0.4081	0.5462	0.5603	0.4474	0.6971	0.2950	0.3549	0.4086	0.4395	0.5045	0.6817	0.3299
NM_001108064	Wiz	0.1648	0.8451	0.2699	0.5585	0.9625	0.1894	0.7534	0.5128	0.6945	0.6146	0.8626	0.2984
NM_001107964	Tmem53	0.9773	0.9865	0.9098	0.8063	0.9980	0.9958	0.9605	0.8147	0.9149	0.9799	0.8998	0.9684
NM_001107519	Nipa1	0.4908	0.4815	0.4695	0.6197	0.1210	0.3577	0.7826	0.2412	0.1762	0.1296	0.2369	0.0252
NM_001107325	Proz	0.1640	0.4378	0.6130	0.5490	0.5284	0.5160	0.3422	0.5402	0.7164	0.3571	0.4988	0.3966
NM_001107321	Htra4	0.7698	0.1342	0.7986	0.6814	0.6405	0.5290	0.8644	0.7466	0.2880	0.3590	0.1732	0.0358
NM_001108209	Tesp2	0.3454	0.1317	0.2410	0.5848	0.3291	0.2574	0.1413	0.3186	0.2367	0.1955	0.1290	0.5129
NM_001107333	Pols	0.9550	0.9950	0.7092	0.6499	0.9812	0.9968	0.9806	0.9971	0.9875	0.9993	0.9954	0.9551
NM_001107341	Susd3	0.6125	0.7453	0.1334	0.7645	0.1164	0.5711	0.8526	0.2590	0.0639	0.3634	0.3543	0.1806
NM_001107376	Me2	0.1436	0.1540	0.2132	0.9028	0.1203	0.3178	0.5243	0.2676	0.0905	0.1854	0.0188	0.4432
NM_001107359	Papd1	0.1749	0.0718	0.4951	0.1341	0.0406	0.3240	0.0180	0.1087	0.1592	0.3255	0.1100	0.8705
NM_001108071	Adamtsl5	0.2805	0.0315	0.0024	0.0150	0.8098	0.8068	0.0041	0.8323	0.0117	0.3258	0.1849	0.7977
NM_001108074	Fzr1	0.8445	0.8050	0.0553	0.6621	0.7827	0.8020	0.9111	0.8674	0.8022	0.8652	0.7413	0.6386
NM_001108073	Tjp3	0.3661	0.7736	0.1643	0.2375	0.3084	0.4103	0.0651	0.2714	0.2588	0.1801	0.2839	0.1377
NM_001107972	Hivep3	0.5641	0.8298	0.7104	0.6282	0.9233	0.0957	0.4891	0.8108	0.9260	0.4359	0.5440	0.8679
NM_001107350	Ripk1	0.7244	0.5207	0.0456	0.7368	0.3045	0.9546	0.3382	0.2371	0.2495	0.5761	0.5038	0.9428
NM_001006954	Coasy	0.8071	0.1919	0.2323	0.0572	0.5908	0.1281	0.2250	0.3076	0.1286	0.6970	0.3218	0.0456
NM_001107512	Tead2	0.7534	0.1512	0.1084	0.0706	0.0963	0.3020	0.4169	0.1788	0.7578	0.2749	0.5464	0.3811

NM_001107337	Nsd1	0.9769	0.9335	0.9669	0.2923	0.9486	0.8038	0.9537	0.8246	0.9511	0.9975	0.9379	0.9286
NM_001108500	Pik3c2a	0.8577	0.5840	0.5481	0.3015	0.7046	0.9489	0.0450	0.3356	0.9675	0.9817	0.9767	0.9029
NM_001108396	Actr8	0.1475	0.2567	0.2376	0.0524	0.1034	0.0653	0.0849	0.1837	0.8246	0.1529	0.4308	0.4995
NM_001107342	Phf2	0.9623	0.4149	0.9012	0.9064	0.9409	0.7681	0.8889	0.9483	0.9380	0.8480	0.8569	0.8199
NM_001107393	Diaph1	0.2136	0.3113	0.8536	0.8944	0.0966	0.8948	0.2677	0.3807	0.4123	0.2746	0.2453	0.4918
NM_001107979	Fhl3	0.6756	0.6119	0.0352	0.1507	0.6871	0.6919	0.1499	0.7796	0.0968	0.8045	0.0512	0.0884
NM_001107355	RGD1560511	0.9162	0.0857	0.8912	0.7183	0.8241	0.9782	0.0548	0.8345	0.1101	0.3023	0.1704	0.8198
NM_001108348	Lhx4	0.0694	0.2261	0.9235	0.4953	0.6682	0.3933	0.5062	0.8800	0.2928	0.5686	0.6140	0.5660
NM_001108276	Trim11	0.6579	0.4772	0.6179	0.0488	0.3437	0.5866	0.6569	0.2327	0.7411	0.6317	0.0599	0.5216
NM_001108213	Rev1	0.9412	0.9911	0.7735	0.2226	0.9866	0.7909	0.4978	0.9455	0.8174	0.9547	0.8355	0.8768
NM_001108287	Prr11	0.2817	0.1258	0.0460	0.6084	0.1212	0.0641	0.1983	0.6837	0.4056	0.1358	0.7128	0.0838
NM_001108280	Inca1	0.1360	0.4607	0.0767	0.4335	0.3050	0.3760	0.1889	0.6775	0.3550	0.0606	0.3999	0.2196
NM_001108331	Ap1s1	0.3536	0.2220	0.4461	0.0820	0.7325	0.4134	0.4270	0.5072	0.4676	0.5910	0.0500	0.7884
NM_001108081	Actr6	0.2722	0.2303	0.0946	0.0144	0.0070	0.0687	0.0101	0.1588	0.0085	0.0037	0.0141	0.0105
NM_001107981	RGD1563072	0.4207	0.4198	0.9816	0.7320	0.3835	0.8670	0.2909	0.5797	0.0802	0.2291	0.0275	0.7332
NM_001108080	Spic	0.5266	0.6153	0.1778	0.2608	0.8380	0.4827	0.8387	0.0500	0.7028	0.7261	0.6123	0.6321
NM_001107313	Cnot7	0.8040	0.9561	0.9755	0.2683	0.9449	0.9213	0.3246	0.9594	0.4553	0.9092	0.9246	0.7246
NM_001107295	Oxnad1	0.9241	0.7443	0.3468	0.3880	0.5679	0.4073	0.8663	0.5761	0.7616	0.8045	0.4030	0.3220
NM_001107345	Tcfap2a	0.7272	0.9936	0.8607	0.8054	0.1578	0.2708	0.9984	0.2375	0.9951	0.8768	0.9904	0.9969
NM_001107529	Prc1	0.9066	0.9387	0.0901	0.6976	0.9320	0.9233	0.9015	0.9219	0.9966	0.9717	0.9629	0.1915
NM_001107524	Rgma	0.7170	0.0432	0.6937	0.9494	0.8286	0.4790	0.3398	0.4477	0.3178	0.1366	0.0515	0.5578
NM_001108086	Eea1	0.6528	0.1868	0.2520	0.4861	0.4956	0.5193	0.3233	0.3746	0.3921	0.2218	0.5998	0.9364
NM_001107523	Chd2	0.0866	0.0669	0.1406	0.1108	0.0308	0.2369	0.0430	0.2003	0.1956	0.0444	0.0328	0.0163
NM_001107543	Ubqln3	0.6570	0.5552	0.5768	0.7259	0.1463	0.3531	0.2814	0.5529	0.5868	0.3413	0.4766	0.5949
NM_001108216	Nck2	0.7784	0.9501	0.5406	0.8780	0.5345	0.1837	0.8871	0.2155	0.9756	0.9888	0.6369	0.8789
NM_001107987	Myom3	0.3827	0.9144	0.5419	0.8267	0.1389	0.2655	0.7659	0.3517	0.6966	0.8714	0.8118	0.5784
NM_001007704	Galm	0.0823	0.4415	0.9065	0.8354	0.1230	0.2781	0.1512	0.2856	0.1560	0.4158	0.3954	0.1506
NM_001108212	Lipt1	0.0135	0.6107	0.4156	0.1424	0.2225	0.4416	0.5113	0.1564	0.2380	0.0157	0.0382	0.7968
NM_001108211	Ankrd23	0.8644	0.6180	0.7030	0.7553	0.9235	0.6908	0.5993	0.7860	0.4934	0.8912	0.8246	0.5577
NM_001108208	Dst	0.2993	0.2515	0.1006	0.2516	0.3517	0.3742	0.2083	0.1658	0.7033	0.2614	0.3835	0.4308
NM_001107366	Mcm10	0.9332	0.9970	0.1545	0.9415	0.9449	0.9104	0.9919	0.9476	0.8193	0.9693	0.8606	0.5983
NM_001107365	RGD1560691	0.4978	0.9237	0.3225	0.1461	0.8434	0.3224	0.9154	0.4989	0.8877	0.8321	0.7355	0.6251
NM_001107550	Sez6l2	0.8054	0.9422	0.8385	0.8863	0.9041	0.9010	0.9298	0.8291	0.8149	0.9222	0.9689	0.9093
NM_001107362	Wdr37	0.8195	0.7552	0.8010	0.4069	0.9753	0.9922	0.5464	0.9446	0.8304	0.9068	0.9312	0.9489
NM_001134563	Zfp7	0.1664	0.0380	0.1631	0.1719	0.0585	0.1352	0.3411	0.0216	0.0146	0.3738	0.3444	0.0647
NM_001108220	Als2cr4	0.2527	0.8082	0.8013	0.6025	0.4887	0.0986	0.3619	0.1663	0.8599	0.6077	0.2119	0.1272
NM_001007628	Cxxc5	0.2619	0.1959	0.1801	0.8763	0.2593	0.2524	0.1890	0.1832	0.1516	0.2705	0.0283	0.2361
NM_001107377	Rkhd2	0.9927	0.9615	0.9849	0.9849	0.9798	0.9897	0.9686	0.9877	0.9512	0.9944	0.9980	0.8562
NM_001108306	Unk	0.7675	0.9438	0.7280	0.4750	0.9816	0.8661	0.6305	0.9809	0.9465	0.9597	0.5435	0.9719
NM_001107989	Mfap2	0.1613	0.3106	0.4313	0.1193	0.4747	0.3773	0.1312	0.3026	0.8865	0.3077	0.0536	0.6691
NM_001108309	Sec14l1	0.9555	0.8456	0.3367	0.0984	0.2378	0.4085	0.0444	0.6546	0.9289	0.9649	0.5886	0.3198
NM_001108346	Nek7	0.3931	0.2164	0.7754	0.8798	0.6254	0.9304	0.7674	0.4262	0.4684	0.6047	0.7005	0.6286
NM_001108344	Ube2t	0.0791	0.8074	0.1270	0.1487	0.8938	0.7263	0.8500	0.7456	0.7422	0.7772	0.8057	0.0461
NM_001108238	Ankrd12	0.6619	0.6870	0.6229	0.7158	0.6033	0.5628	0.5622	0.4930	0.1875	0.6703	0.6686	0.5518
NM_001107991	Lrrc38	0.6391	0.4246	0.4651	0.6395	0.5297	0.4673	0.3797	0.4504	0.4951	0.2636	0.0812	0.6828
NM_001108089	RGD1310270	0.8909	0.5664	0.6859	0.0224	0.9070	0.7985	0.7373	0.8210	0.7597	0.9126	0.8159	0.6697
NM_001108241	Mospd4	0.2164	0.3297	0.1626	0.1486	0.2069	0.5893	0.1439	0.5989	0.4442	0.6048	0.4932	0.5468
NM_001108318	Atp6v1a	0.3001	0.0024	0.9912	0.5069	0.2186	0.9410	0.1940	0.1704	0.1498	0.1639	0.2525	0.7160
NM_001108091	RGD1307051	0.6775	0.5884	0.5732	0.4975	0.5370	0.0591	0.3192	0.1313	0.4153	0.6635	0.7741	0.5548

NM_001108254	Egfl6	0.6408	0.4134	0.7077	0.1993	0.4303	0.1144	0.5378	0.6999	0.7680	0.1935	0.3282	0.0929
NM_001108417	Cul2	0.8061	0.5329	0.7179	0.8124	0.7525	0.8466	0.3996	0.7923	0.6290	0.8050	0.8120	0.6618
NM_001108256	Ocrl	0.8244	0.5143	0.5113	0.9564	0.9029	0.5250	0.7677	0.9906	0.8262	0.5664	0.4653	0.8069
NM_001108252	RGD1562211	0.7180	0.7452	0.6916	0.5580	0.4365	0.1055	0.5969	0.3976	0.9239	0.6132	0.7073	0.0783
NM_001108248	Was	0.0294	0.0275	0.1515	0.1005	0.0588	0.0217	0.5749	0.0061	0.3442	0.0437	0.0547	0.2527
NM_001108247	Wdr13	0.4576	0.1801	0.6598	0.5713	0.4915	0.3002	0.3271	0.8035	0.8081	0.7502	0.7191	0.1574
NM_001108245	Itgb1bp2	0.3730	0.6606	0.0488	0.1258	0.2098	0.3994	0.4894	0.4110	0.8259	0.1716	0.7507	0.7502
NM_001108261	RGD1305823	0.2663	0.0882	0.6286	0.2265	0.0049	0.1537	0.4489	0.0892	0.0200	0.1469	0.2986	0.1511
NM_001108102	Xpot	0.9235	0.8887	0.9089	0.0657	0.9900	0.9154	0.9315	0.9230	0.9603	0.9700	0.9673	0.8919
NM_001108103	Brd1	0.9813	0.7243	0.7616	0.5005	0.9384	0.9438	0.6172	0.9471	0.4795	0.3627	0.4188	0.2698
NM_001108257	Rab33a	0.6279	0.2988	0.5097	0.6724	0.4332	0.5688	0.2452	0.5227	0.7179	0.7180	0.5350	0.4173
NM_001107413	Irx3	0.5043	0.4927	0.9496	0.6637	0.5622	0.1995	0.8120	0.3225	0.4343	0.9215	0.1042	0.3034
NM_001107412	Hmg2l1	0.2281	0.2832	0.1081	0.5531	0.3118	0.4586	0.0969	0.1301	0.0076	0.0833	0.0349	0.2410
NM_001108094	Hrb2	0.5745	0.1929	0.0903	0.2850	0.1527	0.8242	0.0425	0.3512	0.7397	0.1251	0.5780	0.4147
NM_001107995	Phf13	0.3321	0.3579	0.8871	0.2917	0.2593	0.3560	0.6895	0.3028	0.4004	0.2439	0.2596	0.4478
NM_001108258	Gpr101	0.2796	0.2830	0.2289	0.7272	0.3350	0.2566	0.8240	0.3075	0.6623	0.8711	0.7662	0.4021
NM_001107590	Marveld1	0.8822	0.8139	0.7954	0.9149	0.9238	0.8900	0.9744	0.8142	0.7622	0.9132	0.8555	0.9919
NM_001107385	Fbxo38	0.5485	0.5723	0.4922	0.7263	0.4450	0.4166	0.2474	0.4932	0.4224	0.3582	0.6220	0.3273
NM_001107400	Brunol4	0.8735	0.2227	0.5764	0.8657	0.7051	0.7916	0.1472	0.7468	0.0803	0.2570	0.2846	0.4520
NM_001107388	Dmxl1	0.3566	0.5408	0.3134	0.1210	0.8619	0.7883	0.1485	0.7899	0.1087	0.5148	0.6991	0.8954
NM_001108267	Tcfap4	0.6533	0.6944	0.6321	0.8188	0.8048	0.9409	0.9470	0.6380	0.8813	0.4113	0.9404	0.2664
NM_001108270	RGD1311343	0.3702	0.6504	0.5149	0.7503	0.5094	0.8094	0.7123	0.3950	0.5031	0.2534	0.3768	0.7133
NM_001107387	Tnfaip8	0.1049	0.1368	0.8500	0.5098	0.3046	0.5654	0.8542	0.4268	0.0107	0.1141	0.1931	0.0584
NM_001113344	St6gal1	0.9835	0.8917	0.9750	0.9664	0.9976	0.9834	0.9714	0.9879	0.9564	0.9831	0.9948	0.9713
NM_001113223	LOC10013487	0.4516	0.4724	0.1908	0.1986	0.5630	0.2979	0.2025	0.1932	0.4020	0.2839	0.4296	0.2569
NM_001107633	Gcc2	0.7891	0.4567	0.6397	0.5326	0.3242	0.8647	0.4715	0.1650	0.3990	0.7317	0.6365	0.9686
NM_001107394	Taf7	0.4536	0.0377	0.5682	0.2646	0.1969	0.1967	0.0902	0.4138	0.0055	0.2738	0.0554	0.4308
NM_001107669	Plk4	0.9838	0.9574	0.1906	0.7313	0.7327	0.7336	0.8038	0.7700	0.6363	0.9405	0.9049	0.2224
NM_001107647	Utp15	0.4341	0.1808	0.1650	0.3356	0.4705	0.8704	0.0668	0.0859	0.0879	0.2769	0.3638	0.3704
NM_001107541	Art1	0.6020	0.4796	0.6490	0.4774	0.5660	0.4360	0.7705	0.3726	0.3613	0.2887	0.4048	0.4442
NM_001107456	Map3k4	0.3445	0.5080	0.8796	0.8970	0.2077	0.2773	0.9885	0.1997	0.1290	0.7683	0.3335	0.2522
NM_001107571	Klc2	0.5978	0.6266	0.1057	0.5781	0.2315	0.2599	0.0919	0.7707	0.6822	0.4721	0.3157	0.6893
NM_001107405	Mib1	0.7869	0.8728	0.6680	0.5265	0.6671	0.8369	0.8805	0.5324	0.6368	0.7762	0.8137	0.7062
NM_001107569	Bbs1	0.6805	0.7232	0.9147	0.8115	0.9067	0.7723	0.7758	0.6613	0.8011	0.8148	0.9517	0.7817
NM_001107584	Ranbp6	0.4288	0.2698	0.9426	0.9805	0.3270	0.9050	0.2241	0.2432	0.5076	0.3399	0.7034	0.8875
NM_001108117	Copz1	0.1414	0.2114	0.9742	0.3279	0.2272	0.5487	0.4753	0.1774	0.2529	0.0107	0.2140	0.9244
NM_001108462	Mthfd1l	0.0164	0.7842	0.0050	0.2293	0.1811	0.1971	0.8838	0.4188	0.7522	0.6765	0.8194	0.8464
NM_001107440	RGD1565149	0.7376	0.5228	0.3395	0.2401	0.8546	0.9822	0.4736	0.5950	0.0303	0.9024	0.5911	0.5139
NM_001107422	Thap11	0.3201	0.1198	0.4455	0.7448	0.2000	0.9619	0.3146	0.6461	0.7353	0.3285	0.4549	0.8482
NM_001107435	Kcng4	0.3565	0.8499	0.7780	0.4986	0.2227	0.6011	0.7452	0.6573	0.3642	0.4556	0.5330	0.6204
NM_001107454	Nkd2	0.5797	0.6794	0.6120	0.5817	0.6996	0.6037	0.7229	0.7190	0.1879	0.6716	0.7315	0.6379
NM_001107426	Psmd7	0.3937	0.4846	0.1464	0.6432	0.8690	0.7917	0.7715	0.6949	0.3749	0.9176	0.2569	0.6867
NM_001107424	Slc7a6	0.9489	0.8648	0.1034	0.9055	0.9669	0.7601	0.3534	0.9892	0.2721	0.9540	0.6856	0.2998
NM_001108433	Adamts19	0.6121	0.6913	0.7907	0.2902	0.5879	0.4770	0.6736	0.4549	0.8179	0.6980	0.5213	0.5833
NM_001108217	Kdelc1	0.8069	0.9161	0.8158	0.7146	0.9118	0.9184	0.8701	0.8263	0.6906	0.8878	0.9060	0.8579
NM_001108281	RGD1308139	0.4104	0.4886	0.5337	0.2428	0.6043	0.5834	0.5684	0.4713	0.4565	0.6568	0.5355	0.6594
NM_001107417	Neto2	0.9510	0.8088	0.9587	0.9191	0.9568	0.8165	0.4105	0.7867	0.8189	0.9520	0.1838	0.3878
NM_001107691	Trim46	0.9639	0.9816	0.0481	0.9072	0.9237	0.9775	0.8839	0.9921	0.9926	0.8707	0.7254	0.9888
NM_001108128	Chordc1	0.2570	0.3880	0.5146	0.1015	0.2102	0.3582	0.0619	0.0643	0.1037	0.2138	0.3841	0.0645

NM_001108124	Cep57	0.5674	0.9464	0.7606	0.1826	0.8159	0.6321	0.5669	0.6554	0.5778	0.9554	0.8500	0.2998
NM_001108120	Nxt2	0.4200	0.1013	0.9680	0.9280	0.8878	0.8419	0.6595	0.3847	0.4140	0.6706	0.9297	0.6805
NM_001108121	Kbtbd3	0.6704	0.1141	0.0438	0.2266	0.2562	0.2056	0.0311	0.2614	0.1921	0.3591	0.1338	0.4490
NM_001108856	Vamp4	0.2749	0.3630	0.9146	0.8663	0.5180	0.3358	0.2770	0.6202	0.1192	0.3844	0.2309	0.1579
NM_001105809	Dph1	0.9417	0.9100	0.4767	0.9483	0.9645	0.9352	0.8475	0.9575	0.9140	0.8386	0.9075	0.9702
NM_001108351	Sertad4	0.9695	0.9895	0.9702	0.9740	0.9733	0.9830	0.9958	0.9972	0.8454	0.9094	0.9686	0.2439
NM_001108350	Klhdc9	0.6716	0.9090	0.4103	0.7119	0.8614	0.3139	0.6081	0.8329	0.5386	0.5398	0.9323	0.4769
NM_001107439	Snai3	0.1423	0.4018	0.4157	0.1165	0.3494	0.5209	0.4220	0.2330	0.3806	0.5288	0.4777	0.1743
NM_001107438	RGD1561676	0.5533	0.7982	0.7107	0.7261	0.7849	0.6751	0.2947	0.7657	0.3645	0.4496	0.6704	0.2291
NM_001107436	RGD1304884	0.2667	0.2749	0.1695	0.0985	0.2364	0.2105	0.1552	0.2703	0.1039	0.0273	0.1438	0.1988
NM_001108123	Mtmr2	0.3576	0.3383	0.7809	0.9231	0.2475	0.9555	0.5738	0.1013	0.3164	0.2900	0.1706	0.9136
NM_001107594	Nkx2-3	0.3707	0.5413	0.8597	0.3916	0.7393	0.7974	0.2202	0.4600	0.2502	0.5753	0.6711	0.7586
NM_001108125	Sesn3	0.1646	0.7123	0.4499	0.8900	0.2560	0.1537	0.6561	0.3737	0.1396	0.3562	0.4486	0.2438
NM_001108126	Amotl1	0.5004	0.6163	0.5248	0.5516	0.2368	0.2786	0.4135	0.0928	0.5950	0.5363	0.4826	0.7600
NM_001012037	Lias	0.8768	0.3294	0.7910	0.4136	0.7201	0.7712	0.3289	0.1918	0.4538	0.4436	0.6952	0.4314
NM_001012026	Mdm4	0.7643	0.8161	0.4846	0.8551	0.8142	0.8831	0.2885	0.7463	0.5670	0.8184	0.9500	0.7362
NM_001107602	Elovl3	0.2684	0.2886	0.1821	0.1009	0.3164	0.1386	0.4626	0.5106	0.5610	0.6695	0.3058	0.5258
NM_001107448	RGD1305500	0.1409	0.0104	0.7783	0.2206	0.2797	0.0078	0.2006	0.2611	0.3097	0.0155	0.3715	0.1209
NM_001108320	Tprg1	0.3117	0.3443	0.1907	0.1985	0.1857	0.1872	0.2022	0.1930	0.3029	0.4942	0.5206	0.1847
NM_001107447	Rab11fip2	0.1775	0.2216	0.4358	0.7996	0.6921	0.5524	0.6388	0.5877	0.1290	0.8557	0.6395	0.9249
NM_001107445	Hspa12a	0.3095	0.4965	0.7943	0.5774	0.4533	0.6488	0.4109	0.1555	0.8890	0.7851	0.3702	0.3897
NM_001108141	Dscaml1	0.0618	0.3003	0.5859	0.1215	0.0595	0.1373	0.3831	0.1173	0.6675	0.3829	0.2826	0.4449
NM_001108367	Smek2	0.6896	0.6364	0.7237	0.5615	0.5597	0.6794	0.6013	0.6234	0.7203	0.5979	0.6598	0.5318
NM_001107614	Slc26a8	0.3121	0.7996	0.7569	0.7265	0.7183	0.5895	0.9638	0.7952	0.6228	0.6410	0.9508	0.8908
NM_001107460	Zbtb2	0.6768	0.2003	0.7821	0.7640	0.3407	0.5531	0.3448	0.2083	0.5455	0.5890	0.4896	0.4006
NM_001107459	Rgs17	0.8510	0.2206	0.9493	0.8496	0.2109	0.2383	0.1350	0.2818	0.3430	0.5946	0.3167	0.5361
NM_001107812	Map1d	0.5128	0.7459	0.0253	0.8004	0.5420	0.4822	0.1101	0.6103	0.1134	0.5636	0.2516	0.1311
NM_001107793	Acss2	0.0605	0.0768	0.0550	0.1063	0.0533	0.0925	0.1017	0.0844	0.2984	0.0442	0.0111	0.1268
NM_001107453	Brd9	0.9184	0.8252	0.0568	0.2282	0.9488	0.7590	0.3036	0.9390	0.6658	0.5190	0.5756	0.2220
NM_001107852	Plxna4a	0.3081	0.5021	0.8114	0.6082	0.6989	0.9416	0.5080	0.7778	0.7277	0.5847	0.6800	0.3805
NM_001108156	Itga11	0.8736	0.9154	0.9124	0.8019	0.7641	0.9666	0.9867	0.7874	0.9964	0.9951	0.9874	0.9472
NM_001108172	Ttk	0.9907	0.9767	0.1115	0.9145	0.7721	0.8823	0.8583	0.9092	0.5296	0.9948	0.9043	0.0010
NM_001108157	Fem1b	0.7490	0.7194	0.9784	0.0533	0.4696	0.3217	0.8824	0.3735	0.0593	0.9090	0.9055	0.0080
NM_001107624	Arid5b	0.6668	0.9348	0.5942	0.1176	0.4101	0.1501	0.6998	0.4821	0.8978	0.9878	0.5782	0.7458
NM_001107616	Cpne5	0.6253	0.8431	0.7144	0.6801	0.7498	0.5840	0.7415	0.8322	0.7127	0.3150	0.1681	0.5797
NM_001107485	Six5	0.3714	0.7501	0.3876	0.3672	0.9045	0.5511	0.7205	0.9194	0.5246	0.5183	0.1667	0.6930
NM_001107580	Trex2	0.5768	0.5415	0.3279	0.3003	0.6787	0.5278	0.2360	0.1501	0.1924	0.6029	0.5531	0.7463
NM_001107626	Ado	0.6651	0.8285	0.7427	0.8994	0.3205	0.6781	0.7376	0.8973	0.7902	0.8143	0.7580	0.8465
NM_001108389	Rnf219	0.1948	0.3185	0.2786	0.5135	0.5891	0.7871	0.7790	0.3894	0.3828	0.6041	0.3647	0.5749
NM_001108373	Mapk1ip1l	0.5973	0.4318	0.5157	0.5754	0.2709	0.8147	0.8960	0.8052	0.3018	0.8546	0.5996	0.4503
NM_001107503	Cd22	0.3995	0.0631	0.3553	0.2981	0.6396	0.5843	0.3938	0.7452	0.7956	0.4321	0.4921	0.7966
NM_001107500	Zfp84	0.7150	0.2289	0.4251	0.3234	0.3473	0.7075	0.7182	0.4598	0.6614	0.1818	0.6912	0.9024
NM_001108393	Zmiz1	0.6769	0.4516	0.6353	0.5545	0.6497	0.7819	0.9250	0.4589	0.8707	0.5462	0.1144	0.7546
NM_001108560	Zfp364	0.0360	0.1321	0.5956	0.1208	0.0131	0.0092	0.3710	0.0047	0.2884	0.4658	0.0641	0.2331
NM_001108664	RGD1308165	0.9406	0.9480	0.9857	0.9596	0.9583	0.7888	0.9536	0.8731	0.8913	0.9795	0.8167	0.7657
NM_001108661	Ccl19	0.0350	0.0553	0.1450	0.0486	0.1979	0.3021	0.0249	0.5504	0.0161	0.0092	0.0171	0.5925
NM_001108549	Spata5	0.0832	0.1237	0.1090	0.1181	0.1151	0.0655	0.0587	0.0446	0.0504	0.3785	0.0344	0.0173
NM_001108167	Myo5c	0.7693	0.7633	0.8578	0.9083	0.8726	0.8351	0.6343	0.8422	0.8348	0.4507	0.9400	0.5842
NM_001108399	Pbx4	0.7905	0.7448	0.8061	0.5995	0.6421	0.9366	0.7915	0.7983	0.7907	0.9194	0.9185	0.8771

NM_001108431	Dtwd2	0.6751	0.3705	0.5171	0.4182	0.2216	0.4731	0.6044	0.2319	0.1952	0.2166	0.2228	0.2148
NM_001107505	Rhpn2	0.0137	0.3145	0.8703	0.4053	0.2415	0.6380	0.3385	0.3411	0.4060	0.5648	0.7993	0.2540
NM_001108182	Nek11	0.6193	0.5631	0.1919	0.2288	0.2835	0.3128	0.2772	0.5590	0.1054	0.3982	0.1522	0.0296
NM_001108356	LOC360919	0.7867	0.9982	0.7688	0.6329	0.8990	0.8393	0.8896	0.9678	0.8539	0.9624	0.9073	0.1599
NM_001107525	Wdr73	0.3645	0.0563	0.4009	0.0218	0.5566	0.6213	0.1124	0.3624	0.8561	0.3533	0.1480	0.5342
NM_001107522	Slc35d3	0.5655	0.4571	0.6726	0.2590	0.5708	0.2705	0.6081	0.4743	0.5395	0.7025	0.2366	0.4165
NM_001107720	Pap2d	0.5371	0.5761	0.5616	0.5969	0.4242	0.5443	0.2476	0.5913	0.6152	0.6055	0.6243	0.5315
NM_001107717	Fndc7	0.5700	0.8243	0.6700	0.7505	0.4982	0.4761	0.7712	0.8541	0.3563	0.8902	0.8429	0.6397
NM_001108183	Armet	0.6213	0.6750	0.0794	0.3899	0.0243	0.0611	0.9883	0.0593	0.6420	0.7561	0.8367	0.0238
NM_001108181	Acad11	0.7119	0.8866	0.8981	0.9636	0.9056	0.8758	0.9659	0.8847	0.9226	0.8009	0.8664	0.6418
NM_001108459	Rlbp1l2	0.5706	0.4347	0.6470	0.5712	0.4428	0.5757	0.6137	0.1811	0.5927	0.4896	0.3404	0.3375
NM_001108437	Ccdc102a	0.7524	0.1541	0.0120	0.1301	0.3881	0.4114	0.1886	0.2501	0.3344	0.2706	0.4517	0.2414
NM_001108200	Tbcc	0.5881	0.7702	0.0403	0.8740	0.5613	0.0481	0.1900	0.3614	0.6264	0.6560	0.6783	0.0870
NM_001108187	RGD1560778	0.7838	0.8165	0.4234	0.8577	0.5208	0.2298	0.4708	0.4807	0.7920	0.6604	0.8826	0.8247
NM_001108184	Dock3	0.3282	0.6735	0.6374	0.5408	0.7425	0.4607	0.6917	0.4152	0.4696	0.4490	0.6822	0.5365
NM_001108423	Riok3	0.1144	0.2832	0.3225	0.3691	0.2608	0.5529	0.3511	0.3140	0.1727	0.1821	0.3252	0.2973
NM_001108603	Ptprt	0.9486	0.9913	0.9559	0.9224	0.8887	0.8364	0.9940	0.9215	0.9605	0.8487	0.9346	0.8846
NM_001122947	Efhc1	0.1640	0.1656	0.1298	0.0075	0.4649	0.1501	0.0711	0.0651	0.4544	0.2178	0.0562	0.5442
NM_001108192	Glb1	0.0847	0.1508	0.9735	0.4728	0.6559	0.8762	0.1655	0.5474	0.0674	0.2681	0.3225	0.6110
NM_001108334	Ncor2	0.7363	0.6568	0.4713	0.7737	0.8625	0.5746	0.1925	0.9403	0.7242	0.4771	0.3944	0.5873
NM_001108203	Tjap1	0.6881	0.7567	0.0394	0.0939	0.6828	0.9719	0.3592	0.9670	0.9463	0.5604	0.6951	0.8898
NM_001107651	Depdc1b	0.5829	0.9067	0.3963	0.7292	0.2554	0.6430	0.8417	0.2718	0.7637	0.8396	0.7931	0.1674
NM_001106054	Klhl1	0.3368	0.4037	0.8924	0.3028	0.7110	0.4189	0.8538	0.6448	0.8349	0.0820	0.5076	0.5649
NM_001106996	Cyfip2	0.6414	0.4626	0.6065	0.7082	0.6139	0.6482	0.8141	0.9013	0.4425	0.9031	0.8489	0.8842
NM_001108466	Nat14	0.6990	0.6618	0.3665	0.6450	0.5752	0.5677	0.8354	0.7873	0.8394	0.6182	0.7773	0.7719
NM_001107646	Ap3b1	0.6293	0.2066	0.7384	0.7530	0.8005	0.8005	0.7350	0.7758	0.2809	0.5116	0.1861	0.3448
NM_001108478	Ttc9b	0.4343	0.7379	0.6368	0.8644	0.1137	0.4424	0.7663	0.5914	0.4355	0.5771	0.8431	0.9573
NM_001108476	Zfp428	0.5228	0.7408	0.3336	0.9801	0.6381	0.4981	0.7684	0.8011	0.2677	0.3471	0.4638	0.0257
NM_001108452	Cotl1	0.1269	0.6488	0.6303	0.8853	0.1127	0.0556	0.7562	0.2532	0.1629	0.3115	0.9367	0.1764
NM_001108536	Tube1	0.4107	0.9264	0.3459	0.0870	0.9210	0.9107	0.9604	0.7311	0.1905	0.4473	0.1601	0.0656
NM_001108227	Wnt10a	0.0816	0.0997	0.1508	0.4882	0.3526	0.0950	0.2278	0.3962	0.1381	0.2364	0.1508	0.7235
NM_001108225	Znf142	0.9778	0.7882	0.0692	0.1389	0.9742	0.5969	0.4714	0.9922	0.9015	0.6539	0.9153	0.9193
NM_001108470	Mzf1	0.8758	0.8369	0.6066	0.6957	0.8130	0.9592	0.9262	0.7621	0.7906	0.9632	0.8962	0.9334
NM_001108199	Trerf1	0.1309	0.6374	0.0129	0.0468	0.5589	0.0855	0.1384	0.3230	0.7080	0.2850	0.4515	0.1991
NM_001108479	Vstm2b	0.4614	0.6600	0.5159	0.4636	0.7018	0.7056	0.7299	0.2823	0.5578	0.5198	0.5951	0.7668
NM_001107954	Reck	0.2563	0.3263	0.6677	0.2792	0.1214	0.3465	0.3066	0.0425	0.5022	0.2069	0.3233	0.4354
NM_001107521	Il20ra	0.8138	0.8183	0.7961	0.3391	0.5536	0.6864	0.8106	0.6727	0.7783	0.9478	0.8589	0.1311
NM_001108230	Centg2	0.9772	0.9728	0.9863	0.9368	0.9529	0.8023	0.9999	0.8948	0.9860	0.9847	0.9951	0.8176
NM_001108231	Asb18	0.5534	0.5734	0.1387	0.2553	0.7004	0.7067	0.8859	0.5154	0.6288	0.7149	0.3073	0.2605
NM_001108234	Neu4	0.5925	0.7961	0.8100	0.9038	0.7416	0.7872	0.5740	0.7208	0.9380	0.5695	0.7452	0.8609
NM_001108233	Farp2	0.3318	0.1379	0.0562	0.0673	0.2517	0.2358	0.2451	0.0233	0.2952	0.1421	0.2035	0.2915
NM_001108512	Suv420h1	0.9623	0.9874	0.9624	0.9962	0.9849	0.9940	0.9922	0.9890	0.8882	0.9852	0.9984	0.9540
NM_001100579	Uba2	0.6576	0.3431	0.5498	0.5389	0.2866	0.4594	0.4414	0.3458	0.3391	0.4092	0.3277	0.4095
NM_001108242	Slc9a7	0.5580	0.6521	0.6607	0.5484	0.7672	0.3465	0.7822	0.7219	0.8346	0.8436	0.6166	0.8739
NM_001108235	Fbxl17	0.3521	0.0613	0.8343	0.7608	0.3195	0.1820	0.6433	0.3618	0.4271	0.1104	0.3119	0.8893
NM_001108398	Hapln4	0.3987	0.1328	0.2710	0.1634	0.2824	0.3482	0.3636	0.4778	0.5099	0.0708	0.4022	0.2093
NM_001108407	Auh	0.0113	0.0624	0.9828	0.0158	0.0316	0.0283	0.0420	0.0391	0.3124	0.0617	0.3842	0.3824
NM_001108390	Ndfip2	0.3544	0.8792	0.9809	0.9378	0.4792	0.3317	0.5211	0.4618	0.3525	0.1490	0.4564	0.5035
NM_001108262	Zc3h7a	0.9751	0.8900	0.2574	0.3159	0.9260	0.9938	0.2960	0.9808	0.9643	0.8712	0.8904	0.9954

NM_001107531	Mesp1	0.4927	0.1677	0.1294	0.3145	0.8102	0.5237	0.6302	0.0889	0.1525	0.4335	0.3800	0.2802
NM_001108266	RGD1563547	0.3788	0.7497	0.6492	0.6533	0.7235	0.8871	0.8817	0.8424	0.8006	0.6190	0.7393	0.6951
NM_001108265	Nagpa	0.0725	0.1104	0.0784	0.0547	0.0799	0.3750	0.3211	0.1490	0.7432	0.1036	0.0170	0.1934
NM_001108263	RGD1566405	0.6251	0.7907	0.2207	0.4377	0.7413	0.8685	0.5835	0.7587	0.5253	0.4836	0.7715	0.7376
NM_001107530	RGD1311021	0.0987	0.3242	0.8816	0.5617	0.1243	0.2585	0.6219	0.3396	0.1443	0.2065	0.0056	0.3348
NM_001108472	Meis3	0.1010	0.0670	0.0578	0.7656	0.5877	0.7254	0.2965	0.8554	0.2004	0.0172	0.0333	0.1721
NM_001108277	Tom1l2	0.0967	0.2154	0.6658	0.0816	0.7386	0.4951	0.1101	0.5288	0.6314	0.0309	0.0193	0.7290
NM_001108272	Pank3	0.4478	0.6553	0.6027	0.4874	0.9113	0.6701	0.7036	0.6822	0.3549	0.3938	0.6609	0.3820
NM_001108271	RGD1306625	0.2843	0.2801	0.4390	0.4825	0.4822	0.8211	0.5280	0.1586	0.6357	0.5655	0.4744	0.5590
NM_001108269	Arhgdig	0.3406	0.4202	0.5047	0.3032	0.2736	0.6219	0.3157	0.5656	0.4191	0.3740	0.3086	0.3948
NM_001108268	RGD1565744	0.3777	0.6986	0.6770	0.0988	0.4879	0.1571	0.7607	0.8854	0.8175	0.2764	0.7063	0.5005
NM_001108285	Nxn	0.8032	0.9662	0.9311	0.8929	0.8983	0.7846	0.8482	0.9722	0.8829	0.8620	0.8537	0.8870
NM_001108484	Nomo1	0.2998	0.0276	0.1064	0.1603	0.1338	0.3651	0.4075	0.1447	0.4517	0.1311	0.4708	0.6630
NM_001107538	Arhgef17	0.0821	0.6770	0.7750	0.3874	0.3805	0.7544	0.5582	0.8090	0.9785	0.4400	0.0238	0.6442
NM_001107692	Efna4	0.1429	0.1975	0.2906	0.1420	0.0460	0.2191	0.5559	0.0867	0.0100	0.0977	0.0230	0.2990
NM_001107807	Pmepa1	0.9435	0.9798	0.9642		0.8548	0.9999	0.9974	0.9962	0.8739	0.9835	0.9131	0.9992
NM_001107544	Dchs1	0.5319	0.3819	0.1207	0.4150	0.6691	0.4620	0.3316	0.3683	0.5486	0.3936	0.1520	0.5529
NM_001108296	RGD1306682	0.0442	0.1493	0.1424	0.5423	0.0882	0.1119	0.2679	0.8435	0.0275	0.0821	0.1421	0.1009
NM_001108305	Sap30bp	0.9198	0.8779	0.7701	0.3025	0.5993	0.8740	0.6626	0.8966	0.6835	0.9458	0.8502	0.5098
NM_001108304	Gga3	0.4181	0.8144	0.3031	0.1089	0.4955	0.8228	0.2799	0.6016	0.9023	0.2816	0.8712	0.3050
NM_001107552	Fbxl19	0.5579	0.9573	0.8016	0.8700	0.4235	0.8725	0.9508	0.9205	0.9359	0.8530	0.0973	0.7145
NM_001110487	Tssk4	0.5328	0.9207	0.9666	0.6474	0.9854	0.9132	0.9751	0.9015	0.8725	0.6981	0.7537	0.9837
NM_022379	Tfec	0.5971	0.7980	0.6641	0.1315	0.8031	0.7083	0.6899	0.7408	0.7927	0.2709	0.5824	0.7576
NM_001107561	Scgb1c1	0.2459	0.1373	0.6976	0.2861	0.5386	0.4717	0.4145	0.5900	0.5966	0.5872	0.6304	0.1422
NM_001107559	Gpr123	0.6629	0.5255	0.4062	0.8750	0.7750	0.7513	0.3553	0.2434	0.7744	0.4881	0.6001	0.6121
NM_001107577	RGD1564983	0.0583	0.3708	0.4659	0.3844	0.4704	0.2137	0.5513	0.5688	0.3568	0.3415	0.4678	0.4410
NM_001107576	Ints5	0.8389	0.6972	0.0349	0.2316	0.4387	0.0662	0.4486	0.7372	0.3415	0.1239	0.8445	0.1358
NM_001107575	Taf6l	0.1725	0.3090	0.0020	0.1040	0.0100	0.0671	0.0053	0.2178	0.1348	0.1837	0.2645	0.0795
NM_001107705	Wdr3	0.8784	0.8500	0.8618	0.0878	0.8911	0.6808	0.7596	0.7191	0.6807	0.7387	0.8772	0.7849
NM_001107630	Vgll2	0.5072	0.0923	0.3167	0.2877	0.3544	0.2777	0.1929	0.4483	0.5673	0.3556	0.4061	0.6206
NM_001113185	Gria4	0.7104	0.7367	0.7038	0.6882	0.6039	0.5470	0.6477	0.5721	0.6679	0.6945	0.7225	0.6264
NM_001107724	Tram1l1	0.9119	0.9034	0.9642	0.9243	0.6708	0.6928	0.9903	0.7259	0.7433	0.7884	0.9709	0.9738
NM_001108363	Hmx1	0.9223	0.8911	0.9307	0.8406	0.6408	0.8141	0.6324	0.7639	0.9622	0.9773	0.4613	0.8177
NM_001108352	Cdc7	0.8655	0.6419	0.9326	0.7339	0.3745	0.8481	0.2930	0.5532	0.6630	0.6963	0.5946	0.6628
NM_001108312	RGD1563888	0.9678	0.9772	0.9428	0.3984	0.5533	0.6113	0.8813	0.4607	0.7953	0.7896	0.7046	0.2740
NM_001107579	RGD1565857	0.6999	0.3181	0.2252	0.4740	0.6903	0.0392	0.7794	0.7787	0.8679	0.2585	0.5922	0.8311
NM_001108314	RGD1562683	0.3618	0.4888	0.2279	0.2730	0.3960	0.1701	0.0649	0.3062	0.8049	0.2990	0.1636	0.2347
NM_001107578	Ms4a1	0.2122	0.3000	0.0808	0.4185	0.2232	0.3077	0.1952	0.6212	0.1472	0.1870	0.4739	0.2510
NM_001108501	Cp110	0.2648	0.2690	0.7385	0.4487	0.4918	0.4392	0.4460	0.6335	0.3348	0.4119	0.6663	0.5077
NM_001134506	RGD1305932	0.4684	0.0307	0.9271	0.4283	0.4853	0.1300	0.4040	0.3907	0.5773	0.6139	0.2755	0.4500
NM_001108317	Boc	0.0613	0.0391	0.0131	0.1766	0.6930	0.5409	0.0016	0.6866	0.0532	0.0865	0.0784	0.0018
NM_001107849	Stard8	0.5784	0.4033	0.6562	0.7944	0.6667	0.6447	0.4497	0.8136	0.6764	0.7574	0.5271	0.7375
NM_001107696	Scnm1	0.0121	0.2219	0.3331	0.1356	0.0223	0.1581	0.1713	0.1085	0.0634	0.1563	0.0540	0.3120
NM_001107619	Tmprss3	0.5467	0.7146	0.7723	0.5571	0.6249	0.7089	0.4404	0.4540	0.4816	0.6904	0.3087	0.3237
NM_001107586	Cstf2t	0.8950	0.9220	0.9363	0.5040	0.8062	0.0853	0.0650	0.2259	0.8550	0.4011	0.6290	0.6559
NM_001107714	Dennd2d	0.2217	0.7398	0.8792	0.8403	0.6218	0.8831	0.7873	0.7641	0.8553	0.8037	0.7522	0.8938
NM_001108518	Nat11	0.3564	0.2670	0.5014	0.5417	0.4130	0.2491	0.4432	0.4387	0.4222	0.4942	0.2447	0.5333
NM_001107716	Taf13	0.3617	0.9317	0.6635	0.8886	0.6648	0.8405	0.9158	0.7765	0.4548	0.9344	0.8910	0.0414
NM_001001514	Ablim2	0.9369	0.8612	0.8769	0.6097	0.8118	0.8202	0.8629	0.3936	0.6793	0.7401	0.9238	0.7571

NM_001134543	RGD1564085	0.8762	0.7784	0.5137	0.5515	0.7848	0.4744	0.2775	0.6030	0.5900	0.4545	0.7692	0.3758
NM_001108519	Ms4a8a	0.8486	0.8594	0.4323	0.0964	0.2952	0.3196	0.1356	0.8387	0.2207	0.5483	0.6113	0.0821
NM_001107730	Kif5c	0.8074	0.8383	0.7426	0.6437	0.5690	0.6134	0.8445	0.3956	0.8399	0.6987	0.6726	0.5237
NM_001007007	Rnaseh2b	0.8396	0.9169	0.1590	0.8001	0.9525	0.8653	0.8984	0.9325	0.7639	0.7659	0.9275	0.3846
NM_001108538	Cdc40	0.9474	0.8324	0.9532	0.8209	0.9338	0.8746	0.7289	0.6469	0.4483	0.8203	0.7818	0.8502
NM_001107601	Ldb1	0.8660	0.8351	0.7437	0.5700	0.9685	0.6453	0.5203	0.9507	0.9900	0.9802	0.7042	0.7641
NM_001107949	Dnajc6	0.9117	0.9270	0.6765	0.9179	0.5095	0.8909	0.8559	0.6310	0.5472	0.8560	0.9129	0.7020
NM_001107593	Cnnm1	0.9333	0.9383	0.8042	0.8293	0.8066	0.7548	0.8653	0.7020	0.5833	0.3190	0.5139	0.9252
NM_001107177	Jarid1b	0.9581	0.9699	0.8174	0.4993	0.9975	0.9769	0.7870	0.9900	0.9580	0.9916	0.9945	0.8580
NM_001107939	Pappa	0.5831	0.1349	0.1010	0.0692	0.4423	0.6050	0.3308	0.0635	0.2636	0.2759	0.4672	0.2092
NM_001107603	Fbxl15	0.5936	0.3943	0.3155	0.7459	0.4398	0.6728	0.7951	0.4022	0.8259	0.8082	0.6833	0.6991
NM_001108532	Hnrnph3	0.9474	0.9275	0.8739	0.1537	0.9274	0.8634	0.6906	0.8405	0.8243	0.9518	0.9631	0.8516
NM_001107679	Tiparp	0.2836	0.2519	0.7498	0.0718	0.1626	0.2097	0.2976	0.1145	0.3141	0.1399	0.2886	0.2044
NM_001107763	Ltk	0.2590	0.6681	0.4740	0.7674	0.5962	0.4992	0.3304	0.4122	0.5448	0.1447	0.3396	0.0641
NM_001107734	Tlk1	0.9732	0.9571	0.9895	0.9977	0.9933	0.9827	0.9367	0.9896	0.9419	0.9433	0.9263	0.9077
NM_001107729	Ifi44	0.0988	0.0933	0.1382	0.2423	0.1814	0.1797	0.2191	0.0397	0.0519	0.0471	0.2556	0.2721
NM_001108523	Noc3l	0.6661	0.3924	0.7838	0.3846	0.6152	0.4572	0.2893	0.7127	0.4621	0.1540	0.5155	0.6641
NM_001107591	Sfrp5	0.8327	0.5862	0.7994	0.1817	0.5069	0.3939	0.8594	0.7667	0.4261	0.1530	0.8662	0.5437
NM_001108685	Sfrs4	0.6001	0.7097	0.6790	0.4057	0.3730	0.4105	0.2734	0.4616	0.4518	0.6754	0.6476	0.4523
NM_001108679	Fam176b	0.0319	0.0127	0.5112	0.0491	0.0191	0.0453	0.1661	0.0417	0.1093	0.0198	0.1459	0.0075
NM_001108605	Pcif1	0.7839	0.2876	0.4662	0.2051	0.4924	0.3006	0.3038	0.1971	0.5422	0.6061	0.4565	0.3580
NM_001107604	Pdcd11	0.8844	0.8951	0.2759	0.1536	0.9149	0.9089	0.4762	0.9918	0.8339	0.8584	0.9679	0.6267
NM_001107727	Mttp	0.3833	0.0614	0.3817	0.4858	0.3069	0.2666	0.0802	0.3882	0.1917	0.1824	0.5088	0.1023
NM_001108338	Fbxo21	0.6261	0.4966	0.6546	0.7124	0.6073	0.7117	0.6631	0.5564	0.6095	0.6532	0.4885	0.6589
NM_001108521	Tmc1	0.0671	0.0309	0.2321	0.1810	0.1507	0.1472	0.0780	0.1417	0.1850	0.3669	0.0962	0.1420
NM_001108353	Plac8	0.0514	0.1784	0.1645	0.1798	0.0346	0.0577	0.1852	0.0029	0.4562	0.3568	0.5916	0.2273
NM_001108468	Rdh13	0.7823	0.6280	0.4281	0.8401	0.8150	0.3338	0.9221	0.7034	0.6380	0.4695	0.5164	0.7606
NM_001108513	Unc93b1	0.7432	0.4751	0.7419	0.6390	0.6032	0.0802	0.9175	0.8853	0.8314	0.6870	0.6438	0.9591
NM_001108365	Gas2l1	0.5659	0.9781	0.1719	0.7504	0.4400	0.3625	0.5225	0.7101	0.9765	0.9205	0.5829	0.3870
NM_001108364	Mxd4	0.7632	0.9947	0.9431	0.9957	0.9634	0.7390	0.9558	0.9516	0.8382	0.9961	0.9962	0.2311
NM_001108361	Cpeb2	0.7890	0.8929	0.7376	0.9087	0.9882	0.8534	0.8618	0.8754	0.1217	0.7571	0.8511	0.2478
NM_001108360	Zcchc4	0.8555	0.9472	0.6945	0.9198	0.7460	0.8605	0.9358	0.7918	0.7655	0.9508	0.9532	0.9591
NM_001108358	Lnx1	0.3417	0.3796	0.3339	0.3267	0.3664	0.6461	0.5400	0.0384	0.4299	0.2876	0.6374	0.4067
NM_001108357	Mobkl1a	0.5947	0.5709	0.7347	0.7675	0.5194	0.7724	0.7037	0.5005	0.7725	0.4749	0.8452	0.6853
NM_001108355	Cnot6l	0.9837	0.9889	0.9584	0.9792	0.9584	0.9633	0.9856	0.9926	0.9594	0.9897	0.9831	0.9072
NM_001108535	Ccar1	0.9879	0.9935	0.9763	0.8978	0.9555	0.7607	0.5629	0.9573	0.9758	0.9823	0.9444	0.8068
NM_001108572	Ehmt1	0.9966	0.9305	0.4452	0.8087	0.6741	0.9857	0.6878	0.9548	0.8842	0.9882	0.9377	0.8444
NM_001107606	Sh3pxd2a	0.7501	0.0638	0.6600	0.5909	0.3856	0.7684	0.8453	0.6339	0.7590	0.7721	0.5772	0.7704
NM_001107595	Entpd7	0.8747	0.8498	0.8680	0.9246	0.9371	0.9394	0.6576	0.8096	0.5238	0.7887	0.8718	0.8305
NM_001108563	Sypl2	0.6799	0.9104	0.7509	0.8127	0.5414	0.7561	0.8264	0.7347	0.7986	0.5814	0.0984	0.8916
NM_001108553	Tmem154	0.0308	0.0520	0.5113	0.5196	0.0209	0.1909	0.3841	0.4127	0.0987	0.4153	0.1583	0.3914
NM_001108551	Tmem144	0.0417	0.5504	0.4022	0.1049	0.0697	0.3993	0.0479	0.3213	0.2680	0.4735	0.0444	0.0377
NM_001107740	Ctnnd1	0.9592	0.5041	0.9337	0.9915	0.9376	0.9293	0.9926	0.8739	0.6937	0.4036	0.6769	0.8918
NM_001134544	RGD1565545	0.3266	0.7333	0.7531	0.5137	0.8182	0.3389	0.8675	0.4074	0.2306	0.1839	0.1385	0.5352
NM_001108410	Cd83	0.4484	0.5746	0.6636	0.7028	0.2185	0.2036	0.1443	0.6764	0.5410	0.7286	0.4982	0.3770
NM_001107801	Zswim3	0.7672	0.9390	0.2171	0.8025	0.9143	0.9355	0.8299	0.7876	0.8325	0.9039	0.7412	0.8549
NM_001107737	Itga4	0.8845	0.7592	0.7368	0.8860	0.3092	0.7410	0.5880	0.5040	0.8230	0.7208	0.4193	0.7959
NM_001107673	Slc7a11	0.7169	0.8818	0.9118	0.9412	0.7518	0.8569	0.0980	0.4114	0.8634	0.9400	0.6080	0.8859
NM_001107615	Brpf3	0.4856	0.1199	0.3586	0.4712	0.2367	0.2896	0.1551	0.2968	0.1559	0.3947	0.2730	0.3141

NM_001107753	Lrrc4c	0.7655	0.3862	0.2847	0.2771	0.4154	0.3437	0.1841	0.1444	0.3811	0.3522	0.2510	0.2170
NM_001107750	Tspan18	0.2384	0.3651	0.4058	0.1148	0.7592	0.5150	0.1352	0.0890	0.4441	0.2574	0.6588	0.2929
NM_001108587	Ccdc34	0.2168	0.9911	0.1804	0.4034	0.4401	0.0256	0.9747	0.8270	0.7658	0.9708	0.8926	0.0525
NM_001109562	Gpr137	0.2243	0.2781	0.4311	0.2888	0.3733	0.7253	0.2973	0.7206	0.5965	0.6358	0.1810	0.5943
NM_001107618	Mdga1	0.1808	0.5208	0.4094	0.2974	0.5574	0.3324	0.4698	0.5923	0.5719	0.6239	0.4180	0.6620
NM_001108385	Epb4.9	0.1223	0.6528	0.8638	0.8483	0.9292	0.9537	0.9134	0.8065	0.4903	0.7795	0.8758	0.9298
NM_001108406	Sptlc1	0.3883	0.1399	0.2622	0.2278	0.3163	0.5052	0.0932	0.3712	0.3511	0.2603	0.1982	0.5320
NM_001108405	Lect2	0.1243	0.1637	0.0704	0.3406	0.4476	0.3237	0.2694	0.5495	0.1674	0.0919	0.3064	0.0998
NM_001108395	Plac9	0.2402	0.5466	0.2402	0.1388	0.6937	0.4214	0.2407	0.7667	0.7450	0.4863	0.3276	0.4446
NM_001108404	Cdc14b	0.9883	0.9831	0.9463	0.8799	0.9675	0.9914	0.9325	0.9339	0.9955	0.9739	0.9762	0.8438
NM_001108601	Dhx35	0.4029	0.3054	0.3419	0.3154	0.8344	0.8436	0.0903	0.3105	0.4615	0.7803	0.3556	0.9071
NM_001108579	Sh2d3c	0.8500	0.3882	0.2198	0.1026	0.2455	0.8872	0.2319	0.7388	0.5723	0.6590	0.7119	0.6306
NM_001108588	Slc5a12	0.7925	0.5215	0.7772	0.7422	0.8275	0.8496	0.7107	0.9536	0.6725	0.5971	0.7044	0.8742
NM_001108409	Fgd3	0.7661	0.7728	0.6960	0.6867	0.9158	0.5974	0.7583	0.8899	0.5584	0.9002	0.9626	0.7460
NM_001108408	Nol8	0.8606	0.8583	0.3087	0.1759	0.6658	0.4370	0.1424	0.2661	0.9960	0.9372	0.7698	0.7167
NM_001108397	Sfrs14	0.6550	0.9133	0.7511	0.1786	0.8037	0.7100	0.7594	0.5649	0.7549	0.5627	0.8450	0.5958
NM_001107788	Gzf1	0.8798	0.5024	0.8221	0.9278	0.8559	0.8671	0.5098	0.8833	0.9330	0.8199	0.8289	0.4773
NM_001108604	Rbpjl	0.5102	0.7903	0.2029	0.7397	0.8223	0.5113	0.7394	0.7769	0.7896	0.8173	0.6342	0.7904
NM_001107623	Tmem26	0.6571	0.6506	0.4159	0.6909	0.5586	0.2627	0.3768	0.6039	0.5804	0.0788	0.6906	0.1206
NM_001107622	Rhobtb1	0.6117	0.7893	0.2791	0.8709	0.4765	0.6132	0.3873	0.8241	0.7724	0.7941	0.7777	0.2736
NM_001107664	Hps3	0.4546	0.2863	0.6015	0.2780	0.4398	0.8787	0.0390	0.2574	0.2168	0.0556	0.3236	0.7937
NM_001108427	Pfdn1	0.3270	0.4411	0.6982	0.0207	0.2855	0.1810	0.4452	0.0647	0.5353	0.1233	0.0265	0.1544
NM_001108426	Kif20a	0.8058	0.8132	0.0117	0.9760	0.8154	0.9369	0.9409	0.8608	0.8908	0.8362	0.9992	0.0236
NM_001108559	Prpf3	0.9446	0.5720	0.1799	0.1094	0.8256	0.8301	0.3118	0.9024	0.9598	0.8623	0.6327	0.3996
NM_001108592	Snrpb2	0.3643	0.4978	0.4781	0.8318	0.6175	0.1042	0.4450	0.3885	0.1703	0.1883	0.4848	0.1378
NM_001108555	Lrba	0.3151	0.5477	0.5320	0.3045	0.0093	0.3203	0.4218	0.1958	0.3429	0.1748	0.3434	0.9095
NM_001107681	Ppm1l	0.0205	0.3732	0.9822	0.6112	0.3200	0.2156	0.2326	0.0157	0.7792	0.3034	0.0174	0.7892
NM_001107638	Scml4	0.6871	0.5804	0.5690	0.3755	0.6379	0.3953	0.4660	0.5453	0.3833	0.3937	0.7028	0.6746
NM_001107809	Znf512b	0.8896	0.9178	0.9841	0.9103	0.9438	0.8814	0.9500	0.9006	0.9456	0.9755	0.9886	0.9281
NM_001108130	Ccdc151	0.7195	0.8096	0.4798	0.6946	0.8517	0.9237	0.5659	0.7863	0.7691	0.6736	0.4148	0.5652
NM_001107637	Sec63	0.5165	0.7068	0.5576	0.5177	0.4789	0.4255	0.2003	0.5427	0.5494	0.8770	0.4457	0.2300
NM_001107634	Cdc2l6	0.9834	0.8041	0.6648	0.8394	0.9437	0.9575	0.6374	0.9596	0.8493	0.6747	0.9290	0.9587
NM_001107774	Itpa	0.7899	0.6982	0.7455	0.9544	0.9548	0.7535	0.9888	0.9954	0.8938	0.9616	0.7050	0.1791
NM_001108418	Heatr1	0.2422	0.4520	0.3222	0.4699	0.2229	0.2727	0.3501	0.4510	0.2178	0.1889	0.2740	0.3637
NM_001108439	Large	0.8328	0.7919	0.6818	0.7838	0.8929	0.8312	0.8579	0.6766	0.8281	0.6139	0.3629	0.6231
NM_001108438	Dok4	0.0876	0.0929	0.0800	0.2948	0.0882	0.2410	0.3472	0.2005	0.0793	0.3357	0.0627	0.0614
NM_001108436	Fbxo15	0.0723	0.2629	0.0303	0.3658	0.4986	0.1584	0.1005	0.0916	0.2553	0.1126	0.2948	0.0793
NM_001107833	Lrsam1	0.4793	0.7450	0.9342	0.5502	0.8405	0.5705	0.3893	0.3507	0.6017	0.2630	0.4518	0.5279
NM_001107642	Tulp1	0.7516	0.7498	0.7548	0.7157	0.7508	0.5197	0.5376	0.2486	0.7356	0.9031	0.5525	0.9106
NM_001107782	Btbd3	0.8729	0.7807	0.9849	0.8640	0.9484	0.7010	0.9324	0.9959	0.8580	0.9627	0.7674	0.7424
NM_001108114	Tenc1	0.9770	0.2121	0.1016	0.8952	0.9890	0.9659	0.3132	0.9970	0.9398	0.5241	0.4197	0.2643
NM_001108018	Cbll1	0.8475	0.3782	0.5422	0.2671	0.1291	0.1474	0.3499	0.3991	0.9780	0.8005	0.8647	0.6646
NM_001108460	Hddc2	0.4852	0.5350	0.8289	0.9092	0.0744	0.1658	0.6152	0.1730	0.2630	0.6277	0.2250	0.1279
NM_001108458	Ust	0.6115	0.1908	0.2053	0.0647	0.2957	0.1269	0.2233	0.0237	0.0590	0.3640	0.5809	0.0135
NM_001107645	Rhobtb3	0.8769	0.9775	0.9534	0.8958	0.9557	0.9045	0.9527	0.9625	0.9448	0.9588	0.9952	0.9664
NM_001108585	RGD1563222	0.7010	0.9114	0.6639	0.8656	0.3882	0.6160	0.6424	0.8971	0.5277	0.7110	0.6826	0.8345
NM_001108580	Ptrh1	0.1894	0.1746	0.8880	0.8907	0.0245	0.0300	0.8948	0.2033	0.3257	0.4680	0.2656	0.6588
NM_001108636	Ry1	0.0047	0.3283	0.3090	0.0573	0.0829	0.0267	0.0801	0.0424	0.8638	0.4046	0.3868	0.6922
NM_001108833	Sp6	0.3742	0.6113	0.2847	0.8328	0.3911	0.8616	0.7787	0.7489	0.1108	0.5156	0.3582	0.8663

NM_001108630	Ecop	0.5151	0.4488	0.5052	0.4693	0.6308	0.5936	0.6869	0.6302	0.7247	0.6351	0.6716	0.5280
NM_001107653	Slc45a2	0.0527	0.5735	0.4099	0.2719	0.8063	0.5411	0.6958	0.5793	0.5097	0.1752	0.1884	0.2751
NM_001107648	Ptcd2	0.3305	0.1481	0.1277	0.4875	0.5525	0.5753	0.0326	0.2879	0.7855	0.6526	0.6521	0.4459
NM_001107657	Myo10	0.7282	0.5848	0.6249	0.2809	0.8799	0.3396	0.8419	0.6868	0.6083	0.8301	0.8047	0.7961
NM_001108591	Rnf24	0.4294	0.5969	0.3177	0.4700	0.5645	0.3137	0.4353	0.3321	0.0070	0.0621	0.3071	0.3379
NM_001107803	Slc35c2	0.4442	0.8943	0.1133	0.5546	0.8863	0.6867	0.2698	0.6344	0.6752	0.4959	0.5106	0.7476
NM_001108830	Sox15	0.4616	0.8940	0.3344	0.7013	0.5184	0.5435	0.4272	0.5454	0.8899	0.8238	0.9189	0.8230
NM_001108721	Prima1	0.4777	0.3408	0.4747	0.6030	0.8543	0.1526	0.6190	0.3983	0.6058	0.8522	0.3925	0.5668
NM_001108594	Dtd1	0.8484	0.2772	0.9108	0.7832	0.7057	0.8460	0.9190	0.8976	0.1040	0.1467	0.3606	0.1572
NM_001107792	RGD1559748	0.1278	0.0897	0.1572	0.3240	0.5111	0.2661	0.4407	0.4604	0.5272	0.1148	0.0346	0.2894
NM_001107791	Bpil3	0.1770	0.3727	0.1614	0.0184	0.3357	0.2045	0.4805	0.2988	0.3480	0.4371	0.4292	0.5818
NM_001107668	Dcun1d1	0.4447	0.6404	0.9396	0.9677	0.5977	0.7813	0.9711	0.5457	0.8311	0.3059	0.5540	0.1488
NM_001107671	RGD1308448	0.8608	0.2259	0.8652	0.9400	0.4341	0.6822	0.6499	0.0644	0.8926	0.7376	0.8374	0.6547
NM_001134425	Aig1	0.3250	0.1129	0.0412	0.9112	0.8454	0.6879	0.2021	0.7603	0.2534	0.1660	0.1268	0.0965
NM_001108481	Rras	0.0283	0.0155	0.3357	0.1074	0.1117	0.0438	0.0699	0.0775	0.0829	0.1860	0.0351	0.4441
NM_001108480	Bcl2l12	0.4551	0.6339	0.9708	0.9853	0.2901	0.0983	0.5752	0.6092	0.5319	0.7332	0.7806	0.4732
NM_001108477	Hnrnpul1	0.9894	0.9880	0.9068	0.9282	0.9274	0.6110	0.9483	0.9496	0.9843	0.9210	0.9405	0.9054
NM_001047852	Bclaf1	0.9011	0.8780	0.4567	0.5305	0.9471	0.7378	0.6791	0.8484	0.7866	0.9290	0.9555	0.6108
NM_001107806	Npepl1	0.0528	0.0511	0.2876	0.9104	0.3628	0.7107	0.1418	0.2320	0.2325	0.1259	0.0747	0.8234
NM_001109218	RGD1565355	0.3458	0.7707	0.2767	0.6077	0.6291	0.5051	0.5701	0.5506	0.1137	0.7304	0.7340	0.0416
NM_001108607	Cse1l	0.9810	0.7317	0.1788	0.8675	0.8257	0.9671	0.8431	0.7751	0.9006	0.8393	0.9515	0.6843
NM_001107674	Narg1	0.6069	0.9555	0.4690	0.4375	0.4209	0.3136	0.3044	0.2850	0.9016	0.9646	0.7772	0.8497
NM_001107798	Gdap1l1	0.2917	0.5002	0.6437	0.5647	0.6835	0.6642	0.5164	0.7379	0.2395	0.5295	0.1270	0.0765
NM_001107797	Chd6	0.7853	0.7728	0.9159	0.9030	0.8053	0.8765	0.1790	0.7223	0.7336	0.6273	0.9142	0.5564
NM_001108502	Bucs1	0.1129	0.2998	0.2145	0.1602	0.8761	0.5279	0.4069	0.9371	0.2276	0.3617	0.1192	0.2548
NM_001108654	Tox	0.5153	0.4710	0.8755	0.5391	0.6677	0.6033	0.4117	0.4326	0.6907	0.8383	0.4971	0.5929
NM_001107808	Btbd4	0.6223	0.8713	0.5670	0.6035	0.5505	0.5517	0.8503	0.6336	0.9209	0.7903	0.6957	0.7812
NM_001108514	Ankrd13d	0.8250	0.7943	0.9027	0.6956	0.5339	0.4786	0.8648	0.8289	0.8942	0.9042	0.8409	0.8028
NM_001107880	Mtmr14	0.2781	0.0561	0.7477	0.7774	0.5399	0.6088	0.6135	0.4806	0.7506	0.4717	0.6677	0.6880
NM_001107862	Nt5c3	0.7918	0.9598	0.7864	0.9756	0.9035	0.8049	0.7379	0.9616	0.7952	0.6606	0.9036	0.9580
NM_001107682	Nmd3	0.1396	0.0358	0.1110	0.0653	0.0532	0.0362	0.1584	0.0052	0.0257	0.0443	0.0721	0.3603
NM_001108005	Mast2	0.7701	0.7308	0.0053	0.4149	0.8883	0.6423	0.6803	0.7104	0.8327	0.8498	0.7270	0.3758
NM_001108629	Ggct	0.1641	0.8745	0.7066	0.3441	0.0153	0.0875	0.9955	0.2672	0.3657	0.7834	0.3897	0.6771
NM_001108508	Eps8l2	0.0799	0.7746	0.1566	0.8656	0.3042	0.5841	0.5283	0.2734	0.8554	0.4912	0.8053	0.8475
NM_001108619	Impdh1	0.4002	0.8391	0.8782	0.7757	0.8755	0.9368	0.9973	0.9336	0.9052	0.9081	0.9375	0.9451
NM_001108623	Parp12	0.0374	0.1468	0.1415	0.1910	0.1023	0.1039	0.3299	0.1528	0.5626	0.0853	0.3269	0.6446
NM_001108003	Rnf19b	0.0212	0.0351	0.0494	0.0013	0.0129	0.0325	0.0727	0.0452	0.0267	0.0743	0.0918	0.0121
NM_001134507	RGD1309350	0.0308	0.5405	0.3716	0.8263	0.0425	0.2977	0.7463	0.0151	0.6749	0.3969	0.6100	0.3165
NM_001108628	Znrf1	0.7126	0.7274	0.8603	0.9011	0.2629	0.8834	0.4222	0.3990	0.4080	0.8972	0.7746	0.8865
NM_001108525	Cutc	0.6394	0.6861	0.4214	0.0794	0.3102	0.5329	0.3785	0.2222	0.6932	0.4493	0.7904	0.4752
NM_001108681	Zmym6	0.8754	0.9192	0.6139	0.6252	0.9817	0.9155	0.2639	0.8365	0.4798	0.9556	0.5113	0.8206
NM_001108648	Itfg2	0.6923	0.2085	0.3025	0.4654	0.5769	0.5838	0.7025	0.2449	0.4636	0.1959	0.3689	0.2723
NM_001108529	RGD1308722	0.3343	0.0578	0.7787	0.9019	0.8695	0.8178	0.4031	0.8308	0.9464	0.8051	0.6707	0.8813
NM_001108528	RGD1306917	0.8149	0.7234	0.6999	0.6473	0.7761	0.3131	0.6672	0.6697	0.7546	0.8637	0.6882	0.7828
NM_001108527	Sfxn4	0.7888	0.3876	0.8074	0.2554	0.0311	0.7489	0.2049	0.5482	0.1723	0.4389	0.4097	0.6736
NM_001107895	Med21	0.1364	0.6067	0.4651	0.6281	0.1795	0.2879	0.7823	0.2879	0.2460	0.6777	0.7217	0.1553
NM_001107904	Tgs1	0.3269	0.2297	0.8355	0.3360	0.3830	0.8071	0.1669	0.3311	0.2462	0.1128	0.2682	0.7819
NM_001107690	RGD1306107	0.9892	0.2108	0.9160	0.7678	0.8187	0.9556	0.7532	0.8655	0.5690	0.9522	0.8613	0.9513
NM_001108682	Tlr12	0.5848	0.6443	0.4732	0.6901	0.6010	0.8340	0.7903	0.0839	0.5470	0.8031	0.6989	0.8268

NM_001108641	Rpusd3	0.0096	0.3083	0.3894	0.8414	0.0818	0.2620	0.4269	0.0948	0.7579	0.6023	0.6386	0.4826
NM_001108642	LOC362419	0.3574	0.2818	0.4137	0.1979	0.1853	0.4393	0.2016	0.1922	0.1753	0.4489	0.3251	0.1842
NM_001108543	Mrps27	0.0926	0.8767	0.0716	0.0671	0.1337	0.0465	0.2290	0.2972	0.3230	0.5880	0.3082	0.6827
NM_001108646	Gpr162	0.6011	0.7083	0.7300	0.8342	0.4714	0.8476	0.3011	0.8514	0.6451	0.6921	0.8140	0.3168
NM_001108647	RGD1308257	0.8780	0.7393	0.8679	0.4986	0.7831	0.5600	0.7714	0.5866	0.8070	0.6871	0.5583	0.8085
NM_001108699	Pusl1	0.3650	0.6921	0.7703	0.2331	0.2319	0.0956	0.7419	0.1934	0.9525	0.7029	0.5882	0.7537
NM_001108686	Fam76a	0.5972	0.6797	0.7160	0.6325	0.6305	0.6614	0.6821	0.5564	0.6083	0.6282	0.6656	0.6693
NM_001108546	Ythdf3	0.9845	0.9862	0.9831	0.9735	0.9915	0.9283	0.8133	0.7731	0.8440	0.9876	0.8134	0.8840
NM_001108545	Fbxl7	0.3840	0.3550	0.1703	0.4610	0.4026	0.1697	0.4202	0.3023	0.5606	0.3371	0.5338	0.4551
NM_001009653	Mccc1	0.6675	0.5088	0.3281	0.2562	0.5173	0.5633	0.5850	0.7698	0.4363	0.1751	0.5527	0.3301
NM_001107781	Pak7	0.8929	0.7987	0.8380	0.9507	0.7807	0.9104	0.9067	0.5306	0.6697	0.8817	0.8074	0.8246
NM_001108714	Pigh	0.3413	0.3761	0.8754	0.8821	0.9397	0.8747	0.5962	0.8553	0.1639	0.6210	0.4925	0.7843
NM_001108693	Rbp7	0.0976	0.4844	0.5222	0.7597	0.5275	0.5328	0.6688	0.7634	0.7016	0.7397	0.0746	0.5153
NM_001107800	Stk4	0.8230	0.5796	0.5499	0.4917	0.6070	0.7292	0.3635	0.4145	0.6446	0.9358	0.9186	0.8875
NM_001107688	Ubqln4	0.8815	0.9504	0.6143	0.9824	0.9734	0.9323	0.7781	0.7463	0.9973	0.9609	0.7402	0.8820
NM_001107819	RGD1306304	0.3209	0.3175	0.2517	0.3354	0.4897	0.2578	0.3747	0.6023	0.4180	0.5182	0.2884	0.3163
NM_001107702	Fcrls	0.3455	0.2032	0.8156	0.1014	0.4012	0.0591	0.3259	0.2800	0.0484	0.2032	0.3111	0.1202
NM_001107701	RGD1309453	0.5807	0.5314	0.8508	0.1415	0.7642	0.4115	0.1276	0.7943	0.6565	0.8289	0.2442	0.4585
NM_001110782	Syn1	0.5120	0.6022	0.4877	0.4503	0.5774	0.6052	0.6502	0.6677	0.6534	0.4543	0.5749	0.8415
NM_001110492	Phf14	0.4545	0.9547	0.8975	0.9749	0.8139	0.7220	0.6127	0.7219	0.6631	0.7857	0.8926	0.7285
NM_001108558	RGD1562059	0.6630	0.4892	0.1558	0.2252	0.3348	0.4597	0.7769	0.2096	0.5793	0.1952	0.3189	0.2375
NM_001108557	S100a16	0.8530	0.7757	0.9043	0.9523	0.9620	0.9173	0.9302	0.8158	0.8575	0.9765	0.9831	0.8923
NM_001107844	Paxip1	0.2564	0.2265	0.6906	0.5571	0.3991	0.6237	0.2785	0.4888	0.2176	0.6343	0.4546	0.4928
NM_001105827	Mtmr4	0.7484	0.8525	0.8999	0.9777	0.9689	0.9876	0.6554	0.9544	0.9303	0.4152	0.8010	0.8627
NM_001108797	Fam117b	0.4409	0.9706	0.8995	0.9652	0.8866	0.8754	0.8243	0.8947	0.9139	0.9573	0.5102	0.9744
NM_001108562	Cyb561d1	0.3424	0.5367	0.8032	0.9744	0.9383	0.8222	0.9670	0.8182	0.5135	0.7794	0.8769	0.9658
NM_001108741	Appl2	0.3999	0.2915	0.2553	0.2712	0.2685	0.6602	0.1265	0.4073	0.2773	0.3376	0.2492	0.2729
NM_001108706	Efcab10	0.4014	0.4479	0.3134	0.6446	0.1277	0.1987	0.1344	0.0416	0.2200	0.3949	0.1204	0.2267
NM_001107942	Lad1	0.9432	0.8056	0.7378	0.3845	0.8987	0.6240	0.8884	0.8605	0.8955	0.6343	0.8436	0.9200
NM_001107905	Ubxn2b	0.7573	0.6003	0.6208	0.5881	0.4751	0.7886	0.2112	0.3024	0.5116	0.3269	0.7868	0.7227
NM_001107857	Ephb6	0.1559	0.1421	0.0638	0.2574	0.2830	0.5145	0.4171	0.2711	0.3079	0.2785	0.0559	0.8606
NM_001107708	Olfml3	0.9190	0.6081	0.0463	0.9344	0.8594	0.7498	0.8892	0.8507	0.1222	0.8678	0.5602	0.1033
NM_001108581	Epc2	0.9216	0.8901	0.7849	0.9064	0.9220	0.4833	0.8560	0.8518	0.7157	0.7231	0.7467	0.4584
NM_001108749	Fam116b	0.7829	0.6387	0.0859	0.7578	0.6927	0.9049	0.4345	0.9837	0.4975	0.5390	0.8322	0.8573
NM_001107712	Cttnbp2nl	0.2598	0.3694	0.4251	0.6463	0.2279	0.2148	0.2524	0.1156	0.6210	0.3428	0.3473	0.4386
NM_001107719	Sass6	0.3577	0.5935	0.1173	0.1284	0.4254	0.0784	0.1029	0.4432	0.5392	0.5677	0.7207	0.4106
NM_001107715	Gpr61	0.1761	0.3837	0.4530	0.1563	0.1112	0.0771	0.4540	0.3134	0.1334	0.1345	0.1722	0.1733
NM_001107713	RGD1306526	0.2960	0.7878	0.5770	0.3418	0.4522	0.3200	0.9481	0.3921	0.8889	0.7826	0.9820	0.3543
NM_001108751	Bcdin3d	0.7410	0.7889	0.1912	0.1427	0.7539	0.1542	0.7704	0.7152	0.7153	0.6193	0.7713	0.1570
NM_001107728	Rap1gds1	0.4372	0.8697	0.9675	0.9823	0.3454	0.9744	0.7785	0.3701	0.6974	0.9408	0.5556	0.9948
NM_001108690	RGD1310427	0.2694	0.3218	0.5119	0.3316	0.4446	0.2956	0.9584	0.3597	0.9203	0.0813	0.0109	0.3498
NM_017287	Mcm6	0.7750	0.8754	0.1014	0.7904	0.8539	0.9613	0.9453	0.8686	0.7568	0.9399	0.7939	0.0543
NM_001108596	Xrn2	0.9305	0.9710	0.5038	0.8600	0.7404	0.7547	0.8639	0.8995	0.9255	0.9375	0.9602	0.4817
NM_001108606	Trp53rk	0.5137	0.1271	0.1528	0.4301	0.1691	0.1077	0.2494	0.3116	0.2779	0.0962	0.3348	0.1513
NM_001108756	Rp9	0.6949	0.8132	0.4872	0.0544	0.6173	0.0528	0.7945	0.7401	0.6192	0.3384	0.6744	0.0833
NM_001107723	Sec24d	0.9957	0.5295	0.6767	0.7879	0.9026	0.8791	0.8810	0.9591	0.9063	0.9858	0.8484	0.8447
NM_001107722	Bcar3	0.2543	0.1106	0.2839	0.2052	0.1886	0.0711	0.2427	0.0078	0.1856	0.0569	0.1889	0.0958
NM_001108705	Sh3yl1	0.5019	0.3356	0.3387	0.3328	0.2460	0.1864	0.4190	0.1919	0.4564	0.2730	0.4986	0.2737
NM_001108967	Ntsr1	0.2826	0.5462	0.5568	0.3632	0.3423	0.3155	0.4252	0.6026	0.4689	0.3221	0.1587	0.4785

NM_001108876	Lsm1	0.4864	0.5204	0.3871	0.3168	0.5573	0.4025	0.4808	0.6562	0.6775	0.5546	0.7482	0.9079
NM_001108609	Pard6b	0.9383	0.9103	0.9582	0.8156	0.6334	0.6042	0.7281	0.9037	0.8380	0.8263	0.2616	0.8450
NM_001108599	Scand1	0.6459	0.7921	0.5386	0.7209	0.6779	0.0789	0.6584	0.5778	0.3244	0.8739	0.2804	0.0146
NM_001108610	Stx16	0.7726	0.4816	0.5716	0.4495	0.2706	0.4349	0.4291	0.3927	0.3330	0.4388	0.3106	0.4142
NM_001108608	B4galt5	0.3075	0.6423	0.6156	0.4558	0.6433	0.3640	0.6524	0.6012	0.5138	0.7035	0.3570	0.5679
NM_001108598	Dusp15	0.1701	0.6850	0.1243	0.3226	0.5057	0.6887	0.3914	0.4036	0.0930	0.1024	0.0963	0.5188
NM_001107932	Invs	0.9346	0.9831	0.9533	0.9540	0.9368	0.8666	0.9062	0.8968	0.9787	0.9049	0.9693	0.8712
NM_001107864	Smarcad1	0.5572	0.9185	0.9637	0.9917	0.9481	0.9861	0.8692	0.9539	0.9918	0.8364	0.9936	0.9851
NM_001108700	Ccdc75	0.1896	0.5649	0.0257	0.1828	0.4302	0.1453	0.2529	0.0696	0.3889	0.2261	0.3757	0.0162
NM_001108595	Nat5	0.0495	0.0388	0.1284	0.0729	0.2240	0.1412	0.0118	0.1839	0.1348	0.1260	0.1893	0.3830
NM_001107925	Ifnk	0.0230	0.0080	0.1255	0.0594	0.0088	0.1240	0.0686	0.0641	0.0060	0.1147	0.0416	0.0512
NM_001108715	Exdl2	0.4265	0.5595	0.3440	0.6520	0.5318	0.5748	0.2410	0.4272	0.6852	0.2320	0.4435	0.5471
NM_001107731	Neb	0.5004	0.5643	0.3836	0.2547	0.2028	0.2208	0.2503	0.2484	0.5980	0.3843	0.2218	0.3620
NM_001108053	Otub2	0.5499	0.3912	0.6328	0.1281	0.4058	0.9718	0.7885	0.3552	0.4849	0.4771	0.1987	0.6468
NM_001108626	LOC362347	0.3228	0.2884	0.3391	0.1977	0.5643	0.4400	0.2014	0.4481	0.2860	0.4482	0.1807	0.3769
NM_001108624	Ndufb2	0.2393	0.9140	0.9219	0.6895	0.5450	0.1306	0.9764	0.6863	0.8879	0.9831	0.9038	0.8375
NM_001108627	Cul1	0.9755	0.9376	0.9736	0.8852	0.9769	0.7809	0.4997	0.7479	0.9573	0.9526	0.9329	0.8692
NM_001107867	Atp6v1b1	0.7417	0.8567	0.9304	0.9067	0.5248	0.7954	0.5294	0.8034	0.7254	0.5633	0.7278	0.8613
NM_001113521	Arhgef7	0.7039	0.6255	0.6753	0.6622	0.6089	0.6205	0.5775	0.5011	0.6924	0.7161	0.5438	0.8117
NM_001113497	41,161.0000	0.8193	0.7691	0.8543	0.7554	0.5452	0.6312	0.7980	0.9385	0.5991	0.3680	0.8085	0.8354
NM_001108803	Mogat1	0.6686	0.5579	0.2378	0.5914	0.3202	0.4829	0.2534	0.3869	0.7008	0.3661	0.7566	0.9230
NM_001107738	Ssfa2	0.1925	0.1617	0.8745	0.2100	0.1242	0.3422	0.3032	0.2196	0.2001	0.3038	0.2968	0.8469
NM_001108639	Sumf1	0.1622	0.1523	0.5444	0.5607	0.4706	0.1071	0.2927	0.2305	0.0558	0.2252	0.2342	0.2706
NM_001108637	Fgd5	0.3507	0.5406	0.3444	0.6270	0.3893	0.2689	0.3574	0.7339	0.3612	0.0959	0.4511	0.4663
NM_001107741	Med19	0.2561	0.4856	0.2014	0.3377	0.0768	0.2601	0.3518	0.3043	0.3636	0.2945	0.2966	0.3426
NM_001107739	Dusp19	0.1874	0.2919	0.2312	0.4752	0.0892	0.1675	0.9631	0.2629	0.4456	0.1500	0.4747	0.0421
NM_001108778	Rrp9	0.7736	0.7251	0.0078	0.6754	0.6920	0.2680	0.5437	0.6439	0.8655	0.9289	0.9758	0.8557
NM_001108773	Rwdd2a	0.3198	0.3358	0.1338	0.3190	0.4250	0.0595	0.0275	0.3894	0.4719	0.0532	0.0176	0.0085
NM_001108645	RGD1307916	0.4517	0.7285	0.7918	0.5309	0.5144	0.4173	0.1937	0.5664	0.3821	0.4218	0.5149	0.7883
NM_001108644	Mfap5	0.1713	0.0238	0.1779	0.2005	0.6920	0.4400	0.0398	0.1668	0.1228	0.0638	0.1131	0.3020
NM_001107743	Slc43a3	0.4820	0.7412	0.8214	0.4656	0.6545	0.5011	0.6298	0.9445	0.9174	0.6986	0.7470	0.2123
NM_001108650	Loh12cr1	0.2319	0.6256	0.6801	0.4322	0.2961	0.2993	0.9092	0.0254	0.8002	0.8298	0.6221	0.3553
NM_001108649	RGD1306750	0.3145	0.0966	0.2226	0.1631	0.1743	0.0520	0.2196	0.3556	0.1153	0.1376	0.0527	0.5368
NM_001108793	Ccdc115	0.0417	0.0344	0.8838	0.8965	0.0548	0.1100	0.1724	0.1440	0.4073	0.5393	0.2593	0.7500
NM_001108785	Crtap	0.2921	0.0062	0.8413	0.5779	0.4485	0.7062	0.9066	0.2479	0.0355	0.0703	0.1127	0.8065
NM_001108386	Xpo7	0.9613	0.6519	0.9362	0.9329	0.9736	0.9911	0.7638	0.9416	0.4454	0.6611	0.9286	0.9377
NM_001108775	Rab6b	0.8826	0.3035	0.7659	0.2934	0.7979	0.9361	0.8898	0.7849	0.8502	0.8432	0.8853	0.9180
NM_001108744	Plxnc1	0.6524	0.4000	0.7341	0.6638	0.6590	0.6977	0.6036	0.6853	0.6894	0.6826	0.6849	0.6198
NM_001108724	Traf3	0.9641	0.8928	0.4953	0.7241	0.6759	0.9638	0.5393	0.7879	0.6531	0.8607	0.3042	0.7505
NM_001108691	Fbxo42	0.3160	0.5686	0.9128	0.6559	0.4838	0.2190	0.8612	0.5786	0.3585	0.4629	0.2809	0.6666
NM_001108889	Pou4f3	0.7955	0.4511	0.7645	0.8023	0.8279	0.8608	0.8327	0.8237	0.5641	0.7599	0.8217	0.3322
NM_001108880	Barx1	0.5036	0.4894	0.3218	0.0557	0.4409	0.3099	0.0726	0.1146	0.0774	0.3632	0.4119	0.5050
NM_001108804	Fbxo36	0.1474	0.4728	0.8007	0.7805	0.6186	0.2912	0.5690	0.1043	0.2113	0.6386	0.4682	0.7282
NM_001108675	Col9a2	0.6309	0.5737	0.1302	0.2752	0.5400	0.2685	0.3514	0.3647	0.7712	0.2401	0.0995	0.3348
NM_001108673	Med8	0.9475	0.8521	0.8688	0.8305	0.7867	0.4076	0.8400	0.7535	0.9807	0.7900	0.9535	0.5704
NM_001108864	Asb3	0.0925	0.0495	0.8522	0.1479	0.0239	0.0883	0.5762	0.0897	0.2157	0.0974	0.3819	0.3900
NM_001107744	Nup160	0.9489	0.7155	0.4147	0.3225	0.4001	0.6244	0.8891	0.4682	0.1818	0.9396	0.7983	0.7142
NM_001107876	Prickle2	0.9347	0.9622	0.9671	0.9647	0.5413	0.7485	0.5178	0.9621	0.9729	0.8012	0.9866	0.6340
NM_001108815	Tspan7	0.2330	0.8523	0.1952	0.6020	0.1130	0.4413	0.7963	0.1657	0.8641	0.7242	0.5060	0.7718

NM_001107745	C1qtnf4	0.8160	0.8443	0.8255	0.6159	0.7565	0.6904	0.8443	0.8058	0.6579	0.7602	0.7970	0.5828
NM_001134429	Fkbp2	0.5026	0.5489	0.1902	0.7499	0.4766	0.4059	0.2010	0.7859	0.5050	0.8177	0.8131	0.2676
NM_001107751	Ext2	0.7442	0.2734	0.7750	0.8856	0.4734	0.7082	0.7744	0.6661	0.4027	0.1216	0.7646	0.9731
NM_001107748	Slc35c1	0.7064	0.8274	0.7510	0.9943	0.9171	0.6695	0.9664	0.9269	0.9461	0.6818	0.5897	0.8254
NM_001007016	Sspo	0.9325	0.8432	0.2970	0.7030	0.7704	0.8302	0.6292	0.7892	0.6253	0.7366	0.7556	0.6780
NM_001108678	RGD1559909	0.0384	0.3522	0.0828	0.0362	0.1923	0.2790	0.3959	0.2677	0.0080	0.2752	0.1485	0.2388
NM_001108763	Ppcdc	0.8716	0.3702	0.8718	0.7682	0.3362	0.6122	0.9862	0.8055	0.9600	0.5098	0.5022	0.1514
NM_001108683	Sync	0.3104	0.0120	0.0209	0.0831	0.0066	0.6226	0.1671	0.1974	0.2141	0.0398	0.0779	0.4535
NM_001108671	Ror1	0.8560	0.2941	0.7984	0.7783	0.7510	0.6526	0.9427	0.1230	0.6318	0.5325	0.7739	0.3962
NM_001108823	RGD1562673	0.1417	0.5033	0.1846	0.1603	0.1119	0.4600	0.1676	0.2316	0.3771	0.0605	0.3505	0.1966
NM_001108688	Cda	0.0020	0.0202	0.3250	0.0094	0.0868	0.0234	0.0913	0.0131	0.0405	0.0404	0.1018	0.1501
NM_001108828	Rnf222	0.7422	0.4686	0.4139	0.7238	0.8486	0.6544	0.2956	0.3454	0.7510	0.5735	0.6168	0.6671
NM_001108814	Ndp	0.1881	0.7186	0.5042	0.4952	0.5199	0.1431	0.4178	0.0678	0.5559	0.8271	0.4427	0.3427
NM_001108812	Ubxn6	0.5934	0.2383	0.0364	0.8617	0.6166	0.6330	0.2590	0.8458	0.4168	0.1512	0.0222	0.4019
NM_001108684	Pum1	0.9520	0.7751	0.9671	0.7778	0.9307	0.9635	0.4429	0.9434	0.8950	0.9686	0.9555	0.9449
NM_001107746	Kbtbd4	0.6843	0.1400	0.6102	0.4124	0.4487	0.1105	0.2612	0.4258	0.4534	0.3616	0.0601	0.4589
NM_001107772	Zc3h6	0.3184	0.9050	0.7904	0.8809	0.2302	0.1912	0.4335	0.1873	0.4769	0.4707	0.3674	0.4041
NM_001108836	Ccr10	0.1815	0.5936	0.3813	0.6240	0.0699	0.4880	0.3447	0.2543	0.1889	0.3716	0.0408	0.2519
NM_001108774	RGD1309873	0.7387	0.5203	0.7347	0.4812	0.4802	0.4513	0.6366	0.6934	0.3729	0.7444	0.5552	0.6904
NM_001109668	Tollip	0.2499	0.8218	0.7388	0.8642	0.3684	0.5053	0.9031	0.1945	0.7608	0.4741	0.3418	0.8906
NM_032061	Cntnap1	0.3279	0.5525	0.4827	0.6565	0.6772	0.6573	0.6028	0.6487	0.6025	0.4374	0.4597	0.7168
NM_001108849	Cd209d	0.1742	0.1035	0.7040	0.1039	0.5493	0.6379	0.1268	0.2439	0.1477	0.7660	0.6943	0.3631
NM_001108845	Vpreb1	0.3478	0.4716	0.3929	0.3037	0.3431	0.5144	0.6893	0.3707	0.2170	0.4458	0.4415	0.2842
NM_001108844	Map6d1	0.6834	0.7177	0.4291	0.5107	0.9049	0.5855	0.9486	0.8278	0.7048	0.3491	0.2704	0.1846
NM_001107775	Slc4a11	0.7629	0.4648	0.4047	0.3098	0.4305	0.1761	0.4628	0.8488	0.5870	0.7206	0.3580	0.4061
NM_001108695	Thap3	0.7362	0.7157	0.9556	0.0773	0.2298	0.3254	0.9243	0.5790	0.9664	0.9420	0.8490	0.6343
NM_001005246	Dmd	0.4951	0.5137	0.5400	0.6191	0.4648	0.3607	0.3657	0.6325	0.1998	0.5577	0.5026	0.3961
NM_001108850	Trappc5	0.3180	0.0167	0.1149	0.0595	0.2668	0.0193	0.0994	0.3253	0.0603	0.0580	0.0307	0.3049
NM_001107943	Asah3l	0.9646	0.9976	0.9891	0.9708	0.9876	0.9809	0.9860	0.9616	0.9782	0.9773	0.9897	0.9735
NM_001108041	Acot3	0.4162	0.4785	0.6717	0.2637	0.4445	0.5710	0.5436	0.5400	0.6629	0.6219	0.5948	0.5528
NM_001107777	Siglec1	0.2200	0.4293	0.1575	0.4530	0.3322	0.3720	0.6928	0.4271	0.5760	0.2417	0.7483	0.3076
NM_001108111	Wnt10b	0.4925	0.5391	0.1125	0.2556	0.4031	0.3456	0.1541	0.2279	0.1257	0.0874	0.2903	0.1646
NM_001107973	Dem1	0.7447	0.6386	0.7600	0.7754	0.6687	0.4819	0.3627	0.5386	0.9260	0.7344	0.8554	0.6868
NM_001107785	RGD1308385	0.2598	0.0831	0.4539	0.5480	0.2401	0.7299	0.1561	0.4465	0.6784	0.1004	0.7807	0.4558
NM_001107908	Wdtc1	0.1296	0.3014	0.3563	0.8241	0.8437	0.7923	0.2114	0.5462	0.4892	0.4920	0.6430	0.8578
NM_001108711	Snx6	0.4487	0.3371	0.6655	0.5352	0.5002	0.0628	0.6203	0.4771	0.3846	0.1388	0.6271	0.7691
NM_001108717	Angel1	0.0147	0.0060	0.6729	0.4095	0.3608	0.5171	0.7374	0.2605	0.7194	0.3156	0.0539	0.9438
NM_001108716	Wdr21	0.4811	0.0507	0.0704	0.0851	0.1307	0.2527	0.1793	0.5733	0.0219	0.0933	0.2703	0.4010
NM_001108874	Tmem161a	0.7515	0.7219	0.8501	0.8422	0.8790	0.8924	0.8812	0.9693	0.8907	0.8612	0.8202	0.8166
NM_001108861	Yipf7	0.6638	0.4343	0.7680	0.6455	0.5669	0.7393	0.1155	0.8434	0.3776	0.2756	0.8608	0.2586
NM_001108720	Btbd7	0.2839	0.6305	0.3055	0.4602	0.5523	0.5342	0.5791	0.9289	0.7240	0.3589	0.8799	0.4637
NM_001108802	Speg	0.3109	0.0339	0.7721	0.5904	0.4258	0.5559	0.1617	0.3836	0.2115	0.2398	0.5176	0.1841
NM_001108791	Phf3	0.9831	0.4132	0.9599	0.9742	0.9227	0.5220	0.7256	0.5533	0.7801	0.7374	0.9538	0.6729
NM_001108718	Alkbh	0.7773	0.8881	0.5099	0.6472	0.5559	0.8568	0.3407	0.4786	0.7517	0.9271	0.2675	0.6469
NM_001108807	Cops7b	0.6384	0.3407	0.8970	0.9271	0.3728	0.9566	0.8263	0.7626	0.5784	0.5021	0.1664	0.9838
NM_001107858	Epha1	0.4791	0.2461	0.2431	0.3200	0.2719	0.4964	0.4091	0.3908	0.3663	0.4273	0.2009	0.2387
NM_001107836	Cep110	0.7481	0.7638	0.3145	0.0319	0.9147	0.8849	0.3812	0.7898	0.8504	0.6135	0.6944	0.8168
NM_001107789	H13	0.8856	0.9225	0.8441	0.8940	0.7931	0.7299	0.7733	0.7341	0.8054	0.9261	0.9141	0.7824
NM_001108050	Smek1	0.9484	0.2918	0.8714	0.2354	0.6654	0.9026	0.1495	0.6506	0.8492	0.9373	0.7276	0.8531

NM_001004220	Etfb	0.4255	0.6110	0.3462	0.5338	0.0583	0.0073	0.5262	0.8616	0.4417	0.6753	0.1156	0.0420
NM_001108881	Rnf144b	0.7040	0.7136	0.3960	0.7715	0.5286	0.6242	0.7994	0.6619	0.2729	0.7553	0.8252	0.7909
NM_001108887	Sft2d3	0.6589	0.2087	0.8780	0.9137	0.2811	0.1343	0.6325	0.3391	0.5097	0.4618	0.3115	0.3011
NM_001108885	Klhl14	0.9394	0.4747	0.6803	0.2632	0.5425	0.1112	0.8331	0.0602	0.8565	0.8189	0.2660	0.7245
NM_001108768	Pdcd7	0.8371	0.6583	0.7830	0.8097	0.7920	0.8633	0.4729	0.6412	0.7281	0.8864	0.8819	0.4909
NM_001108822	RGD1305537	0.5361	0.4557	0.6252	0.2763	0.4616	0.4495	0.2641	0.2345	0.6778	0.4083	0.5333	0.0748
NM_001108819	RGD1561958	0.7241	0.6653	0.0758	0.3832	0.6478	0.1277	0.6867	0.7472	0.6640	0.8850	0.3711	0.7078
NM_001108813	Ndufa1	0.0377	0.0475	0.0536	0.3461	0.0559	0.0464	0.0233	0.0124	0.0147	0.0345	0.3078	0.0036
NM_001108730	RGD1307722	0.4938	0.5670	0.2282	0.2951	0.4358	0.6715	0.3998	0.3914	0.2509	0.3026	0.2590	0.6050
NM_001108930	Vpreb3	0.0994	0.0840	0.1156	0.0155	0.0750	0.1002	0.2239	0.0198	0.1057	0.0230	0.1354	0.1049
NM_001108733	Dot1l	0.8760	0.6078	0.6733	0.7950	0.8820	0.9068	0.7948	0.6113	0.9287	0.7623	0.3199	0.5677
NM_001108732	Lsm7	0.5484	0.5913	0.0324	0.0952	0.9216	0.0413	0.5622	0.7775	0.1516	0.3260	0.1006	0.2549
NM_001108731	Hmg20b	0.8092	0.7023	0.7709	0.7310	0.9136	0.7259	0.9425	0.8646	0.9589	0.8234	0.7902	0.9344
NM_001108853	Piwil1	0.6064	0.8645	0.7072	0.5787	0.8816	0.3498	0.8096	0.8347	0.5698	0.1763	0.7464	0.3336
NM_001108735	Mbd3	0.6020	0.8791	0.0786	0.8728	0.9380	0.8443	0.8136	0.7684	0.6305	0.6615	0.8339	0.2697
NM_001108014	Smc6l1	0.8161	0.7119	0.2228	0.7311	0.6280	0.9408	0.5925	0.6002	0.5843	0.4301	0.6333	0.7028
NM_001108903	Prdm9	0.1349	0.0359	0.1161	0.1378	0.1433	0.0292	0.0162	0.0918	0.0705	0.0270	0.3377	0.0830
NM_001108742	Slc41a2	0.2241	0.2559	0.1787	0.8498	0.4536	0.3122	0.8936	0.3337	0.2277	0.2009	0.1689	0.1353
NM_001108838	C1ql1	0.5455	0.3947	0.5018	0.6492	0.7934	0.3657	0.7052	0.8394	0.6843	0.0838	0.6281	0.7956
NM_001108835	Hspb9	0.4258	0.1336	0.3550	0.1108	0.5412	0.0645	0.0889	0.3347	0.5075	0.7154	0.8603	0.5314
NM_001107799	RGD1307696	0.9463	0.7628	0.9730	0.9925	0.9978	0.9924	0.9288	0.9773	0.7856	0.9962	0.9966	0.9532
NM_001107810	Pcmtd2	0.9358	0.1497	0.1666	0.3193	0.5653	0.8907	0.2901	0.1256	0.6468	0.4563	0.4125	0.9207
NM_001108075	S1pr4	0.3584	0.7110	0.9071	0.7811	0.5260	0.5189	0.6732	0.2725	0.8451	0.1584	0.8780	0.4598
NM_001134428	Fkbp2	0.5026	0.5489	0.1902	0.7499	0.4766	0.4059	0.2010	0.7859	0.5050	0.8177	0.8131	0.2676
NM_001107814	Il1f5	0.5886	0.5793	0.7095	0.2559	0.6232	0.6992	0.2377	0.2425	0.3673	0.5358	0.2051	0.5664
NM_001107813	Zfp217	0.3800	0.2705	0.6621	0.1102	0.3404	0.3372	0.1178	0.1960	0.3787	0.7332	0.7329	0.1098
NM_001109204	Rad51	0.2248	0.1286	0.1427	0.6841	0.3643	0.9747	0.1553	0.7004	0.1541	0.0058	0.0918	0.0428
NM_001108895	Best2	0.1247	0.5410	0.5302	0.0687	0.2784	0.5646	0.0930	0.6607	0.1277	0.3557	0.3235	0.4310
NM_001107837	Lhx6	0.9534	0.9672	0.9177	0.8056	0.8258	0.9117	0.9318	0.9482	0.9526	0.9716	0.9218	0.8368
NM_001108750	Cpne8	0.6811	0.4976	0.6212	0.6374	0.5572	0.5070	0.1643	0.3143	0.2966	0.4175	0.3519	0.4275
NM_001107832	Ptges2	0.3693	0.0885	0.2250	0.7769	0.0519	0.0213	0.5862	0.1317	0.0174	0.4772	0.0914	0.1613
NM_001107838	Rbm18	0.2668	0.1524	0.6735	0.3194	0.4980	0.2832	0.2662	0.3345	0.1212	0.0357	0.2137	0.4457
NM_001108037	Rdh12	0.8724	0.6636	0.7678	0.3098	0.8730	0.8892	0.7710	0.4050	0.4571	0.5778	0.2500	0.6950
NM_001109210	Cbln4	0.4414	0.5561	0.5409	0.5222	0.3892	0.4831	0.6643	0.5274	0.7123	0.6950	0.6180	0.4904
NM_001108762	Commd4	0.6290	0.7779	0.2479	0.5080	0.8272	0.8441	0.1299	0.9253	0.7368	0.9443	0.8573	0.9122
NM_001108766	RGD1309779	0.8194	0.8267	0.9443	0.7592	0.8707	0.8682	0.9239	0.8620	0.8120	0.9999	0.8509	0.9019
NM_001108767	Nope	0.8960	0.8258	0.8264	0.8219	0.8823	0.7167	0.8920	0.9300	0.4345	0.8360	0.7453	0.7071
NM_001108770	Ccpg1	0.4400	0.3788	0.7604	0.7548	0.2127	0.8380	0.3922	0.3800	0.1759	0.3219	0.3375	0.5543
NM_001108079	Chst11	0.8901	0.4599	0.7858	0.3940	0.6686	0.7243	0.6085	0.6409	0.4937	0.9379	0.6808	0.0984
NM_001108028	L2hgdh	0.8094	0.9354	0.9044	0.6513	0.9052	0.4442	0.7002	0.9600	0.6162	0.9474	0.9690	0.3788
NM_001108025	RGD1307621	0.1353	0.4878	0.2754	0.4268	0.5383	0.7790	0.1311	0.6459	0.7787	0.7491	0.3478	0.9380
NM_001108051	Slc24a4	0.2434	0.3989	0.0625	0.0660	0.6680	0.8027	0.1015	0.4826	0.7446	0.6599	0.0996	0.1264
NM_001108787	Lars2	0.6778	0.7768	0.1090	0.4810	0.0501	0.7808	0.2161	0.8454	0.3331	0.0937	0.2128	0.6662
NM_001108782	Tmem103	0.9024	0.8473	0.8555	0.8484	0.5748	0.8495	0.7806	0.4462	0.9221	0.7778	0.7290	0.5849
NM_001108888	Reep5	0.7613	0.2630	0.3362	0.9891	0.1803	0.9158	0.2135	0.4713	0.2114	0.5222	0.1484	0.9697
NM_001108872	Chmp7	0.4483	0.4163	0.1859	0.4222	0.1723	0.9727	0.3234	0.0497	0.2078	0.5979	0.3039	0.9691
NM_001107843	Lrp1b	0.6588	0.8834	0.5776	0.9182	0.8171	0.7474	0.9061	0.7984	0.8466	0.8813	0.9558	0.6761
NM_001108906	Chmp2a	0.7270	0.1800	0.3669	0.1971	0.4754	0.3120	0.4862	0.5246	0.2610	0.5062	0.5163	0.4681
NM_001107936	Exosc3	0.7775	0.8407	0.1275	0.6970	0.9070	0.6797	0.4113	0.7790	0.7102	0.4145	0.5283	0.4884

NM_001107847	Golga3	0.2238	0.6650	0.5226	0.7889	0.9268	0.8997	0.2921	0.6436	0.3004	0.6350	0.5803	0.4647
NM_001108911	Upk1a	0.6606	0.4613	0.4098	0.0785	0.5870	0.6124	0.1774	0.5279	0.2100	0.2647	0.4318	0.7478
NM_001108790	Tmem14a	0.0025	0.0078	0.4433	0.5440	0.2302	0.2400	0.8623	0.2270	0.0470	0.0374	0.1752	0.3337
NM_001108800	Klf7	0.8665	0.9674	0.9554	0.7623	0.3994	0.9784	0.9864	0.9858	0.9669	0.9957	0.9806	0.9926
NM_001108912	Ffar3	0.4482	0.3029	0.7623	0.5744	0.5081	0.4804	0.9347	0.4926	0.6513	0.5298	0.4534	0.4958
NM_001109011	Marco	0.0897	0.1554	0.1279	0.3309	0.2772	0.1910	0.2060	0.2024	0.0619	0.1655	0.0602	0.4251
NM_001108811	Twsg1	0.3540	0.3077	0.7794	0.4710	0.6991	0.7976	0.5883	0.1358	0.7404	0.8551	0.7237	0.7733
NM_134467	Rnf38	0.0974	0.4422	0.3098	0.3229	0.0447	0.1377	0.2066	0.1236	0.1188	0.5288	0.6926	0.9558
NM_057119	Sfrs10	0.9539	0.9715	0.3689	0.9384	0.9311	0.9863	0.5209	0.9863	0.8811	0.9125	0.9136	0.0898
NM_053985	H3f3b	0.8499	0.8142	0.8094	0.6246	0.9081	0.4939	0.3341	0.8581	0.6727	0.7646	0.8420	0.3909
NM_053974	Eif4e	0.4647	0.9066	0.8258	0.9173	0.8252	0.8348	0.9199	0.9050	0.8434	0.9696	0.8516	0.6933
NM_053484	Gas7	0.9799	0.2269	0.6147	0.7113	0.9501	0.7038	0.9688	0.7081	0.3088	0.1592	0.0038	0.9347
NM_001109027	Prss33	0.1150	0.4369	0.3085	0.6585	0.7884	0.1541	0.4753	0.4760	0.4772	0.3490	0.5073	0.7364
NM_001109014	Tmem164	0.2702	0.7703	0.5484	0.9728	0.8705	0.2832	0.9655	0.4058	0.9900	0.4793	0.5177	0.8202
NM_001109243	Fam176a	0.7745	0.8571	0.7080	0.4273	0.7301	0.3061	0.8057	0.4869	0.2098	0.4432	0.7654	0.2413
NM_001108897	Pskh1	0.4170	0.4945	0.9500	0.8586	0.2682	0.2391	0.3257	0.8505	0.7634	0.4436	0.9372	0.9668
NM_001107560	Tubgcp2	0.1217	0.5007	0.0882	0.0486	0.3938	0.0929	0.8402	0.0725	0.3272	0.5249	0.8056	0.0784
NM_001107950	RGD1564541	0.9176	0.9341	0.9466	0.9235	0.8049	0.8719	0.9180	0.9790	0.8622	0.9785	0.9254	0.7814
NM_001105869	Rfc4	0.3259	0.7442	0.0107	0.6943	0.8183	0.6197	0.7412	0.6880	0.6334	0.7264	0.7432	0.0149
NM_001108046	Ddx41	0.3948	0.0980	0.1137	0.3825	0.0634	0.4185	0.1607	0.3093	0.2673	0.0739	0.4622	0.8262
NM_001107869	Dysf	0.6028	0.8303	0.5417	0.6230	0.9438	0.8077	0.9418	0.7480	0.1497	0.4356	0.7364	0.2024
NM_001107874	Xpc	0.5396	0.3894	0.3802	0.1783	0.4052	0.9215	0.4606	0.1141	0.9008	0.2595	0.8124	0.2831
NM_001108083	Usp44	0.9627	0.8098	0.9104	0.6974	0.6818	0.9024	0.8167	0.8824	0.9715	0.7812	0.6865	0.8754
NM_001107875	Zfyve20	0.7954	0.8756	0.8986	0.8805	0.7650	0.7256	0.8234	0.5894	0.9483	0.9221	0.8992	0.4349
NM_001109102	Fam167a	0.8443	0.6086	0.5610	0.7572	0.8927	0.4335	0.3035	0.5563	0.9505	0.8129	0.7820	0.8608
NM_001109092	Hus1	0.3952	0.9854	0.9097	0.8274	0.6527	0.4735	0.8521	0.7169	0.9231	0.8113	0.9162	0.3511
NM_001108940	Bhlhb5	0.4850	0.4251	0.6238	0.5291	0.6685	0.4880	0.3550	0.5468	0.4512	0.3693	0.5660	0.4175
NM_001127539	RGD1563279	0.1444	0.1636	0.7515	0.6771	0.6038	0.4288	0.7798	0.1148	0.4824	0.0890	0.6752	0.4973
NM_001108905	Znf524	0.7664	0.6720	0.9133	0.8645	0.8176	0.8503	0.8765	0.9551	0.6738	0.8132	0.7116	0.4330
NM_001108904	Znf787	0.0617	0.3738	0.1282	0.4553	0.3637	0.8423	0.6266	0.4560	0.0855	0.3307	0.4301	0.4291
NM_001108860	Srd5a2l2	0.4436	0.3851	0.4371	0.1341	0.4454	0.3445	0.2121	0.3492	0.3605	0.3242	0.4159	0.3533
NM_001011935	Xpo6	0.8556	0.8129	0.3319	0.7435	0.8925	0.8663	0.7914	0.8247	0.5064	0.6019	0.9291	0.6724
NM_001107958	Tal1	0.3517	0.4829	0.3690	0.0452	0.5646	0.4888	0.4858	0.6287	0.4274	0.6005	0.0650	0.3708
NM_001109000	Alg9	0.3595	0.3329	0.9179	0.9811	0.3995	0.6707	0.9180	0.5231	0.3114	0.2429	0.3405	0.3938
NM_001108866	Abhd4	0.1581	0.8125	0.9823	0.3881	0.2896	0.2859	0.8645	0.2130	0.5120	0.7122	0.9301	0.3272
NM_001108865	Dnajc9	0.9232	0.9496	0.7499	0.9490	0.8616	0.8580	0.8572	0.9055	0.7429	0.9725	0.9701	0.3664
NM_001109055	Wdr53	0.4625	0.5494	0.1421	0.0734	0.0604	0.0177	0.0297	0.0580	0.0953	0.3523	0.0122	0.3636
NM_001109054	Tctex1d2	0.1254	0.0166	0.9319	0.7763	0.1624	0.2368	0.7205	0.3498	0.0498	0.0111	0.0226	0.0514
NM_001108953	Zbtb6	0.1650	0.2351	0.2808	0.2512	0.1378	0.0651	0.2196	0.0238	0.1142	0.6448	0.4523	0.8556
NM_001108307	RGD1311078	0.6758	0.8602	0.7390	0.9238	0.7419	0.3205	0.6796	0.8190	0.6527	0.8181	0.6258	0.0782
NM_001108065	Shc2	0.9206	0.9486	0.4185	0.9224	0.7431	0.9487	0.8802	0.9504	0.3371	0.6499	0.5573	0.9170
NM_001108063	Znf124	0.4627	0.4719	0.5637	0.3606	0.5487	0.4298	0.2011	0.6301	0.0577	0.4898	0.5289	0.2661
NM_001109249	Eefsec	0.6719	0.5574	0.6265	0.6075	0.5627	0.4738	0.6843	0.6407	0.5766	0.2619	0.7304	0.6206
NM_001108873	Tnfrsf10b	0.8182	0.5331	0.4154	0.2377	0.8173	0.3243	0.5361	0.0312	0.8631	0.8169	0.7550	0.7447
NM_001108871	Kctd9	0.9185	0.7857	0.1931	0.4596	0.9034	0.9049	0.5248	0.8687	0.7217	0.7899	0.8483	0.3173
NM_001108869	Cideb	0.4133	0.6719	0.4901	0.9355	0.8474	0.3525	0.6004	0.9180	0.8350	0.4222	0.9764	0.6036
NM_001108878	Thsd1	0.4705	0.1461	0.7665	0.0980	0.4575	0.1568	0.7879	0.1348	0.5560	0.1131	0.2135	0.6040
NM_001108919	Ern2	0.7101	0.3480	0.5415	0.5175	0.2510	0.0366	0.6844	0.4126	0.5690	0.3170	0.6621	0.5321
NM_001107967	RGD1311937	0.7898	0.6419	0.8064	0.5830	0.7776	0.9587	0.8093	0.6983	0.5173	0.7516	0.5384	0.8235

NM_001108333	Rabgef1	0.5256	0.2490	0.6227	0.5670	0.7021	0.6426	0.2392	0.3384	0.0730	0.4904	0.0354	0.8979
NM_001109070	Golt1a	0.2244	0.2361	0.2362	0.4804	0.4057	0.2679	0.4180	0.4897	0.6603	0.2371	0.4449	0.4711
NM_001109069	Mfsd4	0.8028	0.9768	0.7094	0.7092	0.7628	0.8274	0.9694	0.8300	0.9729	0.9617	0.9397	0.5308
NM_001107884	Cecr5	0.4604	0.5981	0.5142	0.2824	0.4598	0.8289	0.8659	0.3647	0.3899	0.9280	0.1223	0.5049
NM_001107881	Plxnd1	0.9319	0.7998	0.8183	0.9835	0.8812	0.9944	0.9182	0.8046	0.8838	0.9873	0.7922	0.9831
NM_001109078	Slamf1	0.6088	0.5763	0.1640	0.7705	0.3584	0.8842	0.3492	0.4098	0.8881	0.8483	0.4126	0.8510
NM_001109077	Arhgap30	0.0654	0.2078	0.1569	0.2187	0.0955	0.1437	0.2925	0.1679	0.1873	0.3829	0.1550	0.4038
NM_001109002	Smad6	0.9987	0.9206	0.9568	0.9890	0.9373	0.9285	0.9911	0.9848	0.9556	0.7658	0.8306	0.9948
NM_001108892	Sall3	0.6997	0.7039	0.3434	0.7894	0.4591	0.6235	0.4025	0.6037	0.1105	0.5561	0.6981	0.5037
NM_001108891	Gm672	0.4766	0.5357	0.6325	0.5489	0.5722	0.6664	0.4041	0.8692	0.5924	0.6488	0.7549	0.8329
NM_001108915	Asb7	0.2621	0.8582	0.3514	0.7316	0.6205	0.2890	0.3033	0.4780	0.5389	0.6436	0.5840	0.4191
NM_001109080	Lefty1	0.2705	0.5222	0.8289	0.6243	0.4973	0.1358	0.0962	0.1589	0.2014	0.0751	0.1518	0.7798
NM_001107900	Cpa6	0.6751	0.5081	0.8843	0.7683	0.7719	0.8325	0.7122	0.8021	0.6027	0.9238	0.6021	0.6660
NM_001107889	Mlf2	0.8746	0.1382	0.8227	0.9610	0.3465	0.9347	0.8897	0.9267	0.9645	0.9340	0.8307	0.9958
NM_001109017	Zfx	0.9395	0.8278	0.9600	0.8417	0.9128	0.7578	0.8369	0.8920	0.8426	0.6659	0.8334	0.8438
NM_001109008	Col6a3	0.0024	0.1341	0.1073	0.0187	0.9263	0.7525	0.1012	0.7817	0.1425	0.0025	0.1446	0.0261
NM_001109012	Rp2h	0.2805	0.3360	0.7989	0.6017	0.6467	0.3718	0.2982	0.6761	0.8415	0.7217	0.6103	0.8935
NM_001109010	Nudt12	0.2927	0.3401	0.6428	0.6775	0.3912	0.8259	0.6440	0.6963	0.5496	0.8968	0.9203	0.7040
NM_001109371	Dgat2l3	0.2557	0.4806	0.3649	0.4772	0.7251	0.2409	0.4703	0.5024	0.3454	0.5726	0.4674	0.4748
NM_001109018	Rab9b	0.8163	0.8217	0.9420	0.9046	0.9454	0.8020	0.9578	0.9152	0.8969	0.7358	0.5581	0.4593
NM_001108917	RGD1305211	0.9447	0.8387	0.5524	0.9599	0.9635	0.8969	0.9914	0.7648	0.3582	0.9955	0.8993	0.3157
NM_001108916	Bnc1	0.2719	0.4489	0.6990	0.2999	0.6432	0.3868	0.1804	0.3618	0.4187	0.3013	0.3385	0.5032
NM_001108914	Kcna7	0.9129	0.8780	0.5977	0.8348	0.8492	0.6922	0.9385	0.7615	0.9285	0.7419	0.6425	0.9278
NM_001107901	Rb1cc1	0.4128	0.2989	0.9262	0.1137	0.2796	0.1252	0.2148	0.0688	0.3629	0.1483	0.5347	0.3726
NM_001107893	Atf7ip	0.1976	0.3439	0.1399	0.2868	0.3277	0.1914	0.1057	0.3145	0.1960	0.3261	0.1130	0.1967
NM_001108139	Mll1	0.9687	0.9907	0.3149	0.5850	0.9933	0.9860	0.7461	0.9662	0.9958	0.8331	0.9766	0.9835
NM_001108927	Osbp	0.9660	0.1217	0.9114	0.9678	0.9348	0.9595	0.8159	0.9212	0.9132	0.3181	0.6998	0.9891
NM_001109022	Inmt	0.5885	0.5626	0.7443	0.3747	0.4842	0.7070	0.6724	0.6269	0.6954	0.7432	0.6277	0.7625
NM_001108986	Wibg	0.1218	0.6314	0.4177	0.5084	0.4525	0.3699	0.3113	0.2304	0.2441	0.1075	0.4005	0.6740
NM_001108007	RGD1311939	0.3725	0.5640	0.7015	0.4314	0.2181	0.7275	0.6808	0.2789	0.6026	0.1898	0.6529	0.6840
NM_001108048	Rps6ka5	0.5040	0.8252	0.6406	0.4485	0.6715	0.4765	0.9014	0.1835	0.7728	0.6525	0.8410	0.9129
NM_001108108	Ano6	0.6861	0.5962	0.8230	0.7610	0.5274	0.8691	0.6633	0.8196	0.5649	0.3081	0.4718	0.9302
NM_001107894	Etnk1	0.6194	0.6905	0.7555	0.4509	0.5115	0.7770	0.8105	0.2487	0.6359	0.6033	0.4768	0.4546
NM_001109105	Commd6	0.6274	0.7900	0.3170	0.4373	0.6618	0.7606	0.6035	0.4789	0.3639	0.2188	0.6722	0.5244
NM_001109104	Serp2	0.7392	0.8085	0.4750	0.8832	0.9502	0.9643	0.8677	0.7867	0.7936	0.7634	0.7889	0.9507
NM_001108163	Fam81a	0.8117	0.7975	0.7507	0.7008	0.8202	0.6984	0.8628	0.8500	0.4716	0.8518	0.4489	0.4078
NM_001108939	Hnf4g	0.7072	0.6463	0.7248	0.4178	0.6751	0.7230	0.6071	0.5962	0.5199	0.5701	0.3861	0.2108
NM_001108955	Fjx1	0.2634	0.1015	0.0541	0.8276	0.2588	0.2463	0.1132	0.1752	0.2580	0.4112	0.0365	0.4613
NM_001108954	B3galt1	0.6631	0.6758	0.8080	0.8296	0.8175	0.7645	0.8071	0.1993	0.2290	0.5942	0.8446	0.2167
NM_001108952	Exosc2	0.8018	0.9391	0.0397	0.4748	0.3918	0.8813	0.3374	0.9794	0.8701	0.3179	0.6892	0.2293
NM_001108950	Gnat2	0.0332	0.0019	0.0142	0.8281	0.0197	0.0780	0.1042	0.3952	0.0421	0.0948	0.1413	0.0008
NM_001108949	Hormad1	0.7547	0.8062	0.4450	0.3672	0.1464	0.4869	0.2006	0.3970	0.7883	0.5095	0.3649	0.0894
NM_001108933	Dse	0.9589	0.5327	0.8305	0.9950	0.9945	0.9163	0.8781	0.7510	0.7737	0.5958	0.9541	0.3025
NM_001108966	Rab22a	0.9109	0.4902	0.4967	0.5290	0.8012	0.4860	0.3652	0.1222	0.8762	0.2136	0.4676	0.8089
NM_001109066	RGD1562310	0.3078	0.7609	0.4760	0.3960	0.5440	0.6322	0.5913	0.7118	0.0679	0.4606	0.5175	0.7705
NM_001108964	Spo11	0.2155	0.1388	0.2653	0.1970	0.6250	0.4853	0.0677	0.1309	0.2800	0.2370	0.4493	0.2624
NM_001108963	Slc2a10	0.7069	0.8316	0.5779	0.6837	0.7766	0.6658	0.4052	0.5921	0.3017	0.7311	0.5955	0.7929
NM_001108960	Otor	0.0535	0.3614	0.1648	0.4014	0.0498	0.1345	0.2042	0.0399	0.4999	0.1326	0.3403	0.0849
NM_001109059	RGD1563425	0.2256	0.4834	0.5916	0.4353	0.4194	0.3676	0.4677	0.0290	0.5207	0.5863	0.4827	0.4510

NM_001108958	Rtf1	0.6412	0.5779	0.6617	0.7205	0.6536	0.7115	0.7153	0.6789	0.6478	0.6204	0.7193	0.6661
NM_001109023	Pnn	0.8986	0.9195	0.4136	0.7248	0.9743	0.7222	0.8321	0.9688	0.8160	0.9193	0.8985	0.2587
NM_001109016	Bmx	0.7454	0.2292	0.3403	0.5459	0.8315	0.4187	0.0580	0.2759	0.5799	0.4818	0.2784	0.1879
NM_001109021	Bcl7b	0.5564	0.9529	0.8209	0.9778	0.8146	0.7773	0.8047	0.8495	0.2643	0.9549	0.9336	0.9815
NM_001107873	Mcm2	0.9058	0.9766	0.0869	0.9629	0.9125	0.9251	0.9780	0.9702	0.9143	0.7612	0.7885	0.8352
NM_001107866	Loxl3	0.1189	0.0129	0.0040	0.0122	0.0035	0.0461	0.1146	0.2031	0.1410	0.0053	0.1254	0.2140
NM_001107946	Hook1	0.6536	0.7819	0.7136	0.6134	0.6777	0.3753	0.8009	0.9677	0.4558	0.8503	0.2940	0.2743
NM_001109029	Sgcd	0.5848	0.6219	0.4940	0.9153	0.4547	0.6679	0.7398	0.7337	0.8462	0.4586	0.8358	0.9048
NM_001109033	Slc25a35	0.8017	0.6569	0.5060	0.8804	0.6541	0.8984	0.6787	0.9083	0.7675	0.9544	0.7824	0.8268
NM_001109031	Ccdc69	0.0250	0.1304	0.3971	0.0315	0.1669	0.1253	0.0486	0.2046	0.3965	0.7207	0.2998	0.2926
NM_001109034	Zfp3	0.4020	0.5945	0.8062	0.4513	0.7118	0.6810	0.8273	0.7093	0.5450	0.5675	0.2791	0.6180
NM_001106408	Dimt1l	0.5219	0.5276	0.5226	0.1410	0.1806	0.1294	0.8280	0.0422	0.2412	0.1518	0.5825	0.4333
NM_001109037	Gemin4	0.8033	0.9852	0.7151	0.8237	0.7207	0.8305	0.8605	0.8843	0.8095	0.9593	0.8260	0.8458
NM_001109036	Ovca2	0.9825	0.7369	0.1586	0.9867	0.9491	0.9878	0.7777	0.9992	0.9728	0.9924	0.9521	0.7791
NM_001109035	Spns3	0.7538	0.7555	0.2889	0.5613	0.2094	0.5075	0.6306	0.1104	0.6508	0.1230	0.2889	0.5247
NM_001108924	Odf3	0.0815	0.2948	0.1359	0.0609	0.2058	0.5319	0.1348	0.1703	0.4129	0.0572	0.0891	0.3672
NM_001109020	LOC367994	0.1571	0.4752	0.4171	0.6760	0.1443	0.0908	0.4014	0.2380	0.0926	0.1258	0.1224	0.0326
NM_001109039	Fam117a	0.9139	0.9545	0.9701	0.9727	0.9232	0.8991	0.8801	0.9288	0.9988	0.9800	0.9533	0.5975
NM_001109044	Myeov2	0.3940	0.2889	0.4720	0.3991	0.2193	0.4177	0.3054	0.2846	0.2447	0.3801	0.2619	0.6071
NM_001109025	Rnf5	0.2186	0.2118	0.2316	0.3435	0.5616	0.7055	0.5213	0.3083	0.5096	0.5113	0.4405	0.5545
NM_001109026	Dexi	0.9821	0.6058	0.9237	0.7452	0.1524	0.1512	0.8585	0.8197	0.8030	0.8435	0.9708	0.4644
NM_001109046	Arl5c	0.1799	0.7656	0.1398	0.4559	0.9037	0.0327	0.2463	0.4735	0.6644	0.6663	0.7016	0.8724
NM_001109032	RGD1565284	0.5425	0.2134	0.3340	0.3187	0.2613	0.7779	0.5132	0.2536	0.4186	0.4667	0.0657	0.5287
NM_001106260	Nosip	0.0185	0.2586	0.1769	0.0628	0.3193	0.0096	0.2504	0.2760	0.1434	0.3033	0.0426	0.3442
NM_001106280	Tmem126b	0.0307	0.1045	0.4142	0.1041	0.0116	0.0346	0.1461	0.0340	0.1217	0.1382	0.3568	0.0565
NM_001108946	Fstl5	0.7073	0.6866	0.7029	0.4047	0.6158	0.4754	0.6430	0.4581	0.6677	0.5069	0.7972	0.5594
NM_001109051	Fn3k	0.6369	0.0936	0.0433	0.3010	0.4000	0.0463	0.1095	0.1038	0.4323	0.8705	0.0411	0.6890
NM_001109049	Ccdc103	0.8678	0.3433	0.4283	0.6124	0.2405	0.4239	0.5289	0.6043	0.4953	0.6701	0.6516	0.3362
NM_001109053	Dtx3l	0.1803	0.6057	0.2654	0.7210	0.4167	0.2427	0.8063	0.4731	0.4945	0.3609	0.1857	0.7881
NM_001108957	Nut	0.1058	0.1420	0.0368	0.3274	0.0874	0.5984	0.1006	0.5295	0.4103	0.5239	0.3918	0.4782
NM_001109057	Yeats2	0.9617	0.8947	0.7751	0.4550	0.9316	0.9561	0.7757	0.8878	0.8132	0.8081	0.7513	0.6116
NM_001109062	Gpr146	0.2297	0.1903	0.0960	0.0846	0.2211	0.2322	0.1389	0.0216	0.3406	0.2774	0.1443	0.2228
NM_001108956	Elf5	0.4124	0.6703	0.7103	0.9267	0.3965	0.7799	0.6182	0.9271	0.8064	0.6159	0.2174	0.1324
NM_001109064	Gal3st4	0.8267	0.1028	0.5048	0.3843	0.3731	0.4237	0.2536	0.5092	0.6097	0.4929	0.3988	0.8828
NM_001108965	Rbm38	0.3701	0.8333	0.1332	0.0117	0.1225	0.3159	0.4287	0.1409	0.8397	0.0467	0.4781	0.9589
NM_001109076	Pvrl4	0.9878	0.7260	0.9249	0.9847	0.9692	0.8688	0.9845	0.9180	0.9533	0.8128	0.8808	0.9830
NM_001109071	Arl8a	0.7273	0.6965	0.4185	0.3941	0.9231	0.6444	0.2974	0.9572	0.9858	0.8685	0.7644	0.4162
NM_001108970	Mobkl2b	0.6114	0.7845	0.7636	0.8688	0.8592	0.2517	0.6865	0.1344	0.7061	0.6331	0.5831	0.8540
NM_001108886	Polr2d	0.8749	0.9075	0.5059	0.6699	0.9579	0.7875	0.2742	0.7591	0.0314	0.3181	0.0281	0.1022
NM_001109176	Fyb	0.1805	0.2052	0.4469	0.2986	0.1830	0.4995	0.2825	0.3913	0.1357	0.3158	0.0795	0.0718
NM_001108961	Cst10	0.1389	0.4953	0.4755	0.3863	0.4798	0.4338	0.5601	0.3047	0.4042	0.6081	0.5166	0.3804
NM_001109074	RGD1565283	0.1659	0.2786	0.1401	0.4895	0.4114	0.2670	0.5408	0.2642	0.4566	0.7026	0.4628	0.0769
NM_001109081	Wdr26	0.3112	0.3599	0.8231	0.2921	0.1315	0.6659	0.4579	0.1279	0.5555	0.2973	0.6400	0.3566
NM_001108893	RGD1559772	0.5495	0.3647	0.4972	0.3059	0.1491	0.3466	0.2007	0.7103	0.1335	0.4684	0.4510	0.3186
NM_001108896	Gcdh	0.1271	0.1810	0.8514	0.0778	0.0785	0.0445	0.3589	0.2148	0.0345	0.5555	0.0323	0.4304
NM_001108907	Cabp5	0.4860	0.7569	0.1416	0.6008	0.7869	0.6194	0.4804	0.4153	0.9565	0.1002	0.4515	0.4470
NM_001109084	Fam71a	0.5872	0.0913	0.3073	0.2872	0.3203	0.4014	0.6837	0.2399	0.1624	0.4876	0.7277	0.1323
NM_001109082	Vash2	0.7173	0.9689	0.5558	0.3982	0.8199	0.9766	0.6373	0.9086	0.8724	0.8417	0.7760	0.6398
NM_001108910	Lrfn1	0.2446	0.4937	0.4239	0.4983	0.3783	0.3316	0.3131	0.5804	0.5624	0.2672	0.2997	0.3614

NM_001109013	Praf2	0.3195	0.8829	0.9284	0.7644	0.1314	0.1104	0.8535	0.1234	0.3443	0.4564	0.4809	0.9862
NM_001109100	Tppp2	0.4549	0.6125	0.8631	0.8936	0.2672	0.4516	0.3101	0.3007	0.5541	0.7125	0.2292	0.3471
NM_001109090	Sec14l4	0.5359	0.1835	0.1175	0.0498	0.3246	0.6402	0.4290	0.7161	0.6443	0.3767	0.1278	0.6649
NM_001108989	Kank3	0.8997	0.1612	0.4748	0.5931	0.8677	0.8452	0.5888	0.7926	0.6231	0.2350	0.0635	0.3223
NM_001109130	Tsnaxip1	0.2012	0.6459	0.1555	0.6541	0.7375	0.4923	0.7046	0.8153	0.7444	0.7707	0.8559	0.5868
NM_001109091	Ascc2	0.0692	0.7573	0.0118	0.4881	0.0091	0.0699	0.5055	0.3162	0.7772	0.6998	0.8822	0.1115
NM_001109093	Grb10	0.8866	0.8279	0.6353	0.5467	0.5102	0.7938	0.8336	0.7565	0.8888	0.8760	0.8987	0.8264
NM_001109083	RGD1562052	0.0490	0.4508	0.0418	0.1886	0.2447	0.0801	0.3386	0.0129	0.2933	0.3309	0.0653	0.0129
NM_001108983	RGD1560556	0.1367	0.3733	0.3625	0.1216	0.1613	0.2479	0.3172	0.4740	0.4397	0.1664	0.6484	0.2820
NM_001109094	Etaa1	0.1662	0.1861	0.9546	0.9296	0.6142	0.3163	0.1738	0.2331	0.3517	0.7927	0.7423	0.2309
NM_001108992	Pphln1	0.9050	0.3396	0.1348	0.2487	0.8267	0.8619	0.5284	0.8590	0.4824	0.3226	0.4741	0.3710
NM_001109110	Anxa10	0.3132	0.4338	0.2558	0.4998	0.4922	0.2607	0.3251	0.1913	0.2374	0.4442	0.5060	0.2595
NM_001109099	RGD1559923	0.8766	0.5851	0.9766	0.8834	0.9027	0.9406	0.9660	0.3341	0.5020	0.5177	0.4305	0.9238
NM_001109097	Myoz1	0.4553	0.7006	0.3622	0.5575	0.7459	0.5247	0.5479	0.1023	0.8273	0.2908	0.2678	0.4847
NM_001109096	Gpr75	0.3375	0.1608	0.1189	0.3254	0.2461	0.1614	0.2174	0.0926	0.0568	0.2234	0.2177	0.2556
NM_001109111	Wwc2	0.8565	0.9042	0.3518	0.3651	0.5331	0.3389	0.9362	0.4607	0.5567	0.8839	0.9348	0.3354
NM_001108948	Tuft1	0.7406	0.8291	0.9001	0.9522	0.9435	0.8023	0.9513	0.2100	0.6593	0.8206	0.9236	0.8690
NM_001108945	Trim59	0.2625	0.2990	0.8775	0.9802	0.2484	0.9446	0.8743	0.2079	0.2881	0.6004	0.6623	0.5966
NM_001005553	Git2	0.9725	0.7318	0.8230	0.5934	0.9680	0.9504	0.3719	0.9713	0.8529	0.6846	0.9849	0.7969
NM_001109126	RGD1561039	0.4337	0.1415	0.1901	0.2568	0.3078	0.4759	0.4675	0.5313	0.5016	0.6320	0.1114	0.1786
NM_001109491	Gapt	0.6788	0.4098	0.5166	0.5438	0.6720	0.6208	0.5843	0.4983	0.2123	0.4747	0.6761	0.4278
NM_001109223	Wnt16	0.5644	0.4787	0.6760	0.6268	0.7822	0.7138	0.8819	0.7184	0.5705	0.5720	0.4838	0.6957
NM_001109038	RGD1566149	0.6967	0.3153	0.3687	0.6404	0.4847	0.3631	0.2077	0.0619	0.2140	0.3055	0.3135	0.3495
NM_001109116	Prr7	0.8750	0.8317	0.8208	0.4564	0.9059	0.8922	0.8703	0.8974	0.8664	0.8577	0.9104	0.8373
NM_001109117	Rnf182	0.0168	0.0610	0.2034	0.1295	0.0997	0.0872	0.0535	0.0455	0.0524	0.1048	0.2115	0.0485
NM_001109050	RGD1565033	0.9056	0.9171	0.8827	0.9066	0.9191	0.7971	0.7893	0.8733	0.8708	0.8984	0.9704	0.4599
NM_001109048	Asb16	0.5046	0.4084	0.6083	0.4770	0.5490	0.6365	0.5892	0.6797	0.7271	0.6481	0.8029	0.4529
NM_001109107	Slc35e1	0.3158	0.2898	0.1581	0.0727	0.3470	0.2497	0.4371	0.3996	0.2902	0.2934	0.2763	0.5474
NM_001109119	Tubb2a	0.9299	0.9951	0.1344	0.9245	0.8357	0.7965	0.9238	0.7295	0.9503	0.8857	0.9821	0.0519
NM_001109169	Emx2	0.3292	0.2554	0.5025	0.7748	0.2322	0.1288	0.3023	0.1200	0.2397	0.2566	0.2548	0.3633
NM_001109128	Fbxl8	0.1535	0.6345	0.0615	0.7770	0.4918	0.7359	0.3552	0.7047	0.4797	0.6648	0.1546	0.9652
NM_001109124	Ccdc112	0.9747	0.7122	0.7012	0.0415	0.6380	0.2305	0.3125	0.0881	0.8503	0.8325	0.7891	0.6447
NM_001109114	LOC498685	0.2327	0.6920	0.2423	0.4855	0.2301	0.3343	0.6554	0.4934	0.2991	0.2329	0.2699	0.4516
NM_001109534	Tktl1	0.5694	0.6713	0.3979	0.4331	0.4151	0.5158	0.2494	0.2927	0.4907	0.5695	0.5917	0.2179
NM_001108969	RGD1564200	0.5849	0.6644	0.8124	0.3905	0.7047	0.8043	0.5470	0.7610	0.9293	0.7185	0.7352	0.8956
NM_001108991	Trhde	0.8324	0.5515	0.8958	0.8783	0.6304	0.4537	0.6706	0.8746	0.8301	0.6783	0.8327	0.8287
NM_001109135	Tmem200a	0.4973	0.4865	0.7254	0.5202	0.2643	0.4009	0.1879	0.7907	0.2782	0.3182	0.4616	0.4204
NM_001109133	Spata2L	0.2674	0.5336	0.1618	0.1435	0.7036	0.7072	0.0362	0.5049	0.4625	0.4631	0.5002	0.7595
NM_001109072	RGD1565760	0.4694	0.3838	0.2863	0.3883	0.7800	0.7877	0.7448	0.0642	0.5192	0.3721	0.4736	0.3350
NM_001109129	RGD1561415	0.3955	0.2657	0.4278	0.3895	0.4146	0.1858	0.3702	0.3258	0.2451	0.3907	0.3585	0.2653
NM_001109132	RGD1562056	0.3966	0.1134	0.7319	0.2108	0.3558	0.1927	0.1998	0.8551	0.3066	0.6131	0.0835	0.2829
NM_001109323	F8a1	0.9214	0.8869	0.7593	0.9932	0.8282	0.8726	0.9594	0.9737	0.8145	0.8067	0.8449	0.6923
NM_001109244	Lbx2	0.4636	0.4225	0.4329	0.8359	0.7365	0.7175	0.6470	0.4877	0.2788	0.7485	0.7314	0.3307
NM_001109140	Dmrtc2	0.3675	0.0499	0.2373	0.3596	0.1368	0.0939	0.4386	0.1092	0.1583	0.0906	0.2815	0.5494
NM_001109139	P42pop	0.7137	0.3901	0.5008	0.4846	0.3624	0.4641	0.5417	0.5502	0.2893	0.2008	0.2268	0.7123
NM_001109143	Cldnd2	0.2965	0.4655	0.0819	0.5439	0.3845	0.4989	0.2295	0.1883	0.2421	0.1814	0.5117	0.4089
NM_001109141	Kctd15	0.6441	0.9733	0.8973	0.5528	0.8922	0.9573	0.9419	0.9672	0.9440	0.9464	0.6805	0.9479
NM_001109433	Sdf2l1	0.2259	0.7381	0.0686	0.5813	0.1403	0.0318	0.8409	0.1559	0.3672	0.7924	0.9598	0.0495
NM_001109138	RGD1559613	0.6929	0.5924	0.7816	0.7961	0.6125	0.5751	0.6383	0.7081	0.6306	0.5140	0.9607	0.6385

NM_001108972	Igfbpl1	0.1012	0.2836	0.1072	0.4720	0.0029	0.0875	0.0620	0.0797	0.0702	0.7408	0.0206	0.0253
NM_001109147	Klf13	0.8058	0.7910	0.8634	0.8838	0.8095	0.6995	0.7180	0.9270	0.5657	0.5739	0.8417	0.6319
NM_001109145	Dkkl1	0.7789	0.5629	0.6495	0.3979	0.9789	0.3798	0.6687	0.4937	0.6235	0.1943	0.6326	0.1766
NM_001109148	Mrps11	0.4280	0.5499	0.0613	0.8035	0.6132	0.3727	0.5972	0.4902	0.1585	0.1335	0.0315	0.0565
NM_001109152	Gdpd5	0.9172	0.9670	0.9598	0.1052	0.7811	0.7551	0.9820	0.6794	0.9915	0.9802	0.7086	0.9355
NM_001109154	Apob48r	0.9584	0.9551	0.8931	0.7076	0.8274	0.6059	0.9781	0.9578	0.8713	0.8185	0.6777	0.9666
NM_001109158	Fam24a	0.1584	0.1357	0.5736	0.5453	0.1555	0.1897	0.1013	0.2964	0.3981	0.3891	0.6328	0.3380
NM_001109159	Hcca2	0.1237	0.0772	0.6810	0.1845	0.1341	0.2981	0.4758	0.1976	0.3822	0.1027	0.0185	0.0774
NM_001109162	Zbtb3	0.2205	0.6873	0.0748	0.0953	0.2854	0.3952	0.3385	0.8257	0.2805	0.7412	0.3123	0.5411
NM_001109160	Flrt1	0.9554	0.8180	0.9081	0.9350	0.9611	0.6753	0.9482	0.9428	0.9312	0.9664	0.9660	0.9728
NM_001109073	RGD1560672	0.6680	0.7048	0.6939	0.7838	0.3998	0.7943	0.8657	0.4422	0.5806	0.5886	0.4900	0.5322
NM_001109166	Tlx1	0.5847	0.5033	0.3338	0.2427	0.4286	0.4768	0.6790	0.2728	0.5583	0.4197	0.2655	0.4568
NM_001109165	Fgfbp3	0.5133	0.1996	0.5472	0.6772	0.6845	0.3996	0.1854	0.1388	0.6986	0.0643	0.1993	0.4238
NM_001109163	Fam111a	0.8717	0.9009	0.8534	0.9613	0.9374	0.9979	0.9992	0.9421	0.9495	0.9544	0.8412	0.7453
NM_001109155	RGD1563217	0.2936	0.9425	0.3276	0.1315	0.5155	0.3685	0.1253	0.2521	0.6537	0.5294	0.1074	0.3201
NM_001108995	RGD1565847	0.1853	0.4091	0.4240	0.4207	0.1633	0.3973	0.3222	0.2586	0.2182	0.3912	0.2783	0.2391
NM_001109157	RGD1560958	0.7365	0.7339	0.3782	0.0161	0.3888	0.5592	0.7009	0.3838	0.7721	0.8743	0.6853	0.7611
NM_001108975	Ptch2	0.7725	0.9208	0.5864	0.8616	0.7491	0.8452	0.8424	0.9404	0.9751	0.7780	0.5497	0.7783
NM_001109174	Ankrd34b	0.8314	0.4127	0.4806	0.4213	0.5372	0.1890	0.5654	0.6347	0.4495	0.8832	0.8874	0.6460
NM_001100472	Ccnc	0.5044	0.4793	0.9450	0.7977	0.8528	0.8025	0.4713	0.5594	0.7365	0.9037	0.9773	0.4824
NM_001109178	Fam173b	0.2681	0.3984	0.1971	0.2341	0.7834	0.3080	0.0894	0.3031	0.1760	0.4679	0.4348	0.1043
NM_001109075	RGD1562658	0.3548	0.5889	0.6504	0.7847	0.5878	0.5626	0.5625	0.4664	0.4101	0.3326	0.3787	0.1797
NM_001109171	Lrrc20	0.4033	0.0616	0.8023	0.3925	0.9050	0.7248	0.5517	0.4196	0.7778	0.5480	0.1938	0.5238
NM_001109173	Gpr150	0.6762	0.8091	0.7741	0.7394	0.8095	0.8858	0.7457	0.8821	0.9274	0.7876	0.9505	0.7354
NM_001109186	RGD1564171	0.7438	0.9157	0.3030	0.1810	0.7957	0.9315	0.0326	0.9030	0.9383	0.6226	0.3980	0.1364
NM_001109175	Ccno	0.7373	0.8402	0.5600	0.7078	0.5584	0.6139	0.3892	0.3785	0.3286	0.4145	0.1935	0.5360
NM_001109181	Sox2	0.8302	0.7020	0.5720	0.8434	0.8697	0.7201	0.7165	0.8663	0.5057	0.7888	0.6461	0.7922
NM_001109177	Lmbrd2	0.6996	0.8594	0.4923	0.8075	0.5336	0.3978	0.5053	0.5713	0.7766	0.6313	0.8650	0.7660
NM_001109302	Srf	0.9607	0.4527	0.5158	0.3723	0.8980	0.9340	0.9759	0.8409	0.7449	0.7277	0.5098	0.9535
NM_001109180	Dnajc5b	0.8595	0.8974	0.8405	0.8710	0.6825	0.8761	0.8660	0.6667	0.8193	0.8822	0.9013	0.8616
NM_001109189	Lingo4	0.0546	0.0384	0.2738	0.0341	0.0679	0.0482	0.3849	0.2022	0.3539	0.3286	0.0197	0.2064
NM_001109440	Acot4	0.7969	0.8311	0.3154	0.6702	0.8071	0.8898	0.5958	0.8081	0.2116	0.9003	0.5422	0.1824
NM_001108978	Pik3cd	0.4302	0.4654	0.3412	0.3462	0.4738	0.9331	0.4154	0.0687	0.6758	0.8638	0.3292	0.8122
NM_001034958	Fam104a	0.5593	0.5094	0.6441	0.5431	0.5558	0.6519	0.6968	0.6444	0.7206	0.7226	0.5421	0.5121
NM_001109202	Tcp11l1	0.7994	0.9153	0.7287	0.7663	0.8590	0.4320	0.8593	0.6517	0.9837	0.8860	0.6483	0.8798
NM_001109079	RGD1560572	0.6700	0.5374	0.3743	0.7899	0.7743	0.4732	0.5488	0.5218	0.5093	0.5469	0.3908	0.3454
NM_001109183	Lhfp	0.2797	0.1434	0.8368	0.1455	0.1305	0.1121	0.2058	0.2599	0.3091	0.1448	0.2420	0.1750
NM_001109184	LOC499618	0.7684	0.6248	0.9045	0.5875	0.8222	0.2869	0.8940	0.5225	0.7928	0.8758	0.9595	0.3655
NM_001108981	RGD1309138	0.7986	0.5249	0.5828	0.6803	0.4328	0.3758	0.3992	0.8267	0.8127	0.5152	0.5587	0.7260
NM_001108979	Atp6v1e2	0.3278	0.7983	0.3532	0.6782	0.1043	0.0900	0.5927	0.1907	0.6300	0.7651	0.6904	0.7209
NM_001109201	RGD1563263	0.7496	0.0825	0.4182	0.6535	0.3724	0.5216	0.3129	0.5353	0.4878	0.5985	0.1318	0.1337
NM_001108982	Cfl2	0.8418	0.6158	0.3598	0.3114	0.7633	0.9736	0.8994	0.6965	0.3015	0.4821	0.5683	0.3918
NM_001109245	Wdr54	0.9917	0.9887	0.9636	0.7800	0.9610	0.6896	0.9906	0.9402	0.9922	0.9796	0.9851	0.9802
NM_001109203	Prrg4	0.2197	0.0077	0.3658	0.1554	0.1680	0.1066	0.2177	0.2050	0.0208	0.0155	0.1859	0.3706
NM_001109205	Eid1	0.1322	0.1079	0.9821	0.9430	0.3383	0.3869	0.9930	0.1191	0.3306	0.0831	0.4344	0.6422
NM_001109195	Rnf208	0.2338	0.1719	0.4511	0.0263	0.2473	0.1603	0.0808	0.2444	0.4462	0.2473	0.4499	0.3997
NM_001109276	Rdh14	0.0049	0.0075	0.2044	0.0570	0.0053	0.0266	0.2720	0.1859	0.0351	0.0042	0.0595	0.0540
NM_001109450	Hs3st6	0.5157	0.3023	0.1897	0.3681	0.4210	0.4320	0.2003	0.4023	0.5024	0.3949	0.4464	0.3904
NM_001109197	Tmem141	0.0870	0.0049	0.3276	0.2409	0.0796	0.2414	0.0971	0.1714	0.0041	0.0054	0.0404	0.0244

NM_001109268	Gmeb1	0.7971	0.8718	0.1281	0.0750	0.0727	0.1179	0.4090	0.0253	0.4544	0.7377	0.1406	0.4707
NM_001109615	Gys1	0.6602	0.4636	0.6080	0.2355	0.5751	0.6921	0.6452	0.5522	0.5904	0.6273	0.4671	0.5701
NM_001109331	Dpm3	0.0964	0.3109	0.0181	0.1906	0.0988	0.0029	0.0592	0.0188	0.1160	0.0056	0.1122	0.1156
NM_001109198	LOC499782	0.5171	0.3495	0.2006	0.7419	0.4878	0.4837	0.7442	0.8518	0.3959	0.3614	0.6083	0.7190
NM_001109506	Elmod2	0.6147	0.6878	0.7234	0.7273	0.6651	0.7158	0.6489	0.3833	0.5086	0.6561	0.3503	0.4606
NM_001134624	Lrrc69	0.3422	0.2877	0.4225	0.5160	0.2480	0.3039	0.5047	0.1908	0.1751	0.3163	0.3226	0.1837
NM_001130573	Maff	0.0244	0.3165	0.6390	0.4261	0.3272	0.1928	0.0877	0.2328	0.2288	0.3572	0.0861	0.2095
NM_001109473	Gtf3c4	0.6865	0.6371	0.5978	0.5870	0.7087	0.4866	0.6660	0.6246	0.6319	0.5003	0.7133	0.3062
NM_001109200	Ppp1r1c	0.5386	0.9318	0.9401	0.6655	0.4744	0.8438	0.4979	0.8002	0.6202	0.9414	0.8771	0.4137
NM_001109298	RGD1560940	0.5197	0.3309	0.5809	0.7973	0.4601	0.2247	0.3717	0.8332	0.3977	0.6448	0.6577	0.5630
NM_001109208	Rya3	0.5961	0.1600	0.4233	0.2841	0.2138	0.1224	0.2052	0.0988	0.0625	0.0301	0.4983	0.7540
NM_001109086	RGD1562136	0.9734	0.8962	0.7427	0.8983	0.8951	0.7296	0.9721	0.8475	0.9030	0.8452	0.9625	0.7280
NM_001108985	Adck1	0.3839	0.1879	0.4130	0.1763	0.1981	0.5325	0.4541	0.5365	0.6363	0.3833	0.0223	0.1864
NM_001108984	Rbm25	0.7614	0.8667	0.4576	0.5981	0.8728	0.8297	0.4618	0.9131	0.7884	0.7823	0.6908	0.6174
NM_001109211	Bhlhb4	0.8598	0.3735	0.8987	0.8795	0.7624	0.8372	0.6913	0.9317	0.9009	0.9034	0.8223	0.7059
NM_001107906	Chd7	0.4894	0.6715	0.4866	0.4186	0.1687	0.2315	0.0518	0.5117	0.9030	0.3451	0.7488	0.6545
NM_001109215	Xrcc2	0.7225	0.7418	0.8266	0.4491	0.5683	0.8759	0.4534	0.8980	0.8048	0.4080	0.6697	0.9856
NM_001108987	Diras1	0.1879	0.5694	0.4592	0.3417	0.5543	0.5668	0.2850	0.7048	0.2476	0.5161	0.5143	0.8256
NM_001109149	Nmb	0.9208	0.8429	0.9526	0.7073	0.6675	0.6241	0.7232	0.7021	0.6348	0.4231	0.8950	0.7510
NM_001109217	Nupl2	0.9288	0.9946	0.4164	0.5169	0.8339	0.9266	0.9892	0.9386	0.9937	0.8938	0.7596	0.9768
NM_001005381	Ddx19a	0.3238	0.3688	0.2185	0.4862	0.5555	0.5510	0.4953	0.2338	0.5297	0.2276	0.3900	0.5951
NM_001115045	Ric3	0.1804	0.4389	0.1897	0.1964	0.2585	0.2935	0.2001	0.3843	0.4778	0.3308	0.4177	0.4463
NM_001109227	Tspan33	0.6038	0.9857	0.7519	0.7768	0.3600	0.5219	0.7132	0.3676	0.9140	0.9483	0.4992	0.9307
NM_001109221	LOC500034	0.6561	0.7036	0.4765	0.7303	0.6950	0.4575	0.6511	0.5658	0.6660	0.6219	0.7166	0.4056
NM_001109224	Fezf1	0.9012	0.1726	0.7309	0.5086	0.8225	0.6412	0.4415	0.6946	0.3696	0.6169	0.6682	0.4497
NM_001109228	RGD1559502	0.2381	0.7012	0.4380	0.3565	0.9154	0.7898	0.6859	0.9331	0.9056	0.6664	0.6595	0.8770
NM_001109225	RGD1560157	0.7942	0.9825	0.4900	0.6623	0.8110	0.8729	0.7954	0.3940	0.5235	0.8038	0.6394	0.3384
NM_001108988	Abhd9	0.3044	0.3213	0.3072	0.7635	0.3633	0.3805	0.4669	0.2678	0.5832	0.6589	0.7857	0.7910
NM_001109230	Zfp398	0.4704	0.5731	0.2596	0.5190	0.4340	0.4255	0.2147	0.4174	0.1934	0.4171	0.2743	0.0918
NM_001109233	Hoxa9	0.0366	0.8610	0.0590	0.0318	0.5780	0.1075	0.1022	0.9094	0.7894	0.7320	0.3805	0.5858
NM_001005528	Rps25	0.2004	0.5324	0.2581	0.0926	0.0128	0.3354	0.6868	0.0898	0.0809	0.1393	0.2127	0.6142
NM_001109231	Lrrc61	0.9511	0.9598	0.9447	0.7357	0.9358	0.8233	0.8943	0.9413	0.9658	0.9167	0.9258	0.9108
NM_001109089	RGD1565796	0.4698	0.8874	0.2742	0.0528	0.7971	0.0938	0.4564	0.6884	0.3847	0.1197	0.9327	0.3291
NM_001109241	Atoh8	0.9591	0.9227	0.7737	0.9609	0.8581	0.9721	0.9771	0.9872	0.9953	0.9360	0.9826	0.4452
NM_001109247	Arhgap25	0.8079	0.6959	0.5194	0.4834	0.6765	0.6899	0.6636	0.8428	0.6665	0.7487	0.3482	0.3831
NM_001109248	Dnajb8	0.3537	0.2434	0.2079	0.1461	0.2774	0.2930	0.0987	0.4887	0.6638	0.2570	0.3495	0.1034
NM_001109101	RGD1565222	0.4003	0.2717	0.1713	0.3189	0.3909	0.7157	0.7343	0.8652	0.4331	0.6206	0.2769	0.5499
NM_001109250	Kbtbd8	0.2777	0.8707	0.4401	0.7276	0.4003	0.2506	0.5483	0.6630	0.5395	0.3999	0.4518	0.8594
NM_001109103	RGD1565212	0.4537	0.1251	0.3982	0.5707	0.3985	0.1872	0.1884	0.5978	0.5002	0.0616	0.6202	0.5405
NM_001109095	RGD1562229	0.3450	0.4561	0.2272	0.2388	0.5978	0.5830	0.1094	0.4546	0.5418	0.1628	0.4374	0.6227
NM_001108994	Fam76b	0.6456	0.5132	0.8232	0.6183	0.5556	0.6743	0.3678	0.9447	0.6113	0.3930	0.9322	0.8389
NM_001109253	Clec1a	0.8730	0.5749	0.9462	0.7315	0.8448	0.9451	0.5153	0.4516	0.9393	0.7804	0.9596	0.8505
NM_001109252	Tatdn2	0.6462	0.7678	0.8572	0.5755	0.7406	0.8889	0.5350	0.6830	0.6599	0.1974	0.5790	0.5154
NM_001109254	Gprc5d	0.3845	0.4849	0.1896	0.4593	0.3992	0.2574	0.3105	0.4132	0.4151	0.4216	0.4585	0.4077
NM_001109255	Sspn	0.8292	0.9602	0.7556	0.7814	0.6598	0.8280	0.6823	0.6623	0.8912	0.8103	0.4202	0.7879
NM_001108993	Lass5	0.8865	0.4960	0.1747	0.5566	0.7775	0.5857	0.9038	0.7809	0.9580	0.6062	0.8537	0.5846
NM_001109265	Zbtb8b	0.4833	0.3884	0.4006	0.4789	0.4118	0.5201	0.5765	0.7329	0.4619	0.3215	0.2468	0.5209
NM_001109258	Tmem70	0.0810	0.0455	0.9320	0.7522	0.0277	0.1371	0.1932	0.0330	0.4996	0.0871	0.1688	0.8180
NM_001109532	Mapk11	0.3253	0.3432	0.2947	0.3090	0.4668	0.4084	0.3095	0.3954	0.5815	0.3840	0.3340	0.3705

NM_001109267	Zcchc17	0.9008	0.9493	0.8812	0.9312	0.3732	0.8006	0.9030	0.7630	0.4849	0.8013	0.7235	0.8855
NM_001109269	Lin28	0.1979	0.5642	0.4343	0.5496	0.9306	0.6702	0.7761	0.4579	0.6436	0.5786	0.8368	0.8448
NM_001109670	Gpr172b	0.6195	0.5593	0.7516	0.8447	0.7185	0.4073	0.9719	0.6359	0.8863	0.9226	0.0967	0.9442
NM_001109262	RGD1559493	0.1429	0.1093	0.0801	0.2336	0.2087	0.1136	0.2472	0.1861	0.2979	0.3418	0.0227	0.0447
NM_001108997	Dock6	0.9547	0.9566	0.9584	0.6989	0.9838	0.9549	0.9810	0.9747	0.9725	0.9967	0.8858	0.9408
NM_001109270	Grrp1	0.1427	0.5701	0.7838	0.5138	0.7074	0.4873	0.5342	0.4893	0.4362	0.6174	0.6057	0.3723
NM_001109271	Ldlrap1	0.1192	0.1319	0.3837	0.3542	0.3463	0.2793	0.5939	0.4071	0.2974	0.1265	0.0136	0.4532
NM_001109273	Tmem51	0.4596	0.3240	0.2915	0.1823	0.6238	0.7732	0.7799	0.7092	0.2710	0.6307	0.6046	0.7176
NM_001109282	Gipc3	0.2112	0.3230	0.0851	0.1732	0.1227	0.1908	0.5484	0.3480	0.2761	0.2996	0.0139	0.4501
NM_001109274	Socs5	0.2266	0.2535	0.2708	0.6128	0.1667	0.5831	0.2890	0.3684	0.1722	0.3010	0.2949	0.7311
NM_001109286	Bbs10	0.1906	0.8350	0.2025	0.4679	0.7040	0.5560	0.5916	0.4270	0.2007	0.7627	0.5208	0.6308
NM_001109098	RGD1562356	0.7389	0.8762	0.5676	0.4522	0.4760	0.3120	0.6768	0.1098	0.2011	0.5740	0.4805	0.3442
NM_001109290	Zfp202	0.9759	0.5609	0.7456	0.9660	0.8017	0.8842	0.5317	0.9626	0.9100	0.4262	0.8577	0.8862
NM_001109275	RGD1559683	0.7996	0.0109	0.3587	0.6383	0.1349	0.2669	0.4902	0.3385	0.2838	0.2272	0.1006	0.5558
NM_001109277	LOC500688	0.7264	0.6589	0.4693	0.2177	0.7692	0.7270	0.5357	0.6666	0.7234	0.7256	0.5196	0.7431
NM_001109278	RGD1563216	0.0277	0.0966	0.7228	0.3459	0.1693	0.0428	0.4524	0.1239	0.0628	0.2433	0.2403	0.3799
NM_001109212	Uckl1	0.8319	0.8534	0.8299	0.8974	0.7695	0.8126	0.6668	0.8380	0.9896	0.8255	0.8693	0.8370
NM_001109118	Elovl2	0.0435	0.4391	0.8066	0.9108	0.4032	0.6157	0.7456	0.0519	0.5304	0.0177	0.5369	0.5289
NM_001109300	Cmtm7	0.4186	0.4394	0.4720	0.6772	0.5403	0.5686	0.4127	0.2500	0.2740	0.2159	0.2312	0.6282
NM_001109220	Pex1	0.2959	0.1253	0.4084	0.0377	0.2782	0.2000	0.2105	0.0937	0.5922	0.2250	0.3047	0.8152
NM_001109284	LOC500797	0.0875	0.1588	0.0813	0.0667	0.0573	0.0823	0.5866	0.0471	0.0906	0.1434	0.0451	0.2174
NM_001109291	H2afx	0.9464	0.9688	0.2329	0.9102	0.9198	0.7596	0.9151	0.9974	0.9853	0.7654	0.9704	0.2554
NM_001109287	RGD1565498	0.1729	0.2438	0.6826	0.4605	0.7313	0.5111	0.6143	0.5819	0.0780	0.2810	0.3969	0.1848
NM_001109122	RGD1562552	0.0858	0.0789	0.1469	0.3145	0.2147	0.1375	0.1046	0.1520	0.0385	0.4233	0.1475	0.1760
NM_001109125	RGD1564428	0.0334	0.0278	0.0171	0.0146	0.0709	0.0067	0.1799	0.0686	0.0043	0.3483	0.0863	0.0550
NM_001109304	Cfc1	0.9652	0.5175	0.6193	0.6623	0.8794	0.6735	0.9226	0.8951	0.7502	0.6996	0.8667	0.5183
NM_001109303	Crip3	0.1638	0.3116	0.5285	0.1929	0.4231	0.2470	0.7090	0.5808	0.1043	0.4286	0.4685	0.5781
NM_001109292	RGD1564560	0.4680	0.6338	0.8430	0.9333	0.7955	0.9872	0.6244	0.8875	0.7057	0.5625	0.4317	0.8183
NM_001109151	RGD1559529	0.0542	0.1685	0.5139	0.5790	0.1934	0.5693	0.0459	0.3615	0.0712	0.2012	0.0530	0.1598
NM_001109305	Tmem182	0.4067	0.8111	0.5143	0.7845	0.3563	0.4017	0.2887	0.6475	0.3925	0.7105	0.4525	0.5140
NM_001109297	Tusc2	0.6366	0.6785	0.8474	0.4924	0.6683	0.4246	0.5890	0.6710	0.5786	0.6943	0.6733	0.8757
NM_001109379	Dnd1	0.1684	0.8422	0.5485	0.8135	0.6388	0.7845	0.3846	0.7326	0.7386	0.3382	0.4722	0.8075
NM_001109584	Vangl1	0.2873	0.7615	0.2511	0.2865	0.2663	0.2726	0.7703	0.2562	0.9653	0.8742	0.6272	0.1078
NM_001109153	RGD1559600	0.3202	0.1518	0.0906	0.4691	0.1598	0.5404	0.3240	0.1505	0.4375	0.3254	0.0998	0.4955
NM_001127694	Lrfn1	0.2446	0.4937	0.4239	0.4983	0.3783	0.3316	0.3131	0.5804	0.5624	0.2672	0.2997	0.3614
NM_001109312	Vwc2	0.6464	0.5641	0.7155	0.4143	0.5504	0.3079	0.4968	0.3381	0.5388	0.7830	0.6506	0.6644
NM_001109310	Efhd1	0.2511	0.0722	0.0715	0.7339	0.1178	0.1829	0.5801	0.0739	0.0689	0.1240	0.1462	0.8998
NM_001109146	RGD1562492	0.7569	0.4472	0.4225	0.5780	0.2808	0.2807	0.4207	0.7263	0.7849	0.5051	0.5921	0.6062
NM_001109144	Pth2	0.3300	0.3355	0.3573	0.3074	0.1033	0.4577	0.3122	0.3815	0.0458	0.3719	0.1234	0.2940
NM_001109134	RGD1559896	0.8842	0.1361	0.9173	0.4166	0.7677	0.5501	0.5388	0.6936	0.6282	0.3610	0.8802	0.5985
NM_001107977	Heyl	0.7278	0.2152	0.6764	0.9116	0.3918	0.5888	0.4799	0.6321	0.6121	0.5950	0.5112	0.4337
NM_001109295	Cplx3	0.4156	0.6489	0.6480	0.4711	0.7134	0.3509	0.4924	0.5037	0.5909	0.5303	0.0485	0.4260
NM_001109457	Spinlw1	0.2334	0.2411	0.1894	0.5676	0.2286	0.5585	0.3170	0.5635	0.4861	0.2898	0.4874	0.3483
NM_001109484	Myl6	0.5433	0.1794	0.3702	0.3886	0.2404	0.2871	0.1996	0.3204	0.3253	0.2820	0.3026	0.3017
NM_001109309	Cdk5r2	0.0531	0.3683	0.0645	0.0703	0.1927	0.4046	0.2025	0.6836	0.4227	0.1872	0.1212	0.2704
NM_001109299	Tmie	0.1394	0.0256	0.3785	0.3196	0.2930	0.1988	0.2490	0.2235	0.0798	0.1176	0.3057	0.4754
NM_001109373	Hmg1l1	0.8298	0.8775	0.7353	0.7958	0.7739	0.5554	0.4477	0.5156	0.5914	0.7132	0.7379	0.5707
NM_001109306	Satb2	0.9324	0.9510	0.9803	0.9872	0.9708	0.7427	0.8478	0.8550	0.9277	0.9440	0.9670	0.7669
NM_001109314	Zbtb33	0.8337	0.1966	0.8653	0.8814	0.0888	0.0469	0.0440	0.0599	0.3827	0.0944	0.1137	0.0398

NM_001109308	RGD1565501	0.3855	0.0990	0.2050	0.6793	0.1404	0.4643	0.8624	0.6112	0.4051	0.8197	0.5523	0.5427
NM_001109319	Gspt2	0.6243	0.4804	0.8606	0.7329	0.8140	0.2021	0.7202	0.6532	0.5857	0.7572	0.3486	0.3566
NM_001109316	Rai2	0.8368	0.8794	0.8611	0.8992	0.8779	0.8947	0.9257	0.8452	0.7390	0.9112	0.9613	0.5790
NM_001109320	Fam123b	0.1014	0.3562	0.2016	0.4452	0.4333	0.2880	0.2658	0.4362	0.4112	0.5147	0.2685	0.3268
NM_001109321	Mum1l1	0.4298	0.4284	0.4254	0.5425	0.5209	0.3014	0.3536	0.2086	0.7939	0.1232	0.2959	0.2916
NM_001109156	RGD1566127	0.2575	0.1027	0.0342	0.5198	0.1533	0.5560	0.2996	0.3550	0.0646	0.4382	0.5327	0.1853
NM_001109322	Ctag2	0.5364	0.3296	0.1920	0.1655	0.1174	0.3418	0.3384	0.3576	0.4242	0.2963	0.1228	0.5068
NM_001109324	Fam3a	0.5717	0.5912	0.8763	0.6039	0.7573	0.5153	0.8770	0.6165	0.8698	0.1703	0.4057	0.7853
NM_001109326	Krtap14	0.8021	0.6316	0.7785	0.5452	0.7639	0.3931	0.6508	0.7518	0.7646	0.7223	0.8934	0.6521
NM_001109327	Coro1c	0.9118	0.9918	0.7365	0.9763	0.6372	0.7610	0.9740	0.8869	0.7877	0.6106	0.9657	0.9171
NM_001109161	RGD1560108	0.4523	0.8605	0.6721	0.1704	0.6147	0.6417	0.7056	0.4823	0.5672	0.5244	0.8616	0.8847
NM_001109330	Cbln3	0.3653	0.5770	0.3418	0.2895	0.6148	0.4154	0.5610	0.5285	0.6046	0.1952	0.4306	0.5468
NM_001109313	Sh2d1a	0.6219	0.6024	0.4369	0.5767	0.7383	0.6364	0.5604	0.7950	0.8627	0.7755	0.5773	0.4302
NM_001109332	Dkk4	0.4802	0.5607	0.4135	0.5945	0.4201	0.4487	0.5651	0.2391	0.6818	0.3220	0.4542	0.3754
NM_001109317	RGD1564799	0.6199	0.6778	0.6866	0.6826	0.6207	0.4227	0.5386	0.7045	0.7093	0.7299	0.7170	0.6179
NM_001109311	RGD1563692	0.3912	0.1287	0.5354	0.1354	0.5217	0.0578	0.6316	0.4182	0.8299	0.1158	0.7251	0.5476
NM_001109338	Slc35f1	0.4543	0.4607	0.3749	0.5257	0.4543	0.6325	0.5426	0.1264	0.8063	0.7539	0.2079	0.3224
NM_001109328	RGD1562617	0.0477	0.3125	0.7587	0.7552	0.1776	0.0444	0.4549	0.1259	0.4887	0.3658	0.3813	0.8835
NM_001109164	RGD1560242	0.0251	0.3047	0.6242	0.3425	0.0494	0.0532	0.1716	0.1955	0.3608	0.1819	0.3973	0.0195
NM_001109136	Vnn3	0.3992	0.3853	0.0782	0.5281	0.3613	0.6451	0.6717	0.5095	0.3179	0.5213	0.5333	0.1577
NM_001109407	Dqx1	0.6211	0.5395	0.3070	0.1961	0.3472	0.4396	0.6168	0.5545	0.3281	0.4062	0.2898	0.5344
NM_001109342	Pramel6	0.1172	0.6054	0.2178	0.1680	0.0621	0.3996	0.2723	0.1715	0.2169	0.0758	0.5366	0.0990
NM_001109339	Eif2a	0.6418	0.0959	0.3566	0.1936	0.2584	0.1256	0.1323	0.2166	0.2292	0.6872	0.2144	0.6075
NM_001109179	RGD1565641	0.6255	0.3691	0.7211	0.4320	0.2441	0.3060	0.5249	0.3479	0.3202	0.5112	0.1814	0.6149
NM_001109333	RGD1564978	0.7599	0.5299	0.8941	0.7612	0.3184	0.4781	0.4500	0.1004	0.6514	0.4984	0.5085	0.2263
NM_001109346	Cpa4	0.6984	0.4863	0.4617	0.5079	0.4211	0.5789	0.8937	0.7632	0.6270	0.4663	0.3242	0.1584
NM_001109343	Cst13	0.2365	0.5528	0.2981	0.1217	0.3623	0.0249	0.0741	0.0894	0.1289	0.4517	0.4637	0.1795
NM_001106785	Cpsf6	0.9923	0.9968	0.9444	0.9993	0.9909	0.9712	0.9897	0.9889	0.9936	0.9898	0.9927	0.9017
NM_001107018	Ankfy1	0.4836	0.0690	0.5868	0.9085	0.3747	0.8760	0.3043	0.3533	0.3647	0.0887	0.3575	0.2988
NM_001106384	Zfp280b	0.7473	0.5347	0.6328	0.3771	0.8434	0.4384	0.2833	0.4491	0.6659	0.8351	0.7093	0.3343
NM_001109468	Polr3g	0.6977	0.6540	0.6234	0.7239	0.5834	0.6093	0.5568	0.5240	0.4858	0.6354	0.6895	0.4403
NM_001109350	Prrt3	0.6720	0.3833	0.3677	0.1438	0.0209	0.4385	0.6455	0.4492	0.3279	0.6509	0.6637	0.6983
NM_001109351	H1foo	0.1746	0.2685	0.4908	0.5294	0.1365	0.1128	0.7108	0.2791	0.5444	0.1958	0.2866	0.1591
NM_001109352	Foxj2	0.7035	0.7216	0.5928	0.5366	0.8681	0.8329	0.4933	0.6263	0.6006	0.0934	0.4251	0.9273
NM_001109353	Clec12b	0.1733	0.5545	0.4246	0.6626	0.3755	0.5082	0.6750	0.0757	0.1251	0.5586	0.2188	0.3080
NM_001109354	Clec9a	0.3874	0.5381	0.6596	0.7075	0.5720	0.6841	0.7648	0.2847	0.3463	0.3889	0.8503	0.7042
NM_001109355	Igbp1b	0.6794	0.2828	0.4196	0.4941	0.4435	0.6623	0.3298	0.6506	0.4332	0.4647	0.4798	0.2340
NM_001109239	RGD1560028	0.5394	0.8001	0.6218	0.3106	0.4518	0.6185	0.0416	0.8242	0.3246	0.0350	0.5115	0.6493
NM_001109234	RGD1564419	0.4374	0.8636	0.9263	0.8703	0.8841	0.9235	0.7275	0.9802	0.7553	0.6579	0.9609	0.8708
NM_001109357	Zfp352	0.3309	0.2698	0.1103	0.3682	0.1083	0.0329	0.2865	0.1941	0.2830	0.3940	0.3344	0.1337
NM_001109242	RGD1562515	0.3639	0.1792	0.3138	0.1959	0.1848	0.1854	0.1993	0.5391	0.1749	0.1838	0.4434	0.1835
NM_001109620	Mrpl10	0.3161	0.4002	0.5001	0.6161	0.2233	0.5958	0.5971	0.5320	0.5973	0.5052	0.2962	0.6332
NM_001109425	Znf703	0.3712	0.4454	0.8765	0.7336	0.3601	0.5595	0.7389	0.3438	0.1806	0.5490	0.7545	0.9100
NM_001109422	Bcl3	0.4538	0.0366	0.6232	0.1513	0.3157	0.0423	0.2575	0.0515	0.3157	0.3827	0.0202	0.3660
NM_001109361	Iqcf1	0.2603	0.3607	0.1029	0.0668	0.4917	0.1364	0.2185	0.1461	0.3422	0.0170	0.0416	0.0323
NM_001127304	Ncf4	0.4191	0.0559	0.0884	0.1319	0.3975	0.7543	0.2444	0.5953	0.6620	0.1446	0.2607	0.7508
NM_001109364	Ankrd57	0.9729	0.9677	0.9550	0.8642	0.9773	0.9042	0.9857	0.8947	0.9859	0.9524	0.9781	0.8647
NM_001109335	RGD1564719	0.3290	0.3778	0.3534	0.2995	0.3415	0.4044	0.3191	0.4479	0.1916	0.3139	0.2153	0.4173
NM_001109387	Mad1l1	0.2741	0.5529	0.3274	0.6030	0.3749	0.6734	0.2717	0.6360	0.6765	0.2267	0.3715	0.3793

NM_001109369	LOC678704	0.6074	0.7943	0.6221	0.7117	0.8042	0.6694	0.6275	0.5673	0.6285	0.7363	0.6784	0.5330
NM_001109370	LOC678749	0.5454	0.3003	0.3473	0.3021	0.2353	0.3425	0.2336	0.4961	0.5024	0.2481	0.4071	0.4256
NM_001109372	LOC679566	0.6980	0.4977	0.3667	0.5248	0.4967	0.5445	0.1991	0.5386	0.6320	0.7233	0.6095	0.2597
NM_001109607	Hint1	0.2639	0.2733	0.5147	0.3293	0.3700	0.2700	0.3876	0.3825	0.4432	0.4762	0.3873	0.3012
NM_001109375	LOC679682	0.1575	0.0504	0.4992	0.4906	0.0926	0.1085	0.4927	0.4463	0.0574	0.3782	0.2100	0.0856
NM_001109376	LOC679692	0.7240	0.6691	0.6845	0.6977	0.6983	0.6998	0.7285	0.3482	0.6129	0.6435	0.6876	0.6646
NM_001109377	Clec5a	0.1990	0.2527	0.2933	0.3920	0.6400	0.2216	0.4610	0.4266	0.3852	0.5969	0.2219	0.5638
NM_001109378	LOC679805	0.4968	0.5479	0.4795	0.5465	0.4718	0.3624	0.5717	0.2271	0.6763	0.3960	0.5958	0.4445
NM_001109557	Crygf	0.3430	0.3018	0.3135	0.1958	0.3194	0.4386	0.4338	0.1905	0.3193	0.1690	0.3544	0.3690
NM_001109380	Capns2	0.4309	0.4035	0.1890	0.1956	0.2641	0.3680	0.4378	0.5226	0.3074	0.4750	0.3016	0.3056
NM_001109381	Gpkow	0.3567	0.5546	0.7444	0.2022	0.2454	0.5342	0.3799	0.3323	0.7678	0.7950	0.6467	0.7838
NM_001109382	Vasn	0.5039	0.5717	0.4853	0.4504	0.5176	0.7014	0.5556	0.6029	0.3048	0.5094	0.3038	0.4206
NM_001109383	Angptl1	0.2765	0.3249	0.4615	0.1955	0.2895	0.4171	0.1989	0.1903	0.1749	0.4847	0.4978	0.3812
NM_001109384	Alkbh7	0.2298	0.3391	0.3510	0.4588	0.5766	0.4348	0.3601	0.2887	0.4631	0.4615	0.2658	0.3968
NM_001127299	Fam84a	0.3404	0.5829	0.6429	0.6261	0.8513	0.8046	0.6674	0.5260	0.5501	0.6779	0.8084	0.3216
NM_001127302	RGD1563669	0.4358	0.7884	0.8641	0.9866	0.3153	0.7295	0.9460	0.8292	0.9136	0.3873	0.7671	0.9137
NM_001109386	Gpr183	0.5289	0.3687	0.2281	0.2698	0.2116	0.4458	0.4532	0.4080	0.6347	0.2198	0.2623	0.3433
NM_001109388	LOC680039	0.6030	0.6966	0.6661	0.6574	0.6074	0.6672	0.6309	0.4528	0.6472	0.5281	0.7162	0.6170
NM_001109390	Pcdhb9	0.2601	0.1723	0.4644	0.4863	0.3874	0.3454	0.1212	0.4628	0.3146	0.3599	0.5636	0.3362
NM_001109391	Mab21l2	0.5572	0.3512	0.7290	0.5409	0.6530	0.4771	0.7190	0.5505	0.5106	0.6401	0.2099	0.6638
NM_001109392	LOC680112	0.2336	0.3148	0.3224	0.2869	0.4245	0.2516	0.3049	0.3900	0.4405	0.2298	0.4529	0.3840
NM_001077644	Ptgir	0.0555	0.3308	0.4027	0.6153	0.1334	0.5297	0.8714	0.3073	0.6346	0.6278	0.3653	0.3053
NM_001109395	Pcdhb20	0.4419	0.4130	0.4554	0.3238	0.2008	0.5941	0.7342	0.2297	0.3836	0.1978	0.3312	0.2127
NM_001109396	Dnajb3	0.5241	0.6037	0.4962	0.4629	0.2965	0.4250	0.7302	0.6438	0.2898	0.4034	0.4382	0.2486
NM_001109397	LOC680254	0.2373	0.4709	0.5742	0.7153	0.6872	0.5372	0.4742	0.4588	0.2126	0.5134	0.6006	0.6586
NM_001109398	LOC680308	0.4548	0.9944	0.9111	0.9240	0.8741	0.9380	0.9312	0.9360	0.9329	0.9809	0.9503	0.8703
NM_001109401	LOC680319	0.4830	0.3835	0.5491	0.2698	0.4975	0.3190	0.5133	0.1901	0.6174	0.3514	0.6692	0.4313
NM_001109402	LOC680370	0.1574	0.2294	0.6341	0.6929	0.3848	0.2268	0.1394	0.2260	0.4578	0.4611	0.3869	0.1803
NM_001109403	C1ql3	0.5909	0.3622	0.7157	0.2281	0.3477	0.3059	0.5149	0.4512	0.7697	0.3861	0.8408	0.3772
NM_001109404	Rsu1	0.3766	0.3271	0.4995	0.6900	0.6077	0.4596	0.3272	0.2607	0.2934	0.6619	0.6504	0.4406
NM_001109406	LOC680428	0.2809	0.2334	0.4243	0.3024	0.2156	0.3913	0.5277	0.2954	0.4917	0.2705	0.3601	0.5101
NM_001109408	Arrdc5	0.2883	0.1788	0.1888	0.1955	0.1847	0.4418	0.1988	0.3575	0.1746	0.3120	0.3901	0.1835
NM_001109409	LOC680454	0.2542	0.5385	0.2575	0.2740	0.6498	0.2149	0.5320	0.3557	0.2087	0.3429	0.3302	0.4500
NM_001109410	Trappc6a	0.3843	0.2437	0.6469	0.3578	0.3586	0.3134	0.4451	0.3422	0.2269	0.2531	0.4722	0.4930
NM_001109411	Ppapdc1b	0.0232	0.4316	0.0413	0.3093	0.1467	0.1032	0.8143	0.1128	0.0832	0.1156	0.3764	0.1065
NM_001109413	Lrrc19	0.7037	0.7120	0.6489	0.6321	0.3514	0.6474	0.2407	0.6891	0.5283	0.4347	0.5428	0.5930
NM_001109414	Dazl	0.5626	0.7226	0.6592	0.6447	0.6789	0.5248	0.5215	0.6414	0.5783	0.6102	0.6064	0.3842
NM_001109416	Cd3eap	0.5350	0.2582	0.3042	0.6098	0.2224	0.6572	0.3556	0.6850	0.2147	0.4131	0.5595	0.4127
NM_001109418	LOC680531	0.2233	0.2561	0.3228	0.3521	0.2645	0.3716	0.5439	0.4844	0.3463	0.3717	0.2638	0.4070
NM_001109419	Apoc4	0.1801	0.1787	0.2926	0.5243	0.1846	0.3539	0.1988	0.1901	0.3249	0.3331	0.1797	0.5444
NM_001109420	Rbm41	0.6003	0.4988	0.6449	0.5168	0.2057	0.2106	0.2335	0.2712	0.4433	0.5575	0.5990	0.5400
NM_001109537	Sssca1	0.2269	0.3130	0.4590	0.6284	0.2426	0.3738	0.4658	0.3502	0.4340	0.2279	0.2926	0.5112
NM_001109546	Vsx1	0.9215	0.9217	0.9457	0.8123	0.8725	0.5368	0.7032	0.6986	0.9434	0.9300	0.9050	0.7409
NM_001109424	Krtap16-5	0.3104	0.2941	0.2516	0.4147	0.2670	0.5849	0.3516	0.5021	0.5850	0.1828	0.3067	0.2492
NM_001109565	Hils1	0.1800	0.1786	0.4502	0.1954	0.1844	0.1852	0.4299	0.5429	0.7307	0.1688	0.1796	0.1833
NM_001109426	LOC680723	0.2057	0.7082	0.5710	0.3358	0.6549	0.2206	0.7310	0.2428	0.5609	0.3604	0.5249	0.4573
NM_001109428	Mrpl20	0.7221	0.3287	0.3307	0.6619	0.2347	0.3598	0.4676	0.3996	0.7200	0.4761	0.3762	0.3013
NM_001109429	Timm8a2	0.2084	0.3201	0.4597	0.2509	0.4576	0.3393	0.6386	0.2436	0.5483	0.4516	0.5836	0.3925
NM_001109430	Lrtm2	0.6982	0.2392	0.2167	0.5128	0.6319	0.2919	0.5600	0.6856	0.7040	0.6158	0.6724	0.6700

NM_001109431	LOC680885	0.2909	0.4963	0.2255	0.5002	0.5285	0.4444	0.3916	0.4008	0.5709	0.3560	0.7107	0.6341
NM_001109432	Bmp8a	0.4520	0.5305	0.2271	0.2926	0.6462	0.4094	0.5580	0.2971	0.3740	0.5662	0.4420	0.5565
NM_001109435	Fam55c	0.6917	0.4302	0.6049	0.5605	0.2611	0.6905	0.5248	0.6259	0.5998	0.4940	0.4281	0.3801
NM_001109438	Map7d1	0.6444	0.6838	0.6735	0.7024	0.5716	0.3407	0.4888	0.5732	0.4764	0.6398	0.5545	0.6889
NM_001109439	LOC681325	0.5422	0.5194	0.6196	0.6774	0.6163	0.2102	0.2778	0.4320	0.2003	0.1894	0.6457	0.4121
NM_012638	Pygm	0.5127	0.0676	0.1538	0.0494	0.3657	0.1876	0.2653	0.1504	0.2792	0.1028	0.6557	0.6842
NM_001109441	LOC681383	0.1800	0.1785	0.1886	0.4687	0.1843	0.1851	0.1987	0.1900	0.1745	0.1687	0.5144	0.1832
NM_001109442	Erh	0.5614	0.5764	0.3997	0.3062	0.3425	0.2353	0.2763	0.7146	0.3958	0.6166	0.4631	0.3838
NM_001109443	LOC681418	0.4638	0.1783	0.1886	0.1952	0.3085	0.5649	0.5134	0.4010	0.3030	0.3334	0.1794	0.1831
NM_001109444	LOC681578	0.6186	0.6099	0.4252	0.6503	0.6862	0.6356	0.6437	0.6350	0.5262	0.6778	0.6189	0.4963
NM_001109451	LOC684993	0.3413	0.1781	0.3574	0.1952	0.2702	0.1850	0.1985	0.3473	0.1743	0.2960	0.1793	0.2955
NM_001109453	LOC685046	0.5138	0.4735	0.2692	0.3662	0.3168	0.2688	0.2946	0.2696	0.6012	0.2446	0.3847	0.4634
NM_001109454	Pgm2l1	0.7029	0.5301	0.6874	0.5369	0.4164	0.5098	0.6350	0.4351	0.5214	0.5850	0.5486	0.5106
NM_001109455	Nkx6-3	0.4927	0.3581	0.2129	0.2529	0.3541	0.5691	0.4434	0.5441	0.4791	0.3538	0.2205	0.4332
NM_001109456	LOC685144	0.6919	0.6145	0.6570	0.2560	0.6094	0.7189	0.6548	0.2421	0.5946	0.5656	0.5397	0.5179
NM_001109568	Slc16a5	0.2641	0.1548	0.7184	0.5359	0.6570	0.5776	0.7543	0.8893	0.7628	0.7549	0.4257	0.8788
NM_001109458	LOC685169	0.5066	0.4339	0.4743	0.2659	0.5390	0.5892	0.3889	0.4963	0.4777	0.2049	0.4656	0.4772
NM_001109459	LOC685171	0.6580	0.6265	0.8696	0.3566	0.7629	0.4091	0.5786	0.4334	0.2312	0.2485	0.8348	0.5842
NM_001109460	Stx19	0.1798	0.1781	0.1884	0.1951	0.2919	0.1850	0.1984	0.1897	0.2921	0.3231	0.4047	0.3233
NM_001109461	Wfdc10	0.1797	0.3612	0.5045	0.1949	0.2903	0.4419	0.1982	0.1896	0.1741	0.3452	0.1792	0.1831
NM_001109462	Tal2	0.4815	0.5050	0.5040	0.4482	0.3117	0.5272	0.5821	0.2332	0.5011	0.5439	0.4393	0.6207
NM_001109463	Hmgb4	0.1795	0.1780	0.1883	0.1948	0.4386	0.1849	0.1981	0.1895	0.1741	0.1686	0.5198	0.1830
NM_001109405	Pex10	0.5926	0.6070	0.8541	0.6372	0.0770	0.8187	0.8296	0.1777	0.6807	0.6866	0.6424	0.9376
NM_001109222	Ppp1r3a	0.5032	0.5464	0.7041	0.5483	0.7922	0.4076	0.8750	0.3255	0.6874	0.4787	0.4762	0.0630
NM_001109466	Fmo9	0.1955	0.2146	0.3484	0.6942	0.5506	0.6279	0.2393	0.2252	0.7163	0.5888	0.6871	0.4648
NM_001109467	Emid1	0.8562	0.8208	0.6928	0.5872	0.7835	0.3800	0.9168	0.8882	0.1910	0.9536	0.5620	0.9602
NM_001109571	Polr3gl	0.2380	0.3513	0.2341	0.3627	0.2964	0.2357	0.4636	0.3237	0.2065	0.3723	0.3173	0.6333
NM_001109469	Lrrtm2	0.6820	0.7255	0.6025	0.5922	0.7001	0.4820	0.4880	0.7250	0.6063	0.6672	0.6917	0.5145
NM_001109256	RGD1562899	0.2289	0.3250	0.7466	0.2128	0.5820	0.6729	0.3790	0.7126	0.6298	0.5120	0.2947	0.7842
NM_001109251	RGD1565997	0.5460	0.1403	0.3594	0.8062	0.5070	0.6024	0.9162	0.4372	0.8521	0.8658	0.4411	0.6738
NM_001109471	S100a7a	0.1794	0.2875	0.1882	0.3806	0.2838	0.3570	0.5117	0.3945	0.4554	0.3305	0.5160	0.2662
NM_001109472	Clgn	0.4338	0.2520	0.2499	0.3319	0.3565	0.3835	0.3202	0.5085	0.3196	0.4170	0.2833	0.3363
NM_001109475	RGD1311273	0.5730	0.8623	0.9149	0.9449	0.8535	0.3406	0.8694	0.8839	0.9839	0.9699	0.8998	0.8057
NM_001109476	Pfdn2	0.4164	0.2628	0.2268	0.3802	0.4930	0.4633	0.3098	0.4687	0.4983	0.4396	0.5224	0.3480
NM_001109479	LOC685722	0.4044	0.2871	0.3102	0.1948	0.1843	0.1848	0.1981	0.4120	0.3009	0.4937	0.5202	0.1829
NM_001109480	LOC685756	0.3914	0.3327	0.4497	0.4413	0.0307	0.7662	0.4794	0.3971	0.3816	0.3791	0.3094	0.1079
NM_001109481	Rprml	0.3421	0.2136	0.2510	0.2805	0.4402	0.3263	0.3982	0.5713	0.3824	0.2583	0.4086	0.4937
NM_001109482	Ubxd4	0.6507	0.7181	0.7260	0.6603	0.6594	0.4514	0.5771	0.6630	0.6917	0.6466	0.2322	0.4430
NM_001109483	Ppil5	0.2655	0.3373	0.3374	0.6863	0.2306	0.3276	0.4288	0.3704	0.4329	0.2751	0.2889	0.1828
NM_001109600	Adam30	0.3810	0.5708	0.4689	0.6752	0.6178	0.6270	0.4994	0.7186	0.6138	0.5402	0.5429	0.3526
NM_001109485	Tmem90a	0.6207	0.5359	0.3433	0.4962	0.6289	0.3966	0.4721	0.4299	0.3813	0.2073	0.3955	0.3882
NM_001109486	Myl6b	0.5203	0.2829	0.2374	0.1217	0.5592	0.3356	0.5369	0.4599	0.5225	0.4419	0.4275	0.2581
NM_001109487	Pfn3	0.1793	0.4831	0.3852	0.4087	0.1842	0.1847	0.3985	0.3741	0.4580	0.4462	0.1791	0.2677
NM_001109488	Ccl26	0.3252	0.5065	0.8602	0.5268	0.5746	0.4001	0.4421	0.4851	0.5737	0.1361	0.4069	0.0303
NM_001109489	LOC685964	0.5836	0.6390	0.7756	0.7400	0.6440	0.8214	0.7070	0.8958	0.2155	0.5218	0.6731	0.4913
NM_001109492	B3galt2	0.6365	0.6274	0.7223	0.5629	0.6880	0.5627	0.5110	0.2462	0.1880	0.7278	0.7223	0.2071
NM_001109494	Hmcn2	0.2823	0.6467	0.2451	0.3105	0.2033	0.4421	0.3195	0.3999	0.3412	0.5317	0.4648	0.4141
NM_001109496	LOC688390	0.4525	0.4208	0.7117	0.7270	0.4541	0.5526	0.7077	0.6731	0.6013	0.2099	0.6546	0.6966
NM_001109497	Mab21l1	0.5859	0.5048	0.5556	0.5582	0.6738	0.6540	0.2491	0.3154	0.5326	0.6136	0.6307	0.5223

NM_001109498	Cdk2ap2	0.4822	0.5105	0.5005	0.5023	0.6717	0.3741	0.2608	0.2733	0.5842	0.5240	0.2927	0.6344
NM_001109499	Wbscr27	0.6598	0.3627	0.6171	0.7122	0.6385	0.2110	0.6287	0.2329	0.6067	0.5408	0.3065	0.5269
NM_001109501	Arhgap10	0.3981	0.1777	0.1881	0.1945	0.5097	0.5982	0.1978	0.4405	0.2997	0.3301	0.1790	0.2927
NM_001109502	LOC688459	0.5870	0.2039	0.5841	0.2651	0.3890	0.6044	0.5931	0.5344	0.3635	0.2302	0.5109	0.3612
NM_001109477	Dnal1	0.0348	0.7749	0.6559	0.8842	0.0474	0.0241	0.6826	0.1308	0.3143	0.5304	0.5922	0.5908
NM_001109504	LOC688539	0.4039	0.3946	0.4316	0.1945	0.2317	0.1846	0.4922	0.5668	0.5053	0.2867	0.4704	0.6769
NM_001109505	Atg10	0.3612	0.5345	0.6157	0.2607	0.5580	0.6360	0.5999	0.5736	0.3779	0.6325	0.5626	0.2116
NM_001109579	Lrrn4cl	0.6370	0.6730	0.4989	0.5433	0.6051	0.3843	0.5346	0.6754	0.6319	0.5058	0.5449	0.4888
NM_001109507	LOC688717	0.5107	0.7274	0.6046	0.6179	0.6199	0.5761	0.6577	0.5182	0.7192	0.6550	0.5097	0.5845
NM_001109508	LOC688721	0.2283	0.4691	0.2375	0.3111	0.2175	0.3867	0.4050	0.3876	0.4235	0.7307	0.1789	0.4952
NM_001109509	Gpr84	0.4254	0.2659	0.6414	0.1943	0.3198	0.1846	0.3188	0.1893	0.4170	0.2564	0.2915	0.3468
NM_001109510	Gpr171	0.3463	0.5648	0.2160	0.2803	0.5762	0.6975	0.2479	0.4385	0.3692	0.4782	0.4023	0.3973
NM_001109511	Hdhd3	0.3026	0.2718	0.2838	0.5036	0.2959	0.4934	0.5879	0.3586	0.2362	0.1896	0.2980	0.2958
NM_001109512	LOC688765	0.3894	0.2176	0.6243	0.6630	0.4671	0.2166	0.6364	0.4649	0.3885	0.2389	0.4579	0.3748
NM_001109513	Grpel2	0.5816	0.6821	0.6554	0.6032	0.6349	0.6195	0.5737	0.6524	0.7036	0.6743	0.6169	0.5718
NM_001109514	Pmp2	0.5922	0.3065	0.2846	0.5519	0.5662	0.6400	0.6160	0.5258	0.5935	0.4578	0.6370	0.6231
NM_001109503	Commd2	0.4994	0.9129	0.7416	0.7896	0.4644	0.0263	0.4954	0.3523	0.2745	0.3762	0.3246	0.0209
NM_001109516	LOC688828	0.1793	0.1775	0.1879	0.1942	0.2546	0.2872	0.5113	0.3418	0.3503	0.2952	0.3443	0.1828
NM_001109517	Zbtb32	0.7460	0.8680	0.0207	0.1866	0.8959	0.9069	0.2274	0.9661	0.8088	0.8235	0.7451	0.5428
NM_001109518	Mrps16	0.2679	0.3432	0.3642	0.5170	0.2485	0.3590	0.4167	0.3320	0.3716	0.2818	0.5086	0.3908
NM_001109519	LOC688924	0.2725	0.2672	0.3418	0.4061	0.4160	0.4891	0.4240	0.3251	0.3130	0.4020	0.3275	0.2518
NM_001109520	Kcnk16	0.7056	0.5776	0.8020	0.5140	0.8720	0.7881	0.7522	0.8859	0.9387	0.9248	0.8242	0.9558
NM_001109521	LOC689065	0.3976	0.3103	0.5237	0.3172	0.3230	0.5712	0.7012	0.5953	0.5160	0.2425	0.2196	0.5649
NM_001109522	LOC689081	0.2644	0.2499	0.2642	0.4177	0.7063	0.2695	0.3784	0.3082	0.2644	0.5168	0.3079	0.3755
NM_001109523	Upk2	0.2463	0.4684	0.1877	0.1940	0.3415	0.2564	0.1977	0.4428	0.4735	0.6441	0.2849	0.5130
NM_001109412	LOC680477	0.1792	0.1774	0.1876	0.1939	0.3238	0.3895	0.1977	0.5478	0.1740	0.1684	0.1788	0.1827
NM_001109526	Snx29	0.3470	0.2661	0.1875	0.4570	0.3102	0.7271	0.1976	0.3226	0.3486	0.2669	0.3023	0.4577
NM_001109528	Tmem25	0.4509	0.2339	0.5115	0.5913	0.7164	0.4207	0.3855	0.6860	0.4256	0.4615	0.6203	0.6502
NM_001109529	Kin	0.2965	0.3245	0.5011	0.4043	0.2528	0.2807	0.5114	0.5841	0.3693	0.6243	0.2960	0.1826
NM_001109530	Spdef	0.3257	0.4262	0.2592	0.4625	0.2944	0.6811	0.4645	0.5863	0.2512	0.2255	0.4562	0.5300
NM_001109531	LOC689296	0.8072	0.7267	0.1208	0.4893	0.7102	0.8502	0.6077	0.4026	0.4987	0.5026	0.8096	0.6988
NM_001109533	Chmp1b	0.2492	0.7562	0.9358	0.4197	0.1193	0.2005	0.3219	0.0089	0.7029	0.8809	0.8948	0.8129
NM_001109678	Nadk	0.9411	0.8888	0.8746	0.7617	0.9300	0.8594	0.9544	0.7144	0.7161	0.9667	0.7835	0.9459
NM_001109535	Rab20	0.3903	0.4967	0.5881	0.2504	0.5623	0.6990	0.6048	0.3520	0.5431	0.2011	0.4540	0.3196
NM_001109536	Ptx3	0.6572	0.2105	0.5622	0.4769	0.4984	0.2215	0.2498	0.7066	0.6147	0.5422	0.6896	0.3888
NM_001109538	LOC689412	0.1790	0.3508	0.4924	0.3122	0.2333	0.3574	0.1975	0.3672	0.5825	0.2576	0.2900	0.1825
NM_001109539	LOC689570	0.4707	0.3871	0.2597	0.4525	0.4688	0.3555	0.4514	0.5163	0.6754	0.3101	0.3001	0.3791
NM_001109540	Nkain3	0.5761	0.5991	0.5645	0.4982	0.5185	0.3956	0.6797	0.6405	0.5045	0.5743	0.6582	0.6562
NM_001109515	Slc25a28	0.3654	0.4885	0.3676	0.6069	0.6649	0.3697	0.2794	0.4343	0.2266	0.3042	0.5362	0.3240
NM_001109542	LOC689618	0.2701	0.3305	0.2973	0.4185	0.3496	0.4458	0.4229	0.5316	0.3317	0.2743	0.4680	0.3143
NM_001100520	Ric8a	0.7778	0.7452	0.2606	0.5300	0.2683	0.5285	0.7552	0.6717	0.9084	0.5352	0.8609	0.8886
NM_001109544	Pdcd2l	0.3005	0.1774	0.3345	0.6700	0.2852	0.3064	0.3291	0.1892	0.2491	0.3168	0.3330	0.4672
NM_001109545	Atg2a	0.5095	0.2406	0.7360	0.3284	0.2088	0.5688	0.5063	0.4875	0.5191	0.5717	0.2837	0.4737
NM_001109259	RGD1564053	0.4896	0.3047	0.8669	0.8405	0.3594	0.4216	0.4575	0.6943	0.7013	0.5150	0.5864	0.3583
NM_001109547	Fam101a	0.3933	0.4138	0.6673	0.4173	0.6316	0.5533	0.2424	0.5728	0.6723	0.3596	0.5402	0.2178
NM_001109548	LOC689755	0.5321	0.3150	0.2225	0.2502	0.5910	0.6567	0.2518	0.3122	0.6465	0.2114	0.4474	0.6217
NM_001109549	LOC689766	0.2714	0.2743	0.2674	0.3589	0.1840	0.4229	0.6483	0.1891	0.1739	0.3393	0.5020	0.1824
NM_001109550	Magea4	0.1789	0.1772	0.1875	0.1938	0.1840	0.1845	0.1974	0.1890	0.1738	0.1683	0.1788	0.1823
NM_001109551	Msgn1	0.5065	0.1093	0.0357	0.1672	0.3397	0.3104	0.0687	0.3989	0.3076	0.0530	0.2045	0.0402

NM_001109552	Sfrs1	0.9972	0.9630	0.9973	0.9762	0.9595	0.9649	0.9386	0.9961	0.6592	0.8029	0.8405	0.6358
NM_001109554	Gpr21	0.5432	0.5975	0.6109	0.2823	0.5559	0.4527	0.2465	0.3427	0.4988	0.6028	0.6373	0.5417
NM_001109555	LOC689927	0.4170	0.5168	0.4785	0.1104	0.7380	0.1717	0.1895	0.3887	0.3728	0.0447	0.2537	0.4040
NM_001109556	Osap	0.5122	0.3716	0.4889	0.5590	0.5730	0.3946	0.2802	0.2918	0.2075	0.2605	0.2681	0.2369
NM_001109559	LOC689959	0.2514	0.5491	0.2867	0.4659	0.5677	0.2301	0.4196	0.2470	0.4086	0.4450	0.2560	0.3342
NM_001109560	LOC689963	0.1788	0.1771	0.2771	0.3338	0.7136	0.3821	0.3301	0.4175	0.6624	0.3555	0.1787	0.2993
NM_001109558	Setd7	0.3388	0.3996	0.6701	0.5697	0.6275	0.3757	0.6128	0.5689	0.3218	0.6200	0.5810	0.5471
NM_001109541	LOC689593	0.6063	0.6312	0.6984	0.4586	0.5943	0.4755	0.6902	0.5571	0.5703	0.6426	0.4746	0.5725
NM_001109563	LOC689986	0.5408	0.6536	0.4716	0.3738	0.1839	0.1844	0.1972	0.1890	0.4307	0.4807	0.7278	0.3784
NM_001127298	Wdr91	0.0543	0.2282	0.2851	0.0358	0.3401	0.7785	0.0309	0.7852	0.5912	0.0367	0.1489	0.1449
NM_001109566	LOC690079	0.4238	0.3505	0.2744	0.3407	0.3201	0.2732	0.3283	0.4742	0.3536	0.4716	0.3440	0.2574
NM_001109567	LOC690206	0.1787	0.3240	0.3496	0.1937	0.1837	0.1843	0.1971	0.1888	0.3063	0.1682	0.1786	0.1822
NM_001109561	Nrsn2	0.5407	0.3808	0.3099	0.5444	0.4443	0.4644	0.5811	0.6473	0.5859	0.1907	0.2124	0.6392
NM_001109570	Slmo1	0.3142	0.5493	0.3829	0.5910	0.4764	0.6277	0.4640	0.4719	0.5275	0.2034	0.2456	0.5626
NM_001109654	Gp1ba	0.5180	0.5172	0.2379	0.5853	0.2092	0.2285	0.3355	0.5890	0.2526	0.6328	0.6804	0.5159
NM_001109573	LOC690276	0.1786	0.5768	0.3711	0.2764	0.5465	0.4233	0.5364	0.2643	0.4057	0.3736	0.2494	0.3537
NM_001109572	Tmem179b	0.4243	0.2613	0.4271	0.5936	0.2625	0.3105	0.1970	0.1886	0.2659	0.5276	0.2896	0.4220
NM_001109575	Cox11	0.6340	0.7293	0.6940	0.6343	0.5591	0.7131	0.6718	0.5759	0.6200	0.6284	0.5386	0.6168
NM_001109574	Tmem169	0.3904	0.5907	0.7197	0.6346	0.3145	0.6577	0.7253	0.6925	0.6219	0.5284	0.6419	0.5308
NM_001109578	LOC690326	0.4662	0.4409	0.1873	0.1937	0.2630	0.1841	0.4448	0.1886	0.1738	0.1681	0.3363	0.2959
NM_001109421	Ebi3	0.3418	0.2427	0.7221	0.3421	0.6367	0.3566	0.2356	0.3328	0.2707	0.2386	0.2733	0.2869
NM_001109580	LOC690333	0.5954	0.5822	0.6313	0.6139	0.6640	0.6401	0.6546	0.5788	0.2058	0.6557	0.2557	0.5300
NM_001109581	LOC690349	0.2943	0.5153	0.4212	0.3210	0.3292	0.3300	0.3042	0.5961	0.2481	0.4613	0.2933	0.4409
NM_001109582	LOC690352	0.2538	0.3398	0.3775	0.3897	0.4096	0.2739	0.4326	0.1883	0.3849	0.3209	0.4028	0.4140
NM_001109577	Derl3	0.7034	0.8970	0.6106	0.7536	0.7910	0.7758	0.4649	0.8634	0.8827	0.9285	0.8713	0.7677
NM_001109576	Tsen54	0.2252	0.3855	0.2348	0.4727	0.3798	0.3371	0.6765	0.6377	0.3428	0.2326	0.6344	0.4100
NM_001109653	Spem1	0.1786	0.1770	0.1872	0.1935	0.1836	0.1840	0.1970	0.1882	0.1736	0.1680	0.1785	0.1821
NM_001109586	LOC690386	0.1785	0.1769	0.1871	0.1934	0.3576	0.4112	0.1969	0.3777	0.1735	0.1679	0.1784	0.1820
NM_001109621	LOC691093	0.4046	0.4829	0.4241	0.1934	0.6917	0.3506	0.3175	0.6019	0.5537	0.3439	0.3376	0.3593
NM_001109588	LOC690402	0.4041	0.4025	0.4102	0.4045	0.4118	0.5135	0.3527	0.3099	0.3699	0.3345	0.3668	0.4290
NM_001109564	Ppp1r3d	0.6540	0.6088	0.5907	0.4412	0.5806	0.6855	0.6535	0.6735	0.5794	0.6332	0.5952	0.5436
NM_001109585	Trim47	0.8674	0.9627	0.0714	0.1477	0.7029	0.8965	0.0530	0.8012	0.7775	0.9059	0.7082	0.7279
NM_001109590	LOC690415	0.4700	0.2156	0.2994	0.2645	0.6140	0.3666	0.6927	0.3905	0.2942	0.2305	0.2392	0.5318
NM_001033970	Pip5k1c	0.8889	0.6760	0.6508	0.2445	0.6558	0.7128	0.7125	0.6571	0.6355	0.6194	0.4291	0.6889
NM_001109593	LOC690460	0.1784	0.4866	0.1870	0.1933	0.3892	0.6200	0.1967	0.1879	0.1734	0.1677	0.1782	0.5328
NM_001109594	LOC690470	0.3715	0.5284	0.4411	0.8194	0.8232	0.7365	0.5385	0.6415	0.6447	0.2883	0.6988	0.5195
NM_001109610	H2afj	0.3878	0.7351	0.3917	0.4030	0.1835	0.4125	0.1965	0.1878	0.3255	0.1676	0.5195	0.1819
NM_001109597	LOC690489	0.5196	0.6819	0.5580	0.4436	0.4176	0.4902	0.5434	0.3450	0.3452	0.3153	0.4369	0.4462
NM_001109598	RGD1309707	0.8767	0.7580	0.4825	0.4686	0.7583	0.5480	0.9353	0.6158	0.2726	0.8764	0.9294	0.9402
NM_001109603	Mansc1	0.6384	0.2188	0.2855	0.5331	0.3409	0.2887	0.2843	0.2599	0.3507	0.3092	0.2268	0.4635
NM_001109602	Thoc4	0.4971	0.4487	0.6153	0.5691	0.5079	0.7251	0.6301	0.6975	0.1960	0.5839	0.5674	0.5913
NM_001109592	Rnf186	0.3105	0.2280	0.2639	0.4334	0.6847	0.4380	0.5818	0.3854	0.4133	0.4309	0.3541	0.7189
NM_001109605	LOC690617	0.7067	0.7228	0.6975	0.7376	0.6822	0.8335	0.7468	0.7165	0.7251	0.7268	0.8994	0.7382
NM_001109591	Jsrp1	0.3442	0.1766	0.3140	0.3729	0.4609	0.3381	0.1962	0.3530	0.2798	0.4899	0.5155	0.5336
NM_001100723	Adpgk	0.6575	0.9540	0.8564	0.9506	0.4999	0.4500	0.9800	0.6471	0.9267	0.5372	0.9620	0.7321
NM_001109608	LOC690728	0.3922	0.4397	0.5304	0.3353	0.3623	0.4219	0.3944	0.3164	0.4009	0.3494	0.4431	0.2405
NM_001109606	Fbxo3	0.7186	0.5488	0.6263	0.6543	0.5282	0.6706	0.6943	0.6581	0.6584	0.6393	0.2092	0.6622
NM_001109613	LOC690918	0.5206	0.2065	0.7067	0.2952	0.5171	0.5074	0.4695	0.5170	0.5060	0.4677	0.7129	0.5951
NM_019337	Rgs10	0.4023	0.4923	0.6864	0.3452	0.6959	0.2498	0.4713	0.3127	0.2357	0.2408	0.3006	0.6638

NM_001109478	Nme4	0.2460	0.1682	0.4298	0.4627	0.7213	0.1428	0.4273	0.3309	0.5566	0.2607	0.2498	0.5562
NM_001109616	LOC691024	0.6049	0.5342	0.3253	0.5826	0.5726	0.5080	0.5245	0.6194	0.6073	0.7031	0.5228	0.6097
NM_001109641	Hist3h2bb	0.2305	0.5592	0.4733	0.4054	0.2280	0.6556	0.3769	0.6060	0.2358	0.3732	0.4044	0.4511
NM_001109937	Cdk10	0.5981	0.8468	0.5746	0.3550	0.7466	0.6650	0.2624	0.5029	0.9358	0.5633	0.8005	0.5568
NM_001114656	LOC10013708(	0.7000	0.4736	0.3400	0.2915	0.4819	0.5804	0.2705	0.2660	0.3975	0.2466	0.4963	0.3591
NM_001109622	Tex19.2	0.3271	0.5306	0.5387	0.2793	0.4343	0.2989	0.3181	0.3058	0.2265	0.5846	0.2374	0.2236
NM_001109611	Adat1	0.6017	0.5584	0.6411	0.6754	0.4681	0.5458	0.4980	0.4978	0.6315	0.1866	0.4017	0.5886
NM_001109624	LOC691141	0.6348	0.4812	0.5860	0.3249	0.2069	0.4676	0.5648	0.5946	0.4547	0.5415	0.2631	0.2369
NM_001109625	Capza1	0.6128	0.6190	0.7332	0.6117	0.7032	0.5993	0.6666	0.6397	0.5439	0.7215	0.6725	0.7138
NM_001109629	LOC691162	0.0664	0.5400	0.5324	0.1317	0.0351	0.0731	0.4813	0.0879	0.0920	0.1624	0.5464	0.1373
NM_001109631	LOC691215	0.5132	0.4579	0.5215	0.5462	0.4969	0.2507	0.4328	0.4490	0.4223	0.3706	0.4299	0.2184
NM_001109632	LOC691259	0.0241	0.6444	0.1351	0.1323	0.0304	0.0223	0.0312	0.0507	0.3383	0.2840	0.0762	0.5776
NM_001109633	LOC691277	0.2672	0.3145	0.2619	0.4287	0.2969	0.2718	0.1961	0.1875	0.2621	0.5120	0.7202	0.3665
NM_001109634	LOC691286	0.2952	0.3074	0.3196	0.3892	0.3373	0.2969	0.1959	0.1874	0.2929	0.4651	0.3498	0.2702
NM_001109635	LOC691300	0.2610	0.3800	0.3345	0.3296	0.2804	0.3050	0.4240	0.1872	0.3464	0.2708	0.5382	0.3555
NM_001109604	Tmem86b	0.2241	0.3759	0.5011	0.3544	0.2229	0.2511	0.4615	0.4955	0.5557	0.5441	0.3078	0.4384
NM_001109612	Rps4y2	0.4575	0.4786	0.4250	0.3428	0.3149	0.2839	0.1959	0.4012	0.2640	0.2961	0.3892	0.1818
NM_001109569	Wdr74	0.3847	0.1765	0.1868	0.1932	0.2905	0.3923	0.3967	0.1872	0.3968	0.3085	0.3940	0.2744
NM_001109638	LOC691352	0.5856	0.2101	0.4093	0.2552	0.5635	0.2312	0.2691	0.4160	0.3066	0.4473	0.7208	0.2187
NM_001109618	Tm4sf20	0.3385	0.3221	0.4210	0.4880	0.5546	0.4509	0.5457	0.6032	0.3282	0.1938	0.2346	0.3042
NM_001109640	LOC691448	0.7109	0.2620	0.4307	0.3447	0.3794	0.3778	0.4034	0.3264	0.3077	0.4174	0.2945	0.1817
NM_031555	Bfsp1	0.0992	0.8452	0.2604	0.8032	0.6381	0.3312	0.3169	0.5100	0.8055	0.7314	0.7893	0.7434
NM_001109617	Pramel1	0.6052	0.5379	0.7313	0.4946	0.6221	0.6590	0.6457	0.5745	0.4674	0.7270	0.6787	0.6060
NM_001109636	Tmem202	0.3512	0.5164	0.2574	0.4599	0.4573	0.2558	0.5945	0.5666	0.2052	0.3618	0.4252	0.2195
NM_001107114	Usp25	0.5647	0.3494	0.9868	0.9560	0.1640	0.8410	0.8665	0.2366	0.7274	0.9231	0.9605	0.9685
NM_001109646	LOC691670	0.1782	0.1764	0.1867	0.1931	0.1833	0.1839	0.1958	0.1870	0.1733	0.1675	0.1781	0.1816
NM_001109647	LOC691729	0.3483	0.2669	0.3347	0.1930	0.3907	0.4061	0.1957	0.3644	0.2322	0.6723	0.3028	0.1814
NM_001109648	LOC691750	0.5879	0.5009	0.3698	0.2609	0.2041	0.3869	0.2880	0.5993	0.2029	0.2031	0.6635	0.2153
NM_001109623	Spaca4	0.4589	0.3846	0.3025	0.1930	0.3263	0.2845	0.1956	0.3681	0.2924	0.1674	0.3249	0.4294
NM_001109651	LOC691931	0.3713	0.2539	0.4342	0.6338	0.3467	0.3325	0.1955	0.3600	0.2346	0.2884	0.3480	0.2728
NM_001109652	LOC691952	0.6478	0.2123	0.7212	0.5612	0.4023	0.5502	0.6655	0.2610	0.5330	0.7020	0.6729	0.3311
NM_053287	B4galt1	0.1422	0.0471	0.9726	0.9007	0.2728	0.4332	0.1293	0.0557	0.2346	0.2563	0.1995	0.9485
NM_001109263	RGD1566001	0.5918	0.8705	0.3308	0.9308	0.5191	0.4317	0.8438	0.8303	0.8132	0.7520	0.7680	0.3550
NM_001109260	RGD1561849	0.4199	0.3825	0.6749	0.7386	0.7073	0.6619	0.3894	0.7837	0.4809	0.5178	0.7387	0.8885
NM_001109264	RGD1560436	0.5378	0.3059	0.5949	0.1488	0.6169	0.4070	0.6955	0.6066	0.7535	0.5511	0.5981	0.6856
NM_001109288	RGD1563200	0.1458	0.3644	0.2447	0.2519	0.2942	0.3301	0.3878	0.1432	0.4666	0.6065	0.1140	0.4451
NM_001109283	RGD1562114	0.8231	0.8862	0.0132	0.7326	0.6725	0.1796	0.2732	0.7439	0.0897	0.8856	0.5266	0.0081
NM_001109281	RGD1562342	0.6952	0.3087	0.8201	0.3341	0.7782	0.6012	0.8484	0.4595	0.5239	0.3015	0.0664	0.8985
NM_001109280	RGD1560608	0.2958	0.3801	0.1086	0.1403	0.1412	0.3390	0.5475	0.1455	0.7853	0.0750	0.1002	0.5233
NM_001109279	RGD1561926	0.5524	0.0591	0.7868	0.6592	0.4480	0.8709	0.1257	0.1082	0.6687	0.9579	0.4733	0.9125
NM_001109272	RGD1561421	0.2412	0.0314	0.2231	0.0911	0.5921	0.1162	0.6925	0.1497	0.0455	0.2076	0.4134	0.1620
NM_001109266	RGD1564163	0.0249	0.5673	0.0620	0.0081	0.1009	0.0057	0.6408	0.1986	0.2214	0.5289	0.1041	0.1219
NM_001109637	Lrrc39	0.6608	0.7337	0.5326	0.6048	0.6578	0.5315	0.5855	0.6241	0.6669	0.7197	0.4497	0.6468
NM_001108394	Zcchc24	0.7514	0.5482	0.8054	0.5956	0.8991	0.7358	0.2244	0.8121	0.7988	0.6244	0.9077	0.6445
NM_001109908	Eno1	0.4781	0.2509	0.2522	0.8765	0.3471	0.5819	0.9360	0.4218	0.5500	0.2949	0.3751	0.5884
NM_001109936	Cdk10	0.5981	0.8468	0.5746	0.3550	0.7466	0.6650	0.2624	0.5029	0.9358	0.5633	0.8005	0.5568
NM_001108517	Ccdc88b	0.0880	0.0780	0.2645	0.6729	0.4993	0.7448	0.7921	0.1390	0.3898	0.2243	0.1838	0.4472
NM_001107926	RGD1310773	0.4833	0.7212	0.5887	0.6188	0.6904	0.4388	0.6677	0.7074	0.5849	0.6960	0.6300	0.2192
NM_001108024	Lrfn5	0.6471	0.6364	0.0740	0.3457	0.6181	0.1628	0.3580	0.4737	0.6434	0.2975	0.1162	0.6652

NM_001107924	Slc35a1	0.1843	0.2192	0.8676	0.7736	0.0466	0.7949	0.0671	0.0249	0.0858	0.8268	0.0968	0.4662
NM_001107921	Casp8ap2	0.5920	0.5702	0.6812	0.5329	0.5011	0.5453	0.2484	0.4643	0.1918	0.4906	0.5562	0.2131
NM_001109644	Ttc19	0.1044	0.3552	0.6108	0.4146	0.0072	0.1891	0.4269	0.0143	0.4525	0.3119	0.3264	0.8869
NM_001109643	Snapc5	0.7127	0.2368	0.2491	0.4164	0.2113	0.3928	0.4069	0.5839	0.6247	0.3196	0.2842	0.4138
NM_001108093	RGD1561474	0.4467	0.3739	0.4319	0.2842	0.3037	0.0783	0.3746	0.2548	0.1985	0.4025	0.3788	0.4759
NM_001107993	Ubiad1	0.7749	0.2585	0.6950	0.3540	0.0420	0.2847	0.6164	0.6804	0.5860	0.4616	0.0916	0.3941
NM_001107996	Klhl21	0.6027	0.5126	0.4810	0.3564	0.4631	0.4254	0.5546	0.5390	0.4533	0.4406	0.2279	0.2852
NM_001107927	Nol6	0.5608	0.5278	0.5826	0.3344	0.7401	0.6804	0.4205	0.6965	0.8799	0.7319	0.6314	0.1694
NM_001108090	Ccdc59	0.1173	0.3173	0.6794	0.1842	0.8424	0.4731	0.4462	0.8179	0.0462	0.3378	0.0596	0.0158
NM_001107990	Crocc	0.5894	0.8196	0.0712	0.9067	0.8408	0.6957	0.5755	0.9587	0.6897	0.9719	0.4666	0.1920
NM_001107368	Bmi1	0.1654	0.0331	0.5165	0.5723	0.0822	0.4614	0.1364	0.1185	0.0304	0.0473	0.4249	0.4129
NM_001107930	Zfp189	0.5893	0.2955	0.1726	0.8885	0.1501	0.4135	0.3174	0.3967	0.1210	0.0089	0.3899	0.2847
NM_001107928	Ubap2	0.9098	0.6754	0.3603	0.9575	0.4980	0.8823	0.8263	0.8223	0.9343	0.1926	0.8301	0.9817
NM_001108116	Hoxc5	0.9111	0.8815	0.6336	0.2234	0.8836	0.6588	0.9389	0.9814	0.8092	0.8100	0.7140	0.4571
NM_001107934	Trim14	0.8031	0.8031	0.7603	0.7402	0.9824	0.9045	0.3418	0.7253	0.9083	0.8986	0.3912	0.8486
NM_001108033	Snapc1	0.4541	0.6965	0.2660	0.3969	0.2065	0.2039	0.3729	0.3775	0.0527	0.0403	0.0586	0.4878
NM_001107935	Wdr32	0.9754	0.9777	0.5000	0.9889	0.9522	0.9485	0.8715	0.5935	0.9699	0.9606	0.9897	0.2485
NM_001108034	Mpp5	0.2329	0.7237	0.6078	0.1796	0.0227	0.0734	0.8933	0.0513	0.3751	0.9342	0.9159	0.6494
NM_001108186	Rbm6	0.8783	0.8580	0.6921	0.6859	0.9453	0.8544	0.5921	0.9438	0.7252	0.8155	0.8335	0.1351
NM_001108246	Ddx3x	0.5332	0.4618	0.8754	0.4116	0.3868	0.5206	0.7774	0.3938	0.4479	0.7389	0.5574	0.5158
NM_001108122	Jrkl	0.9604	0.8529	0.9711	0.9272	0.9391	0.8745	0.9639	0.7765	0.9467	0.8867	0.9595	0.7647
NM_001108119	Nckap1l	0.1400	0.1541	0.2433	0.2116	0.1405	0.2189	0.0949	0.0056	0.1816	0.1694	0.0402	0.3308
NM_001108115	Atf7	0.9909	0.7874	0.6823	0.8893	0.9489	0.9355	0.8395	0.9788	0.9216	0.9984	0.9188	0.8816
NM_001107970	Zmynd12	0.1620	0.5008	0.1375	0.1772	0.2363	0.1818	0.4330	0.1747	0.4245	0.4583	0.3133	0.2312
NM_001108036	Plekhh1	0.9142	0.5996	0.6220	0.5655	0.5680	0.8001	0.3296	0.9479	0.8518	0.8990	0.6992	0.8025
NM_001109912	Tsc22d1	0.6969	0.8222	0.7026	0.8754	0.7860	0.8815	0.8125	0.7656	0.7867	0.5088	0.7671	0.7937
NM_001108370	Ttc18	0.0780	0.1016	0.9692	0.5102	0.6312	0.1017	0.1321	0.7554	0.0313	0.1377	0.0487	0.6089
NM_001108488	Fes	0.3799	0.1587	0.5037	0.2490	0.4746	0.1301	0.7019	0.0800	0.4474	0.8474	0.5049	0.5387
NM_001108366	Vrk2	0.0897	0.4895	0.0733	0.5058	0.3928	0.5198	0.7144	0.5506	0.4371	0.8076	0.4388	0.0317
NM_001108378	RGD1309307	0.8748	0.9848	0.5626	0.9209	0.8464	0.8363	0.9602	0.7382	0.6554	0.7477	0.9003	0.8194
NM_001108540	Rfesd	0.7916	0.8163	0.9232	0.3939	0.7209	0.6081	0.8702	0.4758	0.8061	0.7453	0.7377	0.4336
NM_001108537	Gtf3c6	0.1527	0.8732	0.8522	0.7989	0.1865	0.1228	0.6937	0.5544	0.0846	0.0431	0.3850	0.5001
NM_001108516	Dpf2	0.7985	0.4502	0.8296	0.7658	0.5516	0.8662	0.5422	0.4746	0.8001	0.6218	0.9119	0.9191
NM_001108542	Rgnef	0.9782	0.9374	0.8959	0.9168	0.9659	0.9401	0.2390	0.7655	0.9824	0.8571	0.9232	0.9204
NM_001108651	Hebp1	0.1181	0.1378	0.3047	0.8152	0.0031	0.0384	0.5210	0.3259	0.1225	0.0488	0.0294	0.0147
NM_001109315	RGD1560927	0.0136	0.4565	0.0340	0.0724	0.0493	0.0156	0.0860	0.0969	0.8375	0.0206	0.1307	0.0227
NM_001110056	Mln	0.1782	0.1763	0.1865	0.1926	0.1831	0.3639	0.1952	0.5048	0.2779	0.3805	0.1780	0.5238
NM_001108548	Rpl22l1	0.4469	0.6813	0.0373	0.8444	0.4215	0.2597	0.8519	0.4411	0.4254	0.7909	0.7681	0.1603
NM_001108577	Ppp2r4	0.4869	0.2598	0.9291	0.8136	0.9730	0.9668	0.8978	0.5315	0.7141	0.4452	0.6042	0.8460
NM_001108868	Rnf31	0.1904	0.0382	0.0082	0.1288	0.4285	0.0603	0.0585	0.3963	0.7629	0.0411	0.0995	0.3722
NM_001127531	Ap1s2	0.7744	0.0096	0.6731	0.4006	0.8611	0.4243	0.0110	0.5087	0.3035	0.2601	0.2048	0.4025
NM_001108578	RGD1305081	0.2816	0.5164	0.5895	0.4633	0.3008	0.4706	0.7972	0.6468	0.8121	0.2911	0.8759	0.5941
NM_001004075	Rnf34	0.8661	0.9234	0.9461	0.9114	0.9619	0.8963	0.0486	0.7384	0.4276	0.9413	0.9628	0.8216
NM_001108589	Mapkbp1	0.4786	0.5731	0.4695	0.4202	0.1525	0.6910	0.4461	0.2234	0.5365	0.0335	0.0880	0.5546
NM_001002809	Atf6b	0.9427	0.7841	0.8987	0.9442	0.8894	0.7554	0.8983	0.8707	0.9757	0.9097	0.8585	0.8184
NM_001108621	Fam180a	0.3857	0.8743	0.0978	0.2543	0.2193	0.4251	0.5058	0.4839	0.3736	0.2277	0.7867	0.4800
NM_001108618	Krit1	0.9380	0.9254	0.7996	0.1286	0.7899	0.9227	0.0497	0.8446	0.8862	0.9581	0.9417	0.8410
NM_001109664	RGD1307554	0.7936	0.8681	0.1494	0.6528	0.3264	0.6946	0.8671	0.3671	0.9213	0.7618	0.5255	0.9632
NM_001110165	Uchl3	0.4261	0.5721	0.2490	0.3839	0.1961	0.2803	0.3036	0.4400	0.1442	0.5018	0.4014	0.1268

NM_001110489	RGD1566180	0.3636	0.7907	0.3436	0.1995	0.1682	0.3763	0.5331	0.2355	0.7236	0.5946	0.0716	0.3875
NM_001110155	RGD1565611	0.4590	0.3399	0.1863	0.4650	0.4728	0.6304	0.4385	0.1867	0.4013	0.1671	0.4080	0.2617
NM_001107710	Lrig2	0.8298	0.5852	0.6102	0.4055	0.1319	0.2961	0.3476	0.1570	0.9183	0.2199	0.8181	0.9268
NM_001106812	Vsig2	0.1214	0.0298	0.3498	0.5684	0.3904	0.1576	0.2227	0.3686	0.0840	0.0086	0.2341	0.3086
NM_001130541	Colec10	0.9238	0.7220	0.8236	0.0529	0.9508	0.2618	0.8342	0.4509	0.0507	0.8589	0.8653	0.4487
NM_001110367	LOC691895	0.2768	0.4317	0.4667	0.4440	0.3803	0.2664	0.4950	0.4634	0.1732	0.2667	0.3005	0.6875
NM_001110270	Slc14a2	0.8586	0.4332	0.6719	0.6659	0.6607	0.4656	0.5780	0.6930	0.8206	0.6187	0.7294	0.5988
NM_053304	Col1a1	0.9662	0.8700	0.9872	0.9458	0.9649	0.9977	0.9752	0.9620	0.9918	0.9886	0.9986	0.8523
NM_001109665	Ptcd1	0.4524	0.5025	0.7035	0.7328	0.6315	0.7209	0.1568	0.2818	0.2989	0.6706	0.5737	0.3206
NM_001109935	Ctxn1	0.3013	0.4041	0.5699	0.2425	0.6466	0.6015	0.2458	0.4772	0.6500	0.6022	0.4514	0.5988
NM_001110366	Pabpc5	0.6493	0.3795	0.7652	0.8769	0.6844	0.5335	0.6817	0.1322	0.3399	0.7158	0.7615	0.3430
NM_019217	Map1b	0.8221	0.7659	0.8001	0.6521	0.8259	0.7692	0.3702	0.6946	0.7733	0.8024	0.7773	0.7964
NM_001112713	Grk6	0.8080	0.6822	0.6586	0.8931	0.5664	0.8727	0.8825	0.9340	0.7880	0.8790	0.4994	0.5364
NM_001110494	Cog2	0.3367	0.7275	0.5466	0.3266	0.2206	0.5314	0.3610	0.4958	0.3904	0.2098	0.2345	0.3813
NM_001110490	Ldb3	0.5237	0.5845	0.2420	0.2643	0.7717	0.7552	0.3450	0.2410	0.3848	0.8060	0.4209	0.2548
NM_001109892	Fgfr2	0.5342	0.8409	0.6061	0.6133	0.5605	0.6934	0.6703	0.7146	0.4394	0.5809	0.6611	0.6351
NM_001109894	Fgfr2	0.5342	0.8409	0.6061	0.6133	0.5605	0.6934	0.6703	0.7146	0.4394	0.5809	0.6611	0.6351
NM_001109904	Fgfr4	0.0148	0.0304	0.0604	0.2761	0.2567	0.3323	0.0984	0.0259	0.0092	0.1344	0.5776	0.2108
NM_001109893	Fgfr2	0.5342	0.8409	0.6061	0.6133	0.5605	0.6934	0.6703	0.7146	0.4394	0.5809	0.6611	0.6351
NM_001110493	Pid1	0.0104	0.0166	0.5079	0.0154	0.0042	0.0775	0.0066	0.0085	0.1304	0.1884	0.3603	0.6581
NM_001109895	Fgfr2	0.5342	0.8409	0.6061	0.6133	0.5605	0.6934	0.6703	0.7146	0.4394	0.5809	0.6611	0.6351
NM_001109900	Akr1cl1	0.7695	0.0395	0.9327	0.6716	0.8070	0.8030	0.8191	0.8292	0.0429	0.0169	0.0217	0.8164
NM_012986	Nedd4	0.9701	0.3979	0.9547	0.9233	0.7133	0.7672	0.7061	0.8012	0.7331	0.4727	0.5436	0.4275
NM_001109669	Scmh1	0.0757	0.0385	0.1791	0.7300	0.3241	0.2436	0.0154	0.1229	0.9375	0.7867	0.7464	0.8461
NM_001109650	Kctd4	0.6191	0.6843	0.7008	0.5979	0.6902	0.5291	0.5185	0.5013	0.6193	0.6571	0.5826	0.4219
NM_031524	Pdgfb	0.5822	0.8532	0.4758	0.8496	0.7673	0.8739	0.9088	0.5891	0.7760	0.5582	0.3562	0.9157
NM_013193	Nf2	0.7032	0.9508	0.2007	0.9189	0.4821	0.9056	0.4119	0.3233	0.3570	0.3741	0.5506	0.2119
NM_001109911	Hnrpm	0.9026	0.8247	0.5019	0.9249	0.9727	0.8941	0.9625	0.7257	0.8716	0.8156	0.8866	0.4610
NM_019181	Spr	0.0095	0.9704	0.1165	0.9111	0.3304	0.0501	0.9008	0.9409	0.9660	0.9427	0.7999	0.3882
NM_012712	Fgfr2	0.5342	0.8409	0.6061	0.6133	0.5605	0.6934	0.6703	0.7146	0.4394	0.5809	0.6611	0.6351
NM_012802	Pdgfra	0.3024	0.1749	0.7867	0.8985	0.5569	0.7371	0.2632	0.3459	0.1728	0.1700	0.1996	0.2871
NM_057209	Mylk2	0.0807	0.6826	0.5548	0.5237	0.6461	0.3010	0.7073	0.6488	0.6960	0.5132	0.1046	0.2353
NM_001025420	Lsp1	0.4432	0.4183	0.2611	0.3496	0.6253	0.2778	0.3331	0.3157	0.3931	0.4662	0.3011	0.4305
NM_001127337	Brsk1	0.4077	0.1850	0.6280	0.0095	0.3179	0.6152	0.1072	0.7246	0.7311	0.4370	0.6858	0.7975
NM_001112742	Gria3	0.6700	0.6103	0.7305	0.6914	0.6974	0.8620	0.6637	0.7180	0.6963	0.7347	0.7108	0.5558
NM_001111064	Mbnl2	0.6448	0.6303	0.7014	0.6817	0.5035	0.2217	0.7303	0.7242	0.6873	0.7099	0.6863	0.6088
NM_013162	Rbp4	0.4831	0.1294	0.0576	0.7088	0.0924	0.0728	0.6289	0.4605	0.1925	0.1514	0.5570	0.3220
NM_001111269	LOC689064	0.3412	0.2679	0.2982	0.3614	0.4054	0.4414	0.4033	0.3250	0.3288	0.3014	0.5046	0.3081
NM_031547	Chrm4	0.6319	0.4769	0.4441	0.3185	0.8532	0.7897	0.3875	0.6793	0.4053	0.3251	0.8384	0.3206
NM_012918	Cacna1a	0.5255	0.4430	0.3942	0.5706	0.5104	0.3246	0.2228	0.6255	0.1423	0.0993	0.2753	0.9194
NM_001127450	Alkbh6	0.0664	0.0988	0.0730	0.1323	0.0133	0.0663	0.1007	0.0860	0.0325	0.1570	0.1028	0.0558
NM_053671	Tmf1	0.2889	0.3636	0.3858	0.5063	0.1827	0.1838	0.5107	0.3864	0.1731	0.6885	0.5147	0.1812
NM_001130539	Med30	0.1266	0.0149	0.1739	0.0658	0.0184	0.0209	0.2071	0.0257	0.0430	0.0351	0.0262	0.1860
NM_133288	Atp2b3	0.6802	0.8676	0.8365	0.0748	0.9129	0.9158	0.7872	0.9915	0.9916	0.9901	0.9071	0.9570
NM_001113183	Tesb	0.2372	0.3363	0.6993	0.3974	0.4885	0.2512	0.6000	0.2938	0.3075	0.3542	0.2748	0.3032
NM_001100762	Havcr2	0.4102	0.2826	0.2722	0.3963	0.1712	0.3229	0.2615	0.0538	0.2468	0.3370	0.2671	0.1907
NM_001100724	Camp	0.1702	0.2609	0.5427	0.4531	0.1302	0.4798	0.3281	0.1926	0.6652	0.6429	0.4012	0.1512
NM_001100474	Ccnf	0.7054	0.9846	0.1552	0.8049	0.9607	0.9550	0.9933	0.9995	0.7716	0.9867	0.8634	0.4102
NM_057183	Gzmm	0.4557	0.1759	0.4190	0.3448	0.3299	0.2799	0.7279	0.3392	0.4504	0.2864	0.1779	0.1811

NM_019209	Urod	0.0348	0.3393	0.7872	0.2108	0.0058	0.0517	0.3808	0.0253	0.1800	0.2086	0.1075	0.4344
NM_001109896	Fgfr2	0.5342	0.8409	0.6061	0.6133	0.5605	0.6934	0.6703	0.7146	0.4394	0.5809	0.6611	0.6351
NM_001107490	Cic	0.8761	0.8814	0.3045	0.0931	0.3974	0.4149	0.2623	0.3658	0.9574	0.8329	0.7500	0.9994
NM_019138	Cyp7b1	0.4503	0.1384	0.2536	0.4459	0.2493	0.3280	0.1598	0.4948	0.1297	0.5659	0.6577	0.3826
NM_001109294	RGD1562084	0.8183	0.5844	0.7931	0.7964	0.8416	0.7205	0.8041	0.5741	0.9848	0.8145	0.6566	0.6925
NM_001113365	Tbc1d14	0.8925	0.9342	0.8395	0.9266	0.7814	0.7977	0.9370	0.5525	0.7524	0.9558	0.8576	0.8631
NM_001109899	Oaz2	0.2099	0.1971	0.9569	0.2676	0.2738	0.2818	0.1239	0.2443	0.1067	0.2506	0.2876	0.4250
NM_053885	Rere	0.9593	0.2388	0.2315	0.9638	0.9195	0.9654	0.3730	0.8797	0.4130	0.2415	0.3645	0.9626
NM_001107185	Tpr	0.9099	0.8090	0.6472	0.6518	0.8773	0.6873	0.5528	0.8712	0.4748	0.6794	0.6510	0.0399
NM_001034931	Pkhd1l1	0.3970	0.3000	0.4016	0.5105	0.6895	0.6846	0.6249	0.4811	0.0363	0.6745	0.2897	0.1400
NM_001109979	Rab1b	0.6181	0.3225	0.6017	0.5632	0.4282	0.6163	0.5578	0.5205	0.6201	0.4268	0.2489	0.7304
NM_001109671	Gdf3	0.2700	0.3194	0.3068	0.3617	0.4193	0.3125	0.3015	0.4866	0.6177	0.0766	0.3780	0.6316
NM_001109601	Tcl1a	0.5957	0.4833	0.6450	0.5592	0.4935	0.4972	0.5401	0.6765	0.6867	0.2017	0.4190	0.7266
NM_019197	Serpine2	0.0963	0.1248	0.1501	0.2435	0.1191	0.3196	0.1250	0.2557	0.1414	0.1009	0.1519	0.1394
NM_001109337	RGD1559903	0.4470	0.1061	0.1300	0.1018	0.3692	0.4412	0.1985	0.2313	0.4250	0.4764	0.0289	0.0809
NM_001109336	RGD1561870	0.6868	0.6008	0.6972	0.4215	0.2545	0.4034	0.5382	0.4042	0.5613	0.6951	0.8572	0.6920
NM_001037773	Ccnjl	0.3141	0.6084	0.5339	0.8434	0.6949	0.7660	0.6925	0.5328	0.7138	0.1886	0.4167	0.8107
NM_001077660	Urg4	0.4560	0.5606	0.5630	0.3795	0.2168	0.5339	0.3783	0.6851	0.4685	0.3390	0.2349	0.4872
NM_001109334	RGD1561909	0.9459	0.6127	0.6077	0.7162	0.7626	0.7639	0.8483	0.9524	0.7945	0.7204	0.9209	0.6191
NM_001105900	Cggbp1	0.6083	0.9841	0.4149	0.3315	0.2349	0.2464	0.6739	0.2337	0.7830	0.8520	0.9724	0.1162
NM_001113522	Arhgef7	0.7039	0.6255	0.6753	0.6622	0.6089	0.6205	0.5775	0.5011	0.6924	0.7161	0.5438	0.8117
NM_001037782	Cnot4	0.6841	0.7052	0.6215	0.6387	0.5983	0.2530	0.7083	0.5709	0.5763	0.6419	0.6980	0.5686
NM_001107446	Pdzd8	0.7763	0.7406	0.6521	0.9384	0.6287	0.7388	0.3236	0.4971	0.7870	0.3472	0.8438	0.7520
NM_001107197	Igsf9	0.0182	0.6612	0.6419	0.4105	0.0157	0.0574	0.8667	0.1439	0.0761	0.1722	0.0232	0.0590
NM_001110333	Vegfa	0.7331	0.7016	0.8891	0.5415	0.5108	0.5606	0.9797	0.4868	0.5533	0.6890	0.7006	0.9767
NM_001110334	Vegfa	0.7331	0.7016	0.8891	0.5415	0.5108	0.5606	0.9797	0.4868	0.5533	0.6890	0.7006	0.9767
NM_138526	Ccrn4l	0.4235	0.3825	0.4394	0.9332	0.3140	0.0588	0.8685	0.1155	0.9049	0.3130	0.3782	0.8896
NM_001100798	RGD1560286	0.5851	0.5707	0.6206	0.7743	0.6263	0.7245	0.6743	0.6753	0.7999	0.7209	0.6137	0.7105
NM_001113752	Tmem189	0.5539	0.6207	0.8199	0.5762	0.5735	0.3968	0.7755	0.3745	0.7000	0.5990	0.6909	0.9447
NM_001113747	Setd4	0.6491	0.6993	0.1042	0.0357	0.1211	0.6737	0.2882	0.3059	0.7577	0.8980	0.8398	0.8990
NM_001113743	Nrap	0.3789	0.4738	0.6225	0.5547	0.5734	0.6166	0.2941	0.6069	0.3068	0.3254	0.7388	0.6420
NM_001113793	Harbi1	0.4739	0.4014	0.6306	0.3504	0.2267	0.3644	0.5869	0.3486	0.2225	0.5222	0.2869	0.3210
NM_001106270	Map7	0.3875	0.2545	0.4434	0.5534	0.5909	0.7982	0.8791	0.9407	0.8852	0.4196	0.1140	0.4489
NM_001106377	RGD1564450	0.0362	0.0649	0.0406	0.0669	0.0781	0.0048	0.0016	0.0264	0.0170	0.0278	0.0016	0.0633
NM_001113789	Lrrc27	0.2464	0.4279	0.4588	0.4546	0.3523	0.2683	0.6852	0.4135	0.2955	0.2782	0.4926	0.5496
NM_001113790	Il1f9	0.0424	0.3692	0.4007	0.1524	0.2121	0.1097	0.0412	0.2633	0.4789	0.7227	0.4870	0.6680
NM_001113753	Xrra1	0.5345	0.3989	0.5242	0.3484	0.5488	0.3740	0.4422	0.1443	0.5297	0.2748	0.3092	0.0608
NM_001106422	RGD1561817	0.4116	0.0502	0.4330	0.1695	0.1374	0.2630	0.5751	0.1326	0.4218	0.5109	0.5461	0.1411
NM_001107658	Trio	0.8066	0.3861	0.2751	0.2156	0.2857	0.8885	0.3584	0.4541	0.5726	0.3361	0.8224	0.6718
NM_001113777	RGD1306008	0.2963	0.1924	0.3082	0.2963	0.1786	0.2654	0.4793	0.7274	0.2196	0.5681	0.1643	0.3211
NM_001114392	Gpr39	0.1778	0.1755	0.1860	0.1923	0.1824	0.1833	0.1948	0.1862	0.1729	0.1668	0.1777	0.1807
NM_001114401	Cttnbp2	0.4985	0.5409	0.6518	0.6839	0.3822	0.0395	0.3835	0.5106	0.2524	0.1872	0.5868	0.1104
NM_001114402	RGD1308101	0.6667	0.5745	0.7071	0.5998	0.6757	0.6881	0.4419	0.5322	0.7269	0.5615	0.4520	0.6419
NM_001113786	Rbmx2	0.4089	0.1937	0.3206	0.4040	0.1523	0.1089	0.2861	0.1494	0.4520	0.0096	0.2729	0.0907
NM_001113782	RGD1563706	0.6127	0.7057	0.7135	0.7304	0.2695	0.2814	0.3311	0.3891	0.7097	0.2544	0.4991	0.5102
NM_001113792	RGD1565410	0.6740	0.6806	0.6431	0.8435	0.7511	0.8201	0.6834	0.7100	0.4810	0.6129	0.5566	0.8795
NM_001113788	RGD1562024	0.1939	0.8170	0.3657	0.5229	0.6749	0.5116	0.6349	0.7089	0.8331	0.6257	0.8030	0.2417
NM_001113787	RGD1559804	0.5207	0.4893	0.2658	0.5771	0.3768	0.1516	0.6801	0.0209	0.5464	0.4086	0.5075	0.1851
NM_001114606	Akap10	0.7133	0.7250	0.7392	0.6810	0.5154	0.8223	0.7292	0.6540	0.8303	0.8249	0.8061	0.7970

NM_001114502	Ccdc101	0.7412	0.6906	0.2294	0.7947	0.4050	0.2100	0.7551	0.8987	0.6589	0.8603	0.6555	0.1342
NM_013148	Htr5a	0.6410	0.7814	0.2343	0.4939	0.3754	0.6186	0.8046	0.7040	0.7284	0.3062	0.4995	0.6709
NM_013147	Clcn1	0.3377	0.8430	0.6531	0.3785	0.5869	0.0322	0.6619	0.1299	0.4106	0.4837	0.5879	0.4846
NM_019188	Msmb	0.4748	0.2163	0.4407	0.0648	0.1098	0.2323	0.1840	0.5870	0.1240	0.3654	0.1544	0.2625
NM_017343	Mrlc2	0.0918	0.4427	0.9928	0.4391	0.1500	0.4236	0.5922	0.3541	0.4740	0.6475	0.4905	0.8713
NM_012491	Add2	0.6635	0.5058	0.4520	0.5815	0.5684	0.6477	0.9579	0.3561	0.5926	0.6666	0.6728	0.6137
NM_017209	Mecr	0.0778	0.0934	0.0206	0.0805	0.0269	0.3776	0.2631	0.1144	0.2364	0.0489	0.0649	0.1074
NM_017058	Vdr	0.7408	0.8127	0.1552	0.7650	0.6579	0.6764	0.4488	0.4533	0.5977	0.6968	0.8481	0.9569
NM_013152	Slc18a1	0.3621	0.1684	0.2008	0.3063	0.2801	0.0359	0.1032	0.2185	0.1027	0.2296	0.0475	0.3817
NM_017222	Slc10a2	0.8283	0.9144	0.7764	0.8612	0.8812	0.7528	0.6444	0.6057	0.8837	0.9431	0.8242	0.9011
NM_013163	Il2ra	0.8356	0.6512	0.7951	0.3644	0.7779	0.5609	0.7456	0.8507	0.8014	0.6854	0.5755	0.8089
NM_031591	Pecam1	0.6770	0.7104	0.4789	0.5034	0.6248	0.5542	0.4380	0.4113	0.4771	0.5122	0.6549	0.6961
NM_013214	Acot7	0.5099	0.4604	0.3077	0.4450	0.6256	0.7031	0.4105	0.5052	0.5621	0.2759	0.2742	0.6563
NM_013198	Maob	0.7746	0.6343	0.8623	0.7914	0.5112	0.2569	0.5008	0.1301	0.8437	0.6727	0.8781	0.7488
NM_017158	Cyp2c7	0.3482	0.3068	0.1717	0.0828	0.5198	0.1757	0.1539	0.1680	0.4140	0.0142	0.4689	0.1075
NM_017134	Arg1	0.0908	0.0995	0.0979	0.4992	0.0571	0.0490	0.1555	0.1051	0.0045	0.0663	0.0689	0.1115
NM_019345	Slc12a3	0.6900	0.6648	0.4382	0.4836	0.5045	0.7004	0.4030	0.4193	0.5691	0.2195	0.0952	0.7344
NM_001109907	Stau1	0.9053	0.9617	0.9278	0.9776	0.9161	0.9667	0.8989	0.8937	0.9208	0.9752	0.9685	0.9397
NM_021660	Ihpk2	0.9645	0.3828	0.1826	0.1129	0.6769	0.8343	0.0303	0.8341	0.2963	0.5228	0.3905	0.8289
NM_001100634	Tph1	0.2318	0.4365	0.2986	0.5324	0.4278	0.2395	0.3207	0.2956	0.2148	0.2282	0.3147	0.6010
NM_017132	Rcn2	0.4740	0.1340	0.9922	0.9832	0.6252	0.6528	0.7944	0.3038	0.0264	0.2490	0.6525	0.9640
NM_013155	Vldlr	0.5601	0.9437	0.7877	0.8448	0.0900	0.2173	0.8456	0.1253	0.9172	0.7732	0.8544	0.9965
NM_017163	Slc6a18	0.4741	0.8164	0.6539	0.4869	0.6629	0.6105	0.5473	0.2901	0.5012	0.8026	0.6721	0.4203
NM_013168	Hmbs	0.0795	0.8577	0.3568	0.7123	0.0538	0.3967	0.6640	0.2457	0.2338	0.6375	0.5406	0.5333
NM_013223	Eif2ak1	0.7436	0.1393	0.7514	0.2890	0.2262	0.6985	0.0584	0.5837	0.4734	0.5336	0.2001	0.6133
NM_013172	Myf6	0.3637	0.2683	0.6415	0.3709	0.5401	0.2089	0.2004	0.4783	0.0652	0.2468	0.6242	0.0944
NM_013169	Cd3d	0.6893	0.2183	0.4101	0.2954	0.2794	0.1903	0.2849	0.7299	0.6021	0.6530	0.4141	0.6188
NM_017068	Lamp2	0.1080	0.0235	0.8303	0.1836	0.8794	0.4447	0.3167	0.5995	0.0274	0.2276	0.1209	0.8442
NM_017009	Gfap	0.6212	0.6149	0.5488	0.6326	0.6896	0.7781	0.9282	0.9088	0.7542	0.5986	0.5388	0.7472
NM_017099	Kcnj8	0.4990	0.7507	0.5993	0.6928	0.4380	0.6732	0.7537	0.4498	0.6289	0.8783	0.3642	0.7074
NM_017165	Gpx4	0.5142	0.4859	0.6208	0.3050	0.4602	0.4936	0.3046	0.6268	0.2971	0.4050	0.1863	0.5100
NM_017160	Rps6	0.5123	0.4913	0.5997	0.2251	0.7572	0.5928	0.5077	0.6357	0.5911	0.8306	0.8477	0.6134
NM_013176	Tcf12	0.6948	0.1203	0.9266	0.6617	0.1458	0.7561	0.4142	0.2658	0.1337	0.5929	0.7176	0.8619
NM_012525	Cgm4	0.4756	0.5536	0.1593	0.3115	0.2456	0.0994	0.2929	0.0638	0.1438	0.2688	0.5697	0.0721
NM_013180	Itgb4	0.9060	0.8026	0.8532	0.7557	0.9993	0.9713	0.9165	0.9005	0.9975	0.9250	0.8221	0.9795
NM_013181	Prkar1a	0.2487	0.1020	0.7491	0.3508	0.1276	0.0649	0.1439	0.0440	0.1258	0.2182	0.3789	0.2826
NM_013182	Mc5r	0.1016	0.1997	0.1305	0.3218	0.1637	0.2469	0.2701	0.3287	0.0521	0.3425	0.1217	0.3544
NM_017175	Pkn1	0.0198	0.0270	0.0009	0.1205	0.0010	0.0593	0.1289	0.0025	0.3368	0.0178	0.1392	0.4724
NM_017090	Gucy1a3	0.6731	0.4449	0.6129	0.5925	0.0842	0.5128	0.7382	0.1147	0.8867	0.0518	0.4526	0.5068
NM_017019	Il1a	0.3901	0.8023	0.7295	0.1748	0.6960	0.4355	0.5592	0.3527	0.8921	0.8056	0.9108	0.6310
NM_019316	Mafb	0.4223	0.7969	0.6967	0.8568	0.6669	0.8945	0.4819	0.5600	0.7884	0.3758	0.8258	0.6877
NM_020471	Isl2	0.4644	0.3534	0.2920	0.6146	0.4584	0.1246	0.1166	0.1798	0.6437	0.7270	0.5227	0.6675
NM_017007	Gad1	0.6107	0.7729	0.6568	0.1202	0.4226	0.5087	0.1794	0.3588	0.3651	0.2574	0.6420	0.6325
NM_017030	Pccb	0.4491	0.1398	0.5870	0.8431	0.5038	0.0668	0.6263	0.8433	0.1371	0.5360	0.5575	0.0769
NM_017103	Tceb3	0.0996	0.1084	0.1792	0.1398	0.4522	0.0922	0.0660	0.3374	0.1951	0.0162	0.3977	0.7232
NM_017127	Chka	0.5068	0.5013	0.2066	0.1059	0.1995	0.2341	0.2421	0.3378	0.5371	0.3980	0.8487	0.4087
NM_016994	C3	0.4690	0.5252	0.1856	0.3556	0.2939	0.1831	0.1946	0.1860	0.1876	0.1666	0.1777	0.2986
NM_017053	Tacr3	0.3598	0.5244	0.6365	0.4125	0.2560	0.5625	0.4137	0.2061	0.0692	0.4389	0.1812	0.6570
NM_017227	Padi4	0.7546	0.7527	0.6187	0.9052	0.7417	0.8048	0.7721	0.7801	0.5278	0.7396	0.5497	0.9608

NM_017038	Ppm1a	0.2086	0.3894	0.9370	0.3423	0.3875	0.6748	0.5847	0.1037	0.8125	0.3531	0.3435	0.8991
NM_017168	Plcg2	0.0122	0.0750	0.0078	0.0171	0.0365	0.1053	0.0883	0.0324	0.0896	0.1780	0.1477	0.2701
NM_013215	Akr7a3	0.1167	0.1088	0.5622	0.1788	0.0795	0.4378	0.3571	0.1244	0.1627	0.4866	0.1032	0.0563
NM_019906	Frap1	0.8382	0.9365	0.1437	0.9106	0.8862	0.9740	0.6393	0.8183	0.9416	0.8537	0.7726	0.9592
NM_021658	Hcn4	0.5979	0.2742	0.5046	0.7481	0.5585	0.1642	0.5343	0.1504	0.3008	0.1406	0.3455	0.6626
NM_019294	Cacna1e	0.0603	0.0183	0.1285	0.1485	0.3968	0.3500	0.4465	0.0848	0.1422	0.0359	0.0087	0.4876
NM_017233	Hpd	0.0502	0.0756	0.3912	0.2458	0.0566	0.0658	0.0487	0.1592	0.3851	0.0466	0.0353	0.5407
NM_017194	Chrne	0.5538	0.3516	0.2042	0.0010	0.4165	0.8658	0.5288	0.3308	0.6542	0.8539	0.1834	0.6852
NM_017252	Pou3f4	0.3426	0.7352	0.7591	0.8977	0.5875	0.8307	0.5649	0.5103	0.3365	0.2836	0.5305	0.1656
NM_017328	Pgam2	0.0287	0.2449	0.5310	0.4787	0.0561	0.5048	0.4769	0.1694	0.0194	0.2000	0.1178	0.0272
NM_013220	Ankrd1	0.8688	0.0457	0.3176	0.0902	0.1232	0.2094	0.0185	0.1914	0.0569	0.0319	0.3037	0.0032
NM_017292	Gabrr2	0.2399	0.3636	0.1356	0.3142	0.1920	0.1187	0.1270	0.0503	0.0473	0.0730	0.0030	0.2766
NM_017042	Ppp3cb	0.9386	0.8998	0.9715	0.8990	0.9828	0.7834	0.7549	0.7478	0.9275	0.9734	0.7606	0.9650
NM_013226	Rpl32	0.8858	0.6036	0.8274	0.8531	0.6426	0.4812	0.6375	0.9527	0.6283	0.8958	0.6643	0.2611
NM_013225	Cntn6	0.6372	0.2694	0.2806	0.5655	0.2844	0.4281	0.3811	0.1854	0.2436	0.2935	0.3207	0.3358
NM_013413	Rln1	0.2961	0.2739	0.6013	0.6489	0.3048	0.1061	0.1188	0.6511	0.3685	0.2297	0.3987	0.2808
NM_017116	Capn2	0.8458	0.1371	0.8583	0.8896	0.9311	0.8089	0.0363	0.9811	0.4485	0.2383	0.4217	0.4991
NM_017046	Scnn1g	0.6215	0.7550	0.8949	0.5384	0.7902	0.5088	0.7687	0.3809	0.5156	0.8230	0.5711	0.5137
NM_017154	Xdh	0.1149	0.0392	0.7343	0.0208	0.9242	0.7939	0.0571	0.9485	0.1613	0.0655	0.0026	0.8398
NM_017107	Ogt	0.9742	0.9837	0.9977	0.9981	0.9826	0.9907	0.9801	0.9966	0.9443	0.9503	0.9794	0.9581
NM_017002	Epor	0.2780	0.5714	0.0708	0.1262	0.4369	0.1878	0.0191	0.4025	0.5846	0.2432	0.1886	0.1231
NM_021772	Cdkl3	0.0414	0.2297	0.2818	0.7317	0.1610	0.7647	0.2665	0.0610	0.1478	0.5876	0.9335	0.0807
NM_017054	Tbxa2r	0.7866	0.8209	0.2859	0.3230	0.8019	0.3385	0.4099	0.6528	0.7296	0.5149	0.0955	0.7652
NM_001105776	Foxi1	0.9609	0.8500	0.9254	0.9416	0.5479	0.5768	0.7759	0.6210	0.8601	0.8820	0.9728	0.7310
NM_019623	Cyp4f1	0.2962	0.1550	0.0254	0.2135	0.3645	0.0717	0.1974	0.1088	0.3037	0.3349	0.1407	0.3158
NM_017143	F10	0.0917	0.5263	0.1628	0.3555	0.3568	0.5407	0.0336	0.2575	0.6561	0.3723	0.0874	0.3399
NM_016987	Acly	0.9153	0.8435	0.5765	0.5640	0.7113	0.7699	0.6120	0.7904	0.8208	0.9373	0.8733	0.4529
NM_017253	Bcat1	0.5645	0.0060	0.6979	0.9279	0.2250	0.9187	0.5427	0.4397	0.0886	0.2967	0.0282	0.8617
NM_017280	Psma3	0.3501	0.1818	0.7298	0.8569	0.4529	0.2348	0.3632	0.3654	0.4485	0.1206	0.8152	0.0249
NM_017123	Areg	0.1305	0.8495	0.2594	0.1383	0.0045	0.0180	0.0521	0.0013	0.0191	0.0886	0.6763	0.0264
NM_017128	Inhba	0.1859	0.8398	0.1093	0.0038	0.4526	0.7121	0.1304	0.1413	0.3372	0.3700	0.8492	0.6623
NM_020084	Cacna1i	0.1777	0.1752	0.1853	0.1919	0.3445	0.3971	0.1942	0.3595	0.5517	0.3166	0.1776	0.4749
NM_016996	Casr	0.6951	0.8950	0.6018	0.7126	0.2200	0.5675	0.4685	0.8260	0.9297	0.4604	0.8569	0.5491
NM_017221	Shh	0.1594	0.5289	0.6454	0.7857	0.0835	0.2792	0.1755	0.3319	0.6814	0.0540	0.2965	0.1442
NM_017192	S1pr2	0.3879	0.0813	0.2871	0.3020	0.4260	0.5924	0.3365	0.4253	0.0452	0.1130	0.0043	0.3111
NM_017151	Rps15	0.6642	0.2580	0.5550	0.3782	0.5657	0.2926	0.8931	0.7694	0.5054	0.8457	0.5167	0.0717
NM_017027	Mpz	0.6278	0.7733	0.3636	0.6477	0.4177	0.5171	0.8355	0.4053	0.4351	0.6831	0.5293	0.9323
NM_017026	Mbp	0.3217	0.2552	0.5839	0.6952	0.7808	0.2483	0.4483	0.5076	0.5358	0.5647	0.5904	0.5811
NM_017075	Acat1	0.7949	0.7537	0.7475	0.7841	0.9383	0.8241	0.7035	0.7644	0.7586	0.8616	0.8158	0.8515
NM_017241	Grik1	0.9337	0.7699	0.8844	0.6345	0.6973	0.6754	0.7080	0.5252	0.4387	0.8605	0.7533	0.5539
NM_017140	Drd3	0.5636	0.8108	0.7265	0.5358	0.3448	0.5352	0.0976	0.4472	0.6418	0.7792	0.5262	0.5609
NM_017021	Il9r	0.4003	0.6891	0.4205	0.7095	0.7655	0.2591	0.2856	0.6714	0.2232	0.0222	0.4825	0.3588
NM_017110	Cartpt	0.5190	0.1159	0.3497	0.1148	0.4373	0.4508	0.5507	0.0532	0.3586	0.2487	0.3452	0.6719
NM_017147	Cfl1	0.6515	0.5910	0.3273	0.7759	0.2876	0.7641	0.6190	0.4767	0.2909	0.2043	0.1593	0.5591
NM_016992	Avp	0.4496	0.2684	0.3771	0.0961	0.4883	0.2596	0.3180	0.0959	0.1101	0.0386	0.4491	0.8962
NM_017220	Pts	0.9718	0.6988	0.9323	0.9893	0.9780	0.8360	0.9878	0.9981	0.9090	0.6430	0.9852	0.3942
NM_017254	Htr2a	0.1769	0.0679	0.1192	0.0265	0.3096	0.0380	0.0986	0.0202	0.8177	0.0913	0.2081	0.1235
NM_017117	Capn3	0.9166	0.7634	0.7674	0.5615	0.6509	0.7451	0.6887	0.7690	0.8734	0.8209	0.7672	0.3540
NM_017081	Hsd11b2	0.0747	0.6584	0.5816	0.7852	0.3878	0.7912	0.3446	0.8595	0.3913	0.7768	0.6318	0.4257

NM_017092	Tyro3	0.7333	0.9762	0.9113	0.9726	0.7298	0.4005	0.9507	0.6354	0.8524	0.8859	0.9633	0.9765
NM_001003959	Dnmt3b	0.9963	0.9475	0.9843	0.9536	0.9947	0.8931	0.9599	0.9916	0.9908	0.9832	0.9507	0.9205
NM_017036	Prl4a1	0.2221	0.5771	0.4375	0.5553	0.8542	0.7794	0.1303	0.1333	0.8499	0.7459	0.8021	0.3569
NM_017301	S1pr1	0.6234	0.3257	0.7565	0.7460	0.6341	0.4455	0.8194	0.5017	0.6990	0.3293	0.4723	0.5506
NM_017093	Akt2	0.0693	0.1359	0.4605	0.1814	0.1123	0.3794	0.3329	0.2996	0.2878	0.3230	0.1262	0.8718
NM_017201	Ahcy	0.1036	0.9832	0.1789	0.9071	0.8161	0.3703	0.9851	0.8198	0.8545	0.7994	0.8037	0.3541
NM_017223	Slc20a2	0.2983	0.2731	0.2105	0.0410	0.0755	0.1519	0.0863	0.2282	0.2540	0.2402	0.2148	0.5617
NM_017341	Lipf	0.4276	0.3902	0.1858	0.1050	0.1205	0.4174	0.0796	0.0773	0.0471	0.6543	0.1413	0.1980
NM_017094	Ghr	0.8323	0.9013	0.9369	0.7604	0.5582	0.8104	0.7654	0.8496	0.9352	0.7361	0.8762	0.7539
NM_017047	Slc10a1	0.9606	0.9064	0.9780	0.7783	0.6868	0.8107	0.5970	0.9519	0.8192	0.5989	0.8558	0.6186
NM_019351	Timm17a	0.4801	0.8867	0.4929	0.7017	0.8603	0.6542	0.6188	0.7805	0.4620	0.5332	0.8777	0.1148
NM_012632	Prp15	0.5412	0.3750	0.2774	0.5804	0.2616	0.3451	0.0568	0.1515	0.4823	0.2692	0.3794	0.2638
NM_019306	Flt1	0.1709	0.5975	0.8139	0.3592	0.3366	0.3698	0.4700	0.1713	0.4924	0.9571	0.4724	0.3031
NM_019129	Ins1	0.1380	0.1417	0.3137	0.0811	0.1552	0.0333	0.3483	0.2516	0.4369	0.2519	0.4531	0.4276
NM_017056	Tnp1	0.0879	0.0278	0.0621	0.1293	0.2705	0.1496	0.4951	0.2577	0.1387	0.3304	0.3334	0.0509
NM_001109307	Als2cr2	0.7934	0.9495	0.9093	0.2019	0.3174	0.2108	0.7627	0.3754	0.9360	0.8390	0.9402	0.9169
NM_001108132	Tbx20	0.2978	0.2067	0.3509	0.4101	0.3806	0.4635	0.4873	0.0985	0.2840	0.3130	0.5434	0.0405
NM_001108131	Snx19	0.9515	0.6048	0.9715	0.7264	0.9833	0.9144	0.9667	0.8918	0.8772	0.9958	0.9267	0.9246
NM_053393	Cdh8	0.5323	0.3411	0.3185	0.6899	0.6566	0.1186	0.3004	0.1197	0.3438	0.5910	0.4943	0.3980
NM_001108342	Kdsr	0.1490	0.2907	0.2251	0.2787	0.9197	0.9947	0.1116	0.9165	0.1013	0.3391	0.2765	0.9453
NM_001108045	Tmem63c	0.0206	0.9159	0.8967	0.1473	0.3758	0.2476	0.5570	0.1277	0.6672	0.1126	0.2460	0.7486
NM_017258	Btg1	0.4815	0.9242	0.9157	0.5478	0.9063	0.8430	0.2629	0.9730	0.8893	0.8087	0.8853	0.8937
NM_020071	Fgb	0.8262	0.1598	0.5366	0.6371	0.5908	0.5029	0.6026	0.7834	0.8374	0.2763	0.7784	0.8258
NM_020073	Pthr1	0.0220	0.0373	0.5495	0.0554	0.0628	0.0854	0.2471	0.4943	0.0842	0.7445	0.0595	0.2793
NM_017283	Psma6	0.0936	0.4250	0.7601	0.4665	0.1560	0.0683	0.4329	0.3377	0.1447	0.7076	0.6396	0.2635
NM_017236	Pebp1	0.5801	0.9136	0.7400	0.6471	0.6190	0.6630	0.9743	0.7528	0.8808	0.9322	0.8214	0.1338
NM_017111	Slco1a1	0.5246	0.4160	0.4604	0.4422	0.6665	0.2212	0.2599	0.3417	0.4380	0.3104	0.5424	0.4431
NM_017063	Kpnb1	0.7163	0.7147	0.9932	0.8994	0.8288	0.9373	0.8827	0.2112	0.6979	0.8094	0.8783	0.7149
NM_017065	Gabrb3	0.2791	0.1150	0.7819	0.8637	0.0206	0.1728	0.6524	0.2265	0.2587	0.9539	0.1811	0.3923
NM_019140	Ptprd	0.1062	0.0045	0.3167	0.0457	0.0185	0.0506	0.6058	0.0915	0.2716	0.1515	0.0200	0.0956
NM_017251	Gjb1	0.1840	0.1225	0.4161	0.0963	0.3997	0.1591	0.1312	0.4230	0.3013	0.1453	0.4503	0.6288
NM_012657	Serpina3k	0.5653	0.7834	0.2069	0.5111	0.2931	0.2367	0.4652	0.6544	0.2571	0.2283	0.6766	0.4093
NM_017261	Gria2	0.7190	0.6768	0.8462	0.3530	0.7390	0.8537	0.8198	0.7832	0.7492	0.7364	0.8185	0.8528
NM_017245	Eef2	0.8561	0.8678	0.6423	0.7830	0.9867	0.9774	0.9215	0.8976	0.7655	0.9635	0.9351	0.7746
NM_013012	Prkg2	0.4363	0.7211	0.9090	0.3629	0.1076	0.0798	0.7356	0.0791	0.5818	0.6504	0.4359	0.2214
NM_017177	Chkb	0.7349	0.8757	0.0718	0.0579	0.8990	0.8282	0.0352	0.9076	0.4971	0.7874	0.4462	0.3057
NM_017181	Fah	0.0511	0.0446	0.8537	0.8440	0.2509	0.0493	0.5524	0.5699	0.1287	0.1034	0.0270	0.0295
NM_019204	Bace1	0.3408	0.1762	0.1079	0.2200	0.1852	0.5541	0.1881	0.1825	0.0488	0.1237	0.5159	0.6403
NM_017077	Foxa3	0.3451	0.2429	0.5782	0.5416	0.2604	0.7365	0.5766	0.3198	0.7703	0.1798	0.3712	0.4934
NM_017076	PVR	0.0914	0.8375	0.0520	0.8052	0.0111	0.1230	0.0180	0.0901	0.2650	0.4462	0.6183	0.9506
NM_017079	Cd1d1	0.0079	0.3818	0.9523	0.5459	0.0021	0.2622	0.1947	0.0067	0.1902	0.4298	0.2991	0.1172
NM_019169	Snca	0.2451	0.7804	0.7645	0.4221	0.2822	0.3532	0.3778	0.5809	0.4898	0.7904	0.7111	0.4709
NM_017167	Oprk1	0.4474	0.7262	0.3611	0.3298	0.4570	0.1826	0.4997	0.3302	0.3797	0.3813	0.3420	0.3610
NM_017120	Csn2	0.1611	0.0754	0.3824	0.5299	0.4166	0.6255	0.5368	0.4298	0.2670	0.1399	0.1607	0.5038
NM_017082	Umod	0.7920	0.0351	0.6657	0.4123	0.3740	0.7814	0.6217	0.2877	0.2520	0.1623	0.4952	0.4744
NM_019231	Mapk13	0.4435	0.3833	0.5777	0.5236	0.2511	0.5734	0.8574	0.7486	0.5744	0.7613	0.5187	0.1485
NM_019210	Pak3	0.7097	0.7029	0.9539	0.8947	0.7447	0.9414	0.6419	0.7053	0.9935	0.8740	0.9441	0.9871
NM_019380	Nptn	0.9912	0.9842	0.9690	0.9666	0.9473	0.9842	0.9695	0.9866	0.9322	0.9217	0.8958	0.9340
NM_017087	Bgn	0.7513	0.8428	0.9292	0.8993	0.9815	0.9442	0.9844	0.8098	0.6450	0.8501	0.7440	0.6834

NM_017119	Gzmk	0.0048	0.0369	0.0697	0.1366	0.0163	0.0940	0.0410	0.0091	0.0413	0.1308	0.0111	0.1548
NM_017088	Gdi1	0.8972	0.1036	0.9762	0.1919	0.7130	0.8885	0.1616	0.3432	0.2764	0.1090	0.5798	0.9882
NM_017142	Adcy8	0.9460	0.7503	0.7502	0.7462	0.8492	0.7402	0.5423	0.7544	0.7410	0.9045	0.9746	0.8315
NM_017202	Cox4i1	0.0315	0.1990	0.0569	0.3276	0.0990	0.2763	0.0059	0.0333	0.3887	0.0463	0.5548	0.3781
NM_017178	Bmp2	0.7395	0.7517	0.7500	0.7533	0.7175	0.7307	0.6017	0.7447	0.7393	0.7478	0.7498	0.7422
NM_017246	Map2k5	0.6005	0.7676	0.5538	0.7732	0.8463	0.7961	0.9525	0.6141	0.9125	0.6528	0.8566	0.5215
NM_017302	Slc16a7	0.2310	0.5491	0.4823	0.6661	0.3546	0.4684	0.3554	0.2450	0.2507	0.4845	0.2873	0.2951
NM_017340	Acox1	0.3861	0.8365	0.1589	0.8651	0.7408	0.7521	0.8052	0.8660	0.7371	0.7560	0.6038	0.3060
NM_017196	Aif1	0.3342	0.3081	0.5663	0.2222	0.3456	0.5729	0.2150	0.3617	0.2720	0.7464	0.6188	0.4954
NM_017139	Penk1	0.6289	0.8463	0.9580	0.8587	0.4316	0.7779	0.5683	0.4981	0.9369	0.9021	0.5730	0.8653
NM_001131002	Hspb11	0.3749	0.1748	0.1851	0.3429	0.3512	0.1823	0.4123	0.1847	0.3853	0.3948	0.4924	0.1802
NM_017313	Rab3ip	0.9758	0.9371	0.8899	0.7634	0.8204	0.9238	0.7440	0.6568	0.9455	0.9974	0.8216	0.9276
NM_017312	Bok	0.0781	0.1238	0.0453	0.3930	0.0573	0.0224	0.0915	0.1916	0.2315	0.2660	0.0080	0.0492
NM_017089	Efnb1	0.0443	0.9456	0.5513	0.9897	0.9960	0.9962	0.9959	0.9898	0.4144	0.9913	0.9574	0.2553
NM_017112	Hpn	0.6671	0.7863	0.5187	0.5261	0.5444	0.5564	0.2508	0.2624	0.4642	0.1315	0.6742	0.8316
NM_017247	Scn10a	0.5943	0.5983	0.1785	0.3359	0.4976	0.6452	0.5581	0.1902	0.6603	0.3374	0.3441	0.6504
NM_017161	Adora2b	0.2871	0.2386	0.3985	0.2472	0.1305	0.4129	0.0271	0.5244	0.2035	0.3122	0.1634	0.3602
NM_017250	Htr2b	0.8131	0.1100	0.6809	0.8365	0.8918	0.7910	0.4214	0.7315	0.0640	0.4030	0.1261	0.7670
NM_017321	Aco1	0.9780	0.7565	0.0298	0.8141	0.5824	0.9014	0.6738	0.5329	0.8649	0.7225	0.1164	0.2545
NM_017272	Aldh1a7	0.8804	0.8143	0.6192	0.6997	0.8537	0.6083	0.1436	0.8314	0.8994	0.7481	0.9773	0.7599
NM_017195	Gap43	0.9479	0.8615	0.8908	0.7091	0.8320	0.7693	0.8337	0.8863	0.8244	0.9156	0.7879	0.9694
NM_017204	Map6	0.0384	0.0325	0.1297	0.9165	0.1888	0.4589	0.1935	0.0728	0.1197	0.1194	0.1398	0.8485
NM_001108099	Mdm2	0.1171	0.1207	0.2350	0.2567	0.3092	0.0281	0.0718	0.2604	0.0993	0.1424	0.2766	0.9680
NM_001108092	E2f7	0.5322	0.7811	0.3787	0.7241	0.7674	0.3638	0.2472	0.7784	0.6431	0.3932	0.0929	0.4181
NM_001107953	Zcchc11	0.9249	0.4706	0.5608	0.9234	0.9036	0.6643	0.1591	0.8357	0.8719	0.8773	0.8110	0.8270
NM_001108052	Rin3	0.5178	0.4225	0.2208	0.6946	0.3828	0.5130	0.8901	0.4858	0.7594	0.4245	0.6011	0.1922
NM_001108140	Cd3e	0.0989	0.0389	0.3109	0.6987	0.3070	0.0718	0.0426	0.1101	0.2196	0.4352	0.4267	0.6450
NM_001108137	Mfrp	0.1114	0.1096	0.3096	0.1988	0.0510	0.3093	0.3158	0.3210	0.2316	0.1534	0.3747	0.0581
NM_001108204	Polh	0.0705	0.3288	0.2461	0.0670	0.1483	0.1337	0.4509	0.0160	0.1306	0.2511	0.4478	0.1430
NM_001108049	Gpr68	0.4742	0.6223	0.5883	0.4536	0.2458	0.7729	0.6690	0.4889	0.1963	0.4795	0.1621	0.5163
NM_020100	Ramp3	0.7760	0.8914	0.9140	0.7251	0.0373	0.3058	0.8007	0.1581	0.9106	0.6590	0.6921	0.6363
NM_017104	Csf3	0.3610	0.7037	0.5391	0.6994	0.4422	0.4031	0.4614	0.7240	0.9313	0.4408	0.7238	0.1478
NM_017105	Bmp3	0.7124	0.4815	0.5951	0.2628	0.6821	0.2929	0.8856	0.6238	0.3481	0.7844	0.3613	0.6529
NM_016995	C4bpb	0.6227	0.5906	0.3930	0.1738	0.7744	0.2595	0.6318	0.7941	0.5461	0.7965	0.5366	0.5185
NM_017281	Psma4	0.1442	0.6372	0.1566	0.1446	0.1873	0.0377	0.3215	0.1311	0.0994	0.4611	0.6089	0.3005
NM_019244	Bckdk	0.3449	0.2883	0.6012	0.0944	0.8181	0.7701	0.7399	0.8172	0.7756	0.6687	0.8942	0.9682
NM_017303	Kcnab1	0.5843	0.1272	0.5236	0.8661	0.3949	0.4292	0.8289	0.0893	0.4178	0.2712	0.5943	0.7708
NM_017106	Clcn5	0.6230	0.4324	0.5251	0.5388	0.0943	0.7194	0.4178	0.1352	0.8590	0.6643	0.8553	0.7962
NM_017215	Slc1a2	0.4918	0.2181	0.6152	0.3460	0.6767	0.4787	0.6113	0.2641	0.4120	0.5095	0.2439	0.6315
NM_017214	Rgs4	0.7783	0.6225	0.7565	0.3650	0.4563	0.8511	0.2934	0.8277	0.3641	0.6829	0.7941	0.7592
NM_017212	Mapt	0.5678	0.7170	0.5423	0.6973	0.6571	0.7044	0.3318	0.5954	0.6492	0.6113	0.5452	0.7083
NM_017098	Fabp6	0.8132	0.1838	0.6045	0.5045	0.4405	0.2687	0.5509	0.3050	0.5725	0.5928	0.4758	0.2476
NM_017108	Kcnh3	0.3740	0.2086	0.2239	0.1581	0.7237	0.6489	0.6242	0.3924	0.8200	0.3025	0.4413	0.5563
NM_019155	Cav3	0.1661	0.0025	0.0185	0.0289	0.0285	0.2659	0.2666	0.1272	0.0781	0.2225	0.0483	0.7973
NM_017109	Syn3	0.0465	0.5100	0.6233	0.1712	0.3306	0.1258	0.1472	0.0693	0.5164	0.6751	0.6801	0.7675
NM_017198	Pak1	0.1299	0.0926	0.1166	0.2388	0.1606	0.0060	0.1127	0.1370	0.1351	0.2254	0.2457	0.1431
NM_019139	Gdnf	0.1524	0.0846	0.0399	0.0143	0.1126	0.1485	0.0791	0.0704	0.4052	0.1274	0.1398	0.2471
NM_020101	Adap2	0.4163	0.0633	0.1773	0.4247	0.0715	0.1162	0.4333	0.3614	0.0647	0.3649	0.1446	0.5089
NM_017235	Hsd17b7	0.8465	0.8644	0.0617	0.0842	0.9796	0.7594	0.5542	0.8723	0.9045	0.9511	0.9675	0.2093

NM_017113	Grn	0.0144	0.0708	0.4435	0.0247	0.1243	0.0546	0.0206	0.0509	0.0984	0.0926	0.0227	0.8013
NM_017237	Uchl1	0.8910	0.5873	0.7595	0.8785	0.7337	0.4195	0.3741	0.7989	0.3863	0.7386	0.4333	0.8156
NM_017115	Myog	0.8604	0.5424	0.1493	0.1405	0.6984	0.8921	0.4142	0.5401	0.7546	0.7790	0.5913	0.6270
NM_017275	Pnck	0.6152	0.5582	0.8609	0.4393	0.9214	0.8186	0.6931	0.6752	0.6610	0.4978	0.8612	0.8395
NM_017229	Pde3b	0.1895	0.4151	0.1464	0.3091	0.7124	0.5633	0.2916	0.3649	0.4897	0.3271	0.3421	0.2841
NM_017282	Psma5	0.0521	0.7412	0.3396	0.3455	0.2004	0.2261	0.7511	0.8862	0.6831	0.6406	0.6555	0.6789
NM_017266	Ldhc	0.3275	0.2319	0.5379	0.1279	0.5756	0.3467	0.4102	0.1541	0.1867	0.7525	0.5200	0.1467
NM_001123352	Abhd10	0.4997	0.2276	0.3301	0.6567	0.6909	0.6066	0.2815	0.5369	0.3396	0.4877	0.5726	0.3253
NM_001124770	Tac1	0.8379	0.5443	0.8929	0.8376	0.9096	0.8212	0.4845	0.6012	0.3701	0.7514	0.6959	0.4666
NM_001124769	Tac1	0.8379	0.5443	0.8929	0.8376	0.9096	0.8212	0.4845	0.6012	0.3701	0.7514	0.6959	0.4666
NM_017122	Hpca	0.2149	0.0199	0.1832	0.5659	0.5992	0.4839	0.4995	0.7109	0.6090	0.8278	0.4423	0.8318
NM_017124	Cd37	0.0532	0.0730	0.0008	0.1334	0.1385	0.0118	0.0017	0.0248	0.0517	0.0061	0.0005	0.2718
NM_017333	Ednrb	0.0506	0.0271	0.0561	0.0775	0.4317	0.4015	0.0901	0.0563	0.0695	0.4430	0.5192	0.6195
NM_017126	Fdx1	0.0538	0.4633	0.0296	0.1712	0.0101	0.0667	0.0958	0.1572	0.0757	0.0671	0.2734	0.0525
NM_017230	Padi3	0.2493	0.7780	0.8410	0.9428	0.0771	0.0024	0.9653	0.2101	0.9037	0.1333	0.2395	0.2878
NM_017129	Ctf1	0.4476	0.1512	0.1864	0.2518	0.8776	0.5774	0.4567	0.6929	0.3433	0.2575	0.2042	0.3740
NM_017338	Calca	0.5146	0.2222	0.4223	0.7027	0.6452	0.3174	0.2607	0.4353	0.3327	0.4308	0.4268	0.5937
NM_017270	Adh4	0.1252	0.1120	0.0737	0.1925	0.0711	0.0078	0.1145	0.3599	0.2234	0.2661	0.3908	0.1261
NM_019147	Jag1	0.4441	0.9141	0.5720	0.3047	0.2568	0.2415	0.7790	0.0267	0.2146	0.9352	0.1456	0.1992
NM_017327	Gnao1	0.6715	0.9166	0.9353	0.9912	0.1616	0.8929	0.7384	0.2109	0.9109	0.8619	0.6492	0.9878
NM_017137	Clcn2	0.8234	0.8178	0.2957	0.1953	0.6499	0.6030	0.2238	0.5999	0.7222	0.4276	0.6501	0.7831
NM_017144	Tnni3	0.4282	0.5254	0.2697	0.0661	0.4721	0.5239	0.5276	0.6193	0.5479	0.7695	0.8366	0.4284
NM_138507	Ptprc	0.8419	0.9679	0.9525	0.9107	0.9667	0.3282	0.6999	0.3839	0.8020	0.9482	0.9111	0.6854
NM_017149	Meox2	0.5551	0.3429	0.9695	0.9973	0.9132	0.9707	0.9380	0.9978	0.3710	0.3156	0.4116	0.9998
NM_017148	Csrp1	0.7639	0.7998	0.9681	0.8599	0.6515	0.3900	0.9172	0.7892	0.3109	0.6017	0.8177	0.1306
NM_017150	Rpl29	0.6016	0.4952	0.5936	0.4269	0.5800	0.3659	0.3840	0.4582	0.4172	0.4678	0.3939	0.3835
NM_017263	Gria4	0.7104	0.7367	0.7038	0.6882	0.6039	0.5470	0.6477	0.5721	0.6679	0.6945	0.7225	0.6264
NM_017295	Gabra5	0.6482	0.2516	0.5667	0.5801	0.4298	0.3232	0.6608	0.4193	0.3842	0.5554	0.3968	0.1238
NM_017183	Il8rb	0.6106	0.7183	0.5953	0.2577	0.5753	0.7063	0.4428	0.6404	0.6271	0.5546	0.6366	0.2609
NM_019195	Cd47	0.9672	0.3134	0.8772	0.2246	0.8502	0.8347	0.1610	0.6774	0.1681	0.2410	0.0060	0.7992
NM_020097	Extl3	0.2801	0.6156	0.7925	0.7401	0.1059	0.5090	0.7841	0.4874	0.4707	0.8697	0.3596	0.7351
NM_001108062	Ppp1r13b	0.7234	0.6157	0.6732	0.6825	0.4068	0.6406	0.1252	0.8591	0.6647	0.6300	0.7089	0.7643
NM_001108219	RGD1306941	0.6237	0.1894	0.9125	0.6737	0.4212	0.9191	0.3352	0.0986	0.3490	0.5083	0.8193	0.0283
NM_001108218	Hecw2	0.1280	0.0689	0.3097	0.4048	0.1572	0.3556	0.2374	0.4220	0.3162	0.3544	0.8426	0.0953
NM_001108155	Kif23	0.7287	0.7826	0.2974	0.9496	0.8368	0.8812	0.8156	0.8094	0.9907	0.8961	0.9853	0.0253
NM_001108101	Irak3	0.4730	0.0529	0.2878	0.0741	0.4551	0.7229	0.0220	0.6224	0.1312	0.0080	0.1020	0.8129
NM_001108100	Dyrk2	0.2922	0.6787	0.7463	0.2723	0.3933	0.6669	0.9234	0.5015	0.2163	0.5285	0.3868	0.3297
NM_001107984	Pdik1l	0.9174	0.9003	0.9873	0.9719	0.9219	0.9733	0.6682	0.9045	0.8828	0.9794	0.8286	0.9643
NM_001107955	Hint2	0.0806	0.0543	0.0719	0.6403	0.0247	0.0155	0.0094	0.0743	0.0082	0.0038	0.0691	0.2199
NM_020076	Haao	0.1566	0.0414	0.2882	0.2508	0.3763	0.5154	0.2566	0.4305	0.4765	0.5160	0.0569	0.0430
NM_019170	Cbr1	0.6427	0.2703	0.6001	0.8863	0.0913	0.0772	0.9314	0.0812	0.6295	0.7905	0.3461	0.5114
NM_001108168	Bmp5	0.1072	0.3119	0.5950	0.3425	0.6655	0.5721	0.1667	0.9402	0.0255	0.2208	0.2591	0.5428
NM_001108161	Cilp	0.9456	0.1670	0.9560	0.9983	0.8453	0.7722	0.8109	0.9376	0.4087	0.9432	0.7383	0.1046
NM_001108158	Lctl	0.7789	0.6943	0.8943	0.7463	0.7513	0.7399	0.6746	0.8037	0.7978	0.6592	0.6735	0.6593
NM_001107961	Dmbx1	0.7253	0.8061	0.9413	0.4403	0.8844	0.6617	0.8499	0.7878	0.6746	0.6979	0.7910	0.4676
NM_001107960	Mobkl2c	0.9033	0.6911	0.5650	0.3894	0.5650	0.6705	0.4320	0.5827	0.8219	0.5131	0.5948	0.0665
NM_001107959	Atpaf1	0.9341	0.9729	0.9654	0.9814	0.9576	0.9332	0.9775	0.9507	0.8720	0.8754	0.7249	0.9284
NM_020072	Acpp	0.0767	0.5976	0.0967	0.7880	0.3891	0.2624	0.5137	0.3568	0.8383	0.2210	0.1472	0.0262
NM_017162	Gif	0.2697	0.3933	0.2354	0.5879	0.1488	0.5240	0.6308	0.2233	0.1564	0.0158	0.1186	0.1717

NM_017166	Stmn1	0.9427	0.9537	0.5998	0.9712	0.9966	0.9935	0.9242	0.8515	0.9430	0.9233	0.9434	0.4984
NM_017176	Pla2g10	0.4829	0.2714	0.4807	0.3838	0.1976	0.6019	0.6670	0.3405	0.0806	0.5176	0.3175	0.7156
NM_017174	Pla2g5	0.7382	0.2813	0.7688	0.8055	0.8856	0.8682	0.9863	0.9178	0.0898	0.1487	0.1034	0.9870
NM_017164	Capza3	0.0900	0.2636	0.0706	0.1374	0.1465	0.4041	0.4498	0.3211	0.7121	0.2013	0.5411	0.3133
NM_017184	Tnni1	0.5650	0.5280	0.3475	0.4240	0.1396	0.2447	0.0904	0.1722	0.5019	0.5861	0.3508	0.1091
NM_017289	Gabrd	0.2521	0.1232	0.0141	0.2072	0.1025	0.3833	0.5904	0.3496	0.3768	0.0465	0.3322	0.3869
NM_019286	Adh1	0.0560	0.4776	0.6788	0.3244	0.2411	0.1091	0.6683	0.1387	0.2261	0.5114	0.4917	0.6133
NM_017255	P2ry2	0.7659	0.7876	0.8652	0.9915	0.9189	0.7040	0.9124	0.9511	0.9370	0.9115	0.7998	0.8655
NM_017288	Scn1b	0.3176	0.2978	0.2156	0.4739	0.5901	0.3003	0.5812	0.3447	0.2866	0.0646	0.4174	0.7420
NM_017172	Zfp36l1	0.3311	0.2133	0.9780	0.2011	0.2589	0.2720	0.0515	0.1130	0.1341	0.2472	0.1153	0.9737
NM_020102	Mos	0.3289	0.2762	0.3313	0.3637	0.3339	0.3191	0.1934	0.3938	0.4481	0.4255	0.3237	0.3911
NM_019292	Car3	0.7453	0.7534	0.7541	0.7572	0.7462	0.8707	0.7656	0.7567	0.0252	0.7488	0.0011	0.0005
NM_019620	Zfp386	0.8639	0.8858	0.4219	0.3752	0.9027	0.7187	0.1115	0.7228	0.9186	0.8231	0.9488	0.6998
NM_017180	Phlda1	0.8005	0.8562	0.7715	0.7898	0.8101	0.7104	0.8558	0.9491	0.9222	0.8277	0.7635	0.8755
NM_017182	H2afy	0.9524	0.9074	0.3641	0.9169	0.8015	0.2920	0.4633	0.9255	0.9279	0.8419	0.9161	0.2307
NM_017243	Prps1	0.5638	0.5497	0.5804	0.6211	0.6985	0.6008	0.4236	0.5464	0.4835	0.3961	0.6311	0.4187
NM_019165	Il18	0.6566	0.4007	0.5721	0.6598	0.4834	0.7630	0.6535	0.8888	0.2992	0.7085	0.8642	0.6970
NM_017329	Sftpa1	0.8480	0.7346	0.5107	0.4331	0.6359	0.1420	0.4908	0.2149	0.1234	0.2768	0.1502	0.6798
NM_176857	Vcpip1	0.6567	0.1267	0.7520	0.7798	0.2183	0.8159	0.0665	0.3608	0.7709	0.5611	0.4884	0.6293
NM_176856	41,161.0000	0.8193	0.7691	0.8543	0.7554	0.5452	0.6312	0.7980	0.9385	0.5991	0.3680	0.8085	0.8354
NM_176080	Naglt1	0.5524	0.2970	0.3751	0.1605	0.4505	0.3396	0.1746	0.2792	0.2143	0.2172	0.2711	0.4491
NM_176079	Myod1	0.2444	0.0539	0.0104	0.3094	0.3651	0.3032	0.5790	0.1187	0.1345	0.0834	0.0403	0.4184
NM_176078	Clic6	0.4514	0.3684	0.2715	0.1595	0.3403	0.4505	0.1221	0.3271	0.1288	0.2887	0.1216	0.2882
NM_199091	Cct3	0.0504	0.3020	0.0826	0.5390	0.4531	0.4916	0.7591	0.6133	0.5627	0.6521	0.3891	0.9548
NM_176074	C6	0.5511	0.1795	0.0376	0.3814	0.4726	0.3916	0.4763	0.5608	0.4746	0.1275	0.7574	0.7366
NM_175869	Plod2	0.1972	0.2189	0.6084	0.5523	0.5733	0.4925	0.4306	0.2339	0.3121	0.5262	0.7046	0.4391
NM_175846	Ugt1a8	0.3593	0.6897	0.6648	0.6412	0.4868	0.4896	0.4263	0.2858	0.3790	0.4164	0.5318	0.6242
NM_175844	Abra	0.4387	0.7201	0.5429	0.1811	0.3830	0.0954	0.6324	0.0776	0.5248	0.8398	0.6279	0.6075
NM_175843	Sqstm1	0.6568	0.4590	0.4052	0.5100	0.6676	0.5003	0.3327	0.5608	0.4362	0.2988	0.5647	0.2566
NM_181634	Prg-2	0.2314	0.3455	0.1133	0.2977	0.0488	0.3857	0.2602	0.2423	0.1943	0.3284	0.0901	0.2333
NM_181371	Gstk1	0.0167	0.6745	0.5988	0.2447	0.0233	0.0152	0.8032	0.0600	0.0450	0.3416	0.1276	0.0187
NM_181370	Hs3st2	0.5765	0.8492	0.8238	0.7048	0.4573	0.8417	0.9023	0.2827	0.9744	0.9286	0.6633	0.7664
NM_178866	Igf1	0.5304	0.4676	0.6053	0.7230	0.5689	0.6631	0.4305	0.6835	0.2139	0.6217	0.6019	0.5162
NM_177962	Slc14a2	0.8586	0.4332	0.6719	0.6659	0.6607	0.4656	0.5780	0.6930	0.8206	0.6187	0.7294	0.5988
NM_175764	Jmjd1a	0.4979	0.6990	0.6714	0.4606	0.7253	0.6884	0.7301	0.2782	0.1996	0.5586	0.7100	0.5218
NM_017317	Rab27a	0.4417	0.2226	0.8351	0.4778	0.1406	0.7217	0.5269	0.4562	0.1514	0.3288	0.4498	0.9093
NM_017185	Tnni2	0.8254	0.6367	0.3828	0.5067	0.5807	0.4803	0.5885	0.4587	0.6046	0.6642	0.6700	0.8674
NM_017188	Unc119	0.2863	0.4928	0.9329	0.4541	0.0788	0.0091	0.8226	0.1089	0.8276	0.8490	0.1325	0.4046
NM_017186	Gcm1	0.1132	0.2996	0.1955	0.0758	0.4592	0.1195	0.0278	0.1040	0.0727	0.2933	0.1268	0.3178
NM_017190	Mag	0.4773	0.7411	0.0987	0.5988	0.8749	0.8465	0.4369	0.5131	0.9019	0.6675	0.4684	0.4776
NM_019187	Coq3	0.1075	0.0804	0.1900	0.7232	0.0052	0.0034	0.1736	0.1326	0.2270	0.2072	0.1395	0.0613
NM_019247	Pitx3	0.1287	0.2644	0.4156	0.1499	0.5883	0.6027	0.1400	0.7969	0.4979	0.1672	0.5068	0.6001
NM_017189	Asgr2	0.2456	0.0646	0.4560	0.0036	0.4389	0.0347	0.0908	0.4440	0.1138	0.1778	0.0828	0.5243
NM_020088	Odz2	0.2332	0.2589	0.4725	0.4098	0.6519	0.5635	0.1931	0.3008	0.2291	0.5691	0.5929	0.7282
NM_017205	Avpr1b	0.7417	0.3057	0.9334	0.4129	0.5705	0.6739	0.8388	0.8780	0.3050	0.7718	0.2225	0.6087
NM_017206	Slc6a6	0.0293	0.0900	0.2477	0.1660	0.8732	0.7876	0.9492	0.9942	0.0074	0.1164	0.3646	0.0905
NM_017207	Trpv2	0.4370	0.6236	0.7975	0.7364	0.6034	0.5306	0.8691	0.8535	0.4891	0.8409	0.1668	0.7286
NM_017197	Cugbp2	0.3936	0.6237	0.3750	0.7325	0.4218	0.3604	0.4895	0.3658	0.6322	0.3887	0.7018	0.3520
NM_022714	Crhr2	0.0907	0.2186	0.3973	0.0959	0.2557	0.6230	0.6442	0.3036	0.4631	0.2184	0.2508	0.1218

NM_017208	Lbp	0.9176	0.7881	0.8220	0.8102	0.8440	0.8794	0.8551	0.8725	0.7709	0.8052	0.9229	0.9995
NM_017211	Glg1	0.2491	0.3745	0.9644	0.5968	0.8885	0.9723	0.7103	0.9779	0.6766	0.3541	0.5728	0.9882
NM_019258	Cst8	0.7043	0.1592	0.2468	0.0381	0.2331	0.2165	0.2294	0.2523	0.6950	0.1641	0.1553	0.3372
NM_017213	Odf2	0.5565	0.3043	0.5921	0.4643	0.4972	0.5227	0.3942	0.1840	0.4499	0.5523	0.1766	0.7020
NM_019183	Actc1	0.3186	0.6509	0.2783	0.3458	0.4824	0.1819	0.4639	0.4391	0.5800	0.3218	0.3126	0.3836
NM_019211	Rasgrp1	0.1461	0.6122	0.7351	0.4175	0.2466	0.1742	0.6719	0.4059	0.6767	0.0420	0.1503	0.7146
NM_017217	Slc7a3	0.4586	0.5715	0.6343	0.1898	0.7262	0.3201	0.4389	0.2765	0.5145	0.4560	0.3101	0.0789
NM_017216	Slc3a1	0.1262	0.0253	0.0321	0.2629	0.0059	0.0201	0.6173	0.0241	0.0319	0.0873	0.0587	0.0224
NM_019377	Ywhab	0.9313	0.4637	0.8261	0.9561	0.9060	0.9883	0.9626	0.9279	0.5118	0.5048	0.4823	0.8231
NM_017299	Slc19a1	0.7812	0.9292	0.4351	0.8631	0.3970	0.3572	0.9723	0.6609	0.9703	0.7109	0.7570	0.9313
NM_017225	Pctp	0.5257	0.8119	0.6412	0.5848	0.7711	0.6516	0.8785	0.3452	0.7668	0.7856	0.7101	0.3472
NM_019191	Smad2	0.8922	0.9998	0.9615	0.9278	0.7039	0.9929	0.9395	0.9517	0.9674	0.9790	0.9860	0.9984
NM_019907	Cript	0.1485	0.2050	0.6897	0.2434	0.0612	0.4245	0.1271	0.1988	0.2241	0.1562	0.2919	0.0241
NM_022236	Pde10a	0.3861	0.3277	0.3609	0.1839	0.4265	0.0760	0.1393	0.4051	0.1383	0.0271	0.1911	0.2229
NM_017228	Atn1	0.4399	0.2235	0.5190	0.7045	0.6353	0.4709	0.4277	0.2506	0.5749	0.4700	0.4461	0.6460
NM_017224	Slc22a6	0.7562	0.6473	0.8513	0.1965	0.4603	0.2109	0.5128	0.8063	0.7498	0.7716	0.5770	0.1130
NM_017279	Psma2	0.6434	0.5371	0.4867	0.4847	0.6972	0.6424	0.0167	0.5655	0.1634	0.1410	0.5131	0.4223
NM_017278	Psma1	0.2970	0.4764	0.4706	0.1268	0.1516	0.1185	0.5142	0.0355	0.0661	0.2892	0.1557	0.0551
NM_017231	Pitpna	0.3359	0.3149	0.5644	0.9758	0.3041	0.3377	0.9618	0.0993	0.0856	0.7558	0.1708	0.5002
NM_001077680	RGD1563047	0.3439	0.4211	0.9367	0.3914	0.4769	0.7170	0.6517	0.2861	0.5451	0.4232	0.6511	0.8471
NM_019141	Tle4	0.6703	0.7801	0.6694	0.6384	0.2797	0.4087	0.8341	0.1533	0.5241	0.8809	0.6883	0.8860
NM_001108205	RGD1310693	0.0554	0.4050	0.4226	0.6208	0.1179	0.0794	0.1266	0.2781	0.3437	0.5609	0.2303	0.6221
NM_001108492	Sytl2	0.1357	0.2346	0.5121	0.6412	0.0903	0.5899	0.6451	0.1020	0.2051	0.0851	0.2581	0.5926
NM_001108077	RGD1306886	0.2463	0.5383	0.1306	0.4347	0.1223	0.0884	0.3949	0.1553	0.0755	0.2827	0.5889	0.3381
NM_001108076	Myo1f	0.8367	0.2196	0.3988	0.1252	0.7767	0.2540	0.1297	0.6922	0.1945	0.1413	0.2275	0.1004
NM_001107997	Mmel1	0.0470	0.1021	0.5163	0.3109	0.0586	0.1493	0.1510	0.4075	0.3139	0.0318	0.3797	0.2920
NM_001108321	Rtp4	0.1525	0.1017	0.0180	0.1542	0.1329	0.0033	0.2431	0.1052	0.0589	0.0113	0.0048	0.3516
NM_001108244	Klhl4	0.8790	0.3920	0.8697	0.9207	0.4125	0.1357	0.7567	0.6024	0.9080	0.5117	0.6322	0.6391
NM_001107969	RGD1564804	0.3336	0.3472	0.5267	0.3636	0.3374	0.3524	0.8346	0.7735	0.3957	0.0066	0.2952	0.0547
NM_017300	Baat	0.1241	0.3841	0.1503	0.4622	0.4810	0.6141	0.2104	0.3734	0.2945	0.0498	0.3962	0.1586
NM_017334	Crem	0.7323	0.6731	0.6364	0.5832	0.6363	0.6802	0.3971	0.6033	0.7011	0.6441	0.8775	0.6708
NM_017244	Crabp2	0.0060	0.0369	0.0644	0.0571	0.2843	0.0642	0.2015	0.1725	0.3551	0.0712	0.1123	0.0977
NM_017242	Lsamp	0.0963	0.4310	0.5944	0.8392	0.4790	0.5442	0.2032	0.6346	0.6003	0.8993	0.0908	0.5777
NM_017345	L1cam	0.4657	0.1432	0.2064	0.4036	0.5850	0.6266	0.9299	0.4739	0.6194	0.8046	0.4984	0.6694
NM_021594	Slc9a3r1	0.7622	0.7533	0.3243	0.9107	0.8516	0.7896	0.7909	0.8014	0.7931	0.8998	0.8518	0.4320
NM_001108078	Btbd11	0.4412	0.7095	0.5320	0.8368	0.5484	0.7987	0.8065	0.7350	0.8106	0.8350	0.9078	0.6537
NM_001108188	Plxnb1	0.9216	0.7401	0.4036	0.8068	0.5427	0.9777	0.8700	0.9648	0.9484	0.9493	0.9148	0.5929
NM_001108179	Stag1	0.9667	0.3857	0.9635	0.8018	0.6225	0.9628	0.2894	0.7397	0.9499	0.9740	0.9562	0.9369
NM_017259	Btg2	0.5983	0.9272	0.9478	0.1911	0.7492	0.3338	0.9194	0.2319	0.9806	0.9345	0.7753	0.9940
NM_017260	Alox5ap	0.9041	0.7540	0.7284	0.9127	0.9082	0.3806	0.7847	0.7624	0.9023	0.6654	0.7184	0.9464
NM_021696	Serpinb2	0.1521	0.2803	0.0332	0.1897	0.1591	0.0047	0.0063	0.0192	0.1296	0.0608	0.0629	0.0066
NM_017264	Psme1	0.0196	0.1050	0.4564	0.0553	0.0562	0.2046	0.2029	0.0809	0.0373	0.0346	0.0070	0.4943
NM_021764	Rbck1	0.5142	0.2956	0.0803	0.6354	0.8208	0.5455	0.5339	0.8870	0.2310	0.1346	0.0631	0.1541
NM_017271	Nudc	0.1624	0.7162	0.0416	0.6517	0.3955	0.1312	0.5144	0.6642	0.8167	0.7562	0.5858	0.0835
NM_019291	Car2	0.8920	0.8431	0.9202	0.7970	0.9982	0.7450	0.9840	0.8935	0.9431	0.8416	0.9115	0.9356
NM_017306	Dci	0.7818	0.3822	0.8511	0.7948	0.1542	0.0242	0.5607	0.4408	0.0857	0.7294	0.0944	0.1030
NM_017293	Uhmk1	0.7443	0.7300	0.7805	0.4678	0.5803	0.6377	0.6075	0.7871	0.3860	0.7157	0.6223	0.5862
NM_017307	Slc25a1	0.7261	0.2912	0.5467	0.5991	0.1245	0.0227	0.8176	0.3254	0.4078	0.8594	0.8118	0.4016
NM_017276	Gdi2	0.9245	0.8715	0.2183	0.9379	0.9601	0.7335	0.4935	0.6807	0.3631	0.4580	0.9155	0.3635

NM_021669	Ghrl	0.2006	0.0424	0.1677	0.3840	0.0766	0.3141	0.0905	0.1943	0.1818	0.0381	0.0159	0.0955
NM_017322	Mapk9	0.5167	0.3203	0.9136	0.4430	0.8138	0.8981	0.7369	0.3917	0.9478	0.1704	0.4625	0.5487
NM_019152	Capn1	0.7720	0.3797	0.3756	0.7379	0.6531	0.1632	0.6583	0.4915	0.3134	0.6739	0.4446	0.4434
NM_017347	Mapk3	0.1502	0.3206	0.9672	0.7372	0.2509	0.5507	0.5309	0.1871	0.8965	0.7719	0.5498	0.9435
NM_019184	Cyp2c	0.8306	0.9511	0.9317	0.8542	0.9955	0.7452	0.8882	0.8813	0.7823	0.8698	0.8940	0.8097
NM_019230	Slc22a3	0.6730	0.7419	0.3310	0.4888	0.5697	0.6565	0.3494	0.4884	0.5892	0.3574	0.4500	0.6784
NM_019133	Syn1	0.5120	0.6022	0.4877	0.4503	0.5774	0.6052	0.6502	0.6677	0.6534	0.4543	0.5749	0.8415
NM_017362	Chrm5	0.6487	0.3004	0.3052	0.2494	0.5060	0.2544	0.3203	0.3182	0.0923	0.1246	0.6215	0.1054
NM_019387	Hgs	0.3223	0.4182	0.1083	0.0797	0.2192	0.0531	0.0622	0.4098	0.3544	0.6032	0.4361	0.3054
NM_019128	Ina	0.6595	0.5953	0.5531	0.3134	0.6417	0.4507	0.8412	0.5868	0.5078	0.8823	0.3849	0.7560
NM_017294	Pacsin1	0.0889	0.5168	0.1524	0.4544	0.2520	0.5664	0.2261	0.4484	0.5873	0.2974	0.3271	0.7738
NM_017304	Kcnab2	0.0195	0.0451	0.0351	0.0310	0.0118	0.0253	0.0348	0.0249	0.1549	0.0566	0.1427	0.0291
NM_019379	Uso1	0.6238	0.2870	0.4837	0.4370	0.2298	0.0818	0.3858	0.2110	0.6779	0.2885	0.5973	0.2455
NM_017308	Sema6c	0.7616	0.8405	0.4201	0.4242	0.5954	0.3872	0.6762	0.7921	0.3763	0.5291	0.4513	0.4123
NM_017311	Atp5g1	0.3941	0.7830	0.2844	0.4671	0.0334	0.1806	0.8496	0.0424	0.5316	0.3434	0.5530	0.0115
NM_017314	Ubc	0.3677	0.2857	0.2835	0.4968	0.4081	0.3098	0.1926	0.5931	0.1720	0.4111	0.5111	0.2715
NM_001006966	Peci	0.1971	0.3560	0.7899	0.7559	0.0933	0.0585	0.7935	0.0673	0.2702	0.3319	0.1089	0.0415
NM_017316	Slc23a2	0.5494	0.2806	0.3734	0.8388	0.5966	0.8171	0.4251	0.4636	0.4743	0.5367	0.2281	0.6252
NM_001108260	Baz2b	0.8208	0.6027	0.1788	0.0169	0.9236	0.6710	0.0070	0.7203	0.5507	0.2758	0.4946	0.3213
NM_001108275	P4ha2	0.0785	0.2904	0.1663	0.1582	0.0113	0.1759	0.6910	0.1925	0.4280	0.2220	0.1669	0.9036
NM_001107982	Zmym4	0.7824	0.2496	0.6618	0.2619	0.3691	0.9528	0.3432	0.9427	0.9039	0.5901	0.6671	0.8642
NM_021863	Hspa2	0.4662	0.1681	0.2079	0.8645	0.3497	0.8467	0.6519	0.2349	0.0985	0.2260	0.7367	0.0439
NM_017355	Rab4b	0.4494	0.6375	0.5739	0.2315	0.4914	0.4007	0.7358	0.9127	0.2674	0.6091	0.4933	0.2835
NM_017354	Hnt	0.2095	0.2730	0.3614	0.3922	0.2839	0.5844	0.4391	0.6322	0.5938	0.5034	0.2302	0.3855
NM_017353	Slc7a5	0.9237	0.8419	0.3024	0.9497	0.8913	0.9952	0.9686	0.8414	0.9842	0.9841	0.9675	0.3877
NM_017352	Nr4a3	0.6082	0.6055	0.6601	0.7110	0.6174	0.6034	0.7265	0.6534	0.6364	0.6999	0.6580	0.8232
NM_017351	Itih3	0.6822	0.7783	0.8750	0.5799	0.5795	0.8361	0.7251	0.6877	0.6869	0.5925	0.8951	0.5116
NM_019157	Aqp7	0.1372	0.3222	0.3059	0.2599	0.4287	0.1685	0.3827	0.4120	0.0598	0.4338	0.6172	0.1112
NM_019214	Slc26a4	0.1571	0.5250	0.6826	0.4181	0.0481	0.8737	0.4560	0.2174	0.6397	0.7296	0.7950	0.6831
NM_019353	Tpo	0.3003	0.7099	0.7774	0.5978	0.7457	0.1687	0.2194	0.4538	0.3675	0.5406	0.0893	0.1508
NM_021593	Kmo	0.3651	0.3923	0.4004	0.4786	0.3619	0.4857	0.5200	0.2902	0.5839	0.4608	0.7868	0.1229
NM_021687	Erbb4	0.1678	0.7581	0.6893	0.1329	0.5744	0.1470	0.2072	0.7335	0.0268	0.4542	0.4497	0.0569
NM_019304	Dgkb	0.6944	0.6497	0.6711	0.6917	0.7221	0.5370	0.3964	0.4201	0.7040	0.7059	0.4991	0.6190
NM_001135020	Sec61g	0.5470	0.4670	0.2729	0.3907	0.5003	0.2707	0.4971	0.4275	0.1719	0.2849	0.3548	0.2488
NM_019309	Grik2	0.1323	0.3460	0.0471	0.2949	0.3626	0.1935	0.2425	0.1261	0.0682	0.0731	0.0472	0.1124
NM_019168	Arg2	0.6673	0.2782	0.3411	0.4970	0.3128	0.6429	0.4911	0.2485	0.0312	0.5800	0.9440	0.5031
NM_021577	Asl	0.8381	0.8613	0.8293	0.8485	0.5515	0.7375	0.3595	0.7609	0.8452	0.7688	0.6174	0.4344
NM_017339	Isl1	0.4459	0.3547	0.2141	0.0966	0.2211	0.1283	0.1120	0.2859	0.1651	0.2078	0.1030	0.1432
NM_017238	Vipr2	0.6913	0.7862	0.6830	0.8859	0.8997	0.9220	0.8207	0.6591	0.4394	0.8541	0.1562	0.6024
NM_001011897	Alg3	0.1834	0.1843	0.7156	0.4366	0.5333	0.4631	0.2093	0.5714	0.2655	0.4891	0.1850	0.2921
NM_001012036	Utp3	0.0734	0.2097	0.0461	0.0722	0.0814	0.0769	0.0060	0.0871	0.1046	0.2416	0.1955	0.5122
NM_001011895	Trmt2a	0.7778	0.7710	0.1515	0.1566	0.8765	0.7656	0.1722	0.7865	0.7316	0.6756	0.7136	0.7838
NM_017267	Timm44	0.7836	0.8081	0.4442	0.6359	0.7003	0.1051	0.8315	0.9128	0.6892	0.7447	0.4025	0.6426
NM_017240	Myh7	0.4407	0.3388	0.5346	0.7470	0.3578	0.0145	0.4083	0.2138	0.9259	0.4037	0.1381	0.2542
NM_017285	Psmb3	0.2721	0.4777	0.2234	0.2520	0.3634	0.2687	0.2961	0.3357	0.3910	0.1618	0.4955	0.2687
NM_017284	Psmb2	0.4405	0.7939	0.3411	0.7168	0.1090	0.1044	0.1399	0.4931	0.7014	0.6702	0.8849	0.2481
NM_022509	Smn1	0.8479	0.7531	0.8498	0.6586	0.9011	0.6321	0.7362	0.8243	0.8690	0.7550	0.9264	0.8992
NM_017249	Mbc2	0.7467	0.1178	0.0478	0.5908	0.9175	0.9926	0.6641	0.9518	0.7715	0.1475	0.3202	0.8162
NM_019145	Chrng	0.5714	0.1032	0.4437	0.1401	0.1530	0.0497	0.3469	0.1657	0.0125	0.3941	0.0081	0.0904

NM_017257	Psme2	0.2419	0.2232	0.8770	0.0380	0.2868	0.0636	0.2404	0.0116	0.0090	0.1421	0.0718	0.3595
NM_017256	Tgfbr3	0.8913	0.1788	0.9800	0.6568	0.6539	0.8730	0.8416	0.5201	0.3148	0.4369	0.0640	0.6542
NM_019174	Car4	0.1123	0.0207	0.0573	0.0326	0.0268	0.0097	0.1153	0.0372	0.0159	0.0782	0.0214	0.3097
NM_012977	Lgals9	0.0698	0.1661	0.1228	0.1259	0.2732	0.0107	0.3488	0.0195	0.2410	0.3747	0.5995	0.5682
NM_017348	Slc6a8	0.9551	0.7457	0.9951	0.6781	0.9891	0.8913	0.9918	0.8274	0.9696	0.9846	0.9144	0.9962
NM_017262	Grik5	0.8372	0.8334	0.6638	0.4803	0.3880	0.6978	0.6634	0.8344	0.8957	0.6100	0.7616	0.6917
NM_013043	Tsc22d1	0.6969	0.8222	0.7026	0.8754	0.7860	0.8815	0.8125	0.7656	0.7867	0.5088	0.7671	0.7937
NM_019322	Tpsab1	0.3054	0.1419	0.5031	0.4378	0.2622	0.2276	0.3993	0.0945	0.4694	0.1596	0.6371	0.4234
NM_019284	Cspg5	0.5735	0.5324	0.4636	0.5682	0.5162	0.6459	0.4089	0.6815	0.3815	0.3278	0.4045	0.4424
NM_017319	Pdia3	0.7956	0.7775	0.9953	0.9060	0.7387	0.7422	0.8759	0.5204	0.7625	0.9489	0.7723	0.6929
NM_017318	Ptk2b	0.0240	0.3437	0.6963	0.8362	0.2870	0.0968	0.9090	0.1307	0.3112	0.1902	0.4402	0.7363
NM_019193	Sox10	0.2314	0.4434	0.2217	0.1227	0.3815	0.6301	0.2537	0.4238	0.5502	0.3065	0.3355	0.4004
NM_001109885	Fam129b	0.4771	0.8751	0.9628	0.9974	0.3701	0.7668	0.8367	0.8294	0.9491	0.8737	0.6708	0.8964
NM_001110811	Serinc4	0.7185	0.5407	0.8009	0.5801	0.8680	0.2694	0.7958	0.6384	0.2552	0.8659	0.4981	0.5846
NM_001111098	Lyn	0.6960	0.5970	0.6467	0.9221	0.7616	0.2529	0.5782	0.8067	0.7050	0.8500	0.9802	0.8955
NM_080395	Npepps	0.6718	0.9258	0.7708	0.2891	0.3839	0.1941	0.5662	0.3500	0.9687	0.8721	0.3002	0.8751
NM_001110763	Tmem163	0.1556	0.3156	0.3447	0.1098	0.3885	0.4772	0.2492	0.0449	0.2917	0.6128	0.0775	0.1421
NM_001039699	Lrfn2	0.1199	0.4275	0.3479	0.6978	0.2281	0.2231	0.4566	0.5462	0.8656	0.2661	0.1022	0.5680
NM_001025674	Abt1	0.5827	0.8194	0.9351	0.1538	0.1818	0.0307	0.4750	0.1080	0.4037	0.5728	0.2828	0.2453
NM_001110848	Cacna2d1	0.8602	0.5084	0.9762	0.1904	0.8779	0.9975	0.8678	0.8061	0.9493	0.5128	0.7652	0.9930
NM_001110847	Cacna2d1	0.8602	0.5084	0.9762	0.1904	0.8779	0.9975	0.8678	0.8061	0.9493	0.5128	0.7652	0.9930
NM_001013434	Rras2	0.3768	0.5273	0.5171	0.5324	0.1629	0.4760	0.5747	0.4109	0.3823	0.3737	0.3843	0.3309
NM_001001508	Lppr4	0.5270	0.7030	0.6948	0.6673	0.5861	0.6543	0.6410	0.2348	0.6224	0.6909	0.6902	0.5380
NM_001101828	40,979.0000	0.6478	0.3024	0.3891	0.4486	0.9276	0.5429	0.6550	0.6281	0.3137	0.7919	0.5963	0.5282
NM_023967	Gfra4	0.7507	0.4523	0.8705	0.1577	0.5603	0.5906	0.1366	0.7307	0.7784	0.4346	0.2788	0.7829
NM_019202	Pla2g2c	0.6976	0.2779	0.6823	0.1638	0.2235	0.0738	0.1508	0.2254	0.3539	0.2385	0.0654	0.4525
NM_019196	Mpdz	0.8996	0.8873	0.9747	0.4579	0.9580	0.9522	0.3516	0.7938	0.7051	0.7564	0.9755	0.3252
NM_020104	Myl1	0.2325	0.3863	0.1977	0.2084	0.4453	0.4065	0.2927	0.2873	0.5060	0.5950	0.2862	0.2622
NM_019178	Tlr4	0.1619	0.9194	0.2340	0.5518	0.1516	0.8469	0.0124	0.7925	0.7673	0.7889	0.5086	0.6256
NM_019904	Lgals1	0.7385	0.2025	0.1587	0.4915	0.4038	0.2907	0.0360	0.5683	0.7786	0.4204	0.2647	0.1300
NM_017305	Gclm	0.1269	0.9711	0.5517	0.9588	0.5110	0.5464	0.9009	0.9179	0.7730	0.4704	0.9551	0.8339
NM_133537	Expi	0.8625	0.0115	0.1608	0.0975	0.9731	0.6019	0.0642	0.9029	0.0164	0.0464	0.0514	0.0640
NM_133512	Klrk1	0.3166	0.1246	0.1119	0.1338	0.1419	0.4935	0.1024	0.2219	0.5529	0.5962	0.3398	0.0922
NM_020098	Pclo	0.4338	0.7079	0.8178	0.7616	0.5674	0.7020	0.6952	0.4767	0.8324	0.8936	0.5798	0.6351
NM_017309	Ppp3r1	0.9980	0.9481	0.9682	0.9864	0.9896	0.9803	0.9141	0.9703	0.9308	0.8141	0.9803	0.6808
NM_019194	Tef	0.9514	0.9838	0.8911	0.8806	0.9871	0.9794	0.7017	0.9869	0.9836	0.9827	0.9775	0.9081
NM_019287	Apob	0.4631	0.6138	0.3240	0.1593	0.3079	0.2989	0.4608	0.4418	0.1835	0.1481	0.1050	0.0836
NM_017315	Slc23a1	0.8014	0.6258	0.6098	0.6353	0.4581	0.5669	0.5895	0.5675	0.5335	0.5779	0.8155	0.2142
NM_019201	Ctbp1	0.9855	0.9980	0.9716	0.9907	0.9925	0.9828	0.7529	0.9576	0.8530	0.8336	0.9977	0.8926
NM_053593	Cdk4	0.8527	0.8634	0.0969	0.9972	0.8855	0.9156	0.9951	0.8506	0.5339	0.7490	0.9401	0.0576
NM_021576	Nt5e	0.2514	0.2432	0.9544	0.1589	0.1364	0.0075	0.1743	0.3045	0.3268	0.2612	0.1749	0.1158
NM_019362	Stk39	0.9936	0.2015	0.9890	0.8203	0.8221	0.3795	0.7593	0.6620	0.5972	0.7413	0.4883	0.9424
NM_019249	Ptprf	0.0835	0.0583	0.0914	0.2337	0.3469	0.4525	0.3523	0.2837	0.0668	0.1959	0.0519	0.3963
NM_019143	Fn1	0.6978	0.9290	0.4861	0.8555	0.7238	0.8667	0.4311	0.5309	0.9856	0.9848	0.9616	0.5059
NM_019264	Prkar2a	0.4221	0.0831	0.1486	0.1066	0.1655	0.5212	0.4106	0.5582	0.3012	0.4686	0.0318	0.1417
NM_001007235	Itpr1	0.9431	0.8235	0.9939	0.9303	0.9951	0.9980	0.9471	0.9977	0.8468	0.9698	0.8154	0.9879
NM_017330	Prf1	0.1391	0.0766	0.1529	0.1386	0.1372	0.0409	0.1764	0.2830	0.2400	0.0205	0.2672	0.1359
NM_017356	Hpcal1	0.7249	0.5696	0.4372	0.6698	0.7124	0.3938	0.5497	0.2591	0.6941	0.4135	0.2116	0.6926
NM_017323	Nr2c2	0.7938	0.6268	0.6566	0.7626	0.5554	0.4954	0.4604	0.0990	0.4216	0.9155	0.8041	0.7076

NM_017326	Calm2	0.6516	0.7207	0.6750	0.7259	0.5725	0.7093	0.5767	0.3367	0.3601	0.6923	0.6914	0.7161
NM_017325	Runx1	0.8430	0.8393	0.9968	0.9998	0.9653	0.9320	0.8940	0.8386	0.9110	0.9704	0.8224	0.7890
NM_019271	Stch	0.0616	0.0505	0.5151	0.0520	0.0978	0.1572	0.0047	0.1464	0.0307	0.0595	0.0740	0.5920
NM_019208	Men1	0.8505	0.9840	0.5695	0.2906	0.9907	0.7703	0.8713	0.8631	0.8765	0.9316	0.7198	0.9537
NM_017335	Slc6a12	0.0754	0.4548	0.0315	0.0489	0.0516	0.2693	0.2159	0.0227	0.1556	0.1279	0.0384	0.5566
NM_017336	Ptpro	0.4130	0.6347	0.4594	0.3449	0.4650	0.2387	0.3771	0.3859	0.6331	0.4458	0.7247	0.3086
NM_023104	Aacs	0.8524	0.2148	0.1621	0.7477	0.9261	0.9817	0.2963	0.9678	0.0811	0.3824	0.0502	0.5396
NM_017357	Hpcal4	0.0189	0.1732	0.2010	0.1275	0.1537	0.3907	0.1180	0.0689	0.0821	0.0544	0.0324	0.1037
NM_017360	Coil	0.4745	0.8249	0.8189	0.1831	0.4489	0.1435	0.4355	0.7908	0.6207	0.8602	0.0906	0.6195
NM_017361	Nup54	0.8022	0.7454	0.2647	0.0562	0.7774	0.9074	0.2876	0.7625	0.9799	0.7157	0.6029	0.7100
NM_019282	Grem1	0.3110	0.1973	0.2446	0.2879	0.3300	0.7839	0.0861	0.4552	0.2858	0.2134	0.2231	0.1526
NM_017364	Zfp260	0.7880	0.3167	0.9400	0.8470	0.8865	0.9564	0.9131	0.6135	0.6713	0.2527	0.8298	0.9682
NM_021592	Hand1	0.7071	0.4984	0.6845	0.5726	0.5174	0.5284	0.6772	0.6627	0.9618	0.8849	0.7126	0.5402
NM_017365	Pdlim1	0.1614	0.7856	0.4124	0.8558	0.0635	0.2337	0.8602	0.0753	0.1993	0.7542	0.8629	0.4933
NM_019293	Car5a	0.4078	0.0234	0.0642	0.2475	0.0984	0.1766	0.3732	0.0998	0.1175	0.1432	0.3378	0.0595
NM_019131	Tpm1	0.4367	0.7303	0.6371	0.6080	0.4992	0.4851	0.6099	0.4830	0.4713	0.7143	0.3045	0.7344
NM_022179	Hk3	0.0710	0.0634	0.0754	0.0762	0.0252	0.0132	0.2588	0.0024	0.0250	0.0228	0.0297	0.3568
NM_019158	Aqp8	0.2279	0.1376	0.2810	0.6279	0.3383	0.5962	0.2759	0.8583	0.3901	0.5813	0.6543	0.2440
NM_020075	Eif5	0.5895	0.7931	0.8448	0.8919	0.5940	0.6888	0.9490	0.7719	0.7922	0.7825	0.9245	0.7217
NM_022856	Nab1	0.9926	0.9645	0.8144	0.7436	0.8361	0.8461	0.8655	0.8885	0.8845	0.9223	0.9944	0.9915
NM_022388	Fxyd4	0.5303	0.4048	0.0597	0.7809	0.4325	0.1323	0.0718	0.5646	0.3252	0.4304	0.4612	0.5529
NM_021997	Clip2	0.8277	0.8573	0.2458	0.6572	0.5404	0.7317	0.4899	0.3760	0.9241	0.8690	0.6257	0.9628
NM_020078	Adam1a	0.9296	0.8605	0.1337	0.2955	0.8620	0.9119	0.2938	0.9078	0.4671	0.8310	0.2982	0.6580
NM_020082	Rnase4	0.6387	0.0141	0.9282	0.9037	0.8193	0.9336	0.0507	0.7553	0.2185	0.3142	0.4109	0.0539
NM_020302	Adam3a	0.2920	0.7143	0.3796	0.4435	0.5937	0.4858	0.7943	0.2762	0.1035	0.6802	0.6850	0.6742
NM_020080	RGD708545	0.2128	0.3889	0.4235	0.6647	0.2260	0.5157	0.7230	0.2867	0.3875	0.2374	0.2568	0.8062
NM_019295	Cd5	0.3774	0.2671	0.3005	0.4622	0.5029	0.2934	0.2871	0.4240	0.5759	0.2792	0.3745	0.5431
NM_020086	Plvap	0.1295	0.0353	0.4415	0.8103	0.7830	0.9333	0.0873	0.9793	0.1213	0.0081	0.0049	0.9603
NM_019172	Galr2	0.8941	0.0055	0.0513	0.7294	0.7409	0.6805	0.1902	0.8983	0.2502	0.0015	0.2142	0.2524
NM_019329	Cntn3	0.3661	0.7361	0.5471	0.2377	0.4005	0.2254	0.2405	0.5620	0.4443	0.5016	0.6942	0.5550
NM_019299	Cltc	0.9669	0.8702	0.8925	0.8652	0.9721	0.9015	0.9449	0.9501	0.9542	0.9529	0.9341	0.9886
NM_020089	LOC56764	0.8998	0.8517	0.8777	0.8760	0.3370	0.3152	0.4346	0.5370	0.5847	0.8267	0.6632	0.8588
NM_021836	Junb	0.0365	0.0181	0.2825	0.0390	0.0355	0.3043	0.0151	0.4990	0.4541	0.0227	0.0143	0.9726
NM_020091	LOC56825	0.3570	0.0546	0.2548	0.1941	0.4685	0.1460	0.1426	0.0583	0.3285	0.2987	0.6051	0.2330
NM_021688	Kcnk1	0.3312	0.7051	0.4828	0.5016	0.2935	0.0027	0.7365	0.1839	0.5508	0.6883	0.5731	0.2689
NM_020094	Kcnip2	0.5152	0.3958	0.2930	0.6152	0.4791	0.6706	0.5488	0.5655	0.7193	0.4522	0.6099	0.5872
NM_019386	Tgm2	0.5797	0.4267	0.5058	0.7721	0.8010	0.4324	0.9002	0.7618	0.6756	0.7552	0.1997	0.1897
NM_019236	Hes2	0.7878	0.2712	0.8091	0.7997	0.4055	0.7969	0.7357	0.5411	0.6534	0.4618	0.3693	0.1137
NM_020096	Ifit1	0.4588	0.6328	0.5698	0.8466	0.3459	0.7612	0.4915	0.1679	0.3381	0.3426	0.6216	0.3798
NM_020095	Kcnip2	0.5152	0.3958	0.2930	0.6152	0.4791	0.6706	0.5488	0.5655	0.7193	0.4522	0.6099	0.5872
NM_020099	Leprot	0.5723	0.0533	0.6055	0.1813	0.7801	0.8995	0.2171	0.8631	0.3825	0.4670	0.1998	0.9262
NM_001111115	Begain	0.5490	0.7947	0.5364	0.5546	0.8605	0.8033	0.2414	0.6229	0.8378	0.8555	0.3964	0.7247
NM_001100716	Sos1	0.2282	0.5264	0.7615	0.6823	0.7353	0.6422	0.8549	0.8740	0.4085	0.6801	0.7847	0.8606
NM_001100690	Myh14	0.8073	0.7297	0.7147	0.6807	0.7220	0.7385	0.9489	0.6156	0.8128	0.7675	0.4024	0.8483
NM_001110793	Sf1	0.5907	0.3689	0.4891	0.5821	0.8126	0.7412	0.6303	0.7031	0.6174	0.4665	0.8273	0.7336
NM_001126288	Pld4	0.6254	0.3448	0.7820	0.6422	0.3872	0.3340	0.1122	0.3926	0.4890	0.7034	0.5511	0.2991
NM_022681	Adnp	0.9448	0.4480	0.9952	0.9593	0.9860	0.9676	0.8735	0.9465	0.9445	0.8903	0.9082	0.9842
NM_001113357	Npy1r	0.6533	0.5722	0.6769	0.4648	0.7007	0.6285	0.5913	0.4413	0.3676	0.5592	0.7218	0.4376
NM_001110860	Crem	0.7323	0.6731	0.6364	0.5832	0.6363	0.6802	0.3971	0.6033	0.7011	0.6441	0.8775	0.6708

NM_001008381	Ube2f	0.7656	0.4619	0.9369	0.6990	0.3641	0.3327	0.6565	0.3772	0.1097	0.8143	0.7458	0.3945
NM_001111095	Acly	0.9153	0.8435	0.5765	0.5640	0.7113	0.7699	0.6120	0.7904	0.8208	0.9373	0.8733	0.4529
NM_019122	Syt3	0.7692	0.1022	0.9340	0.5716	0.2871	0.8056	0.7194	0.2727	0.7828	0.5246	0.4308	0.7526
NM_019123	St6galnac3	0.7724	0.7696	0.6741	0.6209	0.7927	0.2968	0.4800	0.3902	0.2365	0.5013	0.5655	0.3510
NM_001012003	Orc2l	0.5185	0.4029	0.2181	0.1384	0.6363	0.5771	0.0920	0.5014	0.9194	0.5926	0.7935	0.7094
NM_019125	Pbsn	0.1447	0.3973	0.5535	0.3793	0.1822	0.3879	0.4315	0.4033	0.2704	0.1003	0.2093	0.2862
NM_019124	Rabep1	0.9434	0.9021	0.9541	0.9594	0.8831	0.9677	0.7375	0.7785	0.7484	0.9968	0.9811	0.8694
NM_019224	Rgs9	0.1032	0.4789	0.1828	0.4237	0.0741	0.0902	0.1072	0.4219	0.4320	0.4411	0.6860	0.2027
NM_019185	Gata6	0.9327	0.7674	0.8771	0.1800	0.6574	0.7784	0.9624	0.8768	0.7734	0.9227	0.8861	0.8406
NM_019182	Rnf4	0.0266	0.2287	0.2689	0.0668	0.1060	0.3828	0.2782	0.0336	0.1047	0.1314	0.5417	0.6017
NM_019135	Tnfrsf8	0.7596	0.4025	0.4865	0.2781	0.5577	0.8215	0.7272	0.2407	0.5774	0.6691	0.7927	0.7397
NM_021589	Ntrk1	0.5783	0.1311	0.3799	0.3574	0.0854	0.5862	0.2005	0.7638	0.3837	0.2738	0.7877	0.3431
NM_021595	Ninj2	0.0192	0.0937	0.4345	0.2469	0.8089	0.3015	0.5503	0.3942	0.6019	0.4628	0.7279	0.5420
NM_019137	Egr4	0.7391	0.6132	0.6366	0.2407	0.3572	0.4877	0.6250	0.4236	0.3888	0.4525	0.7351	0.7998
NM_001004084	RT1-Bb	0.5208	0.4368	0.5680	0.4684	0.3874	0.3007	0.2532	0.2637	0.4649	0.6353	0.6937	0.5024
NM_017156	Cyp2b12	0.3202	0.2337	0.1439	0.0817	0.3741	0.1050	0.1843	0.2061	0.4984	0.1440	0.2726	0.2968
NM_001126303	Usp43_predicte	0.4986	0.4883	0.6113	0.6634	0.7343	0.6455	0.5477	0.5930	0.4287	0.4723	0.5673	0.3686
NM_001107986	Srrm1	0.9789	0.8408	0.2891	0.2017	0.8768	0.8697	0.9191	0.6036	0.9773	0.7955	0.7481	0.9176
NM_001108085	Cradd	0.7226	0.8135	0.1542	0.8284	0.8591	0.8529	0.3267	0.8621	0.8604	0.8431	0.8694	0.2395
NM_001107985	Extl1	0.8012	0.7432	0.7572	0.3791	0.6661	0.7171	0.7624	0.3088	0.6820	0.8041	0.8831	0.7432
NM_019226	Dync1h1	0.8291	0.8233	0.3513	0.2517	0.7651	0.8321	0.3120	0.7736	0.7487	0.9713	0.8980	0.8273
NM_001126090	Shfm1	0.3127	0.3168	0.4496	0.3254	0.2819	0.2310	0.6158	0.4502	0.2843	0.5437	0.3243	0.2289
NM_019146	Bsn	0.6248	0.6495	0.4993	0.5284	0.6585	0.6237	0.6783	0.5651	0.6547	0.2004	0.6592	0.7038
NM_019150	Ucn	0.6498	0.3232	0.2676	0.0469	0.2615	0.4616	0.5042	0.2704	0.3455	0.3505	0.3449	0.4346
NM_019148	Strn	0.8462	0.9823	0.8547	0.7071	0.2866	0.4679	0.6995	0.2272	0.1687	0.4558	0.7649	0.7150
NM_019149	Matr3	0.6797	0.5985	0.5684	0.7055	0.6742	0.4977	0.5222	0.4125	0.4645	0.6295	0.6332	0.2073
NM_022219	Fut4	0.3049	0.2740	0.4069	0.7360	0.2416	0.5836	0.4185	0.6800	0.3001	0.2741	0.3217	0.0960
NM_019233	Ccl20	0.0024	0.8626	0.9169	0.2323	0.0737	0.0105	0.2152	0.2763	0.7998	0.9365	0.9197	0.8544
NM_019153	Fbln5	0.8364	0.1030	0.9478	0.6136	0.9986	0.9844	0.1461	0.9058	0.2339	0.0110	0.0254	0.1787
NM_019154	Amelx	0.6175	0.2062	0.6056	0.6116	0.6185	0.0796	0.6050	0.9041	0.2022	0.4060	0.5499	0.3327
NM_019156	Vtn	0.7305	0.8199	0.7223	0.7459	0.4940	0.7015	0.6715	0.3705	0.2101	0.5210	0.6238	0.7511
NM_021262	Acp1	0.5017	0.7094	0.7464	0.5512	0.4777	0.4311	0.3144	0.5889	0.6541	0.7536	0.4315	0.8076
NM_021583	Ptges	0.7301	0.3144	0.4151	0.5912	0.5149	0.2541	0.7383	0.3501	0.3994	0.2835	0.3664	0.4874
NM_019159	Syn2	0.6827	0.7593	0.6898	0.6252	0.6666	0.8793	0.4728	0.4291	0.4994	0.4229	0.5407	0.6417
NM_020538	Aadac	0.4209	0.2719	0.1068	0.3667	0.3601	0.3442	0.1693	0.2538	0.5877	0.3628	0.6097	0.3255
NM_019250	Ralgds	0.8841	0.8509	0.8291	0.4957	0.6314	0.9193	0.6659	0.9223	0.8445	0.4108	0.6053	0.8697
NM_024483	Adra1d	0.6729	0.6045	0.4277	0.3602	0.3381	0.5918	0.7028	0.4977	0.4906	0.5560	0.5405	0.5518
NM_021578	Tgfb1	0.8083	0.7946	0.8628	0.7319	0.8550	0.8915	0.7168	0.7308	0.8266	0.5594	0.8755	0.9304
NM_019257	Sfrs5	0.9812	0.8020	0.7852	0.9675	0.8117	0.8626	0.5120	0.9604	0.8340	0.9729	0.9890	0.8897
NM_019358	Pdpn	0.8359	0.1474	0.0985	0.0256	0.1834	0.1372	0.0533	0.1906	0.0999	0.1319	0.0575	0.1527
NM_019348	Sstr2	0.1378	0.0403	0.1252	0.0818	0.0902	0.0882	0.0135	0.3698	0.3376	0.0646	0.0107	0.2215
NM_019347	Slc14a2	0.8586	0.4332	0.6719	0.6659	0.6607	0.4656	0.5780	0.6930	0.8206	0.6187	0.7294	0.5988
NM_022540	Prdx3	0.9415	0.9773	0.7336	0.3750	0.9702	0.9181	0.7913	0.7716	0.9118	0.9769	0.9940	0.9350
NM_022266	Ctgf	0.9725	0.9991	0.9948	0.4204	0.5373	0.7782	0.8513	0.5614	0.6988	0.9754	0.9436	0.5808
NM_023103	Mug1	0.0825	0.2180	0.4192	0.5715	0.5456	0.2399	0.4085	0.1500	0.4802	0.2734	0.5132	0.2975
NM_022961	Vps33a	0.0221	0.1166	0.1880	0.1007	0.0155	0.0811	0.1051	0.2221	0.3368	0.0450	0.1735	0.6580
NM_019289	Arpc1b	0.4585	0.1404	0.8418	0.3402	0.1825	0.1545	0.0209	0.5587	0.2691	0.0642	0.5238	0.0820
NM_022685	Rem2	0.0458	0.5095	0.0499	0.2858	0.3697	0.3382	0.3401	0.2610	0.1625	0.0812	0.3100	0.0384
NM_021776	Ecel1	0.2894	0.8565	0.5874	0.5022	0.4246	0.4068	0.2352	0.3487	0.7622	0.4364	0.2613	0.7118

NM_021767	Nrxn1	0.2809	0.0777	0.1422	0.3609	0.6085	0.0260	0.5233	0.0339	0.0669	0.6117	0.4650	0.1399
NM_022220	Gulo	0.0716	0.5149	0.3312	0.3205	0.6210	0.6868	0.7559	0.2749	0.4343	0.6793	0.1852	0.3124
NM_021653	Dio1	0.4944	0.5492	0.6067	0.8190	0.9104	0.8136	0.6750	0.8560	0.5901	0.8354	0.8046	0.8118
NM_019162	Tac2	0.3460	0.3531	0.3275	0.6499	0.3148	0.2625	0.5797	0.1448	0.5048	0.5235	0.4929	0.5146
NM_019161	Cdh22	0.3938	0.4644	0.3945	0.5335	0.5676	0.6850	0.6398	0.7050	0.4841	0.4411	0.6485	0.2144
NM_019225	Slc1a3	0.8576	0.9984	0.9916	0.9996	0.9884	0.9742	0.9303	0.7885	0.9987	0.9977	0.9999	0.9714
NM_019164	Chad	0.3118	0.2338	0.1454	0.0916	0.4329	0.4893	0.5304	0.0597	0.3859	0.5484	0.3359	0.2511
NM_019269	Slc22a5	0.9922	0.9063	0.9731	0.9718	0.9885	0.9594	0.9465	0.8440	0.9050	0.7802	0.7280	0.9144
NM_019229	Slc12a4	0.3428	0.7715	0.1697	0.0213	0.4600	0.5863	0.2930	0.7827	0.9321	0.7328	0.7369	0.2864
NM_019166	Syngr1	0.1154	0.6426	0.4298	0.7514	0.3302	0.0331	0.7747	0.2927	0.5300	0.3165	0.6321	0.6674
NM_022240	A4galt	0.4028	0.2485	0.0595	0.2639	0.4248	0.3259	0.6662	0.5560	0.1080	0.2672	0.2974	0.4417
NM_019363	Aox1	0.0197	0.1189	0.0367	0.0780	0.7162	0.9198	0.0471	0.6450	0.1659	0.2666	0.2729	0.1282
NM_019171	Spt1	0.6198	0.5360	0.3655	0.4451	0.5799	0.5638	0.8651	0.4922	0.8123	0.4803	0.3854	0.3822
NM_019173	Galr3	0.1076	0.3154	0.3270	0.4286	0.3013	0.4278	0.3898	0.5844	0.4339	0.1913	0.1380	0.4773
NM_022190	Acan	0.7042	0.7109	0.9200	0.8316	0.6500	0.7881	0.7174	0.5188	0.8602	0.9228	0.6344	0.8493
NM_019177	Sell	0.4789	0.6182	0.8292	0.4713	0.5046	0.5844	0.5997	0.8727	0.5770	0.7216	0.6249	0.7089
NM_022181	Aqp6	0.7875	0.4528	0.2071	0.5488	0.7546	0.6618	0.8564	0.4426	0.4396	0.3832	0.4662	0.2082
NM_023956	Gucy1a2	0.0316	0.0724	0.3996	0.0624	0.4500	0.0944	0.1429	0.1021	0.0941	0.2152	0.0303	0.4942
NM_022926	Galnt7	0.0956	0.0460	0.1333	0.7758	0.0082	0.1351	0.0884	0.1545	0.2217	0.0528	0.0631	0.9683
NM_021840	Hist3h2a	0.4545	0.7813	0.4890	0.8108	0.6450	0.5033	0.7973	0.7292	0.6476	0.6865	0.8303	0.3647
NM_019186	Arl4a	0.5710	0.9403	0.9911	0.1231	0.0290	0.0077	0.9965	0.1108	0.9992	0.8946	0.8132	0.9893
NM_019189	Hapln1	0.3672	0.1716	0.3112	0.4942	0.9142	0.9496	0.0108	0.9478	0.3198	0.1615	0.1139	0.4322
NM_022198	Clcn4-2	0.3291	0.4171	0.5659	0.9295	0.7300	0.7769	0.4615	0.7347	0.3453	0.3210	0.8290	0.2536
NM_022286	Vps33b	0.5968	0.0336	0.2356	0.7349	0.6824	0.4721	0.0313	0.1297	0.1484	0.0277	0.0544	0.7828
NM_021655	Chga	0.6231	0.7168	0.5369	0.8072	0.9514	0.8663	0.6421	0.5181	0.7768	0.4848	0.8337	0.7958
NM_021654	Gja4	0.1020	0.0715	0.2683	0.0307	0.1696	0.2785	0.2356	0.1078	0.1141	0.0943	0.0730	0.0191
NM_019192	Sepp1	0.5034	0.7325	0.7425	0.6884	0.4850	0.4640	0.2519	0.7605	0.5686	0.1956	0.5549	0.5071
NM_019203	Tsx	0.1198	0.4131	0.5697	0.1914	0.8834	0.4422	0.4403	0.5124	0.2975	0.4546	0.1512	0.5917
NM_019621	Dlg4	0.7325	0.3008	0.5470	0.1890	0.9404	0.5798	0.7404	0.8645	0.6422	0.6215	0.4412	0.5291
NM_019221	Tp73l	0.5275	0.6728	0.6041	0.6935	0.5515	0.5759	0.6646	0.2247	0.6482	0.6386	0.7059	0.7205
NM_021858	Gnb3	0.5057	0.7965	0.7685	0.5864	0.9108	0.7394	0.6625	0.8001	0.8586	0.4935	0.4677	0.8191
NM_019205	Ccl11	0.3737	0.5274	0.1675	0.4526	0.2338	0.4182	0.3658	0.4843	0.6297	0.2007	0.3974	0.5288
NM_019305	Fgf2	0.0312	0.1305	0.0426	0.0891	0.0320	0.0060	0.1809	0.0347	0.3263	0.3396	0.6530	0.0918
NM_019288	App	0.0004	0.0005	0.0069	0.0017	0.0004	0.0013	0.0038	0.0019	0.0004	0.0005	0.0013	0.0006
NM_019274	Colq	0.4014	0.2785	0.2805	0.4978	0.7982	0.8928	0.1272	0.3502	0.6452	0.5379	0.1091	0.5408
NM_019265	Scn11a	0.2048	0.5313	0.5104	0.4736	0.4755	0.4197	0.1708	0.2122	0.4271	0.2377	0.0844	0.3773
NM_023022	Rhag	0.2299	0.4278	0.3330	0.5361	0.5731	0.1125	0.0638	0.3065	0.3281	0.1970	0.0508	0.3075
NM_019297	Chrnb2	0.7439	0.7636	0.8400	0.6109	0.4413	0.3407	0.7117	0.8373	0.8137	0.6634	0.3770	0.8942
NM_021843	Kitlg	0.7189	0.6638	0.9273	0.5381	0.6177	0.8483	0.9413	0.8929	0.5929	0.8814	0.9451	0.4588
NM_021748	Nsf	0.1083	0.1366	0.0482	0.0198	0.0022	0.0264	0.2560	0.1333	0.1665	0.2152	0.0598	0.1149
NM_022177	Cxcl12	0.9132	0.6546	0.9875	0.9736	0.9935	0.8409	0.9510	0.9723	0.6400	0.6419	0.4038	0.7140
NM_019340	Rgs3	0.1655	0.3319	0.0248	0.0156	0.0879	0.2271	0.1907	0.0542	0.3265	0.2140	0.1057	0.3744
NM_019212	Acta1	0.0948	0.4652	0.1011	0.1547	0.1474	0.0082	0.4228	0.0565	0.1327	0.0725	0.4041	0.3151
NM_019213	Jtb	0.1899	0.2951	0.1212	0.0298	0.0270	0.1673	0.1141	0.1347	0.3032	0.6523	0.1961	0.8125
NM_019331	Furin	0.6060	0.7021	0.5439	0.7117	0.6261	0.5304	0.6759	0.6337	0.7308	0.3859	0.6948	0.7217
NM_019312	Itpkb	0.0711	0.5124	0.4886	0.4578	0.6046	0.5684	0.4621	0.8681	0.2694	0.2732	0.0501	0.2718
NM_019218	Neurod1	0.5998	0.7358	0.5613	0.7720	0.4659	0.6747	0.8490	0.7419	0.7990	0.8052	0.7646	0.5633
NM_001014251	LOC365778	0.1313	0.5842	0.8005	0.6466	0.4736	0.4930	0.4153	0.4625	0.6176	0.7892	0.7360	0.6283
NM_019220	Aes	0.6140	0.9696	0.9286	0.9537	0.8134	0.8895	0.9724	0.8487	0.9904	0.9893	0.9097	0.9950

NM_019219	Rbbp9	0.8320	0.5827	0.8114	0.7891	0.2535	0.1344	0.2072	0.0364	0.6767	0.5711	0.3272	0.5236
NM_001033715	Cast	0.6583	0.3763	0.9610	0.9512	0.3357	0.6714	0.5842	0.5342	0.6643	0.4498	0.6294	0.6838
NM_019255	Cacng1	0.2150	0.3302	0.5172	0.7234	0.3707	0.1977	0.4892	0.4673	0.6166	0.5044	0.0110	0.6464
NM_031329	Ocln	0.4285	0.6152	0.8210	0.6852	0.3481	0.2960	0.6377	0.6617	0.8357	0.7748	0.3889	0.6493
NM_019222	Coro1b	0.0648	0.1133	0.0329	0.6775	0.0635	0.2596	0.0422	0.0326	0.2722	0.1472	0.0075	0.0883
NM_001037203	Ccl25	0.4884	0.4276	0.0544	0.3325	0.1094	0.1316	0.1721	0.4341	0.2560	0.6677	0.5628	0.0688
NM_022390	Qdpr	0.2394	0.7269	0.6135	0.9736	0.1429	0.1463	0.7898	0.1761	0.8779	0.8863	0.8237	0.1700
NM_020303	Adam5	0.5677	0.4213	0.5745	0.7940	0.6487	0.6999	0.6950	0.2253	0.7724	0.5055	0.7335	0.6400
NM_019268	Slc8a1	0.5363	0.2237	0.4315	0.5208	0.7397	0.7003	0.3987	0.3380	0.5548	0.4933	0.2583	0.0946
NM_019630	Gip	0.1538	0.3890	0.3742	0.5695	0.0624	0.3763	0.6671	0.7411	0.0668	0.1843	0.3550	0.6811
NM_021693	Snf1lk	0.5158	0.8010	0.9915	0.9949	0.0620	0.2585	0.8160	0.0629	0.9071	0.8642	0.8704	0.8304
NM_022245	Cyb5a	0.9366	0.8923	0.4633	0.7256	0.7748	0.7430	0.5502	0.8391	0.4779	0.6259	0.5468	0.1663
NM_022702	Taok2	0.6663	0.1515	0.0107	0.1121	0.8993	0.5389	0.1810	0.3120	0.4009	0.6496	0.5089	0.8599
NM_001127502	Wbp5	0.2854	0.8794	0.9003	0.8867	0.2182	0.2040	0.8676	0.0439	0.8976	0.9166	0.9918	0.6459
NM_019332	Padi1	0.4160	0.4733	0.0230	0.4389	0.1876	0.6350	0.3313	0.3985	0.2428	0.8233	0.5918	0.3195
NM_019234	Dync1i1	0.7615	0.5787	0.4786	0.4196	0.4060	0.3724	0.2131	0.2566	0.3881	0.6115	0.7104	0.0653
NM_019281	Gjd2	0.2457	0.4969	0.5797	0.5364	0.6919	0.6716	0.4066	0.1479	0.3392	0.6991	0.8023	0.5899
NM_019237	Pcolce	0.8255	0.1422	0.9298	0.5649	0.6617	0.6414	0.6886	0.7018	0.0823	0.0268	0.0731	0.1286
NM_019296	Cdc2	0.9401	0.8646	0.0849	0.9276	0.7868	0.9542	0.9109	0.8954	0.6662	0.8091	0.9071	0.0780
NM_019256	P2rx7	0.5414	0.8093	0.8955	0.1837	0.6096	0.7182	0.8907	0.6026	0.7545	0.2075	0.7623	0.6980
NM_019243	Ptgfrn	0.4352	0.4531	0.7330	0.5634	0.8430	0.5730	0.3871	0.9460	0.9702	0.8280	0.4309	0.6157
NM_021775	S1pr5	0.4308	0.0104	0.1662	0.3868	0.0618	0.1169	0.1617	0.4906	0.0765	0.1712	0.0079	0.0877
NM_024400	Adamts1	0.9226	0.3658	0.8593	0.9679	0.9544	0.9384	0.2475	0.8205	0.0320	0.2780	0.3468	0.7267
NM_022669	Scg2	0.8568	0.6004	0.7188	0.7829	0.4553	0.5277	0.2935	0.2399	0.7651	0.7135	0.5801	0.5331
NM_019385	Gorasp1	0.6542	0.5521	0.5182	0.5407	0.5665	0.6216	0.5560	0.4476	0.5037	0.6437	0.2147	0.5166
NM_019251	Bet1	0.1258	0.4791	0.9944	0.2964	0.1369	0.3521	0.6681	0.1857	0.8175	0.7986	0.9137	0.5076
NM_021689	Ereg	0.0945	0.5610	0.1357	0.1495	0.0427	0.0312	0.4153	0.0242	0.0841	0.9421	0.4191	0.4185
NM_019259	C1qbp	0.3772	0.8496	0.6747	0.8292	0.8557	0.4182	0.8324	0.5901	0.7372	0.8376	0.8122	0.7718
NM_022281	Abcc1	0.6081	0.0584	0.6333	0.1692	0.3095	0.8587	0.1104	0.4748	0.1018	0.4093	0.5729	0.8340
NM_019262	C1qb	0.4619	0.2887	0.3539	0.0257	0.3750	0.2964	0.1593	0.4305	0.1340	0.0547	0.0667	0.1518
NM_019261	Klrc2	0.3918	0.5248	0.2773	0.2505	0.4769	0.2421	0.2584	0.4222	0.2844	0.2006	0.5872	0.3531
NM_022235	Kcne3	0.1844	0.1821	0.5355	0.3770	0.1358	0.6689	0.4247	0.6957	0.2998	0.7117	0.1213	0.2231
NM_022539	Metap2	0.8177	0.9333	0.3727	0.7441	0.8599	0.7845	0.9633	0.9656	0.8823	0.9007	0.8366	0.4708
NM_021752	Birc2	0.6209	0.1048	0.6084	0.0429	0.1250	0.1646	0.0332	0.1327	0.4973	0.0129	0.1071	0.5812
NM_019279	Pcsk1n	0.7183	0.1934	0.0104	0.1365	0.6718	0.5994	0.1519	0.6840	0.6683	0.4914	0.2723	0.7989
NM_019278	Resp18	0.7457	0.7386	0.6317	0.0054	0.3971	0.0265	0.4704	0.0668	0.0501	0.7885	0.9002	0.5738
NM_019277	Exoc6	0.0992	0.0829	0.3299	0.5256	0.2203	0.2970	0.1941	0.0191	0.6911	0.4666	0.2513	0.8201
NM_022705	Olah	0.6737	0.6470	0.6961	0.5638	0.7844	0.7992	0.7916	0.7488	0.9261	0.7930	0.9306	0.5844
NM_019374	Pdyn	0.5226	0.7648	0.9557	0.1656	0.6331	0.7482	0.7418	0.6829	0.8407	0.8983	0.8733	0.4642
NM_019273	Kcnmb1	0.3514	0.5388	0.0816	0.5860	0.4036	0.5797	0.6684	0.5250	0.3660	0.5749	0.5629	0.2268
NM_019272	Sema4f	0.9998	0.9992		0.8969	0.9865	0.9961	0.9912	0.9949	0.9607	0.9670	0.9978	0.9907
NM_019315	Kcnn3	0.0038	0.0507	0.0571	0.0226	0.0338	0.0284	0.0926	0.0105	0.1843	0.1428	0.0100	0.1911
NM_019283	Slc3a2	0.8497	0.8289	0.7465	0.8182	0.9493	0.7598	0.7936	0.8171	0.7229	0.8098	0.9034	0.3242
NM_019290	Btg3	0.3693	0.6063	0.4354	0.5182	0.4373	0.3399	0.3216	0.5569	0.3106	0.4792	0.7333	0.1785
NM_022384	Ascl1	0.0409	0.2711	0.4014	0.0417	0.1162	0.1868	0.5891	0.3678	0.3543	0.2414	0.0752	0.1062
NM_032080	Gsk3b	0.1753	0.7481	0.0944	0.2415	0.1328	0.2175	0.1428	0.0355	0.1123	0.5931	0.4839	0.5907
NM_019302	Crk	0.3906	0.2614	0.0485	0.2937	0.3289	0.1175	0.0544	0.1492	0.5945	0.1435	0.0578	0.3420
NM_022290	Tnmd	0.5912	0.1205	0.3623	0.6892	0.2481	0.1414	0.0711	0.6767	0.5858	0.4241	0.1885	0.5924
NM_023977	Golph3	0.6006	0.3593	0.9724	0.9870	0.2404	0.9779	0.9064	0.3318	0.8783	0.4227	0.9817	0.9164

NM_019349	Slk	0.1768	0.4746	0.1841	0.1895	0.2688	0.1811	0.1916	0.1828	0.1712	0.1659	0.1751	0.1784
NM_019303	Cyp2f4	0.9342	0.3037	0.4569	0.5015	0.8165	0.7085	0.6870	0.5444	0.9791	0.3898	0.5892	0.8066
NM_020305	Adam4	0.4504	0.1731	0.1840	0.1892	0.3519	0.3334	0.4937	0.5266	0.3440	0.3316	0.1750	0.3009
NM_031688	Sncg	0.8261	0.3963	0.8863	0.7479	0.9060	0.8552	0.9342	0.9624	0.4638	0.3323	0.3122	0.0502
NM_022269	Cd55	0.0997	0.0085	0.1162	0.0779	0.1021	0.2983	0.0247	0.1408	0.0732	0.0264	0.0124	0.6605
NM_022671	Onecut1	0.9048	0.7485	0.9829	0.9477	0.9427	0.9070	0.9855	0.9832	0.9264	0.9591	0.9482	0.9838
NM_022639	Chrna10	0.4154	0.3152	0.3152	0.4111	0.8517	0.8063	0.8883	0.7902	0.6226	0.8823	0.5671	0.8782
NM_022523	Cd151	0.5984	0.7357	0.2049	0.9671	0.8657	0.9846	0.2940	0.9373	0.7848	0.5131	0.6602	0.1164
NM_023095	Mgat5	0.6780	0.2827	0.1693	0.2380	0.3816	0.5273	0.6119	0.1005	0.1596	0.2587	0.4807	0.6668
NM_022279	Mre11a	0.3689	0.6465	0.3730	0.5397	0.0658	0.0178	0.6728	0.3368	0.5767	0.5196	0.7031	0.2531
NM_020308	Adam15	0.0126	0.4869	0.9965	0.8003	0.3973	0.1199	0.8303	0.0816	0.8067	0.9534	0.7376	0.7682
NM_019343	Rgs7	0.8299	0.2721	0.8165	0.7824	0.6133	0.5162	0.6692	0.1064	0.3668	0.8247	0.7731	0.5018
NM_001109246	Exoc6b	0.7057	0.5986	0.4746	0.5134	0.5464	0.8034	0.0429	0.9053	0.4155	0.5440	0.4493	0.7102
NM_019622	Espn	0.5683	0.6441	0.3950	0.5858	0.6390	0.7518	0.9160	0.5068	0.4664	0.5663	0.5289	0.4176
NM_019372	Ppm2c	0.3889	0.3100	0.8129	0.8781	0.2715	0.5982	0.3918	0.5189	0.2255	0.1327	0.2117	0.7002
NM_019370	Enpp3	0.3528	0.0326	0.1519	0.1363	0.2655	0.2520	0.0129	0.0252	0.0938	0.3051	0.1180	0.1246
NM_019311	Inpp5d	0.0833	0.4721	0.5924	0.2511	0.1095	0.1991	0.1944	0.0558	0.6600	0.3690	0.3689	0.8554
NM_019356	Eif2s1	0.6044	0.9296	0.0638	0.3123	0.1356	0.1502	0.1928	0.0220	0.0667	0.7698	0.4165	0.8078
NM_019360	Cox6c	0.0515	0.0305	0.0709	0.1394	0.0773	0.1940	0.3034	0.1348	0.0035	0.0521	0.1257	0.0099
NM_019317	Madcam1	0.2240	0.4690	0.4946	0.1812	0.2524	0.0675	0.5573	0.4833	0.3702	0.7051	0.4331	0.0718
NM_021664	Dnase2b	0.5135	0.4337	0.3312	0.6179	0.4911	0.1295	0.6346	0.8092	0.1272	0.2653	0.1194	0.4080
NM_019318	Maf	0.9951	0.8985	0.9419	0.9607	0.9076	0.9660	0.9911	0.9944	0.9201	0.9550	0.9839	0.9817
NM_019321	Mcpt4	0.4070	0.6332	0.1293	0.2056	0.0273	0.5921	0.4967	0.4924	0.2590	0.5234	0.5194	0.7934
NM_019323	Mcpt9	0.4289	0.6753	0.5738	0.2881	0.6011	0.6182	0.2316	0.7005	0.8878	0.4840	0.6081	0.3645
NM_019326	Neurod2	0.8119	0.8370	0.9141	0.5766	0.4009	0.5300	0.8284	0.9210	0.8506	0.6569	0.3851	0.8766
NM_019328	Nr4a2	0.8229	0.8677	0.7713	0.5474	0.9615	0.8888	0.8234	0.9602	0.7598	0.5930	0.8093	0.7264
NM_022241	Ptgdrl	0.2467	0.1897	0.2195	0.3352	0.3361	0.4471	0.1052	0.1974	0.2975	0.2825	0.3911	0.1035
NM_022185	Pik3r2	0.1255	0.1877	0.2928	0.5997	0.0765	0.7646	0.0456	0.2876	0.1466	0.0171	0.0430	0.8517
NM_024403	Atf4	0.9911	0.8347	0.6299	0.8165	0.7156	0.8840	0.7458	0.7136	0.7266	0.7431	0.7359	0.6874
NM_022869	Nolc1	0.8542	0.9663	0.6284	0.9684	0.8292	0.8763	0.9847	0.9792	0.9566	0.6595	0.9610	0.3800
NM_019336	Rgs1	0.6170	0.3556	0.7526	0.6663	0.1646	0.1821	0.3294	0.1822	0.3382	0.3123	0.7401	0.0854
NM_031241	Cyp8b1	0.8726	0.5844	0.1354	0.2215	0.4004	0.2492	0.7772	0.2572	0.8738	0.6310	0.5361	0.4858
NM_023091	Gabre	0.2196	0.4363	0.3712	0.4815	0.4594	0.5159	0.4348	0.0657	0.1530	0.3485	0.2435	0.4513
NM_022538	Ppap2a	0.0653	0.3356	0.3133	0.0175	0.0616	0.2864	0.0107	0.1042	0.2944	0.5107	0.0842	0.3191
NM_022500	Ftl	0.0123	0.0009	0.0362	0.0506	0.0267	0.0226	0.0074	0.0275	0.0113	0.0310	0.0534	0.2558
NM_019339	Rgs12	0.7153	0.9658	0.2101	0.9413	0.9019	0.5254	0.5207	0.9033	0.2456	0.7577	0.0720	0.2899
NM_019905	Anxa2	0.1486	0.0682	0.2203	0.9576	0.2855	0.5736	0.1512	0.2088	0.2782	0.5374	0.3158	0.5322
NM_019344	Rgs8	0.2270	0.6869	0.6623	0.4316	0.8160	0.7885	0.8001	0.7946	0.8911	0.9074	0.1453	0.7762
NM_021698	F13a1	0.7923	0.9558	0.9806	0.9880	0.9518	0.7664	0.9933	0.9891	0.9095	0.9798		0.9401
NM_019346	Slc14a1	0.4090	0.6724	0.2950	0.3887	0.5134	0.4188	0.0895	0.8110	0.4467	0.7694	0.5203	0.8018
NM_001108976	Lsm10	0.3785	0.3112	0.5971	0.8513	0.8132	0.5905	0.5471	0.7628	0.2899	0.2567	0.3887	0.3394
NM_001108640	Ttll3	0.6962	0.2776	0.0766	0.1142	0.3812	0.5406	0.1615	0.2878	0.4704	0.4136	0.6388	0.3606
NM_001108769	Fbxl22	0.9354	0.8806	0.2174	0.4400	0.8981	0.6895	0.5054	0.5352	0.9694	0.8511	0.4297	0.5711
NM_001108760	Mpzl3	0.8041	0.8662	0.8884	0.9058	0.7847	0.8853	0.8436	0.9277	0.7080	0.9054	0.9299	0.7297
NM_001108728	Slc39a5	0.1731	0.3806	0.6917	0.6088	0.9516	0.8610	0.3253	0.9021	0.6518	0.5684	0.9127	0.5433
NM_001108633	Reep1	0.4303	0.6351	0.6541	0.5102	0.3790	0.3914	0.3549	0.1422	0.6731	0.7712	0.3304	0.4229
NM_001108777	Pik3r4	0.3171	0.0697	0.2540	0.0515	0.0761	0.3199	0.0562	0.0605	0.6341	0.5879	0.7751	0.6555
NM_001108631	Herc3	0.9656	0.9540	0.9766	0.8677	0.9970	0.9972	0.9894	0.9924	0.9596	0.9499	0.9733	0.9686
NM_021598	Mcpt8	0.6411	0.0852	0.0468	0.2036	0.8892	0.5571	0.0831	0.6958	0.0787	0.0217	0.0467	0.0150

NM_022184	Cask	0.9981	0.9844	0.9498	0.9435	0.9266	0.9362	0.5321	0.9435	0.9721	0.9850	0.9791	0.8849
NM_019368	Bet1l	0.1651	0.2483	0.5742	0.1506	0.1895	0.2741	0.2570	0.3181	0.2504	0.1741	0.4011	0.4708
NM_022260	Casp7	0.9090	0.5750	0.9657	0.6671	0.4779	0.8125	0.7400	0.7252	0.9007	0.9393	0.7056	0.8842
NM_021657	Phlpp	0.9558	0.3966	0.2946	0.7043	0.8390	0.9295	0.6775	0.8884	0.3805	0.4694	0.8627	0.2503
NM_021695	Synpo	0.3207	0.6016	0.2242	0.4887	0.6324	0.3494	0.5049	0.5406	0.3572	0.6615	0.4673	0.1924
NM_021694	Arhgef1	0.2688	0.3774	0.0081	0.1150	0.1549	0.1215	0.2337	0.1214	0.0904	0.3653	0.1809	0.6633
NM_024147	Evl	0.2579	0.2037	0.1039	0.4425	0.1481	0.0952	0.9391	0.1917	0.3092	0.2471	0.1606	0.0881
NM_019350	Syt5	0.1345	0.2628	0.3898	0.4618	0.5082	0.1793	0.6306	0.5342	0.6171	0.5680	0.4508	0.7360
NM_019352	Timm23	0.5273	0.5531	0.8257	0.9305	0.1716	0.3014	0.1159	0.2374	0.4263	0.8394	0.8424	0.2155
NM_021850	Bcl2l2	0.8305	0.8956	0.4017	0.3702	0.7765	0.7742	0.5178	0.8154	0.9766	0.9271	0.7378	0.9049
NM_019359	Cnn3	0.5526	0.7237	0.5625	0.6465	0.6114	0.4937	0.2435	0.7310	0.5397	0.6848	0.6134	0.6432
NM_021261	Tmsb10	0.1400	0.6215	0.7363	0.6575	0.5363	0.5245	0.5440	0.5557	0.6501	0.7056	0.8253	0.7509
NM_021989	Timp2	0.9310	0.6167	0.2881	0.3742	0.9678	0.7644	0.3071	0.9841	0.2720	0.1157	0.3546	0.9404
NM_022248	Dusp12	0.2310	0.7365	0.7416	0.8098	0.7477	0.2027	0.8671	0.8120	0.2122	0.4008	0.8857	0.2433
NM_021750	Csad	0.6062	0.8868	0.9402	0.9813	0.5480	0.2361	0.9528	0.8841	0.4667	0.7285	0.6680	0.3335
NM_022297	Ddah1	0.5816	0.5847	0.8280	0.4305	0.4129	0.1388	0.5287	0.3531	0.9244	0.9417	0.7618	0.5133
NM_022226	Lgmn	0.1883	0.0402	0.3366	0.0116	0.0033	0.0485	0.0770	0.0835	0.0791	0.0076	0.0924	0.1116
NM_021745	Nr1h4	0.6689	0.0640	0.4396	0.5427	0.1534	0.4247	0.4081	0.3799	0.4795	0.0379	0.0682	0.6813
NM_021266	Fzd1	0.0947	0.1992	0.1911	0.9511	0.9540	0.8907	0.2359	0.9806	0.1549	0.1384	0.1415	0.1684
NM_019364	Scfd1	0.6697	0.5095	0.2057	0.0160	0.1730	0.0748	0.1211	0.4192	0.1401	0.5179	0.0920	0.3681
NM_021662	Pold1	0.6507	0.6983	0.0009	0.3687	0.6611	0.6108	0.6994	0.6841	0.6116	0.6730	0.7130	0.0167
NM_019369	Itih4	0.8341	0.2882	0.2602	0.3917	0.1638	0.1465	0.4114	0.8367	0.4253	0.5426	0.8080	0.3598
NM_019371	Egln3	0.4398	0.6601	0.5169	0.6746	0.7215	0.7186	0.6579	0.4513	0.3648	0.5059	0.4800	0.7039
NM_019373	Apom	0.5613	0.3904	0.4713	0.6200	0.7729	0.3365	0.4961	0.6713	0.5196	0.5200	0.5754	0.5945
NM_019376	Ywhag	0.2213	0.3725	0.7116	0.4225	0.5609	0.3818	0.0551	0.1684	0.4493	0.3742	0.3839	0.9261
NM_019378	Snip	0.9077	0.8585	0.8434	0.1013	0.7107	0.8781	0.8878	0.9265	0.9076	0.7929	0.9428	0.9182
NM_022960	Aqp9	0.5214	0.4658	0.4710	0.0933	0.5347	0.4042	0.7899	0.6299	0.5095	0.7587	0.4783	0.2112
NM_022620	Spef2	0.8109	0.8915	0.7828	0.9591	0.5905	0.5616	0.6816	0.6442	0.8723	0.4251	0.8846	0.6573
NM_021690	Rapgef3	0.2400	0.1050	0.1139	0.0700	0.6102	0.1777	0.6794	0.3559	0.0356	0.2388	0.8032	0.1876
NM_019384	Scaf1	0.4039	0.6729	0.0378	0.1743	0.2635	0.3318	0.3237	0.1195	0.9347	0.6673	0.8209	0.8587
NM_021692	Smad5	0.4022	0.0769	0.4760	0.6563	0.0337	0.2030	0.3860	0.1198	0.3618	0.3450	0.8271	0.3711
NM_022606	Ilkap	0.8844	0.8922	0.0598	0.4380	0.8236	0.8057	0.1390	0.8807	0.9749	0.8176	0.7148	0.6307
NM_022280	Lrat	0.6972	0.6577	0.6594	0.6904	0.1989	0.2104	0.5753	0.5373	0.6009	0.6820	0.4043	0.2138
NM_020542	Ccr1	0.1024	0.1559	0.2341	0.1143	0.2402	0.4727	0.0835	0.2868	0.0078	0.0254	0.2113	0.0720
NM_022403	Tdo2	0.3676	0.4836	0.5563	0.7850	0.5232	0.0713	0.4053	0.1952	0.6334	0.1534	0.8425	0.3862
NM_022928	Grk4	0.6004	0.7132	0.3697	0.1057	0.8742	0.8657	0.4731	0.6476	0.9682	0.8237	0.8024	0.0975
NM_021684	Adcy10	0.1767	0.1725	0.3065	0.1884	0.1809	0.1810	0.1915	0.1824	0.1710	0.1657	0.1746	0.1781
NM_022397	Hnrnpf	0.8875	0.9597	0.9698	0.7130	0.8443	0.9277	0.9244	0.8831	0.8546	0.9349	0.9676	0.7430
NM_022298	Tuba1a	0.6747	0.6744	0.6503	0.4084	0.4486	0.7959	0.7845	0.3407	0.7167	0.4475	0.3916	0.1996
NM_022582	Lgals7	0.1236	0.1053	0.4208	0.4498	0.5250	0.7631	0.2945	0.0778	0.0833	0.4469	0.2430	0.0465
NM_022921	H2-M3	0.0372	0.1033	0.8834	0.0306	0.0174	0.0639	0.0310	0.0027	0.2513	0.0249	0.1011	0.8995
NM_022605	Hpse	0.5281	0.8293	0.6475	0.6419	0.6365	0.6335	0.6089	0.4515	0.8307	0.3869	0.6009	0.8311
NM_022250	Qtrt1	0.6869	0.4753	0.3026	0.2352	0.2655	0.4391	0.2589	0.7507	0.6511	0.1790	0.3856	0.4627
NM_021670	Bmp15	0.4818	0.1981	0.2883	0.3584	0.0475	0.0696	0.1548	0.3021	0.0495	0.2830	0.0095	0.0422
NM_021666	Trdn	0.0402	0.1011	0.2052	0.1239	0.9573	0.7774	0.0058	0.9292	0.2319	0.2879	0.2267	0.0035
NM_020976	Tmem27	0.7541	0.6135	0.7815	0.6655	0.5501	0.4792	0.8422	0.3176	0.4048	0.6517	0.3918	0.3439
NM_022264	Kit	0.3366	0.4632	0.5761	0.4346	0.4433	0.5330	0.7995	0.2058	0.6125	0.4682	0.6256	0.7739
NM_022276	Gcnt1	0.7564	0.6148	0.3691	0.4398	0.6002	0.3893	0.8218	0.4818	0.7058	0.5724	0.3524	0.5900
NM_021264	Rpl35a	0.0228	0.0283	0.0119	0.0168	0.0536	0.0083	0.3364	0.0847	0.0157	0.2998	0.0196	0.0511

NM_022243	Hibadh	0.6013	0.6582	0.5662	0.7267	0.5068	0.5964	0.3378	0.4484	0.5628	0.2212	0.5274	0.4058
NM_022296	Xylt2	0.1277	0.1104	0.6732	0.4245	0.0090	0.3496	0.1386	0.2026	0.1287	0.2290	0.0085	0.5404
NM_021579	Nxf1	0.7222	0.3483	0.5023	0.2827	0.4011	0.5004	0.4705	0.2933	0.5153	0.7065	0.2751	0.4796
NM_021581	Sc65	0.0005	0.0005	0.0010	0.2439	0.0293	0.0607	0.1477	0.0476	0.0269	0.0600	0.0249	0.0545
NM_021582	Rpa2	0.8862	0.9693	0.0127	0.2714	0.7455	0.5597	0.9366	0.9570	0.8110	0.8765	0.7719	0.5266
NM_021585	Mca32	0.5481	0.5858	0.1230	0.6287	0.8743	0.3538	0.7975	0.6327	0.7906	0.7066	0.1380	0.5173
NM_021699	Mark2	0.8360	0.9603	0.7830	0.7993	0.8752	0.9700	0.9078	0.9920	0.9405	0.7893	0.9005	0.9981
NM_021586	Ltbp2	0.8373	0.5436	0.4141	0.9887	0.8722	0.8980	0.0260	0.9512	0.5977	0.8291	0.0805	0.9372
NM_021588	Mb	0.1308	0.0426	0.1610	0.2494	0.5224	0.4342	0.2620	0.5132	0.5599	0.2599	0.3155	0.4406
NM_021587	Ltbp1	0.1115	0.0048	0.0915	0.5691	0.1345	0.0692	0.0942	0.0107	0.1766	0.1175	0.2755	0.2815
NM_021766	Pgrmc1	0.4630	0.7978	0.8545	0.2968	0.7067	0.2365	0.5151	0.3674	0.7188	0.9782	0.8294	0.6039
NM_021590	Aipl1	0.5734	0.6334	0.6122	0.6845	0.4676	0.2876	0.8966	0.6297	0.7692	0.5545	0.6423	0.7250
NM_021770	Olig1	0.1167	0.4753	0.5939	0.2360	0.6172	0.2313	0.4931	0.7549	0.8172	0.5248	0.3927	0.8093
NM_021596	Dbil5	0.7975	0.8227	0.0327	0.7887	0.7023	0.7670	0.7905	0.6324	0.4290	0.4797	0.3435	0.0991
NM_022694	Snd1	0.3293	0.6379	0.3605	0.8014	0.3421	0.4090	0.8946	0.4519	0.8681	0.3351	0.8653	0.5517
NM_031009	Agtr1b	0.5426	0.4971	0.4470	0.7010	0.2070	0.4749	0.7100	0.5720	0.1947	0.1910	0.6013	0.3078
NM_021835	Jun	0.9911	0.8219	0.3994	0.8438	0.9465	0.4337	0.9780	0.9886	0.9664	0.9611	0.9703	0.2654
NM_021597	Eif2c2	0.5199	0.5958	0.0653	0.4729	0.2648	0.2932	0.0537	0.8873	0.0211	0.4277	0.8045	0.1806
NM_022592	Tkt	0.1765	0.2810	0.2996	0.3483	0.1808	0.2809	0.3394	0.1819	0.3022	0.4829	0.1744	0.1780
NM_022268	Pygl	0.0581	0.0300	0.7896	0.7932	0.7509	0.7568	0.7274	0.6033	0.0731	0.0459	0.0531	0.8011
NM_021746	Mapk12	0.7187	0.1567	0.3431	0.1226	0.7082	0.1604	0.5201	0.8602	0.8807	0.4680	0.1388	0.1434
NM_022532	Araf	0.8196	0.4581	0.5266	0.6122	0.5844	0.5822	0.6369	0.7391	0.4322	0.3968	0.4016	0.9104
NM_001024261	Trnt1	0.1000	0.3146	0.9245	0.0809	0.1132	0.0991	0.0963	0.1780	0.4665	0.3276	0.5862	0.6554
NM_001108779	Tmem115	0.5570	0.6222	0.3043	0.4761	0.4490	0.6370	0.2506	0.6594	0.6490	0.7160	0.2191	0.4318
NM_001108772	Pgm3	0.4988	0.5353	0.4721	0.2997	0.3797	0.3359	0.2832	0.3505	0.4430	0.2447	0.2582	0.3948
NM_001108908	Nanos2	0.5149	0.4623	0.6146	0.0744	0.2728	0.1881	0.4208	0.1661	0.1440	0.0325	0.4430	0.1747
NM_001109007	Dis3l2	0.7492	0.3436	0.6772	0.3552	0.5818	0.1177	0.8056	0.6352	0.5007	0.7659	0.6660	0.7337
NM_001108761	Sin3a	0.7910	0.4712	0.8333	0.3783	0.5539	0.5237	0.0731	0.9474	0.9591	0.7521	0.7973	0.8722
NM_001108759	Siae	0.1935	0.5098	0.8173	0.4208	0.4437	0.6041	0.5154	0.6652	0.4220	0.8489	0.9716	0.9322
NM_001108758	RGD1304554	0.3851	0.7112	0.6612	0.5798	0.8023	0.5205	0.5409	0.6083	0.8364	0.6035	0.6385	0.4361
NM_001108755	RGD1308026	0.5031	0.8351	0.6865	0.6405	0.8596	0.7507	0.7871	0.9008	0.9204	0.8226	0.6784	0.6424
NM_001108752	Smarcd1	0.7939	0.7723	0.8524	0.9227	0.9809	0.9790	0.9504	0.9529	0.9640	0.9149	0.8060	0.9131
NM_001108858	RGD1309586	0.5462	0.5322	0.6509	0.6135	0.7131	0.3545	0.6180	0.3814	0.4388	0.2431	0.4955	0.6723
NM_001108748	Tubgcp6	0.7788	0.5474	0.2899	0.8377	0.5159	0.5493	0.5418	0.4011	0.3661	0.1975	0.6180	0.8928
NM_001108746	RGD1306474	0.3754	0.1039	0.6690	0.5017	0.3325	0.2192	0.4576	0.1430	0.5187	0.7664	0.4897	0.4321
NM_001108848	Elavl1	0.6481	0.7354	0.2854	0.9806	0.9422	0.1224	0.9191	0.6718	0.8948	0.4884	0.9715	0.1711
NM_001108745	Ppfia2	0.8317	0.3632	0.4626	0.2813	0.0213	0.0732	0.2348	0.4871	0.3608	0.5329	0.3391	0.7612
NM_001108743	Elk3	0.3901	0.0704	0.4197	0.2124	0.1920	0.4167	0.3623	0.1801	0.2953	0.3916	0.2530	0.2978
NM_001004227	Rtcd1	0.0256	0.2564	0.8116	0.4009	0.6936	0.5670	0.4664	0.7661	0.0836	0.5443	0.3129	0.6006
NM_001108740	Ckap4	0.9634	0.9835	0.7247	0.9635	0.7434	0.8511	0.9908	0.7753	0.6159	0.9128	0.9688	0.2830
NM_001108739	RGD1563325	0.7825	0.9899	0.9421	0.9232	0.9784	0.9363	0.9034	0.9827	0.6891	0.9469	0.9510	0.3642
NM_001108738	Ilvbl	0.0624	0.0171	0.8378	0.3588	0.4275	0.7109	0.1765	0.5871	0.0624	0.0227	0.0209	0.1249
NM_001108736	Mum1	0.8688	0.3916	0.4327	0.6660	0.8508	0.8751	0.7823	0.8279	0.6956	0.4909	0.5748	0.2271
NM_001108820	Gla	0.3848	0.2937	0.9573	0.3219	0.2177	0.8500	0.2649	0.1410	0.2285	0.0425	0.1839	0.5675
NM_001108902	Rab32	0.5657	0.5269	0.6900	0.6173	0.6272	0.3752	0.6047	0.6252	0.5844	0.6224	0.2049	0.4620
NM_001108901	Samd5	0.9500	0.9256	0.8803	0.9601	0.8536	0.7800	0.7982	0.5430	0.8885	0.8550	0.9293	0.7754
NM_001108725	Zfyve21	0.6406	0.9085	0.0283	0.0826	0.0467	0.0701	0.6866	0.0711	0.6011	0.8486	0.9192	0.1354
NM_001108862	Rasl10a	0.6600	0.6759	0.4851	0.4971	0.7125	0.4401	0.5413	0.8984	0.4670	0.2330	0.3665	0.6584
NM_001007667	Sat1	0.1642	0.8401	0.9827	0.2298	0.1632	0.2131	0.5254	0.0398	0.9522	0.8997	0.8600	0.8760

NM_001004082	Hsp90ab1	0.6132	0.6976	0.7282	0.7678	0.4498	0.7675	0.7231	0.5173	0.4556	0.3659	0.4576	0.8300
NM_001108796	Slc39a10_pred	0.2747	0.2838	0.8314	0.4257	0.3550	0.4940	0.3800	0.3591	0.7578	0.3688	0.9253	0.8675
NM_001108852	Cnpy4	0.9937	0.6052	0.8925	0.8777	0.7189	0.6520	0.8993	0.7795	0.0974	0.6575	0.5769	0.7041
NM_001108801	Rpl37a_predict	0.8382	0.7329	0.1948	0.9089	0.4540	0.3980	0.2581	0.7864	0.2947	0.3668	0.7655	0.1888
NM_001108786	Axud1	0.7214	0.6566	0.6007	0.5331	0.5512	0.4993	0.5364	0.5060	0.6170	0.6404	0.2120	0.7166
NM_001108709	Etv1	0.7314	0.7183	0.5502	0.6688	0.6912	0.7194	0.7245	0.6187	0.6068	0.6485	0.7315	0.6783
NM_001108708	Snx13	0.8908	0.7085	0.7183	0.3076	0.7280	0.7357	0.5763	0.8076	0.2575	0.2176	0.6579	0.7097
NM_001108698	RGD1308923	0.6208	0.4469	0.7826	0.8557	0.8364	0.3760	0.7394	0.8970	0.3948	0.3163	0.6482	0.0438
NM_001108707	Twistnb	0.2718	0.9646	0.9852	0.9027	0.8668	0.4782	0.9364	0.7682	0.8831	0.8877	0.9624	0.6417
NM_001108697	RGD1308251	0.0544	0.0279	0.0881	0.0752	0.0170	0.0570	0.2779	0.0489	0.0559	0.0991	0.0786	0.0174
NM_001108783	Ccdc12	0.8793	0.7690	0.4620	0.6935	0.6620	0.2105	0.7371	0.8003	0.9027	0.3017	0.5672	0.0612
NM_001108780	Gnat1	0.4669	0.3671	0.0566	0.0981	0.3676	0.5288	0.1586	0.6462	0.4650	0.5632	0.0460	0.5896
NM_001005889	Rdx	0.9312	0.7502	0.8724	0.9928	0.7531	0.4932	0.9207	0.8476	0.4623	0.8162	0.8393	0.8988
NM_001108854	Ikbke	0.0826	0.0300	0.1504	0.1335	0.9249	0.6806	0.2502	0.7117	0.0810	0.1380	0.3287	0.3993
NM_001108703	Rbks	0.1673	0.0665	0.6746	0.7269	0.8404	0.3844	0.0127	0.6865	0.7864	0.2216	0.6741	0.8184
NM_001108702	Spast	0.8883	0.8110	0.4176	0.9726	0.4514	0.7554	0.4879	0.0761	0.8364	0.7687	0.9084	0.7683
NM_001108692	Arhgef19	0.9407	0.1667	0.3601	0.8522	0.7389	0.8859	0.5661	0.7556	0.2775	0.0224	0.2038	0.3279
NM_001108701	Cebpz	0.9423	0.4073	0.1406	0.0127	0.8878	0.8982	0.2804	0.4154	0.8959	0.5510	0.9355	0.8750
NM_001108727	Coq10a	0.4884	0.4720	0.4546	0.5429	0.8143	0.9180	0.8250	0.8396	0.8424	0.6468	0.4368	0.3913
NM_001108722	Glrx5	0.6257	0.6951	0.1519	0.8420	0.6862	0.2634	0.9167	0.9592	0.3220	0.5557	0.4027	0.2789
NM_001108689	Pqlc2	0.6431	0.8847	0.2938	0.7167	0.9096	0.7367	0.9873	0.9162	0.8547	0.8297	0.3438	0.7573
NM_001108832	Mrm1	0.5077	0.5711	0.5371	0.0567	0.6628	0.6278	0.4585	0.1361	0.5468	0.7132	0.2693	0.5933
NM_001108816	Piga	0.4903	0.8584	0.8481	0.9381	0.6749	0.7852	0.9009	0.7273	0.2023	0.9253	0.4790	0.7632
NM_001108754	Mrpl4	0.0846	0.2575	0.2631	0.7483	0.4020	0.7741	0.8532	0.4226	0.2873	0.6259	0.6699	0.5246
NM_001108677	Mtf1	0.7527	0.1691	0.3452	0.2705	0.8204	0.2714	0.4281	0.5703	0.7509	0.8043	0.8835	0.9845
NM_001108676	Trit1	0.9416	0.7659	0.8267	0.2275	0.8965	0.8659	0.8162	0.9493	0.9480	0.7593	0.9763	0.9243
NM_001108747	Rassf3	0.6055	0.4673	0.4987	0.6743	0.4637	0.5608	0.5429	0.7032	0.4137	0.6859	0.7035	0.4929
NM_001108669	Ptplad2	0.9177	0.8657	0.7660	0.7555	0.5046	0.5495	0.8619	0.6195	0.8216	0.9933	0.8982	0.8006
NM_001108668	Akna	0.4000	0.5854	0.6530	0.2644	0.5076	0.4529	0.6642	0.1378	0.4856	0.4327	0.2291	0.7002
NM_001108667	Fktn	0.6771	0.4303	0.8442	0.4120	0.2354	0.9599	0.2158	0.7936	0.9193	0.3232	0.0258	0.8367
NM_001108665	Mrpl50	0.0650	0.1792	0.3132	0.5971	0.1512	0.3017	0.6412	0.0228	0.0071	0.1569	0.0419	0.2487
NM_001108663	RGD1310893	0.1247	0.3124	0.2634	0.3618	0.5322	0.4979	0.4535	0.3155	0.1230	0.2711	0.7282	0.8495
NM_001108660	Ccl27	0.0811	0.0036	0.0806	0.1627	0.7589	0.2232	0.0014	0.4378	0.1001	0.0620	0.1361	0.4645
NM_001108659	Dctn3	0.0857	0.0765	0.6922	0.3860	0.0698	0.0777	0.2301	0.0445	0.1421	0.0965	0.0478	0.1215
NM_001108655	Plekhf2	0.9396	0.6463	0.8586	0.7469	0.8994	0.8732	0.5545	0.4912	0.9129	0.6515	0.6498	0.7826
NM_001108653	Bicd1	0.2626	0.2145	0.5106	0.4362	0.4311	0.6881	0.4594	0.5512	0.3653	0.5167	0.0982	0.1865
NM_001108652	RGD1306151	0.1532	0.2689	0.9040	0.7841	0.3521	0.0551	0.9773	0.3322	0.1191	0.5984	0.9731	0.9189
NM_021686	Cnksr2	0.5744	0.7945	0.7210	0.6321	0.6705	0.6641	0.7154	0.5913	0.4002	0.8211	0.4454	0.4562
NM_022937	Doc2a	0.2507	0.3447	0.2952	0.2883	0.0377	0.1226	0.1020	0.4885	0.4373	0.3306	0.2650	0.2800
NM_021700	Neurog3	0.7577	0.6066	0.6866	0.7751	0.6247	0.9371	0.8325	0.7728	0.6076	0.6935	0.7343	0.7943
NM_022239	Nmu	0.0770	0.2932	0.2668	0.2727	0.2793	0.0759	0.1620	0.0589	0.4407	0.1301	0.2024	0.0093
NM_022683	Cul5	0.3995	0.4011	0.2900	0.1006	0.3390	0.3865	0.4870	0.0587	0.5570	0.3135	0.9353	0.7893
NM_024135	Limk2	0.9995	0.7954	0.9704	0.9831	0.9991	0.9880	0.9937	0.9982	0.9892	0.9979	0.9920	0.9704
NM_021691	Twist2	0.9445	0.9855	0.9315	0.9785	0.9740	0.8050	0.8939	0.8437	0.9248	0.9843	0.9180	0.9675
NM_021701	Ppp3r2	0.5041	0.1192	0.1596	0.5955	0.1355	0.2272	0.1246	0.3101	0.0496	0.1081	0.2097	0.6374
NM_021702	Atxn3	0.8014	0.8954	0.9315	0.8239	0.3018	0.7630	0.7226	0.8855	0.8785	0.2160	0.3824	0.3457
NM_021703	Akap14	0.2892	0.2913	0.4589	0.3478	0.5977	0.2135	0.6903	0.3720	0.7908	0.2116	0.1148	0.0752
NM_022183	Top2a	0.5523	0.3629	0.2249	0.2667	0.6752	0.2300	0.3504	0.4613	0.5004	0.2164	0.4804	0.4813
NM_001108112	Racgap1	0.3915	0.7896	0.0337	0.7670	0.6226	0.8252	0.7645	0.7325	0.1718	0.8278	0.8828	0.0813

NM_001108226	Wnt6	0.5479	0.0301	0.0289	0.8665	0.1498	0.0126	0.4988	0.1833	0.1266	0.1347	0.3569	0.0787
NM_001108134	Pus3	0.4216	0.0680	0.1409	0.3204	0.0811	0.4978	0.4554	0.2834	0.1221	0.1188	0.0660	0.3890
NM_001107999	Acap3	0.3061	0.4784	0.5511	0.8485	0.8838	0.8852	0.9107	0.8207	0.7324	0.9628	0.3193	0.9868
NM_001108387	Sucla2	0.6001	0.0361	0.8894	0.3310	0.3098	0.3240	0.1595	0.0828	0.1411	0.1050	0.1435	0.2517
NM_001108384	Entpd4	0.3419	0.1268	0.0616	0.2702	0.1530	0.5100	0.0267	0.1366	0.1647	0.1888	0.0608	0.6988
NM_001108300	Adam11	0.1897	0.2250	0.0487	0.2480	0.4357	0.3887	0.4379	0.4078	0.1112	0.0546	0.3822	0.7317
NM_001108098	Cpm	0.7912	0.8807	0.8463	0.4163	0.8676	0.9267	0.8710	0.8567	0.6884	0.4384	0.7356	0.1144
NM_001107998	Slc35e2	0.0687	0.0133	0.4257	0.3181	0.0686	0.1348	0.7110	0.0213	0.1083	0.4126	0.4887	0.0631
NM_001108340	Hscb	0.0329	0.1660	0.6443	0.4519	0.0065	0.0053	0.0881	0.0113	0.0027	0.0182	0.1536	0.0071
NM_001108325	Med15	0.1183	0.3421	0.4081	0.7291	0.3885	0.8422	0.7977	0.9844	0.8693	0.6763	0.2674	0.8002
NM_001108337	Med13l	0.9505	0.8816	0.9861	0.8980	0.9209	0.6885	0.9300	0.9565	0.8080	0.9714	0.9885	0.9257
NM_001108107	Pdzrn4	0.8520	0.1599	0.4898	0.3491	0.4813	0.1181	0.5229	0.1522	0.4591	0.4875	0.2111	0.1795
NM_001108096	Lrrc10	0.4917	0.5929	0.7114	0.5357	0.8992	0.8799	0.4815	0.5491	0.7006	0.8134	0.4162	0.5850
NM_001108310	Azi1	0.8721	0.7520	0.6260	0.8060	0.6735	0.3606	0.8699	0.4414	0.8410	0.6757	0.7780	0.1425
NM_001108273	Rasgef1c	0.4210	0.3907	0.3749	0.4408	0.8339	0.7206	0.7990	0.8455	0.3343	0.8080	0.0994	0.5716
NM_001108283	Rpl23a	0.3227	0.7350	0.5297	0.0654	0.5481	0.0680	0.5560	0.2941	0.3640	0.5997	0.5774	0.5086
NM_001108105	Mlc1	0.8549	0.5267	0.7662	0.8943	0.7289	0.6255	0.7136	0.7927	0.4223	0.4804	0.5181	0.5338
NM_001108311	RGD1311925	0.1170	0.0802	0.5543	0.1346	0.2261	0.1615	0.0598	0.0721	0.2156	0.0989	0.0036	0.1621
NM_001108279	Bcl6b	0.4953	0.5051	0.4535	0.5318	0.4147	0.4926	0.2727	0.5211	0.1585	0.2061	0.5720	0.2435
NM_001108297	Gsdma1	0.9191	0.7972	0.5856	0.8431	0.8758	0.6684	0.8180	0.4355	0.1616	0.5789	0.3707	0.3087
NM_001108095	Ptprb	0.6262	0.2077	0.7731	0.5531	0.3127	0.5743	0.1173	0.2450	0.1042	0.5905	0.1222	0.3798
NM_001107994	Spsb1	0.0314	0.0015	0.6585	0.7434	0.2026	0.2730	0.9096	0.3013	0.1038	0.2483	0.0580	0.2261
NM_001108291	Crop	0.9842	0.9897	0.3732	0.9433	0.9966	0.9527	0.3722	0.9547	0.9878	0.9678	0.9683	0.9289
NM_001108288	Trim37	0.8424	0.3937	0.7978	0.7902	0.2051	0.4921	0.8492	0.8078	0.7694	0.7977	0.7438	0.8602
NM_001108237	Lama1	0.6648	0.8169	0.4971	0.4441	0.4279	0.7301	0.8464	0.6990	0.5509	0.1248	0.8144	0.8068
NM_022197	Fos	0.1528	0.2397	0.5985	0.1598	0.8452	0.7443	0.1563	0.4741	0.4736	0.5309	0.1569	0.2228
NM_022194	Il1rn	0.1152	0.2992	0.8402	0.2523	0.1007	0.1122	0.0260	0.2707	0.1031	0.1475	0.1329	0.9813
NM_022178	Myo5a	0.6041	0.8878	0.7944	0.9120	0.7615	0.8062	0.7137	0.6814	0.9882	0.7998	0.9359	0.8362
NM_022231	Xiap	0.5420	0.2908	0.4167	0.7059	0.3296	0.3602	0.7197	0.3693	0.4526	0.4392	0.3211	0.1078
NM_021697	Kcnv1	0.3017	0.1680	0.2090	0.5575	0.3604	0.8402	0.7647	0.5917	0.5205	0.6676	0.4721	0.4504
NM_022533	Pllp	0.3378	0.6844	0.5837	0.7478	0.6576	0.3089	0.6088	0.4766	0.2342	0.7467	0.8377	0.4570
NM_021740	Ptma	0.8336	0.6041	0.8078	0.6830	0.7538	0.6765	0.6117	0.7799	0.7862	0.7601	0.7853	0.7283
NM_021744	Cd14	0.8708	0.7544	0.7861	0.8972	0.8589	0.7555	0.8747	0.9555	0.7937	0.7710	0.8381	0.8731
NM_021742	Nr5a2	0.1516	0.1809	0.2444	0.2483	0.1354	0.3405	0.0997	0.0802	0.4458	0.2937	0.1422	0.1126
NM_022631	Wnt5a	0.8735	0.9893	0.9913	0.9987	0.5120	0.7692	0.9693	0.0604	0.8618	0.7971	0.9452	0.9976
NM_022387	Pafah1b2	0.2720	0.5646	0.8853	0.9301	0.2226	0.2975	0.9387	0.2269	0.2294	0.3460	0.7077	0.0220
NM_022288	Csnk1g1	0.5268	0.4116	0.1008	0.0089	0.1174	0.1724	0.3575	0.0535	0.7051	0.6732	0.3832	0.0582
NM_022389	Dhcr7	0.7567	0.7736	0.2919	0.1923	0.9760	0.6068	0.6797	0.4852	0.9341	0.7766	0.9837	0.1235
NM_021751	Prom1	0.7712	0.8783	0.8992	0.9584	0.8411	0.7961	0.8026	0.9302	0.8903	0.9374	0.8737	0.4731
NM_021757	Plrg1	0.0771	0.0627	0.0684	0.1253	0.0345	0.1475	0.0042	0.0045	0.0344	0.1307	0.1420	0.0685
NM_021754	Nol5	0.8242	0.8145	0.0922	0.2959	0.8413	0.7146	0.1445	0.7158	0.8922	0.9040	0.9121	0.7367
NM_021739	Camk2b	0.6197	0.6376	0.7087	0.5906	0.7241	0.7017	0.6473	0.7210	0.6742	0.5040	0.6041	0.7338
NM_022863	Ireb2	0.9876	0.1038	0.8969	0.5884	0.6908	0.8019	0.5579	0.5862	0.4054	0.2394	0.9329	0.8715
NM_022511	Pfn1	0.7690	0.6359	0.8726	0.9445	0.8041	0.9862	0.8763	0.8558	0.4883	0.8389	0.9101	0.6217
NM_001108798	Raph1	0.9106	0.7378	0.1111	0.7408	0.6728	0.6851	0.7474	0.7212	0.7366	0.7327	0.9063	0.4669
NM_001109001	Arid3b	0.7292	0.5193	0.6612	0.6379	0.6803	0.7257	0.7244	0.5750	0.6651	0.4362	0.2407	0.7169
NM_001108808	Eif4e2	0.4488	0.8341	0.3007	0.8808	0.1111	0.0999	0.8114	0.0454	0.7341	0.6408	0.7559	0.1348
NM_001108805	Spata3	0.4271	0.4259	0.6123	0.3549	0.4306	0.5973	0.3942	0.1689	0.1968	0.2691	0.1570	0.4528
NM_001108795	Tmeff2	0.8405	0.9405	0.9289	0.7994	0.7218	0.9545	0.8800	0.6361	0.9810	0.9069	0.9041	0.8733

NM_001108794	RGD1560909	0.1109	0.0725	0.0242	0.1404	0.0075	0.1115	0.1221	0.0760	0.1098	0.1908	0.1129	0.0267
NM_001108840	Srp68	0.4491	0.2031	0.1057	0.0135	0.2590	0.2362	0.1423	0.1937	0.2607	0.0845	0.5248	0.1248
NM_001108825	Itk	0.4977	0.0158	0.4295	0.3509	0.3060	0.3472	0.2978	0.2869	0.2084	0.5335	0.6518	0.4385
NM_001108837	Meox1	0.8303	0.9060	0.6664	0.9529	0.9429	0.7969	0.9720	0.9191	0.9690	0.6142	0.9479	0.8987
NM_001108834	Stac2	0.3557	0.7314	0.5148	0.6215	0.6719	0.6803	0.6266	0.6631	0.7288	0.4011	0.3972	0.6978
NM_001108923	Inpp5a	0.1608	0.3233	0.3934	0.2028	0.2025	0.2072	0.3593	0.2561	0.3259	0.2646	0.1429	0.8704
NM_001108829	Jmjd3	0.6866	0.0223	0.0404	0.0020	0.3666	0.6324	0.2166	0.7016	0.6117	0.3586	0.3469	0.8734
NM_001108827	RGD1306000	0.0351	0.2293	0.5474	0.0966	0.3237	0.2972	0.4816	0.3356	0.3304	0.1208	0.4321	0.2106
NM_001108826	Tnip1	0.2404	0.3668	0.8944	0.8052	0.2879	0.3468	0.8339	0.0365	0.1437	0.1415	0.2058	0.4146
NM_001108806	Pde6d	0.4068	0.5086	0.6774	0.9876	0.1479	0.0190	0.7964	0.3454	0.3171	0.6233	0.6155	0.2564
NM_001108898	Ddx28	0.1254	0.7619	0.4618	0.8532	0.4761	0.0739	0.7670	0.4424	0.5327	0.1174	0.4046	0.6056
NM_001108824	RGD1562841	0.2368	0.0784	0.5074	0.2018	0.5424	0.4261	0.4005	0.4242	0.0678	0.7073	0.3774	0.3360
NM_001108818	RGD1562502	0.1636	0.0479	0.0961	0.0674	0.0517	0.1130	0.0304	0.2016	0.0158	0.1291	0.1177	0.0172
NM_001108918	Lyrm1	0.5914	0.7127	0.3617	0.8534	0.8656	0.5779	0.9777	0.6557	0.9772	0.7030	0.6527	0.4159
NM_001108821	Abcd1	0.2038	0.3650	0.7285	0.5220	0.3254	0.4436	0.4949	0.4225	0.5108	0.4300	0.3281	0.6202
NM_001108810	Ing5	0.6355	0.9376	0.8019	0.8187	0.9627	0.9140	0.9727	0.5281	0.9527	0.9153	0.8841	0.5579
NM_001109216	Asb10	0.6569	0.5253	0.1392	0.3818	0.3871	0.4515	0.1814	0.2278	0.4796	0.1678	0.3737	0.5304
NM_001108817	Snx12	0.0978	0.0375	0.6532	0.0016	0.0006	0.0020	0.0050	0.0465	0.0010	0.0024	0.0573	0.0058
NM_001113184	Gria4	0.7104	0.7367	0.7038	0.6882	0.6039	0.5470	0.6477	0.5721	0.6679	0.6945	0.7225	0.6264
NM_001111056	Adarb1	0.2532	0.9282	0.7303	0.2920	0.4561	0.6341	0.6847	0.5770	0.6461	0.9299	0.3428	0.7781
NM_001111055	Adarb1	0.2532	0.9282	0.7303	0.2920	0.4561	0.6341	0.6847	0.5770	0.6461	0.9299	0.3428	0.7781
NM_030836	Erap1	0.8713	0.8131	0.2940	0.1093	0.9253	0.9161	0.1416	0.7776	0.8705	0.8090	0.3915	0.5909
NM_021759	Lypd3	0.4024	0.2177	0.4391	0.3420	0.4767	0.2552	0.3016	0.5585	0.2404	0.2028	0.3729	0.6480
NM_024376	Gja3	0.6889	0.2917	0.2682	0.0431	0.3703	0.4615	0.1538	0.2054	0.2400	0.5491	0.4669	0.4326
NM_022302	Necab1	0.1901	0.7467	0.5065	0.1920	0.7369	0.2496	0.0839	0.5117	0.0866	0.3576	0.2795	0.5367
NM_021760	Col5a3	0.5514	0.9400	0.7879	0.4669	0.5019	0.6686	0.8249	0.1816	0.8539	0.8092	0.2260	0.3590
NM_021763	Arfip1	0.1997	0.2792	0.9697	0.4826	0.1441	0.6493	0.2243	0.0727	0.1108	0.0106	0.4266	0.8269
NM_021762	Tsn	0.2303	0.7339	0.9612	0.8698	0.7681	0.6807	0.9038	0.9187	0.0595	0.9546	0.9195	0.0453
NM_053781	Akr1b7	0.4012	0.6581	0.1951	0.8051	0.5957	0.6595	0.4425	0.6142	0.0701	0.8609	0.1769	0.6126
NM_053326	Pdlim5	0.0013	0.0306	0.6517	0.0019	0.0002	0.0042	0.0485	0.0020	0.0253	0.0806	0.0035	0.7118
NM_021769	Sult1d1	0.7267	0.4973	0.4053	0.5309	0.5442	0.2177	0.5152	0.5129	0.1993	0.5524	0.2924	0.4908
NM_022395	Pmpcb	0.0468	0.1697	0.8440	0.0078	0.0659	0.0446	0.0205	0.0842	0.1396	0.0893	0.3630	0.5690
NM_022602	Pim3	0.2560	0.9592	0.1445	0.2507	0.3629	0.3234	0.4871	0.5555	0.8915	0.8907	0.9388	0.6291
NM_022600	Adcy5	0.6823	0.5108	0.8321	0.3469	0.9318	0.7362	0.6885	0.9163	0.6430	0.4320	0.5011	0.2342
NM_024131	Ddt	0.3325	0.6440	0.7009	0.4415	0.1562	0.0845	0.8143	0.6176	0.1256	0.5426	0.3997	0.2842
NM_001108926	Cabp4	0.5519	0.4987	0.7915	0.1304	0.5187	0.1950	0.6384	0.7884	0.6132	0.6121	0.2461	0.8929
NM_001108925	Psmd13	0.0415	0.0500	0.0813	0.0742	0.1436	0.0203	0.3616	0.1167	0.0807	0.1736	0.0437	0.2137
NM_001108851	Znf498	0.3777	0.7014	0.4647	0.4885	0.6265	0.3439	0.1275	0.6189	0.8402	0.5397	0.4432	0.6269
NM_001108847	Ube2l3	0.4760	0.1377	0.1370	0.8187	0.3969	0.5125	0.3856	0.2683	0.1542	0.3818	0.2032	0.3809
NM_001108846	Gscl	0.1433	0.1807	0.1740	0.1649	0.2127	0.6680	0.2879	0.4174	0.5613	0.3626	0.0256	0.2727
NM_001108839	Mettl2	0.5695	0.5527	0.4143	0.7745	0.2684	0.1921	0.8601	0.3276	0.8489	0.7982	0.8340	0.8467
NM_001108842	Arl6	0.2673	0.3595	0.2308	0.3553	0.1350	0.2039	0.0603	0.1999	0.2132	0.0317	0.0443	0.7699
NM_001108870	Scara3	0.8988	0.8502	0.8047	0.6551	0.9871	0.8642	0.8029	0.7678	0.7181	0.9814	0.3857	0.9220
NM_001108947	Slc25a44	0.6647	0.7175	0.6104	0.4765	0.6348	0.5744	0.2355	0.6182	0.5687	0.5878	0.6802	0.6940
NM_001109047	Ccdc56	0.0497	0.0513	0.0096	0.9432	0.1173	0.1326	0.8300	0.0362	0.1161	0.0511	0.0385	0.1089
NM_001108859	Irf6	0.6205	0.5582	0.7584	0.5562	0.3696	0.5203	0.5823	0.3351	0.7430	0.4822	0.4957	0.2227
NM_001108857	Mael	0.1480	0.6449	0.6782	0.0863	0.0459	0.0381	0.3963	0.0351	0.4653	0.4168	0.6133	0.4777
NM_001108929	RGD1561557	0.4673	0.2143	0.2241	0.4781	0.5723	0.2140	0.4393	0.5815	0.6714	0.1998	0.2777	0.4671
NM_001109028	RGD1565784	0.2427	0.3214	0.1303	0.2129	0.2225	0.3690	0.1846	0.3660	0.0281	0.1202	0.0244	0.3341

NM_022596	Golga2	0.9463	0.6204	0.8747	0.8623	0.8721	0.9478	0.8350	0.2956	0.9339	0.7038	0.7933	0.9601
NM_022710	Pde1b	0.0379	0.1767	0.7123	0.6710	0.1532	0.2565	0.7186	0.2937	0.6733	0.1585	0.7683	0.8320
NM_022246	Rad50	0.7097	0.5309	0.7856	0.2292	0.6022	0.5322	0.8025	0.6519	0.8870	0.6492	0.8767	0.5220
NM_022292	Kcnk12	0.1742	0.1610	0.0075	0.0904	0.1651	0.0805	0.0558	0.4006	0.0439	0.3823	0.2841	0.1045
NM_022628	Nphs1	0.2605	0.7663	0.1869	0.5593	0.5417	0.1467	0.3230	0.2077	0.7049	0.5063	0.4377	0.6481
NM_022633	Galp	0.6591	0.5994	0.6721	0.2959	0.4296	0.5399	0.5109	0.6313	0.7117	0.7190	0.5933	0.9249
NM_030857	Lyn	0.6960	0.5970	0.6467	0.9221	0.7616	0.2529	0.5782	0.8067	0.7050	0.8500	0.9802	0.8955
NM_023024	Dync2h1	0.2528	0.2897	0.5575	0.3869	0.2386	0.3074	0.1909	0.3451	0.3768	0.2832	0.3024	0.3095
NM_031504	C4b	0.1763	0.1723	0.1832	0.5197	0.3178	0.2824	0.3899	0.1812	0.2593	0.3646	0.1740	0.4295
NM_022008	Fxyd7	0.3485	0.4157	0.7407	0.3198	0.7551	0.7645	0.3645	0.7743	0.6621	0.3825	0.5516	0.6357
NM_022005	Fxyd6	0.0065	0.9975	0.7536	0.9234	0.0967	0.1812	0.8275	0.2483	0.9552	0.9029	0.0449	0.9607
NM_021869	Stx7	0.9543	0.4981	0.9740	0.9530	0.8979	0.9427	0.9991	0.8811	0.8361	0.8998	0.6568	0.2909
NM_021868	Cttn	0.3218	0.2705	0.9267	0.9093	0.3906	0.9242	0.8094	0.3905	0.7553	0.6027	0.9095	0.8995
NM_024154	Accn2	0.8838	0.6284	0.7948	0.5625	0.7651	0.5207	0.2328	0.8325	0.5432	0.5673	0.4915	0.8482
NM_022499	Pvalb	0.5612	0.6277	0.7834	0.7707	0.3718	0.7244	0.6145	0.5213	0.8181	0.9820	0.5169	0.9503
NM_022399	Calr	0.9124	0.4482	0.5902	0.9204	0.2949	0.1785	0.8776	0.1288	0.5668	0.3750	0.7236	0.3853
NM_022950	C1galt1	0.6926	0.7911	0.8234	0.0535	0.4833	0.7849	0.8244	0.2478	0.7742	0.9318	0.8434	0.7935
NM_022186	Nrbf2	0.9388	0.8267	0.9123	0.8319	0.8597	0.8769	0.5389	0.4791	0.8144	0.9439	0.7960	0.8420
NM_030861	Mgat1	0.6870	0.8860	0.4699	0.0936	0.3695	0.0897	0.2265	0.2951	0.7713	0.7910	0.8539	0.2638
NM_022507	Prkcz	0.8819	0.9432	0.9458	0.6564	0.1370	0.0873	0.3286	0.2680	0.9584	0.9118	0.5667	0.9606
NM_022407	Aldh1a1	0.8830	0.6716	0.6150	0.6340	0.3514	0.6148	0.6259	0.3310	0.8686	0.1108	0.5205	0.6151
NM_173296	V1ra12	0.4281	0.6591	0.5348	0.4589	0.5534	0.4840	0.7631	0.7706	0.5723	0.7721	0.5096	0.4522
NM_173131	Vom2r53	0.2486	0.3442	0.4483	0.3280	0.3942	0.3156	0.3586	0.5541	0.2473	0.3461	0.3683	0.2849
NM_173120	Sels	0.9199	0.9723	0.2613	0.3894	0.7635	0.3789	0.2886	0.3191	0.5741	0.8982	0.8337	0.6595
NM_022521	Oat	0.7394	0.2501	0.8150	0.7739	0.8514	0.8194	0.9181	0.7193	0.3732	0.5922	0.6624	0.6636
NM_022498	Ppp1cc	0.6030	0.6516	0.6137	0.6019	0.6345	0.6430	0.6223	0.5607	0.6197	0.7238	0.6433	0.7301
NM_022954	Fat2	0.6360	0.4678	0.5759	0.4063	0.3983	0.6092	0.6874	0.5597	0.6797	0.6177	0.3402	0.5919
NM_022191	Syt6	0.3055	0.0975	0.4994	0.0569	0.0606	0.1309	0.1330	0.0141	0.1906	0.0516	0.0868	0.0309
NM_052797	Fga	0.6635	0.4756	0.3769	0.4701	0.4927	0.3127	0.4174	0.3722	0.5660	0.2592	0.4164	0.5111
NM_001047854	Chid1	0.8579	0.2930	0.3767	0.6393	0.6893	0.6084	0.8649	0.5523	0.2976	0.3275	0.4381	0.6111
NM_022205	Cxcr4	0.6449	0.8077	0.6050	0.7863	0.4818	0.8522	0.2017	0.7517	0.3059	0.5389	0.6634	0.6569
NM_019263	Minpp1	0.6953	0.3040	0.8554	0.9353	0.7928	0.9167	0.6686	0.8585	0.7396	0.5825	0.5908	0.8912
NM_022519	Serpina1	0.0810	0.0169	0.0016	0.0586	0.0084	0.0270	0.1097	0.0402	0.0135	0.0289	0.0453	0.8451
NM_022204	Exoc5	0.3894	0.0958	0.7886	0.8273	0.0189	0.8431	0.5705	0.0362	0.6239	0.0966	0.6224	0.6717
NM_001109882	Bsg	0.2183	0.6099	0.6185	0.5263	0.6509	0.5722	0.4784	0.5120	0.4160	0.3032	0.2504	0.2851
NM_001109880	Add2	0.6635	0.5058	0.4520	0.5815	0.5684	0.6477	0.9579	0.3561	0.5926	0.6666	0.6728	0.6137
NM_001100838	Prkcbp1	0.9241	0.8736	0.1514	0.0736	0.8043	0.7419	0.0131	0.9224	0.9167	0.9100	0.9009	0.8848
NM_001030020	Prkar2b	0.2510	0.9333	0.8939	0.3859	0.9428	0.9009	0.9229	0.9549	0.7022	0.8788	0.9933	0.0719
NM_001109301	RGD1560271	0.5901	0.0696	0.4640	0.4908	0.4127	0.0640	0.5275	0.2207	0.1006	0.3508	0.2709	0.3536
NM_001109219	RGD1562351	0.8301	0.9954	0.9820	0.7833	0.7768	0.5766	0.5458	0.8070	0.8776	0.7548	0.9711	0.9196
NM_001109289	RGD1561795	0.6587	0.3025	0.6349	0.1081	0.3593	0.3293	0.6956	0.4064	0.5934	0.2226	0.1097	0.3766
NM_001109493	Lce1l	0.6217	0.8412	0.0780	0.0045	0.3077	0.1013	0.4491	0.2602	0.7233	0.4822	0.4834	0.2144
NM_001109341	RGD1564854	0.3296	0.4340	0.3571	0.4561	0.4172	0.7566	0.6170	0.1929	0.7631	0.4838	0.4465	0.0853
NM_001109238	Atoh1	0.6744	0.2924	0.8205	0.4547	0.5698	0.5532	0.5997	0.6030	0.5604	0.4042	0.8981	0.5946
NM_001109235	Plekha8	0.3924	0.7080	0.7602	0.8730	0.0643	0.1079	0.9949	0.1475	0.8527	0.7115	0.7300	0.5715
NM_001109237	Neurod6	0.4900	0.9460	0.8801	0.8981	0.8333	0.1370	0.3949	0.2354	0.0407	0.8820	0.4849	0.5066
NM_001109236	Nod1	0.3436	0.6626	0.6048	0.5433	0.6088	0.9623	0.4438	0.5693	0.8044	0.7440	0.5409	0.8905
NM_001109150	RGD1564695	0.5889	0.3722	0.6502	0.1017	0.3429	0.6324	0.7500	0.5066	0.1359	0.7410	0.5391	0.6845
NM_001109068	Pm20d1	0.3958	0.3468	0.5128	0.4313	0.9135	0.5717	0.8602	0.4255	0.3850	0.6808	0.5856	0.6463

NM_001109109	Lpar2	0.7297	0.3551	0.4019	0.6451	0.4725	0.8870	0.7742	0.5033	0.5157	0.8717	0.6126	0.5285
NM_001108998	Tmprss4	0.5388	0.1574	0.1683	0.3294	0.4061	0.3926	0.4495	0.9115	0.1979	0.5092	0.2936	0.8277
NM_001109115	Ctla2a	0.0435	0.0140	0.1141	0.0726	0.0108	0.0075	0.0858	0.0682	0.0167	0.2248	0.2157	0.0468
NM_001109112	Tnfsf13b	0.2518	0.1220	0.7329	0.7412	0.3599	0.2636	0.8841	0.2978	0.1370	0.0588	0.7786	0.3969
NM_001108968	Ptk6	0.3537	0.6389	0.7166	0.1470	0.2142	0.2519	0.4296	0.8180	0.2978	0.2441	0.3639	0.7457
NM_001108937	Paip1	0.3297	0.3066	0.9508	0.7333	0.3736	0.9447	0.2144	0.2103	0.2208	0.3236	0.2988	0.4564
NM_001108936	Marveld2	0.1259	0.1086	0.1937	0.3009	0.6079	0.2858	0.4873	0.1615	0.3501	0.1489	0.3709	0.0533
NM_001108935	Col4a3bp	0.8377	0.2518	0.7990	0.3683	0.2844	0.9404	0.2953	0.4163	0.4398	0.2207	0.2409	0.9946
NM_001108934	RGD1306703	0.9259	0.8854	0.9567	0.8619	0.9264	0.8152	0.9402	0.4986	0.9278	0.8538	0.9810	0.7387
NM_001108932	Tysnd1	0.8244	0.5676	0.1385	0.5468	0.7653	0.3349	0.4000	0.7038	0.5735	0.2777	0.5441	0.5403
NM_001108931	RGD1560350	0.3990	0.8501	0.0415	0.4189	0.1609	0.5656	0.0168	0.5519	0.2444	0.1728	0.4206	0.0598
NM_001109030	RGD1564036	0.1318	0.2203	0.0738	0.2614	0.3493	0.1070	0.2552	0.1210	0.0222	0.1043	0.0395	0.0297
NM_001108928	Cwf19l1	0.3535	0.8354	0.1414	0.1060	0.6689	0.5695	0.0975	0.4861	0.7249	0.9621	0.6775	0.8384
NM_001108980	Ferd3l	0.6332	0.3742	0.5510	0.6735	0.4886	0.6582	0.2010	0.0824	0.4478	0.5286	0.4682	0.6780
NM_001109192	Gstm6	0.1496	0.3255	0.2000	0.0974	0.8335	0.0512	0.1574	0.3334	0.6374	0.4646	0.2078	0.3493
NM_001108999	Ankk1	0.2994	0.9047	0.5694	0.7878	0.6556	0.8216	0.7859	0.7822	0.7706	0.5337	0.7696	0.8242
NM_001109434	Qsox2	0.6897	0.6480	0.6880	0.5471	0.7246	0.5303	0.7270	0.7245	0.6250	0.6393	0.5972	0.4539
NM_001108920	Tbx6	0.7991	0.4874	0.2478	0.2178	0.6378	0.8435	0.7568	0.6691	0.5802	0.7595	0.5211	0.8633
NM_001108973	Slc1a7_predict	0.3786	0.5140	0.5544	0.6026	0.2254	0.5598	0.7021	0.2517	0.4519	0.5254	0.4528	0.5540
NM_001109006	Slc23a3	0.7152	0.3443	0.0885	0.8706	0.2720	0.7979	0.1654	0.4863	0.1989	0.4319	0.2735	0.6416
NM_001109005	Rab23	0.4088	0.6439	0.6689	0.6320	0.4862	0.7817	0.5069	0.2981	0.5221	0.5255	0.7691	0.6500
NM_001109003	Cpne4	0.5263	0.5761	0.5861	0.5422	0.1048	0.2037	0.3454	0.0577	0.5586	0.6823	0.2361	0.7486
NM_001109019	Zic3	0.2161	0.0607	0.4555	0.8249	0.7615	0.2745	0.6627	0.4437	0.4987	0.1228	0.6565	0.3152
NM_001108900	Ankrd11	0.9591	0.9869	0.1170	0.1043	0.9825	0.9859	0.4659	0.9759	0.9833	0.9637	0.9287	0.9254
NM_001108890	Ticam2	0.2849	0.2950	0.8370	0.7068	0.5071	0.6583	0.3116	0.2890	0.4549	0.2235	0.3213	0.3158
NM_001108884	RGD1311659	0.6919	0.7276	0.5815	0.4747	0.6891	0.0768	0.2533	0.7498	0.4420	0.1323	0.2465	0.6077
NM_001108922	Stk32c	0.4934	0.7045	0.2365	0.2039	0.6867	0.3609	0.1531	0.3927	0.2620	0.1505	0.4289	0.4440
NM_001108843	Lmln	0.3748	0.8216	0.8358	0.4395	0.8395	0.6040	0.8797	0.5590	0.6490	0.4711	0.8314	0.5028
NM_001109009	Aqp12b	0.1479	0.1880	0.0844	0.4169	0.5307	0.2363	0.3285	0.3360	0.3674	0.2508	0.2365	0.2835
NM_001108879	Mrpl36	0.9643	0.8716	0.9260	0.8939	0.8075	0.7866	0.7848	0.8174	0.0620	0.2716	0.5080	0.0849
NM_001108977	Epha2	0.9246	0.9514	0.9156	0.2115	0.9163	0.8994	0.7602	0.8102	0.9650	0.9390	0.9951	0.9637
NM_001109060	Stard13	0.3016	0.4999	0.3590	0.3404	0.5216	0.1929	0.4745	0.5841	0.8212	0.4675	0.4681	0.6963
NM_001109052	RGD1562726	0.5964	0.8469	0.8052	0.7084	0.8039	0.5911	0.6321	0.9146	0.8269	0.9234	0.0616	0.9202
NM_001108944	Rab33b	0.7095	0.2477	0.8321	0.5899	0.6219	0.7948	0.7786	0.7982	0.4990	0.6812	0.8831	0.8503
NM_001109199	Ifih1	0.6236	0.9754	0.9815	0.0387	0.0625	0.1181	0.2876	0.0234	0.8629	0.4253	0.7465	0.7741
NM_022209	Ppp2r2b	0.9431	0.7864	0.9689	0.9838	0.8662	0.9692	0.9431	0.8438	0.9391	0.9292	0.9963	0.9534
NM_024392	Hsd17b4	0.8186	0.0289	0.9062	0.8090	0.7805	0.6549	0.6479	0.7237	0.0297	0.1102	0.0810	0.5767
NM_030851	Bdkrb1	0.5231	0.0070	0.0931	0.1381	0.2523	0.6655	0.3063	0.6379	0.6672	0.6020	0.8486	0.3351
NM_022525	Gpx3	0.1137	0.2847	0.0545	0.2472	0.3379	0.5408	0.3690	0.2228	0.2776	0.5051	0.1099	0.5049
NM_022218	Cmklr1	0.0224	0.1407	0.6588	0.7564	0.9155	0.7280	0.8333	0.8306	0.4589	0.9189	0.0940	0.2283
NM_022224	Pter	0.9898	0.9893	0.8304	0.9620	0.1563	0.2493	0.9628	0.1328	0.7076	0.9696	0.7908	0.9155
NM_022536	Ppib	0.5767	0.6396	0.7110	0.9077	0.3635	0.2111	0.5519	0.5183	0.1485	0.4748	0.1986	0.0758
NM_022234	Accn4	0.8728	0.7700	0.6692	0.5603	0.5275	0.6183	0.5495	0.8710	0.8608	0.6119	0.3728	0.7348
NM_031598	Pla2g2a	0.0260	0.0209	0.7522	0.9345	0.9819	0.9704	0.1786	0.8379	0.0961	0.2215	0.0893	0.9242
NM_022230	Stc2	0.0199	0.0480	0.0428	0.0147	0.4063	0.0552	0.0546	0.1273	0.1372	0.2017	0.5288	0.3992
NM_022865	Gphn	0.9211	0.9137	0.9350	0.9178	0.3606	0.2757	0.7709	0.2922	0.6392	0.9838	0.8038	0.8900
NM_022924	F2	0.4008	0.0760	0.2501	0.1342	0.3228	0.1552	0.3152	0.0096	0.0876	0.0576	0.0764	0.0140
NM_022227	Accn5	0.6966	0.1185	0.8717	0.5597	0.4826	0.2093	0.2239	0.4151	0.2546	0.4470	0.1471	0.2315
NM_020079	Prl8a3	0.0359	0.1153	0.0281	0.3705	0.2046	0.2692	0.1420	0.0931	0.2764	0.2728	0.5021	0.1066

NM_021659	Syt7	0.1575	0.4459	0.1498	0.2374	0.7902	0.0824	0.2487	0.3236	0.5473	0.5454	0.0577	0.1256
NM_021661	Rgs19	0.4967	0.7225	0.0729	0.9697	0.7676	0.2344	0.3474	0.9107	0.5475	0.6031	0.4726	0.1653
NM_021663	Nucb2	0.6879	0.8376	0.5480	0.5200	0.2016	0.0150	0.7219	0.1970	0.8220	0.8801	0.8552	0.0498
NM_022598	Cnbp	0.5248	0.9736	0.4544	0.5360	0.7747	0.8190	0.6607	0.4455	0.9944	0.9108	0.9591	0.8405
NM_022597	Ctsb	0.0959	0.1362	0.4506	0.2563	0.2156	0.2764	0.0829	0.2013	0.0851	0.0051	0.0053	0.8224
NM_022797	Grin2d	0.7343	0.4189	0.7880	0.3548	0.7055	0.2468	0.4993	0.8271	0.6965	0.6625	0.3084	0.5508
NM_021671	Tmem33	0.6122	0.6732	0.9130	0.8593	0.6695	0.3082	0.5347	0.5539	0.6685	0.7249	0.8604	0.2774
NM_021852	Epn2	0.9682	0.9820	0.9962	0.7430	0.9552	0.9680	0.8495	0.8760	0.9496	0.9980	0.9750	0.9887
NM_021676	Shank3	0.6464	0.7088	0.6037	0.5646	0.6928	0.7063	0.6284	0.6648	0.7178	0.5283	0.6508	0.7242
NM_021682	Negr1	0.6252	0.6736	0.4982	0.6023	0.5228	0.3212	0.5348	0.1982	0.4920	0.6109	0.5413	0.6799
NM_021681	Epb4.1l1	0.6922	0.3369	0.8318	0.6706	0.6569	0.6358	0.9396	0.6912	0.7079	0.6692	0.4108	0.6131
NM_022799	Nucks1	0.9558	0.9755	0.3753	0.9827	0.8580	0.9405	0.8265	0.7901	0.9568	0.9737	0.9093	0.1075
NM_022798	Trim17	0.7869	0.7620	0.1373	0.6142	0.6066	0.5762	0.7745	0.8169	0.6516	0.3995	0.7354	0.2856
NM_021679	Nxph3	0.2907	0.1722	0.3470	0.1875	0.3886	0.4600	0.3470	0.3400	0.4463	0.4366	0.1737	0.1776
NM_021680	Nxph4	0.2932	0.2002	0.4903	0.6091	0.5093	0.6788	0.4502	0.2511	0.8769	0.6612	0.8979	0.3475
NM_022301	Pi4ka	0.2374	0.1456	0.7966	0.2587	0.6435	0.4640	0.8034	0.7420	0.5903	0.5693	0.7385	0.5786
NM_021909	Fxyd5	0.0899	0.3858	0.0170	0.6905	0.1236	0.0826	0.0632	0.0762	0.3893	0.0982	0.0603	0.8114
NM_030850	Bhmt	0.9853	0.8886	0.0249	0.0919	0.5678	0.6766	0.3556	0.8532	0.8431	0.3277	0.8923	0.8102
NM_022922	Tpi1	0.1211	0.0889	0.2677	0.4534	0.2780	0.2546	0.9365	0.1082	0.1011	0.0144	0.1740	0.6440
NM_022508	Mthfd1	0.7803	0.7938	0.0187	0.9265	0.8651	0.7487	0.8384	0.9191	0.7681	0.7923	0.8297	0.4701
NM_024001	Mrs2	0.6468	0.9496	0.0624	0.0233	0.7574	0.6870	0.7702	0.6688	0.7436	0.9452	0.7581	0.7012
NM_022952	Ap2s1	0.5597	0.1162	0.3203	0.7819	0.5246	0.6780	0.2462	0.4039	0.4773	0.2453	0.1761	0.2922
NM_022512	Acads	0.1112	0.4164	0.9475	0.2324	0.0102	0.2210	0.5080	0.1186	0.5224	0.0599	0.3102	0.2915
NM_001108376	RGD1308430	0.1319	0.1831	0.1292	0.4876	0.0770	0.1918	0.1381	0.0306	0.3332	0.1211	0.0272	0.0213
NM_022238	Abcb9	0.8054	0.2356	0.8056	0.3144	0.5961	0.6629	0.3392	0.8082	0.6323	0.6391	0.5119	0.3656
NM_024354	Chrna4	0.3993	0.3301	0.3057	0.3275	0.5164	0.0966	0.3114	0.2648	0.6404	0.3000	0.2900	0.1519
NM_024351	Hspa8	0.6558	0.7252	0.2291	0.7638	0.5786	0.4232	0.4972	0.5317	0.6535	0.7420	0.6528	0.4521
NM_022956	Barhl2	0.5770	0.5416	0.2369	0.3487	0.5781	0.2971	0.3600	0.3000	0.7068	0.6346	0.2642	0.8035
NM_021580	Prl8a4	0.5596	0.2316	0.5160	0.1727	0.6044	0.6943	0.5399	0.3769	0.7566	0.7880	0.8347	0.0122
NM_022300	Basp1	0.7691	0.7323	0.7718	0.8459	0.1058	0.0422	0.8489	0.1515	0.7969	0.8273	0.8498	0.6415
NM_022284	Guca2b	0.0302	0.0729	0.0445	0.1435	0.0358	0.1511	0.7565	0.2073	0.2851	0.5185	0.1374	0.3596
NM_022247	Pdcl	0.9578	0.1998	0.9860	0.5119	0.7962	0.9711	0.4387	0.4371	0.8891	0.7328	0.8467	0.9215
NM_022522	Casp2	0.9666	0.9725	0.9435	0.7491	0.9834	0.9500	0.9485	0.9727	0.9955	0.9855	0.9289	0.9878
NM_022252	Slc33a1	0.1040	0.0538	0.8170	0.0471	0.0806	0.1010	0.4709	0.1370	0.4377	0.4214	0.4599	0.7127
NM_022665	Alpi	0.4650	0.4544	0.5670	0.3371	0.3455	0.5365	0.5798	0.6820	0.5144	0.7616	0.0571	0.8809
NM_022249	Khdrbs3	0.1974	0.1187	0.1749	0.1459	0.2487	0.0293	0.0504	0.0125	0.0650	0.1418	0.1008	0.0906
NM_022256	Btc	0.6470	0.3962	0.5717	0.0187	0.0661	0.4531	0.3981	0.2686	0.5201	0.6281	0.6032	0.2573
NM_022254	Gpr85	0.9743	0.9807	0.9216	0.8692	0.9012	0.7370	0.9143	0.9456	0.6556	0.9644	0.9905	0.9711
NM_022257	Masp1	0.5876	0.5155	0.4957	0.6408	0.7064	0.6805	0.6490	0.5628	0.5477	0.6583	0.5964	0.6816
NM_022258	A1bg	0.5136	0.1626	0.0954	0.4342	0.3607	0.3726	0.2094	0.3577	0.2731	0.2909	0.2071	0.5366
NM_022261	Bspry	0.1193	0.4779	0.6870	0.4805	0.5592	0.5883	0.5646	0.4574	0.8287	0.7445	0.8960	0.7416
NM_022623	Fzd4	0.5427	0.5867	0.4105	0.8426	0.2508	0.4149	0.5915	0.4936	0.4056	0.7382	0.6676	0.6980
NM_022588	Mta1	0.9632	0.8184	0.9624	0.9939	0.7281	0.9577	0.9509	0.9181	0.8933	0.7391	0.8088	0.9934
NM_022267	Ccnd2	0.9377	0.4667	0.9082	0.6777	0.3226	0.0915	0.8572	0.6503	0.9166	0.5913	0.8784	0.9250
NM_022282	Dlg2	0.2730	0.2763	0.7250	0.5795	0.4860	0.4453	0.1901	0.1807	0.2378	0.7281	0.5928	0.1774
NM_023990	Six3	0.6986	0.2418	0.1312	0.3622	0.7328	0.5215	0.5315	0.7288	0.7461	0.4719	0.1402	0.5053
NM_001077671	Zc3h12a	0.7008	0.6324	0.1629	0.1201	0.5168	0.5048	0.1685	0.7504	0.8126	0.0420	0.0620	0.6849
NM_001077673	Rad18	0.6653	0.8449	0.3805	0.3080	0.5181	0.7714	0.7411	0.4049	0.9005	0.9209	0.8324	0.5289
NM_031005	Actn1	0.6557	0.2553	0.0637	0.0120	0.0477	0.7300	0.6761	0.1797	0.0401	0.0752	0.2212	0.9706

NM_001108232	Asb1	0.0617	0.3191	0.1355	0.2775	0.2538	0.0394	0.2829	0.3503	0.0877	0.0169	0.3009	0.2028
NM_001108189	Setd2	0.9750	0.7118	0.9181	0.7416	0.9450	0.9820	0.7478	0.9813	0.9460	0.9614	0.6853	0.9544
NM_001108135	Robo3	0.2597	0.2655	0.2815	0.4023	0.6444	0.3891	0.7791	0.3499	0.8481	0.4021	0.7591	0.4975
NM_001108133	Nfrkb	0.9846	0.9632	0.9246	0.9442	0.8128	0.9682	0.8063	0.7598	0.8812	0.6431	0.8969	0.7663
NM_001108339	Gatc	0.8864	0.8122	0.7505	0.3870	0.9042	0.8818	0.4469	0.9609	0.9462	0.6927	0.4792	0.9060
NM_001108129	RGD1309188	0.4085	0.1738	0.3153	0.1261	0.1436	0.4598	0.4441	0.0818	0.5177	0.2430	0.3150	0.3859
NM_022278	Glrx1	0.0191	0.0508	0.9034	0.9730	0.7992	0.9184	0.6802	0.6239	0.0505	0.1167	0.0511	0.4071
NM_021677	Psgb1	0.0290	0.3158	0.0868	0.0136	0.0410	0.1333	0.0950	0.4392	0.2917	0.1011	0.1698	0.1474
NM_022590	Slc5a2	0.3850	0.4538	0.7950	0.5003	0.5117	0.7204	0.6901	0.8769	0.1622	0.3683	0.3640	0.4280
NM_023950	Rab7a	0.3551	0.4828	0.9351	0.2373	0.4958	0.8496	0.2211	0.4293	0.7862	0.3763	0.3246	0.9889
NM_022285	Hapln2	0.7628	0.3530	0.4708	0.4188	0.0451	0.0802	0.2919	0.2368	0.1129	0.4557	0.7989	0.7784
NM_022711	Srd5a2	0.6333	0.5566	0.5078	0.1992	0.7311	0.5643	0.3540	0.2239	0.0770	0.5990	0.3417	0.2143
NM_022287	Slc26a1	0.7885	0.4546	0.7286	0.8798	0.7435	0.6852	0.6259	0.8350	0.7885	0.8726	0.9735	0.9699
NM_022289	Snx16	0.4677	0.5375	0.9383	0.9780	0.7426	0.2865	0.4084	0.4251	0.7997	0.4486	0.3679	0.4411
NM_024159	Dab2	0.2825	0.0042	0.1885	0.0942	0.1670	0.8379	0.1157	0.0407	0.1151	0.1692	0.2561	0.3278
NM_031017	Creb1	0.4578	0.2182	0.3199	0.3654	0.5852	0.3086	0.5895	0.9003	0.1689	0.2044	0.5244	0.3485
NM_024138	Gng7	0.8342	0.6710	0.8733	0.5226	0.8947	0.8428	0.8818	0.8630	0.6782	0.6151	0.7547	0.9159
NM_024136	Lcn5	0.1265	0.0779	0.6211	0.4532	0.4625	0.1754	0.3243	0.1035	0.4172	0.2418	0.0275	0.8989
NM_022303	Card9	0.8671	0.8377	0.8375	0.8583	0.8167	0.8278	0.8631	0.9766	0.8692	0.8279	0.9072	0.7412
NM_022294	Eltd1	0.9239	0.5380	0.7568	0.5448	0.9205	0.8485	0.7410	0.5623	0.6887	0.9246	0.9718	0.7071
NM_022848	Atp1a4	0.3459	0.1226	0.0970	0.5103	0.0783	0.3088	0.0805	0.1849	0.0816	0.0685	0.1373	0.0810
NM_022381	Pcna	0.9224	0.8020	0.1355	0.8030	0.8932	0.8860	0.8640	0.8191	0.7404	0.9321	0.8900	0.7545
NM_022380	Stat5b	0.2418	0.0472	0.4164	0.7668	0.0076	0.0206	0.1214	0.1042	0.0531	0.0984	0.2559	0.0367
NM_022690	Ube2g1	0.7191	0.2869	0.8415	0.9766	0.9434	0.9463	0.8992	0.8010	0.4773	0.8667	0.5094	0.8991
NM_001130568	Rrp7a	0.8247	0.8104	0.3944	0.3196	0.9414	0.4445	0.8505	0.7221	0.7239	0.5918	0.7704	0.4972
NM_022401	Plec1	0.3836	0.4311	0.4460	0.5550	0.5613	0.7166	0.6057	0.7131	0.6694	0.6836	0.6289	0.7135
NM_022383	Cap1	0.2007	0.1228	0.8882	0.4337	0.3375	0.9347	0.3663	0.0897	0.2910	0.1784	0.2762	0.9937
NM_022855	Csnk1g3	0.9195	0.9875	0.9982	0.9910	0.7415	0.6833	0.9937	0.5527	0.9648	0.9929	0.9958	0.9849
NM_022385	Arl1	0.7912	0.0423	0.7447	0.1894	0.1940	0.0278	0.4045	0.3007	0.5861	0.4869	0.2183	0.0329
NM_022701	Flot1	0.3948	0.2109	0.9288	0.8923	0.0577	0.0426	0.5170	0.1057	0.6316	0.6012	0.0740	0.9021
NM_030586	Cyb5b	0.7206	0.6193	0.4109	0.6859	0.5675	0.5036	0.6477	0.2223	0.6745	0.6678	0.2009	0.6421
NM_022676	Ppp1r1a	0.2513	0.2033	0.5866	0.1689	0.3756	0.4757	0.5482	0.4107	0.8221	0.4287	0.3950	0.2354
NM_022684	Bid	0.0112	0.0322	0.9544	0.7863	0.1050	0.3550	0.1245	0.0934	0.0386	0.0467	0.0357	0.1119
NM_022673	Mecp2	0.5805	0.7161	0.4356	0.0018	0.3305	0.0267	0.5571	0.0190	0.7196	0.7106	0.7122	0.6595
NM_022668	Mog	0.2345	0.8053	0.8317	0.8507	0.8162	0.4198	0.9146	0.5434	0.6488	0.9653	0.8663	0.6619
NM_022392	Insig1	0.9987	0.9729	0.3654	0.2820	0.9981	0.9644	0.9802	0.7771	0.9927	0.9825	0.9947	0.8426
NM_022391	Pttg1	0.5331	0.4177	0.4294	0.4425	0.2850	0.1804	0.1899	0.3545	0.3597	0.4394	0.4667	0.2941
NM_023970	Trpv4	0.2022	0.6567	0.5250	0.4338	0.1077	0.1959	0.8782	0.2318	0.4386	0.4331	0.6004	0.6960
NM_022396	Gng11	0.1467	0.1479	0.9664	0.9630	0.2592	0.2665	0.8373	0.1652	0.2440	0.5721	0.3823	0.2992
NM_022394	Safb	0.9951	0.7040	0.1660	0.2001	0.9057	0.7920	0.1347	0.8983	0.6964	0.7023	0.7785	0.4348
NM_022393	Mgl1	0.0746	0.0544	0.4547	0.3741	0.0848	0.1508	0.0784	0.2482	0.1243	0.0396	0.0271	0.0984
NM_024162	Fabp3	0.0063	0.0326	0.0823	0.0755	0.0056	0.0214	0.0242	0.0319	0.0935	0.0499	0.0402	0.0366
NM_022501	Crip2	0.7132	0.5940	0.7971	0.9583	0.5032	0.8044	0.7896	0.8665	0.8544	0.8139	0.8911	0.6918
NM_022503	Cox7a2	0.3085	0.3601	0.2769	0.4229	0.5714	0.1803	0.6972	0.3495	0.2956	0.3387	0.3944	0.2645
NM_022504	Rpl36	0.2764	0.4404	0.3554	0.2112	0.0464	0.0017	0.7099	0.2525	0.1771	0.3483	0.0401	0.3326
NM_030852	Mia	0.6141	0.4786	0.1220	0.1119	0.2867	0.1471	0.6975	0.4820	0.5446	0.5066	0.6248	0.0159
NM_022506	Rpl31	0.3369	0.6804	0.5069	0.1183	0.2657	0.1041	0.4282	0.2899	0.1229	0.2766	0.5909	0.2240
NM_030858	Smad7	0.9678	0.9845	0.9733	0.9969		0.9916	0.9755	0.9973	0.9797	0.9730	0.9903	0.8812
NM_024125	Cebpb	0.1201	0.0053	0.0893	0.0453	0.0244	0.4118	0.1220	0.0755	0.0072	0.2026	0.3093	0.7674

NM_022587	Entpd1	0.0552	0.1582	0.2669	0.0951	0.2050	0.6641	0.2057	0.0307	0.6107	0.1840	0.5956	0.3108
NM_024157	Cfi	0.3162	0.6186	0.3258	0.2875	0.4948	0.3184	0.1845	0.6502	0.0992	0.1479	0.1762	0.4811
NM_024129	Dcn	0.9996	0.0176	0.9616	0.0129	0.9439	0.7736	0.0589	0.8072	0.1534	0.2442	0.0656	0.0251
NM_022510	Rpl4	0.5149	0.3476	0.5184	0.0369	0.4467	0.6013	0.4259	0.4847	0.3277	0.4567	0.2527	0.6383
NM_024377	Gng5	0.6293	0.5339	0.3746	0.5664	0.3931	0.3734	0.6573	0.5975	0.4431	0.5476	0.5116	0.2077
NM_022513	Sult1b1	0.4017	0.5550	0.3647	0.7150	0.5985	0.5492	0.6088	0.7803	0.3960	0.4655	0.0213	0.1744
NM_024364	Hr	0.7747	0.8628	0.0980	0.5593	0.4257	0.5460	0.4231	0.3837	0.8774	0.9056	0.6691	0.5246
NM_022514	Rpl27	0.4837	0.4549	0.4353	0.2710	0.0971	0.1068	0.4416	0.1491	0.4091	0.3662	0.6654	0.2757
NM_022516	Ptbp1	0.9494	0.9769	0.1857	0.9544	0.9990	0.9031	0.9962	0.9988	0.9986	0.7607	0.9894	0.9228
NM_022515	Rpl24	0.0489	0.7192	0.6786	0.1846	0.0265	0.0018	0.7145	0.0206	0.0231	0.5502	0.3239	0.0043
NM_030990	Plp1	0.9670	0.9369	0.8457	0.8038	0.9217	0.7981	0.8205	0.6015	0.9432	0.9784	0.9046	0.9757
NM_024373	Galnt1	0.1760	0.1714	0.3948	0.1868	0.2687	0.3043	0.5166	0.1806	0.4766	0.3291	0.4353	0.1771
NM_022518	Arf1	0.6123	0.3331	0.6587	0.3957	0.3808	0.0341	0.4823	0.3731	0.2077	0.4104	0.6458	0.4155
NM_024384	Thoc6	0.7799	0.7154	0.4997	0.9225	0.7768	0.7777	0.9233	0.8928	0.8232	0.7230	0.8723	0.8318
NM_022548	Zmat3	0.5504	0.7236	0.5362	0.6496	0.7195	0.5179	0.6703	0.6375	0.6574	0.1878	0.4438	0.6387
NM_022584	Txnrd2	0.7721	0.8782	0.2233	0.0169	0.8092	0.9773	0.2767	0.8445	0.8728	0.6444	0.9089	0.1911
NM_022524	Srpx	0.8917	0.8591	0.8585	0.8634	0.8467	0.8559	0.9086	0.8598	0.5677	0.8640	0.8840	0.3064
NM_022526	Dap	0.1301	0.3309	0.6182	0.9524	0.1268	0.1650	0.1380	0.3060	0.3136	0.4956	0.1333	0.5780
NM_022530	Prl7a3	0.2943	0.3211	0.3897	0.4906	0.1425	0.0153	0.2441	0.3243	0.1182	0.3924	0.1580	0.2798
NM_022528	Hif3a	0.1288	0.0655	0.0264	0.0484	0.5300	0.0841	0.1177	0.3243	0.6676	0.3423	0.0700	0.4442
NM_022529	Mrpl23	0.4597	0.8049	0.7521	0.7417	0.7113	0.0911	0.6754	0.8525	0.7149	0.8467	0.6377	0.0226
NM_022531	Des	0.0105	0.8809	0.9929	0.9405	0.9328	0.6628	0.9872	0.7889	0.9649	0.7691	0.7888	0.9500
NM_022860	B4galnt1	0.8467	0.1019	0.1222	0.0107	0.8265	0.7448	0.1492	0.7495	0.1295	0.0784	0.0028	0.2544
NM_022535	Calu	0.7254	0.7912	0.9966	0.8714	0.9853	0.9966	0.9904	0.9843	0.6645	0.7510	0.9369	0.9932
NM_022534	Tcn2	0.7184	0.0259	0.9409	0.0417	0.1948	0.2345	0.2061	0.5301	0.1007	0.4273	0.0347	0.2969
NM_182843	Acbd3	0.9706	0.9078	0.7909	0.3675	0.9624	0.9720	0.4026	0.9936	0.8359	0.9016	0.9497	0.8422
NM_207586	Chia	0.4571	0.4069	0.3287	0.5178	0.3680	0.1799	0.1894	0.1805	0.1706	0.1646	0.4661	0.4603
NM_182825	Rhebl1	0.6959	0.9070	0.0915	0.2395	0.4363	0.4872	0.0966	0.9201	0.9309	0.8201	0.7266	0.7598
NM_182824	Cd276	0.2385	0.4201	0.9079	0.9676	0.4034	0.4695	0.9155	0.6665	0.6218	0.5245	0.3402	0.7957
NM_182821	Pxk	0.9723	0.3691	0.7819	0.2630	0.9275	0.9741	0.6948	0.6754	0.4774	0.8224	0.6455	0.6284
NM_182820	Lyk5	0.1455	0.4329	0.5959	0.2522	0.7862	0.4140	0.7064	0.9450	0.9332	0.4602	0.3367	0.8635
NM_182818	Smptb	0.5239	0.4067	0.0307	0.3346	0.2612	0.1954	0.4362	0.5022	0.5338	0.1606	0.1312	0.2320
NM_182816	Amigo2	0.3928	0.8598	0.8089	0.9582	0.8601	0.6471	0.7557	0.6306	0.6296	0.8063	0.8761	0.8213
NM_182815	Zp3r	0.1542	0.0481	0.2197	0.4897	0.1348	0.3115	0.3931	0.1138	0.1385	0.1374	0.2000	0.0254
NM_212507	Ltb	0.4851	0.0792	0.7747	0.5520	0.4655	0.1806	0.3887	0.1600	0.2274	0.6303	0.5613	0.6776
NM_212506	Pfdn6	0.2935	0.1710	0.3371	0.1865	0.4276	0.3985	0.5033	0.4149	0.1705	0.3420	0.5180	0.3990
NM_182738	Chp2	0.1538	0.1933	0.3157	0.1450	0.2285	0.2171	0.1814	0.1692	0.3448	0.1713	0.2425	0.0667
NM_182950	Tnfaip1	0.7042	0.1172	0.9071	0.8024	0.2755	0.9442	0.4145	0.3127	0.3917	0.6968	0.7968	0.9755
NM_213557	Rps18	0.2980	0.1259	0.5455	0.1886	0.1264	0.1504	0.2654	0.4953	0.3006	0.0969	0.5767	0.0919
NM_182670	RSA-14-44	0.4942	0.7873	0.4127	0.6829	0.7333	0.3653	0.1408	0.4258	0.7645	0.5494	0.7665	0.1197
NM_182668	Nit1	0.2531	0.5182	0.3091	0.3879	0.6050	0.8272	0.3629	0.3897	0.2248	0.5944	0.7041	0.7202
NM_182473	Corin	0.4832	0.4665	0.6681	0.6926	0.5578	0.5697	0.1170	0.5198	0.3260	0.3978	0.5038	0.3735
NM_181822	Ncr3	0.1531	0.2930	0.0543	0.3141	0.4861	0.2126	0.3680	0.1852	0.1021	0.0764	0.3208	0.0740
NM_199501	Cdk2	0.5222	0.6186	0.3723	0.6844	0.5783	0.6196	0.6402	0.4466	0.5290	0.1876	0.6793	0.4864
NM_198751	Eif3h	0.6963	0.0668	0.8531	0.1264	0.3674	0.1889	0.6983	0.8318	0.0282	0.0320	0.1402	0.1345
NM_198684	Ankrd36	0.4146	0.0348	0.3970	0.1220	0.2484	0.4779	0.5567	0.1235	0.3755	0.2037	0.4568	0.2024
NM_198051	Trmt11	0.4574	0.6596	0.7120	0.0071	0.1347	0.4176	0.0500	0.1456	0.6312	0.5493	0.8288	0.7202
NM_181687	Nnat	0.6713	0.6587	0.7219	0.6313	0.5817	0.5505	0.7838	0.4622	0.6909	0.8065	0.7815	0.7816
NM_181639	Slc29a3	0.9938	0.8530	0.4912	0.9562	0.8754	0.8557	0.6098	0.8190	0.8648	0.7662	0.9462	0.3729

NM_181637	Cdc91l1	0.4275	0.3800	0.9204	0.9035	0.4272	0.7623	0.7681	0.8753	0.2750	0.1740	0.1762	0.2010
NM_022537	Prl8a7	0.4779	0.0742	0.4420	0.4838	0.4447	0.5200	0.6710	0.1386	0.6778	0.5401	0.1232	0.5133
NM_022544	Defb4	0.0666	0.3127	0.2269	0.2061	0.1992	0.2178	0.0837	0.0645	0.3299	0.2202	0.2433	0.1476
NM_022542	Rhob	0.9908	0.9551	0.9531	0.7906	0.9043	0.9434	0.9991	0.9660	0.9107	0.8907	0.9909	0.9966
NM_022541	Timm8b	0.4912	0.7060	0.4376	0.2519	0.1032	0.2013	0.2425	0.4054	0.0538	0.2346	0.0671	0.1713
NM_022925	Ptprq	0.3610	0.2448	0.4217	0.3262	0.3857	0.3552	0.3006	0.1802	0.5215	0.3935	0.5137	0.3416
NM_139183	Crhbp	0.7135	0.8898	0.8954	0.9516	0.3094	0.5405	0.6541	0.5265	0.8625	0.9946	0.5426	0.7624
NM_031024	Dbn1	0.1924	0.4371	0.6942	0.9314	0.1356	0.2944	0.8911	0.2300	0.4745	0.0762	0.4639	0.2795
NM_031064	Ndufv2	0.5867	0.0755	0.6466	0.6853	0.1796	0.1107	0.4727	0.0953	0.0271	0.1023	0.0345	0.0107
NM_022583	Insl6	0.1473	0.0758	0.0671	0.0636	0.4458	0.4807	0.2915	0.8031	0.5206	0.2950	0.5558	0.0678
NM_024145	Fgr	0.0949	0.1121	0.3531	0.2269	0.1274	0.0559	0.2966	0.1014	0.2205	0.0591	0.0205	0.2427
NM_022713	Tgm4	0.7250	0.2223	0.4267	0.8682	0.7367	0.2941	0.5308	0.6535	0.4982	0.1603	0.4611	0.6042
NM_022585	Azin1	0.7199	0.3724	0.2628	0.9875	0.9943	0.9169	0.5286	0.9046	0.3615	0.2622	0.3414	0.3591
NM_031141	Pax8	0.5535	0.6041	0.1642	0.8110	0.7840	0.2646	0.3189	0.4822	0.8350	0.8041	0.9420	0.5799
NM_024394	Htr3a	0.3691	0.1234	0.3285	0.1381	0.3779	0.0635	0.0952	0.6290	0.2586	0.1135	0.1242	0.0656
NM_022867	Map1lc3b	0.5091	0.1530	0.4777	0.4083	0.8568	0.9893	0.3212	0.4996	0.7934	0.2714	0.3188	0.9763
NM_022861	Unc13a	0.3988	0.0842	0.6134	0.5732	0.3827	0.4647	0.4948	0.2075	0.4977	0.1539	0.1652	0.6228
NM_022589	Tspan2	0.2224	0.2802	0.2270	0.2664	0.1152	0.2265	0.2683	0.0904	0.2279	0.0020	0.2026	0.0673
NM_022591	Tep1	0.8947	0.8722	0.8650	0.7012	0.8634	0.8439	0.8914	0.9028	0.8071	0.7405	0.9118	0.7439
NM_022593	Tceb1	0.0864	0.4524	0.0573	0.0182	0.1128	0.0335	0.1548	0.0595	0.0075	0.3079	0.5326	0.0041
NM_022603	Fgfbp1	0.6556	0.0722	0.5795	0.2119	0.4946	0.4074	0.0659	0.4265	0.2111	0.3121	0.2976	0.0832
NM_022242	Niban	0.0442	0.0573	0.6372	0.2551	0.1184	0.4096	0.2303	0.1268	0.0588	0.1389	0.3792	0.3312
NM_022244	Arhgap17	0.1326	0.0486	0.3000	0.6223	0.0813	0.9341	0.3010	0.1905	0.0079	0.2417	0.1774	0.4524
NM_022594	Ech1	0.2775	0.6961	0.8797	0.8506	0.7792	0.8105	0.6727	0.8610	0.2341	0.7531	0.6653	0.1443
NM_022595	Pdap1	0.0928	0.5240	0.4359	0.1352	0.0865	0.1975	0.1244	0.2905	0.5015	0.9072	0.1298	0.2451
NM_022800	P2ry12	0.5265	0.1496	0.1311	0.3607	0.3621	0.1064	0.3821	0.4895	0.4070	0.7110	0.1375	0.2926
NM_022604	Esm1	0.3235	0.5171	0.4094	0.0844	0.2044	0.4302	0.3585	0.0417	0.2054	0.3644	0.3577	0.1861
NM_024141	Duox2	0.9755	0.9928	0.9209	0.9839	0.9997	0.9977	0.9718	0.8826	0.7996	0.9401	0.7763	0.9662
NM_030833	Ifitm2	0.2914	0.4620	0.4687	0.1861	0.2492	0.3022	0.5011	0.3254	0.2719	0.3274	0.4439	0.6016
NM_023021	Kcnn4	0.7552	0.8734	0.9151	0.8029	0.9551	0.9548	0.8787	0.7886	0.9515	0.9720	0.8079	0.9857
NM_022607	Ndufv3l	0.5765	0.5346	0.8900	0.5970	0.1885	0.1922	0.6841	0.5415	0.4226	0.4036	0.3759	0.7807
NM_022599	Synj2bp	0.2819	0.4283	0.8511	0.8008	0.9249	0.8809	0.2288	0.1063	0.5417	0.6761	0.1984	0.5586
NM_031328	Bcl10	0.6552	0.6197	0.3672	0.6600	0.5053	0.6508	0.5887	0.4559	0.6021	0.6270	0.6980	0.3249
NM_030835	Serp1	0.9915	0.9828	0.9634	0.9449	0.9650	0.7699	0.9272	0.8704	0.9470	0.9660	0.9686	0.3179
NM_031037	Gnb2	0.4309	0.8537	0.6882	0.8719	0.2218	0.3056	0.8621	0.1132	0.9518	0.6346	0.3046	0.3473
NM_024486	Acvr1	0.8297	0.2057	0.8205	0.7526	0.6245	0.9001	0.7830	0.7088	0.6700	0.8068	0.5398	0.7926
NM_022614	Inhbc	0.4518	0.0469	0.1701	0.0823	0.1579	0.1359	0.1537	0.2087	0.3617	0.0602	0.0241	0.2413
NM_024148	Apex1	0.7128	0.8483	0.0347	0.8975	0.2219	0.6860	0.5522	0.6172	0.8039	0.4013	0.8895	0.7109
NM_022263	Rsd6	0.2804	0.3864	0.3538	0.4253	0.5380	0.5602	0.4397	0.1767	0.5329	0.4364	0.3033	0.3286
NM_024143	Slc27a5	0.2698	0.4262	0.5609	0.6065	0.3320	0.0644	0.4319	0.2347	0.3048	0.6554	0.4772	0.4833
NM_022616	41,159.0000	0.7117	0.6260	0.8974	0.7215	0.7090	0.7285	0.7167	0.7128	0.6930	0.5779	0.6224	0.4042
NM_022618	Akap6	0.8658	0.9560	0.8490	0.8609	0.9342	0.6971	0.9271	0.9650	0.9616	0.5580	0.9264	0.9380
NM_031029	Gabrp	0.6549	0.6747	0.5295	0.5255	0.4511	0.2245	0.5977	0.6358	0.3965	0.5707	0.7484	0.0860
NM_031026	Dync1li2	0.3509	0.3660	0.3696	0.9929	0.5652	0.4011	0.0653	0.3662	0.3942	0.3849	0.3565	0.7224
NM_032612	Stat1	0.7508	0.7091	0.7007	0.6717	0.6358	0.5474	0.5955	0.5909	0.6813	0.5798	0.6060	0.5438
NM_031136	Tmsb4x	0.2189	0.4942	0.2367	0.6918	0.2309	0.6272	0.6189	0.5055	0.2031	0.2592	0.2726	0.7195
NM_031006	Adar	0.2641	0.5075	0.0726	0.3398	0.1014	0.1707	0.4418	0.1508	0.7033	0.2536	0.5231	0.6617
NM_022622	Bard1	0.7028	0.8373	0.0178	0.6505	0.7376	0.6233	0.7577	0.8589	0.5752	0.8452	0.7515	0.0580
NM_022698	Bad	0.3209	0.8426	0.3796	0.7786	0.9722	0.4213	0.8018	0.9667	0.4444	0.6128	0.2434	0.2875

NM_024361	Ndst1	0.6038	0.1690	0.2831	0.6847	0.0929	0.5118	0.2696	0.2788	0.2857	0.1813	0.3395	0.1975
NM_022627	Prkab2	0.3700	0.3239	0.4098	0.7719	0.2506	0.1544	0.2511	0.6695	0.8391	0.3515	0.3700	0.8279
NM_031044	Hnmt	0.3416	0.0534	0.3739	0.7957	0.6030	0.4984	0.1043	0.4800	0.6684	0.1949	0.0507	0.4158
NM_023989	Senp2	0.0035	0.0050	0.6831	0.0364	0.0922	0.7454	0.2789	0.0708	0.3525	0.0683	0.0480	0.6903
NM_022630	Tmprss11d	0.2652	0.5004	0.4242	0.7253	0.3170	0.3070	0.4111	0.9026	0.8928	0.4065	0.5689	0.2831
NM_031330	Hnrnpab	0.9141	0.9528	0.9619	0.9587	0.9243	0.7924	0.7612	0.9755	0.9013	0.9131	0.9943	0.9539
NM_022635	Cml4	0.4924	0.3741	0.8162	0.5520	0.6063	0.4797	0.7112	0.4647	0.4334	0.5375	0.5855	0.1958
NM_024386	Hmgcl	0.2018	0.2079	0.8223	0.0234	0.1913	0.2047	0.3896	0.5037	0.1338	0.0675	0.3622	0.1475
NM_031023	Ctbs	0.0574	0.1061	0.0525	0.0211	0.0968	0.0931	0.0761	0.0103	0.0825	0.0033	0.2632	0.2075
NM_030991	Snap25	0.9478	0.8969	0.8377	0.6420	0.9453	0.6146	0.5390	0.8088	0.7438	0.9385	0.8238	0.8704
NM_031068	Actr3	0.2304	0.2624	0.4955	0.1741	0.7236	0.7495	0.2176	0.4621	0.1373	0.1149	0.2184	0.0504
NM_030988	Tg	0.8849	0.8200	0.8386	0.7332	0.7853	0.9257	0.7425	0.7251	0.8278	0.8322	0.9246	0.9960
NM_022643	Hist1h2ba	0.1758	0.1709	0.1821	0.1856	0.1801	0.1795	0.1890	0.1799	0.3313	0.4968	0.1730	0.4020
NM_022666	Grm4	0.9143	0.9304	0.9886	0.6919	0.9196	0.9409	0.6415	0.9443	0.9256	0.7496	0.7075	0.9385
NM_022667	Slco2a1	0.1982	0.2494	0.2978	0.3495	0.7224	0.4638	0.6510	0.7538	0.7857	0.3623	0.1658	0.3028
NM_023955	Scamp2	0.3086	0.2641	0.4675	0.9828	0.8590	0.9709	0.3883	0.8117	0.5652	0.1317	0.2859	0.9387
NM_024134	Ddit3	0.3942	0.9500	0.5607	0.1854	0.4323	0.3427	0.5441	0.5147	0.9985	0.9605	0.8893	0.5135
NM_023101	Vti1a	0.0834	0.3558	0.6860	0.8110	0.0429	0.0567	0.7587	0.2458	0.0119	0.0710	0.0934	0.2660
NM_024158	Dck	0.0952	0.5918	0.5793	0.1373	0.6479	0.6481	0.6222	0.5338	0.6931	0.2116	0.8889	0.5214
NM_022944	Inppl1	0.7416	0.9168	0.0482	0.2490	0.4087	0.0249	0.3642	0.8674	0.8282	0.7433	0.8849	0.3244
NM_022853	Slc30a1	0.5176	0.6859	0.5384	0.6507	0.1782	0.4305	0.0926	0.5280	0.0083	0.7731	0.6538	0.5020
NM_022847	Pgr	0.2495	0.1819	0.1807	0.0732	0.1318	0.0259	0.0024	0.0335	0.0565	0.1065	0.0715	0.1557
NM_024485	Chrna1	0.4151	0.8051	0.0516	0.6192	0.5792	0.1705	0.1715	0.5912	0.5286	0.4742	0.2787	0.2653
NM_022670	Sct	0.7960	0.7970	0.6223	0.2497	0.5206	0.2501	0.6794	0.3612	0.7846	0.5208	0.4161	0.5378
NM_001108148	Rab39	0.8537	0.6301	0.2399	0.7795	0.2953	0.3674	0.5062	0.8900	0.7565	0.7815	0.6720	0.7086
NM_001108066	Arid3a	0.7525	0.7270	0.7982	0.2603	0.7372	0.6725	0.5547	0.7130	0.7909	0.5965	0.7455	0.3759
NM_001107966	Jmjd2a	0.9814	0.8565	0.8790	0.8955	0.8825	0.9075	0.9806	0.9020	0.9012	0.9527	0.9814	0.9708
NM_001108145	RGD1562252	0.1735	0.4766	0.3483	0.7701	0.5314	0.2785	0.3152	0.3782	0.0890	0.0961	0.6186	0.3121
NM_031832	Lgals3	0.3022	0.1574	0.0210	0.7255	0.7140	0.7851	0.1469	0.6724	0.1443	0.2108	0.2668	0.8140
NM_022700	Arl3	0.1210	0.8511	0.8701	0.0877	0.6941	0.6169	0.7284	0.6173	0.2611	0.0341	0.1387	0.3453
NM_022687	Hes3	0.2827	0.2916	0.3531	0.2850	0.5393	0.7002	0.8227	0.6680	0.1940	0.6632	0.7156	0.1568
NM_022686	Hist1h4b	0.2157	0.4509	0.4104	0.4022	0.6488	0.4800	0.6129	0.4565	0.3925	0.7170	0.3627	0.3569
NM_022691	Exoc7	0.1468	0.0162	0.3266	0.4311	0.0880	0.0741	0.8459	0.1846	0.0914	0.1105	0.2989	0.1445
NM_022936	Ephx2	0.1112	0.5765	0.8467	0.7495	0.0556	0.1664	0.8211	0.0658	0.1395	0.4981	0.6657	0.1426
NM_022703	Sgta	0.8360	0.8535	0.7414	0.9922	0.9296	0.9244	0.7499	0.9064	0.8758	0.8691	0.8284	0.9766
NM_053530	Twist1	0.1622	0.0292	0.6789	0.6462	0.6846	0.7726	0.4778	0.5684	0.2359	0.6349	0.7389	0.6489
NM_032617	Rab11b	0.7852	0.0895	0.0519	0.4587	0.8050	0.2067	0.5430	0.7939	0.2318	0.3324	0.0789	0.1607
NM_022704	Mbl2	0.2042	0.2051	0.0187	0.4776	0.0475	0.0919	0.1326	0.3774	0.3394	0.4022	0.5830	0.6731
NM_022706	Gabarapl2	0.3051	0.2599	0.7767	0.1587	0.2304	0.3373	0.2118	0.1579	0.1418	0.2843	0.3169	0.2984
NM_024353	Plcb4	0.7026	0.7146	0.6834	0.6701	0.6829	0.7050	0.6407	0.6819	0.7035	0.7257	0.6715	0.5639
NM_022707	Pln	0.9477	0.0710	0.9257	0.0614	0.7866	0.9569	0.9057	0.8238	0.1978	0.1145	0.0089	0.9413
NM_022697	Rpl28	0.5729	0.2043	0.5910	0.2661	0.2312	0.3477	0.2359	0.3087	0.7135	0.5022	0.6184	0.2934
NM_024362	Arntl	0.9095	0.8791	0.8216	0.4620	0.6350	0.5517	0.4855	0.7376	0.6615	0.8970	0.9191	0.7990
NM_001008338	Lrrc8d	0.9794	0.8948	0.9626	0.9403	0.8547	0.9855	0.8604	0.7396	0.8621	0.7638	0.8800	0.7603
NM_022699	Rpl30	0.8001	0.0896	0.0216	0.0060	0.8021	0.6407	0.2277	0.6598	0.1380	0.2090	0.1400	0.0578
NM_022402	Arbp	0.6784	0.2401	0.2112	0.1421	0.7889	0.1808	0.0150	0.3549	0.5754	0.3872	0.7391	0.2294
NM_031755	Ceacam1	0.8506	0.6352	0.9150	0.7145	0.6614	0.8986	0.9526	0.9740	0.8316	0.8947	0.8951	0.8638
NM_022404	Gbl	0.9516	0.9554	0.8424	0.9671	0.9554	0.9923	0.9598	0.9867	0.9537	0.8361	0.7284	0.9771
NM_023090	Epas1	0.4187	0.4119	0.7483	0.3992	0.5395	0.4312	0.6278	0.5317	0.6525	0.5831	0.6020	0.3326

NM_022708	Pip	0.4458	0.0659	0.1957	0.4937	0.3394	0.5204	0.8053	0.2023	0.8972	0.7213	0.5551	0.2744
NM_001113335	Slc9a2	0.7634	0.6191	0.8769	0.2473	0.6244	0.2779	0.6790	0.6652	0.7114	0.7379	0.6363	0.8012
NM_019232	Sgk1	0.9977	0.9659	0.9989	0.9781	0.9934	0.9998	0.9985	0.9952	0.8739	0.9980	0.9949	0.9908
NM_001029897	Strn3	0.5339	0.5790	0.9388	0.5392	0.8379	0.8489	0.3643	0.7365	0.2446	0.3497	0.4758	0.5376
NM_001015032	Galnt3	0.9685	0.9961	0.9414	0.7910	0.9965	0.8748	0.9611	0.9926	0.9228	0.9930	0.9907	0.9940
NM_001111057	Adarb1	0.2532	0.9282	0.7303	0.2920	0.4561	0.6341	0.6847	0.5770	0.6461	0.9299	0.3428	0.7781
NM_001037780	Itgb2	0.3617	0.7762	0.3396	0.8627	0.6791	0.7605	0.7703	0.4836	0.7496	0.6877	0.6960	0.8777
NM_012773	Akap11	0.8469	0.7797	0.7619	0.7980	0.2712	0.7330	0.4138	0.8698	0.5771	0.3993	0.1267	0.9366
NM_001110055	RGD1564664	0.8959	0.0028	0.3352	0.1632	0.0397	0.1323	0.3153	0.1290	0.2669	0.2145	0.1631	0.2303
NM_001112716	Grik3	0.2156	0.2057	0.4840	0.4138	0.5136	0.5865	0.4360	0.6643	0.6079	0.3943	0.2201	0.4828
NM_001111117	Grik1	0.9337	0.7699	0.8844	0.6345	0.6973	0.6754	0.7080	0.5252	0.4387	0.8605	0.7533	0.5539
NM_022715	Mvp	0.0194	0.0056	0.8879	0.4653	0.0138	0.0245	0.0689	0.0386	0.1201	0.3921	0.1155	0.8904
NM_023963	Cdx2	0.9660	0.9751	0.8309	0.9765	0.7939	0.9960	0.3513	0.8100	0.9242	0.9551	0.8002	0.9628
NM_023962	Pdgfd	0.2300	0.7640	0.2166	0.4161	0.2779	0.0408	0.1064	0.2416	0.0471	0.7234	0.5567	0.4101
NM_031089	Pth2r	0.5470	0.5215	0.2033	0.7371	0.6310	0.5563	0.8021	0.2815	0.4911	0.8275	0.4166	0.3333
NM_022846	Prl8a2	0.1120	0.4808	0.3699	0.3988	0.2213	0.4398	0.1696	0.2475	0.7007	0.1861	0.1817	0.6609
NM_024356	Gch1	0.0557	0.0876	0.1219	0.0490	0.0001	0.0482	0.0367	0.0009	0.0114	0.0120	0.0180	0.3314
NM_022850	Dpp6	0.2727	0.3276	0.0949	0.2287	0.1757	0.4795	0.3047	0.5866	0.1268	0.2070	0.3017	0.5457
NM_022958	Pik3c3	0.1534	0.0630	0.6535	0.2356	0.3124	0.6164	0.0274	0.0337	0.1204	0.0832	0.1020	0.7180
NM_022854	Fabp9	0.1019	0.0347	0.1280	0.1688	0.0257	0.4793	0.0100	0.0438	0.0631	0.2363	0.3564	0.0145
NM_001047901	Ankle2	0.6413	0.1438	0.6756	0.6337	0.6393	0.7176	0.0740	0.5422	0.3870	0.3559	0.5951	0.6803
NM_022857	N5	0.8271	0.8416	0.7798	0.5880	0.9529	0.6724	0.4349	0.4553	0.5098	0.3202	0.9081	0.2685
NM_023092	Myo1c	0.7039	0.8786	0.9884	0.7844	0.9660	0.9754	0.7667	0.7572	0.7940	0.9496	0.9474	0.9853
NM_023964	Gapdhs	0.0391	0.5063	0.5150	0.4267	0.2673	0.6625	0.6045	0.3736	0.4781	0.4917	0.1269	0.2555
NM_022858	Foxq1	0.3973	0.3207	0.3251	0.5244	0.2914	0.6017	0.4487	0.3331	0.4027	0.4705	0.4114	0.5312
NM_022859	Crisp1	0.2969	0.2487	0.2150	0.6630	0.2895	0.2571	0.2457	0.2390	0.5399	0.2119	0.2689	0.3639
NM_030854	Lect1	0.0338	0.4207	0.1399	0.3299	0.2327	0.3105	0.0508	0.1013	0.0679	0.1429	0.2471	0.0804
NM_024359	Hif1a	0.2115	0.2558	0.0676	0.2718	0.1815	0.2388	0.1317	0.2975	0.2677	0.2498	0.2851	0.8153
NM_022862	Unc13b	0.5390	0.6263	0.6008	0.4997	0.4780	0.5902	0.6404	0.6910	0.2414	0.6955	0.7752	0.5693
NM_022866	Slc13a3	0.5747	0.6689	0.6460	0.6378	0.4260	0.4544	0.8010	0.5604	0.5449	0.6263	0.3195	0.8508
NM_031010	Alox15	0.6584	0.5795	0.3885	0.5554	0.6795	0.7579	0.2454	0.6007	0.8158	0.7964	0.7908	0.8676
NM_030656	Agxt	0.5905	0.3827	0.1319	0.3985	0.4497	0.4334	0.5333	0.2515	0.3685	0.0817	0.4875	0.4289
NM_022864	Cplx1	0.7743	0.8089	0.2017	0.8015	0.8213	0.6299	0.6392	0.8101	0.8844	0.9351	0.8352	0.7787
NM_024160	Cyba	0.2521	0.0925	0.0953	0.0947	0.1310	0.0526	0.0217	0.3520	0.1183	0.1595	0.2286	0.3374
NM_022920	Grm6	0.5760	0.4102	0.7276	0.5110	0.3517	0.6866	0.7315	0.2250	0.3081	0.7354	0.2836	0.4690
NM_031336	Kl	0.3806	0.8951	0.5652	0.7178	0.1196	0.6632	0.8254	0.3750	0.8028	0.6797	0.6162	0.6260
NM_031315	Acot1	0.7396	0.2661	0.6736	0.5306	0.3066	0.5939	0.3432	0.7636	0.5872	0.6124	0.6899	0.5525
NM_031059	Msx1	0.4778	0.5243	0.5546	0.6695	0.1875	0.5764	0.8045	0.3408	0.8118	0.8794	0.7342	0.7470
NM_030845	Cxcl1	0.8989	0.9576	0.9580	0.9606	0.9273	0.8914	0.9941	0.9016	0.7608	0.9998	0.9882	0.8767
NM_031151	Mdh2	0.0606	0.7971	0.0533	0.8493	0.4524	0.3597	0.0871	0.2372	0.3902	0.0347	0.5766	0.2550
NM_031012	Anpep	0.1225	0.0067	0.1392	0.0419	0.1156	0.0429	0.1110	0.3643	0.0335	0.1029	0.0097	0.7911
NM_023023	Dpysl5	0.5980	0.6763	0.7956	0.9723	0.6284	0.8309	0.8180	0.3631	0.7626	0.6946	0.3904	0.5840
NM_023020	Tmeff1	0.9326	0.9769	0.9859	0.9841	0.6857	0.9823	0.6991	0.8764	0.9085	0.9820	0.9656	0.9649
NM_024358	Notch2	0.7818	0.1261	0.7924	0.3691	0.8334	0.5348	0.4687	0.5383	0.4063	0.3828	0.8729	0.6572
NM_031149	Psmc5	0.5898	0.6547	0.0381	0.4604	0.0599	0.0114	0.5715	0.0147	0.1541	0.0362	0.5298	0.0934
NM_031721	Htra1	0.1240	0.2130	0.7019	0.1065	0.7111	0.5488	0.1367	0.5296	0.0584	0.2750	0.3298	0.4113
NM_031631	Vapa	0.5341	0.4304	0.8926	0.5831	0.2428	0.5662	0.2904	0.1012	0.1854	0.1396	0.3881	0.6918
NM_022617	Mpeg1	0.1511	0.5990	0.4069	0.2330	0.1769	0.0442	0.1618	0.0752	0.0754	0.1501	0.4717	0.2865
NM_023991	Prkaa2	0.8706	0.7560	0.7404	0.5129	0.7993	0.8462	0.1527	0.3470	0.7483	0.9042	0.8257	0.7196

NM_023102	Csnk1g2	0.2137	0.4698	0.7759	0.9779	0.2307	0.9085	0.9209	0.7747	0.2000	0.6057	0.7109	0.6359
NM_023025	Cyp2j4	0.4098	0.3349	0.7168	0.2623	0.3333	0.2294	0.4513	0.4761	0.1948	0.3727	0.2951	0.2105
NM_024398	Aco2	0.8311	0.3289	0.7459	0.8999	0.6540	0.2719	0.7643	0.8324	0.1580	0.2994	0.0340	0.2348
NM_023981	Csf1	0.1291	0.2141	0.8354	0.1178	0.7348	0.9810	0.4883	0.5497	0.5083	0.1747	0.2046	0.9915
NM_022927	Mid1	0.3585	0.4679	0.5376	0.4844	0.6633	0.7006	0.5422	0.5314	0.4515	0.4843	0.4380	0.5596
NM_031318	Dynlt1	0.4983	0.7041	0.3825	0.2829	0.4606	0.0617	0.9152	0.7036	0.7600	0.8635	0.4298	0.1793
NM_024381	Gk	0.5777	0.5486	0.6549	0.5015	0.6003	0.6084	0.5138	0.5696	0.6181	0.7300	0.6739	0.5831
NM_022934	Dnaja1	0.8082	0.4199	0.4989	0.1650	0.0858	0.3632	0.0282	0.4022	0.7162	0.4324	0.1766	0.5758
NM_022931	Rims3	0.4351	0.5236	0.2723	0.0504	0.6977	0.3911	0.0813	0.3132	0.4701	0.6361	0.2133	0.4602
NM_031045	Itpka	0.4834	0.3126	0.4297	0.5845	0.7863	0.5913	0.6637	0.6151	0.4489	0.8670	0.8734	0.5167
NM_031025	Dlat	0.8532	0.8263	0.9297	0.9499	0.9127	0.9140	0.9316	0.8748	0.9932	0.9030	0.9522	0.9291
NM_022938	Htr7	0.7904	0.7349	0.5249	0.5517	0.8270	0.2711	0.8177	0.6167	0.8257	0.8492	0.4713	0.0424
NM_022939	Stx12	0.9001	0.1537	0.9855	0.5480	0.3774	0.8369	0.7856	0.3902	0.5497	0.3283	0.5813	0.7263
NM_053362	Dffb	0.1635	0.0569	0.1088	0.0440	0.0242	0.1193	0.3293	0.2576	0.0160	0.0565	0.1420	0.2474
NM_053383	Cd93	0.3163	0.1424	0.6425	0.3316	0.7306	0.5755	0.2098	0.2364	0.5290	0.7723	0.5406	0.8009
NM_031503	Ascl2	0.3558	0.4864	0.3759	0.4744	0.1763	0.8172	0.5544	0.5487	0.6237	0.1433	0.4608	0.5344
NM_031356	Aifm1	0.7854	0.7343	0.8147	0.7890	0.7587	0.7594	0.8034	0.7830	0.8336	0.8729	0.8570	0.8054
NM_024399	Aspa	0.3337	0.5651	0.2601	0.5072	0.8371	0.6106	0.4313	0.5997	0.2081	0.7984	0.1619	0.0090
NM_001113754	Grhpr	0.4192	0.0762	0.4949	0.5797	0.3959	0.1234	0.7319	0.5488	0.0402	0.7303	0.1566	0.2006
NM_030848	Bst1	0.1203	0.0151	0.0758	0.0757	0.0035	0.0592	0.0092	0.0156	0.1974	0.0045	0.1329	0.0106
NM_053436	Stau1	0.9053	0.9617	0.9278	0.9776	0.9161	0.9667	0.8989	0.8937	0.9208	0.9752	0.9685	0.9397
NM_022945	Vegp1	0.4427	0.1707	0.3296	0.3286	0.2398	0.3588	0.5028	0.1791	0.5450	0.3227	0.3617	0.4590
NM_022689	Snap23	0.6111	0.6775	0.4304	0.6925	0.5084	0.6393	0.4316	0.4997	0.5129	0.7097	0.6701	0.5595
NM_022947	Clpb	0.0454	0.0304	0.1531	0.0059	0.0263	0.1000	0.5577	0.0957	0.5499	0.1056	0.2008	0.5017
NM_022946	Dlgap1	0.6536	0.9410	0.9527	0.8532	0.9756	0.8446	0.9865	0.9894	0.4201	0.8379	0.9552	0.2109
NM_022693	Sh3bp4	0.9871	0.8695	0.9558	0.7440	0.8191	0.9778	0.8623	0.9697	0.9795	0.9165	0.7889	0.9823
NM_022951	Ppp1r10	0.9915	0.9689	0.0984	0.9868	0.8909	0.9904	0.6982	0.9610	0.9135	0.9346	0.9748	0.7419
NM_022953	Slit1	0.4031	0.3107	0.3499	0.2399	0.3206	0.4000	0.6062	0.7245	0.3929	0.1139	0.3162	0.6411
NM_031583	Smc3	0.9622	0.9160	0.8911	0.8177	0.8292	0.7581	0.2436	0.9102	0.8932	0.7796	0.8344	0.9708
NM_031558	Star	0.3319	0.5003	0.2725	0.1278	0.4322	0.4746	0.2838	0.6431	0.7260	0.6597	0.5646	0.6676
NM_022949	Rpl14	0.4219	0.7168	0.4395	0.5168	0.2241	0.0575	0.6157	0.5098	0.0197	0.3291	0.1393	0.2097
NM_022948	Sfxn3	0.9399	0.9466	0.8435	0.9391	0.9948	0.8593	0.9977	0.8903	0.9889	0.9732	0.8197	0.7072
NM_031562	Csn3	0.2226	0.4433	0.4112	0.6391	0.5527	0.1534	0.5463	0.4645	0.2343	0.5780	0.4903	0.3094
NM_031599	Eif2ak3	0.9303	0.9660	0.9733	0.2382	0.8008	0.6777	0.8654	0.8695	0.7624	0.9424	0.9506	0.9758
NM_022957	Serpina5	0.7003	0.5530	0.2796	0.5012	0.5764	0.6355	0.2676	0.1690	0.6654	0.3611	0.1115	0.5634
NM_024128	Nsg1	0.7690	0.3178	0.5382	0.4670	0.7150	0.5257	0.6780	0.6597	0.4138	0.3177	0.2725	0.5212
NM_024388	Nr4a1	0.3615	0.0515	0.3701	0.0581	0.0541	0.4809	0.0556	0.0870	0.0566	0.6837	0.3198	0.6938
NM_030855	Lig1	0.8540	0.8296	0.3873	0.6033	0.8759	0.8787	0.8900	0.8583	0.8404	0.8988	0.7904	0.3850
NM_031048	Lifr	0.4976	0.6243	0.2949	0.4128	0.1414	0.3627	0.7026	0.4237	0.3439	0.4913	0.4059	0.6791
NM_031077	Pctk1	0.7290	0.8855	0.7128	0.9315	0.6123	0.9024	0.4340	0.5940	0.9395	0.8963	0.7865	0.9375
NM_022962	Lphn1	0.9671	0.9718	0.9667	0.9878	0.7159	0.9203	0.9924	0.6423	0.8884	0.9427	0.8024	0.9797
NM_030860	Mef2d	0.2814	0.5091	0.0710	0.2235	0.7558	0.1956	0.0788	0.0671	0.8321	0.2448	0.1727	0.7067
NM_024144	Pigm	0.9429	0.8820	0.8678	0.9670	0.9767	0.9777	0.9803	0.9762	0.9639	0.8126	0.9784	0.9671
NM_023987	Birc3	0.8714	0.8223	0.1420	0.0063	0.5446	0.7027	0.2169	0.6518	0.7914	0.8523	0.8065	0.3518
NM_031008	Ap2a2	0.3794	0.8060	0.4414	0.8386	0.6556	0.7308	0.9682	0.9274	0.7488	0.3906	0.8740	0.8775
NM_024368	Frk	0.1526	0.0940	0.3265	0.9362	0.4537	0.5341	0.5513	0.2838	0.1724	0.1348	0.2515	0.1214
NM_023093	Atp5a1	0.4004	0.2106	0.2935	0.6059	0.0490	0.6071	0.0701	0.0300	0.7367	0.1726	0.4566	0.8165
NM_031062	Mvd	0.8800	0.9684	0.0058	0.0221	0.6864	0.9151	0.1235	0.9103	0.8910	0.7489	0.7998	0.0737
NM_023098	Nup62	0.4607	0.3971	0.1246	0.4731	0.4900	0.4986	0.3156	0.3548	0.4537	0.6297	0.6773	0.3983

NM_023096	Kcnk10	0.7007	0.7387	0.4393	0.4823	0.5846	0.4560	0.4720	0.5157	0.6343	0.7559	0.5500	0.7569
NM_024000	Camkv	0.9063	0.8215	0.9894	0.8129	0.9862	0.6560	0.9711	0.9701	0.9798	0.8339	0.8469	0.9751
NM_024002	Secisbp2	0.9045	0.5724	0.3675	0.3200	0.7346	0.6891	0.1930	0.8675	0.6328	0.8314	0.7363	0.8518
NM_031011	Amd1	0.9958	0.9502	0.9646	0.9547	0.9480	0.9912	0.9592	0.9716	0.9769	0.9918	0.9864	0.9525
NM_031616	Zranb2	0.9492	0.9671	0.5591	0.3101	0.9926	0.8492	0.4229	0.9060	0.9693	0.8800	0.9239	0.9835
NM_031621	Sh2b3	0.5887	0.4589	0.7420	0.5502	0.7818	0.6336	0.2217	0.8605	0.9254	0.3911	0.4495	0.5832
NM_030864	Mtr	0.5590	0.8631	0.1823	0.2063	0.8419	0.6103	0.6506	0.7699	0.7344	0.6070	0.7271	0.5481
NM_023957	Arhgef9	0.9848	0.9877	0.9024	0.7789	0.9801	0.9200	0.9881	0.8708	0.9133	0.8652	0.9315	0.9318
NM_024363	Prmt1	0.8362	0.8146	0.6802	0.4800	0.0857	0.5967	0.1714	0.7452	0.7948	0.8066	0.5653	0.3461
NM_023960	Kcnmb4	0.6902	0.8154	0.5908	0.8235	0.6670	0.7446	0.7005	0.7233	0.6464	0.4770	0.8846	0.8312
NM_023958	Impg1	0.6150	0.1926	0.2100	0.5164	0.4355	0.5426	0.7004	0.1426	0.5917	0.5810	0.2293	0.1406
NM_031327	Cyr61	0.4173	0.8916	0.9107	0.0148	0.8426	0.3034	0.9744	0.5939	0.0257	0.8854	0.9742	0.1709
NM_023966	Kcns2	0.6246	0.6292	0.3378	0.4798	0.5643	0.5873	0.5505	0.5047	0.7261	0.4425	0.5788	0.2693
NM_031142	Doc2b	0.5111	0.7033	0.5040	0.4364	0.0828	0.5637	0.4356	0.6585	0.5759	0.5008	0.0493	0.1555
NM_023968	Npy2r	0.2154	0.4170	0.3647	0.3906	0.5042	0.4818	0.2832	0.4822	0.2593	0.2445	0.1191	0.4311
NM_030867	Nfkbib	0.0548	0.1647	0.0160	0.0055	0.0071	0.0173	0.0748	0.0084	0.1156	0.7495	0.1745	0.2594
NM_031015	Opn1sw	0.8288	0.6303	0.5819	0.7703	0.5970	0.8990	0.9570	0.7247	0.7682	0.8780	0.7371	0.7966
NM_023974	Synpr	0.5989	0.5517	0.8138	0.5491	0.5503	0.4702	0.1316	0.8749	0.0949	0.4118	0.8521	0.1087
NM_023976	Prx	0.4851	0.3244	0.4895	0.4282	0.3631	0.2698	0.3258	0.5299	0.7174	0.5639	0.2854	0.2060
NM_023978	Per3	0.4269	0.6137	0.4462	0.5062	0.5028	0.3908	0.3848	0.4206	0.3715	0.6977	0.3551	0.5446
NM_023979	Apaf1	0.9285	0.9241	0.8927	0.2927	0.7971	0.3976	0.5631	0.7421	0.1641	0.8933	0.4670	0.3330
NM_031111	Rps21	0.1702	0.8581	0.0723	0.3523	0.2299	0.0998	0.2414	0.5492	0.0229	0.2311	0.0184	0.3022
NM_024366	Freq	0.7320	0.3866	0.5957	0.2863	0.7135	0.4333	0.6362	0.7241	0.5520	0.6013	0.5529	0.8233
NM_023983	Mcam	0.6482	0.5048	0.6329	0.4866	0.4411	0.5425	0.5177	0.6059	0.4942	0.3772	0.8326	0.6181
NM_031019	Crh	0.9668	0.6714	0.9446	0.9241	0.4803	0.6053	0.6404	0.7424	0.7949	0.5630	0.7382	0.5002
NM_024346	Stmn3	0.4268	0.6568	0.8542	0.6004	0.5822	0.5433	0.6243	0.7899	0.8128	0.7750	0.7774	0.8457
NM_031639	Dlg3	0.9872	0.9465	0.9593	0.8100	0.8408	0.9503	0.9689	0.9344	0.9362	0.9971	0.8963	0.9393
NM_031855	Khk	0.3949	0.8052	0.7621	0.6947	0.2044	0.1432	0.7314	0.1010	0.6636	0.5980	0.9242	0.9031
NM_031841	Scd	0.9740	0.9380	0.3236	0.7157	0.9969	0.9525	0.9748	0.9984	0.6114	0.9774	0.7838	0.3425
NM_031653	Ncan	0.1978	0.4538	0.3404	0.4420	0.6968	0.5506	0.6748	0.6190	0.5955	0.6619	0.2134	0.6303
NM_024126	Odf1	0.1104	0.4247	0.1297	0.6201	0.3677	0.4156	0.2829	0.4474	0.1313	0.0907	0.7984	0.2512
NM_031098	Rock1	0.1279	0.0593	0.8570	0.1637	0.6332	0.9950	0.3371	0.2408	0.1687	0.0392	0.5478	0.6218
NM_030986	Ak2	0.5921	0.6105	0.4642	0.1839	0.2624	0.4950	0.6734	0.6315	0.3237	0.5626	0.4802	0.2963
NM_024130	Dctn1	0.2324	0.3305	0.8798	0.9004	0.8403	0.7932	0.5753	0.7476	0.8985	0.9243	0.6361	0.9063
NM_024132	Faah	0.7990	0.3544	0.1657	0.2914	0.3539	0.8106	0.5498	0.7909	0.5791	0.4858	0.6468	0.2636
NM_024391	Hsd17b2	0.2336	0.2931	0.3221	0.3079	0.2679	0.0229	0.2854	0.0608	0.0259	0.3834	0.3182	0.5251
NM_024390	Hpgd	0.5878	0.2775	0.1686	0.4940	0.2278	0.3362	0.3010	0.4057	0.0606	0.6368	0.0409	0.2769
NM_031054	Mmp2	0.7254	0.7092	0.2951	0.9673	0.6490	0.2584	0.3683	0.9202	0.4303	0.4474	0.6547	0.4068
NM_024142	Mepe	0.5575	0.2342	0.4819	0.5204	0.2175	0.2843	0.4691	0.6000	0.3619	0.3731	0.3898	0.2314
NM_031020	Mapk14	0.8965	0.9478	0.8198	0.7198	0.9805	0.8863	0.9681	0.9767	0.9415	0.9475	0.7442	0.9395
NM_031014	Atic	0.2631	0.5601	0.3820	0.6049	0.3093	0.4541	0.6626	0.3245	0.7326	0.4673	0.6319	0.5340
NM_024139	Chp	0.3951	0.3231	0.2089	0.8963	0.4586	0.3567	0.3505	0.4401	0.6302	0.2927	0.7491	0.8017
NM_024140	Nrgn	0.1458	0.2878	0.4068	0.3520	0.3652	0.6075	0.4577	0.5830	0.5831	0.4960	0.5971	0.5535
NM_024137	Hivep2	0.6678	0.7205	0.6995	0.5947	0.5614	0.4987	0.6580	0.6882	0.5732	0.6201	0.7125	0.7050
NM_031772	Polr1a	0.7229	0.9248	0.7613	0.8591	0.7760	0.9399	0.9384	0.9555	0.9318	0.9191	0.9578	0.8958
NM_024387	Hmox2	0.4061	0.8398	0.8677	0.8802	0.8061	0.1461	0.7300	0.8429	0.7767	0.7665	0.4857	0.6672
NM_024380	Gucy2e	0.8739	0.4643	0.5484	0.1870	0.5859	0.8318	0.7521	0.8028	0.3845	0.1348	0.6931	0.9142
NM_024133	Hap1	0.2525	0.6233	0.7831	0.7368	0.6570	0.6373	0.8008	0.6663	0.6130	0.6146	0.2835	0.8355
NM_031546	Rgn	0.4828	0.3780	0.8372	0.7357	0.5264	0.5259	0.5616	0.5282	0.6653	0.7518	0.6066	0.3208

NM_031345	Tsc22d3	0.2350	0.3352	0.4892	0.3071	0.3317	0.5349	0.5229	0.4395	0.2844	0.4477	0.0841	0.3489
NM_031082	Pggt1b	0.1579	0.1803	0.1715	0.2225	0.0078	0.4575	0.0999	0.0442	0.0409	0.1614	0.2628	0.4396
NM_031016	Chrm2	0.0340	0.5125	0.2719	0.5524	0.0511	0.3210	0.1372	0.1465	0.3421	0.2374	0.2830	0.2886
NM_031105	Rpl36al	0.2790	0.3330	0.1814	0.4411	0.1798	0.2902	0.1883	0.5389	0.3880	0.3366	0.4441	0.1762
NM_024152	Arf6	0.8847	0.9795	0.5203	0.9253	0.7244	0.0625	0.9664	0.8402	0.9933	0.9218	0.8872	0.5009
NM_024151	Arf4	0.9314	0.9925	0.9014	0.8193	0.9282	0.9954	0.9729	0.9266	0.9299	0.9977	0.9889	0.9483
NM_024149	Arf5	0.7927	0.9591	0.9194	0.9487	0.9877	0.9785	0.9551	0.9100	0.8619	0.8914	0.9124	0.4387
NM_024156	Anxa6	0.7502	0.6353	0.4100	0.6965	0.8263	0.2890	0.6110	0.5753	0.2072	0.0446	0.6774	0.3554
NM_024153	Fdxr	0.5776	0.8271	0.0199	0.9226	0.7227	0.3514	0.8820	0.9245	0.7923	0.6914	0.7298	0.5086
NM_031987	Crot	0.2328	0.5917	0.8267	0.8729	0.5416	0.7550	0.1411	0.6914	0.0900	0.5657	0.4598	0.5752
NM_024150	Arf2	0.1454	0.1581	0.3298	0.2839	0.1816	0.2654	0.1856	0.2880	0.1675	0.3116	0.1457	0.2424
NM_024155	Anxa4	0.8419	0.2901	0.9154	0.8702	0.8896	0.9678	0.3110	0.9002	0.7338	0.2956	0.3986	0.8745
NM_030856	Lrrn3	0.0552	0.0752	0.2824	0.0191	0.2829	0.2941	0.0155	0.0318	0.0192	0.0173	0.1795	0.0160
NM_031081	Pdpk1	0.5583	0.7024	0.5379	0.8783	0.7142	0.3687	0.2192	0.6886	0.7599	0.3049	0.6658	0.6406
NM_024161	Dnajc5	0.1533	0.6898	0.8837	0.9434	0.1493	0.0906	0.8376	0.2744	0.6627	0.4720	0.8916	0.4297
NM_024163	Begain	0.5490	0.7947	0.5364	0.5546	0.8605	0.8033	0.2414	0.6229	0.8378	0.8555	0.3964	0.7247
NM_030827	Lrp2	0.5832	0.5364	0.6028	0.4613	0.5928	0.6902	0.5223	0.6273	0.6209	0.6607	0.6428	0.5689
NM_031038	Gnrhr	0.0643	0.2570	0.1024	0.5056	0.5548	0.4833	0.5257	0.3342	0.7010	0.5539	0.2820	0.3264
NM_030834	Slc16a3	0.2891	0.0282	0.1484	0.1348	0.4652	0.4065	0.0777	0.5194	0.3878	0.4172	0.0341	0.4871
NM_024352	Mst1	0.0392	0.0435	0.0093	0.0324	0.0344	0.2117	0.1174	0.1053	0.0440	0.1400	0.1845	0.0851
NM_031557	Ptgis	0.8025	0.8009	0.9310	0.8340	0.0719	0.0424	0.0734	0.1320	0.0394	0.9181	0.8305	0.0102
NM_024355	Axin2	0.2736	0.8223	0.6947	0.7181	0.1287	0.1719	0.7805	0.0528	0.4497	0.5264	0.7708	0.9468
NM_031735	Stk3	0.8773	0.2271	0.8023	0.0681	0.1290	0.0231	0.8617	0.1300	0.1381	0.2168	0.3451	0.3066
NM_031021	Csnk2b	0.3330	0.3661	0.5590	0.3503	0.3109	0.3475	0.3291	0.4094	0.2633	0.4797	0.4271	0.3404
NM_031114	S100a10	0.5919	0.2555	0.5272	0.2976	0.4830	0.2525	0.4928	0.4418	0.2663	0.2405	0.2760	0.3269
NM_030829	Grk5	0.1010	0.1352	0.5651	0.0905	0.0986	0.0133	0.2349	0.0723	0.4806	0.0995	0.2339	0.9828
NM_024360	Hes1	0.8517	0.9600	0.9027	0.5410	0.8907	0.8540	0.9330	0.9099	0.8945	0.8069	0.9979	0.1487
NM_030846	Grb2	0.9149	0.8550	0.4163	0.4295	0.9463	0.9051	0.5338	0.9119	0.3750	0.0881	0.6878	0.9590
NM_030838	Slco1a5	0.0729	0.5922	0.1286	0.6204	0.6235	0.4040	0.5852	0.5174	0.2501	0.0063	0.5348	0.1995
NM_031723	Sec11a	0.9113	0.3852	0.9205	0.3050	0.8665	0.1166	0.3726	0.9691	0.0719	0.3909	0.6834	0.2965
NM_031683	Smc1a	0.7499	0.7758	0.0500	0.5688	0.9193	0.8146	0.7377	0.9438	0.4688	0.2923	0.5042	0.3409
NM_031007	Adcy2	0.3373	0.5054	0.7888	0.8903	0.8268	0.7428	0.1206	0.7882	0.4322	0.2222	0.7416	0.2708
NM_024369	Fstl1	0.9740	0.7953	0.7376	0.8230	0.9587	0.9918	0.9077	0.8992	0.9229	0.8314	0.9586	0.6979
NM_001106240	Sars2	0.6857	0.8504	0.1565	0.6456	0.7484	0.8124	0.8768	0.7849	0.3021	0.5073	0.5988	0.8218
NM_199115	Angptl4	0.2050	0.0619	0.3023	0.1282	0.5273	0.5430	0.3312	0.4687	0.0673	0.2341	0.5124	0.4047
NM_001077672	Cstf3	0.1388	0.4110	0.5483	0.7925	0.5115	0.0504	0.7600	0.6658	0.5121	0.7870	0.3729	0.1600
NM_001079887	Ing4	0.2145	0.6535	0.8021	0.2203	0.2595	0.2467	0.0379	0.1032	0.5894	0.5199	0.7919	0.8915
NM_024374	Mtpn	0.0784	0.3070	0.9957	0.6553	0.1247	0.3747	0.9563	0.2559	0.4061	0.3093	0.8231	0.9514
NM_030849	Bmpr1a	0.9405	0.7712	0.9851	0.9931	0.9322	0.9297	0.9564	0.9911	0.8606	0.9169	0.8739	0.2486
NM_024372	Slc6a11	0.1072	0.3847	0.4674	0.3967	0.5039	0.2003	0.3603	0.5059	0.6410	0.6458	0.1086	0.5390
NM_024371	Slc6a1	0.6853	0.8764	0.5266	0.6107	0.6934	0.7957	0.3691	0.6893	0.7524	0.6971	0.6122	0.7466
NM_024375	Gdf10	0.1935	0.4443	0.4531	0.2814	0.1297	0.1131	0.0116	0.1635	0.2198	0.7271	0.1504	0.4781
NM_030987	Gnb1	0.6338	0.2681	0.2526	0.6363	0.6979	0.3837	0.5408	0.5136	0.5970	0.4785	0.6994	0.5071
NM_024378	Grid1	0.8315	0.4243	0.7601	0.5810	0.6609	0.3265	0.5146	0.5877	0.8697	0.6208	0.7727	0.9567
NM_024379	Grid2	0.9665	0.9792	0.9804	0.8954	0.5162	0.4241	0.9652	0.9272	0.9853	0.8900	0.9584	0.9709
NM_031582	Aoc3	0.1779	0.3523	0.1343	0.1650	0.8094	0.4205	0.2209	0.3822	0.4304	0.1310	0.5835	0.5718
NM_031635	Fut2	0.0828	0.4533	0.4782	0.1861	0.3298	0.2858	0.4790	0.2257	0.2815	0.4465	0.5437	0.4496
NM_031117	Snrpn	0.9112	0.2088	0.9172	0.0007	0.0657	0.0491	0.0065	0.1954	0.0717	0.0666	0.3275	0.7676
NM_024382	Serpind1	0.5082	0.4133	0.8409	0.7245	0.4589	0.3157	0.3189	0.8757	0.6868	0.2110	0.3228	0.7066

NM_031579	Ptp4a1	0.8944	0.8260	0.7914	0.3482	0.1823	0.8270	0.6269	0.2374	0.9768	0.5729	0.9828	0.9390
NM_024383	Hes5	0.1857	0.1083	0.0444	0.2297	0.2518	0.1381	0.0411	0.3543	0.3361	0.1774	0.0715	0.0092
NM_031108	Rps9	0.2831	0.6845	0.1657	0.8229	0.5221	0.0795	0.7700	0.6157	0.1104	0.4972	0.3046	0.0937
NM_024385	Hhex	0.4360	0.5700	0.6677	0.7848	0.1097	0.6238	0.1232	0.4677	0.3648	0.1472	0.5682	0.0233
NM_031002	Inpp4a	0.9847	0.9417	0.9838	0.7875	0.8998	0.6801	0.9297	0.7711	0.9747	0.6591	0.8198	0.9037
NM_031001	RGD69425	0.4010	0.6010	0.4481	0.3170	0.4239	0.1744	0.4692	0.5679	0.5191	0.6594	0.6217	0.7617
NM_031000	Akr1a1	0.8355	0.5038	0.8209	0.9364	0.6710	0.8295	0.6863	0.3633	0.2906	0.8551	0.9107	0.6004
NM_031605	Cyp4a8	0.4720	0.7238	0.2848	0.7613	0.6143	0.5419	0.1296	0.0872	0.5671	0.5921	0.4737	0.2695
NM_030870	Ogg1	0.3488	0.6937	0.0847	0.6241	0.5419	0.2174	0.4550	0.3986	0.7548	0.4572	0.6675	0.5123
NM_030872	Pdk2	0.0490	0.1868	0.9249	0.6510	0.2622	0.1153	0.9148	0.3606	0.7278	0.4059	0.2778	0.8071
NM_031728	Snap91	0.2612	0.2391	0.5899	0.7851	0.0726	0.1731	0.0595	0.1785	0.4451	0.3520	0.2145	0.4104
NM_031034	Gna12	0.6950	0.1866	0.6011	0.5403	0.5322	0.8695	0.4841	0.0909	0.0226	0.4775	0.5217	0.6073
NM_024404	Hnrpd	0.7105	0.7880	0.8604	0.8332	0.6938	0.3275	0.7459	0.6406	0.7968	0.4598	0.7435	0.6802
NM_024405	Axin1	0.9348	0.9281	0.8143	0.5128	0.9288	0.3761	0.9392	0.9307	0.9270	0.7641	0.4578	0.2042
NM_024396	Abca2	0.9425	0.8691	0.9597	0.7470	0.9405	0.8676	0.8461	0.9144	0.9963	0.7245	0.9039	0.9996
NM_031796	Galnt5	0.8366	0.8723	0.9100	0.7812	0.8689	0.9376	0.7892	0.7960	0.9521	0.7555	0.9766	0.8913
NM_031003	Abat	0.2736	0.4167	0.6739	0.5558	0.0957	0.0159	0.0210	0.1755	0.0392	0.5448	0.5987	0.0489
NM_031509	Gsta3	0.1221	0.4889	0.6496	0.4995	0.3437	0.6016	0.8909	0.7019	0.7826	0.6506	0.4601	0.4417
NM_031502	Amy2	0.3074	0.2953	0.2273	0.6161	0.2841	0.1194	0.2019	0.1481	0.3998	0.5735	0.5748	0.1558
NM_024487	Grpel1	0.1513	0.0588	0.0366	0.2769	0.1181	0.1084	0.0349	0.0265	0.0842	0.1581	0.4388	0.0408
NM_024489	Zbtb10	0.4975	0.7128	0.4914	0.5068	0.4667	0.5091	0.6505	0.5920	0.2189	0.6910	0.5311	0.4373
NM_031154	Gstm7	0.3562	0.3414	0.5920	0.3121	0.3464	0.5427	0.7614	0.6644	0.4552	0.4800	0.2321	0.6129
NM_030585	Rabep2	0.5663	0.2818	0.4826	0.6834	0.8949	0.9480	0.6310	0.7738	0.9652	0.7336	0.6698	0.3443
NM_031078	Pde1c	0.0237	0.2617	0.0299	0.0445	0.1681	0.0549	0.1834	0.2466	0.5851	0.0235	0.0532	0.6510
NM_030871	Pde1a	0.0031	0.0210	0.0084	0.0416	0.0195	0.2766	0.0220	0.0170	0.0049	0.0253	0.1653	0.5753
NM_031058	Msh2	0.9975	0.8380	0.1456	0.9122	0.8354	0.9020	0.7290	0.7304	0.7797	0.7133	0.9321	0.7329
NM_030873	Pfn2	0.1689	0.3327	0.9499	0.2025	0.2782	0.2917	0.3359	0.2920	0.2713	0.2981	0.5472	0.6812
NM_031051	Mif	0.0929	0.0903	0.3255	0.4954	0.1003	0.0744	0.6216	0.0387	0.1904	0.0809	0.0098	0.0646
NM_030830	Luzp1	0.9856	0.7541	0.9860	0.9809	0.9926	0.9853	0.9850	0.9204	0.9323	0.7459	0.9953	0.8285
NM_030832	Fabp7	0.3053	0.2032	0.3592	0.6897	0.6049	0.6947	0.3740	0.6629	0.5580	0.7239	0.8571	0.2784
NM_031741	Slc2a5	0.1365	0.6214	0.5317	0.4124	0.0667	0.1449	0.6386	0.1947	0.1399	0.5501	0.2318	0.5423
NM_031052	Mipep	0.0155	0.2815	0.6843	0.6753	0.0660	0.1036	0.4603	0.3379	0.0275	0.0938	0.1005	0.1448
NM_031551	Idh3g	0.8630	0.9289	0.6033	0.9704	0.5178	0.7540	0.8774	0.7042	0.8482	0.9077	0.7090	0.9657
NM_030837	Slc21a4	0.6977	0.9550	0.7130	0.7834	0.9001	0.7032	0.6018	0.6027	0.7664	0.9438	0.9118	0.7552
NM_030841	Nptxr	0.7100	0.5349	0.5237	0.3382	0.5140	0.7173	0.5087	0.4808	0.6152	0.6152	0.5373	0.7262
NM_030842	Itga7	0.5106	0.0052	0.0370	0.0093	0.6503	0.0828	0.1190	0.4817	0.0725	0.0904	0.1555	0.8591
NM_030843	Stxbp5	0.3002	0.1700	0.1813	0.5041	0.2862	0.5961	0.3380	0.3258	0.2714	0.3971	0.5029	0.2600
NM_030844	Ica1	0.1483	0.8166	0.8822	0.7133	0.8484	0.7783	0.8822	0.8121	0.9205	0.8374	0.8937	0.9133
NM_031107	Rps6ka1	0.4076	0.9257	0.2027	0.4791	0.2818	0.1497	0.6378	0.5885	0.9344	0.6957	0.6401	0.8369
NM_031236	Fut1	0.0539	0.4046	0.3957	0.0864	0.2812	0.4066	0.5938	0.4421	0.0432	0.2841	0.1942	0.5126
NM_031043	Gyg1	0.1539	0.0948	0.9693	0.1206	0.1138	0.0123	0.1018	0.0467	0.2228	0.1373	0.0151	0.7068
NM_030847	Emp3	0.9950	0.6992	0.0235	0.7428	0.8896	0.6184	0.4779	0.9414	0.7603	0.5033	0.5169	0.0034
NM_031049	Lss	0.4652	0.4636	0.3478	0.4134	0.4759	0.2465	0.4418	0.6048	0.4579	0.5914	0.2370	0.5572
NM_031039	Gpt	0.8686	0.8054	0.0846	0.0798	0.7871	0.8845	0.2684	0.8420	0.7433	0.8296	0.7658	0.6370
NM_031030	Gak	0.4936	0.4059	0.1373	0.1211	0.3737	0.4124	0.1821	0.1924	0.6421	0.1152	0.7198	0.3907
NM_053485	S100a6	0.8573	0.8216	0.1479	0.8142	0.7524	0.6409	0.2047	0.7956	0.7886	0.9595	0.8915	0.0964
NM_031057	Aldh6a1	0.8358	0.6044	0.7885	0.3721	0.6621	0.5653	0.1192	0.4686	0.6893	0.7437	0.7523	0.8491
NM_031028	Gabbr1	0.1378	0.7114	0.8719	0.0638	0.8125	0.5296	0.2994	0.6552	0.9201	0.7101	0.5595	0.9166
NM_030853	Lat	0.7741	0.5812	0.1969	0.1271	0.6852	0.8925	0.1639	0.9151	0.6703	0.4533	0.7173	0.3668

NM_031242	Cds1	0.1030	0.2755	0.7743	0.0430	0.8615	0.7282	0.1011	0.7748	0.8652	0.7006	0.8522	0.5646
NM_031056	Mmp14	0.6877	0.0808	0.1945	0.8148	0.9547	0.8562	0.4313	0.8238	0.6931	0.1455	0.1340	0.9990
NM_031731	Aldh3a2	0.6162	0.5849	0.3493	0.4028	0.7035	0.6845	0.5238	0.8245	0.5681	0.6312	0.8908	0.4922
NM_031070	Nell2	0.7673	0.7722	0.8861	0.8989	0.7917	0.7479	0.9753	0.7741	0.7523	0.7965	0.5548	0.9688
NM_030862	Marcksl1	0.9863	0.2203	0.9483	0.9049	0.8389	0.7816	0.8389	0.9321	0.3682	0.3786	0.7048	0.2158
NM_030863	Msn	0.0501	0.1844	0.8663	0.8523	0.1921	0.9063	0.7724	0.1810	0.0147	0.1342	0.1468	0.9748
NM_030994	Itga1	0.0172	0.5422	0.4735	0.2417	0.0349	0.5970	0.6845	0.0906	0.0023	0.0497	0.2784	0.0016
NM_031022	Cspg4	0.0018	0.1288	0.1060	0.0098	0.1874	0.0868	0.1726	0.1654	0.1663	0.1630	0.0047	0.1168
NM_030865	Myoc	0.9055	0.8981	0.5771	0.7159	0.5191	0.8212	0.2339	0.6505	0.6133	0.8132	0.8385	0.4903
NM_031133	Thpo	0.7345	0.8707	0.0296	0.8905	0.9534	0.8488	0.7138	0.9878	0.8138	0.8101	0.7811	0.7007
NM_030869	Nrp2	0.1202	0.2699	0.5604	0.1275	0.0678	0.2379	0.5119	0.0875	0.0847	0.4971	0.2597	0.0256
NM_054003	B3gat1	0.2580	0.0695	0.2687	0.1078	0.7614	0.1708	0.1664	0.2929	0.2772	0.5188	0.0739	0.6437
NM_053750	Nppc	0.9543	0.7547	0.9009	0.8103	0.6328	0.9098	0.7666	0.7377	0.7707	0.9889	0.3267	0.8163
NM_001002819	Gfpt2	0.3685	0.4699	0.4203	0.5030	0.3969	0.1729	0.1384	0.2786	0.5488	0.4229	0.2236	0.0634
NM_030868	Nov	0.2645	0.0394	0.5981	0.0264	0.1192	0.3589	0.0565	0.2301	0.0573	0.0650	0.0876	0.1117
NM_031596	Sart1	0.5336	0.7356	0.1013	0.0188	0.0645	0.0062	0.1163	0.1227	0.0562	0.4386	0.3722	0.1559
NM_031061	Musk	0.4732	0.2140	0.5990	0.4489	0.3632	0.2928	0.1451	0.4337	0.3305	0.5982	0.4384	0.4262
NM_031055	Mmp9	0.9161	0.9398	0.7256	0.7468	0.9388	0.8848	0.9249	0.8609	0.8847	0.9784	0.9878	0.9636
NM_031047	Jup	0.6842	0.8423	0.7847	0.8210	0.8772	0.9183	0.9653	0.8842	0.9150	0.9384	0.6206	0.9272
NM_031075	P2rx3	0.6307	0.4847	0.5991	0.6556	0.5319	0.5716	0.5558	0.3740	0.6053	0.3197	0.6336	0.5395
NM_031325	Ugdh	0.6090	0.1441	0.2504	0.5708	0.5308	0.8237	0.0138	0.3774	0.4368	0.1161	0.2897	0.8028
NM_031349	Aplnr	0.3076	0.1711	0.5333	0.8073	0.4208	0.1746	0.9630	0.7231	0.9256	0.2432	0.6790	0.9756
NM_031348	Fcn1	0.0510	0.0446	0.0188	0.0769	0.0297	0.0678	0.1417	0.1039	0.1021	0.1479	0.0415	0.0536
NM_031347	Ppargc1a	0.5789	0.4545	0.5724	0.5741	0.6306	0.5321	0.5756	0.0838	0.5363	0.5830	0.3146	0.4020
NM_031144	Actb	0.3609	0.5590	0.6389	0.8270	0.7850	0.8406	0.3048	0.5164	0.4033	0.4874	0.7932	0.5213
NM_031113	Rps27a	0.5945	0.6321	0.3981	0.2057	0.4456	0.1597	0.4733	0.2094	0.0984	0.0457	0.5103	0.2803
NM_031027	Dpyd	0.7017	0.3261	0.2954	0.5307	0.2536	0.6759	0.2576	0.5774	0.5957	0.6809	0.6997	0.3814
NM_031682	Hsd17b10	0.0599	0.0879	0.4661	0.5975	0.0398	0.0387	0.5012	0.2965	0.0784	0.0717	0.0015	0.0190
NM_031644	Ptgds2	0.4369	0.6033	0.4066	0.4494	0.0613	0.4336	0.4057	0.6008	0.2158	0.1245	0.0431	0.0437
NM_031013	Abcc6	0.0712	0.3646	0.0486	0.5321	0.1194	0.2210	0.2541	0.1219	0.7421	0.5224	0.0903	0.4753
NM_030996	Oprs1	0.8874	0.7253	0.4716	0.8387	0.9309	0.9298	0.8252	0.9110	0.9821	0.9087	0.6640	0.9267
NM_031139	Usf2	0.6269	0.4500	0.3272	0.1505	0.0856	0.4889	0.3727	0.5331	0.5510	0.3124	0.2609	0.7839
NM_031614	Txnrd1	0.8503	0.3758	0.2621	0.1568	0.7429	0.5008	0.9560	0.4946	0.3343	0.0970	0.2875	0.6996
NM_031123	Stc1	0.8694	0.8319	0.8175	0.6726	0.5344	0.6342	0.2619	0.6485	0.5306	0.6585	0.8177	0.7803
NM_030985	Agtr1	0.7374	0.7472	0.4731	0.7467	0.4292	0.4268	0.3247	0.5807	0.8764	0.5420	0.4500	0.4814
NM_031073	Ntf3	0.2376	0.4662	0.0788	0.4099	0.0779	0.0102	0.1055	0.0026	0.3267	0.0506	0.1457	0.8610
NM_053402	Wnt4	0.9099	0.9998	0.8394	0.9791	0.9478	0.8374	0.7565	0.9196	0.9210	0.8267	0.9463	0.9966
NM_031620	Phgdh	0.0645	0.8787	0.0526	0.8690	0.8828	0.8332	0.8598	0.8647	0.7913	0.9415	0.9513	0.0285
NM_031083	Pi4kb	0.0840	0.3583	0.3862	0.6482	0.2004	0.0980	0.9085	0.1079	0.9675	0.2802	0.4987	0.2844
NM_031745	Clip1	0.8993	0.3631	0.9183	0.8936	0.9190	0.8431	0.2519	0.7996	0.1720	0.0988	0.0783	0.9755
NM_031097	Rnpep	0.8681	0.7685	0.7775	0.8996	0.7414	0.7771	0.7103	0.8205	0.5679	0.7037	0.8151	0.6937
NM_031603	Ywhae	0.4293	0.3996	0.7458	0.9141	0.3965	0.2862	0.5907	0.1796	0.8556	0.9245	0.9443	0.3436
NM_031588	Nrg1	0.7610	0.7947	0.3547	0.8739	0.5893	0.6825	0.3923	0.5798	0.3949	0.3441	0.8732	0.4588
NM_031750	Hspb3	0.3773	0.0144	0.4306	0.4280	0.6683	0.0937	0.5511	0.6746	0.3865	0.4103	0.2890	0.0760
NM_031095	Renbp	0.0944	0.0074	0.5253	0.0249	0.1163	0.0237	0.0054	0.0262	0.0701	0.0060	0.0099	0.7532
NM_031090	Rab1	0.4286	0.2700	0.9261	0.9020	0.1701	0.1821	0.6240	0.1232	0.2204	0.2905	0.0406	0.2365
NM_031086	Pros1	0.4480	0.1464	0.9775	0.9667	0.7838	0.9097	0.6221	0.9363	0.0742	0.2217	0.1529	0.2426
NM_031032	Gmfb	0.7709	0.8801	0.8876	0.8991	0.3709	0.3303	0.6579	0.3768	0.8751	0.4569	0.9804	0.8350
NM_031235	Pard3	0.9621	0.8989	0.8753	0.8311	0.9768	0.9348	0.9161	0.9867	0.9910	0.9808	0.8691	0.9926

NM_031040	Grm7	0.7493	0.3414	0.3745	0.5340	0.4780	0.1579	0.3854	0.4109	0.3044	0.5826	0.5398	0.4611
NM_031602	Kcnj10	0.0797	0.1002	0.0267	0.1369	0.4795	0.3091	0.5881	0.2363	0.7495	0.2003	0.6231	0.6583
NM_031601	Cacna1g	0.7559	0.4124	0.3291	0.8299	0.8588	0.8624	0.1459	0.7525	0.4840	0.4943	0.5522	0.3166
NM_175763	Igsf1	0.1796	0.8529	0.6833	0.4573	0.8494	0.1915	0.3204	0.7410	0.2846	0.5940	0.6715	0.2493
NM_031041	Gtf2b	0.1098	0.1823	0.0537	0.1850	0.0917	0.1382	0.0697	0.1090	0.1189	0.0173	0.3249	0.0101
NM_031085	Prkch	0.1504	0.2625	0.0530	0.0056	0.3557	0.5044	0.2650	0.3566	0.1512	0.1010	0.0645	0.4309
NM_031042	Gtf2f2	0.2005	0.6657	0.2330	0.5160	0.4682	0.5482	0.2643	0.2541	0.5466	0.5544	0.5529	0.5309
NM_031237	Ube2d3	0.6347	0.6118	0.6662	0.5658	0.6712	0.5376	0.6134	0.5313	0.3982	0.6229	0.7307	0.7222
NM_031096	Grk1	0.5892	0.6199	0.6343	0.5895	0.2167	0.7234	0.5500	0.6437	0.6217	0.6263	0.8277	0.1878
NM_031632	Casp9	0.8331	0.8310	0.2798	0.7361	0.6341	0.2473	0.8650	0.7358	0.7851	0.8752	0.4237	0.4926
NM_031360	Smpd2	0.6675	0.6886	0.4935	0.5796	0.6694	0.4902	0.5380	0.7041	0.6384	0.9966	0.6806	0.0496
NM_031088	Ptger2	0.3144	0.6435	0.7734	0.8070	0.7502	0.5283	0.8128	0.6935	0.8457	0.7483	0.7355	0.6530
NM_031110	Rps11	0.5812	0.7586	0.0405	0.5176	0.5976	0.1933	0.3127	0.8774	0.3510	0.2662	0.5905	0.1019
NM_031101	Rpl13	0.6340	0.5748	0.6605	0.0556	0.3007	0.3793	0.5533	0.6248	0.3979	0.2839	0.2217	0.3300
NM_031100	Rpl10	0.5295	0.7261	0.4655	0.5960	0.5438	0.4293	0.8633	0.5805	0.4587	0.6425	0.2233	0.5168
NM_031510	Idh1	0.8994	0.6586	0.9981	0.2347	0.6939	0.7798	0.7186	0.7125	0.3168	0.6405	0.5027	0.5631
NM_031092	Rab13	0.0010	0.0020	0.8427	0.6494	0.0575	0.0271	0.0133	0.1068	0.0162	0.0942	0.0019	0.0621
NM_031104	Rpl22	0.0948	0.2359	0.1007	0.1119	0.1998	0.1369	0.6729	0.4480	0.2859	0.1198	0.5146	0.0627
NM_001080149	Ptpn20	0.3031	0.1940	0.3405	0.5228	0.4194	0.3195	0.1340	0.1664	0.2990	0.6121	0.1709	0.3756
NM_030993	Ddn	0.2929	0.3978	0.7274	0.6835	0.7066	0.7389	0.5351	0.7714	0.5474	0.8135	0.6260	0.4771
NM_031103	Rpl19	0.5699	0.6273	0.6709	0.2391	0.4665	0.4842	0.5323	0.1885	0.5480	0.3003	0.6322	0.6952
NM_031578	Tesk1	0.9959	0.7644	0.8766	0.9531	0.9878	0.9425	0.9994	0.9197	0.9616	0.9259	0.9740	0.9809
NM_031094	Rbl2	0.9973	0.9705	0.9902	0.9940	0.9013	0.9558	0.9602	0.9389	0.8771	0.9284	0.9512	0.1562
NM_031517	Met	0.4692	0.3041	0.1808	0.1832	0.3966	0.1786	0.1872	0.4198	0.1695	0.1632	0.1715	0.2734
NM_030997	Vgf	0.2346	0.2592	0.1383	0.1434	0.1122	0.2122	0.2478	0.1294	0.6476	0.3914	0.0260	0.8126
NM_031657	Grk6	0.8080	0.6822	0.6586	0.8931	0.5664	0.8727	0.8825	0.9340	0.7880	0.8790	0.4994	0.5364
NM_024370	Gabrg3	0.0782	0.4475	0.3449	0.6800	0.1375	0.2437	0.5328	0.1789	0.1315	0.1774	0.1537	0.4559
NM_031152	Rab11a	0.7768	0.3839	0.9373	0.9848	0.9783	0.8889	0.4363	0.9706	0.5150	0.2632	0.2636	0.4499
NM_031736	Slc27a2	0.3738	0.2370	0.5945	0.5248	0.4149	0.3454	0.4113	0.2875	0.3008	0.6407	0.3726	0.2318
NM_031148	Slc20a1	0.5830	0.8939	0.2256	0.1635	0.4451	0.8498	0.3187	0.1356	0.9471	0.9467	0.9132	0.9873
NM_031147	Cirbp	0.4407	0.0693	0.8380	0.9086	0.9334	0.9033	0.3564	0.9408	0.2370	0.1865	0.2440	0.1007
NM_031146	Arpc1a	0.6284	0.0433	0.6410	0.9488	0.8663	0.6768	0.6218	0.6676	0.5414	0.2065	0.4698	0.7435
NM_031565	Es22	0.2678	0.3654	0.2992	0.3659	0.1991	0.2690	0.3695	0.1784	0.2337	0.4179	0.4973	0.2816
NM_031564	Epha3	0.1745	0.1699	0.5057	0.3966	0.2561	0.5964	0.1872	0.1782	0.2760	0.1632	0.1715	0.3040
NM_031559	Cpt1a	0.3361	0.1314	0.3986	0.9505	0.9470	0.9424	0.5220	0.9475	0.2282	0.1771	0.2511	0.6995
NM_052980	Nr1i2	0.2522	0.0258	0.3639	0.2815	0.3324	0.2276	0.2826	0.4176	0.0629	0.2919	0.0224	0.1412
NM_031594	P2rx4	0.1138	0.3076	0.6824	0.1101	0.2371	0.6180	0.3539	0.1097	0.2251	0.1792	0.1614	0.6821
NM_031535	Bcl2l1	0.6446	0.5666	0.6885	0.6086	0.5832	0.7071	0.6674	0.6460	0.6880	0.6262	0.4395	0.7091
NM_053407	Asah1	0.0541	0.0963	0.7371	0.7954	0.0534	0.5357	0.0925	0.0139	0.2364	0.1350	0.1597	0.7621
NM_053400	Tle3	0.8899	0.0108	0.8286	0.9317	0.1301	0.3785	0.6312	0.6208	0.3830	0.1552	0.4508	0.3434
NM_031527	Ppp1ca	0.1067	0.3531	0.2170	0.8140	0.4326	0.4920	0.9289	0.3643	0.4573	0.4974	0.6603	0.4751
NM_031530	Ccl2	0.0453	0.0319	0.2869	0.0950	0.0111	0.0589	0.0005	0.0367	0.0187	0.2369	0.2015	0.0108
NM_031521	Ncam1	0.9104	0.7902	0.8404	0.6915	0.9938	0.7150	0.9984	0.8853	0.9889	0.9938	0.8975	0.9722
NM_031135	Klf10	0.2939	0.2588	0.9520	0.2164	0.2831	0.0735	0.0253	0.1702	0.1388	0.2290	0.2278	0.8428
NM_031654	Rabggta	0.7434	0.6612	0.2552	0.5727	0.4092	0.7795	0.4036	0.7501	0.6997	0.7456	0.7205	0.8372
NM_031646	Ramp2	0.8466	0.7449	0.8434	0.7494	0.9930	0.9626	0.8802	0.8751	0.8777	0.8896	0.9394	0.9584
NM_031317	Pdgfc	0.2609	0.1790	0.7403	0.6554	0.1628	0.6188	0.7034	0.1428	0.2683	0.8187	0.7506	0.9798
NM_031604	Atp6v0a1	0.0086	0.3451	0.1510	0.0828	0.8719	0.7640	0.2314	0.8525	0.3580	0.3398	0.0827	0.4163
NM_031515	Kras	0.4808	0.7788	0.7598	0.9401	0.4990	0.4258	0.5608	0.0853	0.4300	0.9336	0.9408	0.5195

NM_031130	Nr2f1	0.9824	0.2233	0.2189	0.2796	0.9395	0.8918	0.1273	0.8230	0.1752	0.3247	0.2901	0.0409
NM_031126	Stxbp2	0.4646	0.3814	0.1412	0.6328	0.2019	0.0488	0.2522	0.6889	0.7851	0.6383	0.6569	0.3716
NM_031125	Stx4	0.6641	0.8930	0.0105	0.4276	0.8456	0.8444	0.3350	0.6252	0.8782	0.7990	0.9057	0.7024
NM_031124	Stx3	0.7160	0.2284	0.6758	0.3212	0.2042	0.3836	0.1955	0.8481	0.6041	0.7340	0.3420	0.4443
NM_031678	Per2	0.9498	0.4077	0.6197	0.3113	0.9882	0.9870	0.8050	0.7643	0.8494	0.6505	0.9798	0.9922
NM_031122	St13	0.3103	0.2804	0.4808	0.1976	0.3270	0.6114	0.6528	0.3349	0.2418	0.1189	0.1465	0.3024
NM_031121	Ssrp1	0.9031	0.8295	0.2051	0.8084	0.9771	0.9821	0.9698	0.9700	0.9538	0.8766	0.7366	0.3396
NM_031119	Ssb	0.3173	0.5182	0.3638	0.3243	0.0335	0.3864	0.0785	0.0974	0.5663	0.7165	0.4752	0.7902
NM_031120	Ssr3	0.3052	0.0147	0.8698	0.3731	0.2729	0.5525	0.4923	0.1153	0.1701	0.3538	0.2871	0.4687
NM_031134	Thra	0.6192	0.5502	0.9192	0.5737	0.7071	0.3927	0.8131	0.3930	0.5615	0.5180	0.5253	0.7681
NM_031116	Ccl5	0.0873	0.4949	0.6228	0.3070	0.1071	0.0634	0.0365	0.0226	0.3117	0.5015	0.4358	0.2489
NM_031131	Tgfb2	0.9935	0.9820	0.9522	0.9953	0.9148	0.9902	0.9998	0.9902	0.7543	0.9921	0.9940	0.9999
NM_031358	Kcnj11	0.2148	0.0401	0.2781	0.0803	0.4191	0.3183	0.1125	0.2845	0.1587	0.2654	0.0817	0.1742
NM_031333	Cdh2	0.6626	0.7206	0.6586	0.5710	0.6060	0.6649	0.5588	0.2606	0.6004	0.7042	0.6558	0.4699
NM_031112	Rps24	0.2665	0.3845	0.1868	0.0463	0.0769	0.0880	0.3378	0.0649	0.0860	0.1079	0.2133	0.1320
NM_031099	Rpl5	0.0056	0.7179	0.0054	0.6573	0.0214	0.2918	0.6265	0.0036	0.6221	0.8300	0.8946	0.6995
NM_001110141	Eif5b	0.5769	0.4351	0.4160	0.2989	0.0953	0.3004	0.1291	0.2751	0.0812	0.3657	0.5820	0.7942
NM_031129	Tceb2	0.4049	0.6492	0.6675	0.9096	0.1770	0.1047	0.7098	0.5550	0.3215	0.4002	0.4184	0.6881
NM_031140	Vim	0.2248	0.5557	0.0293	0.2748	0.5431	0.4655	0.2044	0.2891	0.1258	0.1915	0.4908	0.3162
NM_031316	Prl3c1	0.6837	0.2707	0.1782	0.1091	0.4539	0.2054	0.1326	0.2955	0.2096	0.3501	0.5343	0.3376
NM_031145	Cib1	0.0195	0.0901	0.0427	0.1324	0.1408	0.1843	0.0339	0.0805	0.1829	0.0159	0.0878	0.1968
NM_031539	Cd8b	0.7774	0.5042	0.7501	0.8184	0.7091	0.9083	0.5334	0.7990	0.4678	0.8308	0.5284	0.7813
NM_031538	Cd8a	0.7624	0.2334	0.2442	0.4719	0.2349	0.5910	0.4148	0.3220	0.1565	0.3313	0.0989	0.2204
NM_031050	Lum	0.9280	0.0580	0.8520	0.9065	0.9959	0.8625	0.9884	0.9763	0.0736	0.0665	0.1644	0.0033
NM_031127	Suox	0.6604	0.1886	0.9826	0.7529	0.7904	0.7366	0.9413	0.7503	0.9114	0.7749	0.5376	0.9144
NM_031118	Soat1	0.3746	0.0192	0.6641	0.6748	0.7859	0.6692	0.6484	0.6390	0.5855	0.4110	0.7174	0.7173
NM_030992	Pld1	0.6764	0.9675	0.8787	0.8227	0.8460	0.7434	0.9079	0.9175	0.9814	0.5215	0.9235	0.7811
NM_031751	Shank1	0.9536	0.7466	0.7411	0.4310	0.7994	0.7398	0.9029	0.7481	0.7375	0.8630	0.1682	0.8823
NM_031138	Ube2b	0.3102	0.6055	0.4134	0.5935	0.5024	0.5097	0.5014	0.4436	0.5202	0.6306	0.5453	0.3549
NM_031137	Tpp2	0.9761	0.8717	0.9167	0.6924	0.9618	0.9392	0.9569	0.9445	0.9340	0.9515	0.9789	0.9305
NM_031773	Polr1b	0.8557	0.7375	0.5729	0.9033	0.7075	0.7927	0.6480	0.8084	0.7004	0.4348	0.7508	0.8353
NM_031507	Egfr	0.7535	0.0112	0.8660	0.0261	0.8641	0.7504	0.6363	0.8936	0.2036	0.1796	0.0972	0.9068
NM_031622	Mapk6	0.9730	0.9939	0.9410	0.7243	0.8785	0.8983	0.8361	0.9242	0.9684	0.9835	0.9896	0.9889
NM_031556	Cav1	0.6000	0.7048	0.9661	0.9995	0.6048	0.8550	0.8209	0.6185	0.7073	0.6073	0.3935	0.9769
NM_031533	Ugt2b	0.9576	0.1832	0.6603	0.7776	0.4238	0.1997	0.4633	0.5704	0.7338	0.5653	0.2812	0.5500
NM_031645	Ramp1	0.8601	0.8229	0.8831	0.8840	0.9842	0.8087	0.8682	0.8736	0.9252	0.8862	0.9697	0.8209
NM_031525	Pdgfrb	0.5586	0.6295	0.4701	0.6147	0.7225	0.5269	0.5523	0.6863	0.4775	0.6674	0.2123	0.5643
NM_031079	Pde2a	0.5648	0.4833	0.4778	0.5418	0.5923	0.4626	0.3728	0.4927	0.7069	0.6172	0.2118	0.7330
NM_031065	Rpl10a	0.9015	0.7194	0.6793	0.7395	0.8026	0.7812	0.7507	0.7178	0.6972	0.7188	0.7127	0.6457
NM_031786	Trim3	0.7488	0.1869	0.7663	0.5152	0.0522	0.0867	0.7933	0.3275	0.9504	0.5774	0.3609	0.5800
NM_031066	Fez1	0.1677	0.0219	0.0653	0.0566	0.8151	0.9683	0.3127	0.9584	0.1352	0.1591	0.0896	0.4300
NM_031553	Nfyb	0.9469	0.9635	0.8396	0.4321	0.4579	0.3540	0.7140	0.8613	0.7458	0.8966	0.6199	0.3731
NM_031069	Nell1	0.3386	0.5520	0.4075	0.7676	0.7585	0.4576	0.5930	0.3426	0.5852	0.7368	0.6832	0.2417
NM_031331	Psmd4	0.0060	0.6578	0.0387	0.1250	0.0653	0.0080	0.0509	0.3929	0.6109	0.7736	0.6967	0.0631
NM_031326	Tfam	0.9506	0.9018	0.8033	0.9047	0.6120	0.8896	0.9699	0.6564	0.3983	0.6193	0.9219	0.1398
NM_031132	Tgfbr2	0.4241	0.1751	0.6630	0.4675	0.6493	0.5691	0.8303	0.8110	0.3670	0.6788	0.5887	0.8524
NM_031153	Sharpin	0.4625	0.2656	0.0407	0.1322	0.3696	0.1599	0.2913	0.3402	0.0304	0.3877	0.2261	0.0200
NM_031357	Tpp1	0.0790	0.1210	0.8937	0.2784	0.1657	0.4030	0.0115	0.2841	0.2407	0.0792	0.2344	0.9306
NM_031744	Slc16a8	0.2985	0.2969	0.2117	0.1822	0.2713	0.3502	0.3785	0.3320	0.2498	0.1628	0.3294	0.3440

NM_031337	St3gal5	0.5026	0.3789	0.6426	0.6244	0.6161	0.5611	0.4655	0.6031	0.4525	0.5322	0.2095	0.5593
NM_030998	Amhr2	0.9340	0.7742	0.7201	0.1186	0.7496	0.7801	0.4195	0.8249	0.5259	0.6740	0.5424	0.1327
NM_031143	Dgkz	0.8789	0.9002	0.3116	0.8973	0.8383	0.9374	0.7918	0.8951	0.8825	0.6223	0.8782	0.4892
NM_032065	Slc1a6	0.6612	0.6244	0.3245	0.4172	0.1869	0.5808	0.3425	0.5233	0.5534	0.5145	0.6104	0.2220
NM_031338	Camkk2	0.7465	0.8445	0.5706	0.7619	0.9827	0.8754	0.8938	0.7446	0.3468	0.8198	0.7419	0.8203
NM_031970	Hspb1	0.0264	0.0216	0.0776	0.0332	0.0208	0.0440	0.0026	0.0038	0.2597	0.0005	0.1179	0.0692
NM_031802	Gabbr2	0.5782	0.7493	0.5932	0.4851	0.3764	0.3580	0.7172	0.4640	0.2787	0.5452	0.7835	0.7985
NM_031109	Rps10	0.6966	0.5925	0.8155	0.6295	0.3688	0.1156	0.9245	0.4668	0.4845	0.6147	0.8711	0.3898
NM_031324	Prep	0.7044	0.7441	0.0981	0.9924	0.8231	0.6673	0.9174	0.4537	0.9092	0.7287	0.7695	0.8412
NM_031320	Celsr3	0.3945	0.4393	0.3847	0.7738	0.8470	0.5088	0.6093	0.7957	0.3856	0.5225	0.8280	0.9450
NM_031087	Psen2	0.2788	0.3295	0.3846	0.4585	0.4163	0.2716	0.1869	0.3498	0.4041	0.3338	0.3014	0.2785
NM_030995	Map1a	0.2623	0.3386	0.2240	0.3363	0.2753	0.3693	0.7245	0.2556	0.3844	0.1359	0.3165	0.2741
NM_031093	Rala	0.4549	0.5836	0.0600	0.4535	0.5541	0.3802	0.1257	0.1216	0.0759	0.5069	0.4705	0.6084
NM_030989	Tp53	0.9691	0.9914	0.1766	0.4938	0.9759	0.5252	0.3940	0.9490	0.9449	0.9386	0.7609	0.4393
NM_053347	Nde1	0.7163	0.9056	0.3588	0.8891	0.9387	0.8311	0.9469	0.9567	0.9570	0.9925	0.7755	0.3726
NM_031091	Rab3b	0.0046	0.8162	0.8002	0.7665	0.0387	0.1565	0.8049	0.0984	0.7397	0.4182	0.7167	0.9545
NM_031664	Slc28a2	0.9762	0.8325	0.8923	0.6048	0.7428	0.7094	0.8661	0.7758	0.9122	0.9484	0.7850	0.6066
NM_031106	Rpl37	0.1337	0.5658	0.2144	0.3068	0.0832	0.0860	0.7060	0.0620	0.1399	0.2158	0.4142	0.2584
NM_031339	Parg	0.8902	0.4700	0.1116	0.7575	0.8940	0.7048	0.1807	0.7488	0.4066	0.7345	0.4242	0.8899
NM_031523	Klk1b3	0.8081	0.5955	0.2173	0.0843	0.7040	0.5768	0.0988	0.1973	0.2231	0.6309	0.3649	0.3481
NM_031830	Flot2	0.9107	0.9906	0.7583	0.9793	0.9310	0.9734	0.9540	0.9769	0.9777	0.9258	0.8598	0.9678
NM_031643	Map2k1	0.3402	0.3645	0.3643	0.8334	0.2898	0.6783	0.5539	0.4720	0.2870	0.2674	0.0671	0.9681
NM_031522	Neu1	0.0213	0.7230	0.9385	0.6895	0.1849	0.4493	0.4338	0.9442	0.7389	0.6934	0.7267	0.9977
NM_031115	Sctr	0.6828	0.0155	0.5898	0.8761	0.8919	0.7178	0.9290	0.8186	0.0713	0.0137	0.0769	0.0133
NM_031585	Pla2g1b	0.2885	0.4651	0.1091	0.5581	0.5308	0.2417	0.6182	0.8351	0.6504	0.7995	0.8648	0.7170
NM_031102	Rpl18	0.6303	0.6925	0.3822	0.1606	0.1362	0.1103	0.1597	0.0978	0.2613	0.6323	0.5695	0.0128
NM_031351	Atrn	0.3340	0.2967	0.2438	0.0278	0.2316	0.3024	0.1681	0.2807	0.1603	0.2631	0.2777	0.2460
NM_031838	Rps2	0.2382	0.6318	0.7148	0.7278	0.4587	0.6112	0.7443	0.4969	0.5232	0.5724	0.4788	0.2011
NM_031332	Slc22a8	0.3854	0.4556	0.4610	0.2285	0.5907	0.1235	0.3007	0.1486	0.4599	0.4585	0.3608	0.1723
NM_001003653	Pard6a	0.4279	0.5331	0.3582	0.8013	0.6881	0.2159	0.4193	0.8347	0.5430	0.6988	0.5384	0.6028
NM_031238	Sh3gl3	0.3257	0.5040	0.5893	0.3528	0.1011	0.1766	0.0674	0.0830	0.2004	0.0997	0.1348	0.2230
NM_001003654	Pard6a	0.4279	0.5331	0.3582	0.8013	0.6881	0.2159	0.4193	0.8347	0.5430	0.6988	0.5384	0.6028
NM_031554	Acvr2b	0.5510	0.7582	0.8645	0.6102	0.4943	0.5788	0.7737	0.6647	0.7554	0.7710	0.9536	0.8155
NM_031343	Slc6a2	0.7070	0.7464	0.9346	0.8058	0.5479	0.6276	0.8548	0.7064	0.8747	0.6168	0.9737	0.3600
NM_031972	Aldh3a1	0.7951	0.9221	0.8598	0.9662	0.8923	0.9854	0.9093	0.7470	0.9326	0.8526	0.9425	0.0394
NM_033485	Pawr	0.9092	0.9490	0.9757	0.9799	0.9642	0.8980	0.7967	0.9980	0.6719	0.9595	0.8482	0.4841
NM_053021	Clu	0.7807	0.0609	0.6858	0.0011	0.2520	0.4302	0.1355	0.7692	0.0326	0.0114	0.0352	0.9806
NM_001080147	Anapc5	0.4621	0.7372	0.5876	0.6888	0.7458	0.7842	0.9016	0.8715	0.5878	0.7247	0.7402	0.2015
NM_031321	Slit3	0.4243	0.1064	0.2687	0.2181	0.4169	0.1314	0.1419	0.1690	0.3479	0.3658	0.0853	0.3487
NM_031560	Ctsk	0.7957	0.9045	0.5589	0.0195	0.8943	0.7822	0.0282	0.7226	0.8560	0.6372	0.7238	0.9287
NM_031322	Lrp4	0.9426	0.8270	0.9813	0.9282	0.7475	0.7667	0.9422	0.5891	0.3519	0.6728	0.9183	0.4621
NM_031576	Por	0.8495	0.5692	0.5370	0.6332	0.2409	0.7492	0.4074	0.4486	0.8368	0.0994	0.4631	0.9126
NM_053532	Psmb7	0.0055	0.0395	0.1148	0.2054	0.0389	0.0349	0.0087	0.1463	0.0992	0.3510	0.2453	0.0852
NM_053512	Prdx4	0.2830	0.4467	0.4519	0.5235	0.1794	0.1783	0.1865	0.3855	0.3775	0.4367	0.3457	0.1756
NM_031550	Cdkn2a	0.2990	0.1696	0.4285	0.4381	0.7092	0.2721	0.3760	0.5159	0.4348	0.3752	0.2915	0.3790
NM_031512	Il1b	0.4949	0.3925	0.2549	0.5541	0.3190	0.5557	0.8107	0.6330	0.2830	0.5389	0.9032	0.8257
NM_031341	Slc7a7	0.0812	0.3549	0.0580	0.3735	0.0993	0.0151	0.5810	0.2118	0.2206	0.6102	0.0678	0.0891
NM_031511	Igf2	0.5059	0.3773	0.3461	0.4961	0.4572	0.4238	0.5535	0.4535	0.3987	0.3296	0.2631	0.5541
NM_031729	Ppp5c	0.9473	0.7799	0.0542	0.6657	0.7498	0.9271	0.6966	0.8576	0.6035	0.9355	0.8574	0.4668

NM_031334	Cdh1	0.4563	0.3954	0.4284	0.2365	0.4605	0.6855	0.4004	0.5934	0.3335	0.0698	0.1244	0.2377
NM_031514	Jak2	0.4359	0.0483	0.9746	0.8136	0.0294	0.7914	0.0399	0.0716	0.8059	0.5471	0.8507	0.7945
NM_031335	Polr2f	0.6537	0.0430	0.1712	0.0927	0.7309	0.2657	0.2505	0.7372	0.5602	0.8899	0.0369	0.0425
NM_031720	Dio2	0.7965	0.3736	0.8738	0.5933	0.2273	0.1595	0.5206	0.5215	0.7609	0.2517	0.2084	0.4483
NM_031629	Psmb4	0.0008	0.0443	0.1630	0.1872	0.0184	0.0046	0.0079	0.0043	0.0279	0.0011	0.0017	0.0031
NM_031340	Timeless	0.7031	0.9861	0.0103	0.3629	0.9913	0.9151	0.9546	0.8024	0.9781	0.9216	0.8727	0.1788
NM_031776	Gda	0.0002	0.0010	0.6633	0.0220	0.0276	0.0993	0.3065	0.0014	0.0176	0.0006	0.0014	0.8255
NM_031342	Lypla2	0.2638	0.2184	0.4033	0.9374	0.0982	0.8694	0.6146	0.1723	0.3103	0.0184	0.2366	0.9168
NM_031703	Aqp3	0.2455	0.5893	0.6315	0.3913	0.7856	0.4411	0.3519	0.3820	0.7491	0.2046	0.0313	0.5511
NM_031698	Rpn2	0.9250	0.6342	0.2455	0.4050	0.5710	0.6248	0.8497	0.4849	0.8787	0.9125	0.7110	0.6588
NM_031537	LOC24906	0.9733	0.7196	0.2556	0.4879	0.2795	0.5681	0.9531	0.5757	0.6208	0.8971	0.8161	0.7023
NM_053769	Dusp1	0.2760	0.3025	0.0116	0.3257	0.0178	0.3307	0.3743	0.0428	0.3098	0.3340	0.3520	0.8202
NM_031344	Fads2	0.0080	0.4308	0.1633	0.7539	0.0461	0.2118	0.4407	0.0118	0.4071	0.1120	0.1207	0.1006
NM_031346	Rod1	0.7083	0.4550	0.5758	0.8093	0.3426	0.5491	0.2570	0.1036	0.7873	0.6002	0.4209	0.9045
NM_031561	Cd36	0.7279	0.6187	0.6101	0.8615	0.5991	0.1602	0.4920	0.6915	0.3546	0.4527	0.1464	0.5542
NM_031518	Cd200	0.1734	0.1276	0.2887	0.0500	0.1041	0.2549	0.0285	0.2705	0.0013	0.0099	0.0083	0.0082
NM_031584	Slc22a2	0.0280	0.3928	0.0533	0.4666	0.3860	0.6171	0.6899	0.1000	0.3324	0.2559	0.2971	0.4057
NM_031350	Pex3	0.1477	0.0851	0.3834	0.3929	0.2129	0.0523	0.0122	0.2778	0.3588	0.1271	0.2811	0.2463
NM_031352	Dbnl	0.4962	0.3521	0.3779	0.4951	0.3525	0.4133	0.3473	0.2700	0.3323	0.4267	0.2401	0.6927
NM_031355	Vdac3	0.8097	0.7718	0.9115	0.8697	0.5321	0.6633	0.8293	0.6730	0.5653	0.8434	0.7969	0.6977
NM_031354	Vdac2	0.6961	0.8701	0.4963	0.8528	0.5482	0.7998	0.9373	0.6921	0.9001	0.6613	0.8297	0.8362
NM_031353	Vdac1	0.6646	0.6698	0.8251	0.9817	0.8011	0.4342	0.9660	0.5802	0.8099	0.8453	0.9223	0.5611
NM_031571	Acvr2a	0.7209	0.5838	0.7612	0.6588	0.8632	0.3970	0.8302	0.8670	0.6726	0.5870	0.7979	0.8833
NM_031359	Mkln1	0.0868	0.2822	0.5752	0.0049	0.1341	0.0708	0.1784	0.0023	0.3976	0.5174	0.0857	0.9012
NM_031543	Cyp2e1	0.5939	0.4788	0.4487	0.4507	0.5690	0.4192	0.6094	0.5343	0.4424	0.6332	0.1728	0.4537
NM_031797	Cd82	0.8627	0.0355	0.0241	0.8101	0.0551	0.3112	0.0093	0.3071	0.1520	0.1835	0.1202	0.7805
NM_031737	Nkx6-1	0.8811	0.9239	0.5519	0.4575	0.6213	0.7761	0.8541	0.6947	0.8714	0.5340	0.8241	0.9290
NM_031577	Ghrh	0.3670	0.6613	0.2113	0.2643	0.6734	0.1444	0.5377	0.4610	0.0861	0.1887	0.1736	0.0691
NM_031705	Dpys	0.0162	0.2530	0.2541	0.2301	0.1660	0.1888	0.4885	0.6070	0.6840	0.0398	0.5645	0.1388
NM_031508	Grik5	0.8372	0.8334	0.6638	0.4803	0.3880	0.6978	0.6634	0.8344	0.8957	0.6100	0.7616	0.6917
NM_031548	Scnn1a	0.7641	0.7500	0.3816	0.4997	0.8348	0.7025	0.5800	0.4523	0.9307	0.8999	0.6685	0.9518
NM_033230	Akt1	0.4263	0.5513	0.0441	0.8232	0.0636	0.7516	0.4226	0.0994	0.3739	0.5233	0.9171	0.7753
NM_033097	Vps52	0.7795	0.8103	0.5350	0.6347	0.7895	0.8235	0.9042	0.8383	0.8313	0.8881	0.7313	0.7832
NM_031762	Cdkn1b	0.9780	0.9929	0.9613	0.9230	0.9994	0.9273	0.9403	0.9757	0.9624	0.9818	0.9726	0.7516
NM_031513	Il3	0.0954	0.4258	0.2752	0.2432	0.6028	0.5448	0.2792	0.2102	0.1114	0.0213	0.2922	0.2629
NM_031787	Hipk3	0.0166	0.3420	0.3765	0.1585	0.0875	0.3531	0.1755	0.1466	0.0875	0.4562	0.1489	0.5060
NM_031570	Rps7	0.4214	0.1694	0.2964	0.1817	0.4347	0.1782	0.1859	0.1770	0.1685	0.4987	0.1710	0.1753
NM_031520	Myh10	0.9768	0.9056	0.9900	0.9755	0.9973	0.9929	0.9878	0.9979	0.9991	0.9996	0.9964	0.7614
NM_033237	Gal	0.7836	0.6001	0.7172	0.2020	0.4436	0.5015	0.5923	0.7445	0.7187	0.9296	0.6620	0.7740
NM_031799	Pax4	0.0309	0.1852	0.1180	0.1423	0.0994	0.0686	0.1398	0.1461	0.1898	0.0391	0.0387	0.0139
NM_031665	Stx6	0.1899	0.5989	0.8485	0.3961	0.6196	0.3633	0.4227	0.8985	0.2999	0.1474	0.3153	0.3833
NM_031695	St3gal2	0.5443	0.3980	0.1817	0.9764	0.9398	0.9360	0.6390	0.9147	0.7745	0.5457	0.8413	0.9884
NM_031528	Rara	0.9422	0.1218	0.8773	0.8375	0.8645	0.8264	0.7818	0.9114	0.2251	0.1612	0.8323	0.8852
NM_031531	Serpina3n	0.3315	0.1143	0.1561	0.0091	0.9468	0.9300	0.2864	0.9744	0.0819	0.1410	0.0969	0.9693
NM_031783	Nefl	0.2941	0.4218	0.7791	0.4637	0.7338	0.2382	0.5456	0.4084	0.1393	0.6208	0.3725	0.4429
NM_031572	Cyp2c12	0.0874	0.3936	0.0728	0.0193	0.0229	0.0774	0.2595	0.2353	0.3565	0.0202	0.1166	0.0418
NM_031819	Fat	0.4930	0.6062	0.5197	0.6332	0.6766	0.6481	0.6121	0.7033	0.4640	0.7200	0.6331	0.6083
NM_031536	Smcp	0.5633	0.6746	0.8600	0.7136	0.7487	0.7655	0.9816	0.7547	0.5884	0.6758	0.7328	0.2759
NM_031534	Wt1	0.9364	0.9395	0.9577	0.9517	0.9696	0.7230	0.9613	0.9132	0.7182	0.5106	0.9734	0.9638

NM_183402	Grinl1a	0.5135	0.6019	0.6361	0.4596	0.5887	0.5408	0.1963	0.5833	0.6636	0.5237	0.5817	0.4471
NM_178104	Cpg1	0.6023	0.5077	0.6230	0.2327	0.5219	0.6142	0.6896	0.6452	0.6841	0.6508	0.5633	0.6078
NM_178092	Slc4a10	0.5302	0.4504	0.6159	0.0560	0.2570	0.1908	0.4599	0.5458	0.0630	0.2869	0.4584	0.7967
NM_181823	Rhot2	0.7999	0.7849	0.1609	0.7235	0.8538	0.9293	0.8893	0.7222	0.9168	0.6641	0.6236	0.3183
NM_178091	Insig2	0.0042	0.0243	0.6332	0.0053	0.1029	0.5983	0.0048	0.1235	0.0051	0.0031	0.0024	0.7009
NM_177982	Hap1	0.2525	0.6233	0.7831	0.7368	0.6570	0.6373	0.8008	0.6663	0.6130	0.6146	0.2835	0.8355
NM_201422	Pcdhac2	0.6692	0.6174	0.6186	0.6799	0.7284	0.5106	0.7155	0.7287	0.6429	0.6855	0.6351	0.7078
NM_181372	Klre1	0.3581	0.4181	0.1799	0.4786	0.3820	0.1778	0.1857	0.1769	0.1683	0.1626	0.4813	0.1749
NM_177935	Il1rapl1	0.6300	0.5815	0.7968	0.2439	0.4906	0.4226	0.6104	0.3103	0.3458	0.5309	0.2620	0.1201
NM_001077683	Btbd6	0.3169	0.5698	0.3332	0.6193	0.1792	0.2770	0.4478	0.1766	0.2482	0.3874	0.4417	0.5293
NM_198769	Tlr2	0.7439	0.6325	0.5466	0.5167	0.5458	0.4469	0.4887	0.6732	0.4413	0.3822	0.5665	0.4377
NM_181769	Tnni3k	0.8222	0.5116	0.8627	0.8176	0.7175	0.8449	0.5510	0.6146	0.5777	0.6798	0.5806	0.5465
NM_181695	Acsm4	0.5166	0.0450	0.0874	0.2685	0.2911	0.1110	0.7161	0.3846	0.0541	0.1550	0.0998	0.0962
NM_199092	Orc4l	0.3407	0.7974	0.2944	0.0839	0.0923	0.1150	0.0818	0.2137	0.5270	0.7083	0.7792	0.1604
NM_177930	Vac14	0.1022	0.1283	0.0421	0.0676	0.0107	0.0139	0.2912	0.2048	0.1509	0.3607	0.0218	0.0568
NM_001079886	Cuedc2	0.7953	0.7121	0.6679	0.8641	0.7684	0.5944	0.7538	0.8192	0.3139	0.7692	0.7897	0.4803
NM_182735	Cdkn1c	0.5953	0.4758	0.5969	0.4767	0.4504	0.6506	0.5312	0.2441	0.8198	0.7427	0.5118	0.6519
NM_031542	Brca2	0.4501	0.4114	0.4550	0.3314	0.2504	0.2771	0.4378	0.1766	0.3143	0.2737	0.5584	0.3868
NM_031627	Nr1h3	0.0483	0.1130	0.1953	0.8378	0.8771	0.8449	0.1282	0.6368	0.1535	0.0732	0.1190	0.0315
NM_031573	Phkg1	0.8269	0.3251	0.8127	0.6090	0.6392	0.8038	0.6591	0.8370	0.7434	0.6612	0.5447	0.8288
NM_031821	Plk2	0.7718	0.9716	0.9739	0.6682	0.8974	0.9605	0.7773	0.5878	0.7683	0.8656	0.9588	0.8529
NM_031610	Kcnj3	0.2401	0.5878	0.1248	0.6490	0.0639	0.1484	0.4134	0.1105	0.0680	0.4985	0.4322	0.1999
NM_031687	Uba52	0.6119	0.3787	0.4473	0.5944	0.4108	0.0877	0.6338	0.4913	0.6846	0.4290	0.8497	0.0341
NM_033376	Kcnk3	0.4490	0.8358	0.9748	0.8889	0.5229	0.9509	0.6179	0.9335	0.2223	0.4531	0.4192	0.8658
NM_031761	Figf	0.7672	0.0165	0.3578	0.1199	0.6520	0.6047	0.2141	0.6888	0.1426	0.0296	0.2248	0.1704
NM_031739	Kcnd3	0.3304	0.7397	0.6628	0.4475	0.8553	0.8334	0.4198	0.2820	0.5959	0.6260	0.6124	0.7822
NM_031549	Tagln	0.6525	0.2247	0.0153	0.1719	0.9565	0.7463	0.7270	0.9664	0.0901	0.0598	0.0336	0.1885
NM_031706	Rps8	0.4559	0.2637	0.5455	0.7121	0.4308	0.6601	0.3693	0.2211	0.1994	0.2710	0.4544	0.5418
NM_031552	Add3	0.8501	0.3498	0.1458	0.2790	0.0224	0.1462	0.0954	0.0281	0.3923	0.2602	0.4777	0.2521
NM_033235	Mdh1	0.0694	0.0887	0.5600	0.7132	0.0356	0.1250	0.6102	0.4819	0.0748	0.2665	0.1017	0.2355
NM_031726	Scamp5	0.0912	0.1216	0.0317	0.0196	0.1469	0.2444	0.0786	0.2092	0.0648	0.1162	0.1534	0.7568
NM_031612	Apln	0.9436	0.6674	0.7846	0.9203	0.7918	0.7735	0.9332	0.8770	0.6993	0.8643	0.5209	0.5501
NM_031973	Dpp7	0.1595	0.0061	0.0879	0.0582	0.7087	0.6352	0.0402	0.8134	0.0685	0.0705	0.0907	0.0681
NM_001108143	Rbm7	0.4787	0.2409	0.2561	0.0957	0.1531	0.5387	0.0259	0.0902	0.0621	0.4265	0.5339	0.4871
NM_001108142	Sidt2	0.9336	0.3505	0.6663	0.2754	0.8582	0.5796	0.4067	0.6491	0.8079	0.0697	0.2150	0.9663
NM_031793	Ppig	0.7116	0.7041	0.6503	0.7063	0.5341	0.4866	0.4071	0.5339	0.3994	0.7352	0.7297	0.5186
NM_001108380	Rcbtb1	0.7757	0.8973	0.9222	0.9118	0.9255	0.9483	0.9400	0.9588	0.9743	0.5255	0.8424	0.8544
NM_001108191	Ccrl2	0.4848	0.2861	0.4317	0.5959	0.3121	0.5792	0.3950	0.5368	0.0904	0.4851	0.3428	0.2336
NM_001108201	Abcc10	0.3907	0.5713	0.5725	0.5122	0.5821	0.8008	0.4077	0.4812	0.5908	0.6183	0.7595	0.8161
NM_001108464	Prr18	0.7258	0.4498	0.5722	0.4538	0.3735	0.5895	0.5279	0.6579	0.6406	0.5821	0.6764	0.5927
NM_001108461	Tppp	0.6931	0.8366	0.4236	0.6550	0.8284	0.8525	0.8135	0.6923	0.8735	0.6937	0.3808	0.8032
NM_001108432	Prr16	0.6027	0.6979	0.6862	0.7424	0.7418	0.7780	0.6775	0.3826	0.1146	0.4230	0.7677	0.2502
NM_001134574	LOC361646	0.9065	0.8800	0.7115	0.3538	0.7506	0.9443	0.6400	0.9118	0.9068	0.9700	0.9975	0.9610
NM_001108173	Tbx18	0.2715	0.0837	0.8851	0.6666	0.3266	0.9831	0.2410	0.3611	0.3878	0.1144	0.6719	0.7507
NM_001109120	Mboat1	0.9568	0.9651	0.9970	0.8150	0.8621	0.9795	0.9219	0.9685	0.9812	0.9903	0.9838	0.9482
NM_001108465	Usp29	0.7854	0.8241	0.5627	0.8535	0.3699	0.8603	0.9154	0.7039	0.4110	0.9479	0.8518	0.4668
NM_001108165	RGD1560796	0.8464	0.1955	0.8235	0.8957	0.8565	0.6463	0.0608	0.9489	0.4958	0.7104	0.8534	0.7880
NM_001108162	Rasl12	0.2776	0.6651	0.2186	0.7190	0.2714	0.8183	0.7683	0.3627	0.6257	0.4698	0.6825	0.6522
NM_001108401	Alg11	0.2028	0.4335	0.2211	0.1122	0.0514	0.1494	0.1249	0.0808	0.4546	0.1341	0.0582	0.2231

NM_001109214	En2	0.1677	0.5333	0.5649	0.6812	0.6966	0.6541	0.3115	0.4514	0.4467	0.4064	0.7373	0.4098
NM_001109106	Hesx1	0.4397	0.3774	0.2893	0.3276	0.5749	0.4837	0.2205	0.4170	0.1272	0.4181	0.6061	0.2160
NM_001108883	Suv39h2	0.4757	0.6436	0.8067	0.1173	0.9109	0.8590	0.4993	0.8685	0.9673	0.8845	0.3437	0.6796
NM_001108877	Tpte	0.3464	0.5313	0.3648	0.1566	0.1617	0.2774	0.2040	0.3808	0.2121	0.1152	0.5844	0.3819
NM_001108328	Zfp358	0.7686	0.7158	0.8223	0.9486	0.8623	0.8650	0.8292	0.9194	0.5399	0.8651	0.4856	0.5694
NM_001108324	Ranbp1	0.6510	0.8359	0.0795	0.8185	0.7637	0.2341	0.8954	0.3110	0.8394	0.9658	0.8824	0.4075
NM_001108298	Cdc6	0.9525	0.9675	0.1583	0.9703	0.9776	0.9744	0.7698	0.9980	0.8696	0.8183	0.3970	0.0648
NM_001108295	Ccdc49	0.8592	0.9538	0.9469	0.7951	0.9267	0.9584	0.5770	0.7947	0.4827	0.8835	0.9368	0.8096
NM_001108303	Sdk2	0.3374	0.2763	0.5069	0.1128	0.6907	0.7910	0.7096	0.0243	0.0572	0.1547	0.1193	0.9530
NM_001108160	Punc	0.3525	0.0194	0.0238	0.2207	0.6135	0.2538	0.6027	0.4549	0.1302	0.0373	0.4969	0.3052
NM_001108154	Larp6	0.8143	0.5971	0.1490	0.7772	0.8902	0.5218	0.5900	0.8203	0.3214	0.5700	0.7113	0.6093
NM_001108152	Imp3	0.8696	0.5999	0.7550	0.4493	0.3791	0.2454	0.0869	0.6586	0.2103	0.0899	0.3013	0.1990
NM_001108151	Odf3l1	0.9875	0.8245	0.8387	0.9171	0.8025	0.9634	0.8910	0.8737	0.8098	0.8367	0.9160	0.8831
NM_001108150	Hmg20a	0.6522	0.8575	0.0401	0.3673	0.3831	0.3361	0.2757	0.0490	0.4926	0.7892	0.3337	0.7537
NM_001108149	RGD1312026	0.8779	0.9091	0.8980	0.3283	0.9409	0.8352	0.9108	0.8547	0.9185	0.8816	0.9759	0.9363
NM_031563	Ybx1	0.0799	0.9007	0.7911	0.7343	0.2773	0.5461	0.5167	0.6495	0.4985	0.5432	0.8556	0.6449
NM_031600	Ptprn2	0.7992	0.3320	0.5488	0.7859	0.7217	0.7377	0.6764	0.2632	0.4349	0.7054	0.7079	0.6613
NM_053654	Pafah1b3	0.3176	0.6035	0.5408	0.0024	0.2073	0.0025	0.4782	0.1482	0.1145	0.5874	0.4965	0.0616
NM_031568	Clcn7	0.2821	0.4887	0.0882	0.1033	0.7739	0.6912	0.0631	0.1475	0.7672	0.4909	0.3453	0.9414
NM_053538	Laptm5	0.6722	0.5904	0.5542	0.5120	0.0067	0.1255	0.3479	0.2832	0.7320	0.4519	0.3318	0.5795
NM_031853	Dbi	0.9503	0.7834	0.2734	0.7990	0.9655	0.8904	0.7594	0.7383	0.2993	0.7481	0.8051	0.0339
NM_031587	Pxmp2	0.7815	0.7721	0.7578	0.7743	0.7588	0.8061	0.7370	0.9251	0.7215	0.9915	0.8536	0.6526
NM_031586	Ighmbp2	0.5129	0.1782	0.1148	0.1163	0.1603	0.1392	0.6439	0.4111	0.4860	0.6510	0.4880	0.1526
NM_033299	Pld2	0.1469	0.4664	0.4468	0.4251	0.8247	0.5871	0.5317	0.6666	0.7960	0.8014	0.4980	0.3677
NM_032614	Glrx3	0.3131	0.7370	0.3853	0.0300	0.0253	0.2261	0.3802	0.1981	0.4653	0.3895	0.5860	0.6164
NM_031581	Ppyr1	0.0101	0.3971	0.0454	0.4344	0.1967	0.1638	0.0432	0.1275	0.2508	0.4506	0.1205	0.2173
NM_032416	Aldh2	0.2275	0.4261	0.4444	0.3067	0.2174	0.2879	0.2870	0.5064	0.2165	0.3305	0.4729	0.4769
NM_031663	Slc18a3	0.1890	0.5073	0.3597	0.4824	0.5071	0.3268	0.5228	0.6231	0.4847	0.4106	0.6107	0.8902
NM_001109639	Chst14	0.1248	0.5824	0.7099	0.2027	0.8736	0.8952	0.2307	0.6228	0.6960	0.3788	0.2966	0.2478
NM_001109630	Slc12a6	0.7967	0.5431	0.7728	0.6687	0.8830	0.8185	0.3335	0.6335	0.8420	0.8738	0.9406	0.5848
NM_001109884	Hoxc4	0.5652	0.5710	0.5910	0.6247	0.6300	0.6052	0.5626	0.4706	0.3810	0.3909	0.3379	0.6353
NM_001109883	Abcf1	0.5737	0.2347	0.4920	0.2893	0.6449	0.6170	0.2610	0.4357	0.4831	0.1997	0.2286	0.5011
NM_053587	S100a9	0.5334	0.4532	0.1763	0.2221	0.1016	0.2951	0.4654	0.6805	0.1464	0.5170	0.6722	0.1617
NM_031589	Slc37a4	0.8816	0.7682	0.4495	0.7233	0.7648	0.8675	0.8625	0.6417	0.8332	0.9642	0.7860	0.8571
NM_031662	Camkk1	0.9698	0.4415	0.8744	0.5095	0.1741	0.2916	0.8069	0.0361	0.5147	0.3498	0.1128	0.4697
NM_031590	Wisp2	0.8038	0.2606	0.1756	0.9533	0.9539	0.9717	0.3082	0.9454	0.0912	0.1064	0.0058	0.0829
NM_031659	Tgm1	0.3115	0.4354	0.3974	0.0880	0.5540	0.3767	0.7263	0.7703	0.2327	0.3058	0.6871	0.3235
NM_001108259	Htatsf1	0.7374	0.3913	0.9111	0.0522	0.8888	0.8261	0.1939	0.9184	0.4442	0.4706	0.6955	0.5277
NM_001108223	Shd	0.6085	0.7969	0.5167	0.5894	0.6094	0.9311	0.9187	0.5870	0.7339	0.8215	0.3159	0.7915
NM_001108255	RGD1565844	0.0969	0.2784	0.0031	0.0394	0.4311	0.3842	0.3767	0.2494	0.2149	0.2213	0.1426	0.6446
NM_001108253	RGD1561065	0.8876	0.8460	0.9451	0.9699	0.9688	0.8686	0.9668	0.8898	0.9885	0.9824	0.6617	0.9863
NM_001108382	Ccdc25	0.3732	0.2825	0.0695	0.2188	0.0680	0.6772	0.1730	0.1064	0.7257	0.0805	0.2391	0.2168
NM_001108375	Mrpl52	0.0373	0.0861	0.6925	0.6866	0.5309	0.0604	0.7267	0.8579	0.7106	0.7418	0.4675	0.2126
NM_001108359	Dcun1d4	0.6134	0.6097	0.5586	0.5873	0.4642	0.5672	0.3598	0.0886	0.6233	0.5042	0.7962	0.4259
NM_001134611	RGD1565096	0.7793	0.7838	0.3703	0.2572	0.4611	0.5745	0.8356	0.0487	0.8569	0.2092	0.2625	0.5440
NM_001134591	RGD1561491	0.3060	0.5179	0.8074	0.0241	0.5732	0.3542	0.7409	0.5054	0.3459	0.4508	0.2069	0.5898
NM_001108222	Smarcal1	0.7870	0.6464	0.0602	0.8404	0.7763	0.7005	0.8608	0.7051	0.8834	0.6770	0.8358	0.5370
NM_001108221	Mdh1b	0.6914	0.8007	0.3600	0.6383	0.9735	0.7121	0.4222	0.7889	0.7613	0.7925	0.9104	0.6561
NM_001108210	Hs6st1	0.8814	0.8271	0.8659	0.9644	0.9522	0.8248	0.9812	0.9849	0.9845	0.8942	0.9173	0.9571

NM_001108198	Guca1b	0.5076	0.7840	0.2971	0.7938	0.5288	0.7471	0.6002	0.4957	0.4739	0.7567	0.7173	0.6467
NM_001108207	Tnfrsf21	0.2954	0.2912	0.2610	0.1982	0.2539	0.2046	0.1609	0.1459	0.2895	0.2755	0.0870	0.5953
NM_001108206	Tcte1	0.1341	0.1235	0.5805	0.3847	0.5537	0.6932	0.3443	0.7068	0.2349	0.4280	0.2377	0.4835
NM_001108195	Kbtbd5	0.2222	0.6818	0.2661	0.0817	0.6075	0.3116	0.4520	0.3521	0.4412	0.1413	0.0696	0.6555
NM_001108194	Oxsr1	0.5550	0.4985	0.9765	0.8551	0.3030	0.9728	0.7406	0.4721	0.9760	0.6624	0.9674	0.9504
NM_001108147	Npat	0.7911	0.9073	0.1986	0.2369	0.9707	0.8549	0.3567	0.4920	0.3456	0.6912	0.8215	0.3922
NM_031647	Sfmbt1	0.8946	0.9320	0.9202	0.8589	0.2789	0.6133	0.9934	0.9696	0.7009	0.8903	0.8832	0.8744
NM_031715	Pfkm	0.7050	0.0859	0.4321	0.2945	0.2500	0.0500	0.7078	0.1343	0.0362	0.0163	0.0131	0.0868
NM_031593	Sv2c	0.5444	0.6482	0.7829	0.8223	0.5912	0.6151	0.7325	0.6898	0.8597	0.7773	0.7703	0.9559
NM_053293	Gstt1	0.0923	0.6172	0.4793	0.1843	0.0502	0.0472	0.6834	0.0827	0.3106	0.7205	0.0865	0.0506
NM_031758	Mchr1	0.5172	0.4058	0.1949	0.7426	0.5091	0.7239	0.4902	0.7026	0.3883	0.1477	0.2707	0.3344
NM_031619	Prg2	0.4025	0.8440	0.3999	0.8405	0.9317	0.5889	0.8885	0.3203	0.5863	0.6874	0.9054	0.5354
NM_031851	Phb	0.2868	0.2171	0.3295	0.0754	0.2800	0.1924	0.6208	0.2495	0.3269	0.0164	0.0393	0.1018
NM_031977	Src	0.0526	0.5611	0.7046	0.2852	0.4327	0.4522	0.4132	0.8284	0.8605	0.5399	0.8376	0.4169
NM_031595	Psmc3	0.7766	0.9132	0.0109	0.9422	0.5127	0.4244	0.9133	0.7608	0.4115	0.3698	0.1518	0.0019
NM_053349	Sox11	0.7259	0.7467	0.9168	0.9420	0.8598	0.8505	0.8449	0.8999	0.6323	0.3107	0.7030	0.7522
NM_053355	Ikbkb	0.9061	0.6363	0.5251	0.0593	0.8393	0.9022	0.0870	0.6841	0.9115	0.8787	0.5786	0.8940
NM_031727	Limk1	0.2838	0.2507	0.1001	0.1494	0.0601	0.2618	0.1941	0.2509	0.0551	0.2019	0.2012	0.0882
NM_031777	Usf1	0.7505	0.7291	0.6839	0.8945	0.9158	0.8161	0.9225	0.9345	0.1523	0.4422	0.5144	0.1301
NM_031704	Stx5	0.7737	0.9544	0.5438	0.2079	0.7945	0.3126	0.9523	0.8138	0.9128	0.5364	0.7028	0.7837
NM_031693	Syt4	0.8924	0.5372	0.4516	0.4676	0.5010	0.6014	0.4124	0.7789	0.5364	0.4185	0.2938	0.2907
NM_031613	Tmod2	0.2337	0.5120	0.5815	0.3654	0.7507	0.1640	0.4088	0.2335	0.0801	0.5048	0.1917	0.1507
NM_053465	Fut9	0.6109	0.4943	0.8092	0.2966	0.3551	0.4753	0.6628	0.4037	0.1869	0.8001	0.4715	0.0482
NM_033233	Prl3d4	0.7712	0.1760	0.0389	0.3739	0.0866	0.0583	0.0406	0.0656	0.0709	0.7079	0.3827	0.0889
NM_033021	Sec31a	0.2632	0.4043	0.2097	0.0915	0.0187	0.0832	0.3531	0.2247	0.7845	0.0575	0.4049	0.1891
NM_031749	Gcs1	0.9579	0.8110	0.9407	0.9021	0.7356	0.8381	0.8441	0.8740	0.7982	0.7886	0.7038	0.9275
NM_031759	Naaladl1	0.6190	0.0820	0.6347	0.5131	0.2658	0.5294	0.2073	0.6298	0.3554	0.6503	0.6475	0.5415
NM_031833	Nme2	0.8103	0.7157	0.3923	0.8438	0.2803	0.5823	0.9053	0.4676	0.8630	0.8332	0.5286	0.2938
NM_031709	Rps12	0.3198	0.4572	0.5044	0.5673	0.6295	0.2587	0.6015	0.4662	0.2074	0.4604	0.1826	0.2262
NM_031624	Igbp1	0.9949	0.4742	0.8920	0.9936	0.8953	0.6983	0.8328	0.8667	0.6279	0.6097	0.7687	0.8478
NM_031626	Nr1h2	0.1555	0.0397	0.0675	0.1763	0.6604	0.7868	0.2182	0.9169	0.4333	0.2403	0.3576	0.5629
NM_031628	Nr4a3	0.6082	0.6055	0.6601	0.7110	0.6174	0.6034	0.7265	0.6534	0.6364	0.6999	0.6580	0.8232
NM_031732	Sult1c3	0.8670	0.6990	0.8105	0.9380	0.7273	0.7721	0.6900	0.7293	0.8931	0.8463	0.8263	0.7914
NM_031811	Taldo1	0.0357	0.0542	0.0905	0.0267	0.0493	0.0184	0.0700	0.0507	0.0923	0.0191	0.0917	0.0623
NM_031630	Ddx25	0.5233	0.1188	0.2323	0.3951	0.4565	0.5738	0.0382	0.8618	0.1773	0.4464	0.0240	0.0198
NM_031680	P2ry4	0.8040	0.3993	0.5478	0.0629	0.1859	0.3401	0.1886	0.5719	0.3204	0.7597	0.2847	0.0625
NM_031634	Mefv	0.9461	0.2989	0.5291	0.6081	0.4470	0.7441	0.7731	0.6408	0.8483	0.9624	0.3716	0.6347
NM_031633	Foxm1	0.9154	0.9531	0.1124	0.8930	0.9736	0.9994	0.9464	0.9354	0.9436	0.9945	0.9924	0.5638
NM_031788	Rest	0.3625	0.5230	0.4718	0.4993	0.3996	0.2770	0.5727	0.1696	0.5462	0.7679	0.5924	0.0991
NM_053527	Cdc5l	0.8164	0.4364	0.3734	0.2149	0.2061	0.5546	0.0528	0.2618	0.4365	0.1193	0.2213	0.3489
NM_031642	Klf6	0.8943	0.9694	0.9893	0.8267	0.9656	0.7575	0.9580	0.9635	0.9966	0.7522	0.8936	0.9962
NM_031640	Pgcp	0.0519	0.0827	0.6779	0.0774	0.5247	0.6335	0.0733	0.3447	0.0799	0.0370	0.0184	0.0500
NM_053289	Reg3b	0.2834	0.3533	0.7379	0.7442	0.2324	0.0020	0.0655	0.0071	0.0326	0.1504	0.1573	0.7887
NM_001135013	Ankrd42	0.2732	0.1024	0.2834	0.0363	0.0303	0.0212	0.0013	0.0675	0.0494	0.0729	0.0359	0.1533
NM_001115033	LOC681837	0.3534	0.3944	0.5027	0.5734	0.2418	0.3352	0.3654	0.3058	0.3113	0.5431	0.3295	0.2355
NM_031651	Slc13a1	0.0238	0.6973	0.1010	0.0646	0.3057	0.2660	0.5352	0.0581	0.0978	0.1273	0.2594	0.2242
NM_053336	Ager	0.3628	0.4499	0.1340	0.1706	0.4618	0.5233	0.4776	0.5498	0.5868	0.4101	0.0711	0.7799
NM_031652	Kcnab3	0.2178	0.0839	0.0271	0.0837	0.7931	0.5324	0.0358	0.2653	0.4618	0.3343	0.1039	0.2857
NM_031650	Slco1b2	0.2925	0.2467	0.5929	0.5651	0.7590	0.6535	0.6488	0.4342	0.5194	0.2596	0.8315	0.4272

NM_033351	Fcgrt	0.5987	0.2139	0.8359	0.4394	0.8058	0.6907	0.2814	0.7776	0.3278	0.0263	0.0504	0.4879
NM_031655	Lxn	0.8135	0.8961	0.8729	0.8695	0.7223	0.3350	0.7308	0.7804	0.9187	0.7853	0.7588	0.6534
NM_031656	Stx8	0.1164	0.0516	0.7140	0.6493	0.1260	0.0026	0.0262	0.0175	0.0633	0.0947	0.3158	0.0685
NM_053322	Nup210	0.9227	0.9794	0.9424	0.9612	0.9684	0.9699	0.9792	0.9919	0.8841	0.9093	0.9355	0.9550
NM_052801	Vhl	0.2789	0.2865	0.8991	0.8594	0.3989	0.3648	0.9504	0.4345	0.9161	0.6113	0.5359	0.9133
NM_031658	Msln	0.0830	0.0160	0.0459	0.0543	0.0669	0.0417	0.2189	0.0692	0.0879	0.0203	0.0007	0.7978
NM_031742	Kcnh1	0.5153	0.3836	0.5896	0.1723	0.4811	0.3276	0.2358	0.2189	0.4946	0.3311	0.5589	0.4435
NM_031746	Slc13a2	0.6388	0.8296	0.8867	0.7835	0.9779	0.6603	0.9597	0.9739	0.9509	0.7219	0.9955	0.9566
NM_031719	Clns1a	0.2397	0.0318	0.5760	0.3843	0.0505	0.1614	0.3676	0.0707	0.2622	0.0627	0.3467	0.1773
NM_031730	Kcnd2	0.9525	0.6836	0.5270	0.9443	0.9000	0.7918	0.7343	0.8079	0.9284	0.9610	0.5693	0.0781
NM_031667	Syt11	0.4396	0.5964	0.2314	0.3927	0.4584	0.2526	0.8376	0.8829	0.1196	0.8716	0.7930	0.7198
NM_031669	Prap1	0.6387	0.7877	0.4352	0.5059	0.8583	0.8999	0.4264	0.4832	0.8016	0.3584	0.4460	0.6908
NM_031670	Napsa	0.5670	0.0230	0.2018	0.3851	0.1298	0.4306	0.1729	0.6651	0.1378	0.1392	0.0264	0.0572
NM_031672	Slc15a2	0.5161	0.7117	0.3272	0.8973	0.5905	0.1037	0.6771	0.1130	0.7720	0.5118	0.2096	0.7750
NM_031674	Ctla4	0.6182	0.6213	0.5315	0.1303	0.1523	0.1772	0.3366	0.8720	0.6829	0.6326	0.6974	0.0082
NM_031676	Tagln3	0.5755	0.4251	0.6597	0.4033	0.5526	0.6298	0.5136	0.6396	0.2246	0.2347	0.5409	0.3522
NM_031675	Actn4	0.8496	0.8729	0.1089	0.2704	0.8394	0.9664	0.9355	0.9294	0.9870	0.7218	0.9566	0.9961
NM_031814	Git1	0.0349	0.7203	0.4658	0.2669	0.3773	0.3241	0.6530	0.6381	0.9732	0.8723	0.8586	0.5172
NM_031828	Kcnma1	0.4909	0.4661	0.7383	0.7704	0.4331	0.6283	0.6185	0.7030	0.4147	0.8415	0.5902	0.7272
NM_031984	Calb1	0.8684	0.6026	0.9053	0.6576	0.3948	0.7913	0.5557	0.4331	0.5727	0.8406	0.8156	0.6871
NM_053327	Clcnka	0.2244	0.2514	0.0448	0.4737	0.5610	0.7232	0.8680	0.6681	0.4722	0.2051	0.6049	0.3598
NM_031684	Slc29a1	0.7375	0.0116	0.0285	0.2696	0.9527	0.9255	0.1815	0.9095	0.0150	0.0482	0.0329	0.0609
NM_031685	Gosr2	0.2717	0.4410	0.4054	0.6555	0.5182	0.5421	0.5253	0.2973	0.4419	0.1490	0.8345	0.1892
NM_031686	Scn7a	0.4298	0.4214	0.7488	0.4481	0.7560	0.4374	0.4374	0.5742	0.7092	0.6939	0.5006	0.7952
NM_031691	Itgad	0.6350	0.3792	0.6124	0.4212	0.3307	0.5437	0.4473	0.6995	0.5675	0.0380	0.5057	0.3475
NM_031789	Nfe2l2	0.5439	0.5435	0.8558	0.4135	0.1712	0.7499	0.1813	0.2931	0.9792	0.9693	0.8175	0.7261
NM_031702	Cldn7	0.3726	0.6477	0.3580	0.5451	0.2397	0.2667	0.4523	0.4143	0.2401	0.2615	0.4304	0.2964
NM_032079	Dnaja2	0.5018	0.2230	0.2486	0.3394	0.0733	0.4047	0.1388	0.1137	0.4255	0.4619	0.5616	0.1754
NM_031694	Hsf2	0.7140	0.9549	0.9853	0.3651	0.9257	0.9044	0.7851	0.6374	0.8956	0.9477	0.9843	0.9566
NM_031785	Atp6ap1	0.2537	0.1987	0.3835	0.8154	0.9354	0.6800	0.6284	0.8369	0.1006	0.1202	0.1702	0.5022
NM_032075	Ghsr	0.2132	0.4845	0.3422	0.3451	0.3729	0.2235	0.3168	0.4779	0.5208	0.0668	0.5603	0.2402
NM_031974	Clta	0.3763	0.3217	0.8702	0.9085	0.6560	0.1436	0.7661	0.4968	0.6103	0.7407	0.5235	0.0779
NM_031808	Capn6	0.8897	0.9444	0.9442	0.8321	0.9577	0.4549	0.9478	0.9066	0.7486	0.9758	0.7008	0.9736
NM_031696	Gpr88	0.6884	0.1976	0.9242	0.7213	0.9066	0.7386	0.1766	0.6903	0.2489	0.2211	0.1591	0.4991
NM_001107607	Tnks2	0.6185	0.3038	0.9295	0.5085	0.4846	0.6395	0.8330	0.0311	0.1713	0.3446	0.8370	0.9228
NM_031708	Adrm1	0.1825	0.6655	0.0442	0.0065	0.1217	0.3678	0.6177	0.7360	0.8591	0.5470	0.0231	0.6047
NM_031831	Rtn4	0.3256	0.1820	0.1408	0.3390	0.0766	0.3323	0.2166	0.2864	0.4864	0.1398	0.2479	0.2221
NM_032077	Pon1	0.4117	0.3121	0.8802	0.5819	0.6822	0.4632	0.7146	0.7668	0.9590	0.6997	0.5912	0.5616
NM_001013134	Nek4	0.2431	0.3069	0.4228	0.4027	0.1996	0.4143	0.3774	0.4627	0.2727	0.2598	0.3659	0.2379
NM_053976	Krt18	0.4665	0.4088	0.1795	0.1805	0.4309	0.4062	0.4130	0.1758	0.1680	0.1621	0.1700	0.4326
NM_031712	Pdzk1	0.3297	0.2267	0.4000	0.4749	0.4904	0.5989	0.3199	0.2785	0.6558	0.4218	0.2555	0.2918
NM_031711	Arl2	0.0838	0.1985	0.4862	0.0954	0.1381	0.3147	0.4258	0.0686	0.5664	0.5947	0.1340	0.1864
NM_031699	Cldn1	0.7872	0.8198	0.7586	0.8666	0.3452	0.2878	0.4985	0.5057	0.1385	0.3099	0.4419	0.5358
NM_031714	Hrsp12	0.4328	0.3425	0.8835	0.3580	0.0300	0.1094	0.8833	0.0614	0.0104	0.1014	0.1764	0.0214
NM_031713	Lilrb3	0.6263	0.2113	0.2238	0.2493	0.4085	0.2137	0.4591	0.5906	0.5581	0.3764	0.2169	0.2652
NM_053539	Idi1	0.8525	0.9319	0.0140	0.1984	0.8329	0.7306	0.6864	0.8666	0.8661	0.9340	0.8613	0.0333
NM_031775	Casp6	0.8200	0.4418	0.8834	0.8896	0.6001	0.6613	0.8295	0.6459	0.2638	0.6324	0.5920	0.0797
NM_052798	Zfp354a	0.8482	0.1741	0.2287	0.2291	0.2894	0.5879	0.4506	0.4816	0.0153	0.3743	0.0348	0.2058
NM_031716	Wisp1	0.7687	0.9186	0.9449	0.8497	0.9689	0.9195	0.9947	0.7985	0.9349	0.9013	0.9244	0.9648

NM_032072	Nae1	0.9439	0.6147	0.2037	0.1213	0.6550	0.6376	0.0235	0.7702	0.6353	0.6739	0.1063	0.5523
NM_031969	Calm1	0.8519	0.5895	0.6529	0.9373	0.7685	0.8398	0.5379	0.5538	0.6199	0.5137	0.4891	0.7064
NM_031722	Tmed2	0.9802	0.9035	0.4498	0.6163	0.9200	0.9284	0.3557	0.6364	0.5131	0.5699	0.9702	0.9217
NM_053337	Pias2	0.2811	0.1861	0.6169	0.7321	0.1401	0.3619	0.6811	0.1743	0.0508	0.1212	0.5210	0.0130
NM_032063	Dll1	0.5757	0.3291	0.5584	0.6578	0.6945	0.6030	0.6710	0.7932	0.7771	0.6782	0.7961	0.4297
NM_031725	Scamp4	0.4855	0.1254	0.3069	0.6129	0.8540	0.4193	0.3705	0.9190	0.7800	0.3472	0.2328	0.2870
NM_053420	Bnip3	0.1992	0.1634	0.0150	0.0308	0.1392	0.2297	0.5676	0.1683	0.2247	0.1656	0.2108	0.7963
NM_032073	Kcnq1	0.0600	0.3682	0.1729	0.0840	0.3106	0.2340	0.4064	0.2159	0.5554	0.5637	0.0433	0.2874
NM_001077667	Ccdc68	0.6205	0.9147	0.5048	0.3656	0.4434	0.2788	0.4559	0.4269	0.5309	0.5823	0.2780	0.3548
NM_001077677	Pacrg	0.0808	0.5783	0.3555	0.6306	0.5971	0.5665	0.2091	0.2790	0.1050	0.2941	0.1431	0.2537
NM_181385	Zar1	0.1746	0.1544	0.4447	0.3541	0.0568	0.3435	0.1943	0.0988	0.3100	0.3696	0.3220	0.1552
NM_181381	Abcg2	0.6559	0.3334	0.5008	0.2665	0.5349	0.3341	0.3247	0.4054	0.4752	0.6270	0.5577	0.5451
NM_181380	Rtn4rl2	0.2928	0.0398	0.0732	0.1011	0.1017	0.1952	0.2468	0.0730	0.3950	0.0867	0.1517	0.5452
NM_181378	Ctsm	0.3886	0.4182	0.6687	0.5738	0.4703	0.4710	0.4919	0.6140	0.2566	0.5939	0.6659	0.2017
NM_181377	Rtn4rl1	0.5877	0.4298	0.8471	0.9573	0.6876	0.6146	0.2508	0.6831	0.4909	0.9535	0.3343	0.7713
NM_181376	Spats1	0.6924	0.7964	0.3725	0.4877	0.5701	0.4829	0.7459	0.5775	0.5886	0.6656	0.7348	0.4840
NM_181373	Grik3	0.2156	0.2057	0.4840	0.4138	0.5136	0.5865	0.4360	0.6643	0.6079	0.3943	0.2201	0.4828
NM_199230	Acvr1b	0.1585	0.4288	0.1603	0.1372	0.2390	0.5515	0.0493	0.3706	0.6330	0.3773	0.5101	0.5227
NM_181369	Hrc	0.1884	0.1017	0.0575	0.0857	0.2202	0.1503	0.0618	0.5110	0.2514	0.1517	0.4090	0.0330
NM_181368	Mustn1	0.0154	0.0125	0.1295	0.3833	0.0415	0.0617	0.0436	0.0485	0.2094	0.0272	0.1523	0.0243
NM_181367	Lhx9	0.5990	0.5746	0.5371	0.5387	0.5621	0.1198	0.6295	0.5284	0.4408	0.5263	0.6312	0.5131
NM_181366	Gpr64	0.9979	0.9757	0.9995	0.7801	0.9806	0.9562	0.9733	0.9628		0.9911	0.8091	0.9446
NM_181365	Kcnip4	0.6156	0.0726	0.1854	0.6923	0.5013	0.2805	0.0630	0.5242	0.1726	0.0839	0.2455	0.4505
NM_181364	Trhr2	0.5582	0.2195	0.3747	0.3801	0.1256	0.4108	0.7692	0.4203	0.5748	0.4900	0.3424	0.5609
NM_181363	Nudt6	0.6944	0.5608	0.7251	0.2109	0.8186	0.0098	0.1590	0.6383	0.8008	0.7668	0.3013	0.7165
NM_181362	Cand2	0.5125	0.0590	0.0969	0.7353	0.0602	0.0608	0.1121	0.0162	0.1307	0.0508	0.1647	0.0279
NM_181091	Gmfg	0.0268	0.6752	0.0753	0.6459	0.0805	0.0085	0.6809	0.0183	0.1311	0.5236	0.4220	0.0096
NM_207587	Adipor1	0.1476	0.4539	0.8580	0.3048	0.9424	0.9217	0.4671	0.8456	0.9021	0.5807	0.8749	0.9782
NM_206950	Mid1ip1	0.9946	0.9977	0.2038	0.8791	0.9755	0.9065	0.7255	0.9746	0.9671	0.9741	0.9650	0.9745
NM_181087	Cyp26b1	0.3310	0.9715	0.9878	0.9100	0.2379	0.1208	0.9749	0.2379	0.9952	0.9906	0.9988	0.9973
NM_181086	Tnfrsf12a	0.8648	0.9209	0.0417	0.8322	0.8299	0.9162	0.7725	0.8993	0.7585	0.9989	0.7860	0.9042
NM_181084	Tp53inp1	0.0299	0.4328	0.8617	0.0871	0.0206	0.0170	0.5886	0.0369	0.6154	0.8188	0.6652	0.6384
NM_181083	Arfgef2	0.4037	0.0953	0.0817	0.0373	0.0147	0.1905	0.4382	0.1045	0.6789	0.4831	0.6131	0.1429
NM_201988	Pgpep1	0.9321	0.4875	0.8549	0.2592	0.3956	0.3083	0.7942	0.3413	0.7621	0.7997	0.8591	0.8173
NM_182819	Cox7b	0.0231	0.5946	0.0436	0.0102	0.0540	0.0597	0.1106	0.0370	0.1061	0.0995	0.0790	0.0013
NM_207600	Chst7	0.8444	0.8067	0.9403	0.7590	0.9614	0.8819	0.8706	0.7343	0.8066	0.9592	0.8753	0.5505
NM_001079884	Galntl4	0.1126	0.0569	0.0322	0.0842	0.0220	0.1286	0.0209	0.2910	0.6352	0.0497	0.0125	0.9921
NM_001077675	Trim72	0.6824	0.6350	0.7410	0.8934	0.7855	0.4498	0.7830	0.6803	0.6086	0.5954	0.4918	0.4043
NM_178097	Sla	0.2845	0.6270	0.3808	0.4724	0.1220	0.2731	0.1842	0.7333	0.2080	0.1123	0.3499	0.4901
NM_031826	Fbn2	0.5038	0.5030	0.0986	0.2300	0.3249	0.4871	0.2250	0.4790	0.4966	0.4986	0.2208	0.7707
NM_033350	Plcb3	0.6930	0.2253	0.5885	0.6694	0.6533	0.6098	0.6945	0.6776	0.6614	0.6202	0.5813	0.6688
NM_033231	Acsm3	0.9307	0.3192	0.5619	0.5962	0.6676	0.4910	0.7349	0.0766	0.4445	0.8706	0.8093	0.7700
NM_053370	Timm8a1	0.0116	0.7140	0.1962	0.0096	0.7521	0.4274	0.5156	0.6797	0.4679	0.7296	0.9170	0.0499
NM_053356	Col1a2	0.7921	0.7295	0.3106	0.3178	0.7421	0.2684	0.1112	0.4348	0.8252	0.8633	0.2666	0.1326
NM_031976	Prkab1	0.3682	0.3942	0.4997	0.4724	0.6392	0.6357	0.2368	0.2309	0.1956	0.4804	0.4313	0.3278
NM_033443	Arsb	0.2124	0.0378	0.1987	0.1161	0.6952	0.7360	0.0948	0.7916	0.1822	0.0620	0.1351	0.4636
NM_031798	Slc12a2	0.6143	0.6412	0.7076	0.6937	0.7089	0.6890	0.6063	0.7132	0.6676	0.7167	0.7143	0.7276
NM_031740	B4galt6	0.9897	0.9779	0.9917	0.9543	0.9343	0.9809	0.8154	0.9973	0.9907	0.9914	0.9974	0.9234
NM_031738	Slc29a2	0.5585	0.5496	0.4881	0.2961	0.6430	0.8934	0.6418	0.9012	0.6372	0.6731	0.2654	0.9006

NM_031985	Rps6kb1	0.9932	0.7441	0.6364	0.6581	0.9541	0.9111	0.8193	0.5575	0.7677	0.9157	0.9564	0.7901
NM_031971	Hspa1a	0.3850	0.6815	0.3904	0.2751	0.4564	0.6009	0.3106	0.6320	0.2055	0.2727	0.2618	0.3280
NM_031982	Trpv1	0.3587	0.1616	0.0657	0.1604	0.6815	0.6364	0.3173	0.1561	0.2536	0.3710	0.1098	0.2912
NM_031743	Slc24a2	0.6174	0.2024	0.6405	0.2286	0.6378	0.5985	0.2321	0.6990	0.4961	0.5182	0.1991	0.5966
NM_031967	Ndrg4	0.9776	0.9431	0.9691	0.8139	0.1558	0.1992	0.9926	0.1079	0.9433	0.9512	0.9647	0.9554
NM_031756	Ggcx	0.1077	0.1478	0.7930	0.8716	0.5026	0.4324	0.8511	0.7534	0.2657	0.6943	0.4770	0.1222
NM_031765	Rxrg	0.8647	0.4018	0.8071	0.1599	0.1717	0.0588	0.0298	0.1407	0.5429	0.5405	0.7621	0.7216
NM_031839	Cyp2c23	0.4091	0.3809	0.3389	0.6295	0.5655	0.2316	0.6654	0.7512	0.4488	0.0659	0.1235	0.7297
NM_031771	Thbd	0.2763	0.9343	0.7405	0.8874	0.1030	0.3504	0.4751	0.0229	0.8117	0.8678	0.4742	0.8196
NM_032057	Impa1	0.7164	0.1170	0.7627	0.3043	0.1791	0.4358	0.1223	0.2131	0.0369	0.0653	0.0339	0.1064
NM_031774	Rabac1	0.2526	0.4230	0.7395	0.8962	0.5787	0.1973	0.7247	0.6872	0.5514	0.1953	0.7532	0.4455
NM_031778	Kcns3	0.2369	0.7514	0.0464	0.2154	0.4784	0.2407	0.6992	0.1660	0.7612	0.4441	0.5444	0.3737
NM_053397	Artn	0.5483	0.7638	0.0682	0.7025	0.6763	0.7503	0.8164	0.7354	0.6389	0.7014	0.6988	0.6232
NM_031781	Apba3	0.9212	0.7007	0.1142	0.7139	0.8733	0.6838	0.6161	0.8533	0.8354	0.7750	0.7788	0.8137
NM_031780	Apba2	0.1596	0.6656	0.4990	0.6699	0.2425	0.1335	0.3668	0.3466	0.2112	0.8885	0.5954	0.9687
NM_031782	Slc32a1	0.6310	0.1721	0.3282	0.3805	0.1581	0.5770	0.4683	0.5374	0.1342	0.7390	0.5207	0.6886
NM_053498	Entpd6	0.8737	0.9736	0.7434	0.7685	0.9520	0.9047	0.8289	0.8654	0.7567	0.9587	0.7557	0.8634
NM_053911	Cyth2	0.9605	0.8945	0.5877	0.8834	0.9164	0.9325	0.9709	0.9907	0.8418	0.7685	0.8686	0.8324
NM_053797	Crnkl1	0.0517	0.1999	0.1559	0.2312	0.0232	0.0393	0.0476	0.0348	0.2021	0.0295	0.3611	0.2937
NM_053596	Ece1	0.0439	0.0111	0.9242	0.1257	0.0620	0.0499	0.7890	0.1238	0.3709	0.3105	0.3137	0.9747
NM_031801	Deaf1	0.7553	0.7670	0.2672	0.8900	0.9357	0.9879	0.9295	0.9067	0.9585	0.8105	0.7994	0.9158
NM_031803	Gmeb2	0.0431	0.6793	0.2749	0.4913	0.1622	0.5478	0.2919	0.5143	0.3084	0.4938	0.0663	0.6729
NM_031792	Spag4	0.5873	0.5226	0.6873	0.0343	0.5902	0.4665	0.6213	0.6174	0.1878	0.3942	0.0293	0.5711
NM_001047099	Ythdf2	0.9452	0.7689	0.9184	0.9603	0.8830	0.1923	0.9693	0.7575	0.3786	0.9430	0.7980	0.4830
NM_031747	Cnn1	0.6169	0.9469	0.5028	0.7338	0.5408	0.8480	0.2178	0.3113	0.0850	0.6463	0.3600	0.4680
NM_053306	Pak2	0.1831	0.1188	0.8709	0.6864	0.2200	0.7105	0.8216	0.0384	0.1154	0.0637	0.3592	0.7323
NM_053684	Hcn2	0.4709	0.2294	0.3939	0.3773	0.5053	0.4875	0.0567	0.3641	0.5465	0.4393	0.3536	0.4292
NM_053577	Spp2	0.4897	0.1101	0.2894	0.3608	0.3029	0.5558	0.1541	0.5381	0.7582	0.4669	0.0960	0.6760
NM_053626	Dao	0.4105	0.6576	0.2261	0.5117	0.5428	0.9246	0.7548	0.7112	0.7542	0.9161	0.8548	0.9047
NM_053473	Ppp1r9a	0.2195	0.4104	0.4809	0.2807	0.6850	0.2363	0.2790	0.2840	0.6512	0.5873	0.2539	0.6533
NM_053638	Idh3a	0.4497	0.7925	0.5846	0.3410	0.1684	0.3346	0.4029	0.0780	0.7820	0.7183	0.1259	0.3053
NM_031752	Bcam	0.2919	0.4171	0.8589	0.8425	0.7577	0.5576	0.7625	0.4827	0.6740	0.7972	0.8640	0.5757
NM_052807	Igf1r	0.9514	0.8569	0.0176	0.0695	0.7798	0.1589	0.1053	0.6319	0.9776	0.5658	0.4213	0.4617
NM_031753	Alcam	0.9967	0.9529	0.9427	0.2808	0.9079	0.9202	0.9967	0.7918	0.9372	0.9766	0.9879	0.9002
NM_053398	Gfra3	0.2202	0.1240	0.2529	0.7550	0.3555	0.3042	0.1954	0.2732	0.4127	0.0579	0.5183	0.6043
NM_031795	Ugcg	0.5431	0.4851	0.9073	0.9967	0.8762	0.9801	0.0227	0.8262	0.8987	0.1877	0.1912	0.9913
NM_031763	Pafah1b1	0.8050	0.6505	0.5447	0.9743	0.4457	0.9205	0.8612	0.5026	0.3814	0.8021	0.7126	0.5653
NM_031766	Cpz	0.2227	0.7494	0.7991	0.6899	0.1804	0.3213	0.8718	0.8411	0.6610	0.7527	0.4487	0.4356
NM_031835	Agxt2	0.7979	0.5851	0.6161	0.3867	0.8228	0.5418	0.4401	0.4048	0.6215	0.5197	0.5687	0.3597
NM_053321	Ptafr	0.7322	0.3798	0.0424	0.7878	0.7229	0.8988	0.1614	0.9473	0.2492	0.6899	0.2932	0.2931
NM_031779	Apba1	0.0896	0.3311	0.5947	0.1859	0.3851	0.5230	0.5936	0.8039	0.6249	0.8685	0.3149	0.2778
NM_031784	Pias3	0.9483	0.8845	0.8518	0.8314	0.9546	0.9267	0.9449	0.9966	0.9689	0.9907	0.8867	0.9213
NM_031800	Dedd	0.6517	0.2759	0.7203	0.8418	0.8343	0.7634	0.5611	0.8596	0.1760	0.5423	0.6169	0.2302
NM_033441	Rho	0.2718	0.1040	0.3001	0.1517	0.1353	0.2069	0.0644	0.6939	0.3766	0.2399	0.4854	0.3734
NM_032062	Kalrn	0.6172	0.8994	0.0307	0.5557	0.6761	0.6928	0.6379	0.6870	0.6865	0.6873	0.1364	0.7208
NM_031805	Ank3	0.7210	0.7056	0.4647	0.6855	0.5948	0.5547	0.5921	0.6848	0.7186	0.7348	0.6500	0.6718
NM_031807	Tpbg	0.7676	0.9803	0.9349	0.8908	0.9821	0.8527	0.9027	0.8047	0.9787	0.9319	0.9725	0.7911
NM_053323	Degs1	0.4880	0.2473	0.0369	0.1083	0.7984	0.8545	0.0664	0.8008	0.4471	0.1753	0.3000	0.4322
NM_053303	Cxcr5	0.1643	0.2124	0.4508	0.7988	0.4875	0.4742	0.4903	0.2011	0.6718	0.1320	0.3555	0.4184

NM_053507	Nme3	0.7339	0.2668	0.8171	0.6669	0.7925	0.3330	0.8314	0.8233	0.6098	0.5204	0.8073	0.8773
NM_053574	Vegp2	0.4611	0.2234	0.3365	0.5055	0.1755	0.4218	0.3900	0.5436	0.4196	0.6949	0.6617	0.6977
NM_053504	Prss12	0.3440	0.6530	0.3777	0.7817	0.5502	0.1242	0.7760	0.5584	0.1851	0.4690	0.1493	0.0819
NM_052804	Fmr1	0.9765	0.7390	0.6706	0.3184	0.9631	0.9091	0.1333	0.9053	0.6120	0.7121	0.8319	0.2311
NM_031809	Cngb1	0.4447	0.1686	0.2636	0.4142	0.4383	0.1763	0.3991	0.6135	0.2375	0.2731	0.3736	0.3190
NM_031812	Cd164	0.9884	0.8371	0.9686	0.9941	0.9864	0.9944	0.9915	0.9782	0.9961	0.9715	0.9648	0.9641
NM_053291	Pgk1	0.2903	0.0786	0.1184	0.9140	0.3908	0.5942	0.8441	0.6843	0.2904	0.0380	0.0538	0.7566
NM_031810	Defb1	0.3240	0.2511	0.3456	0.3880	0.0631	0.0406	0.4827	0.5387	0.0480	0.1057	0.2962	0.1190
NM_053394	Klf5	0.9906	0.9955	0.9973	0.9595	0.9900	0.9996	0.8015	0.9862	0.9693	0.9793	0.9942	0.8195
NM_031813	Mybph	0.4847	0.4768	0.1937	0.1417	0.4197	0.5806	0.8890	0.6812	0.8427	0.7471	0.6660	0.2577
NM_053019	Avpr1a	0.8831	0.0002	0.0235	0.0351	0.8503	0.8096	0.0370	0.8656	0.0162	0.0003	0.0004	0.0002
NM_053499	Mterf	0.0523	0.3956	0.8467	0.3057	0.7668	0.6491	0.1310	0.3516	0.3188	0.6315	0.6799	0.6412
NM_031817	Omd	0.7964	0.8668	0.8657	0.9098	0.8732	0.8741	0.8390	0.9614	0.8763	0.8221	0.8977	0.6153
NM_031816	Rbbp7	0.1494	0.5863	0.3661	0.8415	0.7780	0.7598	0.6745	0.1677	0.3062	0.9900	0.7848	0.3959
NM_053586	Cox5b	0.0922	0.4222	0.1094	0.6135	0.0326	0.0911	0.5021	0.1283	0.0996	0.4582	0.7725	0.1984
NM_053623	Acsl4	0.2190	0.3580	0.2356	0.2688	0.3480	0.1621	0.0421	0.2842	0.1841	0.5265	0.3017	0.2584
NM_053511	Fbxo2	0.0187	0.3223	0.8028	0.0537	0.0906	0.2992	0.7984	0.0681	0.3318	0.4269	0.2562	0.0683
NM_031820	Dvl1	0.9393	0.9557	0.0984	0.0425	0.8787	0.9951	0.9734	0.9641	0.9679	0.9509	0.7744	0.8827
NM_031823	Wfs1	0.1320	0.0602	0.0306	0.0180	0.0369	0.2744	0.0897	0.0966	0.3095	0.7833	0.4913	0.3015
NM_053359	Atox1	0.0898	0.1255	0.6263	0.7747	0.0694	0.1854	0.6241	0.6123	0.0478	0.0761	0.0316	0.1612
NM_001108170	Klhl31	0.5379	0.0984	0.8687	0.7827	0.6901	0.4822	0.5313	0.6587	0.5008	0.0897	0.2311	0.1122
NM_001108106	Plxnb2	0.7620	0.4730	0.5802	0.3638	0.2550	0.9499	0.0617	0.2926	0.2291	0.4356	0.9140	0.9822
NM_198767	Pde8a	0.6885	0.0649	0.5873	0.6590	0.7617	0.7834	0.7874	0.8653	0.6668	0.4860	0.2941	0.8131
NM_207609	Naprt1	0.4566	0.0218	0.0126	0.0771	0.6505	0.9299	0.0293	0.5700	0.0089	0.0720	0.0919	0.0239
NM_207610	Ube4a	0.9971	0.9796	0.9039	0.9393	0.9939	0.9885	0.2028	0.9606	0.9009	0.9542	0.9715	0.7863
NM_031825	Fbn1	0.0150	0.0222	0.1301	0.1318	0.1169	0.0649	0.1560	0.1103	0.0907	0.2596	0.1599	0.8519
NM_053567	Ftcd	0.3116	0.1069	0.2757	0.3516	0.6216	0.2629	0.2883	0.5517	0.1017	0.6982	0.2143	0.3676
NM_053339	Acox3	0.7931	0.6971	0.7440	0.5052	0.8259	0.9157	0.9472	0.6775	0.8815	0.7488	0.6154	0.8614
NM_032066	Hsd17b12	0.9674	0.3445	0.1750	0.9574	0.9362	0.9606	0.5873	0.9758	0.2016	0.3795	0.1053	0.1491
NM_031834	Sult1a1	0.3620	0.4334	0.8774	0.5624	0.6318	0.1592	0.3704	0.4379	0.2415	0.4056	0.8100	0.3504
NM_053388	Gjb6	0.8243	0.8674	0.8500	0.5867	0.7091	0.5687	0.5100	0.4956	0.5402	0.7926	0.8440	0.7836
NM_145096	Zdhhc2	0.9467	0.9610	0.7396	0.9093	0.5649	0.7539	0.9971	0.8626	0.7810	0.9976	0.9351	0.7613
NM_032071	Synj2	0.9289	0.8790	0.9575	0.8633	0.7276	0.9402	0.9832	0.8164	0.9118	0.7186	0.8839	0.8685
NM_031837	41,161.0000	0.8193	0.7691	0.8543	0.7554	0.5452	0.6312	0.7980	0.9385	0.5991	0.3680	0.8085	0.8354
NM_001108169	Fam83b	0.3886	0.0882	0.8819	0.6999	0.1586	0.2753	0.3022	0.5009	0.7256	0.7576	0.6355	0.3283
NM_001108166	Pigb	0.2085	0.5355	0.3797	0.3352	0.4190	0.5682	0.2764	0.4683	0.4378	0.5293	0.5753	0.2232
NM_001108428	RGD1563159	0.7958	0.6499	0.6095	0.3083	0.2768	0.1272	0.3236	0.2581	0.5579	0.6769	0.8500	0.6591
NM_001108301	Map3k14	0.7078	0.7458	0.1825	0.2889	0.5953	0.9485	0.8951	0.8638	0.7839	0.9464	0.8814	0.9478
NM_001108290	Spag9	0.2902	0.2144	0.3512	0.3956	0.1446	0.3158	0.2840	0.2881	0.1279	0.2949	0.2831	0.5569
NM_001108289	Mrps23	0.2350	0.7310	0.2996	0.0487	0.0897	0.1363	0.0755	0.2041	0.0533	0.0283	0.0094	0.0055
NM_001134609	RGD1562044	0.2889	0.1055	0.1985	0.3440	0.4296	0.1825	0.6074	0.0510	0.0408	0.0321	0.1868	0.1886
NM_001108413	Cdkal1	0.1998	0.2556	0.2777	0.3134	0.4814	0.3062	0.2520	0.6954	0.1803	0.1333	0.4756	0.3093
NM_001108251	Ubqln2	0.9452	0.9612	0.9685	0.9136	0.9637	0.6793	0.9966	0.8794	0.7755	0.8930	0.9929	0.9265
NM_001108250	Foxp3	0.3175	0.2430	0.6785	0.2107	0.8602	0.6117	0.0752	0.8147	0.4214	0.7291	0.4184	0.4180
NM_001039003	Mtrr	0.6224	0.2662	0.3832	0.2220	0.0340	0.3915	0.1470	0.3256	0.0925	0.4066	0.6760	0.8583
NM_001109666	Abcf2	0.1988	0.7575	0.1370	0.7463	0.6429	0.8919	0.3775	0.7458	0.9520	0.7822	0.8101	0.9987
NM_033483	Crygc	0.3957	0.3709	0.3691	0.5092	0.3762	0.3923	0.4320	0.5204	0.1673	0.6922	0.7443	0.1741
NM_001109887	Ptprc	0.8419	0.9679	0.9525	0.9107	0.9667	0.3282	0.6999	0.3839	0.8020	0.9482	0.9111	0.6854
NM_001109875	Crygb	0.6851	0.2207	0.3239	0.8105	0.5692	0.5901	0.4542	0.2708	0.1836	0.6043	0.5726	0.3064

NM_001109649	Mrp63	0.7096	0.4703	0.5295	0.6583	0.4394	0.3266	0.3419	0.7217	0.5895	0.2625	0.2987	0.2424
NM_001100659	Cdc23	0.4891	0.6238	0.9344	0.5990	0.2955	0.9582	0.8845	0.2923	0.1094	0.4949	0.8774	0.9293
NM_001109587	Pla2g2f	0.5110	0.5823	0.7543	0.4978	0.6127	0.8933	0.9304	0.3374	0.8395	0.4871	0.6881	0.8214
NM_001100470	Nup153	0.9878	0.9713	0.3203	0.2510	0.9747	0.8372	0.6679	0.9559	0.9911	0.9354	0.9928	0.8453
NM_032081	Pnliprp1	0.4814	0.5237	0.4170	0.7140	0.4844	0.1003	0.6198	0.4447	0.3989	0.3200	0.5392	0.2971
NM_053316	Ihpk1	0.8820	0.4778	0.5683	0.2516	0.6955	0.6118	0.0641	0.8614	0.7719	0.7590	0.9398	0.8212
NM_032060	C3ar1	0.4087	0.5915	0.5357	0.6015	0.6567	0.2439	0.4510	0.3985	0.5307	0.9633	0.5188	0.5635
NM_032058	Eif2b2	0.1634	0.6061	0.0316	0.1179	0.4125	0.3366	0.2821	0.4431	0.2877	0.2105	0.3967	0.0373
NM_032056	Tap2	0.1070	0.1330	0.0245	0.2021	0.1526	0.1596	0.2399	0.0932	0.3926	0.4654	0.1515	0.6329
NM_033099	Ptprv	0.0248	0.6190	0.5154	0.0088	0.0649	0.0204	0.7477	0.0926	0.3663	0.7656	0.1416	0.8185
NM_033096	Ppm1b	0.9089	0.5429	0.7518	0.6290	0.7673	0.2129	0.7783	0.5779	0.9087	0.4887	0.7188	0.5082
NM_032082	Hao2	0.0632	0.5630	0.1257	0.4783	0.0326	0.0169	0.4556	0.1050	0.3157	0.4999	0.4046	0.0618
NM_053477	Mlycd	0.2947	0.7890	0.3247	0.8325	0.0039	0.2829	0.6910	0.0418	0.6987	0.3290	0.5257	0.6490
NM_032070	Hmga2	0.4591	0.2790	0.1728	0.7525	0.5350	0.1647	0.0512	0.5873	0.6307	0.9514	0.7420	0.0132
NM_032067	Ralbp1	0.9156	0.9089	0.9839	0.9110	0.8792	0.8683	0.5614	0.8848	0.9340	0.8878	0.8050	0.9730
NM_053448	Hdac3	0.0092	0.1223	0.0146	0.1901	0.1085	0.0373	0.2825	0.1265	0.1092	0.0057	0.0195	0.0697
NM_053333	Retnla	0.2268	0.1064	0.0451	0.4815	0.0521	0.0660	0.1803	0.3135	0.2901	0.1494	0.3232	0.2269
NM_032069	Grip1	0.1225	0.1464	0.2623	0.0283	0.7586	0.7628	0.1509	0.4742	0.0093	0.2825	0.3310	0.9957
NM_052803	Atp7a	0.3519	0.7707	0.0508	0.1106	0.7050	0.8010	0.1578	0.5507	0.8792	0.5974	0.8955	0.1131
NM_032462	Kcnip3	0.0225	0.4330	0.5294	0.5888	0.0495	0.2058	0.2033	0.0074	0.1012	0.0464	0.5313	0.1686
NM_133415	Necab2	0.8245	0.8070	0.4692	0.4777	0.6842	0.1535	0.5211	0.8998	0.5328	0.3160	0.8834	0.7503
NM_053557	Prmt3	0.9397	0.9311	0.2425	0.9313	0.2139	0.3885	0.8518	0.8577	0.2712	0.7203	0.2110	0.1580
NM_032085	Col3a1	0.7752	0.2534	0.2746	0.0300	0.8386	0.8753	0.0015	0.7470	0.1076	0.2359	0.0604	0.2284
NM_053936	Lpar1	0.2462	0.9216	0.9915	0.8946	0.9572	0.7252	0.1774	0.9681	0.2942	0.9008	0.1609	0.2889
NM_001135015	Gabpb1l	0.8393	0.7579	0.7530	0.5311	0.5006	0.3759	0.8336	0.5356	0.7062	0.6531	0.6439	0.7776
NM_053295	Cast	0.6583	0.3763	0.9610	0.9512	0.3357	0.6714	0.5842	0.5342	0.6643	0.4498	0.6294	0.6838
NM_032074	Irs3	0.4996	0.3598	0.1182	0.1144	0.1287	0.5485	0.0448	0.4891	0.1721	0.1295	0.3732	0.2039
NM_053297	Pkm2	0.3313	0.0452	0.0106	0.5789	0.2039	0.3890	0.2322	0.0620	0.3146	0.1932	0.4675	0.6932
NM_031975	Ptms	0.8651	0.8253	0.9333	0.8002	0.9010	0.8359	0.9196	0.8086	0.9820	0.9392	0.9198	0.9582
NM_032076	Ptger4	0.2923	0.0598	0.0110	0.0664	0.1024	0.0288	0.1025	0.0208	0.0937	0.1458	0.0699	0.0320
NM_001037347	Sfrs15	0.9527	0.7069	0.3626	0.7434	0.8521	0.8207	0.7516	0.8736	0.4399	0.4867	0.6512	0.1358
NM_001108625	RGD1311823	0.5790	0.0830	0.4978	0.5763	0.7171	0.5947	0.4947	0.5616	0.5918	0.2321	0.1389	0.2873
NM_001108622	Hipk2	0.7703	0.6211	0.7653	0.2581	0.7975	0.1143	0.7372	0.7270	0.3940	0.4146	0.6297	0.3100
NM_001108634	Pole4	0.9709	0.9731	0.9464	0.9277	0.9835	0.9571	0.9678	0.9206	0.9411	0.7883	0.9768	0.9035
NM_001108657	Runx1t1	0.8256	0.7435	0.6201	0.5809	0.8570	0.5540	0.6970	0.8594	0.4529	0.4540	0.3897	0.4413
NM_001108656	Ccne2	0.9196	0.8427	0.9594	0.9905	0.9543	0.8863	0.9341	0.9853	0.9357	0.9519	0.9391	0.8532
NM_001108713	Trmt5	0.7683	0.8365	0.4543	0.8696	0.7203	0.8903	0.1546	0.8071	0.8341	0.5822	0.3366	0.6515
NM_001108687	Man1c1	0.5813	0.6524	0.4484	0.2335	0.6502	0.6288	0.7164	0.6986	0.6541	0.4789	0.2166	0.5026
NM_001108530	Ube2d1	0.9751	0.8118	0.9819	0.9900	0.8936	0.8378	0.6705	0.9168	0.2360	0.6954	0.5911	0.3462
NM_001108511	Tnfrsf26	0.6203	0.4295	0.5233	0.6497	0.8769	0.2522	0.5956	0.4636	0.8960	0.7920	0.9260	0.8522
NM_001108510	Dusp8	0.3423	0.8000	0.0476	0.4626	0.3115	0.1930	0.7277	0.5684	0.6102	0.8837	0.7323	0.8787
NM_001108498	Nrip3	0.7280	0.8735	0.6703	0.8301	0.7689	0.9129	0.9428	0.8945	0.9243	0.8965	0.9718	0.8103
NM_001108507	Pwwp2b	0.5314	0.5432	0.1784	0.6640	0.6225	0.4724	0.8865	0.5874	0.6078	0.8458	0.5271	0.9672
NM_001108505	Bccip	0.6363	0.6812	0.6665	0.8205	0.6501	0.6503	0.6156	0.7023	0.7321	0.8911	0.6688	0.7973
NM_001108547	Ect2	0.9704	0.9864	0.2607	0.9365	0.9272	0.9984	0.8741	0.7011	0.5344	0.9202	0.9066	0.1380
NM_001108493	RGD1561459	0.1283	0.0790	0.7424	0.7698	0.5296	0.1008	0.7864	0.9300	0.0939	0.1210	0.1168	0.0344
NM_001108491	Me3	0.3849	0.5550	0.4606	0.6367	0.4314	0.6733	0.7438	0.5892	0.7120	0.7958	0.4996	0.4244
NM_001108494	Fam168a	0.8226	0.6829	0.5746	0.4713	0.9023	0.5718	0.9383	0.2724	0.4940	0.8964	0.9080	0.6438
NM_001108515	Fbxl11	0.9685	0.6370	0.4476	0.6594	0.7786	0.9689	0.4738	0.8713	0.9742	0.8615	0.5940	0.9773

NM_001108483	Snrp70	0.9278	0.8442	0.5756	0.8111	0.9649	0.2697	0.9460	0.9370	0.9981	0.9776	0.8816	0.4948
NM_053642	Sc5dl	0.3164	0.4024	0.0871	0.0356	0.0230	0.1955	0.2327	0.0718	0.1827	0.5855	0.5380	0.4581
NM_032084	Chn2	0.5616	0.4448	0.8371	0.8667	0.5311	0.5506	0.6351	0.5586	0.5992	0.8427	0.6024	0.2007
NM_032083	Chn1	0.5969	0.7790	0.7350	0.8245	0.8078	0.0879	0.9747	0.1178	0.4258	0.3665	0.4440	0.2316
NM_031983	Smarcd2	0.0089	0.1315	0.2294	0.2941	0.2145	0.2842	0.3243	0.1704	0.1136	0.2109	0.2495	0.2590
NM_053343	Dclk1	0.9016	0.4406	0.2593	0.3753	0.2620	0.4490	0.2796	0.3798	0.2616	0.3865	0.1250	0.3026
NM_053318	Hpx	0.8986	0.8343	0.0056	0.1552	0.7594	0.4454	0.1514	0.6669	0.7811	0.6790	0.0858	0.2104
NM_031981	Nsfl1c	0.7508	0.8720	0.1624	0.4471	0.4577	0.6510	0.2698	0.8657	0.7729	0.6755	0.6984	0.9094
NM_033352	Abcd2	0.8235	0.8773	0.8858	0.5285	0.7219	0.8319	0.9241	0.5141	0.4567	0.9461	0.9285	0.7021
NM_031979	Csda	0.1759	0.8972	0.9240	0.8384	0.5319	0.3408	0.7266	0.2425	0.8018	0.9196	0.9446	0.9904
NM_031978	Psmd1	0.1888	0.1459	0.0136	0.1494	0.1067	0.0537	0.0174	0.2719	0.0457	0.0774	0.0009	0.0282
NM_053572	Pcdh21	0.2249	0.0057	0.0099	0.0589	0.2037	0.0183	0.0344	0.0115	0.2690	0.1836	0.1111	0.7620
NM_053571	Sec16b	0.9165	0.8348	0.1581	0.8272	0.9494	0.9003	0.2613	0.9435	0.5700	0.8033	0.8454	0.5323
NM_031986	Sdcbp	0.5020	0.2735	0.8287	0.4703	0.5737	0.7212	0.5623	0.6286	0.0493	0.3138	0.2668	0.7517
NM_213627	Zdhhc23	0.6176	0.9367	0.2297	0.7563	0.8372	0.3846	0.7839	0.8623	0.5172	0.5102	0.0526	0.7442
NM_053421	Arid4b	0.8335	0.3215	0.7362	0.5289	0.7555	0.9598	0.3484	0.4720	0.2709	0.6511	0.3422	0.3184
NM_053622	Pom121	0.9839	0.9708	0.3892	0.9873	0.9141	0.9228	0.9020	0.9795	0.9086	0.9630	0.7940	0.3511
NM_053612	Hspb8	0.1087	0.3227	0.1497	0.4276	0.0249	0.6379	0.9451	0.2174	0.3028	0.3563	0.3771	0.8803
NM_053582	Tinagl1	0.0038	0.4232	0.0088	0.0251	0.2162	0.0317	0.4724	0.0112	0.6111	0.5071	0.2845	0.3562
NM_032613	Lasp1	0.4240	0.3212	0.8562	0.4771	0.5754	0.4870	0.7352	0.6095	0.4191	0.4503	0.3993	0.4923
NM_053428	Fgf13	0.6145	0.8891	0.6825	0.6736	0.8307	0.7176	0.2275	0.5881	0.8139	0.7642	0.8090	0.3915
NM_133529	Cabp1	0.4206	0.6933	0.4965	0.2674	0.5075	0.6721	0.3467	0.7277	0.4284	0.2332	0.2445	0.6339
NM_032616	Lsr	0.9194	0.6590	0.0791	0.9267	0.0555	0.0045	0.8480	0.0880	0.8474	0.4650	0.8360	0.0548
NM_053785	Tm4sf4	0.7397	0.8805	0.9026	0.9849	0.7324	0.9180	0.9398	0.7680	0.0998	0.9347	0.9967	0.3413
NM_053766	Wbp4	0.7014	0.4291	0.8548	0.1139	0.1228	0.1004	0.1318	0.0151	0.8306	0.7931	0.5851	0.6071
NM_032990	Gria3	0.6700	0.6103	0.7305	0.6914	0.6974	0.8620	0.6637	0.7180	0.6963	0.7347	0.7108	0.5558
NM_053625	Gfm1	0.0407	0.0562	0.0762	0.0461	0.0639	0.2527	0.0745	0.2212	0.2071	0.0327	0.0655	0.5687
NM_001108391	Zic5	0.7680	0.6983	0.8295	0.4455	0.8132	0.8109	0.8570	0.6899	0.4318	0.9456	0.6628	0.6209
NM_001108440	Brd7	0.9138	0.8689	0.0864	0.1675	0.8046	0.8349	0.1535	0.7594	0.5293	0.5237	0.7054	0.0675
NM_001108299	Etv4	0.6634	0.6673	0.1455	0.8200	0.9531	0.9259	0.3242	0.9346	0.4628	0.4824	0.5609	0.6070
NM_001108308	RGD1307394	0.4230	0.3784	0.8141	0.5069	0.2215	0.9591	0.5055	0.2030	0.5264	0.5323	0.5304	0.9190
NM_001108446	Abce1	0.3601	0.8863	0.2918	0.2486	0.4893	0.1637	0.2990	0.5742	0.7323	0.7245	0.2888	0.1602
NM_001108294	Copz2	0.3012	0.3098	0.4224	0.5313	0.4449	0.2828	0.7262	0.1452	0.4126	0.6938	0.5299	0.4300
NM_001108274	Tbc1d9b	0.9079	0.4392	0.9424	0.9253	0.9406	0.9087	0.5868	0.9617	0.9869	0.9129	0.9757	0.9661
NM_001108435	Mppe1	0.9148	0.6615	0.8519	0.8998	0.8195	0.9057	0.0662	0.8808	0.5443	0.6973	0.8144	0.3508
NM_001108302	Ccdc44	0.5204	0.6611	0.3054	0.7252	0.7295	0.5971	0.9248	0.9785	0.7324	0.5361	0.6415	0.5521
NM_001108292	Itga3	0.0630	0.4417	0.1507	0.1572	0.1494	0.5612	0.3053	0.2454	0.5217	0.5253	0.5439	0.9764
NM_001108425	Mocos	0.1361	0.0215	0.0238	0.0049	0.0343	0.0446	0.0737	0.0040	0.2759	0.0383	0.3137	0.0933
NM_053462	Nfs1	0.2359	0.0337	0.8095	0.2541	0.0786	0.3257	0.5568	0.1752	0.5668	0.3019	0.1910	0.7319
NM_053458	Rab9a	0.7779	0.6708	0.9296	0.7089	0.2880	0.0328	0.3282	0.0380	0.3426	0.8180	0.8852	0.1671
NM_033095	Crygd	0.3579	0.0697	0.1632	0.0231	0.1153	0.3342	0.2253	0.1919	0.1709	0.0647	0.1133	0.1874
NM_053437	Dgat1	0.5858	0.5809	0.0136	0.8699	0.7446	0.5731	0.1040	0.9111	0.5620	0.5313	0.5759	0.8624
NM_033098	Tapbp	0.3869	0.6852	0.7794	0.2459	0.3585	0.2243	0.6342	0.4924	0.7759	0.7638	0.7093	0.8424
NM_053433	Fmo3	0.4633	0.3967	0.4479	0.1795	0.1778	0.1756	0.5135	0.3648	0.2641	0.3151	0.1689	0.2797
NM_053406	Pomt1	0.9710	0.9382	0.6216	0.8374	0.7143	0.7346	0.8085	0.7327	0.8645	0.6377	0.6836	0.7316
NM_052983	Slc5a5	0.3820	0.6083	0.7628	0.6968	0.6283	0.4276	0.5576	0.6132	0.7085	0.9231	0.6078	0.4619
NM_053842	Mapk1	0.0850	0.1745	0.6088	0.7393	0.2842	0.4970	0.0120	0.0352	0.8484	0.5585	0.4757	0.9015
NM_057103	Akap12	0.8854	0.5725	0.7863	0.3727	0.7106	0.6773	0.8847	0.7066	0.6321	0.6376	0.7996	0.7327
NM_053669	Sh2b2	0.5834	0.5599	0.2471	0.3418	0.0980	0.3487	0.3160	0.7826	0.6566	0.5705	0.5868	0.8737

NM_033236	Psmc2	0.3267	0.6779	0.1905	0.8116	0.7848	0.5813	0.4602	0.6207	0.6056	0.6718	0.8106	0.5624
NM_053424	Slc4a4	0.2309	0.4276	0.3698	0.4600	0.2559	0.1056	0.2417	0.3443	0.3717	0.2501	0.2644	0.7184
NM_053871	Srp54a	0.0818	0.1909	0.4531	0.0540	0.0897	0.1298	0.0515	0.1184	0.0529	0.2571	0.4081	0.3982
NM_033234	Hbb	0.4570	0.3643	0.0083	0.1439	0.4099	0.4756	0.0552	0.0822	0.3450	0.3833	0.3959	0.0971
NM_053415	Cxcr3	0.7363	0.0972	0.7873	0.6103	0.7109	0.9150	0.7598	0.0935	0.8355	0.8719	0.5072	0.4174
NM_033298	Yes1	0.1828	0.4839	0.5462	0.6809	0.0831	0.2134	0.5695	0.4655	0.2491	0.1289	0.3413	0.4908
NM_053445	Fads1	0.8142	0.2872	0.2799	0.1160	0.8954	0.9531	0.3400	0.7935	0.1853	0.2465	0.1160	0.0587
NM_001107279	Pcdh17	0.3543	0.5214	0.3373	0.7873	0.2922	0.3812	0.0604	0.5591	0.3961	0.5386	0.5562	0.2377
NM_001107247	Dlg5	0.9281	0.9542	0.8620	0.6819	0.8293	0.7944	0.9187	0.1836	0.9861	0.8441	0.4036	0.8576
NM_053460	Pick1	0.9360	0.7987	0.6908	0.6491	0.9032	0.9073	0.7646	0.9795	0.6686	0.5877	0.6239	0.8683
NM_052809	Cdo1	0.8776	0.6362	0.8361	0.7976	0.7682	0.4540	0.5971	0.4547	0.8841	0.8974	0.6985	0.6984
NM_198763	Slc1a4	0.1465	0.8016	0.8935	0.3686	0.5709	0.6975	0.9283	0.7821	0.2840	0.9357	0.3380	0.8598
NM_198762	Kcnt2	0.8120	0.7613	0.6986	0.6220	0.7422	0.5222	0.5905	0.6478	0.8832	0.7878	0.8394	0.7694
NM_212522	Sptb	0.3787	0.1495	0.4807	0.8092	0.8215	0.5524	0.8403	0.8112	0.0852	0.7648	0.7663	0.8637
NM_199393	Galnt11	0.9881	0.3374	0.8324	0.8962	0.0671	0.4151	0.9039	0.4867	0.3782	0.3489	0.3357	0.6647
NM_198785	Usp48	0.9784	0.6018	0.4378	0.9767	0.9841	0.9432	0.8664	0.9682	0.8288	0.8318	0.9414	0.6712
NM_198756	Mrps18a	0.0165	0.2760	0.0820	0.0536	0.0682	0.1937	0.3967	0.0893	0.2060	0.1478	0.1273	0.1233
NM_199491	Fut7	0.0672	0.1273	0.1655	0.1138	0.2392	0.4773	0.1645	0.2116	0.0500	0.3875	0.3304	0.2957
NM_199087	Spint2	0.3717	0.2814	0.6606	0.4478	0.3185	0.4341	0.2820	0.4393	0.2426	0.2649	0.2805	0.6526
NM_198754	Cmtm8	0.8009	0.9994	0.8893	0.9406	0.9814	0.9462	0.7528	0.8368	0.9867	0.8365	0.8855	0.9475
NM_198752	KIFC2	0.9468	0.9462	0.8969	0.7574	0.8087	0.9651	0.9216	0.7829	0.9041	0.7708	0.9619	0.9920
NM_001039611	Nsmce1	0.4040	0.6859	0.8846	0.6463	0.5808	0.4653	0.7886	0.1485	0.6324	0.7116	0.8655	0.6373
NM_198750	Cry1	0.1371	0.9351	0.1269	0.0852	0.1380	0.0890	0.7536	0.0169	0.4249	0.5448	0.7137	0.1377
NM_053329	Igfals	0.4202	0.3909	0.5829	0.2141	0.2075	0.4327	0.7689	0.5114	0.6259	0.2726	0.4466	0.1528
NM_033359	Ngb	0.7005	0.4689	0.7979	0.9493	0.7990	0.7112	0.3820	0.8730	0.8110	0.7677	0.1828	0.8427
NM_053328	Bhlhb2	0.4043	0.2642	0.9960	0.4582	0.9640	0.9433	0.2577	0.9911	0.3423	0.2869	0.3278	0.9993
NM_053617	Cpb2	0.5052	0.6892	0.0458	0.6795	0.6163	0.5221	0.4814	0.5764	0.3155	0.8422	0.5297	0.4126
NM_053765	Gne	0.7802	0.8455	0.6073	0.7594	0.6985	0.6587	0.7608	0.8244	0.9003	0.7308	0.8478	0.7900
NM_033442	Gata2	0.3530	0.3507	0.1985	0.1750	0.2029	0.1440	0.2394	0.3836	0.2015	0.1485	0.3103	0.1108
NM_052802	Kap	0.0856	0.3140	0.2686	0.7501	0.6165	0.6022	0.3497	0.8044	0.3734	0.5315	0.7802	0.2671
NM_040669	Hps1	0.7811	0.7740	0.9376	0.8114	0.9177	0.9028	0.7999	0.8961	0.9817	0.8917	0.9173	0.9449
NM_033499	Scrg1	0.0737	0.1249	0.0064	0.0632	0.7734	0.4848	0.0613	0.0987	0.0936	0.0355	0.0278	0.0155
NM_053646	Asah2	0.1196	0.4792	0.6890	0.2470	0.0893	0.2942	0.2999	0.2217	0.4907	0.4825	0.6414	0.0259
NM_053439	Ran	0.7306	0.9942	0.2364	0.9479	0.8640	0.8893	0.7480	0.8922	0.8962	0.8954	0.8133	0.0878
NM_053862	Lgals8	0.9580	0.2923	0.7012	0.8724	0.8975	0.9470	0.3864	0.7062	0.1945	0.5142	0.0285	0.5823
NM_053752	Suclg1	0.0207	0.1392	0.8465	0.8128	0.2007	0.1016	0.6222	0.2231	0.0359	0.0331	0.0809	0.1180
NM_053774	Usp2	0.1550	0.7480	0.0955	0.0998	0.4202	0.0231	0.7188	0.3332	0.1178	0.6993	0.7543	0.2484
NM_053826	Pdk1	0.0640	0.0532	0.8691	0.8043	0.0483	0.0969	0.8414	0.2370	0.0605	0.0220	0.1155	0.6514
NM_053578	Atp6v0e1	0.0440	0.2757	0.4679	0.0756	0.1123	0.1218	0.1013	0.1292	0.0922	0.0391	0.1800	0.0780
NM_052808	Psp	0.1738	0.4961	0.0220	0.3571	0.2837	0.6176	0.2549	0.1498	0.1436	0.0562	0.0315	0.1746
NM_052829	Rims1	0.5335	0.3622	0.4191	0.3883	0.6345	0.3083	0.5926	0.3196	0.2825	0.6814	0.5342	0.0940
NM_053506	Hrh3	0.9387	0.2710	0.9094	0.5857	0.7993	0.8326	0.7571	0.4079	0.9274	0.3718	0.4829	0.8409
NM_053591	Dpep1	0.5134	0.0410	0.5336	0.3327	0.5729	0.6137	0.6630	0.3272	0.0122	0.0139	0.0687	0.2208
NM_052979	A	0.1775	0.3685	0.1790	0.0448	0.0912	0.5444	0.1905	0.2592	0.0606	0.1302	0.1556	0.0072
NM_052981	Ccnh	0.0562	0.0286	0.5300	0.0005	0.0920	0.1022	0.0069	0.0062	0.0141	0.0244	0.0310	0.0577
NM_053718	Mllt3	0.2194	0.6949	0.9280	0.8175	0.6801	0.8649	0.9150	0.5443	0.4943	0.9755	0.8229	0.1093
NM_053288	Orm1	0.3710	0.0244	0.3738	0.1896	0.2387	0.1266	0.1268	0.3189	0.6788	0.4883	0.6035	0.5651
NM_212499	G7c	0.9688	0.7510	0.8594	0.9751	0.9139	0.9889	0.9956	0.8305	0.9844	0.8834	0.9582	0.9780
NM_053919	Ceacam9	0.2784	0.0233	0.1202	0.0068	0.0908	0.3987	0.0749	0.0914	0.0105	0.0549	0.0409	0.0817

NM_053301	Hfe	0.5091	0.1293	0.2358	0.0989	0.5384	0.7915	0.0580	0.6155	0.5427	0.8883	0.1718	0.8569
NM_053429	Fgfr3	0.8990	0.8758	0.3350	0.6646	0.0729	0.2035	0.6795	0.2158	0.3321	0.4805	0.3872	0.8548
NM_053497	Cnga1	0.5054	0.1795	0.2070	0.1051	0.4345	0.3825	0.3596	0.2952	0.2092	0.5284	0.7876	0.0384
NM_053311	Atp2b1	0.3722	0.0209	0.1345	0.1339	0.0716	0.6717	0.2481	0.1512	0.1975	0.3276	0.2380	0.9490
NM_053352	Cxcr7	0.1344	0.0603	0.9782	0.9136	0.8639	0.9278	0.0773	0.8381	0.0184	0.1934	0.1198	0.0083
NM_053332	Cubn	0.7516	0.7594	0.5505	0.9043	0.8441	0.7287	0.6957	0.7750	0.8694	0.9184	0.7429	0.6046
NM_053801	Sec14l2	0.7607	0.7299	0.7242	0.4107	0.5511	0.3424	0.7993	0.6997	0.8950	0.7255	0.4410	0.6265
NM_053299	Ubd	0.5252	0.7416	0.7019	0.2663	0.1198	0.0400	0.0620	0.0588	0.5334	0.5434	0.3587	0.5107
NM_053309	Homer2	0.9965	0.9568	0.2432	0.9834	0.8091	0.8471	0.8965	0.6970	0.9496	0.6579	0.8700	0.2281
NM_053310	Homer3	0.0282	0.0082	0.0591	0.1105	0.1002	0.0737	0.0232	0.1708	0.0377	0.1811	0.0153	0.2903
NM_053597	Rps27	0.6168	0.4989	0.4917	0.4410	0.6272	0.2825	0.6562	0.5138	0.3915	0.2450	0.2964	0.5319
NM_053592	Dut	0.7263	0.2319	0.3543	0.3664	0.4015	0.4578	0.2858	0.2798	0.5088	0.4593	0.4232	0.1737
NM_053314	Kcnj16	0.9575	0.5450	0.9031	0.8479	0.8319	0.7016	0.2317	0.8157	0.0544	0.3412	0.6445	0.0733
NM_053313	F2rl2	0.2292	0.4079	0.2320	0.2835	0.2187	0.5550	0.6315	0.2804	0.4991	0.2312	0.3184	0.3522
NM_053317	Nr0b1	0.7713	0.8202	0.4749	0.5901	0.4835	0.4491	0.4864	0.8343	0.2462	0.7620	0.8322	0.6074
NM_053319	Dynll1	0.2737	0.6415	0.1363	0.8036	0.9670	0.4404	0.4770	0.4701	0.4853	0.8623	0.6845	0.2516
NM_053320	Hgfac	0.7001	0.1108	0.3992	0.4001	0.2148	0.5480	0.2959	0.7201	0.5442	0.1931	0.3928	0.4726
NM_053516	Nol3	0.0097	0.1594	0.4469	0.0159	0.0637	0.0352	0.2352	0.1240	0.1552	0.2687	0.0396	0.3170
NM_053844	Tff2	0.0300	0.7838	0.0003	0.1046	0.0582	0.2580	0.0277	0.0005	0.6258	0.2014	0.0977	0.9562
NM_053821	Ralb	0.8884	0.0217	0.9465	0.7711	0.7990	0.7613	0.9089	0.8582	0.2369	0.1155	0.2472	0.1976
NM_053324	Syt9	0.4970	0.4156	0.0588	0.4443	0.1793	0.5384	0.5079	0.3870	0.4848	0.3963	0.4009	0.4186
NM_053503	Jub	0.8867	0.9812	0.8466	0.9022	0.9766	0.9967	0.8886	0.9818	0.8363	0.9454	0.9696	0.9973
NM_053487	Pex11a	0.2547	0.2565	0.9646	0.6487	0.7666	0.8983	0.5979	0.2108	0.1378	0.2807	0.1952	0.3484
NM_053380	Slc34a2	0.6748	0.7474	0.6273	0.1344	0.6888	0.7837	0.6433	0.3072	0.5013	0.2821	0.8462	0.5689
NM_053904	Oplah	0.0366	0.0002	0.0006	0.0002	0.0145	0.0011	0.0722	0.0016	0.0358	0.0002	0.0003	0.0125
NM_053330	Rpl21	0.4315	0.4697	0.3474	0.3643	0.7649	0.2668	0.3314	0.4487	0.4036	0.7816	0.4153	0.2776
NM_053469	Hamp	0.3230	0.4102	0.3376	0.1199	0.2141	0.3585	0.2729	0.2647	0.5603	0.1763	0.1187	0.8039
NM_053331	Txn2	0.4452	0.0886	0.3861	0.4184	0.3354	0.1977	0.5127	0.1577	0.0766	0.3041	0.0355	0.0730
NM_053581	Idh3B	0.0679	0.3478	0.5477	0.8772	0.1063	0.1570	0.5272	0.0839	0.1278	0.2145	0.1893	0.4633
NM_053493	Hacl1	0.6095	0.2283	0.7073	0.8391	0.9956	0.6250	0.7278	0.8850	0.8636	0.2723	0.8333	0.8141
NM_053772	Pkia	0.7832	0.4326	0.8200	0.2400	0.3827	0.3974	0.5269	0.4880	0.9175	0.7120	0.5372	0.2096
NM_053453	Rgs2	0.0005	0.0081	0.0037	0.0070	0.0148	0.0003	0.0009	0.0006	0.1218	0.1244	0.0024	0.3460
NM_053546	Angpt1	0.0129	0.0984	0.1753	0.1422	0.0497	0.3414	0.1665	0.0908	0.0919	0.1001	0.2548	0.6830
NM_053524	Nox4	0.3939	0.0498	0.0193	0.5495	0.1654	0.7106	0.3149	0.5653	0.0316	0.1267	0.3491	0.0318
NM_053334	Camlg	0.9147	0.9835	0.0829	0.1514	0.3825	0.0225	0.7728	0.1415	0.3630	0.8320	0.9651	0.1670
NM_053335	Ctbp2	0.8129	0.9069	0.9506	0.4285	0.3252	0.6543	0.8761	0.4970	0.7587	0.8011	0.6954	0.7751
NM_053338	Rrad	0.1523	0.0795	0.0760	0.1312	0.0878	0.0657	0.0578	0.1296	0.0744	0.0093	0.0002	0.0642
NM_053806	Kcnk6	0.6698	0.4331	0.2149	0.4176	0.4444	0.2152	0.5300	0.5724	0.4052	0.4726	0.5841	0.4353
NM_053624	Pitx1	0.5863	0.7430	0.4811	0.7713	0.6101	0.3772	0.6769	0.7500	0.4763	0.1689	0.4819	0.7913
NM_053441	Slco1c1	0.2386	0.5344	0.4925	0.3291	0.4876	0.2334	0.2079	0.4973	0.1598	0.4247	0.8606	0.4910
NM_053588	Rnf138	0.5380	0.3920	0.8277	0.3108	0.2637	0.2501	0.3078	0.2756	0.5119	0.6197	0.2671	0.4323
NM_053464	Srm	0.9528	0.9052	0.0546	0.7982	0.8099	0.3902	0.9662	0.9627	0.8750	0.8423	0.9006	0.0185
NM_053340	Ogfr	0.2364	0.3137	0.4051	0.2821	0.3183	0.2886	0.2894	0.5134	0.3871	0.2263	0.2606	0.4977
NM_053341	Gipc1	0.2747	0.9504	0.0897	0.7823	0.7186	0.3940	0.9916	0.8649	0.8843	0.9747	0.2337	0.6368
NM_053963	Mmp12	0.7324	0.6909	0.8683	0.7033	0.2654	0.8121	0.6103	0.6950	0.6317	0.7506	0.7515	0.7721
NM_053348	Fetub	0.8498	0.9563	0.8053	0.7212	0.8917	0.7789	0.8645	0.8366	0.7302	0.9064	0.9739	0.7984
NM_053811	Slc9a3r2	0.6354	0.7325	0.8529	0.9289	0.9447	0.9937	0.7657	0.9153	0.6757	0.6803	0.7767	0.9785
NM_053351	Cacng2	0.1245	0.0065	0.1548	0.0434	0.1546	0.1176	0.0922	0.0611	0.3339	0.1412	0.0889	0.1849
NM_053427	Slc17a6	0.3096	0.2934	0.3126	0.2905	0.3339	0.2561	0.1483	0.1061	0.4132	0.3352	0.0788	0.5771

NM_053674	Phyh	0.4016	0.2512	0.2337	0.7343	0.2589	0.1701	0.7748	0.6466	0.0311	0.0756	0.0374	0.0969
NM_053657	Cdc42bpa	0.2061	0.3970	0.5013	0.4303	0.0376	0.3862	0.7423	0.4622	0.2086	0.5871	0.3917	0.2908
NM_053883	Dusp6	0.9945	0.8150	0.9399	0.9995	0.9118	0.9473	0.7877	0.9856	0.9078	0.8699	0.2609	0.9001
NM_053849	Pdia4	0.7474	0.8096	0.8479	0.8755	0.6082	0.7688	0.9969	0.6398	0.7684	0.8246	0.9265	0.7646
NM_053616	Nup88	0.7479	0.8556	0.7004	0.7077	0.5245	0.7129	0.3825	0.3359	0.6762	0.5668	0.6295	0.6237
NM_053342	Cxxc4	0.6131	0.5160	0.6561	0.1643	0.6602	0.4023	0.4471	0.5039	0.7055	0.9065	0.8099	0.5504
NM_080478	Apbb1	0.1602	0.0113	0.0312	0.8173	0.3169	0.2911	0.3653	0.0636	0.2099	0.2021	0.0360	0.5259
NM_053995	Bdh1	0.1695	0.9842	0.0162	0.5165	0.0921	0.0169	0.7023	0.0764	0.7053	0.9154	0.9523	0.6544
NM_053846	Nrxn2	0.3468	0.4080	0.7462	0.5777	0.4178	0.5630	0.7464	0.7654	0.4154	0.6693	0.8441	0.7370
NM_053870	Kcnj4	0.3212	0.5300	0.8190	0.4350	0.7680	0.4163	0.8281	0.7351	0.9307	0.3986	0.0674	0.6608
NM_053869	Phox2a	0.5390	0.5356	0.1018	0.4760	0.6156	0.1692	0.9416	0.6536	0.7171	0.5338	0.1419	0.8506
NM_053358	Ssbp3	0.3758	0.5310	0.5335	0.4746	0.3500	0.5313	0.5838	0.6859	0.6077	0.5300	0.2489	0.6908
NM_053360	Sh3kbp1	0.0595	0.5544	0.4895	0.7532	0.1322	0.0799	0.2964	0.2643	0.8683	0.5518	0.4877	0.4002
NM_053824	Csnk2a1	0.1222	0.0390	0.7922	0.5504	0.1209	0.3581	0.8121	0.4606	0.2791	0.0083	0.2614	0.7373
NM_053372	Slpi	0.0657	0.6847	0.4011	0.7109	0.1410	0.0418	0.1122	0.0246	0.7530	0.8240	0.8809	0.8030
NM_053363	Clcn3	0.4826	0.2476	0.5175	0.9029	0.2676	0.3937	0.2080	0.2717	0.6303	0.3691	0.2449	0.9432
NM_053364	Prl7d1	0.5732	0.2053	0.4792	0.2828	0.5702	0.1751	0.4496	0.5680	0.6033	0.4529	0.4899	0.0118
NM_138842	Sftpb	0.3056	0.1435	0.2306	0.5677	0.1585	0.0747	0.2860	0.1283	0.7394	0.4961	0.2166	0.3140
NM_053365	Fabp4	0.0325	0.0031	0.6678	0.7805	0.6370	0.8060	0.0703	0.6326	0.0951	0.0711	0.0291	0.8644
NM_053373	Pglyrp1	0.8069	0.8405	0.7542	0.5158	0.8995	0.8306	0.9045	0.8333	0.9077	0.7016	0.6629	0.8075
NM_053371	Fxc1	0.2270	0.8671	0.0329	0.8979	0.1294	0.2308	0.8056	0.4833	0.6585	0.6467	0.4609	0.1100
NM_053369	Tcf4	0.1491	0.0429	0.0023	0.1759	0.0935	0.0601	0.0594	0.0042	0.0948	0.1360	0.1821	0.0140
NM_053374	Il18bp	0.0151	0.0071	0.0125	0.0141	0.3725	0.3851	0.0166	0.1159	0.1058	0.1353	0.1044	0.1319
NM_053495	Cnga3	0.5825	0.1348	0.7685	0.6501	0.6168	0.6762	0.7481	0.4564	0.7833	0.3751	0.4171	0.6128
NM_053381	Atp1b4	0.5867	0.6514	0.6219	0.8866	0.3863	0.5119	0.7732	0.1471	0.8318	0.7019	0.7709	0.4369
NM_053385	Prelp	0.0093	0.0145	0.0647	0.0316	0.1484	0.1383	0.0009	0.7121	0.1321	0.1058	0.0322	0.0369
NM_053390	Il12a	0.5294	0.4312	0.8193	0.5846	0.5944	0.5214	0.7070	0.5622	0.5273	0.8582	0.3941	0.7850
NM_053894	Jdp2	0.0610	0.6637	0.8903	0.7277	0.6270	0.7012	0.5673	0.1033	0.2363	0.8580	0.8017	0.4786
NM_053877	Cdc123	0.9253	0.5859	0.7033	0.7408	0.8148	0.7259	0.6751	0.6089	0.1915	0.2832	0.3060	0.1024
NM_053389	Sip1	0.8239	0.8839	0.6385	0.3039	0.8921	0.9416	0.1162	0.7707	0.3852	0.7452	0.7846	0.6872
NM_053401	Ngfrap1	0.6732	0.4631	0.7998	0.7199	0.1570	0.0447	0.8634	0.0806	0.1150	0.7273	0.8609	0.2758
NM_053688	Pde6h	0.0026	0.0083	0.1028	0.0864	0.0311	0.0323	0.0060	0.1551	0.1199	0.1370	0.0055	0.0338
NM_053723	Lancl1	0.0382	0.0809	0.8670	0.1574	0.0430	0.1097	0.6187	0.0027	0.7836	0.5058	0.6481	0.8643
NM_053403	Grb7	0.5411	0.8688	0.5308	0.6148	0.5797	0.2419	0.8912	0.4187	0.5905	0.7537	0.9645	0.9613
NM_053395	Smpx	0.4185	0.4154	0.8192	0.5424	0.4903	0.3782	0.5934	0.5480	0.8360	0.9200	0.4857	0.2393
NM_053551	Pdk4	0.0071	0.5189	0.8898	0.1827	0.5798	0.6411	0.9842	0.6639	0.1225	0.4487	0.8681	0.3359
NM_053478	Plcb2	0.7687	0.5862	0.7167	0.9216	0.4067	0.8122	0.7199	0.7082	0.8662	0.6230	0.7211	0.7534
NM_053550	Pip4k2b	0.4123	0.2173	0.5459	0.6981	0.5040	0.7156	0.6907	0.3883	0.4385	0.4988	0.3889	0.8221
NM_053399	Nrtn	0.2041	0.5853	0.1438	0.1122	0.2506	0.5326	0.4250	0.4935	0.2500	0.5635	0.4270	0.3088
NM_053409	Maged1	0.2528	0.0727	0.6457	0.0264	0.1996	0.1264	0.9307	0.0288	0.0339	0.0574	0.1145	0.0071
NM_053733	Bcl2l10	0.6056	0.8474	0.0896	0.7788	0.7025	0.7490	0.6164	0.8180	0.7560	0.5683	0.3670	0.5417
NM_053411	Snx1	0.2312	0.2054	0.3927	0.9369	0.1656	0.0929	0.0231	0.4298	0.1578	0.2327	0.0816	0.2373
NM_053412	Ilf3	0.2198	0.9263	0.3692	0.3975	0.1278	0.1432	0.8566	0.5640	0.1029	0.5216	0.6712	0.1690
NM_053414	Ddx1	0.1419	0.5055	0.0900	0.1998	0.0278	0.0904	0.0438	0.3304	0.1382	0.0451	0.2417	0.0809
NM_053798	Sacm1l	0.8775	0.9245	0.3471	0.2631	0.6516	0.6579	0.2865	0.5349	0.7659	0.8892	0.5256	0.6229
NM_053416	Strbp	0.2211	0.9780	0.9312	0.9526	0.1361	0.1157	0.9760	0.1792	0.9342	0.9886	0.9754	0.7656
NM_053521	Slc5a7	0.8153	0.8394	0.8460	0.4018	0.7058	0.6214	0.7115	0.6635	0.5342	0.8711	0.9133	0.7930
NM_053880	Dync1i2	0.9077	0.4566	0.6783	0.9901	0.3150	0.3232	0.4588	0.0603	0.3410	0.5124	0.3340	0.2787
NM_053590	Psmb1	0.2547	0.0741	0.1017	0.1889	0.0172	0.1214	0.3235	0.0174	0.1056	0.3228	0.0742	0.0053

NM_053425	Ccs	0.1356	0.5808	0.6836	0.8023	0.7070	0.0511	0.8107	0.7065	0.5003	0.8901	0.2692	0.1223
NM_053928	Ube2n	0.3729	0.6332	0.5145	0.9104	0.9370	0.9035	0.9170	0.8699	0.1665	0.5291	0.6341	0.8004
NM_053926	Pip4k2a	0.9595	0.9562	0.9162	0.8789	0.8499	0.9524	0.9758	0.9338	0.7399	0.5520	0.9449	0.7298
NM_053430	Fen1	0.9836	0.9361	0.2188	0.9897	0.7355	0.9766	0.7961	0.9391	0.8818	0.9650	0.9973	0.1449
NM_053576	Prdx6	0.0211	0.1704	0.4436	0.0013	0.0224	0.1318	0.8922	0.1058	0.1283	0.0518	0.2664	0.7841
NM_054006	Csde1	0.2080	0.2478	0.8649	0.8351	0.2962	0.2927	0.8979	0.3807	0.2326	0.3113	0.1988	0.1759
NM_053435	Xrcc1	0.5105	0.6907	0.7667	0.3064	0.7669	0.4330	0.2661	0.8389	0.8122	0.6172	0.7573	0.4287
NM_001108449	Cog4	0.2117	0.5451	0.2261	0.4011	0.2095	0.6682	0.2741	0.3350	0.3528	0.4127	0.4522	0.3393
NM_001108443	Zfp330	0.0805	0.2860	0.0631	0.0109	0.0926	0.6179	0.0635	0.1768	0.2432	0.1501	0.2264	0.1257
NM_053440	Stmn2	0.4251	0.5881	0.4308	0.3025	0.9239	0.1574	0.5621	0.6650	0.9597	0.5482	0.8709	0.3994
NM_053438	Rnf103	0.8437	0.7026	0.8732	0.6539	0.6522	0.8634	0.9145	0.6047	0.8875	0.9377	0.6413	0.7500
NM_053558	Trpc1	0.6029	0.5683	0.5131	0.7258	0.6730	0.4189	0.2670	0.4626	0.6290	0.5739	0.5035	0.6571
NM_053442	Slc7a8	0.6620	0.8804	0.2406	0.4746	0.4294	0.8181	0.5459	0.8604	0.6533	0.2191	0.4330	0.9046
NM_053962	Sds	0.2647	0.3843	0.1333	0.2683	0.2971	0.2873	0.5129	0.1501	0.5279	0.3295	0.0587	0.4266
NM_053664	Sardh	0.1484	0.4361	0.0438	0.5371	0.0442	0.3589	0.3333	0.0850	0.0813	0.0478	0.1344	0.0974
NM_053595	Pgf	0.7722	0.9302	0.9356	0.8398	0.8274	0.9753	0.9632	0.9122	0.4915	0.6795	0.8885	0.3618
NM_053594	Ptprr	0.6773	0.5142	0.9688	0.6484	0.5854	0.5375	0.5284	0.6098	0.4551	0.7071	0.5431	0.9502
NM_053455	Fgl2	0.7621	0.2800	0.6597	0.5245	0.7723	0.5499	0.5106	0.4991	0.2888	0.1816	0.4132	0.5970
NM_053648	Bcmo1	0.0389	0.2715	0.0700	0.1213	0.0744	0.2139	0.3917	0.1402	0.2900	0.3347	0.2764	0.0672
NM_053457	Cldn11	0.5108	0.3252	0.7439	0.8353	0.6711	0.5058	0.5633	0.4910	0.5781	0.6372	0.4182	0.2103
NM_053456	Plcl1	0.9414	0.9384	0.9121	0.7058	0.2541	0.1479	0.8198	0.1183	0.8239	0.7867	0.9649	0.6686
NM_053467	Tmed10	0.0742	0.3872	0.4742	0.4529	0.1630	0.1745	0.3885	0.3141	0.1767	0.1812	0.1802	0.4327
NM_053822	S100a8	0.0891	0.4370	0.4568	0.3551	0.1836	0.1355	0.4221	0.5027	0.5745	0.1022	0.1875	0.3169
NM_053604	Mgat2	0.8691	0.3324	0.6004	0.6526	0.3301	0.1623	0.3965	0.2970	0.3418	0.2978	0.4824	0.0293
NM_053472	Cox4i2	0.4132	0.5625	0.1781	0.5065	0.5249	0.5202	0.1832	0.4784	0.4247	0.2642	0.4842	0.7181
NM_053652	Flt4	0.2405	0.3228	0.2476	0.4883	0.3708	0.4029	0.5335	0.4055	0.4850	0.3588	0.3132	0.4912
NM_053554	Picalm	0.0043	0.0008	0.8106	0.4709	0.0267	0.6591	0.0872	0.0028	0.0138	0.0229	0.0378	0.7693
NM_053459	Rab27b	0.4871	0.5251	0.2654	0.1440	0.0073	0.0158	0.4586	0.0133	0.4264	0.3473	0.0904	0.3365
NM_053463	Nucb1	0.1876	0.1671	0.9234	0.0928	0.3004	0.0785	0.5736	0.1697	0.9157	0.0737	0.6900	0.9855
NM_053471	Sema6b	0.4728	0.1071	0.3651	0.1025	0.1303	0.1231	0.5324	0.3992	0.2240	0.7935	0.4620	0.5431
NM_053474	Ppp1r9b	0.5841	0.3280	0.8337	0.4911	0.5984	0.3751	0.6066	0.4749	0.5867	0.3931	0.4915	0.4129
NM_053607	Acsl5	0.5193	0.7505	0.0674	0.0596	0.5803	0.7840	0.1002	0.6230	0.8584	0.8522	0.3394	0.5143
NM_053698	Cited2	0.1338	0.1437	0.2300	0.2078	0.1499	0.0197	0.2045	0.3090	0.1780	0.2086	0.1438	0.2609
NM_053602	Atp5j	0.2991	0.3262	0.2247	0.2182	0.0915	0.0577	0.0807	0.0282	0.0246	0.1737	0.0754	0.0434
NM_053480	Pola2	0.8351	0.6106	0.0872	0.9180	0.8135	0.8022	0.8447	0.8639	0.6474	0.7372	0.6288	0.2035
NM_001110099	Ret	0.9914	0.6441	0.9497	0.8543	0.5628	0.5902	0.9333	0.8131	0.9162	0.6801	0.7398	0.9820
NM_147139	Itgb5	0.1551	0.1001	0.0787	0.2913	0.0126	0.2617	0.2488	0.1534	0.0464	0.0147	0.0242	0.1188
NM_001109996	Apoc1	0.4334	0.1913	0.6094	0.2132	0.3529	0.6462	0.3789	0.4441	0.3804	0.2913	0.1904	0.4319
NM_001109994	Cox6a2	0.4544	0.9142	0.8347	0.4790	0.7349	0.7889	0.6391	0.8466	0.7987	0.7779	0.8378	0.8140
NM_053853	Nat1	0.4680	0.4861	0.3385	0.4069	0.4889	0.2163	0.7030	0.3367	0.5104	0.3886	0.5554	0.3886
NM_053850	Blvra	0.0378	0.1428	0.0358	0.7983	0.0734	0.0628	0.8318	0.1719	0.0232	0.1672	0.0710	0.0007
NM_053854	Nat2	0.3962	0.4400	0.3211	0.3680	0.4018	0.3138	0.1831	0.1741	0.2653	0.2555	0.5789	0.2834
NM_053483	Kpna2	0.9856	0.9732	0.2210	0.9680	0.8525	0.9536	0.7494	0.7375	0.9179	0.9710	0.8377	0.0943
NM_053486	Kif3c	0.0098	0.5510	0.0777	0.1425	0.1210	0.0094	0.1823	0.3072	0.9510	0.4664	0.5469	0.8158
NM_053500	Slc25a27	0.9616	0.9402	0.9101	0.5778	0.9722	0.9492	0.9049	0.9873	0.9708	0.9895	0.8932	0.9407
NM_053501	Slc25a14	0.8265	0.9817	0.9837	0.8420	0.9075	0.9372	0.6201	0.6710	0.7963	0.6969	0.9540	0.8977
NM_053492	Cdw92	0.7218	0.5101	0.8357	0.9485	0.6167	0.6931	0.6239	0.5232	0.8863	0.7039	0.7500	0.3854
NM_053907	Dnase1l3	0.1981	0.6200	0.5340	0.5966	0.4831	0.5723	0.4638	0.2090	0.2283	0.3679	0.6916	0.2202
NM_053502	Abcg1	0.4505	0.0999	0.1425	0.2862	0.1869	0.1781	0.1108	0.1446	0.0664	0.0493	0.1726	0.1500

NM_053946	Magt1	0.4416	0.1676	0.3507	0.5866	0.3411	0.3707	0.1829	0.1739	0.3211	0.3712	0.5808	0.1729
NM_053494	Slc2a8	0.8953	0.7901	0.7778	0.8347	0.9495	0.8009	0.9202	0.9777	0.9747	0.5828	0.9344	0.7240
NM_053496	Cnga4	0.2031	0.4611	0.5560	0.7004	0.5435	0.5790	0.5845	0.2705	0.3096	0.2847	0.2904	0.3988
NM_053949	Kcnh2	0.8201	0.7154	0.5306	0.6995	0.4953	0.6505	0.9193	0.7422	0.9627	0.7898	0.9067	0.8669
NM_053568	Pcyt2	0.9693	0.7760	0.0992	0.4361	0.9434	0.9243	0.1335	0.8849	0.9265	0.7670	0.6696	0.9975
NM_053508	Tekt1	0.7719	0.6800	0.7016	0.6693	0.6526	0.2860	0.8696	0.4277	0.7342	0.8688	0.8404	0.6248
NM_078622	Pcyt1a	0.7046	0.3105	0.7374	0.5797	0.5325	0.5579	0.7217	0.3605	0.7003	0.9362	0.3245	0.7769
NM_057197	Decr1	0.6484	0.7350	0.9137	0.8861	0.9098	0.6722	0.8758	0.8591	0.6605	0.8146	0.8349	0.8060
NM_053951	Mcf2l	0.2666	0.7747	0.1781	0.4004	0.4393	0.3603	0.7194	0.5716	0.6938	0.6272	0.8497	0.6959
NM_053517	Shc1	0.5870	0.3880	0.2723	0.4595	0.6126	0.4945	0.3103	0.2784	0.6039	0.3896	0.6964	0.7093
NM_053515	Slc25a4	0.8087	0.8826	0.9727	0.8951	0.6894	0.8110	0.9420	0.6790	0.7414	0.7779	0.8935	0.6741
NM_053520	Elf1	0.5168	0.1505	0.9726	0.9257	0.0271	0.7751	0.0307	0.0655	0.0293	0.2041	0.8309	0.6670
NM_053522	Rhoq	0.3778	0.4750	0.5255	0.4121	0.5188	0.6247	0.8507	0.4179	0.3670	0.4221	0.4272	0.7820
NM_053891	Cdk5r1	0.3143	0.7336	0.5614	0.4279	0.3452	0.2908	0.3410	0.6317	0.1668	0.1614	0.3590	0.1727
NM_057114	Prdx1	0.0839	0.2390	0.0360	0.2636	0.3387	0.2800	0.1757	0.2010	0.1661	0.1491	0.2855	0.2132
NM_053523	Herpud1	0.8350	0.8969	0.8968	0.6226	0.0093	0.2867	0.8398	0.2396	0.9629	0.9494	0.7020	0.8297
NM_053589	Rab14	0.1548	0.5016	0.7186	0.4423	0.1999	0.1278	0.9323	0.0059	0.2253	0.6015	0.9145	0.0411
NM_133514	Mmp10	0.2271	0.1306	0.4113	0.4217	0.0046	0.0910	0.4463	0.2652	0.0382	0.7775	0.0864	0.3009
NM_133409	Ilk	0.4771	0.1983	0.2396	0.7083	0.1508	0.1418	0.7852	0.1208	0.3556	0.3112	0.2230	0.4793
NM_053525	Ddx52	0.8903	0.8046	0.3090	0.7629	0.9048	0.8269	0.3698	0.7276	0.1312	0.1292	0.4078	0.0094
NM_001126080	Ufm1	0.5814	0.2275	0.3446	0.4923	0.4371	0.6684	0.5076	0.2386	0.4889	0.3785	0.4990	0.2350
NM_057123	Psmc1	0.2601	0.6857	0.0927	0.1480	0.2374	0.1862	0.1465	0.2490	0.3987	0.3702	0.3756	0.2580
NM_001115025	Mbd2	0.7019	0.5688	0.4837	0.4269	0.3713	0.2457	0.6430	0.7142	0.4872	0.5757	0.5460	0.6955
NM_053984	Gjb4	0.3623	0.3921	0.5247	0.3072	0.7239	0.5542	0.3905	0.4880	0.5344	0.1890	0.5074	0.6175
NM_053569	Mbtps1	0.1085	0.3099	0.1600	0.3225	0.9691	0.6973	0.3273	0.9319	0.9314	0.2854	0.4010	0.4886
NM_057130	Hrk	0.6133	0.4897	0.8322	0.6961	0.7056	0.7576	0.8556	0.1421	0.1769	0.1523	0.6597	0.6358
NM_053965	Slc25a20	0.1251	0.0912	0.7716	0.0296	0.2564	0.0933	0.4840	0.0588	0.0766	0.0824	0.0189	0.3263
NM_053570	Cxadr	0.0563	0.0735	0.0165	0.0924	0.2674	0.0770	0.3043	0.0831	0.1412	0.1053	0.0420	0.0542
NM_053534	Gpr175	0.1728	0.1675	0.1777	0.1781	0.3673	0.4439	0.1827	0.4164	0.1666	0.1613	0.1679	0.1726
NM_053536	Klf15	0.8411	0.0878	0.4885	0.1897	0.4770	0.1899	0.0751	0.5187	0.2919	0.3356	0.2918	0.3444
NM_053609	Bat3	0.7611	0.9839	0.1926	0.2006	0.3627	0.2881	0.2859	0.1952	0.6199	0.4471	0.6595	0.6869
NM_053537	Slc22a7	0.1870	0.3705	0.4342	0.1048	0.4695	0.5512	0.3978	0.0846	0.5216	0.4910	0.4561	0.0806
NM_053716	Fbp2	0.0271	0.2771	0.5516	0.4024	0.0282	0.6004	0.3352	0.2836	0.2582	0.2443	0.0744	0.1368
NM_080585	Napa	0.0103	0.1672	0.5215	0.0937	0.2033	0.1133	0.0995	0.1108	0.1654	0.0117	0.2288	0.1887
NM_053529	Ciita	0.7922	0.6637	0.2827	0.2601	0.3223	0.7589	0.8028	0.5738	0.4787	0.6796	0.7436	0.7465
NM_053542	Gna15	0.7297	0.6980	0.1462	0.6066	0.8230	0.4434	0.9044	0.7467	0.8898	0.5788	0.1469	0.6721
NM_053541	Lrp3	0.7677	0.7892	0.1828	0.4167	0.6239	0.6357	0.7583	0.8354	0.2790	0.5964	0.6522	0.4537
NM_053549	Vegfb	0.7174	0.6707	0.3709	0.2500	0.6153	0.2227	0.6880	0.4112	0.5413	0.6925	0.6398	0.3264
NM_053544	Sfrp4	0.9544	0.0151	0.9918	0.7810	0.9899	0.8807	0.1241	0.7931	0.0818	0.1106	0.1053	0.7725
NM_053807	Gripap1	0.6784	0.6796	0.1534	0.1320	0.4670	0.1450	0.2483	0.8356	0.8226	0.7948	0.7617	0.9553
NM_053552	Tnfsf4	0.5173	0.8637	0.8014	0.4974	0.6054	0.4885	0.3661	0.6042	0.5814	0.5660	0.7846	0.7878
NM_053621	Magi2	0.7532	0.6289	0.8569	0.7252	0.9846	0.8959	0.9227	0.9948	0.9536	0.9764	0.7953	0.9330
NM_057147	Sec22a	0.4563	0.2123	0.8392	0.3781	0.4813	0.1758	0.7045	0.1487	0.2054	0.4316	0.8430	0.6117
NM_057139	Hnrnpu	0.9829	0.8771	0.9038	0.7689	0.9717	0.7994	0.8434	0.9729	0.9540	0.9142	0.9872	0.9232
NM_053817	Nrxn3	0.7162	0.4603	0.6044	0.3909	0.2035	0.4589	0.2841	0.4314	0.3110	0.4472	0.4112	0.4859
NM_057184	Chrna6	0.3555	0.2756	0.2905	0.1779	0.3568	0.1748	0.3847	0.3381	0.4469	0.2876	0.4700	0.3445
NM_057109	Barhl1	0.3481	0.3181	0.4269	0.0896	0.2700	0.3582	0.1801	0.2265	0.4046	0.3203	0.0319	0.3477
NM_057204	Ptpn23	0.7739	0.5455	0.0699	0.0322	0.0642	0.1093	0.0161	0.3287	0.8024	0.0819	0.1448	0.0653
NM_053560	Chi3l1	0.0229	0.4527	0.1752	0.6779	0.0707	0.4399	0.4454	0.2433	0.3500	0.7530	0.0464	0.6126

NM_053561	Nap1l1	0.9940	0.9380	0.9555	0.9961	0.9799	0.9530	0.9988	0.9944	0.8973	0.9138	0.9630	0.9195
NM_053562	Rpe65	0.9356	0.9783	0.8909	0.9184	0.6858	0.2099	0.9815	0.9301	0.8202	0.9826	0.7473	0.8136
NM_053563	Ddx39	0.7275	0.9234	0.0689	0.4580	0.8170	0.9269	0.1425	0.8991	0.7897	0.7276	0.8743	0.8334
NM_053610	Prdx5	0.0142	0.1485	0.0427	0.0477	0.0098	0.0303	0.0004	0.0022	0.1796	0.1032	0.1043	0.1390
NM_053598	Nudt4	0.1900	0.2925	0.2472	0.1555	0.1568	0.3704	0.2486	0.2000	0.0225	0.2089	0.2207	0.2513
NM_053605	Smpd3	0.7433	0.6666	0.8825	0.9138	0.8359	0.9187	0.8140	0.5680	0.7031	0.8369	0.1584	0.7490
NM_057192	Wipf1	0.6624	0.1075	0.3886	0.4953	0.5243	0.6317	0.3591	0.4131	0.3437	0.0407	0.1687	0.7137
NM_057212	Tmem158	0.7370	0.0020	0.7441	0.7444	0.7287	0.7337	0.7499	0.7405	0.0005	0.0303	0.0216	0.0022
NM_001077669	Slc39a4	0.5833	0.8564	0.5480	0.6335	0.7010	0.8426	0.8014	0.6625	0.8648	0.3336	0.7108	0.7998
NM_053796	F11r	0.9991	0.7750	0.9785	0.9850	0.9776	0.9864	0.9990	0.9880	0.9501	0.7667	0.9846	0.9260
NM_053573	Olfm1	0.8278	0.2608	0.9934	0.7211	0.1081	0.1205	0.9585	0.1530	0.4090	0.1946	0.1974	0.9758
NM_053608	Kcnj13	0.2769	0.1006	0.6437	0.0829	0.4110	0.0362	0.0782	0.4826	0.2935	0.0449	0.1929	0.3594
NM_053575	Cabin1	0.8281	0.4737	0.7030	0.8181	0.9223	0.9920	0.9122	0.9943	0.8941	0.8573	0.6112	0.4617
NM_053702	Ccna2	0.7455	0.9883	0.1551	0.9254	0.8463	0.8092	0.8662	0.9664	0.9069	0.9267	0.9585	0.0186
NM_053580	Slc27a1	0.1317	0.6742	0.4829	0.2392	0.3639	0.4142	0.8027	0.6670	0.2695	0.2865	0.1349	0.8325
NM_053565	Socs3	0.1385	0.6017	0.1379	0.5644	0.4171	0.3345	0.0826	0.5135	0.1359	0.0384	0.0938	0.5275
NM_053543	Ncdn	0.9308	0.8889	0.9551	0.9907	0.7797	0.8002	0.9477	0.7516	0.9880	0.8512	0.9869	0.9964
NM_134417	Ipmk	0.9561	0.6645	0.8512	0.5277	0.8476	0.6077	0.7985	0.7216	0.7558	0.8587	0.3667	0.9415
NM_053583	Zfp423	0.9383	0.7284	0.8313	0.9293	0.3032	0.4891	0.8312	0.7435	0.8535	0.8940	0.9206	0.8454
NM_133404	Lonp1	0.2172	0.2597	0.0383	0.3753	0.0166	0.1587	0.1887	0.2443	0.7995	0.4550	0.3290	0.8642
NM_053584	Gosr1	0.9690	0.3429	0.8070	0.9161	0.7160	0.9493	0.8980	0.9453	0.5988	0.9341	0.9260	0.9013
NM_053748	Dpp3	0.6194	0.2638	0.0841	0.1157	0.1904	0.4588	0.3972	0.6858	0.1226	0.4962	0.4079	0.3892
NM_053735	Pi4k2a	0.7256	0.1385	0.4417	0.1927	0.2551	0.5631	0.1150	0.1083	0.4794	0.0449	0.3231	0.1834
NM_053585	Madd	0.4265	0.1354	0.7765	0.6885	0.6827	0.9381	0.0928	0.3865	0.0604	0.4629	0.5425	0.8122
NM_053825	Atp5c1	0.1771	0.0447	0.1471	0.1532	0.1699	0.1015	0.6972	0.0144	0.0419	0.3931	0.0735	0.0023
NM_053620	Cdc42bpb	0.9747	0.9494	0.7674	0.5753	0.7631	0.5943	0.5617	0.6818	0.6768	0.9279	0.0681	0.9335
NM_053600	Fez2	0.1410	0.0046	0.8159	0.0326	0.0476	0.7460	0.1093	0.0203	0.2574	0.1635	0.0187	0.8217
NM_053601	Nnat	0.6713	0.6587	0.7219	0.6313	0.5817	0.5505	0.7838	0.4622	0.6909	0.8065	0.7815	0.7816
NM_053734	Ncf1	0.9664	0.7365	0.9126	0.8991	0.9652	0.9280	0.9890	0.8551	0.9171	0.9947	0.9374	0.9520
NM_053603	Clic5	0.0996	0.6059	0.8194	0.7471	0.3117	0.2665	0.2715	0.2819	0.7529	0.9478	0.2879	0.9958
NM_133511	Adcyap1r1	0.7738	0.9099	0.6319	0.1636	0.8235	0.3796	0.7883	0.4295	0.4303	0.5538	0.5514	0.6292
NM_053656	P2rx2	0.5350	0.9719	0.5877	0.8966	0.8708	0.7383	0.6815	0.2455	0.6829	0.5968	0.6246	0.7925
NM_053635	St14	0.4378	0.2014	0.3679	0.7920	0.2234	0.1333	0.0911	0.4181	0.1441	0.4856	0.4555	0.2592
NM_053643	Cds2	0.4329	0.2952	0.5942	0.3210	0.0319	0.5061	0.0554	0.1864	0.8052	0.3692	0.2544	0.4917
NM_053754	Abcg5	0.3934	0.8871	0.6024	0.6370	0.7193	0.9112	0.7652	0.5695	0.6028	0.8469	0.5036	0.9073
NM_053606	Mmp23	0.3457	0.3272	0.0636	0.2680	0.4442	0.2401	0.1432	0.3094	0.3735	0.3843	0.6395	0.2501
NM_001108471	Ube2m	0.3708	0.4917	0.7952	0.8179	0.5384	0.2909	0.8335	0.6937	0.8881	0.7633	0.3811	0.4554
NM_001108467	Eps8l1	0.5018	0.8740	0.8740	0.5997	0.6636	0.7382	0.6449	0.5974	0.4785	0.9416	0.8210	0.8941
NM_001108620	Nup205	0.9913	0.7247	0.2417	0.6070	0.8889	0.8081	0.6655	0.6621	0.7802	0.8451	0.8188	0.7981
NM_001108457	Ppil4	0.6200	0.6043	0.7090	0.5251	0.5618	0.6333	0.6694	0.6034	0.6926	0.6527	0.6645	0.5769
NM_001108576	Mrps2	0.9485	0.6929	0.4803	0.9705	0.6993	0.9609	0.4204	0.9458	0.9979	0.6704	0.4750	0.9612
NM_001108454	Cpne7	0.4400	0.1666	0.3207	0.4018	0.3672	0.1331	0.3646	0.5537	0.5678	0.2096	0.1763	0.5004
NM_001108447	Nfatc3	0.9069	0.7345	0.9972	0.9460	0.9558	0.9983	0.9849	0.9440	0.9141	0.9507	0.9881	0.9664
NM_001108445	Anapc10	0.1697	0.0281	0.1597	0.7196	0.4274	0.4389	0.0321	0.6739	0.1407	0.0286	0.1027	0.0060
NM_001108503	Polr3e	0.9517	0.9926	0.5375	0.4385	0.8627	0.8538	0.5415	0.9062	0.7098	0.9365	0.8711	0.5750
NM_001108388	Scel	0.3520	0.2381	0.3061	0.3911	0.7069	0.3103	0.1165	0.2445	0.3620	0.0669	0.5834	0.3204
NM_001108434	Fech	0.1302	0.7141	0.9745	0.8749	0.5494	0.5991	0.9683	0.5986	0.4022	0.5229	0.6339	0.6109
NM_001108672	Bend5	0.7223	0.0871	0.6515	0.3116	0.2926	0.3063	0.0733	0.3333	0.4336	0.0482	0.1624	0.7575
NM_001108567	Rpl34	0.5255	0.7201	0.3032	0.5539	0.3423	0.2856	0.3024	0.5789	0.3051	0.3675	0.5135	0.1550

NM_001108565	Pla2g12a	0.3656	0.3714	0.9652	0.2642	0.2444	0.4377	0.4238	0.2923	0.4360	0.4725	0.4567	0.8169
NM_001108444	Gab1	0.5857	0.5786	0.8580	0.9729	0.6898	0.9220	0.8425	0.7565	0.6962	0.3753	0.8383	0.8403
NM_001108430	Sema6a	0.9029	0.9730	0.8815	0.9971	0.9088	0.7962	0.9166	0.4324	0.5836	0.9137	0.9644	0.9742
NM_001106243	Map4k1	0.0788	0.0261	0.1233	0.1151	0.0107	0.0006	0.0889	0.0322	0.0366	0.0802	0.0765	0.4853
NM_001108632	Rpia	0.8269	0.8827	0.9132	0.9815	0.8109	0.9714	0.9096	0.8361	0.8654	0.8987	0.9862	0.8996
NM_001108614	Lime1	0.2616	0.2794	0.4071	0.3494	0.3554	0.3508	0.1819	0.3251	0.2430	0.4495	0.3071	0.2675
NM_001108612	Dido1	0.4515	0.8005	0.4591	0.1224	0.7410	0.8274	0.5772	0.4886	0.7512	0.0855	0.4385	0.5814
NM_001108429	Rbm27	0.9721	0.5338	0.9754	0.9519	0.9533	0.9906	0.5192	0.7759	0.9053	0.9186	0.9047	0.5424
NM_001108533	Spock2	0.8165	0.9249	0.9638	0.5445	0.9870	0.7776	0.8067	0.9486	0.9864	0.9761	0.7144	0.9916
NM_001108531	Bicc1	0.1229	0.1466	0.4158	0.3222	0.6619	0.4948	0.4032	0.3563	0.4926	0.3120	0.2846	0.1558
NM_001108552	Trim2	0.5590	0.5316	0.5141	0.5047	0.7791	0.9149	0.8352	0.4850	0.4984	0.8008	0.4703	0.7650
NM_001108424	Rnf125	0.5941	0.8796	0.0602	0.1868	0.7749	0.6035	0.0653	0.5858	0.1955	0.5833	0.4595	0.4381
NM_001108611	Col9a3	0.2237	0.7963	0.7650	0.7054	0.3066	0.6580	0.5480	0.3371	0.1902	0.8508	0.7980	0.2884
NM_001108416	Svil	0.9907	0.3766	0.9888	0.9194	0.9773	0.8298	0.8885	0.9634	0.8617	0.9036	0.4355	0.6478
NM_001108485	Gtf2h1	0.4648	0.6622	0.4897	0.2174	0.0710	0.4280	0.3766	0.4223	0.5403	0.2814	0.4349	0.3774
NM_001108415	Elmo1	0.9677	0.9577	0.9985	0.9842	0.9998	0.9875	0.9777	0.9913	0.9996	0.9731	0.9653	0.9991
NM_001108414	Hist1h2bm	0.1726	0.1673	0.1774	0.1773	0.1777	0.1747	0.1818	0.1734	0.1664	0.1610	0.1677	0.1724
NM_001108412	Dusp22	0.5835	0.7845	0.5818	0.7750	0.5158	0.8622	0.5910	0.3723	0.9717	0.6421	0.3203	0.7926
NM_001108411	Tbc1d7	0.7771	0.6848	0.7320	0.9125	0.2669	0.7531	0.8392	0.3958	0.1913	0.7144	0.7987	0.9859
NM_001108582	Dapl1	0.8655	0.7853	0.6029	0.7669	0.7395	0.7092	0.7889	0.6196	0.7458	0.9762	0.7849	0.8864
NM_001108566	Dc2	0.7251	0.9956	0.7535	0.8471	0.8135	0.8612	0.7891	0.8927	0.8477	0.9013	0.7716	0.2254
NM_001108564	Agl	0.2680	0.3418	0.4939	0.5990	0.2701	0.2628	0.2647	0.2640	0.4438	0.4945	0.5870	0.9475
NM_001108497	RGD1306959	0.7545	0.9638	0.8262	0.2344	0.1306	0.2723	0.9807	0.0531	0.8566	0.5868	0.9040	0.7334
NM_001108506	Ebf3	0.3792	0.2862	0.6444	0.8010	0.8567	0.3687	0.5299	0.4544	0.4041	0.6658	0.5468	0.7388
NM_057101	Cyp21a1	0.4045	0.7469	0.2031	0.1276	0.4096	0.2637	0.8296	0.4825	0.7314	0.7578	0.4238	0.4682
NM_053835	Cltb	0.7072	0.7485	0.9054	0.8768	0.3065	0.1400	0.9813	0.2421	0.8413	0.8471	0.8020	0.8533
NM_053618	Bbs2	0.2323	0.0099	0.0114	0.0540	0.1114	0.0318	0.1144	0.0359	0.0972	0.0246	0.1291	0.1701
NM_053940	Pcdha12	0.8764	0.7019	0.7783	0.8913	0.6978	0.5642	0.8783	0.8239	0.8058	0.7841	0.6105	0.7905
NM_053828	Il13	0.4804	0.4200	0.3236	0.6315	0.2739	0.1387	0.2170	0.1815	0.1570	0.0052	0.3090	0.0479
NM_053633	Egr2	0.1109	0.8141	0.3517	0.9351	0.2214	0.1330	0.1788	0.2362	0.8362	0.5991	0.7466	0.0927
NM_053819	Timp1	0.0425	0.0004	0.0251	0.0693	0.3022	0.0002	0.0002	0.0007	0.0003	0.0004	0.0845	0.0144
NM_053970	Nln	0.8255	0.5972	0.6030	0.8747	0.9009	0.9055	0.9707	0.9834	0.1316	0.3771	0.1412	0.1804
NM_053639	Ltc4s	0.0380	0.0184	0.0347	0.1221	0.0998	0.0103	0.2230	0.0798	0.0901	0.1669	0.0990	0.0947
NM_080888	Bnip3l	0.4378	0.2844	0.9024	0.9919	0.7495	0.8798	0.5638	0.5579	0.3144	0.3048	0.3051	0.4200
NM_053994	Pdha2	0.6136	0.3167	0.5037	0.4442	0.0695	0.2521	0.1402	0.4056	0.2160	0.4153	0.1157	0.5420
NM_080776	Mmp16	0.9588	0.9125	0.9061	0.9050	0.4151	0.4354	0.7739	0.7438	0.8082	0.9849	0.6755	0.5174
NM_053704	Bik	0.3899	0.7429	0.6158	0.3322	0.8875	0.7934	0.7312	0.7797	0.4555	0.6543	0.9217	0.8929
NM_053631	Banf1	0.0268	0.6573	0.2163	0.5622	0.1316	0.2810	0.7616	0.3280	0.3044	0.1042	0.2177	0.2305
NM_053714	Ankh	0.7311	0.2060	0.0595	0.9567	0.7724	0.9281	0.0951	0.6877	0.1285	0.2356	0.0563	0.2233
NM_053630	Kcnh4	0.5284	0.6331	0.4824	0.6072	0.6337	0.5765	0.8033	0.5582	0.3482	0.3454	0.4271	0.7353
NM_053902	Kynu	0.3511	0.5532	0.5811	0.4957	0.7364	0.3680	0.1625	0.2305	0.3498	0.3176	0.3309	0.3657
NM_053634	Fcnb	0.5776	0.6126	0.4851	0.7074	0.4427	0.1443	0.0071	0.1359	0.1274	0.6084	0.4035	0.1308
NM_053637	Stxbp3	0.9382	0.6669	0.7687	0.5230	0.5947	0.9947	0.1082	0.5477	0.8798	0.6107	0.8299	0.7130
NM_053703	Map2k6	0.7998	0.9355	0.6994	0.8441	0.6696	0.2325	0.5373	0.2811	0.4714	0.6040	0.6664	0.8826
NM_133624	Gbp2	0.0183	0.3005	0.4723	0.2625	0.0296	0.0049	0.1328	0.0863	0.0516	0.0189	0.0125	0.2128
NM_053729	Tdg	0.9864	0.9805	0.4666	0.9942	0.9375	0.9793	0.9736	0.9917	0.9829	0.9959	0.9604	0.1084
NM_053644	Cdh23	0.3197	0.3977	0.5502	0.5153	0.6394	0.6986	0.4975	0.3793	0.6942	0.3970	0.3762	0.4955
NM_001106923	Cul3	0.4145	0.5554	0.7281	0.9421	0.5092	0.9424	0.3945	0.6078	0.4008	0.2149	0.7951	0.4694
NM_053768	Uox	0.8206	0.4982	0.8503	0.9482	0.9394	0.9417	0.7841	0.6606	0.8188	0.9236	0.7246	0.7580

NM_053647	Cxcl2	0.7112	0.5389	0.9687	0.8583	0.8551	0.7862	0.3801	0.3430	0.6545	0.7388	0.9345	0.5478
NM_053762	Zp3	0.0146	0.3342	0.1009	0.0886	0.4997	0.0686	0.0980	0.0223	0.0919	0.1650	0.0696	0.0311
NM_053650	Pdlim3	0.7931	0.7130	0.8096	0.7725	0.8361	0.5000	0.8515	0.7429	0.3357	0.5301	0.8148	0.4578
NM_053649	Kremen1	0.4886	0.8277	0.5655	0.4356	0.4624	0.7165	0.5839	0.7623	0.5761	0.8459	0.6369	0.6966
NM_080889	Il2rg	0.7900	0.8546	0.6848	0.4011	0.4330	0.6354	0.2599	0.5567	0.5625	0.6161	0.6809	0.9564
NM_080900	Afap1	0.2277	0.2352	0.2997	0.3014	0.4110	0.2462	0.2868	0.1730	0.4649	0.2303	0.3915	0.7139
NM_053651	Nkx2-5	0.5024	0.5235	0.3458	0.4862	0.6527	0.5190	0.3552	0.7800	0.8004	0.2979	0.1977	0.4143
NM_053653	Vegfc	0.0522	0.1304	0.1516	0.0034	0.0324	0.0261	0.2149	0.0274	0.0053	0.0084	0.0337	0.0005
NM_053837	Ap2m1	0.8477	0.7570	0.1075	0.8548	0.4088	0.5411	0.3403	0.2262	0.9502	0.7177	0.9228	0.9820
NM_053896	Aldh1a2	0.5297	0.5917	0.7825	0.5711	0.6250	0.0542	0.1114	0.1067	0.3586	0.4635	0.3120	0.6678
NM_053660	Gng10	0.7227	0.3458	0.7876	0.7856	0.2713	0.1683	0.7193	0.5818	0.0741	0.3876	0.5300	0.1195
NM_053887	Map3k1	0.0869	0.3643	0.3606	0.5522	0.2018	0.6495	0.1357	0.2217	0.1068	0.2677	0.4922	0.1778
NM_053655	Dnm1l	0.3868	0.2027	0.8681	0.8925	0.1237	0.7608	0.3957	0.3505	0.4944	0.8478	0.6830	0.6815
NM_053758	Plce1	0.5088	0.0237	0.7267	0.3165	0.3993	0.3961	0.5587	0.7733	0.3678	0.1766	0.6861	0.2384
NM_053756	Atp5g3	0.4444	0.4192	0.5711	0.7473	0.1583	0.1163	0.2097	0.3340	0.3703	0.7150	0.4481	0.1318
NM_053599	Efna1	0.4381	0.7621	0.9332	0.5271	0.6486	0.8110	0.4930	0.3828	0.8034	0.5571	0.8235	0.9710
NM_053720	Aatf	0.7543	0.9081	0.0924	0.7892	0.8674	0.2010	0.8089	0.4873	0.8373	0.7010	0.8275	0.1460
NM_053611	Nupr1	0.1401	0.1087	0.0423	0.1174	0.2054	0.1612	0.0289	0.3416	0.1159	0.1125	0.0280	0.0996
NM_080769	Lta	0.4489	0.6019	0.5437	0.3976	0.7819	0.5708	0.2291	0.8649	0.2496	0.2888	0.9482	0.8257
NM_053614	Lenep	0.4799	0.9593	0.3316	0.0513	0.9418	0.7437	0.1050	0.9789	0.8289	0.6917	0.7009	0.9382
NM_053662	Ccnl1	0.9988	0.9838	0.8088	0.5961	0.9638	0.8703	0.2325	0.8754	0.8456	0.9932	0.9387	0.7993
NM_053814	Mprip	0.7589	0.4098	0.9364	0.9706	0.9929	0.9551	0.8811	0.9467	0.9849	0.8714	0.8974	0.8484
NM_053779	Serpini1	0.6868	0.7101	0.9904	0.9653	0.8038	0.8585	0.9405	0.6870	0.9928	0.9634	0.9176	0.8595
NM_053675	Spata2	0.7288	0.5644	0.5840	0.7568	0.6107	0.3510	0.5627	0.8060	0.7535	0.2227	0.4822	0.2853
NM_080892	Selenbp1	0.8538	0.0332	0.8831	0.1439	0.0051	0.0953	0.8987	0.1203	0.0057	0.0302	0.0465	0.0664
NM_057206	Pnliprp2	0.2852	0.2773	0.4396	0.2449	0.2180	0.3950	0.2159	0.2357	0.3195	0.7517	0.1598	0.1881
NM_053999	Ppp2r2a	0.9385	0.8240	0.9880	0.9194	0.3187	0.8129	0.9678	0.3893	0.6851	0.9089	0.9075	0.9977
NM_001108372	Rpp14	0.2026	0.6623	0.6627	0.9886	0.8030	0.5659	0.8860	0.8663	0.6134	0.6253	0.4844	0.5587
NM_053667	Lepre1	0.8236	0.5718	0.6849	0.6750	0.1292	0.8304	0.9267	0.4857	0.2278	0.6942	0.6111	0.1121
NM_053665	Akap1	0.0014	0.0009	0.3241	0.3213	0.0179	0.0451	0.0196	0.0811	0.0042	0.0765	0.0008	0.2606
NM_053680	Insl3	0.5703	0.1333	0.4738	0.1182	0.6029	0.6663	0.1285	0.8234	0.8876	0.7894	0.3553	0.5409
NM_053681	S100a3	0.5014	0.9475	0.3120	0.8545	0.0436	0.1749	0.7809	0.5510	0.7677	0.7350	0.7340	0.8377
NM_053679	Dffa	0.1529	0.4519	0.2923	0.6730	0.2792	0.1974	0.5681	0.1737	0.2409	0.1224	0.7118	0.7988
NM_053670	Crcp	0.9263	0.7936	0.9456	0.7837	0.8870	0.8412	0.9069	0.8075	0.9583	0.7174	0.9485	0.8364
NM_053771	Lim2	0.7942	0.1462	0.4577	0.2325	0.5695	0.4991	0.6964	0.1096	0.4267	0.1219	0.6864	0.1540
NM_053666	Dll3	0.6973	0.5138	0.6303	0.6253	0.4620	0.6159	0.6644	0.7403	0.6854	0.7601	0.7689	0.5447
NM_053686	Trpv6	0.3897	0.3842	0.2389	0.0960	0.2469	0.4639	0.1133	0.5255	0.4119	0.8627	0.5715	0.5151
NM_053917	Inpp4b	0.8149	0.8404	0.7119	0.8172	0.9324	0.8967	0.5665	0.3760	0.5608	0.6840	0.7536	0.7671
NM_053700	Ccl28	0.4267	0.7320	0.7495	0.2613	0.4092	0.6045	0.5662	0.0674	0.8798	0.8262	0.8361	0.3438
NM_053690	Dnajc14	0.7495	0.5157	0.9617	0.8100	0.8863	0.7827	0.9915	0.7834	0.4613	0.6512	0.6851	0.6378
NM_054004	Cand1	0.9960	0.9368	0.4382	0.9322	0.8427	0.8539	0.9928	0.9545	0.4289	0.9329	0.9407	0.5625
NM_053687	Slfn3	0.9840	0.9789	0.2346	0.1441	0.7187	0.1417	0.0049	0.4284	0.8939	0.8468	0.9965	0.9216
NM_053682	Yme1l1	0.7897	0.3665	0.9845	0.0623	0.1070	0.2826	0.1085	0.0701	0.9038	0.4073	0.3551	0.8457
NM_053693	Dmtf1	0.6795	0.8783	0.9720	0.7011	0.6831	0.9797	0.6557	0.8314	0.9362	0.8517	0.8737	0.9319
NM_053859	Slc17a7	0.5139	0.8260	0.5230	0.2978	0.7770	0.3774	0.5914	0.6881	0.4811	0.6760	0.8036	0.2107
NM_053706	Dmrt1	0.8546	0.6994	0.9084	0.6308	0.5133	0.3012	0.6842	0.7754	0.5821	0.7981	0.7673	0.6687
NM_057135	Pfkfb3	0.0904	0.1552	0.2538	0.0881	0.0588	0.1513	0.1536	0.0722	0.2056	0.1068	0.4071	0.1277
NM_053699	Cited4	0.1395	0.5229	0.2355	0.3005	0.6065	0.4460	0.3167	0.8400	0.5250	0.5648	0.4659	0.6715
NM_053786	Rfc2	0.3323	0.8642	0.0777	0.8064	0.5760	0.3168	0.8401	0.6108	0.5228	0.7422	0.5845	0.1220

NM_053707	Hdgf	0.8834	0.7606	0.1112	0.7392	0.9704	0.9995	0.9458	0.7740	0.9926	0.7786	0.9864	0.9614
NM_080903	Trim63	0.1957	0.1180	0.2894	0.0138	0.7415	0.9475	0.1301	0.8557	0.2331	0.1107	0.0864	0.1016
NM_053713	Klf4	0.2163	0.0664	0.1901	0.2847	0.0391	0.1395	0.1656	0.2198	0.1073	0.1717	0.1384	0.1855
NM_053749	Aurkb	0.9127	0.8909	0.1047	0.9937	0.9537	0.7730	0.9640	0.9640	0.8251	0.9099	0.9217	0.3624
NM_053715	Slc5a3	0.0437	0.0100	0.0176	0.0106	0.0423	0.0454	0.0003	0.0204	0.0418	0.2799	0.1300	0.7322
NM_053719	Emb	0.2515	0.0122	0.1588	0.1891	0.0081	0.0868	0.1456	0.0784	0.1144	0.0261	0.1286	0.0427
NM_053810	Snap29	0.7065	0.4852	0.7106	0.6794	0.6891	0.6547	0.6568	0.4101	0.5666	0.2179	0.7140	0.4260
NM_053726	Slc7a10	0.6336	0.6597	0.1991	0.3256	0.5899	0.1468	0.3206	0.0751	0.0738	0.1951	0.4201	0.0308
NM_001108347	RGD1566099	0.9499	0.7525	0.7629	0.8965	0.7991	0.7903	0.9264	0.8991	0.6827	0.8376	0.7082	0.6209
NM_001108374	RGD1310110	0.0333	0.0152	0.0930	0.0659	0.0012	0.1531	0.1516	0.0299	0.1323	0.0023	0.2249	0.4360
NM_001108369	Chchd1	0.0134	0.0574	0.0382	0.1484	0.0284	0.0176	0.1489	0.1154	0.0457	0.0830	0.2171	0.0522
NM_053727	Nfil3	0.1182	0.1126	0.0574	0.0600	0.0246	0.1304	0.0475	0.0703	0.1476	0.1941	0.0183	0.9120
NM_057190	Nelf	0.8127	0.9644	0.8111	0.9202	0.8059	0.9436	0.8927	0.9945	0.9523	0.9296	0.9987	0.9457
NM_053730	Stag3	0.0622	0.1938	0.0850	0.1599	0.3003	0.1366	0.2108	0.2183	0.0239	0.2174	0.2727	0.1664
NM_053990	Ptpn2	0.3016	0.2244	0.8703	0.1141	0.1581	0.0933	0.0855	0.1316	0.2388	0.2082	0.5961	0.8260
NM_057195	Smu1	0.1810	0.0992	0.2062	0.3443	0.2282	0.0563	0.2640	0.0784	0.5629	0.2760	0.4463	0.6238
NM_053857	Eif4ebp1	0.1739	0.8332	0.7256	0.8028	0.8748	0.1367	0.9716	0.9149	0.9056	0.9033	0.8278	0.3420
NM_053908	Ptpn6	0.9638	0.8692	0.8622	0.8879	0.7998	0.8049	0.7212	0.8295	0.8632	0.8753	0.7216	0.8881
NM_053751	Wap	0.8145	0.7759	0.6978	0.7623	0.7144	0.7565	0.9281	0.3445	0.7779	0.7175	0.5477	0.6322
NM_053747	Ubqln1	0.3304	0.5570	0.5486	0.8020	0.1585	0.1745	0.6853	0.0031	0.3441	0.3694	0.9892	0.6487
NM_053744	Dlk1	0.4991	0.2212	0.3440	0.2256	0.2995	0.1541	0.2739	0.0991	0.1126	0.1537	0.5202	0.7766
NM_053743	Cdc37	0.4386	0.4407	0.0861	0.8320	0.1461	0.2821	0.2601	0.0434	0.1325	0.3735	0.2336	0.3632
NM_053742	Pitpnb	0.6592	0.9110	0.6659	0.2171	0.3950	0.3847	0.5817	0.8480	0.9706	0.9481	0.8965	0.5113
NM_053741	Rap2a	0.7696	0.9516	0.0301	0.3282	0.8164	0.6844	0.5543	0.8260	0.0578	0.1315	0.0824	0.8470
NM_130413	Skap2	0.0566	0.0618	0.9799	0.8851	0.1710	0.3771	0.0823	0.5642	0.3213	0.1119	0.3878	0.0585
NM_057141	Hnrnpk	0.9686	0.9109	0.9842	0.9798	0.9936	0.9382	0.9754	0.9574	0.9074	0.9940	0.9337	0.9727
NM_053738	Wif1	0.5771	0.9746	0.8031	0.7826	0.7852	0.5298	0.9651	0.8038	0.8790	0.9098	0.6558	0.7111
NM_053736	Casp4	0.0133	0.0408	0.7761	0.0173	0.7506	0.8082	0.3480	0.6942	0.1199	0.0382	0.1151	0.2421
NM_001108455	Fanca	0.8551	0.7816	0.0918	0.4692	0.7862	0.9751	0.7814	0.6906	0.9692	0.8896	0.8620	0.8193
NM_001134545	RGD1565772	0.6553	0.0807	0.0675	0.5785	0.5422	0.5069	0.0096	0.9075	0.5180	0.0618	0.1920	0.0743
NM_001108323	RGD1310348	0.6876	0.7037	0.8316	0.8998	0.1711	0.1286	0.9826	0.6270	0.8152	0.6112	0.7311	0.8545
NM_001108322	Tbx1	0.3388	0.0686	0.3426	0.5528	0.1453	0.0959	0.2527	0.3654	0.2799	0.2498	0.2121	0.2497
NM_001108319	Rabl3	0.3527	0.5634	0.8842	0.2939	0.1039	0.4618	0.5141	0.0293	0.4161	0.3126	0.7310	0.7671
NM_001134610	RGD1563574	0.8615	0.7959	0.0581	0.4148	0.6182	0.6738	0.3754	0.0501	0.5467	0.5712	0.6197	0.4282
NM_001108586	Rcn1	0.8888	0.9894	0.9918	0.9718	0.9820	0.9857	0.9909	0.9871	0.5937	0.9880	0.9423	0.2494
NM_001108568	Dapp1	0.1987	0.0592	0.1138	0.2732	0.1121	0.1952	0.2618	0.0617	0.3073	0.1764	0.2053	0.2065
NM_001108495	Relt	0.2024	0.7772	0.1628	0.7547	0.2557	0.5174	0.2686	0.6852	0.0611	0.2968	0.0875	0.6046
NM_001108504	Mettl10	0.8659	0.2315	0.3905	0.9387	0.7968	0.7401	0.1641	0.9101	0.4231	0.1698	0.4942	0.0274
NM_001108313	Ifngr2	0.1748	0.0858	0.5036	0.1901	0.1625	0.2314	0.0636	0.3336	0.1354	0.2580	0.1481	0.1880
NM_001108293	Nfe2l1	0.1572	0.1424	0.6009	0.0467	0.1485	0.0222	0.1830	0.3862	0.1770	0.0545	0.0591	0.1971
NM_001108442	Ndufb7	0.0001	0.0629	0.0493	0.1353	0.0134	0.0684	0.0023	0.0516	0.2124	0.0434	0.1642	0.0014
NM_001109889	Ptprc	0.8419	0.9679	0.9525	0.9107	0.9667	0.3282	0.6999	0.3839	0.8020	0.9482	0.9111	0.6854
NM_001109888	Ptprc	0.8419	0.9679	0.9525	0.9107	0.9667	0.3282	0.6999	0.3839	0.8020	0.9482	0.9111	0.6854
NM_080399	Ddit4l	0.2049	0.2260	0.0326	0.0133	0.0137	0.0309	0.0105	0.0154	0.0021	0.0049	0.0492	0.0004
NM_053753	Clec4f	0.2484	0.3484	0.2821	0.3614	0.4007	0.3560	0.1812	0.6001	0.3758	0.1846	0.3523	0.5894
NM_053755	Slc26a3	0.6665	0.6866	0.4475	0.8372	0.6637	0.5574	0.4982	0.7086	0.8030	0.8359	0.8821	0.6835
NM_053818	Slc6a9	0.9904	0.9884	0.8892	0.8667	0.9818	0.9260	0.7599	0.9584	0.9886	0.9646	0.9887	0.9080
NM_053838	Npr2	0.9843	0.5835	0.8559	0.5877	0.8932	0.9100	0.8872	0.9207	0.7063	0.7902	0.6246	0.9391
NM_053757	Scye1	0.0100	0.2896	0.0708	0.1093	0.0784	0.0652	0.0121	0.0842	0.4691	0.6564	0.7417	0.0931

NM_080479	Maged2	0.1443	0.2344	0.6819	0.1447	0.5544	0.2936	0.8168	0.6808	0.3792	0.9933	0.7475	0.0604
NM_053831	Gucy2f	0.7413	0.7463	0.6914	0.7653	0.5532	0.4429	0.7927	0.2794	0.5942	0.4420	0.7171	0.7393
NM_057196	Baiap2	0.1240	0.0365	0.1075	0.0702	0.0751	0.1453	0.1763	0.0769	0.0844	0.0983	0.0009	0.2709
NM_053764	Rgs14	0.4694	0.6228	0.1315	0.3148	0.8097	0.6708	0.4164	0.9355	0.8370	0.6069	0.3731	0.6108
NM_001127447	Fam173a	0.1079	0.0919	0.3264	0.7671	0.4689	0.1024	0.3408	0.7369	0.1156	0.4829	0.0263	0.2292
NM_001100651	Pigx	0.2060	0.0866	0.1836	0.2607	0.0899	0.0104	0.0377	0.0612	0.0662	0.0246	0.1639	0.0049
NM_130756	Acot8	0.1339	0.1504	0.2873	0.8679	0.1421	0.0086	0.1455	0.1399	0.1691	0.0362	0.1272	0.0580
NM_053851	Cacnb2	0.9910	0.8910	0.9613	0.5916	0.9842	0.9041	0.9114	0.9034	0.9947	0.9673	0.9665	0.9500
NM_053770	Argbp2	0.7978	0.9649	0.9769	0.3573	0.3059	0.1610	0.7120	0.2734	0.9898	0.9560	0.9893	0.9901
NM_053739	Becn1	0.9401	0.9260	0.9801	0.9987	0.9693	0.7572	0.7621	0.7806	0.7484	0.9791	0.9774	0.9297
NM_053947	Mark1	0.9864	0.9737	0.9289	0.7773	0.9606	0.7936	0.9880	0.8127	0.7478	0.7820	0.7571	0.8181
NM_130420	Trim9	0.6992	0.4057	0.4775	0.6843	0.5098	0.3451	0.4627	0.5627	0.7491	0.6961	0.5145	0.6755
NM_053773	Tjp2	0.4581	0.6755	0.3763	0.9869	0.2253	0.5601	0.0629	0.2080	0.4055	0.6214	0.9109	0.8555
NM_053778	Ipo13	0.6139	0.6298	0.0620	0.5037	0.2300	0.4499	0.6437	0.3915	0.8204	0.6931	0.6195	0.9147
NM_053776	Dnajc2	0.7814	0.7133	0.2330	0.6342	0.7826	0.5534	0.5851	0.8516	0.6823	0.7943	0.7857	0.1059
NM_053847	Map3k8	0.2875	0.4921	0.8905	0.0910	0.0241	0.1158	0.0086	0.1053	0.8097	0.7545	0.7213	0.7315
NM_053783	Ifngr1	0.7901	0.2403	0.2498	0.0530	0.7267	0.6755	0.0311	0.8289	0.0956	0.0721	0.1748	0.3334
NM_053836	Il2	0.4572	0.3677	0.6342	0.2727	0.2438	0.1651	0.3862	0.2171	0.3419	0.6573	0.3482	0.0266
NM_053787	Trpv5	0.6130	0.6279	0.5557	0.5935	0.7012	0.4784	0.2952	0.0951	0.5250	0.5992	0.5973	0.5686
NM_130426	Tnfrsf1b	0.7239	0.6676	0.4842	0.4886	0.5193	0.4835	0.5848	0.7041	0.4784	0.5000	0.2054	0.5382
NM_053890	Ppp1r12a	0.1736	0.0835	0.1448	0.1838	0.2764	0.4435	0.0722	0.0646	0.1228	0.0924	0.0808	0.3222
NM_053800	Txn1	0.0257	0.1565	0.0102	0.4231	0.0767	0.0524	0.0686	0.0500	0.0180	0.0537	0.0347	0.0077
NM_131910	Dynlrb1	0.0446	0.1448	0.9722	0.3510	0.1259	0.0767	0.0149	0.1574	0.2281	0.0192	0.4188	0.6431
NM_053791	Prl2a1	0.5821	0.5669	0.1162	0.4611	0.4438	0.3294	0.2797	0.3629	0.6509	0.4551	0.2881	0.5982
NM_053777	Mapk8ip1	0.9656	0.9118	0.9218	0.9061	0.9754	0.9989	0.9976	0.9252	0.9070	0.8977	0.9528	0.9034
NM_131909	Hrh4	0.0321	0.0748	0.4221	0.1526	0.3757	0.2426	0.3334	0.1854	0.3761	0.2214	0.7220	0.0728
NM_001110295	Lypd4	0.4618	0.4811	0.1772	0.1769	0.4271	0.1741	0.1806	0.1721	0.2808	0.4661	0.1672	0.1720
NM_053805	Snai1	0.1063	0.3534	0.0156	0.2578	0.0728	0.0769	0.4126	0.1223	0.2968	0.1093	0.1017	0.8414
NM_053795	Kidins220	0.2190	0.3267	0.4932	0.2647	0.2527	0.3640	0.1167	0.0221	0.8732	0.4676	0.4247	0.8473
NM_053856	Scg3	0.5375	0.6525	0.8794	0.1533	0.8400	0.8792	0.6949	0.5534	0.7943	0.3863	0.4729	0.2271
NM_138511	Gpc2	0.6631	0.2129	0.3502	0.5130	0.3987	0.3198	0.5403	0.4090	0.3085	0.3157	0.4144	0.5052
NM_138506	Adra2c	0.1004	0.2422	0.3646	0.3774	0.4143	0.2450	0.2968	0.5673	0.0626	0.5757	0.5621	0.0391
NM_057138	Cflar	0.6460	0.7808	0.7795	0.8696	0.3251	0.4483	0.9101	0.5644	0.7803	0.6415	0.8835	0.6031
NM_057137	Ebp	0.0439	0.0608	0.0326	0.6631	0.6047	0.0509	0.2095	0.5444	0.1392	0.0566	0.0825	0.1435
NM_053967	Spam1	0.5254	0.4396	0.9354	0.1614	0.5428	0.5422	0.8591	0.5539	0.5772	0.3057	0.8282	0.2319
NM_001008888	Uqcrfs1	0.0179	0.0291	0.7043	0.1149	0.0617	0.0340	0.3098	0.1162	0.1455	0.1755	0.3753	0.1039
NM_001009391	Enoph1	0.2630	0.8649	0.3520	0.0994	0.1635	0.0836	0.7588	0.3753	0.6866	0.7372	0.9553	0.7802
NM_001007607	Pigq	0.2135	0.2371	0.4654	0.9474	0.3802	0.7445	0.9120	0.7277	0.2939	0.2670	0.1648	0.7551
NM_138505	Adra2b	0.8518	0.3981	0.3850	0.8107	0.5959	0.4884	0.4655	0.4951	0.8406	0.7901	0.7708	0.5762
NM_133573	Gper	0.7751	0.6393	0.7608	0.9026	0.6269	0.7859	0.5586	0.8267	0.7748	0.8767	0.2795	0.7631
NM_133296	Slc6a20	0.6558	0.8151	0.6595	0.5149	0.6069	0.5775	0.5731	0.6308	0.6574	0.7269	0.5724	0.7513
NM_053820	Ebf1	0.9307	0.9250	0.8789	0.8648	0.7486	0.8566	0.9208	0.7177	0.8784	0.9584	0.9115	0.8818
NM_053830	Nup107	0.9181	0.9285	0.0282	0.1537	0.9941	0.8702	0.4309	0.8159	0.7087	0.7819	0.6028	0.0385
NM_053923	Pik3c2g	0.9201	0.4353	0.9207	0.7632	0.6804	0.6111	0.9013	0.7877	0.7572	0.8245	0.8437	0.7737
NM_057104	Enpp2	0.7847	0.1584	0.8841	0.8767	0.8277	0.7858	0.5092	0.7138	0.0821	0.1624	0.4533	0.5655
NM_080480	Pip4k2c	0.3834	0.1883	0.2283	0.1361	0.3901	0.9645	0.8427	0.1783	0.8165	0.2588	0.3853	0.8388
NM_019325	Myh4	0.8334	0.6807	0.7183	0.4971	0.4183	0.3921	0.3465	0.4315	0.7518	0.5976	0.5125	0.6659
NM_053816	Calcr	0.6574	0.6123	0.7032	0.7262	0.7249	0.4590	0.7658	0.4708	0.8241	0.6804	0.5363	0.6839
NM_053868	Nlgn1	0.1163	0.4210	0.4711	0.5351	0.2451	0.1007	0.5095	0.4707	0.4110	0.5194	0.7032	0.3455

NM_133320	Ndel1	0.9238	0.8787	0.7381	0.3878	0.4873	0.9245	0.7832	0.6073	0.9233	0.9552	0.9813	0.9762
NM_053901	Dlgap2	0.3791	0.7160	0.4309	0.3281	0.4082	0.3332	0.4011	0.4327	0.2211	0.4091	0.1671	0.6603
NM_053812	Bak1	0.8353	0.9476	0.9947	0.9772	0.9983	0.9529	0.9511	0.9496	0.9808	0.9837	0.9328	0.9982
NM_053843	Fcgr3	0.5792	0.6201	0.3728	0.5328	0.4016	0.3283	0.5103	0.2646	0.2036	0.2173	0.2457	0.2235
NM_001079889	Sephs2	0.9096	0.7985	0.8686	0.9151	0.8869	0.9327	0.9582	0.9561	0.8637	0.8263	0.8870	0.5598
NM_183334	Ldhal6b	0.5977	0.3782	0.2640	0.5951	0.1513	0.2791	0.2679	0.7652	0.2749	0.5271	0.9243	0.5669
NM_054010	Neu3	0.6831	0.7809	0.9866	0.9375	0.9788	0.8645	0.9631	0.6656	0.9791	0.7325	0.5751	0.9201
NM_054007	Hsd17b3	0.7441	0.7326	0.7523	0.8393	0.8083	0.7328	0.6670	0.6408	0.7793	0.6582	0.8415	0.8675
NM_053834	Kcnj9	0.0214	0.7661	0.6593	0.5363	0.3841	0.0675	0.3105	0.5157	0.6211	0.7722	0.7624	0.7356
NM_053964	Ptf1a	0.5733	0.3669	0.6872	0.7286	0.5602	0.6113	0.1093	0.3951	0.7569	0.4244	0.4861	0.4402
NM_053909	Nfasc	0.3622	0.2891	0.3053	0.3946	0.2819	0.3046	0.3809	0.1720	0.2545	0.5411	0.3981	0.2694
NM_053848	Opcml	0.8586	0.9084	0.6584	0.8873	0.9413	0.6670	0.9616	0.9387	0.9649	0.9140	0.8466	0.9233
NM_001077428	Gtf2h2	0.7581	0.4553	0.1661	0.0113	0.1762	0.7237	0.0043	0.2774	0.5115	0.3814	0.7250	0.1551
NM_001039539	Pax9	0.4716	0.3953	0.9325	0.5929	0.8323	0.5084	0.7719	0.5131	0.4029	0.7726	0.9053	0.8385
NM_080885	Cdk5	0.5272	0.7328	0.6378	0.8132	0.8815	0.8310	0.9198	0.9068	0.8505	0.6226	0.7918	0.7436
NM_080787	Dgka	0.0041	0.0875	0.0733	0.0642	0.1018	0.0361	0.0570	0.0176	0.0378	0.0064	0.0317	0.4623
NM_053988	Calb2	0.9763	0.7726	0.7108	0.8358	0.9062	0.8256	0.8189	0.8407	0.8019	0.6734	0.7344	0.8099
NM_203493	Dmp1	0.9711	0.7073	0.8544	0.9239	0.9847	0.6991	0.8951	0.9121	0.9671	0.9555	0.9542	0.4226
NM_144737	Fmo2	0.9717	0.0457	0.7437	0.0443	0.7229	0.7349	0.0384	0.7986	0.0038	0.0612	0.0707	0.1433
NM_138895	Ubb	0.7058	0.4840	0.3297	0.6452	0.5468	0.6750	0.0907	0.2589	0.4108	0.2804	0.7085	0.4959
NM_053863	Slc28a1	0.2088	0.1044	0.2571	0.0819	0.2056	0.2691	0.5667	0.1070	0.1148	0.2636	0.4143	0.5029
NM_053924	Abcc5	0.9332	0.4484	0.0295	0.2598	0.9811	0.9984	0.2319	0.9903	0.6204	0.1643	0.1822	0.8613
NM_053865	Rtn1	0.4993	0.6997	0.6432	0.8347	0.8477	0.4420	0.6340	0.3484	0.2664	0.8131	0.9707	0.8348
NM_053866	Plaa	0.1564	0.1527	0.0189	0.1001	0.0366	0.1182	0.0267	0.0095	0.1030	0.1104	0.2719	0.7764
NM_053867	Tpt1	0.1455	0.2465	0.5554	0.5313	0.2710	0.7307	0.5496	0.4323	0.2047	0.4930	0.3117	0.3117
NM_053864	Vcp	0.4765	0.1325	0.1159	0.9074	0.1389	0.3756	0.4990	0.7363	0.8026	0.1339	0.6464	0.2860
NM_080482	Dbc1	0.2685	0.1272	0.3227	0.0598	0.1159	0.4913	0.0809	0.4317	0.1894	0.3701	0.3118	0.2772
NM_133424	Actn3	0.1096	0.0187	0.3455	0.2946	0.1154	0.1406	0.1966	0.2729	0.0723	0.1597	0.0729	0.3505
NM_053878	Cplx2	0.4287	0.5914	0.1771	0.3442	0.4245	0.3094	0.1805	0.6076	0.2340	0.2722	0.3140	0.4837
NM_138530	Pbld	0.7892	0.1643	0.7315	0.3065	0.5864	0.5032	0.4839	0.8176	0.3528	0.5629	0.8825	0.6600
NM_133397	Erg	0.7279	0.7739	0.2097	0.5663	0.7804	0.4543	0.1768	0.6063	0.4690	0.4330	0.5110	0.4473
NM_053876	Hnrpm	0.9026	0.8247	0.5019	0.9249	0.9727	0.8941	0.9625	0.7257	0.8716	0.8156	0.8866	0.4610
NM_139252	Ppap2c	0.1051	0.5903	0.2001	0.8564	0.1567	0.3425	0.1731	0.3922	0.1503	0.1697	0.2387	0.1492
NM_053879	Cntn4	0.8774	0.5742	0.4111	0.7192	0.4460	0.7088	0.4939	0.1107	0.6081	0.5527	0.7619	0.4338
NM_133583	Ndrg2	0.3934	0.0497	0.4501	0.3110	0.8976	0.8876	0.6326	0.9810	0.0686	0.1432	0.2332	0.2633
NM_057099	Psmb6	0.0576	0.5109	0.0308	0.0198	0.0872	0.0010	0.3128	0.0102	0.2174	0.7705	0.3726	0.0242
NM_053882	Ecm1	0.1724	0.1668	0.1770	0.3600	0.3303	0.4349	0.3901	0.4294	0.4169	0.1945	0.3809	0.2846
NM_053884	Atp6v1f	0.0475	0.0883	0.6578	0.3140	0.1378	0.0354	0.3414	0.0606	0.0545	0.3050	0.2238	0.2297
NM_053886	Lman1	0.0774	0.1137	0.9099	0.1707	0.0910	0.1027	0.9944	0.1935	0.1014	0.7343	0.6170	0.0432
NM_053998	Rab8a	0.4772	0.1243	0.8958	0.6824	0.4442	0.5826	0.5986	0.2461	0.0895	0.2101	0.2251	0.4962
NM_057207	Sv2b	0.5333	0.6834	0.5142	0.9111	0.9241	0.9096	0.4557	0.8994	0.8687	0.7460	0.7148	0.9162
NM_053888	Myt1l	0.4178	0.8345	0.9920	0.7749	0.3237	0.9733	0.6246	0.5692	0.7142	0.8343	0.7894	0.6703
NM_054000	Kcnb2	0.2982	0.4022	0.0469	0.4159	0.3161	0.1680	0.4946	0.2941	0.1089	0.0492	0.4110	0.2551
NM_054001	Scarb2	0.7520	0.8043	0.9933	0.9229	0.9485	0.4404	0.8470	0.5660	0.8339	0.9190	0.8232	0.5398
NM_133533	Cd79b	0.5472	0.7891	0.6991	0.3794	0.5468	0.3236	0.8703	0.1263	0.6448	0.7728	0.6935	0.5659
NM_057100	Gas6	0.2201	0.5231	0.3549	0.2694	0.5121	0.5025	0.2731	0.4149	0.2311	0.6870	0.2958	0.2576
NM_054002	Zbtb7a	0.8707	0.9460	0.9911	0.9687	0.9477	0.9842	0.8272	0.9327	0.9881	0.9613	0.8282	0.9942
NM_057210	Sv2a	0.3234	0.1423	0.3602	0.3491	0.4653	0.8991	0.2172	0.3919	0.5406	0.2708	0.0659	0.0928
NM_057120	Nudt1	0.3852	0.1374	0.0892	0.4145	0.4656	0.5749	0.3134	0.3945	0.0508	0.2230	0.1409	0.1176

NM_001115039	LOC683402	0.4858	0.6679	0.7010	0.5301	0.6149	0.5600	0.4239	0.2691	0.3159	0.2247	0.5017	0.6909
NM_053903	Efna5	0.8116	0.6427	0.7189	0.7138	0.9212	0.7059	0.5632	0.7052	0.7472	0.5995	0.6694	0.9970
NM_053893	Sdc3	0.8219	0.8389	0.5810	0.6862	0.7824	0.7114	0.9424	0.9164	0.7043	0.3784	0.4009	0.5700
NM_133300	Bat1	0.8788	0.8598	0.6924	0.9624	0.8886	0.8041	0.8259	0.9369	0.8057	0.9973	0.9369	0.9384
NM_131911	Anp32b	0.5251	0.8998	0.3848	0.9759	0.8189	0.1743	0.7834	0.5574	0.9647	0.8219	0.5857	0.2580
NM_130823	Atp6v0c	0.2888	0.3926	0.2374	0.6712	0.0522	0.7087	0.1528	0.3298	0.8000	0.3109	0.5171	0.9769
NM_130400	Dhfr	0.7123	0.7384	0.4072	0.7892	0.6988	0.7405	0.7925	0.7693	0.7042	0.4603	0.7856	0.3137
NM_057105	Ugt1a6	0.3595	0.6902	0.6651	0.6415	0.4871	0.4898	0.4265	0.2860	0.3791	0.4166	0.5320	0.6245
NM_054005	Cuzd1	0.4715	0.6874	0.7471	0.5776	0.5970	0.4793	0.7447	0.7430	0.3845	0.3330	0.2628	0.5888
NM_053905	Lmnb1	0.5603	0.3608	0.1768	0.5234	0.3979	0.3151	0.1803	0.3860	0.1662	0.1607	0.1671	0.1718
NM_053895	Frag1	0.7359	0.8541	0.7438	0.8482	0.9203	0.9731	0.9184	0.8667	0.7921	0.8989	0.8371	0.8945
NM_057107	Acsl3	0.9223	0.9985	0.9834	0.9385	0.8556	0.9496	0.9529	0.7881	0.9879	0.7686	0.9861	0.8909
NM_133516	Svs5	0.7172	0.8321	0.6577	0.8705	0.6056	0.6952	0.7466	0.7836	0.8021	0.6824	0.4414	0.8188
NM_053897	F2rl1	0.9158	0.9748	0.7389	0.7345	0.9820	0.8911	0.9642	0.8503	0.9476	0.9942	0.7219	0.9967
NM_133549	Hdgfl1	0.8345	0.4115	0.2266	0.2901	0.6837	0.7309	0.3520	0.6353	0.7211	0.7962	0.2860	0.1391
NM_080397	Chst10	0.3093	0.6069	0.6943	0.2800	0.7368	0.6550	0.6714	0.4020	0.8677	0.9412	0.8649	0.8464
NM_057108	Serpinb5	0.6649	0.3983	0.5419	0.4819	0.4773	0.1723	0.2175	0.3756	0.5408	0.2901	0.6740	0.0746
NM_053982	Rps15a	0.3255	0.0337	0.3839	0.2274	0.1229	0.0984	0.1152	0.1059	0.0482	0.0245	0.1403	0.2717
NM_053968	Mt3	0.6249	0.6251	0.7489	0.5395	0.5796	0.4874	0.5743	0.7187	0.7019	0.4862	0.5860	0.3031
NM_133420	Chrna2	0.8184	0.7694	0.2145	0.5333	0.4643	0.1315	0.0463	0.1262	0.8470	0.6648	0.0060	0.5013
NM_053899	Cgrrf1	0.6485	0.7145	0.7533	0.3595	0.8678	0.5937	0.5521	0.6188	0.1526	0.6952	0.3772	0.0355
NM_130406	Faf1	0.0451	0.1530	0.2224	0.4012	0.8795	0.8875	0.0315	0.7461	0.5753	0.2354	0.0668	0.2315
NM_134364	Atp5b	0.1174	0.2034	0.0883	0.7281	0.0593	0.5476	0.0088	0.0119	0.7215	0.2221	0.1060	0.3296
NM_057124	P2ry6	0.2954	0.3586	0.4833	0.4632	0.2711	0.5641	0.7629	0.2316	0.3707	0.3509	0.2350	0.1280
NM_080783	Gale	0.7601	0.9016	0.4986	0.8773	0.9469	0.7545	0.9657	0.9714	0.9554	0.7863	0.8133	0.5588
NM_054011	Sh3bp5	0.7087	0.1453	0.8551	0.9484	0.8310	0.1483	0.7968	0.5659	0.0347	0.3519	0.1765	0.1645
NM_053912	Cyth3	0.9718	0.3237	0.9693	0.9753	0.9624	0.9745	0.9210	0.9912	0.9454	0.4481	0.9295	0.9952
NM_138920	Zc3h14	0.6351	0.7010	0.4858	0.3332	0.6027	0.6917	0.6102	0.6278	0.5611	0.6765	0.5475	0.3679
NM_001008863	Usp54	0.3097	0.6388	0.5686	0.6263	0.6654	0.2901	0.2983	0.4680	0.3408	0.4075	0.4445	0.4980
NM_001108482	Prrg2	0.0665	0.0509	0.2620	0.0110	0.0064	0.0131	0.0078	0.0202	0.0289	0.0191	0.0186	0.4437
NM_001004234	Pyroxd1	0.8472	0.8072	0.1505	0.0266	0.7430	0.1495	0.5073	0.7547	0.5419	0.4238	0.8810	0.7345
NM_133289	Scn9a	0.6970	0.6275	0.7216	0.8132	0.4855	0.6647	0.6401	0.2403	0.7607	0.3114	0.5703	0.4566
NM_133550	Fcer2	0.5605	0.3392	0.4759	0.6288	0.4996	0.5183	0.5670	0.7015	0.8022	0.3666	0.5420	0.6212
NM_057142	Lrrc7	0.5845	0.4698	0.5076	0.7090	0.6785	0.6826	0.3630	0.5197	0.4384	0.3381	0.6925	0.2515
NM_053921	Pex12	0.0593	0.1623	0.1003	0.0606	0.0529	0.2840	0.0977	0.3378	0.3294	0.1602	0.3244	0.1102
NM_133297	41,167.0000	0.1615	0.3177	0.5378	0.3497	0.1832	0.3567	0.1370	0.1480	0.0562	0.0885	0.3121	0.1005
NM_053948	Polr2g	0.0644	0.0916	0.1273	0.0441	0.0936	0.1075	0.1031	0.0435	0.8040	0.1497	0.0985	0.4638
NM_053959	Bin1	0.2055	0.3899	0.2525	0.2654	0.4699	0.6044	0.3731	0.5702	0.4293	0.2015	0.2550	0.5851
NM_133422	Znf483	0.2119	0.3923	0.2268	0.3163	0.2066	0.2292	0.4525	0.3596	0.1967	0.5041	0.2708	0.6294
NM_053920	Trip10	0.8604	0.6780	0.0865	0.1111	0.7586	0.8079	0.0076	0.8275	0.7979	0.9089	0.7224	0.8922
NM_053918	Cga	0.6178	0.8417	0.9656	0.4964	0.7875	0.9302	0.1169	0.7781	0.8168	0.5692	0.6471	0.8743
NM_057140	Ppp2r1a	0.8370	0.6598	0.1069	0.8769	0.6327	0.7423	0.6789	0.8946	0.8372	0.5960	0.8890	0.9271
NM_057129	Tff1	0.2594	0.8018	0.2126	0.0511	0.2415	0.0981	0.0231	0.5175	0.1563	0.2323	0.3933	0.9268
NM_057115	Ptpn12	0.5124	0.2174	0.8664	0.7411	0.3352	0.7670	0.3436	0.4998	0.4480	0.3069	0.4547	0.7377
NM_053927	Epb4.1l3	0.9997	0.9511	0.9797	0.9964	0.9609	0.9364	0.9943	0.9957	0.9893		0.9983	0.9929
NM_080400	Chek1	0.5907	0.3572	0.4952	0.4568	0.3818	0.3360	0.3670	0.5763	0.9855	0.8118	0.1379	0.4661
NM_053929	Slc7a9	0.1731	0.1156	0.2180	0.1754	0.2230	0.5926	0.3265	0.0385	0.0820	0.2617	0.1163	0.1562
NM_053931	41,157.0000	0.2267	0.6764	0.6854	0.2361	0.6108	0.7215	0.6601	0.7216	0.6828	0.4844	0.2088	0.5728
NM_053933	Pcdha4	0.9786	0.9489	0.9590	0.9578	0.9588	0.8771	0.9538	0.9742	0.9680	0.9902	0.9657	0.9311

NM_053934	Pcdha13	0.6688	0.6169	0.6183	0.6794	0.7280	0.5102	0.7150	0.7283	0.6425	0.6852	0.6345	0.7073
NM_053937	Kcnh6	0.1977	0.8176	0.0400	0.4905	0.0200	0.1101	0.2697	0.0235	0.2313	0.4754	0.7838	0.2668
NM_053939	Pcdha10	0.8047	0.6663	0.3389	0.8296	0.8833	0.6666	0.8520	0.7091	0.4875	0.8361	0.6176	0.6320
NM_053941	Pcdha3	0.6774	0.6885	0.4031	0.7409	0.7923	0.3144	0.4854	0.6568	0.6520	0.5036	0.6446	0.4788
NM_053943	Pcdhgc3	0.5354	0.6749	0.2439	0.6036	0.5913	0.6875	0.6248	0.7114	0.7021	0.5366	0.5037	0.7211
NM_053942	Pcdha8	0.9334	0.9463	0.9227	0.9027	0.9136	0.6438	0.9553	0.7303	0.3812	0.9810	0.8292	0.9619
NM_133324	Mak10	0.3244	0.4373	0.2148	0.1437	0.0651	0.0322	0.0462	0.1152	0.6416	0.1493	0.3886	0.5209
NM_080477	Pfkfb2	0.5721	0.5921	0.7296	0.5564	0.6767	0.7084	0.8268	0.8376	0.7158	0.6081	0.7405	0.5834
NM_133555	Csf2rb	0.3505	0.1668	0.3194	0.1762	0.4231	0.1739	0.1797	0.3961	0.3895	0.1606	0.1668	0.5667
NM_053945	Rims2	0.6767	0.8673	0.5490	0.7156	0.5263	0.4209	0.7041	0.8020	0.6734	0.7370	0.7795	0.5301
NM_058211	Slc4a7	0.0412	0.0713	0.2544	0.0327	0.0791	0.0088	0.0104	0.0763	0.2539	0.5921	0.0551	0.4651
NM_053996	Slc6a7	0.1263	0.1629	0.2232	0.2023	0.1789	0.0337	0.2526	0.0401	0.2135	0.1948	0.0784	0.2246
NM_057185	Folh1	0.6096	0.4039	0.4954	0.3890	0.5279	0.5971	0.5653	0.5840	0.2350	0.3210	0.6289	0.5425
NM_053950	Eif2b4	0.8830	0.3062	0.1169	0.0179	0.0693	0.1417	0.0800	0.0296	0.1575	0.4524	0.5068	0.3748
NM_133390	Zfp347	0.8000	0.2852	0.2056	0.2810	0.8961	0.7863	0.0381	0.8317	0.0388	0.0718	0.1147	0.0189
NM_133603	Kcne2	0.7716	0.4450	0.5281	0.7869	0.5017	0.1341	0.6052	0.4166	0.4576	0.7267	0.6669	0.5449
NM_133557	Itfg1	0.9762	0.8540	0.9853	0.8357	0.9875	0.8562	0.8121	0.7404	0.9766	0.9690	0.9581	0.9723
NM_053961	Erp29	0.8240	0.9111	0.1518	0.8317	0.7505	0.9973	0.8012	0.9842	0.8579	0.8027	0.9102	0.4967
NM_053960	Ccr5	0.2561	0.0844	0.6020	0.7218	0.6537	0.3331	0.6281	0.8254	0.3386	0.0354	0.3128	0.0349
NM_057133	Nr0b2	0.8044	0.6112	0.7992	0.6213	0.5778	0.6155	0.8581	0.6255	0.3872	0.8682	0.1983	0.4659
NM_053958	Ccr3	0.5051	0.5085	0.1118	0.0176	0.1799	0.3072	0.4725	0.0996	0.5231	0.5375	0.6212	0.5053
NM_053957	Apbb3	0.9564	0.8021	0.5084	0.9074	0.9977	0.7983	0.3500	0.8253	0.7790	0.8870	0.8844	0.8843
NM_053954	Kcns1	0.8795	0.7477	0.9283	0.7206	0.9089	0.7922	0.8611	0.7491	0.9569	0.4328	0.8813	0.9453
NM_053953	Il1r2	0.0930	0.0168	0.0410	0.1219	0.0140	0.0671	0.1923	0.1731	0.0557	0.0458	0.0235	0.0767
NM_053952	Nup155	0.9358	0.7044	0.0053	0.1376	0.4459	0.8464	0.5410	0.7359	0.7790	0.2618	0.4042	0.6838
NM_080767	Psmb8	0.7612	0.6310	0.0075	0.0573	0.0715	0.8266	0.1288	0.4346	0.7817	0.7507	0.1423	0.7299
NM_133601	Cblb	0.3253	0.1577	0.2083	0.0438	0.8015	0.7134	0.0186	0.8722	0.2684	0.0486	0.0384	0.8883
NM_057188	Gmpr	0.0800	0.0123	0.8353	0.1017	0.2098	0.0849	0.3112	0.2781	0.0404	0.1040	0.0256	0.5598
NM_053971	Rpl6	0.4847	0.4100	0.6310	0.4356	0.3587	0.4725	0.5963	0.1759	0.4597	0.7486	0.6063	0.5040
NM_053972	RragB	0.6180	0.7977	0.4174	0.8344	0.5246	0.5570	0.8161	0.4589	0.4663	0.9242	0.7468	0.5108
NM_133609	Eif2b3	0.4807	0.5896	0.1082	0.0689	0.0602	0.0722	0.1663	0.0602	0.7782	0.8609	0.6002	0.6714
NM_053973	Rraga	0.0827	0.0650	0.7239	0.1612	0.0503	0.0191	0.0216	0.0178	0.0191	0.2954	0.1173	0.4582
NM_053978	Rab28	0.1877	0.8508	0.9325	0.7593	0.4773	0.8972	0.1720	0.3625	0.5788	0.8119	0.8905	0.4869
NM_053977	Cdh17	0.0315	0.0528	0.9290	0.9714	0.0513	0.1998	0.1704	0.0420	0.1109	0.2262	0.2263	0.4921
NM_133421	Lkap	0.9779	0.8753	0.9536	0.1596	0.9523	0.9448	0.4168	0.9303	0.9651	0.9884	0.9481	0.9790
NM_139337	Macrod1	0.3384	0.2091	0.7218	0.8616	0.7233	0.7892	0.6397	0.9216	0.7084	0.7746	0.8116	0.9174
NM_053981	Kcnj12	0.4345	0.4821	0.4768	0.8492	0.1672	0.2193	0.5022	0.1246	0.7834	0.9608	0.0574	0.6439
NM_053979	Arl5a	0.7402	0.0547	0.9430	0.6470	0.9087	0.8262	0.9411	0.8423	0.4125	0.1381	0.6318	0.9645
NM_053980	Arfrp1	0.0690	0.0428	0.1323	0.2801	0.2132	0.1582	0.1213	0.0876	0.0456	0.0031	0.0549	0.0583
NM_053983	Cd52	0.3763	0.5624	0.2829	0.4895	0.6480	0.6467	0.6946	0.6094	0.3469	0.6228	0.4832	0.1416
NM_053986	Myo1b	0.4093	0.0845	0.0035	0.0006	0.0118	0.0016	0.0097	0.0010	0.0015	0.0039	0.1584	0.0395
NM_057136	Epn1	0.7504	0.2128	0.5051	0.3450	0.0439	0.7671	0.6742	0.1834	0.3231	0.0479	0.5649	0.8379
NM_080396	Trpc4	0.5285	0.3200	0.2890	0.6126	0.4836	0.4345	0.1795	0.3909	0.2679	0.4261	0.6151	0.6737
NM_057186	Hadh	0.2769	0.4681	0.8704	0.9878	0.2571	0.2205	0.7869	0.5749	0.5866	0.0919	0.1878	0.5980
NM_053992	Nlgn2	0.5211	0.1347	0.5575	0.3819	0.7756	0.1019	0.4078	0.3522	0.6601	0.6401	0.5642	0.9052
NM_080576	Apoa5	0.3522	0.1414	0.3851	0.5314	0.4111	0.6062	0.4935	0.2039	0.4543	0.5618	0.6683	0.4111
NM_053997	Kcnc3	0.9491	0.9399	0.9299	0.9205	0.8506	0.5990	0.7827	0.7913	0.8856	0.6452	0.5606	0.9183
NM_078623	Echs1	0.4390	0.9344	0.7605	0.9807	0.6773	0.9454	0.9516	0.9004	0.7504	0.7309	0.5153	0.1767
NM_172039	Hdlbp	0.9230	0.8962	0.0667	0.6469	0.7859	0.9296	0.6604	0.9784	0.8037	0.9926	0.8126	0.4253

NM_057199	Ncr1	0.4438	0.6066	0.3860	0.0624	0.2012	0.5544	0.4913	0.4429	0.7350	0.8569	0.5600	0.4650
NM_057194	Plscr1	0.2506	0.1596	0.0619	0.5855	0.2424	0.2330	0.0698	0.0795	0.0096	0.1558	0.1625	0.0258
NM_057102	Slc25a5	0.6479	0.8619	0.3920	0.4655	0.5342	0.4619	0.6488	0.6041	0.5276	0.7589	0.5414	0.6205
NM_130755	Cs	0.0051	0.0368	0.3321	0.3408	0.0690	0.0525	0.7533	0.0136	0.0580	0.3484	0.4073	0.1401
NM_057213	Atp6v1b2	0.2929	0.2563	0.6705	0.4518	0.1528	0.2978	0.1086	0.2214	0.1132	0.0797	0.2330	0.8282
NM_057097	Vamp3	0.4769	0.8883	0.9507	0.9746	0.6756	0.7228	0.9635	0.9362	0.8317	0.9480	0.9300	0.9184
NM_078619	Slc8a2	0.4178	0.2441	0.3287	0.3978	0.5177	0.3248	0.7337	0.3039	0.7086	0.2813	0.4846	0.5628
NM_057098	Tcea2	0.7506	0.5597	0.8084	0.8427	0.7306	0.7500	0.8003	0.7885	0.2197	0.9449	0.6451	0.7591
NM_133534	Cx3cr1	0.2614	0.2650	0.1185	0.0686	0.1317	0.3355	0.0478	0.2531	0.1195	0.1657	0.1417	0.0663
NM_080586	Gabrg1	0.5956	0.9151	0.5962	0.7882	0.6820	0.5129	0.8778	0.8835	0.7699	0.4763	0.7222	0.6657
NM_080689	Dnm1	0.6927	0.2799	0.3624	0.0442	0.9340	0.5080	0.0859	0.7861	0.1279	0.1443	0.0899	0.4664
NM_080481	Atp5i	0.0180	0.1654	0.0322	0.7912	0.0350	0.0115	0.3061	0.0270	0.1432	0.2470	0.0457	0.0100
NM_078620	Slc8a3	0.7046	0.5156	0.8729	0.7714	0.6697	0.9333	0.7332	0.9540	0.8601	0.8055	0.6432	0.3668
NM_133535	Cbwd1	0.5493	0.4858	0.8762	0.4950	0.6325	0.7854	0.7212	0.3607	0.8390	0.5273	0.6911	0.6937
NM_057118	Cntn1	0.2277	0.0163	0.0702	0.0564	0.1093	0.1030	0.2216	0.1742	0.0048	0.4500	0.3907	0.0763
NM_133621	Hopx	0.0497	0.9424	0.2105	0.2552	0.0333	0.1755	0.0891	0.0472	0.3055	0.7176	0.9313	0.0067
NM_057122	Psmc4	0.5666	0.8186	0.0497	0.0206	0.5220	0.4487	0.4709	0.6728	0.9847	0.7918	0.6129	0.1537
NM_130749	Mark3	0.8837	0.7066	0.3163	0.2908	0.9690	0.4164	0.3569	0.7779	0.7819	0.9251	0.9519	0.5972
NM_001039505	Irx2	0.9187	0.8384	0.8329	0.4696	0.7726	0.2604	0.4546	0.6368	0.8164	0.1863	0.7711	0.5409
NM_133615	Taf9b	0.1009	0.7543	0.5127	0.4892	0.2534	0.3956	0.3003	0.3560	0.2535	0.5220	0.4060	0.1533
NM_057125	Pex6	0.6334	0.5859	0.1917	0.8094	0.8495	0.6349	0.2821	0.7677	0.8896	0.8191	0.8924	0.8617
NM_134413	Nacc1	0.5170	0.8947	0.4790	0.6479	0.8452	0.4224	0.5266	0.7416	0.7291	0.0772	0.1352	0.3628
NM_057127	Slc26a2	0.6349	0.3642	0.4268	0.4684	0.2693	0.1699	0.7055	0.5554	0.1752	0.3792	0.2834	0.1870
NM_134360	Cd40	0.4789	0.3755	0.1625	0.3040	0.2573	0.3578	0.4255	0.3102	0.1232	0.5902	0.3526	0.3797
NM_057131	Prpsap2	0.6825	0.4493	0.7484	0.8660	0.6688	0.7532	0.7952	0.9718	0.1949	0.1000	0.5329	0.1965
NM_057200	Kif1b	0.5269	0.3019	0.6966	0.4548	0.6276	0.3111	0.3227	0.6328	0.3002	0.5166	0.2899	0.4211
NM_133651	Cav1	0.6000	0.7048	0.9661	0.9995	0.6048	0.8550	0.8209	0.6185	0.7073	0.6073	0.3935	0.9769
NM_133597	Chrnb3	0.6998	0.3988	0.7139	0.4577	0.3441	0.4082	0.5372	0.4147	0.3080	0.3214	0.1362	0.2298
NM_133558	Nat8b	0.8084	0.9316	0.6851	0.7176	0.7126	0.6770	0.7652	0.7981	0.8444	0.7339	0.9269	0.4239
NM_080886	Sc4mol	0.9899	0.9932	0.2868	0.0174	0.9350	0.9953	0.2395	0.7183	0.8116	0.9530	0.9921	0.0827
NM_133416	Bcl2a1d	0.1121	0.1301	0.1079	0.3563	0.5926	0.5172	0.0602	0.3871	0.0804	0.0773	0.5882	0.1139
NM_133563	Giot1	0.7962	0.3323	0.2078	0.1169	0.8939	0.7869	0.0393	0.8319	0.0238	0.1007	0.1135	0.0116
NM_057143	Park7	0.2146	0.6910	0.7134	0.9016	0.2263	0.0612	0.8311	0.5787	0.1811	0.5262	0.8551	0.1064
NM_057144	Csrp3	0.0234	0.1995	0.2727	0.0384	0.0068	0.0272	0.7388	0.0448	0.2030	0.5238	0.0615	0.0869
NM_057146	C9	0.3541	0.7513	0.3445	0.2317	0.7385	0.1543	0.0708	0.2706	0.0706	0.1566	0.1686	0.6216
NM_057148	41,154.0000	0.5385	0.5819	0.6485	0.5940	0.6057	0.5702	0.2364	0.4815	0.4846	0.6874	0.4624	0.3926
NM_057152	Keap1	0.1204	0.6281	0.3461	0.7019	0.3125	0.9715	0.2660	0.5455	0.2777	0.5376	0.2363	0.7210
NM_057155	Xpnpep2	0.3554	0.0263	0.3604	0.2848	0.1417	0.3302	0.3525	0.2199	0.2389	0.2139	0.2214	0.3159
NM_080775	Smgb	0.0934	0.1171	0.0627	0.1835	0.1204	0.2031	0.0857	0.2199	0.3341	0.0689	0.2306	0.0620
NM_130747	Acot12	0.0508	0.1947	0.2469	0.1298	0.0364	0.0745	0.0868	0.2976	0.2087	0.2486	0.0435	0.0729
NM_080906	Ddit4	0.8943	0.9227	0.9655	0.7936	0.9431	0.9147	0.8591	0.8066	0.9654	0.9963	0.9511	0.9122
NM_080894	Pde7b	0.5211	0.1390	0.4448	0.4439	0.1167	0.5377	0.0935	0.6674	0.5839	0.4733	0.7645	0.8219
NM_057187	Grifin	0.4640	0.2789	0.3346	0.3642	0.3048	0.3652	0.3686	0.3779	0.3243	0.4548	0.4365	0.4695
NM_172036	Gabarap	0.0730	0.2574	0.8962	0.9431	0.3650	0.2284	0.6001	0.2280	0.0970	0.0733	0.0620	0.1525
NM_152936	Spink1	0.5661	0.0839	0.3702	0.7987	0.1690	0.4456	0.2062	0.2655	0.2882	0.3032	0.3682	0.0986
NM_080587	Gabra4	0.4059	0.3111	0.0860	0.2122	0.0362	0.4150	0.0305	0.2248	0.2252	0.2970	0.0855	0.0873
NM_080582	Abcb6	0.7945	0.9169	0.0795	0.8598	0.8920	0.8900	0.9116	0.8371	0.7935	0.9327	0.8824	0.8031
NM_133295	Ces3	0.2753	0.1664	0.1762	0.4203	0.2313	0.1737	0.4094	0.3158	0.3056	0.5205	0.6821	0.1714
NM_057201	Gpr37	0.7487	0.6660	0.7883	0.4086	0.8027	0.7425	0.8187	0.1126	0.8226	0.6850	0.4920	0.6506

NM_057191	Kbtbd10	0.0035	0.2676	0.0339	0.1915	0.1887	0.1079	0.0234	0.0381	0.0020	0.0036	0.1560	0.1504
NM_173308	Fut11	0.4844	0.0059	0.3987	0.5745	0.0344	0.1275	0.0503	0.1330	0.1192	0.0126	0.1303	0.0078
NM_080910	Paics	0.7458	0.7770	0.7941	0.7963	0.8507	0.8474	0.9870	0.7493	0.9249	0.9892	0.7843	0.8558
NM_080887	Txnl1	0.0631	0.2094	0.6459	0.0907	0.0280	0.1863	0.0671	0.0302	0.1672	0.4097	0.3595	0.7493
NM_080777	Sncb	0.5906	0.2853	0.5564	0.1280	0.5854	0.3080	0.1486	0.0568	0.4099	0.4936	0.5353	0.7395
NM_057203	Ccl22	0.1597	0.2623	0.2807	0.3869	0.5517	0.6087	0.5212	0.2277	0.3546	0.1559	0.3710	0.4740
NM_057193	Il10ra	0.6263	0.4854	0.2879	0.5434	0.4346	0.2931	0.1817	0.5088	0.5789	0.3963	0.4097	0.4340
NM_080893	Pde11a	0.6026	0.4951	0.5636	0.4383	0.6647	0.6564	0.2556	0.2359	0.6838	0.5937	0.7256	0.4342
NM_057208	Tpm3	0.4340	0.8411	0.3841	0.7059	0.7718	0.9282	0.6150	0.5394	0.7313	0.8614	0.7812	0.9466
NM_130407	Ugt1a7c	0.3589	0.6895	0.6644	0.6410	0.4867	0.4895	0.4261	0.2855	0.3788	0.4163	0.5316	0.6240
NM_080766	Nras	0.0935	0.5602	0.9379	0.8995	0.5239	0.9750	0.5295	0.4060	0.8381	0.8112	0.8961	0.9752
NM_130399	Ada	0.3688	0.1890	0.3509	0.7918	0.3292	0.0235	0.6065	0.0363	0.0231	0.1574	0.0648	0.0635
NM_138510	Akr1c18	0.8552	0.0406	0.0085	0.0874	0.2615	0.4033	0.0005	0.6801	0.0052	0.1290	0.0856	0.0987
NM_138547	Akr1c14	0.9925	0.0466	0.9925	0.5919	0.9643	0.8602	0.4755	0.7943	0.2922	0.1261	0.0427	0.1522
NM_057211	Klf9	0.2219	0.9176	0.9682	0.3246	0.1980	0.3145	0.6073	0.0786	0.7243	0.5282	0.8898	0.9543
NM_133284	Pgc	0.3392	0.5206	0.3960	0.5527	0.4078	0.1389	0.3090	0.4919	0.5696	0.6058	0.1773	0.4937
NM_134384	Spata19	0.0400	0.0595	0.0204	0.0569	0.0094	0.0005	0.0085	0.1179	0.0308	0.0544	0.0006	0.0983
NM_133518	Rph3a	0.1783	0.1401	0.3089	0.5310	0.5704	0.6099	0.5040	0.6908	0.1602	0.2507	0.4457	0.4809
NM_058210	Sf1	0.5907	0.3689	0.4891	0.5821	0.8126	0.7412	0.6303	0.7031	0.6174	0.4665	0.8273	0.7336
NM_080581	Abcc3	0.7645	0.9232	0.6189	0.9096	0.7571	0.9198	0.7298	0.9827	0.7965	0.8324	0.9682	0.7900
NM_078617	Rps23	0.4466	0.5546	0.3278	0.2676	0.5758	0.5152	0.4504	0.5376	0.4922	0.5060	0.4239	0.4166
NM_133589	Ppp6c	0.2591	0.6837	0.6975	0.1877	0.3367	0.3316	0.3133	0.0900	0.3705	0.5080	0.5993	0.8586
NM_133411	Abcc4	0.1698	0.4713	0.4622	0.4717	0.6286	0.5619	0.3891	0.9122	0.4206	0.6544	0.4893	0.6369
NM_080688	Plcd4	0.7006	0.9515	0.8407	0.7885	0.8728	0.8655	0.8743	0.8281	0.8740	0.7972	0.8459	0.7634
NM_078621	Ccbp2	0.3658	0.2920	0.2450	0.3089	0.5988	0.4808	0.4593	0.3875	0.4725	0.3020	0.2728	0.4208
NM_058208	Socs2	0.3111	0.5189	0.2045	0.3709	0.0895	0.0758	0.6441	0.7302	0.0525	0.3131	0.4235	0.6345
NM_134363	Slc12a5	0.9964	0.9721	0.9490	0.9337	0.9957	0.8960	0.9968	0.9733	0.9923	0.9629	0.7569	0.9951
NM_134327	Cd69	0.3235	0.7011	0.3105	0.2432	0.3572	0.6675	0.4036	0.8358	0.7822	0.3403	0.4241	0.3991
NM_133299	Pecr	0.1404	0.0467	0.9978	0.5598	0.2440	0.1479	0.6905	0.6253	0.0748	0.2348	0.1142	0.0282
NM_130428	Sdha	0.0704	0.1645	0.8256	0.0395	0.0756	0.0221	0.0327	0.0294	0.0112	0.0112	0.2068	0.4925
NM_130414	Abcg8	0.7485	0.6695	0.5206	0.6757	0.1096	0.6699	0.1540	0.4633	0.5590	0.7136	0.1390	0.3580
NM_134334	Ctsd	0.0046	0.0039	0.7848	0.0559	0.0984	0.1884	0.2781	0.2869	0.2983	0.0840	0.0517	0.6934
NM_080411	Gpr83	0.2062	0.4924	0.6946	0.5396	0.6841	0.6346	0.5859	0.6873	0.4314	0.4588	0.5337	0.5576
NM_080394	Reln	0.9416	0.7605	0.8878	0.8192	0.3525	0.3586	0.7929	0.2825	0.6629	0.6362	0.7245	0.4717
NM_133306	Olr1	0.2295	0.3069	0.7953	0.2033	0.7146	0.7564	0.4107	0.7436	0.7654	0.4532	0.2653	0.8566
NM_080782	Cdkn1a	0.2808	0.6001	0.7139	0.2223	0.2552	0.1712	0.2989	0.3019	0.8581	0.6283	0.7956	0.7748
NM_130408	Cyp26a1	0.4713	0.5764	0.5375	0.7285	0.4274	0.3395	0.5218	0.6610	0.8576	0.8337	0.8137	0.4885
NM_134390	Tmem176b	0.4982	0.0284	0.5601	0.4593	0.4771	0.6609	0.0666	0.5006	0.0744	0.0176	0.0129	0.5464
NM_080577	Nploc4	0.2390	0.4321	0.5731	0.8020	0.1826	0.5986	0.1494	0.2055	0.4038	0.3329	0.0845	0.8609
NM_080580	Rab3d	0.8974	0.9556	0.7699	0.9566	0.7947	0.6657	0.9740	0.8856	0.9388	0.7950	0.9090	0.9373
NM_131914	Cav2	0.1463	0.2147	0.3291	0.2526	0.0825	0.0038	0.2389	0.0747	0.2041	0.2330	0.1201	0.1257
NM_130432	Rps13	0.7915	0.9005	0.6813	0.5206	0.6000	0.3793	0.8771	0.5924	0.9060	0.8172	0.8941	0.5348
NM_080579	Gpr19	0.1343	0.7792	0.0419	0.1985	0.8843	0.6001	0.5091	0.6084	0.7025	0.8755	0.9014	0.1517
NM_134369	Cyp2t1	0.1578	0.4145	0.5888	0.3507	0.4496	0.4452	0.7390	0.1856	0.4668	0.5435	0.2821	0.4922
NM_080583	Ap2b1	0.9480	0.3072	0.5094	0.9097	0.9351	0.9148	0.4092	0.9554	0.8519	0.4018	0.3256	0.6511
NM_133546	Ppp1r15a	0.0612	0.4542	0.1908	0.0032	0.0016	0.0012	0.0148	0.0020	0.5824	0.1061	0.4482	0.4565
NM_133394	Zdhhc7	0.0398	0.2576	0.2737	0.0176	0.3223	0.3628	0.2351	0.2584	0.4183	0.0857	0.1246	0.5383
NM_080905	Siah1a	0.8921	0.2765	0.9080	0.8976	0.3941	0.9833	0.8062	0.1243	0.4309	0.8648	0.9751	0.9078
NM_130779	Adcy3	0.6493	0.6945	0.4786	0.5315	0.3678	0.3287	0.6248	0.7755	0.6977	0.1647	0.2474	0.2136

NM_001135698	RGD1564579	0.9128	0.1230	0.4541	0.4797	0.8691	0.1981	0.7795	0.4647	0.7580	0.3625	0.2814	0.8148
NM_080690	Caskin1	0.5543	0.6179	0.6177	0.1355	0.9221	0.9343	0.5515	0.9448	0.8905	0.9195	0.7435	0.3482
NM_134379	UST4r	0.5117	0.6635	0.7949	0.0978	0.6032	0.6494	0.8192	0.1366	0.1482	0.5360	0.2290	0.2249
NM_134380	Ust5r	0.1846	0.3306	0.5026	0.3235	0.4588	0.3927	0.2088	0.0977	0.4147	0.3660	0.5538	0.5284
NM_153731	Zfp709	0.7505	0.8019	0.8551	0.7693	0.8541	0.6836	0.8709	0.8330	0.9572	0.8169	0.8817	0.4394
NM_080698	Fmod	0.1519	0.0213	0.1714	0.9911	0.3710	0.1336	0.4860	0.2627	0.2497	0.0674	0.1731	0.2211
NM_080697	Dynll2	0.3254	0.3446	0.8933	0.9710	0.9005	0.4025	0.9647	0.8365	0.6085	0.3615	0.6589	0.3319
NM_138522	Cxcl3	0.0358	0.3987	0.2415	0.2807	0.4548	0.1785	0.2396	0.1641	0.4185	0.8154	0.1683	0.1269
NM_134443	Creb1	0.4578	0.2182	0.3199	0.3654	0.5852	0.3086	0.5895	0.9003	0.1689	0.2044	0.5244	0.3485
NM_080778	Nr2f2	0.0395	0.0290	0.7208	0.0743	0.2334	0.2632	0.0089	0.0115	0.0244	0.0299	0.0277	0.5733
NM_131912	Kcnh7	0.7663	0.0924	0.2436	0.5322	0.1694	0.3011	0.0591	0.0555	0.0622	0.2514	0.5734	0.4850
NM_133543	Rdh7	0.3908	0.5891	0.7001	0.3130	0.7277	0.0545	0.9153	0.4459	0.5872	0.3493	0.6459	0.3339
NM_134396	Efha1	0.7203	0.6140	0.4473	0.5360	0.2006	0.3858	0.5354	0.2519	0.3819	0.2083	0.5751	0.5081
NM_130424	Tmprss2	0.9081	0.4986	0.8783	0.7377	0.9296	0.8668	0.7401	0.9278	0.9231	0.9649	0.8505	0.8475
NM_134399	Mk1	0.8603	0.7070	0.1443	0.7172	0.6401	0.7465	0.9987	0.9142	0.9957	0.7959	0.9435	0.7528
NM_138504	Osgin1	0.0056	0.0473	0.0319	0.0820	0.0032	0.0070	0.1501	0.2702	0.0088	0.0082	0.0612	0.0340
NM_080780	P2rx5	0.0052	0.0144	0.0431	0.2970	0.2283	0.2861	0.3287	0.2359	0.2599	0.1712	0.0045	0.2338
NM_080781	Copb1	0.9126	0.7651	0.8263	0.1907	0.2555	0.6382	0.0522	0.3272	0.8476	0.2770	0.5365	0.4715
NM_080786	Slco2b1	0.7057	0.7509	0.3745	0.3703	0.7469	0.4109	0.4822	0.5268	0.5228	0.4022	0.9018	0.8130
NM_080890	As3mt	0.8626	0.4615	0.8433	0.4785	0.7566	0.1945	0.0767	0.5720	0.6947	0.5572	0.5377	0.1616
NM_133427	Cyb5r4	0.3513	0.7631	0.8845	0.7771	0.2935	0.4113	0.8905	0.2119	0.1792	0.3587	0.2327	0.3153
NM_133283	Map2k2	0.8064	0.8762	0.8802	0.8830	0.9112	0.8427	0.8517	0.9591	0.8747	0.7362	0.9116	0.8443
NM_133567	Adap1	0.1141	0.0198	0.5322	0.0424	0.3618	0.3516	0.4405	0.1779	0.2549	0.1092	0.3530	0.5204
NM_080901	Rcvrn	0.3603	0.0265	0.1163	0.6922	0.3819	0.0413	0.3076	0.2608	0.2245	0.3807	0.1263	0.5875
NM_139192	Scd1	0.9659	0.9973	0.1912	0.2002	0.3910	0.7907	0.0806	0.4494	0.9595	0.9950	0.9880	0.1752
NM_139060	Csnk1d	0.3960	0.4033	0.9756	0.2035	0.1386	0.2534	0.7808	0.0690	0.5292	0.2194	0.2308	0.9117
NM_138849	Syt17	0.5501	0.4801	0.5867	0.5811	0.4416	0.5882	0.5996	0.6590	0.5210	0.5600	0.6071	0.6511
NM_138840	Tgoln1	0.5104	0.2510	0.9380	0.9810	0.3295	0.9457	0.9201	0.1250	0.6516	0.2422	0.3547	0.9733
NM_134419	Hspbap1	0.2291	0.4729	0.0018	0.1112	0.5771	0.0160	0.0037	0.5634	0.8787	0.1449	0.8002	0.0057
NM_138855	Spdya	0.6793	0.6261	0.6012	0.6175	0.3068	0.1554	0.1864	0.0857	0.6040	0.5499	0.4337	0.4090
NM_080902	Higd1a	0.2164	0.2823	0.1460	0.4302	0.0893	0.2414	0.7185	0.0460	0.0155	0.2025	0.2874	0.0828
NM_080895	Faim	0.1161	0.6247	0.6393	0.9417	0.7338	0.4997	0.2370	0.6037	0.5750	0.6551	0.5994	0.6093
NM_080904	Arf3	0.2193	0.2484	0.2989	0.3743	0.2558	0.3072	0.3822	0.1251	0.2215	0.2626	0.1654	0.1769
NM_133307	Prkcd	0.2040	0.0552	0.9515	0.0268	0.1091	0.1896	0.9286	0.0101	0.4638	0.2006	0.1769	0.7553
NM_080896	Hnrph1	0.9994	0.9339	0.6795	0.6503	0.9472	0.8866	0.1966	0.9859	0.6070	0.7550	0.7246	0.4433
NM_080897	Bnip1	0.0770	0.1277	0.0929	0.1909	0.1097	0.0373	0.3099	0.0219	0.1163	0.0693	0.1019	0.1044
NM_080907	Ppp4r1	0.8504	0.9819	0.6248	0.9063	0.9535	0.9474	0.2974	0.9817	0.9727	0.9970	0.9822	0.9933
NM_001108456	Afg3l1	0.3883	0.6397	0.8363	0.1592	0.4377	0.8327	0.9676	0.3789	0.5151	0.1730	0.4199	0.8527
NM_001108286	RGD1311564	0.8293	0.7858	0.7513	0.9094	0.6863	0.8471	0.8814	0.9096	0.6084	0.8645	0.3160	0.9154
NM_001107868	Zfml	0.3835	0.8522	0.1811	0.0716	0.4029	0.3992	0.0343	0.6071	0.4082	0.5568	0.1479	0.7014
NM_001107507	RGD1310358	0.7194	0.6423	0.6278	0.5910	0.7190	0.6539	0.5068	0.6201	0.4832	0.5961	0.6375	0.7307
NM_133304	Heph	0.9984	0.8106	0.8647	0.9100	0.9889	0.7649	0.9957	0.7939	0.2334	0.8663	0.9747	0.0300
NM_001108343	Etnk2	0.5546	0.8292	0.4509	0.6034	0.7978	0.4741	0.7260	0.9271	0.6337	0.4000	0.9148	0.7915
NM_001108473	Pnmal1	0.7636	0.7281	0.7899	0.6238	0.7451	0.7843	0.8975	0.6644	0.6983	0.8152	0.6523	0.6342
NM_001108336	Sdsl	0.7471	0.0842	0.7097	0.6784	0.7815	0.9016	0.2638	0.8178	0.0544	0.2047	0.0947	0.0304
NM_001108534	Aifm2	0.5947	0.5012	0.4430	0.9260	0.6607	0.5194	0.9762	0.6113	0.8051	0.6204	0.9273	0.8755
NM_001108583	Bbs5	0.1460	0.2325	0.9087	0.0502	0.0627	0.1624	0.0329	0.1127	0.0602	0.0939	0.0701	0.7402
NM_001108575	Brd3	0.3547	0.5323	0.1932	0.3789	0.2344	0.2324	0.7875	0.7077	0.3276	0.6176	0.6657	0.3609
NM_001108332	Wbscr16	0.9219	0.8001	0.1037	0.4563	0.8382	0.9263	0.3628	0.9531	0.9059	0.3989	0.1064	0.0433

NM_001108330	RGD1309735	0.0766	0.0631	0.1439	0.1829	0.4467	0.0406	0.1046	0.4330	0.0767	0.0789	0.0057	0.0270
NM_001108403	Nsun2	0.9771	0.9867	0.3744	0.6031	0.5118	0.9426	0.7861	0.1287	0.9951	0.7558	0.9923	0.9909
NM_001108371	Ube2e2	0.7303	0.8329	0.5464	0.4860	0.7497	0.2621	0.8307	0.8193	0.1265	0.2790	0.5873	0.8565
NM_001108327	Prkdc	0.9700	0.9943	0.2360	0.0279	0.9723	0.9957	0.4884	0.9934	0.9942	0.9845	0.9360	0.9494
NM_001108284	Ksr1	0.2670	0.5831	0.7255	0.3764	0.2093	0.1781	0.4177	0.5562	0.8947	0.0180	0.4545	0.6604
NM_001108469	Cdc42ep5	0.7896	0.6668	0.0111	0.7201	0.8007	0.0979	0.6466	0.6733	0.6112	0.8408	0.5214	0.1006
NM_080909	Rtn3	0.4649	0.7028	0.6980	0.5273	0.6450	0.5498	0.7143	0.3673	0.4830	0.4491	0.4610	0.7242
NM_080899	Ikbkap	0.7745	0.7195	0.5174	0.5812	0.8606	0.7891	0.4415	0.7686	0.8180	0.4765	0.7682	0.8036
NM_138881	Rsad2	0.2474	0.2007	0.6455	0.2421	0.6083	0.7255	0.6083	0.5429	0.6759	0.2021	0.6033	0.6114
NM_130401	Pdzk1ip1	0.5630	0.7558	0.8654	0.6373	0.7684	0.5513	0.6358	0.8465	0.7502	0.5569	0.5507	0.8628
NM_139336	Uxs1	0.4989	0.7881	0.8595	0.9079	0.6775	0.8767	0.9819	0.9530	0.1049	0.5550	0.7937	0.0153
NM_130403	Ppp1r14a	0.5642	0.5771	0.5152	0.6352	0.0519	0.2041	0.4957	0.5410	0.0745	0.6037	0.8448	0.5362
NM_130405	Khdrbs1	0.9268	0.9473	0.9558	0.9984	0.9921	0.9312	0.7598	0.9886	0.9116	0.8748	0.6097	0.8834
NM_133310	Icmt	0.0463	0.4174	0.3695	0.6140	0.0560	0.2196	0.4886	0.7721	0.6900	0.3240	0.1280	0.5111
NM_138853	Ubxn11	0.0045	0.2336	0.8521	0.0202	0.0677	0.0788	0.2133	0.2620	0.3873	0.5311	0.0610	0.6355
NM_130409	Cfh	0.9563	0.9611	0.7859	0.9951	0.8670	0.7754	0.8313	0.8543	0.7764	0.9533	0.8541	0.7409
NM_138537	LOC171573	0.0938	0.1080	0.3523	0.0854	0.1288	0.0147	0.0358	0.1298	0.0646	0.0085	0.0813	0.0471
NM_130410	Il23a	0.5503	0.9543	0.7474	0.3634	0.0353	0.3642	0.5597	0.7236	0.7191	0.7720	0.9350	0.4729
NM_138838	Pou3f1	0.8829	0.9395	0.8581	0.9733	0.9648	0.8021	0.9635	0.8743	0.5820	0.8610	0.9365	0.9238
NM_133595	Gchfr	0.0413	0.0598	0.0202	0.3727	0.0828	0.2316	0.0136	0.0177	0.0377	0.2272	0.0738	0.1899
NM_130411	Coro1a	0.1497	0.2713	0.6440	0.5596	0.2859	0.2336	0.7071	0.3004	0.2302	0.5498	0.2740	0.6127
NM_130412	Sdf4	0.2376	0.0600	0.7339	0.4174	0.0150	0.0729	0.2911	0.0366	0.1090	0.0417	0.0215	0.3896
NM_139099	Atp5e	0.2782	0.5044	0.2841	0.5088	0.3217	0.4018	0.4646	0.1710	0.2557	0.4005	0.3392	0.3834
NM_138544	Fat3	0.7437	0.5807	0.7781	0.9921	0.8574	0.8093	0.7894	0.8627	0.4283	0.8385	0.9430	0.3602
NM_138860	Opn4	0.4844	0.7078	0.5517	0.4038	0.5163	0.8263	0.6723	0.7579	0.8405	0.4813	0.8418	0.9634
NM_133610	Kcnh5	0.4203	0.2358	0.3582	0.0505	0.0908	0.2356	0.0833	0.2595	0.5513	0.4217	0.5938	0.4563
NM_130415	Slc36a1	0.1491	0.7820	0.0493	0.4723	0.7440	0.4511	0.2582	0.2191	0.6234	0.4422	0.8372	0.5825
NM_130416	Anxa7	0.0680	0.0370	0.9162	0.4284	0.0742	0.5689	0.0359	0.0400	0.1439	0.0580	0.0505	0.7736
NM_138892	Xtp3tpa	0.0986	0.5555	0.0294	0.0682	0.0299	0.2295	0.3700	0.1996	0.0694	0.6838	0.5823	0.1382
NM_130419	Dcbld2	0.2363	0.1221	0.0446	0.0631	0.0837	0.8416	0.0064	0.0193	0.2181	0.1169	0.0809	0.0165
NM_130421	Lcp2	0.9328	0.4128	0.7722	0.5814	0.4926	0.6795	0.8360	0.2299	0.8143	0.6835	0.9133	0.7093
NM_133386	Sphk1	0.5811	0.4757	0.1794	0.7342	0.6267	0.5610	0.1558	0.6226	0.0331	0.0577	0.0096	0.0100
NM_131907	Atp2c1	0.3726	0.8907	0.9832	0.9440	0.4429	0.8204	0.9738	0.2028	0.9005	0.9736	0.7732	0.8729
NM_130422	Casp12	0.0999	0.0018	0.4551	0.0056	0.0428	0.0398	0.0036	0.0736	0.0087	0.0880	0.0150	0.4099
NM_178330	Tmem184c	0.0806	0.0555	0.0338	0.0245	0.0857	0.2343	0.0201	0.0049	0.0107	0.0522	0.1444	0.3599
NM_153469	Pkig	0.8121	0.1312	0.7710	0.4349	0.7760	0.8720	0.5899	0.8529	0.1866	0.2936	0.0114	0.9116
NM_133523	Mmp3	0.0843	0.7139	0.6560	0.4309	0.0036	0.0110	0.3151	0.0250	0.5385	0.5019	0.5250	0.4789
NM_130430	Psmd9	0.0344	0.3285	0.0225	0.0437	0.0904	0.0321	0.4476	0.1472	0.0442	0.0150	0.0132	0.0417
NM_001106441	Robld3	0.1466	0.5448	0.7683	0.6375	0.6090	0.3756	0.5420	0.4979	0.5035	0.6483	0.1064	0.5603
NM_130734	Gnb2l1	0.5015	0.0783	0.5741	0.3535	0.1074	0.8504	0.0544	0.7055	0.1364	0.5756	0.3364	0.6508
NM_130739	Acsl6	0.6475	0.2785	0.0336	0.2621	0.1334	0.3181	0.3487	0.6786	0.0908	0.5670	0.5051	0.2944
NM_133381	Crebbp	0.6679	0.5321	0.6620	0.5394	0.6805	0.5133	0.6467	0.6532	0.4667	0.6550	0.7095	0.6100
NM_130736	Slco1a6	0.7285	0.5020	0.0989	0.3153	0.7986	0.5379	0.7359	0.2395	0.5020	0.9435	0.5784	0.6813
NM_130735	Sycp2	0.2153	0.7307	0.1837	0.1580	0.1145	0.6426	0.3093	0.7181	0.5981	0.8446	0.7117	0.6523
NM_001004271	Ugt2b36	0.6791	0.4136	0.5715	0.6271	0.3694	0.3262	0.6450	0.4068	0.2739	0.3327	0.2758	0.6086
NM_001004266	RGD1303144	0.3755	0.5354	0.2229	0.3964	0.1152	0.3304	0.5004	0.7870	0.7513	0.6553	0.6849	0.1944
NM_183054	Rhbg	0.4678	0.0906	0.5791	0.3688	0.2924	0.1114	0.3108	0.2998	0.2487	0.2874	0.1853	0.4022
NM_183053	Rhcg	0.2477	0.2011	0.1278	0.0489	0.2020	0.0643	0.2596	0.4044	0.0276	0.1305	0.3148	0.4951
NM_172031	Plunc	0.5481	0.0894	0.4660	0.6996	0.4176	0.5503	0.3149	0.3084	0.8906	0.8257	0.5309	0.6657

NM_001105741	Csn1s2a	0.0817	0.3815	0.0779	0.4837	0.1593	0.1167	0.0956	0.4507	0.0220	0.2064	0.4108	0.1722
NM_133387	Tmlhe	0.0042	0.1345	0.0919	0.5493	0.0190	0.0146	0.0586	0.0700	0.0500	0.0531	0.0500	0.0987
NM_130740	Pacsin2	0.3694	0.8172	0.2298	0.9470	0.3998	0.7276	0.9080	0.6771	0.7262	0.8710	0.6781	0.5670
NM_130738	Snurf	0.9178	0.0733	0.9258	0.0802	0.0071	0.0158	0.0836	0.0337	0.0439	0.0377	0.1508	0.8366
NM_139102	Dmgdh	0.5788	0.1268	0.6681	0.5689	0.6195	0.2244	0.3545	0.1028	0.4967	0.6932	0.0470	0.3351
NM_131913	Xpnpep1	0.2576	0.4036	0.0596	0.2085	0.1800	0.6136	0.1581	0.1260	0.8477	0.7048	0.8115	0.8391
NM_130741	Lcn2	0.0083	0.0087	0.1070	0.0675	0.1027	0.1598	0.1014	0.0005	0.1865	0.0872	0.0295	0.4866
NM_133315	Slc40a1	0.8572		0.9809	0.9929	0.1160	0.0248	0.7882	0.2261	0.8046	0.9872	0.9956	0.8173
NM_130744	Cygb	0.9569	0.9223	0.8936	0.9340	0.8568	0.7501	0.9496	0.9264	0.9286	0.5362	0.9126	0.9184
NM_134394	Atg3	0.0479	0.0334	0.1447	0.1787	0.0896	0.1068	0.0615	0.0471	0.0663	0.0292	0.3386	0.0238
NM_130746	Slc5a6	0.9026	0.6674	0.0629	0.4667	0.8901	0.3520	0.2470	0.9787	0.7717	0.9235	0.7982	0.8435
NM_138865	RGD621352	0.3533	0.4174	0.8474	0.8849	0.4750	0.7224	0.8358	0.1152	0.3142	0.4169	0.7832	0.0500
NM_130748	Slc38a4	0.6339	0.5500	0.8526	0.8222	0.7095	0.5264	0.9706	0.8391	0.0118	0.4443	0.4530	0.0202
NM_130750	Asz1	0.1064	0.6357	0.3093	0.4296	0.1647	0.0839	0.3150	0.3497	0.1796	0.2223	0.2894	0.0988
NM_134326	Alb	0.5571	0.3485	0.4216	0.3061	0.4806	0.5820	0.1825	0.2545	0.2695	0.5513	0.1362	0.2532
NM_130812	Cdkn2b	0.7469	0.6823	0.4073	0.6348	0.4851	0.5897	0.7860	0.3960	0.8210	0.5412	0.8031	0.5483
NM_133392	Stk17b	0.2358	0.0571	0.6775	0.2759	0.3142	0.6728	0.0482	0.1817	0.1356	0.0035	0.0705	0.0173
NM_133309	Capn8	0.0779	0.2247	0.1453	0.1109	0.0043	0.0100	0.0254	0.1963	0.3199	0.0780	0.0762	0.0993
NM_134398	P34	0.1130	0.1257	0.1168	0.1310	0.0431	0.2754	0.3502	0.0678	0.1529	0.7678	0.1240	0.4264
NM_130813	Kcnk15	0.2643	0.8658	0.5240	0.7747	0.8972	0.5986	0.8754	0.9183	0.8761	0.6972	0.7430	0.6138
NM_130820	Pnma1	0.5597	0.9158	0.2432	0.4972	0.2945	0.5717	0.4578	0.3307	0.5694	0.0905	0.6841	0.7541
NM_130819	Dhrs9	0.4453	0.0066	0.2925	0.5353	0.0330	0.7472	0.0427	0.1217	0.0737	0.0089	0.2943	0.9328
NM_130822	Lphn3	0.7486	0.5882	0.7732	0.7943	0.6084	0.6061	0.7489	0.6411	0.8201	0.8602	0.8762	0.8984
NM_133528	Mobkl3	0.2264	0.3469	0.9768	0.4497	0.3181	0.3281	0.3946	0.4019	0.1881	0.2844	0.3104	0.1246
NM_138885	Golgb1	0.7472	0.1977	0.7146	0.7001	0.0217	0.6965	0.0992	0.5939	0.2873	0.0547	0.1406	0.7440
NM_130824	Rasgrp4	0.6422	0.5968	0.7895	0.7314	0.5999	0.3978	0.7754	0.5396	0.9361	0.7498	0.6380	0.8748
NM_133393	Lfng	0.9776	0.8885	0.7000	0.9424	0.8797	0.9526	0.9739	0.9432	0.7054	0.7126	0.7535	0.8927
NM_130829	Palm	0.8794	0.4521	0.2708	0.0898	0.9791	0.8247	0.8278	0.8973	0.7312	0.7787	0.4926	0.7464
NM_130828	Nphs2	0.8181	0.4745	0.5975	0.6073	0.5334	0.3578	0.8563	0.3296	0.2649	0.3083	0.5927	0.3707
NM_138708	Rabggtb	0.4796	0.9929	0.0687	0.1530	0.8397	0.5777	0.2487	0.8921	0.7349	0.7394	0.6472	0.6249
NM_138531	Stambp	0.2104	0.5469	0.7473	0.3518	0.1545	0.1201	0.9704	0.3851	0.9463	0.4816	0.4431	0.6634
NM_131902	Cdkn2c	0.7777	0.4554	0.0411	0.8658	0.9915	0.9275	0.7815	0.9777	0.7225	0.7944	0.9347	0.0716
NM_131905	Cldn16	0.3828	0.4627	0.4822	0.6506	0.3111	0.1875	0.4617	0.5219	0.4199	0.0639	0.1572	0.6366
NM_134459	Cd99l2	0.6416	0.3281	0.6608	0.6881	0.5823	0.7099	0.4297	0.6890	0.1877	0.6373	0.6872	0.4464
NM_153296	Aurka	0.6886	0.9120	0.1683	0.7431	0.9145	0.9617	0.9962	0.7427	0.2006	0.7856	0.9396	0.1070
NM_145089	Asrgl1	0.0681	0.1679	0.4973	0.3127	0.5632	0.4677	0.0596	0.6124	0.0019	0.0049	0.1167	0.0074
NM_181383	Lgsn	0.3750	0.1444	0.1120	0.4040	0.4121	0.4903	0.5989	0.2290	0.0644	0.6539	0.1104	0.7355
NM_172335	Gm2a	0.4097	0.2022	0.8922	0.8714	0.8683	0.4294	0.7939	0.7940	0.1494	0.2390	0.0698	0.2022
NM_133517	Atp12a	0.8406	0.8859	0.7724	0.6552	0.3461	0.5064	0.7314	0.2066	0.8680	0.6495	0.6563	0.0602
NM_130894	Mfn2	0.7993	0.2199	0.2032	0.9715	0.3191	0.5375	0.4897	0.3755	0.8105	0.1644	0.0976	0.7706
NM_133578	Dusp5	0.2012	0.1130	0.3762	0.7949	0.7200	0.1879	0.3637	0.5041	0.2046	0.3208	0.0105	0.2880
NM_133401	Abcb1	0.5316	0.3859	0.6172	0.6594	0.2064	0.2130	0.5897	0.4276	0.4651	0.7281	0.6050	0.3118
NM_133322	Kcnq2	0.4738	0.6848	0.3925	0.6485	0.3913	0.7674	0.8413	0.4335	0.5527	0.6976	0.6540	0.7757
NM_133574	Lnpep	0.5293	0.7601	0.6154	0.5888	0.5673	0.8532	0.3356	0.7949	0.2895	0.4147	0.4643	0.5366
NM_133396	Tesk2	0.7771	0.9076	0.4770	0.5993	0.8643	0.6677	0.9329	0.8470	0.8989	0.9706	0.9540	0.5989
NM_172326	Elac2	0.1424	0.2573	0.1533	0.1287	0.0670	0.0202	0.4973	0.1416	0.6276	0.3629	0.1613	0.2162
NM_133290	Zfp36	0.3635	0.2493	0.2156	0.0073	0.4916	0.3483	0.1594	0.4869	0.1320	0.1446	0.3410	0.7008
NM_001007144	Adfp	0.1651	0.1026	0.1444	0.0278	0.0037	0.0656	0.0207	0.1930	0.0014	0.0034	0.0686	0.0380
NM_133302	Adarb2	0.7230	0.7743	0.7169	0.7521	0.5217	0.5413	0.7364	0.6400	0.5801	0.4889	0.6115	0.4655

NM_173311	Skap1	0.1306	0.5157	0.2670	0.5644	0.8561	0.7512	0.5764	0.3555	0.6596	0.6364	0.7235	0.4468
NM_181081	Myst2	0.9850	0.9860	0.5299	0.9495	0.9400	0.9486	0.8786	0.6547	0.9334	0.9641	0.8617	0.9777
NM_133547	Sult1c2	0.7024	0.2044	0.7058	0.7296	0.7176	0.2141	0.5296	0.2267	0.3280	0.4918	0.5354	0.5014
NM_133562	Inpp5j	0.8706	0.8617	0.9382	0.8242	0.9121	0.9521	0.8893	0.8202	0.7815	0.8062	0.9334	0.9111
NM_133294	Hemgn	0.8995	0.6743	0.6688	0.8689	0.7037	0.5060	0.5171	0.8336	0.6135	0.4171	0.7303	0.8016
NM_133308	Grin3b	0.6937	0.1806	0.3067	0.4557	0.1832	0.0494	0.0488	0.0927	0.3070	0.2305	0.1699	0.0520
NM_133406	Agpat4	0.9258	0.7616	0.7324	0.8201	0.5277	0.7908	0.7917	0.5723	0.6313	0.8194	0.1107	0.9279
NM_133311	Il24	0.1953	0.5268	0.2565	0.1991	0.8711	0.3345	0.5149	0.3610	0.8146	0.5057	0.8587	0.9139
NM_133551	Pla2g4a	0.1264	0.1932	0.1051	0.0377	0.8037	0.7343	0.0118	0.5622	0.1804	0.2883	0.1009	0.2063
NM_139100	Slc25a3	0.9830	0.1934	0.8233	0.6060	0.6709	0.8654	0.9176	0.4407	0.2924	0.1283	0.0602	0.4122
NM_133305	Ap3m2	0.6975	0.8314	0.5414	0.0784	0.6501	0.4265	0.4877	0.7189	0.4976	0.7234	0.5725	0.2658
NM_134455	Cx3cl1	0.2802	0.1872	0.4953	0.9419	0.4381	0.7943	0.0057	0.2893	0.2258	0.0019	0.0054	0.3287
NM_133429	Zfp384	0.9937	0.9787	0.5288	0.9209	0.9855	0.8887	0.7694	0.9318	0.9602	0.9908	0.9435	0.9496
NM_133298	Gpnmb	0.1476	0.0211	0.4607	0.8383	0.7160	0.1679	0.1800	0.5782	0.5574	0.0358	0.4151	0.0119
NM_133312	Prdm4	0.7312	0.4930	0.1899	0.1075	0.4863	0.8252	0.5026	0.3134	0.2834	0.1901	0.8485	0.7665
NM_133313	Atxn10	0.1374	0.1126	0.0586	0.0250	0.0130	0.0507	0.0375	0.0483	0.0095	0.0238	0.0695	0.5281
NM_139084	Magi3	0.8438	0.9965	0.9987	0.9914	0.9787	0.9493	0.7954	0.5908	0.9638	0.9992	0.9959	0.9956
NM_133314	Steap3	0.5891	0.4121	0.5779	0.4336	0.5118	0.4670	0.4686	0.5553	0.1650	0.3611	0.4989	0.3816
NM_133553	B3galt4	0.8596	0.6510	0.4744	0.6953	0.7877	0.7582	0.8353	0.8612	0.8367	0.8442	0.3767	0.7468
NM_133545	Ptpn21	0.9398	0.9027	0.8979	0.9900	0.9367	0.9143	0.7008	0.9463	0.9121	0.9496	0.9446	0.5271
NM_133318	Khdrbs2	0.7674	0.4524	0.7510	0.6488	0.7080	0.2756	0.5402	0.6056	0.6203	0.7260	0.8953	0.4202
NM_139112	Lmo1	0.9340	0.9891	0.8338	0.7214	0.2464	0.5208	0.9442	0.1086	0.9401	0.8276	0.9389	0.8090
NM_139107	Tmem150	0.7527	0.8206	0.9366	0.9331	0.9457	0.9770	0.9973	0.9712	0.9535	0.9668	0.8871	0.9622
NM_133323	Zfp111	0.7907	0.8496	0.2086	0.9013	0.9500	0.6721	0.8693	0.9022	0.7125	0.6438	0.8184	0.7658
NM_138826	Mt1a	0.0228	0.0460	0.1376	0.1285	0.8587	0.1770	0.0253	0.6572	0.0108	0.0236	0.0956	0.0360
NM_138837	Pou3f3	0.5837	0.3545	0.7467	0.1915	0.4613	0.6065	0.3491	0.6220	0.3292	0.4407	0.4694	0.6907
NM_001135720	LOC688452	0.3839	0.2872	0.1759	0.4691	0.3562	0.4395	0.1782	0.3571	0.1645	0.3478	0.1660	0.2711
NM_134376	Clstn3	0.1717	0.1657	0.1759	0.1752	0.1765	0.1730	0.1781	0.1707	0.1644	0.1599	0.1660	0.1708
NM_133385	Ucn2	0.5612	0.7822	0.5344	0.5510	0.6823	0.7494	0.7800	0.6351	0.6141	0.4418	0.6806	0.6380
NM_001042505	Pitx2	0.4986	0.5690	0.7241	0.9550	0.7959	0.8541	0.2889	0.8691	0.5648	0.6746	0.8601	0.2922
NM_138827	Slc2a1	0.7580	0.1979	0.2074	0.5736	0.5839	0.6677	0.4538	0.7256	0.6521	0.7085	0.2641	0.2137
NM_133321	Kcnj15	0.4701	0.8067	0.6105	0.7606	0.8972	0.5440	0.8209	0.7590	0.7680	0.7301	0.7567	0.7518
NM_134340	Clk3	0.8251	0.9306	0.6728	0.1694	0.6220	0.9011	0.5227	0.3609	0.8663	0.7979	0.7548	0.3887
NM_133380	Il4ra	0.4511	0.9072	0.9008	0.3094	0.5992	0.3988	0.4707	0.8747	0.8986	0.1370	0.6457	0.8866
NM_134395	Tmem14c	0.2840	0.7264	0.8278	0.1974	0.3054	0.4185	0.7066	0.2882	0.6243	0.8821	0.9137	0.3129
NM_133519	Il11	0.2288	0.3865	0.3420	0.2588	0.3659	0.2264	0.4166	0.3833	0.3180	0.4349	0.3885	0.4522
NM_133383	Scpep1	0.0825	0.0904	0.5009	0.0256	0.3393	0.1430	0.0513	0.0729	0.2381	0.1605	0.1299	0.1739
NM_139185	Gng8	0.1294	0.1815	0.3038	0.3849	0.2718	0.2421	0.2555	0.6982	0.1669	0.6748	0.5631	0.0962
NM_133402	Nap1l3	0.9582	0.9766	0.3995	0.8778	0.6323	0.7277	0.8119	0.7445	0.4200	0.9674	0.5401	0.4877
NM_139331	Lrit1	0.4403	0.6663	0.7412	0.7688	0.4008	0.6986	0.2937	0.3443	0.5006	0.5488	0.7653	0.7565
NM_133605	Camk2g	0.8093	0.0161	0.1358	0.1269	0.7597	0.8865	0.0526	0.9228	0.1210	0.0250	0.5278	0.7024
NM_139255	P76	0.0868	0.0262	0.9234	0.1947	0.1675	0.9074	0.1945	0.7721	0.2940	0.0216	0.0173	0.8332
NM_139115	Coro6	0.6244	0.2439	0.3798	0.1468	0.9436	0.5469	0.3009	0.3988	0.7479	0.6433	0.8769	0.8640
NM_133395	Tpo1	0.6562	0.7235	0.6557	0.4553	0.6578	0.5870	0.6924	0.6533	0.6821	0.6630	0.6158	0.6668
NM_133405	Cry2	0.6390	0.2945	0.4422	0.7022	0.8150	0.7321	0.7578	0.3683	0.3743	0.8868	0.4881	0.8442
NM_139184	Sgp158	0.3616	0.5167	0.5597	0.2433	0.5728	0.1552	0.4440	0.5111	0.4205	0.8725	0.3535	0.6409
NM_133571	Cdc25a	0.6850	0.9682	0.3516	0.9453	0.9590	0.9776	0.7416	0.9755	0.3601	0.8243	0.7293	0.7947
NM_133618	Hadhb	0.2470	0.2065	0.6604	0.9934	0.7976	0.7091	0.6224	0.6328	0.7499	0.3491	0.3504	0.8682
NM_133398	Mtdh	0.8812	0.6914	0.8459	0.9080	0.6606	0.7895	0.6409	0.8962	0.2297	0.9005	0.8940	0.5075

NM_133594	Sumo2	0.5899	0.6227	0.6752	0.8487	0.8478	0.3608	0.7580	0.3823	0.4852	0.4767	0.3261	0.4017
NM_134349	Mgst1	0.0044	0.0387	0.0068	0.0697	0.0180	0.0108	0.0417	0.0527	0.1141	0.0871	0.0725	0.0045
NM_133412	Slco6b1	0.8312	0.0316	0.1835	0.5035	0.2171	0.2232	0.1835	0.5214	0.2378	0.4801	0.2180	0.2764
NM_133414	Clcc1	0.9470	0.8853	0.8204	0.9274	0.9747	0.8161	0.5787	0.9064	0.9336	0.6184	0.5540	0.5935
NM_133418	Slc25a10	0.7756	0.4155	0.0167	0.3202	0.1890	0.7305	0.8632	0.8087	0.9420	0.5818	0.2482	0.5869
NM_133417	Syf2	0.8139	0.5788	0.9314	0.1855	0.6900	0.4099	0.3714	0.2520	0.6925	0.7306	0.6298	0.8349
NM_139257	Ly6b	0.7361	0.5697	0.6970	0.8954	0.2975	0.2813	0.6176	0.2673	0.7402	0.9419	0.4094	0.8841
NM_133423	Yt521	0.9423	0.8980	0.7710	0.3949	0.9955	0.8764	0.1675	0.9382	0.9646	0.9662	0.8160	0.8984
NM_139261	Hspbp1	0.0399	0.0490	0.4478	0.7950	0.2385	0.1052	0.1294	0.4073	0.0534	0.1608	0.3164	0.4998
NM_133425	Ppp1r14c	0.2595	0.2805	0.0956	0.1502	0.1668	0.1374	0.1612	0.0058	0.3001	0.2585	0.2886	0.8264
NM_133572	Cdc25b	0.6050	0.8570	0.2085	0.6407	0.9672	0.9316	0.8729	0.8907	0.9736	0.7355	0.8966	0.4135
NM_133426	Kcng3	0.4754	0.4350	0.6794	0.3052	0.5923	0.3807	0.4596	0.3185	0.5347	0.5799	0.4136	0.6758
NM_138863	Ptgr1	0.0171	0.0250	0.0203	0.0495	0.0336	0.0721	0.0011	0.0412	0.0416	0.0918	0.0243	0.0487
NM_133428	Hrg	0.4758	0.2423	0.0122	0.0432	0.3769	0.0121	0.1119	0.0303	0.1171	0.1075	0.2790	0.1105
NM_133441	Shank2	0.8295	0.8358	0.8412	0.8812	0.6399	0.8180	0.7678	0.6648	0.8974	0.7852	0.4408	0.7264
NM_134351	Mat2a	0.3462	0.3097	0.7947	0.9737	0.7247	0.8201	0.2272	0.8218	0.5949	0.5017	0.8373	0.8417
NM_133440	Shank2	0.8295	0.8358	0.8412	0.8812	0.6399	0.8180	0.7678	0.6648	0.8974	0.7852	0.4408	0.7264
NM_139339	Slc36a2	0.5716	0.4720	0.2107	0.6596	0.4312	0.2329	0.7256	0.5451	0.4978	0.3148	0.6897	0.3547
NM_133513	Muc10	0.1050	0.0175	0.0717	0.2731	0.0462	0.0562	0.1181	0.1295	0.0704	0.3722	0.0424	0.1642
NM_133515	Akap5	0.1607	0.4702	0.5440	0.5863	0.1116	0.2392	0.4942	0.4920	0.4164	0.5533	0.4604	0.4600
NM_145770	Acox2	0.1371	0.0523	0.1200	0.3027	0.6873	0.0978	0.2285	0.1771	0.2104	0.2530	0.4169	0.1498
NM_133522	Sstr3	0.3529	0.3638	0.7279	0.4349	0.5581	0.5550	0.7325	0.5643	0.6127	0.3449	0.6052	0.6989
NM_138886	Ick	0.4800	0.3389	0.3891	0.1749	0.2631	0.3817	0.1780	0.1705	0.7218	0.5909	0.3572	0.2620
NM_133526	Tspan8	0.9389	0.9558	0.8063	0.9551	0.8966	0.8772	0.8860	0.7431	0.7901	0.8096	0.8402	0.8393
NM_133525	RGD620382	0.1008	0.8507	0.4488	0.7044	0.7799	0.6368	0.7395	0.6925	0.7196	0.8769	0.8063	0.5078
NM_133524	Tcfe2a	0.8033	0.7013	0.4874	0.4137	0.5227	0.3850	0.5069	0.9718	0.9249	0.6417	0.8848	0.5399
NM_147206	Cyp3a9	0.0471	0.8837	0.0074	0.0962	0.0027	0.0368	0.1162	0.0444	0.1454	0.2399	0.0837	0.0087
NM_133527	Folr1	0.6078	0.1728	0.6949	0.5339	0.7794	0.6199	0.5829	0.5309	0.5731	0.7737	0.4813	0.5303
NM_138539	Dnase2a	0.0139	0.1063	0.6848	0.0762	0.0914	0.0205	0.0460	0.1933	0.1355	0.2545	0.0182	0.0687
NM_138502	Mgll	0.7351	0.8842	0.8820	0.7391	0.8688	0.9942	0.9656	0.9466	0.8052	0.8588	0.7390	0.7378
NM_175757	Cryl1	0.4332	0.3293	0.8184	0.1443	0.1336	0.0749	0.2801	0.0313	0.0579	0.2742	0.1495	0.0823
NM_175595	Cacna2d3	0.7850	0.4298	0.5089	0.3335	0.5359	0.1898	0.8311	0.3645	0.5845	0.4095	0.3534	0.6597
NM_133536	Rab3c	0.1312	0.1584	0.2381	0.3887	0.1949	0.1625	0.3494	0.2308	0.1702	0.3825	0.1353	0.3940
NM_133538	Il13ra2	0.0533	0.0692	0.1232	0.1546	0.5315	0.6193	0.1717	0.0505	0.4021	0.4079	0.1548	0.2465
NM_133539	Mrpl17	0.6206	0.8940	0.8022	0.8042	0.7675	0.5362	0.9456	0.7888	0.6976	0.7333	0.8020	0.1473
NM_133540	Nkg7	0.2407	0.3029	0.1467	0.3056	0.2115	0.3576	0.2137	0.3713	0.1848	0.0801	0.1260	0.3972
NM_001115021	Qk	0.3348	0.5273	0.6761	0.5114	0.3612	0.6109	0.4680	0.7080	0.5484	0.5025	0.6118	0.4511
NM_177482	Psbpc1	0.8197	0.3086	0.3634	0.0378	0.6737	0.4802	0.3706	0.2874	0.5159	0.6733	0.4391	0.3402
NM_177928	Nampt	0.8237	0.9604	0.9148	0.8717	0.9461	0.9792	0.7627	0.9503	0.9857	0.8664	0.9235	0.8498
NM_133541	Gtf3c1	0.8055	0.8973	0.0613	0.1835	0.9201	0.7294	0.7053	0.7728	0.6877	0.6744	0.9123	0.9674
NM_138884	Akr1d1	0.8364	0.4910	0.6743	0.5253	0.8935	0.4906	0.5650	0.4501	0.6472	0.4069	0.0983	0.7511
NM_133542	Igsf6	0.5668	0.4582	0.4037	0.1352	0.8438	0.0289	0.0973	0.5651	0.8320	0.3841	0.1988	0.0201
NM_145088	Rmt1	0.1023	0.3430	0.3560	0.5819	0.6079	0.8714	0.9195	0.4356	0.7282	0.5614	0.0980	0.4622
NM_138900	C1s	0.0741	0.2258	0.0413	0.0512	0.1838	0.1213	0.1192	0.0207	0.2335	0.2890	0.0372	0.1515
NM_134332	Gzmc	0.5410	0.2425	0.1341	0.3078	0.1439	0.3294	0.5240	0.6064	0.3183	0.4160	0.3055	0.3899
NM_133544	Usmg5	0.4161	0.4436	0.1181	0.0031	0.0802	0.0876	0.0033	0.0461	0.1333	0.2543	0.0581	0.0798
NM_134359	Ppp4c	0.4813	0.0872	0.5454	0.9304	0.3958	0.1373	0.7870	0.7669	0.4725	0.1301	0.7019	0.1618
NM_133548	Hdgfrp2	0.6973	0.6782	0.0266	0.0038	0.1038	0.0710	0.0035	0.4087	0.2150	0.2306	0.0093	0.0114
NM_133586	Ces2	0.0246	0.0729	0.5798	0.5648	0.0234	0.0124	0.3773	0.0580	0.0964	0.0349	0.1113	0.1276

NM_138710	Dab2ip	0.9256	0.9167	0.9340	0.9820	0.2742	0.3297	0.9505	0.2854	0.9696	0.7807	0.8431	0.7305
NM_133554	Slc17a1	0.5096	0.8632	0.4607	0.6336	0.3894	0.1577	0.6778	0.3057	0.6351	0.6546	0.8115	0.7228
NM_134366	Rac1	0.0854	0.4452	0.8310	0.8682	0.0133	0.1152	0.1392	0.2467	0.0510	0.4332	0.7448	0.8020
NM_133556	Atp5g2	0.8476	0.4239	0.8398	0.8742	0.7105	0.5426	0.7207	0.8441	0.3521	0.4743	0.3724	0.1053
NM_173317	Vom2r40	0.3158	0.7054	0.4873	0.5449	0.3236	0.3193	0.5448	0.4394	0.2043	0.6141	0.5474	0.2428
NM_173127	LOC286911	0.2448	0.1446	0.0501	0.4149	0.8245	0.3882	0.0747	0.6498	0.3184	0.3662	0.5814	0.1361
NM_181693	Adam28	0.8233	0.6682	0.4549	0.3865	0.6104	0.3430	0.5442	0.0457	0.6864	0.7063	0.6233	0.6409
NM_147211	Wipf3	0.6925	0.7081	0.6401	0.6642	0.6232	0.5935	0.4640	0.7214	0.6585	0.3406	0.6648	0.6573
NM_133559	Pcsk4	0.5426	0.6609	0.6874	0.6500	0.5872	0.7939	0.9452	0.6801	0.8260	0.8198	0.6609	0.3751
NM_133561	Brp44l	0.1850	0.8865	0.1061	0.4878	0.1279	0.0725	0.5735	0.1308	0.4693	0.6142	0.3169	0.1469
NM_133606	Ehhadh	0.6456	0.6476	0.2119	0.2368	0.6108	0.7265	0.3927	0.6826	0.5593	0.6925	0.5743	0.4198
NM_133566	Cst6	0.2667	0.3568	0.3268	0.5145	0.2040	0.2550	0.1996	0.4065	0.3919	0.0702	0.5611	0.3769
NM_145679	Lrrc3	0.0906	0.0617	0.0952	0.4407	0.3062	0.1227	0.1750	0.2537	0.1248	0.0294	0.0425	0.1069
NM_133568	Rasd2	0.6124	0.4866	0.8197	0.5405	0.0851	0.4008	0.4768	0.1740	0.4489	0.7798	0.1905	0.3478
NM_133569	Angptl2	0.9456	0.6830	0.7990	0.2261	0.0251	0.0090	0.8669	0.0771	0.9788	0.7354	0.8915	0.9223
NM_133570	Grp	0.9282	0.7164	0.8458	0.5162	0.7461	0.7648	0.5444	0.8163	0.6723	0.9681	0.5272	0.3508
NM_134330	Keg1	0.9647	0.7084	0.6205	0.5000	0.5781	0.0813	0.4278	0.6742	0.1069	0.8744	0.2161	0.5000
NM_134329	Adh7	0.3783	0.6709	0.5413	0.5816	0.4629	0.2214	0.5416	0.6401	0.6920	0.2697	0.7075	0.2086
NM_134331	Epha7	0.0066	0.1451	0.0029	0.0027	0.7147	0.6305	0.0002	0.8767	0.0001	0.0258	0.1126	0.3239
NM_173124	Cyp4f5	0.1091	0.1914	0.1757	0.5218	0.3021	0.4060	0.3453	0.3491	0.2573	0.3815	0.1049	0.1662
NM_134345	Cox8a	0.1451	0.0136	0.1074	0.0191	0.2082	0.0141	0.3342	0.0671	0.0290	0.0514	0.0156	0.3405
NM_133577	P2ry14	0.8835	0.8083	0.6462	0.6750	0.8458	0.4629	0.3753	0.6152	0.5195	0.2121	0.4901	0.8336
NM_133575	Il1rl2	0.5987	0.4395	0.5468	0.7000	0.4772	0.5055	0.8152	0.3055	0.6701	0.5997	0.4980	0.6180
NM_133580	Rab26	0.2110	0.6300	0.6193	0.4090	0.3705	0.6404	0.5446	0.2780	0.6092	0.1969	0.4847	0.7526
NM_133582	Blcap	0.9952	0.9795	0.8882	0.9935	0.9667	0.9208	0.7707	0.9547	0.8895	0.9692	0.9971	0.9746
NM_133581	Wfdc1	0.8817	0.7967	0.7853	0.8234	0.9906	0.8940	0.8807	0.9234	0.8277	0.9267	0.8089	0.9275
NM_173339	Ceacam10	0.0905	0.7067	0.1431	0.0323	0.0306	0.0308	0.7223	0.2179	0.7464	0.6463	0.7779	0.8844
NM_134389	Acsbg1	0.4171	0.1024	0.5489	0.4840	0.5167	0.6123	0.5067	0.4644	0.1711	0.5444	0.1343	0.2977
NM_134367	Ppp3cc	0.6114	0.2649	0.4240	0.3933	0.2380	0.3509	0.3130	0.3396	0.2349	0.3128	0.2937	0.4212
NM_138882	Pla1a	0.1135	0.1455	0.2885	0.1079	0.0609	0.0355	0.3338	0.1056	0.1100	0.1021	0.2981	0.0942
NM_138875	Jund	0.2426	0.9407	0.6064	0.9745	0.1739	0.6933	0.8777	0.3114	0.7359	0.7996	0.7453	0.9861
NM_138836	Prss8	0.6943	0.5032	0.6654	0.5077	0.7570	0.4322	0.6082	0.8049	0.4776	0.6939	0.6429	0.1212
NM_134391	Defb22	0.3744	0.2400	0.1325	0.0608	0.6006	0.3242	0.1336	0.0829	0.5036	0.3256	0.7537	0.4002
NM_133600	Slc31a1	0.7049	0.4947	0.0395	0.9154	0.3347	0.0533	0.7857	0.1289	0.6833	0.8296	0.6495	0.0478
NM_133587	Dscam	0.0941	0.2270	0.2706	0.2173	0.4651	0.3018	0.2784	0.1010	0.5246	0.4004	0.5375	0.2900
NM_145087	Spag11b	0.2908	0.1092	0.5705	0.4119	0.3903	0.2413	0.3774	0.8208	0.8248	0.7885	0.6580	0.5329
NM_133591	Rph3al	0.3204	0.6503	0.4593	0.6206	0.3477	0.4020	0.3756	0.4398	0.0670	0.3884	0.1998	0.6318
NM_145786	Iiig9	0.0626	0.3105	0.2201	0.6153	0.5719	0.1525	0.3456	0.3536	0.4409	0.6478	0.1939	0.1332
NM_133602	Khsrp	0.1862	0.4190	0.3269	0.2624	0.1022	0.4360	0.4183	0.4004	0.4484	0.5939	0.2187	0.3652
NM_134371	Trpm8	0.6323	0.2745	0.1607	0.4916	0.6925	0.9093	0.4470	0.1757	0.2582	0.1662	0.6059	0.2588
NM_133593	Ap3m1	0.3236	0.5948	0.8340	0.9478	0.6262	0.9710	0.7539	0.5602	0.7099	0.4331	0.4936	0.7408
NM_145790	Lipogenin	0.8282	0.9847	0.0082	0.0665	0.6887	0.8783	0.9692	0.7069	0.7951	0.9207	0.8508	0.5918
NM_134356	Ptprg	0.1162	0.0544	0.0155	0.5958	0.0613	0.1468	0.0021	0.0667	0.0202	0.0218	0.0933	0.0126
NM_001110335	Vegfa	0.7331	0.7016	0.8891	0.5415	0.5108	0.5606	0.9797	0.4868	0.5533	0.6890	0.7006	0.9767
NM_001107312	Mtmr7	0.8003	0.8849	0.2774	0.2121	0.1339	0.8598	0.7662	0.6549	0.7155	0.9004	0.7071	0.9526
NM_001115035	Eif3i	0.4426	0.7611	0.3309	0.3689	0.0419	0.0861	0.8854	0.3363	0.6066	0.4774	0.5308	0.1179
NM_001108894	Nkd1	0.6777	0.6398	0.4500	0.4505	0.5457	0.4883	0.7833	0.2622	0.6281	0.8212	0.5906	0.1325
NM_001107178	Ppp1r12b	0.2551	0.7718	0.8731	0.6398	0.2089	0.7747	0.6550	0.1638	0.7760	0.8448	0.4362	0.3391
NM_001105967	Lmx1a	0.5746	0.5924	0.4183	0.5115	0.6741	0.2290	0.9275	0.4564	0.7424	0.5468	0.9052	0.7648

NM_147142	LOC257650	0.9433	0.9246	0.4559	0.2466	0.9987	0.9076	0.7837	0.9173	0.9856	0.5251	0.6737	0.7442
NM_133596	Ugcgl1	0.8394	0.9205	0.7149	0.9579	0.7504	0.9722	0.9848	0.8738	0.9378	0.7932	0.9060	0.9137
NM_138828	Apoe	0.2687	0.0043	0.1086	0.0028	0.5595	0.5821	0.0291	0.5637	0.0514	0.1053	0.0046	0.2860
NM_133598	Gcsh	0.0034	0.8816	0.0431	0.6611	0.6296	0.5333	0.8620	0.3840	0.5398	0.9619	0.8198	0.1733
NM_133608	Slco4a1	0.7450	0.4269	0.1465	0.1426	0.3807	0.0797	0.1739	0.2843	0.1292	0.2740	0.0688	0.3464
NM_147207	Vof16	0.8620	0.7752	0.2019	0.2928	0.8652	0.8113	0.2512	0.9209	0.9860	0.9875	0.9224	0.6299
NM_147212	LOC259244	0.2419	0.4292	0.3280	0.1742	0.6476	0.2559	0.3606	0.4027	0.6543	0.3689	0.3748	0.2972
NM_147213	LOC259245	0.2642	0.4313	0.2679	0.1741	0.6529	0.2758	0.4235	0.3691	0.4165	0.4779	0.3074	0.3302
NM_133599	Lgals2	0.2143	0.4307	0.0061	0.5668	0.4635	0.4350	0.1308	0.3923	0.6618	0.6580	0.1700	0.0576
NM_138532	Nme7	0.1774	0.4764	0.9700	0.3684	0.4979	0.5706	0.2562	0.6280	0.1803	0.2878	0.0944	0.2019
NM_133611	Slc2a13	0.5966	0.7277	0.1520	0.5769	0.5633	0.4402	0.4569	0.8838	0.8292	0.8208	0.2293	0.2126
NM_133614	Slc25a21	0.0853	0.6953	0.6413	0.6566	0.9346	0.3766	0.7379	0.4530	0.6122	0.9096	0.7146	0.5406
NM_133617	Serpina10	0.5555	0.2211	0.0551	0.5139	0.1683	0.4279	0.6639	0.0140	0.9394	0.5650	0.3220	0.0586
NM_153306	Rbm45	0.9040	0.6714	0.3259	0.8536	0.7771	0.8611	0.4519	0.6451	0.2753	0.3419	0.1318	0.1213
NM_134466	Stau2	0.9866	0.9922	0.9993	0.9988	0.9995	0.9973	0.9495	0.9766	0.8819	0.9431	0.9996	0.9910
NM_153466	Gzmf	0.1300	0.0637	0.0798	0.0077	0.0640	0.0951	0.0948	0.1668	0.2724	0.0708	0.0987	0.1540
NM_153476	Krt9	0.1710	0.1654	0.1751	0.1739	0.1761	0.1726	0.1775	0.1701	0.1640	0.1599	0.1657	0.1703
NM_133619	Gpha2	0.2896	0.2200	0.7146	0.5927	0.4739	0.5556	0.3847	0.4927	0.1424	0.6445	0.1675	0.5374
NM_133623	Slc6a13	0.4220	0.7765	0.4821	0.4567	0.4120	0.4568	0.4969	0.4452	0.2076	0.5169	0.1399	0.6818
NM_144745	Gpsm1	0.7982	0.8882	0.9502	0.6745	0.8739	0.8791	0.8726	0.7159	0.8412	0.5383	0.7316	0.8488
NM_138905	Ppap2b	0.9769	0.9993	0.9877	0.9944	0.9968	0.9988	0.9981	0.9950	0.9865	0.9998	0.9995	0.9953
NM_173123	Cyp4f4	0.3929	0.4420	0.0475	0.5398	0.4196	0.5312	0.6696	0.5003	0.3095	0.5115	0.6745	0.0689
NM_138883	Atp5o	0.1289	0.2839	0.1344	0.2812	0.1231	0.0988	0.0045	0.1170	0.0209	0.0439	0.0675	0.0093
NM_138512	Cyp2c22	0.9102	0.8136	0.7633	0.8948	0.8470	0.3781	0.5724	0.6722	0.8775	0.8201	0.7572	0.6222
NM_138521	Ppp1r1b	0.7987	0.7492	0.5626	0.5720	0.6439	0.3764	0.7721	0.5687	0.8423	0.8426	0.8777	0.7602
NM_138508	Scp2	0.1489	0.0757	0.9031	0.9616	0.2793	0.1768	0.1287	0.2709	0.0734	0.1846	0.1751	0.2976
NM_138876	Ampd1	0.1615	0.6875	0.1304	0.0684	0.1930	0.0569	0.2716	0.4809	0.5441	0.2540	0.5108	0.1842
NM_147205	St6gal1	0.9835	0.8917	0.9750	0.9664	0.9976	0.9834	0.9714	0.9879	0.9564	0.9831	0.9948	0.9713
NM_134353	Pabpc1	0.1351	0.2243	0.0582	0.1774	0.8500	0.6711	0.7678	0.1228	0.2584	0.1221	0.3949	0.4318
NM_134432	Agt	0.6171	0.5281	0.7649	0.6836	0.1753	0.0366	0.1191	0.1509	0.8543	0.5637	0.8107	0.6713
NM_134350	Mx2	0.3748	0.0478	0.2212	0.0906	0.0122	0.1061	0.0331	0.2177	0.3027	0.2404	0.2329	0.0939
NM_138538	Dnm3	0.4954	0.4692	0.5737	0.2639	0.4070	0.4225	0.2652	0.2912	0.5293	0.3325	0.6062	0.4242
NM_134336	Nlgn3	0.5244	0.6264	0.4717	0.8244	0.5990	0.5334	0.3159	0.4925	0.2307	0.6683	0.1288	0.8313
NM_138877	Cyb5r3	0.7170	0.3380	0.8414	0.7222	0.7132	0.3629	0.9031	0.6881	0.3565	0.3105	0.1289	0.7831
NM_153628	Sec11c	0.0017	0.5401	0.0051	0.0086	0.0633	0.0375	0.4359	0.0034	0.6338	0.7263	0.4231	0.2141
NM_138823	Ppp1r2	0.2750	0.9652	0.9649	0.3123	0.1946	0.6703	0.5175	0.1355	0.9399	0.9809	0.9913	0.9776
NM_138543	Pde9a	0.1276	0.0287	0.3669	0.1848	0.0688	0.0068	0.6470	0.1150	0.2236	0.3043	0.3795	0.2850
NM_138910	Dad1	0.1848	0.0635	0.5130	0.5828	0.2633	0.1307	0.8923	0.5261	0.0838	0.1578	0.0362	0.0715
NM_138901	Pigl	0.9251	0.8627	0.4167	0.6731	0.8415	0.6072	0.8456	0.9423	0.4635	0.6246	0.5191	0.6698
NM_153316	Slc35e4	0.0070	0.9291	0.1766	0.2004	0.7950	0.7470	0.1176	0.8912	0.7701	0.6459	0.7442	0.8162
NM_153630	Nalcn	0.4885	0.6081	0.6281	0.5681	0.6138	0.2125	0.6396	0.6218	0.6365	0.7002	0.5708	0.4758
NM_134365	Atp5f1	0.4021	0.3571	0.3857	0.4956	0.4300	0.4072	0.3794	0.5042	0.5548	0.3481	0.2667	0.2257
NM_138514	Cyp2c13	0.2379	0.0682	0.1318	0.2525	0.2757	0.4517	0.0053	0.1464	0.2706	0.1590	0.0836	0.0573
NM_153471	Rnf40	0.5459	0.6378	0.5923	0.9041	0.8399	0.6334	0.9074	0.8889	0.5960	0.8815	0.6723	0.9195
NM_153724	Rcan1	0.0719	0.1081	0.1673	0.0682	0.1993	0.1304	0.1397	0.0304	0.1164	0.1287	0.1411	0.1413
NM_134377	Clstn2	0.5286	0.5506	0.6734	0.8566	0.7360	0.4570	0.6752	0.7510	0.8546	0.8124	0.8501	0.8489
NM_138913	Oas1a	0.2316	0.5900	0.1072	0.0295	0.0087	0.1340	0.2703	0.0016	0.1567	0.6026	0.5018	0.6565
NM_138908	Slc22a25	0.6485	0.3287	0.4685	0.5332	0.2804	0.5229	0.3745	0.2301	0.6811	0.2186	0.6084	0.2150
NM_134374	Rnf39	0.7248	0.2706	0.3540	0.0986	0.3982	0.4851	0.4855	0.7599	0.8554	0.3867	0.3977	0.1057

NM_134373	Avpi1	0.0170	0.3138	0.2267	0.1807	0.0491	0.0165	0.5157	0.2928	0.2760	0.1355	0.2348	0.1951
NM_134378	Sulf1	0.0084	0.1850	0.1296	0.0865	0.2817	0.2756	0.0170	0.2992	0.0660	0.2111	0.0076	0.3140
NM_153623	Cdig1l	0.5025	0.5830	0.2213	0.3579	0.4590	0.5837	0.5924	0.6993	0.2037	0.1892	0.2144	0.4851
NM_134385	Prl8a9	0.6187	0.4147	0.5665	0.3271	0.5608	0.3891	0.4024	0.0227	0.1301	0.1520	0.6571	0.0419
NM_134383	Elovl6	0.5546	0.8363	0.8453	0.6189	0.7127	0.8143	0.7856	0.5078	0.5319	0.8234	0.5245	0.6788
NM_134388	Tnnt1	0.1789	0.0703	0.2685	0.6277	0.3855	0.1590	0.5108	0.6706	0.5139	0.3401	0.0840	0.5343
NM_153734	Cst12	0.8558	0.7558	0.5405	0.6121	0.7929	0.7214	0.8562	0.7812	0.8583	0.5751	0.7984	0.7399
NM_181632	Syt15	0.5367	0.4629	0.7226	0.5292	0.6460	0.7787	0.8614	0.6277	0.2667	0.3080	0.3354	0.6119
NM_181631	Fbxo11	0.9886	0.9915	0.9936	0.9895	0.9940	0.9849	0.9334	0.9905	0.6776	0.9891	0.9919	0.6733
NM_134400	Dnttip1	0.4921	0.5005	0.2483	0.3639	0.2249	0.2812	0.3008	0.4738	0.4148	0.4264	0.3230	0.4367
NM_138524	A3galt2	0.9021	0.3727	0.5390	0.9383	0.4973	0.5976	0.7804	0.4211	0.4143	0.4404	0.1771	0.9024
NM_138515	Cyp2d4v1	0.6380	0.7451	0.4334	0.5542	0.3721	0.5094	0.3014	0.1046	0.5544	0.8784	0.8287	0.7684
NM_153726	Ly49s3	0.2151	0.2155	0.3421	0.3948	0.2609	0.2279	0.2637	0.4185	0.4034	0.3341	0.2481	0.4149
NM_134392	Spata6	0.1536	0.1309	0.8638	0.2155	0.5433	0.6767	0.4530	0.0708	0.0614	0.0364	0.7282	0.3191
NM_153730	Klhl12	0.1368	0.8336	0.8203	0.4566	0.7370	0.5189	0.6693	0.5235	0.7226	0.9358	0.8013	0.7855
NM_134402	Bzw2	0.7146	0.6771	0.7281	0.7164	0.7198	0.7023	0.7190	0.7225	0.8087	0.9344	0.7233	0.8063
NM_134393	Pmfbp1	0.1054	0.8073	0.1022	0.2120	0.2295	0.0573	0.4739	0.5925	0.1545	0.4617	0.3919	0.7765
NM_134403	Abtb2	0.2966	0.0833	0.2652	0.0119	0.2985	0.3170	0.2341	0.1413	0.1208	0.1803	0.0113	0.7609
NM_138878	Nedd8	0.5507	0.3520	0.8403	0.9036	0.5782	0.2091	0.6347	0.6611	0.8721	0.6830	0.7649	0.5519
NM_134404	Svop	0.4344	0.2541	0.1681	0.6906	0.5235	0.3536	0.5241	0.6701	0.3848	0.3639	0.2545	0.4939
NM_134407	Akr7a2	0.8808	0.7885	0.8736	0.8230	0.7482	0.5346	0.7339	0.8297	0.7549	0.8808	0.7318	0.2559
NM_134406	Pacs1	0.8664	0.1759	0.9639	0.8668	0.8858	0.8827	0.9025	0.6020	0.5478	0.0617	0.1286	0.9943
NM_134408	Lphn2	0.8546	0.1655	0.9810	0.5437	0.9694	0.9910	0.2745	0.9983	0.0697	0.1787	0.2872	0.3809
NM_134409	Zg16	0.2391	0.4877	0.4211	0.1515	0.1505	0.5199	0.4417	0.5964	0.5252	0.8729	0.3488	0.4091
NM_134410	Mosc2	0.5663	0.7888	0.9930	0.7975	0.9178	0.7609	0.8279	0.6914	0.4755	0.8616	0.7598	0.6758
NM_138513	Calcb	0.0075	0.4800	0.5428	0.3341	0.2890	0.0808	0.1897	0.0049	0.6143	0.8110	0.5483	0.7396
NM_138833	Snrk	0.3982	0.4364	0.7053	0.4702	0.1044	0.8775	0.4476	0.6388	0.5790	0.2535	0.7427	0.9028
NM_138548	Nme1	0.2274	0.7798	0.0272	0.9220	0.5399	0.1832	0.2716	0.8195	0.6340	0.6786	0.8103	0.0639
NM_139103	Cd48	0.2124	0.0774	0.8349	0.3010	0.1391	0.5802	0.0107	0.0999	0.0040	0.0348	0.1144	0.0363
NM_139037	Tex101	0.0104	0.0677	0.0583	0.0580	0.0378	0.1505	0.4323	0.1928	0.2347	0.1363	0.0398	0.1151
NM_138509	Mapre1	0.2761	0.2330	0.9995	0.8252	0.2737	0.6826	0.2627	0.2205	0.2517	0.1616	0.2204	0.3482
NM_138843	Mpst	0.7618	0.8045	0.8525	0.7111	0.9832	0.6997	0.8689	0.8682	0.7539	0.8049	0.8719	0.7051
NM_138899	Cdipt	0.3703	0.5288	0.2115	0.7414	0.1658	0.6223	0.3870	0.0431	0.4312	0.1388	0.7887	0.9078
NM_175838	Eef1a1	0.2295	0.3610	0.5102	0.4811	0.5303	0.1725	0.4447	0.5440	0.4403	0.4769	0.7098	0.3712
NM_138518	Crispld2	0.6321	0.6692	0.6106	0.6018	0.6076	0.5057	0.5981	0.4167	0.4232	0.6275	0.2151	0.6946
NM_138520	Klc3	0.4615	0.4381	0.4586	0.2029	0.4857	0.5648	0.5642	0.1763	0.5479	0.5321	0.5047	0.3292
NM_138519	Dkk3	0.5532	0.7447	0.8516	0.9456	0.7084	0.7841	0.8889	0.1970	0.9685	0.8722	0.7988	0.7577
NM_172017	Yif1	0.4127	0.8768	0.2505	0.3715	0.1448	0.0027	0.4890	0.1518	0.1682	0.7148	0.6405	0.0242
NM_172021	Tbkbp1	0.5188	0.2791	0.4193	0.2168	0.7662	0.6845	0.3909	0.5411	0.6407	0.3488	0.8679	0.5813
NM_172022	Prosapip1	0.2030	0.7143	0.2396	0.2363	0.2688	0.5710	0.2085	0.0556	0.5302	0.2678	0.2070	0.8260
NM_134415	Cdk105	0.3630	0.6766	0.3894	0.0598	0.0757	0.1104	0.3269	0.0133	0.1756	0.3072	0.0838	0.4679
NM_134414	Exoc2	0.9565	0.5679	0.6659	0.1968	0.5257	0.6835	0.1611	0.8703	0.4040	0.4502	0.2880	0.2843
NM_172032	Txndc9	0.0777	0.0487	0.8591	0.0275	0.1046	0.0206	0.2396	0.0373	0.2602	0.0388	0.0793	0.2887
NM_134418	Gp2	0.2855	0.2443	0.5496	0.4138	0.2291	0.3957	0.4425	0.3136	0.6086	0.3447	0.3692	0.3275
NM_138872	Smad9	0.3474	0.5843	0.4510	0.3723	0.5145	0.0889	0.2023	0.6321	0.9356	0.2414	0.7834	0.1508
NM_138867	Hyou1	0.9714	0.8776	0.6214	0.8074	0.9299	0.7807	0.9519	0.5859	0.9481	0.4057	0.6329	0.9377
NM_134449	Prkcdbp	0.8935	0.7914	0.0539	0.9734	0.9129	0.7683	0.7354	0.8388	0.6998	0.8708	0.4702	0.0698
NM_173319	Vom2r31	0.0106	0.2756	0.7408	0.4105	0.2240	0.2716	0.2192	0.3659	0.3004	0.0453	0.1498	0.1304
NM_173099	Pcdhb12	0.5224	0.2265	0.5026	0.5367	0.4105	0.4865	0.4393	0.4051	0.2131	0.2254	0.5067	0.2195

NM_138915	Caly	0.0939	0.2379	0.5524	0.1642	0.4150	0.1308	0.3485	0.4130	0.0474	0.0504	0.4255	0.6967
NM_134452	Col5a1	0.7342	0.7327	0.6537	0.9016	0.7065	0.9991	0.7434	0.7030	0.9779	0.7313	0.7358	0.8876
NM_134457	Siah2	0.8484	0.9960	0.9365	0.9476	0.8885	0.6857	0.8789	0.9025	0.9867	0.8554	0.8943	0.6913
NM_134458	Psmc3ip	0.5795	0.8474	0.0828	0.7014	0.4682	0.9522	0.7805	0.8908	0.7555	0.8478	0.6518	0.8006
NM_153740	Sclt1	0.6471	0.3207	0.1720	0.2384	0.4684	0.4110	0.4215	0.4403	0.6064	0.3985	0.3902	0.1762
NM_138866	Eif2b5	0.1738	0.3563	0.1617	0.2305	0.1915	0.2882	0.5000	0.0409	0.1020	0.1450	0.1994	0.2791
NM_134461	Capn5	0.4355	0.6677	0.6554	0.5563	0.6653	0.7408	0.8144	0.7506	0.8179	0.7974	0.5007	0.8021
NM_172064	Prpg1	0.0573	0.0130	0.0085	0.0271	0.2276	0.0072	0.0918	0.0145	0.0047	0.3314	0.0205	0.1396
NM_134472	Kif2c	0.6964	0.8685	0.2184	0.7753	0.8635	0.9981	0.8439	0.9330	0.7084	0.9122	0.7554	0.4902
NM_138536	Ttl	0.0140	0.0620	0.1995	0.0749	0.6892	0.1540	0.4176	0.2578	0.1433	0.2299	0.0579	0.4216
NM_139036	Lhx5	0.7427	0.8711	0.7399	0.7103	0.7480	0.6328	0.8858	0.7866	0.6883	0.7121	0.9038	0.8142
NM_138902	Ear11	0.0361	0.1343	0.0268	0.0861	0.0385	0.0181	0.0371	0.0947	0.0296	0.1119	0.0927	0.0122
NM_138889	Cdh13	0.1985	0.2874	0.0335	0.2773	0.1714	0.1523	0.1791	0.2721	0.2120	0.1225	0.1003	0.2917
NM_001100821	Ccne1	0.0851	0.2459	0.1108	0.6291	0.2707	0.3788	0.9791	0.6616	0.7840	0.8919	0.4595	0.3437
NM_001109898	Qsox1	0.9931	0.9394	0.8940	0.7035	0.8908	0.9570	0.9871	0.9860	0.9969	0.7674	0.9824	0.8981
NM_001109890	Ptprc	0.8419	0.9679	0.9525	0.9107	0.9667	0.3282	0.6999	0.3839	0.8020	0.9482	0.9111	0.6854
NM_138523	Prpf18	0.7972	0.8255	0.8176	0.9293	0.8745	0.9066	0.8308	0.8771	0.9698	0.2686	0.7688	0.9906
NM_170787	Erc2	0.5728	0.4760	0.5373	0.5679	0.5279	0.4788	0.5163	0.4204	0.0784	0.3695	0.5221	0.4055
NM_138916	Crkrs	0.8825	0.9533	0.6086	0.6430	0.7702	0.7264	0.4901	0.8125	0.8688	0.9369	0.7244	0.5860
NM_138517	Gzmb	0.4402	0.5008	0.3560	0.4966	0.1548	0.3220	0.7667	0.4545	0.0865	0.5450	0.4323	0.6034
NM_138525	Mucdhl	0.1244	0.5366	0.0005	0.0163	0.9103	0.6094	0.0008	0.9027	0.7507	0.0991	0.2528	0.6352
NM_138527	Prl5a1	0.4074	0.1387	0.4404	0.3004	0.7447	0.4078	0.6487	0.1213	0.3604	0.2487	0.4459	0.4161
NM_138528	Ero1l	0.2173	0.0652	0.8259	0.2846	0.0801	0.6390	0.9114	0.1549	0.1991	0.2716	0.1747	0.9423
NM_138907	Acot2	0.3326	0.7421	0.8373	0.9366	0.9181	0.4106	0.2381	0.9623	0.3606	0.7700	0.8720	0.9653
NM_138904	Gls2	0.9070	0.8305	0.7803	0.7876	0.9584	0.9186	0.9977	0.8299	0.9977	0.9118	0.9638	0.9788
NM_173318	Vom2r18	0.6654	0.5680	0.5754	0.5208	0.2350	0.2169	0.2706	0.2268	0.5770	0.4745	0.6101	0.2203
NM_172324	Elavl3	0.0275	0.0347	0.1188	0.5157	0.2769	0.4473	0.0868	0.1346	0.3225	0.1524	0.2315	0.3605
NM_138535	Grip2	0.5517	0.3878	0.2892	0.1234	0.2337	0.1522	0.4818	0.1193	0.8642	0.0588	0.4879	0.5841
NM_139042	Gucy2g	0.1531	0.3338	0.3407	0.3137	0.1432	0.2475	0.3480	0.1177	0.5715	0.1168	0.2857	0.3630
NM_145776	Slc38a3	0.8402	0.2840	0.1850	0.4295	0.8891	0.8288	0.8110	0.7560	0.5811	0.5244	0.4967	0.8700
NM_138897	Gabrr3	0.3225	0.1488	0.5221	0.7354	0.5348	0.3319	0.5666	0.6415	0.3160	0.3996	0.6315	0.2412
NM_170789	Cd247	0.9370	0.9129	0.8241	0.9642	0.7191	0.9218	0.6346	0.9784	0.8706	0.8595	0.9729	0.8794
NM_170788	Erc1	0.6329	0.3712	0.5156	0.4473	0.7655	0.7911	0.3007	0.1691	0.7746	0.3953	0.0894	0.3503
NM_138541	Tacstd1	0.7528	0.3917	0.4458	0.4244	0.4391	0.4778	0.5091	0.1578	0.5159	0.2673	0.8006	0.3857
NM_138542	Rhov	0.3864	0.5297	0.7277	0.7438	0.6785	0.4138	0.8491	0.7818	0.7918	0.2805	0.1188	0.8917
NM_173301	LOC286960	0.1143	0.1480	0.1490	0.1879	0.1228	0.3692	0.0316	0.3677	0.5340	0.3818	0.3303	0.3582
NM_138549	Gpsn2	0.1095	0.1310	0.4393	0.1571	0.7542	0.6862	0.0132	0.8559	0.4972	0.1121	0.0140	0.3122
NM_138613	Zfp179	0.2519	0.3908	0.1724	0.3652	0.1738	0.0914	0.3319	0.1285	0.1486	0.1016	0.2577	0.3579
NM_138832	Slc38a1	0.0217	0.2807	0.0286	0.0096	0.1180	0.0989	0.5106	0.2204	0.3801	0.1985	0.0862	0.1370
NM_138831	Slc16a10	0.1720	0.5964	0.3127	0.6293	0.3535	0.1160	0.0929	0.0646	0.4834	0.5324	0.4732	0.4034
NM_144754	Cant1	0.5309	0.5653	0.9605	0.6502	0.1763	0.5005	0.8987	0.5179	0.4158	0.8572	0.8471	0.9766
NM_138922	Nos1ap	0.7449	0.8297	0.5319	0.4399	0.7179	0.7830	0.7752	0.7602	0.6520	0.4735	0.4253	0.7623
NM_145669	Fhl1	0.6911	0.7219	0.9625	0.5510	0.9715	0.9663	0.2366	0.9986	0.3474	0.6532	0.7460	0.9802
NM_145091	Pdp2	0.0796	0.0585	0.0605	0.0396	0.3083	0.1086	0.1082	0.0242	0.0380	0.0307	0.0149	0.0178
NM_138835	Syt12	0.9458	0.7927	0.6740	0.8088	0.8201	0.7616	0.8136	0.6528	0.7596	0.9715	0.6869	0.8931
NM_145184	Usp15	0.8622	0.9451	0.1852	0.0588	0.4466	0.1189	0.4070	0.1432	0.4470	0.8269	0.7229	0.0413
NM_145085	Pcyox1	0.0871	0.5510	0.9955	0.9741	0.9287	0.9040	0.9629	0.9833	0.7508	0.9411	0.6408	0.9206
NM_138839	Tmem49	0.9236	0.8003	0.4405	0.2650	0.9920	0.9009	0.0140	0.9603	0.8801	0.9329	0.6875	0.9338
NM_145673	Mafk	0.5338	0.8175	0.4418	0.0230	0.2847	0.2364	0.1313	0.3108	0.8514	0.7409	0.3809	0.9836

NM_145674	Ggta1	0.2002	0.0782	0.0755	0.0099	0.1261	0.0275	0.0071	0.0952	0.0227	0.1253	0.2917	0.0777
NM_001011908	Spta1	0.2466	0.2092	0.5774	0.3167	0.2063	0.7095	0.6350	0.2757	0.2286	0.2010	0.3778	0.2152
NM_138844	Unc13d	0.1303	0.1701	0.5917	0.5051	0.3530	0.3025	0.4959	0.3567	0.5231	0.3860	0.0329	0.7325
NM_138846	Gpm6b	0.6502	0.7241	0.2169	0.5349	0.7231	0.4941	0.7113	0.4883	0.6973	0.5047	0.5736	0.4160
NM_138847	Nip7	0.1334	0.5451	0.1155	0.0804	0.4614	0.7234	0.0256	0.6186	0.6720	0.9052	0.0939	0.7329
NM_147215	Obp3	0.2498	0.4680	0.2506	0.2140	0.6626	0.4298	0.4033	0.3716	0.6553	0.3945	0.7159	0.2910
NM_145774	Rab38	0.3195	0.0982	0.0559	0.3811	0.1054	0.2090	0.0916	0.0450	0.0267	0.4365	0.1336	0.0478
NM_145724	Znf394	0.7926	0.8307	0.7742	0.2583	0.6255	0.8707	0.5237	0.7298	0.7911	0.7732	0.9320	0.9617
NM_172019	Ifi47	0.0500	0.3276	0.3305	0.5451	0.3668	0.2397	0.0246	0.2076	0.0584	0.0133	0.1778	0.5886
NM_172010	Fgl1	0.3708	0.7818	0.8495	0.2077	0.1185	0.3431	0.9435	0.2499	0.8113	0.7532	0.8716	0.4624
NM_138854	Slc38a5	0.3830	0.3618	0.4578	0.3601	0.3758	0.4705	0.1850	0.3437	0.3397	0.4925	0.4289	0.4962
NM_138857	Prom2	0.2645	0.2486	0.1080	0.5009	0.2490	0.1054	0.1446	0.1716	0.3759	0.0846	0.2348	0.7349
NM_138858	Slc9a5	0.5787	0.8797	0.0220	0.1764	0.9662	0.8966	0.5808	0.9962	0.9886	0.7939	0.8472	0.7696
NM_152849	Homez	0.5871	0.2572	0.1274	0.4662	0.0915	0.2181	0.3828	0.7606	0.0863	0.0866	0.3989	0.0505
NM_138861	Prl2b1	0.5463	0.4693	0.3018	0.4708	0.4083	0.5477	0.1524	0.3118	0.7320	0.0806	0.6045	0.3940
NM_138864	Ccdc5	0.6391	0.9834	0.1112	0.4889	0.4220	0.9064	0.7736	0.9248	0.6470	0.3680	0.4727	0.3238
NM_177421	Slc22a17	0.2228	0.2285	0.2437	0.3118	0.7226	0.3529	0.7038	0.4657	0.5104	0.2273	0.2710	0.4265
NM_138848	Podxl	0.8872	0.7789	0.2453	0.5731	0.2365	0.1704	0.8637	0.3603	0.6261	0.9697	0.9646	0.9824
NM_138862	Spata7	0.0759	0.1141	0.3814	0.0090	0.4132	0.5803	0.1860	0.0106	0.7351	0.4826	0.8455	0.3172
NM_139080	Xrcc6	0.1754	0.7086	0.1006	0.1508	0.0675	0.0746	0.5331	0.3831	0.1172	0.5079	0.0669	0.2837
NM_172040	Hyal2	0.1464	0.6978	0.0041	0.0951	0.1166	0.0762	0.2315	0.2665	0.0166	0.0296	0.1106	0.1584
NM_153294	Npw	0.7858	0.5998	0.0280	0.8082	0.6164	0.5877	0.0623	0.4750	0.2935	0.6597	0.6537	0.0003
NM_139097	Scn3b	0.1441	0.1029	0.2445	0.0551	0.2022	0.0023	0.2018	0.0067	0.1963	0.1909	0.1166	0.3284
NM_170667	Rln3	0.5812	0.4314	0.5961	0.7603	0.5330	0.5466	0.8316	0.5812	0.5481	0.5359	0.7628	0.1677
NM_138871	Tdrd7	0.1617	0.0843	0.1560	0.4214	0.1515	0.3626	0.0276	0.0295	0.4927	0.0679	0.5640	0.6123
NM_138874	Csn1s1	0.5058	0.7394	0.4513	0.7487	0.9561	0.5092	0.8262	0.5245	0.4884	0.4638	0.5264	0.6587
NM_139041	Muc13	0.5962	0.5055	0.4133	0.4583	0.3634	0.4447	0.2583	0.6424	0.2091	0.6794	0.3342	0.5165
NM_138898	Phlpb	0.1560	0.1953	0.3243	0.2357	0.0834	0.0853	0.3139	0.2740	0.5879	0.4702	0.2273	0.1136
NM_139101	Alg10b	0.8609	0.7089	0.5618	0.0465	0.8044	0.8329	0.4079	0.8009	0.5890	0.3392	0.8058	0.8766
NM_139106	Atp5d	0.0759	0.1107	0.3040	0.7874	0.0221	0.0439	0.7595	0.0470	0.1026	0.0798	0.3109	0.0562
NM_172065	Prb1	0.5761	0.5059	0.8041	0.9325	0.6128	0.7416	0.1017	0.4453	0.7981	0.8519	0.7805	0.2762
NM_171994	Cdc42	0.8110	0.3950	0.5331	0.8773	0.1952	0.3054	0.3405	0.3345	0.4894	0.2189	0.7257	0.2674
NM_138879	Sele	0.2600	0.4405	0.4599	0.3360	0.3139	0.3483	0.8446	0.1552	0.5087	0.2837	0.6398	0.2209
NM_139256	Man2c1	0.4313	0.0147	0.2519	0.8040	0.0546	0.1752	0.8246	0.2712	0.4143	0.0578	0.1208	0.7865
NM_138888	Phf5a	0.7414	0.4662	0.2811	0.1267	0.4854	0.1769	0.8874	0.8200	0.2849	0.2284	0.1410	0.1933
NM_138887	Hspb6	0.2471	0.2980	0.0683	0.7825	0.9594	0.6128	0.4587	0.7261	0.2503	0.2799	0.3260	0.2493
NM_138890	Ehd3	0.0637	0.5192	0.3334	0.5559	0.3343	0.2635	0.2898	0.0484	0.3255	0.3383	0.1079	0.0696
NM_139342	Ripk3	0.3441	0.8719	0.0145	0.6861	0.0089	0.1009	0.3117	0.0515	0.5140	0.7697	0.6747	0.1402
NM_144730	Gata4	0.2396	0.1289	0.1320	0.1850	0.1773	0.0894	0.3725	0.1449	0.1299	0.2552	0.2578	0.4235
NM_173106	Csn1s2b	0.6848	0.5779	0.2566	0.3583	0.7643	0.5727	0.6244	0.7839	0.3841	0.6476	0.6287	0.6328
NM_138891	Gpr149	0.8529	0.8330	0.8854	0.9115	0.6369	0.8143	0.8048	0.7712	0.8674	0.7840	0.4969	0.4904
NM_138903	Obp1f	0.3972	0.3061	0.6436	0.6571	0.8006	0.4301	0.2248	0.6291	0.3374	0.3346	0.3761	0.5245
NM_138893	Myo16	0.3678	0.3648	0.3361	0.3547	0.5153	0.3654	0.1351	0.3700	0.5109	0.3455	0.7199	0.4049
NM_138894	Grasp	0.2771	0.8027	0.4702	0.7935	0.5624	0.8917	0.7764	0.6349	0.8104	0.3989	0.4194	0.4078
NM_175766	Cyp2j3	0.6344	0.8397	0.6317	0.6538	0.6582	0.6393	0.9415	0.8952	0.8666	0.8909	0.6730	0.6767
NM_175759	Klks3	0.2576	0.5234	0.6304	0.1504	0.2427	0.0065	0.5264	0.7666	0.6680	0.3505	0.3931	0.0228
NM_139090	Acvr1c	0.5622	0.8583	0.5572	0.4154	0.2680	0.0550	0.1217	0.1583	0.2801	0.2657	0.7203	0.3694
NM_173098	Slc9a4	0.7594	0.4761	0.3990	0.9372	0.7591	0.1403	0.5851	0.3101	0.2986	0.5548	0.6257	0.5958
NM_144561	Fmo4	0.3187	0.5663	0.3663	0.5890	0.5278	0.6561	0.6425	0.4522	0.6709	0.5044	0.4591	0.5355

NM_138906	Adam6	0.7754	0.8907	0.6959	0.7681	0.7378	0.7108	0.8767	0.7299	0.5320	0.9066	0.6961	0.1322
NM_173133	Phax	0.6945	0.1821	0.2599	0.6118	0.2448	0.4664	0.1263	0.3552	0.0653	0.1955	0.3820	0.2430
NM_173117	Dpy30	0.3667	0.2770	0.1468	0.6528	0.8693	0.4845	0.5864	0.8876	0.1446	0.2741	0.2585	0.0403
NM_138896	Pja2	0.4749	0.2571	0.9395	0.2991	0.2233	0.3841	0.2203	0.1650	0.3483	0.2946	0.3266	0.8440
NM_138873	Nbn	0.7596	0.9402	0.5643	0.8463	0.7584	0.7580	0.7226	0.8409	0.1281	0.7178	0.7461	0.3325
NM_138909	Foxe1	0.5691	0.2911	0.3949	0.3224	0.3186	0.3000	0.1260	0.3180	0.7156	0.2831	0.4912	0.2282
NM_173149	Havcr1	0.6814	0.5365	0.7070	0.6256	0.6279	0.6267	0.6113	0.5300	0.6485	0.1894	0.2449	0.4315
NM_138911	Stip1	0.0760	0.4543	0.0393	0.1050	0.1785	0.1828	0.0880	0.2312	0.4899	0.3367	0.2296	0.4584
NM_173320	Vom2r75	0.4072	0.2126	0.4820	0.2735	0.1750	0.3780	0.1874	0.6143	0.2589	0.3125	0.4293	0.5555
NM_153314	Ugt2b5	0.3281	0.4078	0.3072	0.3820	0.3644	0.3571	0.4352	0.1694	0.2272	0.6518	0.3240	0.1693
NM_153313	Cyp2d1	0.0647	0.0599	0.0162	0.0810	0.1627	0.2681	0.0024	0.0187	0.0612	0.0667	0.1184	0.1086
NM_144748	Acsm2	0.6606	0.3204	0.0697	0.5126	0.2130	0.2888	0.0820	0.3003	0.4272	0.0956	0.3538	0.4079
NM_138919	Unc50	0.9900	0.7791	0.8619	0.4612	0.6438	0.6495	0.6112	0.8587	0.7881	0.7017	0.8623	0.7482
NM_138918	Ss18l1	0.5475	0.3829	0.4002	0.2923	0.0934	0.2458	0.0769	0.0271	0.1568	0.7891	0.1521	0.9152
NM_173322	Pnrc1	0.9683	0.4832	0.9825	0.8343	0.9591	0.6499	0.6540	0.4731	0.3499	0.8534	0.5542	0.7693
NM_153732	Znf597	0.3219	0.5351	0.3519	0.1486	0.1956	0.1901	0.0102	0.3241	0.2765	0.1643	0.3763	0.4806
NM_153721	Ppbp	0.3493	0.2417	0.0993	0.5123	0.2335	0.5815	0.5477	0.2437	0.0743	0.4363	0.0839	0.5548
NM_139038	Rin1	0.2627	0.2433	0.0429	0.6652	0.4550	0.1207	0.1552	0.2967	0.4011	0.3953	0.1729	0.1363
NM_153472	Mnat1	0.5938	0.2905	0.8535	0.0303	0.0392	0.3055	0.2702	0.4744	0.0277	0.1024	0.4176	0.1406
NM_139040	RGD621098	0.0732	0.2399	0.8311	0.1552	0.3779	0.8768	0.1343	0.6744	0.5779	0.2187	0.1296	0.7767
NM_145084	Retsat	0.6494	0.4822	0.7082	0.8779	0.6374	0.6610	0.6994	0.6854	0.7091	0.5777	0.0445	0.7159
NM_138912	Ppp1r3b	0.9804	0.9967	0.9940	0.9475	0.9198	0.7437	0.9931	0.8348	0.9974	0.8661	0.9877	0.9442
NM_144562	Fmo4	0.3187	0.5663	0.3663	0.5890	0.5278	0.6561	0.6425	0.4522	0.6709	0.5044	0.4591	0.5355
NM_139043	Exoc8	0.7476	0.8788	0.6955	0.2821	0.5661	0.4651	0.3535	0.6623	0.3432	0.4537	0.7675	0.6143
NM_138921	Eml2	0.0955	0.1313	0.7814	0.0130	0.2152	0.0095	0.0760	0.4829	0.8646	0.2095	0.2565	0.6841
NM_175708	Car11	0.3969	0.3141	0.3537	0.1730	0.2659	0.1720	0.4109	0.4705	0.3164	0.2930	0.7308	0.7015
NM_138975	Wbp2	0.6071	0.5104	0.6128	0.6041	0.4424	0.6036	0.4863	0.6200	0.6604	0.5772	0.5130	0.5746
NM_145680	Gimap5	0.3020	0.2873	0.4325	0.3926	0.2371	0.3301	0.3881	0.3581	0.4372	0.4626	0.6596	0.2832
NM_144741	Retn	0.2837	0.1946	0.3794	0.3923	0.4059	0.7054	0.6353	0.6285	0.7124	0.6134	0.1482	0.5445
NM_139330	Sipa1l1	0.3327	0.0991	0.2730	0.8449	0.9322	0.8470	0.7976	0.7044	0.7469	0.1300	0.7097	0.5657
NM_201425	Ugt1a9	0.1399	0.5699	0.8903	0.7085	0.3611	0.3095	0.5397	0.1884	0.1187	0.2633	0.3998	0.5359
NM_138979	Bsnd	0.8046	0.4595	0.7870	0.7929	0.6870	0.7755	0.8150	0.8468	0.5838	0.7871	0.6893	0.4974
NM_139083	Rpl41	0.7055	0.4807	0.4301	0.7062	0.9744	0.2802	0.0898	0.7743	0.8695	0.7936	0.7782	0.0838
NM_139082	Bambi	0.7260	0.8766	0.8153	0.6933	0.0548	0.0614	0.7259	0.0002	0.7245	0.8937	0.7242	0.8649
NM_139081	Oaz1	0.3865	0.0889	0.5881	0.7515	0.1974	0.5270	0.0504	0.5123	0.3333	0.2952	0.3846	0.3300
NM_139085	Cst11	0.2807	0.3927	0.4712	0.0999	0.4546	0.6204	0.3092	0.6425	0.3346	0.4401	0.3201	0.2048
NM_139087	Cgref1	0.3711	0.8101	0.5735	0.8345	0.5894	0.0497	0.8945	0.4997	0.5155	0.6462	0.8130	0.1260
NM_139089	Cxcl10	0.1807	0.3050	0.1236	0.2009	0.0927	0.0163	0.0465	0.1155	0.2257	0.0733	0.4647	0.1159
NM_139088	Impg2	0.7623	0.5144	0.7141	0.5269	0.6209	0.6210	0.0970	0.4652	0.2433	0.4381	0.5259	0.2420
NM_173324	Tox4	0.4283	0.4617	0.9293	0.5609	0.3160	0.2016	0.1292	0.5400	0.8293	0.8287	0.5935	0.8360
NM_153307	Cyp4a10	0.2032	0.2425	0.4338	0.2861	0.6690	0.2236	0.4076	0.3193	0.1982	0.2289	0.7045	0.5304
NM_207594	Glo1	0.8810	0.9358	0.9275	0.8467	0.7473	0.9179	0.9147	0.8285	0.7540	0.7608	0.7769	0.4151
NM_139091	Nupl1	0.6079	0.4371	0.3056	0.4384	0.9603	0.9202	0.4115	0.8617	0.1825	0.3760	0.6908	0.4379
NM_144739	Fmo5	0.5155	0.5956	0.5979	0.6281	0.5625	0.3192	0.7451	0.7958	0.8059	0.8774	0.2118	0.5933
NM_139093	Phrf1	0.7401	0.7023	0.7679	0.8517	0.7556	0.7302	0.5826	0.7974	0.9310	0.6990	0.7088	0.6210
NM_139104	Egfl7	0.6847	0.6038	0.6037	0.8846	0.7866	0.4618	0.9173	0.8659	0.2247	0.4811	0.1944	0.8459
NM_139105	Rnh1	0.3664	0.4946	0.1036	0.8117	0.4148	0.6132	0.4022	0.8025	0.0600	0.2927	0.3945	0.0393
NM_139094	Rbm16	0.9601	0.4651	0.9185	0.9256	0.9259	0.9688	0.8595	0.7508	0.7127	0.8641	0.6357	0.8188
NM_153303	Dyt1	0.5372	0.8696	0.8785	0.7081	0.2757	0.5255	0.3073	0.5230	0.4113	0.8732	0.4358	0.6038

NM_176076	S100vp	0.2878	0.5317	0.4034	0.5024	0.4069	0.2749	0.3303	0.1688	0.2321	0.3076	0.5070	0.2801
NM_175578	Rcan2	0.9420	0.3791	0.9790	0.9840	0.3281	0.3464	0.8894	0.1675	0.3425	0.5447	0.5509	0.7927
NM_139096	Lgals3bp	0.9052	0.7990	0.8190	0.8478	0.0970	0.0200	0.5117	0.0340	0.9031	0.9574	0.7911	0.7784
NM_139108	Commd5	0.8280	0.0626	0.0584	0.2258	0.3747	0.0795	0.1995	0.6130	0.2393	0.0597	0.1466	0.0307
NM_139098	Ddx46	0.9733	0.8978	0.5832	0.6232	0.5634	0.9665	0.1149	0.2084	0.9827	0.9910	0.8356	0.7431
NM_139109	Xab2	0.5830	0.3456	0.0038	0.0051	0.1172	0.0698	0.0067	0.7045	0.1942	0.1459	0.0077	0.4767
NM_139111	Cklf	0.0003	0.0524	0.0005	0.0005	0.0393	0.0009	0.0006	0.1072	0.0661	0.0489	0.0296	0.0552
NM_139110	Gpr116	0.2461	0.6040	0.5136	0.4409	0.0167	0.1435	0.8016	0.0146	0.4342	0.1331	0.2670	0.3808
NM_139114	Rpl15	0.8623	0.8625	0.8417	0.8519	0.8237	0.8532	0.9100	0.7749	0.8266	0.7448	0.9363	0.6089
NM_139113	Nr2f6	0.8850	0.7674	0.8693	0.9464	0.9765	0.8606	0.8514	0.7823	0.8225	0.8654	0.7783	0.8331
NM_177929	Sdccag8	0.0169	0.2329	0.3801	0.7351	0.1407	0.0420	0.1259	0.5248	0.0741	0.0247	0.0300	0.0976
NM_139116	Il11ra1	0.2388	0.1137	0.2494	0.3678	0.1399	0.0462	0.0283	0.5124	0.1167	0.0138	0.0399	0.0847
NM_175603	Hnrnpr	0.9615	0.9067	0.8082	0.9542	0.9671	0.1867	0.9726	0.5152	0.8310	0.8689	0.8904	0.0835
NM_022545	Prpsap1	0.7652	0.7711	0.7836	0.8547	0.9215	0.8729	0.7110	0.9579	0.7531	0.7817	0.7851	0.8753
NM_139187	Klrh1	0.8002	0.4869	0.6718	0.8262	0.8447	0.3130	0.6553	0.8328	0.5810	0.6708	0.8582	0.7618
NM_139186	Mki67ip	0.6286	0.4217	0.6899	0.4673	0.6137	0.4841	0.4490	0.7857	0.7749	0.6562	0.4468	0.1314
NM_139189	Lmbrd1	0.8289	0.1439	0.9100	0.2495	0.1384	0.3512	0.0123	0.2796	0.0942	0.1272	0.0929	0.6898
NM_139188	Otos	0.6821	0.6133	0.1934	0.4733	0.2892	0.2134	0.7380	0.4696	0.8150	0.7425	0.5557	0.6300
NM_139190	Calcoco1	0.2102	0.1174	0.8101	0.0699	0.8112	0.3677	0.2391	0.6169	0.1890	0.0094	0.0507	0.9325
NM_172222	C2	0.5954	0.7995	0.7364	0.7686	0.5661	0.6653	0.6555	0.7638	0.8789	0.5319	0.4535	0.7640
NM_152242	Gpr56	0.2882	0.5855	0.6387	0.3858	0.2138	0.2327	0.3440	0.0123	0.7036	0.1480	0.1409	0.2411
NM_139217	Kcnc2	0.6453	0.8109	0.6965	0.6808	0.7034	0.6921	0.6875	0.7279	0.7443	0.6450	0.6411	0.3910
NM_139216	Kcnc2	0.6453	0.8109	0.6965	0.6808	0.7034	0.6921	0.6875	0.7279	0.7443	0.6450	0.6411	0.3910
NM_139193	Prlhr	0.3760	0.5695	0.4074	0.5587	0.6770	0.6615	0.7771	0.3974	0.5197	0.6101	0.4179	0.5315
NM_172062	P4ha1	0.1888	0.0106	0.7656	0.2098	0.1177	0.0315	0.8351	0.0358	0.0832	0.2579	0.0296	0.7104
NM_172030	Entpd2	0.7913	0.5553	0.6672	0.6790	0.7720	0.5412	0.5175	0.8530	0.5175	0.5466	0.3389	0.6953
NM_178093	Mtus1	0.9476	0.9806	0.9441	0.9198	0.9748	0.6457	0.9857	0.9817	0.9695	0.9864	0.9835	0.9886
NM_147177	Ruvbl1	0.5085	0.9751	0.5755	0.8282	0.7273	0.7277	0.5688	0.9154	0.8310	0.8782	0.8250	0.7164
NM_172224	Impa2	0.0322	0.1232	0.1970	0.1084	0.9147	0.8169	0.7049	0.9383	0.0835	0.0837	0.1701	0.4306
NM_145771	Miox	0.3064	0.5082	0.2843	0.3199	0.5447	0.6829	0.6375	0.5611	0.6263	0.3880	0.4858	0.3718
NM_175604	Yrdc	0.2051	0.1332	0.1231	0.2125	0.1326	0.6420	0.0290	0.1188	0.2269	0.1420	0.4691	0.1961
NM_139263	Fgd4	0.6762	0.2251	0.9000	0.3156	0.8725	0.5415	0.9189	0.8569	0.6336	0.9234	0.7942	0.8585
NM_139262	Ctsq	0.2723	0.5411	0.4506	0.2904	0.1855	0.1913	0.2668	0.0851	0.2939	0.3539	0.7886	0.1569
NM_184045	Srpk3	0.1967	0.2550	0.7727	0.0527	0.0726	0.2472	0.4431	0.3030	0.1545	0.1122	0.4187	0.1424
NM_182953	Nek6	0.5010	0.2664	0.7786	0.7599	0.3359	0.7507	0.2563	0.5345	0.7476	0.7455	0.4223	0.8968
NM_178103	Fam12b	0.6733	0.6788	0.5954	0.3744	0.5518	0.4956	0.5706	0.5330	0.5361	0.1970	0.7317	0.4282
NM_139253	Mcfd2	0.3306	0.0260	0.3630	0.3228	0.0944	0.1606	0.3269	0.2341	0.1430	0.2079	0.1549	0.6058
NM_177927	Serpinf1	0.8128	0.8799	0.7331	0.7939	0.8781	0.7878	0.7216	0.9135	0.7673	0.8193	0.6894	0.2861
NM_139260	Il3ra	0.8039	0.6402	0.2258	0.2763	0.3400	0.4240	0.6393	0.6516	0.7843	0.6541	0.6570	0.7749
NM_139258	Bmf	0.2533	0.2783	0.8913	0.1364	0.5549	0.3723	0.7633	0.8438	0.3908	0.7852	0.0413	0.9777
NM_212490	Atp6v1g2	0.9653	0.7566	0.9504	0.8339	0.9944	0.9234	0.4651	0.9513	0.9366	0.9456	0.9208	0.9937
NM_212458	Agpat1	0.9062	0.8905	0.8605	0.7570	0.8671	0.8016	0.9779	0.9347	0.9569	0.4207	0.7656	0.5313
NM_001007756	Gemin8	0.3013	0.6091	0.6837	0.9930	0.8881	0.1202	0.8239	0.3982	0.2287	0.4137	0.8557	0.0801
NM_139324	Ehd4	0.1887	0.0597	0.3083	0.9192	0.2079	0.1547	0.0744	0.1880	0.4558	0.1976	0.1048	0.9295
NM_178334	Egln1	0.8724	0.3628	0.8514	0.9600	0.9787	0.9886	0.9940	0.9470	0.6873	0.1235	0.8428	0.8758
NM_153725	Slc17a8	0.0750	0.5768	0.2500	0.0948	0.5493	0.3983	0.2922	0.1975	0.3939	0.3840	0.5431	0.4900
NM_153629	Hspa4	0.9044	0.2017	0.4631	0.8304	0.6371	0.5110	0.6181	0.6845	0.5701	0.6317	0.5727	0.9402
NM_175765	Psip1	0.4953	0.1687	0.8642	0.9762	0.6259	0.8950	0.5157	0.3949	0.3102	0.2057	0.3788	0.5458
NM_139329	Hsd3b7	0.0397	0.0063	0.0913	0.1307	0.1877	0.2821	0.0159	0.2266	0.2025	0.0791	0.0290	0.5658

NM_201991	Gpn3	0.1946	0.0311	0.1537	0.0672	0.0596	0.3611	0.0377	0.0726	0.1318	0.0755	0.1629	0.0889
NM_148891	Nmt1	0.3741	0.1494	0.1435	0.1483	0.0236	0.3565	0.1980	0.0065	0.1656	0.0166	0.0849	0.4378
NM_153318	Cyp4f6	0.1870	0.0011	0.0071	0.0003	0.5286	0.4563	0.0020	0.3743	0.0035	0.0009	0.0707	0.0011
NM_139333	Prpf19	0.9835	0.8846	0.9178	0.8732	0.7659	0.5771	0.9407	0.8783	0.8157	0.9929	0.4357	0.9143
NM_139332	Tpcn1	0.2926	0.4814	0.9412	0.7849	0.8515	0.9053	0.5278	0.9257	0.9303	0.7631	0.6157	0.9071
NM_139338	Slc34a3	0.1724	0.6968	0.4802	0.6333	0.8271	0.6652	0.8647	0.4898	0.4637	0.6457	0.4151	0.4944
NM_139341	Slc15a3	0.1189	0.1144	0.5334	0.0558	0.1807	0.0973	0.4774	0.1712	0.4592	0.4071	0.4259	0.7406
NM_199106	Galnt13	0.3522	0.3543	0.1910	0.4455	0.3785	0.0852	0.1854	0.0706	0.6022	0.6948	0.7064	0.1648
NM_153315	Dhrs4	0.8456	0.9693	0.7712	0.8924	0.9856	0.9464	0.8780	0.8310	0.7736	0.7688	0.8659	0.1510
NM_177932	Pafah2	0.3779	0.4418	0.4253	0.0209	0.4189	0.1263	0.4737	0.4675	0.2243	0.0952	0.5140	0.0926
NM_172066	Slc30a4	0.5778	0.6168	0.6551	0.6860	0.7127	0.5320	0.2423	0.2540	0.6522	0.6276	0.3929	0.6013
NM_153312	Cyp3a2	0.6844	0.0977	0.0606	0.2916	0.0686	0.2482	0.6252	0.4030	0.2891	0.0652	0.4004	0.0700
NM_144740	Arhgap4	0.3631	0.2903	0.2635	0.8508	0.5640	0.5018	0.8245	0.2594	0.6858	0.3882	0.1916	0.7108
NM_171983	Spna2	0.3600	0.4286	0.3318	0.5677	0.4104	0.5347	0.5120	0.5375	0.5063	0.5500	0.4646	0.6558
NM_144743	Ces6	0.4228	0.4194	0.2646	0.3725	0.2109	0.3544	0.4048	0.1686	0.3209	0.1853	0.4712	0.2853
NM_145880	Lhx1	0.5451	0.7137	0.8621	0.7370	0.2675	0.0280	0.7135	0.6288	0.2415	0.2693	0.8328	0.4577
NM_144746	Ppp2r2d	0.7367	0.8198	0.6870	0.4333	0.5915	0.5827	0.7512	0.4684	0.8026	0.7061	0.9623	0.5877
NM_144747	Slc45a1	0.7734	0.3870	0.7230	0.5229	0.5793	0.4847	0.7829	0.6612	0.4646	0.2735	0.8253	0.3097
NM_145782	Cyp3a18	0.4998	0.4546	0.5352	0.8294	0.6086	0.6190	0.5432	0.5077	0.3945	0.4070	0.6488	0.6614
NM_144752	Oas1b	0.3644	0.6327	0.6878	0.7454	0.8079	0.6530	0.2454	0.6372	0.5522	0.8150	0.5923	0.7904
NM_181379	Ppp2r5b	0.5537	0.2304	0.0130	0.2105	0.4065	0.8336	0.3013	0.5772	0.7384	0.5462	0.4801	0.9606
NM_144753	Fev	0.1446	0.5468	0.2546	0.6841	0.8288	0.4794	0.2374	0.4543	0.4744	0.7251	0.5820	0.4602
NM_144755	Trib3	0.2082	0.8107	0.8365	0.8241	0.8827	0.8536	0.8798	0.9622	0.9161	0.9232	0.8311	0.8595
NM_145683	Ptpn7	0.0356	0.8134	0.7590	0.5980	0.4977	0.3019	0.8283	0.6632	0.7468	0.2815	0.3994	0.4882
NM_181382	Gldn	0.7541	0.6924	0.6258	0.6962	0.5969	0.4810	0.6197	0.6576	0.9373	0.2360	0.2969	0.9324
NM_199388	Dpagt1	0.1386	0.1245	0.4439	0.8146	0.2651	0.0487	0.0342	0.0733	0.0746	0.0460	0.0257	0.0682
NM_144756	Faim2	0.6322	0.4898	0.6002	0.4967	0.8511	0.3087	0.4308	0.4347	0.1028	0.1833	0.5590	0.6495
NM_144762	Baalc	0.5339	0.7667	0.2770	0.2614	0.5974	0.2810	0.3486	0.6254	0.4157	0.6484	0.7747	0.1094
NM_144758	Slc15a4	0.5720	0.1988	0.3058	0.2535	0.4131	0.3997	0.1848	0.0251	0.0722	0.2100	0.2005	0.5796
NM_145081	Optn	0.2197	0.1487	0.2091	0.2919	0.1560	0.1803	0.0561	0.1514	0.0642	0.2947	0.1704	0.4303
NM_145086	Serbp1	0.7432	0.6093	0.7717	0.6424	0.8106	0.8218	0.1898	0.5177	0.9223	0.9973	0.9445	0.8603
NM_181473	Klhl24	0.3925	0.2647	0.9881	0.1551	0.2644	0.2843	0.4035	0.2560	0.4023	0.2294	0.6835	0.9277
NM_181474	Tfb1m	0.9631	0.7514	0.6270	0.7067	0.8321	0.3109	0.7528	0.7285	0.9929	0.8088	0.9871	0.9010
NM_145090	Arfgap1	0.7465	0.5307	0.6698	0.2066	0.5880	0.4411	0.9098	0.1467	0.6495	0.6784	0.7566	0.4139
NM_181478	Rdh10	0.1902	0.0047	0.0771	0.0082	0.0095	0.2281	0.0730	0.0803	0.0846	0.0134	0.0216	0.0541
NM_001113403	Lnpep	0.5293	0.7601	0.6154	0.5888	0.5673	0.8532	0.3356	0.7949	0.2895	0.4147	0.4643	0.5366
NM_203337	St3gal4	0.0743	0.2023	0.8860	0.6763	0.0664	0.0176	0.0335	0.1513	0.0839	0.0265	0.1653	0.8759
NM_145092	Tor1aip1	0.9413	0.8173	0.8569	0.9770	0.9774	0.7605	0.5131	0.9937	0.8538	0.6750	0.6463	0.9911
NM_145093	Aard	0.5528	0.5031	0.6271	0.5770	0.4607	0.3909	0.4531	0.7434	0.3328	0.4800	0.9064	0.5103
NM_145094	Rab31	0.3930	0.0909	0.9926	0.9388	0.1517	0.8089	0.7391	0.1405	0.2058	0.1460	0.3635	0.5697
NM_145095	Kcnh8	0.4165	0.4491	0.6666	0.5437	0.5066	0.4760	0.8103	0.7339	0.4267	0.5975	0.6809	0.0816
NM_181630	LOC299271	0.3882	0.0568	0.5186	0.5215	0.0719	0.0822	0.4150	0.4612	0.4759	0.2527	0.3511	0.5155
NM_181387	Lhfpl4	0.7749	0.6161	0.5875	0.7956	0.4283	0.3402	0.5786	0.4603	0.6032	0.5654	0.5806	0.2507
NM_173116	Sgpl1	0.0669	0.0908	0.4676	0.0814	0.0846	0.4343	0.2200	0.1389	0.0237	0.0271	0.1775	0.7044
NM_145097	Serpina4	0.3556	0.3458	0.1523	0.1581	0.2982	0.5066	0.3667	0.5093	0.4315	0.3061	0.6255	0.1601
NM_144744	Adipoq	0.8203	0.8971	0.7369	0.8143	0.6443	0.8147	0.9227	0.9328	0.7893	0.8343	0.9191	0.8500
NM_001077356	Clca4l	0.2661	0.2817	0.3807	0.5097	0.4569	0.2875	0.1758	0.3227	0.4260	0.5449	0.5008	0.2846
NM_173839	Tph2	0.7740	0.6246	0.7054	0.3522	0.7436	0.8365	0.6536	0.3800	0.6192	0.5164	0.2486	0.5551
NM_147209	Ppm1g	0.7941	0.9286	0.1941	0.8950	0.8761	0.7397	0.9493	0.9618	0.8111	0.9897	0.9438	0.4984

NM_001034157	Bat4	0.2524	0.3269	0.2616	0.5636	0.2498	0.3138	0.3244	0.3666	0.2371	0.2621	0.3784	0.2895
NM_206849	Rxrb	0.9136	0.8740	0.8728	0.4835	0.8714	0.7445	0.7496	0.6256	0.8660	0.9082	0.7831	0.7421
NM_145672	Cxcl9	0.7967	0.0816	0.0434	0.1715	0.6638	0.2386	0.3796	0.0946	0.1658	0.2131	0.1169	0.0606
NM_145671	Klhl17	0.4448	0.6184	0.3671	0.6144	0.4892	0.5873	0.4967	0.8150	0.6172	0.8321	0.5322	0.4988
NM_181694	Rsb66	0.5099	0.1217	0.6355	0.3972	0.1706	0.1480	0.4701	0.0773	0.0797	0.3407	0.6111	0.2749
NM_172336	Atf5	0.9220	0.8454	0.7296	0.8776	0.8347	0.8780	0.8575	0.8979	0.8563	0.8783	0.8938	0.8624
NM_153821	Prrx1	0.1048	0.0449	0.1405	0.0167	0.9331	0.5955	0.2225	0.9124	0.1688	0.0064	0.1646	0.1439
NM_145677	Slc25a25	0.6158	0.8480	0.3410	0.0884	0.1713	0.3853	0.2068	0.4461	0.9937	0.6604	0.7273	0.4273
NM_001113409	Syngap1	0.2210	0.2111	0.5356	0.7066	0.6507	0.5767	0.5593	0.8565	0.6096	0.7077	0.5133	0.7322
NM_147165	Gpx6	0.1669	0.3163	0.5081	0.8654	0.1829	0.2422	0.3618	0.3680	0.2303	0.4789	0.5671	0.5305
NM_145682	Filip1	0.4140	0.4188	0.2750	0.1188	0.2525	0.5459	0.2375	0.3874	0.1079	0.2385	0.2861	0.4168
NM_145721	Cdk5rap1	0.4242	0.3935	0.6251	0.1120	0.6647	0.5259	0.1567	0.4994	0.1209	0.3442	0.4818	0.2918
NM_145723	Stx17	0.3811	0.3254	0.5029	0.8933	0.0891	0.9358	0.7365	0.5398	0.5266	0.0784	0.7462	0.9383
NM_145098	Nrp1	0.9809	0.9655	0.9942	0.9339	0.9998	0.9125	0.9378	0.9996	0.9773	0.9786	0.8048	0.8774
NM_145767	Prrxl1	0.2806	0.3436	0.3006	0.2137	0.4380	0.3398	0.4825	0.1699	0.2419	0.4382	0.1229	0.3876
NM_145768	Mamdc4	0.6835	0.2910	0.5318	0.5601	0.6577	0.4407	0.6435	0.5781	0.7342	0.7703	0.7693	0.6243
NM_172075	Thop1	0.8300	0.9013	0.8591	0.9307	0.9822	0.9606	0.8775	0.8750	0.9092	0.8562	0.8766	0.9984
NM_145772	Dync1li1	0.0050	0.1330	0.0968	0.0566	0.0457	0.1894	0.0417	0.0046	0.0618	0.1010	0.1405	0.0398
NM_182817	Smagp	0.9041	0.9340	0.8722	0.7724	0.6097	0.6626	0.9712	0.9842	0.9180	0.9840	0.8837	0.9818
NM_145773	Mxd3	0.8719	0.8522	0.0195	0.7962	0.8682	0.9740	0.9267	0.9935	0.9714	0.7966	0.9865	0.3265
NM_182673	Rnasel	0.1156	0.1163	0.0297	0.0294	0.0808	0.1709	0.0275	0.3980	0.0628	0.1625	0.1255	0.0701
NM_145775	Nr1d1	0.6933	0.5857	0.6174	0.4841	0.4951	0.3715	0.2918	0.1683	0.8333	0.6066	0.5069	0.9536
NM_173095	Kcna1	0.2475	0.4150	0.6935	0.4793	0.4282	0.5946	0.5094	0.5299	0.4010	0.2584	0.5276	0.7817
NM_145777	Olfm3	0.2067	0.2014	0.6812	0.3498	0.6702	0.4382	0.2683	0.4854	0.0932	0.2897	0.2858	0.5290
NM_145778	Tubg1	0.1384	0.7579	0.1059	0.4109	0.5395	0.6574	0.8736	0.4658	0.7589	0.1461	0.3143	0.0616
NM_145780	Nr2c1	0.7530	0.5762	0.6433	0.5484	0.9046	0.7325	0.8849	0.9057	0.5257	0.6892	0.7781	0.9067
NM_145779	Pzp	0.2656	0.4113	0.7039	0.2170	0.3990	0.2158	0.3718	0.8425	0.7408	0.0570	0.7540	0.7755
NM_145781	Timm13	0.2456	0.8427	0.0660	0.0507	0.1898	0.3480	0.3225	0.8048	0.4601	0.1276	0.1513	0.1386
NM_173093	Cyp2d3	0.2827	0.3696	0.2956	0.5223	0.2759	0.4047	0.1752	0.3442	0.3471	0.4915	0.3332	0.1691
NM_145785	Hdgfrp3	0.9074	0.4081	0.4891	0.9704	0.9360	0.9498	0.4408	0.8527	0.4297	0.4199	0.4068	0.7011
NM_145789	Il13ra1	0.4585	0.3034	0.6217	0.6006	0.5911	0.1362	0.6760	0.3782	0.4886	0.0878	0.4882	0.8480
NM_171990	Bpnt1	0.0085	0.4233	0.1715	0.2979	0.6127	0.4636	0.9309	0.4734	0.8223	0.3621	0.4615	0.0925
NM_144750	LOC246266	0.1567	0.4835	0.1262	0.0478	0.1134	0.1509	0.1490	0.0545	0.3092	0.3462	0.2317	0.5897
NM_145787	Mrga10	0.7062	0.6627	0.7611	0.7127	0.8926	0.7991	0.8749	0.6249	0.4913	0.6078	0.9499	0.7609
NM_198766	Ccdc127	0.1772	0.0969	0.4367	0.1872	0.0492	0.1018	0.0461	0.0235	0.0102	0.2403	0.1015	0.2775
NM_198749	Rab15	0.2773	0.1692	0.3150	0.4182	0.1649	0.3965	0.3872	0.1181	0.2841	0.2923	0.2798	0.5351
NM_145877	Kif1c	0.1632	0.8117	0.8776	0.9886	0.3617	0.3050	0.8329	0.6743	0.9613	0.6164	0.3082	0.6020
NM_145881	Rims2	0.6767	0.8673	0.5490	0.7156	0.5263	0.4209	0.7041	0.8020	0.6734	0.7370	0.7795	0.5301
NM_147140	Slc35a4	0.4797	0.3471	0.0772	0.8842	0.1447	0.2967	0.0828	0.3843	0.1036	0.2773	0.2115	0.1758
NM_147137	LOC257643	0.3100	0.3457	0.3116	0.3607	0.3124	0.2799	0.1751	0.5327	0.2655	0.4833	0.3911	0.2582
NM_198731	Chdh	0.6337	0.8631	0.9059	0.7115	0.8931	0.5981	0.5637	0.9249	0.8816	0.5920	0.9057	0.9365
NM_147141	Cacna1b	0.8236	0.2734	0.5619	0.4722	0.6398	0.4352	0.9350	0.8413	0.3093	0.8606	0.8736	0.7897
NM_147144	Casc3	0.6100	0.5930	0.2812	0.7507	0.2862	0.8384	0.3945	0.2308	0.0828	0.6211	0.6282	0.5369
NM_147145	Dclre1c	0.3646	0.4540	0.2020	0.3220	0.7706	0.6300	0.0419	0.8849	0.5031	0.5583	0.6372	0.6745
NM_184050	Ermp1	0.2263	0.3409	0.9335	0.3517	0.1298	0.2211	0.9789	0.2600	0.0065	0.2803	0.3305	0.2699
NM_153738	Prl7b1	0.1609	0.0345	0.0169	0.3035	0.0777	0.0122	0.0886	0.0284	0.3992	0.0948	0.5035	0.2905
NM_172323	Has1	0.5826	0.5661	0.5500	0.8606	0.5442	0.3403	0.3752	0.3017	0.1174	0.3262	0.5493	0.2468
NM_172319	Has3	0.7167	0.2971	0.8946	0.5404	0.7562	0.0548	0.3018	0.5997	0.2806	0.1468	0.4300	0.1895
NM_153814	Cacna1h	0.8024	0.8231	0.5495	0.8453	0.8459	0.0786	0.7650	0.6615	0.9196	0.8829	0.6979	0.9860

NM_001113422	Nr1d1	0.6933	0.5857	0.6174	0.4841	0.4951	0.3715	0.2918	0.1683	0.8333	0.6066	0.5069	0.9536
NM_198728	LOC288913	0.0656	0.3415	0.0722	0.0267	0.6843	0.3167	0.0264	0.6655	0.2778	0.0551	0.0248	0.0676
NM_198729	Vcsa2	0.2436	0.0067	0.4338	0.3600	0.5497	0.3182	0.3437	0.3363	0.7830	0.6928	0.8488	0.4246
NM_198730	Psmd6	0.1086	0.0449	0.0902	0.0015	0.0446	0.1429	0.0748	0.0745	0.1801	0.0347	0.0953	0.3828
NM_198732	Commd3	0.6826	0.6271	0.8546	0.7039	0.6429	0.7397	0.3276	0.9078	0.5179	0.5731	0.7869	0.4028
NM_001040271	Dut	0.7263	0.2319	0.3543	0.3664	0.4015	0.4578	0.2858	0.2798	0.5088	0.4593	0.4232	0.1737
NM_198768	Igsf10	0.4931	0.6700	0.5316	0.3301	0.5022	0.2402	0.3840	0.5603	0.2010	0.6170	0.6177	0.3250
NM_212532	Ddah2	0.7961	0.7364	0.1202	0.9269	0.7322	0.8979	0.5309	0.9186	0.8298	0.8121	0.9643	0.8294
NM_199463	Ripk5	0.2677	0.2356	0.9806	0.9681	0.9380	0.9482	0.3970	0.9392	0.1468	0.3245	0.0722	0.6201
NM_198765	Bicd2	0.6744	0.9285	0.7174	0.7163	0.6923	0.5990	0.8005	0.6721	0.4751	0.5206	0.8042	0.6427
NM_198743	Bwk1	0.3640	0.3527	0.1501	0.8884	0.5328	0.1748	0.1060	0.4102	0.2644	0.4055	0.0642	0.6147
NM_170718	Kcnj14	0.8958	0.9199	0.3878	0.9113	0.7523	0.8500	0.9228	0.9431	0.8788	0.6537	0.5932	0.8907
NM_170668	Slc13a5	0.4916	0.4444	0.6742	0.2350	0.4697	0.4621	0.6941	0.5867	0.4410	0.5481	0.2165	0.6895
NM_182672	Cbr4	0.2987	0.0384	0.8787	0.8574	0.1008	0.0208	0.8828	0.0921	0.0397	0.0877	0.2073	0.4122
NM_001044239	Ap1m1	0.8088	0.6468	0.6360	0.9504	0.9225	0.8992	0.7921	0.8128	0.9428	0.8613	0.7539	0.9474
NM_182669	Micalcl	0.7021	0.7782	0.8708	0.7622	0.7448	0.9264	0.6602	0.5899	0.7393	0.7689	0.8827	0.7251
NM_183056	Stk40	0.2660	0.5507	0.5224	0.1260	0.5772	0.5608	0.8290	0.3407	0.9235	0.0231	0.6000	0.7369
NM_183325	Adprh	0.1113	0.0256	0.0273	0.1283	0.0652	0.0079	0.0981	0.1202	0.0334	0.0920	0.0158	0.0711
NM_001134508	RGD1307603	0.5651	0.2952	0.5551	0.1997	0.3510	0.5475	0.2669	0.1478	0.4896	0.0611	0.1304	0.8868
NM_182736	Ccdc50	0.0219	0.1073	0.4814	0.0365	0.0106	0.0218	0.1315	0.0011	0.0033	0.1947	0.4157	0.0176
NM_172008	Canx	0.2942	0.3528	0.9704	0.9410	0.2745	0.8015	0.8958	0.2962	0.4459	0.4109	0.9780	0.9980
NM_172011	Ppp1r14d	0.7595	0.5131	0.0868	0.1559	0.3019	0.6501	0.5204	0.4962	0.2603	0.5567	0.0776	0.0904
NM_182823	Zfp472	0.8238	0.6840	0.0886	0.0601	0.6227	0.8052	0.2434	0.8803	0.0435	0.4691	0.0811	0.1444
NM_182822	Fam70a	0.7123	0.8225	0.2103	0.6635	0.4141	0.8632	0.7082	0.6783	0.1416	0.6370	0.8809	0.4189
NM_172029	Eif2b1	0.9694	0.8261	0.7226	0.5463	0.8337	0.5714	0.5256	0.9133	0.7208	0.8232	0.7657	0.5231
NM_198753	Rpl3	0.6983	0.3143	0.7406	0.8920	0.5681	0.7978	0.9047	0.5600	0.5242	0.6357	0.3200	0.4464
NM_172035	Fzd2	0.2798	0.4209	0.4416	0.1718	0.2502	0.4637	0.3321	0.6403	0.3625	0.4073	0.3825	0.5200
NM_181092	Syngap1	0.2210	0.2111	0.5356	0.7066	0.6507	0.5767	0.5593	0.8565	0.6096	0.7077	0.5133	0.7322
NM_172033	Plekhb1	0.3659	0.8903	0.1924	0.7511	0.8758	0.5241	0.5706	0.7813	0.6361	0.3957	0.0477	0.5880
NM_173151	Pcyt1b	0.2132	0.1534	0.4471	0.4716	0.3900	0.5374	0.1475	0.5058	0.0128	0.6222	0.0755	0.3023
NM_172243	Ppif	0.4380	0.7504	0.6465	0.2048	0.3549	0.8985	0.1914	0.6088	0.7900	0.2181	0.0276	0.8986
NM_198758	Rap2ip	0.7879	0.7314	0.7657	0.7484	0.9298	0.9058	0.6891	0.7622	0.9500	0.9082	0.8999	0.9707
NM_173115	Fam5b	0.7494	0.9138	0.7308	0.8371	0.8239	0.3031	0.8043	0.5520	0.2535	0.7789	0.6368	0.6080
NM_172067	Spon1	0.8375	0.8520	0.7639	0.9166	0.9293	0.5416	0.2148	0.2439	0.7708	0.7381	0.4627	0.8220
NM_173294	Cyp2b3	0.8357	0.4657	0.4680	0.6233	0.4897	0.8738	0.7943	0.4641	0.5733	0.5983	0.2368	0.6187
NM_172042	Kcnk2	0.6918	0.5877	0.7587	0.8462	0.6181	0.6754	0.7267	0.8725	0.7232	0.7237	0.5340	0.8355
NM_172041	Kcnk2	0.6918	0.5877	0.7587	0.8462	0.6181	0.6754	0.7267	0.8725	0.7232	0.7237	0.5340	0.8355
NM_173295	Ugt2b17	0.4027	0.5384	0.8268	0.3717	0.6762	0.7801	0.5731	0.6389	0.6712	0.4946	0.1264	0.7898
NM_183055	Cox8c	0.4349	0.0398	0.6569	0.2232	0.1953	0.7163	0.8597	0.4506	0.7961	0.4243	0.4093	0.4583
NM_172045	Ppp1r14b	0.6529	0.8156	0.0014	0.8125	0.8802	0.7874	0.1194	0.9692	0.8954	0.9500	0.7071	0.9237
NM_173330	Znf689	0.6614	0.9559	0.8576	0.8997	0.8253	0.7652	0.7497	0.8228	0.8574	0.9586	0.5458	0.9805
NM_173147	Vps54	0.6033	0.6136	0.6929	0.5982	0.7154	0.6969	0.7283	0.7125	0.4277	0.7187	0.4835	0.6448
NM_172055	Cited1	0.8063	0.5522	0.5553	0.4304	0.3335	0.4327	0.3640	0.7399	0.5504	0.3385	0.7855	0.2431
NM_172047	Eaf2	0.1674	0.1854	0.4778	0.4216	0.3688	0.7960	0.5003	0.2228	0.6240	0.4434	0.4875	0.4550
NM_172063	Pex14	0.3178	0.0881	0.7631	0.8287	0.1495	0.4423	0.3687	0.1744	0.5079	0.1181	0.0468	0.9598
NM_198772	MGC72974	0.0320	0.2736	0.0191	0.1333	0.5078	0.6554	0.0082	0.4615	0.0050	0.0787	0.1449	0.7116
NM_198774	Tmem110	0.1588	0.2363	0.8699	0.8663	0.5691	0.4109	0.2392	0.7368	0.3761	0.2918	0.4379	0.2089
NM_181082	Ms4a14	0.5680	0.6746	0.7887	0.5044	0.6028	0.7076	0.6987	0.4851	0.4275	0.6308	0.5471	0.2688
NM_172068	Surf1	0.1655	0.1907	0.2585	0.0042	0.0235	0.0009	0.0044	0.0790	0.2244	0.1620	0.0245	0.1457

NM_198779	RGD735140	0.9037	0.9424	0.9038	0.8649	0.7564	0.8042	0.1610	0.8336	0.5126	0.6286	0.9284	0.7100
NM_175577	Cd6	0.0340	0.4268	0.6447	0.0809	0.5547	0.5222	0.5540	0.7536	0.3707	0.3698	0.3487	0.1494
NM_001112712	Grk6	0.8080	0.6822	0.6586	0.8931	0.5664	0.8727	0.8825	0.9340	0.7880	0.8790	0.4994	0.5364
NM_172073	Tpbpa	0.7500	0.6670	0.2363	0.8759	0.5999	0.6909	0.7756	0.5517	0.7776	0.7961	0.7486	0.8048
NM_172074	Timm10	0.0450	0.5360	0.3476	0.2160	0.0930	0.0442	0.1136	0.2547	0.7722	0.0623	0.3353	0.4457
NM_183403	Gpx2	0.0876	0.3337	0.7429	0.8174	0.7168	0.1152	0.3712	0.3263	0.5458	0.1760	0.6449	0.2075
NM_172077	Reg3a	0.5620	0.8587	0.7366	0.5473	0.7467	0.5930	0.7088	0.3203	0.2793	0.3925	0.6802	0.8208
NM_178101	Plod3	0.2044	0.0202	0.6060	0.4354	0.4019	0.7707	0.1550	0.3620	0.7971	0.1093	0.2043	0.7907
NM_001077650	Edn3	0.1706	0.1640	0.1732	0.1714	0.2614	0.4389	0.1747	0.1678	0.1629	0.3793	0.3466	0.2066
NM_175761	Hsp90aa1	0.3039	0.8059	0.3010	0.3085	0.4806	0.9173	0.6053	0.1417	0.2480	0.4200	0.1539	0.8060
NM_173119	Hsbp1	0.1412	0.2517	0.0804	0.7200	0.9456	0.7440	0.4749	0.9653	0.0127	0.2817	0.2162	0.2180
NM_198790	Rgsl2h	0.5681	0.3087	0.3451	0.4890	0.2918	0.5640	0.4604	0.0755	0.5672	0.4130	0.5045	0.2238
NM_175594	Igf2bp1	0.6882	0.3896	0.8444	0.6155	0.8210	0.5581	0.9736	0.6004	0.5982	0.8351	0.6486	0.8838
NM_174864	Ncstn	0.7136	0.0759	0.9565	0.5979	0.9326	0.9585	0.3109	0.6259	0.3532	0.2167	0.3134	0.9256
NM_172090	Epb4.1l1	0.6922	0.3369	0.8318	0.6706	0.6569	0.6358	0.9396	0.6912	0.7079	0.6692	0.4108	0.6131
NM_176075	Ppargc1b	0.2583	0.1412	0.0755	0.0868	0.0264	0.0171	0.0874	0.0107	0.0681	0.0729	0.5416	0.1796
NM_175756	Fcgr2b	0.1029	0.1729	0.3686	0.1693	0.3805	0.4716	0.4058	0.6126	0.0893	0.0835	0.2920	0.1711
NM_172091	Gcgr	0.4030	0.2395	0.2563	0.4850	0.3579	0.3859	0.5029	0.3524	0.4427	0.2848	0.3507	0.6851
NM_171991	Ccnb1	0.7214	0.3096	0.3653	0.4780	0.2964	0.2962	0.1745	0.3301	0.4461	0.4820	0.1645	0.3258
NM_172092	Gcgr	0.4030	0.2395	0.2563	0.4850	0.3579	0.3859	0.5029	0.3524	0.4427	0.2848	0.3507	0.6851
NM_199090	Nudt22	0.1706	0.1639	0.1731	0.1713	0.1756	0.1713	0.1745	0.1675	0.1628	0.3780	0.3991	0.1687
NM_199094	Tubb2c	0.3434	0.4054	0.3261	0.5630	0.4220	0.4097	0.4876	0.4005	0.4353	0.4333	0.4663	0.3852
NM_172317	Fxyd3	0.7976	0.7692	0.6739	0.8099	0.6344	0.5957	0.6168	0.5328	0.8710	0.6191	0.6360	0.8721
NM_199100	Tor1aip2	0.4782	0.4296	0.5349	0.3891	0.4995	0.4982	0.3490	0.6222	0.2698	0.5608	0.2464	0.3703
NM_172157	Arid1b	0.8786	0.8748	0.8098	0.8770	0.5699	0.9048	0.8613	0.8885	0.7156	0.7933	0.9184	0.8224
NM_199102	MGC72560	0.1953	0.2893	0.7580	0.9258	0.1986	0.0536	0.7950	0.1560	0.1748	0.1206	0.4085	0.2135
NM_172223	Pxmp4	0.0341	0.1025	0.2392	0.3720	0.1078	0.3108	0.3275	0.3061	0.0487	0.2427	0.2871	0.5995
NM_173142	Stk16	0.5347	0.6615	0.7672	0.8510	0.8209	0.9631	0.8554	0.7773	0.7618	0.7771	0.8439	0.7903
NM_178102	Mapkapk2	0.8961	0.8037	0.1676	0.5120	0.7283	0.6546	0.9621	0.8751	0.6458	0.9247	0.8331	0.4889
NM_172321	Slc6a15	0.3875	0.6425	0.2650	0.3865	0.6237	0.3898	0.4317	0.5200	0.6519	0.6972	0.8180	0.2418
NM_178106	Entpd3	0.9254	0.7026	0.9373	0.7618	0.9524	0.9002	0.7778	0.9533	0.9578	0.9490	0.8702	0.9462
NM_172329	Ccr9	0.5219	0.2420	0.2865	0.4367	0.3259	0.1486	0.1769	0.3409	0.5453	0.0781	0.2428	0.2344
NM_172328	Tac4	0.1439	0.0700	0.0774	0.2647	0.4072	0.1379	0.0788	0.0541	0.5577	0.2337	0.3460	0.2049
NM_172327	Aip	0.3704	0.1739	0.1229	0.5252	0.1970	0.7046	0.0374	0.8398	0.0747	0.3327	0.3041	0.4493
NM_177931	Orc1l	0.7141	0.7289	0.0004	0.6711	0.7161	0.7088	0.6888	0.7157	0.7096	0.7036	0.9468	0.4802
NM_172330	Zp4	0.3510	0.8638	0.2730	0.4829	0.3862	0.1237	0.5303	0.6700	0.5747	0.6117	0.3631	0.7862
NM_172333	Cthrc1	0.1673	0.2215	0.6408	0.3676	0.6323	0.3378	0.4481	0.4018	0.2466	0.1423	0.5157	0.0211
NM_172332	Wrnip1	0.3246	0.0949	0.8443	0.0426	0.0253	0.0207	0.2282	0.0186	0.1459	0.1627	0.5066	0.6984
NM_199116	Tmbim4	0.0912	0.0795	0.0762	0.0973	0.0730	0.0004	0.0408	0.0018	0.0158	0.0214	0.0860	0.0056
NM_172334	Fibp	0.0090	0.0435	0.2279	0.4896	0.2450	0.0093	0.1199	0.0267	0.0375	0.2341	0.0410	0.0523
NM_173130	Vom2r27	0.4960	0.5390	0.4794	0.7674	0.5761	0.4569	0.5707	0.6082	0.5632	0.7361	0.5265	0.5085
NM_173045	Zc3hav1	0.0849	0.5102	0.0634	0.3400	0.0712	0.1456	0.3684	0.0712	0.6832	0.1595	0.2176	0.6257
NM_001040180	Tbcb	0.4228	0.2994	0.2134	0.7812	0.4511	0.0277	0.1484	0.7034	0.1169	0.5074	0.3360	0.1431
NM_173101	Myo1e	0.7791	0.5951	0.7492	0.4376	0.5509	0.9005	0.5535	0.6604	0.8219	0.7324	0.1922	0.8615
NM_199233	Doxl1	0.3832	0.1200	0.4340	0.6457	0.2290	0.4720	0.5375	0.4917	0.1843	0.6944	0.5717	0.1602
NM_173305	Hsd17b6	0.4767	0.1577	0.6452	0.5494	0.7735	0.7783	0.5993	0.5004	0.2661	0.4720	0.6268	0.6524
NM_173102	Tubb5	0.9404	0.7701	0.1610	0.9532	0.9633	0.9684	0.9446	0.9925	0.9962	0.9726	0.9888	0.6146
NM_173304	Cyp2d5	0.5834	0.0667	0.0824	0.1490	0.3859	0.2891	0.0081	0.0446	0.2913	0.0478	0.2406	0.1090
NM_173103	Clcnkb	0.3817	0.0317	0.1032	0.0611	0.1130	0.7515	0.0560	0.3361	0.0116	0.0371	0.0089	0.1381

NM_173105	Aqp11	0.2387	0.0657	0.1741	0.0883	0.1817	0.2837	0.1161	0.2166	0.0522	0.1362	0.1551	0.0225
NM_001039914	Akirin2	0.3067	0.4495	0.9193	0.9446	0.3062	0.1986	0.2521	0.2533	0.1420	0.4219	0.2913	0.4375
NM_207601	Plp2	0.3431	0.7973	0.0711	0.5011	0.3999	0.5124	0.0517	0.9399	0.9486	0.3672	0.8398	0.6667
NM_212526	Kcne4	0.4538	0.2412	0.5181	0.4144	0.2542	0.3982	0.4747	0.4105	0.4079	0.2664	0.0115	0.2366
NM_199397	Panx1	0.2649	0.6346	0.2712	0.6229	0.1376	0.2328	0.2845	0.2559	0.5706	0.6261	0.4335	0.4971
NM_001107589	Ankrd2	0.1448	0.3058	0.4714	0.1671	0.2629	0.0624	0.2995	0.2921	0.2995	0.1044	0.1236	0.2386
NM_173096	Mx1	0.0473	0.1364	0.2413	0.2972	0.1501	0.1001	0.0977	0.1571	0.5539	0.1235	0.1930	0.2508
NM_173109	Wfdc2	0.8211	0.6297	0.5024	0.8070	0.9835	0.2043	0.5613	0.9488	0.7380	0.8187	0.7874	0.7918
NM_201271	Prg-3	0.4535	0.1586	0.2796	0.0712	0.2196	0.2708	0.0187	0.1998	0.2565	0.0293	0.0082	0.0635
NM_173110	Prl8a5	0.4556	0.0343	0.0907	0.5793	0.4842	0.4586	0.2453	0.2091	0.6069	0.2293	0.2424	0.0674
NM_173111	Tpm3	0.4340	0.8411	0.3841	0.7059	0.7718	0.9282	0.6150	0.5394	0.7313	0.8614	0.7812	0.9466
NM_173114	Cipar1	0.6345	0.8038	0.7277	0.6235	0.0315	0.0516	0.6030	0.0004	0.9216	0.8516	0.8227	0.5128
NM_173113	Vnr1	0.6985	0.7257	0.7850	0.7646	0.6790	0.7021	0.7810	0.8335	0.5984	0.8217	0.6346	0.6958
NM_001079707	Gtsf1	0.6103	0.1785	0.6288	0.1481	0.6818	0.6538	0.5105	0.0453	0.6196	0.6634	0.5664	0.3235
NM_201416	Zc3h18	0.5216	0.9037	0.0535	0.2402	0.9131	0.6055	0.1413	0.8546	0.5512	0.5495	0.3723	0.1185
NM_173118	Npc2	0.0239	0.0880	0.0409	0.0158	0.0020	0.1774	0.0804	0.0724	0.0029	0.2061	0.1271	0.1191
NM_173312	Gcnt3	0.8979	0.9605	0.9820	0.9962	0.8223	0.9837	0.9036	0.8490	0.8656	0.7767	0.7602	0.9086
NM_178105	Gpm6a	0.9962	0.9940	0.9977	0.9659	0.9914	0.7488	0.9677	0.9453	0.9818	0.9958	0.9854	0.9783
NM_199379	RGD735065	0.5965	0.6413	0.8965	0.6626	0.7455	0.6656	0.9405	0.8013	0.7984	0.6440	0.6928	0.6191
NM_173121	Fam5c	0.2335	0.2970	0.0936	0.5447	0.4207	0.2263	0.0329	0.5475	0.1223	0.0279	0.2412	0.0813
NM_173323	LOC286989	0.3226	0.1275	0.0798	0.2367	0.0944	0.4425	0.2733	0.1039	0.4065	0.1332	0.4911	0.1071
NM_173327	Taok1	0.6613	0.8325	0.3524	0.8743	0.3592	0.9889	0.8101	0.4267	0.6425	0.5328	0.9560	0.9289
NM_173125	Pdlim7	0.8259	0.7698	0.0365	0.2970	0.8016	0.8265	0.8194	0.8729	0.0449	0.4819	0.5918	0.1884
NM_173126	Nid67	0.0337	0.1054	0.1486	0.9510	0.1079	0.3254	0.1079	0.0700	0.1239	0.1129	0.1454	0.0712
NM_199387	Mterfd1	0.9507	0.6899	0.8182	0.7406	0.6692	0.6872	0.5353	0.6545	0.7777	0.6649	0.8811	0.5016
NM_001042619	Hsd3b	0.5992	0.2256	0.2779	0.2745	0.2730	0.6703	0.4607	0.2730	0.3485	0.3988	0.6278	0.3544
NM_199389	RGD735194	0.1053	0.2163	0.0657	0.2461	0.0518	0.3556	0.3879	0.3578	0.1071	0.4414	0.1078	0.0104
NM_199390	Flcn	0.9350	0.9735	0.8108	0.5043	0.9795	0.9310	0.1849	0.9522	0.7382	0.9834	0.9969	0.8297
NM_199391	RGD735112	0.8887	0.4284	0.3200	0.9398	0.6971	0.4711	0.6241	0.6811	0.5710	0.5233	0.4123	0.4109
NM_173132	Testin	0.8365	0.8592	0.7553	0.7480	0.8018	0.8744	0.9573	0.6531	0.8258	0.8791	0.9678	0.7124
NM_173139	Gnat3	0.5828	0.5568	0.7289	0.4779	0.3714	0.2251	0.5199	0.2808	0.3312	0.4185	0.6668	0.3169
NM_173138	Dlgap3	0.1547	0.1490	0.0480	0.0578	0.0745	0.0893	0.0204	0.2461	0.4169	0.3602	0.0449	0.5761
NM_173128	Krt20	0.5666	0.0079	0.1250	0.5626	0.1272	0.2352	0.5777	0.3425	0.0287	0.2174	0.5291	0.6326
NM_001047857	Ube2e3	0.9136	0.9355	0.9738	0.9903	0.8999	0.9380	0.9586	0.8566	0.8765	0.9302	0.9828	0.1908
NM_173129	Olr1278	0.2472	0.4537	0.5015	0.7634	0.6723	0.3943	0.8666	0.2381	0.8077	0.4992	0.6077	0.4933
NM_199403	Dd25	0.2133	0.0296	0.0649	0.0131	0.0795	0.0835	0.1388	0.0592	0.3527	0.2577	0.1341	0.1614
NM_203335	Vkorc1	0.1891	0.0951	0.7559	0.7389	0.0958	0.2991	0.2437	0.0841	0.0873	0.0678	0.1975	0.0169
NM_173331	Mapk15	0.2354	0.1372	0.4198	0.4227	0.6615	0.1494	0.5943	0.5229	0.1099	0.5849	0.4195	0.5088
NM_199406	Cxadrl1	0.4972	0.0791	0.0333	0.5042	0.1387	0.1464	0.3468	0.2948	0.1562	0.4584	0.6259	0.4421
NM_001034925	Cpt1c	0.8136	0.0663	0.0056	0.8472	0.7208	0.7339	0.3018	0.7975	0.8153	0.4207	0.7301	0.6431
NM_173141	Tfpi2	0.3572	0.5659	0.9339	0.5455	0.1119	0.7161	0.9384	0.6624	0.2164	0.4246	0.8453	0.6452
NM_173140	Cryba2	0.3497	0.4517	0.2970	0.1337	0.1604	0.4559	0.5790	0.3389	0.7559	0.4375	0.2496	0.1145
NM_001079711	Rad51ap1	0.2054	0.3131	0.9092	0.2576	0.7377	0.3645	0.5542	0.0837	0.6800	0.3870	0.0942	0.2110
NM_173145	Dlgap4	0.8954	0.8682	0.7866	0.8571	0.8077	0.7250	0.7859	0.8493	0.8918	0.8194	0.8260	0.8104
NM_173143	Abi2	0.6899	0.7240	0.6442	0.7274	0.6361	0.5832	0.4080	0.6895	0.6551	0.5928	0.5926	0.4906
NM_199412	Cbara1	0.3891	0.8209	0.9102	0.9177	0.4336	0.3349	0.7066	0.2064	0.8306	0.7586	0.7511	0.4580
NM_207596	Nudt2	0.1270	0.4618	0.7549	0.9513	0.5233	0.0293	0.8518	0.7562	0.1265	0.1800	0.1568	0.1529
NM_173146	Unc13c	0.5376	0.8976	0.3665	0.5579	0.4455	0.4869	0.5624	0.7417	0.8157	0.7600	0.5258	0.4458
NM_199486	Pcdha11	0.9165	0.8300	0.8817	0.8845	0.8450	0.4549	0.9304	0.7224	0.7753	0.3547	0.8652	0.6986

NM_182814	Cct4	0.0675	0.1690	0.0302	0.0318	0.1254	0.5987	0.0537	0.0877	0.1462	0.1303	0.2506	0.8245
NM_001085353	Gpr177	0.6380	0.2196	0.7472	0.7340	0.3932	0.5417	0.5820	0.6087	0.6800	0.3540	0.5599	0.4843
NM_173153	Gimap4	0.4764	0.7001	0.3872	0.3072	0.5179	0.7037	0.4303	0.3796	0.4757	0.1299	0.2046	0.4329
NM_173154	Asam	0.9529	0.9526	0.9927	0.8147	0.9950	0.9877	0.9995	0.9786	0.8870	0.9830	0.9911	0.6099
NM_173300	Olr1271	0.4346	0.0995	0.1424	0.1104	0.0468	0.2155	0.1480	0.2351	0.0897	0.0868	0.3975	0.2068
NM_173289	Cryge	0.0520	0.1344	0.1188	0.0314	0.3024	0.3187	0.4210	0.0598	0.1185	0.0892	0.1927	0.1309
NM_173293	Olr59	0.7790	0.2112	0.5825	0.4480	0.5879	0.3875	0.6233	0.7000	0.3473	0.6447	0.7798	0.8115
NM_173303	Cox6c1	0.3469	0.4247	0.5574	0.0970	0.0733	0.1006	0.3565	0.0595	0.0402	0.4908	0.0592	0.0540
NM_173309	Elavl2	0.7082	0.6835	0.6941	0.6330	0.6942	0.6099	0.6073	0.6464	0.6480	0.7130	0.7261	0.5640
NM_199499	Lgi4	0.3024	0.6739	0.8838	0.1584	0.1269	0.4102	0.2376	0.2554	0.3809	0.6414	0.8906	0.6282
NM_173148	Obp2b	0.1603	0.2997	0.1499	0.2110	0.0381	0.5713	0.0944	0.1639	0.4161	0.2310	0.0104	0.1462
NM_203334	Slc6a5	0.8296	0.4185	0.2797	0.5683	0.8392	0.3620	0.7811	0.4032	0.5988	0.4885	0.2398	0.5708
NM_173291	Klra22	0.2558	0.3406	0.3693	0.1708	0.4831	0.4728	0.5370	0.3899	0.2488	0.4632	0.4082	0.4051
NM_182842	Camk1g	0.1569	0.6323	0.2726	0.5334	0.6397	0.6315	0.3724	0.4096	0.8236	0.2509	0.3824	0.3468
NM_181386	Sgms1	0.1717	0.0853	0.6649	0.4778	0.0778	0.1569	0.6526	0.3425	0.5824	0.6126	0.2562	0.2612
NM_207589	Selk	0.0158	0.2159	0.0817	0.1483	0.0482	0.0513	0.1904	0.2883	0.3557	0.0524	0.0589	0.1140
NM_181629	Sgtb	0.8326	0.8438	0.6545	0.5491	0.9271	0.7856	0.5070	0.6664	0.7974	0.1745	0.1741	0.8960
NM_198770	Zmynd19	0.8884	0.9902	0.4044	0.9158	0.6652	0.7643	0.8605	0.8165	0.7980	0.7873	0.9181	0.7624
NM_152790	Carhsp1	0.4042	0.3662	0.1567	0.2855	0.2512	0.3225	0.3528	0.2726	0.4431	0.4198	0.4210	0.9900
NM_177425	Csrp2	0.8376	0.8368	0.8799	0.5672	0.7499	0.2138	0.8707	0.6106	0.5889	0.9777	0.8889	0.0175
NM_148892	Ostf1	0.0641	0.0570	0.1744	0.4457	0.0444	0.0300	0.0272	0.2463	0.1112	0.0648	0.0489	0.0799
NM_198132	Hnrnpa3	0.6721	0.7197	0.6348	0.6428	0.5666	0.5794	0.6072	0.6750	0.5619	0.6974	0.7147	0.6606
NM_152845	Brs3	0.6795	0.7473	0.6283	0.5339	0.4908	0.0045	0.1634	0.4692	0.2822	0.4963	0.7368	0.5537
NM_145783	Cox5a	0.1714	0.4556	0.4010	0.8576	0.0240	0.0681	0.0943	0.0866	0.1155	0.2101	0.1395	0.0837
NM_172072	Vps45	0.2215	0.0350	0.2534	0.6972	0.8351	0.6332	0.5657	0.5938	0.0724	0.0992	0.4433	0.0708
NM_152847	Snx27	0.9260	0.9824	0.8945	0.6851	0.7928	0.6837	0.9705	0.6538	0.9532	0.9220	0.9661	0.9090
NM_173097	Reg3g	0.6047	0.4193	0.6441	0.4375	0.3460	0.4153	0.7437	0.5399	0.1332	0.4499	0.6144	0.7678
NM_201421	Fgfr1op2	0.8494	0.6070	0.7413	0.3707	0.9305	0.9685	0.2545	0.9864	0.9409	0.8803	0.9587	0.9651
NM_152848	Ly49i2	0.2076	0.3867	0.2416	0.3633	0.5723	0.3150	0.3984	0.4673	0.3706	0.2045	0.5566	0.2188
NM_201424	Ugt1a3	0.3588	0.6894	0.6642	0.6409	0.4866	0.4894	0.4260	0.2854	0.3787	0.4162	0.5315	0.6236
NM_199120	Ift81	0.7792	0.1952	0.9238	0.9984	0.8774	0.6277	0.6825	0.7583	0.7113	0.1862	0.7007	0.7972
NM_152935	Tomm20	0.8403	0.7703	0.4629	0.4289	0.5035	0.8356	0.1242	0.1521	0.2789	0.7619	0.8055	0.7195
NM_152861	Rbm10	0.7446	0.8388	0.5775	0.7125	0.7817	0.9580	0.9209	0.8212	0.9273	0.7855	0.7717	0.6860
NM_001100942	Pomp	0.0016	0.0817	0.0012	0.0276	0.0024	0.0013	0.0027	0.0035	0.0026	0.0028	0.0158	0.0024
NM_173100	Bdkrb2	0.5488	0.4954	0.7445	0.6128	0.7445	0.7894	0.6656	0.5739	0.2233	0.9237	0.5555	0.3435
NM_172331	Vps24	0.1261	0.4325	0.8527	0.8225	0.1854	0.3534	0.8049	0.7925	0.4487	0.5793	0.8268	0.0845
NM_001013901	RGD1305592	0.3030	0.6448	0.4083	0.9218	0.9366	0.1315	0.2799	0.9638	0.5136	0.6474	0.4906	0.1221
NM_017037	Pmp22	0.7018	0.7471	0.9474	0.9946	0.9660	0.9812	0.8934	0.9217	0.1116	0.9387	0.4889	0.9705
NM_001105908	Pms2	0.8733	0.7772	0.0846	0.7801	0.8654	0.7539	0.4727	0.8745	0.8761	0.7714	0.7958	0.7766
NM_177933	Sel1l	0.2936	0.4019	0.4409	0.6399	0.2565	0.2042	0.9929	0.3341	0.5492	0.9078	0.9017	0.3606
NM_153302	Dcps	0.3920	0.4273	0.4432	0.8292	0.0372	0.0948	0.5873	0.2592	0.6298	0.8499	0.5758	0.1441
NM_153305	Fzd9	0.0802	0.1891	0.3883	0.0956	0.2984	0.4694	0.1575	0.4017	0.3416	0.5516	0.5915	0.4722
NM_184049	Stk25	0.5654	0.0806	0.1313	0.1290	0.4350	0.6226	0.0420	0.6741	0.6110	0.0397	0.7160	0.8534
NM_153739	Duox1	0.2434	0.1555	0.2392	0.2312	0.9630	0.8976	0.2151	0.7766	0.2485	0.0059	0.0221	0.0924
NM_153297	Cops2	0.7265	0.9074	0.9357	0.9147	0.4016	0.7705	0.6857	0.2892	0.7878	0.8514	0.4404	0.8087
NM_153308	Grina	0.0967	0.0001	0.0002	0.0001	0.0154	0.0781	0.0453	0.0009	0.0002	0.0002	0.0002	0.0038
NM_153298	Sfxn5	0.1752	0.3184	0.5658	0.0395	0.5693	0.3721	0.0527	0.0869	0.7717	0.3350	0.6068	0.6079
NM_203410	Ifi27l	0.2503	0.2959	0.3061	0.3258	0.3914	0.3924	0.3833	0.4340	0.2691	0.3036	0.2944	0.2445
NM_153309	Mina	0.8113	0.6049	0.0239	0.5728	0.8431	0.7278	0.4184	0.2078	0.4035	0.8992	0.8804	0.4863

NM_203512	LOC298109	0.2867	0.4265	0.2872	0.2573	0.6436	0.2159	0.3806	0.4158	0.6905	0.3463	0.3493	0.3126
NM_001047916	Syncrip	0.7010	0.6488	0.4731	0.6849	0.6754	0.6511	0.5061	0.7010	0.6921	0.5081	0.6627	0.6558
NM_153310	St18	0.8078	0.8622	0.7964	0.7274	0.9023	0.4344	0.9608	0.7708	0.8183	0.9543	0.9637	0.9422
NM_153311	Tmprss5	0.9068	0.0862	0.8446	0.8212	0.7567	0.9308	0.7970	0.7115	0.7968	0.3411	0.7661	0.8402
NM_172034	Fntb	0.4043	0.4466	0.1256	0.7381	0.3043	0.6778	0.9355	0.0813	0.5964	0.6186	0.6244	0.8040
NM_153728	Soat2	0.1933	0.2585	0.5799	0.3942	0.2269	0.4037	0.3141	0.5558	0.3217	0.7877	0.1831	0.6568
NM_152937	Fadd	0.0569	0.8052	0.0631	0.6811	0.2691	0.4316	0.6270	0.0821	0.7024	0.3622	0.6690	0.2906
NM_173094	Hmgcs2	0.0129	0.1239	0.4879	0.2588	0.2324	0.5160	0.5814	0.1964	0.6094	0.0194	0.3368	0.4700
NM_153473	Myo7a	0.3034	0.1406	0.0256	0.3440	0.3216	0.3573	0.3361	0.2544	0.1640	0.1352	0.1287	0.1293
NM_173137	Fads3	0.1700	0.1635	0.1723	0.1704	0.1751	0.1708	0.1737	0.1667	0.1625	0.1581	0.1640	0.1681
NM_171992	Ccnd1	0.9689	0.9647	0.9909	0.9882	0.9700	0.9802	0.9993	0.9955	0.9441	0.9917	0.9827	0.9315
NM_206847	Pfkp	0.2458	0.0850	0.0228	0.8612	0.0581	0.1980	0.4131	0.0720	0.3560	0.2123	0.1514	0.7809
NM_198760	Slc16a6	0.5531	0.5452	0.5118	0.4822	0.6265	0.8142	0.7561	0.7846	0.5730	0.2696	0.4984	0.7802
NM_153722	Mrgprf	0.0934	0.0032	0.2465	0.1166	0.2163	0.2640	0.0376	0.0496	0.2211	0.0053	0.1038	0.3217
NM_153626	Npas4	0.3071	0.1320	0.1323	0.3311	0.0579	0.0844	0.0261	0.1401	0.0827	0.0644	0.1924	0.1408
NM_153625	Slc12a8	0.3863	0.1409	0.4258	0.4502	0.3221	0.6843	0.5870	0.3151	0.4788	0.0495	0.4795	0.1472
NM_175580	Gnl3	0.8669	0.9620	0.2543	0.1421	0.7800	0.8581	0.6300	0.8702	0.7012	0.7182	0.8738	0.7107
NM_181626	Isca1	0.3311	0.3375	0.9787	0.9268	0.2348	0.1716	0.9542	0.1527	0.3150	0.2372	0.5148	0.1080
NM_153733	Serpinb10	0.0317	0.0089	0.0685	0.0385	0.0453	0.0002	0.0577	0.0358	0.0615	0.0409	0.0177	0.0483
NM_153729	V1ra16	0.1282	0.0282	0.3545	0.2521	0.4977	0.2776	0.1065	0.0683	0.3472	0.3503	0.1960	0.1573
NM_207611	Bhlhb9	0.5930	0.8315	0.6488	0.7449	0.6112	0.3787	0.4955	0.3916	0.3725	0.7046	0.5835	0.1955
NM_207599	Hyal3	0.3414	0.0671	0.1321	0.5033	0.5332	0.4843	0.3069	0.5857	0.5301	0.8202	0.4785	0.5620
NM_153736	Prl3a1	0.1703	0.2528	0.0761	0.1684	0.1235	0.4959	0.2122	0.2018	0.0632	0.3793	0.1837	0.0361
NM_153735	Nptx1	0.6043	0.6887	0.6528	0.2296	0.7294	0.6895	0.6881	0.7037	0.4706	0.5641	0.6756	0.7031
NM_207614	LOC362855	0.9035	0.1702	0.1577	0.0751	0.0850	0.2957	0.5437	0.0540	0.0570	0.1153	0.0976	0.0486
NM_153737	Sostdc1	0.5300	0.8367	0.4799	0.6452	0.8136	0.4264	0.5135	0.6434	0.5370	0.5679	0.7886	0.7413
NM_207617	Iqsec3	0.4262	0.9293	0.8341	0.4617	0.2568	0.0063	0.4637	0.3567	0.7442	0.4031	0.7653	0.5585
NM_212459	Arl9	0.6864	0.4214	0.8298	0.7733	0.1510	0.5054	0.5679	0.6375	0.7197	0.7505	0.5236	0.7068
NM_001079899	Aup1	0.3027	0.1631	0.2839	0.1703	0.1749	0.2923	0.4906	0.1665	0.6592	0.4653	0.3837	0.7048
NM_175579	Wnk4	0.6416	0.6142	0.9231	0.7029	0.8277	0.6049	0.3869	0.5992	0.8585	0.8618	0.6649	0.8407
NM_212488	Btnl7	0.5701	0.0888	0.2911	0.0708	0.1894	0.1410	0.0830	0.1479	0.0038	0.4134	0.7180	0.0431
NM_173307	Abca5	0.8533	0.7202	0.1963	0.5004	0.7593	0.4520	0.6680	0.8901	0.7276	0.7012	0.5585	0.7763
NM_173314	Zfp455	0.7917	0.6457	0.7452	0.7681	0.8224	0.8718	0.2774	0.8245	0.8416	0.8532	0.8286	0.6535
NM_001079891	Magee1	0.1211	0.1072	0.3069	0.2420	0.0207	0.0809	0.0194	0.0533	0.1647	0.2435	0.0442	0.0559
NM_212497	Dom3z	0.6333	0.7430	0.1054	0.0227	0.3002	0.1457	0.2671	0.4458	0.7557	0.7899	0.6028	0.5273
NM_212498	RGD1303066	0.3941	0.6255	0.4722	0.1331	0.4753	0.0350	0.1264	0.4410	0.1253	0.7736	0.4248	0.1882
NM_173315	Vom2r32	0.1962	0.6551	0.3146	0.6430	0.4671	0.3964	0.6462	0.5172	0.6529	0.4054	0.4784	0.5533
NM_173299	Np4	0.1544	0.3585	0.1429	0.4058	0.2825	0.0995	0.1217	0.0205	0.3444	0.2778	0.2956	0.3621
NM_175707	Ppil3	0.7575	0.9797	0.0809	0.2591	0.5025	0.2523	0.2690	0.5023	0.3040	0.7975	0.3804	0.0351
NM_175755	Ppm1f	0.5567	0.4535	0.1563	0.8214	0.3752	0.5208	0.3749	0.3316	0.7638	0.0387	0.8631	0.5602
NM_175760	Cyp4a3	0.8287	0.5136	0.3007	0.5669	0.3554	0.6392	0.4501	0.5256	0.6690	0.2861	0.5166	0.5006
NM_212514	Tomm22	0.4218	0.0987	0.5952	0.7965	0.1541	0.5246	0.5675	0.2488	0.1024	0.0146	0.1798	0.3123
NM_212517	Ndufa11	0.0673	0.5341	0.1226	0.7864	0.1176	0.0307	0.6512	0.0172	0.3659	0.6250	0.6428	0.0327
NM_001079894	Plekha1	0.3730	0.7745	0.7655	0.6255	0.6672	0.8954	0.4095	0.4178	0.9251	0.4521	0.7702	0.8947
NM_173329	Defa	0.1744	0.1846	0.0628	0.0977	0.2449	0.4063	0.3060	0.1477	0.1376	0.4220	0.0694	0.1483
NM_173328	Lgr4	0.8702	0.0832	0.9552	0.0379	0.5802	0.8856	0.7412	0.7016	0.0560	0.2345	0.5612	0.1765
NM_001044233	Clec2l	0.9175	0.4140	0.6153	0.5837	0.9250	0.4533	0.3791	0.6121	0.6358	0.7103	0.5019	0.7587
NM_173340	Rpl13a	0.4225	0.0478	0.8276	0.3008	0.3539	0.7767	0.6906	0.6061	0.1882	0.3433	0.1499	0.6598
NM_173338	Slco6c1	0.2485	0.4998	0.5845	0.0541	0.3637	0.3279	0.6709	0.4036	0.3427	0.2526	0.4618	0.6238

NM_173337	Camk2n1	0.0133	0.1510	0.7769	0.0688	0.0159	0.0233	0.0258	0.0269	0.1052	0.0152	0.0417	0.0831
NM_198972	Gkn1	0.1284	0.1395	0.2592	0.1878	0.0723	0.0916	0.5025	0.1315	0.5253	0.1827	0.0704	0.2733
NM_198792	Txndc3	0.6995	0.3618	0.1719	0.1701	0.2494	0.4121	0.3836	0.5180	0.3140	0.2922	0.1638	0.2402
NM_198791	Tlr3	0.7511	0.2764	0.7554	0.7496	0.7218	0.5929	0.1079	0.4584	0.3581	0.8413	0.7380	0.0086
NM_198789	Bzw1	0.9519	0.9573	0.9833	0.9898	0.4186	0.6819	0.8829	0.5630	0.7648	0.4272	0.7793	0.8664
NM_198788	Sdhd	0.1811	0.5826	0.8877	0.4644	0.0194	0.3145	0.6199	0.0199	0.6680	0.7272	0.5637	0.7722
NM_198786	Mal2	0.6809	0.5514	0.8140	0.7673	0.6592	0.6488	0.6495	0.6109	0.6905	0.0908	0.6020	0.6973
NM_001077642	Cfd	0.0449	0.0040	0.8594	0.6221	0.4103	0.1607	0.2972	0.4855	0.0039	0.0875	0.0027	0.0026
NM_001039606	Gmds	0.3058	0.4034	0.4844	0.4766	0.3528	0.3647	0.5857	0.3364	0.2920	0.4483	0.4919	0.4416
NM_198784	Mup4	0.2849	0.4196	0.3541	0.1701	0.4304	0.2651	0.3309	0.4212	0.6370	0.3402	0.2950	0.2707
NM_198783	Hrpap20	0.4122	0.3824	0.2387	0.1409	0.3294	0.0427	0.0557	0.1948	0.5555	0.8416	0.1313	0.0152
NM_198781	Tmub1	0.4697	0.8760	0.7648	0.9197	0.6990	0.9477	0.9071	0.8780	0.8983	0.7337	0.5651	0.8047
NM_001077659	Mrps24	0.8890	0.8489	0.8965	0.7416	0.8338	0.7864	0.8271	0.9324	0.8131	0.9625	0.9639	0.5803
NM_178095	Abca1	0.0602	0.0254	0.0991	0.0815	0.0196	0.0474	0.0434	0.0650	0.0714	0.0551	0.0471	0.0449
NM_198780	Pck1	0.7131	0.5371	0.6668	0.5648	0.5184	0.4971	0.1187	0.1396	0.3569	0.6487	0.8001	0.7036
NM_001079898	RatNP-3b	0.3356	0.4578	0.2531	0.1697	0.3422	0.1706	0.1733	0.3525	0.2452	0.4200	0.3227	0.3753
NM_212548	Rdbp	0.3363	0.3560	0.4527	0.3855	0.3269	0.3797	0.4560	0.5243	0.3583	0.1579	0.4464	0.2819
NM_001077679	Med22	0.7574	0.0624	0.7656	0.7508	0.3114	0.8074	0.8027	0.5229	0.6216	0.1365	0.2198	0.3092
NM_001077682	Spesp1	0.1848	0.2607	0.1950	0.5191	0.2846	0.0859	0.0193	0.5968	0.4560	0.5258	0.4018	0.2669
NM_178094	Itpkc	0.3606	0.0089	0.7753	0.2299	0.3135	0.3458	0.2878	0.0862	0.3609	0.2123	0.0219	0.8971
NM_176077	G6pc3	0.2288	0.2675	0.7641	0.5782	0.0509	0.1115	0.9696	0.1282	0.8432	0.9151	0.6776	0.3589
NM_201270	Il4	0.0096	0.0075	0.0262	0.0683	0.1188	0.1672	0.2780	0.1231	0.0235	0.0461	0.2824	0.2795
NM_175582	Id4	0.7142	0.6248	0.6522	0.6470	0.5448	0.4606	0.4160	0.6510	0.5165	0.2639	0.6933	0.3545
NM_175581	Ctsr	0.5759	0.6407	0.6301	0.5259	0.4776	0.1017	0.4723	0.7407	0.3477	0.7865	0.7630	0.0945
NM_213610	RGD1302996	0.8284	0.9114	0.7322	0.8961	0.9089	0.9265	0.7989	0.8696	0.8541	0.8995	0.9114	0.9336
NM_175592	Cacna2d2	0.5908	0.4305	0.6427	0.3356	0.0177	0.5126	0.2700	0.4399	0.0366	0.8118	0.3648	0.6254
NM_001079885	Vwa3a	0.4637	0.1256	0.1145	0.1433	0.2827	0.1828	0.0740	0.3068	0.3970	0.1821	0.1281	0.4999
NM_175593	Tpsg1	0.5595	0.3243	0.7045	0.5095	0.4112	0.4524	0.2036	0.4381	0.3867	0.6287	0.4140	0.6627
NM_199266	P22k15	0.3613	0.5266	0.6766	0.4636	0.4297	0.4861	0.5882	0.2978	0.6935	0.4596	0.0677	0.1366
NM_199256	Sec61a1	0.7098	0.9061	0.5002	0.8792	0.2223	0.8034	0.9438	0.3399	0.9245	0.7463	0.7272	0.7917
NM_199253	Pcsk9	0.7974	0.7935	0.7536	0.7651	0.6679	0.8401	0.7895	0.8980	0.9545	0.6053	0.9051	0.3874
NM_199409	Panx2	0.5383	0.8656	0.5443	0.6945	0.7954	0.3320	0.5932	0.7362	0.8613	0.8842	0.5292	0.9647
NM_201419	Clca4	0.4017	0.4996	0.3066	0.2034	0.2731	0.0895	0.5781	0.5291	0.6034	0.8466	0.4586	0.0600
NM_001106937	Tbx22	0.8140	0.5067	0.6110	0.6350	0.8521	0.3979	0.0814	0.9035	0.8119	0.4920	0.8577	0.1705
NM_001115027	RGD1562416	0.9057	0.9218	0.9660	0.9416	0.9222	0.9705	0.9918	0.9638	0.9871	0.9171	0.9461	0.9703
NM_001115044	LOC685157	0.4792	0.2578	0.6138	0.2595	0.3201	0.3886	0.3438	0.6116	0.4878	0.3053	0.5944	0.3317
NM_001122658	Fam178b	0.0915	0.0006	0.0011	0.0004	0.0002	0.0007	0.0010	0.1291	0.0002	0.0529	0.0013	0.0020
NM_001114391	Rpl7a	0.7648	0.4818	0.5088	0.2693	0.2469	0.3255	0.5696	0.2683	0.1920	0.4621	0.3882	0.1588
NM_001126081	Leap2	0.3100	0.5520	0.6630	0.4492	0.1281	0.4944	0.6609	0.5435	0.5235	0.3809	0.2831	0.5203
NM_001126079	Acbd7	0.0163	0.4474	0.8848	0.5221	0.0086	0.0084	0.6826	0.0002	0.1508	0.5507	0.8262	0.5260
NM_001126272	Znf692	0.8330	0.8943	0.0505	0.0493	0.8186	0.8571	0.5008	0.9497	0.9630	0.9750	0.9398	0.7932
NM_001126285	Slc22a13	0.1830	0.3961	0.0612	0.5040	0.5435	0.4262	0.2765	0.4881	0.1408	0.2959	0.5126	0.3835
NM_001130547	Gpihbp1	0.3443	0.2305	0.6492	0.7547	0.7379	0.6186	0.5953	0.6924	0.0101	0.3272	0.7482	0.7042
NM_001100810	Rnpc3	0.4785	0.4628	0.3676	0.4073	0.3946	0.3523	0.4938	0.3553	0.3362	0.7210	0.5108	0.2394
NM_001115026	RGD1565316	0.4382	0.5902	0.1952	0.1086	0.5486	0.5193	0.4759	0.0540	0.4629	0.4425	0.2436	0.4838
NM_001115023	Ddx49	0.2565	0.7279	0.1117	0.0009	0.0143	0.0572	0.1113	0.0031	0.5939	0.6344	0.2255	0.4594
NM_001013900	Ccdc95	0.7916	0.7456	0.9243	0.7412	0.8757	0.8000	0.8730	0.3112	0.9609	0.6196	0.9565	0.3155
NM_022935	Abp1	0.4573	0.2323	0.3402	0.4407	0.3186	0.2844	0.3472	0.1685	0.1966	0.4105	0.3446	0.1707
NM_138880	Ifng	0.1197	0.0173	0.1155	0.0835	0.0215	0.0196	0.2708	0.0525	0.2382	0.1895	0.1428	0.0733

NM_133316	Mutyh	0.2607	0.1148	0.0027	0.3694	0.5715	0.4485	0.0039	0.6284	0.1483	0.4345	0.0259	0.4981
NM_144757	Zfp180	0.8539	0.9973	0.3101	0.7152	0.9712	0.9835	0.6863	0.9857	0.7731	0.8644	0.8254	0.8250
NM_001114330	Grm1	0.7193	0.5535	0.6226	0.6718	0.7964	0.6303	0.2344	0.8374	0.6530	0.6983	0.5260	0.5095
NM_001110336	Vegfa	0.7331	0.7016	0.8891	0.5415	0.5108	0.5606	0.9797	0.4868	0.5533	0.6890	0.7006	0.9767
NM_001108776	Dnajc13	0.9962	0.9866	0.9604	0.9509	0.9904	0.9798	0.9430	0.9967	0.9427	0.9896	0.9906	0.8511
NM_001113740	41,159.0000	0.7117	0.6260	0.8974	0.7215	0.7090	0.7285	0.7167	0.7128	0.6930	0.5779	0.6224	0.4042
NM_001111294	Hnrnpa3	0.6721	0.7197	0.6348	0.6428	0.5666	0.5794	0.6072	0.6750	0.5619	0.6974	0.7147	0.6606
NM_001126302	LOC691556	0.4126	0.6252	0.3115	0.5568	0.5357	0.5742	0.5293	0.3006	0.3555	0.3132	0.1631	0.4691
NM_001007149	Stau2	0.9866	0.9922	0.9993	0.9988	0.9995	0.9973	0.9495	0.9766	0.8819	0.9431	0.9996	0.9910
NM_001007150	Stau2	0.9866	0.9922	0.9993	0.9988	0.9995	0.9973	0.9495	0.9766	0.8819	0.9431	0.9996	0.9910
NM_001115031	RGD1563046	0.3784	0.5587	0.3234	0.4877	0.1415	0.5423	0.4336	0.2849	0.2422	0.1905	0.0253	0.6954
NM_001115024	Lman2	0.2452	0.3370	0.3709	0.1042	0.0886	0.1083	0.6080	0.0064	0.6407	0.6726	0.4928	0.1189
NM_001115047	LOC687694	0.3790	0.4173	0.1716	0.6641	0.2642	0.5934	0.1728	0.3811	0.3022	0.3939	0.3195	0.2334
NM_001025270	Mc3r	0.6367	0.5921	0.5351	0.3888	0.1557	0.0704	0.4110	0.3978	0.3505	0.3778	0.4526	0.2598
NM_001100549	Mrps9	0.1522	0.1834	0.8179	0.0607	0.0798	0.1555	0.7626	0.4685	0.3958	0.2882	0.1616	0.6027
NM_001122644	Galnt9	0.4175	0.7148	0.5091	0.2418	0.7622	0.5131	0.7109	0.3144	0.5923	0.2054	0.5240	0.6466
NM_001008326	Rexo2	0.7243	0.8320	0.0330	0.9162	0.8721	0.7641	0.4738	0.9134	0.2578	0.7449	0.6770	0.3572
NM_001009535	Pms1	0.8102	0.7459	0.7525	0.7573	0.5979	0.8742	0.6272	0.7853	0.6469	0.8428	0.8794	0.6255
NM_001008324	Eif4b	0.9727	0.8623	0.9436	0.6628	0.9944	0.9970	0.9328	0.9449	0.9831	0.8085	0.9037	0.7949
NM_001012035	Cdkl2	0.3135	0.3424	0.3637	0.4008	0.2597	0.3288	0.2236	0.3687	0.4986	0.4504	0.3412	0.0972
NM_001013174	Laptm4b	0.8946	0.0736	0.9871	0.8925	0.3297	0.1624	0.7408	0.6102	0.9968	0.7093	0.1486	0.6268
NM_001008317	Txndc4	0.5986	0.1659	0.7932	0.6226	0.1133	0.0100	0.3780	0.0258	0.2049	0.1549	0.1482	0.1925
NM_001008316	Plag1	0.0132	0.1730	0.2333	0.0260	0.1768	0.0936	0.1933	0.2563	0.1280	0.2143	0.1211	0.0969
NM_001008314	Mkrn2	0.7372	0.8663	0.8711	0.6675	0.8036	0.8597	0.5628	0.6447	0.3041	0.4709	0.4886	0.9441
NM_001008312	Serinc3	0.8378	0.2745	0.8966	0.5829	0.7077	0.6498	0.0191	0.2770	0.3235	0.3767	0.3826	0.9362
NM_001008309	Btf3	0.4569	0.9277	0.8644	0.7185	0.4678	0.7110	0.7991	0.8512	0.3240	0.6880	0.7425	0.6602
NM_001009706	Lhpp	0.0306	0.2581	0.8496	0.9425	0.3326	0.2072	0.6589	0.3928	0.2815	0.0067	0.1544	0.2751
NM_001008298	Stard3nl	0.9421	0.0427	0.9699	0.0995	0.8707	0.9529	0.0746	0.7859	0.0843	0.0426	0.1089	0.6067
NM_001008297	RGD1305689	0.9612	0.9314	0.5202	0.8831	0.6475	0.7338	0.7903	0.7756	0.6528	0.7747	0.7438	0.4264
NM_001012217	Ssh3	0.1639	0.4451	0.5204	0.5844	0.4119	0.2288	0.2353	0.1065	0.9412	0.9806	0.1633	0.8098
NM_001008295	Fip1l1	0.0854	0.0242	0.5891	0.0354	0.0228	0.0806	0.0013	0.0653	0.0352	0.0876	0.0849	0.3063
NM_001008293	Tfb2m	0.4600	0.1145	0.6519	0.6011	0.8822	0.6375	0.0469	0.6550	0.9236	0.3014	0.2033	0.7475
NM_001008303	Snrpa	0.8120	0.8485	0.7245	0.9669	0.8285	0.9882	0.8481	0.8978	0.9155	0.7874	0.9268	0.2398
NM_001008292	Diablo	0.5266	0.8172	0.7736	0.6869	0.1653	0.1857	0.8567	0.4471	0.9046	0.6186	0.9756	0.3340
NM_001008291	Tfip11	0.5372	0.7392	0.2528	0.0923	0.1935	0.3917	0.1216	0.6931	0.9042	0.4117	0.4806	0.4035
NM_001025720	Dhtkd1	0.5234	0.5797	0.4279	0.8597	0.7575	0.2010	0.5353	0.5706	0.7575	0.8893	0.2060	0.7631
NM_001013875	Rhbdd3	0.6144	0.6384	0.0358	0.0288	0.9451	0.2201	0.2547	0.8045	0.8821	0.8718	0.8330	0.7948
NM_001008286	Mtx2	0.0396	0.5879	0.6391	0.6612	0.0918	0.1005	0.2777	0.2471	0.2927	0.5943	0.9049	0.1515
NM_001008284	Crkl	0.8459	0.4694	0.4917	0.9333	0.9022	0.9566	0.8551	0.5636	0.5193	0.3908	0.5218	0.2589
NM_001009637	Lars	0.8354	0.9228	0.8025	0.8621	0.7662	0.9539	0.9593	0.6871	0.7586	0.7932	0.9092	0.7540
NM_001008281	Psmd3	0.8049	0.6515	0.1614	0.5133	0.9467	0.8442	0.8361	0.8922	0.5170	0.4360	0.7651	0.4795
NM_001008280	Lrrc59	0.9602	0.9903	0.1407	0.9945	0.8962	0.9815	0.9896	0.8629	0.9459	0.9878	0.9968	0.9403
NM_001010966	Pigv	0.9551	0.6881	0.8947	0.8005	0.8244	0.4461	0.8397	0.9283	0.7605	0.6036	0.7099	0.5921
NM_001009965	Tsku	0.3251	0.1952	0.1635	0.0203	0.2414	0.2225	0.2923	0.2848	0.6345	0.6157	0.3790	0.9310
NM_001008277	Tbl3	0.6270	0.5052	0.0007	0.0258	0.4645	0.8072	0.1496	0.4631	0.2068	0.3214	0.5193	0.5030
NM_001011923	Prpf4b	0.8189	0.5699	0.8217	0.6466	0.3743	0.4464	0.3509	0.5815	0.1384	0.4969	0.5755	0.5027
NM_001011927	Atp6v0d1	0.4462	0.1986	0.7797	0.4017	0.2342	0.3854	0.6848	0.3392	0.3626	0.1115	0.2758	0.5039
NM_001007801	Yipf3	0.9330	0.7952	0.5562	0.9658	0.8775	0.5439	0.9641	0.7433	0.7702	0.2553	0.7480	0.2615
NM_001007759	40,971.0000	0.0643	0.0321	0.1623	0.2270	0.0780	0.2621	0.2311	0.0086	0.1959	0.1532	0.1445	0.1299

NM_001007758	Nufip1	0.5550	0.7189	0.1576	0.2778	0.6484	0.4117	0.1246	0.2225	0.1341	0.6759	0.6559	0.1012
NM_001007757	Armcx6	0.5317	0.7110	0.5521	0.0722	0.7870	0.6719	0.3761	0.7653	0.4859	0.1375	0.2885	0.4228
NM_001009419	Cmas	0.2361	0.8555	0.7190	0.0383	0.0572	0.0504	0.8816	0.2886	0.6483	0.7102	0.7413	0.7680
NM_001007753	Cyb561d2	0.0608	0.0427	0.3921	0.0476	0.0171	0.1559	0.5603	0.0486	0.0595	0.1903	0.0162	0.0352
NM_001010945	Tmem120a	0.2525	0.3462	0.3290	0.3971	0.3896	0.2717	0.6001	0.3183	0.3020	0.1577	0.1630	0.4337
NM_001009679	Psph	0.7619	0.8838	0.0994	0.8150	0.8412	0.8644	0.6615	0.8572	0.8400	0.8826	0.8453	0.3150
NM_001013982	Orai1	0.0486	0.1318	0.0147	0.0189	0.0115	0.0172	0.0007	0.0573	0.1350	0.0682	0.2277	0.4213
NM_001013886	Tubb2b	0.7388	0.3466	0.1135	0.2876	0.1698	0.3225	0.1092	0.4877	0.3245	0.6443	0.1206	0.2068
NM_001011902	Rabl5	0.0708	0.3721	0.7346	0.7994	0.3051	0.0987	0.3145	0.5158	0.0655	0.2316	0.1655	0.1237
NM_001007744	Tor2a	0.8037	0.6619	0.9900	0.8539	0.8033	0.5251	0.9497	0.9728	0.4758	0.7406	0.7871	0.1852
NM_001013177	Sult1c2a	0.7016	0.8312	0.8008	0.6257	0.8216	0.7650	0.7501	0.5153	0.6501	0.0660	0.5608	0.2679
NM_001007741	Isg20l2	0.1990	0.0967	0.1847	0.5033	0.0117	0.1419	0.6478	0.0182	0.4626	0.2926	0.4021	0.7404
NM_001013863	Ydjc	0.8590	0.6226	0.6994	0.9611	0.8790	0.7570	0.9005	0.8777	0.5065	0.7451	0.8173	0.3736
NM_001012189	Lpcat3	0.3322	0.4583	0.3186	0.8673	0.6403	0.3256	0.4921	0.2773	0.4829	0.8153	0.5000	0.9160
NM_001007738	RGD1359349	0.3447	0.1605	0.3577	0.6728	0.5713	0.3438	0.2416	0.5388	0.2884	0.4655	0.7694	0.6642
NM_001007736	Tmem109	0.8583	0.9515	0.8938	0.8437	0.8446	0.4371	0.9874	0.8905	0.9655	0.9467	0.7620	0.9053
NM_001011967	Fastk	0.5160	0.8099	0.8036	0.8227	0.8530	0.7439	0.9337	0.7820	0.8812	0.7581	0.7823	0.6258
NM_001007731	Golga7	0.1812	0.3272	0.6444	0.2854	0.3122	0.2530	0.2776	0.2294	0.2353	0.2850	0.5296	0.8853
NM_001007730	Cabp7	0.2955	0.0968	0.2790	0.0289	0.2870	0.3221	0.8405	0.3967	0.3252	0.2492	0.6293	0.2655
NM_001013854	Fam44b	0.1836	0.9441	0.0886	0.0215	0.0987	0.0946	0.2682	0.0718	0.8488	0.9152	0.6650	0.6722
NM_001007728	Mpzl1	0.9793	0.9701	0.9770	0.7275	0.9987	0.9536	0.7651	0.9799	0.9472	0.8754	0.9785	0.5297
NM_001007725	Icam2	0.4339	0.2847	0.3210	0.7598	0.2760	0.5309	0.6107	0.6768	0.6025	0.3339	0.0585	0.5046
NM_001007721	Emp2	0.3222	0.0322	0.1711	0.2405	0.4375	0.2644	0.3596	0.2989	0.3496	0.3264	0.2779	0.3262
NM_001011928	Dpep2	0.6895	0.1606	0.7481	0.4088	0.3921	0.4326	0.6498	0.4473	0.5835	0.5819	0.0624	0.5478
NM_001007720	Gorasp2	0.9540	0.6799	0.8090	0.9323	0.7138	0.6651	0.7444	0.6137	0.6666	0.8657	0.8187	0.3519
NM_001007714	Morf4l2	0.8553	0.9740	0.4446	0.3330	0.8350	0.4968	0.1749	0.8763	0.9368	0.9760	0.9795	0.3708
NM_001007711	Gtf2f1	0.2192	0.3032	0.0013	0.5694	0.1928	0.0053	0.1863	0.6091	0.2227	0.4334	0.5358	0.0232
NM_001012020	Papolb	0.8701	0.5733	0.6697	0.7528	0.8493	0.8300	0.6226	0.5326	0.4128	0.7403	0.6460	0.5676
NM_001011912	Polm	0.2092	0.5277	0.2937	0.2708	0.3447	0.0419	0.0743	0.8303	0.7804	0.5610	0.6971	0.6051
NM_001012127	Mapkapk3	0.9598	0.7898	0.7233	0.4985	0.8827	0.9694	0.6427	0.8355	0.8031	0.9064	0.8686	0.7155
NM_001007696	mrpl9	0.0701	0.1747	0.7446	0.0433	0.1320	0.5750	0.9476	0.3063	0.3885	0.3708	0.2344	0.7601
NM_001007694	Ifit3	0.2510	0.5535	0.4492	0.4818	0.4968	0.2439	0.5301	0.3404	0.3122	0.2879	0.1862	0.4486
NM_001007692	Nfatc2ip	0.5423	0.4149	0.4453	0.1526	0.5385	0.2021	0.6267	0.0526	0.3397	0.0490	0.8089	0.3567
NM_001009676	Anks3	0.7830	0.9589	0.0916	0.2083	0.8554	0.8217	0.4181	0.8124	0.6139	0.8371	0.7900	0.6635
NM_001007691	Prss23	0.2246	0.1948	0.9784	0.9755	0.6360	0.5816	0.8292	0.8187	0.1964	0.0121	0.1901	0.2100
NM_001007700	M6pr	0.6089	0.7662	0.7121	0.4935	0.7753	0.5520	0.6641	0.6847	0.5775	0.4737	0.2280	0.8271
NM_001007690	Fbxo30	0.8693	0.8487	0.9606	0.6706	0.3566	0.2705	0.9082	0.5666	0.8528	0.4776	0.9317	0.1766
NM_001011910	Lap3	0.4660	0.8153	0.7327	0.7493	0.2433	0.0191	0.1010	0.0329	0.9329	0.5725	0.7428	0.0245
NM_001007686	Cln8	0.2568	0.3328	0.7318	0.9300	0.0642	0.1555	0.8642	0.4369	0.0623	0.0329	0.2181	0.0387
NM_017234	Pxmp3	0.6163	0.0354	0.8749	0.9908	0.4070	0.3920	0.6054	0.3365	0.0726	0.1046	0.6940	0.7428
NM_001007681	Zfp219	0.9508	0.8965	0.8044	0.9242	0.8798	0.9598	0.4590	0.9487	0.9950	0.8701	0.9548	0.9150
NM_001007680	Abhd6	0.6312	0.0857	0.6761	0.0229	0.7737	0.1885	0.4974	0.9362	0.0908	0.1789	0.3035	0.1728
NM_001009673	Fastkd2	0.1919	0.6283	0.0654	0.0201	0.0446	0.1106	0.1199	0.0633	0.2783	0.3542	0.2323	0.1838
NM_001007677	Lyl1	0.8169	0.4065	0.0067	0.0447	0.8569	0.9700	0.3420	0.7906	0.7267	0.7194	0.1772	0.2626
NM_001007676	RGD1359380	0.0943	0.1555	0.0870	0.0321	0.1031	0.2418	0.0555	0.2573	0.0792	0.2308	0.4940	0.0269
NM_001007675	C1qtnf1	0.8635	0.4483	0.2651	0.3833	0.6476	0.2424	0.0692	0.3896	0.7652	0.7540	0.3957	0.6552
NM_001007674	Slc25a19	0.0215	0.0915	0.0233	0.0145	0.1268	0.3551	0.4167	0.0884	0.0406	0.3040	0.0733	0.4770
NM_001007670	Odf4	0.0995	0.3142	0.3185	0.0518	0.7135	0.4304	0.6547	0.3997	0.1712	0.4131	0.0751	0.2002
NM_001007666	Bcs1l	0.7734	0.8977	0.0938	0.6990	0.7740	0.7436	0.5935	0.6489	0.7452	0.5535	0.6526	0.2021

NM_001007664	Abhd14b	0.7671	0.3687	0.9846	0.9167	0.8477	0.8910	0.9591	0.9803	0.5188	0.2627	0.5579	0.6076
NM_001007662	Arcn1	0.9716	0.6493	0.9109	0.4155	0.7085	0.7725	0.9456	0.4191	0.4367	0.6099	0.8470	0.6271
NM_001007656	Mapre3	0.2951	0.9059	0.6235	0.7987	0.3703	0.2240	0.7166	0.4199	0.8479	0.9922	0.9708	0.7126
NM_001010970	Amy1a	0.3059	0.1544	0.4796	0.3710	0.4748	0.4847	0.5412	0.4609	0.2080	0.1430	0.4260	0.2384
NM_001007653	Mrps15	0.8200	0.3018	0.2188	0.1492	0.7750	0.5811	0.7908	0.1756	0.5281	0.6369	0.8138	0.1089
NM_001013231	Pea15a	0.3584	0.3717	0.9224	0.9715	0.8727	0.8123	0.9211	0.9193	0.6344	0.3582	0.4490	0.4682
NM_001007648	Cdca3	0.3559	0.8361	0.0474	0.8488	0.8057	0.9016	0.7258	0.7074	0.7619	0.9660	0.9251	0.0409
NM_001009646	Qprt	0.3835	0.3910	0.3778	0.8258	0.6511	0.4742	0.5553	0.6525	0.1594	0.1724	0.3708	0.3528
NM_001009641	Pepd	0.2885	0.0715	0.6615	0.0686	0.1551	0.5582	0.1779	0.0635	0.0078	0.0693	0.0351	0.4069
NM_001007643	Tmx2	0.0149	0.0349	0.6918	0.5536	0.1807	0.2345	0.0357	0.1643	0.3886	0.4274	0.3024	0.7735
NM_001009639	Tppp3	0.2892	0.6012	0.4247	0.5317	0.4044	0.5663	0.5438	0.6401	0.6958	0.0980	0.0887	0.8993
NM_001007641	Rnd3	0.2925	0.0726	0.9956	0.2971	0.2651	0.2478	0.8766	0.1149	0.2980	0.1920	0.1665	0.2732
NM_001009636	Prelid1	0.1667	0.6095	0.6034	0.9865	0.3964	0.3585	0.8834	0.7970	0.6163	0.9043	0.9438	0.8000
NM_001007637	mrpl24	0.2153	0.3112	0.2784	0.1924	0.2726	0.2423	0.1588	0.2491	0.0549	0.1604	0.2605	0.8365
NM_001007636	S100a1	0.0429	0.1006	0.7271	0.1321	0.2575	0.0005	0.0020	0.1814	0.0016	0.0610	0.0238	0.1343
NM_001007633	Mocs2	0.5281	0.2137	0.5549	0.5917	0.3850	0.3845	0.4582	0.4691	0.5503	0.5730	0.4502	0.2117
NM_001009633	Ccdc98	0.1819	0.3264	0.0033	0.1490	0.0663	0.1817	0.0505	0.1112	0.1623	0.4413	0.1224	0.0485
NM_001012183	Cryz	0.2500	0.4080	0.2587	0.8038	0.7193	0.6989	0.4862	0.6242	0.0868	0.2225	0.2848	0.2683
NM_001008861	Usp11	0.2071	0.1299	0.6894	0.4178	0.7531	0.4152	0.3232	0.5657	0.2411	0.3477	0.0979	0.2416
NM_001017511	Klhl36	0.0286	0.0573	0.2214	0.0235	0.0431	0.0210	0.0353	0.0001	0.1017	0.0690	0.1492	0.7425
NM_001012149	Uchl5	0.6154	0.7439	0.4122	0.5924	0.6727	0.4751	0.4162	0.1261	0.8185	0.8962	0.8773	0.4828
NM_001007622	Pdlim2	0.2245	0.7280	0.2401	0.4680	0.4107	0.4277	0.4017	0.2694	0.2073	0.3993	0.2738	0.2252
NM_001007621	Dhrs1	0.3021	0.3331	0.7064	0.5714	0.7545	0.8951	0.5750	0.3714	0.8603	0.5680	0.4948	0.6390
NM_001007619	Tmed5	0.3028	0.0798	0.8034	0.6230	0.1621	0.1641	0.4016	0.1726	0.2628	0.1095	0.1105	0.0762
NM_001007618	Rchy1	0.9738	0.3067	0.4444	0.2451	0.9711	0.6164	0.2604	0.8258	0.7630	0.3003	0.6622	0.0923
NM_001014127	U1snrnpbp	0.7443	0.2177	0.6973	0.9215	0.8325	0.1926	0.4412	0.8688	0.0417	0.1823	0.0298	0.0111
NM_001007616	Wsb2	0.1186	0.7823	0.8307	0.7870	0.0486	0.2568	0.8739	0.3455	0.8823	0.8002	0.7332	0.8261
NM_001007615	Wipi2	0.3080	0.8665	0.4942	0.6561	0.0876	0.3291	0.1144	0.5000	0.8214	0.9677	0.8192	0.4131
NM_001009536	Trim25	0.5914	0.8828	0.0232	0.1170	0.2723	0.4242	0.6522	0.2872	0.3854	0.6252	0.6831	0.3780
NM_001007599	Rpl23	0.8153	0.5687	0.8348	0.0345	0.2434	0.2120	0.3672	0.1781	0.3470	0.1526	0.3896	0.2444
NM_001007610	Wdr79	0.6402	0.6089	0.0805	0.3543	0.5188	0.5350	0.7613	0.5353	0.5017	0.8687	0.6268	0.2878
NM_001007609	Mfap3	0.3489	0.5828	0.1433	0.3695	0.1771	0.4785	0.3332	0.1635	0.7993	0.5146	0.8238	0.7806
NM_001012021	Zscan21	0.9715	0.9334	0.9528	0.8309	0.8407	0.8299	0.2573	0.8199	0.8203	0.8798	0.8989	0.8058
NM_001011557	Tmem126a	0.6717	0.7294	0.6253	0.5076	0.5132	0.2183	0.4473	0.6679	0.6059	0.6705	0.6338	0.6754
NM_001009720	Sfrs2	0.9953	0.9634	0.1453	0.0375	0.7823	0.9353	0.8086	0.9238	0.8572	0.9011	0.8952	0.7368
NM_001009714	Mitd1	0.4357	0.1158	0.0658	0.3793	0.5065	0.3233	0.0405	0.1537	0.3224	0.1213	0.0726	0.0670
NM_001007604	Rplp1	0.4394	0.3546	0.0971	0.5193	0.3173	0.2748	0.1044	0.2009	0.3938	0.4215	0.1376	0.1465
NM_001011916	Rec8	0.4582	0.1212	0.0959	0.0949	0.3548	0.0155	0.0601	0.1776	0.2287	0.0769	0.2584	0.0802
NM_001011900	Tssk1	0.3096	0.4383	0.2922	0.3623	0.3204	0.1532	0.0091	0.5200	0.2586	0.6454	0.4344	0.7555
NM_001010962	Rps6kb2	0.3895	0.5114	0.2559	0.4280	0.8227	0.6691	0.1290	0.1617	0.5697	0.5917	0.6615	0.1944
NM_001011938	Scyl1	0.7447	0.7603	0.5526	0.4539	0.6641	0.2552	0.5506	0.4982	0.7348	0.5785	0.7424	0.4620
NM_001007558	Emr4	0.5915	0.6162	0.5571	0.4483	0.5785	0.1886	0.5073	0.6011	0.2159	0.3777	0.5172	0.6086
NM_001007557	Emr1	0.6287	0.8308	0.7361	0.6648	0.5364	0.2899	0.8790	0.5947	0.8094	0.6892	0.5628	0.3844
NM_001007556	Lefty2	0.1502	0.5245	0.0108	0.1583	0.4680	0.0613	0.1608	0.4144	0.1899	0.0950	0.2970	0.0756
NM_001007554	Fblim1	0.3755	0.6834	0.7262	0.4496	0.9295	0.6514	0.0993	0.6467	0.4093	0.3817	0.8540	0.7148
NM_001009349	Mthfs	0.2141	0.5799	0.4678	0.9920	0.9573	0.9249	0.2877	0.7220	0.3798	0.1776	0.3751	0.8537
NM_001009246	RGD1309906	0.0878	0.2876	0.4109	0.0824	0.2799	0.0566	0.0676	0.0691	0.3099	0.3565	0.1316	0.1772
NM_001012649	Klri1	0.3048	0.3376	0.2865	0.3182	0.1742	0.3336	0.4309	0.4131	0.1621	0.3826	0.3170	0.1671
NM_001009628	Kng1l1	0.8057	0.9015	0.8485	0.9188	0.7182	0.7930	0.9737	0.8762	0.8326	0.8863	0.9271	0.7320

NM_001007148	Btrc	0.0258	0.3570	0.4055	0.1265	0.2767	0.1681	0.5824	0.0638	0.4669	0.6514	0.3282	0.5755
NM_001007147	Unc84a	0.0785	0.1570	0.8328	0.3591	0.0246	0.4145	0.0933	0.0037	0.2546	0.3991	0.4540	0.8198
NM_001007146	Tob2	0.9343	0.5484	0.7677	0.3712	0.8565	0.9656	0.9649	0.5210	0.7858	0.8084	0.4450	0.9563
NM_001033899	Sms	0.8263	0.2584	0.3900	0.1431	0.1943	0.4661	0.2450	0.3731	0.0149	0.0891	0.0373	0.2409
NM_001006999	Xrcc4	0.2100	0.3118	0.7127	0.0287	0.1902	0.6274	0.0839	0.2522	0.3679	0.0289	0.1739	0.2094
NM_001009697	Mtfmt	0.7792	0.9162	0.8081	0.0788	0.1687	0.2163	0.8134	0.0122	0.7015	0.7979	0.8230	0.7704
NM_001009492	Oas1e	0.5727	0.1851	0.8354	0.7361	0.7315	0.6448	0.7328	0.4287	0.6483	0.6524	0.1199	0.6527
NM_001009486	Klra2	0.3011	0.2974	0.2732	0.1113	0.0045	0.0042	0.1584	0.0100	0.1128	0.0224	0.2031	0.1111
NM_001006993	Sgcg	0.0371	0.1074	0.2192	0.2393	0.0563	0.1243	0.0112	0.0676	0.0539	0.1054	0.0052	0.1850
NM_001009691	Dclk2	0.9655	0.9427	0.9971	0.9713	0.9790	0.9932	0.9907	0.9740	0.9873	0.9502	0.7750	0.9780
NM_001006990	LOC304000	0.1670	0.0031	0.1580	0.0777	0.1522	0.0431	0.0455	0.1532	0.1252	0.1989	0.0535	0.6985
NM_001006987	Spg21	0.3697	0.0239	0.9862	0.9341	0.1129	0.8751	0.6692	0.0852	0.1990	0.3241	0.4154	0.7469
NM_001006985	Mrpl13	0.1484	0.0484	0.0223	0.1802	0.0405	0.0358	0.0989	0.0905	0.0348	0.1820	0.0390	0.0002
NM_001006984	Vezt	0.6736	0.2098	0.7377	0.0888	0.9146	0.9735	0.0853	0.9100	0.0425	0.2847	0.0414	0.4573
NM_001011993	Pycrl	0.4213	0.3777	0.1758	0.2719	0.0952	0.1425	0.8200	0.3691	0.1953	0.0571	0.0758	0.2646
NM_001006980	Bcap29	0.2074	0.1065	0.3317	0.4451	0.1580	0.0949	0.0143	0.2128	0.2175	0.1861	0.2824	0.2337
NM_001006979	Matn1	0.5880	0.8158	0.8416	0.5006	0.0380	0.1676	0.6389	0.0147	0.8944	0.1279	0.2483	0.8299
NM_001009709	Tmem140	0.1806	0.0293	0.9300	0.5973	0.2378	0.1090	0.8827	0.2097	0.2000	0.0048	0.1378	0.1329
NM_001006977	Adad1	0.5575	0.3999	0.0093	0.1454	0.4187	0.3892	0.4006	0.1826	0.4000	0.6761	0.0925	0.1175
NM_001012084	Adh6	0.3690	0.6233	0.4288	0.2103	0.2212	0.1510	0.5392	0.6079	0.1324	0.0771	0.1436	0.2693
NM_001009627	Yars2	0.2636	0.1595	0.0547	0.1206	0.0396	0.1058	0.0214	0.0044	0.3548	0.6178	0.2249	0.2443
NM_001006975	Ms4a6b	0.4116	0.5757	0.3327	0.2563	0.6420	0.2472	0.2581	0.4159	0.5247	0.6903	0.5661	0.4798
NM_001006973	mrpl11	0.1599	0.9423	0.5364	0.8045	0.3180	0.0584	0.7807	0.6948	0.2069	0.8763	0.1110	0.1770
NM_001006972	Ndufv1	0.1142	0.0441	0.0857	0.4390	0.3171	0.1183	0.4806	0.0680	0.2226	0.0116	0.4328	0.3675
NM_001012034	Art3	0.3072	0.4176	0.3500	0.3349	0.3037	0.5073	0.5431	0.5975	0.5028	0.5419	0.5093	0.1605
NM_001009626	Apoh	0.1310	0.1614	0.5262	0.2000	0.2400	0.3904	0.2208	0.4729	0.2067	0.2126	0.0179	0.1619
NM_001006970	Uqcrc2	0.5353	0.6706	0.3414	0.2934	0.4689	0.2296	0.2630	0.2750	0.5930	0.2967	0.4100	0.4062
NM_001009705	Atp5sl	0.8408	0.8262	0.9595	0.9533	0.9739	0.9351	0.9388	0.8998	0.9192	0.8158	0.6155	0.3701
NM_001009385	Apon	0.3057	0.4462	0.8339	0.5746	0.2287	0.3032	0.4237	0.0783	0.8112	0.1974	0.4644	0.0667
NM_001008380	Dis3l	0.7198	0.6702	0.3033	0.7842	0.3405	0.5364	0.6960	0.2004	0.8178	0.5741	0.9002	0.6807
NM_001009539	B4galt3	0.4097	0.0503	0.2125	0.0798	0.8252	0.8183	0.2078	0.9409	0.0477	0.0788	0.1047	0.8559
NM_001006965	Mrpl34	0.8839	0.1326	0.3180	0.8238	0.7725	0.0898	0.8113	0.8298	0.0520	0.1442	0.1713	0.0758
NM_001006963	Itm2b	0.9088	0.1501	0.9944	0.9580	0.8624	0.9260	0.1283	0.5578	0.2457	0.1555	0.1009	0.4733
NM_001006962	Tinf2	0.0188	0.5368	0.7758	0.9424	0.0549	0.1397	0.5982	0.1636	0.2209	0.1869	0.3068	0.0325
NM_001006959	RGD1359460	0.7077	0.9802	0.1707	0.1068	0.6605	0.4617	0.8097	0.7937	0.7388	0.8668	0.7106	0.9753
NM_001006958	Sdad1	0.3146	0.7766	0.1602	0.7056	0.2993	0.1970	0.0838	0.5237	0.9599	0.8467	0.8696	0.8499
NM_001009409	Nanp	0.7709	0.6575	0.7634	0.0972	0.0826	0.6428	0.5473	0.3231	0.2756	0.5326	0.4657	0.1278
NM_001006955	Cldnd1	0.9809	0.8884	0.9432	0.9070	0.9045	0.8862	0.9338	0.9170	0.9508	0.9465	0.8313	0.9569
NM_001007020	Hmgn3	0.9887	0.9774	0.6881	0.1980	0.8687	0.7696	0.4458	0.4342	0.9324	0.9871	0.9752	0.8357
NM_001011955	Chst1	0.6475	0.2981	0.6634	0.3239	0.5304	0.5244	0.5158	0.4171	0.6609	0.3596	0.2016	0.5299
NM_001037496	Otud5	0.9201	0.4195	0.8847	0.2003	0.9420	0.8778	0.2327	0.6443	0.7838	0.2076	0.3859	0.9492
NM_001007004	Tuba4a	0.1103	0.7074	0.0504	0.5834	0.1468	0.0602	0.6619	0.0907	0.2069	0.5446	0.2441	0.0933
NM_001007002	Mxra8	0.9442	0.9668	0.7824	0.7404	0.9530	0.9978	0.9381	0.9907	0.9681	0.9576	0.7259	0.9620
NM_001007001	Ift74	0.0471	0.0338	0.0534	0.6604	0.1911	0.2180	0.0423	0.1923	0.0348	0.0634	0.1360	0.6169
NM_001039019	Kif18b	0.6591	0.2768	0.2733	0.8743	0.3485	0.7999	0.8485	0.6996	0.1436	0.3225	0.6642	0.8276
NM_001005899	Clecsf6	0.3133	0.1621	0.3913	0.1690	0.4236	0.3094	0.3793	0.5368	0.2997	0.3321	0.4250	0.3953
NM_001005908	Ghitm	0.0919	0.9770	0.8438	0.4490	0.0373	0.1727	0.8649	0.0406	0.7538	0.8180	0.9027	0.7888
NM_001005898	Omg	0.3123	0.5877	0.4974	0.4160	0.3838	0.6576	0.2286	0.6435	0.2089	0.2268	0.3650	0.3402
NM_001009831	Spg3a	0.1590	0.3258	0.3498	0.0930	0.7819	0.7659	0.6572	0.1594	0.7564	0.3368	0.5849	0.3889

NM_001005907	Efemp2	0.1298	0.0003	0.1550	0.2227	0.0015	0.1072	0.0446	0.0828	0.0005	0.0511	0.0498	0.0406
NM_001012120	Naga	0.2176	0.0732	0.8030	0.7458	0.0162	0.5389	0.3986	0.3053	0.1247	0.0745	0.0440	0.5857
NM_001005905	Cct2	0.6425	0.5270	0.1041	0.2963	0.2640	0.7806	0.1506	0.2263	0.7705	0.1343	0.3373	0.0755
NM_001005903	Capzb	0.3718	0.2167	0.9196	0.9769	0.2338	0.2337	0.9583	0.5572	0.0703	0.5460	0.5097	0.6862
NM_001005892	Sfpi1	0.5112	0.5476	0.4673	0.6074	0.5023	0.5310	0.1592	0.7026	0.6909	0.4775	0.8920	0.8197
NM_001005902	Abtb1	0.8645	0.9915	0.8376	0.9325	0.9224	0.7686	0.9524	0.7905	0.9993	0.9813	0.7756	0.9743
NM_001005900	Hcst	0.0011	0.2450	0.4494	0.0576	0.2815	0.0758	0.2221	0.4321	0.1356	0.6408	0.2162	0.0496
NM_001008352	Pmvk	0.0562	0.0140	0.0437	0.3594	0.1171	0.0496	0.0219	0.0887	0.1083	0.0841	0.2325	0.1482
NM_001012225	Mgat4a	0.2340	0.1777	0.1146	0.1054	0.0881	0.1388	0.0689	0.1568	0.1060	0.1522	0.0830	0.2942
NM_001012071	Gsto2	0.0809	0.2479	0.1341	0.0012	0.0460	0.1028	0.0817	0.4021	0.5117	0.0118	0.0932	0.0538
NM_001005885	Dnajb13	0.6157	0.5593	0.7194	0.8226	0.7206	0.4826	0.4140	0.9049	0.7059	0.2337	0.2195	0.3860
NM_001009658	Thnsl2	0.0695	0.0107	0.2665	0.7584	0.0380	0.0945	0.0319	0.0529	0.0718	0.0275	0.0165	0.0240
NM_001005878	Kifc1	0.8383	0.7383	0.0455	0.8758	0.8652	0.8989	0.7341	0.8498	0.9994	0.9627	0.9573	0.4856
NM_001005875	Psmd12	0.1159	0.1486	0.0086	0.0555	0.0996	0.0751	0.1468	0.1270	0.1775	0.1066	0.2130	0.0219
NM_001007747	Pomgnt1	0.6121	0.7557	0.5433	0.5238	0.9332	0.9191	0.9399	0.8714	0.9940	0.8791	0.9349	0.9833
NM_001007743	Cdk9	0.5687	0.8609	0.9543	0.9024	0.6290	0.7823	0.9278	0.7604	0.7175	0.6292	0.8750	0.9611
NM_001005871	Atp2b4	0.2301	0.5128	0.4653	0.5986	0.6932	0.5667	0.7024	0.8133	0.4624	0.1035	0.4234	0.4657
NM_001009264	Mrfap1	0.6755	0.5576	0.6323	0.3180	0.4228	0.2981	0.4847	0.6802	0.3890	0.7168	0.6384	0.3991
NM_001005762	Sorbs3	0.1802	0.0402	0.1064	0.1330	0.4025	0.5332	0.1562	0.3847	0.1314	0.5790	0.2599	0.5147
NM_001005761	Mtmr9	0.5267	0.5873	0.0950	0.1158	0.7647	0.7584	0.7469	0.8154	0.8183	0.7733	0.9128	0.6053
NM_001005564	Thap4	0.3695	0.0920	0.0490	0.3813	0.2599	0.3025	0.3400	0.0933	0.0697	0.1304	0.0537	0.0142
NM_001005563	Fbxl6	0.8114	0.8552	0.1391	0.0640	0.2274	0.2730	0.1446	0.2687	0.6792	0.2378	0.5239	0.6018
NM_001005562	Creb3l1	0.9664	0.8572	0.7620	0.9109	0.9793	0.9538	0.9843	0.7132	0.7667	0.8424	0.9140	0.3875
NM_001025728	Smarcb1	0.7770	0.7462	0.8678	0.9236	0.8854	0.7411	0.8163	0.8269	0.9057	0.9819	0.9635	0.8525
NM_001005557	Akap3	0.3084	0.3325	0.1745	0.0666	0.1015	0.1670	0.6151	0.2946	0.4886	0.3705	0.5809	0.9420
NM_001005555	Ptbp2	0.8633	0.8292	0.8161	0.7881	0.7861	0.8416	0.8274	0.9020	0.8836	0.9307	0.8464	0.8779
NM_001005554	Tm9sf2	0.9314	0.7401	0.8030	0.6921	0.7288	0.7350	0.0590	0.7793	0.9096	0.6812	0.5479	0.9630
NM_001014063	Acnat2	0.6361	0.4884	0.3833	0.4461	0.3167	0.4283	0.4376	0.4233	0.3024	0.1851	0.3070	0.3108
NM_001013234	Rnaseh2a	0.1052	0.0180	0.0094	0.0123	0.0016	0.0580	0.3094	0.1119	0.2073	0.1538	0.1326	0.2561
NM_001005552	MGC95208	0.3394	0.8922	0.6632	0.9812	0.7437	0.8230	0.1147	0.8107	0.2097	0.9312	0.7922	0.1095
NM_001012091	Foxs1	0.2616	0.0090	0.2884	0.0526	0.1912	0.1792	0.0472	0.3616	0.0066	0.0087	0.0146	0.0056
NM_001007719	Hsd3b1	0.6783	0.6051	0.7310	0.6636	0.4742	0.3177	0.5985	0.0550	0.3634	0.6695	0.9316	0.6566
NM_001007710	Acpl2	0.9163	0.5603	0.9395	0.9275	0.5901	0.8063	0.9960	0.7910	0.6352	0.9019	0.9261	0.9885
NM_001005549	Tinag	0.1756	0.3138	0.4614	0.7945	0.0904	0.1248	0.1934	0.6492	0.1626	0.4826	0.6704	0.1052
NM_001005547	Tspan3	0.8480	0.6108	0.9719	0.7798	0.6884	0.9357	0.8151	0.8809	0.8245	0.8236	0.3926	0.8071
NM_001005543	Nasp	0.4767	0.8256	0.5101	0.4193	0.4281	0.6961	0.8524	0.5251	0.4612	0.6847	0.4846	0.4792
NM_001009176	Trim10	0.1815	0.3814	0.5086	0.0637	0.2859	0.8978	0.8853	0.4100	0.4838	0.5676	0.5658	0.0966
NM_001009172	Zbtb22	0.8964	0.8342	0.9537	0.9558	0.9807	0.8279	0.9467	0.9868	0.7360	0.9350	0.7813	0.9109
NM_001008885	Slc39a7	0.2033	0.3603	0.3533	0.3167	0.5124	0.4551	0.3928	0.4939	0.2950	0.5690	0.2773	0.2328
NM_001005539	Smpdl3a	0.8578	0.0653	0.7813	0.0747	0.0339	0.0101	0.0402	0.0163	0.0346	0.0313	0.1979	0.0001
NM_001013880	Isyna1	0.7398	0.0928	0.0059	0.0532	0.7102	0.5484	0.0082	0.8340	0.4089	0.2833	0.2615	0.4810
NM_001009643	RGD1310111	0.3133	0.3379	0.2530	0.1024	0.1373	0.1660	0.0502	0.2983	0.2861	0.2530	0.1350	0.1224
NM_001005537	Ik	0.1366	0.0694	0.1475	0.1905	0.0641	0.0338	0.1064	0.1312	0.1177	0.7684	0.2360	0.4255
NM_001005536	Srfbp1	0.0217	0.6562	0.2046	0.0286	0.0371	0.0594	0.0051	0.0008	0.2786	0.7075	0.3180	0.6167
NM_001005535	Ddx59	0.3326	0.6688	0.1213	0.0057	0.6523	0.4636	0.1949	0.8693	0.1205	0.5768	0.4833	0.0294
NM_001005534	Sdhc	0.2470	0.1526	0.1457	0.3232	0.4359	0.3410	0.3210	0.3170	0.1690	0.0790	0.1817	0.2783
NM_001005529	Ttc1	0.1041	0.0639	0.4509	0.0296	0.2972	0.2970	0.1486	0.2111	0.0454	0.0304	0.1289	0.0660
NM_001008328	Parp3	0.2572	0.6471	0.8904	0.6055	0.0205	0.0363	0.7422	0.1383	0.6832	0.2097	0.7776	0.8763
NM_001109436	Mogat2	0.4460	0.4244	0.4843	0.3569	0.4894	0.3084	0.6515	0.4360	0.4720	0.1996	0.3628	0.6824

NM_001011907	Ndufs2	0.4066	0.4611	0.2914	0.3622	0.2924	0.2668	0.3763	0.4382	0.3951	0.4689	0.4104	0.2392
NM_001007623	Mettl6	0.4940	0.3651	0.5615	0.5830	0.3790	0.7005	0.4503	0.6556	0.1338	0.1593	0.4480	0.0273
NM_001012076	Phyhipl	0.4642	0.3975	0.8423	0.5497	0.6544	0.4758	0.7163	0.7703	0.4289	0.7765	0.2612	0.4315
NM_012674	Spink3	0.4606	0.1620	0.3972	0.7219	0.3536	0.3112	0.4679	0.5277	0.1618	0.1576	0.3579	0.1668
NM_012656	Sparc	0.7963	0.9054	0.9689	0.9294	0.9067	0.9427	0.9718	0.9457	0.9281	0.9751	0.9857	0.9359
NM_001012025	Ubxn4	0.4794	0.1370	0.8327	0.3392	0.8445	0.8596	0.7396	0.9846	0.8525	0.6379	0.3365	0.5025
NM_001010965	Rage	0.0430	0.0613	0.5134	0.0064	0.3183	0.2241	0.2411	0.1642	0.9449	0.5404	0.0875	0.6399
NM_001009713	Slc17a5	0.9114	0.8410	0.8143	0.4645	0.8217	0.8277	0.9319	0.3877	0.9660	0.9711	0.8033	0.9180
NM_001004448	Dear	0.2349	0.2135	0.2577	0.1771	0.2014	0.2055	0.4019	0.5109	0.5639	0.1394	0.5075	0.0941
NM_001004446	Smarca2	0.6657	0.6867	0.6660	0.5543	0.4846	0.5468	0.6060	0.7310	0.5543	0.7081	0.3531	0.5906
NM_001004444	Zbtb1	0.9541	0.6254	0.4561	0.9980	0.9901	0.7231	0.9433	0.9708	0.9158	0.2557	0.9432	0.7358
NM_001004424	Tacc3	0.2695	0.2636	0.4356	0.3250	0.2699	0.3313	0.1712	0.3483	0.3230	0.2807	0.5014	0.3256
NM_001004418	Tacc2	0.9877	0.9941	0.9585	0.8511	0.8692	0.9336	0.8070	0.8880	0.7615	0.9158	0.8173	0.9065
NM_001004415	Tacc2	0.9877	0.9941	0.9585	0.8511	0.8692	0.9336	0.8070	0.8880	0.7615	0.9158	0.8173	0.9065
NM_001007706	RGD1359191	0.3913	0.9329	0.1204	0.7120	0.6017	0.7433	0.8813	0.4374	0.8459	0.6637	0.8316	0.6259
NM_001007687	Cndp1	0.8926	0.8820	0.6543	0.9906	0.3817	0.2949	0.9902	0.5746	0.5110	0.9824	0.9729	0.1686
NM_001004280	RGD1303272	0.3056	0.1155	0.1636	0.4102	0.2889	0.4530	0.7092	0.4439	0.1221	0.0935	0.1890	0.0466
NM_001007742	Ubac1	0.1408	0.8285	0.1173	0.3058	0.2750	0.7241	0.5032	0.4716	0.1927	0.5968	0.5151	0.5865
NM_001007701	Tram1	0.9265	0.5287	0.7240	0.9798	0.9604	0.9646	0.9559	0.7761	0.5505	0.5119	0.9418	0.4944
NM_001004274	Igfbp4	0.8624	0.8566	0.9529	0.8638	0.9373	0.7369	0.9284	0.8652	0.9548	0.8216	0.9366	0.7686
NM_001004269	Jam3	0.2001	0.1569	0.0184	0.2759	0.1076	0.0727	0.1482	0.2295	0.0009	0.2454	0.1964	0.0373
NM_001009603	Acy3	0.1570	0.5120	0.8142	0.7080	0.8633	0.7921	0.0447	0.9158	0.6643	0.2963	0.3653	0.0264
NM_001009480	Uqcrh	0.1152	0.0296	0.2538	0.0628	0.1771	0.0848	0.0723	0.1110	0.0324	0.0269	0.0379	0.0460
NM_001009239	Rps3	0.1958	0.2590	0.5342	0.1530	0.2571	0.2486	0.1102	0.0464	0.0216	0.0664	0.3686	0.0248
NM_001012019	Tssk2	0.3238	0.1275	0.2457	0.3851	0.3338	0.3235	0.7072	0.3435	0.0315	0.3871	0.4769	0.3737
NM_001011992	Atp6v1c1	0.3139	0.0865	0.6923	0.0562	0.2531	0.5397	0.2009	0.0378	0.1555	0.3796	0.1449	0.6650
NM_001004258	Nudt19	0.6235	0.6239	0.3993	0.8302	0.6465	0.1807	0.1224	0.2913	0.0815	0.4343	0.1295	0.5012
NM_001008847	H2-Ea	0.6639	0.7922	0.2705	0.1661	0.6483	0.4199	0.4160	0.7607	0.5963	0.6891	0.3586	0.7697
NM_001004254	Mtif2	0.5803	0.5404	0.0843	0.0040	0.6305	0.6450	0.0012	0.5819	0.4870	0.4209	0.5884	0.6219
NM_001004253	Syap1	0.5557	0.1789	0.9037	0.0675	0.0061	0.1313	0.4360	0.0825	0.0777	0.2638	0.4693	0.7529
NM_001008386	LOC493574	0.5939	0.4962	0.5604	0.7639	0.9281	0.5940	0.3376	0.5650	0.9182	0.1901	0.2782	0.2755
NM_001008296	Fam158a	0.0390	0.0117	0.8682	0.6319	0.0974	0.2257	0.1712	0.1438	0.0115	0.3430	0.0029	0.4233
NM_001008802	Krt1	0.2861	0.1166	0.4552	0.4891	0.6441	0.6317	0.0798	0.5272	0.6203	0.1272	0.5033	0.4008
NM_001004245	Esam	0.1363	0.5014	0.5640	0.5312	0.3847	0.7832	0.5950	0.3483	0.3194	0.3388	0.3085	0.5673
NM_001004241	Samm50	0.6609	0.6007	0.0231	0.8411	0.1046	0.2974	0.9831	0.7322	0.1546	0.7669	0.6034	0.0573
NM_001008829	RT1-A2	0.5447	0.4701	0.5218	0.5572	0.6496	0.2603	0.5383	0.5896	0.4500	0.3927	0.2354	0.3174
NM_001004239	Dctn2	0.5503	0.0405	0.8390	0.8009	0.1509	0.6092	0.9128	0.4624	0.1716	0.2059	0.4310	0.1734
NM_001004238	Rab21	0.2758	0.1601	0.0912	0.2452	0.2600	0.1290	0.2846	0.2438	0.0025	0.2483	0.2052	0.3069
NM_001004236	Tspan1	0.8036	0.7836	0.8734	0.8027	0.8904	0.9141	0.9742	0.8587	0.8727	0.9795	0.8901	0.8402
NM_001004235	Mrpl37	0.2676	0.8503	0.3494	0.7990	0.0422	0.1035	0.5819	0.3138	0.6145	0.8655	0.7529	0.8158
NM_001008826	H2-T24	0.2755	0.1149	0.5546	0.0270	0.3522	0.0800	0.0199	0.3727	0.1170	0.0933	0.0816	0.4746
NM_001004232	Fscn3	0.7706	0.7688	0.0496	0.3829	0.6897	0.3658	0.1330	0.9724	0.7469	0.4069	0.3948	0.5406
NM_001004231	Npdc1	0.4659	0.0172	0.4959	0.1716	0.0293	0.0137	0.4743	0.0715	0.6850	0.0828	0.3416	0.9134
NM_001004229	Nup35	0.4137	0.9613	0.0715	0.1696	0.0965	0.0842	0.2438	0.2176	0.3102	0.2357	0.8004	0.0342
NM_001004228	Emcn	0.5021	0.0948	0.3175	0.3778	0.5014	0.7160	0.3064	0.7023	0.5632	0.0559	0.3591	0.3415
NM_001007612	Ccl7	0.0734	0.2936	0.2817	0.1035	0.0028	0.0537	0.0200	0.1881	0.0062	0.0041	0.0675	0.0346
NM_001004226	RGD1303130	0.1592	0.0344	0.1846	0.6583	0.0570	0.1567	0.1028	0.0298	0.0214	0.0195	0.0685	0.2084
NM_001004225	RGD1303003	0.8563	0.6434	0.9329	0.7839	0.6854	0.8565	0.9162	0.9043	0.7107	0.8445	0.5777	0.8737
NM_001004224	Bcap31	0.4035	0.4030	0.7958	0.8565	0.2788	0.4935	0.7105	0.4113	0.3927	0.1594	0.3657	0.6462

NM_001004222	Arfip2	0.8772	0.8381	0.6298	0.4032	0.9460	0.9279	0.9638	0.8477	0.9575	0.9935	0.7613	0.9963
NM_001013951	Alg13	0.5664	0.6790	0.4366	0.2346	0.6236	0.2202	0.5219	0.6809	0.5985	0.4979	0.3563	0.3585
NM_001009399	Nsdhl	0.8709	0.9546	0.0095	0.6237	0.7898	0.8994	0.8176	0.7418	0.7873	0.9022	0.9640	0.3954
NM_001011946	Hexb	0.2761	0.3166	0.6309	0.2956	0.2597	0.3621	0.3969	0.5122	0.4245	0.3258	0.2213	0.5298
NM_001008282	Galk1	0.0343	0.1291	0.7291	0.6980	0.2309	0.0235	0.8764	0.0035	0.0318	0.1228	0.3432	0.3104
NM_001004212	Tusc3	0.1993	0.1919	0.8012	0.3730	0.3550	0.5660	0.1004	0.4688	0.0294	0.1737	0.1230	0.5882
NM_001004211	Ddx56	0.0544	0.8751	0.0180	0.1079	0.2605	0.1621	0.2075	0.0601	0.5617	0.7850	0.7190	0.0661
NM_001004210	Xbp1	0.8744	0.9279	0.9492	0.9656	0.7742	0.9367	0.9462	0.8264	0.9497	0.9881	0.9448	0.8561
NM_001004199	Tax1bp1	0.1350	0.1169	0.1159	0.0214	0.0260	0.0371	0.0890	0.0266	0.0089	0.0766	0.1334	0.1224
NM_001004208	Cacybp	0.1995	0.1535	0.6399	0.1674	0.1887	0.1221	0.0616	0.0883	0.3724	0.2142	0.1102	0.8520
NM_001004198	Tbp	0.8331	0.6876	0.9514	0.6785	0.8401	0.8572	0.9078	0.6772	0.6294	0.6084	0.5676	0.3177
NM_001004206	Pa2g4	0.2907	0.8213	0.0143	0.3945	0.6726	0.6539	0.7965	0.3141	0.9685	0.8347	0.7948	0.9972
NM_001004203	Mcm7	0.8861	0.8952	0.0186	0.8956	0.7986	0.9046	0.7311	0.9365	0.8685	0.8676	0.7306	0.7473
NM_001004277	Pla2g15	0.2534	0.0340	0.7077	0.1013	0.3364	0.9787	0.2610	0.1210	0.0814	0.3201	0.1748	0.9398
NM_001004133	Shank2	0.8295	0.8358	0.8412	0.8812	0.6399	0.8180	0.7678	0.6648	0.8974	0.7852	0.4408	0.7264
NM_001009671	Coq10b	0.1352	0.5519	0.1737	0.3055	0.0520	0.1411	0.0256	0.0321	0.4614	0.2349	0.4867	0.5453
NM_001009681	Oasl	0.2505	0.1489	0.0063	0.0014	0.2313	0.0056	0.1204	0.0569	0.0990	0.0040	0.0005	0.1682
NM_001004098	Cenpc1	0.7075	0.8472	0.4862	0.2625	0.6145	0.8376	0.8009	0.5771	0.6607	0.9429	0.7839	0.4390
NM_001004107	Tacc1	0.2689	0.0216	0.5133	0.9492	0.2447	0.5001	0.2616	0.0489	0.2239	0.0664	0.0711	0.4392
NM_001004092	Txndc8	0.1120	0.2175	0.1594	0.1294	0.1529	0.3594	0.4357	0.3147	0.5695	0.1966	0.3615	0.3238
NM_001012014	Ftsj3	0.0773	0.4896	0.1411	0.3170	0.4086	0.4775	0.1838	0.1410	0.7258	0.4100	0.1101	0.5918
NM_001004090	Tspan5	0.2398	0.2646	0.1178	0.2130	0.2258	0.1620	0.2203	0.1225	0.2808	0.2085	0.2533	0.8180
NM_001012208	Pycr2	0.5959	0.3497	0.6704	0.6441	0.4121	0.5366	0.2690	0.4018	0.3725	0.2916	0.2357	0.5784
NM_001004087	Pcdh7	0.1695	0.1615	0.1710	0.1685	0.1740	0.1698	0.1709	0.1646	0.1614	0.1575	0.1622	0.1667
NM_001012018	B4galt4	0.9303	0.5166	0.9460	0.9667	0.9358	0.9726	0.7690	0.9719	0.2850	0.5748	0.8661	0.0127
NM_001004083	Egln2	0.7497	0.9322	0.4949	0.8321	0.8910	0.9411	0.8719	0.9693	0.8510	0.8441	0.8059	0.6054
NM_001029900	Mrpl12	0.2415	0.6106	0.5164	0.2567	0.3175	0.4092	0.6852	0.2385	0.5685	0.4571	0.6151	0.5339
NM_001012352	Dusp26	0.1190	0.3765	0.2142	0.5930	0.2582	0.8936	0.3739	0.2298	0.4935	0.0900	0.2622	0.4741
NM_001008524	C1qc	0.3796	0.5591	0.5535	0.5125	0.7330	0.1825	0.2985	0.5521	0.5261	0.6099	0.2211	0.1791
NM_001004079	Bpi	0.4283	0.4143	0.2400	0.6804	0.1893	0.1409	0.5906	0.4837	0.5644	0.5138	0.4006	0.5809
NM_001003978	Gspt1	0.8477	0.9922	0.9180	0.8211	0.5169	0.0547	0.9125	0.8449	0.9969	0.9613	0.9901	0.9253
NM_001008557	Znf667	0.6611	0.7205	0.6173	0.4709	0.4205	0.4931	0.7866	0.5549	0.4573	0.5988	0.7690	0.6922
NM_001008523	Aox4	0.6012	0.5036	0.5346	0.7328	0.6660	0.2154	0.4350	0.5546	0.3383	0.5812	0.5969	0.2120
NM_001007655	Ube2j2	0.2492	0.5931	0.3840	0.2503	0.0810	0.3208	0.5644	0.1785	0.0778	0.8580	0.2244	0.0048
NM_001008522	Aox3l1	0.4046	0.3364	0.2130	0.3981	0.6748	0.3807	0.4134	0.5848	0.0468	0.3512	0.5335	0.2823
NM_001004068	Rffl	0.3699	0.7131	0.7118	0.6339	0.2971	0.3416	0.7812	0.1646	0.6889	0.2036	0.8523	0.2647
NM_001009353	Pla2g7	0.0324	0.3274	0.0446	0.0767	0.1105	0.1650	0.0998	0.0807	0.6287	0.0686	0.3407	0.2637
NM_001012110	Znf513	0.7521	0.7904	0.3831	0.8626	0.7314	0.8070	0.4217	0.7524	0.9298	0.8465	0.9166	0.8951
NM_001008521	Twf1	0.9128	0.9152	0.3408	0.1614	0.8088	0.8677	0.3805	0.9750	0.7415	0.9815	0.7463	0.2511
NM_001008398	Gimap9	0.4179	0.1391	0.7619	0.6903	0.4661	0.7058	0.6552	0.4870	0.6387	0.0484	0.4953	0.3851
NM_001011925	Nup93	0.2285	0.2021	0.2487	0.8576	0.2270	0.3571	0.6465	0.6142	0.4550	0.5227	0.5735	0.2408
NM_001003708	Trappc4	0.3247	0.0668	0.8487	0.0043	0.0531	0.1195	0.0824	0.0949	0.1233	0.0096	0.0266	0.1564
NM_001003707	Clec4d	0.7115	0.0704	0.4935	0.0859	0.3856	0.3528	0.5099	0.4177	0.3649	0.2581	0.2555	0.4108
NM_001006995	Acat2	0.8513	0.8893	0.4060	0.2648	0.5601	0.8839	0.7237	0.6759	0.5133	0.9151	0.8656	0.2628
NM_001003404	Il22ra2	0.6678	0.9121	0.9433	0.8584	0.8685	0.8660	0.9708	0.7745	0.9755	0.8844	0.9515	0.8138
NM_001011711	Fam89a	0.4094	0.5317	0.2874	0.6692	0.7170	0.5851	0.8405	0.2771	0.2345	0.9572	0.7168	0.2218
NM_001003401	Enc1	0.9941	0.8692	0.9908	0.9559	0.9528	0.9956	0.9904	0.7875	0.8685	0.9682	0.9873	0.9978
NM_001002854	Tanc1	0.8029	0.8616	0.8011	0.9380	0.8706	0.8492	0.8929	0.6703	0.7280	0.8099	0.9332	0.7524
NM_001002851	Nenf	0.8460	0.8337	0.7270	0.8525	0.8318	0.7241	0.7081	0.9293	0.7962	0.9136	0.7628	0.1188

NM_001009599	Clp1	0.3635	0.5881	0.2263	0.3702	0.4627	0.4217	0.3541	0.7017	0.2033	0.2094	0.5733	0.3556
NM_001002830	Rasl11b	0.1688	0.1475	0.2072	0.1851	0.0754	0.0937	0.2369	0.0861	0.2469	0.3436	0.3486	0.1698
NM_001009657	Hat1	0.3099	0.5018	0.0611	0.4341	0.0941	0.4056	0.2089	0.1075	0.1214	0.0929	0.4998	0.6658
NM_001008333	Amn1	0.0751	0.6421	0.7809	0.6056	0.4462	0.6935	0.8304	0.6857	0.7793	0.7256	0.6973	0.7597
NM_001006998	Aldh3b1	0.0364	0.1031	0.6117	0.1620	0.0817	0.0595	0.2523	0.1199	0.2039	0.0151	0.1948	0.3248
NM_001007642	Phospho2	0.7865	0.8639	0.9480	0.7856	0.8647	0.9305	0.8793	0.8773	0.8964	0.8979	0.7809	0.1969
NM_001002815	Ece2	0.7714	0.3678	0.8860	0.7064	0.9460	0.8285	0.5204	0.9668	0.6276	0.7872	0.6362	0.8335
NM_001002813	Ctsql2	0.3867	0.2399	0.6526	0.2145	0.3154	0.4465	0.6564	0.5513	0.2166	0.2361	0.5079	0.1748
NM_001007015	Rnase2	0.0479	0.5913	0.6004	0.8427	0.0314	0.0886	0.8363	0.3037	0.6327	0.1996	0.7762	0.1155
NM_001005881	Lppr2	0.4086	0.1059	0.2995	0.5091	0.4776	0.7291	0.1344	0.5115	0.8124	0.1369	0.0496	0.6831
NM_001009534	Eapa2	0.6951	0.5083	0.8060	0.6368	0.2877	0.6604	0.7465	0.4004	0.6291	0.8017	0.7928	0.3212
NM_001006997	Smpd1	0.0319	0.0260	0.3761	0.2534	0.0751	0.0921	0.2246	0.1379	0.0854	0.0153	0.0204	0.5788
NM_001002807	Clic1	0.5917	0.8464	0.0951	0.8940	0.5635	0.0609	0.8291	0.4145	0.8707	0.8853	0.7516	0.0591
NM_001002806	Capn11	0.2281	0.1628	0.1446	0.0713	0.2743	0.1679	0.0991	0.5596	0.1610	0.1445	0.3644	0.4508
NM_001002805	C4-2	0.1200	0.0026	0.0002	0.1257	0.0932	0.5280	0.0467	0.0122	0.0298	0.0373	0.0010	0.1436
NM_001006991	Nudt9	0.0588	0.5899	0.0797	0.1373	0.2910	0.0286	0.1243	0.6237	0.4712	0.7267	0.5697	0.1908
NM_001008348	Frmd8	0.9737	0.4609	0.0244	0.2838	0.9173	0.9461	0.4964	0.9580	0.6378	0.3739	0.5883	0.1652
NM_001025281	Slc9a8	0.4418	0.8254	0.9211	0.5397	0.6995	0.8314	0.4284	0.2754	0.6454	0.1221	0.8170	0.8808
NM_001008345	Mesdc2	0.1206	0.1346	0.5555	0.1304	0.1854	0.2238	0.2788	0.2883	0.1481	0.0773	0.2669	0.0754
NM_001008327	Fam96a	0.1151	0.1177	0.3028	0.6885	0.0937	0.0650	0.1742	0.1654	0.1692	0.0282	0.0935	0.0065
NM_001001972	Ly6g6e	0.2331	0.5967	0.5469	0.1034	0.1475	0.8933	0.2110	0.9113	0.5271	0.1363	0.5344	0.6739
NM_001001969	Ly6g6c	0.8436	0.7595	0.0509	0.2148	0.9092	0.6384	0.4687	0.8521	0.2977	0.8372	0.7905	0.8327
NM_001002022	Enth	0.6857	0.3084	0.6594	0.2323	0.1465	0.1654	0.5465	0.5167	0.3364	0.4727	0.5301	0.2130
NM_001002016	Lmna	0.9886	0.9978	0.8186	0.9322	0.9517	0.9567	0.8716	0.9940	0.9518	0.9951	0.9500	0.9388
NM_001001800	rnf141	0.0788	0.0526	0.3381	0.5670	0.0127	0.0435	0.0683	0.0012	0.1065	0.0314	0.1824	0.0912
NM_001008308	Zufsp	0.7556	0.7394	0.1360	0.8546	0.8019	0.8497	0.4882	0.8823	0.6279	0.7854	0.6431	0.6171
NM_001029923	Oscp1	0.0724	0.2150	0.0740	0.6419	0.1385	0.1472	0.7246	0.1302	0.0384	0.0596	0.0936	0.4100
NM_001008305	Eif1ad	0.5029	0.7986	0.2468	0.3818	0.6174	0.3189	0.3976	0.8798	0.9390	0.8553	0.9916	0.8338
NM_001001519	Lcn6	0.1633	0.0527	0.1114	0.5414	0.6691	0.4796	0.4767	0.1820	0.3590	0.3189	0.3156	0.3766
NM_001009667	RGD1308031	0.0408	0.1116	0.0115	0.4514	0.1472	0.1225	0.7723	0.4400	0.2469	0.2103	0.7808	0.4345
NM_001008279	Flii	0.9372	0.8744	0.7707	0.9181	0.8684	0.7186	0.6076	0.7809	0.8162	0.8312	0.7946	0.8404
NM_001008278	Cdkn2aipnl	0.1949	0.1538	0.2659	0.4644	0.1275	0.2953	0.1937	0.2960	0.2907	0.0470	0.2226	0.2118
NM_001029901	Csf1r	0.2001	0.6522	0.3265	0.3997	0.7002	0.5124	0.7034	0.2538	0.5023	0.6144	0.7212	0.5078
NM_001001513	Tnfsf12	0.1411	0.0393	0.4753	0.6218	0.0096	0.1530	0.2036	0.0408	0.1349	0.0100	0.2040	0.2724
NM_001001510	Klhl10	0.3345	0.6479	0.2244	0.8805	0.2929	0.1999	0.3767	0.5926	0.0220	0.1697	0.8964	0.1335
NM_001001507	Oit3	0.6935	0.1734	0.3758	0.3774	0.3056	0.3792	0.2793	0.5472	0.4025	0.5429	0.0713	0.1108
NM_001001504	Gtf2ird1	0.8666	0.8259	0.7571	0.9461	0.8032	0.8508	0.8257	0.6842	0.5311	0.7493	0.6824	0.2399
NM_001007749	Atp5s	0.1251	0.1698	0.5594	0.1355	0.2124	0.5784	0.0648	0.0562	0.3420	0.0694	0.0771	0.2809
NM_001007740	Vps26a	0.0304	0.0646	0.4775	0.2476	0.0890	0.0243	0.0643	0.1516	0.0475	0.0523	0.0617	0.1181
NM_001007669	Znf672	0.6633	0.5159	0.2770	0.9557	0.3486	0.5637	0.8241	0.7526	0.0773	0.1498	0.6006	0.2675
NM_001007665	Tex264	0.2134	0.0232	0.9647	0.9646	0.7636	0.7314	0.5216	0.7929	0.5634	0.3193	0.3247	0.1167
NM_001007654	Agtrap	0.8433	0.9564	0.9058	0.9700	0.9156	0.8267	0.9849	0.9940	0.9770	0.8939	0.8289	0.9020
NM_012702	Ceacam3	0.5398	0.6699	0.2459	0.2606	0.7307	0.2157	0.6605	0.4702	0.6185	0.4380	0.5635	0.3917
NM_001079938	Bbx	0.1516	0.4414	0.0943	0.1584	0.0304	0.1014	0.0068	0.2032	0.1432	0.7956	0.2493	0.7056
NM_001037315	Nat1	0.4680	0.4861	0.3385	0.4069	0.4889	0.2163	0.7030	0.3367	0.5104	0.3886	0.5554	0.3886
NM_001037310	Taf9	0.7092	0.5150	0.5446	0.4458	0.3787	0.5867	0.4531	0.5445	0.3405	0.5574	0.2840	0.2471
NM_001079941	Gtf3c5	0.2846	0.6517	0.4204	0.5790	0.8782	0.9370	0.4011	0.7462	0.7319	0.9134	0.4126	0.9578
NM_001013206	Mrps26	0.3872	0.7495	0.7209	0.6408	0.1348	0.0779	0.7333	0.6272	0.6062	0.5076	0.6198	0.0522
NM_001024237	Farsa	0.4458	0.4480	0.2709	0.4092	0.2690	0.2553	0.3239	0.5735	0.3986	0.4252	0.3377	0.2825

NM_001014043	Sgms2	0.6180	0.2225	0.4265	0.5411	0.6889	0.4850	0.1704	0.3080	0.5146	0.5146	0.6998	0.6380
NM_001013039	Art5	0.5232	0.3164	0.4869	0.6787	0.7353	0.3719	0.2989	0.0942	0.4813	0.5904	0.1610	0.5994
NM_001011915	Fermt2	0.6278	0.5200	0.7842	0.5393	0.9464	0.7374	0.3534	0.9333	0.8088	0.7092	0.8708	0.8836
NM_001009669	Uba5	0.7542	0.7899	0.6459	0.0618	0.2372	0.2321	0.7210	0.0464	0.5324	0.8529	0.8646	0.4268
NM_001008562	Lmcd1	0.7876	0.9280	0.9067	0.9541	0.9999	0.9204	0.9954	0.9229	0.9697	0.8548	0.9431	0.9524
NM_001008373	Sfrs12ip1	0.3347	0.2340	0.8936	0.9231	0.4909	0.4927	0.6285	0.8143	0.5707	0.3502	0.4283	0.6532
NM_001009665	Ebag9	0.3837	0.5935	0.4538	0.1576	0.3311	0.4033	0.1431	0.4768	0.3165	0.9016	0.2603	0.2140
NM_001003965	Dpcr1	0.1694	0.1610	0.1708	0.1683	0.3244	0.3258	0.1703	0.6599	0.3355	0.1574	0.4649	0.2932
NM_001012504	Set	0.9294	0.9631	0.8274	0.9678	0.9893	0.9697	0.9964	0.9285	0.9280	0.9062	0.9526	0.8966
NM_001012227	Xk	0.3380	0.3883	0.5105	0.3081	0.3804	0.4196	0.6193	0.5524	0.5999	0.4098	0.2463	0.5911
NM_001005383	Acy1	0.0559	0.1307	0.2519	0.3734	0.0008	0.0377	0.7641	0.0003	0.1122	0.1069	0.6043	0.0973
NM_001107236	Cobl	0.3719	0.2858	0.4715	0.9248	0.1665	0.3632	0.3455	0.0261	0.6898	0.4559	0.0780	0.9178
NM_001107223	Znf509	0.9101	0.8903	0.5255	0.8788	0.2321	0.9037	0.5404	0.6645	0.6316	0.8948	0.7918	0.9635
NM_001107163	Zswim4	0.2863	0.2727	0.2265	0.3655	0.2094	0.4296	0.2447	0.5221	0.4198	0.5290	0.0362	0.2146
NM_001109172	Col13a1	0.2703	0.5218	0.2382	0.2690	0.0471	0.0621	0.0702	0.0834	0.2634	0.7696	0.8127	0.1933
NM_001108127	Slc36a4	0.3602	0.4562	0.2701	0.2947	0.3360	0.2948	0.1128	0.0731	0.7779	0.8251	0.4653	0.5373
NM_001105991	Atp6v1g3	0.6149	0.2856	0.2703	0.3443	0.6632	0.2780	0.4702	0.6500	0.5755	0.6127	0.8636	0.1114
NM_001106120	Usp6nl	0.9219	0.8548	0.8271	0.9152	0.6346	0.8699	0.6453	0.9923	0.9642	0.9682	0.8983	0.9711
NM_001106117	RGD1561038	0.3890	0.8250	0.8250	0.4638	0.6333	0.8238	0.8762	0.6427	0.4438	0.9551	0.9237	0.8477
NM_001106298	Bcl7c	0.8322	0.7212	0.6840	0.8607	0.9226	0.8120	0.7281	0.9595	0.8778	0.7216	0.7145	0.0684
NM_001107676	RGD1309403	0.6393	0.3813	0.3490	0.2463	0.4571	0.8234	0.3348	0.4014	0.5038	0.8599	0.7471	0.7031
NM_001109257	Klhdc5	0.9227	0.7699	0.7270	0.9595	0.5201	0.9190	0.8584	0.8337	0.8871	0.9309	0.8423	0.9113
NM_001107962	Mmachc	0.7614	0.4673	0.8421	0.9031	0.5148	0.7942	0.8836	0.0911	0.2298	0.8365	0.9180	0.9138
NM_001130582	Xpnpep3	0.6652	0.4975	0.4864	0.2776	0.6021	0.3967	0.4803	0.3866	0.4072	0.2942	0.5099	0.5412
NM_001005873	Dhx40	0.8728	0.8074	0.9213	0.8941	0.6603	0.9637	0.9961	0.5973	0.8191	0.9857	0.9929	0.8601
NM_001122782	Mapk1ip1	0.9150	0.1793	0.0557	0.0344	0.8880	0.5400	0.3988	0.8111	0.6902	0.3694	0.5769	0.4148
NM_001107532	Ap3b2	0.0814	0.0612	0.0231	0.1451	0.1189	0.1404	0.1131	0.0663	0.6232	0.0736	0.0824	0.2360
NM_001108882	Lyzl1	0.2344	0.3956	0.1264	0.0733	0.3545	0.1359	0.1525	0.6633	0.1414	0.7513	0.2722	0.5831
NM_001109004	Traip	0.9097	0.9908	0.0893	0.7451	0.9121	0.8290	0.7753	0.7988	0.7149	0.8401	0.8888	0.1049
NM_001126266	Utp23	0.9080	0.6028	0.7538	0.0399	0.4052	0.1206	0.2917	0.1832	0.3241	0.4010	0.6475	0.0751
NM_001123469	Hist2h4	0.6820	0.3905	0.2917	0.5138	0.2953	0.2663	0.0939	0.5278	0.1370	0.1282	0.3336	0.2571
NM_001007712	Sdpr	0.1370	0.2132	0.1530	0.3581	0.4366	0.2510	0.2923	0.5577	0.0886	0.0835	0.0166	0.0095
NM_001013119	Gna13	0.4269	0.0494	0.0879	0.0449	0.0484	0.2519	0.0477	0.0547	0.1495	0.0985	0.1342	0.1930
NM_001010967	Naaa	0.4502	0.5302	0.4131	0.1857	0.5500	0.4132	0.2177	0.4676	0.3409	0.5722	0.1335	0.4428
NM_001010920	Cndp2	0.3437	0.1056	0.4780	0.1349	0.3151	0.2836	0.3990	0.1017	0.4374	0.1782	0.1036	0.1499
NM_001008379	Prr13	0.0499	0.0664	0.7792	0.8271	0.2497	0.1575	0.2656	0.1084	0.0489	0.0740	0.0199	0.3460
NM_001008366	Cenpn	0.2179	0.9886	0.2789	0.2596	0.8304	0.9274	0.8214	0.4338	0.9713	0.9179	0.7142	0.0880
NM_001007678	Rabif	0.8514	0.9633	0.5273	0.9392	0.9549	0.8445	0.8467	0.9437	0.2543	0.5018	0.6889	0.0980
NM_001007668	Fam100a	0.5751	0.2498	0.9564	0.5143	0.8937	0.1869	0.8087	0.5175	0.9624	0.8400	0.9622	0.9075
NM_001008311	Ermn	0.7099	0.7116	0.1224	0.7726	0.2753	0.6135	0.7576	0.4190	0.0788	0.2050	0.8700	0.5410
NM_001008302	Sft2d1	0.3624	0.1758	0.6534	0.0213	0.1756	0.1408	0.0483	0.5782	0.3369	0.2624	0.0336	0.1497
NM_001011903	Tpst1	0.3961	0.4841	0.5750	0.0406	0.2308	0.6974	0.1799	0.6289	0.1607	0.2373	0.2948	0.1251
NM_001012002	Zap70	0.5154	0.1115	0.6077	0.7245	0.4520	0.5271	0.8689	0.6821	0.4522	0.5436	0.4316	0.6014
NM_001007651	Pef1	0.0162	0.2039	0.1400	0.0400	0.2476	0.0498	0.2614	0.2403	0.2700	0.0180	0.3297	0.2567
NM_001007732	Serpinb9	0.0652	0.0068	0.0062	0.1527	0.2161	0.1074	0.0693	0.0922	0.1981	0.0435	0.0261	0.0617
NM_001007640	Ints12	0.0848	0.0324	0.4545	0.0008	0.1752	0.1098	0.8523	0.0094	0.1880	0.2016	0.3020	0.0554
NM_001007634	Pelo	0.1068	0.4382	0.0661	0.1418	0.3368	0.2088	0.9076	0.0823	0.2161	0.3605	0.4139	0.0125
NM_001007629	Nutf2	0.3736	0.6175	0.5593	0.0466	0.8675	0.6599	0.5398	0.9176	0.7292	0.7127	0.3752	0.9739
NM_001007630	Zfand6	0.4331	0.3985	0.9409	0.5350	0.6750	0.7438	0.1227	0.2997	0.2008	0.7845	0.8148	0.4331

NM_001007627	Stard6	0.4633	0.6060	0.3844	0.7815	0.4221	0.6761	0.6303	0.8456	0.2484	0.4701	0.5279	0.1038
NM_001007625	Epdr1	0.9302	0.0674	0.9587	0.9694	0.8850	0.9391	0.0893	0.9651	0.1369	0.0208	0.0042	0.5600
NM_001010951	Prepl	0.0420	0.0648	0.0447	0.2880	0.0074	0.0162	0.1574	0.1159	0.0943	0.0365	0.0423	0.0744
NM_001007611	Fam101b	0.7618	0.9733	0.7997	0.8059	0.8899	0.9500	0.9823	0.9893	0.9740	0.7866	0.9970	0.9105
NM_001009687	Riok2	0.6292	0.1607	0.1706	0.6527	0.4188	0.3822	0.5757	0.1639	0.3145	0.2775	0.3215	0.3889
NM_001108789	Xpo5	0.8612	0.5673	0.8346	0.5411	0.9605	0.3252	0.8879	0.5796	0.6981	0.9736	0.8362	0.9788
NM_001007265	Os9	0.6936	0.1182	0.6180	0.7518	0.8102	0.9468	0.5717	0.9456	0.8794	0.0186	0.2540	0.9515
NM_001007695	Tcp11	0.3937	0.4871	0.0937	0.3589	0.5226	0.3985	0.5381	0.3998	0.2368	0.1452	0.4708	0.1128
NM_001007703	Gltpd1	0.5144	0.8730	0.6778	0.8260	0.5244	0.2743	0.7960	0.5869	0.9946	0.9269	0.7154	0.8635
NM_001006994	Gpr180	0.6102	0.9703	0.5440	0.5382	0.1977	0.3352	0.7767	0.3688	0.7916	0.9366	0.8632	0.2337
NM_001006986	Ecsit	0.0289	0.3557	0.6062	0.6052	0.0238	0.0618	0.5651	0.0492	0.1567	0.6135	0.1045	0.0761
NM_001008507	Dhrs7b	0.1094	0.6117	0.0997	0.1501	0.0225	0.0599	0.1697	0.0923	0.5740	0.8547	0.4890	0.2278
NM_001006978	Sec13	0.7457	0.8579	0.7361	0.6070	0.0894	0.0409	0.9246	0.2872	0.9346	0.9407	0.8913	0.1466
NM_001006957	Eif4h	0.9256	0.8561	0.9566	0.9414	0.7638	0.9535	0.8830	0.6193	0.9623	0.9482	0.9694	0.9264
NM_001033675	Cabp1	0.4206	0.6933	0.4965	0.2674	0.5075	0.6721	0.3467	0.7277	0.4284	0.2332	0.2445	0.6339
NM_001033700	Lass2	0.4277	0.3578	0.4086	0.9441	0.3004	0.8358	0.1053	0.2596	0.2255	0.0945	0.2355	0.8067
NM_001033676	Cabp1	0.4206	0.6933	0.4965	0.2674	0.5075	0.6721	0.3467	0.7277	0.4284	0.2332	0.2445	0.6339
NM_001014258	Crls1	0.4384	0.3992	0.8878	0.1110	0.1477	0.3113	0.8973	0.2365	0.6005	0.5510	0.9270	0.8468
NM_001034014	Accn1	0.4310	0.4189	0.2804	0.3756	0.2264	0.4054	0.7649	0.3018	0.7473	0.5791	0.3381	0.5724
NM_001007014	Rbm4b	0.3300	0.3186	0.3218	0.3590	0.5196	0.2441	0.3328	0.6558	0.3656	0.3018	0.2977	0.3202
NM_001006602	Pigs	0.3083	0.1757	0.8778	0.9653	0.2172	0.8821	0.6537	0.3214	0.1494	0.3159	0.1780	0.8822
NM_001005897	Clec4e	0.5409	0.5448	0.4000	0.7150	0.1206	0.2570	0.2035	0.5128	0.3838	0.5525	0.1428	0.4881
NM_001005896	Clec4b2	0.3533	0.1327	0.3640	0.3036	0.1199	0.7069	0.3190	0.0857	0.3868	0.0525	0.1331	0.3893
NM_001011975	Aldh1b1	0.7717	0.6355	0.4275	0.5356	0.7702	0.2578	0.0911	0.6136	0.0395	0.7782	0.8806	0.6911
NM_001005559	Txndc2	0.1691	0.1606	0.1704	0.1681	0.1736	0.1693	0.1701	0.1637	0.1610	0.1839	0.1618	0.1660
NM_001005558	Fam151a	0.0855	0.0919	0.1958	0.0643	0.0559	0.4035	0.1730	0.0320	0.0658	0.2147	0.0380	0.1166
NM_001005545	Myg1	0.3468	0.2058	0.5858	0.3597	0.6425	0.1173	0.7062	0.6508	0.1019	0.1858	0.2055	0.0113
NM_001007660	Ccdc82	0.7204	0.6532	0.6604	0.6164	0.6580	0.5165	0.5644	0.2283	0.1977	0.2132	0.6427	0.4873
NM_001004449	Smgc	0.8955	0.8380	0.6575	0.6941	0.7837	0.8182	0.5330	0.1212	0.1303	0.1283	0.8607	0.6461
NM_001004445	Rnf113a2	0.5027	0.9431	0.7889	0.8166	0.6992	0.2778	0.9540	0.6816	0.3258	0.6356	0.8703	0.7374
NM_001004283	Eif3d	0.1147	0.4855	0.0252	0.1491	0.0569	0.1526	0.1698	0.4765	0.2550	0.1308	0.1071	0.1682
NM_001004273	Ggnbp2	0.9673	0.5428	0.2759	0.1981	0.5766	0.3855	0.0224	0.5631	0.6694	0.9058	0.3518	0.6969
NM_001009642	Pop4	0.1160	0.2440	0.0391	0.2987	0.0528	0.0135	0.1256	0.2104	0.2687	0.0816	0.0885	0.0134
NM_001004270	Snupn	0.8167	0.7387	0.7117	0.7457	0.7727	0.1789	0.6329	0.5000	0.1971	0.1243	0.4562	0.3952
NM_173122	LOC500901	0.2367	0.7591	0.8623	0.3229	0.7331	0.4157	0.6968	0.4567	0.5212	0.6466	0.2534	0.3322
NM_001010958	Slc25a29	0.7083	0.4608	0.5421	0.1073	0.3638	0.3888	0.5255	0.2993	0.5717	0.4342	0.1109	0.3166
NM_001009632	G0s2	0.3383	0.6570	0.3768	0.4117	0.3240	0.6932	0.5941	0.5626	0.5544	0.2992	0.7289	0.1535
NM_001004257	Hspa14	0.8692	0.4946	0.0854	0.7376	0.6543	0.6088	0.1202	0.7165	0.5635	0.7015	0.6999	0.1998
NM_001013933	Ube2a	0.6089	0.9045	0.8635	0.5014	0.6026	0.9384	0.4812	0.8950	0.6392	0.6030	0.8532	0.7152
NM_001004247	Ubl7	0.0820	0.0985	0.2856	0.3320	0.0315	0.0353	0.0409	0.0429	0.4390	0.0649	0.0078	0.0463
NM_001007647	Rnf181	0.2231	0.4106	0.9417	0.8543	0.2388	0.1861	0.8565	0.1613	0.8303	0.7221	0.5128	0.9091
NM_001008383	Dpep3	0.6420	0.6313	0.6209	0.6975	0.7796	0.4470	0.5026	0.6483	0.7832	0.7159	0.5891	0.2089
NM_001007613	Ddx5	0.9542	0.9591	0.0799	0.8565	0.8324	0.8366	0.0289	0.6608	0.8671	0.7156	0.5668	0.5948
NM_001004230	Edem2	0.0794	0.0341	0.0133	0.9604	0.0003	0.0216	0.8036	0.0015	0.0123	0.0537	0.0528	0.0878
NM_001008321	Gadd45b	0.7319	0.9521	0.8872	0.0492	0.8481	0.3327	0.2687	0.8967	0.9665	0.8328	0.7562	0.9718
NM_001004076	Abcg3l1	0.1690	0.1603	0.1701	0.1678	0.1733	0.1691	0.1700	0.1634	0.1609	0.1573	0.1615	0.1658
NM_001008384	Rac2	0.2105	0.8188	0.3277	0.2172	0.5598	0.4495	0.7892	0.5084	0.7244	0.0423	0.2427	0.5443
NM_001008370	Rab1b	0.6002	0.4356	0.9264	0.8622	0.6774	0.8802	0.8919	0.8053	0.2218	0.7397	0.4536	0.9292
NM_001008362	Znf655	0.7161	0.5484	0.7269	0.6875	0.6212	0.4740	0.7228	0.7160	0.6934	0.6420	0.6668	0.4722

NM_001008342	Akr1cl2	0.4325	0.6475	0.6510	0.2668	0.6817	0.8740	0.7426	0.9619	0.7503	0.8210	0.5757	0.7092
NM_001037555	Rassf1	0.9419	0.9458	0.7227	0.1676	0.8004	0.9504	0.9412	0.7924	0.8670	0.9122	0.7602	0.9772
NM_001004022	Krt15	0.1078	0.0609	0.7605	0.2450	0.0047	0.0870	0.5273	0.0213	0.4078	0.2156	0.0267	0.3787
NM_001003709	Ufc1	0.1397	0.6335	0.2215	0.3465	0.9052	0.8656	0.4422	0.8377	0.1158	0.4622	0.2613	0.1973
NM_001003705	Slc38a7	0.5200	0.5303	0.0304	0.8012	0.1094	0.6925	0.1123	0.6383	0.6510	0.7368	0.4042	0.8417
NM_001002798	Top1mt	0.7409	0.8369	0.6404	0.6431	0.5429	0.5049	0.2659	0.3981	0.3932	0.5539	0.4290	0.2722
NM_001009652	Znf622	0.4520	0.4726	0.4410	0.3760	0.3513	0.6916	0.3583	0.5286	0.3105	0.1571	0.3933	0.2495
NM_001007264	Ugt2b37	0.5563	0.5670	0.6931	0.5539	0.4219	0.5497	0.4042	0.2414	0.4895	0.3465	0.4551	0.4399
NM_001005540	Ppm1j	0.0261	0.6919	0.1810	0.3363	0.0543	0.1339	0.0832	0.0639	0.5251	0.4731	0.0327	0.3449
NM_001003958	Dnmt3a	0.7159	0.9958	0.9887	0.9784	0.9085	0.8749	0.9836	0.9783	0.9790	0.9640	0.8396	0.9595
NM_001024316	Gata5	0.3155	0.6808	0.0818	0.3671	0.4156	0.4799	0.7095	0.3467	0.6370	0.2941	0.3876	0.2716
NM_017239	Myh6	0.1811	0.2301	0.1381	0.5136	0.0287	0.0250	0.1629	0.0576	0.5712	0.1696	0.0859	0.5937
NM_001126048	Ccdc72	0.7094	0.2241	0.3628	0.5402	0.4828	0.6227	0.2709	0.2789	0.3778	0.4341	0.2828	0.4712
NM_001126083	Cks2	0.8593	0.9808	0.0198	0.0232	0.7896	0.8678	0.9996	0.9297	0.9074	0.8022	0.8897	0.0012
NM_001109527	Ccdc87	0.1688	0.1603	0.4474	0.1675	0.2798	0.1689	0.3751	0.1633	0.3003	0.4892	0.1611	0.3089
NM_001126085	Lsm6	0.3427	0.9962	0.1574	0.3575	0.9489	0.8956	0.4325	0.8772	0.5968	0.9727	0.8245	0.2966
NM_001126084	Mt4	0.4300	0.2796	0.5886	0.3400	0.4046	0.3543	0.3838	0.4960	0.4822	0.2616	0.1609	0.2477
NM_001126093	Pcp4l1	0.5817	0.5154	0.6292	0.6697	0.5109	0.6558	0.4371	0.4156	0.5997	0.3608	0.2206	0.5741
NM_001126092	Sf3b5	0.2242	0.4600	0.3879	0.4804	0.4384	0.4107	0.5902	0.2940	0.3899	0.2443	0.2700	0.3171
NM_001126091	Snrpf	0.9173	0.0533	0.0470	0.4542	0.7016	0.5953	0.6367	0.3509	0.6942	0.3185	0.2194	0.1456
NM_001105925	Mmp17	0.4997	0.8220	0.1336	0.4071	0.8783	0.4599	0.8399	0.7227	0.9565	0.8894	0.2372	0.8200
NM_001007603	Rps20	0.3243	0.3403	0.3563	0.2248	0.2677	0.0372	0.4305	0.1334	0.1032	0.3106	0.2242	0.0534
NM_001126099	Triap1	0.6280	0.4656	0.2245	0.4843	0.5593	0.6178	0.6321	0.6266	0.4912	0.5440	0.6697	0.4195
NM_001126098	Elof1	0.3697	0.4957	0.3914	0.2592	0.5270	0.5481	0.5033	0.2939	0.5188	0.1988	0.3168	0.6820
NM_001126097	Uqcr	0.3857	0.3487	0.2436	0.3910	0.2776	0.3367	0.2924	0.5066	0.2172	0.6772	0.7293	0.6850
NM_001126096	Lyrm2	0.7928	0.2811	0.3562	0.9916	0.9256	0.7290	0.0378	0.9104	0.3445	0.1719	0.2870	0.2904
NM_001126095	Srp9	0.9325	0.6361	0.7983	0.8983	0.8241	0.8316	0.9776	0.5972	0.4474	0.8290	0.7055	0.5779
NM_001126094	Mrps21	0.2899	0.1602	0.3208	0.4676	0.1731	0.3962	0.1699	0.5324	0.3783	0.2823	0.1608	0.3209
NM_001126276	Znf579	0.9378	0.8486	0.9969	0.7861	0.7851	0.9458	0.8973	0.9089	0.9761	0.8874	0.7870	0.9504
NM_001126086	Morn2	0.1999	0.2770	0.5073	0.3734	0.2468	0.3830	0.1697	0.1632	0.4278	0.2779	0.4744	0.4370
NM_001126088	Gtf2h5	0.2008	0.8187	0.7547	0.6827	0.8124	0.6569	0.9859	0.5419	0.1403	0.5815	0.5744	0.1632
NM_001109240	RGD1562720	0.8164	0.6791	0.7344	0.5952	0.5599	0.6280	0.8626	0.5777	0.9067	0.8861	0.8422	0.6183
NM_001126082	Anapc11	0.1766	0.3900	0.5309	0.2988	0.3967	0.3127	0.2050	0.3611	0.5971	0.2938	0.2953	0.2720
NM_001106624	Dusp16	0.9110	0.7812	0.7125	0.3782	0.5434	0.6634	0.7165	0.3538	0.8987	0.5124	0.5718	0.5536
NM_001126100	Nola3	0.3994	0.4955	0.2518	0.5514	0.3498	0.6681	0.5407	0.4087	0.3667	0.3403	0.5151	0.3916
NM_001008765	Igtp	0.3813	0.2075	0.4670	0.6881	0.1167	0.5614	0.4907	0.3010	0.2817	0.1354	0.0442	0.3615
NM_001126089	LOC691031	0.7298	0.5865	0.6204	0.6117	0.6627	0.6960	0.6792	0.6943	0.6802	0.6627	0.6071	0.6929
NM_001107965	B4galt2	0.1040	0.3509	0.5261	0.3128	0.2255	0.5706	0.4620	0.6975	0.4436	0.3674	0.0624	0.5108
NM_001106346	Ubl4	0.6741	0.6499	0.8450	0.8794	0.8437	0.9162	0.6004	0.8910	0.7426	0.8636	0.8685	0.9002
NM_001127201	Tbpl1	0.3871	0.3810	0.5361	0.1734	0.0449	0.3479	0.1089	0.3303	0.3191	0.1063	0.3072	0.1589
NM_001047100	Tmprss9	0.3439	0.7516	0.4200	0.2006	0.4691	0.7042	0.4916	0.5931	0.4085	0.3864	0.4286	0.4938
NM_019160	Uts2	0.1230	0.5995	0.2927	0.2791	0.3727	0.0763	0.4200	0.3751	0.0975	0.0642	0.0553	0.2798
NM_019367	Ppt2	0.3090	0.1886	0.9060	0.9335	0.1612	0.3444	0.9627	0.2020	0.1672	0.4573	0.0161	0.4706
NM_020306	Adam17	0.3353	0.2602	0.2152	0.2687	0.1418	0.1394	0.1126	0.2469	0.2267	0.3500	0.1246	0.7406
NM_001127301	RGD1563853	0.9642	0.2214	0.8887	0.7828	0.9482	0.9693	0.8744	0.8554	0.5124	0.1384	0.6635	0.8573
NM_001126269	Slc25a17	0.2226	0.2567	0.8523	0.4039	0.5650	0.5275	0.8751	0.5838	0.3935	0.1439	0.2925	0.6335
NM_001126271	RGD1561552	0.0383	0.4512	0.4155	0.2414	0.1658	0.4094	0.0424	0.3261	0.7383	0.0366	0.2093	0.1414
NM_001126270	Ndc80	0.8111	0.8708	0.0402	0.6739	0.7178	0.7184	0.7237	0.9384	0.6927	0.8713	0.8748	0.1954
NM_001127319	Ephb2	0.0728	0.4099	0.2985	0.3784	0.3646	0.1653	0.4258	0.7532	0.1394	0.3411	0.3213	0.5882

NM_001100172	Kctd8	0.3974	0.7259	0.8602	0.6139	0.3052	0.4022	0.4482	0.5536	0.8249	0.5082	0.6182	0.4725
NM_001100171	Noxa1	0.7252	0.0318	0.2138	0.6448	0.2760	0.1668	0.4364	0.2625	0.7146	0.4398	0.2284	0.5901
NM_001126275	Ccm2	0.6862	0.3440	0.6543	0.4056	0.4628	0.3253	0.5531	0.3598	0.3838	0.2232	0.3632	0.5152
NM_001126299	Esco1	0.5736	0.9096	0.8885	0.9554	0.9241	0.8961	0.0333	0.6503	0.6683	0.5553	0.9222	0.8445
NM_001126297	RGD1563714	0.5215	0.6892	0.3875	0.6224	0.4161	0.3936	0.3399	0.3730	0.6748	0.4316	0.5584	0.5381
NM_001126296	Tra2a	0.9569	0.9987	0.9285	0.4512	0.9441	0.9907	0.7907	0.9884	0.9983	0.9997	0.9855	0.8732
NM_001126295	RGD1564379	0.9045	0.6182	0.4207	0.5583	0.8051	0.8139	0.7710	0.9218	0.8263	0.5464	0.7325	0.5199
NM_001127373	Adnp2	0.4895	0.1893	0.3551	0.1424	0.1883	0.4985	0.1108	0.2898	0.0273	0.1640	0.3777	0.9082
NM_001126291	Flrt3	0.9600	0.9744	0.9921	0.1164	0.0283	0.3010	0.8416	0.2264	0.9581	0.9811	0.8670	0.7362
NM_001126289	LOC363326	0.4036	0.7405	0.5518	0.4868	0.5607	0.3909	0.7250	0.1226	0.4711	0.1424	0.4607	0.2322
NM_001126290	RGD1564319	0.6555	0.6272	0.5596	0.4433	0.8335	0.4387	0.5043	0.8009	0.8611	0.4444	0.5635	0.5589
NM_001126373	Hdac8	0.5652	0.4021	0.9901	0.9507	0.6351	0.2883	0.7844	0.5662	0.4818	0.4065	0.0479	0.3230
NM_001126287	RGD1309543	0.4156	0.5352	0.0467	0.6331	0.3390	0.5979	0.0867	0.4344	0.6131	0.4878	0.5705	0.9358
NM_001127552	Lsm14a	0.5221	0.9387	0.9125	0.4944	0.7251	0.3420	0.3837	0.6699	0.2784	0.9270	0.6250	0.7722
NM_001126284	Mon1a	0.0158	0.1337	0.0305	0.0002	0.0697	0.0965	0.0754	0.0099	0.5100	0.1897	0.3138	0.1798
NM_001126282	RGD1305138	0.7756	0.1446	0.1999	0.6633	0.6809	0.8090	0.5957	0.3472	0.0608	0.1209	0.0656	0.0537
NM_001126281	LOC314600	0.8554	0.1475	0.8245	0.8049	0.7233	0.7127	0.7792	0.6509	0.8899	0.8044	0.8886	0.6548
NM_001126280	RGD1310269	0.5382	0.7254	0.7939	0.8401	0.9004	0.6319	0.6784	0.8658	0.7199	0.8420	0.9008	0.8632
NM_001126279	RGD1309308	0.8951	0.7031	0.7892	0.3676	0.4174	0.3714	0.9346	0.2152	0.7924	0.9721	0.6561	0.9700
NM_001126278	RGD1564894	0.5517	0.7198	0.5935	0.4666	0.3658	0.6514	0.2000	0.3348	0.7573	0.7004	0.3990	0.5619
NM_001126277	Ccdc114	0.6892	0.2851	0.3773	0.4345	0.6233	0.1361	0.3210	0.0940	0.2736	0.2683	0.2598	0.6224
NM_001100493	Gnptg	0.7761	0.6395	0.8718	0.8006	0.9733	0.9838	0.8089	0.8073	0.9355	0.7544	0.8451	0.7948
NM_001126273	Alkbh2	0.0027	0.3469	0.2305	0.3716	0.0274	0.0035	0.6253	0.0879	0.4032	0.3637	0.2254	0.0037
NM_001107644	Rtn4ip1	0.4735	0.6002	0.2612	0.1175	0.2335	0.1137	0.7460	0.6201	0.6527	0.0706	0.0925	0.1471
NM_001106945	Pdzd11	0.1166	0.1004	0.0934	0.4234	0.2717	0.6966	0.2226	0.2546	0.7427	0.0538	0.3145	0.3046
NM_001126372	LOC362710	0.1687	0.5098	0.2589	0.3152	0.4027	0.3412	0.1694	0.5084	0.5575	0.3749	0.3697	0.6653
NM_001126375	LOC680177	0.1685	0.1599	0.1696	0.1670	0.1729	0.1683	0.4597	0.1630	0.1605	0.1570	0.5267	0.1657
NM_001126374	Zc4h2	0.6580	0.3365	0.8408	0.6870	0.4882	0.2433	0.2028	0.2214	0.3216	0.1527	0.0609	0.4116
NM_001126371	RGD1309829	0.0602	0.5716	0.0168	0.0789	0.1440	0.0315	0.1735	0.0831	0.0126	0.7262	0.1174	0.0046
NM_001127246	LOC288526	0.7354	0.7529	0.0512	0.1246	0.7180	0.7081	0.0600	0.9202	0.8753	0.7921	0.8169	0.8344
NM_019285	Adcy4	0.3935	0.0809	0.1130	0.1519	0.0956	0.1260	0.1365	0.0824	0.3248	0.0757	0.2235	0.3189
NM_001014223	Dnase1l1	0.0970	0.3334	0.0161	0.1780	0.0970	0.2188	0.0871	0.1778	0.0178	0.0161	0.0544	0.1017
NM_130433	Acaa2	0.7392	0.2725	0.8344	0.8053	0.9391	0.7824	0.9731	0.9262	0.9592	0.4967	0.6526	0.1157
NM_001012150	Enah	0.0700	0.6740	0.5525	0.5066	0.0759	0.4669	0.7243	0.1519	0.5457	0.5822	0.6299	0.5187
NM_001012147	Pxn	0.6563	0.5444	0.5122	0.8349	0.7166	0.4806	0.5724	0.6987	0.6461	0.5530	0.2060	0.8211
NM_001012061	Cnksr3	0.9485	0.9307	0.9511	0.9871	0.8379	0.9508	0.9877	0.8095	0.9619	0.8852	0.9997	0.8972
NM_001012022	Cldn4	0.7313	0.4771	0.5530	0.2632	0.1416	0.5696	0.4775	0.2585	0.8710	0.1278	0.5669	0.7951
NM_001010953	Rnd2	0.6833	0.0651	0.9214	0.8458	0.1352	0.2863	0.5290	0.6566	0.1268	0.1693	0.0202	0.3234
NM_001009680	Oas1i	0.1021	0.0415	0.2032	0.0787	0.2603	0.0887	0.0164	0.0860	0.0540	0.2313	0.0116	0.1351
NM_001013432	Tmed1	0.0411	0.6721	0.0531	0.9586	0.0901	0.0905	0.5957	0.8866	0.0768	0.7842	0.6695	0.1081
NM_001011962	Cercam	0.1185	0.2653	0.1199	0.6968	0.4017	0.3663	0.9028	0.7977	0.8653	0.9685	0.5311	0.7151
NM_001009268	Actr2	0.2435	0.0012	0.0267	0.0126	0.1239	0.5912	0.0019	0.2430	0.1215	0.0012	0.0872	0.1270
NM_001007005	Arhgdia	0.8131	0.7936	0.1942	0.7049	0.4856	0.8243	0.5671	0.7824	0.9020	0.8908	0.8333	0.9173
NM_001007733	Nudt5	0.5071	0.6675	0.6289	0.8753	0.6177	0.2301	0.8842	0.7005	0.7034	0.8846	0.8597	0.0194
NM_001007723	Nt5c3l	0.2139	0.7208	0.5919	0.2658	0.3798	0.6676	0.4189	0.5310	0.2531	0.3783	0.2523	0.5231
NM_001008776	Serpina11	0.1684	0.1597	0.1694	0.1668	0.1727	0.1682	0.1692	0.1629	0.1603	0.1567	0.1606	0.1655
NM_001108635	Mrpl53	0.0347	0.4060	0.0858	0.1021	0.0151	0.2151	0.2633	0.0246	0.4700	0.2989	0.2245	0.1220
NM_001126267	Fam49b	0.6856	0.5777	0.6528	0.3768	0.4928	0.4989	0.6381	0.5737	0.7205	0.6299	0.6406	0.4705
NM_001126268	Tigd5	0.6070	0.8322	0.6257	0.3133	0.8505	0.6819	0.5382	0.6763	0.8571	0.9170	0.7269	0.7772

NM_001126293	Fam170a	0.1418	0.1715	0.2629	0.2569	0.4402	0.1446	0.3258	0.2405	0.4672	0.7334	0.4764	0.2184
NM_001109437	Dbr1	0.0745	0.1573	0.3192	0.5730	0.1099	0.0817	0.0208	0.0415	0.1298	0.3064	0.2551	0.0115
NM_001109363	Rab17	0.2237	0.0420	0.3705	0.2829	0.4392	0.4019	0.1278	0.5711	0.3799	0.1462	0.0888	0.2070
NM_001126286	RGD1565685	0.2710	0.1046	0.3643	0.0304	0.1563	0.5685	0.0664	0.3758	0.0913	0.0351	0.0926	0.1355
NM_001108236	Lpin2	0.4751	0.9338	0.7889	0.6979	0.7958	0.5907	0.6252	0.5171	0.8295	0.9315	0.4512	0.4628
NM_001024263	Prkd3	0.9587	0.2529	0.9987	0.9806	0.8655	0.9965	0.9535	0.7428	0.6498	0.3594	0.9569	0.8592
NM_001127480	Pde11a	0.6026	0.4951	0.5636	0.4383	0.6647	0.6564	0.2556	0.2359	0.6838	0.5937	0.7256	0.4342
NM_001114181	Mettl5	0.1747	0.1942	0.4665	0.6674	0.5897	0.3160	0.1228	0.4009	0.6715	0.7018	0.5617	0.0867
NM_001009424	Eps15	0.8210	0.8249	0.8983	0.9783	0.6113	0.2262	0.3080	0.5755	0.6853	0.2997	0.8064	0.6517
NM_001008694	Rcn3	0.8400	0.5682	0.3557	0.5505	0.7608	0.9012	0.9368	0.9620	0.6774	0.5394	0.7754	0.2966
NM_001007754	Rassf1	0.9419	0.9458	0.7227	0.1676	0.8004	0.9504	0.9412	0.7924	0.8670	0.9122	0.7602	0.9772
NM_001024992	Tmem199	0.1364	0.1285	0.0148	0.0067	0.0564	0.1441	0.0449	0.0176	0.4074	0.0861	0.0523	0.4132
NM_001007679	Tmem206	0.1093	0.0374	0.5311	0.0730	0.0300	0.0294	0.3463	0.0882	0.3404	0.1646	0.0729	0.3829
NM_001127390	Wash2	0.2988	0.0431	0.2182	0.0917	0.6796	0.9398	0.1491	0.9185	0.3061	0.2163	0.7003	0.6477
NM_001127379	Api5	0.9243	0.4547	0.4316	0.2279	0.3049	0.3388	0.1822	0.2753	0.4613	0.2939	0.5310	0.9605
NM_001127449	Actg1	0.8386	0.8461	0.3735	0.8413	0.8294	0.8651	0.7094	0.6777	0.7471	0.8492	0.8969	0.8581
NM_001127456	LOC300191	0.0028	0.1052	0.2490	0.6939	0.3951	0.7569	0.6443	0.3353	0.1553	0.2765	0.2253	0.9639
NM_001127446	Dlc1	0.9471	0.6417	0.3453	0.4248	0.8113	0.9334	0.0241	0.8543	0.9722	0.2685	0.4130	0.6788
NM_001127452	RGD1305587	0.0213	0.0093	0.3565	0.2180	0.2735	0.1287	0.2928	0.0453	0.2640	0.2792	0.1984	0.7651
NM_001110345	Ube2v1	0.5121	0.5979	0.6110	0.5680	0.5733	0.6799	0.7259	0.6749	0.6036	0.7211	0.2083	0.6750
NM_001127455	Tsta3	0.8013	0.8197	0.8118	0.7155	0.7478	0.8100	0.8230	0.8029	0.9929	0.7401	0.6717	0.7389
NM_001127490	Rfx7	0.9805	0.9977	0.4683	0.4495	0.9762	0.9544	0.4173	0.9145	0.9914	0.9944	0.9788	0.4512
NM_001127485	RGD1306520	0.8853	0.8013	0.6256	0.2404	0.6760	0.6575	0.4449	0.0982	0.4174	0.6493	0.5042	0.2223
NM_001127484	RGD1305793	0.2408	0.3357	0.3189	0.1228	0.2072	0.8154	0.0192	0.3593	0.3345	0.1210	0.4311	0.9087
NM_001127483	Dis3	0.6854	0.8415	0.8160	0.3433	0.5594	0.2257	0.3440	0.6514	0.7684	0.7448	0.7151	0.6150
NM_001127659	Mars	0.9274	0.7535	0.5890	0.3701	0.6564	0.8722	0.8540	0.6826	0.7130	0.9589	0.9211	0.6825
NM_001127529	Glb1l	0.0677	0.0849	0.9004	0.0583	0.1156	0.6926	0.7321	0.0264	0.1502	0.1217	0.0049	0.7685
NM_001127522	Armc7	0.4291	0.6047	0.4570	0.3800	0.4525	0.4789	0.5688	0.5626	0.3434	0.4854	0.2179	0.5791
NM_001127492	Sphkap	0.2355	0.6528	0.2716	0.6143	0.7797	0.1994	0.1518	0.3652	0.6145	0.3265	0.6732	0.5731
NM_001127494	Stk24	0.1683	0.1595	0.1691	0.1665	0.7321	0.5302	0.1689	0.6119	0.2885	0.1566	0.1604	0.4482
NM_001127481	Pde11a	0.6026	0.4951	0.5636	0.4383	0.6647	0.6564	0.2556	0.2359	0.6838	0.5937	0.7256	0.4342
NM_001127497	Agk	0.2791	0.0797	0.3585	0.0414	0.1618	0.9329	0.1432	0.6367	0.3584	0.1770	0.4516	0.1962
NM_001127495	Rgs20	0.4587	0.4997	0.5254	0.5225	0.2993	0.4300	0.2264	0.4082	0.5854	0.6240	0.2718	0.2773
NM_001006969	Irf3	0.4687	0.1593	0.1690	0.3952	0.1726	0.3054	0.1687	0.1628	0.2734	0.1565	0.5232	0.3474
NM_001127488	Snx33	0.1125	0.4023	0.6386	0.4102	0.3463	0.8755	0.2499	0.1473	0.8480	0.2618	0.4183	0.8504
NM_001127526	RGD1311605	0.0200	0.0564	0.2494	0.4392	0.1083	0.0395	0.5027	0.1206	0.0518	0.1192	0.1339	0.0215
NM_001034007	Ncoa4	0.6166	0.6882	0.6331	0.7221	0.7292	0.5121	0.4832	0.6044	0.4116	0.6737	0.6950	0.5853
NM_001034073	Tpm1	0.4367	0.7303	0.6371	0.6080	0.4992	0.4851	0.6099	0.4830	0.4713	0.7143	0.3045	0.7344
NM_001127597	Rnf168	0.6807	0.5183	0.6306	0.6828	0.5737	0.6381	0.6997	0.2292	0.5657	0.3517	0.6827	0.5610
NM_001127549	Llgl2	0.7308	0.5271	0.6728	0.6550	0.8001	0.9232	0.7878	0.6848	0.3916	0.4468	0.8171	0.5631
NM_001127548	Traf7	0.5643	0.4588	0.3682	0.6141	0.4399	0.4678	0.2648	0.3825	0.6160	0.3360	0.2322	0.5072
NM_001127547	Fbln1	0.9270	0.8812	0.8552	0.7849	0.7996	0.5613	0.9853	0.8584	0.8405	0.9129	0.9468	0.6784
NM_001127540	RGD1307439	0.2447	0.2922	0.7647	0.5064	0.1043	0.4898	0.3068	0.7929	0.4369	0.6587	0.0847	0.0751
NM_001127533	Mgat4b	0.6080	0.1515	0.9236	0.6414	0.0879	0.4466	0.4644	0.1940	0.3411	0.4607	0.5849	0.8574
NM_001127532	Txndc11	0.3124	0.6901	0.8590	0.5688	0.5721	0.5876	0.8569	0.5884	0.8261	0.1919	0.3754	0.5486
NM_001127534	Nle1	0.8189	0.9233	0.4105	0.3269	0.8119	0.5260	0.8525	0.8043	0.7561	0.8509	0.7546	0.3389
NM_001127537	RGD1309501	0.5556	0.4162	0.5058	0.7659	0.6890	0.6046	0.5538	0.5928	0.2659	0.6540	0.3527	0.7144
NM_001127536	Ildr1	0.3551	0.6629	0.4682	0.3803	0.6661	0.2197	0.7000	0.2676	0.3415	0.1922	0.4853	0.2470
NM_001127546	Kdelr3	0.9371	0.3205	0.8369	0.7583	0.0479	0.7097	0.8773	0.2688	0.0445	0.8496	0.6727	0.8411

NM_001127545	Rnf139	0.9005	0.2673	0.4336	0.7121	0.1518	0.6288	0.1479	0.7102	0.3086	0.5953	0.3166	0.0859
NM_001127544	Slc25a24	0.1840	0.1085	0.3189	0.9394	0.2131	0.3854	0.2830	0.1825	0.0722	0.0696	0.2503	0.5912
NM_001127543	Fermt3	0.4768	0.1359	0.2897	0.2556	0.6931	0.8561	0.3568	0.5425	0.7568	0.0654	0.7564	0.8220
NM_001127542	Peli3	0.6046	0.6381	0.6222	0.9376	0.3132	0.6281	0.7928	0.7616	0.4536	0.2048	0.2973	0.3944
NM_001127541	Efcab4a	0.7583	0.8391	0.7738	0.8731	0.8429	0.7856	0.7998	0.8634	0.9913	0.8576	0.8081	0.7919
NM_001127538	Spire2	0.2058	0.5186	0.1722	0.2467	0.1538	0.3026	0.7407	0.5347	0.5564	0.3769	0.1886	0.6882
NM_001127524	Aadacl1	0.1478	0.1954	0.1793	0.4300	0.0836	0.2933	0.1839	0.1131	0.0343	0.0240	0.1856	0.6002
NM_001127564	RGD1560481	0.7178	0.2373	0.4411	0.3331	0.3336	0.1794	0.0606	0.2275	0.2928	0.3552	0.2013	0.1238
NM_001127528	Tmprss13	0.7112	0.6550	0.6346	0.5141	0.8242	0.6192	0.5975	0.1548	0.5905	0.8650	0.2780	0.5743
NM_001127535	Falz	0.6623	0.2627	0.9088	0.0413	0.3583	0.7227	0.0173	0.4373	0.5298	0.8142	0.3602	0.9825
NM_001127554	Nudt16	0.2651	0.2019	0.7765	0.8516	0.0700	0.5793	0.1963	0.1119	0.6356	0.0598	0.2967	0.7542
NM_001127551	Lrch4	0.6488	0.8991	0.9063	0.0219	0.2975	0.4010	0.8748	0.2310	0.9337	0.7443	0.6613	0.9508
NM_001127557	Rtdr1	0.7293	0.6531	0.6715	0.4046	0.1068	0.7423	0.7472	0.4908	0.3531	0.5429	0.1525	0.4279
NM_001100766	Cno	0.3380	0.8268	0.0991	0.3958	0.2611	0.5692	0.3738	0.1243	0.4715	0.5602	0.5838	0.1366
NM_001127558	Zfp2	0.8499	0.6408	0.6822	0.8278	0.6707	0.4719	0.4214	0.0983	0.6961	0.6477	0.6585	0.6049
NM_001127556	RGD1563815	0.3379	0.2658	0.2352	0.2672	0.8052	0.2615	0.7176	0.0777	0.3058	0.3545	0.6667	0.5224
NM_001127563	RGD1564114	0.5197	0.3280	0.5003	0.3413	0.4591	0.4280	0.5379	0.1006	0.6156	0.6008	0.2908	0.8784
NM_001127562	RGD1565059	0.4971	0.2919	0.8859	0.6855	0.5137	0.3963	0.1463	0.5955	0.5025	0.0707	0.3088	0.1060
NM_001127561	Lass3	0.8216	0.8025	0.9089	0.6062	0.8022	0.6187	0.4936	0.6522	0.8029	0.7074	0.8083	0.8126
NM_001127560	Sytl3	0.6839	0.6154	0.6357	0.4299	0.5202	0.3121	0.5320	0.7003	0.7022	0.3018	0.6664	0.7701
NM_001127574	LOC691431	0.2306	0.3746	0.4958	0.6402	0.4252	0.3381	0.6394	0.7230	0.7150	0.2406	0.5563	0.2278
NM_001127523	RGD1310778	0.1432	0.8044	0.0848	0.4360	0.7912	0.5292	0.8196	0.8421	0.0561	0.9114	0.7679	0.0179
NM_001127559	RGD1563863	0.6781	0.2001	0.3284	0.8549	0.4574	0.7210	0.0300	0.3592	0.3514	0.1409	0.2105	0.5674
NM_001127521	RGD1308134	0.1158	0.0728	0.7268	0.4920	0.5242	0.4028	0.5122	0.6772	0.2688	0.2500	0.2541	0.3217
NM_001127572	LOC688393	0.2342	0.4513	0.6684	0.4581	0.5885	0.7150	0.5366	0.5254	0.1976	0.1990	0.2315	0.6993
NM_001127569	LOC683514	0.3108	0.5600	0.7017	0.5364	0.2763	0.2616	0.6876	0.5140	0.3632	0.4505	0.3293	0.3627
NM_001127568	RGD1562161	0.7381	0.9340	0.8243	0.1333	0.9245	0.7652	0.2197	0.5195	0.7279	0.7032	0.9870	0.8308
NM_001127567	RGD1561648	0.2016	0.3635	0.0743	0.2130	0.1634	0.1771	0.0766	0.3715	0.0415	0.5318	0.6551	0.2904
NM_001127566	RGD1566112	0.8007	0.5583	0.6787	0.2277	0.7423	0.6775	0.7288	0.4025	0.6667	0.3974	0.5662	0.7726
NM_001127565	RGD1562988	0.3214	0.6144	0.1492	0.4192	0.6014	0.4120	0.4518	0.1606	0.3877	0.1495	0.5220	0.3919
NM_001127575	Calml4	0.6271	0.3680	0.2615	0.1664	0.3474	0.3799	0.4642	0.2768	0.6236	0.4752	0.5495	0.4418
NM_001127573	LOC689226	0.3431	0.6020	0.7448	0.8151	0.3801	0.3356	0.4451	0.2034	0.4733	0.6749	0.3680	0.5456
NM_001127527	Yeats4	0.5714	0.8879	0.0999	0.8460	0.7472	0.8528	0.3323	0.9200	0.7768	0.4378	0.5710	0.0443
NM_001127530	LOC302022	0.7715	0.9009	0.9635	0.6690	0.9080	0.8790	0.6473	0.9282	0.9457	0.8154	0.6230	0.9222
NM_001127525	Tmem50a	0.8610	0.1796	0.8881	0.5538	0.3547	0.5479	0.2299	0.6575	0.8334	0.6241	0.3468	0.2332
NM_001127555	Irak1	0.6357	0.5820	0.6248	0.0293	0.9183	0.7821	0.6446	0.8844	0.7541	0.5520	0.7685	0.8237
NM_001127579	RGD1565712	0.7017	0.6693	0.5493	0.2330	0.6642	0.2976	0.6840	0.2636	0.4540	0.6534	0.2222	0.5948
NM_001127599	RGD1311730	0.7334	0.9562	0.4188	0.7761	0.9107	0.9780	0.9432	0.8788	0.8267	0.9919	0.8638	0.8772
NM_001108496	Stim1	0.9958	0.9412	0.8953	0.9959	0.9979	0.9962	0.9805	0.9690	0.9557	0.8127	0.8790	0.9880
NM_001127594	Zfp786	0.4529	0.2054	0.2327	0.2756	0.4839	0.1678	0.3300	0.5882	0.2455	0.7173	0.3020	0.4419
NM_001127581	Sh3bp5l	0.2027	0.4492	0.5762	0.2316	0.6756	0.6583	0.7274	0.6572	0.6739	0.5242	0.5947	0.5777
NM_001127607	Ttc30b	0.1682	0.1591	0.1688	0.1663	0.1725	0.1677	0.1686	0.1628	0.1601	0.1564	0.1602	0.1653
NM_001127606	LOC10015822[illegible]	0.6256	0.6579	0.5412	0.2661	0.4365	0.4459	0.6892	0.4992	0.5062	0.3664	0.2501	0.3697
NM_001127590	Slc25a42	0.5630	0.3225	0.6500	0.3937	0.6134	0.7039	0.6772	0.3161	0.5394	0.1443	0.5656	0.8626
NM_001127657	Spin2a	0.1111	0.1394	0.3174	0.0305	0.0383	0.1710	0.0453	0.0132	0.0195	0.1758	0.3264	0.0332
NM_001127604	Klf16	0.6991	0.5952	0.4719	0.6233	0.5450	0.6841	0.5060	0.6938	0.1913	0.3883	0.4062	0.5995
NM_001127638	Usp21	0.5628	0.2310	0.5430	0.5569	0.2135	0.3702	0.2813	0.4338	0.3172	0.4736	0.3192	0.6756
NM_001127578	Fam91a1	0.7217	0.7234	0.7021	0.6439	0.5799	0.5062	0.7239	0.6588	0.7011	0.7024	0.7162	0.4973
NM_001127601	Rpgr	0.9538	0.8689	0.9194	0.9751	0.8504	0.8158	0.8423	0.7196	0.7690	0.9947	0.9113	0.9810

NM_001127655	Chchd8	0.8512	0.9583	0.9250	0.9213	0.7950	0.7447	0.8437	0.9707	0.8302	0.9707	0.8744	0.8820
NM_053431	Qsox1	0.9931	0.9394	0.8940	0.7035	0.8908	0.9570	0.9871	0.9860	0.9969	0.7674	0.9824	0.8981
NM_001127658	Amac1	0.3023	0.4015	0.5264	0.3026	0.2452	0.2556	0.3079	0.3622	0.2464	0.2387	0.2937	0.2712
NM_001108867	Prmt5	0.9675	0.9657	0.1123	0.7430	0.8801	0.3498	0.8269	0.9564	0.9710	0.8521	0.8246	0.3691
NM_001127342	Tp73l	0.5275	0.6728	0.6041	0.6935	0.5515	0.5759	0.6646	0.2247	0.6482	0.6386	0.7059	0.7205
NM_001127339	Tp73l	0.5275	0.6728	0.6041	0.6935	0.5515	0.5759	0.6646	0.2247	0.6482	0.6386	0.7059	0.7205
NM_001127343	Tp73l	0.5275	0.6728	0.6041	0.6935	0.5515	0.5759	0.6646	0.2247	0.6482	0.6386	0.7059	0.7205
NM_001100704	Extl2	0.3415	0.2812	0.9906	0.9982	0.6650	0.9501	0.8532	0.9299	0.1123	0.0541	0.1372	0.1503
NM_001127654	Tm6sf2	0.2520	0.3535	0.3467	0.3083	0.5160	0.2592	0.2984	0.3419	0.3270	0.2478	0.3554	0.7032
NM_001127653	Nkx2-6	0.3393	0.4124	0.4347	0.6022	0.0553	0.2498	0.4906	0.3554	0.4237	0.3578	0.0842	0.2955
NM_001127635	Zfp9	0.6683	0.6486	0.7235	0.4226	0.4827	0.2846	0.6808	0.2396	0.2835	0.4403	0.6903	0.3931
NM_001100709	Lck	0.3782	0.2799	0.4312	0.4218	0.2667	0.4410	0.5702	0.3528	0.4383	0.4620	0.5761	0.3043
NM_001127642	Slc35a2	0.1984	0.6710	0.6268	0.2849	0.6343	0.6994	0.6463	0.5107	0.3662	0.3047	0.2977	0.6274
NM_001127680	Erich1	0.4428	0.5265	0.0922	0.0545	0.7833	0.5471	0.0225	0.6444	0.3506	0.2348	0.2702	0.2530
NM_001127682	Sh3bgr	0.1469	0.4817	0.6684	0.0790	0.4937	0.3870	0.7930	0.6160	0.4702	0.4599	0.4939	0.5434
NM_001127681	Fam45a	0.6222	0.5286	0.6842	0.9501	0.6882	0.2647	0.6894	0.9420	0.2336	0.3464	0.3937	0.2165
NM_001127683	Hsf2bp	0.3548	0.7150	0.7200	0.6992	0.3574	0.3665	0.2174	0.1712	0.6413	0.6446	0.2829	0.6124
NM_001106491	RGD1309730	0.2480	0.1199	0.6513	0.0050	0.0136	0.0475	0.3317	0.1911	0.0973	0.5189	0.1248	0.0744
NM_001127684	Ndufa4	0.2048	0.2491	0.5753	0.2837	0.4664	0.4817	0.6212	0.2530	0.3161	0.5762	0.4007	0.2151
NM_001127690	Ubtf	0.3140	0.8115	0.4424	0.7240	0.8561	0.8382	0.4358	0.8779	0.4826	0.9666	0.2195	0.8236
NM_001108712	Mbip	0.8404	0.9596	0.7822	0.8758	0.9120	0.9113	0.8023	0.7002	0.7732	0.9966	0.9020	0.9212
NM_001135566	LOC691921	0.2997	0.3236	0.5385	0.3811	0.1723	0.4347	0.1684	0.1626	0.1600	0.4757	0.3212	0.1651
NM_001127689	Il1rl1	0.4577	0.4260	0.3171	0.1662	0.4241	0.3903	0.5096	0.3478	0.4136	0.3074	0.4198	0.3671
NM_001106486	Dnajc10	0.3761	0.3352	0.5204	0.2701	0.4093	0.6149	0.4475	0.3070	0.3169	0.1683	0.1966	0.4507
NM_058209	Zfp37	0.5455	0.5710	0.7181	0.5269	0.7108	0.5720	0.2476	0.2322	0.7078	0.4224	0.7189	0.2061
NM_001108016	Mboat2	0.7208	0.7802	0.9622	0.7965	0.8821	0.5790	0.9119	0.9780	0.9011	0.7387	0.8310	0.8728
NM_001037783	LOC314328	0.8860	0.6121	0.8329	0.8602	0.7471	0.7788	0.6201	0.7828	0.6796	0.8317	0.8068	0.6700
NM_001109293	RGD1563941	0.1680	0.1589	0.1687	0.3871	0.1721	0.1672	0.1683	0.1623	0.4602	0.3506	0.1602	0.3409
NM_001105962	Prg4	0.8485	0.1363	0.2014	0.5479	0.9396	0.1559	0.0030	0.7271	0.3173	0.1830	0.1308	0.3767
NM_001037980	Tada1l	0.9273	0.9189	0.9931	0.8454	0.8064	0.9230	0.9607	0.8489	0.8635	0.9475	0.9566	0.7860
NM_001100698	Zfp131	0.8880	0.7654	0.3181	0.8499	0.8950	0.8976	0.3067	0.7336	0.7120	0.8476	0.4060	0.9384
NM_032078	Ryr2	0.6723	0.3402	0.2123	0.2832	0.2473	0.3066	0.1352	0.3529	0.3379	0.2397	0.2373	0.5048
NM_001017380	Cyld	0.0713	0.2169	0.0133	0.0360	0.1802	0.1246	0.0921	0.0328	0.0664	0.0352	0.1511	0.0902
NM_022441	Acvrl1	0.7188	0.6474	0.5341	0.2314	0.5886	0.4887	0.5411	0.5899	0.9668	0.7117	0.7362	0.8961
NM_001100672	Tspan6	0.9577	0.7561	0.9776	0.9944	0.9016	0.9240	0.9654	0.9627	0.7489	0.9679	0.9790	0.7639
NM_001127580	Oxct1	0.5923	0.7731	0.4126	0.9135	0.9172	0.4368	0.5672	0.7157	0.7439	0.7290	0.6228	0.5608
NM_001025689	Psmd14	0.2724	0.5043	0.5450	0.3955	0.5058	0.5295	0.3057	0.5599	0.3026	0.5084	0.2749	0.3246
NM_001107820	Ssna1	0.5277	0.4919	0.3088	0.6925	0.3690	0.2738	0.4619	0.3178	0.4504	0.2996	0.5013	0.1271
NM_001107916	Tmem67	0.7827	0.8024	0.9377	0.8199	0.4478	0.8998	0.0609	0.5002	0.3062	0.0900	0.6846	0.6689
NM_001039025	Tln1	0.7463	0.1862	0.2386	0.7050	0.8308	0.8338	0.6332	0.9775	0.2167	0.3643	0.3961	0.4596
NM_012979	Man2a1	0.8233	0.8232	0.5131	0.9447	0.8262	0.6699	0.7271	0.9707	0.7591	0.5155	0.8098	0.5074
NM_001128079	Ctdsp1	0.6226	0.5910	0.7939	0.4976	0.8243	0.8083	0.6147	0.7519	0.7400	0.6994	0.1346	0.4718
NM_001100700	Sfrp2	0.8848	0.7425	0.5771	0.8248	0.6532	0.6445	0.0238	0.4749	0.3943	0.4884	0.5504	0.3106
NM_001037650	Nphp4	0.2187	0.0488	0.3385	0.1624	0.0402	0.0977	0.2021	0.3829	0.1196	0.3005	0.0850	0.0844
NM_001013091	Gba2	0.9707	0.8532	0.1137	0.8050	0.8074	0.5830	0.6839	0.8940	0.8513	0.8598	0.7862	0.9210
NM_001127712	Bco2	0.6177	0.8140	0.4041	0.8489	0.7412	0.6245	0.7514	0.5045	0.4938	0.4294	0.3068	0.8240
NM_001100732	Nudt18	0.1320	0.0272	0.0198	0.4669	0.1072	0.1236	0.5084	0.0042	0.3328	0.0504	0.1119	0.1281
NM_001100510	Osgep	0.6912	0.7099	0.1855	0.8650	0.7199	0.7404	0.5187	0.7914	0.7467	0.7566	0.8164	0.5099
NM_001134849	Fam25a	0.0893	0.7383	0.8706	0.7354	0.0188	0.0515	0.9530	0.0843	0.7331	0.7379	0.9548	0.8449

NM_001100969	Appbp2	0.9134	0.9758	0.4484	0.3708	0.4138	0.3813	0.4698	0.3232	0.5265	0.7968	0.5104	0.4675
NM_001128192	LOC684322	0.5823	0.2708	0.4941	0.4067	0.5441	0.6666	0.5297	0.3732	0.5906	0.5856	0.2512	0.5461
NM_001100532	Anapc2	0.0370	0.1951	0.0291	0.3277	0.2364	0.4484	0.0394	0.2150	0.4729	0.2175	0.3144	0.3950
NM_001129879	Psmg4	0.2841	0.1588	0.4013	0.1659	0.2731	0.1671	0.1679	0.1622	0.1597	0.1563	0.3345	0.1650
NM_001100669	Smap2	0.8051	0.4731	0.9075	0.9025	0.8976	0.9089	0.4781	0.9819	0.5260	0.3917	0.4288	0.7420
NM_001100778	E2f1	0.2619	0.9278	0.1172	0.9625	0.9591	0.4557	0.9613	0.9865	0.2522	0.8872	0.8669	0.3542
NM_001100542	Eif2s3x	0.2206	0.3652	0.9827	0.9969	0.4502	0.4947	0.9910	0.3835	0.4327	0.5211	0.2904	0.8769
NM_001129882	Pcgf5	0.5182	0.5968	0.5958	0.4218	0.5815	0.4675	0.1679	0.7106	0.4864	0.4724	0.5663	0.6326
NM_001128083	Trim8	0.8722	0.9019	0.8961	0.9877	0.9707	0.9239	0.9667	0.8965	0.6108	0.8089	0.8315	0.7896
NM_001100750	Suclg2	0.5417	0.7185	0.6614	0.5486	0.2047	0.4777	0.4467	0.6583	0.6148	0.4903	0.6214	0.3981
NM_001128078	Fam32a	0.1952	0.7379	0.7567	0.0139	0.1841	0.3228	0.4975	0.4928	0.2585	0.5382	0.3687	0.5211
NM_001130492	Vps28	0.0774	0.1410	0.0897	0.1225	0.0432	0.0298	0.1545	0.4754	0.0890	0.0631	0.1177	0.0161
NM_001025632	Ewsr1	0.7603	0.9522	0.1068	0.6302	0.7068	0.8097	0.2625	0.6968	0.8901	0.9986	0.9760	0.6968
NM_001100496	Spop	0.2833	0.1799	0.1883	0.1981	0.8905	0.5301	0.2026	0.9245	0.1923	0.2663	0.2808	0.3730
NM_144738	Pnpla7	0.2403	0.4327	0.3004	0.1657	0.2222	0.2451	0.5226	0.2919	0.3436	0.3458	0.5711	0.3638
NM_001013216	Hkr3	0.8266	0.9912	0.0693	0.0272	0.7331	0.1177	0.5499	0.8916	0.8932	0.5008	0.7742	0.5801
NM_001130508	Scx	0.5865	0.5565	0.2357	0.4674	0.4406	0.2610	0.2839	0.4247	0.6878	0.2170	0.2553	0.4347
NM_053547	Rfc1	0.3111	0.5037	0.4593	0.3149	0.2758	0.9481	0.1622	0.1676	0.2517	0.8016	0.5805	0.8736
NM_053426	Sf3b1	0.9412	0.1756	0.9997	0.9122	0.8963	0.9755	0.9356	0.9951	0.9489	0.9538	0.9671	0.9675
NM_134358	Snrpb	0.2499	0.3422	0.4641	0.6356	0.4630	0.4593	0.4596	0.5306	0.2284	0.4053	0.6755	0.4298
NM_001128099	Ly6al	0.2332	0.2192	0.4523	0.5996	0.5358	0.6463	0.5802	0.2741	0.4728	0.6004	0.3463	0.5644
NM_001128155	Tmem88	0.4507	0.1827	0.9213	0.7589	0.7386	0.3400	0.7118	0.4917	0.6479	0.3174	0.7551	0.8171
NM_053696	Rbm3	0.9676	0.8711	0.9300	0.9747	0.8934	0.9900	0.9329	0.8936	0.8759	0.9766	0.9493	0.6078
NM_024357	Htt	0.5852	0.7058	0.1275	0.2834	0.4397	0.5000	0.4749	0.3667	0.9097	0.7794	0.5423	0.8481
NM_001128152	Rwdd3	0.2508	0.1906	0.2624	0.7397	0.0238	0.1045	0.1478	0.1336	0.1928	0.2588	0.1129	0.0925
NM_001128138	Lcn12	0.0564	0.0229	0.1245	0.0538	0.3591	0.1566	0.0295	0.0224	0.0060	0.0216	0.0225	0.1893
NM_001128137	Lcn10	0.6650	0.5655	0.5927	0.6251	0.6811	0.5683	0.5221	0.8538	0.6366	0.6800	0.2635	0.7768
NM_001128139	Lcn13	0.1679	0.3129	0.1686	0.4219	0.2661	0.3713	0.3512	0.5133	0.5377	0.2659	0.1600	0.4474
NM_001100515	Slc25a46	0.2630	0.3911	0.9683	0.3840	0.1650	0.1377	0.5234	0.0980	0.1857	0.7704	0.2431	0.4428
NM_001100647	Fxr2	0.5477	0.5897	0.2130	0.0173	0.7957	0.5317	0.0350	0.3200	0.4902	0.3983	0.2241	0.8646
NM_001128184	B3gat3	0.1233	0.0170	0.1434	0.1561	0.0348	0.2676	0.1873	0.1137	0.1734	0.0160	0.2514	0.2784
NM_001100739	Syvn1	0.9085	0.9517	0.6075	0.9429	0.9492	0.9396	0.8460	0.9687	0.9883	0.9849	0.9990	0.9655
NM_138516	Polk	0.3997	0.3122	0.8325	0.0382	0.6473	0.6938	0.0591	0.7645	0.4096	0.6143	0.3863	0.9110
NM_001128494	Lyc2	0.6242	0.5619	0.4565	0.6099	0.4329	0.3622	0.6786	0.2529	0.4755	0.3566	0.4588	0.3245
NM_134460	Ap1g1	0.2713	0.2245	0.1988	0.8372	0.3540	0.3738	0.4866	0.2810	0.3754	0.5750	0.4519	0.5448
NM_022271	LOC64038	0.5711	0.5539	0.5386	0.9237	0.3084	0.1539	0.2249	0.4150	0.3818	0.4291	0.0285	0.3010
NM_001128183	Lcn8	0.5368	0.7506	0.5922	0.5400	0.6771	0.9315	0.6056	0.7841	0.9103	0.3975	0.1247	0.6846
NM_001128196	Agxt2l2	0.2646	0.3419	0.3375	0.4827	0.3736	0.3441	0.4044	0.6542	0.1596	0.4368	0.4102	0.1649
NM_001128185	RGD1311499	0.8290	0.6031	0.7177	0.8718	0.7088	0.6757	0.7680	0.8464	0.5303	0.6066	0.4407	0.2104
NM_001015014	Surf6	0.8683	0.8238	0.3915	0.2715	0.5410	0.7221	0.9118	0.9227	0.8173	0.7252	0.5926	0.9209
NM_001128188	RGD1562135	0.0817	0.3196	0.3046	0.1301	0.1603	0.3852	0.1366	0.2287	0.2431	0.4513	0.8460	0.2290
NM_001128195	Bag2	0.0793	0.2272	0.3786	0.1832	0.2293	0.6115	0.1253	0.2752	0.1715	0.1691	0.1369	0.7661
NM_144759	Asmt	0.1535	0.1431	0.1500	0.3000	0.6399	0.3518	0.7067	0.4007	0.3324	0.5993	0.2741	0.0750
NM_001007709	Smcr7l	0.7537	0.7052	0.0888	0.9284	0.7493	0.7622	0.9006	0.9125	0.8939	0.9364	0.6262	0.7734
NM_001005546	Ttc36	0.3045	0.3783	0.7461	0.4418	0.4947	0.4811	0.1602	0.6198	0.7831	0.5688	0.4860	0.5094
NM_001004099	Gjb2	0.7587	0.4559	0.8977	0.2564	0.9056	0.6615	0.8215	0.3966	0.7789	0.5029	0.8654	0.9761
NM_001003403	Apold1	0.9557	0.7872	0.6821	0.6747	0.8533	0.2673	0.7493	0.9630	0.7982	0.4295	0.3054	0.4133
NM_001128311	Abcg3	0.6198	0.6082	0.5804	0.3480	0.3158	0.5359	0.2847	0.3330	0.2865	0.4281	0.4704	0.2349
NM_001100740	Mta2	0.7906	0.6502	0.3869	0.1130	0.6231	0.1592	0.3576	0.7370	0.3390	0.9618	0.4872	0.1538

NM_001128187	Bnipl	0.6904	0.5412	0.8437	0.8659	0.8895	0.6398	0.8055	0.5685	0.5504	0.4259	0.2531	0.3207
NM_001130550	Pop1	0.7440	0.7933	0.0139	0.8159	0.8362	0.7772	0.6545	0.8312	0.2207	0.8575	0.9618	0.1203
NM_001130990	Dnlz	0.5087	0.7476	0.5053	0.2546	0.3878	0.5575	0.6851	0.7306	0.3867	0.6917	0.4602	0.5836
NM_001008300	Nedd4l	0.7399	0.9641	0.5693	0.0705	0.4528	0.1257	0.7867	0.5592	0.9301	0.8627	0.9494	0.7166
NM_001128493	Magebl1	0.1915	0.8386	0.6401	0.4201	0.4896	0.3505	0.2221	0.3159	0.2193	0.5256	0.7828	0.1336
NM_001129775	RGD1304924	0.7635	0.4010	0.7577	0.7112	0.9403	0.9172	0.8794	0.9897	0.7405	0.8810	0.6060	0.6295
NM_001130441	Hras	0.4505	0.2277	0.5219	0.2594	0.2137	0.4303	0.6812	0.7001	0.5835	0.2239	0.4318	0.6744
NM_001098241	Hras	0.4505	0.2277	0.5219	0.2594	0.2137	0.4303	0.6812	0.7001	0.5835	0.2239	0.4318	0.6744
NM_001108392	Zic2	0.6369	0.6111	0.9020	0.8223	0.6836	0.5586	0.5472	0.6682	0.6130	0.7378	0.8959	0.6413
NM_001129878	Hoxa10	0.8246	0.9315	0.8748	0.6459	0.4662	0.6292	0.8496	0.6745	0.4111	0.5581	0.1561	0.5377
NM_001128194	Kctd7	0.4498	0.5415	0.3162	0.4108	0.5061	0.7154	0.5344	0.4691	0.3677	0.3039	0.3258	0.2180
NM_001129997	Ehbp1l1	0.2657	0.4589	0.3812	0.3385	0.2393	0.2562	0.3581	0.4333	0.5659	0.3012	0.4711	0.6395
NM_001005886	Dagla	0.8065	0.7142	0.5515	0.3483	0.5156	0.8506	0.7079	0.7442	0.2356	0.5620	0.0569	0.3995
NM_001011985	Mknk2	0.9234	0.6839	0.3145	0.9771	0.9897	0.9787	0.9945	0.9770	0.7574	0.9528	0.9635	0.3463
NM_001008335	Eif4a2	0.9554	0.9036	0.5125	0.4047	0.9547	0.8221	0.4528	0.5109	0.8488	0.7416	0.9664	0.8478
NM_001007620	Pdhb	0.1820	0.8201	0.7554	0.2615	0.1571	0.6742	0.5741	0.3469	0.6716	0.6022	0.7682	0.7268
NM_001004080	Gsn	0.9363	0.0628	0.3151	0.5765	0.7981	0.6943	0.7987	0.8311	0.6161	0.3806	0.3833	0.7813
NM_001003964	Dnmt3l	0.2754	0.2778	0.4534	0.3109	0.4793	0.2963	0.5116	0.4040	0.4260	0.4614	0.3806	0.4868
NM_001007650	Tssk3	0.6282	0.6414	0.3822	0.1944	0.1685	0.0529	0.0707	0.1963	0.2437	0.8373	0.0986	0.9127
NM_001007626	Ggps1	0.8099	0.5547	0.9570	0.8206	0.7982	0.5469	0.8846	0.9422	0.6876	0.4695	0.2438	0.0592
NM_001007602	Gsto1	0.0030	0.6011	0.0887	0.3432	0.3829	0.0360	0.1198	0.1556	0.7030	0.4634	0.1322	0.0532
NM_001008301	Usp14	0.5809	0.8133	0.6121	0.4080	0.7258	0.9165	0.5236	0.5259	0.9485	0.6339	0.9706	0.9444
NM_001001718	Rai12	0.0467	0.1206	0.0563	0.1668	0.1602	0.1561	0.2055	0.0041	0.0310	0.0280	0.0431	0.0286
NM_001007726	Dnai2	0.1433	0.1885	0.2146	0.1232	0.2529	0.1174	0.0551	0.1751	0.7418	0.5832	0.1488	0.1122
NM_001008359	Fam75a4	0.2864	0.1963	0.3694	0.7042	0.2166	0.1042	0.1661	0.1399	0.2449	0.4581	0.2921	0.4755
NM_001113544	Wtap	0.9811	0.9628	0.9886	0.7917	0.8805	0.6479	0.9574	0.7922	0.9117	0.9436	0.9845	0.6997
NM_147135	Sbk1	0.9137	0.8856	0.7779	0.4318	0.7557	0.8432	0.4209	0.8717	0.8430	0.8475	0.2806	0.8108
NM_001114405	Ptp4a3	0.5982	0.1186	0.3035	0.8530	0.5938	0.6435	0.1415	0.5761	0.1180	0.0141	0.0420	0.8800
NM_001130039	Dhx32	0.2081	0.3617	0.4850	0.2515	0.4038	0.6222	0.3052	0.4264	0.3153	0.6265	0.6140	0.6235
NM_022215	Gpd1	0.8662	0.3216	0.4073	0.7905	0.6866	0.7884	0.7027	0.8122	0.4724	0.8568	0.5871	0.4626
NM_001130061	RGD1308380	0.8179	0.7553	0.6902	0.6789	0.5542	0.6848	0.0857	0.0689	0.5460	0.3502	0.8531	0.7758
NM_001130062	Dok7	0.7763	0.1348	0.7646	0.1155	0.7915	0.7651	0.7622	0.7395	0.8343	0.4302	0.5958	0.7962
NM_001130077	Tcerg1l	0.3657	0.2471	0.4423	0.1846	0.5972	0.2077	0.4840	0.0913	0.4124	0.2377	0.6911	0.8383
NM_001130097	Atxn2l	0.9823	0.9211	0.1066	0.1209	0.9337	0.9418	0.3762	0.9534	0.9956	0.9079	0.8742	0.8780
NM_001130098	Aof2	0.4600	0.2348	0.5432	0.5422	0.2189	0.6222	0.4018	0.3632	0.3973	0.7045	0.2588	0.4167
NM_057153	Oxr1	0.7139	0.3397	0.9802	0.7859	0.4991	0.7752	0.6993	0.9305	0.4514	0.3598	0.7864	0.9646
NM_001130491	Cyc1	0.0589	0.0560	0.0555	0.0132	0.1691	0.2850	0.6049	0.0239	0.1085	0.1188	0.2475	0.1242
NM_001130551	RGD1308195	0.3804	0.2537	0.5866	0.6053	0.6327	0.7543	0.8199	0.3025	0.4428	0.6641	0.4501	0.8097
NM_001134882	Meig1	0.4964	0.2701	0.2738	0.4292	0.2436	0.1669	0.4207	0.1621	0.4426	0.5226	0.4367	0.2588
NM_001130548	Col14a1	0.9362	0.9576	0.9686	0.8389	0.7691	0.8679	0.9730	0.9901	0.2786	0.8765	0.7636	0.0731
NM_001130552	Ly6d	0.1293	0.3942	0.5252	0.1567	0.4388	0.2308	0.1759	0.2582	0.2376	0.1741	0.2774	0.3700
NM_001130553	Gsdmd	0.7989	0.5656	0.5271	0.9288	0.9159	0.0539	0.9437	0.6698	0.1632	0.3235	0.8478	0.0515
NM_001130501	Zfpm2	0.9704	0.5146	0.7690	0.9845	0.9514	0.9540	0.9429	0.9666	0.5702	0.6181	0.7518	0.9546
NM_001130565	Fam83h	0.5002	0.7470	0.4957	0.5903	0.5086	0.1964	0.7978	0.3685	0.4319	0.4731	0.6392	0.7886
NM_001130564	Efr3a	0.8342	0.7242	0.4766	0.2831	0.1458	0.5629	0.6616	0.2670	0.6716	0.8605	0.8403	0.8455
NM_001130542	Sntb1	0.1354	0.1381	0.3229	0.1926	0.3775	0.1134	0.4487	0.2490	0.4311	0.0795	0.1301	0.3649
NM_001130556	Tmprss6	0.5522	0.7778	0.5972	0.5345	0.6796	0.8296	0.9126	0.9125	0.5837	0.5209	0.4312	0.6916
NM_001130555	Mei1	0.1645	0.2365	0.1644	0.1789	0.2875	0.3768	0.2514	0.3532	0.0988	0.1419	0.2598	0.0770
NM_001130567	Dmc1	0.8840	0.8772	0.8279	0.7128	0.7022	0.7790	0.5809	0.7297	0.8990	0.7456	0.8022	0.5207

NM_001130566	Ppp1r16a	0.8977	0.7322	0.5529	0.8614	0.7485	0.7997	0.8001	0.8113	0.7571	0.7776	0.8562	0.6911
NM_001130583	Parvg	0.4350	0.5103	0.2583	0.2931	0.6376	0.5440	0.5794	0.5303	0.7167	0.1879	0.5807	0.5604
NM_001130581	LOC685444	0.6883	0.7719	0.7412	0.7134	0.6414	0.3732	0.7023	0.6968	0.8217	0.6247	0.6646	0.4865
NM_001130580	Eny2	0.6937	0.6761	0.7161	0.4927	0.6203	0.6584	0.5271	0.5825	0.6555	0.4205	0.6436	0.6871
NM_001130579	Serhl2	0.4334	0.2309	0.9772	0.5874	0.3910	0.6993	0.9052	0.8969	0.5910	0.6067	0.1515	0.9511
NM_001130509	LOC680799	0.3709	0.3807	0.4392	0.1653	0.1716	0.1668	0.1673	0.1620	0.2615	0.3345	0.3518	0.1649
NM_001134933	Crip	0.2622	0.3873	0.3965	0.4840	0.3064	0.3270	0.2505	0.4824	0.5869	0.2335	0.3319	0.4405
NM_001134495	Gsdmc1	0.1232	0.8606	0.4788	0.7365	0.7141	0.7720	0.7741	0.7600	0.6663	0.6961	0.4951	0.4730
NM_001130557	R3hdm2	0.9316	0.5259	0.0249	0.3982	0.9351	0.8708	0.9203	0.8059	0.2771	0.8326	0.5511	0.9217
NM_001130560	Rnf19a	0.7843	0.6048	0.8689	0.7680	0.8524	0.7850	0.5881	0.9326	0.8196	0.6500	0.7265	0.6215
NM_001130559	Npal2	0.8621	0.2081	0.2422	0.0413	0.9887	0.9489	0.0181	0.7230	0.0069	0.2068	0.0075	0.1706
NM_001130558	Stac3	0.4931	0.4470	0.0722	0.1479	0.8327	0.7339	0.2969	0.3545	0.3447	0.3673	0.3446	0.2286
NM_001130563	Mtss1	0.7371	0.2447	0.6390	0.4510	0.4441	0.3643	0.9306	0.3880	0.3739	0.7150	0.8865	0.8191
NM_001130562	Samd12	0.5069	0.8038	0.7450	0.6059	0.6731	0.7123	0.3948	0.1221	0.3542	0.5133	0.8225	0.4351
NM_001008372	Papd4	0.0537	0.4586	0.6561	0.0053	0.0544	0.0526	0.0737	0.0900	0.6608	0.7824	0.1038	0.5150
NM_001130494	Recql4	0.1407	0.0849	0.1963	0.1010	0.3491	0.5537	0.0758	0.7194	0.3719	0.0960	0.5543	0.0059
NM_001130495	Rabl4	0.5827	0.8446	0.3791	0.7930	0.7810	0.8570	0.9986	0.6773	0.5826	0.7241	0.7608	0.0595
NM_001130571	Cpsf1	0.2783	0.3491	0.1685	0.4309	0.2553	0.3814	0.1672	0.1618	0.3752	0.3067	0.1596	0.2614
NM_001130569	Mpped1	0.5810	0.5004	0.5463	0.6767	0.5459	0.3269	0.5046	0.2938	0.6342	0.1177	0.1213	0.5373
NM_001130570	Scrt1	0.0524	0.0178	0.0398	0.1410	0.7547	0.1827	0.4104	0.7852	0.1912	0.0105	0.2020	0.4317
NM_001134862	Pbx1	0.9130	0.8214	0.6222	0.6197	0.9665	0.8711	0.8791	0.5528	0.9635	0.9537	0.9410	0.9890
NM_001130572	Nfkbil2	0.7932	0.7929	0.0135	0.0639	0.8207	0.7823	0.7432	0.9106	0.5362	0.5037	0.7966	0.7385
NM_001130575	Rspo2	0.8534	0.7940	0.4480	0.6796	0.6807	0.3033	0.5118	0.4243	0.3353	0.7335	0.4442	0.2446
NM_001130574	Tcf20	0.7544	0.5675	0.5276	0.1008	0.5238	0.4528	0.7881	0.9580	0.8892	0.9853	0.9120	0.4469
NM_001130496	Gtpbp1	0.6722	0.1072	0.5879	0.0255	0.1379	0.3986	0.1883	0.0546	0.0121	0.3672	0.3857	0.2447
NM_001130502	RGD1562089	0.2252	0.4906	0.3364	0.0649	0.7151	0.4832	0.3143	0.3727	0.4656	0.6548	0.3813	0.4911
NM_001130503	Adsl	0.7862	0.9260	0.0424	0.7490	0.8577	0.8524	0.3956	0.8224	0.9285	0.5737	0.7906	0.6011
NM_001130510	Dnajb7	0.4113	0.2367	0.4215	0.4542	0.3930	0.3049	0.4786	0.4978	0.3577	0.7084	0.4212	0.2285
NM_001130546	Lynx1	0.5382	0.5677	0.6307	0.1737	0.5941	0.7532	0.4429	0.8868	0.6265	0.7543	0.7715	0.4660
NM_001130545	Lypd2	0.3588	0.1037	0.0435	0.2747	0.3015	0.2639	0.7014	0.4706	0.4435	0.4449	0.0652	0.3501
NM_001130544	Slurp1	0.8387	0.8152	0.4841	0.8481	0.1377	0.0623	0.9372	0.6855	0.7825	0.8440	0.8882	0.7675
NM_001130543	Rrm2b	0.3153	0.1492	0.1160	0.3919	0.2837	0.3575	0.4711	0.5168	0.0849	0.4296	0.4096	0.1546
NM_001130504	Cenpm	0.2549	0.4076	0.3217	0.4887	0.3706	0.5095	0.1670	0.4404	0.3627	0.2400	0.3623	0.3999
NM_001130511	Fam109b	0.3975	0.6430	0.4180	0.3533	0.6974	0.5764	0.4656	0.5993	0.5057	0.2875	0.5768	0.6567
NM_001130505	Ndufa6	0.0498	0.0899	0.0959	0.0009	0.0275	0.0635	0.7310	0.0191	0.1045	0.1668	0.0484	0.0792
NM_001130506	Poldip3	0.7106	0.4774	0.8484	0.7978	0.3288	0.2092	0.6919	0.4993	0.1051	0.5067	0.3161	0.4343
NM_001130497	Pnpla5	0.6669	0.2217	0.6026	0.1314	0.6599	0.8448	0.2574	0.7499	0.5457	0.0573	0.6343	0.9089
NM_001130578	Rps19bp1	0.7694	0.5127	0.0387	0.2538	0.0227	0.0860	0.6873	0.0475	0.0201	0.4494	0.0524	0.0886
NM_001130577	Cyth4	0.7727	0.7150	0.5709	0.9238	0.6260	0.8387	0.8672	0.5678	0.4584	0.6779	0.9409	0.7740
NM_001130507	Upk3a	0.1232	0.3588	0.3458	0.4449	0.1247	0.2141	0.5153	0.0908	0.6006	0.4921	0.6554	0.6832
NM_001130498	Smc1b	0.3532	0.4103	0.0390	0.5792	0.2150	0.1694	0.1603	0.1117	0.8428	0.5882	0.4700	0.2260
NM_001130499	Ttc38	0.3220	0.5093	0.3143	0.8754	0.6385	0.5037	0.6043	0.4493	0.6641	0.7417	0.4213	0.7417
NM_001130500	Gtse1	0.9785	0.7988	0.0442	0.0575	0.8306	0.9506	0.7183	0.8340	0.7538	0.8165	0.8857	0.2780
NM_031764	Ddr2	0.5539	0.3190	0.2557	0.0225	0.9180	0.4478	0.1301	0.4653	0.7868	0.7994	0.6159	0.2901
NM_001014124	Dnajc28	0.2307	0.0748	0.1102	0.0339	0.0144	0.0964	0.0482	0.0215	0.2970	0.4161	0.0282	0.2768
NM_001130680	Phf21b	0.5182	0.2403	0.5666	0.7096	0.0692	0.7900	0.9359	0.4251	0.6633	0.7738	0.6601	0.4790
NM_001130695	Zc3h7b	0.9298	0.2949	0.8615	0.9821	0.9929	0.9931	0.9620	0.9644	0.3796	0.3456	0.2995	0.3912
NM_001130696	RGD1306001	0.2346	0.1930	0.0214	0.5430	0.7194	0.3411	0.7316	0.7642	0.6575	0.1940	0.5095	0.2525
NM_001130717	RGD1565672	0.7661	0.4735	0.0511	0.1417	0.8301	0.3552	0.1571	0.7183	0.1444	0.2424	0.6046	0.0182

NM_001130728	Whdc1	0.2423	0.1473	0.4132	0.3938	0.6258	0.5044	0.5615	0.5595	0.2601	0.3946	0.3393	0.0863
NM_001008508	Tpst2	0.7310	0.9364	0.7743	0.7474	0.9415	0.8926	0.9275	0.7993	0.9688	0.8556	0.7784	0.7775
NM_001012039	Efemp1	0.6454	0.5485	0.5708	0.5376	0.7753	0.5961	0.5520	0.3476	0.2866	0.8931	0.1366	0.5093
NM_001008217	Psma7	0.3005	0.7754	0.0802	0.8337	0.1224	0.5951	0.2215	0.0693	0.1501	0.4211	0.5991	0.7974
NM_001006967	Kars	0.0916	0.4473	0.0735	0.0499	0.1741	0.0356	0.5713	0.5710	0.7273	0.7756	0.3871	0.1027
NM_001007006	Dusp13	0.2860	0.2407	0.2457	0.4254	0.2339	0.5340	0.2949	0.3795	0.2553	0.5217	0.4811	0.3109
NM_001005906	Chpf	0.8395	0.7479	0.6375	0.0406	0.8354	0.9848	0.9361	0.9631	0.7944	0.8956	0.8976	0.9343
NM_001005888	Galc	0.4790	0.5815	0.6682	0.5735	0.2029	0.5104	0.4953	0.1846	0.6936	0.5065	0.6754	0.6987
NM_001008558	Ndn	0.8787	0.5503	0.8102	0.3943	0.6443	0.4362	0.3737	0.4029	0.8036	0.5418	0.7530	0.7225
NM_001007755	Scly	0.8581	0.9717	0.8058	0.9752	0.9115	0.9632	0.9213	0.9221	0.9620	0.8355	0.7408	0.9051
NM_001007697	Prune	0.0281	0.1708	0.0599	0.0045	0.1382	0.1574	0.1251	0.2192	0.0448	0.0681	0.0774	0.3904
NM_001011710	Crisp2	0.3227	0.4650	0.3034	0.2826	0.3943	0.5240	0.3433	0.6734	0.2152	0.3195	0.2380	0.2234
NM_001004252	Farsb	0.7403	0.6507	0.2381	0.8787	0.8877	0.9445	0.8503	0.8261	0.9018	0.7699	0.9961	0.8380
NM_001004215	Ppic	0.8631	0.8863	0.8721	0.9640	0.9329	0.8906	0.8142	0.9295	0.8463	0.8991	0.9784	0.8303
NM_001009362	Pask	0.9059	0.8180	0.0966	0.6380	0.7713	0.8468	0.7884	0.8326	0.8279	0.7914	0.8388	0.7016
NM_001007617	Nuak2	0.9512	0.3756	0.8201	0.9632	0.9162	0.9992	0.7539	0.9689	0.8567	0.5892	0.9106	0.8381
NM_001100489	Wnt5b	0.6879	0.5541	0.7099	0.4695	0.6302	0.5700	0.7417	0.2370	0.8929	0.7128	0.5438	0.9156
NM_001004086	Pon3	0.9792	0.9428	0.8583	0.8039	0.3863	0.0821	0.8974	0.3201	0.9468	0.7136	0.6777	0.8604
NM_001007682	Thtpa	0.9105	0.9107	0.9464	0.9039	0.9542	0.9464	0.9767	0.9140	0.9546	0.9055	0.7592	0.9804
NM_001007652	Pole3	0.3155	0.7239	0.5018	0.3103	0.1439	0.4536	0.8145	0.3331	0.3628	0.2787	0.3962	0.4358
NM_001007606	Sars	0.3085	0.8449	0.8285	0.7765	0.5997	0.4859	0.9944	0.7099	0.8449	0.9654	0.7864	0.3228
NM_001008870	Spink2	0.0892	0.9434	0.6181	0.6276	0.0566	0.0066	0.7401	0.0594	0.6394	0.5893	0.9543	0.8186
NM_001008364	Snx24	0.1029	0.1334	0.8828	0.6423	0.4135	0.4057	0.4103	0.9173	0.7176	0.4116	0.4635	0.9022
NM_001001511	Gcnt2	0.9013	0.8060	0.9879	0.4646	0.9389	0.8660	0.9547	0.4534	0.9406	0.9085	0.9022	0.9056
NM_080584	Phkg2	0.5047	0.1358	0.0311	0.0838	0.7382	0.7052	0.4868	0.8041	0.9288	0.7576	0.7345	0.9013
NM_053845	Upb1	0.0304	0.2601	0.1718	0.1236	0.0313	0.0476	0.1280	0.0045	0.4770	0.0864	0.4029	0.2657
NM_053827	Plod1	0.0522	0.0037	0.1452	0.0367	0.0610	0.6765	0.7768	0.2731	0.1886	0.0944	0.0065	0.4169
NM_013217	Mllt4	0.9849	0.3605	0.9586	0.8554	0.9258	0.9674	0.9549	0.8259	0.9874	0.9667	0.9856	0.9635
NM_001017960	Thra	0.6192	0.5502	0.9192	0.5737	0.7071	0.3927	0.8131	0.3930	0.5615	0.5180	0.5253	0.7681
NM_001025277	Bud13	0.1316	0.1992	0.0669	0.1474	0.0988	0.0962	0.0178	0.1190	0.1115	0.0583	0.2063	0.0471
NM_001130939	RGD1566403	0.5381	0.2323	0.2541	0.2825	0.5512	0.4912	0.2905	0.4617	0.1883	0.4402	0.6304	0.3384
NM_012538	Cyp11b2	0.1042	0.8936	0.2548	0.8041	0.3536	0.4200	0.0061	0.3086	0.4516	0.8212	0.0562	0.0930
NM_001131000	LOC10017490[illegible]	0.3902	0.4781	0.4482	0.6280	0.2437	0.3247	0.1668	0.6768	0.3127	0.4672	0.5838	0.1647
NM_001131013	Arvcf	0.3232	0.6533	0.3256	0.1308	0.0608	0.1774	0.4367	0.2377	0.5848	0.5186	0.9152	0.7515
NM_001131006	Spcs1	0.3803	0.2290	0.5426	0.2941	0.1316	0.1461	0.0705	0.0951	0.0131	0.1948	0.1670	0.1092
NM_001131003	LOC10017491[illegible]	0.5626	0.5062	0.5072	0.2818	0.7326	0.2194	0.4796	0.4211	0.5410	0.3312	0.6456	0.4190
NM_012602	Muc1	0.5101	0.0785	0.0736	0.0371	0.0873	0.1184	0.1205	0.1437	0.0511	0.0619	0.0593	0.5126
NM_001131012	Mier1	0.4199	0.3172	0.9676	0.9740	0.4998	0.9890	0.8450	0.4104	0.4400	0.2788	0.4835	0.9575
NM_001130999	Ap4s1	0.2343	0.4066	0.3662	0.4702	0.4484	0.2598	0.3462	0.3080	0.3523	0.4032	0.4050	0.4084
NM_001131001	Fcer1g	0.5762	0.0386	0.0260	0.3698	0.9065	0.8355	0.2439	0.8562	0.8954	0.4412	0.4580	0.0840
NM_001008304	Ptov1	0.9222	0.9033	0.0718	0.7582	0.9211	0.9789	0.8351	0.9682	0.8433	0.7769	0.7738	0.3619
NM_001007705	Ubr7	0.9797	0.9308	0.4873	0.3668	0.9623	0.9631	0.9001	0.9696	0.9704	0.8922	0.9568	0.9443
NM_001007600	Rps4x	0.5856	0.2807	0.7913	0.1595	0.4843	0.8154	0.5007	0.5951	0.6247	0.8377	0.4979	0.4566
NM_001014220	Sp100	0.4923	0.3051	0.3578	0.2534	0.3026	0.6329	0.5029	0.2457	0.2019	0.5260	0.3383	0.4780
NM_001006960	Mtp18	0.2770	0.2046	0.5313	0.4685	0.3071	0.6958	0.4002	0.2340	0.4005	0.6542	0.5614	0.4905
NM_001004259	Pnkp	0.2429	0.3006	0.4429	0.4963	0.4182	0.7717	0.5720	0.7872	0.5809	0.5907	0.6293	0.6044
NM_053466	Jak1	0.9507	0.9541	0.9999	0.9056	0.2582	0.9757	0.8507	0.0871	0.6280	0.8886	0.7764	0.9555
NM_001134341	Nyw1	0.9207	0.1632	0.8845	0.2046	0.5089	0.7759	0.3675	0.7268	0.3201	0.1457	0.1577	0.8910
NM_001134502	RGD1311892	0.3122	0.8538	0.5389	0.5750	0.3490	0.9420	0.6610	0.4903	0.5651	0.7236	0.8213	0.7852

NM_001134503	RGD1310950	0.8347	0.6602	0.4728	0.3389	0.9007	0.9601	0.3337	0.9126	0.1847	0.4469	0.4208	0.7977
NM_001134501	RGD1311526	0.2690	0.2324	0.1063	0.0735	0.4587	0.9453	0.2372	0.7098	0.7593	0.5514	0.7962	0.5682
NM_001134540	Copa	0.7393	0.7522	0.2924	0.5308	0.9904	0.7725	0.5902	0.8263	0.5064	0.8747	0.7676	0.8572
NM_001135087	LOC681033	0.4483	0.4740	0.3191	0.4628	0.3950	0.2952	0.4838	0.5161	0.2423	0.2749	0.5204	0.4460
NM_053432	Tec	0.8725	0.0711	0.8506	0.2905	0.9458	0.8975	0.2512	0.9401	0.1310	0.0494	0.1126	0.0722
NM_001134639	RGD1565192	0.4858	0.5982	0.7430	0.6267	0.4923	0.4027	0.7924	0.6331	0.4936	0.7248	0.2314	0.5317
NM_001134604	Cytl1	0.5405	0.1039	0.5731	0.4448	0.2987	0.2582	0.1368	0.2619	0.3832	0.2283	0.3512	0.3678
NM_001134558	RGD1306371	0.7704	0.5357	0.7503	0.4743	0.2968	0.0296	0.5876	0.1201	0.5821	0.4656	0.8863	0.5446
NM_001013049	Smtn	0.8521	0.8065	0.1129	0.2431	0.7935	0.9438	0.9576	0.9987	0.9232	0.8838	0.9125	0.2147
NM_001012005	Nid2	0.7419	0.8642	0.7588	0.6007	0.8328	0.6101	0.9461	0.7028	0.4605	0.5046	0.8401	0.8682
NM_022933	Chd8	0.7093	0.8904	0.2227	0.4076	0.9339	0.9836	0.1627	0.8918	0.9479	0.9012	0.9842	0.9798
NM_001134571	RGD1308117	0.5401	0.5015	0.4373	0.1156	0.9693	0.8616	0.6634	0.8491	0.5979	0.6075	0.0429	0.7501
NM_001134414	Tmtc4	0.9159	0.8685	0.9509	0.9979	0.7846	0.8331	0.8492	0.8471	0.9716	0.9498	0.9672	0.8563
NM_001134467	Fam116a	0.9034	0.3637	0.9459	0.2225	0.9602	0.9476	0.1501	0.9151	0.3843	0.3440	0.4828	0.2876
NM_001134605	RGD1561145	0.3308	0.8539	0.7288	0.8906	0.8700	0.6174	0.9854	0.8431	0.7693	0.5197	0.9437	0.8033
NM_001134416	Ints10	0.5428	0.3751	0.5451	0.0918	0.3088	0.7597	0.4802	0.3228	0.1276	0.5371	0.6848	0.4783
NM_001134606	RGD1565972	0.6214	0.5744	0.6092	0.5550	0.6961	0.4506	0.5006	0.4971	0.5864	0.7145	0.7678	0.6882
NM_001134572	RGD1306058	0.1959	0.5285	0.0566	0.4894	0.0465	0.0959	0.1554	0.0168	0.5312	0.5015	0.4522	0.0148
NM_001134417	RGD1308872	0.6343	0.9049	0.4761	0.8874	0.7881	0.6638	0.2037	0.7999	0.8295	0.6781	0.9362	0.6473
NM_001134607	RGD1562608	0.6399	0.6841	0.8342	0.7106	0.6581	0.9027	0.7953	0.8897	0.7067	0.7635	0.9450	0.9342
NM_001134542	Pcyox1l	0.5418	0.9518	0.1195	0.9980	0.7279	0.8824	0.8158	0.9205	0.3495	0.6626	0.8693	0.0650
NM_001134538	RGD1563628	0.6472	0.6369	0.6498	0.3515	0.5064	0.5915	0.6022	0.8896	0.8434	0.7342	0.9100	0.7827
NM_001134421	RGD1308706	0.1576	0.1410	0.4128	0.1700	0.1978	0.2057	0.2043	0.1806	0.0586	0.1186	0.1569	0.0277
NM_001134539	RGD1308221	0.3293	0.3341	0.7232	0.4982	0.7880	0.7736	0.4008	0.8471	0.5523	0.9238	0.5235	0.8930
NM_139328	Slc7a6os	0.2897	0.6741	0.9876	0.7419	0.2505	0.3100	0.4444	0.3596	0.0513	0.2269	0.0936	0.1413
NM_001134608	RGD1559505	0.3220	0.5057	0.4556	0.4315	0.6374	0.8345	0.8917	0.3590	0.4642	0.5844	0.6267	0.2656
NM_001134573	RGD1306192	0.3571	0.2752	0.8447	0.1912	0.1870	0.1320	0.6895	0.0322	0.0213	0.1451	0.1594	0.0798
NM_001134510	RGD1561161	0.4403	0.1270	0.3756	0.3272	0.3410	0.3745	0.1927	0.4097	0.3594	0.6524	0.3942	0.4086
NM_001134548	RGD1305938	0.8894	0.9491	0.5851	0.3923	0.4552	0.9175	0.2068	0.1436	0.7592	0.9014	0.9700	0.8756
NM_001134436	C1qtnf3	0.6138	0.5963	0.4664	0.5432	0.3285	0.5050	0.5692	0.2350	0.5508	0.2550	0.5915	0.2091
NM_001134614	RGD1565000	0.0534	0.0092	0.0380	0.1522	0.4420	0.0646	0.0067	0.1099	0.0179	0.0101	0.0020	0.0564
NM_001134615	LOC499587	0.8095	0.5291	0.8220	0.8361	0.4362	0.8864	0.1857	0.9398	0.8820	0.8811	0.7780	0.9584
NM_001107666	Ttc14	0.7237	0.8696	0.0457	0.9264	0.7538	0.6988	0.1239	0.7572	0.7095	0.8525	0.9682	0.1295
NM_001134640	RGD1560220	0.0813	0.2083	0.0688	0.6323	0.3768	0.1059	0.6644	0.3014	0.5373	0.4058	0.1108	0.0445
NM_181768	Acad9	0.1325	0.3195	0.2177	0.6845	0.0661	0.2490	0.8130	0.4142	0.3352	0.9227	0.4155	0.4779
NM_001134511	RGD1307595	0.4718	0.0186	0.1734	0.1255	0.0864	0.2283	0.0579	0.1204	0.0300	0.0241	0.0728	0.2601
NM_001037185	Smc4	0.8401	0.6773	0.0035	0.0347	0.6674	0.6660	0.6314	0.5937	0.4910	0.8140	0.9015	0.0260
NM_001134409	Serpini2	0.2417	0.5419	0.4178	0.0782	0.2942	0.3814	0.3599	0.5725	0.1828	0.2962	0.5714	0.2782
NM_001134577	Slc39a1	0.5573	0.3949	0.8125	0.8160	0.5453	0.7988	0.6448	0.8123	0.6735	0.5512	0.8191	0.6965
NM_001134593	RGD1566380	0.0447	0.0033	0.8540	0.8810	0.7622	0.7791	0.1839	0.5312	0.3158	0.0893	0.1188	0.5566
NM_001134512	RGD1309110	0.6151	0.5309	0.5931	0.5680	0.3873	0.0040	0.6210	0.5669	0.2042	0.0925	0.2358	0.0753
NM_001134578	RGD1309139	0.8718	0.4848	0.1322	0.8170	0.8824	0.7760	0.6230	0.7269	0.8332	0.4530	0.8902	0.6052
NM_001134513	RGD1307816	0.4261	0.8813	0.6461	0.4837	0.7893	0.7557	0.8953	0.4578	0.6578	0.8528	0.4630	0.6095
NM_001134579	RGD1309170	0.5740	0.4327	0.4934	0.3479	0.4314	0.5436	0.3943	0.6911	0.6597	0.7521	0.6606	0.2914
NM_001134616	RGD1560065	0.7561	0.4555	0.8668	0.1512	0.7627	0.7680	0.1897	0.6876	0.1929	0.6764	0.7643	0.3184
NM_001100833	Mtch1	0.3350	0.1966	0.9104	0.6644	0.4214	0.0642	0.0892	0.5351	0.0963	0.8497	0.0800	0.4649
NM_001134576	RGD1306739	0.0953	0.1639	0.2140	0.3361	0.4307	0.1563	0.1334	0.5710	0.0580	0.2858	0.3936	0.4763
NM_001034128	Pald	0.4622	0.4601	0.5096	0.2746	0.8406	0.8652	0.7912	0.5178	0.2794	0.7514	0.6649	0.2827
NM_001134547	RGD1304770	0.6445	0.3447	0.6267	0.5713	0.1680	0.5673	0.4661	0.7331	0.3121	0.6644	0.0951	0.4448

NM_001134447	Spopl	0.5209	0.2070	0.4026	0.4472	0.0977	0.1063	0.3379	0.4845	0.2569	0.1977	0.5269	0.3922
NM_001134516	Uap1l1	0.2328	0.0886	0.1078	0.2385	0.0050	0.1210	0.0812	0.0984	0.2145	0.0908	0.1220	0.1660
NM_001134617	RGD1565512	0.5190	0.5860	0.7667	0.3186	0.5068	0.2335	0.3508	0.0090	0.3892	0.4515	0.7287	0.2866
NM_001134517	RGD1306074	0.3978	0.2394	0.0904	0.0462	0.0948	0.1992	0.3748	0.3672	0.1367	0.6434	0.3691	0.0069
NM_001134518	LOC296637	0.4430	0.5939	0.6688	0.8540	0.2100	0.5031	0.3578	0.8251	0.4476	0.4404	0.6036	0.7756
NM_001134580	Lypd6b	0.9135	0.6883	0.9224	0.8145	0.9574	0.7931	0.9517	0.3783	0.6484	0.8552	0.6963	0.8150
NM_001134504	Klhl2	0.9092	0.9184	0.9070	0.9689	0.9641	0.9559	0.6323	0.9721	0.3031	0.9553	0.0299	0.4548
NM_001134550	RGD1565257	0.7822	0.7961	0.9030	0.7390	0.9507	0.8621	0.5903	0.5357	0.5066	0.4575	0.8812	0.1150
NM_001134514	Sestd1	0.6506	0.7895	0.3847	0.7929	0.6266	0.3040	0.2046	0.3873	0.5287	0.7743	0.4287	0.5555
NM_001100527	Frzb	0.5469	0.9082	0.8716	0.9075	0.7840	0.7442	0.6364	0.8735	0.8674	0.9067	0.6130	0.7224
NM_001134515	RGD1566226	0.2177	0.1604	0.3726	0.6168	0.4901	0.3660	0.1300	0.0653	0.0438	0.3798	0.5317	0.6540
NM_001134618	RGD1561852	0.0404	0.1930	0.1930	0.4813	0.3575	0.4160	0.1557	0.4099	0.0690	0.0356	0.0974	0.3038
NM_001037350	LOC296235	0.1691	0.0708	0.5577	0.5259	0.7200	0.1458	0.6195	0.4207	0.1898	0.2718	0.0718	0.3259
NM_001134619	RGD1562582	0.6081	0.9154	0.8297	0.7279	0.7153	0.3636	0.9007	0.7176	0.4313	0.5867	0.8646	0.2419
NM_001134620	RGD1564340	0.7015	0.7720	0.4557	0.4694	0.4609	0.5459	0.5397	0.4529	0.7047	0.2410	0.1170	0.2466
NM_001134553	RGD1310722	0.9590	0.9937	0.6589	0.2348	0.9874	0.9974	0.3445	0.9639	0.9129	0.9859	0.9634	0.9551
NM_001134621	RGD1566107	0.3668	0.7993	0.6015	0.7194	0.3152	0.5456	0.4931	0.4535	0.6843	0.6514	0.8550	0.5871
NM_001134622	RGD1565731	0.2844	0.5459	0.2248	0.3434	0.1538	0.1486	0.4754	0.0390	0.3197	0.0727	0.2072	0.1245
NM_001134554	RGD1304792	0.6874	0.9060	0.4771	0.0620	0.5195	0.9229	0.2783	0.9675	0.8414	0.7275	0.7508	0.7220
NM_001134623	RGD1560289	0.4737	0.2461	0.0532	0.2575	0.3430	0.2071	0.4014	0.6178	0.4730	0.6230	0.2584	0.4940
NM_173332	Anubl1	0.2696	0.5433	0.1934	0.6786	0.5747	0.6249	0.5428	0.4465	0.4923	0.3355	0.2767	0.6208
NM_001134581	RGD1304592	0.9019	0.7732	0.2832	0.8373	0.6110	0.8692	0.3545	0.9111	0.8805	0.8629	0.8705	0.9005
NM_001134519	Ttc39a	0.6392	0.9025	0.7946	0.8947	0.8093	0.8377	0.7336	0.7457	0.8134	0.9202	0.5394	0.3458
NM_001134626	RGD1562911	0.1058	0.3729	0.4700	0.3313	0.3929	0.7536	0.1231	0.6819	0.1927	0.0889	0.2313	0.5574
NM_001134627	RGD1563015	0.5416	0.0620	0.1002	0.4639	0.3879	0.5256	0.5414	0.6163	0.4006	0.7841	0.0950	0.4409
NM_001134521	RGD1563049	0.0931	0.1315	0.5678	0.2933	0.4827	0.7285	0.0595	0.2367	0.7808	0.0326	0.1007	0.2434
NM_001134628	RGD1564943	0.1910	0.3576	0.6634	0.1466	0.2128	0.7761	0.6832	0.3853	0.5814	0.4923	0.5644	0.8166
NM_001134629	RGD1561149	0.9453	0.8966	0.9805	0.9675	0.9402	0.9951	0.9835	0.8572	0.9536	0.9671	0.9615	0.9995
NM_001134556	RGD1309198	0.6221	0.6594	0.8991	0.8536	0.7308	0.9197	0.8539	0.7961	0.3481	0.7819	0.9749	0.6082
NM_001134522	RGD1308878	0.6999	0.6350	0.7317	0.3305	0.4791	0.5841	0.4430	0.4377	0.5528	0.3585	0.5307	0.6451
NM_001134523	RGD1311019	0.7814	0.7309	0.8820	0.9733	0.4605	0.9388	0.8720	0.8851	0.9641	0.7859	0.6010	0.7030
NM_001134557	RGD1562284	0.8048	0.8870	0.8320	0.7108	0.0399	0.0351	0.5682	0.1643	0.5622	0.6785	0.9433	0.6822
NM_001134463	RGD1310453	0.3641	0.3278	0.9050	0.9038	0.1544	0.1775	0.7696	0.0366	0.7481	0.4052	0.6477	0.9445
NM_001134524	RGD1561792	0.7643	0.7423	0.0471	0.7706	0.9947	0.8268	0.8652	0.9296	0.5072	0.6495	0.9395	0.0136
NM_001134630	RGD1562540	0.3188	0.5423	0.2676	0.0693	0.2417	0.3379	0.1363	0.3069	0.2668	0.2477	0.4583	0.2322
NM_001134631	RGD1560978	0.9562	0.9097	0.3906	0.3815	0.7396	0.4477	0.8525	0.8943	0.8234	0.9744	0.5721	0.7448
NM_001134525	RGD1307392	0.2212	0.6501	0.7837	0.1814	0.6656	0.1038	0.8562	0.7724	0.5155	0.7089	0.7769	0.3383
NM_001134559	RGD1308907	0.5924	0.3555	0.9907	0.4716	0.6240	0.4887	0.5744	0.6964	0.3798	0.2266	0.0860	0.7179
NM_001134632	RGD1560932	0.8481	0.8778	0.9435	0.7931	0.8491	0.9121	0.8317	0.9247	0.6140	0.8510	0.7220	0.7484
NM_001134526	RGD1305298	0.1353	0.2895	0.1050	0.7236	0.5047	0.6010	0.1232	0.4786	0.4738	0.8694	0.2686	0.4677
NM_001134560	RGD1305627	0.4753	0.4935	0.1061	0.6187	0.1860	0.3378	0.6095	0.2832	0.8205	0.6357	0.1440	0.7394
NM_001134562	RGD1309019	0.1274	0.4642	0.0677	0.0337	0.0078	0.1196	0.0230	0.0308	0.6596	0.3160	0.0100	0.9486
NM_001134464	RGD1308261	0.5882	0.2614	0.2488	0.1221	0.0731	0.1861	0.1068	0.1981	0.5716	0.6652	0.3111	0.5286
NM_001134595	RGD1564851	0.7064	0.9646	0.7960	0.8653	0.8578	0.8587	0.9575	0.9768	0.6702	0.5424	0.7959	0.8969
NM_001134527	Grhl2	0.2255	0.2741	0.3883	0.2236	0.4782	0.6953	0.0724	0.2516	0.4862	0.4025	0.1088	0.1597
NM_001033864	Cflar	0.6460	0.7808	0.7795	0.8696	0.3251	0.4483	0.9101	0.5644	0.7803	0.6415	0.8835	0.6031
NM_001135085	LOC681031	0.3148	0.3706	0.4918	0.3653	0.2608	0.2803	0.4462	0.4209	0.2505	0.3169	0.1592	0.3068
NM_001134529	RGD1311154	0.5594	0.0998	0.4847	0.6433	0.1170	0.5903	0.8816	0.2079	0.3423	0.8980	0.1024	0.8693
NM_001134528	RGD1305928	0.1676	0.4257	0.4087	0.1647	0.3116	0.2892	0.1660	0.1614	0.1590	0.4254	0.1591	0.2777

NM_001013106	Mcrs1	0.9187	0.7989	0.1046	0.8147	0.9393	0.9702	0.9798	0.9568	0.9258	0.5727	0.8988	0.0407
NM_001134633	Fam186b	0.4795	0.3015	0.5672	0.3419	0.6419	0.5977	0.3793	0.2671	0.3388	0.2999	0.3548	0.2253
NM_001134583	RGD1565411	0.0193	0.3832	0.1251	0.0216	0.1982	0.0460	0.1523	0.3749	0.5553	0.0613	0.0637	0.6112
NM_001134454	Csad	0.6062	0.8868	0.9402	0.9813	0.5480	0.2361	0.9528	0.8841	0.4667	0.7285	0.6680	0.3335
NM_001134530	Mmp1a	0.1091	0.4248	0.4888	0.6908	0.4963	0.2407	0.4201	0.0591	0.5907	0.5804	0.3867	0.2323
NM_001134584	RGD1309410	0.9070	0.9112	0.9998	0.8660	0.9852	0.9077	0.9649	0.9154	0.8703	0.7881	0.6890	0.2667
NM_001134634	RGD1565419	0.7935	0.7180	0.5265	0.3776	0.7612	0.8281	0.3097	0.8211	0.5797	0.7086	0.3314	0.8139
NM_001134585	RGD1564241	0.7662	0.6377	0.9592	0.5148	0.9041	0.9294	0.3217	0.7012	0.9752	0.9020	0.8722	0.9493
NM_001134636	RGD1564937	0.4933	0.7530	0.7976	0.7070	0.6814	0.7328	0.8728	0.8119	0.7077	0.8975	0.4853	0.4072
NM_001134469	Lrrc49	0.5081	0.3657	0.4998	0.9014	0.3659	0.7477	0.4552	0.7848	0.3643	0.2241	0.5900	0.7604
NM_001134564	RGD1564964	0.8712	0.9901	0.7885	0.1905	0.5291	0.8970	0.6856	0.7910	0.7133	0.9188	0.9593	0.9538
NM_001134531	RGD1561074	0.2183	0.3110	0.5093	0.4773	0.2102	0.4190	0.4493	0.4630	0.6029	0.4739	0.5668	0.5327
NM_001134472	RGD1309079	0.4676	0.5735	0.3516	0.7760	0.3569	0.3767	0.5966	0.5372	0.5714	0.8395	0.4975	0.4489
NM_001134520	Rad54l2	0.3114	0.6385	0.8425	0.7976	0.4679	0.4757	0.6288	0.3291	0.5930	0.8434	0.6245	0.8932
NM_001100548	Rbm5	0.9051	0.0910	0.2464	0.2192	0.8488	0.8387	0.0495	0.9322	0.1691	0.0828	0.2164	0.6999
NM_001134532	RGD1311095	0.7346	0.4178	0.7810	0.1263	0.2653	0.6089	0.8216	0.8089	0.4687	0.8110	0.5521	0.1403
NM_001134565	RGD1307844	0.8785	0.9194	0.4231	0.0129	0.6040	0.2465	0.8746	0.6365	0.8615	0.7776	0.5526	0.5678
NM_001134567	RGD1564811	0.7529	0.3394	0.1654	0.4454	0.3328	0.3744	0.4225	0.0826	0.5919	0.8035	0.2056	0.0725
NM_001134596	RGD1308299	0.1200	0.6452	0.2990	0.1724	0.2453	0.5571	0.8606	0.3518	0.1262	0.8190	0.7842	0.0850
NM_001008320	Rhoj	0.3856	0.1426	0.7055	0.7775	0.9946	0.8855	0.2099	0.8933	0.0467	0.0972	0.0332	0.1405
NM_001007598	Rpl9	0.5863	0.6295	0.6408	0.5659	0.4280	0.3960	0.6067	0.5378	0.1165	0.4582	0.4324	0.2956
NM_001134597	Fam135a	0.2656	0.2933	0.8610	0.1610	0.1470	0.1140	0.1283	0.0066	0.6709	0.4353	0.3748	0.8302
NM_001134642	RGD1560421	0.4197	0.2842	0.6623	0.4527	0.1917	0.6939	0.4242	0.2195	0.5948	0.4864	0.2300	0.2436
NM_001134586	RGD1563955	0.6184	0.8023	0.9220	0.5232	0.5794	0.7245	0.9393	0.6139	0.8107	0.1082	0.7201	0.6726
NM_001134587	RGD1311447	0.4076	0.6252	0.2756	0.3023	0.4128	0.8067	0.4257	0.5630	0.1343	0.7568	0.2155	0.5932
NM_001134566	LOC316124	0.2867	0.4727	0.3868	0.1646	0.5361	0.3716	0.2333	0.5171	0.3315	0.5678	0.2992	0.3638
NM_001134533	LOC301124	0.0370	0.1181	0.2272	0.9090	0.1214	0.0762	0.2313	0.3076	0.0777	0.0668	0.1955	0.1217
NM_001134637	LOC501283	0.2230	0.4956	0.4927	0.6984	0.1909	0.4771	0.6033	0.4295	0.7429	0.3724	0.0236	0.3745
NM_001134500	LOC288978	0.7151	0.8281	0.1668	0.8525	0.7997	0.6964	0.5728	0.9147	0.1392	0.8587	0.7407	0.0009
NM_001134598	RGD1563155	0.4352	0.7414	0.6599	0.3934	0.6115	0.4181	0.7638	0.7780	0.8268	0.8831	0.8548	0.7192
NM_001134588	RGD1563952	0.2258	0.9566	0.3002	0.3436	0.4472	0.3775	0.0171	0.1468	0.9093	0.9275	0.5129	0.1475
NM_001134589	RGD1566265	0.5436	0.3734	0.5328	0.3283	0.2684	0.2331	0.2705	0.2556	0.2731	0.2062	0.6714	0.4526
NM_001134691	LOC100188932	0.3912	0.5011	0.5541	0.1645	0.5088	0.5186	0.1658	0.3279	0.6101	0.4080	0.5508	0.7115
NM_001134690	LOC100188932	0.3912	0.5011	0.5541	0.1645	0.5088	0.5186	0.1658	0.3279	0.6101	0.4080	0.5508	0.7115
NM_001134599	NEWGENE_15	0.9230	0.9548	0.1449	0.6543	0.8346	0.7903	0.8182	0.9220	0.8184	0.8007	0.8057	0.8684
NM_001106215	Smoc2	0.3286	0.3607	0.5773	0.4426	0.5159	0.3249	0.2415	0.0460	0.2757	0.2907	0.2627	0.6148
NM_001107467	Zfp51	0.2445	0.5197	0.3788	0.1709	0.6021	0.2017	0.5142	0.3788	0.4855	0.2541	0.7882	0.5758
NM_001106224	Ube2s	0.3138	0.7660	0.4529	0.7972	0.6945	0.5243	0.9289	0.6760	0.6112	0.7430	0.9117	0.4372
NM_001109399	Snrpd2	0.3043	0.5465	0.0300	0.3241	0.1573	0.0977	0.6743	0.0819	0.2576	0.0558	0.1452	0.1365
NM_001108475	Vasp	0.4760	0.9453	0.3036	0.9089	0.2673	0.3221	0.4572	0.2089	0.4627	0.6387	0.5541	0.5472
NM_001134760	Hnrnpl	0.8593	0.9216	0.4209	0.8591	0.9040	0.9806	0.6319	0.6065	0.7412	0.9573	0.8480	0.8436
NM_032619	Hnrnpl	0.8593	0.9216	0.4209	0.8591	0.9040	0.9806	0.6319	0.6065	0.7412	0.9573	0.8480	0.8436
NM_001134726	RGD1563239	0.5618	0.8857	0.8039	0.5192	0.6975	0.6787	0.4651	0.9098	0.8281	0.7570	0.7548	0.7483
NM_001106261	Rasip1	0.0660	0.3504	0.6572	0.3804	0.1450	0.0962	0.0470	0.2432	0.2595	0.2572	0.1320	0.0860
NM_001106278	Eed	0.8425	0.6816	0.3231	0.4078	0.8317	0.7816	0.2103	0.8496	0.6871	0.3068	0.9163	0.3924
NM_001134735	Taf10	0.2186	0.5134	0.4804	0.6959	0.2979	0.0416	0.7208	0.3535	0.3587	0.4714	0.7252	0.0929
NM_001134694	LOC691909	0.2509	0.3039	0.1211	0.0431	0.1141	0.0021	0.0055	0.0344	0.1750	0.0007	0.0053	0.4373
NM_001107570	Tmem151a	0.6979	0.2159	0.5004	0.7270	0.7522	0.6906	0.8092	0.4542	0.6982	0.6418	0.4889	0.6842
NM_001108520	Patl1	0.9487	0.6686	0.7102	0.2709	0.9592	0.7863	0.6092	0.9000	0.8425	0.8384	0.4421	0.4784

NM_001037793	Dock8	0.7856	0.2483	0.1777	0.4546	0.8372	0.9036	0.1530	0.8953	0.0776	0.2556	0.4325	0.3713
NM_001105778	Rnf145	0.8490	0.2145	0.7401	0.8154	0.9859	0.9236	0.6167	0.9820	0.3911	0.4766	0.3384	0.7423
NM_001134737	Trim41	0.8412	0.8747	0.8929	0.9918	0.6573	0.8167	0.8183	0.8601	0.9806	0.9275	0.8575	0.9740
NM_001134729	LOC686506	0.5480	0.6593	0.2110	0.6506	0.6600	0.3489	0.6382	0.5964	0.4257	0.1865	0.3749	0.6770
NM_001107029	Rad51l3	0.7274	0.8984	0.8096	0.5793	0.5037	0.9272	0.7439	0.5276	0.8114	0.6271	0.6466	0.9868
NM_001134699	Ankrd40	0.5184	0.4169	0.9387	0.9696	0.4663	0.6475	0.9091	0.5446	0.4376	0.6887	0.7172	0.8333
NM_001107056	Cyb561	0.9904	0.9905	0.9679	0.9991	0.9958	0.9928	0.9989	0.9143	0.9719	0.7650	0.7816	0.9721
NM_001011899	Gart	0.7923	0.7637	0.1839	0.8867	0.8829	0.9176	0.8343	0.7833	0.8975	0.8144	0.9086	0.4918
NM_001107090	Osbpl11	0.2691	0.1445	0.4371	0.3983	0.2646	0.3809	0.3966	0.1558	0.2390	0.3212	0.3668	0.7785
NM_001107078	Slc7a4	0.8059	0.6987	0.6888	0.6599	0.5629	0.8260	0.7043	0.8887	0.8241	0.8332	0.7569	0.6474
NM_001108326	Lztr1	0.6518	0.4882	0.4433	0.7984	0.6229	0.6086	0.7763	0.7687	0.5263	0.1386	0.7493	0.4670
NM_001108990	Caps2	0.5648	0.6077	0.2545	0.5217	0.5757	0.1657	0.6125	0.2348	0.1954	0.6093	0.5332	0.2122
NM_001107146	Rfc5	0.6359	0.9669	0.0190	0.9399	0.8579	0.8841	0.9917	0.9969	0.9438	0.8020	0.8657	0.0655
NM_001134739	Tdrd5	0.5311	0.3060	0.5786	0.4828	0.3616	0.3432	0.3708	0.4282	0.2985	0.4888	0.5154	0.1640
NM_001134740	Tdrd5	0.5311	0.3060	0.5786	0.4828	0.3616	0.3432	0.3708	0.4282	0.2985	0.4888	0.5154	0.1640
NM_001108354	RGD1306105	0.6176	0.5315	0.4834	0.4290	0.7317	0.6537	0.5316	0.6125	0.6308	0.6752	0.3108	0.7141
NM_001105997	Mrpl1	0.1234	0.6906	0.8145	0.8324	0.2251	0.0067	0.4767	0.2553	0.2154	0.7492	0.7744	0.3592
NM_001105725	Ccng2	0.9556	0.0938	0.9737	0.9627	0.9780	0.9832	0.9846	0.9542	0.5597	0.8103	0.9282	0.8788
NM_001109087	Shisa3	0.4999	0.3573	0.4960	0.2749	0.1001	0.0679	0.3209	0.5108	0.2889	0.4258	0.2762	0.4014
NM_001107228	Fam53a	0.3795	0.9937	0.0500	0.1243	0.8188	0.8111	0.2361	0.8362	0.4998	0.7356	0.7392	0.0706
NM_001135006	Nrn1l	0.2931	0.2738	0.3876	0.5155	0.2692	0.2765	0.4690	0.3563	0.4501	0.3527	0.5955	0.2733
NM_001134702	Meis1	0.9368	0.2938	0.2410	0.3060	0.8522	0.4512	0.1507	0.8860	0.3152	0.2125	0.3105	0.3008
NM_001134730	Hspc159	0.8938	0.8030	0.9892	0.8540	0.9318	0.9729	0.7762	0.9761	0.9450	0.5797	0.9614	0.9712
NM_001106026	RGD1306063	0.7284	0.1131	0.0996	0.8864	0.6673	0.5424	0.3027	0.5806	0.2180	0.0584	0.5443	0.1693
NM_001134757	Gnpnat1	0.1993	0.9349	0.9713	0.2410	0.3963	0.6871	0.6965	0.3984	0.7214	0.5971	0.9620	0.5119
NM_001134756	Gnpnat1	0.1993	0.9349	0.9713	0.2410	0.3963	0.6871	0.6965	0.3984	0.7214	0.5971	0.9620	0.5119
NM_001107256	Socs4	0.6560	0.5589	0.3579	0.2716	0.1674	0.1308	0.5393	0.2072	0.1985	0.8195	0.7900	0.0867
NM_001134727	Lrch1	0.4383	0.5393	0.6629	0.3207	0.4256	0.3871	0.6557	0.4538	0.1003	0.2828	0.3882	0.5940
NM_001107302	Nxnl1	0.4149	0.3346	0.4195	0.3320	0.3466	0.2994	0.4735	0.4197	0.2389	0.3374	0.2820	0.5450
NM_001134686	Slc7a2	0.4866	0.2273	0.7136	0.2898	0.5524	0.6700	0.6959	0.4303	0.5598	0.6715	0.3755	0.6141
NM_022619	Slc7a2	0.4866	0.2273	0.7136	0.2898	0.5524	0.6700	0.6959	0.4303	0.5598	0.6715	0.3755	0.6141
NM_001106088	Erlin2	0.9353	0.9804	0.9783	0.9722	0.9668	0.9400	0.7996	0.9655	0.9826	0.9879	0.9740	0.9405
NM_001134747	Zmat4	0.6378	0.6688	0.6275	0.6939	0.6242	0.6696	0.6514	0.2759	0.5068	0.5442	0.2006	0.4172
NM_001134744	Agpat5	0.7716	0.3997	0.9281	0.8547	0.3899	0.9031	0.9459	0.3248	0.7332	0.4437	0.7209	0.1001
NM_001106094	Fam70b	0.4406	0.4586	0.3985	0.5671	0.3702	0.7348	0.1045	0.1457	0.5795	0.4244	0.4214	0.5087
NM_001109121	Stam	0.7089	0.6935	0.6707	0.5304	0.6524	0.6253	0.6939	0.2626	0.5137	0.7341	0.6225	0.5887
NM_001108422	Plxdc2	0.9549	0.9020	0.9950	0.9144	0.7762	0.7667	0.9980	0.8234	0.8467	0.9687	0.9788	0.9850
NM_001107397	Epb4.1l4a	0.2020	0.0422	0.0580	0.0185	0.5551	0.4815	0.0559	0.0573	0.1465	0.1906	0.0576	0.0790
NM_001134746	Lrrtm4	0.4866	0.3050	0.5688	0.5561	0.8291	0.7990	0.6933	0.3716	0.5628	0.7735	0.8165	0.8042
NM_001107148	Hps4	0.5169	0.3385	0.0113	0.1021	0.1201	0.9082	0.1500	0.1002	0.7495	0.3985	0.5261	0.5766
NM_001134696	Ctxn3	0.3739	0.3574	0.4383	0.5710	0.4010	0.4211	0.2428	0.4218	0.5658	0.6894	0.2257	0.3044
NM_001134748	Fam151b	0.4893	0.4638	0.0522	0.1207	0.8295	0.7122	0.1424	0.8125	0.0152	0.3492	0.1828	0.1056
NM_001134761	Ccdc125	0.4127	0.3822	0.2012	0.5365	0.5638	0.5894	0.4719	0.6134	0.4787	0.7049	0.9172	0.0607
NM_001106428	Hspa4l	0.1629	0.9492	0.7768	0.3467	0.2851	0.3447	0.1433	0.2330	0.9948	0.9260	0.7605	0.9583
NM_001106458	Bcas2	0.1889	0.2086	0.3014	0.2311	0.1332	0.2256	0.0792	0.2047	0.2120	0.2458	0.1949	0.3558
NM_001033902	Ccdc76	0.8702	0.9747	0.1684	0.1198	0.8122	0.7838	0.0147	0.7752	0.9931	0.7125	0.7495	0.8044
NM_001134708	Odf2l	0.1509	0.2767	0.0807	0.4404	0.6513	0.9307	0.2862	0.2216	0.1273	0.5437	0.8204	0.5137
NM_001134736	Wdr63	0.2554	0.3016	0.2628	0.3687	0.1709	0.5077	0.3213	0.1609	0.4406	0.4738	0.4725	0.5054
NM_001134695	LOC10018893	0.3453	0.1580	0.3207	0.1639	0.4225	0.4673	0.4247	0.3439	0.4509	0.3923	0.3188	0.1639

NM_001134755	Znf76	0.9939	0.8821	0.8914	0.9863	0.9714	0.9019	0.9717	0.9751	0.9278	0.7772	0.9922	0.8934
NM_001134705	Cox7c	0.2683	0.2727	0.3022	0.3680	0.2706	0.1657	0.3249	0.1608	0.2906	0.2703	0.5395	0.2469
NM_001107815	Traf2	0.8414	0.3897	0.0962	0.1071	0.0792	0.4175	0.3475	0.7509	0.7809	0.7999	0.6078	0.2748
NM_001025040	LOC499754	0.0493	0.1617	0.0877	0.0474	0.1102	0.1142	0.0062	0.2154	0.1838	0.5320	0.1180	0.2726
NM_001107754	Traf6	0.2166	0.0275	0.3615	0.3796	0.2091	0.4131	0.3561	0.5442	0.1512	0.2798	0.4471	0.3417
NM_001108590	Epb4.2	0.5458	0.1632	0.1705	0.2506	0.4749	0.1124	0.7196	0.6086	0.4268	0.3994	0.3719	0.4569
NM_001108638	Slc25a26	0.4824	0.7523	0.5528	0.6606	0.6541	0.4862	0.9445	0.7143	0.7153	0.5651	0.1116	0.1936
NM_001106525	Snph	0.4932	0.8902	0.2707	0.6739	0.5892	0.7765	0.7011	0.7584	0.8872	0.8089	0.8504	0.9114
NM_001106527	Tspyl3	0.8306	0.7483	0.7016	0.4977	0.4649	0.6626	0.4204	0.2669	0.5796	0.4674	0.3453	0.1204
NM_001109348	Znf777	0.2604	0.3411	0.3627	0.5335	0.1359	0.2417	0.1503	0.6849	0.4429	0.3847	0.0833	0.3289
NM_001106851	Nck1	0.8415	0.6071	0.6155	0.7636	0.6745	0.8051	0.6412	0.6437	0.2260	0.7630	0.7158	0.7983
NM_001106665	Ttc39b	0.4129	0.7830	0.7112	0.7581	0.2764	0.7267	0.7018	0.2851	0.4472	0.1708	0.7468	0.7773
NM_001109232	Ccdc126	0.3265	0.1621	0.9795	0.2940	0.3033	0.4469	0.7750	0.3314	0.5233	0.4233	0.7519	0.8466
NM_001134718	Ptcd3	0.7478	0.9740	0.8107	0.9357	0.8353	0.9383	0.8285	0.9720	0.8936	0.8545	0.9780	0.7616
NM_001134700	Fam19a4	0.6183	0.6728	0.6212	0.2342	0.4891	0.6179	0.6339	0.6724	0.6065	0.4658	0.6378	0.4188
NM_001134713	Shq1	0.2640	0.4767	0.7321	0.1601	0.3384	0.0388	0.4592	0.5775	0.7837	0.2257	0.4238	0.5824
NM_001134716	Clec12a	0.2220	0.2526	0.5688	0.5560	0.6586	0.5539	0.2746	0.2775	0.2324	0.4911	0.4654	0.3479
NM_001025274	Sfrs18	0.9992	0.9924	0.6420	0.9857	0.9991	0.9946	0.2806	0.7845	0.9895	0.9882	0.9882	0.9353
NM_053545	Tie1	0.1965	0.4464	0.6670	0.7549	0.7469	0.8433	0.8108	0.5605	0.3703	0.3250	0.4220	0.6675
NM_001107978	RGD1559610	0.0081	0.0136	0.1935	0.0605	0.2053	0.0185	0.0218	0.1068	0.0977	0.0909	0.1125	0.1068
NM_001107912	Rbbp4	0.5994	0.4989	0.8260	0.3912	0.3337	0.6433	0.8757	0.0598	0.2964	0.1545	0.4711	0.1438
NM_001134703	LOC641315	0.4703	0.1578	0.1679	0.1635	0.3502	0.3280	0.4684	0.4787	0.1588	0.1553	0.1590	0.1638
NM_001134698	LOC641316	0.5960	0.4191	0.5586	0.3812	0.6013	0.6116	0.5734	0.6517	0.7230	0.6486	0.2185	0.7126
NM_001108010	Supt7l	0.8321	0.6266	0.7367	0.7710	0.6359	0.7867	0.7445	0.8489	0.8992	0.9071	0.8838	0.3764
NM_001134754	Seli	0.8019	0.5221	0.9002	0.9539	0.3364	0.9739	0.9376	0.2451	0.5715	0.7998	0.5120	0.8980
NM_001108015	Trib2	0.9071	0.9712	0.9473	0.9826	0.4835	0.7849	0.9860	0.5961	0.8487	0.9647	0.9884	0.9918
NM_001106719	Itgb1bp1	0.3475	0.1652	0.0154	0.0128	0.1158	0.0209	0.3089	0.0634	0.0607	0.2451	0.2191	0.4821
NM_001106730	RGD1305089	0.3344	0.1136	0.1919	0.2499	0.4712	0.2430	0.1058	0.1709	0.0256	0.2770	0.0556	0.1591
NM_001105732	Sec23a	0.4974	0.7402	0.2290	0.5791	0.6840	0.4164	0.6414	0.5844	0.7601	0.4806	0.6574	0.0235
NM_001106741	Churc1	0.1396	0.2364	0.3803	0.1726	0.1629	0.0365	0.4096	0.3021	0.2273	0.1965	0.2505	0.0997
NM_001134731	Ttc9	0.8042	0.9897	0.9760	0.9367	0.2636	0.1087	0.3362	0.3098	0.9748	0.9602	0.9010	0.9951
NM_001108719	Ttc7b	0.3679	0.4825	0.7959	0.3568	0.7103	0.0670	0.6507	0.5077	0.6564	0.4637	0.6090	0.5413
NM_001108060	Rcor1	0.5935	0.3931	0.8473	0.8627	0.4809	0.3876	0.3660	0.7720	0.1785	0.8943	0.3607	0.8735
NM_001108734	Mobkl2a	0.4819	0.2545	0.5570	0.5884	0.5520	0.7029	0.5367	0.1486	0.2697	0.3086	0.1365	0.3016
NM_001130561	Nudcd1	0.5346	0.2474	0.0698	0.1047	0.0198	0.1839	0.2457	0.0247	0.0665	0.2117	0.0421	0.1284
NM_001108109	Asb8	0.3246	0.1053	0.6828	0.9327	0.3260	0.2312	0.5611	0.2446	0.2936	0.2628	0.2929	0.1478
NM_001134714	Tcfcp2	0.9039	0.9177	0.6144	0.5976	0.9123	0.9847	0.7429	0.8923	0.9176	0.8873	0.9721	0.7410
NM_001134715	Slc44a2	0.6010	0.0299	0.7757	0.4468	0.6292	0.5403	0.4410	0.7390	0.8017	0.6389	0.0203	0.7999
NM_001134749	Stt3a	0.2274	0.4126	0.9777	0.8775	0.2366	0.2219	0.4524	0.4001	0.3052	0.3480	0.3327	0.6682
NM_001106849	Spsb4	0.4182	0.5655	0.7105	0.4042	0.5250	0.7273	0.7054	0.7083	0.7134	0.4399	0.5470	0.5618
NM_001112737	Limd1	0.2170	0.1830	0.2260	0.7423	0.2977	0.9061	0.4653	0.2307	0.4931	0.6413	0.4948	0.8900
NM_001134762	Tbc1d5	0.9481	0.7920	0.4357	0.1432	0.9012	0.8786	0.5546	0.5864	0.8768	0.5068	0.8610	0.6878
NM_001134710	Cnpy3	0.1960	0.6824	0.2164	0.4578	0.5155	0.4491	0.6029	0.2603	0.5581	0.5869	0.2122	0.6955
NM_001108799	Eef1b2	0.7102	0.8281	0.8419	0.5557	0.4421	0.6175	0.6871	0.6073	0.5894	0.5295	0.8034	0.6366
NM_001134728	Spag16	0.3655	0.2099	0.4263	0.4898	0.4142	0.3622	0.1652	0.3830	0.3896	0.4076	0.3958	0.3695
NM_001134753	Pnkd	0.5008	0.6373	0.6202	0.4816	0.6700	0.6308	0.6423	0.7282	0.7200	0.5957	0.3786	0.6194
NM_001134751	Pnkd	0.5008	0.6373	0.6202	0.4816	0.6700	0.6308	0.6423	0.7282	0.7200	0.5957	0.3786	0.6194
NM_001134750	Pnkd	0.5008	0.6373	0.6202	0.4816	0.6700	0.6308	0.6423	0.7282	0.7200	0.5957	0.3786	0.6194
NM_001108224	Vil1	0.7212	0.4253	0.7423	0.3163	0.0323	0.1450	0.4255	0.3436	0.4806	0.0564	0.1358	0.2380

NM_001106922	Stk11ip	0.8188	0.0988	0.0224	0.1755	0.7757	0.7964	0.1494	0.5426	0.3403	0.5011	0.2624	0.6081
NM_001109673	Ccdc94	0.8389	0.7810	0.4038	0.4157	0.6203	0.3618	0.4958	0.5502	0.4900	0.7181	0.2817	0.7311
NM_001134688	Ccdc40	0.3606	0.3532	0.2133	0.2743	0.5856	0.4295	0.5291	0.2406	0.6540	0.3854	0.7274	0.4614
NM_001134835	Nat9	0.2234	0.6768	0.8811	0.0759	0.0275	0.0369	0.8228	0.0494	0.6710	0.9387	0.6736	0.5090
NM_001134706	Nsbp1	0.5233	0.5478	0.6656	0.6806	0.3023	0.5394	0.6590	0.5547	0.6214	0.2055	0.5747	0.3079
NM_001108243	Srpx2	0.9205	0.7889	0.8685	0.8252	0.9300	0.8836	0.9325	0.8399	0.7520	0.8009	0.9460	0.9411
NM_001100802	Aplp1	0.0476	0.1068	0.4051	0.1825	0.2068	0.1257	0.5451	0.0999	0.4167	0.3938	0.0496	0.4024
NM_001100782	Nudt16l1	0.0707	0.0213	0.1526	0.1097	0.1871	0.0414	0.0115	0.0162	0.2631	0.1032	0.0063	0.2113
NM_001115030	Gltpd2	0.3569	0.0828	0.1549	0.4116	0.1577	0.2641	0.6121	0.4046	0.4929	0.0308	0.1743	0.1556
NM_001098727	Rprd1b	0.5796	0.8244	0.7245	0.8131	0.9471	0.4406	0.9089	0.7819	0.8605	0.7868	0.9304	0.7424
NM_001035222	N4bp2l1	0.9228	0.9366	0.6861	0.7330	0.8153	0.5367	0.6229	0.7234	0.6647	0.9204	0.8955	0.5557
NM_001134763	Hip1r	0.8892	0.8776	0.4042	0.6608	0.8520	0.8224	0.6914	0.5586	0.5822	0.8666	0.2262	0.5575
NM_031234	Hip1r	0.8892	0.8776	0.4042	0.6608	0.8520	0.8224	0.6914	0.5586	0.5822	0.8666	0.2262	0.5575
NM_001134766	Ccdc62	0.5637	0.5637	0.6863	0.2545	0.6068	0.2450	0.2920	0.5476	0.3859	0.5885	0.4763	0.3595
NM_001107609	Kif20b	0.3877	0.9506	0.0017	0.6458	0.7906	0.7380	0.9132	0.5571	0.7847	0.8056	0.8879	0.1073
NM_001134777	Pex19	0.8978	0.8383	0.7705	0.9049	0.9698	0.9926	0.8476	0.9656	0.9567	0.8996	0.9288	0.8943
NM_032059	Prkci	0.5251	0.4083	0.9391	0.5282	0.5792	0.4080	0.8116	0.5766	0.4096	0.3683	0.8519	0.6214
NM_001134781	Ankib1	0.9434	0.7641	0.9811	0.4967	0.9857	0.7787	0.9056	0.9717	0.9376	0.9265	0.9571	0.8920
NM_001100781	Nanog	0.7603	0.8396	0.7599	0.5226	0.8601	0.5073	0.1493	0.9260	0.7961	0.5328	0.6137	0.7748
NM_001134765	Oc90	0.4859	0.4184	0.2718	0.3466	0.2727	0.2811	0.1650	0.6236	0.6435	0.5783	0.2819	0.4277
NM_001134780	Parvb	0.7119	0.5559	0.7659	0.7561	0.8384	0.7517	0.1183	0.4555	0.3839	0.1170	0.4947	0.7606
NM_181085	Lhfpl1	0.0075	0.0854	0.4503	0.0253	0.2488	0.2694	0.3270	0.4468	0.1275	0.0941	0.0400	0.0738
NM_012541	Cyp1a2	0.2738	0.2746	0.4331	0.1254	0.4578	0.4811	0.3349	0.3850	0.7854	0.1099	0.4455	0.4112
NM_012540	Cyp1a1	0.3559	0.6131	0.7142	0.2670	0.3582	0.7444	0.6192	0.2346	0.5605	0.5715	0.5561	0.3380
NM_175837	Cyp4a1	0.4464	0.4385	0.7574	0.1162	0.8331	0.1635	0.6659	0.3734	0.2660	0.2180	0.7158	0.5489
NM_001100736	Fam108c1	0.9217	0.5283	0.9488	0.9651	0.2111	0.3271	0.9704	0.1781	0.7020	0.5682	0.6171	0.9218
NM_001008879	Znf292	0.6426	0.4630	0.7358	0.8885	0.5968	0.8100	0.4372	0.9047	0.6556	0.7146	0.6910	0.8667
NM_001113749	Hif1an	0.2394	0.1568	0.1824	0.4829	0.0426	0.0730	0.1754	0.0717	0.0613	0.1236	0.2928	0.5802
NM_001100551	RGD1304587	0.7025	0.6514	0.9309	0.5544	0.9679	0.8395	0.8792	0.3848	0.8795	0.9420	0.5679	0.5722
NM_001108831	Kctd11	0.4593	0.6302	0.9078	0.9054	0.7514	0.8993	0.9725	0.8114	0.5435	0.9504	0.9059	0.9129
NM_001100787	Hoxb4	0.7414	0.9134	0.2567	0.9522	0.9058	0.9937	0.9003	0.9985	0.9195	0.9590	0.9592	0.9700
NM_001127550	Snx4	0.5171	0.8553	0.9576	0.9363	0.9270	0.9679	0.7405	0.8220	0.8271	0.9453	0.9464	0.9140
NM_001134789	Polr2h	0.8611	0.6786	0.4091	0.4545	0.4201	0.5268	0.7345	0.7963	0.6424	0.7053	0.6975	0.3497
NM_001134788	Vpreb2	0.3187	0.5984	0.2159	0.3232	0.1719	0.6521	0.8947	0.1494	0.1293	0.6237	0.4591	0.1710
NM_001024969	RGD1305455	0.3563	0.3948	0.1495	0.0719	0.2394	0.2195	0.0427	0.3100	0.1705	0.2462	0.2108	0.1635
NM_001134792	RGD1564614	0.1031	0.3803	0.1603	0.3102	0.2429	0.2449	0.3421	0.0697	0.1483	0.8114	0.2224	0.4471
NM_001109667	Tiprl	0.3831	0.1678	0.9848	0.1987	0.6294	0.9043	0.0294	0.2001	0.1933	0.1799	0.4091	0.7824
NM_001012031	Brdt	0.8641	0.6659	0.5850	0.6212	0.3505	0.1132	0.5604	0.5794	0.2178	0.2309	0.1467	0.1929
NM_001126274	Tmem129	0.5386	0.9165	0.3439	0.8711	0.7784	0.9543	0.8704	0.4070	0.9553	0.9212	0.5082	0.8976
NM_022608	Sec14l3	0.1060	0.7075	0.5396	0.6916	0.4410	0.4704	0.6837	0.4593	0.3197	0.5215	0.3400	0.3937
NM_001011982	Nt5c1b	0.4141	0.3345	0.3123	0.1633	0.1709	0.1655	0.5189	0.1607	0.1586	0.1552	0.4745	0.1637
NM_001013051	Sugt1	0.0715	0.0329	0.5504	0.0940	0.0005	0.0008	0.0612	0.0013	0.1551	0.2465	0.0413	0.0775
NM_001134797	Dydc2	0.3194	0.2716	0.5129	0.3227	0.3408	0.2698	0.5173	0.3771	0.2696	0.3100	0.3333	0.3821
NM_001134790	Dda1	0.0156	0.1173	0.8428	0.9355	0.1251	0.4126	0.7303	0.0781	0.2305	0.4882	0.3089	0.7791
NM_001134841	Car6	0.2441	0.7909	0.5307	0.1003	0.6758	0.2354	0.8340	0.5875	0.7597	0.6470	0.4072	0.7940
NM_001134796	Mast3	0.5048	0.5151	0.6464	0.6524	0.5312	0.6548	0.6332	0.6127	0.6118	0.5189	0.2253	0.6962
NM_134454	Angpt2	0.5844	0.8084	0.2512	0.1457	0.8760	0.6968	0.5677	0.2621	0.6792	0.3796	0.2057	0.2098
NM_001134798	Dcun1d2	0.2333	0.6500	0.4875	0.3598	0.5355	0.2671	0.6209	0.4137	0.3081	0.3897	0.2329	0.5125
NM_001115029	RGD1565425	0.4375	0.2621	0.2727	0.4145	0.3844	0.5530	0.4484	0.5094	0.3807	0.7163	0.3321	0.4085

NM_001100666	Schip1	0.8015	0.3623	0.7835	0.3898	0.9119	0.9152	0.2592	0.7364	0.1094	0.4062	0.2524	0.6508
NM_001100708	Nrf1	0.7977	0.7382	0.0027	0.1825	0.5967	0.3592	0.0474	0.4841	0.2816	0.2027	0.7915	0.0872
NM_080884	Cml5	0.1946	0.1576	0.4451	0.4258	0.1708	0.4159	0.1649	0.1605	0.3572	0.3920	0.3661	0.1636
NM_001134555	C1r	0.1556	0.0034	0.9365	0.1085	0.1112	0.2727	0.0151	0.0539	0.1206	0.1528	0.1004	0.7573
NM_001100752	Ndufa9	0.2896	0.1575	0.3100	0.1632	0.2617	0.1652	0.1648	0.3801	0.7299	0.3187	0.3613	0.3521
NM_001100534	Rpl7	0.5732	0.1923	0.7870	0.0983	0.6018	0.6492	0.6575	0.4257	0.4465	0.1715	0.3197	0.8589
NM_001044234	Osbpl9	0.3333	0.6056	0.4773	0.2792	0.5343	0.4496	0.3590	0.5510	0.4962	0.4893	0.6156	0.5453
NM_001100540	Iah1	0.1473	0.8424	0.8630	0.1082	0.2081	0.0116	0.6062	0.3840	0.5092	0.5667	0.3426	0.0515
NM_001109672	Ccnk	0.5509	0.8746	0.8998	0.7712	0.8650	0.8249	0.8466	0.8931	0.8473	0.7640	0.5638	0.7398
NM_001100757	Pias4	0.9744	0.9174	0.7550	0.9390	0.9988	0.9385	0.8727	0.9156	0.9701	0.9877	0.9465	0.9969
NM_001100719	Ap3d1	0.8566	0.5376	0.1792	0.8274	0.8674	0.9402	0.4244	0.7896	0.6625	0.1919	0.7585	0.4543
NM_053855	Akap8	0.7436	0.9048	0.2698	0.0889	0.8030	0.9835	0.3898	0.8597	0.6755	0.9162	0.7403	0.8078
NM_001134800	Zbed4	0.7949	0.7065	0.6284	0.8620	0.8712	0.6808	0.8019	0.7076	0.8726	0.6461	0.8687	0.8659
NM_001126283	Ankrd49	0.5548	0.5617	0.2353	0.9534	0.3218	0.2209	0.1392	0.7123	0.2818	0.4222	0.9053	0.3837
NM_001113791	RGD1562618	0.8308	0.6722	0.8874	0.8736	0.4423	0.1222	0.8874	0.3290	0.8958	0.6950	0.8988	0.6381
NM_031804	Cish	0.7340	0.8979	0.3197	0.1432	0.4954	0.5355	0.8998	0.7772	0.6369	0.8871	0.8342	0.8263
NM_001106271	Akap13	0.8192	0.6034	0.3522	0.6429	0.8086	0.8531	0.7339	0.8650	0.9123	0.6787	0.7501	0.8668
NM_001134833	Ccdc89	0.5713	0.5933	0.6602	0.6123	0.7512	0.4783	0.4765	0.1081	0.7993	0.9048	0.8752	0.2948
NM_001100662	Eif3c	0.8426	0.6734	0.0831	0.6709	0.8442	0.8958	0.6801	0.8892	0.8981	0.8898	0.8285	0.7834
NM_053767	Ptpre	0.2845	0.0328	0.3257	0.0216	0.3640	0.2997	0.4010	0.1315	0.2026	0.0098	0.4185	0.0450
NM_001134826	Sgcd	0.5848	0.6219	0.4940	0.9153	0.4547	0.6679	0.7398	0.7337	0.8462	0.4586	0.8358	0.9048
NM_001134843	Myo1g	0.4920	0.1755	0.3392	0.4436	0.2989	0.3216	0.2334	0.7588	0.0783	0.0951	0.4473	0.0776
NM_001134840	RGD1308009	0.7868	0.2959	0.3826	0.5815	0.9398	0.9340	0.7550	0.7469	0.5843	0.1468	0.7770	0.8973
NM_001134834	Fahd2a	0.2976	0.0823	0.6951	0.0679	0.0126	0.1595	0.0795	0.2152	0.0241	0.1132	0.1355	0.3077
NM_001100538	Pabpc4	0.7864	0.7510	0.8445	0.9469	0.8203	0.8886	0.8381	0.9515	0.9998	0.9119	0.9164	0.9893
NM_001135184	Iars2	0.5878	0.7059	0.5444	0.6547	0.6797	0.6859	0.6658	0.6883	0.6001	0.6867	0.5995	0.6354
NM_001134861	Cerk	0.1568	0.2079	0.2643	0.9853	0.9907	0.9675	0.3090	0.7592	0.2600	0.1912	0.1158	0.1794
NM_001134837	Trps1	0.9628	0.4518	0.4632	0.0892	0.8147	0.9420	0.1672	0.7380	0.3774	0.3437	0.1649	0.1135
NM_001134842	Wdr67	0.6662	0.5605	0.0015	0.0617	0.2519	0.6473	0.8853	0.0971	0.4507	0.7987	0.5390	0.2317
NM_001134839	Ly6h	0.1830	0.6073	0.2949	0.2359	0.7973	0.4382	0.5425	0.3702	0.7754	0.2747	0.8875	0.2009
NM_001044236	LOC301128	0.5819	0.6846	0.4525	0.4252	0.4489	0.6709	0.2701	0.6892	0.6272	0.5324	0.5606	0.4997
NM_001130989	Ptges3	0.9038	0.9745	0.8989	0.9591	0.9558	0.7357	0.9308	0.9471	0.9416	0.9767	0.9667	0.0826
NM_001134858	Synm	0.5951	0.8802	0.9562	0.7107	0.8737	0.8718	0.9820	0.6217	0.9725	0.9620	0.9471	0.9493
NM_001107554	Inpp5f	0.9405	0.4646	0.8163	0.3828	0.8681	0.9248	0.9291	0.6397	0.5111	0.4297	0.6536	0.6897
NM_001134859	Sec23ip	0.8408	0.1679	0.7929	0.0766	0.2547	0.5793	0.3758	0.0382	0.7595	0.4950	0.5999	0.6990
NM_001037197	Ankrd15	0.7184	0.7941	0.7890	0.2284	0.9382	0.9873	0.2440	0.9544	0.9282	0.9198	0.7866	0.9332
NM_001108368	Dupd1	0.2023	0.3492	0.6905	0.3012	0.0219	0.0788	0.6648	0.2418	0.1133	0.5111	0.2112	0.5696
NM_001134857	Heatr2	0.6981	0.0531	0.8218	0.5458	0.3434	0.4712	0.1619	0.3240	0.9227	0.2698	0.5451	0.9140
NM_001134867	R3hdm1	0.4073	0.2615	0.1843	0.3416	0.9725	0.7921	0.1771	0.3711	0.9456	0.2489	0.3838	0.6367
NM_001135007	Nrbp2	0.2465	0.4616	0.5397	0.3679	0.4994	0.3889	0.4563	0.4576	0.5447	0.4276	0.2228	0.6892
NM_001105796	Acap1	0.0064	0.0445	0.9056	0.4122	0.4536	0.2123	0.9005	0.1100	0.1404	0.1049	0.1220	0.9163
NM_001081634	Fbxw9	0.7337	0.0471	0.1878	0.1996	0.6909	0.9210	0.0418	0.7307	0.2713	0.1223	0.0234	0.1664
NM_001108420	Asb13	0.6514	0.9108	0.8852	0.1523	0.9676	0.7797	0.3229	0.8056	0.9290	0.9257	0.7917	0.9042
NM_001134863	Stox2	0.9014	0.9386	0.9375	0.7991	0.6786	0.8533	0.9054	0.6281	0.6341	0.9283	0.6379	0.6101
NM_001134864	Afg3l2	0.2711	0.2674	0.3688	0.0140	0.0595	0.5842	0.0286	0.1414	0.5304	0.3966	0.0714	0.8874
NM_001107700	Prcc	0.6116	0.8488	0.9601	0.8076	0.9379	0.9831	0.9351	0.7750	0.7291	0.7165	0.7558	0.9282
NM_001134856	Cdc14a	0.7531	0.2991	0.9706	0.9860	0.9278	0.9909	0.2542	0.6219	0.4554	0.5480	0.2470	0.8974
NM_001107718	Cdc14a	0.7531	0.2991	0.9706	0.9860	0.9278	0.9909	0.2542	0.6219	0.4554	0.5480	0.2470	0.8974
NM_001134854	Smox	0.8843	0.8340	0.9064	0.9426	0.8343	0.9431	0.9254	0.7768	0.6860	0.9402	0.9210	0.8815

NM_001134865	Zc3h3	0.5077	0.4338	0.1659	0.4633	0.6257	0.4816	0.3604	0.7723	0.5916	0.5327	0.7884	0.7231
NM_001134860	Exosc4	0.2979	0.2546	0.0540	0.0780	0.0617	0.0326	0.2081	0.0868	0.1579	0.3782	0.0422	0.0976
NM_001134845	LOC688613	0.2987	0.2712	0.3021	0.2974	0.4785	0.3975	0.7328	0.3126	0.3518	0.5959	0.1586	0.6620
NM_001134866	Pkdrej	0.3604	0.2737	0.1554	0.4727	0.0973	0.0104	0.1766	0.0750	0.0720	0.3687	0.4228	0.5229
NM_001122781	Letmd1	0.7242	0.5681	0.4763	0.6508	0.5545	0.4547	0.6983	0.6299	0.6403	0.3592	0.6194	0.4348
NM_001128065	Rpl36a	0.4257	0.6537	0.6072	0.4549	0.1338	0.6386	0.4084	0.2650	0.2821	0.6805	0.4660	0.2186
NM_001009533	MGC72567	0.4879	0.0177	0.4587	0.7224	0.7687	0.4714	0.1325	0.4375	0.1406	0.3052	0.0187	0.0882
NM_001015024	Osbpl5	0.5601	0.6915	0.5717	0.4635	0.5873	0.5346	0.5405	0.6312	0.6229	0.6199	0.5442	0.6337
NM_001100900	Ss18	0.9702	0.9532	0.2563	0.8813	0.9596	0.9450	0.0860	0.9686	0.8551	0.9428	0.9691	0.7690
NM_001004088	Lrrc36	0.2356	0.5022	0.0034	0.0475	0.0408	0.1870	0.2309	0.2105	0.5341	0.0748	0.1056	0.3813
NM_001013198	Ddx50	0.9966	0.8549	0.9569	0.3670	0.7828	0.7654	0.8201	0.9684	0.8521	0.7976	0.9266	0.8192
NM_001134881	Psd4	0.8101	0.7490	0.3149	0.9358	0.8496	0.8296	0.4977	0.7633	0.8135	0.8882	0.4784	0.8144
NM_001013159	Fnbp4	0.9788	0.8097	0.2297	0.2655	0.9872	0.9124	0.1224	0.8580	0.7347	0.8439	0.7740	0.9153
NM_001126087	Znf706	0.9806	0.9375	0.9662	0.9831	0.8860	0.9948	0.9426	0.9969	0.8010	0.9757	0.6962	0.9585
NM_001129880	LOC683626	0.6008	0.4592	0.6962	0.6008	0.6356	0.6608	0.6979	0.2402	0.5631	0.3716	0.5345	0.5532
NM_001012186	Vps39	0.4584	0.7601	0.0664	0.4013	0.9509	0.9378	0.2744	0.4444	0.3561	0.6455	0.4958	0.9075
NM_001024784	Nfx1	0.9169	0.6856	0.9665	0.9256	0.9512	0.8929	0.9674	0.9927	0.9887	0.8591	0.6486	0.9009
NM_001012102	Kif12	0.4698	0.5769	0.1038	0.2662	0.2479	0.2461	0.3895	0.4173	0.3536	0.2203	0.1231	0.1068
NM_001134873	Ctps	0.7617	0.7835	0.7048	0.5548	0.5195	0.5368	0.8631	0.2303	0.8044	0.9563	0.9473	0.6969
NM_001013169	Phc2	0.9018	0.9315	0.6014	0.9738	0.9622	0.9964	0.7553	0.9889	0.9606	0.9536	0.9392	0.8463
NM_001012114	Rexo1	0.7522	0.8600	0.1127	0.1087	0.8510	0.9391	0.2134	0.9699	0.6447	0.3958	0.5352	0.9097
NM_001134887	Nt5dc3	0.1171	0.0758	0.4407	0.3469	0.1262	0.5615	0.1038	0.5792	0.0760	0.0366	0.3034	0.6022
NM_001134874	Nab2	0.5541	0.7962	0.3480	0.0872	0.8952	0.7484	0.0317	0.6515	0.3045	0.4019	0.0133	0.5559
NM_001134886	Cohh1	0.7943	0.8465	0.5012	0.4751	0.2921	0.5760	0.8394	0.3793	0.7039	0.6209	0.5832	0.3937
NM_001134883	Lrp12	0.5324	0.2723	0.3576	0.3371	0.2810	0.6144	0.6667	0.1330	0.0824	0.3294	0.3286	0.5964
NM_001134879	Atad2	0.4319	0.0266	0.2748	0.3013	0.4853	0.1028	0.0941	0.3095	0.7739	0.3676	0.2402	0.6715
NM_001134880	Chrac1	0.0792	0.1901	0.2175	0.1785	0.7441	0.0358	0.1690	0.0616	0.5189	0.5297	0.1656	0.2520
NM_001134884	Scube1	0.8404	0.8327	0.4335	0.0641	0.8346	0.7567	0.3874	0.9576	0.7850	0.9712	0.7631	0.9814
NM_001134871	Yaf2	0.2208	0.1737	0.9398	0.2603	0.2105	0.5680	0.7536	0.3178	0.1157	0.3002	0.3938	0.4960
NM_001135833	Lyzl6	0.5186	0.4425	0.6655	0.1959	0.6091	0.4513	0.4982	0.6334	0.4731	0.2891	0.7010	0.2518
NM_001134994	Tpk1	0.8828	0.5946	0.7993	0.6115	0.7378	0.9137	0.9095	0.4602	0.8255	0.5734	0.8807	0.9358
NM_001134885	Usp40	0.9526	0.9352	0.6563	0.8734	0.4619	0.8491	0.8242	0.6118	0.9327	0.9722	0.8671	0.5416
NM_001040155	Zmym3	0.8648	0.5199	0.8868	0.8026	0.9589	0.9471	0.4676	0.9807	0.8154	0.1017	0.2978	0.9653
NM_001134962	Ttll13	0.3753	0.3769	0.4611	0.3516	0.3543	0.2500	0.3666	0.5026	0.3667	0.5277	0.3370	0.2748
NM_001134970	LOC691036	0.6137	0.5353	0.4909	0.2930	0.4784	0.6842	0.4631	0.3729	0.4675	0.7325	0.6178	0.7005
NM_001013237	Cd19	0.7944	0.4738	0.9260	0.9262	0.8659	0.3153	0.9700	0.8706	0.6803	0.7861	0.7017	0.7920
NM_001106375	Papss2	0.2728	0.1967	0.2568	0.0315	0.3227	0.2658	0.0355	0.0155	0.6651	0.2030	0.0915	0.1496
NM_001134912	Kcnk18	0.3267	0.1571	0.3048	0.1628	0.1707	0.2949	0.1646	0.1603	0.1584	0.4318	0.1585	0.2900
NM_001134961	Pdxdc1	0.8763	0.5295	0.8933	0.8533	0.8448	0.8599	0.8479	0.9238	0.9134	0.9518	0.5569	0.9118
NM_001134958	Srgap2	0.5306	0.5473	0.2262	0.5106	0.4783	0.6832	0.7367	0.4669	0.8060	0.6984	0.3427	0.6199
NM_001107219	Lgi2	0.0721	0.3234	0.1595	0.1913	0.1010	0.1626	0.5217	0.5153	0.4269	0.0104	0.4006	0.0710
NM_001134971	Man2b2	0.2911	0.3053	0.3430	0.5087	0.1705	0.3702	0.1644	0.1601	0.2958	0.4648	0.3948	0.2468
NM_001106047	Loxl2	0.9980	0.8193	0.8493	0.7283	0.1285	0.8066	0.8834	0.5927	0.8579	0.8431	0.7228	0.8322
NM_001134974	Trim27	0.9736	0.8569	0.4524	0.2170	0.8638	0.9657	0.5474	0.9475	0.6974	0.4931	0.4053	0.7215
NM_001109452	LOC684996	0.4272	0.6634	0.6666	0.6756	0.4139	0.3202	0.4932	0.2493	0.3972	0.4451	0.4695	0.2171
NM_001100524	Pcdh18	0.6118	0.1140	0.2959	0.4958	0.2248	0.1095	0.2065	0.4672	0.1635	0.1465	0.1891	0.5556
NM_001134959	Pear1	0.7560	0.5950	0.2061	0.5877	0.7182	0.4689	0.4584	0.5873	0.5407	0.3312	0.4356	0.4755
NM_001100770	Arrdc1	0.7250	0.7806	0.7980	0.8715	0.7011	0.8060	0.9063	0.9598	0.9634	0.6815	0.8493	0.8903
NM_001100901	Snta1	0.1176	0.1417	0.3949	0.6773	0.3641	0.3295	0.3825	0.8307	0.3550	0.0220	0.1124	0.2265

NM_001134955	Elmo2	0.0682	0.2384	0.1842	0.3326	0.3345	0.3549	0.0775	0.4793	0.4350	0.3348	0.5162	0.6944
NM_001106578	Sema3c	0.4009	0.5091	0.7918	0.7827	0.3936	0.2398	0.2713	0.1115	0.9027	0.8647	0.3280	0.7122
NM_001134908	Zfp637	0.9335	0.8727	0.8983	0.9183	0.8405	0.6903	0.8085	0.9395	0.1501	0.4597	0.8039	0.3232
NM_001134963	Crispld1	0.4775	0.5633	0.3589	0.4565	0.2981	0.6121	0.6494	0.6338	0.3773	0.3080	0.3900	0.1950
NM_001135583	Fa2h	0.2592	0.7328	0.5780	0.3698	0.5324	0.6130	0.7842	0.6001	0.6266	0.5108	0.3980	0.3315
NM_001134969	Ankrd6	0.7578	0.4064	0.8241	0.8022	0.7553	0.2053	0.5247	0.8523	0.9348	0.8445	0.9029	0.8668
NM_001134960	Rad54l	0.5192	0.9420	0.0328	0.2927	0.8710	0.7899	0.8274	0.6983	0.9409	0.6397	0.6945	0.0704
NM_001015033	Col16a1	0.8371	0.0235	0.2870	0.2903	0.0077	0.2761	0.7604	0.0495	0.0413	0.0226	0.0543	0.0137
NM_001134956	Ahdc1	0.4818	0.5750	0.2093	0.2708	0.5330	0.5927	0.1798	0.4065	0.5055	0.7057	0.5649	0.6704
NM_001100756	Ttc7	0.1156	0.6968	0.5128	0.3053	0.3439	0.7409	0.0347	0.2502	0.5206	0.2972	0.3741	0.9321
NM_001037791	Wdr43	0.8113	0.9697	0.3242	0.1758	0.8163	0.9322	0.7619	0.1129	0.9949	0.9656	0.9800	0.9460
NM_001134910	Anxa13	0.0318	0.7307	0.5353	0.5946	0.5544	0.1137	0.7868	0.2672	0.1566	0.6133	0.1678	0.6748
NM_001134957	Zfat	0.8704	0.4873	0.0875	0.5007	0.8910	0.4918	0.1600	0.6514	0.6487	0.2873	0.5508	0.8278
NM_001108178	Pls1	0.7808	0.8841	0.9306	0.8186	0.6802	0.7642	0.9173	0.6557	0.8186	0.5243	0.7115	0.7400
NM_001012202	Ppp2r3a	0.4157	0.3515	0.2603	0.1654	0.4672	0.9102	0.6126	0.3457	0.4352	0.4188	0.2634	0.8973
NM_001100859	Abpa	0.2953	0.1970	0.2569	0.1326	0.3273	0.0555	0.4151	0.6204	0.4121	0.3223	0.3727	0.6748
NM_001013225	Gtpbp2	0.6140	0.4252	0.3706	0.1528	0.8490	0.8192	0.6034	0.6279	0.9181	0.9691	0.7510	0.9552
NM_001134978	Leng4	0.1449	0.7747	0.5222	0.2749	0.5099	0.1497	0.0708	0.1166	0.3795	0.3761	0.5919	0.5015
NM_001134986	Rnf180	0.6216	0.4426	0.3938	0.4211	0.4585	0.3245	0.5655	0.6455	0.2472	0.5105	0.6205	0.4493
NM_001134983	RGD1564927	0.6655	0.9411	0.8300	0.8069	0.5631	0.8769	0.7264	0.5945	0.9398	0.8824	0.6220	0.7897
NM_001134979	Ezh2	0.9633	0.9224	0.3267	0.9904	0.9150	0.8370	0.7923	0.8444	0.7610	0.9792	0.9157	0.8437
NM_001134982	Mpp6	0.9565	0.9252	0.9705	0.9626	0.9006	0.7666	0.9995	0.7933	0.9365	0.8876	0.9324	0.8762
NM_001134980	Cyp2j10	0.2070	0.2381	0.2512	0.1240	0.1700	0.3832	0.2256	0.5234	0.4575	0.4644	0.2114	0.1227
NM_001134987	Eapp	0.1826	0.7827	0.3937	0.7367	0.1878	0.3643	0.2079	0.1978	0.4233	0.1723	0.1842	0.4180
NM_001134981	Trip4	0.2452	0.0335	0.8813	0.0107	0.0607	0.1645	0.3674	0.0072	0.0353	0.3757	0.0478	0.3540
NM_001134984	Tmem146	0.3038	0.3130	0.4681	0.5018	0.4064	0.3618	0.4158	0.5708	0.2604	0.4040	0.3937	0.4210
NM_001134998	LOC10018898	0.6231	0.5280	0.6266	0.3862	0.6771	0.5804	0.3685	0.2479	0.5323	0.4094	0.2564	0.3083
NM_001135008	Pabpn1	0.6750	0.5548	0.5994	0.5577	0.6298	0.5382	0.7012	0.6714	0.6580	0.6422	0.4664	0.7231
NM_001135010	Mcpt8l2	0.4593	0.3488	0.2836	0.3931	0.6459	0.2965	0.3523	0.3634	0.4545	0.3096	0.3658	0.1635
NM_001134990	N6amt2	0.6405	0.7865	0.8463	0.7110	0.9152	0.1919	0.8692	0.6787	0.5416	0.8167	0.6384	0.0464
NM_001135009	Col4a1	0.9977	0.8595	0.9959	0.0304	0.2954	0.1393	0.8190	0.3873	0.9402	0.8100	0.9276	0.7010
NM_001037210	Gipc2	0.0311	0.0672	0.1494	0.2757	0.8480	0.7743	0.0263	0.8987	0.0063	0.0267	0.0923	0.0475
NM_001134993	Car13	0.4057	0.5869	0.9009	0.4884	0.5814	0.6248	0.8697	0.4952	0.6230	0.2396	0.8930	0.3329
NM_001134991	Ng35	0.0291	0.6219	0.1742	0.7884	0.3487	0.6502	0.6092	0.3044	0.2413	0.2868	0.1021	0.2162
NM_001135002	LOC689425	0.4522	0.5890	0.3060	0.1624	0.4098	0.6950	0.4453	0.1599	0.2987	0.2789	0.6471	0.6902
NM_001106559	Glt6d1	0.6045	0.2107	0.8167	0.4029	0.2931	0.3727	0.7801	0.4536	0.5992	0.1633	0.5953	0.6200
NM_001136124	Ifitm3	0.0650	0.0204	0.4776	0.0663	0.2160	0.1476	0.0762	0.1912	0.0099	0.2365	0.1077	0.0876
NM_031623	Grb14	0.4910	0.2377	0.3429	0.4706	0.1704	0.4091	0.5730	0.1598	0.1582	0.2591	0.5287	0.1634
NM_001135003	Cwf19l2	0.2262	0.0105	0.3595	0.2781	0.4161	0.4743	0.0075	0.0413	0.2859	0.0018	0.0078	0.0376
NM_001135836	Pdzd4	0.7676	0.7066	0.9620	0.4842	0.3979	0.4286	0.9264	0.5466	0.9967	0.7183	0.6348	0.7893
NM_001134992	Sash3	0.7307	0.6335	0.6314	0.5242	0.3474	0.5956	0.5775	0.5029	0.8496	0.0783	0.5546	0.8306
NM_001135011	Rftn1	0.9188	0.3656	0.9019	0.9911	0.9252	0.9533	0.9206	0.9504	0.0959	0.4873	0.8506	0.1357
NM_001135014	Ankrd39	0.8868	0.0355	0.3968	0.8560	0.2018	0.1760	0.8947	0.5288	0.1309	0.5851	0.6120	0.0957
NM_001135018	Zfp426l	0.9492	0.5997	0.6939	0.8898	0.9478	0.9704	0.5517	0.5216	0.6509	0.9256	0.6556	0.9518
NM_001135016	Tmem86a	0.1035	0.5740	0.6946	0.4084	0.4079	0.5224	0.7668	0.6206	0.3567	0.0129	0.1087	0.6424
NM_053294	Adora2a	0.3486	0.2127	0.8288	0.4415	0.0297	0.5997	0.8431	0.1659	0.6594	0.8927	0.8433	0.3428
NM_053302	Gpr182	0.2671	0.8885	0.6768	0.5637	0.9431	0.4309	0.8229	0.5652	0.8344	0.7953	0.4166	0.6322
NM_001135017	RGD1309537	0.7909	0.3133	0.2555	0.4003	0.3896	0.6029	0.3451	0.1195	0.2407	0.1411	0.4678	0.7057
NM_001135030	Tctex1d1	0.2225	0.6890	0.7823	0.7728	0.5981	0.4564	0.1753	0.8648	0.7581	0.8115	0.6393	0.4686

NM_001135029	Tmigd1	0.2037	0.6437	0.4365	0.2477	0.4493	0.2794	0.5002	0.1529	0.0554	0.7952	0.3257	0.4714
NM_001008322	Shmt2	0.7257	0.9608	0.1194	0.2817	0.7709	0.8308	0.9643	0.7782	0.8164	0.7041	0.9326	0.3401
NM_001134850	Dph3	0.6402	0.5460	0.5914	0.6324	0.7334	0.6074	0.6804	0.7258	0.6971	0.6848	0.6462	0.4366
NM_001135039	Utp18	0.9692	0.7263	0.8675	0.8341	0.7854	0.5500	0.5961	0.9284	0.4317	0.4927	0.6359	0.5132
NM_001135033	Trim16	0.4552	0.4386	0.3213	0.4864	0.9165	0.7310	0.6551	0.5516	0.6377	0.2343	0.5388	0.8315
NM_001135084	Zfp322a	0.5932	0.6438	0.5718	0.6357	0.6906	0.2121	0.6283	0.5747	0.4921	0.4859	0.4780	0.2079
NM_133292	Sval1	0.2756	0.5225	0.2656	0.1623	0.2786	0.3166	0.1642	0.3514	0.3955	0.2761	0.4995	0.2995
NM_001135094	Klf14	0.8449	0.8433	0.2324	0.7359	0.6510	0.8604	0.7031	0.6904	0.8675	0.8558	0.6273	0.5527
NM_001135088	Zfp385a	0.4155	0.4380	0.6799	0.5984	0.5409	0.5388	0.7003	0.6393	0.5042	0.6966	0.2868	0.7054
NM_001135089	Zfp385a	0.4155	0.4380	0.6799	0.5984	0.5409	0.5388	0.7003	0.6393	0.5042	0.6966	0.2868	0.7054
NM_001109470	Zfp385a	0.4155	0.4380	0.6799	0.5984	0.5409	0.5388	0.7003	0.6393	0.5042	0.6966	0.2868	0.7054
NM_001105828	Supt4h1	0.2674	0.0424	0.4062	0.3803	0.1747	0.1860	0.1410	0.1233	0.0762	0.0520	0.0220	0.1133
NM_001135119	Angel2	0.7149	0.6534	0.7321	0.6738	0.6614	0.5441	0.5322	0.4526	0.6439	0.6626	0.6875	0.5484
NM_001135157	Myh2	0.0586	0.3417	0.4020	0.6406	0.5128	0.1599	0.0182	0.3171	0.1457	0.0079	0.0018	0.3115
NM_001135158	Myh1	0.3129	0.4770	0.6384	0.5151	0.3739	0.1649	0.4891	0.1596	0.2510	0.3800	0.1582	0.4834
NM_017363	Prl3d1	0.5748	0.1570	0.2792	0.4120	0.1702	0.1648	0.1640	0.1595	0.5226	0.4027	0.5090	0.3285
NM_001083940	Prl3d2	0.6759	0.4741	0.3911	0.4959	0.5811	0.2377	0.3430	0.6203	0.8212	0.5933	0.5260	0.5671
NM_001103354	LOC10012536	0.6709	0.6597	0.6555	0.6536	0.6585	0.6281	0.6957	0.5197	0.2959	0.1857	0.6811	0.6064
NM_001007750	Chpt1	0.7319	0.2505	0.8988	0.8911	0.1363	0.4339	0.8332	0.8181	0.1147	0.3454	0.1537	0.6786
NM_001008353	Mkks	0.8222	0.7410	0.5103	0.9485	0.8806	0.9013	0.8320	0.9034	0.8330	0.6906	0.5646	0.8399
NM_001004250	Uqcrc1	0.3042	0.7847	0.1417	0.5657	0.0149	0.1882	0.4957	0.0032	0.8668	0.8776	0.7319	0.7558
NM_001100565	Peli1	0.4731	0.2747	0.8001	0.5254	0.6189	0.6923	0.5009	0.7546	0.2044	0.5158	0.6706	0.6286
NM_001001517	Rnf114	0.0957	0.2049	0.8957	0.7197	0.1140	0.3462	0.1339	0.2281	0.1520	0.0070	0.2651	0.2771
NM_001134844	Cyp2b1	0.3724	0.2789	0.5539	0.4609	0.4075	0.5781	0.4108	0.3051	0.3393	0.2141	0.2775	0.3369
NM_001135562	Acp1	0.5017	0.7094	0.7464	0.5512	0.4777	0.4311	0.3144	0.5889	0.6541	0.7536	0.4315	0.8076
NM_001106485	Fkbp7	0.7988	0.5773	0.8743	0.8507	0.8804	0.8936	0.7101	0.9239	0.6523	0.8449	0.7604	0.2183
NM_001135174	Tomm7	0.2916	0.4324	0.4938	0.5080	0.1701	0.2905	0.6122	0.1595	0.4387	0.4535	0.4092	0.1633
NM_001013165	Lrrc23	0.5615	0.6735	0.3251	0.4960	0.6036	0.5573	0.8544	0.4527	0.2969	0.3814	0.5625	0.5235
NM_001106736	Fkbp3	0.3712	0.4144	0.1021	0.0714	0.3128	0.1605	0.3019	0.1104	0.1250	0.0462	0.0846	0.1307
NM_022229	Hspd1	0.6385	0.6124	0.4182	0.4775	0.7891	0.5010	0.0936	0.1920	0.6049	0.6059	0.3397	0.2902
NM_031074	Nup98	0.8804	0.9326	0.7977	0.2349	0.9713	0.8018	0.8820	0.5795	0.8227	0.8200	0.5804	0.3775
NM_001135252	LOC688495	0.6519	0.6545	0.5946	0.6055	0.5499	0.6241	0.5816	0.3020	0.4559	0.5996	0.7013	0.5213
NM_001135238	LOC684352	0.2573	0.4211	0.3349	0.2705	0.2326	0.2377	0.4668	0.4279	0.2637	0.5526	0.3864	0.4478
NM_001135253	LOC689933	0.2996	0.3821	0.2257	0.2929	0.2084	0.2350	0.3003	0.2632	0.3953	0.2083	0.4169	0.3978
NM_001135250	LOC685072	0.3352	0.7316	0.6375	0.7188	0.6447	0.7065	0.5720	0.6605	0.6732	0.6936	0.2210	0.7209
NM_001135249	LOC685072	0.3352	0.7316	0.6375	0.7188	0.6447	0.7065	0.5720	0.6605	0.6732	0.6936	0.2210	0.7209
NM_001135260	Tmem167b	0.0226	0.0793	0.7754	0.7877	0.1449	0.0616	0.8852	0.2193	0.0872	0.1675	0.4831	0.0771
NM_001135259	Cmc1	0.0375	0.1435	0.3884	0.1064	0.0704	0.0784	0.7540	0.0017	0.0337	0.0300	0.1768	0.0009
NM_001135561	Sos2	0.7996	0.9739	0.8863	0.8942	0.6498	0.9533	0.9722	0.5729	0.8800	0.9197	0.9509	0.9787
NM_001008315	Ltbr	0.0894	0.1669	0.6178	0.6196	0.4078	0.8281	0.8705	0.9198	0.2839	0.3730	0.8188	0.7128
NM_001007608	Skp1	0.9790	0.8629	0.9045	0.9860	0.8881	0.9716	0.6257	0.9526	0.7770	0.9689	0.8486	0.9103
NM_001008553	Dhodh	0.0765	0.7487	0.1181	0.9044	0.8234	0.7711	0.9710	0.7947	0.8113	0.8923	0.5308	0.7673
NM_001005530	Slc16a13	0.8878	0.5971	0.5263	0.9573	0.5522	0.2445	0.8179	0.6926	0.3283	0.5848	0.6857	0.3221
NM_001004265	Spint1	0.7148	0.2552	0.4836	0.5207	0.7895	0.4690	0.5968	0.7190	0.7325	0.6563	0.7582	0.3465
NM_001004260	Slc22a18	0.0204	0.0191	0.6815	0.8652	0.0622	0.0001	0.3558	0.0488	0.2676	0.1357	0.1297	0.8253
NM_001135584	Sec1	0.2496	0.1159	0.2337	0.3612	0.4929	0.2342	0.7728	0.5822	0.8083	0.2611	0.1013	0.2715
NM_001014012	Hars2l	0.6718	0.5542	0.6236	0.2446	0.6640	0.5444	0.4342	0.5593	0.7122	0.5994	0.4137	0.4362
NM_001135582	Zmat2	0.2390	0.0921	0.8592	0.5328	0.3761	0.0569	0.3694	0.2363	0.1347	0.1187	0.0667	0.2239
NM_001135576	LOC690806	0.2606	0.2626	0.2775	0.3915	0.3386	0.4193	0.5363	0.3242	0.4344	0.3901	0.3356	0.2388

NM_001109190	Tnrc4	0.3231	0.5472	0.7801	0.7347	0.3665	0.3679	0.1050	0.8517	0.5230	0.4957	0.9158	0.8952
NM_001107783	Kif16b	0.4278	0.3209	0.9230	0.9503	0.1766	0.2702	0.4390	0.4753	0.4827	0.2745	0.7100	0.8356
NM_001135603	Brunol5	0.9827	0.9918	0.9813	0.9698	0.9699	0.9874	0.9784	0.8474	0.9987	0.7605	0.7797	0.9983
NM_001135596	Agfg1	0.8863	0.9814	0.9864	0.6162	0.3070	0.6119	0.9623	0.3702	0.9798	0.9315	0.9738	0.9737
NM_001135607	Rhox3	0.3652	0.2901	0.1672	0.1621	0.2800	0.3902	0.1639	0.4966	0.1581	0.1548	0.1581	0.2682
NM_001007601	Pcbd1	0.5554	0.3775	0.3856	0.1472	0.6105	0.5496	0.7128	0.5151	0.5340	0.1992	0.1624	0.5217
NM_001135612	Hps5	0.4254	0.5644	0.3854	0.4360	0.7345	0.7181	0.6395	0.6282	0.1885	0.3371	0.5285	0.3724
NM_001024233	Bles03	0.7053	0.7515	0.8588	0.8256	0.5396	0.1406	0.5451	0.4551	0.2856	0.6920	0.7534	0.3944
NM_001033959	Obp3	0.2498	0.4680	0.2506	0.2140	0.6626	0.4298	0.4033	0.3716	0.6553	0.3945	0.7159	0.2910
NM_053447	Hdac2	0.9868	0.9497	0.9164	0.9088	0.9533	0.8803	0.9093	0.7353	0.7985	0.7608	0.8545	0.8719
NM_001135173	Klk1c10	0.7982	0.4047	0.3216	0.2389	0.7054	0.4862	0.3594	0.2337	0.4449	0.7911	0.4031	0.5874
NM_212547	Rgl2	0.9594	0.9921	0.9096	0.9773	0.7968	0.8946	0.7987	0.8545	0.9488	0.9988	0.9149	0.9225
NM_001135666	Lrrc47	0.5406	0.1497	0.2854	0.3197	0.2822	0.7573	0.0457	0.0514	0.9198	0.3009	0.2283	0.7027
NM_001135708	Urb2	0.4055	0.7777	0.3918	0.3392	0.7644	0.7955	0.4179	0.4651	0.5171	0.8515	0.6284	0.9351
NM_130423	Ggt7	0.2751	0.0115	0.4388	0.1067	0.0671	0.1043	0.4087	0.4246	0.2938	0.3098	0.3750	0.4538
NM_001135718	RGD1306534	0.1215	0.1425	0.4421	0.2353	0.0161	0.0891	0.2268	0.0654	0.8091	0.1097	0.3458	0.1287
NM_001135709	RGD1562679	0.3285	0.6404	0.1195	0.2106	0.6734	0.7147	0.3602	0.5855	0.1028	0.1995	0.2792	0.5597
NM_001135719	RGD1563550	0.1956	0.1110	0.7401	0.0071	0.0398	0.0138	0.1341	0.0137	0.0031	0.2467	0.1574	0.1011
NM_001135712	RGD1563037	0.5321	0.2191	0.9368	0.7350	0.3905	0.9568	0.7065	0.3534	0.4024	0.2650	0.5598	0.7234
NM_001135169	Tmem14c	0.2840	0.7264	0.8278	0.1974	0.3054	0.4185	0.7066	0.2882	0.6243	0.8821	0.9137	0.3129
NM_001007646	Fkbp9	0.7794	0.7027	0.9338	0.9592	0.9138	0.9472	0.9609	0.8956	0.9292	0.9323	0.8698	0.7984
NM_001006971	Cln3	0.1764	0.2946	0.5679	0.4979	0.5946	0.6581	0.0598	0.7340	0.1125	0.3967	0.0059	0.4446
NM_001007012	Alx3	0.9279	0.9198	0.9191	0.7960	0.6624	0.8414	0.7010	0.8372	0.5526	0.8209	0.8247	0.8119
NM_001005876	Hn1	0.9019	0.5433	0.2302	0.8429	0.9446	0.9216	0.7974	0.8351	0.5956	0.3846	0.5161	0.3471
NM_001005765	Rap1a	0.2361	0.3116	0.8554	0.8911	0.7009	0.4736	0.5699	0.9195	0.1232	0.2701	0.2431	0.3838
NM_001005541	Vps16	0.1059	0.1665	0.0866	0.0024	0.0735	0.2577	0.0155	0.0849	0.4357	0.3363	0.6514	0.6304
NM_001004284	Zmynd10	0.7406	0.6543	0.3254	0.0768	0.7098	0.9769	0.0322	0.8678	0.6304	0.6896	0.8494	0.8871
NM_001004255	Dek	0.5464	0.6975	0.8059	0.5549	0.3681	0.7980	0.3574	0.6049	0.2551	0.7636	0.8891	0.2639
NM_001004249	Tmed3	0.6256	0.8589	0.8565	0.3512	0.3020	0.0673	0.9074	0.2570	0.4416	0.5670	0.6864	0.3995
NM_001004240	Gpaa1	0.0570	0.0016	0.0001	0.0022	0.0846	0.0489	0.0933	0.0522	0.0306	0.0014	0.0016	0.0231
NM_001004233	Mkrn1	0.0781	0.1646	0.9818	0.8984	0.1202	0.4252	0.2477	0.1310	0.5415	0.1199	0.1906	0.8795
NM_001004078	Cct5	0.8997	0.9569	0.8281	0.8527	0.8461	0.8054	0.8796	0.9314	0.8379	0.8827	0.8860	0.8094
NM_001004071	Ano7	0.0723	0.2225	0.4029	0.3908	0.1028	0.3990	0.1036	0.3509	0.3321	0.2720	0.0708	0.2977
NM_001002827	Notch4	0.3692	0.1082	0.2782	0.2859	0.3346	0.6408	0.0781	0.7736	0.4020	0.0964	0.2907	0.6664
NM_001002821	H2-T3	0.5518	0.7827	0.7874	0.7531	0.1075	0.0449	0.5093	0.2241	0.2916	0.2177	0.0224	0.1697
NM_001002803	Btnl1	0.0941	0.3465	0.2147	0.3445	0.4278	0.7180	0.8154	0.1029	0.4370	0.7670	0.1189	0.7780
NM_001002253	Atp6v0e2	0.1393	0.2414	0.8525	0.8430	0.1490	0.3486	0.9377	0.1647	0.9227	0.7473	0.7206	0.9857
NM_001135742	Irgq	0.7355	0.8882	0.9589	0.8971	0.8739	0.9115	0.8723	0.1887	0.8338	0.6409	0.8828	0.7846
NM_001135743	Wbscr22	0.2056	0.3771	0.6777	0.8269	0.7018	0.6645	0.7006	0.1735	0.5459	0.4671	0.6490	0.5397
NM_001135819	Pdxp	0.3684	0.5800	0.6590	0.2333	0.6036	0.5636	0.6174	0.4834	0.6124	0.5117	0.4767	0.6152
NM_001135744	RGD1559604	0.3424	0.2548	0.2909	0.3646	0.3231	0.6818	0.4445	0.3043	0.2320	0.4327	0.1579	0.3738
NM_001135757	RGD1560916	0.0067	0.0107	0.1745	0.0850	0.1636	0.0106	0.0688	0.1850	0.0934	0.0999	0.1309	0.2318
NM_001135749	RGD1561797	0.4917	0.9389	0.3108	0.9819	0.9188	0.7529	0.9783	0.9671	0.8982	0.9890	0.9848	0.1617
NM_001135754	RGD1562865	0.9281	0.5328	0.9119	0.6514	0.9686	0.5084	0.9773	0.7681	0.5807	0.9526	0.5337	0.4542
NM_001135758	Macf1	0.9874	0.3421	0.3520	0.6790	0.9740	0.9752	0.4047	0.9233	0.3023	0.7096	0.5877	0.1737
NM_001135755	RGD1311640	0.7681	0.3063	0.3424	0.3476	0.8107	0.8039	0.1155	0.9660	0.1202	0.4527	0.3004	0.2237
NM_001135759	Col4a3	0.0438	0.1296	0.3113	0.1497	0.1124	0.3170	0.3288	0.0056	0.5244	0.2944	0.2542	0.4757
NM_001135767	LOC690825	0.3611	0.2767	0.3008	0.4335	0.2440	0.3053	0.7351	0.4217	0.4421	0.3198	0.3654	0.2805
NM_001135778	Prodh	0.4239	0.4008	0.3399	0.2894	0.2117	0.3110	0.3912	0.6263	0.3812	0.4715	0.2744	0.4205

NM_054008	Rgc32	0.5164	0.4465	0.2277	0.5116	0.5075	0.5931	0.6940	0.3574	0.3385	0.1337	0.5846	0.1893
NM_001135779	Gabra2	0.1482	0.7486	0.4487	0.5892	0.2462	0.1585	0.4539	0.1765	0.6332	0.6670	0.2679	0.0193
NM_001135777	LOC680149	0.3446	0.2837	0.2768	0.3290	0.2785	0.1644	0.4143	0.1591	0.1578	0.3881	0.3678	0.1631
NM_001135780	RGD1304693	0.8375	0.7107	0.2584	0.6190	0.5370	0.8254	0.5196	0.4287	0.3978	0.2781	0.5704	0.1199
NM_001135782	RGD1564061	0.0740	0.4602	0.2596	0.3203	0.1313	0.6225	0.6861	0.3968	0.6156	0.6120	0.3112	0.0322
NM_001135781	RGD1305350	0.3322	0.6336	0.8176	0.9411	0.3309	0.4363	0.7699	0.3789	0.8478	0.8121	0.4406	0.7918
NM_001135835	Dpy19l3	0.3614	0.5549	0.2225	0.1849	0.6207	0.5739	0.1953	0.5125	0.1620	0.5240	0.4645	0.2794
NM_001135798	Adck5	0.8523	0.4749	0.0377	0.2204	0.7532	0.8728	0.8695	0.8166	0.7950	0.0959	0.3529	0.3818
NM_001135834	Mrap	0.0999	0.1964	0.1082	0.7433	0.0824	0.7481	0.3040	0.4800	0.0434	0.2842	0.2580	0.3282
NM_001135866	Rft1	0.0253	0.0468	0.8637	0.7443	0.1972	0.3076	0.1876	0.2245	0.2062	0.0221	0.1377	0.1736
NM_001135840	Gtpbp6	0.7806	0.6026	0.0565	0.0890	0.6834	0.6648	0.3975	0.6909	0.6586	0.6780	0.7766	0.3663
NM_001135804	Fam134c	0.9816	0.5691	0.9878	0.9852	0.9902	0.9751	0.9889	0.9840	0.5661	0.5598	0.9231	0.3254
NM_001135809	Lcn11	0.3015	0.1564	0.1669	0.3567	0.2532	0.2836	0.1636	0.4002	0.3375	0.4873	0.3464	0.3146
NM_001135802	Dlgap5	0.8333	0.8009	0.0875	0.3307	0.9116	0.8481	0.8642	0.6928	0.8291	0.8751	0.8972	0.0400
NM_001135801	Ddx27	0.8987	0.5446	0.8347	0.4903	0.7207	0.5782	0.9598	0.5780	0.4737	0.5193	0.3326	0.4849
NM_001135803	Dus4l	0.9179	0.8123	0.8587	0.8468	0.9405	0.9276	0.9102	0.7188	0.6101	0.7365	0.8511	0.8937
NM_001135814	Mgat4c	0.8666	0.4731	0.9093	0.3463	0.8270	0.7592	0.0403	0.3849	0.4930	0.5492	0.4537	0.1289
NM_001135813	Med31	0.3983	0.8995	0.6172	0.1187	0.4838	0.1165	0.8634	0.1669	0.2931	0.3552	0.3152	0.2613
NM_001135799	Bmper	0.9767	0.9763	0.9882	0.9933	0.9566	0.9839	0.9867	0.9480	0.9529	0.9853	0.9829	0.9333
NM_001032285	Prrt1	0.5568	0.5759	0.5108	0.5749	0.6357	0.4481	0.5205	0.5881	0.6665	0.1890	0.6901	0.5968
NM_001135807	H2afy2	0.8257	0.5848	0.7417	0.8880	0.4740	0.5898	0.7104	0.7814	0.8317	0.9175	0.8633	0.3320
NM_001135837	Ccdc22	0.2670	0.4336	0.3899	0.2542	0.3925	0.5058	0.5362	0.6593	0.5140	0.5109	0.6030	0.2133
NM_001135839	Ard1a	0.8175	0.7285	0.2448	0.6632	0.8054	0.8434	0.8968	0.9118	0.9429	0.7186	0.7230	0.5993
NM_001134704	LOC10018893(	0.3696	0.2291	0.4385	0.6304	0.3789	0.2192	0.4141	0.7085	0.6967	0.4993	0.5578	0.5699
NM_001135858	Tmtc3	0.3915	0.3569	0.9707	0.9572	0.8867	0.9422	0.2401	0.4288	0.9202	0.3678	0.8558	0.9616
NM_001135857	Tmco3	0.7491	0.1494	0.9744	0.3288	0.1932	0.8914	0.1820	0.1559	0.5951	0.7034	0.6207	0.7437
NM_001135855	Scara5	0.6433	0.3210	0.6055	0.1413	0.6744	0.6095	0.7005	0.7120	0.7855	0.3397	0.4830	0.4774
NM_001135849	Saps1	0.8488	0.3779	0.3363	0.4250	0.9726	0.8543	0.4694	0.9502	0.9642	0.7859	0.8628	0.5392
NM_001135871	Ppa2	0.5240	0.7087	0.8362	0.8765	0.7783	0.5013	0.8587	0.6168	0.6840	0.8030	0.8261	0.3229
NM_001134972	Plekhg2	0.8174	0.7536	0.4344	0.5808	0.8893	0.7713	0.8478	0.9353	0.7905	0.8494	0.7141	0.8622
NM_001135868	Slc45a3	0.2099	0.4596	0.2822	0.6979	0.4987	0.5109	0.8061	0.6932	0.7144	0.6009	0.7494	0.7660
NM_001135867	Serpinb12	0.0719	0.2387	0.5648	0.7077	0.0455	0.7509	0.1137	0.6305	0.4511	0.2137	0.1686	0.6355
NM_001135845	Rpusd2	0.4539	0.6564	0.6762	0.5278	0.5890	0.3749	0.6496	0.4415	0.7246	0.6399	0.6473	0.6777
NM_001135875	Ranbp10	0.4079	0.2738	0.1485	0.3338	0.6709	0.3595	0.1662	0.8874	0.5662	0.4367	0.3172	0.2479
NM_001135879	Tmem56	0.0313	0.7030	0.7506	0.7365	0.3648	0.6328	0.8463	0.1603	0.8770	0.3959	0.6086	0.7415
NM_001135874	Ston2	0.1216	0.0632	0.0796	0.0391	0.2704	0.5579	0.0468	0.2649	0.1916	0.2603	0.2675	0.1271
NM_001135859	Txlnb	0.7698	0.6430	0.2496	0.2036	0.4499	0.2633	0.5406	0.5921	0.7644	0.7587	0.3820	0.6485
NM_001135872	Rsbn1l	0.9049	0.6508	0.8714	0.7066	0.6799	0.8180	0.6061	0.9261	0.9945	0.8973	0.8068	0.7733
NM_001135877	Taf7l	0.1098	0.4803	0.1235	0.1443	0.3598	0.1347	0.2903	0.8638	0.1824	0.5000	0.5084	0.0203
NM_001135846	Rrn3	0.3926	0.8361	0.7898	0.5175	0.3560	0.7219	0.1442	0.6350	0.1869	0.4007	0.0542	0.1330
NM_001135876	Trmu	0.0980	0.5684	0.0361	0.0616	0.1906	0.0924	0.0960	0.3773	0.0333	0.0979	0.0605	0.0454
NM_001135873	Upf3b	0.9870	0.9535	0.1665	0.3706	0.9500	0.9669	0.3084	0.9665	0.8564	0.8410	0.9583	0.6762
NM_001135893	Usp9x	0.6786	0.7218	0.8625	0.7166	0.6625	0.8030	0.8990	0.6750	0.9075	0.6535	0.9344	0.9317
NM_001135901	RGD1561517	0.4560	0.1563	0.1668	0.1615	0.3405	0.4855	0.1635	0.6341	0.2678	0.2993	0.4937	0.3506
NM_001135878	Plxnb3	0.4724	0.0702	0.4040	0.3531	0.0764	0.8579	0.4583	0.7151	0.2338	0.2912	0.3043	0.7000
NM_001135899	RGD1312038	0.0069	0.2404	0.4018	0.2213	0.1300	0.2133	0.0338	0.2609	0.4639	0.0682	0.0260	0.1415
NM_001135897	RGD1311188	0.1644	0.1693	0.6237	0.0514	0.2444	0.0417	0.5404	0.1425	0.0542	0.2060	0.0911	0.0598
NM_001135895	Wdr48	0.8126	0.6064	0.3014	0.1223	0.5335	0.3348	0.8072	0.3579	0.7813	0.4088	0.5275	0.8730
NM_001135894	Wdr25l	0.7411	0.3965	0.2598	0.2600	0.1909	0.4697	0.3640	0.4509	0.3288	0.3222	0.3442	0.3529

NM_001135903	RGD1562665	0.8939	0.8418	0.4018	0.7427	0.1856	0.3236	0.9165	0.7951	0.6621	0.9576	0.1150	0.2813
NM_001135896	RGD1308720	0.8659	0.6733	0.6115	0.5323	0.8986	0.5801	0.8962	0.8594	0.8335	0.8455	0.6511	0.6598
NM_001135922	Ttll12	0.9043	0.8349	0.7086	0.5986	0.8297	0.8801	0.7383	0.7594	0.9247	0.8914	0.7558	0.8908
NM_001011979	Tardbp	0.9974	0.9811	0.9943	0.3014	0.8686	0.9786	0.9942	0.9675	0.9165	0.9865	0.9777	0.9465
NM_001135923	Usp9x	0.6786	0.7218	0.8625	0.7166	0.6625	0.8030	0.8990	0.6750	0.9075	0.6535	0.9344	0.9317
NM_001135918	Gng13	0.8095	0.9297	0.0246	0.1336	0.8703	0.8645	0.7084	0.9359	0.8474	0.8481	0.8615	0.1464
NM_001135916	LOC689801	0.1671	0.1562	0.1667	0.1613	0.1697	0.1643	0.1635	0.1588	0.1576	0.1544	0.1577	0.1631
NM_001135915	LOC688553	0.7074	0.3616	0.4526	0.6696	0.3197	0.3542	0.4051	0.6099	0.6588	0.2762	0.3985	0.5712
NM_001135913	Enthd1	0.6739	0.6399	0.7852	0.8366	0.7290	0.9675	0.8895	0.7542	0.7357	0.7583	0.3430	0.8880
NM_001009624	Fam33a	0.9074	0.9267	0.3120	0.9459	0.7972	0.8794	0.9072	0.7378	0.7103	0.7021	0.6976	0.0045
NM_001008306	Calhm2	0.9648	0.9758	0.7634	0.9642	0.8303	0.7695	0.9157	0.9577	0.8531	0.9675	0.8026	0.8053
NM_001006983	Fam108a1	0.0021	0.1076	0.1637	0.0151	0.0737	0.0380	0.2446	0.1772	0.0555	0.0656	0.0854	0.0827
NM_001007699	Cept1	0.3239	0.0253	0.6021	0.3585	0.3215	0.0453	0.0765	0.0555	0.1807	0.3191	0.0931	0.5736
NM_001106579	Sema3e	0.7364	0.0718	0.6074	0.3240	0.5568	0.6306	0.4138	0.0220	0.4446	0.0722	0.4020	0.3515
NM_001108526	Sema4g	0.3115	0.1293	0.9187	0.4604	0.5620	0.8196	0.3615	0.5731	0.1642	0.2981	0.1571	0.3783
NM_001135992	LOC498276	0.9501	0.1824	0.0973	0.5893	0.9051	0.8474	0.1286	0.7987	0.0017	0.0027	0.0062	0.0281
NM_001100517	Aars	0.4144	0.9303	0.1377	0.8412	0.9725	0.8263	0.7789	0.8798	0.9835	0.9612	0.7964	0.7171
NM_001100681	Pbx1	0.9130	0.8214	0.6222	0.6197	0.9665	0.8711	0.8791	0.5528	0.9635	0.9537	0.9410	0.9890
NM_001106902	Sema4c	0.8284	0.9179	0.4622	0.9775	0.8212	0.7188	0.7670	0.9511	0.6188	0.8352	0.9415	0.9146
NM_001108185	Sema3f	0.1314	0.0636	0.7840	0.4767	0.1718	0.3654	0.4598	0.1745	0.1969	0.0475	0.2864	0.6104
NM_001108153	Sema7a	0.9376	0.3571	0.2728	0.5968	0.8941	0.3367	0.9673	0.8390	0.8993	0.4429	0.3474	0.1810
NM_001135997	RGD1563226	0.8824	0.7908	0.9322	0.8820	0.7501	0.6120	0.8401	0.4446	0.4598	0.8241	0.6819	0.5217
NM_001006981	Dlst	0.5558	0.8174	0.2338	0.5377	0.2851	0.2435	0.8506	0.2598	0.9524	0.9463	0.9393	0.9460
NM_001006976	Tars	0.8191	0.7173	0.7275	0.7809	0.3261	0.4521	0.6444	0.4117	0.7398	0.5611	0.6653	0.2780
NM_001005560	Pla2g6	0.5557	0.0226	0.0890	0.1575	0.9332	0.8578	0.4199	0.9822	0.2199	0.3432	0.0753	0.1395
NM_001004443	Hexa	0.0888	0.0485	0.6117	0.0262	0.0718	0.0605	0.0758	0.0787	0.0288	0.0196	0.0271	0.8034
NM_001004085	Crat	0.4530	0.4917	0.6191	0.4051	0.0952	0.3512	0.6492	0.2460	0.8640	0.2647	0.3513	0.5773
NM_001004081	Mpi	0.0487	0.0267	0.0891	0.1504	0.0018	0.0311	0.0766	0.0387	0.0444	0.0387	0.0391	0.2005
NM_001003673	Pmpca	0.1488	0.4359	0.1289	0.2372	0.0762	0.1020	0.1460	0.1519	0.2477	0.1967	0.3532	0.4220
NM_019227	Itsn1	0.2938	0.3203	0.5400	0.6887	0.4551	0.4437	0.3680	0.3238	0.3646	0.3417	0.2809	0.7203
NM_001136098	Hook3	0.5970	0.4900	0.5550	0.5231	0.5222	0.2226	0.3963	0.7057	0.3120	0.5612	0.5539	0.5560
NM_057198	Ppat	0.8025	0.9972	0.1557	0.1366	0.6738	0.8091	0.9261	0.9882	0.9962	0.8890	0.8806	0.9773
NM_053509	Zp1	0.7787	0.5926	0.2454	0.4506	0.5056	0.6073	0.8022	0.5682	0.5546	0.8241	0.7395	0.0470
NM_001004218	Fuca2	0.2479	0.8773	0.7161	0.8909	0.1390	0.0130	0.7022	0.3015	0.7807	0.7501	0.9194	0.7446
NM_001004207	Dhps	0.1006	0.0863	0.0039	0.8827	0.1498	0.1024	0.0597	0.0427	0.0555	0.2428	0.0597	0.0204
NM_053991	Vip	0.7045	0.8436	0.9781	0.9450	0.7708	0.4340	0.8722	0.8068	0.7497	0.7113	0.7620	0.4246
NM_053916	Trim28	0.7679	0.7992	0.0247	0.8221	0.9387	0.8213	0.9232	0.8415	0.7419	0.7727	0.8668	0.7868
NM_134375	Nlrp6	0.1849	0.4442	0.1241	0.5088	0.1797	0.1546	0.0333	0.2054	0.1693	0.3479	0.2010	0.5977
NM_001011939	Ehd1	0.7283	0.6456	0.2905	0.5665	0.5038	0.6568	0.5940	0.7175	0.2672	0.3516	0.8751	0.4488
NM_134370	Psd	0.3242	0.2335	0.3793	0.6043	0.6339	0.5379	0.2861	0.7006	0.6119	0.0854	0.3943	0.7557
NM_053476	Synj1	0.8650	0.4461	0.2845	0.2302	0.6348	0.8486	0.1450	0.3453	0.4622	0.3822	0.7499	0.9667
NM_053760	Trafd1	0.4508	0.4238	0.0646	0.1549	0.3112	0.2792	0.0682	0.1814	0.3533	0.4044	0.0588	0.6643
NM_145784	Gpr37l1	0.8925	0.7077	0.6240	0.7516	0.9137	0.9369	0.7958	0.9412	0.8559	0.7615	0.9534	0.8958
NM_001100539	Sdhb	0.0393	0.0864	0.8323	0.2585	0.0673	0.0268	0.8230	0.0617	0.1048	0.0779	0.0607	0.2755
NM_019176	Stmn4	0.6073	0.3611	0.8063	0.6245	0.7742	0.4611	0.6461	0.3622	0.5931	0.7592	0.2030	0.6302
NM_001100682	Fcrla	0.1074	0.0501	0.1046	0.1241	0.0218	0.0436	0.0001	0.1181	0.6167	0.0831	0.0056	0.0155
NM_022212	Insrr	0.6882	0.2240	0.7967	0.7407	0.8139	0.2428	0.4292	0.4935	0.5928	0.6235	0.3888	0.6362
NM_001100531	Dlx1	0.9990	0.9569	0.9633	0.8794	0.9952	0.9329	0.8850	0.9999	0.9904	0.9676	0.9937	0.8933
NM_001100529	Txndc13	0.4057	0.2722	0.7891	0.5173	0.3780	0.8153	0.3639	0.2060	0.6722	0.2206	0.7844	0.4966

NM_053761	Zyx	0.2045	0.4724	0.4904	0.4649	0.5952	0.5062	0.7141	0.4923	0.2572	0.5186	0.5502	0.5623
NM_001100840	Txndc12	0.0861	0.1060	0.7766	0.0254	0.2373	0.2304	0.3059	0.0206	0.2002	0.0676	0.0112	0.0686
NM_001100791	LOC498662	0.2239	0.2751	0.1472	0.1428	0.5239	0.8188	0.3138	0.3199	0.1997	0.1694	0.2275	0.3013
NM_001100779	aicda	0.8768	0.5588	0.4282	0.4867	0.7232	0.8292	0.7520	0.6362	0.6785	0.8847	0.8142	0.8038
NM_001012117	Fbxo43	0.0550	0.1239	0.0838	0.1778	0.0231	0.0340	0.0439	0.2286	0.2913	0.8138	0.4977	0.0202
NM_199396	Prickle1	0.1426	0.4843	0.6645	0.3362	0.2078	0.1533	0.1240	0.2661	0.1097	0.3494	0.6862	0.2999
NM_001136141	Treh	0.7860	0.8011	0.3148	0.2412	0.4040	0.4776	0.6334	0.3973	0.7354	0.6885	0.5089	0.6396
NM_001006968	Tsen34	0.7894	0.1115	0.9006	0.8266	0.7069	0.6472	0.8638	0.7402	0.8121	0.9726	0.0882	0.8293
NM_001006956	Katnal1	0.7705	0.9126	0.6451	0.8307	0.6581	0.5338	0.6503	0.5255	0.7640	0.6792	0.8196	0.3413
NM_001005883	Pi4k2b	0.4651	0.7714	0.8109	0.5803	0.0395	0.9653	0.8899	0.0770	0.8517	0.8679	0.8699	0.7081
NM_001005879	Gfpt1	0.6617	0.8700	0.0391	0.6028	0.1234	0.1353	0.6191	0.0081	0.7389	0.7728	0.8233	0.3742
NM_001009666	Dnal4	0.3492	0.2544	0.5082	0.7818	0.5072	0.4769	0.5089	0.2243	0.3509	0.0361	0.2620	0.5225
NM_001005561	Vrk3	0.2036	0.1264	0.4007	0.6723	0.1614	0.4990	0.1001	0.3032	0.1471	0.0306	0.0737	0.3901
NM_001008299	Rnmt	0.8637	0.7681	0.9343	0.1711	0.8028	0.7364	0.0821	0.0664	0.5530	0.7284	0.7580	0.8699
NM_001005550	Ndufs1	0.1037	0.1957	0.4866	0.1988	0.0259	0.0187	0.2906	0.0406	0.5077	0.4154	0.9247	0.7608
NM_001004279	Ppid	0.6643	0.8511	0.4287	0.1745	0.2861	0.2080	0.2977	0.2052	0.2982	0.6488	0.5170	0.5170
NM_001004275	Cops4	0.8739	0.8580	0.7823	0.0248	0.7885	0.2555	0.5086	0.7587	0.6787	0.6154	0.4040	0.1719
NM_001004256	Sirt5	0.2856	0.6194	0.8469	0.0704	0.2881	0.0126	0.1476	0.0885	0.6248	0.5858	0.3963	0.7414
NM_001004072	Pdha1	0.4188	0.5138	0.7857	0.4171	0.2247	0.4128	0.5189	0.2746	0.5591	0.5444	0.4351	0.6730
NM_001002831	Rpp21	0.6954	0.9078	0.0122	0.8771	0.8185	0.2456	0.7102	0.6896	0.8263	0.7201	0.7155	0.0703
NM_001002808	Cpa5	0.2815	0.6418	0.7191	0.4263	0.7897	0.6640	0.7849	0.6117	0.5258	0.4410	0.7751	0.7715
NM_022187	Plk3	0.2293	0.3246	0.6919	0.3593	0.4932	0.4676	0.7147	0.4792	0.3921	0.3112	0.6700	0.6711
NM_001136151	Nrg2	0.1768	0.2622	0.3607	0.7787	0.4425	0.4153	0.0229	0.3163	0.1259	0.5723	0.1992	0.4632
NM_001106513	Pank2	0.3959	0.8810	0.8397	0.6165	0.5340	0.7502	0.9321	0.4982	0.9778	0.9229	0.9514	0.9789
NM_001100727	Rufy1	0.8156	0.2337	0.1998	0.8346	0.7528	0.8404	0.3697	0.6967	0.5756	0.1323	0.2389	0.7269
NM_001100584	Pign	0.1711	0.3428	0.6393	0.4236	0.2744	0.3568	0.3839	0.1354	0.0817	0.2238	0.3205	0.4613
NM_001100501	Usp16	0.8833	0.8114	0.0999	0.4233	0.7358	0.7376	0.5101	0.7991	0.7727	0.4186	0.8851	0.6609
NM_001100482	Slc5a11	0.1565	0.0734	0.4078	0.1646	0.0496	0.2831	0.0308	0.1188	0.1507	0.0056	0.0485	0.0307
NM_001136162	Ttc13	0.8008	0.3472	0.1752	0.7776	0.9499	0.8518	0.3567	0.8067	0.5647	0.4302	0.3561	0.5675
NM_022624	Slc22a23	0.6477	0.4031	0.7827	0.5696	0.8526	0.7972	0.5198	0.8077	0.4808	0.6819	0.4219	0.8135
NM_031084	Pls3	0.3147	0.2968	0.9842	0.2053	0.8696	0.9894	0.1009	0.6226	0.2329	0.1795	0.2047	0.4225
NM_053468	Rag1	0.8907	0.9255	0.7965	0.7322	0.9506	0.8595	0.7238	0.8492	0.7182	0.8503	0.8657	0.3366
NM_031767	Sort1	0.9009	0.9140	0.7995	0.9462	0.6410	0.7765	0.9684	0.6366	0.6505	0.4847	0.9424	0.3929
NM_031638	Pdcd2	0.0088	0.0864	0.3039	0.0104	0.0631	0.0044	0.0162	0.0263	0.0322	0.0701	0.1836	0.1103
NM_053759	Six1	0.0575	0.2321	0.7961	0.3745	0.9513	0.9122	0.1129	0.9995	0.2106	0.1190	0.1900	0.2810
NM_053505	Slc24a3	0.9518	0.9613	0.9241	0.1360	0.9582	0.7192	0.9404	0.9611	0.9320	0.7426	0.5572	0.9435
NM_134368	Smarca4	0.8224	0.8118	0.0671	0.8118	0.9419	0.8114	0.5508	0.8542	0.6048	0.8380	0.9846	0.5697
NM_133319	Taf2	0.7631	0.2909	0.5689	0.3295	0.7809	0.6313	0.6586	0.2690	0.2396	0.1525	0.4002	0.4540
NR_003527	Dzip1-ps1	0.6404	0.6858	0.6269	0.3807	0.6584	0.7133	0.6335	0.6705	0.6241	0.4038	0.2091	0.5737
NR_002703	Rmrp	0.4259	0.3673	0.3043	0.1609	0.5125	0.3003	0.4643	0.7218	0.4792	0.3282	0.4488	0.5385
NM_001014139	RGD1308626	0.8122	0.5706	0.8318	0.8780	0.4168	0.6464	0.7787	0.8765	0.7264	0.8249	0.8444	0.6858
NM_183331	F8	0.6173	0.2119	0.5368	0.2339	0.6191	0.5177	0.6939	0.5185	0.5876	0.4463	0.2039	0.3267
NM_152938	Slc4a9	0.7758	0.8276	0.8661	0.9690	0.7571	0.6980	0.9609	0.8085	0.9573	0.8489	0.8157	0.8108
NR_002705	LOC252890	0.6409	0.1560	0.2927	0.1608	0.1695	0.2922	0.1633	0.1585	0.1574	0.5706	0.1575	0.1628
NM_001003957	Dnmt3a	0.7159	0.9958	0.9887	0.9784	0.9085	0.8749	0.9836	0.9783	0.9790	0.9640	0.8396	0.9595
NR_002156	AA926063	0.5109	0.5557	0.2319	0.3414	0.2258	0.2854	0.2872	0.3034	0.2192	0.2363	0.2761	0.6486
NR_002154	E230034O05Ri	0.6640	0.6241	0.6091	0.2538	0.6243	0.6056	0.6973	0.3207	0.6649	0.6746	0.5729	0.4844
NR_002151	RT1-T24-2	0.6576	0.6344	0.2116	0.2287	0.6150	0.6344	0.5722	0.7027	0.6705	0.1832	0.2004	0.5262
NR_002149	E030032D13Ri	0.1966	0.5133	0.4189	0.2403	0.4622	0.5535	0.5466	0.6979	0.4389	0.6724	0.2129	0.7270

NR_024073	Dnlz	0.5087	0.7476	0.5053	0.2546	0.3878	0.5575	0.6851	0.7306	0.3867	0.6917	0.4602	0.5836
NM_030875	Scn1a	0.6526	0.8664	0.5870	0.5590	0.2049	0.4691	0.5889	0.3802	0.3317	0.6138	0.5894	0.3635
NM_020087	Notch3	0.6511	0.5139	0.5111	0.0405	0.4144	0.1023	0.2738	0.5862	0.3954	0.0500	0.5991	0.8764
NR_002707	Defb16-ps	0.3634	0.5569	0.7085	0.4278	0.3120	0.3934	0.4361	0.4029	0.4337	0.6165	0.3834	0.1627
NR_024118	Tnxa	0.2014	0.4743	0.6696	0.2523	0.4995	0.5588	0.7019	0.6618	0.6227	0.6096	0.2111	0.5526
NM_130737	Gucy2d	0.4920	0.5437	0.8798	0.5280	0.4385	0.6361	0.5844	0.2835	0.6415	0.6383	0.5023	0.8817
NM_001100730	Nipsnap1	0.4825	0.8238	0.9278	0.7387	0.7483	0.3374	0.8457	0.8092	0.7604	0.5713	0.5760	0.7871
NM_001136183	LOC10019220[illegible]	0.4854	0.4795	0.3998	0.3512	0.2790	0.1639	0.1631	0.5382	0.4067	0.1544	0.3559	0.2791
NM_001100860	Slc25a16	0.8247	0.8699	0.6458	0.3379	0.4020	0.1767	0.5636	0.3888	0.2138	0.9449	0.6301	0.9004
NM_134463	Sgk2	0.1092	0.1974	0.3843	0.0336	0.2430	0.4230	0.4399	0.3358	0.5126	0.6130	0.8611	0.2181
NM_001134687	Slc35e3	0.7530	0.1105	0.4408	0.4142	0.6489	0.7381	0.2818	0.9671	0.3226	0.1670	0.0117	0.3036
NM_001107062	Cog1	0.8680	0.6346	0.1350	0.9409	0.7960	0.6858	0.6629	0.8569	0.5346	0.7250	0.4504	0.1740
NM_001107335	Dapk1	0.6067	0.8230	0.8530	0.6092	0.4370	0.5617	0.6785	0.4475	0.6953	0.5173	0.7286	0.5155
NM_001012197	Tra1	0.9777	0.9087	0.2943	0.0297	0.6982	0.5921	0.9950	0.3458	0.8951	0.9253	0.8990	0.8170
NM_001108214	Npas2	0.3687	0.2803	0.1925	0.0274	0.3277	0.3989	0.0451	0.0256	0.1765	0.3466	0.2766	0.1566
NM_001136241	Ngef	0.3189	0.5818	0.3110	0.2037	0.4716	0.3818	0.5525	0.2772	0.2711	0.1560	0.7829	0.3493
NM_001136261	LOC10019231[illegible]	0.6701	0.6930	0.7283	0.2345	0.7000	0.6032	0.7063	0.4775	0.6916	0.4447	0.6977	0.5272
NM_001136470	Usp49	0.8641	0.5600	0.4431	0.4591	0.7541	0.7762	0.6016	0.8398	0.4215	0.5113	0.3361	0.6057
NM_012896	Adora3	0.3045	0.8650	0.3552	0.5993	0.7364	0.1120	0.9408	0.6715	0.4708	0.8182	0.6529	0.5605
NM_001107747	Arhgap1	0.9287	0.6805	0.9758	0.9324	0.5033	0.9467	0.9149	0.3283	0.4644	0.5357	0.2224	0.9848
NM_001025702	Phf20l1	0.9739	0.9969	0.7031	0.9427	0.9911	0.9319	0.2704	0.9849	0.9354	0.9578	0.9981	0.9770
NM_001035000	Hdac10	0.2587	0.3011	0.3128	0.5109	0.2514	0.1639	0.3261	0.5625	0.2542	0.2724	0.1573	0.3623
NM_001107794	Myh7b	0.6681	0.6532	0.6087	0.8746	0.7384	0.7965	0.7556	0.8696	0.9283	0.7790	0.8035	0.7796
NM_001137561	Rnasek	0.0688	0.2525	0.5789	0.8907	0.1933	0.3647	0.4710	0.3699	0.3132	0.3111	0.0268	0.6435
NM_001137562	Urm1	0.7638	0.6960	0.7642	0.5823	0.2791	0.1916	0.7249	0.5902	0.5280	0.2910	0.1729	0.5263
NM_001137564	Mt2A	0.5088	0.1559	0.1664	0.1607	0.2681	0.4276	0.1630	0.5262	0.3357	0.2801	0.5044	0.2575
NM_001009622	Sar1b	0.0118	0.3300	0.7093	0.7445	0.0220	0.0521	0.0712	0.1116	0.3301	0.2154	0.0961	0.3290
NM_001009537	Zpf799	0.9744	0.7932	0.8336	0.8078	0.7713	0.8554	0.7543	0.8612	0.9314	0.8662	0.8594	0.8042
NM_001007739	Sar1a	0.9143	0.5654	0.8788	0.7921	0.9335	0.8525	0.8438	0.8144	0.8393	0.8715	0.9602	0.7795
NM_001003974	Gpsm3	0.4602	0.2939	0.1838	0.8149	0.1157	0.1750	0.2050	0.2925	0.3595	0.6586	0.6260	0.4914
NM_001002828	Pbx2	0.7642	0.8149	0.5479	0.7781	0.6251	0.8286	0.9094	0.8316	0.9296	0.9273	0.4428	0.6464
NM_001137626	E2f3	0.0845	0.4357	0.6316	0.3827	0.4152	0.4142	0.6912	0.3694	0.7822	0.7482	0.6859	0.6030
NM_001137622	Adamts2	0.8127	0.8205	0.7696	0.8987	0.8608	0.8163	0.8465	0.8467	0.7728	0.8165	0.8829	0.9341
NM_001137647	Sh3bgrl2	0.4330	0.5808	0.2404	0.6207	0.5007	0.3251	0.4593	0.3971	0.5118	0.3309	0.4773	0.1111
NM_001137646	Zfp259	0.0668	0.3044	0.2193	0.3018	0.4376	0.9088	0.1073	0.7593	0.0118	0.5884	0.4105	0.8729
NM_001137645	Fgd6	0.7782	0.9813	0.1973	0.1242	0.2472	0.4698	0.6200	0.4593	0.3489	0.4017	0.3875	0.0263
NM_001137644	Tnfrsf25	0.8991	0.7287	0.3589	0.8394	0.6061	0.9242	0.7476	0.7392	0.8851	0.7350	0.8862	0.7882
NM_001137643	Gstt3	0.3019	0.2210	0.1326	0.3438	0.3867	0.2182	0.4792	0.3582	0.7423	0.5816	0.0867	0.7132
NM_001137642	Kif18a	0.2734	0.0642	0.2009	0.1350	0.2357	0.2326	0.2046	0.0904	0.1327	0.1921	0.1853	0.3115
NM_001137641	Lmtk2	0.9644	0.9629	0.9747	0.9219	0.9078	0.9784	0.9414	0.9256	0.9220	0.9468	0.9074	0.9717
NM_001025011	RGD1305464	0.6103	0.4995	0.6126	0.5507	0.4756	0.5875	0.5700	0.4662	0.4641	0.5967	0.5979	0.5602
NM_001137633	Tnfaip2	0.8178	0.3093	0.9226	0.5866	0.8255	0.9613	0.8878	0.6453	0.5112	0.2929	0.2689	0.9718
NM_001139494	RGD1561509	0.3801	0.2816	0.1330	0.4385	0.5421	0.3613	0.6904	0.1290	0.3089	0.3011	0.3229	0.4870
NM_001139506	RGD1310429	0.7669	0.6643	0.3094	0.4742	0.1032	0.8953	0.5319	0.3064	0.3641	0.3134	0.5349	0.8924
NM_001139484	Znf644	0.8157	0.4193	0.9663	0.9803	0.8080	0.8318	0.9362	0.8685	0.9073	0.8402	0.8778	0.8861
NM_001139491	LOC680200	0.2918	0.1556	0.4504	0.1606	0.4288	0.4393	0.1628	0.1584	0.1572	0.3803	0.4087	0.1625
NM_001139486	Gpr89	0.5327	0.4695	0.4746	0.6837	0.6035	0.7373	0.6273	0.9091	0.6261	0.8034	0.7699	0.5230
NM_001139483	Aifm2	0.5947	0.5012	0.4430	0.9260	0.6607	0.5194	0.9762	0.6113	0.8051	0.6204	0.9273	0.8755
NM_001139493	Qser1	0.8931	0.7790	0.8547	0.2254	0.8473	0.5164	0.8543	0.3288	0.1344	0.7682	0.9193	0.9418

NM_001139487	LOC10012536‹	0.2208	0.2640	0.1664	0.2919	0.5282	0.3948	0.3782	0.3573	0.2057	0.3978	0.3525	0.1624
NM_001139507	Hcfc1	0.9503	0.8739	0.3811	0.2501	0.9813	0.7880	0.9398	0.8570	0.9921	0.9594	0.9736	0.9484
NM_024127	Gadd45a	0.0635	0.3157	0.5377	0.0853	0.0048	0.1421	0.2483	0.0518	0.0067	0.1826	0.1082	0.0553
NM_021844	Kitlg	0.7189	0.6638	0.9273	0.5381	0.6177	0.8483	0.9413	0.8929	0.5929	0.8814	0.9451	0.4588
NM_001141935	Atp10a	0.8992	0.7675	0.7532	0.2689	0.5883	0.4249	0.7376	0.5433	0.6770	0.8749	0.9376	0.8278
NM_001106389	Asf1a	0.9865	0.9827	0.9467	0.8030	0.7640	0.3495	0.8064	0.3764	0.6169	0.7791	0.9256	0.7933
NM_001141929	Rpp14	0.2026	0.6623	0.6627	0.9886	0.8030	0.5659	0.8860	0.8663	0.6134	0.6253	0.4844	0.5587
NM_001108381	Ebpl	0.7861	0.2625	0.0292	0.1028	0.1997	0.2609	0.6161	0.5490	0.1811	0.2969	0.1455	0.1638
NM_001100895	Atf1	0.5649	0.2228	0.9147	0.4453	0.7528	0.9580	0.5633	0.3506	0.1767	0.2754	0.9150	0.4436
NM_019300	Cpa3	0.2618	0.6062	0.5735	0.3424	0.6200	0.1622	0.2681	0.5086	0.3974	0.5571	0.4778	0.1328
NM_001100505	Asna1	0.0638	0.1101	0.6856	0.2410	0.2345	0.5096	0.1024	0.5879	0.3794	0.5168	0.1218	0.3019
NM_001100533	Btbd14a	0.9773	0.8684	0.9471	0.8580	0.9797	0.9711	0.9951	0.9543	0.6612	0.8794	0.6984	0.7638
NM_001100671	Arl6ip2	0.8851	0.8423	0.6609	0.2890	0.6745	0.6259	0.3625	0.1381	0.5875	0.9382	0.9594	0.7912
NM_001100685	Clybl	0.1937	0.1695	0.6523	0.3998	0.5579	0.2712	0.4809	0.6170	0.8729	0.6601	0.4651	0.7951
NM_001100710	Alg2	0.9611	0.1878	0.9883	0.9498	0.2086	0.8300	0.4062	0.2812	0.3037	0.1961	0.3470	0.9239
NM_001100728	Ccdc43	0.3629	0.3286	0.8690	0.8826	0.2722	0.2532	0.9320	0.2524	0.3402	0.1917	0.7456	0.8953
NM_001100794	Bxdc5	0.0441	0.5944	0.2351	0.1070	0.4445	0.5896	0.3377	0.1574	0.1858	0.2118	0.3174	0.7795
NM_001100903	Brd4	0.8428	0.5868	0.4727	0.3339	0.4535	0.8195	0.2816	0.4557	0.9177	0.9425	0.0144	0.3466
NM_031717	Amotl2	0.3156	0.4375	0.6841	0.5837	0.6518	0.5577	0.5564	0.6507	0.7115	0.4907	0.6493	0.6129
NM_001100735	Lpcat1	0.3417	0.0978	0.2909	0.9528	0.2067	0.0882	0.2827	0.0549	0.3030	0.2229	0.2926	0.8057
NM_001100519	Gga2	0.3885	0.6320	0.9510	0.3772	0.4185	0.9196	0.8851	0.3542	0.3918	0.8649	0.3793	0.9726
NM_001100556	Gtf2e1	0.1722	0.4165	0.7368	0.3245	0.2634	0.2351	0.0575	0.3041	0.1488	0.2645	0.5550	0.2973
NM_001100512	Gpld1	0.7887	0.1286	0.6920	0.7398	0.3947	0.8127	0.7575	0.7531	0.7178	0.3098	0.6434	0.6456
NM_001100504	Gadd45gip1	0.1507	0.8712	0.0089	0.2762	0.1068	0.2861	0.1858	0.0275	0.2258	0.2295	0.1308	0.0818
NM_001100518	Hs2st1	0.4472	0.4227	0.9347	0.9717	0.8805	0.9621	0.9194	0.8817	0.9351	0.7734	0.9556	0.7271
NM_001100771	Esf1	0.8734	0.4968	0.4826	0.4943	0.3030	0.4474	0.0212	0.4052	0.1127	0.1631	0.1937	0.7637
NM_001100780	Hyal4	0.7997	0.8318	0.7051	0.5653	0.6943	0.8238	0.9503	0.7962	0.6417	0.8941	0.9031	0.7830
NM_053875	Exoc4	0.0775	0.1214	0.2556	0.5594	0.1703	0.3708	0.4424	0.2853	0.0358	0.0901	0.3831	0.1283
NM_001100536	Magoh	0.0882	0.5102	0.0101	0.1195	0.3455	0.1284	0.1449	0.0991	0.2356	0.8117	0.5693	0.0047
NM_001100887	Glt8d3	0.3735	0.4691	0.2626	0.6166	0.4405	0.7521	0.0169	0.0873	0.6928	0.5558	0.7002	0.0816
NM_001100722	Lingo1	0.5492	0.4501	0.2850	0.1445	0.2284	0.3458	0.1331	0.5917	0.2145	0.4524	0.3265	0.7177
NM_001100725	Exosc7	0.6112	0.8877	0.5067	0.8071	0.5140	0.1466	0.5846	0.8894	0.8023	0.8363	0.7766	0.1646
NM_053384	Ihh	0.2022	0.4229	0.6547	0.3618	0.3252	0.6023	0.7048	0.6786	0.3754	0.1750	0.4693	0.5166
NM_053366	Rab6a	0.9638	0.8878	0.8483	0.9134	0.9323	0.9224	0.9520	0.8636	0.8756	0.9395	0.9559	0.5250
NM_001129777	Rad51c	0.3770	0.3785	0.9344	0.4735	0.4431	0.8303	0.7331	0.6144	0.5322	0.1312	0.1823	0.5717
NM_001100834	Ppa1	0.6249	0.8822	0.0420	0.1028	0.8489	0.7601	0.7306	0.7248	0.7213	0.7504	0.9853	0.3027
NM_001100831	Psmd8	0.3959	0.6477	0.0144	0.1080	0.0919	0.0930	0.0563	0.0976	0.1124	0.8994	0.2756	0.1685
NM_001100582	Ppfibp2	0.9091	0.9648	0.6300	0.9608	0.3685	0.2713	0.2874	0.6683	0.7105	0.9403	0.8869	0.7555
NM_001100898	Mtf2	0.9572	0.9416	0.2099	0.1371	0.5382	0.8255	0.5733	0.6555	0.9022	0.9462	0.9429	0.6878
NM_001100508	Oxsm	0.4196	0.6410	0.4124	0.5673	0.7341	0.3097	0.2382	0.5803	0.3273	0.4982	0.2256	0.7060
NM_001100509	Psmc6	0.5824	0.6797	0.6816	0.8888	0.6076	0.6551	0.5034	0.8747	0.5115	0.7474	0.4425	0.4931
NM_001100570	Myst3	0.5605	0.9330	0.6847	0.3291	0.6956	0.8813	0.7323	0.7450	0.9514	0.8020	0.9274	0.8860
NM_031080	Pde7a	0.9844	0.9984	0.9954	0.9998	0.9482	0.9998	0.9852	0.9188	0.9628	0.9922	0.9998	0.9991
NM_001100749	Mxd1	0.9027	0.8401	0.9139	0.8889	0.8800	0.9351	0.9238	0.7582	0.9635	0.9256	0.9824	0.9362
NM_133407	Map4k3	0.7130	0.2890	0.3003	0.3347	0.3073	0.2686	0.2223	0.2761	0.2189	0.3425	0.2343	0.1158
NM_013093	Nkx2-1	0.5800	0.4424	0.8081	0.7005	0.8928	0.3374	0.4093	0.5977	0.3133	0.2729	0.6750	0.6624
NM_001100886	Mbrl	0.7120	0.9071	0.0250	0.2208	0.7733	0.7478	0.0266	0.9929	0.7456	0.9224	0.4258	0.6333
NM_133530	Mmp13	0.9488	0.9311	0.9256	0.6386	0.4414	0.9072	0.6233	0.1753	0.8767	0.7055	0.7638	0.1129
NM_001139465	Tmem59	0.6788	0.3953	0.6772	0.5920	0.2951	0.2266	0.2592	0.6095	0.5049	0.6304	0.5854	0.4134

NM_001108097	Frs2	0.7844	0.7216	0.8963	0.6312	0.9252	0.8138	0.7743	0.8960	0.8146	0.4029	0.8580	0.4253
NM_001109976	Map3k7ip1	0.8834	0.9384	0.7079	0.9084	0.8900	0.5226	0.8756	0.7734	0.9562	0.6510	0.6625	0.7933
NM_053354	Dnmt1	0.8531	0.9028	0.0926	0.8469	0.9626	0.9892	0.9755	0.9878	0.9423	0.9587	0.9567	0.8645
NM_001013108	Arih1	0.9252	0.9885	0.7703	0.9855	0.2923	0.3505	0.8190	0.3129	0.9731	0.9735	0.9108	0.9041
NM_001108809	Atg16l1	0.8895	0.6521	0.4360	0.2441	0.8483	0.8472	0.3138	0.3485	0.8201	0.6145	0.5634	0.8512
NM_001142371	Pnpt1	0.7490	0.5961	0.6082	0.3022	0.0478	0.0133	0.1100	0.0161	0.8692	0.9395	0.4629	0.6503
NM_173107	Stox2	0.9014	0.9386	0.9375	0.7991	0.6786	0.8533	0.9054	0.6281	0.6341	0.9283	0.6379	0.6101
NM_001142366	Aqp4	0.9700	0.3676	0.9416	0.6294	0.9581	0.7459	0.9348	0.9595	0.9736	0.6845	0.9772	0.8967
NM_012825	Aqp4	0.9700	0.3676	0.9416	0.6294	0.9581	0.7459	0.9348	0.9595	0.9736	0.6845	0.9772	0.8967
NM_001100702	Wdr47	0.8826	0.5531	0.8888	0.5284	0.7829	0.9362	0.5415	0.8250	0.4820	0.6160	0.9216	0.4736
NM_001013100	Abcd4	0.6125	0.5311	0.2690	0.3218	0.8198	0.6268	0.6724	0.9405	0.4675	0.4257	0.8641	0.4986
NM_001108104	Alg12	0.3069	0.8652	0.3450	0.1224	0.6134	0.3337	0.8896	0.0212	0.8869	0.3705	0.3952	0.7778
NM_001142562	Hexdc	0.6450	0.4460	0.3561	0.2770	0.7222	0.5139	0.6777	0.4596	0.4963	0.7138	0.2454	0.4631
