# Supplementary material for: KSHV MicroRNAs Mediate Cellular Transformation and Tumorigenesis by Redundantly Targeting Cell Growth and Survival Pathways
Source: PLoS Pathog. 2013 Dec 26;9(12):e1003857. doi: 10.1371/journal.ppat.1003857 (PMC3873467; doi:10.1371/journal.ppat.1003857)
Supplement: Table S5 — Expression fold change (log2) of MTKi, MutCl and WT cells vs. MTVt cells. (PDF) [file ppat.1003857.s016.pdf]

Table S5. Expression fold change (log2) of MTKi, MutCI and WT cells vs. MTVt cells

Refseq	Gene symbol	K1	K2	K3	K4	K5	K6	K7	K8	K9	K10	K11	K12	CL	WT
NM_001024872	2610020o08rik	0.50619	0.1328	0.29829	0.32954	0.58787	0.47555	0.24774	0.29888	0.23291	0.21355	0.30952	0.19356	0.29543	0.40167
NM_052979	A	0.16002	0.23367	0.06813	0.4131	0.19914	0.04591	0.07762	0.38038	0.29372	0.17674	0.08929	0.36467	0.0265	0.07158
NM_022258	A1bg	-0.0337	0.09966	0.26514	-0.0858	0.02836	0.03948	0.01577	0.0987	0.11813	0.02268	0.22273	-0.0569	0.00106	-0.0319
NM_012488	A2m	-0.3116	-0.3878	-0.4165	-0.495	-0.4254	-0.3561	-0.4445	-0.4571	-0.3912	-0.0811	-0.3725	-0.3833	-0.4571	-0.4732
NM_138524	A3galt2	-0.1909	0.02366	0.01324	-0.9876	0.02333	0.0154	-0.6944	0.00548	0.11929	-0.0175	0.05282	-0.2137	-0.0618	-0.0596
NM_022240	A4galt	0.15303	0.11331	0.17839	0.02583	0.01005	0.10787	-0.0109	-0.1286	0.14877	0.121	0.08634	0.03149	0.02283	0.0311
NR_002156.1	AA926063	-0.2198	-0.0755	0.16435	-0.2421	-0.0668	-0.0454	-0.0351	-0.2006	0.07509	0.06829	0.08862	0.26914	0.0556	0.0504
NM_001106795	Aaas_predicted	0.05223	-0.4654	0.97695	0.31937	-0.1991	-0.246	-0.2899	-0.4115	-0.2522	0.00986	-0.41	0.21613	0.22875	0.33509
NM_023104	Aacs	-0.2351	0.30768	0.62893	-0.3166	-0.2555	-0.475	0.17888	-0.3069	0.11977	0.13727	0.1728	0.04004	0.40139	0.486
NM_020538	Aadac	0.08664	0.10218	0.19993	0.09692	0.05916	0.08907	0.03565	0.0527	0.06346	0.0687	-0.0392	0.05441	0.18991	0.03273
NM_001127524	Aadacl1_predicted	0.65039	0.7314	0.17541	-0.0267	0.40383	0.1605	0.46965	0.69764	0.55258	0.86683	0.37625	-0.0278	0.56081	0.48821
NM_017193	Aadat	1.0623	0.60284	0.55145	0.67084	0.88528	0.8592	2.4008	0.8536	0.52535	0.4445	0.66648	0.19589	0.85171	0.68664
NM_001106920	Aamp_predicted	0.09036	0.11632	0.09358	0.03723	0.13404	0.07705	0.06473	0.04439	-0.1341	0.04309	-0.0843	0.32663	0.08311	-0.0483
NM_012818	Aanat	0.02173	0.13657	0.02992	0.15787	0.17605	0.09726	0.04473	-0.0627	0.09622	0.02151	-0.0049	0.02079	0.05818	0.07361
NM_145093	Aard	0.0421	0.05815	0.00915	0.00669	0.09209	0.10708	-0.0605	-0.0825	0.09947	0.13535	-0.0932	-0.0058	-0.0114	-0.0237
NM_001100517	Aars	0.06372	-1.0802	0.27708	-0.5135	-0.5042	-0.5737	-0.4004	-0.2903	-0.5029	-0.6979	-0.6903	-0.068	0.23684	0.20721
NM_001106891	Aarsl_predicted	-0.2141	-0.2293	0.09351	0.11713	-0.0404	-0.0504	-0.0661	0.03809	0.03687	-0.1843	-0.0944	0.01732	-0.056	0.00039
NM_001106798	Aasdhppt_predicted	-0.5235	-0.3631	-0.2578	-0.5122	-0.4743	-0.382	-0.4573	-0.2667	-0.1458	-0.1918	-0.2578	-0.4554	-0.112	-0.4012
NM_001100963	Aass_predicted	-0.0337	0.08427	-0.09	-0.0994	-0.1775	-0.2076	0.16135	-0.1629	-0.047	-0.1623	-0.204	0.08618	-0.2285	0.06556
NM_053720	Aatf	-0.1269	-0.4705	0.09959	-0.3702	-0.1272	0.25391	-0.1808	-0.1337	-0.3425	-0.1769	-0.3481	0.12725	0.26069	0.27126
NM_031003	Abat	0.05204	0.01753	-0.0774	-0.0655	0.17873	0.34677	0.2393	0.32591	0.27069	0.01013	-0.099	0.23723	-0.0108	-0.0862
NM_178095	Abca1	1.6601	1.4867	0.66817	2.3006	2.1416	1.9516	1.665	2.0853	1.1721	1.2666	1.132	0.23462	0.36223	0.24716
XM_237242.4	Abca12_predicted	0.12091	-0.118	-0.0942	0.00087	-0.2113	0.06793	-0.1195	-0.1841	-0.152	-0.2367	-0.0874	-0.0826	-0.1941	-0.1644
NM_001106020	Abca13_predicted	0.00172	0.12845	-0.2733	0.17785	0.0377	-0.0108	-0.2431	-0.0782	-0.0016	-0.101	-0.0462	-0.0899	-0.0575	-0.2545
NM_001106293	Abca15_predicted	-0.0297	-0.1554	-0.0118	0.0205	-0.1304	0.05308	-0.0231	-0.139	-0.1594	-0.2257	-0.2143	-0.1475	-0.1645	-0.0405
NM_024396	Abca2	-0.6141	-0.4479	-0.3193	-0.4008	-0.3	-0.1962	-0.5229	-0.2823	-0.6182	-0.442	-0.6092	-1.077	-1.1195	-0.8753
XM_001054650.1	Abca3	0.13225	0.24963	0.44782	0.25909	0.20768	-0.0041	0.41328	0.0349	-0.2426	0.03803	0.24285	-0.3571	0.3311	0.35731
NM_001107721	Abca4_predicted	0.00221	0.23073	0.12381	0.10629	0.56477	0.64262	0.16026	0.11665	0.40078	0.28931	-4E-05	0.09212	0.019	0.13371
NM_173307	Abca5	-0.4516	-0.1943	0.10536	-0.0003	-0.2378	0.10615	-0.3672	-0.3132	-0.2923	-0.2474	-0.0512	-0.3081	-0.068	-0.3166
XM_001081607.1	Abca6_predicted	0.06152	0.03982	-0.0317	0.0329	0.11199	0.02495	0.04631	-0.068	0.05352	0.04171	-0.0348	0.09357	0.0093	0.02387
NM_207598	Abca7	-0.0916	-0.1912	0.66452	1.2944	0.10934	0.23748	0.50874	0.05155	0.02551	0.16407	0.03809	0.70801	-0.0275	-0.293
XM_001081603.1	Abca8a_predicted	0.01657	0.07397	-0.0681	0.06593	0.11772	0.03721	0.07929	-0.0016	0.03895	-0.0828	0.26315	-0.0552	0.08746	-0.0074
XM_001081601.1	Abca8b_predicted	-0.0719	0.01998	0.02709	-0.0025	0.19328	-0.0361	-0.0431	-0.1098	-0.064	0.0802	0.0125	0.01937	-0.012	0.10161
XM_001081601.1	Abca8b_predicted	-0.2266	-0.2169	-0.3289	-0.1181	-0.0326	-0.1689	-0.2146	-0.2588	-0.2123	-0.1172	-0.3122	-0.0595	-0.1572	-0.1496
XM_221101.3	Abca9_predicted	0.11542	0.04439	-0.0295	-0.1967	0.01165	-0.1422	-0.1512	0.04851	-0.0687	-0.0519	-0.2025	-0.1589	-0.042	0.05676
NM_001012166	Abcb10	-0.2203	0.42089	0.16144	0.07509	0.29653	-0.2919	0.11627	0.04495	0.24864	0.15336	0.22038	0.07438	0.1491	0.16476
NM_001012166	Abcb10_predicted	-0.0798	-0.0636	0.05903	-0.1047	-0.0441	0.12033	0.00689	0.22308	0.06221	-0.0047	0.20047	-0.0435	-0.0295	0.03615
NM_031760	Abcb11	0.06963	0.15411	-0.1203	-0.232	-0.0702	-0.0459	-0.1387	0.20432	-0.1123	-0.0872	-0.1225	-0.0951	-0.1067	0.01558
NM_031760	Abcb11	0.28675	-0.0194	0.13524	0.05635	0.19407	0.18921	-0.0232	-0.0661	-0.1445	0.06959	-0.0072	-0.0423	0.08698	0.02411
NM_012623	Abcb1b	1.1774	0.10098	0.68705	2.4863	1.1138	1.1405	1.6426	0.90274	0.55417	0.33892	0.21669	0.68795	0.537	0.47576
NM_012690	Abcb4	0.18447	0.13596	0.04594	-0.0248	0.1002	0.28323	-0.0309	-0.0496	0.08603	0.05224	-0.029	-0.0071	0.08875	-0.0458
NM_012690	Abcb4	0.01024	0.076	-0.0812	-0.0928	-0.1528	0.13923	-0.1384	0.14404	-0.2065	0.01151	-0.1021	0.08086	-0.1225	-0.0426
NM_080582	Abcb6	-1.1665	-1.0743	0.35446	-0.4895	-1.0073	-0.9972	-0.7359	-0.9578	-0.9578	-1.1113	-0.7581	-0.3071	-0.4524	-0.3827
NM_212518	Abcb7	0.32444	0.08122	0.14794	-0.1537	0.48802	0.54047	0.06398	0.47015	0.29926	-0.0739	-0.2299	0.03468	0.40321	0.3811
NM_001007796	Abcb8	-0.1707	-0.1472	0.12912	-0.1366	-0.5786	-0.5085	-0.2459	-0.648	-0.3539	-0.2995	-0.3895	-0.3145	-0.4075	-0.1733

NM_022238	Abcb9	-0.0406	0.10656	-0.0812	-0.0035	0.05043	-0.0743	0.12912	-0.0544	0.00538	-0.0015	-0.1067	0.12417	-0.0666	0.16582
NM_022281	Abcc1	-0.1892	0.17603	-0.1901	0.19961	-0.0054	-0.2159	0.21745	0.03152	0.24312	0.06365	-0.1231	-0.2509	-0.0436	-0.0597
NM_001108201	Abcc10_predicted	0.02261	-0.0262	0.02083	-0.0874	0.06211	-0.1055	-0.0048	0.02618	0.01064	0.08553	-0.0642	0.13258	-0.0645	-0.1143
NM_001108201	Abcc10_predicted	-0.1252	-0.1701	0.1785	-0.2276	-0.2586	-0.1409	-0.1648	-0.2058	-0.0263	-0.2938	-0.1001	-0.3095	-0.2586	-0.2644
NM_199377	Abcc12	-0.0876	0.0439	0.09175	-0.0417	0.03246	-0.1242	-0.2542	0.03365	0.01156	0.01251	-0.1107	0.00411	0.2663	-0.0326
NM_012833	Abcc2	0.43273	0.07523	0.39333	0.19433	0.26762	0.53088	0.28064	0.25627	0.44488	0.62765	0.09061	0.66005	0.25136	0.27967
NM_080581	Abcc3	-0.93	-0.4737	-0.1889	-0.3736	-1.0705	-0.8529	-0.3061	-1.0354	-0.5379	-0.3516	-0.3208	-0.4574	-0.6361	-0.7362
NM_133411	Abcc4	0.15206	0.1031	0.06102	0.02241	-0.0279	-0.0242	-0.0648	-0.1174	-0.0532	0.04751	0.00837	-0.0155	0.0849	0.0086
NM_053924	Abcc5	-0.4676	0.21761	1.165	0.58019	-0.9027	-0.7983	1.0032	-1.0204	-0.0197	0.40942	0.30696	-0.1644	-0.7731	-0.9064
NM_031013	Abcc6	0.2978	0.08469	0.29743	-0.0217	0.70452	0.44178	0.31094	0.24719	-0.0127	-0.0532	0.462	0.04422	-0.0477	-0.0288
NM_013039	Abcc8	0.16146	0.00739	0.27595	0.05671	0.0969	0.01132	0.12247	0.1179	-0.0609	0.10312	0.35089	0.1853	0.11139	0.23901
NM_013040	Abcc9	-0.1155	-0.0804	-0.0862	0.57997	-0.0966	-0.1076	-0.0882	-0.2061	-0.0314	-0.1892	-0.0573	0.34247	-0.0644	0.0681
NM_033352	Abcd2	-0.0678	-0.1121	-0.1437	0.12744	-0.015	-0.1128	-0.1768	0.11867	0.14185	-0.2358	-0.1439	-0.006	-0.2045	-0.1546
NM_012804	Abcd3	-0.1038	-0.444	-0.0545	-0.4285	-0.3417	-0.0553	-0.3551	-0.3359	-0.1299	-0.286	-0.1911	0.39684	-0.182	-0.3687
NM_001013100	Abcd4	-0.43	-0.2364	0.88	0.68549	-0.2976	-0.0299	-0.1605	-0.3752	-0.0854	-0.43	-0.2852	0.55515	-0.2825	-0.0465
NM_001013100	Abcd4	-0.1344	0.07375	-0.446	-0.4121	0.07131	-0.3653	-0.1445	-0.1487	0.10859	0.04782	-0.4009	-0.2439	0.14703	-0.0175
NM_001108446	Abce1	0.25892	-0.0937	0.60054	0.71136	0.15154	0.73949	0.22618	0.08062	-0.0124	-0.0496	0.19274	0.65434	0.25889	0.72455
NM_001109666	Abcf2_predicted	0.03642	-0.2773	0.43025	-0.0327	0.06379	-0.576	0.05528	0.0093	-0.481	-0.0945	-0.1291	-0.5398	0.42926	0.58707
NM_001011896	Abcf3	0.05598	0.23747	0.57741	0.50167	0.35495	0.35451	0.45339	0.13477	0.0665	0.2333	0.19669	0.02078	0.2101	0.32166
NM_053502	Abcg1	0.11436	2.1184	0.79688	0.82142	0.56975	0.90748	2.3662	0.85896	1.4129	1.7465	1.545	0.53119	0.11864	0.13989
NM_181381	Abcg2	-0.1669	0.02169	-0.1252	-0.0084	-0.0941	-0.0433	-0.0326	0.00855	-0.0381	-0.1192	-0.0854	-0.1038	0.02221	0.11458
NM_001014133	Abcg3l1	-0.3754	0.17359	-0.2215	0.13231	-0.4749	-0.4567	0.02767	-0.1975	0.1205	0.15613	-0.0682	-0.2097	-0.3332	-0.3061
NM_001106816	Abcg4	-0.3311	-0.3408	-0.0011	0.36919	-0.1588	-0.0659	-0.1344	-0.498	-0.4538	0.04335	-0.2787	0.05493	-0.2656	-0.3038
NM_053754	Abcg5	0.04824	-0.2433	-0.1023	-0.1865	-0.23	-0.2175	-0.154	-0.1058	-0.1642	-0.167	-0.0394	-0.1226	-0.1479	-0.1802
NM_130414	Abcg8	-0.0104	0.04737	0.09995	0.05	0.37226	0.03051	0.08425	0.08244	0.04969	0.01337	0.06011	0.09036	0.04734	-0.0292
NM_001008520	Abhd1	-0.3283	-0.4538	-0.4265	-0.393	-0.5718	-0.3924	-0.4853	-0.565	-0.3697	-0.3426	-0.3137	-0.5248	-0.2345	-0.2894
NM_001008520	Abhd1	-0.0268	0.17978	0.09964	0.20737	-0.0492	0.05091	-0.0442	0.08767	0.05144	-0.1059	-0.0913	-0.1643	0.00357	0.16345
NM_001009670	Abhd14a	0.01309	0.10821	0.04902	0.36538	0.31296	0.71511	0.13427	0.3215	0.01327	0.10784	0.04396	0.1019	-0.051	0.09945
NM_001007664	Abhd14b	-0.5088	-0.0013	-0.4942	-0.9362	-0.4592	-0.4778	-0.504	-0.4459	-0.0457	-0.0004	0.04966	0.02358	-0.6728	-0.6337
NM_001106275	Abhd2_predicted	-0.4686	-0.5087	-0.1047	-0.4535	-0.1717	-0.0102	-0.3935	-0.0917	-0.4195	-0.4565	-0.4316	-0.0858	0.08765	0.03573
NM_001106162	Abhd3_predicted	0.81877	0.31807	0.43024	2.1228	1.5758	1.38	0.64515	1.6202	0.34777	0.66066	0.73725	0.03725	0.59749	0.60292
NM_001108866	Abhd4_predicted	0.12838	-0.0949	-0.4352	0.14439	0.21309	0.35233	-0.1456	0.28532	0.09791	-0.2405	-0.2428	0.18355	-0.1794	-0.2431
NM_212524	Abhd5	1.0358	1.2115	0.24172	1.7805	1.3021	0.92618	0.81053	1.4141	0.86401	1.036	0.61908	0.24033	1.328	1.351
NM_001007680	Abhd6	0.03909	0.63198	0.05444	0.52292	-0.036	0.14449	0.07356	-0.1711	0.34213	0.28392	0.56679	0.29091	-0.4134	-0.351
NM_001105994	Abhd7_predicted	0.1973	-0.0051	0.13793	0.14378	0.13896	0.09101	0.13682	0.14949	-0.0757	0.19466	0.17399	-0.2235	0.0184	0.03362
NM_001107301	Abhd8_predicted	0.38448	0.17343	-0.5057	0.1989	0.4707	0.48842	-0.3631	0.45899	0.37209	0.17247	0.25161	0.43327	-0.1632	-0.2461
NM_001108988	Abhd9_predicted	0.03935	-0.0046	0.07716	-0.1241	0.0255	0.02004	-0.0404	0.10724	-0.1127	-0.2153	-0.2214	-0.1103	0.14286	0.16361
NM_024397	Abi1	-0.3522	0.26258	0.36981	0.0558	-0.0326	-0.8261	0.52711	0.01114	-0.0223	0.01151	-0.2192	-0.4732	0.2062	0.29283
NM_001013118	Abi3	-0.1356	-0.1706	-0.1374	-0.2076	-0.1521	-0.1775	-0.0861	-0.2139	-0.1296	-0.0392	-0.1237	0.26915	-0.0219	-0.2169
XM_231137.4	Abl1_mapped	-0.5867	0.05649	0.15885	0.02403	-0.4474	-0.6926	0.09963	-0.4286	0.02074	-0.2933	-0.0951	-0.0971	-0.0984	0.00239
NM_001107186	Abl2	0.02221	-0.0876	-0.1628	-0.2024	-0.2439	0.01671	0.00317	-0.2928	-0.1968	-0.0668	-0.2231	-0.1883	0.01001	0.05461
NM_001001514	Ablim2	-0.1863	-0.1912	-0.0932	0.00139	-0.3055	-0.1347	-0.1326	0.149	-0.0861	-0.1301	-0.2197	-0.0639	0.05881	-0.2967
NM_023094	Abo	0.07282	0.23218	0.01671	0.09192	0.09379	0.02248	-0.052	0.00178	0.23608	0.2723	0.12084	0.49518	-0.038	0.2473
XM_215986.4	Abo_predicted	-0.0546	0.03757	0.13537	-0.0491	0.08495	-0.0758	0.00886	0.14247	0.07506	0.19375	0.01651	0.19438	-0.0025	-0.043
NM_022935	Abp1	-0.0779	0.03938	-0.0071	0.17339	0.09992	0.07041	-0.0026	0.12909	0.07127	-0.0356	-0.0032	0.11376	0.23821	-0.1005
NM_001100859	Abpa_predicted	0.10427	0.06158	-0.0096	0.05327	-0.0466	0.15551	0.00114	-0.1025	-0.038	0.05163	0.05745	-0.1352	0.25982	0.2575
NM_001105814	Abr_predicted	0.22299	0.53896	0.83854	-0.5425	-0.1893	-0.3437	0.28882	-0.165	0.11065	0.32573	0.20499	0.11719	0.3583	0.55909
NM_175844	Abra	0.00256	-0.238	0.00186	0.03532	0.03635	0.41745	-0.0269	0.12872	-0.1554	-0.2865	-0.1133	-0.2174	-0.062	-0.0317
NM_001025674	Abt1	-0.019	-0.0819	-0.1751	0.08748	0.28398	0.38972	0.01075	0.23555	0.11751	-0.1621	0.00556	0.0297	0.08095	0.04458

NM_001005902	Abtb1	-0.5677	-0.6552	-0.6449	-0.8135	-0.6774	-0.1883	-0.4267	-0.7372	-0.7082	-0.5716	-0.5042	-0.5605	-1.1002	-0.8667
NM_134403	Abtb2	1.0266	0.8413	0.30605	0.64084	1.0055	0.96605	0.79466	0.80701	0.47332	1.0214	0.66656	-0.0243	0.3688	0.13081
NM_012489	Acaa1	-0.0548	0.16787	0.10304	-0.4654	-0.1387	0.00652	-0.1536	-0.2777	0.17645	0.06477	0.29339	-0.0574	-0.5652	-0.3796
NM_130433	Acaa2	-0.4149	0.13452	-1.0341	-0.7605	-0.1473	-0.1381	-0.4328	-0.508	-0.2361	-0.0662	-0.1061	0.12059	-0.877	-0.5822
NM_022193	Acaca	0.09424	0.07963	0.12018	0.28909	0.16604	0.23087	0.32959	0.17021	0.41191	-0.0429	0.11407	-0.0255	0.18928	0.38258
NM_181768	Acad9	0.11235	0.1512	0.18589	-0.2722	0.15548	0.04085	-0.0949	0.07267	0.2246	-0.135	-0.0509	-0.0416	0.42362	0.30926
NM_012819	Acadl	0.1126	-0.1375	-0.4613	-0.7135	-0.1006	-0.2721	-0.2662	-0.069	-0.0461	-0.1038	-0.1629	-0.3076	-0.3913	-0.4192
NM_016986	Acadm	0.78152	0.22931	-0.1579	-0.0174	0.05488	-0.1415	0.29843	-0.111	0.27656	0.36152	0.22124	-0.1319	-0.1739	-0.2046
NM_022512	Acads	0.29137	0.02609	-0.4054	0.17973	0.47397	0.48279	-0.0808	0.36796	-0.1222	0.15556	0.07514	0.15333	-0.2712	-0.4042
NM_013084	Acadsb	0.17973	0.17977	-0.0557	-0.0906	0.01471	-0.1304	-8E-05	-0.1041	0.15312	0.01578	-0.0613	0.0836	0.16155	0.01696
NM_012891	Acadvl	0.14322	0.3283	-0.4478	-0.7209	0.1441	0.40417	-0.2749	0.06599	0.27812	0.40133	0.09923	0.07219	-0.3327	-0.5313
NM_022190	Acan	-0.5389	-0.5297	-0.2789	-0.4384	-0.2093	-0.2564	-0.479	-0.0521	-0.2808	-0.4571	-0.2629	-0.361	-0.4078	-0.4447
NM_017075	Acat1	-0.477	-0.1455	-0.4384	-0.6801	-0.5938	-0.8373	-0.3978	-0.5072	-0.1618	-0.3273	-0.3022	-0.1539	-0.2989	-0.0655
NM_001006995	Acat2	-0.9616	-1.1291	1.8203	0.31091	-0.9399	-0.9231	-0.1514	-1.2004	-0.0731	-0.7494	-0.751	0.73397	0.23317	0.12132
NM_182843	Acbd3	-0.3467	-0.1627	-0.0994	0.43953	-0.4962	-0.6757	0.09903	-0.4871	-0.0938	-0.1458	-0.168	-0.178	-0.0561	-0.0391
NM_001012013	Acbd4	-0.1041	0.04711	0.04174	0.07601	0.03137	0.24674	0.11282	-0.1055	0.09633	-0.0387	0.12226	-0.0196	0.06262	0.16755
NM_001012013	Acbd4	-0.381	-0.5354	-0.3985	-0.2613	-0.2698	-0.0596	-0.4753	-0.3228	-0.3415	-0.3668	-0.1512	0.1317	-0.6869	-0.6874
NM_001011906	Acbd6	-0.4263	-0.5896	-0.7629	-0.9409	-0.4905	-0.5916	-0.4378	-0.6734	-0.5445	-0.8626	-0.6616	-0.6167	-0.6532	-0.6317
NM_001034014	Accn1	0.07209	0.04764	0.03667	0.04381	0.20397	0.07934	-0.0053	0.11193	-0.0249	-0.0259	0.07874	-0.0065	0.10692	0.08563
NM_024154	Accn2	-0.154	-0.0279	-0.0193	0.0393	0.00023	0.15349	0.12692	-0.072	0.08885	-0.0265	0.03963	-0.0552	-0.0203	-0.1307
NM_173135	Accn3	-0.216	-0.1283	-0.1776	-0.0723	-0.0983	-0.1598	-0.1253	-0.1082	-0.1659	0.05229	-0.1076	-0.0823	-0.0686	-0.2731
NM_022234	Accn4	-0.0879	-0.0898	-0.1184	0.01989	-0.0439	-0.0327	-0.0579	-0.1447	-0.1286	-0.0549	-0.0413	-0.0223	-0.0554	-0.0557
NM_022227	Accn5	-0.1015	0.09199	-0.0696	0.01396	-0.0767	0.08052	0.14548	0.03715	0.1021	0.07239	0.11932	0.02992	-0.0366	0.11961
NM_001037193	Acd	0.27308	-0.0309	0.00027	-0.1094	-0.0146	-0.5005	0.46392	0.29497	0.34186	-0.052	0.16502	-0.2859	0.19179	0.14076
NM_012544	Ace	0.26486	0.30757	0.03226	0.16383	0.03589	-0.0599	0.00917	-0.1091	0.05114	0.23805	0.05996	0.17049	0.40895	0.0489
NM_001012006	Ace2	0.07884	-0.2037	-0.1306	-0.034	0.02842	0.32822	-0.2481	-0.0157	-0.0788	0.07961	-0.1365	-0.1029	-0.1518	-0.2971
NM_001012006	Ace2	-0.079	0.04252	-0.0069	-0.0732	0.08591	-0.032	0.0917	0.08696	-0.0507	0.05308	-0.054	-0.0385	0.02076	0.06772
NM_133400	Acf	0.11337	0.12263	0.0367	0.07751	-0.0159	0.04197	-0.0101	0.06322	-0.0376	0.07697	-0.0796	0.07834	0.16551	0.13204
NM_001170468	Acin1	-0.6506	-0.4374	0.56954	0.10104	-0.6423	-0.5143	0.3374	-0.5783	-0.2856	-0.714	-0.377	0.14526	-0.2731	-0.1495
NM_001111095	Acly	-0.1185	-0.4276	0.83567	0.05191	-0.0875	-0.1056	-0.0142	-0.0784	-0.2995	-0.1896	-0.0826	0.23172	0.01123	-0.0216
NM_134372	Acmsd	0.06359	0.14139	0.21412	0.06846	0.07598	0.00518	0.0855	0.15006	0.19183	0.04074	0.24249	0.01977	0.08664	0.01161
NM_001047914	Acn9	0.81481	0.18045	-0.0696	0.37895	0.52363	0.67903	-0.1896	0.60412	0.1371	0.26014	0.27975	0.12072	0.19109	0.09331
NM_017321	Aco1	-0.4286	-0.1518	0.43592	-0.4226	-0.0294	-0.1659	-0.195	0.11078	-0.2425	-0.1778	0.17856	0.18684	0.07755	0.19817
NM_024398	Aco2	-0.0832	0.28296	-0.17	-0.3319	-0.2345	0.21934	-0.3114	-0.125	0.21981	0.47112	0.2138	0.32269	0.05778	-0.1308
NM_031315	Acot1	-0.0408	0.03736	-0.1043	-0.0753	0.08445	-0.0696	0.03666	-0.0904	-0.0985	-0.0906	-0.0783	-0.1677	0.08818	0.05973
NM_130747	Acot12	0.15665	0.01505	-0.0002	0.06195	0.19336	0.13234	0.09459	-0.0292	0.00857	0.07966	0.15272	0.12235	0.06507	-0.0739
NM_138907	Acot2	0.3336	-0.0687	-0.2722	-0.3758	-0.1399	0.0166	0.14092	-0.3379	0.07003	-0.2335	-0.1894	-0.261	-0.1587	-0.0363
NM_001146061	Acot7	0.23952	-0.1113	0.58649	-0.9067	0.14551	0.18608	-0.1959	0.09279	-0.2524	-0.0596	-0.2145	0.12138	0.334	0.62683
NM_130756	Acot8	0.15825	0.0864	-0.0172	-0.5483	0.33998	0.66599	0.12835	0.26423	0.07725	0.19594	0.16068	0.23466	0.24555	0.01763
NM_017340	Acox1	0.0064	-0.3605	0.08813	-0.2441	-0.2988	-0.2382	-0.357	-0.4934	-0.1952	-0.2345	-0.0181	0.06403	-0.1651	-0.0828
NM_145770	Acox2	0.0915	0.22757	0.1107	0.09854	0.01765	0.15397	0.16594	0.09612	0.10168	0.08463	0.04498	0.16362	0.01215	0.04059
NM_053339	Acox3	-0.2267	-0.1482	-0.1032	0.07539	-0.1937	-0.2856	-0.1503	-0.099	-0.1127	-0.1688	-0.1732	-0.3025	-0.2152	-0.3249
NM_001106508	Acoxl_predicted	-0.1215	0.12311	-0.0468	-0.0539	-0.0574	-0.1538	0.05415	-0.1928	-0.0442	-0.0387	-0.1428	-0.0061	-0.1613	-0.2155
NM_021262	Acp1	-0.0501	-0.229	0.03032	0.10564	-0.1087	-0.0274	0.00152	-0.0935	-0.0893	0.03061	-0.096	-0.2056	0.15094	0.04331
NM_016988	Acp2	-0.5237	0.35117	0.1016	-0.7908	-0.1346	-0.1371	0.3459	-0.088	0.29177	0.28382	0.29538	0.18426	0.53611	0.38203
NM_001031645	Acp6	0.28225	0.03945	-0.2436	0.0774	0.06604	0.33796	-0.1553	0.17473	0.08569	0.30857	0.17144	-0.1765	-0.222	-0.1588
NM_001007710	Acpl2	-0.4679	0.02011	-1.2279	-0.9543	0.02177	-0.0327	-0.539	-0.0137	-0.121	-0.1602	-0.2397	-0.4294	-0.7739	-0.7295
NM_020072	Acpp	0.48681	-0.0202	0.27344	-0.2648	0.03686	0.06893	-0.0964	-0.0096	-0.0556	0.02154	0.07579	0.28968	-0.1161	0.12874
NM_001107510	Acpt_predicted	0.3123	-0.112	-0.0202	-0.03	0.13812	-0.2783	-0.0408	0.07594	0.00078	-0.1604	-0.0837	0.10054	-0.0884	-0.0147

NM_012490	Acr	0.18816	0.07626	-0.0109	0.12553	0.20607	0.22697	0.03677	0.07644	0.08625	0.21318	0.1367	0.2659	0.23216	0.16751
NM_021747	Acrv1	-0.1311	-0.2166	-0.2983	0.10874	-0.1546	-0.1819	-0.1809	-0.3396	-0.2529	-0.2041	-0.2844	-0.3098	-0.251	-0.0941
NM_134389	Acsbg1	0.11694	0.2438	0.03423	0.03438	0.09858	0.09175	-0.0814	0.18662	0.18404	0.05031	0.33474	0.16005	0.20126	0.01096
NM_012820	Acsl1	0.67948	0.22467	0.49771	0.61166	0.48846	0.3659	-0.2489	0.61649	-0.0197	0.16397	-0.0786	0.1464	0.32064	0.40607
NM_057107	Acsl3	-0.8632	-0.6477	-0.5881	-0.7159	-1.2584	-1.1501	-0.8201	-1.006	-0.3075	-0.7231	-0.73	-0.1907	-0.7757	-0.5896
NM_053623	Acsl4	0.73598	0.40182	0.93362	0.93355	0.3373	0.72393	0.87085	0.68547	0.48418	0.1393	0.56367	0.42341	0.15546	0.08544
NM_053607	Acsl5	0.14654	-0.0647	0.59528	0.2901	-0.151	-0.2069	0.32119	-0.137	-0.2889	-0.0995	-0.0042	-0.0996	0.67157	0.61818
NM_130739	Acsl6	-0.0357	0.01982	0.27482	0.15472	0.09909	0.15502	0.01446	-0.106	0.18266	0.01203	0.04628	0.11383	0.04612	0.08999
NM_144748	Acsm2a	-0.009	0.13134	0.17343	0.14435	0.1435	0.05736	0.14951	0.00207	0.02018	0.1832	0.16094	0.03373	0.06895	0.05389
NM_033231	Acsm3	-0.1476	0.07595	-0.0398	-0.1451	-0.1352	0.02054	-0.2765	0.18293	-0.0495	-0.3404	-0.1105	-0.1571	-0.1954	-0.283
NM_181695	Acsm4	0.02576	0.27444	0.30396	0.0929	0.00326	0.19401	-0.0579	-0.0049	0.25428	0.33768	0.19248	0.28085	0.05531	0.07329
NM_001107793	Acss2_predicted	0.76028	0.95888	0.60703	1.0057	0.96835	0.84836	0.34174	0.75447	0.2244	0.70251	0.39488	0.32409	-0.0529	-0.1566
NM_019212	Acta1	0.5333	0.08161	0.79515	1.4517	1.2983	1.9875	-0.0743	1.6537	0.10191	0.16966	0.25976	0.08453	0.33925	0.2275
NM_031144	Actb	-0.0804	-0.0368	0.04436	-0.032	-0.1827	-0.2136	0.06742	-0.0219	-0.1725	-0.0831	-0.309	0.04695	-0.0815	-0.3102
NM_031144	Actb	-0.2201	-0.1256	-0.2939	-0.6352	-0.3103	-0.9039	-0.1096	-0.351	-0.5332	-0.3572	-0.4197	-0.1443	0.07107	0.18936
NM_031144	Actb	0.08148	-0.0355	-0.2543	-0.6732	-0.2356	-1.1463	-0.0403	0.11458	0.14235	0.13821	-0.1906	0.01643	0.49103	0.55326
NM_031004	Actc1	0.12558	0.26605	0.00607	0.35121	0.02875	0.19923	0.18949	0.12836	0.09663	0.10519	0.08163	0.10567	0.17054	0.24148
NM_001127449	Actg_predicted	-0.2337	-0.1394	0.21292	-0.367	-0.1511	-0.4606	-0.1181	-0.0977	-0.5676	-0.2274	-0.4477	-0.172	0.04117	0.1498
NM_001127449	Actg1	-0.0797	-0.0849	0.00918	-0.1005	-0.1255	-0.1009	0.08401	-0.197	0.251	-0.0852	-0.088	-0.172	-0.0669	-0.0823
NM_012893	Actg2	-0.0044	-0.1257	-0.2624	-0.3458	0.20028	0.32197	-0.2337	-0.1095	-0.2212	-0.0613	0.02195	-0.2406	0.21141	0.17698
NM_001039033	Actl6a	-0.1671	-0.4077	0.42759	0.47188	-0.1201	-0.514	0.0363	-0.0583	-0.2577	-0.2977	-0.4334	-0.2072	0.34187	0.34685
NM_001105917	Actl6b_predicted	-0.0181	0.16769	0.20918	0.14179	0.29032	0.21722	0.28541	-0.0307	0.1309	0.25102	0.26796	0.26257	0.07553	0.27824
NM_001011973	Actl7a	-0.0382	-0.1423	-0.077	0.09608	0.03889	-0.1618	-0.1561	-0.0441	-0.05	0.00658	-0.0407	-0.0674	-0.0026	-0.0124
NM_001025417	Actl7b	-0.1231	-0.2881	-0.0777	-0.0085	-0.086	-0.1825	-0.0118	-0.2823	-0.0725	-0.2339	-0.1483	-0.2447	0.082	-0.1763
NM_031005	Actn1	-0.3337	-0.0085	0.54048	0.3751	0.32053	-0.3387	-0.3332	0.28859	0.34617	0.10201	0.00359	-0.5629	0.4607	0.7322
NM_001170325	Actn2_predicted	-0.0603	0.05331	0.35633	0.07096	-0.0435	-0.0221	0.07645	0.14329	0.26185	0.12812	0.1934	0.15473	0.01256	0.14677
NM_133424	Actn3	0.17064	0.22997	0.02732	-0.0236	0.14916	0.11642	0.12585	0.09204	0.2819	0.1398	0.2606	0.14544	0.05929	0.22872
NM_031675	Actn4	-0.3841	-0.5378	0.24574	0.26258	-0.2985	-0.5582	-0.516	-0.4622	-0.667	-0.4649	-0.5619	-0.9781	-0.275	-0.1347
NM_001009602	Actr10	0.18747	0.25342	0.09199	0.23506	0.18933	0.07722	0.35878	0.21172	0.2836	0.34856	0.23084	0.03827	0.08203	0.05167
NM_001106364	Actr1a_predicted	0.03613	-0.1099	-0.1288	0.06562	0.01933	0.04573	-0.0914	-0.0993	-0.125	-0.1099	0.02792	-0.071	0.00819	-0.146
NM_001106364	Actr1a_predicted	0.22881	0.23536	0.21075	-0.034	0.39058	0.2459	-0.2505	0.30533	0.01594	0.11763	0.02797	0.20632	0.21778	0.42993
NM_001009268	Actr2	0.23537	0.62483	0.20793	0.30965	0.06481	-0.2965	0.53985	0.21015	0.41279	0.58699	0.39594	0.13462	0.35228	0.40321
NM_031068	Actr3	0.5804	0.90672	0.10692	0.44646	0.00315	-0.0002	0.43028	0.08155	0.38741	0.70687	0.58568	0.31482	0.79297	0.67093
NM_001108600	Actr5_predicted	-0.3173	-0.5369	0.08035	0.1763	-0.2486	-0.4519	-0.2514	-0.3198	-0.3337	-0.3628	-0.2765	-0.3852	-0.4233	-0.3781
NM_001108081	Actr6_predicted	0.65451	1.1341	0.30536	1.6185	0.64734	0.44563	1.1748	0.83718	0.95589	1.1749	1.0852	0.45595	0.55703	0.39082
NM_001108396	Actr8_predicted	0.11956	0.04915	0.0498	0.69888	0.13937	0.3742	0.11122	0.15357	-0.4104	0.18117	0.05107	-0.1186	-0.4426	-0.2713
NM_001013961	Actrt1	0.20592	0.25685	0.13737	0.11798	0.225	0.19698	0.10415	0.08058	0.47595	0.16225	0.35118	0.13321	0.21424	0.35921
NM_001013937	Actrt2	-0.0132	-0.1512	0.01385	-0.043	-0.1424	-0.2903	0.03591	0.00655	-0.046	-0.2213	-0.1691	-0.0755	-0.1861	0.04893
NM_024486	Acvr1	-0.4181	0.01364	-0.4784	-0.1261	-0.1875	-0.7019	-0.2255	-0.1591	-0.1544	-0.2259	-0.1636	-0.1745	0.05596	-0.013
NM_199230	Acvr1b	0.09474	0.05434	0.28623	0.09021	0.24357	0.02075	0.26517	0.2684	-0.0036	0.10417	-0.0284	0.05213	0.0607	0.13697
NM_139090	Acvr1c	-0.0007	-0.0578	-0.0547	-0.0036	0.05112	0.21421	0.05896	0.03456	0.00056	0.15426	-0.0928	0.02507	0.02578	0.05211
NM_031571	Acvr2	-0.0137	0.09541	0.01332	-0.0045	-0.1515	0.30927	-0.0867	-0.1117	0.0013	0.10222	-0.0112	-0.1294	-0.0295	0.04063
NM_031554	Acvr2b	-0.3388	-0.3058	-1.0994	-0.8047	-0.0853	-0.487	-0.5086	-0.221	-0.4429	-0.4107	-0.399	-0.8826	-1.0546	-0.9457
NM_031554	Acvr2b	-0.0995	-0.209	-0.333	-0.1368	-0.2138	-0.1491	-0.0708	-0.0606	-0.1948	-0.0474	-0.1842	-0.0044	-0.3354	-0.296
NM_022441	Acvrl1	-0.1129	-0.0047	0.08732	0.03083	0.05247	0.09816	0.03684	0.02071	-0.1972	-0.0133	0.00964	-0.2216	0.09268	0.21769
NM_001005383	Acy1	0.4543	0.04463	0.20354	0.04285	0.77289	0.52685	-0.1365	1.1825	0.17501	0.14327	-0.0625	0.26004	0.71031	0.49692
NM_001107922	Acy1l2_predicted	0.13085	-0.0551	0.0491	0.05615	-0.0809	0.08113	0.08383	0.09767	0.23494	-0.0563	-0.0303	0.28411	0.19838	0.04767
NM_001009603	Acy3	0.10905	-0.0801	-0.538	-0.8209	-0.9191	-0.7496	0.59341	-0.8158	0.00136	0.03151	-0.0656	0.64645	-0.6626	-0.6111
NM_001106746	Acyp1_predicted	-0.105	0.03297	0.6607	0.54378	-0.0966	0.41283	0.08212	-0.1626	0.25183	0.18579	0.29678	0.59604	0.2327	0.18381

XM_344275.2	Acyp2	-0.0778	-0.1269	-0.2041	-0.0777	-0.013	-0.1268	-0.1366	0.02538	0.02648	-0.035	-0.0097	0.05877	-0.0358	0.00679
NM_130399	Ada	0.02699	0.0561	0.41031	-0.4396	0.50935	1.0711	-0.0293	0.61119	0.29291	0.12419	0.2074	0.78662	0.50248	0.54997
NM_001006977	Adad1	-0.1879	-0.0152	0.32177	0.04512	-0.0919	-0.0781	-0.0748	0.01867	-0.0808	-0.1021	0.23963	0.0714	0.23582	0.12845
XM_001054737.1	Adam10	-0.283	-0.0078	-0.7052	-0.6561	-0.2829	-0.4379	-0.4059	-0.048	-0.4188	-0.1364	-0.4197	-0.675	-0.2842	-0.1937
XM_001054737.1	Adam10	-0.1767	-0.1021	-0.4417	-0.1965	-0.2712	-0.4879	-0.1967	-0.2091	-0.3156	-0.2337	-0.0926	-0.086	-0.3664	-0.363
NM_001108300	Adam11_predicted	0.11431	0.03806	0.23046	0.00458	0.05623	0.10277	-0.0337	-0.0008	0.12938	0.13409	0.02563	0.02024	-0.0276	0.11746
XM_001055685.1	Adam12_predicted	0.01053	-0.1465	0.03024	-0.1874	-0.1772	-0.1028	-0.1512	-0.0667	-0.282	-0.1537	-0.2164	-0.0379	0.0192	0.11756
NM_020308	Adam15	0.56751	-0.0205	-0.5978	-0.4666	0.09213	0.31702	-0.2181	0.23372	-0.3862	-0.2325	-0.0894	-0.5532	-0.6771	-0.4022
NM_020306	Adam17	0.55309	0.53705	0.49314	0.46618	1.3526	1.0668	0.93589	1.1934	0.07796	0.24204	0.18313	-0.0047	0.26999	0.31829
XM_001072121.1	Adam18	0.03425	-0.0025	0.0841	0.21024	0.18922	-0.0088	0.16027	0.23684	0.32021	0.5189	0.07582	0.19017	0.2057	0.16477
NM_020078	Adam1a	-0.1786	-0.2157	0.52471	0.02983	-0.4867	-0.631	0.41578	-0.4141	-0.0148	-0.4319	0.0298	-0.1059	-0.4158	-0.1843
NM_020077	Adam2	0.0716	0.01278	-0.117	0.0262	-0.1347	0.03535	0.27499	-0.0097	-0.1204	-0.1702	-0.0923	-0.1429	0.10692	0.05321
NM_001029899	Adam23	-0.0777	-0.0579	-0.1483	-0.0952	-0.1029	-0.1787	-0.1389	-0.1422	-0.024	0.03896	0.0514	-0.0301	-0.0605	-0.1426
XM_001064767.1	Adam24_predicted	0.12289	-0.0037	0.20485	0.19905	-0.0819	-0.1849	-0.0201	0.09242	0.26493	0.05774	-0.1509	0.04312	0.09597	0.08216
NM_181693	Adam28	-0.2205	-0.1979	-0.0257	-0.067	-0.0762	0.02597	-0.0621	0.17704	-0.2137	-0.1762	0.015	0.0539	-0.1562	-0.059
NM_020302	Adam3	-0.0217	-0.0682	-0.053	-0.0236	-0.0284	-0.0513	-0.1228	-0.0137	0.23773	-0.0688	-0.0142	-0.1581	-0.1378	-0.0109
NM_001170582	Adam32	0.34641	0.01261	0.35518	1.2388	-0.073	0.09014	0.59183	0.25267	0.17496	0.03403	0.16811	-0.1042	0.17504	0.08992
NM_001107776	Adam33_predicted	-0.2809	-0.0957	0.01252	-0.3389	-0.5974	-0.67	0.28081	-0.5861	-0.3266	-0.0421	-0.2054	-0.6029	-1.0942	-0.6821
NM_138906	Adam6	-0.3398	-0.3305	-0.1843	-0.191	-0.4809	-0.3623	-0.329	-0.2496	-0.0663	-0.3969	-0.0829	0.0595	-0.2671	-0.2061
NM_020301	Adam7	-0.322	-0.2629	-0.2817	-0.1136	-0.0231	-0.0639	-0.2262	-0.2463	-0.1807	-0.23	-0.2588	0.03412	-0.1223	-0.1034
NM_001107560	Adam8_predicted	0.22335	-0.0668	0.35237	0.18264	0.00644	0.20756	-0.2792	0.14168	0.01428	-0.042	-0.284	0.41549	0.16144	0.30582
NM_001106046	Adamdec1_predicted	0.0203	0.11998	-0.0032	-0.0102	0.01671	0.10373	0.03673	0.05348	-0.0054	-0.0056	0.14037	-0.0535	0.01504	0.01917
NM_024400	Adamts1	-0.4985	0.31028	-0.2347	-0.629	-0.8171	-0.6798	1.2613	-0.5095	0.20976	0.22024	0.33009	-0.4111	-0.2777	-0.2683
NM_001106420	Adamts12_predicted	-0.4835	-0.3103	-0.2842	-0.3477	-0.3703	-0.3531	-0.185	-0.3718	-0.3915	-0.2006	-0.3826	-0.3053	-0.1221	-0.3008
XM_342395.3	Adamts13_predicted	0.07483	0.03835	0.12325	-0.1583	-0.1415	-0.1749	0.04906	-0.1401	0.13219	-0.0534	0.12083	0.07118	-0.0603	-0.0756
NM_001107636	Adamts14_predicted	0.03644	-0.0502	-0.1474	-0.0961	-0.0787	-0.0592	-0.0613	-0.0398	0.08221	0.01588	-0.0628	0.05358	-0.1034	-0.0678
NM_001106810	Adamts15_predicted	-0.0038	-0.292	-0.0286	-0.1696	-0.241	-0.0205	-0.166	-0.2415	-0.2384	-0.2686	-0.0737	-0.0606	-0.2899	0.08528
NM_001107332	Adamts16_predicted	-0.0511	-0.0351	-0.0181	0.06308	-0.0545	0.15948	-0.1494	-0.0989	0.18118	0.07997	-0.0538	-0.0018	0.15295	-0.0667
XM_218753.4	Adamts17_predicted	-0.0241	-0.0094	-0.0643	-0.0206	-0.0336	0.00208	-0.131	0.00821	0.17931	-0.082	-0.0244	-0.1276	0.00586	0.03515
NM_001108433	Adamts19_predicted	0.08063	0.04414	-0.0095	0.18087	0.0859	-0.0022	0.01829	0.20943	-0.0268	0.03182	0.11713	0.0248	0.12258	0.03633
XM_001058144.1	Adamts20_predicted	-0.309	-0.2929	-0.1679	-0.3805	-0.2468	-0.337	-0.2309	-0.0056	-0.335	-0.2378	-0.4043	-0.2558	-0.421	-0.3259
NM_001107212	Adamts3_predicted	-0.0924	-0.1199	-0.1768	-0.0897	-0.1274	-0.0448	-0.0679	-0.1289	0.09782	-0.1348	-0.0711	-0.034	0.01428	-0.1115
NM_023959	Adamts4	0.12995	0.18335	0.11101	0.40833	0.3215	0.55977	0.28852	0.2731	0.02932	0.39549	0.21257	0.12506	0.30536	0.2783
NM_198761	Adamts5	0.0143	-0.0888	0.09613	0.11416	0.05205	-0.015	-0.0278	0.15607	0.03585	0.0314	0.11641	0.20158	0.14078	0.06532
NM_001108544	Adamts6_predicted	-0.1791	0.14822	0.16432	0.65438	-0.1315	-0.0419	0.31308	-0.2156	-0.1643	-0.2573	-0.0948	-0.3012	0.27691	0.10535
NM_001047101	Adamts7_predicted	1.1859	0.819	1.0541	1.2019	1.8758	2.0399	0.4367	1.861	0.35008	0.68404	0.63234	0.03375	0.22125	0.24875
NM_001106811	Adamts8_predicted	1.1864	0.3929	0.74319	0.35928	0.88732	0.83573	0.89501	0.81033	0.3534	0.22252	0.4645	-0.0796	0.56157	0.30877
NM_001107877	Adamts9_predicted	-0.6128	-0.8955	-0.8482	-0.6318	-0.6094	-0.8444	-0.8742	-0.781	-0.1354	-0.7951	-0.6789	0.56082	-0.8833	-0.9247
NM_001034012	Adamtsl4	0.33241	0.3046	0.24847	0.3251	0.00985	0.23191	0.40094	-0.0269	0.10038	0.2295	0.2645	0.00774	-0.3184	-0.0687
NM_001034012	Adamtsl4	0.43232	0.42144	0.29144	0.26845	-0.0453	0.21131	0.45029	-0.0972	-0.0208	0.2701	0.35565	-0.0667	-0.3453	-0.2602
NM_001108071	Adamtsl5_predicted	0.12619	0.19609	0.70846	0.37489	-0.3138	-0.4237	0.66407	-0.2815	0.30154	-0.0405	0.27323	-0.2358	0.00667	0.17391
NM_031006	Adar	-0.0111	-0.1324	0.2241	-0.0387	0.16001	0.03851	0.00944	0.04663	-0.1276	0.06175	-0.0243	-0.1665	-0.0313	0.08332
NM_001111057	Adarb1	0.00592	-0.2651	0.07624	0.00192	0.08933	0.16323	0.20473	0.03166	0.13396	-0.1508	-0.0345	-0.0229	-0.0408	0.08167
NM_133302	Adarb2	-0.2075	-0.082	-0.1183	-0.1729	-0.0426	-0.1172	-0.1195	-0.0483	-0.0958	-0.0726	-0.0217	-0.0645	0.00228	-0.2983
NM_001108985	Adck1_predicted	0.24119	0.05379	0.07332	0.06743	0.13881	-0.0528	0.13008	-0.0453	-0.0121	0.09669	0.35093	0.1767	0.21706	0.0988
NM_001107855	Adck2_predicted	-0.0481	0.0842	0.06733	-0.2679	-0.1195	-0.0611	-0.3214	-0.344	-0.2952	-0.0464	-0.1963	0.08371	-0.2991	-0.3496
NM_001012065	Adck4	-0.2192	0.04278	-0.2957	-0.1357	-0.2835	-0.4178	-0.45	-0.2943	-0.3774	-0.4703	-0.0116	-0.4657	-0.4359	-0.7329
NM_001012065	Adck4_predicted	-0.0471	-0.1516	0.02468	-0.1068	-0.1422	-0.2555	-0.126	-0.0895	-0.2486	-0.0977	-0.2638	-0.0407	-0.1446	-0.1773
NM_001135798	Adck5	-0.5383	-0.0303	0.49121	0.00381	-0.5483	-0.7724	-0.2502	-0.5557	-0.1173	0.20904	0.07685	0.01321	-0.5639	-0.3509

NM_001107239	Adcy1_predicted	-0.0609	0.03914	0.00539	-0.0356	-0.0442	-0.0343	0.12197	0.05404	0.01919	-0.0279	0.02855	-0.0035	-0.0248	0.01926
NM_031007	Adcy2	0.31694	0.06718	-0.0617	-0.1574	-0.0942	-0.0841	0.0991	-0.1387	0.05629	0.15728	-0.0479	0.21321	-0.1871	-0.1041
NM_130779	Adcy3	0.05972	0.03832	0.09248	-0.0109	0.00077	0.17288	0.0566	-0.0095	-0.029	0.19668	0.02558	0.19069	0.0062	0.02088
NM_019285	Adcy4	0.06207	0.0902	0.16906	0.22958	0.16731	0.37625	0.50616	0.10621	0.07039	0.09515	0.21699	0.12668	0.31728	0.06562
NM_022600	Adcy5	-0.0394	-0.1031	-0.135	-0.0146	-0.1338	-0.0739	-0.1299	-0.1676	-0.0751	0.08959	-0.1043	0.23138	0.00864	-0.148
NM_012821	Adcy6	0.1385	0.16379	0.01534	-0.112	0.02799	-0.1808	0.34546	0.07966	-0.0104	0.1354	0.04086	-0.1116	0.17461	-0.1307
NM_053396	Adcy7	-0.1928	0.08894	-0.4524	-0.1459	7E-05	-0.3287	-0.1062	0.09545	-0.127	0.23037	-0.2218	-0.4113	-0.3139	-0.17
NM_017142	Adcy8	-1.2972	-1.5031	-1.3086	-1.1016	-1.3621	-1.5557	-0.1493	-1.7854	-1.5674	-1.542	-1.224	-1.314	-1.082	-1.015
NM_001106980	Adcy9_predicted	-0.0383	-0.0292	-0.1033	0.22796	0.28862	0.04516	0.19872	0.05938	0.26487	0.00343	0.02569	0.06803	-0.0917	0.07232
NM_016989	Adcyap1	-0.0979	-0.1165	-0.0233	-0.0665	-0.2288	-0.1034	-0.0535	-0.2164	-0.3913	-0.1878	-0.2332	-0.3	-0.3164	-0.3155
NM_133511	Adcyap1r1	-0.079	-0.1169	-0.0454	0.08335	-0.0683	0.03176	-0.1292	0.14393	0.10068	-0.1621	-0.0281	-0.0141	0.20826	0.05561
NM_016990	Add1	-0.0178	-0.0092	-0.3629	-0.4593	-0.0649	-0.678	-0.1673	-0.0182	-0.1562	-0.0399	-0.1148	-0.4428	0.13794	0.0861
NM_001109880	Add2	-0.1635	-0.1306	-0.0742	-0.199	0.02479	-0.0976	-0.1628	0.21609	0.28704	-0.1059	-0.2132	-0.0632	-0.0706	-0.2236
NM_031552	Add3	-0.0493	0.05885	0.14554	0.17816	0.25335	0.15732	0.08864	0.24292	0.0339	0.11174	0.06029	-0.0009	0.12322	0.0345
NM_001007144	Adfp	1.9162	1.4729	0.59339	0.70259	1.4868	1.2638	0.8602	1.5148	1.2361	1.2378	1.5234	0.23998	0.52926	0.55391
NM_019286	Adh1	0.19242	0.02546	-0.0626	-0.0035	0.27066	0.60528	-0.0279	0.11078	0.01662	0.03427	-0.0959	0.07351	-0.1419	-0.0019
NM_017270	Adh4	0.34604	0.06979	0.3413	0.15536	0.12966	0.48797	0.22708	0.12934	0.04711	0.1478	-0.0687	0.17117	0.22568	0.14331
NM_001012084	Adh6	0.12545	0.04287	0.06319	0.06053	0.0179	0.06758	0.02047	0.08285	0.33834	0.11911	0.11257	0.2153	0.09072	0.0238
NM_001106475	Adh6a_predicted	0.03336	0.0407	-0.0888	0.01159	0.19716	-0.0543	0.23933	0.11037	0.06371	-0.0079	0.13412	0.01838	0.04051	0.0336
NM_001025423	Adhfe1	0.47736	0.49712	-0.2228	0.37231	0.66279	0.39723	0.15751	0.55409	0.25493	0.57469	0.54475	-0.0118	-0.3786	-0.1949
NM_199097	Adi1	0.10185	-0.0132	0.06136	-0.3243	0.39757	0.02496	-0.2082	-0.1501	-0.0645	0.10381	-0.2385	0.26185	0.18024	-0.0589
NM_144744	Adipoq	-0.3675	-0.6303	-0.278	-0.5494	-0.374	-0.295	-0.4775	-0.5303	-0.645	-0.4284	-0.4874	-0.6058	-0.4734	-0.6135
NM_207587	Adipor1	0.27908	0.04881	-0.2346	0.12825	-0.3246	-0.281	0.08512	-0.2788	-0.3771	0.03044	-0.1539	-0.4145	-0.0283	-0.0295
NM_001037979	Adipor2_predicted	0.50814	0.46774	0.29399	-0.0369	0.08559	0.34617	0.23252	-0.0225	-0.1342	0.12644	0.16054	-0.1439	-0.427	-0.1552
NM_012895	Adk	-0.2018	-0.6772	-0.4904	-0.7403	-0.4657	-0.5554	-0.7394	-0.33	-0.5874	-0.6794	-0.762	-0.2875	-0.1704	-0.1853
NM_012715	Adm	1.5802	1.8643	-0.3915	0.53657	2.2603	2.2563	-0.1446	2.1258	1.9687	1.7138	1.767	0.79397	-0.0747	0.03366
NM_201426	Adm2	-0.2563	0.34206	0.50132	0.26436	0.00524	-0.0076	0.10407	-0.1583	0.47436	0.15965	0.2085	0.29178	0.35018	0.33154
NM_053302	Admr	0.062	-0.1145	-0.1373	-0.1205	-0.1484	0.19424	-0.1297	-0.0101	-0.0513	-0.0326	0.01149	0.07876	0.02338	-0.0946
NM_022681	Adnp	-0.4283	0.20909	-0.4843	-0.5203	-0.4488	-0.8887	-0.1174	-0.4339	-0.2635	-0.1378	-0.2758	-0.4765	-0.1159	-0.0786
NM_017155	Adora1	-0.1005	0.02496	-0.0042	0.04115	0.37949	0.26341	-0.0265	0.49934	0.10284	-0.0614	0.0113	0.07127	-0.0009	-0.1206
NM_053294	Adora2a	0.11486	0.32939	-0.071	-0.0481	0.26523	0.08633	-0.0942	0.16213	-0.0692	-0.2179	-0.1785	0.09488	-0.0765	-0.1866
NM_017161	Adora2b	0.48923	0.78498	0.13568	0.16697	0.15979	0.09749	0.69031	0.10112	0.52552	0.71087	0.67791	0.07395	0.15484	0.50253
NM_012896	Adora3	0.02479	-0.1209	0.01723	-0.1292	-0.0835	0.12967	-0.1318	-0.1388	-0.0071	-0.0422	-0.1025	0.00207	-0.1874	0.14283
NM_001100723	Adpgk	-0.29	-0.4507	-0.1214	-0.2266	0.0155	0.23	-0.2934	-0.0058	-0.278	-0.1485	-0.3833	0.01573	-0.1763	-0.0822
NM_183325	Adprh	0.4734	0.81292	0.57337	0.61413	0.44967	0.42718	0.73301	0.4287	0.64291	0.86639	0.76884	0.54705	0.78659	0.81998
NM_001013054	Adprhl1	-1.0274	-1.2616	-1.0033	-1.4046	-1.268	-1.2276	-1.3097	-1.336	-1.3385	-1.2404	-1.1701	-1.1743	-1.0207	-1.1096
NM_001108680	Adprhl2_predicted	-0.0858	-0.4531	-0.3688	-0.0541	-0.3596	-0.2598	-0.1669	-0.4633	-0.3265	-0.3525	-0.4394	-0.2741	-0.3308	-0.413
NM_001017375	Adprtl1	0.49409	0.68903	-0.069	0.21496	0.59428	0.79119	0.44864	0.64501	0.53663	0.68066	0.65119	0.51119	0.47735	0.50698
NM_017191	Adra1a	-0.459	-0.0868	-0.0741	0.0313	-0.1221	-0.3648	-0.1688	-0.1535	-0.1184	-0.1262	-0.123	0.14543	-0.1201	-0.0298
NM_016991	Adra1b	0.21409	0.19627	0.13293	-0.0037	0.31479	0.21222	0.14274	0.15789	0.0652	-0.0114	0.15641	-0.3569	0.02977	0.13646
NM_138505	Adra2b	-0.1066	0.03819	0.0287	-0.1098	0.04836	0.00906	0.0593	-0.0628	-0.1167	-0.0815	-0.1241	-0.002	-0.1068	0.13375
NM_138506	Adra2c	0.16789	0.10846	0.21123	0.0378	-0.0073	0.13097	0.05659	0.00556	0.16812	0.00723	-0.1857	0.34213	-0.0029	-0.0274
NM_012701	Adrb1	0.23193	0.09304	0.15162	0.14278	0.07184	-0.0251	-0.1287	0.48876	-0.0098	0.12754	-0.0687	-0.1585	-0.0155	-0.0522
NM_012492	Adrb2	-0.1664	-0.1003	-0.1699	-0.1049	-0.0381	-0.3392	-0.1057	-0.2975	-0.3434	-0.1859	-0.303	-0.183	-0.2684	-0.2381
NM_012492	Adrb2	-0.7146	-0.8572	-0.8049	-0.8325	-1.0032	-0.8964	-0.8521	-0.8709	-0.8669	-1.0639	-0.6814	-0.825	-1.3351	-1.1299
NM_013108	Adrb3	-0.257	-0.3591	-0.0617	-0.1755	-0.2434	-0.3402	-0.2799	-0.4322	-0.1184	-0.5318	-0.325	0.5565	-0.3012	-0.4047
NM_012776	Adrbk1	-0.1493	0.09876	0.00659	-0.4057	0.05507	-0.2319	0.24723	0.07924	-0.3214	-0.2474	-0.2417	0.08572	-0.031	-0.0398
NM_012897	Adrbk2	-6E-05	0.24262	0.08062	0.08991	0.01134	0.19677	0.29607	0.20381	0.06731	0.2049	0.24543	0.18838	0.03057	0.24953
NM_031708	Adrm1	0.11374	-0.1269	0.53616	0.47306	0.15303	0.00966	-0.0846	-0.1325	-0.2019	-0.0841	0.20745	-0.1268	0.02438	0.23534

NM_001130503	Adsl_predicted	-0.2303	-0.1184	0.39495	-0.1305	-0.4394	-0.3943	0.01541	-0.4003	-0.2121	-0.1601	-0.1489	-0.0451	0.33505	0.18026
NM_001105975	Adss_predicted	0.10683	0.26754	0.0674	0.25378	0.13245	-0.092	0.91741	0.16963	0.02764	0.1994	0.16325	-0.6034	0.13242	0.0064
NM_001100970	Aebp1_predicted	0.81736	0.69907	1.2428	1.2482	0.7849	0.59987	1.0084	0.40927	0.76199	0.65692	0.82938	0.6823	0.19643	0.64166
NM_001106626	Aebp2_predicted	-0.1428	-0.0049	0.03114	0.09612	-0.0919	-0.5062	0.24254	-0.0801	0.00854	0.18029	-0.2087	-0.1558	0.25072	0.04168
NM_001009502	Aer61	0.16723	-0.0081	-0.0026	0.09315	0.03165	0.10523	-0.108	0.10314	-0.0143	-0.2741	0.01953	0.13238	-0.0558	-0.095
NM_019220	Aes	-0.2193	-0.2539	-0.5978	-0.6387	-0.2812	-0.666	-0.7291	-0.2231	-0.4245	-0.3736	-0.26	-0.5017	-0.2769	-0.1639
XM_001054673.1	Aff2_predicted	0.00999	0.0318	-0.0619	0.11537	-0.0184	0.11057	0.00634	-0.0246	-0.0044	0.0063	0.03904	-0.0054	-0.0156	-0.0151
XM_001058147.1	Aff3_predicted	0.16866	0.16607	0.14429	0.23622	0.03912	0.30577	0.16804	-0.0881	0.00692	-0.2757	0.02012	-0.0705	0.05655	0.18266
NM_001107001	Aff4_predicted	-0.2868	-0.0618	-0.5183	0.33912	-0.1587	-0.3604	0.02982	0.10846	-0.3899	-0.2822	-0.3837	-0.4909	-0.1112	0.12651
NM_001108456	Afg3l1_predicted	0.10415	0.0258	-0.0434	0.28516	0.13887	-0.0438	-0.2182	0.29832	0.12955	0.03497	0.20105	-0.087	0.05683	0.13576
NM_001134864	Afg3l2	0.15945	0.2152	0.098	0.78514	0.22331	0.06124	0.35382	0.53525	-0.0166	0.14994	0.18694	-0.536	-0.5143	-0.2939
NM_172320	Afm	0.20271	0.17644	0.13748	0.38521	0.3239	0.14646	0.31718	0.27775	0.10113	0.15241	0.19346	0.05771	0.28455	0.26484
NM_012493	Afp	0.27067	0.09834	0.04813	0.01296	1.0966	1.7067	0.17808	1.1789	0.04097	0.2054	0.07665	-0.0358	-0.0484	-0.0525
NM_001031641	Aga	0.76436	0.89831	0.37111	0.44575	0.32332	0.2273	0.98764	0.6243	0.96785	0.78797	0.93277	0.23201	0.26378	0.10404
XM_001077842.1	Agbl2_predicted	-0.2508	-0.14	-0.1336	-0.1857	-0.2214	-0.0144	-0.1626	-0.1462	-0.2931	-0.0419	-0.1568	-0.3036	0.04391	-0.1785
NM_053336	Ager	-0.0013	-0.0102	0.34301	0.25223	-0.0143	-1E-04	-0.0349	-0.0824	-0.0337	-0.0083	0.10674	-0.0048	-0.1861	-0.1038
XM_226709.4	Aggf1	0.1217	-0.0709	-0.0704	-0.3232	-0.0834	-0.2888	-0.0288	-0.0441	-0.1033	-0.0201	0.02101	-0.0584	0.15298	-0.0078
NM_001108564	Agl_predicted	0.48154	0.24264	0.109	-0.0103	0.6202	0.56534	0.38913	0.49616	0.04532	0.04379	0.04074	-0.4116	0.00088	0.12546
NM_001048185	Agmat	-0.4084	0.59421	-0.143	0.10935	-0.3857	-0.1449	-0.2573	-0.0618	0.26168	0.60754	0.53118	0.16299	-0.1903	-0.3166
NM_212458	Agpat1	-0.2302	-0.2877	-0.4307	-0.6723	-0.1395	-0.2763	-0.4005	-0.2344	-0.2479	0.12106	-0.069	0.06291	0.16994	-0.17
NM_001107821	Agpat2_predicted	0.32528	0.07133	0.24807	-0.4779	0.46582	0.71007	-0.3644	0.05027	0.00534	0.24816	0.02518	-0.0859	0.17617	0.11878
NM_001106378	Agpat3_predicted	-0.2617	-0.4123	-0.3961	-0.2402	-0.2561	-0.1886	-0.1982	-0.1966	-0.4013	-0.201	-0.4013	-0.3964	-0.2449	0.12422
NM_133406	Agpat4	-0.278	-0.2228	-0.167	-0.1082	-0.0839	-0.1576	-0.1936	0.03984	-0.1333	-0.2321	0.10471	-0.1532	-0.2217	-0.251
NM_001134744	Agpat5_predicted	-0.0449	0.16471	-0.3528	-0.0674	0.19102	-0.1748	-0.5559	0.13519	-0.026	0.15167	-0.0244	0.3307	0.24935	0.23098
NM_001047849	Agpat6	-0.0356	-0.3237	0.49098	0.21876	-0.0641	-0.0888	-0.1104	0.08363	-0.0847	-0.0548	-0.2958	0.08293	0.20508	0.25912
NM_001106494	Agpat7_predicted	0.13721	0.20179	0.06313	-0.0089	0.2876	0.26515	0.2893	0.20146	0.1762	0.50104	0.02239	-0.0653	-0.0333	0.0928
NM_053350	Agps	0.02189	0.03178	0.08833	0.14733	0.23229	0.24225	0.08663	-0.0528	0.00494	0.01911	0.11173	0.0786	0.05457	0.18332
NM_001106725	Agr2_predicted	-0.2518	-0.2105	-0.2637	-0.296	-0.2207	-0.2417	-0.1841	-0.1307	-0.1684	-0.1394	-0.1887	-0.3308	-0.2453	0.11523
NM_175754	Agrn	-0.3147	0.1988	0.06302	0.28486	-0.2156	-0.3375	-0.1716	-0.2769	0.101	0.11427	0.17681	-0.0767	-0.4862	-0.4156
NM_033650	Agrp	0.2046	0.38066	0.30488	-0.0407	0.13052	0.12606	0.12246	0.11914	0.11245	0.00053	-0.0446	0.31698	0.23698	0.17487
NM_134432	Agt	-0.192	-0.1557	-0.1683	-0.0064	0.04213	0.41429	0.12465	0.26191	-0.3154	-0.1396	-0.2826	-0.1182	-0.1089	-0.182
NM_001106100	Agtpbp1_predicted	-0.6665	-0.5105	0.20271	-0.1286	-0.6136	-0.8752	-0.1428	-0.6892	-0.5109	-0.6509	-0.6332	-0.855	-0.186	-0.2047
NM_030985	Agtr1a	-0.0451	-0.116	-0.0388	-0.0697	-0.0148	-0.016	-0.009	-0.1288	-0.0854	0.10259	0.1902	-0.0744	0.00024	-0.1259
NM_021866	Agtr1b	0.14865	0.26588	0.47308	0.12953	0.05809	0.25489	0.947	0.02519	0.30453	0.24656	0.25998	0.13504	-0.0204	0.04868
NM_012494	Agtr2	0.0351	0.00283	0.07373	-0.0111	0.29437	-0.054	-0.0638	0.00587	-0.154	0.0175	-0.1209	0.16204	-0.162	0.07508
NM_001007654	Agtrap	-0.3033	-0.4423	-0.5909	-0.6957	-0.4925	-0.7281	-0.669	-0.5172	-0.5841	-0.1449	-0.6378	-0.2845	-0.6442	-0.2031
NM_031349	Agtrl1	0.17302	0.04411	0.10941	-0.0815	0.25596	0.58322	-0.2517	-0.0155	-0.1676	0.06836	-0.3018	-0.2563	-0.0554	-0.0885
NM_030656	Agxt	-0.1216	0.01694	0.2236	0.00441	0.10242	0.06547	-0.0763	-0.0044	-0.0218	0.14544	-0.0486	0.08121	-0.1143	-0.0593
NM_031835	Agxt2	-0.1508	-0.1005	-0.1422	0.09279	-0.1273	-0.0376	-0.0401	0.00406	-0.2088	-0.1163	0.08254	-0.0263	-0.1473	0.10632
XM_341161.3	Ahctf1_predicted	-0.881	-0.6598	0.16784	-0.6966	-1.3895	-1.779	-0.1227	-1.5401	-0.7542	-0.6089	-0.5715	-0.3306	-0.3701	-0.4935
NM_017201	Ahcy	0.13117	-0.3043	0.33846	-0.2539	-0.0877	0.16733	-0.476	-0.1056	-0.3758	-0.235	-0.3575	0.18231	0.40102	0.41785
NM_001108561	Ahcyl1_predicted	0.01114	0.11604	-0.592	0.73979	-0.2754	-0.4663	-0.5488	-0.0031	-0.2286	-0.263	-0.5626	-0.8231	-0.3827	-0.3295
NM_001134956	Ahdc1_predicted	0.12412	-0.0075	0.25028	0.19313	0.14779	0.07803	0.05548	0.2991	0.06032	-0.0712	0.0704	0.01338	0.0716	0.23771
NM_001002277	Ahi1	0.64344	0.32904	0.67502	0.697	0.6765	0.35742	0.73548	0.85517	0.66696	0.44265	0.27104	0.10771	0.5	0.42263
XM_574618.1	Ahnak	-0.119	-0.3344	0.04306	0.03861	-0.134	-0.3076	0.13814	-0.2902	-0.1677	-0.0125	0.01175	-0.2008	-0.313	0.06612
NM_013149	Ahr	-0.0738	1.4312	-0.6034	0.43426	0.14281	-0.5503	1.3644	0.23679	0.99572	1.2624	1.006	-0.3422	-0.1176	-0.0791
NM_001024285	Ahrr	0.04628	-0.4781	-0.5315	0.56824	0.54219	0.53761	-0.4081	0.54975	-0.5097	-0.5432	-0.5823	-0.3853	-0.5017	-0.3628
NM_012898	Ahsg	0.10569	0.03312	0.01425	0.09648	-0.0976	0.03346	-0.042	0.10354	-0.0697	-0.054	0.26129	-0.0035	-0.0059	-0.0388
NM_001100779	aicda	-0.0845	0.07029	0.1104	-0.0896	-0.0571	-0.1125	-0.0188	0.02588	0.02373	-0.1329	-0.0277	-0.0621	0.01544	-0.0128

NM_017196	Aif1	0.03473	0.02012	-0.0868	0.01734	-0.0035	-0.0387	0.11929	0.01669	0.03464	-0.0894	-0.0878	-0.0123	0.05468	0.07176
NM_031356	Aifm1	-0.4869	-0.8164	-0.2858	-0.4513	-0.2334	-0.3908	-0.3264	-0.2985	-0.6695	-0.6618	-0.8049	-0.5431	-0.2042	-0.1051
XM_001067657.1	Aim1_predicted	0.17494	0.27915	-0.1854	-0.122	1.0894	0.47099	-0.0588	0.98694	-0.09	-0.2865	-0.2454	-0.1382	-0.3533	-0.1334
XM_216539.4	Aim1l_predicted	0.12971	0.11642	0.13672	0.03083	0.08215	-0.049	-0.0738	0.04279	0.12696	-0.1579	0.04247	0.11675	-0.1675	0.28313
XM_222949.3	Aim2_predicted	-0.085	-0.1178	-0.066	-0.1515	0.04917	0.13063	-0.0287	-0.0505	-0.014	-0.0252	-0.1628	-0.0861	-0.1768	-0.1641
NM_172327	Aip	-0.0139	0.28007	0.15141	0.10782	0.20343	0.01967	0.2532	-0.0837	0.17215	-0.0243	0.19429	0.19895	0.24354	0.01446
NM_021590	Aipl1	-0.1469	-0.0924	-0.1623	0.04889	-0.0978	0.40462	-0.2095	-0.1705	-0.0718	-0.1058	-0.1672	-0.0873	0.32175	-0.0298
NM_001106379	Aire_predicted	0.31279	0.18273	0.08659	0.20365	0.21521	0.12822	-0.0047	0.19934	0.51127	0.22608	-0.0028	0.21444	0.22368	0.12784
NM_001033967	Ak2	0.93737	0.4062	0.35079	1.5378	1.101	1.4543	0.28543	1.0859	0.35913	0.46913	0.29652	0.47321	0.33329	0.53003
NM_013218	Ak3	0.41514	0.36211	-0.3052	-1.0212	0.05437	0.03914	0.35632	-0.1063	0.27125	0.12891	0.25069	-0.1215	-0.1508	-0.1086
NM_017135	Ak3l1	0.61885	0.55663	-0.1428	-0.0559	0.42642	0.11276	-0.5426	0.2892	0.3851	0.46151	0.41245	-0.6563	0.05599	0.01159
NM_001108055	Ak7_predicted	-0.0806	0.15432	0.17494	0.19732	-0.1818	-0.0164	-0.0724	-0.1774	0.25405	-0.3453	-0.0627	-0.2236	-0.0922	-0.029
NM_053665	Akap1	0.62839	0.87386	-0.0324	0.21242	0.68162	0.45745	0.2371	0.59931	0.40707	0.90285	0.86275	-0.0053	0.56306	0.44411
NM_001114606	Akap10_predicted	-0.109	-0.0093	0.11362	0.10266	-0.0836	-0.3894	0.10865	-0.1776	-0.1524	-0.3277	-0.0515	-0.5659	-0.1471	-0.205
NM_001114606	Akap10_predicted	0.0416	-0.065	-0.0605	-0.0811	-0.0077	0.01968	-0.1068	-0.0103	0.02574	0.06938	0.00739	0.02656	-0.0274	-0.0379
NM_001033653	Akap12	-0.1429	-0.0745	-0.0117	0.24565	0.52322	0.66456	-0.2264	0.49326	-0.0038	-0.0228	-0.0365	0.38446	-0.1355	-0.0045
NM_001106271	Akap13	0.01801	0.01536	0.02962	0.03805	-0.0063	0.08793	-0.152	-0.1184	-0.1319	-0.1096	-0.0532	-0.1724	0.06319	0.08591
NM_001106271	Akap13	-0.1021	-0.0324	0.06348	0.03054	-0.12	-0.4038	0.14134	-0.1682	-0.1379	0.11314	-0.1242	-0.2022	-0.2928	-0.3122
NM_021703	Akap14	0.01992	-0.0232	-0.0464	-0.0413	-0.0983	0.01836	-0.0532	-0.0593	-0.1608	0.06138	0.19322	0.12576	-0.1466	-0.0075
NM_001011974	Akap2	1.1001	1.5398	0.95843	0.98211	1.8958	1.8531	0.61907	1.6474	1.3244	1.5517	1.2261	0.5513	1.2212	1.1676
NM_001005557	Akap3	0.14186	0.25049	0.03002	0.12687	0.1549	0.13274	-0.0456	0.16299	0.0316	0.13623	-0.024	-0.1894	-0.1335	-0.0042
NM_024402	Akap4	0.02017	0.00547	0.13964	0.02708	0.09496	0.36622	-0.0237	0.0089	0.34721	0.04147	-0.0295	-0.0102	0.10111	0.05574
NM_133515	Akap5	0.03431	-0.113	-0.1638	-0.2364	0.07902	0.00116	-0.1268	-0.1393	0.01477	-0.1908	-0.1147	0.00873	-0.0589	-0.1832
NM_022618	Akap6	-0.2607	-0.38	-0.1708	-0.1232	-0.1873	0.01559	-0.314	-0.3015	-0.1838	0.06617	-0.1499	-0.2687	-0.1178	-0.2599
NM_001001801	Akap7	-0.1227	0.01139	-0.1766	0.18089	0.22601	-0.209	0.16792	0.19782	0.21616	-0.0847	-0.0234	0.02168	0.0875	-0.0806
NM_053855	Akap8	-0.6597	-0.1332	0.71271	0.76934	-0.6863	-0.7105	0.20873	-0.5034	-0.0852	-0.235	0.00298	-0.0358	-0.0949	-0.1004
NM_001013946	Akap8l	-0.2042	0.26124	0.07817	0.36951	-0.1886	-0.0621	0.13839	-0.3249	-0.3029	-0.1354	0.25755	0.2736	-0.1754	-0.0607
NM_001037093	Akap9	0.06833	0.01734	-0.0366	0.12708	0.22205	0.17877	0.15271	0.21576	0.05798	-0.0054	0.04186	0.03572	0.03168	0.04157
NM_001037093	Akap9	-0.1771	-0.4114	-0.28	0.24547	0.04239	-0.0427	0.10943	0.25686	-0.4111	-0.3166	-0.3482	-0.4852	-0.2683	-0.3615
NM_001037093	Akap9	-0.1464	-0.1492	-0.3382	0.08673	-0.0254	-0.1482	-0.145	0.0072	-0.023	-0.2688	-0.0805	-0.0982	-0.0103	-0.1095
NM_001037093	Akap9	-0.0603	0.01795	-0.0024	-0.0027	-0.0166	0.25381	-0.073	-0.0845	0.14847	-0.1236	0.29386	0.11411	0.02644	-0.0311
NM_001108668	Akna_predicted	0.31888	0.05856	-0.0022	0.06697	0.00043	-0.0908	0.00567	0.11271	0.14826	0.0626	0.01773	-0.0555	0.10003	0.25461
XM_237353.1	Akp5_predicted	0.27764	0.17391	0.21356	0.27065	0.02906	0.58486	0.13057	0.31799	0.06847	0.22293	0.26036	0.22675	0.08963	0.12586
NM_031000	Akr1a1	-0.1588	-0.0407	-0.5521	-0.7075	-0.2439	-0.5531	-0.3696	0.02816	0.07778	-0.2053	-0.2082	-0.1415	-0.0647	-0.2178
NM_012498	Akr1b1	0.72253	0.0427	0.04814	1.1708	0.62692	0.59178	0.95486	0.72074	-0.0729	-0.1305	0.10377	-0.1278	0.00476	0.13642
NM_001013084	Akr1b10	0.03641	0.16067	-0.0969	0.03957	-0.0048	0.07415	0.01682	-0.0335	0.02961	-0.0127	-0.0678	-0.0301	-0.0446	-0.0111
NM_053781	Akr1b7	-0.0471	-0.1043	0.06219	-0.1158	-0.1073	-0.0968	0.02642	-0.0777	0.20845	-0.1225	0.09205	-0.1306	-0.0558	-0.0479
NM_173136	Akr1b8	1.7902	0.70938	0.22648	0.75849	1.8592	1.8881	2.0733	1.6146	0.66691	0.45247	0.48311	0.43018	0.02902	0.09249
NM_001170342	Akr1c12_predicted	1.1435	1.2671	0.79796	0.6648	0.99202	1.1217	1.3103	1.0011	0.813	1.2175	1.4676	0.05149	0.88516	0.67445
NM_138510	Akr1c18	-0.064	3.5625	1.2936	4.0632	0.01609	0.00484	1.3186	-0.1146	2.9137	3.2277	3.4169	0.45792	1.5781	1.5374
NM_001013057	Akr1c21	-0.0847	0.06044	0.15904	0.13357	0.10306	0.13826	0.13634	0.07643	0.07147	0.01993	0.26816	0.00616	-0.0073	0.16005
NM_001109900	Akr1cl1_predicted	-0.2427	1.3493	-1.9689	-0.0933	-2.2947	-2.1029	-0.6902	-2.0731	0.74406	1.0606	0.9351	-1.8264	-2.0082	-2.1421
NM_138884	Akr1d1	-0.1044	0.12379	0.0509	0.15272	-0.173	0.09002	0.07521	-0.0734	0.04734	0.12125	0.36532	-0.0419	-0.0959	-0.0344
NM_001008342	Akr1e1	-0.0463	-0.2213	-0.2077	0.03926	-0.3547	-0.3287	-0.2924	-0.2911	-0.4165	-0.2988	-0.1201	-0.2061	-0.7105	-0.5133
NM_134407	Akr7a2	-0.2506	-0.3755	-0.4895	-0.8032	-0.2906	0.00142	-0.8646	-0.2023	-0.1954	-0.3511	-0.1381	0.13918	-0.3632	-0.4057
NM_013215	Akr7a3	0.14831	0.16703	-0.0722	0.09552	0.18754	0.12978	0.01567	0.19566	0.07385	-0.0567	0.07851	0.27218	-0.0029	-0.081
NM_033230	Akt1	0.00846	-0.0699	0.36082	-0.631	0.20188	-0.1715	-0.0082	0.27844	-0.0219	-0.1619	-0.3754	-0.3519	-0.0192	0.17626
NM_001106259	Akt1s1_predicted	-0.3628	0.01729	-0.454	-0.1895	-0.384	-0.953	0.06007	-0.0559	-0.3995	-0.1323	-0.2468	-0.6219	-0.0817	-0.1171
NM_017093	Akt2	0.20006	0.22032	0.0566	0.18762	0.16146	0.02722	0.06957	0.13405	0.06678	0.00269	0.13097	-0.2358	0.15297	-0.0594

NM_031575	Akt3	0.65541	0.58212	-0.1143	0.02305	0.63977	0.35624	0.11353	0.49834	0.23284	0.35595	0.1601	0.22945	0.45053	0.6497
NM_012899	Alad	0.63204	0.81743	0.07943	-5E-06	0.71388	0.96196	0.0604	0.66944	0.58529	0.62858	0.42112	0.35041	0.08619	0.27482
NM_013197	Alas2	-0.0546	-0.0281	0.05953	0.02985	0.32041	0.04673	0.02713	-0.0106	0.20504	0.02516	0.08999	0.11837	-0.0486	0.02817
NM_134326	Alb	-0.0981	-0.0422	-0.1372	-0.0157	-0.1609	0.15282	0.03328	-0.0175	0.03588	-0.1212	0.1774	-0.0693	0.01252	0.05568
NM_134326	Alb	-0.0934	-0.06	-0.0363	-0.0433	0.02271	0.03626	0.01122	-0.0028	-0.0041	0.08295	0.08106	0.23896	0.07562	0.00926
NM_031753	Alcam	-0.5491	-0.52	-0.8582	0.36709	-0.142	-0.5598	-0.5363	-0.0962	-0.6011	-0.4162	-0.7193	-1.067	-0.6223	-0.4623
NM_001008694	Aldh16a1	-0.8049	-0.2444	0.12741	-0.8899	-0.573	-0.7744	-0.4119	-0.693	-0.3397	-0.3252	-0.3677	0.09918	0.06427	0.23535
NM_022407	Aldh1a1	-0.0775	-0.0843	-0.0271	0.03099	-0.0294	0.00483	0.01503	0.05367	-0.113	0.09597	0.07126	-0.0215	0.07544	0.12481
NM_053896	Aldh1a2	0.02387	0.03608	-0.0097	0.03546	0.05332	0.22699	0.07045	0.14205	0.08429	-0.0295	0.09885	0.02354	0.03872	0.01558
NM_153300	Aldh1a3	-0.1492	-0.0664	0.08676	-0.1129	-0.1555	-0.1637	0.02783	-0.0854	-0.1945	0.06769	-0.1522	-0.0443	0.08538	-0.0379
NM_017272	Aldh1a7	-0.0974	-0.1299	-0.1209	-0.1052	-0.2674	-0.1857	0.08875	-0.3229	-0.1016	-0.4664	-0.261	-0.3635	-0.1107	-0.0741
NM_001011975	Aldh1b1	-0.2135	-0.0745	0.03264	-0.1031	-0.2059	0.22368	0.25282	-0.0645	0.29522	-0.0812	-0.1516	-0.2868	-0.186	0.02923
XM_235005.4	Aldh1l2_predicted	0.01955	-0.0079	0.07601	0.20729	0.33894	0.05076	0.05432	0.05351	-0.0479	0.13746	-0.0298	-0.0948	0.15354	0.19579
NM_031972	Aldh3a1	-1.5459	-1.2158	-1.3404	-1.5718	-1.4698	-1.5709	-1.1799	-1.7176	-0.6036	-1.6747	-1.321	0.47666	-1.753	-1.7591
NM_031731	Aldh3a2	0.16821	0.16058	0.28029	0.35672	0.06095	-0.0856	0.30221	0.0659	0.16177	0.16366	-0.2653	0.39692	0.15764	0.18055
NM_031731	Aldh3a2	-0.1193	-0.1397	-0.0542	-0.2291	-0.1376	-0.035	-0.0212	-0.2762	0.00856	-0.3722	-0.0811	-0.1353	-0.1179	-0.081
NM_001006998	Aldh3b1	0.32491	0.32006	0.0924	0.11277	0.39664	0.71068	0.13208	0.42739	0.10305	0.41098	0.47092	0.2486	0.02349	0.11843
XM_001062926.1	Aldh5a1	-0.009	0.54884	-0.7432	-0.7434	0.56357	0.91714	-0.3905	0.79477	0.55838	0.39109	0.42153	0.44649	-0.1134	-0.0298
NM_031057	Aldh6a1	-0.153	0.04571	-0.022	0.13141	-0.0029	-0.081	0.17318	0.0254	-0.0029	-0.0454	-0.0168	-0.1379	-0.0318	0.09475
XM_001059375.1	Aldh7a1	-0.1536	0.08882	0.18862	-0.114	-0.0812	0.12465	-0.2496	-0.0627	0.22533	-0.0513	0.09863	0.23412	0.2677	0.29092
NM_022273	Aldh9a1	0.30029	0.55363	-0.2468	0.00737	-0.0512	-0.8994	0.09751	-0.2622	0.31163	0.33655	-0.0116	-0.0593	0.52429	0.81599
NM_012495	Aldoa	0.83421	0.90327	-0.2435	-0.3551	0.86238	0.26476	-0.3321	1.0121	0.56646	0.80702	0.59375	-0.32	0.40102	0.29443
NM_012495	Aldoa	0.72272	0.78131	0.52221	0.38217	0.80013	0.63606	-0.2032	0.67974	0.1274	0.77267	0.81437	-0.2608	0.23346	0.29212
NM_001013943	Aldoal1	0.10548	0.11338	0.13018	0.01823	0.1028	0.01731	0.14656	0.24265	0.22989	0.10457	0.13853	0.05376	0.03877	0.24027
NM_012496	Aldob	0.30441	0.26499	0.1772	0.26964	0.02162	-0.0113	0.39189	0.17038	0.18397	0.13722	0.34943	0.02705	0.16914	0.14102
NM_012497	Aldoc	0.26911	-0.1632	-0.193	-0.2467	-0.0158	-0.0282	-0.3147	-0.1244	-0.2065	-0.0646	-0.2804	-0.0704	-0.0589	-0.1695
NM_001108264	Alg1_predicted	0.46562	0.12912	0.25886	0.02658	0.15146	-0.323	0.32492	-0.3933	-0.0093	0.03155	0.21093	0.28188	-0.0532	0.21297
NM_001108104	Alg12_predicted	0.08215	-0.1165	0.23533	0.22054	0.05671	0.08893	-0.1447	0.52461	-0.21	-0.0296	0.02369	-0.2131	-0.0323	0.27609
NM_001100710	Alg2	-0.2039	0.50507	-0.4756	-0.2429	0.247	-0.1266	0.03346	0.3017	0.19952	0.30967	0.316	-0.5772	0.47449	0.40461
NM_001011897	Alg3_predicted	0.04649	0.21489	-0.0346	0.22644	0.02279	0.0526	0.14314	-0.0731	0.09212	0.05828	0.39887	0.06991	-0.0078	-0.0295
NM_001025407	Alg5	-0.1985	0.15086	0.0783	-0.5862	-0.0656	-0.1421	0.31372	-0.1367	0.15577	0.51309	0.17876	0.06252	0.15342	0.23629
NM_001033709	Alg6	-0.001	-0.1872	-0.2541	-0.294	-0.1135	-0.3503	-0.1603	-0.1506	-0.3521	-0.2514	-0.4188	-0.5473	-0.1579	-0.1864
NM_001034127	Alg8	-0.6956	-0.8097	0.71305	-0.233	-0.9859	-1.0062	-0.1857	-0.8818	-0.6498	-0.7432	-0.5452	-0.2993	0.03444	-0.2221
NM_001109000	Alg9_predicted	0.21254	0.23051	-0.1098	-0.7015	0.25641	0.02444	-0.2204	0.04058	0.12928	0.23518	0.19158	0.09055	0.39832	0.26716
NM_001169101	Alk	0.13393	0.1212	-0.1717	0.02721	0.89288	0.57089	0.03882	0.86543	0.0345	0.00814	-0.0642	-0.0879	0.00315	0.15972
NM_001108718	Alkbh_predicted	-0.0245	-0.2134	0.04904	0.02664	0.11448	-0.2698	0.22671	0.1715	-0.0493	-0.2433	0.01929	-0.0039	0.16857	0.27367
NM_001126273	Alkbh2_predicted	0.48088	-0.0533	0.00361	-0.0637	0.21692	0.5893	-0.2764	0.09822	-0.0836	-0.0679	0.12648	0.42201	0.08437	-0.0224
NM_001014180	Alkbh3	0.72439	0.46359	0.0358	1.0871	1.2735	1.3595	0.31098	1.0637	0.18779	0.26836	0.56564	0.23496	0.13123	0.27472
NM_001105920	Alkbh4_predicted	0.00361	0.11783	-0.0542	0.019	0.23405	-0.0603	0.23081	-0.0029	-0.0961	0.42734	0.17269	-0.0142	0.20337	-0.0071
XM_220525.4	Alkbh5_predicted	0.56946	0.51523	0.13687	0.45319	0.36321	0.05666	0.14681	0.44125	0.06871	0.3719	0.16988	0.02637	0.51856	0.38168
NM_001013037	Allc	0.49625	-0.0434	0.1185	-0.1469	1.0513	1.0037	0.31168	0.87877	0.04374	0.1266	0.2157	-0.0119	-0.0389	0.00832
NM_001106604	Alms1_predicted	-0.7193	-0.7939	-0.1031	-0.5559	-0.9623	-0.9594	-0.6998	-1.1132	-0.7572	-0.8158	-0.6783	-0.2346	-0.3421	-0.5435
NM_001105798	Alox12_predicted	-0.216	-0.3088	-0.3095	-0.3788	-0.3287	-0.3547	-0.4115	-0.3983	-0.6436	-0.4621	-0.2189	-0.1359	-0.198	-0.0487
NM_001039377	Alox12b	0.14185	-0.1499	-0.1118	-0.044	0.1281	0.30639	0.19288	0.22103	0.02043	-0.017	0.13635	0.03357	0.08463	0.08517
NM_001107014	Alox12e_predicted	0.89186	0.05062	0.08968	-0.0194	0.10104	-0.0616	0.24981	0.21976	0.16613	0.18809	0.01669	0.20444	-0.0948	-0.0433
NM_031010	Alox15	-0.08	-0.1308	0.00777	0.06951	-0.209	-0.078	0.07756	-0.0125	-0.2177	-0.1484	-0.1539	-0.2492	-0.0745	-0.3265
NM_012822	Alox5	-0.0913	0.05428	-0.1801	-0.0056	-0.0327	-0.235	-0.138	-0.1863	0.10021	-0.1873	-0.025	0.0513	-0.1123	-0.1496
NM_017260	Alox5ap	-0.1494	-0.143	-0.1598	-0.266	-0.1014	0.00077	-0.0902	-0.2165	-0.2587	-0.2539	-0.0305	-0.2421	-0.2694	-0.2736
NM_001105793	Aloxe3_predicted	0.02116	-0.0803	0.15124	-0.0543	-0.0831	0.24642	-0.0727	-0.0398	-0.2191	-0.18	-0.1104	-0.1603	-0.0976	-0.0461

NM_022665	Alpi	-0.0801	0.14856	-0.1413	-0.0075	0.11702	0.05501	-0.0085	-0.0615	0.09841	-0.0787	0.17658	-0.1425	0.05441	-0.0176
NM_022680	Alpi2	-0.232	-0.0542	-0.1387	-0.2136	0.1501	-0.1631	0.00922	-0.3132	-0.3906	-0.3257	-0.1427	-0.1553	-0.0706	-0.1982
XM_344916.3	Alpk3_predicted	-0.32	-0.1775	-0.154	-0.2571	-0.2836	-0.1571	-0.4366	-0.3363	-0.1575	-0.1091	-0.1995	0.20243	-0.3623	-0.106
NM_013059	Alpl	0.00575	-0.5135	-0.4072	-0.3925	-0.4314	-0.2714	-0.4825	-0.57	0.01211	-0.5948	-0.2721	0.34738	-0.6508	-0.3035
NM_001013413	Als2	-0.2925	-0.1321	0.42472	0.35446	-0.4395	-0.1592	0.09577	-0.5453	-0.1467	-0.0509	-0.3272	0.21199	0.10601	0.07691
NM_001024363	Als2cr11	-0.1628	-0.1813	-0.1628	-0.1643	-0.0927	-0.1972	-0.1934	0.01492	-0.1977	-0.3075	-0.255	-0.0755	0.0704	-0.1811
NM_001014101	Als2cr12	0.37864	0.11761	0.17071	0.28356	0.29533	0.12148	-0.0912	0.471	0.21231	-0.0052	0.0598	-0.0339	0.26264	0.29071
NM_001108797	Als2cr13_predicted	0.04505	-0.3241	-0.1058	-0.2306	-0.2285	-0.1685	-0.09	-0.1179	-0.1687	-0.228	0.09547	-0.2425	-0.2889	-0.1982
NM_001108220	Als2cr4_predicted	0.05894	-0.0954	-0.0386	0.01745	-0.1022	0.14128	-0.0079	0.14129	-0.2158	0.01301	0.12774	0.13861	-0.1192	0.02979
XM_001069526.1	Als2cr7_predicted	0.01151	0.04673	0.09199	0.26117	0.09629	0.02517	0.36108	0.10005	0.1559	0.04137	0.09853	0.07875	0.25776	0.14065
NM_012921	Alx1	0.06922	0.02061	-0.0079	0.13837	0.21935	0.13229	-0.0178	0.09381	0.20585	0.2675	0.09632	0.06081	-0.1461	0.1764
NM_001007012	Alx3	-0.1669	-0.2079	-0.2456	-0.0285	-0.337	-0.2006	-0.1667	-0.1605	-0.1514	-0.2684	-0.243	-0.1603	-0.111	-0.3171
NM_001106553	Alx4_predicted	-0.2354	-0.2019	-0.2871	-0.092	-0.2392	-0.1984	-0.1085	-0.1574	-0.1905	-0.3113	-0.1011	-0.1472	-0.3139	-0.3401
NM_012816	Amacr	-0.1846	-0.3876	-0.3326	-0.2299	-0.5721	-0.4652	0.15152	-0.6207	-0.337	-0.4787	-0.3959	-0.0126	-0.4511	-0.4174
NM_012900	Ambn	-0.0105	-0.0507	-0.1092	0.10154	0.22255	0.01071	-0.0131	0.1038	0.03771	0.00115	-0.0888	-0.0459	0.23572	-0.0403
NM_012901	Ambp	0.0364	0.17609	-0.0425	0.11189	0.12705	0.1316	0.01841	0.34729	0.0892	0.08335	-0.0626	0.02536	-0.0002	0.1514
NM_031011	Amd1	-0.4757	-0.8283	-0.2645	-0.593	-0.9253	-0.9195	-0.8439	-1.0042	-0.4598	-0.6033	-0.8147	-0.1976	-0.0294	-0.0304
XM_235065.4	Amdhd1_predicted	0.11589	0.07042	0.08662	0.11033	0.06374	-0.0559	0.1354	0.04235	-0.005	0.01541	0.03	0.05051	0.02004	-0.0361
NM_019154	Amelx	0.05352	0.08987	-0.0827	-0.0344	-0.0695	0.13228	-0.0786	-0.0918	0.1461	0.25491	-0.0702	-0.0349	-0.0245	0.00681
XM_001062954.1	Amfr_predicted	0.14226	0.57303	-0.0093	-0.0429	0.12426	-0.6686	0.27817	0.18478	0.5962	0.68927	0.37987	-0.2263	0.49726	0.56339
NM_012902	Amh	-0.0699	-0.011	0.11298	0.19498	0.11212	0.02744	0.15957	0.3727	0.00094	0.02923	0.17878	0.00765	0.00898	0.17454
NM_030998	Amhr2	-0.252	-0.4813	-0.2	0.22485	-0.1811	-0.3505	-0.0764	-0.228	-0.0759	-0.1667	-0.0232	0.11629	-0.0902	-0.5223
XM_001067982.1	Amica1_predicted	-0.1033	-0.0468	-0.0709	-0.0955	-0.1048	-0.0516	-0.0737	0.06785	-0.0525	-0.0369	-0.0322	-0.0647	0.09636	0.05961
NM_001139483	Amid_predicted	-0.0641	0.01023	-0.0192	-0.2912	0.11489	0.32097	-0.4984	0.17636	-0.1125	0.0218	-0.1396	-0.2457	-0.1622	-0.2169
NM_206881	Amigo1	-0.1369	-0.0849	0.07197	-0.2401	-0.1692	-0.2194	-0.124	-0.2023	-0.1437	-0.0997	-0.1587	-0.2726	-0.2614	-0.0712
NM_182816	Amigo2	-0.0474	-0.1249	-0.1459	-0.2814	-0.193	-0.1541	-0.1533	-0.2149	0.04169	-0.2356	-0.1454	-0.1949	-0.2648	0.09022
NM_178144	Amigo3	0.02384	0.07849	0.12374	0.41157	0.25031	-0.0744	0.14933	0.00154	0.04152	0.17568	0.17354	0.25008	0.15058	-0.01
NM_001108061	Amn_predicted	0.06579	0.01629	0.04826	-0.1587	-0.0197	-0.0114	-0.1128	-0.0459	0.0683	0.01948	-0.1455	-0.0542	-0.025	-0.0569
NM_001108126	Amotl1_predicted	-0.0245	-0.0488	0.13154	-0.0231	0.11389	0.0803	-0.0655	0.18429	-0.0787	-0.1363	-0.0895	-0.1064	-0.1294	-0.0317
NM_138876	Ampd1	0.03362	-0.1169	0.18994	0.12778	0.01899	0.16005	-0.016	-0.1314	-0.1778	-0.0165	-0.1487	0.02636	-0.1309	-0.0404
NM_031544	Ampd3	1.2233	0.22879	0.00398	0.06344	0.44513	0.1821	0.03267	0.39866	-0.0608	0.09273	0.07502	-0.1527	0.05583	-0.0526
NM_022217	Amph	0.49238	1.285	0.79823	0.28654	-0.0436	-0.2467	0.45932	-0.1319	0.95826	0.89214	1.0842	0.77762	0.87914	0.94408
NM_001014004	Amt	-0.5222	0.05958	0.1209	0.77076	-0.2735	-0.2288	0.31377	-0.4572	-0.1205	-0.009	-0.2227	0.0608	-0.2686	-0.4983
NM_001010970	Amy1	-0.0328	0.03061	-0.1171	-0.0619	-0.1286	-0.1483	-0.168	-0.1208	0.00738	0.03579	-0.0957	5E-05	-0.0458	-0.1388
NM_031502	Amy2	0.01483	0.01791	-0.0075	-0.0672	-0.0467	0.11891	0.04998	0.04873	0.01755	0.20219	-0.1433	0.36603	-0.0146	0.00112
NM_031502	Amy2_predicted	-0.0465	-0.0278	-0.022	-0.014	0.1205	0.14392	-0.0403	0.05708	-0.0106	-0.0208	0.22398	-0.076	-0.0781	0.15874
NM_001014121	Amz2	-0.1554	-0.1686	0.00395	-0.0563	-0.0553	0.11269	0.13794	-0.0979	0.14344	-0.115	-0.1851	0.01163	-0.0607	-0.1233
NM_001107771	Anapc1_predicted	-0.8791	-0.648	-0.4707	-0.2908	-0.2546	-0.0985	-0.8716	-0.4488	-0.5564	-0.6681	-0.8257	-0.0123	-0.4515	-0.5031
NM_001108445	Anapc10_predicted	0.68675	0.42208	0.17257	-0.0682	-0.0093	0.08731	0.41573	-0.2288	0.18361	0.28072	0.54659	0.38312	0.38553	0.44252
NM_001100532	Anapc2	0.32493	0.07218	0.43001	0.08819	0.06013	-0.0384	0.15898	0.18013	0.05531	0.07862	0.22788	0.10027	0.17055	0.15413
NM_001107220	Anapc4	-0.5433	-0.6761	0.26958	0.38611	-0.7684	-0.8633	0.23912	-0.7693	-0.4643	-0.4791	-0.5796	-0.3025	-0.3047	-0.3638
NM_001080147	Anapc5_predicted	-0.0383	-0.2023	-0.1963	-0.5456	-0.3114	-0.489	-0.2831	-0.3722	-0.2156	-0.1954	-0.1494	0.12697	0.0993	0.08095
NM_001107142	Anapc7_predicted	0.5313	0.39764	-0.0054	0.89065	0.71743	0.69477	0.31545	0.60917	0.35404	0.15431	0.03815	0.05287	-0.0434	0.10489
NM_012718	Andpro	0.17367	0.27154	0.19076	-0.0263	0.20669	0.18599	0.02754	0.12718	0.12435	0.03841	0.1848	0.02861	-0.0694	0.0898
NM_001006992	Ang1	0.61853	0.48432	0.06801	1.1122	1.022	1.345	0.60113	0.92365	0.82092	0.67701	0.70396	0.59861	0.34698	0.04376
NM_001108717	Angel1_predicted	0.48572	0.69005	-0.1462	0.23375	0.3663	0.15674	-0.0395	0.55683	0.01281	0.26974	0.35044	-0.1642	-0.0281	0.00114
XM_001068063.1	Angel2_predicted	-0.7918	-0.1927	-0.1078	0.16323	-1.3142	-1.4332	0.01981	-1.4683	-0.408	-0.2442	-0.3455	-0.0919	-0.2006	-0.2153
NM_053546	Angpt1	0.30946	0.2178	0.14403	0.19203	0.25626	0.00271	0.14627	0.10362	0.1373	0.23203	0.16523	-0.0805	0.08895	0.04448
NM_134454	Angpt2	-0.065	-0.0524	0.13807	0.31901	-0.0947	-0.0852	-0.0253	0.09902	-0.0133	0.10423	0.06522	0.03428	0.163	0.14207

NM_001106526	Angpt4_predicted	0.5782	0.35579	0.20775	0.09087	0.34471	0.11612	-0.0347	0.26111	0.32902	0.40272	0.42756	0.26131	0.65421	0.57412
NM_133569	Angptl2	-0.4866	-0.25	-0.9229	0.08835	1.5787	1.1763	-0.5008	1.4382	-0.6442	-0.2894	-0.3868	-1.161	-1.0952	-0.9036
NM_199115	Angptl4	-0.158	0.10867	-0.0241	0.33082	-0.0775	-0.0735	0.09168	0.07515	0.03137	-0.0573	-0.0869	-0.136	0.00838	0.08407
NM_199115	Angptl4	0.40965	1.4336	0.09107	1.6475	0.25444	0.38042	0.9828	0.50019	1.0588	1.1821	1.0794	0.83223	-0.3527	-0.2499
NM_001106702	Angptl6_predicted	0.02105	0.23169	0.10516	0.32201	0.15008	0.19806	0.43153	0.05002	0.04783	0.34815	0.19564	-0.0311	0.08707	0.19117
NM_053714	Ank	-0.4476	0.41857	0.17079	-0.8429	-0.4057	-0.3781	0.98669	-0.3018	0.37508	0.23562	0.19641	0.22062	-0.1649	-0.3221
NM_001107322	Ank1_predicted	1.1956	0.39855	0.69212	0.19031	1.3125	1.0748	0.71752	1.2502	0.12639	0.17029	0.49818	0.11624	0.15739	0.2658
XM_001076082.1	Ank2	-0.2257	0.02816	0.0558	0.08247	-0.2384	-0.0656	-0.0814	-0.0814	0.05341	-0.0452	0.17831	0.05983	0.10948	0.07951
XM_001076082.1	Ank2	0.14832	0.13894	0.24242	0.14482	0.17593	0.08188	0.06997	0.41867	0.29739	-0.026	-0.0166	0.23204	0.14749	0.35505
NM_001107018	Ankfy1_predicted	0.10554	0.12894	0.08977	-0.1313	0.2114	-0.0753	0.34667	0.34285	0.22629	0.22929	0.29434	0.14826	0.08527	-0.0726
NM_207595	Ankra2	-0.2638	-0.0641	-0.0631	0.00278	0.04201	0.10937	0.08644	0.08722	0.11739	-0.2023	-0.1695	-0.0678	-0.022	-0.0471
NM_013220	Ankrd1	-0.0936	0.94988	0.26213	0.94708	0.91986	0.90421	0.41312	0.82787	0.74756	0.91939	0.71608	1.0631	0.0555	0.20883
XM_341466.3	Ankrd10	-0.312	-0.2785	0.39666	0.32444	-0.5726	-0.6452	0.03072	-0.4557	-0.2333	-0.3928	-0.2684	-0.103	-0.4119	-0.5147
NM_001108900	Ankrd11_predicted	-0.2527	-0.2776	0.54239	0.82461	-0.3694	-0.4645	0.06409	-0.5384	-0.4775	-0.2374	-0.2924	-0.1823	-0.479	-0.4411
NM_001012148	Ankrd13a	0.29582	0.24946	0.32577	0.1503	0.35697	0.05669	0.34552	0.48953	0.25847	0.2052	0.27262	0.13378	0.37539	0.57678
NM_001108514	Ankrd13d_predicted	-0.41	-0.5813	-0.917	-0.0386	-0.1	0.15874	-0.6823	-0.1465	-0.9974	-0.7286	-0.7223	-0.8625	-1.1831	-0.9418
NM_001037197	Ankrd15	-0.0566	-0.0562	-0.093	0.32333	-0.4488	-0.6449	0.11724	-0.4208	-0.2949	-0.1319	-0.1728	-0.5392	-0.7643	-0.6963
NM_001033698	Ankrd16	-0.4391	-0.1908	-0.0134	0.07304	-0.1045	-0.0036	0.03102	-0.4384	-0.1743	0.23687	-0.0177	-0.0375	-0.47	-0.4575
NM_001107589	Ankrd2_predicted	0.04442	0.10265	-0.0157	0.14597	0.05495	0.33975	-0.0283	0.16792	0.00995	0.28938	0.05273	0.12836	-0.0458	0.27661
XM_220065.4	Ankrd22_predicted	0.11635	0.12175	-0.0872	0.21114	0.07773	0.13998	0.20775	0.09983	0.24974	0.07184	-0.1079	-0.0197	0.43513	-0.012
NM_001108211	Ankrd23_predicted	-0.0906	-0.0008	-0.1169	-0.0766	-0.2173	-0.0294	0.05692	-0.0655	-0.0575	-0.1575	-0.0881	0.10007	-0.0713	-0.106
NM_001106771	Ankrd24_predicted	-0.132	-0.3358	0.11863	-0.9562	-0.4976	-0.3863	-0.2444	-0.8504	-0.1645	-0.2816	-0.3805	0.15886	-0.1803	0.06158
XM_225482.4	Ankrd26_predicted	-0.2156	-0.1278	-0.0765	0.00542	-0.199	-0.0404	-0.061	-0.1726	-0.1637	-0.1183	0.0678	-0.0006	0.15397	-0.1774
XM_341843.2	Ankrd27_predicted	-0.0681	-0.0299	0.23687	0.39799	-0.0582	0.03941	0.41089	0.04688	-0.0134	-0.1137	-0.0899	-0.0626	-0.1236	-0.1367
NM_001106400	Ankrd32_predicted	-0.1247	-0.0753	0.00445	0.02685	-0.0389	0.09382	-0.1121	0.12788	0.08045	-0.0147	-0.1809	0.30857	-0.0343	-0.1442
NM_001106400	Ankrd32_predicted	0.06875	0.52739	-0.184	0.12522	0.017	-0.1475	-0.0576	0.13607	0.04352	-0.0134	-0.1377	-0.0778	0.10791	0.25504
NM_198684	Ankrd36	-0.0338	0.26162	0.02719	0.27003	0.05874	0.0278	-0.0943	0.19728	-0.0318	0.04456	0.0958	0.04852	-0.0052	0.13739
NM_001135014	Ankrd39_predicted	-0.1477	0.22212	0.07892	-0.0634	0.11259	0.10197	-0.1025	-0.0212	0.27333	-0.1445	0.0151	0.33952	0.24717	0.12091
NM_001135013	Ankrd42_predicted	0.18232	0.62581	0.20467	0.93201	0.30585	0.30126	0.94246	0.26764	0.18329	0.53101	0.37545	0.13059	-0.1757	-0.2601
XM_213908.4	Ankrd45_predicted	0.61506	0.08855	0.17759	0.26287	-0.038	0.49391	0.12896	0.02562	-0.1468	-0.0507	0.15429	0.34061	0.35122	0.26845
NM_001013948	Ankrd46	0.1183	0.93867	-0.1884	0.17317	0.26166	0.10548	-0.0059	0.51761	0.36133	0.41331	0.56438	0.02702	0.0386	0.14479
NM_001108989	Ankrd47_predicted	-0.5714	0.19565	-0.0121	-0.1119	-0.2164	-0.2959	-0.0174	-0.4784	0.07199	0.1537	0.22834	0.03554	-0.4201	-0.3985
NM_001126283	Ankrd49_predicted	0.03959	0.04837	0.29502	-0.1978	0.15042	0.08799	0.12009	0.01286	0.21054	0.23753	-0.1092	0.21993	0.235	0.29359
NM_001106516	Ankrd5_predicted	0.091	-0.0949	-0.0137	-0.0175	0.05152	0.00896	-0.0642	-0.0866	-0.0503	-0.0225	-0.0272	0.01749	-0.0727	-0.0625
XM_215553.4	Ankrd50_predicted	0.30984	0.21673	-0.0233	0.22828	0.3969	0.12692	0.16196	0.17533	0.00597	0.21869	0.41654	0.12621	-0.0435	0.0631
XM_215553.4	Ankrd50_predicted	-0.2641	-0.1945	-0.0781	-0.0489	0.13964	-0.0433	-0.2346	0.1438	-0.0868	-0.2925	-0.418	-0.0564	-0.4851	-0.1806
XM_343139.3	Ankrd52_predicted	-0.0179	-0.0207	0.08363	-0.1136	-0.064	0.0363	-0.0609	0.00597	-0.0896	-0.0054	-0.114	-0.1065	-0.0531	-0.0057
XM_343139.3	Ankrd52_predicted	-0.1775	-0.0153	-0.1121	0.01195	-0.1132	-0.1846	0.11154	-0.2046	-0.073	-0.1543	-0.1147	0.1671	-0.0281	-0.048
NM_001012112	Ankrd9	-0.48	-1.0305	0.1703	-0.0477	-0.591	-0.4089	-0.7414	-0.7078	-0.7907	-0.3443	-0.9171	-0.606	-0.4527	-0.3039
NM_001107613	Anks1_predicted	0.14465	0.08034	0.00393	0.10735	-0.0238	0.01548	0.13266	0.07992	0.46431	0.00127	0.15198	-0.0022	0.2142	0.00628
NM_001009676	Anks3	-0.2683	-0.4124	0.21007	0.07733	-0.5086	-0.4292	0.06317	-0.2466	-0.1608	-0.5133	-0.3863	-0.1687	-0.4546	-0.2719
NM_001015028	Anks6	-0.2068	0.07871	0.33816	0.21472	0.03596	-0.1425	-0.0277	-0.3829	-0.2467	0.38713	-0.2084	0.08912	0.10892	-0.0436
NM_001014219	Ankzf1	-0.1438	-0.1467	0.09074	0.71655	-0.1404	0.19383	0.21896	-0.0045	-0.0845	-0.3269	-0.3263	-0.4041	-0.6568	-0.4142
XM_219687.3	Anln_predicted	0.38179	-0.3915	0.21373	-0.1311	0.37767	-0.1913	-0.3421	0.57445	0.03794	-0.2136	-0.4125	0.07524	1.3201	1.1846
NM_012903	Anp32a	-0.8852	-1.0554	-0.7925	-1.0338	-0.9248	-0.6309	-1.1704	-1.0877	-0.8587	-1.4615	-1.0372	-0.5562	-0.7756	-0.8013
NM_131911	Anp32b	0.01859	-0.2674	0.1146	-0.3987	-0.2887	0.38081	-0.4157	0.08335	-0.1997	-0.2027	-0.067	0.44051	0.19029	0.0643
NM_001013200	Anp32e	0.63037	0.0085	0.12241	-0.4109	0.30558	0.426	-0.2441	0.15174	0.02295	-0.0064	-0.1804	0.48166	0.66011	0.54183
NM_031012	Anpep	0.54207	0.82829	0.08271	0.94895	0.25565	0.28192	0.17862	0.17834	0.88115	0.98764	0.73216	-0.0742	-0.5636	-0.2062
NM_001044249	Antxr1_predicted	0.15068	0.1147	-0.0011	0.04929	-0.0412	-0.0761	-0.0524	-0.0207	-0.0934	-0.0312	0.02365	-0.0356	-0.0084	-0.1095

NM_173332	Anubl1	0.11799	-0.1758	0.13149	-0.0433	-0.2309	-0.4012	-0.0555	-0.1146	-0.0728	-0.0539	0.06559	-0.258	0.05188	0.09396
NM_012904	Anxa1	0.9071	0.60566	0.5587	0.64438	0.86212	0.55169	0.79248	1.2203	0.73151	0.58482	0.78456	-0.0143	0.7069	0.30872
NM_001011918	Anxa11	0.21893	0.34715	-0.123	-0.543	0.52885	0.00043	-0.2798	0.48937	0.03573	0.21124	0.03716	-0.5834	-0.5382	-0.4035
NM_001011918	Anxa11_predicted	-0.1565	-0.1791	-0.2136	-0.1007	0.03396	-0.1648	-0.235	-0.1283	-0.246	0.14205	0.05059	-0.3086	-0.0481	-0.121
NM_001134910	Anxa13_predicted	0.27095	-0.0733	-0.0191	0.07477	-0.0167	0.11314	-0.0074	0.34932	0.18529	0.01581	0.12215	-0.0456	0.00926	0.03963
NM_019905	Anxa2	0.48581	0.15796	0.35542	-0.8306	0.16682	-0.0743	0.17744	0.09134	0.03178	0.00518	0.01201	0.07682	0.25878	0.24966
NM_012823	Anxa3	-0.6056	-0.7196	-0.5049	-1.0106	0.47771	0.35322	-1.3872	0.40325	-0.486	-0.509	-0.7505	0.14194	-0.0954	-0.0821
NM_024155	Anxa4	-0.1717	0.29198	-0.2728	-0.4063	-0.2233	-0.4725	0.26859	-0.1888	-0.0819	0.35557	0.12242	-0.2525	-0.161	-0.3033
NM_013132	Anxa5	0.49516	0.47782	-0.2093	-0.27	0.06572	0.02485	0.38823	-0.1456	-0.0555	0.17068	-0.0385	0.41048	-0.0244	0.12331
NM_024156	Anxa6	-0.0618	-0.2214	0.07275	-0.1874	-0.0572	0.04524	-0.1304	-0.1264	0.16694	0.18779	-0.0495	0.28172	0.19722	0.18605
NM_130416	Anxa7	0.39926	0.58541	-0.2465	-0.0317	0.19246	0.00023	0.51735	0.39776	0.06382	0.50806	0.25144	-0.6246	0.14376	-0.0079
NM_001031654	Anxa8	-1.8604	-3.8575	-2.3552	-3.1431	-1.569	-1.7459	-2.9368	-1.8655	-3.2406	-3.0666	-2.3138	-2.9042	-3.6126	-3.6016
NM_031582	Aoc3	0.10902	0.05377	0.24225	0.20415	-0.0543	0.10599	0.15659	0.27619	0.08745	0.15327	0.07399	0.04611	0.17351	0.00428
NM_001107343	Aof1_predicted	0.17749	0.07101	0.20258	0.06875	0.23011	-0.156	-0.0762	0.16902	-0.0669	0.01365	-0.0876	-0.0287	0.16791	0.1037
NM_019363	Aox1	0.52422	2.2981	0.75773	1.3484	-0.3871	-0.6102	1.2393	-0.3189	1.8369	2.1362	1.8101	0.09768	-0.5252	-0.5015
NM_001008527	Aox3	0.04231	0.05347	0.09918	0.00022	0.19802	0.0626	0.10276	0.06455	0.03881	0.03451	0.06624	0.24231	0.01615	0.01284
NM_001008522	Aox3l1	-0.0321	0.12567	0.11231	0.04135	-0.0596	0.03538	0.04964	-0.1215	0.19656	0.03176	-0.0232	0.04101	-0.031	-0.1689
NM_017277	Ap1b1	-0.1578	-0.2769	0.29031	-0.5681	-0.2292	-0.0392	-0.1293	-0.4183	-0.3054	-0.0018	-0.2076	0.26522	0.01663	0.07825
NM_134460	Ap1g1	0.49746	0.13678	0.40631	-0.0503	0.37425	0.45256	0.10957	0.47036	0.04763	0.08062	0.13311	0.11716	0.60655	0.57921
XM_214197.3	Ap1g2_predicted	0.91293	0.82785	1.1302	0.39903	1.6247	1.3787	1.0435	1.5439	0.67607	1.071	0.64959	0.11935	0.9268	0.77355
NM_053419	Ap1gbp1	-0.4719	-0.0396	-0.2286	-0.578	0.03098	-0.4473	-0.2759	-0.1541	-0.0704	0.04872	-0.0616	-0.4326	-0.3535	-0.0064
NM_001044239	Ap1m1	-0.5499	-0.2271	-0.0709	-0.9024	-0.6924	-0.7895	-0.6222	-0.744	-0.2943	-0.4241	-0.348	-0.1599	-0.3516	-0.3935
NM_001108331	Ap1s1_predicted	0.2334	0.01965	0.20749	0.26199	-0.0347	0.0676	0.02847	-0.0315	0.04829	0.03378	0.1958	-0.0201	0.00289	0.02385
NM_001127531	Ap1s2_predicted	-0.0194	0.58228	-0.0331	0.11783	-0.1066	0.18878	0.61528	0.10882	0.39999	0.5661	0.61153	0.12384	0.65355	0.45864
NM_001107511	Ap2a1_predicted	-0.2435	-0.3035	-0.1196	-0.1343	-0.2554	-0.5387	-0.2891	-0.2493	-0.2144	-0.1711	-0.1992	-0.3103	-0.1363	0.00834
NM_031008	Ap2a2	-0.0116	-0.1456	-0.0029	-0.8869	-0.0993	-0.4133	-0.2181	-0.1885	-0.3272	-0.0456	-0.3669	-0.3624	0.05953	0.0649
NM_080583	Ap2b1	-0.2795	0.27375	0.06302	-0.2677	-0.308	-0.348	0.14321	-0.3938	-0.0603	0.22897	0.15487	0.03379	-0.1399	-0.2038
NM_053837	Ap2m1	-0.2282	-0.0014	0.40223	-0.2944	0.14965	-0.0867	0.12965	0.11873	-0.3334	-0.3584	-0.3176	-0.2939	-0.0625	0.01634
NM_022952	Ap2s1	-0.1019	0.06462	0.04075	-0.5282	-0.0522	-0.0143	-4E-05	0.04842	-0.0139	0.11632	0.14772	0.12677	-0.0141	-0.1536
NM_001107646	Ap3b1_predicted	-0.2325	0.02867	-0.1474	-0.5313	-0.0364	-0.0667	-0.3196	-0.0224	0.00789	-0.0103	0.18025	0.21354	0.0663	0.2405
NM_001107532	Ap3b2_predicted	0.39446	0.41034	0.38578	0.2976	0.22586	0.37815	0.52836	0.27703	0.0009	0.32987	0.27919	0.23543	-0.115	-0.235
NM_001100719	Ap3d1	-0.2281	-0.0262	0.46095	-0.0527	-0.0594	-0.1884	0.07361	-0.2512	-0.1033	0.06186	0.00755	0.19748	0.16461	0.15553
NM_133593	Ap3m1	0.17289	0.08708	-0.1095	-0.3404	0.0599	-0.2721	-0.1828	0.06832	0.02747	0.21996	0.10599	0.02761	0.10237	0.26786
NM_133305	Ap3m2	-0.1969	-0.2212	-0.0789	0.11504	-0.1959	-0.0992	-0.1217	-0.0911	-0.0315	-0.0512	-0.198	-0.0089	-0.1749	-0.2946
NM_001106933	Ap3s1_predicted	-0.146	0.19031	-0.6497	-0.2981	-0.2642	-0.4383	-0.387	-0.1549	0.03722	0.0078	-0.006	-0.1003	-0.1185	-0.0924
NM_001107709	Ap4b1_predicted	-0.1428	-0.1672	0.01581	-0.2754	-0.1148	-0.2162	-0.0173	-0.0769	-0.2048	-0.3626	-0.2124	-0.2262	-0.1863	-0.1267
NM_001037977	Ap4m1	0.01409	-0.1912	-0.2615	-0.4198	-0.3494	-0.4956	-0.3362	-0.5285	-0.2412	-0.4713	-0.407	-0.117	-0.3829	-0.1727
NM_023979	Apaf1	-0.2102	-0.1853	-0.0838	0.49074	-0.0408	0.03487	-0.0114	-0.091	0.26371	-0.1418	0.03835	0.32863	-0.1101	-0.2213
NM_031779	Apba1	0.1366	0.02438	0.01088	0.05942	0.1584	0.01851	0.00016	-0.0431	0.0321	-0.0769	0.069	0.18631	0.19306	0.05815
NM_031780	Apba2	0.03604	-0.1299	-0.0661	-0.0813	0.12658	0.12612	0.01305	0.12091	0.0602	-0.3453	-0.1194	-0.22	0.27428	0.11509
NM_001098724	Apba2bp_predicted	0.02881	-0.0198	0.36445	0.28346	-0.157	-0.1015	0.05148	-0.1824	-0.0325	0.35802	0.04964	-0.2822	0.06936	-0.1114
NM_031781	Apba3	-0.4096	-0.2347	0.18458	-0.0666	-0.8524	-0.6256	-0.2347	-0.8399	-0.4322	-0.3705	-0.3111	-0.2644	-0.4189	-0.406
NM_080478	Apbb1	0.07866	0.47244	0.27793	-0.1071	0.28322	0.07792	0.06938	0.30378	0.29224	0.50095	0.32287	0.10419	-0.0137	0.19932
NM_001100577	Apbb1ip	-0.186	0.28332	0.45983	0.08148	0.371	0.14881	0.39484	0.40507	0.14828	0.55092	0.41953	0.07088	0.33018	0.43758
NM_053957	Apbb3	-0.7569	-0.4868	-0.1341	-0.413	-0.9246	-1.0844	0.02779	-0.8236	-0.7368	-0.5522	-0.6827	-1.1847	-1.4457	-1.1581
NM_012499	Apc	0.0162	-0.0545	0.10866	0.08271	-0.0647	-0.0513	0.03678	-0.067	0.16558	-0.0774	-0.0314	0.20357	-0.0021	0.13464
NM_001106769	Apc2_predicted	-0.0917	-0.0016	0.01869	-0.0362	-0.004	-0.0598	-0.0335	-0.1359	0.21627	-0.0666	0.07159	0.05196	-0.1143	0.08821
NM_017170	Apcs	0.0799	-0.0132	0.06601	0.15898	0.19021	0.26727	-0.0038	0.09081	0.02131	0.08697	0.2735	0.14207	0.03687	0.21622
NM_001034160	Apeg3	0.00209	-0.1059	-0.1682	-0.0503	-0.0045	0.06916	-0.1118	-0.0873	0.05335	-0.1022	0.11302	-0.1937	-0.0582	-0.0722

NM_012500	Apeh	0.21297	0.06237	0.37416	-0.2657	0.14997	-0.0575	-0.397	0.24911	-0.0005	0.21961	0.09554	-0.2219	0.00332	0.03229
NM_024148	Apex1	-0.0557	-0.5465	0.33882	-0.5338	0.13127	-0.2737	-0.0768	-0.0971	-0.2682	-0.0294	-0.4993	-0.0864	0.48443	0.51613
XM_001059968.1	Apex2_mapped	0.01156	0.00929	-0.0589	0.06627	0.0136	-0.0308	0.25112	0.09447	0.10085	-0.0616	-0.003	-0.0415	0.06456	0.06426
NM_001014255	Aph1a	0.22785	0.03919	-0.3344	-0.7776	-0.2689	-0.4084	-0.1343	-0.1528	-0.1359	-0.1415	0.06657	-0.1922	-0.0067	0.08966
NM_001047090	Aph1b	0.22969	0.48867	-0.1769	-0.5541	0.16797	0.03581	0.11894	0.16934	0.56339	0.4523	0.46501	0.17285	0.4033	0.29243
NM_001127379	Api5_predicted	-0.1485	0.13504	0.18989	0.69664	0.71764	0.38575	0.42554	0.84449	0.13982	0.27984	0.11132	-0.2292	0.40658	0.4299
NM_001106492	Apip_predicted	0.75581	0.567	0.28645	0.13184	0.74368	1.0066	0.25533	0.69515	0.27118	0.53376	0.43211	0.56507	0.48628	0.35043
NM_031612	Apln	-0.1889	-0.2507	-0.1596	-0.1583	-0.2889	-0.0035	-0.1895	-0.18	-0.0683	-0.0994	-0.1303	0.01812	-0.1479	-0.1335
XM_001056214.1	Aplp2	1.0333	1.6075	-0.2949	0.80606	1.0662	0.72191	0.79842	1.1982	1.3213	1.3439	1.2779	0.39682	0.55799	0.47692
XM_001056214.1	Aplp2	-0.0857	-0.1918	0.01551	-0.0532	-0.0655	-0.0522	-0.1206	0.0355	-0.1019	-0.1221	-0.1631	0.07477	-0.2384	-0.1418
NM_001106440	Apoa1bp_predicted	0.95216	0.61516	-0.2823	0.4306	0.88552	0.91215	0.34533	0.64131	0.57137	0.42044	0.59843	0.05822	-0.1076	-0.0585
NM_013112	Apoa2	-0.0647	-0.1597	-0.128	-0.0036	-0.1307	0.08863	-0.0938	-0.198	-0.0838	-0.1023	-0.191	-0.1303	-0.0624	-0.1677
NM_080576	Apoa5	0.05274	0.25983	0.0907	0.0784	0.09543	0.07181	0.15766	0.36867	0.06185	0.01372	-0.0086	0.21703	0.22361	0.07319
NM_019287	Apob	0.08699	0.01909	0.05652	0.09144	0.2155	0.03899	0.0493	0.04168	0.03812	0.08691	0.20502	0.14284	0.02294	0.03344
NM_012907	Apobec1	0.95772	-0.1027	-0.0708	0.0981	0.3163	0.13025	0.01997	-0.0596	0.06625	0.05647	0.14146	0.16906	-0.1518	0.02536
NM_001106883	Apobec2_predicted	0.12955	0.55046	0.19509	-0.114	0.07167	-0.1158	0.02486	-0.2267	0.18156	-0.0052	0.59439	0.15887	-0.122	-0.0987
NM_001033703	Apobec3	-0.7457	-0.3002	0.02417	0.2095	-0.5303	-0.2281	-0.1596	-0.7335	-0.2992	-0.3359	-0.3043	-0.0824	-0.4612	-0.4295
NM_001017492	Apobec4	0.16026	0.1014	-0.0088	0.1347	0.1043	0.12791	0.00785	0.21916	0.10489	-0.0052	0.15713	0.11948	0.11901	-0.1076
NM_001109996	Apoc1	0.16156	0.03829	-0.0401	0.01542	0.07543	-0.0791	-0.0574	0.00891	0.08003	0.04681	0.04427	0.03116	0.10354	0.0076
NM_001085352	Apoc2_predicted	-0.0514	0.04347	-0.0246	0.06077	0.01953	-0.1065	0.1429	0.10835	-0.0288	0.16259	0.00359	-0.2109	-0.0911	0.06858
NM_138828	Apoe	0.04322	0.43028	0.41075	0.62	-0.1271	-0.0997	0.19303	-0.0746	0.24674	0.38546	0.36509	0.11754	-0.1547	-0.0727
NM_001024351	Apof	0.0334	0.00722	-0.0161	0.08144	0.01008	0.1515	0.05527	0.02266	0.11257	0.14934	0.12694	0.0441	0.09221	0.02909
NM_001009626	Apoh	0.11699	0.10352	-0.0228	0.01718	0.03465	0.05508	0.08226	0.06011	0.05102	0.10731	0.21784	0.03599	0.03312	0.14859
XM_001075828.1	Apol2_predicted	0.01183	0.07383	0.03471	-0.0077	0.06014	0.11391	0.03697	0.1875	0.03705	-0.0147	0.17029	0.16184	0.05112	-0.0458
NM_001013175	Apol3_predicted	0.29497	0.20672	0.03088	0.19698	-0.0511	-0.0465	0.36288	-0.0902	0.23219	0.41952	0.0851	-0.0537	-0.0563	-0.1122
NM_001025066	Apol9a	0.7527	0.8828	0.60144	0.82219	1.8546	1.8504	0.97457	1.8939	0.58194	0.79844	0.72885	0.43096	0.92629	0.96657
NM_019373	Apom	-0.0603	-0.0664	0.01969	-0.0549	-0.1816	0.01287	-0.1175	-0.1598	-0.153	-0.0668	-0.1919	-0.0869	-0.0594	0.03191
NM_001009385	Apon	0.03735	-0.0637	-0.1001	-0.0654	0.17165	0.09116	0.00156	0.19292	-0.0636	0.03619	-0.0648	0.21365	-0.0074	-0.0595
NM_001014105	Apool	0.19875	-0.0969	-0.6569	0.21886	0.73138	0.44838	-0.0488	0.75094	-0.0024	-0.0108	-0.1359	-0.0559	-0.1788	-0.1071
NM_019288	App	0.93642	1.2619	0.41036	1.0534	1.1586	0.96057	0.56528	1.1958	1.2314	1.125	0.90923	1.0114	0.76437	0.97507
NM_032072	Appbp1	-0.2603	-0.1279	0.06567	0.3833	-0.1579	-0.1774	0.22511	-0.2263	-0.2819	-0.1694	0.1951	-0.1102	-0.0201	-0.2774
NM_001100969	Appbp2	-0.166	-0.2398	0.19341	0.28092	0.18795	0.13178	0.14805	0.33567	0.04401	-0.0164	0.02448	0.18957	0.31047	0.30429
NM_001102383	Aprin_predicted	-0.2113	-0.3723	0.09042	-0.2241	-0.4039	-0.334	-0.109	-0.3727	0.02508	-0.0754	-0.1831	0.25549	0.10452	0.16648
NM_001013061	Aprt_predicted	0.71612	0.7278	-0.1108	0.53001	0.36794	0.92174	0.02575	0.11862	0.36112	0.52755	0.63833	0.21925	-0.0172	-0.097
NM_148889	Aptx	0.43433	0.20391	0.21672	-0.1209	0.37475	0.42516	0.06017	0.41907	0.1125	-0.0623	0.1011	0.2167	0.22425	0.29822
NM_001047893	Apxl	-0.1408	-0.1447	-0.0946	-0.1515	0.00309	-0.0239	-0.1463	0.04533	0.09492	0.05938	-0.1107	-0.0196	-0.1532	-0.0047
NM_012778	Aqp1	3.9801	3.7913	3.1094	3.3722	3.2875	3.3107	3.5925	3.1468	3.1379	3.7071	3.8085	0.94162	1.3427	0.97334
NM_173105	Aqp11	0.95086	0.84561	0.2909	0.3739	0.92534	1.1986	0.23373	0.99967	0.84437	0.75321	0.82732	0.53403	0.74145	0.77035
NM_012909	Aqp2	-0.0134	-0.0406	-0.0501	0.02267	-0.1645	0.07679	0.01412	-0.1004	-0.0882	0.09388	-0.101	0.07662	0.05378	-0.096
NM_031703	Aqp3	0.08217	0.05679	0.01769	0.02479	-0.0559	0.03013	0.06122	0.07671	-0.0966	0.21215	0.22318	0.10408	0.14479	0.01192
NM_012825	Aqp4	-0.3352	-0.0423	-0.1546	-0.1957	-0.272	-0.0277	-0.246	-0.3586	-0.2515	0.08149	-0.3946	-0.1536	-0.3537	-0.1948
NM_012779	Aqp5	0.14501	0.13332	0.13814	0.26325	0.12832	0.01547	0.01049	0.11827	0.10437	0.12893	0.14475	0.1141	0.3097	0.02825
NM_022181	Aqp6	-0.0806	-0.0014	0.13581	-0.0656	-0.0627	-0.0118	-0.067	-0.0401	0.01156	-0.0436	-0.0694	0.14246	0.00447	-0.0467
NM_019157	Aqp7	0.09466	0.02567	0.02995	0.05471	-0.0585	0.16314	0.03844	-0.0167	0.29554	-0.048	-0.122	0.22831	-0.0182	-0.0061
NM_019158	Aqp8	0.06574	0.13696	0.14008	0.00665	0.03687	0.09531	0.01761	-0.0647	0.09	0.03561	-0.0038	0.05175	0.06955	0.21323
NM_022960	Aqp9	-0.0674	-0.0061	-0.0395	0.33229	-0.0934	0.08285	-0.0568	-0.0025	-0.0916	-0.4141	-0.0782	0.09656	-0.0683	-0.1353
NM_001100987	Aqr_predicted	-0.033	0.2132	0.22315	0.43484	0.08349	0.23033	0.26666	0.21459	-0.3307	0.12607	-0.0782	-0.1949	-0.0787	0.19219
NM_012502	Ar	-0.2693	-0.1366	-0.2198	-0.1211	-0.1402	-0.0616	-0.1265	-0.0491	-0.2832	-0.0121	-0.0328	-0.1037	0.19822	-0.1841
NM_001033663	Araf	-0.0709	0.17611	0.23272	0.19989	0.1663	0.20017	0.24962	-0.0149	0.00467	0.23566	0.40135	-0.0968	-0.1924	-0.0394

NM_022402	Arbp	-0.147	-0.0026	0.16591	0.05624	-0.1887	0.10778	0.28351	-0.0526	-0.2017	-0.0761	-0.0726	0.10965	-0.039	-0.2014
NM_019361	Arc	0.01971	0.12261	0.14406	0.02714	0.17605	0.15334	0.07769	0.01547	0.09684	-0.0828	0.12923	0.23557	0.12627	0.12642
NM_001007662	Arcn1	-0.2724	-0.0127	-0.214	0.09148	0.02634	-0.0226	-0.2207	0.1809	0.1554	0.03728	-0.1779	0.06752	-0.0733	-0.0914
NM_001135839	Ard1_predicted	-0.78	-1.1064	0.29045	-0.5081	-0.9838	-0.3765	-0.4858	-1.0422	-0.8645	-0.786	-0.9174	-0.0942	0.13551	0.12225
NM_017123	Areg	0.98309	-0.2587	0.36185	1.1903	1.5142	1.8207	0.51254	1.6547	0.36716	0.24626	-0.0287	0.95628	1.002	0.8861
NM_022518	Arf1	0.01183	0.07081	-0.002	0.16589	0.3029	0.38156	-0.0472	0.38636	0.12525	0.22664	-0.0801	0.23796	0.05808	-0.0264
NM_024150	Arf2	0.92812	0.97696	0.57985	0.54306	1.8511	1.4888	0.71804	1.8693	0.71058	0.74604	0.8653	0.51686	1.2529	1.1399
NM_080904	Arf3	0.47268	0.94914	0.34466	0.23473	0.7474	1.0278	0.28622	0.80249	0.70574	0.63276	0.87251	0.63189	0.56458	-0.0039
NM_024151	Arf4	-0.5433	-0.5307	-0.5	-0.0406	-0.513	-0.7472	-0.2553	-0.4362	-0.2185	-0.5602	-0.6436	-0.1656	-0.4476	-0.3454
NM_001107052	Arf4l_predicted	-0.0148	0.12952	0.08604	0.16921	0.12757	0.4693	-0.1481	0.21029	-0.004	-0.0396	0.1792	0.43107	-0.2468	-0.2056
NM_024149	Arf5	-0.3276	-0.5373	-0.9248	-0.9913	-0.5719	-0.3376	-0.5674	-0.4574	-0.3405	-0.3939	-0.6076	0.17808	-0.8796	-0.7692
NM_024152	Arf6	-0.2153	-0.2983	0.01996	-0.1408	-0.0536	0.22369	-0.3398	-0.1562	-0.3788	-0.2572	-0.1335	0.1538	0.0554	-0.0012
NM_145090	Arfgap1	-0.1277	-0.1405	-0.0651	-0.1008	0.49585	0.49729	-0.2902	0.37902	-0.1531	-0.5061	-0.3708	0.28294	0.21531	0.14312
XM_001066125.1	Arfgef1_predicted	-0.5801	-0.2363	0.00097	-0.1694	-0.514	-0.6244	0.12952	-0.477	-0.1844	-0.4603	-0.3495	-0.1554	-0.325	-0.3653
NM_181083	Arfgef2	0.23209	0.26336	0.54603	0.63397	0.39783	0.49587	0.07985	0.48419	-0.2332	0.01165	0.03776	0.18961	0.27291	0.17248
NM_021763	Arfip1	0.46379	0.65756	-0.5991	0.08257	0.71503	-0.227	0.07002	1.0818	0.28646	0.42798	0.0943	-0.149	0.47017	0.49503
NM_001004222	Arfip2	-0.6381	-0.4732	0.06777	0.28623	-0.3002	-0.1517	-0.212	-0.2513	-0.4893	-0.5688	-0.3676	-0.466	-0.8015	-0.6098
NM_053980	Arfrp1	0.66886	0.51759	0.12194	0.18309	0.9958	1.3534	0.17034	0.90446	0.46507	0.45481	0.58057	0.39834	0.42524	0.39733
NM_017134	Arg1	1.1616	1.2072	0.72544	0.12935	0.73802	0.84742	0.75039	0.61576	0.70559	0.7307	0.96209	0.89839	0.66168	0.57715
NM_019168	Arg2	0.0209	0.12327	0.0924	-0.0597	0.0703	-0.0484	0.00753	-0.0005	0.25727	0.09605	-0.231	-0.0084	0.02436	-0.0457
NM_053770	Argbp2	-0.1048	-0.5369	-0.327	0.35945	0.60519	0.67781	-0.005	1.0335	-0.6978	-0.3084	-0.317	-0.9262	-0.8243	-0.5953
NM_001107747	Arhgap1_predicted	-0.4968	-0.0244	-1.1038	-0.6184	0.08869	-0.8416	-0.1025	0.22541	-0.0001	0.03603	0.02621	-0.4067	0.13899	0.16301
NM_001107357	Arhgap12_predicted	-0.0334	0.25552	0.27954	0.30829	0.27518	0.15738	0.66086	0.44101	0.22258	0.31666	0.33931	0.18054	0.3069	0.17267
NM_022244	Arhgap17	0.41961	0.74988	0.60806	-0.0281	0.13551	-0.2609	0.33792	0.20946	0.4378	0.46894	0.56901	0.05855	0.24959	0.25077
NM_001170575	Arhgap18_predicted	0.08632	0.44087	-0.3047	-0.6449	-0.3935	-0.4818	-0.1996	-0.3924	0.21218	0.17117	0.03084	0.01797	-0.3997	-0.3879
XM_001074395.1	Arhgap21_predicted	-0.93	-0.6616	-0.4055	-1.221	-0.9878	-1.124	-0.6302	-0.8907	-0.5742	-0.6903	-0.8363	-0.4277	-0.3108	-0.4863
NM_001107297	Arhgap22_predicted	-0.5101	-0.3153	0.34569	-0.6706	-0.4902	-0.4719	-0.0557	-0.8558	-0.0691	-0.3576	-0.2129	0.40278	-0.3216	-0.2564
XM_220918.4	Arhgap23_predicted	-0.147	-0.0548	-0.0034	-0.1693	-0.1191	-0.0764	-0.026	-0.0812	0.00919	-0.0315	0.02265	-0.0442	0.09824	-0.0813
NM_001012032	Arhgap24	0.07639	-0.0044	0.04459	0.1667	0.13214	0.06178	0.0992	0.1606	0.08402	-0.0877	0.17273	-0.0172	0.06047	0.14218
NM_001107389	Arhgap26_predicted	-0.1191	0.06501	-0.1454	-0.0126	0.15228	-0.2172	-0.115	-0.1636	-0.2317	-0.0867	0.01546	0.02015	-0.1707	-0.0792
NM_198759	Arhgap27	-0.0715	0.15427	0.16721	0.18861	-0.0771	-0.0821	-0.0179	0.0255	0.07673	-0.0731	0.0378	0.43351	0.09529	-0.0921
NM_001009405	Arhgap29	0.16255	1.0431	-0.4597	0.33982	0.02147	-0.2129	-0.0383	0.0079	0.57759	0.62048	0.45201	0.09112	0.12811	-0.0712
NM_144740	Arhgap4	-0.0181	0.01239	0.14714	-0.2179	-0.2048	-0.0919	-0.182	0.13483	-0.216	-0.0097	0.08571	-0.091	0.09433	-0.3225
NM_001047869	Arhgap5	-0.252	0.0557	-0.4541	-0.5152	0.24056	-0.1378	-0.0416	0.18074	0.04559	-0.0686	-0.2341	-0.2149	0.09026	0.08133
NM_001004242	Arhgap8	1.5089	-0.4964	0.22022	0.13955	2.125	2.1264	0.60561	2.0406	-0.2993	-0.0904	-0.1247	0.20899	0.0131	0.00739
NM_001012121	Arhgap8_predicted	-0.0639	0.03636	0.06335	-0.005	0.06275	0.18318	-0.0051	0.00637	0.18668	-0.041	-0.0733	-0.0711	0.04012	-0.0951
NM_001080789	Arhgap9	-0.076	-0.2089	-0.2452	-0.0952	-0.2554	-0.0929	-0.1959	0.0123	-0.0822	-0.1343	-0.0568	-0.1913	-0.1674	-0.2657
NM_001080789	Arhgap9	0.15775	0.33734	0.2806	0.12125	0.31192	0.2585	0.32052	0.34297	-0.0624	0.17495	0.45803	0.03432	0.24978	0.36058
NM_001080789	Arhgap9	0.15839	-0.1164	0.55787	0.64064	0.04767	0.22143	-0.0375	0.17085	-0.1966	-0.2574	-0.0388	0.2588	-0.3393	-0.1162
NM_001007005	Arhgdia	-0.1269	-0.0769	0.46185	-0.3265	0.06864	-0.085	0.0661	-0.0297	-0.1252	-0.1316	-0.0761	-0.106	0.21534	0.43123
NM_001009600	Arhgdib	-3.1272	-3.3021	-3.4592	-3.4312	-3.3386	-3.3318	-3.3716	-3.2531	-3.3921	-3.4089	-3.385	-3.372	-3.4303	-3.4129
NM_001108269	Arhgdig_predicted	0.08632	0.15704	0.07505	-0.0048	0.11293	-0.0774	0.06795	0.06904	-0.0664	0.05264	0.12132	0.0642	-0.0244	0.00091
NM_021694	Arhgef1	0.08517	0.17359	0.72738	0.82442	0.52321	0.75665	0.66524	0.53641	0.12788	0.29122	0.27212	-0.0999	-0.2223	-0.2241
NM_023982	Arhgef11	-0.0533	-0.3561	-0.0363	-0.0552	-0.2747	-0.6636	-0.0794	-0.5253	-0.2228	-0.3199	-0.327	-0.4273	-0.3396	-0.4019
NM_001013246	Arhgef12	0.21781	-0.1077	0.15703	0.16314	0.13078	-0.104	0.04632	0.27289	-0.0235	0.12462	0.0364	-0.0896	0.35229	0.51752
NM_001105789	Arhgef15_predicted	0.01776	0.10684	0.03739	0.23961	0.14889	0.15165	0.00331	0.32091	0.05148	0.10298	0.00971	0.09453	0.20678	0.1049
NM_001107538	Arhgef17_predicted	0.23602	0.02683	-0.03	0.19275	0.29436	-0.0429	0.08396	-0.0326	-0.2817	0.16436	0.24189	0.05901	0.25445	0.07115
NM_001107115	Arhgef18_predicted	-0.0012	0.08473	0.12896	0.09987	0.03925	0.37416	0.04857	0.01081	0.06522	0.08712	-0.0721	0.07246	0.08161	0.09036
NM_001108692	Arhgef19_predicted	-0.1578	0.33175	0.11138	-0.3474	-0.5615	-0.3532	0.04677	-0.5681	0.19023	0.35211	0.31874	0.37816	0.28566	-0.0978

NM_001012079	Arhgef2	0.08543	-0.4951	0.11431	-0.8689	-0.4797	-0.7154	-0.3474	-0.5217	-0.269	-0.6675	-0.4388	-0.3554	-0.6929	-0.4941
NM_001106061	Arhgef3_predicted	-0.0364	-0.0863	-0.1327	-0.0487	0.24866	-0.002	0.04581	-0.1269	-0.0985	0.28637	0.15509	0.00616	-0.0611	-0.0615
XM_001073085.1	Arhgef5	-0.2828	-0.5333	-0.2953	-0.542	-0.0815	-0.1578	-0.5803	0.02793	-0.8418	-0.6856	-1.0225	-0.4535	-0.719	-0.5507
NM_001005565	Arhgef6	0.09908	0.39257	-0.2441	-0.0624	-0.0096	-0.0852	0.19425	-0.1894	0.03743	0.21304	0.18849	-0.0972	0.00844	0.09042
NM_001113521	Arhgef7	0.1779	0.06915	-0.0703	0.15684	0.23673	0.10415	0.13462	0.21119	0.01818	0.15192	0.03885	-0.0396	0.02425	0.00265
NM_023957	Arhgef9	-0.4112	-0.2802	-0.2888	-0.051	-0.3232	-0.6628	-0.3564	-0.2448	-0.3625	-0.1783	-0.1971	-0.3529	-0.4696	-0.591
NM_001106635	Arid1a_predicted	-0.3472	-0.3133	-0.0864	-0.8259	-0.4951	-0.8443	-0.3628	-0.4818	-0.4044	-0.3689	-0.4401	-0.4047	-0.3594	0.00497
XM_345867.2	Arid2_predicted	0.26721	0.22025	0.38785	0.03779	0.19548	0.20415	-0.0114	0.31653	0.29229	0.13854	0.2056	0.31902	0.3271	0.15965
NM_001108066	Arid3a_predicted	-0.0564	-0.0031	-0.0164	0.1591	-0.027	-0.0064	0.10607	0.02237	-0.0063	0.02863	0.00381	0.17347	-0.0206	-0.0256
NM_001108029	Arid4a_predicted	-0.3196	0.23916	-0.0239	-0.4883	0.01952	-0.4046	-0.0122	-0.2453	0.28848	0.08533	0.25482	-0.223	-0.1248	0.01955
NM_053421	Arid4b	-0.0515	0.1565	-0.034	0.14143	-0.1264	-0.3475	0.15357	0.07538	0.20573	0.05015	0.21341	0.27733	0.10014	-0.1619
NM_001034934	Arid5a	-0.4419	-0.3266	-0.2308	-0.4891	-0.0031	-0.4692	0.14927	-0.0888	-0.2711	-0.364	-0.5139	-0.6058	-0.3409	-0.4002
NM_001107624	Arid5b_predicted	-0.2252	-0.1687	-0.0755	0.37396	0.00771	0.45544	-0.1173	0.08078	-0.4145	-0.3572	-0.1384	-0.1885	-0.0097	-0.1946
NM_001013108	Arih1	-0.1649	-0.3217	-0.034	-0.3492	0.34899	0.15366	-0.0256	0.51174	-0.4332	-0.2162	-0.5249	-0.1994	0.31094	0.24305
NM_022385	Arl1	-0.2049	0.27061	-0.2623	0.32587	0.19834	0.47598	0.00415	0.06498	0.08721	-0.0757	0.05484	0.47809	0.17172	0.10876
NM_207165	Arl10	-0.3382	-0.2011	-0.4552	-0.2646	-0.0498	-0.3892	-0.236	0.04602	-0.1311	0.03951	-0.4156	-0.5542	0.16013	-0.0809
NM_001013433	Arl11	-0.1237	-0.1796	-0.2516	-0.1842	-0.0804	-0.1938	-0.234	-0.3207	-0.2168	-0.1369	-0.1714	-0.2431	-0.1469	-0.1605
NM_001107046	Arl12_predicted	-0.4317	-0.4204	-0.2819	-0.3152	-0.2719	-0.3298	-0.2638	-0.2214	-0.3108	-0.204	-0.404	-0.1663	-0.2722	-0.0051
NM_031711	Arl2	0.47959	0.18799	-0.0262	0.3192	0.37381	0.97371	-0.0022	0.26147	0.0126	0.02476	0.31877	0.33856	-0.1156	-0.1665
NM_022700	Arl3	0.12669	-0.1807	-0.435	0.25087	-0.3394	-0.0431	-0.1542	-0.0738	0.08994	0.2002	0.10251	0.26784	-0.4218	-0.2144
NM_019186	Arl4a	-0.1528	-1.6115	-0.7198	0.36528	0.98733	1.0512	-0.6201	1.0804	-1.3652	-1.3335	-1.3535	-1.0837	-0.8063	-0.7535
NM_053979	Arl5a	-0.0395	0.39206	-0.4619	-0.1563	-0.2592	-0.4677	-0.6188	-0.2503	0.08711	0.15126	-0.1008	-0.2982	-0.3424	-0.1943
NM_001015031	Arl5b	0.02669	-0.0143	-0.1002	0.13073	0.04948	-0.0687	-0.0544	-0.0049	0.14388	-0.1776	-0.2612	-0.0313	0.524	0.01233
NM_001108842	Arl6_predicted	0.24082	0.24838	0.01026	0.15478	0.13456	0.23648	0.47623	0.26183	0.13036	0.48287	0.35293	-0.2424	-0.047	-0.1925
NM_001100671	Arl6ip2	-0.08	-0.0772	0.04439	0.88781	-0.0857	-0.1182	0.10176	0.18766	-0.0498	-0.4416	-0.2108	-0.0003	0.03403	0.0061
NM_023972	Arl6ip5	0.88067	0.36709	-0.194	-0.6585	0.35764	0.38893	0.01315	0.50119	0.246	0.18304	0.27965	0.0443	0.01219	0.10269
NM_001024310	Arl6ip6	-0.0643	0.12837	-0.1412	-0.1662	-0.2119	-0.6957	0.02327	-0.0632	0.26949	0.17469	0.01984	-0.1586	0.23188	0.29096
NM_212459	Arl9	-0.1346	-0.0895	-0.2362	-0.1038	0.22626	-0.1654	-0.192	-0.1257	-0.1189	-0.255	-0.0661	-0.2113	0.05946	-0.2615
NM_001106425	Armc1_predicted	0.15244	0.25672	0.24564	0.38553	0.25596	0.09113	0.39778	0.34166	0.51939	0.35361	0.28379	0.34328	0.40923	0.46227
XM_225610.3	Armc3_predicted	0.02789	-0.0038	0.05319	-0.0295	0.15696	-0.048	0.00368	0.05862	-0.0722	-0.0037	-0.0642	-0.0028	-0.0224	0.0652
XM_225474.4	Armc4_predicted	0.01933	0.02025	0.07899	0.1702	0.07569	0.06971	0.0779	0.01911	0.04016	0.133	0.01328	0.1338	0.30564	0.0851
NM_001009455	Armc5	-1.585	-1.3488	-0.6065	-1.3016	-1.7538	-1.7581	-0.9753	-1.6106	-1.2279	-1.3619	-1.0811	-1.1322	-1.495	-1.3886
XM_236599.4	Armc8_predicted	0.03691	0.06701	0.01758	0.14492	0.03218	0.24304	0.08293	0.0041	0.20075	0.07314	0.27232	0.23307	0.07727	0.02941
XM_236599.4	Armc8_predicted	-0.2911	0.02645	0.24303	-0.2881	-0.265	-0.315	0.03058	-0.1308	-0.1167	-0.0765	-0.1466	0.13747	0.1602	0.19625
NM_001109663	Armc9_predicted	-0.1753	0.87715	0.51205	0.00207	-0.1025	-0.1195	0.87351	-0.1683	0.62947	0.52722	0.39442	0.46876	0.57812	0.50897
NM_001024367	Armcx1	-0.3843	-0.6967	-1.0388	0.41297	-0.131	-0.0542	-0.6136	-0.0324	-0.4963	-0.5612	-0.4364	-0.5588	-0.6084	-0.6154
NM_001014274	Armcx2	0.19338	0.79517	0.18015	1.0114	-0.3347	-0.3637	0.43722	-0.3701	0.42462	0.71745	0.74181	0.47105	0.31537	0.2338
NM_001014273	Armcx3	-0.2801	-0.563	-0.569	-0.3019	0.1289	-0.1011	-0.5672	0.10887	-0.5201	-0.4557	-0.4945	-0.1036	-0.0665	-0.3388
NM_001007757	Armcx6	-0.0509	-0.0762	-0.0367	0.2309	-0.1321	-0.1134	0.20454	-0.0022	-0.0698	0.1211	-0.0028	-0.0059	-0.0628	0.01398
NM_001108183	Armet_predicted	-0.1109	-0.1978	0.25676	0.00802	0.51371	1.2064	-0.4026	0.83295	-0.1701	-0.257	-0.2178	0.41415	0.61774	0.72377
NM_001037543	Armetl1_predicted	0.23087	0.49354	0.03072	0.08667	0.17128	0.3206	0.13826	0.28898	0.4207	0.52956	0.58813	0.1068	0.23598	0.45506
NM_012780	Arnt	0.11856	0.08824	0.12789	0.61206	0.29447	-0.0518	0.19303	0.14987	0.00705	-0.0726	-0.0523	-0.2618	-0.1641	-0.0353
NM_012781	Arnt2	0.00178	0.02208	-0.1175	-0.2934	0.10158	-0.0966	-0.1663	0.32505	0.07109	-0.0019	0.05966	-0.2176	-0.0073	-0.1293
NM_024362	Arntl	-0.2701	-0.0903	-0.1527	0.10216	-0.2407	-0.0125	0.04655	-0.1573	-0.0969	-0.1549	-0.1107	-0.0571	-0.5148	-0.3906
NM_031146	Arpc1a	-0.1888	0.25122	-0.1735	-0.5742	-0.347	-0.5676	-0.0167	-0.4122	-0.1286	0.16474	-0.0537	-0.3267	-0.2145	-0.1841
NM_019289	Arpc1b	-0.0657	0.1836	-0.0752	-0.0373	0.05003	0.2889	0.78662	-0.0535	0.10848	0.37479	0.02316	0.29515	-0.2006	-0.2665
NM_001106919	Arpc2_predicted	0.18627	-0.0021	0.17651	0.24209	0.25257	-0.0227	0.31273	0.21538	0.02926	-0.0159	0.13781	0.02696	0.18088	-0.0532
NM_001105933	Arpc3_predicted	0.33684	0.26459	-0.244	0.36737	0.55223	0.44995	0.54953	0.26601	0.32894	0.3963	0.28236	0.02042	-0.0887	0.15807
NM_001106615	Arpc4_predicted	0.3434	0.12227	-0.0609	-0.8748	0.08509	-0.1249	-0.4151	-0.1269	0.06994	0.02124	-0.0314	-0.1026	0.07319	0.1236

NM_001025717	Arpc5	-0.2017	-0.3434	-0.3442	-0.6521	-0.7271	-0.8548	-0.2007	-0.832	-0.19	-0.4179	-0.404	-0.368	-0.2196	-0.1932
NM_001037767	Arpc5l_predicted	-0.3776	-0.4068	-0.1317	-0.4958	-0.5361	-0.2076	-0.0209	-0.5071	-0.2435	-0.2982	-0.4794	-0.475	-0.3613	-0.5682
NM_031660	Arpp19	0.16477	-0.2256	-0.2066	0.32558	0.43959	0.73234	-0.3106	0.31469	-0.5689	-0.3947	-0.4292	-0.0673	0.06435	0.00256
NM_012910	Arrb1	0.01345	0.13351	-0.0849	0.04455	0.03537	-0.0122	-0.0136	-0.0752	0.00141	0.21668	0.25761	-0.1124	0.01993	-0.0251
NM_012911	Arrb2	-0.0362	0.09424	0.11104	-0.0555	-0.0539	0.03724	0.0015	0.15568	0.0221	0.02206	0.04155	0.13877	-0.0757	0.10983
NM_001100770	Arrdc1	-0.3746	-0.382	-0.3382	-0.319	-0.3748	-0.351	-0.2334	-0.4451	-0.3884	-0.2385	-0.3554	-0.3784	-0.3688	-0.4092
NM_001007797	Arrdc3	0.09343	0.34627	0.08857	0.00821	0.33031	0.10768	0.09271	0.20151	0.17395	0.07561	-0.0569	0.06279	0.12273	0.11296
NM_001047853	Arrdc4	0.15908	0.0215	0.03639	0.05458	0.0481	0.17112	0.10664	0.14126	0.03611	0.06689	0.17542	0.1155	-0.0198	-0.0792
NM_001034933	Arsa_predicted	0.41208	0.47893	0.49	0.38688	0.31351	0.23007	0.26022	0.45786	0.26689	0.3993	0.59842	0.09533	0.3225	0.22191
NM_033443	Arsb	0.14046	0.39428	0.15897	0.16987	-0.2477	-0.4433	0.44171	-0.2859	0.20537	0.32072	0.37381	0.11382	0.11252	0.16181
NM_001047885	Arse	0.10563	0.11822	0.08448	-0.0142	-0.0799	-0.2868	-0.0829	-0.5092	-0.0687	-0.087	-0.1086	-0.0101	-0.3726	-0.2865
NM_001047877	Arsg	-0.0293	-0.205	-0.0449	0.02619	-0.186	-0.1104	-0.0204	-0.0602	0.10934	-0.0697	-0.0413	0.06723	0.05575	-0.0793
NM_001047887	Arsj	-0.068	-0.3235	-0.3349	-0.5829	-0.1023	-0.1034	-0.2482	-0.139	-0.1888	-0.5035	-0.3187	-0.5896	0.22174	-0.0527
NM_001047917	Arsk	-0.0589	0.25019	-0.0097	-0.097	0.01965	-0.229	0.39782	0.22949	0.47167	0.30839	0.42305	0.02894	0.27476	0.21494
NM_001107541	Art1_predicted	-0.0429	-0.0168	0.02845	-0.0127	-0.0991	0.0847	-0.0794	-0.0176	0.02739	-0.0038	-0.0389	-0.0567	0.1302	0.02125
NM_198735	Art2b	0.25055	0.05712	0.07909	0.03324	0.15735	0.11597	-0.0038	0.03801	0.18797	0.21855	0.02386	0.00096	0.03213	0.07412
NM_198735	Art2b	-0.0044	0.02851	-0.0697	0.0281	-0.1024	-0.0109	0.02433	-0.0423	-0.0333	-0.0337	0.06362	0.07747	0.03993	-0.0614
NM_001012034	Art3	0.0873	0.08562	-0.1157	-0.07	0.08705	-0.1933	-0.0921	-0.078	0.05214	-0.315	0.14042	0.086	-0.0269	0.09039
NM_001012034	Art3_predicted	0.0972	-0.0741	0.07968	0.10347	-0.1052	-0.0369	0.02925	-0.0574	-0.1018	0.35479	-0.0092	0.1041	0.07685	-0.0433
XM_001072607.1	Art4_predicted	-0.1249	-0.013	-0.1116	-0.1749	-0.0204	0.09495	0.09354	-0.0983	-0.1064	0.22093	0.1527	0.02188	-0.0932	-0.0117
NM_001013039	Art5	-0.0152	-0.002	0.04225	-0.1209	-0.2516	-0.0204	0.03391	0.19675	-0.0344	-0.1412	0.10088	0.00073	-0.0944	-0.0065
NM_053397	Artn	-0.0694	-0.1757	0.28972	-0.275	-0.3537	-0.0741	-0.3107	-0.3037	-0.0945	-0.3267	-0.2603	-0.1769	-0.2791	-0.0955
NM_030836	Arts1	-0.0839	-0.0271	0.44898	0.59788	-0.2537	-0.2432	0.46896	-0.0835	-0.1352	-0.0495	0.13621	0.03434	0.18921	0.04142
NM_001106197	Arv1_predicted	-0.6384	-0.3233	0.07828	1.1272	-0.2478	-0.0112	0.38441	-0.1608	-0.388	-0.1873	-0.2618	-0.2605	-0.5116	-0.5194
NM_001131013	Arvcf_predicted	0.01578	-0.2098	0.11332	0.5855	0.54046	0.9558	-0.0524	0.71526	-0.1846	-0.0806	-0.1298	-0.3922	-0.2936	-0.3806
NM_080890	As3mt	-0.0794	-0.0649	-0.1069	-0.0517	-0.1547	0.08569	0.17269	-0.0543	-0.0321	-0.1455	-0.1182	0.13135	-0.0698	-0.0707
NM_053407	Asah1	0.43139	0.35192	-0.4002	-0.0877	0.28569	0.02239	0.53598	0.64049	0.00596	0.37763	0.46051	-0.704	-0.0113	-0.3479
NM_053646	Asah2	0.14429	0.00244	-0.0003	0.13853	0.19397	-0.0237	-0.0241	0.00695	-0.0262	-0.042	-0.0576	0.22499	0.09912	0.10298
NM_001107943	Asah3l_predicted	-0.5944	-0.6931	-0.9209	-0.8506	-0.7193	-0.4439	-0.7734	-0.7129	-0.773	-0.896	-0.6218	-0.6443	-1.0888	-0.98
NM_001010967	Asahl_predicted	0.0539	-0.0491	0.03047	0.06317	0.08618	0.09675	0.10803	-0.0759	-0.0306	-0.0075	0.0864	0.03585	0.00849	-0.0174
NM_173154	Asam	-1.8582	-1.2333	-1.6499	-2.1958	-1.8546	-1.8317	-1.5859	-1.7393	-0.6724	-1.2019	-0.9272	0.05587	-1.7253	-1.4014
NM_001108232	Asb1_predicted	0.18388	0.1277	0.18281	0.1186	0.33712	0.50505	0.04313	0.30025	0.24563	0.31147	0.05651	0.1143	0.11939	0.26859
NM_001106962	Asb11_predicted	0.41357	-0.0185	0.11373	0.11153	0.02137	-0.0037	0.3462	0.1362	0.11338	-0.1273	0.05969	-0.028	-0.0329	-0.0456
NM_001108420	Asb13_predicted	-0.2746	-0.173	-0.2637	0.09206	-0.2833	-0.1549	0.15795	-0.0829	-0.2177	-0.2729	-0.2072	-0.2644	-0.1873	-0.1378
XM_216108.4	Asb15	0.12056	-0.0035	0.0457	0.0784	-0.0156	-0.0332	-0.1404	0.00955	0.01548	0.04573	0.09643	0.14519	-0.0614	0.16115
NM_001108231	Asb18_predicted	-0.0768	-0.1137	0.10969	0.07698	-0.1883	-0.1608	-0.1715	0.16365	-0.1167	-0.121	-0.0287	0.02965	-0.1441	-0.1244
NM_001011984	Asb2	-0.0443	-0.0073	0.05488	-0.143	0.3495	-0.0273	0.14713	0.05612	0.23754	0.10305	-0.0081	0.0739	-0.1292	0.07381
NM_001011984	Asb2_predicted	0.01198	-0.1088	0.01316	0.01547	0.02293	0.08411	0.04025	0.18225	0.13135	-0.0355	0.14303	0.31047	0.00697	0.08345
NM_001108864	Asb3_predicted	0.34777	0.36239	-0.0623	0.52934	0.487	0.39972	-0.0842	0.53793	0.18189	0.19599	0.15578	-0.0431	0.00393	0.09056
NM_001011963	Asb6	0.10737	0.03589	-0.1981	-0.002	0.53607	0.07455	0.06189	0.04771	0.07624	0.00801	-0.0684	-0.175	0.05065	0.04653
NM_001108915	Asb7_predicted	0.07724	-0.1249	0.03746	-0.1264	-0.0193	0.103	-0.0045	-0.1135	-0.0117	-0.0864	-0.0864	-0.0748	-0.0023	0.21123
NM_001108109	Asb8_predicted	0.04492	0.39585	-0.031	-0.3009	0.37487	0.38332	-0.0157	0.34201	0.40788	0.27658	0.30958	0.25492	0.05751	0.18449
NM_001007632	Ascc1	-0.3558	-0.4414	-0.296	-0.6977	-0.1792	0.02581	-0.5317	-0.3776	-0.4205	-0.2912	-0.2898	-0.023	-0.1314	-0.0637
NM_022384	Ascl1	0.28171	0.17889	0.0436	0.19504	0.10863	0.06856	-0.0258	0.03782	0.03032	0.03533	0.24024	0.14285	0.12036	0.06055
NM_031503	Ascl2	-0.0375	-0.0893	0.02108	0.04242	0.12764	-0.0812	-0.0621	0.03911	-0.0907	0.05662	-0.0774	-0.0196	-0.1287	0.17832
XM_344947.3	Ascl3	-0.1256	-0.0164	-0.1465	-0.064	0.00504	-0.1127	0.21493	0.07343	0.07434	0.07712	0.07342	-0.0517	0.14192	-0.0108
XM_235013.3	Ascl4_predicted	-0.0389	0.13457	0.02633	0.07723	-0.0427	-0.0857	-0.0436	0.2294	-0.0285	0.03631	0.11882	-0.0825	0.02104	-0.0389
NM_001106389	Asf1a_predicted	-0.3708	-0.3654	-0.1628	-0.1667	-0.0006	0.09688	-0.0476	-0.0304	0.06082	-0.1175	-0.157	-0.0665	-0.1148	0.07237
NM_001107160	Asf1b_predicted	0.6556	-0.6309	1.2278	-0.0178	-0.4459	-0.4536	-0.4737	-0.6023	-0.2918	-0.3336	-0.1076	0.30151	1.0793	1.1252

NM_012503	Asgr1	-0.0613	0.55262	-0.0064	-0.1548	-0.1456	-0.0422	-0.1125	-0.2072	0.75119	0.72516	0.76958	0.7609	-0.0808	-0.0419
NM_017189	Asgr2	0.08679	0.17821	0.03702	0.71106	0.03452	0.34618	0.16703	-0.0106	0.09299	0.20207	0.1601	0.11011	0.05345	0.10649
NM_001107689	Ash1l_predicted	-0.0919	0.10094	0.22897	-0.118	-0.0618	-0.3029	0.33697	-0.062	-0.2335	-0.1301	0.1937	-0.1266	0.00448	-0.0492
NM_001106089	Ash2l_predicted	-0.2323	0.42836	0.09013	0.03977	0.02081	-0.0316	0.18527	-0.0161	0.25361	0.17376	0.28825	0.26372	0.53018	0.4657
NM_021577	Asl	-0.2365	-0.4339	-0.2174	-0.4754	-0.0612	-0.3643	-0.0428	-0.2115	-0.3648	-0.2209	-0.238	-0.0402	-0.2944	-0.2486
NM_144759	Asmt	0.3516	0.09316	0.22743	0.0977	-0.091	0.00655	-0.1294	-0.0874	-0.0182	-0.1448	0.03099	0.21715	-0.1851	0.09334
NM_001105915	Asmtl_predicted	-0.1044	-0.1263	-0.1603	-0.0718	-0.097	0.42834	0.2001	-0.0834	-0.1132	-0.152	-0.0913	0.1321	-0.5427	-0.4863
NM_001100505	Asna1	0.19025	0.13201	-0.0107	0.31807	0.13636	0.09253	0.15112	-0.0114	0.27357	0.1288	0.21664	0.4809	0.07651	0.01032
NM_013079	Asns	-0.3451	-1.0421	-0.5571	-0.4137	-0.2375	-0.519	-0.9978	-0.1928	-0.9297	-0.9767	-1.0056	-0.6793	-0.6842	-0.5847
NM_024399	Aspa	0.27632	0.07341	0.01998	0.12438	-0.2417	-0.1185	0.16869	-0.1489	0.02266	-0.0558	0.25656	0.81821	0.2768	0.06221
NM_001098239	Asph_predicted	-0.1672	0.01844	-0.2251	-0.4337	-0.0036	-0.23	-0.3169	-0.3009	0.00368	0.00535	-0.3847	-0.2569	-0.0337	-0.0648
NM_001009716	Asphd2	0.04698	-0.0887	0.19821	-0.0488	-0.04	0.21282	0.10012	-0.0383	0.25062	0.02292	0.12417	0.30679	0.18387	0.18357
NM_001105955	Aspm_predicted	-0.3984	-1.0195	0.74291	0.57912	-1.3186	-1.4572	-0.585	-1.2761	-0.2586	-0.994	-0.6633	0.15897	0.43524	0.47289
NM_001014008	Aspn	-1.4792	1.6235	-0.8593	0.60136	-2.1406	-2.0441	-0.5219	-1.8672	1.6192	1.5136	1.238	1.133	-1.4376	-1.6722
NM_145089	Asrgl1	0.41877	0.68431	0.16386	0.24282	-0.1294	-0.0374	0.23664	-0.1818	0.83811	0.58505	0.81759	1.1089	0.57038	0.38885
NM_145089	Asrgl1	0.15483	0.13076	-0.0517	0.0954	-0.0144	0.07739	0.0635	0.03768	0.36774	0.27726	0.05657	0.22527	0.15361	0.18435
NM_013157	Ass	0.42287	-0.0095	0.05085	0.00797	1.9127	2.0946	0.07471	2.0185	-0.0842	-0.0292	0.15964	0.04022	-0.0049	0.19369
NM_001040156	Aste1	-0.1498	0.0115	0.00036	0.31348	0.12938	0.11574	0.09042	-0.0418	-0.2362	-0.065	-0.1537	-0.108	-0.3024	-0.101
NM_001170603	Astn1	-0.003	-0.0226	0.03309	0.09868	0.22808	0.14344	0.00711	0.1243	0.13343	0.08239	-0.0535	0.10535	0.13376	0.16172
NM_001170603	Astn1	-0.1267	-0.2135	-0.1465	-0.1271	-0.1849	-0.2089	-0.1281	-0.2561	-0.2085	-0.128	-0.1922	-0.1867	-0.139	-0.186
NM_001108011	Asxl2_predicted	-0.1049	0.09684	-0.1947	0.3891	-0.0441	-0.319	0.14492	-0.1305	-0.0034	0.24068	0.13587	-0.3523	0.053	0.22656
NM_130750	Asz1	0.13807	-0.0051	0.14355	0.06561	0.10019	0.11816	0.04105	-0.0012	0.27784	0.03625	0.14034	0.25246	-0.008	0.19874
NM_001035002	Atad1	0.04943	0.63688	-0.5773	-0.3595	0.2806	-0.5345	0.00477	0.47697	-0.0067	0.06542	-0.2604	-0.1416	0.40191	0.32247
NM_001134879	Atad2_predicted	0.12853	0.25018	0.13005	0.56997	0.07544	0.13359	0.17113	0.1134	-0.0649	0.13775	0.21549	-0.0072	0.05621	0.20884
NM_001034922	Atad3a	-0.9486	-0.8324	0.1676	-0.6889	-1.1479	-1.2228	-0.7377	-1.2464	-0.8117	-0.7368	-0.7122	-0.5706	-0.0974	-0.2111
NM_001040190	Atcay_predicted	-0.1546	-0.0627	-0.1071	0.16014	-0.1366	-0.1662	-0.0262	0.05659	0.02812	-0.1383	0.02295	-0.1328	0.04843	0.04072
NM_001106300	Ate1_predicted	0.2627	0.15018	0.51446	0.49974	0.40903	0.5803	0.14753	0.33402	0.24352	0.40974	0.02428	0.41873	0.70084	0.66458
NM_001100895	Atf1	0.01327	0.58202	-0.391	0.21412	0.00404	-0.3501	-0.1293	0.32093	0.40392	0.34084	-0.1091	-0.0161	0.47868	0.43577
NM_031018	Atf2	0.15412	0.08459	0.05676	0.34622	0.04354	0.18111	0.06521	0.12449	-0.0308	-0.1031	0.00427	0.32853	0.11114	-0.0371
NM_012912	Atf3	-0.1315	-0.0138	0.12514	0.06224	0.32318	0.20599	0.0023	0.28787	0.16444	0.10572	0.28606	0.25376	0.0191	-0.0365
NM_024403	Atf4	-0.3783	-1.0183	-0.1277	-0.2538	-0.747	-0.6325	-0.8078	-0.6511	-0.7082	-1.1638	-0.7609	-0.3706	-0.8104	-0.653
NM_172336	Atf5	-0.6639	-1.7824	-0.9254	-1.9414	-1.8622	-1.6551	-1.3157	-1.5727	-1.0908	-1.2859	-1.3395	-0.5955	-0.3875	-0.4952
NM_001107196	Atf6_predicted	-0.0156	-0.0623	0.12302	-0.0033	0.23583	0.02825	-0.0181	-0.12	-0.0917	0.04476	-0.1153	-0.0766	0.21239	0.08722
NM_001108115	Atf7_predicted	-0.761	-0.5927	0.01154	-0.1294	-0.9125	-0.9799	-0.1126	-0.9409	-0.4898	-0.6813	-0.4928	-0.1407	-0.7538	-0.5176
NM_001107893	Atf7ip_predicted	0.25767	0.5135	0.47272	1.6242	0.46287	0.27827	0.57057	0.46077	0.36902	0.54556	0.44101	0.33423	0.12522	0.33922
NM_001017471	Atf7ip2	0.02088	0.03566	-0.0604	0.13944	-0.0586	-0.0568	-0.0339	0.13307	-0.047	0.00723	0.00288	-0.0319	0.25418	0.06658
NM_001038495	Atg12	0.16263	0.28671	-0.3665	0.99245	0.32136	0.49865	0.30197	0.30853	-0.0201	0.20765	0.24397	-0.0065	-0.16	0.09209
NM_001108809	Atg16l1_predicted	-0.1298	0.00238	0.10752	0.42886	-0.176	-0.1136	0.29201	0.29099	-0.0278	0.07663	-0.1502	-0.3088	0.01724	0.17123
NM_134394	Atg3	0.56365	0.32549	0.18289	0.75365	0.41125	0.88134	0.3664	0.52734	0.60322	0.42858	0.44022	0.36214	0.53491	0.18912
NM_001025711	Atg4b	0.42118	0.36514	0.26108	0.32505	0.62389	0.78579	0.2625	0.48368	0.2636	0.3787	0.20872	0.4337	0.21449	0.28517
NM_001012097	Atg7	0.65493	0.15142	0.19425	0.03672	0.38842	0.53993	0.10223	0.43558	0.19723	0.27472	0.20751	0.12202	-0.1272	0.17676
NM_001014218	Atg9a	-0.7763	-0.3138	-0.0611	-0.0492	-0.4359	-0.611	-0.1465	-0.6689	-0.4228	-0.426	-0.5726	-0.2544	-0.5491	-0.4958
XR_007957.1	Athl1_predicted	0.03986	-0.021	-0.0506	0.07751	0.00276	0.13512	-0.0445	-0.0481	0.03494	0.00784	-0.0719	0.22133	-0.0699	0.09021
NM_031014	Atic	0.20476	0.19127	0.50199	0.10746	0.46795	0.30199	0.2041	0.27219	0.1422	0.38725	0.35662	0.36729	0.68366	0.53123
NM_001106821	Atm_mapped	-0.0358	0.02814	-0.0716	0.29586	-0.014	-0.01	0.19625	0.12236	0.04645	0.09161	0.05465	0.08055	0.13925	0.00257
NM_001170482	Atoh7_predicted	-0.2288	-0.2444	-0.2578	-0.2501	-0.0383	-0.015	-0.2945	-0.2594	-0.2643	-0.1318	-0.2433	-0.2373	-0.2247	-0.1573
NM_053359	Atox1	0.15494	0.21949	-0.0964	-0.3809	0.20034	1.1084	-0.2477	-0.0273	0.45637	0.2726	0.48132	1.1398	-0.0752	-0.0658
NM_001141935	Atp10a	-0.3661	-0.4319	-0.0671	0.02107	-0.2032	-0.0711	-0.3486	-0.126	-0.2962	-0.1041	-0.3276	-0.329	-0.3571	-0.0037
NM_001107324	Atp11a_predicted	0.03836	-0.2426	0.35893	-0.1638	0.12342	0.04479	-0.0438	0.29145	-0.0184	0.09354	0.01362	0.13321	-0.0998	0.11424

NM_133517	Atp12a	-0.2128	-0.1301	-0.0286	-0.0072	-0.033	-0.1264	-0.046	0.05626	-0.173	-0.1234	-0.121	0.26379	0.31088	0.07652
NM_001106079	Atp13a1_predicted	-0.7442	-0.4885	0.47681	0.12445	-0.637	-0.7513	-0.019	-0.6473	-0.7115	-0.6335	-0.3456	-0.5289	-0.2186	-0.2734
XM_342962.3	Atp13a2_predicted	-0.3767	-0.2581	-0.1992	0.03597	-0.0279	-0.4288	-0.4695	-0.4656	-0.5246	-0.3871	-0.2692	-0.3809	-0.5725	-0.2874
NM_012504	Atp1a1	0.41185	-0.022	0.15882	-0.1906	0.872	0.95551	0.07134	0.912	-0.1975	-0.0941	-0.145	-0.0492	-0.4732	-0.6524
NM_012505	Atp1a2	0.12187	0.06263	0.05201	0.03187	0.18717	0.13999	0.15762	0.05488	0.04136	0.2153	0.07899	0.20336	0.14705	0.01677
NM_012506	Atp1a3	-0.0176	0.10636	0.08184	0.12757	-0.0049	-0.0267	0.1025	0.07499	0.36932	0.1144	0.13076	0.4684	0.00395	0.10241
NM_022848	Atp1a4	-0.0449	0.13611	0.35535	-0.0287	0.14982	0.09613	0.33933	0.12336	0.13105	0.17529	0.16276	0.11727	0.17971	0.15684
NM_013113	Atp1b1	-0.33	-0.6674	-0.4276	0.63008	-0.5881	-0.5408	-1.3528	-0.6058	-0.7055	-0.543	-0.7374	-1.0206	-0.6687	-0.5689
NM_012507	Atp1b2	-0.2378	-0.0986	-0.1544	-0.2017	-0.3316	0.04271	-0.246	-0.168	-0.0704	0.00139	-0.2412	-0.0746	0.16834	-0.4701
NM_012913	Atp1b3	0.51243	0.98852	0.2789	0.49939	0.7823	0.59788	1.0551	0.68341	0.63414	0.91156	0.80067	0.26157	0.33773	0.47062
NM_053381	Atp1b4	-0.0328	0.04551	0.07452	-0.0996	0.10631	0.09236	-0.0272	0.07202	-0.064	0.01955	-0.0146	0.16237	-0.1099	-0.0366
NM_001110139	Atp2a2	0.18218	0.17452	0.28988	0.6553	0.93568	0.75251	0.5932	1.0156	0.17422	0.16074	0.14069	-0.1957	0.00598	-0.1058
NM_012914	Atp2a3	-0.665	-0.7517	-0.5148	-0.8352	-0.7393	-0.6925	-0.7729	-0.8284	-0.7443	-0.8205	-0.5502	-0.7266	-0.7547	-0.78
NM_053311	Atp2b1	0.30726	0.33236	0.74997	0.87888	0.26549	-0.1442	1.1204	0.44525	0.14844	0.17618	0.35472	-0.1577	0.52844	0.9118
NM_012508	Atp2b2	-0.0575	-0.0389	-0.368	-0.1295	-0.2607	-0.1416	-0.2329	-0.2539	-0.3338	-0.1376	-0.1709	-0.194	-0.0047	-0.2164
NM_133288	Atp2b3	-0.346	-0.5191	-0.2497	0.17306	-0.3209	-0.3741	-0.4233	-0.5061	-0.6127	-0.527	-0.3875	-0.1908	-0.292	-0.4387
NM_001005871	Atp2b4	0.06553	-0.0624	0.15193	-0.0521	-0.0687	-0.011	-0.232	-0.0941	0.06363	0.15196	-0.0527	0.08308	-0.0343	-0.1384
NM_131907	Atp2c1	0.29403	-0.0796	-0.3063	-0.7087	0.18252	-0.1093	-0.2139	0.31979	-0.2352	-0.2328	-0.013	-0.4271	-0.1253	-0.093
NM_012509	Atp4a	0.03517	0.03673	-0.0683	0.09744	0.08088	0.01149	-0.0011	-0.0042	-0.137	-0.0535	-0.0442	-0.0205	-0.037	0.04755
NM_012510	Atp4b	-0.0374	0.09031	-0.0048	-0.0056	0.17165	0.18489	0.03214	0.19061	0.0365	-0.0443	0.11359	-0.026	0.07761	-0.0628
NM_023093	Atp5a1	-0.0726	0.01064	-0.0171	-0.2535	0.16543	-0.147	0.12068	0.23802	-0.1412	0.12934	-0.1077	-0.228	-0.2081	-0.0116
NM_134364	Atp5b	0.25163	0.09692	0.37041	-0.0741	0.25682	-0.0941	0.31674	0.36547	-0.0449	0.08349	0.18731	0.02053	0.59385	0.53721
NM_053825	Atp5c1	0.1016	0.26556	0.25811	0.04359	0.28031	0.50077	-0.1173	0.33129	0.27342	0.06367	0.33183	0.50514	0.24096	0.25257
NM_139106	Atp5d	0.47137	0.30741	-0.0267	-0.42	0.53246	0.6157	-0.009	0.55412	0.37403	0.2931	0.33876	0.39666	0.36848	0.38394
NM_017311	Atp5g1	-0.0186	-0.3095	0.17515	-0.0726	0.34568	0.58319	-0.3005	0.12498	-0.1692	-0.0309	-0.0569	0.60016	0.22478	0.29928
NM_133556	Atp5g2	-0.1765	0.19437	-0.5206	-0.7678	-0.2497	-0.056	-0.5403	-0.3356	0.55294	0.19573	0.1824	0.38523	-0.3645	-0.2799
NM_053756	Atp5g3	0.10044	0.24993	0.00186	-0.1024	0.20477	0.30881	0.08324	0.09726	0.05257	-0.1672	-0.0259	0.20653	0.15429	0.05984
NM_019383.1	Atp5h	0.29983	0.0186	-0.0783	-0.3358	0.23444	0.80458	-0.2505	0.36289	0.4349	0.05528	0.25447	0.47536	0.31782	0.26861
NM_080481	Atp5i	0.26701	0.08905	0.44738	-0.3565	0.3913	0.37621	0.21028	0.23933	0.0401	-0.0129	0.1474	0.31832	0.22827	0.20562
NM_053602	Atp5j	0.27216	0.22489	0.21681	0.20315	0.78828	0.66426	0.24081	0.57638	0.41256	0.22854	0.44228	0.21049	0.2477	0.19971
NM_138883	Atp5o	0.14198	-0.0248	0.1467	-0.0192	0.35587	0.39767	0.42669	0.37708	0.22121	0.14829	0.10866	0.32409	0.29163	0.25871
NM_001007749	Atp5s	0.5557	0.49639	0.06178	0.19211	0.09436	0.10202	0.16954	0.2226	0.43867	0.4763	0.43849	0.03659	0.21308	0.19864
NM_001009705	Atp5sl	-0.2266	-0.1796	-0.3512	-0.5121	-0.529	-0.5101	-0.3809	-0.4834	-0.146	-0.1303	-0.0195	0.1003	-0.1928	-0.3241
NM_031785	Atp6ap1	0.25408	0.36224	0.10337	-0.1575	-0.327	-0.4155	-0.1605	-0.2824	0.42258	0.49957	0.34276	0.08801	0.42628	0.14543
XM_217592.4	Atp6ap2	0.72225	0.69158	0.23827	1.3203	0.75071	0.82889	1.0682	0.99239	0.94945	0.6317	0.7153	0.41486	0.70594	0.53758
NM_031604	Atp6v0a1	0.50586	0.32096	1.0349	0.52874	-0.1709	-0.0235	0.43978	-0.1592	0.28476	0.37972	0.53526	0.23667	0.12549	-0.0618
NM_001106591	Atp6v0a4_predicted	0.01091	0.10685	-0.1211	0.01563	0.08716	-0.0716	-0.1541	-0.0743	0.04991	0.05765	-0.0959	0.03221	-0.0151	-0.0712
NM_130823	Atp6v0c	0.58843	0.15264	0.34336	-0.2469	0.30259	0.00324	0.20983	0.30409	-0.0236	0.19744	0.05993	-0.5041	0.13396	0.33005
NM_001011927	Atp6v0d1	0.36675	0.32463	-0.1504	-0.321	0.01508	0.10454	0.10923	0.00381	0.12827	0.06456	0.09783	0.30267	0.2686	0.14622
NM_001011927	Atp6v0d1_predicted	-0.2218	0.03897	-0.1339	0.22212	0.02472	-0.071	-0.0683	-0.0214	0.02029	0.15575	-0.041	-0.0039	0.19149	-0.0071
NM_001011972	Atp6v0d2	0.13449	0.08065	0.04819	0.26166	-0.0351	0.10523	0.16645	0.15835	-0.0566	0.02476	-0.0507	0.03813	-0.0227	0.05479
NM_053578	Atp6v0e	0.62623	0.52892	0.04578	0.29011	0.71289	0.84657	0.73365	0.78173	0.59673	0.59385	0.61202	0.7831	0.47404	0.30037
NM_001002253	Atp6v0e2	0.1018	0.09327	-0.5485	-0.2765	0.21678	0.20062	-0.1245	0.56023	-0.1889	-0.0491	-0.0716	-0.6475	0.00505	-0.1868
NM_001108318	Atp6v1a1_predicted	0.91809	1.5308	-0.3579	0.09601	0.77761	-0.2328	0.37807	0.84916	0.80296	1.1606	0.56427	0.00719	0.53052	0.8477
NM_001107867	Atp6v1b1_predicted	-0.1897	-0.1669	-0.3852	-0.2225	-0.1427	-0.1407	0.07176	-0.335	-0.2077	-0.171	-0.1508	-0.2024	-0.2643	-0.273
NM_057213	Atp6v1b2	0.62694	0.38006	0.04512	0.1199	0.60929	0.37404	0.23129	0.52458	0.10102	0.35605	0.25107	-0.0501	0.33481	0.34933
NM_001011992	Atp6v1c1	0.24114	0.33664	0.35507	0.61744	0.06159	-0.1806	0.53506	0.20063	0.24387	0.15828	0.46831	-0.1227	0.45893	0.24026
NM_001011992	Atp6v1c1_predicted	0.68712	0.89566	-0.617	0.14934	0.61041	0.13035	0.24092	0.7861	0.53368	0.79666	0.11326	0.17475	0.62876	0.78392
NM_001014199	Atp6v1c2	-0.1025	0.24748	0.59505	-0.125	-0.2803	-0.1167	0.61043	-0.1521	0.09297	0.25403	0.09801	0.13503	0.24418	0.27632

NM_199386	Atp6v1d	0.553	0.21132	-0.1572	0.94006	0.50033	0.34419	0.69524	0.44458	0.11159	0.16399	0.11581	0.12659	0.18419	0.1683
NM_198745	Atp6v1e1	0.67769	0.62125	0.07984	0.2115	0.26973	0.3794	0.47057	0.33348	0.55285	0.70116	0.76411	0.59799	0.14599	0.1044
NM_001108979	Atp6v1e2_predicted	-0.0005	-0.098	0.00774	-0.0577	0.12993	0.20312	-0.0877	0.09468	-0.0243	-0.1202	-0.1398	-0.0974	-0.0968	-0.1474
NM_053884	Atp6v1f	0.81477	0.20209	-0.3139	0.0756	0.50652	0.92323	0.01698	0.32684	0.35289	0.04839	0.31595	0.11211	-0.0522	0.02965
NM_001106660	Atp6v1g1_predicted	0.06049	-0.1279	-0.0294	0.38171	0.18922	0.28422	0.44588	0.1164	0.16947	-0.1882	0.06892	0.46382	-0.0896	0.10943
NM_212490	Atp6v1g2	-0.6256	-0.4549	-0.2255	-0.3055	-0.5741	-0.1961	-0.0258	-0.5224	-0.4778	-0.5527	-0.5674	-0.5745	-0.7173	-0.6855
NM_001105991	Atp6v1g3_predicted	-0.0396	0.18391	0.13411	0.13426	-0.0056	0.15534	0.01595	-0.02	-0.0126	-0.1739	-0.1213	0.35284	-0.0844	-0.1958
NM_001013929	Atp6v1h	0.72688	1.012	0.34334	1.0393	1.1	0.89605	0.68475	0.98423	0.7101	0.84659	0.88133	0.30696	0.55534	0.49964
NM_052803	Atp7a	-0.0108	-0.1389	0.26853	0.39302	-0.194	-0.4862	0.13844	-0.0858	-0.3227	0.00386	-0.1075	0.08117	-0.099	-0.0771
NM_012511	Atp7b	-0.1288	-0.0912	-0.0444	0.02929	0.01991	-0.012	-0.1167	-0.0837	0.0039	-0.0833	-0.106	0.0425	0.15678	-0.0885
XM_223390.4	Atp8a1_predicted	0.0096	0.21535	0.02964	0.09298	0.16016	0.04503	0.06209	-0.0475	0.19681	0.31744	0.17566	0.15963	0.00589	-0.1044
XM_223390.4	Atp8a1_predicted	0.10008	0.11712	-0.0085	-0.0089	0.12071	0.04907	0.00058	0.02432	0.27076	0.15056	0.00074	0.03843	0.15732	0.102
NM_001106140	Atp8b1_predicted	-0.022	0.03941	-0.1512	0.00355	-0.1036	0.29609	-0.09	-0.0972	0.32439	-0.096	0.08155	0.10857	-0.1686	-0.1447
XM_001076355.1	Atp8b3_predicted	-0.0845	0.1109	0.01138	-0.0977	-0.0769	-0.1223	-0.0617	0.04807	0.16494	-0.1064	0.01207	0.19224	-0.0868	-0.086
XM_001055184.1	Atp8b4	-0.2089	-0.0592	-0.0328	-0.052	-0.1338	-0.117	-0.0723	-0.0723	-0.0735	0.03392	-0.1158	-0.1147	0.04644	-0.1122
NM_001107959	Atpaf1_predicted	-0.3114	-0.296	-0.2268	-0.3369	-0.459	-0.3568	-0.2316	-0.2244	-0.0638	-0.4314	-0.0759	-0.3226	-0.2059	-0.2314
NM_001107006	Atpaf2_predicted	-0.2675	-0.2823	-0.1521	-0.3489	-0.2649	-0.1515	-0.4259	-0.1352	-0.0533	-0.1806	-0.1468	-0.328	-0.4753	-0.6215
XM_342932.3	Atpbd1b_predicted	-0.0442	-0.125	0.03796	0.11723	-0.0689	0.13261	-0.2258	-0.0931	-0.1951	-0.2888	-0.1768	0.23356	0.32343	0.2182
NM_201991	Atpbd1c	0.32328	0.30057	0.08126	0.39836	0.41344	0.51004	0.612	0.31928	0.07537	0.25961	0.18869	0.39024	0.16633	0.25484
NM_001106251	Atpbd3_predicted	-0.2035	-0.1415	0.07755	-0.1965	-0.3745	-0.1027	-0.2478	-0.2363	-0.0998	-0.2489	-0.0826	-0.2062	-0.2467	-0.2113
NM_012915	Atpi	-0.0006	-0.1155	0.18624	-0.0332	-0.1762	-0.0774	0.26165	0.01776	-0.062	-0.0107	-0.1567	-0.1065	-0.1416	-0.0893
NM_031351	Atrn	0.26491	0.7092	0.67773	0.35391	1.189	0.91154	1.2534	1.0379	0.51726	0.672	0.55272	0.365	0.58603	0.94252
NM_001105757	Atrx	0.03393	0.32183	0.15231	0.07192	0.32255	0.0593	0.2268	0.11134	0.06717	-0.0219	0.21846	0.01764	0.10354	0.30981
NM_012726	Atxn1	0.04706	0.04556	0.00146	0.09145	0.03364	0.01871	-0.028	-0.0459	-0.0249	0.01376	0.1353	0.12774	0.13624	0.02082
NM_133313	Atxn10	0.3119	0.55467	0.18014	0.76086	0.93278	0.4357	0.36597	0.79569	0.50967	0.33674	0.26177	-0.1068	0.17153	0.25731
XM_213779.4	Atxn2_predicted	-0.1117	0.0762	-0.2741	0.22481	-0.3137	-0.6755	0.17728	-0.2576	0.02401	0.2113	0.00666	-0.2851	-0.1807	-0.1555
NM_001130097	Atxn2l_predicted	-0.3187	-0.6168	0.74645	0.31335	-0.3338	-0.4063	0.00565	-0.513	-0.4772	-0.1639	-0.4009	-0.0751	-0.3752	-0.2452
NM_021702	Atxn3	-0.0856	-0.1007	-0.1774	-0.0641	0.30877	-0.1016	0.02671	-0.1087	-0.3005	0.02284	0.18611	-0.0274	0.17095	0.03753
NM_001108407	Auh_predicted	0.71876	0.40939	-0.2821	0.9316	1.093	1.2301	0.4186	1.0025	0.21134	0.25468	0.26753	-0.0067	-0.4146	-0.4882
NM_001004237	Aurkaip1	-0.0338	-0.3531	-0.3149	-0.387	-0.3017	0.32042	-0.3897	-0.2692	-0.0381	-0.391	-0.0733	0.37307	-0.2345	-0.2546
NM_053749	Aurkb	-0.4263	-1.6212	0.58907	-0.7897	-1.2172	-1.346	-0.956	-1.0883	-0.6785	-1.1909	-0.8885	-0.0283	0.51675	0.4479
NM_001106221	Aurkc_predicted	-0.2998	-0.2683	0.34756	0.10855	-0.8046	-0.8039	0.44432	-0.824	-0.4341	-0.3501	-0.2094	-0.1953	-0.5828	-0.4008
NM_001107136	Auts2_predicted	-0.1831	0.04313	0.05393	-0.0988	0.16926	-0.1023	0.08615	0.03436	0.05226	0.00156	-0.0097	-0.048	0.11692	-0.1154
NM_001107136	Auts2_predicted	0.07821	0.07095	-0.0305	0.11819	0.18294	0.05768	0.01019	0.28367	0.16688	0.13511	0.13633	0.19641	0.10549	0.09206
NM_001107757	Aven_predicted	-0.1684	-0.3986	0.22499	-0.9429	-0.4596	-0.3068	-0.4829	-0.5485	-0.1158	-0.2901	-0.254	0.06643	0.40594	0.42202
NM_016992	Avp	0.03003	0.0367	-0.0509	0.10419	-0.063	-0.003	-0.0248	0.3721	0.06951	0.16766	0.04501	-0.0847	0.17122	-0.0737
NM_134373	Avpi1	0.54356	-0.0012	0.08614	0.18034	0.47948	0.80685	0.00145	0.30638	0.18421	0.08959	0.04744	0.10665	0.13892	0.08305
NM_053019	Avpr1a	-1.3647	1.5326	0.63435	1.7063	-1.5383	-1.2741	0.78577	-1.4828	1.5109	1.4427	1.3353	1.3873	1.675	1.6832
NM_017205	Avpr1b	-0.2287	-0.0279	-0.2357	0.02403	-0.2063	-0.1914	-0.2387	-0.2741	0.07225	-0.1129	0.00427	-0.1038	-0.3056	-0.0456
NM_019136	Avpr2	-0.0376	-0.1226	0.02096	-0.0654	0.0888	-0.1003	-0.0272	0.04702	-0.0798	0.05444	-0.1166	-0.1338	0.31765	-0.0874
NM_024405	Axin1	-0.3374	-0.2245	-0.1401	-0.086	-0.1415	0.03255	-0.1784	-0.3107	-0.2874	-0.1292	-0.0848	0.22785	-0.2005	-0.1761
NM_024355	Axin2	0.07332	-0.0415	-0.0073	-0.0447	0.42027	0.20638	-0.0609	0.30265	0.03199	-0.0002	-0.0146	-0.1512	-0.0931	0.01698
NM_001013147	Axl	0.55164	0.99421	0.32534	0.22186	0.81942	0.05599	0.63661	0.79396	0.54644	0.86529	0.48913	0.11479	0.94332	0.97854
NM_001013147	Axl	0.53138	1.1481	0.31516	0.24272	0.80037	0.13422	0.91949	0.80755	0.9083	1.0883	0.75557	0.30182	1.1402	1.3164
NM_001012087	Axot_predicted	-0.1365	-0.1216	-0.194	-0.2094	0.23423	-0.1561	-0.0412	-0.1179	-0.1134	-0.1726	-0.0887	-0.0901	-6E-05	-0.0492
NM_001100735	Aytl2_predicted	0.27781	0.40077	0.39236	-0.2787	0.33877	0.14186	0.1083	0.27238	0.16812	0.34358	0.27472	-0.047	0.46683	0.58567
NM_012826	Azgp1	0.03676	0.05656	0.05696	-0.0689	0.14641	0.17774	0.00949	0.14404	-0.0139	0.07924	0.07967	0.25985	0.22272	0.07429
NM_001108310	Azi1_predicted	-0.477	-0.2701	-0.1587	-0.442	-0.2881	0.00655	-0.3838	0.00311	-0.1642	-0.2254	-0.2323	0.11815	-0.5451	-0.4046
NM_001025705	Azi2	-0.5501	-0.2922	-0.4807	-0.4112	-0.2856	-0.3171	-0.0561	-0.2945	-0.4608	-0.5035	-0.2803	-0.3492	-0.5528	-0.5602

NM_022585	Azin1	0.01957	0.28591	0.58844	-0.3952	-0.7115	-0.7248	0.09362	-0.3891	0.17376	0.26597	0.3506	0.25521	0.80472	0.72503
NM_012512	B2m	0.75899	0.61003	0.19926	0.8482	0.67773	0.86056	0.88317	0.80375	0.48644	0.50603	0.50437	0.3976	0.3878	0.49975
NM_001013158	B3galnt1	0.10544	1.3828	0.18053	-0.5419	-0.2628	-0.4775	0.84171	-0.2299	1.0841	1.1318	1.1179	0.61524	0.17673	0.45788
NM_001108954	B3galt1_predicted	-0.0263	-0.0207	-0.0244	-0.0464	-0.0408	-0.0402	-0.0639	0.04747	0.00983	0.08115	-0.0754	0.34177	-0.0058	0.00889
NM_133553	B3galt4	-0.3928	-0.1396	-0.0503	-0.2091	-0.2409	-0.2036	-0.1583	-0.3807	-0.459	-0.27	-0.0682	-0.2465	-0.1303	-0.1948
NM_001105887	B3galt5_predicted	-0.0315	0.01833	0.26475	0.09921	0.1928	-0.0247	0.03569	0.02514	0.00425	0.20855	0.09344	-0.0705	-0.0274	0.2506
NM_001106699	B3galt6_predicted	0.04809	-0.0241	-0.1276	0.25843	0.11747	-0.116	-0.1258	0.08142	0.05103	0.25089	0.09619	-0.0677	0.26604	0.28361
NM_001107492	B3galt7_predicted	0.02034	-0.041	0.08308	0.04323	0.00307	-0.0574	0.0772	0.13987	0.25338	-0.0589	0.15017	0.01269	-0.0754	-0.2218
NM_054003	B3gat1	0.20599	0.24697	0.07966	0.13993	-0.0395	0.19735	0.16226	0.4884	0.23916	0.07393	0.41625	0.03479	0.13555	0.08281
NM_001128184	B3gat3_predicted	0.6938	0.38991	0.58642	0.66318	0.52846	0.66665	0.583	0.44605	0.12244	0.39437	0.32546	0.34604	0.63384	0.65498
NM_001107240	B3gnt1_predicted	1.0216	1.0762	-0.1789	0.35934	1.2805	0.20888	0.64944	1.3681	0.65045	0.81744	0.68361	-0.5634	0.50152	0.43474
NM_001106068	B3gnt3_predicted	-0.2472	-0.2041	-0.1655	0.07112	-0.0724	-0.3652	-0.1248	-0.2715	-0.113	-0.1952	-0.0831	-0.08	-0.1176	-0.0014
NM_001105938	B3gnt4_predicted	-0.0291	-0.0553	0.1233	0.00654	0.21147	-0.0519	0.01133	-0.2405	0.04679	-0.0907	0.30321	0.29275	0.05131	0.01655
NM_053932	B3gnt5	0.07967	0.18806	0.12044	0.0662	-0.126	-0.0127	0.12158	-0.0876	0.16238	0.2156	-0.045	0.19474	-0.0745	0.02129
NM_001106324	B3gnt6_predicted	0.54714	0.66465	-0.6096	-0.3823	0.6427	0.59515	0.02591	0.66958	0.64438	0.76668	0.78755	0.14672	-0.3855	-0.5189
NM_001012134	B3gnt7	0.06423	0.38022	0.13029	0.02071	0.35279	0.14573	-0.0434	0.13074	0.09825	-0.1565	-0.0283	0.18891	0.17947	-0.0253
NM_001015035	B3gntl1	0.10338	-0.0357	0.00487	0.32038	-0.1256	-0.1698	-0.0518	-0.1431	0.02166	-0.1044	-0.0383	-0.1408	-0.1216	0.11098
NM_022860	B4galnt1	-0.2868	0.74534	0.61685	0.39037	-0.3701	-0.6308	0.66302	-0.4654	0.83403	0.78514	0.52192	0.92861	0.88215	1.0344
NM_053287	B4galt1_mapped	0.33886	0.57628	-0.301	-0.1612	0.81049	0.24612	0.17136	1.019	0.2813	0.28783	0.31291	-0.4217	0.74009	0.58834
NM_001107965	B4galt2_predicted	0.11448	0.13959	0.10083	0.18413	0.26315	0.07703	0.17765	0.00491	0.07889	0.08332	0.1575	0.05946	0.10919	-0.0789
NM_001009539	B4galt3	0.15157	0.65246	0.39457	0.72471	-0.141	-0.1297	0.68888	-0.4071	0.45105	0.57392	0.58312	-0.0451	0.15508	0.04492
NM_001012018	B4galt4	-0.385	0.03769	-1.278	-1.2743	-0.8299	-0.867	-0.4138	-0.9047	0.16694	-0.0352	-0.1271	0.40743	-1.1201	-1.1824
NM_031740	B4galt6	-2.1642	-2.6925	-2.1141	-3.3455	-2.1347	-2.0499	-2.6526	-1.8608	-2.4982	-2.4899	-2.6016	-2.364	-2.5387	-2.7469
NM_001031661	B4galt7	0.70075	0.28392	0.35363	0.74348	0.88592	1.0294	0.36345	0.73241	0.35555	0.0876	0.1178	0.30499	0.25641	0.07164
NM_144762	Baalc	0.13596	-0.0128	0.12344	0.21685	0.04597	0.28051	0.06917	0.02013	0.04831	-0.0331	-0.0366	0.27087	0.08686	0.13987
NM_017300	Baat	0.14635	-0.0184	0.12167	-0.0022	0.04426	-0.1248	0.38617	0.02811	0.08061	0.21653	-0.0124	0.07318	0.18668	0.16881
NM_019204	Bace1	0.15906	0.27427	0.13247	0.07649	0.15785	-0.0695	0.0788	0.21736	0.20163	0.10293	-0.0809	0.00544	0.12706	-0.0557
NM_001002802	Bace2	-0.0209	0.08433	0.09328	0.35993	0.18735	0.14638	0.05239	0.22539	0.02144	0.18373	-0.0937	0.03952	-0.1992	0.20364
NM_001107113	Bach1_predicted	-0.1839	-0.0809	-0.255	-0.1828	-0.0293	-0.0796	0.00693	-0.0908	-0.2136	-0.2326	-0.1968	-0.2944	-0.1953	-0.1781
NM_022698	Bad	-0.0176	-0.224	0.05138	-0.6097	-0.2434	-0.0577	-0.6631	-0.3756	0.00965	-0.1228	0.02574	0.32879	0.23928	0.26202
NM_001106647	Bag1_predicted	-0.1242	-0.3953	0.20024	-0.5949	-0.0299	-0.024	-0.2488	0.03894	-0.4332	-0.3686	-0.3124	0.27763	0.16051	0.27495
NM_001011936	Bag3	0.15153	0.73224	-0.1947	-0.3936	0.09329	-0.222	-0.135	0.13463	0.21063	0.63343	0.42385	-0.3462	0.33967	0.24393
NM_001011936	Bag3_predicted	0.17504	0.03218	0.0793	0.10097	0.12896	0.37021	-0.0388	0.05383	0.06299	0.01632	0.05463	0.04645	0.0509	0.08991
NM_001008526	Bag5	-0.303	-0.5625	-0.2976	-1.2987	-0.1187	-0.123	-0.275	-0.2671	-0.5579	-0.2587	-0.2119	-0.2652	-0.2698	-0.2618
NM_001170597	Bai1_predicted	0.03519	0.06153	0.17422	0.03332	0.11563	0.04944	0.00858	-0.0036	0.11322	0.10546	-0.0292	0.10922	-0.0956	0.02092
NM_001107914	Bai2_predicted	0.16759	0.06061	-0.0379	0.17058	0.09692	0.16621	-0.0529	0.04595	-0.0669	0.01465	0.06911	0.03706	0.09156	-0.0027
NM_001106898	Bai3_predicted	-0.0974	-0.1023	0.01869	-0.0201	0.01562	-0.03	0.00874	-0.002	-0.1184	0.00736	-0.0744	-0.0631	-0.0377	-0.0795
XM_216228.3	Baiap1_predicted	-0.2773	-0.1596	-0.2736	-0.2019	-0.3325	-0.4479	-0.1605	-0.2215	-0.1916	-0.3644	-0.281	-0.0379	-0.4935	-0.2218
NM_057196	Baiap2	0.30616	0.30939	1.255	0.51841	0.5013	0.59743	1.0448	0.26387	0.38809	0.74101	0.84771	0.50314	0.99378	0.97979
NM_053812	Bak1	-0.6012	-0.839	-0.8247	-1.4014	-0.656	-0.8714	-1.1192	-0.778	-0.7407	-0.8658	-1.1212	-0.6198	-0.472	-0.4175
NM_139082	Bambi	-0.0179	-0.6594	-0.7482	-0.6559	1.5908	1.4781	-0.7293	1.5117	-0.8414	-0.7193	-0.771	-0.4216	-0.8077	-0.8853
NM_053631	Banf1	0.2984	0.0358	0.22085	-0.1112	0.36041	0.56515	-0.1718	0.3218	0.07638	0.1031	0.27816	0.50354	0.40144	0.37747
NM_001106191	Banp_predicted	-0.3778	-0.3425	0.0648	0.74481	-0.6302	-0.413	-0.1675	-0.3984	-0.4991	-0.5872	-0.3307	-0.2782	-0.1806	-0.3875
NM_001107292	Bap1_predicted	-0.151	-0.2574	0.0124	-0.2036	-0.0313	-0.5029	-0.0048	-0.2067	-0.0057	-0.2557	0.10192	-0.2188	-0.1936	-0.0297
NM_022622	Bard1	-0.0654	-0.2767	0.66941	-0.1107	-0.3791	-0.3475	-0.2371	-0.3979	-0.1281	-0.2549	-0.2228	0.24544	0.43328	0.46488
NM_057109	Barhl1	0.11929	0.30738	0.18898	0.15657	0.25145	0.05644	0.11885	0.23401	0.08092	-0.0186	0.23379	0.20777	0.22879	0.13953
NM_022956	Barhl2	-0.1567	-0.0542	0.0298	0.00074	-0.0802	0.03362	-0.0442	-0.025	-0.0677	-0.0743	-0.0134	-0.0393	-0.0762	-0.0237
NM_001108880	Barx1_predicted	-0.0338	-0.0032	0.1288	0.27111	0.03843	0.01812	0.17881	0.18821	0.53257	0.05274	0.00756	0.01928	0.40168	0.05206
NM_022300	Basp1	-0.3066	-0.751	-0.1278	-0.8633	0.48463	0.20767	-1.1091	0.50777	-0.2818	-0.26	-0.5525	-0.1173	0.1924	0.33336

NM_133300	Bat1a	-1.2241	-0.793	-0.2341	-0.9603	-1.4722	-1.3382	-0.6076	-1.3252	-0.791	-0.8354	-0.8404	-0.4995	-0.6802	-0.597
NM_212462	Bat2	-0.6646	-0.5922	0.21143	0.00734	-0.7238	-0.7312	-0.4917	-0.6623	-0.7588	-0.3027	-0.5494	-0.3866	-0.5386	-0.3909
NM_053609	Bat3	-0.188	-0.2649	0.30349	0.05404	0.15928	0.22311	-0.0199	0.01914	-0.1207	-0.1111	-0.1381	0.05566	-0.2249	-0.094
NM_212531	Bat5	-1.2586	-0.9711	-0.252	-0.6406	-1.1873	-1.2251	-0.3577	-0.9716	-1.1484	-1.145	-1.0293	-0.6415	-1.1165	-0.9119
NM_001106748	Batf_predicted	-0.7738	-1.3794	-0.9125	-0.8619	0.32325	0.54409	-0.6898	0.46026	-1.1768	-1.0038	-0.5649	-1.5723	-0.8983	-0.9082
NM_017059	Bax	0.02623	-0.3861	-0.1714	0.14798	-0.1936	0.47485	-0.3289	-0.2814	-0.2512	-0.0746	-0.2114	0.13069	-0.5146	-0.4796
NM_001170568	Baz1a_predicted	0.03278	-0.0735	0.21098	0.27055	0.02488	-0.105	0.1783	0.0842	0.24864	0.26141	-0.1053	0.20603	0.80271	0.76388
XM_001077467.1	Baz1b	-0.0523	-0.1492	-0.2577	-0.0789	-0.1647	-0.1897	-0.0136	-0.0359	-0.0388	-0.0725	-0.1054	0.03809	-0.033	-0.0091
NM_001107158	Baz2a_predicted	-0.0057	-0.0057	0.16277	0.08436	0.0204	-0.0051	0.16941	-0.0637	-0.0882	-0.1537	-0.1489	-0.0114	0.22186	0.10447
NM_001107158	Baz2a_predicted	0.04166	0.3251	0.1939	0.37202	0.096	-0.1151	0.09462	-0.0517	0.14026	0.13713	0.13522	0.11779	0.2985	0.23672
NM_001108260	Baz2b_predicted	-0.4252	-0.2247	0.03488	0.3338	-0.5667	-0.5096	0.40705	-0.6971	-0.0364	-0.0221	-0.129	0.07167	-0.2091	-0.3499
NM_022629	Bbox1	0.15024	0.20898	0.10108	0.16375	0.04125	0.10716	0.17943	0.03735	0.30514	0.03277	0.18581	0.02994	0.02744	0.28231
NM_001107569	Bbs1_predicted	-0.0256	-0.082	-0.1906	-0.1288	-0.0925	-0.0819	-0.1454	0.02151	-0.0516	-0.027	-0.1679	-0.0497	-0.0335	-0.2319
NM_053618	Bbs2	0.32118	0.65209	0.61136	0.9722	0.54041	0.38778	0.4278	0.49061	0.61834	0.45155	0.64659	0.53982	0.09375	0.18723
NM_001106826	Bbs4_predicted	-0.334	0.06976	-0.1012	-0.3034	-0.187	-0.1681	-0.0132	-0.0273	0.00963	0.12657	-0.1076	-0.4738	-0.159	-0.1156
NM_001108583	Bbs5_predicted	0.23715	0.38681	-0.4776	0.98993	0.19167	0.21051	0.50323	0.25818	0.24701	0.37797	0.4558	-0.2513	-0.1683	-0.4046
NM_001012180	Bbs7	0.83386	0.45173	0.89354	1.802	1.0438	0.95567	0.69933	1.1317	0.45804	0.36746	0.36728	0.31066	0.4424	0.37098
NM_001079938	Bbx_predicted	0.46731	-0.1006	0.18486	0.63672	0.42128	0.09321	0.32128	0.11943	0.03804	-0.1418	0.06564	-0.1799	0.03401	0.15507
NM_001107947	BC060737_predicted	-0.3678	-0.3292	-0.3642	-0.3136	-0.3435	-0.3886	-0.26	-0.2955	-0.3695	-0.0952	-0.0795	0.02191	-0.2571	-0.4383
NM_031752	Bcam	0.01337	-0.0602	-0.075	-0.1219	-0.0802	0.06199	-0.2233	0.03888	-0.033	-0.0297	-0.1844	0.11284	-0.0869	-0.0471
NM_012916	Bcan	-0.0235	-0.1309	-0.1012	0.00684	-0.1487	-0.0768	-0.1149	-0.1416	-0.0289	0.2289	-0.1617	-0.1417	-0.16	0.00696
NM_001006980	Bcap29	0.51283	0.55558	0.10463	0.14716	0.28419	0.1473	0.89764	0.31577	0.51313	0.53566	0.60565	0.15669	0.58948	0.38105
NM_001004224	Bcap31	0.00419	0.05742	-0.6965	-0.2569	0.1027	0.13324	-0.2107	0.03021	0.23892	0.0883	0.01161	-0.02	0.14123	0.14869
NM_012931	Bcar1	0.73854	0.6697	1.0586	0.92467	0.92581	0.74938	0.70547	0.8182	0.60278	0.71904	0.51786	0.52813	0.51726	0.7716
NM_001107722	Bcar3_predicted	0.4402	0.31641	0.29873	0.26679	0.82648	0.50104	0.42149	0.54243	0.36399	0.44398	0.30593	0.35379	0.07197	0.20325
NM_145670	Bcas1	-0.3652	-0.3206	-0.1711	-0.34	-0.3438	-0.3985	-0.2095	-0.3105	-0.4122	-0.2756	-0.4097	0.00386	-0.3213	-0.1412
NM_001106458	Bcas2_predicted	0.41274	0.04948	0.0792	0.21085	0.08039	0.31216	0.20954	0.11965	0.03111	0.018	0.18207	-0.0255	-0.0488	-0.1861
NM_017253	Bcat1	0.10417	0.5822	-0.1297	-0.5145	0.21441	-0.2907	-0.0701	0.0803	0.51456	0.50885	0.2525	-0.0552	0.64391	0.70405
NM_022400	Bcat2	0.15178	0.24654	-0.2188	-0.0664	0.4036	0.47104	-0.1117	0.26535	0.07935	0.12668	0.19166	0.0841	-0.2448	-0.1745
NM_001108505	Bccip_predicted	-0.1154	-0.1815	-0.3437	-0.3621	0.03787	0.06312	-0.3502	-0.0158	-0.3209	-0.2181	-0.3318	-0.2689	-0.2853	-0.4057
NM_001108505	Bccip_predicted	-0.3564	-0.2968	0.14406	-0.2673	-0.2132	-0.3128	-0.0072	-0.3783	-0.228	-0.1623	-0.3861	-0.2459	-0.0208	0.06399
NM_001127712	Bcdo2	-0.049	-0.0953	-0.0423	-0.0583	-0.1479	-0.0051	-0.1159	-0.1006	0.00362	-0.0778	0.04048	-0.1219	-0.1052	-0.1368
NM_022942	Bche	-0.0356	-0.1232	-0.0659	0.09345	-0.1604	-0.2079	-0.0924	-0.0675	-0.0587	-0.1095	-0.0451	-0.2361	-0.0711	-0.1869
NM_012782	Bckdha	0.12459	0.14224	-0.688	-0.1349	0.38366	0.52128	-0.0749	0.47716	0.20091	0.15606	0.24403	-0.1661	-0.3862	-0.442
NM_019267	Bckdhb	-0.0953	-0.2662	-0.2336	-1.0774	0.28374	0.09255	-0.4203	-0.0084	-0.0275	0.01111	-0.1618	0.08055	0.27163	0.14559
NM_019244	Bckdk	0.00898	0.0645	-0.1061	0.17943	-0.3085	-0.0743	-0.2267	-0.2099	-0.2571	-0.0754	-0.1591	-0.3152	0.01191	-0.0142
NM_022297	Bcl10	0.34097	0.23486	-0.0979	0.52285	0.14509	0.21955	0.69928	0.15732	-0.1117	-0.1076	0.10739	-0.0371	0.02344	0.07669
XM_223693.3	Bcl11a_predicted	-0.064	0.25816	0.14712	-0.0992	-0.126	-0.1701	-0.0214	-0.1796	0.06918	0.00715	0.11514	-0.0818	0.01064	0.01547
NM_001108057	Bcl11b_predicted	0.30432	-0.0105	0.0249	0.06942	0.2023	-0.0283	0.45163	-0.0295	-0.0741	0.02093	0.02063	-0.0179	0.16526	0.19763
NM_016993	Bcl2	-0.0384	0.03997	-0.1054	-0.1539	-0.1029	-0.0975	-0.1133	0.04194	0.05398	-0.166	0.14413	-0.0898	-0.0256	-0.1376
NM_133416	Bcl2a1	0.21416	0.29591	0.1636	0.11066	-0.0569	-0.0116	0.31772	0.10278	0.14708	0.34078	-0.0427	0.12231	-0.0054	0.19884
NM_001033671	Bcl2l1	-0.0028	0.0759	0.32202	0.26781	0.50749	0.04145	0.21432	-0.1086	-0.0418	-0.0603	-0.139	0.086	0.06509	0.24846
NM_001033671	Bcl2l1	0.1621	0.12107	0.06033	-0.0912	0.04744	0.0976	-0.0651	0.05736	0.05741	0.17842	-0.0132	-0.0385	0.13926	-0.1184
NM_053733	Bcl2l10	-0.1329	-0.0637	0.13341	-0.094	-0.0269	-0.046	-0.062	-0.0585	-0.0998	-0.1182	-0.0323	-0.0423	0.06818	-0.0282
NM_171988	Bcl2l11	0.12615	-0.0004	0.05477	-0.1457	-0.1204	0.03158	0.07383	0.03067	-0.0153	0.01948	0.04338	0.36523	0.0776	0.12621
NM_171988	Bcl2l11	0.14023	0.05643	0.22122	0.00704	0.06498	0.01525	0.08088	0.05216	0.09496	-0.0632	0.06509	-0.0756	0.09254	0.00646
NM_001108480	Bcl2l12_predicted	-0.0542	-0.1095	-0.3984	-0.4495	-0.0097	0.30989	-0.1304	-0.16	-0.0571	-0.1933	-0.0557	0.02629	-0.3281	-0.0716
NM_001107885	Bcl2l13_predicted	0.01027	-0.1242	-0.3669	-0.5361	0.0766	0.37541	-0.2787	-0.0859	-0.2582	0.14774	0.01733	0.05447	0.08419	0.11725
NM_001024338	Bcl2l14	0.58626	-0.2712	-0.1192	0.20001	-0.0347	0.04376	-0.4905	-0.2293	-0.0776	-0.4735	-0.2178	-0.1019	-0.4689	-0.32

NM_021850	Bcl2l2	-0.0942	0.03186	0.2882	0.49301	0.3376	0.54851	0.58964	0.33127	-0.1756	-0.0313	-0.0776	-0.0927	-0.4916	-0.2422
NM_001107084	Bcl6_predicted	0.49483	0.68841	-0.4222	0.61306	0.43587	-0.0003	0.49186	0.5303	0.2836	0.76712	0.39354	-0.6656	-0.3739	-0.2577
XM_341081.2	Bcl7a_predicted	-0.1394	-0.0675	0.05894	0.2833	0.37104	0.29353	0.0437	0.15121	0.08461	0.22345	0.21286	-0.028	-0.0691	-0.0266
NM_001106298	Bcl7c_predicted	-0.6465	-0.8379	-0.4316	-1.5882	-1.0233	-0.7538	-0.8769	-1.2442	-0.427	-0.7872	-0.6813	0.12495	-0.0861	-0.0976
NM_001107703	Bcl9_predicted	-0.0291	-0.0217	-0.167	-0.2094	0.01702	-0.1132	-0.132	-0.0139	-0.0185	0.00032	-0.1326	-0.0982	0.05181	0.10139
NM_001106817	Bcl9l_predicted	-0.0865	0.03764	0.21713	-0.0266	-0.0091	0.30529	0.14351	-0.0169	0.00803	-0.0793	0.20617	0.15873	0.07124	0.20831
NM_001047852	Bclaf1	-0.942	-0.6127	0.05951	-0.1451	-1.1888	-1.2052	-0.0595	-1.1553	-0.5177	-0.5334	-0.3674	-0.2157	-0.4915	-0.6376
NM_053648	Bcmo1	0.18454	0.06944	0.12623	0.0707	0.21469	0.01484	0.07889	0.26569	0.02347	0.02035	0.15505	0.13208	0.22982	0.13169
XR_009547.1	Bcor_predicted	-0.1189	-0.2105	0.05634	0.08856	-0.0126	0.11888	-0.0814	0.03243	0.17523	-0.012	-0.3119	0.14005	-0.0309	-0.1832
XM_228091.4	Bcr_predicted	0.07944	-0.041	0.24639	-0.102	0.01352	0.25538	0.28333	0.1356	-0.0584	0.03802	-0.0885	0.13117	-0.0609	-0.0417
NM_001007666	Bcs1l	-0.195	-0.4311	0.5462	-0.7176	-0.2626	-0.3143	-0.224	-0.3911	-0.2689	-0.1937	-0.3345	0.07351	0.80691	0.60437
NM_053995	Bdh1	0.40613	-0.6328	0.3776	-0.0849	0.84005	0.53144	-0.2207	0.89883	-0.3581	-0.6239	-0.5299	-0.0467	0.1338	0.15532
NM_001106473	Bdh2_predicted	0.26337	0.33467	0.03656	-0.31	-0.2203	0.16459	0.37551	-0.4667	0.4549	0.62632	0.32575	0.66915	0.2706	0.26081
NM_030851	Bdkrb1	-0.0562	0.36725	0.24913	0.16248	0.03239	-0.0486	0.08472	-0.0783	-0.1134	-0.0669	-0.1818	0.01312	0.1091	0.26306
NM_173100	Bdkrb2	-0.1262	-0.115	-0.2469	-0.108	0.02332	-0.0832	-0.1803	-0.0909	-0.0459	-0.2374	-0.284	-0.0568	-0.0701	-0.0382
NM_012513	Bdnf	1.7078	1.2885	0.90052	0.9111	1.8187	1.7886	2.0222	2.0011	1.0011	1.2838	1.253	0.73627	1.4988	1.4294
NM_001106403	Bdp1_predicted	0.06309	-0.1382	-0.118	-0.1845	-0.0154	0.00486	-0.1775	-0.0869	-0.1438	-0.0234	-0.0617	-0.0414	0.02602	0.04231
NM_001034117	Becn1	-0.8868	-0.7582	-0.7035	-1.1898	-1.0132	-0.9425	-0.4148	-0.7443	-0.6516	-0.754	-0.6237	-0.8236	-0.7179	-0.7761
NM_024163	Begain	-0.0168	-0.0863	0.06824	-0.0147	-0.1549	-0.1149	0.1139	-0.1573	-0.0618	-0.1573	-0.0501	0.16548	-0.0964	-0.1413
NM_001003928	Bem46l3	0.21637	0.08151	0.10758	0.15953	0.05438	0.08225	-0.028	0.12718	0.0972	0.17922	0.11919	0.0904	0.13553	0.05481
NM_001011940	Best1	0.19565	-0.1597	0.14488	0.02563	0.29249	0.27311	0.04768	0.16331	0.02797	0.07809	0.05238	-0.1599	-0.0289	0.01931
NM_019251	Bet1	0.42494	0.15924	-0.6449	0.65007	0.62719	0.53833	-0.0746	0.74809	-0.1087	-0.1125	-0.167	0.02171	-0.1009	-0.2453
NM_019368	Bet1l	0.36019	0.45258	0.07177	0.12204	0.44788	0.36445	0.21291	0.50945	0.33231	0.26368	0.21546	0.11458	0.41019	0.41681
NM_031555	Bfsp1	0.1577	-0.2158	0.04156	-0.2812	-0.1363	0.00545	0.06036	-0.1398	-0.389	-0.1975	-0.0554	-0.1522	-0.2585	-0.1598
NM_013414	Bglap	-0.2614	-0.2085	0.12922	-0.0378	-0.2698	-0.2695	-0.193	0.01363	-0.2501	-0.1988	-0.2498	-0.1728	-0.2006	-0.1216
NM_017087	Bgn	-0.7972	-0.089	-0.7636	-0.7425	-0.8093	-0.4643	-0.708	-0.673	0.04342	-0.0779	-0.0212	0.00797	-0.9617	-0.7575
NM_053328	Bhlhb2	0.05241	0.66038	-0.7333	0.14114	-0.8476	-1.1439	0.22101	-0.8479	0.25383	0.39872	0.15948	-1.1124	-0.5119	-0.4353
NM_133303	Bhlhb3	0.55673	0.41631	0.19216	0.23685	0.96099	1.0647	0.16534	1.051	0.612	0.51087	0.48029	0.23332	0.21088	0.2673
NM_001108940	Bhlhb5_predicted	0.07734	0.16783	0.01914	0.02131	0.00185	0.11884	0.11559	0.13398	0.15163	0.13343	0.05329	0.07877	0.20857	0.00802
NM_207611	Bhlhb9	0.00546	-0.0742	0.02346	-0.0188	-0.0123	0.02778	0.03644	-0.0394	-0.0514	-0.0138	0.00355	0.04299	-0.0478	-0.0095
NM_030850	Bhmt	-0.367	-0.2526	0.29304	0.26283	-0.1776	-0.246	0.05443	-0.2591	-0.2572	0.01109	-0.1935	-0.1373	-0.0724	-0.3084
NM_001108531	Bicc1_predicted	0.08526	0.1506	0.11634	-0.0349	-0.0339	0.00369	0.12823	0.1701	0.07238	0.18617	0.05359	0.06512	0.18184	0.07896
NM_001108653	Bicd1_predicted	0.52788	0.28608	0.06241	0.22271	0.19746	0.01684	0.181	0.088	0.24187	0.12224	0.26741	0.41365	0.34119	0.24171
NM_001033674	Bicd2	0.05137	-0.191	0.42753	0.53971	0.63879	0.58714	-0.0401	0.79491	0.17037	0.026	-0.0558	0.07548	0.10245	-0.009
NM_022684	Bid	0.63656	0.89231	-0.4155	-0.4609	0.55511	-0.0152	0.30625	0.52462	0.77698	0.58835	0.38226	0.20056	0.49933	0.4256
NM_053704	Bik	0.07504	-0.2308	-0.1673	0.01132	-0.2326	-0.1769	-0.1804	-0.1601	-0.0299	-0.0623	-0.2039	-0.1061	-0.1554	-0.173
NM_001012223	Bin2	-0.0231	0.11727	0.24738	0.0416	0.24952	0.74807	0.17979	-0.0735	0.14807	-0.0083	0.2221	-0.1317	-0.2319	-0.1351
NM_001009524	Bin2a	0.02462	0.19039	-0.0612	0.01128	0.27192	0.38797	0.13707	0.21243	-0.1568	0.12537	-0.1438	0.02621	0.23637	0.20942
XM_226742.4	Birc1b	-0.118	-0.1886	-0.1594	0.13475	-0.1626	-0.1535	-0.1074	-0.0473	0.04427	0.32261	-0.155	0.00709	-0.1277	-0.1866
NM_021752	Birc2	-0.2142	0.15808	-0.0212	1.2327	0.27087	0.30033	0.52924	0.1911	0.00384	0.33747	0.11763	-0.1238	0.26152	-0.0213
NM_023987	Birc3	-0.2402	-0.1539	0.15141	0.50112	-0.1305	-0.1658	0.17629	-0.105	-0.0953	-0.2602	-0.4075	0.06143	0.13871	-0.1341
NM_022231	Birc4	0.05648	0.13699	0.23563	0.00331	0.10261	0.05121	0.01747	-0.0309	0.00909	0.10099	0.11495	0.12307	0.07578	0.09364
NM_001170596	Birc6_predicted	-0.5362	-0.349	-0.6203	-0.4079	-0.5206	-0.8519	-0.4426	-0.1737	-0.4282	-0.5441	-0.2363	-0.6319	-0.3866	-0.5318
XM_237103.3	Bivm_predicted	-0.2936	0.46969	-0.6033	-0.4499	-0.3239	-0.7511	-0.1261	-0.2324	0.09147	0.21185	-0.185	-0.4337	0.01638	-0.1854
NM_133582	Blcap	-0.6792	-0.6441	-0.1166	-0.7731	-0.5328	-0.2086	-0.2318	-0.4735	-0.5991	-0.5619	-0.5936	-0.3674	-0.219	-0.3878
NM_001024233	Bles03	-0.3888	-0.16	-0.2408	-0.13	0.01047	0.29835	-0.0451	0.05934	0.21675	-0.0582	-0.1387	0.1776	-0.0552	0.16376
NM_001025751	Blk	-0.0774	-0.2209	-0.0827	-0.1935	-0.0461	0.06328	0.06329	-0.2963	-0.109	0.08809	-0.144	-0.1988	-0.0111	-0.0481
NM_001107526	Blm_predicted	-0.2112	-0.7967	0.61497	0.10269	-0.5386	-0.718	-0.2247	-0.7566	-0.1801	-0.2426	-0.4111	0.31855	0.10914	0.11067
NM_001034163	Blmh	0.12536	0.14595	0.07976	-0.3168	-0.0308	0.13452	0.09712	-0.06	0.07227	0.11986	0.3503	0.38771	0.31222	0.23726

NM_001025767	Blnk	0.54504	0.2519	0.24061	0.09205	1.4333	1.6594	0.40862	1.3348	0.56093	0.22084	0.34209	0.63381	0.16239	0.35262
NM_001105941	Bloc1s1_predicted	-0.2008	-0.2344	-0.1946	-0.4724	-0.6782	-0.2735	-0.3751	-0.9694	0.1221	-0.0818	0.09859	0.44205	-0.2174	-0.2527
NM_001037349	Bloc1s2	0.5865	0.06063	0.31181	0.53788	0.54051	0.57339	0.3768	0.37108	0.05297	0.05689	0.14982	0.06205	0.1162	0.13204
NM_053303	Blr1	0.03378	0.00836	-0.0941	-0.1449	-0.0523	-0.0489	0.00393	0.01655	-0.1164	0.0463	-0.0557	-0.0338	-0.1289	-0.0061
NM_053850	Blvra	0.39147	0.27373	0.18804	-0.0476	0.21919	0.51263	-0.1535	0.25072	0.58422	0.12631	0.28344	0.74199	0.10089	0.28666
NM_001106236	Blvrb_predicted	0.51561	-0.058	-0.1634	0.21697	0.69065	1.0022	-0.1473	0.52394	0.33047	0.38442	0.258	0.04418	0.17948	0.20053
NM_001017494	Blzf1	-0.1867	0.14271	-0.1833	0.23578	-0.4259	-0.4844	0.27056	-0.1586	0.12493	0.14699	0.11551	-0.2207	-0.0645	-0.2171
NM_139258	Bmf	0.2391	0.20959	-0.1682	0.09699	0.01621	0.30331	0.00998	-0.1224	0.15152	-0.0391	0.18971	-0.2415	-0.1023	-0.1089
NM_031323	Bmp1	-0.3875	0.44177	0.44917	0.4251	0.52977	0.49769	0.42764	0.61786	0.33086	0.34927	0.44516	0.28343	-0.3681	-0.2556
NM_021670	Bmp15	0.00286	0.0171	-0.0139	0.03262	0.17007	0.14623	0.04224	0.188	0.14575	-0.025	0.3024	0.18119	0.1009	0.09867
NM_017178	Bmp2	-1.2156	-1.6743	-1.2967	-1.5357	-0.7541	-1.056	-0.2028	-1.0649	-1.2148	-1.6456	-1.4518	-0.8826	-1.0026	-1.183
XM_573559.2	Bmp2k	-0.4663	-0.346	-0.1645	-0.3668	-0.0605	-0.3208	-0.5044	0.02001	-0.3127	-0.6082	-0.4258	-0.4396	-0.2563	-0.0681
XM_573559.2	Bmp2k	0.0052	-0.0162	-0.0496	0.05234	-0.0879	0.33756	-0.0032	0.09252	-0.1166	-0.0812	0.19762	-0.0066	0.09082	0.28222
NM_017105	Bmp3	-0.1052	-0.1133	-0.1963	0.12317	-0.1693	-0.0185	-0.2566	-0.2729	-0.049	-0.2157	0.00195	-0.1641	-0.2943	-0.2984
NM_012827	Bmp4	-0.0954	-0.0711	-0.2081	-0.0396	-0.1569	-0.2108	-0.3285	-0.2438	0.1533	-0.1334	-0.0432	-0.1096	-0.1773	-0.2665
NM_001108168	Bmp5_predicted	0.16198	0.34386	0.10354	0.14097	0.0264	-0.0312	0.22241	-0.1919	0.45915	0.60908	0.37174	0.0442	0.03358	-0.0467
NM_013107	Bmp6	-0.1867	0.04573	-0.2268	-0.2879	-0.2797	0.02427	-0.1179	-0.2294	-0.1521	-0.3347	-0.2651	-0.2543	-0.1852	-0.1644
XM_001053727.1	Bmp7	0.15686	0.01394	-0.0003	-0.0715	0.91139	0.74407	-0.0944	0.84942	0.09642	-0.0355	0.06414	-0.067	-0.1079	-0.0543
NM_001135799	Bmper_predicted	-2.211	-1.5931	-2.7708	-2.1385	-1.7638	-2.2557	-1.7214	-1.6082	-1.8279	-1.8002	-1.9962	-2.7932	-1.7044	-1.6989
NM_030849	Bmpr1a	-0.225	-0.0456	-0.4274	-0.8642	-0.5964	-0.4461	-0.2427	-0.7492	-0.0644	-0.157	-0.1702	0.13023	-0.1599	-0.0458
NM_001024259	Bmpr1b	0.11527	0.10305	0.18514	0.1819	-0.0228	-0.1315	-0.0358	0.16744	0.22831	0.37704	0.29649	0.22566	0.11871	0.05373
NM_080407	Bmpr2	-0.6578	-0.4497	-0.3142	-0.1839	-0.4741	-0.5019	-0.4731	-0.134	-0.3033	-0.434	-0.5894	0.08148	-0.1111	0.06817
NM_001100751	Bms1l	-0.1324	-0.4016	-0.0377	-0.0065	-0.7312	-0.3546	-0.3646	-0.8257	-0.3505	-0.5568	-0.4905	0.04332	-0.0726	-0.3063
NM_001013163	Bmyc	-0.0539	0.04413	-0.04	-0.0158	-0.0778	-0.0958	-0.0656	0.06056	-0.1	-0.0364	-0.0532	-0.0695	-0.036	0.01424
NM_001108916	Bnc1_predicted	0.31206	0.15665	0.04042	0.2422	0.05214	0.11985	0.11294	0.21651	0.08635	0.15607	0.16447	0.08266	0.17122	0.15138
NM_080897	Bnip1	0.56588	0.16725	0.22136	0.34728	0.49412	1.0071	0.11226	0.58986	0.39077	0.20206	0.35606	0.61238	0.06057	0.15006
NM_001106835	Bnip2_predicted	-0.1807	0.09023	0.34119	0.18894	-0.1384	-0.3456	0.55979	-0.2432	-0.1865	-0.1295	0.21424	-0.2736	-0.0654	-0.0251
NM_053420	Bnip3	1.5837	1.0934	0.3615	0.35403	0.88988	0.97562	0.02206	1.0357	0.94546	0.96045	1.162	-0.4852	0.43408	0.28315
NM_080888	Bnip3l	0.16553	0.5707	-0.1248	-0.4722	-0.0218	-0.1131	0.06538	-0.0127	0.56973	0.47592	0.57042	0.05767	-0.3179	-0.1667
NM_001128187	Bnipl_predicted	0.00178	-0.13	-0.1224	-0.0654	-0.1439	-0.0531	-0.0891	-0.1411	0.06306	0.00585	0.0161	0.01798	-0.0715	0.10325
NM_001108317	Boc_predicted	1.2911	2.7458	2.0297	1.1579	-0.278	-0.1836	2.8156	-0.1356	2.2988	2.7613	2.515	0.6381	-0.1042	0.0771
NM_017312	Bok	1.0376	0.9492	1.0473	0.13465	0.70852	0.54324	0.80285	0.56413	0.83067	0.87464	1.0214	0.68695	0.91567	1.0395
NM_001071776	Bola1_predicted	0.10033	0.01644	0.12318	-0.0107	0.03682	0.11813	0.06484	0.15436	0.38282	0.24203	0.08867	0.25803	0.07045	0.33138
NM_001106974	Boll_predicted	0.20756	0.81778	0.48488	0.24267	0.33173	0.60235	0.52469	0.45285	0.48699	1.1684	1.7784	0.02599	0.0656	0.11725
NM_001024250	Bop1	-0.1878	-0.4994	0.8676	0.49869	0.15891	0.01251	-0.066	0.17835	-0.319	-0.2679	-0.12	0.13625	0.3338	0.14275
NM_199382	Bpgm	0.05487	-0.1626	-0.7365	0.25153	0.98948	1.006	1.666	0.88718	-0.3926	-0.4591	-0.3765	-0.8549	-0.9038	-0.9699
NM_001037206	Bphl	-0.3728	-0.2603	-1.3616	-1.6559	-0.9182	-1.0617	-0.7041	-0.9713	-0.0798	-0.1533	-0.3498	-0.2018	-0.996	-1.0393
NM_001004079	Bpi	-0.0581	0.01673	0.0303	-0.042	0.14173	0.18382	-0.1174	-0.0284	-0.1517	-0.0371	0.00838	-0.0373	-0.0421	-0.1277
NM_001106531	Bpil1_predicted	-0.0144	-0.0928	-0.0038	-0.002	-0.0802	-0.1225	0.02634	0.00217	0.01577	0.17425	0.10612	-0.0053	0.17392	-0.0708
NM_001107791	Bpil3_predicted	0.25107	-0.0033	0.08854	0.33579	-0.0113	0.08813	-0.0126	0.10532	0.00778	0.04262	0.01297	-0.0058	0.07597	0.0128
NM_171990	Bpnt1	0.46192	0.07512	0.0765	0.22298	-0.0548	0.09839	-0.2011	-0.0037	-0.0473	0.17574	0.0772	0.40291	0.12194	0.29481
NM_001106070	Bpy2ip1_predicted	-0.0147	0.25699	0.27685	-0.5509	0.14047	-0.3845	0.09849	0.01033	0.07021	-0.0703	0.2131	-0.5212	0.22762	0.05078
XM_001070228.1	Braf	-0.1233	-0.7567	-0.3905	-0.7652	-0.5106	-0.912	-0.4836	-0.7337	-0.6812	-0.5921	-0.5768	-0.5334	-0.684	-0.7506
NM_001108103	Brd1_predicted	-0.5816	0.01538	0.01134	0.06899	-0.4085	-0.3806	0.06477	-0.303	0.05648	0.12433	0.23919	0.27383	-0.1352	-0.2862
NM_212495	Brd2	-0.6582	-0.3988	0.07892	-0.1958	-0.9637	-0.5802	-0.5578	-1.0383	-0.4707	-0.8069	-0.4776	-0.4587	-0.5297	-0.4061
NM_001108575	Brd3_predicted	0.13963	0.07156	0.18804	0.20018	0.02343	0.23041	-0.0284	0.02554	0.1628	0.07445	0.02198	0.10411	0.10054	0.08142
NM_001100903	Brd4	-0.0813	0.08307	0.15998	0.58246	0.21701	-0.0539	0.61297	0.15025	-0.0974	-0.1578	0.30934	0.36322	0.48	0.38945
NM_001108440	Brd7_predicted	-0.4263	-0.1399	0.48943	0.18913	-0.3563	-0.1819	0.10622	-0.2161	-0.0125	-0.0973	-0.0663	0.19005	0.00714	0.07976
NM_001008509	Brd8	-0.6425	-0.6916	0.06278	-0.5163	-1.1022	-1.0601	-0.1376	-1.0893	-0.2443	-0.5935	-0.4809	-0.2129	-0.3521	-0.306

NM_001107453	Brd9_predicted	-0.6416	-0.0863	0.37291	0.1747	-0.7826	-0.8548	0.08172	-0.8684	-0.2441	-0.0656	-0.1462	0.09535	-0.244	-0.2115
NM_001012031	Brdt	-0.0758	-0.1174	-0.1436	-0.0641	0.03287	0.14894	-0.0947	-0.0488	0.0251	0.08772	0.18926	0.05473	0.00709	-0.1268
NM_199270	Bre	0.60727	0.78861	-0.0297	-0.2562	0.5696	0.56094	0.34133	0.54052	0.48626	0.63761	0.60183	0.30623	0.28395	0.35136
NM_001106761	Brf1_predicted	-0.3883	-0.2644	0.59895	-0.0835	-0.197	0.08959	0.45902	-0.185	-0.3686	0.00015	-0.2739	0.18323	-0.1109	0.00833
NM_001106761	Brf1_predicted	-0.1497	-0.2355	0.07539	-0.2691	-0.2017	-0.3603	0.03869	-0.249	0.04038	-0.1343	-0.1521	-0.4074	-0.1413	-0.1938
NM_001024773	Brf2	-0.0655	0.03721	0.3553	0.39703	-0.062	-0.0032	0.48774	0.08809	-0.0195	0.27619	0.27019	-0.0505	-0.0326	0.07764
NM_001009604	Bri3	-0.1323	0.04192	-0.3136	-0.4805	-0.0999	-0.3972	0.28638	-0.4383	0.09792	0.0408	-0.0705	0.23666	-0.0066	0.02962
NM_001017487	Bri3bp	0.05192	-0.5261	-0.5385	-0.2681	-0.0546	0.17377	-0.5049	-0.114	-0.1743	-0.3069	-0.3	-0.2132	-0.2156	-0.2373
NM_173115	Brinp2	-0.0154	-0.1837	-0.0765	-0.0591	-0.1582	-0.0065	-0.1174	-0.1042	0.01129	-0.0779	-0.219	0.0658	-0.1612	-0.0411
NM_173121	Brinp3	0.37155	1.15	0.99254	0.07801	0.15416	0.13905	1.2159	0.03158	0.99157	1.1357	1.4322	0.30337	0.5199	0.36617
XM_340869.3	Brip1_predicted	-0.4688	-0.4909	-0.0442	-0.0657	-0.5364	-0.2356	-0.4678	-0.5231	-0.3591	-0.4325	-0.3051	0.20171	0.20894	0.08424
NM_001009605	Brms1	0.23397	0.27982	-0.1437	0.26198	0.49659	0.61166	0.11064	0.26498	0.05205	0.01197	0.15789	-0.0221	0.2098	0.50126
NM_001106731	Brms1l_predicted	0.25958	0.18932	-0.2316	-0.1931	-0.1203	-0.7249	-0.2539	-0.2814	-0.0337	-0.0547	0.01673	-0.4137	0.19544	0.05853
NM_001007707	Brp16	-0.6089	-0.6974	0.26246	-0.3624	-0.5981	-0.5265	-0.3361	-0.7236	-0.3083	-0.3705	-0.0737	-0.0806	0.3842	0.2404
NM_133561	Brp44l	0.39644	-0.2059	0.11595	0.03479	0.17088	0.38465	0.0452	0.11875	0.01854	0.06866	-0.0019	0.17679	0.08527	0.25053
NM_001107615	Brpf3_predicted	-0.0154	0.0648	0.02944	0.00903	0.00308	0.03448	0.04708	0.05568	0.03574	-0.0767	-0.0138	0.01133	-0.0797	0.05364
NM_152845	Brs3	-0.0756	-0.1064	-0.1292	-0.0404	-0.0401	0.55037	0.02796	-0.1246	0.09345	-0.0079	-0.1413	-0.1771	-0.2031	-0.0338
XM_219498.4	Brsk2	0.06672	0.11698	0.01875	9E-05	-0.0537	0.12061	0.23548	-0.0542	0.05567	-0.0159	-0.0796	0.17294	-0.0048	0.01914
NM_001107400	Brunol4_predicted	-0.0677	0.40824	0.01442	-0.0638	-0.0147	-0.0427	0.50864	0.00361	0.30937	0.53256	0.30585	0.11124	-0.0424	-0.0751
NM_001106827	Brunol6_predicted	-0.2187	-0.2363	-0.2738	-0.1195	-0.0948	-0.0915	0.05058	0.07	-0.0644	-0.1324	-0.0229	-0.1608	-0.0178	-0.0088
NM_001107106	Brwd1_predicted	-0.2511	-0.369	-0.1214	0.33022	-0.3646	-0.09	0.06153	-0.2518	-0.0132	-0.3122	-0.1435	-0.1625	-0.5378	-0.5667
XM_001054667.1	Brwd3_predicted	-0.1702	-0.1497	-0.1222	-0.1247	-0.1234	0.05359	-0.0136	-0.145	-0.1942	-0.1225	0.06828	-0.01	-0.1992	-0.1546
NM_012783	Bsg	0.51687	1.0684	-0.1827	0.58857	0.28526	0.51821	0.17238	0.35265	0.79937	0.78602	1.005	0.27516	-0.0726	-0.0294
NM_138979	Bsnd	-0.0464	0.09608	-0.0116	-0.0186	-0.043	-0.0053	-0.0382	-0.1191	0.0742	-0.0581	0.01313	0.03725	-0.0096	0.07381
NM_022261	Bspry	0.1017	0.14755	0.03971	-0.0782	-0.0209	-0.0492	0.06585	0.09691	-0.1158	-0.0911	-0.0863	-0.0353	0.01055	0.09452
NM_030848	Bst1	0.76927	0.75869	1.6288	1.8407	1.6132	1.4256	2.1855	1.615	0.85351	0.89673	1.0833	0.60225	1.4511	1.3718
NM_198134	Bst2	0.0376	-0.2382	-0.0446	0.01379	0.01901	0.24373	-0.0303	-0.0295	-0.0932	-0.0943	-0.0512	0.11201	-0.2343	-0.0393
NM_001011932	Btbd1	0.74692	0.56733	-0.2791	0.29761	0.98039	1.0432	0.12608	1.0103	0.74637	0.58127	0.71682	0.17831	-0.0535	0.02493
NM_001014022	Btbd10	0.11819	-0.0339	0.38684	0.57276	0.35612	0.17134	0.44408	0.36323	-0.2379	0.14619	0.07059	-0.0593	0.2021	0.16537
NM_001108078	Btbd11_predicted	0.04058	-0.0108	0.10846	-0.0436	0.10088	-0.0154	-0.0462	0.01148	-0.0411	-0.1217	-0.1445	0.07011	-0.0105	-0.2064
NM_001100533	Btbd14a	-0.4693	-0.0994	-0.158	-0.1032	-0.5354	-0.3712	-0.4584	-0.6741	0.0195	-0.1378	-0.2287	0.00417	-0.2503	-0.2914
NM_134413	Btbd14b	0.04323	-0.1767	0.09188	-0.1696	-0.1861	0.06603	-0.0853	-0.0931	-0.0867	0.13697	0.09368	0.16286	0.16055	0.32219
NM_001017464	Btbd16	0.15763	0.15799	-0.021	0.27784	0.19127	0.12644	0.24754	0.15737	0.04563	0.31486	0.21393	-0.0608	-0.0678	0.09704
NM_001107782	Btbd3_predicted	-0.3118	-0.4254	-0.5286	-0.4917	-0.3628	-0.4409	-0.3137	-0.5354	-0.4062	-0.4289	-0.4901	-0.1869	-0.1015	-0.228
NM_001107808	Btbd4_predicted	-0.3241	-0.1904	-0.0511	-0.2568	-0.1673	-0.0034	-0.3848	-0.2478	-0.2892	-0.1502	-0.1823	-0.1999	-0.1529	-0.1545
NM_001107808	Btbd4_predicted	0.14649	-0.0624	0.18692	0.01386	0.15649	0.07558	-0.0835	0.05493	-0.0448	0.13811	-0.0161	-0.0167	0.00169	0.05121
NM_001106735	Btbd5_predicted	-0.158	-0.1586	-0.3069	-0.2766	-0.1936	-0.1707	-0.0502	-0.2388	-0.0614	0.01446	-0.2387	0.10077	-0.0672	-0.1963
NM_001108720	Btbd7_predicted	0.15464	0.02735	0.25759	-0.0717	0.04744	-0.0287	0.04375	-0.1535	-0.0294	0.14258	-0.0819	0.13202	0.33872	0.11407
NM_001013073	Btbd9	0.08913	0.10165	0.23988	-0.1111	0.23801	0.13499	0.24816	0.11088	0.22452	0.26106	0.07201	0.47787	0.22104	0.24855
NM_022256	Btc	-0.1257	-0.0016	-0.1795	0.30387	0.13458	-0.1126	-0.0636	-0.0115	-0.0259	-0.2786	-0.0595	-0.0016	0.23433	0.02884
NM_001012047	Btd	-0.6164	-0.1065	-0.0793	-0.6495	-0.8144	-0.6042	-0.4832	-0.8934	-0.3812	-0.279	-0.1712	0.08518	-0.7242	-0.3186
NM_001012047	Btd_predicted	-0.0657	-0.0869	0.1042	0.03446	-0.0087	-0.0407	-0.0926	0.00885	-0.1143	-0.0496	-0.1447	0.07836	0.06689	0.02404
NM_001008309	Btf3	-0.2872	-0.3084	-0.5049	-0.3429	-0.1435	-0.3171	-0.1061	-0.1778	-0.1856	-0.2617	-0.3162	-0.1099	-0.1661	-0.095
NM_017258	Btg1	0.09045	-0.1355	-0.3369	0.02892	-0.5941	-0.7169	0.31823	-0.5721	-0.0721	-0.0947	-0.2001	-0.1413	-0.753	-0.7775
NM_017259	Btg2	-0.0231	-0.1938	-0.3109	0.3001	-0.06	0.62162	-0.1978	0.20347	-0.4064	-0.1754	-0.3096	-0.5389	-0.3379	-0.5273
XM_346415.2	Btg3_predicted	0.2541	0.0899	-0.0232	1.1049	0.69759	0.36601	0.51698	0.77985	0.12513	-0.2127	0.21544	-0.0649	-0.096	0.00999
NM_001013176	Btg4	0.06237	-0.2288	-0.0502	0.02117	-0.0603	-0.0682	-0.1623	0.03961	0.05123	0.33891	-0.0895	0.14779	0.00829	-0.0591
NM_001007798	Btk	0.23267	-0.0741	-0.144	-0.0843	0.07858	0.12195	-0.0024	-0.171	0.15324	0.35697	0.01996	-0.2301	-0.0508	0.19799
NM_213630	Btla	0.05302	0.07868	-0.0364	0.15095	-0.0586	0.0749	0.03217	-0.0916	0.03878	-0.0533	-0.0124	-0.1401	0.26793	0.08752

NM_001170337	Btn1a1	-0.1158	-0.069	0.03115	-0.0213	-0.0567	0.00307	-0.0085	-0.223	-0.0661	-0.1643	-0.0241	-0.0777	0.02825	-0.2662
XM_001072372.1	Btn2a2_predicted	0.23072	0.34903	0.48494	0.77908	0.56537	0.3311	0.29966	0.39858	-0.0006	0.18924	0.09654	-0.0722	0.39648	0.48349
NM_053815	Btnl2	-0.0425	-0.1316	0.06269	-0.035	-0.0695	0.18264	0.10006	0.11529	0.00739	0.04846	-0.0219	0.0071	0.08048	-0.1187
NM_001002803	Btnl3	0.11918	0.15274	0.03492	0.02033	0.078	-0.067	-0.0198	0.14612	0.0986	-0.0628	0.05316	-0.1051	0.00043	-0.0949
NM_212488	Btnl7	0.03109	0.2917	0.58225	1.1888	0.16682	0.25379	0.6913	0.17855	0.40271	0.00252	-0.1143	0.16858	0.07639	0.17615
NM_001166344	Btnl8	-0.2932	-0.3115	0.15176	-0.1707	-0.3483	0.03878	-0.1907	-0.2091	-0.2649	-0.2718	-0.0351	-0.0816	-0.3412	-0.3701
NM_001007148	Btrc	0.32196	0.11713	-0.0028	0.4412	0.76834	0.52524	0.03286	0.60943	0.08046	0.0188	0.0043	0.11291	-0.23	-0.031
NM_001106507	Bub1_predicted	-0.576	-0.8296	0.88598	-0.0279	-0.7352	-0.933	-0.3633	-0.8619	-0.2299	-0.6364	-0.7232	0.20179	0.54103	0.53694
XM_342494.3	Bub1b	-0.7	-0.9828	0.55807	0.03579	-0.822	-1.1929	-0.9148	-0.8439	-0.2932	-0.6892	-0.6177	-0.0136	0.23091	0.22721
NM_001047906	Bub3_predicted	-0.0294	-0.0101	0.03735	0.06956	-0.0253	0.10747	0.03754	0.0501	0.0309	-0.0465	0.02441	-0.0325	0.14513	-0.0128
NM_001108502	Bucs1_predicted	0.54643	0.50833	0.49859	0.1689	-0.1065	-0.1456	0.25084	-0.2235	0.4191	0.07527	0.19266	0.50782	-0.0315	0.07583
NM_001025277	Bud13	0.23274	0.04873	0.25141	0.28686	0.32906	0.30534	0.22425	0.37513	0.10666	0.27639	0.07885	0.22888	0.50714	0.27281
NM_001077590	Bves	-0.222	-0.1782	-0.0639	0.05218	-0.1057	-0.0305	-0.028	-0.0166	-0.249	-0.1088	0.15703	-0.226	-0.022	-0.1393
NM_198743	Bwk1	0.06291	0.34038	0.31287	-0.0954	0.10883	0.29761	0.22389	0.1418	0.15206	0.10764	0.15351	0.09123	0.31968	0.4568
NM_001106391	Bxdc1_predicted	-0.3144	-0.5727	-0.0396	-0.0634	-0.3509	-0.1153	-0.183	-0.5043	-0.4258	-0.054	-0.2871	-0.19	0.09469	-0.037
NM_001029915	Bxdc2	0.01549	0.11257	-0.2462	0.13521	0.16185	-0.1723	0.36538	0.14026	-0.0753	0.25543	0.18385	-0.1331	0.38929	0.16653
NM_001100794	Bxdc5	0.17616	-0.0827	0.06916	-0.0868	-0.2218	-0.1431	-0.0919	-0.1716	0.09617	0.23695	0.04533	-0.1834	0.10125	0.18568
NM_001100794	Bxdc5	0.43052	0.05551	0.09664	1.229	0.42655	0.1438	0.43727	0.40085	0.08082	-0.0518	-0.014	-0.2254	-0.0052	0.29439
XM_001081125.1	Bzrap1	-0.1663	-0.1183	-0.1941	-0.0825	0.01154	0.13427	-0.029	-0.125	-0.1241	0.00354	-0.1379	-0.0316	-0.0996	0.16998
NM_198789	Bzw1	-0.171	-0.1997	-0.2974	-0.5033	0.13829	-0.0383	-0.2967	0.04516	-0.1212	-0.0144	-0.4374	-0.2993	-0.0558	0.13901
NM_134402	Bzw2	-0.7201	-0.4276	-0.8348	-0.8565	-0.905	-0.8039	-0.6274	-0.9464	-0.53	-0.5935	-0.7528	-0.5832	-0.9765	-0.7599
NM_198778	C11orf8h	-0.0227	-0.0587	-0.0567	0.07682	0.01673	-0.0389	0.04813	-0.0527	0.00943	-0.098	0.17224	0.06515	-0.0315	-0.0947
NM_022950	C1galt1	-0.1563	-0.2785	-0.3394	0.1644	-0.0614	-0.1832	-0.4946	0.06284	-0.2766	-0.4432	-0.5298	-0.3339	-0.1599	0.04208
NM_001030033	C1galt1c1	0.47136	0.34547	0.17753	0.74309	0.33726	0.41196	0.75229	0.48553	0.68556	0.47904	0.42176	0.36076	0.52114	0.54449
NM_001008515	C1qa	0.2638	0.21442	0.16911	0.10905	0.12959	-0.0167	0.10791	0.05265	0.12998	0.13684	0.12036	0.06698	0.0626	0.11627
NM_019262	C1qb	0.03505	0.03038	0.06416	0.25394	0.07021	0.06024	0.11664	0.04209	0.08857	0.34901	0.11754	0.18086	0.25905	0.28766
NM_019259	C1qbp	0.04299	-0.3356	-0.2157	-0.6637	-0.0904	-0.0431	-0.5924	-0.1091	-0.4009	-0.4091	-0.365	-0.1215	0.06074	0.31578
NM_001008524	C1qc	-0.0103	-0.1093	-0.0748	-0.0238	-0.0273	0.06462	0.20822	-0.0355	-0.1186	-0.1194	0.06134	0.11836	0.057	-0.0401
NM_001108838	C1ql1_predicted	-0.1422	0.10062	0.03699	-0.1933	-0.1105	-0.0327	-0.157	-0.2191	-0.0574	0.1344	-0.1814	-0.0885	-0.1793	-0.0781
XM_235645.4	C1ql4_predicted	0.04875	-0.0374	0.09292	-0.0269	-0.0721	0.1989	-0.1183	-0.0385	0.1635	0.0349	-0.0964	-0.0214	0.01815	-0.1016
NM_053383	C1qr1	-0.0247	0.0563	0.03212	-0.0134	-0.0465	0.03502	0.16387	0.22271	0.09172	-0.0486	0.08694	-0.0271	-0.0102	0.01354
NM_001007675	C1qtnf1	-0.1491	-0.0732	0.18016	0.00498	-0.0294	0.04855	0.21621	-0.0312	-0.1676	-0.1588	0.01374	-0.0413	-0.0787	-0.0304
NM_001107745	C1qtnf4_predicted	-0.3337	-0.1778	-0.2297	-0.2487	-0.2646	-0.2141	-0.2972	-0.1261	-0.2099	-0.3131	-0.2075	-0.0148	-0.4198	-0.1325
NM_001012123	C1qtnf5	0.37877	0.74142	-0.5717	-0.2824	-0.7279	-0.5352	0.03876	-0.7729	0.70177	0.58563	0.55269	0.44218	0.17393	0.07154
NM_001034932	C1qtnf6	0.04222	0.37029	-0.373	-0.2838	0.28659	-0.2618	0.16493	0.29733	0.90229	0.70525	0.42003	0.00608	0.46545	0.46934
NM_001107221	C1qtnf7_predicted	-0.1111	-0.0808	-0.041	-0.0378	0.0726	-0.0748	0.01656	-0.0071	0.04858	-0.1649	0.00632	0.31362	-0.1202	0.23735
NM_001134555	C1r	0.75211	1.0508	-0.4988	1.12	0.37833	0.22403	0.61746	0.55137	0.65503	0.87331	0.72746	-0.5834	-0.8204	-0.8549
NM_001002804	C1rl	0.85745	0.30467	-0.0068	0.23226	0.43843	0.27946	0.4202	0.58863	0.20462	0.0714	0.32049	-0.2494	-0.1722	-0.1493
NM_138900	C1s	1.2359	2.147	1.2434	1.9857	0.88032	0.74416	2.5257	1.1668	1.721	2.1232	2.1072	0.05687	0.20878	0.17054
NM_172222	C2	-0.0554	-0.2253	-0.1916	-0.1497	-0.0443	-0.2786	-0.1536	-0.1985	-0.2846	-0.0839	-0.019	-0.3612	-0.101	-0.1819
NM_001025769	C20orf165	-0.1161	0.10211	-0.169	0.09182	0.18478	0.08365	-0.0868	0.22868	0.17034	0.03655	0.03954	-0.1192	-0.0686	0.05547
NM_001025769	C20orf165	0.16091	0.12275	0.15869	0.01748	0.11112	0.00334	0.12023	0.13779	0.13583	0.06738	0.05872	0.07212	0.21468	0.25232
NM_032060	C3ar1	0.11078	-0.1644	-0.0528	-0.0487	-0.0725	0.08177	-0.0512	0.13163	-0.072	-0.2558	-0.006	-0.0611	-0.298	-0.152
NM_001002805	C4-2	0.06395	0.52415	1.2611	0.83591	0.21659	-0.0978	1.9426	0.3366	0.35543	0.75546	0.69313	0.24035	-0.0713	0.01684
NM_012516	C4bpa	0.06882	0.05344	0.44444	0.12768	0.05854	0.38456	0.19071	0.13971	0.0753	0.09412	0.16094	0.02588	0.07315	-0.0067
NM_016995	C4bpb	-0.0968	-0.0597	0.01269	0.03936	-0.1817	-0.0032	-0.2392	-0.2697	-0.0078	-0.1291	0.01891	-0.1358	-0.0692	-0.2579
XM_345342.3	C5	-0.0284	0.04938	0.26487	-0.0209	0.13359	0.19481	-0.0276	0.07045	0.12426	0.03896	0.07151	-0.0056	0.05197	0.11062
XM_345342.3	C5	0.29219	-0.0702	0.08989	0.02815	0.2179	0.18696	0.035	0.51097	0.23531	0.04098	0.05264	0.05864	0.07241	-0.1653
NM_176074	C6	-0.0163	0.09687	0.3027	-0.0559	-0.0729	-0.0308	-0.0191	0.00635	0.0617	0.17741	-0.273	-0.1544	0.11328	-0.1046

XM_226803.4	C7	-0.0767	0.0091	0.07355	0.05085	0.08448	0.1335	0.08276	0.01534	-0.044	0.39158	0.18859	0.19775	0.15211	-0.1174
NM_001106670	C8a_predicted	-0.0055	-0.1102	0.02296	0.00052	0.09932	-0.0936	-0.049	0.04423	-0.1132	0.00112	0.0012	-0.1287	-0.0112	-0.0903
XM_001058539.1	C8b_mapped	0.02838	0.09522	-0.1431	0.06529	-0.0691	0.00037	0.00867	0.14043	0.01545	0.17361	0.06183	-0.0695	0.14101	0.05217
NM_001106555	C8g_predicted	0.29185	-0.0014	-0.3128	-0.2518	-0.0114	0.37287	-0.4344	-0.0497	-0.1765	0.13489	-0.2901	-0.1201	-0.3791	-0.1791
NM_057146	C9	-0.0308	0.00912	0.01295	0.05349	0.01544	0.18614	0.23428	0.09704	0.14264	0.06307	0.09411	-0.0555	0.32415	0.08773
NM_019291	Ca2	-0.7151	-0.7666	-0.1233	-0.3295	-0.7623	-0.818	-0.6987	-0.632	-0.6041	-0.8884	-0.7063	-0.5591	-0.6787	-0.8595
NM_019292	Ca3	-3.655	-3.6663	-2.4951	-4.6528	-4.7746	-4.6174	-4.708	-4.8795	0.21355	-2.6817	1.1119	1.473	-2.1235	-1.9074
NM_019174	Ca4	3.1163	0.41904	0.26522	4.2089	3.0575	3.3604	0.44138	3.1898	0.41869	0.63903	0.76874	0.11007	0.17299	0.02512
NM_019293	Ca5a	0.21646	0.38175	0.19148	0.14309	0.12352	0.07578	0.24306	0.18196	0.19592	0.19865	0.29761	0.39531	0.19201	0.36829
NM_001005551	Ca5b	-0.3077	0.11185	-0.995	-0.9272	-0.3605	-0.3644	-0.3152	-0.3514	-0.4353	-0.245	-0.2289	-0.8253	-1.2814	-1.4014
NM_001106924	Cab39_predicted	-0.371	-0.0858	-0.1084	-0.1294	-0.3025	-0.3567	0.18801	-0.3393	0.10635	-0.2093	-0.1218	-0.0044	-0.1171	-0.134
NM_001011917	Cab39l	0.36298	-0.0503	-0.2569	0.3448	0.3564	0.48165	-0.4442	0.39177	0.0898	0.01058	-0.0699	0.29043	-0.0964	0.00478
NM_001011917	Cab39l_predicted	-0.196	-0.2087	-0.0448	0.17639	-0.3362	0.04087	0.10011	0.00013	-0.0708	0.02251	-0.1437	-0.0843	-0.2038	-0.1479
NM_001013185	Cabc1	-0.371	-0.6069	-1.0746	-0.4328	-0.5641	-0.412	-0.9946	-0.4083	-0.8737	-0.5311	-0.6837	-0.6422	-1.0349	-0.9262
NM_053575	Cabin1	-0.7374	0.10007	-0.1602	-0.4754	-0.9793	-0.7559	-0.2648	-1.1553	-0.1505	-0.1998	-0.0445	0.03191	-0.5332	-0.4302
NM_001107404	Cables1_predicted	0.01924	-0.0435	-0.0468	0.02617	-0.0228	-0.0882	-0.0809	-0.0315	-0.185	-0.1043	0.05242	-0.085	-0.1551	-0.1262
XM_001059501.1	Cables2_predicted	0.2503	0.27006	0.1581	0.09115	0.32245	0.35699	0.15422	0.16671	0.06213	0.27655	0.04406	-0.079	0.12291	0.18715
NM_133529	Cabp1	0.18786	-0.0043	0.04637	0.28386	0.15791	0.16049	0.05461	0.27999	0.10572	0.20037	0.25125	0.10469	0.11884	0.10158
NM_001108926	Cabp4_predicted	-0.0914	-0.02	-0.1676	0.25432	-0.0532	0.04472	-0.1329	-0.1384	-0.0796	-0.1015	0.02004	-0.1794	-0.1663	-0.054
NM_001108907	Cabp5_predicted	0.11142	-0.1042	0.07497	0.04361	-0.0134	0.01628	0.16827	-0.0509	-0.1564	0.24743	0.06808	0.03546	0.02271	0.21188
NM_001007730	Cabp7	0.17664	0.25277	0.20064	0.35847	0.20295	0.70401	-0.0748	0.23563	0.22386	0.27177	0.04225	0.47594	0.31764	0.22898
XM_001056642.1	Cachd1_predicted	-0.0131	-0.111	0.22182	0.02739	-0.1863	0.07448	-0.0986	-0.0586	-0.2419	-0.0225	-0.0323	-0.1839	-0.1501	0.00631
NM_012918	Cacna1a	-0.1497	0.06619	-0.0598	-0.1868	-0.1451	-0.0338	0.01308	-0.0703	0.04422	0.07674	-0.0156	-0.1111	-0.0265	0.00947
NM_147141	Cacna1b	-0.0943	-0.0041	0.06679	-0.0832	-0.0722	0.08017	-0.1397	-0.0577	0.2476	-0.0718	-0.1123	-0.2046	0.01142	-0.0309
NM_012517	Cacna1c	-0.6536	-0.4965	0.0317	-0.0079	-0.2697	-0.4103	-0.1755	-0.3866	-0.7523	-0.336	-0.3617	-0.3731	-0.4232	-0.4627
NM_017298	Cacna1d	-0.1626	-0.1556	-0.0539	-0.1146	-0.1038	-0.0782	-0.0753	-0.1766	-0.023	-0.0395	-0.0877	-0.2084	-0.0787	-0.0998
NM_019294	Cacna1e	0.14711	0.2493	0.07046	0.0545	-0.0185	0.00426	-0.012	0.12003	0.17786	0.17735	0.32291	0.02494	0.43523	0.15218
NM_031601	Cacna1g	-0.283	0.04453	0.11283	-0.149	-0.205	-0.1054	0.46637	-0.1986	-0.0379	-0.0687	-0.1074	0.3674	-0.1991	-0.0399
NM_153814	Cacna1h	-0.2199	-0.1014	-0.0542	-0.1651	-0.0593	0.32058	-0.1029	-0.0318	-0.3005	-0.0795	-0.0566	-0.3298	-0.1857	-0.0401
NM_012919	Cacna2d1	-0.8817	0.05724	-0.354	0.5314	-1.0319	-1.3138	-0.1587	-1.1088	-0.2401	-0.0223	-0.0555	-1.0791	-0.5678	-0.5288
NM_175592	Cacna2d2	-0.2288	-0.0941	-0.2909	-0.0445	0.268	-0.1718	-0.017	-0.1075	0.19456	-0.2963	-0.0641	-0.1237	-0.1846	0.05713
NM_175595	Cacna2d3	-0.2964	-0.0923	-0.0598	-0.0417	-0.1748	0.02361	-0.1555	-0.0622	-0.2176	0.01006	-0.0569	-0.3174	-0.1746	-0.3236
NM_017346	Cacnb1	-0.1314	-0.3145	-0.0452	-0.2553	-0.3577	-0.3338	-0.1625	-0.1615	-0.0919	-0.2323	0.08352	-0.3004	-0.1409	-0.2516
NM_053851	Cacnb2	-0.4488	-0.1345	-0.2314	-0.043	-0.5693	-0.4481	-0.209	-0.2365	-0.4871	-0.4452	-0.5074	-0.4469	-0.7448	-0.6347
NM_012828	Cacnb3	-0.2249	0.38267	0.63248	0.26772	-0.0714	-0.2274	-0.0035	-0.0551	0.12542	0.00891	0.21088	0.23157	-0.1278	-0.0258
NM_001105733	Cacnb4	-0.0122	0.01115	-0.1208	0.18508	0.10978	0.09845	-0.0967	-0.0374	0.0009	0.04973	-0.0591	0.02013	-0.0221	-0.0859
NM_019255	Cacng1	0.1317	0.00372	-0.0517	-0.0712	-0.0599	0.02572	-0.1125	-0.0307	-0.0728	-0.0791	0.28334	-0.1438	0.10821	0.01906
NM_053351	Cacng2	0.06315	0.36223	0.15683	0.17799	0.14581	0.08007	0.38344	0.1501	0.05898	0.03923	0.08683	0.36219	0.07285	0.03432
NM_080691	Cacng3	0.03511	-0.0153	0.045	-0.0355	0.12317	0.02072	0.00229	0.0653	0.11273	0.00676	0.11478	0.04626	0.09053	-0.0702
NM_080692	Cacng4	0.04016	0.05161	-0.0184	0.03428	0.11022	0.13363	0.02865	0.03523	0.10021	-0.0126	0.0565	0.12704	0.06047	0.07744
NM_080693	Cacng5	0.01216	-0.0673	-0.1095	-0.0815	-0.2241	-0.1435	-0.2104	-0.1197	-0.1268	-0.0033	-0.1401	-0.223	-0.2837	-0.1862
NM_080694	Cacng6	0.08929	0.35222	0.11022	0.06836	0.27532	-0.0291	0.23055	0.27263	0.22004	-0.0281	0.04947	0.17289	0.2213	0.12332
NM_080695	Cacng7	0.21256	0.02881	0.12416	0.08088	0.11522	0.1715	0.08843	0.10404	0.13603	0.29732	0.16934	0.21527	0.13652	0.00696
NM_080696	Cacng8	0.1369	0.07831	0.02443	-0.1	-0.0376	-0.1113	-0.0536	-0.0299	0.09063	-0.0199	-0.0091	0.01263	0.12868	-0.0842
NM_001004208	Cacybp	0.67498	0.19782	-0.104	0.62703	0.68834	0.42727	1.1136	0.74952	-0.0017	0.3143	0.14833	-0.4538	-0.1528	-0.102
NM_001105710	Cad_mapped	-0.8426	-1.1761	0.66761	-0.2352	-1.0849	-0.6278	-0.7736	-0.993	-0.6607	-0.9897	-0.8214	-0.2311	-0.266	-0.2241
NM_001012201	Cadm1	-0.0703	-0.0368	-0.0981	-0.0752	-0.0051	0.11035	0.03072	0.02623	-0.0122	0.03875	0.04797	0.01784	-0.0729	0.14766
NM_001012201	Cadm1	-0.526	-1.0664	-0.4633	-0.5261	-0.5108	-0.6389	-0.7754	-0.4948	-0.451	-0.7586	-1.0106	-0.4151	0.23344	0.18488
NM_013219	Cadps	-0.2605	-0.1251	-0.2733	-0.4187	-0.2975	-0.265	-0.1713	-0.1577	-0.1577	-0.1343	-0.1726	-0.2926	-0.3295	-0.207

NM_001012052	Cage1	0.20479	0.30189	0.2559	0.64208	0.3433	0.50415	0.29897	0.34513	0.02001	0.31312	0.39192	0.20066	0.00083	0.18068
NM_031984	Calb1	-0.1021	0.03924	-0.1255	-0.1616	0.09855	-0.1185	-0.0813	0.01409	-0.0391	-0.0971	-0.0679	-0.0797	-0.0731	-0.0944
NM_053988	Calb2	-0.3795	-0.2897	-0.1439	-0.1564	-0.3259	-0.2554	-0.0982	-0.1835	-0.1132	-0.2922	-0.3174	-0.0615	-0.2385	-0.2264
NM_017338	Calca	0.40865	0.07389	0.03722	-0.036	1.9881	2.2963	0.0898	1.6204	0.29234	0.12183	-0.0112	-0.1399	0.32198	-0.0264
NM_138513	Calcb	2.8163	-0.0859	-0.1143	-0.0421	3.0695	3.1533	0.0683	2.6347	-0.0531	-0.1281	-0.0089	-0.1114	-0.0455	-0.0346
NM_139190	Calcoco1	0.15464	0.49365	-0.1866	0.90097	-0.0722	0.09824	0.48688	-0.073	0.25837	0.40791	0.32191	-0.1277	-0.4584	-0.664
NM_053816	Calcr	0.09474	0.07153	0.04025	0.11998	0.1684	0.13907	-0.0367	0.13592	-0.075	0.06309	0.09461	0.20552	0.0785	-0.0123
NM_012717	Calcrl	0.0035	0.09212	0.06653	-0.0419	-0.1646	-0.1492	-0.0419	-0.0521	0.04554	0.15487	-0.0308	0.00045	0.10228	-0.0614
NM_013146	Cald1	0.78455	1.2644	-1.6043	-0.1335	0.32476	-0.5772	-0.8741	0.5389	0.47779	0.78672	0.1736	-0.1306	0.71714	0.7677
NM_031969	Calm1	-0.1759	0.30919	-0.243	-1.081	-0.3225	-0.6316	-0.0506	-0.4603	0.13206	0.18989	0.26555	-0.1556	-0.272	-0.2625
NM_031969	Calm2	0.0121	-0.1768	0.14639	-0.1425	0.10081	-0.2309	0.08355	0.21333	-0.1137	-0.0131	-0.1135	-0.1305	0.16749	0.18212
NM_031969	Calm3	-0.0911	0.46381	0.29023	-0.6337	-0.2517	-0.2899	-0.0334	-0.3524	0.2581	0.38241	0.32763	0.47825	0.38207	0.40752
XM_344627.2	Calm4_predicted	0.14031	0.21644	0.08097	0.08396	-0.035	0.0366	0.04511	-0.0124	0.23442	0.26976	0.20155	0.15497	-0.1059	0.0542
NM_001077201	Caln1_predicted	0.04615	-0.0507	-6E-05	-0.0018	-0.031	0.11506	0.00815	0.12239	0.02853	0.09262	0.01579	0.07821	0.01676	0.1506
NM_022399	Calr	-0.3461	-0.0757	-0.1617	-0.4231	0.2783	0.38204	-0.3399	0.411	-0.011	0.10483	-0.1442	0.23982	0.45471	0.27977
NM_001012212	Calr3	0.14073	0.16652	0.04405	0.06917	0.27406	0.29656	0.07326	0.15799	0.22067	0.48068	0.10991	0.49342	0.25338	0.36037
NM_001012212	Calr3_predicted	0.25913	0.14392	0.02504	0.30413	0.11545	0.0099	0.24868	0.24699	0.02554	-0.0336	0.21206	0.24259	0.45864	0.50673
NM_001033898	Calu	0.09411	-0.0168	-1.1786	-0.3237	-0.4688	-1.3436	-0.4889	-0.4896	0.08708	-0.0576	-0.1663	-0.9067	-0.2522	-0.0225
NM_134468	Camk1	1.213	0.84445	-0.4269	-0.3887	0.91581	0.69882	0.45114	0.71901	0.7887	0.81135	0.73025	-0.0741	0.0247	0.2416
NM_182842	Camk1g	0.09214	-0.0122	0.2187	-0.0156	0.03624	-0.0525	0.09455	0.25057	-0.0702	0.20852	0.09928	0.1608	-0.0501	0.04405
NM_012920	Camk2a	-0.1103	-0.101	-0.0467	-0.0184	-0.0944	-0.2513	-0.0147	0.02593	-0.0964	0.13455	0.08705	0.05114	0.03336	0.0194
NM_001042356	Camk2b	0.02811	0.41869	0.18515	0.23797	0.17396	0.10713	0.14038	0.18011	0.486	0.43971	0.36232	0.3192	-0.1535	0.17861
NM_012519	Camk2d	-0.0705	0.16526	-0.0389	-0.1526	-0.4834	-0.4289	-0.1228	-0.6988	0.10526	0.18032	0.24341	-0.0532	-0.2463	-0.1423
NM_133605	Camk2g	-0.4314	0.25137	0.27793	0.78147	-0.2885	-0.4247	0.49499	-0.2237	0.11703	0.20185	0.10143	-0.4914	-0.3738	-0.2467
NM_173337	Camk2n1	0.86554	1.5947	-0.0502	0.67254	1.645	1.3935	1.2287	1.5063	1.3286	1.5396	1.4783	0.70997	0.0355	0.09363
NM_021678	Camk2n2	-0.1267	-0.2026	-0.2154	-0.1431	-0.2575	-0.2243	-0.177	-0.364	-0.1915	-0.0833	-0.1104	-0.0408	-0.2043	-0.1131
NM_012727	Camk4	0.05282	-0.0159	0.20522	0.41587	0.08121	0.28426	-0.0323	0.01935	-0.1212	-0.2186	-0.1012	0.1851	-0.0649	0.04283
NM_031662	Camkk1	-0.4569	0.11556	-0.0733	0.15454	0.44878	0.32039	-0.0352	0.29264	0.13021	0.20709	0.08352	0.1971	-0.1736	0.04755
NM_031338	Camkk2	-0.3332	-0.2232	-0.0662	-0.1412	-0.3436	-0.0799	-0.1933	-0.1497	-0.0171	-0.3179	-0.2614	-0.1672	-0.018	0.06646
NM_024000	Camkv	-0.2558	-0.1462	-0.4373	-0.3618	-0.3817	0.01909	-0.2851	-0.3296	-0.2297	-0.0984	-0.3078	-0.2595	-0.2903	-0.1434
NM_053334	Camlg	-0.2237	-0.3422	0.16421	0.5768	0.31012	0.43994	-0.3267	0.30543	0.09918	-0.1332	-0.2068	0.24875	0.0928	0.30499
NM_001100724	Camp	0.23106	-0.0092	-0.1503	-0.0945	0.0661	-0.1299	-0.0282	0.02678	-0.0618	-0.2973	-0.0726	0.11696	-0.0157	-0.0504
NM_001105801	Camta2_predicted	-0.6174	-0.1888	0.06741	-0.3337	-0.6558	-0.7481	0.04055	-0.6147	-0.2582	-0.2645	-0.189	-0.2769	-0.7654	-0.6877
NM_054004	Cand1	-0.5621	-0.5156	0.23571	-0.3147	-0.4818	-0.6387	-0.4348	-0.3097	0.00835	-0.2263	-0.3006	-0.1037	0.13007	-0.0427
NM_181362	Cand2	0.01582	0.83101	0.72234	-0.0657	1.1351	1.0271	0.51482	0.71431	0.6818	0.70087	0.63356	0.62688	0.74192	0.75446
NM_144754	Cant1	-0.0539	0.02976	-0.3319	-0.2414	0.33186	0.0756	-0.2219	0.06985	0.08306	-0.1697	-0.1495	-0.4258	-0.1419	-0.0718
NM_172008	Canx	0.48454	0.43379	-1.0176	-0.1657	0.67137	-0.1575	-0.1999	0.83477	0.15539	0.17822	-0.2576	-0.5004	0.55344	0.63605
NM_022383	Cap1	0.47276	1.0268	-0.2692	0.10993	0.63075	-0.5449	0.36901	0.76617	0.56433	0.62185	0.42245	-0.4884	0.80938	0.58622
XM_341136.3	Cap350	-0.1686	-0.3293	-0.0246	0.27902	-0.1594	-0.2992	-0.197	-0.2145	-0.3437	-0.3143	-0.0179	-0.2845	-0.2265	-0.0398
NM_001013086	Capg	0.47794	0.0659	-0.5789	-0.7187	0.65639	0.49758	-0.2191	0.5875	0.01727	-0.255	-0.0143	-0.1692	-0.4005	-0.3053
NM_019152	Capn1	-0.131	0.04503	0.03246	-0.4116	-0.0003	0.06835	-0.2081	-0.0868	0.1379	-0.0676	-0.0763	0.07162	-0.015	-0.1942
NM_001002806	Capn11	0.1106	0.26965	0.17039	0.24968	0.03055	0.06636	0.07564	0.01641	0.24064	0.08782	0.03046	0.04986	-0.0387	0.09435
NM_001110808	Capn12_predicted	0.13615	0.24434	0.03724	0.0365	-0.061	0.14551	0.08519	-0.0366	0.07107	-0.0018	0.28163	0.194	0.00456	0.15287
NM_001025133	Capn13	-0.1365	-0.1557	0.03163	-0.1845	-0.1414	-0.1951	-0.111	-0.0977	0.02104	-0.1882	-0.1965	0.2898	-0.0963	-0.1828
NM_017116	Capn2	-0.2209	0.17694	-0.2152	-0.5914	-0.3124	-0.2391	0.28735	-0.3893	0.06685	0.17832	0.07656	-0.0138	-0.2492	-0.2793
NM_017117	Capn3	-0.2757	-0.1635	-0.2276	-0.068	-0.1691	-0.1815	-0.0562	-0.1525	-0.219	-0.1101	-0.3392	0.3921	-0.1444	-0.2713
NM_134461	Capn5	-0.0934	-0.1878	-0.3139	-0.1917	-0.2256	-0.1775	-0.135	-0.1996	-0.2022	-0.2047	-0.1415	-0.211	-0.1424	-0.1389
NM_031808	Capn6	-0.1658	-0.1598	-0.2172	-0.1865	-0.3254	0.20868	-0.1961	-0.1933	-0.1564	-0.3962	-0.3004	-0.2699	-0.1648	-0.0999
NM_001030037	Capn7_predicted	-0.1134	0.02556	-0.4272	-0.2953	-0.2846	-0.5294	0.04323	-0.0724	-0.173	-0.208	-0.1878	-0.2969	-0.0043	-0.1806

NM_133309	Capn8	0.60436	0.07622	1.0403	0.54582	2.5489	2.7796	0.81323	2.6664	-0.0128	0.23206	0.46921	0.24421	0.08101	0.00837
XM_001054278.1	Capn9	-0.0269	-0.0567	-0.0432	-0.0879	-0.04	-0.1337	0.03963	-0.078	-0.009	-0.2179	0.13192	-0.0766	-0.0365	-0.217
NM_017118	Capns1	0.52477	0.75343	-0.5804	-0.7543	0.36052	-0.1464	-0.2686	0.1809	0.65045	0.61462	0.41857	-0.1845	0.31199	0.46762
NM_001106417	Capsl_predicted	0.1452	0.14205	-0.0218	0.14767	0.97021	1.0418	0.02613	0.92646	-0.1407	0.01368	0.16361	-0.0004	-0.0626	0.12715
NM_001009180	Capza2	0.40631	0.40686	-0.6372	-0.4193	0.24738	-0.3832	-0.2097	0.0931	0.22052	0.27961	-0.0788	-0.1128	0.03114	-0.0011
NM_017164	Capza3	0.15753	0.05114	0.20107	0.08419	0.08193	0.00918	0.03302	0.14949	0.02958	0.05452	-0.0012	0.06246	0.15304	-0.0015
NM_001005903	Capzb	0.26986	0.41975	-0.2632	-0.6111	0.44983	0.28864	-0.5291	0.11362	0.32172	0.10256	0.05187	0.00787	-0.039	0.00022
NM_001107660	Car1_predicted	-0.2086	-0.1602	-0.1769	-0.1946	0.0439	-0.0898	-0.0545	0.06525	-0.0005	0.02617	-0.1263	0.1154	0.05728	0.02372
NM_001080756	Car12	0.03754	-0.0091	0.20142	-0.0145	0.38649	-0.0057	0.06688	0.07256	0.09732	-0.0389	0.1686	0.39542	-0.0579	-0.1051
XM_342297.2	Car14_predicted	0.04797	0.03439	0.00043	-0.0111	0.04973	0.16212	0.16697	0.07097	0.16654	-0.0608	-0.0692	0.10393	-0.0632	0.09198
XM_342297	Car14_predicted	-0.1449	-0.0638	0.17626	-0.0866	-0.0797	-0.0601	-0.0292	0.00687	0.0651	-0.0099	-0.0464	0.0034	-0.0143	-0.0187
NM_001105901	Car15_predicted	0.07581	-0.0975	-0.0892	-0.0169	0.04098	0.00708	-0.1247	0.03546	-0.1468	0.08296	0.00083	-0.1554	-0.1048	-0.0023
NM_001134841	Car6	0.0187	-0.1641	0.12445	0.17237	-0.0663	0.03328	-0.1527	-0.1298	-0.1969	-0.2205	-0.0433	-0.176	-0.0956	0.26019
NM_001106165	Car7_predicted	-0.0226	0.08761	-0.073	0.06877	-0.1623	-0.0044	-0.0708	-0.0114	-0.0918	0.03428	-0.1809	-0.0319	-0.1172	-0.0203
NM_001009662	Car8	-0.0777	-0.0726	-0.0682	-0.0507	-0.0874	0.07961	0.00869	-0.0295	0.14454	0.03976	0.17588	0.17925	0.09096	0.1876
NM_001009662	Car8	-0.0436	-0.0064	-0.0168	0.03645	-0.0064	0.02322	0.05721	0.00319	-0.022	-0.0094	0.05372	0.14417	0.03217	-0.0556
NM_001107956	Car9_predicted	0.46009	1.24	-0.9103	0.02001	-0.0697	-0.5897	-0.688	0.05075	0.69872	1.1276	0.67464	-0.3615	0.15682	0.1054
NM_001130554	Card10_predicted	-0.0827	0.06189	-0.0913	-0.0722	-0.0357	0.11696	-0.1301	0.04122	-0.1423	-0.1099	-0.0999	-0.0875	-0.0874	-0.1349
XM_001073551.1	Card11_predicted	-0.2776	-0.2729	-0.2422	-0.2097	-0.2354	-0.3502	-0.279	-0.3277	-0.1751	-0.2649	-0.2063	-0.1366	-0.145	-0.2824
NM_001106707	Card12_predicted	-0.0734	-0.1482	-0.2585	-0.1448	-0.1499	-0.1381	-0.0868	-0.2319	-0.0673	-0.2347	-0.2744	-0.2782	-0.0866	-0.0586
NM_001106707	Card12_predicted	-0.1905	-0.0281	-0.0566	-0.0815	-0.0631	-0.024	-0.1482	-0.1326	0.01722	-0.0305	-0.0516	0.05773	0.00656	-0.0042
NM_001106172	Card15_predicted	-0.2268	-0.0651	0.0781	-0.1206	0.34075	0.39253	-0.1243	0.09354	0.20496	-0.0453	0.22641	-0.174	0.15546	-0.0106
NM_001106413	Card6_predicted	0.06697	-0.0276	-0.2228	-0.5813	0.10701	-0.319	-0.5578	0.27605	-0.4726	-0.0917	-0.3077	-0.3393	-0.4698	-0.3223
NM_022303	Card9	-1.1024	-0.8448	-1.0934	-0.949	-0.8756	-0.7716	-1.1516	-0.9266	-0.9816	-1.0946	-0.7034	-1.0074	-0.9618	-0.8048
NM_001106915	Carf_predicted	-0.132	-0.1462	-0.2769	-0.3895	-0.2307	-0.3108	-0.2266	-0.2089	-0.2131	-0.2961	-0.2591	-0.0359	-0.2852	-0.2033
NM_152790	Carhsp1	0.19932	0.17431	0.4161	0.56475	0.62721	0.47067	0.39108	0.5923	0.01136	0.09064	0.23327	-0.4119	0.49006	0.34952
NM_001106319	Cars_predicted	-0.0415	-0.6399	0.18636	-0.4666	-0.1184	0.06147	-0.7234	-0.2979	-0.7504	-0.9421	-0.5016	-0.0772	-0.3841	-0.2326
NM_017110	Cart	-0.0693	0.08556	0.13783	0.13038	0.00408	0.10008	-0.0079	0.29769	0.07324	0.18625	0.08033	0.0044	0.03809	0.0876
NM_001106627	Casc1_predicted	-0.1189	0.04904	-0.2101	0.54578	-0.0881	-0.1721	-0.0684	-0.2095	-0.07	-0.0019	-0.1169	-0.193	-0.0742	-0.2423
NM_001106627	Casc1_predicted	0.02896	-0.0838	-0.1769	-0.0334	0.00679	-0.2786	-0.0876	0.01051	0.02513	-0.2793	-0.2051	0.01146	-0.2364	-0.2243
NM_147144	Casc3	-0.1072	-0.0813	0.02468	-0.1103	-0.0251	-0.2141	0.00966	0.10669	0.29144	-0.1304	-0.0953	-0.1026	-0.1613	0.09728
XM_001077041.1	Casc4_predicted	0.26301	0.44148	0.05198	0.65632	0.5142	0.19352	0.27511	0.47717	0.35257	0.29728	0.29282	0.20355	0.49239	0.31055
NM_022184	Cask	-0.927	-0.3448	-0.3967	-0.494	-0.3348	-0.5557	-0.1067	-0.282	-0.1972	-0.3535	-0.4589	-0.3657	-0.5388	-0.6248
NM_080690	Caskin1	-0.1185	-0.1355	0.06769	0.09377	-0.2313	-0.1586	0.00367	-0.1624	-0.1019	-0.1793	-0.0382	0.31998	-0.1781	-0.1882
NM_001107065	Caskin2_predicted	0.22238	-0.0115	0.09163	0.08632	-0.0253	0.10656	-0.0799	0.0943	-0.0722	-0.0125	0.12596	0.08055	-0.0232	0.08515
NM_012762	Casp1	1.5548	1.4433	0.25825	0.74943	-0.5614	-0.776	1.46	-0.4716	1.4807	1.5616	1.4343	0.51858	-0.0968	-0.0611
NM_053736	Casp11	0.72573	0.15808	-0.4399	0.29871	-0.5577	-0.4469	0.42374	-0.583	0.15483	0.28515	0.1611	0.0009	-0.692	-0.7833
NM_130422	Casp12	0.17093	0.62928	-0.0986	0.47091	0.41308	0.20566	0.47009	0.54964	0.31759	0.6541	0.23675	-0.0772	-0.1167	0.03006
XM_234878.4	Casp14_predicted	-0.0138	0.07028	-0.0291	0.00905	-0.0481	0.34545	0.02105	0.05008	0.03238	-0.0345	0.05209	-0.1178	-0.0106	-0.0351
NM_022522	Casp2	-0.8324	-0.6574	-0.3228	-0.5665	-1.0383	-1.1754	-0.4623	-1.0386	-0.7649	-0.6662	-0.6804	-0.605	-0.7434	-0.6025
NM_012922	Casp3	0.46589	1.0575	0.3212	0.05013	-0.0584	-0.1937	0.74946	-0.0838	0.81354	0.9458	0.98314	0.65552	0.27455	0.30684
NM_031775	Casp6	-0.073	-0.0752	-0.4712	-0.4467	-0.0493	-0.167	-0.2691	-0.2145	0.15442	-0.098	-0.1022	0.57998	-0.3689	-0.2933
NM_022260	Casp7	-0.3271	-0.1656	-0.3654	-0.2706	0.03579	-0.2364	-0.4026	-0.0022	-0.2951	-0.2628	-0.3497	-0.4034	-0.1199	-0.2017
NM_022277	Casp8	-0.0121	-0.0836	-0.272	-0.3869	-0.3903	-0.5148	0.21036	-0.0457	-0.0432	-0.5214	-0.2171	-0.39	-0.069	-0.0545
NM_031632	Casp9	-0.1516	-0.1736	0.1746	-0.4999	-0.0422	0.11708	-0.278	-0.0502	-0.1332	-0.2736	-0.0656	0.13662	-0.0374	0.36703
NM_017131	Casq2	0.23341	0.09725	0.09039	0.1741	-0.2242	0.34966	-0.1244	0.67042	-0.2356	0.25095	0.1479	-0.1418	-0.0324	-0.1562
NM_016996	Casr	-0.2467	-0.237	-0.1556	-0.2021	0.07554	-0.1096	-0.0176	-0.2268	-0.2177	-0.0638	-0.2243	-0.0569	-0.1437	-0.1466
NM_053295	Cast	0.33634	0.36084	-0.3288	-0.2218	0.59185	0.58967	1.1909	0.59989	0.09183	0.28297	0.01501	-0.2914	0.05792	0.07207
NM_012520	Cat	0.68226	0.41482	-0.3756	-0.1915	0.98432	0.7963	0.15133	0.27214	0.35743	0.27191	0.27421	1.3026	0.67838	0.51912

NM_001106101	Catsper3_predicted	-0.0571	-0.0442	0.05693	0.07912	-0.097	-0.0673	0.14324	-0.0718	0.08988	0.04067	-0.0327	0.23891	-0.0436	-0.1054
NM_133651	Cav1	0.87596	1.1396	-1.2555	-1.1303	1.1003	-0.34	0.11112	1.5069	0.15737	0.83122	-0.0994	-0.9924	0.93475	0.8436
NM_133651	Cav1	0.96021	0.72102	-1.1672	-1.5138	1.3532	-0.1181	-0.3969	1.7173	0.23141	0.54714	-0.0166	-0.843	1.1984	1.0568
NM_131914	Cav2	1.76	1.3136	0.59025	0.93675	1.7943	1.2663	1.191	1.9167	1.2715	1.5035	1.0045	0.58088	1.2714	1.229
NM_019155	Cav3	2.1222	1.7732	0.75143	1.0359	2.6961	2.7231	1.4778	2.7896	1.4527	1.7287	1.6765	-0.1023	1.0396	1.0679
NR_002316.1	CB741658	-0.0407	-0.0655	0.1044	0.14229	-0.1183	-0.0274	-0.0216	0.08917	0.05049	-0.034	-0.141	0.15417	0.00141	-0.007
NM_199412	Cbara1	0.03197	-0.2964	-0.4561	-0.4024	0.11654	0.4192	-0.1492	0.17112	-0.3043	-0.0825	-0.5357	0.11996	-0.2757	-0.4785
NM_001108657	Cbfa2t1_predicted	-0.0424	0.00862	0.07878	0.06041	-0.0849	0.01898	0.00163	-0.1417	0.17887	0.16282	0.23892	0.11186	-0.0113	-0.102
NM_001168542	Cbfa2t2_predicted	0.04098	-0.0462	-0.2872	0.13618	0.00649	-0.2861	-0.1251	-0.2241	0.09859	-0.0234	-0.1376	0.12424	-0.0016	0.11575
NM_001108453	Cbfa2t3_predicted	-0.2743	-0.1555	-0.0278	-0.1988	-0.1535	-0.185	-0.2587	-0.0948	-0.3352	0.07117	-0.2104	-0.2446	-0.1147	-0.1196
NM_001013191	Cbfb	0.20755	0.53676	-0.6465	-0.7344	-0.9737	-1.3354	-0.2155	-0.9081	0.10963	0.42301	0.40183	0.20309	-0.2265	-0.263
NM_138845	Cbl27	-0.189	-0.1576	0.18635	0.09065	-0.28	-0.4318	-0.0862	0.00201	-0.3853	-0.1902	-0.1052	0.39836	-0.2347	-0.0644
NM_133601	Cblb	0.28421	0.55849	0.46017	1.7342	-0.1491	-0.0138	0.47678	-0.2642	0.18873	0.71099	0.49267	-0.0923	-0.0166	0.19406
NM_001034920	Cblc_predicted	-0.004	0.03583	-0.0903	0.18281	0.06558	-0.0523	-0.0803	0.0679	0.05207	-0.1357	0.01216	-0.0952	-0.0183	-0.0523
NM_001108018	Cbll1_predicted	-0.0453	0.27764	0.08858	0.73382	0.26753	0.28442	0.32952	0.29419	-0.2091	-0.0556	-0.1056	-0.094	0.14527	0.18721
NM_001012740	Cbln2	0.2975	0.27185	0.12725	0.09265	0.06756	0.066	0.21679	0.32868	0.13246	0.17778	0.04551	0.02682	0.21825	0.05288
NM_001012740	Cbln2	-0.0708	0.01543	0.14627	0.03868	-0.0801	-0.0344	-0.1051	-0.0644	0.35602	0.18474	0.05232	-0.0824	0.03085	0.04399
NM_019170	Cbr1	-0.1551	-0.014	-0.2017	-0.406	0.5355	0.24063	-0.5275	0.30824	-0.1377	-0.246	0.029	-0.0175	-0.0671	-0.0118
NM_001107110	Cbr3_predicted	-0.228	0.25513	-0.1554	-0.4098	0.13599	0.06067	-0.1128	-0.0266	0.34825	0.05201	0.16704	-0.0681	-0.0925	-0.0586
NM_182672	Cbr4	0.33439	0.24354	-0.4702	-0.3415	0.37482	0.48986	-0.249	0.55296	0.20455	0.16278	0.1753	-0.0489	-0.1745	-0.0689
NM_012522	Cbs	-0.099	-0.0948	0.16951	-0.3374	0.03311	0.20395	-0.2494	0.14015	0.07894	0.15044	-0.0764	-0.0248	-0.1796	0.01722
NM_133535	Cbwd1	-0.1018	-0.0343	-0.3626	-0.0136	-0.0722	-0.2243	-0.1008	0.0243	-0.288	-0.0822	-0.1917	-0.1449	-0.3665	-0.1804
XM_340885.3	Cbx1_predicted	-0.1906	-0.0822	-0.4804	-0.2142	-0.0539	0.25955	-0.4617	0.04163	0.34397	0.00406	0.11381	0.60315	-0.021	0.04879
NM_001008313	Cbx3	-0.0715	-0.4866	-0.5755	-0.795	-0.4894	-0.8915	-0.693	-0.6196	-0.4692	-0.4493	-0.4704	-0.3084	-0.1863	-0.2934
NM_001106797	Cbx5_predicted	-0.32	-0.0988	0.01036	-0.6939	-0.3502	-0.3687	-0.3763	-0.3424	0.26459	0.02399	-0.0393	0.58668	0.1706	0.162
NM_001012119	Cbx6	-0.1488	0.31802	-0.6282	0.13823	0.1529	-0.9313	0.19469	-0.0803	0.2145	0.22652	0.16047	-0.4922	0.08648	0.19604
NM_001012119	Cbx6_predicted	-0.2942	-0.1884	-0.427	-0.3168	0.00184	-0.095	0.04375	0.12787	-0.0853	-0.0396	0.14227	-0.2834	-0.0226	0.0394
NM_199117	Cbx7	-0.2075	-0.0344	-0.2711	-0.0078	0.11483	-0.1057	0.06329	-0.1196	-0.2634	-0.0947	0.11309	-0.1798	-0.8267	-0.9013
NM_001034078	Cbx8	-0.1797	-0.2346	-0.1167	-0.4132	0.04257	0.12088	0.02759	-0.1326	0.01366	-0.0929	0.03651	0.10286	0.19125	0.23649
NM_145676	Cby1	-0.2155	-0.1667	-0.3932	-0.1087	-0.0623	-0.0959	-0.1729	0.27817	-0.0968	-0.2486	-0.0937	-0.203	-0.1032	-0.1112
NM_001013869	Cc2d1a	0.16885	-0.143	0.93511	0.31292	-0.1692	-0.6385	0.25703	-0.5097	-0.0004	-0.0447	0.24909	-0.1967	-0.2402	-0.0467
XM_001067280.1	Cc2d1b	-0.3783	-0.2937	0.309	-0.1954	-0.2688	-0.3729	0.3405	-0.3335	-0.3641	-0.0745	-0.3393	-0.2299	-0.134	0.19741
XM_001065428.1	Ccbe1_predicted	0.16042	0.15814	0.10401	0.23882	0.36497	0.46332	0.12716	0.33289	0.33702	-0.078	0.007	0.03117	0.05738	0.20762
NM_001013164	Ccbl1	0.30238	0.13462	0.13568	-0.2386	0.4433	0.275	-0.1667	0.17361	0.34415	0.27095	0.31745	0.47004	-0.0222	-0.1405
NM_001024866	Ccdc104	-0.0197	0.20193	-0.1351	-0.0748	-0.2523	-0.0153	-0.2696	-0.1719	0.21992	0.10483	0.27389	0.63659	-0.3374	-0.1009
NM_001017502	Ccdc117	-0.1655	-0.115	0.00764	-0.2106	-0.156	-0.09	-0.1467	-0.0683	-0.1335	-0.0499	-0.1252	-0.0511	-0.1042	-0.0559
NM_001017502	Ccdc117	0.19887	-0.0511	-0.0022	0.04634	0.30163	0.12742	0.11132	0.21787	-0.1258	-0.2663	-0.2206	-0.1217	0.09085	0.12077
NM_001108783	Ccdc12_predicted	-0.5445	-0.4616	-0.0236	-0.1887	-0.3416	0.233	-0.1398	-0.568	-0.1817	-0.0111	-0.0633	0.36493	-0.2053	-0.221
NM_198766	Ccdc127	0.65254	0.49649	-0.0593	0.23653	0.48241	0.28175	0.2858	0.63596	0.45568	0.3092	0.46826	0.10271	0.56245	0.74254
NM_001037644	Ccdc130	0.32634	0.24436	0.28845	0.32868	0.41982	0.11947	0.37898	0.14244	0.10598	0.27184	0.22957	0.00409	0.39251	0.4073
NM_001024355	Ccdc134	-0.4673	-0.5785	0.52538	0.01728	-0.1572	0.00184	-0.1727	-0.2043	-0.1899	-0.2656	-0.1153	0.08742	-0.2695	-0.2843
NM_001037360	Ccdc16	-0.191	-0.2707	-0.2484	-0.252	0.1115	-0.0466	0.04889	-0.0871	-0.1588	-0.1633	-0.1952	-0.2209	-0.1698	-0.0988
NM_001024882	Ccdc19	-0.2082	-0.1567	-0.1469	-0.3135	-0.1663	0.06378	0.07608	-0.1103	-0.1934	-0.0487	-0.0965	-0.0708	0.10234	-0.166
NM_001080151	Ccdc21	-0.0921	0.0874	0.70611	0.50567	-0.3554	-0.3385	0.46936	-0.1216	-0.052	-0.2128	0.0449	0.07213	-0.3795	-0.0672
NM_001038992	Ccdc23	0.26338	0.72232	0.19117	0.79198	0.05118	0.62846	-0.0715	0.10586	0.86119	0.54805	0.83769	1.1871	0.17809	0.23319
NM_001024245	Ccdc32	0.04106	0.12727	-0.3497	0.01449	0.11859	0.14328	0.00529	0.12454	0.15196	0.1087	0.01478	-0.2137	-0.016	0.06646
XM_001076288.1	Ccdc37_predicted	0.53914	0.55032	0.18277	0.06619	-0.3929	-0.3923	-0.0247	-0.4943	0.44329	0.62069	0.71256	0.62071	-0.2723	-0.2635
NM_001107667	Ccdc39_predicted	-0.4101	0.00399	-0.4105	-0.0963	-0.3414	-0.3566	-0.0481	-0.1751	-0.3045	-0.1054	-0.218	-0.3436	-0.3246	-0.1361
NM_001014266	Ccdc41	-0.2092	-0.0148	0.45839	0.954	-0.5257	-0.5281	0.35683	-0.4298	-0.0346	-0.164	0.13704	0.21171	-0.1403	-0.0584

NM_001107009	Ccdc42_predicted	0.04418	0.2665	0.14599	0.13431	0.21692	0.13648	-0.0815	0.38717	0.16747	0.14131	0.24476	0.04898	0.2479	0.05343
NM_001100728	Ccdc43	0.30618	0.17058	-0.1147	-0.1436	0.40348	0.35248	-0.1366	0.46536	0.13225	0.26132	-0.0143	-0.2669	0.40251	0.32067
NM_001013862	Ccdc45	-0.4194	-0.1094	0.43251	0.80317	-0.271	-0.1142	0.47127	-0.3285	-0.3496	-0.3227	-0.1356	-0.1068	-0.1692	-0.2104
NM_001108295	Ccdc49_predicted	-0.0776	-0.1787	-0.2901	-0.0528	-0.1727	-0.2421	0.07674	-0.0669	0.05184	-0.2168	-0.1538	-0.0881	0.01864	-0.0966
NM_138864	Ccdc5	-0.2393	-0.4107	0.2013	0.02193	0.09376	-0.1664	-0.0756	-0.292	-0.0535	0.14449	0.00998	0.16916	0.01114	-0.1275
NM_182736	Ccdc50	0.65045	0.06665	-0.1174	0.82015	0.98253	0.78755	0.08149	0.96506	0.41854	0.16861	-0.0154	0.25755	0.07757	0.05203
NM_001014098	Ccdc51	0.48176	0.19907	0.09881	0.30188	0.41869	0.69433	0.28042	0.43471	0.18248	0.26694	0.33536	-0.2339	0.37143	0.28708
NM_001008285	Ccdc52	-0.0217	-0.3425	0.22752	0.35473	-0.3766	-0.286	-0.196	-0.1351	-0.2794	-0.0378	-0.1369	-0.1836	-0.2077	-0.2965
NM_001106776	Ccdc53_predicted	0.06914	0.00857	-0.1033	-0.2329	0.06998	0.3856	0.10946	0.11087	0.38501	0.18795	0.29818	0.55756	0.03352	-0.0048
NM_001105875	Ccdc58_predicted	0.37245	-0.0761	0.55646	0.35777	0.19181	0.55722	0.02816	0.11321	0.09976	-0.0791	-0.0409	0.43456	0.45517	0.37925
NM_001108090	Ccdc59_predicted	0.09198	0.21523	-0.0363	0.08772	-0.1719	-0.0063	0.00652	-0.3559	0.36944	0.04184	0.2605	0.38498	0.39331	0.2116
NM_001014203	Ccdc65	0.04663	0.35029	-0.0233	-0.0779	-0.0822	0.10868	0.27659	0.03817	0.38772	0.6168	0.29919	0.40009	0.18971	0.08571
NM_001014087	Ccdc67	0.17316	0.09172	0.1005	-0.0414	0.00444	0.14031	-0.0274	-0.0043	-0.0097	0.09559	0.0868	0.02027	0.32146	0.1662
NM_001011561	Ccdc7	-0.1966	-0.2464	-0.0566	-0.197	-0.0009	-0.2226	-0.2617	-0.1022	-0.2988	-0.309	-0.3104	-0.2681	-0.2497	-0.0141
NM_001024904	Ccdc71	0.23455	0.11588	-0.0465	-0.3538	0.36964	0.03263	0.18878	0.19192	0.34476	-0.158	-0.2374	-0.163	-0.0438	0.13163
NM_001024885	Ccdc90b	0.01306	0.11839	-0.4659	-0.4187	0.0168	-0.2289	0.21414	-0.0536	0.41219	0.39627	0.22945	-0.0618	-0.1663	-0.4733
NM_001014061	Ccdc91	0.28245	-0.0729	-0.374	-0.5141	-0.0061	-0.4849	0.07398	-0.2109	0.09688	-0.0236	0.06696	-0.0877	-0.3156	-0.1829
NM_001024997	Ccdc93	-0.0882	0.06243	0.24535	-0.0259	-0.0934	-0.3443	0.01673	-0.1637	0.22594	0.10908	0.19719	0.33392	0.04314	0.21151
NM_001013900	Ccdc95	-0.1525	-0.0466	-0.1438	0.01688	-0.1288	-0.0237	-0.1018	0.05358	-0.1778	-0.0134	-0.2319	0.35047	-0.1007	-0.0462
NM_001009633	Ccdc98	0.37532	0.02907	0.46561	0.74332	0.34191	0.39533	0.72629	0.3482	0.09083	0.00227	0.31513	0.33765	0.16521	0.15209
NM_001034138	Ccdc99	-0.0264	-0.1471	0.90723	0.19684	0.03841	-0.0498	0.03714	-0.2171	0.03754	-0.1241	-0.2289	0.24828	0.72605	0.55091
NM_001005904	Ccin	-0.1945	-0.0925	-0.1646	-0.1693	-0.1908	-0.1794	-0.0349	-0.1824	0.09815	-0.1525	-0.0726	0.00653	-0.0921	-0.1492
NM_012829	Cck	-0.0573	0.00092	-0.2315	-0.2541	1.291	1.6327	-0.1707	1.0183	-0.0393	-0.0711	0.1015	-0.1921	-0.0378	-0.3596
NM_012688	Cckar	0.04377	0.01228	0.23263	0.22359	0.13446	0.13344	0.0294	0.02749	0.1697	0.33912	0.33373	0.25606	0.00422	0.09231
NM_013165	Cckbr	-0.2108	-0.2692	-0.2771	-0.1101	-0.2557	-0.273	-0.1423	-0.2869	-0.098	-0.1943	-0.3062	0.00593	-0.1817	-0.2529
NM_019205	Ccl11	0.03018	-0.0126	0.15424	0.01592	0.09379	-0.0002	0.07374	-0.0042	-0.0072	0.29233	0.13977	0.10923	0.21324	0.09044
NM_001105822	Ccl12_predicted	0.15349	-0.0435	0.03041	0.30053	0.32711	0.07986	0.13734	0.05347	0.07704	-0.0473	0.06671	0.12416	0.0753	0.206
NM_057151	Ccl17	-0.0935	0.25098	0.27146	0.01341	0.18533	0.3682	0.19214	0.19061	-0.15	-0.1502	0.04882	-0.0085	0.15201	0.07109
NM_001108661	Ccl19_predicted	2.5804	2.383	0.47685	0.40279	0.05176	0.09681	1.61	0.02666	2.2529	2.2914	2.3956	-0.0963	0.35715	0.28044
NM_031530	Ccl2	1.8934	0.81938	3.1167	2.0323	1.4643	1.7031	3.0333	1.6495	0.56918	1.2661	0.95097	0.52868	2.0482	1.9681
NM_019233	Ccl20	1.2237	-0.8007	-0.6768	2.4535	0.60516	0.90478	0.92556	0.76739	-0.8829	-0.5469	-0.847	-2.3743	-1.0992	-1.1089
NM_001008513	Ccl21b	0.03038	0.10973	0.01385	0.08601	0.04258	0.00244	0.46141	0.03261	0.34053	0.27438	0.22305	0.00274	0.22134	0.28237
NM_057203	Ccl22	0.38052	0.39108	0.06382	0.10564	-0.0033	0.03223	0.09108	0.19017	0.37759	0.47272	0.18304	0.15165	-0.0854	0.07398
NM_001037203	Ccl25	0.05966	0.24958	0.18941	0.1849	0.1153	0.34503	0.34686	0.18723	0.1703	0.02879	0.04705	0.1785	0.01548	0.07193
NM_001108660	Ccl27_predicted	0.29245	0.5292	0.11864	0.04298	-0.1857	0.01415	1.1969	-0.0938	0.32271	0.34905	0.54433	0.00688	-0.086	0.00989
NM_053700	Ccl28	0.11907	-0.0083	-0.0127	0.12706	0.10633	-0.0012	-0.1073	0.22698	-0.0906	-0.0568	-0.1598	0.21176	0.15059	-0.1688
NM_013025	Ccl3	0.13978	0.07628	0.09071	0.09379	0.14876	0.19715	0.11292	0.16004	0.29523	0.21898	-0.0424	0.13633	0.16167	0.15011
NM_053858	Ccl4	-0.0451	0.11271	0.02218	-0.0032	-0.026	0.02284	-0.0178	0.08386	-0.0221	-0.0005	0.02007	0.14883	0.01466	0.04236
NM_031116	Ccl5	0.20005	-0.0202	-0.0778	0.0674	1.1885	1.6082	0.49743	1.335	-0.0023	-0.092	0.01099	0.16316	0.37766	0.45681
NM_001004202	Ccl6	0.06727	-0.1003	-0.0955	-0.0914	-0.0672	0.06627	-0.0335	-0.0749	0.00374	-0.087	-0.0083	-0.0934	-0.0257	0.07466
NM_001007612	Ccl7	1.2135	0.58791	1.9575	2.1236	1.812	1.9695	1.9692	1.5625	0.56899	0.93808	0.93828	0.44125	1.2878	1.4417
NM_001012357	Ccl9	0.03672	0.07771	0.10049	0.20637	0.10098	0.10619	0.00919	-0.0365	0.06215	0.07445	0.0144	-0.1107	-0.0534	0.09004
NM_001011949	Ccna1	0.72444	0.38289	-0.0653	0.31012	0.52556	0.62337	0.05683	0.50471	0.44179	0.58903	0.47767	0.36957	-0.0816	-0.0241
NM_053702	Ccna2	-0.4869	-1.4843	0.69865	-0.7564	-1.3239	-1.2984	-1.079	-1.3331	-0.5133	-0.8769	-0.9183	0.25909	0.72938	0.80873
NM_001025141	Ccnb1ip1	0.21451	0.05104	-0.0328	0.0864	-0.0364	0.02746	0.04686	0.03626	-0.0147	-0.1588	-0.1874	-0.1458	0.0026	-0.1336
NM_001025141	Ccnb1ip1	-0.182	-0.1271	-0.2952	-0.0798	-0.1239	-0.1544	-0.1329	-0.1392	-0.3266	-0.2782	-0.0822	-0.195	-0.084	-0.1616
NM_001009470	Ccnb2	-0.1408	-1.4347	0.86603	-0.4622	-1.1112	-0.9809	-0.8517	-1.2166	-0.2347	-1.1166	-0.8483	0.80132	0.48181	0.49099
XM_001064448.1	Ccnb3_predicted	-0.0325	-0.0834	-0.1049	-0.0799	-0.0818	0.03194	-0.0648	-0.0561	-0.0782	0.18395	-0.008	-0.0606	0.06103	0.06064
NM_001100472	Ccnc	0.1315	0.08091	-0.2701	-0.0115	-0.1528	-0.3604	-0.0729	-0.1506	-0.0237	-0.1755	-0.3404	0.13484	-0.0551	-0.1297

NM_171992	Ccnd1	-0.2546	-0.3352	-0.38	-0.9731	-0.7004	-0.4794	-0.6988	-0.8701	-0.26	-0.4471	-0.3773	-0.2246	-0.4407	-0.3625
NM_022267	Ccnd2	-0.3196	0.10439	-0.2873	-0.1889	0.22211	0.15693	-0.0675	-0.0804	-0.3045	-0.1844	-0.0837	-0.1927	-0.3635	-0.1389
NM_001013204	Ccndbp1	0.09197	0.48166	-0.2706	0.03164	0.21899	0.1553	0.18156	0.25989	0.26602	0.28296	0.27705	0.28494	-0.0827	-0.1245
NM_001100821	Ccne1	0.22325	0.03726	0.30498	-0.1775	0.03959	0.32713	-0.5302	-0.0153	-0.1341	-0.1446	-0.0758	0.21545	0.46059	0.44821
NM_001108656	Ccne2_predicted	-0.2367	-0.2581	-0.1888	-0.7206	-0.7403	-1.066	-0.7263	-0.4803	-0.4359	-0.2353	-0.4698	-0.2352	0.4481	0.29806
NM_001100474	Ccnf	-0.2009	-0.7555	0.62669	-0.2083	-0.8491	-0.9169	-0.5147	-0.8277	-0.4345	-0.7368	-0.4791	0.14264	0.22079	0.19304
NM_012923	Ccng1	0.3105	0.32985	-0.1273	0.37485	0.23878	0.04338	0.24295	0.34686	0.13092	0.23933	0.28176	-0.4063	0.28727	0.31074
NM_001105725	Ccng2_predicted	-0.4291	0.46133	-1.5395	-0.7264	-0.7365	-1.2288	-0.5615	-0.7207	0.06486	-0.0533	-0.1684	-0.7127	-0.6251	-0.7618
NM_052981	Ccnh	0.45375	0.40731	0.08417	1.3194	0.40342	0.40215	0.36197	0.49896	0.61728	0.52177	0.62322	0.14647	0.52725	0.31962
NM_001105998	Ccni_predicted	-0.1418	0.48298	-0.9368	-0.6462	-0.6077	-0.8159	-0.6155	-0.3487	-0.0099	0.27452	-0.1714	-0.2698	-0.3533	-0.6966
NM_001106369	Ccnj_predicted	0.06642	-0.0774	-0.0723	0.06962	0.32994	-0.0673	0.09627	-0.0328	0.18109	-0.0769	0.08342	-0.038	0.05974	0.10434
NM_053662	Ccnl1	-1.4131	-1.2264	-0.4016	0.0036	-1.2562	-1.4029	0.30865	-1.1907	-1.3727	-1.1259	-1.0358	-1.2751	-1.2052	-1.4807
NM_001013094	Ccnl2_predicted	-0.7575	-0.6318	0.24273	1.405	-0.9347	-0.9139	0.23598	-0.7873	-0.7219	-0.9335	-0.5564	-0.8565	-1.6449	-1.696
NM_001108110	Ccnt1_predicted	0.00257	0.113	0.22723	0.11503	0.25127	0.37821	-0.0531	-0.1044	0.22242	-0.088	0.13163	-0.0155	0.01383	0.12867
NM_001107171	Ccnt2_predicted	-0.6699	-0.5364	-0.1239	0.21559	-0.6294	-0.7446	0.08319	-0.5476	-0.6187	-0.2943	-0.4653	-0.1624	-0.3725	-0.3767
NM_001108770	Ccpg1_predicted	0.12587	0.01021	0.31879	-0.0659	0.08795	-0.218	0.05763	-0.1193	-0.0185	-0.061	0.21691	0.06518	0.06563	0.14175
NM_001108770	Ccpg1_predicted	0.37963	0.93346	-0.7498	0.07819	0.40419	0.06704	0.42206	0.49069	0.78909	0.78543	0.67154	-0.0495	-0.3749	-0.184
NM_020542	Ccr1	2.6114	2.4499	1.5774	0.69629	0.11308	-0.0503	2.8464	0.32807	2.0172	2.1252	1.9469	0.21846	0.60464	0.6151
NM_001106872	Ccr1l1_predicted	0.10957	0.32023	0.06388	-0.0363	0.00412	0.04593	0.16315	0.19432	0.14771	0.03927	-0.1574	0.13302	0.01286	0.21184
NM_021866	Ccr2	-0.0115	-0.0156	0.19492	-0.1249	-0.1683	-0.1245	-0.1788	-0.1417	0.2283	0.18245	0.14393	0.58783	0.0137	-0.0325
NM_053958	Ccr3	-0.1406	-0.1375	0.08002	0.29118	0.02604	-0.031	-0.111	0.09088	-0.1603	-0.1763	-0.2589	-0.1338	0.07153	-0.0314
NM_133532	Ccr4	0.12661	-0.0141	0.1474	0.0842	-0.0263	0.15285	0.05229	0.06129	-0.2234	0.00748	0.07671	0.09984	0.07032	-0.038
NM_053960	Ccr5	0.13846	0.11238	-0.036	-0.026	-0.0386	0.13486	0.01732	-0.0466	0.09914	0.21222	0.19688	0.25937	0.02232	0.05136
NM_001013145	Ccr6	0.20719	0.09263	-0.1639	-0.1062	-0.08	0.1478	-0.1868	-0.0245	0.13669	-0.0285	0.15895	-0.0711	-0.0441	0.04644
NM_199489	Ccr7	-0.1401	-0.3474	-0.0085	0.337	-0.0334	0.1452	-0.1456	-0.1305	0.03469	0.07372	-0.0201	0.11228	0.19039	-0.146
XM_236704.2	Ccr8_predicted	0.00031	0.07928	0.10904	0.14219	-0.0067	-0.0522	0.08949	0.01699	-0.0279	-0.0422	-0.0587	0.11066	0.17155	0.02442
NM_172329	Ccr9	0.06253	0.13918	0.00059	0.11454	0.12306	0.34091	0.23833	-0.0145	0.06025	0.35046	0.39051	0.21027	0.1385	0.1677
NM_001025752	Ccrk	-0.2477	-0.1498	-0.117	-0.044	-0.3174	0.13164	-0.321	0.02311	-0.1873	-0.13	-0.0789	-0.3574	0.04131	0.01605
NM_001108191	Ccrl2_predicted	0.03266	0.27081	-0.0492	-0.0611	0.13449	-0.1273	0.0941	-0.0045	0.30205	-0.0965	0.14593	0.12042	-0.0127	-0.1034
NM_053425	Ccs	0.10077	-0.0206	-0.3913	-0.4934	-0.1461	0.36981	-0.1815	-0.0876	-0.0378	-0.3057	0.12979	0.12452	0.02658	0.19219
NM_001005905	Cct2	-0.2064	0.12652	0.51338	0.72579	0.00992	-0.1723	0.03449	0.07864	-0.0874	0.17151	0.20392	0.16827	0.30826	0.41839
NM_199091	Cct3	0.25052	0.04492	0.16663	-0.0804	-0.0736	-0.083	-0.0438	-0.2533	-0.0285	-0.0108	-0.0174	-0.1701	0.26833	0.35194
NM_182814	Cct4	0.16742	0.29825	0.29077	0.45958	0.33231	-0.0359	0.26687	0.50391	0.29315	0.25585	0.20813	-0.4011	0.22324	0.0932
NM_001004078	Cct5	-0.564	-0.657	-0.2771	-0.492	-0.3902	-0.8495	-0.6288	-0.5195	-0.48	-0.5436	-0.5059	-0.4997	-0.0839	-0.0849
NM_001033684	Cct6a_predicted	-0.2084	-0.5151	-0.0204	-0.1518	-0.2935	-0.4678	-0.0105	-0.3136	-0.3152	-0.1239	-0.505	-0.3832	0.18015	0.19598
NM_001106603	Cct7_predicted	0.16942	-0.4314	-0.0389	-0.9225	-0.1478	-0.3665	-0.4002	-0.3119	-0.2839	-0.3368	-0.4077	-0.4706	-0.2238	0.04637
NM_001108771	Cd109_predicted	0.12607	0.03226	0.11734	-0.2212	-0.1761	-0.0909	-0.0852	-0.1819	0.02711	-0.152	-0.2269	-0.2221	-0.1747	-0.2031
NM_021744	Cd14	-0.8766	-0.3932	-1.5851	-2.3061	-0.3171	-0.3761	-0.83	-0.3055	-0.2776	-0.5	-0.552	-0.729	-1.3595	-1.2575
NM_022523	Cd151	-0.0478	-0.0646	0.32158	-0.872	-0.47	-0.3816	0.09646	-0.3768	-0.1615	0.14671	0.05554	0.19393	0.16741	0.2684
NM_001107887	Cd163_predicted	0.05893	0.12036	0.11529	0.22717	0.17357	-0.0119	0.12581	0.16493	-0.0189	0.06148	0.11163	0.11922	0.15138	-0.0919
NM_031812	Cd164	-0.5038	-0.3459	-0.3998	-0.5288	-0.3805	-0.7295	-0.3959	-0.3567	-0.3911	-0.3829	-0.422	-0.6037	-0.5082	-0.5149
NM_001013237	Cd19_predicted	-0.3905	-0.0727	-0.35	-0.3809	-0.2077	0.16817	-0.2704	-0.3642	-0.0177	-0.3494	-0.2645	-0.1507	-0.2344	-0.2158
NM_017079	Cd1d1	0.81953	-0.0008	-0.4313	0.0236	2.2916	2.3157	1.393	2.4438	0.29212	0.14431	0.40979	0.42214	0.26913	0.32914
NM_031518	Cd200	0.65205	0.40735	0.39537	1.6617	0.87286	0.82612	0.82086	0.93162	0.92332	0.88164	0.61776	0.52765	0.80893	0.62143
NM_023953	Cd200r1	-0.4066	-0.6779	-0.4715	-0.6753	-0.5535	-0.5652	-0.5447	0.02581	-0.7966	-0.3721	-0.5861	-0.6095	-0.5924	-0.297
NM_001105904	Cd209a_predicted	0.02128	-0.1312	-0.1338	-0.0138	0.13303	0.11766	-0.1829	-0.0569	0.09746	-0.1669	-0.0379	0.08085	0.1296	0.0619
NM_001170397	Cd209b	0.09356	0.15532	-0.1508	0.10776	0.06418	0.07761	-0.0163	-0.1657	-0.0517	-0.234	-0.2079	-0.0263	-0.1184	-0.0888
NM_001107503	Cd22_predicted	0.02307	0.15763	0.12297	0.05729	-0.033	0.0555	0.15498	-0.0481	-0.0443	0.21069	-0.077	-0.0549	0.21284	0.07831
NM_001107370	Cd226_predicted	0.08531	0.05604	0.28715	0.07736	0.0924	0.09715	0.07928	0.14479	0.09246	0.24973	0.04251	0.23584	0.18435	0.12692

NM_012752	Cd24	-1.0777	-2.3098	0.34181	-0.6073	1.0933	1.1619	-0.1818	1.0638	0.24659	-1.1348	-0.4114	1.4642	0.70535	0.6635
NM_022259	Cd244	-0.0039	0.02919	-0.0826	-0.0729	0.00428	-0.0562	-0.0185	0.1069	-0.1035	-0.0767	0.03764	-0.0107	-0.0284	0.01823
NM_170789	Cd247	-0.2978	-0.2499	-0.3281	-0.3317	-0.1596	-0.1609	-0.1428	-0.2833	-0.0699	-0.2828	-0.3764	-0.1785	-0.2898	-0.3039
NM_001106325	Cd248_predicted	-0.2909	-0.2071	-0.0675	0.03284	-0.2566	-0.2443	-0.3752	-0.2739	-0.3172	-0.2178	-0.2961	-0.1675	-0.3743	-0.3325
NM_001024335	Cd27	0.02139	0.05879	0.10968	0.06585	0.093	0.03776	0.26953	-0.0743	0.06704	0.14258	0.07018	-0.0209	0.07839	0.00442
NM_182824	Cd276	0.00855	0.21341	-0.1506	-0.5291	0.19829	0.21375	-0.1228	-0.2825	0.02898	0.09651	0.11618	-0.0762	-0.4247	-0.5311
NM_013121	Cd28	0.27963	0.04469	0.18928	0.16016	0.17413	0.28763	0.19197	0.35588	0.10034	0.33484	0.28345	0.19319	0.23919	0.16529
NM_001106297	Cd2bp2_predicted	-0.0999	-0.3169	0.33954	0.45085	-0.2145	-0.2973	0.01137	-0.149	-0.1198	0.07281	0.05975	0.12997	0.05177	-0.1759
XR_009489.1	Cd300le_predicted	0.12527	0.53523	0.03893	0.22549	0.04723	0.11657	0.30229	0.06725	0.12796	0.17309	-0.0325	0.0874	-0.0846	0.05024
NM_001025111	Cd300lf	0.06064	-0.0249	0.06583	0.04234	0.02975	0.07872	0.08532	-0.0255	0.12765	0.06207	0.0383	0.10327	0.01049	-0.0502
NM_001013916	Cd302	0.09765	0.0493	0.08638	0.02618	-0.0138	0.01982	0.31012	-0.0202	0.34317	0.14258	0.13683	-0.0171	0.05486	0.00821
NM_001014201	Cd320	-0.1833	-0.39	0.13828	0.16943	-0.2308	-0.1343	-0.2342	-0.3443	0.04118	-0.237	-0.2698	0.28994	0.05935	0.33666
NM_001107202	Cd34_predicted	-0.0207	0.02756	-0.0105	0.02314	-0.1664	-0.1859	-0.0597	-0.0355	-0.0302	0.09792	0.03747	0.0804	0.50738	0.04024
NM_031561	Cd36	-0.0878	-0.0814	-0.0498	-0.1848	-0.1433	0.16482	0.20256	-0.0743	0.1377	0.08761	0.262	-0.1967	-0.0892	-0.0894
NM_017124	Cd37	2.1092	1.7831	1.3767	0.48289	1.3843	1.3678	1.2707	1.479	1.8356	1.9926	2.0334	0.91659	1.3729	1.369
NM_013127	Cd38	0.358	0.26181	0.28483	0.33612	-0.0249	0.3859	0.04581	0.07275	0.00953	0.13391	0.09496	0.06262	-0.0204	-0.0207
NM_013169	Cd3d	-0.0833	0.1031	-0.0219	-0.0163	-0.0171	0.02973	-0.013	-0.1263	-0.0339	-0.0456	-0.0798	-0.0913	-0.0572	0.04468
NM_001108140	Cd3e_predicted	0.23393	0.22511	0.00069	-0.1194	0.85378	0.76565	0.35864	0.63709	0.20361	0.08075	-0.0177	0.05721	0.0142	0.24311
NM_001077646	Cd3g	-0.0132	-0.2025	-0.0229	-0.1411	-0.1835	-0.2509	-0.2427	-0.1025	-0.1664	-0.2014	-0.131	-0.2883	-0.0065	-0.1197
NM_012705	Cd4	0.09218	-0.0787	-0.1736	-0.0263	-0.0224	0.25979	-0.2447	0.00337	-0.1568	-0.2203	0.09237	-0.1026	-0.0495	-0.0385
NM_134360	Cd40	0.10905	0.08648	0.09181	0.01864	0.03291	0.03294	-0.0197	0.16929	0.09729	-0.0243	0.06676	-0.0328	0.08092	0.21282
NM_053353	Cd40lg	0.10878	0.07552	0.14351	0.00215	-0.0513	0.03448	-0.0217	0.19696	0.08866	0.11934	0.16156	0.1412	0.16032	0.21849
NM_012924	Cd44	2.3024	1.6551	0.74753	1.5494	1.7875	1.5432	1.6902	2.0996	1.1648	1.7413	1.3032	-0.0379	1.5666	1.5268
NM_019190	Cd46	-0.3691	-0.2669	0.01058	-0.2368	-0.5104	0.0254	-0.1012	-0.1208	-0.1283	-0.3274	0.17764	0.06961	0.10203	-0.0964
NM_019195	Cd47	-0.4449	0.67274	-0.1201	0.15749	-0.1288	-0.2784	0.42358	-0.281	0.58921	0.45501	0.67937	-0.0052	0.07732	-0.0397
NM_139103	Cd48	0.2532	0.63434	-0.1633	0.17095	0.1705	-0.0209	0.68063	0.33663	0.57049	0.56945	0.3688	0.23464	0.28721	0.30215
NM_019295	Cd5	0.04857	0.00262	0.00501	0.01975	0.00946	0.24584	0.05073	-0.0386	-0.035	0.00686	0.07478	0.0154	0.07483	0.09306
NM_053983	Cd52	0.05687	0.01711	0.05535	0.03767	-0.0904	-0.1017	-0.1184	-0.0427	0.11819	-0.1506	-0.0414	0.09137	-0.1498	0.16662
NM_012523	Cd53	-0.2066	-0.1456	-0.209	-0.1717	-0.1097	-0.2699	-0.1106	0.04484	-0.2088	-0.2255	-0.1422	-0.2386	-0.187	-0.074
NM_012925	Cd59	3.3425	2.1722	-0.0073	0.56742	4.0467	3.9186	0.27049	3.9586	1.8143	1.8638	1.8641	0.59821	0.27713	0.31598
NM_175577	Cd6	0.23432	0.05789	-0.0371	0.16823	0.0785	0.15466	0.02749	-0.1127	0.03636	-0.0513	0.07328	0.48366	0.03895	0.1871
NM_017125	Cd63	0.26002	0.25723	0.15406	0.15108	-0.0503	0.09846	0.60255	0.00708	0.24804	0.33037	0.40292	0.51161	0.18242	0.07233
NM_001031638	Cd68	0.45938	0.69751	-0.0542	0.98199	0.47605	0.83862	0.11911	0.20543	0.42385	0.73627	0.69017	-0.04	-0.2675	-0.3654
NM_134327	Cd69	0.11116	-0.0677	0.1536	0.17564	0.0878	-0.1035	-0.0138	-0.1099	-0.1236	0.11498	0.01742	0.02944	0.07217	-0.0847
NM_001107074	Cd7_predicted	0.02463	-0.0394	-0.0568	0.08133	0.08455	0.18817	0.00528	0.31363	0.08494	0.03723	0.2093	0.29441	-0.0105	0.28734
NM_001015016	Cd72	-0.1912	-0.034	-0.2337	-0.229	-0.1129	-0.2262	-0.3518	0.06286	-0.3173	-0.2011	-0.148	-0.0231	-0.2023	-0.3116
NM_013069	Cd74	0.01597	0.06098	0.18741	-0.0983	0.13276	-0.0442	-0.0027	-0.0351	-0.0803	0.00176	0.13623	-0.06	-0.0709	0.02563
NM_133533	Cd79b	-0.0923	-0.234	-0.1365	0.00157	0.0808	0.16429	-0.1751	0.11371	0.00169	-0.2357	-0.0471	-0.0551	-0.0521	-0.1636
NM_012926	Cd80	0.23974	-0.0637	-0.0119	0.23956	0.03551	0.00103	0.00697	-0.0002	-0.017	0.0078	0.16675	-0.0033	0.06481	0.00628
NM_013087	Cd81	0.01389	-0.6642	-0.5034	-0.71	0.60954	0.97169	-0.7738	0.61402	-0.2971	-0.6834	-0.3763	-0.7106	-0.8373	-0.7005
NM_031797	Cd82	-0.2206	0.66509	0.32167	-0.6098	0.46117	0.37666	0.70836	0.23986	0.42496	0.50111	0.69085	-0.0515	-0.08	-0.0559
NM_001108410	Cd83_predicted	0.0981	0.08367	0.02051	0.0177	0.1405	0.40407	0.09943	-0.0896	-0.0211	-0.007	-0.0288	-0.0414	0.04303	0.1002
NM_020081	Cd86	0.10548	0.14315	0.16752	0.05666	0.1597	0.13288	0.09456	0.35436	0.17262	0.26141	-0.1271	0.13939	0.18864	0.23683
NM_031538	Cd8a	-0.0444	0.14898	0.06028	0.06168	0.03946	0.00508	0.09972	0.10911	0.05142	0.1446	0.11396	0.18911	0.34819	0.05767
NM_031539	Cd8b	-0.1302	-0.1069	-0.0972	-0.0577	-0.0384	-0.3076	-0.0623	-0.1281	0.02302	-0.194	-0.0485	-0.1596	-0.1093	-0.1665
NM_001134481	Cd96	0.06783	-0.079	-0.046	-0.1035	-0.0824	-0.0073	-0.1199	-0.1397	0.07834	0.04315	0.05185	0.11258	0.00181	-0.1758
NM_001012164	Cd97	0.09374	-0.5451	0.06929	-0.2058	0.06028	0.14644	-0.0968	0.11139	-0.2706	-0.1007	-0.0446	-0.1437	-0.2409	-0.2912
NM_001012164	Cd97_predicted	0.15433	-0.0092	0.05484	0.16208	-0.0342	-0.0941	-0.0358	-0.0424	-0.0305	-0.108	0.07824	-0.0339	0.04634	0.02328
NM_001100804	Cd99	0.37948	0.56999	0.26738	0.36575	0.04491	0.39209	0.56813	-0.1783	0.49258	0.53537	0.58245	0.80199	0.38299	0.25542

NM_001108688	Cda_predicted	1.6604	1.143	0.54173	1.3679	1.8897	2.3103	0.54972	1.772	0.75874	1.0692	1.1488	0.45467	0.57451	0.75183
NM_133557	Cda08	-0.4685	-0.0899	-0.5957	-0.8369	-0.6311	-0.7964	-0.1194	-0.4973	-0.2239	-0.2142	-0.329	-0.3571	-0.6342	-0.4806
NM_001012156	Cdadc1	-0.3234	0.11651	-0.3189	-0.8015	-0.5643	-0.801	0.14475	-0.4888	-0.0944	0.08945	-0.2785	-0.0568	0.01902	0.11746
NM_001012156	Cdadc1_predicted	-0.632	-0.1716	0.17325	-0.549	-0.593	-0.546	-0.0727	-0.7954	-0.3786	-0.222	-0.2145	0.02503	-0.3528	-0.2443
NM_001107765	Cdan1_predicted	-0.0856	-0.1611	0.11783	-0.0417	-0.293	-0.2284	-0.1518	-0.1702	-0.1062	-0.0042	0.06864	0.13471	-0.1231	0.14263
NM_001134856	Cdc14a_predicted	0.01416	0.76544	-0.5103	-0.4941	-0.2882	-0.6327	0.01425	-0.0532	0.43351	0.42913	0.38447	-0.1959	-0.104	-0.0061
NM_001108404	Cdc14b_predicted	-0.4392	-0.5125	-0.1819	-0.1356	-0.2853	-0.5293	-0.1583	-0.2095	-0.3792	-0.4155	-0.4373	-0.154	-0.412	-0.4014
NM_001024744	Cdc16	-0.3428	-0.5669	-0.1214	-0.0833	-0.4973	-0.2163	-0.3519	-0.4909	-0.3846	-0.5424	-0.4001	0.38191	-0.317	-0.2124
NM_001024744	Cdc16	-0.4866	-0.6027	-0.2675	-0.2581	-0.7366	-0.5736	-0.341	-0.7208	-0.4576	-0.7039	-0.7348	0.01257	-0.3193	-0.2702
NM_171993	Cdc20	-0.6547	-1.5459	0.92385	-0.539	-0.9808	-0.7885	-1.0857	-1.1961	-0.364	-0.7662	-1.0201	0.83128	0.56771	0.64633
NM_001100659	Cdc23	0.04757	-0.0278	-0.267	-0.1731	0.19388	-0.3198	-0.1806	0.16415	0.20555	0.03298	-0.4435	-0.5711	0.17179	0.24057
NM_133571	Cdc25a	-0.0645	-0.3775	0.1402	-0.3081	-0.2113	-0.4495	-0.2642	-0.3221	0.19429	-0.1687	-0.0326	-0.1145	0.05351	0.06532
NM_133572	Cdc25b	-0.0852	-0.2137	0.32735	-0.0567	-0.4071	-0.4508	-0.2687	-0.4734	-0.3695	-0.4762	-0.3374	0.14975	0.08395	0.08078
NM_001107396	Cdc25c_predicted	-0.224	-0.322	-0.0768	-0.1852	-0.276	-0.4439	-0.2714	-0.1781	-0.2444	-0.43	-0.4122	-0.1372	-0.0235	-0.0425
NM_001013240	Cdc26	0.19841	0.22072	-0.2346	-0.2753	0.18799	0.0966	0.02002	0.28967	0.20369	0.0742	-0.0252	0.34152	0.37547	0.40543
NM_001024793	Cdc27	-0.0678	0.32425	-0.3218	0.01784	0.01522	-0.0706	-0.2377	-0.0715	-0.0626	0.03384	-0.1489	-0.2557	0.32869	0.2695
NM_019296	Cdc2a	-0.2104	-1.1156	0.19729	-0.6736	-1.015	-1.1047	-1.3751	-1.0874	-0.3381	-0.9086	-0.8024	0.21068	0.18958	0.25013
XM_001053539.1	Cdc2l5	-0.7012	-0.1096	-0.2285	0.10496	-0.463	-0.5466	0.00927	-0.7151	-0.0917	-0.2773	-0.2296	-0.128	-0.452	-0.2495
NM_001107634	Cdc2l6_predicted	-0.399	-0.0917	-0.2493	-0.167	-0.2289	-0.4117	0.05084	-0.2218	-0.0809	0.0146	-0.1612	-0.3372	-0.217	-0.211
NM_053743	Cdc37	0.01212	0.08085	0.14565	-0.1662	0.49768	0.29851	0.41601	0.40303	0.28291	0.08755	0.1473	0.21443	0.11937	0.15525
NM_001011941	Cdc37l1	0.2178	0.20619	0.08867	-0.3264	-0.0518	-0.0819	0.31657	-0.087	0.20133	0.08245	-0.0197	-0.0832	0.08573	0.13893
NM_001108538	Cdc40_predicted	-0.3189	-0.0353	-0.2092	-0.0253	-0.3293	-0.2897	0.14556	-0.0346	0.13285	0.01282	0.05534	-0.0555	-0.2574	-0.3977
NM_001108538	Cdc40_predicted	-0.3853	-0.2083	-0.3987	-0.1276	-0.2086	-0.5611	-0.084	-0.3532	-0.3029	-0.5973	-0.2423	-0.4292	-0.2042	-0.3761
NM_171994	Cdc42	-0.1451	0.08783	-0.0239	-0.2184	0.12189	0.00718	0.00703	0.12899	0.11901	0.09992	-0.0389	0.20154	0.32828	0.09954
NM_053657	Cdc42bpa	0.16378	0.09651	-0.0074	0.07352	0.23171	0.10971	-0.0379	0.0995	0.05878	-0.0363	0.08234	0.47044	-0.0356	0.11889
NM_053620	Cdc42bpb	-0.337	-0.3652	-0.0741	-0.0434	-0.1028	0.10331	-0.0583	-0.0074	-0.0019	-0.157	0.13741	-0.139	-0.1305	-0.2022
NM_001079700	Cdc42ep1_predicted	-0.574	-0.1747	0.57892	-0.1811	-0.1662	-0.5355	-0.4431	-0.1436	0.10717	-0.0097	-0.4816	0.23499	0.03895	-0.0863
NM_001009689	Cdc42ep2	0.43292	0.7027	0.0307	-0.4007	0.19606	0.20767	0.29216	0.12865	0.71452	0.65892	0.45659	0.50221	-0.1292	-0.1514
NM_001048044	Cdc42ep3_predicted	-0.7857	-0.7211	-0.9091	-0.6855	-0.4588	-0.6855	-1.0333	-0.5585	-0.8554	-0.9244	-0.9553	-0.5309	-0.5622	-0.6853
NM_001107063	Cdc42ep4_predicted	-0.1068	-0.2427	-0.1376	-0.5218	-0.2179	0.03043	-0.3554	-0.2818	-0.5713	-0.3517	-0.4258	-0.0297	-0.2849	-0.5692
NM_001108469	Cdc42ep5_predicted	-0.2681	-0.3737	0.31273	-0.8679	-0.2932	0.10086	-0.2995	-0.4793	-0.0364	-0.4308	-0.1564	0.5021	0.66131	0.3402
NM_001039044	Cdc42se1	0.71458	0.17425	0.51383	-0.7667	0.22067	0.23525	0.01104	0.13314	0.04235	0.09677	0.07724	0.43123	0.5135	0.59975
NM_053527	Cdc5l	-0.306	-0.0064	0.11408	0.54193	0.06864	-0.0222	0.1425	0.06555	0.0674	0.21988	0.14877	0.00064	0.19479	0.19062
NM_001108298	Cdc6_predicted	-0.3029	-0.4565	1.1581	-0.4693	-0.7643	-0.6054	-0.443	-0.8235	-0.0948	-0.3128	-0.0523	0.17179	0.907	0.99881
NM_001108352	Cdc7_predicted	-0.127	-8E-05	-0.1652	-0.1672	0.0839	-0.2451	0.00516	0.0149	-0.0376	-0.218	-0.1613	-0.251	-0.0805	0.11016
NM_181637	Cdc91l1	-0.0475	0.02667	-0.2978	-0.4122	-0.0639	-0.0682	-0.1169	-0.1253	0.0929	0.12094	0.07794	0.25139	0.01594	-0.1097
NM_001012028	Cdca1_predicted	-0.3247	-1.335	1.1556	-0.1011	-1.5353	-1.4567	-0.6206	-1.5368	-0.5404	-0.7887	-0.7957	0.6102	0.89946	0.81606
NM_001107273	Cdca2	-0.2091	-0.8178	0.44282	-0.1783	-0.5193	-0.556	-0.6576	-0.6729	-0.2686	-0.5446	-0.5124	0.54646	0.5273	0.53602
NM_001007648	Cdca3	0.07932	-0.9566	0.34788	-0.5112	-0.5671	-0.5854	-0.8337	-0.7116	-0.1511	-0.6121	-0.7284	0.48805	0.65734	0.48264
NM_001037214	Cdca4	-0.6118	-0.7413	-0.0039	-0.4039	-0.6446	-0.8284	-0.669	-0.7323	-0.5664	-0.6568	-0.5805	-0.3806	-0.1984	-0.097
NM_001025693	Cdca7	-0.3945	-0.3721	-0.2406	-0.2886	-0.2678	-0.4004	-0.3833	-0.3775	-0.3045	-0.2231	-0.2881	-0.17	-0.1056	-0.3026
NM_001025693	Cdca7	-1.0129	-1.4916	-0.8975	-1.2019	-1.492	-1.6128	-1.5776	-1.4739	-1.0704	-1.2884	-1.4457	-1.0693	-0.7081	-0.5861
NM_001025050	Cdca8	-0.0377	-0.759	0.56833	-0.2431	-0.2429	-0.0687	-0.6745	-0.3692	-0.1208	-0.751	-0.5698	0.51507	0.81078	0.73083
NM_001106869	Cdcp1_predicted	-0.0442	-0.1294	0.12031	-0.0252	0.21355	0.09588	-0.0262	0.11332	0.00985	-0.2507	0.00427	0.25591	-0.0344	-0.0796
NM_001105879	Cdgap_predicted	-0.047	-0.1895	-0.1091	-0.2672	-0.2482	-0.0202	-0.3441	-0.2119	-0.1062	-0.4047	-0.3352	-0.1757	0.00621	-0.1116
NM_031334	Cdh1	0.17587	0.13528	0.09379	0.2496	0.06989	-0.0361	0.12101	0.0744	0.05359	0.26985	0.0835	0.15043	0.04356	0.24053
NM_001168631	Cdh10	0.07858	0.08572	0.18192	0.03859	0.04278	0.0955	0.66616	0.0882	0.13182	0.37926	0.33612	0.05108	0.18509	-0.0138
NM_053392	Cdh11	-0.3024	0.81165	0.19059	0.03739	-0.2341	-0.2284	0.73766	-0.3388	0.6852	0.67375	0.73724	0.42771	0.19749	-0.0063
NM_138889	Cdh13	2.5889	1.3429	1.1422	1.6863	1.907	1.8926	1.1473	1.7828	1.2579	1.1599	1.4963	1.1028	0.73854	0.67638

NM_207613	Cdh15	-0.0544	0.01465	-0.0009	0.04199	0.06715	0.07326	0.01188	0.03653	-0.0117	0.09646	0.27605	0.25955	0.18144	-0.0533
NM_001012055	Cdh16	-0.0693	0.30516	0.29684	0.10039	0.1153	0.14681	0.26048	0.10536	0.09732	0.18587	0.32282	-0.1035	0.17885	0.06646
NM_053977	Cdh17	1.308	1.8402	-0.1849	-0.3974	0.32499	0.35518	1.2092	0.29273	1.4909	1.6239	1.524	0.14038	-0.5105	-0.4627
NM_001107656	Cdh18_predicted	0.19695	-0.1709	-0.0466	0.14266	0.3609	0.70102	0.3547	0.42973	-0.0344	-0.0446	-0.1375	-0.0888	0.17886	0.32191
NM_001009448	Cdh19	-0.1903	0.07078	0.08982	0.29066	-0.0033	0.281	-0.0281	0.0407	0.31389	-0.1047	-0.167	0.24176	0.23089	0.15274
NM_001012748	Cdh20	-0.1813	-0.0301	-0.2059	-0.142	-0.1738	0.05788	-0.151	-0.0702	-0.0747	-0.0826	-0.0428	-0.0865	-0.0949	-0.2091
XM_230942.2	Cdh26_predicted	-0.1381	-0.1752	-0.1798	0.1673	-0.1583	0.02742	-0.0121	-0.3357	-0.3271	-0.1125	-0.2765	0.18742	0.06345	-0.2211
NM_053938	Cdh3	0.55206	0.34936	0.64722	0.44605	0.46419	0.77082	-0.0426	0.2556	0.50946	0.63405	0.30329	0.73348	0.4967	0.35415
NM_001107407	Cdh5_predicted	-0.1279	-0.0868	-0.0474	-0.0693	0.05728	-0.0928	0.12497	-0.023	0.0913	0.0222	0.14478	0.09274	-0.0101	0.04562
NM_001012737	Cdh7	0.20299	0.10008	0.06226	0.3359	0.03107	0.15088	0.16027	8E-05	0.14637	0.16494	0.21268	0.1666	0.12374	0.06963
NM_053393	Cdh8	0.11691	-0.0044	0.14097	0.0305	-0.0253	0.08417	0.02925	0.15957	0.03422	0.03454	-0.0077	0.1033	-0.0248	0.04603
NM_001168630	Cdh9_predicted	0.08405	3.7231	0.00541	0.01952	0.09956	0.16427	1.8111	0.14885	2.9675	3.511	3.2166	0.08742	-0.0382	-0.0951
NM_153624.1	Cdig2	0.35934	0.17283	0.33407	0.32335	0.43239	0.48201	0.50225	0.33804	0.01323	0.07177	0.1813	0.26377	0.06514	0.22369
NM_138899	Cdipt	0.11282	-0.0696	0.12137	-0.4511	0.31378	0.00665	0.12897	0.33789	0.09476	0.12957	-0.1333	-0.2222	0.15905	0.13284
NM_001109937	Cdk10	-0.1895	-0.1659	-0.1079	0.18091	-0.3433	-0.0507	0.19342	-0.0699	-0.1832	0.00801	-0.1985	0.00704	-0.5261	-0.3897
NM_134415	Cdk105	0.61825	0.20088	0.00107	0.28858	0.53204	0.36892	0.13177	0.59047	0.51273	0.25567	0.47467	-0.0708	0.24331	0.31984
NM_001113751	Cdk2ap1_predicted	-0.4349	-0.646	-0.1947	-0.6573	-0.3563	-0.2972	-0.3086	-0.2782	-0.2016	-0.5393	-0.3191	0.16261	-0.0083	0.19939
NM_053593	Cdk4	-0.3708	-0.3689	0.14591	-0.7947	-0.5502	-0.5394	-0.6741	-0.7351	-0.1254	-0.3263	-0.1736	0.49452	0.3634	0.38659
NM_080885	Cdk5	-0.1035	-0.2185	-0.1711	-0.659	-0.3824	-0.4235	-0.3546	-0.4537	-0.3171	-0.2774	-0.3562	-0.1062	-0.5433	-0.5047
NM_145721	Cdk5rap1	0.00346	-0.0199	-0.0861	0.07578	-0.0469	-0.0751	0.03261	-0.0559	0.05534	0.00174	-0.0541	0.02664	-0.0895	0.12243
NM_173134	Cdk5rap2	-0.7121	0.26099	-0.4052	-1.1533	-1.024	-1.1537	-1.1449	-1.0176	0.54196	0.06007	0.32384	0.81525	-0.409	-0.3074
XM_342638.3	Cdk6	0.26404	0.06768	0.26327	-0.2966	0.03029	0.35236	0.22787	-0.0819	0.03924	-0.0512	-0.1584	0.11208	0.43015	0.56665
XM_215467.4	Cdk7	-0.2133	-0.0711	0.06673	0.51458	0.11504	0.08699	0.03087	0.00817	0.153	0.0675	0.01027	0.08529	0.04736	0.27384
NM_001007743	Cdk9	-0.0491	-0.3939	-0.2351	-0.1605	-0.0416	-0.1838	-0.2931	-0.0585	-0.1217	-0.199	-0.41	-0.3371	-0.1842	-0.2676
NM_001108413	Cdkal1_predicted	0.11623	0.07222	0.04763	0.0874	-0.0053	0.05158	0.08596	-0.0012	0.07913	0.15549	0.05418	0.05857	0.11607	-0.0211
NM_001025121	Cdkl1_predicted	0.13562	-0.0011	0.11792	0.03747	0.07339	0.05632	0.18783	0.11179	0.11219	-0.0129	0.05532	-0.0141	0.1284	0.14719
NM_001012035	Cdkl2	-0.0954	-0.2078	0.05836	-0.1871	0.07703	-0.2619	0.00023	-0.0061	-0.1484	-0.148	-0.2806	0.06499	-0.1955	-0.1205
NM_001012035	Cdkl2_predicted	0.15789	0.24178	-0.0583	-0.0054	-0.015	0.08332	0.00406	0.08432	0.02537	0.14051	0.07661	0.32328	0.15148	0.08176
NM_021772	Cdkl3	0.38873	0.05043	0.06744	-0.1671	0.12194	-0.1968	0.07234	0.1608	0.07219	-0.05	-0.2357	0.19576	0.15103	-0.1721
NM_080782	Cdkn1a	0.29269	-0.1282	-0.0615	1.0608	0.54352	1.1683	0.47021	0.293	-0.2144	-0.0051	-0.191	-0.0007	-0.1688	0.0359
NM_031762	Cdkn1b	-0.387	-0.5224	-0.608	-0.177	-0.9855	-0.7331	-0.4715	-0.8161	-0.3659	-0.2624	-0.3132	-0.0966	-0.7258	-0.7539
NM_182735	Cdkn1c	0.32033	0.21467	0.28829	0.02537	0.29273	0.32714	0.24286	0.13907	-0.044	-0.0144	0.17787	0.06389	0.1322	0.21779
NM_001014000	Cdkn2aip	-0.1034	0.23597	0.19932	0.1648	-0.0638	-0.0506	0.73815	-0.0353	0.35018	0.35094	0.13128	0.25504	0.20131	0.18103
NM_001008278	Cdkn2aipnl	0.96857	0.94965	0.43895	0.14014	0.89042	0.76759	0.83006	0.90912	0.58675	0.71105	0.78264	0.25488	0.56511	0.41704
NM_130812	Cdkn2b	-0.03	-0.0149	0.04232	0.01544	0.11533	0.0815	-0.0201	-0.047	-0.0681	0.11198	-0.0504	-0.0283	0.07119	-0.0291
NM_131902	Cdkn2c	-0.53	-0.0044	0.23471	-0.189	-0.7333	-0.7274	-0.4743	-0.8193	-0.1828	-0.2041	-0.4076	0.33231	-0.1391	-0.0383
NM_001106028	Cdkn3_predicted	0.19971	0.19902	0.11902	0.14706	0.10895	0.16404	0.06266	-0.0355	-0.0614	0.0618	0.06533	-0.07	0.06043	0.01965
NM_052809	Cdo1	-0.248	-0.211	-0.0825	-0.2178	-0.1332	0.00583	-0.0705	-0.0532	-0.2596	-0.2236	-0.3104	-0.2733	-0.2399	-0.2048
NM_001025682	Cdr2	0.06103	0.05904	0.41374	0.97463	0.64187	0.41097	-0.2792	0.90118	-0.0966	-0.1779	-0.2218	0.10711	0.54826	0.47793
NM_031242	Cds1	0.45328	0.00579	-0.0212	0.51783	-0.1718	-0.0219	0.15951	-0.0555	-0.2312	-0.1899	-0.0972	0.06784	-0.103	-0.1403
NM_053643	Cds2	-0.0341	0.09453	-0.1203	0.08354	0.20905	0.0842	0.13609	0.11126	-0.06	0.08249	0.17368	-0.0731	0.02905	0.06005
NM_001033852	Cdw92	0.10317	0.11549	-0.0822	-0.1835	0.29546	0.26083	0.07899	0.12396	-0.0803	0.09595	0.00377	0.21885	-0.0751	-0.0053
XM_344691.3	Cdx1_predicted	-0.0766	0.1606	0.08324	0.15034	0.12492	0.24852	0.18061	0.13135	0.08145	0.00058	0.28069	0.1643	0.37828	0.10277
NM_023963	Cdx2	-0.7297	-0.9095	-0.1894	-1.0725	-0.9589	-0.6631	0.11713	-0.9619	-0.6018	-0.6198	-0.8953	-0.5277	-0.6415	-0.5532
NM_001106942	Cdx4_predicted	-0.1003	-0.0474	0.18782	-0.024	0.06756	0.09556	0.17684	0.1306	0.01614	0.06391	0.12125	0.07177	0.04273	0.04235
NM_001014145	Cdyl	0.54528	0.44987	-0.2301	0.28778	1.0276	0.5856	0.2937	1.1562	0.23966	0.39965	0.20718	0.05623	0.47223	0.44052
NM_001106189	Cdyl2_predicted	0.83761	0.60455	0.66508	0.40058	0.57235	0.6244	0.6922	0.51863	0.32768	0.14362	0.27921	0.59105	0.92471	0.79624
NM_001033861	Ceacam1	-0.1605	-0.233	-0.2114	-0.1955	-0.0849	-0.1201	-0.1705	-0.2967	-0.0601	-0.214	-0.1935	-0.1369	-0.144	-0.011
NM_173339	Ceacam10	0.1549	-0.1017	0.12382	0.20523	0.39408	0.46865	-0.1909	0.12361	-0.248	-0.1536	-0.229	-0.0964	-0.2164	-0.1666

NM_001025404	Ceacam11	-0.016	-0.0309	-0.0657	0.02271	-0.017	0.00371	-0.0091	0.20417	0.04061	-0.0046	-0.0302	-0.1363	-0.0241	0.08046
NM_001108474	Ceacam12_predicted	-0.2856	-0.0124	0.12624	-0.145	-0.0808	0.06088	0.08203	0.01823	-0.0485	-0.0591	0.0099	0.16898	-0.1734	-0.0756
NM_001170324	Ceacam20_predicted	-0.2287	0.09	-0.1776	-0.1528	-0.3043	-0.061	-0.2718	-0.1715	-0.2838	-0.2934	-0.2469	-0.2326	-0.293	-0.0721
NM_001025679	Ceacam3	0.09021	0.02096	0.07038	-0.0487	0.09457	0.0473	-0.0172	0.03457	-0.0063	0.12382	-0.0375	0.05669	0.17866	0.10191
NM_053919	Ceacam9	0.33085	0.35632	0.21816	0.43809	0.13354	0.03026	0.21727	0.20488	0.768	0.19477	0.44486	0.26744	0.08635	0.1105
NM_012524	Cebpa	-0.1385	-0.1801	0.05203	-0.0756	0.00019	-0.1052	-0.064	-0.0824	-0.0696	-0.2841	0.04225	-0.2348	-0.3096	-0.1592
NM_024125	Cebpb	0.46891	0.81811	0.3876	0.70813	0.66988	0.33888	0.82665	0.67514	0.46634	0.49778	0.55655	-0.0824	0.1772	0.2706
NM_017095	Cebpe	0.01983	-0.0618	-0.1699	-0.1803	-0.2558	0.01604	0.10404	-0.1738	-0.1243	-0.0241	0.09912	0.02044	0.20555	0.06239
NM_012831	Cebpg	-0.5226	-1.2008	-0.8322	-0.8125	-0.5358	-0.3082	-0.9271	-0.717	-0.876	-1.2419	-1.0527	-0.5389	-0.8701	-0.6024
NM_001108701	Cebpz_predicted	-0.3458	-0.0522	0.18799	0.63708	-0.2363	-0.5357	0.2349	-0.0292	-0.1952	0.00335	-0.2612	-0.4845	-0.0463	0.05389
NM_001107884	Cecr5_predicted	0.0534	-0.0018	-0.0297	0.21474	0.04493	-0.2682	-0.0507	0.00427	0.03647	-0.1633	0.08431	-0.0648	-0.2715	-0.2266
NM_001011962	Ceecam1	0.08306	0.0221	0.09331	-0.0434	0.06759	0.14025	-0.2562	-0.0549	-0.2171	-0.3798	0.00226	-0.0407	-0.2221	-0.1113
NM_016997	Cel	0.01068	-0.0009	0.17871	0.13546	0.08322	-0.0661	0.21136	0.1634	0.02546	0.08283	-0.0012	0.0622	0.18696	-0.0837
XM_001070474.1	Celsr1	0.00834	0.11149	0.12972	0.11079	0.26566	0.18503	0.057	0.1507	0.23017	0.22678	0.04259	0.2835	0.12826	0.1215
XM_001076220.1	Celsr2	-0.227	0.06316	0.09983	0.00741	-0.0341	0.02267	0.09023	0.07457	-0.1338	0.08157	-0.0283	0.02764	-0.0793	-0.1579
NM_031320	Celsr3	0.25285	0.14002	0.14882	-0.0475	-0.1219	0.17725	0.04877	-0.0454	0.29701	0.03618	-0.037	-0.161	0.03607	0.08829
NM_001014163	Cend1	-0.2419	-0.196	-0.0813	-0.1839	-0.1274	-0.1595	-0.0953	-0.1054	-0.251	-0.2595	-0.1486	-0.3655	-0.2314	-0.1856
XM_001081194.1	Cenpb_predicted	-0.1271	-0.4824	0.05687	-1.2159	-0.051	0.16425	-0.4239	-0.3238	-0.2169	-0.4442	-0.3684	0.03073	0.22253	0.07195
NM_001004098	Cenpc1	-0.0742	-0.3664	0.00547	0.44851	-0.2232	-0.3907	-0.2668	-0.0967	-0.1301	-0.3611	-0.1201	-0.0551	0.12235	0.1049
XM_001077739.1	Cenpe_predicted	-0.0852	-0.4411	0.99191	0.107	-0.7958	-1.0634	-0.28	-0.7079	-0.1273	-0.3342	-0.4996	0.52179	0.39458	0.5213
XM_001077739.1	Cenpe_predicted	-0.1315	-0.1236	0.25846	0.27492	-0.0971	-0.143	0.17303	-0.2472	-0.2253	-0.143	-0.0631	0.00368	-0.0201	0.1928
NM_001100827	Cenpf	0.00616	-0.5713	0.07731	-0.0986	-0.6537	-1.0534	-0.5053	-0.6503	-0.2371	-0.4587	-0.1669	0.1499	0.42091	0.35377
NM_012955	Cenpi	0.12933	-0.5122	0.96395	-0.0294	-0.4569	-0.5502	-0.1291	-0.1693	0.01581	-0.3895	0.05785	0.54922	0.6432	0.67177
NM_001107265	Cenpj_predicted	0.03823	-0.1082	0.32564	0.92128	-0.131	-0.3647	0.44667	0.02388	-0.1668	-0.037	-0.022	-0.2019	-0.2764	-0.0687
NM_001033061	Cenpl	0.10639	-0.0826	0.55346	0.7951	-0.3769	-0.3136	-0.1021	-0.461	0.0627	-0.1407	-0.0713	0.31247	0.83877	0.81012
NM_001008366	Cenpn	0.16394	-0.4084	0.34035	0.19566	-0.3099	-0.3082	-0.3054	-0.051	-0.2319	-0.5022	-0.1113	0.20051	0.27137	0.25439
NM_001024257	Cenpt	-0.1994	-1.3782	1.3935	0.46578	-1.3573	-1.315	-0.4911	-1.2745	-0.6565	-1.1152	-0.7711	0.70514	0.54681	0.52595
NM_133567	Centa1	0.32694	0.28484	0.10733	0.24959	0.19843	0.55341	0.21811	0.30244	0.41331	0.08821	0.23147	0.07493	0.00568	0.17463
NM_020101	Centa2	-0.031	0.14256	0.12967	0.14689	0.1599	0.20032	0.05478	0.30214	0.18529	0.10949	0.06601	0.02384	0.05102	0.04881
NM_001105796	Centb1_predicted	0.92315	0.57864	-0.3736	-0.0256	0.17544	0.4249	-0.3115	0.14387	0.34089	0.55088	0.63915	-0.3622	-0.4043	-0.3317
NM_001034006	Centb2	-0.0041	-0.0608	-0.1471	0.0126	-0.0859	0.21922	-0.1035	-0.0194	-0.0427	-0.1058	-0.0369	-0.0462	-0.0297	-0.0062
NM_001107999	Centb5_predicted	0.24349	-0.089	-0.0036	-0.1058	-0.171	-0.1552	-0.1276	-0.1388	-0.0202	-0.2816	-0.0023	-0.4534	-0.2307	-0.0974
NM_001107216	Centd1_predicted	0.2618	0.02862	0.02515	-0.0345	0.35744	0.04731	0.08135	0.10746	0.04534	0.16249	0.06677	0.18732	0.17186	0.18714
XM_001066901.1	Centd2	-1.1626	-0.9074	-0.7084	-1.1709	-0.9745	-1.2299	-0.9281	-0.9411	-1.092	-1.0558	-0.8375	-1.4422	-1.1126	-1.3111
XM_001066167.1	Centd3_predicted	-0.0386	0.01113	-0.0144	-0.0398	0.12096	-0.0279	0.09716	-0.0744	-0.1367	-0.1004	-0.0624	0.32837	0.09764	-0.0435
NM_023026	Centg1	0.2218	0.12707	-0.1184	-0.1151	-0.062	0.08132	-0.0038	0.01174	0.00944	0.03874	0.0924	0.10452	-0.0329	-0.0418
NM_001108230	Centg2_predicted	-0.4464	-0.4423	-0.3849	-1.1579	-0.2468	-0.1568	-1.0654	-0.098	-0.2954	-0.4863	-0.5211	-0.0292	-0.147	-0.095
NM_001108616	Centg3_predicted	-0.2336	-0.4221	0.07167	-0.298	-0.5853	-0.5686	-0.2199	-0.6488	-0.5275	-0.6947	-0.5202	-0.5651	-0.3293	-0.4081
NM_001107836	Cep1_predicted	0.01233	-0.0295	0.40512	0.32924	-0.1305	-0.2563	0.26184	-0.0395	-0.1838	-0.0202	-0.0111	-0.1678	-0.0185	-0.0457
XR_009444.1	Cep152_predicted	-0.3496	-0.0646	0.92925	0.60044	-0.0566	-0.2475	0.44677	-0.0315	-0.1073	0.0004	0.06682	-0.079	-0.1009	0.27525
NM_001025646	Cep55	0.65372	-0.5245	0.59206	0.08471	-0.6041	-0.5314	-0.3081	-0.1184	-0.1756	-0.1918	-0.5211	0.1142	0.67499	0.70199
NM_001108124	Cep57	-0.1209	-0.2598	-0.1228	0.07015	-0.3836	-0.0851	-0.1115	-0.3269	0.02827	-0.3379	-0.259	0.03396	0.29798	-0.2643
XM_001059836.1	Cep68_predicted	-0.0767	-0.1834	0.11945	-0.0332	-0.067	-0.0997	-0.0056	-0.0641	0.1739	-0.0273	0.12088	0.16565	0.10385	-0.0949
NM_001017470	Cep70	-0.1113	-0.1404	-0.1865	-0.0134	-0.2597	-0.2223	-0.208	-0.1823	-0.0072	-0.205	-0.0402	-0.166	-0.3081	-0.1842
NM_001017470	Cep70	0.46996	0.24658	-0.0463	0.60471	0.26709	-0.2247	0.07092	0.26241	0.00298	0.25769	0.14229	-0.2901	-0.2568	-0.3238
NM_001100514	Cep76	0.45074	-0.0017	0.13169	-0.0063	0.59794	0.07588	-0.0548	0.59481	0.06373	0.19948	-0.1063	-0.076	0.92705	0.70496
NM_001007699	Cept1	0.46459	0.49708	0.08305	0.52904	0.35982	0.29431	0.80132	0.38289	0.38448	0.34789	0.24694	-0.0255	0.01715	-0.0333
NM_001134861	Cerk_predicted	0.3926	0.81675	0.64031	-0.8148	-0.7446	-0.8252	0.32408	-0.7367	0.74492	0.65371	0.79528	0.55422	0.19311	0.12258
NM_133586	Ces2	1.3161	0.4616	-0.0341	-0.2	1.0975	1.139	0.34286	0.99917	0.39685	0.37693	0.32367	0.10494	0.70455	0.70171

NM_001013889	Ces3	0.2897	0.17151	0.20939	0.25759	0.04338	0.23482	0.09373	0.59155	0.11474	0.04687	0.07368	0.1447	0.15785	0.21746
NM_001012056	Ces7	-0.0988	-0.1609	-0.1674	-0.1355	0.07801	-0.1318	-0.1714	-0.0045	-0.1388	0.002	-0.1507	0.02163	0.0445	-0.0946
NM_053461	Cetn1	-0.1083	-0.0105	-0.0607	-0.0596	6E-05	0.02044	0.13316	0.08625	0.03026	0.09533	0.0019	0.04802	0.02035	-0.112
XM_001053739.1	Cetn2	0.10131	0.13198	-0.4187	-0.9554	-0.1851	-0.0073	-0.1591	-0.1687	0.18263	0.03053	0.14511	0.3226	-0.1274	-0.207
XM_342168.3	Cetn3	0.04126	-0.4458	-0.3854	0.34365	0.11265	0.11421	-0.1861	-0.0689	-0.1269	-0.1645	-0.256	-0.0452	-0.172	-0.3
XM_342234.2	Cetn4_predicted	0.12358	-0.1372	-0.0694	-0.1857	-0.0809	-0.113	-0.1153	-0.0476	0.05772	-0.0814	-0.0825	0.11889	0.00812	0.07294
NM_212466	Cfb	0.24544	0.09651	0.02138	0.05326	0.40233	0.34197	0.13495	0.55066	0.20151	-0.0208	0.01352	-0.0141	0.04947	0.13555
NM_001077642	Cfd	0.16557	0.42345	-0.2751	-0.3198	0.02292	0.03806	-0.0282	-0.139	0.40003	0.42928	0.43628	0.45804	-0.1092	-0.2404
NM_199378	Cfdp1	0.46522	0.07584	0.25908	0.30056	-0.227	-0.2941	0.28529	-0.1614	0.12352	0.0377	0.11482	0.1167	0.20813	0.05124
NM_130409	Cfh	-3.8767	-3.6066	-3.8641	-3.3962	-3.8651	-3.8293	-3.3875	-3.7342	-2.9529	-3.6657	-3.608	-3.0531	-3.9716	-3.9866
NM_001044227	Cfhl1_predicted	-0.0755	-0.2102	-0.0302	-0.1031	-0.186	-0.2086	-0.1008	-0.1017	-0.1386	-0.2219	-0.1609	-0.1775	-0.0745	-0.0253
NM_024157	Cfi	0.02426	-0.0468	-0.0304	0.05785	-0.0302	0.00777	0.02694	-0.0947	0.20811	0.14028	0.1218	-0.0419	0.11396	0.11357
NM_017147	Cfl1	-0.0194	-0.0048	0.30487	-0.1517	0.18763	-0.1145	-0.0729	0.05169	0.11424	0.15725	0.05836	0.12769	0.41058	0.51559
NM_001108982	Cfl2_predicted	-0.1347	0.03957	0.16714	0.4894	-0.1057	-0.4989	-0.0863	-0.0419	0.14185	0.09477	0.06506	0.06111	0.40591	0.30269
NM_001033864	Cflar	0.01183	-0.3841	-0.1839	-0.3816	0.1302	0.31776	-0.2843	0.25033	-0.4639	-0.3213	-0.5004	-0.2356	-0.0989	-0.2821
NM_031506	Cftr	0.01029	0.12295	-0.0305	0.20098	0.00895	0.14934	0.21229	0.15509	0.10481	0.16276	0.08467	0.08571	-0.0188	0.10322
NM_031506	Cftr	-0.1248	-0.1495	-0.0611	-0.1264	0.03767	0.008	-0.1512	-0.0834	-0.0173	0.1323	-0.0277	-0.0142	-0.1139	-0.1152
NM_053918	Cga	-0.148	-0.173	-0.2379	-0.1336	-0.2548	-0.2775	0.14233	-0.2695	-0.27	-0.1125	-0.1379	-0.2016	-0.0901	-0.0155
NM_001105900	Cggbp1_predicted	0.06251	-0.2824	0.26661	0.36911	0.8467	1.1056	0.00477	0.84489	-0.0573	-0.0904	-0.2621	0.5691	0.43717	0.47399
NM_001014028	Cgi67l	0.51771	0.61312	-0.3811	-0.7336	0.45585	0.10779	0.83929	0.46687	-0.0147	0.53341	0.27597	-1.0299	-0.0849	-0.1947
NM_012525	Cgm4	0.15656	0.08206	0.07777	0.06723	0.28983	0.27123	0.20437	0.20924	0.20958	-0.0006	-0.0253	0.279	0.05737	0.21795
XM_001059265.1	Cgn_predicted	0.30779	0.15235	-0.0031	0.10657	0.08257	0.264	0.29082	0.04158	-0.0632	0.24155	-0.0045	0.08422	0.18592	0.11574
NM_139087	Cgref1	0.04379	-0.356	-0.1185	-0.3579	-0.1889	0.25694	-0.512	0.0104	-0.1194	-0.203	-0.2587	0.59478	0.2262	0.15553
NM_053899	Cgrrf1	-0.1944	0.00828	-0.3199	0.04247	-0.4391	-0.1608	-0.1697	-0.2908	0.07421	-0.1137	0.03232	0.19404	-0.2354	-0.3031
NM_001025415	Ch25h	-0.052	-0.0067	0.05687	-0.055	-0.0417	-0.0139	0.0082	0.04252	0.00183	0.06711	0.00299	-0.057	0.00712	0.0137
XM_342497.3	Chac1_predicted	0.49455	-0.6082	-0.0881	-0.3128	0.36191	0.26562	-0.1297	0.41046	-0.5975	-0.4378	-0.7681	-0.4126	-0.0323	-0.0408
NM_019164	Chad	0.09643	0.22023	0.2657	0.15514	0.085	-0.0256	0.10131	0.25601	0.23716	0.00302	-0.0204	0.3106	0.15748	0.1834
NM_001024741	Chaf1b	-0.2301	-1.0103	0.30212	-0.7961	-0.5046	-0.4105	-0.99	-0.6848	-0.0133	-0.8601	-0.6885	0.11602	0.22678	0.16403
NM_001170593	Chat_predicted	-0.0889	0.00329	-0.0806	0.03427	0.1682	0.0948	-0.1067	0.06849	-0.0476	0.16097	-0.0434	-0.0377	-0.019	-0.1684
NM_001108369	Chchd1_predicted	0.74039	0.20242	0.62195	0.41783	0.55621	0.82972	0.30812	0.39577	0.36444	0.32187	0.4456	0.60518	0.59523	0.56099
NM_001106588	Chchd3_predicted	0.32625	-0.1442	-0.4488	-0.9824	0.07851	0.13826	-0.1703	0.10171	-0.1287	-0.2734	-0.2297	-0.1019	-0.1242	-0.1428
NM_001106588	Chchd3_predicted	0.19732	-0.2056	-0.4681	-0.6331	0.09205	0.0985	-0.372	0.01008	-0.032	-0.3144	-0.2608	-0.2473	-0.2823	-0.1899
NM_001013431	Chchd4	0.47571	-0.0437	0.11438	0.29121	0.35636	0.43317	-0.0315	0.35787	-0.0088	-0.004	0.05736	-0.1828	0.25956	0.18858
NM_001106509	Chchd5_predicted	0.39011	0.04977	-0.0732	-0.0476	0.21819	0.68381	-0.4343	0.20606	0.52326	0.19061	0.19581	0.45261	0.09271	0.22893
NM_001106608	Chchd6_predicted	0.35207	-0.6154	-0.2761	-0.7355	0.05937	0.27159	-0.9599	0.02256	-0.3362	-0.319	-0.3056	0.20592	-0.0355	-0.1813
NM_001107465	Chd1_predicted	-0.0496	0.07569	-0.1241	0.24127	-0.1232	-0.2132	0.23887	-0.0068	0.00962	0.27609	-0.1837	0.00296	0.13208	0.24457
NM_001107704	Chd1l_predicted	0.4471	0.07702	0.02601	-0.1227	0.07609	0.33405	-0.1495	-0.2152	-0.0654	-0.0649	0.11052	0.05358	-0.0434	0.05421
NM_001107523	Chd2_predicted	0.1069	0.11883	0.06323	0.55559	0.22345	0.00998	0.18147	0.02502	0.0191	0.15689	0.187	0.29397	0.21415	0.15409
XM_220602.4	Chd3	-0.3889	-0.1607	0.45535	0.20443	0.12856	-0.4729	0.33829	-0.3152	0.24199	-0.1603	0.18575	0.16734	0.29666	0.19273
XM_001063352.1	Chd4	-0.043	-0.1569	-0.1952	-0.6822	-0.0187	-0.1417	-0.0819	-0.1147	-0.1182	-0.3183	-0.2144	-0.3885	-0.2527	-0.3559
NM_001107797	Chd6_predicted	0.08678	-0.0087	-0.0429	0.01674	0.10406	-0.0562	0.17231	-0.0615	-0.0814	-0.0599	-0.0615	0.00587	-0.013	-0.1092
NM_001107797	Chd6_predicted	-0.2011	0.01039	-0.2849	-0.5071	-0.1942	-0.1557	0.02695	-0.1853	0.13092	0.09993	-0.1631	0.46925	-0.4728	-0.5029
NM_001107906	Chd7_predicted	0.11917	-0.0105	-0.0213	0.03449	0.16576	0.06585	0.15666	0.02991	-0.1576	0.10216	-0.1026	-0.2784	-0.2069	-0.0426
NM_022933	Chd8	-0.4085	-0.3814	0.21131	0.12695	-0.4212	-0.5383	0.18423	-0.634	-0.6233	-0.6664	-0.3688	-0.5712	-0.4147	-0.3463
NM_198731	Chdh	-0.0085	-0.2095	-0.1795	-0.2235	-0.1841	-0.0381	-0.0677	-0.2602	-0.1996	-0.1995	-0.1936	-0.2167	-0.1012	-0.2329
NM_080400	Chek1	0.06092	0.22646	0.10116	0.20044	0.12852	0.29339	0.08753	0.10171	-0.2784	-0.0788	0.28024	0.00482	0.43451	0.3776
NM_053677	Chek2	-0.5761	-1.1853	0.144	-0.3025	-0.8596	-0.7573	-0.3803	-0.6874	-0.4246	-1.076	-0.6386	0.07759	-0.1885	-0.2857
NM_001106064	Cherp_predicted	-0.407	-0.0601	0.50575	0.34917	-0.3462	0.0106	-0.107	-0.236	-0.339	-0.0962	-0.1504	-0.3243	-0.2327	0.00551
NM_001106064	Cherp_predicted	0.04568	0.19822	0.12945	0.03425	-0.0254	0.14041	0.42001	0.02083	0.28372	0.32132	0.22598	0.31707	-0.1514	-0.0093

NM_001108047	Ches1_predicted	-0.0294	0.28534	0.17321	-0.0828	0.01108	0.05103	0.22869	0.03378	0.05801	0.22227	0.14288	-0.0878	0.02823	0.03984
NM_001009258	Chfr	0.04949	-0.272	-0.2413	0.09474	0.04275	0.28414	0.22365	0.17184	-0.1421	-0.1452	-0.2152	-0.0607	-0.0729	-0.0919
NM_021655	Chga	-0.1722	-0.019	-0.0527	-0.1761	-0.1951	-0.1997	-0.1278	-0.0625	-0.1921	-0.0964	-0.0817	-0.1286	0.12141	-0.1012
NM_012526	Chgb	2.2746	1.045	1.1877	0.62649	1.5082	1.6258	1.6006	1.4813	0.9196	1.1159	1.8027	0.93817	0.63788	0.8345
NM_053560	Chi3l1	0.34342	-0.0278	0.05807	-0.1164	0.15759	0.01814	0.07873	0.10236	0.00132	-0.0781	0.21336	0.08032	-0.0658	0.06612
XM_227566.3	Chi3l3_predicted	-0.0427	-0.1501	-0.1494	-0.0789	-0.1476	-0.0594	0.04335	0.08503	0.25831	-0.0811	-0.0938	-0.018	-0.1241	-0.0333
XM_227567.4	Chi3l4_predicted	-0.0274	-0.2189	-0.2096	-0.2719	-0.1974	-0.2779	-0.1854	-0.1655	-0.1164	-0.12	-0.2007	-0.142	-0.1696	-0.2088
NM_001105736	Chic2_predicted	-0.1266	0.307	-0.2514	0.19321	0.38016	0.3111	0.50722	0.4785	0.30272	-0.0341	0.00339	0.13526	-0.0009	0.11566
NM_001079689	Chit1_predicted	0.72846	0.53633	-0.2082	0.22997	-0.1366	-0.0478	-0.0181	-0.0955	0.1615	0.56298	0.48493	-0.1104	-0.105	-0.1352
NM_017127	Chka	0.02931	0.05313	0.83033	1.2169	0.37453	0.55593	0.54179	0.44283	0.10168	0.12762	-0.146	0.09762	0.05341	-0.0876
NM_017177	Chkb	-0.3853	-0.33	0.77116	0.56347	-0.573	-0.5543	0.47207	-0.6833	-0.0956	-0.3969	0.02772	0.06225	-0.5549	-0.4515
NM_017067	Chm	0.24704	0.21686	0.13409	0.32918	0.37285	0.57667	0.24146	0.22049	0.35858	0.50521	0.32081	0.43893	0.11787	0.24019
NM_001025410	Chmp5	0.46544	0.36007	-0.0485	0.61185	0.54343	0.28028	0.73359	0.57379	0.08805	0.16566	0.25736	-0.1375	-0.0888	-0.1882
NM_001105856	Chmp6_predicted	0.18981	-0.262	-0.1513	-0.7218	-0.0475	-0.2142	-0.3798	-0.3244	-0.405	-0.5396	-0.3795	-0.2127	0.25371	0.14123
NM_001108872	Chmp7_predicted	-0.0472	0.26934	0.59637	0.15887	0.31654	-0.5589	0.43947	0.25511	0.22278	0.05568	0.03773	-0.2685	-0.0658	0.09291
NM_032083	Chn1	0.09847	-0.075	-0.0583	-0.0421	-0.1375	0.48199	-0.377	0.11459	0.2127	0.0595	0.10632	0.97958	0.68927	0.80693
NM_032084	Chn2	-0.1863	-0.0969	-0.2854	-0.4356	-0.1668	-0.2097	-0.2659	-0.1951	-0.2334	-0.3123	-0.2292	0.0206	-0.2595	-0.1945
NM_001105894	Chodl_predicted	0.08992	0.02461	0.1021	0.09191	0.01141	-0.0394	0.02086	0.03538	0.17945	0.01156	0.02632	-0.0696	0.17663	-0.0051
NM_001108128	Chordc1_predicted	0.32365	0.33297	0.10014	0.89678	0.67862	0.49243	0.68062	0.78892	0.19227	0.36575	0.20591	0.22055	0.52643	0.6051
NM_024139	Chp	0.17421	0.28127	0.15025	-0.3084	0.05666	0.26894	0.3304	0.20933	0.19528	0.29027	0.00551	-0.0264	0.32093	0.28653
NM_182738	Chp2	0.11201	0.04162	0.08036	0.13037	0.03168	0.11713	0.15523	0.08869	-0.018	0.06696	0.11005	0.17794	-0.0164	0.0225
NM_001005906	Chpf	-0.2846	-0.352	0.00841	0.3401	-0.2798	-0.5027	-0.1934	-0.3356	-0.3609	-0.2622	-0.403	-0.4773	-0.3673	-0.266
NM_001007750	Chpt1	-0.1245	0.05713	-0.2678	-0.2743	0.17104	0.25547	-0.2573	-0.1811	0.09709	0.06194	0.10374	-0.2813	0.24967	-0.0177
NM_001134880	Chrac1_predicted	0.19127	0.17119	0.1635	0.1269	-0.1262	0.3224	0.15774	0.23831	0.027	-0.0218	0.23075	0.38891	-0.0973	-0.1283
NM_057134	Chrd	-0.6137	-0.3199	-0.2233	-0.4451	0.4114	0.33582	-0.3805	0.35536	-0.285	-0.4003	-0.2755	-0.4471	-0.7783	-0.649
NM_199502	Chrdl1	-0.0449	0.04128	-0.0622	0.0274	-0.1064	-0.1022	-0.1777	0.03318	0.01044	0.0612	0.01453	-0.0182	0.00249	0.04043
NM_001107537	Chrdl2_predicted	-0.094	-0.1043	-0.0732	-0.058	0.21126	-0.0591	-0.0816	0.03466	-0.0245	-0.0177	0.07643	0.08735	-0.0725	0.00227
NM_080773	Chrm1	0.16805	0.08937	0.07975	0.07726	0.03715	0.01611	0.07684	0.16475	0.09994	0.22782	0.24386	0.05132	0.09091	0.08402
NM_031016	Chrm2	0.23654	0.10238	0.15821	0.04233	0.18883	0.11384	0.14527	0.09681	-0.0225	0.03632	0.07729	0.07933	0.04075	0.23328
NM_012527	Chrm3	0.17504	-0.1169	0.09452	0.26114	0.30278	0.0432	0.01421	-0.1666	0.03288	0.11796	0.03441	-0.0355	0.04787	0.08675
NM_031547	Chrm4	0.02022	-0.0758	0.04172	0.02929	-0.085	-0.0808	0.12716	-0.0347	0.05282	0.11755	-0.1336	0.47102	0.17535	-0.0664
NM_017362	Chrm5	-0.1262	0.04575	0.06996	0.06536	-0.0323	0.01105	0.0335	-0.0316	0.08422	0.11187	-0.0758	0.2193	0.09323	-0.0582
NM_024485	Chrna1	-0.0125	-0.0481	0.21182	-0.0468	-0.0731	0.08568	0.13655	0.05336	-0.0176	-0.0677	0.01165	0.11546	0.07839	-0.0545
NM_022639	Chrna10	-0.0579	0.08476	0.00804	0.10594	-0.1436	-0.0627	-0.2628	-0.0939	-0.0175	-0.1736	-0.1386	-0.3082	-0.0308	-0.0088
NM_133420	Chrna2	-0.1782	-0.165	0.14394	-0.0522	0.08246	0.32971	0.15295	0.17521	-0.339	-0.0803	0.33863	-0.0519	-0.2639	-0.251
NM_052805	Chrna3	0.04982	0.0607	-0.0687	-0.0302	0.00019	0.01817	0.01757	-0.0924	-0.0851	-0.1292	0.09815	0.13994	-0.1219	0.05982
NM_024354	Chrna4	-0.0096	0.1983	0.00666	0.03306	-0.0287	0.26998	0.01818	0.04759	-0.0569	0.06209	0.02559	0.08284	0.09722	0.04532
NM_017078	Chrna5	0.07214	0.04388	0.08678	0.19422	0.13612	0.10601	0.2094	0.17189	0.04524	0.08207	0.08481	0.09277	-0.0218	0.02127
NM_012832	Chrna7	-0.0915	-0.1329	0.01134	-0.2336	0.03715	0.03546	-0.1078	-0.0793	0.07318	-0.0934	-0.115	-0.0047	-0.3108	-0.0232
NM_022930	Chrna9	0.03275	-0.0552	0.05057	0.01722	-0.0826	-0.0788	0.12567	-0.1579	-0.1029	-0.0379	0.2305	0.05681	-0.0864	0.11348
NM_012528	Chrnb1	0.1436	-0.2286	0.25923	0.33353	0.31877	0.74107	0.08605	0.20164	-0.1653	-0.2691	0.36182	-0.0793	0.12611	0.07949
NM_019297	Chrnb2	-0.0738	-0.0069	-0.2665	-0.1575	-0.063	-0.0197	-0.0896	-0.292	-0.3097	-0.2479	0.04834	-0.2143	0.06057	-0.1762
NM_133597	Chrnb3	-0.0188	0.19348	-0.0042	-0.0305	0.15362	0.007	0.06254	0.16852	0.11729	-0.0263	0.21883	0.18992	0.03424	0.11762
NM_052806	Chrnb4	-0.1083	-0.27	-0.1038	-0.0128	-0.1509	0.39895	-0.317	-0.2169	-0.3117	-0.2689	0.08481	-0.4456	-0.1935	-0.2346
NM_019298	Chrnd	-0.2705	-0.0311	-0.1121	0.03925	0.03428	0.08974	0.06372	-0.0216	0.17716	0.21408	-0.0644	0.25221	-0.3158	-0.0069
NM_017194	Chrne	-0.0899	-0.0466	0.11342	1.6858	-0.0456	-0.0782	-0.1381	-0.037	-0.0959	-0.2022	0.022	-0.1152	-0.0062	0.00411
NM_019145	Chrng	-0.029	0.34913	-0.0314	0.14415	0.15409	0.39306	0.08362	0.18131	0.28499	0.0195	0.33556	0.1552	0.39754	0.34226
NM_001011955	Chst1	0.04651	0.09236	0.00866	0.14972	0.08741	0.12262	0.11675	0.16504	-0.015	0.3056	0.04401	0.13971	-0.0221	0.0374
NM_080397	Chst10	0.14859	-0.0925	0.03403	0.00843	-0.0763	-0.1134	-0.0655	0.06002	-0.1535	-0.3021	-0.1523	-0.1348	-0.0936	-0.0344

NM_001108079	Chst11_predicted	-0.1099	-0.0814	-0.0587	0.08745	-0.0027	-0.1596	-0.1548	-0.211	-0.1158	-0.162	0.00525	0.39132	0.08637	-0.1524
NM_001037775	Chst12	0.32068	0.55736	0.24104	0.74075	0.85494	1.015	0.7036	0.65714	0.6845	0.89837	0.53148	0.52082	0.65874	0.46367
XM_345970.3	Chst2_predicted	-0.2016	-0.1095	0.33874	-0.406	-0.2238	-0.4269	-0.0013	-0.4395	-0.0806	0.07396	-0.2426	0.13871	0.21276	0.17943
NM_053408	Chst3	-0.0374	-0.1174	-0.1654	0.03122	-0.0437	-0.0002	-0.1842	-0.0583	0.3202	-0.1704	0.029	-0.2152	0.1018	-0.0286
NM_001107427	Chst4_predicted	-0.155	-0.1458	0.04842	-0.1602	-0.0801	-0.1256	-0.1035	-0.1395	-0.0417	-0.1242	-0.1617	-0.0418	-0.0986	-0.1516
NM_207600	Chst7	-0.2168	-0.1643	-0.2152	-0.087	-0.2571	-0.1675	-0.1582	-0.1042	-0.2779	-0.2422	-0.2606	-0.1402	-0.0612	-0.2025
NM_001107504	Chst8_predicted	-0.1465	0.02887	-0.1832	-0.044	-0.1692	-0.0675	-0.2043	0.07842	-0.1598	-0.1858	-0.0098	-0.1674	-0.0948	-0.1151
NM_001107504	Chst8_predicted	-0.0727	-0.1428	-0.193	-0.0123	-0.0777	-0.1667	-0.2274	-0.2238	-0.2524	-0.1613	-0.2593	-0.2568	0.05539	-0.2466
NM_001170470	Chst9_predicted	-0.1152	-0.4056	-0.4392	-0.3322	-0.3367	-0.1018	-0.223	0.00661	-0.3469	-0.2605	-0.2761	-0.2634	-0.389	-0.1088
NM_001106268	Chsy1_predicted	-0.4411	-0.4049	-0.8045	-0.7311	-0.5131	-0.9743	-0.6558	-0.2629	-0.4159	-0.7224	-0.9198	-0.6948	-0.3243	-0.4726
NM_001107588	Chuk_predicted	0.181	0.12891	-0.0343	0.09828	0.16596	0.00098	-0.1165	0.1732	-0.0865	0.28657	-0.0616	0.12443	0.17457	0.29742
NM_001106741	Churc1_predicted	0.20799	0.05046	-0.0631	0.53473	0.56919	0.56109	-0.0827	0.14494	0.09675	0.10773	0.11842	0.18578	0.08014	0.00959
NM_001169128	Chx10	0.08969	0.18551	0.05423	0.16559	0.03578	0.35585	0.11542	0.15071	0.09142	0.15074	0.04648	0.20565	0.17504	0.16404
NM_001008766	Ciao1	0.23541	0.21924	0.18195	0.12149	0.64597	0.84051	0.28855	0.3435	0.00488	-0.0838	0.13787	0.2615	0.22704	0.29206
NM_001007689	Ciapin1	0.57179	0.70498	0.84597	0.81876	0.82255	0.54498	0.48901	0.78758	0.46094	0.79806	0.60669	0.34482	0.68512	0.75825
XM_220513.4	Cias1_predicted	0.23075	-0.0007	0.22645	0.54911	0.12729	0.15372	0.04583	0.34723	-0.1533	0.12887	-0.0073	0.44592	0.33017	0.32856
NM_031145	Cib1	0.91389	0.38851	0.49443	0.5454	1.0933	1.6343	0.6253	1.2328	0.3198	0.57905	0.32884	0.45472	0.37279	0.48856
NM_001015010	Cib2	0.0219	0.03148	0.05985	0.03105	-0.0259	0.00842	-0.0295	0.11487	-0.0402	0.00508	-0.0539	0.14015	0.00102	-0.0069
NM_001015010	Cib2	0.02323	-0.0816	-0.225	0.33135	0.7009	0.83242	-0.2214	0.70565	0.14401	-0.1353	0.04493	0.3451	-0.3038	-0.3043
NM_001107490	Cic_predicted	-0.1789	-0.1444	0.81897	0.69108	0.04372	0.07114	0.48757	0.1008	-0.2682	-0.1489	-0.2606	-0.6475	-0.332	-0.3275
NM_001170467	Cidea_predicted	1.1506	0.82921	-0.2104	0.24035	1.0441	1.4476	0.46855	1.2247	0.46109	0.57567	0.56303	0.24205	0.12877	-0.3118
NM_001108869	Cideb_predicted	-0.0354	0.00183	-0.0814	-0.2203	-0.0586	0.04085	-0.1195	-0.1535	-0.0544	-0.0687	-0.2383	-0.1631	-0.001	-0.0214
NM_001024333	Cidec	0.03302	-0.1713	0.03739	0.04513	-0.1223	-0.2059	-0.1212	-0.1114	-0.1624	0.16992	-0.0082	0.17338	0.03552	-0.1255
NM_053529	Ciita	-0.0848	-0.0342	0.06196	0.02742	0.10088	-0.0885	-0.0804	-0.0276	0.08614	-0.0898	-0.0436	-0.0613	-0.0687	-0.0559
NM_001108161	Cilp_predicted	-0.748	0.17999	-1.4311	-1.1127	-1.3358	-1.4922	-1.0896	-1.3463	0.08219	-0.2317	-0.3511	0.33726	-1.334	-1.2605
NM_001107307	Cilp2_predicted	-0.0779	-0.031	-0.007	-0.117	-0.0241	-0.0382	-0.0565	0.02544	-0.0129	-0.0297	0.08015	0.03242	0.05809	-0.1427
NM_001033070	Cip29	-0.4733	-0.0114	-0.5282	-0.4346	-0.657	-0.0473	-0.5633	-0.3552	-0.1763	-0.2698	-0.5999	0.17499	-0.566	-0.5765
NM_181088	Cip98	-0.2227	-0.255	-0.3336	-0.2135	0.06603	-0.1704	-0.1926	-0.2965	-0.2765	-0.1665	-0.1827	-0.3291	0.00374	-0.101
NM_173114	Cipar1	-0.1161	-0.3656	-0.2345	-0.3205	1.2389	1.5925	-0.2343	1.3596	-0.3689	-0.4363	-0.1889	-0.0648	0.14546	0.11966
NM_001007799	Cir	0.31188	0.24434	-0.111	0.54955	0.34676	0.71849	0.34697	0.10873	0.38972	0.09453	0.34425	0.61338	0.14393	0.101
NM_031147	Cirbp	0.12955	0.41682	-0.123	-0.6805	-0.5141	-0.1643	0.05307	-0.7087	0.45224	0.34621	0.38136	0.46767	-0.1948	-0.0889
NM_001009640	Cirh1a	0.43603	0.48286	0.71171	1.1525	0.23637	0.2658	0.64701	0.0891	0.15563	0.33386	0.53937	0.36538	0.73679	0.75622
NM_031804	Cish	-0.0513	-0.1227	0.06902	0.21734	0.06275	0.08477	-0.2326	-0.1682	-0.001	-0.1688	-0.1977	-0.0741	-0.0367	-0.1038
NM_001029911	Cit	-0.0222	0.12131	-0.133	0.03289	0.0803	-0.0298	-0.0289	-0.0019	0.22177	0.41897	-0.0325	-0.126	0.1275	-0.2703
NM_172055	Cited1	-0.1023	-0.0851	-0.1756	-0.0926	-0.047	-0.0301	-0.0543	-0.0401	-0.0972	-0.0537	-0.106	0.05948	-0.062	-0.1461
NM_053698	Cited2	1.1537	0.66515	0.43652	0.77995	0.57037	0.44932	1.4243	0.6889	0.69805	0.45871	0.78782	0.58015	1.2035	1.2482
NM_053699	Cited4	0.15068	-0.1491	0.06102	0.04053	-0.1768	0.07056	-0.0309	-0.0957	-0.0035	-0.0115	-0.1165	-0.0995	-0.0217	0.01748
NM_001106568	Ciz1_predicted	0.35101	0.58893	0.59103	0.93841	0.21351	0.30941	0.35543	0.33785	0.42203	0.3525	0.26594	0.5647	0.34755	0.27175
NM_001040180	Ckap1_predicted	0.00249	0.03065	0.11049	-0.1434	-0.024	0.2474	0.15779	-0.1835	0.10839	0.01533	0.2771	0.58459	0.18745	0.335
NM_001108740	Ckap4_predicted	-0.5702	-0.3383	-0.0819	-0.6011	-0.6506	-0.3704	-0.9994	-0.7246	-0.1765	-0.2773	-0.6451	0.50196	0.39024	0.38993
XM_001068836.1	Ckap5	-0.456	-0.023	0.29125	0.03277	-0.1181	-0.5734	-0.0874	0.00617	-0.0613	-0.1063	-0.2961	-0.0339	0.16205	0.18547
NM_012529	Ckb	-0.8097	-0.5893	-0.7982	-0.7564	-0.7113	-0.7048	-0.7024	-0.7472	-0.5937	-0.8426	-0.7859	-0.5876	-0.7459	-0.7461
NM_139111	Cklf	0.82645	0.54098	0.88033	1.0584	0.82052	0.95626	0.99593	0.61091	0.93409	0.56859	1.1277	0.92589	1.1356	1.1523
NM_001106034	Cklfsf5_predicted	-0.0889	0.16057	-0.0108	0.02357	-0.0504	0.24813	0.09677	0.05379	0.07388	0.0547	0.15349	0.22074	0.09528	0.00519
NM_012530	Ckm	0.21618	0.06848	0.2117	0.2271	0.13917	0.09568	0.18859	0.02455	0.13454	-0.0114	0.17024	0.04824	0.06007	0.01735
XM_574829.1	Ckmt2	-0.0447	-0.0211	-0.0465	0.03091	0.04957	-0.1044	0.04062	-0.0346	-0.089	0.21169	0.03138	0.18032	0.17655	-0.0948
XM_001053715.1	Clasp1	0.00228	0.14008	-0.1096	0.10191	0.0244	-0.0183	-0.0847	-0.0032	-0.0171	-0.1461	-0.0277	0.09965	0.06442	0.29651
NM_053722	Clasp2	-0.2291	-0.3957	-0.0098	0.45168	0.33799	0.29478	-0.1402	0.36313	-0.423	-0.2391	-0.3837	-0.3194	-0.2057	-0.139
NM_001013202	Clca2	0.05479	-0.0483	0.17816	0.01575	-0.0644	0.04229	-0.0029	0.11161	-0.0073	0.11527	0.04067	0.01111	-0.0122	0.04676

NM_001107450	Clca2_predicted	-0.0163	0.00098	-0.092	0.06718	0.10312	0.08998	-0.0646	0.06184	-0.079	-0.0315	-0.0881	-0.0014	-0.1376	0.18095
NM_001107449	Clca3_predicted	0.06348	0.30328	0.21852	-0.0171	0.13084	0.07725	0.08612	0.0415	0.04716	0.00742	0.17439	-0.0348	-0.0463	0.04939
NM_201419	Clca4	-0.0772	-0.1346	-0.0287	0.01539	-0.0196	0.22672	-0.2031	-0.1765	-0.2452	-0.2917	-0.1157	0.13839	-0.165	-0.0573
NM_133414	Clcc1	-0.1654	-0.1234	-0.0775	-0.21	-0.2852	-0.118	0.03325	-0.5993	-0.1339	0.04971	0.10704	-0.066	-0.5225	-0.4669
NM_207615	Clcf1	0.17204	0.40141	0.61989	0.03191	-0.2176	-0.2936	0.3041	-0.1394	0.58823	-0.1051	-0.0567	-0.1277	0.45336	0.21775
NM_013147	Clcn1	0.01212	-0.2349	-0.0633	-0.0542	-0.1531	0.25335	-0.0445	0.1592	-0.0375	-0.0355	-0.2023	-0.0671	0.11558	0.02686
NM_017137	Clcn2	-0.3935	-0.3364	0.01411	0.18942	-0.2256	-0.1217	0.2352	-0.0273	-0.3491	0.02426	-0.0645	-0.0077	-0.2912	-0.2807
NM_053363	Clcn3	0.16296	0.21617	0.14454	-0.1297	0.3215	0.3476	0.19352	0.40045	0.00041	0.04986	0.24748	-0.3043	0.2129	0.24715
NM_022198	Clcn4-2	0.07308	0.13306	0.00264	-0.1953	0.01542	-0.0832	0.1148	-0.0575	0.29855	0.24195	-0.111	0.16299	-0.0894	-0.1224
NM_017106	Clcn5	-0.0482	-0.0865	-0.0644	-0.0624	0.10038	-0.1612	-0.0072	0.06179	-0.0778	-0.1027	-0.1601	-0.2123	-0.0757	0.04174
NM_001106479	Clcn6_predicted	0.24799	-0.1811	-0.0368	0.0543	-0.0528	-0.1192	0.0539	0.03151	0.11754	-0.0527	-0.008	-0.0535	-0.1869	-0.1053
NM_031568	Clcn7	0.00576	0.0601	0.30605	0.2418	-0.0127	-0.1303	0.33751	0.1357	-0.265	-0.0277	0.08193	-0.2028	0.02651	0.08131
NM_053327	Clcnka	0.03243	0.12591	0.16871	-0.047	-0.0711	-0.0646	-0.117	-0.1143	0.11816	0.08798	-0.093	-0.0032	-0.0943	0.06058
NM_173103	Clcnkb	-0.017	0.67136	0.08653	0.59472	0.26853	-0.0048	0.30887	0.15064	0.59902	0.79464	0.28261	0.20693	-0.3667	-0.5112
NM_031699	Cldn1	-0.0803	-0.0464	-0.0431	-0.1173	0.22509	0.25005	0.10616	0.16035	0.22155	-0.0214	0.18824	-0.1022	-0.0957	0.07309
NM_001106058	Cldn10_predicted	-0.1303	0.04002	-0.1369	-0.1363	-0.0443	-0.1231	0.05553	0.02209	-0.1263	-0.15	0.09442	-0.184	0.14315	-0.0288
NM_053457	Cldn11	0.16066	0.23847	-0.0409	-0.0529	-0.0396	0.04358	0.0245	0.02643	0.08947	-0.0051	0.07065	0.26186	0.03729	0.1667
NM_001100815	Cldn12_predicted	0.68841	0.03265	0.21664	0.78717	0.71995	0.81302	0.2342	0.86757	-0.0013	0.05091	-0.0743	0.2032	0.35093	0.5348
NM_001107135	Cldn15_predicted	-0.1967	-0.1088	-0.0927	-0.1074	-0.1396	-0.1527	-0.1768	-0.0849	-0.0082	-0.0457	-0.1277	-0.0326	-0.1454	-0.1096
NM_131905	Cldn16	0.00307	-0.0828	0.0913	-0.0752	-0.0202	0.26374	0.00148	-0.0315	-0.0738	0.16112	0.10723	-0.0112	0.07375	0.00668
NM_001107112	Cldn17_predicted	-0.0584	-0.0148	0.07389	-0.2125	0.12698	0.28051	-0.0661	-0.1133	-0.1266	-0.046	-0.0049	-0.098	0.03418	-0.2565
NM_001008514	Cldn19	0.11439	-0.0025	0.16976	0.21565	0.10696	0.35521	0.06366	0.19046	0.09405	0.03962	0.2023	0.40147	0.32406	-0.1103
NM_001106846	Cldn2_predicted	-0.1231	0.15131	-0.1481	-0.0588	-0.0148	-0.0849	-0.1287	-0.1533	-0.0606	-0.0193	-0.0927	-0.1686	-0.2042	-0.1334
NM_001110143	Cldn22_predicted	-0.161	0.13694	-0.3197	-0.1041	0.09527	-0.0731	-0.2303	-0.0723	-0.2935	-0.2432	-0.0002	-0.1861	-0.1466	-0.2514
NM_031700	Cldn3	-0.1655	-0.0361	-0.1597	-0.0085	-0.2039	-0.1135	-0.1142	-0.165	-0.0264	0.02197	-0.1687	0.14056	0.21709	-0.0241
NM_001012022	Cldn4	-0.0375	-0.0288	0.00067	0.24937	0.31808	0.09	0.03006	0.02152	-0.117	0.35732	0.06512	-0.0932	0.06315	0.10308
NM_031701	Cldn5	-0.1743	-0.0772	-0.0196	-0.0064	-0.1131	0.29962	0.13149	0.09197	0.00799	-0.0048	-0.0512	0.31225	0.05651	-0.0553
NM_001102364	Cldn6_predicted	-0.2674	-0.2262	-0.1858	-0.1366	-0.3274	0.00521	-0.3487	-0.1662	-0.1167	-0.2661	-0.2366	-0.2255	-0.1886	-0.3251
NM_001037774	Cldn8	0.02921	0.17871	0.05315	0.07443	0.03064	0.04798	-0.061	0.31795	-0.0754	0.02777	-0.0733	0.0052	-0.058	0.05031
NM_001011889	Cldn9	0.17275	0.05371	0.20663	0.48041	0.11979	0.11265	0.13662	0.12822	-0.0068	0.01681	0.08536	0.0677	0.08547	0.48353
NM_001006955	Cldnd1	-0.4604	-0.2929	-0.6981	-0.1798	-0.2407	-0.2583	-0.7019	-0.3992	-0.3779	-0.2633	-0.2857	-0.3443	-0.8178	-0.8374
NM_001012459	Clec11a	0.18616	-0.0967	0.20736	0.15052	0.03287	-0.0524	0.07784	0.1694	-0.0396	-0.0893	0.14704	0.00068	-0.0944	0.07957
NM_001014077	Clec14a	-0.0432	0.04203	0.05552	0.11135	-0.165	0.33693	0.0051	-0.0858	0.01316	0.0161	-0.1627	0.19442	-0.0238	-0.1752
NM_001005890	Clec4a1	0.03621	0.06221	0.18537	0.05426	0.15527	0.07569	0.11746	0.23848	0.04838	0.10873	0.13465	0.09623	-0.0759	0.1043
NM_001005891	Clec4a3	0.01655	-0.0622	-0.1091	0.47651	0.04713	-0.049	0.23486	0.05061	-0.0092	0.09855	-0.2307	0.08841	-0.1861	-0.1048
NM_001005896	Clec4b2	0.01153	0.11621	0.00458	-0.0327	0.17242	-0.1147	0.03629	0.10159	-0.0115	0.13046	0.1428	-0.0144	-0.0063	-0.0476
NM_001003707	Clec4d	-0.0882	0.10151	-0.0546	0.0996	-0.0737	-0.0587	-0.0116	-0.094	-0.0635	-0.0196	-0.0163	-0.0806	-0.0061	0.03551
NM_001005897	Clec4e	-0.1722	-0.0658	-0.0728	-0.0786	0.06864	-0.0093	0.00916	-0.1638	-0.0727	-0.0873	0.03743	-0.1248	-0.0983	-0.0698
NM_001108899	Clecsf1_predicted	0.20361	0.09257	-0.0712	-0.0899	0.1902	-0.0858	-0.0532	0.06014	-0.0674	0.16698	0.11898	0.01704	-0.0652	-0.0428
XM_001067454.1	Clecsf2_predicted	0.15525	0.03576	0.0958	0.36608	0.20949	0.28295	0.18479	0.22807	0.06465	0.27462	0.0672	0.14262	0.26692	0.16168
NM_001002807	Clic1	-0.0155	-0.3679	0.1433	-0.5907	-0.147	0.56286	-0.3553	0.03934	-0.2402	-0.5301	-0.2261	0.39898	0.16847	0.13215
NM_001009651	Clic2	1.411	1.6738	1.2905	1.2801	1.002	0.91515	1.769	1.0946	1.5925	1.4842	1.8096	1.0577	0.53541	0.4275
NM_001013080	Clic3	0.17217	-0.0854	0.27977	0.11375	0.01567	0.38998	-0.0787	0.03545	0.16165	0.08105	0.31154	0.10539	-0.0201	0.16605
NM_031818	Clic4	0.20032	0.37524	-0.5332	-1.2324	0.37904	-0.2462	-0.2738	0.4648	0.2426	0.60963	-0.0955	-0.2136	0.90519	0.72165
NM_053603	Clic5	1.5003	0.00711	-0.6156	-0.6308	1.0984	1.123	0.61413	0.85513	-0.7139	-0.1928	-0.0122	-2.4023	-0.1336	0.23243
NM_176078	Clic6	0.02973	0.04026	0.03323	0.04574	0.00068	-0.0009	0.06849	0.07235	0.10272	0.052	0.06152	0.02864	0.23529	0.12864
NM_031745	Clip1	-0.5342	0.38603	-0.6118	-0.6114	-0.4919	-1.231	0.03268	-0.4243	0.41227	0.40438	0.39413	-0.4494	-0.1078	-0.1621
NM_021997	Clip2	-0.2082	-0.133	0.25734	0.00586	0.06667	-0.1323	0.06375	0.24997	-0.1608	-0.2745	0.04047	-0.4089	0.25993	-0.0019
NM_001106913	Clk1	-0.692	-0.3106	0.07077	1.0153	-0.8732	-0.8617	0.65584	-0.7299	-0.5826	-0.4234	-0.5177	-0.5256	-1.0735	-1.0747

NM_001014254	Clk2	-0.6672	-0.6577	-0.0412	0.21789	-0.9665	-0.8381	0.03771	-0.8616	-0.5449	-0.5732	-0.3615	-0.4092	-0.9891	-0.7157
NM_134340	Clk3	-0.2479	-0.4402	0.04456	0.17217	-0.2133	-0.1828	-0.0028	-7E-05	-0.2741	-0.3191	-0.2867	-0.0077	-0.2421	-0.2421
NM_001013041	Clk4	-0.7232	-0.2889	0.02194	0.60287	-0.6758	-0.9606	0.24634	-0.5695	-0.3435	-0.1906	-0.2514	-0.5428	-0.8532	-0.6846
NM_001106755	Clmn_predicted	0.19595	0.11969	0.12123	0.17416	0.14787	0.13801	0.22069	0.02401	0.01745	0.18495	-0.0294	0.04787	0.00682	0.04569
NM_001006971	Cln3	0.14092	0.3319	-0.0315	-0.0231	-0.1545	-0.1856	0.51958	-0.2632	0.30111	-0.0323	0.31442	0.12145	-0.1198	-0.0846
XM_224477.3	Cln5_predicted	-0.1855	-0.0245	-0.1685	-0.0406	-0.1594	-0.0847	0.02622	-0.1598	-0.1214	-0.1663	0.01042	0.01131	-0.04	-0.0355
XM_236325.3	Cln6_predicted	-0.7027	0.11791	-0.5657	-1.6926	-1.0156	-0.5242	-0.1368	-1.0239	0.69036	-0.0321	0.04224	1.4798	-0.7917	-0.6802
NM_001007686	Cln8	0.17802	0.03909	-0.1073	-0.4346	0.34847	0.47274	-0.1496	0.19712	0.26418	0.21284	0.14542	0.46458	0.20202	-0.0723
NM_031719	Clns1a	0.25685	0.37533	-0.0047	0.29003	0.32955	0.33753	0.28041	0.401	0.09664	0.24822	0.19629	0.37511	0.28316	0.304
NM_021856	Clock	0.21612	0.25148	0.17088	0.39788	0.2827	0.38452	0.15052	0.27729	0.39235	0.43128	0.22351	0.2232	0.3225	0.29861
NM_001025136	Clp1	0.50984	0.59073	0.23629	0.64016	0.85395	0.73679	0.57826	0.83628	0.46424	0.53172	0.59819	0.36813	0.53929	0.49348
NM_022947	Clpb	0.5562	0.18868	0.25938	0.51022	0.81633	1.0143	-0.1669	0.9242	-0.0109	0.24202	0.2458	-0.0695	0.16774	0.1427
NM_013139	Clps	0.15684	-0.1256	0.07044	-0.0278	-0.1735	0.08654	0.00911	0.19208	-0.0638	-0.071	-0.1037	0.17898	-0.1667	-0.1193
NM_001106232	Clptm1_predicted	-0.08	0.11097	0.20749	0.33893	-0.0919	0.05523	0.31684	-0.1656	-0.0988	0.15138	0.18464	-0.2631	0.04524	0.25524
NM_001007803	Clpx	0.0537	0.00929	0.10877	-0.0549	0.06174	0.07814	0.24063	0.24314	0.09546	-0.0236	0.01194	0.13359	0.04051	0.05529
NM_001007803	Clpx	0.29476	0.02885	-0.2745	-0.031	0.27077	0.18983	0.08364	0.47879	-0.001	-0.1436	0.04768	0.01981	-0.0481	0.05392
NM_001014026	Clrn3	-0.0704	-0.1415	-0.1412	0.08844	-0.1211	-0.1254	-0.1409	-0.0336	0.02506	-0.0793	-0.0034	-0.1431	-0.107	0.00893
NM_001106687	Clspn_predicted	-0.2786	-0.3206	-0.0466	-0.0473	-0.3297	-0.3156	-0.2086	-0.4394	-0.1628	-0.1384	-0.213	-0.1883	-0.1331	0.22434
XM_243040.4	Clstn1	-0.4639	0.33487	-0.4839	-0.329	0.04105	-0.3728	-0.2398	0.17251	0.09609	0.1717	0.1525	-0.2401	0.04107	0.10246
NM_134377	Clstn2	-0.1593	-0.1762	-0.3747	-0.3885	-0.2839	-0.1216	-0.3754	-0.2021	-0.3295	-0.3189	-0.1866	-0.3228	-0.2388	-0.2518
NM_031974	Clta	0.0793	0.09241	-0.2458	-0.6646	-0.0662	0.22099	-0.1707	-0.1385	-0.0218	0.01992	0.08302	0.54022	0.0504	0.10326
NM_053835	Cltb	-0.2332	-0.4072	-0.2441	-0.8479	0.24024	0.2936	-0.6	0.05454	-0.237	-0.3453	-0.1239	-0.5294	-0.2547	-0.2283
NM_019299	Cltc	-0.2163	-0.1116	-0.2324	-0.3333	-0.3167	-0.4737	-0.1484	-0.2426	-0.4201	-0.1732	-0.3309	-0.3658	-0.1317	-0.1679
NM_053021	Clu	-0.8798	1.0894	-0.3871	1.0513	0.05137	-0.0506	0.85837	-0.006	0.89676	1.0428	0.90834	-1.3746	-1.2834	-1.4616
NM_001033071	Clul1	0.13103	0.0473	0.10877	-0.0222	0.04739	0.01613	-0.0242	0.1211	0.02677	0.08074	-0.0067	0.0904	0.02419	0.03831
NM_001100685	Clybl_predicted	0.08003	0.06874	-0.1277	0.05483	0.03886	0.05595	-0.0447	-0.0584	-0.0932	-0.0865	-0.051	-0.1015	-0.0339	0.10258
NM_013092	Cma1	0.12213	0.09239	0.114	0.018	0.15903	0.08396	0.06612	0.10217	-0.0171	0.02206	-0.0571	0.00049	0.09346	-0.0121
NM_001024273	Cmah	0.07872	0.15625	0.42553	0.1935	-0.0526	0.10436	0.22878	0.04311	0.10959	0.04628	0.03308	0.05533	0.12325	0.08445
NM_001009419	Cmas	0.38707	-0.1173	-0.0765	0.52492	0.17766	0.21961	-0.1724	0.10785	-0.077	-0.093	-0.1059	-0.2413	0.14522	0.06502
NM_022218	Cmklr1	0.28461	0.18136	-0.044	-0.1457	-0.153	0.02044	-0.1563	-0.0358	0.08542	-0.2169	0.18875	0.27158	0.12937	0.00468
NM_133558	Cml1	-0.1329	-0.3436	-0.1649	-0.1843	-0.2188	-0.0625	-0.1318	-0.1594	-0.2159	-0.2065	-0.4371	-0.0253	-0.071	-0.0987
NM_021668	Cml2	-0.1139	-0.0094	0.18716	-0.0882	-0.2986	-0.1462	-0.1471	-0.2113	-0.0655	0.02316	-0.3136	-0.0605	-0.1458	-0.139
XM_001074100.1	Cml3	0.13413	0.32669	0.1669	0.09427	0.08418	0.4202	0.13252	0.07269	0.01154	-0.1513	0.15536	-0.0317	0.08712	-0.0317
NM_022635	Cml4	-0.0594	#######	-0.1726	-0.175	-0.1156	-0.0065	-0.1209	-0.112	-0.0021	-0.0557	-0.2042	0.09476	-0.0965	-0.1038
NM_001025655	Cmpk1	-0.3107	-0.0832	-0.3818	-0.522	-0.4289	-0.4753	-0.5505	-0.3717	-0.1689	-0.1364	-0.0473	-0.1341	-0.3544	-0.3443
NM_001029914	Cmtm1	0.01407	-0.2729	-0.2365	-0.0711	-0.0989	-0.2791	-0.3235	0.09987	-0.3817	-0.337	-0.0892	-0.0357	0.05177	-0.0765
NM_001013142	Cmtm2a	-0.0464	-0.0881	-0.2343	-0.0752	-0.1524	-0.1729	0.06952	0.02272	-0.2962	-0.1899	-0.159	-0.0611	-0.0979	-0.1484
NM_001106164	Cmtm3_predicted	-0.2336	0.04486	-0.1768	-1.4179	-0.9042	-0.6867	-0.116	-1.1219	0.08257	0.17057	0.26039	0.56824	0.11825	0.12705
NM_001007802	Cmtm6	-0.0624	0.07341	-0.4114	-0.4206	-0.2472	-0.5234	-0.3755	-0.0657	-0.071	0.04565	-0.502	-0.6283	-0.1146	-0.2077
NM_001007802	Cmtm6	-0.3219	-0.405	-0.1784	-0.0418	-0.1929	-0.0978	-0.3002	-0.243	-0.3067	-0.2651	-0.1838	-0.0701	-0.2247	-0.2606
NM_198754	Cmtm8	-2.4997	-2.4221	-2.2802	-2.5697	-2.6025	-2.5788	-2.2	-2.2854	-2.4097	-2.6584	-2.5414	-2.1986	-2.4224	-2.3347
XM_236702.4	Cmya1_predicted	-0.0312	0.09721	0.23893	-0.0659	0.0146	0.19585	-0.0123	0.24156	0.14782	0.04323	-0.0525	0.14575	0.1552	0.06003
NM_022598	Cnbp	0.10066	-0.2519	0.17454	0.06926	-0.162	-0.4451	0.01615	-0.0533	-0.4452	-0.2066	-0.3256	-0.2196	0.13635	0.02775
NM_001007687	Cndp1	-0.2154	-0.2253	-0.103	-0.5585	0.22009	0.11447	-0.3991	0.03256	-0.0631	-0.2819	-0.3268	0.41454	0.04798	0.16431
NM_001010920	Cndp2	-0.0112	-0.0291	-0.0124	-0.0261	-0.002	-0.045	-0.0863	0.01548	0.01083	0.02996	0.11061	0.09653	-0.0422	0.26222
NM_001010920	Cndp2	0.33482	0.54258	0.90944	0.59298	0.7357	0.81397	0.37002	0.60096	0.26089	0.37614	0.4194	0.45206	0.5281	0.76891
NM_053497	Cnga1	-0.054	0.10588	0.09057	0.34661	-0.0547	-0.069	0.03119	0.05089	0.04976	0.00804	-0.1103	0.32138	0.14726	0.08298
NM_012928	Cnga2	0.16049	-0.0705	-0.1404	0.02676	-0.0605	-0.0209	-0.0889	-0.2533	-0.0302	0.01082	-0.096	-0.0309	-0.1729	-0.0585
NM_053495	Cnga3	-0.1125	0.0743	-0.1461	-0.1823	-0.2273	-0.1658	-0.1886	-0.0493	-0.0626	-0.038	-0.0405	-0.0977	-0.1862	-0.0944

NM_001106029	Cnih_predicted	0.00837	-0.1807	-0.1733	-0.2674	-0.7127	-0.8009	-0.342	-0.4426	-0.1221	-0.1305	-0.2794	-0.3473	-0.2698	-0.2337
NM_001025132	Cnih2	0.20748	0.55606	0.28173	-0.0341	0.33659	0.49825	0.39951	0.26473	0.08048	0.29867	0.36542	0.49716	0.31437	0.08355
NM_001039011	Cnksr1	0.07005	0.16426	-0.1542	-0.1873	-0.0683	-0.1803	-0.0727	-0.0656	-0.048	0.08281	-0.1754	-0.2704	-0.1499	-0.2705
NM_021686	Cnksr2	0.10288	-0.0127	0.01602	0.02153	-0.0202	0.0251	0.03037	0.05057	0.05179	-0.075	0.15898	0.0329	0.11183	0.15598
NM_001012061	Cnksr3	-1.4164	-2.3228	-2.2145	-1.7399	-0.776	-0.5779	-1.7204	-0.5177	-1.7935	-2.2271	-1.9952	-1.3134	-1.8898	-1.9006
NM_031747	Cnn1	-0.0328	-0.2126	0.12823	-0.044	0.01117	-0.1164	0.05171	0.11768	0.32328	0.03981	0.12038	0.07479	-0.1742	0.00759
NM_001107593	Cnnm1_predicted	-0.2758	-0.2971	-0.3166	-0.1932	-0.2276	-0.2657	-0.1624	-0.2957	-0.1694	-0.0271	-0.1162	-0.2389	-0.1933	-0.2073
NM_001011942	Cnnm2_predicted	-0.0149	0.24741	-0.1331	-0.4158	-0.1963	-0.4774	0.34264	-0.3825	0.17368	0.14892	0.11473	-0.3531	-0.0964	0.13529
NM_001106901	Cnnm3_predicted	-0.0476	-0.1468	0.2909	0.13291	0.10071	-0.127	-0.2575	-0.1319	-0.0606	-0.1832	-0.097	-0.0447	0.25683	-0.1136
NM_001106901	Cnnm3_predicted	-0.2522	-0.1702	0.38603	0.06402	-0.2354	-0.0807	0.16698	-0.1996	-0.3233	-0.324	-0.0155	-0.0967	-0.1194	0.00625
NM_001100766	Cno	0.14008	-0.06	0.2903	0.07291	0.00657	-0.0865	-0.0256	0.10599	-0.0112	0.02521	-0.0556	0.08275	0.22479	0.31566
NM_001007003	Cnot10	-0.2235	-0.179	0.11932	0.64423	-0.284	-0.2606	0.16611	-0.2708	-0.16	-0.1097	-0.0154	-0.2204	-0.198	-0.4771
NM_001011988	Cnot2	-0.1277	0.30687	-0.1039	0.2137	0.06878	-0.3251	0.18256	0.18547	-0.219	0.13002	-0.067	0.02032	0.25378	0.1502
NM_001011988	Cnot2_predicted	-0.4022	-0.1826	0.10335	0.41924	-0.3055	-0.3688	-0.0374	-0.4839	-0.0859	-0.2414	-0.0366	0.13008	-0.0552	-0.0959
NM_001107471	Cnot3_predicted	-0.2856	0.12087	0.15643	0.03185	-0.2386	-0.1038	-0.0579	-0.0292	-0.081	-0.0105	0.06037	0.01885	-0.2791	-0.2356
NM_001013856	Cnot6	0.05539	0.34661	-0.4436	-0.3156	0.15042	-0.4626	-0.0722	0.07763	0.30538	0.3987	0.23561	-0.213	0.25686	0.16174
NM_001108355	Cnot6l_predicted	-0.3313	-0.5002	-0.2404	-0.3632	-0.4641	-0.362	-0.3663	-0.4322	-0.2424	-0.4154	-0.5159	-0.3971	-0.2292	-0.2947
NM_001107313	Cnot7_predicted	-0.0262	-0.1711	-0.243	0.60891	-0.5276	-0.556	-0.0082	-0.3699	0.08469	-0.1449	-0.1253	-0.0302	-0.1714	-0.4027
NM_001008382	Cnot8	-0.4268	-0.1518	-0.1439	0.29691	-0.1949	-0.1645	0.00723	-0.0689	-0.1267	0.03407	-0.0884	-0.0669	-0.4684	-0.5067
NM_012809	Cnp	0.53901	0.57042	-0.1444	0.11375	0.73538	0.72067	0.24264	0.71639	0.28092	0.4249	0.29609	0.12361	0.33445	0.40596
NM_012784	Cnr1	0.13718	-0.0642	-0.1224	0.04521	-0.1064	-0.1163	0.02386	0.16522	0.02425	-0.1443	0.03524	-0.1289	0.11477	0.06132
NM_020543	Cnr2	0.00061	-0.0623	0.0085	-0.0036	0.01407	0.00142	0.13152	0.07757	0.01765	-0.0046	-0.0209	0.06089	0.06796	-0.047
NM_001014232	Cnrip1	0.36232	-0.0201	0.12618	0.03346	0.16269	0.17126	0.13728	0.20907	-0.0301	0.11893	0.09787	0.1549	0.03754	0.17382
NM_013166	Cntf	-0.0605	0.19566	0.27038	0.25029	-0.0761	-0.0018	0.27025	-0.0807	-0.002	0.04545	0.04496	0.07508	-0.1499	-0.06
NM_001003929	Cntfr	-0.1077	0.16094	0.0433	0.08709	0.09071	0.32286	0.21434	0.073	0.19573	-0.0011	-0.0617	0.02408	0.11006	0.0354
NM_057118	Cntn1	0.11266	0.25185	0.31326	0.14784	0.08238	0.09479	0.00621	0.02728	0.38444	-0.0228	-0.0745	0.11892	0.05272	0.11968
NM_012884	Cntn2	-0.0236	-0.097	-0.0494	-0.0963	-0.0657	-0.1304	-0.167	-0.0721	-0.0959	-0.1906	-0.1045	0.00505	-0.1196	-0.1027
NM_053879	Cntn4	-0.069	0.10071	0.106	0.03009	0.09111	0.03052	0.03966	0.16819	-0.0176	0.10133	0.00986	0.10662	-0.0208	-0.0462
NM_053746	Cntn5	0.07247	0.04774	-0.1079	-0.1772	-0.0626	-0.1443	-0.0037	-0.0119	-0.091	-0.119	-0.1244	-0.1569	0.06734	-0.1214
NM_001107432	Cntnap4_predicted	0.37759	0.35765	0.22487	0.23247	0.2006	0.08102	0.27571	0.29711	0.18995	0.11141	0.15573	0.25227	0.15555	0.19634
NM_001134645	Cntrob_predicted	-0.5162	-0.2324	0.67494	0.01548	-0.4571	-0.711	-0.2504	-0.5808	-0.3623	-0.24	-0.1056	0.24994	-0.2947	-0.0623
NM_001006954	Coasy	-0.0346	0.08198	0.16348	0.34207	-0.0088	0.12224	0.24723	0.01566	0.07384	-0.0415	0.0645	0.2163	0.11033	-0.0103
NM_001107236	Cobl_predicted	0.10661	0.65386	0.06147	-0.2577	0.3183	0.19439	0.25682	0.47241	0.00669	0.15164	0.22325	-0.2146	0.00782	0.36226
NM_001107733	Cobll1_predicted	0.35287	0.42137	0.19129	0.14043	0.39327	0.24103	0.19883	0.3637	0.22377	0.34996	0.23878	-0.0879	0.48834	0.41203
NM_001108710	Coch_predicted	-0.019	-0.0519	-0.1251	-0.1719	0.0392	0.11758	-0.0124	-0.095	-0.0279	-0.1108	-0.0229	0.20795	-0.0067	0.00835
NM_001107062	Cog1_predicted	-0.7265	-0.312	0.05493	-0.9076	-0.8234	-0.7346	-0.3837	-0.8166	-0.0967	-0.2164	-0.1152	0.08757	-0.1904	-0.3294
NM_001012157	Cog3	-0.0423	0.09129	0.09494	0.03552	-0.0878	-0.1807	0.04107	-0.1671	0.05501	0.17722	-0.0339	-0.1594	0.13797	0.00669
NM_001004262	Cog6	0.14537	0.21352	-0.1162	0.7571	0.29298	-0.0993	0.25044	0.34911	0.20559	0.12698	-0.1245	-0.1159	-0.0971	-0.1704
NM_001033889	Cog7	0.09857	0.12069	-0.1112	-0.3885	-0.3243	-0.0665	-0.2152	-0.2621	-0.0763	0.26	0.09351	0.17198	0.06588	-0.006
NM_001106182	Cog8_predicted	-0.2533	-0.1002	0.06177	0.16328	-0.1786	0.29068	-0.0117	-0.1001	-0.315	0.1476	-0.0414	0.25435	-0.0695	-0.095
NM_001134886	Cohh1_predicted	-0.0562	-0.0736	0.10212	0.12201	0.07124	0.05607	-0.1153	-0.0396	-0.0464	0.07748	-0.0027	0.03628	0.10233	0.01106
NM_017360	Coil	-0.0394	-0.0674	-0.0274	0.27721	0.06354	0.16387	0.03422	-0.0926	-0.0806	-0.0934	0.27	0.00928	0.16233	0.14751
XM_342325.3	Col11a1	0.23063	0.01884	0.11602	0.15948	0.12245	0.02184	0.02912	0.21085	0.12983	0.23867	0.21345	0.2202	0.10279	0.11001
XM_342325.3	Col11a1	0.44908	0.01397	-0.4049	0.81017	0.13869	-0.16	-1.3284	0.10072	0.60297	0.06876	-0.1576	1.005	0.22306	0.03799
NM_212528	Col11a2	-0.2318	-0.0905	0.49616	0.0312	0.07004	0.04458	0.16242	0.13356	-0.0275	-0.0467	-0.0851	0.12976	0.39032	0.52561
XM_243912.4	Col12a1	-0.8576	-0.2708	0.13472	-0.5229	-0.892	-0.8341	-0.6649	-1.1	0.34629	-0.0455	-0.5519	0.89236	-0.5936	-0.7083
NM_001130548	Col14a1_predicted	-0.586	-0.6265	-0.7174	-0.6827	-0.6523	-0.7072	-0.5497	-0.7159	0.15831	-0.5877	-0.6682	1.009	-0.6964	-0.541
XM_001066530.1	Col15a1	-0.1022	0.12589	-0.0456	0.06761	-0.0031	-0.0558	0.19561	-0.0556	-0.0452	0.19922	-0.0219	-0.1157	0.05894	-0.0176
NM_001015033	Col16a1	-0.1073	0.57848	0.19817	0.2371	1.1116	0.7738	-0.2621	0.86068	0.60088	0.46381	0.58583	0.90182	-0.4822	-0.2304

NM_001106366	Col17a1_predicted	0.39708	0.55614	0.27981	0.44103	0.55043	0.60042	0.2758	0.4885	0.20338	0.38604	0.52243	-0.0107	0.29956	-0.0082
NM_053489	Col18a1	2.1408	1.8789	1.3821	2.8425	2.4761	2.3911	1.4308	2.6976	1.9658	1.9572	1.7931	0.89383	0.93601	0.83966
XM_001072067.1	Col19a1_predicted	0.03827	0.14191	-0.0866	0.07099	-0.0467	-0.0938	-0.0263	0.06239	-0.0354	0.1841	0.03848	-0.0289	-0.1487	-0.1129
NM_053304	Col1a1	-3.3673	-1.663	-0.578	-2.2439	-1.8272	-1.8086	-2.3953	-1.6337	-0.7666	-1.7023	-1.9405	-0.0786	-1.9355	-1.6421
NM_053356	Col1a2	-0.0648	-0.0763	0.12116	0.02564	-0.0517	0.04053	0.34516	0.12367	-0.2529	-0.0778	0.06454	0.23305	-0.0926	-0.109
NM_181636	Col23a1	-0.0082	0.33582	0.0247	0.06347	0.04905	-0.0525	-0.0727	-0.0862	0.10448	0.56461	0.30086	0.17474	-0.1984	0.0439
NM_198747	Col27a1	0.3274	0.29182	0.53933	0.82569	0.21989	0.5572	1.4306	0.41023	0.14507	0.33612	0.74675	0.35404	0.39335	0.13444
NM_012929	Col2a1	-0.0218	0.03413	0.1276	0.04786	0.11509	0.19349	0.01283	-0.0347	-0.0051	-0.0419	0.01122	-0.0279	-0.0028	-0.0195
NM_032085	Col3a1	-0.9619	1.1174	0.62802	0.87177	-2.4613	-2.3884	0.84649	-2.5522	0.72255	0.94373	0.9516	0.7893	-0.1545	-0.201
NM_001135009	Col4a1	-0.9682	-0.2364	-0.7511	0.32067	0.36335	0.65428	-1.7112	0.31123	-0.262	-0.0588	-0.2849	-0.0336	-0.435	-0.5908
XM_225043.4	Col4a2_predicted	-0.3117	-0.2579	-0.3252	-0.0107	-0.1114	-0.3692	-0.6025	0.02269	-0.1846	-0.0163	-0.183	-0.3797	-0.1583	-0.1447
NM_001108935	Col4a3bp_predicted	-0.0477	0.47465	-0.0297	0.2328	0.15565	-0.2905	0.80258	0.25589	0.1448	0.50369	0.49212	-0.4561	0.37845	0.41222
NM_001008332	Col4a4	-0.0458	-0.1713	-0.3358	-0.197	-0.3076	-0.0201	-0.1874	-0.114	-0.1939	-0.2979	-0.1991	-0.2394	-0.2159	-0.2668
XM_343778.3	Col4a5_predicted	-0.0402	0.16748	0.00494	0.07076	-0.0113	0.10316	0.09108	0.08616	0.20789	0.11585	0.05803	0.02775	0.00619	0.04359
NM_134452	Col5a1	-1.6054	-0.9769	-0.3106	-0.942	-0.7281	-0.9831	-1.4334	-0.6884	-0.946	-0.9526	-1.1249	-0.6939	-1.1834	-1.0253
NM_053488	Col5a2	-0.1035	0.1768	0.36776	0.78845	-0.317	-0.7331	0.41905	-0.1699	0.03358	0.14676	0.23002	0.30442	0.26875	-0.0527
NM_021760	Col5a3	-0.0802	-0.2143	-0.0863	-0.0389	0.02281	-0.0823	-0.0568	0.21214	-0.1629	-0.0994	0.11279	0.20927	-0.2227	-0.2535
XM_215375.4	Col6a1_predicted	1.496	1.0326	1.5708	1.2319	1.4166	1.1412	0.97931	1.372	0.89134	1.2395	1.0535	0.89792	0.95088	0.72286
NM_001100741	Col6a2_predicted	0.123	0.11416	0.10159	-0.0345	0.0087	-0.1426	0.02468	-0.1389	-0.0479	-0.0419	-0.2578	0.01357	-0.1581	0.01911
NM_001109008	Col6a3_predicted	2.9813	3.4425	3.4871	3.3949	-0.1523	-0.3974	1.9666	-0.148	2.8557	3.3926	3.2645	1.1559	0.55233	0.36532
NM_001106858	Col7a1_predicted	-0.0253	-0.0036	0.09528	-0.0442	-0.1595	-0.0012	0.07764	0.02138	-0.0192	-0.0306	0.05726	0.19851	-0.0046	-0.0636
NM_001107100	Col8a1_predicted	0.22293	0.6306	0.38767	0.31026	0.54041	-0.0274	0.8463	0.38585	0.5437	0.6891	0.17574	0.42864	0.2116	0.19978
XM_233542.3	Col8a2_predicted	-1.2899	-0.6643	-1.4181	-0.9512	-1.5321	-1.883	-1.4172	-1.5611	-0.6133	-0.6666	-0.7411	-0.6431	-1.0572	-1.0537
NM_001100842	Col9a1	-0.1666	-0.1203	0.09967	-0.2104	-0.1323	-0.0206	-0.2876	-0.257	-0.1458	-0.1296	-0.2532	-0.1779	-0.0178	-0.2918
NM_001108675	Col9a2_predicted	-0.0008	-0.0641	0.08873	0.11969	0.13197	0.3061	0.14404	0.0098	-0.029	0.01006	0.16448	0.14926	0.09048	0.06877
NM_001108611	Col9a3_predicted	0.24809	0.03907	-0.1543	-0.0734	0.0691	0.0783	-0.0775	0.13949	0.08172	-0.0792	-0.0433	0.04868	-0.0567	-0.0646
NM_001108611	Col9a3_predicted	0.00047	-0.0549	-0.0449	0.01137	-0.1075	-0.0643	0.12584	0.1154	0.0725	-0.0796	-0.0115	-0.0517	0.10635	-0.0418
NM_001130541	Colec10_predicted	-0.1347	-0.0447	-0.0551	0.21207	-0.1792	0.34419	-0.0722	-0.0792	0.15697	-0.2147	-0.106	0.114	-0.0507	0.07309
NM_001025721	Colec12	0.23998	0.10438	0.11022	0.00865	0.17906	0.17922	0.19288	-0.059	-0.0353	-0.0109	0.02163	-0.0268	0.17588	0.23186
NM_019274	Colq	0.15901	0.04926	0.15164	0.01099	-0.0619	-0.1106	0.5573	0.10755	0.03327	-0.0166	0.08847	0.05519	-0.0203	0.22835
NM_001004276	Commd10	-0.0331	-0.1415	-0.1153	-0.2173	-0.1313	-0.2424	-0.2183	-0.1465	0.03714	-0.1909	0.02748	0.05418	0.30087	0.25389
NM_198732	Commd3	-0.2742	-0.1864	-0.2041	-0.2451	-0.1132	-0.1972	0.01686	-0.2446	-0.0566	0.09496	-0.1679	0.08819	-0.1808	-0.0952
NM_001108762	Commd4_predicted	-0.1456	-0.3326	0.06686	0.0072	-0.6421	-0.2272	0.0831	-0.7089	-0.316	-0.7016	-0.212	-0.1379	-0.4708	-0.4969
NM_139108	Commd5	-0.209	0.2623	0.23328	0.06264	0.01912	0.44229	0.16465	-0.1413	0.22202	0.22254	0.14464	0.72671	-0.1032	-0.2071
NM_001030029	Commd7_predicted	-0.002	-0.1019	-0.0339	0.31376	-0.0251	-0.2669	-0.0944	-0.3037	-0.1131	-0.1331	-0.1333	-0.0263	-0.3272	0.1345
NM_001106004	Commd8_predicted	0.2699	-0.0966	-0.0627	0.02102	0.11706	0.08817	0.03034	-0.0311	0.08829	-0.1487	-0.1106	0.04197	-0.0092	0.21691
NM_001033692	Commd9	0.39002	0.08344	0.07961	0.40293	0.31712	0.52164	0.09772	0.21719	0.25594	-0.0455	0.23503	0.31843	0.2077	0.13186
NM_012531	Comt	0.12323	0.28731	0.04903	-0.3552	1.0224	1.3573	0.11479	0.97202	0.25375	0.00869	0.28594	0.03062	0.3671	0.34313
NM_001107249	Comtd1_predicted	0.1121	-0.0392	-0.1557	-0.346	0.24994	0.51644	-0.0112	0.24654	0.34697	0.06542	-0.1936	-0.0279	-0.3167	-0.1752
NM_001134540	Copa_predicted	-0.599	-0.1027	0.01619	0.14457	-0.6862	-0.7329	-0.0382	-0.4215	-0.1227	-0.2366	-0.3628	-0.2552	-0.2522	-0.3909
NM_080781	Copb1	-0.2834	-0.0666	-0.2221	0.54371	0.21322	-0.2156	0.21839	0.09696	-0.2097	0.04377	0.06108	-0.0184	-0.0792	-0.0895
NM_080781	Copb1	-0.2436	-0.0592	-0.0652	0.38534	0.00665	-0.1145	0.08533	0.22419	-0.1207	-0.0356	-0.1078	0.04729	-0.075	-0.0976
NM_001106076	Cope_predicted	0.62067	0.36954	0.2633	0.70692	1.0713	1.4233	0.11893	0.9166	0.43205	0.34511	0.31484	0.78054	0.54009	0.58747
NM_001031822	Copg	-0.2079	-0.3732	-0.1997	-0.7104	-0.0484	-0.2696	-0.3624	0.01533	-0.3377	-0.2505	-0.3902	-0.3689	0.01691	-0.1126
NM_153297	Cops2	-0.1442	-0.1146	-0.1863	-0.2548	0.14297	-0.01	0.03125	0.14183	-0.0166	-0.0789	0.09408	-0.2104	0.07528	0.13267
NM_001004200	Cops3	-0.1884	-0.226	0.04994	0.12075	0.06433	0.2279	0.03883	0.11044	-0.0156	-0.1665	-0.2727	0.03106	0.01238	0.09655
NM_001004275	Cops4	-0.1858	-0.2088	-0.1602	0.66383	-0.0994	0.03088	-0.0399	-0.1649	-0.2715	-0.0188	-0.0012	0.06865	-0.161	-0.1262
NM_001025695	Cops5	0.19409	0.11336	0.09394	0.6065	0.46956	0.16507	0.31331	0.24966	0.14734	-0.1037	0.05963	0.03959	0.20671	0.32989
NM_001107129	Cops6_predicted	0.08688	-0.0173	-0.1557	-0.1878	0.20255	0.37344	-0.0317	0.28201	0.18904	-0.0381	0.03796	0.2151	0.04876	0.05621

NM_001108807	Cops7b_predicted	-0.0339	0.18134	-0.5316	-0.1976	0.21522	-0.473	-0.0692	-0.0675	0.03725	0.14812	0.0581	-0.4511	0.25432	0.28737
NM_001013227	Cops8	0.3994	0.3828	0.06932	0.38147	0.15499	0.28806	0.06106	-0.0201	0.20946	0.4391	0.23863	0.4591	0.30445	0.40604
NM_001108117	Copz1_predicted	0.34743	0.70743	-0.4925	0.13757	0.74973	0.07147	0.03176	0.64744	0.30504	0.521	0.16933	-0.1308	0.57041	0.77944
NM_001108294	Copz2_predicted	0.07774	0.00094	-0.047	-5E-05	0.10754	0.03066	-0.0167	0.12491	0.02156	-0.0896	-0.0218	0.0034	-0.0594	0.06238
NM_001108727	Coq10a_predicted	-0.0476	0.04557	0.05006	0.07631	-0.5427	-0.1606	-0.2651	-0.4302	-0.1107	-0.0863	-0.0664	0.06051	-0.2762	-0.0902
NM_001009671	Coq10b	0.24279	0.04525	0.39601	0.88985	0.36929	0.36356	0.61807	0.48798	0.05819	0.22942	0.08965	-0.0667	0.52108	0.52187
NM_019187	Coq3	0.38222	0.16488	0.11396	-0.7256	0.50609	0.76795	0.30697	0.41197	0.1011	0.1828	0.34864	0.47569	0.01701	0.30027
NM_001031662	Coq4	0.17689	0.08288	-0.005	-0.0709	-0.0174	0.21301	-0.1079	0.30832	0.09673	-0.0228	-0.0367	0.1091	0.04035	0.13781
NM_001011983	Coq6	0.10155	0.17992	0.18042	0.60945	0.38534	0.55425	0.25301	0.57067	0.3018	0.07517	0.21269	0.42589	-0.028	0.02902
NM_001011983	Coq6	0.0832	0.13705	0.17988	0.7833	0.45744	0.54063	0.50257	0.40319	0.43092	0.14023	0.39894	0.36971	0.03465	-0.3722
NM_012785	Coq7	0.14828	-0.236	0.1293	-0.1307	0.07621	0.39412	-0.1817	0.0541	-0.0846	-0.0069	-0.0961	0.47252	0.07414	0.25705
NM_182473	Corin	-0.0143	-0.0628	-0.0953	-0.0626	-0.0883	-0.1193	0.20947	-0.0736	0.02313	0.13217	-0.0303	-0.0295	-0.1737	-0.0596
NM_130411	Coro1a	0.04297	-0.0196	-0.087	-0.0044	-0.0237	0.06561	-0.0629	-0.0307	0.1133	-0.0023	0.15779	-0.0181	0.08832	-0.0886
NM_019222	Coro1b	0.54798	0.20929	0.2801	-0.1069	0.46476	0.75364	0.36537	0.45986	0.17511	0.13274	0.33967	0.42335	0.14644	0.07629
NM_001107154	Coro1c_predicted	-0.0128	-0.0755	0.5241	0.85991	-0.0278	0.19235	0.19966	0.03358	0.1193	-0.057	0.05869	0.02717	-0.329	-0.2191
NM_001012101	Coro2a	0.04682	-0.0647	-0.1709	-0.3317	0.01995	0.04451	-0.3897	-0.0204	-0.2484	-0.3361	-0.2379	-0.0173	0.0184	-0.2302
NM_001012101	Coro2a_predicted	0.13788	0.09699	0.12222	0.40435	0.85799	0.99174	0.17953	0.86266	-0.1451	0.15686	-0.061	-0.1798	-0.0122	0.14496
NM_139115	Coro6	-0.1787	0.15892	0.083	0.10729	-0.203	0.0535	-0.0007	0.14234	-0.0171	-0.0456	-0.086	-0.148	-0.1598	-0.1765
XM_001077234.1	Coro7	0.41737	0.26375	-0.0263	0.1362	0.52028	0.4222	-0.1918	0.64496	0.1175	0.01503	0.11736	-0.0249	-0.1854	-0.0895
XM_001077234.1	Coro7	-0.0155	-0.0607	0.00618	0.00151	0.06106	-0.0185	-0.0593	0.15645	-0.1012	0.03001	-0.0009	0.00981	0.10237	0.03262
NM_012835	Cort	-0.0989	-0.2634	-0.0017	-0.1039	0.10867	0.06599	-0.2085	-0.1068	-0.1363	-0.2573	-0.1786	-0.1648	-0.0336	-0.2243
NM_001108452	Cotl1_predicted	1.0016	-0.0185	-0.2011	-0.2802	0.66924	0.87957	-0.3952	0.48824	0.261	0.02888	-0.2245	0.58834	0.20648	-0.0078
XM_001060535.1	Cova1_predicted	-0.6265	-0.1824	-0.2766	-0.1369	-0.2009	-0.4245	-0.3074	-0.4843	-0.1571	-0.2448	-0.2029	-0.261	-0.2588	-0.2812
XM_213433.3	Cox11_predicted	0.18935	-0.1648	-0.0174	0.07482	0.05192	-0.1065	0.13585	-0.0886	0.17293	-0.0252	-0.201	-0.099	-0.1068	0.25136
NM_001033699	Cox15_predicted	0.38513	0.32041	0.11743	0.67037	0.23817	0.20881	-0.0779	0.2662	0.2086	0.34668	0.29551	-0.0022	-0.2703	-0.2039
NM_053540	Cox17	0.86365	0.26049	0.30061	0.86794	0.69045	1.3164	0.15236	0.40494	0.06875	0.18716	0.14762	0.35645	0.24611	0.27794
NM_017202	Cox4i1	0.46629	0.11378	0.28443	0.06368	0.4497	0.41241	0.4613	0.23637	0.03335	0.15238	-0.0782	0.33351	0.34109	0.29115
NM_001012165	Cox4nb	0.38311	-0.1609	0.31515	-0.1821	-0.2096	0.17917	-0.0193	-0.3564	0.01481	0.03503	-0.0027	0.82317	0.36085	0.46556
NM_145783	Cox5a	0.48797	0.05863	0.00842	-0.1915	0.65143	0.74583	0.23306	0.60167	0.11615	0.13526	0.12044	0.23573	0.27856	0.35076
NM_053586	Cox5b	0.37288	0.11287	0.1703	-0.1051	0.41572	0.70944	-0.1276	0.3444	0.15066	0.01552	-0.0963	0.3033	0.36733	0.45602
NM_012814	Cox6a1	0.3282	0.15483	0.09059	0.4613	0.27553	0.60341	0.41644	0.46208	0.3849	0.23422	0.38157	0.31217	0.33195	0.45792
NM_001109994	Cox6a2	0.81632	-0.2975	-0.6022	-0.1198	-0.2722	-0.2809	-0.2752	-0.2853	-0.3812	-0.2956	-0.1589	-0.6526	-0.4533	-0.6428
NM_001108911	Cox6b_predicted	-0.0753	-0.0774	0.0613	0.25173	0.01897	0.04112	0.14473	-0.1086	0.24345	0.08427	0.09758	-0.1298	0.07835	-0.0698
NM_019360	Cox6c	0.30123	0.36632	0.41367	0.51276	0.37043	0.74137	0.10267	0.67906	0.45502	0.25497	0.33592	0.34165	0.35056	0.14052
NM_173303	Cox6c1	-0.0139	0.02351	0.04562	0.36827	0.63535	0.57599	0.00879	0.43965	0.43674	-0.0022	0.26614	0.53038	0.4597	0.14217
NM_001106704	Cox7a2l_predicted	0.46642	0.6956	-1.0416	-0.1876	0.34784	-0.0153	-0.3389	0.21823	0.62057	0.60299	0.62554	-0.0005	-0.2322	-0.2643
NM_182819	Cox7b	0.71067	-0.0389	0.82558	0.37756	0.43871	0.4907	0.62968	0.21017	0.28834	0.3619	0.20977	0.58574	0.2797	0.32643
NM_134345	Cox8a	0.74071	0.65329	0.27952	0.27431	0.91763	0.96862	0.48985	0.90327	0.72206	0.76107	0.87889	0.29066	0.50077	0.5664
NM_183055	Cox8c	0.05872	0.15592	-0.16	0.07146	0.04469	-0.1424	-0.2859	-0.0112	-0.173	-0.0831	0.04206	-0.067	-0.0687	0.11851
NM_012786	Cox8h	-0.2061	-0.1813	-0.1745	-0.1842	-0.1089	-0.0452	-0.0369	-0.0144	-0.0994	-0.2117	-0.1083	-0.1877	-0.1353	-0.0346
NM_016998	Cpa1	0.06246	0.01838	-0.0703	-0.0283	0.11976	0.24287	0.05142	0.06914	-0.1313	0.11219	-0.0079	-0.0526	-0.0749	0.05937
NM_001013083	Cpa2_predicted	-0.0288	-0.1167	-0.15	-0.1804	-0.224	-0.0457	-0.0937	-0.025	-0.1389	-0.0856	-0.0232	-0.0579	-0.2406	-0.1315
NM_019300	Cpa3	0.0286	-0.0404	-0.1546	0.07405	-0.0391	0.13051	0.06372	-0.166	-0.0378	-0.0226	0.06816	0.31769	-0.075	-0.1171
NM_001002808	Cpa5	0.00044	-0.021	-0.0738	0.02535	-0.0181	0.04299	-0.081	-0.0904	0.00634	-0.0728	-0.0783	-0.1058	-0.1403	-0.0036
NM_001107900	Cpa6_predicted	-0.306	-0.0984	-0.2591	-0.2749	-0.1978	-0.1269	-0.2311	-0.2	-0.1967	-0.1569	-0.1583	-0.2316	-0.1743	-0.2456
NM_012533	Cpb1	0.1328	-0.0175	0.1667	-0.0046	0.12966	0.26738	-0.0119	0.05552	0.01355	0.04107	0.12398	-0.064	0.08573	0.20772
NM_053617	Cpb2	-0.0531	-0.119	0.16563	-0.0561	-0.0352	-0.0821	0.00731	-0.0531	-0.036	-0.0381	0.00138	-0.018	0.13032	-0.0046
NM_012836	Cpd	-0.0052	0.56153	0.05399	0.22841	0.03819	-0.5415	0.09357	-0.0793	-0.1001	0.00414	0.23704	-0.4482	-0.4999	-0.1655
NM_013128	Cpe	1.0458	1.3549	-1.1208	-0.3148	1.0973	0.26288	0.24046	1.3579	0.576	0.92633	0.54651	-0.3718	-0.2932	-0.1504

NM_001106276	Cpeb1_predicted	0.24163	-0.1391	0.12706	-0.1106	-0.1144	-0.071	0.00425	-0.1512	-0.0316	-0.001	-0.1677	-0.1304	0.09931	-0.0702
NM_001108361	Cpeb2_predicted	-0.0363	-0.1295	0.0297	-0.1032	-0.4597	-0.1052	-0.0464	-0.0793	0.36943	0.01469	-0.0677	0.61062	0.04646	-0.0686
NM_001106992	Cpeb4_predicted	0.92192	0.7352	0.56858	0.95995	0.83102	0.64966	1.3215	1.0099	0.57798	0.85272	0.67132	0.17005	0.67665	0.4503
NM_001029909	CPG2	0.13992	0.04672	0.0757	0.02272	0.04571	0.01393	-0.0473	-0.0842	0.02528	0.11792	0.02224	0.07065	0.07778	-0.0169
NM_022864	Cplx1	-0.0636	-0.1776	0.03618	-0.0478	-0.0271	0.03302	-0.0625	-0.059	-0.1581	-0.1416	-0.038	-0.0482	-0.1264	-0.1302
XM_341625.3	Cplx4_predicted	0.1598	0.06831	-0.0253	-0.0478	0.11207	-0.0059	-0.1153	0.01626	-0.1333	0.05106	0.03729	0.11597	0.15052	0.26725
NM_001108098	Cpm_predicted	-0.0153	-0.1011	-0.0573	0.18772	-0.1519	-0.1454	-0.0609	-0.1104	-0.0684	-0.0706	0.0047	0.09777	0.09823	-0.1873
NM_053526	Cpn1	-0.1539	0.02383	0.02998	0.00866	0.07138	0.0195	-0.0306	0.04482	-0.0737	-0.1775	-0.0236	-0.0316	0.37617	0.07405
XM_342555.2	Cpne1_predicted	-0.2993	0.23658	-0.1195	-0.4197	-0.2427	-0.1752	-0.1382	0.23371	-0.1406	-0.129	-0.2158	-0.0997	-0.0228	0.3271
NM_001107917	Cpne3_predicted	0.11632	0.1928	-0.2161	-0.28	0.23082	0.18569	-0.0279	0.18274	0.13947	0.08815	0.24696	0.18932	0.01612	0.24417
NM_001109003	Cpne4_predicted	0.12927	0.06096	0.03558	-0.0254	0.85134	0.75022	-0.0075	0.90349	0.01286	0.00456	0.18423	-0.0556	0.14696	-0.0125
NM_001107616	Cpne5_predicted	0.01008	-0.0239	0.1031	0.01279	0.01667	0.14658	0.04508	-0.0028	0.05037	0.01955	0.07446	0.05481	0.12068	-0.0638
NM_001108454	Cpne7_predicted	0.07212	0.04499	-0.0119	0.01224	0.0907	0.10753	0.16865	0.04982	0.02646	0.2701	0.05259	-0.0085	-0.0357	0.00286
NM_001108750	Cpne8_predicted	0.14832	0.15662	0.13892	0.05394	0.27297	0.08711	0.21845	0.1078	0.21093	0.14403	0.07454	0.21913	0.35247	0.15271
NM_001024982	Cpne9	0.02908	-0.0091	0.01977	-0.0248	0.00051	0.02401	-0.1096	-0.0605	-0.0055	0.00469	0.06861	-0.125	-0.1193	-0.0106
NM_001037095	Cpox	0.43577	0.34574	0.03298	0.36342	0.57997	0.10764	-0.1384	0.4246	0.10732	0.36319	0.22645	-0.244	0.13617	0.21422
NM_017072	Cps1	0.01843	-0.0363	0.00231	-0.0352	0.03726	-0.0887	-0.1251	0.03117	-0.0031	-0.0299	0.15684	-0.1136	0.37946	0.08063
NM_001106753	Cpsf2_predicted	0.24541	0.05532	0.16418	0.08931	0.2953	-0.214	0.21851	0.29236	0.1096	0.08218	0.18605	0.10321	0.74113	0.64734
NM_001030030	Cpsf3	-0.0452	-0.0441	0.18054	-0.7203	-0.0208	0.06159	-0.154	-0.0061	-0.1463	0.03868	-0.0202	-0.0338	0.21079	0.16718
NM_001033892	Cpsf3l	0.4111	0.23328	0.2712	0.3529	1.0133	0.94229	0.21994	0.95559	0.48521	0.41224	0.34192	0.28236	0.52007	0.52597
NM_001106785	Cpsf6_predicted	-0.9877	-0.8944	-0.1494	-0.8593	-0.9178	-1.2647	-0.4352	-0.831	-0.571	-0.7149	-0.6694	-0.3862	0.01679	-0.0068
NM_031559	Cpt1a	0.30377	0.80276	0.15837	-0.5884	-0.471	-0.6303	0.06514	-0.2689	0.6863	0.78748	0.55139	0.02267	0.12876	0.22001
NM_013200	Cpt1b	-0.2426	-0.2701	0.42103	0.17699	-0.3501	-0.2728	0.20476	-0.2425	-0.077	-0.2692	-0.2611	-0.1031	-0.0722	-0.205
NM_001034925	Cpt1c	-0.2935	0.28447	0.39355	-0.1933	-0.2788	-0.2746	0.1811	-0.3355	-0.3859	0.02254	-0.0609	-0.063	0.09914	-0.0276
NM_012930	Cpt2	0.1563	0.11944	-0.1657	-0.7535	0.31385	0.23628	-0.5214	0.22692	0.11431	0.26315	0.18879	-0.0401	-0.0292	0.02471
NM_001106511	Cpxm1_predicted	-0.1636	0.05653	-0.0032	0.00613	-0.067	0.06677	-0.1173	-0.1162	-0.1764	-0.05	0.02665	-0.1531	-0.0486	0.18473
NM_001106306	Cpxm2_predicted	1.0913	1.3662	0.97224	0.67568	3.1575	3.3251	0.95965	3.2926	1.087	1.347	1.2595	0.05913	-0.0559	0.00228
NM_031766	Cpz	0.00508	-0.2265	-0.208	-0.1667	0.06638	0.04833	-0.2191	-0.1877	-0.0411	-0.2935	-0.0245	-0.0326	-0.243	-0.1581
NM_001105989	Cr2_predicted	0.14386	0.04492	0.05807	0.0852	0.17338	0.16225	0.02091	0.20996	0.12054	-0.0334	0.05906	0.06291	0.23934	0.17799
NM_001105716	Crabp1_mapped	0.13663	0.08631	-0.0033	0.25638	-0.0781	-0.0577	0.18687	-0.1177	0.01821	-0.1395	-0.1464	0.35035	-0.2601	-0.0215
NM_017244	Crabp2	1.2406	0.30159	0.90661	0.4481	0.09148	0.28551	0.23177	0.16159	0.14665	0.33977	0.39028	0.56929	0.75975	0.57825
NM_001108085	Cradd_predicted	-0.7142	-0.6101	0.20279	-0.3614	-1.1153	-1.0172	0.05226	-1.1447	-0.2482	-0.3728	-0.2676	0.12379	-0.3176	-0.3394
NM_001004085	Crat	0.14701	0.14109	0.29189	-0.177	0.24764	0.33749	-0.1225	0.01179	-0.0849	0.15612	0.04511	0.12468	-0.2148	0.12853
NM_001004085	Crat	-0.025	0.01882	-0.0784	0.06695	0.05284	-0.0284	0.00067	0.05472	-0.0647	0.0467	-0.0289	-0.0691	-0.0452	0.11077
NM_001107182	Crb1_predicted	0.23651	0.14406	-0.0212	0.13664	-0.0472	0.10725	0.15803	0.0535	-0.015	0.03019	0.09074	0.06201	0.06111	0.00703
NM_001025661	Crb3	-0.1486	-0.0167	-0.0522	-0.1388	-0.0211	-0.1379	0.21586	-0.1452	-0.1295	0.25997	-0.0435	0.37396	0.15336	-0.0547
NM_001015003	Crbn	0.64222	0.771	-0.6099	0.59456	0.9351	0.63154	0.35353	0.9029	0.27052	0.46938	0.0858	-0.1562	-0.1369	0.12087
NM_053670	Crcp	-0.936	-0.4466	-0.5988	-0.7463	-0.8212	-0.7529	-0.1091	-0.7709	-0.3828	-0.449	-0.4817	-0.5879	-0.6248	-0.6195
NM_031017	Creb1	-0.2527	-0.0937	-0.0173	-0.2514	-0.1641	-0.2656	0.05113	-0.2617	0.00141	-0.1597	-0.0167	-0.2187	0.1415	0.04494
NM_031017	Creb1	0.20706	0.11414	0.21283	0.06468	-0.0878	0.12747	0.15203	-0.1282	0.23348	0.20024	0.11386	0.0755	0.30758	0.49974
NM_001013092	Creb3	0.06055	0.02562	0.20797	0.06706	0.28416	0.41097	0.09181	0.28798	-0.0067	0.22905	0.14565	0.22328	0.47931	0.41575
NM_001005562	Creb3l1	-0.7705	-0.311	-0.4345	-1.2223	-0.4269	-0.4589	-0.8635	-0.2217	-0.0523	-0.222	-0.6497	0.29502	0.01752	0.16549
NM_001012188	Creb3l2	-0.2684	-0.3362	0.14933	-0.0741	-0.2226	-0.0814	-0.2395	0.0088	-0.2048	-0.1767	-0.0604	0.27352	0.33348	0.49279
NM_001012115	Creb3l3	0.16727	0.0304	0.00992	0.05657	0.17123	0.2057	0.02475	0.1737	0.11964	-0.0707	0.1151	0.398	-0.1093	0.10579
NM_001007093	Creb3l4	0.23492	-0.0241	-0.0651	-0.0042	0.10755	0.11357	0.06043	-0.0338	0.07329	0.23542	0.194	-0.1757	-0.0388	0.25671
NM_001002809	Crebl1	-0.4851	-0.8109	-0.2977	-1.0029	-0.4675	-0.7804	-0.429	-0.5197	-0.6028	-0.7852	-0.5458	-0.7471	-0.5634	-0.5663
NM_001015027	Crebl2	0.09073	0.35602	-0.0092	0.01809	0.01968	-0.0917	-0.0421	-0.1096	-0.094	-0.015	-0.1097	0.07677	-0.0724	-0.0899
NM_001105966	Creg_predicted	0.16729	0.15316	0.01477	0.27963	0.15859	0.22648	0.102	-0.0579	-0.1259	0.1571	-0.1578	-0.0508	0.06415	-0.0388
NM_001024783	Creld1	-0.779	-0.6713	0.26415	0.17464	-0.9471	-0.7639	-0.0954	-1.0586	-0.7982	-0.6625	-0.6442	-0.6792	-0.8968	-0.8772

NM_001037208	Creld2	-0.2312	-0.1415	0.78457	0.48182	0.47912	0.74885	-0.2914	0.72014	-0.0973	0.13167	0.10434	0.3839	0.73231	0.83255
NM_017334	Crem	0.03306	-0.2835	-0.1046	0.05908	-0.1733	-0.4502	0.09397	-0.1391	-0.0306	-0.4934	-0.1012	-0.0989	-0.188	-0.177
NM_017334	Crem	-0.2535	-0.0921	-0.4457	-0.7943	-0.4167	-0.4547	0.30692	-0.3223	-0.1841	-0.0956	-0.3327	-0.1088	0.03757	-0.1477
NM_017334	Crem	0.14734	0.17576	-0.6275	-0.0771	-0.1654	-0.1504	0.19615	-0.3472	0.12847	-0.1249	-0.2647	0.06154	0.10846	-0.0825
NM_031019	Crh	-0.2793	-0.2426	-0.2891	-0.1251	-0.0322	-0.2193	-0.1725	-0.2283	-0.324	-0.0378	-0.1157	-0.0994	-0.0893	-0.1207
NM_139183	Crhbp	-0.4173	-0.4504	-0.497	-0.3912	0.1263	0.01168	-0.1493	-0.1078	-0.6313	-0.4494	-0.0405	-0.6576	-0.43	-0.5114
NM_030999	Crhr1	-0.0065	0.2755	-0.0182	0.08292	-0.0728	-0.0341	0.11929	-0.071	-0.1027	-0.1501	-0.0291	0.05904	0.01985	-0.0332
NM_022714	Crhr2	0.24095	0.05395	0.04523	0.10358	0.20443	-0.1621	-0.0841	-0.0249	0.17577	0.21347	-0.0074	0.205	-0.029	-0.0645
NM_001169103	Crim1_predicted	0.67613	0.62419	0.36701	0.65554	1.2628	0.92011	0.53959	1.2248	0.56027	0.68979	0.54312	0.74738	1.2418	1.2412
NM_022501	Crip2	-0.6292	-0.3229	-0.1172	-0.8254	-0.581	-0.656	-0.5464	-0.5862	-0.3737	-0.3654	-0.4661	-0.5517	-0.3799	-0.3188
NM_019907	Cript	0.32649	0.63281	0.0196	0.41222	0.31526	0.23566	0.48627	0.37503	0.90521	0.66653	0.73862	0.63207	0.57679	0.53388
NM_031240	Crisp2_predicted	-0.0455	-0.129	-0.0275	-0.0451	0.22075	-0.0654	0.03304	-0.0524	-0.0523	0.01507	-0.0868	-0.0638	-0.0875	0.01524
NM_031240	Crisp2_predicted	-0.2209	-0.1068	-0.1646	-0.0731	-0.1301	-0.0387	0.05982	0.00531	-0.1245	-0.0624	0.18009	-0.242	-0.3189	-0.0401
NM_019302	Crk	0.30824	0.0691	0.29884	0.09635	0.53775	0.6265	0.34108	0.38192	-0.0863	0.34913	0.30807	0.33291	0.36245	0.40643
NM_001008284	Crkl	-0.2259	0.11775	0.0982	-0.3632	-0.1782	-0.2185	-0.2344	-0.1516	0.02498	0.18557	0.08094	0.13626	0.26594	0.29288
NM_138916	Crkrs	-0.0907	-0.2665	0.32213	0.15848	-0.0616	-0.3032	0.23012	-0.16	-0.2746	-0.2125	-0.1316	-0.1252	-0.0242	-0.212
NM_001106074	Crlf1_predicted	-0.0397	0.97272	0.08555	0.04291	0.21084	0.12713	0.29773	0.18855	0.74875	1.0451	1.0499	0.83632	-0.3897	-0.356
NM_001014258	Crls1	0.1779	0.23196	-0.4546	0.14061	0.31736	0.05826	-0.219	0.56355	0.00095	0.01154	-0.2017	-0.4875	0.04153	0.15963
NM_012932	Crmp1	0.14233	0.05044	-0.0159	0.0715	0.01326	0.00955	-0.0578	0.17382	-0.0572	-0.0521	-0.0628	0.17635	0.02724	0.13301
NM_053797	Crnkl1	0.31877	0.51288	0.46042	0.52042	0.62288	0.49041	0.61596	0.79602	0.23193	0.42464	0.33766	0.07671	0.57368	0.26146
XM_227367.4	Crnn_predicted	-0.4421	-0.3077	0.07701	-0.4918	0.43525	0.49952	0.08905	0.42404	-0.3462	-0.4664	-0.6396	-0.4125	-0.3407	-0.4949
NM_001107990	Crocc_predicted	0.0154	-0.0264	0.16017	-0.1058	-0.0572	0.01202	-0.1416	-0.1935	0.03709	-0.2294	-0.0966	0.42437	-0.2254	0.00106
NM_031987	Crot	0.30998	-0.0344	-0.3807	-0.3497	-0.1102	-0.472	0.38193	-0.0699	0.3138	0.07143	0.05633	-0.1322	-0.3605	-0.439
NM_017096	Crp	0.34815	0.12543	0.15115	0.17623	0.25685	0.18016	0.34685	0.1411	-0.0121	0.16471	-0.1028	0.02921	0.20801	0.28079
NM_001005330	Crry	0.67153	0.73065	0.61849	-0.326	-0.1592	-0.3083	1.1618	-0.1691	0.40012	0.71454	0.47364	0.0274	0.66482	0.66429
XM_001057683.1	Crsp3	-0.3755	0.55214	0.48652	0.34143	-0.1788	-0.6901	0.99914	-0.2878	0.31323	0.26528	0.25588	-0.2519	-0.2004	-0.1372
NM_001106801	Crsp6	-0.4657	-0.2587	0.39633	0.68518	-0.0232	-0.1808	0.4245	-0.2726	-0.0956	-0.1492	-0.1895	-0.3234	0.27353	-0.1132
NM_134401	Crtac1	0.03524	0.12285	0.06458	0.02532	0.05803	-0.0613	0.09503	-0.0348	0.03662	0.09411	0.10116	0.21529	0.00218	0.07063
NM_001106813	Crtam_predicted	-0.166	-0.1929	-0.1612	-0.1488	-0.0564	-0.0337	-0.006	-0.252	-0.113	-0.0453	-0.1439	-0.1381	-0.0979	-0.0542
NM_021855	Crx	-0.0053	-0.0436	-0.06	0.05691	-0.073	0.15405	0.03225	-0.0136	-0.0598	-0.1068	-0.0497	0.04806	0.02598	0.06995
NM_198750	Cry1	0.46733	-0.429	0.41224	0.99161	1.2487	0.95707	-0.1315	1.2036	0.07013	-0.0287	-0.3237	0.27282	0.40295	0.28714
NM_133405	Cry2	-0.0891	0.04977	-0.0252	-0.0812	-0.2999	-0.122	-0.2665	0.12108	-0.0443	-0.4545	-0.0152	-0.3559	-0.1943	-0.1826
NM_012935	Cryab	2.6844	1.4093	1.417	1.4727	1.7465	2.0013	1.3337	1.6451	1.3003	1.4355	1.3467	1.313	2.0639	2.2206
XM_340846.3	Cryba1	0.06684	0.23636	0.01731	0.25973	-0.0899	0.19604	-0.0712	-0.1155	0.0609	0.55741	-0.1009	0.00251	-0.1183	0.05748
NM_173140	Cryba2	0.08506	-0.1042	-0.0245	0.05601	0.03706	-0.1225	-0.0385	0.07202	-0.1899	0.03851	-0.0124	0.19323	0.30635	0.10211
NM_001109875	Crygb_mapped	-0.1153	0.00443	-0.0313	-0.0178	-0.1285	-0.0794	-0.0979	0.06845	0.02087	-0.1089	-0.0614	0.0878	-0.0434	0.22215
NM_001081660	Crygc	-0.0197	0.0619	0.10287	-0.0267	-0.0101	0.08634	-0.0705	-0.0334	0.09275	-0.0346	-0.0916	0.14551	-0.1614	0.13555
NM_033095	Crygd	0.0519	0.32681	0.14822	0.25891	0.13146	0.02979	0.0063	0.02101	0.13567	0.11272	0.1802	0.28185	0.12996	0.01779
NM_173289	Cryge	0.2939	0.16174	0.18424	0.20333	0.04902	0.03326	0.06874	0.14509	0.17908	0.24503	0.24443	0.16497	0.16535	0.15479
NM_175757	Cryl1	0.24691	-0.0163	-0.0681	0.14674	0.36124	0.3367	0.01897	0.43472	0.26043	0.11046	0.07372	0.61595	0.55883	0.43005
NM_001012183	Cryz	0.17147	0.16949	0.08512	-0.0401	0.00838	0.02671	0.04144	-0.1736	0.3834	0.33781	0.13764	0.17636	0.24724	0.12561
NM_001013044	Cryzl1	-0.2456	-0.3496	-0.3634	-0.3814	-0.1905	-0.034	-0.3823	-0.458	-0.2188	-0.2181	-0.2081	-0.4019	-0.4503	-0.3291
NM_130755	Cs	0.43732	0.64289	0.00105	-0.1399	0.5726	0.0792	-0.1564	0.48447	0.13541	0.36557	0.11551	-0.0093	0.90338	0.66611
NM_001134454	Csad	-0.1984	-0.0805	-0.5163	-0.6034	-0.1544	0.16072	-0.7647	-0.1882	-0.0209	-0.0438	-0.0253	0.10634	-0.5464	-0.5511
NM_031979	Csda	0.3149	-0.2573	-0.1771	-0.0891	0.10701	0.09152	-0.3052	0.2834	-0.8369	-0.3036	-0.4491	-0.7613	-0.4615	-0.3716
NM_001170542	Csdc2	0.51431	1.2519	0.58598	0.70138	0.21954	0.32416	0.39259	0.28399	1.0242	0.90625	1.1539	0.33636	0.04067	0.00553
NM_054006	Csde1	0.46728	0.28673	-0.1682	-0.0442	0.17174	0.45048	-0.2073	0.34023	0.34592	0.16259	0.62359	0.24162	0.44473	0.16085
NM_001108607	Cse1l_predicted	-0.461	-0.4563	0.2323	-0.2303	-0.355	-0.5467	-0.2445	-0.411	-0.2847	-0.1487	-0.2748	-0.0425	0.21886	0.11467
NM_023981	Csf1	0.45579	0.43526	-0.067	0.77438	0.02099	-0.342	0.1422	0.09418	0.10149	0.43428	0.37197	-0.5751	0.15128	0.05171

XM_340799.3	Csf2	-0.0508	0.21118	0.11157	0.0817	0.05234	0.25133	0.08014	0.03621	0.28934	0.06457	0.18912	0.19223	0.05496	0.07338
NM_017104	Csf3	0.01991	-0.0781	0.03015	-0.0642	0.0469	0.13211	0.016	0.0204	-0.1179	-0.02	-0.0847	0.22926	-0.0646	-1E-05
NM_001106685	Csf3r_predicted	0.01538	0.0466	-0.175	0.0591	0.09378	-0.0708	0.10402	-0.0582	-0.0717	0.01368	0.22091	-0.0875	0.21653	0.22816
NM_001030039	Csk_predicted	-0.0868	-0.4202	-0.0777	-0.0869	0.04364	0.41528	-0.2924	0.19977	-0.4527	-0.3588	-0.3316	-0.1137	-0.0341	-0.0162
NM_001037327	Csmd1	-0.0202	0.12453	0.18551	0.0361	0.18389	0.03983	0.00672	-0.0675	0.16189	-0.0587	0.15544	0.50263	0.25302	-0.0101
NM_001037327	Csmd1	-0.0498	0.08644	-0.0413	0.05731	-0.0438	0.08716	-0.0706	0.16752	-0.1277	0.25914	0.0735	0.18309	0.00664	0.04636
NM_138874	Csn1s1	-0.1108	-0.0866	0.06345	-0.1846	-0.1792	-0.12	-0.247	-0.0176	-0.1092	-0.0891	-0.0081	-0.0826	-0.0429	-0.2326
NM_173106	Csn1s2b	-0.1401	-0.0846	0.01171	-0.0544	-0.0377	-0.0954	-0.0719	-0.1586	-0.0384	-0.1172	0.04743	-0.145	0.21967	-0.1276
NM_017120	Csn2	0.05763	0.13775	0.09818	0.10348	0.08938	-0.0961	0.12634	0.01553	0.06991	0.2834	0.33871	-0.0557	0.02495	0.33599
NM_031562	Csn3	0.04036	-0.019	-0.071	-0.0372	-0.0473	0.04882	-0.0671	-0.046	0.03084	-0.0224	-0.0558	0.03781	0.09973	0.09105
NM_001105741	Csng	0.15388	0.02249	0.20479	0.07892	0.06395	0.14394	0.16048	0.05047	0.41572	0.09554	0.08692	0.08066	0.28678	0.12376
NM_053615	Csnk1a1	0.38111	0.40223	-0.2355	0.14727	0.24349	0.26252	-0.2734	0.45082	0.3564	0.21437	0.01579	0.03526	0.18007	0.10813
NM_139060	Csnk1d	0.16622	0.26577	-0.3033	0.5424	0.88034	0.56804	-0.0028	0.79642	-0.0185	0.38462	0.13547	-0.1291	-0.0733	0.01794
NM_022288	Csnk1g1	0.00579	0.02263	0.19607	0.42742	0.1509	0.28805	0.09407	0.1728	-0.1508	-0.0006	0.11615	0.1492	-0.0883	0.00738
NM_001033870	Csnk1g2	0.23606	0.30722	-0.6986	-0.7913	0.13458	-0.5119	-0.1867	-0.0246	0.22056	0.13209	0.06629	-0.1047	0.08955	0.1776
NM_022855	Csnk1g3	-0.3398	-0.3029	-0.6034	-0.6283	-0.0274	-0.0517	-0.4422	0.08904	-0.2926	-0.4664	-0.4697	-0.313	-0.3634	-0.4678
NM_053824	Csnk2a1	0.22733	0.29023	-0.3319	0.0135	0.48221	0.09764	-0.0788	0.22494	0.09313	0.39446	0.146	0.01045	0.24518	0.32715
NM_001107409	Csnk2a2_predicted	0.07434	-0.0152	0.13998	-0.0606	0.3745	0.18992	0.04281	0.47375	0.10136	0.10953	-0.0066	0.02578	0.52087	0.29542
NM_053663	Cspg2	0.18123	1.0468	0.72333	-0.1676	-1.8365	-1.9627	1.479	-1.8223	0.78156	0.71373	1.0452	0.81188	0.80851	0.7865
NM_031022	Cspg4	2.4121	2.0237	2.6005	1.1609	2.198	2.2695	2.3121	2.0691	1.5909	2.0834	2.0738	1.1815	1.9568	1.9396
NM_133652	Cspg5	-0.0799	-0.044	-0.1796	-0.146	0.03408	0.30183	-0.2299	0.01182	-0.029	-0.1647	0.03807	0.00699	0.01024	0.03278
NM_133652	Cspg5	-0.2528	-0.2598	-0.4777	-0.4562	-0.247	-0.4536	-0.1999	-0.233	-0.4019	-0.1544	-0.1912	-0.5968	-0.0199	-0.132
NM_031583	Cspg6	-0.2109	-0.3022	-0.0724	-0.2684	-0.5443	-0.7439	0.04186	-0.4418	-0.513	-0.3671	-0.2164	-0.3746	-0.179	-0.078
XM_237360.3	Csprs_predicted	0.08331	-0.0996	-0.1574	0.32558	-0.3711	0.12977	0.0062	-0.1614	-0.1643	0.06186	-0.1199	0.14699	-0.1398	0.04294
NM_017148	Csrp1	-0.0966	-0.1293	-0.336	-0.1001	-0.0489	0.16462	-0.6588	-0.0564	0.1491	-0.041	-0.0574	0.59667	-0.1581	-0.3386
NM_177425	Csrp2	-0.4299	-1.0095	-0.2565	-0.0507	-0.3115	0.05943	-1.1869	-0.2837	0.01401	-0.7467	-0.6225	0.86913	-0.4083	-0.2278
XM_001054440.1	Csrp2bp_predicted	-0.0028	0.2149	0.52385	0.12197	-0.0092	-0.09	0.05415	0.18445	0.08221	0.44715	0.26662	-0.1579	0.46069	0.09815
NM_057144	Csrp3	1.0325	0.06245	0.04474	0.52144	1.4039	1.8717	-0.0549	1.272	0.09881	-0.0944	0.26235	0.17892	0.009	0.02039
NM_001108961	Cst10_predicted	0.10682	-0.0795	-0.0244	0.02146	0.05819	-0.059	-0.0149	0.08879	0.08006	-0.0864	-0.0365	-0.0307	-0.0354	-0.0003
NM_139085	Cst11	-0.0205	-0.0734	-0.1101	0.08834	-0.0412	-0.0582	-0.0307	-0.0438	-0.0472	-0.1091	-0.0422	0.01647	0.0894	-0.0935
NM_153734	Cst12	-0.1801	-0.2169	-0.0768	-0.0857	-0.3247	-0.2473	-0.2078	-0.3048	-0.185	-0.0738	-0.1527	-0.2727	-0.1942	-0.4519
NM_012837	Cst3	1.0518	1.9602	0.48104	1.2833	0.37427	0.46859	1.4771	0.33747	1.7654	1.7967	1.9008	0.41188	0.8851	0.83845
NM_133566	Cst6	0.1601	0.01128	0.0871	-0.0114	0.0132	0.01182	0.13772	0.04177	-0.0305	0.22285	-0.0388	-0.0175	0.01392	0.00374
NM_001106523	Cst7_predicted	-0.1375	-0.1074	-0.1835	-0.0773	0.05546	0.01949	-0.0699	-0.0076	0.04056	0.02978	0.01997	-0.1367	-0.0655	-0.0139
NM_019258	Cst8	-0.0193	0.10623	0.04883	0.20881	0.00327	0.11486	0.00898	0.08006	-0.0775	0.15085	0.03704	0.18465	0.0629	0.07735
NM_001108597	Cst9_predicted	0.0396	0.15662	0.18285	0.15714	0.0896	0.13185	0.04913	0.26339	0.16488	0.19248	0.29794	0.10556	0.00674	0.10637
NM_001105876	Csta_predicted	0.03241	-0.148	-0.197	-0.0675	0.21248	0.23946	-0.1058	0.23264	-0.1611	-0.1429	-0.0529	-0.1344	-0.0872	0.03366
NM_012838	Cstb	0.17652	-0.0804	0.06726	0.19688	-0.1427	-0.0832	0.45413	-0.4097	0.03815	-0.0144	0.19447	-0.098	-0.1013	-0.1968
NM_001107586	Cstf2t_predicted	-0.1422	-0.1103	-0.1431	0.09077	-0.1159	0.16226	0.17625	0.03341	-0.0622	0.17157	0.0112	-0.0111	-0.2026	-0.0286
NM_001077672	Cstf3_predicted	0.14195	-0.0779	-0.0479	-0.393	-0.1499	0.18258	-0.1858	-0.4048	-0.1487	-0.1267	0.00846	0.17605	0.02287	0.11681
NM_001106522	Cstl1_predicted	0.19704	0.09851	0.12162	0.14109	0.17657	0.17936	0.05081	0.13551	0.0526	-0.0182	0.05749	0.27681	0.02261	0.06378
NM_001106522	Cstl1_predicted	0.05733	-0.0826	0.00457	-0.008	0.02194	-0.0022	0.07976	-0.0829	-0.0893	0.00418	0.19677	0.05461	-0.026	-0.1094
NM_019201	Ctbp1	-0.8659	-0.9728	-0.2408	-1.5292	-0.9581	-1.2353	-0.4123	-1.1057	-0.6919	-0.9068	-0.8861	-0.2526	-0.4417	-0.2497
NM_053335	Ctbp2	-0.1539	-0.1675	-0.5586	0.01182	0.07376	-0.0441	-0.1494	0.1029	-0.2655	-0.3051	-0.2303	-0.5301	-0.4736	-0.3875
NM_031023	Ctbs	1.9323	1.3287	0.2929	0.95822	1.1021	1.2067	1.1312	1.4251	0.99492	0.96809	1.0907	0.14812	0.5957	0.59528
NM_031824	Ctcf	-0.4932	-0.4941	0.18506	-0.12	-0.6442	-1.0666	0.06808	-0.746	-0.2119	-0.3564	-0.1756	-0.2775	-0.0823	0.0631
XM_230900.3	Ctcfl_predicted	-0.1294	-0.0532	0.06743	-0.0077	-0.1031	-0.1452	-0.1168	-0.0923	-0.1798	0.0715	-0.0864	0.02134	-0.1165	-0.2632
NM_001106131	Ctdp1_predicted	-0.263	-0.4368	0.06594	-0.2293	-0.2293	-0.1016	-0.1496	0.1514	-0.3376	-0.4056	-0.2112	-0.038	-0.147	-0.0314
NM_001128079	Ctdsp1_predicted	0.05985	0.056	-0.0828	-0.0875	-0.0849	-0.0202	0.06855	-0.0245	-0.0353	-0.0247	0.11277	0.14045	-0.0336	0.09358

NM_001106865	Ctdspl_predicted	-0.0366	-0.3753	0.03124	-0.3876	-0.1908	-0.249	-0.4187	-0.7148	0.15552	-0.4165	-0.1739	0.15653	0.20572	0.15259
NM_001014048	Ctdspl2	0.33288	0.51695	0.06967	0.08961	0.33101	-0.016	0.30936	0.6104	0.10819	0.54544	0.10201	0.1571	0.69545	0.4562
NM_017129	Ctf1	-0.0097	0.17037	0.08005	0.02689	-0.0994	-0.0528	0.04984	0.01161	0.10002	0.17649	0.27939	0.16263	0.3512	0.21554
NM_001135800	Ctf2_predicted	-0.1499	-0.0765	-0.0335	-0.0193	-0.0224	0.0302	0.15279	0.08064	-0.0442	-0.0529	-0.0034	0.07452	-0.0145	-0.0002
NM_022266	Ctgf	-0.2647	-0.784	-0.6393	0.12696	0.03817	-0.0214	-0.136	0.11617	-0.3737	-0.7815	-0.6582	-0.1582	-0.1611	-0.127
NM_017074	Cth	0.23063	0.21933	0.47733	0.71909	0.04376	-0.0667	1.0323	0.16872	0.15195	0.42368	0.27002	0.28576	-0.0274	0.14557
NM_172333	Cthrc1	0.07234	0.22696	-0.0384	0.03503	-0.1107	-0.0143	0.07461	0.10646	0.13433	0.09991	0.14028	0.3726	-0.0198	0.02765
NM_031674	Ctla4	-0.1379	-0.1333	-0.0403	0.06466	0.2409	0.03307	-0.0377	-0.4989	-0.0989	-0.0618	-0.1326	0.35916	0.168	-0.0611
NM_001007145	Ctnna1	-0.4589	-0.1744	-0.3461	-0.5724	-0.1657	-0.3858	-0.1547	-0.1338	-0.3492	-0.0357	-0.256	0.10365	-0.1415	-0.1804
NM_001106598	Ctnna2_predicted	-0.106	0.05723	0.03862	-0.0668	0.20699	0.34842	0.08694	0.17703	-0.083	-0.0669	0.17608	0.19012	0.16786	-0.0344
NM_001106598	Ctnna2_predicted	0.17847	-0.0674	-0.0138	0.15753	0.13416	0.12864	0.10414	0.03103	0.04346	0.10393	0.11835	0.18113	-0.0126	0.09479
NM_001106649	Ctnnal1_predicted	-0.4912	-0.3679	-0.132	-0.0055	-0.3535	-0.5562	-0.0634	-0.1572	-0.4754	-0.3058	-0.4335	-0.5282	-0.5617	-0.4514
NM_053357	Ctnnb1	-0.3108	0.62244	-0.7695	-0.6807	-0.0136	-0.5082	-0.2844	0.15766	0.36532	0.38639	-0.0073	0.02995	0.2598	0.10458
NM_001024870	Ctnnbl1	-0.0752	-0.1221	0.14789	-0.0105	0.11636	0.15208	0.04592	0.21698	-0.1021	0.03374	-0.3281	-0.0658	0.26527	0.09662
NM_001107740	Ctnnd1_predicted	-0.6754	0.10969	-0.2642	-0.5038	-0.3302	-0.4395	-0.4059	-0.3189	-0.0413	0.15608	-0.0731	-0.1055	-0.5076	-0.4447
XM_001064375.1	Ctnnd2	0.18068	0.02875	0.02803	0.01081	0.64602	0.67331	0.35148	0.72406	-0.1876	0.00659	0.00866	-0.0796	0.15257	0.19033
XM_220649.4	Ctns_predicted	0.32843	0.11406	0.1643	-0.0039	0.28357	0.17053	0.14128	-0.03	0.02632	-0.032	-0.1361	0.16166	0.23051	0.12281
NM_001134873	Ctps_predicted	0.03773	-0.4496	0.43026	-0.0398	0.45394	0.10959	0.01565	0.57202	-0.4686	-0.4256	-0.3516	-0.6097	-0.1022	0.06867
NM_012536	Ctrb1	0.24666	0.07829	0.17049	0.14032	-0.042	0.19501	0.10115	0.12	0.02008	-0.0694	0.1544	0.0252	0.33429	0.44244
NM_001077649	Ctrc	0.12245	-0.0674	0.04105	0.0371	0.14171	0.39297	0.15181	0.01326	0.02254	0.07627	0.07621	0.04814	0.00751	0.06899
NM_054009	Ctrl	0.03744	0.02064	0.67113	0.5338	0.11564	0.19137	0.04526	0.1208	0.1104	-0.0389	0.2195	0.02242	0.12909	0.07649
NM_001106099	Cts7_predicted	0.06888	0.12585	-0.0301	-0.0339	0.00515	-0.1097	-0.0523	-0.047	-0.1322	0.01545	-0.0162	-0.1148	-0.0587	-0.0694
XM_001065250.1	Cts8_predicted	-0.0505	0.09106	0.1173	0.26161	0.07609	0.29676	-0.1119	-0.0579	0.00993	0.08354	-0.0697	0.00389	0.11769	0.03493
NM_022597	Ctsb	0.56147	0.74377	0.01658	0.87565	1.0476	0.83517	0.39584	1.1106	0.48375	0.78575	0.71011	-0.1333	0.24393	0.32599
NM_017097	Ctsc	-0.2674	0.14016	-0.1178	-0.1165	-0.1698	-0.1288	-0.0233	-0.1143	0.24677	0.01623	0.02598	-0.1295	-0.2713	-0.1492
NM_134334	Ctsd	0.79867	0.8464	-0.3893	0.69762	0.73913	0.69264	0.50975	0.79427	0.4258	0.41741	0.61847	-0.0635	-0.0192	0.05966
NM_012938	Ctse	-0.0917	0.0301	-0.0453	0.01948	-0.1328	-0.0398	-0.1757	-0.1474	-0.1349	-0.1056	0.01118	0.05402	0.01315	-0.0483
NM_001106041	Ctsg_predicted	0.08504	-0.0361	0.04499	0.22407	0.02067	-0.0755	0.05583	0.01262	0.02989	-0.0839	-0.0265	0.02684	0.02024	-0.0591
NM_012939	Ctsh	-0.0753	-0.1009	-0.1594	-0.1859	-0.1009	-0.2026	-0.1175	-0.2016	-0.1697	-0.3346	-0.2008	-0.2167	-0.0199	-0.1932
NM_031560	Ctsk	-0.2062	-0.3032	0.01716	1.0561	-0.3106	-0.424	0.41838	-0.2971	-0.1526	-0.215	-0.1085	-0.1888	-0.4932	-0.6843
NM_013156	Ctsl	0.68421	0.57641	0.03971	0.42098	0.83228	0.80105	0.85684	0.94222	0.51745	0.55877	0.39158	0.13934	0.03976	0.13959
NM_139262	Ctsq	-0.0174	-0.0556	-0.0002	-0.0239	0.05894	0.04117	-0.0148	0.10593	0.05089	0.04162	-0.0117	0.17568	-0.0086	0.03498
NM_001002813	Ctsql2	0.12776	0.03272	0.0621	0.07374	-0.0104	-0.0512	-0.013	0.00696	0.02988	0.06959	-0.0755	0.08038	0.08951	-0.0186
NM_175581	Ctsr	-0.074	0.01252	-0.0955	0.0775	-0.0706	0.17384	0.02618	-0.1188	-0.0074	-0.1286	-0.0861	0.0938	-0.1005	0.0833
NM_017320	Ctss	0.04321	-0.0076	0.06174	0.19802	0.07346	-0.004	0.2781	0.2562	0.04062	0.03503	0.1087	0.11491	0.16548	0.26855
NM_001024242	Ctsw	-0.3797	-0.2479	-0.4231	-0.3117	-0.2787	-0.2685	-0.3341	-0.3809	-0.1606	-0.402	-0.2638	-0.0809	-0.2548	-0.2902
NM_021868	Cttn	0.38012	0.07994	-0.2739	-0.1759	0.33291	-0.1564	-0.0354	0.10331	-0.0652	0.01703	-0.2189	-0.3511	0.0937	0.07561
NM_001114401	Cttnbp2	0.16659	-0.0262	-0.0496	-0.0261	0.09513	0.26302	0.14194	-0.0441	0.08541	0.23385	0.06572	0.10308	0.05219	-0.0694
NM_001107712	Cttnbp2nl_predicted	0.57739	0.21247	0.20997	0.05216	0.62025	0.45066	0.40667	0.72574	0.0387	0.25601	0.27715	0.07132	0.38524	0.25566
NM_053332	Cubn	-0.1004	-0.2115	-0.0518	-0.138	-0.2487	-0.054	-0.0974	-0.2385	-0.2438	-0.3518	-0.2423	-0.1386	-0.0793	-0.2848
NM_001013971	Cuedc1	-0.3613	0.06375	0.00156	-0.4692	-0.3242	-0.0941	0.00601	-0.6246	0.03256	-0.112	0.19143	0.34412	-0.1927	-0.1493
NM_001079886	Cuedc2_predicted	-0.1276	-0.2569	-0.1921	-0.1529	-0.1757	-0.1152	-0.207	-0.2972	0.00643	-0.3542	-0.3583	-0.0315	0.17608	-0.1256
NM_001025421	Cugbp1	-0.3597	-0.1736	0.06356	-0.2861	0.06925	-0.136	-0.332	0.36071	-0.0542	-0.1755	-0.404	-0.2187	0.59902	0.49
NM_017197	Cugbp2	0.00705	-0.1004	0.01665	0.09291	0.14088	-0.0179	-0.112	-0.0548	-0.1085	0.05264	-0.1967	0.1578	-0.0503	-0.1248
NM_001108627	Cul1_predicted	-0.3484	-0.4607	-0.5269	-1.0958	-0.3442	-0.2877	-0.0772	-0.3168	-0.4167	-0.3483	-0.2461	-0.3336	-0.4448	-0.5584
NM_001108417	Cul2_predicted	-0.012	-0.0151	0.21902	0.00564	0.11008	0.08916	0.11848	0.15125	0.21408	0.12033	0.07885	0.23573	0.13128	0.04521
NM_001108417	Cul2_predicted	-0.2391	0.13251	-0.3289	-1.0361	-0.4744	-0.3135	-0.1258	-0.4284	-0.163	-0.2792	-0.1745	-0.0384	-0.0035	-0.0193
NM_001106923	Cul3_predicted	0.22633	0.10844	0.02516	-0.2326	0.08455	-0.2444	0.1487	-0.0277	0.23125	0.24541	-0.0448	0.17678	0.25907	0.19657
NM_001106951	Cul4b_predicted	-0.2419	-0.5803	-0.3795	-0.0446	-0.4241	-0.3552	-0.2922	-0.2348	-0.3316	-0.3744	-0.5437	-0.432	-0.367	-0.4775

NM_022683	Cul5	0.0421	-0.0376	0.06562	0.36417	0.44309	0.08963	-0.112	0.80985	0.01602	0.13496	-0.233	-0.2408	0.16189	0.15282
NM_001164706	Cuta	-0.0946	-0.4439	-0.1662	-0.5489	-0.2899	0.00406	-0.5656	-0.3686	-0.3116	-0.5643	-0.3956	-0.0008	0.07298	0.08224
NM_001108525	Cutc_predicted	-0.1367	-0.115	0.15341	0.21527	0.02425	-0.0101	0.18252	0.12848	-0.1061	-0.0218	-0.2244	0.00113	0.00776	-0.0488
XM_001070482.1	Cutl1	-0.5101	-0.3367	0.12977	-0.3842	-0.1525	-0.5584	-0.1119	-0.3632	-0.5436	-0.5096	-0.4297	-0.6757	-0.4699	-0.2095
XM_001070482.1	Cutl1	-0.0043	0.04951	0.16961	0.04279	-0.013	0.11017	0.08663	0.14102	0.08206	-0.0123	-0.019	-0.0213	0.2401	0.23937
XM_347163.3	Cutl1	-0.0729	-0.0591	-0.1282	-0.1803	0.05502	-0.1986	-0.0624	-0.1808	-0.2672	-0.1776	-0.3733	-0.1884	-0.1308	-0.2077
NM_001105931	Cutl2_predicted	0.11216	0.11206	0.04513	0.36057	0.35072	0.18284	0.2065	0.23145	0.05419	0.15619	0.25642	0.22496	0.21649	0.07259
NM_054005	Cuzd1	-0.0271	-0.1463	-0.1269	-0.0725	-0.0946	-0.042	-0.069	-0.1145	-0.0365	0.04826	0.04942	-0.1455	-0.1771	-0.1785
NM_001024987	Cwc15	0.2749	0.1136	0.07188	0.39158	0.18018	0.59515	0.0449	0.1632	0.29085	0.23174	0.02273	0.4767	0.38472	0.12821
NM_001108928	Cwf19l1_predicted	0.03964	-0.0315	0.31191	0.13255	-0.0103	-0.1639	0.1261	0.10545	-0.2003	-0.3022	-0.2571	-0.3226	0.34236	-0.115
NM_001135003	Cwf19l2_predicted	0.38011	0.58328	0.42711	0.8122	0.17398	-0.1095	0.79442	0.34157	0.50481	0.87001	0.62858	0.22292	0.33011	0.18438
NM_134455	Cx3cl1	0.62336	2.9667	0.13009	-0.2442	0.13018	-0.0378	2.4453	0.36038	2.3139	2.4345	2.5423	0.21486	-0.3311	-0.3687
NM_133534	Cx3cr1	0.05178	0.07992	0.14045	0.12947	0.29845	0.0486	0.33416	0.15236	0.23414	0.19222	0.10682	0.22569	0.0751	0.31486
NM_053570	Cxadr	0.20314	0.73821	0.40862	0.86993	0.80345	0.82948	0.48691	0.89232	0.47966	0.47038	0.65021	0.37895	0.15488	0.07798
NM_199406	Cxadrl1	0.02513	0.29805	0.189	-0.0021	0.24726	0.17139	0.04879	0.04911	0.07261	-0.0553	-0.1049	-0.0515	0.06411	-0.0713
NM_030845	Cxcl1	-0.715	-2.8745	-0.3716	-1.1899	-0.8303	-0.6844	-1.1119	-0.8106	-1.8122	-1.8593	-2.3656	-0.9856	-1.6045	-1.6429
NM_139089	Cxcl10	1.0122	0.08825	0.97531	0.32262	1.2026	1.5126	0.22842	1.0885	0.72773	0.12329	-0.0694	1.5475	1.3564	1.5462
NM_182952	Cxcl11	0.17876	0.34559	0.25381	0.18057	0.99401	0.77286	0.58808	0.79423	0.11028	0.12774	-0.1224	0.36281	0.12077	0.2958
NM_022177	Cxcl12	-0.2041	1.0748	-0.6955	-2.1698	-2.7421	-2.8607	-1.2795	-2.6775	1.1614	1.1374	0.67562	0.83439	-0.1203	-0.3641
NM_001017496	Cxcl13	0.00863	-0.0239	0.15501	0.21429	-0.0174	0.13039	0.01307	0.00714	0.0144	0.0141	0.26738	0.10397	0.06573	0.08558
NM_001017478	Cxcl16	0.56601	0.49088	0.3592	0.38006	0.3735	0.4207	0.94027	0.42261	0.68769	0.44182	0.36717	0.53821	0.21033	0.14816
NM_001017478	Cxcl16	0.00419	0.0774	-0.1756	0.21317	-0.0021	-0.0323	-0.0891	0.07025	-0.1812	-0.1087	0.02879	0.18061	-0.2221	0.02472
NM_053647	Cxcl2	-0.0036	0.04458	-0.2236	-0.0552	-0.2096	-0.1462	-0.0271	0.14248	-0.2535	-0.0569	-0.2127	-0.0077	-0.0536	-0.1039
NM_138522	Cxcl3	0.26031	0.05116	0.02601	0.0289	0.09408	0.07147	0.04227	0.22178	0.15468	-0.0323	0.06867	0.14166	0.05137	-0.0166
NM_022214	Cxcl5	0.01618	-0.0207	-0.0023	0.06618	-0.0932	0.21342	-0.0603	0.0596	0.14788	0.06296	-0.066	-0.0381	0.01131	-0.0691
NM_145672	Cxcl9	-0.1712	0.09209	0.16458	0.13049	-0.1215	0.00076	0.0311	0.3518	0.02688	0.1084	0.2588	0.2826	0.02732	0.0357
NM_053415	Cxcr3	-0.1572	0.11612	-0.0628	-0.0445	-0.184	-0.2771	-0.3569	0.1916	-0.0704	-0.3798	-0.0883	-0.0429	-0.367	-0.2441
NM_022205	Cxcr4	0.04515	-0.0467	0.0868	-0.0559	-0.0274	-0.1051	0.04674	-0.0254	0.13676	-0.1111	0.04044	-0.0271	0.11036	-0.0342
NM_053352	Cxcr7	0.48981	1.5374	-0.3632	-1.0878	-1.1752	-0.8243	0.80469	-0.9975	1.3843	1.2558	1.6355	0.80873	0.31452	-0.0658
NM_001079698	Cxxc1	-0.2955	-0.4989	0.11473	-0.1041	-0.0301	-0.3997	0.10966	-0.2616	-0.354	-0.5483	-0.1876	-0.3651	-0.4593	-0.2767
NM_053342	Cxxc4	-0.1307	-0.0459	-0.102	0.03793	-0.188	0.00415	0.01113	-0.1462	-0.0285	-0.167	-0.2004	-0.1699	-0.2667	-0.0931
NM_001007628	Cxxc5	0.69002	0.71125	0.40627	-0.0881	0.39607	0.58067	0.17485	0.4752	0.90779	0.73539	0.75945	1.323	0.43076	0.60773
NM_001107643	Cxxc6_predicted	-0.0466	-0.0049	0.0136	0.08098	0.05587	0.17619	-0.0708	-0.0014	-0.0156	-0.0292	-0.0413	-0.0358	0.24624	0.05239
NM_022245	Cyb5	-0.4833	-0.3234	0.13058	-0.7632	-0.7289	-0.3331	-0.0908	-0.901	0.06536	-0.0498	-0.0662	0.69485	0.1898	0.14533
NM_001107056	Cyb561_predicted	-0.4326	-1.0177	-1.5222	-1.4377	-0.7197	-0.6534	-0.6635	-1.0555	-1.0519	-1.365	-0.8968	-1.7636	-1.8391	-1.6734
NM_001108562	Cyb561d1_predicted	-0.0348	-0.0033	-0.3205	-0.6121	-0.1633	-0.0408	-0.3853	-0.1239	0.07217	-0.0617	-0.0871	-0.4171	-0.2481	-0.0677
NM_001007753	Cyb561d2	0.20449	0.31136	0.10589	0.63593	0.43725	0.81365	0.0509	0.3881	0.44168	0.29949	0.22515	0.79048	0.2498	0.27732
NM_001007671	Cyb5d2	0.38381	0.41137	0.041	-0.0971	0.59217	0.48045	0.2517	0.2666	-0.2618	0.01202	0.2674	0.04367	0.34124	-0.0625
NM_001013126	Cyb5r1	-0.085	-0.3169	-0.4712	-0.2401	-0.3846	-0.5578	-0.0498	-0.3429	-0.3696	-0.6795	-0.0133	-0.2688	0.10014	-0.2896
NM_001014244	Cyb5r2	0.01744	0.05199	0.11762	0.03003	-0.0277	-0.0325	0.07401	-0.026	-0.0571	0.01489	0.21137	0.0495	0.01467	-0.0058
NM_138877	Cyb5r3	-0.2228	0.25774	-0.3266	-0.1055	-0.0501	0.17113	-0.1493	-0.0817	0.13901	0.32388	0.34952	-0.1503	-0.629	-0.5414
NM_133427	Cyb5r4	0.24551	-0.0009	-0.3536	-0.2026	0.24191	0.24483	-0.1899	0.17203	0.16335	0.23691	0.13479	0.03466	0.07851	0.04781
NM_024160	Cyba	0.36311	0.18593	0.29535	0.28537	0.12507	0.34886	0.23673	-0.0505	0.14262	0.15266	0.20263	0.04941	0.43003	0.16969
NM_023965	Cybb	0.07969	0.03735	0.09802	0.08232	0.02285	0.06466	0.00184	0.19357	0.12013	0.21512	0.08675	0.10753	0.04784	0.09731
NM_001011954	Cybrd1_predicted	0.02922	0.00329	0.27603	0.20037	0.05563	0.03233	-0.0295	0.1753	0.26804	0.15877	-0.0335	0.03623	0.31313	0.04711
NM_001130491	Cyc1_predicted	0.50937	0.3192	0.52856	0.66932	0.54279	0.74471	0.07324	0.6316	0.32998	0.35643	0.40814	0.41358	0.49422	0.65978
NM_012839	Cycs	0.37434	-0.2633	0.31979	-0.4611	0.12693	0.02285	-0.3353	0.21757	0.08054	0.02427	-0.0919	0.17475	0.60533	0.27039
NM_012840	Cyct	-0.0836	-0.0736	-0.1909	-0.0679	-0.0955	0.14228	-0.0365	-0.236	-0.1574	-0.0472	-0.1412	0.03307	-0.2426	-0.2282
NM_001107517	Cyfip1_predicted	-0.6242	-0.0188	-0.1814	-0.73	-0.4343	-0.4826	-0.0594	-0.5107	-0.1683	-0.1315	-0.3748	-0.0012	-0.2322	-0.175

NM_001106996	Cyfip2_predicted	-0.122	0.17178	0.07744	-0.009	-0.1555	-0.1154	-0.0483	-0.1588	0.13972	-0.219	-0.0789	-0.0903	0.04148	-0.0524
NM_130744	Cygb	-0.2106	-0.2521	-0.2594	-0.1767	-0.1209	-0.0713	-0.283	-0.2161	-0.1282	0.07823	-0.1786	-0.2072	-0.1115	-0.2561
NM_001025122	Cyhr1	-0.5505	-0.3109	-0.1784	-0.4542	-0.5284	-0.5526	-0.1901	-0.2803	-0.0133	-0.2467	-0.1329	0.10277	-0.5337	-0.5374
NM_017286	Cyp11a1	0.1923	-0.0701	0.01881	-0.0566	0.15313	0.24032	-0.1494	0.07273	-0.0331	0.0682	-0.0304	-0.1138	-0.0664	-0.0573
XM_579335.1	Cyp11b1	-0.2983	-0.3869	-0.0964	-0.3367	0.17212	0.05851	1.2377	0.07119	-0.0352	-0.4137	-0.1705	0.44609	-0.3675	-0.2578
NM_012537	Cyp11b1	-0.278	0.22996	0.5197	0.09248	-0.6279	-0.609	1.7064	-0.6728	0.81165	0.13653	0.59161	1.2417	0.46596	0.36714
NM_012538	Cyp11b2	0.21081	-0.1838	0.24974	-0.0518	0.10537	0.17342	1.1639	0.35991	0.16994	-0.1223	0.1665	0.64944	-0.2422	-0.1329
NM_181824	Cyp11b3	0.1458	-0.0256	0.09969	0.33992	0.23083	0.16095	0.01348	-0.0158	0.08951	-0.0559	-0.0074	-0.145	0.11906	0.03572
NM_012753	Cyp17a1	-0.2854	-0.3015	-0.1633	-0.2028	-0.1177	-0.2917	-0.1641	-0.0337	-0.2153	-0.1624	-0.2034	-0.3065	-0.2552	-0.3203
NM_017085	Cyp19a1	-0.147	0.00758	-0.1055	0.07575	-0.1186	0.06481	0.0964	-0.0978	-0.1335	0.1912	-0.0361	0.15705	-0.2663	0.09462
NM_012540	Cyp1a1	0.05188	0.01938	-0.034	0.05575	0.01827	-0.0457	-0.0104	0.04871	0.02599	0.01421	0.04409	0.01465	-0.0418	0.15462
NM_012541	Cyp1a2	0.01022	0.01062	-0.0487	0.20529	-0.0242	-0.0316	0.0077	0.02271	-0.0036	0.1063	-0.0264	0.01012	0.13444	0.01946
NM_012940	Cyp1b1	0.68997	1.1439	0.2687	0.71383	-0.1804	-0.2227	1.083	-0.1251	0.93624	1.1119	1.0083	0.34108	0.79498	0.70972
NM_199401	Cyp20a1	-0.0213	0.10749	-0.5957	-0.5233	-0.2549	-0.1681	-0.161	-0.2829	0.10016	0.02994	-0.0243	0.48182	0.65521	0.71728
NM_057101	Cyp21a1	0.26918	-0.0701	0.12519	0.1203	0.04295	0.01365	-0.072	-0.071	-0.1364	-0.083	-0.0908	0.19512	-0.2471	-0.1285
NM_201635	Cyp24a1	0.34457	0.58489	2.9787	2.1218	0.58464	0.71525	2.6108	0.42042	0.34147	0.58843	0.83676	0.47178	0.94701	0.73395
NM_130408	Cyp26a1	-0.0628	0.03375	-0.0288	-0.0233	-0.0617	-0.0486	-0.0027	-0.0927	-0.194	-0.2277	-0.2062	-0.0799	-0.1418	-0.0053
NM_181087	Cyp26b1	0.37391	-1.2267	-0.9169	-1.3795	1.0484	1.0053	-1.0306	1.1371	-1.3026	-1.0137	-0.8224	-0.9947	-0.6299	-0.5379
XM_217935.3	Cyp26c1_predicted	0.01018	-0.1062	-0.0035	-0.0437	-0.1819	0.02648	-0.0262	-0.0974	-0.1472	0.01667	-0.06	0.11373	-0.0105	-0.0135
NM_178847	Cyp27a1	0.02786	0.37717	-0.2784	-0.5349	-0.0107	-0.1142	-0.1328	-0.1879	0.36741	0.48069	0.11776	-0.1177	-0.1811	-0.2068
NM_012692	Cyp2a1	0.15634	-0.0702	0.2299	-0.0023	0.0867	-0.0403	-0.0468	0.06169	0.05204	-0.0814	-0.0011	0.08277	-0.0303	-0.1312
NM_012693	Cyp2a2	-0.1472	0.14696	-0.1561	-0.1643	-0.0968	-0.2265	0.07144	-0.1912	0.1123	-0.2413	-0.3246	-0.0678	0.17261	-0.2254
NM_012542	Cyp2a3a	0.10444	0.14998	0.18549	-0.0414	0.01854	-0.0169	-0.0114	0.05499	-0.0124	0.05346	-0.0083	0.0238	-0.0267	0.07805
NM_017156	Cyp2b15	0.03673	0.03727	0.02686	0.18827	-0.0346	0.2507	-0.0074	0.01749	0.00331	0.04711	0.17679	0.01381	0.05883	0.19204
NM_017156	Cyp2b15	0.75823	0.3786	0.41463	0.12942	0.60097	0.71389	0.23614	0.48035	0.4825	0.23403	0.486	0.28452	0.58798	0.35948
NM_198733	Cyp2b21	-0.0017	0.13813	0.00444	0.05668	0.10703	0.07326	0.01088	0.13082	-0.0125	0.03371	0.038	0.1196	0.1482	-0.0165
NM_173294	Cyp2b3	-0.1542	-0.0679	-0.0227	-0.1414	-0.0654	-0.1179	-0.1347	-0.0439	-0.069	-0.0353	0.04586	-0.1545	-0.1163	-0.1462
NM_019184	Cyp2c	-0.7431	-0.3572	-0.8948	-0.8798	-0.827	-0.6546	-0.8982	-0.8116	-0.8784	-0.7993	-0.5799	-0.773	-0.68	-0.8203
NM_031572	Cyp2c12	0.22922	0.18401	0.44546	0.28963	0.3693	0.32824	0.14325	0.10387	0.02244	0.47273	0.12531	0.35111	0.27317	0.11127
NM_138514	Cyp2c13	0.35145	0.16427	0.12298	0.05385	0.09541	0.07361	0.3833	0.41895	0.06837	0.16538	0.12727	0.19665	0.22719	0.06981
NM_138512	Cyp2c22	-0.1784	-0.1762	-0.2243	-0.1124	-0.1389	-0.0214	-0.0964	-0.1468	-0.2267	-0.1657	-0.1881	-0.1468	-0.1132	-0.0905
NM_031839	Cyp2c23	0.00208	-0.04	-0.0152	-0.1632	-0.0782	0.03274	-0.1731	-0.1687	0.03084	0.1968	0.09446	-0.0686	0.02493	-0.2135
XM_001063361.1	Cyp2c37	0.00987	0.08709	0.00854	0.01512	-0.0705	0.15508	0.05576	0.11035	0.02986	0.01054	-0.0079	0.04532	0.14339	0.0139
XM_217906.4	Cyp2c55_predicted	0.2035	0.05849	0.07546	0.09869	0.13243	0.03341	0.24019	0.16592	0.01813	0.12519	-0.1357	0.07426	-0.0412	0.05394
XM_574666.1	Cyp2c6	0.11499	0.02592	0.06086	-0.0009	0.13847	0.11963	-0.0163	0.20141	0.04329	0.01871	0.19435	0.01744	0.05515	0.15771
XM_219933.3	Cyp2c65_predicted	-0.1512	0.0147	-0.0297	-0.1853	-0.1512	-0.0642	-0.1745	-0.068	-0.0491	-0.1556	-0.0329	-0.0775	-0.2632	-0.1547
NM_017158	Cyp2c7	0.02169	0.15391	0.23552	0.5427	-0.0129	0.08499	0.90104	0.16114	0.07513	0.70505	0.06661	0.35284	0.14831	0.39087
NM_153313	Cyp2d1	0.30939	0.3567	0.2626	0.33392	0.03581	0.13515	0.55685	0.28555	0.70866	0.31003	0.881	0.0826	0.44915	0.51391
NM_012730	Cyp2d2	0.29773	0.09425	0.23074	0.48492	0.32444	0.38862	0.3892	0.52666	0.2093	0.21601	0.35763	0.10979	0.03897	0.43543
NM_138515	Cyp2d22	-0.0517	-0.2268	-0.0266	-0.1897	-0.0022	-0.0738	0.17288	0.38291	-0.1878	-0.1517	-0.2645	-0.2308	-0.3126	-0.2407
NM_173304	Cyp2d5	-0.0928	0.31189	0.10741	0.15549	-0.0726	0.05751	0.31146	0.17745	0.08211	0.48251	0.19696	0.08155	-0.0964	-0.0198
NM_031543	Cyp2e1	-0.055	0.02456	-0.0916	-0.1003	-0.103	0.01016	-0.074	-0.0504	-0.0383	-0.1412	0.10501	-0.0531	0.0087	-0.0886
NM_019303	Cyp2f4	-0.1568	0.03094	-0.0369	0.00068	-0.1472	-0.1964	-0.1676	-0.079	-0.2339	-0.0214	-0.119	-0.1906	-0.1221	-0.1784
NM_012787	Cyp2g1	0.22169	0.16592	0.09857	0.00433	0.0076	-0.1078	0.15334	0.01824	0.14742	0.22181	-0.0002	0.01965	0.08551	0.00381
NM_175766	Cyp2j3	-0.163	-0.2831	-0.1679	-0.104	-0.1778	-0.2243	-0.1367	-0.336	-0.3041	-0.276	-0.1889	-0.2341	-0.374	-0.2007
NM_001108499	Cyp2r1_predicted	0.04071	-0.0701	-0.1176	-0.2096	-0.0675	0.209	-0.0102	0.11904	0.08425	-0.0664	-0.0907	0.23926	0.17742	0.05014
NM_001107495	Cyp2s1	1.7724	1.0248	0.46942	0.55362	1.2834	0.95137	1.0541	1.6227	0.68096	1.0813	1.0575	0.25371	1.4743	1.5267
NM_134369	Cyp2t1	0.09857	0.02749	-0.1115	0.09327	-0.0632	-0.1135	-0.0711	0.11071	-0.0507	-0.0438	0.07708	-0.0731	-0.0871	-0.0515
NM_001024779	Cyp2u1	0.01774	-0.0841	0.01939	0.0729	0.08707	-0.0498	-0.0184	-0.0717	0.21222	-0.0259	0.06049	0.07672	-0.0627	0.05273

XM_001074391.1	Cyp2w1_predicted	-0.0218	-0.1868	-0.0122	-0.0454	-0.0091	0.00297	0.04351	0.03627	0.01961	-0.0931	0.00464	0.20174	-0.0468	-0.137
NM_001106893	Cyp39a1_predicted	0.06842	0.12393	0.13378	0.1281	-0.0318	0.03681	0.05893	0.07845	0.02527	0.24186	0.10471	0.01177	0.04252	0.23085
NM_013105	Cyp3a1	-0.1058	0.11852	-0.152	-0.095	-0.1763	-0.1238	-0.1179	-0.1294	-0.1515	0.06145	-0.1301	0.01477	-0.1166	-0.0216
NM_147206	Cyp3a13	0.73432	-0.6049	2.1282	1.1463	3.0098	3.0492	1.9238	3.0909	0.08456	0.02266	2.1264	0.57612	1.9706	1.8887
NM_145782	Cyp3a18	-0.0858	0.07984	-0.0463	-0.1459	-0.0861	-0.0933	-0.1032	-0.0919	-0.0586	-0.0614	-0.0994	-0.0762	-0.0102	-0.0925
NM_153312	Cyp3a2	#######	0.12071	0.17805	0.07196	0.15573	0.05448	0.07311	0.12389	0.05708	0.15479	-0.0409	0.16196	0.01397	0.00688
NM_013105	Cyp3a3	-0.0757	-0.0018	0.07492	-0.1273	-0.2073	-0.1528	-0.1235	-0.0998	0.08034	0.10232	-0.1542	-0.162	-0.1189	0.04732
NM_001108723	Cyp46a1_predicted	-0.0198	-0.0957	-0.2112	0.04373	-0.2162	-0.0211	-0.0289	-0.1583	-0.1348	-0.1188	0.02053	-0.1832	-0.1612	-0.244
NM_175837	Cyp4a1	-0.0406	-0.0745	-0.0812	0.06271	0.02343	-0.033	-0.0123	-0.0365	0.02832	0.22024	-0.0278	-0.043	0.0662	-0.016
NM_175837	Cyp4a10	-0.1265	-0.0385	-0.2126	0.33687	-0.1868	0.21858	-0.2321	0.04365	-0.0338	-0.1034	0.07498	0.10008	-0.0622	-0.0974
NM_175760	Cyp4a3	-0.3326	-0.2263	-0.1789	-0.1369	-0.0301	-0.0922	-0.2088	-0.2455	-0.1671	-0.1191	-0.2	-0.1037	-0.0742	-0.0447
NM_031605	Cyp4a8	0.01037	-0.0116	0.11928	-0.0881	-0.006	0.04286	0.08583	0.19026	0.01753	0.01944	0.01506	0.12203	0.12262	0.04915
NM_016999	Cyp4b1	0.94421	0.0032	0.27317	0.16801	0.54085	0.39589	0.28714	0.62275	0.00316	-0.1735	-0.0495	0.14736	-0.0016	0.01145
NM_019623	Cyp4f1	-0.0077	0.34304	0.39835	0.10152	0.26639	0.1904	0.26979	0.23573	0.1248	0.02041	0.31337	0.17992	0.21199	0.51646
NM_001033686	Cyp4f18_predicted	0.02728	0.13271	0.09221	-0.0492	-0.1066	0.09515	-0.0523	-0.1778	-0.0397	-0.1893	-0.0129	-0.1143	-0.1196	0.06011
NM_173123	Cyp4f4	0.03426	-0.0623	0.23808	0.06497	0.09864	-0.075	-0.0125	-0.0436	0.02041	0.04195	-0.0151	0.28829	0.08334	0.07303
NM_173124	Cyp4f5	0.30184	0.23845	0.13448	-0.2118	0.12269	0.1143	0.29697	0.14067	0.21822	0.21125	0.45405	0.38467	0.11128	0.16442
NM_153318	Cyp4f6	0.21086	0.70448	0.36326	1.1172	-0.168	-0.1218	0.59215	-0.0684	0.41535	0.68547	0.38261	0.55898	-0.4652	-0.4959
NM_012941	Cyp51	-0.5524	-0.6717	0.52778	0.24973	-0.7761	-0.5308	-0.1081	-0.6331	-0.3382	-0.854	-0.7284	0.56577	0.02486	-0.1649
NM_012942	Cyp7a1	0.01923	0.25558	0.27912	-0.0401	0.00074	0.14731	-0.0207	-0.0706	0.03231	-0.0235	0.09972	-0.0177	0.11906	0.15977
NM_019138	Cyp7b1	0.09286	0.13328	0.35265	0.04691	0.14425	0.11218	0.26685	0.01725	0.25746	0.01927	0.05868	0.04162	0.217	0.02072
NM_031241	Cyp8b1	-0.087	-0.0338	0.09244	0.05374	0.20863	0.10206	-0.0544	0.16138	-0.1042	-0.1126	-0.1148	0.03229	0.01308	0.1536
NM_031327	Cyr61	0.06539	-0.7046	-0.7408	1.0242	-0.1832	0.52719	-1.9967	-0.0578	0.23885	-0.1624	-0.3982	0.94012	0.15146	-0.0805
NM_053641	Cysltr1	0.04845	0.18216	0.27186	0.05949	0.16074	0.20415	0.23671	0.06376	0.27367	0.46377	0.17698	0.11033	0.21605	0.11849
NM_133413	Cysltr2	-0.1109	-0.0622	-0.0859	0.04821	0.14131	0.03832	0.00607	-0.0323	0.03846	0.28923	0.0958	0.12796	-0.0423	0.02825
NM_198685	Cyss	0.12249	1.409	-0.1563	0.02085	-0.0844	-0.0817	-0.0284	0.13428	0.97442	1.3027	1.1832	-0.0017	0.10984	-0.0777
NM_001013980	Cyyr1	-0.0338	-0.0009	-0.0633	0.004	0.10394	0.04817	-0.1277	0.0296	-0.0005	-0.0925	0.11326	-0.1018	-0.0907	-0.0382
NM_053877	D123	-0.2994	-0.1044	-0.0999	-0.0642	-0.2709	-0.3657	-0.0531	-0.2662	0.0853	0.01465	-0.0343	0.14637	-0.0744	0.09013
NM_001108030	Daam1_predicted	-0.0244	-0.2725	0.00433	0.12818	0.01286	0.07618	-0.2662	-0.0541	-0.1541	-0.0678	0.02233	0.11982	0.28594	-0.0409
XM_236909.4	Daam2_predicted	0.1099	0.13878	-0.0084	-0.0175	0.11196	0.06239	0.00225	0.23238	0.19274	0.07089	0.03831	0.31746	0.10632	0.12391
NM_153621	Dab1	-0.0917	0.19037	-0.0457	-0.0408	0.05704	-0.0843	0.05606	0.01492	0.01606	-0.0349	0.13319	0.22636	0.15239	0.12217
NM_024159	Dab2	0.53801	0.91022	0.4293	0.80915	0.2899	-0.1856	1.0481	0.4218	0.77377	0.91856	0.64527	0.06474	-0.0099	0.08229
NM_138710	Dab2ip	-0.1745	-0.2638	-0.2517	-0.4621	0.56887	0.54204	-0.2802	0.61877	-0.2933	-0.2866	-0.0672	0.02806	-0.6455	-0.2363
NM_001107464	Dact2_predicted	-0.0477	-0.1337	-0.1171	-0.2025	-0.2211	-0.1107	0.01314	-0.266	-0.2007	0.04112	-0.2263	-0.165	-0.0983	-0.1452
NM_138910	Dad1	0.058	0.16548	-0.0905	-0.1161	0.09713	0.29998	-0.3507	0.09406	0.18488	0.10538	0.30442	0.37345	0.24558	0.48191
NM_022269	Daf1	0.95497	0.51341	0.88069	0.20955	0.32743	0.15114	0.87979	0.42637	0.35433	0.34441	0.42771	-0.2316	0.9634	1.1545
XM_343483.3	Dag1	0.26005	0.66026	0.22148	0.76292	0.39607	0.35376	0.22928	0.38153	0.27104	0.62275	0.39148	-0.2242	0.1869	0.18309
NM_001005886	Dagla	-0.0167	-0.058	0.08523	0.20742	0.07668	-0.1061	0.01226	-0.0199	0.16805	0.05189	0.15484	0.2525	0.09925	0.01069
NM_053626	Dao1	-0.0559	-0.0512	0.13312	-0.0885	-0.0493	-0.2766	-0.1294	-0.167	-0.2028	-0.1228	-0.2275	-0.0955	-0.3227	-0.0493
NM_022526	Dap	0.5021	0.39515	-0.1286	-0.6859	0.1615	0.24881	0.08048	0.25153	0.12978	0.11585	0.08134	0.02953	-0.0468	0.02624
NM_001011950	Dap3	0.12573	-0.1043	0.32146	-0.2301	-0.2719	-0.1372	0.06794	-0.26	0.01688	-0.0619	0.16089	0.31517	-0.0557	0.0132
NM_001107335	Dapk1_predicted	0.05992	-0.1078	-0.0457	0.03673	0.11779	0.00957	0.03869	0.15081	-0.0085	0.08856	-0.0934	0.04835	-0.048	-0.0211
NM_001108568	Dapp1_predicted	2.2516	0.63605	0.41464	1.605	1.2276	1.714	1.1141	1.4213	0.68635	0.59763	0.78515	0.52635	0.7974	0.59081
NM_053799	Dars	0.01866	0.18522	0.10946	-0.1437	-0.3034	-0.7732	-0.1455	-0.2954	-0.0074	0.11955	0.14999	-0.4249	0.00992	0.07917
NM_001034143	Dars2	0.398	0.24443	0.09947	0.22895	0.13234	0.0364	0.04011	-0.3304	0.06172	0.26262	0.30958	-0.1972	0.17184	0.22057
NM_001108612	Datf1_predicted	0.13526	-0.0098	0.04424	0.18715	0.01754	-0.04	0.07077	-0.0999	-0.054	0.10291	0.1125	0.02938	0.12247	0.02955
NM_080891	Daxx	-0.5393	-0.4452	-0.1553	-0.2873	-0.3271	-0.4771	-0.1608	-0.5369	-0.3959	-0.2095	-0.5401	-0.0135	-0.255	-0.4631
NM_001025742	Dazap1_predicted	-0.6077	-0.7131	0.41143	-0.059	-0.7206	-0.5285	-0.257	-0.8853	-0.5273	-0.5181	-0.5042	-0.0902	-0.2741	-0.2836
NM_080482	Dbc1	0.05214	0.05165	-0.0321	0.14341	0.15884	-0.0419	0.10297	-0.0969	0.06757	0.02442	-0.0377	0.03356	0.04403	0.00531

NM_013158	Dbh	-0.0896	-0.1309	-0.0658	-0.0509	0.20047	0.30145	0.01615	0.07693	-0.0898	-0.1576	0.14785	-0.0962	0.08893	-0.0854
NM_031853	Dbi	-0.5788	-0.5457	0.44376	-0.523	-1.2566	-1.0218	-0.056	-1.2504	0.04919	-0.4022	-0.5522	0.5612	0.11271	-0.0024
NM_021596	Dbil5	-0.1797	-0.1823	0.20674	-0.3751	-0.3006	-0.3453	-0.3163	-0.294	-0.0933	-0.0309	0.04181	0.09683	0.11848	-0.099
NM_031024	Dbn1	0.22007	0.11415	-0.1019	-0.2688	0.40875	0.48428	-0.2298	0.54028	0.09636	0.26752	0.15094	0.52021	0.34708	0.42153
NM_001014156	Dbndd1	-0.279	-0.1388	-0.147	-0.332	-0.2899	-0.0784	-0.1114	-0.1689	-0.0674	-0.0721	-0.2748	-0.1738	-0.2622	-0.1888
NM_012543	Dbp	-0.1622	-0.2275	-0.2634	0.01985	-0.0703	-0.1469	-0.1208	-0.0721	0.26209	-0.0435	0.3357	-0.2075	-0.2988	-0.1814
NM_053312	Dbt	0.37131	0.06347	0.07166	0.36454	0.29021	0.3336	0.03266	0.27287	0.32935	0.15746	0.31358	-0.0759	0.36601	0.01342
NM_001009644	Dbx1	0.15512	-0.0264	-0.1322	0.1135	-0.0255	0.07308	0.01534	-0.0432	0.12708	-0.1364	0.02099	0.09933	0.0106	0.03026
XM_001053826.1	Dbx2	-0.0277	0.04065	-0.096	-0.0489	-0.0315	-0.0968	0.07202	0.20967	0.00705	-0.0955	0.00812	-0.0195	-0.0206	-0.0009
NM_001007724	Dcakd	-0.1971	0.40911	-0.5656	-0.6082	-0.3284	-0.6533	-0.6335	-0.357	0.40908	0.29195	0.28412	-0.2051	-0.0102	0.01254
XM_236661.4	Dcamkl3_predicted	0.12939	0.49224	0.09419	0.06402	0.23798	0.21942	0.05563	0.2059	0.47974	0.45007	0.55097	0.19492	0.02495	0.06407
XM_001058071.1	Dcbld1_predicted	-0.1471	0.04672	-0.0116	0.09483	0.0612	0.15207	-0.0321	0.10586	0.22356	-0.0055	0.07213	0.01381	0.07884	0.05394
XM_001058071.1	Dcbld1_predicted	-0.204	-0.3378	-0.2813	-0.1767	-0.302	-0.3453	-0.2848	-0.2883	-0.2435	-0.1845	-0.3709	-0.282	-0.2287	-0.2442
NM_130419	Dcbld2	-0.0015	0.05439	0.3891	0.13674	0.10866	-0.045	0.36345	0.28457	0.00363	0.05688	0.09318	0.26819	0.37237	0.16357
NM_012841	Dcc	-0.0694	-0.0286	-0.151	-0.0852	-0.1214	-0.1324	-0.1786	-0.1243	-0.0794	-0.0678	-0.0492	0.08922	-0.0029	0.00877
NM_001106110	Dcdc2_predicted	-0.1199	-0.1725	-0.0683	-0.2145	0.03064	-0.1459	0.06778	-0.2018	-0.2138	0.00722	-0.0986	0.11154	0.07673	-0.0053
NM_001107544	Dchs1_predicted	-0.0295	0.12021	0.09716	0.09399	-0.0332	0.04689	0.13985	0.17177	0.02724	0.08648	0.22277	0.07608	0.1105	0.12514
XR_008777.1	Dchs2_predicted	0.26695	0.1745	0.21257	0.09341	0.21034	0.39117	0.35445	0.07896	0.12918	0.11975	0.14008	0.23104	-0.0198	0.31056
NM_017306	Dci	-0.1552	-0.0147	-0.5068	-0.3639	0.09311	0.2912	0.00268	0.11629	0.23164	-0.0863	0.1964	0.21037	0.08296	-0.0062
NM_001005880	Dcir2	0.05782	0.28595	-0.1067	0.01487	-0.022	-0.0403	0.08451	-0.0146	-0.1634	-0.0326	-0.0465	-0.0608	-0.0769	0.04806
NM_024158	Dck	0.17241	0.03053	-0.1076	0.17807	-0.1899	-0.1023	-0.2354	-0.0535	-0.0976	0.05883	-0.1883	-0.1406	0.52101	0.12795
NM_053343	Dclk1	-0.1347	0.12976	0.31195	0.30496	0.13658	0.24435	0.46313	0.33507	0.25466	0.19753	0.43808	0.57827	-0.0709	0.17735
NM_001009691	Dclk2	-0.6203	-0.5309	-0.493	-0.5407	-0.2938	-0.4507	-0.5668	-0.3122	-0.5421	-0.6121	-0.6012	-0.4486	-0.5463	-0.5436
NM_001009691	Dclk2	-0.9709	-1.032	-1.0516	-0.9704	-0.5487	-0.8829	-1.1116	-0.6704	-1.0769	-0.9897	-1.0622	-0.9025	-0.9066	-0.859
NM_001106201	Dclre1a_predicted	-0.5065	-0.2757	0.45083	-0.3547	-0.8437	-0.8588	0.18383	-0.69	-0.4054	-0.314	-0.2427	-0.1016	-0.3455	-0.2643
NM_001106201	Dclre1a_predicted	-0.266	-0.1199	0.36982	-0.202	-0.6623	-0.3827	0.17966	-0.4989	-0.3757	-0.2365	-0.0706	-0.1151	-0.1964	-0.278
NM_001025687	Dclre1b	-0.0418	0.05673	0.29653	0.37919	-0.0971	0.51445	0.16963	0.16291	0.0092	0.08097	0.26082	0.2579	0.20804	0.19923
NM_147145	Dclre1c	-0.0565	-0.105	0.01708	0.06889	-0.4405	-0.4025	0.15774	-0.4668	-0.1455	-0.1969	-0.2915	-0.3632	-0.323	0.02191
NM_147143.1	Dcm5	-0.0088	-0.024	0.06515	0.02555	-0.0076	0.10513	0.0208	0.0327	0.08549	0.39051	0.0244	0.07083	0.01662	0.13571
NM_024129	Dcn	-2.3108	5.3289	-1.0649	0.83573	-2.2401	-2.4521	0.21883	-2.3377	4.9404	5.2807	4.9897	1.5005	-1.9779	-2.3026
XM_001063126.1	Dcp1a_predicted	0.21033	0.16	0.35727	0.28519	0.10898	0.35976	0.01067	0.21668	-0.0413	0.07903	0.09358	0.66309	0.41875	0.30296
NM_001170469	Dcp2_predicted	-0.1008	0.00871	-0.0666	0.08777	-0.1081	-0.2263	0.0568	0.3012	-0.1384	0.13315	-0.0797	-0.0764	0.02015	0.1392
NM_153302	Dcps	0.09985	-0.0421	-0.0016	-0.2701	0.27722	0.31646	-0.0905	0.05721	-0.2823	-0.3237	-0.0784	0.18316	-0.0929	0.22706
XM_001074533.1	Dcst1_predicted	-0.2021	-0.146	-0.0799	0.02891	-0.1548	-0.0638	-0.2223	-0.0522	-0.122	-0.0792	-0.1095	-0.0692	-0.0888	-0.031
NM_001161512	Dctd	0.20555	0.01496	0.70424	0.30837	-0.3981	-0.6568	0.0282	-0.6897	-0.0243	-0.2507	-0.2912	-0.0811	0.38917	0.29616
NM_024130	Dctn1	0.02374	0.14732	-0.6515	-0.8553	-0.2948	-0.3571	-0.1837	-0.3482	-0.246	-0.2156	-0.0668	-0.422	-0.2511	-0.1837
NM_001004239	Dctn2	-0.0578	0.2229	-0.1454	-0.2711	0.21092	0.003	-0.1966	0.20707	0.27993	0.28203	0.13128	0.29043	0.14116	0.16451
NM_001108659	Dctn3_predicted	0.57737	0.33235	0.07029	0.00657	0.47929	1.1257	0.14672	0.28801	0.33874	0.45225	0.35717	0.63754	0.09069	0.00596
NM_053404	Dctn4	0.28442	0.66529	0.05153	0.10941	0.58073	0.21861	0.35459	0.61853	0.32699	0.46471	0.14487	0.0477	0.64037	0.62577
NM_001037778	Dctn5	0.29712	0.54022	-0.3603	0.01014	0.72175	-0.0639	0.24984	0.45875	0.55997	0.38228	0.21896	0.30352	0.54605	0.45809
NM_001106085	Dctn6_predicted	0.3196	0.01887	0.02415	0.1634	0.30102	0.52128	0.08053	0.18148	0.12918	0.26024	0.16482	0.38071	0.40033	0.109
NM_001107668	Dcun1d1_predicted	0.08014	-0.0003	-0.4433	-0.4822	0.06352	-0.0626	-0.2455	-0.1335	-0.0617	0.16658	-0.1094	0.21907	0.16397	0.19354
NM_001024886	Dcun1d3	-0.4602	-0.106	-0.2795	-0.0971	0.43661	0.25791	-0.1779	-0.0549	-0.0834	-0.1828	-0.1801	-0.0257	-0.0589	-0.0237
NM_001108359	Dcun1d4_predicted	-0.0082	-0.0461	0.03297	-0.1095	0.07532	-0.0204	0.07617	0.04692	-0.0853	0.06453	-0.0858	0.032	0.25951	0.00445
NM_001108359	Dcun1d4_predicted	0.26755	0.24091	0.26361	0.67668	0.24891	0.63705	0.21773	0.47849	0.22415	0.1915	0.06518	0.4169	0.15551	0.05385
NM_001009696	Dcun1d5	-0.0327	-0.2925	0.22614	-0.0876	-0.2428	-0.1372	-0.0544	-0.1484	0.00904	-0.0415	-0.1899	0.11218	0.11667	0.17718
NM_199403	Dd25	0.2894	0.18137	0.28576	0.31826	0.241	0.20827	0.14379	0.14633	0.15261	0.03562	0.19989	0.03734	0.27337	0.00325
XM_576252.2	Dd5	-0.2866	0.06722	0.42913	0.94218	0.08764	-0.1245	0.25372	0.15626	-0.1031	0.0999	0.13581	-0.1015	0.12231	0.07761
NM_022297	Ddah1	-0.3678	-0.9007	-0.0003	0.02531	1.4155	1.0051	-0.8181	1.3853	-0.2261	-0.6755	-0.7188	0.30418	0.65367	0.65242

NM_212532	Ddah2	-1.338	-1.1883	0.29778	-0.5255	-1.3351	-1.5962	0.03237	-1.424	-1.0497	-0.9199	-1.062	-1.0553	-1.597	-1.6714
NM_171995	Ddb1	-0.4477	-0.0913	0.2251	0.248	0.11105	0.08259	-0.2284	0.19288	-0.2405	-0.1679	-0.0877	-0.1352	-0.2848	-0.1276
XM_242065.3	Ddb2_predicted	-0.3485	-0.1243	-0.4441	-0.3162	-0.1373	-0.1767	-0.344	0.0505	-0.1523	-0.0697	0.20133	-0.2553	-0.4092	-0.2057
NM_012545	Ddc	0.09205	-0.0193	0.01258	0.05413	0.15937	0.01636	0.08912	-0.0193	-0.0288	0.06873	-0.0103	0.0776	0.05959	0.0216
NM_001017481	Ddc8	-0.1627	-0.1282	0.52559	0.06319	-0.0293	-0.0851	-0.012	-0.036	-0.2854	-0.1519	-0.122	-0.1778	0.04646	0.15681
NM_001044245	Ddef1_predicted	-0.7496	-0.4919	-0.1453	-0.2463	-0.0914	-0.0577	-0.4188	-0.1132	-0.6289	-0.52	-0.314	-0.4194	-0.3518	-0.2337
XM_001074649.1	Ddef2_predicted	-0.2616	-0.3336	-0.4246	-0.6581	-0.0499	-0.1699	-0.7794	0.03862	-0.1434	-0.504	-0.3827	-0.4259	-0.2549	-0.4816
NM_001109986	Ddit3	0.64659	-0.9123	0.29561	1.7801	0.88682	1.9552	0.01791	1.1619	-0.4601	-0.7168	-0.7281	0.26623	-0.0998	-0.0535
NM_080906	Ddit4	-0.7928	-0.5961	-1.0364	-1.0623	-0.8476	-0.66	-1.1176	-1.16	-0.8981	-0.7071	-0.792	-0.3695	-0.956	-1.072
NM_080399	Ddit4l	3.6443	1.7689	1.8354	2.6893	2.7639	2.9197	3.0616	2.7182	1.6711	1.8325	2.128	0.8358	1.3209	1.3697
NM_030993	Ddn	0.16107	0.12358	0.01426	0.01806	0.00758	-0.015	0.09719	0.00168	0.04106	-0.0526	0.00411	0.13175	0.01352	0.06211
XM_215402.3	Ddo_predicted	-0.1121	-0.1072	-0.0946	-0.0497	-0.067	0.01358	0.06051	0.0959	-0.0185	-0.1212	-0.0604	-0.0998	-0.016	0.00719
NM_001012104	Ddost_predicted	0.06036	0.38864	-0.5141	-0.3517	0.53909	0.09102	-0.5545	0.41701	0.13355	0.13047	0.15753	-0.3374	0.09903	0.00755
NM_001166022	Ddr1	-0.371	-0.3289	-0.1491	-0.3295	-0.3037	-0.5241	-0.1522	-0.1442	-0.2838	-0.3201	-0.3592	-0.6065	-0.3118	-0.1187
NM_024131	Ddt	0.10338	-0.0735	-0.2237	-0.0169	0.1564	0.66952	-0.4917	-0.1286	0.2656	-0.0498	0.03181	0.58164	-0.3491	-0.1984
NM_053414	Ddx1	0.3154	0.1181	0.51509	1.0505	0.6346	0.46702	0.37744	0.66858	0.23659	0.33121	0.17899	0.11439	0.64697	0.70053
NM_001106820	Ddx10_predicted	0.25409	0.21805	0.14721	0.58206	0.1894	-0.0568	0.41152	0.5831	0.05851	0.3937	0.20388	-0.3959	0.40099	0.28744
XM_001070646.1	Ddx11_predicted	-0.239	-0.512	0.18371	-0.1604	-0.5322	-0.2498	-0.4917	-0.1565	-0.1946	-0.645	-0.4189	-0.0728	-0.1636	-0.2107
XM_001070646.1	Ddx11_predicted	-0.0023	-0.0072	0.12675	-0.015	0.01912	0.08492	-0.1142	-0.1491	-0.1667	0.22243	-0.0951	-0.012	-0.0718	0.0095
NM_001015018	Ddx17	-0.286	-0.0967	-0.0546	-0.1772	-0.2498	-0.4042	0.02554	-0.4052	-0.0274	-0.0594	0.10724	0.22239	-0.372	-0.2496
NM_001006996	Ddx18	-0.1591	-0.4433	0.50037	0.52668	-0.3364	-0.7612	0.06942	-0.6724	-0.108	-0.1121	-0.0577	-0.1768	0.52342	0.53845
XM_001066705.1	Ddx20	-0.1708	-0.4154	0.60721	0.11177	-0.1959	-0.1784	0.18048	-0.0358	-0.0096	-0.1011	0.00587	-0.0005	0.25633	0.07553
NM_001037201	Ddx21a_predicted	-0.0162	-0.0429	0.14473	0.12401	-0.2524	0.40103	0.15971	0.03785	-0.1434	-0.0637	-0.1646	-0.0938	-0.0423	-0.1068
NM_001106793	Ddx23_predicted	0.04527	0.11699	0.40725	0.33911	0.22637	-0.4636	0.17293	0.1609	0.19552	-0.103	-0.0335	-0.0573	0.32842	0.27358
NM_199119	Ddx24	0.03916	0.03923	-0.0082	-0.3747	0.45145	0.04988	0.08988	0.37579	-0.1464	-0.1142	-0.0122	-0.1543	0.02418	0.04893
NM_031630	Ddx25	-0.094	0.18023	0.3376	0.10003	-0.0718	-0.2422	0.17889	-0.3675	0.02347	0.04079	0.2022	0.46808	-0.0593	0.03184
NM_001047904	Ddx26_predicted	0.25967	0.34452	0.03879	0.02799	0.14328	0.2642	0.01421	0.39051	0.03572	0.36978	0.40296	0.12079	0.50865	0.07132
NM_001135801	Ddx27_predicted	-0.1652	-0.0318	-0.1724	-0.1396	-0.16	-0.1631	-0.2113	-0.2426	-0.019	-0.1091	-0.0023	-0.0233	-0.079	-0.1213
NM_001108898	Ddx28_predicted	0.25332	-0.1296	-0.0212	-0.0948	-0.046	0.1959	-0.0209	0.18312	-0.1278	0.25565	-0.0341	-0.1382	0.22294	0.13498
NM_001107824	Ddx31_predicted	-0.3661	-0.4461	0.04909	-0.372	-0.5427	-0.6024	-0.3194	-0.607	-0.4849	-0.5646	-0.0801	-0.3288	-0.069	0.10174
NM_053563	Ddx39	-0.1589	-0.8648	1.2156	0.21765	-0.9119	-0.9422	0.14561	-0.8841	-0.6042	-0.7874	-0.4825	-0.1868	0.37857	0.61479
NM_001108246	Ddx3x	0.14775	0.1892	-0.0593	0.30642	0.27065	0.11555	0.00018	0.26818	0.12649	-0.0029	0.1103	0.12418	0.22235	0.35865
NM_001108046	Ddx41_predicted	0.09781	0.4985	0.14982	0.30114	0.28842	-0.1056	0.4098	0.20967	0.21384	0.22854	0.19041	-0.3821	-0.2535	-0.0911
NM_001107059	Ddx42_predicted	0.0473	0.21384	-0.4335	-0.0717	-0.1305	-0.051	-0.0448	-0.0993	0.03653	0.2013	0.22881	0.00311	0.06771	-0.0056
NM_139098	Ddx46	-0.4273	-0.3038	0.08466	-0.0588	-0.0691	-0.3887	0.14245	0.00946	-0.3434	-0.6188	-0.3738	-0.34	-0.3536	-0.3573
NM_001015005	Ddx47	-0.5018	-0.3602	-0.1902	0.03404	-0.2846	-0.377	-0.0573	-0.3204	-0.3154	-0.517	-0.2671	-0.2707	-0.47	-0.459
NM_001115023	Ddx49_predicted	0.41319	-0.0224	0.15044	0.8426	0.85573	1.0475	0.06537	0.60389	-0.0143	-0.0356	-0.0035	-0.0425	0.31336	0.48499
NM_001007613	Ddx5	-0.5066	-0.2323	0.42554	-0.3258	-0.64	-0.9793	0.5017	-0.4552	-0.3227	-0.1289	0.05211	-0.1428	-0.0725	-0.1578
NM_001013198	Ddx50	-0.8609	-0.7565	-0.6428	0.00467	-0.5419	-0.5132	-0.5617	-0.4389	-0.6575	-0.71	-0.6342	-0.6946	-1.0125	-0.9093
NM_001107150	Ddx51_predicted	-0.0097	0.01897	0.4312	0.32513	0.00172	-0.1469	0.48034	0.10078	0.28809	0.34238	0.07552	-0.0941	0.04415	0.47917
NM_053525	Ddx52	-0.5235	-0.1656	0.21759	-0.4656	-0.48	-0.4104	0.10994	-0.3194	0.3604	0.07833	-0.0332	0.59388	0.54558	0.37526
XM_222149.3	Ddx55_predicted	0.09974	0.08559	0.29296	0.19365	0.1644	0.10051	0.74491	0.12825	-0.1114	0.02036	0.15702	-0.0077	0.35045	0.37915
NM_001004211	Ddx56	0.41477	-0.3197	0.64046	0.48538	1.0029	1.2365	0.0727	0.80895	-0.0914	-0.4013	-0.226	0.26747	0.63142	0.7421
NM_001106645	Ddx58_predicted	0.13678	0.06703	-0.1198	0.14708	0.37504	0.15565	0.05385	0.27085	0.27611	-0.0723	0.11391	0.08255	0.06718	0.09669
NM_001005535	Ddx59	0.03733	-0.1077	0.28404	0.41747	-0.1798	0.09686	0.04006	-0.2045	0.11984	-0.1077	-0.0266	0.47609	0.34551	0.31484
XM_236192.2	Ddx6	0.28392	0.31852	-0.6064	-0.202	0.08065	-0.7093	0.00667	0.51798	0.23068	0.27275	-0.2753	-0.4441	0.0571	0.16644
NM_001115028	Deadc1_predicted	-0.0207	0.23037	-0.3468	-0.5244	-0.0531	-0.1745	0.12788	-0.1727	0.14322	0.13989	0.19213	-0.2401	0.07238	0.08926
NM_031801	Deaf1	-0.6476	-0.608	0.0326	-0.3385	-0.6175	-0.5442	-0.3894	-0.8294	-0.6828	-0.961	-0.6496	-0.3372	-0.6187	-0.3736
NM_001004448	Dear	0.07574	0.04695	0.11366	0.14286	0.09573	0.06072	0.01628	0.02795	0.01756	0.2286	0.14862	0.14073	0.26589	-0.0874

NM_057197	Decr1	-0.3258	-0.1267	-1.3534	-2.1429	-0.5222	-0.5572	-0.8019	-0.4606	-0.3366	-0.3091	-0.2879	-0.7228	-0.9798	-0.9735
NM_001109478	Decr2	0.54256	0.46855	0.05817	-0.1476	0.27144	0.27534	0.24449	0.2074	0.27726	0.4423	0.24702	0.12902	-0.1968	-0.2315
NM_031800	Dedd	-0.3319	0.02758	-0.6199	-0.8265	-0.6128	-0.5313	-0.0095	-0.716	0.06493	-0.0777	-0.2464	0.05438	-0.2508	-0.1344
XM_001078315.1	Def6_predicted	-0.3525	-0.3145	0.17125	-0.2825	-0.5178	0.10845	-0.2576	-0.4774	-0.7907	-0.4873	-0.4867	-0.3006	-0.4993	-0.3675
NM_001024774	Def8	-0.1791	-0.1527	-0.008	-0.2358	-0.1599	-0.5141	-0.2086	-0.0503	-0.4246	-0.1097	-0.3088	-0.593	-0.2151	-0.2622
NM_173329	Defa	0.02427	0.08585	0.12932	0.08846	0.17775	0.02513	0.09409	0.04078	0.09822	0.00463	0.10355	0.20861	0.40314	0.03825
NM_031810	Defb1	0.02009	0.07444	0.0307	0.05686	0.1371	0.20762	-0.0237	-0.0088	0.34565	0.06711	0.04588	0.0748	0.14167	0.01732
NM_134391	Defb22	-0.0608	-0.0055	0.06157	0.13999	-0.1112	0.17676	0.05076	0.11061	-0.0596	-0.0462	-0.1555	0.03363	-0.0972	-0.0237
NM_022544	Defb4	0.22665	0.05813	0.01152	0.02157	0.01811	0.01561	0.10492	0.1491	0.02121	0.07629	0.06497	0.14127	0.03235	0.00518
NM_001037524	Defb52	-0.0113	-0.0196	-0.0086	0.11898	-0.151	-0.022	-0.0068	0.168	0.03068	0.08307	0.0275	0.21154	0.23454	-0.1183
NM_001013053	Defcr4_predicted	-0.1011	-0.0298	-0.0699	-0.1468	-0.1108	0.02026	0.01906	-0.0974	-0.1423	0.05709	-0.0513	-0.0113	-0.0093	-0.1731
NM_053323	Degs1	0.10066	0.36923	0.24213	0.14458	-0.2171	-0.1781	0.19348	-0.0731	0.06788	0.30493	0.24431	0.09344	0.54073	0.59969
NM_001017457	Degs2	0.24717	-0.0869	-0.0538	-0.0542	0.40447	0.46236	-0.1477	0.19998	-0.1395	-0.1379	0.15155	0.16677	0.07628	-0.0035
NM_001004255	Dek	0.05301	-0.011	-0.0389	0.11856	0.04654	-0.0593	0.06926	0.00043	0.20692	-0.0304	-0.0821	0.00786	0.02753	0.11868
NM_001107714	Dennd2d_predicted	0.03513	-0.2542	-0.384	-0.4195	-0.1268	-0.4581	-0.4588	-0.155	-0.4943	-0.1296	-0.3956	-0.5046	0.01025	-0.468
XM_001080406.1	Depdc1a_predicted	-0.1585	-0.2954	0.17751	0.21424	-0.2681	-0.1832	0.03185	-0.0668	-0.0639	-0.1166	0.00158	-0.103	0.32893	0.44943
NM_001107651	Depdc1b_predicted	0.08369	-0.1809	0.15635	-0.1365	0.04394	-0.0284	-0.1891	0.07229	-0.1145	-0.0673	-0.0784	0.11142	0.09769	0.28315
NM_001107899	Depdc2_predicted	0.35311	-0.0241	0.19612	0.04906	0.2974	0.25633	0.2372	0.08463	0.0351	-0.0361	0.11723	0.01291	0.03968	-0.0713
NM_001107229	Depdc5_predicted	-0.0152	-0.0363	0.01303	-0.0542	-0.1117	-0.07	-0.0929	-0.0144	-0.1079	0.02852	0.1037	-0.1692	-0.0239	-0.0796
NM_001029916	Depdc7	0.12769	-0.0973	0.1398	0.51783	-0.0015	-0.1498	-0.1032	-0.1035	0.15652	0.26697	0.05982	0.26148	0.38714	0.46024
NM_022531	Des	0.41656	-1.5166	-2.0843	-1.3595	-0.3751	-0.3603	-2.79	-0.3365	-1.9534	-1.4401	-1.8064	-3.5887	-2.9385	-2.7918
NM_053679	Dffa	0.3273	0.10692	0.1511	-0.123	0.48193	0.53075	0.07342	0.43595	0.34703	0.22935	0.01459	-0.1093	0.08237	0.1819
NM_053362	Dffb	0.19774	0.47587	0.32071	0.4131	0.3382	0.6178	0.05405	0.08154	0.37228	0.19186	0.214	0.13717	0.08104	-0.0457
XM_231759.4	Dfna5h	0.00932	-0.1398	0.00773	-0.273	-0.0469	-0.0849	-0.3047	-0.115	0.07406	-0.0359	-0.0307	0.17406	-0.1002	-0.0209
XM_231759.4	Dfna5h	0.45987	0.22113	-0.0173	-0.0861	0.22826	0.01445	0.03986	0.15364	0.11095	0.30931	0.21586	-0.0982	0.0812	0.04853
NM_053437	Dgat1	-0.1492	-0.0553	0.6193	-0.2748	-0.0603	-0.124	0.3367	-0.193	-0.1391	-0.1048	-0.1984	-0.1974	-0.1254	0.20358
NM_001012345	Dgat2	0.01971	-0.1113	0.03764	0.10827	-0.1759	0.12184	0.16227	0.065	0.05275	-0.0564	0.05926	0.14787	-0.0815	0.01093
NM_001012345	Dgat2	-0.769	-0.4692	-1.1201	-0.5816	-0.7109	-0.4775	-1.3197	-0.3024	-0.6848	-0.5907	-0.7584	-0.5037	-0.9465	-1.0392
XM_001069359.1	Dgat2l4_predicted	0.13249	0.05371	-0.1582	0.10612	0.13148	0.29947	0.03589	-0.1025	-0.0187	-0.2252	0.08911	0.09567	-0.0452	0.09773
NM_001012472	Dgcr14	0.4737	-0.0586	0.23595	0.44314	0.67234	0.45762	-0.1029	0.57343	0.06667	0.28066	-0.0197	0.61659	0.20233	0.16245
NM_001012146	Dgcr2	-0.3703	-0.3411	-0.3118	-0.4462	-0.3336	-0.4532	-0.2088	-0.1752	-0.3107	-0.4856	-0.4719	-0.419	-0.1582	-0.1232
NM_001107080	Dgcr6_predicted	0.56803	0.15757	0.11448	0.15975	0.59635	0.987	0.08835	0.41505	0.37353	0.29148	0.12516	0.5237	0.28819	0.21603
NM_001105865	Dgcr8_predicted	-0.1093	0.14186	0.09869	-0.0155	0.01468	-0.1908	0.30519	-0.1523	0.11562	0.09208	0.1219	-0.3026	0.07732	0.36991
NM_080787	Dgka	1.2423	1.1641	0.5783	1.7659	0.96001	0.98183	0.78571	1.0363	0.62345	1.0297	0.85512	0.13787	-0.1871	-0.2722
XM_223739.3	Dgkq_predicted	-0.004	0.12438	0.33462	0.39337	-0.0404	-0.0501	0.1291	-0.0022	0.10524	0.20509	0.14487	0.07583	0.00622	0.15246
NM_031143	Dgkz	-0.3269	-0.2252	0.12724	-0.4028	-0.303	-0.4943	-0.1497	-0.4597	-0.3478	-0.221	-0.4928	0.14797	0.30066	0.03148
NM_001080148	Dhcr24	-2.3176	-2.4429	1.0467	-0.7016	-2.021	-1.9038	-1.1808	-2.0102	-1.0752	-1.7506	-1.9671	0.20923	-0.8549	-0.7962
NM_022389	Dhcr7	-0.2261	-0.8218	1.3801	0.58821	-0.2607	-0.2041	-0.0152	0.00126	-0.2593	-0.899	-0.5692	0.27316	-0.0688	0.01462
NM_001011978	Dhdds	0.05103	0.43904	-0.0178	0.35781	0.18271	0.47049	0.22977	0.29126	0.32206	0.35295	0.1488	0.43179	0.22196	0.25584
NM_130400	Dhfr	-0.0405	-0.4376	0.03182	-0.5505	-0.435	-0.4339	-0.7222	-0.315	-0.3098	-0.5333	-0.4193	0.19157	0.21651	0.24231
NM_053367	Dhh	0.39778	-0.019	0.27038	0.52011	0.03886	-0.0936	0.23155	0.24147	0.13024	-0.0018	0.28951	0.00289	0.01601	0.14791
NM_001008553	Dhodh	0.1578	-0.236	0.44872	-0.5795	-0.5026	-0.6955	-0.499	-0.642	-0.563	-0.4671	-0.0932	-0.5785	0.3444	0.61989
NM_001004207	Dhps	0.15397	0.08264	0.41451	-0.2824	0.2661	0.40341	0.1177	0.17352	0.25895	-0.0139	0.54614	0.62061	0.4221	0.33683
NM_001007621	Dhrs1	0.02198	-0.0033	-0.1326	-0.1019	-0.0968	-0.1701	-0.0982	0.12608	-0.1143	-0.0674	-0.0668	-0.1324	0.22239	0.18155
NM_153315	Dhrs4	-1.1827	-0.79	-0.1444	-1.3936	-0.8952	-1.4152	-0.6157	-1.3204	-0.1835	-0.5963	-0.3375	0.18339	0.21176	0.13275
NM_001013098	Dhrs7	-0.2692	-0.2456	-0.0409	-0.3524	-0.0773	-0.2086	-0.1955	-0.201	-0.1917	-0.1143	-0.275	-0.0767	-0.1121	-0.0818
NM_001013098	Dhrs7_predicted	0.11917	0.15226	0.04598	0.10419	0.05744	0.20309	0.19374	0.18516	0.2354	0.05563	0.11063	-0.0391	0.24282	0.15796
NM_001008507	Dhrs7b	0.2349	-0.1627	0.20066	0.1267	0.38669	0.41171	0.1995	0.20538	-0.0989	-0.2572	-0.0397	0.05045	0.23786	0.23295
XM_001078936.1	Dhrs7c_predicted	-0.1355	-0.116	0.07858	0.00848	0.08447	0.03554	-0.092	-0.0733	0.07641	-0.0875	-0.0609	-0.0845	0.00734	-0.0466

NM_130819	Dhrs9	0.04708	0.60356	0.00145	-0.0896	0.35139	-0.0078	0.33382	0.3714	0.40442	0.62208	0.31607	-0.1367	0.1742	0.05669
NM_001105914	Dhrsx_predicted	-0.6045	-0.5787	0.36275	-0.4619	-0.9484	-0.2706	-0.1075	-1.0478	-0.706	-0.6629	-0.3741	0.16054	-0.5373	-0.5011
NM_001025720	Dhtkd1	0.11729	0.0041	0.09432	-0.0567	-0.0348	0.11083	0.07865	0.07118	-0.0377	-0.0811	0.32695	-0.1457	0.07745	0.10617
XM_001054651.1	Dhx15_predicted	-0.2197	-0.5397	0.23153	0.13987	-0.2528	-0.3712	0.29339	-0.3315	-0.3186	-0.4379	-0.4001	0.03997	0.07605	0.11066
NM_212496	Dhx16	-0.6753	-0.6727	-0.4093	-0.3271	-0.5236	-0.4813	-0.5062	-0.5984	-0.5706	-0.685	-0.6303	-0.6057	-0.751	-0.5617
XM_215481.3	Dhx29_predicted	0.00559	-0.2251	0.26211	0.39075	0.07359	-0.0636	-0.0272	0.20617	0.05049	-0.0886	-0.0376	0.11922	0.24559	0.33881
NM_001013249	Dhx30	-0.2269	-0.0992	0.53779	0.21701	-0.3851	-0.1987	0.239	-0.2436	-0.0599	-0.1668	-0.2721	-0.0347	-0.1295	-0.1961
NM_001105802	Dhx33_predicted	-0.0503	-0.0009	-0.0223	0.09387	-0.0653	-0.126	-0.0708	-0.1646	-0.0302	0.14236	0.03905	0.09474	0.17492	-0.0024
NM_001108601	Dhx35_predicted	0.14276	0.18798	0.17185	0.52126	-0.1294	-0.0482	0.23481	0.0584	-0.0027	-0.056	0.12216	-0.3001	-0.4627	-0.2373
NM_001107678	Dhx36_predicted	-0.1991	0.08281	-0.2317	0.18524	0.14311	-0.1467	0.12324	0.39358	0.02714	0.08524	0.11132	-0.1363	0.28471	0.02697
NM_001105926	Dhx37_predicted	-0.321	-0.4462	0.45318	0.59545	-0.2331	-0.1675	-0.0129	-0.364	-0.1141	-0.2922	-0.2307	0.15596	-0.1974	0.1445
NM_001106185	Dhx38_predicted	-0.0246	-0.0132	0.34802	0.09837	-0.3246	-0.1171	0.05622	-0.1876	-0.3095	-0.2167	-0.0307	-0.0386	0.01252	-0.0361
NM_001005873	Dhx40	-0.2377	-0.4007	-1.13	-0.398	-0.3053	-0.388	-0.6361	-0.1072	-0.5373	-0.6145	-0.3999	-0.5859	-0.8769	-0.8337
XM_001062787.1	Dhx57	-0.6235	-0.301	-0.0743	0.29041	-0.5877	-0.5585	-0.065	-0.7921	-0.239	-0.2588	-0.2043	-0.1553	-0.8778	-0.6948
NM_001047844	Dhx8	0.01869	-0.0432	-0.2492	-0.095	-0.0195	-0.3696	0.06198	-0.0586	0.0665	-0.3649	0.06855	-0.2994	-0.1735	-0.1365
NM_001107184	Dhx9_predicted	-0.5028	-0.5047	-0.1545	-0.8584	-0.8808	-0.4935	-0.5734	-0.9803	-0.0864	-0.3167	-0.3266	0.35276	-0.083	-0.2158
NM_001008292	Diablo	0.00389	-0.1236	-0.2965	0.03822	0.13116	0.57201	-0.3567	0.21099	-0.1723	0.03207	-0.2331	0.17116	-0.0354	-0.0152
NM_001107393	Diap1_predicted	0.19998	0.08419	-0.2235	-0.1996	0.16841	-0.1094	0.07996	0.26797	0.17875	0.12184	0.21406	0.1795	-0.0777	0.00042
XM_001066898.1	Diap2_predicted	0.11984	0.05369	0.25171	-0.001	-0.1263	-0.0443	0.13783	-0.1306	-0.1746	0.00022	0.08762	0.019	-0.062	0.05923
XM_001069041.1	Dicer1	-0.3792	-0.4025	0.03408	-0.7963	-0.4657	-0.2776	-0.3754	-0.4764	-0.3881	-0.3953	-0.2923	-0.1589	-0.156	-0.1422
NM_021653	Dio1	-0.1155	-0.0797	-0.1717	-0.0474	-0.1328	-0.1846	0.01553	-0.1494	-0.0143	-0.1573	-0.0673	-0.0709	-0.0654	0.10979
NM_031720	Dio2	-0.1233	0.00463	-0.0653	-0.0452	0.05091	0.16459	-0.0565	-0.078	-0.1162	0.00629	0.13632	-0.0771	0.1328	-0.1897
NM_017210	Dio3	-0.0404	0.00937	0.0726	-0.1086	-0.162	0.24713	-0.0939	-0.1364	-0.0425	0.19297	0.12876	-0.0196	-0.1462	-0.0659
NM_001108987	Diras1_predicted	0.12773	0.10126	0.05828	0.10072	0.06636	0.10723	0.00467	0.01469	0.25224	-0.0331	0.03108	-0.0207	0.0309	-0.0178
NM_001169578	Diras2_predicted	-0.1771	-0.0941	-0.1569	-0.1838	-0.1622	-0.1721	0.14225	-0.1418	0.05691	-0.1256	0.20783	-0.2063	-0.185	-0.0801
NM_001012017	Dirc2	0.04183	0.1086	-0.4876	-0.8048	0.04808	-0.0787	0.02967	-0.0674	0.14689	0.06937	-0.1144	-0.1221	-0.0565	-0.1036
NM_001008380	Dis3l	-0.094	-0.4075	-0.0281	-0.1715	-0.0502	-0.202	-0.4907	0.01115	-0.247	-0.2144	-0.3343	-0.3981	0.07745	-0.2037
NM_175596	Disc1	-0.2484	-0.1617	-0.0907	-0.2126	-0.0579	-0.2712	-0.1906	-0.0343	-0.0695	-0.2737	-0.2173	-0.1641	-0.0612	-0.2228
NM_001105983	Disp1_predicted	0.03815	0.31389	0.27515	0.197	0.09652	0.23318	0.20743	0.31862	-0.1314	0.02715	0.04491	0.21999	0.0775	0.19363
NM_001107759	Disp2_predicted	-0.1674	-0.1583	-0.0961	0.03034	-0.1105	0.06488	-0.0442	-0.0239	-0.0785	-0.0141	-0.0719	0.05928	-0.106	0.04213
NM_001037654	Dixdc1	-0.0996	0.02709	-0.1814	-0.0235	0.04403	-0.1182	0.16002	-0.2624	-0.2664	-0.1292	-0.2385	-0.1943	0.04124	-0.1022
NM_001106350	Dkk1_predicted	0.05716	-0.0126	0.00323	-0.0475	0.06697	-0.088	-0.0287	-0.1462	-0.044	-0.0754	-0.0367	-0.0506	0.08473	-0.1511
NM_138519	Dkk3	-0.1498	-0.1096	-0.0955	-0.1901	-0.0679	-0.1514	-0.222	0.08565	-0.3284	-0.291	-0.1225	0.0036	-0.2384	-0.2758
NM_001106472	Dkk4_predicted	0.34764	-0.0158	0.04808	0.06647	1.5552	1.2326	0.07283	1.7384	0.13575	0.20155	0.11671	0.0748	0.27672	0.00937
NM_031025	Dlat	-0.3994	-0.2785	-0.406	-0.747	-0.444	-0.2663	-0.6256	-0.4032	-0.3831	-0.2331	-0.3078	-0.733	-0.4673	-0.5682
NM_001127446	Dlc1	-0.2516	-0.2256	0.49963	0.27647	-0.4028	-0.2314	0.35754	-0.3404	-0.242	0.05183	-0.0607	-0.0046	-0.3795	-0.3097
NM_199385	Dld	0.19594	0.40049	-0.1403	0.1086	-0.0605	-0.4246	0.20571	0.3326	0.14523	0.28278	0.17704	-0.2878	0.21607	0.18818
XM_001062041.1	Dlec1	-0.0651	-0.2152	-0.0239	-0.0062	-0.0399	8E-05	-0.1524	-0.231	-0.1005	-0.2401	-0.0688	-0.1908	-0.2191	-0.0178
NM_001106043	Dleu7_predicted	-0.1079	-0.1177	-0.1323	-0.1107	-0.1292	-0.0534	-0.0455	-0.1119	-0.236	-0.0314	-0.1961	-0.1061	-0.1144	-0.1687
NM_031639	Dlg3	-0.5719	-0.3498	-0.4447	-0.4596	-0.0704	-0.5592	-0.3357	-0.3753	-0.3655	-0.4813	-0.1934	-0.3845	-0.5556	-0.3419
NM_019621	Dlg4	-0.1046	-0.0144	0.01861	0.12737	-0.196	-0.0155	0.01723	-0.1063	-0.0459	0.02928	-0.0742	0.10906	-0.1165	-0.0088
NM_001107247	Dlg5_predicted	-0.2427	-0.1834	-0.204	0.04544	-0.0643	-0.0777	-0.1714	0.04899	-0.28	-0.0614	0.15034	-0.0471	-0.084	-0.1062
NM_001135802	Dlg7_predicted	-0.3973	-1.3444	0.97144	0.01926	-0.5957	-0.7421	-0.8193	-0.7043	-0.6874	-0.7385	-0.7329	0.51465	0.48563	0.48607
NM_022946	Dlgap1	-0.0142	-0.1623	-0.5455	-0.3037	-0.7964	-0.2519	-0.3712	-0.4518	0.12181	-0.1743	-0.3361	0.64148	-0.5276	-0.6367
NM_173138	Dlgap3	0.19754	0.11034	0.15475	0.14152	0.11835	0.10966	0.22163	0.08129	0.12636	0.07394	0.14614	-0.0137	0.16226	0.21719
NM_173145	Dlgap4	-0.6173	-0.6747	-0.3056	-1.4083	-1.0121	-1.3965	-0.317	-1.1766	-0.8081	-0.857	-0.4962	-0.7054	-0.3956	-0.2776
NM_012788	Dlgh1	0.24027	0.2424	-0.5225	-0.1145	0.29801	-0.487	-0.0974	0.55546	0.04785	0.19568	-0.1811	-0.1274	-0.0577	-0.0425
NM_053744	Dlk1	0.00815	0.06871	0.03614	0.00888	0.01453	0.21974	0.0742	0.25692	0.1292	0.03107	0.10658	0.00115	0.05393	0.07467
NM_032063	Dll1	-0.0803	0.05684	0.00231	-0.1802	-0.09	0.07961	-0.1264	-0.1026	-0.2498	-0.1124	-0.1125	0.01527	0.09228	-0.0315

NM_053666	Dll3	-0.1932	-0.0899	-0.142	-0.1127	-0.0447	-0.1799	-0.1499	-0.2741	-0.1055	-0.3588	-0.2263	-0.0924	0.00105	-0.1218
NM_001107760	Dll4_predicted	0.02655	0.06298	0.06625	0.13115	0.08614	0.00755	0.08622	0.09557	0.22516	0.10859	0.14595	0.2066	0.15718	0.19549
NM_001006981	Dlst	0.09319	-0.0935	0.43888	0.09502	0.50235	0.41032	-0.1167	0.44273	-0.2422	-0.3013	-0.2803	-0.1998	0.23147	0.21189
NM_001100531	Dlx1	-1.144	-0.5842	-0.1957	-0.8167	-0.867	-0.7133	-0.4333	-1.0306	-0.8543	-0.5844	-0.4696	-0.1905	-0.1995	-0.2264
XM_001060785.1	Dlx2	-0.2488	-0.2896	-0.2643	-0.2874	-0.0461	0.01034	-0.1644	-0.2586	-0.3531	-0.2049	-0.1964	-0.155	-0.0577	-0.1268
NM_001105832	Dlx3_predicted	1.3886	0.75865	1.1874	0.90719	1.6278	1.6623	0.97056	1.6184	0.32134	0.86054	0.87658	0.82703	1.024	1.2003
NM_012943	Dlx5	-1.0654	-0.9386	-1.0064	-0.7303	-0.775	-0.9612	-1.0115	-0.9353	-0.721	-1.1155	-1.1501	-0.6433	-1.0578	-0.9103
NM_022849	Dmbt1	-0.0971	-0.0385	-0.04	-0.0204	-0.1758	0.33209	-0.1244	0.12256	-0.233	-0.2011	-0.116	-0.1623	-0.0697	-0.1958
NM_022849	Dmbt1	-0.0789	0.05929	0.00723	-0.0604	-0.1774	0.20428	-0.1214	-0.2231	0.06584	0.2186	-0.1497	0.13734	0.1545	0.03017
NM_001107961	Dmbx1_predicted	-0.0459	-0.1241	-0.2109	0.03883	-0.0999	0.00075	-0.0624	-0.0537	0.01451	-0.0391	-0.1427	0.13853	-0.182	-0.0962
NM_001130567	Dmc1h_predicted	-0.106	-0.1266	-0.1157	-0.0565	-0.0603	-0.0801	-0.1469	-0.075	-0.0979	-0.1147	0.00294	0.13645	-0.1655	-0.1664
NM_001130567	Dmc1h_predicted	-0.0925	-0.176	-0.0932	0.0596	-0.1601	-0.2647	0.13193	-0.1153	-0.1013	0.1355	-0.0925	-0.0833	0.03942	0.0342
NM_001005244	Dmd	0.22905	0.46184	0.09073	0.6274	0.1144	0.10375	0.22009	0.16648	0.17037	0.28613	0.59601	0.09999	0.09904	0.40163
NM_139102	Dmgdh	0.02803	0.14132	0.04724	0.03409	0.07317	0.0381	0.03939	0.1538	0.04028	0.00558	0.23838	0.24092	0.00772	0.0834
NM_001134858	Dmn	-0.0848	-0.1213	-0.6041	-0.0069	-0.1195	-0.1773	-0.2741	0.08306	-0.338	-0.3428	-0.231	-1.042	-0.8004	-0.7263
NM_203493	Dmp1	-0.4975	-0.3444	-0.3532	-0.3702	-0.467	-0.3634	-0.1912	-0.4359	-0.3351	-0.384	-0.5226	0.00786	-0.4928	-0.394
NM_001107484	Dmpk_predicted	1.1742	1.6978	0.80371	0.31334	-0.0536	-0.2494	1.3619	0.18388	1.1395	1.3402	1.4059	0.17817	-0.0169	0.14051
NM_053706	Dmrt1	-0.2209	-0.2115	-0.2542	-0.0201	-0.1538	0.04616	-0.1432	-0.3239	-0.1359	-0.4132	-0.3086	-0.1936	-0.2788	-0.018
NM_001107597	Dmrt2_predicted	-0.4501	-0.3672	0.2502	0.56169	-0.7579	-0.6217	-0.2235	-0.495	0.3778	-0.3212	-0.2158	0.70668	0.04346	0.17557
NM_001106358	Dmrt3_predicted	-0.4393	-0.6951	-0.5162	-0.71	-0.5762	-0.5431	-0.6186	-0.622	-0.6071	-0.4602	-0.1802	-0.6458	-0.4473	-0.6114
NM_001107945	Dmrta1_predicted	0.10812	0.16881	0.03435	0.1352	0.04175	0.24884	0.19186	-0.0543	0.05207	-0.0256	0.06596	0.03363	0.06491	0.11542
NM_001107951	Dmrta2_predicted	-0.1554	0.0506	0.19518	0.07291	0.04506	-0.0802	0.04227	0.07618	-0.0752	0.077	-0.0286	0.36948	0.25637	0.2201
NM_001025288	Dmrtc1a	0.27524	0.14519	0.21039	0.13395	0.1227	0.0842	-0.0256	0.2415	0.25702	0.04857	0.21211	0.06762	0.03503	0.24046
NM_001014222	Dmrtc1c	-0.1198	-0.1377	0.03974	0.00027	-0.2429	-0.0926	-0.0455	-0.0805	-0.007	-0.1447	-0.2526	-0.1731	-0.0266	-0.0133
NM_053693	Dmtf1	-0.1749	-0.1924	-0.4019	0.26851	-0.3384	-0.4689	0.25506	-0.2634	-0.3367	-0.2413	-0.3172	-0.4174	-0.7007	-0.8313
NM_053693	Dmtf1	-0.0013	0.01376	-0.2014	-0.2545	-0.0244	-0.2659	-0.1274	-0.1092	-0.1035	-0.0759	-0.1697	-0.3076	-0.1822	-0.148
NM_001024748	Dmwd	-0.0372	0.09602	0.02376	0.03261	0.07836	0.2439	0.18288	0.03501	0.15473	0.10759	0.16236	0.28268	0.10439	0.03151
NM_001107388	Dmxl1_predicted	-0.0173	-0.0202	0.21118	0.62343	-0.1993	-0.5505	0.19383	-0.4307	0.12641	-0.0894	-0.1945	-0.389	-0.1729	-0.1259
XM_001071882.1	Dnah10	0.03275	0.192	0.27716	0.0934	0.14811	0.18076	-0.0533	0.07076	-0.0178	-0.0355	0.08273	0.2034	0.10444	-0.011
XM_001061747.1	Dnah11	-0.05	-0.0622	-0.119	-0.1939	0.01086	0.09125	0.28311	-0.0923	-0.0453	0.0096	0.05787	-0.1968	-0.1826	-0.141
XM_001079121.1	Dnah3_predicted	0.1666	-0.0814	-0.0528	-0.1093	-0.1151	-0.0681	-0.1506	-0.1659	-0.1372	-0.2339	0.10787	0.04702	-0.1079	0.02428
XM_001079121.1	Dnah3_predicted	-0.0355	-0.125	0.03043	-0.1468	-0.0892	-0.0244	-0.1877	-0.0822	-0.2712	-0.2118	-0.0472	-0.1828	0.02645	-0.1119
XM_342709.3	Dnah6	0.00957	0.09886	-0.0383	0.07474	0.0437	0.07759	0.0538	0.14553	-0.0046	0.05269	0.13103	0.00801	0.10628	0.0542
XM_342709.2	Dnah6	0.10821	0.23161	0.02827	0.05488	0.05654	0.01536	-0.0095	0.14193	0.18045	-0.0133	0.17227	0.08437	-0.0244	0.02146
XM_001065965.1	Dnah7	-0.0178	0.47951	0.06857	0.04289	0.04663	0.27668	0.19362	0.05161	0.28188	0.29283	0.28832	0.1884	0.1873	0.13421
XM_228058.4	Dnah8	0.02485	0.70941	0.25176	0.11808	-0.0272	0.28407	0.07725	0.09729	0.63909	0.46811	0.53108	0.68061	0.43689	0.71049
XM_001078646.1	Dnah9	0.35112	0.16918	-0.0711	-0.1535	0.00236	-0.0997	-0.1287	-0.1052	0.02943	-0.091	-0.1755	-0.2089	-0.0961	0.08674
XM_213354.4	Dnah9	-0.0481	0.07759	0.11758	0.2821	0.07215	0.13157	0.00536	0.16024	-0.1011	-0.1364	-0.0909	-0.1165	-0.002	-0.0491
NM_001033655	Dnahc1	-0.0256	0.05563	-0.0931	-0.0323	-0.0032	-0.1514	0.0081	-0.0942	-0.2196	-0.0764	0.30433	0.13015	0.01392	-0.1575
XM_213534.4	Dnahc11	-0.2135	-0.2017	-0.2699	-0.0334	-0.0986	-0.0988	0.00436	-0.0192	-0.2866	-0.1794	-0.111	-0.2851	-0.1836	-0.2609
XM_226891.4	Dnahc5	-0.0289	0.06623	0.2253	0.15013	0.01533	0.09972	0.01399	0.0603	0.21983	-0.0041	0.23548	0.03595	0.10666	0.06506
NM_001024342	Dnaic1	-0.0203	0.24178	0.13565	0.22087	0.21229	0.30473	0.22789	-0.0284	0.05923	-0.1117	0.1004	-0.0726	0.05953	-0.0512
NM_001007726	Dnaic2	0.20672	0.12408	0.22087	0.11416	0.25916	0.16351	0.19487	0.19261	-0.1123	0.06208	0.06515	0.27936	-0.0155	0.01889
NM_022934	Dnaja1	-0.0338	0.08061	0.10088	0.69606	0.25411	0.30505	0.4861	0.22333	-0.0732	0.04434	0.11729	-0.0176	0.32481	0.24633
NM_032079	Dnaja2	0.13125	0.28526	0.10777	0.66339	0.32808	0.24348	0.47529	0.46487	0.18971	0.06096	0.05411	0.11543	0.23694	0.29383
NM_001038595	Dnaja3	0.37126	0.05068	-0.1955	-0.0698	0.47977	0.2562	-0.1638	0.4682	-0.0478	0.04157	0.08946	-0.2514	0.00884	-0.0688
NM_001025411	Dnaja4	0.04256	-0.3041	-0.405	-0.3296	0.08022	-0.0946	-0.4643	0.18333	0.02451	-0.4939	-0.209	-0.2135	0.18717	0.10693
NM_001108441	Dnajb1_predicted	0.26455	0.02446	0.09585	-0.3449	-0.2859	-0.1727	-0.4976	-0.3263	0.0056	-0.0695	0.07556	0.06751	0.21472	0.28225
NM_001015021	Dnajb11	0.14266	0.06579	0.39938	0.13415	0.23837	0.04618	0.1763	0.09046	0.3918	0.02335	-0.0299	0.12336	0.03964	0.42316

NM_001015021	Dnajb11	-0.2316	-0.4922	-0.0472	-0.2728	-0.0249	0.25465	-0.4201	0.15191	-0.2794	-0.3368	-0.4475	-0.08	0.15131	0.27609
NM_001013907	Dnajb12	-0.0689	-0.0435	-0.0325	0.05652	0.18529	-0.0861	-0.0704	0.02455	-0.0767	0.11767	0.13482	-0.0068	-0.1051	0.18487
NM_001005885	Dnajb13	-0.0373	0.01679	-0.084	-0.0683	-0.0898	0.03802	0.13452	-0.0982	-0.0125	0.08575	0.25302	0.07569	-0.0458	0.03516
NM_001013076	Dnajb4	1.478	1.1641	-0.3823	1.2071	1.1983	0.48534	0.64902	1.1522	0.90574	0.75186	0.64006	-0.0134	1.303	1.4599
NM_001108004	Dnajb5_predicted	-0.0561	-0.4064	0.1422	-0.3451	0.16481	0.67621	-0.2675	0.25499	-0.2066	-0.1526	-0.0949	0.17101	0.19703	0.06489
NM_001013209	Dnajb6	0.02355	-0.323	-1.0351	-1.375	-0.4863	-0.2485	-0.535	-0.3692	-0.4197	-0.5133	-0.4475	-0.1907	-1.1129	-0.8763
NM_012699	Dnajb9	0.35486	0.30434	-0.4204	0.40983	0.97205	0.95511	0.23883	1.2657	0.25269	0.30154	0.23283	-0.1618	0.54993	0.22362
XM_214522.3	Dnajc1_predicted	-0.6649	-0.3085	-0.2312	-0.2617	-0.4014	-0.3524	-0.4255	-0.5961	-0.4719	-0.3828	-0.3536	-0.0129	-0.3183	-0.1132
NM_001106486	Dnajc10	0.33748	0.42974	0.05762	0.38064	0.12587	0.00015	0.10714	0.19474	0.29314	0.3843	0.41292	0.17925	0.3488	0.29045
NM_001108694	Dnajc11_predicted	-0.0102	0.06041	0.05445	-0.252	0.33552	0.33389	-0.3266	0.09299	-0.0567	-0.1256	0.04644	0.0998	0.28375	0.1232
NM_001108776	Dnajc13_predicted	-0.6265	-0.3009	-0.1832	-0.403	-0.5206	-0.5029	-0.136	-0.6387	-0.2695	-0.3433	-0.3344	-0.068	-0.3614	-0.3619
NM_053690	Dnajc14	-0.2338	0.03644	-0.2736	-0.096	-0.445	-0.5046	-0.4584	-0.447	0.02832	-0.0881	-0.023	0.06318	-0.0816	-0.2644
NM_001014194	Dnajc16	-0.0412	0.04467	0.01764	0.04554	-0.086	0.15019	0.01762	-0.1327	0.10818	0.00599	-0.0055	0.11933	-0.0526	-0.0623
XM_230468.3	Dnajc17_predicted	-0.273	-0.2552	-0.0435	0.24455	0.18026	0.23572	0.11182	-0.0677	-0.2014	-0.1132	-0.2229	0.00027	-0.1293	-0.0051
NM_001013887	Dnajc18	1.1186	0.8403	0.64739	0.76799	1.9886	2.2799	0.59653	2.0506	0.98077	0.9283	0.85669	0.89233	0.48788	0.36748
NM_053776	Dnajc2	-0.1805	-0.6145	0.34395	-0.3305	-0.5624	-0.2168	-0.2021	-0.5704	-0.4049	-0.3487	-0.2607	0.08679	0.11544	0.1904
NM_022232	Dnajc3	-0.0749	-0.1522	0.57764	0.12483	0.52546	0.48004	0.01405	0.67202	-0.1092	-0.1473	-0.0887	-0.0092	0.89306	0.98989
NM_024161	Dnajc5	0.25509	-0.0913	-0.1891	-0.4113	0.50093	0.28745	-0.3153	0.39686	-0.1205	0.07001	-0.2375	0.24182	0.04791	0.1487
NM_001013242	Dnajc5g	-0.052	0.06381	0.03743	0.32587	0.05404	0.04141	-0.0407	0.05028	0.1838	-0.0431	0.08202	0.11535	0.14934	0.12869
NM_001107949	Dnajc6_predicted	-0.1273	-0.1541	0.05976	-0.368	0.13678	-0.2107	-0.1342	-0.0526	-0.138	-0.0713	-0.1061	-0.3271	-0.1388	0.09465
NM_213625	Dnajc7	-0.2238	0.04057	-0.0985	0.15624	0.06553	-0.0851	0.32474	0.24367	-0.1024	0.3563	-0.1023	-0.2895	0.08737	-0.0465
NM_001013168	Dnajc8_predicted	-0.1557	-0.1393	-0.0934	-0.008	0.07297	0.02863	-0.1703	-0.0052	0.00798	0.11205	0.06673	-0.1067	0.03223	-0.2137
NM_001108865	Dnajc9_predicted	-0.4325	-0.5243	-0.0905	-0.6598	-0.7808	-0.9136	-0.4095	-0.7075	-0.2741	-0.443	-0.5268	0.02487	0.33158	0.29978
NM_001106050	Dnajd1_predicted	-0.6381	-0.9121	-0.9456	-1.1086	-0.5216	-0.3417	-0.8732	-1.049	-0.6352	-0.9358	-0.8046	-0.4064	-0.4152	-0.6451
NM_001009666	Dnalc4	0.06016	0.23582	0.16254	-0.328	0.05336	0.33526	-0.1438	-0.1277	0.25411	0.28506	0.32978	0.12145	-0.504	-0.5496
NM_001031647	Dnali1	-0.2823	-0.3003	-0.3307	-0.0895	-0.1262	-0.0893	-0.1747	-0.2937	-0.0706	-0.2874	-0.2187	-0.0357	0.05607	-0.1964
NM_013097	Dnase1	0.25904	0.15753	0.03638	0.11473	-0.0066	0.15904	0.35376	0.05176	0.01318	0.19075	0.13403	0.05853	0.05033	-0.0424
NM_001014223	Dnase1l1	0.44886	0.53371	0.42641	0.2443	0.24039	0.41866	0.31485	0.12006	0.41446	0.54261	0.57825	0.23319	0.13498	0.0583
NM_053907	Dnase1l3	0.08815	-0.0562	-0.0527	-0.1207	-0.0983	-0.0279	-0.0452	0.06957	0.03253	0.00827	-0.125	0.04236	-0.1767	-0.0679
NM_138539	Dnase2a	0.29884	0.30893	-0.4074	0.47946	0.26862	0.59265	0.33089	0.16761	0.16317	0.35842	0.66064	0.39446	0.00945	0.18875
NM_021664	Dnase2b	-0.0984	-0.0397	-0.0043	-0.03	0.00992	0.12727	-0.1362	-0.0899	0.11003	0.06616	0.11867	-0.0422	-0.0489	0.00994
NM_019226	Dnch1	-0.2835	-0.2055	-0.1478	-0.0441	-0.2653	-0.1936	-0.1373	-0.0114	-0.2456	-0.1566	-0.1448	-0.2097	-0.2339	-0.1934
NM_001109379	Dnd1_predicted	0.07949	-0.1628	-0.0385	-0.1269	-0.0626	-0.0964	-0.0163	-0.0142	-0.2246	0.04646	0.01797	-0.0231	-0.1164	-0.1164
NM_080689	Dnm1	-0.033	0.74139	0.36656	0.43495	-0.1533	0.02208	0.55786	-0.015	0.47027	0.62245	0.77283	0.19498	-0.6736	-0.6972
NM_053655	Dnm1l	-0.0155	0.08564	-0.2149	-0.1637	0.12315	-0.2842	-0.0086	0.0751	-0.0178	-0.1693	-0.1521	-0.3758	-0.001	0.11423
NM_013199	Dnm2	0.21746	0.60704	0.17354	0.0122	0.60203	0.23371	0.32115	0.64782	0.23953	0.2494	0.29092	0.05245	-0.0446	0.03508
NM_053354	Dnmt1	-0.2351	-0.1835	0.85486	-0.0768	-0.4792	-0.972	-0.306	-0.5347	-0.3466	-0.2103	-0.3868	-0.1878	0.52077	0.51578
NM_001003957	Dnmt3a	-0.4257	-0.7428	-0.5704	-0.3043	-0.4444	-0.2872	-0.6936	-0.2806	-0.5816	-0.6548	-0.7272	-0.6142	-0.6582	-0.7204
NM_001003959	Dnmt3b	-1.1823	-0.6729	-0.7793	-0.9497	-0.917	-0.9841	-0.4171	-0.7532	-0.6145	-0.6844	-0.5612	-0.2561	-1.0042	-0.9096
NM_001003964	Dnmt3l	0.00318	0.00716	-0.0252	0.08998	0.04151	0.09361	-0.0505	-0.0702	-0.0787	-0.196	-0.1676	-0.051	-0.0718	-0.1062
NM_001003964	Dnmt3l	0.0167	0.02893	0.03602	-0.0284	-0.034	0.07972	0.11215	0.02815	-0.0315	0.019	-0.0048	0.06187	0.04111	0.05043
NM_001024879	Dnpep	-1.5831	-1.2403	-1.6395	-2.246	-1.3328	-1.5646	-1.343	-1.4657	-1.6021	-1.3343	-1.37	-1.6539	-1.8242	-1.6535
NM_001012461	Dntt	-0.2403	0.07511	-0.2768	-0.2187	-0.1135	0.00956	-0.2892	-0.2201	-0.3666	-0.3812	-0.237	-0.2271	-0.3619	-0.2232
NM_022937	Doc2a	0.15652	-0.0042	0.11738	0.07032	0.31866	0.14606	0.21605	0.01455	0.16535	0.04035	0.12134	0.12983	0.073	0.16418
NM_031142	Doc2b	-0.1167	0.00387	-0.0648	-0.0397	0.13506	0.01834	0.03799	-0.2092	0.08524	0.04403	0.14629	0.22354	0.15462	-0.0063
NM_001011937	Doc2g	-0.0889	-0.1798	-0.1213	-0.0299	-0.2734	-0.0946	-0.1114	-0.2545	-0.0473	0.03153	-0.1074	-0.0506	-0.2675	-0.3713
XM_219421.3	Dock1_predicted	-0.0535	-0.0582	-0.1339	-0.1581	-0.0836	-0.1655	-0.0205	-0.1377	-0.1342	-0.1847	-0.1723	-0.0579	-0.0956	-0.0982
XM_001061988.1	Dock11	0.13181	0.05418	0.0626	0.01606	-0.0236	0.0665	0.17823	0.16037	-0.057	-0.0276	0.02608	0.08415	-0.1204	0.12131
XM_001061988.1	Dock11	1.2188	1.3409	0.86993	0.68387	1.7186	1.5629	1.3093	1.5934	1.2589	1.0979	1.217	1.058	0.99521	0.9305

NM_001108184	Dock3_predicted	0.28972	0.02807	0.05862	0.1162	0.00204	0.13986	-0.0219	0.2475	0.14536	0.1683	0.02204	0.13019	-0.0192	0.05937
NM_001107274	Dock5_predicted	-0.0126	0.01284	-0.0207	0.05312	0.08454	0.08406	-0.1436	-0.0444	-0.1558	-0.2102	-0.1681	0.00351	0.13185	0.20435
NM_001108997	Dock6_predicted	-0.3701	-0.3076	-0.2509	-0.3437	-0.3502	-0.2559	-0.298	-0.321	-0.341	-0.5403	-0.2811	-0.2301	-0.9407	-0.9598
NM_001105759	Dock9	-0.2253	0.09832	-0.4967	-0.147	0.05575	-0.1812	-0.2999	-0.1738	-0.1801	-0.1797	-0.0121	-0.3485	-0.4487	-0.4473
NM_001025416	Dok1	1.355	0.93264	0.99787	1.1049	0.93795	1.2128	1.0822	0.86413	0.90942	0.81021	0.98709	0.98732	0.88883	0.83298
NM_001106048	Dok2_predicted	0.56755	0.16999	-0.0214	-0.0812	0.06541	-0.1003	0.41384	-0.1998	0.303	0.30563	0.00295	0.14047	-0.2583	-0.0444
NM_001107336	Dok3_predicted	-0.1525	-0.2174	-0.0412	-0.1138	0.02747	-0.1655	-0.161	-0.1748	-0.1481	-0.1715	-0.1393	-0.1434	-0.1751	-0.1138
NM_001108438	Dok4_predicted	0.18767	0.09069	0.45159	0.41514	0.0153	0.29252	0.22048	-0.0812	0.07814	0.10374	0.39439	0.31755	0.12878	0.07475
NM_001108438	Dok4_predicted	0.47825	0.23886	0.14934	0.04181	0.71297	0.93758	0.00435	0.69652	0.26899	0.38394	0.38358	0.51564	0.16902	0.19396
NM_001106567	Dolpp1_predicted	-0.1496	-0.2665	0.04369	-0.1666	0.05735	-0.2304	-0.3416	-0.1668	-0.3581	0.00596	-0.3386	-0.092	-0.209	-0.0749
NM_212497	Dom3z	-0.053	-0.1598	0.3419	0.24214	0.09382	0.28258	0.05383	-0.0102	-0.1162	-0.1293	-0.1013	-0.0238	-0.1859	0.14467
NM_001008287	Donson	-0.012	-0.5411	0.59476	0.53713	-0.1607	-0.252	-0.1811	0.02028	-0.2349	-0.2976	-0.494	0.17309	0.61969	0.43327
XM_001055500.1	Dopey2_predicted	-0.0448	0.11581	0.34607	0.14631	0.16554	0.12692	0.02572	0.16308	-0.1097	-0.2182	-0.0581	-0.1003	-0.297	-0.1921
NM_001108070	Dos_predicted	-0.112	-0.0913	0.31592	-0.1379	-0.207	-0.1393	-0.2356	0.0149	-0.2099	-0.2382	-0.0577	-0.1921	-0.075	-0.2297
NM_001108733	Dot1l_predicted	-0.1658	0.02049	-0.0074	-0.1544	-0.0718	-0.2243	-0.0794	0.05144	-0.2644	-0.1699	0.10882	0.04026	-0.0707	-0.1082
NM_199233	Doxl1	-0.0678	0.05566	-0.089	-0.108	0.23531	-0.0458	-0.072	-0.005	0.05766	-0.2049	-0.0967	0.20764	0.09676	-0.0033
NM_199291	Doxl2	0.02199	0.11634	0.11407	-0.1122	0.06386	0.30267	-0.0203	-0.0594	-0.0358	0.07035	-0.0065	0.20163	-0.0488	0.00469
NM_199388	Dpagt1	0.36192	0.49217	0.15954	-0.2451	0.17077	0.27408	0.3572	0.21215	0.34437	0.21442	0.30747	0.25372	0.25188	0.30592
XM_001070301.1	Dpde1	-0.2046	-0.2809	-0.1425	-0.0439	-0.299	-0.0855	-0.0946	-0.1714	-0.2019	-0.3429	-0.2281	-0.1187	-0.1293	-0.1097
NM_053591	Dpep1	-0.0849	0.85382	-0.0274	0.12449	-0.0885	-0.123	-0.0747	-0.0417	0.54689	0.9381	0.56773	0.20432	-0.0601	0.09961
NM_001011928	Dpep2_predicted	-0.0381	0.1044	-0.1558	0.00553	0.1132	0.03417	-0.0994	-0.1121	-0.0859	-0.0748	0.11488	-0.0116	0.08694	-0.0024
NM_001008383	Dpep3	-0.3234	-0.297	-0.2574	0.02722	-0.2208	-0.1187	-0.1389	-0.3956	-0.2127	-0.1709	-0.0598	0.15595	-0.0032	0.01902
NM_001108516	Dpf2_predicted	-0.0759	0.10238	-0.0779	-0.0395	0.13185	-0.1966	0.0785	0.12277	-0.0114	-0.0097	-0.1725	-0.1157	-0.0731	-0.0669
XM_238462.4	Dpf3_predicted	0.17648	0.01093	0.05016	0.05693	0.00808	-0.013	0.04689	-0.0086	0.07675	0.26412	-0.0134	0.24001	0.10761	0.03758
NM_001015007	Dph2	-0.1004	-0.2621	-0.0431	0.01209	-0.0904	-0.1868	-0.3196	-0.2111	-0.1502	-0.116	-0.0226	-0.1708	0.05282	0.21792
NM_001017449	Dph5	0.77255	-0.0206	0.49365	0.32977	0.17842	0.19704	0.17994	0.08398	-0.073	0.20551	0.26764	-0.0004	0.55652	0.50966
NM_001106544	Dpm1_predicted	0.37099	0.26658	-0.1798	0.45183	0.46202	0.57502	0.43569	0.77861	0.39833	0.37597	0.12626	0.05655	-0.1764	-0.1318
NM_001012205	Dpp10	0.01467	0.19855	0.11419	-0.0181	-0.0346	0.10575	0.03288	0.03349	0.15121	-0.0259	0.27884	-0.0328	0.07977	0.01738
NM_053748	Dpp3	-0.1027	0.30392	0.39187	0.23141	0.11412	0.16568	0.10093	-0.0195	0.17548	0.15606	0.32448	0.02267	-0.2606	-0.0394
NM_012789	Dpp4	-0.0054	0.15363	0.22711	0.22423	0.09941	0.00064	0.19075	0.13228	0.07249	0.30568	0.08199	0.04713	0.12943	0.08713
NM_022850	Dpp6	-0.0144	0.03257	0.10065	0.01135	0.202	-0.1345	-0.0232	-0.0668	0.05415	0.07441	-0.0303	-0.0298	0.00992	-0.0429
NM_031973	Dpp7	0.21064	0.38283	0.6332	0.73079	-0.2686	-0.0475	0.58863	-0.3471	0.17135	0.26104	0.29116	0.19322	0.08656	0.34318
NM_001108159	Dpp8_predicted	-0.1093	-0.2081	0.02405	-0.8058	0.03183	0.22259	0.08621	0.0798	0.17219	0.05949	-0.1353	0.3162	0.22608	0.10085
NM_001105905	Dppa1_predicted	0.12601	-0.0274	0.06998	-0.0666	0.02202	-0.0218	0.04764	0.05446	-0.0051	0.01059	0.09385	0.07247	-0.0036	0.02756
NM_001047864	Dppa3	0.16787	0.19567	0.05622	0.07887	-0.1226	0.21411	-0.0142	0.05596	0.1692	-0.0999	-0.0503	0.13772	0.0577	-0.0459
XM_001059859.1	Dppa5_predicted	0.50285	0.05583	-0.0849	0.25573	0.25298	0.49258	0.15081	0.12515	0.14069	-0.032	-0.0693	-0.0812	-0.0555	0.06743
NM_001105965	Dpt_predicted	-0.3176	3.2898	0.0802	0.24481	-0.281	-0.2237	0.01461	-0.3079	2.7683	3.2306	3.0979	-0.3009	-0.1536	-0.0682
NM_001135835	Dpy19l3_predicted	0.00478	0.03728	0.07072	0.08962	-0.0284	-0.0609	0.16983	-0.0341	0.14212	-0.0041	-0.0578	0.08474	0.03076	0.13254
NM_173117	Dpy30	0.04208	0.00901	0.35447	-0.1515	-0.2081	0.05454	-0.0926	-0.3166	0.13382	0.03251	0.03753	0.3491	0.25414	0.30102
NM_031705	Dpys	0.40186	0.03873	0.21457	0.16319	0.26785	0.26471	0.14094	-0.0102	-0.1422	0.20747	0.06442	0.60291	0.03692	0.23113
NM_001105717	Dpysl2	-0.0467	0.0264	-0.0267	0.15174	-0.0083	0.01196	0.01796	0.0848	-0.0208	0.15968	0.03456	-0.0447	0.09804	0.04472
NM_012934	Dpysl3	-0.1124	-0.2149	-0.1566	-0.7484	0.36233	0.09322	-0.2721	0.38583	-0.0423	-0.347	-0.4611	0.33301	0.19081	0.24652
NM_012933	Dpysl4	-0.0723	-0.1518	-0.1659	0.05226	0.07603	0.02739	-0.0883	-0.165	0.01831	-0.0789	-0.1226	-0.1633	0.23945	-0.2623
NM_023023	Dpysl5	0.06021	0.03019	-0.0735	-0.2429	0.08191	-0.0522	-0.0472	0.2927	0.00282	-0.0328	0.19649	0.10363	-0.1279	0.07374
NM_001011914	Dr1	-0.1864	-0.1437	-0.0202	-0.2465	0.15868	-0.1163	-0.1566	0.20159	-0.1545	-0.1202	-0.1091	-0.0565	0.23398	0.04258
NM_001077668	Drap1_predicted	0.66682	0.76653	0.42081	-0.0005	0.44786	0.86894	0.12164	0.51916	0.56533	0.77877	0.72419	0.74792	0.62634	0.59841
NM_012546	Drd1a	-0.1812	-0.0345	-0.0662	-0.2138	-0.1162	-0.1883	0.02263	0.05372	-0.1325	-0.0212	-0.0707	0.42295	-0.0576	-0.1353
NM_138915	Drd1ip	0.23125	0.0361	-0.03	0.32713	0.10038	0.18703	0.0096	-0.0089	0.20844	0.23512	-0.0063	0.03893	0.04142	0.16967
NM_012547	Drd2	-0.0604	0.09226	-0.0463	0.26654	0.04207	0.08513	-0.1486	0.0214	-0.0577	0.01258	-0.0974	0.24361	-0.0826	0.13285

NM_017140	Drd3	-0.183	-0.1563	-0.1179	-0.1544	-0.047	-0.1808	0.09209	-0.0967	-0.2029	-0.2179	-0.1506	-0.085	-0.2018	-0.123
NM_012944	Drd4	-0.1534	-0.0438	-0.0983	-0.0477	0.06223	-0.0515	-0.1847	-0.0799	-0.0973	-0.1078	0.09815	-0.1591	-0.1125	0.01625
NM_012768	Drd5	0.19849	-0.0396	0.02727	-0.0921	-0.085	-0.0755	-0.2561	0.12215	-0.0889	-0.0735	-0.0271	-0.216	0.06025	-0.1347
NM_001009685	Drg1	-0.4433	-1.0976	-0.3207	-0.7658	-0.3729	-0.3661	-0.3058	-0.4115	-0.8062	-0.6962	-0.765	-0.0265	0.12033	0.11573
NM_001106161	Dsc1_predicted	0.01458	-0.0529	-0.0438	0.19021	-0.1493	0.2521	-0.0317	0.2722	0.09943	0.00102	-0.0183	0.18317	-0.026	-0.1532
NM_001033688	Dsc2	-0.0258	-0.0277	-0.0804	-0.16	-0.0835	0.01712	-0.0515	0.05581	-0.1498	0.04951	-0.1675	0.05924	0.15317	-0.089
NM_001107402	Dsc3_predicted	0.05227	0.09994	0.1156	0.05033	0.88696	0.95309	-0.1414	1.2435	0.03048	0.35391	0.04721	-0.0261	-0.109	-0.1105
NM_133587	Dscam	0.26644	0.10189	-0.0111	0.01096	-0.0663	0.12799	0.08055	0.29709	-0.0213	-0.0213	-0.0327	-0.0198	0.00755	-0.0333
NM_001108141	Dscaml1_predicted	0.16272	0.08542	0.06914	0.2996	0.251	0.25396	-0.0248	0.16752	-0.0366	0.13979	0.18475	0.22044	0.34783	0.18293
NM_001105891	Dscr2_predicted	0.02741	-0.3066	0.20901	-0.3855	-0.3426	0.01624	-0.2204	-0.391	-0.0616	-0.5377	-0.479	0.3542	0.02266	0.09452
NM_001108316	Dscr3_predicted	0.42707	0.84105	-0.0084	-0.5603	0.7257	-0.0827	0.67504	0.81475	0.58583	0.65432	0.43471	-0.1577	0.19875	-0.0811
NM_001105892	Dscr6_predicted	0.03262	0.11215	0.18195	0.11745	0.17624	0.06899	0.10869	0.18727	0.35126	-0.0547	0.0989	0.00681	0.26169	-0.0355
XM_001054396.1	Dsg2_predicted	-0.0066	-0.0206	0.13418	0.22064	0.05255	0.22759	0.09653	0.32169	0.31944	0.05293	0.03728	-0.0128	0.29104	0.06061
XM_001054396.1	Dsg2_predicted	0.00436	0.12724	0.03703	0.06955	0.19269	0.09709	0.01789	0.11225	0.00786	0.03011	0.06097	0.01219	0.0606	0.09773
NM_199490	Dsg4	-0.0666	0.04502	-0.1173	-0.1342	-0.0945	-0.0473	0.03844	0.19811	-0.0402	0.04594	0.08336	-0.0932	-0.0747	-0.0313
NM_001037215	Dsm-1	0.11594	0.09162	0.06667	-0.1976	0.11747	0.34463	-0.2889	0.13327	0.08084	0.07151	0.16233	0.41248	0.10832	0.36139
XM_225259.4	Dsp	0.13175	-0.6625	-1.1523	-0.8486	0.71501	0.60363	-0.8823	0.78009	-0.9604	-0.5202	-0.9316	-0.885	-1.1251	-0.8216
NM_001108088	Dspg3_predicted	-0.2865	-0.0538	-0.2439	-0.261	0.05147	-0.1526	-0.2673	0.13306	0.00674	-0.1564	-0.1624	0.04671	-0.2338	-0.2425
NM_012790	Dspp	-0.0471	0.08406	0.21468	0.01682	-0.0093	-0.0675	0.02203	0.00142	0.03147	-0.0353	-0.0821	0.01489	0.29277	0.05808
NM_001108208	Dst_predicted	0.40307	0.42586	0.73165	0.68451	0.45251	0.32507	0.49329	0.2675	0.0249	0.33793	0.23104	0.17897	0.17161	0.32921
NM_001033666	Dstn_predicted	0.32177	-0.0962	-1.3467	0.61423	0.93994	0.07157	-0.3207	1.0047	-0.4425	0.01451	-0.3449	-1.1267	-0.1343	-0.2599
NM_001012191	Dtnb	-0.039	0.03242	0.19656	0.18675	0.02534	-0.0885	0.2187	-0.0546	0.0834	0.27779	0.08294	0.28345	0.02339	0.15001
NM_001012191	Dtnb_predicted	0.12667	0.02056	-0.0873	-0.0187	0.01455	-0.0712	0.07916	0.0355	-0.0126	-0.079	-0.0282	0.03049	-0.0763	0.02466
NM_001013921	Dtwd1	0.37397	-0.1382	0.29664	0.92574	0.41629	0.4216	0.63146	0.31183	0.16626	-0.2264	0.13948	0.13604	0.08458	0.10873
NM_001107157	Dtx2	-1.2137	-0.8125	-0.9751	-1.1241	-1.0491	-1.3306	-0.8981	-1.1969	-0.7338	-0.8395	-0.7652	-0.891	-1.2527	-1.2676
NM_001047855	Dtx4	-0.3968	-0.4447	-0.2969	-0.3194	-1.0576	-0.8832	-0.6364	-1.0268	-0.4327	-0.311	-0.4717	-0.2041	-0.2554	-0.7315
NM_001106925	Dtymk_predicted	-0.0731	-0.1675	0.03897	-0.328	-0.4854	-0.4244	-0.0836	-0.4988	-0.063	-0.3342	-0.2515	0.15194	-0.081	0.09482
XM_218781.3	Dufd1_predicted	0.02194	0.0401	0.0287	0.0398	-0.0238	0.01121	0.07877	0.00199	0.10499	0.12219	0.12885	0.01293	0.03934	-0.0657
NM_153739	Duox1	0.02956	0.83825	0.79922	0.18885	-0.5817	-0.4126	0.7933	-0.3241	0.48873	0.73723	0.61298	0.57074	0.26518	0.29199
NM_024141	Duox2	-3.8909	-3.9771	-2.7949	-2.9092	-4.2056	-4.3887	-3.3857	-4.2521	-3.8223	-3.8108	-3.5043	-3.6734	-3.9093	-3.9459
NM_001108368	Dupd1_predicted	0.09654	0.05352	-0.0037	0.1265	0.34204	0.26088	-0.0574	0.18799	0.08499	-0.053	0.05338	-0.0253	-0.043	-0.0089
NM_001106181	Dus2l_predicted	0.63765	0.34677	1.0276	0.5336	0.17524	0.27936	0.85594	0.15671	0.55289	0.46184	0.61549	0.19297	0.6283	0.60519
NM_001034923	Dus3l	-0.9297	-0.5	0.24646	0.50254	-0.8714	-0.8741	-0.012	-0.9635	-0.1732	-0.548	-0.3748	-0.0929	-0.3543	-0.4631
NM_001135803	Dus4l_predicted	-0.172	-0.2657	-0.2573	-0.471	-0.5408	-0.6073	-0.2191	-0.3783	-0.1884	-0.3441	-0.286	-0.469	-0.0803	-0.1341
NM_053769	Dusp1	0.73587	0.18897	0.45504	0.3424	0.45023	0.06424	0.00178	0.53853	0.17392	0.23938	0.28878	-0.0185	0.26945	0.38047
NM_001105734	Dusp10_predicted	0.59226	0.19811	0.60103	0.65872	0.8253	0.7032	0.16651	0.45338	0.26001	0.28427	0.26152	0.24247	0.26531	0.25939
NM_001025650	Dusp11_predicted	-0.2221	-0.6004	-0.1485	-0.9066	-0.5112	-0.2394	-0.6233	-0.313	-0.7109	-0.7747	-0.8016	-0.2428	-0.0374	0.23661
NM_022248	Dusp12	0.14241	-0.3307	-0.2138	-0.4165	-0.0882	0.15132	-0.1116	-0.2731	0.065	-0.0403	-0.1452	0.03679	0.2893	0.01192
NM_001162408	Dusp13	0.21801	-0.1154	0.22483	0.16661	-0.0123	-0.0709	-0.0114	0.11239	0.19458	-0.1868	0.15733	0.02443	0.13669	-0.0693
NM_001079893	Dusp14_predicted	-0.2373	-0.3044	-0.2789	-0.1903	-0.2706	-0.2589	-0.2417	-0.2163	-0.1904	-0.1303	-0.3372	-0.3213	-0.2117	-0.2871
NM_001108598	Dusp15_predicted	0.08097	-0.0264	0.15829	0.01679	-0.001	-0.0065	0.10034	0.02945	0.13183	0.27833	0.14175	0.02786	-0.0432	0.01429
NM_001106624	Dusp16_predicted	-0.1385	-0.0797	-0.0371	0.18045	0.05813	0.02641	-0.0106	-0.027	-0.105	0.07723	0.08869	0.0833	-0.0222	0.10189
NM_001013128	Dusp18	-0.0907	-0.0805	0.04247	0.05389	-0.1955	-0.3409	-0.1341	-0.2407	0.07887	-0.1538	-0.1262	-0.1387	-0.2795	0.08939
NM_001107739	Dusp19_predicted	0.22009	0.04812	0.1457	0.1308	0.33998	0.59316	-0.1729	0.47139	-0.0909	0.34957	0.10478	0.21204	0.04803	-0.0007
NM_001012089	Dusp2	-0.0536	0.04481	0.29871	0.02393	0.02829	0.08943	0.11114	0.24705	-0.0174	0.02262	0.18202	-0.0268	0.16611	0.09405
NM_001108412	Dusp22_predicted	-0.009	-0.1731	0.00574	-0.1907	-0.0137	-0.0914	-0.0565	0.00046	-0.2099	-0.2045	0.00884	-0.1515	-0.008	-0.1737
XM_341156.3	Dusp23_predicted	-0.0373	-0.0105	-0.0831	0.25666	0.11495	-0.014	-0.0308	-0.0088	0.16504	0.13962	0.13596	-0.0024	-0.1095	-0.001
NM_001012352	Dusp26	0.15766	0.09097	0.21022	-0.1122	0.1495	-0.1253	0.2515	0.03315	0.00357	0.10371	0.01619	0.16984	0.02729	0.13278
NM_022199	Dusp4	-0.0346	-0.0237	0.09845	-0.0926	0.02273	0.07058	-0.0147	0.15142	0.13203	-0.0696	0.01177	-0.0528	0.20987	0.2519

NM_133578	Dusp5	0.53829	0.18325	0.3032	-0.0791	0.00676	0.26308	0.23842	0.11427	0.37612	0.39167	0.36619	0.3144	0.2665	0.09677
NM_053883	Dusp6	-0.6747	-0.2103	-0.6799	-2.066	-2.0069	-1.9319	-0.0866	-2.0471	-0.1026	-0.1877	0.07543	-0.2531	-0.7423	-0.7219
XM_001070874.1	Dusp7	0.04365	0.0254	0.03603	0.10811	0.04945	0.16435	0.12182	-0.0323	-0.1158	-0.0182	-0.1468	-0.0011	0.11573	-0.1395
NM_001108510	Dusp8_predicted	0.1517	-0.4328	0.33916	0.18723	0.6127	0.3565	-0.1458	0.70669	-0.3401	-0.3093	-0.527	-0.4512	0.14372	0.28707
NM_001108510	Dusp8_predicted	-0.0195	-0.3662	0.15191	0.00296	-0.1039	0.05617	-0.1608	-0.1778	-0.1254	-0.046	-0.2148	-0.2255	-0.1402	-0.0246
NM_001037973	Dusp9	-0.0646	0.12416	0.08811	0.05279	0.27062	0.27605	0.00406	0.36682	0.13929	0.17225	0.17539	-0.0402	-0.0381	0.07788
NM_031820	Dvl1	-1.0878	-0.8495	0.38977	0.39356	-1.1123	-0.9757	-0.4341	-1.2027	-1.2363	-1.1121	-1.3078	-0.8834	-1.2356	-1.0749
NM_001107081	Dvl3_predicted	-0.1425	-0.3	-0.3359	-0.3783	-0.18	-0.2947	0.12817	-0.2586	-0.2695	-0.2421	-0.2853	-0.1988	-0.1557	-0.1276
NM_001106133	Dym_predicted	-0.1281	-0.2858	0.04658	-0.1007	-0.0224	0.2317	-0.4462	-0.0429	-0.4385	-0.3102	-0.4125	0.10615	-0.1135	-0.1036
NM_019226	Dync1h1	-0.7854	-0.2878	0.14877	0.22957	-0.297	-0.7493	0.25258	-0.3601	-0.6555	-0.3091	-0.425	-0.6724	-0.5997	-0.5168
NM_019234	Dync1i1	-0.0106	0.0346	0.02839	0.27646	0.17236	0.07917	0.09926	0.32239	0.08615	-0.0101	0.02594	0.28266	0.04534	0.054
NM_053880	Dync1i2	-0.3199	0.14534	-0.178	-0.5508	0.14172	0.02983	0.02034	0.20657	0.08035	-0.0235	0.15327	0.05726	0.05367	0.13338
NM_145772	Dync1li1	0.40197	0.3228	0.33522	0.40407	0.49757	0.10446	0.47	0.51872	0.2282	0.2078	0.47835	0.41819	0.52584	0.41347
NM_031026	Dync1li2	0.04006	0.20248	0.26158	-0.6152	0.11341	0.24021	0.18992	0.06603	0.1671	0.30877	0.28392	-0.1825	0.12505	-0.0102
NM_001013940	Dync2li1	0.11995	0.24678	-0.6544	0.01558	-0.0759	0.19882	0.15613	-0.1836	0.12218	0.42136	0.06621	-0.2295	-0.9418	-0.8495
NM_053319	Dynll1	0.17054	-0.1144	0.2528	-0.6946	-0.2438	-0.0356	-0.0508	-0.0572	-0.0361	-0.182	-0.1742	0.19819	0.2432	0.42658
NM_080697	Dynll2	0.27145	0.09653	-0.2667	-0.2619	-0.157	0.33857	-0.5411	-0.0917	0.0744	0.22626	0.01654	0.29877	-0.0414	0.1054
NM_131910	Dynlrb1	0.24978	0.22105	-0.3085	0.1746	0.2358	0.58946	0.26844	0.2352	0.08433	0.38084	0.13981	0.00714	-0.2619	-0.2937
NM_001108451	Dynlrb2_predicted	0.00134	-0.015	-0.2509	-0.1019	0.09589	0.29647	-0.0779	0.12127	-0.225	-0.2123	-0.152	0.2002	0.00879	-0.1672
NM_031318	Dynlt1	-0.0122	-0.2913	-0.0328	0.0356	-0.009	0.49474	-0.6453	-0.2618	-0.1002	-0.0663	-0.0461	0.38095	-0.0851	-0.0946
NM_001013228	Dynlt3	0.56039	0.46958	-0.7814	0.42984	0.65972	0.48869	-0.0043	0.72577	0.32042	0.41103	0.30118	-0.1107	-0.2265	-0.5053
NM_012791	Dyrk1a	0.04557	-0.2128	0.02151	0.31628	-0.1084	-0.1747	0.09173	0.09834	-0.3671	-0.2839	-0.2663	0.10626	-0.2857	-0.0942
NM_001107496	Dyrk1b_predicted	-0.2809	0.00703	-0.1676	-0.1249	-0.0177	-0.108	-0.1903	0.42643	-0.1726	-0.0861	-0.0485	-0.0193	-0.0282	-0.1995
NM_001108100	Dyrk2_predicted	0.35191	0.0247	0.00202	1.0763	0.21803	0.00682	-0.3415	0.05466	0.1759	0.11065	0.17266	0.42766	0.46252	0.38438
NM_001024767	Dyrk3	0.87987	0.60271	0.38014	1.1307	0.59545	0.79143	0.44098	0.71232	0.31887	0.47287	0.23545	0.01959	0.05215	-0.0221
NM_001107869	Dysf_predicted	-0.1497	-0.0933	-0.1017	-0.086	-0.1718	-0.133	-0.1316	-0.1896	0.10537	-0.0582	-0.088	0.19338	-0.1801	-0.1798
NM_153303	Dyt1	-0.0261	-0.1428	-0.0729	-0.2452	-0.0007	0.08694	0.12039	-0.0516	0.0554	-0.1161	0.03706	0.0554	0.12413	0.03632
NM_001007010	Dyx1c1	0.16992	-0.0948	0.09176	0.28847	0.00706	-0.2235	0.11585	-0.2239	0.18629	-0.2946	0.07409	0.02661	-0.2413	-0.0396
NM_001007010	Dyx1c1	0.08183	0.06097	0.24093	0.09736	0.16871	0.21201	-0.0375	0.05402	0.07165	0.04206	-0.0047	0.01625	0.10667	0.01676
XM_001078447.1	Dzip1	-0.4451	-0.1797	-0.0796	-0.1959	-0.3237	-0.3208	-0.2883	-0.5636	-0.0948	-0.1473	-0.0352	0.18721	-0.1722	-0.2548
NM_001014095	Dzip1l	0.09204	-0.1131	0.28815	0.13697	-0.013	0.12409	-0.0814	-0.0489	0.06822	0.05822	-0.0521	0.51614	0.05353	0.12934
NM_001166020	E030032D13Rik	0.07892	-0.0427	0.03288	0.35194	0.01075	-0.0636	-0.0035	0.1054	0.12699	0.10156	0.12734	0.03299	0.02205	0.08125
NR_002154.1	E230034O05Rik	-0.0233	0.02703	0.045	-0.0134	-0.0365	-0.0101	0.03285	0.13796	-0.0449	0.06076	-0.1045	-0.0675	0.01255	-0.0979
NM_001100778	E2f1	0.20513	-0.1815	0.48802	-0.4717	-0.4032	0.13156	-0.4012	-0.4031	0.07604	-0.0902	-0.0676	0.21128	0.65419	0.53132
XM_226441.3	E2f4_predicted	-0.0829	-0.0959	0.49065	0.17778	-0.2785	-0.4562	0.15853	-0.5611	-0.3787	-0.2963	-0.2471	-0.165	-0.0646	-0.2689
XM_574892.2	E2f5	0.09395	0.11571	0.84608	0.44418	-0.0565	-0.1754	0.7597	0.01142	0.27401	0.33046	0.27951	0.47915	0.40956	0.38574
NM_001100717	E2f6	0.628	0.46376	0.00861	0.61481	0.60612	0.12404	0.359	0.54875	0.26559	0.40959	0.38209	-0.3279	0.6556	0.61599
NM_001108092	E2f7_predicted	0.03445	-0.0665	0.19688	-0.0042	0.00648	0.19553	0.01935	-0.0025	0.02478	0.22622	0.10406	0.09508	0.15437	0.20492
XM_001080259.1	E2f8	-0.3871	-1.1422	0.44418	-0.5921	-1.0346	-0.7758	-0.9824	-0.8468	-0.3294	-0.5922	-0.6572	-0.0858	0.14978	0.31327
XM_001080259.1	E2f8	-0.387	-0.3358	0.01201	-0.2306	-0.2765	-0.4359	-0.2343	-0.4089	-0.2309	-0.0933	-0.3237	-0.3013	-0.4051	-0.3493
NM_001107293	Eaf1_predicted	-0.1121	-0.0141	0.34589	0.0573	0.20823	-0.003	-0.0484	0.05962	-0.1174	0.05351	0.06215	-0.0235	0.2548	0.4578
NM_172047	Eaf2	0.18355	0.06383	-0.0347	0.02779	-0.0061	-0.2057	-0.1341	0.08895	-0.0906	-0.0121	-0.0188	-0.1023	0.07479	0.17769
NM_001009534	Eapa2	-0.1714	-0.0928	-0.2435	0.05296	0.06822	-0.2357	-0.1647	0.05182	-0.2114	-0.2565	-0.2581	0.07859	-0.2265	-0.1091
NM_138902	Ear11	2.1161	3.2483	0.3974	1.8258	4.8031	4.7684	1.484	4.585	2.7808	3.0998	3.043	0.879	-0.0843	-0.1848
NM_001007015	Ear4	0.74608	-0.1235	-0.1286	-0.3097	0.3711	0.21038	-0.1639	0.2546	-0.1229	0.08257	-0.1758	0.24619	-0.0252	-0.2226
NM_001009665	Ebag9	0.31592	-0.003	0.11523	0.24001	0.14965	0.04676	0.27713	0.07504	0.27602	-0.1027	0.38566	0.60487	0.41688	0.39256
NM_053820	Ebf1	-0.6042	-0.5799	-0.617	-0.6766	-0.8108	-0.9296	-0.2109	-0.8045	-0.5128	-0.4536	-0.5961	-0.6353	-0.8812	-0.6635
NM_001108383	Ebf2_predicted	-0.0945	-0.0599	-0.0972	-0.0263	-0.1674	-0.1722	-0.107	-0.081	0.12758	-0.0641	-0.1154	0.06379	-0.1974	-0.1236
NM_001108506	Ebf3_predicted	0.20036	0.1587	0.05396	-0.041	-0.0851	0.37272	-0.0037	0.18441	0.23971	0.05271	0.11753	-0.0023	-0.0594	-0.006

NM_001008721	Ebna1bp2	-0.347	-0.7378	0.0988	-0.2981	-0.4816	-0.0385	-0.3899	-0.2084	-0.2972	-0.5602	-0.3368	0.07622	0.11528	0.16163
NM_057137	Ebp	0.24691	0.1734	1.1845	-0.1064	-0.0211	0.27568	0.26155	-0.0623	0.17872	0.21825	0.35901	0.42105	0.54682	0.61846
NM_001108381	Ebpl_predicted	-0.122	0.22161	0.2912	0.3071	0.20747	0.14732	0.02803	-0.0794	0.08962	0.13864	0.28918	0.06242	0.04949	0.05072
NM_053596	Ece1	0.20839	0.45829	-0.5798	0.16794	0.77994	1.0555	-0.5445	0.82911	0.27228	0.26831	0.37358	-0.4626	-0.6866	-0.8256
NM_001002815	Ece2	-0.2747	0.03264	-0.3185	-0.2175	-0.2552	-0.132	-0.0937	-0.4117	-0.0773	-0.1732	-0.2114	-0.1133	-0.2511	-0.1694
NM_021776	Ecel1	-0.0132	-0.0878	0.01585	-0.1022	0.15071	0.25818	0.05046	-0.0286	-0.0148	-0.091	0.13769	0.03429	0.18608	-0.0352
NM_001002816	Ecg2	0.07981	-0.0482	-0.0022	0.03757	0.09047	0.01084	0.16483	0.14167	0.1189	0.07563	0.00885	0.0357	-0.0335	0.0256
NM_022594	Ech1	0.04606	-0.1118	-0.6601	-1.0682	-0.5038	-0.2711	-0.3283	-0.5107	0.11173	-0.286	-0.1292	0.17517	-0.5205	-0.4149
NM_001007734	Echdc1	-0.3388	-0.4897	-0.2857	0.24571	-0.6262	-0.4826	-0.0761	-0.2325	-0.4809	-0.4545	-0.3223	-0.2126	-0.5286	-0.3319
NM_001007734	Echdc1	0.02423	0.19308	0.05668	0.04983	0.00633	0.06614	-0.0066	0.134	-0.1168	-0.0875	0.181	0.34605	-0.0356	0.06819
NM_001106675	Echdc2_predicted	-0.0063	-0.0255	0.09882	0.07463	0.05969	0.07516	0.0592	0.06475	0.08382	0.10346	0.1738	0.09111	0.09251	0.10042
NM_078623	Echs1	0.07074	-0.2805	-0.2105	-0.8258	-0.2064	-0.3407	-0.6888	-0.4075	-0.1723	-0.2497	-0.0918	0.06774	-0.0187	-0.182
NM_001134593	Ecm1	0.67734	0.44602	-0.0452	-0.3362	-0.2813	-0.2698	0.01647	-0.1836	-0.0398	0.50718	0.23223	-0.0378	-0.7484	-0.5965
XM_214443.3	Ecm2_predicted	-0.176	0.19952	-0.0127	-0.0272	-0.1122	-0.0455	0.02479	-0.1395	-0.0507	0.00728	0.03144	0.18479	-0.0298	-0.1388
NM_001006986	Ecsit	0.21041	-0.0575	-0.2333	-0.284	0.23306	0.58894	-0.1931	0.16296	0.02923	-0.0264	0.17583	0.11557	-0.0826	-0.1185
NM_001108547	Ect2_predicted	-0.2968	-0.3002	0.10368	-0.156	-0.4935	-1.0991	-0.4433	-0.3599	0.06109	-0.1434	-0.2573	0.26067	0.84798	0.8845
XM_001068241.1	Eda2r_predicted	-0.0356	0.08287	0.08315	0.04221	0.05297	0.01819	-0.2784	-0.1462	-0.2055	-0.0683	-0.0374	-0.0323	0.04599	-0.0182
NM_001004230	Edem2	0.38635	0.39544	0.27483	-0.3083	0.85551	0.8897	-0.3238	0.72831	0.26559	0.36263	0.34775	0.09983	0.26299	0.09997
NM_001106557	Edf1_predicted	0.50379	0.1323	-0.1772	0.11458	0.89285	0.71515	0.09906	0.42471	0.38929	0.36335	0.2899	-0.2083	0.41437	0.35206
NM_017301	Edg1	0.02666	0.13421	0.01639	-0.0218	0.0255	0.11616	-0.0265	0.01691	0.01857	0.03208	0.16358	0.0702	0.02407	0.0154
NM_053936	Edg2	0.1961	-0.1705	-0.5174	-0.0936	-0.2093	-0.6514	0.41936	-0.3028	0.27651	-0.1114	0.15727	0.22158	0.19	0.04946
NM_001108399	Edg4_predicted	-0.2287	-0.336	-0.7135	-0.0691	-0.233	-0.2956	-0.3717	-0.3184	-0.6212	-0.6282	-0.5721	-0.5028	-0.7941	-0.5224
NM_017192	Edg5	0.13868	0.59595	0.38854	0.18965	0.15982	0.05954	0.2888	0.03972	0.62151	0.65466	0.71806	0.34398	0.109	0.17194
NM_001108075	Edg6_predicted	0.14828	-0.0725	-0.2209	-0.1642	-0.0751	-0.0682	-0.076	0.02834	-0.088	0.17571	-0.0976	0.05849	-0.1023	0.02897
XM_346646.2	Edg7_predicted	0.43147	-0.1701	-0.2036	-0.0771	-0.3551	-0.2612	-0.26	-0.1708	-0.1999	-0.2495	-0.0246	0.13922	-0.1577	-0.1591
NM_021775	Edg8	0.11223	0.44049	0.78668	0.13641	0.16014	0.26822	0.74185	0.12852	0.28863	0.47197	0.53414	0.389	0.38456	0.44593
NM_012548	Edn1	0.35431	0.29599	0.05634	0.30396	1.2549	0.97766	-0.0099	1.2788	0.12114	-0.0005	0.3156	0.52864	0.63811	0.37526
NM_012549	Edn2	0.1516	0.30339	0.22837	0.35209	0.27935	0.21132	0.15264	0.20627	0.23138	0.13332	0.17389	0.19278	0.15717	0.06722
NM_017333	Ednrb	0.1957	0.26947	0.1907	0.20614	0.03258	-0.051	0.395	0.25865	0.25334	0.14093	0.13768	-0.1287	0.12586	0.14023
NM_001108086	Eea1_predicted	0.06119	0.48488	0.39116	0.18714	0.14728	-0.0248	0.32307	0.4012	0.17012	0.82223	0.07576	-0.2837	0.91285	0.79863
NM_001106278	Eed_predicted	-0.1556	-0.2303	0.1818	0.13162	-0.217	-0.3274	0.17796	-0.215	-0.033	0.1409	-0.2376	0.11066	0.2438	0.13446
NM_033539	Eef1a1	-0.0718	-0.01	-0.0323	-0.1149	-0.1775	-0.1814	-0.0367	-0.0358	-0.0023	-0.1723	-0.132	-0.0725	-0.0962	-0.0626
NM_033539	Eef1a1	-0.1777	-0.1158	0.19711	-0.0043	0.07667	0.19241	0.26492	0.08867	-0.2135	-0.2945	-0.2058	0.27417	-0.1569	0.03954
NM_033539	Eef1a1	0.01001	0.45945	-1.7427	-1.2591	-0.0554	-1.0762	-0.8624	0.2244	0.24466	0.35038	-0.0321	-0.7151	-0.1059	-0.0368
NM_012660	Eef1a2	0.24602	0.05444	0.26349	0.19717	0.27196	0.13566	0.07377	0.27315	-0.024	0.11082	0.03672	0.04737	0.01297	0.21992
NM_033539	Eef1a2l1	-0.0813	-0.001	0.00162	-0.0483	-0.102	-0.268	-0.0602	-0.0151	-0.0349	-0.1097	0.01419	-0.0775	-0.0172	-0.0824
NM_033539	Eef1a2l1	-0.131	-0.1918	0.01187	-0.0638	-0.1283	-0.2367	-0.0609	0.01553	-0.1915	-0.3832	0.02342	-0.0301	0.03744	-0.1269
NM_001108799	Eef1b2_predicted	-0.2062	-0.4035	-0.5018	-0.2027	-0.0894	-0.3797	-0.4765	-0.2926	-0.2299	-0.0749	-0.113	-0.2896	-0.1697	-0.0585
NM_001013104	Eef1d	-0.3133	-0.1153	0.08171	0.03626	0.05113	0.18282	-0.4991	-0.0243	-0.2309	-0.0609	-0.1422	0.2457	0.24244	0.38916
NM_001106106	Eef1e1_predicted	0.18198	-0.1235	0.29	0.13214	-0.076	0.32944	-0.06	-0.2007	-0.0275	0.00443	-0.1457	0.29586	0.49604	0.54717
NM_017245	Eef2	-0.5483	-0.2975	-0.191	-0.4489	-0.7756	-0.7841	-0.6749	-0.7936	-0.3529	-0.414	-0.2725	-0.5359	-0.7497	-0.5282
NM_012947	Eef2k	-0.827	-0.0795	-0.1354	-0.231	-1.11	-1.1284	-0.0645	-0.767	-0.1461	-0.1594	-0.305	-0.2699	-0.6389	-0.3078
NM_001105977	Efcab2_predicted	-0.035	-0.1369	-0.1585	-0.2045	-0.4349	-0.0646	0.03825	-0.2523	0.15185	-0.0735	-0.2998	-0.0133	-0.217	-0.1429
NM_022302	Efcbp1	0.02163	-0.0398	0.0298	0.02896	-0.1132	0.00048	0.10566	-0.0486	0.17869	0.0274	-0.0194	-0.0791	-0.0701	-0.0144
NM_133415	Efcbp2	-0.1989	-0.2013	-0.105	-0.1178	-0.2178	0.20858	-0.1428	-0.2604	-0.052	0.08626	-0.2135	-0.1476	-0.1007	-0.2025
NM_001012039	Efemp1	-0.1639	-0.1259	-0.0491	-0.0251	-0.1232	-0.1071	0.0056	0.05758	0.01505	-0.2005	0.31324	-0.0414	0.04415	-0.0477
NM_001005907	Efemp2	0.21149	0.95535	0.5022	0.3519	0.66121	0.44327	0.43871	0.57538	0.72854	0.99669	0.85652	0.22167	0.42687	0.65412
XM_001065080.1	Efha2	0.14885	0.11502	-0.0857	0.07501	0.22023	-0.0376	0.13533	0.11075	0.11044	0.18923	-0.1221	-0.0104	0.12809	-0.1586
NM_001106879	Efhb_predicted	0.22953	-0.0069	-0.0786	-0.0118	-0.0024	0.12032	-0.0959	-0.0153	0.08315	-0.0543	-0.1293	0.07056	0.07679	0.18907

NM_001031648	Efhd2_predicted	0.17508	-0.2061	0.24877	-0.1898	0.54775	0.28167	-0.2538	0.67962	-0.1467	-0.2927	-0.0529	-0.1113	0.55167	0.28876
NM_053599	Efna1	0.02312	-0.0323	-0.2268	0.13879	0.02293	-0.0886	0.09502	0.0351	-0.1022	-0.1576	-0.0353	-0.2784	0.01053	-0.2641
NM_001168670	Efna2	-0.185	0.05327	0.05448	-0.0316	-0.1871	-0.1746	0.09899	-0.2668	-0.0053	-0.0825	-0.1033	-0.0796	-0.0016	-0.0893
XM_001072657.1	Efna3	-0.3906	-0.3215	-0.2622	-0.2664	0.00022	0.36965	-0.2277	0.26561	-0.0868	-0.431	-0.3177	-0.1089	-0.2984	-0.3479
NM_001107692	Efna4_predicted	0.35802	0.28258	0.14825	0.18961	0.60645	0.27588	0.09086	0.6065	0.5743	0.17794	0.31424	0.55526	0.12178	0.01386
NM_053903	Efna5	-0.7932	-0.3068	-0.6445	-0.7756	-0.6356	-0.8169	-0.1806	-0.6765	-0.145	-0.2395	-0.3678	-0.4978	-0.4233	-0.3104
NM_017089	Efnb1	1.4287	-0.4232	0.06004	-0.5711	-1.1094	-0.7587	-0.5734	-1.0571	0.20639	-0.4322	-0.5522	1.0162	-0.6203	-0.5195
NM_001107328	Efnb2_predicted	0.21227	-0.0804	-0.2806	-0.0011	-0.3576	0.08447	0.02302	0.01809	-0.4643	-0.1679	-0.1111	-0.2403	0.11165	-0.0593
NM_001100980	Efnb3_predicted	-0.1051	-0.0773	0.23768	0.01157	-0.1536	-0.2745	0.08158	-0.1196	-0.0234	-0.0655	0.04927	-0.0981	-0.1162	-0.1461
NM_001106033	Efs_predicted	-0.0236	-0.1399	0.27281	-0.2043	-0.2283	-0.1573	0.01109	-0.1846	-0.0142	0.13369	-0.1123	-0.1897	-0.052	0.20998
NM_001107534	Eftud1_predicted	0.11641	0.12295	0.10098	0.0691	0.1616	0.11359	-0.002	0.09605	0.07408	-0.0318	0.19759	-0.3101	0.12541	0.04168
NM_001107534	Eftud1_predicted	0.1555	0.15516	-0.1347	-0.1617	0.19029	-0.072	0.00883	0.23045	0.02549	-0.0085	-0.041	-0.1299	-0.0833	0.36677
XM_213492.4	Eftud2	-0.631	-0.3947	0.42714	-0.7468	-0.6079	-0.5812	-0.2713	-0.6244	-0.5454	-0.3696	-0.2887	-0.1243	0.22024	0.21436
NM_012842	Egf	-0.0389	-0.2294	0.44905	0.29611	-0.3416	-0.2513	0.4999	-0.2934	-0.1327	-0.1523	-0.0382	-0.2304	-0.1738	-0.3433
XM_001077319.1	Egfl4	-0.0133	0.14182	0.04188	0.01861	-0.0058	0.16318	0.26429	0.09251	-0.036	0.10095	0.13736	0.16024	-0.0233	-0.0143
NM_001107940	Egfl5_predicted	-0.1178	0.037	-0.1742	-0.1557	-0.1119	-0.1257	-0.0976	0.0507	0.09203	0.07128	0.02195	-0.2137	-0.1068	-0.0872
NM_001108254	Egfl6	-0.008	-0.0268	-0.0271	0.06428	0.00875	0.26291	0.05497	0.03039	-0.006	0.10282	0.10925	0.17278	-0.0454	0.08969
NM_139104	Egfl7	-0.0177	-0.0418	0.03291	-0.3766	-0.1008	0.1046	-0.3344	-0.4594	0.05658	-0.0847	0.04965	-0.2039	0.31989	0.289
NM_031507	Egfr	-0.3949	0.48979	-0.0801	0.24016	-0.416	-0.3221	3E-05	-0.2229	0.09384	0.27002	0.35759	-0.1536	-0.2543	-0.1214
NM_178334	Egln1	-0.2004	0.37445	-0.1833	-1.1719	-0.4038	-0.9399	-0.4296	-0.5329	-0.0654	0.26286	-0.1388	-0.5338	-0.1934	-0.0334
NM_001004083	Egln2	-0.7143	-0.259	0.1405	-0.1812	-0.4475	-0.1708	-0.1256	-0.5963	-0.1812	-0.1818	-0.1802	-0.0834	-0.2387	-0.1769
NM_012551	Egr1	-0.105	0.32844	-1.7453	-1.659	-1.1491	-1.2264	-0.4692	-1.1153	0.13339	0.3336	0.10344	0.14212	-1.596	-1.7288
NM_053633	Egr2	0.48465	-0.0773	0.19248	-0.152	0.54477	1.0754	0.25245	0.36479	-0.0973	0.08969	-0.0351	0.56206	0.25822	0.41539
NM_017086	Egr3	0.16785	0.16898	0.07816	0.06869	0.22453	0.01345	0.081	0.24822	0.1006	0.08212	0.32175	-0.0066	0.38235	0.07262
NM_019137	Egr4	0.0036	-0.0201	0.00075	0.03884	0.04491	0.02791	0.01092	-0.0471	-0.0061	0.09183	-0.0179	-0.0996	0.10889	-0.0236
XM_001056760.1	Ehbp1_predicted	0.2273	0.14075	-0.0092	0.16053	0.13851	-0.0266	-0.0071	0.03809	0.23223	0.11371	0.15226	0.15839	0.00684	0.1917
NM_001011939	Ehd1_predicted	0.06783	-0.0745	0.03908	0.02075	-0.0151	0.00799	-0.1282	-0.1891	0.12868	0.07983	-0.1114	0.0038	-0.1859	-0.0327
NM_001011939	Ehd1_predicted	-0.0146	0.01229	-0.0285	0.09493	0.13009	0.12117	0.28953	0.05455	0.2126	0.40314	-0.0514	0.39625	-0.0145	-0.1341
NM_001007598	Ehd2	0.12977	0.36693	-0.5653	-1.0489	0.37094	-0.1708	-0.104	0.39943	0.2516	0.24884	0.0838	-0.3793	0.39315	0.47281
NM_138890	Ehd3	0.19079	-0.0405	-0.084	-0.2208	0.23628	0.16482	0.15442	0.31392	-0.0417	0.26237	-0.0203	0.05002	0.13806	0.44451
NM_139324	Ehd4	0.69194	0.32374	0.64478	-0.7436	1.1206	0.85467	0.74725	1.1815	0.0731	0.41046	0.31133	-0.4065	0.88545	0.98281
NM_001106493	Ehf_predicted	-0.0437	0.14069	-0.0156	0.10022	-0.0669	0.18113	0.24036	0.03947	0.10721	0.0472	0.06872	0.05458	0.24415	0.09484
NM_001108572	Ehmt1_predicted	-0.5842	-0.3157	0.11095	-0.0611	-0.3737	-0.5956	-0.0616	-0.5535	-0.2643	-0.3761	-0.293	-0.2584	-0.4195	-0.2018
NM_212463	Ehmt2	-0.3611	-0.3233	-0.0839	-0.3141	-0.4031	-0.5151	-0.1916	-0.3631	-0.2772	-0.4216	-0.4807	-0.3818	-0.4391	-0.4119
NM_001025660	Ei24	0.47286	0.72055	0.20848	0.21223	-0.0154	-0.2233	0.35235	-0.0333	0.56829	0.56342	0.76031	0.09506	0.11626	0.25006
NM_001106963	Eif1a	0.25361	0.72443	-0.3337	0.68973	0.62084	0.05875	0.41961	0.89997	0.26508	0.45119	0.18943	-0.1449	0.68715	0.63141
NM_001008305	Eif1ad	0.15462	-0.2526	0.31948	0.1376	-0.0767	0.10779	-0.0398	-0.1414	-0.3062	-0.1527	-0.338	-0.2777	0.1452	0.05791
NM_001106867	Eif1b_predicted	0.33929	-0.1316	0.3871	-0.1269	-0.07	0.14702	0.12421	-0.0746	0.2843	0.10328	0.24562	0.63886	0.29779	0.27913
NM_013223	Eif2ak1	-0.1526	0.13391	-0.2619	0.47406	0.24349	-0.3608	0.25757	0.10926	0.08034	-0.0242	0.28692	-0.1543	-0.0596	0.1581
NM_019335	Eif2ak2	0.13908	0.27687	-0.0728	0.34455	0.3782	-0.3109	0.08608	0.31373	0.25241	0.04926	0.08702	-0.3509	0.17738	0.24621
NM_031599	Eif2ak3	-0.1834	-0.4973	-0.2417	0.37488	-0.1495	-0.1811	-0.1766	-0.1241	-0.2827	-0.3361	-0.519	-0.3033	-0.319	-0.3816
NM_001105744	Eif2ak4_predicted	-0.5116	-0.3189	-0.0547	0.25847	-0.5862	-0.4849	0.14781	-0.3126	-0.0773	-0.2345	-0.0489	-0.1454	-0.509	-0.701
NM_172029	Eif2b1	-0.4504	-0.5727	-0.2117	-0.1159	-0.4184	0.00885	-0.0974	-0.3133	-0.3889	-0.3242	-0.1368	-0.0316	-0.2326	-0.1813
NM_032058	Eif2b2	0.18614	-0.0309	0.5817	0.1414	0.21503	0.21174	0.05537	0.11765	0.08016	0.04694	-0.0287	0.34835	0.33909	0.35115
NM_133609	Eif2b3	-0.0342	-0.0872	0.08447	0.47328	0.28199	0.53881	0.03002	0.14903	-0.4767	-0.4076	-0.2306	-0.1016	-0.0121	0.02647
NM_053950	Eif2b4	-0.3689	-0.0327	0.07492	0.29051	0.36222	0.31005	0.26775	0.22857	0.03098	-0.0169	0.03217	0.04705	0.07764	0.01381
NM_138866	Eif2b5	0.43515	0.43825	0.20844	0.27551	0.46477	0.30974	0.03136	0.38575	0.31803	0.36748	0.34183	0.23251	0.37916	0.44897
XM_001058138.1	Eif2c1_predicted	-0.0559	0.26833	-0.0101	-0.0435	0.10897	-0.0156	0.05797	-0.1329	-0.0107	0.02834	-0.0663	-0.0384	0.01727	-0.0319
NM_021597	Eif2c2	-0.147	-0.2171	0.25169	0.13929	-0.0122	-0.0199	0.14665	-0.2266	0.22707	-0.0968	-0.2329	0.03271	0.27139	0.2001

XM_001058231.1	Eif2c3_predicted	0.24199	0.45627	0.17705	0.18085	0.25438	-0.032	0.06192	0.13874	0.11815	0.10684	0.1439	0.11078	0.11335	0.29955
NM_019356	Eif2s1	0.03984	-0.2233	0.22973	0.67141	0.28346	0.13776	0.33879	0.58993	0.30695	-0.1247	0.08355	-0.2887	0.25954	0.28491
NM_199380	Eif2s2	0.28918	-0.4292	0.04932	0.02969	0.1015	0.15631	-0.121	0.22465	-0.338	-0.2976	-0.3191	-0.2373	0.05019	0.07672
NM_001100542	Eif2s3x	0.10385	0.07959	-0.4111	-0.6718	0.17622	0.07502	-0.3757	0.03074	0.14267	0.09101	0.1708	-0.0935	0.03592	-0.19
NM_001004283	Eif3d	0.13818	0.02379	0.68174	0.86404	0.51042	0.35284	0.28763	0.11329	0.03206	0.15956	0.2095	0.16315	0.34047	0.38273
NM_001011990	Eif3e	-0.1584	0.32979	-0.3014	0.26382	-0.3111	-0.4832	-0.2263	-0.1526	0.29918	0.13877	0.17778	-0.157	0.16422	0.31722
NM_198751	Eif3h	0.02323	0.26139	-0.3289	0.21453	-0.0165	0.27153	-0.4056	-0.1411	0.46808	0.43178	0.36204	0.34071	0.00674	0.03816
NM_001047087	Eif3s10	-0.4517	-0.2033	-0.0326	-0.1785	-0.093	-0.318	0.05953	-0.1732	-0.147	-0.2309	-0.2598	-0.2703	0.14053	-0.0839
NM_001106242	Eif3s12_predicted	-0.173	-0.1511	-0.288	-0.4047	-0.04	0.13304	-0.1725	-0.2469	-0.3747	-0.236	-0.1445	-0.0447	0.20853	-0.0251
NM_001106292	Eif3s5_predicted	-0.0455	0.45435	-0.528	-0.0479	0.16465	-0.1061	-0.4087	0.23314	0.15125	0.29346	0.3688	0.02707	-0.2067	-0.1865
NM_001100662	Eif3s8	-0.4098	-0.4389	0.22133	-0.5531	-0.3706	-0.6524	-0.4435	-0.4008	-0.3569	-0.287	-0.4265	-0.4628	-0.1356	-0.0462
NM_199372	Eif4a1	-0.8461	-0.7395	-0.0222	-0.9393	-0.9537	-1.0772	-0.5774	-1.0748	-0.529	-0.6613	-0.3933	-0.2114	-0.0156	-0.1443
NM_001008335	Eif4a2	-0.3183	-0.5162	0.10901	0.18235	-0.2436	-0.1548	0.17006	-0.1018	-0.5742	-0.3504	-0.4248	-0.4533	-0.6617	-0.6566
NM_001008324	Eif4b	-0.8694	-0.1216	-0.6061	0.04154	-0.6159	-0.6866	-0.6957	-0.6773	-0.3531	-0.1645	-0.1821	-0.1161	-1.0032	-0.9207
NM_053974	Eif4e	0.20263	-0.0973	-0.0673	-0.2003	-0.0856	-0.0678	-0.1869	-0.0981	-0.121	-0.2748	-0.0764	0.01701	0.09739	0.15369
NM_001108808	Eif4e2_predicted	0.07452	-0.3711	0.07346	-0.8389	0.43102	0.62981	-0.4333	0.34556	-0.0539	-0.0732	-0.2875	0.22499	0.37702	0.5243
NM_053857	Eif4ebp1	0.15322	-0.6088	-0.192	-0.267	-0.36	0.19292	-0.8001	-0.6202	-0.2915	-0.5448	-0.6499	0.11808	-0.3825	-0.1465
NM_001033069	Eif4ebp2	0.03719	-0.0068	-0.2835	-0.1445	0.11609	-0.023	0.0549	0.01395	0.21677	-0.1308	-0.1417	-0.1659	-0.0573	0.19406
XM_001060756.1	Eif4g1	-0.0385	-0.0632	0.05196	-0.0093	0.21044	-0.0225	0.18181	0.01282	0.11046	-0.05	0.05048	-0.0132	-0.0802	0.27141
NM_001106693	Eif4g3_predicted	-0.4565	-0.1646	0.25556	-0.3246	-0.1631	-0.4898	0.20355	-0.3546	-0.4888	-0.2618	-0.06	-0.52	-0.907	-0.6077
NM_001006957	Eif4h	-0.201	-0.2583	-0.5433	-0.2024	-0.0653	-0.2786	-0.0862	0.05946	-0.1796	-0.2253	-0.3079	-0.396	-0.4319	-0.1578
NM_020075	Eif5	-0.2159	-0.2677	-0.4346	-0.6578	-0.2743	-0.3229	-0.3429	-0.1013	-0.2196	-0.3481	-0.3415	-0.013	0.25871	-0.196
NM_001033681	Eif5a	-0.0685	-0.4769	-0.3877	-0.6645	0.08967	-0.1915	-0.5953	-0.2847	-0.0601	-0.4002	-0.2849	-0.1961	0.11184	0.12381
XM_226974.4	Eif5a2_predicted	0.49688	0.35048	-0.0958	-0.0303	0.65545	0.50437	-0.0103	0.56588	0.36502	0.22642	0.17563	-0.0986	0.58637	0.41189
NM_012552	Ela1	-0.2475	-0.2439	-0.0228	-0.275	-0.1677	0.10927	-0.0777	-0.2746	-0.1328	-0.1416	-0.1589	0.04035	-0.1747	-0.2201
NM_001106767	Ela2_predicted	0.0434	0.11072	0.0606	0.00136	0.14236	0.03928	0.06484	0.04663	-0.0357	0.06953	0.10147	0.14233	-0.0111	0.06362
NM_012553	Ela2a	0.12073	-0.0119	-0.0839	-0.0439	-0.0385	-0.0453	0.1204	0.31073	0.14983	-0.0367	-0.1034	0.0837	0.0932	0.05276
NM_001106692	Ela3b_predicted	-0.0242	0.04393	-0.0092	-0.0003	0.07084	0.00372	0.34133	0.30119	0.0288	-0.0194	-0.0036	-0.0511	0.17162	0.18503
NM_001107406	Elac1_predicted	0.08651	0.12725	0.22829	0.1332	0.18814	0.07091	0.09901	0.22454	0.25243	0.17763	0.08959	0.19728	0.33602	0.23038
NM_172326	Elac2	0.11004	0.01628	0.46442	0.2014	0.40025	0.46569	0.02016	0.17759	-0.2167	0.10836	0.12006	0.28387	0.29749	0.12574
NM_001108848	Elavl1_predicted	-0.0496	-0.0575	0.22024	-0.3673	-0.2217	0.09693	-0.2023	-0.1112	-0.0734	0.04456	-0.3456	0.34312	0.33496	0.4507
NM_172324	Elavl3	0.28503	0.22434	0.16061	0.06934	0.12682	0.03722	0.19095	0.1055	0.1722	0.05353	0.07324	0.14979	0.22782	0.16753
NM_053520	Elf1	0.04345	0.59486	-0.3381	-0.3523	0.2815	-0.4482	0.19313	0.26278	0.33692	0.38019	-0.1008	-0.1583	0.28473	0.34673
NM_001012181	Elf2	-0.2505	0.06604	0.03593	-0.7129	-0.4874	-0.1234	-0.2275	-0.4259	-0.1201	0.0052	-0.0085	0.22922	-0.1564	0.03375
NM_001012181	Elf2	-0.3655	0.01117	-0.3706	-0.768	-0.675	-0.565	-0.1849	-0.6897	-0.1889	-0.0425	-0.0843	0.21073	-0.107	-0.046
NM_001024768	Elf3	0.00229	0.01054	0.15492	0.02342	0.04556	-0.0698	0.14472	0.39894	-0.0643	0.03772	0.09122	0.22456	0.07054	0.14496
XM_001059821.1	Elf4_predicted	0.35054	0.41267	0.42634	0.44889	0.36987	0.34518	0.26452	0.40655	0.55865	0.14072	0.36546	0.11792	0.40123	0.58273
NM_001108956	Elf5_predicted	0.14117	-0.0373	0.03899	-0.1469	-0.0584	-0.0934	-0.0129	-0.2023	-0.0787	0.01519	0.03482	0.33492	-0.1402	-0.0965
NM_001108059	Elk1	-0.0724	0.04534	-0.4133	-0.0694	-0.2286	-0.3836	-0.1516	0.23711	-0.1542	0.05049	-0.1264	-0.3834	-0.022	0.16509
NM_001108743	Elk3_predicted	0.19353	0.35598	0.2529	0.55444	0.46275	0.25057	0.25878	0.1822	0.30809	0.2813	0.29168	0.45238	0.58067	0.87397
NM_001107173	Elk4_predicted	0.05684	0.12985	0.05033	-0.025	0.04251	0.02589	0.02845	-0.0872	0.0181	0.07467	0.01211	0.12834	0.38935	0.43446
NM_001107304	Ell_predicted	0.45377	0.85885	0.30507	1.1664	0.40131	-0.0121	0.58621	0.54233	0.21845	0.66282	0.45372	-0.4978	-0.0143	0.11567
XM_001054852.1	Ell2	-0.8527	-0.9166	-1.1488	-0.6953	-0.9131	-0.8005	-0.9516	-0.7218	-0.5068	-0.8164	-0.9871	-0.5108	-0.7698	-0.8283
NM_001011957	Ell3	-0.1794	-0.1633	-0.258	-0.2629	-0.1786	-0.2598	-0.1017	-0.2861	-0.0701	-0.1953	-0.2189	-0.1686	-0.2111	-0.2047
NM_001108415	Elmo1_predicted	-0.9451	-0.8848	-0.8088	-0.9077	-1.1033	-0.9456	-0.8489	-0.9911	-1.0651	-1.0628	-0.8327	-0.8658	-1.1009	-0.7713
NM_001134955	Elmo2	0.29398	0.30721	0.42933	0.57958	0.42368	0.28931	0.15413	0.19448	0.15704	0.25931	0.13046	0.03237	-0.0651	0.05371
NM_001030028	Elmo3	-0.4297	-0.5573	0.75066	1.3507	-0.8921	-0.7818	0.56827	-0.7132	-0.4676	-0.5395	-0.5912	-0.0947	-0.6929	-0.6089
XR_008801.1	Elmod1_predicted	-0.141	0.02726	0.04828	-0.0672	-0.0928	0.12343	0.27972	0.13295	-0.1214	0.08368	0.10741	-0.0043	0.00893	-0.0459
NM_012722	Eln	-0.136	1.2838	0.60235	0.40412	-0.1619	-0.1231	0.85388	-0.2122	1.0985	1.0952	1.1181	-0.0503	-0.0734	-0.1594

NM_001109118	Elovl2_predicted	0.19678	-0.0769	-0.0992	-0.128	0.18648	-0.0662	-0.0012	0.22755	0.09434	0.31482	0.07589	0.06174	0.1697	-0.0066
NM_001107602	Elovl3_predicted	-0.001	0.27392	0.16062	0.18231	0.21322	0.13902	0.07535	0.15735	-0.0111	-0.0264	0.07789	0.07575	0.00991	0.01351
XM_001062735.1	Elovl4_predicted	0.08746	-0.1245	-0.2096	-0.073	0.22368	0.46575	-0.2349	0.43049	-0.0156	0.04486	-0.1398	-0.0443	0.2027	0.0826
NM_134382	Elovl5	-0.0432	0.63044	-0.3948	-0.0549	0.10876	-0.3031	-0.2778	0.30509	0.26516	0.50332	-0.3648	-0.2742	0.08995	-0.1752
NM_134383	Elovl6	0.08408	-0.0902	-0.1156	-0.0215	-0.0505	-0.0784	-0.0273	0.06821	-0.0691	-0.0427	0.06374	-0.0337	-0.0913	-0.0198
XM_001065454.1	Elovl7_predicted	-0.0796	-0.0436	-0.0986	0.1445	-0.1853	-0.0634	-0.0923	-0.0496	0.11224	-0.0096	0.15376	0.09532	-0.0703	-0.1197
NM_001034145	Elp2	-0.3406	-0.0851	0.0346	0.18652	-0.5033	-0.444	0.09329	-0.3567	-0.1538	0.03584	-0.2306	-0.0427	-0.4107	-0.38
NM_022294	Eltd1	-0.2102	-0.1254	0.01003	0.04078	-0.1484	-0.1707	-0.1285	0.00149	-0.1113	-0.1348	-0.2303	-0.0897	-0.2401	-0.0425
NM_053719	Emb	0.36827	0.61784	0.21657	0.68662	0.59779	0.84296	0.41187	0.49734	0.56991	0.82891	0.73509	0.39182	0.25812	0.27427
NM_001004228	Emcn	-0.0286	0.10275	0.0766	0.1184	0.02224	-0.0504	0.19683	-0.0538	-0.0435	0.1571	0.03931	0.18988	0.19382	0.08415
NM_012948	Emd	-0.1268	-0.173	0.03736	0.60591	0.14027	0.20711	-0.1831	-0.0099	-0.145	-0.1467	-0.1158	0.03705	-0.0179	0.02933
NM_001105830	Eme1_predicted	-0.2335	-0.3225	0.31954	-0.2184	-0.2768	-0.3073	-0.2983	-0.214	0.0487	-0.2437	0.01017	-0.0433	0.26513	0.19766
NM_001107888	Emg1_predicted	0.1107	-0.731	-0.1299	-0.8888	-0.3438	-0.1979	-0.9647	-0.3916	-0.7259	-0.596	-0.5625	0.16275	0.30399	0.40899
NM_001106710	Emilin1_predicted	-0.9178	0.52368	-0.5583	-0.4817	-1.1897	-1.5399	-0.576	-1.3072	0.26896	0.44276	0.44013	-0.3608	-0.9489	-0.7649
XM_237520.3	Emilin2_predicted	-0.4495	0.00745	0.03371	-0.2367	-0.326	-0.1909	-0.1373	-0.2527	0.22439	-0.1614	0.48129	-0.1541	0.04893	-0.0105
NM_001109901	Emilin3_predicted	-0.008	0.0029	0.10994	0.01094	0.24688	-0.0586	-0.1208	-0.0201	0.13972	0.07393	0.14939	0.15309	-0.1044	0.01696
NM_001025741	Eml1	-0.1399	-0.1682	-0.1238	0.16798	-0.3105	-0.1072	-0.1203	0.07594	-0.1663	-0.1201	-0.0761	-0.0885	-0.2224	-0.1232
NM_138921	Eml2	0.33819	0.15525	-0.276	0.32251	0.10211	0.40069	0.3878	-0.1341	-0.3651	0.00354	-0.0153	-0.4011	-0.5071	-0.4363
NM_001108008	Eml4_predicted	0.08829	0.16067	0.19086	0.13798	0.13448	-0.0551	0.30307	-0.0797	0.22039	0.24795	0.15606	0.07014	0.30006	0.25017
XM_001064022.1	Eml5	-0.0098	-0.2126	-0.0835	-0.1412	-0.1272	-0.111	-0.0671	0.01605	-0.1103	-0.1072	-0.2148	-0.029	-0.0131	-0.1249
NM_012843	Emp1	0.5517	-0.005	-0.5863	-0.888	0.061	-0.3967	0.8388	0.24336	-0.417	-0.0589	-0.4029	-1.0464	-0.2402	-0.3114
NM_001007721	Emp2	0.54203	0.43226	0.35883	0.28114	0.25403	0.31291	0.36024	0.50882	0.37283	0.21251	0.36208	0.42311	0.4792	0.19439
NM_030847	Emp3	-0.4428	-0.4908	1.0564	-1.1626	-0.5687	-0.3385	-0.1088	-0.7681	-0.2774	-0.145	-0.147	0.79646	0.66157	0.87608
NM_001007557	Emr1	-0.1001	-0.2057	-0.1786	-0.1273	-0.1338	0.0036	-0.2075	-0.1493	-0.1135	-0.1793	-0.0669	0.01421	-0.161	-0.1527
NM_001007558	Emr4	-0.2308	-0.0955	-0.1795	-0.1068	-0.2328	0.02226	-0.1419	-0.2699	0.07363	-0.0761	-0.1565	-0.1323	-0.0658	-0.0207
NM_001012150	Enah	0.51732	0.08028	0.32711	0.61316	0.63108	0.42971	0.10314	0.60116	0.09177	0.20268	0.09649	0.0993	0.54439	0.45687
NM_001012150	Enah_predicted	0.03321	-0.0876	-0.0101	-0.2	0.15041	0.04904	-0.0559	0.14158	0.01954	-0.226	-0.1291	0.14669	0.12825	-0.1487
NM_001106001	Enam_predicted	-0.0303	-0.0447	-0.0284	0.07264	0.06188	0.04858	0.12994	0.0306	0.08355	-0.0797	-0.0555	-0.0739	0.02938	-0.015
NM_001003401	Enc1	-0.6747	-0.437	-0.6506	-0.3842	-0.4981	-0.6052	-0.4855	-0.4015	-0.8005	-0.3598	-0.6191	-0.6292	-0.4599	-0.5468
NM_001106866	Endogl1_predicted	-0.1242	-0.0839	0.00734	-0.156	-0.1309	-0.2669	-0.1334	-0.0352	-0.0745	0.12931	-0.2185	-0.586	0.13232	0.30272
NM_012554	Eno1	0.20708	0.61396	0.28077	-0.5705	0.42304	0.38366	-0.7193	0.39068	0.56899	0.42423	0.52967	0.04928	0.48506	0.5627
NM_139325	Eno2	1.6874	1.3153	0.78838	1.5001	1.1108	1.1528	0.26935	0.86345	1.1594	1.1194	1.2941	0.3224	0.2125	0.18506
NM_012949	Eno3	-0.8079	-0.8313	0.60495	-1.2341	-1.1215	-0.9883	-0.5559	-1.4078	-0.3031	-0.9097	-0.6802	0.50827	0.35684	0.49461
NM_001009391	Enoph1	0.24294	-0.2438	0.23498	0.33737	0.54708	0.48096	-0.301	0.27607	-0.2698	-0.043	-0.2664	-0.0695	-0.0352	0.08351
NM_022251	Enpep	0.05455	0.25243	-0.0272	0.00985	-0.2005	-0.2624	-0.2354	-0.2122	-0.0609	0.02432	-0.366	-0.1147	0.11036	0.01054
NM_053535	Enpp1	-1.1665	0.17107	-1.6384	-0.1546	-3.0891	-3.051	-0.5821	-3.2805	-0.2417	-0.2071	-0.168	-0.9098	-1.7428	-1.6171
NM_057104	Enpp2	-0.0848	0.34245	-0.1737	-0.083	-0.1714	-0.0903	-0.078	-0.0795	0.32082	0.28863	0.1174	-0.0297	0.03776	-0.0172
NM_019370	Enpp3	1.717	3.9356	2.1576	2.0078	1.5569	1.4503	2.9859	1.5975	3.0095	3.6207	3.4307	0.39906	1.7518	1.6691
NM_019370	Enpp3	0.00721	0.06498	-0.037	0.12136	-0.0785	0.00122	0.17557	0.12868	0.30784	0.03515	0.10879	-0.0673	-0.0425	0.24607
NM_001106892	Enpp4_predicted	-0.0361	-0.2703	0.04658	-0.113	-0.079	0.0869	-0.0756	-0.1692	-0.0817	-0.1798	-0.1103	-0.1666	-0.0859	-0.1615
NM_001012744	Enpp5	-1.3394	-1.6307	-1.6248	-1.5786	-1.519	-1.5799	-1.5229	-1.3951	-1.6658	-1.2725	-1.6607	-1.5989	-1.4765	-1.6744
NM_001107311	Enpp6_predicted	0.07528	-0.0773	0.16162	-0.045	-0.0117	0.0963	0.07611	-0.0769	0.01038	-0.0193	0.00527	0.09846	-0.0177	-0.0387
NM_001012466	Enpp7	0.10348	0.10508	0.01014	0.12075	0.06381	-0.01	0.06422	-0.1829	0.15887	0.06437	0.32077	0.18391	0.27061	0.12321
NM_001002022	Enth	-0.088	0.02998	-0.0801	0.05559	0.07114	0.09673	-0.0016	0.01735	0.20032	-0.0104	-0.0791	0.0572	0.38154	0.04024
NM_022587	Entpd1	0.15436	0.27591	0.04476	0.10189	0.14968	0.0576	0.01707	0.23686	0.019	0.08189	-0.0083	0.01209	0.06573	0.11774
NM_172030	Entpd2	-0.111	-0.0917	-0.0985	-0.0773	-0.1856	-0.1405	-0.0264	-0.1208	-0.0773	-0.1268	-0.0104	-0.1	0.02396	-0.1268
NM_178106	Entpd3	-0.2516	-0.2103	-0.4142	-0.3375	-0.2978	-0.2838	-0.3426	-0.3994	-0.3351	-0.484	-0.1775	-0.5567	-0.3429	-0.2547
NM_001108384	Entpd4_predicted	0.49999	0.58629	0.17821	0.89916	0.58182	0.07527	0.43811	0.65061	0.48313	0.24132	0.71898	-0.0259	0.33418	0.16501
NM_199394	Entpd5	1.2864	0.76381	0.68234	0.82738	1.0509	0.89433	1.0008	1.0015	0.72922	0.64332	0.52912	0.15429	0.31148	0.53249

NM_053498	Entpd6	-0.2019	-0.3984	-0.051	-0.0651	-0.3932	-0.4149	-0.1872	-0.3807	-0.5023	-0.4045	-0.4515	-0.2411	-0.1139	-0.1967
NM_001107595	Entpd7_predicted	-0.1153	-0.1879	-0.2605	-0.216	-0.3345	-0.1622	-0.051	-0.1309	-0.0576	-0.4222	-0.2324	-0.1591	-0.1944	-0.2324
XM_001076610.1	Ep300	0.02899	0.06483	0.347	0.14942	0.00445	0.04165	0.22002	0.01586	0.07079	0.0362	-0.0188	0.00183	0.04201	0.00847
NM_001107149	Ep400	-0.5454	-0.4043	0.10424	0.07159	-0.4716	-0.2323	-0.0013	-0.6425	-0.46	-0.478	-0.2979	-0.1477	-0.4203	-0.2591
NM_023090	Epas1	-0.0809	-0.0779	-0.1116	-0.0676	-0.1746	-0.097	-0.2451	-0.1634	-0.3136	-0.2154	-0.2234	-0.0363	-0.2953	-0.1445
NM_021681	Epb4.1l1	0.04234	-0.0545	0.00531	-0.0896	-0.1185	0.09573	-0.0381	0.00924	0.01797	0.19793	0.02758	0.00965	0.13751	-0.0938
NM_021681	Epb4.1l1	1.0193	0.38664	-0.837	-0.6871	0.19818	-0.1369	-0.5791	0.2599	-0.1167	0.11111	0.19178	-0.5691	-0.1374	-0.1541
NM_021681	Epb4.1l1	0.60427	-0.2156	-0.2145	-0.6995	0.00676	0.26027	-0.765	-0.0514	0.2598	0.229	0.09557	0.32176	0.41718	0.28556
NM_053927	Epb4.1l3	-2.4787	-2.5363	-1.955	-2.0399	-1.9675	-1.9528	-1.5002	-2.0859	-2.4619	-2.5425	-1.7405	-2.3414	-2.3396	-2.4165
NM_001107397	Epb4.1l4a_predicted	0.39694	0.76367	0.948	0.80802	0.04799	-0.033	0.41173	0.30742	0.86823	0.53925	0.76244	0.83464	0.82516	0.62999
NM_001108590	Epb4.2_predicted	-0.121	0.07779	0.12089	0.14242	-0.0045	0.3194	-0.1239	-0.1468	-0.0307	-0.0529	-0.0245	0.02974	0.21642	-0.0813
NM_001108385	Epb4.9_predicted	0.27852	-0.001	-0.1417	-0.1191	-0.2379	-0.2353	-0.1573	-0.0835	0.15794	-0.1449	-0.1654	-0.1713	-0.0326	0.02598
NM_001100972	Epc1_predicted	-0.8218	-0.4439	0.28522	-0.2966	-0.868	-0.6638	0.21633	-0.9775	-0.0882	-0.2732	-0.0371	0.10658	-0.2235	-0.2568
NM_001108581	Epc2_predicted	-0.0553	-0.2291	-0.0902	-0.2252	-0.1971	-0.1636	-0.2676	-0.2806	-0.1385	-0.1378	-0.1157	-0.2563	-0.279	-0.3058
NM_001007625	Epdr1	-0.2912	0.86829	-0.3739	-0.8739	-0.4872	-0.6518	0.95001	-0.644	0.52432	0.82285	0.66292	-0.0532	-0.122	-0.1744
NM_001108977	Epha2_predicted	-0.6018	-0.602	-0.4087	0.19141	-0.4243	-0.7162	-0.4752	-0.2605	-0.6341	-0.5513	-0.6407	-0.8838	-0.6744	-0.7022
NM_024367	Epha5	-0.1693	-0.2101	-0.0538	-0.0627	-0.1361	-0.1462	-0.0928	-0.2508	-0.0727	0.00689	-0.0778	-0.1727	-0.1234	-0.2688
XM_221595.4	Epha6_predicted	-0.1321	-0.3233	-0.0848	-0.2407	-0.0207	-0.2294	-0.1908	-0.2082	-0.0603	-0.0069	0.0214	-0.014	-0.023	-0.236
XM_221595.4	Epha6_predicted	0.09636	0.15965	0.14153	0.11947	0.08729	0.03862	0.06674	-0.022	0.07011	0.03464	0.00452	0.17646	0.00643	0.08327
NM_134331	Epha7	0.37813	1.4115	0.50395	0.62722	-0.3067	-0.3974	1.2825	-0.299	1.0845	1.2936	1.2168	0.44349	1.045	1.0057
XM_342952.3	Epha8	0.00146	0.0384	0.28968	0.0822	0.16721	0.21619	0.08637	-0.0339	0.15885	0.2147	0.00718	0.1067	0.35589	0.01596
NM_001104528	Ephb1	-0.2093	-0.2353	-0.2392	-0.0117	-0.0522	-0.071	-0.2846	-0.1095	-0.1918	0.09809	0.02268	0.02186	0.23169	-0.1755
NM_001127319	Ephb2_predicted	0.11562	-0.0847	-0.0268	-0.0675	0.02331	0.0344	-0.0903	-0.2187	0.04015	-0.0566	-0.0445	-0.2143	-0.1179	-0.0792
NM_001105868	Ephb3_predicted	-0.2935	-0.2796	-0.2093	-0.1564	-0.0391	-0.2454	-0.3487	-0.3602	0.21203	-0.0257	-0.2415	-0.1184	-0.2068	-0.2059
NM_001107857	Ephb6	0.14447	0.08938	0.1996	0.16544	0.11366	-0.0508	-0.0674	0.04363	0.08598	0.0135	0.25631	-0.0608	0.21215	0.24613
NM_001034090	Ephx1	-0.2036	1.8237	-0.2201	1.0041	-0.2327	0.00346	0.18664	-0.1336	1.5004	1.7488	1.5399	1.0536	-0.0751	-0.1555
NM_022936	Ephx2	0.62846	-0.0723	-0.592	-0.2633	0.40923	0.32198	-0.4472	0.2678	0.16977	0.00444	-0.1933	0.24038	-0.1867	-0.1359
NM_012748	Epim	-0.3551	-0.3085	-0.1191	-0.2058	0.08483	-0.3952	-0.1783	-0.138	-0.0908	-0.3253	-0.2674	-0.2158	-0.068	0.13785
XM_217039.4	eplin	-0.0827	-0.0075	-0.1639	-0.1456	0.02256	0.01228	0.04113	-0.1205	-0.0603	-0.0828	-0.132	-0.2084	0.24578	0.05216
XM_236659.3	Epm2aip1_predicted	-0.2418	-0.0906	-0.1611	-0.1722	0.07246	-0.1326	-0.1499	-0.1693	-0.1559	-0.1342	-0.1222	-0.0588	0.42407	-0.0736
NM_057136	Epn1	-0.0958	0.00671	0.00514	0.18643	0.28463	-0.1926	-0.0184	0.31334	0.00644	0.14117	-0.0276	-0.2301	-0.0379	0.08472
NM_001033914	Epn2	-0.7714	-0.3588	-0.6429	-0.0322	-0.4036	-0.4435	-0.0743	-0.3162	-0.4566	-0.6179	-0.3784	-0.8319	-0.7363	-0.6771
NM_001024791	Epn3	0.04973	-0.002	-0.1302	-0.1158	0.00479	0.21537	-0.0816	0.20846	-0.1252	-0.0219	-0.1327	-0.1434	0.36112	-0.1751
NM_017001	Epo	-0.1082	-0.0711	-0.0879	-0.0343	-0.0219	-0.1171	-0.0781	-0.1081	-0.1827	-0.0729	0.03114	-0.0369	-0.0533	-0.1102
NM_017002	Epor	0.07752	0.00417	0.20302	0.4615	-0.0516	0.08032	0.2663	0.07569	-0.0054	0.08652	0.13062	0.44122	0.1725	0.18637
NM_001105786	Eppb9_predicted	-0.1704	-0.282	-0.2728	-1.668	-0.2296	0.11789	-0.7149	-0.403	0.02282	-0.5088	-0.2039	-0.0362	-0.4256	-0.512
NM_001024238	Eprs	-0.3863	-0.7399	-0.1235	-0.0183	-0.6283	-0.609	-0.6791	-0.6788	-0.5043	-0.8388	-0.6172	-0.2206	-0.3429	-0.3864
NM_001009424	Eps15	-0.0576	-0.1375	-0.0883	-0.3211	-0.0009	0.07748	0.07493	-0.0697	-0.2612	0.09291	-0.1077	-0.1332	-0.1275	-0.0252
NM_001029921	Eps15l1	-0.1868	-0.0878	-0.1411	0.0162	-0.122	-0.186	-0.1932	-0.1474	0.07962	0.00396	-0.2048	0.14741	-0.0319	-0.0487
XM_001072957.1	Eps8_predicted	0.73924	0.00537	0.45075	0.40416	0.2805	0.11264	0.03474	0.42893	-0.1099	0.10955	0.42059	-0.1103	0.04931	0.28216
NM_001108467	Eps8l1_predicted	-0.1131	-0.1657	-0.1411	-0.0425	-0.044	-0.0173	0.00261	-0.0251	0.11923	-0.1388	-0.0594	-0.0931	-0.0651	0.05548
NM_001108508	Eps8l2_predicted	0.27838	-0.1677	0.43161	-0.334	0.17609	0.04737	0.04208	0.22708	-0.2121	-0.0006	-0.0533	-0.1347	0.29703	0.07414
NM_001106463	Eps8l3_predicted	0.1277	0.00174	-0.0495	-0.1855	-0.0245	-0.0398	0.09454	0.04365	0.04216	-0.0596	0.20744	-0.0339	0.30564	-0.1546
NM_001107037	Epx_predicted	-0.12	-0.0162	-0.1257	-0.064	-0.0873	-0.0317	-0.1397	-0.0065	-0.0448	-0.07	-0.0781	-0.0998	-0.0537	-0.0975
NM_001106299	Eraf_predicted	-1.7163	-1.6268	-1.9516	-1.6718	-1.1965	-0.876	-1.7309	-1.2893	-1.6118	-1.7507	-1.6382	-1.7911	-2.0755	-1.8396
NM_001013229	Eral1	0.26407	0.17539	-0.1828	-0.1859	0.11801	-0.0042	-0.062	0.43215	0.37664	0.16028	0.08697	-0.0998	0.09835	0.03126
NM_017003	Erbb2	-0.3763	-0.0413	0.09409	0.14891	0.04611	-0.3778	0.14354	-0.2668	-0.0141	0.12755	-0.0027	-0.0187	-0.0291	-0.096
NM_017218	Erbb3	0.08588	0.23465	0.19191	0.2085	0.11416	0.05396	-0.0055	0.13422	0.1324	0.14041	-0.087	0.30934	0.06249	0.11171
NM_021687	Erbb4	0.03368	-0.2394	-0.19	0.17369	0.01481	0.13429	0.01784	-0.1692	0.2066	-0.1113	0.07879	0.3532	-0.0909	-0.1279

NM_170788	Erc1	-0.0233	0.0207	-0.0445	0.10112	-0.011	-0.0415	0.09841	0.35612	-0.0604	0.08613	0.14056	0.14279	0.07031	-0.0251
NM_170787	Erc2	-0.205	-0.1167	-0.1586	-0.2076	-0.1668	-0.1409	-0.1455	-0.0924	0.09673	-0.0698	-0.1581	-0.0756	-0.0661	0.02162
NM_001106228	Ercc1_predicted	0.6987	0.2718	0.56984	-0.5802	0.56633	0.48665	0.77208	0.36601	-0.0984	-0.2263	0.06774	0.14867	0.77909	0.78227
NM_001031644	Ercc3	-0.0561	-0.0439	0.20709	0.10644	-0.043	0.4063	0.06174	-0.1457	-0.1391	0.08891	0.08572	0.25635	-0.1299	0.037
XM_340741.2	Ercc4_predicted	0.0487	0.03907	0.07337	0.0402	-0.0975	-0.0938	-0.0471	0.08521	-0.0319	0.08189	-0.0484	0.04321	0.01057	-0.0891
NM_001106910	Ercc5_mapped	-1.229	-0.857	-0.4044	-0.5805	-1.2662	-1.1459	-0.0972	-1.0393	-0.6611	-0.8039	-0.8117	-0.725	-1.3104	-1.2493
NM_001107296	Ercc6_predicted	0.02594	-0.0594	-0.0974	0.17465	-0.0856	0.01601	0.00986	-0.0981	0.01195	0.04206	0.12824	0.03176	-0.1203	-0.1574
NM_001107650	Ercc8_predicted	0.26189	-0.3095	-0.0261	0.32797	0.1423	0.35197	-0.0831	-0.0407	-0.3726	-0.247	-0.2561	-0.0716	-0.0742	0.071
NM_021689	Ereg	0.52952	-0.1074	0.32712	0.85994	0.95139	1.6992	0.05539	1.1079	0.18078	-0.1523	0.03349	-0.0767	0.22581	0.31591
NM_001170335	Erf_predicted	-0.6033	-0.5553	-0.328	-0.539	-0.5492	-0.4152	-0.5766	-0.4923	-0.4519	-0.4076	-0.6549	-0.2079	-0.2888	-0.0324
NM_133397	Erg	-0.0896	-0.1049	0.24784	-0.1069	-0.1401	0.00153	0.07099	0.0772	0.01814	-0.0346	0.1243	-0.0695	0.14172	-0.1523
NM_001106533	Ergic3_predicted	-0.197	0.19945	-0.713	-0.4371	-0.1192	-0.1039	0.16094	-0.1875	0.27078	0.24652	0.02831	0.03427	-0.4411	-0.3881
XM_001073566.1	Ermap_predicted	0.15804	0.09836	0.03186	-0.0353	0.18643	0.20398	0.04352	0.41421	0.07627	0.43571	0.04506	0.07584	0.13896	-0.0421
NM_184050	Ermp1	0.94166	0.5161	-0.3704	0.50641	0.71825	0.77699	-0.8782	0.65417	0.44607	0.44035	0.1815	0.81081	0.08858	-0.0336
NM_001108919	Ern2_predicted	-0.118	0.02858	-0.0759	-0.1349	-0.0037	0.23556	-0.1013	-0.0764	-0.1908	0.04315	-0.0367	-0.1435	-0.0797	0.04939
NM_138528	Ero1l	0.57882	0.67165	-0.1443	0.4031	0.26044	-0.0729	-0.284	0.42441	0.11284	0.7452	0.37343	-0.754	-0.1363	-0.1493
NM_053961	Erp29	-0.4425	-0.7717	0.10787	-1.3071	-1.0862	-0.8673	-0.4188	-1.1835	-0.2001	-0.2907	-0.4221	0.00163	0.08213	0.04034
NM_001014071	Errfi1	-0.0188	-0.1775	-0.1724	0.10989	0.03972	0.07389	-0.2232	0.38469	-0.2593	0.11652	-0.0765	-0.2866	-0.3173	-0.3428
NM_017004	Es1	0.01481	0.09348	0.11001	0.26492	0.05169	0.40965	0.18381	-0.0045	0.04576	0.079	0.11229	0.19781	-0.1344	0.06655
NM_001103359	Es22	0.23553	0.08259	0.10094	-0.0237	0.09679	0.10494	0.10244	0.13122	-0.0247	0.10708	0.046	0.35283	0.15995	0.13646
NM_001004245	Esam	0.20776	-0.0154	-0.0284	0.0165	0.05432	-0.0115	0.01682	0.04502	0.30289	0.01332	0.06345	-0.0359	0.02843	0.10989
NM_001106051	Esd_mapped	0.14557	0.27009	0.02067	0.27009	0.23398	0.32004	0.28873	0.27671	0.26356	0.4192	0.24974	0.20029	0.14494	0.10525
NM_022604	Esm1	0.06572	0.07423	0.27921	1.117	0.14587	0.0683	0.23463	0.42709	0.11428	0.23251	0.11337	0.21652	0.33137	0.2912
NM_001170602	Espl1_predicted	-0.6079	-1.0407	1.3684	0.07107	-1.0855	-1.0889	-0.6044	-0.8714	-0.2083	-0.9255	-0.5459	0.43185	0.10464	0.2731
NM_001170602	Espl1_predicted	-0.2446	-0.0245	-0.0787	-0.081	-0.2398	-0.0914	-0.0069	-0.0918	-0.0509	0.16123	-0.0722	-0.1339	-0.1512	-0.0372
NM_019622	Espn	-0.0802	-0.1031	-0.0314	-0.1113	-0.0267	0.00735	-0.1544	0.02163	0.07862	-0.0761	-0.0891	0.00021	0.0207	0.00916
NM_012689	Esr1	-0.0717	-0.0926	-0.1048	-0.1249	-0.0325	0.1546	-0.0612	0.04411	-0.1102	-0.0598	-0.0772	-0.0204	-0.028	-0.0832
NM_012754	Esr2	0.59966	0.25362	0.13916	0.17586	0.18571	0.11596	0.09434	0.36619	0.26542	0.00606	0.19188	0.32412	0.24382	0.48356
NM_001008516	Esrrb	-0.0479	-0.1025	-0.1552	-0.0586	-0.0434	-0.0568	0.03692	-0.1927	-0.0762	0.05215	-0.0521	0.08048	-0.1718	-0.1146
NM_203336	Esrrg	-0.0863	0.0146	-0.0485	0.00742	-0.0574	0.03715	-0.0946	-0.0783	0.10132	-0.0902	-0.059	-0.0082	-0.0478	0.09642
NM_001008344	Etf1	0.10362	0.01612	0.22224	0.45711	0.58131	0.43463	-0.1662	0.58418	0.05501	0.10313	-0.118	-0.0262	0.44003	0.35271
NM_001009668	Etfa	0.35572	-0.0354	-0.4801	-0.1221	0.07189	0.16462	-0.233	0.28338	0.40279	0.09201	0.0244	-0.0257	-0.1444	-0.265
NM_001004220	Etfb	-0.081	-0.2278	0.0832	-0.1488	0.3504	0.52078	-0.1333	-0.2047	0.02052	-0.1107	0.06832	0.31218	0.04103	-0.1086
NM_198742	Etfdh	0.39925	0.48148	-0.2796	-0.206	0.5204	0.55154	0.18241	0.40647	0.49453	0.38312	0.47773	0.05273	-0.0762	-0.221
NM_001106234	Ethe1_predicted	-0.1427	0.03934	-1.0824	0.10476	0.45229	0.72336	-0.2654	0.18616	-0.2531	-0.0309	-0.1944	-0.273	-0.7402	-0.7044
NM_001107894	Etnk1_predicted	0.08283	0.02986	0.00553	0.07605	0.1824	0.04367	0.02926	-0.0111	-0.0252	0.00584	0.09514	0.04675	0.11454	0.20756
NM_001107894	Etnk1_predicted	0.07705	0.00803	0.02825	0.04032	0.01546	-0.0426	-0.1102	0.07666	0.14603	0.13048	0.16773	0.0931	0.11343	0.22711
NM_001108343	Etnk2_predicted	-0.0088	-0.0686	-0.0549	-0.1339	-0.0549	-0.0806	-0.1728	-0.1954	-0.1246	-0.03	-0.1061	-0.0831	0.06945	-0.0721
NM_012555	Ets1	-0.1937	-0.4487	-0.4082	-0.1119	0.02187	-0.169	-0.1988	-0.1962	-0.251	-0.4634	-0.225	-0.4867	-0.2965	-0.2645
NM_001107107	Ets2_mapped	-0.2343	0.00568	0.02846	-0.1512	-0.1333	-0.47	-0.1243	-0.2406	-0.2599	-0.3153	-0.1711	-0.4027	-0.3043	0.04634
XM_341830.3	Etsrp71_predicted	-0.1862	-0.2233	-0.1662	0.00312	-0.1105	-0.0963	-0.0649	-0.0919	-0.2648	-0.2762	-0.1345	-0.0596	-0.1943	-0.2792
NM_001163156	Etv1_predicted	0.42195	1.1547	0.21781	0.57485	0.24281	-0.0605	0.74538	0.04093	1.0108	0.99207	1.0224	0.65465	0.22062	0.37758
NM_001108299	Etv4_predicted	-0.1151	-0.2013	0.48663	-0.2559	-0.443	-0.7564	0.44797	-0.5047	0.12831	-0.1091	-0.139	0.0099	-0.0592	-0.0562
NM_001106818	Eva_predicted	0.03096	0.06939	0.02844	0.0884	-0.0617	0.05564	0.03626	0.03112	-0.0748	0.00865	0.0559	-0.0242	0.03567	0.10335
NM_001106818	Eva_predicted	0.04161	0.23302	0.00844	0.13558	0.33145	0.21461	-0.1212	0.43277	0.0515	0.12145	-0.0062	0.17965	0.35261	0.00047
NM_001170439	Evc	0.25239	0.05219	0.15479	0.32576	-0.0401	0.08358	-0.0169	0.13288	0.26689	0.37667	-0.0372	-0.0625	0.08679	0.00713
NM_001170439	Evc	-0.0588	-0.0972	-0.0714	-0.1344	-0.048	0.03944	0.04311	-0.1475	0.0477	-0.0545	-0.1254	0.11174	0.16105	-0.0356
NM_001106012	Evc2_predicted	-0.3459	-0.2732	0.06497	-0.1207	-0.3069	-0.0855	0.02428	-0.1401	-0.3408	-0.4752	-0.3609	-0.0182	-0.0689	0.0157
NM_001106423	Evi1_predicted	0.22	0.06663	0.06813	0.08127	0.09979	0.11539	0.0981	0.01559	0.11556	0.21996	0.17211	0.24361	0.16233	0.17928

NM_001106423	Evi1_predicted	-1.2798	0.0277	-0.2258	-1.2774	-1.655	-1.9869	-0.318	-1.8473	0.2176	-0.0305	-0.0715	0.22111	-0.2087	-0.0742
NM_024147	Evl	0.25401	0.26578	0.19146	0.0999	0.44761	0.80954	-0.5587	0.4149	0.45204	0.10258	0.42154	1.337	0.4471	0.26226
NM_001107066	Evpl_predicted	-0.1077	-0.0922	0.05065	-0.116	-0.0834	0.16428	0.06839	-0.0598	-0.0667	0.05672	0.04251	-0.0254	0.14629	-0.0089
XM_221512.3	Evx2_predicted	0.10467	-0.1892	-0.1971	-0.0404	0.1498	0.11052	-0.0866	-0.014	-0.1273	-0.1621	0.06016	0.02429	-0.0906	0.09196
NM_001025632	Ewsr1	-0.4728	-0.9313	0.47536	-0.062	-0.7875	-0.8054	-0.0163	-0.7095	-0.6257	-0.7745	-0.7065	-0.2266	-0.1324	-0.0239
NM_001107761	Exdl1_predicted	0.06205	0.05952	0.03579	-0.0628	0.00865	-0.0204	0.04051	-0.0639	-0.0478	0.06918	-0.0603	-0.1022	-0.0333	0.06226
NM_001107198	Exo1_predicted	-0.4877	-0.7719	0.77512	-0.4597	-1.1312	-1.2181	-0.4787	-0.94	-0.2057	-0.0683	-0.3827	0.16137	0.47942	0.46062
NM_134414	Exoc2	-0.1948	-0.0411	0.02802	0.62866	0.01977	-0.0673	0.33973	-0.166	0.08239	0.10042	0.57679	0.19207	-0.185	-0.2981
NM_001024964	Exoc3	0.59312	0.46272	0.00402	0.62768	0.89689	0.51977	0.34419	0.77324	0.37777	0.43077	0.43171	-0.149	0.44367	0.52518
NM_053875	Exoc4	0.21851	0.14591	0.13311	-0.0369	0.26538	0.24099	0.1807	0.22757	0.25701	0.10654	0.11101	0.13917	0.40829	-0.0033
NM_022204	Exoc5	0.04056	0.12905	-0.2245	-0.1652	0.40218	-0.2697	-0.003	0.47484	0.04533	0.13156	-0.1303	-0.1928	0.29362	0.19472
NM_019277	Exoc6	0.24135	0.14505	0.02098	-0.0771	0.00893	-0.0216	0.02245	0.31585	-0.1584	0.02764	0.06432	-0.8595	-0.0303	-0.2019
NM_022691	Exoc7	0.27678	0.26401	-0.031	0.07658	0.37282	0.42942	-0.2828	0.44105	0.20872	0.16003	0.05812	0.1046	-0.2129	-0.1024
NM_139043	Exoc8	-0.3398	-0.1761	-0.2313	-0.0203	-0.0449	-0.0689	-0.049	-0.1482	0.07205	-0.0569	-0.2843	-0.1229	-0.3232	-0.3125
NM_001108952	Exosc2_predicted	-0.2203	-0.4846	0.69981	-0.0727	-0.0535	-0.3144	0.07657	-0.4261	-0.1103	0.08993	-0.2736	0.22255	0.56883	0.46592
NM_001107936	Exosc3_predicted	-0.0855	-0.2281	0.33363	-0.1495	-0.2752	-0.1943	0.09766	-0.3924	-0.215	-0.0239	0.03583	0.15532	-0.0448	0.13419
NM_001134860	Exosc4_predicted	0.26549	0.29196	0.21143	0.41036	0.52432	0.70178	0.15344	0.5354	0.19258	0.14969	0.25687	0.28022	0.25424	0.42694
NM_001107493	Exosc5_predicted	-0.3552	-0.4529	-0.0706	-0.9685	-0.3307	0.29219	-0.5388	-0.6546	-0.1202	-0.4458	-0.2649	0.18707	-0.0049	-0.0927
XM_001077520.1	Exosc6_predicted	0.32639	0.00219	0.17679	-0.1186	0.09711	0.12353	0.1337	0.16281	0.49418	0.08284	0.48911	0.2305	0.76801	1.1998
NM_001100725	Exosc7	-0.1298	-0.599	-0.0436	-0.4684	-0.079	0.13953	-0.2238	-0.2391	-0.3245	-0.4092	-0.2065	0.15694	-0.1343	-0.1437
NM_001106432	Exosc8_predicted	-0.0017	-0.2704	0.53785	0.70318	-0.2362	0.05866	0.16842	-0.1792	-0.1166	-0.4548	-0.2576	0.29111	0.37398	0.37329
NM_001106432	Exosc8_predicted	-0.3675	-0.4138	0.65824	0.96807	-0.4682	-0.2878	0.3431	-0.4869	-0.1509	-0.3613	-0.0337	-0.1699	-0.3348	-0.3651
NM_001025406	Exosc9	0.69917	0.65073	0.26197	1.2532	0.82333	0.68377	1.0552	0.90457	0.89354	0.58398	0.80875	0.19504	0.08952	0.45645
XM_236272.4	Exph5_predicted	0.19971	-0.0641	0.02973	-0.0926	0.08228	-0.0974	-0.1005	0.20882	0.0383	0.23759	-0.0693	0.12642	-0.0417	0.09526
NM_001107751	Ext2_predicted	-0.6698	0.24523	-1.3	-0.5119	-0.0957	-0.4568	-0.4735	0.02626	0.03964	0.0751	-0.0238	-0.4486	-0.5254	-0.4553
NM_001107985	Extl1_predicted	-0.1127	-0.041	-0.0251	-0.0186	-0.005	-0.0144	-0.0688	0.11635	-0.1325	-0.1147	-0.122	-0.1028	-0.1055	-0.0587
NM_001100704	Extl2	0.47974	0.68635	-0.3504	-1.1048	-0.1315	-0.3028	-0.1375	-0.2694	0.53717	0.46393	0.4029	0.18528	0.09563	0.24728
NM_020097	Extl3	0.23417	-0.1445	-0.1293	-0.1954	0.12503	-0.0064	-0.1319	-0.0793	-0.023	-0.1579	0.11363	-0.2375	0.01261	0.33091
NM_130427	Eya2	-0.1016	-0.1113	0.18097	-0.1291	-0.0803	-0.1877	0.05333	-0.0241	-0.1262	0.02496	0.09332	-0.0445	-0.1514	-0.1469
NM_001107910	Eya3_predicted	0.0643	-0.0836	0.14175	-0.0099	-0.0206	-0.0917	0.49241	0.21843	0.10463	0.07827	-0.0501	0.04637	0.06343	0.2417
XM_217715.4	Eya4_predicted	0.27544	0.14685	-0.0182	0.00807	0.12668	0.27718	0.27193	0.33663	0.04499	0.01569	0.18067	-0.1671	0.0353	0.06135
NM_001107051	Ezh1_predicted	-0.1289	-0.1819	-0.1816	0.38111	-0.0317	-0.2293	0.00242	-0.2948	-0.2055	-0.1036	-0.0771	-0.284	-0.0456	-0.3019
NM_001107051	Ezh1_predicted	-0.2191	-0.0118	0.18977	-0.3385	-0.2818	-0.189	-0.041	-0.2168	-0.3527	-0.0721	0.10383	-0.2976	-0.3509	-0.3826
NM_017143	F10	0.10034	-0.1444	0.04462	-0.0429	-0.0096	-0.1871	0.2019	-0.0037	-0.0536	-0.0669	0.09389	0.08381	0.09521	-0.0335
NM_001047848	F11	-0.0978	0.01194	0.09766	-0.1263	-0.069	0.12842	-0.1439	-0.109	0.14275	0.06676	0.07014	0.26726	-0.0377	0.22226
NM_053796	F11r	-2.0709	-2.3335	-2.0471	-2.3915	-2.2624	-2.2305	-2.2948	-2.2492	-2.4636	-2.5274	-2.5877	-2.3118	-2.3054	-2.3748
NM_001014006	F12	0.0994	0.10176	0.20703	0.09551	0.29343	0.1227	0.14501	0.02727	0.09133	0.3532	0.1905	0.22362	0.11742	0.25715
NM_021698	F13a1	-2.5734	-2.7405	-2.5662	-2.6712	-2.7166	-2.2651	-2.097	-2.6931	-2.7739	-2.123	-1.9427	-2.5881	-2.7223	-2.6345
NM_001105956	F13b_predicted	-0.1162	0.02166	-0.0196	0.1716	0.04988	0.28042	-0.079	-0.1087	-0.1054	0.08309	0.29782	0.0748	-0.1282	0.18365
NM_022924	F2	0.00316	0.19597	0.14508	0.16532	0.09798	0.18754	0.37264	0.44338	0.14451	0.12817	0.10224	0.29736	0.33847	0.07655
NM_012950	F2r	-0.5766	-0.0614	-0.628	-0.0552	-0.0544	-0.1523	-0.7819	-0.1201	-0.257	-0.186	0.04592	-0.1194	-0.2691	-0.4219
NM_053897	F2rl1	-0.2399	-0.4015	-0.0821	-0.4781	-0.3531	-0.3432	-0.2969	-0.1914	-0.2611	-0.4375	-0.26	-0.5127	-0.3722	-0.4755
NM_013057	F3	-0.589	-0.6871	-0.7604	2.0994	-0.5533	-0.4466	-0.498	-0.6576	-0.7079	-0.2453	-0.9044	-0.8505	-0.3705	-0.236
NM_001047878	F5_mapped	0.21897	0.10574	0.12341	0.20468	1.5319	1.1917	0.14949	1.2544	0.21847	0.13381	0.22042	0.14383	0.09582	0.15231
NM_152846	F7	-0.1071	-0.0574	0.10954	0.15205	-0.173	-0.1429	-0.0075	0.13492	-0.187	-0.1436	-0.154	-0.1767	0.07198	-0.042
NM_031540	F9	-0.0114	-0.1196	-0.0317	0.07749	-0.1539	-0.1644	-0.096	0.06075	0.09409	-0.0931	-0.1277	0.03685	-0.0417	0.04483
NM_024132	Faah	-0.0687	0.08807	0.18276	0.00427	0.15868	-0.0525	0.07855	-0.0288	0.0305	0.08281	0.04127	0.18529	0.00901	-0.0941
NM_012556	Fabp1	-0.0184	-0.0761	-0.1994	-0.1121	-0.1171	-0.0521	-0.1014	-0.2656	-0.1397	-0.2623	-0.1042	0.00889	-0.0703	0.17438
NM_013068	Fabp2	-0.0578	-0.089	-0.0306	-0.0225	0.19175	-0.1176	-0.1739	-0.0987	-0.1262	0.11975	-0.1397	0.17464	-0.1234	-0.1492

NM_024162	Fabp3	1.7646	0.75685	0.72956	0.48335	1.1297	1.5962	0.81947	0.96957	0.56807	0.59083	0.87296	0.70795	0.679	0.57136
NM_053365	Fabp4	0.98844	0.52717	-0.3388	-0.3291	-0.3417	-0.3659	0.51504	-0.3147	0.32316	0.56767	0.76062	-0.4476	-0.2743	-0.4916
NM_017098	Fabp6	-0.1865	0.12433	-0.066	-0.1285	-0.099	0.08876	-0.0296	-0.0252	-0.1154	-0.0878	-0.1173	0.00354	-0.0927	-0.0826
NM_030832	Fabp7	0.03971	0.06121	-0.0042	-0.0386	-0.1015	-0.1404	-0.053	-0.1513	-0.118	-0.0884	-0.2083	-0.0123	-0.128	0.08388
NM_022854	Fabp9	0.21008	0.40731	0.24648	0.13332	0.23704	0.18546	0.32998	0.19471	0.19372	0.1521	0.10072	0.2923	0.22379	0.07761
NM_152937	Fadd	0.46091	-0.348	0.32238	-0.5761	0.08056	0.01694	-0.0775	0.10986	-0.0984	0.00157	-0.3813	0.0255	0.41653	0.44518
NM_053445	Fads1	-0.1056	0.30151	1.8703	0.50522	-0.296	-0.2866	0.25689	-0.2971	0.74625	0.5351	0.38927	1.2463	0.40701	0.38284
NM_031344	Fads2	0.37167	-0.0038	0.21479	-0.0881	0.29804	0.50651	0.1391	0.38469	0.04811	0.1618	0.05926	1.0044	0.375	0.2387
NM_001107064	Fads6_predicted	-0.1618	-0.0607	-0.1991	-0.281	-0.1876	-0.1775	-0.3382	-0.1524	-0.092	-0.0615	0.08364	-0.1322	-0.2073	-0.2257
NM_130406	Faf1	0.16815	0.09287	0.05178	-0.0733	-0.3235	-0.1141	0.18775	-0.144	-0.0419	0.09849	0.11146	0.19778	0.17036	0.4623
NM_017181	Fah	0.27418	0.15787	-0.4921	-0.1825	0.03389	0.49344	-0.0225	0.01233	0.20733	0.17691	0.20023	0.2208	-0.2517	-0.0936
NM_001024991	Fahd1	-0.6678	-0.1486	-0.0837	-0.1273	-0.2851	-0.2734	0.1179	-0.2107	0.04983	0.02111	-0.16	0.40952	-0.2187	-0.2169
NM_001134834	Fahd2a_predicted	0.56228	0.66744	-0.5107	0.12114	0.72844	0.71325	0.27828	0.47765	0.42884	0.31714	0.41459	0.07197	-0.3925	-0.3867
NM_080895	Faim	0.06942	-0.2741	-0.1377	-0.3953	-0.2609	-0.1564	-0.0011	-0.2648	-0.0999	-0.3369	-0.0332	-0.1292	-0.1482	-0.168
NM_144756	Faim2	-0.1197	-0.0701	-0.0291	-0.0491	-0.0861	0.05616	-0.0442	0.04607	0.09652	0.12501	-0.0753	0.01286	-0.0799	-0.059
NM_001127535	Falz_predicted	-0.1512	0.08891	-0.486	0.43127	0.01906	-0.3293	0.23182	0.1341	-0.0927	-0.115	0.05189	-0.4339	-0.0204	-0.1378
NM_001007668	Fam100a	-0.1764	0.14677	-0.2453	0.01106	-0.1023	0.27143	-0.1176	0.03508	-0.2631	-0.0937	-0.2731	-0.117	0.0558	0.01527
NM_001007611	Fam101b	-2.3842	-1.9565	-2.102	-2.6636	-1.8147	-1.9003	-2.7417	-1.9547	-1.8815	-1.9632	-1.8186	-1.7041	-1.612	-1.5675
NM_001037648	Fam105a	-0.6338	-1.6995	-1.7266	-1.4719	0.97616	0.28918	-0.8861	0.74427	-2.0388	-1.4659	-2.0089	-1.982	-1.5589	-1.4865
NM_001025034	Fam107b	-0.2381	-0.1738	-0.4438	-0.1521	-0.1211	-0.079	-0.4718	-0.1146	-0.2082	-0.3396	-0.4192	-0.193	-0.1882	-0.408
NM_001006983	Fam108a1	0.51364	0.3107	0.16533	0.28011	0.48316	0.48727	0.05355	0.25927	0.32176	0.48996	0.25772	0.32933	0.09234	0.22011
NM_001014050	Fam110a	0.49341	0.3437	0.5447	0.46877	0.63036	0.69759	0.53842	0.54787	0.25807	0.67057	0.30465	0.62173	0.59283	0.61725
NM_001024341	Fam110b	-0.2865	0.28228	0.04151	-0.0444	0.03246	0.19201	0.22416	0.028	0.4619	0.20772	0.23148	0.61744	0.0792	-0.0493
NM_001025051	Fam110c	0.26742	0.01625	0.15335	0.05553	-0.0053	0.03009	0.06209	0.0015	0.14331	-0.0957	0.16192	0.048	0.07774	-0.0938
NM_001039454	Fam113b	0.1066	-0.147	-0.0027	0.12423	-0.0009	0.14025	0.02377	0.30267	0.04513	0.11081	0.02263	-0.062	-0.0102	0.09342
NM_001014029	Fam122a	0.01885	-0.1005	-0.15	-0.1161	-0.1357	-0.1341	-0.0218	-0.0741	-0.0385	-0.114	-0.1191	-0.11	0.10503	0.09574
NM_199375	Fam125a	0.49463	0.78406	-0.3755	-0.2002	0.41783	0.32237	0.10849	0.21247	0.7236	0.58865	0.84103	0.26567	-0.0412	-0.0679
NM_001025046	Fam131b	0.04218	0.02171	-0.1462	-0.2251	-0.0602	-0.1529	-0.0069	0.08441	-0.0028	0.1677	-0.1088	0.14077	-0.0614	-0.1305
NM_001013878	Fam149b1	-0.3852	-0.0615	-0.2091	-0.0998	-0.3801	-0.4885	-0.1294	-0.4894	-0.1762	-0.1022	-0.2605	-0.2881	-0.7084	-0.4627
NM_001005558	Fam151a	0.09898	0.07923	0.01861	0.12857	0.14758	-0.0035	0.02231	0.20943	0.11176	4E-05	0.15661	0.07171	0.31864	0.09478
NM_001013873	Fam152a	-0.0797	-0.2006	-0.1468	-0.1923	-0.2776	-0.3379	-0.3674	-0.2929	-0.1181	-0.1905	-0.1123	0.13816	0.08354	0.04188
NM_001008306	Fam26b	-1.1476	-0.6489	-0.7125	-1.6758	-1.3575	-1.4099	-0.8554	-1.2412	-0.5608	-0.9195	-0.9095	-0.6184	-0.3602	-0.5399
XR_005430.1	Fam29a_predicted	-0.0845	-0.1167	0.07653	-0.0748	-0.0901	-0.042	-0.1501	-0.1873	-0.1792	-0.0395	-0.0667	-0.1194	-0.0888	-0.153
XM_222678.3	Fam31b_predicted	-0.3633	-0.1109	-0.1233	-0.0376	-0.1165	-0.1666	0.00797	0.09137	-0.2338	-0.1303	-0.098	-0.0147	-0.1613	-0.1406
NM_001105976	Fam36a_predicted	0.15153	-0.0697	0.3844	-0.0052	-0.1391	-0.1197	0.03038	-0.0365	0.12218	0.12994	0.29915	-0.0609	0.30003	0.16438
NM_001077200	Fam38a_predicted	0.04237	-0.1794	1.4589	0.72907	0.13544	-0.0219	0.7771	-0.0207	-0.5075	-0.32	-0.1789	-0.2049	-0.1859	0.03215
NM_198771	Fam3c	-0.3029	-0.5518	-0.5696	-1.41	-0.8301	-0.5803	-0.989	-0.7649	-0.3982	-0.5932	-0.6875	-0.3249	-0.1538	-0.1182
NM_001039002	Fam43a	-0.1387	-0.1699	-0.3285	-0.0653	-0.2592	-0.204	-0.2657	-0.2507	-0.1384	-0.2218	-0.2566	-0.0929	-0.3359	-0.2579
NM_001013854	Fam44b	0.31694	-0.1478	0.45801	0.5025	0.21333	0.27719	0.50846	0.22935	-0.2837	-0.1919	-0.2421	-0.254	0.10074	0.29714
NM_001013935	Fam54b	0.15214	-0.0391	-0.4952	-0.0872	0.35452	0.45162	-0.5291	0.25939	-0.0525	0.04306	0.03387	0.05395	-0.4729	-0.3474
NM_001025412	Fam58b	-0.0224	0.10743	-0.0575	0.05721	0.04825	-0.0778	0.06367	0.04276	0.01159	-0.0674	-0.0342	0.00789	-0.0622	0.00953
NM_001025412	Fam58b	0.92407	0.50923	0.59859	0.13008	0.48483	0.72739	0.51377	0.38582	0.57895	0.56077	0.61587	0.6037	0.54945	0.71013
NM_001025118	Fam63a	0.0429	0.19348	0.01869	-0.434	-0.0442	-0.1795	0.0472	-0.2123	-0.0321	-0.0741	0.18164	-0.375	-0.308	-0.171
NM_001037200	Fam82a	0.02307	0.02681	0.49126	-0.07	-0.0971	-0.1886	0.15141	-0.2664	0.00982	-0.0667	0.27684	0.26909	0.15886	0.27773
NM_001031663	Fam82b	0.66663	0.81817	-0.0962	-0.0402	0.20131	0.26749	0.58249	0.348	0.49659	0.70296	0.5921	0.28709	-0.0131	-0.1915
NM_001008327	Fam96a	0.85141	0.60009	0.21205	-0.0551	0.88576	1.0666	0.25978	0.67498	0.59273	0.64537	0.6069	0.74838	0.86761	0.65178
NM_001014073	Fam98a	0.38266	-0.072	0.53341	0.7451	0.87699	0.92694	0.06279	0.76972	0.09683	0.19682	0.149	0.32082	0.65356	0.54645
NM_001108455	Fanca_predicted	-0.6338	-0.6301	0.71155	-0.0026	-0.5474	-0.5238	-0.3589	-0.5628	-0.4787	-0.5726	-0.4697	-0.0485	0.13848	0.19168
NM_012557	Fancc	0.03918	0.09514	0.19315	-0.1233	-0.0646	0.19852	-0.1352	-0.1385	0.10274	0.0732	-0.008	0.26748	-0.0735	-0.1324

NM_001001719	Fancd2	-0.3241	-0.6038	0.48371	-0.2105	-0.7744	-0.8251	-0.6295	-0.397	-0.4056	-0.4308	-0.2679	-0.1507	0.3456	0.24495
XM_223701.3	Fancl_predicted	-0.0489	0.15522	0.0206	-0.3524	-0.3651	-0.3934	-0.2942	-0.3188	0.14025	0.04621	0.09124	0.02024	-0.3282	-0.2316
NM_001008347	Fank1	-0.2591	-0.3778	-0.1352	-0.3315	-0.1982	-0.3797	-0.3027	-0.2165	-0.3286	-0.0798	-0.2944	-0.3282	-0.0469	-0.0408
NM_138850	Fap	0.08555	-0.0287	0.03712	-0.0608	0.12186	0.06735	0.02661	-0.0647	0.0199	0.08719	-0.0417	0.01756	0.03076	0.08249
NM_001107287	Farp1_predicted	-0.1796	-0.1776	0.12979	0.20771	-0.1064	0.35351	0.15755	-0.069	-0.025	0.16819	-0.074	0.09311	-0.0161	-0.1973
NM_001107287	Farp1_predicted	-0.4938	-0.0598	0.20483	-0.1624	-0.1548	-0.2693	-0.3425	-0.2996	-0.0297	0.02245	-0.0659	0.03084	0.13459	-0.0053
NM_001108233	Farp2_predicted	0.39085	0.74818	0.53695	0.19335	0.54222	0.56547	0.28194	0.45848	0.65565	0.53232	0.57855	0.21769	0.58178	0.40559
NM_001013139	Fars2	-0.0037	0.04646	0.38371	-0.012	-0.0169	0.2105	0.02762	0.22786	0.00808	0.07755	0.01801	0.25172	0.17548	0.06785
NM_001004252	Farsb	-0.0584	-0.2004	0.23808	-0.4359	-0.3071	-0.3933	-0.2747	-0.3115	-0.2681	-0.1712	-0.4447	-0.244	0.52148	0.59264
NM_139194	Fas	-0.2552	0.01438	-0.4592	-0.4617	-0.5648	-0.651	-0.2194	-0.5347	-0.2286	-0.1193	-0.1809	-0.5971	-0.6462	-0.5318
NM_012908	Fasl	-0.2489	-0.2287	0.21144	-0.1726	-0.1433	-0.1285	-0.2199	-0.0869	-0.2204	-0.0248	-0.081	-0.0583	-0.2494	-0.091
NM_001011967	Fastk	-0.1008	-0.2738	-0.2471	-0.3878	-0.5448	-0.392	-0.7293	-0.4297	-0.4337	-0.3768	-0.3755	-0.0193	-0.5854	-0.5727
NM_001009673	Fastkd2	0.0706	-0.1448	0.24055	0.25735	0.16831	0.08271	0.32937	0.13793	0.05918	0.01073	0.19215	0.0252	0.2522	0.04863
NM_138544	Fat3	-0.4982	0.06542	-0.2107	-0.8047	-0.5099	-0.3886	-0.6384	-0.2819	-0.0463	-0.0751	-0.2458	-0.0312	-0.7668	-0.5928
NM_001105854	Fbf1_predicted	-0.2088	-0.3216	0.4105	-0.1117	-0.207	0.19122	-0.0695	-0.4381	-0.2893	-0.5696	-0.1153	-0.2567	0.09312	-0.3146
NM_001025643	Fbl	-0.5851	-1.0915	0.1829	-0.6794	-0.875	-0.628	-0.8596	-0.847	-0.9536	-0.9219	-0.7646	0.0049	0.06964	0.26928
NM_001007554	Fblim1	0.0464	-0.235	-0.1904	0.03341	-0.3009	-0.1274	0.14823	-0.1805	-0.036	-0.0398	-0.2713	-0.1889	-0.2328	-0.2922
NM_001127547	Fbln1_predicted	-0.898	-1.0176	-0.8628	-0.9753	-0.3065	0.03811	-0.9546	-0.3124	-0.5357	-0.638	-0.7963	-0.0145	-0.9645	-1.0219
XM_001073317.1	Fbln2	0.14285	-0.1902	0.08777	-0.2235	0.35105	0.24317	-0.0542	0.07296	-0.1538	-0.2261	0.18108	-0.0484	-0.0585	0.00232
NM_019153	Fbln5	-2.6519	1.3672	-0.62	0.07342	-2.8514	-2.8415	1.1572	-2.9367	0.99505	1.2498	1.0393	0.22957	-0.3485	-0.1832
NM_031825	Fbn1	0.52604	3.2294	1.2127	1.1791	1.9083	1.4365	2.3713	2.0101	2.7549	3.1311	3.1507	-0.1855	1.2591	1.2296
NM_031826	Fbn2	-0.0686	-0.0813	0.17841	0.14988	0.03113	-0.1835	0.03735	-0.1651	-0.0284	-0.0923	0.27807	0.03627	0.01494	-4E-05
NM_012558	Fbp1	0.00156	0.20081	0.08403	0.04302	0.25414	-0.0412	0.20669	-0.1446	0.01468	0.2254	0.11392	0.39731	-0.0079	0.08178
NM_053716	Fbp2	0.22237	0.10723	0.07438	0.05897	0.33305	-0.0824	0.01455	0.357	0.05349	0.0644	0.26432	0.15791	-0.0302	0.02581
NM_001108515	Fbxl11_predicted	-0.3679	-0.0093	0.11428	0.04821	-0.1498	-0.2845	0.10365	-0.1311	-0.3654	-0.0742	0.07017	-0.3739	-0.1514	-0.1782
NM_001025700	Fbxl12	0.17877	0.11779	-0.0105	0.20286	0.15186	-0.1759	0.29045	0.14379	0.28906	0.01992	0.33165	0.24662	-0.0118	0.00606
NM_138523	Fbxl14_predicted	-0.182	-0.1028	-0.2522	-0.2829	-0.2622	-0.3493	-0.1969	-0.3181	-0.3463	-0.14	-0.0354	-0.3942	-0.0726	-0.2012
NM_001009504	Fbxl16	-0.1305	-0.0896	-0.1579	-0.0158	-0.1061	-0.1725	0.01028	0.01055	-0.0073	-0.1858	-0.1707	-0.2927	-0.1242	-0.3162
NM_001108235	Fbxl17_predicted	0.16123	0.25926	-0.186	-0.0742	0.12007	0.17838	-0.0539	0.18561	0.12569	0.0791	0.02102	-0.149	0.21116	0.01856
NM_001107552	Fbxl19_predicted	-0.1588	-0.3349	-0.0736	-0.2819	0.07717	-0.2252	-0.1982	-0.1524	-0.1863	-0.101	0.10802	0.02321	-0.2815	-0.2072
NM_022272	Fbxl20	-1.3828	-0.8888	-0.5868	0.05676	-1.3766	-1.3737	-0.1899	-1.2716	-1.1424	-0.9347	-0.8624	-1.3627	-1.4286	-1.5286
NM_001100568	Fbxl3	-0.1452	0.06467	-0.0628	-0.0896	-0.0217	0.06675	0.22071	0.02086	0.32625	0.01568	0.0222	0.06317	0.27922	0.31252
NM_001107919	Fbxl4_predicted	0.06935	0.18422	-0.2803	-0.0841	-0.0124	0.04132	0.34395	0.10265	0.02742	0.04991	-0.0189	-0.0879	-0.0332	-0.2108
NM_001107222	Fbxl5_predicted	0.50358	0.55077	0.67688	1.7159	1.0245	1.0053	1.4839	1.1663	0.48201	0.55844	0.41284	0.16423	0.36527	0.28834
NM_001005563	Fbxl6	-0.385	-0.0785	0.13108	0.75061	0.00058	-0.0166	0.03808	-0.0199	-0.4099	0.07123	0.01126	-0.0901	-0.5503	-0.3332
NM_001108545	Fbxl7_predicted	0.07115	0.17259	0.18801	0.17501	0.22444	0.17001	0.17363	0.20051	0.02207	0.24377	-0.0017	0.13304	0.28564	0.12942
XM_001071167.1	Fbxo10_predicted	0.10902	0.07916	0.13893	0.08109	0.07617	0.09469	0.23779	0.19393	0.07364	0.07146	-0.0126	0.38573	0.28735	0.08419
NM_181631	Fbxo11	-0.6807	-0.7079	-0.6802	-0.4364	-0.7727	-0.6728	-0.1977	-0.5717	0.01051	-0.3098	-0.4028	0.02398	-0.4637	-0.497
NM_001108436	Fbxo15_predicted	0.25031	0.20759	0.22168	0.27529	0.17076	0.32923	0.20784	0.31151	0.13197	0.38283	0.04699	0.12454	0.09072	0.1565
NM_001013132	Fbxo16	0.09592	0.16673	0.36653	0.06814	0.1943	0.17169	0.15245	0.24587	0.0918	0.1092	0.05809	0.09978	0.27404	0.199
NM_001013064	Fbxo17	-0.1269	0.0084	0.06588	-0.1777	-0.0347	-0.2088	-0.1242	-0.0736	-0.0173	-0.2082	-0.2084	-0.1355	-0.3608	0.05301
NM_001106119	Fbxo18_predicted	-0.4642	0.01506	0.11696	-0.2544	-0.2825	-0.3802	0.21346	-0.3193	-0.0193	-0.1185	-0.1194	-0.0211	-0.0332	-0.1431
NM_053511	Fbxo2	0.5149	0.05693	-0.1531	0.28358	0.94762	0.8721	-0.3669	0.88064	0.16981	-0.0448	0.13286	0.37909	-0.2979	-0.3829
NM_001037770	Fbxo22	-0.2564	-0.1476	0.0694	-0.4008	-0.4004	-0.4362	-0.1145	-0.5174	-0.0885	-0.055	-0.1699	-0.0631	-0.1316	-0.0062
NM_001107130	Fbxo24_predicted	0.05675	0.28806	0.26623	-0.0117	0.20746	0.27013	0.09258	-0.0874	0.19879	0.08725	0.1676	0.41242	0.39096	0.0873
NM_001107203	Fbxo28_predicted	0.13567	0.17107	0.03227	0.09826	0.12196	-0.0088	0.12339	0.27001	-0.1493	0.1217	0.00885	-0.0024	0.24621	0.31532
NM_001007690	Fbxo30	-0.1398	-0.0866	-0.3413	-0.1029	0.06875	0.17959	-0.2907	-0.1284	-0.058	0.06946	-0.1268	0.02865	0.37753	0.15907
NM_133521	Fbxo32	-0.1225	0.2558	-0.11	-0.0361	0.28389	0.16343	-0.0799	0.36379	-0.1205	0.00161	0.0165	-0.135	-0.0939	0.03327
NM_001108023	Fbxo33_predicted	0.31428	-0.2517	-0.1297	-0.2741	0.68608	0.5074	0.24265	0.37353	0.12986	0.00073	-0.0503	0.22023	0.16726	0.21701

NM_001107257	Fbxo34_predicted	-0.2685	-0.0497	-0.1952	0.30741	0.03228	0.02314	-0.1726	-0.1228	-0.2619	-0.5055	-0.2504	-0.3134	-0.0475	-0.2488
NM_001108804	Fbxo36_predicted	0.13253	0.02045	-0.1517	-0.2761	0.06425	0.1407	-0.0574	0.25257	0.14406	-0.1377	-0.0152	-0.1745	-0.3202	-0.1656
NM_001039018	Fbxo39	-0.2861	-0.3355	-0.1761	-0.0725	0.10992	0.23053	-0.2208	-0.2121	-0.3642	-0.1877	-0.1171	-0.1984	0.00024	-0.1399
NM_001107672	Fbxo4_predicted	0.32868	0.46486	-0.5559	-0.3687	0.48478	0.07099	-0.0432	0.46668	0.19142	0.15297	0.06338	-0.0975	-0.0707	-0.0215
XM_001070422.1	Fbxo40_predicted	0.04767	0.14234	0.00781	-0.021	0.07705	0.13566	-0.0036	0.17638	0.04796	0.0178	0.01635	0.0244	0.20338	0.28753
NM_001108691	Fbxo42_predicted	0.12669	0.05992	-0.1276	0.05537	0.12824	0.32087	-0.0559	0.09086	0.14786	0.19702	0.30312	0.06119	-0.0126	0.12666
NM_001012117	Fbxo43	0.21379	0.09512	0.42881	0.28135	0.53565	0.58889	0.33478	0.09755	0.14242	-0.209	-0.0323	0.65579	0.15677	0.08076
NM_001025642	Fbxo46	-0.013	0.24037	0.2707	0.03339	-0.0054	-0.1159	0.00367	-0.0893	-0.1606	0.01108	0.00727	-0.0196	0.29293	-0.0115
NM_001106206	Fbxo5_predicted	-0.593	-1.0659	0.62626	-0.7264	-1.2461	-1.6555	-0.655	-1.3905	-0.4874	-1.0796	-0.8586	0.21207	0.86693	0.92457
NM_138917	Fbxo6b	0.01811	-0.0212	-0.0873	-0.6352	0.141	0.29903	-0.0643	-0.0253	0.32261	0.20165	0.0745	0.30734	-0.2081	-0.0759
NM_001012222	Fbxo7	0.40264	0.65113	0.67788	0.86206	0.8953	0.80442	0.66121	0.8953	0.45957	0.45705	0.71638	0.28539	0.53759	0.38467
NM_001012050	Fbxo8	0.37008	0.38452	-0.337	-0.1282	0.50273	0.33129	0.05901	0.41117	0.22997	0.37118	0.1256	0.1977	-0.0077	-0.0662
NM_001012050	Fbxo8_predicted	0.68105	0.42067	-0.2408	-0.2576	0.56216	0.57212	0.16582	0.72928	0.62834	0.4994	0.34497	0.47669	0.33202	0.17184
NM_001011998	Fbxo9	-0.2729	-0.2582	0.17536	0.11849	-0.3299	-0.4584	0.018	-0.4467	-0.1794	-0.126	-0.064	-0.1452	-0.2394	-0.1547
NM_001011998	Fbxo9_predicted	-0.0403	-0.0548	0.06834	0.00123	-0.0719	-0.1773	0.00141	-0.2875	-0.0269	-0.1672	-0.0702	-0.2481	-0.0779	-0.1327
NM_001106993	Fbxw11_predicted	0.12336	0.18487	-0.0413	0.09138	-0.0779	0.16044	0.05955	-0.056	-0.1277	0.04163	0.10848	-0.052	0.14645	0.27658
NM_001106993	Fbxw11_predicted	-0.0214	0.0286	-0.1167	-0.0234	0.04893	0.05256	-0.0362	0.19688	0.04544	0.09234	0.06354	-0.2671	0.26919	0.46368
NM_001107835	Fbxw2_predicted	-0.2465	-0.0739	-0.0127	-0.2838	0.01609	0.15163	-0.2012	-0.1799	-0.085	0.21184	0.05247	0.04478	-0.0876	-0.2912
NM_001107600	Fbxw4_predicted	-0.2338	-0.1304	-0.7795	0.21368	-0.4333	-0.4259	-0.1407	-0.4358	-0.2899	-0.3114	-0.2149	-0.3989	-1.1096	-1.0091
NM_001025730	Fbxw5	0.52361	0.58061	-0.1081	0.23387	0.50894	0.04532	0.16686	0.55284	0.29008	0.42241	0.16073	0.09126	0.09592	0.12389
NM_001107145	Fbxw8_predicted	0.03957	0.26124	-0.2381	-0.3922	0.18142	-0.3866	0.11913	0.3635	-0.0374	-0.0012	-0.0004	-0.0523	0.09201	0.19187
NM_001081634	Fbxw9	-0.1756	0.18924	0.09042	0.47639	-0.1066	-0.2858	0.31692	-0.0563	0.20241	0.36112	0.34335	0.12285	-0.1368	-0.2045
NM_201992	Fcar	-0.1167	-0.2214	-0.2322	-0.1169	-0.0875	0.00488	-0.2885	-0.1122	-0.0769	-0.1881	0.03981	-0.0991	-0.1365	-0.2612
NM_012724	Fcer1a	-0.1974	-0.2264	-0.2279	-0.2745	-0.0827	-0.0028	-0.0563	-0.0944	-0.1529	-0.098	0.00171	-0.237	-0.0712	-0.0175
NM_133550	Fcer2a	0.05123	-0.0278	0.05847	-0.0295	0.07321	0.02701	-0.0809	0.10728	-0.0127	0.07778	0.13675	0.06381	0.05898	0.25141
NM_001100836	Fcgr1	0.32763	0.06966	-0.0513	0.05414	0.03817	-0.0221	-0.0397	-0.0306	-0.0958	-0.0727	-0.0114	0.11924	0.36112	0.10893
NM_175756	Fcgr2b	0.07344	0.13839	0.04464	-0.0391	0.04567	-0.0128	-0.0749	0.02901	0.18954	0.02497	-0.0523	0.05021	0.01586	0.04635
NM_175756	Fcgr3	0.45019	1.7116	0.10515	0.49247	-0.1919	-0.2318	0.70303	-0.1034	1.1765	1.2503	1.183	0.82302	0.16844	0.6229
NM_207603	Fcgr3a	0.07848	-0.0648	-0.061	-0.1894	0.11884	-0.2436	-0.1343	-0.2277	-0.1189	-0.0541	-0.1043	-0.0555	-0.0192	-0.0262
NM_033351	Fcgrt	-0.1646	0.4442	-1.1607	-0.0925	-0.3364	-0.3407	0.05877	-0.2822	0.51373	0.54443	0.28813	0.05599	-0.6444	-0.524
NM_001107392	Fchsd1_predicted	0.11618	0.12968	0.21616	0.11156	0.28273	0.5812	0.2192	0.10089	0.09347	0.38067	0.1013	0.16433	0.08743	0.30858
NM_001107539	Fchsd2_predicted	0.07887	-0.0557	-1.2839	-0.346	-0.5178	-0.3813	-0.2031	-0.5622	0.01874	-0.0907	-0.1344	-0.3212	-0.8355	-0.991
NM_001108667	Fcmd_predicted	-0.0504	0.24399	-0.2507	0.23268	0.27813	-0.3444	0.20614	-0.0293	-0.0983	0.2572	0.28741	-0.4379	-0.0457	0.17781
NM_031348	Fcna	2.2465	2.1983	0.58962	0.5989	2.4847	2.7482	2.0045	2.3623	2.1274	2.3318	2.3095	0.33826	0.4669	0.45344
NM_053634	Fcnb	-0.0323	-0.0689	-0.0632	-0.1	-0.065	0.20088	0.34988	0.14933	0.16768	-0.0673	0.03319	0.15556	0.07724	-0.0229
NM_019238	Fdft1	-0.3178	-0.657	0.60143	0.66844	-0.1032	-0.1218	-0.2448	0.07669	-0.3272	-0.5811	-0.5563	0.39879	-0.2142	0.0043
NM_031840	Fdps	-0.8929	-1.4707	1.0561	-0.1059	-0.5713	-0.8114	-0.3754	-0.7837	-0.6459	-1.4454	-1.0825	0.35664	-0.1926	-0.2861
NM_017126	Fdx1	0.71774	0.0342	0.3734	0.63733	1.0172	1.1687	0.09423	0.95861	0.34567	0.22022	0.19513	0.41978	0.50503	0.55266
NM_024153	Fdxr	-0.1026	-0.1775	0.5044	-0.4587	-0.1833	0.02449	-0.4055	-0.4377	-0.0667	-0.2697	-0.1533	-0.0979	0.18322	0.14033
NM_001108434	Fech_predicted	0.13007	0.10764	-0.4328	-0.3967	0.0596	-0.3198	-0.2723	0.03297	0.29578	-0.0492	0.05639	-0.0188	-0.2457	-0.158
NM_001108434	Fech_predicted	0.13846	-0.1451	-0.5136	-0.2796	-0.2443	-0.1151	-0.2484	0.09319	0.0841	-0.3305	0.03039	0.00626	-0.2805	-0.3826
NM_001025706	Fem1a	-0.0571	-0.0549	-0.0665	-0.6262	0.16393	-0.0657	-0.0919	0.06378	-0.2839	-0.284	-0.0906	-0.8585	-0.2085	-0.3392
NM_001108157	Fem1b_predicted	-0.2797	-0.5528	-1.0067	0.63332	-0.1159	0.04792	-1.2051	-0.0557	0.13082	-0.35	-0.3364	0.39183	-1.0922	-1.1512
NM_001106932	Fem1c_predicted	-0.1426	0.09556	-0.1228	-0.154	-0.0124	-0.4073	0.00155	0.12314	0.20559	0.17124	0.2527	-0.0836	0.33194	0.28888
NM_053430	Fen1	-0.4838	-0.6645	0.27332	-0.9549	-0.549	-0.6247	-1.0778	-0.7534	-0.1611	-0.4807	-0.5362	0.28462	0.72592	0.69123
NM_001106534	Fer1l4_predicted	0.02085	0.2189	-0.0001	0.16563	0.03207	0.17055	-0.0627	-0.1876	-0.0655	0.00182	-0.0925	0.33961	0.04872	-0.1208
NM_001108980	Ferd3l_predicted	-0.0803	0.14049	-0.0341	-0.0891	-0.0108	-0.1804	0.18445	0.2006	0.00928	-0.1203	-0.0229	-0.0045	0.09601	-0.0324
NM_053348	Fetub	-0.894	-0.8852	-0.9199	-0.835	-0.7852	-0.7253	-0.8153	-0.7957	-0.8813	-0.7871	-0.8944	-0.9081	-0.9321	-0.8445
NM_144753	Fev	0.09649	0.03278	0.01985	0.00091	-0.038	0.06553	0.084	0.0043	0.09662	0.00075	0.00604	0.12799	0.07876	0.06382

NM_031066	Fez1	0.6005	0.79748	0.20398	0.77906	-0.5012	-0.4311	0.27661	-0.4213	0.8736	0.89182	0.87662	0.1565	-0.21	-0.2242
NM_053600	Fez2	0.12045	0.44204	-0.5664	0.20992	0.42345	-0.3713	0.22872	0.28185	0.16468	0.25672	0.22383	-0.3688	0.18175	0.19464
NM_153304	Ffar1	0.01917	-0.0175	-0.0135	-0.0292	0.01082	-0.0043	0.32958	0.2717	0.18402	-0.1247	-0.0149	0.05929	-0.0846	0.02244
NM_001005877	Ffar2	0.00818	0.0678	0.1042	-0.0041	-0.0012	0.19075	-0.0456	0.11442	0.0636	0.09069	-0.0072	0.13526	0.03273	0.06972
NM_020071	Fgb	-0.2185	0.08945	-0.1466	-0.0254	-0.1552	-0.1502	-0.2039	-0.1443	-0.0805	0.05141	0.00025	-0.0813	0.02365	-0.2104
NM_001037546	Fgd1	0.00749	0.28543	0.08327	0.08475	0.00798	0.0739	0.05988	0.00599	-0.0072	0.0573	0.14263	0.29634	-0.0594	0.1615
NM_001108409	Fgd3_predicted	-0.24	-0.204	-0.2056	-0.2565	-0.2111	0.02843	-0.1057	-0.2043	0.03192	-0.2618	-0.2349	-0.163	-0.2039	-0.2083
NM_139263	Fgd4	-0.0486	0.34774	-0.1422	0.20828	-0.1553	0.01057	-0.2366	-0.3739	0.00933	-0.209	-0.0617	-0.1329	-0.1409	-0.0341
NM_001108637	Fgd5_predicted	0.17446	0.0738	0.10486	0.05076	0.08748	0.31821	0.18452	0.0245	0.2256	0.28615	0.06151	0.03565	0.26839	0.05106
NM_012846	Fgf1	-0.7845	-0.7581	-0.8026	-0.8789	-0.5966	-0.7572	-0.7932	-0.7837	-0.7225	-0.9128	-0.7918	-0.57	-0.8471	-0.705
NM_012951	Fgf10	0.09363	-0.0453	0.07705	0.35131	0.10818	-0.0059	0.04222	0.05298	0.06266	0.04014	-0.0403	-0.0028	0.15909	0.04149
NM_130816	Fgf11	0.02673	-0.1389	-0.0405	-0.0716	-0.1068	0.01155	-0.1416	-0.076	-0.1127	0.02631	0.0391	-0.1307	-0.0875	-0.0973
NM_130814	Fgf12	-0.0345	-0.1114	-0.0464	-0.1702	-0.0904	-0.0402	-0.1273	-0.1535	-0.0507	0.04691	-0.0632	-0.0873	-0.11	-0.0942
NM_053428	Fgf13	-0.0512	-0.092	0.02784	0.02015	-0.0876	-0.0527	0.09072	0.04171	-0.0372	-0.0046	-0.0352	0.16004	0.05051	-0.0567
NM_022223	Fgf14	-0.0259	-0.0953	-0.0001	-0.1311	0.02223	-0.1018	-0.034	0.16659	-0.0542	-0.1092	0.0102	0.07787	-0.0462	-0.0674
NM_130753	Fgf15	0.05437	-0.0274	0.23021	0.19744	-0.0078	0.09505	0.06349	0.03743	0.01039	0.11434	0.08736	0.03578	0.02591	0.05348
NM_021867	Fgf16	-0.0946	-0.211	-0.4941	-0.0927	-0.2715	-0.0733	-0.089	-0.4075	-0.1802	-0.7902	0.04103	-0.2026	-0.3204	-0.287
NM_019198	Fgf17	0.08781	-0.093	-0.0512	0.01147	-0.077	0.0358	0.01718	-0.0636	-0.0272	-0.0812	-0.061	0.09862	0.0009	-0.0217
NM_019305	Fgf2	0.21349	0.0531	0.17868	0.29906	0.21495	0.53102	0.02972	0.22855	-0.0389	0.02963	-0.0688	0.25978	0.04452	0.16548
NM_023961	Fgf20	-0.0014	0.0244	0.04705	0.01019	-0.0346	0.07543	0.08897	0.01235	-0.0635	0.11783	0.02438	-0.0001	-0.0154	0.17678
NM_130752	Fgf21	-0.0242	-0.0777	-0.0387	0.07811	-0.0866	0.02769	-0.0087	-0.037	0.10591	-0.0166	-0.0331	-0.078	-0.0215	-0.0538
NM_130751	Fgf22	-0.0392	-0.0201	-0.0579	-0.0798	-0.033	-0.0184	-0.1102	-0.056	0.02554	0.05859	0.00175	0.0743	0.04821	0.06613
NM_130754	Fgf23	-0.0534	-0.101	-0.1609	-0.0533	-0.1029	-0.0684	0.14684	-0.0925	-0.0717	0.01067	0.03666	-0.0862	-0.0924	-0.1271
NM_130817	Fgf3	0.12935	0.12023	0.06279	-0.0925	0.17861	-0.1275	-0.0859	0.04515	-0.0458	-0.0772	-0.1214	-0.1203	0.20881	-0.0234
NM_053809	Fgf4	-0.3035	-0.2171	-0.3475	-0.1923	-0.348	-0.2455	-0.2189	-0.1208	-0.087	-0.2902	-0.0317	-0.187	-0.1836	-0.3007
NM_022211	Fgf5	0.02272	0.0231	0.23841	0.11594	0.25187	0.20153	0.17746	0.32269	0.08589	0.32804	0.07209	0.0717	0.16023	0.27978
NM_131908	Fgf6	0.04301	0.06808	-0.0303	0.14215	0.01835	-0.0038	-0.0508	0.04176	0.12849	0.04198	0.09664	0.02112	-0.0257	0.1434
NM_022182	Fgf7	0.07231	0.54461	-1.7848	-0.5409	-1.9122	-2.1412	-0.2378	-1.634	0.13742	0.23018	0.1169	-0.6187	-0.0865	-0.1061
NM_133286	Fgf8	-0.014	-0.211	0.03726	-0.0725	-0.0064	0.30877	-0.2517	0.11534	0.0265	-0.2214	0.08756	0.04654	-0.1871	-0.1076
NM_012952	Fgf9	0.00439	-0.0449	-0.0551	-0.0938	-0.0175	-0.1319	-0.0696	-0.0778	-0.1439	0.21778	0.04528	-0.0948	-0.1413	0.0821
NM_022603	Fgfbp1	0.00162	0.34867	-0.0378	0.10459	0.14351	0.10282	0.22306	0.25529	0.07898	0.02782	0.09481	0.20246	0.0683	0.01207
NM_201421	Fgfr1op2	-0.117	-0.1917	-0.0439	0.26158	-0.465	-0.5306	0.1154	-0.5367	-0.2151	-0.2798	-0.268	-0.6024	-0.6586	-0.5152
NM_001109892	Fgfr2	0.02624	-0.0474	-0.0014	0.06268	0.00321	-0.0332	0.1388	-0.0193	0.10861	0.17512	0.04316	0.04633	-0.0725	0.51974
NM_053429	Fgfr3	-0.2215	-0.1313	0.05196	-0.1261	0.44602	0.78273	-0.1774	0.43981	0.04846	0.05058	0.05458	-0.2086	-0.0433	0.07669
NM_001109904	Fgfr4	0.43525	0.45852	0.31487	0.25213	0.2906	0.2967	0.29017	0.42393	0.46657	0.45772	0.09418	0.56418	0.31545	0.43466
NM_199114	Fgfrl1	0.55244	0.70401	-0.0526	0.60936	0.90423	0.91582	0.53064	0.7687	0.79195	0.86854	0.57167	0.39228	0.21239	0.20011
NM_012559	Fgg	0.46622	1.957	0.93608	2.2612	0.46492	0.33556	2.0946	0.69171	1.8938	2.0181	1.9145	1.395	0.83041	0.76078
NM_172010	Fgl1	0.13868	-0.0957	-0.1753	0.08178	0.19855	0.36885	-0.1995	0.23295	-0.1699	-0.122	-0.1677	-0.0656	-0.1987	0.07648
NM_053455	Fgl2	-0.0242	0.3787	-0.0287	0.08812	-0.0869	0.02969	0.03634	0.01884	0.32506	0.54062	0.22793	-0.0214	0.31669	-0.1169
NM_024145	Fgr	0.1522	0.24459	0.25294	0.41385	0.34646	0.50065	0.25601	0.56237	0.25772	0.14916	0.40241	0.32114	0.12669	0.24135
NM_017005	Fh1	0.06037	-0.168	0.51779	0.25884	-0.0705	-0.2726	0.04147	-0.1416	-0.1107	0.12271	-0.1825	0.03998	0.53164	0.5431
NM_001033926	Fhl1	0.19549	0.40354	-0.658	1.6559	-1.1773	-1.657	0.16031	-0.8781	0.22631	-0.0283	-0.0287	-0.6267	0.5031	0.5098
NM_031677	Fhl2	1.4184	1.6868	0.72667	2.34	1.6744	1.3272	1.56	1.4285	1.2159	1.5287	1.4757	0.29864	1.325	1.3346
NM_001107979	Fhl3_predicted	-0.3887	-0.0978	0.48834	0.05543	-0.2458	-0.2873	0.18128	-0.3302	0.2594	-0.1873	0.15085	0.45555	-0.0639	-0.2277
NM_001013172	Fhl4	-0.3074	0.0146	-0.1823	-0.1468	-0.2521	-0.0282	-0.3351	-0.4419	0.07857	-0.1021	-0.258	-0.2464	0.03419	0.20291
NM_001013088	Fhl5	0.10021	0.47099	0.3192	0.19821	0.23159	0.25758	0.10145	0.21535	0.31504	0.1384	0.20798	0.14822	0.0126	0.31707
NM_001107829	Fibcd1_predicted	-0.2098	-0.1247	-0.0088	-0.0944	-0.1887	-0.1179	-0.1791	-0.0791	-0.0737	-0.1236	-0.0252	-0.0529	0.03269	-0.079
NM_172334	Fibp	0.34318	0.47302	0.00595	-0.1324	0.26617	0.39963	0.05933	0.23358	0.45175	0.18192	0.15374	0.4448	-0.2239	-0.1164
NM_001010946	Ficd	0.16084	-0.195	-0.2407	-0.2588	0.24002	-0.0497	-0.0991	0.33514	-0.0983	-0.2208	-0.1544	-0.1951	-0.0639	-0.03

NM_031761	Figf	-0.2003	0.49229	0.02922	0.51858	-0.1228	-0.1563	0.12263	-0.1341	0.22669	0.32751	0.33212	0.09216	0.36415	0.2923
NM_001106484	Fign_predicted	0.10798	-0.0791	0.23272	0.44648	-0.0915	0.0372	0.45216	0.07239	0.03298	0.09709	-0.0676	-0.0431	0.45415	0.29716
NM_001011913	Fignl1	-0.3936	-0.3668	-0.2577	-0.6302	-0.4991	-0.4076	-0.5285	-0.4974	-0.4281	-0.5417	-0.534	-0.0646	-0.0847	-0.0776
NM_145682	Filip1	0.10185	0.12319	0.06962	0.13291	0.14838	0.04655	0.11571	0.05399	0.31659	0.14031	0.07555	0.047	0.24552	0.18984
NM_001008295	Fip1l1	0.53382	0.63657	0.00046	0.74863	0.85614	0.25856	0.6881	0.97669	0.72463	0.69896	0.4347	0.05398	0.7348	0.71713
NM_001105919	Fis1	-0.3058	0.10378	-0.6769	-0.7399	-0.0183	0.31278	0.22665	-0.15	0.22826	0.25413	0.0964	0.44578	-0.1604	0.03421
NM_001108955	Fjx1_predicted	0.13545	0.10313	0.64977	-0.0393	0.24985	0.46624	0.36116	0.23792	0.14774	0.11581	0.22165	0.03255	0.68123	0.58042
NM_001014120	Fkbp10	1.2973	1.6533	1.0036	1.6674	1.5322	1.092	1.6866	1.5523	1.7598	1.9005	1.6717	1.0719	1.3475	1.2697
NM_013102	Fkbp1a	-0.1844	-0.0124	0.07143	-0.7173	-0.3514	0.06957	-0.3351	-0.1618	-0.1178	0.05198	-0.2426	0.3085	0.05787	0.30722
NM_001134429	Fkbp2_predicted	-0.0736	-0.092	0.22901	-0.0819	0.00536	0.24216	0.08992	-0.2011	-0.0845	-0.2855	-0.168	0.42488	0.14654	0.2768
NM_001106736	Fkbp3_predicted	0.18751	0.21731	0.11573	0.97982	0.14638	0.29345	0.1309	0.48348	0.51805	0.19733	0.26333	0.32712	0.17746	0.19868
XM_001066628.1	Fkbp4	-0.1064	0.0902	-0.1045	-0.1176	-0.1322	-0.094	-0.1154	0.01246	-0.1248	-0.1257	0.21932	0.06208	0.173	0.06144
NM_001012174	Fkbp5	1.1693	0.74308	0.49991	0.45187	0.86313	0.81203	0.17528	1.1799	0.66007	0.76809	0.58752	0.34416	0.65676	0.68216
NM_001105922	Fkbp6_predicted	-0.0011	0.1376	0.1912	-0.1305	0.03423	-0.0706	0.02403	0.00914	-0.0633	0.00114	0.13734	0.05919	0.06604	-0.0875
NM_001106485	Fkbp7_predicted	-0.9886	-0.2166	-1.0799	-1.1565	-1.0433	-1.0115	-0.6608	-1.087	-0.1153	-0.4018	-0.2618	0.09274	-0.4544	-0.5949
NM_001037180	Fkbp8	0.15812	0.16034	0.27581	-0.0175	0.27501	0.09052	0.16294	0.04058	0.35941	0.22652	0.28044	-0.0428	-0.4234	-0.2317
NM_001007646	Fkbp9	-0.2497	-0.0929	-1.0333	-0.5733	-0.907	-1.1197	-1.1723	-0.8823	-0.1606	-0.3791	-0.4663	-0.186	-1.0129	-1.0051
NM_001002818	Fkbpl	0.64925	0.04924	-0.1007	-0.1025	0.68173	0.83809	0.14439	0.45267	0.43717	0.20622	0.21695	0.37501	0.18265	0.06401
NM_001012091	Fkhl18	0.18227	0.82369	0.22189	0.14775	0.04526	0.18105	0.31776	-0.0001	0.88604	0.86706	0.89341	1.1079	0.61802	0.53695
NM_001025678	Fkrp	0.00685	0.07993	0.09156	-0.1886	-0.0888	-0.2592	0.00429	-0.1757	0.11607	0.31536	-0.1244	-0.0101	-0.1088	0.07437
NM_199390	Flcn	-0.3313	-0.3883	-0.1428	0.04894	-0.3186	-0.5637	0.16594	-0.4061	-0.4508	-0.3088	-0.4914	-0.1909	-0.2073	-0.414
XM_238235.4	Flg	0.08202	0.11862	-0.1154	-0.0019	-0.126	-0.0457	-0.0072	0.01315	0.02112	0.3208	-0.067	0.09577	-0.0451	-0.1498
NM_001017381	Fli1	-0.1911	-0.0144	-0.0249	-0.1188	-0.0999	0.0721	-0.133	-0.0229	-0.1645	-0.1053	-0.0107	-0.0651	-0.0523	-0.059
NM_001008279	Flii	-0.4396	-0.1681	-0.286	-0.5994	-0.2763	-0.3583	-0.2465	-0.365	-0.4802	-0.4543	-0.3078	-0.4487	-0.5666	-0.4627
NM_001106928	Flk	-0.2015	-0.1643	-0.2077	-0.1777	-0.1033	-0.0378	0.00082	-0.0326	-0.1766	-0.1526	-0.1331	-0.0812	-0.2037	-0.2096
NM_053760	Fln29	0.09487	0.0798	0.55509	1.2127	0.07525	0.42795	0.95377	0.21801	-0.0425	0.077	0.17305	0.00728	-0.1791	-0.207
NM_001134599	Flna_predicted	-0.8991	-0.4603	0.50763	-0.0483	-0.8269	-1.1611	-0.6747	-0.7524	-0.3759	-0.6783	-0.4362	-0.1313	0.18993	0.1045
NM_001107288	Flnb_predicted	-0.1316	-0.2195	-0.0697	-0.4057	-0.1443	-0.414	-0.2428	-0.0968	-0.0446	-0.3323	0.08051	-0.1563	-0.2997	-0.0329
XM_342653.3	Flnc_predicted	0.36116	0.47074	0.02437	-0.6164	0.4351	0.27851	-0.1028	0.27205	0.34969	0.60159	0.2559	0.04755	0.41669	0.31542
NM_022701	Flot1	-0.0378	0.1172	-0.9362	-0.4052	0.19675	0.39165	-0.1345	0.20109	-0.199	-0.1101	0.20651	-0.3603	-0.5358	-0.6858
NM_031830	Flot2	-0.4423	-0.4387	-0.1681	-0.5739	-0.4699	-0.5411	-0.478	-0.6626	-0.4122	-0.3096	-0.2603	-0.7026	-0.7092	-0.645
NM_001106750	Flrt2_predicted	0.10884	-0.0232	-0.3237	0.03681	0.09519	0.15259	-0.0049	0.13313	0.14735	0.00387	0.09184	-0.023	0.24452	0.1975
NM_001126291	Flrt3_predicted	-0.8594	-0.9404	-0.9786	0.84419	0.6995	0.55054	-0.4269	0.88284	-0.6705	-0.9791	-1.0667	-0.3966	-0.9713	-0.932
NM_019306	Flt1	0.33705	-0.177	-0.1327	0.19235	0.26625	0.04837	0.06089	0.42715	-0.0432	-0.2203	0.0957	0.1294	0.21626	-0.2298
NM_001100822	Flt3	-0.0183	0.03402	0.00537	0.07378	-0.1484	-0.0124	-0.1413	0.1442	-0.0467	0.09508	-0.0934	0.02985	0.01291	0.04247
NM_199109	Flvcr2	-0.0718	-0.1891	-0.1359	-0.2705	-0.2177	-0.2649	-0.1609	-0.2053	-0.0792	-0.2645	-0.2409	-0.1797	-0.0942	0.01096
XM_001058601.1	Fmn2_predicted	-0.0045	0.0196	0.03791	-0.0616	-0.0072	-0.0791	-0.077	0.09938	-0.044	0.02967	0.13316	-0.0019	-0.0419	-0.0478
NM_001105846	Fmnl1_predicted	-0.1249	-0.1932	-0.0283	-0.1214	-0.1439	-0.0104	-0.0887	-0.1783	-0.0172	-0.2226	-0.1749	-0.0407	-0.1738	-0.2081
NM_012792	Fmo1	0.51113	4.959	2.391	2.9407	-0.1883	-0.0769	2.2116	0.00681	4.4295	4.8399	4.8666	0.19636	0.37149	0.46761
XM_001075840.1	Fmo13_predicted	-0.0701	0.00436	-0.0448	-0.1145	0.04747	0.26372	0.03362	-0.2332	-0.0573	-0.0833	0.05155	0.22786	-0.0259	0.04798
NM_144737	Fmo2	-0.5698	2.2112	-0.3581	0.49054	-0.6203	-0.5498	0.46624	-0.2919	1.5599	1.9361	1.6601	0.57992	-0.6907	-0.5674
NM_144562	Fmo4	0.07871	1.6104	0.55846	1.2419	-0.1198	-0.1213	0.64084	-0.0127	1.4869	1.8757	1.4654	0.63755	0.08618	-0.0958
NM_144562	Fmo4	-0.2056	1.9258	0.29931	1.1142	-0.2411	-0.0929	0.65225	-0.2039	1.3487	1.6389	1.4961	0.69375	-0.1907	-0.3745
NM_144739	Fmo5	-0.1511	-0.2295	-0.2152	-0.1544	-0.0907	-0.0031	-0.2036	-0.1533	-0.1961	-0.2376	0.00346	-0.1455	-0.258	-0.1044
NM_080698	Fmod	0.31359	0.57528	0.12518	-0.4661	0.20449	0.46368	0.16664	0.26334	0.29059	0.40237	0.35124	0.33608	0.3732	0.2514
NM_052804	Fmr1	-0.4436	-0.0211	0.0573	0.70845	-0.6964	-0.6597	0.59495	-0.4037	0.00828	0.03099	-0.1346	0.16735	0.04609	-0.2322
XM_001055042.1	Fmr1nb_predicted	0.68369	0.58135	0.51881	0.88091	0.40014	0.50301	0.56435	0.27648	0.5766	0.81725	0.59069	0.55424	0.4474	0.53055
NM_019143	Fn1	-0.1692	-0.2847	0.00191	-0.352	-0.0876	-0.226	0.22167	-0.051	-0.3665	-0.3359	-0.5493	0.12771	-0.1327	-0.1483
NM_138914	Fnbp1	-0.0601	0.09433	0.0267	0.01781	-0.0561	0.07748	-0.2261	0.07714	-0.0367	-0.0945	-0.0457	-0.1269	-0.2617	-0.2235

NM_001013159	Fnbp4	-0.5263	-0.0742	0.41089	0.46913	-0.5578	-0.3591	0.09473	-0.2777	-0.3484	-0.2307	-0.1622	-0.2158	-0.1095	-0.3306
NM_001038615	Fndc1_predicted	0.31486	0.15091	0.18499	-0.016	0.18196	-0.1448	0.12457	0.0634	0.28163	0.42732	0.25863	-0.0229	-0.0218	0.03144
NM_001107278	Fndc3a_predicted	0.78874	0.94193	-0.1545	0.25103	0.44789	0.55154	1.4023	0.53293	0.9565	0.97983	1.0026	-0.128	-0.1211	-0.2918
XM_226988.4	Fndc3b_predicted	-0.0494	0.14687	-0.396	-0.3489	0.07364	-0.0389	0.40556	0.34628	-0.1724	-0.0097	0.06032	-0.6096	-0.261	-0.2683
XM_226988.4	Fndc3b_predicted	0.09861	-0.0032	0.22623	0.19523	0.24198	0.0847	0.14031	0.25283	0.03552	0.16646	-0.0646	0.00426	0.19768	0.05093
XM_001060505.1	Fndc5	0.02371	-0.2202	-0.0965	0.16323	-0.0149	0.11083	-0.0562	0.24695	-0.3603	-0.2825	-0.1387	0.07099	-0.007	-0.1402
NM_001109051	Fnsk_predicted	-0.0477	0.08805	0.1828	-0.0173	0.01752	0.50236	0.27754	0.23993	-0.0933	-0.2241	0.16822	-0.3602	-0.0177	-0.2053
NM_012847	Fnta	0.13238	0.12592	-0.0382	0.43195	0.23932	0.06512	0.29131	0.2791	0.03691	0.10057	0.19187	0.03505	-0.0072	0.15392
NM_172034	Fntb	0.23985	0.1935	0.36481	-0.4954	0.11863	0.04852	-0.5711	0.15617	0.01202	0.07505	0.05936	-0.0114	-0.1026	0.1276
NM_057185	Folh1	-0.0233	0.00456	-0.044	-0.024	-0.0564	-0.006	0.05133	-0.0641	0.00727	-0.0263	0.03915	-0.0193	0.00481	-0.0023
NM_133527	Folr1	-0.1336	0.07464	-0.2082	-0.0129	-0.2372	-0.1759	-0.0833	-0.009	-0.0759	-0.118	-0.0014	-0.0783	-0.1414	0.00245
NM_001106283	Folr2_predicted	1.1915	0.15746	0.07811	-0.0785	2.7091	2.9214	0.08147	2.7236	0.19159	0.06683	0.2904	0.09998	0.03464	-0.0196
NM_053748	Folr4_predicted	-0.0694	-0.2242	0.1578	0.11047	0.16795	-0.0111	-0.1442	-0.0664	0.15742	0.12193	-0.0936	0.31478	0.14253	-0.043
NM_022197	Fos	0.30263	0.23095	-0.0354	0.09493	-0.2597	-0.1783	0.12594	-0.0611	-0.0646	-0.0884	0.17931	0.11715	-0.2467	-0.1733
NM_001013146	Fosb	-0.0788	0.11817	0.04777	0.00253	0.07199	-0.0132	0.0237	-0.0311	-0.0522	0.08413	0.01503	0.0436	0.11926	-0.0323
NM_012742	Foxa1	-0.067	-0.1643	-0.0105	0.02426	-0.0748	-0.1385	-0.1549	-0.1202	-0.0366	-0.0498	-0.1299	-0.1209	-0.0294	-0.0812
NM_012743	Foxa2	-3.4368	-3.8945	-3.8839	-3.526	-2.2958	-2.2926	-3.2603	-2.5125	-3.518	-3.6405	-3.8845	-3.2268	-3.7656	-3.5407
NM_012743	Foxa2	-0.2783	-0.2585	-0.2561	-0.2635	-0.2937	-0.3063	-0.1882	-0.3202	0.00624	-0.2395	-0.187	-0.2191	-0.1558	-0.3354
NM_017077	Foxa3	0.17715	0.02014	-0.057	0.05519	0.06517	-0.1434	-0.0828	0.12088	-0.0786	0.05496	0.04693	-0.0919	-0.0278	-0.0055
XM_233422.3	Foxd2_predicted	0.35334	0.1328	0.24004	0.21468	0.44319	0.16504	0.21008	0.27575	0.13036	0.29654	0.24944	0.21273	0.19567	0.30179
XM_575873.2	Foxd3	0.0587	0.06566	0.07102	0.04972	0.34296	0.2123	0.2823	0.03562	-0.072	0.111	0.08387	-0.0314	-0.0197	0.01495
XM_574644.1	Foxd4	-0.6868	-0.132	-0.5054	-0.3453	-0.4901	-0.4513	-0.7155	-0.4174	-0.2674	-0.0726	-0.2508	-0.459	-0.456	-0.6633
NM_138909	Foxe1	0.05444	0.07721	0.08182	0.04434	0.15074	0.10922	0.23947	0.10921	0.01506	0.12197	0.1035	0.33728	0.14642	0.11884
XM_001069443.1	Foxe3	-0.0626	-0.104	-0.0411	-0.0181	-0.0629	0.13783	-0.0388	-0.0724	-0.052	-0.0314	0.11297	0.00993	-0.0457	-0.0503
NM_012560	Foxg1	-0.0388	-0.0255	0.0204	0.05544	-0.0522	-0.053	0.06821	0.12152	-0.0037	0.01299	-0.0342	0.00564	0.05662	0.09972
NM_001130493	Foxh1_predicted	-0.0414	-0.0551	-0.1289	-0.0258	-0.1027	0.13001	-0.1045	0.03892	-0.2476	-0.0393	0.0127	-0.1258	-0.3446	-0.1776
NM_001105776	Foxi1_predicted	-0.2593	-0.2555	-0.2276	-0.2495	-0.0474	-0.1748	-0.2294	-0.1841	-0.2502	-0.2076	-0.2437	-0.0268	0.04372	-0.1666
XM_001056035.1	Foxi2	-0.0842	0.01858	0.06628	-0.0867	0.16675	0.05488	-0.0241	0.16851	0.32509	0.25696	-0.0104	0.09324	0.13665	0.0019
NM_001107971	Foxj3_predicted	0.27442	0.12125	0.104	0.10342	0.01976	0.11094	0.11293	-0.106	0.16709	-0.1362	0.09475	-0.1343	0.17141	0.48014
NM_001107075	Foxk2_predicted	0.07691	0.05533	0.18646	-0.2559	0.1585	-0.0771	-0.0088	0.1966	0.00054	-0.0885	-0.1297	0.00564	0.33423	0.23349
XM_345975.2	Foxl2_predicted	0.17143	0.00756	0.09371	0.00404	0.11431	0.16878	-0.0159	0.08001	0.11971	0.07398	0.09226	0.1623	0.01965	0.23724
NM_031633	Foxm1	-0.1184	-0.971	0.49559	-0.6983	-0.9757	-1.1977	-0.5505	-0.9311	-0.4548	-0.7305	-0.7134	0.04556	0.28644	0.2246
NM_001100648	Foxn1_mapped	-0.0322	-0.2655	-0.1117	-0.0906	-0.0655	0.04559	-0.0975	-0.2514	-0.1623	-0.0972	-0.2637	-0.2356	-0.1607	-0.2077
NM_001105935	Foxn4_predicted	-0.0406	0.11323	-0.0748	0.05011	-0.191	-0.0228	-0.0463	-0.0343	-0.0368	-0.108	0.00827	-0.0218	0.16947	0.02029
XM_001056726.1	Foxo1a	-0.1133	0.04062	0.10214	-0.0841	0.13761	-0.0768	0.02418	0.13109	0.03476	0.05853	-0.1136	-0.047	-0.0429	0.01979
NM_001106395	Foxo3_predicted	0.04894	0.11651	-0.0132	-0.192	0.03455	-0.049	-0.0357	-0.0753	-0.1288	0.17916	0.0199	-0.0373	-0.0032	-0.0237
NM_001034131	Foxp1_predicted	-0.6031	-1.0525	-0.5595	-0.945	-0.6347	-0.5042	-0.9024	-0.5287	-0.914	-1.0545	-0.9749	-0.4251	-0.1989	-0.0413
NM_001108250	Foxp3_predicted	0.18347	0.35379	-0.006	0.05023	-0.054	0.01073	0.28136	-0.0229	0.14395	-0.0007	0.20125	0.20929	-0.0382	0.09119
NM_001108788	Foxp4_predicted	-1.2419	-0.6936	-0.6452	-0.8621	-0.808	-0.9725	-0.7303	-1.1038	-0.939	-0.7009	-0.8746	-0.4001	-0.7776	-0.7871
NM_199494	Fpgt	0.44006	0.12872	-0.0454	1.1698	0.54171	-0.0175	0.1633	0.57963	-0.0162	0.23893	-0.2827	-0.1534	0.21791	0.10906
NM_001106216	Fpr1_predicted	-0.1308	-0.1815	-0.0392	-0.0905	-0.1308	-0.187	-0.0196	-0.1541	0.07553	-0.0201	0.00696	0.11155	0.17567	-0.1496
NM_001169140	Fpr-rs3_predicted	-0.3006	-0.0639	-0.2148	-0.3339	-0.2767	-0.0795	-0.3394	-0.051	-0.0961	-0.205	-0.3629	-0.2484	-0.3198	-0.1817
NM_053895	Frag1	-1.6841	-1.7999	-1.9364	-1.802	-1.8801	-1.6218	-1.981	-1.9885	-1.9554	-2.0039	-1.7535	-2.1148	-2.1644	-2.3156
NM_019906	Frap1	-0.2859	-0.2504	0.46069	-0.1647	-0.144	-0.2947	-0.1757	-0.1164	-0.2337	-0.2097	-0.2035	-0.2815	-0.069	-0.0492
XM_001062222.1	Fras1_predicted	0.05906	0.06197	-0.0143	0.0487	0.01564	0.05542	0.17422	0.1177	0.0765	0.03762	0.05323	0.00054	0.25224	-0.0506
XM_001057556.1	Frem2_predicted	-0.2269	-0.0955	-0.1485	-0.2593	0.01798	-0.1984	-0.0193	0.00441	-0.2523	-0.2545	-0.2783	-0.1237	-0.0767	0.07344
NM_024366	Freq	-0.1451	0.10897	0.01491	0.27989	-0.059	0.24898	-0.0097	0.02219	0.03206	0.05072	-0.0395	-0.0239	0.00035	0.09803
XM_001064153.1	Frg1_predicted	0.23816	0.11664	-0.2892	0.3618	0.38447	0.06487	0.22678	0.33429	0.41216	0.36626	0.31397	0.17303	0.14634	0.13006
NM_024368	Frk	0.62662	0.33943	0.33752	-0.3304	0.13551	0.12298	0.05939	0.133	0.35764	0.31182	0.40323	0.33775	0.14722	0.13706

NM_001106662	Frmd3_predicted	0.18079	-0.0658	-0.03	0.07493	0.06824	0.1392	0.02887	-0.0719	0.03325	-0.1496	-0.1447	0.01039	0.09344	0.05795
NM_001008348	Frmd8	-0.3615	0.08367	0.33288	0.31961	-0.3078	-0.1823	-0.0648	-0.2254	0.05659	0.03694	-0.1735	0.15557	-0.0311	-0.1031
NM_001107937	Frmpd1_predicted	-0.0502	-0.0861	-0.1392	-0.0381	-0.0486	-0.0394	-0.049	-0.1023	-0.1182	-0.1007	-0.0288	0.08489	0.16666	-0.1126
NM_001108097	Frs2_predicted	-0.0976	0.00867	-0.094	0.0542	-0.179	-0.0332	-0.012	-0.1091	-0.0228	0.16248	-0.0984	0.13673	0.09752	-0.1211
NM_001017382	Frs3	-0.113	0.01596	0.09212	-0.0418	0.03084	-0.2409	0.09687	-0.0957	-0.1196	0.02873	0.07766	-0.1391	-0.0288	0.00359
NM_001100527	Frzb	0.08619	-0.1614	-0.1219	-0.166	-0.1459	-0.0156	0.01115	-0.1835	-0.1896	-0.1023	0.01108	-0.0372	-0.0487	-0.0987
NM_001107072	Fscn2_predicted	0.17509	0.0954	0.00541	-0.0715	0.073	0.15907	-0.0441	0.25826	-0.0257	0.1991	0.00169	0.14135	-0.1123	0.17792
NM_001004232	Fscn3	-0.0502	-0.3926	0.25128	0.28892	-0.1348	0.06245	0.04778	-0.3511	-0.3838	-0.0292	-0.0061	-0.0862	-0.4048	-0.2335
XM_218860.4	Fsd2_predicted	0.11439	0.01941	0.09784	0.03917	0.09242	-0.0726	-0.0681	0.13142	0.03969	-0.0198	0.19017	0.07128	-0.1533	-0.0443
NM_001007597	Fshb	0.00729	-0.1765	0.02986	-0.1258	-0.0907	-0.1494	-0.0405	0.06633	-0.2343	-0.1724	-0.1247	-0.2219	-0.2208	0.07535
NM_199237	Fshr	-0.177	-0.0981	-0.1471	-0.062	0.18924	-0.1161	-0.1781	-0.2267	-0.0821	-0.0917	0.04488	-0.0654	-0.0185	-0.043
NM_001013078	Fsip1	-0.1391	-0.1998	-0.2782	-0.3193	-0.2422	-0.1663	-0.2916	-0.3263	-0.1803	-0.3035	-0.286	-0.2766	-0.1585	-0.1714
NM_012561	Fst	0.28427	-0.6141	0.69862	1.2283	0.95669	0.79677	0.66533	0.75069	0.07607	-0.559	0.5624	0.98594	-0.2884	-0.2527
NM_024369	Fstl1	-0.4792	-0.1002	-0.047	-0.6208	-0.2018	-0.7217	-0.1379	-0.1506	-0.2551	-0.0636	-0.2801	-0.1239	0.04828	-0.0599
NM_053629	Fstl3	1.1619	0.6998	0.11314	-0.1186	1.1862	1.1483	0.6605	1.3628	0.5308	0.70325	0.59561	0.43779	0.64833	0.61031
NM_001107000	Fstl4_predicted	0.22213	0.16795	0.09919	-0.0042	0.22283	0.20849	0.16777	0.18717	0.04718	0.1245	0.18991	0.2785	0.21439	0.0447
NM_001108946	Fstl5_predicted	-0.0201	0.04367	0.03759	0.22782	0.07844	0.14314	0.06117	0.16617	-0.0066	0.10697	-0.0277	0.06202	0.07229	0.05983
NM_053567	Ftcd	-0.0012	0.11293	0.05981	0.04024	-0.0412	0.13881	0.08164	-0.0609	0.12681	-0.0449	0.00463	-0.0224	0.07653	0.0266
NM_012848	Fth1	0.46042	0.58043	0.37684	0.07253	0.5394	0.6643	0.43856	0.52864	0.39408	0.61105	0.62553	0.37591	0.60165	0.45118
NM_022500	Ftl1	1.6606	1.2049	1.0926	1.1884	1.3979	1.7695	0.30277	1.4541	0.75035	1.0706	0.98515	0.99292	1.0965	1.2606
NM_001106136	Ftmt_predicted	-0.0611	-0.0679	-0.1993	-0.155	-0.0968	0.0271	-0.0064	-0.3068	-0.1601	-0.2134	-0.28	-0.1663	-0.2326	-0.1501
NM_001011926	Fts	0.69377	0.96952	-0.3955	0.16713	0.61489	0.2438	0.10012	0.54073	0.44913	0.75565	0.50604	0.00086	0.62512	0.52048
NM_001011926	Fts_predicted	-0.0501	0.02448	-0.0649	-0.0801	-0.1084	-0.1455	-0.1302	-0.0742	0.11118	0.20402	0.03202	-0.1	0.00618	-0.0272
XM_001062916.1	Ftsj1_predicted	0.14788	0.06994	0.45483	0.21693	0.27546	0.0911	0.23418	0.27967	-0.0292	0.15146	0.21442	0.17862	-0.0735	0.50377
NM_001107125	Ftsj2_predicted	0.00997	0.06009	-0.12	0.00096	-0.213	-0.0611	-0.1356	-0.0416	-0.0288	0.09792	0.02039	-0.0294	0.04597	-0.015
NM_001012014	Ftsj3	0.15185	-0.0882	0.29363	0.01981	-0.0427	0.16528	0.09835	0.04565	-0.2985	-0.0477	0.13917	-0.0314	0.22007	0.14242
NM_001037653	Fubp1	0.04896	-0.0677	0.17473	-0.09	0.18472	0.35582	0.09026	0.50645	0.4696	-0.005	-0.0298	0.29754	0.32139	0.48394
NM_001039337	Fubp3_predicted	-0.3499	-0.3282	-0.2798	0.0967	-0.4677	-0.2221	-0.0913	-0.3053	-0.3589	-0.3854	-0.3252	0.1013	-0.1501	-0.177
NM_012562	Fuca	0.35851	0.46099	-0.5425	0.32234	0.50789	0.41379	0.1053	0.43666	0.11755	0.50203	0.3923	-0.0434	-0.5419	-0.281
NM_001004218	Fuca2	0.05784	-0.3383	-0.7554	-0.3274	0.10792	0.33338	-0.5821	0.06296	-0.3534	-0.1734	-0.3836	-0.3245	-0.3276	-0.3664
NM_001107429	Fuk_predicted	0.61157	0.60632	0.31082	0.58108	0.51652	0.59132	0.30821	0.37439	0.46115	0.48907	0.60335	0.52899	0.01221	0.14965
NM_001107429	Fuk_predicted	0.54948	0.18167	0.43758	0.68856	0.33768	0.25985	0.2848	0.59871	0.07482	0.10231	0.10049	0.1421	-0.3278	-0.1566
NM_001025027	Fundc1	0.32544	0.20287	-0.2744	0.39012	0.96692	0.7825	0.07863	0.97523	0.38935	0.20707	0.00609	0.35375	-0.1769	-0.1872
NM_001025027	Fundc1	0.51669	0.44022	0.03013	0.18601	0.96781	0.70035	-0.1438	1.2853	0.41577	0.4761	0.16362	0.25759	0.21916	0.33023
NM_001012137	Fus	-0.3322	-0.341	0.62196	-0.0995	-0.9293	-0.5372	0.09758	-0.9786	-0.3993	-0.5334	-0.3149	0.25631	0.08073	0.03712
NM_001025738	Fusip1	-0.233	-0.0844	-0.1566	-0.2092	-0.2502	-0.3595	-0.1557	-0.2129	-0.0855	0.16038	-0.3253	0.02459	0.20693	0.25971
NM_031236	Fut1	0.22136	0.08312	0.1347	0.15789	0.05269	0.12901	0.06743	0.07543	0.18127	0.10164	0.25452	0.07541	0.15085	0.07889
NM_173308	Fut11	-0.1261	0.39161	-0.0683	-0.2133	0.20156	0.0681	0.14263	0.05561	0.05809	0.27291	0.04952	0.35178	0.33083	0.29177
NM_031635	Fut2	0.35572	0.06817	0.13078	0.18121	0.16353	0.21244	0.14313	0.19241	0.1292	0.05502	0.07875	0.08934	0.09694	0.03785
NM_022219	Fut4	0.24797	0.46345	0.2096	0.00089	0.12504	-0.2117	0.15877	0.00242	0.65521	0.58138	0.4127	0.46369	0.39948	0.58776
NM_199491	Fut7	0.18644	0.12781	0.1337	0.12333	0.01809	0.11445	0.1776	0.04493	0.16968	0.05668	0.0224	0.15759	0.28566	0.104
NM_001002289	Fut8	-0.0014	-0.0888	-0.0051	0.04231	0.00753	-0.0768	0.00126	-0.052	-0.0099	0.00357	0.07688	0.18153	0.12758	0.12497
NM_001002289	Fut8	-0.9435	-0.5678	-1.7916	-1.5832	-0.8196	-1.4397	-1.3523	-0.8445	-0.7149	-0.8018	-0.9505	-1.0189	-0.8981	-0.8455
NM_053465	Fut9	0.05229	-0.0489	-0.0856	0.25239	0.14539	-0.0097	-0.047	0.07034	0.0306	-0.0374	0.14475	0.29836	0.15053	0.21418
NM_001037646	Fuz	-0.2219	-0.1249	0.30337	-0.1844	-0.013	-0.1243	0.05226	-0.2414	-0.2528	-0.1079	-0.2702	-0.0679	-0.0477	-0.1902
NM_001108342	Fvt1_predicted	0.54065	0.61131	0.91712	0.32503	-0.1832	-0.585	0.57555	-0.2658	0.39329	0.39789	0.44942	-0.401	0.67799	0.78742
NM_053371	Fxc1	0.03971	-0.2718	0.60392	-0.2929	0.11279	0.58427	-0.4437	-0.0708	-0.152	-0.2224	-0.0563	0.33204	0.69928	0.6236
NM_001012179	Fxr1	0.2278	0.72318	-0.4556	0.65168	0.68511	0.02608	0.46595	0.634	0.54669	0.80677	0.35175	-0.2479	0.20792	0.17847
NM_001012179	Fxr1h_predicted	0.34016	0.33212	-0.1519	0.16581	0.18632	0.10154	-0.007	0.39522	0.07169	0.26024	0.22288	-0.1415	0.22979	0.14796

NM_001100647	Fxr2h_predicted	-0.0007	0.06277	0.39072	0.58826	-0.0439	0.01255	0.32333	0.12779	0.06519	0.10831	0.02316	-0.051	0.29771	0.15999
NM_031648	Fxyd1	0.0343	-0.1825	-0.0671	0.21662	-0.0521	0.02911	-0.0087	-0.1644	-0.0013	-0.004	-0.0402	0.06612	-0.0537	0.06253
NM_145717	Fxyd2	4.6054	1.2545	2.2938	1.6559	5.2576	5.1109	2.5027	5.08	1.1265	1.8401	1.9944	1.0499	2.4267	2.1813
NM_145717	Fxyd2	4.0592	0.84398	1.8377	1.2176	4.8431	4.7916	2.1098	4.665	0.81306	1.3138	1.7602	0.62835	1.1691	1.217
NM_172317	Fxyd3	-0.3022	-0.1448	-0.1494	-0.1232	-0.0998	-0.1518	-0.115	-0.1779	-0.2833	-0.1534	-0.1061	-0.1926	-0.0377	0.1351
NM_022388	Fxyd4	-0.0132	-0.0256	0.2175	-0.0793	-0.0474	0.28339	0.25902	0.00778	0.08294	-0.0331	-0.0443	-0.1169	-0.0392	0.21659
NM_021909	Fxyd5	0.61846	0.01779	0.79569	-0.5289	0.72721	1.0635	0.54034	0.48601	-0.047	0.14819	0.12692	-0.184	0.19501	0.41615
NM_022005	Fxyd6	0.91044	-0.7048	-0.0508	-0.3581	2.6947	2.8309	-0.1194	2.4752	-0.6849	-0.5305	0.19096	-0.5961	-0.5672	-0.5904
NM_022008	Fxyd7	0.03262	-0.0462	-0.0305	0.0949	-0.072	-0.0686	0.26907	-0.0302	-0.0367	-0.0082	0.03534	0.0635	0.13214	-0.0838
XM_226812.4	Fyb_predicted	-0.1314	-0.0083	-0.0961	0.27953	-0.0715	-0.0251	0.12215	-0.0535	-0.0392	0.3388	-0.1698	0.17595	-0.0856	-0.2143
NM_001106870	Fyco1_predicted	0.04977	-0.0048	-0.1106	0.14878	0.30649	-0.1826	0.11504	-0.0448	-0.0571	0.07994	-0.2562	-0.2585	-0.0441	-0.1191
NM_012755	Fyn	0.27801	-0.0329	0.24233	-0.0469	0.11193	0.31846	-0.0168	0.30436	0.05137	0.13843	0.16603	0.13235	0.2074	0.03617
NM_001047899	Fyttd1	0.17085	0.11022	0.04102	0.26667	-0.0987	-0.3354	0.19072	0.03408	-0.2868	-0.2499	0.06967	-0.0819	-0.0968	0.04876
NM_021266	Fzd1	0.78061	1.7197	0.49713	-0.3135	-0.1696	-0.3142	1.4668	-0.3896	1.6443	1.755	1.6898	0.73246	0.73776	0.7989
NM_022623	Fzd4	-0.165	-0.0661	0.02022	-0.0933	0.05022	-0.0858	-0.196	0.05129	0.05312	-0.2357	-0.0398	-0.1851	-0.1658	-0.2206
NM_173838	Fzd5	-0.4015	-0.4307	-0.2905	-0.1176	-0.1946	-0.3689	-0.3779	-0.2296	-0.2762	0.0617	-0.3961	-0.2197	-0.469	-0.4116
XM_237191.4	Fzd7_predicted	0.18317	0.11008	0.16424	0.01981	0.17761	0.04142	0.20678	0.12472	0.17608	0.35564	0.03109	0.45132	0.17867	0.1974
XM_237191.4	Fzd7_predicted	0.45158	0.95248	0.04302	0.29298	0.05714	-0.1059	0.30799	0.25156	0.57691	0.87315	0.63308	0.22258	0.73108	0.54706
NM_153305	Fzd9	0.25083	0.29558	0.23554	0.57401	0.14372	-0.0562	0.44761	0.13317	0.1284	-0.1295	0.04028	0.10208	0.20541	0.2834
NM_001108074	Fzr1_predicted	-0.3267	-0.6036	0.2499	-0.3475	-0.4693	-0.7377	-0.5747	-0.7357	-0.5119	-0.7284	-0.7491	-0.072	-0.0843	-0.0416
NM_001009632	G0s2	-0.0447	-0.0713	0.13171	-0.0327	-0.0549	0.04429	-0.0386	-0.1372	-0.0614	0.01721	-0.072	-0.0733	-0.1545	-0.005
NM_001009632	G0s2	0.00482	0.03959	0.04996	-0.0202	0.00998	-0.0276	0.06995	0.11793	0.20495	0.06252	-0.0031	0.45804	-0.0143	0.00388
NM_053556	G10	0.0005	-0.4142	0.01444	-0.1239	0.09886	0.22406	0.30209	-0.0213	-0.2213	-0.1491	-0.124	0.25805	0.02335	0.10681
NM_001106700	G1p2_predicted	-0.1354	-0.0094	0.11724	-0.2594	-0.2464	-0.0589	0.0458	-0.172	-0.0981	-0.1998	-0.1114	-0.1855	-0.0318	-0.3764
NM_133565	G3bp	-0.1475	-0.3186	-0.0364	-1.0504	-0.6425	-0.7286	-0.438	-0.512	-0.2557	-0.0245	-0.4058	-0.2907	0.18188	0.28582
NM_001013989	G3bp2	0.0233	0.39425	-0.0825	-0.399	0.19621	-0.0164	0.06149	0.42433	0.28744	0.42268	0.09229	0.16589	0.71103	0.66904
NM_001013989	G3bp2	-0.216	-0.1567	-0.4334	-0.302	-0.4168	-0.2758	-0.3885	-0.3644	-0.3316	-0.135	-0.1766	-0.5743	-0.4342	-0.3923
NM_001003975	G4	0.18306	-0.0185	0.04431	0.00817	0.13237	-0.0648	0.1129	0.05178	-0.2502	0.05554	0.10075	0.38419	0.16231	0.11951
NM_001003976	G6b	0.04627	0.00428	0.06308	0.00986	-0.0076	0.1215	-0.0617	-0.0043	0.05037	-0.0998	-0.0039	-0.0434	-0.0665	0.10421
NM_013098	G6pc	-0.1126	-0.054	0.07102	0.03466	0.02038	0.05469	-0.0471	0.0395	-0.0512	0.08971	-0.0419	0.18918	-0.0883	-0.0462
NM_176077	G6pc3	0.13113	0.01476	-0.2684	-0.1108	0.21224	0.40247	-0.6094	0.17352	-0.3178	-0.1442	-0.1192	0.07208	0.1445	0.11463
NM_017006	G6pdx	0.29774	0.45788	0.56047	0.09697	0.46416	0.36333	0.15244	0.43717	-0.0426	0.09865	0.25998	-0.3137	-0.2717	-0.0807
NM_212499	G7c	-0.3364	-0.6201	-0.3771	-0.4019	-0.4874	-0.4261	-0.439	-0.2191	-0.4286	-0.4932	-0.4284	-0.4026	-0.5751	-0.3846
NM_199118	Gaa	0.73954	0.89812	0.1084	0.99165	0.77503	0.49284	0.64799	0.77494	0.6668	0.61919	0.55133	-0.0891	0.01118	0.21554
NM_001108444	Gab1_predicted	0.09605	0.05667	-0.1053	-0.3206	-0.0531	-0.1557	-0.0878	-0.0054	0.00948	0.19307	-0.0385	-0.1477	-0.1645	-0.0195
NM_053417	Gab2	0.15085	-0.0171	0.2234	-0.0669	0.19113	0.21515	0.2518	-0.0835	0.08501	-0.0104	0.05078	-0.0391	0.12601	-0.1013
NM_172036	Gabarap	0.15836	0.22173	-0.2701	-0.4034	0.29325	0.20694	0.01373	0.33755	0.58872	0.30307	0.22759	0.22827	0.35148	0.13644
NM_022706	Gabarapl2	0.51732	0.56674	0.07326	0.36559	0.07729	0.30319	0.44743	0.47241	0.5648	0.48241	0.62496	0.45654	0.20895	0.04275
NM_031028	Gabbr1	0.09299	-0.0061	-0.1486	0.18753	-0.0561	0.08772	0.24606	0.02649	-0.2441	-0.0321	0.01244	-0.2026	0.24878	-0.0402
NM_031802	Gabbr2	0.10255	0.00822	0.02294	0.12507	0.22448	0.48391	0.02851	0.19968	0.34268	0.07449	-0.0242	-0.0119	-0.0046	-0.0373
NM_001108841	Gabpa_predicted	0.16571	0.06695	0.14443	-0.7346	0.22851	0.00229	0.01332	0.28317	-0.049	0.04719	-0.0489	-0.042	0.51035	0.41667
NM_001135015	Gabpb1_predicted	-0.463	-0.269	-0.0713	0.37154	-0.3299	-0.4958	-0.1781	-0.2216	-0.4915	-0.2196	-0.2975	-0.3244	0.1258	-0.0859
NM_183326	Gabra1	-0.2069	0.04851	0.00464	-0.0471	-0.1149	-0.063	-0.1071	-0.18	0.14008	0.24404	-0.0441	-0.1354	0.07027	0.19404
NM_017069	Gabra3	-0.074	-0.0896	0.03331	0.04339	0.01328	-0.0369	-0.0631	-0.1596	0.08848	0.02658	-0.0261	-0.0582	-0.0419	-0.0365
NM_080587	Gabra4	0.04902	0.04506	0.26447	0.01462	0.1942	0.05133	0.19064	0.20241	0.14106	0.05735	0.23214	0.10419	0.06058	0.07874
NM_017295	Gabra5	0.0472	0.11559	-0.003	-0.0373	0.11072	0.31137	-0.0391	0.16945	0.162	-0.0466	0.05759	0.21172	0.27061	0.06908
NM_021841	Gabra6	-0.0312	-0.0521	-0.161	-0.0013	-0.1203	-0.0367	-0.1026	-0.0636	-0.128	-0.099	0.02084	-0.0477	-0.1075	0.07194
NM_012956	Gabrb1	-0.0594	-0.2112	0.14841	0.10017	-0.1039	0.06791	0.01214	0.18292	-0.077	-0.09	-0.0592	-0.0684	-0.1037	-0.0431
NM_012957	Gabrb2	0.21917	0.0248	0.06369	0.23212	0.34945	0.30936	0.28489	-0.0018	0.08253	0.19368	0.03096	0.36562	0.26887	0.40995

NM_017065	Gabrb3	0.09445	0.26552	-0.0666	-0.1397	0.3628	0.05915	-0.1863	0.12834	0.2593	-0.2157	0.07748	0.05313	0.05665	-0.0115
NM_017289	Gabrd	0.15951	0.19825	0.47987	0.06063	0.11433	0.08715	0.09294	0.22843	0.06314	0.19788	0.22368	0.10048	0.2344	0.24812
NM_023091	Gabre	0.00867	-0.0903	-0.0495	-0.0082	-0.1117	-0.0693	-0.081	0.14926	0.03561	-0.0545	-0.0048	-0.0936	-0.04	-0.1122
NM_080586	Gabrg1	-0.1301	-0.2498	-0.2102	-0.3349	-0.192	-0.1675	-0.2614	-0.1805	-0.2158	-0.068	-0.2076	-0.3294	-0.1691	-0.0722
NM_183327	Gabrg2	0.19739	0.07124	-0.2109	-0.0546	0.02913	0.31505	0.12131	0.08456	0.08043	0.20255	0.22417	0.06749	0.34187	0.36848
NM_024370	Gabrg3	0.11389	0.03895	0.00399	-0.0602	0.10593	0.0822	-0.0399	0.02815	0.11127	0.08929	0.03579	-0.0413	0.03972	0.05802
NM_031029	Gabrp	-0.3333	-0.3825	-0.1439	-0.1496	-0.1066	0.01278	-0.2061	-0.305	-0.0751	-0.2007	-0.1855	0.11009	-0.1495	-0.2226
NM_031733	Gabrq	-0.0657	-0.1524	-0.1381	-0.1212	-0.2405	-0.0235	-0.1453	0.05189	0.14721	-0.045	-0.142	-0.2317	-0.2088	-0.2074
NM_017291	Gabrr1	-0.0867	0.00117	0.23672	0.03576	-0.0705	0.05055	0.00453	-0.0913	-0.1234	-0.0904	-0.1039	0.07487	0.06435	0.21997
NM_017292	Gabrr2	0.12487	0.03649	0.22728	0.13358	0.1448	0.41213	0.15643	0.40567	0.3459	0.24015	0.55856	0.13735	0.16294	0.17443
NM_138897	Gabrr3	0.01566	0.09906	-0.0453	-0.1067	-0.0787	0.15904	-0.0313	-0.0401	0.17237	-0.0443	-0.0174	0.09508	-0.0463	-0.0455
NM_017007	Gad1	0.0712	-0.0318	0.04046	0.10998	0.21111	0.11492	0.05051	0.32425	0.15171	0.17648	-0.0224	0.07496	0.18861	0.15249
NM_012563	Gad2	-0.1566	-0.0384	-0.0273	-0.1817	0.03581	-0.1302	-0.1259	-0.0253	0.02681	-0.0079	-0.1138	-0.1802	-0.1725	-0.0515
NM_024127	Gadd45a	1.4057	0.72128	0.08609	2.0662	1.0515	0.8384	0.09717	0.97632	0.59864	0.78772	0.69201	0.38068	-0.3337	-0.286
NM_001008321	Gadd45b	-0.5705	-0.5508	-0.672	1.1825	-0.37	0.0768	0.02261	-0.3891	-0.736	-0.4251	-0.4986	-0.2428	-0.7326	-0.7595
NM_001077640	Gadd45g	-0.4011	-1.0221	-0.0843	1.5095	0.18608	0.86804	-0.2368	0.45365	-0.9491	-0.4242	-0.8696	-0.611	-0.4508	-0.3778
NM_001100504	Gadd45gip1	1.1724	-0.2171	0.582	0.26411	0.55069	1.1273	0.21399	0.62621	0.4551	0.15594	0.57976	0.84994	0.60447	0.66056
NM_031030	Gak	0.10216	-0.003	0.3023	0.45109	0.07196	0.25984	0.38801	0.08777	-0.1417	0.08819	-0.1224	0.07163	-0.1004	-0.1363
NM_033237	Gal	-0.2417	-0.146	-0.165	0.09582	-0.0598	-0.047	-0.2029	-0.2666	-0.2411	-0.1743	-0.1654	-0.1319	0.01528	-0.0565
NM_001024290	Gal3st3	0.11889	-0.0545	0.10797	-0.0223	-0.0112	0.127	0.00738	-0.069	-0.0646	-0.0268	0.11439	-0.0688	-0.1706	-0.017
NM_001005888	Galc	-0.1257	-0.2198	-0.3804	-0.2306	0.01134	-0.1732	-0.1356	0.01575	-0.481	-0.1552	-0.415	-0.5126	-0.3025	-0.2977
NM_080783	Gale	-0.7202	-1.0202	-0.0361	-0.5809	-1.0148	-0.7064	-0.7652	-0.8099	-0.7286	-0.8733	-0.8399	-0.0322	-0.5997	-0.7499
NM_001008282	Galk1	0.56266	0.10326	-0.1074	-0.7187	0.87544	0.93196	-0.9036	0.56562	0.39452	0.13771	0.0934	0.14992	0.21333	0.3863
NM_001007704	Galm	0.89945	0.14454	-0.2482	-0.0896	0.67774	0.65445	0.06905	0.62315	0.44932	0.11183	0.20662	0.45617	0.22465	0.23064
NM_173310	GalNAc4S6ST	-0.1855	0.21723	-0.0118	-0.1388	-0.1799	-0.1547	0.08752	-0.0673	-0.009	-0.0709	0.22244	-0.2261	0.13545	0.07828
NM_130742	Galnt10	0.17462	0.05985	-0.105	-0.3694	-0.0162	-0.2856	-0.0453	-0.3922	-0.1083	-0.2136	-0.1655	-0.2357	0.07796	-0.0376
NM_199393	Galnt11	-0.8909	0.112	-0.3275	-0.1312	0.15421	0.17083	-0.3293	0.07412	0.10619	0.30688	-0.0034	-0.2455	-0.6001	-0.7526
XM_223905.3	Galnt12_predicted	-0.0073	-0.149	-0.1172	0.04068	-0.0545	0.08237	-0.1048	0.22015	-0.163	0.42261	0.04333	-0.2225	-0.0739	0.04537
NM_199106	Galnt13	0.15412	0.31667	0.12425	0.14582	-0.0481	0.50223	0.23419	0.25912	-0.0596	0.00846	-0.0305	0.06086	0.11369	0.14847
NM_001012109	Galnt14	-0.1869	-0.0481	-0.1124	0.05827	0.07338	-0.0627	-0.0731	0.17445	-0.0826	-0.0214	-0.1581	-0.0677	0.37845	0.13921
NM_001106196	Galnt2_predicted	0.78339	0.81922	0.53988	0.57826	1.0351	0.65164	0.87786	0.87293	0.87124	0.76131	0.72126	0.02607	0.53988	0.62703
NM_001015032	Galnt3	-1.0428	-1.5797	-1.754	-1.4549	-2.0398	-1.9641	-2.0031	-1.7623	-1.6083	-1.5242	-1.7652	-2.2674	-1.6755	-1.7101
NM_031796	Galnt5	-0.3769	-0.6405	-0.4562	-0.4224	-0.5007	-0.4473	-0.5476	-0.7023	-0.7125	-0.5633	-0.4282	-0.5803	-0.5106	-0.5502
NM_022926	Galnt7	1.2585	0.80044	0.29641	-0.1455	1.2404	0.37037	0.48316	1.5424	0.25512	0.64519	0.62502	-0.2631	0.7448	0.72446
NM_001107151	Galnt9_predicted	0.07173	0.1843	0.16105	0.12057	-0.0322	0.21602	-0.0313	0.03967	0.06734	0.02301	-0.0557	0.00252	0.00443	0.03038
XM_001053416.1	Galntl1	-0.3106	-0.1109	-0.1637	-0.5008	-0.4096	-0.0417	-0.3929	-0.4308	0.37376	-0.2443	-0.1657	0.59197	-0.511	-0.2398
XM_001061731.1	Galntl2_predicted	0.3823	0.11887	0.19995	0.18261	0.18302	0.04851	-0.0074	0.11753	-0.0232	0.20283	0.09458	-0.0171	0.18238	0.02904
NM_001079884	Galntl4_predicted	0.48628	0.20395	0.87595	0.33483	1.5831	1.5023	0.7129	1.4479	-0.1617	0.27123	0.86863	-0.4435	-0.3474	-0.47
NM_001025148	Galntl5	-0.1152	-0.0517	-0.2038	-0.1803	-0.1489	-0.0452	-0.2015	-0.0595	-0.023	-0.0704	-0.1099	-0.1026	-0.1553	-0.1359
NM_022633	Galp	-0.2137	-0.0989	-0.1573	0.16729	-0.065	-0.1901	-0.1286	-0.1478	-0.1435	-0.1983	-0.122	-0.1489	-0.0083	-0.2027
NM_012958	Galr1	0.07136	0.10894	0.06171	0.37788	0.23098	-0.0988	0.06822	-0.0884	0.10029	0.19327	0.03699	-0.0112	0.05331	0.1373
NM_019172	Galr2	-0.4296	0.44629	0.16076	-0.7969	-0.3486	-0.5633	0.19854	-0.4334	0.105	0.62164	0.23843	0.00049	0.69369	0.73591
NM_019173	Galr3	0.08301	-0.0324	0.04711	-0.0809	-0.0277	-0.0934	-0.0577	-0.0555	0.02091	0.01443	0.04846	-0.0222	-0.1247	0.34467
NM_001013089	Galt	0.48443	0.30433	0.56808	0.19853	0.58332	1.2106	1.5752	0.77346	0.62601	0.28923	0.39458	1.0677	0.48061	0.33962
NM_012793	Gamt	-0.1472	0.21653	0.13526	-0.2888	0.0572	0.50469	-0.0658	0.01944	0.40487	0.34456	0.39024	0.5216	0.20661	0.17889
NM_001107434	Gan_predicted	-0.0076	-0.045	0.12618	-0.1124	-0.3205	0.26303	-0.1456	0.01917	-0.0764	-0.1	0.03173	0.10698	0.10655	-0.1324
NM_001106334	Ganab_predicted	-0.1213	0.23224	0.38597	0.33128	-0.2385	0.02253	0.31821	-0.1523	-0.2445	-0.1206	0.03971	-0.2153	-0.1449	-0.0731
NM_017195	Gap43	-0.4732	-0.1315	-0.0758	-0.2978	-0.2176	-0.271	-0.1274	-0.2864	-0.0863	-0.2349	-0.3277	-0.3827	-0.379	-0.319
NM_001037190	Gapdh	0.29509	0.04173	0.16765	-0.5484	0.22432	-0.0126	-0.1638	0.32495	0.07367	0.04153	0.14076	-0.0205	0.42684	0.36969

NM_001037190	Gapdh	0.6051	-0.0138	0.1944	-0.3129	0.36911	0.60172	-0.444	0.46505	0.17539	0.05185	0.15508	0.3034	0.60732	0.25499
NM_001037190	Gapdh	0.57405	0.11011	0.26288	-0.2511	0.26967	0.7335	-0.4266	0.48426	0.31684	0.01701	0.26894	0.25327	0.29342	0.40919
NM_023964	Gapdhs	0.18803	0.01311	-0.0543	-0.0819	0.08206	-0.0844	-0.1021	0.08061	-0.0769	-0.0337	0.05538	0.10405	-0.0107	0.01584
XM_231161.4	Gapvd1_predicted	-0.2058	-0.2793	0.24691	0.02907	0.06372	-0.0855	-0.1342	-0.2662	-0.1613	-0.4831	-0.0144	-0.1506	0.10145	-0.1392
NM_020083	Garnl1	-0.0933	0.01352	-0.3532	-0.3087	-0.007	-0.666	0.01073	-0.0049	-0.1784	-0.0748	-0.3272	-0.6072	-0.2225	-0.287
NM_001107019	Garnl4_predicted	0.07639	-0.0659	-0.1032	0.03941	0.00224	-0.0465	-0.0085	-0.0026	0.02904	0.08384	0.04351	-0.1232	0.00866	-0.0154
NM_001170434	Garp_predicted	-0.0496	-0.066	0.00952	0.0927	0.06686	0.09636	0.14586	0.02439	0.12635	0.15381	0.07852	0.2726	0.11286	0.08745
XM_216152.4	Gars	-0.3555	-1.098	-0.2455	-0.5334	-0.4195	-0.5916	-0.9332	-0.6017	-1.1934	-1.0529	-1.2716	-0.6281	-0.6353	-0.4221
NM_001011899	Gart	-0.5289	-0.4866	0.11882	-0.5824	-0.4103	-0.2666	-0.4639	-0.4627	-0.7154	-0.4297	-0.7533	-0.0859	-0.0622	-0.2274
NM_001108365	Gas2l1_predicted	0.02413	-0.4458	0.46964	-0.2707	0.12675	0.48215	0.1363	-0.019	-0.3554	-0.3629	-0.169	0.10965	-0.0954	-0.1148
XM_220783.3	Gas2l2_predicted	-0.0871	-0.1547	-0.1466	0.11169	-0.0964	-0.2252	-0.1072	-0.0025	-0.0323	0.00664	-0.1374	-0.0557	-0.1515	-0.2063
XM_235046.3	Gas2l3_predicted	0.36166	0.00451	0.01221	-0.0053	-0.2248	-0.2253	-0.2119	-0.0565	-0.1818	-0.0236	-0.1686	0.00151	0.20088	0.17162
NM_053484	Gas7	-0.5086	0.82843	-0.1929	-0.4066	-0.2527	0.02027	-0.4098	-0.3442	0.57167	0.6565	0.8188	-0.2479	-0.2398	-0.3604
NM_001039030	Gas8	-0.2645	0.22606	-0.0401	-0.1396	-0.2132	-0.2249	0.07746	-0.2918	0.00205	0.1465	0.10179	-0.0021	-0.1444	0.08481
NM_012849	Gast	-0.0058	-0.115	0.03834	0.08378	-0.1058	0.07088	0.01632	0.08131	0.09605	0.27587	0.13846	-0.054	-0.013	-0.0941
NM_012764	Gata1	-0.0612	-0.067	-0.0426	0.03654	-0.0036	-0.1029	-0.0619	0.00156	-0.0239	-0.0501	-0.0973	-0.1323	0.02626	-0.0809
NM_033442	Gata2	0.30766	0.14888	0.87002	0.17888	0.40694	0.68624	1.1853	0.33479	0.40381	0.2337	0.49895	0.87573	0.10677	0.09676
NM_133293	Gata3	-0.0225	0.09693	0.68625	0.41612	0.05703	-0.0913	0.51824	0.1713	0.15857	0.33232	-0.0282	0.06623	0.01599	0.22619
NM_144730	Gata4	0.67804	0.5828	0.39581	0.58741	1.2209	1.0361	0.14713	0.97999	0.22101	0.48495	0.21403	0.12025	0.35034	0.40822
NM_001024316	Gata5	-0.017	-0.0576	0.14178	0.07072	0.14219	-0.0098	-0.0663	0.10551	0.06739	0.17093	0.12151	0.13661	0.14041	-0.0171
NM_019185	Gata6	-0.4191	-0.2533	-0.5329	0.12039	-0.3002	-0.2991	-0.3187	-0.3289	-0.1759	-0.3777	-0.3144	-0.2954	-0.4695	-0.3591
NM_001013881	Gatad2a	-0.0629	-0.113	0.69321	-0.0081	0.0153	0.23557	-0.1119	-0.2038	-0.0541	-0.0235	-0.165	0.09905	0.21672	0.15073
NM_001013881	Gatad2a	0.03535	0.06148	0.09684	0.06138	0.08111	0.10462	0.15327	0.08632	0.05886	0.08086	0.30257	-0.0174	0.05324	0.04018
NM_001024888	Gatad2b	0.24566	-0.4292	-0.1324	-0.5422	0.08445	0.06803	-0.1327	0.21248	-0.4518	-0.0221	-0.4201	-0.0356	0.21246	0.12156
NM_031031	Gatm	0.22406	0.23404	0.30827	0.21496	0.45238	0.21451	0.17147	0.14038	0.3918	0.38485	0.25742	0.23834	0.44293	0.21337
NM_031031	Gatm	0.1554	0.02208	0.02027	0.12414	0.1615	0.14114	0.2061	0.07803	0.08604	0.27266	0.21829	-0.0112	0.25157	0.10017
NM_001100561	Gats	-0.0579	0.03648	0.05658	0.089	0.08212	0.0341	-0.0526	-0.0922	0.05923	-0.002	-0.1191	0.18813	0.15277	-0.1145
NM_001013091	Gba2	-0.4778	-0.3659	0.18424	-0.2383	-0.4835	-0.1712	-0.0727	-0.4651	-0.5066	-0.2985	-0.261	-0.1669	-0.4873	-0.3001
NM_001106010	Gba3_predicted	0.11513	-0.0288	0.12597	0.06175	0.12165	0.19568	0.1166	0.19512	0.24667	0.17412	0.11246	0.17838	0.03335	0.01226
NM_001017486	Gbas	-0.0396	0.22771	-0.1837	-0.0729	0.28251	-0.1547	0.14463	0.06574	0.40254	0.29362	-0.0908	-0.179	-0.0958	0.15538
NM_001017486	Gbas	0.04799	0.14227	0.06631	0.05613	0.0653	0.09606	-0.0034	0.03902	0.02615	0.01254	-0.0343	0.0634	0.01194	0.04143
NM_001100502	Gbe1	0.662	0.79491	0.30212	-0.4243	-0.0476	-0.2066	-0.4006	-0.0684	0.36563	0.52026	0.49197	-0.2103	0.27209	0.12156
NM_022404	Gbl	-0.8756	-0.2826	-0.1116	-0.9157	-0.8236	-1.1324	-0.1793	-0.6869	-0.2727	-0.2802	-0.4032	-0.4077	-0.6013	-0.4951
XM_001078913.1	Gbp1_predicted	0.1637	0.1173	0.27082	-0.0252	-0.0602	0.05937	0.31101	0.0356	-0.099	-0.031	-0.048	0.17129	0.03221	0.07828
NM_133624	Gbp2	1.4371	1.1405	0.00461	0.51079	1.6336	1.597	1.5511	1.4291	1.0459	1.0257	1.0934	0.1306	0.59288	0.57468
XM_001078892.1	Gbp4_predicted	0.30777	0.52402	0.08079	0.01573	0.17918	0.27505	0.09866	0.58076	0.37295	0.23991	0.0563	0.26805	0.22479	0.32753
NM_001108569	Gbp5_predicted	-0.169	-0.1042	-0.0302	-0.2816	-0.1555	0.02348	0.11044	-0.1096	-0.1936	-0.2406	0.10551	-0.0303	-0.2678	-0.1072
NM_053708	Gbx2	-0.0038	0.0433	0.0736	0.10618	0.07361	0.23833	0.06312	0.07417	0.07424	0.07094	0.06349	0.1827	0.05139	0.06631
NM_012564	Gc	0.23142	0.0471	0.1306	0.0223	0.18637	0.1529	0.08683	-0.0275	0.25976	0.40923	0.1542	0.3292	-0.0063	0.09638
NM_001106483	Gca_predicted	-0.315	-0.3011	-0.3606	-0.0597	-0.0129	-0.0645	-0.0757	-0.1308	-0.3083	-0.2507	-0.1436	-0.2032	-0.3042	-0.1849
NM_001108896	Gcdh_predicted	0.16732	0.25828	-0.3207	0.20147	0.19499	0.27126	-0.011	0.21753	0.25734	0.11456	0.28579	-0.0513	-0.1209	-0.3716
NM_012707	Gcg	-0.1881	-0.1775	-0.1362	0.08382	-0.1301	-0.0447	-0.1673	-0.1915	-0.107	-0.0144	-0.1878	-0.1423	-0.0428	-0.2526
NM_172091	Gcgr	-0.0632	0.01505	0.1428	-0.0411	-0.0719	0.04397	0.00566	-0.0408	0.02362	0.09376	0.2674	-0.1467	0.16371	-0.096
NM_172091	Gcgr	-0.029	0.1974	-0.066	-0.0054	-0.0879	-0.1777	-0.0276	0.05179	0.04546	0.03143	0.15158	-0.0793	0.13674	-0.059
NM_172091	Gcgr	-0.0355	0.16205	0.10261	0.0889	0.0598	-0.0034	-0.0942	0.08362	0.19534	0.02347	0.07752	-0.0037	0.11116	-0.0405
NM_024356	Gch1	2.2499	1.5915	1.4312	1.8986	3.0584	3.3427	1.3255	3.1269	1.3141	1.4744	1.3884	-0.0038	0.98035	1.0581
NM_133595	Gchfr	1.2514	1.1845	0.55818	0.04233	1.2664	1.4938	1.0939	1.0758	0.99475	1.2008	1.1643	0.57677	-0.0414	-0.1121
NM_012565	Gck	-0.0788	-0.274	-0.0684	-0.2444	-0.1335	-0.1932	-0.1638	-0.13	-0.1002	-0.262	-0.1717	-0.1991	-0.2156	-0.111
NM_012815	Gclc	-0.6757	-0.7298	-0.1517	0.13073	-1.0294	-1.1295	-0.614	-0.9887	-0.7452	-0.6512	-0.7797	-0.6863	-0.5595	-0.5925

NM_017305	Gclm	0.40832	-0.2554	0.07193	-0.5906	0.00555	-0.0963	-0.2726	-0.1281	-0.2422	-0.0009	-0.2615	-0.4616	0.14011	0.28059
NM_017186	Gcm1	0.07765	-0.0258	0.02712	0.13037	0.02132	0.22993	0.22165	0.09475	0.24051	-0.0283	0.05772	0.04471	-0.001	0.14623
NM_001106105	Gcm2_predicted	0.16923	0.19941	0.06977	0.12895	0.03455	0.03938	0.22475	0.21574	0.05316	0.0649	0.18506	0.12509	-0.0142	0.08861
NM_001107050	Gcn5l2_predicted	-0.7869	-0.6147	0.2035	-0.0126	-0.9739	-0.6087	-0.2562	-1.1023	-0.6025	-0.5193	-0.379	-0.3077	-0.4332	-0.285
NM_022276	Gcnt1	-0.0157	0.05078	0.20519	0.15974	-0.0363	0.11642	-0.0595	-0.0533	0.0257	0.10455	-0.0377	-0.0469	0.05406	0.03932
NM_001001511	Gcnt2	-0.0156	0.10658	-0.2376	0.06285	-0.2044	-0.0905	-0.1614	-0.0717	-0.208	-0.0794	-0.0244	-0.1313	-0.126	-0.2114
NM_001001511	Gcnt2	-0.6077	-0.2881	-0.5847	0.00148	-0.1873	-0.6477	-0.2387	-0.0907	-0.3843	-0.3637	-0.4774	-0.2938	-0.7091	-0.4786
NM_173312	Gcnt3	-0.5304	-0.5902	-0.6588	-0.6906	-0.6268	-0.7535	-0.7001	-0.6874	-0.6388	-0.7239	-0.4451	-0.6069	-0.6089	-0.6465
NM_031749	Gcs1	-0.3916	-0.4934	-0.4583	-0.408	-0.1698	-0.4839	-0.6722	-0.3372	-0.5982	-0.4711	-0.4978	-1.0892	-0.7291	-0.7671
NM_133598	Gcsh	0.75297	-0.2345	0.52388	-0.1155	-0.0331	-0.0033	-0.0737	-0.0305	0.03201	-0.1875	-0.1877	0.20695	0.54473	0.67657
NM_031776	Gda	0.99734	0.92431	-0.3194	0.26922	1.063	0.59851	1.1214	1.1023	0.72793	0.83722	0.7813	-0.3912	-0.481	-0.1845
NM_001107897	Gdap1_predicted	-0.184	-0.1571	-0.305	-0.1717	-0.2399	-0.0297	-0.13	-0.0163	-0.0171	-0.2244	0.07196	-0.0589	-0.2675	-0.1072
NM_001107798	Gdap1l1_predicted	-0.02	-0.1227	-0.0833	-0.176	-0.0937	-0.184	-0.1258	-0.1399	-0.0024	-0.161	0.05965	0.12857	0.01092	-0.1761
NM_001013201	Gdap2	0.49693	0.34917	0.37504	0.63881	0.91407	0.69945	0.26468	0.8243	0.40674	0.29611	0.38044	0.19301	0.5139	0.40575
NM_001044240	Gdf1_predicted	0.03036	0.07479	0.05374	0.03457	0.17615	0.15362	0.10819	0.11073	-0.0267	0.08567	0.04237	0.01808	0.03717	0.03817
NM_001044240	Gdf1_predicted	-0.4461	-0.2874	0.23832	0.25183	-0.5557	-0.1025	-0.0299	-0.2714	-0.1145	-0.2243	-0.2119	0.12137	0.01373	0.00693
NM_001044240	Gdf1_predicted	-0.1406	0.55768	-0.0095	-0.0422	-0.1997	-0.2016	0.22397	-0.3644	0.622	0.47459	0.58642	0.49853	-0.113	-0.0171
NM_024375	Gdf10	0.76277	0.13806	0.15816	0.08564	0.94215	1.0228	0.75156	0.88384	0.07215	-0.0684	0.05789	0.06134	0.11658	0.0216
XM_343148.3	Gdf11	0.01213	0.1261	0.12192	-0.0797	0.09862	0.07106	0.05298	0.26446	0.16781	-0.0009	0.21277	0.21779	0.28113	0.34332
NM_019216	Gdf15	1.8776	0.16431	0.01847	3.1263	2.0261	2.3565	0.52123	1.9175	0.01579	0.15665	-0.0914	0.51315	-0.221	-0.1405
NM_001013038	Gdf6	-0.0717	0.09812	0.09049	0.09254	0.14731	0.00626	0.42481	0.05034	0.05391	0.04139	0.10374	-0.1053	-0.0118	0.00611
NM_001170350	Gdf7	0.17294	-0.009	0.12059	-0.0197	0.00033	0.35402	0.20011	0.21095	0.11396	0.0842	0.07481	0.10974	-0.0248	-0.0868
NM_021672	Gdf9	0.01508	0.08177	-0.059	0.05067	0.02903	-0.0971	-0.0824	0.06385	-0.0648	-0.0623	-0.0503	0.17477	-0.0286	0.0438
NM_017088	Gdi1	-0.357	0.5541	-0.2909	0.61209	-0.0496	-1.0602	0.76124	0.20382	0.29422	0.29429	-0.0051	-1.0563	-0.413	-0.5481
NM_017276	Gdi2	-0.389	-0.066	0.19686	-0.8443	-0.3635	-0.6	-0.0333	-0.2948	0.09274	0.08005	-0.193	0.20088	0.42487	0.24135
NM_019139	Gdnf	0.04442	0.09535	0.18502	0.36352	0.08239	0.0548	0.11476	0.14176	0.02933	0.05246	0.0481	0.05926	0.11402	0.05666
NM_001106944	Gdpd2_predicted	-0.2973	0.99977	-0.2719	-0.0359	-0.3524	-0.3395	0.1898	-0.1484	0.75059	0.65351	0.8091	-0.1692	-0.0843	-0.4023
XR_009334.1	Gdpd3_predicted	-0.0673	-0.2149	-0.1101	-0.0938	-0.2037	0.20286	-0.0541	-0.1357	-0.2417	-0.1157	-0.1312	-0.038	-0.2374	-0.0404
NM_199395	Geft	-0.0179	0.64151	0.6394	0.33505	-0.2849	-0.1723	0.54415	-0.4683	0.36039	0.57578	0.42131	0.77163	0.24751	0.25703
NM_001106637	Gem_predicted	-0.0065	0.00842	-0.034	-0.0948	-0.0303	-0.0809	0.01949	0.00232	0.20407	-0.0334	-0.0067	-0.0966	-0.0333	-0.1175
NM_001009466	Gemin6	0.09908	-0.5586	0.28813	0.74042	0.54784	0.71511	-0.3376	0.82087	0.03278	-0.3788	-0.2331	0.63474	0.79902	0.56308
NM_001007756	Gemin8	0.21343	-0.1295	-0.0844	-0.5663	-0.1804	0.15007	-0.8074	0.14505	0.09214	-0.0149	-0.2257	0.29036	0.25482	0.41952
NM_017009	Gfap	-0.1573	-0.1893	0.02307	-0.2129	0.00592	-0.0417	-0.246	-0.1138	-0.0313	-0.029	-0.1406	0.0193	-0.0195	0.00064
NM_012566	Gfi1	0.09236	0.27737	0.04452	-0.0455	0.13752	0.01604	0.31	0.21903	0.23787	0.04035	0.40961	-0.0083	-0.0628	0.26105
NM_001107823	Gfi1b_predicted	-0.1083	0.02479	-0.0355	-0.0929	-0.0301	-0.1847	-0.1655	-0.208	-0.2165	-0.1519	-0.1541	0.01855	-0.2568	-0.1831
NM_053625	Gfm1	0.26744	0.27074	0.25068	0.40583	0.18948	0.09709	0.37484	0.06715	0.05197	0.33898	0.21895	-0.1297	0.17372	0.27552
XM_226707.4	Gfm2	0.16716	0.15722	0.06154	-0.113	0.01989	-0.1353	-0.0499	0.18883	-0.0081	0.27447	-0.081	-0.2775	0.17085	0.10805
NM_001170334	Gfod1_predicted	0.03253	-0.0044	0.1679	0.04936	0.18272	0.14384	0.31886	0.18939	0.05274	0.10377	0.00829	0.04563	0.08352	0.10841
NM_001005879	Gfpt1	-0.0709	-0.3191	0.16578	-0.0137	0.13227	0.30028	-0.2753	0.37657	-0.2179	-0.3364	-0.4898	-0.0637	0.38664	-0.0932
NM_001002819	Gfpt2	0.2649	0.06375	0.13227	0.09519	0.22601	0.30854	0.34246	0.41511	0.11709	0.23751	0.19585	0.28544	0.22874	0.2545
NM_012959	Gfra1	0.15215	-0.179	-0.0007	-0.1105	0.07564	0.05662	-0.0029	0.03145	-0.0494	-0.191	0.01291	0.29545	-0.0811	0.11286
NM_012750	Gfra2	0.28365	0.03547	0.11127	0.13499	1.36	1.2952	0.00992	1.5394	-0.0946	0.01785	-0.1165	0.17745	0.0302	0.00781
NM_053398	Gfra3	0.20726	0.26614	0.17781	0.0049	0.23636	0.13677	0.13238	0.32899	0.15929	0.21072	0.00667	0.05312	0.29914	0.25275
NM_023967	Gfra4	-0.139	-0.0967	-0.2125	0.3612	-0.1279	-0.153	0.12655	-0.25	-0.2022	0.0879	0.03094	-0.225	-0.2634	-0.2319
NM_001011994	Gga1	-0.4375	-0.0168	0.58409	0.57227	-0.3596	-0.3535	0.26182	-0.5402	-0.2275	-0.2023	-0.0212	0.09585	-0.6105	-0.43
NM_001011994	Gga1_predicted	0.36537	0.09376	0.30677	0.14256	0.11012	0.13422	0.2557	0.31681	0.33311	0.33208	0.1741	0.16899	0.20735	0.36464
NM_001100519	Gga2	0.05433	0.06301	-0.2243	0.2008	0.10694	-0.1369	-0.1416	0.21883	0.1489	-0.0728	-0.0392	-0.3281	0.02773	-0.0903
NM_001108304	Gga3_predicted	0.029	-0.0538	0.90357	1.3673	0.17311	-0.038	0.41469	0.02885	-0.1534	0.12626	-0.1955	0.17724	0.01017	0.08797
NM_031756	Ggcx	0.10404	0.13734	-1.3267	-0.5492	-0.1131	0.04673	-0.7638	-0.1621	0.01595	-0.2463	0.00607	0.16534	-0.4092	-0.3806

NM_012960	Ggh	-0.5662	-0.8481	-1.4834	-1.9146	-0.9333	-0.8742	-1.1825	-0.9163	-0.6392	-0.765	-0.7282	-0.9539	-0.9898	-0.9888
NM_001013065	Ggn	-0.0278	-0.0698	-0.0002	0.37113	-0.0211	-0.0104	0.03336	-0.0472	0.23865	-0.0446	0.12045	0.07106	-0.0595	-0.0161
NM_001009972	Ggnbp1	0.16674	0.15836	0.46705	0.10751	-0.0008	0.0973	0.05083	0.09024	-0.0021	-0.001	0.20302	0.16482	0.30252	-0.0309
NM_001007626	Ggps1	-0.0408	0.02219	-0.2134	-0.3264	-0.1389	0.04801	-0.1886	-0.2974	-0.0351	-0.1007	0.22128	0.28803	0.35617	0.06229
NM_001002820	Ggt6	0.04412	-0.0524	0.12967	-0.1901	-0.1399	0.40395	-0.1418	0.19513	0.00223	0.13319	0.38885	0.2812	0.13856	0.19783
NM_130423	Ggt7	0.17504	0.47431	0.17607	0.23221	0.28986	0.46793	0.16477	0.14233	0.09509	0.25629	0.08651	0.21104	-0.2272	-0.0278
NM_145674	Ggta1	0.19639	0.28956	0.24577	0.35623	0.30432	0.63394	0.33188	0.30176	0.21174	0.0831	0.21609	0.18911	0.13747	0.27599
NM_001005908	Ghitm	0.50429	-0.241	-0.4386	0.07579	0.36747	0.12399	-0.2704	0.50411	-0.4392	-0.2659	-0.4877	-0.456	-0.2709	-0.3628
NM_017094	Ghr	-0.1264	-0.1792	-0.2164	-0.0078	-0.0624	-0.1795	-0.0551	-0.0594	-0.1456	-0.1093	-0.0683	-0.2195	-0.2188	0.06174
NM_031577	Ghrh	0.03065	-0.045	0.09324	0.12932	-0.029	0.1274	0.06694	-0.0208	0.34924	0.01417	0.02372	0.20852	-0.0897	-0.125
NM_012850	Ghrhr	-0.1048	-0.0361	-0.0353	-0.0729	-0.1536	0.16645	0.14741	-0.1533	-0.074	-0.0693	0.02998	0.01524	-0.0291	-0.0334
NM_021669	Ghrl	0.12914	0.1633	0.11458	0.04482	0.25839	0.26725	0.09942	0.10379	0.06561	0.48147	0.25021	0.29485	0.30987	0.31541
NM_032075	Ghsr	0.04601	-0.0778	-0.0041	0.02829	-0.0215	0.19196	0.02717	-0.0034	-0.1185	0.16717	-0.0769	0.02899	0.03294	-0.0371
NM_017162	Gif	0.12942	0.013	0.09415	-0.0567	0.09358	-0.0585	-0.0479	0.01133	0.09461	0.26188	0.06398	0.03869	0.045	-0.0245
NM_173153	Gimap4	-0.031	-0.1202	0.0134	0.07079	-0.0335	-0.0334	-0.0411	0.15274	0.07873	0.13955	0.10808	0.04773	-0.0006	0.00439
NM_001033913	Gimap5	0.47231	0.01631	0.05621	0.02791	0.1536	0.29214	-0.04	0.25184	-0.0327	0.0336	-0.0148	0.05143	-0.0246	0.00954
NM_001011968	Gimap6	0.18901	0.05783	0.03901	0.14094	0.27557	0.22011	0.14017	0.06416	0.02232	-0.0394	0.09466	0.09854	0.06815	0.28132
NM_001033923	Gimap8	-0.1187	0.05423	0.34145	0.07504	-0.0502	0.12995	-0.101	0.08939	-0.2931	-0.1789	0.00902	-0.184	-0.1124	-0.075
NM_001008398	Gimap9	0.15371	0.0626	-0.067	0.04245	0.00223	-0.0224	-0.026	-0.072	-0.0265	0.16574	0.00386	0.01358	0.03472	-0.0835
NM_001030027	Gins4	-0.0083	-0.6818	0.7206	0.05997	-0.1604	-0.2412	-0.4356	-0.1397	-0.2695	-0.6048	-0.4902	0.35108	0.69325	0.79305
NM_133563	Giot1	-0.9259	0.2004	0.96464	0.18151	-1.6391	-1.8122	1.6467	-1.5588	0.49163	0.17982	0.62917	0.61007	-0.0773	-0.3701
NM_019630	Gip	0.24192	0.01667	0.03949	-0.0511	0.3029	-0.011	-0.0418	-0.0748	0.20112	0.11396	0.01083	-0.1696	-0.1541	-0.0722
NM_053341	Gipc1	0.45285	-0.3267	0.12971	-0.378	-0.1504	0.08597	-0.4488	-0.0935	-0.2722	-0.2276	0.02293	0.00357	0.36804	0.33437
NM_012714	Gipr	-0.1	-0.0946	-0.1334	-0.0167	0.13785	-0.039	-0.2402	-0.1672	-0.1661	-0.0119	-0.1442	-0.1245	-0.0114	0.04628
NM_031814	Git1	0.32933	-0.1568	0.17664	0.03576	0.12782	0.29595	0.00112	0.0284	-0.2136	-0.1252	-0.0751	0.08256	0.16053	0.09176
NM_001005553	Git2	-0.7393	-0.1258	-0.1868	-0.0353	-0.7784	-0.9788	-0.0233	-0.6605	-0.2532	-0.3107	-0.3672	-0.4632	-0.6545	-0.6555
NM_001009292	Giyd2	-0.259	0.18362	0.30749	0.19383	-0.2298	-0.2967	0.05101	-0.213	-0.3129	-0.4716	-0.1619	-0.0302	-0.049	-0.2302
NM_012567	Gja1	0.30433	0.39391	0.05336	-0.0285	-0.6963	-0.9009	0.34884	-0.5594	0.50302	0.49073	0.35042	0.65645	0.35739	0.45875
XM_001063402.1	Gja10	0.04031	0.13205	0.28499	0.27749	0.24041	0.30278	0.19139	0.27525	0.28063	0.0794	0.1709	0.04001	0.141	0.32975
XM_573100.2	Gja12_predicted	-0.1668	-0.0031	0.1768	-0.1961	-0.1393	-0.1573	-0.0567	-0.1176	-0.0842	-0.0161	-0.149	-0.0666	-0.0795	-0.1078
XM_573100.2	Gja12_predicted	-0.2431	-0.2274	-0.2159	-0.3585	-0.265	-0.2432	-0.225	-0.1212	-0.2617	-0.3032	-0.0427	-0.2565	-0.2439	-0.1888
NM_024376	Gja3	-0.0874	0.0268	-0.0055	0.18776	0.0537	-0.1181	0.22671	0.01766	0.09073	-0.0026	-0.0306	-0.0419	0.05941	0.03058
NM_021654	Gja4	1.5371	1.8281	1.1139	0.94383	0.71538	0.69285	1.114	0.72277	1.5079	1.7441	1.9596	1.5059	1.2201	1.323
NM_019280	Gja5	-0.3819	0.10819	-0.2975	-0.6018	-0.5146	-0.4878	0.15884	-0.4797	-0.1225	0.01462	-0.0032	-0.4286	-0.2643	-0.5596
NM_019308	Gja6	0.04597	0.0641	-0.0518	-0.0911	-0.0827	0.01311	-0.0859	-0.1595	0.08133	-0.0627	0.02551	-0.1119	-0.0334	0.00954
NM_019281	Gja9	0.06716	0.06523	-0.0807	0.07689	-0.0162	-0.0477	-0.03	0.10272	0.1179	0.00982	-0.0679	0.00432	0.03981	0.16341
NM_017251	Gjb1	0.09315	0.22909	0.06621	0.15915	0.01395	0.28164	0.24941	0.06424	0.23318	0.36873	0.0714	0.03554	0.29495	0.12938
NM_001004099	Gjb2	-0.0748	-0.012	-0.1519	0.26701	-0.1701	-0.0384	-0.0738	0.10055	-0.1472	-0.0284	-0.086	-0.2598	-0.1156	-0.0283
NM_019240	Gjb3	-0.0193	0.02178	-0.048	-0.3098	0.00973	-0.2594	-0.0967	-0.1683	-0.2055	-0.1821	-0.1195	-0.1152	0.04179	0.21246
NM_053388	Gjb6	-0.1597	-0.1782	-0.1991	0.04956	-0.1882	-0.1328	-0.0407	-0.0532	-0.1407	-0.1203	-0.1102	-0.2279	-0.2493	-0.1832
XM_343965.3	Gjc1	-0.1115	-0.1177	-0.0538	-0.0129	-0.0774	0.0322	-0.1966	-0.1258	0.0499	-0.0738	-0.1746	0.00098	-0.0283	0.0045
XM_001075553.1	Gje1	-0.081	-0.058	-0.0821	-0.0379	-0.1253	0.08169	-0.0698	-0.1269	-0.1215	-0.2286	-0.0087	-0.0034	-0.0722	0.05923
NM_001003977	Gk11	0.096	-0.0005	-0.0416	0.04868	0.04434	-0.0378	0.02271	-0.107	-0.0113	0.00351	0.12355	0.05499	0.17671	-0.0006
NM_001004077	Gk2	-0.007	-0.0699	0.07548	-0.0756	-0.0298	-0.0862	-0.2012	0.17	0.27034	0.08246	-0.0905	-0.0722	0.08416	-0.0304
NM_001012160	Gkap1	-0.3908	-0.2804	-0.7424	-0.1373	-0.0584	0.03219	0.12354	-0.0152	-0.5118	-0.3904	-0.5268	-0.8325	-0.8664	-0.7964
NM_198972	Gkn1	0.26783	0.17245	0.16994	0.41643	0.37044	0.33772	0.00244	0.17578	0.05621	0.1165	0.28006	0.06974	0.09816	0.29397
NM_001039686	Gkn2	-0.041	-0.0758	0.01348	-0.0418	-0.1097	-0.1382	0.02127	-0.0804	0.07368	-0.1135	-0.1647	-0.1469	-0.121	-0.0907
NM_134341	Gk-rs1_predicted	0.02384	0.20019	0.0909	0.12551	0.03817	0.09245	0.04378	0.06718	0.20158	0.06648	0.12922	0.23135	0.14718	0.20828
NM_001108820	Gla_mapped	0.27438	0.17247	-0.2019	0.01813	0.2116	-0.1181	0.01032	0.25864	0.37555	0.18153	0.14502	-0.0351	0.21428	0.10259

NM_001108192	Glb1_mapped	0.35845	0.40266	-0.4564	0.17624	-0.0177	-0.3213	0.06129	0.09863	0.25976	0.26259	0.28165	-0.1745	-0.5128	-0.1819
NM_001127529	Glb1l_predicted	0.2442	0.42063	-0.5864	0.13772	0.15133	-0.161	-0.1651	0.2281	0.20849	0.4421	0.34045	-0.2342	0.00054	-0.1371
NM_001024358	Glb1l3	-0.0737	0.0397	-0.1998	0.03028	0.08325	0.03351	-0.0633	-0.0196	-0.3003	-0.0836	-0.2341	-0.1494	-0.145	-0.0235
NM_001107583	Gldc_predicted	-0.1761	-0.1111	-0.1841	-0.2583	-0.2741	-0.119	-0.091	-0.0458	-0.3603	-0.1849	-0.284	-0.0787	-0.0525	-0.1924
NM_181382	Gldn	0.00682	-0.0512	-0.1083	-0.0586	-0.0654	-0.0442	-0.0108	-0.0245	-0.1415	0.1001	0.09182	-0.1339	0.00161	-0.0727
NM_001025731	Gle1	0.32134	-0.0036	0.26095	0.74664	0.32272	0.52185	0.24928	0.4795	0.21619	0.15607	0.15563	0.03892	0.24327	0.16096
NM_017211	Glg1	0.30025	0.36512	-0.5257	-0.0442	-0.1428	-0.8731	-0.3068	-0.2711	0.01663	0.19117	0.0793	-0.6502	-0.3381	-0.3463
NM_001107169	Gli2_predicted	-0.1471	-0.1535	-0.1749	-0.2613	-0.2933	-0.2961	-0.2391	-0.2522	-0.2175	-0.0328	-0.2794	0.11499	-0.1284	-0.0848
NM_080405	Gli3	0.07797	0.08019	0.05303	0.19461	0.01704	0.13131	0.11936	0.12107	0.08288	0.07051	0.18536	0.03298	0.06427	0.21271
NM_080405	Gli3	-0.1426	-0.1758	-0.1254	-0.1353	-0.0716	-0.1266	-0.1773	-0.1608	-0.1088	-0.1107	-0.1413	-0.2434	-0.2188	-0.1951
NM_001011987	Glipr1	0.3783	0.86064	0.81088	0.91639	0.38598	0.7069	0.89756	0.29351	0.74605	0.60483	0.78963	0.91011	0.91709	0.88365
NM_001106703	Glis1_predicted	0.08228	0.31205	0.07365	0.06534	0.30156	0.09433	0.16482	0.31845	0.20825	0.35769	-0.0534	0.32804	0.14149	0.25218
NM_001106978	Glis2_predicted	-0.1618	0.10089	0.27494	0.2622	-0.0782	-0.4392	0.34646	-0.0469	0.12947	0.44483	0.17598	-0.1678	0.04657	0.10722
NM_001105993	Glmn	-0.3397	-0.2954	-0.0382	-0.1069	-0.5858	-0.2932	-0.3642	-0.3553	-0.3042	-0.2921	-0.278	-0.2084	-0.0642	-0.2924
NM_207594	Glo1	-0.4199	-0.3909	-0.883	-0.6467	-0.2822	-0.2409	-1.047	-0.354	-0.2702	-0.5702	-0.5076	-0.0514	-0.2738	-0.2694
NM_001014227	Glod4	0.18068	0.23093	-0.542	-0.3381	0.17635	-0.1596	-0.2509	0.10316	0.14967	-0.132	0.00026	-0.5493	-0.0413	-0.0308
NM_012728	Glp1r	-0.1295	-0.0914	0.34023	0.64874	0.04772	-0.0864	-0.0417	-0.1561	-0.1141	-0.0799	0.14198	-0.1066	-0.1934	-0.0105
NM_021848	Glp2r	0.07379	-0.1683	0.0352	-0.311	-0.1551	-0.1457	-0.3252	0.15989	-0.0662	-0.1335	0.13193	-0.1124	-0.0418	0.08412
NM_013133	Glra1	-0.3914	-0.1966	-0.1046	-0.1328	-0.2862	0.15956	-0.1594	-0.0735	0.08419	-0.0867	-0.288	0.18162	-0.1643	-0.271
NM_012568	Glra2	-0.0314	0.05096	-0.0198	-0.0207	0.21603	-0.0131	-0.0484	-0.041	-0.0102	0.02132	0.03039	-0.0114	-0.0399	-0.0493
NM_053724	Glra3	0.26304	0.20559	0.0213	0.05096	0.18797	0.19767	0.05098	0.08648	0.06515	0.21541	0.26528	0.1464	0.10018	0.0756
NM_053296	Glrb	0.13828	0.09813	0.02748	0.11817	-0.0025	0.12156	0.02615	0.01789	0.06761	0.15425	-0.0437	0.06912	-0.0245	0.03839
NM_001107675	Glrp1_predicted	-0.0047	0.20076	0.08438	0.07733	0.23456	0.09414	0.28677	0.11712	0.25084	-0.0103	0.15114	0.50728	0.26505	0.29085
NM_022278	Glrx1	0.47245	0.86797	-0.1932	-0.3804	-0.2385	-0.4413	-0.0305	-0.2037	0.88077	0.99748	0.80505	0.10591	-0.0295	0.06103
NM_001013034	Glrx2	0.47649	-0.1869	0.00979	0.07971	-0.2653	-0.0593	0.04632	-0.0806	0.26013	0.00747	-0.1837	-0.0292	0.14787	0.16329
NM_032614	Glrx3	0.086	-0.2276	-0.0248	0.81514	0.38447	0.19125	0.0346	0.275	-0.0819	-0.0178	0.00205	-0.0772	0.05244	0.01455
NM_001108722	Glrx5_predicted	0.07082	-0.0634	0.06468	-0.3382	0.0472	0.32218	-0.2092	-0.1965	0.42989	0.1051	0.07463	0.60386	0.0811	0.22127
NM_012569	Gls	-0.0022	0.35004	0.60854	1.3842	-0.8281	-0.6285	0.73501	-0.7722	0.01548	0.16909	0.37891	0.15499	-0.3291	0.0042
NM_138904	Gls2	-1.0112	-0.7512	-1.0583	-0.5244	-1.4397	-1.1484	-1.4832	-1.4227	-0.7008	-0.7471	-0.9955	-0.4958	-1.6462	-1.5308
XM_222147.4	Glt1d1_predicted	0.06171	-0.0467	0.10512	0.10497	0.00079	0.06278	0.06763	0.24062	0.05517	0.12439	0.06939	0.11498	0.19432	0.17186
NM_001106067	Glt25d1_predicted	-0.047	0.25007	-0.5523	-0.2479	0.00576	-0.1602	-0.1933	0.45507	0.01735	0.03121	0.0443	-0.2847	0.10344	-0.018
XM_222718.4	Glt25d2_predicted	-0.0294	0.01333	0.14119	0.18387	0.23869	0.44769	-0.0816	0.05486	-0.0503	0.12036	0.17729	0.04283	0.15322	0.13627
NM_001106559	Glt6d1_predicted	-0.0208	0.05983	-0.1886	-0.0826	-0.0306	-0.0702	-0.1757	-0.0361	-0.1578	0.1065	-0.1173	-0.1657	-0.07	-0.1069
NM_001007683	Glt8d1	-0.4244	-0.2627	-0.5557	-0.8908	-0.6764	-0.7051	-0.4704	-0.6291	-0.1219	-0.1771	-0.3096	-0.184	-0.777	-0.9126
NM_001134413	Gltp_predicted	-0.3915	-0.3655	-0.2575	-1.4352	-0.6604	-0.617	-0.5742	-0.7541	-0.2785	-0.3125	-0.4558	0.05889	-0.1577	-0.1086
NM_001007703	Gltpd1	0.09546	-0.2037	0.01014	-0.651	0.06425	0.21941	-0.4625	-0.0935	-0.3948	-0.3749	-0.3866	-0.1482	-0.0267	-0.0563
NM_001106226	Gltscr1_predicted	0.26587	0.29803	0.07926	-0.0299	0.23742	0.29802	0.02815	0.22365	0.06185	0.42467	0.16221	0.23361	0.20704	0.14241
NM_207591	Gltscr2	-0.0746	-0.0987	-0.4785	0.20529	-0.1845	-0.1874	-0.5257	-0.1897	0.14248	-0.0526	0.07207	-0.4306	-0.7643	-0.6771
NM_012570	Glud1	-0.4121	-0.108	-0.2582	-0.5831	-0.3307	-0.3417	-0.0151	-0.3599	-0.1517	-0.0025	-0.0809	-0.3576	-0.4099	-0.5425
NM_181383	Gluld1	0.03294	0.14266	0.32466	0.0336	0.02555	0.00934	0.0454	0.23601	0.11569	0.06156	0.1707	0.01755	0.08915	0.11884
NM_001009648	Glyat	0.11136	0.00783	0.08347	0.02076	0.08719	-0.0032	0.10871	0.18176	0.2244	0.08614	0.09169	0.31478	-0.0315	0.14728
NM_012794	Glycam1	-0.075	-0.0519	0.06171	0.10821	0.03493	0.09187	-0.056	-0.054	-0.0822	-0.0967	-0.0431	0.16849	0.16375	0.09767
NM_001099471	Gm1961_predicted	0.1228	0.15715	0.05382	0.00908	0.13292	-0.0174	0.09228	0.06743	0.05466	0.12167	0.01847	0.0381	0.07222	-0.038
NM_172335	Gm2a	0.23933	0.73638	-0.4199	-0.7889	-0.2534	-0.0744	-0.6662	-0.2836	0.45939	0.79605	0.60127	0.43893	-0.2827	-0.2894
NM_001014771	Gm52_predicted	-0.0668	0.28765	0.17473	0.02214	0.05443	0.09884	0.09621	0.22234	0.21025	0.13208	0.09289	0.20808	0.07403	0.01419
NM_001108891	Gm672_predicted	-0.1133	-0.1498	-0.1708	-0.0453	-0.118	-0.2288	-0.0454	-0.3136	-0.1519	-0.3024	-0.2508	-0.2389	-0.1544	-0.1676
NM_001106327	Gm963_predicted	0.14682	0.18347	0.01034	0.17706	-0.0301	-0.0218	0.0147	0.01383	0.11717	-0.0476	-0.0207	0.04336	-0.0754	-0.0047
NM_001010956	Gmcl1	0.07585	-0.0142	0.19036	0.01586	0.0852	0.08477	0.07838	-0.0491	0.01983	0.33893	0.10616	0.25896	0.13395	0.06419
NM_031803	Gmeb2	0.26101	-0.0532	0.137	-0.0122	0.08829	0.0999	0.07626	-0.0381	-0.0064	0.03549	0.11419	-0.051	0.12111	0.2122

NM_031032	Gmfb	-0.0555	-0.0653	-0.1827	-0.2506	0.17126	0.19297	-0.0515	0.34055	-0.1742	0.11219	-0.4446	-0.1386	0.41215	0.21244
NM_181091	Gmfg	0.2273	-0.3954	0.49206	-0.0735	0.18742	0.44189	-0.0756	0.3084	0.10991	-0.0594	-0.0104	0.33351	0.38311	0.18945
XM_235422.2	Gml_predicted	0.05293	-0.0648	0.07002	0.2764	-0.0349	-0.0871	0.25562	0.22505	0.15422	0.09412	-0.0896	0.08826	0.12094	0.13552
NM_001106112	Gmnn_predicted	0.88997	1.0053	0.70699	0.34987	-0.185	0.06818	1.0941	-0.0855	1.0439	1.0655	1.079	0.44208	0.6909	0.61995
NM_001025056	Gmppa	0.11756	0.13581	0.07176	0.00423	0.13069	-0.0256	-0.2944	0.21091	0.11364	-0.004	-0.1368	-0.0244	-0.2979	-0.4721
NM_001108781	Gmppb_predicted	0.12811	-0.4581	0.53258	0.07472	0.26012	0.36193	-0.227	0.51361	-0.1603	-0.2047	-0.5099	-0.0207	0.17672	0.48829
NM_057188	Gmpr	0.45782	0.52853	-0.4707	0.6379	0.42625	0.5094	0.02669	0.22951	0.51899	0.45975	0.42695	0.03457	-0.6052	-0.4436
NM_001024754	Gmps	-0.2744	-0.2903	0.24768	-0.3654	-0.2303	-0.1884	-0.1546	-0.3164	-0.328	-0.1255	-0.1476	-0.1464	0.37718	0.40158
NM_031033	Gna11	-0.1015	-0.3662	-0.0531	-0.4459	-0.1635	-0.1779	-0.2664	-0.3504	-0.1295	-0.3223	-0.256	-0.1607	0.13527	0.15545
NM_031034	Gna12	-0.0655	0.14621	-0.1146	-0.0326	-0.0431	-0.496	-0.0485	0.32609	0.39732	0.03965	0.00999	-0.1199	0.04226	0.3437
NM_001013119	Gna13	0.05039	0.29867	0.09968	0.16263	0.16002	0.13896	0.13982	0.1528	0.17185	0.18647	0.21244	0.02011	0.22717	0.04158
NM_001013151	Gna14	0.04822	-0.0579	0.05706	0.22977	0.04987	0.02734	0.04057	0.05468	-0.0686	0.21206	-0.0016	0.26856	0.04385	0.15872
NM_001108019	Gna14_predicted	-0.1148	0.00802	-0.3969	-0.4344	0.11269	-0.5654	0.00628	-0.0914	0.15304	-0.2347	0.12189	-0.4081	-0.1132	0.05372
NM_053542	Gna15	-0.0173	-0.1309	0.08197	-0.0677	-0.1645	-0.0612	-0.1298	-0.147	-0.1771	-0.0506	0.09766	-0.0473	-0.1523	-0.0021
NM_013145	Gnai1	-0.1639	-0.2832	-0.1647	0.01626	0.13349	0.06244	-0.3291	0.48288	-0.0544	-0.406	-0.0909	-0.1096	0.51749	-0.008
NM_031035	Gnai2	0.10113	0.02191	-0.1186	-0.649	0.16277	0.14851	-0.0226	0.14109	-0.3317	-0.0482	-0.216	0.19831	0.05251	0.09636
NM_013145	Gnai3	0.2797	0.03289	-0.0279	0.15945	0.03369	-0.1682	-0.0064	0.16676	-0.0134	0.01225	0.03501	-0.2126	0.10664	0.05523
XM_341626.3	Gnal	0.00863	0.00965	-0.1879	0.03751	-0.0582	0.01479	-0.0897	-0.1176	-0.255	-0.2664	-0.1976	-0.0789	-0.1539	-0.2806
NM_017327	Gnao	-0.0149	-0.3338	-0.3812	-0.5077	0.30056	-0.1389	-0.6544	0.38453	-0.5544	-0.5123	-0.0613	-0.6624	-0.54	-0.5114
NM_031036	Gnaq	0.00843	0.22694	-0.1892	0.20766	-0.0232	-0.2087	0.0729	0.13226	0.1792	0.19671	0.05134	0.0973	0.35614	0.36113
NM_019132	Gnas	0.63519	0.09341	0.25188	0.16551	0.17679	0.00476	-0.0422	0.11755	0.32263	0.08795	0.27839	0.12967	0.03445	0.02924
NM_001108780	Gnat1_predicted	0.07829	0.11531	0.17779	0.18474	0.10041	0.05285	0.20047	0.00789	0.16516	0.04004	0.18022	0.09669	0.09829	0.0298
NM_001108950	Gnat2_predicted	0.55231	0.81649	1.1913	-0.2474	0.28177	0.23967	0.84802	0.04901	0.8632	0.96552	1.0595	0.97765	0.53661	0.40394
NM_013189	Gnaz	-0.254	-0.0952	-0.0947	-0.2147	-0.0686	-0.1026	-0.2774	0.14671	-0.1516	-0.0827	0.05187	-0.0309	-0.0123	-0.2389
NM_001013910	Gnb1	-0.0171	0.0129	0.08753	-0.5203	0.038	0.09859	-0.1894	0.08308	0.20512	-0.027	0.15485	0.14805	0.02816	-0.0281
NM_031037	Gnb2	-0.0277	-0.1421	-0.1541	-0.1253	0.12353	0.12395	-0.2207	0.39709	-0.1832	0.0115	0.07995	0.23884	-0.0343	-0.2648
NM_130734	Gnb2l1	0.01228	0.09589	-0.1904	0.04961	0.08357	-0.4377	0.13454	-0.049	0.0437	-0.0184	0.04407	-0.2977	-0.0803	0.00245
NM_021858	Gnb3	-0.1021	-0.1187	-0.1492	-0.1382	-0.1028	-0.1692	-0.1848	-0.1283	-0.2101	-0.0472	-0.0488	-0.1011	-0.0922	-0.1076
NM_001013910	Gnb4	0.07372	0.16354	0.0539	0.08	0.02328	0.06101	0.24053	0.1424	0.06851	0.1836	0.13974	0.16708	0.0297	0.07235
NM_031770	Gnb5	0.42354	0.84675	-0.113	0.06986	0.74869	0.43696	0.22377	0.62545	0.54599	0.82646	0.43333	-0.0839	0.1934	0.25028
NM_053765	Gne	-0.3132	-0.4252	-0.1841	-0.3755	-0.3812	-0.2164	-0.1523	-0.2537	-0.2632	-0.3295	-0.4833	-0.0923	-0.1286	-0.3521
NM_053660	Gng10	-0.1375	0.41352	-0.2575	-0.3377	-0.1966	0.03218	-0.2134	-0.1465	0.30824	0.17896	0.05201	0.40839	-0.014	0.14614
NM_053660	Gng10	0.09054	0.12085	0.13462	0.1041	0.20222	0.0914	0.09309	0.03572	0.22247	0.07741	0.0654	0.11509	0.1802	0.03178
NM_022396	Gng11	0.88238	0.10817	-0.2522	-1.1653	0.93079	1.1884	-0.2592	0.874	0.20753	-0.0726	0.22209	0.47886	-0.2156	-0.193
XM_578287.1	Gng12	-0.1583	-0.1401	-0.0181	0.10865	-0.1303	0.06265	-0.2023	-0.1757	-0.1971	0.09358	-0.1444	0.12889	0.11589	0.13671
NM_024138	Gng7	-0.0979	-0.1828	-0.1145	-0.1117	-0.1048	-0.1089	-0.1312	-0.1209	0.03105	-0.0842	-0.0628	-0.1052	-0.0282	-0.0761
NM_139185	Gng8	0.30945	0.0864	0.01824	0.0143	0.04656	0.0503	0.0841	-0.1169	0.12901	-0.1394	-0.0816	0.14916	0.06607	-0.0892
NM_212500	Gnl1	-0.2792	-0.5087	-0.2241	-0.2194	-0.1474	-0.0352	-0.3593	-0.0953	-0.2825	-0.3804	-0.4626	-0.2334	-0.2584	-0.2036
NM_001025736	Gnl2	-0.2875	-0.0758	0.09036	0.61041	0.16032	-0.0336	0.07115	0.37597	-0.0762	-0.1532	0.07468	-0.5913	0.19858	0.0735
NM_175580	Gnl3	-0.4641	-0.9909	0.27309	0.21267	-0.5487	-0.552	-0.0278	-0.6226	-0.5309	-0.7218	-0.5669	-0.5653	-0.3325	-0.0805
NM_017084	Gnmt	0.1938	0.15027	0.06373	0.10466	0.31056	0.51892	-0.0362	0.09207	0.33033	0.04858	0.04511	0.28455	0.08937	-0.0656
NM_001170605	Gnn	-0.0572	0.0075	-0.0838	-0.1133	-0.1372	-0.136	-0.0889	-0.041	-0.0468	0.01689	-0.0899	0.10729	-0.0219	0.11351
NM_053410	Gnpat	0.20658	0.47908	0.06643	0.74541	0.23999	-0.0237	-0.2043	0.16591	0.48521	0.4521	0.33913	0.30233	0.16963	0.12124
NM_001106005	Gnpda2_predicted	-0.2587	0.24979	-0.1234	0.35729	0.20491	0.22328	0.23578	0.32121	0.32956	-0.0836	0.35514	0.02798	-0.5046	-0.6262
NM_001100493	Gnptg	-0.6891	-0.1842	-0.4137	-0.5746	-0.9375	-0.8885	-0.5063	-0.5745	-0.4713	-0.274	-0.348	-0.4551	-0.4249	-0.2992
NM_012767	Gnrh1	-0.7847	-0.4405	0.64529	1.0356	-0.9316	-1.1487	0.18181	-0.9928	-0.3981	-0.4104	-0.4579	-0.2668	-0.6056	-0.5873
NM_031038	Gnrhr	0.1527	0.00981	0.11708	0.0266	0.08782	0.027	0.02009	-0.0108	0.00827	0.07041	0.0147	0.12364	-0.0128	0.03716
NM_001011989	Gns_predicted	-0.1412	0.67197	-0.125	0.42615	-0.1524	-0.4116	0.00958	-0.1058	0.49255	0.63514	0.58626	0.16474	0.36027	0.40019
NM_001107842	Golga1_predicted	-0.3596	-0.3959	-0.1412	-0.1084	-0.2482	-0.4761	-0.0484	-0.2553	-0.1484	-0.1556	-0.3876	-0.1389	-0.3208	-0.0924

NM_022596	Golga2	-0.1854	0.01385	-0.2538	-0.1641	-0.2663	-0.3667	-0.1906	0.12427	-0.4289	-0.0863	-0.3199	-0.4401	-0.3303	-0.3779
NM_001107847	Golga3_predicted	0.01975	-0.0276	-0.0547	-0.1594	-0.139	-0.2163	0.13815	0.01188	0.15857	0.03086	0.00688	-0.0835	0.1492	0.31381
NM_001033065	Golga5	0.05994	0.18274	-0.1902	-0.1699	0.12968	-0.0002	0.52889	-0.0313	-0.0678	0.06536	0.09851	-0.633	-0.1994	-0.1381
NM_001007731	Golga7	0.44648	0.32275	-0.0288	0.2905	0.45671	0.38935	0.22197	0.47362	0.17893	0.27314	0.12527	-0.0706	0.10972	0.09354
NM_138885	Golgb1	-0.0508	0.2353	0.04177	-0.154	0.35662	-0.0914	0.31846	-0.0574	-0.0057	0.20327	0.10322	-0.2033	-0.4388	-0.2206
NM_023977	Golph3	0.02545	0.35553	-1.5295	-0.8065	0.34014	-0.8693	-0.601	0.53618	-0.128	0.15461	-0.4825	-0.902	0.04449	-0.005
NM_001007698	Golph3l	0.29101	0.12227	-0.0369	-0.1212	0.21694	0.12554	0.25303	0.05366	0.00217	-0.0057	0.05738	0.04917	0.23538	0.07957
NM_001113783	Golt1b_predicted	-0.2563	-0.428	-0.118	0.41679	-0.0082	0.30144	-0.1308	-0.1064	-0.201	-0.2498	-0.3498	0.16354	0.26121	0.30011
NM_001107631	Gopc_predicted	0.04207	-0.0882	-0.2333	0.11086	-0.0423	-0.1495	-0.1664	-0.1245	-0.0614	-0.1142	0.01402	-0.0911	-0.1472	-0.1491
NM_001007720	Gorasp2	-0.3336	-0.0547	-0.3351	-0.5088	-0.1132	-0.0123	-0.126	-0.0502	-0.0959	-0.4326	-0.1794	0.14648	-0.0652	-0.1458
NM_001007720	Gorasp2	-0.1036	-0.0529	0.18349	-0.3697	0.17562	0.16994	0.18417	0.06466	0.25263	0.06845	0.10133	0.31112	0.36847	0.35138
NM_053584	Gosr1	-0.2132	0.11659	-0.1304	-0.3726	-0.0669	-0.328	-0.0932	-0.1878	0.02978	-0.1883	-0.2702	-0.2738	0.26336	0.12599
NM_031685	Gosr2	0.22672	0.11033	0.14317	-0.0018	0.1594	0.0696	0.05257	0.1099	0.12649	0.27317	-0.0519	0.34444	0.34076	0.32689
NM_012571	Got1	0.42788	0.01314	-0.0744	-0.3141	0.37993	0.16848	0.20389	0.4077	-0.0374	0.11055	-0.0876	-0.2685	-0.4796	-0.2794
NM_013177	Got2	0.59207	0.35901	0.1893	-0.402	0.68454	0.33616	-0.1648	0.75391	0.25517	0.41992	0.29974	-0.4682	0.34067	0.40618
NM_053930	Gp1bb	-1.2512	-0.5659	-0.3983	-0.515	-0.804	-1.2024	-0.7835	-0.9464	-0.6257	-0.316	-0.4449	-0.2762	-0.8429	-0.9561
NM_012795	Gp5	0.06227	0.01926	0.20646	0.19827	0.13428	0.06782	0.00999	0.01377	-0.0498	-0.067	0.272	0.01455	-0.0863	0.0953
NM_001031825	Gp9	-0.0684	-0.1063	-0.1098	-0.1647	-0.124	-0.0786	-0.1126	-0.0988	0.11269	-0.1103	-0.0356	-0.0535	-0.0724	-0.125
XM_341149.3	Gpa33_predicted	-0.0187	0.05307	-0.1757	-0.0524	-0.0295	-0.0406	0.06224	-0.1419	0.00964	-0.0536	-0.1647	0.12428	0.06953	0.09196
NM_001004240	Gpaa1	0.14185	0.57046	1.0557	0.6064	0.30658	0.42865	0.88665	0.15262	0.45356	0.52853	0.55064	0.45299	0.52192	0.83157
NM_017274	Gpam	0.0805	-0.1134	0.03723	0.12576	0.10512	0.27863	0.11169	0.09938	0.03521	0.46137	-0.1094	0.09581	-0.0088	0.2319
NM_001106246	Gpatc1_predicted	0.08951	-0.1026	0.0168	0.27438	0.03093	0.37956	0.02974	0.11965	-0.1094	0.199	-0.0412	-0.0325	0.03035	0.13792
NM_001011909	Gpatc2_predicted	0.1553	0.04901	0.03959	-0.0257	0.27072	0.16906	0.09274	0.01452	0.02753	0.12328	0.04131	0.0446	0.08513	0.11934
NM_001024979	Gpatch4	0.20721	-0.5465	0.4928	-0.1355	-0.1894	-0.428	0.16101	-0.2437	-0.2663	-0.3075	-0.1391	-0.2172	0.42116	0.67349
NM_177936	Gpbar1	0.30678	0.05103	0.05225	0.0206	0.17474	-0.0182	-0.0281	0.19673	0.04751	0.15361	0.12964	0.15469	-0.0621	0.10277
NM_012774	Gpc3	-0.0988	-0.032	-0.041	0.16508	0.1035	0.06763	0.27485	0.4319	0.19517	0.15184	0.17441	0.28102	-0.0932	-0.0905
NM_001014108	Gpc4	-0.0832	-0.0139	-0.0071	0.12679	0.09063	-0.0176	-0.0873	0.16053	0.06718	-0.0602	-0.0197	0.16746	-0.0274	-0.0156
NM_001107285	Gpc5_predicted	-0.2842	-0.2694	-0.1953	-0.2658	-0.2264	-0.0982	-0.1953	-0.2719	-0.0214	-0.1151	-0.1544	-0.2187	-0.3124	0.00819
NM_001037295	Gpcr12	0.08189	-0.0236	0.03485	0.12903	0.02291	0.01529	-0.0235	0.15786	-0.0485	-0.0156	0.03059	0.05365	0.01445	-0.0171
NM_022215	Gpd1	-0.2484	-0.0203	0.08755	-0.1873	-0.0261	-0.0422	-0.0419	-0.0428	0.14278	-0.1622	0.08124	0.13775	-0.1274	-0.1294
NM_012736	Gpd2	-0.2098	-0.2337	0.03008	-0.4906	-0.0626	-0.2818	-0.4078	-0.1996	-0.5146	-0.4386	-0.4231	-0.4367	-0.2173	-0.363
NM_133573	Gper	-0.1804	-0.2998	-0.3457	-0.3811	-0.1899	-0.2794	-0.1753	-0.3184	-0.182	-0.273	-0.0232	-0.3358	-0.2587	-0.0746
NM_133619	Gpha2	0.06053	0.13281	-0.0512	0.0108	0.04243	-0.0109	-0.0638	-0.0088	0.1274	-0.1648	0.02398	-0.0783	0.25806	0.08017
NM_001007013	Gphb5	0.1182	0.06794	0.0053	0.19344	0.34792	0.06775	0.11619	0.0615	-0.0023	0.28771	-0.0039	0.03749	0.12633	0.07388
NM_022865	Gphn	-0.1379	-0.2541	-0.3031	-0.2845	0.28315	0.38482	-0.1835	0.56545	0.00089	-0.2746	-0.1012	-0.6096	-0.3564	-0.4417
NM_207592	Gpi	0.17064	0.54351	0.24378	-0.3381	0.16861	-0.1689	-0.5916	0.09108	0.00328	0.43618	0.58588	-0.264	0.24344	0.09356
NM_001012185	Gpiap1	0.01496	0.22695	-0.1584	-0.0251	-0.0324	0.04562	-0.0478	-0.0099	-0.0039	-0.0146	-0.0277	-0.0558	0.47394	0.30522
NM_001100512	Gpld1	-0.116	0.0805	-0.2114	-0.1886	0.11306	-0.2497	-0.2458	-0.1877	0.01785	0.14797	-0.24	-0.038	0.02424	-0.299
NM_178105	Gpm6a	-0.492	-0.4674	-0.5593	-0.4425	-0.6512	-0.2894	-0.2712	-0.4722	-0.4917	-0.5207	-0.3856	-0.6487	-0.3486	-0.551
NM_133298	Gpnmb	0.31461	0.41186	-0.0581	-0.1183	-0.0471	0.21939	0.19948	-0.0961	0.00661	0.30228	0.05487	0.4201	-0.0835	0.02337
NM_012961	Gpr1	0.16464	0.05096	0.03265	0.05162	0.08032	0.13315	0.02972	0.14801	0.01077	0.0614	-0.0417	0.10711	0.10843	0.08207
NM_001108258	Gpr101_predicted	0.11813	0.04662	0.06476	-0.0304	-0.0782	0.18907	-0.0893	0.00744	-0.1899	-0.113	-0.0066	0.11407	0.15271	0.04989
NM_001108258	Gpr101_predicted	-0.1156	0.0034	-0.0025	0.03769	0.01657	-0.0159	-0.1495	0.00917	-0.0737	-0.159	-0.1125	-0.0032	-0.0393	-0.0158
NM_198199	Gpr103	-0.1847	-0.3471	-0.3648	-0.2987	-0.3332	-0.4264	-0.2749	-0.2857	-0.2951	-0.5314	-0.3389	-0.4309	-0.4858	-0.3444
NM_001107828	Gpr107_predicted	0.36173	0.23623	0.22692	0.85055	0.57263	0.65588	0.40382	0.21487	0.04322	0.21376	0.18083	0.1335	0.16857	0.22619
NM_199399	Gpr108	-0.0825	-0.2432	0.12753	-0.0822	-0.2161	0.30438	0.24587	-0.5002	-0.2995	0.04643	-0.0758	-0.1465	-0.2913	-0.3175
NM_181476	Gpr109a	0.20232	-0.0516	-0.0143	-0.0516	-0.0023	0.24528	-0.0349	-0.093	-0.0136	0.0951	0.00496	0.18333	0.08255	0.1687
NM_001106894	Gpr110_predicted	-0.2183	-0.2333	-0.2543	-0.0263	-0.1949	-0.2451	-0.0282	0.01202	-0.2132	0.03974	-0.1377	-0.1122	-0.1141	0.01018
XM_229201.4	Gpr112_predicted	0.00744	-0.1065	0.34641	0.04599	0.2672	0.06205	-0.0974	0.11568	-0.0542	-0.0349	0.29797	-0.0797	-0.0636	-0.026

NM_001106712	Gpr113_predicted	-0.0251	0.0364	0.03289	-0.0014	0.04758	0.25211	0.02902	0.11613	0.03021	0.12442	0.15479	0.04642	0.12757	0.04853
NM_001107410	Gpr114_predicted	-0.1338	0.02093	-0.0977	-0.1487	-0.1684	-0.1094	-0.1692	-0.1545	-0.1395	-0.0387	-0.1677	-0.1485	-0.1993	-0.0955
XM_236958.4	Gpr115_predicted	-0.0866	-0.0786	-0.0413	-0.0766	-0.0105	-0.0458	0.19048	-0.0642	-0.0429	0.06686	-0.1416	-0.0713	-0.047	-0.0424
NM_139110	Gpr116	0.19373	0.00755	-0.0496	-0.0523	0.69729	0.53222	-0.0948	0.67661	0.06119	0.05993	0.00835	0.10516	0.05548	-0.049
NM_181770	Gpr119	-0.1084	-0.0766	0.00105	0.09042	-0.0616	-0.1573	-0.1462	0.12685	-0.0209	-0.1036	0.05441	-0.1493	0.31997	0.13691
NM_001047088	Gpr120_predicted	0.10465	0.05796	0.06669	0.13703	0.21228	0.14809	0.08513	0.06303	0.08393	-0.045	0.06915	0.04546	0.01894	0.02391
NM_001107559	Gpr123_predicted	0.00184	0.08804	0.01495	-0.1131	-0.0501	-0.0148	0.09799	0.03671	-0.0309	0.15304	-0.0651	-0.0828	0.03059	0.02158
XM_240449.3	Gpr124_predicted	0.18736	0.01612	0.10074	0.06256	0.26034	0.15366	0.15329	0.29522	0.02024	0.16231	0.11136	-0.0465	0.01956	0.27261
NM_001107218	Gpr125_predicted	-0.1829	-0.197	-0.052	0.20079	-0.0852	-0.0553	0.07562	-0.236	0.43873	0.09404	0.07895	0.58364	0.14229	0.14403
XM_001071417.1	Gpr126_predicted	0.09066	0.54351	0.67228	0.37617	0.465	0.43165	0.8685	0.56228	0.2543	0.57366	0.47327	-0.2881	0.66872	0.79851
NM_001107098	Gpr128_predicted	-0.1891	-0.332	-0.1912	-0.2988	0.06633	-0.1618	-0.3019	-0.0465	-0.0648	-0.1142	-0.0523	-0.3302	-0.0446	-0.3384
NM_001170595	Gpr132_predicted	0.08961	-0.1677	0.03854	0.20648	0.08347	0.19852	0.18958	-0.1002	-0.0814	-0.0136	0.01366	-0.0212	-0.1173	-0.0599
NM_181771	Gpr135	-0.094	-0.1279	-0.086	-0.0784	-0.2345	0.07366	-0.0584	0.24342	-0.1542	-0.0698	-0.1932	0.0138	0.00243	-0.3343
NM_001024241	Gpr139	0.14498	-0.029	0.0352	-0.1793	-0.1504	0.01653	0.00786	0.08829	0.04124	-0.0064	-0.116	0.09567	-0.1089	-0.1142
NM_001024241	Gpr139	-0.0307	-0.032	-0.0377	0.02762	0.07148	-0.0011	-0.035	0.10395	0.01792	0.03733	0.0403	-0.023	0.02976	-0.0461
NM_001106958	Gpr143_predicted	-0.0624	-0.0494	-0.1487	0.02137	-0.0171	-0.0052	0.05756	-0.1514	-0.064	-0.1179	-0.1701	-0.0369	-0.0273	-0.1118
NM_138891	Gpr149	-0.1026	-0.1251	-0.1941	-0.1093	-0.0995	-0.1672	-0.1683	-0.179	-0.1369	-0.0809	-0.0363	-0.0347	-0.0657	-0.1116
NM_001105890	Gpr15_predicted	0.01108	0.04362	0.05603	-0.0406	-0.0156	-0.0028	-0.0848	0.02068	0.05527	0.05624	0.04212	0.03006	0.27818	0.10805
NM_181633	Gpr151	-0.2023	-0.1506	-0.1425	-0.1571	-0.2126	-0.2336	-0.1391	-0.1301	-0.1946	-0.2166	-0.1781	-0.2437	-0.222	0.02946
NM_001107811	Gpr155_predicted	-0.0168	-0.0498	-0.0148	-0.0938	0.02936	-0.0693	-0.0541	-0.0801	-0.0861	0.10116	-0.0134	-0.0194	0.11926	-0.0811
NM_153295	Gpr156	0.07573	-0.0406	-0.0321	0.02119	-0.0624	0.04633	-0.016	-0.078	-0.0653	0.06663	-0.0601	-0.0307	-0.0818	-0.0679
NM_001012107	Gpr157_predicted	0.13929	0.1682	0.22167	0.08525	-0.0051	0.02277	0.08832	0.1113	0.04614	0.02774	0.12798	0.09815	0.08189	-0.0227
NM_001108646	Gpr162_predicted	-0.1793	-0.1908	-0.1432	-0.428	0.00926	-0.2937	0.13035	-0.2886	0.06555	-0.0878	-0.1491	0.07667	-0.3192	-0.24
NM_022255	Gpr173	0.00368	-0.027	-0.0321	0.04226	-0.0021	-0.1162	-0.1011	-0.1644	-0.1004	-0.1083	-0.0868	-0.0755	-0.1966	0.11784
XM_342493.3	Gpr176	0.36162	0.65967	0.41354	0.9183	0.06923	0.16328	0.53313	0.06585	0.75671	0.62261	0.52936	0.70202	0.14508	0.38887
NM_199408	Gpr177	-0.0289	0.36978	-0.7355	-0.6672	0.22324	0.05974	0.06692	0.4335	0.16253	0.22728	0.14464	-0.099	-0.2114	-0.351
NM_001006994	Gpr180	-0.0634	-0.2268	0.08505	-0.0151	0.06142	0.04922	-0.1368	-0.0215	-0.1445	-0.3154	-0.2896	0.03375	0.24817	0.31255
NM_080579	Gpr19	0.33782	-0.4207	0.35735	0.56954	-0.2199	-0.3029	-0.1372	-0.0194	-0.196	-0.2773	-0.3204	0.08813	0.18035	0.34033
NM_001108836	Gpr2_predicted	0.14202	0.0365	0.15805	0.04702	0.26759	0.05194	0.1234	0.08337	0.16693	0.16341	0.3006	0.15236	0.22303	0.16353
NM_022216	Gpr20	0.1027	0.07489	0.17086	0.04635	0.00124	0.04352	0.21646	0.11677	0.20348	0.09466	0.17191	0.03514	0.12342	0.0132
NM_001107841	Gpr21_predicted	-0.0371	-0.1745	-0.4494	0.13537	-0.0174	-0.4172	0.01257	0.08806	-0.115	-0.1931	0.10031	-0.4361	-0.2499	-0.074
NM_001106722	Gpr22_predicted	0.00902	0.00171	0.00509	-0.0067	0.13086	0.19283	-0.0849	-0.0082	0.01189	-0.0087	-0.0351	0.0214	0.02468	-0.0044
XM_001064036.1	Gpr25_predicted	-0.0071	-0.0495	-0.0886	0.01996	-0.0793	0.34767	-0.1555	0.09594	0.24172	0.12911	-0.0078	0.14414	0.05705	0.13383
NM_023099	Gpr27	0.00818	-0.2052	-0.1073	-0.0514	0.06639	-0.0152	-0.0689	-0.1637	-0.0477	0.02465	-0.1162	0.00324	0.05375	-0.1754
NM_153727	Gpr3	0.23722	-0.0201	0.08196	-0.107	-0.0021	-0.0876	-0.0212	0.04106	-0.0345	-0.0649	0.04218	0.1066	0.21612	0.03603
NM_001169132	Gpr31_predicted	0.13088	0.04816	0.11465	0.19827	0.09482	0.22018	0.30956	0.13096	0.3344	0.26583	0.16076	0.31007	0.08781	-0.0018
NM_001031823	Gpr33_predicted	-0.0477	0.14925	-0.0413	0.00463	-0.0016	-0.0287	0.06447	0.02332	0.03281	0.06582	-0.0258	0.05919	0.07235	-0.0154
NM_001037359	Gpr35_predicted	0.19354	0.2119	0.04446	0.12218	-0.0155	-0.0832	0.18033	0.25314	-0.0461	0.25571	0.07305	0.18965	0.13456	-0.0023
NM_057201	Gpr37	-0.06	-0.0101	-0.0663	0.07164	-0.0616	-0.1082	-0.0839	0.12553	-0.1612	-0.0761	-0.092	-0.0795	0.00915	-0.0118
NM_145784	Gpr37l1	-0.2503	-0.1397	0.02293	-0.1201	-0.2405	-0.1976	-0.1393	-0.2762	-0.2487	-0.0724	-0.3532	-0.3135	-0.1739	-0.0747
NM_001100943	Gpr39_predicted	0.19227	0.37133	0.01459	0.25078	0.28562	0.13682	0.17665	0.36412	0.01802	0.24775	0.30921	0.34918	0.31283	0.22974
NM_001025680	Gpr4	0.139	-0.0232	0.13673	0.16908	0.0857	0.10446	0.12793	-0.0273	0.06456	0.03242	0.19326	0.07243	0.27718	-0.081
NM_001108912	Gpr41_predicted	-0.0942	-0.0254	-0.193	0.03594	-0.1426	0.00998	-0.2367	0.02798	-0.1261	-0.0531	-0.101	0.15564	-0.1643	0.02669
NM_001012070	Gpr44	-0.0506	0.09832	-0.0763	0.27094	0.01213	0.0385	-0.0118	-0.0567	0.10962	-0.0565	0.05936	0.00342	0.07186	0.16072
NM_001106906	Gpr45_predicted	-0.0568	0.10226	0.04157	0.06427	0.01047	0.06227	0.16962	-0.0962	0.16864	0.00135	-0.0158	0.27569	-0.0919	0.03537
XM_346374.3	Gpr50	-0.1733	-0.142	-0.0745	-0.2104	-0.0323	-0.0097	0.01706	0.21217	-0.2003	0.01537	-0.1468	-0.1013	-0.1087	-0.0189
XM_576605.2	Gpr55_predicted	-0.0719	-0.0237	-0.0686	-0.0547	-0.1331	-0.0673	-0.0737	-0.2236	-0.0934	-0.1407	-0.2102	-0.0553	-0.1335	0.11413
NM_152242	Gpr56	0.4576	0.01816	-0.0766	0.14443	1.8256	1.7783	0.36136	1.9436	-0.0387	0.24274	0.20525	0.29152	0.04897	0.04819
NM_001107715	Gpr61_predicted	0.07153	0.04705	-0.0108	0.05033	0.08504	0.26566	0.19474	0.13644	0.08866	0.13126	0.20138	0.14321	0.12262	0.16381

NM_001106640	Gpr63_predicted	0.1215	0.03913	0.0558	0.16689	0.25014	0.08602	0.06909	0.05924	0.04675	0.00957	-0.0623	0.06442	0.05205	0.11522
NM_181366	Gpr64	-1.4386	-1.2901	-1.3495	-1.3402	-1.2176	-1.3451	-1.4743	-1.4386	-1.4169	-1.1268	-1.5634	-1.2889	-1.5064	-1.244
NM_001106751	Gpr65_predicted	0.23933	0.0425	-0.0389	0.06776	0.16393	0.10685	0.34074	0.2397	-0.0099	-0.0728	-0.0387	0.06957	0.06158	0.10118
NM_001108049	Gpr68_predicted	0.09026	-0.0098	0.0045	0.13584	0.15622	-0.0395	0.01569	0.13151	0.08878	-0.0033	0.05249	-0.0037	0.09451	0.05113
NM_022254	Gpr85	-0.2624	-0.289	-0.2117	-0.0822	-0.1796	0.00529	-0.1444	-0.2627	-0.1786	-0.1953	-0.34	-0.3289	-0.1412	-0.2345
NM_001107677	Gpr87_predicted	0.01373	-0.003	0.13727	0.06504	-0.0226	0.19687	-0.0779	0.07394	0.12336	0.23995	0.19147	-0.0207	0.07312	0.04503
NM_031696	Gpr88	0.04208	1.7652	-0.1323	-0.02	-0.1811	-0.0706	0.61465	0.03269	1.3888	1.6198	1.4836	0.10656	-0.2997	-0.3143
NM_001139486	Gpr89_predicted	-0.1248	-0.1047	-0.0309	-0.0395	-0.041	-0.0631	-0.0537	-0.0995	-0.0972	-0.1104	-0.1212	-0.1056	-0.1845	-0.1042
NM_001139486	Gpr89_predicted	0.17615	-0.0451	0.11443	-0.1864	-0.2091	-0.1212	-0.3622	-0.3239	-0.3407	0.00773	-0.2227	0.17864	-0.1266	-0.1367
XM_226243.4	Gpr97_predicted	-0.0088	0.07722	0.06857	-0.0918	0.07546	-0.0423	0.01993	0.03953	0.11703	0.193	-0.039	-0.0247	0.07651	-0.0276
NM_134386	Gprasp1	0.03336	0.23654	0.13788	0.34629	0.03209	-0.1935	0.31504	0.11709	0.35752	-0.0022	0.19414	0.08532	0.15448	0.05662
NM_001079890	Gprc5a	-0.2154	-0.4164	-0.5251	-0.4782	0.47353	0.36961	-0.1698	0.35374	-0.6071	-0.4575	-0.4254	-0.6474	-0.7432	-0.5338
NM_001106304	Gprc5b_predicted	0.30333	0.32136	0.09345	0.60464	0.28512	0.4182	0.21254	0.49671	0.12472	0.2605	0.19898	0.0375	0.09509	-0.0689
XM_001081664.1	Gprc5c	-0.0664	0.11244	-0.131	-0.1446	0.08211	-0.0209	-0.1566	0.00398	0.13437	-0.2299	0.03075	0.15292	0.2219	0.15308
XM_001059232.1	Gprc6a	-0.4936	-0.4913	-0.3625	-0.4145	-0.2071	-0.4106	-0.5314	-0.4374	-0.3944	-0.4749	-0.5102	-0.6527	-0.4438	-0.5622
XM_001070221.1	Gprin1_predicted	-0.2428	-0.0594	-0.1108	-0.2515	-0.0071	-0.2816	0.07029	-0.2311	-0.1414	-0.2191	0.00425	-0.0139	-0.2193	0.16504
NM_001112713	Gprk6	-0.192	-0.388	0.22425	-0.4151	-0.0855	-0.2655	-0.3862	-0.2371	-0.3598	-0.2604	-0.0783	0.37505	0.24612	0.2591
NM_001017477	Gps2	-0.1411	0.18469	-0.0906	-0.1785	-0.0577	0.19625	-0.1764	-0.1216	0.00682	-0.0856	0.01141	0.15249	0.14862	0.09157
NM_001107013	Gps2_predicted	-0.2763	-0.36	0.30928	-0.0322	-0.3371	-0.3612	0.08541	-0.7575	-0.5549	-0.6891	-0.2158	-0.2239	-0.6232	-0.4234
NM_144745	Gpsm1	-0.1723	-0.389	-0.2357	-0.2104	-0.3892	-0.1134	-0.1571	-0.3228	-0.2001	-0.1356	-0.3087	-0.1585	-0.3328	-0.2096
NM_001003974	Gpsm3	-0.082	0.0414	0.05098	-0.0759	0.10112	0.0592	0.18	0.0222	0.05365	-0.0003	-0.1494	0.05333	-0.0484	-0.1231
NM_138549	Gpsn2	0.4546	0.14488	0.18921	0.16743	-0.2879	-0.3173	0.18148	-0.2458	-0.1285	0.24615	0.24509	-0.0095	0.12866	0.21176
NM_031039	Gpt1	-1.0432	-0.5928	0.11149	0.11937	-1.2136	-1.0993	-0.0101	-0.7783	-0.3404	-0.7109	-0.1645	-0.176	-1.0846	-0.898
NM_030826	Gpx1	0.83934	0.64255	-0.1829	0.74852	0.66814	0.67318	0.17915	0.54948	0.69159	0.74713	0.79535	0.17087	-0.1613	0.18061
NM_183403	Gpx2	0.14011	0.04191	-0.0355	-0.0507	0.01687	0.46677	0.08363	0.01029	0.02701	0.05161	-0.0277	0.15797	0.12877	0.1229
NM_022525	Gpx3	0.26573	0.15465	0.24598	0.14863	0.04876	-0.0347	0.06364	0.22006	0.15027	0.0589	0.09847	0.15618	0.05111	0.06184
NM_017165	Gpx4	0.04477	0.00295	0.06901	0.09449	0.31253	0.15639	0.47536	0.18284	0.14905	0.01399	0.2335	0.25106	0.22735	0.2148
NM_001105738	Gpx5	0.08433	0.07738	-0.0492	0.03826	0.10771	-0.0118	0.16438	0.12191	0.01719	0.35091	0.22565	0.37155	-0.0465	0.01246
NM_147165	Gpx6	0.20814	0.09637	-0.1087	-0.0785	0.31548	0.02982	-0.0085	0.00432	0.08903	0.17976	-0.0187	-0.0189	0.15246	0.13999
NM_001106673	Gpx7_predicted	-0.9334	-0.5993	0.17595	-1.2826	-0.7217	-0.895	-0.5657	-0.7555	0.0416	-0.1656	-0.5311	0.60039	0.20419	0.48285
NM_001014160	Gramd1a	-0.4415	-0.5045	-0.0646	0.36802	-0.514	-0.4056	-0.1323	-0.4341	-0.4429	-0.596	-0.2868	-0.3595	-0.7016	-0.8221
XM_001059472.1	Gramd1b_predicted	2.0106	1.0901	0.63809	0.84394	2.2102	2.205	1.134	2.2363	1.069	1.0023	0.95937	0.34903	0.58847	0.64097
NM_001025749	Grap	-0.031	-0.0415	0.14526	0.04815	0.01408	-0.0368	0.00946	-0.0228	0.17656	0.06022	0.02487	0.08751	0.0868	0.03355
NM_001034944	Grap2	-0.0364	0.02573	-0.0052	-0.0559	0.02584	-0.0462	-0.1477	-0.0204	-0.1458	-0.0026	-0.1262	-0.0368	-0.003	0.10044
NM_138894	Grasp	0.01168	-0.1739	-0.0345	-0.1437	-0.1281	-0.2398	-0.1155	-0.1157	-0.3153	-0.0299	-0.0314	0.08807	-0.2381	-0.2333
NM_030846	Grb2	-0.2471	-0.0494	0.08558	0.08036	-0.2381	-0.1175	0.0561	-0.1945	0.17109	0.10356	-0.0108	-0.2009	-0.1448	0.14314
NM_053403	Grb7	-0.0729	-0.1765	-0.0868	-0.1436	-0.1823	0.16011	-0.1925	0.02961	-0.062	-0.0857	-0.2706	-0.2178	-0.1065	-0.2512
NM_001012189	Grcc3f_predicted	-0.121	-0.1397	-0.0337	-0.0827	-0.1168	-0.1137	-0.0987	-0.1781	-0.1454	-0.2503	-0.1146	-0.095	-0.2689	-0.1555
NM_001012189	Grcc3f_predicted	-0.2784	-0.2017	0.04466	-0.116	-0.1409	0.02219	-0.0076	-0.1465	-0.1342	-0.4267	-0.3181	0.02329	-0.158	-0.0899
NM_019282	Grem1	0.87973	1.8976	1.4633	0.7204	0.1023	-0.1488	2.7557	0.1573	1.5023	1.7107	1.6877	1.0109	1.4648	1.4822
NM_001105974	Grem2_predicted	-0.0191	0.13389	0.04571	0.1155	0.2478	0.05346	0.12761	-0.0381	0.08787	-0.0244	0.02231	-0.0984	0.07913	0.17469
NM_001083811	Gria2	0.08282	0.07015	-0.0609	0.12599	-0.0079	-0.1154	-0.0367	-0.1107	-0.0654	-0.0067	-0.0895	-0.0533	-0.111	-0.064
NM_032990	Gria3	0.1291	0.14692	0.27972	0.39374	0.35017	-0.0944	0.16574	0.20559	0.13101	0.09283	0.27821	0.26979	0.22002	0.13283
NM_017263	Gria4	0.17638	0.11374	0.09694	0.18057	0.16519	0.27679	0.22036	0.30904	0.13648	0.09296	0.17302	0.22394	0.17261	0.19422
NM_024378	Grid1	-0.1903	-0.0482	-0.1674	-0.1107	-0.0398	0.02338	-0.0642	-0.0426	-0.2814	-0.0027	-0.3301	-0.17	-0.146	-0.1385
NM_024379	Grid2	-0.3612	-0.2444	-0.3328	-0.1	0.12041	0.09368	-0.193	-0.1714	-0.2972	-0.0998	-0.2374	-0.2698	-0.0897	-0.2261
NM_001105910	Grid2ip_predicted	-0.1418	0.0013	-0.0337	-0.0685	-0.0874	0.1208	-0.1126	-0.0323	-0.2243	-0.2543	-0.0206	-0.0126	-0.0265	-0.1343
NM_017241	Grik1	-0.2382	-0.1481	-0.2723	-0.1576	-0.1106	-0.1528	-0.1514	-0.1535	-0.0541	-0.167	-0.318	-0.1254	-0.2635	-0.0709
NM_019309	Grik2	0.05904	-0.0445	0.16938	-0.0171	-0.0558	0.02748	0.10128	0.06962	0.24034	0.2714	0.15404	0.13987	0.12972	0.09621

NM_001112716	Grik3	0.03702	0.13379	0.0259	0.11959	0.02473	0.23004	0.04884	0.0833	0.09583	0.16928	0.03338	0.1032	0.25471	-0.0449
NM_012572	Grik4	-0.0257	-0.064	0.20572	0.57666	0.09917	0.02576	0.40535	-0.0699	-0.0063	0.19787	0.02505	0.12637	0.14598	0.02154
NM_017262	Grik5	-0.2961	-0.3299	-0.2896	-0.2755	-0.1324	-0.1189	-0.2578	-0.3785	-0.2075	-0.2723	-0.1216	0.0049	-0.3108	-0.3295
NM_017262	Grik5	-0.1207	-0.0649	0.10602	-0.0308	0.08297	-0.0554	0.03425	0.06722	-0.1107	0.09369	-0.2019	0.07112	-0.1308	-0.0878
NM_017010	Grin1	-0.0402	0.11137	0.01814	0.14283	-0.1368	0.24453	0.3679	0.21141	0.14804	0.27491	0.23483	0.01924	0.13012	0.02596
NM_012573	Grin2a	-0.0786	-0.0803	-0.0381	0.24003	-0.0205	-0.0018	0.0168	-0.0064	-0.1235	0.10338	0.06316	-0.0302	-0.1078	-0.0479
NM_012574	Grin2b	-0.1171	-0.0334	-0.0169	-0.0603	-0.1031	-0.0485	-0.0588	-0.0558	-0.079	-0.0625	0.00667	-0.0515	-0.1043	0.09608
NM_012575	Grin2c	0.20449	0.38485	0.14719	0.1842	0.00868	0.07395	0.10508	0.04861	0.01456	0.02332	0.17998	0.15402	0.25868	0.26322
NM_022797	Grin2d	-0.1198	-0.0812	-0.0296	0.16004	0.00585	0.02292	0.03966	-0.0507	-0.0773	0.01155	-0.0046	0.13055	-0.054	-0.0812
NM_133308	Grin3b	-0.076	0.10629	0.02176	0.01083	0.1853	0.17705	0.14428	0.09821	0.05907	0.18486	0.29312	0.15228	0.28867	0.20372
NM_153308	Grina	0.79706	1.5049	1.2237	1.4479	1.1859	1.4055	1.2595	1.0899	0.90272	1.2413	1.2996	0.43193	0.48363	0.90107
NM_183402	Grinl1a	-0.0479	-0.0216	-0.4243	-0.4026	0.08162	-0.0668	-0.1065	0.04978	-0.2195	-0.2027	-0.2459	-0.1313	-0.0211	0.1207
NM_032069	Grip1	0.53323	0.48868	0.60667	0.91573	-0.0137	-0.3026	1.104	0.15247	0.37048	0.69409	0.52543	-0.4634	0.40929	0.33851
NM_138535	Grip2	0.10553	0.09209	-0.0002	0.10199	1.2305	0.91118	0.11939	1.179	-0.0554	0.61012	0.06351	0.04744	0.03543	0.04792
NM_053807	Gripap1	-0.0116	-0.0587	0.34662	0.26605	-0.0321	0.32762	0.12546	-0.4006	-0.1822	-0.0978	-0.0514	-0.1741	-0.1082	-0.2531
NM_031096	Grk1	-0.1551	-0.0896	-0.1346	-0.2255	0.06531	-0.1706	-0.0039	-0.1513	-0.113	-0.1726	-0.1935	0.0647	-0.1509	-0.1789
NM_022928	Grk4	-0.0373	-0.548	0.01108	0.09204	-0.4762	-0.5079	-0.0292	-0.3278	-0.1823	-0.4455	-0.2184	0.31194	-0.5116	-0.3648
NM_030829	Grk5	0.39936	0.38249	0.08577	0.31967	1.229	1.1712	0.69501	1.3783	-0.0855	0.38075	0.16241	-0.4704	0.01439	-0.0033
XM_224781.3	Grlf1_predicted	0.04504	0.16749	0.07927	0.27766	0.0974	0.35828	0.00674	-0.106	0.23261	0.19513	-0.0183	0.0281	-0.0663	0.09487
NM_001114330	Grm1	0.69852	0.54697	0.12033	0.19183	-0.0258	-0.0008	0.52855	-0.0756	0.39538	0.61487	0.35345	0.13987	0.06901	0.12743
NM_001105711	Grm2	0.05182	0.08836	0.14687	-0.0103	-0.0428	0.15762	0.00408	-0.0652	-0.0457	-0.1206	0.06356	0.08312	-0.099	-0.0755
NM_022666	Grm4	-0.3137	-0.2182	-0.3333	-0.0775	-0.2282	-0.3797	-0.177	-0.3257	-0.3214	-0.1856	-0.2995	-0.2807	-0.1997	-0.3051
NM_017012	Grm5	-0.0507	0.06639	-0.0784	0.01001	0.20391	-0.0054	0.14115	0.01748	0.4568	0.22713	0.00973	0.20348	0.11192	0.25429
NM_031040	Grm7	-0.0127	-0.0145	-0.0237	0.08249	0.11517	0.20888	0.01581	-0.025	0.15105	0.03854	0.0425	0.05211	0.00415	-0.0866
NM_022202	Grm8	0.00727	0.01169	-0.0247	0.11134	0.00163	-0.0235	0.02767	0.04597	8E-06	-0.0383	0.15214	0.01572	-0.0205	-0.029
NM_017113	Grn	0.67521	0.83343	0.08052	0.68118	0.55799	0.44411	0.59141	0.36304	0.41461	0.66936	0.68344	-0.1321	-0.2889	-0.1496
NM_133570	Grp	-0.2981	-0.1828	-0.1966	-0.019	-0.0806	-0.4009	-0.0923	-0.1817	-0.2166	-0.2334	-0.0987	0.02204	-0.1767	-0.1783
NM_181440	Grpca	0.1871	0.19247	0.05055	0.2429	0.04576	0.23368	0.16034	0.26724	0.08604	0.14209	0.40438	0.05376	0.2086	0.33965
NM_024487	Grpel1	0.95461	0.17669	0.2721	1.0401	1.2999	1.6192	0.67629	1.259	0.36165	0.16634	0.20946	0.81519	0.53192	0.36863
NM_012706	Grpr	-0.071	0.13242	0.06676	-0.0622	0.10386	0.07856	0.22154	0.06249	0.20244	0.23335	0.07325	0.23258	0.16974	0.06367
NM_001100890	Grsf1	-0.1962	0.30921	-0.3246	-0.0453	0.06559	-0.0073	-0.1499	0.26355	0.11159	0.14979	0.17975	0.0003	0.07643	-0.0726
NM_001031813	Grtp1_predicted	0.04432	-0.1781	0.01797	-0.4278	-0.2525	-0.086	0.03299	-0.3216	-0.1318	0.16077	-0.1009	0.06589	0.0055	0.18314
NM_138856	Gs3	0.07283	-0.128	0.15547	-0.0676	0.20548	0.45618	0.17808	0.37973	-0.0141	0.0191	0.00253	-0.3861	0.17147	0.19806
NM_153467	Gsbs	-0.086	-0.0582	-0.0666	-0.136	-0.1268	-0.2455	-0.1721	-0.1088	-0.1097	-0.146	-0.0591	0.09394	-0.1101	0.32629
XM_001067894.1	Gsc	-0.0229	0.01383	0.09815	0.04071	0.23491	0.08752	0.05932	0.18602	-0.0608	-0.0372	0.07367	0.15669	0.28222	0.26769
NM_001108846	Gscl_predicted	0.39896	0.04634	0.13679	0.0804	0.10101	-0.0719	0.01499	0.16604	-0.1271	-0.0167	0.38003	0.34239	0.08072	-0.0559
NM_001130553	Gsdmdc1_predicted	-0.6929	-0.0186	-0.0811	-0.789	-0.2887	0.16048	-0.4476	-0.1295	0.13179	0.03997	-0.1952	0.47659	-0.0361	-0.1038
NM_001013166	Gsg1	-0.063	-0.1702	-0.2103	-0.0874	-0.1206	-0.0494	-0.1274	0.00761	0.04392	-0.1675	-0.0018	-0.0121	-0.1001	-0.0966
XM_001069419.1	Gsh1_predicted	0.02886	0.11855	0.00629	0.02643	0.02023	0.03897	0.07953	0.05516	0.10663	0.03341	0.15844	0.13172	0.08528	0.01034
NM_001137563	Gsh2_predicted	-0.0574	0.07009	0.10694	0.03404	-0.0345	-0.0937	-0.0092	0.02873	-0.0388	0.10856	-0.009	-0.158	-0.0492	-0.0916
NM_017344	Gsk3a	0.1138	-0.0238	-0.0376	0.02223	0.15045	-0.1507	0.03132	0.22534	0.03581	-0.1676	-0.0705	-0.143	-0.0308	0.28158
NM_032080	Gsk3b	0.02888	-0.0866	0.10249	0.1719	0.26847	0.09398	0.051	0.22555	0.1967	0.09739	-0.0028	-0.1324	0.24426	0.26402
NM_001004080	Gsn	-0.3299	0.18641	0.00682	-0.1053	-0.3435	-0.2077	-0.135	-0.3826	-0.0448	-0.0266	-0.0746	-0.1708	-0.9653	-1.0181
NM_001003978	Gspt1	-0.3615	-0.5174	-0.1475	-0.3689	0.0441	0.40539	-0.497	-0.052	-0.5259	-0.3981	-0.4096	-0.19	0.17866	0.01469
NM_053906	Gsr	0.49168	0.29768	-0.2345	-0.0667	0.33237	-0.0734	-0.2828	0.35643	0.02668	0.32694	-0.0456	-0.4168	0.19508	0.2332
NM_012962	Gss	0.18147	0.04104	0.33144	0.01806	0.69288	0.86306	0.2272	0.63572	-0.1162	-0.0851	-0.1484	0.17068	0.2996	0.21288
NM_017013	Gsta2	0.69686	-0.1011	-0.1177	0.02673	-0.0842	-0.0685	0.00944	0.0124	-0.1089	0.05127	0.0933	-0.0653	-0.0594	-0.0548
NM_031509	Gsta3	0.18182	0.01448	-0.0611	-0.0559	0.00279	-0.1007	-0.1309	-0.065	-0.0266	-0.0854	-0.0437	-0.0608	-0.0845	-0.0693
NM_001106840	Gsta4	0.02021	0.15226	-0.0809	0.72465	-0.3833	-0.4518	0.52781	-0.3002	0.22726	-0.1105	-0.0216	0.24991	0.16593	0.26695

NM_181371	Gstk1	0.57379	-0.0765	-0.133	0.79811	0.42915	0.88503	-0.2466	0.40292	0.33625	0.00512	0.201	0.49921	-0.0038	0.19541
NM_017014	Gstm1	0.68673	0.14716	-0.2134	-0.2351	0.11857	0.59321	-0.4101	0.09895	0.51341	0.37594	0.38634	0.63895	0.44104	0.1504
NM_177426	Gstm2	-0.0279	0.40219	-1.4888	-1.035	-0.7259	-0.6434	-1.031	-0.9799	0.40724	0.13408	0.15918	0.26055	-0.3007	-0.4952
NM_031154	Gstm3	0.05961	-0.1909	-0.072	0.09513	0.03478	0.35704	-0.2182	-0.0231	-0.1112	0.02945	-0.0342	0.0375	-0.0991	-0.2686
NM_031154	Gstm4	-0.1145	0.08142	-0.0409	-0.0474	0.00369	-0.1533	-0.0306	-0.2061	-0.2117	-0.1723	0.26226	0.07513	0.06723	-0.2765
NM_172038	Gstm5	0.10112	-0.2071	-1.2894	-0.7621	-0.6511	-0.3894	-1.1795	-0.7371	-0.406	-0.3436	-0.3374	0.04187	-1.015	-1.1572
NM_001106464	Gstm6_predicted	0.02333	-0.114	-0.1248	-0.0966	-0.0108	-0.2296	-0.1555	0.0745	-0.2938	-0.1067	-0.1104	-0.2637	-0.0365	0.02816
NM_001007602	Gsto1	0.46551	-0.2492	0.46668	0.07819	-0.0282	0.20836	0.05572	0.03224	0.02213	-0.1252	0.15439	0.23786	0.27951	0.09726
NM_001012071	Gsto2	0.28995	0.10603	0.27269	0.75788	0.25993	0.41379	0.34402	0.03791	-0.0975	0.27623	0.17624	0.2202	-0.1299	0.12548
NM_012577	Gstp1	0.89602	0.96431	-0.5747	0.24234	0.04196	0.16514	-0.7803	0.00242	0.84079	0.83463	0.63586	0.6973	-0.2516	-0.1857
NM_012577	Gstp2	1.0393	0.81316	-0.2432	0.25864	0.29946	0.83644	-0.5165	0.30404	1.2152	0.81214	1.0243	1.1706	-0.1436	-0.4212
NM_053293	Gstt1	0.5085	-0.0986	-0.0112	0.17962	0.27835	0.67232	-0.0716	0.38196	0.03802	-0.1388	0.32066	0.43832	0.14561	-0.1611
NM_012796	Gstt2	0.71718	0.30715	-0.5814	0.55552	0.99093	1.2183	-0.2879	0.9753	0.44545	0.3254	0.22519	0.18116	-0.833	-0.8824
NM_001024274	Gtdc1	-0.0059	-0.2902	-0.0641	0.05456	0.06462	-0.1919	-0.1522	-0.148	0.10583	-0.1461	-0.1399	0.52079	-0.0379	0.02439
NM_022208	Gtf2a1	-0.2323	-0.0948	-0.0283	-0.1037	0.12906	-0.0304	0.00705	0.00193	-0.1368	0.42629	0.09408	0.0409	0.22161	0.21597
NM_053345	Gtf2a2	-0.129	-0.0028	0.05668	0.1925	-0.2876	-0.2035	-0.0105	-0.1279	0.13514	0.04002	0.09447	0.08206	-0.0503	-0.056
NM_031041	Gtf2b	0.79956	0.54274	0.21573	0.30833	0.38379	0.44001	0.69258	0.31289	0.24392	0.51932	0.42021	0.331	0.39127	0.40453
NM_001100556	Gtf2e1	0.4226	0.22117	0.01255	0.2485	0.24836	0.12025	0.29397	0.31728	0.15512	0.21694	0.11304	0.2584	0.4485	0.58396
NM_001107318	Gtf2e2_predicted	0.46625	0.07768	-0.0031	0.0362	0.1715	0.11124	0.18951	0.31671	0.06386	0.09836	-0.0322	0.04975	0.27625	0.14546
NM_001007711	Gtf2f1	0.17812	-0.0343	0.61433	-0.216	0.01783	0.49063	0.01736	-0.2842	-0.0002	-0.1054	-0.1707	0.22984	0.41127	0.26104
NM_001108485	Gtf2h1_predicted	0.02945	0.04527	0.04973	0.69212	0.1734	0.17964	0.2351	0.1556	0.06728	0.20953	0.11139	0.08245	0.25268	0.10709
NM_001077428	Gtf2h2_predicted	-0.0251	-0.1036	0.08523	0.35132	0.02842	-0.1348	0.44988	-0.0197	-0.1509	-0.0096	-0.0436	0.04452	-0.3608	-0.217
NM_001024236	Gtf2h3	-0.3771	-0.2629	-0.195	-0.7717	-0.6992	-0.5287	0.0321	-0.7084	-0.3596	-0.1086	-0.3295	-0.386	-0.322	-0.1902
NM_212501	Gtf2h4	0.24412	-0.1149	0.09124	-0.2935	0.18421	-0.1671	-0.2551	-0.2465	-0.3182	-0.1762	-0.1897	-0.1898	-0.2244	-0.1223
NM_001001512	Gtf2i	-0.6871	-0.2924	-0.2676	-0.4805	-0.5353	-0.6026	0.02925	-0.4527	-0.3725	-0.5965	-0.2834	-0.2315	-0.3239	-0.4602
NM_001001504	Gtf2ird1	-0.5797	-0.1332	-0.1942	-0.5194	-0.4077	-0.347	-0.2728	-0.5844	-0.0285	-0.1212	-0.0686	-0.0136	-0.2516	-0.3563
NM_001001504	Gtf2ird1	-0.7178	-0.4711	-0.3919	-0.7217	-0.6117	-0.5999	-0.4478	-0.6031	-0.0921	-0.3993	-0.3086	0.14546	-0.0553	-0.0651
NM_133541	Gtf3c1	-0.3043	-0.2182	0.18357	0.64421	-0.4014	-0.3705	-0.1551	-0.4204	-0.2828	-0.2746	-0.2662	-0.3429	-0.6486	-0.5722
NM_001079941	Gtf3c5_predicted	0.03512	-0.1132	0.04208	-0.1314	-0.1251	-0.474	0.1388	-0.1141	-0.0062	-0.2387	-0.0649	-0.2134	0.04847	0.32774
NM_001037978	Gtl3	0.57813	0.1754	0.32202	0.34432	0.25173	0.14733	-0.0081	0.28401	0.33112	0.25254	0.40086	0.12671	0.04736	0.24257
NM_001170541	Gtlf3b_predicted	0.06864	-0.2367	-0.1532	-0.3972	-0.2592	-0.1648	-0.3279	0.19782	-0.2783	-0.2336	-0.195	-0.108	-0.3426	-0.068
NM_001130496	Gtpbp1_predicted	-0.0666	0.06413	-0.2121	0.21803	0.05229	-0.0857	0.01449	0.15107	0.26613	-0.0707	0.01656	-0.003	0.48812	-0.0569
NM_001013225	Gtpbp2	0.01674	-0.0451	-0.021	0.71308	-0.0461	-0.0366	0.06785	-0.0045	-0.3486	-0.3385	-0.1845	-0.2347	-0.7501	-0.5089
NM_001011919	Gtpbp3	0.1061	0.1743	0.12911	-0.034	0.06037	0.17198	0.0485	-0.0451	0.01663	0.16819	0.07299	0.14758	-0.0519	0.11261
NM_001011919	Gtpbp3_predicted	-0.0297	-0.1261	-0.0941	0.03561	0.12072	0.15981	0.01314	-0.0039	0.03212	-0.0051	0.11554	-0.0322	0.14919	-0.0567
NM_001013924	Gtpbp5	-0.0587	-0.0043	-0.0347	0.10023	-0.1762	0.03176	-0.0644	0.08734	-0.3176	-0.156	-0.1219	-0.218	-0.2765	-0.0958
NM_001135840	Gtpbp6_predicted	-0.4101	-0.2537	0.28945	0.09297	-0.598	-0.5896	-0.0773	-0.6869	-0.3543	-0.442	-0.1574	0.00151	-0.4984	-0.511
NM_001025015	Gtpbp8	0.25897	-0.0383	-0.4757	-0.2262	0.07757	-0.0066	0.33922	0.05739	0.22145	0.11642	-0.0143	-0.0086	0.03882	0.13763
NM_001106887	Guca1a_predicted	-0.1657	-0.0321	-0.179	-0.0728	0.12963	-0.1673	-0.1241	0.03973	0.03186	-0.1381	-0.1097	-0.1587	0.09793	-0.1138
NM_001108198	Guca1b_predicted	-0.1213	-0.075	0.06101	-0.085	0.00227	0.01344	0.03723	-0.097	0.03709	-0.0997	-0.0191	0.0155	0.06745	-0.0882
NM_013118	Guca2a	-0.0774	-0.0627	-0.0407	0.2252	-0.1311	-0.0869	-0.0375	-0.0178	-0.0544	-0.0977	0.05601	-0.0987	-0.1297	-0.1175
NM_022284	Guca2b	0.21598	0.10718	0.27696	0.1675	0.29447	0.10719	-0.0945	0.53578	0.12291	-0.0905	0.16357	0.05808	0.17056	0.14425
NM_023956	Gucy1a2	0.21243	0.23655	-0.0635	0.14839	-0.0399	0.25814	0.22077	0.09648	0.14706	0.20382	0.18809	0.16965	0.10452	0.02766
NM_012770	Gucy1b2	-0.051	-0.2449	0.03032	0.19951	-0.0613	-0.062	-0.0812	-0.1545	-0.0547	-0.0958	0.01171	-0.0774	-0.2251	-0.138
NM_012769	Gucy1b3	0.11134	0.17632	-0.0691	0.02226	0.02622	0.04778	0.08036	0.12122	0.03265	0.07262	0.01687	0.00055	0.25444	0.07157
NM_013170	Gucy2c	0.09826	0.08987	0.11413	0.04991	0.09185	0.09704	0.13976	-0.0549	0.25771	0.02379	0.12843	0.17111	0.21314	-0.0039
NM_130737	Gucy2d	-0.0017	-0.1008	-0.2278	0.01645	0.01273	0.01015	-0.2235	0.15767	-0.0894	-0.1113	-0.1486	-0.224	-0.2144	-0.0241
NM_024380	Gucy2e	-0.0741	0.03431	-0.0946	0.03017	-0.1615	-0.1323	-0.1671	-0.1269	0.0065	0.10061	-0.1188	-0.1284	-0.141	-0.0578
NM_053831	Gucy2f	-0.1259	-0.1121	-0.2043	-0.0869	-0.1327	-0.0505	-0.1504	0.12702	-0.1437	0.00168	-0.1747	-0.1726	0.11114	-0.0859

NM_139042	Gucy2g	0.10078	0.09114	0.00386	0.04919	0.11578	-0.0033	0.04828	0.06716	-0.0145	0.17644	0.05973	0.01267	0.13647	0.107
NM_001013115	Guk1_predicted	0.2584	0.35775	-0.0086	0.1621	-0.1019	0.02279	0.1476	0.15065	0.01027	0.26749	0.19191	0.32751	0.16803	0.03145
NM_022220	Gulo	0.1415	0.03087	0.17234	0.06771	-0.0307	0.00678	-0.0049	0.11376	0.1222	-0.0216	0.0687	0.1184	0.22514	0.09966
NM_001013171	Gulp1	-0.1675	0.97293	0.02345	0.33522	-0.0857	0.06309	0.58607	-0.0385	0.94982	0.83751	0.92408	0.60871	0.0072	-0.2536
NM_001106868	Gup1_predicted	0.15696	-0.0236	0.13397	0.09005	0.10345	0.13508	0.06322	0.15037	-0.0294	0.12807	0.14553	0.23057	0.1399	0.06688
NM_017015	Gusb	-0.0282	0.34963	-0.1651	-0.5505	-0.1477	-0.4556	0.18697	-0.1359	0.28744	0.46203	0.27031	0.00195	-0.1746	-0.1849
NM_031043	Gyg1	0.65796	0.94993	-0.4584	0.2271	0.72863	0.58323	0.12094	0.5621	0.51397	0.90628	0.66766	-0.2261	0.12276	0.11315
NM_199107	Gyltl1b	0.00548	0.03463	0.05374	-0.0576	-0.0369	0.16606	0.19622	0.05665	0.0815	0.26847	0.08365	-0.002	0.04154	-0.0176
NM_001013233	Gypc	0.29247	1.1037	0.1096	0.26603	0.51791	0.28096	0.82829	0.57882	0.85846	1.0141	0.97982	0.39289	0.27221	0.06369
NM_001013233	Gypc	0.31159	0.0155	0.07419	0.12968	0.32178	0.1371	0.05454	0.05814	0.17761	0.07622	0.00231	0.06601	0.191	0.02338
NM_013089	Gys2	0.02489	-0.0933	-0.1399	-0.0365	-0.1528	-0.0548	0.03066	-0.129	-0.0908	-0.1392	-0.0009	-0.0047	-0.073	-0.0876
NM_153468	Gzma	0.16282	0.15707	0.06087	0.1518	0.26225	0.5114	0.17519	0.28454	0.19986	0.40436	0.18859	0.29364	-0.011	0.24264
NM_138517	Gzmb	-0.059	-0.0068	-0.0131	-0.0599	0.25298	-0.0016	-0.1511	-0.0477	0.12039	-0.1177	0.02403	-0.0841	0.13536	-0.199
NM_134332	Gzmc	0.01578	0.12041	0.14955	0.1218	0.03707	0.00554	-0.1154	-0.1132	-0.0209	-0.0521	0.32073	-0.1062	0.11151	-0.1153
NM_153466	Gzmf	0.31343	0.21598	0.24211	0.3874	0.3089	0.58294	0.34153	0.1852	0.2436	0.37682	0.32488	0.11865	-0.0246	0.24386
NM_017119	Gzmk	0.45121	0.37199	0.28084	0.58554	0.30061	0.39993	0.6404	0.48339	0.3929	0.22568	0.28588	0.14477	0.53357	0.26901
NM_001107789	H13_predicted	-0.5888	-0.6676	-0.4052	-1.1357	-0.8648	-0.9566	-0.1125	-0.7634	-0.3968	-0.7204	-0.4156	-0.5793	-0.0008	0.12907
XR_000314.1	H19	0.16555	-0.0013	0.04052	0.02553	0.15918	-0.0186	0.21751	0.03892	0.02716	0.06835	0.25916	0.01353	0.06044	-0.0122
XR_000314.1	H19	-0.2083	-0.1918	-0.1177	-0.1157	-0.1028	0.11261	-0.2804	-0.2687	-0.1928	-0.17	-0.2687	-0.139	-0.2035	-0.1414
NM_012578	H1f0	-0.2542	0.18473	0.02717	0.45701	-0.0915	0.10401	0.2106	-0.0631	0.4477	0.14596	0.29741	0.83467	-0.1183	-0.1779
NM_001024356	H1fnt	0.0294	0.02164	0.13476	0.03178	0.01596	0.07625	-0.0588	0.07809	0.30692	0.16532	-0.1153	0.07209	0.02488	0.154
NM_017182	H2afy	-0.9122	-0.305	0.02775	-0.2539	-0.0407	0.03524	-0.0958	-0.2809	-0.1317	-0.2763	-0.2747	0.3255	-0.192	-0.2734
NM_022674	H2afz	-0.2223	-1.1901	0.55655	-0.4205	-0.9854	-1.1546	-0.6671	-1.0973	-0.5141	-0.8426	-0.81	0.16834	0.2146	0.58289
NM_001002821	H2-T18	-0.1848	-0.1714	-0.1772	-0.0814	0.07959	0.45109	-0.1487	0.13038	0.0938	0.06815	0.19503	0.0849	-0.0473	0.18254
NM_053985	H3f3b	-0.4063	-0.1089	-0.7661	-0.4419	-0.8924	-0.9352	0.24046	-1.143	-0.382	-0.2963	-0.2029	-0.0574	-0.4641	-0.3572
NM_001106698	H6pd_predicted	-0.0503	0.0279	-0.0537	-0.1478	-0.04	-0.2375	-0.1246	-0.1885	-0.1231	0.21942	0.07963	-0.0812	0.09198	-0.1262
NM_020076	Haao	0.06525	0.22831	0.19021	0.10541	0.07241	0.02542	0.03901	0.05072	-0.0228	0.11298	0.21501	0.40521	0.24644	0.1851
NM_001001505	Habp2	0.07538	0.24571	0.14335	0.36725	0.13456	0.15299	0.02955	0.16773	-0.0422	0.06782	0.1503	-0.0185	-0.018	0.22948
NM_001079940	Habp4_predicted	-0.043	0.03738	-0.1135	0.23761	0.58761	0.57021	-0.2286	0.58221	-0.0212	0.08418	0.06627	-0.1214	-0.2699	-0.1424
NM_001108539	Hace1_predicted	-0.6232	-0.3627	-0.4037	-0.281	-0.4076	-0.4367	-0.3794	-0.3966	-0.4352	-0.531	-0.522	-0.4505	-0.5091	-0.411
NM_053493	Hacl1	-0.1159	0.04765	-0.5165	-0.5354	-0.6094	-0.3653	-0.6615	-0.6229	-0.2598	0.06086	-0.1738	-0.254	-0.4081	-0.4324
NM_057186	Hadh	0.50788	0.10355	-0.4862	-0.4892	0.114	0.35611	-0.4437	0.09041	0.04967	0.10823	0.33881	0.02931	-0.2499	-0.073
NM_130826	Hadha	0.41568	0.60181	-0.2452	-0.6321	-0.0808	-0.0958	-0.1725	-0.1345	0.74611	0.5855	0.58239	0.62372	-0.2604	-0.2424
NM_133618	Hadhb	0.29251	0.37597	-0.0915	-0.6473	-0.0778	-0.2675	-0.0921	-0.0803	-0.068	0.16985	-0.0237	-0.1774	-0.1995	-0.0428
NM_033349	Hagh	0.77625	0.33139	0.85173	1.0019	0.69633	1.2514	0.61306	0.6624	0.26716	0.51216	0.7071	0.56704	0.51285	0.57397
NM_001013114	Haghl	0.40527	0.04374	0.2038	0.06936	0.1976	0.19462	0.27923	-0.1169	-0.0681	-0.178	0.17369	-0.0479	-0.2326	-0.1372
NM_017159	Hal	-0.0252	-0.0023	-0.06	0.05252	-0.0263	0.02089	-0.0168	-0.1694	0.03533	0.16259	0.14366	-0.0255	0.08205	0.02537
NM_053469	Hamp	0.04471	-0.0798	-0.037	0.07206	0.06766	-0.0013	-0.0139	-0.0133	-0.0726	0.15669	0.28043	-0.0159	-0.1607	-0.0061
NM_021592	Hand1	-0.1372	-0.0453	-0.1317	-0.1928	-0.042	-0.1143	-0.1302	-0.1449	-0.2744	-0.0706	-0.1593	-0.1022	-0.2089	0.04039
NM_022696	Hand2	0.70617	0.35787	0.00809	-0.1913	0.50536	0.31044	0.48807	0.39999	0.44529	0.11659	0.5754	0.20919	0.65336	0.57245
NM_001107780	Hao1_mapped	-0.1959	-0.0756	-0.1404	-0.0183	-0.0469	0.02138	-0.1741	-0.0123	-0.0962	-0.0753	-0.2273	-0.1339	-0.1533	-0.1198
NM_032082	Hao2	0.32891	-0.0573	0.11486	0.09205	0.25872	0.5501	0.02549	0.15027	0.13176	0.01033	0.04027	0.35347	-0.0041	0.18848
NM_024133	Hap1	0.79562	0.287	0.07424	0.31915	0.83127	1.0764	0.11274	0.90369	0.29355	0.25121	0.26397	0.07711	-0.0175	0.11194
NM_024133	Hap1	-0.1539	-0.2042	-0.3729	-0.2502	-0.2311	-0.1737	-0.2645	-0.1107	-0.2458	0.21197	-0.3146	-0.2975	-0.2116	-0.2734
NM_019189	Hapln1	0.35387	1.188	0.06592	0.09871	-0.243	-0.3372	0.58097	-0.3478	0.5094	0.66884	0.77234	-0.0674	1.2586	1.1314
NM_022285	Hapln2	-0.1503	-0.0489	-0.0601	0.07354	0.31405	0.31214	0.08558	0.09484	0.21146	-0.0514	-0.0104	-0.1374	-0.0967	-0.1033
NM_001008559	Hapln3	0.02817	-0.3696	-0.1796	0.33808	0.33481	0.37123	-0.1229	0.37912	-0.4883	-0.0062	-0.2757	-0.2558	0.1956	-0.1876
NM_001108398	Hapln4_predicted	0.15702	0.38132	0.25037	0.1975	0.07551	0.21826	0.13862	0.10001	0.09108	0.12144	0.16944	0.38966	0.12052	0.26797
NM_001100540	Harpb64	0.14853	-0.2156	-0.3336	0.07593	0.17639	0.34674	-0.1083	0.03503	-0.0983	-0.0279	0.02381	0.23603	0.11112	0.16448

NM_001108594	Hars2_predicted	-0.0739	0.00024	-0.7011	-0.7632	-0.0631	-0.1363	-0.1859	-0.379	0.19088	0.34013	0.14304	0.06572	-0.2867	-0.24
NM_172323	Has1	-0.0922	-0.1315	-0.0184	-0.1694	-0.1298	-0.0145	0.14895	0.14109	0.1503	0.01545	0.01588	0.02672	0.21348	-0.0197
NM_013153	Has2	0.01338	-0.0729	0.10135	-0.0617	-0.0444	0.21812	0.04457	-0.0943	-0.0157	0.04439	-0.0706	-0.022	0.07695	0.00334
NM_172319	Has3	-0.1147	0.0638	-0.0933	0.02735	-0.043	0.26549	0.11305	0.06022	0.05378	0.0529	0.16924	0.22994	0.02424	0.1469
NM_001009657	Hat1	0.16191	0.15364	0.62352	0.06498	0.14589	-0.0094	0.00688	0.2689	0.13393	0.18607	0.15496	-0.1371	0.65982	0.68719
NM_001100762	Havcr2	0.50824	0.51414	0.16621	0.61464	0.7602	0.52785	0.82985	0.76898	0.65895	0.40722	0.48101	0.35896	0.65777	0.62799
NM_001100762	Havcr2	0.13012	0.06888	0.12148	0.15233	0.17467	0.15084	0.11981	0.27963	0.05716	0.10705	0.10033	0.02862	0.04174	0.16917
NM_013096	Hba-a2	-0.1429	-0.0131	-0.1831	-0.0818	-0.0499	-0.0514	-0.1608	-0.1165	-0.071	-0.1397	-0.1696	-0.1493	-0.1578	-0.0826
NM_033234	Hbb	0.02439	0.07636	0.35653	0.05233	0.11197	-0.0545	0.13744	0.11587	0.05501	-0.0156	0.0412	0.15016	-0.028	0.23344
NM_001008890	Hbe1_predicted	0.12773	-0.0516	0.06998	0.07621	0.1411	0.12823	0.04359	-0.0666	-0.006	-0.0966	0.23651	0.04366	0.27024	0.0866
NM_001024805	Hbe2_predicted	0.15103	0.1978	0.18747	0.03194	0.17471	0.00222	0.11562	0.0314	0.07287	0.11312	0.16823	0.2766	0.2302	0.00663
NM_012945	Hbegf	0.26122	0.15606	-0.0313	0.15714	0.51335	0.55198	-0.4221	0.68918	-0.0826	0.53952	-0.1181	0.07516	0.22132	0.23933
NM_172093	Hbg1	0.21694	0.18148	0.30126	0.38899	-0.0314	0.37588	0.36047	0.30052	0.31381	0.42668	0.06568	-0.0403	0.34708	0.05859
NM_001109278	Hbld1_predicted	0.20927	0.16624	0.01305	0.30625	0.03748	0.17431	0.18035	0.16893	0.12242	0.0944	0.03269	0.22032	0.11151	0.34934
NM_013221	Hbp1	-0.0497	0.37929	-0.5929	-0.0346	0.09333	-0.0586	0.23488	0.05768	-0.0385	0.18137	0.14975	-0.1938	-0.7143	-0.3275
XM_347266.2	Hbq1_predicted	-0.2571	-0.1855	-0.1116	-0.1876	-0.1371	-0.0666	-0.2088	-0.2299	-0.0701	0.11058	-0.1158	-0.174	-0.1529	-0.2106
NM_001011934	Hbs1l	0.13578	0.04253	0.04957	0.47088	0.2639	0.30792	0.20268	0.31667	-0.2469	0.06958	-0.0283	0.15936	0.09987	0.10815
NM_001011934	Hbs1l_predicted	0.11326	0.09765	0.11597	0.16539	0.08059	-0.0213	0.00898	0.00711	-0.0339	-0.0073	0.00501	0.04841	-0.058	0.08494
XM_001064125.1	Hbxap_predicted	0.11884	0.06369	-0.1401	0.24447	0.0827	0.14183	0.13657	0.37727	0.08585	0.3091	-0.2992	0.10697	-0.0413	-0.1034
NM_001106462	Hbxip_predicted	0.93898	0.64253	-0.3318	0.53673	0.68187	1.2681	0.20082	0.71844	0.77391	0.719	0.56522	0.92751	0.65585	0.60787
XM_001065465.1	Hccs_predicted	0.04642	-0.1282	0.20196	0.57624	-0.0043	0.2779	-0.0078	0.20692	0.15381	-0.1109	0.01913	0.41416	0.46027	0.14922
NM_001139507	Hcfc1_predicted	-0.1944	-0.366	0.29052	0.28081	-0.2881	-0.1276	-0.2097	-0.5221	-0.484	-0.2092	-0.3421	-0.1737	0.00921	-0.1219
XM_001055621.1	Hcfc1r1	0.09021	0.02106	-0.755	0.0883	0.03951	0.25935	-0.3529	-0.0204	-0.1999	-0.0343	0.01346	-0.2645	-1.0482	-0.9484
NM_001008357	Hcfc2	0.06297	-0.0369	0.5386	0.63272	0.50826	0.32359	0.32076	0.42736	0.0842	0.0094	0.1681	0.36126	0.5005	0.57565
NM_013185	Hck	-0.1287	-0.1321	-0.1148	-0.1166	-0.3619	-0.1759	-0.1399	0.00494	-0.1942	-0.0744	-0.1674	-0.074	-0.0016	-0.0217
NM_001011898	Hcls1	-0.2229	-0.2837	-0.1313	-0.1771	-0.0254	-0.2075	-0.1669	-0.0805	-0.0024	-0.0831	-0.0701	0.05477	-0.1544	-0.1411
NM_053375	Hcn1	0.30619	0.76271	0.02771	0.02866	0.1879	0.44125	0.16676	0.27956	0.75017	0.72396	0.42476	0.00812	0.36702	0.29711
NM_053684	Hcn2	-0.1152	0.00121	-0.0637	-0.0595	-0.1473	-0.1449	0.13641	-0.0587	-0.1768	-0.106	-0.0549	-0.0842	-0.0268	-0.113
NM_053685	Hcn3	-0.1234	-0.047	-0.0072	-0.078	-0.2756	-0.005	-0.0494	-0.0453	-0.0279	-0.0761	0.0488	-0.121	-0.1727	-0.0244
NM_021658	Hcn4	-0.0743	0.1244	0.01723	-0.0595	0.02652	0.04399	-0.0411	0.0499	0.06655	0.13834	0.01986	0.05084	-0.0085	0.10996
NM_013179	Hcrt	-0.1697	-0.1753	0.04013	-0.0118	-0.1094	-0.0787	-0.0689	-0.0661	0.03776	-0.0295	-0.0638	-0.0198	-0.0439	-0.1787
NM_013064	Hcrtr1	0.02087	-0.1927	-0.0723	-0.2247	-0.1655	-0.0931	-0.186	-0.1192	-0.1345	-0.1354	-0.1442	-0.1667	-0.1318	-0.069
NM_013074	Hcrtr2	0.12047	0.18298	0.04524	0.0773	0.05477	0.08065	0.20396	-0.0021	0.29016	0.19073	0.03911	0.30964	-0.0132	0.06232
NM_001005900	Hcst	0.61158	-0.0094	-0.0252	0.14057	0.16298	0.53304	0.36013	0.00325	0.18655	-0.0731	0.12127	0.3295	-0.1122	0.03223
NM_001025409	Hdac1	-0.4437	-0.3802	-0.2595	-0.3592	-0.3387	-0.1825	-0.3641	-0.355	-0.1462	-0.2056	-0.3511	-0.019	-0.4958	-0.3318
NM_001025409	Hdac1	0.06436	0.24569	-0.2233	-0.0404	0.26352	-0.0689	0.06853	0.30332	-0.0777	-0.0677	0.27557	0.1281	-0.0864	0.02508
NM_001025409	Hdac1_predicted	0.03188	0.08791	-0.1849	-0.2247	0.06525	0.35815	0.10091	0.11319	0.01788	0.08602	0.24716	0.51224	-0.0472	0.12041
NM_001106610	Hdac11_predicted	-0.1228	-0.1446	-0.2015	-0.1894	0.02118	-0.0535	-0.186	-0.0098	-0.0287	-0.1099	-0.0334	0.02829	-0.0792	-0.1847
NM_053447	Hdac2	-0.5246	-0.6289	-0.4205	-0.2894	-0.4862	-0.5671	-0.5714	-0.391	-0.3599	-0.7136	-0.5087	-0.4245	-0.2949	-0.1209
NM_053448	Hdac3	0.78679	0.69236	0.42206	0.33408	0.7535	0.77082	0.74813	0.8204	0.70711	0.63895	0.91958	0.44069	0.35589	0.31516
XM_001067733.1	Hdac4_predicted	0.17975	0.32898	0.43371	0.45887	0.16365	0.22841	0.12859	0.23071	-0.1729	-0.1143	0.09649	-0.1209	0.22264	0.11646
NM_053450	Hdac5	-0.2642	0.09844	-0.2093	0.73942	-0.0645	-0.2144	0.43888	-0.272	-0.3392	-0.0799	-0.0702	-0.53	-0.8237	-0.7424
NM_001109375	Hdac6	0.18992	0.21791	0.04933	0.04627	0.25346	0.38671	0.00919	0.00386	0.19121	0.12281	0.09934	0.24861	0.03899	0.18883
XM_345868.3	Hdac7a	-0.5247	-0.2069	0.42501	0.24976	-0.4366	-0.3044	-0.1951	-0.8108	-0.3779	-0.3546	-0.204	-0.0114	-0.8978	-0.7137
NM_001126373	Hdac8_predicted	-0.0041	0.27157	-0.5697	-0.4301	0.06522	0.49232	-0.0099	0.11644	0.04632	0.25205	0.20079	0.25905	-0.2101	-0.1473
NM_017016	Hdc	0.01732	0.06133	-0.1137	-0.1767	-0.255	-0.0051	-0.1634	0.0188	-0.0164	-0.077	-0.0018	-0.0445	-0.082	-0.1359
NM_001108460	Hddc2_predicted	0.00011	-0.0181	-0.0629	-0.3212	0.25353	0.41561	-0.1368	0.18597	0.07947	-0.0785	0.05524	0.17354	-0.4229	-0.0881
NM_001107528	Hddc3_predicted	0.31975	0.08602	-0.1846	-0.5901	-0.2586	0.09395	-0.2222	-0.1561	0.17188	0.07333	0.13916	0.25179	0.14185	-0.134
NM_053707	Hdgf	-0.3014	-0.8845	0.14632	-0.5083	-0.8235	-1.1544	-0.4599	-1.0551	-0.6264	-0.6871	-0.7292	-0.3447	-0.1426	-0.0069

NM_133549	Hdgfl1	-0.0383	-0.0208	0.25254	0.42035	0.02243	0.00224	0.23388	0.00796	-0.0072	-0.0659	0.04684	0.24852	-0.0182	0.0318
NM_133548	Hdgfrp2	0.03611	-0.0628	0.91132	0.56665	0.13553	0.70717	0.46861	0.20849	0.061	0.09783	0.28391	0.62769	0.41793	0.34714
NM_145785	Hdgfrp3	-0.1238	0.11738	0.11743	-0.3005	-0.2222	-0.3543	0.19743	-0.0738	0.19197	0.16523	0.25084	-0.056	0.27094	-0.0153
NM_024357	Hdh	-0.0129	-0.0097	0.47594	0.15	0.10918	0.0665	0.03764	0.08235	-0.1211	-0.2488	-0.1143	-0.2034	0.3123	0.08949
NM_001106146	Hdhd1a_predicted	-0.049	0.22431	0.09753	0.00674	0.05013	0.10619	0.00291	0.05499	0.11072	0.05731	0.06038	0.18675	0.07316	0.00523
NM_172039	Hdlbp	-0.8321	-0.4342	0.18113	-0.1732	-0.4691	-0.7198	-0.1592	-0.3983	-0.2758	-0.4063	-0.5094	0.12564	0.2237	0.0657
NM_001001509	Hdmcp	0.06058	-0.0268	0.04203	-0.0841	0.06556	0.00889	0.17842	-0.0772	0.03942	0.01458	-0.0566	0.19041	0.03784	0.15431
NM_001108651	Hebp1_predicted	0.96897	0.33692	0.17875	-0.1777	0.63011	0.74871	-0.0638	0.46912	0.58689	0.67314	0.60962	0.30072	0.63777	0.60173
NM_001107515	Hebp2_predicted	0.7941	0.2035	0.07181	0.03517	0.45581	0.53602	0.08017	0.30756	0.69694	0.22845	0.3703	0.78557	-0.006	0.16967
NM_001107514	Heca_predicted	0.20687	-0.1479	-0.0397	-0.074	-0.1723	-0.0669	0.02253	-0.0178	-0.1066	-0.0856	-0.0868	-0.0548	0.00788	0.01073
NM_001107608	Hectd2_predicted	0.07665	0.02917	-0.0628	0.17948	0.1128	0.01949	0.00745	0.08852	0.04781	0.09748	-0.0141	0.40023	0.05356	0.06852
NM_001105848	Helz_predicted	-0.0964	0.33401	0.49596	0.07704	0.19133	0.0826	0.21688	0.16954	0.20823	0.2308	0.26422	0.01263	-0.1146	0.06026
NM_133294	Hemgn	-0.1895	-0.011	-0.0404	-0.0981	-0.0409	-0.1203	-0.0176	-0.0481	-0.1448	-0.0559	-0.0337	-0.0292	-0.1214	-0.1515
NM_001106853	Hemk1_predicted	0.08578	-0.056	0.07444	0.04984	0.07192	-0.0605	0.14147	-0.0387	0.10063	-0.0268	0.1251	0.21342	0.06822	0.03048
XM_213675.3	Hemk2_predicted	0.23252	-0.25	0.00976	0.19245	0.07054	0.44467	0.06923	-0.1017	-0.1463	-0.2982	0.02019	0.01222	-0.151	-0.1461
NM_133304	Heph	-0.823	-0.2955	-1.6071	-0.2378	-1.9142	-1.5846	-0.7391	-1.6521	0.1488	-0.4977	-0.3801	0.2628	-1.0817	-1.1211
XM_236362.3	Herc1_predicted	-0.2969	-0.0617	0.32606	0.04917	-0.1882	-0.7347	-0.0415	-0.361	-0.628	-0.1458	-0.1754	-0.336	-0.2498	-0.0242
NM_001107520	Herc2_predicted	-0.3045	-0.3005	0.18317	-0.1405	-0.2574	-0.6188	0.19763	-0.4611	-0.331	-0.0889	-0.0818	-0.125	-0.0868	-0.2047
NM_001108631	Herc3_predicted	-0.9512	-0.2194	-0.4732	-0.4061	-1.1725	-1.2551	-0.572	-1.0305	-0.5579	-0.3171	-0.4169	-0.8578	-0.9442	-0.9935
NM_001012074	Herc4	-0.2408	-0.0915	-0.2363	0.42374	-0.2176	-0.4854	-0.0134	-0.4032	0.00295	0.20057	-0.2429	-0.3927	-0.4571	-0.4944
NM_001012074	Herc4_predicted	-0.1125	-0.0251	-0.1982	0.35223	-0.5384	-0.6067	0.04526	-0.0537	-0.5528	-0.354	-0.2483	-0.4206	-0.4746	-0.5773
XM_342700.3	Herc6	0.79875	0.8652	0.35382	1.1905	0.84622	0.85718	1.1808	0.8877	0.67588	0.80071	0.46398	0.21829	0.15041	0.36076
NM_053523	Herpud1	-0.0896	-0.3735	-0.3416	-0.1298	0.49817	0.97833	-0.8844	0.79798	-0.2838	-0.298	-0.2169	-0.1258	-0.3348	-0.3256
NM_001024988	Herpud2	-0.0254	0.19487	-0.327	0.4289	-0.0119	0.06524	0.09513	0.09607	0.04659	0.01754	0.12291	-0.1696	-0.3354	-0.5348
NM_001024988	Herpud2	-0.02	0.21423	-0.489	0.0071	0.24332	-0.4323	-0.0761	0.04193	-0.08	0.07259	-0.0976	-0.4407	-0.1053	-0.1705
NM_024360	Hes1	-1.0423	-0.6505	-0.647	-0.0426	-0.4832	-0.489	-0.7592	-0.1093	-0.4076	-0.8706	-0.9087	0.07683	-1.0175	-0.8538
NM_019236	Hes2	-0.2797	0.04152	-0.1286	-0.124	0.08297	-0.0577	-0.1713	0.01733	-0.1578	-0.116	-0.059	0.08394	-0.1266	0.09203
NM_022687	Hes3	0.1073	0.03728	0.02688	0.0701	-0.031	-0.1901	-0.1533	-0.1439	0.05055	-0.1726	-0.0924	0.08003	-0.0465	-0.0618
NM_024383	Hes5	0.20983	0.06992	0.17196	0.25331	0.13061	0.15746	0.174	0.14936	0.09247	0.02038	0.10745	0.34282	0.07637	0.18482
NM_001013179	Hes6	-0.6734	-0.5752	-0.1923	-0.056	-0.8926	-0.4945	-0.3492	-0.7804	-0.3666	-0.5453	-0.4349	-0.0748	-0.7904	-0.918
NM_001105792	Hes7_predicted	-0.0103	-0.0091	0.05144	-0.1229	0.14374	-0.1707	0.1287	-0.0563	0.07919	0.02354	-0.0833	-0.0458	-0.0379	0.00616
NM_001004443	Hexa	0.72376	1.0101	-0.1447	1.151	0.38386	0.55099	0.53945	0.32849	0.34481	0.66319	0.71311	-0.1961	-0.1557	-0.0658
NM_001011946	Hexb	0.14108	0.40832	-0.0014	1.1845	0.2222	0.05251	0.25038	0.22859	0.19711	0.34371	0.30237	-0.3097	0.12968	-0.0291
NM_001011946	Hexb	-0.013	0.01583	-0.0274	0.26246	0.06982	-0.0334	-0.0645	0.13907	-0.0252	0.20134	0.00537	-0.0981	-0.0623	0.08394
NM_001011946	Hexb_predicted	-0.0137	0.08607	-0.014	-0.1233	0.06778	-0.0469	-0.1423	-0.1191	-0.0665	0.00646	-0.0115	-0.1277	-0.1059	-0.0023
NM_001107054	Hexim2_predicted	0.01825	0.16118	0.43439	0.28355	0.40591	0.03028	0.51486	-0.0287	0.52612	0.16074	0.31914	0.70398	0.39353	0.28377
XM_342216.3	Hey1	0.21282	0.01369	0.05009	-0.128	-0.0454	0.18648	-0.095	0.31564	-0.0389	-0.1931	-0.0325	-0.0987	0.04208	-0.1292
NM_130417	Hey2	0.04279	0.00674	-0.108	0.0368	0.35477	0.13169	0.09772	0.36211	0.15703	0.20081	0.07586	0.0376	0.14564	-0.0387
NM_001107977	Heyl_predicted	-0.0159	0.02131	-0.015	-0.1265	0.19087	0.07337	0.11908	0.00931	-0.0136	0.06098	-0.124	0.12999	-0.11	-0.0106
NM_053301	Hfe	-0.1167	0.28472	0.15425	0.29872	0.12915	-0.1831	0.61367	-0.167	-0.1001	-0.0948	0.05264	-0.1884	0.18992	0.11983
NM_001012080	Hfe2	1.6043	-0.2901	-1.0618	-1.1344	-0.9009	-0.9387	-0.8463	-1.0625	-0.2407	-0.3064	-0.0596	-0.6303	-0.9631	-0.9004
NM_001012145	Hgd	-0.0916	0.12846	-0.1214	0.14034	-0.0022	0.20172	-0.0653	0.06783	0.26717	0.15401	0.10479	0.14623	0.26428	0.04571
NM_017017	Hgf	-0.0993	-0.1715	0.08914	0.01898	0.15445	0.15074	0.13281	-0.0355	-0.021	-0.0293	0.3166	0.47147	0.02331	-0.0191
NM_053320	Hgfac	-0.087	0.23408	#######	-0.0714	0.15591	-0.022	-0.0231	-0.0452	-0.0589	0.08199	-0.074	-0.0219	0.12174	0.06408
NM_019387	Hgs	0.16694	0.17729	0.3021	0.21033	0.07765	0.21075	0.19577	0.05867	0.32351	0.08366	0.04764	0.13099	0.30538	0.36581
XR_008636.1	Hhat_predicted	-0.0877	-0.0533	0.06576	-0.0404	0.00575	0.25424	-0.0592	0.03657	-0.1108	0.06277	0.1062	-0.0252	0.11753	0.03986
NM_024385	Hhex	0.01563	0.03577	0.02314	-0.0061	0.09837	-0.0586	0.09133	0.10433	-0.0246	0.06805	0.06683	0.29581	0.08922	-0.0007
XM_001067587.1	Hiat1_predicted	-0.4617	-0.5839	0.01711	-0.161	-0.6243	-0.6534	-0.0759	-0.5545	-0.2505	-0.5708	-0.4053	-0.4198	-0.286	-0.4457
NM_001013112	Hibch	0.20647	0.32348	-0.4313	-0.1397	0.17292	0.39899	-0.0095	0.08794	-0.0151	0.2331	0.15063	0.0155	-0.4194	-0.3803

NM_001107021	Hic1_predicted	-0.0485	0.02259	0.25809	0.22456	0.09817	-0.0275	0.09272	0.02812	-0.0261	0.0656	0.01964	0.19601	0.02895	0.05485
NM_001105862	Hic2_predicted	0.13842	0.12778	-0.0234	0.06702	0.02823	0.2567	-0.013	0.08281	0.0679	0.10981	0.05506	0.03257	0.04084	0.17612
NM_024359	Hif1a	1.0905	1.7784	0.63756	1.6242	1.3918	1.0879	1.0253	1.5292	0.93969	1.2166	1.2405	-0.055	0.92723	1.0221
NM_001113749	Hif1an_predicted	0.17694	0.07488	0.15927	-0.0271	0.63183	0.50383	0.1182	0.42736	0.25748	0.49307	0.08701	0.0749	0.68652	0.44217
NM_022528	Hif3a	0.06166	0.11551	0.22676	0.17355	0.01521	0.12333	0.07009	0.06303	-0.112	0.10288	0.11092	-0.0011	0.09133	0.13708
NM_080902	Hig1	1.0818	0.71792	1.1309	0.27329	1.2013	1.6196	-0.0034	1.078	1.0944	0.79586	1.1309	0.46908	0.89054	0.71618
NM_001105844	Higd1b_predicted	-0.138	-0.0882	0.13252	0.12501	0.08228	0.23222	0.24682	-0.017	0.01295	-0.166	-0.0513	0.14969	-0.0065	0.06747
NM_001106102	Higd2a_predicted	-0.0798	0.06803	0.21411	-0.2284	0.00466	0.22341	0.13012	0.18448	0.20594	-0.0981	0.19997	0.73316	0.118	0.00798
XM_231925.3	Hint1_predicted	-0.0239	-0.0727	0.138	-0.0346	0.10223	0.05624	0.00949	0.1056	-0.0158	-0.0283	0.08702	0.06506	0.01864	0.0962
NM_001107955	Hint2_predicted	0.66573	0.2815	0.21625	-0.311	1.4802	1.6828	0.58712	1.2498	0.34048	0.45389	0.53875	0.09464	0.95728	0.74231
XM_341742.2	Hint3	0.81668	0.24475	0.2869	0.41536	0.70806	0.69845	0.41989	0.48475	0.23087	0.30622	0.41569	0.28336	0.28016	0.10456
NM_001100475	Hip1	-0.1827	-0.0223	0.54226	0.64465	0.36049	0.36176	0.75399	0.29137	-0.2694	0.00274	0.02739	-0.401	0.00994	-0.0374
NM_001100475	Hip1	-0.4465	-0.1793	0.29988	0.29082	-0.0618	-0.1558	0.70583	-0.209	-0.1628	-0.2536	-0.2348	-0.4435	0.04165	-0.0603
NM_001134763	Hip1r	-0.3063	-0.2431	0.05664	0.01825	-0.1218	-0.1203	-0.0036	-0.0115	-0.0546	-0.323	0.01864	0.02457	-0.0089	-0.1447
NM_001106006	Hip2_predicted	-0.2832	-0.124	-0.164	-0.4633	-0.4236	-0.5361	-0.1957	-0.3813	-0.1699	-0.0973	-0.3374	-0.4514	-0.0215	0.01938
NM_001100986	Hipk1_predicted	0.05017	-0.0246	0.17677	-0.1842	-0.058	-0.0262	0.08208	0.0526	0.19188	0.11538	0.09208	0.00586	-0.0789	0.00579
NM_001108622	Hipk2_predicted	-0.1234	-0.04	-0.0377	0.15951	-0.0738	0.25642	-0.0181	-0.0273	0.12967	0.05237	0.02611	0.19077	0.26519	-0.0398
NM_031787	Hipk3	0.28056	0.044	-0.0542	0.15311	0.10551	-0.0527	0.02901	0.04858	0.08745	0.04337	0.12934	-0.1306	0.50129	0.2026
NM_001024776	Hipk4	-0.0438	0.05724	0.18303	0.0441	-0.0176	0.06823	0.09999	-0.0677	-0.0939	-0.0347	0.04467	0.056	-0.0618	0.13009
NM_001025725	Hirip3	-0.1127	-0.7761	0.78523	0.17812	-0.5461	-0.4686	-0.1574	-0.6127	-0.5046	-0.6007	-0.5715	0.07255	0.281	0.38048
NM_001106606	Hirip5_predicted	0.03382	-0.0892	0.13283	0.06422	0.21655	0.26361	0.20968	-0.0177	0.29687	0.17553	0.33164	0.29144	0.20104	0.06556
NM_001106113	Hist1h1a_predicted	-0.0647	0.13542	-0.129	-0.1317	0.17788	0.16376	-0.0201	0.15947	-0.1206	-0.0132	-0.0084	-0.0587	-0.0357	-0.0528
NM_012579	Hist1h1t	0.18709	0.02471	0.03212	-0.0651	0.2525	-0.0267	0.12237	0.46035	0.03041	0.10025	-0.027	0.15539	-0.0399	0.10608
NM_021839	Hist1h2aa	-0.0146	-0.0299	-0.0645	-0.04	-0.1173	-0.1456	0.02387	0.19646	0.01074	0.13907	-0.2021	-0.0518	-0.085	0.03981
NM_001013056	Hist1h2ai_predicted	0.01257	-0.051	0.10445	-0.0161	0.12324	0.0688	0.04155	0.04515	0.07292	0.03924	0.08774	0.11646	0.11604	0.0058
NM_021840	Hist1h2an_predicted	0.10846	-0.0051	0.25851	0.12287	-0.003	0.08235	0.06248	0.1262	0.1593	0.09568	0.01904	-0.0444	-0.0574	0.0354
XM_344599.2	Hist1h2ao_predicted	-0.7732	-0.6149	0.49075	0.26742	-0.6399	-0.7309	-0.0639	-0.3443	0.11411	-0.1877	-0.0749	0.27412	-0.1004	-0.0803
NM_001107352	Hist1h2bl	-0.2539	-0.3281	-0.1377	-0.3864	-0.2526	-0.2629	-0.047	-0.2454	-0.1885	0.12814	-0.3366	0.0701	-0.155	-0.1981
NM_001106114	Hist1h2bm_predicted	-0.681	-0.5128	0.14626	-0.3828	-0.1613	-0.097	-0.362	-0.2382	0.20937	-0.1565	-0.032	0.86028	-0.2861	-0.2054
NM_001106114	Hist1h2bm_predicted	-0.0861	0.02303	0.02879	0.0015	0.03655	-0.081	0.1257	0.02469	-0.0448	0.08371	0.0107	-0.1201	-0.0009	0.04181
NM_001106114	Hist1h2bn_predicted	-0.5003	-0.4355	-0.0253	0.27675	-0.3292	-0.3112	-0.3648	-0.3854	0.10222	-0.3209	-0.2715	0.61661	-0.5843	-0.4268
XM_001061682.1	Hist1h2bp_predicted	-0.8299	-0.3309	-0.0222	0.56781	0.45899	0.45442	0.26572	0.08687	0.24275	0.11113	-0.0039	0.78419	-0.49	-0.5231
NM_001013056	Hist1h4a_predicted	-0.517	-0.4946	0.39563	-0.3544	-0.3324	-0.2834	-0.4775	-0.0255	0.25571	-0.0139	-0.1905	0.91155	-0.0437	0.16777
NM_001123469	Hist1h4m_predicted	-0.0421	-0.1449	0.36803	0.21798	0.28769	0.69554	0.26446	0.39551	0.26813	0.07576	-0.0153	0.97765	0.00252	-0.2283
XM_345255.2	Hist2h2aa_predicted	0.04707	-0.1167	0.10454	-0.1575	0.03395	0.03602	-0.0136	0.00998	0.15284	0.03574	-0.0691	-0.1083	0.03702	0.02443
NM_001013056	Hist2h3c2_predicted	-1.1915	-1.1362	0.16546	-1.003	-0.7566	-0.5792	-0.9832	-1.0506	0.12202	0.33754	-0.7882	1.1872	-0.1695	-0.3069
NM_001123469	Hist2h4_predicted	-0.0298	-0.1112	-0.1168	-0.0282	-0.0673	0.16113	-0.0731	-0.2506	0.07543	0.19317	-0.0905	-0.0893	-0.0265	-0.1008
NM_001111127	Hist3h2ba_predicted	0.04519	-0.0628	0.04342	-0.0713	0.24474	-0.1412	-0.0373	0.1124	0.03371	-0.0502	0.06381	0.0684	0.09467	0.01258
NM_001105751	Hivep1	-0.1889	-0.3129	-0.1666	0.68324	-0.0858	0.31459	-0.2648	-0.1338	-0.5655	-0.3162	0.01763	-0.3745	-0.4132	-0.3995
NM_001107972	Hivep3_predicted	-0.0773	-0.1295	0.01278	-0.2017	-0.1201	0.15307	-0.0062	-0.1123	-0.1656	0.03814	-0.1318	-0.1125	0.04501	-0.2201
NM_012735	Hk2	0.27556	0.10957	-0.2072	-0.3621	-0.5617	-0.7124	-0.4007	-0.1408	0.06998	0.01851	0.13382	-0.7148	0.44466	0.49189
NM_022179	Hk3	1.7659	1.1476	0.801	0.75266	1.1262	1.1082	1.5293	1.0634	0.78085	1.0601	0.83771	0.05238	0.57254	0.37528
XM_218295.2	Hkr2_predicted	-0.0556	-0.0229	0.12577	-0.0159	-0.0777	-0.0821	0.06293	-0.0814	-0.1886	0.14111	-0.0445	-0.0144	0.17556	0.31011
NM_001013216	Hkr3	-0.438	-0.3274	0.23589	0.21298	-0.2737	0.07452	-0.1798	-0.24	-0.4587	-0.1504	-0.1662	-0.1176	-0.2508	-0.2449
NM_198741	Hla-dma	0.37234	-0.0489	0.20874	0.54549	0.72043	0.97773	0.11904	0.62611	0.29695	0.09345	0.35957	0.73174	0.00735	0.09438
NM_198740	Hla-dmb	-0.1525	-0.1112	0.03368	0.17636	0.11463	-0.16	-0.1283	-0.2271	-0.0892	-0.1003	-0.2607	-0.0606	-0.1015	-0.3593
NM_001100635	Hlals_mapped	-0.1607	-0.1432	-0.1458	-0.178	-0.292	-0.2074	0.09884	-0.104	-0.1619	0.12324	-0.1639	0.11624	-0.1637	0.04684
XM_001081149.1	Hlf	-0.0044	-0.0013	0.04293	0.26809	0.03957	0.00596	0.02276	-0.0032	0.07274	0.08492	0.02742	0.09073	0.05853	-0.021
NM_001077674	Hlx1_predicted	0.11968	-0.0006	0.00891	-0.1105	0.41117	0.45087	0.19666	0.32741	0.43063	0.19521	0.08626	0.14941	0.08741	0.0306

NM_013168	Hmbs	0.2327	-0.1845	0.36248	-0.1634	0.21663	0.01022	-0.1493	0.17376	0.01979	-0.2077	-0.0903	0.01209	0.38985	-0.0163
XM_222716.4	Hmcn1_predicted	0.20267	0.18951	0.03517	0.79999	0.11862	-0.0216	-0.0305	0.00486	0.05522	0.00225	-0.0591	0.01974	0.07999	0.15148
NM_001108150	Hmg20a_predicted	0.0062	-0.1104	0.21517	0.16879	0.3019	0.65514	0.31999	0.23501	0.13728	-0.1042	0.22718	0.01733	0.12362	0.17613
NM_001108731	Hmg20b_predicted	-0.4436	-0.3405	-0.3626	-0.743	-0.34	-0.5587	-0.274	-0.2216	-0.4704	-0.4855	-0.2214	-0.4425	-0.1921	-0.6548
NM_139327	Hmga1	0.08013	0.02574	-0.0649	0.0401	0.16044	-0.105	0.26568	-0.0396	0.04023	0.24737	-0.1254	-0.1907	0.01093	0.13036
NM_032070	Hmga2	-0.1069	0.10751	0.0349	-0.1013	-0.0664	0.03898	0.66413	-0.0889	0.00556	-0.2773	-0.1186	0.29823	0.24481	0.27842
NM_001109373	Hmgb1	-0.3514	-0.2957	0.21719	-0.2193	-0.3106	-0.4664	-0.1383	-0.1825	-0.2834	-0.3575	-0.3733	-0.2474	-0.0663	-0.1531
NM_017187	Hmgb2	-1.5111	-1.9827	0.4595	-0.9103	-1.9525	-1.8346	-1.4424	-2.0777	-0.8031	-1.3781	-1.4456	-0.018	-0.1051	0.08396
NM_001107412	Hmgb2l1_predicted	0.13707	0.67617	0.22419	0.07086	0.21092	-0.015	0.42999	0.21985	0.58894	0.24174	0.38729	0.03682	0.29034	0.35762
NM_024386	Hmgcl	0.21137	0.03261	-0.1885	0.59103	0.11745	0.51653	0.0574	0.16068	0.16264	0.14705	-0.0232	0.2724	-0.236	-0.5482
NM_013134	Hmgcr	-1.0482	-1.0377	1.2006	0.33165	-1.1689	-1.2305	0.0195	-0.947	-0.3946	-0.7089	-0.6223	0.06764	-0.139	-0.0437
NM_017268	Hmgcs1	-2.1288	-2.1025	1.3831	0.78304	-1.8108	-1.6623	-0.4547	-1.6772	-0.6413	-1.8651	-1.819	0.58986	-0.7945	-0.8958
NM_173094	Hmgcs2	0.47977	0.09267	0.17414	0.03357	0.08261	0.07955	0.02945	0.0628	0.05215	0.3413	0.21611	0.06679	0.14172	0.2559
NM_001013184	Hmgn1	0.03107	-0.4769	0.01679	-0.5224	-0.6972	-0.7405	-0.4213	-0.5261	-0.2386	-0.174	-0.2744	0.09795	-0.1462	-0.2639
NM_001025624	Hmgn2	-1.5132	-1.5667	0.00967	-2.0324	-2.4595	-2.6196	-1.3818	-2.6183	-0.7224	-0.9251	-1.0008	0.25498	0.18417	0.10169
NM_001007020	Hmgn3	-0.3953	-0.317	0.02141	1.1239	-0.1009	-0.0499	0.07206	0.23365	-0.5012	-0.391	-0.3834	-0.0497	-0.6008	-0.3352
NM_001108067	Hmha1_predicted	0.11532	-0.1022	0.13438	0.28824	0.18171	0.38726	-0.1857	0.28941	0.00185	0.09527	0.24875	0.10571	0.15997	0.10241
NM_012964	Hmmr	0.32978	-0.2343	0.65423	0.60652	-0.1555	-0.3691	-0.1813	0.10287	-0.1357	-0.1045	-0.3026	0.21596	0.98796	0.87597
NM_012580	Hmox1	0.06658	0.26282	0.55389	-0.3893	-0.005	0.32753	0.02349	0.03132	0.50298	0.41385	0.40492	1.03	1.3965	1.0991
NM_024387	Hmox2	-0.0174	-0.1148	-0.243	-0.4376	-0.0188	0.24477	-0.0245	-0.1274	-0.0695	-0.0704	-0.0986	-0.0926	0.00925	0.13493
NM_001108363	Hmx1_predicted	-0.2849	-0.3151	-0.3048	-0.2246	-0.1337	-0.3268	-0.1558	-0.066	-0.2363	-0.2911	-0.1055	-0.2004	-0.0273	-0.0542
NM_001005876	Hn1	-0.4426	0.0095	0.1716	-0.3034	-0.5733	-0.4654	-0.1773	-0.6949	0.07257	-0.0597	0.05814	0.17766	0.32597	0.38554
NM_012669	Hnf1a	-0.0312	-0.011	-0.0835	0.05677	0.12936	0.04973	-0.0749	0.05122	0.02136	0.06629	0.22655	0.05716	0.22631	0.22719
NM_013103	Hnf1b	-0.0556	-0.0453	-0.0293	0.18406	-0.0066	0.01176	0.13308	0.11982	0.0005	0.12909	0.05562	0.39131	-0.0547	0.19768
NM_022180	Hnf4a	-0.0954	0.04766	-0.1045	-0.1095	-0.0678	0.02068	0.01771	-0.0569	-0.1002	-0.0138	-0.018	-0.0505	-0.0665	-0.0588
NM_031044	Hnmt	0.0003	0.18428	0.02111	-0.0872	-0.1169	-0.0998	0.1944	-0.1224	-0.0151	0.24483	0.23163	-0.0512	-0.1195	-0.0427
NM_017248	Hnrnpa1	-0.4843	-0.486	-0.1877	-1.2607	-0.4122	0.07336	-1.0659	-0.5282	-0.1094	-0.269	-0.129	0.47771	0.44704	0.30516
NM_175603	Hnrnpr	-0.2186	-0.229	-0.0421	-0.2119	-0.2223	0.1248	-0.2347	-0.0135	-0.2434	-0.2085	-0.2195	0.15818	-0.2846	-0.017
NM_001104613	Hnrpa2b1_predicted	-0.1836	-0.57	-1.1459	-1.3751	-0.7772	-1.1349	-0.2128	-0.6609	-0.8606	-0.5969	-0.823	-0.3234	-0.1864	-0.1109
NM_001111295	Hnrpa3	-0.3859	-0.5919	-0.2215	0.05194	-1.0542	-1.0406	-0.4318	-0.732	-0.2337	-0.5725	-0.4895	-0.1879	-0.7736	-0.838
NM_031330	Hnrpab	-0.2751	-0.7953	-0.684	-0.661	-0.4577	-0.3138	-0.4153	-0.676	-0.516	-0.4549	-0.691	-0.4223	-0.3791	-0.5335
NM_001082541	Hnrpd	-0.1127	-0.0865	-0.4147	-0.4583	-0.1613	-0.0091	-0.2912	-0.2771	-0.0932	-0.0051	-0.1281	0.18104	0.00766	-0.1382
NM_001033696	Hnrpdl_predicted	-0.6305	-0.3127	0.13908	0.27405	-0.7449	-0.6037	0.26102	-0.5834	-0.2982	-0.3653	-0.204	-0.2301	-0.3419	-0.3448
NM_001037286	Hnrpf	-0.3293	-0.6667	-0.3291	0.07454	-0.4781	-0.8525	-0.3868	-0.3146	-0.4155	-0.3904	-0.6397	-0.4876	0.0933	-0.2701
NM_080896	Hnrph1	-0.6574	-0.124	-0.0091	0.02751	-0.7648	-0.9776	0.51554	-0.5256	-0.0712	-0.0091	-0.0326	-0.0725	-0.0316	-0.246
NM_001014019	Hnrph2	-0.47	-0.3903	-0.1982	-0.4482	-0.6436	-0.6708	-0.5148	-0.5206	-0.1874	-0.4409	-0.6467	-0.4482	-0.2368	-0.2095
NM_001108532	Hnrph3_predicted	-0.6661	-0.3517	-0.1089	0.48483	-0.4653	-0.2939	0.0037	-0.4014	-0.3297	-0.4703	-0.6183	-0.1686	-0.8092	-0.8258
NM_057141	Hnrpk	-0.4199	-0.5067	-0.519	-0.7569	-0.4302	-0.5986	-0.5786	-0.3348	-0.5459	-0.4094	-0.3638	-0.3271	-0.0374	-0.2285
NM_001134760	Hnrpl	-0.3888	-0.4593	0.07278	-0.3952	-0.4218	-0.6182	-0.1482	-0.2907	-0.0893	-0.3729	-0.3681	-0.2996	-0.1551	-0.0854
XM_001063027.1	Hnrpll_predicted	-0.3977	-0.2781	-0.3799	-0.5625	-0.4213	-0.6518	-0.1233	-0.2158	-0.2094	-0.3671	-0.3876	0.04752	0.15035	0.07221
NM_053876	Hnrpm	-0.6693	-0.4627	0.40969	-0.4906	-1.0134	-1.2614	-0.1843	-0.959	-0.3371	-0.3718	-0.2898	-0.1033	-0.1033	-0.1813
NM_057139	Hnrpu	-0.3336	-0.1969	-0.0915	-0.7671	-0.5952	-0.6435	-0.0965	-0.5897	-0.1603	-0.1845	-0.2936	-0.1525	-0.0158	0.05271
NM_001108477	Hnrpul1_predicted	-0.3924	-0.4873	-0.1115	-0.1324	-0.4294	-0.0251	-0.3242	-0.4919	-0.3522	-0.4322	-0.2472	-0.2351	-0.097	-0.1776
NM_053309	Homer2	-0.8488	-0.4003	0.32964	-0.4458	-0.1232	-0.0959	-0.4983	-0.2987	-0.1983	-0.0211	-0.4364	0.20255	0.1471	-0.0663
NM_053310	Homer3	0.21905	0.33799	0.14121	0.33135	0.15471	0.28438	0.22341	0.14852	0.39064	0.01699	0.23969	0.09247	0.28033	0.10912
NM_152849	Homez	-0.0236	0.16643	0.13106	-0.115	0.09843	0.12687	-0.063	-0.1444	0.17091	0.08496	0.08441	0.2678	0.02374	0.07904
NM_001107946	Hook1_predicted	0.0231	-0.1188	0.02049	0.05813	-0.0054	0.27084	-0.0093	-0.2218	-0.0002	-0.0552	0.16269	0.00242	-0.1094	0.07696
NM_001100562	Hook2_predicted	0.05682	0.17502	0.11408	0.00975	0.14786	-0.0912	-0.0097	-0.2097	0.01122	-0.1511	0.17298	-0.1716	-0.0376	-0.2026
NM_133621	Hopx	2.3826	-0.2816	1.8637	2.7287	2.6395	2.8341	1.8919	2.7028	0.26975	-0.0558	-0.2839	1.1329	0.41608	0.21195

NM_013075	Hoxa1	0.03595	0.01084	-0.0253	0.01771	0.01548	0.08223	0.01455	-0.0542	-0.0189	0.00056	0.10171	0.04106	-0.0472	-0.0784
NM_012581	Hoxa2	0.12995	-0.1305	-0.046	-0.3941	-0.1816	-0.2821	-0.3659	-0.3302	-0.1933	-0.2443	-0.2931	-0.182	-0.211	-0.293
NM_001109233	Hoxa7_mapped	0.45495	-0.1677	0.49838	0.96563	-0.0928	0.19679	0.0894	-0.6469	-0.4539	-0.2517	0.03577	-0.1922	-0.1517	-0.3669
XM_220896.3	Hoxb1_predicted	-0.1225	0.04567	-0.0115	0.04102	-0.2494	-0.2815	-0.1887	0.06298	-0.2831	0.14107	-0.1426	-0.0233	0.08925	-0.0444
NM_001107041	Hoxb13_predicted	0.02129	0.10651	0.03829	0.12072	0.12807	0.14905	0.03256	0.05228	0.07017	0.04244	-0.1107	0.01239	0.13076	-0.0983
NM_001047091	Hoxb2_predicted	-0.1617	-0.3328	0.08292	-0.2331	-0.1271	-0.2369	-0.3084	-0.2088	-0.1552	0.04608	-0.1246	-0.2531	-0.2534	-0.1143
NM_001107042	Hoxb3_predicted	-0.2866	-0.1988	0.12831	-0.0285	-0.3111	-0.3808	-0.0522	-0.4422	-0.1773	-0.2936	-0.193	-0.1755	-0.2404	-0.1792
NM_001017480	Hoxb7	-0.3843	0.07361	-0.0192	-0.8765	-0.7254	-0.6725	0.12875	-0.9769	-0.0066	0.14849	0.187	0.37857	-0.0055	-0.0256
XM_220888.3	Hoxb8_mapped	-0.5346	-0.5143	-0.456	-0.6646	-0.394	-0.3591	-0.5438	-0.3826	-0.2143	-0.2799	-0.4524	0.06688	-0.2132	-0.6433
NM_001100497	Hoxb9_predicted	-0.1264	-0.1891	-0.1185	-0.1535	-0.2084	-0.2563	-0.0935	-0.0039	-0.2093	0.04912	-0.1932	-0.2564	0.08625	-0.004
XM_001068573.1	Hoxc10	-0.3025	-0.1554	-0.1065	0.23762	0.0522	-0.0981	0.02081	0.03649	0.26943	-0.1084	-0.0192	0.17119	0.02417	-0.0164
NM_001106796	Hoxc12_predicted	-0.1349	-0.0167	-0.1322	-0.2016	-0.1317	-0.0062	-0.1773	-0.0976	0.11056	0.00144	-0.0587	-0.0315	-0.0079	-0.0882
NM_001108116	Hoxc4_mapped	-0.3084	-0.1513	-0.0326	0.74493	-0.168	0.02232	-0.1668	-0.3026	-0.0809	-0.1885	-0.0073	0.09833	-0.0467	-0.1771
XM_001069410.1	Hoxc6	-0.2804	0.24968	0.91165	0.86748	-0.3426	-0.1073	0.68857	-0.326	-0.0724	-0.147	0.11056	0.4896	-0.1089	0.26919
XM_347335.3	Hoxc8_mapped	0.01572	0.08558	0.07428	0.05978	0.32217	0.09199	0.09841	-0.1224	0.06452	0.01096	0.09095	0.28577	0.08196	0.06258
XM_217059.3	Hoxc9_predicted	-0.7961	-0.783	-0.1674	-1.0861	-0.4811	-0.3477	-0.3029	-0.8361	-0.196	-0.4825	-0.5553	0.48666	-0.281	-0.3193
NM_001105884	Hoxd1_predicted	0.124	0.06583	0.09599	-0.0002	0.09206	-0.1208	0.10884	0.17887	0.01064	-0.1116	-0.1766	-0.0186	0.1494	-0.101
NM_001107094	Hoxd10_predicted	-0.4962	-0.3516	-0.3229	-0.2431	-0.4562	-0.4771	0.00588	-0.6536	0.10825	-0.1975	-0.2054	0.28627	0.01841	-0.0294
XM_001063522.1	Hoxd12_predicted	0.34152	-0.0135	-0.0078	0.27493	-0.091	0.24744	0.09471	-0.0076	-0.0233	-0.0244	-0.0154	0.25878	0.03342	-0.0296
NM_001105886	Hoxd13_predicted	-0.0839	0.24567	-0.3887	-0.2814	-0.2132	-0.3401	-0.1308	-0.1913	0.03252	-0.1309	-0.2813	-0.2644	-0.2119	-0.235
XM_213633.4	Hoxd3_mapped	-1.483	-1.2209	-0.2381	-0.979	-1.6271	-1.5094	-0.4167	-1.698	-1.1339	-1.3499	-1.2534	-0.6187	-1.0173	-1.1207
NM_001105885	Hoxd4_predicted	-0.1361	0.07685	0.23076	-0.1505	-0.1908	-0.0739	0.07497	-0.0141	-0.019	-0.1847	-0.3082	0.05113	0.02213	-0.0244
NM_012582	Hp	0.45587	-0.172	0.48357	0.54523	-0.1402	-0.2546	0.13761	-0.2766	-0.2057	-0.3886	-0.0808	-0.2966	-0.2475	-0.0955
NM_199108	Hp1bp3	-0.2891	-0.1773	-0.0298	-0.5945	-0.5087	-0.6821	-0.1208	-0.6313	-0.2052	-0.2907	-0.1846	-0.0728	-0.2505	-0.2674
NM_017122	Hpca	0.02334	0.29013	0.05492	-0.1164	0.01738	0.05856	0.04096	-0.0594	0.00165	-0.0634	0.0125	-0.0895	-0.0052	0.00576
NM_017357	Hpcal4	0.27747	0.02821	0.11184	0.0709	0.04592	0.02814	0.07193	0.14374	0.09606	0.13496	0.18163	0.09322	0.08996	0.0597
NM_017233	Hpd	0.25764	0.17296	-0.0588	0.08173	0.31855	0.42259	0.1605	0.42382	0.28941	0.15172	0.17845	-0.0562	-0.1539	0.01759
NM_001014068	Hpdl	-0.1026	-0.1947	-0.0438	-0.3036	-0.0248	-0.1644	-0.3169	-0.155	-0.2146	-0.1877	-0.3318	-0.1081	0.13957	0.2752
NM_024390	Hpgd	-0.0478	0.03023	0.15252	-0.0373	0.01423	-0.0066	0.00026	0.09905	0.1409	0.04528	0.2266	0.04241	0.15326	0.04167
NM_017112	Hpn	-0.1053	-0.182	-0.049	0.00895	-0.0908	-0.0188	0.05356	0.10163	-0.0659	0.08519	-0.1298	-0.0266	0.08415	0.00394
NM_012583	Hprt1	0.491	0.66052	0.11598	0.73689	0.70942	0.51694	0.15115	0.81136	0.45724	0.40374	0.52854	0.00653	0.47408	0.61142
NM_040669	Hps1	-1.0935	-1.0257	-1.2084	-1.8719	-0.7597	-0.8671	-0.6153	-0.8127	-1.2791	-1.2704	-1.0771	-1.3605	-0.9657	-0.9045
NM_001107664	Hps3_predicted	0.11189	0.37284	0.00989	0.29127	0.17213	-0.1777	0.62953	0.27741	0.33431	0.28537	0.31542	-0.1022	0.19752	-0.0312
NM_001107148	Hps4_predicted	-0.1107	0.17795	0.53545	0.28553	0.20585	-0.2417	0.54303	0.08248	-0.1286	-0.0327	-0.1004	-0.1405	-0.0634	-0.1133
NM_181432	Hps6	-0.1143	0.10264	-0.1383	-0.5357	0.17565	0.03736	0.01963	0.1409	-0.0285	-0.0624	-0.0751	-0.1817	-0.0196	0.04249
NM_022605	Hpse	0.09189	-0.0521	-0.0491	0.04415	-0.0467	-0.0317	0.04841	-0.062	-0.0967	0.09001	-0.0233	-0.0892	-0.0665	0.01861
NM_053318	Hpx	-0.5418	-0.2898	0.42805	0.13878	-0.1329	-0.1118	0.36503	-0.4258	-0.3621	-0.4106	0.22521	0.09779	0.17026	0.1688
NM_024364	Hr	-0.1765	-0.1053	0.11711	0.11384	0.19672	0.12189	0.13654	0.13039	-0.0741	-0.1254	-0.0333	0.05857	-0.0668	0.05291
NM_001105871	Hrasls_predicted	-0.0087	0.01396	-0.1785	0.03193	-0.0695	-0.0116	0.05479	0.06905	0.15144	0.00859	0.16322	-0.1345	-0.1183	-0.0957
NM_017060	Hrasls3	2.1871	1.3966	1.0377	0.89626	0.60684	1.1144	3.4936	0.36806	1.2936	1.7358	1.9825	0.66553	1.7716	1.7752
NM_001108094	Hrb2_predicted	0.02524	0.49338	0.216	0.49922	0.12423	-0.227	0.40687	0.07288	0.0223	0.08587	-0.0341	0.06972	0.32986	0.29595
NM_001107131	Hrbl_predicted	0.02118	0.01455	-0.0268	0.01044	-0.0575	-0.1522	-0.0452	0.14537	-0.1753	-0.0651	-0.0332	0.21457	-0.1358	-0.1465
NM_181369	Hrc	0.02045	0.0756	0.14421	0.10828	0.08515	0.23853	0.12972	-0.0338	0.07775	0.03284	0.08387	0.2033	0.07496	0.05492
NM_133428	Hrg	0.02159	0.07593	0.29566	0.17403	-0.0672	0.36543	0.06774	0.22481	0.12609	0.24977	0.16094	0.08078	0.13854	0.04289
NM_017018	Hrh1	0.10751	-0.0188	0.10011	0.13279	-0.0636	-0.054	-0.1084	0.01817	-0.1067	-0.2727	-0.007	-0.0533	0.09786	-0.055
NM_012965	Hrh2	-0.0651	-0.041	0.10136	0.00397	0.01342	0.06107	-0.0173	0.07596	0.00988	0.14573	0.08522	0.05846	0.01867	-0.0116
NM_053506	Hrh3	-0.1839	0.09518	-0.1022	-0.0426	-0.0646	-0.135	-0.271	0.12543	-0.1904	-0.0513	-0.1007	-0.0441	0.03398	-0.0172
NM_131909	Hrh4	0.26251	0.17031	0.08013	0.21715	0.09575	0.06986	0.02957	0.06805	0.03734	0.02363	0.00539	0.15957	-0.0375	-0.0763
NM_057130	Hrk	0.03988	0.12946	-0.0656	0.02006	0.03784	-0.03	-0.063	0.07861	0.03465	0.04206	0.03157	0.02145	-0.0218	-0.0691

NM_001106466	Hrmt1l6_predicted	-0.1498	-0.1411	-0.0564	-0.1001	-0.1902	0.11934	-0.068	-0.2815	-0.1256	-0.0564	0.05804	0.22815	-0.0383	-0.1409
NM_198783	Hrpap20	0.25882	0.04103	0.38287	0.81237	0.4121	0.58039	0.59202	0.57911	0.05538	-0.1247	0.20132	0.37949	0.31357	0.3596
NM_001024769	Hrpt2_predicted	0.1363	-0.1251	-0.0457	-0.1375	-0.2041	-0.0217	0.23209	-0.3343	-0.2304	0.00578	0.20957	0.13361	0.36601	0.3638
NM_031714	Hrsp12	0.17285	0.12052	-0.4901	0.07688	0.26571	0.41687	-0.396	0.24179	0.4219	0.38195	0.44613	0.33064	-0.1862	-0.1457
NM_001100518	Hs2st1	0.23082	0.14718	-0.1309	-0.3038	-0.1522	-0.2892	-0.1876	-0.0998	-0.1839	-0.0061	-0.1862	-0.018	-0.08	-0.051
NM_053391	Hs3st1	1.059	-0.1461	0.13483	0.60561	0.99208	1.0318	0.88379	0.97209	0.25381	0.00787	0.34138	-0.0845	-0.1579	0.11217
NM_181370	Hs3st2	0.06184	-0.1599	-0.1299	-0.2677	0.03301	-0.1356	-0.0931	0.08526	-0.2101	-0.1563	-0.2065	-0.0931	-0.0329	-0.1978
XM_220557.3	Hs3st3b_predicted	0.08843	0.15857	-0.0524	0.12356	0.13037	0.17206	0.06768	0.15101	0.31838	0.02319	0.15319	0.17833	0.02938	0.03496
NM_001106392	Hs3st5_predicted	-0.0666	0.12573	0.09163	0.05914	0.05163	-0.0056	-0.0452	0.03742	0.06654	-0.0947	-0.0102	0.04071	-9E-05	0.11599
NM_001108210	Hs6st1_predicted	-0.3114	-0.139	-0.2307	-0.4426	-0.2441	-0.1499	-0.4121	-0.4055	-0.2742	-0.2077	-0.1211	-0.2231	-0.2476	-0.154
XM_001078574.1	Hs6st3_predicted	0.05189	0.09739	-0.1198	0.16987	0.18272	0.0379	0.05176	0.18054	0.25549	0.15095	0.08006	0.44442	-0.0451	0.09189
NM_173119	Hsbp1	0.25189	0.30324	0.36875	0.01267	-0.2019	-0.1381	0.01922	-0.4186	0.54456	0.48196	0.49965	0.41996	-0.0229	-0.1702
NM_017080	Hsd11b1	0.86151	3.8471	1.5477	1.429	-1.5747	-1.3626	4.16	-1.2604	3.5634	3.821	3.5997	0.77198	1.7234	1.8557
NM_017081	Hsd11b2	0.1523	0.00831	-0.0033	-0.0295	0.09193	-0.0616	0.02003	-0.1441	0.1172	-0.0981	-0.0724	0.06397	0.01679	0.05061
NM_012851	Hsd17b1	-0.5124	-0.2353	-0.0455	-0.3375	-0.5115	-0.2713	-0.2246	-0.0402	-0.2965	-0.1481	-0.4619	-0.5401	-0.2697	-0.2143
NM_031682	Hsd17b10	0.32921	0.37991	-0.1013	-0.065	0.33062	0.58742	-0.1246	0.36835	0.66668	0.62957	0.76571	0.57644	0.47827	0.19419
NM_001004209	Hsd17b11	-0.1554	-0.0096	-0.4514	-1.0517	-0.6129	-0.5178	-0.2068	-0.6942	-0.0379	-0.0224	-0.094	-0.1747	-0.253	-0.0679
NM_032066	Hsd17b12	-0.5364	0.03937	0.47376	-0.8604	-0.601	-0.5954	0.06497	-0.5893	0.27129	0.09581	0.37309	0.20941	0.5571	0.43814
NM_001009684	Hsd17b13	-0.4371	-0.2879	0.36797	0.62374	-0.3575	-0.3391	0.0407	-0.08	-0.3045	-0.4438	-0.2201	-0.1467	-0.5395	-0.2846
NM_024391	Hsd17b2	0.00193	-0.0249	0.06685	-0.0257	0.09788	0.29089	0.05102	0.42916	0.42958	0.07667	0.09061	-0.1436	0.03626	-0.0473
NM_054007	Hsd17b3	-0.291	-0.2748	-0.2659	-0.2594	-0.3385	-0.1606	-0.1484	-0.3372	-0.443	-0.3408	-0.3027	-0.282	-0.3917	-0.3116
NM_024392	Hsd17b4	-0.0585	0.31601	-0.965	-0.2302	-0.3697	-0.4587	-0.1629	-0.2984	0.2555	0.24551	0.30233	-0.1491	-0.4827	-0.4132
NM_173305	Hsd17b6	-0.088	0.07753	-0.1373	0.03117	-0.1476	-0.2033	-0.1231	0.062	0.09635	-0.0833	-0.0997	-0.1168	0.11516	-0.317
NM_017235	Hsd17b7	-0.3468	-0.4972	0.87961	0.18053	-0.3165	-0.1382	-0.0822	-0.5663	-0.1739	-0.3234	-0.3457	0.15411	-0.0674	-0.1756
NM_212529	Hsd17b8	0.67365	0.15067	-0.715	0.26756	0.55498	0.62997	0.06545	0.45694	0.10168	0.19775	0.26864	-0.9143	-0.6877	-0.5295
NM_012584	Hsd3b	0.33565	0.0349	0.00223	-0.0485	0.01721	0.37407	0.01274	0.19032	0.10595	-0.1116	-0.0844	0.01493	0.24642	0.09014
NM_017265	Hsd3b1	-0.0462	0.06187	-0.1156	-0.0018	0.03805	-0.1679	-0.2352	0.0902	-0.0496	-0.005	-0.1845	-0.0938	-0.0155	-0.0937
NM_001007719	Hsd3b1	-0.1674	-0.1729	-0.2294	-0.1914	0.01908	0.38371	-0.0328	0.295	-0.0042	-0.1582	-0.2049	-0.1107	0.04472	-0.1504
NM_139329	Hsd3b7	0.5394	0.59161	0.16485	0.24419	0.73041	0.77616	0.59339	0.70627	0.39255	0.45078	0.54432	0.02397	-0.0428	-0.0731
NM_031694	Hsf2	0.01374	-0.5691	-0.372	0.15162	-0.3936	-0.4749	-0.7046	-0.0441	-0.1329	-0.3675	-0.4113	-0.2999	-0.2418	-0.3681
NM_001106177	Hsf4_predicted	0.03865	-0.0892	0.20483	-0.1109	0.03846	0.06707	-0.0569	0.03624	-0.0317	-0.0056	-0.0338	-0.0151	-0.0846	0.12199
NM_001012132	Hsfy2	0.04557	-0.0671	0.00532	-0.059	0.00103	0.09042	-0.0561	0.03226	-0.0177	0.52875	-0.0499	-0.0325	0.05895	-0.0218
NM_001002823	Hsn2	0.07865	-0.0155	-0.0591	-0.0553	0.18023	0.11133	-0.0069	-0.0934	-0.0071	-0.0148	-0.0886	-0.0759	0.06456	-0.0182
NM_001011901	Hsp105_predicted	0.10474	0.05691	0.11405	0.0802	0.09893	0.2603	0.00694	0.11065	-0.0478	0.08724	0.25796	0.07271	0.22008	0.06121
NM_175761	Hsp90aa1	0.16256	-0.1169	0.68934	0.90317	-0.007	-0.1905	-0.2364	0.49743	0.13894	-0.0558	0.08113	-0.1172	0.6404	0.47091
NM_001107445	Hspa12a_predicted	0.04304	0.11998	-0.0133	0.08567	0.19231	-0.0059	0.08576	0.06674	-0.0961	0.00125	0.16816	0.14271	0.07131	0.14264
NM_001107778	Hspa12b_predicted	0.17688	0.02284	0.12348	0.01374	-0.0197	0.01352	0.09067	0.19312	0.03898	0.02278	-0.0273	0.05089	0.06605	0.13468
NM_001004257	Hspa14	-0.246	-0.0336	0.43998	-0.1546	-0.4249	-0.3405	0.3737	-0.1397	-0.0233	-0.1509	-0.0657	0.017	0.09923	0.16644
NM_212504	Hspa1b	-0.0962	0.00173	-0.2817	-0.0093	-0.1524	-0.204	-0.179	-0.2659	-0.1759	-0.1613	-0.2582	-0.2515	-0.0909	-0.1829
NM_021863	Hspa2	0.18731	0.16657	0.23185	-0.1036	0.11258	-0.2093	0.00985	0.03604	0.23792	0.22455	-0.1489	0.21723	0.36856	0.21478
NM_153629	Hspa4	-0.2133	0.18055	0.0854	-0.1227	-0.0261	0.06163	0.05026	-0.1986	0.09491	0.07015	0.07868	-0.3568	-0.1706	0.10627
NM_001106428	Hspa4l_predicted	0.76883	-0.2772	-0.0309	0.50956	1.0433	0.60458	0.51476	1.1407	-0.4502	-0.1807	-0.0153	-1.1981	-0.2681	-0.0917
NM_013083	Hspa5	-0.5231	-0.5146	0.08479	0.282	0.40276	0.49763	-0.9506	0.59158	-0.5059	-0.5545	-0.6438	-0.2019	0.34659	0.05402
NM_001100658	Hspa9a_predicted	0.11121	-0.3546	-0.102	-0.0103	-0.1188	-0.1878	-0.1847	-0.0562	-0.2374	-0.167	-0.3017	-0.5102	-0.0201	-0.2078
NM_031970	Hspb1	0.82034	0.92637	0.20189	1.1218	0.78695	0.77051	0.65018	0.66937	0.75737	0.95079	0.7032	0.38305	0.23577	0.50375
NM_130431	Hspb2	0.21371	0.18876	0.05411	0.12908	0.18951	0.2226	-0.0011	0.06398	0.22041	0.05651	0.17538	0.46584	0.17441	0.14392
NM_031750	Hspb3	0.07083	0.27954	-0.0339	-0.0836	-0.0628	0.13172	0.05704	-0.0403	0.01154	-0.0063	0.11473	0.12314	0.08976	0.02795
NM_138887	Hspb6	0.50664	0.28712	0.27264	-0.7622	-0.3	-0.2535	-0.0635	-0.4091	0.30362	0.48901	0.37572	0.38592	0.70614	0.80997
NM_031607	Hspb7	3.2987	1.7082	2.9336	1.8704	2.2165	2.6745	2.0987	2.2394	1.5474	2.0719	1.7768	0.87997	2.5745	2.4971

NM_053612	Hspb8	0.18582	0.41032	0.16747	0.19162	0.28462	-0.029	-0.1637	0.05177	0.09205	0.14807	0.06752	-0.2024	0.31346	0.30909
NM_001108835	Hspb9_predicted	-0.0812	0.11341	-0.0391	0.09096	-0.0199	0.15895	0.10517	0.12456	-0.1406	-0.1236	-0.1755	-0.1425	-0.1054	-0.0249
NM_134419	Hspbap1	0.11179	-0.1145	0.60305	1.1328	-0.0945	0.32966	0.46252	-0.2058	-0.2765	0.108	-0.222	0.38342	-0.2817	-0.0597
NM_139261	Hspbp1	0.18009	0.14008	-0.0541	-0.1032	0.39425	0.31349	0.22324	0.15589	0.20988	0.07918	0.09391	0.05254	0.31909	0.22553
XM_216334.3	Hspcal3_predicted	0.05963	-0.2643	0.68345	0.9636	0.06423	-0.2068	0.02194	0.25502	-0.1191	0.10118	0.03827	-0.0083	0.61511	0.44143
NM_001004082	Hspcb	0.13104	-0.1443	-0.2931	-0.0722	-0.018	-0.1752	-0.089	-0.0782	-0.163	0.25542	-0.014	-0.2299	0.00187	-0.0649
NM_001004082	Hspcb	-0.2021	-0.0092	-0.2143	-0.245	-0.0242	-0.2285	-0.2437	-0.0017	0.04539	-0.203	-0.024	0.08135	-0.1724	-0.1145
NM_022229	Hspd1	-0.178	-0.4548	0.4505	-0.463	-0.3325	-0.5795	0.05526	-0.0241	-0.195	-0.1577	-0.2869	-0.4162	0.50313	0.28012
NM_022229	Hspd1	0.15109	0.14895	0.36997	0.23798	0.13618	0.29042	0.24388	0.21212	0.05457	-0.0426	0.0264	0.20445	0.06153	0.32905
NM_012966	Hspe1	0.14728	-0.2514	0.14475	-0.0955	-0.0925	0.07025	-0.2064	-0.1996	-0.24	0.01702	-0.0594	0.39924	0.46401	0.43891
NM_001011901	Hsph1	0.07842	0.1441	0.0922	0.01128	0.04227	0.0275	0.23353	0.01133	-0.0239	0.05175	0.02264	-0.0192	0.0924	-0.0584
NM_001005872	Htatip	-0.6696	-0.343	0.19596	-0.2678	-0.6446	-0.5762	0.16022	-0.8738	-0.4925	-0.3992	-0.379	-0.2227	-0.1554	-0.2268
NM_001106263	Htatip2_predicted	0.14824	-0.0089	-0.1245	0.81308	0.45874	0.78954	-0.1614	0.37339	0.11511	-0.0829	-0.0869	0.28651	-0.1795	0.04185
NM_001108259	Htatsf1_predicted	-0.1304	0.11046	-0.2685	0.29972	-0.3342	-0.3799	0.0641	-0.3022	0.10366	0.09119	-0.0879	-0.0993	-0.1812	-0.402
NM_001011895	Htf9c	-0.2642	-0.3966	0.28074	0.14831	-0.4122	-0.3387	0.10967	-0.3512	-0.1764	-0.1889	-0.1772	-0.352	-0.202	-0.5632
NM_012585	Htr1a	0.15772	0.06799	0.12878	0.06394	0.01511	0.22199	0.05045	0.00253	0.27286	-0.036	0.12026	0.02915	0.34222	0.1491
NM_022225	Htr1b	-0.0915	0.01843	-0.2695	-0.2223	-0.2538	-0.1452	-0.161	-0.1716	-0.2203	0.09095	-0.2459	-0.1228	-0.1461	0.01483
NM_012852	Htr1d	0.07193	0.07201	0.18813	0.2875	0.20347	0.45029	0.1906	0.09821	0.12696	0.06858	0.22512	0.14122	0.06294	0.14679
NM_021857	Htr1f	-0.0318	0.10021	0.03913	0.13694	-0.0455	0.02562	0.19285	-0.1103	-0.055	0.02362	-0.1001	0.03833	-0.046	-0.0367
NM_017254	Htr2a	0.02842	0.19877	0.18775	0.25437	-0.0318	0.47265	0.23158	0.31348	-0.0195	0.17721	0.08817	0.11984	0.15036	-0.0402
NM_017250	Htr2b	-0.199	0.18433	-0.2094	-0.1329	-0.2136	-0.2684	0.04121	-0.1651	0.32773	0.00651	0.16602	-0.1304	-0.2276	-0.3073
NM_012765	Htr2c	0.05362	-0.1181	0.00031	-0.1258	0.13482	-0.1171	0.01618	0.12542	-0.138	-0.0719	0.05343	0.04411	0.08631	-0.1368
NM_024394	Htr3a	0.16271	0.11397	-0.0108	0.21125	0.20849	0.21331	0.21899	0.00293	0.04301	0.14253	0.33485	0.36363	0.19331	0.03666
NM_022189	Htr3b	0.13046	0.11141	0.1124	0.02001	-0.0344	0.06628	0.03429	0.18605	0.17447	0.40002	0.03342	0.084	0.1105	0.14097
NM_012853	Htr4	-0.1934	-0.1272	-0.1099	-0.1879	-0.1748	-0.078	-0.1144	-0.1163	-0.1518	-0.1669	-0.0291	-0.0568	-0.1636	-0.171
NM_013148	Htr5a	-0.0807	-0.1061	0.14072	-0.0838	0.06531	-0.0237	-0.0158	-0.1687	-0.0919	0.05644	-0.1075	-0.0464	-0.037	-0.1109
NM_024395	Htr5b	0.1263	0.25151	0.08926	0.14987	0.29203	0.01788	0.25738	-0.0119	0.21267	0.19651	0.0461	-0.0506	0.06027	0.34265
NM_024365	Htr6	-0.0504	0.00777	0.0061	0.19573	0.55827	0.09735	0.05532	0.1118	0.10434	0.23277	0.23658	-0.007	-0.0405	0.18115
NM_022938	Htr7	-0.2089	-0.1051	-0.0653	-0.1757	-0.2591	-0.0097	-0.2571	-0.2702	-0.1922	-0.15	-0.1166	0.40705	0.12639	0.00854
NM_031721	Htra1	0.21844	0.42631	-0.1008	0.49711	-0.1829	-0.0253	0.48497	0.06803	0.39676	0.34532	0.34634	0.20553	0.23304	0.219
XM_001058037.1	Htra3_predicted	-0.4631	-0.3602	-0.4199	-0.5253	-0.3904	-0.3917	-0.4517	-0.2523	-0.2729	-0.3514	-0.3058	-0.4147	-0.607	0.0219
NM_001107321	Htra4_predicted	-0.3113	0.10482	-0.1605	-0.2941	-0.1831	-0.1442	-0.163	-0.1344	0.20361	-0.0157	0.20381	0.33559	-0.1274	-0.3361
NM_207616	Hyal1	-0.0393	0.1446	-0.0275	0.09143	0.01217	0.0742	0.18738	0.11698	0.20125	0.10144	0.10662	0.2564	-0.0069	0.01644
NM_172040	Hyal2	0.36873	-0.1367	0.45126	0.17655	0.43482	0.2545	0.79683	0.4577	0.33793	0.29833	0.32911	0.16663	0.27346	0.33871
NM_207599	Hyal3	-0.0468	0.10656	0.0603	-0.1438	-0.1744	-0.1472	-0.0308	-0.2428	-0.0537	-0.1476	-0.1295	-0.1859	-0.1795	-0.2096
NM_001100780	Hyal4	-0.0857	-0.0693	-0.0136	0.03849	-0.0811	-0.1614	-0.2374	-0.2473	-0.0166	-0.1153	-0.1246	-0.0318	-0.0888	-0.0049
NM_001024321	Hyal5	0.31545	-0.0292	0.01885	-0.0673	-0.0657	0.45227	0.22207	0.00198	0.17559	-0.0921	0.00812	-0.0339	0.05581	0.10441
NM_001034028	Hyou1	-0.2451	-0.1882	-0.1959	-0.1799	-0.238	-0.1023	-0.2982	0.08309	-0.3101	0.00165	-0.2056	-0.2902	-0.1928	0.01511
NM_012586	Iapp	-0.1095	0.16367	0.01679	0.27118	-0.0808	-0.0715	0.11765	0.08617	-0.014	0.10703	-0.175	0.28851	-0.0969	0.0552
NM_001100572	Iars_predicted	-0.1589	-0.8716	-0.0426	-0.4396	-0.3495	0.15634	-0.8406	0.16244	-0.5049	-0.5412	-0.7589	-0.0068	-0.0881	-0.2431
XM_001065536.1	Iars2_predicted	-0.0019	-0.1608	-0.422	0.27742	-0.0309	-0.4565	-0.006	-0.2786	-0.2	-0.0984	-0.0835	-0.3045	-0.0886	-0.2467
NM_001106204	Ibrdc1_predicted	-0.3041	-0.5691	-0.54	-1.0108	0.23884	0.18465	-0.7614	0.244	-0.3323	-0.2897	-0.4815	-0.1047	-0.2371	-0.1744
NM_001108881	Ibrdc2_predicted	0.03382	0.00204	0.22913	-0.0543	0.08209	0.00861	-0.0124	0.0216	0.11686	-0.048	-0.0357	-0.0585	-0.0536	-0.0278
NM_001108003	Ibrdc3_predicted	0.67127	1.2049	1.5141	1.8055	0.7629	0.90779	0.9615	0.69402	0.80195	1.2082	1.0835	0.61603	0.81317	0.82007
NM_012587	Ibsp	0.15216	-0.0032	-0.0183	-0.1956	0.00469	-0.2275	-0.1644	0.05418	0.0932	0.03116	-0.0028	-0.2585	-0.0247	0.17026
XM_001062352.1	Ibtk_predicted	-0.1318	0.0243	-0.3871	-0.1941	0.02501	-0.5146	-0.2021	-0.1801	-0.0925	0.04225	-0.1991	-0.3863	-0.02	0.17566
NM_030844	Ica1	0.21334	-0.7337	-0.6345	-0.7045	-0.3641	-0.5719	-0.5592	-0.2663	-0.8932	-0.8446	-0.9666	-0.9619	-0.4211	-0.4846
NM_199400	Ica1l	0.03236	-0.0385	0.1202	-0.1037	-0.0124	0.00169	-0.0427	-0.081	-0.092	-0.138	0.103	0.10193	0.09145	-0.0523
NM_012967	Icam1	1.9227	1.9792	0.73178	2.3064	2.0127	1.7148	1.7894	1.9848	1.391	1.6992	1.6294	0.27294	0.77315	0.92025

NM_001007725	Icam2	-0.0185	0.06122	0.01357	-0.0782	0.03768	-0.0017	-0.0579	-0.0476	-0.1085	0.09281	0.1213	-0.0388	-0.0015	0.05151
NM_001172079	Icam5_predicted	0.12926	0.65712	0.01047	-0.0976	0.49802	0.3743	0.17458	0.41385	0.58467	0.67508	0.52279	0.49777	-0.1181	-0.0746
NM_133310	Icmt	0.20511	0.09892	0.16909	-0.154	0.2018	0.18626	0.16372	-0.0496	-0.0023	0.07991	0.07426	-0.0034	0.14925	0.35769
XM_001081688.1	Ict1_predicted	0.13351	-0.0169	0.19322	0.06987	-0.0151	0.26261	-0.1944	-0.2758	-0.1628	0.01784	-0.1051	0.29415	0.27653	0.01581
NM_012797	Id1	-1.5894	-2.0112	-1.4083	-2.9698	-2.7946	-2.4161	-1.8227	-2.8321	-1.1365	-1.4996	-1.5155	-0.2645	-1.2247	-1.1445
NM_013060	Id2	0.31136	-1.2684	-0.3471	-1.4951	-1.3093	-1.0777	-0.5338	-1.1336	-0.3702	-0.7979	-0.5646	0.20838	-0.0421	-0.2545
NM_013058	Id3	0.27896	-0.32	0.08844	-0.2486	-0.1334	0.01294	-0.2933	-0.038	0.02895	-0.0205	0.01924	0.54206	0.17129	0.2545
NM_013159	Ide	0.6915	0.51735	-0.4627	-0.4122	-0.0268	-0.6615	-0.0934	0.07659	0.25111	0.43053	0.12705	-0.6742	0.14334	0.36292
NM_031510	Idh1	-0.3638	0.04405	-0.5573	0.21992	-0.4229	-0.5122	-0.2606	-0.0626	-0.0227	-0.0227	-0.0439	-0.0716	-0.5939	-0.7332
NM_001014161	Idh2	0.01956	0.00642	0.21483	0.03341	0.039	0.03572	0.01684	0.08145	-0.0371	0.03801	0.00152	-0.0124	0.01896	0.00953
NM_001014161	Idh2	0.93268	0.59417	-0.6681	-0.2352	0.88588	0.38482	-0.1296	0.78229	0.6778	0.67434	0.53487	0.17748	0.02559	-0.191
NM_053638	Idh3a	0.19361	-0.1221	-0.0289	-0.0003	0.26428	0.22923	0.03697	0.32683	-0.0003	-0.177	0.23237	0.17345	0.10028	-0.1242
NM_053581	Idh3B	0.24401	0.223	-0.033	-0.3297	0.65571	0.38702	0.01306	0.1944	0.10817	0.17953	0.113	0.02195	0.17957	0.1434
NM_031551	Idh3g	-0.3085	-0.421	-0.2114	-0.3876	-0.0881	-0.1379	-0.4936	-0.1108	-0.5611	-0.5519	-0.5177	-0.2207	-0.235	-0.1399
NM_053539	Idi1	-1.3013	-1.6275	0.99371	0.29382	-1.3933	-1.2945	-0.3828	-1.2514	-0.3642	-1.1631	-0.9894	0.7435	-0.2365	-0.2471
XM_225508.3	Idi2_predicted	0.01746	-0.0758	0.05204	0.16442	-0.0268	0.0192	-0.0275	0.0963	0.14786	0.0708	-0.0402	0.09326	-0.0063	0.23883
NM_001009541	Ier2	1.1217	0.70317	0.90843	0.58673	0.12586	0.44131	1.1134	-0.0501	0.45795	0.52743	0.58315	0.48794	0.39057	0.5992
NM_212505	Ier3	-0.0331	-0.5458	-0.3987	0.81205	-0.1316	0.13815	-0.5076	-0.1017	-0.592	-0.4212	-0.481	-0.5737	-0.6529	-0.6414
NM_001025137	Ier5	0.27295	0.25254	0.14093	1.0971	0.07955	0.32195	0.38013	0.12778	0.27676	0.23056	0.39685	0.0732	0.02159	0.18611
NM_001025041	Ier5l	-0.946	0.07061	0.13549	-0.8642	-0.643	-0.3025	0.41022	-0.361	-0.2963	-0.1605	-0.1304	0.08665	0.24231	0.31112
NM_001012029	Ifi204	3.9806	3.8996	3.8999	4.293	2.8243	2.8033	4.4693	2.9762	3.4575	3.9949	3.8087	0.41512	3.7849	3.7747
NM_001012029	Ifi204	0.21649	0.34453	0.30278	0.41959	0.15553	0.35922	0.33884	0.22092	-0.0004	-0.0067	0.28312	-0.0044	0.30985	0.33604
NM_130743	Ifi27l	0.60737	0.84088	0.13753	0.73878	1.5144	1.4087	1.3197	1.6206	0.69278	0.66808	0.70099	0.44657	0.34563	0.3171
NM_130743	Ifi27l	0.92936	0.9828	0.56477	1.1394	1.6436	1.7638	1.488	1.6692	0.67534	0.94513	0.87022	0.4955	0.59087	0.52025
NM_001030026	Ifi30	-0.6122	-0.5612	-0.5942	-1.0242	-1.1559	-0.8063	-0.7955	-0.9273	-0.8143	-0.8006	-0.9653	-0.4883	-1.0515	-0.8108
NM_001009625	Ifi35	0.88467	0.86993	0.21636	0.67012	1.3453	1.6751	0.53097	1.3317	0.71899	1.0573	0.91352	0.86714	0.93953	0.78694
NM_001107729	Ifi44	1.0646	0.88399	0.77656	0.79072	1.8656	1.1645	1.4773	1.9737	0.56286	0.75564	0.85611	0.23346	1.2598	1.5524
NM_172019	Ifi47	0.32688	0.49251	0.06643	-0.0207	0.02799	0.15884	0.93517	0.06718	0.1784	0.48939	0.09506	-0.1449	-0.0439	0.38321
NM_020096	Ifit1	-0.0038	-0.0361	0.03027	-0.1126	-0.0336	-0.0521	-0.0819	0.0678	0.15203	0.07032	-0.0343	0.12822	-0.0142	-0.0157
XM_220058.2	Ifit1_predicted	-0.0938	0.14994	0.15223	0.13912	0.05156	0.12965	0.03186	-0.0949	0.27246	0.19287	0.17823	0.26	0.07072	0.1854
NM_001024753	Ifit2	0.16481	0.08224	0.24775	0.10697	0.21378	0.18674	0.01278	0.14867	0.03104	0.0706	0.09397	-0.0456	0.16001	0.12897
NM_001007694	Ifit3	0.07858	-0.0218	0.02404	-0.0245	0.17476	0.04335	0.03781	0.0922	-0.0193	0.11306	0.03985	0.04225	0.03225	0.06223
NM_001106314	Ifitm1_predicted	1.149	1.3237	0.61229	1.3856	0.66673	0.89367	1.0503	0.64147	1.4552	1.0925	1.39	1.2076	1.1372	1.0412
NM_001136124	Ifitm3	0.25404	1.3117	-0.0429	0.75864	0.09128	0.23996	1.2793	0.10082	0.89484	1.2107	0.97678	0.23919	0.01474	-0.0208
XM_215126.2	Ifitm5_predicted	-0.0312	-0.1578	-0.1058	0.16874	-0.1317	-0.1502	-0.0573	0.04013	-0.0751	0.02704	0.14934	-0.1243	-0.0901	-0.106
XM_219476.3	Ifitm6_predicted	-0.053	0.04648	0.16773	0.04123	0.01623	0.20961	0.18606	0.10482	0.13139	0.1202	-0.1185	-0.0009	0.26089	0.00227
XM_221637.1	Ifitm7_predicted	0.03812	0.39904	0.08166	0.359	0.09291	0.44183	0.21047	0.1339	0.59642	0.50888	0.3344	0.09276	0.20884	-0.0947
NM_001014786	Ifna1	-0.043	-0.1673	-0.0936	-0.0171	-0.1451	-0.0431	-0.0184	-0.0126	-0.021	2E-06	-0.0149	0.00022	0.04267	-0.0545
NM_001014786	Ifna1	-0.076	-0.1468	0.17004	-0.0755	-0.0685	0.06326	-0.1807	0.05201	0.21223	-0.1768	-0.0333	-0.2269	-0.0113	-0.2328
XM_233150.3	Ifna11_predicted	0.20914	0.02245	0.02071	0.05905	0.01887	0.01012	-0.009	0.16808	0.27433	-0.0455	0.01169	-0.0557	0.13568	-0.1624
XM_001054353.1	Ifna11_predicted	0.02901	-0.1585	-0.0493	-0.1723	-0.1805	-0.1992	-0.0237	-0.1252	0.09367	0.0539	0.01039	-0.1722	-0.1999	0.01771
NM_001106667	Ifna4_predicted	-0.0047	-0.0277	-0.2356	-0.0793	0.24557	0.0989	-0.1614	-0.305	0.08658	-0.3356	-0.154	-0.1869	-0.0147	-0.0667
NM_001105893	Ifnar1_predicted	0.06563	0.04173	0.0312	0.30898	0.13771	0.03944	-0.0267	0.04025	0.07303	0.10193	0.26167	-0.0611	0.09456	0.07157
NM_019127	Ifnb1	-0.3049	-0.2056	-0.1504	-0.1294	-0.1487	-0.1535	-0.0707	-0.2475	-0.1373	-0.0675	-0.2042	-0.0441	0.00939	-0.2374
NM_138880	Ifng	0.23543	0.23779	0.22984	0.10326	0.25104	0.2951	0.06876	0.15826	-0.007	0.01132	0.0381	0.11905	0.10561	0.12436
NM_053783	Ifngr1	-0.1461	0.29966	0.45416	0.51315	-0.1874	-0.086	0.36108	-0.406	0.18898	0.14377	0.19926	0.24331	0.59796	0.35823
NM_001108313	Ifngr2_predicted	0.55555	0.69741	0.12034	0.51431	0.55035	0.72894	0.64853	0.38355	0.47595	0.59655	0.56976	0.38429	0.27811	0.26363
NM_001107925	Ifnk_predicted	0.25417	0.38191	0.07494	0.16223	0.41894	0.08133	0.13238	0.15878	0.38925	0.06355	0.16754	0.16616	0.08235	0.40986
NM_001047871	Ifrd2_predicted	-0.5822	-0.8514	0.06664	-0.5935	-0.3388	-0.2602	-0.4815	-0.5447	-0.9732	-1.0377	-0.9876	-0.593	-0.2612	-0.1804

NM_001107093	Ift57_predicted	0.3008	0.07624	-0.6015	0.01855	0.25355	0.26006	-0.371	0.16124	-0.1088	-0.1757	-0.2114	-0.1219	-0.3434	-0.2999
NM_001007001	Ift74	0.27457	0.44414	0.24416	-0.0765	0.05308	0.00977	0.44031	0.15624	0.41417	0.36755	0.35943	-0.0148	0.31583	0.1787
NM_199120	Ift81	-0.0408	0.26766	-0.9553	-0.905	-0.1263	0.049	-0.2076	-0.0673	0.02048	0.14426	-0.0295	-0.2048	-0.5364	-0.7097
NM_031624	Igbp1	-0.7265	-0.0631	-0.4458	-0.5668	-0.8	-0.7141	-0.3934	-0.7974	-0.0622	-0.1691	-0.0985	-0.1651	-0.3242	-0.2425
XM_216802.3	IgE FE-3	-0.1406	-0.0716	0.0456	-0.0716	0.03699	-0.0998	-0.056	0.01735	-0.2125	-0.1863	-0.0512	-0.1245	-0.0689	0.01267
NM_052807	Igf1r	-0.4921	-0.0664	0.63717	0.99599	-0.0267	0.29923	0.40267	-0.0129	-0.2725	-0.0471	-0.0434	0.02307	0.47454	0.45659
NM_031511	Igf2	0.03599	0.17122	0.11959	0.14071	0.20846	0.07128	0.08321	0.16057	0.20836	0.22958	0.41625	0.08139	0.16416	0.12011
NM_175594	Igf2bp1	-0.1872	-0.0725	-0.17	-0.1131	-0.2363	-0.1211	-0.2059	-0.0755	-0.1546	-0.296	-0.1051	-0.326	0.0041	-0.0749
NM_001047888	Igf2bp3	0.08855	-0.0411	-0.4726	-0.0558	-0.1859	-0.271	-0.4637	-0.2096	-0.1225	0.01315	-0.1626	-0.3298	-0.167	-0.1536
NM_012756	Igf2r	-0.582	-0.4687	-0.2579	0.15954	-1.1112	-0.8976	-0.2951	-1.1421	-0.3095	-0.2978	-0.2892	0.01991	-0.8366	-0.9201
NM_053329	Igfals	-0.0057	0.05304	-0.039	0.1468	0.13686	-0.0411	-0.0746	-0.0334	-0.0136	-0.0123	-0.0346	0.08888	0.02746	0.05284
NM_013144	Igfbp1	0.09303	-0.2773	-0.0418	0.04588	0.53542	0.52041	-0.0579	0.23335	-0.3274	-0.1751	-0.0386	0.02536	-0.2316	-0.1903
NM_013122	Igfbp2	0.07655	0.09025	0.00391	0.01419	0.04956	0.12279	0.18054	0.04011	-0.0649	-0.1294	0.08435	0.02543	-0.0879	-0.1571
NM_012588	Igfbp3	-0.1322	-0.0098	-0.0596	-0.0794	-0.0823	0.05939	-0.0977	-0.1084	-0.0441	0.01317	-0.0302	-0.0494	-0.0303	0.08839
NM_001004274	Igfbp4	-0.1817	-0.2388	-0.1737	-0.1958	-0.248	-0.133	-0.2029	-0.2263	-0.2498	-0.2794	-0.2962	-0.0552	-0.2187	-0.3703
NM_012817	Igfbp5	0.07713	0.06331	0.09214	0.12955	-0.0366	0.02514	0.26564	0.10195	-0.0547	0.0535	0.17235	0.16169	-0.0138	0.03129
NM_013104	Igfbp6	-0.4534	1.6215	0.67589	0.44273	-1.6714	-1.5001	1.2971	-1.885	1.2519	1.0662	1.1107	1.1754	0.42829	0.76282
NM_001013048	Igfbp7	-0.0184	0.89119	-0.0239	0.60583	-0.4529	-0.4426	0.20472	-0.5299	0.79957	0.61859	0.66839	0.81215	-0.2207	-0.1007
NM_001108972	Igfbpl1_predicted	0.39479	0.01125	0.40367	0.16767	1.6282	1.8183	1.1046	1.724	0.13827	-0.1049	0.4143	0.30405	0.1954	0.48919
XM_216800.3	Igh-1a_predicted	0.02454	0.19897	0.14426	0.07608	0.02049	0.18512	0.01112	0.02716	0.2821	0.20579	0.10203	0.12706	0.09748	0.09467
NM_001013945	Ighg	0.13168	0.15809	0.16425	0.15021	-6E-05	0.13092	0.06092	0.09018	-0.0685	-0.0843	0.03607	0.04529	-0.0113	0.06506
NM_031586	Ighmbp2	-0.0641	0.19018	0.18376	0.32436	0.09762	0.10951	-0.1369	-0.0111	-0.0564	-0.0149	0.00598	0.0937	-0.0072	0.13056
XM_341195.2	Igj_predicted	0.02394	0.00954	-0.1496	-0.0219	-0.1121	-0.0817	-0.1042	-0.0957	-0.0079	-0.0484	-0.0692	-0.0306	0.07898	-0.1338
XM_578317.1	Igkv28	0.17248	0.10565	0.18875	0.03888	0.08376	0.06732	0.24483	0.08863	-0.0569	-0.0855	0.09151	0.24679	-0.1207	-0.0718
NM_175763	Igsf1	0.06527	-0.1649	-0.0733	-0.0009	-0.2402	0.13376	0.02023	-0.3129	0.02282	-0.0612	-0.1061	0.13259	-0.002	-0.0736
NM_001013120	Igsf11	0.29888	-0.0372	-0.046	0.03576	-0.067	-0.0454	0.02117	0.10108	0.21881	0.05048	0.43336	0.01716	-0.0589	0.07779
XM_227554.3	Igsf2_predicted	0.07139	-0.0214	0.48233	0.19152	0.24121	0.03069	0.29012	0.15224	0.22396	0.03519	0.24266	0.53716	0.30205	0.07156
NM_001106455	Igsf3_predicted	-0.1591	-0.4713	-0.7343	-0.3297	0.08465	-0.3041	-0.5337	0.01892	-0.0596	-0.4986	-0.2979	0.11845	-0.445	-0.1418
NM_001047103	Igsf4b_predicted	-0.0024	-0.0641	0.79879	1.7791	0.02706	0.09423	0.18828	-0.058	0.02313	-0.0104	0.09238	0.08395	-0.1043	0.00796
NM_001047107	Igsf4c_predicted	0.91209	1.4661	0.47889	0.72317	2.923	3.3348	1.1581	2.8325	1.5466	1.5253	1.8072	1.1224	1.6677	1.3201
NM_001047102	Igsf4d_predicted	0.04	0.08768	-0.0572	0.16253	-0.0689	0.24408	0.25967	0.05196	0.08172	0.10891	0.11167	0.0602	0.01305	0.18243
NM_133542	Igsf6	0.05591	0.05442	0.2269	0.30488	-0.1122	0.3329	0.11562	-0.0262	-0.0508	0.15793	0.02891	0.46297	-0.3202	0.08139
XM_340957.2	Igsf7	0.0208	0.05364	0.1287	0.0815	0.15155	0.13586	0.09481	0.19785	0.05063	0.19314	0.30849	0.10423	0.19052	0.27212
NM_001107197	Igsf9_predicted	0.43706	-0.1364	-0.0796	0.12073	0.53118	0.54609	-0.154	0.37937	0.15204	0.05554	0.42046	0.25001	-0.1841	-0.2105
NM_001008765	Igtp	0.18026	0.10775	-0.0266	-0.0759	0.31683	-0.1568	0.12578	0.03432	0.10921	0.07637	0.24251	0.12978	0.02295	0.30764
NM_053384	Ihh	0.06579	-0.0624	0.00569	0.09664	0.20924	-0.0049	-0.0473	0.22384	0.19635	0.21986	-0.1109	0.02722	0.07342	-0.026
NM_053384	Ihh	0.01044	0.08448	0.0883	0.18879	-0.0469	0.11305	-0.0054	-0.07	0.05408	0.07285	-0.0329	0.2411	0.1318	0.07585
NM_053316	Ihpk1	-0.4117	-0.0445	-0.0616	0.20112	-0.2835	-0.163	0.12666	-0.336	-0.2898	-0.2113	-0.2907	-0.167	-0.6666	-0.4054
NM_021660	Ihpk2	-0.2239	0.08543	0.16683	0.27928	-0.0978	-0.3247	0.21358	-0.0784	0.10322	0.06854	0.06743	-0.1018	-0.334	-0.4462
NM_145786	Iiig9	0.13474	0.01713	0.00998	-0.0685	-0.0416	0.04611	0.02125	0.02363	-0.0382	-0.0094	0.01122	0.14827	0.13885	0.0616
NM_001017450	Iip45	-0.0903	-0.5267	0.4598	0.0406	-0.1707	-0.1041	-0.3757	-0.3649	-0.1365	-0.1964	-0.0127	0.38005	0.16048	-0.0573
NM_001005537	Ik	0.22312	0.16837	0.32191	0.59685	0.3744	0.40538	0.43406	0.59725	0.18276	-0.0963	0.22656	-0.0439	0.22418	0.23509
NM_080899	Ikbkap	-0.4056	-0.1732	0.00467	0.00552	-0.233	-0.2233	0.05514	-0.3097	-0.4668	0.05805	-0.4162	-0.1978	-0.1449	-0.2769
NM_053355	Ikbkb	-0.2098	0.01097	0.02233	1.165	-0.1066	-0.1835	0.41313	-0.0712	-0.2479	-0.2002	-0.165	-0.1501	-0.4479	-0.728
NM_001108854	Ikbke_predicted	0.35187	0.70319	0.41048	0.10552	-0.2389	-0.2918	0.51438	-0.1207	0.45597	0.56458	0.39667	0.16188	-0.4173	-0.3223
NM_199103	Ikbkg	-0.4073	-0.246	-0.3709	-0.5028	-0.2447	-0.2849	-0.233	0.12252	-0.5541	-0.2799	-0.2504	-0.468	0.2814	-0.0128
NM_012854	Il10	1.9022	-0.0176	-0.077	1.0046	1.2317	1.2294	1.2833	1.1166	-0.0546	-0.1378	-0.1288	-0.0906	-0.0194	-0.1426
NM_057193	Il10ra	-0.1402	-0.1219	-0.0181	-0.0345	-0.0278	0.03212	0.11618	-0.0433	-0.0553	-0.0836	-0.0843	-0.0326	-0.1344	0.02966
NM_139116	Il11ra1	0.21087	0.2897	0.06488	0.04419	0.20844	0.28977	0.57825	0.12262	0.24052	0.50709	0.2623	0.25352	-0.1216	-0.077

NM_053390	Il12a	-0.0928	-0.091	-0.1508	-0.0172	-0.1353	-0.0853	-0.0647	-0.0806	-0.075	-0.2153	-0.075	-0.1704	0.01239	-0.141
NM_022611	Il12b	-0.1014	-0.076	-0.144	-0.1208	-0.006	0.18804	0.07848	0.01061	-0.1302	-0.1363	-0.135	-0.1518	-0.0832	-0.168
NM_001170604	Il12rb1	-0.0376	0.11481	0.13816	-0.0614	0.12441	0.3013	-0.1491	0.02353	0.06429	-0.039	0.11749	0.1565	0.12084	0.21855
NM_001170604	Il12rb1	-0.188	-0.0525	-0.0696	-0.044	-0.2277	0.00185	-0.1196	-0.2213	-0.2316	-0.1938	-0.0872	-0.0991	-0.1157	0.02758
XM_231873.4	Il12rb2	0.18004	0.05086	0.13061	0.14161	0.09725	0.09183	0.14079	0.10352	0.0878	0.32875	0.06483	0.16051	0.12828	0.10356
NM_053828	Il13	-0.0289	0.05705	0.11685	-0.0334	0.032	0.15506	0.00876	0.12194	0.13638	0.37248	-0.0362	0.16185	0.10788	-0.0368
NM_145789	Il13ra1	0.10993	0.32186	0.0706	0.04602	0.10154	0.09096	-0.0575	0.24107	0.02592	0.36585	0.08078	-0.0618	0.08673	0.09543
NM_133538	Il13ra2	0.25126	0.23697	0.18482	0.13066	0.01197	-0.113	0.02683	0.16647	-0.0391	0.05787	0.15935	0.04825	0.01489	0.15411
NM_013129	Il15	2.3654	0.87942	1.8729	2.221	1.9427	2.0178	2.4622	2.0724	0.38836	0.75008	1.4195	0.06325	0.06852	0.04766
NM_001105749	Il16_mapped	-0.0425	0.00964	0.06485	-0.0423	-0.1002	-0.0509	0.0335	0.06643	0.11403	0.03065	-0.0671	2E-06	-0.0863	0.01072
NM_053789	Il17b	0.24774	0.27601	0.2109	0.08137	0.10764	-0.0296	0.03098	0.03406	0.15743	0.10982	-0.0684	0.08298	0.26944	0.13238
NM_001107883	Il17r_predicted	0.2457	0.33009	1.0578	0.73139	0.16625	0.0492	1.0483	0.01645	0.28724	0.41258	0.32488	-0.1976	0.41692	0.53767
NM_001107290	Il17rb_predicted	-0.1503	-0.1386	-0.0267	0.24825	-0.2167	-0.0736	-0.073	-0.1731	-0.2991	-0.0869	-0.0531	-0.1185	-0.0904	-0.3858
NM_001107290	Il17rb_predicted	-0.2374	0.02853	-0.0145	-0.192	-0.1654	0.07473	0.0902	0.01007	-0.1311	-0.1145	-0.1286	0.24547	-0.1838	-0.1185
NM_001170565	Il17rc_predicted	-0.1042	-0.4251	-0.0939	-0.5605	-0.0607	-0.1461	-0.183	-0.0473	-0.3363	-0.0806	-0.2054	-0.9168	-0.4205	-0.2994
NM_001170565	Il17rc_predicted	0.20514	0.0994	-0.5404	-0.5094	-0.0121	-0.2921	-0.3066	0.0424	-0.1671	0.0229	-0.0519	-0.5157	0.03482	-0.1209
NM_001004091	Il17re	0.10511	0.46787	0.37791	-0.8423	0.11419	0.12325	-0.1099	-0.218	0.01045	0.40921	0.36869	-0.3452	0.19094	0.22285
NM_019165	Il18	-0.1888	0.01736	-0.1771	-0.1412	-0.0062	-0.3161	-0.2746	-0.2548	0.11843	-0.1391	-0.2899	-0.4078	0.1385	-0.1926
NM_053374	Il18bp	0.29077	0.34843	0.30029	0.3272	0.06066	0.0619	0.26111	0.21365	0.27676	0.13073	0.37291	0.06156	0.09861	-0.0395
NM_001106905	Il18r1_predicted	-0.0274	-0.1726	0.06719	-0.0028	-0.0114	0.11792	-0.0915	-0.0496	-0.133	0.00186	-0.0115	-0.1907	-0.1787	-0.1769
NM_184047	Il18rap	-0.1053	-0.149	0.00181	-0.093	-0.0375	-0.0375	-0.057	-0.0375	0.03544	0.02894	-0.0808	-0.1224	-0.0216	-0.0252
XM_237089.3	Il18rap_predicted	-0.0665	0.24477	-0.0604	0.12303	0.21666	-0.0107	0.24658	0.12526	0.06553	0.08674	0.12395	-0.0319	-0.0177	0.15942
NM_017019	Il1a	0.28467	-0.1794	-0.096	0.07786	0.01028	-0.0144	0.00609	0.05974	-0.1722	-0.1215	-0.1671	-0.2048	-0.1359	-0.132
NM_031512	Il1b	-0.0566	-0.0471	0.03513	-0.0465	0.03247	-0.1416	-0.0892	-0.0783	0.13035	-0.0387	-0.1079	-0.1408	0.02572	-0.0085
NM_001108571	Il1f10_predicted	0.35081	0.02795	0.09825	0.0392	0.37675	0.5798	0.03618	0.24439	0.21944	0.12922	0.20837	0.03677	0.10038	0.16595
NM_001106554	Il1f6_predicted	0.10971	0.16446	-0.0077	0.06619	-0.0509	-0.0666	0.01363	-0.0697	0.01891	0.27462	0.02231	0.11184	-0.0497	-0.0309
NM_001108570	Il1f8_predicted	1.0022	0.11406	0.14233	0.51264	0.67623	0.52198	0.24061	0.5524	0.18749	0.12338	0.22497	0.1298	0.17344	0.20318
NM_013123	Il1r1	0.00194	-0.1202	-0.164	-0.1526	-0.0912	0.02021	-0.109	-0.0865	-0.1074	-0.1099	-0.1648	-0.1844	-0.02	-0.1295
NM_053953	Il1r2	0.09381	0.24942	0.17205	0.06906	0.30658	0.14237	0.01732	0.02787	0.13	0.14665	0.20037	0.11852	0.0124	0.05537
NM_001167840	Il1rap	0.0057	0.17174	0.0028	-0.0096	0.19728	-0.0318	0.03398	0.00138	0.18289	0.19818	0.13734	0.03428	0.05967	0.12359
NM_177935	Il1rapl1	-0.1465	0.02949	-0.1263	0.00322	0.03375	-0.0313	-0.0927	0.13945	0.00655	-0.1682	-0.0142	0.07399	-0.0225	-0.0585
NM_001166342	Il1rapl2_predicted	0.07533	0.13799	-0.1733	0.15282	0.18727	0.3928	0.18435	0.20695	0.06313	-0.0508	-0.0501	-0.0111	0.16427	0.14577
NM_013037	Il1rl1	1.2036	1.8051	1.6978	4.1191	1.3161	1.5721	1.2577	1.3557	1.3174	1.8325	1.5494	0.53539	1.9538	1.8934
NM_133575	Il1rl2	-0.0607	-0.0175	-0.0991	-0.0579	-0.042	-0.0439	-0.1567	0.03191	-0.0546	0.00179	-0.07	-0.09	-0.0673	-0.0159
NM_022194	Il1rn	2.4919	0.62609	-0.1598	1.1006	3.1573	2.0588	1.4595	2.7638	1.0255	0.69273	1.2051	-0.4977	1.2516	1.3902
NM_053836	Il2	0.05403	0.08801	0.03558	0.12003	0.06459	0.03781	0.00415	0.08028	0.03067	0.0159	0.06926	0.42216	0.24423	-0.0639
NM_001107521	Il20ra_predicted	-0.2263	-0.2041	-0.156	0.01414	-0.1116	-0.2094	-0.3243	-0.205	-0.2163	-0.1506	-0.277	0.09884	-0.3157	-0.2992
NM_001012469	Il21r	-0.0372	-0.007	0.00603	-0.0603	-0.0559	-0.127	-0.0416	-0.1124	-0.0785	0.06777	0.04548	-0.0621	0.01934	-0.0075
NM_001003404	Il22ra2	-0.3196	-0.2698	-0.2471	-0.1986	-0.2673	-0.2181	-0.2153	-0.1073	-0.3292	-0.1716	-0.2055	-0.1633	-0.1479	-0.2454
NM_130410	Il23a	0.05978	-0.223	-0.0111	0.12787	0.23445	-0.0275	0.00562	-0.0515	-0.3782	-0.1716	-0.2794	-0.0812	-0.0586	-0.0116
NM_133311	Il24	0.15014	-0.1156	0.19509	0.11185	-0.2167	0.02656	-0.0605	-0.06	-0.2444	-0.1066	-0.1415	-0.2768	-0.3105	0.08315
NM_001105943	Il27ra_predicted	0.26225	-0.0346	0.25896	0.32285	0.02509	0.24341	0.00017	0.0333	0.09922	-0.0024	0.12634	0.28774	-0.0242	0.0067
NM_013163	Il2ra	-0.1412	-0.0709	-0.1103	0.0463	0.00029	-0.1408	-0.1376	-0.2113	-0.0639	-0.0796	-0.1548	-0.1495	0.01437	-0.1199
NM_080889	Il2rg	-0.0904	-0.2688	-0.2672	-0.028	-0.0737	-0.2748	0.02338	-0.0236	-0.0211	-0.2048	-0.2181	-0.3773	-0.1288	-0.1082
NM_031513	Il3	0.0928	-0.0864	-0.0104	0.00388	-0.05	-0.0295	-0.0154	0.01329	0.06631	0.22777	-0.0273	-0.0049	-0.0092	-0.0354
NM_001014166	Il33	-0.6479	0.55616	-0.7504	-0.8175	-0.8998	-0.7799	-0.6029	-0.7669	0.22819	0.58665	0.30941	-0.7513	-0.7331	-0.962
NM_001014166	Il33	0.14504	0.01764	0.07435	0.01569	0.10127	0.15377	-0.0062	-0.0104	0.05102	0.05876	0.00127	0.31778	0.05295	0.0518
NM_139260	Il3ra	-0.1703	-0.3079	0.00718	-0.0176	-0.0491	-0.0983	-0.2916	-0.3884	-0.1492	-0.141	-0.3401	-0.2497	-0.2098	-0.3575
NM_201270	Il4	0.33653	0.32706	0.21245	0.12502	0.07119	0.03451	-0.0209	0.06096	0.20728	0.14352	-0.0269	-0.017	0.05163	0.13194

NM_133380	Il4ra	-0.0862	-0.1165	-0.2235	0.00027	-0.0695	0.11939	0.13971	-0.1476	-0.1075	0.24275	-0.2144	-0.0737	-0.1012	-0.1415
NM_021834	Il5	-0.3522	-0.2259	-0.3177	0.00278	-0.2654	0.16884	-0.0585	-0.1869	-0.0198	-0.1579	-0.2154	-0.2306	0.11294	-0.2593
NM_053645	Il5ra	-0.0908	0.05134	-0.0737	-0.0465	-0.0935	-0.0272	-0.0929	-0.053	-0.1593	-0.0104	-0.0462	-0.2593	0.0277	-0.1435
NM_012589	Il6	0.29457	0.10337	0.05526	0.51016	0.25205	0.16583	0.3823	0.20529	0.26614	0.17309	0.11762	0.16231	0.20398	0.27789
NM_017020	Il6ra	0.13682	0.28014	0.23788	-0.0554	0.31564	0.09355	0.12621	-0.0576	0.25892	0.26842	0.24076	-0.2941	0.08352	-0.13
NM_001008725	Il6st	-0.1755	-0.2655	-0.1511	-0.2614	-0.1501	-0.2771	-0.1508	-0.2029	-0.0901	0.04149	-0.1096	-0.1024	-0.1085	-0.0947
NM_001106418	Il7r_predicted	-0.0716	-0.2616	-0.1907	-0.1869	-0.2422	-0.1763	-0.1139	-0.2223	-0.2417	-0.2142	-0.1464	-0.1417	-0.2062	-0.0413
NM_019310	Il8ra	0.08461	0.0721	-0.1421	-0.0082	-0.0447	0.11114	-0.0761	-0.0688	0.09875	-0.0994	0.04686	0.31466	-0.1485	0.13612
NM_017183	Il8rb	-0.1284	-0.1569	-0.0827	0.0836	-0.1451	-0.1985	-0.0841	-0.1071	-0.0234	-0.0127	-0.0622	0.06509	-0.0932	-0.0414
NM_001105747	Il9	0.4212	0.05308	0.23912	0.05189	0.45921	0.40599	0.25851	0.17417	-0.0488	-0.0723	0.07185	0.44586	0.09906	-0.1411
NM_017021	Il9r	0.17529	-0.0259	0.17558	-0.1231	-0.0048	0.15301	0.18534	-0.0449	0.21794	0.32959	0.10373	0.13042	-0.0974	0.12043
NM_053412	Ilf3	0.14006	-0.2231	0.116	0.22292	0.14461	0.4997	-0.3107	-0.075	0.27724	-0.0328	-0.0659	0.44742	0.18164	0.06548
NM_133409	Ilk	0.0682	0.48932	0.38845	-0.0013	0.33912	0.34837	0.13079	0.38604	0.17909	0.19648	0.18223	0.10478	0.10723	0.15615
NM_133409	Ilk	-0.1187	0.08481	0.06423	-0.1353	0.21685	0.34168	-0.0023	0.32383	0.03346	0.10401	0.02668	0.19622	0.11175	0.40015
NM_133409	Ilk	-0.2845	0.14439	0.00943	-0.4071	0.17269	-0.0109	-0.1346	0.22833	0.14404	0.13794	0.12753	0.10973	-0.016	0.03968
NM_022606	Ilkap	-0.8442	-0.3583	0.27097	-0.0001	-1.0496	-0.9498	0.05387	-1.1578	-0.6548	-0.5895	-0.527	-0.2485	-0.1892	-0.1815
NM_001108738	Ilvbl_predicted	0.20268	0.51226	-0.102	0.00517	-0.0359	-0.3979	0.27137	-0.017	0.4052	0.44408	0.54477	0.18339	0.33838	0.16475
NM_001034928	Immt_predicted	0.16361	-0.0603	0.22601	-0.495	0.03043	-0.1705	0.10308	0.10064	-0.0081	-0.0112	-0.0178	-0.217	0.12707	0.11423
NM_001108152	Imp3_predicted	-0.2121	-0.0397	-0.0695	-0.041	-0.0274	0.03654	0.31362	-0.2007	0.09211	0.12848	0.01013	0.15975	-0.2303	-0.0851
NM_001009700	Imp4	0.18763	-0.0796	0.20502	0.09948	0.10284	0.07242	0.08697	0.13915	-0.004	-0.0957	-0.2099	-0.2168	0.15698	0.39633
NM_032057	Impa1	-0.0133	0.51475	-0.0255	0.16909	0.21576	0.04147	0.42343	0.34436	0.36432	0.37759	0.36963	0.39793	0.40172	0.38384
NM_172224	Impa2	0.74184	0.68775	0.16123	0.5576	-0.3393	-0.3228	-0.1134	-0.4845	0.43024	0.63586	0.6996	0.16364	-0.0371	-0.0025
XM_001069864.1	Impad1	-0.224	-0.1848	-0.3071	-0.5997	0.2444	-0.5242	-0.097	0.16214	-0.0541	-0.2886	-0.3535	-0.421	0.1277	0.15224
NM_001108619	Impdh1_predicted	0.0743	-0.0798	-0.6343	-0.7204	-0.0394	-0.0999	-0.8695	-0.0681	-0.1877	-0.2556	-0.3527	-0.2545	-0.3135	-0.2943
NM_001108619	Impdh1_predicted	-0.3251	-0.3232	-0.5871	-0.7512	-0.2936	-0.5795	-1.0335	-0.3219	-0.3808	-0.2952	-0.3818	-0.5798	-0.4921	-0.6762
NM_199099	Impdh2	-0.426	-0.2828	-0.1745	-0.437	-0.6604	-0.716	-0.7038	-0.743	-0.3704	-0.1276	-0.2095	-0.3004	-0.2297	-0.2409
NM_023958	Impg1	-0.0487	0.05051	0.04613	0.00091	-0.0737	-0.1427	-0.068	0.11358	-0.0047	-0.0804	0.23981	0.13263	0.10171	-0.0972
NM_139088	Impg2	-0.104	-0.0167	-0.0525	-0.0003	-0.1607	-0.0278	0.13415	-0.0701	0.01176	-0.0249	-0.1119	0.02397	0.14955	0.17529
NM_001106335	Incenp_predicted	-0.1452	-0.5652	0.55867	-0.1149	-0.353	-0.0664	-0.5044	-0.5394	-0.2658	-0.3666	-0.1847	0.43187	0.52536	0.38115
NM_023973	Indo	0.09365	0.14506	0.20648	0.40317	0.43626	0.41816	0.20938	0.036	0.11115	-0.1778	0.12886	0.24979	-0.0242	0.22338
NM_019128	Inexa	0.00375	0.04926	0.0433	0.25365	-0.0307	0.00995	-0.0927	0.06915	0.11808	-0.0751	0.12087	-0.0018	0.3176	0.07412
NM_001038591	Ing1_predicted	0.12654	-0.3816	-0.1946	-0.1893	-0.2682	-0.3255	-0.184	-0.128	-0.0413	0.14954	0.27866	0.03096	-0.0694	-0.3198
NM_001106083	Ing1l_predicted	-0.0171	-0.0202	0.05747	-0.4637	-0.4496	-0.5004	-0.0829	-0.6725	-0.1095	-0.0864	-0.2091	0.35998	-0.1207	0.02255
NM_001034107	Ing3	0.11673	-0.0261	0.05949	-0.1114	-0.2	-0.3101	-0.0593	-0.1064	-0.1375	-0.0843	-0.1362	-0.0861	0.10462	0.265
NM_001079887	Ing4	0.26962	-0.0005	-0.0659	0.70037	0.38513	0.56215	0.25625	0.27522	-0.1314	-0.0083	-0.2111	-0.089	-0.5379	-0.4776
NM_001108810	Ing5_predicted	-0.0085	-0.214	-0.0819	-0.1425	-0.2299	-0.2135	-0.2368	0.00267	-0.1947	-0.1774	-0.1691	0.04581	0.21006	0.16305
NM_012590	Inha	-1.2528	-0.77	-0.4628	-0.9931	-1.2101	-1.1529	-0.6627	-1.1179	-0.8437	-0.9706	-0.9175	-0.4901	-1.3337	-1.1337
NM_017128	Inhba	0.1254	-0.2017	0.40716	0.71648	-0.0318	-0.1577	0.50888	0.15401	0.0006	0.01193	-0.2074	-0.0921	0.51271	0.01583
NM_080771	Inhbb	0.0606	0.23203	0.10718	0.03251	0.12703	-0.0769	-0.0327	0.26595	0.10844	0.06116	0.13969	0.01814	0.04084	0.32526
NM_080771	Inhbb	-0.2242	-0.1273	-0.0547	-0.2428	-0.1567	-0.2865	-0.2254	-0.1129	-0.2519	-0.1161	-0.1753	-0.2374	-0.2113	-0.2433
NM_022614	Inhbc	0.0043	0.37098	0.32272	0.18794	0.30221	0.3157	0.42889	0.17101	0.34286	0.16736	0.48396	0.43934	0.19239	0.1123
NM_001012131	Inpp1_predicted	0.03533	-0.0062	0.05593	0.10482	0.01029	0.14248	0.05394	0.2608	0.15535	0.06012	0.05483	0.06684	0.09999	0.15031
NM_031002	Inpp4a	-0.3152	-0.2069	-0.3171	-0.0355	-0.1105	-0.004	-0.3047	-0.2881	-0.2649	-0.0974	-0.0508	-0.1943	-0.8494	-0.8837
NM_053917	Inpp4b	-0.0399	-0.0482	-0.0016	-0.0576	-0.1755	-0.1461	-0.039	0.24146	-0.0452	-0.0098	-0.0141	-0.0929	-0.0879	-0.1577
NM_001108923	Inpp5a_predicted	1.1028	0.53398	0.23882	0.91393	1.5097	1.4432	0.23569	1.4984	0.39671	0.60461	0.45475	-0.0857	0.32797	0.24606
NM_001100755	Inpp5b	-0.4467	0.22108	0.20612	0.58319	-0.2107	-0.5065	0.4247	-0.105	-0.1234	0.18299	-0.0156	-0.3018	-0.4563	-0.3872
NM_019311	Inpp5d	0.51591	-0.0929	-0.0496	0.93076	0.95077	1.1561	0.42772	1.0829	-0.0092	0.11286	0.32425	-0.1816	0.0391	-0.0814
XM_342391.2	Inpp5e	-0.0492	0.14694	0.12181	0.34302	-0.096	-0.1231	0.41845	0.0123	0.18313	0.01872	0.16072	-0.0526	-0.1606	0.00616
NM_001107554	Inpp5f_predicted	-0.2453	0.08719	-0.3725	0.15303	-0.1681	-0.2444	-0.217	-0.1667	0.01832	0.20251	0.03001	-0.0424	-0.0338	-0.0762

NM_022944	Inppl1	-0.2371	-0.3031	0.62774	0.19619	0.05072	0.4761	0.13225	-0.3048	-0.3734	-0.4508	-0.1979	0.04795	-0.435	-0.5042
NM_019129	Ins1	0.11859	0.04415	0.04653	0.12336	0.04549	0.24649	-0.0374	-0.0013	0.03593	0.1547	0.05689	0.02541	0.00122	-0.0728
NM_019130	Ins2	0.03417	0.00343	-0.1103	0.03081	0.03482	0.07245	-0.0147	0.13494	-0.0668	-0.0686	-0.0848	0.11063	-0.0799	-0.116
NM_178091	Insig2	0.46866	0.7046	-0.2577	0.53493	0.08969	-0.2911	0.44434	0.06745	0.3881	0.47393	0.53754	-0.4499	-0.2794	-0.2639
NM_053680	Insl3	-0.0716	0.09082	-0.0651	0.72141	-0.1154	-0.2721	0.14161	-0.3408	-0.1794	-0.24	-0.0036	-0.0553	-0.4012	-0.4316
NM_022583	Insl6	0.21705	0.10302	0.13363	0.14454	-0.0067	-0.0173	-0.0188	-0.1181	-0.0016	0.05209	-0.0764	0.13492	0.37066	-0.0257
XM_001055419.1	Insm1_predicted	-0.0031	-0.1561	-0.1267	0.12325	0.22519	0.02561	0.13883	0.04299	-0.0016	-0.0197	-0.1221	-0.1352	0.14214	-0.0048
XM_001079190.1	Insm2_predicted	0.2618	0.07552	0.17835	0.13214	0.15483	0.20786	0.23225	0.16108	0.13062	0.17769	0.18084	0.28932	0.27081	0.10899
NM_017071	Insr	0.12913	-0.0275	-0.0449	-0.1854	-0.0716	0.04757	-0.0585	0.08791	0.29289	-0.0065	-0.0277	-0.0381	0.06444	-0.01
NM_022212	Insrr	-0.1367	0.01935	-0.0364	-0.0984	-0.1496	0.13256	-0.0154	0.04886	0.0582	0.01765	-0.0197	0.07093	-0.1681	-0.1268
NM_001007640	Ints12	0.45115	0.17149	-0.1028	0.8513	0.22588	0.44493	-0.2463	0.37908	0.08737	0.09952	0.04387	0.14349	0.3771	0.42355
NM_001170799	ipcef1	0.0444	0.13156	0.04411	0.16179	0.01736	0.1101	0.15749	0.14693	0.01208	0.15897	0.28897	-0.0341	0.17009	0.14542
NM_134417	Ipmk	-0.2708	0.12305	0.07983	0.23203	-0.037	0.22594	0.06962	-0.0394	0.01811	0.06648	0.02735	-0.121	-0.0022	-0.0029
XM_226752.3	Ipo11_predicted	-0.3356	-0.3327	-0.3767	-0.2601	-0.1348	-0.128	-0.141	-0.1235	-0.1139	-0.2169	-0.2407	-0.1409	-0.3163	-0.2479
NM_053778	Ipo13	-0.2608	-0.2794	0.44493	-0.1386	0.0621	0.02718	-0.2867	-0.0755	-0.4489	-0.4866	-0.2552	-0.3028	-0.2413	-0.0695
NM_001106038	Ipo4_predicted	-0.1331	-0.3605	0.51326	0.21743	-0.1146	0.06798	-0.258	-0.0561	-0.1491	-0.2786	-0.2924	-0.2355	0.19084	0.23371
NM_001107545	Ipo7_predicted	0.33182	0.22159	0.11079	0.47153	0.56105	0.63893	0.16456	0.48029	0.05958	-0.1396	-0.0032	-0.0804	0.29988	0.02705
NM_001107180	Ipo9_predicted	-0.0414	-0.2623	-0.3637	-0.4322	-0.293	0.11212	-0.1231	-0.289	-0.1742	-0.3731	-0.0077	0.44004	0.4022	0.32847
NM_001107180	Ipo9_predicted	0.09329	-0.0524	-0.0037	-0.0022	-0.01	-0.1263	0.00224	-0.0484	-0.0687	-0.0201	0.01016	0.11059	0.00655	-0.0838
NM_001106679	Ipp_predicted	-0.0185	-0.0083	0.10736	0.03224	0.20394	-0.1397	-0.0232	0.0693	0.15069	0.19854	0.11978	-0.059	0.20793	0.10539
NM_001008556	Ippk	-0.1269	0.20434	-0.0814	0.02655	-0.0797	-0.0574	-0.0244	0.04042	0.16489	-0.021	0.07705	-0.0046	0.04392	0.26357
NM_001107092	Iqcb1_predicted	-0.0574	-0.1779	-0.134	0.41551	0.13788	-0.2072	0.03685	0.03616	-0.2407	-0.1673	-0.1702	-0.1089	-0.346	-0.0991
XM_001073653.1	Iqce_predicted	-0.3377	-0.2668	-0.1726	-0.1936	-0.2343	-0.0182	0.15526	-0.1429	-0.0442	-0.1388	-0.0778	-0.2168	-0.1169	-0.0066
NM_001014230	Iqcg	-0.1038	-0.0199	-0.1057	0.11923	0.02041	-0.0189	0.00691	0.03715	-0.1025	-0.1046	-0.1368	0.02753	0.0466	-0.0816
NM_001108489	Iqgap1_predicted	-0.3503	0.18999	-0.9274	-0.4901	0.11708	-0.4683	-0.3891	0.34806	-0.1229	0.00959	-0.4139	-0.7291	0.11537	0.09231
XM_227396.4	Iqgap3_predicted	-0.2466	-0.9176	1.0908	-0.3177	-0.8498	-1.0072	-0.4127	-0.6484	-0.3019	-0.606	-0.4495	0.10727	0.52497	0.62408
XR_008556.1	Iqsec2_predicted	0.19373	0.29791	0.33618	0.33043	0.13492	-0.4712	-0.0709	0.40402	0.25288	0.53501	0.50417	0.19857	0.29561	0.24124
NM_207617	Iqsec3	0.13649	-0.131	-0.108	0.18263	0.51054	0.7178	0.07988	0.41945	0.00451	-0.0552	-0.0408	0.11236	0.13944	0.07421
NM_001034130	Iqub	-0.1563	-0.1089	-0.0733	0.11107	-0.0988	-0.168	0.05406	0.15182	-0.1187	0.28153	-0.1021	-0.089	0.0089	-0.0978
NM_001127555	Irak1_predicted	-0.2087	-0.1211	-0.0411	0.43279	-0.4014	-0.5434	-0.0347	-0.3271	-0.0326	-0.1463	-0.105	-0.328	-0.0082	0.02749
NM_001106843	Irak1bp1_predicted	0.17198	-0.1017	-0.2521	0.1387	-0.1337	0.01208	-0.0367	-0.1658	0.1308	-0.0998	0.01507	-0.0626	-0.1888	-0.2595
NM_001025422	Irak2	0.33672	0.33037	0.27941	0.11019	0.33139	0.18683	0.37347	0.19505	0.24572	0.05212	0.33962	-0.0682	0.0221	0.09664
NM_001108101	Irak3_predicted	0.03619	0.66443	0.35462	0.24403	0.08971	-0.1753	0.8119	0.00292	0.2393	0.46329	0.32506	-0.1414	0.58149	0.10063
NM_001106791	Irak4_predicted	-0.1026	0.14683	0.05098	-0.1195	0.05988	0.15821	0.00594	-0.0486	0.40219	0.36716	0.10072	0.05628	0.22503	0.15118
NM_022863	Ireb2	-0.369	0.52171	-0.2741	-0.2169	-0.188	-0.563	-0.0623	-0.0231	0.05374	0.18776	-0.3038	-0.0959	0.25969	0.14183
NM_012591	Irf1	-0.4063	0.04734	0.08135	0.17702	-0.0344	-0.1148	0.69457	-0.0031	0.00169	0.05351	0.07688	-0.0823	-0.0456	-0.0304
NM_001047086	Irf2_predicted	-0.0304	-0.1031	-0.1626	-0.2113	-0.1186	0.08502	-0.1821	-0.2362	-0.1032	-0.178	-0.2169	-0.1597	0.00709	-0.1441
NM_001107483	Irf2bp1_predicted	-0.3825	0.13112	-0.1976	-0.2498	-0.3462	-0.8165	-0.1182	-0.3242	-0.0083	-0.0599	0.068	-0.4426	0.09609	0.153
NM_199088	Irf3	-0.6115	-0.4777	0.07709	0.05474	-1.0357	-0.6983	0.15577	-0.9321	-0.4559	-0.4043	-0.3273	-0.2106	-1.0468	-1.0345
NM_001106108	Irf4_predicted	0.04975	0.11145	0.07146	0.0485	-0.0012	0.11277	0.01924	0.09406	0.02254	-0.0264	0.04046	-0.0156	0.01723	0.00678
NM_001106586	Irf5_predicted	0.09587	0.388	0.17141	-0.0632	0.22662	0.32311	0.25707	0.08168	0.05911	0.35339	0.11538	0.58677	0.10469	0.01764
NM_001108859	Irf6_predicted	0.03044	0.07915	-0.0059	-0.0526	0.16428	0.12628	0.02304	0.30782	-0.0399	0.09769	0.09648	0.25663	0.20915	0.02385
NM_001033691	Irf7	-0.0523	-0.1966	0.13777	-0.2918	-0.0519	0.00198	0.1726	-0.0493	-0.1667	-0.3184	-0.2698	-0.2519	0.30755	0.31359
NM_001008722	Irf8	0.29469	0.24147	0.02414	-0.0587	0.01524	-0.0327	-0.0176	-0.0389	-0.0796	0.11402	-0.0903	0.02277	-0.0289	-0.0536
NM_001012041	Irf9	-0.4401	-0.1096	0.03037	0.04324	-0.019	0.11496	0.4384	0.25663	-0.0583	-0.1187	-0.0494	-0.4128	0.03146	-0.0276
NM_001012007	Irgm	0.05695	0.17152	-0.0069	-0.3171	0.21369	0.19458	0.24631	-0.0012	0.26486	0.31756	0.06594	0.22446	0.16364	0.0219
NM_001012007	Irgm	-0.0461	-0.0312	-0.0271	-0.0031	-0.0841	-0.0508	-0.0357	-0.033	0.02221	-0.0849	-0.0839	0.0235	-0.0328	-0.1006
NM_012969	Irs1	0.07222	0.06973	-0.0481	-0.2486	0.04964	-0.1604	-0.1098	-0.0881	-0.2016	-0.1089	-0.2412	-0.2742	-0.393	-0.1973
NM_001168633	Irs2	0.07709	-0.0709	0.1374	-0.1293	-0.0934	-0.1566	-0.0595	-0.1399	-0.1539	-0.0159	-0.1556	-0.1151	-0.1011	-0.1823

NM_032074	Irs3	-0.094	0.02306	0.23564	0.31702	0.10245	0.09501	0.15948	-0.014	0.10567	0.17056	0.03038	0.24661	0.12781	-0.0082
XM_235721.4	Irs4_predicted	0.02376	0.04152	-0.0461	-0.0698	0.00559	0.29312	-0.0071	-0.0644	0.0733	0.02242	-0.1245	-0.053	-0.0715	0.0811
NM_001107331	Irx1_predicted	-0.216	-0.1707	-0.1594	-0.1767	-0.2134	-0.0102	-0.2642	-0.1549	-0.2515	-0.1777	-0.1573	-0.1735	-0.1659	-0.1567
NM_001107331	Irx1_predicted	-0.0694	0.0518	0.00826	0.05232	-0.0673	0.03049	-0.0511	0.00525	-0.1202	0.00526	-0.0469	-0.0677	0.01346	-0.0583
NM_001039505	Irx2	-0.1175	-0.0477	-0.0346	0.07546	-0.085	0.25901	0.0163	0.00232	-0.0826	0.04882	-0.0774	0.02529	-0.1445	0.10917
NM_001107413	Irx3_predicted	-0.0222	-0.09	-0.2114	-0.1753	-0.03	0.27379	-0.0745	0.00788	-0.072	-0.1425	0.26736	0.48836	-0.0346	0.073
NM_001107330	Irx4_predicted	-0.0866	-0.1041	-0.2052	-0.0041	0.49534	0.21334	-0.1194	0.19569	-0.2002	-0.0391	-0.0092	-0.0825	-0.1182	-0.0243
NM_181626	Isca1	0.27491	0.26833	-0.3137	-0.1993	0.82921	1.0485	-0.1903	0.63575	0.19962	0.06846	0.08108	0.44905	0.48382	0.35962
NM_206846	isg12(b)	2.158	0.21459	0.61597	1.5227	3.6293	3.6908	0.41094	3.4998	0.85103	0.31186	0.51868	1.2971	0.69767	0.58698
NM_001008510	Isg20	-0.9251	-1.3519	-0.9623	0.97498	-0.8037	-0.5816	-0.6723	-0.7283	-1.2109	-1.444	-1.0891	-1.0811	-1.4343	-1.2424
NM_001108487	Isg20l1_predicted	-0.386	-1.1547	-0.4436	0.34152	-0.1813	-0.3029	-0.2492	-0.1071	-0.7712	-0.7741	-0.8119	-0.8085	-0.1491	-0.1921
NM_001007741	Isg20l2	0.33674	0.33147	-0.1657	-0.1441	0.29435	0.05252	-0.1017	0.20607	0.10108	0.08104	-0.1076	-0.1465	0.32058	-0.0312
NM_017339	Isl1	-0.0943	-0.0486	0.01831	0.10245	0.00879	0.07354	0.07732	-0.0177	0.0309	0.00807	0.07775	0.05818	0.04643	0.03739
NM_020471	Isl2	0.04696	-0.0471	-0.0143	-0.0346	0.05857	0.07917	0.17471	0.03187	-0.0842	-0.0898	0.01246	-0.023	0.14231	0.05345
NM_001014242	Isoc1	0.60183	0.42386	0.03862	0.31181	0.453	0.33204	-0.2046	0.44231	0.49425	-0.059	0.21781	0.56084	0.16866	0.29036
NM_001008367	Isoc2b	-0.1033	0.21841	0.20065	-0.1028	-0.045	0.26785	-0.4205	-0.0771	-0.0998	0.0767	-0.2509	0.32645	-0.0699	-0.045
NM_001014188	Isy1	-0.0714	-0.3998	-0.6036	-0.3271	-0.2656	-0.1639	-0.4902	-0.3484	-0.1615	-0.3264	-0.3762	-0.1298	-0.391	-0.1622
NM_001013880	Isyna1	-0.2444	0.04804	0.31249	0.15317	-0.2298	-0.2955	0.34812	-0.2827	-0.0216	0.20548	0.21985	0.17432	-0.1381	-0.2031
NM_001013880	Isyna1	-0.2167	0.11301	0.49166	0.14137	-0.2855	-0.1658	0.26902	-0.1044	-0.0269	-0.0917	0.1341	0.04156	-0.0068	0.07894
NM_001005887	Itch	0.20867	0.53599	-0.0699	0.07353	0.55635	-0.0203	0.15126	0.63776	0.14221	0.17256	0.16869	0.04	0.42959	0.34062
NM_001009701	Itfg3	0.44166	0.5061	0.2664	0.49126	0.66516	0.56266	0.14881	0.54609	0.21415	0.36641	0.39781	-0.0929	0.07879	-0.0237
NM_030994	Itga1	0.28044	-0.1624	-0.1064	0.00556	0.20215	-0.2864	-0.3675	0.10702	0.49929	0.14024	-0.0198	0.58016	-0.1426	-0.1406
NM_001107699	Itga10_predicted	0.13269	0.14946	0.10715	0.08812	0.79621	0.75235	0.32456	0.50649	0.07878	0.22135	0.11447	0.3779	0.15666	0.0683
NM_001108156	Itga11_predicted	-2.1949	-1.5387	-2.8717	-2.6704	-2.9446	-2.6599	-3.9694	-2.6891	-0.8225	-1.4915	-1.8563	-0.2748	-3.2756	-3.4729
XM_345156.3	Itga2	-0.1343	0.10149	-0.0217	-0.1257	-0.1515	-0.0869	-0.1724	-0.0171	-0.2614	-0.1043	-0.1524	-0.1783	-0.1664	-0.1401
NM_001108292	Itga3_predicted	0.5297	-0.0558	0.26402	0.28529	0.73002	-0.0184	0.19655	0.33641	0.13122	0.11632	-0.0653	-0.2943	0.13428	0.30977
NM_001107737	Itga4_mapped	-0.2519	-0.2469	-0.1732	-0.1244	0.02465	-0.2651	-0.127	-0.1321	-0.2732	0.02525	-0.0408	-0.2792	-0.1523	-0.2444
NM_001108118	Itga5_mapped	0.74697	0.90829	1.1645	1.2258	1.2158	0.79622	0.82302	0.97738	0.58073	0.52188	0.60251	0.19105	0.10324	0.44968
XM_001059528.1	Itga6	-0.4178	-0.6304	-0.3975	-0.8726	0.24562	-0.4866	-0.5801	0.37203	-0.733	-0.6793	-0.485	-0.5653	-0.5156	-0.538
NM_030842	Itga7	0.08244	0.88828	1.4744	1.2978	-0.033	0.16273	1.7669	-0.0691	0.32343	0.82057	0.58654	-0.2392	-0.0884	-0.0128
NM_031691	Itgad	-0.1401	0.00641	-0.1023	-0.0814	0.07438	-0.0731	0.05452	-0.0685	-0.0691	0.1647	-0.1408	-0.0049	-0.1069	-0.022
NM_001033998	Itgal	0.01845	-0.111	-0.0149	-0.0037	-0.0453	0.08414	-0.0442	-0.1052	0.04355	-0.043	-0.0413	0.01213	0.07037	0.00574
NM_012711	Itgam	0.02014	0.06667	0.02807	0.19608	0.09635	-0.0471	0.24038	0.06278	0.02248	0.17306	0.16864	0.05103	-0.0708	-0.0512
NM_001106549	Itgav_predicted	0.51552	0.80298	0.24331	1.2994	1.0343	0.84932	0.57352	1.1846	0.56531	0.6854	0.80086	0.07019	0.65278	0.52217
NM_017022	Itgb1	-0.3717	0.58514	-1.0855	-0.3169	0.01418	-0.4786	-0.3486	-0.0247	0.12246	0.25471	0.01891	-0.3747	-0.078	-0.2313
NM_001106719	Itgb1bp1_predicted	0.46073	0.2049	0.44024	0.96666	0.49629	0.51448	0.49727	0.45946	0.14663	0.07331	0.19495	-0.088	0.38439	0.45396
NM_001108245	Itgb1bp2_predicted	-0.0317	0.00484	0.44627	0.3074	0.15066	0.28559	-0.0645	0.05781	-0.3264	0.05592	-0.2136	-0.0145	-0.0424	0.08167
NM_001037780	Itgb2	0.19748	-0.2166	0.15947	-0.1416	-0.005	-0.2153	-0.0125	-0.087	-0.045	-0.1907	-0.0272	-0.2026	0.08175	-0.0726
NM_001013213	Itgb3bp	0.29414	-0.0322	0.27603	0.01474	-0.3304	-0.2916	0.17877	-0.3751	0.51507	0.13533	0.32234	0.70651	0.3148	0.17865
NM_013180	Itgb4	-1.435	-1.3564	-2.0607	-1.8976	-1.7626	-2.0149	-1.4139	-1.7846	-1.2691	-1.4038	-1.5198	-1.3702	-2.7372	-2.6285
NM_001004263	Itgb6	-0.2274	-0.2107	-0.1381	-0.1999	-0.2917	-0.2517	-0.2836	-0.2684	-0.2027	-0.0785	-0.2434	-0.1421	-0.0952	-0.1954
NM_013171	Itgb7	0.31464	-0.1052	0.0498	0.00885	0.07532	0.04868	0.01577	-0.1674	-0.1256	-0.1071	0.17162	0.03275	0.07809	-0.1106
NM_001108726	Itgb8_predicted	0.16783	0.25579	0.02082	0.80796	-0.0252	0.00728	0.35636	0.01334	0.30817	0.03838	0.28871	-0.1486	-0.1005	-0.1191
NM_001017505	Itgbl1	0.87935	2.4617	0.18649	2.1348	0.04832	0.19903	0.17285	0.00154	2.3507	2.3942	2.3552	0.61668	-0.3879	-0.0623
NM_001107291	Itih1_predicted	0.04997	-0.0059	-0.1566	-0.0876	0.00583	-0.0827	0.04957	-0.0169	0.05356	-0.156	-0.204	0.01061	0.00885	0.01223
NM_017351	Itih3	-0.1614	-0.2676	-0.2346	-0.1992	-0.2196	-0.1271	-0.1482	-0.1647	-0.2113	-0.0759	-0.2484	-0.1306	-0.092	-0.2188
NM_019369	Itih4	-0.0423	-0.0218	0.07972	0.01151	0.07117	0.1071	-0.0688	-0.0602	0.02627	-0.0796	-0.0783	0.00773	-0.005	0.13154
NM_001108825	Itk_predicted	0.15495	0.36309	0.13513	0.3283	0.73335	0.55636	0.21931	0.7304	0.18263	0.08929	0.05052	0.12777	0.01467	0.03359
NM_001025712	Itm2a	-0.134	-0.1772	-0.0742	-0.1729	0.18426	-0.1182	-0.2037	0.00759	-0.0181	-0.077	-0.2899	-0.2842	-0.1006	-0.2067

NM_001006963	Itm2b	-0.1211	0.86069	-1.1875	-0.4951	-0.1703	-0.272	0.09256	0.11252	0.28543	0.73506	0.3954	0.13375	-0.7552	-0.6469
NM_001009674	Itm2c	-0.1651	0.51172	-0.878	0.71141	-0.234	-0.1464	0.20168	-0.1592	0.41174	0.27745	0.39731	0.16728	-0.6422	-0.7547
NM_001107774	Itpa_mapped	-0.7297	-0.1522	-0.5007	-0.5171	-0.5974	-0.4337	-0.5405	-0.6922	-0.3066	-0.4951	-0.2886	0.18564	-0.1217	-0.0834
NM_031045	Itpka	-0.0692	0.0644	-0.0559	-0.1298	-0.1871	-0.1904	-0.093	-0.1566	-0.0134	-0.2299	-0.2666	0.13534	-0.1788	-0.0491
NM_019312	Itpkb	0.23102	0.13117	-0.0636	-0.0092	-0.0042	-0.0333	0.17571	-0.0605	0.22363	0.20201	0.18375	0.11293	0.06569	-0.0078
NM_178094	Itpkc	0.04374	0.4691	-0.0038	0.2836	0.3423	0.58389	0.39881	0.39998	0.02275	0.40115	0.2613	-0.2445	-0.0879	-0.0192
NM_001007235	Itpr1	-0.3079	-0.0362	-1.3233	-0.217	-0.7896	-0.8778	-0.8998	-0.6954	-0.0803	-0.2095	-0.1525	-0.5934	-1.1325	-1.0788
NM_031046	Itpr2	0.67297	0.87093	0.44353	0.10607	0.1956	0.22246	0.47312	0.25142	0.52116	0.64567	0.63528	-0.1201	0.31835	0.1901
NM_013138	Itpr3	-0.0667	0.24634	0.17177	0.05529	0.068	0.18326	0.25194	0.00228	0.31636	0.20869	0.16976	0.12348	-0.1064	0.0203
NM_019227	Itsn1	0.09276	0.08956	0.10264	-0.0424	0.10396	-0.0428	0.09275	0.19799	0.03695	0.10846	0.08061	-0.0137	-0.0278	0.13996
NM_012592	Ivd	-0.3042	-0.4822	-1.0059	-1.6203	-0.6412	-0.6097	-0.9776	-0.6363	-0.4642	-0.6108	-0.5177	-0.1194	-0.4832	-0.5838
NM_022195	Ivl	0.04775	0.33712	0.2475	-0.0387	0.28292	0.01331	0.08852	0.08748	0.32219	0.20189	0.1249	0.08091	0.43021	0.01091
NM_001047085	Ivns1abp_predicted	-0.9795	-1.0233	0.29007	-0.867	-1.3603	-1.3416	-0.5324	-1.2498	-0.3429	-0.7875	-0.8265	0.1574	0.31426	0.23514
NM_001034918	Iws1	-0.0528	-0.1303	0.23505	0.93931	0.33881	0.21274	0.21767	0.1538	0.19538	0.16971	0.00661	0.11673	0.39641	0.25637
NM_001106237	Ixl_predicted	0.56324	0.025	0.31836	0.06319	0.35809	0.84781	-0.0291	0.25081	0.34738	0.12803	0.07595	0.8602	0.5749	0.76235
NM_001017514	Izumo1	-0.0247	0.00996	-0.0761	-0.0149	-0.0224	0.11727	-0.1818	0.10686	-0.018	0.00805	0.0208	-0.0549	-0.0399	-0.0845
NM_019147	Jag1	0.20427	-0.1763	0.10402	0.76835	0.43831	1.0476	-0.0055	0.67445	0.2979	-0.1721	0.48216	0.99308	0.33436	0.07971
XM_001073124.1	Jag2	0.15983	0.00157	0.14681	0.06953	0.07259	0.03473	0.16999	-0.0565	0.18243	-0.0611	-0.0216	0.07785	-0.017	0.19185
NM_053466	Jak1	-0.4381	-0.3383	-0.9177	-0.5542	0.31737	-0.3885	-0.159	0.31871	-0.2371	-0.4217	-0.2673	-1.0247	-0.314	-0.3906
NM_031514	Jak2	0.08911	0.14464	-0.4364	-0.5063	0.36048	-0.2756	0.45518	0.50677	-0.1756	-0.1064	-0.2223	-0.6937	-0.1473	-0.023
NM_012855	Jak3	-0.523	-0.6814	-0.8924	-0.8182	-0.5079	-0.7566	-0.4412	-0.5329	-1.0064	-0.6761	-0.725	-1.6875	-1.2703	-1.2865
NM_001033894	Jakmip1	0.13926	0.02449	-0.0427	-0.1314	0.14463	0.24219	0.06473	0.18826	-0.0248	-0.0823	0.20845	0.11841	-0.0047	0.0582
NM_001004269	Jam3	1.4148	1.4888	0.80898	3.4202	3.7543	3.4347	2.3472	3.7438	1.5143	1.6491	1.8351	0.94708	0.89857	0.91365
NM_021865	Jdp1	-0.0082	-0.0443	-0.1003	-0.0174	-0.0161	-0.0324	-0.0058	0.36048	0.0587	0.03451	-0.0438	0.15281	-0.0183	0.05986
XM_001080424.1	Jmjd1c	-0.4784	-0.4549	-0.1227	-0.1707	-0.5507	-0.3362	-0.3089	-0.5004	-0.5678	-0.2	-0.3071	-0.6342	-0.2122	-0.392
XM_001080424.1	Jmjd1c	-0.1978	0.12112	-0.0545	0.29996	-0.2614	-0.5173	0.21375	-0.4065	-0.1097	0.04864	-0.0112	-0.3434	-0.2526	-0.3517
NM_001107966	Jmjd2a_predicted	-0.4178	-0.281	-0.0713	-0.1531	-0.1391	-0.2811	-0.277	-0.1956	-0.2732	-0.2056	-0.3553	-0.4112	-0.1023	-0.3522
XM_001067369.1	Jmjd2c_predicted	-0.1171	0.53014	-0.3088	0.3132	0.17843	0.16743	0.20099	0.46707	0.09377	0.20637	0.22794	-0.0722	0.02875	-0.1533
NM_001108829	Jmjd3_predicted	-0.1091	0.22015	0.18202	0.94553	0.08373	-0.1987	0.07153	0.00779	-0.0697	0.18479	0.00449	-0.2182	-0.585	-0.3058
NM_001105784	Jmjd4_predicted	-0.109	-0.2041	0.05223	-0.2985	0.05857	-0.1673	0.01019	0.04692	-0.0704	-0.1301	-0.0964	-0.2403	-0.0503	0.14592
NM_001025009	Josd1	-0.0952	0.20363	-0.2672	-0.0148	0.06569	-0.086	0.15489	-0.0037	0.03374	0.08792	0.12242	-0.1875	0.05419	0.00955
NM_001106256	Josd2_predicted	-0.002	0.0731	-0.0947	0.31577	-0.2371	0.15214	-0.358	-0.0264	-0.4261	-0.111	0.00602	-0.0207	-0.2941	-0.1607
NM_001014207	Josd3	-0.4936	-0.3794	0.59029	0.36838	-0.4926	-0.3363	0.37322	-0.2722	-0.2009	-0.2562	-0.2401	0.06461	0.0706	0.10521
NM_001106630	Jph1_predicted	-0.1148	-0.1212	-0.012	-0.074	0.02848	-0.0564	-0.0459	-0.0174	-0.0752	-0.0462	-0.1592	-0.0992	-0.1202	-0.1296
NM_001037974	Jph2	0.02163	0.19065	0.11999	-0.2592	0.39833	0.03145	0.15052	0.18167	0.13968	-0.0868	0.0951	-0.1382	0.04285	-0.0242
NM_001003711	Jph4	-0.2818	-0.3732	-0.2	-0.389	-0.2909	-0.1047	-0.1871	-0.2674	-0.1277	-0.1194	-0.3056	-0.1703	-0.0708	-0.1856
NM_001104612	Jrk_predicted	0.00389	0.08206	0.02657	-0.1267	0.00906	0.12317	0.20285	-0.0326	0.01843	0.08498	0.00956	-0.1708	0.08048	-0.1407
NM_001108122	Jrkl_predicted	-0.4182	-0.6392	-0.5753	-0.7771	-0.5066	-1.094	-0.4206	-0.6511	-0.7603	-0.6427	-0.615	-0.7761	-0.8292	-0.6356
NM_019213	Jtb	0.58069	0.17989	0.49175	0.64898	0.30763	0.06832	0.52214	0.19919	0.18156	-0.1889	0.08768	-0.1816	0.29666	0.42189
NM_053503	Jub	-1.4826	-1.2642	-0.182	-0.5687	-1.2934	-1.4042	-0.9193	-1.375	-1.0994	-0.9927	-0.919	-0.8143	-0.46	-0.5602
NM_021835	Jun	-0.6415	-0.6473	0.13314	-0.7246	-0.3313	0.11985	-0.4494	-0.3808	-0.2544	-0.531	-0.2838	0.35664	0.02649	0.04847
NM_021836	Junb	0.63685	0.82409	0.16051	0.33865	0.26909	0.00779	0.80013	0.03746	0.1636	0.64528	0.82456	-0.5856	-0.2657	-0.2391
NM_138875	Jund	0.13382	-0.2997	-0.0164	-0.9645	0.39628	-0.3523	-0.4498	0.42246	-0.1085	-0.3779	-0.3611	-0.3399	0.04215	0.12471
NM_053894	Jundm2	0.14004	-0.3587	-0.5044	-0.9245	-0.0934	-0.7587	-0.182	0.24052	-0.0043	-0.2314	-0.27	-0.0478	-0.4158	-0.1414
NM_031047	Jup	-0.1198	-0.2655	-0.0861	-0.214	-0.1682	-0.2646	-0.2548	-0.152	-0.2373	-0.1678	-0.1931	-0.2434	-0.1147	-0.1307
NM_001008750	Ka11	0.10426	-0.0265	-0.0135	0.06491	0.00102	0.1336	0.09293	0.11227	0.25248	-0.0153	0.08825	-0.0673	-0.0043	-0.0719
NM_133537	Kal1	-1.4568	4.0631	1.1135	0.39319	-0.4542	-0.1763	5.1362	-0.5535	4.1339	4.1066	4.1254	1.2186	0.54779	0.56034
NM_032062	Kalrn	-0.2629	-0.3795	0.2056	-0.1838	-0.499	-0.6787	-0.2691	-0.5234	-0.4114	-0.4465	0.0465	-0.1471	-0.3299	-0.3454
NM_052802	Kap	0.23609	-0.0353	0.02778	-0.2804	-0.1342	-0.1077	0.03423	-0.2394	-0.0296	-0.1046	-0.326	0.0261	-0.176	-0.1747

NM_001006967	Kars	0.28276	-0.0296	0.31024	0.60139	0.07138	0.40545	0.00274	-0.0962	-0.2317	-0.212	0.01952	0.13701	0.06755	0.0522
NM_001004217	Katna1	0.01686	-0.4137	0.13856	0.70022	-0.0347	0.24985	0.19211	0.13858	-0.1787	-0.23	0.13043	-0.1032	-0.0202	-0.1446
NM_001006956	Katnal1	-0.1433	-0.3499	0.03238	-0.2795	-0.459	-0.2044	-0.3478	-0.1819	-0.3713	-0.4485	-0.3993	0.00468	-0.0291	-0.0955
NM_001024746	Katnb1_predicted	0.05697	0.0502	0.03686	-0.106	0.08425	-0.1826	-0.0104	0.32686	0.13405	0.12762	0.28715	0.02301	-0.0491	0.03615
NM_001033064	Kazald1	-1.3364	-1.2091	-1.4812	-2.0814	-1.9072	-1.7903	-1.3902	-2.2149	-0.5637	-1.225	-1.1216	0.53021	-1.8714	-1.533
NM_001008802	Kb1	0.05072	0.12077	-0.0712	0.08713	0.02393	-0.0274	0.16205	0.08618	-0.0958	0.09214	-0.0221	0.1083	0.09243	-0.0382
NM_001008825	Kb15	0.00337	0.12408	-0.0594	0.04775	0.13006	0.02824	-0.0348	-0.0933	0.07128	0.02964	0.01216	-0.0512	0.0356	0.01927
NM_001008814	Kb21	0.09198	0.20406	0.1326	0.13616	0.09614	0.06071	0.08556	0.04474	0.17547	0.08616	0.05267	0.02068	-0.019	0.09473
NM_001008813	Kb23	-0.2057	0.01114	-0.3491	-0.2452	-0.0522	-0.2793	-0.053	-0.1574	-0.2762	-0.2436	-0.1466	-0.1874	-0.2709	-0.0511
NM_057191	Kbtbd10	2.0204	0.7478	2.2882	2.8218	0.77201	0.69519	0.70515	1.129	1.1012	1.0363	1.169	0.23835	1.5578	1.5042
NM_001107861	Kbtbd2_predicted	0.12144	0.54292	-0.3715	-0.0515	-0.1156	-0.6022	0.28797	0.10418	0.08689	0.3347	0.28527	-0.5124	0.28269	0.32403
NM_001108121	Kbtbd3_predicted	-0.1176	0.13758	0.18247	0.07913	0.03991	0.05585	0.21812	0.12718	0.12556	0.02283	0.20671	-0.0618	0.10479	0.11347
NM_001107746	Kbtbd4_predicted	-0.3188	0.17299	-0.0368	0.10144	-0.0146	0.34236	0.20296	0.04697	-0.0886	-0.0144	0.19424	0.03108	-0.1536	-0.104
NM_001108195	Kbtbd5_predicted	0.0217	-0.0158	0.569	0.17423	0.08656	0.11468	0.05292	0.34311	0.03758	0.33138	0.16015	0.03259	0.12663	0.17657
NM_001012045	Kbtbd7	-0.0964	-0.0996	-0.0061	0.07171	-0.0545	-0.0492	0.07708	0.04948	0.04602	0.10973	-0.0636	0.01329	0.08269	-0.1039
NM_001106713	Kbtbd9_predicted	-1.3549	-1.1913	-1.0566	-1.1099	-1.0922	-1.1151	-0.495	-0.8368	-0.1435	-1.3009	-1.1417	0.62084	-0.5511	-0.5408
NM_173095	Kcna1	0.05552	-0.0104	-0.1915	0.03528	0.00719	-0.0403	0.00673	-0.1743	-0.0053	0.10722	0.03122	-0.1732	-0.0325	0.22677
XM_227577.4	Kcna10_predicted	-0.055	-0.1508	0.00318	-0.0421	0.08911	0.07597	0.03061	0.02065	-0.1711	-0.1769	-0.0089	-0.0812	-0.0848	0.02824
XM_227577.4	Kcna10_predicted	-0.0179	0.08773	0.08932	-0.1131	0.03374	0.24891	0.00634	0.00682	0.03445	-0.0997	-0.117	0.04063	-0.1514	-0.0206
NM_012970	Kcna2	-0.0685	-0.1066	-0.0121	-0.0499	-0.0347	-0.0494	0.19237	0.07886	0.03896	-0.1032	-0.0079	-0.0751	-0.1018	-0.1191
NM_019270	Kcna3	0.03253	-0.0578	0.06432	0.06612	0.11344	0.03557	0.1053	-0.0299	0.15378	0.18079	-0.0606	0.02045	0.00972	-0.1152
NM_012971	Kcna4	0.05758	-0.0832	0.31657	0.05166	0.17826	0.22721	0.11448	0.08828	0.01743	0.11692	0.04359	0.44752	0.24568	0.15192
NM_012972	Kcna5	-0.0643	0.01341	0.25401	0.02759	0.03685	0.21722	-0.0594	0.04147	0.11785	-0.0199	0.12876	0.01175	-0.0644	-0.0072
NM_023954	Kcna6	-0.0895	-0.3009	-0.5049	-0.3704	0.20155	-0.2704	-0.062	0.26877	-0.3061	-0.2945	-0.4198	-0.5333	0.00042	-0.1028
NM_001108914	Kcna7_predicted	-0.1663	-0.0823	-0.093	-0.1787	-0.1803	-0.0749	-0.1977	-0.1528	-0.1558	-0.0356	-0.1374	-0.1418	-0.1581	-0.0285
NM_017303	Kcnab1	0.10408	0.22634	0.06342	-0.1213	0.21314	0.2773	-0.101	0.3882	0.15539	0.1987	0.04471	-0.0711	0.1251	-0.1174
NM_017304	Kcnab2	0.27382	0.15589	0.20124	0.23243	0.35272	0.28428	0.1962	0.27271	0.03506	0.13064	0.04556	0.2221	0.06604	0.24723
NM_031652	Kcnab3	0.08213	0.12603	0.29572	0.17	-0.0134	0.05686	0.28661	0.15366	0.03834	0.11921	0.23453	0.32713	0.13861	0.18242
NM_013186	Kcnb1	-0.0616	-0.1604	-0.1171	-0.2149	-0.1443	-0.1733	-0.159	0.08148	-0.1387	0.03529	-0.2271	-0.1033	-0.2082	-0.1822
NM_054000	Kcnb2	0.05017	0.14027	0.26471	0.04877	0.04451	0.14675	-0.0056	0.09256	0.14346	0.25648	0.25098	0.02652	0.14544	0.19138
NM_012856	Kcnc1	0.04441	-0.06	-0.0044	-0.1202	-0.0329	0.07662	0.19282	-0.0155	0.11418	-0.0682	-0.048	0.08351	0.04749	0.05078
NM_139217	Kcnc2	-0.0446	-0.2577	0.0521	-0.1433	0.0159	-0.1764	-0.0418	0.00206	-0.1106	-0.2508	-0.208	-0.0237	-0.0672	0.13239
NM_139217	Kcnc2	0.04536	0.02608	0.10216	0.154	0.09568	0.26377	0.0379	-0.0689	0.00645	0.11069	0.08812	0.37294	0.11217	0.01391
NM_139217	Kcnc2	-0.006	0.01122	0.04719	-0.0549	-0.0379	0.04954	0.07818	0.01774	-0.068	-0.0338	0.0465	-0.0466	0.02205	0.03432
NM_053997	Kcnc3	-0.2558	-0.1802	-0.1353	-0.2163	-0.0552	0.01859	-0.0133	-0.0244	-0.1098	0.0227	-0.1323	-0.1163	-0.0016	-0.1259
NM_001105748	Kcnd1	-0.0079	1.3529	0.64249	0.21324	1.2006	1.2676	0.97601	1.0586	1.1068	1.3087	1.241	0.77615	-0.2649	-0.2331
NM_031730	Kcnd2	-0.2105	0.04541	0.09195	-0.2189	-0.1315	-0.0663	-0.0029	-0.1403	-0.1747	-0.2855	0.10102	0.24054	0.06963	-0.0429
NM_031739	Kcnd3	-0.0276	-0.0428	0.01855	0.18701	-0.1214	-0.0664	0.08667	0.07216	0.05197	-0.0087	-0.0195	-0.0045	0.04024	-0.0181
NM_012973	Kcne1	-0.0306	0.02999	0.02572	-0.0862	0.10767	0.23752	0.00628	0.431	0.18224	-0.1363	-0.1588	0.13645	0.11584	0.02004
NM_133603	Kcne2	-0.1294	0.03736	0.04391	-0.0593	0.00151	0.13055	-0.0382	-0.0591	0.02228	-0.0538	-0.0129	-0.0435	0.0985	0.03986
NM_022235	Kcne3	0.05952	0.20288	0.03024	0.03228	0.17549	-0.0417	0.07927	-0.0127	-0.0172	-0.1053	0.32101	0.06967	0.10372	-0.0509
NM_212526	Kcne4	-0.1052	-0.0073	0.00402	-0.0834	-0.0112	0.00129	-0.1163	-0.0892	0.00964	-0.022	0.25834	0.05385	0.06391	-0.0182
NM_001169104	Kcnf1	-0.038	-0.0677	0.15475	0.0294	0.06239	0.0014	0.06577	0.09655	0.05518	0.13046	-0.0373	0.0634	-0.0231	-0.057
NM_001106545	Kcng1	0.33945	0.17336	0.24885	0.24642	0.49752	0.55355	0.31093	0.45601	0.07446	0.1075	0.04561	0.19578	0.16919	0.37218
NM_001107372	Kcng2	0.17349	0.01801	-0.1422	0.33989	0.44359	0.16437	-0.1478	0.1943	0.12207	-0.1274	0.00976	-0.2111	0.0816	0.01209
NM_133426	Kcng3	0.00354	0.11568	-0.0976	0.07769	-0.0107	0.03893	-0.0275	0.01965	0.00824	0.14317	0.04857	-0.0191	0.08542	-0.1084
NM_001107435	Kcng4_predicted	0.21029	-0.0771	-0.0365	0.1282	0.18335	0.09514	-0.0037	0.05854	0.30568	0.10101	0.09183	0.05634	-0.058	0.23449
NM_031742	Kcnh1	0.07405	0.07718	0.09364	0.26707	0.14886	0.10716	0.02413	0.10838	0.0974	0.08956	0.05129	0.11073	0.13155	0.00225
NM_053949	Kcnh2	-0.1456	-0.188	-0.0721	-0.0618	-0.0121	-0.0718	-0.1899	-0.2112	-0.347	-0.323	-0.1283	-0.1223	-0.1868	-0.1797

NM_017108	Kcnh3	-0.0008	0.14604	0.22984	0.30172	-0.1386	0.01434	-0.0301	0.02241	-0.0902	-0.0046	-0.0183	-0.0257	0.06508	-0.0127
NM_053630	Kcnh4	0.01597	-0.0269	-0.0828	-0.0014	-0.0533	-0.0012	-0.0251	-0.0289	0.06151	0.04122	0.03306	-0.0205	0.05388	-0.088
NM_133610	Kcnh5	0.19309	0.08851	-0.0127	0.34314	0.13197	0.21217	0.09828	0.05425	0.00967	0.07318	0.04959	-0.0387	0.01989	0.21614
NM_053937	Kcnh6	0.24855	-0.2144	0.3038	-0.0769	0.34656	0.35021	0.1205	0.55505	0.01165	-0.1035	-0.1865	0.1938	-0.1559	0.08566
NM_131912	Kcnh7	-0.1788	0.08162	0.05692	-0.1658	0.11925	-0.0286	0.12924	0.15943	0.27187	0.05045	-0.1994	-0.055	0.07862	0.09034
NM_145095	Kcnh8	0.06202	-0.0356	-0.0408	0.01279	-0.0003	0.02162	-0.027	-0.0228	-0.0307	-0.007	-0.0017	0.14855	0.26581	-0.0442
NM_022929	Kcnip1	-0.0099	-0.1056	-0.0669	-0.1122	0.00035	-0.08	-0.1181	0.0275	0.25507	-0.083	0.02145	0.05262	-0.038	0.19591
NM_020094	Kcnip2	0.10418	0.08455	0.01683	0.03124	0.05653	0.04064	0.24299	0.0924	0.26211	0.12973	0.10154	0.00167	0.02601	0.17045
NM_032462	Kcnip3	0.24522	-0.0911	-0.0191	-0.2316	0.5192	0.19664	0.01551	0.47742	0.07356	0.14736	-0.1616	0.03658	-0.113	-0.197
NM_181365	Kcnip4	-0.1639	0.46285	0.04305	-0.0634	-0.0706	-0.0159	0.12831	-0.0084	0.52005	0.32912	0.37426	-0.0386	0.15245	0.02256
NM_017023	Kcnj1	0.18097	-0.0141	0.0619	0.04738	0.08814	-0.0432	0.11728	0.03794	0.45504	0.04445	0.03598	0.21432	0.09995	0.0398
NM_031602	Kcnj10	0.32852	0.22996	0.22363	0.05986	0.11475	0.27095	-0.0594	0.06714	-0.148	0.06105	-0.0657	0.04615	-0.0758	-0.1102
NM_031358	Kcnj11	0.25486	0.19341	0.18104	0.38669	0.11974	0.6225	0.38872	0.44255	0.16831	0.40331	0.11719	0.5582	0.10855	0.24869
NM_053981	Kcnj12	-0.0757	-0.0599	-0.0842	-0.1274	0.3295	0.43815	0.0777	0.27223	-0.0709	-0.1927	0.27657	-0.0839	-0.0646	-0.0591
NM_053608	Kcnj13	0.12813	0.15852	-0.046	0.10954	0.19144	0.21895	0.10658	0.00452	0.04594	0.14861	0.09386	-0.0504	0.08067	0.11217
NM_170718	Kcnj14	-0.1291	-0.229	0.08439	-0.1955	-0.1521	-0.1184	-0.1084	-0.5112	-0.2517	-0.0986	-0.1509	-0.4927	-0.0039	-0.2934
NM_133321	Kcnj15	0.01513	-0.2142	-0.2327	-0.0983	-0.1823	-0.0138	-0.1786	-0.1563	-0.2185	-0.1782	-0.2032	-0.1102	-0.1974	-0.087
NM_053314	Kcnj16	-0.4156	0.03213	-0.2547	-0.3053	-0.4363	-0.4187	0.13024	-0.2182	0.16081	-0.0313	-0.0022	0.61381	-0.1194	-0.371
NM_017296	Kcnj2	-0.1871	-0.0388	0.05055	-0.03	-0.0578	-0.227	0.03636	-0.113	-0.0387	-0.1637	-0.1676	-0.1507	0.02785	-0.1291
NM_031610	Kcnj3	0.03773	0.01223	0.14253	0.0224	0.29818	0.18602	0.05319	0.18491	0.24006	-0.0813	0.15576	0.26829	0.11083	-0.019
NM_053870	Kcnj4	0.13621	0.02859	-0.0254	0.03346	-0.1433	0.14183	-0.0966	-0.1281	-0.1171	0.14297	0.16307	-0.0262	0.05838	0.08976
NM_017297	Kcnj5	-0.0062	0.04233	0.01545	0.01468	0.05019	0.08627	0.18932	0.10468	0.03859	-0.0404	-0.0677	0.06067	-0.0317	0.1089
NM_017099	Kcnj8	-0.0204	-0.0815	-0.1995	-0.1491	-0.0869	-0.1856	-0.1613	-0.0225	-0.1591	-0.2167	-0.0551	-0.1238	-0.2186	-0.1555
NM_053834	Kcnj9	0.36218	-0.1711	-0.0539	0.07955	-0.0376	0.27513	0.01918	-0.0901	-0.0662	-0.3515	-0.1256	-0.0443	-0.2168	-0.1739
NM_021688	Kcnk1	0.65577	-0.0658	0.11044	0.06007	2.9682	2.6685	0.00422	3.0808	0.08585	-0.0241	0.01784	0.23367	-0.1566	0.08069
NM_023096	Kcnk10	-0.0537	0.01089	0.10839	0.1176	0.10923	0.08619	0.06778	0.15183	0.02415	-0.0917	0.06963	-0.0137	-0.1306	0.16015
NM_022292	Kcnk12	0.16496	0.03398	0.39558	0.41052	0.03756	0.2535	0.14334	0.11421	0.27724	0.15326	0.06624	0.52333	0.36204	0.42449
NM_022293	Kcnk13	-0.0655	-0.1259	-0.1179	-0.2221	-0.2024	0.07989	-0.1376	0.06773	-0.1001	0.01221	-0.1272	-0.1594	0.04067	-0.1761
NM_130813	Kcnk15	0.09965	-0.1445	-0.0655	-0.1155	-0.0888	0.02819	-0.1015	-0.3048	-0.1232	-0.0296	-0.0156	0.04531	-0.186	-0.2112
NM_001003820	Kcnk18	0.2888	0.5235	-0.0296	-0.1074	0.01492	-0.0017	-0.0123	0.29847	0.35917	0.19059	0.25037	-0.0719	-0.0803	-0.0087
NM_172041	Kcnk2	-0.1136	-0.1925	-0.1225	-0.1967	-0.158	0.07329	-0.1114	-0.1994	0.00153	-0.1195	-0.1676	-0.2794	-0.0825	-0.0162
NM_033376	Kcnk3	-0.0684	-0.2281	-0.2669	-0.2736	-0.132	-0.247	-0.163	-0.1963	0.10308	-0.0135	-0.0608	-0.1526	0.02611	-0.2446
NM_053405	Kcnk9	-0.0352	0.14455	-0.067	-0.1311	-0.1023	0.07752	-0.1064	-0.1463	0.05514	0.03383	-0.0673	-0.0136	0.00012	-0.0796
NM_031828	Kcnma1	-0.0421	0.08785	-0.0455	-0.0721	0.09157	-0.0041	0.00947	-0.0479	0.18179	-0.0435	0.04643	0.00075	-0.0443	0.18219
NM_019273	Kcnmb1	-0.0133	0.03447	0.30312	-0.0413	-0.0392	-0.1277	-2E-05	-0.0764	0.21384	-0.1553	-0.1129	0.12373	0.23972	0.08454
NM_176861	Kcnmb2	0.14311	-0.1491	-0.0159	-0.0179	1.32	1.217	0.67159	1.3088	-0.3674	-0.0308	0.10552	-0.2542	-0.328	-0.0571
NM_001104560	Kcnmb3_predicted	0.04253	0.10414	0.10768	0.0182	-0.0853	-0.1262	-5E-05	-0.1478	-0.0045	0.014	0.06364	0.07779	0.1183	-0.007
NM_023960	Kcnmb4	-0.1276	-0.081	-0.1448	-0.0966	-0.3092	-0.1249	-0.1598	-0.1128	-0.239	-0.0689	-0.3125	-0.0684	-0.1078	-0.2217
NM_019313	Kcnn1	0.21462	0.20855	0.02558	0.24979	0.25257	0.31362	0.22231	0.12369	0.10473	0.17455	0.11821	0.17045	0.18944	0.21221
NM_019314	Kcnn2	0.16886	0.03511	-0.0653	0.04156	-0.0085	0.11178	0.08231	0.12856	0.08642	0.03205	0.10861	0.24181	0.39107	-0.0892
NM_019315	Kcnn3	0.49493	0.1439	0.14875	0.2804	0.2109	0.26666	0.09666	0.4302	0.08181	0.19145	0.29374	0.26974	0.27043	0.08267
NM_023021	Kcnn4	-0.885	-0.9839	-1.0316	-1.4494	-0.5177	-0.3556	-0.5619	-0.865	-1.3105	-1.0525	-1.03	-2.0096	-1.1947	-1.0496
NM_032073	Kcnq1	0.19023	0.15279	0.21215	0.24652	0.21623	0.19307	0.15211	0.56926	-0.0003	-0.0002	0.22656	0.28229	0.25563	0.05825
NM_133322	Kcnq2	0.0427	0.01055	0.11893	-0.0359	0.0562	-0.0202	-0.0568	0.05972	0.10249	-0.0046	0.03896	-0.0721	0.01969	0.02168
NM_031597	Kcnq3	0.19458	0.02584	0.09913	0.017	0.15802	0.04162	0.06754	0.02879	0.00894	-5E-05	-0.0989	0.10207	0.11187	0.09744
XM_001071249.1	Kcnq5	-0.3367	-0.13	0.32855	-0.1321	-0.6808	-0.386	-0.2794	-0.564	0.1136	-0.2395	-0.0642	0.67585	0.03214	0.32004
XM_001071249.1	Kcnq5	-1.5409	-0.6868	-0.3695	-1.0423	-1.7373	-1.6936	-0.6024	-1.5957	-0.1737	-0.5838	-0.7548	0.50952	-0.2464	-0.3168
XM_224261.2	Kcnrg_predicted	0.09337	-0.1355	-0.1565	-0.0254	-0.0441	-0.1568	-0.1159	-0.139	-0.1236	-0.164	-0.1705	-0.1201	-0.2101	-0.117
NM_053954	Kcns1	-0.224	-0.2112	-0.2336	-0.1269	-0.2989	-0.3281	-0.1282	-0.1978	-0.1554	-0.084	-0.1834	-0.2493	-0.2841	-0.1022

NM_023966	Kcns2	-0.2696	-0.2696	-0.0354	-0.1159	-0.1978	-0.2656	0.00101	-0.1424	-0.1234	-0.107	-0.1972	-0.007	0.15374	0.05564
NM_031778	Kcns3	0.21548	-0.0679	0.21138	0.09938	-0.1006	0.06345	-0.0914	0.08323	-0.0394	0.05171	0.0172	0.0297	-0.1707	0.08062
NM_021853	Kcnt1	-0.1452	0.02901	-0.1763	-0.1278	-0.1875	-0.1669	-0.2531	-0.0091	-0.1724	0.10775	-0.0216	-0.1393	0.10138	-0.042
NM_198762	Kcnt2	-0.1851	-0.214	-0.1999	-0.2943	-0.228	-0.1753	-0.154	-0.3518	-0.2914	-0.3492	-0.2047	-0.1678	-0.1909	-0.2065
NM_021697	Kcnv1	-0.0101	0.27469	0.13928	-0.0361	0.08471	-0.058	-0.0379	-0.0843	-0.0406	0.016	0.012	-0.0759	0.16671	-0.0736
NM_001106370	Kcnv2_predicted	0.16431	-0.0029	0.00846	-0.0881	-0.0437	0.28118	-0.0527	-0.0923	-0.0874	-0.129	0.08034	-0.1396	0.01508	-0.0729
XM_001062225.1	Kctd1	0.23602	0.10068	0.09725	-0.1117	-0.1171	-0.1825	-0.1779	-0.0106	0.20071	0.07197	0.37508	0.01569	0.0203	0.20099
NM_001108831	Kctd11_predicted	-0.0878	-0.2197	-0.2269	-0.5777	-0.3631	-0.5216	-0.2922	-0.1806	0.03909	-0.3911	-0.1531	-0.2955	-0.3489	-0.2977
XM_344450.2	Kctd12_predicted	0.13134	0.16907	0.054	0.09387	0.29239	0.26875	0.0706	0.05078	0.14883	0.12946	0.2619	0.20351	0.13588	0.07772
XM_344450	Kctd12_predicted	0.05954	0.09115	0.06297	0.21274	0.00703	0.0745	0.04916	0.01165	0.21542	0.34512	0.32178	0.15343	0.06484	0.25962
NM_198736	Kctd13	1.4614	0.68934	0.19402	2.0536	0.89008	0.82244	0.32379	0.88478	0.76717	0.83401	0.53682	0.12537	0.10553	0.21312
XM_001064247.1	Kctd14_predicted	-0.2899	-0.2083	0.12202	-0.8354	-0.2418	-0.3116	-0.2129	-0.2353	-0.1747	-0.37	-0.2159	0.21342	0.17102	0.21659
XM_001075666.1	Kctd19_predicted	0.48694	0.36359	0.82088	0.51372	0.14285	0.67021	0.59724	0.12066	0.2886	0.65631	0.56565	0.53971	0.27345	0.20966
NM_001107199	Kctd3	-0.225	-0.1315	-0.2414	0.03265	-0.4277	-0.4077	-0.353	-0.3567	-0.0562	-0.2907	-0.3952	-0.232	-0.1556	0.07698
NM_001105768	Kctd5_predicted	0.32009	0.27901	0.03638	-0.0211	0.30915	0.16061	-0.0603	0.0541	0.35153	0.19218	0.26453	-0.1246	0.0059	0.20414
NM_001107253	Kctd6_predicted	-0.5149	-0.4533	-0.1324	-0.5028	-0.3031	-0.5201	-0.5083	-0.3558	-0.2496	-0.6799	-0.3644	0.15042	-0.2792	-0.2719
NM_001100172	Kctd8_predicted	0.2672	-0.0843	-0.0802	0.07558	0.17037	0.2151	0.07845	0.11324	-0.1176	0.0301	0.07558	-0.0876	0.12472	-0.0938
NM_001108871	Kctd9_predicted	-0.1141	-0.0494	0.44659	0.10068	-0.2753	-0.1929	-0.1084	-0.078	0.02342	-0.1293	-0.0649	0.09962	0.00097	-0.0707
NM_001108217	Kdelc1	-0.1513	-0.151	-0.0912	-0.1176	-0.1298	-0.1722	-0.1016	-0.1882	-0.0909	-0.0924	-0.1304	-0.1422	-0.0656	-0.1233
NM_001017385	Kdelr1	-0.3712	-0.1286	-0.4869	-0.7156	-0.2525	-0.3595	-0.3838	-0.2297	-0.2472	-0.0982	-0.1582	0.04042	-0.3941	-0.3669
NM_001127546	Kdelr3_predicted	-0.3967	-0.0168	-0.4834	-0.569	0.28413	-0.5118	-0.8169	0.23605	0.26063	-0.1706	-0.1215	-0.2386	0.21841	0.22724
NM_001164718	Ke2	0.21785	-0.1489	0.07352	-0.0869	0.20774	1.0519	0.04313	0.2157	0.05969	-0.26	-0.006	0.30914	-0.2017	-0.0442
NM_057152	Keap1	0.14524	-0.1001	0.06372	-0.6829	0.07459	-0.3997	0.06553	-0.0057	0.00303	-0.0449	0.07613	-0.2695	0.06708	0.22847
NM_134330	Keg1	-0.2073	-0.0242	-0.0361	-0.0087	-0.0484	0.15968	-0.05	-0.0579	0.21705	-0.1487	0.10893	-0.0362	-0.0826	0.07049
NM_001108087	Kera_predicted	-0.1302	-0.089	0.03326	-0.1326	0.02528	-0.1614	-0.2106	0.03247	-0.0278	-0.1738	-0.1872	-0.0646	-0.0467	-0.0366
NM_130405	Khdrbs1	-0.2138	-0.2586	-0.3834	-1.0237	-0.5764	-0.5549	-0.3496	-0.4338	-0.1399	-0.0676	0.0192	-0.1706	0.1394	-0.0358
NM_133318	Khdrbs2	-0.0074	-0.0213	-0.0352	-0.0124	0.01051	0.07147	0.01761	-0.0223	-0.0673	-0.0237	-0.097	0.05996	-0.168	-0.0063
NM_022249	Khdrbs3	0.49183	1.0503	0.9664	0.55113	1.325	1.3326	0.64823	1.1801	1.3146	1.0522	1.0203	1.5234	1.0535	1.0385
NM_031855	Khk	0.04922	-0.1173	-0.2271	-0.1509	0.10171	0.13093	-0.1783	0.64845	-0.2274	-0.1381	-0.4763	-0.4126	0.26545	-0.2852
NM_133602	Khsrp	0.01978	-0.086	-0.0348	-0.0094	0.08839	-0.107	-0.0814	-0.0816	0.00651	-0.2337	0.00123	-0.0087	0.10131	-0.1657
NM_001037220	Kiaa0415	-0.1816	-0.0399	0.63295	0.09444	-0.0266	0.07022	0.43624	0.11523	-0.2519	0.10356	-0.0351	-0.2627	-0.1927	-0.2909
NM_053795	Kidins220	0.27745	0.13195	0.04848	0.43098	0.35793	0.06824	0.38135	0.49071	-0.1288	0.07169	0.15957	-0.2752	-0.1706	-0.1825
NM_001169112	Kif11	-0.0901	-0.1178	-0.1423	-0.2428	-0.163	-0.0938	-0.2394	-0.0588	0.00612	-0.1276	-0.2475	-0.2479	0.28243	0.24677
NM_001169112	Kif11	-0.4508	-1.045	0.56778	-0.3438	-1.0916	-1.3573	-0.9894	-1.3211	-0.2793	-0.9756	-0.6013	0.43885	0.55404	0.58841
NM_001012102	Kif12	0.09512	-0.0277	0.17965	-0.0163	0.04902	0.20752	0.11363	0.13807	0.0666	0.08246	0.25901	0.47124	0.13334	-0.0181
NM_001107462	Kif13a_predicted	0.07144	-0.0342	-0.0214	0.03284	0.10877	-0.0035	0.0959	0.22326	0.13538	0.29134	0.01288	0.04356	0.30887	0.18957
NM_213626	kif13B	-0.1062	-0.1228	-0.1091	-0.0625	-0.1127	0.1587	-0.0281	-0.0423	-0.0671	0.28134	0.16396	0.01792	-0.028	-0.063
NM_001108345	Kif14_predicted	0.08995	-0.091	0.12088	0.00921	-0.0754	-0.0968	-0.0719	-0.1084	-0.0377	0.16173	-0.1225	0.00681	0.36984	0.45229
NM_181635	Kif15	-0.2149	-0.2474	0.51477	0.19758	-0.5799	-0.751	-0.1667	-0.5752	0.0013	-0.0721	-0.1043	-0.0366	0.40833	0.35573
NM_001107783	Kif16b_predicted	0.14497	0.2521	-0.1198	-0.2137	0.23861	0.19134	-0.0463	0.18359	-0.0045	0.32871	0.02047	-0.0931	-0.0909	0.095
XM_343630.2	Kif1a_predicted	0.04324	0.01501	-0.1063	0.18559	-0.009	-0.1123	-0.0996	-0.0652	0.02876	0.13286	-0.0199	-0.0102	-0.0741	0.10227
NM_012835	Kif1b	0.16343	-0.3116	-0.0574	0.18181	0.51157	0.50382	-0.1469	0.5274	-0.1157	-0.3694	-0.2347	-0.402	0.03047	-0.1009
NM_145877	Kif1c	0.05902	-0.3037	-0.1029	-0.4317	0.1179	0.18951	-0.0486	-0.0294	-0.338	-0.0201	-0.0094	-0.004	0.1615	-0.0944
XM_001065173.1	Kif2	-0.2101	-0.1075	-0.0611	0.07175	-0.2082	-0.1499	-0.2116	-0.1375	-0.1272	0.02391	-0.3543	-0.1182	-0.1167	0.03631
NM_001108426	Kif20a_predicted	-0.4345	-1.3559	0.85262	-0.6132	-1.1264	-1.1475	-0.9478	-1.2199	-0.5541	-1.0314	-0.7787	0.4611	0.74416	0.82132
NM_001106790	Kif21a_predicted	0.25541	0.37292	0.20157	0.34457	0.23736	0.02856	0.11685	0.02415	-0.0025	0.00742	0.25153	0.50925	0.09184	0.03935
NM_001009645	Kif22	-0.2155	-0.6716	0.89743	0.04258	-0.7198	-0.4152	-0.0348	-0.6895	-0.3101	-0.2974	-0.2166	0.22081	0.42011	0.42943
NM_001108155	Kif23_predicted	-0.4644	-1.1966	0.78156	-0.4154	-1.0391	-1.2275	-0.9573	-1.1117	-0.4622	-0.9376	-0.7012	0.29933	0.36149	0.46604
XM_001081262.1	Kif2b	0.06072	-0.0376	0.05679	0.05783	0.15162	0.03281	0.00919	0.07793	0.12665	0.36199	0.09434	0.0189	-0.0342	0.03199

NM_134472	Kif2c	-0.374	-0.9335	0.61943	-0.1556	-0.7327	-1.0534	-0.4755	-0.8425	-0.369	-0.5293	-0.4435	0.26904	0.54295	0.54821
NM_001106529	Kif3b_predicted	0.16459	0.14947	0.0336	-0.246	0.19674	-0.0025	0.31687	0.03511	-0.0308	0.04318	0.10775	0.01309	0.01184	0.03996
NM_053486	Kif3c	0.46167	-0.0308	0.55031	0.1122	0.72239	0.67415	0.53491	0.52218	-0.2397	-0.0963	-0.1433	-0.0893	0.10768	0.37385
XM_343797.3	Kif4	-0.7928	-0.7165	0.60329	-0.8148	-1.4182	-1.4228	-1.1058	-1.3467	-0.1959	-0.4487	-0.5093	0.00074	0.48184	0.36043
NM_212523	Kif5a	-0.207	-0.2541	-0.2196	0.08798	-0.2082	-0.1655	-0.0938	-0.2371	-0.3197	0.09948	-0.2476	-0.1714	-0.2034	-0.1533
NM_057202	Kif5b	-0.6328	-0.0823	-0.9174	-0.6086	-0.5232	-0.9462	-0.4388	-0.134	-0.0516	-0.0674	-0.2949	-0.444	-0.0221	0.23204
NM_001107730	Kif5c_predicted	-0.1916	-0.0835	-0.0484	-0.2679	-0.0685	-0.1088	-0.2322	-0.0053	-0.2277	-0.235	-0.0446	-0.2029	0.01984	-0.1653
NM_001107730	Kif5c_predicted	0.05345	-0.059	-0.0283	0.21512	0.03664	0.08239	0.00149	0.13353	0.10427	0.13713	-0.0109	0.04688	0.06645	-0.0236
XM_343524.3	Kif6	-0.1959	0.04442	-0.1676	-0.0168	-0.1434	0.05205	0.03254	-0.0833	0.09443	-0.2623	-0.0125	-0.0154	-0.101	-0.0211
XM_218828.4	Kif7_predicted	0.18046	0.09373	0.17552	0.00455	0.11732	0.04969	0.14639	0.25189	-0.0082	0.16661	0.36436	0.1752	0.12523	0.00012
XM_218828.4	Kif7_predicted	-0.2264	-0.0365	0.38009	-0.1446	-0.395	-0.5786	0.29731	-0.5292	0.14503	0.0731	-0.1429	0.22621	-0.3508	-0.3382
NM_001108189	Kif9_predicted	-0.8515	-0.3424	-0.1337	-0.0687	-0.5609	-0.5626	-0.0532	-0.5368	-0.3619	-0.3429	-0.1919	-0.2062	-0.3564	-0.3344
NM_001105964	Kifap3_predicted	0.4784	0.67668	0.003	0.73405	-0.0271	-0.1892	0.31219	-0.0279	0.31837	0.59057	0.27039	0.21836	0.04904	-0.0323
NM_001005878	Kifc1	-1.0176	-1.7918	0.58288	-0.6177	-1.5747	-1.7915	-1.0254	-1.4723	-0.7124	-1.3036	-1.0515	-0.0337	0.23604	0.37826
NM_198752	KIFC2	-1.0539	-0.9571	-0.2509	-0.1614	-1.3064	-0.7285	-0.1105	-1.0913	-0.6126	-0.6053	-0.4391	-0.4825	-1.0168	-1.1743
NM_001103352	Kifc3	-0.4978	-0.6501	-0.096	-0.5267	-0.0643	-0.009	-0.8404	-0.085	-0.3896	-0.4929	-0.3344	-0.4	-0.2871	-0.4064
NM_181479	Kir3dl1	-0.2763	-0.0373	-0.2936	-0.2921	-0.3052	-0.265	-0.0995	-0.0691	-0.0362	-0.2279	-0.2004	-0.1184	-0.2715	-0.2117
NM_207606	Kirrel	0.04715	0.13427	0.15552	0.07132	0.09698	0.13068	0.06352	0.04447	0.0621	0.09954	0.15383	0.26993	0.11937	0.05447
XM_218486.3	Kirrel2_predicted	-0.1282	-0.0792	-0.0025	-0.0833	0.06354	0.04325	0.15643	-0.0516	0.07756	0.05735	-0.0085	-0.1094	0.14754	-0.0887
NM_001048215	Kirrel3_predicted	0.03504	0.13188	0.16035	0.09532	-0.0455	0.05847	0.07744	0.00239	0.10494	-0.0084	-0.0666	0.08271	0.04414	0.08743
NM_181692	Kiss1	0.07963	-0.003	0.27727	-0.1367	-0.1276	0.07112	0.04991	-0.0402	-0.1622	0.11989	0.02263	-0.0413	-0.0625	-0.0023
NM_023992	Kiss1r	0.22625	0.26461	0.03985	0.21134	0.08717	0.23618	0.09725	0.04033	0.04579	0.05522	-0.002	0.10669	0.14218	0.06072
NM_022264	Kit	0.23139	-0.084	0.01955	-0.0119	0.21002	0.08567	-0.0318	0.22417	0.03781	0.04579	-0.0326	-0.0536	-0.0019	0.05872
NM_021844	Kitl	0.12803	-0.0156	-0.1186	0.19273	-0.0444	-0.1145	-0.1635	-0.1347	0.084	-0.0948	-0.1442	0.00935	-0.0562	0.00499
NM_031336	Kl	0.03232	-0.1688	-0.1103	-0.2123	0.26977	-0.1112	-0.0263	0.02849	-0.071	-0.0481	-0.0572	-0.127	-0.2077	-0.1227
XM_001078178.1	Klb_predicted	-0.0653	0.03391	0.00959	-0.1188	-0.0484	0.05235	0.06886	0.12557	-0.0203	-0.0635	-0.1066	0.01212	0.04135	-0.0817
NM_001107571	Klc2_predicted	-0.0621	-0.0526	0.09573	-0.0685	0.26401	0.13469	0.10297	-0.0755	-0.0852	-0.0711	0.11458	-0.0237	0.09332	0.25771
NM_138520	Klc3	-0.1106	-0.0965	-0.1017	0.01769	-0.1344	-0.0408	-0.185	0.02494	-0.1816	-0.173	-0.144	-0.0384	-0.24	-0.2229
NM_001009601	Klc4	0.1554	0.57784	-0.1478	0.7417	0.29666	0.1589	0.1429	0.26105	0.19897	0.49306	0.55695	-0.1259	-0.3604	-0.428
NM_001107164	Klf1_predicted	0.01672	0.03151	0.05259	0.14515	0.15332	0.29181	0.14887	0.03709	0.04423	0.1376	0.18125	0.11635	-0.0965	0.1071
NM_031135	Klf10	0.27534	0.94703	-0.2454	0.79481	0.68617	0.51226	0.32998	0.54793	0.52714	0.80361	0.87311	-0.2253	-0.0153	0.11596
NM_001107281	Klf12_predicted	-0.0226	0.03981	-0.1007	0.0999	-0.0683	0.03442	0.04813	-0.0169	0.07901	-0.042	0.18911	0.26902	-0.0896	-0.0403
NM_053536	Klf15	-0.2199	0.47273	0.12522	0.28232	0.17746	0.37932	0.31676	0.14845	0.40095	0.36061	0.22135	0.36579	-0.0703	-0.0723
XM_345792.2	Klf16_predicted	0.21596	-0.0352	-0.0944	-0.251	-0.0196	-0.1675	-0.0766	-0.0771	-0.0162	-0.1055	-0.1491	-0.3067	-0.1678	0.20725
NM_001007684	Klf2_predicted	0.44498	1.106	0.52142	0.6444	0.72406	0.03343	0.07395	0.3966	0.97449	1.0695	0.90696	0.75528	0.70391	0.96246
NM_001105742	Klf3_mapped	0.28061	0.05326	-0.1705	0.31704	0.24997	0.16287	-0.0885	-0.0755	0.0892	0.01495	0.13106	0.07476	-0.1536	0.10753
NM_053713	Klf4	1.3581	0.64625	1.5206	0.89316	1.3302	1.4552	1.74	1.2146	0.74919	1.0085	0.85253	0.92733	1.1318	1.0582
NM_053394	Klf5	-3.1903	-4.0235	-3.3489	-3.0653	-3.2314	-3.0829	-3.4169	-3.2457	-3.9056	-3.7323	-3.9895	-4.1312	-3.4521	-3.1757
NM_031642	Klf6	-0.9486	-0.8928	-0.3548	-1.0583	-1.0237	-1.1904	-0.683	-1.0695	-0.8199	-0.6428	-0.5509	-0.941	-0.4106	-0.634
NM_001108800	Klf7_predicted	-0.4378	-0.5277	-0.5029	0.00776	0.17016	-0.4952	-0.2779	-0.3564	-0.2664	-0.5407	-0.3901	-0.3522	-0.3716	-0.2789
NM_057211	Klf9	0.24572	-0.1866	-0.8877	0.47836	0.73739	0.45772	0.04204	0.85285	0.01746	0.09148	-0.1044	-0.6253	-0.5159	-0.3901
NM_001108027	Klhdc1_predicted	0.30127	0.22107	-0.076	0.91373	0.17522	0.02888	0.18964	0.18669	-0.0599	-0.0817	-0.0409	-0.3321	-0.3959	-0.5251
XR_008781.1	Klhdc7a_predicted	0.03225	0.0816	0.021	0.02408	0.00178	-0.0253	-0.0659	-0.0055	-0.0391	-0.0686	-0.1358	0.13093	0.02052	-0.036
NM_001100683	Klhdc8a	-0.9212	-0.232	0.00117	-1.6627	-2.3901	-2.8452	-0.3311	-2.4197	0.08673	-0.208	-0.3578	0.14188	-0.5776	-0.7038
NM_001007685	Klhdc8b	-0.3768	-0.2486	-0.4639	0.42069	-0.1583	-0.2217	-0.1471	-0.2598	-0.1209	-0.4152	-0.3122	-0.5512	-0.9531	-0.6519
NM_001106054	Klhl1_predicted	0.19147	0.10231	-0.2033	0.2348	-0.0274	0.29041	-0.0992	0.05931	-0.1215	0.40338	0.04481	-0.0061	-0.0782	0.02865
NM_001001510	Klhl10	0.00458	-0.1159	0.00623	-0.1101	-0.0293	0.01733	-0.0636	-0.0934	0.21111	0.1094	-0.0804	0.11753	-0.0251	0.01094
NM_001105838	Klhl11_predicted	0.12147	0.19943	0.17558	0.05027	0.08795	-0.0444	0.00328	0.0229	0.15949	-0.0612	0.05788	0.37999	0.09498	0.12112
NM_153730	Klhl12	0.1511	-0.1895	-0.1509	-0.1095	-0.2456	-0.0202	-0.1102	-0.1674	-0.2621	-0.3339	-0.2331	-0.1937	0.10523	-0.2803

NM_001107944	Klhl13	1.2264	0.88444	0.49073	-0.3257	0.86539	0.80165	0.37122	0.88352	0.80254	0.91482	0.73382	1.2883	1.135	0.95004
NM_001108021	Klhl15_predicted	-0.2606	-0.1099	-0.2529	-0.0523	-0.1855	-0.1973	0.15559	-0.0119	-0.0383	-0.1778	-0.1003	-0.0991	-0.2097	0.15388
NM_145671	Klhl17	-0.0998	-0.119	-0.0536	-0.2965	-0.0468	-0.2804	-0.1302	-0.2843	-0.1224	-0.3407	-0.0468	-0.0628	-0.1875	-0.221
XM_001073589.1	Klhl2_predicted	0.07715	0.16814	0.36994	0.2257	0.12085	0.24325	0.25315	0.37012	0.32669	0.2842	0.2152	0.2955	0.70743	0.70908
XM_001073589.1	Klhl2_predicted	-0.0011	0.11477	0.39768	0.38228	-0.0517	0.2497	-0.0405	-0.0104	-0.0209	0.2877	-0.0101	-0.1056	0.3294	0.36682
NM_001107996	Klhl21_predicted	0.03275	0.08764	0.08358	0.15641	0.17274	0.14531	0.10807	0.0458	0.10569	0.07398	0.28628	0.18513	0.11123	0.07066
NM_001107079	Klhl22_predicted	0.26653	0.04425	-0.1075	0.0958	0.39853	0.50105	-0.4385	0.4354	-0.0636	0.1194	0.06224	-0.0284	0.06	0.14732
NM_001134504	Klhl23_predicted	-0.2036	-0.1804	-0.2358	-0.4356	-0.3645	-0.6858	-0.1826	-0.5796	0.15643	-0.3011	0.23095	-0.0028	-0.0004	0.05802
NM_181473	Klhl24	0.23346	0.43575	-0.6601	1.1886	0.59514	0.17005	0.27606	0.52126	0.19341	0.02237	0.04225	-0.273	-0.5078	-0.3294
NM_001039006	Klhl25	0.08264	0.29972	0.22274	0.10042	0.27752	0.03621	0.31517	0.07694	0.30484	0.17852	0.14201	0.19221	0.1394	0.07923
NM_001039006	Klhl25	0.01065	-0.0842	0.13732	-0.2539	0.33	-0.1008	0.00734	-0.126	0.13945	0.28868	0.01371	-0.0755	0.15493	0.01526
NM_001106075	Klhl26_predicted	0.07909	0.07736	0.07266	-0.0457	0.12076	0.00971	-0.0088	-0.0349	0.06661	-0.0283	0.07415	-0.0254	-0.024	-0.0527
NM_001047093	Klhl5	-0.2056	-0.047	-0.3954	0.29604	-0.0623	-0.0729	-0.3783	0.03687	-0.161	0.07568	-0.0376	-0.1043	-0.2738	-0.4076
NM_001105867	Klhl6_predicted	-0.355	-0.3255	-0.2822	-0.2278	-0.2901	-0.3501	-0.2106	-0.3109	-0.2101	-0.2837	-0.3306	-0.3415	0.0547	-0.1394
NM_001012187	Klhl7	0.19849	0.25275	-0.7685	-0.0928	-0.5043	-0.6621	-0.1612	-0.2609	0.19458	0.43971	0.08192	-0.1052	-0.3857	-0.5386
NM_001012187	Klhl7_predicted	0.08057	0.22862	0.02355	0.02486	0.39759	0.13475	0.05296	0.11913	-0.0452	-0.0757	-0.0422	-0.1009	0.36145	-0.0451
NM_001105995	Klhl8_predicted	-0.3066	0.13065	0.2042	0.13089	-0.363	-0.3602	0.06563	-0.4252	0.13382	0.11233	-0.0133	0.15203	-0.2002	-0.2501
NM_001107944	Klhl9_predicted	0.37177	0.67873	-1.5534	-1.1476	0.76902	-0.0533	-0.1288	0.45637	0.65016	0.44955	0.59668	0.62665	0.82392	0.59788
NM_001005382	Klk1	-0.0027	-0.1581	-0.1739	-0.1211	-0.0639	-0.0538	-0.1406	-0.0983	-0.0812	-0.1129	-0.1516	-0.2041	-0.1315	-0.0806
NM_001004100	Klk10	-1.5711	-1.5519	-1.5053	-1.3851	-1.5737	-1.6795	-1.6267	-1.6594	-1.3553	-1.5174	-1.6004	-1.461	-1.3916	-1.7024
NM_001106252	Klk11_predicted	-0.2053	-0.1131	-0.2453	0.03844	-0.0413	-0.0314	-0.1444	-0.119	-0.0259	-0.1192	-0.0855	-0.1531	-0.1239	-0.2324
NM_001107508	Klk12_predicted	0.16168	0.03515	-0.0373	0.01994	-0.046	0.00677	-0.0512	-0.1425	-0.0976	0.24376	-0.086	0.02293	0.28809	0.13169
NM_001170405	Klk13_predicted	-0.0464	0.03956	-0.0089	0.06102	0.21539	-0.1467	-0.1151	-0.205	-0.2028	-0.1368	-0.0939	-0.1206	-0.0874	-0.161
NM_001170405	Klk13_predicted	-0.1148	-0.0558	0.0541	0.02447	0.12333	0.13216	-0.0794	0.15937	0.07857	-0.1918	-0.2064	-0.0402	-0.0241	-0.1782
XM_001080394.1	Klk14_predicted	-0.1837	-0.1675	-0.1458	0.12484	-0.0828	0.06101	-0.2146	-0.159	-0.1231	-0.2404	-0.035	-0.0516	-0.0133	0.01546
XM_001080538.1	Klk15_predicted	-0.0208	-0.1011	-0.0246	-0.0163	-0.0152	-0.0575	0.13028	-0.0504	-0.0087	-0.0624	0.22849	0.18399	-0.0852	0.09097
NM_001135173	Klk1c10	-0.1953	-0.3008	-0.3132	-0.252	-0.3794	-0.2544	-0.2652	-0.1262	-0.1194	-0.3383	-0.3215	-0.4662	-0.1048	-0.2611
NM_001135173	Klk1c10	-0.1345	0.02449	0.11865	0.21731	-0.0879	-0.0916	0.05403	0.10997	-0.0995	-0.1927	0.02513	0.02065	0.07298	0.01395
NM_001004101	Klk4	-0.0836	-0.0764	0.11223	0.01145	0.00446	0.00522	0.02465	0.0139	-0.0142	-0.1201	0.01098	0.11156	0.00585	-0.1073
NM_001004101	Klk4	-0.0385	-0.0951	0.24789	-0.0461	-0.055	-0.1218	-0.1137	-0.0843	0.10518	0.15378	0.03475	-0.1596	-0.0438	-0.1024
XM_001080498.1	Klk5	-0.0672	-0.0246	-0.0109	-0.0373	-0.0262	-0.1337	0.08458	-0.0142	-0.0572	0.22309	0.20687	0.12852	0.04019	0.00105
NM_019175	Klk6	0.08165	-0.044	0.09527	-0.1002	0.10657	0.03528	0.16649	0.11496	0.35932	0.27239	-0.0564	0.01001	-0.1464	-0.0533
NM_001013067	Klk6_predicted	0.12872	0.05513	0.24908	0.22784	0.05737	0.00094	0.10391	0.1591	0.06405	0.11417	0.10707	0.01578	0.12506	0.23285
NM_012593	Klk7	0.06905	0.15772	0.37213	0.11418	0.16113	0.03681	0.09507	0.09302	0.28993	0.21231	0.04385	0.02989	0.14766	0.23436
NM_012593	Klk7_predicted	-0.2321	-0.0779	-0.1159	-0.1838	-0.2198	-0.2461	-0.2328	-0.1725	-0.0741	-0.272	-0.1859	-0.1988	-0.11	0.11245
NM_001107509	Klk8	-0.1064	-0.1041	0.02691	0.21743	0.04296	-0.0736	0.00046	-0.0801	0.03725	0.0556	-0.1484	0.17334	-0.0779	-0.0946
NM_001106253	Klk9_predicted	0.14736	0.23299	0.22009	0.24636	0.25336	0.11943	0.12909	0.00726	0.16109	0.07173	-0.0274	-0.0959	0.15516	-0.0156
NM_012725	Klkb1	-0.0007	0.10622	-0.0473	0.01448	0.15403	-0.0242	-0.0647	0.11555	-0.0168	0.03792	0.06648	0.09695	0.16502	-0.0391
NM_175759	Klks3	0.14334	-0.152	-0.1363	0.04304	0.08978	0.45729	-0.1535	-0.084	-0.177	0.02549	-0.0766	0.23424	0.05992	-0.0499
NM_001009486	Klra2	0.35024	0.09253	0.21758	0.12462	1.0285	1.1271	0.38972	1.0003	0.33475	0.26802	0.27734	0.19656	-0.0117	0.22339
NM_198746	Klra5	0.02776	-0.0173	0.03334	-0.0817	-0.0752	-0.0244	-0.1043	-0.0757	-0.0315	-0.032	-0.0338	-0.0395	0.02134	0.02337
NM_001010964	Klrb1a	-0.0812	-0.0879	0.08765	0.06887	-0.0257	0.08279	-0.014	0.03617	0.07984	0.0703	0.05758	-0.0615	0.07251	0.00708
NM_173292	Klrb1b	0.17442	0.12	0.37236	-0.0016	-0.0009	0.09781	0.13139	0.05684	0.07168	-0.0226	0.08656	0.11905	0.03265	0.10102
NM_001029908	Klrc2	0.05273	0.14382	0.01534	0.04631	0.08338	0.09802	0.11355	0.16663	0.116	0.21913	0.12085	0.19656	0.11885	0.11391
NM_001029908	Klrc3	0.10946	-0.0115	-0.0877	-0.0241	0.18245	0.09047	-0.0118	0.11125	0.05627	0.18922	-0.1305	0.2517	-0.0582	0.34147
NM_012745	Klrd1	-0.1258	-0.098	-0.0848	-0.0946	-0.0652	-0.0594	-0.0961	-0.0261	-0.1321	-0.0835	-0.0127	-0.0445	-0.0617	-0.098
NM_012745	Klre1	0.00092	0.03285	-0.0649	-0.0009	0.2839	0.14617	0.10116	0.02708	-0.0319	-0.0811	0.04999	0.03143	0.20643	0.21249
NM_031649	Klrg1	0.06539	0.08151	0.0194	0.05671	0.11317	0.20566	0.01195	0.2342	0.12466	0.05035	0.03666	0.30209	-0.0251	0.02685
NM_139187	Klrh1	-0.2295	-0.0376	-0.3726	-0.2166	-0.3057	-0.0353	-0.0328	-0.179	-0.0961	-0.1703	-0.3513	-0.3062	-0.1334	-0.2013

NM_133512	Klrk1	0.07972	0.07992	0.11589	0.11517	0.07228	-0.0763	0.13339	0.05877	0.00116	0.0179	0.06792	0.27133	0.2007	-0.024
NM_021593	Kmo	-0.0204	0.11017	-0.0635	0.03797	-0.003	-0.0822	-0.0497	-0.0195	-0.0446	-0.0641	-0.0502	0.13015	0.02303	-0.0261
NM_012696	Kng1	-3.5805	-3.0264	-4.1967	-3.2934	-1.8985	-1.4873	-2.9991	-2.0443	-3.8211	-3.192	-3.2832	-4.0122	-4.2088	-4.0103
NM_012696	Kng1	-1.0278	-1.1381	-1.2811	-1.2424	-0.4503	-0.6256	-1.2181	-0.6391	-1.1283	-1.2038	-1.0627	-1.1657	-1.2891	-1.2034
NM_001081973	Kns2	-0.1544	0.14257	0.32813	0.17612	-0.2458	-0.5478	0.15421	-0.1828	-0.2203	0.03199	0.01078	-0.5088	-0.4733	-0.3061
NM_001107140	Kntc1_predicted	-0.5372	-1.6172	0.39052	-0.7107	-1.2304	-1.2512	-1.1446	-1.3429	-0.632	-1.2449	-0.9798	0.11801	0.19291	0.12744
NM_001126270	Kntc2_predicted	-0.551	-1.0022	0.32013	-0.5552	-0.955	-1.1865	-0.792	-1.1042	-0.4721	-0.8332	-0.8316	0.10685	0.54568	0.60218
NM_198726	Kpna1	0.05365	0.04594	0.14921	0.42683	0.4434	0.26479	0.05597	0.37934	-0.0245	-0.0345	-0.2404	-0.1494	0.2802	0.36203
NM_001014792	Kpna3	-0.0132	0.28094	-0.1718	-0.2579	0.12988	-0.4699	-0.2023	0.26062	-0.0882	-0.0224	-0.1914	-0.4152	0.48476	0.32417
NM_001014793	Kpna4	0.30667	0.73368	-0.2718	0.1082	0.66748	0.11781	0.20531	0.41547	0.2642	0.56043	0.27928	-0.036	0.72153	0.62488
NM_001025113	Kpna5	0.23624	0.0097	0.19887	-0.0058	-0.0614	-0.1057	-0.0077	-0.0865	0.01344	0.14839	-0.0132	-0.0313	-0.0225	-0.06
NM_017063	Kpnb1	-0.2795	-0.3065	-0.381	-0.8851	-0.196	-0.7669	-0.4794	0.0171	-0.2725	-0.0953	-0.4661	-0.2081	0.69702	0.65898
XM_224534.3	Kpnb3_predicted	0.20514	-0.1088	0.07555	0.30801	0.09777	0.10684	0.1659	0.25981	0.16906	0.20682	-0.1184	0.02313	0.52487	0.6046
NM_001002290	Kprp	-0.2011	-0.2766	1.1445	1.3509	0.34832	0.62191	-0.1236	0.38227	0.14074	-0.1145	-0.2818	0.62553	1.4923	1.1227
NM_001107457	Kptn_predicted	-0.3586	0.03377	0.13996	-0.2715	-0.1139	0.01679	0.07012	-0.0385	-0.372	-0.2786	-0.1236	-0.3058	-0.1356	-0.3356
NM_031515	Kras	0.00742	-0.2941	-0.2951	-0.1861	0.07128	-0.0949	-0.1715	0.29678	-0.0027	-0.2141	-0.1383	-0.0527	-0.0027	0.07924
NM_001009413	Krcc1	-0.3723	-0.467	-0.5441	-2.0727	-1.1372	-1.1918	-0.5037	-1.1474	-0.6027	-0.7099	-0.5733	-0.6991	-0.3155	-0.5107
NM_053649	Kremen1	-0.1083	-0.0699	-0.1393	0.05387	-0.047	-0.1241	0.08039	-0.1505	0.08105	-0.1754	-0.1086	-0.0071	-0.2099	0.03824
NM_001105767	Kremen2_predicted	-0.0186	-0.1955	-0.1628	-0.0088	-0.1094	-0.074	0.00046	-0.1151	-0.1123	0.03634	-0.1314	-0.0305	-0.1866	-0.2205
NM_001008804	Krt10	-0.0587	-0.0922	0.1038	-0.0039	-0.0472	0.02373	-0.1335	-0.0747	-0.0616	-0.0961	0.01843	0.05521	0.20155	-0.1561
NM_001008761	Krt12	0.12139	-0.0325	0.11831	-0.0352	-0.0245	-0.1006	-0.0903	0.09978	-0.092	-0.1108	0.11587	0.0596	-0.1149	-0.0212
NM_001004021	Krt13	-0.0916	-0.0736	-0.1869	-0.0798	0.17266	0.28479	-0.1852	0.51797	-0.1094	0.09416	0.05895	-0.1039	-0.0662	-0.0949
NM_001008751	Krt14	0.23103	-2.2093	-2.2553	-1.9027	3.1111	3.3171	-2.1995	3.0159	-1.8752	-1.2963	-1.5452	-1.8029	-1.8194	-2.0068
NM_001004022	Krt15	0.15318	0.21947	-0.0698	0.06439	1.3549	1.4368	0.01715	1.4631	0.03516	0.09679	0.18272	0.03098	0.1273	-0.0636
NM_001008759	Krt1-5	0.17399	0.10642	0.06464	0.00138	-0.0066	0.21547	0.12196	0.21115	-0.0349	-0.0694	0.36172	-0.0598	0.01581	0.21797
NM_001008752	Krt16	0.21949	-0.0325	0.0203	-0.0934	-0.0133	0.01467	-0.068	-0.0562	-0.0741	-0.0297	-0.0869	0.03315	-0.0183	0.07028
NM_212545	Krt17	-0.3665	-0.3157	-0.2248	-0.1064	-0.194	0.28703	-0.2336	-0.2771	-0.2756	-0.056	-0.1532	-0.1976	0.05619	-0.0285
NM_199498	Krt19	3.1843	-0.1578	-0.0749	-0.0152	6.2987	5.9629	-0.0516	6.1713	-0.0776	0.6296	-0.1343	-0.0745	-0.2615	-0.0532
NM_001008899	Krt2	0.14019	0.01058	-0.1057	-0.0508	-0.1283	-0.1152	-0.0958	-0.0845	-0.1274	-0.0979	-0.057	-0.0776	-0.1321	-0.0749
NM_173128	Krt20	0.10087	0.32563	0.11884	-0.0409	0.16334	0.04936	-0.0825	0.0479	0.45978	0.05205	-0.0374	-0.1059	0.1894	0.07814
NM_001008753	Krt23	1.2116	0.25058	0.66335	1.4204	3.4697	3.4712	-0.0051	3.3941	0.12622	0.12765	1.5447	-0.1039	0.13979	0.18104
NM_001004131	Krt24	-0.1394	-0.1521	-0.1288	-0.0755	-0.1582	-0.0488	-0.2368	-0.1346	-0.0578	0.12815	-0.114	-0.0019	0.04997	-0.1173
NM_001008822	Krt25	-0.0094	0.03414	0.19668	-0.0249	0.06044	-0.1743	-0.0464	-0.0126	-0.1598	-0.0353	-0.0103	-0.1568	-0.1147	0.10993
NM_001008823	Krt26	-0.1517	-0.1559	-0.0114	-0.133	-0.2368	-0.2214	-0.1417	-0.0603	-0.2147	-0.2397	-0.113	-0.1281	-0.187	-0.2085
NM_001008824	Krt27	0.05831	0.07823	-0.0955	-0.037	0.65331	0.90845	-0.1766	0.51368	0.19358	-0.0652	-0.1543	-0.1881	-0.2062	-0.1489
NM_001047870	Krt2-7	0.42687	0.16007	0.26144	0.45166	0.61334	0.86733	-0.0972	0.93671	-0.101	-0.1378	-0.041	-0.125	0.20128	0.18848
NM_001008760	Krt28	0.62747	0.62033	0.43564	0.51147	0.62725	0.56342	0.87533	0.52556	0.68808	0.75118	0.87976	0.72745	0.3575	0.32481
NM_001008817	Krt31	-0.0186	0.16721	-0.0277	-0.0278	0.06725	0.00323	0.0597	0.35703	0.06858	0.15586	0.19049	0.22793	0.02762	-0.0089
NM_001008818	Krt32	-0.2269	-0.099	-0.0324	-0.1245	-0.2147	-0.1589	-0.1891	-0.1059	-0.2005	-0.067	-0.1928	-0.1613	-0.0442	-0.0889
NM_001008757	Krt33a	0.03462	-0.1647	-0.1161	0.09138	-0.0243	-0.0082	-0.078	0.01746	0.14724	-0.1217	-0.0941	-0.1005	-0.0284	-0.0859
NM_001008819	Krt33b	-0.0188	0.04453	-0.0107	-0.0617	-0.115	-0.0368	-0.0648	0.00939	0.02223	-0.0118	-0.0767	0.09257	-0.1693	0.09223
NM_001008758	Krt34	-0.1632	-0.159	-0.2429	-0.2041	-0.1571	-0.2418	-0.1478	0.00735	-0.2071	-0.1953	-0.2365	-0.1868	-0.1219	-0.2206
NM_001008820	Krt35	-0.0772	-0.0977	-0.0593	-0.03	-0.1272	-0.0752	-0.124	-0.0122	-0.2063	-0.1307	0.39937	-0.0196	-0.0076	0.12929
NM_001004130	Krt39	-0.0498	-0.0216	-0.0326	0.22987	0.01715	0.07063	-0.062	0.06131	-0.0079	-0.0126	0.01505	0.01508	-0.0189	0.12887
NM_001008806	Krt4	0.05264	-0.0302	-0.047	-0.0379	-0.0899	-0.098	-0.0112	-0.0001	-0.2038	-0.0217	-0.1847	0.07249	0.12997	-0.0341
NM_001008821	Krt40	0.07764	0.04256	0.05937	0.26952	-0.0041	0.03778	0.02881	0.24439	0.02265	0.26079	0.12785	0.1049	0.11341	0.20957
NM_001008816	Krt42	-0.2315	-0.0583	0.01853	0.03768	-0.1508	-0.0647	-0.1762	-0.0845	-0.009	0.00028	0.01228	-0.1578	0.07752	0.0188
NM_001008809	Krt72	-0.0379	0.00146	0.13255	-0.1848	0.10254	-0.242	-0.1743	-0.0223	0.02503	0.27372	-0.0825	-0.2101	-0.165	-0.1355
NM_001008828	Krt75	0.07131	-0.1448	-0.1959	-0.1407	0.12973	0.39851	-0.021	-0.0682	-0.1369	-0.0547	-0.0448	-0.179	-0.0326	0.08297

NM_001008805	Krt76	0.09634	0.21547	0.07298	-0.0793	-0.0117	0.53711	-0.1549	-0.0047	0.11612	0.33492	0.06885	0.19713	0.33737	0.1831
NM_001008807	Krt77	0.16273	0.13892	-0.0497	0.00663	0.2046	0.01526	0.11632	0.33086	0.19696	0.07359	0.0817	0.23324	0.04182	0.18126
NM_199370	Krt8	-0.8654	-0.5257	0.13357	-1.2016	-0.563	-0.837	-0.3562	-0.5538	-0.2773	-0.2859	-0.4556	0.18812	-0.1805	-0.1551
NM_001008815	Krt80	-0.1162	-0.13	-0.2057	-0.1885	-0.1142	-0.0647	-0.085	-0.11	-0.1487	-0.1146	-0.0796	-0.1698	-0.0533	0.08934
NM_001008803	Krt82	0.06246	0.10517	0.02832	0.01947	0.03772	0.14882	0.16117	0.1356	0.25647	0.14749	0.11117	0.10542	0.11048	0.02979
NM_001008812	Krt84	-0.0649	-0.093	-0.1257	0.04039	-0.0183	-0.0885	0.0513	-0.105	-0.0843	0.0262	-0.0507	-0.102	0.07703	-0.1078
NM_001008810	Krt86	-0.0735	0.14718	-0.0392	0.03477	0.00469	0.10883	0.14377	-0.0108	0.00552	0.02106	-0.0728	-0.0435	0.04652	0.05511
XM_344003.2	Krtap14_predicted	0.0421	0.14108	-0.096	0.10725	0.01474	-0.0079	-0.0462	0.01639	0.13605	0.12715	0.01585	0.11047	-0.0007	0.13836
NM_001105896	Krtap15_predicted	-0.0986	0.13778	0.00199	0.028	0.24044	0.09951	-0.0267	-0.0844	0.01022	0.02516	-0.0349	-0.013	0.23132	0.06614
NM_001106444	Krtcap2_predicted	0.21824	-0.3866	-0.294	-0.9397	-0.3136	0.17632	-0.7063	-0.4203	-0.0451	-0.3072	-0.1537	0.25526	-0.0853	-0.0074
NM_001108284	Ksr_predicted	0.09259	0.06534	-0.0081	0.23987	0.22427	0.05222	0.1284	0.10268	-0.0808	0.28523	-0.0825	0.05298	0.18499	0.08057
XM_341305.3	Ktn1_predicted	-0.2905	0.05316	-0.1434	1.095	-0.2973	-0.2424	0.18116	-0.2449	0.07647	0.26246	-0.0585	-0.3527	0.11812	-0.0636
XM_341305.3	Ktn1_predicted	0.21265	0.43662	-0.0921	0.93704	0.16501	0.08168	0.2456	0.2136	0.33075	0.29153	-0.0129	-0.2311	0.5542	0.53656
NM_001113752	Kua_predicted	-0.292	-0.3344	-0.1374	-0.3014	-0.2165	-0.1295	-0.3328	-0.1854	-0.1134	-0.1708	-0.3793	-0.275	-0.1878	-0.2809
NM_001113752	Kua_predicted	0.16299	-0.2277	-0.0657	-0.5176	0.41582	0.53996	-0.4059	0.36453	-0.2407	-0.0422	-0.1086	-0.1592	0.68168	0.76139
NM_001108180	Ky_predicted	0.18855	0.20092	0.2453	0.08988	0.11908	0.24256	0.01443	0.18912	0.21066	0.19565	0.15631	-0.0037	-0.0662	0.12627
NM_053902	Kynu	0.00367	0.00518	-0.0228	0.02959	-0.0796	0.06128	0.08444	0.06237	-0.0194	0.16856	0.00057	0.06872	0.05446	0.00037
NM_017345	L1cam	0.0639	0.11177	0.111	0.16715	0.01247	0.0865	-0.1459	0.05426	0.07634	-0.0256	0.04424	0.0473	0.21974	0.07229
NM_001108028	L2hgdh_predicted	-0.0383	-0.1584	-0.1676	0.01982	-0.316	-0.0866	-0.2817	-0.2371	-0.1068	-0.1497	-0.2815	0.10616	-0.0697	0.2846
NM_001033695	L3mbtl2	-0.2906	-0.0685	0.64181	1.4628	-0.1876	-0.2221	0.50267	-0.0387	-0.3488	-0.1318	-0.1428	-0.0679	-0.2605	-0.1116
NM_001106833	Lactb_predicted	0.08712	0.30759	0.52855	0.44825	0.09348	0.13728	0.20972	0.09707	0.42672	0.28451	0.41355	0.31462	0.34756	0.29861
NM_001024247	Lactb2_predicted	-0.1616	0.16714	0.19462	-0.1465	0.09733	-0.2259	0.46837	0.08048	0.32177	0.3546	0.36947	0.19421	0.53231	0.43545
NM_001107942	Lad1_predicted	-0.2044	-0.2957	-0.2715	-0.0313	-0.2732	-0.0997	-0.2736	-0.2979	-0.2894	-0.2233	-0.3087	-0.3745	-0.2423	-0.2917
NM_212513	Lag3	-0.2103	-0.0591	-0.0047	-0.0547	-0.2675	-0.1279	-0.1573	-0.0611	-0.258	-0.1411	0.10553	-0.3486	-0.2921	-0.0784
NM_012594	Lalba	-0.1972	-0.229	-0.1402	-0.1387	-0.1709	-0.182	-0.1671	-0.0446	-0.1614	-0.1155	-0.1275	-0.121	-0.1845	-0.0201
NM_001108237	Lama1_predicted	-0.1134	-0.121	0.05225	-0.0307	-0.0191	-0.1806	-0.1139	-0.0549	0.03734	0.17327	-0.1265	-0.3015	-0.0899	-0.0709
XM_001062273.1	Lama2_predicted	-0.1631	0.61225	0.12715	0.33903	0.10947	0.49729	2.6317	0.21028	0.39291	0.47562	0.55362	0.1983	0.47376	0.24387
XM_001060527.1	Lama5	-0.6091	-0.6627	-0.0992	0.141	-0.6008	-0.491	-0.1659	-0.8717	-0.7526	-0.6129	-0.3075	-1.1096	-1.1581	-0.9867
NM_001106721	Lamb1_predicted	0.72487	1.0321	0.34959	0.80115	0.39853	0.36374	0.64619	0.51632	0.85976	1.1885	1.023	0.18603	0.41118	0.26968
NM_012974	Lamb2	-1.3703	-0.887	-0.1786	-0.3074	-1.4528	-1.0767	-0.3547	-1.6028	-1.1767	-0.989	-0.8815	-0.9524	-1.4706	-1.4515
NM_053966	Lamc1	-0.2957	-0.1524	-0.2591	-0.3553	-0.2898	-0.2186	-0.2559	-0.211	-0.2569	0.18894	0.02501	-0.2055	-0.1753	-0.0067
NM_001100640	Lamc2	2.2482	1.3465	0.7835	1.7685	1.9042	1.7404	1.6626	1.8799	0.93519	1.353	1.3252	-1.1996	0.44479	0.39404
NM_001107830	Lamc3_predicted	0.14269	0.07622	0.16445	0.09753	0.10548	0.1731	0.09904	0.23116	0.06566	0.1112	0.15702	0.04471	0.18511	0.09919
NM_012857	Lamp1	0.2124	0.19043	0.2609	0.27685	-0.1219	-0.1839	0.96915	-0.2895	-0.0343	0.23252	0.1023	-0.2954	0.42355	0.26491
NM_017068	Lamp2	0.12522	0.64791	-0.5389	0.16541	-0.0926	-0.0758	0.51312	-0.0065	0.25938	0.47379	0.22103	-0.627	-0.1382	-0.2538
NM_001012015	Lamp3	0.08592	-0.246	0.10278	-0.1845	-0.2107	-0.0524	-0.1425	0.09044	-0.3002	-0.1289	-0.0345	-0.0077	0.06733	0.16919
NM_001012015	Lamp3_predicted	0.02719	-0.0947	-0.0179	0.27635	-0.1487	-0.1831	-0.1414	0.02758	-0.1575	-0.1101	0.0083	-0.0913	-0.1099	-0.1082
NM_053723	Lancl1	0.36843	0.18636	-0.9558	0.42096	0.50589	0.24087	-0.2419	0.77704	-0.1393	-0.0145	-0.0922	-0.7806	-0.7166	-0.7443
XM_216158.3	Lancl2_predicted	-0.7	-0.7434	-0.228	-1.4972	-1.3033	-1.3168	-0.7012	-1.2625	-0.6004	-0.5751	-0.6303	-0.1853	-0.1445	-0.2249
XM_228739.4	Lancl3_predicted	0.12768	-0.0165	0.08307	0.15737	0.09342	0.03768	0.18136	-0.055	0.18286	0.27213	0.17089	0.18185	0.1168	0.18579
NM_001106682	Lao1_predicted	0.02672	-0.0618	-0.0412	-0.0818	0.09395	0.02824	0.2374	0.00016	-0.1029	0.10175	0.00808	-0.0649	-0.1203	0.05592
NM_001011910	Lap3	0.08353	-0.0467	0.00438	-0.2645	0.39053	0.44384	0.12587	0.36746	-0.3627	-0.1017	-0.1391	0.30171	-0.0352	0.15848
NM_199384	Laptm4a	-0.102	0.31264	-0.0382	-0.1498	0.05861	0.02439	0.15462	0.02914	0.3933	0.19092	0.21418	0.21475	0.043	0.04264
NM_001013174	Laptm4b	-0.3339	0.12786	-0.4103	-0.2547	0.17175	0.19634	-0.3951	0.05361	-0.4126	-0.4169	0.05692	-0.0483	-0.2624	0.00395
NM_053538	Laptm5	-0.0742	0.02285	0.03004	0.1282	0.77748	0.787	0.04251	0.58816	-0.0072	0.06023	0.14279	0.00634	-0.0902	0.0915
NM_001108439	Large_predicted	0.03885	0.05283	0.07437	0.02075	-0.0961	0.1549	0.07649	0.12556	0.05118	0.05102	0.01038	0.06469	0.18318	0.13097
NM_001108439	Large_predicted	-0.1549	-0.0953	-0.0294	-0.2018	-0.2048	-0.784	-0.263	-0.0379	-0.1243	0.12236	-0.0798	0.06199	0.21154	0.40271
XM_220446.4	Larp1_predicted	-0.5382	-0.7521	-0.3848	-0.4719	-0.6383	-0.8701	-0.4939	-0.7167	-0.3165	-0.6771	-0.538	-0.5195	-0.1796	-0.3172
NM_001107361	Larp5_predicted	-0.2061	-0.0489	-0.4234	0.02037	-0.2042	-1.214	-0.1631	-0.1814	-0.2954	-0.2876	-0.1862	-1.0243	-0.1615	-0.1102

NM_001108154	Larp6_predicted	-0.0939	0.02623	0.11905	0.00182	-0.1782	0.0232	-0.1414	-0.215	0.05423	0.00198	-0.0085	-0.025	-0.2081	-0.0058
NM_001009637	Lars	-0.3832	-0.3951	-0.1888	-0.4102	-0.5323	-0.4962	-0.4336	-0.6004	-0.4285	-0.2974	-0.3342	-0.2453	-0.5513	-0.3683
NM_001108787	Lars2_predicted	-0.1694	-0.0483	0.35748	-0.0076	0.22872	-0.1667	0.07286	-0.1216	0.03542	0.29572	0.0706	-0.0442	-0.064	-0.0742
NM_001170590	Las1l_predicted	-0.1263	-0.3363	0.38313	0.04857	-0.1032	0.21421	0.02787	0.00561	-0.0614	-0.1884	-0.2009	0.24174	0.35271	0.36431
NM_032613	Lasp1	0.13431	0.17719	-0.0905	0.03044	0.04519	0.1826	0.02404	0.08449	0.09888	0.06586	0.11642	0.13682	0.19237	0.12898
NM_001033700	Lass2_predicted	0.19985	0.30025	-0.0133	-0.4723	0.19726	-0.2807	0.42678	0.20034	0.21121	0.4798	0.23265	-0.1598	0.35644	0.1102
NM_001107117	Lass4_predicted	-0.0332	-0.1614	0.00361	0.16934	0.0981	-0.3877	-0.3314	-0.3174	0.0972	-0.0621	0.30233	0.12994	0.05487	-0.1132
NM_001108993	Lass5_predicted	-0.4356	-0.0102	0.26789	0.03317	-0.1566	0.03438	-0.195	-0.1406	-0.2533	0.02569	-0.1586	-0.0049	0.07591	-0.0478
NM_030853	Lat	-0.1811	-0.059	0.18892	0.49973	-0.3053	-0.4929	0.15613	-0.3633	-0.1653	-0.0659	-0.1293	0.24159	-0.1297	-0.255
NM_173840	Lat2	0.03906	0.00688	0.08214	-0.0187	0.01417	-0.0307	-0.0611	-0.0093	-0.0476	-0.0724	-0.0037	0.14766	0.04417	-0.0617
NM_001107267	Lats2_predicted	0.21102	0.34484	0.28281	0.24474	0.20015	-0.2436	0.09313	0.20884	0.20663	0.11658	-0.043	-0.0187	0.47717	0.42796
NM_001017491	Lax1	0.08515	0.26828	0.35514	0.30597	0.30148	0.20139	0.00993	0.17117	0.09082	0.07502	0.40929	0.298	0.31146	0.17938
NM_017208	Lbp	-2.0501	-1.4108	-1.6237	-2.2979	-2.8773	-3.2106	-0.3862	-3.1835	-1.7554	-1.4599	-1.5314	-3.3746	-2.5064	-2.4731
XM_001075793.1	Lbxcor1_predicted	0.17206	0.00545	0.14743	0.01662	0.14589	0.29455	0.22541	0.24783	-0.0088	-0.0928	-0.013	0.0133	-0.0105	0.18103
NM_001013954	Lca5	-0.1088	-0.0269	-0.0545	0.06383	-0.0867	0.04798	-0.0348	0.04835	0.06745	0.15316	-0.0583	0.04773	0.09698	0.05368
NM_001017484	Lca5l	0.04329	0.07868	0.42959	0.14651	0.18376	-0.0075	0.17601	0.18667	0.06856	0.13502	0.04303	0.35563	0.12895	0.0136
NM_017024	Lcat	-0.7663	-1.0376	0.61225	1.2414	-1.2779	-1.045	0.46777	-1.2541	-0.8783	-1.1479	-0.6393	-0.4057	-1.3543	-1.3065
NM_001100709	Lck_mapped	0.07472	0.19265	0.07298	0.047	0.10323	-0.0445	-0.0319	0.01803	0.1874	-0.0103	-0.1206	0.1037	0.12184	0.00902
NM_199405	Lcmt1	-0.1265	-0.0417	0.03053	-0.0925	0.00281	0.45914	-0.2298	-0.0452	0.21143	0.0414	-0.0472	0.30245	-0.1533	-0.1876
NM_001011956	Lcmt2	-0.3898	-0.3532	-0.3567	-1.2139	-0.6275	-0.8757	-0.486	-0.6493	-0.3344	-0.3081	-0.3928	-0.2572	0.10706	0.01955
NM_130741	Lcn2	1.6798	0.9483	0.13968	0.57425	1.6409	2.0595	1.2707	1.6246	0.64577	1.1302	0.71484	0.09146	0.01888	0.16071
XM_231034.3	Lcn3_predicted	-0.0209	0.08814	0.21125	0.07894	0.15463	-0.0081	0.0487	-0.0737	0.06298	-0.0087	-0.0659	-0.096	0.13626	0.06563
NM_024136	Lcn5	0.15326	0.10048	-0.0669	0.0608	-0.034	0.12046	0.1164	0.23557	0.17511	0.14275	0.1955	-0.0993	-0.0214	0.22053
NM_001001519	Lcn6	0.10366	0.22951	0.17553	0.00953	0.04555	0.11042	0.04472	0.1632	0.06864	0.10711	0.02682	0.07997	0.51776	0.16936
NM_001106560	Lcn9_predicted	-0.1188	-0.1391	-0.1894	0.09177	-0.0786	-0.1453	-0.0145	0.04007	-0.1527	0.11677	-0.0006	-0.009	0.0849	0.01269
NM_001012044	Lcp1	0.89617	0.10206	0.08828	0.07421	1.6505	1.4494	0.4323	1.6765	-0.0838	0.14551	0.13563	0.08841	-0.0767	0.23411
NM_001012044	Lcp1_predicted	-0.0549	0.02551	0.21136	-0.1097	-0.1698	-0.0972	0.04688	0.01689	-0.2167	-0.1394	-0.2598	0.21766	-0.0592	-0.0014
NM_130421	Lcp2	-0.1518	0.20179	-0.0208	0.02195	-0.0374	0.04036	-0.0309	0.02703	-0.0408	-0.0173	-0.1701	-0.0227	0.00957	-0.0672
NM_053841	Lct	-0.1558	-0.169	-0.1624	-0.0149	-0.0679	-0.1164	-0.0655	0.01296	-0.0188	0.00025	0.06643	-0.0601	-0.1026	-0.1134
NM_001108158	Lctl_predicted	-0.3358	-0.174	-0.3719	-0.2774	-0.1006	-0.1863	-0.1055	-0.2915	-0.2615	-0.3275	-0.1632	-0.244	-0.2708	-0.1327
NM_001107601	Ldb1_predicted	-0.551	-0.0987	-0.2328	-0.1303	-0.5909	-0.0404	-0.0794	-0.3447	-0.2933	-0.5896	-0.2776	0.01268	-0.0523	-0.3437
NM_001106009	Ldb2_predicted	-0.032	-0.131	0.10524	-0.1504	-0.0761	-0.08	0.16447	-0.0725	-0.0277	0.191	-0.0399	0.27643	0.09019	-0.0771
NM_017025	Ldha	0.57621	0.6752	0.6336	0.00151	0.59903	0.26609	0.1332	0.43934	0.35913	0.79115	0.44848	-0.143	0.63586	0.67686
NM_183334	Ldhal6b	-0.0629	0.19301	0.04075	-0.0774	0.13263	0.0502	0.06813	-0.0543	0.05082	-0.0686	-0.1181	-0.1263	-0.0058	-0.0707
NM_012595	Ldhb	-0.0889	0.29996	-1.17	-0.7422	-0.8228	-0.8807	-0.2326	-0.8339	0.02062	0.24323	0.26389	-0.7127	0.00286	-0.033
NM_017266	Ldhc	0.09447	0.10869	-0.1479	0.23416	-0.041	0.00611	-0.0667	0.04617	0.07687	-0.098	-0.1458	0.05613	0.1422	0.17674
NM_001008893	Ldhd	1.0157	0.99946	-0.0847	0.7476	0.81767	1.1626	-0.1406	0.87358	0.50475	0.80321	0.75595	0.50658	-0.2129	-0.0015
NM_175762	Ldlr	-0.2661	-0.3856	-0.0092	0.29507	-0.1451	-0.2815	-0.0793	-0.1321	-0.3837	-0.3161	-0.197	-0.1501	0.09794	-0.1278
NM_030854	Lect1	0.29959	0.2443	0.2289	0.08108	0.03391	0.02783	0.14923	0.17937	0.31527	0.21656	0.09059	0.20346	0.05892	0.08095
NM_001108405	Lect2_predicted	0.28884	0.07849	0.21344	0.08144	0.09318	0.13904	0.0441	0.00124	0.08941	0.17028	0.01474	0.13513	0.18204	0.14181
NM_130429	Lef1	-0.5747	-0.787	-0.5449	-0.6996	-0.5326	-0.6271	-0.6952	-0.6724	-0.6078	-0.7783	-0.6973	-0.3337	-0.6194	-0.7616
NM_001007556	Lefty2	0.04058	0.14234	0.30101	0.14908	0.1792	0.29302	0.19922	0.15051	0.25471	0.18791	0.12734	0.31254	0.00035	0.14693
NM_001039032	Lemd2	0.06424	0.03279	-0.3245	0.27137	0.15622	-0.2981	0.05932	0.17987	-0.0874	0.0956	-0.2152	-0.6523	0.10721	-0.1668
NM_053614	Lenep	-0.0857	-0.336	-0.0082	0.28147	-0.3938	-0.6688	0.13169	-0.3307	-0.4157	-0.1087	-0.2526	-0.6444	-0.5989	-0.477
NM_001106218	Leng1_predicted	0.10056	-0.25	0.36574	0.7731	0.26183	0.61421	-0.0112	0.12407	-0.0264	-0.2466	0.07347	0.38306	0.20796	0.2398
NM_001134978	Leng4_predicted	0.17506	-0.0795	0.06986	0.19294	0.14914	0.07291	0.17577	0.12581	0.21333	0.13055	0.01467	0.00735	0.11126	0.18988
NM_001037790	Leng8	-0.1315	-0.087	-0.3183	-1.1956	-0.0765	-0.1955	-0.3643	-0.5553	-0.1895	-0.1115	-0.1933	-0.0217	0.02557	0.04904
NM_001037790	Leng8	0.02368	0.10177	0.12204	0.11949	-0.0053	-0.0325	0.08546	-0.1184	1E-04	0.02285	0.21435	0.0674	-0.0842	0.04679
NM_001005548	Leo1	-0.4054	-0.2865	-0.289	-0.748	-0.5873	-0.7692	-0.337	-0.6283	-0.5169	-0.4827	-0.6132	-0.37	-0.2239	-0.2221

NM_013076	Lep	0.1136	-0.059	0.05704	0.04315	0.11177	0.12938	-0.011	0.00932	-0.0673	0.0587	-0.0503	-0.0221	-0.065	-0.0313
NM_012596	Lepr	0.01664	0.12389	0.15291	0.12759	0.11945	0.12784	0.01226	-0.0196	-0.0192	0.00993	0.15343	-0.0309	-0.0275	0.02839
NM_053667	Lepre1	-0.3131	-0.0403	-0.2471	-0.2098	0.14671	-0.1208	-0.3367	0.12402	0.09488	-0.0293	-0.097	0.1859	-0.0782	-0.1427
NM_001025627	Leprel1	2.7091	1.1315	0.62613	1.3103	3.2267	2.8472	-0.0614	3.0957	0.81862	0.83456	0.89158	0.21645	0.69414	0.80581
NM_001106620	Leprel2_predicted	-0.6542	0.50666	-0.2748	-0.0418	-0.4945	-0.5505	0.30182	-0.5801	0.365	0.28831	0.26365	0.33593	-0.2344	-0.0883
NM_020099	Leprot	0.04318	0.45676	-0.0308	0.26538	-0.1603	-0.2767	0.61837	-0.3784	0.19187	0.13073	0.26589	-0.2371	-0.5423	-0.3653
NM_001013188	Leprotl1	0.58228	0.52713	-0.4914	-0.1871	0.61602	0.50379	0.05448	0.72418	0.51888	0.11874	0.44479	0.2636	0.55277	0.3299
NM_001005884	Letm1	0.1093	0.13493	0.24315	0.07553	0.04962	0.10441	0.08878	0.08975	0.23088	0.18915	0.25995	0.09995	0.1781	0.17025
NM_001005884	Letm1	-0.5865	-0.698	-0.1255	-0.3911	0.04184	-0.2962	-0.026	0.01756	-0.8055	-0.598	-0.6831	-0.421	0.0146	0.18134
NM_001012158	Letm2	-0.2012	-0.3328	-0.0183	-0.1768	-0.1646	-0.3171	-0.1772	-0.474	-0.5143	-0.406	-0.4417	-0.3112	-0.2362	0.02472
NM_133393	Lfng	-0.3401	-0.2709	-0.1126	-0.751	-0.2509	-0.624	-0.2174	-0.6012	-0.0153	-0.1424	-0.5177	-0.3562	0.20977	0.18642
NM_019904	Lgals1	-0.0254	0.01574	0.047	-0.1196	-0.0214	0.07337	0.1924	-0.1941	-0.3032	-0.0925	-0.0126	0.23287	0.08047	-0.1243
NM_001106333	Lgals12_predicted	0.1238	0.06342	0.02845	0.26408	0.04406	0.0014	0.14241	0.05283	-0.0078	0.10607	0.12687	0.39688	0.11701	0.03377
NM_133599	Lgals2	0.05767	-0.037	0.39382	-0.0738	0.01251	0.05046	0.05231	-0.0772	-0.0489	-0.1025	0.12385	0.56066	0.43569	0.19334
NM_031832	Lgals3	0.13011	0.17784	0.58199	-0.8042	-0.3207	-0.5653	0.04567	-0.4267	0.08893	0.41809	0.26892	-0.3946	0.61825	0.63166
NM_139096	Lgals3bp	-0.9079	-0.3247	-1.0593	-0.5615	0.67198	0.54893	0.03325	0.50073	-0.4705	-0.5283	-0.4898	-0.5955	-0.2786	-0.1961
NM_012975	Lgals4	-2.693	-2.6578	-2.7037	-2.7772	-2.7575	-2.6843	-2.7638	-2.7641	-2.6146	-2.6728	-2.7807	-2.7346	-2.619	-2.6389
NM_012976	Lgals5	0.40927	0.22176	0.47634	0.17683	0.50939	0.28476	0.03597	0.31209	0.12236	0.15152	0.20813	-0.0183	0.29734	0.22721
NM_022582	Lgals7	0.23217	0.22953	0.01391	-0.0167	-0.0863	-0.3624	0.06273	0.12698	0.31382	0.04494	0.18658	0.17095	0.21976	0.15811
NM_053862	Lgals8	-0.2273	0.33789	-0.0401	-0.0821	-0.2645	-0.4325	0.05117	-0.3027	0.29058	0.14275	0.4039	0.04206	-0.159	-0.082
NM_012977	Lgals9	0.30588	0.21112	0.11394	0.37978	0.74331	0.75441	-0.0049	0.66668	0.1985	-0.0437	-0.0284	-0.0353	0.03209	0.25362
NM_145769	Lgi1	0.17894	-0.0703	0.04352	0.22747	-0.0774	-0.0237	0.11772	-0.1358	0.13741	-0.0041	0.12988	0.1554	0.01762	-0.0736
NM_001107219	Lgi2_predicted	0.24681	0.03596	0.0368	0.10897	0.0857	0.03461	-0.0252	0.03738	0.03926	0.28149	-0.0079	0.46457	0.08266	0.23034
NM_001107277	Lgi3_predicted	-0.0233	0.18377	0.15133	0.23212	0.10899	0.01903	0.05287	0.00853	0.07896	0.38244	0.07945	0.28057	0.0692	0.04849
NM_199499	Lgi4	0.05403	-0.0621	-0.0762	0.22295	0.60117	0.18024	0.1897	0.27552	-0.0553	-0.2173	-0.1117	-0.0227	0.05251	0.13143
NM_022226	Lgmn	1.1858	1.9853	0.37494	1.2609	1.3282	1.046	1.6687	1.2145	1.6262	1.9079	1.8201	0.33449	0.39902	0.45169
NM_173328	Lgr4	-0.3094	0.24156	-0.2592	0.89262	-0.0657	-0.3541	-0.1468	-0.0273	0.15837	0.15417	-0.0374	0.37199	0.61076	0.41993
NM_001106784	Lgr5	0.21778	-0.0826	0.08277	0.12792	0.26281	-0.0923	0.20471	0.12672	0.2361	0.30274	0.04792	0.31086	0.08184	0.14096
XM_001076874.1	Lgtn_predicted	-0.0409	-0.0885	0.08262	0.49482	0.04983	0.42564	0.1951	0.01218	-0.0644	-0.1652	0.10078	0.15714	-0.1133	-0.1284
NM_001033975	Lhb	0.30151	0.02503	0.10667	0.01088	0.09892	0.06474	0.18906	-0.0924	-0.0235	0.16048	-0.0931	-0.0189	-0.0803	0.00722
NM_012978	Lhcgr	-0.0953	-0.0851	-0.2419	-0.1141	-0.1738	-0.1786	-0.2694	-0.0673	-0.1691	-0.1706	-0.2011	0.1016	-0.1597	-0.2675
NM_181085	Lhfp	0.35072	0.08387	-0.1018	0.21755	-0.0102	-0.0166	-0.0426	-0.1153	0.04839	0.07516	0.15155	0.11614	-0.0936	0.0804
NM_001106402	Lhfpl2_predicted	-0.1664	0.21864	-0.1072	-0.0729	-0.141	-0.1377	0.01241	-0.1281	-0.1781	-0.0216	0.03252	-0.1728	0.16925	-0.2291
NM_181387	Lhfpl4	-0.1688	-0.0653	-0.2057	-0.1913	-0.0477	-0.0505	-0.2008	-0.1183	-0.1335	-0.2034	-0.0773	-0.0034	-0.2333	-0.2002
NM_001009706	Lhpp	0.20177	0.43331	-0.4945	-0.5571	-0.0821	0.08412	-0.2507	0.1717	0.6915	0.39286	0.52555	0.27053	-0.0166	0.0412
NM_001009706	Lhpp	0.32063	0.7885	-0.5033	-0.5789	0.27947	0.24162	-0.1795	0.25603	0.80712	0.67472	0.64055	0.41744	0.22152	0.19303
NM_145880	Lhx1	-0.1753	-0.1995	-0.2974	-0.1315	-0.0164	0.24827	-0.1643	-0.3242	-0.0078	-0.0229	-0.1479	-0.1034	-0.1794	-0.2812
XM_231083.3	Lhx3	0.01858	0.073	0.18626	-0.0862	-0.1132	-0.0016	-0.1638	-0.0089	0.05893	-0.0145	-0.0685	0.1713	0.00188	0.12283
NM_001108348	Lhx4_predicted	0.15362	0.03221	-0.1856	0.07158	-0.059	0.22271	0.01598	-0.0812	0.18011	0.09183	-0.0555	-0.0052	-0.2379	-0.0954
NM_139036	Lhx5	-0.206	-0.3487	-0.3542	-0.0879	-0.2846	-0.2486	-0.2559	-0.0306	-0.2988	-0.3257	-0.1997	-0.3805	-0.0513	-0.3227
NM_001107837	Lhx6_predicted	-0.2625	-0.2629	-0.1463	-0.1917	-0.0508	-0.2655	-0.1584	-0.1676	-0.1595	-0.2348	-0.151	-0.0355	-0.1688	-0.1911
XM_575064.1	Lhx8	0.17234	0.06219	0.08095	0.18183	0.09474	0.41121	0.11934	0.21702	0.15794	0.08401	0.28458	0.03769	0.18616	0.29706
NM_181367	Lhx9	-0.2321	-0.1974	-0.1533	-0.1679	-0.1968	0.07842	-0.2544	-0.1652	-0.1017	-0.1641	-0.2677	-0.1367	-0.0477	-0.1426
NM_001012037	Lias_predicted	-0.1778	0.07691	-0.0328	-0.2049	-0.0817	-0.1917	0.03132	0.05184	-0.0212	-0.067	-0.1465	-0.0183	-0.1834	-0.0626
NM_001012037	Lias_predicted	0.00546	-0.0986	-0.0283	0.10981	0.06821	-0.0727	-0.0016	0.07429	0.04793	-0.0206	-0.0543	-0.106	-0.1968	-0.0217
NM_022196	Lif	0.09141	0.02683	0.06296	0.13007	-0.0222	0.28498	-0.0158	-0.109	-0.0681	-0.1125	-0.1883	-0.033	0.14841	-0.1331
NM_031048	Lifr	-0.1313	-0.001	-0.0179	-0.0747	0.12493	-0.0564	-0.051	-0.0882	-0.0482	0.04879	-0.0779	-0.1084	-0.1879	0.02827
NM_030855	Lig1	-0.3197	-0.9419	1.0039	-0.2478	-0.7996	-0.5572	-0.4858	-0.6964	-0.5856	-0.7758	-0.3544	0.23242	0.41182	0.47834
NM_001012011	Lig3	-0.3434	-0.1391	0.06458	-0.2956	-0.3129	-0.1598	-0.32	-0.1784	-0.2739	-0.3636	-0.5074	-0.428	0.05321	0.02302

NM_001106095	Lig4_predicted	-0.332	-0.3345	-0.5977	0.11043	-0.1405	-0.4944	-0.0855	-0.0048	-0.3167	-0.1285	-0.1677	-0.1781	-0.4678	-0.3473
NM_053771	Lim2	-0.1421	0.07761	-0.0629	0.06133	0.00082	-0.1005	-0.1836	0.15757	-0.0669	0.09373	-0.1124	0.12174	0.01932	-0.1029
NM_001112737	Limd1_predicted	0.33193	0.29206	0.32549	-0.0951	0.44152	-0.2515	0.1157	0.35385	0.02132	0.0583	0.11366	-0.1958	0.24604	0.23466
NM_001025715	Limd2	0.14545	0.20025	-0.0026	-0.1207	-0.0965	0.45172	0.10394	0.04608	0.02946	0.38717	0.1041	0.58311	0.02993	0.20531
NM_031727	Limk1	0.77331	0.34695	0.95825	0.64315	0.77273	1.0035	0.59208	0.42076	0.42446	0.66792	0.90592	0.86766	0.53565	0.86375
NM_024135	Limk2	-1.4979	-1.2873	-0.6966	-1.3235	-1.2002	-1.3771	-1.1492	-1.2271	-1.1779	-1.3201	-1.4281	-1.0314	-0.9322	-0.9221
NM_001107633	Lims1_predicted	-0.0504	0.02497	0.06554	-0.0121	-0.0131	-0.1412	0.02784	0.06306	0.24467	-0.1005	0.04899	-0.3138	0.12763	0.07397
NM_001012163	Lims2_predicted	-0.0459	0.15762	0.19941	0.0665	0.17317	0.25138	0.03594	0.14026	-0.1146	0.12999	-0.0087	-0.0655	0.02627	0.1905
XM_233546.3	Lin28_predicted	0.09038	0.00461	0.2045	0.08519	0.15801	0.17835	0.12926	0.03515	0.05586	0.11078	0.04297	0.05809	0.03053	0.09734
NM_053514	Lin7a	-0.005	-0.0322	0.02179	0.15474	0.00578	-0.0019	-0.0028	0.07912	-0.0339	-0.014	0.07048	0.03249	-0.0334	-0.0005
NM_021758	Lin7b	0.17171	0.00752	-0.0817	0.13216	0.10337	0.10815	0.24339	0.26072	-0.0862	0.03086	-0.1393	0.00582	-0.0488	-0.1581
NM_021851	Lin7c	0.47781	-0.1337	0.03409	0.62101	0.86294	0.86998	0.23203	0.80755	0.41962	0.08208	0.08984	0.33779	0.52295	0.48499
NM_001014100	Lincr	0.00725	-0.029	0.23004	-0.1255	0.00631	0.0594	0.44204	0.00321	0.00933	-0.0323	0.07608	-0.0005	-0.009	0.13873
XM_218748.3	Lins2_predicted	0.06403	-0.1718	0.16107	0.04043	-0.3208	-0.1405	-0.1153	-0.3024	-0.2249	-0.318	-0.1897	0.05925	-0.1029	0.03991
NM_012732	Lipa	-0.1817	0.22554	-0.4615	-0.0321	0.04857	-0.2244	-0.2182	-0.1954	0.03666	-0.132	-0.1036	-0.2932	-0.018	-0.034
NM_012597	Lipc	-0.2535	-0.2786	-0.218	-0.1837	-0.2132	-0.3244	-0.2699	-0.3486	-0.3326	-0.2038	-0.1818	-0.0929	-0.2971	-0.3322
NM_012859	Lipe	0.02531	0.10991	0.21799	0.05475	0.06852	0.07468	-0.0121	0.0882	0.0093	0.05519	0.06847	-0.0051	0.08074	0.18181
NM_017341	Lipf	0.13361	0.19722	0.07696	0.24252	0.12596	-0.0244	0.24781	0.13	0.23498	-0.05	0.14416	0.0728	0.20184	0.36479
NM_001012741	Lipg	0.03634	0.04739	-0.0779	-0.1618	-0.2073	-0.2407	-0.2006	-0.0934	-0.0763	-0.052	-0.0023	0.0057	-0.0383	0.24001
NM_001105899	Liph_predicted	0.24887	0.35192	0.24946	0.16762	0.11916	0.26522	0.22429	-0.0331	0.12665	0.30905	0.1397	0.22531	0.35096	0.37278
NM_001106374	Lipl2_predicted	0.16497	0.04931	-0.0545	-0.0628	-0.0907	-0.0732	-0.008	-0.0689	0.08998	-0.1255	-0.067	-0.168	0.08593	-0.0411
XM_220066.3	Lipl3_predicted	0.13715	-0.0002	0.02395	-0.012	0.1324	0.15902	-0.0239	0.03669	0.07781	-0.0124	0.15276	0.1115	0.05192	0.00819
NM_145790	Lipogenin	-0.4525	-0.5517	0.34597	0.13085	-0.5808	-0.6014	-0.3589	-0.7154	-0.5195	-0.4338	-0.5151	-0.1254	-0.3175	-0.3734
NM_001108212	Lipt1_predicted	0.36938	0.08662	0.15216	0.11102	0.54936	0.13173	0.07655	0.60393	0.14063	0.34604	0.19958	-0.0546	0.23647	0.07383
NM_001105735	Litaf	0.30162	0.54777	0.18635	-0.0376	0.64102	0.39074	0.24761	0.62869	0.43203	0.50067	0.36608	0.11433	0.17356	0.23062
NM_001106214	Lix1_predicted	-0.0178	-0.0464	0.11131	0.14636	0.12125	-0.0768	-0.0419	0.13304	-0.0094	-0.0861	0.08565	0.07048	0.01099	0.0133
NM_133421	Lkap	-0.3258	-0.1077	-0.2551	0.10146	-0.1891	-0.28	0.15356	-0.208	-0.252	-0.3078	-0.1715	-0.3437	-0.644	-0.4933
NM_053886	Lman1	0.17611	0.11286	-0.4376	0.67105	0.60997	0.56889	-0.6022	0.54721	0.11971	-1E-05	-0.094	0.28125	0.05348	0.07118
NM_001012465	Lman1l	-0.2025	-0.1387	-0.1603	0.01045	-0.0869	-0.1914	-0.1102	0.1564	0.27276	-0.1762	-0.0122	-0.2375	-0.16	-0.1125
NM_001115024	Lman2_predicted	0.06041	0.01595	0.01671	0.3394	0.69591	0.61126	-0.0035	0.44225	-0.0582	-0.1959	-0.0048	0.11841	0.07563	0.18613
NM_001106900	Lman2l_predicted	0.03789	-0.0204	-0.0639	-0.0535	-0.0384	-0.0652	-0.0473	0.23685	0.151	0.00403	0.03726	-0.0447	-0.0943	-0.0191
NM_139189	Lmbrd1	-0.0388	0.30199	-0.1562	0.6241	0.32742	0.11867	0.59489	0.38151	0.317	0.28237	0.28672	-0.2986	0.03628	-0.0461
NM_001008562	Lmcd1_predicted	-2.6348	-2.5294	-3.5212	-3.059	-1.3706	-1.1581	-0.96	-1.1122	-2.8193	-2.5417	-2.8749	-4.3759	-3.6377	-3.6543
NM_001108843	Lmln_predicted	-0.041	-0.0557	-0.0567	0.03508	-0.0892	0.03596	-0.1215	-0.0321	0.05241	0.13487	-0.2176	-0.093	0.01213	-0.0643
NM_001002016	Lmna	-0.6071	-0.6775	-0.0455	-1.1079	-0.5635	-0.2508	-0.1538	-0.8735	-0.352	-0.4729	-0.2337	-0.1419	-0.3735	-0.5953
NM_139112	Lmo3	-0.4141	-0.5335	-0.2059	-0.8959	0.2177	-0.0195	-0.2166	0.39503	-0.3124	-0.2617	-0.2953	-0.1394	0.00132	-0.1542
NM_001009708	Lmo4	-0.8046	-1.2219	-0.8916	-1.1235	-1.0057	-1.2484	-1.0389	-0.922	-0.806	-1.1495	-1.2653	-0.7178	-1.066	-0.9812
NM_001001515	Lmo7	-0.693	-0.3727	-0.2775	-0.8258	-1.1683	-1.4611	-0.228	-1.1365	-0.192	-0.3269	-0.2124	-0.4219	-0.4484	-0.2996
NM_001107179	Lmod1_predicted	0.20131	0.37474	0.19507	0.29902	0.25099	0.23523	0.21442	0.14894	0.32	0.43526	0.229	0.09199	0.14731	0.20504
NM_001105967	Lmx1a_predicted	0.0355	0.02836	0.08243	0.11013	-0.0192	0.20899	-0.1157	0.08172	-0.0089	0.09789	-0.1635	-0.0523	-0.054	-0.071
XM_342419.2	Lmx1b	-0.0447	-0.0083	-0.0091	-0.0806	0.01614	0.08539	-0.0824	-0.0865	-0.134	-0.0575	-0.0724	0.21442	-0.1173	0.01685
NM_001077429	Lnp_predicted	0.9042	0.05672	0.25856	0.37446	0.68229	0.66467	0.13896	0.82282	0.05615	0.39021	0.28498	0.20231	0.83495	0.67651
NM_001113403	Lnpep	-0.1605	-0.1642	-0.1696	-0.2449	-0.2107	-0.219	-0.0422	-0.1435	0.03763	-0.0845	-0.0597	-0.0919	0.09724	-0.0559
NM_001108358	Lnx1_predicted	0.03322	-0.0363	-0.017	0.01214	-0.0225	-0.1145	0.0149	0.35705	0.00057	0.13337	0.00621	-0.0506	-0.0264	0.19905
NM_001108329	Lnx2_predicted	0.1236	-0.0165	0.03954	-0.0386	-0.0419	0.03219	0.07663	0.05668	0.06435	0.12249	0.1915	0.05408	0.09658	0.15786
NM_138537	LOC171573	0.37142	0.59821	-0.0139	0.44981	0.36497	0.46044	0.70219	0.1772	0.7479	0.85177	0.66226	0.599	0.16633	-0.1221
NM_138547	LOC191574	-1.3041	1.7287	-0.6733	-0.024	-1.3861	-1.7313	0.04127	-1.4971	1.0044	1.373	1.1205	0.23365	-0.9539	-1.211
NM_139093	LOC245925	-0.3176	-0.2371	-0.1157	-0.6366	-0.458	-0.3873	-0.2059	-0.391	-0.4248	-0.028	-0.1265	-0.163	-0.3048	-0.1564
NM_139101	LOC245960	-0.2065	-0.041	0.10152	1.1193	-0.4338	-0.2058	0.14991	-0.2498	-0.193	0.06987	-0.1951	-0.3477	-0.3448	-0.321

NM_139255	LOC246120	0.2603	0.46968	-0.6676	0.10455	0.0557	-0.3033	0.64957	-0.1454	0.13767	0.30756	0.41148	-0.2434	-0.4597	-0.3336
NM_139328	LOC246187	0.74522	0.00637	-0.3563	-0.3656	0.24912	0.25124	-0.0266	0.1475	0.34846	0.14812	0.26676	0.35104	0.23077	0.22855
NM_144750	LOC246266	0.24403	0.04133	1.0219	0.29968	0.21277	0.38521	0.53188	0.33244	0.21966	-0.0136	0.01731	0.02549	0.00946	0.14657
XM_575306.1	LOC246270	0.10079	0.06109	-0.0978	0.32108	0.21448	0.17976	0.00443	0.05046	0.07836	0.36278	-0.2626	0.26249	0.0354	0.25657
NM_145082	LOC246295	0.35613	0.23346	0.05499	0.64821	-0.044	0.31624	-0.1225	0.40486	-0.1604	0.06105	-0.0933	-0.1429	0.24445	0.05878
NM_031537	LOC24906	-0.2546	-0.0942	0.01906	-0.0853	0.09751	-0.185	-0.1575	-0.0085	0.04597	-0.3506	-0.2177	-0.1325	-0.0579	-0.2831
NM_147142	LOC257650	-0.5345	-0.4761	0.04334	0.42369	-0.6968	-0.7165	-0.0503	-0.7469	-0.7712	-0.1432	-0.1034	-0.2167	-0.5537	-0.5084
NM_203512	LOC259244	0.25385	0.12991	0.11243	0.13721	0.31885	0.11079	0.13055	0.266	0.08642	0.1663	0.16068	0.11763	0.13598	0.17675
NM_203512	LOC259244	0.0296	0.0741	0.09707	-0.1361	0.27788	0.03183	-0.0789	0.18768	-0.0518	0.0223	0.19338	-0.1268	0.01405	-0.0481
NM_203512	LOC259245	0.1204	0.25275	0.15405	0.02881	0.22037	0.38434	0.15038	0.12918	0.19755	0.10419	0.12317	0.1024	0.2522	0.10928
NM_203512	LOC259246	-0.0294	0.02195	0.04593	0.04314	0.00788	0.20026	0.05399	0.17921	0.0452	0.13614	0.0244	0.04134	0.11541	0.06313
NM_203512	LOC259246	0.0057	-0.0153	0.06452	0.23142	0.00917	0.05393	0.12102	0.06536	0.07654	-0.0196	0.03044	0.12157	-0.0199	0.09192
NM_001135600	Loc266761	0.18518	0.31354	0.02992	0.07141	0.18151	0.22218	0.08871	0.13281	0.23478	0.46516	0.33165	0.15987	0.52498	0.2577
NM_153729	LOC266771	0.1775	0.34794	0.19901	0.04387	0.03863	0.01898	0.13469	0.30176	0.25928	0.16785	0.0951	0.06779	0.25912	0.15288
NM_173127	LOC286911	0.05562	0.12949	0.15355	-0.0818	-0.0289	-0.0749	0.10666	-0.0902	0.01884	0.00221	-0.0886	0.15264	-0.0694	-0.194
NM_173130	LOC286914	-0.1832	-0.149	-0.0627	-0.1626	-0.1122	0.07159	-0.1973	-0.1842	-0.1169	-0.1994	-0.1444	-0.0954	-0.2196	-0.2341
NM_173301	LOC286960	0.13775	0.03482	0.24831	0.02445	0.11823	0.02477	0.18276	0.09189	0.12599	-0.0136	0.12071	0.05108	0.16989	-0.098
NM_001099659	LOC286981	-0.1667	-0.1831	-0.1564	-0.1248	-0.1803	-0.1739	-0.2519	-0.2257	-0.4491	-0.1324	-0.2771	-0.31	0.02796	-0.3012
NM_173316	LOC286982	-0.0152	0.08976	0.04978	0.15394	0.08303	0.10634	0.01931	0.10822	0.06637	0.17405	0.23153	0.05136	0.04853	0.32237
NM_001099512	LOC286983	-0.1682	0.03281	0.0792	-0.2517	-0.0906	-0.0408	-0.0353	-0.1183	-0.1272	-0.0914	-0.1477	0.00698	-0.0069	-0.0434
NM_001099491	LOC286984	0.23029	-0.1018	-0.0597	0.32051	-0.0907	-0.1198	-0.0272	-0.0409	-0.0098	0.01937	-0.0467	0.07443	0.11868	-0.0868
NM_173319	LOC286985	0.44367	0.42854	-0.1057	0.0289	0.24768	0.54432	0.53849	0.11381	0.61317	0.44549	0.42795	0.69293	0.26836	0.04076
NM_173320	LOC286986	0.05567	-0.0539	0.04131	0.32858	0.06799	0.11524	0.03339	-0.1228	0.30067	0.29005	0.12491	-0.0004	-0.0267	-0.057
NM_173323	LOC286989	0.41436	0.10816	0.48157	0.12905	0.16889	-0.0029	0.14782	0.28072	-0.0005	0.07792	-0.081	0.11992	0.07275	0.03104
XM_237748.2	LOC287028	-0.0477	-0.3004	-0.2836	-0.1852	-0.1079	-0.4043	0.10371	0.12592	-0.116	-0.0841	-0.127	-0.0313	0.04215	-0.0687
XM_237751.2	LOC287036	-0.1211	-0.173	-0.1187	-0.1628	-0.1747	-0.1172	-0.0903	-0.1127	-0.3119	-0.1671	-0.2292	-0.1353	-0.0556	0.16902
XM_213245.2	LOC287111	0.17355	-0.1458	0.9658	0.59245	-0.0469	0.12751	-0.0191	-0.0752	-0.0986	-0.2919	-0.2201	0.34547	0.53051	0.84759
NM_001031637	LOC287112	0.24198	0.40633	0.1333	-0.1511	0.20949	0.13858	0.33548	0.36455	0.40142	0.11993	0.42297	0.05838	0.06742	0.44876
XM_213241.3	LOC287132	-0.1527	-0.2117	-0.1729	0.28968	-0.0105	0.41428	-0.058	-0.0856	-0.1434	-0.1605	-0.2751	0.0974	0.07548	0.13858
NM_001105774	LOC287148	0.39951	0.19262	0.25036	0.43069	0.31951	0.39018	0.01354	0.24774	0.04756	0.06597	0.11379	0.25398	0.19069	0.0804
NM_001013853	LOC287167	0.09665	0.17863	0.28308	-0.014	0.17419	-0.0136	0.10547	0.01167	-0.0136	-0.0957	-0.014	0.05845	-0.0133	0.07059
XM_220323.3	LOC287211	0.16006	-0.0057	0.0166	0.27332	-0.0842	0.03448	-0.1661	-0.1177	-0.0734	-0.1903	0.08638	0.04904	-0.0629	0.21409
XM_220355.3	LOC287228	0.205	0.20365	0.25668	0.08984	0.12394	0.13719	0.25846	0.09161	0.29245	0.32568	0.18081	0.12267	0.2007	0.29113
XM_220416.2	LOC287248	-0.445	-0.2258	-0.3793	-0.0953	-0.2328	-0.2293	-0.3444	-0.3393	-0.1316	-0.3823	-0.4321	-0.3101	-0.2769	-0.3265
NM_001024965	LOC287274	0.42899	-0.0156	-0.0159	-0.3759	0.00018	0.17219	0.11577	0.03119	0.40902	0.10769	0.16818	0.09478	0.23267	0.16003
XM_220490.3	LOC287338	-0.1587	-0.1195	-0.2123	-0.1353	-0.0654	0.10417	-0.0369	0.05576	-0.1186	-0.1355	-0.0845	-0.0703	-0.1784	0.0021
XM_213322.3	LOC287360	-0.2431	-0.0465	-0.19	-0.2518	-0.1897	-0.1038	-0.0941	-0.128	-0.1676	-0.0267	-0.1652	0.03927	-0.0591	-0.1371
XM_220624.2	LOC287403	-0.08	-0.1789	-0.0265	0.15398	-0.0196	-0.0106	-0.3006	-0.1886	-0.0793	-0.0675	0.22085	8E-05	0.21774	-0.1476
XM_220572.3	LOC287459	0.07269	0.11489	0.15305	0.23207	-0.0522	0.06533	0.13705	0.10471	0.08417	0.07974	0.0767	0.27818	0.23253	-0.0049
XM_220690.2	LOC287518	-0.0446	-0.1777	-0.3648	0.17236	-0.1981	-0.2021	-0.037	-0.2089	-0.2004	-0.208	0.23728	-0.2351	-0.0864	-0.1772
NM_001105808	LOC287522	-0.0958	0.02303	-0.0072	0.10178	-0.2471	-0.1793	-0.1616	-0.1546	-0.1907	-0.3293	0.07099	-0.1025	-0.1805	-0.1159
NM_021264	LOC287558	0.26462	0.2431	0.5037	0.24241	0.06325	0.57732	0.1188	0.0761	0.35409	-0.2203	0.23777	0.11344	0.40095	0.53866
NM_001134410	LOC287564	-0.0546	0.00521	-0.0898	0.07424	-0.126	0.00069	-0.0598	-0.0585	-0.0302	-0.017	-0.0263	0.00761	-0.1021	-0.066
XR_005445.1	LOC287569	0.04549	-0.0196	-0.0741	-0.0535	-0.1243	0.12486	-0.0344	0.0894	0.07595	-0.1869	0.03629	0.06471	-0.1251	0.04419
XM_220861.3	LOC287622	-0.0022	-0.0154	0.04755	-0.0481	0.00481	0.21085	0.07641	-0.0163	-0.0984	0.07218	-0.0955	0.27235	-0.1827	0.00497
NM_001024966	LOC287637	0.31063	0.1679	0.11631	0.13213	0.07938	0.17246	0.02941	0.05023	0.50765	0.1398	0.11717	-0.0457	0.01365	0.50876
XM_220913.1	LOC287651	0.01229	0.05671	0.10127	-0.0302	0.05756	0.06937	-0.0329	-0.0865	0.10638	-0.1157	-0.0009	-0.0571	0.02692	0.00067
XM_213493.3	LOC287734	-0.0803	0.13352	-0.0411	-0.0809	-0.0699	-0.07	-0.0283	-0.1217	-0.0906	0.01186	-0.0503	0.1343	-0.3491	-0.033
XM_213491.3	LOC287736	0.08923	0.17348	0.03466	-0.0839	-0.0606	0.01228	-0.0448	-0.0545	0.14091	-0.0632	0.02587	-0.0637	0.04058	0.06015

NM_001105849	LOC287776	0.11504	-0.0264	0.06768	0.17198	0.02302	0.13158	0.07813	0.17785	-0.0568	-0.0678	-0.0257	0.13856	0.02446	0.20702
NM_031051	LOC287782	-0.0281	-0.0108	0.10865	0.27474	0.09789	0.15533	0.0041	0.21794	0.00509	0.14652	0.13323	0.18197	0.42081	0.29166
NM_001134498	LOC287847	-0.3074	-0.2846	-0.1955	-0.1774	-0.1846	-0.196	-0.3282	-0.195	-0.1809	-0.1728	-0.2268	0.13664	-0.2698	-0.1569
XM_213536.3	LOC287866	-0.3077	-0.1832	-0.1733	-0.176	-0.3117	-0.0586	-0.0778	-0.2049	-0.2838	-0.0385	-0.1466	-0.2432	-0.2183	-0.194
NM_001134499	LOC287871	0.16186	0.05664	0.23637	0.18697	0.2235	-0.0413	-0.056	-0.1817	-0.1719	-0.0364	0.10224	-0.2018	0.04418	0.17885
XM_221315.3	LOC287990	0.30899	-0.1467	0.01356	-0.1434	-0.2522	-0.0197	-0.1495	-0.29	0.12499	0.08338	0.18104	0.09848	0.02523	0.00859
XM_237832.3	LOC287992	0.05054	-0.0946	-0.1159	-0.147	0.00314	-0.0901	0.02512	-0.2055	-0.2638	0.04695	-0.0581	-0.0364	-0.0035	0.03679
XM_212689.3	LOC287996	0.13277	-0.3318	0.03636	-0.2739	-0.0892	-0.1014	-0.1917	-0.0176	0.02733	-0.2807	-0.2203	0.06015	-0.2004	-0.1094
NM_001013864	LOC288010	-0.0624	0.04744	0.00418	0.06147	0.01873	-0.1545	0.06787	-0.0802	-0.0468	-0.1431	-0.0053	-0.1419	-0.144	0.26759
XM_221412.2	LOC288071	-0.0764	-0.1179	0.06576	0.02376	0.07378	0.18169	0.02763	0.03967	0.14187	-0.0068	0.02469	0.24117	-0.0343	-0.04
NM_001100653	LOC288109	0.00245	-0.2022	0.06214	0.06233	-0.057	-0.0304	-0.1382	-0.063	-0.2415	-0.1545	-0.0939	-0.1155	-0.0913	-0.0712
NM_001100653	LOC288109	-0.109	0.21507	0.07795	0.00042	0.07057	-0.0331	0.08268	0.03746	0.16036	-0.0053	0.06088	0.02314	0.04854	-0.0302
XM_221491.2	LOC288136	0.11257	0.0713	-0.0194	0.06137	-0.0377	0.0527	0.08899	0.00774	-0.0702	-0.0449	0.00567	0.01189	0.01692	-0.0076
NM_001047846	LOC288165	-0.311	-0.0697	0.00385	0.02264	-0.5126	-0.6432	0.14397	-0.4194	0.02909	-0.0309	0.14286	-0.0914	-0.2928	-0.3875
NM_001034126	LOC288174	-0.6093	-0.5293	-0.1903	-0.8973	-0.8055	-0.6874	-0.7438	-0.8222	-0.6415	-0.7835	-0.5238	-0.3189	-0.5251	-0.6428
XM_212694.3	LOC288178	0.29818	0.3597	-0.3961	0.01359	0.4135	-0.0877	0.0473	0.66047	0.20943	0.16693	0.25397	0.0021	0.1819	0.13596
NM_001099480	LOC288396	0.08922	0.28513	0.06458	0.34759	-0.0347	0.3635	0.15815	0.09031	0.24736	0.25767	0.1181	0.22535	0.19671	0.32578
NM_001100942	LOC288455	0.5713	0.09126	0.62597	0.21763	0.60265	0.79093	0.50918	0.58001	0.4467	0.44357	0.22874	0.47351	0.98173	0.79092
XM_221885.1	LOC288457	-0.1026	-0.1268	-0.0239	-0.1037	0.01528	0.0887	-0.2639	-0.1616	0.04257	-0.1306	-0.0937	-0.1197	-0.2367	-0.1072
XM_221895	LOC288469	0.05244	0.05354	0.01454	0.02948	0.00896	-0.0396	0.00956	0.01812	0.03726	-0.0991	0.00461	0.03044	0.03108	0.04979
XM_213721.1	LOC288501	-0.1152	-0.2764	-0.0206	0.03441	-0.1644	0.2414	-0.0962	-0.3889	-0.1342	0.0673	-0.152	-0.2517	-0.0974	-0.1142
XM_213717.3	LOC288514	-0.7968	-0.4893	0.11542	-1.0268	-0.8947	-1.2357	-0.1073	-1.145	-0.4218	-0.5272	-0.3639	-0.2564	-0.3135	-0.2337
XM_221956.3	LOC288515	-0.8016	-0.8201	-0.3364	1.1493	-0.3004	-0.4053	-0.3302	-0.3178	-0.5567	-0.5025	-0.6988	-0.5165	-0.942	-1.0257
NM_001013868	LOC288521	0.13968	0.0013	0.03284	-0.0225	-0.0075	-0.0744	0.09378	-0.0255	-0.0024	0.23031	-0.0156	0.02588	-0.0499	0.1723
NM_001127246	LOC288526	-0.3562	-0.3562	0.76428	0.62135	-0.3557	-0.265	0.60641	-0.4169	-0.2189	-0.4083	-0.1058	-0.1819	-0.4308	-0.221
XM_222014.3	LOC288549	0.07308	-0.0476	-0.0378	-0.0629	0.18639	-0.0428	-0.0574	-0.059	-0.0636	0.08831	-0.0021	-0.0824	-0.1162	-0.1269
XR_007334.1	LOC288574	0.12919	0.02777	0.08478	0.07223	0.06732	0.03953	-0.0209	0.19655	0.01684	0.08093	0.00567	-0.0022	0.04969	0.02496
XM_213751.3	LOC288589	0.0247	0.04907	-0.0719	0.13234	0.04463	0.12025	0.02769	0.00776	-0.031	-0.063	0.06015	0.15902	-0.0714	-0.1131
XM_213758.3	LOC288604	-0.1633	-0.2126	-0.1801	-0.2082	0.01866	-0.2693	-0.2153	-0.2335	-0.2019	-0.1057	-0.0869	-0.1381	-0.1059	-0.133
XM_222124.3	LOC288633	-0.4121	0.89321	1.7377	1.6566	-0.5824	-0.4906	2.6403	-0.3598	0.97911	1.2245	1.1282	0.74844	0.96619	1.016
NM_001170554	LOC288654	0.02604	-0.2233	0.43608	0.19262	0.02206	-0.054	0.28088	-0.0172	-0.0798	-0.1957	0.05818	0.092	0.15002	0.34073
NM_001105927	LOC288657	-0.0441	-0.1188	-0.1017	0.01135	-0.0568	-0.075	-0.105	0.0268	0.01823	-0.0263	0.02935	-0.0227	0.02625	-0.062
XM_222160.2	LOC288662	0.12592	-0.2308	-0.3058	-0.2985	-0.2266	0.47662	-0.1744	-0.1905	-0.0523	-0.3326	-0.2062	0.44991	0.14093	0.07277
XM_222183.3	LOC288664	-0.1068	-0.1409	-0.2441	-0.0051	0.04251	0.1388	0.21143	0.1216	-0.2731	0.15118	0.02708	0.10842	0.0173	-0.045
XM_222246.2	LOC288715	0.20102	-0.1145	-0.0248	-0.0815	0.03306	0.10596	-0.0136	-0.0705	0.07228	-0.0482	0.01895	-0.0996	0.06223	-0.1826
XM_237883.3	LOC288748	-0.1842	-0.2303	-0.0969	-0.0898	-0.1577	-0.0423	-0.102	-0.2278	-0.1466	-0.1394	-0.1784	-0.1158	-0.1435	-0.1333
XM_222295.3	LOC288758	0.2006	0.07296	-0.0613	-0.0462	0.09523	0.07472	-0.061	-0.0114	0.26602	0.02075	0.04544	-0.0422	-0.0968	0.23689
XM_213822.1	LOC288762	-0.0395	-0.2746	-0.421	-0.0982	0.04133	0.05105	-0.2373	-0.0633	-0.2777	-0.253	-0.4105	-0.2197	-0.2703	-0.2806
NM_198728	LOC288913	0.51776	0.05952	0.64163	0.97861	-0.1145	0.57153	0.42818	-0.2213	0.23642	0.18479	0.64591	0.7935	0.06878	-0.0394
XM_222529.2	LOC288959	0.09302	0.23728	0.10016	0.03297	0.10194	0.0421	0.10319	0.226	0.1337	0.20571	0.17832	-0.0326	0.09942	0.18852
NM_001134500	LOC288978	-0.7996	-0.4247	0.11009	-0.2381	-0.9117	-0.8162	-0.2049	-0.9938	0.10071	-0.2533	-0.205	0.56591	0.04959	0.04232
NM_001105949	LOC288979	-0.0219	-0.0364	0.28092	0.16331	0.07484	-0.0038	-0.0075	0.14793	-0.0442	-0.0994	-0.0558	0.02596	0.18261	-0.0488
XM_222652.3	LOC289038	0.11794	0.0808	0.14622	0.02888	0.1521	0.05418	0.0022	0.07097	0.1147	0.02182	0.12377	0.06492	0.16186	0.0886
NM_001105959	LOC289084	0.21043	0.19124	0.01279	-0.0217	0.22208	-0.0978	0.31679	-0.0125	0.09118	-0.0563	-0.0306	0.05612	0.20777	0.16196
XM_222968.3	LOC289281	-0.1255	-0.2157	-0.1777	-0.0109	-0.127	-0.2312	-0.111	-0.2499	-0.003	-0.0498	-0.196	-0.1759	0.00055	0.02654
XM_222975.3	LOC289288	0.17746	0.10072	0.07098	0.22187	0.02428	0.05035	0.08839	0.00667	0.05898	0.02795	0.02965	0.04939	0.03775	0.02
XM_223007.3	LOC289329	-0.0404	0.08464	-0.0763	-0.1302	0.02617	-0.0724	-0.162	-0.0842	-0.1761	-0.1882	-0.1101	-0.0814	-0.0533	-0.0656
XM_237909.2	LOC289332	-0.0123	0.13158	-0.0746	-0.0185	-0.1695	-0.1288	-0.1512	-0.1135	-0.0969	-0.1162	-0.0096	-0.075	0.12211	0.16378
NM_001170428	LOC289334	0.0264	-0.137	-0.0635	0.10568	-0.1827	-0.1255	-0.1263	-0.1175	-0.264	-0.0393	-0.0366	0.01156	0.06483	-0.1482

NM_001105987	LOC289378	0.07224	-0.1931	-0.0236	-0.1648	-0.5432	-0.5258	0.09644	-0.4647	0.05204	-0.0633	-0.012	-0.0857	-0.1078	-0.0058
XM_212735.3	LOC289401	-0.1417	-0.0226	-0.0077	0.26023	-0.0446	-0.0334	-0.3476	-0.0487	0.0608	0.07259	0.04074	-0.0345	-0.313	-0.3606
XM_214002.4	LOC289471	0.04406	-0.218	0.1844	-0.1577	-0.0217	0.13056	0.00212	-0.3151	-0.0192	-0.2538	-0.3144	-0.013	-0.2029	0.1399
NM_001024742	LOC289482	0.04265	0.138	-0.1547	0.02596	-0.03	-0.2208	0.0527	-0.1045	0.02513	-0.0607	-0.0188	0.06466	-0.0867	-0.1555
XM_223265.3	LOC289494	-0.1169	0.12161	0.16653	-0.0225	-0.0154	0.09191	0.20972	0.16193	0.01005	-0.0694	0.10905	0.04759	0.0097	-0.0266
NM_001106000	LOC289522	0.08706	0.39549	0.06726	0.06774	0.26043	0.08329	0.0502	0.14251	-0.06	0.03687	0.109	-0.032	0.42994	-0.1735
XM_223286.1	LOC289529	-0.0765	0.01494	-0.0522	0.07244	0.09898	0.15532	0.03247	0.08878	0.17178	0.10775	0.15103	-0.0027	-0.0637	0.04127
NM_001135779	LOC289606	0.49794	-0.0141	0.09691	0.08547	0.13552	0.11111	-0.0654	0.27907	0.05532	-0.014	0.33108	0.3295	0.34895	0.16883
XM_223426.3	LOC289633	-0.3406	-0.0759	-0.1848	-0.025	-0.0763	0.12307	-0.1895	-0.1571	-0.0412	-0.1242	-0.1147	-0.1254	-0.2132	-0.1574
XM_223453.2	LOC289656	-0.1775	-0.0364	-0.0224	0.03108	-0.0292	0.24641	-0.0211	-0.2467	-0.1144	-0.131	-0.0244	-0.0559	-0.1094	-0.0765
XM_223504.2	LOC289701	0.129	0.24156	-0.0252	0.12082	0.16664	0.02266	0.07457	0.09201	0.1597	0.73572	0.3331	0.14927	0.1733	0.09551
NM_031106	LOC289715	0.06029	-0.0933	0.04295	0.10237	0.10438	0.20826	0.0316	0.05778	0.12085	0.00558	0.18412	0.03165	0.1549	-0.0883
NM_001044228	LOC289740	0.14187	-0.0676	-0.5801	-0.1872	0.37002	-0.3985	-0.2091	0.29952	0.32157	-0.3324	-0.1016	-0.2773	0.17093	0.26448
XM_223612.2	LOC289781	0.0086	0.05441	-0.0171	0.00023	0.05954	0.21192	0.15177	-0.0533	0.23148	0.19525	-0.05	-0.0131	-0.0828	0.03675
NM_001100655	LOC289786	0.44167	0.31155	0.40254	0.43652	0.63572	0.74523	0.17592	0.48325	0.17537	0.18487	0.1691	0.47987	0.01761	0.01423
NM_001100655	LOC289786	-0.0849	-0.1191	0.12591	0.10563	0.13423	0.1184	-0.0673	0.30951	0.1232	0.11118	0.07256	0.24822	0.19293	0.10878
XM_223678.1	LOC289836	-0.0465	-0.0848	-0.0224	0.03354	-0.0637	0.00028	0.06758	0.01098	-0.0617	-0.1375	-0.0663	0.08	-0.1276	-0.0114
XM_223723.3	LOC289855	-0.1736	-0.1876	-0.0686	-0.1003	-0.037	-0.0944	-0.0723	-0.039	-0.0598	-0.1818	-0.2088	-0.1127	-0.0973	-0.1995
NM_001099517	LOC289876	-0.1321	-0.1504	-0.1925	-0.1302	-0.1767	-0.2828	-0.1527	-0.1868	-0.1264	-0.108	-0.0735	-0.2258	-0.1214	-0.1998
XM_214125.3	LOC289879	-0.0918	0.04002	0.09499	0.18717	-0.2161	0.13562	-0.1532	-0.0134	-0.1046	0.00614	-0.213	0.00202	0.23169	0.00374
XM_223807.3	LOC289918	-0.0031	0.04418	-0.2309	0.0683	-0.0634	-0.1451	-0.2538	-0.0498	-0.1449	-0.1555	-0.0225	-0.0375	-0.1799	0.06093
XM_223830.3	LOC289926	0.06655	-0.0064	0.06502	0.0838	0.01028	0.00857	-0.113	-0.0974	0.15646	-0.0718	-0.0545	0.15199	0.00671	0.04388
XM_237935.2	LOC289937	-0.0202	-0.1293	-0.135	-0.1321	-0.1495	-0.0554	0.0301	-0.0298	-0.0303	-0.1354	-0.1632	0.36394	-0.0995	-0.111
XM_223966.3	LOC290033	0.08311	0.04869	0.23074	0.40451	0.0326	0.37112	0.12735	0.14118	0.04222	0.38612	-0.0176	0.31449	0.1342	0.02589
XM_224025.3	LOC290071	-0.0425	-0.0438	0.00657	0.0949	0.01068	0.21408	-0.0038	0.14402	0.34527	0.30407	-0.1416	0.01812	0.29182	0.12968
NM_001039204.1	LOC290071	-0.1447	-0.2364	-0.0109	-0.2968	-0.196	-0.1043	-0.2225	-0.179	-0.2999	0.07628	-0.1731	-0.2583	-0.0436	0.01414
XM_224033.3	LOC290078	0.14248	0.1401	0.02288	0.00079	-0.01	-0.0111	0.01522	-0.0278	0.04734	0.18703	-0.0074	-0.0216	-0.0016	0.13218
XM_214167.3	LOC290086	0.12449	-0.091	-0.045	0.16245	-0.0659	0.01808	-0.1187	-0.0682	0.23093	0.00508	-0.1424	-0.1384	-0.0073	0.07811
XM_224053.2	LOC290098	-0.1596	-0.1513	-0.0939	-0.2049	-0.208	0.1696	-0.0579	-0.0272	-0.1005	-0.1977	0.04816	-0.203	-0.0869	-0.1028
XM_224063.3	LOC290108	0.18421	0.03103	0.18126	0.14086	0.22313	-0.0043	0.07841	0.31952	0.16497	0.07767	0.09165	0.05479	0.21385	0.19457
XM_224063.3	LOC290108	-0.0201	0.0529	-0.0535	-0.0043	-0.0405	0.04941	0.01135	0.01729	-0.1129	0.08011	0.29059	-0.089	0.0106	0.06393
XM_224108.3	LOC290150	-0.3256	0.12124	-0.1917	-0.0483	-0.1605	-0.003	-0.1165	0.19239	0.12002	-0.0649	0.07141	-0.1867	-0.1487	-0.0159
XM_224117.2	LOC290157	0.00754	-0.1026	0.25436	0.03388	-0.101	-0.1113	-0.0173	0.16543	0.01621	0.00286	-0.0687	0.09902	0.09118	-0.1517
XM_224118.3	LOC290158	0.0239	0.09906	0.08481	0.20887	0.37566	0.18192	0.13685	0.30212	0.07239	0.06011	0.02276	0.20091	0.12237	0.0761
XM_224121.2	LOC290161	-0.049	0.11309	-0.0561	0.1681	0.31032	0.16024	-0.1262	0.02733	0.09147	0.04578	-0.0986	-0.0253	0.04171	0.04659
XM_224125.3	LOC290165	0.06631	0.31048	0.00901	0.1448	0.15431	0.22684	0.06538	0.24343	0.17727	0.38208	0.01781	0.07059	0.02694	0.29787
XM_224131.3	LOC290170	0.01312	-0.1945	0.08328	0.00696	-0.0866	-0.09	-0.1164	0.05335	0.05858	-0.0055	0.06387	0.02108	0.17113	0.13912
XM_224133.1	LOC290172	0.04152	-0.0065	0.01527	0.09535	0.09016	0.22314	0.15726	0.12849	0.10268	0.22751	0.06564	0.26122	0.10716	0.28203
XM_224138.3	LOC290177	0.07366	0.112	0.12803	-0.0873	-0.0097	0.07699	-0.0976	-0.0027	-0.1335	-0.2399	0.0307	-0.138	-0.0709	-0.0457
XM_214174.3	LOC290195	-0.203	-0.0937	-0.1475	0.14916	-0.1305	-0.0588	-0.1756	-0.2567	-0.0463	0.18418	-0.0685	0.09301	-0.2026	-0.1081
XM_224155.3	LOC290196	-0.0667	0.12834	-0.0012	0.02144	0.08771	0.09792	0.04172	0.05138	0.06853	-0.1319	0.07833	-0.0246	0.20612	0.18545
XM_224159.3	LOC290199	0.23823	-0.082	-0.0694	-0.1632	-0.1014	0.13979	-0.0211	0.15882	0.10787	-0.078	0.19312	0.18171	0.31355	0.22589
XM_224224.2	LOC290262	-0.0452	-0.121	-0.074	-0.1128	-0.1506	-0.1337	-0.0562	-0.1106	-0.0723	-0.0461	0.02494	0.18324	-0.1716	0.0142
NM_001105790	LOC290273	0.1163	-0.0068	0.1726	0.17197	0.14876	0.25039	0.13246	0.23208	0.45362	0.02102	0.34487	0.3127	0.25071	0.06318
XM_214188.3	LOC290282	-0.0304	-0.1038	-0.1029	-0.0596	-0.1613	0.05087	0.18334	0.0618	-0.08	-0.1958	-0.082	-0.2455	-0.1227	-0.1419
XM_224270.3	LOC290295	-0.2859	-0.1915	-0.0725	-0.0365	-0.193	-0.1645	-0.238	-0.1533	-0.3166	-0.0181	-0.2164	-0.1527	-0.252	-0.2474
XM_214221.3	LOC290339	-0.0962	0.08431	0.10305	0.05875	0.0292	0.04778	0.24366	0.10773	0.12106	0.15769	0.02023	0.1409	-0.1163	0.04123
XR_008528.1	LOC290341	-0.1714	0.29544	-0.6274	-0.4441	0.02962	-0.2859	-0.2384	0.14994	0.14431	0.18649	-0.3983	-0.3023	0.0967	0.13751
NM_001024239	LOC290348	-0.0525	-0.06	-0.01	0.17118	0.10803	-0.0838	-0.0252	0.00385	-0.0228	0.03863	0.13889	-0.0603	-0.0279	-0.0279

XM_224348.3	LOC290362	-0.0055	-0.1256	0.00243	-0.0264	-0.0769	0.09525	-0.075	-0.0661	-0.0888	-0.0346	0.01993	-0.062	0.03034	-0.0451
XM_001074393.1	LOC290396	0.11605	-0.1877	0.27292	-0.1445	-0.2081	-0.3678	-0.4718	0.01621	0.4236	-0.2518	0.01712	0.25461	0.92936	0.87063
XM_224392.3	LOC290396	0.01785	-0.0442	0.08518	0.03266	0.01822	-0.0206	0.133	-0.0143	0.03425	-0.0555	-0.0155	0.13055	0.13974	0.07812
XM_224405.3	LOC290404	0.08554	0.04095	-0.0014	-0.0518	0.00832	0.04403	-0.0004	-0.077	0.04694	-0.0621	-0.0455	0.02414	0.08861	0.09345
NM_207589	LOC290549	0.4167	0.2911	0.42314	1.0146	0.6404	0.96588	0.48404	0.76422	0.33999	0.32194	0.55896	0.56067	0.58156	0.44682
NM_001131006	LOC290555	0.37332	0.36608	-0.1109	0.17695	0.2834	0.41881	0.37253	0.50938	0.51595	0.4006	0.50667	0.2334	0.42218	0.22052
XM_224650.3	LOC290579	0.17531	0.15983	0.10808	-0.2112	0.02478	-0.11	-0.1461	-0.0833	-0.0187	0.00828	0.13391	-0.1154	0.17666	0.22281
XM_224653.3	LOC290580	-0.1248	-0.0328	-0.1383	-0.0544	-0.1649	-0.117	-0.2598	-0.1348	-0.171	-0.1437	-0.1037	-0.1994	-0.1191	-0.1125
XM_224663.3	LOC290589	0.13839	0.09654	0.00774	0.00844	-0.047	0.12108	0.10091	0.10005	0.00747	0.1352	0.08467	0.08807	0.07413	-0.0417
NM_001106063	LOC290595	0.67665	0.58567	0.53385	0.53684	0.7014	0.85398	0.22898	0.73117	0.76796	0.56053	0.59129	0.1489	0.79737	0.71858
XM_214338.3	LOC290704	-0.4163	0.76103	-0.6943	-0.6429	-0.2647	-0.8844	-0.4904	-0.4459	0.48195	0.55087	0.34653	-0.096	-0.153	-0.034
XM_224826.3	LOC290710	0.082	0.09372	0.02366	0.06252	0.17729	0.05035	0.00939	0.06203	0.07905	0.14646	0.01036	0.25497	0.07064	0.04799
NM_001169119	LOC290763	-0.132	0.05099	0.03514	0.11457	-0.1477	-0.0733	-0.2189	-0.0263	-0.0578	0.19134	-0.0211	-0.0835	0.05504	-0.0383
XM_224918.3	LOC290805	-0.1782	-0.0669	-0.0324	-0.0797	-0.1463	0.10849	0.03089	-0.1261	-0.1303	0.12739	-0.1068	0.01344	-0.0729	-0.1171
XM_224967.3	LOC290836	-0.0182	-0.0908	0.26098	-0.0857	0.05936	0.0583	0.07772	-0.0994	-0.0661	0.01928	-0.0988	-0.0749	-0.0492	-0.1191
XM_237985.3	LOC290847	0.17808	0.08104	0.22278	0.027	-0.0626	0.02169	0.00625	0.0352	0.05751	0.07025	0.08008	0.06928	0.17315	0.16876
NM_001106093	LOC290864	-0.3481	-0.2822	0.32846	-0.1175	0.02137	-0.0988	0.15031	-0.049	-0.1787	-0.0842	-0.0718	-0.1391	-0.222	-0.1175
NM_001037182	LOC290876	-0.1469	-0.1868	-0.3409	0.00241	-0.0723	-0.2303	-0.2468	-0.0191	-0.2002	-0.3653	-0.0583	-0.1484	-0.1625	-0.2647
XM_225046.3	LOC290908	0.12022	0.03515	-0.0433	0.04488	0.17504	0.0827	0.12573	0.0549	0.05548	0.2078	0.00709	0.11134	0.04305	0.05133
NM_001106096	LOC290921	-0.0398	0.01914	-0.0483	-0.1149	-0.1096	-0.044	0.00742	-0.0925	0.10106	0.10037	0.15855	-0.2576	0.12914	0.08568
XM_237994.2	LOC290933	0.05745	-0.0372	-0.0542	-0.0373	0.25657	-0.0713	-0.1068	0.43174	-0.0201	-0.1525	0.05802	-0.0469	0.07439	-0.0365
XM_214409.3	LOC290964	0.77962	0.29209	-0.1888	1.7122	1.3006	1.3979	0.2408	1.5419	0.20268	0.25482	0.27259	-0.0171	-0.2493	-0.2261
NM_001106102	LOC290999	-0.0521	-0.0449	-0.3334	0.92437	0.78335	1.2193	0.00063	1.0778	0.4956	0.19561	0.37489	0.42258	-0.0025	-0.0174
XM_225194.3	LOC291014	-0.1055	-0.2771	-0.0491	-0.1129	-0.2033	0.05198	-0.2128	0.04416	-0.1816	0.05701	-0.2278	-0.2294	-0.0554	-0.0586
NM_001106117	LOC291209	0.04525	-0.2144	-0.1879	-0.0202	-0.1853	-0.229	-0.1945	-0.0911	-0.0703	-0.1841	-0.2228	-0.2961	-0.1561	-0.1172
XM_238009.1	LOC291218	-0.2247	-0.2491	-0.0925	-0.1329	-0.2231	-0.1885	-0.2454	0.2484	-0.2806	-0.1394	-0.273	-0.197	-0.1895	-0.2148
XM_225493.3	LOC291244	-0.0382	-0.1183	-0.1471	-0.1683	-0.2297	-0.1806	-0.102	0.00523	-0.1521	-0.1538	-0.0973	-0.0628	-0.0191	-0.0369
NM_053689	LOC291276	0.10332	0.15971	0.25807	0.07926	0.09918	0.16477	0.13821	0.11807	0.15004	0.28405	0.13039	0.0616	0.04183	0.05466
XM_225571.3	LOC291313	-0.023	0.16429	0.00275	0.06358	-0.0441	-0.0404	0.00863	-0.0334	-0.0065	-0.035	-0.0193	0.00573	0.2258	0.1418
XM_225591.3	LOC291332	0.06146	-0.0297	0.07015	-0.0332	-0.0242	-0.0688	-0.0384	-0.0594	0.00465	-0.0624	-0.0311	0.04755	-0.0512	0.03264
XM_225619.3	LOC291345	-0.0264	-0.0362	-0.1843	-0.0662	-0.1022	-0.0817	-0.1438	-0.0252	-0.0704	-0.0863	-0.1605	-0.0045	-0.0344	-0.1294
XM_225620.2	LOC291346	0.20598	0.18782	0.15522	0.18757	0.21981	0.44373	0.13713	0.15734	0.0465	0.19441	0.10426	0.14062	0.33521	0.10867
XM_225691.3	LOC291406	0.08239	0.12407	0.22755	0.2902	0.02692	0.08753	-0.0227	0.10878	-0.0837	0.17206	0.10806	0.17315	0.05726	0.23777
NM_001007598	LOC291407	-0.1285	-0.0831	-0.052	-0.0214	-0.0475	-0.0977	-0.0857	-0.0598	-0.0482	-0.0176	0.0013	-0.0322	0.03887	-0.0182
NM_001106130	LOC291411	-0.369	-0.2624	-0.5242	-0.1747	-0.5408	-1.2351	-0.2585	-0.9649	-0.4412	-0.3558	-0.3908	-1.0668	-0.1671	-0.3642
NM_134415	LOC291419	0.01131	0.04188	0.06613	0.03119	0.00571	0.08093	0.07753	0.17955	0.02963	0.05831	0.15104	0.07481	0.06637	-0.0017
XM_214536.3	LOC291448	0.04594	0.14573	0.02284	-0.2667	0.05766	-0.0835	-0.1017	-0.0784	0.02604	0.19834	0.11835	0.31272	0.26698	0.25619
XM_225800.4	LOC291480	-0.0095	-0.0144	0.01516	-0.0024	0.05741	0.04175	0.04779	0.076	0.00481	0.08164	0.02652	-0.0756	0.0619	0.01237
XM_225817.2	LOC291496	0.04053	0.33281	0.08466	0.0847	0.07706	0.15985	0.18006	0.05538	0.03477	0.14585	0.14301	0.03065	0.04021	0.06521
XM_225832.3	LOC291510	0.12321	0.06439	0.02437	-0.0066	0.01269	0.06788	0.01528	-0.0939	0.03973	0.13655	0.06046	0.03423	0.10348	0.07592
XR_008809.1	LOC291521	-0.3068	0.42765	0.86273	0.68506	-0.289	-0.3303	1.1767	-0.254	0.36061	0.40181	0.44006	0.04023	0.48824	0.61226
NM_001077667	LOC291530	-0.1004	-0.1695	-0.0337	0.08698	-0.0087	0.1343	-0.0439	-0.0119	-0.0792	-0.1625	0.02024	-0.027	-0.095	-0.1202
XM_212827.3	LOC291561	-0.1319	-0.1133	-0.1377	-0.0885	0.15284	0.03107	-0.0395	0.05572	-0.0946	-0.0296	-0.0717	0.13366	-0.0922	-0.0697
XM_225993.2	LOC291616	0.03398	-0.0394	0.15698	0.11034	0.09875	0.0423	0.00822	-0.03	0.02588	0.12467	-0.0881	0.04723	0.1511	-0.0204
XM_238028.2	LOC291665	0.25898	0.62039	-0.1701	1.6806	0.85847	1.4014	0.57654	0.93428	0.76422	0.34505	0.78769	-0.013	-0.3505	-0.1094
XM_226020.3	LOC291669	0.04776	0.04731	0.15205	0.08691	0.00496	-0.0096	0.05189	0.1767	-0.0427	-0.0252	0.05707	-0.0394	-0.0317	0.05678
NM_001025636	LOC291676	-0.0389	-0.2161	-0.3155	-0.2552	-0.2183	0.01574	-0.441	-0.295	-0.0844	-0.2415	-0.2892	-0.0948	-0.1839	-0.4257
XM_226123.2	LOC291762	-0.0053	-0.2925	-0.2295	0.14492	-0.2824	-0.4418	0.26306	-0.1147	-0.4129	-0.3305	-0.4668	-0.4835	-0.4586	-0.627
XM_226146.3	LOC291773	0.12665	0.13141	-0.0449	-0.0957	0.15383	-0.0222	-0.0125	0.17431	0.05603	-0.1062	-0.0137	-0.0492	0.00507	0.11263

NM_001106163	LOC291794	-0.0328	0.06194	0.08567	0.01553	0.02938	0.1938	-0.001	0.12209	-0.0928	0.02099	0.0013	0.12295	-0.021	-0.0851
XM_226183.3	LOC291802	-0.1195	0.20681	-0.0868	-0.0277	-0.1283	0.18097	0.01916	-0.0733	0.31069	-0.0169	-0.1334	-0.0857	0.10063	-0.0503
XM_226241.3	LOC291814	0.01935	0.09168	0.28601	0.16834	0.12489	-0.0085	0.00824	0.15861	-0.0026	0.01469	-0.0015	0.03245	0.20863	0.01277
XM_226249.3	LOC291815	-0.0546	-0.1153	0.06884	0.1308	-0.1934	-0.1274	0.02397	-0.0991	-0.0175	-0.0156	-0.01	-0.1062	0.1018	-0.1493
NM_001003705	LOC291840	-0.1101	-0.1119	0.29244	-0.1608	0.14222	-0.2661	0.32777	-0.0936	-0.0038	-0.2025	0.0403	-0.317	0.31553	-0.1109
NM_001013889	LOC291863	-0.0083	-0.1871	-0.1641	-0.1561	-0.1829	-0.0866	-0.0126	-0.16	0.12949	-0.1018	-0.2084	-0.1101	-0.1116	-0.1456
XM_238040.2	LOC291900	0.51004	0.96068	0.92381	0.42521	1.4111	1.8412	0.417	1.3979	1.0955	1.3069	1.4345	0.292	0.87902	0.90584
NM_001039713	LOC291905	-0.0591	0.2047	-0.4699	0.02949	0.40529	0.62506	-0.3399	0.15041	0.07267	0.20688	0.09938	0.58887	-0.1547	-0.0526
NM_001106173	LOC291914	0.08994	0.39284	-0.6922	-0.5971	0.3775	-0.449	0.11394	0.42614	0.27941	0.20093	0.09204	-0.2634	0.29634	0.22704
NM_001110810	LOC291926	0.06862	0.01524	0.01302	-0.0276	-0.0402	0.0601	-0.0917	-0.1399	0.01952	-0.0922	0.0325	0.06271	-0.0513	-0.0691
XM_226382.1	LOC291934	-0.0095	0.04356	0.02443	0.08935	0.07393	-0.0086	-0.0231	0.01664	0.03121	0.24344	0.0953	0.09056	0.00244	-0.1003
XM_226370.2	LOC291943	-0.2703	-0.2088	-0.3931	-0.2586	-0.3146	-0.2944	-0.3168	-0.2609	-0.3142	-0.2369	-0.3168	-0.1634	-0.1742	-0.2829
XM_001072430.1	LOC291964	0.32478	0.31317	0.84519	0.31783	-0.5168	-0.2031	0.83775	-0.4694	0.11817	0.31596	0.35713	0.36285	-0.0428	-0.1426
XM_226456.2	LOC292014	0.14235	-0.2271	-0.244	-0.0969	-0.0671	-0.0633	-0.0665	-0.0743	0.13453	-0.0621	-0.1772	-0.1034	-0.018	0.01174
XM_001077360.1	LOC292017	-0.0379	0.02822	-0.1183	0.31828	-0.0787	-0.051	-0.0676	-0.0291	0.49562	0.15979	-0.0085	-0.0459	-0.0811	-0.1328
XM_226489.1	LOC292030	0.17859	0.10887	0.14542	0.1108	0.02677	-0.0361	0.05165	0.16526	-0.0672	0.26974	0.03391	0.38848	0.01254	0.10637
NM_001106189	LOC292045	0.00973	0.05282	-0.0365	0.02902	0.06757	0.01059	0.01939	0.14933	0.1343	0.06732	0.14685	0.06098	0.10975	0.19945
XM_226521.3	LOC292053	-0.0589	0.03408	-0.0506	0.06417	-0.0711	-0.0271	-0.0238	0.01705	-0.0534	-0.073	-0.0662	0.0518	-0.0434	-0.0138
NM_001037094	LOC292069	-0.2064	-0.5156	0.30387	0.18506	-0.5548	-0.508	-0.1263	-0.3894	-0.3367	-0.7475	-0.3433	0.03313	-0.0039	-0.0796
XM_214704.1	LOC292072	0.43703	0.40617	-0.0496	0.4583	0.25672	0.43471	0.44678	0.22763	0.15044	0.33471	0.31959	0.01952	-0.1906	0.04224
NM_001047851	LOC292073	0.55351	0.47465	0.31559	0.19123	0.48269	0.80032	0.01617	0.60804	0.40715	0.14278	0.52828	0.00174	0.23393	0.37094
NM_001106193	LOC292074	1.1796	0.8303	-0.0077	0.62934	0.94543	1.0118	0.26988	0.85785	0.89312	0.7722	0.80504	0.62603	0.55003	0.51444
XM_214707.3	LOC292082	-1.0638	-0.1785	0.55588	1.2418	-0.5409	-0.6497	0.56245	-0.5985	-0.2585	-0.2187	-0.1513	-0.3952	-0.7045	-0.692
NM_001105788	LOC292088	0.45717	0.15771	-0.1082	0.47322	0.3443	0.90511	-0.2466	0.46037	-0.1331	-0.1672	0.31503	0.21663	0.08109	0.02102
XM_226568.2	LOC292104	0.0256	0.24328	0.04643	0.11085	-0.0652	0.35684	-0.0247	0.15399	-0.1412	0.13929	0.07756	0.17646	0.20579	0.27311
XM_214728.2	LOC292116	0.03822	-0.0082	0.26964	-0.0269	0.30019	0.09241	0.13706	0.07255	0.15271	-0.2837	0.16142	-0.1397	0.1431	0.35695
XM_238062.2	LOC292128	0.23252	-0.1176	0.12281	-0.0245	0.06277	-0.1088	-0.0912	-0.0806	-0.1846	-0.123	-0.1226	0.04898	0.03634	-0.0536
XM_217693.3	LOC292158	0.1202	0.03545	-0.0076	0.114	-0.0362	0.0161	0.07226	0.24784	0.17359	0.03449	-0.0698	-0.1868	0.15895	0.18885
XM_238066.4	LOC292209	-0.0555	0.04195	0.14317	-0.034	-0.0486	0.00062	-0.0144	-0.0244	0.0519	-0.0298	0.07957	-0.0161	-0.0513	-0.0572
XM_217809.3	LOC292247	-0.1244	-0.0925	0.00629	0.00893	-0.0022	0.05386	-0.1168	0.14372	0.13403	0.0203	-0.041	-0.0876	0.06679	0.02773
XM_214760.3	LOC292266	0.03963	-0.0347	0.08032	0.32529	-0.0015	0.06582	0.15203	0.14562	0.10483	0.07308	0.17413	0.11043	0.00748	0.22983
NM_001040128	LOC292268	0.03656	0.26783	0.01712	0.0239	-0.0569	0.35913	0.14306	0.03239	0.09738	-0.0753	0.39837	0.14135	0.0408	0.17405
XM_214765.2	LOC292282	0.41377	0.3748	-0.442	-0.4201	0.12143	0.57985	-0.3623	-0.0245	0.34255	0.11684	0.44703	0.46684	-0.0119	-0.0615
XM_217957.3	LOC292367	0.24949	-0.0664	0.19239	-0.0188	-0.0284	0.13103	0.07853	0.3646	0.17884	0.37964	0.22807	0.09774	0.13373	0.08267
XR_007133.1	LOC292393	-0.0755	-0.0889	-0.0785	0.03459	-0.0353	-0.0413	-0.0467	-0.083	-0.054	0.11174	0.08463	0.19995	-0.015	0.01071
XM_218050.2	LOC292455	0.04705	-0.0158	0.0622	0.04601	0.00486	0.06915	0.07513	0.21002	0.36537	-0.0041	0.1542	0.23781	-0.046	0.03104
NM_001106290	LOC292474	0.35149	0.55249	0.61621	0.66587	0.3499	0.41451	1.0838	0.38939	0.10724	0.22721	0.40106	0.28589	0.17728	0.12782
XR_009620.1	LOC292498	-0.1439	-0.0891	-0.1635	-0.0653	-0.0918	-0.1487	0.02689	-0.1502	-0.1967	-0.0187	0.04909	-0.0474	-0.043	-0.0915
XR_007939.1	LOC292510	-0.1527	-0.1693	-0.2272	-0.0379	-0.1594	-0.1716	-0.2111	-0.119	-0.0939	-0.0385	-0.0751	-0.1049	-0.1828	-0.1829
NM_001013893	LOC292516	0.21804	0.21041	0.10386	0.25978	0.14316	0.35787	0.01943	0.37874	0.21627	0.21087	0.20389	0.33388	0.45056	0.47926
NM_001025639	LOC292543	0.07336	0.34018	0.08422	0.14648	0.04948	-0.0245	0.05541	-0.0424	0.21061	0.05594	-0.01	0.17669	0.04321	0.04043
NM_001099472	LOC292547	0.01743	0.09506	0.253	0.04239	0.13983	0.18192	0.09012	-0.0332	0.09339	0.18232	-0.0627	-0.0993	-0.0228	0.1228
XM_238077.1	LOC292565	-0.0386	0.0276	0.12429	0.16055	0.11221	0.08656	-0.0156	-0.0171	0.11476	-0.0084	0.02889	-0.0331	-0.0218	0.06176
XM_218275.2	LOC292602	0.11763	0.14727	0.18429	0.21942	0.22067	0.15868	0.39052	0.01238	0.00345	0.13651	0.0895	0.08894	0.04255	0.05101
NM_147135	LOC292603	-0.2492	-0.2063	-0.0571	-0.1913	-0.1856	-0.2349	-0.0192	-0.2014	-0.1378	-0.2561	-0.0831	-0.0895	-0.0765	-0.2379
XM_218282.2	LOC292609	0.08081	-0.154	-0.0991	-0.1456	-0.0943	-0.141	-0.2382	0.11509	0.00806	-0.0219	-0.0031	-0.039	0.16804	0.15993
NM_001099473	LOC292615	-0.1187	-0.0792	0.146	-0.1277	-0.1606	-0.0505	0.09084	0.04867	0.04337	0.01912	-0.2766	0.03248	-0.2486	-0.1118
XM_218301.3	LOC292630	-0.0154	0.05411	0.18932	0.01953	-0.0333	-0.0018	0.10418	0.07261	0.12192	0.44787	0.03125	0.36407	0.08651	-0.0097
XM_218323.3	LOC292645	-0.1703	-0.161	-0.3212	-0.168	-0.1884	-0.3022	-0.3653	-0.0672	-0.2106	-0.2573	-0.1282	-0.1339	-0.1644	-0.2764

NM_001025641	LOC292666	0.08202	0.04845	0.00445	-0.065	-0.0014	0.03296	0.04033	0.09915	-0.0541	0.04131	0.36653	-0.0769	0.02072	-0.1041
NM_001106230	LOC292687	-0.1	-0.1279	-0.0953	-0.0795	0.01253	-0.0561	-0.0604	-0.0043	-0.1464	-0.1499	-0.09	0.15316	-0.0269	-0.1148
NM_001135742	LOC292708	-0.0184	-0.1107	-0.2873	-0.1788	-0.251	-0.1926	-0.056	0.05376	-0.0661	-0.251	-0.2017	-0.002	-0.1622	0.03824
NM_001127450	LOC292780	0.92796	0.33525	0.26228	0.65512	0.66588	1.1752	0.08958	0.41857	0.32296	0.15433	0.28377	0.2522	0.0915	-0.0751
NM_001127599	LOC292811	-0.6014	-0.5807	0.15204	-0.4367	-0.6553	-0.6525	-0.5003	-0.7427	-0.3507	-0.4795	-0.5759	-0.0998	-0.34	-0.3707
XM_214903.3	LOC292812	0.03197	-0.073	-0.0416	-0.1591	-0.0514	-0.1502	-0.1079	0.08704	0.03918	0.01866	-0.0488	-0.2002	0.04308	0.07008
XM_218619.3	LOC292884	-0.3395	-0.2759	-0.2685	-0.2185	-0.2962	-0.2962	-0.319	-0.2901	-0.3896	-0.2272	-0.0741	-0.3614	-0.2109	-0.2077
XM_218674.3	LOC292959	0.23871	-0.0932	0.00648	-0.1071	-0.1423	0.06462	-0.1204	-0.1684	-0.09	-0.1264	-0.0243	-0.0417	-0.0202	-0.0656
NM_001134563	LOC293044	0.13258	0.17652	0.22248	0.0434	0.15253	0.06907	0.13482	0.31549	0.28073	0.00109	0.04972	0.40161	0.10756	0.01742
NM_001130728	LOC293057	0.10857	0.2376	0.24856	0.14945	-0.0183	0.02192	0.00928	0.06366	0.25182	0.14425	-0.0081	0.1512	0.1103	0.04913
NM_001024749	LOC293059	-0.2737	-0.2417	-0.2812	-0.3209	-0.2395	-0.2357	-0.1948	-0.2666	-0.2544	-0.1667	-0.2052	-0.1796	-0.2061	-0.1704
NM_001013897	LOC293103	0.20444	0.05671	0.07994	-0.3013	-0.0609	0.06374	-0.246	-0.3226	-0.1702	0.08295	0.0605	0.02622	0.24962	0.2075
NM_001106285	LOC293166	0.08	0.09978	0.04312	0.11952	0.03129	0.11455	0.1332	0.05656	0.00404	0.21513	0.17278	-0.0623	-0.001	0.2462
XM_219368.3	LOC293521	0.12076	0.09415	0.03855	-0.1416	-0.0441	-0.0952	-0.0986	0.11839	0.05807	-0.1212	0.10309	0.19107	0.05213	0.0068
NM_001106311	LOC293589	0.09718	-0.1573	-0.2404	-0.0285	-0.4317	-0.0633	-0.1267	-0.6706	-0.053	-0.0065	0.01717	-0.0383	-0.2095	-0.1772
NM_001106311	LOC293589	0.30613	0.2538	0.08646	0.47843	0.40774	0.50792	0.20584	0.23849	-0.1185	-0.0334	0.29498	0.09216	-0.1383	0.0465
NM_001106317	LOC293623	-0.0791	-0.0046	0.14456	-0.024	0.09898	-0.0633	-0.0324	0.02817	0.06904	-0.0057	-0.0188	0.21654	0.17648	0.2327
XM_219505.3	LOC293641	0.03672	0.08541	0.08288	0.09092	0.0372	0.32773	0.15926	0.10801	0.04276	0.01483	0.09845	-0.0184	0.12357	0.13183
XM_215142.2	LOC293679	-0.3555	-0.5389	-0.0826	-0.5666	-0.1229	-0.1709	-0.6496	-0.2771	0.03282	-0.4765	-0.5441	0.58082	0.07849	0.02927
NM_001130013	LOC293693	0.04169	-0.1854	-0.1152	-0.0596	-0.1991	-0.2016	-0.2402	-0.2102	-0.1552	-0.1765	-0.2111	0.12463	-0.1553	-0.2056
NM_001039007	LOC293711	0.07067	0.00357	0.05803	-0.0155	0.12083	0.05972	0.07393	0.10373	0.01787	0.0423	0.00727	0.10449	0.07761	0.04222
XM_215147.3	LOC293723	-0.3343	-0.0041	-0.0678	-0.6017	-0.2948	-0.4955	-0.0154	-0.3723	0.0161	-0.0594	0.02838	-0.2813	-0.3086	-0.3941
XM_219592.3	LOC293745	-0.1276	-0.0382	0.14755	0.23392	-0.0164	0.27639	0.21897	0.099	-0.16	-0.0448	-0.1446	0.02845	-0.1255	0.07127
NM_001106339	LOC293746	0.44447	0.23738	0.08525	0.20097	-0.0348	0.16372	0.15368	0.17748	0.42354	0.26448	0.25472	0.11766	0.42346	0.47226
XM_219677.3	LOC293822	-0.0805	0.18366	-0.0825	0.08314	0.12206	0.03113	0.33049	0.165	0.17026	0.32446	0.17053	-0.0135	0.27271	0.09539
NM_001106342	LOC293840	0.1376	-0.0719	0.20956	0.08489	0.00212	0.02114	0.09936	0.12379	-0.1265	-0.176	-0.1672	-0.1938	-0.0431	-0.0486
NM_001024974	LOC293871	0.24159	0.18072	0.27752	0.30774	0.0031	-0.2646	-0.0769	0.35558	0.1028	0.19379	0.06038	-0.0072	0.27403	0.26902
XM_219773.3	LOC293882	0.1775	0.12531	0.11385	0.22438	0.03404	0.19381	0.04027	0.00103	0.12948	0.32144	0.17044	0.10013	0.07436	0.02249
XM_219865.2	LOC293944	-0.0453	0.08672	0.05164	0.1612	-0.0664	0.01869	0.1393	-0.1655	-0.2279	0.35059	-0.1703	-0.0154	-0.1869	0.00605
XM_219896.2	LOC293963	0.17356	0.33985	0.32747	0.17577	0.39361	-0.082	0.32922	0.25306	0.03801	0.2122	0.19575	0.21864	0.15678	0.29905
XM_220002.3	LOC294052	-0.071	-0.038	0.1509	0.0563	0.18338	0.22199	0.03585	-0.0395	0.17178	-0.1378	0.26299	-0.1187	0.05293	-0.1259
XM_220029.3	LOC294072	-0.09	-0.1288	-0.0516	0.04659	0.16238	0.16299	-0.0953	-0.0686	0.32276	-0.1338	0.09978	-0.1164	-0.1296	-0.079
NM_031709	LOC294077	0.04162	-0.0433	0.0479	0.04986	-0.0106	0.04519	-0.1189	-0.0475	-0.0386	-0.1021	-0.0024	-0.0613	0.17847	-0.0725
XM_228129.3	LOC294368	0.00537	0.08797	0.01976	-0.0751	0.13293	-0.1803	0.11555	0.12092	-0.1062	0.05083	-0.0309	-0.0961	-0.1259	-0.0259
XM_215399.3	LOC294412	-0.2361	-0.1804	-0.1591	0.08598	-0.0231	-0.1536	-0.1268	-0.0555	-0.1723	-0.1927	-0.2512	-0.1678	-0.195	-0.0138
XM_228202.3	LOC294435	-0.0012	0.04486	0.03214	-0.1199	0.10043	0.08334	0.06845	0.0213	0.18913	-0.0008	0.00433	0.06903	-0.0419	-0.0273
XM_228242.3	LOC294473	0.23754	0.11387	0.26224	0.25499	0.27164	0.23846	0.12826	0.19299	0.22291	0.02392	0.14876	0.08815	0.17298	0.16502
XM_228242.1	LOC294473	-0.199	-0.0802	-0.1084	-0.0827	-0.1731	0.09805	0.09417	-0.0914	-0.076	-0.1948	-0.1798	-0.042	-0.133	0.01036
XM_228268.4	LOC294497	0.10957	0.07913	0.04954	0.06746	-0.0281	0.0346	0.03013	0.25969	0.0452	0.13634	0.03065	0.13148	0.1192	-0.0036
XM_001054117.1	LOC294557	0.14915	0.07363	-0.0252	0.2106	0.18815	0.0868	0.00655	0.12714	0.1576	0.15543	0.02095	0.04203	0.19931	0.00566
NM_001106398	LOC294560	-0.2597	-0.2414	-0.5886	-0.7139	-0.1357	-0.1928	-0.1936	-0.0536	-0.1861	-0.0515	-0.4862	-0.4662	-0.1642	-0.185
XM_226616.3	LOC294603	0.05586	0.04098	0.18868	0.08342	0.22133	0.06202	0.00732	0.07943	0.17972	0.12876	0.07194	0.08936	0.16576	0.11719
XM_238208.3	LOC294611	0.10558	0.1072	0.07457	0.10602	0.07684	0.13313	0.13795	0.24662	0.25666	0.11389	0.15912	0.08467	-0.0417	-0.0333
XM_226650.2	LOC294622	0.21478	-0.0706	0.0959	0.2158	-0.0213	-0.0898	0.10523	0.12785	0.10565	-0.0745	0.19527	0.03787	0.08957	0.08969
XM_226700.3	LOC294668	-0.2082	-0.1974	-0.2691	-0.1004	-0.2259	-0.2058	-0.1362	-0.1465	-0.2374	-0.2107	-0.0489	0.08182	-0.2666	-0.245
XM_226742.1	LOC294690	-0.007	-0.1513	0.01557	0.09888	0.03082	0.06724	-0.0707	-0.0248	-0.0644	-0.1026	0.02498	0.00978	-0.0209	0.04713
XM_226802.2	LOC294726	-0.0742	-0.2899	-0.0624	0.27163	0.02244	0.12634	0.19188	0.14267	0.11283	-0.0795	0.13708	-0.0285	-0.0327	-0.1343
XM_001068789.1	LOC294762	-1.8105	-1.5518	-1.599	-1.3273	-1.5061	-1.5632	-1.1251	-1.3257	-1.5949	-1.8448	-1.3831	-1.5158	-2.0993	-2.2665
XM_212926.3	LOC294781	-0.047	-0.3882	-0.5096	-0.1049	-0.199	0.14175	-0.6911	0.05538	-0.1114	-0.5103	-0.0746	-0.131	-0.4463	-0.2815

XM_215498.3	LOC294789	0.05827	0.35424	0.21247	0.65324	0.22151	-0.0273	0.07642	0.10572	-0.0164	-0.0021	0.23487	0.31641	0.509	0.26556
XM_215517.3	LOC294862	-0.1388	-0.0727	0.02196	-0.1909	0.0482	-0.1953	-0.1502	-0.1983	-0.0266	0.03293	-0.144	-0.0369	-0.1419	-0.0843
XM_238220.2	LOC294936	0.05307	-0.1277	-0.0424	-0.0716	-0.0463	-0.108	-0.0335	-0.0889	-0.1526	-0.1334	-0.0523	0.08929	-0.1642	0.05221
XM_215538.3	LOC294941	0.22119	0.02547	0.01132	0.05274	0.0402	-0.1367	-0.1576	0.09818	0.01284	-0.092	0.00315	-0.0019	0.11067	-0.1311
XM_215541.2	LOC294942	1.2255	3.362	0.63703	1.436	-0.2547	-0.191	2.6151	-0.1925	2.8459	3.2795	2.8923	0.06976	0.4372	0.59238
XM_238225.2	LOC294998	-0.2965	-0.2933	-0.1937	-0.0597	-0.1574	-0.2396	-0.138	-0.0715	-0.0834	-0.2182	-0.0343	-0.2436	-0.2487	-0.2141
XM_227132.4	LOC295015	-0.2461	-0.0084	-0.0592	0.06941	-0.0299	0.04076	0.3379	0.20534	-0.181	0.10125	-0.1163	0.0246	-0.1467	-0.0247
NM_001024869	LOC295062	-0.1496	0.40166	-0.088	-0.5829	0.14608	-0.0349	0.0723	0.24237	0.24943	0.19831	-0.0388	0.17301	0.47831	0.4496
XM_227200.3	LOC295083	1E-04	0.01791	-0.0802	0.01831	0.01339	-0.063	0.06799	-0.0898	0.0785	-0.0866	0.00278	0.02466	-0.102	0.03763
NM_001099502	LOC295091	-0.115	-0.0118	0.07411	-0.0331	-0.0213	-0.0818	-0.224	-0.0031	0.15371	-0.0399	-0.0405	-0.0258	-0.0628	-0.1365
XM_227212.3	LOC295092	-0.1009	-0.1373	-0.1261	-0.1717	0.08468	-0.0959	-0.0787	0.16134	-0.2263	-0.2579	-0.0324	-0.1302	0.1154	-0.1903
XM_227324.3	LOC295167	-0.1372	-0.0552	0.01831	0.14334	-0.0162	-0.0347	-0.1235	-0.0798	0.08458	-0.0887	-0.0715	-0.0402	-0.1632	-0.1755
XM_227332.3	LOC295175	0.18327	0.0185	-0.0384	0.26009	0.33846	0.07914	0.24104	0.08441	-0.0593	0.50116	0.23753	0.03339	-0.0384	-0.0075
XM_227355.1	LOC295199	0.15788	-0.0098	0.18156	0.0231	-0.0228	0.00024	0.11463	-0.0187	-0.0714	0.26185	0.04795	-0.1048	-0.0113	0.07204
XM_227358.2	LOC295201	-0.14	-0.2577	-0.214	-0.0289	-0.0692	0.0108	-0.1872	-0.0058	-0.2064	-0.017	-0.2529	-0.1694	-0.0524	-0.074
NM_001100667	LOC295241	-0.0488	0.08303	0.07108	-0.0643	0.00687	-0.0043	-0.0177	0.00505	0.10137	0.02294	-0.0834	-0.047	-0.0348	-0.0093
XM_227418.3	LOC295247	-0.0376	0.02472	-0.115	0.21061	-0.0947	-0.0647	-0.0044	-0.0283	-0.0283	-0.1989	-0.1248	0.00573	-0.1316	-0.0905
XM_227424.2	LOC295254	-0.2157	0.25762	-0.1777	0.19655	0.12394	-0.1086	-0.0252	0.07438	-0.0293	0.02508	-0.092	-0.002	0.04968	-0.1097
XM_227433.3	LOC295258	0.02039	0.05281	0.04861	0.06224	-0.0093	0.10354	-0.1993	-0.0159	0.35325	-0.07	-0.1722	0.19535	0.03498	-0.0417
XM_227532.3	LOC295333	0.1601	-0.0498	0.05919	-0.1029	0.00328	0.00607	-0.1218	-0.0168	0.09281	-0.1611	-0.1144	-0.1022	-0.0068	0.15837
XM_227546.3	LOC295340	0.22091	0.139	0.17735	0.04596	-0.0428	0.17964	-0.0666	-0.0246	0.36254	0.25203	0.1744	0.57643	0.10109	0.18618
XM_227564.3	LOC295350	-0.0332	0.21575	0.2173	0.05759	0.03223	0.01775	0.02892	0.15417	0.02605	-0.0054	0.11167	0.09663	-0.0408	-0.0813
NM_031014	LOC295406	-0.1582	-0.3052	-0.2497	-0.2292	-0.1669	-0.3233	-0.2759	-0.3455	-0.3243	-0.1128	-0.3202	-0.1734	-0.3727	-0.2481
XM_215688.3	LOC295419	0.07154	-0.3702	0.0753	0.24475	-0.0593	0.24017	0.30875	0.23544	0.23522	-0.0978	0.06566	-0.4353	-0.0235	-0.0038
XM_227654.3	LOC295420	0.24064	-0.1277	0.17102	-0.2971	0.2104	0.46164	-0.4578	0.3453	-0.045	-0.0681	-0.1394	0.21819	0.11326	0.1897
XR_009150.1	LOC295452	0.48442	0.2187	0.25591	-0.8766	0.17436	-0.2683	-0.4021	0.31478	0.01986	0.25943	0.09716	-0.5628	0.42726	0.59346
XM_227709.3	LOC295470	-0.1232	0.10609	0.20623	-0.0294	0.0341	-0.0302	-0.0673	-0.0537	-0.0591	-0.0204	0.18703	-0.1494	0.14544	0.09248
XR_009184.1	LOC295487	-0.012	0.03972	0.09117	0.00942	0.14063	0.0481	0.00704	-0.0462	0.164	0.06528	0.10064	0.17786	0.16067	-0.0201
NM_001044232	LOC295496	0.29306	-0.2745	0.04764	1.0426	0.20028	0.13874	-0.1744	0.21191	-0.0981	0.0069	-0.3961	0.05466	-0.0158	0.00886
XM_001080231.1	LOC295528	0.31742	0.23608	0.20049	0.12935	0.16934	0.01888	0.25144	0.25956	0.26101	0.05903	0.1504	0.17347	0.10375	0.04578
XM_229992.2	LOC295653	-0.2072	-0.1555	-0.2354	0.08953	-0.0049	-0.0567	-0.0478	-0.1437	-0.1596	0.00613	-0.0876	-0.1297	-0.1235	-0.1026
XM_230018.2	LOC295671	-0.0195	0.11079	-0.2022	-0.1562	-0.0476	-0.1473	-0.166	-0.0491	0.12144	0.0955	0.03172	0.03048	-0.0196	-0.0051
XM_238275.3	LOC295976	-0.3186	-0.3105	-0.1951	-0.3026	-0.2799	-0.2099	-0.2555	-0.2213	-0.3203	-0.1807	-0.2762	-0.3896	-0.3482	-0.3234
NM_001024981	LOC295985	-0.2292	-0.0205	-0.2377	-0.1019	-0.0817	-0.1092	-0.0737	-0.0179	-0.0547	-0.2385	-0.2604	-0.3346	-0.1014	0.09624
NM_001168527	LOC296115	0.131	-0.0229	0.05685	-0.0136	0.02151	-0.0103	0.36527	0.12929	0.02037	0.04883	0.02377	0.01492	0.07652	-0.0067
NM_001037766	LOC296126	-0.1288	0.26593	0.40528	0.54768	0.01748	-0.1474	-0.0002	0.10708	0.03811	0.0399	0.03117	-0.2962	-0.1354	0.05768
NM_001106520	LOC296207	0.31061	0.36396	0.46301	0.27361	-0.1193	0.12239	0.49926	-0.1981	0.40848	0.28718	0.39475	0.63616	0.75107	0.45389
NM_001037350	LOC296235	0.0422	0.61532	0.11568	0.08435	-0.0842	0.20582	-0.0305	0.11953	0.4693	0.48408	0.74173	0.13083	0.24345	0.00817
XM_215910.1	LOC296318	0.35961	0.42074	0.11931	0.51306	0.44759	0.72126	0.21296	0.46861	0.06169	0.21175	0.19759	0.36521	0.04175	0.03036
XM_230802.3	LOC296323	0.34944	-0.0421	-0.0977	0.09494	0.11909	0.21096	0.00578	0.31732	0.31772	0.13667	0.2663	0.15711	0.18013	-0.0124
XM_230831.3	LOC296351	-0.2398	-0.2508	-0.1499	-0.2655	-0.3793	0.11064	-0.2176	-0.2991	-0.1509	-0.1311	-0.1827	-0.2289	-0.3196	-0.3554
XM_238298.2	LOC296404	0.0454	-0.0108	0.0396	0.03639	0.02547	-0.0219	0.08853	-0.0847	0.05004	-0.0593	0.14101	0.03774	-0.0363	-0.0772
XM_230936.3	LOC296449	-0.1902	-0.1463	-0.1064	0.06977	-0.1258	-0.1638	-0.048	-0.049	0.11001	-0.1208	0.02226	0.03081	-0.1243	0.12541
XM_231000.2	LOC296507	-0.1317	-0.1329	-0.055	-0.1058	-0.031	0.13361	-0.039	-0.0898	-0.0208	0.18912	0.23333	-0.0003	-0.0322	-0.0817
XM_231006.3	LOC296512	-0.0433	0.30561	0.24331	1.0928	-0.1175	0.12208	0.00272	0.35415	0.22916	0.30016	0.16814	0.06807	0.35718	0.29544
XM_231013.3	LOC296516	-0.1559	-0.1296	-0.1109	0.0114	-0.0268	-0.0222	-0.0909	-0.1792	-0.0438	-0.1169	-0.1419	-0.0286	-0.1771	-0.1572
XM_238309.3	LOC296529	0.13348	0.20385	0.09051	-0.0143	0.07453	-0.0277	0.0201	-0.072	-0.0013	0.01513	0.11687	-0.0174	0.0218	0.03317
XM_231081.3	LOC296582	0.15336	-0.1725	-0.3162	-0.7384	0.0973	0.07319	-0.836	-0.158	0.09239	-0.2438	-0.1181	0.29307	0.18384	0.18876
NM_001106561	LOC296599	-0.1561	0.07347	-0.3795	-0.3148	0.27199	-0.2572	-0.3147	0.40765	-0.0646	0.02345	-0.094	-0.2432	-0.2266	-0.2227

NM_001134518	LOC296637	0.07108	0.04718	0.04912	-0.0651	0.20722	0.18407	0.21244	-0.0477	0.0259	0.17167	0.06201	-0.0576	0.04846	0.06773
XM_212982.3	LOC296724	-0.1115	0.01612	-0.616	-1.117	-0.1694	-0.7798	-0.569	-0.2531	0.34	0.28095	-0.238	-0.0505	0.76193	0.60717
XM_216071.3	LOC296777	-0.1016	0.70732	0.33144	0.22694	0.4536	0.55616	0.17817	0.25769	0.27592	0.29525	0.70191	0.08704	-0.0332	0.03886
XM_216075.3	LOC296782	0.22762	-0.1827	0.19491	0.04238	0.13521	-0.1815	0.20055	-0.066	0.12549	0.14672	0.20931	0.09116	0.23868	-0.0606
NM_001037186	LOC296813	0.11668	-0.0713	-0.2958	-0.1237	-0.169	-0.5265	-0.385	-0.4269	-0.0139	-0.0868	-0.4302	-0.0721	-0.1759	-0.0586
NM_001106582	LOC296866	0.31408	-0.1084	-0.0118	0.26056	-0.0256	0.12036	-0.027	0.02857	0.22148	0.22755	0.08665	0.0742	-0.0243	0.04044
XM_216096.3	LOC296884	0.21976	0.15486	-0.3418	0.27655	-0.1026	-0.0997	0.03872	-0.1305	0.13306	0.21125	-0.0187	0.15904	0.01416	0.02553
NM_001100964	LOC296935	0.10936	0.11197	-0.0313	-0.0287	0.29863	-0.0164	0.13295	-0.0313	0.2904	-0.0712	-0.0845	-0.1356	-0.0231	0.03785
XM_231561.3	LOC296952	0.05875	-0.0722	0.16483	0.06818	-0.0278	-0.0698	-0.0031	0.06404	0.02186	-0.1156	-0.1074	-0.0218	0.02727	-0.0821
NM_001039008	LOC297077	-0.2446	-0.2681	-0.2116	-0.1726	-0.3706	-0.2302	-0.2005	-0.2593	-0.3726	-0.4099	-0.2416	-0.1346	-0.3449	-0.3061
XM_216149.3	LOC297099	-0.5754	-0.5862	0.31209	0.38618	-0.299	-0.6083	0.10526	-0.6271	-0.4104	-0.7671	-0.2137	-0.3772	-0.2981	-0.5576
XM_238352.1	LOC297110	0.0514	0.0594	0.0777	0.03337	0.2325	-0.0208	0.02766	0.05694	0.27671	0.02623	0.0349	0.11724	0.06343	0.0056
XM_231999.3	LOC297260	-0.0369	-0.0562	-0.0668	0.02377	-0.0157	0.00786	-0.0533	-0.0912	0.13179	-0.01	-0.004	0.01582	-0.0943	0.02469
XM_232111.3	LOC297380	-0.1294	-0.1199	-0.2263	-0.1156	-0.2201	-0.2417	-0.0606	-0.3563	-0.265	-0.0854	-0.2046	-0.2213	-0.2035	-0.1647
NM_001106612	LOC297481	0.60445	-0.2037	-1.1761	-0.2912	0.79258	0.65567	-1.0546	0.65791	-0.2753	-0.2058	0.00286	-1.6175	-0.8925	-0.9782
NM_001134713	LOC297483	0.01986	0.12513	-0.0879	0.13948	-0.0206	0.32725	-0.049	-0.1051	-0.2095	0.1029	0.06646	-0.0718	-0.0219	0.03752
NM_001037975	LOC297568	0.01174	-0.0401	-0.0491	0.16316	-0.1072	-0.0399	-0.0488	-0.0115	-0.0177	0.0976	0.06259	0.01293	0.26831	-0.1259
NM_001106618	LOC297570	0.29228	0.19574	-0.1359	-0.2148	0.18753	-0.0911	-0.1806	0.00322	-0.0384	-0.1229	-0.124	-0.4235	-0.0013	-0.1951
NM_001099503	LOC297590	-0.1533	-0.084	0.01898	-0.1448	0.06131	-0.1845	-0.1861	-0.1219	0.06895	-0.1233	-0.2405	-0.1595	-0.205	0.03311
XM_216272.2	LOC297591	-0.369	-0.2521	-0.9972	-0.8119	-0.7386	-0.658	-0.5721	-0.881	-0.3389	-0.2958	-0.2762	-0.269	-1.208	-1.0869
XM_232590.2	LOC297763	-0.0592	0.13861	-0.0322	-0.0411	-0.0527	-0.0433	0.01315	0.00597	-0.0746	0.00421	-0.053	0.03019	0.09758	0.08868
XM_232669.3	LOC297816	0.05229	0.03756	0.04576	0.04539	-0.0471	0.06835	-0.0875	-0.0012	0.028	-0.1046	0.02599	-0.1987	0.12867	0.0937
NM_001047865	LOC297832	-0.1755	-0.131	-0.2072	-0.0239	-0.0375	-0.0608	-0.2444	-0.185	-0.1382	-0.1726	0.04092	-0.1667	-0.1754	-0.0114
NM_001047865	LOC297846	-0.093	-0.1652	-0.0277	-0.0109	0.00797	-0.0681	0.01267	-0.0959	-0.0023	0.01141	-0.0192	-0.0714	-0.0361	-0.0266
XM_232716.3	LOC297865	-0.0854	0.0594	0.14263	-0.0172	-0.1299	0.23922	-0.1056	0.12091	-0.0435	0.01346	0.09967	0.09321	-0.0448	0.04518
XM_232769.1	LOC297892	-0.0431	0.08392	0.05492	-0.0597	-0.1075	0.09781	0.00341	0.07401	-0.1155	0.00626	0.14786	0.01746	0.34387	0.03613
XM_232777.3	LOC297899	0.3528	0.38106	0.24793	0.13795	0.15345	0.33569	0.73345	0.08998	0.25163	0.03748	0.19182	0.21691	0.01977	0.24285
XM_232791.3	LOC297908	-0.0239	-0.1618	0.03081	-0.0582	-0.0032	0.00372	-0.1222	-0.0883	-0.1129	-0.0313	-0.005	-0.0852	-0.0296	-0.1414
XM_232841.3	LOC297949	0.05379	0.16686	0.16112	0.0571	0.16324	-0.0166	0.01599	0.08266	-0.0212	0.09189	-0.033	-0.1101	0.01402	0.01004
XM_232844.2	LOC297951	0.08455	0.05507	0.22377	0.08571	0.02131	0.07946	0.11033	0.19417	0.12141	0.20727	0.0373	0.36429	0.09868	0.0307
NM_001039914	LOC297968	0.19572	0.21423	-0.2191	-0.1797	0.44088	0.36609	0.19246	0.42813	0.30057	0.15141	0.26732	0.17081	0.17344	0.33643
XM_216369.3	LOC297970	-0.0996	0.02022	0.032	-0.0399	-0.0777	-0.0775	-0.2475	-0.0827	-0.1521	-0.0928	0.06603	-0.0861	-0.1173	-0.0518
XM_232926.3	LOC298008	0.05004	0.07595	0.10382	0.01297	0.00185	0.04175	0.07897	0.12015	0.17165	0.27939	0.0727	0.18842	0.13032	-0.0441
NM_001025275	LOC298012	0.73215	0.55398	0.62411	0.65033	1.2478	1.3509	0.37356	1.3814	0.63679	0.47653	0.52416	0.5812	0.78993	0.82429
NM_001106651	LOC298033	-0.0126	0.14754	0.10433	-0.129	0.01351	0.05576	-0.1106	0.07511	-0.092	0.13539	-0.1215	0.05399	0.25556	0.05348
XM_216406.3	LOC298076	-0.0548	-0.3014	-0.2406	-0.2262	-0.2432	-0.1763	-0.2099	-0.0597	-0.2383	-0.376	-0.2159	-0.1206	-0.3585	-0.2224
NM_001106661	LOC298104	0.0711	0.14126	0.13069	0.15512	0.04616	0.00207	0.09486	0.05652	-0.0893	-0.0168	0.02438	-0.0802	-0.0722	0.15885
NM_203512	LOC298111	-0.0173	0.04237	0.0143	-0.0377	-0.0984	0.08952	-0.1164	0.00679	-0.0613	0.08072	0.00116	-0.0734	-0.0852	-0.0607
XM_233031.3	LOC298113	0.14106	0.00201	0.08662	-0.0504	0.15924	0.04435	0.06495	0.02462	-0.0702	0.05684	0.00137	0.07028	0.06752	-0.0023
NM_203512	LOC298116	-0.0175	0.03883	-0.024	-0.0472	-0.0216	0.28106	0.04464	0.01447	0.05201	0.1167	-0.0521	0.10944	-0.0067	0.03224
XM_216425.3	LOC298120	-0.0922	-0.0915	0.04267	-0.0158	-0.1419	-0.1464	-0.0357	0.04066	-0.0694	-0.1091	-0.0071	-0.1278	-0.0516	-0.1244
XM_233051.2	LOC298135	0.15485	0.1648	-0.0779	0.05393	0.11986	0.07623	-0.0973	0.01281	0.03663	0.19584	0.14774	0.17293	0.08986	-0.0794
NM_001013930	LOC298139	-0.132	-0.1046	-0.078	-0.0148	-0.0528	0.22039	0.03411	0.03326	0.03208	-0.0089	0.20024	0.01609	0.16828	0.02018
XM_233195.3	LOC298251	-0.2389	-0.1917	-0.1601	-0.0913	0.0153	-0.0088	-0.045	-0.1172	9E-05	0.13017	-0.0987	-0.1823	-0.0058	-0.1112
XM_233230.3	LOC298270	0.04494	-0.1516	-0.1701	-0.058	-0.1868	-0.1294	-0.1457	-0.1247	0.02566	-0.0242	-0.1187	-0.1451	0.0155	-0.0082
NM_001037544	LOC298340	0.19615	0.17787	0.08372	0.16035	0.14124	0.40057	0.06625	0.11385	0.12089	0.03445	-0.0521	0.15812	-0.0426	0.0539
NM_001024873	LOC298346	0.15856	0.22476	0.10721	0.08314	0.09116	0.00167	0.25064	0.32587	0.18788	-0.0802	0.01305	0.0005	0.09991	0.05852
NM_001079896	LOC298349	-0.0719	0.09588	0.06573	0.02551	0.16088	0.06158	-0.0014	-0.087	0.00786	0.16084	-0.1326	-0.0341	-0.0539	0.07555
NM_001025654	LOC298353	0.09697	0.09871	0.14493	0.16222	0.3318	0.12926	0.1297	0.1398	0.35778	0.35791	0.0775	0.06051	0.14784	0.19846

XM_233355.2	LOC298383	-0.1141	0.16457	0.05946	-0.0489	-0.1168	0.06962	-0.1292	0.16941	-0.0859	-0.0303	-0.1792	-0.0469	-0.106	-0.0222
XM_216496.3	LOC298409	-0.1268	-0.0458	-0.0993	-0.1575	-0.1204	-0.2041	-0.0444	-0.1486	-0.1386	0.05376	-0.1415	-0.0518	0.07531	0.04717
NM_001025656	LOC298442	-0.5599	-0.3252	0.21352	0.58718	-0.4636	-0.5293	-0.3083	-0.5088	-0.5726	-0.4146	-0.4823	0.00309	-0.37	-0.1257
XM_213045.2	LOC298495	0.3061	-0.066	0.1236	0.41734	0.52859	0.84246	-0.1808	0.31862	0.5617	0.11238	0.52789	0.63382	0.01274	-0.1105
XM_233477.3	LOC298496	0.09857	-0.0343	0.04305	0.84091	0.18242	0.06653	-0.0394	0.27274	-0.0127	-0.2089	-0.0107	0.0477	0.11885	0.18268
XM_216537.3	LOC298536	0.29422	0.31523	-0.0457	0.33629	0.589	-0.0275	-0.0959	0.50991	-0.1977	0.07739	0.0607	-0.2035	-0.0886	-0.0194
XM_233593.3	LOC298582	0.02629	0.06874	-0.0597	0.00168	-0.127	0.41345	-0.0913	-0.1253	-0.1238	0.23226	-0.1163	-0.078	-0.0063	0.05213
XM_238429.3	LOC298605	-0.199	-0.3801	-0.1333	-0.0853	-0.2957	-0.0832	-0.0742	-0.3039	0.21663	-0.0727	-0.2254	0.0997	-0.2277	-0.2988
XM_233643.2	LOC298627	-0.0733	0.00597	-0.2172	-0.0556	-0.0644	-0.0108	-0.041	-0.2333	-0.1973	-0.3147	-0.2542	-0.277	-0.1917	-0.1026
XM_238433.2	LOC298658	-0.0252	-0.1226	-0.002	-0.0413	-0.041	0.06581	-0.0726	0.03974	0.03767	0.02448	0.0799	0.18721	0.10703	0.07268
NM_001039012	LOC298675	0.01676	-0.0532	-0.155	-0.2362	-0.0875	-0.2509	-0.2463	-0.1924	-0.3339	-0.0681	-0.0442	-0.101	0.14837	0.18882
XM_233715.3	LOC298684	0.0612	0.05588	0.1555	0.26733	0.07644	0.11309	0.04044	0.10181	0.26093	0.2896	0.24259	0.06212	0.20514	0.27751
XM_233854.3	LOC298793	-0.1341	0.16896	-0.1347	0.13488	-0.2212	-0.0949	0.08687	0.1588	-0.2384	-0.127	-0.341	-0.3807	-0.046	-0.0625
NM_001013941	LOC298795	0.01568	0.0731	0.08944	-0.0201	0.14831	0.13618	0.17703	0.28848	-0.0749	-0.0266	-0.0779	0.24294	0.11472	-0.0579
XM_233901.2	LOC298820	-0.0009	0.04926	0.25018	0.01343	0.10019	0.02997	-0.049	0.17677	0.14852	0.09392	0.21087	0.01038	0.03273	0.0934
XM_216656.3	LOC298842	-0.1202	0.19431	-0.2972	-0.5297	0.09611	0.03888	-0.0585	0.23782	0.37571	0.30822	0.15627	0.18982	0.3834	0.35751
XM_216649.3	LOC298844	-0.2102	-0.1613	-0.0888	0.11313	0.0294	0.09453	-0.1628	-0.0214	0.07023	-0.1864	-0.02	-0.0728	0.00658	-0.1475
NM_001106711	LOC298850	-0.0466	-0.0263	-0.1542	-0.1359	-0.1934	0.11236	-0.0438	0.03128	-0.1959	-0.0648	-0.1286	-0.0436	-0.0163	-0.0192
XM_233972.3	LOC298871	0.02512	-0.0641	0.02122	0.30043	-0.106	0.10623	0.19577	-0.1249	-0.1178	0.09955	0.34038	-0.0704	0.14217	0.10435
XM_233981.1	LOC298903	-0.3287	0.03251	-0.0846	0.06332	-0.1647	-0.0999	-0.1957	-0.1535	-0.0303	0.34646	0.20781	0.19216	-0.1053	-0.0156
XM_233992.2	LOC298910	0.04441	-0.0355	-0.0468	-0.0808	-0.1086	0.09516	0.04524	-0.0589	0.07291	-0.0109	-0.0552	-0.0618	-0.0296	-0.1172
XM_234023.3	LOC298931	-0.1451	-0.0407	-0.0677	-0.0348	0.04764	-0.106	-0.036	-0.1188	-0.1702	-0.0119	0.08202	-0.1936	-0.1182	-0.1559
XM_213066.3	LOC298973	-0.0824	-0.2176	-0.0406	0.19042	-0.0967	0.01978	-0.0831	0.04858	-0.0114	0.0067	-0.2132	-0.0113	0.11785	-0.0869
XM_216701.4	LOC298977	-0.0089	0.18204	-0.1186	0.2909	0.16026	0.11364	0.09059	0.26215	0.06401	-0.0609	0.11868	-0.0209	-0.281	-0.0679
XM_234081.3	LOC298980	-0.0121	0.25997	0.23984	0.55979	-0.143	-0.1777	0.46845	0.0178	0.09105	0.23375	0.24038	-0.0669	-0.0522	0.2274
XM_238451.3	LOC298998	-0.0254	0.16017	0.10843	0.0276	0.06858	0.21159	0.11013	0.02995	0.10773	0.02003	0.00971	-0.0195	0.04591	0.10237
NM_001106726	LOC299002	-0.282	-0.5085	0.18969	-0.1624	-0.4109	-0.7907	-0.1289	-0.4097	-0.3134	-0.2302	-0.2874	-0.0416	0.49439	0.33972
XM_216715.3	LOC299105	-0.3688	-0.1975	-0.0523	-0.2077	-0.2482	-0.1004	-0.1228	-0.2939	-0.3066	-0.2681	-0.168	0.08724	-0.1874	-0.2183
XM_216730.3	LOC299123	-0.1487	0.0552	0.44239	0.42598	0.04108	-0.0004	0.1934	-0.1039	0.05672	-0.2204	-0.063	0.32284	0.21185	-0.0417
XM_234407.2	LOC299177	-0.1243	-0.0997	-0.1432	-0.386	-0.0431	-0.0464	-0.1556	-0.1546	-0.1415	0.0761	-0.0432	-0.0148	-0.1841	-0.1053
XM_216763.3	LOC299179	0.20589	0.05845	0.1105	0.22422	0.46639	0.73424	0.0862	0.23047	0.45819	0.06214	0.27364	0.61619	0.54963	0.69759
XM_238465.3	LOC299262	0.6426	0.3509	0.48618	1.0993	0.82861	0.88975	0.60566	0.64866	0.5297	0.43666	0.52883	0.47184	0.7735	0.65076
XM_216764.2	LOC299264	0.21469	0.11806	0.14677	0.64397	0.11735	0.09383	-0.0887	0.187	-0.0414	-0.0385	0.13967	0.17018	0.05034	0.13726
NM_181630	LOC299271	0.06108	0.12365	-0.072	0.05505	0.12269	0.1189	0.05214	-0.1189	-0.0342	-0.0164	0.10889	-0.1149	-0.0718	0.03682
XM_234496.3	LOC299279	-0.0172	-0.0256	-0.1091	0.0654	-0.1668	0.33068	-0.1026	-0.0356	-0.0705	-0.0582	0.02436	0.01925	-0.0051	-0.0831
NM_182474	LOC299282	-0.0284	0.0569	0.01167	0.1856	-0.0262	0.18149	0.00072	0.04824	0.14511	0.10198	-0.0436	0.05202	-0.0677	0.07124
XM_238470.2	LOC299297	-0.0993	-0.2307	-0.1355	-0.2063	-0.102	-0.1536	-0.165	-0.16	-0.0407	-0.1878	-0.0904	-0.1784	-0.118	-0.0918
NM_147177	LOC299306	0.05557	-0.2553	0.02274	-0.0062	-0.1561	-0.0227	-0.0819	-0.1391	-0.0746	-0.1949	-0.1372	-0.1081	-0.0356	0.1917
XM_238474.3	LOC299315	0.13489	-0.2191	0.29249	-0.7909	-0.3086	0.15533	-0.3067	-0.3142	0.01477	-0.0794	0.09358	0.77571	0.21149	0.35762
NM_001137633	LOC299339	-0.1147	0.21684	-0.4047	-0.0157	-0.1869	-0.2778	-0.0744	-0.2298	0.06767	0.24013	0.19131	-0.3731	-0.2569	-0.0462
NM_001106762	LOC299356	0.22388	-0.0711	-0.1877	-0.1297	-0.2116	-0.3232	0.20369	-0.1482	-0.0636	-0.3101	-0.05	0.15099	0.1788	0.20608
XM_234629.1	LOC299390	-0.0086	0.08351	-0.0372	-0.07	0.1045	0.00396	-0.0465	0.21192	-0.0329	0.01993	0.05359	-0.0342	0.06381	-0.1008
XM_234649.2	LOC299406	0.06223	-0.0947	-0.0876	-0.1629	-0.0688	-0.078	-0.1209	-0.0945	-0.0027	-0.0134	-0.0698	-0.1348	-0.0729	-0.1213
XM_234654.3	LOC299411	0.06665	0.04132	-0.0095	-0.0138	0.06398	-0.0738	-0.0744	-0.1063	0.08892	0.00546	-0.0439	0.10856	-0.0272	0.14009
XM_234680.2	LOC299434	-0.0135	-0.0221	-0.1305	0.00084	0.07682	-0.0643	-0.0741	0.02734	-0.0332	-0.1675	-0.0428	-0.1093	-0.0779	-0.1161
XM_234686.3	LOC299440	0.24371	0.07146	0.10004	0.47841	0.12224	0.35939	0.09814	0.09213	0.14483	0.08414	0.16925	0.29434	0.01374	0.24184
XM_234734.2	LOC299458	0.0673	0.06401	-0.1888	-0.1068	0.08169	-0.1748	-0.233	-0.1083	-0.2791	-0.0093	0.17648	0.27163	-0.2094	-0.083
XM_234734.1	LOC299458	0.11548	0.0695	0.22742	0.18933	0.11496	0.30923	0.28362	0.19517	0.12385	0.00484	0.1408	-0.0041	0.0313	0.10452
XM_234736.2	LOC299460	-0.1609	0.0433	0.01151	-0.04	0.04298	0.09566	-0.0129	0.03274	0.43709	-0.0014	0.1591	0.0632	0.14898	-0.0671

XM_234742.2	LOC299464	0.28935	0.13841	0.39186	0.00782	0.23911	0.16239	0.14452	0.30962	0.15859	-0.0463	0.16642	0.06802	0.10016	0.17991
XM_234744.3	LOC299466	-0.279	-0.2326	-0.2988	-0.2814	-0.1351	-0.2927	-0.2736	-0.3499	-0.2447	-0.3002	-0.2459	-0.2243	-0.2022	-0.0371
XM_234745.3	LOC299467	0.04584	0.17036	-0.0354	0.02051	0.00639	0.03988	-0.0326	0.29636	-0.0457	-0.0247	-0.0552	-0.0352	-0.077	0.14731
XM_234749.2	LOC299469	-0.0579	0.03641	-0.0534	0.08715	-0.0491	0.00921	0.05371	-0.0188	-0.128	-0.0216	-0.1818	-0.077	0.05976	0.00447
XM_234757.2	LOC299478	0.33687	0.10574	0.26065	0.18603	0.23868	0.30729	0.18544	0.31314	0.17177	0.03289	-0.0534	0.14113	-0.0147	0.0608
XM_234709.3	LOC299488	-0.3554	-0.235	-0.0226	-0.1583	-0.2365	-0.3098	-0.2519	-0.1926	-0.398	-0.1573	-0.3124	-0.0851	-0.1853	-0.2532
XM_234830.2	LOC299551	0.17439	0.11126	0.18178	-0.0011	0.00842	-0.0875	0.03827	-0.0045	-0.0347	0.08195	0.17408	-0.0187	0.20538	0.11444
XM_216840.2	LOC299598	0.07232	0.02611	0.11994	0.23594	-0.0288	0.22272	0.03734	0.08605	0.02413	0.20188	0.19221	0.06869	0.03756	0.07348
XM_216850.3	LOC299625	-0.7354	-0.9308	0.97264	-0.6082	-1.4149	-1.2475	-0.4457	-1.5822	-0.8422	-1.1461	-0.5644	-0.1396	-0.1271	0.25096
XM_234965.3	LOC299662	-0.0719	0.07039	-0.0862	-0.0885	-0.1527	-0.0598	-0.0705	0.04496	0.12282	-0.0229	-0.0286	-0.0198	0.0872	0.15283
NM_001099474	LOC299683	0.19798	0.03789	0.0657	0.38695	0.22486	0.00392	0.19621	0.21255	0.26667	0.15994	0.00081	0.19709	0.0635	0.19075
XM_234990.3	LOC299690	0.2657	0.15053	0.00414	0.13115	0.13668	0.11858	-0.1048	0.06495	0.19028	0.15337	0.24272	0.42424	0.14901	0.17255
XM_235015.3	LOC299701	-0.2491	-0.2107	-0.1005	-0.1065	-0.256	-0.1517	-0.1569	0.00329	-0.1147	-0.1637	-0.14	-0.2004	-0.1405	-0.0277
NM_001106780	LOC299737	0.07407	0.09263	0.06297	0.12104	0.06543	0.05224	0.058	-0.1746	-0.0615	0.06058	0.08114	-0.1253	0.18825	0.16835
XM_213087.3	LOC299740	-0.0107	-0.3687	0.13144	0.30784	0.24262	0.21442	-0.3259	-0.0351	0.01691	-0.2576	-0.0704	-0.1323	0.07892	0.00077
XM_238492.2	LOC299741	0.15071	-0.0211	0.06161	0.02309	0.05361	-0.0239	-0.0137	0.18746	-0.0459	-0.0035	0.11808	0.09906	-0.0204	-0.0069
NM_130755	LOC299750	0.38535	0.0614	-0.0763	0.03092	0.01014	0.38863	-0.1182	0.25616	0.1168	-0.2314	0.01806	0.13512	0.09509	0.13246
XM_235088.3	LOC299759	0.04958	-0.1011	0.00854	0.00347	-0.0576	0.23517	0.0442	-0.0812	-0.0125	0.31795	0.10327	-0.0066	0.45028	-0.0504
NM_001171177	LOC299762	0.02812	0.07934	-0.0069	-0.037	0.03027	0.0661	0.09742	0.20009	-0.0222	0.05581	0.04166	0.03852	0.1017	0.05813
XM_216900.3	LOC299821	0.0133	0.07654	0.12477	0.0384	0.17742	0.25608	0.07615	0.24196	0.10699	0.16092	0.07807	0.19522	0.03247	0.11784
NM_001106786	LOC299827	-0.2784	0.24553	0.06153	-0.0196	-0.3668	-0.5382	0.04517	-0.2784	0.06037	0.05371	0.00636	0.03276	0.0533	0.14336
NM_001134466	LOC299828	-0.2333	0.20548	-0.2497	1.3492	-0.0231	0.03501	0.19358	-0.0711	0.29693	0.07909	0.22145	0.17191	-0.6782	-0.526
XM_238499.2	LOC299898	0.79046	0.43357	0.41251	1.3346	0.9811	1.1596	0.47953	0.88177	0.56611	0.26912	0.60349	0.85496	0.51773	0.61945
NM_001130540	LOC299907	-0.2838	0.02222	0.22315	-0.1749	0.53766	0.49771	-0.3356	0.59109	0.2604	-0.0901	0.30432	0.4754	0.39997	0.56905
XM_235301.1	LOC299915	-0.2189	-0.0234	-0.0231	0.019	-0.1805	0.112	-0.0477	-0.198	-0.1494	-0.122	-0.0893	-0.031	-0.1751	-0.2617
XM_235288.3	LOC299919	-0.1118	-0.1727	-0.1611	-0.2793	-0.0343	0.08251	-0.038	0.26044	-0.0598	-0.1442	0.14733	-0.1637	-0.062	0.06778
NM_001025659	LOC299920	0.05984	0.06375	0.13446	0.15814	0.06302	0.09189	0.21054	0.01224	0.08827	0.02221	0.20152	0.07374	0.01657	0.23842
NM_001134842	LOC299949	-0.0116	-0.1919	0.56716	0.7364	0.01991	-0.1038	-0.2359	0.23066	-0.0744	-0.1757	-0.0014	0.15941	0.09363	0.04591
XM_213095.3	LOC299961	0.14024	0.1753	0.34534	-0.0827	0.11695	0.28973	0.01382	-0.0299	0.03043	0.04555	0.1433	-0.1119	0.14469	0.25447
XM_235352.3	LOC299964	-0.2011	-0.0511	-0.0141	-0.0491	-0.2034	-0.0035	0.03802	-0.1444	0.00145	-0.1301	-0.096	0.00231	-0.2046	-0.0068
XM_235358.2	LOC299968	0.08757	0.03072	0.00998	-0.0134	0.16719	0.04091	-0.0087	0.20929	0.02419	0.11411	0.09754	0.00157	0.07522	-0.0238
XM_238503.2	LOC299981	0.27609	-0.1075	0.0493	-0.036	0.13533	-0.0868	0.05459	-0.0267	0.02219	-0.1007	0.11344	-0.0501	-0.1436	-0.0107
XM_238510.2	LOC300013	0.12821	0.05803	-0.1171	-0.005	-0.0431	0.13503	0.24281	-0.0371	0.02232	0.02009	0.00902	0.13582	-0.0537	-0.0024
XM_216959.2	LOC300024	-0.1993	-0.0267	-0.1951	-0.1267	0.08253	-0.0968	-0.1535	0.15404	0.02456	-0.163	0.01417	-0.18	-0.102	-0.0376
XM_216959.2	LOC300024	0.20389	-0.1572	-0.1841	0.00715	0.6159	0.55491	0.05019	0.35687	-0.1304	-0.1353	0.11339	-0.1259	-0.3175	0.04923
XM_238508.2	LOC300041	0.22146	0.08949	0.27217	-0.116	-0.0387	0.15188	-0.0372	0.05689	-0.065	0.05552	0.10661	0.08221	0.14605	0.01749
XM_216963.3	LOC300043	0.06487	0.83586	0.23477	0.29261	0.52423	0.58624	0.73785	0.38766	0.47232	0.82905	0.64942	0.36586	-0.0993	-0.1508
XM_216945.3	LOC300049	-0.0651	0.30883	0.05752	0.16158	0.07641	-0.2496	0.10407	0.1378	0.0718	-0.095	-0.0693	-0.1759	0.07758	0.13271
XM_235514.2	LOC300097	0.09668	-0.0743	-0.1126	0.00854	0.00666	-0.0479	-0.1469	-0.0595	0.00099	-0.0477	0.14741	0.06071	0.00078	-0.0496
NM_001135922	LOC300105	-0.3186	-0.227	-0.2466	-0.1543	-0.1663	-0.2648	-0.2285	-0.5112	-0.3727	-0.3935	-0.382	-0.2547	-0.4032	-0.6068
NM_001130497	LOC300108	-0.1265	0.11628	-0.0006	0.10462	-0.0501	-0.1748	0.16426	-0.0742	0.00269	0.19868	-0.0634	-0.1822	0.042	-0.1073
XM_235542.3	LOC300114	0.01847	-0.0442	0.1149	0.10281	0.1639	0.11282	0.13227	0.26	-0.0459	-0.0766	0.0484	-0.0424	0.06483	0.04642
NM_001130680	LOC300117	-0.0186	0.07259	-0.1082	-0.1742	0.20681	-0.2217	-0.214	-0.1002	-0.0955	-0.089	-0.15	-0.12	-0.0735	-0.1395
NM_001100887	LOC300173	0.07881	0.10791	0.4906	0.05353	0.15421	-0.0327	0.41274	0.14147	0.01783	0.08636	0.01109	0.14686	0.10079	0.0978
NM_001127456	LOC300191	0.76395	0.66659	0.21503	-0.3294	0.13764	-0.3254	0.03712	0.36436	0.69811	0.2703	0.42908	-0.4671	0.32002	0.46981
XM_217032.1	LOC300201	-0.0381	-0.0334	-0.0233	-0.12	-0.0436	0.05009	0.02857	-0.0191	-0.0227	-0.0058	0.03992	0.06564	-0.1021	-0.0272
XM_235733.3	LOC300289	-0.2057	0.15327	0.35362	0.08209	-0.1692	-0.1745	0.0869	0.27328	0.02328	-0.1017	0.21005	0.10591	-0.0982	-0.0915
NM_012992	LOC300303	-0.2135	-0.0336	-0.2336	-0.1839	-0.1328	-0.0602	-0.0792	-0.0884	-0.2397	-0.1101	-0.1911	-0.1328	-0.1913	-0.0896
XM_235761.1	LOC300308	0.05265	0.09968	0.03622	0.02365	0.05676	0.05436	0.0965	0.04372	0.07414	-0.2089	3E-05	0.06203	-0.0032	-0.0383

XM_238532.3	LOC300314	-0.1557	-0.2337	-0.3092	0.07479	-0.122	-0.2304	-0.2717	-0.2592	-0.2762	-0.265	-0.3092	-0.2124	-0.1438	-0.1448
XM_235797.1	LOC300342	0.09668	0.02184	0.09552	-0.0434	0.08404	-0.0021	0.02269	0.05319	0.02178	0.19879	0.04672	0.05954	-0.0515	-0.0214
XM_235808.3	LOC300350	-0.8212	-0.7309	-0.678	-0.4832	-0.7002	-0.645	-0.8341	-0.7627	-0.5499	-0.7845	-0.3942	-0.5093	-0.7352	-0.7895
XM_217084.1	LOC300351	-0.0251	-0.1158	-0.0139	0.18528	-0.034	0.12956	0.03468	0.02082	-0.1037	-0.1201	0.11758	-0.0706	-0.0488	-0.0395
XM_235850.3	LOC300373	0.03354	0.10856	0.11716	-0.0654	-0.1581	-0.0342	0.18123	0.03876	0.02227	-0.0067	0.06246	-0.1222	0.07886	-0.0707
XM_238535.3	LOC300439	-0.128	-0.0844	-0.012	-0.0757	-0.1739	-0.0108	-0.0262	0.09857	-0.0849	0.02762	-0.2071	0.16137	-0.0565	-0.0742
XM_235972.3	LOC300461	0.1235	0.33663	0.17491	0.13914	0.18662	0.07415	0.23742	0.23032	0.34397	0.41687	0.10346	0.29426	0.14022	0.15847
NM_001106809	LOC300472	0.61349	0.43772	0.31708	0.41035	1.0384	0.73693	-0.1886	1.0248	0.25342	0.73494	0.26388	0.36585	0.68747	0.57764
XM_235992.3	LOC300490	-0.1636	-0.1543	-0.1703	-0.1419	-0.2324	-0.0488	-0.3297	-0.095	-0.2061	-0.3398	-0.2845	-0.1098	-0.0143	-0.3417
NM_001127528	LOC300682	-0.0454	0.00333	-0.0505	0.02096	-0.0327	0.03958	0.02783	0.32556	0.00983	-0.0607	-0.0069	-0.0478	-0.0783	-0.0408
XM_236241.1	LOC300694	0.02119	-0.1499	-0.0213	-0.0381	-0.1661	0.0273	0.15983	-0.0884	-0.2179	-0.1064	0.01003	-0.0966	-0.212	0.0581
XM_213128.2	LOC300708	-0.1132	-0.1233	-0.0085	-0.0345	-0.0854	-0.0991	-0.0544	-0.074	-0.0218	-0.0123	-0.0076	0.09608	-0.0764	0.10046
NM_053330	LOC300731	-0.4635	-0.2081	-0.2665	-0.304	-0.3746	-0.1107	-0.3218	-0.0333	-0.0075	-0.147	-0.1612	-0.0012	-0.1715	-0.5314
XM_236289.3	LOC300742	-0.0352	-0.0847	-0.1352	-0.1854	-0.2606	-0.0701	-0.1994	0.00625	-0.0579	-0.091	0.15554	0.21892	-0.1593	0.01174
XM_236308.3	LOC300760	-0.5024	-0.61	-0.7273	-0.7168	-0.7762	-0.4987	-0.4907	-0.6904	-0.0017	-0.2997	-0.351	0.19186	-0.5375	-0.5018
XM_217183.3	LOC300768	-0.3133	-0.0452	-0.2181	-0.0479	0.38164	-0.2374	0.01326	0.04363	0.02239	0.01101	0.06597	-0.234	-0.0776	0.13152
XM_236355.3	LOC300793	0.15722	0.16641	0.14967	0.06518	0.39591	0.22821	-0.1121	0.36948	0.06968	0.17171	0.25991	0.2978	0.07092	-0.0161
XM_236440.3	LOC300864	0.09999	-0.0098	-0.0754	-0.0617	-0.0244	-0.093	-0.0524	0.0016	-0.1117	-0.0149	-0.0734	0.02859	-0.0474	-0.1351
XM_217220.3	LOC300874	-0.0177	0.02764	-0.1232	-0.0469	0.0063	-0.0318	-0.0926	-0.0793	-0.0262	0.15714	0.16587	0.10539	-0.0416	0.01259
NM_001037772	LOC300963	-0.4066	-0.5106	-0.1968	-0.4012	-0.4966	-0.4762	-0.5284	-0.5521	-0.7067	-0.4836	-0.6598	-0.3114	-0.561	-0.3476
NM_001134532	LOC301004	-0.0349	0.02287	-0.1158	0.40189	0.20926	-0.0179	-0.0553	-0.0623	0.06602	-0.0284	0.05151	0.25096	-0.0357	0.02014
XM_238561.3	LOC301044	-0.1758	-0.0936	-0.1583	-0.3915	-0.1101	-0.0927	-0.1921	0.08398	-0.0255	-0.1288	-0.1555	0.0679	-0.2196	-0.2933
NM_001030032	LOC301067	-0.7101	-0.6798	-0.3523	-0.766	-0.9675	-0.9634	-0.5596	-0.8559	-0.756	-0.7363	-0.7041	-0.5276	-0.4689	-0.4553
XM_217306.3	LOC301105	-0.34	-0.5006	-0.1878	-0.0371	-0.321	-0.5399	-0.0351	-0.2231	-0.595	-0.2574	-0.2545	-0.3857	-0.3518	-0.2125
NM_001106873	LOC301113	-0.043	-0.0626	-0.0149	0.00565	-0.0417	0.00173	-0.0692	-0.0668	0.07646	-0.0922	0.02573	-0.1202	0.07349	-0.0369
NM_001106874	LOC301114	-0.1194	-0.155	-0.0445	0.12129	0.01043	0.12961	-0.2298	-0.0885	-0.1996	0.11371	0.16067	-0.0094	-0.0462	-0.1599
XM_001055676.1	LOC301117	0.27642	0.09451	0.02889	0.5837	0.53637	1.0763	0.01436	0.48674	0.10215	0.01357	0.04971	0.18203	0.44842	0.44638
NM_001106875	LOC301118	0.04412	0.06682	0.23475	0.17246	0.16209	0.02236	0.2729	0.13501	0.01137	0.08142	0.05624	0.15212	0.10421	0.12098
NM_001106876	LOC301119	-0.5326	-0.784	-0.0857	-0.8266	-0.3795	-0.6701	-0.4955	-0.4517	-0.4268	-0.7394	-0.5268	-0.2201	-0.1491	-0.3114
NM_001080096	LOC301120	0.20718	0.09563	0.31117	0.13914	0.03495	0.15377	0.03955	0.10353	0.21028	0.25057	0.24431	0.38606	0.3181	0.39431
NM_001106877	LOC301121	0.03124	0.12878	0.37071	-0.033	0.07596	0.0454	0.11701	0.1621	-0.0118	0.09015	0.03919	0.02837	0.27486	0.25773
NM_001134533	LOC301124	0.46897	0.31114	0.26704	-0.1562	0.37871	0.80809	0.25113	0.32475	0.44139	0.41621	0.42646	0.62974	0.74889	0.69359
XR_007870.1	LOC301126	-1.0117	-0.4136	0.52951	0.17898	-1.1648	-1.191	0.20717	-1.1593	-0.4075	-0.4217	-0.2374	-0.1455	-0.651	-0.4954
XM_001061312.1	LOC301130	-0.3638	0.06056	0.34439	0.77315	-0.2425	-0.486	0.35579	0.03206	-0.0182	-0.2113	-0.0893	-0.471	-0.0883	-0.0005
NM_001106878	LOC301132	0.00477	0.00696	0.04386	-0.1115	0.0655	-0.068	0.22039	0.11383	0.01907	-0.2244	0.0947	0.045	0.30302	0.12682
XM_001059278.1	LOC301133	-0.0795	-0.0728	-0.0981	-0.0825	-0.1713	0.04967	-0.0934	-0.0807	-0.1237	-0.043	-0.1092	-0.0677	-0.0265	-0.153
NM_001099505	LOC301154	-0.038	-0.0785	0.0734	-0.0987	0.00227	-0.0472	0.05355	-0.1003	-0.0785	-0.0442	-0.0547	-0.086	0.28809	-0.0489
NM_001025662	LOC301165	0.00183	0.089	0.21988	0.35438	0.26704	0.12006	0.09977	0.19096	0.02324	0.0596	0.05432	0.05346	0.14337	-0.0113
XM_236868.3	LOC301188	0.30928	0.14153	-0.0827	-0.0684	-0.0605	-0.1068	-0.059	-0.0704	0.17219	0.02981	0.15564	0.10408	-0.0153	0.04079
XM_236873.3	LOC301193	-0.1527	-0.0386	-0.1127	-0.1629	-0.1355	-0.1248	-0.0612	-0.1856	0.10922	-0.1089	-0.0555	0.1321	0.23362	-0.1901
XM_217332.3	LOC301215	0.09664	-0.0213	-0.0119	0.02665	0.03647	-0.0076	-0.0483	0.01184	0.05041	0.03048	-0.0324	-0.0066	0.00099	0.10185
XM_238578.1	LOC301267	0.02949	0.02092	-0.0239	-0.0349	-0.0153	0.19157	-0.013	-0.0285	-0.0262	-0.0437	-0.0601	0.01869	0.12251	0.07467
XM_236967.3	LOC301278	-0.0901	-0.0529	0.0005	-0.066	0.05917	-0.0484	0.00303	-0.1313	-0.0601	-0.0175	-0.0875	-0.0848	-0.0623	-0.0522
XM_236973.2	LOC301282	0.08039	-0.0638	0.04144	0.21847	-0.0208	0.10179	0.10567	0.06165	0.03303	0.08746	0.027	0.06901	0.06576	-0.0437
NM_001122947	LOC301295	0.08587	0.17711	0.07129	0.51882	-0.0692	0.05624	0.25056	0.40807	-0.0359	0.27427	0.2646	0.06134	0.23523	0.18182
XM_237049.3	LOC301334	-0.0638	0.05872	-0.0009	-0.1039	-0.0378	-0.0601	0.00304	-0.0012	-0.0562	0.07188	0.08078	-0.0164	-0.0705	-0.1254
NM_001007698	LOC301376	0.08888	0.05974	0.11604	0.06754	0.14189	0.02042	0.02892	0.12512	0.03471	0.10001	-0.0577	0.27235	0.08246	0.30001
NM_031853	LOC301444	-0.5157	-0.5656	0.14451	-0.507	-0.9425	-0.601	-0.4193	-1.0459	-0.0387	-0.1284	-0.2943	0.85235	-0.0174	-0.1361
XM_217414.3	LOC301445	-0.1399	0.15695	-0.0748	0.08631	-0.0199	0.11085	-0.0008	0.11019	0.05331	-0.0228	-0.0189	0.01053	-0.0429	0.31229

XM_217410.2	LOC301448	-0.3043	-0.1209	-0.3288	-0.6976	-0.6914	-0.9551	-0.3185	-0.6957	-0.1722	-0.2804	-0.2004	-0.2948	-0.4217	-0.2809
XM_237215.4	LOC301455	0.1516	-0.0129	-0.0883	-0.1309	0.02567	-0.0104	-0.2082	0.03404	0.04596	-0.1316	0.11506	0.04934	0.06201	-0.0462
XM_217430.3	LOC301497	0.06631	-0.0066	0.20623	-0.0449	0.03902	0.0465	0.17311	0.05396	0.029	0.08881	-0.0167	0.08305	0.04242	-0.0149
NM_001106918	LOC301506	0.73866	1.0309	0.28124	0.13776	1.0059	1.2106	0.88283	0.99841	0.94346	0.95356	1.0404	0.51856	0.19907	0.08636
XM_237286.4	LOC301509	0.15526	0.4675	0.36044	-0.1504	0.06817	0.27763	0.39866	0.2851	0.21779	0.45489	0.36709	0.09308	-0.1063	-0.1637
XM_217457.3	LOC301556	0.0164	0.00616	0.02986	0.06923	0.12462	-0.076	-0.0134	0.12938	0.02343	-0.0103	-0.0056	0.01846	0.06516	0.05144
NM_001047873	LOC301650	-0.0166	0.18633	-0.0209	0.16324	0.13626	0.27037	0.07719	-0.0034	0.10402	0.21809	0.43181	0.1852	0.10565	0.2271
XM_213162.3	LOC301658	0.0449	-0.0462	-0.0442	-0.1624	-0.0154	-0.1262	-0.0911	-0.1433	-0.1856	-0.111	-0.0487	-0.0804	-0.1573	-0.0946
NM_212511	LOC301725	0.15561	0.17052	0.10455	0.0929	-0.032	0.47015	0.03816	0.00425	0.02561	0.46824	0.25532	-0.1381	0.15903	0.04988
XM_229238.3	LOC301772	-0.3082	-0.0343	-0.2134	-0.0209	-0.2425	-0.1818	-0.1058	-0.008	0.00526	-0.0467	-0.1876	-0.2161	0.00831	0.08703
XM_229255.3	LOC301786	0.1299	0.15533	-0.0148	-0.0034	0.04142	0.13676	0.02789	-0.0019	0.30758	0.0149	-0.0305	0.09119	0.08741	0.0428
XM_229279.1	LOC301803	-0.0188	0.34284	0.45389	0.38267	0.02971	0.24738	0.59801	0.06654	0.06873	0.13646	0.15467	0.05024	0.16881	-0.0466
XM_229292.4	LOC301816	0.06176	0.08001	0.12285	0.2181	0.14431	0.17905	0.1916	0.24448	0.01828	0.10697	0.20455	0.06371	0.34652	0.07935
XM_229303.3	LOC301824	-0.0156	0.162	0.18257	0.16133	0.00718	-0.0035	0.07952	-0.0087	-0.0312	0.11469	0.08445	0.33446	-0.0056	0.11552
XM_229322.3	LOC301843	-0.2277	1.0268	1.5892	1.0309	-0.2892	-0.2112	2.0839	-0.3261	0.73727	1.108	1.0406	0.72704	0.85645	0.72358
XM_229338.3	LOC301858	-0.0666	-0.145	0.02991	0.09443	0.00415	0.05424	0.12861	0.08414	0.06913	-0.1569	-0.1008	0.05312	0.0032	0.01309
XM_229360.2	LOC301878	0.02346	-0.1484	0.11392	0.0104	0.11288	0.3307	0.03864	0.07138	0.07917	0.13588	-0.043	0.13576	0.37172	-0.0139
XR_009224.1	LOC301879	-0.0061	0.06454	0.06996	0.16036	-0.0077	0.06313	-0.0011	-0.0268	-0.0238	0.13605	0.01295	-0.025	0.41027	0.04939
XM_229363.3	LOC301881	-0.0261	0.00723	0.03139	0.01301	0.06271	0.1187	-0.0261	-0.0141	0.09238	-0.0025	-0.0283	-0.0662	0.05745	-0.026
XM_229369.3	LOC301887	0.09915	-0.0077	-0.0075	0.00855	0.09509	0.1476	0.10781	0.03369	0.07727	0.10993	0.11741	0.21421	0.07417	0.02953
XM_229375.3	LOC301893	-0.103	-0.0726	-0.0984	-0.0541	0.01324	-0.1362	-0.0467	-0.0398	-0.0661	0.08089	-0.0715	-0.0872	-0.0999	0.19994
XM_229383.3	LOC301899	-0.1433	-0.0457	-0.0566	0.02408	0.12299	-0.1111	-0.2226	0.37547	-0.1568	0.04491	-0.1602	0.15732	0.09913	0.02464
XM_217507.3	LOC301906	0.19149	0.01139	0.20959	0.00971	0.06706	0.11748	0.19582	0.23176	0.00726	-0.0303	0.07518	0.15312	0.10343	0.22378
XM_229422.3	LOC301926	0.03476	-0.0008	-0.0077	0.13881	0.1944	0.2737	-0.0966	0.12529	0.18597	0.02731	-0.0151	0.10219	0.10958	0.06081
XM_229431.3	LOC301934	0.10255	0.43741	0.58763	0.316	-0.0411	0.1387	0.70621	-0.0334	0.35152	0.37397	0.49878	0.17254	0.35224	0.3153
NM_001106930	LOC301957	-0.1881	-0.0768	-0.1014	0.03395	0.10012	0.00769	0.02273	-0.0324	0.01223	-0.1128	0.11937	-0.0619	-0.0843	0.07698
XM_217508.4	LOC301959	0.07386	0.08683	0.12682	0.08909	0.3047	0.15622	0.01734	0.05894	0.05239	0.11256	0.02381	0.01032	0.03724	0.15236
XM_229476.3	LOC301966	0.08743	0.13482	0.37393	0.31481	0.07095	0.24845	0.76168	0.09677	0.06157	0.26409	0.30867	0.13972	0.14354	0.30832
NM_001014221	LOC301977	0.34805	0.78881	0.21651	0.44099	0.51828	0.67077	0.46315	0.20741	0.45187	0.63948	0.86825	0.696	0.08264	0.21185
XM_217512.3	LOC301978	-0.0247	-0.1225	-0.0336	-0.1504	0.136	-0.1337	-0.1793	0.04643	-0.0777	0.19987	-0.1816	-0.0183	-0.0222	-0.1525
XM_217515.3	LOC301986	-0.0107	-0.1127	-0.0868	-0.1047	-0.0121	0.02252	-0.0143	0.0906	-0.0804	0.13979	-0.0925	-0.0415	-0.0642	-0.0073
NM_001011701	LOC302000	0.09532	-0.005	-0.1733	-0.1333	-0.1052	-0.0051	-0.1574	-0.0346	0.08461	0.0427	-0.0373	0.23752	-0.1219	-0.1086
XM_229530.2	LOC302005	0.08264	0.01835	0.39996	0.28524	-0.0806	0.04518	0.6675	-0.0225	0.10102	0.00088	0.21902	0.10027	0.13494	0.18975
NM_001127530	LOC302022	-0.0808	-0.0882	-0.2099	-0.0516	-0.106	-0.2674	-0.0118	-0.1431	-0.2073	-0.1382	-0.0634	-0.2061	-0.2941	-0.1935
XM_217530.3	LOC302085	0.37363	0.21042	0.60427	0.33795	0.401	0.17985	0.70093	0.25776	0.46521	0.07646	0.11891	0.11984	0.21496	0.19609
XM_229665.3	LOC302091	0.26435	-0.079	0.01876	-0.0138	-0.0852	0.16935	-0.0441	-0.0493	-0.0887	0.0367	-0.1291	-0.0041	-0.0538	0.0475
XM_217531.3	LOC302093	-0.0252	0.00608	-0.0042	-0.0019	-0.0425	0.10821	-0.0274	0.0703	-0.0478	-0.0165	0.02	-0.0169	-0.0349	-0.004
XM_229683.2	LOC302112	-0.4589	-0.4737	-0.423	-0.4431	-0.4068	-0.4012	-0.3129	-0.3383	-0.3841	-0.5463	-0.4293	-0.5652	-0.2434	-0.3933
XM_229684.3	LOC302113	0.05572	0.08044	0.091	0.06231	0.17097	0.14029	-0.0758	-0.2009	0.15839	0.16996	0.25308	-0.1882	0.03303	0.07501
XM_229690.2	LOC302118	0.02739	0.10318	0.03291	0.07266	0.17487	0.06022	-0.0578	0.01517	0.20465	0.11276	0.23089	0.09944	0.12735	0.18523
XM_229695.2	LOC302122	-0.4509	-0.5587	-0.4142	-0.5985	-0.4163	-0.5237	-0.4203	-0.5031	-0.4133	-0.6531	-0.3846	-0.4897	-0.4202	-0.4352
XM_229717.3	LOC302140	0.40267	0.16382	0.36372	0.0945	0.06487	0.10955	0.54285	0.1572	0.25036	0.24356	0.24502	0.08887	0.11142	0.33358
XM_229736.3	LOC302157	-0.0359	-0.1225	-0.0672	-0.0499	0.02262	-0.0491	0.01762	-0.1539	-0.0298	-0.0256	-0.0754	-0.0662	-0.0761	-0.0748
NM_001025755	LOC302192	0.07019	0.0323	0.175	-0.0521	0.09975	-0.1113	-0.1331	0.08297	-0.085	-0.1566	-0.016	-0.2029	-0.14	-0.0762
NM_001014221	LOC302199	1.0885	0.95917	0.62384	0.85371	0.67876	0.78032	0.92656	0.57517	1.0261	0.9686	1.2245	0.864	0.37948	0.43381
XM_217546.3	LOC302210	0.00585	-0.2402	-0.1797	-0.2312	-0.1111	-0.3463	-0.3013	-0.2823	0.15346	-0.1461	-0.2804	0.11936	-0.1832	-0.1513
XM_229842.3	LOC302228	-0.1026	0.01308	0.04973	0.00926	0.24102	-0.0392	0.01166	0.08796	0.10826	0.01109	0.10184	-0.0257	0.11578	0.0601
XM_217554.3	LOC302264	0.13773	0.10137	-0.0271	0.10096	0.32046	0.07913	-0.1101	-0.0413	0.14691	0.15745	-0.0329	0.21992	-0.0108	0.17505
XM_229904.3	LOC302280	0.12433	0.0933	0.05884	0.08992	0.20057	0.26432	0.18035	0.14672	0.01512	0.1422	0.23754	0.12197	0.05529	0.18171

XM_229908.3	LOC302282	-0.0761	0.33097	-0.1584	-0.1417	-0.1026	-0.2092	-0.0353	-0.0893	-0.2223	-0.2086	-0.1676	0.01317	0.0369	-0.0161
XM_228410.3	LOC302297	0.03998	0.10532	0.19023	0.14642	0.05314	0.10478	0.07842	0.0963	0.22858	0.20922	0.15071	0.16653	0.16214	0.29497
XM_228445.3	LOC302324	-0.187	-0.1209	-0.1112	-0.2546	-0.2074	-0.0403	-0.1728	-0.0771	-0.2193	0.04776	-0.1814	0.01924	-0.1444	-0.1474
XM_228451.3	LOC302327	0.04588	0.09472	-0.0687	0.09775	0.05779	-0.0112	-0.0238	-0.017	0.11229	0.11499	-0.0567	0.05706	-0.0084	0.11622
NM_001106934	LOC302328	-0.2341	-0.239	-0.0495	-0.2259	-0.2248	-0.1873	-0.232	-0.2187	0.03545	-0.2118	-0.0657	-0.04	-0.249	-0.2415
XM_228455.2	LOC302331	-0.1653	0.02039	-0.1619	-0.0697	-0.107	-0.1518	-0.0073	-0.1884	0.04504	-0.0948	-0.0352	-0.1549	-0.0988	-0.1139
XM_228458.3	LOC302333	-0.0458	0.11857	0.10174	0.14548	-0.0635	0.0638	-0.0437	-0.0766	0.11757	-0.1013	0.01557	0.26804	0.03029	-0.0313
XM_228468.3	LOC302340	-0.2028	0.02149	0.00383	-0.1789	-0.2023	0.08495	0.00229	-0.2449	-0.0559	-0.0214	-0.0079	-0.2304	0.06389	0.18014
NM_001106937	LOC302369	-0.0467	0.126	0.0015	0.05847	-0.0545	-0.0603	0.14786	-0.1126	-0.0867	0.09446	-0.1053	0.05321	-0.0506	0.03134
NM_001106940	LOC302378	0.00026	0.21516	0.04818	0.08899	0.04025	0.14207	0.09773	0.12282	0.08188	0.0374	0.06941	0.00449	0.17345	0.32776
XM_217568.3	LOC302396	0.33767	0.00678	-0.0302	-0.0565	0.16842	0.02659	-0.0563	0.29214	0.07755	0.08793	0.11255	-0.0312	-0.0787	0.01229
XM_228571.3	LOC302402	1.2841	1.0065	0.71727	0.17445	0.99135	1.35	0.04228	0.84925	0.57342	0.77523	0.97127	0.16167	0.45741	0.79047
XM_228576.2	LOC302405	0.05486	0.13657	-0.0409	0.11566	0.18487	0.2266	0.03649	0.15447	-0.0008	0.02921	-0.0367	0.18006	0.04816	-0.0087
XM_228548.3	LOC302412	0.00363	-0.16	0.00449	-0.1253	-0.1242	0.11862	-0.1888	-0.0849	-0.148	0.06047	-0.1879	-0.0836	-0.0462	-0.041
NM_001106943	LOC302415	-0.2247	-0.225	-0.0501	-0.1102	-0.3922	-0.2176	-0.1731	-0.252	-0.126	-0.1134	-0.2252	-0.0292	-0.3061	-0.1374
XM_228600.3	LOC302436	-0.0012	-0.0133	-0.0561	-0.0908	0.00516	-0.0039	-0.0834	-0.0535	-0.1247	-0.0029	0.10854	-0.0249	-0.101	-0.0152
XM_228611.3	LOC302447	0.08243	-0.0577	0.02179	-0.0562	0.06427	-0.2017	-0.1795	-0.0677	-0.0444	-0.0641	-0.1138	0.10951	-0.1612	-0.0393
XM_217578.2	LOC302457	-0.1331	-0.075	-0.1573	0.01135	-0.0425	-0.0199	0.02254	0.14934	0.01722	-0.0464	-0.0491	-0.102	-0.3104	0.02112
NM_001106949	LOC302492	-0.001	-0.0673	0.00282	-0.0775	0.01347	-0.0517	-0.0767	0.11295	0.10276	-0.0737	-0.0616	0.01444	0.10204	0.10783
NM_031065	LOC302497	-0.5914	-0.6658	-0.6794	-1.1825	-0.6864	-0.1828	-1.3924	-0.7382	-0.4996	-0.6536	-0.4226	-0.1651	-0.5961	-0.3216
NM_001106952	LOC302507	0.11778	-0.0818	0.04929	-0.0722	-0.1583	0.09697	-0.0925	-0.1735	-0.2242	-0.1634	-0.1677	-0.0557	-0.0751	-0.1897
XM_228795.3	LOC302573	0.27929	0.15658	0.04609	-0.0035	-0.0337	0.00578	-0.0203	0.02094	-0.0891	0.22363	0.11256	-0.0046	-0.0628	-0.0134
XM_228805.3	LOC302582	0.11037	0.15869	0.14953	0.04768	-0.1204	-0.0001	0.13471	0.01021	0.04822	0.02436	0.32949	0.32092	0.21287	0.23519
XM_217607.3	LOC302612	-0.3466	-0.1329	0.09721	0.17396	-0.2821	-0.3148	0.29943	-0.3218	-0.3986	-0.2509	-0.0162	-0.3769	-0.3359	-0.2841
NM_001013960	LOC302640	0.26461	0.09268	-0.0425	0.1252	0.57351	0.5458	0.23881	0.87793	0.20037	0.18206	0.20851	0.33842	0.05699	0.01566
XM_228895.2	LOC302645	-0.0025	-0.01	0.1649	0.15156	0.00071	0.31831	0.00738	-0.01	0.37999	0.11352	0.01003	0.32038	0.23468	0.24136
XM_217612.3	LOC302670	-0.2046	-0.0398	-0.049	-0.1468	0.01002	0.06865	-0.1232	0.06725	-0.1532	-0.2152	-0.0541	-0.1369	-0.1421	-0.0664
NM_001037187	LOC302680	-0.0218	-0.1519	-0.0858	-0.0592	-0.1541	-0.0961	-0.2359	-0.1251	-0.1095	0.0816	-0.0709	-0.1925	0.01957	-0.1199
XM_228943.2	LOC302687	-0.1782	0.02287	-0.0811	-0.115	-0.1428	-0.1142	-0.0526	-0.0585	-0.1196	0.1663	-0.1389	-0.1849	-0.039	0.02347
XM_241848.3	LOC302692	0.1439	0.09205	0.04497	0.00385	0.11037	0.12136	-0.1052	0.0497	-0.0465	0.04341	0.07947	-0.0376	0.20871	-0.1606
NM_001106963	LOC302697	-0.0753	-0.1057	0.07301	-0.0684	0.0072	-0.0541	-0.076	0.11253	0.10186	-0.0276	0.06751	0.08535	-0.011	0.08899
XM_228993.1	LOC302718	-0.0474	-0.1592	-0.0604	-0.1099	-0.0415	-0.0344	-0.1147	-0.1374	-0.0429	0.01668	0.0881	0.02051	-0.0491	-0.1531
XM_229001.1	LOC302724	0.09964	0.08368	0.05475	0.02466	0.01298	0.0607	0.06479	0.05033	0.04539	0.16164	-0.0309	0.26827	-0.03	0.04638
XM_229054.3	LOC302756	0.01884	0.10145	0.07318	-0.0934	-0.0908	0.0955	0.09348	0.02564	-0.0775	-0.0757	0.02861	0.0465	0.06348	0.1329
NM_001110366	LOC302768	-0.0567	0.09367	-0.0003	-0.0977	-0.0179	0.04441	-0.0092	0.32907	0.22267	-0.0671	-0.045	0.07382	0.11426	-0.064
NM_022229	LOC302797	0.05855	0.133	0.04775	0.08235	-0.015	-0.0022	0.21483	0.06057	0.00457	-0.0087	0.11357	0.04745	0.18276	0.16806
XM_238631.2	LOC302832	0.0927	-0.0359	0.21573	0.0624	0.36605	0.12192	0.25295	0.2851	0.12611	0.02389	0.24573	0.04633	0.04927	0.26975
NM_001013962	LOC302845	1.3872	1.2275	1.044	1.3958	0.96226	0.98331	1.0695	0.94937	1.0441	1.2474	1.2352	1.1748	1.5071	1.4889
NM_001127532	LOC302899	0.0403	-0.0864	-0.1804	-0.2196	-0.2257	-0.2897	-0.1857	-0.092	-0.187	0.14966	0.01953	-0.1762	-0.1813	-0.0484
NM_001106972	LOC302913	-0.1792	-0.0018	-0.2922	0.15614	-0.1197	-0.1892	0.1857	-0.0842	-0.1527	0.20985	0.06529	-0.2594	-0.1349	-0.2825
XM_220196.3	LOC302961	0.02259	0.10024	0.15128	0.16314	0.01224	-0.0446	0.05931	0.20653	-0.0095	0.08128	0.09668	-0.0186	0.04633	-0.0259
XM_220252.3	LOC302996	0.40107	0.13975	-0.0065	0.12998	0.34721	-0.0326	0.02539	0.31619	-0.0287	0.14194	0.13706	0.47681	-0.1136	-0.0971
XM_220257.2	LOC302999	0.00039	-0.0357	-0.1179	-0.1447	-0.1354	0.30779	-0.0878	-0.1668	-0.0867	-0.1587	-0.2262	-0.216	-0.2075	-0.1175
XM_220273.3	LOC303016	0.28944	0.47907	-0.0498	0.06067	0.34501	0.15856	0.16829	0.28473	0.25842	0.26547	0.10546	0.00247	0.15948	0.31846
NM_001142652	LOC303019	-0.0076	0.07188	-0.0567	0.12602	0.25457	0.05741	0.05556	-0.0315	0.23599	0.11493	0.15691	0.00386	0.18209	-0.0144
NM_001100550	LOC303057	0.38709	0.26795	0.16537	0.57336	0.33375	0.11323	0.56944	0.42655	0.22441	0.24506	0.3733	-0.3164	-0.0717	0.10277
NM_001037773	LOC303059	0.14786	-0.0367	0.06319	-0.0674	-0.0252	-0.0293	0.0208	0.05064	-0.0428	0.29195	0.03343	-0.0353	0.14482	-0.0749
XM_220427.4	LOC303140	0.14221	0.03822	0.03534	0.18163	0.15419	0.12037	0.07535	0.25904	0.09082	0.28316	0.09194	0.16828	0.07104	0.1236
XM_220442.3	LOC303155	0.24155	0.2353	0.06706	0.11137	0.10151	0.02849	0.00627	0.19368	0.0559	0.26534	0.02741	0.17044	0.18277	0.05168

NM_001013434	LOC303202	0.06042	0.03537	0.0103	0.01234	0.13555	0.05579	-0.0918	0.07525	0.05455	0.20046	0.22962	-0.0221	0.23242	0.109
XM_001077639.1	LOC303215	-0.0951	-0.0935	0.08803	0.1771	-0.1541	-0.1021	0.19126	-0.1148	-0.0644	0.05519	-0.1372	-0.1322	-0.011	0.01715
XM_220588.3	LOC303222	0.15854	0.13691	0.11027	0.2045	0.15618	0.11812	0.21638	0.24615	0.04296	0.12443	0.10666	0.06806	0.02161	0.35132
XM_220714.3	LOC303291	0.14489	-0.1597	-0.0089	0.07397	-0.0195	0.14415	0.03535	-0.1029	0.04178	-0.1511	-0.02	0.12597	-0.0133	-0.0293
NM_001024992	LOC303332	0.34168	-0.0396	0.33971	1.1059	0.5369	0.60152	0.49581	0.49039	0.27715	0.03864	0.15736	0.2558	0.17033	0.22335
NM_001024992	LOC303332	0.59365	0.41126	0.46122	1.387	0.75441	0.88412	0.52901	0.59712	-0.0736	0.23682	0.26471	0.15459	0.35336	0.27842
NM_001107023	LOC303337	0.00736	0.14746	0.12209	0.19758	0.18713	-0.0148	-0.0454	0.08058	0.02054	0.21626	0.07913	-0.0136	0.03321	0.15659
NM_001044238	LOC303407	0.36046	0.44746	-0.4258	-0.1701	-0.2663	-0.305	0.058	-0.5057	0.2927	0.39559	0.36943	-0.2156	-0.3447	-0.3432
XM_220846.3	LOC303437	-0.0762	-0.0809	-0.0184	-0.1197	-0.0436	-0.0411	-0.0129	0.07963	-0.1698	-0.0891	0.01635	0.04102	-0.0886	0.02701
NM_001037190	LOC303448	0.20705	0.00744	0.1121	-0.3022	0.15283	0.24376	-0.2313	0.30393	0.11108	-0.04	0.15634	-0.1694	0.37567	0.32802
NM_001135039	LOC303456	-0.4525	-0.0984	-0.057	-0.4426	-0.1819	-0.1494	-0.0668	-0.3011	-0.009	0.04081	0.01663	0.04777	0.60037	0.55123
NM_001004279	LOC303466	-0.2669	-0.288	-0.2165	-0.1796	-0.1128	0.05716	-0.3128	0.04881	-0.0318	-0.0067	-0.187	-0.2473	-0.0154	-0.0539
XM_220874.2	LOC303471	-0.4781	-1.7582	0.32258	-1.3738	-1.6109	-1.5249	-1.1614	-1.5505	-0.8691	-1.2116	-1.0832	-0.0377	0.26324	0.48098
XM_239336.4	LOC303515	0.04725	0.16196	-0.0395	-0.1228	0.22875	-0.0595	-0.0369	0.07299	-0.0515	-0.1292	0.01837	-0.1619	-0.0091	-0.0018
NM_001024881	LOC303517	-0.1079	-0.0976	-0.07	-0.0456	-0.1878	-0.0869	-0.0889	0.27919	0.11812	0.00589	-0.1279	-0.0043	0.2982	0.08263
NM_001017988	LOC303566	-0.3625	-0.2877	0.36152	-0.4334	-0.3345	-0.4345	-0.3103	-0.5564	-0.3122	-0.4737	-0.418	0.00096	0.13109	0.10489
NM_001039019	LOC303575	-0.087	-0.0039	0.16715	-0.2492	0.15751	-0.1601	-0.205	-0.0485	0.31794	0.00162	0.001	-0.1473	0.02841	-0.1366
NM_001107061	LOC303614	0.34067	0.09997	0.08367	0.38707	0.18037	0.02313	0.10903	0.06386	0.11118	0.02502	-0.0878	0.2494	0.09082	-0.1433
NM_001168284	LOC303666	0.11808	-0.3013	0.40768	-0.1101	0.1456	0.28683	-0.2052	0.08458	0.09573	-0.2414	-0.1463	0.01743	0.24626	0.12978
NM_001135714	LOC303684	-0.2229	-0.2886	-0.3078	-0.1323	-0.2981	-0.066	-0.275	-0.1688	0.04238	-0.1347	-0.2839	-0.1722	-0.2738	-0.1675
NM_001107071	LOC303730	-0.0741	0.17363	0.06816	0.62501	0.03111	0.25327	-0.1703	0.03669	-0.0486	-0.0577	0.00644	0.29158	0.18636	0.09436
XM_221188.4	LOC303734	0.10966	0.21159	0.19572	0.21804	0.07262	-0.007	-0.0101	0.20962	-0.0528	0.0992	-0.0695	-0.1137	0.03345	0.25693
NM_001013978	LOC303823	0.0144	0.07985	-0.02	-0.0181	0.05432	0.0904	0.10631	0.19146	0.13365	-0.0061	-0.0474	0.0676	0.1211	0.04497
NM_001107083	LOC303835	-0.1097	0.03617	0.00401	0.11498	-0.0278	0.41809	0.07486	0.17091	-0.0132	0.06943	-0.1169	-0.0083	0.00342	0.05875
XM_221357.3	LOC303860	-0.1516	-0.1515	-0.1456	-0.1351	-0.2418	-0.0865	-0.0856	-0.2948	-0.207	-0.0168	-0.0539	-0.0029	-0.1995	-0.174
XM_221419.3	LOC303889	-0.1194	-0.1334	-0.0852	-0.0136	0.01414	0.14711	-0.0329	-0.2217	-0.0928	-0.1043	0.04536	0.00992	0.0562	-0.0066
XM_221433.3	LOC303919	0.06087	0.15438	0.25865	0.204	0.10988	-0.0423	0.00474	0.20282	0.0116	-0.1362	0.0179	0.02238	-0.1326	-0.0448
NM_001007598	LOC303930	-0.0821	0.10668	0.00854	-0.1287	0.06781	-0.0539	-0.0845	-0.0229	0.14093	0.18879	-0.0766	-0.0025	0.12481	-0.0947
NM_001006990	LOC304000	0.86441	1.1849	0.36017	0.17558	1.5466	1.4813	1.0122	1.6186	0.97581	1.3673	1.1585	-0.1229	0.98902	0.94154
XM_221586.3	LOC304031	0.07496	0.19736	0.02088	0.08862	0.12435	0.06554	0.071	0.03055	0.08603	0.27389	-0.0283	0.0002	0.0303	-0.0238
NM_001114391	LOC304035	0.12737	0.23546	-0.0633	0.1016	0.19687	0.05788	-0.0949	0.22107	0.19346	0.00232	0.18791	0.00673	0.06688	0.10051
NM_001107101	LOC304037	-0.3026	-0.2217	-0.2009	0.11188	-0.5356	-0.7265	0.13953	-0.4434	-0.521	-0.2129	-0.111	-0.2322	-0.5994	-0.2432
XM_221669.3	LOC304104	-0.6751	-0.1503	0.48146	0.11333	-0.8178	-0.7385	0.0665	-0.496	-0.5062	-0.5537	-0.4822	-0.7214	-0.391	-0.536
NM_001107112	LOC304125	0.03624	0.12462	0.18353	-0.0646	-0.0309	0.36603	-0.0298	-0.1935	0.1182	-0.0536	-0.1601	0.25042	0.0701	0.04619
NM_001013979	LOC304131	0.14131	-0.3908	-0.4771	-0.7754	-0.9063	-0.748	-0.9439	-0.9883	-0.2984	-0.1737	-0.2434	-0.3602	-0.6439	-0.4516
XM_221810.2	LOC304199	0.08883	-0.032	0.03403	0.05556	-0.0064	-0.0696	-0.0287	-0.0204	-0.0179	-0.0727	-0.0989	-0.0767	0.04339	0.06027
XM_239589.3	LOC304237	-0.0272	0.04009	-0.0983	-0.0464	-0.1369	-0.0292	0.07338	-0.1522	-0.0968	-0.1681	-0.0729	0.01874	-0.1392	0.18193
NM_001170399	LOC304280	0.35701	0.13051	-0.1071	0.10635	-0.0813	0.31079	0.09071	0.02301	0.1554	0.13267	0.14757	0.11666	0.05465	0.07525
NM_001137641	LOC304286	-0.3281	-0.2657	-0.2599	-0.3553	-0.1362	-0.3191	-0.1968	-0.144	-0.1336	-0.3674	-0.2888	-0.2911	-0.3337	-0.3325
XM_221978.3	LOC304338	-0.0972	0.1761	-0.0831	-0.1234	0.09814	-0.1477	-0.1237	-0.0598	-0.1167	-0.0661	-0.0378	0.0867	-0.1427	-0.1087
XM_221996.3	LOC304353	-0.1617	-0.0929	-0.1391	-0.0584	-0.0814	0.17015	0.03059	-0.1086	-0.008	-0.1291	-0.0433	-0.0394	0.03423	0.07594
NM_001013981	LOC304396	-0.2148	0.00091	0.14238	-0.1267	-0.0974	-0.0912	0.37089	-0.0621	0.03601	0.21239	0.00277	-0.0305	-0.0507	-0.0934
NM_001013981	LOC304396	-0.1625	-0.1207	0.07355	0.1676	-0.1157	-0.0773	0.02016	-0.3025	-0.2208	0.06675	-0.1516	-0.2509	-0.1308	-0.2413
XM_222128.3	LOC304438	-0.1866	0.42573	1.4361	1.4023	-0.0768	-0.1507	1.6153	0.12877	0.51982	0.70969	0.80144	0.46249	0.77642	0.75218
XM_222140.3	LOC304462	0.09231	-0.0503	-0.1577	0.00901	0.18179	-0.1438	0.01097	0.21585	0.11936	-0.0998	-0.059	0.01941	-0.0179	0.27808
XM_222162.3	LOC304479	0.11227	0.39298	0.23452	0.28616	0.18956	0.06326	0.09391	0.33706	0.05251	0.18054	0.13697	0.09971	0.14755	0.31089
NM_001025665	LOC304484	0.00752	0.12673	-0.0419	0.19276	-0.0942	0.21053	0.00932	-0.0653	0.16461	0.12645	0.11861	-0.0002	-0.2334	-0.0494
XM_222179.3	LOC304497	-0.0323	-0.008	0.07465	0.13544	0.05991	0.36361	0.24968	-0.033	-0.116	-0.0148	0.04021	-0.147	0.03314	0.08449
NM_001134537	LOC304500	-0.5068	-0.5369	-0.9487	-0.607	-0.4447	-0.4993	-0.6299	-0.4507	-0.4674	-0.5799	-0.6446	-0.6756	-1.1043	-1.0767

XM_222198.3	LOC304509	-0.1811	-0.1885	0.19628	-0.2446	-0.2388	-0.3863	-0.3241	-0.1356	-0.2542	-0.1504	-0.3769	-0.4123	-0.1944	-0.2329
NM_001024254	LOC304530	-0.0743	-0.1356	-0.339	-0.1507	-0.0556	-0.0656	0.03114	-0.159	-0.121	-0.0667	0.10133	-0.0643	-0.036	-0.0802
XM_222232.3	LOC304547	-0.0284	0.0797	-0.1442	0.07759	0.15883	0.0139	-0.1849	0.24804	-0.0445	-0.0667	-0.0958	0.00102	-0.1205	-0.0546
XR_009380.1	LOC304551	-0.043	-0.1696	-0.2592	-0.117	-0.1427	0.20333	-0.1508	-0.1794	-0.0208	-0.2188	-0.0641	-0.1407	-0.2185	-0.0805
XM_001080633.1	LOC304558	0.03618	0.17188	0.15556	0.09369	0.17275	-0.0542	-0.099	0.1188	0.07848	0.01673	0.05434	0.05315	0.12038	0.00819
XM_222254.3	LOC304572	-0.0403	0.1329	-0.085	-0.1256	-0.1319	-0.0764	-0.0952	-0.0614	-0.0418	0.11934	-0.1402	0.10431	0.02375	-0.0622
NM_001170403	LOC304592	-0.3324	-0.4088	-0.274	-0.4201	-0.4621	-0.3947	-0.0424	-0.323	-0.1832	-0.5048	-0.0052	-0.2875	-0.1739	-0.3602
NM_001025666	LOC304646	0.03367	0.08335	0.2596	0.65	0.02627	-0.0184	0.23627	0.18925	0.05725	0.09198	0.23865	0.05256	0.22829	0.09337
NM_001025740	LOC304657	0.3593	0.22184	-0.1672	-0.1519	0.18566	-0.3452	-0.3785	0.24799	0.00073	0.13732	-0.3389	-0.2718	0.85219	0.85405
NM_001135846	LOC304714	0.23899	-0.0757	-0.0571	0.06568	0.19138	-0.0366	0.09494	0.0291	0.15948	0.13417	0.20436	0.55361	0.01135	-0.1079
NM_001134961	LOC304721	-0.0753	0.0443	-0.1853	-0.0569	-0.1253	-0.2138	-0.1042	-0.167	-0.2371	-0.1671	0.0172	-0.2291	0.05619	0.04877
XR_007922.1	LOC304725	-0.1992	-0.1971	-0.2544	-0.228	-0.093	-0.1152	-0.2141	-0.2479	0.13527	-0.3232	-0.0889	-0.1157	-0.1937	-0.1257
XM_222750.1	LOC304872	-0.0436	-0.0039	0.05995	0.09206	-0.0595	0.02076	-0.0381	-0.1029	-0.0042	-0.0917	0.17462	0.06381	0.03825	-0.036
NM_001107192	LOC304920	0.07347	-0.1174	-0.2474	0.2163	-0.2078	-0.3441	0.12302	-0.0343	-0.4661	0.1162	-0.0176	-0.5152	-0.3526	-0.1164
NM_001100563	LOC304923	0.13542	0.19116	-0.3207	0.61044	-0.2714	-0.4037	0.44342	-0.3576	0.13461	0.23092	0.04423	0.10511	-0.2736	-0.3918
NM_001083338	LOC304971	0.09963	0.21713	-0.2591	0.19891	0.09114	-0.1146	-0.355	-0.1674	-0.1219	-0.1769	-0.0814	-0.1047	-0.1775	-0.3095
NM_001013986	LOC305076	-0.4575	-0.262	0.33616	-0.0786	-0.7456	-0.5626	0.15152	-0.7286	-0.3442	-0.388	-0.3077	-0.1602	-0.2029	-0.2711
NM_001100841	LOC305078	0.01246	-0.369	0.10378	-0.2471	-0.1181	0.03847	-0.3107	-0.1705	-0.1458	0.16369	-0.1427	-0.14	-0.3755	-0.2816
XM_223094.3	LOC305087	0.04054	0.11473	0.07316	0.13482	0.13252	0.25713	-0.0782	0.01926	-0.044	-0.002	-0.0178	0.00247	0.00149	0.15278
XM_223116.3	LOC305101	0.0853	0.0293	0.25786	0.00821	-0.1334	-0.1786	-0.0988	-0.1004	-0.0346	-0.1494	0.00214	-0.0123	0.06702	0.04368
NM_001164726	LOC305103	-0.1214	-0.1401	-0.1275	0.03593	0.06595	-0.1499	-0.0441	-0.1094	0.0623	-0.0374	0.02458	-0.1248	0.10539	-0.127
NM_001107204	LOC305135	-0.0239	-0.1517	-0.0802	0.25175	0.02501	-0.1205	0.19633	0.27303	-0.0295	0.04438	0.00306	-0.0076	-0.0658	-0.0768
NM_001025670	LOC305166	-0.0854	0.1888	0.28372	-0.042	0.15275	0.03621	0.20943	0.09697	-0.0646	0.15812	-0.0003	0.18953	-0.0598	0.21011
XM_223203.3	LOC305185	-0.175	-0.123	-0.1133	0.0424	0.18116	-0.1148	-0.2298	-0.2303	-0.092	-0.1156	0.12582	-0.1291	-0.0118	-0.0995
XM_223330.3	LOC305284	-0.0523	0.12287	-0.0109	0.04738	0.04849	0.03359	-0.0126	0.00654	0.01084	-0.0067	0.11511	0.23759	0.0821	0.17541
XM_223397.3	LOC305332	-0.0348	0.11791	0.06237	0.33552	0.17955	0.2757	0.1564	0.31321	0.15259	0.05538	0.0153	0.22258	0.15507	0.08682
NM_001024785	LOC305350	-0.4259	-0.068	-0.3813	-0.9853	-0.4841	-0.8257	-0.3287	-0.5065	-0.3196	-0.3625	-0.4865	-0.4966	-0.1802	-0.0946
XM_223442.3	LOC305375	0.20635	0.02713	0.24106	0.06325	0.15785	0.49581	-0.1028	0.46227	0.07511	-0.0294	0.15016	0.71453	0.31679	0.38215
XM_240008.3	LOC305453	-0.099	-0.0489	-0.2518	-0.2863	0.22617	0.01801	-0.0473	0.26379	0.06772	-0.3472	-0.4642	-0.1555	0.37155	0.33796
XM_223586.3	LOC305466	-0.3799	-0.0965	-0.2338	-0.4301	-0.0183	-0.2667	0.11837	-0.0814	-0.2255	-0.0569	-0.1648	-0.233	-0.1753	-0.0585
NM_001107233	LOC305476	0.11857	0.12506	0.13256	0.12816	0.27975	0.01432	0.11847	0.10577	0.14809	0.09434	0.34156	0.24649	0.18175	0.19674
XM_223579.2	LOC305492	0.02347	0.06126	-0.0525	-0.0767	-0.0175	-0.0715	-0.0888	-0.0958	-0.0638	0.0407	-0.0774	0.18164	0.05429	0.03179
NM_001127537	LOC305552	-0.0027	-0.0294	-0.1378	-0.1439	-0.0553	-0.3302	-0.1833	-0.0526	0.0179	-0.1816	0.02864	-0.1725	-0.1026	0.10865
XM_223671.3	LOC305567	0.06031	-0.0187	-0.0341	0.15423	-0.0993	-0.0265	0.12421	0.00425	-0.108	0.10491	0.10988	0.1654	0.06457	-0.054
NM_001100971	LOC305579	0.10638	0.12001	0.19289	0.66979	0.23479	0.18506	0.11196	0.32573	0.2075	0.26171	0.05091	0.19556	0.16821	0.1427
NM_001025278	LOC305583	-0.3408	-0.1262	-0.0321	0.33509	-0.676	-0.7372	0.03491	-0.526	-0.4897	-0.3974	-0.3752	-0.6502	-0.5669	-0.7013
XM_001069109.1	LOC305633	0.57805	0.77066	0.10763	-0.1233	0.71204	0.55393	0.55672	0.6649	0.31452	0.49841	0.64589	-0.0309	0.16938	0.34391
XM_223775.1	LOC305667	0.01902	0.02798	0.06789	0.03713	0.00598	0.19568	-0.0027	-0.0926	-0.1043	0.21895	0.07557	0.14237	-0.0227	-0.0118
NM_001013993	LOC305698	-0.0871	0.01592	-0.2292	-0.1046	-0.13	-0.1156	-0.0121	-0.1527	0.0047	0.08789	-0.1232	0.00598	-0.1012	-0.1611
XM_223815.3	LOC305710	0.0164	-0.0603	-0.0438	-0.0313	-0.0051	-0.0486	-0.1182	-0.1278	0.01282	0.29415	0.02263	-0.0108	0.07516	0.18225
XM_223854.3	LOC305764	-0.2084	-0.1109	-0.1585	-0.0975	0.30494	-0.2545	-0.1039	-0.0196	-0.0523	-0.1452	0.02239	-0.2361	0.19192	-0.0126
XM_240182.1	LOC305894	0.03727	0.11664	-0.0442	0.06996	-0.0879	-0.0367	-0.1318	-0.0021	-0.0045	-0.1719	-0.0856	-0.0386	-0.1008	-0.1628
XM_224235.3	LOC305911	0.19848	0.22825	0.23537	0.10089	-0.0119	0.33834	0.04246	-0.0332	0.21489	0.16024	0.02328	0.19655	0.25055	0.22283
XM_001060431.1	LOC305913	-0.7713	-0.2138	-0.351	0.1698	-0.5466	-0.634	0.05554	-0.4324	-0.6341	-0.3653	-0.6098	-0.2396	-0.5597	-0.6922
NM_001170471	LOC305955	-0.0621	-0.0373	-0.0453	-0.1293	0.11389	-0.186	-0.0861	-0.212	-0.0174	-0.0187	-0.1294	-0.0232	-0.2099	-0.069
NM_001135855	LOC305974	0.00749	-0.022	-0.0577	0.0844	-0.0002	-0.0924	0.02333	-0.0013	-0.0342	0.06639	-0.066	0.00897	-0.0027	0.08223
XM_224366.3	LOC306038	-0.045	0.01974	0.12894	-0.1071	-0.1024	-0.047	-0.0424	0.37699	-0.0125	0.04227	0.01098	-0.072	-0.2187	-0.0355
NM_001047879	LOC306079	-0.0949	-0.0184	-0.125	-0.0848	-0.1172	-0.0663	-0.125	-0.1883	0.00155	0.19533	-0.2438	-0.1	0.02909	-0.2639
XM_224510.3	LOC306137	-0.3947	-0.4697	-0.3893	-0.2966	-0.5378	-0.8033	-0.3156	-0.6487	-0.5929	-0.4556	-0.4721	-0.6593	-0.3997	-0.4178

XM_224520.3	LOC306166	-0.0924	-0.1454	-0.1334	-0.1444	-0.1498	-0.1693	-0.2167	-0.0907	0.04942	-0.0852	-0.1554	0.22489	-0.1194	-0.1149
NM_138892	LOC306223	0.05877	0.03651	0.09479	0.03261	0.10825	0.02839	0.11543	0.10945	0.05077	0.05638	0.04484	0.12393	0.12551	0.0676
XM_224652.3	LOC306294	0.07936	-0.0937	-0.0929	0.00393	0.11039	0.07129	-0.0678	-0.082	-0.0281	-0.0528	0.01022	0.05048	-0.1348	-0.0148
XM_224667.3	LOC306298	-0.013	0.01881	0.12266	-0.0311	0.0806	0.15189	0.06789	-0.0292	0.01355	0.0399	0.06538	0.19534	0.06265	0.0182
XM_001065941.1	LOC306312	-0.0772	-0.1041	-0.0939	0.01142	-0.1206	-0.0498	-0.1459	0.11492	-0.0926	0.30082	0.01559	-0.0486	0.0143	-0.0424
NM_001169127	LOC306324	-0.5329	-0.756	-0.5747	-0.5846	-0.338	-0.9809	-0.5936	-0.3781	-0.9486	-0.8775	-1.1173	-1.1451	-0.815	-0.7498
XM_001070347.1	LOC306346	-0.1525	-0.0641	-0.2113	0.00709	0.11437	-0.0738	-0.0474	-0.0116	-0.0159	0.18987	0.13276	-0.1018	0.03655	-0.0931
XM_224750.3	LOC306365	-0.114	-0.003	0.59774	0.79226	0.13604	0.11019	0.96036	0.06533	0.01998	-0.0517	0.38292	0.24615	0.16135	0.16088
XM_224759.3	LOC306380	0.02766	-0.0379	0.27307	0.14603	-0.0141	-0.0096	-0.0365	0.19125	0.04716	0.14593	0.31136	-0.0783	0.07453	0.12868
XM_224793.3	LOC306407	-0.0236	0.01575	0.01625	-0.0572	0.07389	-0.1567	0.03546	-0.0588	-0.1073	0.00197	-0.0293	0.08924	-0.0858	0.15595
XM_224824.3	LOC306428	-0.4028	-0.3403	-0.0861	0.10013	-0.5297	-0.7037	-0.7842	-0.4082	-0.5871	-0.342	-0.2793	-0.6023	0.26733	0.36932
XM_224840.3	LOC306450	-0.1426	0.09515	-0.0743	-0.0559	-0.0124	0.02755	-0.1686	0.09702	0.09501	-0.1175	-0.1119	0.03413	-0.0691	-0.1353
NM_001025673	LOC306464	0.18841	0.17311	0.09365	0.12428	0.03227	0.1806	0.06249	0.08714	0.00878	0.06618	0.12255	0.06434	0.15926	0.14974
NM_001107314	LOC306504	0.11974	0.2633	0.24669	0.13052	0.1904	0.13086	0.06091	0.03471	0.27783	0.10862	0.11338	0.35338	0.20082	0.03826
NM_001007598	LOC306511	-0.184	-0.0889	-0.212	-0.157	-0.3664	-0.0101	-0.0895	0.06574	0.01478	-0.4668	-0.2868	-0.1684	-0.1768	-0.0648
XM_224972.3	LOC306577	-0.0877	0.02207	0.00033	0.01432	0.18311	-0.0286	0.13103	0.02937	-0.0299	0.0668	0.00037	0.08015	0.05665	0.06856
NM_001107331	LOC306659	-0.0853	0.14314	0.06609	0.05029	0.05976	0.13732	-0.011	-0.0081	-0.0748	-0.1476	-0.1629	0.10575	0.14464	-0.0758
NM_001014007	LOC306766	0.03282	0.13875	0.10601	0.01176	0.13561	0.43186	0.10831	0.06208	0.14352	-0.0715	0.2422	0.22482	0.08037	0.05002
NM_001014007	LOC306766	0.15766	0.24047	-0.33	0.01944	0.4472	0.44965	0.00351	0.4704	0.15921	0.12129	0.07049	0.01507	0.04839	0.00611
XM_225216.4	LOC306792	-0.0632	0.044	0.2847	0.05243	0.17339	0.05678	0.00512	-0.1003	0.07929	-0.1166	0.04765	0.04563	0.0681	0.01957
XM_225274.3	LOC306896	-0.0512	-0.1074	-0.0254	-0.2076	-0.0976	0.21394	-0.0712	-0.0753	0.02524	0.0157	-0.1347	0.12192	-0.0424	-0.1781
XM_225320.2	LOC306926	0.03035	0.1178	0.09319	0.13229	0.0653	0.33886	-0.021	0.05574	-0.0984	-0.016	0.16452	0.16917	0.3497	-0.1862
NM_021840	LOC306962	-0.0216	0.09419	-0.0113	0.11639	0.1477	-0.0236	3E-05	0.05504	-0.0533	-0.0632	-0.0265	0.00931	-0.0554	0.15289
XM_225472.3	LOC307029	-0.0163	0.0585	0.18934	0.35665	0.04009	-0.0367	0.161	0.0629	0.04634	0.20469	0.06178	-0.0015	0.36047	0.20068
NM_001106290	LOC307049	-0.2967	-0.1462	-0.2125	-0.2528	-0.1078	0.17768	0.01742	-0.0911	-0.1916	-0.246	-0.2847	-0.1871	-0.1755	-0.2228
XM_225479.3	LOC307056	-0.0154	-0.2506	-0.1086	-0.0158	-0.1984	-0.1753	-0.3035	-0.1808	-0.254	-0.2168	-0.3306	0.21065	-0.1103	-0.2362
XM_225481.2	LOC307057	0.00491	0.33397	-0.0164	0.30092	0.13257	0.51005	-0.0068	-0.0304	-0.0073	0.06918	-0.015	0.20712	0.27808	0.00734
XM_225528.3	LOC307073	-0.0368	-0.0069	-0.1547	-0.107	-0.2446	-0.2001	-0.2195	0.0334	0.02635	-0.093	-0.2706	0.01028	-0.0795	-0.042
XM_240734.3	LOC307128	-0.1579	0.11195	0.13195	0.06301	-0.1234	-0.0532	0.12249	-0.0257	0.01	0.01837	0.09728	0.18489	-0.1141	0.01996
XM_225618.3	LOC307168	0.31322	0.23718	0.10052	0.19938	0.1055	0.08245	0.20925	0.08019	0.14368	0.07112	0.12402	0.16778	0.27006	0.0938
XM_225655.2	LOC307202	0.14111	0.03523	0.14493	0.37052	-0.0744	0.20423	0.08791	0.03306	-0.0279	-0.168	0.08632	0.05796	-0.0629	0.09295
XM_225713.3	LOC307231	-0.1402	-0.2948	-0.1908	-0.0805	-0.2666	0.03407	-0.3649	-0.2996	-0.1934	-0.0523	-0.2705	-0.2408	-0.2021	0.04462
XM_225740.3	LOC307239	-0.1288	-0.1028	-0.1403	-0.1341	0.07155	-0.0283	-0.0814	-0.0317	-0.0957	0.08074	-0.0087	-0.0643	-0.0799	0.07197
XM_225708.3	LOC307255	-0.1872	-0.1387	-0.0185	-0.106	-0.1253	-0.2614	-0.0859	-0.2213	-0.0176	0.0495	0.04295	-0.2447	-0.2003	-0.2186
XM_225779.3	LOC307315	-0.045	0.09733	0.15361	-0.0319	-0.0236	0.00168	0.08095	-0.053	0.0663	-0.0287	0.14131	0.15707	-0.0045	0.09309
XM_225836.2	LOC307328	-0.207	1.1684	1.1892	1.0459	-0.1514	-0.2445	1.9217	-0.2012	0.80799	0.81135	0.9773	0.51979	0.93249	0.65953
XM_225841.2	LOC307332	0.07929	-0.1503	-0.3645	-0.0854	0.30906	0.23389	0.05362	0.44392	0.17523	-0.0665	-0.1207	-0.0952	-0.0394	0.02417
XR_005455.1	LOC307347	-0.1151	0.08124	-0.3769	-0.1475	-0.258	-0.3766	-0.0244	-0.1241	0.11707	0.25827	-0.068	-0.3506	0.18811	0.35218
XR_005455.1	LOC307347	-0.652	-0.4728	0.53834	0.20735	-0.6136	-0.71	0.04613	-0.8102	-0.0227	-0.1835	-0.1205	0.01581	0.0262	-0.0076
XM_225887	LOC307393	-0.1406	-0.1712	-0.0739	0.19275	-0.0046	-0.2349	-0.1373	-0.0517	-0.1085	-0.0751	0.05335	-0.2174	-0.0518	0.10868
XM_226009.3	LOC307493	-0.0156	0.16143	0.09226	-0.1034	0.05711	-0.0505	0.00485	-0.0421	0.0145	-0.0539	0.0252	-0.0833	-0.0634	0.05976
NM_173306	LOC307582	-0.1099	-0.1265	-0.1664	-0.0543	-0.0857	0.0468	-0.0749	-0.1376	-0.0628	-0.1541	-0.173	-0.068	-0.1413	-0.1973
XM_226231.3	LOC307636	0.10353	0.09069	0.06123	0.11875	0.21138	0.07321	-0.0511	-0.1038	0.05887	-0.0571	0.19854	0.10592	0.27873	-0.1307
NM_001012056	LOC307660	0.12868	0.02245	0.0077	0.08033	-0.0398	-0.0315	0.11352	0.11559	0.01598	0.01301	0.1238	0.01307	0.04931	0.0881
XM_226278.3	LOC307671	0.26058	0.37691	0.39903	0.14895	0.35384	-0.039	-0.0823	0.07013	0.05176	0.42989	0.22876	0.20583	0.12151	0.00887
XM_226288.3	LOC307681	-0.0164	0.16273	0.16206	0.01571	-0.0357	-0.0654	0.06091	0.11575	-0.0412	0.11747	0.12207	0.1056	0.16471	0.15798
NM_183402	LOC307706	-0.0626	-0.1128	0.00224	-0.0557	-0.1531	0.11109	-0.0563	-0.1329	0.03898	0.10144	-0.0245	-0.0299	-0.0624	-0.1271
NM_001047882	LOC307727	-0.1073	-0.0109	0.05039	-0.2423	-0.1447	-0.1151	-0.1974	-0.1309	-0.11	-0.056	-0.2285	-0.1738	-0.0932	-0.2065
XM_226326.3	LOC307731	0.73049	0.95972	0.54576	-0.3398	0.76476	-0.0334	-0.1644	0.75574	0.70889	0.70149	0.55428	-0.4324	0.5868	0.92263

NM_001135583	LOC307855	-0.0002	-0.0438	0.07777	0.09443	0.14529	0.03022	-0.013	0.03341	0.0368	0.02482	0.08859	0.36304	0.22345	0.03588
XM_226525.3	LOC307901	0.07156	-0.0881	-0.0845	-0.1374	-0.0361	-0.016	-0.1632	0.15119	0.21075	-0.0372	-0.0801	0.01788	-0.059	0.02414
NM_001107436	LOC307907	1.9465	1.7194	1.4841	0.9066	0.87263	0.86584	1.11	0.93404	1.2621	1.7553	1.5683	0.50726	0.47019	0.64103
NM_001107441	LOC307926	-0.154	-0.1787	-0.0813	-0.3763	-0.2541	-0.3198	-0.3208	-0.2571	-0.2253	-0.2295	-0.1661	-0.1386	-0.1093	0.13694
XM_226573.3	LOC307951	0.05639	0.10402	-0.0369	0.22342	-0.1436	0.19581	0.17519	-0.0201	0.16425	0.04286	0.38053	-0.0698	0.18997	0.05414
XM_217724.3	LOC308053	-0.0118	-0.1206	-0.0883	0.05952	0.07677	0.37667	-0.1082	0.00729	0.04553	0.0409	0.2193	0.198	0.31387	0.13077
NM_001025000	LOC308129	0.12029	-0.056	0.35248	0.05409	0.17256	0.09554	0.07929	-0.0432	0.04837	0.13062	0.10837	0.15441	-0.0586	0.12146
XM_217910.3	LOC308183	0.2302	0.18432	0.21617	0.1278	0.18573	0.08699	0.1463	0.2287	-0.0054	0.10122	0.14507	0.00258	-0.0083	0.02848
XM_217925.3	LOC308188	0.07045	0.07409	0.11754	0.01538	0.16534	0.12759	0.0791	0.07903	0.06243	0.05085	-0.0138	0.27395	0.01605	-0.0117
XM_217963.3	LOC308198	-0.069	0.062	-0.0842	-0.0151	-0.0552	-0.0708	0.25701	0.24156	0.02747	-0.0469	-0.0382	-0.0294	-0.0384	-0.0233
XM_218008.3	LOC308222	-0.0795	0.08453	0.45657	0.36397	0.00718	-0.0186	0.33606	-0.0593	0.09864	0.0541	0.06667	0.18027	-0.0267	-0.1604
XM_218114.3	LOC308253	-0.0575	-0.07	-0.0937	-0.0476	0.10159	-0.1581	-0.041	-0.0882	0.00613	-0.039	-0.0088	0.05644	0.24301	0.02108
NM_001107469	LOC308266	-0.1042	0.03352	-0.0443	-0.0792	-0.0685	-0.1245	-0.1664	-0.1665	-0.1211	0.05653	-0.1171	-0.1581	-0.0661	-0.1024
NM_001107472	LOC308320	0.38301	0.15303	-0.0489	0.16908	0.36692	0.18165	-0.0449	0.21274	0.43896	0.49955	0.17455	0.14211	-0.0497	-0.1156
NM_001145401	LOC308344	-0.3271	-0.2857	-0.284	-0.2403	-0.2746	-0.3065	-0.2423	-0.2924	-0.1735	-0.0089	-0.0751	-0.4202	-0.3359	-0.2656
XM_218261.3	LOC308350	0.18585	0.31322	0.20482	0.14655	0.05749	0.30984	0.10128	0.12372	0.12904	0.20739	0.02946	0.18313	0.03157	0.04281
NM_001025679	LOC308394	-0.3176	-0.152	-0.3362	-0.2644	-0.3333	-0.2625	-0.164	-0.2439	-0.3889	-0.3342	-0.1805	-0.3091	-0.1924	-0.2682
NM_001107482	LOC308398	-0.4308	-0.1069	-0.4721	-0.2572	-0.3911	-0.3822	-0.1896	-0.369	-0.2774	-0.2692	-0.3519	-0.3551	-0.4413	-0.3746
XM_218473.3	LOC308488	-0.3508	-0.3724	-0.3376	-0.286	-0.3946	-0.347	-0.4448	-0.3854	-0.1746	-0.337	-0.4534	-0.3055	-0.3298	-0.4515
XM_238783.3	LOC308494	0.18085	0.19789	0.13287	0.17764	0.07438	0.37601	0.12998	0.00458	0.19326	0.17842	-0.014	0.00699	0.09012	0.15757
XM_218493.3	LOC308503	-0.5945	-0.8526	-0.0553	-0.5422	-0.5826	-0.3322	-0.5965	-0.3237	-0.5911	-0.8782	-0.5299	-0.1554	0.09368	0.14626
XM_218517.2	LOC308525	0.09909	0.1659	0.14991	0.16835	0.15328	0.19165	-0.0139	0.30839	0.38642	0.06028	0.25968	0.16357	-0.0064	0.17596
NM_001099487	LOC308551	-0.1865	-0.1733	-0.186	-0.061	-0.2086	-0.1963	-0.1293	-0.2364	-0.1986	-0.2575	-0.2631	-0.2441	-0.1947	-0.1588
XM_218633.3	LOC308556	-0.2207	-0.1889	-0.1712	-0.3344	0.27833	0.26458	-0.091	0.60048	-0.1359	-0.1734	-0.207	-0.2728	-0.1569	-0.2733
XM_218679.3	LOC308637	-0.1477	-0.1162	-0.0174	-0.128	-0.1187	-0.0477	-0.0814	0.11406	-0.1651	-0.0217	0.0345	-0.0314	-0.0476	-0.1319
XM_218779.2	LOC308719	0.48977	-0.0588	0.17146	-0.1809	0.78294	1.5931	-0.3524	1.0557	0.37468	0.38863	-0.0276	1.1806	1.0927	1.0856
XM_218795.3	LOC308742	-0.236	-0.0513	-0.2163	-0.0733	-0.072	-0.0051	-0.161	-0.2425	-0.3048	-0.0665	-0.1274	0.17434	-0.0379	-0.1712
NM_001037194	LOC308775	-0.2132	-0.3631	-0.3819	-0.8465	-0.2648	-0.5708	-0.3298	-0.4495	-0.1217	-0.1801	-0.4432	-0.0397	-0.2962	0.08949
NM_001107536	LOC308846	-0.2126	0.11899	-0.1543	-0.0434	0.19782	-0.0316	-0.037	-0.102	0.0822	0.10584	0.03977	0.05869	-0.0955	-0.1402
XM_219202.3	LOC308919	0.0166	-0.1593	-0.036	-0.2154	-0.2647	0.03909	-0.0288	0.07234	-0.073	-0.2071	-0.0835	-0.1922	-0.0202	-0.0954
XM_219235.3	LOC308929	0.0073	0.02062	0.06783	0.25578	-0.0005	-0.0235	0.15679	0.09959	0.0239	0.00906	0.03978	0.05959	0.04724	0.03116
XM_219240.3	LOC308934	0.08001	-0.3769	-0.1646	-0.0162	-0.2804	0.11931	-0.1553	-0.1462	-0.0677	0.08428	0.14488	-0.2035	-0.0154	0.04924
NM_001024777	LOC308954	-0.0037	0.10142	0.01431	-0.1994	-0.047	-0.2705	-0.0701	0.25138	0.34046	0.23056	0.08475	-0.271	0.03866	-0.0829
NM_001024777	LOC308954	0.03803	-0.0011	0.02535	-0.0272	0.07194	-0.0536	0.03822	0.15429	-0.0836	0.01023	0.05506	-0.0309	0.08996	-0.0524
XM_219290.3	LOC308956	0.1328	0.21969	0.15888	0.09696	0.14025	0.01614	0.47514	0.12131	0.06636	0.19062	-0.0002	0.22238	0.18849	0.1365
NM_001037196	LOC308976	-0.0011	-0.1003	0.1328	0.06118	-0.0026	0.0174	0.08051	0.14132	0.01438	-0.0441	-0.0822	0.2175	-0.0184	0.01978
NM_001025001	LOC308990	-0.7597	-0.6865	-0.5093	-0.7621	-0.8129	-0.8643	-0.0349	-0.8139	-0.7521	-0.6692	-0.4067	-0.6494	-0.819	-0.5272
XM_219350.3	LOC308996	0.05514	-0.0257	0.05738	-0.0163	-0.1311	-0.0683	0.03796	-0.2925	-0.1017	0.02484	-0.1534	0.13452	-0.0178	0.01392
XM_219364.3	LOC309003	0.32219	0.165	0.12736	0.16875	-0.0019	0.06445	0.06083	0.41974	0.28836	0.09198	0.02698	0.06067	0.07897	0.18944
XM_001080483.1	LOC309016	-0.3625	-0.0988	-0.0745	-0.4798	-0.3483	-0.381	-0.0673	-0.4974	-0.0398	-0.0433	-0.0736	-0.1327	-0.2419	-0.371
NM_001024886	LOC309035	0.13714	0.10244	-0.0534	0.01899	0.16861	0.55676	-0.096	0.18072	0.23484	0.12793	0.1117	0.16088	0.26895	0.08336
NM_001079706	LOC309036	0.09209	0.16424	0.0365	0.0126	0.26924	-0.245	-0.0579	0.07151	0.09532	-0.0523	-0.0138	-0.1318	0.26415	-0.0471
XM_219392.3	LOC309038	-0.0262	-0.283	-0.0449	-0.2187	-0.1129	-0.1871	-0.0407	0.17348	-0.0617	-0.2239	0.06115	0.02921	-0.0589	-0.001
NM_001127540	LOC309108	0.12862	0.0238	-0.1335	0.01818	0.08341	0.00542	-0.0329	-0.1081	-0.0091	-0.0844	0.2257	0.11586	-0.055	-0.0046
XM_219503.3	LOC309129	-0.083	0.08952	-0.1063	-0.127	-0.1454	-0.1672	-0.1787	-0.1035	-0.0133	-0.1083	-0.1015	-0.0571	0.0002	-0.1136
XM_219511.3	LOC309162	-0.1915	-0.1122	-0.0781	-0.088	-0.1936	-0.2049	-0.2038	-0.1919	-0.2172	-0.1845	-0.1666	-0.1551	-0.2274	-0.2258
NM_001025117	LOC309169	0.33495	0.27708	0.60546	0.12287	0.07232	0.13344	0.45253	0.08713	-0.0862	0.0059	0.07732	0.17595	0.1439	0.30586
XM_219521.2	LOC309173	-0.2529	0.03355	-0.2358	-0.1196	-0.0671	-0.0453	-0.1123	-0.0891	0.0047	-0.0409	0.0369	0.08786	-0.1235	-0.0622
XM_219560.3	LOC309197	-0.0991	-0.1161	-0.1701	-0.1933	0.186	0.69247	-0.3071	0.02417	0.03878	-0.2007	-0.1701	0.37258	-0.0302	-0.1174

XM_219819.3	LOC309300	0.12268	-0.059	0.03619	-0.0203	0.03435	0.05921	0.29077	0.07645	0.34318	0.05438	-0.0096	0.4151	0.09679	0.06359
NM_001107582	LOC309304	0.01919	-0.112	0.07204	0.20923	0.0067	0.21428	0.10186	-0.0367	-0.0916	0.05629	-0.0575	0.17648	-0.0613	0.1818
XM_219782.1	LOC309309	0.19787	-0.1574	0.00713	0.08069	0.13595	0.27622	-0.0398	0.18597	-0.2465	0.10689	-0.1543	-0.356	-0.0598	-0.0052
NM_001107587	LOC309349	0.12643	0.26205	0.03739	0.09928	0.14152	0.14344	0.25914	-0.0287	0.08299	0.21912	0.14508	0.02915	0.07001	0.25784
NM_001107588	LOC309361	0.10444	-0.0652	-0.0216	0.19558	-0.0641	-0.0403	0.06121	-0.0435	0.16644	0.01771	-0.1655	0.0792	-0.1436	-0.0949
XM_001060754.1	LOC309362	-0.3356	-0.3344	-0.0714	-0.0264	-0.512	-0.6243	-0.3917	-0.1796	-0.5517	-0.2986	-0.3674	-0.8367	-0.6213	-0.6465
XM_219889.3	LOC309378	0.12116	0.05704	0.01811	-0.0316	-0.0393	-0.0077	-0.1649	-0.1088	-0.024	-0.2185	-0.0695	-0.1057	-0.127	0.00081
NM_001135713	LOC309433	-0.066	0.0524	-0.0895	-0.0642	-0.3163	-0.2411	-0.0189	0.17685	0.09601	0.0065	-0.041	-0.2521	0.03369	0.057
XM_219953.1	LOC309451	0.18369	0.04792	0.04581	0.05585	0.22964	0.1854	0.15613	0.09515	-0.0653	0.10764	0.05158	0.07676	0.02153	0.11326
NM_001107603	LOC309453	-0.1326	-0.0108	0.01117	-0.2103	-0.0511	-0.1887	-0.1934	0.01571	-0.2326	-0.1967	-0.1714	-0.1444	-0.2015	-0.2203
XM_220013.3	LOC309475	-0.016	0.3636	-0.4666	-0.3865	-0.5385	-0.465	-0.0581	-0.4513	0.1537	0.43158	0.30837	-0.2404	-0.6185	-0.6265
NM_001030038	LOC309478	-0.5941	-0.5514	-0.6788	-0.4372	-0.5017	-0.5439	-0.5414	-0.5633	-0.4692	-0.6479	-0.4414	-0.4909	-0.5953	-0.5231
XM_220043.3	LOC309510	0.16355	0.08828	-0.0838	-0.0145	0.02209	0.26958	0.19656	-0.0242	-0.0018	0.00511	0.1018	-0.0107	0.1441	-0.0263
XM_220117.3	LOC309557	-1.0987	-0.0558	-0.6161	-0.3905	-0.7013	-0.7248	-0.729	-0.6989	-0.1906	-0.0634	-0.4063	-0.397	-0.7246	-0.7694
XM_228025.3	LOC309637	0.00258	0.16433	0.03727	0.07899	0.20296	0.05024	0.11593	0.28804	0.11031	0.16083	0.19272	0.25362	0.04431	0.12747
XM_228032.3	LOC309643	-0.1239	-0.4783	-0.2969	0.02051	-0.0775	-0.4466	-0.109	-0.0736	-0.3978	-0.1861	-0.1973	-0.2453	-0.551	-0.3641
NM_001107616	LOC309650	-0.0845	-0.1625	-0.1762	-0.0468	-0.0732	0.02609	-0.0184	-0.1058	0.01367	-0.0846	0.09247	0.02623	-0.0011	-0.1084
XM_228184.3	LOC309796	0.04288	-0.0782	0.10679	-0.0239	-0.0423	0.04918	0.04106	-0.0068	-0.0282	0.03683	-0.0633	0.23847	0.07741	-0.0171
XM_228300.3	LOC309854	0.05141	0.03837	-0.0808	0.11576	-0.1519	0.26909	-0.0436	-0.1903	0.10606	0.19439	0.03119	-0.2485	0.01333	0.11563
NM_001107637	LOC309858	0.11146	0.01374	0.09302	0.11585	0.11896	0.11687	0.28065	0.05626	0.09521	-0.0666	0.16454	0.19376	0.03289	0.02499
NM_001104640	LOC309860	-0.0003	0.11743	-0.0254	0.02821	-0.0601	0.11031	-0.0349	0.00402	0.08653	-0.0955	-0.0544	0.04264	-0.1586	0.08791
XM_226612.3	LOC309928	0.22997	0.02551	-0.0292	0.13767	0.03761	0.05355	0.01384	-0.0943	-0.0074	-0.0767	-0.0235	-0.0387	-0.0525	0.0008
XM_226706.4	LOC310013	-0.051	-0.193	0.0023	-0.1217	-0.0613	-0.0309	-0.2432	-0.2262	-0.1072	-0.1731	-0.1026	-0.2508	-0.2616	-0.0209
XM_226733.3	LOC310045	-0.0938	-0.0894	-0.0649	0.24805	-0.0088	0.16687	0.08259	0.15522	-0.0194	0.36352	-0.0937	0.07952	-0.079	-0.0739
NM_001168000	LOC310086	-0.6414	-0.3265	-0.5785	-0.7118	-0.418	-0.2865	-0.3082	-0.267	-0.3347	-0.4388	-0.2865	-0.3225	-0.3701	-0.5848
NM_001035251	LOC310091	0.07786	0.18048	0.00851	0.18379	0.11061	0.10371	0.23402	0.3248	0.03039	0.03588	0.20297	0.32252	0.23089	0.17832
NM_001024987	LOC310177	0.22844	0.08032	0.10745	0.13295	0.26748	0.60602	0.17442	0.40511	0.22843	0.03276	0.15696	0.35296	0.08213	0.1627
XM_226926.2	LOC310212	-0.3133	-0.1635	-0.0649	-0.0437	-0.1655	-0.1553	-0.3786	-0.3245	-0.0298	-0.0594	-0.2766	-0.1202	-0.0847	-0.1494
XM_226929.3	LOC310214	0.1101	0.11581	0.10994	0.12762	0.03794	0.25171	0.04233	0.11141	0.19589	0.17484	0.04311	0.10699	0.21787	-0.004
NM_001107661	LOC310244	0.09392	0.12161	0.06937	0.10848	0.08495	-0.078	-0.1002	-0.069	0.05449	-0.0034	-0.0708	0.03047	0.13023	0.01531
NM_001107663	LOC310269	0.25185	0.04234	-0.1261	-0.0913	0.02466	-0.1192	0.04481	0.01027	-0.1467	-0.0723	-0.0409	-0.1132	0.03469	0.09663
XM_227080.3	LOC310360	-0.0418	0.18811	-0.9554	-0.4128	-0.4021	-0.2071	-0.8758	-0.2831	0.01084	-0.0832	-0.1752	-0.0642	-0.4437	-0.4149
XM_227096.3	LOC310382	-0.3234	-0.4423	0.55595	-0.3446	-0.3692	-0.5139	-0.4206	-0.5666	0.02129	-0.5583	-0.2683	0.66016	0.34836	0.31177
NM_138526	LOC310395	0.21389	0.07392	0.13118	-0.4853	0.37831	0.46461	-0.1015	0.35303	-0.146	0.3599	0.16982	-0.1967	0.1233	0.02635
XM_227169.3	LOC310436	0.11902	0.2816	-0.0198	-0.1063	0.18922	0.06804	-0.0616	0.02284	-0.0583	0.01396	-0.0756	0.0726	0.00914	-0.0205
XM_227179.3	LOC310445	0.01958	0.07363	0.21586	0.06367	0.07835	0.08192	0.06613	0.10371	0.13116	0.0471	0.02335	0.00987	0.09838	0.216
XM_227251.2	LOC310510	0.18518	0.06031	0.05411	0.17958	0.12903	0.27587	0.13691	0.11433	0.16815	0.18969	0.14385	0.01758	0.05915	0.24687
NM_001135716	LOC310544	-0.0807	0.18661	-0.0701	0.13167	0.16274	0.02062	0.01291	-0.02	0.11005	0.14011	0.06654	0.02108	0.14334	0.12189
XM_227368.3	LOC310586	0.13324	0.01991	-0.0508	-0.0563	0.2055	0.24952	0.10278	0.2494	0.03625	0.00525	0.06989	-0.0533	-0.0879	0.0849
XM_227369.3	LOC310587	0.10075	0.05233	-0.0343	-0.0057	-0.0237	0.34135	0.11916	-0.0467	0.00905	0.21643	0.11817	-0.0084	-0.074	0.0159
XM_227345.3	LOC310590	0.06615	0.0286	-0.0358	0.01981	-0.048	0.16616	-0.0418	-0.1632	0.06398	0.09386	-0.0078	0.01544	-0.0235	-0.0431
NM_001047886	LOC310612	0.1891	0.04906	0.93827	0.88521	-0.1348	-0.0303	1.2008	0.15453	-0.1954	-0.1501	0.00612	0.13302	-0.0722	0.10723
XM_227387.1	LOC310613	0.00067	0.01207	0.0796	0.01074	0.18678	0.05984	0.00141	0.09138	-0.0036	0.02708	-0.0441	0.31523	0.02647	0.11048
NM_001033895	LOC310615	-0.219	-0.1494	-0.064	-0.0154	-0.5387	-0.8034	0.13156	-0.5414	-0.3298	-0.2772	-0.3034	-0.6773	-0.4889	-0.3016
XM_227403.3	LOC310631	-0.0106	0.03693	-0.0638	0.12783	0.02992	0.10772	0.1445	-0.0836	0.11346	-0.0764	0.00921	-0.1129	-0.1568	-0.1772
XM_227422.1	LOC310647	-0.061	0.04465	-0.1044	-0.0919	0.07967	0.13912	-0.0201	-0.0322	-0.3131	-0.2381	-0.1057	0.1997	-0.1997	-0.0532
XM_227427.3	LOC310660	0.2105	0.08478	0.20284	0.27856	0.09178	0.43961	0.13152	0.06284	0.2216	0.09539	-0.0117	0.46327	0.08704	0.19678
NM_001107697	LOC310677	-0.0615	-0.0245	-0.1011	-0.0179	-0.0035	0.10089	-0.1552	-0.2091	-0.1025	-0.1337	-0.0885	-0.1379	-0.1042	-0.2099
NM_001014041	LOC310721	-0.2976	-0.1364	-0.2792	-0.2728	-0.1315	0.08582	0.02054	0.12341	-0.0522	-0.1001	-0.31	-0.0372	-0.1674	-0.1367

NM_001107711	LOC310756	-0.0036	-0.0774	-0.005	-0.1913	0.32774	0.61377	-0.1715	-0.0232	-0.0444	-0.0055	-0.1197	-0.0915	-0.147	-0.1165
XM_227557.3	LOC310761	0.08256	0.19438	0.24344	0.15957	-0.0323	0.1458	0.166	0.06988	0.11618	0.08876	0.09196	0.1028	0.13751	0.04943
NM_001039609	LOC310839	-0.0633	-0.1125	-0.0801	-0.3324	-0.2623	-0.0882	-0.2276	-0.0829	-0.1744	-0.1956	-0.107	0.11265	0.09706	-0.1351
XM_227715.3	LOC310879	0.00864	-0.0073	0.08535	0.0572	-0.0122	0.03986	0.04124	0.16759	0.01806	0.30997	0.03728	0.00851	0.01116	-0.0098
XM_227717.3	LOC310880	-0.2924	-0.2468	0.06169	-0.0422	-0.0529	-0.1289	-0.2361	-0.0419	-0.1888	-0.1021	-0.1914	0.16649	-0.0246	-0.2738
XR_009220.1	LOC310902	-0.0475	-0.1212	0.01508	0.09272	0.0237	-0.1021	0.09044	-0.0436	-0.1097	-0.0428	0.00999	0.10376	-0.104	-0.0447
NM_001025002	LOC310926	0.18617	0.01401	-0.6514	-0.1151	-0.0312	-0.0159	0.47159	0.02942	-0.0226	-0.0995	-0.0747	-0.4767	-0.4438	-0.2958
NM_001034926	LOC310946	0.43913	0.11727	0.23039	0.34013	0.18557	-0.1283	-0.0371	0.16595	-0.0615	0.18851	0.05957	-0.1997	0.17181	-0.0093
NM_001134549	LOC310958	-0.2668	-0.0726	-0.3835	-0.3236	-0.1405	-0.459	-0.264	-0.3925	-0.3151	-0.3017	-0.4712	-0.5289	0.04532	0.15157
NM_001134549	LOC310958	-0.6486	-0.9396	-0.4103	-0.2752	-0.9968	-0.9377	-0.414	-0.8793	-0.6037	-0.7745	-0.567	-0.4893	-0.3685	-0.4597
XM_227820.4	LOC310968	0.2516	0.02178	0.07077	0.2555	0.21776	0.04789	0.03303	0.17228	0.05184	0.06892	0.21901	0.06386	0.21485	0.08468
NM_001100509	LOC310988	0.06834	-0.1377	0.03823	-0.061	0.02215	0.02595	-0.0281	-0.0511	0.03282	0.0192	0.14367	-0.0071	0.16069	0.017
NM_001107732	LOC311051	0.15541	0.05876	0.11482	0.08989	0.02695	0.30144	0.13132	0.01862	0.06322	0.1027	0.11285	0.08768	0.0198	0.03817
XM_229993.3	LOC311093	0.05581	0.03618	0.10684	-0.0068	-0.0513	0.10905	-0.0986	-0.1041	-0.0783	0.04056	-0.0462	0.02585	0.06459	-0.0336
NM_001134550	LOC311115	-0.0437	-0.0727	-0.1108	-0.0209	-0.248	-0.5236	0.00776	-0.1231	-0.041	0.03405	-0.1543	0.09963	-0.3186	0.08254
NM_001122975	LOC311171	-0.1379	0.01954	-0.0323	-0.026	-0.0896	0.00188	0.1438	0.02414	0.08136	-0.0087	0.17396	-0.0722	0.14821	0.20925
NM_001044242	LOC311254	0.4255	0.26421	0.06237	0.50836	0.44782	0.45736	0.30451	0.36152	-0.0632	0.24055	0.08715	0.06182	0.52514	0.17892
XM_230530.3	LOC311323	-0.0132	-0.0889	-0.0976	-0.0903	-0.1177	0.11028	-0.0346	-0.1281	0.0616	0.04715	-0.0933	-0.117	0.03079	0.22164
NM_001033999	LOC311338	-0.0093	-0.0087	0.24706	-0.0063	-0.0309	-0.1285	0.02563	-0.0188	-0.1215	-0.2025	-0.1222	-0.0567	-0.0558	-0.1652
NM_001014047	LOC311352	-0.2028	-0.0502	0.08053	-0.2641	-0.1393	-0.2942	0.00799	-0.2798	0.04009	0.00613	-0.0983	-0.0456	0.04485	-0.0163
XM_242139.3	LOC311372	0.19899	0.01705	0.17649	0.51592	0.01854	0.22946	0.02865	0.33871	0.07898	0.1125	0.22662	0.06148	0.03108	0.13998
XM_230560.2	LOC311382	0.40148	-0.0085	-0.0146	0.05226	0.13127	0.6988	-0.3174	0.12981	0.19777	-0.3117	0.16494	0.69234	0.49272	0.29796
NM_001107770	LOC311401	-0.0868	-0.1752	-0.221	-0.0629	-0.216	-0.1412	-0.0627	-0.1354	-0.2431	-0.2018	0.01935	-0.0199	-0.1832	0.03862
NM_001025691	LOC311428	0.0616	-0.0497	-0.1285	-0.0536	-0.0062	-0.0145	-0.0626	0.10055	0.22884	-0.0175	0.14694	0.0444	-0.0492	0.02775
XM_230651.3	LOC311496	-0.0021	-0.0577	-0.1499	-0.1155	-0.1493	-0.087	-0.0441	0.12412	-0.093	-0.1312	-0.1335	-0.0084	-0.1134	-0.1281
XM_230710.3	LOC311527	0.09258	-0.0396	-0.0846	-0.0064	0.00552	-0.0141	0.01412	-0.0492	-0.0731	-0.0325	0.11132	0.00288	0.07443	0.01893
NM_001037198	LOC311536	0.10924	-0.0033	-0.0093	-0.0224	-0.0669	-0.0232	0.04103	0.25522	-0.0311	0.15792	-0.007	0.0069	0.07067	0.01303
XM_230743.3	LOC311550	-0.0048	-0.0361	-0.1263	0.12777	-0.0051	-0.0121	0.00899	0.05113	0.19237	0.00974	-0.0342	0.05549	-0.0351	0.00691
XM_001067659.1	LOC311578	-0.0771	-0.1137	0.25715	-0.0288	0.01332	-0.1462	-0.0031	-0.092	-0.0777	0.05786	-0.0204	-0.0555	-0.1265	-0.1188
XM_230844.3	LOC311629	0.0589	0.20286	-0.0973	-0.2787	0.0401	-0.0065	-0.0951	0.19209	0.18011	0.02489	0.12207	-0.0477	-0.0093	-0.0414
NM_001134551	LOC311692	0.16888	0.11875	0.14011	0.01303	-0.0203	-0.1134	0.13207	-0.096	-0.1539	0.01884	0.33596	-0.012	0.27973	0.16729
XM_230955.3	LOC311710	-0.0557	-0.1702	-0.2455	-0.0019	-0.1536	-0.0867	-0.0199	-0.1259	-0.0622	-0.0015	0.00715	0.00785	-0.0837	0.24066
NM_145090	LOC311716	-0.4123	-0.0155	-0.2074	0.87032	-0.252	0.02273	-0.1771	0.17085	-0.25	-0.1703	-0.3335	-0.32	-0.0709	-0.3891
NM_001134552	LOC311760	0.07796	0.01842	0.2501	0.09449	-0.0213	0.18682	-0.0381	0.10222	0.02086	-0.0095	0.16014	0.07488	-0.0149	0.0349
XM_231028.3	LOC311772	-0.3467	-0.9632	-0.7423	-0.4436	-0.6854	-0.843	-0.8239	-0.9139	-0.286	-0.957	-0.7691	-0.1486	-0.5444	-0.2934
XM_231125.3	LOC311827	-0.0585	0.20155	0.19965	-0.0571	-0.0706	0.20928	0.02826	-0.0431	0.01527	0.00512	-0.0211	-0.0563	-0.0746	0.06287
NM_001100979	LOC311852	0.02575	0.10922	0.07188	0.07648	0.19288	0.17853	0.12766	0.08278	-0.016	0.08086	0.07533	0.33593	0.11315	-0.0504
XM_231169.2	LOC311889	-0.0399	-0.287	-0.1587	-0.2482	-0.1371	-0.2019	-0.1888	-0.0488	-0.1656	-0.299	-0.1621	-0.0668	-0.2676	-0.2862
XM_231245.3	LOC311908	0.03375	0.0215	0.05712	0.16571	0.00619	0.0598	0.05675	0.11673	0.18829	-0.096	0.04093	0.11576	0.10487	-0.0308
NM_001107845	LOC311984	0.11782	0.00364	0.05851	0.04042	0.15895	-0.0077	0.17099	0.02887	0.09631	-0.0199	0.03044	0.25701	-0.0298	0.04258
XM_231361.3	LOC312030	-0.0465	-0.1108	-0.0212	-0.0378	-0.0664	-0.0709	-0.0874	-0.1605	-0.1325	0.17443	-0.0279	-0.095	0.26675	-0.1422
XM_231564.3	LOC312192	-0.0462	0.00577	-0.0352	0.08937	-0.0077	-0.0277	0.09556	-0.0472	-0.0395	-0.0675	-0.0433	0.01355	0.03751	-0.0037
NM_001017456	LOC312199	0.11475	0.02745	0.00626	0.15876	0.04296	-0.1345	-0.0253	0.25901	-0.1423	0.09155	0.05467	-0.2046	0.13696	0.09555
XM_231601.3	LOC312226	-0.1998	-0.3016	-0.2254	0.00313	-0.0493	-0.0812	-0.0917	-0.2153	0.22218	-0.0542	-0.1349	-0.1138	0.06435	-0.1335
NM_001107856	LOC312273	-0.0219	-0.2172	-0.1703	0.03973	-0.1144	0.05074	-0.0542	-0.1682	-0.0115	-0.1593	-0.2323	-0.228	-0.274	-0.2131
NM_001134979	LOC312299	-0.3693	-0.5981	0.08651	-0.5354	-0.8563	-0.8856	-0.5432	-0.8725	-0.0553	-0.5567	-0.4876	-0.0829	0.42531	0.36099
XM_231787.3	LOC312365	-0.3735	0.02003	-0.1312	-0.2609	-0.0626	0.17633	-0.3265	-0.1267	-0.1419	-0.2577	-0.0903	-0.1492	-0.0257	-0.011
XM_001073801.1	LOC312502	-0.0702	0.00648	0.00523	0.15942	0.15401	0.1952	-0.0506	0.00884	0.52071	0.12372	-0.0093	0.34034	0.08484	-0.0284
XM_232287.3	LOC312654	-0.1249	-0.2222	-0.3835	-0.6348	-0.1029	-0.2737	-0.4541	-0.2932	-0.1662	0.03701	-0.007	-0.176	-0.4799	-0.1732

XM_232319.3	LOC312667	-0.2357	-0.5772	-0.2831	-0.1022	-0.5442	-0.7821	-0.4799	-0.5717	-0.7145	-0.8514	-0.8244	-0.5202	-0.517	-0.5799
XR_005442.1	LOC312678	-0.0443	0.03733	-0.1049	-0.0445	-0.012	0.05383	0.18898	0.07613	0.1158	0.21056	0.04776	0.03937	0.09135	-0.0174
XM_232253.3	LOC312683	0.05265	-0.081	0.07742	0.11553	0.45628	-0.2499	0.11546	0.0467	-0.1977	-0.1382	0.19947	-0.1844	-0.0175	0.15335
NM_001014058	LOC312688	1.3931	0.89272	0.03836	0.25892	1.39	0.9975	0.50841	1.7662	0.58458	0.69154	0.32888	-0.1636	1.0727	0.99589
XM_232658.2	LOC312959	0.18561	0.08329	0.03479	0.01059	0.02207	0.04588	0.04245	0.02749	0.20893	0.02201	0.05392	0.07561	0.02519	0.34605
XM_232753.3	LOC313040	0.05264	-0.0039	0.21423	0.10813	0.06534	0.08695	0.00205	-0.0357	-0.0042	0.03778	-0.0341	0.2079	0.11698	0.20514
XM_001060201.1	LOC313045	-0.0758	0.00146	-0.212	-0.0893	-0.0822	0.10361	-0.014	0.17001	-0.2025	0.01094	-0.0032	-0.1264	-0.0534	0.02476
XM_232779.3	LOC313060	-0.2602	-0.2691	-0.2383	-0.139	0.05481	-0.2352	-0.0247	-0.2712	-0.0943	-0.1427	-0.1968	-0.1749	-0.199	-0.1471
NM_001107916	LOC313067	-0.1092	-0.0396	-0.3	-0.2	0.02852	-0.1749	0.18926	-0.1036	0.17232	0.37968	0.02085	-0.1006	-0.0212	-0.1859
XM_232840.3	LOC313111	0.16462	0.04625	0.37764	0.10698	0.17288	0.00418	-0.0259	0.45247	0.30793	0.1775	0.04636	0.17041	0.54271	-0.061
NM_001135754	LOC313125	-0.3769	0.06258	-0.2609	0.06575	-0.2666	-0.0566	-0.4398	-0.2892	0.04233	-0.2876	0.04543	0.10114	-0.2206	-0.2433
NM_001025697	LOC313200	-0.089	-0.0179	0.01407	0.06109	-0.057	-0.1134	-0.1556	-0.0813	-0.1253	-0.0939	0.04891	-0.0948	-0.1154	-0.1354
XM_232943.3	LOC313202	0.10471	0.2983	0.20624	0.03494	0.09095	-0.1368	0.05629	-0.0109	0.1445	0.12815	0.00792	-0.0656	0.03879	0.17598
XM_232947.3	LOC313204	-0.0945	-0.0309	-0.0123	0.16461	-0.05	-0.0443	-0.132	-0.0698	0.149	-0.0069	0.23019	0.03969	-0.0783	-0.0837
XM_232988.3	LOC313233	0.00032	0.03399	0.1434	-0.0232	0.04049	0.01561	-0.0438	0.02006	0.05791	0.07958	0.10539	0.07153	0.27381	0.07938
NM_001107938	LOC313245	-0.2549	-0.3023	0.05608	-0.0517	-0.2061	-0.3373	-0.1028	-0.2214	-0.1876	-0.4501	0.01103	-0.0385	-0.0984	0.11621
XM_233106.2	LOC313303	0.08645	0.05336	0.13628	0.25557	-0.0233	-0.0133	0.06024	0.04909	0.23324	-0.0018	0.17008	-0.0174	0.03057	0.18628
XM_233139.3	LOC313340	0.24876	0.20321	-0.2681	0.29684	0.25489	-0.3405	0.08472	0.33895	0.3086	0.09668	0.3568	-0.2738	0.09589	-0.0829
NM_001107948	LOC313391	0.0087	0.34621	-0.3689	-0.4236	-0.306	-0.4201	-0.0394	-0.0142	0.10671	0.11773	-0.0912	-0.1936	0.30478	0.16181
NM_001107950	LOC313433	-0.2636	-0.1859	-0.1571	-0.2554	-0.3059	-0.2005	-0.163	-0.354	-0.1048	-0.4008	-0.2321	-0.0254	-0.2578	-0.0213
XM_233356.3	LOC313484	-0.0238	-0.0976	0.09004	-0.2207	0.01498	-0.1749	-0.1299	0.05705	-0.1567	-0.0866	-0.1161	-0.1774	0.21345	-0.1906
XM_233379.3	LOC313493	-0.1846	-0.057	-0.0549	0.08069	-0.172	-0.2188	-0.1237	0.01775	-0.0665	-0.1845	-0.053	-0.1977	0.16672	-0.0876
XM_233435.3	LOC313535	-0.0704	0.16531	0.1133	-0.0868	0.06622	-0.0824	-0.0051	0.14471	0.11051	0.01971	0.01493	0.0488	0.23252	-0.0109
NM_001034081	LOC313615	0.16241	0.03816	0.10406	0.17992	-0.0037	-0.1032	-0.026	0.23783	0.09464	0.25342	-0.008	-0.0278	-0.0334	0.0464
XR_005454.1	LOC313641	0.05601	-0.0536	0.00525	0.00541	0.05794	0.04574	0.03764	0.01672	0.20008	0.24761	0.15832	0.19013	0.07844	0.00998
NM_001039026	LOC313658	-0.5444	-0.0694	0.02815	0.44632	-0.3302	-0.2342	-0.122	-0.21	-0.3856	-0.2814	-0.2495	-0.3043	-0.6308	-0.5512
NM_001034149	LOC313668	0.16538	0.16213	0.06012	0.1089	0.0626	0.17534	0.17876	0.07804	0.114	0.02869	0.14582	0.12721	0.25087	0.05747
NM_001014070	LOC313672	-0.0986	-0.2676	-0.048	-0.1182	-0.1921	-0.0843	-0.039	-0.1787	-0.0718	-0.1474	-0.2782	-0.0567	-0.1715	-0.2222
NM_001014070	LOC313672	0.00142	0.06091	0.232	-0.0171	0.07933	0.01383	0.17458	0.01572	0.01839	0.19087	0.28309	0.04084	0.06489	0.02738
XM_233684.2	LOC313720	0.13976	0.06268	-0.0005	-0.1024	0.05055	-0.1086	-0.1234	-0.1249	-0.1252	-0.1636	-0.0461	0.20641	0.14652	0.17924
NM_001037650	LOC313749	0.10899	0.18959	0.41432	0.26771	0.27	0.32596	0.2111	0.08619	0.29675	0.11589	0.33167	0.40547	0.22879	0.16112
XM_233707.2	LOC313760	0.17015	0.03893	0.13095	0.19133	0.2128	0.32013	0.0702	0.04551	0.00037	0.33856	0.25007	0.13363	0.09909	-0.0081
NM_001034949	LOC313824	-0.0433	0.25743	0.10833	-0.3078	-0.1202	-0.4077	-0.0661	-0.423	0.03758	-0.0243	-0.0158	0.09515	0.07049	0.28636
XM_233802.3	LOC313831	-0.2769	-0.1658	-0.1967	-0.1848	-0.192	-0.381	-0.1802	-0.2605	-0.1836	-0.1933	-0.1135	0.06328	-0.0781	-0.2841
XM_001067157.1	LOC313934	-0.0455	0.19456	0.00294	-0.078	-0.039	-0.3624	0.35188	-0.266	-0.0714	0.18629	0.03451	-0.2679	-0.236	-0.2071
NM_001014074	LOC313936	-0.0307	0.01774	0.13267	0.08515	0.00963	0.2354	-0.2093	0.08263	0.08712	-0.0823	0.0042	0.24208	-0.0461	-0.0723
XM_233953.3	LOC313940	-0.4544	-0.2767	-0.2372	0.39213	-0.3696	-0.1447	0.40975	-0.3637	-0.4774	-0.4902	-0.3386	-0.24	-0.4734	-0.4045
XM_234034.3	LOC313967	0.11094	0.27721	0.05563	0.30448	-0.0258	-0.014	-0.1261	0.01566	-0.1579	0.00061	0.10316	0.43292	0.01282	0.05677
XM_234042.3	LOC314030	0.13225	0.07269	-0.0211	-0.0963	-0.0604	-0.0084	-0.1232	-0.0761	0.1157	-0.0717	-0.0157	-0.0503	0.20575	0.19758
XM_234218.3	LOC314162	0.09733	0.21593	0.13379	0.13608	-0.1729	0.12322	0.016	0.13305	-0.0281	-0.1116	-0.0775	-0.0194	-0.0786	0.13444
XM_234301.3	LOC314232	-0.143	-0.1687	-0.1677	0.08585	-0.2968	0.03471	-0.2482	-0.1664	-0.2028	-0.1571	0.08896	0.02527	0.13742	-0.0053
NM_212522	LOC314251	0.03038	0.12921	-0.112	-0.2153	-0.1365	-0.1301	-0.169	-0.118	0.34482	-0.1893	-0.2177	-0.2594	0.05489	-0.0149
NM_001033967	LOC314258	-0.0961	-0.2224	-0.0204	-0.2445	-0.048	-0.1798	-0.2402	-0.3412	-0.1496	-0.1353	-0.1178	-0.1691	-0.0492	-0.0257
XM_234410.3	LOC314311	-0.105	-0.2227	-0.1861	-0.243	-0.1037	-0.212	-0.2232	-0.1548	-0.1584	-0.1688	-0.1943	-0.0931	-0.2542	-0.1012
NM_001134559	LOC314325	-0.0228	0.4292	-0.4028	0.12366	0.07467	0.10201	-0.1237	-0.001	0.13185	0.0085	0.1653	-0.0285	0.03049	-0.083
NM_001037783	LOC314328	-0.469	-0.265	-0.3449	-0.3553	-0.28	-0.5199	-0.269	-0.1486	-0.218	-0.2748	-0.3813	-0.3641	-0.1658	-0.2748
XM_234491.2	LOC314394	0.04095	0.02777	0.07157	0.01238	-0.0505	-0.0305	0.00479	-0.0485	0.02213	-0.0774	-0.0196	-0.0521	-0.0357	-0.057
NM_001108058	LOC314431	-0.0664	-0.0428	-0.009	-0.0514	0.12891	-0.0157	-0.056	0.15707	0.11647	-0.0541	0.04143	0.01161	-0.0303	-0.1322
NM_001135894	LOC314443	-0.1353	0.07285	-0.0439	-0.0127	0.0791	0.11666	0.12005	-0.0481	-0.048	-0.1057	-0.0279	0.13744	0.00484	-0.1315

NM_024351	LOC314468	-0.2669	-0.2575	-0.19	-0.3008	-0.3339	-0.0666	-0.2286	-0.4882	-0.4254	-0.2942	-0.2729	0.0001	-0.1487	-0.1213
NM_001126280	LOC314472	0.0285	-0.1215	-0.1048	-0.183	-0.1042	-0.0371	-0.0329	-0.0745	-0.1393	-0.1986	-0.1534	-0.0981	-0.0809	-0.1109
XM_234597.2	LOC314492	0.0204	-0.0724	-0.0134	-0.0167	0.05063	0.00874	-0.0843	-0.0186	-0.1102	-0.0601	0.0757	-0.1329	0.08423	-0.0193
XM_234602.3	LOC314494	-0.169	-0.0442	-0.1233	-0.1781	-0.104	-0.094	-0.1628	-0.1227	0.01464	-0.0537	-0.0874	-0.1708	-0.0864	-0.1198
XM_234622.3	LOC314501	0.22286	-0.0357	0.02181	0.03498	0.11468	0.10919	-0.0499	0.02223	0.05194	-0.0031	0.07907	0.17154	-0.0754	-0.0583
XM_234661.3	LOC314509	0.04281	0.1695	0.18788	-0.0186	0.02518	-0.0784	0.08489	0.07215	0.02227	0.06992	0.00833	0.02051	0.07984	0.11174
XM_234676.2	LOC314512	0.19224	0.07279	0.14464	0.53273	0.21408	0.02871	0.12306	0.0928	0.05694	0.16933	0.04269	0.06768	0.02827	0.2328
XM_234747.2	LOC314521	0.33135	0.15985	0.33295	0.19195	0.33595	0.20282	0.35914	0.47014	0.41897	0.36114	0.22024	0.20743	0.43655	0.12395
XM_234810.3	LOC314570	0.15275	0.00594	-0.075	-0.0921	-0.1071	0.12014	-0.0964	-0.1633	-0.0898	0.01803	0.1146	0.21291	-0.0624	0.00125
NM_001134561	LOC314584	0.07438	1.0155	0.76686	0.87501	-0.0522	-0.1252	1.2991	0.04014	0.93349	0.82649	0.89613	0.89352	0.48331	0.55183
XM_234852.3	LOC314592	0.12122	0.03961	0.04018	0.0011	0.20481	0.04126	-0.0366	0.21197	0.26303	0.01761	0.24026	0.10191	0.15298	-0.0338
NM_001126281	LOC314600	-0.1821	0.22677	-0.3161	-0.1579	-0.2052	-0.2424	-0.0822	-0.2105	-0.296	-0.0311	-0.2882	-0.0665	0.04752	-0.1806
NM_001135603	LOC314647	-1.1607	-1.2685	-1.3573	-1.3302	-1.647	-1.7294	-1.4	-1.4822	-1.5402	-1.4091	-1.3941	-1.6789	-1.5504	-1.5331
XM_234980.3	LOC314670	0.02024	-0.0643	0.01043	0.0022	0.02935	0.07829	0.06514	0.02304	0.11409	0.05596	0.07548	0.00424	0.01946	-0.0096
NM_031353	LOC314782	-0.1884	-0.1829	-0.0828	-0.1929	-0.3388	-0.0405	-0.1369	-0.2861	-0.1654	-0.1965	-0.1439	0.04363	-0.176	0.02661
XM_235115.3	LOC314814	0.02724	0.0434	-0.0417	0.05231	0.11281	0.11324	-0.0003	-0.0067	0.0765	0.19688	0.07521	0.02366	0.13464	-0.0137
XM_235171.3	LOC314858	0.10777	-0.0704	-0.0426	-0.0976	-0.0337	-0.1296	-0.1861	0.04632	-0.1021	-0.0571	-0.1266	-0.203	-0.0072	-0.067
XM_235213.3	LOC314903	0.15187	0.09528	0.01058	0.01917	0.16439	0.1549	0.07493	0.21005	0.26377	0.04147	0.06291	0.13223	0.11254	0.03289
NM_001025702	LOC314964	-0.9303	-0.4758	-0.01	-0.2662	-0.9115	-1.212	0.16695	-0.7117	-0.2824	-0.3256	-0.5034	-0.4701	-0.0987	-0.1331
NM_001025007	LOC314992	0.13847	0.10888	0.01147	0.18931	0.16916	0.066	0.10064	0.27631	0.1633	0.18823	0.16696	0.23559	0.26258	0.13931
NM_001025008	LOC314996	-0.1811	-0.1663	-0.2133	-0.2883	-0.3448	-0.2071	-0.1796	-0.0598	0.3488	-0.3315	-0.027	-0.1435	-0.1207	-0.1007
NM_031112	LOC315035	-0.021	0.01011	0.03067	-0.0168	0.17407	0.11699	0.03834	0.0338	0.14182	0.15465	-0.066	-0.0493	-0.0207	0.03341
NM_017150	LOC315051	-0.0061	-0.0397	0.01333	0.00132	-0.0265	-0.0055	-0.0097	-0.0754	0.1718	0.05583	-0.0548	-0.11	-0.0393	-0.076
XM_235402.3	LOC315059	0.03232	0.3276	0.26638	0.41449	0.31347	-0.1325	0.08109	-0.0018	0.07032	0.29216	0.26165	-0.1492	0.0467	0.00179
XM_235459.3	LOC315106	0.81489	0.7403	0.72637	1.3938	1.9373	1.2011	1.1027	1.8951	0.39066	0.61735	0.48396	-0.0816	0.66674	0.41804
XM_235476.3	LOC315117	-0.0146	-0.0082	-0.088	0.04992	-0.0406	-0.0443	-0.0739	-0.0209	-0.0646	0.03424	-0.1207	-0.1014	0.23814	0.10252
NM_001130502	LOC315145	0.11143	0.07539	0.20863	0.18863	-0.046	0.10836	-0.0037	0.2259	0.09756	0.00481	0.09566	0.08389	0.24311	0.17185
NM_001025703	LOC315160	0.16867	-0.0065	0.22403	0.05099	0.18951	0.02182	-0.2524	0.39372	-0.0452	-0.0248	0.0319	-0.2436	0.29815	0.1367
NM_001037190	LOC315205	0.0466	-0.0227	-0.0462	-0.1105	-0.1224	-0.0036	-0.0105	-0.1175	-0.0107	0.02242	-0.0475	-0.0202	-0.0987	-0.1192
XM_235649.3	LOC315297	0.13696	-0.1006	0.14513	0.3481	0.047	0.13151	0.24865	-0.1053	0.05494	0.03627	-0.0013	0.28948	-0.1462	-0.0845
XM_235695.3	LOC315337	-0.5361	-0.5089	-0.3918	-0.0401	-0.2402	0.06266	-0.3802	-0.2823	-0.1033	-0.1409	-0.5168	0.66965	-0.4057	-0.4165
XM_235746.3	LOC315372	0.0211	-0.0124	-0.0417	0.03858	0.086	0.01649	0.07808	-0.0055	0.05772	-0.0432	0.05132	0.10031	0.00989	0.10619
XM_235805.3	LOC315409	-0.1198	-0.1445	-0.0827	0.02056	-0.1175	-0.0458	-0.154	0.11691	-0.257	-0.2354	-0.2269	0.09064	-0.1457	0.02292
XM_235970.3	LOC315496	-0.529	-0.1208	-0.615	-0.801	-0.5244	-0.5879	-0.3267	-0.6847	-0.1102	0.03398	-0.3662	-0.3112	-0.459	-0.3471
NM_138890	LOC315499	0.08649	-0.1121	0.15966	-0.17	-0.0718	-0.2299	-0.3013	0.09581	0.03717	-0.0908	0.20374	0.25377	0.17195	-0.1109
XM_235959.3	LOC315510	-0.0695	0.17156	-0.1353	0.14626	0.19462	0.15098	0.11027	0.00385	-0.0011	0.07234	0.10466	-0.1182	-0.0159	0.1607
XM_235988.2	LOC315547	-0.0278	0.22607	0.74749	0.66793	0.26537	0.19246	0.2753	0.4061	0.16465	-0.0291	-0.0553	0.38045	0.13474	0.4653
NM_001108134	LOC315554	-0.0062	0.16811	0.1486	0.02463	0.21561	-0.008	-0.0347	0.13899	0.08726	0.10447	0.22038	-0.0235	-0.0095	0.00454
XM_243842.3	LOC315651	-0.0645	-0.0108	-0.0495	0.07266	0.08886	0.18775	0.11989	-0.0137	0.01903	-0.0203	-0.1183	-0.0247	0.00644	0.05044
NM_001025123	LOC315664	0.16749	0.221	-0.1934	-0.3157	0.43721	0.16228	-0.3504	0.52555	0.10786	0.16368	0.39541	-0.093	0.24905	0.37985
NM_001108149	LOC315686	-0.2419	-0.1269	-0.2486	0.34392	-0.1754	-0.181	-0.2474	-0.2235	-0.2581	-0.2622	-0.3167	-0.3873	-0.0918	-0.3022
NM_001014091	LOC315712	-0.0247	-0.0278	-0.0155	-0.0748	-0.0333	0.00517	-0.054	0.02059	-0.102	-0.1535	0.12993	0.14543	-0.0299	0.15818
XM_236312.3	LOC315728	-0.0852	0.14288	0.12695	0.01803	-0.0735	0.06343	-0.0621	0.04656	-0.1275	-0.1035	0.09157	0.04875	-0.0402	-0.0147
XM_236339.3	LOC315732	-0.0648	0.13266	0.08929	0.23486	0.04928	0.37986	0.02354	0.36058	-0.1569	-0.096	-0.1015	-0.1666	0.00728	0.07659
XM_236358.4	LOC315766	-0.0072	0.016	0.00801	0.04439	0.01634	0.02721	-0.0027	-0.0658	-0.005	-0.0064	0.0757	0.08241	0.06776	-0.0049
XM_236524.3	LOC315907	-0.0866	-0.1074	0.21472	-0.0169	0.11845	-0.0844	-0.0542	0.09425	0.04042	-0.0701	-0.0668	-0.1408	0.0526	0.07495
XM_236532.2	LOC315910	0.15249	0.13222	0.07919	0.06861	0.07843	0.0923	0.25045	0.21767	0.14875	0.03448	0.0406	0.04429	0.1957	0.04381
NM_001037784	LOC315920	2.186	0.35477	0.1087	0.25998	0.01146	0.01221	0.1228	0.15837	-0.109	0.29478	0.15184	0.03622	0.15387	0.14672
NM_001108181	LOC315973	0.06631	-0.3485	-1.4876	-0.9025	-0.6077	-0.8213	-0.6601	-0.4777	-0.2069	-0.2627	-0.4113	-0.4913	-1.011	-0.9363

XM_236593.3	LOC315979	0.05439	0.14397	0.05895	0.02585	0.15846	0.25555	0.17831	0.07325	0.10613	0.26011	0.08223	0.03027	0.01076	0.15358
XM_236612.3	LOC315987	-0.1258	0.22829	-0.3859	-0.3813	-0.0532	-0.0592	-0.0437	0.03627	-0.015	0.2078	-0.0461	-0.0867	-0.003	-0.0642
XM_236647.3	LOC316012	0.05313	-0.0657	0.06101	0.01777	0.09669	-0.2759	-0.1118	-0.0901	-0.1056	0.03271	-0.019	-0.198	-0.0785	0.08488
XM_236683.2	LOC316048	0.0531	0.09703	0.09253	-0.0431	0.14011	0.0655	0.11713	0.03648	0.21486	0.2721	0.16704	0.05637	0.15758	0.02189
NM_001134565	LOC316085	-0.1501	-0.2744	0.0764	0.61603	0.06223	0.50134	-0.2211	0.05779	-0.1426	-0.0755	0.02032	0.09657	-0.2717	-0.3619
XM_236722.3	LOC316087	-0.0494	0.22076	0.10118	0.05228	-0.1697	-0.0274	0.13848	0.0217	0.10744	-0.1318	0.21694	-0.0415	-0.1146	-0.0885
XM_001061015.1	LOC316130	0.04749	0.14083	0.11539	-0.7277	0.45194	0.05071	-0.0301	0.20468	0.06471	0.28899	0.19512	-0.1918	-0.1094	-0.0087
XM_236876.3	LOC316186	0.05855	0.12239	0.00729	0.43915	0.15121	0.15681	0.24294	0.03719	0.15222	0.16213	0.0347	0.10974	0.19602	0.08026
XM_236887.3	LOC316192	0.48425	0.25004	0.18776	0.19823	0.05948	0.00664	0.14148	0.10943	0.20216	-0.0378	0.11959	0.11964	-0.0941	0.29926
XM_236901.3	LOC316207	-0.2226	0.15311	-0.0702	0.07601	-0.0928	-0.0696	-0.1011	-0.0239	0.11188	0.13566	-0.0971	-0.0891	-0.0103	-0.0596
XM_244043.3	LOC316229	0.1887	0.07479	0.32781	0.01036	-0.0448	0.19394	0.14216	-0.1222	-0.0275	0.17678	0.28816	0.18549	0.00435	0.09161
XM_237081.3	LOC316353	-0.1044	-0.015	-0.0391	-0.0388	0.08313	-0.0738	0.07348	-0.161	0.0648	0.2078	-0.0815	-0.0087	0.10788	-0.0024
NM_001108216	LOC316369	-0.1325	-0.1316	0.14439	0.00169	-0.0256	-0.0066	0.04237	0.05775	-0.1251	-0.2518	-0.0863	-0.1658	-0.1712	-0.0135
NM_001109373	LOC316373	-0.1183	-0.4339	-0.0374	-0.168	-0.3754	0.13437	-0.1074	-0.0746	0.03986	-0.4253	-0.2028	0.34619	0.40512	0.42349
NM_001108218	LOC316395	0.20364	0.29704	0.09985	0.28841	0.08389	0.28398	0.42658	0.10606	0.17045	0.19171	-0.0741	0.48787	0.05366	0.1541
NM_001025710	LOC316415	0.13853	-0.01	0.02121	0.32916	-0.0425	-0.0839	0.16052	0.03678	-0.1377	0.22239	-0.0792	-0.0768	-0.1197	-0.0722
XM_237220.3	LOC316440	0.31822	-0.0052	0.05885	0.16844	0.25556	0.12612	0.0818	-0.0903	0.10814	0.16756	0.01002	-0.0148	-0.0007	0.12151
XM_237217.4	LOC316457	-0.0144	0.06381	0.03176	-0.0481	-0.0933	-0.1323	-0.1242	-0.0178	-0.0258	-0.0865	-0.1449	-0.2181	0.04745	-0.0163
NM_001134963	LOC316482	0.08745	0.0445	0.14031	0.17861	0.31859	0.04435	0.06177	0.01444	0.18016	0.34572	0.20085	0.31315	0.32978	0.1493
NM_001108223	LOC316507	-0.0926	-0.2173	-0.0776	-0.2296	-0.2717	-0.3376	-0.253	-0.2262	-0.2641	-0.3073	0.09079	-0.2589	-0.1062	-0.1799
XM_237326.2	LOC316550	-0.4327	0.16654	-0.6197	-0.4332	-0.3441	-0.5991	-0.1141	-0.1858	-0.0406	-0.1719	0.17258	-0.3064	-0.4246	-0.3953
XM_001077059.1	LOC316573	2.0681	1.4007	1.6751	2.0637	2.3752	2.0042	1.4985	2.4118	1.0161	1.4318	1.3213	0.62786	2.2541	2.2377
XM_237386.3	LOC316616	0.26806	0.33895	0.2423	0.02394	0.09496	0.51675	0.1555	0.31007	0.21618	0.36299	0.30441	0.21153	0.21471	0.19547
NM_182671	LOC316632	0.40599	0.53094	0.17524	-0.155	0.39899	0.49105	0.12654	0.46997	0.58201	0.61683	0.53421	0.33704	0.38364	0.40923
XM_237451.3	LOC316653	0.00517	0.11721	-0.162	-0.1197	-0.1369	-0.1417	-0.1756	-0.1529	-0.104	-0.0578	0.04775	0.01924	0.13178	-0.1049
NM_013065	LOC316717	-0.095	-0.1036	-0.1076	-0.1399	-0.1074	-0.1249	-0.1258	-0.0228	-0.1488	0.09635	-0.1295	-0.1525	-0.0193	-0.1006
XM_237548.3	LOC316764	0.06758	0.20175	-0.0024	-0.0828	0.05262	-0.0649	0.14992	-0.0391	-0.0361	0.09158	-0.0019	-0.0522	-0.1769	-0.0653
XM_229258.2	LOC316825	-0.0025	0.1637	0.02352	0.08548	0.16747	0.0007	0.20574	0.16685	0.05664	-0.0398	-0.0208	0.0249	0.00215	0.22217
XM_229259.3	LOC316826	-0.1192	-0.009	0.22862	0.29056	0.30788	0.51125	0.00679	0.21852	0.19636	0.01385	0.24794	0.05816	0.2581	0.35844
XM_229299.2	LOC316846	-0.0305	1.2853	1.9386	1.7225	-0.0178	0.08071	2.7011	0.14884	1.1717	1.6231	1.5533	0.55583	1.0987	1.1301
XM_229302.3	LOC316848	-0.2434	0.53926	1.242	1.0958	-0.2441	-0.2236	1.5829	0.05063	0.45353	0.77124	0.57397	0.36881	0.70763	0.57097
XM_229366.3	LOC316856	-0.1117	-0.1515	-0.1868	0.03074	-0.5363	-0.2913	-0.1256	0.08255	-0.1762	-0.0737	0.00773	-0.159	-0.496	-0.2771
NM_001014221	LOC316919	-0.395	-0.205	-0.2005	-0.1508	-0.1872	-0.0622	-0.0853	-0.215	-0.271	-0.3221	-0.1429	0.24131	-0.2869	-0.2077
XM_229489.3	LOC316920	-0.1149	-0.0852	-0.0454	-0.1672	-0.0296	0.18797	0.02328	0.26434	0.02952	-0.077	0.09862	0.12728	-0.1497	0.14492
NM_001011701	LOC316931	0.01244	-0.0317	0.08736	0.02748	0.01533	-0.0074	-0.0498	-0.0486	-0.0992	-0.0277	-0.0422	0.01853	0.04176	-0.04
NM_001011701	LOC316933	0.0503	0.16697	0.24634	0.04981	0.1593	0.10707	-0.0206	0.14168	0.17069	0.30511	0.17726	0.19866	0.18615	0.2625
NM_001011701	LOC316935	0.03255	-0.0108	0.16327	0.30335	0.04862	0.17083	0.21815	-0.0182	-0.1131	0.20594	0.12418	0.43408	0.32653	-0.0336
NM_001011701	LOC316935	-0.0393	-0.1236	-0.0754	-0.1386	-0.0965	-0.1816	-0.1691	0.01911	-0.1503	-0.2562	0.01188	0.06582	0.08019	-0.1503
XM_229658.3	LOC317026	-0.0094	0.48933	0.26717	0.18912	0.35126	0.23455	0.08394	0.07316	0.31447	0.41568	0.36711	0.26412	0.34829	0.21188
XM_229742.3	LOC317043	-0.0784	0.04981	-0.0163	0.29567	0.07213	-0.1618	0.26095	-0.0026	-0.1475	0.18018	-0.0435	-0.109	0.205	0.14142
XM_229754.3	LOC317051	0.15078	0.16252	0.15189	-0.012	0.25099	0.23047	0.06559	0.22676	-0.0829	0.16126	0.23113	0.32703	0.24227	0.24181
XM_229775.4	LOC317070	0.02601	0.29322	0.06114	0.48212	0.01751	0.16888	0.88878	0.34279	-0.1636	0.15061	0.39226	0.0243	-0.069	-0.1399
XM_229793.3	LOC317085	0.07204	0.04298	0.07638	-0.0244	-0.0116	0.15412	-0.1305	0.02007	-0.0668	-0.0275	-0.0412	-0.039	-0.0141	-0.1054
NM_183402	LOC317106	0.1721	-0.041	-0.0144	0.33449	0.28193	0.54867	0.25934	0.21411	-0.0163	0.04757	0.1377	0.34643	0.17935	0.29799
XM_229834.3	LOC317124	0.00625	0.08454	0.13801	0.03144	0.04034	0.03315	0.22543	-0.0081	0.32958	0.05864	0.04328	-0.0699	0.19863	0.04721
NM_001047892	LOC317165	-0.0007	0.00027	0.07662	0.50195	-0.0004	-0.0032	0.06097	-0.0857	-0.0698	0.08411	0.09498	0.02083	-0.0334	-0.0594
XM_228473.3	LOC317203	0.23217	-0.0126	0.03577	-0.1358	-0.0228	0.02446	0.02646	-0.225	0.40864	0.19589	0.13367	-0.232	0.272	-0.1897
XM_228615.3	LOC317275	-0.3828	-0.0504	-0.0953	0.11136	-0.3046	-0.3545	0.07244	-0.2497	-0.1751	-0.2422	-0.0543	-0.1773	-0.1481	-0.1667
XM_228678.3	LOC317323	0.12352	0.07	0.11768	0.03422	-0.0581	0.08795	-0.0619	0.16777	0.02033	0.14102	0.14081	0.01778	0.18517	0.08286

NM_001126286	LOC317344	0.05805	0.09935	0.08691	0.2001	0.20208	0.09528	0.38404	0.03469	0.18786	0.29087	0.13644	0.14546	-0.0218	0.05698
XM_228733.1	LOC317352	-0.1778	-0.1557	-0.1031	0.15092	-0.0179	-0.2421	0.02196	0.06211	-0.0986	-0.0428	-0.1615	-0.2269	-0.1976	-0.0197
NM_001172103	LOC317366	0.52585	0.17842	0.3503	1.0898	0.51205	0.72205	0.1601	0.61318	-0.0831	0.35434	0.11581	-0.0232	0.46471	0.03927
NM_031840	LOC317394	0.01247	0.31547	-0.0327	0.11075	-0.0045	0.23945	-0.0668	-0.0614	-0.0896	0.01737	0.09162	-0.0774	0.09712	0.04863
XM_228817.3	LOC317402	-0.006	-0.0105	0.03547	-0.0291	-0.0489	-0.1159	-0.0352	0.08562	-0.1492	-0.0065	-0.0759	-0.0272	0.02346	-0.0678
XM_228818.2	LOC317403	-0.4399	-0.2629	-0.4012	-0.0606	-0.0866	-0.2205	-0.0487	0.01713	-0.1652	-0.228	-0.1689	-0.1723	-0.2858	-0.3277
NM_001024267	LOC317409	-0.3049	-0.2792	-0.0364	-0.3253	-0.2952	-0.2991	-0.182	-0.2415	-0.1606	-0.0099	-0.2137	-0.2023	-0.2757	-0.2092
XM_228832.3	LOC317414	-0.046	-0.1973	0.21729	-0.0518	0.05588	0.11746	-0.0778	-0.1129	0.00038	0.06527	-0.193	-0.0802	-0.0597	-0.2122
NM_001115027	LOC317418	-0.1703	-0.2262	-0.3229	-0.2418	-0.1277	-0.3781	-0.409	-0.3021	-0.3233	-0.1493	-0.1797	-0.2478	0.07125	0.12603
XM_228915.1	LOC317464	-0.0545	-0.1756	-0.0893	-0.0611	-0.1257	-0.118	-0.0222	-0.0918	-0.2122	-0.0107	-0.1561	0.00595	0.1059	0.03443
NM_001014112	LOC317471	-0.0359	-0.0436	-0.0377	-0.0235	0.00259	-0.0289	-0.0911	0.09681	0.08918	-0.0168	-0.0204	0.1062	-0.1012	-0.0953
XM_228942.3	LOC317494	-0.0017	0.07442	0.03883	0.19534	0.19254	0.05257	0.15272	0.05965	0.12922	0.19904	-0.0632	-0.0978	0.0323	0.00522
XM_228973.3	LOC317508	-0.0079	-0.0776	0.05029	0.13447	-0.0182	0.08618	-0.0528	0.14769	-0.0826	0.01028	0.2122	0.04702	0.08677	0.213
XM_229078.3	LOC317568	0.0789	-0.2456	0.1759	0.05706	0.087	-0.1144	-0.0451	-0.0225	0.03398	0.0335	-0.003	-0.0504	0.11965	0.03796
XM_229142.3	LOC317588	-0.0175	0.0475	-0.1966	-0.0662	-0.1895	0.00754	-0.1913	-0.0411	-0.1955	-0.176	-0.2131	-0.014	-0.1115	-0.2062
XM_229162.2	LOC317593	0.11508	0.16234	-0.0321	0.07298	-0.0741	-0.0679	0.22245	0.09083	0.0463	-0.0024	0.08477	-0.0794	0.04249	-0.0899
XM_229173.3	LOC317599	-0.1327	-0.0953	-0.2352	-0.1752	-0.0819	-0.1515	-0.1832	0.16963	-0.2055	-0.156	-0.1661	-0.068	0.06003	-0.2402
XM_229183.3	LOC317605	-0.1373	-0.1035	-0.1649	0.12494	-0.1742	0.09384	-0.1842	0.03708	-0.1907	0.06325	0.32615	-0.2299	-0.2445	-0.1487
XM_229223.3	LOC317626	-0.003	-0.0795	0.02127	-0.0231	0.02239	0.01472	-0.0711	-0.0059	0.04777	-0.0419	0.05582	-0.0143	0.14169	0.01278
NM_001008359	LOC353306	0.05712	0.06817	-0.0065	-0.0647	0.00535	0.08967	0.02444	0.0453	0.05098	-0.0088	0.03675	0.17524	0.0852	0.17754
NM_001003706	LOC360228	0.1354	0.81987	0.24964	0.05208	-0.1471	0.38713	5.1089	0.08421	1.0069	0.81626	0.84996	0.44635	0.03584	0.18005
XM_346394.1	LOC360236	-0.1222	-0.1091	-0.1102	0.19414	-0.0188	-0.0748	-0.0559	0.01538	-0.0716	-0.1267	-0.1161	-0.0515	0.22695	0.04344
XM_346405.2	LOC360244	0.4594	0.07065	0.29336	0.24555	0.13503	0.47106	0.3253	0.34151	0.20556	0.20771	0.1957	0.11979	0.05561	0.21645
XM_346435.1	LOC360258	-0.7318	-0.2077	-0.8789	-0.6785	-0.6099	-1.293	-0.5677	-0.5865	-0.0449	-0.2579	-0.3239	-0.3787	-0.4293	-0.413
XM_346531.2	LOC360303	-0.1002	-0.1207	0.57599	0.4524	-0.4459	0.26077	0.34797	-0.277	-0.048	-0.3565	-0.1057	0.25151	-0.2522	-0.3042
XM_346568.1	LOC360318	-0.2767	-0.1964	-0.1749	-0.2225	-0.1272	-0.2189	-0.1942	-0.0351	-0.0576	0.03952	-0.1839	-0.1872	-0.1513	-0.1169
XM_346642.2	LOC360354	0.53387	0.09333	0.22041	0.16993	0.27845	0.10076	-0.0628	0.14524	0.11703	0.12566	0.03607	-0.0189	0.08204	0.04117
XM_346675.2	LOC360370	-0.0617	0.25186	0.06216	0.05913	-0.0511	-0.0929	0.36733	-3E-05	-0.1257	0.08825	-0.0093	-0.0907	-0.0023	0.11442
XM_346666.2	LOC360372	0.14248	0.03622	0.02269	0.04569	0.09771	0.05957	0.01966	0.22964	0.01467	0.06315	0.1363	0.07458	0.01994	0.25564
NM_203332	LOC360395	-0.1143	-0.1038	-0.1171	-0.1169	-0.018	0.09671	-0.1798	-0.0975	0.07246	-0.1906	0.06529	-0.2585	-0.1296	-0.0225
XM_346731.1	LOC360398	-0.1106	-0.1549	-0.0897	-0.086	-0.0614	-0.0788	-0.138	-0.2351	-0.0875	0.20088	-0.1258	-0.1009	0.02249	-0.1301
XM_346749.2	LOC360407	-0.0524	-0.0993	-0.109	-0.2558	-0.2887	-0.3392	-0.188	-0.3141	-0.4944	-0.2493	-0.3004	-0.2665	-0.2139	-0.1841
XM_346805.2	LOC360425	-0.0341	0.02363	0.19225	0.04079	-0.0063	0.05751	0.07442	-0.0626	-0.0975	0.00931	0.12887	0.01853	-0.0096	0.01776
XM_346819.1	LOC360435	1.6977	0.48124	1.9055	0.70062	2.3494	2.1615	1.3296	2.2906	0.74537	0.79514	0.52337	1.0066	1.7603	1.7368
XM_346825.2	LOC360437	0.1031	0.23194	0.17956	0.44019	0.00292	0.14703	0.37458	0.08211	0.31731	0.20717	0.14433	0.04485	0.18603	0.13805
XM_346841.2	LOC360443	0.04022	0.28637	-0.0448	0.0205	0.00386	-0.0231	0.00571	0.04881	0.16728	-0.0012	0.14042	-0.0014	0.0404	0.2643
XM_346849.1	LOC360446	1.8381	0.48728	0.06321	0.7673	0.58606	0.04922	0.66985	0.16566	-0.0696	-0.1272	0.08373	-0.1022	0.29634	-0.156
XM_346882.2	LOC360485	-0.4199	-0.3105	-0.3648	-0.2808	-0.312	-0.1382	-0.1648	-0.3579	-0.3426	-0.3252	-0.3185	0.03284	-0.2684	-0.2883
XM_340769.2	LOC360495	0.17858	0.01636	-0.1484	-0.1145	-0.0549	-0.0637	-0.0459	-0.097	-0.1123	-0.1775	-0.1201	-0.2454	0.02917	-0.1474
XM_340783.2	LOC360509	0.45197	0.49098	0.2056	0.28845	0.41976	0.24673	0.31644	0.50423	0.09493	0.09751	0.05964	0.12431	-0.008	0.31677
XM_340800.2	LOC360527	-0.0111	0.14194	-0.1872	0.20271	-0.0234	-0.0324	0.20601	-0.0442	-0.1009	-0.1246	-0.1598	-0.2531	0.05358	-0.0069
XM_340837.2	LOC360560	0.10345	0.18386	0.36763	0.1053	0.27286	0.16776	0.09111	0.3075	0.4628	0.12217	0.08424	-0.0395	0.05168	0.20784
XM_340843.2	LOC360566	0.10725	0.02806	-0.0706	0.06726	0.2008	0.0409	-0.0599	0.24842	-0.0067	-0.0567	0.04253	-0.0283	0.08388	0.04007
XM_346894.2	LOC360567	-0.0686	0.02102	0.03952	-0.0289	-0.0837	-0.0296	0.02595	-0.0583	0.06976	-0.0101	0.02153	-0.0907	-0.0203	-0.0493
XM_340844.3	LOC360568	-0.2752	-0.3833	-0.0825	-0.0739	-0.3731	-0.3871	-0.1739	-0.126	-0.4257	-0.4249	-0.4387	-0.1662	-0.0316	-0.0981
NM_001172137	LOC360570	-0.1334	0.20591	0.02535	-0.0031	-0.0279	-0.1004	0.04786	0.10724	-0.0726	-0.1087	-0.0099	0.18496	0.13026	-0.0951
XM_340858.2	LOC360578	0.11425	0.05522	0.10304	0.16425	0.12402	-0.0519	0.05882	0.38485	0.31617	0.1679	0.02808	0.09079	0.13426	0.17974
XM_340878.2	LOC360599	0.00329	0.09549	-0.0029	0.01374	0.02576	0.0945	-0.02	-0.0702	0.02114	0.05478	0.22417	-0.0027	0.17501	-0.0603
NM_001047897	LOC360618	0.03978	-0.1429	-0.7354	-0.2518	0.13512	0.29167	-0.3135	-0.0576	-0.0983	-0.0173	-0.1193	-0.1207	-0.0962	-0.1001

NM_001108297	LOC360619	-0.1145	-0.0417	-0.067	-0.1413	-0.1511	-0.0532	-0.0416	0.1469	0.24928	0.04908	0.15579	0.08815	-0.0742	-0.1326
XM_346903.2	LOC360644	-1.6288	-1.049	-0.5347	-1.6169	-1.5068	-1.2807	-0.7783	-1.6279	-0.5814	-0.9734	-0.6978	0.00028	-0.9489	-1.1003
NM_001108302	LOC360645	-0.0389	0.04761	0.02687	-0.182	-0.1182	-0.1295	-0.1486	-0.2815	-0.151	-0.0208	-0.1409	-0.1267	-0.2162	-0.1112
XM_340933.2	LOC360656	-0.08	-0.0349	-0.1938	0.04748	-0.1483	-0.0799	-0.0138	-0.1028	0.05626	-0.0636	-0.1585	-0.0299	0.20301	0.05721
NM_001127549	LOC360661	-0.1354	0.04384	-0.1967	-0.0709	-0.2894	-0.1506	-0.238	-0.213	0.01459	0.03202	-0.1867	0.00881	0.13796	-0.07
XM_346906.2	LOC360669	0.30118	0.43429	0.18003	0.40256	0.31432	0.08741	0.1832	0.34363	0.12735	0.31752	0.35795	0.43631	0.17652	0.26468
XM_340956.2	LOC360684	0.109	0.02207	0.09253	-0.1065	-0.0108	0.17018	0.17396	0.26626	-0.1718	-0.1322	-0.0972	-0.0364	-0.0801	-0.0137
XM_346908.2	LOC360685	-0.1872	-0.1354	-0.1953	0.09976	0.14378	0.06973	-0.2133	-0.2881	0.04596	-0.2253	0.05025	0.22364	0.29563	-0.2621
XM_340960.2	LOC360689	-0.0126	-0.0829	0.02229	-0.0305	0.1423	0.08051	0.08564	-0.004	0.15439	0.09388	-0.0046	0.03858	0.08487	0.17688
XM_340961.2	LOC360690	-0.6679	-0.5426	0.02419	0.05603	-0.9512	-0.689	0.18714	-0.6696	-0.1551	-0.5414	-0.2215	-0.6178	-0.7738	-0.5458
XM_346909.2	LOC360696	0.02335	-0.141	0.34794	-0.0565	-0.0689	-0.0749	-0.0143	-0.0487	0.11758	0.13596	0.18608	0.03263	0.29773	0.33238
NM_001014124	LOC360699	-0.0012	0.09568	0.07724	0.18913	0.28498	0.09803	0.13707	0.25128	-0.0316	0.04375	0.17832	-0.0172	0.09574	0.03805
XM_340981.1	LOC360710	-0.1484	0.04006	0.16237	-0.1797	-0.0761	-0.3317	-0.2583	-0.0751	-0.3226	-0.447	-0.2228	-0.3438	-0.291	-0.2114
NM_001172090	LOC360712	0.14071	-0.026	0.01683	0.06447	0.04107	0.0071	0.04972	0.02668	0.0143	-0.0012	0.0871	0.06877	0.03351	0.02775
XM_001066275.1	LOC360713	0.02695	-0.2049	0.13929	-0.2653	-0.126	-0.0067	-0.1731	-0.0118	-0.1633	-0.3878	-0.1786	-0.6468	-0.0993	0.02318
XM_340988.2	LOC360717	-0.0519	-0.0489	-0.0137	-0.0355	0.0181	0.14744	0.0505	-0.0156	-0.0784	-0.0894	0.00426	0.08275	-0.0215	-0.2199
NM_001109369	LOC360728	-0.1503	-0.2754	-0.1223	-0.16	-0.1648	-0.2492	-0.118	-0.0347	-0.123	-0.0569	-0.1684	-0.1452	-0.1127	-0.1519
XM_341008.2	LOC360735	-0.0007	-0.092	0.03082	-0.0905	-0.0604	-0.0417	0.3488	-0.0589	-0.0482	0.10465	0.00758	0.12201	0.06517	-0.0532
XM_346913.1	LOC360758	-0.1877	0.02598	-0.1359	-0.0997	-0.1868	-0.1741	-0.1919	-0.1576	-0.2062	-0.05	-0.1295	-0.1119	0.12505	-0.0422
XM_001068449.1	LOC360760	-0.8921	-0.4995	-0.1928	-0.1111	-0.845	-0.8627	0.05988	-0.7541	-0.9179	-0.6476	-0.6844	-0.7361	-0.9167	-0.9202
XM_341049.2	LOC360782	0.00717	-0.0592	-0.0318	0.00079	0.07806	-0.0475	0.07907	-0.0944	0.0981	0.05818	0.16659	-0.0382	0.00776	-0.0755
XM_346917.1	LOC360786	-0.2918	-0.2025	-0.1582	-0.1311	-0.1373	0.10601	-0.0393	-0.1376	-0.0516	-0.2496	-0.2052	-0.121	-0.177	-0.1978
XM_341056.2	LOC360790	-0.5406	-0.4931	-0.3078	-0.5473	-0.4529	-0.3122	-0.4161	-0.5004	-0.473	-0.5503	-0.5702	-0.392	-0.3903	-0.3431
XM_341060.2	LOC360794	-0.0561	0.03631	0.0604	0.00475	-0.0295	-0.0364	0.16961	0.05038	-0.0535	-0.0438	0.03566	0.08116	0.13269	0.02881
NM_001025125	LOC360800	0.76017	0.43575	0.04647	-0.3514	0.70924	0.79694	0.23935	0.16496	0.29504	0.12847	0.31816	0.4601	0.4794	0.32081
NM_001108334	LOC360801	0.1223	0.03284	0.04109	0.04686	0.10542	0.3683	0.1016	-0.1421	0.01355	0.08472	-0.0961	0.14479	-0.0721	0.17017
NM_001014128	LOC360807	-0.5213	-0.2337	-0.1944	-0.0044	-0.4635	-0.592	0.23801	-0.2688	-0.2402	-0.4534	-0.1227	-0.2565	-0.5207	-0.6588
XM_573400.1	LOC360815	-0.0631	-0.2817	0.04053	0.01096	-0.2302	-0.2638	0.20826	-0.2377	-0.1439	-0.1188	-0.3236	0.14979	-0.0135	0.27217
NM_001135743	LOC360830	-0.0454	0.28693	0.14424	0.04005	-0.0031	0.08815	0.13032	0.07732	0.0116	-0.0157	0.21144	0.02425	0.18183	0.21612
NM_001025126	LOC360860	0.19248	-0.1377	-0.096	-0.0774	-0.0999	-0.0346	-0.0297	-0.1864	0.07351	0.18068	0.16831	-0.0154	0.03497	-0.0231
NM_001034011	LOC360868	-0.2227	-0.1015	-0.0775	-0.2238	-0.1042	-0.152	-0.0512	-0.1193	-0.1062	0.28767	-0.1469	-0.1267	-0.233	-0.1188
XM_346923.1	LOC360875	-0.2419	-0.2738	-0.0229	-0.1934	-0.3407	-0.2368	-0.2333	0.09157	-0.2717	-0.2579	-0.2366	-0.1677	-0.1688	-0.2528
NM_001127449	LOC360883	0.06657	-0.1028	-0.0141	-0.0865	0.11017	-0.0185	-0.2363	-0.1874	-0.0174	0.06519	-0.1963	-0.1817	0.54839	0.30097
XM_341160.2	LOC360885	0.59209	0.14035	0.35567	2.4343	0.76337	0.77393	0.51867	0.93696	0.32534	0.21191	0.18484	0.38398	0.43575	0.44846
XM_001061895.1	LOC360888	-0.0456	-0.3357	-0.1661	0.22219	-0.1824	-0.6086	-0.2834	-0.4051	-0.0532	-0.3667	-0.3713	-0.0427	0.27355	0.41323
NM_001014133	LOC360910	0.24126	0.37579	0.44199	0.95625	0.17211	0.1223	0.66285	0.39239	0.53983	0.6706	0.62473	0.45121	0.33615	0.1927
NM_001014134	LOC360912	-0.0092	-0.0738	-0.0253	-0.2012	-0.0089	-0.0784	-0.0637	-0.264	0.01671	-0.2633	-0.1561	-0.2009	-0.2926	-0.0371
NM_001108356	LOC360919	-0.4837	-0.997	-0.2161	-0.1639	-0.6131	-0.1404	-0.7943	-0.6929	-0.5807	-0.6036	-0.5355	0.12669	-0.5419	-0.5978
XM_346926.2	LOC360925	0.06355	-0.0689	0.10254	-0.0062	0.03641	0.33758	0.25698	-0.304	-0.0694	0.09613	0.05662	0.23115	-0.1013	-0.0462
XR_009136.1	LOC360932	0.22885	0.84291	0.29012	0.42689	0.1622	-0.0714	0.567	0.27621	0.33103	0.58736	0.35035	-0.0478	0.47386	0.41898
NM_001047903	LOC360933	0.17525	0.05277	0.15784	0.10758	0.11566	-0.0768	0.01284	-0.1139	0.23599	0.25174	-0.0224	-0.0443	0.18634	0.16108
XM_341218.2	LOC360941	-0.3832	-0.3516	0.86745	0.25831	-0.1648	0.52904	-0.0731	-0.0093	-0.2205	-0.3481	-0.3029	0.47414	0.37669	0.4153
XM_341232.2	LOC360954	-0.14	0.16509	0.10625	0.02351	-0.0486	-0.0928	0.02993	-0.0237	0.0533	-0.0671	0.03874	-0.049	0.18168	-0.1198
NM_001017461	LOC360975	0.24658	0.01014	0.22216	0.14927	0.22094	-0.0052	0.11825	0.22057	-0.1307	-0.0609	-0.3648	-0.2565	-0.0005	0.06032
XM_341254.2	LOC360978	-0.0313	-0.0269	-0.2147	0.09245	-0.1588	-0.2782	-0.0341	-0.0598	0.10894	-0.1497	-0.034	-0.1225	-0.1314	-0.131
XM_341256.2	LOC360980	-0.0504	-0.0405	-0.0444	0.09931	-0.2129	-0.0037	-0.1922	0.25139	0.02885	-0.2258	0.00927	-0.1715	-0.1969	0.02015
XM_346932.2	LOC360987	-0.2027	-0.0611	-0.153	-0.0318	-0.0338	-0.1799	-0.1759	-0.2231	-0.2368	-0.1744	-0.2475	-0.0431	-0.0842	-0.2248
XM_341262.2	LOC360988	0.0067	0.1557	0.00041	0.39465	0.2234	-0.1336	0.16293	0.24154	0.17014	0.17033	0.21686	0.07993	-0.099	-0.2004
XR_009611.1	LOC360990	-0.5445	-0.1812	-0.0914	0.18272	-0.6369	-0.5609	-0.0726	-0.5872	-0.1964	-0.246	-0.1081	-0.1849	-0.3959	-0.3205

XR_009611.1	LOC360990	-0.3607	-0.1944	0.15744	0.03035	-0.1595	-0.2919	-0.1499	-0.2046	-0.25	-0.2682	-0.0229	-0.1414	-0.1294	-0.2442
NM_001014133	LOC360997	0.04385	-0.1041	0.38957	0.1916	0.06616	0.09209	0.12327	0.04054	-0.2113	-0.0329	-0.035	-0.044	-0.2028	0.11391
NM_001014133	LOC360997	-0.0847	0.00102	0.11945	-0.1668	-0.0196	0.17517	0.00556	-0.1399	0.18275	0.01977	0.10871	-0.0523	-0.0445	0.09999
XM_341273.2	LOC360998	-0.0859	-0.0286	-0.0409	-0.0595	-0.0621	0.08533	-0.0328	-0.0706	0.14668	0.10657	-0.0222	-0.0032	0.00357	-0.062
XM_341274.2	LOC360999	-0.1152	-0.1284	-0.1851	-0.1402	-0.1199	-0.0272	-0.0898	0.03571	-0.0578	-0.2325	-0.1638	-0.079	-0.0847	-0.141
NM_001014136	LOC361014	-0.4524	-0.1745	-0.2378	-0.718	-0.3968	-0.8146	-0.1318	-0.5203	-0.0335	-0.1541	-0.0548	-0.2344	-0.3401	-0.2694
NM_001014137	LOC361016	-0.3001	-0.3404	-0.265	-0.343	-0.2672	-0.3454	-0.1151	-0.262	-0.2229	-0.4165	-0.2418	-0.3594	-0.1533	-0.1298
NM_001014137	LOC361016	0.05976	0.0169	0.11159	0.1814	0.05517	-0.0354	0.18651	0.02525	0.22148	-0.0573	0.1193	0.03347	-0.0046	0.07147
XM_341294.2	LOC361019	-0.0921	-0.1265	0.0822	0.0181	-0.102	0.00142	0.0524	-0.0795	0.20107	-0.0602	0.01844	-0.0886	-0.1421	-0.0697
XM_341296.2	LOC361021	0.03131	0.15152	-0.0163	-0.0062	-0.1082	0.07192	-0.0461	0.04469	-0.0271	0.03555	0.30649	0.15187	0.01672	0.26222
XM_341302.2	LOC361026	-0.062	0.06125	-0.0087	0.01873	0.0604	0.04799	-0.07	-0.0613	-0.1645	-0.2838	0.00226	0.09097	-0.0924	-0.024
XM_341309.2	LOC361034	0.13896	0.08362	0.36489	0.17815	0.15509	-0.026	0.13424	0.1228	0.13803	0.18803	0.12686	0.24274	0.13965	0.1812
XM_341311.1	LOC361036	0.13493	0.05251	0.0421	0.08651	0.12832	0.01055	0.18462	0.03875	0.23216	0.12733	0.00767	0.29739	0.03465	0.07599
XM_341316.2	LOC361039	0.15099	0.37127	-0.1372	0.22031	0.18746	0.10543	0.02635	-0.1061	0.04619	0.30166	0.06217	0.14699	0.20733	0.23619
XM_341328.2	LOC361048	0.6682	0.33406	0.04395	0.87765	0.90115	0.70028	0.27185	0.86927	0.23696	0.05459	0.28748	0.02895	0.2978	0.19251
NM_001108382	LOC361059	0.15351	0.41592	0.16004	0.16743	0.45553	0.01026	0.39264	0.28972	-0.0589	0.54566	0.52803	0.1417	0.52796	0.51759
NM_001134571	LOC361066	-0.141	-7E-05	0.21358	0.3501	-0.2985	-0.3601	-0.0031	-0.2712	-0.0594	0.05001	0.2104	-0.1524	-0.3437	-0.468
XM_346935.2	LOC361075	0.03608	-0.1786	-0.1074	-0.1165	-0.1985	-0.1263	-0.1362	-0.0293	-0.0728	-0.1082	-0.0043	0.02062	0.12675	-0.1616
XM_341358.2	LOC361076	-0.2275	-0.1493	-0.2303	-0.1042	-0.107	-0.1207	-0.0852	-0.2343	-0.0628	-0.0691	0.04911	0.15869	-0.0903	-0.1257
XM_341365.2	LOC361082	0.01931	0.00817	-0.1038	-0.0883	0.19685	0.00211	-0.034	-0.1289	-0.0689	-0.2091	-0.1215	0.39077	0.01118	-0.16
XM_341369.2	LOC361085	0.02323	-0.0448	0.01684	-0.0211	0.1985	0.06303	-0.0172	0.04908	-0.1336	-0.0469	0.00516	0.04078	-0.031	-0.0468
XM_341376.2	LOC361091	-0.4128	-0.3833	-0.7378	-0.8983	-1.147	-1.3883	-0.7263	-1.035	-0.063	-0.4285	-0.4794	-0.2028	-0.7342	-0.8381
XM_341387.2	LOC361101	-0.0618	-0.1746	-0.1792	-0.0531	-0.0165	-0.155	-0.0808	0.02176	-0.093	-0.0813	-0.1563	-0.2075	-0.0102	-0.0205
XM_341394.2	LOC361108	-0.1307	-0.2001	-0.0746	-0.0497	-0.1773	0.01345	-0.2358	0.01146	-0.1911	-0.0752	-0.0852	-0.0275	-0.0988	-0.1311
XM_346937.2	LOC361113	0.19639	-0.1086	-0.1012	0.18167	0.01679	-0.086	-0.0206	0.01427	-0.0035	-0.0827	-0.0387	-0.0237	0.11091	-0.0709
XM_341405.2	LOC361117	0.13401	0.00235	0.45927	0.87252	0.0279	0.11477	1.1719	0.24268	0.05391	0.04876	0.09568	0.10247	-0.0372	0.12648
XM_346938.2	LOC361119	-0.1467	0.04189	-0.0972	0.07407	-0.0352	-0.1344	0.01739	-0.1292	-0.0336	-0.112	0.13618	-0.0588	-0.0031	0.03133
XM_341408.2	LOC361121	0.1187	0.04247	0.06168	0.04096	0.04726	0.00175	0.00081	0.16722	0.11699	0.18504	0.0914	0.07795	0.13288	-0.031
NM_001047104	LOC361128	0.14882	0.26178	0.17414	-0.3202	-0.0736	-0.3109	-0.1719	-0.1178	0.11694	0.4316	0.34139	-0.4346	0.22169	0.00953
XM_346939.2	LOC361133	-0.2481	-0.1553	-0.2384	-0.2365	-0.1086	-0.062	-0.2291	-0.3243	-0.1496	-0.2344	-0.1668	-0.0749	-0.2957	-0.1255
XM_341426.2	LOC361140	-0.2361	-0.2311	0.02438	0.42321	-0.191	0.07364	-0.0944	-0.0763	0.02648	-0.3599	-0.0304	0.02409	-0.1239	-0.0437
XM_346940.2	LOC361143	-0.0542	-0.0534	0.36332	-0.0007	0.26587	0.12089	0.13242	0.08377	0.08555	0.18776	0.35737	0.07011	0.17725	0.11908
XM_341437.2	LOC361152	0.03343	-0.0579	-0.0249	0.11009	-0.053	0.01636	0.0314	-0.0046	-0.0356	-0.0668	0.14283	0.27329	-0.0102	-0.0877
XM_341443.2	LOC361157	-0.1642	0.01477	-0.0708	0.2598	-0.0629	0.04945	0.22315	-0.0688	-0.3492	0.0229	-0.3394	-0.0141	-0.2308	-0.2081
XM_341449.2	LOC361163	0.11669	-0.1974	0.28812	0.12693	-0.1987	0.12135	0.08659	-0.1347	-0.1707	0.02428	0.04633	0.07997	-0.1137	-0.0166
XM_346942.2	LOC361164	0.04809	0.13879	0.34244	0.04483	0.13957	-0.1505	0.09773	0.18348	-0.0896	0.06381	0.20173	0.01051	0.15164	0.20315
XM_341450.2	LOC361165	0.06724	0.01317	-0.0219	-0.1072	-0.0204	-0.0055	-0.0175	-0.0778	0.16356	0.02186	-0.0011	0.0913	0.09113	-0.1041
XM_341458.2	LOC361172	0.33718	0.17542	-0.3263	-0.3308	-0.2208	-0.3053	-0.1375	-0.0554	0.1106	0.29723	0.10566	-0.2042	0.01079	0.15401
NM_001044247	LOC361187	0.51374	1.0969	0.20139	1.0061	-0.4619	-0.7021	0.73143	-0.3934	0.67515	1.066	0.90239	0.00608	0.65055	0.63948
XM_341470.3	LOC361188	0.1501	0.0685	0.01253	0.01489	0.05498	-0.0125	0.07882	-0.0475	0.01728	0.04329	0.09345	0.08552	0.02716	0.05288
XM_341485.2	LOC361204	-0.2591	0.04183	-0.2135	-0.1286	-0.1867	0.12627	-0.0093	-0.1116	-0.105	-0.1687	-0.2048	-0.2011	-0.083	-0.1635
XM_341502.2	LOC361220	0.09396	0.065	0.30113	0.16944	0.01678	-0.0191	0.01567	0.14561	0.01056	0.07242	0.34695	0.04143	0.01774	-0.0017
XM_346948.1	LOC361225	-0.1211	-0.0857	-0.1256	-0.0802	-0.0467	0.10834	-0.08	0.033	-0.1263	-0.1352	-0.0721	-0.0804	-0.1085	-0.0139
XM_346949.2	LOC361229	-0.2724	-0.1907	-0.273	-0.2952	-0.2338	-0.1796	-0.1586	-0.2988	-0.096	-0.2867	-0.2586	0.00111	-0.1061	-0.1063
NM_001008774	LOC361230	0.02577	0.0651	-0.0498	-0.1072	0.0001	0.17955	0.08551	0.37019	0.01048	0.07691	-0.0537	0.12683	-0.0468	0.07372
XM_346950.1	LOC361234	-0.1874	-0.0177	-0.0637	0.02802	-0.0783	-0.0338	0.02682	-0.0731	0.18975	-0.0828	-0.0465	-0.0517	-0.1178	-0.0563
XM_341552.2	LOC361269	-0.8082	-0.6642	-0.0751	0.07077	-0.9814	-1.181	-0.0143	-0.64	-0.3434	-0.1193	-0.27	-0.1956	0.00375	-0.2413
XM_341560.2	LOC361275	0.13883	-0.1399	-0.0685	-0.0317	-0.1587	0.11516	-0.0236	-0.1171	0.00247	0.0097	-0.0976	-0.1385	-0.1177	-0.0765
XM_341568.2	LOC361283	-0.322	-0.0092	-1.1063	-0.745	-0.593	-0.8447	-0.1079	-0.3803	-0.0646	-0.034	-0.4074	-0.3955	-0.6719	-0.8281

XM_341569.1	LOC361284	0.25239	0.31496	0.14509	0.29527	0.14805	0.27589	0.20343	0.25215	-0.019	0.28229	0.08826	0.239	-0.0116	0.10199
NM_001135757	LOC361288	0.60566	0.5881	0.15665	0.41187	0.58555	0.76817	0.61933	0.63154	0.54763	0.79673	0.61068	0.34759	0.08306	-0.0002
XM_341576.2	LOC361291	0.05469	0.07755	0.01667	0.43686	-0.0405	0.29568	-0.0156	-0.0819	0.14592	0.0837	-0.0267	0.00614	0.11878	-0.0508
XM_341577.2	LOC361292	-0.0806	-0.124	-0.0519	0.07991	0.05483	-0.1128	-0.0502	0.37922	-0.0244	-0.0846	0.01756	0.07898	-0.023	-0.1371
XM_346953.1	LOC361298	0.01474	0.22762	0.01793	0.02239	0.06926	0.0928	0.03858	0.07233	0.16538	0.39932	0.07743	-0.0101	0.19718	-0.0393
NM_001014150	LOC361315	0.12293	0.02326	-0.0995	0.32029	0.34113	0.2191	0.13833	0.42806	0.2794	-0.0374	-0.006	0.17744	0.18074	0.11778
XM_341620.2	LOC361335	0.07045	-0.0809	0.08112	0.09203	0.03997	-0.201	-0.029	0.15856	0.00362	0.07312	0.14347	-0.174	-0.0745	0.17774
XM_346960.1	LOC361337	-0.0248	0.03431	0.02111	0.12477	0.10216	0.26627	0.10757	-0.0213	0.47297	0.33242	0.15433	0.01969	0.05725	-0.0279
XM_341623.2	LOC361340	0.2029	-0.0053	-0.0633	0.15486	-0.1619	0.03596	0.09735	0.11551	-0.087	0.22694	0.0144	0.13741	-0.0678	-0.0232
NM_001017462	LOC361346	0.16465	0.32943	0.64151	1.4318	-0.3655	-0.5921	0.70425	-0.3518	0.23597	0.41953	0.33184	0.32453	0.63254	0.45995
XM_346964.2	LOC361349	0.19856	-0.1074	-0.0057	-0.0456	1.0368	1.0987	0.13059	1.1209	-0.1157	-0.0336	-0.1378	-0.043	-0.0802	-0.0291
XM_346965.2	LOC361350	0.39329	0.61775	0.14573	0.24094	0.33806	0.61484	0.5904	0.14197	0.15477	0.06139	-0.0191	0.07635	0.48026	0.2176
XM_341638.2	LOC361358	-0.0361	-0.0975	-0.0887	-0.1373	-0.0079	-0.1271	-0.0443	0.00249	0.15144	-0.156	0.11058	0.28837	-0.0748	-0.1338
XM_341652.2	LOC361373	0.07411	-0.0044	0.05611	-0.0044	0.23953	0.05945	0.16059	0.16337	0.05093	0.14698	0.11702	0.12612	0.13226	0.10011
XM_346971.1	LOC361380	-0.0434	0.17828	-0.0758	-0.0638	0.01933	-0.1108	-0.0565	0.04442	0.12792	0.27575	-0.0709	-0.107	0.28949	0.21307
XM_346972.2	LOC361382	-0.0462	0.06203	-0.1372	0.05939	-0.094	0.05058	-0.0802	-0.1035	-0.0984	-0.1156	0.04399	-0.0287	-0.0377	-1E-05
NM_001033068	LOC361399	0.02375	-0.0395	0.14153	0.04114	-0.0577	0.00459	0.15084	0.20192	-0.0474	0.03987	0.08062	-0.142	-0.1122	0.10669
XM_346974.1	LOC361417	-0.0711	0.02475	0.46962	0.01862	0.73131	0.82754	0.20672	0.6408	-0.1685	0.40654	0.50503	0.24147	0.18037	0.07085
NM_001024896	LOC361418	0.39086	0.27952	0.34463	0.22148	-0.0537	-0.3064	0.38449	-0.0915	-0.1122	0.18578	0.12696	-0.0925	0.22089	0.20666
XM_341725.2	LOC361446	-0.1649	0.12149	0.0707	0.09728	-0.212	0.06879	0.06123	0.03245	0.24318	0.21994	-0.0523	-0.1742	-0.0521	-0.2331
XM_341739.2	LOC361460	-0.4285	-0.207	-0.0281	-0.1718	-0.4204	-0.6053	0.09049	-0.3582	-0.1228	-0.1189	-0.2551	-0.3329	-0.2816	-0.2891
XM_346976.2	LOC361469	-0.0183	-0.1283	-0.1229	-0.0711	-0.0731	0.15859	-0.0593	-0.1014	-0.0695	0.02928	-0.1429	-0.11	-0.1347	-0.095
XM_346977.1	LOC361480	0.06082	-0.0339	0.2016	-0.0626	0.09689	0.02643	-0.1279	-0.1124	-0.0942	0.05976	-0.1165	0.05894	0.03164	-0.0606
XM_341759.2	LOC361482	-0.4227	-0.2754	-0.5437	-0.4588	-0.4025	-0.3245	-0.4058	-0.371	-0.3585	-0.4269	-0.2718	-0.4178	-0.2887	-0.3494
XM_341762.2	LOC361485	-0.4288	-0.3094	0.28057	0.42173	-0.4761	-0.5711	0.33857	-0.5204	-0.2293	-0.2155	-0.3876	-0.2546	-0.6073	-0.6192
XM_341768.2	LOC361489	0.00647	-0.0833	0.03997	-0.1684	-0.0292	0.01489	0.18376	0.02466	0.06339	-0.1272	-0.0813	0.21601	0.15167	-0.1003
XM_341769.2	LOC361490	-0.1388	0.00515	-0.1787	-0.0623	-0.1173	-0.2534	0.01497	-0.2156	-0.1469	-0.1811	-0.1285	-0.1947	-0.1122	-0.215
NM_001025131	LOC361510	0.24636	-0.0896	-0.1247	-0.1132	-0.0405	0.01905	-0.0849	-0.1094	0.27496	-0.0196	-0.0484	-0.0055	-0.0002	-0.1494
XM_341832.2	LOC361546	0.30852	-0.1593	0.75329	0.40726	-0.1485	0.00741	0.2544	0.16557	-0.1689	-0.0259	-0.009	0.61101	0.36037	0.40117
XM_341833.2	LOC361547	0.09834	-0.0294	0.21963	-0.0365	-0.0057	-0.0793	0.04265	0.06086	0.29532	-0.0432	0.0337	-0.035	0.09253	0.17201
XM_341835.2	LOC361548	0.01718	0.04768	0.22908	0.07841	0.11062	-0.082	0.03378	0.05445	-0.0046	-0.0237	0.11563	0.08613	0.08697	-0.0291
NM_001127552	LOC361554	0.15273	-0.1595	-0.1407	0.09134	-0.0838	0.31871	-0.0344	0.01837	0.12453	-0.1829	0.02607	-0.0822	-0.1133	-0.2003
XM_341844.2	LOC361556	-0.3597	-0.2887	-0.0653	-0.0651	-0.1672	-0.2739	-0.151	-0.382	-0.3097	-0.2456	-0.3946	-0.154	-0.1571	-0.2082
XM_346983.2	LOC361558	0.02909	-0.118	0.26644	-0.0309	-0.0748	0.12271	0.16947	0.0774	-0.0423	0.3744	0.16116	0.48276	0.09419	-0.002
XM_341866.2	LOC361584	-0.0398	-0.0649	-0.0593	0.03586	-0.0714	-0.0205	0.03054	-0.0346	-0.0815	-0.0769	0.07778	-0.0338	0.06368	-0.08
XM_346989.2	LOC361593	0.27347	-0.1255	0.03559	0.281	0.08377	0.34232	-0.0696	0.06018	0.18884	0.49015	0.2393	0.01341	-0.2633	0.042
XM_346990.2	LOC361595	0.05592	-0.0327	-0.036	0.21986	0.08401	-0.0176	0.02017	0.09277	0.04922	0.06392	0.12896	0.09618	0.06639	0.09363
XM_346991.2	LOC361599	-0.1889	-0.1129	-0.1579	-0.0415	-0.0501	-0.1478	-0.0803	-0.1008	-0.0809	-0.0305	-0.1363	-0.0277	-0.0233	-0.0115
XM_341898.2	LOC361620	0.13811	-0.0588	0.13279	-0.042	-0.0713	0.01352	-0.0515	-0.0446	-0.1402	0.11348	-0.0654	-0.0566	0.00624	0.06061
XM_341901.2	LOC361623	0.28262	0.22914	0.29843	0.19775	0.0542	0.08539	0.14628	0.41487	0.36473	0.04355	0.11219	0.01458	0.03585	0.03934
NM_001017463	LOC361635	-0.188	0.11112	0.0237	-0.4521	0.03748	-0.1445	0.16696	0.13016	0.03417	0.18568	0.27254	-0.2106	0.20241	0.21518
XM_341918.2	LOC361639	0.05958	-0.1905	-0.1517	-0.3562	-0.1372	0.02053	-0.0113	-0.0116	-0.1891	-0.0048	0.27802	-0.0985	0.11225	0.02206
NM_001134574	LOC361646	-0.3829	-0.4047	-0.0616	0.04802	-0.2512	-0.2469	-0.0055	-0.4366	-0.5446	-0.3818	-0.5499	-0.2672	-0.6913	-0.6194
NM_001035517	LOC361654	-0.1799	-0.0205	-0.0134	-0.3622	0.09874	-0.1384	-0.0762	-0.1205	-0.3238	-0.2813	-0.2356	-0.3225	-0.1831	-0.147
XM_341956.2	LOC361672	-0.1028	-0.0151	-0.301	-0.17	-0.1143	-0.2602	-0.1107	-0.0775	-0.1178	-0.136	-0.0603	-0.141	-0.1532	-0.1784
XM_342020.1	LOC361727	0.05454	0.50172	0.15503	-0.6609	-0.0914	0.32661	0.21038	-0.0206	0.3188	0.32815	0.48226	0.16232	-0.0362	-0.0657
XM_342043.2	LOC361750	0.1347	0.20896	-0.1591	0.02424	0.27024	0.41147	-0.2814	0.3176	0.18548	0.12925	0.29163	0.07225	0.33095	0.19433
XM_577864.1	LOC361754	0.21236	-0.1438	-0.0733	-0.0745	-0.027	-0.0971	-0.1432	0.03553	-0.1171	-0.072	-0.0544	-0.0715	-0.1417	-0.0096
XM_342060.2	LOC361768	0.27886	-0.0252	-0.0575	0.01612	0.09427	0.05921	0.07155	0.05546	0.04037	0.19258	0.27638	0.17826	0.20095	0.03846

NM_001024899	LOC361769	-0.0833	-0.0177	0.08001	0.02644	0.02977	-0.1574	-0.2332	-0.1119	0.05249	-0.0243	0.11862	0.17875	0.11968	0.00051
NM_001014169	LOC361776	0.82527	0.48107	0.05052	-0.1351	0.17664	0.35619	-0.0436	0.26232	0.20236	0.64767	0.3129	-0.0468	-0.2067	0.14171
XM_342075.2	LOC361780	-0.1735	-0.216	-0.2129	-0.116	-0.1407	-0.156	-0.0254	0.10718	-0.1351	-0.2123	-0.1816	-0.0642	0.05812	-0.0743
XM_342077.2	LOC361781	0.10457	-0.1347	-0.061	0.03232	-0.0809	0.16308	-0.0944	0.06711	-0.0653	-0.0222	0.05456	-0.1469	-0.1363	-0.1225
XM_347000.2	LOC361786	-0.4116	-0.3117	-0.0272	-0.2257	-0.4296	-0.3589	0.01763	-0.4448	-0.1672	-0.2959	-0.3664	-0.2376	0.07149	-0.0949
NM_001108528	LOC361805	-0.3456	-0.1747	-0.4003	-0.3126	-0.4657	0.06261	-0.2858	-0.0897	-0.2504	-0.3694	-0.3763	-0.4112	-0.3201	-0.2682
NM_001039455	LOC361828	0.45797	0.21982	0.35575	0.08563	0.12039	0.17467	-0.0065	0.14427	0.28224	0.26206	0.23723	0.39535	0.17407	0.3473
XM_342133.2	LOC361839	-0.0487	-0.0715	0.02666	-0.0227	-0.0132	-0.082	0.16534	0.02551	-0.1135	-0.0953	0.28795	0.13022	0.15846	-0.002
NM_001037190	LOC361841	0.42044	0.30331	-0.2225	-1.102	0.27312	-0.5168	-0.5473	0.27877	0.02516	0.16878	-0.0572	-0.6043	0.54044	0.69316
XM_342147.2	LOC361853	0.14666	-0.0013	0.61446	0.47904	0.3953	0.43931	0.43868	0.37907	-0.099	0.15657	0.06543	-0.0833	0.21429	-0.0816
XM_347006.2	LOC361860	0.141	0.02499	0.19735	0.14458	0.06977	0.17839	0.01554	0.32016	0.2248	0.09306	0.0323	0.11288	0.29198	0.33198
XM_342159.2	LOC361865	0.06183	0.06256	0.17055	0.01945	-0.0458	-0.0964	0.0059	-0.0667	0.05634	-0.1166	0.04101	0.20427	-0.0965	0.1191
NM_001037190	LOC361872	0.17834	-0.1165	-0.0444	-0.1372	-0.035	-0.0149	-0.0362	0.08901	-0.1917	0.0477	-0.1012	0.02045	0.06704	-0.0362
XM_342170.2	LOC361876	0.199	0.22446	0.1076	0.20737	0.07344	0.10788	0.11439	0.08496	0.09062	0.05329	0.25403	0.03861	0.04066	0.13999
XM_342182.2	LOC361885	-0.2454	0.07814	0.55014	0.55651	-0.5134	-0.4354	1.0384	-0.2187	0.10838	-0.1206	0.19265	-0.141	-0.1837	0.01175
XM_347009.2	LOC361892	0.2056	-0.1026	-0.0675	0.01762	-0.0514	-0.0671	-0.0439	-0.0905	-0.0166	0.07069	0.00517	-0.0588	-0.0313	-0.0382
XM_347010.2	LOC361911	0.05425	0.08073	0.12503	0.28947	0.07075	0.25946	0.25205	0.33406	0.12232	0.32335	0.05757	0.23383	0.34217	0.13046
XM_342207.2	LOC361912	-0.9259	-0.2028	-0.4389	-0.2034	-1.6476	-1.2147	0.31976	-1.0015	-0.417	-0.2999	-0.1667	-0.5033	-1.1579	-1.5361
NM_001017465	LOC361914	-0.1782	-0.1082	-0.027	0.04858	-0.317	-0.0754	0.04192	-0.1931	-0.0582	-0.1717	-0.2292	-0.076	-0.0229	-0.0651
XM_342211.2	LOC361915	0.03344	0.03442	0.02101	0.05418	0.04395	0.00845	-0.0677	0.07636	0.05197	0.15327	0.02065	-0.0591	0.1201	0.00318
NM_001108548	LOC361923	-0.1371	-0.7134	0.3177	-0.5801	0.06154	0.05934	-0.3484	-0.3217	-0.1388	-0.444	-0.364	0.19415	0.03004	-0.1169
XM_342228.3	LOC361929	-0.5059	-0.2722	-0.3798	0.32234	-0.2873	-0.6982	0.00483	-0.3393	0.05491	-0.1574	-0.0755	-0.6148	-0.376	-0.4757
XM_342241.2	LOC361942	-0.6573	0.021	-0.2054	0.081	-1.5028	-1.1478	0.8693	-1.115	-0.505	0.05967	-0.4184	-0.4135	-1.3266	-1.2444
XM_347013.2	LOC361960	0.2546	-0.0515	0.05998	0.13688	0.10605	-0.0767	0.0965	-0.0153	-0.0468	-0.0605	-0.0905	-0.0663	-0.0073	-0.0084
XM_342261.2	LOC361962	-0.0162	0.0936	0.12982	0.11544	0.11726	0.11783	0.06238	-0.0405	0.02583	0.07019	0.13275	0.08096	0.00186	0.00705
NM_001029922	LOC361964	-0.17	-0.1602	-0.231	-0.0694	-0.0626	-0.0466	-0.1083	-0.156	-0.0517	0.18055	-0.0713	-0.2068	-0.1315	-0.0247
NM_001014175	LOC361990	-0.2169	0.04909	-0.0836	0.59807	-0.0534	0.00682	0.06892	-0.2192	-0.0404	-0.0108	-0.1455	-0.0493	-0.0248	-0.1679
NM_001014175	LOC361990	-0.3839	-0.3799	-0.3377	-0.0832	-0.5686	-0.5957	-0.3586	-0.7639	-0.5206	-0.6145	-0.289	-0.3426	-0.3753	-0.3998
XM_347017.2	LOC362000	0.02862	0.01627	0.02837	0.06884	-0.0019	0.21683	0.10165	0.04367	0.11942	0.14943	0.09821	0.21036	0.08374	-0.0428
XM_347019.2	LOC362009	0.20847	0.10982	0.17792	0.30387	0.4589	0.38992	0.3135	0.17307	0.20247	0.13685	0.4691	0.08789	0.30628	0.05145
XM_342309.2	LOC362010	0.25656	0.22184	0.61221	0.2361	0.23136	0.21222	0.14971	-0.0617	0.06734	0.14658	0.22018	0.27116	0.07595	0.36253
XM_342311.3	LOC362012	-0.0327	-0.1788	0.17813	0.19835	-0.3434	-0.1517	0.04605	-0.3674	-0.1117	-0.1441	-0.0565	-0.0665	-0.2416	-0.0966
NM_001101681	LOC362015	0.15774	0.16646	0.17088	-0.2799	0.26169	-0.0725	0.10171	0.27985	0.13458	0.05627	0.14516	-0.1069	0.04071	0.00044
XM_342327.2	LOC362026	-0.1933	0.18821	-0.4264	-0.0336	-0.5436	-0.309	0.07846	-0.4016	-0.0427	0.14099	0.16796	-0.0979	-0.6173	-0.6665
XM_342335.2	LOC362034	0.07436	-0.0877	0.40286	0.5069	0.0686	0.41569	0.49974	0.04571	0.09794	0.25866	0.05656	0.31819	0.47905	0.06928
XM_342361.2	LOC362058	0.08023	0.107	-0.0386	0.12753	0.13673	-0.1125	0.1003	0.08696	0.06177	0.05784	0.00572	-0.0513	0.19648	0.02123
XM_347024.2	LOC362059	-0.0361	-0.1188	0.07464	0.04331	0.0299	0.05219	0.02534	-0.1238	-0.0797	-0.0877	-0.1057	0.04707	0.10386	0.10416
XM_342362.2	LOC362060	0.07458	0.08659	-0.1104	0.1268	0.07369	0.19649	0.30951	0.14675	0.16419	0.0566	0.19675	0.36902	0.04096	0.01915
XM_001056150.1	LOC362068	0.10831	0.03256	-0.0885	-0.0385	0.00945	-0.0812	0.12866	0.05902	-0.0427	-0.1141	-0.0552	-0.1178	-0.1061	-0.2723
XM_342369.2	LOC362068	0.12304	0.11106	0.05657	0.03577	0.00989	0.06083	0.10703	0.00621	0.07321	0.04499	0.1736	0.28116	0.21379	0.38528
XM_342371.2	LOC362069	0.1653	-0.1163	0.13616	-0.1094	-0.0004	-0.0482	0.03892	-0.0982	0.0109	-0.0211	-0.0185	-0.0826	-0.1199	-0.0556
XM_342373.2	LOC362071	-0.154	-0.2209	-0.0815	0.01567	-0.1845	-0.149	-0.2125	-0.0954	-0.0985	-0.1331	-0.1055	-0.1874	-0.2188	-0.3294
XM_342374.2	LOC362072	0.20886	0.09661	0.00424	0.06484	0.27817	-0.0108	0.07799	0.01138	0.19118	0.12827	0.15735	0.21287	0.01995	0.03373
XM_347026.2	LOC362074	-0.0181	0.01967	0.06341	-0.0081	0.11985	-0.0035	0.02572	0.13359	0.01339	-0.0129	0.15394	0.065	0.00167	0.02249
NM_001109885	LOC362115	-0.0998	-0.2847	-0.3067	-0.9835	0.22496	-0.0642	-0.1465	-0.0656	-0.2679	-0.3127	-0.2599	-0.6232	-0.3883	-0.1758
XM_342420.2	LOC362118	-0.0481	0.05934	-0.0032	0.08719	0.02032	-0.0188	-0.0295	0.15611	0.07791	0.00864	0.05251	0.4295	0.2345	0.14879
XM_342423.2	LOC362121	0.11839	-0.1703	0.23827	-0.2947	-0.1582	-0.238	-0.4067	-0.1096	-0.0434	-0.2922	-0.1018	0.1116	0.55353	0.60651
XM_342428.2	LOC362127	0.0136	0.03928	0.27284	0.14439	-0.0102	-0.0089	0.19567	-0.0137	-0.0173	-0.0478	-0.0272	-0.1635	-0.0893	0.18812
XM_342428.2	LOC362127	-0.0832	-0.14	0.0111	0.19387	-0.0509	0.15094	0.19927	-0.167	-0.1704	0.03247	-0.2493	-0.1441	-0.0552	-0.3057

XM_347030.1	LOC362130	-0.0864	0.00409	0.25594	-0.0967	-0.0148	-0.0033	0.27048	-0.0964	0.07481	-0.0959	-0.0031	0.07623	0.01901	0.08879
XM_342441.1	LOC362141	0.05489	0.00682	0.04684	0.09144	0.07859	0.11054	0.12823	0.0429	-0.0385	0.04491	-0.1197	0.05011	-0.0113	0.13122
XM_342447.2	LOC362148	0.1184	0.02477	0.23982	-0.008	-0.0803	-0.0747	-0.3903	-0.0183	-0.2326	0.03934	0.23879	-0.0885	-0.2081	-0.1193
XM_347034.2	LOC362174	-0.1179	0.16308	0.07587	0.12089	0.02575	-0.0651	0.06735	0.45682	-0.0171	-0.0063	-0.0245	0.28855	0.24273	0.27771
XM_342480.2	LOC362181	-0.0543	-0.0017	-0.3241	-0.0196	-0.1121	-0.1199	-0.1314	-0.0142	-0.1021	-0.0058	-0.1262	-0.3285	-0.1312	-0.214
NM_001137642	LOC362186	0.09921	0.18476	0.10075	0.11676	0.10006	0.25332	0.0664	0.18859	0.17978	0.14315	0.13896	0.08713	0.05759	0.12243
XM_342526.2	LOC362221	0.20644	0.05608	0.11805	0.19849	0.14389	0.11693	0.07205	0.15229	0.09971	0.22388	0.1021	0.0061	-0.0263	-0.0176
XM_342539.2	LOC362232	0.10735	0.19052	-0.0503	0.02682	0.03176	-0.0186	0.08491	-0.0129	0.17764	0.24937	0.17118	-0.0132	0.00239	0.00365
XM_347036.2	LOC362234	0.07444	0.00428	0.03298	-0.0563	0.06987	-0.038	0.13105	0.1157	0.04044	0.15726	0.22155	0.09151	-0.0968	-0.0587
XM_342562.2	LOC362257	0.03767	0.02735	-0.0837	0.26402	0.39376	0.17823	-0.0657	0.3504	0.09755	0.00764	0.07537	-0.2853	0.38755	0.04777
XM_342573.1	LOC362266	0.20646	0.13645	0.0192	0.147	0.06333	0.11186	-0.0022	0.00209	0.026	0.01055	-0.0546	-0.1684	0.17203	0.14273
XM_342604.2	LOC362290	0.12121	0.19853	-0.8876	-0.1977	0.15469	-0.2072	-0.4262	-0.0427	0.32231	0.16554	0.08222	-0.3515	-0.2414	-0.0788
XM_342608.2	LOC362295	-0.0525	0.0005	-0.119	0.08657	-0.0651	-0.2049	0.23089	-0.2263	-0.2191	-0.1394	0.04271	-0.1573	0.16662	-0.1544
XM_342610.2	LOC362297	-0.0767	-0.0173	-0.0181	0.05971	-0.0928	-0.088	0.01013	0.00577	0.08713	0.22468	-0.0502	-0.1344	0.2595	0.04449
XM_347040.1	LOC362299	-0.0208	-0.0947	-0.0254	-0.1493	-0.059	-0.0754	0.05946	0.00687	0.03886	0.03794	-0.0356	-0.0874	0.13297	0.08894
XM_342620.2	LOC362307	0.17632	0.11179	0.04392	-0.0892	0.13141	-0.1623	-0.0371	-0.0748	0.00529	0.0237	-0.0439	-0.115	0.10121	0.00186
XM_342621.2	LOC362308	0.22203	0.09647	-0.1181	-0.042	0.02827	-0.0982	-0.0667	0.0031	-0.0641	0.10331	0.14678	0.10887	0.02635	0.12117
XM_342631.2	LOC362315	-1.0245	-0.2609	-0.6033	-0.3257	-1.7702	-1.3693	0.47912	-1.38	-0.9103	-0.4358	-0.7181	-0.7109	-1.5641	-1.6106
NM_001108618	LOC362317	-0.6	-0.3807	-0.0938	0.82042	-0.4527	-0.8659	0.59953	-0.4292	-0.2967	-0.4736	-0.325	-0.4699	-0.6379	-0.7498
XM_347041.2	LOC362337	-0.1826	-0.1616	0.03562	-0.2411	-0.0551	-0.1202	-0.1745	-0.0961	0.11388	-0.1227	0.02006	-0.1589	-0.212	-0.0987
XM_342661.2	LOC362341	0.19094	-0.0255	-0.0796	-0.0021	0.31022	0.27446	-0.0327	-0.0096	0.04206	-0.0778	0.06618	-0.1628	0.17449	0.16488
XM_342673.2	LOC362350	-0.1637	-0.1534	-0.0781	-0.034	-0.1773	-0.0062	-0.127	-0.0491	-0.1197	-0.1118	0.06946	-0.2353	0.016	-0.2139
XM_347044.2	LOC362362	0.43143	-0.005	0.03378	-0.1005	0.5494	0.36564	-0.0718	0.64184	0.10879	0.14764	0.06506	-0.1387	-0.0196	-0.1809
XM_342688.2	LOC362363	-0.0023	0.13754	-0.1348	-0.0379	-0.0344	0.00169	-0.0286	-0.0751	0.05285	-0.0678	-0.0864	-0.0817	-0.0092	-0.0077
NM_001037190	LOC362373	0.03601	0.1226	0.05968	-0.0646	0.03149	0.05157	0.01463	-0.0498	0.02765	0.13622	0.06951	0.00073	-0.0483	0.01712
XM_342706.2	LOC362382	-0.129	-0.0521	-0.0984	0.05445	-0.0995	-0.1241	-0.086	-0.0745	-0.1012	-0.087	-0.0484	-0.0525	0.21561	-0.0386
NM_001108633	LOC362384	0.17669	0.06295	0.05033	0.1015	0.20824	0.31549	0.25462	0.57152	0.00817	-0.1141	0.21635	0.23632	0.13039	0.17374
XM_342730.2	LOC362408	-0.029	0.0197	0.09384	-0.1104	-0.0257	0.00104	-0.0192	0.11005	0.00701	0.00265	0.02168	0.05498	-0.0291	-0.0817
XM_342740.2	LOC362420	0.57105	-0.3485	-0.0842	-0.0332	-0.1287	0.30909	-0.221	-0.1504	-0.2473	-0.0506	-0.2844	-0.3506	-0.1786	-0.2593
NM_001108643	LOC362424	0.07431	0.09966	0.04267	-0.1143	-0.0933	-0.1578	0.01311	0.01666	-0.2043	-0.1265	-0.0522	-0.1853	-0.0075	0.04509
NM_001108647	LOC362437	-0.2063	-0.117	-0.348	0.00287	-0.0932	0.04914	-0.2542	-0.1398	-0.181	-0.0608	-0.1362	-0.1381	-0.2965	-0.2326
XM_347053.2	LOC362439	-0.0905	0.10825	0.14069	-0.0482	-0.0908	0.0198	0.05702	0.02451	-0.0401	0.071	-0.0714	-0.0412	0.29731	0.27577
XM_342764.2	LOC362442	0.03305	-0.0456	-0.0031	0.15601	-0.028	0.04298	-0.0459	0.11762	0.15288	0.04184	0.0315	0.08152	0.0828	0.14614
NM_001048075	LOC362447	-0.1248	-0.082	-0.0637	-0.131	-0.1728	-0.022	-0.1964	-0.1131	-0.1148	-0.1319	-0.0295	-0.2119	-0.1133	-0.1591
XM_342787.3	LOC362464	-0.0407	-0.0478	-0.0133	0.06187	0.0608	-0.0658	0.31396	0.26339	0.03762	0.06442	-0.13	-0.1999	0.00298	0.04644
NM_001127495	LOC362477	0.05844	0.10806	0.02318	0.00592	0.08139	-0.068	0.04439	0.18666	-0.0251	0.06044	0.02717	0.18034	0.1208	0.05843
XM_347058.2	LOC362480	-0.075	-0.2814	-0.1934	0.02517	-0.2563	-0.122	-0.0747	-0.2174	-0.3585	-0.2516	-0.2077	-0.2691	-0.2116	-0.3263
XM_342805.1	LOC362486	0.2157	0.12257	0.16122	0.13065	0.0193	0.55021	0.11996	0.20037	0.1716	0.21244	0.10295	0.25919	0.33139	0.09243
XM_342805.2	LOC362486	0.11045	-0.0494	0.19296	0.09878	0.20448	0.39281	0.02274	0.12763	-0.1723	0.07841	0.22661	0.23941	-0.0163	-0.0694
NM_001025021	LOC362526	0.02444	-0.1371	0.0301	0.24072	-0.0778	0.2431	0.16797	0.3763	-0.0896	-0.2036	-0.1231	-0.2617	0.40549	0.45849
XM_347061.2	LOC362536	-0.1268	-0.1379	-0.0941	0.08271	0.10801	-0.0704	0.01624	-0.2258	-0.081	-0.0634	-0.0496	-0.0641	-0.08	-0.1507
NM_001108669	LOC362540	-0.3246	-0.3665	-0.5735	-0.3618	-0.1016	-0.1221	-0.3284	-0.1558	-0.476	-0.5804	-0.6005	-0.2766	-0.4909	-0.3119
XM_342864.2	LOC362543	-0.8835	-0.2376	-0.5296	-0.3203	-1.6378	-1.3578	0.56742	-1.4676	-0.7999	-0.1905	-0.3768	-0.594	-1.4907	-1.5181
XM_342868.1	LOC362547	0.11683	0.2306	0.01601	0.07109	0.16101	0.18871	-0.0119	0.07284	0.02625	-0.088	-0.0572	0.06577	-0.0611	0.07539
XM_342871.2	LOC362551	0.0028	-0.0097	-0.0733	-0.086	-0.278	0.15164	-0.1252	-0.0094	0.01192	-0.078	-0.2178	-0.2072	-0.1196	-0.1207
NM_001025023	LOC362557	-0.0519	-0.1299	0.11808	0.05061	0.07806	0.0348	-0.0603	0.12732	-0.2199	-0.0688	-0.1631	-0.0597	0.15373	0.24567
NM_001108672	LOC362564	-0.0145	0.36612	-0.0864	0.03584	0.03196	0.07931	0.20281	0.01238	0.07189	0.2493	0.13587	-0.128	0.06187	0.02573
NM_001135758	LOC362587	-0.362	0.14261	0.23637	0.03308	-0.5089	-0.6758	0.22759	-0.4853	0.18594	0.02083	0.07841	0.17935	-0.0857	-0.0448
XM_347064.1	LOC362603	0.12471	-0.0908	0.14811	0.13288	0.05453	0.09986	-0.0896	0.12868	-0.1301	-0.1291	-0.118	-0.0103	-0.0534	-0.1725

NM_001108682	LOC362604	-0.1606	-0.194	-0.0667	-0.0687	-0.1949	-0.1226	-0.0971	0.21531	-0.0629	-0.1653	-0.1414	-0.1611	-0.1009	-0.1379
XM_342933.2	LOC362615	-0.1218	-0.1115	0.06425	-0.1572	-0.0328	-0.1233	-0.1058	-0.0586	0.0648	0.07236	-0.1065	-0.1057	0.1812	0.51867
XM_342973.2	LOC362655	0.04453	-0.0461	-0.0111	0.01892	0.13912	0.06139	0.08223	0.11868	0.27262	-0.0604	0.0784	0.14382	0.04995	0.30715
XM_342978.2	LOC362659	-0.1632	-0.1149	-0.0596	0.06128	0.05443	0.25841	-0.0624	-0.2287	-0.1439	0.03109	-0.1405	-0.2253	0.04317	-0.0531
XM_342982.2	LOC362665	-0.1364	-0.0387	-0.6916	-0.3529	-0.3795	0.0053	-0.4151	-0.5168	0.14936	0.06741	-0.0114	0.38188	-0.7901	-0.712
XM_347070.2	LOC362670	-0.1517	0.1654	-0.2026	0.06674	-0.0744	-0.0044	-0.2036	0.08582	-0.2238	-0.1608	-0.0612	0.15202	-0.064	0.12373
XM_347072.1	LOC362677	-0.2231	-0.1327	0.02549	-0.4385	-0.6257	-0.3186	0.09892	-0.621	0.29207	-0.3806	0.25787	0.92579	-0.0765	0.19098
XM_347073.2	LOC362680	0.1412	0.02417	0.24347	0.06792	0.16739	0.30233	0.23433	0.35829	0.5301	0.14136	0.2577	0.15217	0.05073	0.15562
NM_001108699	LOC362681	0.41721	-0.0574	-0.1875	0.36588	0.78784	0.79509	-0.3385	0.51339	-0.2445	-0.0587	-0.081	-0.1861	0.12757	0.08608
XM_342997.2	LOC362682	-0.0654	-0.0214	-0.0277	-0.0373	-0.0162	0.03782	-0.0683	0.09842	0.00573	0.05065	-0.0281	-0.1095	-0.0445	-0.0157
XR_008142.1	LOC362684	0.00131	0.07815	-0.1473	-0.2705	0.0807	-0.2162	-0.1455	0.02003	0.13809	0.00543	0.00354	0.01013	-0.1311	-0.0119
XM_347074.2	LOC362690	0.12448	0.01528	0.03755	-0.013	-0.0292	0.13584	0.0756	0.11571	0.35812	-0.0689	-0.0115	0.0894	0.02514	0.10956
XM_343011.2	LOC362694	0.00652	0.0386	-0.0758	0.05652	-0.044	-0.0209	0.01048	-0.0399	-0.0533	-0.0635	0.0594	0.01063	0.02117	-0.0373
NM_001037791	LOC362703	-0.0492	-0.3016	0.18674	0.50564	-0.0287	-0.2149	0.00035	0.11991	-0.3492	-0.4139	-0.2647	-0.7257	0.14476	0.30327
XM_343047.2	LOC362725	-0.4501	-0.1912	-0.4998	-0.1246	-0.743	-0.6687	0.33321	-0.5747	-0.0425	-0.1322	-0.3925	-0.2982	-0.6182	-0.7026
XM_343047.2	LOC362725	-0.459	-0.1342	-0.3883	0.19222	-0.3665	-0.2837	-0.0265	-0.1939	-0.4719	-0.3108	-0.085	-0.1109	-0.517	-0.2204
XM_343048.2	LOC362726	-0.081	0.05423	-0.1818	-0.1194	0.10502	-0.1249	-0.2025	-0.0201	-0.1097	-0.1909	-0.1262	-0.1481	-0.141	-0.0095
NM_001135899	LOC362732	0.90762	0.16633	0.1747	0.12392	0.08959	0.26916	0.30962	0.02246	0.1399	0.13686	0.29436	0.14209	0.16142	0.33116
NM_001108711	LOC362738	-0.0613	0.19775	0.11556	0.08074	0.06201	0.12429	-0.1142	-0.0417	0.00051	-0.0132	-0.1032	-0.0332	-0.0013	-0.0851
XM_343073.2	LOC362748	0.18649	-0.053	-0.0163	-0.1585	-0.0621	0.0383	-0.0537	-0.0429	-0.1357	0.04642	0.19779	-0.0591	0.02628	0.10361
XM_347076.1	LOC362753	0.12535	0.04759	0.25191	0.11101	0.1246	0.09464	0.0024	0.06702	0.03357	0.07017	0.23507	0.13492	0.26693	0.15193
XM_343089.2	LOC362763	-0.0898	0.02639	-0.1938	0.02582	-0.0181	-0.1122	0.06937	-0.071	0.01805	-0.0786	-0.1195	-0.1537	-0.1097	-0.0246
XM_347078.2	LOC362780	0.07529	0.03862	0.02941	0.01413	0.10155	0.10804	0.25834	0.08555	0.03407	0.12789	0.12826	0.06452	0.06933	-0.0114
NM_001126288	LOC362792	-0.0948	0.03776	-0.079	-0.0877	-0.0534	0.14189	0.15516	-0.0239	0.1543	-0.0526	-0.0225	0.09761	-0.1178	0.09698
XM_343121.2	LOC362795	0.35818	0.10698	-0.1915	0.17456	0.25904	0.28232	0.15017	0.02026	0.09256	-0.0397	0.14481	-0.0176	0.14148	0.07245
XM_343122.2	LOC362796	0.03416	0.02554	-0.0609	0.0769	-0.1033	0.07001	-0.0706	0.07984	0.04417	-0.0567	-0.0647	-0.1163	-0.0576	-0.096
XM_343123.1	LOC362797	0.06046	0.12521	0.28513	0.07395	0.15619	0.29383	0.07147	0.07485	0.12796	0.16224	0.07944	0.18155	0.0941	0.04117
XM_001069822.1	LOC362801	0.14345	-0.1058	-0.0549	-0.0893	-0.0628	-0.0345	0.15802	0.11197	-0.0303	0.1607	0.1663	-0.0247	0.13835	0.03688
XM_343130.2	LOC362803	-0.0285	-0.0379	-0.1798	-0.2537	-0.1147	-0.1424	0.52433	-0.2666	0.16279	-0.1462	0.22163	0.13002	0.12443	0.00198
XM_347079.2	LOC362805	0.19802	0.0171	0.0903	0.15432	-0.0392	0.05484	0.25184	0.01683	0.04922	0.03706	-0.0295	-0.0662	0.13841	0.05465
NM_001130989	LOC362809	-0.5303	-0.4601	-0.2635	-0.2927	-0.7501	-0.6394	-0.3121	-1.0277	-0.2597	-0.5315	-0.2799	0.137	0.07549	-0.1033
NM_001163062	LOC362845	0.2311	0.45684	0.28719	0.25153	0.29965	0.04425	0.55188	0.11382	0.70774	0.51656	0.42117	0.02907	0.06308	0.34397
NM_001127573	LOC362852	0.08259	0.03331	-0.0001	-0.3745	-0.0348	0.01718	-0.0201	-0.1667	0.09853	-0.0473	0.05263	0.17827	0.17171	0.0173
NM_001127573	LOC362852	0.21217	0.16444	-0.2771	-0.6417	0.24424	0.06616	0.17403	0.20156	0.03359	0.00563	0.11626	-0.17	0.01518	0.08612
NM_207614	LOC362855	-0.1621	0.08575	0.36569	0.63817	0.47865	0.29541	0.0959	0.28417	0.18045	0.11046	0.17342	0.20405	0.44659	0.63879
XM_347084.2	LOC362870	-0.0676	-0.123	-0.0989	-0.0643	-0.2061	-0.0263	-0.0416	-0.0966	-0.0951	-0.1864	-0.2571	-0.0591	-0.17	-0.2511
XM_343208.2	LOC362879	-0.13	-0.0089	-0.1581	-0.0183	-0.207	0.2281	0.02639	-0.2188	-0.071	-0.0852	-0.281	-0.0602	-0.2114	-0.2288
XM_347085.2	LOC362880	0.05081	-0.058	-0.0918	0.10163	-0.0448	-0.0638	-0.1594	-0.1319	-0.0819	0.13203	0.10997	0.01027	-0.2176	-0.053
NM_001108746	LOC362881	0.06028	0.09354	0.0325	0.07132	0.01948	0.25445	-0.0014	0.36721	0.01397	-0.06	-0.0136	0.09309	0.02258	0.05306
XM_343210.1	LOC362882	0.21663	-0.0401	-0.0516	0.00153	0.02303	0.09812	0.01486	-0.006	-0.0383	-0.0205	0.04604	0.10715	0.02407	0.08614
NM_001008877	LOC362901	0.25865	0.07966	0.17245	0.2269	0.30452	0.22381	0.14999	0.40797	0.17235	0.19165	0.14529	0.14477	0.12942	0.19931
XM_343249.2	LOC362919	0.16925	0.48645	0.40217	0.35799	0.29882	-0.1171	0.23374	0.1422	0.37582	0.21642	0.42584	0.36412	0.40902	0.32349
NM_001008878	LOC362921	0.09882	0.04553	0.04611	0.15716	0.09986	0.18103	0.15076	0.09041	0.14604	0.10963	0.2009	0.13853	0.16534	0.08055
XM_343257.2	LOC362927	-0.0007	0.06832	0.10989	0.22408	0.21374	0.10574	0.0824	0.00135	-0.0512	0.11792	0.27802	0.45663	0.03999	0.17895
NM_001135896	LOC362945	-0.3251	-0.1689	-0.2478	-0.1793	-0.3699	-0.2763	-0.2702	-0.498	-0.2146	-0.2178	-0.3426	-0.3421	-0.2549	-0.484
XM_343295.2	LOC362964	0.00442	-0.1743	-0.2637	-0.0771	-0.1799	-0.231	0.14843	-0.0819	-0.1085	0.05313	-0.1267	-0.0513	-0.0441	0.0123
XM_001078083.1	LOC362972	0.21417	0.11422	0.48369	0.15841	0.29022	-0.0293	0.24198	0.43995	0.20517	0.00544	0.05887	0.14972	0.23196	0.1892
XM_347090.2	LOC362984	-0.0763	0.10138	-0.2642	-0.0803	0.11136	-0.6095	0.09187	-0.3206	-0.4042	-0.2791	0.00714	-0.3829	-0.0455	-0.2648
XM_347091.2	LOC362985	-0.0851	0.07743	0.00794	0.11362	-0.2284	0.16179	-0.1614	-0.089	-0.167	0.04796	-0.1487	0.16962	-0.158	-0.1298

XM_343333.2	LOC363003	-0.1388	-0.057	-0.0312	-0.0093	-0.2555	-0.2272	-0.1065	0.04521	-0.1587	-0.2426	0.00848	-0.1945	-0.1953	0.10716
NM_001108753	LOC363009	0.06766	0.07237	0.30005	0.04612	0.07081	0.10325	0.07307	0.14086	0.05314	0.00232	0.05156	0.0745	0.05586	-0.0409
XM_343343.2	LOC363010	-0.2594	-0.064	-0.3014	-0.3294	-0.1978	-0.0704	-0.3225	-0.4388	-0.2301	-0.0224	-0.1832	-0.1057	-0.2288	-0.0387
XM_343351.2	LOC363018	-0.0167	-0.1564	0.33128	0.22969	-0.3517	0.14069	0.08393	-0.4277	-0.1018	-0.2605	-0.2005	0.37889	0.10039	0.28796
XM_347095.2	LOC363021	-0.1235	0.02111	-0.0791	0.05396	0.10444	-0.1023	-0.1091	-0.1716	-0.2309	0.21106	0.09743	0.07887	-0.0385	0.03012
XM_343361.2	LOC363030	-0.0148	0.02116	-0.1695	-0.0414	-0.1234	-0.1044	-0.1009	0.13764	-0.1516	-0.0522	-0.1481	-0.208	0.01929	-0.2199
XM_343362.2	LOC363031	0.17367	-0.1549	-0.1245	-0.163	0.29147	-0.1396	0.00108	0.22458	0.19626	-0.1218	-0.1306	-0.0611	0.68556	0.8164
NM_001034942	LOC363035	-0.1949	-0.0413	-0.0741	-0.0144	-0.1627	-0.0262	-0.0678	0.19489	-0.0024	-0.0143	-0.0367	0.13898	-0.1912	-0.0456
XM_347097.2	LOC363036	-0.125	0.088	-0.1482	-0.1697	-0.0767	-0.059	-0.1762	-0.2139	-0.2557	-0.1181	-0.1705	-0.2793	-0.118	-0.2278
XM_343374.2	LOC363046	0.01282	0.05099	0.06183	-0.0239	0.0081	-0.0122	-0.03	-0.0886	-0.0989	-0.0798	-0.0612	0.0786	-0.0245	-0.0902
XM_343383.2	LOC363055	-0.0547	0.05817	-0.0567	0.04619	0.08239	0.20385	0.03749	-0.0025	-0.0649	0.0638	0.1199	-0.1829	-0.1257	0.06961
XM_343384.2	LOC363056	-0.0751	0.03698	-0.0592	-0.1898	0.08066	-0.2328	0.05037	-0.2619	0.13823	-0.1775	-0.2232	0.06022	-0.136	0.04361
XM_347104.1	LOC363057	-0.0592	-0.0215	-0.0513	-0.1413	-0.1392	-0.0954	-0.1637	0.05671	0.21874	-0.0995	-0.1436	-0.0695	-0.0907	-0.1239
NM_001014209	LOC363060	-0.6584	-0.791	-0.7222	-0.6784	-0.0531	0.19067	-0.7774	-0.016	-0.8803	-0.8875	-0.8038	-0.7415	-0.7286	-0.6698
NM_001108764	LOC363070	0.06649	0.17784	-0.0483	0.01522	0.1487	0.22557	0.148	0.10515	0.18358	-0.166	0.00233	-0.0286	0.14087	0.13754
NM_001025287	LOC363076	0.01066	-0.0201	0.0559	-0.0399	0.00027	0.10443	0.05914	0.16465	0.03126	0.02407	0.0758	0.04014	0.34266	-0.0238
XM_343448.2	LOC363115	0.07027	0.03772	0.00253	0.02539	0.00832	0.08353	-0.1783	0.00679	0.04392	0.24823	0.31211	-0.0291	0.11774	-0.1503
XM_343481.2	LOC363144	0.00813	-0.1044	-0.0447	-0.0575	0.05547	0.03306	-0.1565	-0.0421	0.08067	0.0808	0.00126	-0.185	-0.1382	0.28142
NM_001135046	LOC363153	-0.0058	0.02182	-0.0472	0.13819	-0.0055	0.09983	0.17676	-0.0127	0.05702	0.08125	0.0052	0.05188	0.2098	0.20933
XM_343498.2	LOC363159	-0.0488	-0.0297	-0.085	0.05953	0.00031	-0.0672	-0.0292	-0.0143	-0.0092	-0.0573	-0.0518	-0.0577	0.172	-0.0132
NM_001110838	LOC363169	-0.0205	-0.2286	-0.1089	0.28511	-0.0836	-0.0942	-0.0273	0.01023	-0.1451	-0.1463	-0.1256	-0.0465	-0.1354	0.01812
XM_001065750.1	LOC363174	-0.0913	-0.2522	0.28199	-0.0767	-0.2838	-0.633	-0.3525	-0.4122	0.08085	-0.2117	-0.3246	-0.0129	0.68274	0.73999
XM_343518.2	LOC363178	0.01771	0.17206	-0.0229	-0.0307	-0.0618	-0.0009	-0.0641	-0.0468	-0.0074	0.1672	0.03797	0.0659	0.00485	-0.046
XM_343520.2	LOC363180	0.03581	0.19163	0.02089	-0.0178	0.00172	-0.0165	0.06155	-0.0997	0.11467	0.03171	0.02184	0.02123	0.07132	0.24728
XM_001061883.1	LOC363181	0.22278	0.00181	-0.0142	0.0687	0.04931	0.16872	0.05327	0.07979	0.04858	0.20973	-0.0089	0.13233	0.05064	-0.0165
XM_001061883.1	LOC363181	0.19004	-0.0911	0.03757	0.1466	0.11633	0.06362	0.03537	-0.0377	0.06865	0.18959	-0.1088	-0.0272	0.00157	-0.0747
XM_347110.2	LOC363186	0.12765	0.24354	0.1714	0.2742	0.17317	0.11603	0.45107	0.15971	0.095	0.08522	0.13821	-0.1123	0.0636	-0.0506
XM_001061882.1	LOC363188	-0.8671	0.10557	0.17398	-0.4729	-1.0848	-1.5347	0.16021	-1.0395	-0.2262	-0.0087	-0.1952	-0.6323	-0.4344	-0.4108
XM_001061882.1	LOC363188	-0.3153	-0.1728	0.40535	0.0954	-0.0904	-0.4425	-0.0703	-0.4216	-0.2147	-0.2436	-0.4333	-0.4074	-0.1972	-0.0241
NM_001108792	LOC363212	0.08325	0.18886	0.14592	0.24953	0.32007	0.18788	0.26576	0.12199	0.09829	-0.0063	0.2519	-0.1159	0.02684	0.05303
XM_343552.2	LOC363214	-0.1518	-0.1349	-0.1434	-0.1341	-0.0894	-0.1533	-0.0509	-0.1983	-0.097	0.06231	-0.0264	0.15379	-0.1012	-0.0837
XM_343556.2	LOC363218	0.09455	-0.0317	-0.0067	0.07047	0.04702	0.04298	0.06415	0.02992	0.22121	-0.073	-0.032	-0.0378	-0.06	-0.0509
NM_001108794	LOC363221	0.22478	0.27239	0.23809	0.15319	0.43207	0.3047	0.15136	0.29804	0.21359	0.09534	0.15609	0.36953	0.24991	0.31284
XM_347115.2	LOC363240	-1.1645	-0.9842	-0.4585	-0.7689	-1.2891	-1.4058	-0.6346	-1.2373	-0.9804	-1.2404	-0.9598	-0.5097	-0.8912	-0.9632
XM_347116.2	LOC363242	-0.0045	-0.0009	-0.0772	0.09996	0.1457	-0.0704	-0.047	-0.026	0.1424	-0.0619	0.20282	-0.1051	0.10739	0.04439
NM_001108802	LOC363256	0.27689	0.22371	-0.1565	-0.0037	0.20488	0.00717	0.27439	0.01433	0.36754	0.14997	0.11998	0.27037	0.05474	-0.0238
XM_343597.2	LOC363257	-0.2309	0.10595	-0.0106	-0.5821	-0.5152	-0.5691	-0.0149	-0.5749	0.02798	-0.0848	0.0513	0.14292	-0.723	-0.6706
XM_343599.2	LOC363259	-0.9325	-0.7111	0.23068	-1.4071	-1.49	-1.3428	-0.6405	-1.4457	-0.4416	-0.4009	-0.6289	0.20377	-0.2556	-0.1744
NM_001135759	LOC363265	0.16561	0.04394	-0.0324	0.0383	0.07365	-0.041	0.08428	0.43273	-0.1667	-0.035	-0.0174	-0.1193	-0.0097	-0.0531
NM_001135596	LOC363266	-0.2265	-0.2674	-0.4075	0.07408	0.19427	-0.0088	-0.2698	0.39867	-0.2563	-0.1428	-0.405	-0.4176	-0.3962	-0.3419
NM_001025025	LOC363267	-0.1405	0.52438	-0.1531	0.72652	-0.028	0.06978	0.14793	-0.0154	0.4806	0.41579	0.49081	-0.1257	-0.0084	-0.248
XM_347118.2	LOC363271	-0.3183	-0.1803	-0.2902	-0.1259	-0.1486	-0.0786	-0.1962	-0.1414	-0.3139	-0.1707	-0.2143	-0.111	-0.3433	-0.1757
XM_347119.2	LOC363298	0.04604	-0.1084	-0.0066	-0.0051	-0.0777	0.12691	-0.0576	0.00173	-0.0965	0.10158	-0.0821	0.04174	-0.0079	-0.1186
NM_001014221	LOC363301	-0.1017	-0.1138	-0.049	-0.1955	-0.2331	-0.1402	-0.1913	0.01068	-0.0623	-0.1186	0.03742	-0.1283	0.03746	-0.1619
XM_343647.2	LOC363306	0.13705	-0.0315	-0.1289	0.10648	0.03385	-0.1034	0.26237	0.07036	-0.1085	0.04232	-0.0138	0.00853	-0.0198	-0.0545
XM_343649.3	LOC363309	-0.5024	-0.3305	-0.1183	-0.2474	0.00603	-0.0251	-0.2599	-0.0041	-0.1753	-0.1563	-0.2584	0.21139	-0.0986	-0.2234
NM_001014221	LOC363313	0.1181	0.18561	0.138	-0.0475	-0.0111	0.06485	0.07876	-0.0084	0.1441	-0.0496	0.27913	0.28464	0.24598	0.24931
XM_343654.2	LOC363314	0.18322	-0.0856	0.06976	-0.0233	0.03767	-0.0894	0.00392	-0.0672	0.0856	-0.0443	-0.1044	0.08577	-0.0555	0.06908
XM_343656.2	LOC363316	0.17847	-0.0299	0.11686	0.19638	0.31522	0.30172	-0.0122	0.06185	0.15233	-0.0508	0.0564	-0.0064	0.04418	0.01248

XM_343657.2	LOC363317	0.04685	-0.1155	0.00563	0.18822	0.07551	0.04278	0.09778	0.3605	0.09937	0.08144	-0.0059	-0.0046	0.02326	0.05797
XM_343658.2	LOC363318	0.01329	0.04527	-0.1159	-0.029	0.02645	0.11946	0.07719	-0.065	0.02281	0.06194	6E-05	0.04453	0.02315	0.17719
XM_343660.3	LOC363320	0.04103	-0.0108	0.3311	0.35277	0.08907	0.19214	0.1651	0.38496	0.14295	0.07608	0.03021	-0.0148	0.00567	0.1182
XM_343660.3	LOC363320	-0.1121	-0.1929	-0.0514	-0.1732	-0.0388	-0.025	-0.1377	-0.1982	0.07025	-0.0673	-0.0379	-0.1804	-0.1722	0.03504
XM_343662.2	LOC363322	-0.0707	0.06688	-0.0119	-0.1611	-0.263	0.07525	0.06342	-0.0062	-0.2072	-0.1813	0.00053	-0.1248	0.04608	0.12752
XM_343663.2	LOC363324	0.10397	-0.0199	-0.1276	-0.129	-0.0985	0.08175	0.04882	0.05721	0.02656	0.08623	0.14429	-0.0466	-0.0159	0.03574
XM_347125.2	LOC363325	-0.033	0.09431	0.02111	0.07641	-0.1275	-0.031	-0.039	-0.0029	-0.0265	-0.0161	-0.109	0.20409	0.02822	-0.0029
NM_001126289	LOC363326	0.00412	-0.2318	-0.04	-0.0257	-0.0704	-0.0801	-0.144	0.05931	-0.0158	0.03544	-0.1186	0.09955	-0.2557	-0.1174
XM_343667.2	LOC363328	-0.0117	-0.048	0.38114	-0.0631	0.16761	0.61821	-0.0366	0.18507	0.18596	-0.1573	0.18269	0.00049	0.06919	0.09788
XM_343669.2	LOC363331	0.21593	-0.1145	0.06821	-0.1436	0.01847	-0.0548	-0.0659	0.01654	0.19151	-0.0779	0.0968	-0.0275	0.0094	-0.069
NM_001108812	LOC363332	-0.0503	0.26866	0.37483	-0.3209	-0.2184	-0.2336	0.03192	-0.4357	0.08629	0.10793	0.25402	0.07801	-0.0587	-0.0586
XM_001061702.1	LOC363333	-0.1755	-0.2573	0.84218	-0.4722	-0.1432	-0.4946	-0.2663	-0.354	-0.4157	-0.3322	-0.2289	0.23134	0.46127	0.32052
NM_001014221	LOC363336	0.3895	0.24666	0.19997	0.08519	0.39905	0.38228	0.18365	0.14131	0.387	0.19782	0.43758	0.08182	0.41354	0.23004
NM_001014221	LOC363337	0.33204	1.6841	1.1752	0.67096	0.63177	0.9752	1.0094	0.65352	1.3557	1.3029	1.8809	0.50254	0.61935	0.53307
XM_343681.2	LOC363343	-0.1003	1.0069	-0.091	-0.1569	0.13117	-0.1222	0.18064	-0.0042	0.54463	1.3106	0.67586	-0.1747	-0.1537	-0.0224
XM_343682.2	LOC363345	0.30864	0.2763	0.0486	0.15298	0.04367	0.2746	0.20383	0.14798	0.09542	0.29531	0.03053	0.29332	0.27004	0.06217
NM_001014221	LOC363351	0.02578	-0.0831	-0.0538	-0.0784	-0.0466	-0.0561	-0.0566	0.10274	0.01002	-0.1123	0.20776	0.01177	0.053	-0.0434
NM_001014221	LOC363354	-0.1124	0.05141	-0.0802	-0.1703	-0.1536	-0.0924	-0.1465	0.05129	-0.1975	0.11768	0.16785	-0.1756	-0.007	-0.0862
XM_343696.2	LOC363361	0.02603	0.24078	-0.0583	-0.0425	0.13412	0.09254	-0.0544	-0.0325	0.11794	0.00787	0.03135	0.02299	0.00586	0.22263
NM_001014221	LOC363363	0.21531	0.03423	0.10884	0.24605	0.20351	0.03816	0.19588	0.11678	0.10824	0.18009	0.10884	0.14694	0.04458	0.11847
XM_343700.3	LOC363366	0.02378	0.07342	0.15991	-0.0685	0.13268	0.04282	0.01747	0.05831	-0.0219	-0.042	0.11034	0.30191	0.0739	0.0755
XM_343704.2	LOC363369	-0.224	-0.069	0.04676	0.05448	-0.0468	0.24784	-0.0229	-0.1841	-0.1354	0.08847	0.31014	-0.1636	-0.1039	0.01734
XM_343712.2	LOC363377	-0.2877	-0.1992	0.22109	-0.1191	-0.3746	-0.2407	0.22542	-0.3628	-0.2207	-0.301	-0.1871	0.07185	-0.2518	-0.201
XM_343715.2	LOC363380	-0.0681	-0.0315	-0.0765	0.04384	-0.0794	-0.0732	-0.0589	-0.0252	-0.064	-0.0697	-0.0418	-0.073	0.0514	-0.1171
XM_343715.2	LOC363380	0.18694	0.14421	0.01712	0.21769	0.06645	-0.0157	0.00749	0.00314	0.0048	-0.1088	0.15815	0.29143	0.11208	0.19772
XM_343720.2	LOC363385	0.19648	0.08834	0.20598	0.1544	0.12768	0.26094	0.26149	0.39573	0.22672	0.4332	0.23679	0.06647	0.249	-0.0143
XM_343722.1	LOC363387	0.03338	-0.0694	-0.0509	0.00214	-0.0303	0.0425	-0.0664	-0.0015	-0.1111	-0.0785	-0.1134	0.02941	0.07509	0.16835
XM_347132.2	LOC363391	-0.0567	-0.1029	-0.1239	-0.1122	-0.1341	0.05553	0.10528	-0.1147	0.02559	-0.018	0.03357	-0.1101	-0.0268	-0.0471
XM_347133.2	LOC363393	0.05673	0.14923	0.00808	0.12252	0.13215	0.50755	0.03805	0.064	-0.005	0.41535	-0.0544	0.06071	0.05062	0.05399
XM_343725.2	LOC363395	-0.017	0.18755	0.06781	-0.0218	0.03407	-0.0043	0.03988	-0.0453	-0.073	-0.0147	0.02828	0.00526	0.25093	0.28234
XM_343727.2	LOC363397	0.07533	0.06953	0.22486	0.05724	-0.0213	0.11586	0.13443	-0.0738	0.01671	0.10707	-0.091	0.09871	-0.0938	-0.0631
XM_347135.2	LOC363403	0.00883	-0.1104	-0.2042	-0.2124	-0.1054	-0.1147	-0.1919	-0.1808	-0.1851	0.02998	0.0019	-0.0759	-0.2843	-0.0105
XM_343732.2	LOC363404	0.0098	0.13823	0.18172	0.03936	0.19527	0.17576	0.11601	0.1709	0.08194	-0.0446	0.02912	0.24012	-0.0783	-0.0102
XM_347136.2	LOC363405	-0.0759	-0.0491	0.0875	-0.0223	-0.0464	0.04852	0.00196	0.07912	0.04486	0.13463	0.14269	0.09742	-0.0522	0.0584
XM_576854.1	LOC363408	-0.0776	-0.0843	-0.0826	-0.1669	-0.0796	0.03579	-0.0325	0.09834	0.14925	0.00488	-0.0441	-0.0081	0.1026	-0.062
XM_343736.2	LOC363410	0.11887	0.04394	0.08806	0.0665	1.0563	0.84788	0.04503	0.83974	0.01768	0.16731	0.02882	-0.0289	0.04207	-0.0235
XM_343737.2	LOC363411	0.01615	0.08103	-0.0598	0.07618	0.04147	0.10696	0.0713	0.15946	0.1161	0.1013	-0.0659	0.15039	-0.0362	-0.0582
XM_343739.2	LOC363413	0.21147	-0.0709	-0.2203	-0.0355	0.21669	0.17118	0.01187	0.15701	-0.0642	-0.175	0.15457	-0.0901	-0.1743	-0.1608
XM_343740.2	LOC363416	-0.5025	-0.7746	1.1468	0.09888	-0.9647	-0.9254	-0.1085	-0.7803	-0.4805	-0.0604	-0.4547	0.17981	-0.1821	-0.2421
XM_001056019.1	LOC363418	-0.2855	-0.1968	-0.562	0.04486	-0.0184	-0.1249	-0.3082	-0.0385	0.0329	-0.1626	-0.0122	-0.0055	-0.2892	-0.3625
XM_343743.2	LOC363420	0.09102	0.0748	0.06177	-0.0322	0.02882	-0.0174	-0.0115	-0.004	0.04994	0.08671	-0.0322	0.13256	-0.0167	0.10011
XM_343746.2	LOC363423	0.05384	0.00878	0.05087	0.12619	-0.0669	0.06989	-0.0325	0.18025	0.02794	0.02496	0.00245	-0.0512	-0.0353	0.03007
NM_001014221	LOC363424	0.06246	-0.0171	0.21791	0.1184	0.05658	-0.0508	-0.0094	0.24699	0.07668	0.03776	0.06092	0.14004	0.03972	-0.0434
XM_343751.1	LOC363429	-0.0475	0.11685	0.07513	0.00197	-0.0916	0.2887	-0.0071	-0.02	-0.0203	0.14245	-0.1343	0.14864	0.03409	0.21968
XM_343754.2	LOC363432	0.1543	-0.0975	-0.1067	-0.0706	-0.0546	0.15898	0.12212	-0.0732	-0.1494	-0.1427	-0.1653	0.34735	0.00547	0.07469
XM_343755.3	LOC363433	-0.1678	-0.1062	-0.0391	0.02837	-0.0955	-0.11	-0.0651	-0.0776	0.02739	-0.0653	0.09076	-0.0307	0.01947	-0.1728
XM_343756.3	LOC363434	0.21177	-0.0082	0.01892	0.01962	0.15862	0.0741	0.07796	0.03782	0.13925	0.25576	0.1115	-0.0559	0.15265	0.01877
NM_001025746	LOC363439	0.17261	0.27037	0.26311	-0.0571	0.00884	0.25121	0.17714	0.18208	0.34242	0.14048	0.19059	-0.0419	0.52215	0.60232
NM_001108814	LOC363443	0.12299	-0.0896	0.0816	0.06642	0.03689	0.27411	0.02528	0.21145	-0.0177	-0.0947	0.2106	0.07558	0.19549	0.34072

XM_343775.2	LOC363453	-0.099	-0.145	-0.1444	0.31377	-0.2317	0.15708	-0.2543	0.09851	-0.1347	-0.0367	-0.0379	-0.0918	-0.2562	-0.0753
XM_347141.2	LOC363459	-0.1716	0.04819	-0.7917	-0.7837	-0.3422	-0.0924	-0.2286	-0.4118	0.07605	-0.0955	0.01076	0.27121	-0.3986	-0.4601
XM_343798.2	LOC363477	0.24697	0.04707	0.1529	0.17094	0.15372	0.02896	0.05936	-0.0157	0.0006	-0.0308	0.24127	-0.0321	0.09379	-0.0002
XM_343815.2	LOC363492	-0.9921	-0.1133	-0.5612	-0.3014	-1.8816	-1.4443	0.7324	-1.3547	-0.8138	-0.1555	-0.5148	-0.7021	-1.4495	-1.6876
XM_343818.2	LOC363495	-2.3245	-2.2929	-2.127	-1.4477	-2.2425	-2.296	-2.1792	-2.0871	-1.9869	-2.0997	-2.3916	-1.5409	-2.1012	-2.3572
NM_001077435	LOC363498	0.01046	0.03951	-0.0057	-0.046	0.03038	0.29137	0.12006	-0.0469	0.01315	-0.0554	0.20677	0.06942	-0.0217	-0.034
XM_343830.2	LOC363506	-0.0346	-0.0711	-0.1392	0.00812	-0.017	0.01219	-0.0596	-0.0648	0.06559	-0.1828	-0.1092	-0.1451	-0.0826	-0.0364
NM_001108822	LOC363528	0.0279	0.04939	-0.067	-0.1143	-0.0005	0.30475	-0.1218	0.08414	-0.0649	0.21821	0.00454	0.05381	0.09001	0.21781
XM_343851.2	LOC363532	0.2225	-0.0651	0.19869	0.10894	0.19109	0.06352	0.05742	-0.0647	-0.0375	0.05446	0.13197	0.26427	0.15491	0.17946
XM_343853.2	LOC363534	0.22943	0.07449	0.10509	-0.0801	0.20092	0.21309	0.04543	0.12622	0.0589	0.00897	0.14065	0.11561	0.05883	0.01188
XM_343859.2	LOC363542	-0.1756	-0.1006	-0.0368	-0.0753	0.05087	0.01888	0.00957	-0.0179	-0.0979	0.01645	-0.1292	0.29582	-0.1712	-0.0298
XM_343863.2	LOC363547	0.05742	0.28168	-0.001	0.30599	0.06519	0.11703	0.10343	0.35662	0.02544	-0.0464	0.07366	0.02357	-0.0273	0.06928
XM_573066.1	LOC363562	-0.0306	0.09954	0.03783	0.17405	0.06641	-0.0404	-0.0227	-0.0322	-0.0267	0.09848	-0.0835	0.02551	-0.1218	-0.0207
XM_343875.2	LOC363564	0.0986	-0.0188	0.15597	0.11701	0.11242	0.1051	-0.0436	0.23372	-0.0596	-0.072	0.05895	0.0238	0.00976	0.05449
XM_343879.2	LOC363576	-0.0909	0.1953	0.10937	0.17542	0.05144	0.10478	-0.0323	0.42289	0.35131	-0.0026	0.04272	0.05418	-0.0996	0.14327
NM_080882	LOC363588	0.15498	0.209	0.43149	0.12251	-0.0032	0.00635	0.04392	0.03795	0.10343	0.03359	0.14492	0.10425	0.14705	0.15135
XM_577090.1	LOC363591	0.1531	0.12694	0.12582	0.04734	0.08791	0.12284	0.22834	0.07281	-0.0293	0.04106	0.28434	0.18642	0.09496	0.19408
XM_577091.1	LOC363596	-0.0081	0.07459	0.06139	0.00412	-0.0349	-0.0429	0.14568	0.01576	0.01461	0.02214	0.10318	-0.0168	0.16034	0.04476
NM_001172067	LOC363618	-0.0222	-0.0645	-0.0451	-0.0502	0.05463	-0.0172	-0.0725	0.0306	0.15067	0.02269	0.05151	0.10738	-0.0472	-0.0612
XM_577105.1	LOC363623	0.00376	0.19842	0.13398	-0.0445	0.05349	0.15555	-0.0011	0.34276	0.11369	0.19252	0.03888	0.09399	0.39572	0.24021
XM_577108.1	LOC363626	-0.0544	0.0041	0.11266	-0.084	-0.0542	0.0593	-0.0799	-0.1559	-0.1794	-0.0468	-0.0008	-0.0804	0.01953	-0.0872
XM_343946.2	LOC363657	-0.1188	-0.0803	-0.0281	0.00918	-0.1262	-0.0239	-0.0763	-0.1201	-0.1939	0.05499	0.01605	-0.1523	0.05341	-0.0463
NM_001108832	LOC363661	0.06345	0.06834	0.11976	0.23108	0.01149	0.05324	0.06837	0.08339	0.08383	-0.0353	0.15263	0.02202	0.03534	0.2151
XM_343952.2	LOC363662	-0.0109	0.13552	-0.0637	-0.1207	0.14054	-0.1513	0.09584	0.1282	0.18204	0.23199	0.04749	-0.041	0.01238	0.12646
XM_343955.2	LOC363666	-0.0782	-0.1427	0.02794	-0.0373	-0.0395	-0.1222	-0.1517	-0.1715	-0.0054	-0.0789	-0.03	-0.0812	-0.0426	-0.1366
XM_343960.2	LOC363670	0.01728	0.01636	0.04334	0.08065	0.05725	0.05958	-0.0397	0.13628	-0.0148	0.06877	-0.0096	-0.0312	0.00282	0.37544
XM_343979.2	LOC363696	0.15777	0.02839	0.20661	0.06072	0.01906	0.33842	0.08424	0.00303	0.05046	0.09243	0.13314	0.04578	0.049	0.16644
XM_577135.1	LOC363697	0.17438	-0.0308	-0.0084	0.05705	0.09635	0.03061	0.00852	0.01915	-0.0196	0.06345	0.05912	0.18696	0.1377	-0.0369
XM_577140.1	LOC363701	-0.0605	0.16453	0.25905	0.59554	0.04947	0.26009	0.18882	0.01666	0.01309	-0.163	0.15254	0.03771	-0.0116	0.13757
XM_343990.3	LOC363711	0.04604	0.27345	-0.1098	-0.0295	-0.0634	0.33097	0.11192	0.09972	-0.0631	-0.1291	-0.0169	-0.0039	0.1376	-0.0065
XM_343991.2	LOC363712	-0.1794	0.19448	-0.0732	-0.1543	-0.0076	0.07583	-0.1405	0.00489	0.01082	-0.0714	-0.0313	-0.2254	-0.0756	-0.0031
XM_343992.2	LOC363713	-0.176	-0.1962	-0.0765	-0.0958	-0.1751	-0.1133	-0.019	-0.0454	-0.1466	-0.0715	-0.0537	-0.1008	-0.1007	-0.0644
XM_344000.2	LOC363729	-0.1614	-0.0937	-0.1408	0.14344	-0.1869	-0.0918	-0.1113	-0.044	-0.0417	-0.1362	-0.0215	0.02951	-0.1117	-0.0896
XM_344001.2	LOC363733	-0.0128	-0.2332	-0.161	-0.2031	-0.1123	-0.1747	-0.1692	-0.1082	-0.1764	-0.1702	-0.2301	-0.2454	-0.2173	-0.1533
NM_001145002	LOC363741	-0.0949	-0.081	-0.1105	-0.1388	-0.149	-0.0651	-0.2112	-0.2244	-0.0129	-0.0047	-0.1614	-0.2168	-0.1773	-0.2309
XM_573263.1	LOC363747	0.01289	0.05341	-0.2607	0.04697	-0.1208	-0.1298	-0.15	0.09396	-0.0301	-0.1667	0.03058	0.01651	0.10236	0.11482
NM_017285	LOC363748	-0.1641	-0.1826	-0.2014	-0.2142	-0.1503	-0.2159	0.09028	-0.1732	-0.0313	-0.0298	-0.1836	-0.1215	-0.0871	0.03227
XM_577174.1	LOC363751	0.01472	-0.0292	0.03299	-0.0605	-0.0638	0.00098	-0.0145	-0.0694	-0.0273	-0.0281	-0.0748	0.10168	-0.0815	-0.087
XM_577176.1	LOC363755	-0.0293	0.04969	0.18529	0.16934	-0.0629	0.02096	0.10865	-0.1204	0.0421	0.29543	-0.0035	-0.0292	0.17435	0.32175
XM_344008.2	LOC363759	-0.0378	-0.0107	0.04171	0.13159	0.02166	0.0194	0.03164	0.07021	0.09774	0.14031	-0.0205	-0.0557	0.13492	0.03492
XM_577180.1	LOC363775	-0.1842	-0.1182	-0.1136	-0.1124	-0.1542	-0.1789	-0.0446	-0.0006	-0.2181	0.15956	-0.1808	-0.1772	-0.1416	-0.1682
NM_001134481	LOC363781	0.56902	0.14312	0.02027	-0.0409	-0.0043	-0.0457	0.07062	-0.0772	0.06926	0.06269	0.26003	0.00907	0.04945	0.05269
XM_344019.2	LOC363782	0.0461	0.03166	0.28802	0.27025	0.0987	0.06444	0.03974	-0.0135	0.15962	0.03905	0.22324	0.04787	0.10774	0.04811
XM_344021.2	LOC363784	0.08651	-0.0036	0.07456	0.08945	0.0543	0.07857	-0.0105	0.13118	0.03726	0.22721	0.08323	0.21421	0.03448	0.02945
XM_577183.1	LOC363787	0.22453	-0.0103	-0.0443	0.08968	0.10707	0.31661	0.13123	0.30799	-0.018	0.28246	0.04136	0.54346	0.28583	0.22608
XM_577186.1	LOC363801	-0.1722	-0.0992	-0.2327	-0.1951	-0.1351	-0.2103	-0.0648	-0.2065	-0.2298	-0.0888	-0.2937	-0.0532	-0.185	-0.2284
XM_344031.2	LOC363804	0.2694	0.51881	0.18827	0.39615	0.33639	0.25483	0.21058	0.44386	0.13787	0.2244	0.23179	0.37802	-0.0868	0.21507
XM_344032.2	LOC363806	-0.2536	0.15104	-0.1102	-0.3714	-0.3436	-0.269	-0.2776	-0.0877	-0.3967	-0.3518	-0.3103	0.0471	-0.1913	-0.1619
XM_573320.1	LOC363827	-0.02	-0.0372	-0.0085	-0.0089	0.04316	0.02928	0.08294	-0.0064	0.02147	-0.0072	-0.0061	0.1653	0.05521	0.06659

XM_344042.2	LOC363828	-0.1088	-0.1006	-0.0315	-0.0514	0.16466	-0.0773	-0.0962	-0.0611	-0.1238	-0.0574	0.04768	-0.0856	-0.0827	0.06402
XM_344050.2	LOC363840	0.21287	-0.1194	-0.0017	-0.1497	0.14272	0.03091	0.13643	-0.0033	-0.0823	-0.149	-0.1082	0.01282	-0.1661	0.0626
XM_344052.2	LOC363843	0.04982	-0.0067	0.01964	0.00922	-0.0381	0.01808	0.14547	0.00907	0.01781	-0.0317	0.01046	0.17315	0.08393	0.11377
XM_344053.1	LOC363844	-0.0467	0.18068	0.23971	0.14144	0.04103	0.06249	0.0952	0.28252	-0.0162	-0.004	0.16272	0.02485	0.05172	0.16455
XM_344054.2	LOC363845	0.07518	-0.0927	-0.1479	-0.0102	-0.0037	-0.1251	-0.0495	-0.0792	-0.0676	0.04943	-0.1644	0.00912	0.0328	-0.1477
XM_344055.2	LOC363846	-0.0559	0.14243	0.0165	0.03592	-0.0577	-0.0527	0.14138	-0.1072	-0.0146	0.02658	-0.0517	-0.2034	-0.2274	-0.0042
NM_001135760	LOC363849	-0.7674	-0.3702	0.07739	-0.5535	-0.9351	-1.3379	-0.1675	-0.9566	-0.4473	-0.3649	-0.6083	-0.5829	-0.1141	-0.127
XM_344060.1	LOC363851	-0.0789	-0.0084	-0.0203	-0.0897	-0.075	0.01602	-0.0451	0.00124	0.02443	-0.0619	0.05071	-0.0687	0.05033	-0.1281
XM_344062.2	LOC363853	0.10887	-0.0655	-0.0782	-0.1824	-0.1903	-0.0389	-0.1676	-0.1859	0.07232	0.11139	-0.1417	-0.0985	-0.0508	-0.1929
NM_001025425	LOC363855	-0.0095	0.00619	0.14105	0.03503	-0.0174	-0.1043	0.26906	-0.0098	0.04249	0.14659	0.38327	0.21083	0.22103	0.07165
NM_017150	LOC363861	-0.0868	-0.1595	-1.0053	-0.4717	-0.0852	-0.0415	-0.9331	-0.0793	-0.0347	-0.25	-0.2321	-0.1898	-0.7726	-0.5632
XM_344070.1	LOC363862	0.04956	0.01513	-0.0056	0.17397	-0.0516	0.06185	0.06665	0.00788	0.07075	-0.04	0.15883	-0.056	-0.045	-0.0246
XM_344072.2	LOC363865	0.41031	0.0861	-0.4115	0.37094	0.19995	0.29068	-0.0811	0.36917	0.20549	0.29893	0.29974	0.03457	-0.048	0.18725
XM_344074.2	LOC363868	-0.1978	0.1195	-0.1714	0.34487	0.12335	0.09391	0.01772	0.00177	-0.1212	-0.1403	-0.0723	-0.1613	-0.023	0.02219
XM_344086.2	LOC363887	0.09893	-0.0911	0.17818	0.07349	-0.0302	0.00951	-0.1006	0.10779	-0.1161	-0.0893	0.00794	0.00264	-0.0681	-0.0126
XM_344090.2	LOC363892	-0.0142	0.01273	0.03554	-0.0299	0.00911	-0.0701	-0.0529	0.04011	0.15854	0.09136	0.03063	0.10599	0.11148	-0.0012
XM_344092.2	LOC363894	0.00034	0.06443	0.00601	0.15598	0.0973	-0.0101	0.11387	0.05364	0.21567	-0.054	0.13541	-0.0224	0.08778	0.04012
NM_134346	LOC363897	0.35647	0.5565	-0.1248	0.53452	0.44997	0.10605	1.3981	0.42136	0.59109	0.33828	0.46626	0.26613	0.31999	0.36021
XM_344095.2	LOC363898	0.01508	0.19798	0.04762	0.02154	0.09042	-0.0267	0.08203	0.111	0.2012	0.02134	-0.0041	0.04462	0.00802	0.0052
XM_344096.2	LOC363899	-0.0445	-0.0562	-0.0644	0.02138	-0.0854	0.11053	0.28733	0.11122	-0.0864	-0.01	-0.0202	-0.0606	-0.0796	-0.0783
XM_344098.1	LOC363901	-0.1739	-0.376	-0.3436	-0.2893	-0.3576	-0.2357	-0.2768	-0.1516	-0.1387	-0.3055	-0.4301	-0.3909	-0.3217	-0.234
XM_344104.1	LOC363911	0.24225	0.09892	-0.0337	0.17369	-0.1034	0.09489	0.0831	0.1639	0.09247	0.06742	0.03108	0.41503	0.03063	0.16624
XR_009243.1	LOC363915	0.04014	0.00196	0.07011	-0.0565	0.10463	0.06865	-0.0365	-0.1011	-0.0087	0.10355	0.14912	0.16975	0.01317	0.10144
XM_344109.2	LOC363920	0.19048	0.07417	0.18419	0.10918	0.11226	0.08154	-0.0265	0.20685	0.38134	0.0255	0.09779	0.1	-0.0196	0.05972
NM_001035252	LOC363925	-0.2208	-0.1338	-0.0745	-0.2056	0.01665	-0.1352	0.13821	-0.0503	0.15284	-0.1194	-0.0714	-0.0141	-0.22	-0.0201
XM_344116.2	LOC363928	0.02905	-0.1211	-0.1131	-0.1079	-0.189	-0.061	-0.2035	-0.1913	-0.2673	-0.2489	-0.1776	-0.2021	-0.0938	-0.0715
XM_344122.2	LOC363942	0.41589	0.31016	0.37996	-0.0646	0.35257	0.36438	0.44848	0.51832	0.31471	0.16759	0.55757	0.31218	0.05662	0.38248
XM_344132.2	LOC363971	-0.0907	0.08046	-0.0134	0.06471	-0.1011	0.15198	-0.1988	-0.1953	0.0878	-0.0479	0.06777	-0.1392	-0.1387	0.17836
XM_344137.1	LOC363981	0.03218	-0.0386	0.10806	0.10516	0.09976	-0.0046	0.09815	0.31719	0.01524	-0.0281	0.02707	0.08201	0.11932	0.07022
XM_344144.2	LOC363991	0.19671	0.11505	0.2662	0.15347	0.15161	0.53855	0.35231	0.12193	0.16356	0.14745	0.08832	0.15114	0.05131	0.10388
XM_344147.2	LOC363995	0.1252	-0.3654	-0.3473	-0.3125	-0.3085	-0.2652	-0.3256	-0.1257	-0.3147	-0.1782	-0.2221	-0.2664	-0.2864	-0.306
XM_344149.2	LOC364004	0.0419	0.13708	-0.0543	0.13761	0.1155	0.32796	-0.0213	0.28634	0.0361	0.15079	0.04922	0.05877	0.135	0.0256
XM_344152.1	LOC364012	-0.0073	0.05918	0.01048	-0.0091	0.04439	-0.0265	-0.0479	0.04862	-0.0238	0.02594	0.07926	0.17117	0.06585	0.23513
XM_577274.1	LOC364015	0.17751	0.09493	0.35916	0.09073	0.22368	-0.0472	-0.0207	0.12281	0.15712	0.3636	0.25834	0.20079	0.0534	0.13478
XM_344157.2	LOC364020	-0.0798	-0.0111	0.02365	0.01163	-0.0923	-0.1187	-0.0163	0.14329	-0.1234	-0.0682	-0.0611	0.01853	0.07452	0.02676
XM_344165.2	LOC364029	-0.1439	-0.0968	-0.1388	-0.152	-0.1254	-0.1977	0.0781	-0.0805	-0.1302	0.00802	-0.142	-0.1567	-0.1947	-0.0224
XM_573491.1	LOC364035	-0.14	-0.1052	-0.0729	-0.1209	-0.1044	-0.0867	-0.1673	-0.0667	-0.1853	-0.1269	-0.147	-0.081	-0.0835	-0.1254
XM_577286.1	LOC364044	-0.208	-0.1014	-0.2479	-0.254	-0.1798	-0.1127	-0.2982	-0.1664	-0.254	-0.0767	-0.2338	0.02572	-0.0896	-0.1302
XM_577289.1	LOC364048	0.66704	0.51731	0.02811	-0.2043	0.57122	1.1396	-0.9036	0.2196	0.54588	0.49611	0.69531	0.35408	0.16909	0.12202
XM_344183.2	LOC364067	-0.1428	0.2042	-0.0573	0.0552	-0.134	0.01903	0.21825	-0.1084	0.05237	0.14512	0.11635	-0.1347	0.10281	-0.2657
XM_344193.2	LOC364080	0.07417	0.06219	0.13093	0.23863	-0.0289	0.24705	0.05279	0.09826	-0.0938	0.16965	-0.1053	0.02909	0.01593	0.13161
XM_577300.1	LOC364082	-0.0384	-0.1055	0.01265	-0.0213	-0.0085	0.16442	-0.1452	0.10568	-0.169	-0.305	0.04758	0.16988	0.21363	0.17286
XM_344196.2	LOC364084	-0.0802	0.01378	-0.0965	-0.0838	-0.1193	-0.0485	-0.1051	-0.1191	-0.0608	-0.0702	0.00291	-0.0128	-0.1103	-0.1167
XM_344206.2	LOC364096	0.0891	0.03914	0.04546	0.0156	0.32227	0.04717	0.14161	-0.0304	0.01994	0.06648	0.0862	0.03525	-0.0306	0.18625
XM_344211.2	LOC364102	-0.0156	-0.058	-0.089	0.04697	0.04744	0.18603	0.10535	0.08401	0.11669	0.196	0.0233	0.24524	-0.0217	0.00798
XM_344212.2	LOC364104	-0.1198	-0.144	0.22904	0.0031	0.05368	0.11314	-0.1443	0.21597	-0.1262	-0.0971	0.13443	0.09874	-0.1247	-0.0427
XR_008399.1	LOC364105	-0.0747	-0.0948	-0.287	0.14317	0.19585	0.70225	-0.5897	-0.0178	-0.074	-0.3277	0.06774	0.58763	-0.1785	-0.0494
XM_577310.1	LOC364111	-0.1803	-0.1637	0.12061	-0.1376	-0.0802	-0.2044	0.05008	-0.0192	-0.0792	-0.1167	0.09611	-0.0503	-0.1874	-0.0828
XM_577311.1	LOC364115	-0.0223	0.11323	-0.0456	0.04089	0.01015	-0.0219	-0.0375	-0.0761	0.06335	0.15348	0.18224	0.01481	0.13381	0.14049

XM_577322.1	LOC364117	-0.081	-0.1917	-0.1526	-0.1415	-0.1335	-0.0776	0.13436	-0.1358	-0.1198	0.16378	0.01502	0.02491	0.17248	-0.0491
XM_573564.1	LOC364121	0.30744	0.0533	0.06789	0.2888	0.08488	0.43276	0.26268	0.14138	0.01444	0.27101	0.12459	0.18338	0.05701	0.0642
XR_008918.1	LOC364127	-0.3623	-0.4681	-0.2351	0.26465	-0.1458	-0.2985	-0.1414	-0.1431	-0.5014	-0.6883	-0.2437	-0.1838	-0.1779	-0.562
NM_053330	LOC364139	-0.2456	-0.2631	-0.7009	-0.5099	-0.5324	-0.1553	-0.4256	-0.1986	-0.249	-0.4707	-0.253	-0.1554	-0.3142	-0.4842
NM_001006995	LOC364149	0.12363	0.01563	0.14959	0.10668	-0.0356	-0.0046	-0.0089	-0.0115	-0.0349	-0.0107	0.07858	0.10212	0.09303	0.05804
XM_573601.1	LOC364155	-0.2746	-0.1432	-0.1637	0.00943	-0.0789	-0.1616	-0.0257	-0.3116	0.03302	-0.2012	-0.1682	-0.2	-0.1014	-0.14
XM_344247.2	LOC364164	-0.0962	-0.0059	0.06468	-0.0861	-0.0916	0.27388	0.16757	-0.0558	0.06889	0.00554	0.02727	0.268	0.15179	-0.0716
XM_573666.1	LOC364200	0.02645	0.08435	0.23158	0.05857	0.27475	0.15306	0.08644	0.05504	0.01642	-0.0138	0.15421	-0.0293	0.11235	-0.0054
NM_001108863	LOC364205	0.00913	0.08799	0.06139	0.04258	0.01965	0.00167	0.10508	0.01159	0.23628	0.02972	0.17292	0.01212	0.12153	0.1122
XM_344272.1	LOC364215	0.03447	0.0979	0.13274	0.10964	0.16147	-0.0071	0.21419	0.18691	-0.047	-0.1174	0.11749	-0.0419	0.16141	-0.0038
NM_001037346	LOC364236	-0.0849	-0.1684	-0.2971	-0.071	-0.2373	0.02139	-0.2111	-0.1307	-0.2424	0.10683	0.00728	-0.0827	-0.3281	-0.3562
XM_344285.2	LOC364239	-0.2583	0.04933	-0.1367	-0.1851	-0.0816	-0.1574	-0.2626	-0.1895	0.09371	-0.0372	-0.2324	-0.1196	-0.4031	-0.1702
NM_001039516	LOC364241	0.12465	0.22776	0.1036	0.16561	0.00932	0.24011	0.14701	0.31894	0.15363	0.20009	0.15071	0.10404	0.18184	0.46233
XM_344293.2	LOC364248	-0.039	0.00735	-0.1057	0.0021	0.05284	0.12479	0.04832	0.15937	0.03418	0.03814	0.06375	0.22893	0.20884	-0.1092
NM_001011701	LOC364250	-0.2893	0.1711	-0.1957	-0.215	0.0019	-0.0711	0.18629	0.0684	-0.0926	-0.1418	-0.0538	0.02141	-0.0167	-0.1027
XM_344296.1	LOC364253	0.16959	0.4997	-1.061	-0.4029	0.04905	-0.0153	-0.0344	0.16308	0.3805	0.39646	0.31779	0.29903	-0.1581	-0.1791
XM_577389.1	LOC364276	-0.0494	-0.1302	-0.0792	-0.0983	-0.126	-0.1723	-0.0652	0.00992	-0.0393	-0.1093	-0.1612	-0.0028	0.09806	-0.035
XM_344326.2	LOC364300	-0.0173	0.23545	0.19012	0.22801	0.0733	0.05163	0.09942	0.11109	-0.0184	0.47471	0.0329	0.28736	0.14399	0.06092
XM_344333.2	LOC364308	-0.1219	-0.2141	-0.1667	-0.1437	-0.158	-0.3226	-0.3179	-0.2635	-0.385	-0.3383	-0.0675	-0.1429	-0.2572	-0.383
XM_344336.2	LOC364311	-0.0325	0.09436	0.01971	-0.0096	-0.0313	0.11092	-0.1643	0.13868	-0.0105	0.3465	0.28443	-0.1336	0.36048	0.00337
XM_344337.2	LOC364312	0.04363	0.14201	0.19036	0.04334	0.07015	0.06524	0.11426	0.08999	0.21107	0.26441	0.16665	0.22195	0.03824	0.00488
XM_344338.2	LOC364313	0.20873	0.09086	-0.0529	0.09516	-0.0671	-0.0186	0.16146	0.17995	-0.02	0.14096	-0.0225	-0.0623	0.16337	0.14399
XM_344343.1	LOC364318	-0.2113	-0.1474	-0.1245	-0.1468	-0.1521	-0.2141	-0.1546	-0.0876	0.03276	-0.1805	-0.1832	-0.1967	-0.2215	-0.159
XM_344344.1	LOC364319	0.18605	0.02092	0.25691	-0.0455	0.09176	0.01122	-0.0311	0.08803	0.02059	0.00807	0.11145	0.32909	-0.0923	0.13081
XM_344346.2	LOC364321	-0.1827	-0.1064	0.04673	0.05098	0.05948	0.07449	-0.1663	-0.035	-0.2718	-0.0628	-0.1457	0.09545	-0.2102	-0.2909
XM_344355.2	LOC364331	0.22423	0.04408	-0.0162	0.10428	0.19974	0.10769	0.19727	0.19783	0.02825	0.07021	0.09319	0.26904	0.08613	0.15889
XM_344357.2	LOC364333	-0.1858	-0.19	-0.0862	-0.1765	-0.1322	-0.1948	-0.2499	-0.3181	-0.0554	-0.0524	-0.1419	0.06051	-0.2733	0.13853
XM_344359.2	LOC364335	-0.028	-0.0707	0.09085	0.29485	0.09881	0.04846	0.00965	0.07051	-0.0279	0.00444	0.07242	-0.0262	0.14114	0.22264
XM_344360.2	LOC364336	0.02473	0.00933	-0.0082	0.04858	0.00543	-0.0521	-0.0519	0.01985	-0.0324	0.13583	0.11273	0.01601	0.03531	0.14597
XM_344361.2	LOC364337	0.12649	0.16245	0.1014	0.07949	-0.0054	0.01317	0.05129	-0.0682	-0.015	0.08338	0.02176	0.01647	0.04355	-0.0326
XM_344361.2	LOC364337	-0.0822	0.01044	0.15257	-0.1397	-0.0984	0.14414	0.12469	-0.0366	0.03661	0.07001	0.00997	0.1109	-0.0364	-0.0607
XM_344366.2	LOC364343	0.03806	-0.1145	-0.0892	0.70916	0.0057	0.11747	-0.032	0.15846	0.06792	-0.1292	0.09897	0.24212	-0.0241	-0.018
XM_344369.2	LOC364346	0.01215	-0.1535	-0.1891	-0.2208	-0.1632	-0.1671	-0.2111	-0.0263	-0.1026	0.12658	-0.1114	-0.0545	-0.1471	-0.1902
XM_344371.1	LOC364348	0.21762	0.22989	-0.0149	-0.0716	-0.0044	-0.0824	0.03652	0.01598	-0.0989	0.03448	-0.0629	0.16928	0.03466	-0.0323
XM_344375.2	LOC364352	0.3479	0.22427	0.39684	0.41117	0.25559	0.34472	0.30446	0.3584	0.13126	0.37182	0.05519	0.39302	0.19066	0.45507
XM_344375.2	LOC364352	0.05782	-0.1171	0.21612	-0.037	-0.0101	0.12006	0.08142	-0.0958	-0.0484	-0.0915	0.05619	0.2773	-0.0655	0.03
XM_344375.2	LOC364352	-0.1069	-0.1112	-0.061	0.14217	-0.0982	0.1998	-0.0025	0.00971	-0.0838	-0.0397	-0.0183	-0.1152	-0.0832	0.00964
XM_344380.2	LOC364357	0.03186	-0.2539	-0.2347	0.06609	0.02234	-0.1409	-0.1961	-0.2624	-0.1547	-0.0406	0.04144	-0.0893	-0.1184	-0.177
XM_344382.2	LOC364359	-0.1773	0.09952	-0.0778	-0.0985	-0.1181	-0.1904	-0.1878	-0.1609	-0.1882	0.00105	-0.2168	-0.2107	-0.2365	-0.1905
XM_344384.2	LOC364361	-0.1007	-0.0954	-0.1136	-0.0642	0.03905	-0.1022	-0.0734	-0.114	0.19635	-0.0363	0.10143	-0.0523	-0.0676	-0.057
XM_344388.2	LOC364365	0.34107	0.03664	0.30096	0.11576	-0.0127	0.18765	0.07178	0.19004	0.19331	0.29945	0.18128	0.24548	0.11258	0.20625
XM_344389.2	LOC364366	0.13382	-0.0678	0.0504	0.12795	-0.0569	-0.0587	0.05243	-0.0788	0.07302	-0.0786	0.00479	-0.0144	-0.0467	0.01245
XM_344390.2	LOC364367	-0.0607	-0.1273	0.03579	0.07997	0.07671	-0.0485	-0.165	-0.1044	-0.0235	-0.0746	0.01902	0.07412	0.02548	-0.0505
XM_344393.2	LOC364370	0.02412	-0.18	-0.1818	-0.1388	0.02162	-0.1313	-0.0475	0.02829	-0.0831	0.02499	-0.1402	-0.0313	0.0503	0.08324
XM_344399.2	LOC364376	-0.0399	0.14259	-0.1182	0.09352	0.06732	0.24989	0.06696	0.23178	-0.1373	0.14093	-0.0414	0.20355	-0.1276	0.25636
XM_344400.2	LOC364377	0.03846	-0.1305	0.01273	-0.0611	0.06812	0.17728	-0.0645	0.10767	-0.012	-0.0804	0.31734	0.20839	-0.0021	-0.0845
XM_344408.2	LOC364385	0.1148	0.09252	0.02598	0.10915	0.00472	0.19408	0.0057	0.19156	0.1066	0.1337	0.29585	0.05042	0.166	0.12625
XM_344424.2	LOC364408	0.00628	0.05449	0.02707	0.11931	0.24583	0.31704	-0.0545	0.15147	0.11289	0.13451	0.22868	-0.0156	0.11467	0.19524
XM_344425.2	LOC364409	-0.0949	-0.0209	0.00778	-0.028	-0.0835	-0.0932	-0.0554	-0.0756	-0.092	-0.1561	0.00219	-0.0654	-0.0818	-0.0288

XM_344435.2	LOC364426	0.01625	0.11128	0.22263	0.12807	0.08978	0.15967	0.01609	0.01285	-0.0142	0.10462	0.10068	0.05514	0.03687	-0.0685
NM_001024803	LOC364427	0.03149	0.16456	-0.0094	-0.0165	-0.002	-0.0434	-0.0325	0.05843	0.01343	0.08366	0.02945	-0.0865	0.04152	0.0911
XM_577451.1	LOC364429	-0.1803	-0.0029	-0.0199	-0.1318	-0.1341	-0.027	-0.1153	-0.133	-0.164	0.01112	-0.1249	-0.0088	0.14554	0.07918
XM_344439.2	LOC364431	0.1868	0.09463	0.14263	0.18544	0.10145	0.1584	0.13827	0.12854	0.11524	-0.0625	0.0508	0.06716	0.35292	-0.0216
NM_134415	LOC364468	0.29317	-0.0432	-0.1599	0.21487	0.45291	0.24252	0.0205	0.4889	0.35055	0.21137	0.31533	-0.0784	0.35306	0.24268
XM_344466.2	LOC364487	0.09647	-0.0089	0.01621	0.06076	0.10856	0.05012	0.07669	0.04113	0.09233	0.0542	0.02898	0.06664	-0.0252	0.25627
XM_344469.2	LOC364490	0.02447	0.15566	-0.0282	-0.041	0.01625	-0.0901	-0.079	-0.0068	-0.1014	-0.0758	-0.0072	0.20024	0.07486	0.13618
XM_344471.2	LOC364493	-0.0031	-0.1351	-0.0336	-0.1392	-0.1072	0.01657	0.0336	-0.0701	-0.1309	0.00278	-0.1576	-0.0788	-0.1328	-0.0959
XM_344472.2	LOC364495	0.16166	0.08537	1.1948	2.1312	0.01212	0.20683	0.38715	0.37584	0.18769	-0.0989	0.32914	0.34246	-0.2287	0.04284
NM_001025028	LOC364514	0.31964	-0.0793	-0.3252	0.14131	0.34541	-0.0925	0.1216	0.31401	-0.0287	-0.0105	0.13009	-0.2273	-0.1826	-0.038
XM_344487.2	LOC364517	-0.0417	-0.0178	0.09326	0.10817	0.11802	0.26002	0.00511	-0.0304	-0.0423	0.12133	0.04062	-0.0078	0.19869	-0.0301
XM_573869.1	LOC364519	-0.0777	-0.0129	-0.0413	-0.1352	-0.079	-0.1372	-0.1252	-0.1585	-0.1422	-0.1035	-0.1185	-0.111	-0.0912	-0.0647
XM_344492.2	LOC364533	-0.0478	0.04152	-0.0348	0.17696	0.07746	-0.1074	0.12844	-0.1139	0.01099	-0.0074	-0.0428	0.05703	0.22098	0.02954
XM_577504.1	LOC364542	-0.1388	-0.0548	-0.115	-0.0531	0.0389	-0.1097	-0.1249	0.12335	-0.1707	0.02549	-0.1174	0.00904	0.09185	0.01355
XM_577506.1	LOC364549	-0.1318	0.46827	0.79223	0.51542	-0.0007	0.03874	0.83096	-0.0627	0.21779	0.08084	0.29497	0.0408	0.21036	0.12969
XM_573906.1	LOC364577	0.31739	0.10731	0.11196	0.05406	0.041	0.20966	0.1054	-0.0191	-0.0458	0.12878	0.10325	0.06342	0.10131	0.08747
XM_577517.1	LOC364582	0.18745	0.30522	0.01109	0.14263	0.13785	0.35244	-0.143	0.09765	-0.0299	0.13064	0.33505	0.36107	0.20452	-0.0966
XM_344527.2	LOC364607	0.07215	-0.058	-0.1096	-0.1176	-0.1371	0.21808	-0.0842	0.01131	-0.1761	0.11757	0.0023	-0.0481	0.04928	-0.0749
NM_053330	LOC364608	0.02701	-0.0005	0.29822	0.12684	-0.086	-0.0332	-0.1119	0.06632	-0.0586	0.06407	-0.0795	-0.0295	-0.1202	0.01979
XM_577529.1	LOC364610	0.08317	0.02866	-0.0943	-0.0298	-0.1726	-0.1141	-0.1123	-0.1508	-0.1073	-0.1125	-0.0512	-0.138	-0.1081	0.12272
XR_009128.1	LOC364647	0.02334	-0.059	-0.0664	-0.0549	0.15323	-0.0468	-0.0535	-0.0614	-0.1141	-0.0066	-0.032	0.21514	-0.0051	-0.0668
XM_344553.2	LOC364650	-0.0522	-0.2269	-0.2718	0.03428	-0.0647	0.02334	-0.156	-0.1584	-0.2873	-0.0992	-0.19	0.34034	0.08302	-0.1186
XM_344557.3	LOC364653	0.01382	-0.0184	-0.0423	-0.1104	0.05231	0.04579	0.04673	0.04886	-0.1401	-0.2433	0.07251	-0.139	-0.0335	0.02653
XM_001061874.1	LOC364654	0.06043	-0.1143	-0.4586	-0.3834	-0.1356	-0.0234	-0.1806	-0.0319	0.1878	0.2255	-0.1428	0.26697	0.36867	0.11916
XM_344561.2	LOC364657	-0.0157	-0.0616	0.07845	-0.0418	-0.044	0.11969	-0.0547	-0.0412	0.05869	0.03186	0.08681	0.08592	0.09816	-0.1035
XM_344562.2	LOC364660	-0.0445	0.21564	0.03141	0.24653	0.08648	0.16684	0.05073	-0.0753	-0.0621	0.28129	0.16216	0.20526	0.00924	-0.0393
XM_573978.1	LOC364670	0.18189	0.0661	0.01226	0.07555	0.15273	0.12475	0.24499	0.07834	0.05723	0.05694	0.17622	0.15468	0.07786	0.0131
XM_344566.2	LOC364671	-0.1486	-0.1191	0.01278	-0.0374	-0.0549	-0.0823	0.07891	-0.1502	-0.0507	-0.0983	-0.0903	0.02205	0.03883	-0.113
XM_344578.2	LOC364683	0.11437	0.42706	0.09224	0.27817	0.06968	0.15197	0.13922	0.23933	0.05803	0.01593	0.11927	0.08183	0.05523	0.07812
XM_344579.2	LOC364684	0.154	0.29294	0.19508	0.23123	0.2364	-0.0416	0.00993	0.25116	-0.0225	0.35723	0.07134	0.05132	0.05912	0.2148
XM_577562.1	LOC364685	0.10944	-0.0207	-0.0388	0.10943	-0.0073	0.07376	0.07683	0.19756	0.07055	0.11067	0.15306	0.18401	0.07617	-0.0085
XM_577567.1	LOC364698	0.25418	0.06114	0.11716	0.10947	0.37612	0.11125	-0.0317	-0.0131	0.12938	0.02122	0.1702	0.16572	0.06869	0.07727
XM_344588.2	LOC364699	0.01828	0.0303	-0.0022	0.08478	0.00769	0.05293	-0.0122	0.02534	-0.0748	0.05218	0.00979	0.14849	0.12961	0.16706
XM_344591.2	LOC364703	0.06454	0.16342	0.1641	-0.0387	0.07781	0.04188	0.25546	0.08917	0.04801	0.02806	-0.0095	0.06542	0.08718	0.09495
NM_001107442	LOC364718	-0.1196	0.07053	0.15626	0.45429	-0.0174	0.16921	0.27221	0.02548	-0.0613	0.12768	-0.0746	0.05243	0.06251	0.16427
NM_001107352	LOC364724	-0.1981	-0.1325	-0.0109	-0.1657	-0.1584	-0.1457	-0.1852	-0.1958	-0.0306	-0.0584	-0.0033	-0.0262	-0.0522	0.04906
XM_344608.2	LOC364741	-0.112	-0.2256	-0.2453	-0.0332	-0.2932	-0.205	-0.2014	-0.1424	-0.1169	-0.2364	-0.1628	-0.0562	-0.0992	-0.0765
XM_344619.2	LOC364759	-0.0136	0.2408	-0.0817	-0.1186	0.13819	0.18172	-0.0775	0.19797	0.01009	0.23269	-0.0434	-0.1019	-0.0271	0.01178
XM_344620.1	LOC364762	-0.0404	0.09761	-0.0405	-0.0549	0.06319	0.08833	-4E-05	0.14294	-0.0704	0.10465	0.14554	0.22387	-0.0696	0.05536
XM_344621.2	LOC364763	0.02959	0.18917	0.02383	0.22929	0.36533	-0.1209	0.18614	0.48226	-0.1748	-0.077	0.00528	-0.5848	0.40848	0.61842
NM_001014240	LOC364773	-0.9891	-1.0324	-1.7115	-1.5312	-1.173	-0.8495	-0.6366	-0.8993	-0.6548	-1.2205	-0.7704	-0.693	-1.4781	-1.414
NM_001014240	LOC364773	-0.9845	0.94679	-0.7646	0.07898	-1.2479	-1.4929	-0.4895	-1.5432	0.53927	0.8813	0.85008	0.27481	-0.1248	-0.1003
XM_574077.1	LOC364779	-0.0612	-0.0965	-0.1872	-0.0902	-0.0944	-0.1637	0.00132	-0.1867	-0.0772	-0.0289	-0.1216	-0.0774	-0.0657	0.03858
XM_577600.1	LOC364787	0.0135	0.02293	-0.082	-0.1016	0.12809	0.02687	-0.1058	0.02705	0.03359	0.03535	0.23081	0.33769	0.00194	0.07427
XM_344641.2	LOC364800	0.02438	0.0201	-0.0536	0.04385	-0.0299	-0.0033	0.013	0.02193	0.00614	0.24544	-0.0254	-0.1011	0.10757	-0.0615
NM_001108827	LOC364802	0.22808	0.14242	0.02782	0.11798	0.06489	0.0926	0.00344	0.24626	0.16841	0.04559	0.03011	0.32672	0.0083	0.10987
XM_344654.1	LOC364825	0.2071	0.08564	-0.0627	-0.0137	0.01439	0.2091	0.01596	0.0228	0.06201	0.10601	0.08625	0.11248	0.21536	0.13528
XM_577616.1	LOC364861	0.0646	0.01212	0.00823	-0.025	0.00737	0.27015	-0.1431	-0.0957	-0.0787	0.03384	-0.0846	-0.0763	0.03759	-0.0353
XM_574142.1	LOC364863	-0.0288	-0.0997	0.15143	0.09064	0.02076	-0.0826	0.1786	-0.0539	0.00097	0.06049	0.05783	-0.1326	-0.0768	-0.0515

NM_001109373	LOC364873	-0.23	-0.1447	-0.1752	-0.0591	-0.0457	-0.1683	-0.2358	-0.1034	-0.0198	-0.1771	-0.2621	-0.0499	-0.1054	-0.0646
XM_344705.1	LOC364905	-0.1611	0.10882	0.0872	0.07038	-0.0358	-0.0805	0.04076	0.01846	-0.1416	-0.1072	0.08061	0.05955	-0.0559	-0.0357
XM_344713.2	LOC364917	-0.0241	0.08032	0.07904	0.15916	0.16347	0.13678	0.05794	0.12431	0.38084	0.41575	0.14637	0.29342	0.09974	0.22109
XM_577643.1	LOC364937	-0.0579	0.08465	-0.0397	-0.0763	-0.0667	0.01359	-0.0942	-0.0476	-0.0293	-0.139	0.17403	0.00982	-0.0983	-0.1624
XM_344732.2	LOC364958	0.05361	0.00986	0.03571	0.13528	0.23621	0.07495	0.05453	0.1045	0.03556	0.42935	-0.0061	0.00359	0.1639	0.28078
XM_344739.2	LOC364969	0.18181	0.09393	0.20399	0.08164	0.01675	0.15393	-0.0256	0.12298	0.17971	0.22352	0.06482	0.03554	0.06199	0.01378
XM_344740.2	LOC364971	-0.1278	-0.0505	0.09051	-0.1093	0.05163	0.23746	-0.044	-0.0753	0.10592	-0.1173	-0.2346	0.05612	-0.1192	0.29375
XM_344752.2	LOC364985	0.10528	-0.0329	-0.2349	-0.1184	0.34323	0.11568	-0.1211	0.10759	-0.1384	0.04599	-0.0092	0.12094	-0.1082	0.06087
XM_344758.2	LOC364991	0.10041	0.03749	-0.0017	0.00098	-0.0048	0.17015	0.1461	0.14796	-0.1091	-0.1108	-0.0427	-0.1148	-0.1023	0.07016
XM_344769.2	LOC365005	0.06412	0.12217	-0.0319	0.03605	0.24266	-0.0777	-0.0746	-0.2226	0.12343	0.10766	0.03292	-0.2027	0.17922	0.34578
XM_577671.1	LOC365025	-0.2078	-0.5533	0.59074	-0.5849	-0.2541	-0.2198	-0.7752	-0.1089	-0.1069	-0.6161	-0.3564	0.40587	0.89489	0.71578
XM_344788.3	LOC365028	-0.1468	-0.0655	0.02379	-0.0638	-0.1274	0.13483	-0.0013	-0.157	0.10562	-0.0543	-0.0155	0.03708	-0.1879	-0.1045
XM_344799.2	LOC365058	0.0812	-0.1535	-0.1811	-0.152	-0.0052	-0.0955	-0.1266	-0.1098	-0.1429	-0.0877	-0.0708	-0.0823	0.01909	-0.0688
XR_007974.1	LOC365059	-0.0927	-0.2739	-0.2645	-0.1423	-0.0857	0.01685	-0.206	-0.0404	0.09578	-0.1341	0.16518	0.08945	-0.1819	-0.4095
XM_344801.2	LOC365063	0.15843	0.14555	0.19141	0.02919	0.02462	0.00642	-0.0339	0.24359	0.15867	0.05881	0.19597	0.12177	0.10247	0.10587
XM_344804.1	LOC365068	-0.1233	0.10631	0.25719	-0.0746	0.00175	0.0249	-0.0152	-0.0477	0.02767	-0.0875	0.01506	0.05618	-0.0854	0.02049
XM_574287.1	LOC365076	-0.3395	-0.2989	-0.3002	-0.2842	-0.2899	-0.1946	-0.1399	-0.1025	-0.3808	-0.3217	-0.2683	-0.221	-0.2429	-0.3096
XM_344808.2	LOC365087	0.19625	0.25734	0.05054	0.06637	0.1946	0.02668	0.13237	0.18269	0.11608	0.19491	-0.013	0.29271	0.10207	0.01869
XM_344810.2	LOC365092	-0.0218	0.08074	0.11957	0.06866	0.10264	0.05511	0.19232	0.12672	-0.0191	0.27718	0.10374	-0.065	0.00025	0.01377
XM_344812.2	LOC365096	-0.0465	-0.023	-0.0223	0.03674	-0.0557	0.07495	0.03933	-0.0098	0.13921	-0.0298	0.0132	0.2041	0.07805	-0.0084
XM_344817.2	LOC365104	-0.132	0.03713	0.08515	-0.0869	-0.2357	-0.0223	-0.1215	-0.192	-0.0468	-0.0747	-0.1192	-0.1372	0.1238	0.0808
XM_344820.2	LOC365110	0.0056	0.11544	0.05711	0.05797	-0.1884	0.26409	-0.2299	0.22618	-0.2095	0.03985	-0.0957	-0.3067	-0.23	0.21834
XM_344821.2	LOC365117	0.01573	0.06073	-0.0345	0.03791	-0.0107	0.21663	0.0915	0.27838	-0.0106	0.00697	-0.0198	0.00526	0.05046	-0.0068
XM_344822.2	LOC365118	-0.0732	0.01	0.08609	-0.0088	0.07711	-0.081	0.02509	0.06413	-0.0233	-0.0399	-0.0605	-0.0108	0.33128	0.09621
XM_344829.2	LOC365128	-0.0315	0.0017	0.00369	0.33281	-0.0391	0.02196	-0.1586	-0.1097	-0.0037	0.05684	-0.0973	-0.0323	0.07085	0.12179
XM_344830.2	LOC365129	0.1267	-0.0376	0.14904	0.07063	-0.0113	0.11704	0.23031	0.2529	-0.0387	-0.0223	0.18871	0.14407	0.08792	0.04605
XM_577718.1	LOC365135	0.02772	0.11209	0.06434	0.0604	0.10589	0.1066	0.06302	0.08049	0.12408	0.02967	0.03307	0.01755	0.01655	0.10481
XM_344835.2	LOC365139	-0.0817	-0.0067	-0.0607	-0.0804	-0.0046	-0.0098	0.13669	-0.048	-0.0168	-0.0141	0.06272	0.03113	-0.0467	-0.0067
XM_344839.2	LOC365143	-0.009	0.03303	-0.1362	-0.027	-0.0519	-0.064	0.10501	-0.1215	-0.0105	-0.198	0.06358	-0.0485	-0.1207	-0.0822
XM_577722.1	LOC365145	-0.1845	-0.0242	-0.2154	-0.2246	-0.0912	-0.0491	-0.2207	-0.1698	-0.0156	-0.1688	-0.1671	0.17978	-0.1575	-0.0311
XM_344856.2	LOC365184	0.14305	0.08004	0.14227	0.14349	0.06958	0.15582	0.32163	0.24527	0.06154	-0.0703	0.04443	-0.0383	0.09766	0.09983
XM_344858.2	LOC365189	-0.0684	0.09887	0.15974	0.14095	0.04704	-0.012	0.02208	0.0019	-0.0096	-0.0123	-0.0987	0.26555	0.04843	0.05508
XM_577766.1	LOC365196	-0.1642	0.09792	-0.0285	0.03028	-0.0787	0.12098	-0.13	-0.1978	0.0213	-0.1362	0.14305	0.10632	-0.1434	-0.0099
XM_344864.2	LOC365202	-0.24	-0.1512	-0.1654	-0.1989	-0.0065	-0.0837	-0.1195	-0.0913	-0.0063	-0.0348	-0.2168	-0.1584	-0.0747	-0.1428
XM_344866.2	LOC365207	0.10814	-0.1309	0.03058	0.07264	-0.0259	0.18338	-0.079	0.1046	0.12402	-0.0548	0.06657	0.09341	0.06206	-0.1957
XM_574381.1	LOC365214	0.30928	0.62351	-0.7003	0.18438	0.43713	-0.2101	0.19465	0.42824	0.42769	0.24568	0.08448	-0.268	0.36595	0.22591
XM_001078974.1	LOC365226	-0.0899	-0.101	-0.0251	0.0266	-0.1481	-0.114	-0.0858	-0.0125	-0.0201	0.07138	-0.1341	-0.0664	-0.0934	-0.1271
XM_344891.2	LOC365243	-0.1006	-0.1014	0.02041	0.12058	0.04213	0.03165	0.09371	-0.0775	-0.1033	-0.0724	-0.0975	0.02388	-0.0572	-0.0625
XM_344897.2	LOC365257	-0.1955	-0.0642	-0.1375	0.10084	-0.1658	0.0896	-0.0316	-0.1145	-0.0397	-0.0359	-0.1335	-0.0225	-0.128	0.0006
XM_574464.1	LOC365271	0.12819	0.30454	0.22055	-0.0045	0.06075	0.17109	0.18791	0.45394	0.0872	0.09551	0.28233	0.22206	0.23127	0.01197
XM_344904.2	LOC365276	-0.0605	-0.1119	-0.2052	-0.1979	-0.0613	0.00795	-0.1632	0.0265	0.00942	0.35693	-0.0077	-0.1103	-0.05	-0.0859
XM_344906.1	LOC365278	-0.0828	0.29491	-0.0223	0.08076	0.13034	-0.0408	-0.0022	-0.0436	-0.0305	0.05542	0.0795	0.24648	-0.0167	-0.0073
XM_577817.1	LOC365303	-0.2894	-0.0635	0.01879	-0.2327	-0.1016	-0.059	0.01825	-0.0018	-0.0008	-0.1461	-0.0136	-0.025	-0.1023	-0.0361
XM_577820.1	LOC365306	-0.0314	-0.0428	-0.1402	-0.1067	-0.035	-0.1383	-0.1525	-0.1594	-0.1524	-0.1695	-0.1517	-0.1735	-0.003	-0.1045
XM_344921.2	LOC365308	-0.0282	-0.049	0.05361	-0.0369	-0.2252	-0.084	-0.0994	-0.2721	-0.0793	-0.0816	-0.1308	0.007	-0.1875	0.01762
XM_344926.2	LOC365316	0.06139	0.071	0.10383	0.05471	0.18523	0.11524	0.17732	0.02317	0.11637	0.13522	0.03217	0.0687	0.17666	0.1381
NM_001037190	LOC365321	0.37141	0.36567	0.26034	-0.1623	0.26294	0.65353	-0.2076	0.49767	-0.1289	0.03013	0.06544	-0.0804	0.30251	0.41351
XM_344952.2	LOC365353	-0.0479	0.03014	0.01839	0.05139	-0.003	-0.0295	-0.0482	0.06289	0.11743	0.01298	0.01856	0.09377	0.01365	0.07292
NM_001007598	LOC365386	-0.0662	0.03706	-0.025	-0.0169	0.10153	0.25549	0.03889	0.00117	0.03804	0.02548	-0.0247	0.09066	0.15297	0.02886

XM_344988.2	LOC365402	-0.0722	-0.1385	-0.0371	-0.0033	-0.1298	-0.1118	-0.064	-0.1065	-0.0514	0.01415	-0.046	-0.0761	-0.0022	-0.0499
XM_344990.2	LOC365405	-0.1354	-0.3188	-0.196	-0.1003	-0.2901	-0.2965	-0.1886	-0.1491	-0.2859	-0.321	-0.1779	-0.0714	-0.2591	-0.274
XM_344994.2	LOC365409	0.02797	-0.1155	-0.1769	-0.0392	-0.1388	-0.1123	-0.0433	-0.1661	0.02086	0.11163	-0.106	-0.1594	-0.1108	0.06167
XM_345004.1	LOC365423	-0.1658	-0.0169	0.11314	-0.0938	0.00905	0.0493	-0.1389	-0.2508	-0.277	-0.014	0.03297	-0.0085	-0.0331	-0.1248
XM_345007.2	LOC365426	0.03516	-0.1646	-0.2984	-1.0127	-0.3623	-0.1743	-0.449	-0.2005	0.07142	0.08062	0.05313	0.0467	0.22925	0.02404
NM_001037190	LOC365427	0.1348	-0.0152	0.03064	-0.0165	0.04367	0.31698	-0.0273	0.13591	0.09056	0.04918	0.04461	0.17343	0.17888	0.26549
XM_345009.2	LOC365430	-0.0246	0.07844	-0.0522	-0.0269	0.01027	0.08262	0.01776	0.12338	0.02525	-0.0317	0.13666	0.09421	-0.0002	0.0541
XM_345010.2	LOC365431	-0.0728	-0.0435	-0.135	-0.1307	-0.1679	-0.1023	-0.1758	-0.0206	-0.2908	-0.1023	-0.1292	-0.0701	-0.1388	-0.1814
XM_577860.1	LOC365436	-0.0193	-0.3117	0.20285	-0.0657	0.00348	0.0116	0.07144	-0.022	0.16954	-0.0203	-0.2592	0.07092	0.53193	0.55098
XM_345013.2	LOC365437	0.03789	-0.0952	-0.0178	-0.0073	0.1751	0.48247	0.1108	0.18661	0.14179	-0.0144	0.15844	0.3557	0.03286	0.02222
XM_345016.2	LOC365442	-0.1127	-0.0571	-0.1319	-0.1442	-0.2285	-0.1976	-0.1096	-0.0732	-0.1451	-0.2307	-0.1026	-0.0053	-0.2432	-0.0448
XM_345018.2	LOC365446	-0.03	0.05741	0.03576	0.02927	0.20847	-0.0615	0.01987	0.14206	0.2408	0.05248	0.08081	0.01936	0.01352	0.10748
XM_345033.2	LOC365462	0.13393	-0.1268	0.17123	0.00928	0.2716	0.21762	-0.0378	0.05929	0.09435	-0.0868	-0.1238	0.09543	0.12543	0.10447
XM_577867.1	LOC365463	0.09293	0.00141	-0.1801	-0.1824	0.12975	0.33866	-0.1299	0.03151	-0.048	-0.1519	0.12918	-0.0556	0.11894	-0.0701
XM_001060079.1	LOC365476	-0.1207	0.29255	0.03646	-0.0381	-0.0681	0.04166	0.20013	0.04559	0.50897	0.22849	0.31039	0.20241	-0.2061	-0.1334
XM_574689.1	LOC365484	0.2575	0.03828	-0.0495	0.09422	0.13386	-0.0393	0.14681	0.12268	0.28504	0.03805	-0.1241	-0.0344	0.11168	-0.1029
XM_345051.2	LOC365491	0.03082	-0.2237	-0.2018	-0.2369	-0.0594	-0.177	-0.1867	-0.1922	-0.1547	-0.0762	-0.081	-0.1908	-0.0518	-0.2195
XM_577874.1	LOC365495	0.00551	0.15258	0.00423	0.05005	0.05944	0.21052	0.09264	0.0838	0.01133	0.0456	0.05223	0.11422	0.12892	0.00794
XM_001053352.1	LOC365499	0.1093	-0.0298	-0.319	0.02568	-0.1786	-0.209	-0.0706	0.06123	-0.0625	-0.1143	-0.1909	-0.2797	-0.1928	-0.1873
XM_345057.2	LOC365500	-0.3171	-0.2938	-0.2565	-0.0969	-0.2885	-0.2051	-0.2031	-0.1757	-0.1433	-0.3141	-0.144	-0.2789	-0.2838	0.15809
XM_345058.1	LOC365502	0.03267	0.09527	-0.0073	0.00548	-0.1062	0.4458	-0.0195	-0.0213	0.05404	0.04171	-0.1213	-0.1071	0.24635	0.04533
XM_345061.3	LOC365506	-0.3018	-0.1376	-0.2257	-0.2881	-0.208	-0.5431	-0.32	-0.3364	-0.4636	-0.0624	-0.2452	-0.0175	-0.5923	-0.3052
XM_345061.3	LOC365506	-0.0242	0.12187	0.17993	0.02888	-0.0625	0.08447	-0.0125	-0.043	0.1944	-0.0469	0.05515	-0.0377	-0.023	0.04916
XM_345062.3	LOC365507	-0.0171	0.04444	0.23077	0.03597	0.10954	0.04034	0.03267	0.05595	-0.0045	0.17361	0.15305	0.33702	0.16847	0.06917
XM_345063.2	LOC365509	-0.1241	-0.0579	-0.1549	0.08856	-0.0242	0.04918	-0.1051	-0.15	-0.1887	-0.1448	-0.0536	-0.0018	-0.1839	-0.0789
XM_345064.1	LOC365510	-0.2465	-0.0147	-0.0134	0.1546	-0.1825	-0.324	0.39933	-0.2168	-0.1451	0.09294	-0.2163	-0.0331	0.14405	-0.0482
XM_001075949.1	LOC365511	-0.2914	-0.3428	-0.0468	-0.0992	-0.4789	-0.4287	-0.144	-0.249	-0.412	-0.3521	-0.3247	-0.242	-0.2006	-0.1226
XM_345066.1	LOC365512	0.03253	0.28121	0.17974	0.24448	0.1883	0.24591	0.08853	0.33506	0.34615	0.15679	0.20018	0.11123	0.0049	0.21305
XM_345067.2	LOC365513	-0.0861	0.03905	-0.0596	-0.0864	-0.0061	0.01096	-0.1386	-0.1694	-0.2217	-0.1634	-0.1033	-0.0504	-0.0568	-0.0728
XM_345069.2	LOC365516	-0.22	0.04499	-0.137	-0.0564	-0.3163	-0.2317	-0.1701	0.11374	-0.0663	-0.0734	-0.2396	0.13247	0.08918	-0.0701
XM_345087.2	LOC365544	0.41247	-0.0548	-0.1736	-0.2864	-0.2624	-0.3883	-0.1001	-0.4361	-0.1863	-0.2803	0.16341	-0.2816	-0.2024	-0.3702
XM_345089.2	LOC365546	0.12253	-0.1267	0.11075	0.25888	0.31167	0.02266	0.11184	0.10692	0.32272	0.01819	0.06377	0.00581	0.05714	-0.0546
NM_001127556	LOC365549	0.07335	0.12051	0.05603	0.07813	-0.1274	0.06566	-0.1115	0.36637	0.3012	-0.0155	-0.1299	-0.0251	-0.1159	-0.0833
NM_001007598	LOC365553	-0.1312	-0.0997	0.04272	-0.2131	-0.2402	-0.0951	-0.1387	-0.2236	0.23099	-0.1301	0.01877	-0.0706	-0.0488	-0.0693
XM_574743.1	LOC365555	0.6731	0.57329	-0.0664	-0.1179	0.61509	0.2154	-0.0106	0.58607	0.49402	0.63756	0.33624	0.14703	0.50363	0.56486
XM_345097.2	LOC365556	-0.1639	0.26748	0.02613	-0.0796	0.12285	-0.087	0.05841	-0.0997	-0.0785	-0.0218	-0.1115	0.04575	-0.0778	0.01909
XM_574750.1	LOC365566	0.05013	-0.3887	0.3805	0.3793	-0.0025	0.25591	-0.2525	-0.0779	0.14017	-0.491	-0.3319	0.36571	0.30425	0.51807
XM_577904.1	LOC365579	0.13723	0.01657	0.22231	0.22193	0.08224	0.09647	0.03021	0.13602	0.01704	0.02529	0.09015	0.05154	0.04178	0.25201
XM_345111.2	LOC365584	-0.0128	-0.0288	-0.0789	0.10852	-0.0592	0.01802	0.02918	0.07331	0.05296	0.07323	0.02835	0.33739	0.03443	0.27992
XM_345112.2	LOC365585	0.12353	0.2017	0.04057	0.02658	0.03603	0.00884	0.13737	0.04709	0.15098	0.0805	0.09478	0.03997	0.05499	0.02867
XM_345121.2	LOC365599	-0.0869	-0.1913	-0.0522	-0.0134	-0.2462	-0.1931	-0.1053	-0.1461	-0.0227	-0.2444	-0.2012	-0.1618	-0.0739	-0.2879
NM_001014250	LOC365601	-0.2448	-0.4367	-0.5428	-0.5441	-0.2504	-0.2641	-0.5364	-0.2042	-0.2739	-0.5601	-0.7062	-0.4357	-0.1989	-0.3574
XM_577963.1	LOC365602	-0.0171	-0.1239	-0.0564	0.10917	0.0006	-0.1271	-0.0003	0.08525	0.03237	-0.0269	-0.0812	-0.079	-0.0421	-0.0561
XM_345124.2	LOC365604	0.05389	0.12639	0.01745	0.10938	0.17818	0.44928	0.06807	0.12839	0.05274	-0.0237	0.05266	0.04787	0.05746	0.01303
XM_345125.2	LOC365606	-0.0074	0.08454	-0.0428	0.03636	-0.0536	0.11346	0.15221	0.23614	-0.0548	0.11657	0.01677	-0.015	0.34169	0.29242
XM_574805.1	LOC365607	-0.1322	-0.2277	0.04903	-0.0123	-0.2105	-0.1789	0.1357	-0.2054	-0.0873	-0.0139	0.04044	0.03697	-0.0172	-0.1999
XM_345126.2	LOC365608	0.13315	0.06056	0.03637	-0.0056	0.13513	0.01109	0.11185	-0.0572	0.00881	0.03531	-0.1096	-0.0264	0.02946	0.00312
XM_345127.2	LOC365609	0.01666	0.03436	-0.0335	0.00988	0.08906	-0.1338	-0.0111	0.00693	0.18309	0.01112	-0.0453	-0.0496	0.01261	0.14508
XM_345128.2	LOC365610	0.17048	0.08743	0.19737	0.0693	-0.0613	0.18198	0.14721	0.3264	-0.1073	-0.0666	0.46351	0.04398	-0.055	0.09553

XM_345129.2	LOC365611	-0.0925	-0.1054	-0.0494	-0.1385	0.1183	-0.0927	0.07961	-0.0316	0.01455	-0.0451	-0.0569	-0.1078	0.09512	0.07332
XM_345130.1	LOC365612	0.24364	-0.0403	5E-05	-0.0701	0.02852	-0.1517	0.07059	-0.0301	0.17365	0.0941	-0.1274	0.0838	0.17536	-0.0331
XM_577974.1	LOC365617	0.14353	0.19117	0.10537	0.24447	0.18735	0.19393	0.01643	0.11043	0.2043	0.03021	0.40908	0.22927	0.17803	0.1493
NM_001108934	LOC365627	-0.1865	-0.2671	-0.5425	-0.2711	-0.247	-0.4504	-0.2642	-0.0907	-0.4963	-0.2465	-0.6341	-0.3972	-0.4483	-0.297
XM_345142.2	LOC365651	0.08104	0.04912	0.39666	0.10879	-0.0174	0.06401	0.25482	-0.0058	-0.0021	0.07006	0.02436	-0.068	0.0744	0.17623
NM_001034093	LOC365668	-0.0121	-0.0851	0.03052	0.19509	-0.0784	-0.0377	-0.0649	-0.067	-0.0008	0.03783	0.18938	-0.0978	0.00514	0.02604
XM_345155.1	LOC365672	0.14879	0.15763	-0.0091	-0.1495	0.42716	-0.1446	0.02196	0.24003	0.03525	0.0676	0.22586	0.04795	0.31438	0.03486
XM_577989.1	LOC365675	0.10456	0.18907	0.15909	-0.0562	-0.0738	0.08811	0.12536	-0.0534	0.36367	0.09526	-0.0177	0.07911	0.13962	0.10001
XM_345157.2	LOC365677	0.10231	-0.0083	0.03287	0.00597	0.05835	0.06261	0.14011	0.07958	0.12849	0.10911	0.12196	0.04079	0.03458	0.14994
XM_345161.2	LOC365686	0.02522	0.04132	0.14211	-0.1167	-0.082	0.06195	-0.0501	0.16801	0.12019	0.15077	0.15575	0.05178	-0.0331	-0.0713
XM_577991.1	LOC365689	-0.0306	-0.0967	-0.0792	0.05617	0.03045	0.17721	-0.0458	0.01635	-0.0614	0.07036	0.08598	-0.0382	0.04896	0.16463
XM_345171.2	LOC365704	0.0693	-0.0182	-0.004	0.18458	0.19568	0.15593	-0.0115	0.18313	0.06757	-0.0392	0.04794	-0.0136	0.16286	0.17361
XM_345174.1	LOC365710	-0.0229	0.0704	-0.1185	-0.2128	-0.067	-0.0498	-0.1028	0.03649	-0.2816	-0.1974	0.01558	-0.0769	-0.0511	-0.1775
XM_345189.2	LOC365745	0.09746	-0.0054	-0.0208	-0.0267	0.1083	0.00124	0.02329	0.08337	-0.0147	0.09153	0.01798	0.00985	0.00396	0.12872
NM_001108943	LOC365769	0.01079	0.07072	0.09275	0.05245	0.10923	0.1632	-0.0395	0.00214	0.09235	0.03466	0.02884	0.08262	0.01955	0.10009
XM_578013.1	LOC365775	-0.2068	-0.1145	0.11624	-0.1482	-0.2909	-0.0539	-0.2785	-0.1646	-0.0548	-0.0932	-0.176	-0.0722	-0.1654	-0.2282
NM_001014251	LOC365778	0.13154	0.05559	-0.0698	-0.0064	0.17931	0.04982	0.13282	0.04979	0.0268	-0.0339	-0.0674	-0.0145	0.08399	0.06004
XM_345206.2	LOC365787	0.22381	0.0345	0.28543	0.09931	0.18374	-0.0676	0.0548	0.3363	0.05113	0.11184	0.13339	0.11185	0.01104	0.1061
NM_001014252	LOC365791	-0.187	-0.0672	-0.0998	-0.1342	0.01728	-0.17	-0.0228	-0.1195	-0.0378	-0.0092	-0.0838	-0.0138	-0.0419	0.08177
NM_001128065	LOC365800	-0.0622	-0.273	-0.0572	-0.1582	-0.1786	-0.296	-0.1635	-0.147	0.08621	-0.2362	-0.0982	-0.0848	-0.2507	-0.4068
NM_001099506	LOC365807	-0.4635	-0.2923	-0.2359	-0.3313	-0.2691	0.04053	-0.2196	-0.3913	-0.3737	-0.4318	-0.3305	-0.2386	-0.5113	-0.3489
XM_345219.2	LOC365808	0.05646	0.0191	-0.0021	0.04633	0.05931	0.30921	-0.0672	-0.0467	-0.1004	-0.1933	0.00063	-0.1353	0.00296	-0.1522
XM_578024.1	LOC365814	0.69695	0.18609	-0.2456	0.02118	0.55055	0.1228	-0.2474	0.57589	0.08051	0.10195	-0.0875	-0.3774	0.28585	0.10929
XM_345226.2	LOC365832	0.24561	0.28197	0.03408	0.12313	0.06348	0.11781	0.07649	0.1872	0.01465	0.06038	0.11483	0.40269	0.13931	0.10323
XM_578062.1	LOC365856	0.01841	-0.0146	0.08798	0.12916	0.09437	0.08388	0.02448	-0.0029	0.05186	0.00533	0.09213	0.2048	0.17667	0.42338
XR_006636.1	LOC365861	-0.13	-0.1024	-0.0622	0.13989	0.08471	-0.0288	0.07922	0.19988	0.09348	0.07186	0.07174	0.01361	-0.1436	-0.0601
NM_001108949	LOC365868	-0.0967	-0.0515	0.06861	0.20042	0.06761	0.08909	0.05836	0.08062	-0.0476	-0.0873	0.12234	0.25185	0.05313	0.02274
XM_345249.2	LOC365869	-0.062	-0.2751	0.03156	0.08023	-0.1373	0.08069	-0.053	-0.4801	-0.069	-0.1819	-0.2545	-0.2898	-0.0171	-0.0121
XM_345252.1	LOC365873	0.05685	-0.0352	0.07707	0.02109	-0.055	0.01148	0.29139	0.00928	-0.0393	-0.0658	-0.1618	0.00938	-0.0314	0.07206
XM_345258.2	LOC365881	-0.0709	-0.0148	0.13913	0.04087	0.02708	0.02911	-0.0006	0.04506	0.05785	-0.0265	0.11075	0.0847	0.0972	0.45459
XM_345262.2	LOC365888	0.03498	0.06968	0.06713	0.20297	0.13122	0.03399	0.12297	0.12772	0.13552	0.30106	0.00218	0.07231	0.11067	0.13019
NM_022298	LOC365889	-0.0085	0.08996	0.27779	0.04435	-0.0152	0.01514	0.05562	-0.0178	0.07841	-0.0111	0.1435	0.09082	-0.0026	0.16095
NM_001077436	LOC365909	-0.0525	0.00181	-0.0417	-0.0838	0.06565	0.48471	-0.051	0.02551	0.05797	0.04344	0.11927	-0.0033	0.2249	0.08722
XM_575023.1	LOC365916	0.0693	0.07347	0.04887	0.0986	0.00883	0.08762	-0.0172	-0.069	0.04193	0.02561	0.25342	-0.0446	0.14874	0.0208
NM_001135879	LOC365924	0.40353	-0.2823	-0.083	-0.084	0.13053	-0.1969	-0.2078	0.10067	-0.3924	-0.0866	-0.1393	-0.1782	-0.0165	0.13089
XM_345285.2	LOC365925	0.07124	0.05925	-0.0595	0.05511	0.17203	0.07113	0.01977	-0.0698	-0.1129	-0.0557	-0.1426	0.00413	-0.0891	-0.1196
XM_345286.2	LOC365928	-0.0565	-0.0326	-0.048	-0.1055	-0.0537	0.02676	-0.0849	-0.1668	0.19815	-0.1238	-0.0307	-0.0004	0.07714	-0.0499
XM_345288.2	LOC365936	0.15056	0.35812	-0.0616	-0.1532	0.15025	0.03072	0.047	0.06619	0.105	0.06176	0.13878	-0.1141	-0.1255	-0.2337
XM_345291.2	LOC365944	0.07661	0.12226	0.05706	-0.1187	0.1532	-0.1178	0.19887	0.00827	-0.1538	-0.0436	0.41101	0.13641	-0.0673	0.09903
XM_345294.2	LOC365947	-0.0381	0.03569	-0.0672	0.03425	-0.1147	0.01241	-0.0401	0.00952	-0.0555	0.04178	-0.0335	0.03566	-0.0797	0.12903
XM_001077928.1	LOC365948	-0.156	-0.0335	-0.0261	-0.3225	-0.1041	0.04499	-0.0995	0.02372	-0.2193	-0.2444	-0.1368	-0.2941	0.08275	-0.2345
XR_009210.1	LOC365949	0.0295	0.00373	-0.0067	0.03174	0.11297	0.03791	0.24513	0.13843	0.14436	-0.1047	0.08208	0.04121	0.06463	0.0458
NM_024351	LOC365951	0.081	0.07927	0.12919	0.1157	-0.0219	0.23428	-0.0043	0.10991	0.11012	0.03086	0.10165	-0.0193	0.55563	0.22169
NM_001037190	LOC365954	0.89789	0.2733	0.46068	-0.5688	0.40269	0.41611	-0.5364	0.26644	0.17638	0.12524	0.20802	-0.1954	0.45652	0.60616
NM_001037210	LOC365960	1.048	0.60674	1.4728	0.10183	-0.4024	-0.3505	0.51878	-0.5321	0.69295	0.71901	0.83749	0.9965	1.2386	1.379
XM_345301.2	LOC365961	0.02456	0.14253	0.05913	0.17029	-0.0758	0.00267	0.04559	0.2102	0.09333	-0.0324	-0.0395	0.06089	0.12603	0.08603
XM_345306.2	LOC365969	-0.0499	0.08561	0.03009	0.02809	0.01612	0.06433	-0.0073	0.05153	0.14239	-0.107	0.10885	-0.0033	0.12659	0.1287
XM_345308.2	LOC365973	0.10684	0.14531	-0.0345	-0.1341	0.30129	0.16958	-0.1164	0.17502	-0.1432	-0.103	-0.0572	0.15894	0.30708	0.21177
XM_345309.2	LOC365974	0.0375	0.04756	-0.0557	0.08828	-0.0055	-0.031	0.11868	-0.058	-0.0087	-0.0507	0.01611	0.41721	0.09288	-0.0612

NM_001079893	LOC365977	0.14841	-0.0513	0.13109	0.13806	-0.0663	0.02391	-0.0225	0.06152	0.10807	0.01814	0.09206	0.14244	-0.081	0.06429
NM_001079893	LOC365977	-0.0364	0.10902	-0.0353	-0.0385	0.12511	-0.0022	-0.0563	-0.012	0.18468	-0.0314	0.00484	0.09688	0.21281	0.15389
NM_001079893	LOC365977	-0.0448	-0.0218	-0.0165	-0.012	0.03549	-0.0371	0.01267	-0.1659	0.10022	0.18106	0.01159	-0.1185	0.11723	0.04264
XM_345315.3	LOC365981	-0.006	0.0463	-0.0428	-0.0487	-0.0725	-0.0638	-0.0843	0.00885	0.27305	0.11171	0.00621	-0.0737	-0.0585	0.00702
XM_345316.1	LOC365982	0.13013	-0.0174	-0.1165	-0.1257	0.22187	0.06198	-0.0656	-0.0032	0.13615	-0.0943	-0.0099	0.28811	0.03521	0.10585
XM_345317.2	LOC365983	0.11694	0.05677	0.11812	0.014	0.16624	0.07499	0.09161	0.18794	0.0897	0.08067	0.22163	0.16415	0.22977	0.15972
NM_001108951	LOC365985	-1.4797	-1.5896	-1.597	-1.3215	-1.5107	-1.5539	-1.3578	-1.623	-1.4361	-1.4541	-1.5942	-1.5124	-1.3938	-1.6671
XM_345321.2	LOC365987	-0.1011	-0.2598	-0.1664	-0.035	-0.1269	-0.0631	-0.0071	-0.2383	-0.2277	0.19331	-0.0021	-0.2755	-0.0866	-0.2336
XM_345325.2	LOC366003	0.23965	-0.1167	-0.131	-0.0572	0.0057	0.1537	0.12276	0.01807	-0.0234	0.00148	0.09537	-0.1897	-0.1013	0.14767
NM_001128183	LOC366008	-0.0894	-0.238	-0.2158	-0.1839	-0.2246	-0.3078	-0.2475	-0.2407	-0.2054	-0.0868	0.04994	-0.1676	-0.1193	-0.0718
XM_345341.2	LOC366022	-0.2326	-0.0757	0.0434	-0.1907	-0.1488	-0.0294	-0.1847	-0.2494	-0.1215	-0.1727	-0.2523	-0.0958	-0.0776	-0.1873
XM_345346.2	LOC366030	0.05488	0.08576	0.07913	0.08651	0.10697	0.02006	0.1578	-0.0469	0.04831	0.13237	0.07449	0.09149	0.09204	0.05944
XM_345355.2	LOC366046	-0.2017	-0.0641	0.06699	0.02333	-0.1073	0.12421	-0.0548	-0.0518	0.02567	0.04649	0.01269	0.07086	-0.0605	0.0642
XM_578122.1	LOC366058	0.08916	0.05742	0.06377	0.10166	0.06444	0.12362	-0.0097	0.1201	0.07717	-0.0856	0.01757	0.11381	-0.0187	-0.0277
XM_345369.2	LOC366074	0.1102	0.10053	0.10527	0.11714	0.17986	-0.058	0.15161	0.29435	0.04797	0.18178	0.09773	0.31197	0.22287	0.09713
XM_345374.2	LOC366083	-0.5146	-0.1642	-0.7135	-0.6967	-0.5052	-0.9188	-0.4823	-0.4166	-0.2435	-0.1534	-0.2404	-0.387	-0.2594	-0.369
XM_345376.2	LOC366086	-0.1548	0.10338	-0.0631	-0.0411	-0.2421	-0.1196	0.00493	-0.1741	-0.2248	0.10899	-0.167	0.06151	-0.0358	-0.1348
NM_001126290	LOC366089	0.05596	-0.0032	0.04663	-0.0457	-0.1003	-0.0527	-0.095	-0.0709	-0.2583	-0.0187	-0.1332	-0.0188	-0.0583	0.04171
XM_345386.1	LOC366107	-0.2121	-0.1539	-0.0554	0.21481	0.182	-0.206	0.07046	-0.1252	0.02177	0.08615	-0.119	-0.0822	-0.0994	-0.0362
NM_031103	LOC366139	-0.1952	-0.0733	-0.1411	-0.1229	-0.1439	-0.0042	-0.0896	-0.0414	-0.189	0.01148	0.06408	-0.2145	-0.1645	-0.0819
XM_345413.2	LOC366146	0.3878	0.12212	0.32128	0.3245	0.05437	0.69332	0.17145	0.24099	0.74403	0.57168	0.38792	0.34873	0.3313	0.38678
XM_345433.2	LOC366191	-0.1519	-0.0121	0.03147	0.05826	-0.058	0.10575	-0.0456	-0.1243	-0.0063	-0.1619	-0.0653	0.12181	0.08546	-0.0392
XM_345452.2	LOC366221	0.10958	0.08364	0.06586	0.06922	0.11522	0.05814	0.11569	0.01703	0.06629	0.01642	0.03859	0.01958	0.19016	0.11964
XM_345460.2	LOC366237	-0.0143	0.27224	0.19915	0.02782	0.1239	0.04207	0.13551	-0.0667	0.12146	0.19875	0.19936	0.12677	0.16193	0.16063
XM_578191.1	LOC366254	0.14307	-0.0499	0.39947	0.22188	-0.0352	0.11223	0.18732	0.2816	-0.0566	-0.0538	0.01948	-0.0819	-0.11	0.00992
XM_578192.1	LOC366255	0.0182	-0.0108	-0.0589	0.00853	-0.0777	0.06263	-0.1048	-0.1175	0.11108	-0.1159	-0.0896	-0.0418	-0.1056	0.12097
XM_575291.1	LOC366258	0.26568	0.08748	-0.5709	-0.0849	0.09557	0.51082	-0.7562	-0.0447	0.02291	-0.0106	0.1153	0.18846	-0.1748	-0.2235
XM_345488.2	LOC366279	-0.0819	-0.0622	0.07776	-0.0008	0.06606	-0.1058	0.1204	-0.1437	-0.0358	-0.0128	0.18385	-0.0335	0.12285	-0.0267
XM_345492.2	LOC366286	0.11771	0.0874	-0.0922	0.14657	0.1902	0.507	-0.1462	-0.1225	-0.065	-0.1081	-0.0373	0.46916	-0.1043	-0.0397
XM_345493.2	LOC366287	-0.0296	0.04546	0.07792	0.20624	0.06176	0.04463	0.42539	0.16191	0.12331	0.03353	0.514	-0.0783	0.18779	0.03861
XM_001069481.1	LOC366304	-0.0309	0.08409	0.06697	0.13028	0.04208	0.26563	-0.046	-0.1086	-0.1118	-0.0828	0.12887	-0.0856	-0.092	0.01814
XM_575760.1	LOC366305	0.04336	0.15435	0.09712	0.10165	0.08873	0.17111	0.08525	0.04891	0.09611	0.13455	0.06419	0.05487	-0.0773	0.09604
XM_578444.1	LOC366313	-0.1096	0.13781	-0.1293	-0.0732	-0.1549	0.00739	0.08278	-0.1102	-0.0303	-0.0676	0.05277	0.01435	0.2127	0.0892
XM_345503.1	LOC366315	0.22614	0.20664	0.10515	0.59815	0.19566	0.52833	0.08729	0.24169	0.28556	0.34254	0.2756	0.3897	0.10725	0.01291
XM_345513.1	LOC366342	-0.0469	0.01497	0.09554	0.01633	0.04257	0.14384	0.02705	-0.0643	0.18115	-0.0159	-0.12	0.04747	-0.0313	-0.0332
XM_345519.1	LOC366354	0.09299	0.01634	-0.0084	0.00122	0.08171	0.14017	0.0465	-0.0122	-0.0253	0.04321	-0.1029	0.09586	0.01171	0.1282
XM_345533.1	LOC366373	-0.1277	0.31636	0.12761	0.08412	0.03273	0.11365	0.0078	0.03497	-0.019	0.34472	0.13389	-0.0726	0.13821	0.12182
XM_345534.2	LOC366374	0.05178	0.13092	0.15399	0.07205	0.18483	0.16311	-0.1132	0.11809	0.08644	0.01211	0.09076	0.0045	-0.0141	-0.0478
XM_345538.2	LOC366379	-0.1313	-0.1314	-0.0901	-0.1811	0.04053	0.01953	-0.1754	-0.1654	0.14804	-0.0293	-0.1868	-0.0335	-0.2656	0.00716
NM_203512	LOC366380	-0.2639	-0.2403	-0.1872	-0.4106	-0.2885	-0.472	-0.3493	-0.2676	-0.5396	-0.3684	-0.4307	-0.0383	-0.3666	-0.3336
XM_345545.2	LOC366389	-0.1958	-0.2678	-0.1669	-0.2305	-0.182	-0.2208	-0.2011	-0.2432	-0.2246	-0.2731	-0.2337	-0.0644	-0.2651	-0.2192
XM_345546.1	LOC366392	-0.1804	-0.2022	-0.0802	-0.1705	-0.1495	-0.0802	-0.2083	-0.1702	-0.1676	-0.1014	-0.1742	-0.1816	-0.0886	-0.0219
XM_345551.2	LOC366406	-0.0531	-0.0786	-0.1423	-0.1054	-0.0333	-0.1392	-0.0865	-0.0242	0.00829	0.06204	-0.1113	0.11075	0.06417	-0.1363
XM_345571.2	LOC366454	-0.0483	0.01434	0.23124	0.13388	0.00156	-0.0575	0.08645	-0.0046	0.07736	0.20009	0.01622	0.16775	0.1404	0.04725
XM_345579.2	LOC366465	0.03833	-0.0041	-0.0155	0.03218	0.06118	0.03312	-0.0293	-0.0536	-0.0551	-0.021	0.03264	-0.0158	0.0986	0.07436
NM_001108976	LOC366468	-0.1429	-0.0244	-0.099	-0.8532	-0.1854	0.01269	-0.1085	-0.2921	0.09826	0.13439	-0.0076	0.40987	-0.0651	-0.2696
NM_001108976	LOC366468	0.0415	0.00525	-0.0912	-0.5805	-0.2557	0.08263	-0.0872	-0.305	0.12132	-0.0217	-0.0606	0.1611	-0.1969	-0.1795
XM_578493.1	LOC366476	0.1173	0.2185	0.21212	0.161	0.12693	0.07558	0.19409	0.35936	0.05216	0.35994	0.10884	0.20328	0.15961	0.1807
XM_345592.2	LOC366486	0.07771	0.05625	0.1364	0.04641	0.30897	0.03016	0.00519	-0.037	0.33278	0.14765	0.07049	0.01303	0.00847	0.19136

XM_345594.2	LOC366489	-0.1364	-0.3028	-0.2412	-0.2123	-0.2901	-0.3474	-0.217	-0.2384	-0.2543	-0.0391	-0.2681	-0.2662	-0.1907	0.00875
XM_345598.2	LOC366498	-0.2278	-0.2646	0.1827	0.12496	-0.0068	0.15268	-0.3049	-0.2227	-0.0555	-0.0052	-0.1863	-0.0947	-0.2039	-0.2478
XM_578510.1	LOC366502	-0.0743	-0.0029	-0.0605	-0.0402	0.09624	-0.0061	0.03468	-0.0155	0.02752	0.08512	-0.0363	-0.0227	0.00453	-0.0168
XM_345602.2	LOC366503	-0.234	-0.2538	-0.0142	-0.082	-0.0809	-0.2103	-0.1194	-0.2381	-0.2045	0.11065	-0.0556	0.10594	-0.0686	-0.1658
XM_345603.2	LOC366504	0.12335	-0.1771	0.11775	0.05398	0.43504	0.03568	-0.2075	0.28242	0.01526	-0.2424	-0.2448	-0.189	-0.1673	-0.3763
XM_345604.2	LOC366505	-0.1794	-0.6549	0.61184	-0.4383	-0.7579	-0.42	-0.9612	-0.8419	-0.618	-0.8123	-0.4098	0.51936	0.54757	0.48222
XM_578511.1	LOC366506	-0.0922	0.04074	0.06661	-0.0199	0.13335	-0.1419	-0.0403	0.1151	-0.0409	0.01692	-0.1037	0.09766	0.09629	0.26362
XM_345610.2	LOC366512	-0.0455	-0.0542	0.00147	0.03808	-0.1286	-0.1282	-0.213	-0.2216	0.06314	0.01535	0.06697	-0.143	-0.1299	-0.0799
XM_345612.2	LOC366514	-0.0597	-0.109	0.32415	-0.0054	-0.1267	-0.0806	-0.0413	0.05726	0.09853	0.10091	-0.0853	-0.1087	-0.108	-0.0143
XM_345620.2	LOC366523	0.25518	0.10077	-0.0129	0.06116	0.03902	0.05873	0.15802	0.03619	0.034	0.14386	0.11782	0.30696	0.08822	0.10724
XM_345621.2	LOC366524	0.17222	-0.0971	0.0769	0.1938	0.04037	0.23349	0.24354	0.6246	0.35414	0.27796	0.27398	0.31711	0.26686	0.32876
XM_345622.2	LOC366527	-0.0933	0.04015	-0.0823	-0.0273	-0.1107	-0.1098	-0.1007	0.09242	-0.0774	-0.0139	-0.0941	-0.0892	0.10542	-0.1189
XM_345634.2	LOC366550	-0.0575	-0.0522	0.26064	0.14347	0.00362	-0.0371	0.11944	-0.1207	0.0889	-0.1517	0.11486	0.04325	-0.0806	-0.069
XM_345638.1	LOC366554	0.1079	-0.0661	0.03509	-0.1858	-0.2964	0.311	-0.2118	-0.1909	-0.0285	-0.1628	-0.0573	0.56476	-0.0515	-0.0884
XM_578529.1	LOC366557	0.20716	0.32296	0.35873	0.17617	0.23689	0.20022	0.06792	0.17039	0.23711	0.13925	0.0974	0.3177	0.27629	0.15431
XM_578534.1	LOC366573	-0.033	0.03361	-0.05	0.07176	-0.1195	-0.1566	0.04406	-0.1202	-0.0804	0.11683	-0.1358	-0.0448	-0.0289	-0.0775
XM_345647.1	LOC366576	0.11147	-0.0656	0.13526	0.09744	-0.0829	-0.0397	0.01969	-0.0551	0.05923	-0.0555	0.05507	-0.0315	-0.0419	-0.0179
XM_345652.1	LOC366588	0.28373	0.4542	0.13758	0.18865	0.2997	0.30856	0.09012	0.16608	0.31421	0.3752	0.52655	0.19613	0.00075	0.25133
XM_345657.2	LOC366596	0.07642	-0.0545	0.05606	-0.0138	0.03373	0.00964	-0.0043	-0.0943	0.06685	0.40283	0.15354	0.18528	0.14434	0.00836
XM_576019.1	LOC366604	-0.0737	-0.0812	-0.313	-0.1624	-0.0186	-0.2757	-0.2723	-0.2317	-0.1346	-0.0469	-0.2346	-0.0591	0.01372	-0.1159
XM_345662.2	LOC366605	0.00173	-0.0839	0.02408	-0.1399	-0.0883	-0.0627	-0.1715	0.10208	0.01493	-0.25	-0.0677	0.09329	0.06316	0.01615
XM_345665.2	LOC366608	-0.1667	0.97231	0.59729	1.1407	0.20937	-0.0087	1.1095	0.3981	0.73746	1.0097	0.73066	-0.0094	0.41779	0.31567
XM_578543.1	LOC366625	0.14298	0.01895	0.09122	0.02246	0.12061	0.20114	0.0482	0.17443	0.09919	0.08255	0.29865	0.18653	0.10278	0.06457
XM_345675.2	LOC366629	0.01086	0.13146	-0.1515	-0.1619	-0.1582	0.07574	-0.0285	-0.002	0.09557	-0.1947	0.11899	0.10471	0.27294	-0.0204
NM_001108983	LOC366631	0.1497	-0.0561	0.02331	0.07972	0.04216	0.0037	-0.0226	-0.1116	-0.0975	0.02785	-0.0651	0.02901	0.07746	-0.0098
XM_345678.2	LOC366634	-0.0661	0.04847	0.10274	-0.0794	-0.0175	-0.0522	0.03634	-0.0611	-0.0647	0.03571	0.24253	-0.0505	0.01363	0.02972
XM_345680.1	LOC366639	-0.1279	-0.1231	-0.0662	-0.1687	-0.1735	-0.1566	0.12567	-0.0342	-0.1181	-0.0605	-0.0795	-0.1282	-0.0656	-0.2323
XM_345685.2	LOC366654	0.20087	0.19977	0.09264	0.12863	0.14109	0.19251	0.17207	0.09079	-0.0263	0.0751	0.02668	0.04257	0.14815	0.24793
XM_001080795.1	LOC366669	-0.5269	-0.1456	-0.0799	-0.2958	-0.348	-0.4125	-0.2077	-0.6291	0.08158	-0.1625	-0.1972	-0.2349	-0.3258	-0.2405
XM_001080795.1	LOC366669	0.17299	0.07912	-0.1311	-0.0517	-0.0206	-0.1874	0.06657	-0.0453	-0.1549	0.20801	0.22984	-0.128	0.22914	0.17283
XM_345692.2	LOC366673	0.25265	0.02974	0.02547	-1.1028	-0.1444	-0.2621	0.07231	-0.2963	0.07551	-0.0808	0.37401	0.06493	-0.0336	0.04402
XM_578562.1	LOC366678	-0.0718	-0.1751	-0.1334	-0.1548	-0.0701	-0.2616	-0.2515	-0.1861	0.06133	-0.1769	-0.1408	-0.1503	-0.1982	-0.2599
XM_576064.1	LOC366682	-0.201	-0.1921	0.01849	-0.1477	-0.2201	0.03295	-0.0287	-0.1395	-0.2437	-0.1693	-0.0037	-0.0695	-0.1967	-0.1238
XM_345723.2	LOC366730	-0.0078	-0.0688	0.10763	-0.0362	0.32599	0.02613	0.06929	-0.0386	0.01836	0.04591	0.00751	0.02893	0.0796	-0.0192
XM_345730.2	LOC366742	-0.0116	-0.0379	-0.0097	-0.0149	0.10713	0.01864	0.01032	0.06661	0.00728	0.1681	-0.0131	0.10344	-0.0201	0.026
XM_345734.2	LOC366746	-0.1188	-0.0609	0.34458	0.02742	-0.0695	-0.0975	-0.1677	0.05515	-0.1166	-0.0277	0.19972	0.04282	0.08246	0.06138
XM_345736.2	LOC366748	0.08943	0.05656	0.0519	0.03657	0.03868	0.17041	0.07469	0.19129	0.30572	0.20053	0.17881	0.08047	0.18904	0.09302
XM_576117.1	LOC366749	0.04673	-0.1824	0.08724	0.08395	-0.1483	0.04219	0.00344	-0.0822	-0.0954	0.02828	-0.1234	-0.2254	-0.1105	-0.0813
XM_345738.2	LOC366751	0.05602	0.02929	0.01002	0.10812	0.24676	0.08388	0.05392	-0.0246	0.07577	0.04347	0.00204	0.01382	0.00769	-0.0246
XM_345739.1	LOC366752	-0.055	0.01455	-0.1525	0.0902	-0.0932	0.02627	-0.1872	0.01379	0.156	-0.1905	0.12944	-0.0837	0.20142	-0.1153
XM_345743.2	LOC366756	-0.0286	-0.0735	0.09609	0.0493	-0.0715	-0.0795	0.15584	0.02866	-0.121	0.10022	0.07337	-0.0603	0.00894	0.03989
XM_345744.2	LOC366757	0.02986	-0.0724	0.00174	-0.1001	0.07355	0.00433	0.15755	-0.0415	0.07103	0.04869	0.19274	0.11422	0.15801	0.02834
XM_345745.2	LOC366758	0.00624	0.02337	0.04473	0.07839	0.1127	-0.0053	0.05211	0.03455	0.01459	-0.0157	0.00845	0.03327	0.09164	0.0756
XM_345750.2	LOC366763	0.06447	0.05465	0.03232	0.07078	0.11291	0.04547	0.11548	0.18585	0.05814	0.27693	0.07345	-0.0378	0.23236	0.14452
XM_345751.2	LOC366764	-0.0197	-0.0308	-0.0796	-0.0287	-0.0823	-0.0229	0.0141	-0.2124	0.03337	-0.0081	-0.1798	0.06459	0.17486	-0.1532
XM_345752.2	LOC366765	-0.0435	0.00093	-0.0369	0.14547	0.09009	0.09537	0.0532	0.06788	-0.062	0.12362	0.28287	0.05148	0.05993	0.05423
XM_345753.1	LOC366766	0.09278	-0.1075	0.16925	0.06593	0.039	0.19204	-0.0232	-0.2627	-0.0383	0.11025	0.0253	0.20987	0.09601	0.02936
XM_345754.2	LOC366767	0.07555	0.01396	0.078	-0.0388	-0.0534	0.03474	0.04346	0.11521	-0.0047	0.03426	0.01275	0.14621	0.08771	-0.0142
XM_345755.2	LOC366768	0.00449	0.12432	0.15363	0.20599	-0.0101	0.15049	0.1456	0.1856	0.1465	0.16454	0.31711	0.0779	0.09681	0.07607

XM_345756.1	LOC366769	0.09441	0.02246	0.01023	0.01775	0.1291	-0.0189	-0.0242	-0.0295	0.16239	-0.0159	0.25871	-0.0058	0.11213	0.04686
XM_345757.1	LOC366770	0.00876	0.10817	-0.0463	-0.0018	0.08007	0.02502	-0.0553	0.08343	0.12743	0.14945	0.21122	0.08854	-0.025	0.05204
XM_001070902.1	LOC366780	-0.1979	-0.0326	0.03468	-0.1541	0.27403	0.27096	-0.2761	-0.2956	0.10068	0.19492	-0.1118	0.32203	0.12738	0.18068
XM_345767.2	LOC366784	0.23953	-0.0792	0.11897	0.03873	-0.206	0.51853	-0.1334	0.12592	0.26347	-0.0582	-0.0239	0.34083	-0.0661	-0.0904
NM_001108986	LOC366790	0.1096	0.02071	0.12011	0.06807	0.11316	0.29644	0.20196	0.04236	0.30272	0.27932	0.0469	-0.0137	0.16369	0.03066
XM_345778.1	LOC366800	-0.17	-0.2593	-0.1985	-0.1877	-0.1916	-0.1665	-0.3232	-0.3582	0.09426	-0.1523	-0.0075	-0.1998	-0.3396	-0.1631
XM_576175.1	LOC366819	0.16163	0.05973	-0.0417	0.29136	0.02047	0.11317	0.10073	-0.0041	0.13303	0.04627	0.03548	0.0319	0.20912	0.0529
XM_578651.1	LOC366862	0.13821	0.1351	-0.0996	-0.0067	0.0985	0.12662	-0.2708	0.21401	0.1835	0.32136	0.09884	-0.2379	0.06724	0.02083
XM_345816.2	LOC366873	-0.0779	-0.0501	-0.0773	0.23477	-0.1737	-0.0529	-0.0728	0.11426	-0.0451	0.15983	-0.1259	0.06592	-0.0283	-0.1198
XM_345821.2	LOC366890	-0.0454	0.06541	0.0331	-0.0733	0.11996	-0.0826	-0.0042	0.01206	0.06387	0.0541	0.0031	0.4317	0.01447	-0.0168
XM_345826.2	LOC366899	0.0578	0.018	0.07849	-0.0527	0.06429	0.17367	-0.0598	0.05975	0.06399	0.02971	0.01338	-0.0606	0.12097	0.00407
XM_345827.2	LOC366902	0.13986	0.16412	0.15696	0.00878	0.07488	0.15634	0.14295	0.01333	0.14882	-0.0035	-0.0187	0.09276	0.01714	0.04162
XM_345828.2	LOC366906	-0.0875	-0.0223	0.06723	-0.122	-0.0848	-0.1155	0.2844	-0.2046	-0.1627	-0.2572	-0.1172	-0.1032	0.02598	-0.01
XM_345829.2	LOC366907	0.09554	0.03045	0.1164	0.32674	0.12061	0.3345	0.05166	0.25023	0.01581	0.04252	0.20258	0.48306	0.07961	0.13973
XM_345831.2	LOC366910	0.45315	0.12415	0.24521	-0.2516	0.38735	0.58346	-0.4588	0.18917	0.09583	0.09773	0.25209	0.04905	0.31309	0.39127
XM_345841.2	LOC366937	0.14965	0.02869	0.25143	0.00124	0.14541	0.08569	-0.0177	0.07008	0.16231	-0.0139	0.24271	-0.0023	0.03325	0.1094
XM_345864.2	LOC366968	0.29274	0.6428	-0.0954	0.32667	0.33199	0.35562	0.32057	0.44774	0.52654	0.6509	0.51022	0.254	-0.2044	-0.1102
XM_345879.2	LOC366994	-0.0889	0.2197	0.13729	-0.1641	-0.0268	-0.4088	0.10965	-0.2108	-0.0293	-0.2597	0.19372	-0.2384	-0.2373	0.14775
XM_345881.2	LOC366996	0.0231	0.15118	0.12796	0.07568	-0.0295	0.06734	-0.0338	-0.0354	0.12048	-0.0139	0.18623	0.02354	0.04905	0.31658
XM_345886.2	LOC367002	0.04601	0.01796	-0.0966	-0.1023	-0.1975	-0.076	-0.0547	-0.277	-0.1556	0.00223	-0.068	-0.0808	-0.0911	-0.114
XM_345890.2	LOC367006	0.34839	0.20871	0.0245	0.05574	0.19915	0.18413	-0.05	0.09288	0.24743	0.03741	0.24497	0.26659	0.06118	0.26036
XM_345903.2	LOC367028	0.17391	-0.008	0.1089	-0.028	0.04863	-0.0368	-0.0736	0.0336	-0.0886	0.11167	-0.0035	0.08323	0.257	0.06645
XM_345903.1	LOC367028	-0.0417	0.04447	0.03549	-0.0207	-0.1375	0.24664	-0.0218	-0.2065	-0.1044	-0.0461	-0.0078	0.09044	-0.104	-0.0206
NM_001108995	LOC367033	0.02503	-0.0729	-0.0726	-0.0726	0.04071	-0.0718	-0.0245	-0.0028	0.0081	-0.0751	-0.0162	0.00724	0.05795	0.03758
NM_001004223	LOC367047	0.03507	0.24236	-0.4813	-0.4302	0.21262	-0.2011	-0.5273	0.16162	0.11549	-0.2784	0.01135	-0.2882	-0.1553	-0.1862
XM_345916.3	LOC367050	0.04404	0.31981	-0.045	0.04641	0.15868	0.21339	0.01315	0.06696	0.02762	0.14458	0.32888	0.05021	-0.1179	0.13915
XM_345920.2	LOC367059	0.11743	0.1079	0.03083	-0.0097	0.00395	0.23259	0.23583	0.27444	0.06294	0.04617	-0.0233	0.13141	-0.0522	0.15812
XM_345923.2	LOC367063	0.03882	0.07769	0.01672	-0.0502	-0.1064	0.20039	-0.0114	0.0333	0.05517	-0.0503	-0.0576	0.01569	0.05456	-0.1201
XM_578729.1	LOC367076	-0.0026	-0.0555	0.02843	-0.0254	0.00514	-0.0875	-0.0157	0.02907	0.04695	0.0186	-0.0945	-0.0444	-0.0527	-0.0827
XM_345934.2	LOC367078	0.26961	0.28775	0.0633	0.09197	-0.0102	0.4715	-0.0667	0.16552	0.0553	0.25668	0.12162	-0.0417	0.18052	-0.0016
XM_345942.2	LOC367088	0.00773	-0.0385	0.24601	0.23558	0.16608	0.56816	0.01134	0.15571	-0.0913	-0.1807	-0.0052	0.61241	0.13092	0.08599
XM_345943.2	LOC367089	0.02239	-0.0482	0.23164	-0.0839	0.08553	-0.0524	0.01737	-0.0284	-0.0903	-0.0875	-0.0274	0.08037	-0.0266	-0.0246
XM_345951.2	LOC367109	-0.1015	0.00546	-0.0929	0.04032	-0.0643	-0.0265	0.03947	0.08785	-0.0548	-0.1082	-0.0056	0.0982	-0.1261	0.10309
NM_001047919	LOC367117	-0.1205	0.02854	-0.1776	0.1099	-0.0344	-0.1824	-0.0763	-0.1226	-0.2091	-0.1081	-0.1843	0.06628	-0.1161	-0.1146
NM_053867	LOC367122	-0.0219	-0.1258	-0.0036	-0.1285	-0.021	0.00613	0.02716	-0.0903	-0.1325	0.07905	-0.1307	0.01269	-0.0296	0.09579
XM_345960.2	LOC367123	0.03725	-0.1332	-0.0164	0.04985	-0.2305	-0.0777	0.09957	-0.0332	-0.1181	-0.0535	-0.0423	-0.0405	0.16195	0.21797
XM_345964.2	LOC367131	0.03698	0.06008	-0.0015	-0.1636	-0.0477	-0.1427	0.01353	0.20616	-0.0692	0.01604	0.0908	-0.0978	0.08853	-0.0189
NM_134417	LOC367135	-0.1725	-0.2673	-0.2391	-0.1769	-0.1239	-0.144	-0.1769	-0.3138	-0.0542	-0.2394	-0.1361	-0.3763	-0.1018	-0.0063
XM_345966.2	LOC367136	0.03152	-0.0087	-0.0562	-0.0064	-0.082	-0.11	0.1322	-0.0597	-0.0269	-0.0882	-0.0326	-0.1423	-0.0348	-0.1195
XM_576450.1	LOC367149	0.04896	-0.0439	0.00084	-0.1079	-0.0174	-0.0847	0.0699	-0.0272	0.00413	-0.0597	-0.1334	0.05805	-0.142	-0.13
NM_022514	LOC367155	-0.2376	-0.1116	-0.0995	-0.201	-0.184	-0.1283	-0.1208	-0.1622	-0.2209	-0.0382	-0.2212	-0.1839	-0.058	-0.1405
XM_345983.1	LOC367170	0.07825	-0.0848	-0.1909	-0.7127	0.18826	0.36484	-0.2013	-0.1359	-0.0238	-0.0404	-0.0341	0.40074	0.65914	0.43358
NM_001024278	LOC367171	-0.391	-0.1371	0.0516	0.03868	-0.4557	0.09137	-0.3542	-0.2777	-0.3139	-0.2161	-0.0715	-0.2092	-0.6582	-0.6628
XM_345990.2	LOC367176	-0.0369	0.01925	0.04193	-0.1336	0.04426	0.01558	-0.1447	-0.0458	-0.0555	0.04821	-0.0192	0.05239	0.24072	-0.1138
XM_345993.2	LOC367181	0.11124	0.05574	0.06748	0.25292	0.07146	0.09693	0.05798	0.07903	0.06049	0.25739	0.0944	0.04508	0.17664	0.09142
XM_345995.2	LOC367184	0.0225	0.07675	-0.0931	0.05731	0.06418	0.07149	-0.0021	-0.0483	0.25414	-0.0042	0.05833	0.04025	0.08269	0.07296
XR_009207.1	LOC367191	-0.1393	0.37907	0.14201	-0.1376	-0.1091	-0.5083	-0.4672	-0.1503	0.28323	0.49529	0.25001	0.14488	-0.0334	-0.18
NM_001047920	LOC367195	0.10873	0.04757	0.24173	0.10823	0.03708	0.00458	0.15992	-0.1025	0.35628	0.19461	0.155	0.42428	-0.0069	-0.0335
XM_346004.2	LOC367197	-0.0425	0.00955	-0.0628	-0.0799	0.08919	-0.0772	0.09187	-0.0639	0.1658	-0.0299	0.19352	0.04991	-0.0112	0.06581

XM_346005.3	LOC367198	0.00654	-0.2466	0.03895	0.7362	0.04485	-0.1053	0.02138	-0.0222	-0.0508	0.07608	-0.2035	-0.4451	0.0623	0.04127
XM_346007.2	LOC367201	-0.02	-0.0379	-0.1972	0.17609	-0.051	0.12309	-0.1476	-0.1426	-0.2374	0.01231	-0.0022	0.0345	-0.1726	-0.0319
XM_346010.2	LOC367208	-0.0783	0.08161	0.11822	-0.0064	-0.061	0.04418	-0.0434	0.24102	-0.0051	0.03598	0.16622	0.04301	0.1053	-0.0494
NM_001134596	LOC367214	0.14431	-0.2034	0.11376	0.32545	0.08945	-0.0741	-0.157	0.01784	0.13862	-0.2288	-0.0121	0.20187	0.18367	0.08118
XM_346015.2	LOC367216	0.04819	0.20261	0.31575	0.3796	0.34201	0.39462	0.21404	-0.0284	0.14814	0.40183	0.355	0.1855	0.36181	0.08541
XM_346024.2	LOC367226	-0.0613	0.1484	-0.0813	0.16295	0.10185	-0.1054	-0.1246	0.11675	-0.0684	0.1153	-0.0241	0.13556	0.15291	-0.0506
XM_578778.1	LOC367229	0.30851	-0.0896	-0.127	-0.4255	0.4008	0.14243	0.17296	0.10912	-0.0347	-0.1306	-0.1396	-0.1064	-0.1599	0.01043
XM_346031.2	LOC367238	-0.1263	-0.2209	-0.3112	-0.2238	-0.0759	-0.273	-0.0514	-0.1058	-0.3268	-0.2482	-0.1867	-0.2815	-0.054	-0.114
XM_578783.1	LOC367239	0.07857	-0.1064	-0.3031	-0.2442	-0.143	0.2093	-0.0783	-0.037	0.24376	-0.0362	-0.1041	-0.1727	-0.0505	-0.131
XM_346035.2	LOC367244	0.37926	0.19979	0.11146	0.01512	0.20431	0.43426	0.01082	0.17808	0.03516	0.20416	0.23032	0.05272	0.17246	0.21569
XM_576541.1	LOC367246	-0.2244	-0.1147	-0.1244	0.02349	-0.1607	-0.2022	-0.2681	-0.2345	-0.1291	-0.1157	-0.1815	-0.0756	-0.235	-0.1682
NM_001114391	LOC367250	0.05681	-0.1854	-0.1742	0.12568	0.04338	0.30855	-0.3607	-0.2157	0.05792	-0.0483	0.08589	0.15322	-0.24	0.09183
NM_001007599	LOC367265	-0.063	0.2115	-0.0378	0.22965	0.00279	0.33453	0.0774	-0.1078	0.1712	-0.0596	-0.0269	0.35391	0.00145	0.35433
XM_346058.3	LOC367289	-0.2923	-0.0801	-0.5632	-0.2693	-0.339	-0.6504	0.00616	-0.4547	-0.1798	-0.122	-0.2271	-0.4392	-0.3317	-0.113
XM_346058.3	LOC367289	0.57916	0.67505	0.12227	0.96953	0.66587	0.52267	0.72201	0.85477	0.49334	0.62604	0.4991	0.24782	0.72116	0.44271
XM_346060.2	LOC367293	-0.0942	0.03784	-0.1039	0.04369	-0.038	0.03176	-0.0724	0.18009	-0.1616	0.01224	0.01146	0.0685	0.08714	-0.0281
XM_346067.2	LOC367305	0.03062	0.19678	-0.0301	0.02871	0.44141	0.21774	0.11585	0.10084	0.01897	0.09638	0.12035	0.23521	0.01748	-0.0392
XM_346070.2	LOC367308	-0.1245	0.11505	-0.0803	0.23454	0.08194	-0.138	-0.062	0.01724	0.04263	0.16015	-0.1274	0.21685	-0.1283	0.07779
NM_001014269	LOC367314	-0.5544	0.23198	0.02303	0.07727	-0.7426	-0.8129	-0.0901	-0.5481	0.0017	0.26113	0.20575	-0.464	-0.7939	-0.8278
NM_001109009	LOC367316	0.17893	0.06376	0.22437	-0.0094	-0.0258	0.16773	-0.0109	0.26977	-0.0147	0.05723	0.16559	0.0215	0.22736	0.13587
XM_346081.2	LOC367324	0.2347	-0.0975	0.07974	0.0835	-0.2722	-0.0033	0.10409	-0.1391	0.07246	0.00185	-0.0061	0.20128	0.25466	0.16357
XM_346089.2	LOC367335	0.15169	-0.0283	0.09762	0.35035	0.08074	0.09299	0.28964	0.22637	0.19103	-0.0435	0.01038	0.2922	0.19969	-0.0071
XM_346091.2	LOC367337	-0.1299	-0.1044	-0.0281	-0.068	-0.127	-0.0941	-0.0115	-0.0582	0.23585	-0.074	-0.0096	0.056	0.07189	-0.104
XM_346096.2	LOC367343	-0.0954	-0.1635	-0.0959	-0.2191	0.08353	-0.3161	-0.0038	-0.1734	-0.0097	-0.1281	0.02912	-0.1499	-0.0889	-0.0414
XM_346102.1	LOC367350	0.12015	0.03791	0.23625	0.3794	0.08054	0.03269	0.2455	0.20022	0.1414	-0.06	0.17279	0.00771	0.24456	0.14653
XR_007715.1	LOC367352	-0.1644	-0.211	-0.1625	-0.1995	-0.1771	-0.1689	-0.1392	-0.1776	-0.1837	-0.1907	-0.229	-0.239	-0.1906	-0.1568
XM_346104.2	LOC367357	-0.0698	0.09363	0.08014	0.02612	-0.0093	-0.0694	0.018	-0.0338	0.00578	-0.0861	-0.0204	-0.0576	0.04053	-0.0434
XR_009117.1	LOC367360	-0.0555	0.0743	-0.036	0.2066	-0.0164	-0.0152	0.00207	-0.0967	0.23314	0.05893	0.02737	-0.001	-0.1542	-0.0994
XM_346105.2	LOC367363	-0.0749	-0.0176	-0.0089	0.38734	-0.0355	-0.0228	0.24038	0.14259	0.13994	-0.1943	0.28228	0.0873	-0.1468	0.01552
XM_346110.2	LOC367372	-0.0562	0.00597	-0.0437	-0.1326	-0.0199	-0.1187	-0.0206	-0.0103	0.01986	-0.0917	-0.0573	0.11231	-0.0907	-0.0694
XM_346113.2	LOC367375	-0.1224	-0.0749	-0.1143	0.07971	0.01287	-0.2171	0.02825	0.04777	-0.2406	-0.1974	-0.2401	-0.2267	-0.2539	-0.2429
XM_346114.2	LOC367378	0.01369	0.11106	-0.0369	0.13944	0.00366	-0.0289	-0.0423	-0.0081	0.11418	0.02567	-0.0661	-0.0387	0.21265	0.25402
XM_346115.2	LOC367379	0.07689	0.02878	0.12193	0.00382	-0.1028	-0.1586	-0.0487	-0.2914	0.12719	-0.2776	-0.0533	0.06218	-0.034	-0.0455
XM_576810.1	LOC367381	0.04935	-0.0281	0.06167	-0.0682	-0.1372	0.32502	0.14234	-0.0333	-0.0326	-0.0461	0.01593	0.05211	-0.0831	0.05492
XM_576810.2	LOC367381	-0.013	0.03053	0.0085	0.00365	0.02157	-0.0027	0.05077	-0.0148	-0.057	0.12385	-0.0685	0.03768	0.01885	-0.0292
XM_576810.2	LOC367381	-0.0294	0.11144	-0.1871	0.0575	0.17249	-0.2931	-0.0171	-0.1103	-0.0932	-0.1291	-0.1191	-0.216	-0.2254	-0.1877
XM_578828.1	LOC367385	-0.1168	0.04216	0.02639	0.16348	-0.0287	0.1393	0.49	0.01561	0.02544	-0.1558	-0.0922	0.00356	0.42543	0.16905
XM_578829.1	LOC367387	0.18121	0.04106	-0.0017	0.00595	0.07898	0.15251	0.05989	-0.0162	-0.1056	-0.0577	0.07892	-0.104	-0.1373	-0.041
NM_001014221	LOC367390	0.08814	0.13224	0.13736	0.09643	0.16453	0.19983	0.027	0.07852	-0.0161	-0.015	0.07019	0.11259	0.15666	0.0684
NM_001109011	LOC367391	0.22233	0.03839	0.14333	0.06993	0.02799	0.22059	0.21208	0.10068	0.19213	0.24313	0.13208	-0.0363	0.1483	0.14492
NM_201415	LOC367398	0.18547	-0.1179	-0.0774	0.22323	-0.0056	-0.0207	-0.2199	0.05364	-0.0429	-0.1751	0.02285	-0.2378	0.0595	-0.1749
XM_346127.2	LOC367400	0.12751	-0.0408	-0.0543	-0.0113	0.0705	0.01254	0.06622	0.21352	0.04579	-0.1416	0.28778	0.14147	0.0574	0.36264
XR_007623.1	LOC367402	-8E-05	0.0254	0.20014	0.13092	-0.0573	0.00114	0.01729	0.04368	0.00134	-0.0307	0.14122	-0.0004	0.05273	0.01662
XM_001053976.1	LOC367415	0.16679	0.05818	-0.0236	0.00073	0.06008	-0.0494	0.02634	0.14401	-0.0275	0.01181	0.1719	0.09611	0.07475	0.09816
XM_576678.1	LOC367422	0.13368	0.16249	0.23412	0.30101	0.09835	0.15967	0.3078	0.04283	-0.0654	0.09263	0.12769	0.07234	0.05687	0.35
XM_346138.2	LOC367436	0.04514	-0.0808	-0.0705	-0.0251	-0.0848	-0.047	-0.0744	-0.0443	0.11598	-0.1598	0.09546	-0.1128	-0.1109	-0.0574
XM_346139.2	LOC367438	0.06716	0.25347	0.10524	0.10847	0.06847	0.08434	0.18339	0.39927	0.15723	0.40423	0.19566	-0.0803	0.28972	0.06518
XM_346141.2	LOC367448	-0.0022	0.03003	0.08052	0.00624	0.21531	-0.1129	-0.136	0.05916	0.05269	-0.1272	-0.1296	0.0694	-0.0213	0.01085
XM_578863.1	LOC367462	0.11546	0.09078	0.13884	-0.0311	0.06176	0.01901	0.04773	-0.089	0.07726	0.09634	0.02864	0.04693	0.07232	0.42051

XM_346152.2	LOC367471	0.21134	0.71545	1.482	1.2689	-0.0727	-0.1994	1.5865	-0.1449	0.85696	1.1885	1.1065	0.14898	0.76105	0.8531
XM_576707.1	LOC367472	-0.2126	0.27516	1.319	0.91187	-0.0382	-0.2418	1.4431	-0.113	0.74075	0.70952	0.71239	0.64934	0.57211	0.71342
XM_346154.2	LOC367477	-0.0088	-0.0559	-0.1972	-0.0527	-0.1591	-0.1315	-0.1266	-0.1737	-0.1122	-0.1095	0.00933	-0.0403	-0.017	-0.0681
NM_001099659	LOC367480	0.34872	0.14631	0.0834	0.21243	0.1273	0.06297	0.07861	0.2117	0.16698	0.05999	0.04089	0.19008	0.24624	0.1116
XM_346160.2	LOC367484	-0.1257	-0.1048	-0.3226	-0.2576	-0.261	-0.1769	-0.2592	-0.1223	-0.1479	0.06396	0.10392	-0.1959	0.01266	-0.1808
NM_183402	LOC367485	0.06869	0.16831	-0.1821	-0.119	-0.0522	0.16169	0.10859	0.09749	-0.1188	0.25008	-0.0581	0.20676	-0.1609	-0.1696
XM_346175.2	LOC367501	-0.0923	0.07313	0.05164	-0.0241	0.01695	0.21713	0.05181	-0.0191	0.05538	-0.0266	0.06575	0.40148	-0.0647	0.10221
XM_346184.2	LOC367513	-0.0849	-0.0544	0.23459	-0.0025	0.02753	-0.0613	0.04161	0.00559	0.07458	-0.0568	0.01207	0.04647	0.00945	-0.0681
NM_001014221	LOC367516	0.05346	0.24395	0.22337	0.12958	0.06059	-0.0309	0.17762	0.04131	0.16811	0.32693	0.17559	0.17162	0.16074	-0.0042
XM_346193.3	LOC367523	0.03203	-0.0865	-0.0262	0.04503	-0.022	0.12794	0.04867	-0.0638	-0.0013	-0.0853	0.01782	0.11502	-0.0122	0.05062
XM_346193.2	LOC367523	0.14686	-0.0599	-0.0143	0.19811	0.00603	0.09707	0.00392	0.27038	0.02205	0.1944	0.05639	0.11073	0.35578	0.19684
XM_346196.2	LOC367526	0.36688	0.07556	0.05563	0.0772	0.18373	0.10823	0.16217	0.05045	0.0441	0.18342	0.03474	0.0364	0.20781	0.09848
XR_009504.1	LOC367536	-0.0695	0.18885	0.01908	0.04836	-0.0265	0.02702	0.21322	-0.0468	-0.0673	-0.0152	-0.0162	0.04537	0.13496	-0.0233
XM_578879.1	LOC367537	0.18477	0.29861	0.08985	0.28987	0.10287	0.11519	0.03668	0.15858	0.15114	0.14441	0.1602	0.14791	0.0166	0.00983
XM_001063146.1	LOC367539	-0.0727	-0.113	-0.0031	-0.0137	0.00337	-0.0679	-0.2374	-0.0502	-0.0836	-0.1155	-0.1834	-0.1649	-0.118	-0.0808
XM_346204.1	LOC367543	0.11588	0.22706	0.23255	0.12031	0.13258	0.093	0.29815	0.19156	0.0016	0.3058	0.12734	0.08526	0.16239	0.12241
XM_578884.1	LOC367544	-0.0379	-0.0026	-0.0446	-0.0941	-0.0516	-0.1536	-0.1424	0.03557	0.00285	-0.0871	-0.017	-0.1001	0.17267	-0.1163
XM_578887.1	LOC367548	0.11364	-0.0381	0.06555	0.09814	-0.0285	0.02208	0.07361	0.0523	0.14283	0.11518	0.03362	0.00444	-0.0125	0.24786
XM_346206.2	LOC367550	-0.151	-0.0641	-0.038	-0.0389	0.1003	-0.0399	-0.0017	-0.1652	-0.0385	0.11262	-0.0968	-0.0206	0.01157	0.01767
XM_346216.2	LOC367576	0.17597	0.08783	0.17659	-0.0783	0.08447	-0.013	0.00581	0.01308	0.0638	0.30432	-0.1362	-0.0354	0.17563	-0.0082
XR_009491.1	LOC367582	-0.124	0.7384	1.2961	1.1605	-0.2926	-0.0049	1.6434	-0.2981	0.6947	0.91063	1.0654	0.29609	0.66897	0.86847
XM_578908.1	LOC367582	0.04324	1.3016	1.4929	1.1824	-0.0189	-0.1751	1.8196	0.0508	0.77126	1.0601	1.2578	0.47654	1.1097	1.0074
XM_346221.2	LOC367591	0.1378	-0.1651	0.11419	0.10349	-0.0116	0.08709	0.00134	-0.0173	-0.0302	0.1601	0.06343	-0.0866	-0.0681	0.20456
XM_346222.1	LOC367592	0.0635	0.02732	0.11908	0.25505	-0.0958	0.11388	0.16496	-0.0702	-0.0231	0.10129	-0.0059	0.18807	0.17466	0.02885
XM_346223.2	LOC367594	0.00692	0.08515	0.16937	0.38881	0.24023	0.35476	0.08982	0.36695	0.09234	0.09864	0.14969	0.11014	0.12141	0.21822
XM_346224.2	LOC367607	-0.1876	-0.0934	0.01449	0.04026	0.11078	-0.0173	0.08222	-0.0745	-0.0795	-0.0105	-0.0987	-0.0308	-0.0646	-0.0991
XM_576850.1	LOC367609	0.13717	-0.0047	-0.1171	0.04496	-0.087	0.17394	0.03776	0.08479	-0.1133	0.11684	0.1284	-0.1203	-0.0364	0.23804
XM_578926.1	LOC367613	-0.4219	-0.5077	-0.5127	-0.2386	-0.315	-0.5161	-0.3483	-0.4753	-0.496	-0.3152	-0.3973	-0.2392	-0.6003	-0.4713
XM_346233.1	LOC367631	0.19062	0.00588	0.145	0.23226	0.02989	-0.088	-0.0431	0.07552	0.14375	0.32033	-0.0036	0.16091	0.22962	0.1558
XM_346234.2	LOC367632	-0.1646	-0.1072	0.02583	-0.1075	-0.0768	0.42903	0.15991	-0.0505	-0.0143	0.29027	0.24222	-0.0812	-0.0176	-0.0694
XM_578941.1	LOC367638	0.03697	0.13391	-0.0044	0.00908	-0.0328	0.07513	-0.0228	0.24284	0.01717	0.03049	0.07765	0.15945	0.0832	-0.0169
XM_578942.1	LOC367640	-0.0793	-0.1149	-0.2247	0.10562	-0.239	-0.2391	-0.2479	-0.1864	-0.2542	-0.0144	-0.18	-0.1598	-0.0248	-0.2213
NM_001044253	LOC367645	0.0006	-0.0217	0.08307	0.11531	0.02996	0.20598	0.07754	0.06396	0.1438	0.13953	0.22477	0.20525	0.19626	0.2942
XM_346238.2	LOC367646	0.1002	0.14053	0.02675	0.09782	0.13092	0.1183	0.04079	0.05387	0.09363	0.10408	0.19073	0.02113	0.03989	0.17045
XM_346239.2	LOC367648	0.15323	0.12647	0.09349	0.25068	0.21682	0.22204	0.09459	0.02577	0.07445	0.10312	0.12489	0.27479	0.09122	0.25713
XM_346242.2	LOC367662	-0.0025	0.02304	0.02564	0.09981	0.05176	0.26169	0.05078	0.02781	-0.0036	0.21474	0.38287	-0.064	-0.0336	0.08303
XM_346244.2	LOC367667	0.01484	0.06239	0.03589	-0.0964	0.01638	-0.0897	-0.0233	0.09548	0.07539	0.21131	0.16241	-0.0164	-0.1026	0.14969
XM_346250.2	LOC367676	0.03346	0.13854	0.02404	0.09595	0.0628	0.01444	0.26566	0.06007	0.02681	0.03691	0.04926	-0.0037	0.02146	0.09943
XM_346262.2	LOC367701	-0.1025	-0.0687	-0.139	0.05399	-0.0063	0.02937	-0.0479	-0.0119	-0.0707	-0.0926	0.19615	0.10425	-0.0407	0.1847
NM_001109012	LOC367714	0.18732	0.2019	-0.0542	0.08962	-0.0035	0.16888	0.13829	0.03617	-0.0378	-0.0131	0.06096	-0.1367	0.23984	0.14183
XM_578970.1	LOC367731	0.01505	0.2372	-0.1325	-0.2691	0.01437	0.0247	-0.0532	0.05484	-0.0435	0.17257	0.09612	0.20216	0.14004	-0.0348
NM_001127601	LOC367733	-0.2939	-0.3203	-0.2994	-0.3158	-0.1962	-0.2676	-0.0472	-0.3853	-0.3456	-0.4126	-0.2647	-0.4051	-0.314	-0.2673
XM_346277.2	LOC367747	0.02293	0.05963	0.06825	0.10249	0.03291	0.18935	0.07759	0.17956	0.0144	0.06699	0.14896	-0.0542	0.07159	0.20525
XM_346281.2	LOC367754	0.0018	-0.0173	0.04065	-0.0447	0.09056	0.02775	-0.0327	0.11549	-0.0087	0.13389	-0.0682	0.14237	0.06922	-0.0172
XM_576936.1	LOC367759	-0.0234	-0.0103	-0.0926	0.00653	-0.1394	-0.0201	0.01955	0.00188	-0.0178	0.06569	0.09154	0.04323	0.04456	-0.0726
XM_578976.1	LOC367771	-0.091	0.03361	-0.1181	0.09344	0.09104	-0.0394	-0.0181	-0.0004	-0.0369	-0.0226	0.04231	0.0612	0.06734	0.00617
XM_578977.1	LOC367773	0.08287	0.07467	0.05732	-0.006	-0.1079	0.04997	-0.0348	-0.0902	0.01644	-0.0237	-0.0072	-0.1859	-0.0484	0.06078
NM_133399	LOC367779	-0.3629	0.16827	-0.6669	0.2788	-0.0972	-0.3627	-0.0717	-0.1836	-0.06	-0.1709	-0.2958	-0.3227	-0.0343	-0.0939
XM_346299.2	LOC367783	0.18169	0.08713	0.10623	0.1534	0.37926	0.27469	0.15077	0.25341	0.10084	0.00614	0.224	-0.0287	0.08748	0.21538

XM_578981.1	LOC367788	-0.1315	0.07592	-0.1063	-0.0553	-0.2002	0.06549	-0.1713	-0.0653	-0.2011	-0.0062	-0.0326	-0.1089	0.04091	-0.0772
XM_346304.2	LOC367793	0.36006	-0.0222	-0.0073	0.04532	0.00402	0.15176	-0.0551	-0.0637	0.11006	0.20363	0.12446	0.00505	-0.0074	0.06344
XM_346305.2	LOC367795	0.13125	0.06337	-0.0366	0.19203	-0.0045	0.08592	0.02586	-0.0104	-0.0052	-0.0054	-0.0586	-0.011	-0.006	0.01419
XM_578988.1	LOC367810	0.10455	0.05135	-0.0767	0.09649	-0.0762	0.11608	0.18336	-0.0584	-0.0685	-0.0334	0.06745	0.10599	0.04455	0.03951
XM_346315.1	LOC367829	0.07823	0.05933	0.19124	0.01073	0.13894	0.17598	0.00619	-0.0413	0.02981	0.00747	0.04757	0.16456	0.04637	0.04514
XM_346315.2	LOC367829	-0.025	0.09492	0.06244	0.06344	-0.0827	0.0304	0.0369	-0.0283	0.04205	-0.1619	-0.0184	0.09489	-0.0604	-0.1372
NM_001037211	LOC367830	0.00732	0.02276	0.02978	-0.1259	0.10289	-0.0432	-0.0195	0.12313	-0.0836	-0.1199	-0.0954	-0.0496	0.0643	0.00384
XM_346319.2	LOC367835	0.40842	0.39315	-0.3187	0.02492	0.43186	0.61805	-0.2274	0.37847	0.46531	0.00966	0.32809	0.69019	0.13582	-0.0042
XM_346321.1	LOC367840	0.05814	-0.0404	0.03187	-0.0163	-0.0412	0.05252	-0.0247	-0.0488	0.02749	-0.0798	-0.0427	0.00092	-0.0466	-0.0024
XM_346327.2	LOC367855	0.06777	0.12216	0.1834	0.17481	0.03137	0.10424	0.09496	0.10312	0.10552	0.33384	0.25631	0.24216	0.05628	0.01449
XM_346328.2	LOC367857	0.01342	-0.0432	-0.322	0.46878	0.35582	0.5215	-0.4573	0.34765	-0.242	-0.0595	-0.1852	0.0787	0.01978	-0.168
XM_346332.2	LOC367865	-0.0166	0.10339	0.00426	0.176	0.14375	0.0447	0.12372	0.07235	-0.0167	0.21685	0.17917	0.09351	0.18496	-0.0432
XM_346339.2	LOC367874	-0.0938	-0.5721	-0.0704	-0.326	0.09947	0.37069	-0.5013	-0.0736	-0.3438	-0.3282	-0.3421	0.0998	0.12985	0.05414
XM_579653.2	LOC367880	0.09815	0.07591	-0.026	-0.1187	0.03652	-0.0074	-0.085	-0.129	-0.0265	-0.1447	0.12217	-0.0859	0.07506	-0.1345
XM_579009.1	LOC367891	-0.2166	-0.0537	-0.2761	-0.1132	-0.1457	-0.1036	-0.3383	-0.3264	-0.1543	-0.1979	-0.1897	-0.1853	-0.0161	-0.2183
XM_579012.1	LOC367898	-0.0004	0.08335	0.06161	0.0187	0.08587	0.0938	0.16291	0.14014	0.21775	0.18066	0.05791	0.03756	-0.0114	0.05916
XM_579020.1	LOC367906	-0.1841	-0.1292	-0.0404	0.00984	-0.0373	0.00039	-0.181	-0.2966	-0.1425	-0.1033	-0.2397	-0.2227	-0.1615	-0.2293
NM_001108283	LOC367923	0.0437	-0.3709	-0.2853	0.19907	-0.1464	0.0059	-0.4531	-0.0635	-0.2338	-0.7266	-0.1334	-0.2097	-0.5718	-0.4353
XM_579030.1	LOC367924	-0.0282	-0.0224	-0.0836	0.1296	-0.0321	-0.0188	0.06995	0.06808	0.04937	0.19271	-0.0706	0.22652	0.11632	0.02728
XM_346354.1	LOC367927	-0.0353	0.07354	0.18056	0.02441	0.02118	0.10522	0.14586	0.07444	0.12049	0.01391	0.08634	-0.0111	0.094	0.1359
NM_001109019	LOC367944	0.02636	0.19543	0.03634	-0.0812	-0.0909	0.01128	-0.0016	-0.0022	0.04182	0.44034	0.03446	0.25913	0.15852	0.21285
XM_346363.2	LOC367945	0.05831	-0.2004	-0.2222	-0.0322	-0.1482	-0.1317	-0.091	-0.0648	-0.0753	-0.1205	-0.1129	-0.1427	-0.0801	-0.0262
XM_346364.2	LOC367946	0.24219	-0.0709	-0.0265	-0.1337	0.16373	0.06606	0.0473	0.07188	-0.1104	-0.1559	0.17145	0.0423	0.11522	-0.2195
XM_346368.1	LOC367956	0.41658	0.13203	0.48709	-0.166	-0.0274	0.03381	0.42312	0.05146	0.31028	0.17356	0.238	0.38865	0.41008	0.37606
XM_346373.1	LOC367968	0.01379	0.13308	0.23475	0.49509	0.03762	0.2556	-0.0659	0.18899	-0.0373	0.02769	0.12992	0.13894	0.61337	0.05213
NM_001079896	LOC367975	-0.0237	0.05982	0.04569	0.09234	0.19579	-0.1374	-0.0169	0.02897	-0.0092	-0.124	0.17272	-0.1092	0.01794	0.02118
XM_346381.2	LOC367976	0.05154	-0.0241	-0.1924	-0.118	-0.1508	0.51797	-0.1818	-0.3106	-0.1071	-0.0928	-0.0415	0.11649	-0.0799	-0.2283
NM_001109020	LOC367994	0.08137	-0.0215	-0.0337	-0.0714	0.09178	0.21382	0.06492	0.06988	0.13615	0.35774	0.24443	0.2137	0.03594	0.07005
NM_001109021	LOC368001	-0.0182	-0.3225	-0.2336	-0.4288	-0.408	-0.5888	-0.1406	-0.1997	0.09729	-0.3267	-0.1889	-0.3969	0.22597	-0.0473
NM_001134781	LOC368062	-0.1515	0.00918	-0.3557	0.17789	-0.391	-0.081	-0.2528	-0.2801	-0.2221	-0.2248	-0.3261	-0.1471	-0.1873	0.13926
NM_001109022	LOC368066	-0.1518	-0.1176	-0.1921	0.05142	0.02575	-0.194	-0.1624	-0.1112	-0.2743	-0.1203	-0.1074	-0.1815	-0.2827	-0.0465
NM_001109023	LOC368070	-0.2927	-0.3289	0.08174	-0.0559	-0.5198	-0.5799	-0.1052	-0.4888	-0.2245	-0.3721	-0.1508	0.02802	-0.1173	-0.1175
NM_001135743	LOC368084	0.14846	-0.3803	-0.8737	-1.0197	0.07592	-0.668	-0.1948	0.1265	-0.0646	0.06493	-0.078	-0.2959	0.64075	0.66101
XM_577642.1	LOC368112	0.05172	0.0614	-0.0361	-0.0285	-0.0965	-0.0341	0.04934	0.05276	-0.068	-0.0606	0.04233	0.04989	-0.0026	0.02701
XM_001079648.1	LOC368120	0.16827	0.16566	0.23351	0.04688	0.12756	0.13644	0.0437	0.04566	-0.0007	0.09152	0.16605	0.04327	0.21057	0.05247
NM_001135762	LOC368128	-0.079	-0.0704	-0.0768	-0.0846	0.04494	0.0179	0.02135	-0.0485	-0.02	0.12831	-0.1075	0.08521	0.0711	-0.0843
XM_001058675.1	LOC368158	-0.0659	0.02946	0.0401	-0.1315	-0.1072	-0.0062	0.05673	-0.1872	-0.058	0.06399	0.20094	-0.0259	-0.1659	0.17073
NM_001109024	LOC368190	0.09843	-0.1836	-0.2253	-0.3786	-0.0847	0.09136	0.00412	-0.0545	-0.1661	0.00579	0.05504	-0.1967	0.04933	-0.1372
XM_001052967.1	LOC474147	-0.2722	-0.263	0.29297	-0.2576	0.05606	-0.1517	-0.2569	0.03712	-0.0813	-0.2625	-0.1513	-0.0574	-0.128	0.26184
NM_001008386	LOC493574	-0.1012	0.00659	0.0637	-0.1188	-0.2241	-0.2248	0.00403	-0.1207	-0.1032	0.03972	0.19045	0.0547	0.12131	0.00013
NM_001009505	LOC494224	0.06228	-0.0449	0.0069	-0.0741	0.27784	0.11824	0.09342	0.2057	0.03206	0.11696	-0.0079	-0.028	0.0894	0.18278
NM_001010921	LOC494499	0.52121	-0.28	-0.2442	-0.2695	-0.1559	-0.0605	-0.0993	-0.2676	-0.1935	-0.2574	-0.0749	-0.2185	-0.3463	-0.1735
XM_577783.2	LOC494528	0.10695	0.2059	0.18196	0.00494	0.08799	0.16422	0.15842	0.04557	0.4202	0.01718	0.12937	0.32993	-0.0252	0.02958
NM_001009977	LOC494539	0.03662	-0.4488	-0.1756	-0.2354	-0.1647	0.0589	-0.2235	-0.2957	-0.0176	0.04411	-0.0852	-0.3782	0.04079	-0.302
NM_001011560	LOC497040	-1.2433	-1.0713	-0.9565	-0.6991	-1.1681	-1.3859	-1.0053	-1.1851	-0.8189	-1.3071	-1.0457	-1.1766	-1.0986	-1.3031
NM_001012228	LOC497083	-0.1598	-0.5803	0.70222	-0.3281	-0.9241	-1.0394	-0.265	-1.0397	-0.2037	-0.1944	-0.2479	0.4067	0.64124	0.48555
XM_579619.1	LOC497663	-0.8586	-0.7894	-0.2806	-0.4793	-0.5968	-0.4131	-0.2005	-0.4593	-0.2455	-0.4772	-0.5153	-0.29	-0.4367	-0.4555
XM_579443.1	LOC497664	-0.0773	-0.0118	0.04769	-0.117	-0.0289	-0.0351	-0.0255	-0.1102	-0.0733	-0.0889	-0.1387	-0.1438	0.03152	-0.1252
XM_579701.1	LOC497665	0.28315	0.25004	0.10596	0.12394	0.05757	0.22081	0.15538	0.20854	0.16968	-0.0785	0.04862	-0.0725	-0.0371	0.02738

XM_579569.1	LOC497666	0.09203	-0.1019	-0.0707	0.11227	-0.0691	0.06713	0.15298	0.19075	0.03777	0.02318	-0.1103	-0.0496	0.31541	0.17231
XM_579667.1	LOC497667	-1.0343	-1.1482	-1.1806	-1.1323	-1.1032	-1.1436	-1.2309	-1.0974	-1.1363	-1.2438	-1.201	-1.2472	-1.3629	-1.1758
XM_579473.1	LOC497668	0.51278	0.17919	0.13426	0.33356	0.83265	0.83196	0.18397	0.61915	0.02614	-0.1152	0.48556	-0.0796	0.20613	0.01914
XM_579679.1	LOC497669	-0.0951	-0.1341	0.22545	-0.0791	-0.0312	-0.3128	0.15491	-0.0566	-0.3187	-0.0177	0.00925	-0.117	0.01438	0.11337
XM_579162.1	LOC497671	0.22913	0.06665	0.13086	0.1984	0.21324	0.09553	0.11373	-0.0098	0.17158	-0.0349	0.18722	-0.0008	0.39774	0.034
NM_012514	LOC497672	-0.1253	-0.1539	-0.1141	-0.0116	-0.1215	-0.1987	-0.1344	-0.1298	0.02309	-0.1109	-0.0077	-0.0719	0.03787	0.06663
XM_579691.1	LOC497673	-1.2522	-0.4359	0.04565	-0.948	-1.194	-1.3026	-1.0842	-1.1862	-0.3207	-0.4999	-0.5296	-0.0903	-0.5232	-0.4621
XM_579401.1	LOC497674	-0.1036	0.62309	-0.4474	-0.3358	0.45318	0.21078	-0.1332	0.45205	0.02015	0.19869	0.40659	-0.3042	-0.5176	-0.5244
XM_579686.1	LOC497675	0.03719	-0.0558	-0.0852	-0.1291	-0.025	-0.0895	-0.0869	-0.1685	-0.1108	-0.0351	-0.0385	-0.1744	0.06275	-0.0655
XM_579515.1	LOC497676	-0.0571	0.07412	-0.0024	-0.0318	0.00785	-0.0606	-0.0518	-0.0656	-0.0592	-0.0784	-0.0388	-0.0534	0.0206	-0.0221
XM_579737.1	LOC497677	0.00933	-0.103	0.04871	0.13469	0.06484	-0.1162	-0.0072	-0.1373	0.04972	-0.0157	-0.0076	0.11363	0.19295	0.0875
XM_579381.1	LOC497678	-0.0309	0.04637	-0.0394	0.03696	0.1481	-0.0114	0.00418	0.02119	0.00123	0.01129	0.18465	0.09223	0.12966	-0.0993
XM_579739.1	LOC497679	0.08216	0.13074	0.12576	0.11488	-0.024	0.08811	0.06251	0.06796	-0.0096	0.15136	-0.0666	0.11504	0.23129	0.06528
XM_579452.1	LOC497680	0.10041	0.21811	0.16271	0.10419	0.0511	0.00096	0.05708	0.01482	0.13045	-0.0351	0.36834	0.15311	0.15908	0.05602
XM_579374.1	LOC497681	-0.0358	0.11621	0.07753	0.00256	-0.0208	0.04087	0.02814	-0.0305	0.0578	0.00181	0.23766	0.05038	-0.0002	0.01042
XM_579511.1	LOC497682	0.0533	-0.6968	0.70084	0.30799	-0.4429	-0.2639	-0.1629	-0.3608	-0.3565	-0.6647	-0.4606	0.09261	-0.3235	-0.0669
XM_579547.1	LOC497685	-0.7943	-0.4652	-1.1407	-1.3034	-0.8684	-0.7585	-0.9068	-0.8947	-0.4252	-0.4911	-0.6356	-0.3435	-1.0483	-0.9162
XM_579585.1	LOC497687	0.07694	0.20939	0.22568	-0.0694	0.14982	0.16512	0.07168	-0.0867	0.04088	0.34587	0.15003	0.27889	0.11181	-0.0276
XM_579556.1	LOC497689	-2.095	1.7621	-0.5954	0.29071	-4.0926	-4.2393	0.80104	-4.0897	1.8552	1.4454	1.5668	1.2849	-1.125	-1.1013
XM_579446.1	LOC497690	-0.1398	-0.0643	-0.0992	-0.0213	-0.0841	0.01514	-0.0197	-0.1711	0.01494	-0.0627	-0.1838	-0.0684	-0.1343	-0.0008
XM_579639.1	LOC497691	-0.1425	0.81906	0.08573	0.39725	-0.3175	-0.6408	0.83441	-0.1449	0.4736	0.67143	0.43715	0.06078	0.20915	0.15932
XM_579641.1	LOC497692	-0.0718	-0.2109	-0.5047	-0.5048	-0.5591	-0.6375	-0.1274	-0.3675	-0.0221	-0.075	-0.438	0.15413	0.12249	0.33292
XM_579635.1	LOC497693	0.49905	0.39492	-0.2889	0.35355	0.55862	0.59835	-0.0053	0.2975	0.20946	0.33602	0.19974	0.33916	-0.1326	0.06637
XM_579482.1	LOC497694	-2.6139	-2.5202	-2.5857	-2.6428	-2.6615	-2.6833	-2.4929	-2.6834	-2.5727	-2.6852	-2.771	-2.3747	-2.7461	-2.7415
XM_579325.1	LOC497695	0.02943	0.04986	-0.0524	0.03475	-0.0052	0.08112	0.16154	0.13473	0.32891	0.05446	-0.0585	0.04712	-0.0584	0.17304
XM_579449.1	LOC497696	-0.0509	0.08443	0.00563	-0.063	0.11056	0.05342	0.03709	-0.0101	0.01951	0.16885	-0.0417	0.0881	-0.0003	-0.0313
XM_579609.1	LOC497697	0.26473	0.15935	-0.0103	0.00041	-0.0624	-0.0011	-0.1018	0.16488	-0.0051	0.07154	0.1134	-0.0305	0.04832	-0.0034
XM_579523.1	LOC497698	0.28356	-0.3989	0.00589	-0.0515	0.49465	0.28571	-0.1787	0.38478	-0.0535	-0.3998	-0.5826	0.21153	0.57106	0.74133
XM_579700.1	LOC497699	-0.0433	0.19697	-0.196	-0.3712	-0.3825	-0.5461	0.21428	-0.2299	0.147	0.02293	0.36346	-0.2275	0.00359	-0.3387
XM_579642.1	LOC497701	-0.1801	-0.0239	-0.039	-0.2458	0.00262	-0.082	-0.1106	0.09015	-0.0642	-0.1543	-0.3159	-0.1934	-0.1998	-0.3488
XM_579713.1	LOC497702	-0.1205	-0.1139	0.02142	0.05616	-0.1656	0.15984	-0.0419	-0.0893	-0.0903	0.0481	-0.1486	0.18906	-0.1216	-0.0885
XM_579683.1	LOC497703	-0.0192	0.16491	0.13067	0.05431	0.10998	-0.0631	0.16637	0.07029	0.20518	0.13021	0.01704	-0.0938	0.00617	0.05548
XM_579521.1	LOC497704	0.0758	0.10193	0.08949	0.00679	0.01612	0.04625	0.14363	-0.0069	-0.0973	0.13585	0.02468	-0.0313	0.0357	0.05788
XM_579538.1	LOC497706	-0.5416	-0.5681	-1.0582	-0.5822	-1.3981	-1.7172	-0.3862	-1.1832	-0.6543	-0.7355	-0.8203	-0.9035	-0.7405	-0.6337
XM_579601.1	LOC497707	0.1249	0.15828	0.16218	0.04928	0.24555	0.15306	0.23677	0.00441	0.09709	0.14349	0.18481	0.15084	0.13739	0.21482
XM_579483.1	LOC497708	2.0926	0.0743	0.81466	1.7172	2.3459	1.9639	0.98561	2.2617	0.04027	0.15941	0.70506	0.20808	0.11283	0.09574
XM_579662.1	LOC497709	0.08631	0.05595	-0.0042	0.06797	0.11764	0.13946	0.22931	0.14951	0.29723	0.424	0.13747	0.184	0.19583	0.0348
XM_579705.1	LOC497710	-0.1236	-0.0306	-0.1482	-0.0298	-0.1097	-0.1759	-0.2087	-0.048	0.12822	-0.1204	0.14046	-0.0929	-0.142	0.04676
XM_579745.1	LOC497711	-0.0268	-0.0864	0.02838	0.09504	0.001	0.07686	-0.07	0.13546	-0.0296	-0.1457	-0.0201	0.15096	-0.1101	-0.0666
XM_579055.1	LOC497712	0.24172	0.45927	-0.0464	-0.1187	0.32112	0.4835	0.20929	0.69019	0.22852	0.5393	0.27463	-0.1038	-0.0516	0.13711
XM_579466.1	LOC497713	-0.1011	0.00356	-0.191	0.00596	-0.1818	-0.1068	-0.0438	0.23024	-0.2182	-0.0963	-0.0908	-0.2126	-0.1037	0.21127
XM_579730.1	LOC497714	0.00269	0.00046	0.00441	0.00097	-0.0414	0.00139	0.09375	-0.0402	0.02847	0.09883	0.0791	0.15475	0.01344	0.22423
XM_579560.1	LOC497715	-0.6723	0.41313	-0.6567	-0.3035	-0.3801	-0.8688	-0.0407	-0.1307	0.08483	0.22642	-0.1911	-0.7552	-0.3099	-0.3809
XM_579571.1	LOC497716	0.12062	-0.0104	-0.1993	0.00779	0.16118	-0.3371	0.31344	0.07963	-0.0384	0.43593	0.01036	-0.2053	0.56307	0.46158
XM_579724.1	LOC497717	0.01164	0.12303	0.06295	0.04637	0.12659	-0.0952	-0.0859	0.04663	0.15789	0.16279	-0.0818	-0.1484	0.12395	0.11672
XM_579587.1	LOC497719	0.17252	0.50326	0.27025	0.00718	0.34696	0.10616	-0.0075	0.19968	0.35405	0.47771	0.58627	0.3166	0.13	0.26891
XM_579423.1	LOC497720	0.15884	0.33203	-0.097	0.48399	0.20199	-0.3089	-0.0546	0.41199	0.29565	0.42958	-0.1392	-0.1715	0.0696	-0.0361
XM_579695.1	LOC497721	-0.0715	0.01026	0.06554	-0.0634	-0.1576	-0.0616	-0.0732	-0.2169	-0.0173	0.0777	0.10681	0.18204	-0.1551	-0.1093
XM_579583.1	LOC497722	-0.4013	-0.6767	-0.1561	-0.3932	-1.0678	-0.6997	-1.2193	-0.93	-0.2362	-0.6599	-0.6801	0.55484	0.65896	0.69757

XM_579534.1	LOC497723	0.13969	0.33271	0.03275	-0.5233	0.50721	0.58034	0.13134	0.69429	0.51208	0.25464	0.38223	0.63932	-0.036	-0.1592
XM_579510.1	LOC497724	1.1651	2.039	1.6661	0.83036	1.4028	1.2604	1.9213	1.4604	1.5441	1.7769	1.8373	1.1866	1.2843	1.1764
XM_579536.1	LOC497725	0.02414	-0.1313	0.10395	0.21058	0.1123	-0.0037	0.24237	-0.0362	0.11172	0.03994	0.00687	0.15398	0.01076	-0.0342
XM_579646.1	LOC497726	-0.0794	-0.0458	-0.0204	0.19096	0.06517	0.21935	0.00312	0.33528	0.15713	0.09902	-0.0934	0.03751	0.2588	0.0556
XM_579313.1	LOC497727	-0.142	0.03927	-0.2681	-0.0959	-0.0221	0.10443	-0.1295	0.03208	-0.16	-0.1521	-0.1746	-0.0816	-0.1607	-0.1608
XM_579481.1	LOC497728	-0.6007	0.17835	0.29455	0.53791	-0.3998	-0.4077	1.1136	-0.402	0.01087	0.07059	0.1237	-0.1774	-0.109	0.06978
NM_172157	LOC497729	-0.0905	0.03179	0.04834	0.07846	-0.0688	-0.023	-0.0706	-0.0081	0.02462	-0.1288	-0.0445	0.03487	0.01776	0.09753
NM_172157	LOC497729	-0.6195	-0.53	-0.3264	-1.481	-0.7001	-0.7774	-0.2894	-0.7901	-0.5548	-0.3833	-0.4688	-0.2174	-0.3961	-0.3897
XM_579480.1	LOC497731	0.04217	0.0703	-0.0061	0.00875	-0.0865	0.05497	0.1349	0.08727	-0.1484	-0.1319	0.02728	0.00702	0.08472	-0.0809
XM_579578.1	LOC497732	-0.2414	-0.4826	0.53846	-0.0265	-0.6338	-0.1507	-0.0619	-0.4852	-0.3358	-0.337	-0.3036	0.18493	-0.2341	-0.3875
XM_579432.1	LOC497733	-0.9938	-0.5267	0.01914	-0.418	-0.6297	-0.7577	0.01048	-0.921	-0.4354	-0.2432	-0.4187	-0.5879	-0.6602	-0.5343
XM_579559.1	LOC497734	0.02567	-0.0318	-0.138	0.0956	0.06513	-0.0465	-0.0282	0.12647	0.06339	-0.0843	-0.0871	0.03517	-0.0911	-0.0609
XM_579514.1	LOC497735	-0.0056	-0.1569	0.32991	-0.3422	-0.1226	-0.2263	-0.1993	-0.2051	-0.2435	-0.3312	-0.1658	0.19971	0.3641	0.37644
XM_579656.1	LOC497736	0.08811	0.3451	0.09189	-0.0463	0.24308	0.1885	0.09809	-0.0249	0.19001	-0.0667	-0.0179	0.27916	0.1731	0.23123
XM_579640.1	LOC497737	0.08528	0.02628	0.08185	-0.0585	0.07622	0.16547	0.066	-0.0764	-0.0761	0.13162	-0.0747	-0.0276	0.10027	0.12884
XM_579444.1	LOC497739	-0.6792	-0.3638	0.2281	-0.1121	-0.8648	-0.6525	0.01095	-0.7481	-0.5686	-0.3767	-0.4211	-0.1735	-0.4946	-0.4579
XM_579710.1	LOC497740	-0.2774	0.42694	0.79396	0.20733	0.4397	0.44284	0.37347	0.23916	0.35945	0.70096	0.57199	0.67984	0.41248	0.24227
XM_579138.1	LOC497742	0.21272	0.14696	-0.0887	0.37748	-0.0926	-0.0135	0.11822	-0.0132	0.35814	0.0789	-0.1501	-0.0473	-0.1471	-0.0104
XM_579435.1	LOC497743	0.32413	0.58727	-0.2311	-0.4727	0.26534	-0.1942	0.11	0.44843	0.42554	0.51379	0.16943	-0.1942	0.63417	0.42157
XM_579111.1	LOC497744	0.00265	0.11671	0.04484	0.07668	0.06452	0.09588	0.08313	0.06405	0.01673	0.18467	0.15447	0.10456	0.07966	0.08821
XM_579502.1	LOC497745	-0.2601	-0.3735	1.1696	0.00356	-0.1594	-0.3356	0.10727	-0.0771	-0.003	-0.6444	-0.3373	0.15958	-0.1714	-0.0373
XM_579568.1	LOC497746	-0.1009	0.12655	0.05453	-0.0428	-0.0178	0.02747	-0.1117	-0.1897	-0.127	-0.0162	-0.0353	-0.0716	0.107	0.05099
XM_579620.1	LOC497747	-0.2103	0.51785	-0.3595	-0.5466	-0.391	-0.4787	-0.2353	-0.4693	0.10246	-0.1375	-0.2696	-0.4185	-0.2717	-0.2113
XM_579551.1	LOC497748	-0.0125	-0.0541	-0.1405	-0.1727	-0.0361	-0.0826	-0.0916	-0.0389	-0.1495	0.04417	-0.0921	-0.0868	-0.1083	-0.055
XM_579689.1	LOC497749	-0.2924	-0.0317	-0.0843	-0.2046	-0.2262	-0.1482	-0.1715	-0.2043	-0.1312	-0.2307	0.08811	-0.1261	-0.0042	0.05945
XM_579364.1	LOC497750	-0.2561	0.24339	-0.5451	-0.3479	-0.4258	-0.5167	-0.2484	-0.3168	-0.0052	0.0352	-0.1679	-0.4687	-0.2917	-0.3108
XM_579360.1	LOC497752	-0.1535	-0.1152	-0.1339	-0.0915	-0.1982	-0.1394	0.0769	-0.1445	-0.11	-0.0254	-0.0993	0.197	-0.0649	-0.1103
XM_579371.1	LOC497753	-0.1352	-0.2128	-0.1268	-0.1479	-0.0369	-0.0655	0.12364	-0.1438	-0.2175	-0.0845	-0.1513	-0.1832	-0.1283	-0.013
XM_579458.1	LOC497754	-0.4296	-0.1825	-0.3524	0.30944	-0.3447	-0.2944	0.05953	-0.3388	-0.232	-0.1715	-0.2787	-0.204	-0.3402	-0.25
XM_579728.1	LOC497755	0.10582	0.05148	0.12933	-0.0566	0.04119	-0.0177	0.01047	0.02863	0.24287	0.19337	0.03644	0.23145	0.11584	0.2528
NM_013061	LOC497756	0.02879	0.00632	0.05177	0.17779	-0.0223	0.01062	0.10729	-0.0105	0.12697	0.07702	0.28039	0.12023	0.01299	0.04043
NM_017090	LOC497757	0.05311	0.10368	-0.0664	-0.1421	0.13698	-0.1229	-0.1785	0.12945	-0.2151	0.47803	-0.0378	-0.1023	0.00126	0.03584
XM_579454.1	LOC497758	-0.0241	-0.0394	-0.0384	-0.08	-0.0354	0.1019	0.00684	-0.0502	0.04742	0.15893	-0.0194	0.04447	0.08371	-0.1005
XM_579456.1	LOC497760	-0.0052	-0.0783	-0.06	-0.0935	-0.1212	-0.1441	-0.0884	-0.0627	-0.1134	-0.0114	-0.0869	-0.099	-0.1041	-0.0141
NM_012830	LOC497761	-0.1261	-0.176	-0.3164	-0.1912	-0.0969	-0.2199	-0.1452	-0.1114	0.22128	-0.127	-0.1841	-0.1715	-0.1973	-0.1774
XM_579738.1	LOC497762	0.22675	0.19738	0.0463	0.2291	-0.0053	0.02573	-0.0032	-0.007	0.23134	-0.0378	-0.0514	-0.1243	0.0164	0.13469
XM_579052.1	LOC497763	0.12256	0.08328	-0.0035	-0.0824	0.02269	-0.0547	0.07166	-0.0693	0.00587	0.04272	0.19925	0.00879	0.14088	0.11972
XM_579499.1	LOC497764	-0.0105	-0.0225	0.03721	0.03044	0.0103	0.06723	-0.0645	-0.0735	0.27824	-0.0879	-0.1206	-0.114	0.00673	-0.1683
XM_579597.1	LOC497765	-0.1426	-0.0687	-0.0193	-0.085	-0.091	-0.1614	-0.1225	-0.0472	-0.0381	-0.1585	-0.1178	0.0077	-0.1196	-0.0605
XM_579688.1	LOC497766	0.23815	0.30146	0.47522	0.7445	0.64178	0.24479	0.20125	0.69099	-0.1356	0.09899	0.20334	-0.3779	-0.1856	-0.101
XM_579388.1	LOC497767	1.3158	1.3659	1.024	1.4231	1.2527	0.92373	1.5847	1.4984	1.0167	1.3482	1.3896	0.29966	0.75165	0.74662
XM_579674.1	LOC497768	0.39928	0.67383	0.10709	1.4312	0.72881	0.48489	0.95178	0.92613	0.58672	0.46281	0.87027	0.28705	0.90394	0.85615
XM_579520.1	LOC497769	0.17013	0.5485	-0.0337	0.89807	-0.1523	0.05733	0.31362	-0.1766	0.11974	0.46817	0.47365	-0.7439	0.21179	0.62096
NM_013119	LOC497770	0.03108	0.01744	0.06382	-0.0784	-0.0115	-0.109	0.06909	0.08212	-0.0785	0.17377	-0.0206	-0.1119	0.07494	0.01274
XM_579736.1	LOC497771	-0.0769	0.02783	0.03276	0.02193	0.01144	0.05376	-0.003	-0.0401	0.05229	0.21738	-0.0227	0.04995	-0.0165	-0.0114
XM_579493.1	LOC497772	0.10828	0.07227	0.11351	0.14146	0.26496	0.074	-0.0186	0.04426	0.06107	0.31995	0.104	0.11194	0.23819	0.03554
XM_579532.1	LOC497773	-0.1579	-0.1418	-0.2179	-0.2169	-0.0813	-0.1324	-0.2838	-0.1677	-0.0248	-0.0629	-0.0323	-0.1478	-0.0815	-0.15
XM_579505.1	LOC497774	0.10861	0.47036	0.28689	0.01865	0.07173	0.08561	0.18684	-0.0009	0.14508	-0.0335	0.05336	0.39271	0.08109	0.0966
XM_579406.1	LOC497775	0.03488	0.06688	0.0833	-0.0056	0.12633	0.15187	0.0053	0.16174	-0.0929	0.10074	0.06291	0.10986	0.00271	0.09283

XM_579692.1	LOC497777	0.55465	0.5612	0.11737	-0.1774	1.072	0.82768	1.0885	1.2375	0.27217	0.52776	0.33208	-0.5534	0.28917	0.24821
XM_579526.1	LOC497779	0.04981	0.05513	0.19583	-0.0076	0.05886	0.09842	-0.0155	0.08498	-0.0223	0.03527	0.11731	0.10675	0.08443	0.00194
XM_579486.1	LOC497780	-0.7919	-0.9102	-0.6612	-0.4593	-1.492	-1.4975	0.32595	-1.3131	-0.6701	-0.7685	-0.9334	-0.7087	-0.9977	-1.0447
XM_579699.1	LOC497781	0.02928	-0.1239	-0.1085	0.29177	-0.0355	-0.1714	-0.0789	-0.003	-0.1913	-0.0831	0.08131	-0.0356	-0.0509	-0.0888
XM_579621.1	LOC497782	0.01156	-0.1366	-0.1706	0.01975	-0.004	0.00403	-0.0913	0.04684	-0.0476	-0.0667	-0.1058	-0.2638	-0.1614	-0.1596
XM_579509.1	LOC497783	0.13242	0.75772	-0.0785	0.02695	0.13643	-0.0423	0.10315	0.09363	0.15536	0.28236	0.03894	-0.0833	0.48157	0.36422
XM_579367.1	LOC497784	-0.0957	0.1243	-0.0033	0.13642	-0.0722	-0.1426	0.07717	0.15352	0.09729	0.0907	-0.0187	0.27641	0.08216	0.02358
XM_579677.1	LOC497785	-0.2085	-0.2464	-0.1967	-0.2683	-0.2556	-0.0809	0.04788	-0.254	-0.0129	-0.2524	-0.1469	-0.0447	-0.2196	-0.2455
NM_001008971	LOC497787	-0.0052	0.02034	0.04866	0.042	0.08526	0.05583	-0.0648	0.03635	0.15162	0.06917	0.00338	0.00535	0.028	-0.0028
XM_579697.1	LOC497788	0.00969	-0.0187	0.06708	0.04514	1E-05	-0.0442	0.09893	0.14499	0.09994	0.18794	-0.0216	0.21755	0.04422	0.24251
XM_579570.1	LOC497789	0.1961	0.02905	0.19847	0.32967	0.12589	0.17434	-0.006	0.1404	-0.0326	0.15887	-0.0703	0.02033	0.2026	0.22617
XM_579431.1	LOC497790	0.08741	0.07565	0.03445	0.16049	-0.0171	0.03746	0.10429	0.21609	0.09702	0.01994	0.07967	0.09218	0.03603	0.00515
XM_579382.1	LOC497791	-0.0852	-0.0346	-0.0021	0.11089	-0.0905	0.0446	-0.0752	0.09985	-0.0353	-0.0319	-0.018	0.05825	-0.0073	-0.1103
XM_579732.1	LOC497793	0.05326	-0.1888	-0.126	-0.0431	-0.3591	-0.3585	-0.0197	-0.1716	-0.2106	-0.2638	-0.4244	-0.5135	-0.4478	-0.4057
NM_023103	LOC497794	0.19009	0.00897	-0.005	-0.0611	-0.0339	0.07676	-0.0012	0.04916	-0.0184	0.08215	0.03196	0.02356	0.07992	-0.0051
XM_579630.1	LOC497795	0.07547	0.24746	-0.0581	0.01356	0.17653	0.0199	0.23108	0.04138	-0.0349	0.21211	-0.0155	0.12722	-0.0379	0.14875
NM_001009497	LOC497796	0.08358	0.13792	-0.0275	0.04854	-0.0053	-0.1125	-0.1162	-0.0897	-0.0093	0.14802	0.00473	0.07722	-0.0411	0.04176
XM_579680.1	LOC497797	-0.228	-0.059	-0.214	-0.1519	-0.2041	-0.0679	-0.2561	-0.1461	-0.0891	-0.108	-0.0332	-0.0761	-0.0423	-0.0745
XM_579394.1	LOC497799	-0.0204	0.00577	0.00805	0.0331	-0.027	0.04498	0.2117	-0.0392	-0.0211	0.01047	-0.0615	-0.0119	0.12909	0.12425
XM_579398.1	LOC497800	0.2083	0.03256	0.11445	0.03689	0.04215	-0.0309	0.12604	0.01848	0.17824	0.01191	0.02447	0.12988	0.19217	0.05627
XM_579608.1	LOC497802	0.33811	0.53281	0.04203	-0.2329	0.68012	-0.1868	0.1214	0.87637	0.25288	0.43791	-0.1274	-0.2033	0.80251	0.61644
XM_579529.1	LOC497804	-0.2427	-0.2038	-0.1822	-0.283	-0.126	-0.1265	-0.0272	-0.1934	-0.1002	-0.1229	-0.2134	-0.1863	-0.1557	-0.2242
XM_579513.1	LOC497805	0.18584	-0.0692	0.19809	0.00301	0.15673	0.13682	0.07107	0.17266	0.20559	0.2134	-0.0332	0.01345	0.02119	0.00996
XM_579702.1	LOC497806	-0.8018	-0.873	-0.7202	-0.741	-0.6529	-0.7082	-0.4752	-0.7614	-0.8101	-0.7989	-0.8874	-0.4427	-0.8236	-0.8092
XM_216338.2	LOC497807	0.11999	0.03874	0.34891	0.19691	0.39492	0.4989	0.35435	0.61284	0.55763	0.11408	0.22171	0.25013	0.2087	0.08696
NM_017154	LOC497811	0.27029	0.56101	-0.6873	0.29981	-1.1008	-1.1706	0.56919	-1.1557	0.15353	0.64411	0.58184	-1.1283	-1.2646	-1.3383
XM_579659.1	LOC497812	0.34921	0.16432	0.21897	0.14477	0.16336	0.19551	0.49295	0.38107	0.32177	0.23159	-0.0206	0.45328	0.06889	0.03391
NM_001024280	LOC497813	0.11345	0.09902	-0.0825	0.07365	0.07777	-0.0367	-0.0502	-0.1525	-0.0626	0.26982	0.07376	-0.1957	0.08322	0.08358
XM_579429.1	LOC497814	-0.1134	0.05912	-0.0375	0.04309	-0.0565	-0.0773	0.05718	-0.0355	0.12202	-0.1204	-0.0561	0.05025	0.16482	-0.1421
XM_579438.1	LOC497816	-0.7565	-0.7473	-1.0421	-0.983	-0.2161	-0.2784	-0.8784	-0.3754	-0.9086	-0.7368	-0.8618	-0.9543	-0.9898	-1.0827
XM_579579.1	LOC497817	0.12225	0.08198	0.08428	0.06614	-0.0097	0.02458	0.00883	0.32431	0.10554	0.08135	-0.0381	0.0473	0.06295	0.06429
XM_579714.1	LOC497818	-0.6927	-0.5423	0.09986	-0.0665	-0.621	-0.8218	-0.0044	-0.5783	-0.4078	-0.427	-0.2091	-0.3788	-0.3019	-0.4918
XM_579469.1	LOC497819	-0.0368	-0.1439	-0.0753	2.7072	-0.0569	-0.1159	0.19142	-0.0337	0.03678	-0.1103	0.06742	-0.1213	-0.0181	0.05371
XM_579408.1	LOC497820	-0.1233	0.39505	0.25512	0.3467	-0.0861	-0.183	0.56373	-0.0949	0.18933	0.39349	0.40007	0.20055	0.12492	0.13169
XM_579537.1	LOC497821	0.23929	-0.1302	0.24131	0.08169	0.07885	0.2086	-0.1305	-0.1602	0.05281	-0.1614	-0.022	0.51393	0.37529	0.18113
XM_579625.1	LOC497822	-0.0515	0.05623	-0.032	-0.0073	-0.0899	-0.0654	0.07492	-0.0643	0.46479	-0.0665	-0.009	0.11289	-0.0327	-0.0332
XM_579623.1	LOC497823	0.04505	0.29922	0.13959	0.14904	0.11415	0.03563	0.10463	0.18676	-0.0526	0.42285	0.34697	0.2328	0.17764	0.18039
XR_009115.1	LOC497824	-0.1269	-0.0504	0.19364	-0.0174	-0.0379	-0.0513	0.02172	-0.0719	-0.1592	-0.1315	-0.1608	-0.1109	-0.1672	-0.0909
XM_579363.1	LOC497825	-0.0068	0.12483	0.31132	0.10153	0.23557	0.01234	0.16154	-0.0481	0.16864	0.1263	0.26995	0.26685	0.35266	0.30944
XM_579475.1	LOC497827	0.06445	0.15062	0.09864	0.13222	0.09417	-0.0074	0.13821	0.05726	0.18789	-0.0312	0.1366	-0.116	0.11719	0.19386
XM_579605.1	LOC497828	-0.0328	-0.0187	0.0015	0.03086	0.00359	0.08975	-0.0019	0.11957	0.12554	-0.0197	-0.1814	-0.0224	-0.1204	0.01414
XM_579744.1	LOC497830	0.13542	-0.1473	0.11247	-0.0452	-0.0029	-0.0741	-0.0388	-0.0145	0.11521	0.03142	-0.1133	0.06271	-0.0467	-0.1005
XM_579562.1	LOC497832	0.1725	0.16902	-0.11	0.28097	0.35236	0.12838	0.03558	0.48709	0.04025	0.14973	-0.0493	-0.3432	-0.3148	-0.3398
XM_579421.1	LOC497833	-0.0188	0.00669	0.03727	-0.0767	0.03783	0.01616	0.12164	0.06062	0.24338	0.0119	-0.0833	-0.0148	0.01495	0.1336
XM_579503.1	LOC497834	0.06334	-0.6031	0.59966	0.81091	-0.4035	-0.177	-0.1155	-0.5167	-0.5046	-0.6972	-0.5276	-0.3073	-0.2584	-0.3406
XM_579720.1	LOC497836	0.39203	-0.1205	0.31831	-0.0887	0.75049	0.73816	0.05936	0.62241	0.16	0.15031	-0.0006	0.21378	0.56165	0.25571
XM_579192.1	LOC497839	0.03172	0.20042	0.14616	0.08228	0.04403	-0.0404	0.0639	-0.0965	0.22138	-0.0462	0.08963	0.13507	0.02648	0.0386
XM_579384.1	LOC497841	0.04698	-0.156	-0.0935	-0.0347	-0.0848	0.13424	0.03617	-0.0636	-0.0694	-0.1767	0.04661	-0.0846	-0.0782	-0.2662
XM_343722.3	LOC497842	-0.1298	0.09389	-0.0406	-0.1062	-0.0022	0.01755	-0.0021	-0.1541	-0.1033	-0.1419	-0.0775	-0.1188	-0.0332	-0.2655

XM_579572.1	LOC497843	0.11278	-0.0662	0.07961	-0.0583	0.0235	-0.0961	0.13394	0.02615	0.02019	0.10415	0.06127	-0.0834	0.01572	0.04166
XM_579655.1	LOC497844	-0.1585	-0.0928	-0.1519	-0.1548	-0.1554	-0.0729	-0.0983	-0.1359	-0.0314	-0.0913	-0.1577	-0.1906	-0.1398	-0.2186
XM_579490.1	LOC497845	0.09058	-0.0178	-0.0586	-0.1012	-0.0109	0.00364	0.01088	-0.0752	0.1159	-0.0055	0.02723	0.05747	0.09381	-0.0649
XM_579586.1	LOC497846	0.23336	0.12809	-0.849	-0.5173	-0.1264	-0.8095	-0.1383	0.30532	-0.1049	-0.2501	-0.4855	-0.7894	0.29923	0.32138
XM_579479.1	LOC497847	0.04771	0.00061	0.01628	0.10922	-0.0331	0.06959	0.03996	-0.0569	0.08905	0.02945	-0.0363	0.02473	0.13452	0.20831
XM_573032.1	LOC497851	0.04919	-0.0083	-0.0884	-0.075	0.14135	-0.1034	-0.0194	-0.1049	-0.142	0.01069	-0.0203	0.00534	-0.0206	-0.1016
XM_573034.1	LOC497853	0.07157	0.14588	0.32109	0.81196	0.23113	-0.0338	0.57156	0.27137	0.4139	0.31111	0.38081	-0.389	-0.0269	0.08125
XM_573035.1	LOC497854	-0.259	-0.0823	-0.1594	-0.1565	-0.1653	-0.1836	-0.0309	-0.1828	-0.2144	-0.164	-0.1262	-0.2935	-0.2675	-0.254
XM_573036.1	LOC497855	0.16111	0.49535	-0.0117	0.16442	0.28911	0.10994	0.31556	0.12654	0.18472	0.12871	0.06478	0.01066	0.07959	0.12259
XM_573039.1	LOC497858	0.12346	-0.0724	-0.0553	-0.0863	0.04692	-0.0389	0.09757	0.13367	-0.1064	0.09544	-0.1202	0.02157	0.09511	0.18896
XM_573041.1	LOC497860	-0.1866	0.08213	-0.0419	-0.1223	0.00445	-0.0728	-0.1628	-0.111	-0.0908	0.01283	0.04497	-0.1322	-0.2146	-0.0336
XM_579753.1	LOC497864	-0.1671	-0.1107	-0.208	-0.5993	-0.1592	-0.1076	-0.1967	-0.2007	-0.0525	-0.1482	0.01843	-0.2587	-0.0034	-0.0661
XM_573046.1	LOC497866	0.06128	-0.1745	-0.2795	-0.1116	-0.1113	0.00133	-0.0536	-0.2462	0.00825	-0.2186	-0.1985	0.11607	-0.2451	0.01479
NM_001100782	LOC497867	0.4551	0.56045	0.42623	0.54464	0.6003	0.36378	0.71887	1.1799	0.75878	0.54822	0.68714	0.20885	0.38228	0.34113
XM_579754.1	LOC497872	0.20838	-0.0014	-0.0835	-0.1191	-0.0876	-0.0761	-0.0528	-0.2791	0.23353	0.09288	-0.0603	0.0871	-0.1038	-0.1501
NM_001109027	LOC497873	0.09798	0.18527	0.06015	-0.0227	-0.0327	0.16104	0.02204	-0.0728	0.05103	0.05009	-0.0419	-0.0292	-0.0135	-0.0447
NM_001106991	LOC497876	0.16287	0.08246	0.00118	-0.1789	0.2918	0.86146	-0.1569	0.39355	0.07938	-0.0407	0.01332	0.27313	0.05391	0.2418
NM_001034152	LOC497878	0.03151	0.48598	0.00447	0.17058	0.10311	0.17879	0.16612	-0.0231	0.11906	0.05101	0.1954	-0.0645	0.07623	0.14019
XM_579755.1	LOC497880	0.01443	-0.1783	-0.1111	-0.0394	0.03636	0.05032	0.03342	0.07227	0.11623	0.11578	0.05756	-0.0587	-0.0282	0.25103
NM_001109373	LOC497883	-0.0355	-0.1062	0.05336	-0.1099	-0.0526	-0.0556	-0.0357	-0.0933	0.06774	0.56532	-0.1351	-0.0188	0.01418	-0.0478
XM_573069.1	LOC497884	0.00212	0.07002	0.09373	0.37611	-0.0494	-0.1479	0.36846	0.20577	-0.1414	-0.1313	0.10701	0.0049	-0.086	-0.1239
XM_573072.1	LOC497887	-0.0421	-0.0795	0.02873	-0.0802	0.00118	0.06393	0.02991	-0.0521	-0.0653	0.05977	-0.0266	-0.0187	0.27414	0.07234
NM_001025031	LOC497888	0.16254	0.01651	-0.0315	0.04843	0.03175	0.11066	0.03393	0.07433	0.06522	-0.0153	-0.0024	-0.0208	0.04243	-0.0197
NM_001025031	LOC497888	-0.2699	-0.1351	-0.0194	0.00363	0.10024	0.57014	-0.2673	-0.1362	-0.0313	-0.1211	-0.0982	-0.1517	-0.0654	0.15438
XM_573074.1	LOC497889	-0.0258	0.05328	0.03984	-0.0803	0.04134	0.04817	0.14753	-0.0225	-0.0359	-0.01	-0.0247	0.0199	-0.0814	-0.0303
NM_001109029	LOC497892	0.44499	-0.2004	-0.0403	-0.2733	-0.0148	-0.3423	-0.326	-0.0849	-0.3068	0.12795	-0.1818	-0.3361	-0.2617	-0.0233
XM_573077.1	LOC497893	0.0196	0.03217	0.28521	0.04803	0.04231	0.07691	0.1304	0.22003	0.20921	0.15494	0.31073	0.04454	0.12726	0.14865
NM_001134600	LOC497896	-0.2116	-0.1738	-0.1507	-0.0501	-0.0444	-0.2471	0.00865	-0.2195	0.00754	0.26244	-0.0806	-0.0831	-0.2175	0.00281
NM_001017472	LOC497899	0.14802	0.01763	-0.0109	-0.0218	-0.0071	0.08932	-0.0804	0.16626	0.08285	0.15787	-0.0485	0.07427	0.33792	-0.0124
NM_001017472	LOC497899	0.66435	0.26724	0.31047	-0.0212	0.55508	0.7984	0.15901	0.52117	0.37603	0.29005	0.21498	0.18578	0.24595	0.55567
NM_001126081	LOC497901	-0.0342	-0.1799	-0.3502	-0.107	0.0624	-0.1562	-0.3504	-0.1886	-0.1631	-0.0772	-0.0271	-0.1482	-0.0398	-0.2226
NM_001114391	LOC497905	0.00267	-0.0992	-0.0607	0.04475	-0.1673	-0.0129	-0.1719	-0.0062	-0.0677	-0.1256	-0.0652	-0.0013	0.08764	-0.0648
XM_579756.1	LOC497908	0.0252	0.26558	0.18153	0.17427	0.11626	-0.079	-0.0402	0.15844	0.11712	0.02559	0.47555	0.03478	0.25582	0.0625
XM_573099.1	LOC497912	-0.0831	-0.3644	-0.2032	-0.2258	-0.1537	-0.1327	-0.0796	-0.1978	-0.2543	-0.3494	-0.1837	-0.2595	-0.2832	-0.3091
XM_573102.1	LOC497915	-0.1115	0.08859	-0.0352	-0.1403	-0.1122	-0.1479	0.09563	-0.1933	-0.136	-0.2012	-0.1644	-0.2163	-0.1054	-0.1802
XM_579758.1	LOC497920	0.08857	-0.5179	0.19327	0.60388	-0.5734	-0.0773	0.15254	-0.5525	-0.3528	-0.2169	-0.3852	0.26387	-0.5288	-0.5215
XM_573107.1	LOC497921	0.05896	0.00187	0.07812	0.02942	0.16862	-0.0714	-0.0225	-0.0396	-0.0548	-0.0038	-0.0052	-0.0172	0.31811	-0.0293
XM_573109.1	LOC497922	0.12452	0.01526	0.47267	-0.183	0.19333	0.01322	-0.0271	0.03549	0.07669	0.0345	-0.0112	-0.2135	0.76907	1.069
XM_573110.1	LOC497923	-0.2941	-0.1625	-0.2406	-0.0891	-0.1854	-0.2647	-0.2333	-0.1806	-0.1667	-0.2681	-0.2291	-0.291	-0.1261	-0.1836
XM_573111.1	LOC497924	-0.3065	-0.1728	-0.0399	-0.064	-0.1355	-0.1322	-0.2208	-0.221	-0.0328	-0.2582	-0.1239	-0.2918	-0.177	-0.2389
XM_579759.1	LOC497925	-0.0064	0.05365	-0.0982	0.13324	-0.0482	0.37646	-0.0945	-0.0477	-0.0821	-0.0403	-0.0481	0.01194	0.0333	-0.039
XM_573113.1	LOC497926	-0.0959	-0.0368	0.01755	-0.1239	0.10894	-0.0404	-0.1164	0.04392	-0.1195	-0.0478	-0.1817	-0.074	-0.0129	-0.1163
NM_001109033	LOC497933	-0.1304	-0.2161	-0.0667	-0.2006	-0.0457	-0.2688	-0.2021	-0.1678	-0.0887	-0.2277	-0.2519	-0.0342	0.13928	0.01078
NM_001017474	LOC497934	0.01317	-0.1678	-0.2002	-0.1666	-0.0149	-0.1538	-0.1664	0.2651	0.06415	0.20004	0.02362	0.20171	0.03859	-0.0155
NM_001017474	LOC497934	0.44091	0.32685	-0.0035	0.15913	0.50574	0.84502	0.36051	0.53753	0.13595	0.09357	0.39072	0.12318	-0.2616	0.00616
NM_001128155	LOC497936	0.00052	0.03632	-0.1093	-0.0926	-0.0132	0.05763	-0.0056	0.06645	-0.0258	0.06345	0.01278	-0.0612	0.01483	0.09213
NM_001017475	LOC497938	0.0904	-0.0453	-0.083	-0.0052	-0.0123	0.07527	0.00431	-0.0476	0.01501	0.12482	-0.0022	0.0278	-0.013	-0.0089
NM_001017475	LOC497938	-0.0371	-0.0455	0.09269	-0.1837	-0.0501	-0.1316	0.18872	0.11447	0.09399	-0.1641	-0.0888	0.06691	-0.0039	-0.0634
NM_001017476	LOC497940	-0.157	0.01605	0.09533	-0.1082	-0.0985	0.01594	-0.1157	-0.2191	-0.1124	-0.2205	-0.0473	-0.0788	-0.1434	-0.176

XM_579760.1	LOC497945	0.24951	-0.1058	-0.1632	-0.1806	-0.0811	-0.0987	-0.179	0.05947	0.03352	-0.0092	-0.04	-0.2097	-0.1391	0.24597
XM_579761.1	LOC497947	0.26611	-0.0292	0.11734	0.12513	0.49037	0.22094	0.11979	-0.0542	0.0751	0.2759	0.29087	-0.0447	0.20226	0.02539
XM_573137.1	LOC497951	-0.216	-0.1322	-0.1909	-0.1145	-0.1432	-0.2973	-0.2208	-0.1879	-0.2655	-0.0521	-0.1805	-0.2029	-0.1807	-0.0617
NM_001025758	LOC497959	-0.074	-0.136	-0.1703	-0.1981	-0.0508	-0.0657	-0.2017	-0.116	-0.1226	-0.2231	-0.191	0.02268	-0.2643	-0.1196
NM_053290	LOC497965	-0.0408	0.0766	-0.0168	0.07939	0.02021	0.34447	0.09694	0.02792	0.24252	0.08944	0.04438	0.08097	0.1416	0.29874
XM_573158.1	LOC497966	-0.1278	-0.2602	-0.1317	-0.0114	-0.0803	0.0905	-0.0791	0.05206	-0.0546	-0.1201	0.02134	-0.1875	0.13784	-0.0701
XM_579763.1	LOC497968	-0.4492	-0.2567	-0.1793	-0.3883	-0.1917	-0.1542	0.19174	-0.1671	-0.2549	-0.1747	-0.146	0.16493	-0.2746	-0.1205
XM_573163.1	LOC497971	0.19557	0.03794	0.16887	0.03459	0.20356	0.10203	0.01781	0.17386	0.12582	0.13671	0.11078	0.08613	0.11021	-0.0157
XM_573167.1	LOC497973	-0.0337	0.02722	0.20683	0.19589	0.09855	0.07434	0.09027	0.07337	-0.0073	0.11924	0.05918	0.1912	0.05306	0.04438
NM_001129777	LOC497976	0.12246	0.06049	-0.2062	-0.0511	0.02348	-0.1091	-0.0731	0.0508	0.10656	0.55879	0.36086	0.01427	0.27211	0.29308
NM_001039341	LOC497978	-0.1586	-0.1854	-0.2909	-0.0688	-0.1236	-0.1566	-0.2974	-0.1762	-0.3199	-0.2984	-0.1871	-0.2465	-0.3112	-0.2865
XM_579764.1	LOC497982	-2.9918	-0.9558	-0.7999	-1.7477	-3.2377	-3.1018	-1.1266	-3.0699	-0.6288	-1.0035	-0.382	-0.1796	-2.1259	-2.1237
NM_001080207	LOC497984	0.10685	0.00903	-0.0024	0.01382	0.00036	0.04064	-0.0398	0.02016	0.11222	0.03309	0.00939	-0.0119	0.01518	-0.005
XM_579765.1	LOC497989	0.24836	0.08919	0.22435	-0.0971	0.03971	-0.0838	0.29336	-0.0523	-0.0698	0.03895	-0.0673	-0.1102	0.20503	-0.0334
NM_001134361	LOC497991	-0.0095	-0.0009	-0.0471	-0.0969	0.09259	-0.0399	0.05102	-0.0504	0.15741	0.00594	0.10815	-0.0836	-0.0291	0.07199
NM_001134361	LOC497991	-0.422	-0.0828	-0.5832	0.08142	-0.2777	-0.7344	0.0057	-0.3357	-0.3168	-0.1397	-0.3765	-0.3304	0.2364	0.28218
XM_573189.1	LOC497992	0.1948	0.29974	0.22574	0.22496	0.29432	0.39277	0.31599	0.33641	0.41415	0.2595	0.25763	0.3613	0.16462	0.09975
NM_001025135	LOC497995	-0.2337	-0.0382	-0.0417	-0.0329	0.07982	-0.1249	-0.0361	0.12854	0.17913	-0.2575	0.27912	-0.1681	-0.3213	-0.0081
XM_573193.1	LOC497996	-0.0227	-0.1818	-0.1997	0.10126	0.01192	-0.0174	-0.0746	-0.1552	0.2119	-0.0201	-0.0149	0.14771	0.08615	-0.1192
XM_579766.1	LOC497997	0.19655	0.045	0.1477	0.07028	0.08048	0.04207	0.30724	0.08344	-0.0046	0.22769	-0.0196	0.08179	0.04876	-0.0066
XM_579767.1	LOC498007	0.18124	0.32672	0.06032	0.01447	0.02985	-0.0749	0.01045	-0.0022	0.13704	-0.0175	0.24582	-0.1779	0.07821	0.15729
NM_001025136	LOC498008	-0.6121	-0.5735	-0.2258	-0.1579	-0.6116	-0.3952	-0.0462	-0.5759	-0.3055	-0.158	-0.0242	-0.2623	-0.1042	-0.2937
XM_579768.1	LOC498009	-0.0284	0.01944	-0.1046	-0.0266	-0.0437	0.01343	-0.0227	0.05704	-0.07	-0.0476	-0.0377	-0.0191	0.13307	0.03898
XM_579769.1	LOC498010	-0.1799	-0.3613	-0.2429	-0.3404	-0.1958	-0.1953	-0.138	-0.1744	-0.0874	-0.2486	-0.2145	-0.2801	-0.2925	-0.0195
XM_573213.2	LOC498015	-0.0713	0.22467	0.29674	0.01643	-0.1423	-0.3168	0.52826	-0.1519	-0.1642	0.3597	0.10205	0.32015	0.16281	0.1244
XM_579770.1	LOC498016	0.1291	0.01655	0.08983	0.08943	0.02342	0.14054	0.09009	-0.0028	0.01481	0.0049	-0.0556	0.19464	0.02917	0.2086
XM_579771.1	LOC498017	0.00034	-0.0886	-0.026	0.23109	0.25011	0.00469	0.03509	0.15064	0.13094	0.39712	0.03171	-0.085	0.10365	-0.0015
NM_001168285	LOC498022	-0.0084	0.10767	-0.0418	0.00318	0.12673	0.00429	-0.0773	0.03284	0.00826	-0.02	0.06198	0.05132	-0.0276	0.11752
NM_057102	LOC498023	0.16492	-0.0353	-0.0553	0.11567	-0.0216	0.10012	-0.0456	-0.0931	-0.0212	0.31613	0.31433	0.02231	0.04361	0.12827
XM_573228.1	LOC498027	0.30289	0.41883	0.70323	0.59939	0.45695	0.48135	0.28109	0.41869	0.50633	0.27919	0.57154	0.58096	0.72517	0.74759
XR_005461.1	LOC498029	0.16049	0.15065	-0.0492	0.26617	0.30977	0.1229	-0.1304	0.23745	0.18767	0.18729	0.18292	0.19543	0.03372	-0.2206
XM_573243.1	LOC498041	-0.1703	-0.1255	-0.2002	-0.3229	-0.3535	-0.1017	-0.3687	-0.2426	-0.3303	-0.2877	-0.331	-0.0752	-0.1859	-0.0952
NM_024351	LOC498045	-0.3824	-0.2374	0.11176	-0.206	-0.4851	-0.5621	-0.0528	-0.525	-0.2644	-0.0825	-0.2227	-0.1514	0.06352	0.1038
XM_579773.1	LOC498046	0.15914	-0.1439	0.3238	-0.0565	0.06756	0.63307	0.0409	0.0449	0.39706	-0.1269	0.10141	0.34529	0.20578	0.00704
XM_573248.1	LOC498048	-0.9274	-0.2744	-0.4332	-0.3221	-1.7637	-1.4132	0.65333	-1.2602	-0.8235	-0.5377	-0.6234	-0.7113	-1.5958	-1.5667
XM_573249.1	LOC498049	0.0309	-0.0004	0.14142	-0.1097	-0.0118	-0.0128	-0.036	-0.1025	-0.0038	0.20202	-0.1157	-0.0824	0.02083	-0.0355
XM_573252.1	LOC498052	0.12121	0.20541	-0.027	-0.1251	-0.1379	-0.069	-0.0229	0.14092	-0.0113	0.1834	0.20565	-0.07	0.0874	0.07465
XM_573253.1	LOC498053	0.13234	0.078	0.23178	0.01347	0.11102	0.09523	0.02645	-0.0022	0.24304	0.04923	0.1683	0.34223	0.15978	0.09533
XM_573254.1	LOC498055	0.08394	0.10012	0.29152	0.17275	0.19906	-0.0015	0.12827	0.16117	0.06244	-0.0097	0.0526	0.07529	0.04115	0.17428
XM_579775.1	LOC498056	0.18653	0.1155	-0.0558	0.09779	-0.078	-0.0764	0.155	-0.0972	-0.1348	0.00186	-0.093	-0.0026	0.10602	0.02102
XM_579776.1	LOC498057	-0.2452	0.06718	-0.1001	-0.1936	-0.1975	-0.1348	-0.1694	-0.1312	-0.1873	-0.2416	-0.08	0.21853	-0.0634	-0.1965
XM_579777.1	LOC498059	-0.2158	-0.0453	-0.1977	-0.1081	-0.2848	-0.1656	-0.066	-0.0285	-0.125	0.11729	-0.1153	-0.1133	0.3179	-0.164
XM_579778.1	LOC498061	0.17255	0.16676	0.10058	0.17481	0.19866	0.21956	0.2652	0.28541	0.02478	0.25333	0.21801	0.15117	0.30261	0.23787
XM_573260.1	LOC498062	-0.2896	-0.4112	-0.4615	-0.4475	-0.5007	-0.0115	-0.4959	-0.6288	-0.5825	-0.6971	-0.6168	0.11382	-0.6499	-0.6521
NM_001017483	LOC498063	0.23667	-0.0869	-0.0073	-0.0541	0.04319	0.01855	-0.0759	0.03201	-0.1718	-0.0777	0.00165	-0.1	-0.0822	0.06699
XM_579779.1	LOC498064	-0.0507	-0.2074	-0.0457	0.01321	-0.0985	0.25449	0.05119	0.0314	-0.0252	-0.1691	0.03386	0.16946	-0.0721	-0.0951
XM_579780.1	LOC498071	0.13656	0.17252	-0.1557	-0.1632	0.1544	0.30254	-0.0127	0.1424	0.07593	-0.1351	-0.073	0.08659	0.21487	0.15948
NM_017187	LOC498072	-1.2655	-1.688	0.20303	-0.7254	-1.5726	-1.5445	-1.4045	-1.7564	-1.0707	-1.3642	-1.1843	0.04301	0.01012	0.07657
XM_573278.1	LOC498076	-0.0099	0.32855	0.7923	1.1013	0.00598	0.15516	1.3792	0.25112	0.07866	0.0531	0.11433	0.19057	-0.0714	0.25536

XM_573279.1	LOC498077	0.06926	0.02307	0.11868	0.01545	-0.1091	0.33073	-0.0369	0.01162	0.09658	0.1589	0.16397	0.01206	-0.004	0.47988
XR_008281.1	LOC498078	-0.1033	-0.2963	-0.4918	-0.1736	-0.2866	0.12611	-0.8285	-0.0262	-0.0794	-0.4379	-0.053	-0.1595	-0.7067	-0.4606
XM_579781.1	LOC498083	0.07774	-0.0332	-0.0636	0.07444	-0.0587	-0.0514	0.01861	0.13959	0.0554	0.06274	0.03203	0.01202	0.00537	-0.0611
NM_001047922	LOC498084	0.01668	0.19178	0.10575	0.11253	-0.1247	0.23574	0.02304	0.09615	0.02566	0.01256	0.13306	0.09917	0.09328	0.1728
NM_001109053	LOC498089	0.1685	0.00214	0.14541	0.01085	0.15442	0.18553	-0.0626	0.19316	0.09911	0.05978	0.04638	-0.048	0.07652	0.2187
XM_573296.1	LOC498090	0.18628	0.1255	0.36959	0.34365	0.21008	0.26988	0.41875	0.13894	0.00409	-0.0293	0.07494	0.12058	0.17427	-0.0477
XM_579782.1	LOC498092	-0.1367	-0.1959	-0.3108	-0.202	-0.2134	-0.1998	0.00985	-0.0969	-0.2656	-0.034	-0.1405	-0.1202	-0.2298	-0.0002
NM_001109054	LOC498095	0.34916	0.43023	-0.8149	-0.6585	0.1262	0.05421	-0.4532	0.2229	0.61986	0.53599	0.3412	0.28463	-0.1655	-0.4239
XM_579783.1	LOC498098	0.07242	-0.0311	0.10851	-0.0648	0.18414	0.10939	0.22411	0.28751	0.09914	0.32692	0.04645	0.22241	0.26773	0.04725
XM_579784.1	LOC498101	0.04769	0.17464	0.11259	0.1354	0.24421	0.04041	0.0315	0.08721	0.21791	0.00584	0.09349	0.0796	-0.0058	-0.0104
XM_573308.1	LOC498104	-0.2046	-0.1028	-0.1672	-0.1201	-0.1402	-0.4048	-0.0643	-0.1882	0.05547	-0.1088	-0.1364	0.0343	-0.0152	-0.2634
XM_573309.1	LOC498105	0.15228	0.31752	0.71049	1.1159	-0.0308	0.39684	1.2301	0.40678	0.08583	0.3531	0.47817	0.20162	0.13932	0.4634
XM_579786.1	LOC498108	0.13776	0.20065	0.04515	0.10543	0.18588	0.48971	0.25625	0.09904	0.1248	0.08517	0.08286	0.20744	0.12621	0.17952
NM_001109057	LOC498112	-0.2592	-0.1104	-0.0199	0.08965	-0.277	-0.2262	-0.0543	-0.1332	-0.0617	-0.1964	-0.0704	-0.0396	-0.2313	-0.2192
XM_573321.1	LOC498113	-0.0452	0.0695	-0.0345	-0.0874	0.01793	-0.0724	-0.0399	-0.0373	-0.0554	0.16194	0.0235	-0.0436	0.09553	0.05535
XM_573323.1	LOC498114	0.32888	0.17335	0.23821	0.03349	0.08349	0.03398	0.1936	0.17945	0.01992	0.35838	0.16006	-0.0405	0.23851	-0.0068
XM_573329.1	LOC498117	0.05921	-0.1018	-0.1007	-0.0187	0.11259	-0.0761	-0.0223	0.00422	-0.0141	-0.099	-0.1113	0.04717	-0.039	-0.1183
XM_579788.1	LOC498118	0.37688	0.09901	-0.0193	-0.1831	0.20743	0.44499	-0.0077	0.22397	-0.1501	0.23423	0.04622	0.09964	-0.0591	-0.0826
NM_001109058	LOC498122	0.15363	0.11789	-0.0065	0.45502	0.37537	0.47445	0.10831	0.28953	0.07942	0.34867	0.08754	0.15849	-0.08	-0.0063
XM_573334.1	LOC498123	0.44623	-0.1551	-0.1532	-0.5098	0.06877	-0.1224	-0.5875	0.17599	-0.0654	0.17092	-0.0997	-0.3404	0.06938	0.16991
XM_573335.1	LOC498124	0.19677	0.23892	-0.0461	0.04998	0.22995	0.23143	0.15355	0.2686	0.14214	0.11169	0.00268	-0.0256	0.04368	0.08796
NM_001035222	LOC498131	-0.1168	-0.2111	0.00205	-0.0007	-0.1027	-0.0375	0.026	0.00755	0.02501	-0.1789	-0.117	-0.0492	-0.2173	-0.1209
NM_001100989	LOC498136	0.17332	0.07314	-0.055	0.06651	-0.0965	-0.0357	0.02342	-0.0247	-0.0741	-0.0621	-0.266	-0.2482	-0.0962	-0.0618
XM_573349.1	LOC498138	-0.406	-0.6097	-0.3258	-0.5206	-0.448	-0.0236	-0.0911	-0.4997	-0.6625	-0.7896	-0.5571	-0.2649	-0.7042	-0.4243
XM_579789.1	LOC498139	0.0132	-0.0402	-0.0579	-0.1056	-0.0935	-0.0446	-0.1389	-0.0519	-0.1368	0.16012	0.04865	-0.0169	0.06795	0.04228
NM_001109061	LOC498140	-0.2273	-0.0481	-0.0687	0.18456	-0.0895	0.29901	0.03405	0.12015	-0.036	0.2205	0.02691	-0.0716	0.17957	0.08081
XM_573351.1	LOC498141	0.12424	0.23445	0.01365	0.19544	0.16516	0.14512	0.15998	0.05609	0.00487	0.07179	0.05699	0.28914	0.2685	0.25506
XM_573352.1	LOC498142	-0.0245	-0.0171	0.41005	0.14689	-0.0755	0.11539	0.28035	0.19659	-0.0124	0.02407	0.1387	0.34303	0.23793	0.33342
NM_001017485	LOC498145	0.13665	0.35801	-0.236	0.16523	0.27509	0.20826	0.259	0.23504	0.10372	0.13212	0.25461	0.12843	0.26584	0.25827
XM_573358.1	LOC498148	-0.1148	-0.043	-0.1288	-0.0516	-0.1213	-0.0787	-0.0782	-0.033	-0.0936	-0.1147	-0.154	-0.0917	-0.07	0.11941
XM_573362.1	LOC498151	-0.0136	0.16629	0.13561	0.00899	0.03376	0.01323	0.04512	-0.124	0.12671	0.08841	0.10636	0.08119	-0.0905	-0.1167
NM_001047923	LOC498152	0.11952	0.05441	0.22658	0.12689	0.0746	0.20232	0.16249	0.10868	0.17222	0.23246	0.31942	0.16382	0.12473	0.40131
NM_001025033	LOC498154	-0.0502	-0.4933	0.06751	-0.0968	-0.4053	-0.0852	0.11392	-0.6871	-0.4148	-0.3712	-0.2792	0.20791	-0.1287	-0.2902
NM_001047924	LOC498155	0.13314	0.17204	0.08141	0.09584	0.17953	0.04785	0.1354	0.27152	0.19581	0.24576	0.15328	0.0378	0.01606	0.34266
XM_573371.1	LOC498158	0.09205	0.11485	0.10319	0.06487	0.12317	0.12187	0.28837	0.05939	0.01993	0.36163	0.346	0.06984	0.16585	0.09961
XM_573376.1	LOC498161	-0.0267	0.15821	0.02024	-0.1326	-0.0434	-0.0448	0.02131	-0.1586	0.03035	0.25004	0.07289	-0.0329	-0.17	0.00419
XM_573378.1	LOC498163	0.2958	0.32549	0.15535	0.06728	0.29876	0.42446	0.11182	0.49707	0.22862	0.51352	0.32164	0.35067	0.32599	0.13349
XM_573379.1	LOC498164	-0.1981	-0.2316	-0.2061	-0.2395	-0.2474	-0.154	-0.1695	-0.2921	-0.2096	-0.1849	-0.1786	-0.1997	-0.225	-0.2084
XM_573383.1	LOC498168	-0.0262	-0.0935	0.10429	-0.0922	-0.0406	-0.0607	-0.1191	-0.0827	-0.0909	0.05761	-0.0641	0.18068	-0.0379	-0.0988
NM_001098793	LOC498171	-0.6651	-0.4486	-0.0777	0.52329	-0.5819	-0.3782	0.37668	-0.5925	-0.3513	-0.279	-0.431	-0.3947	-0.7008	-0.8286
XM_579790.1	LOC498175	0.153	0.03133	0.00464	0.02635	0.09992	-0.0601	0.08799	0.06183	0.27117	0.18075	0.04341	-0.0223	0.25992	0.04524
XM_573393.1	LOC498177	0.21235	0.18383	-0.0797	-0.0402	0.28423	0.49036	0.06279	0.3829	0.1893	-0.1666	0.13474	0.0008	0.24996	0.39925
XM_573396.1	LOC498180	0.17135	0.25609	0.12019	-0.0139	0.10223	0.26718	-0.1332	0.20447	-0.0577	0.03749	-0.1374	-0.0051	0.33461	0.26115
XM_573397.1	LOC498182	0.07165	0.03942	0.04268	0.08136	-0.0355	0.20439	0.06328	-0.0407	0.06809	0.05336	-0.0264	-0.0421	-0.0285	-0.0556
NM_001037361	LOC498184	0.18347	0.06137	-0.0439	0.07854	-0.132	-0.0434	-0.1017	-0.1552	-0.0657	-0.0956	-0.1066	-0.2212	0.03301	-0.1624
XM_579792.1	LOC498187	-0.0751	-0.0025	0.00239	-0.0731	-0.0289	0.07467	-0.0727	0.04986	0.11305	0.23304	-0.0495	0.11377	-0.0975	0.12579
NM_001034945	LOC498190	0.01495	0.27119	0.31141	0.14715	0.02552	0.09662	0.15622	-0.2094	-0.0971	-0.0805	0.06563	0.23659	-0.1479	0.17081
XM_573408.1	LOC498191	-0.042	-0.1616	-0.263	0.02725	-0.086	-0.1436	-0.2167	0.07174	0.07038	0.20378	0.02587	-0.2421	0.09387	0.0379
XM_579793.1	LOC498192	-0.0019	0.30581	0.72531	0.56916	0.23499	0.28473	1.0917	0.21358	0.19011	0.2059	0.41265	0.19196	0.48845	0.51373

XM_573413.1	LOC498194	-0.0659	-0.0087	-0.0798	-0.0822	-0.18	0.14262	0.10268	0.01484	0.07034	0.4644	-0.0484	-0.0546	-0.0965	0.0148
XM_579794.1	LOC498195	0.09462	0.05322	0.04139	0.19536	0.0661	0.03506	0.09531	0.05056	0.07178	0.16677	0.11652	0.06305	0.06514	0.1614
XM_001070664.1	LOC498196	-0.01	0.21262	0.28091	0.10081	0.21641	0.27158	0.06931	0.21761	0.29414	0.1982	0.18875	0.05008	-0.0212	0.24869
XM_573415.1	LOC498197	-0.4225	0.05962	0.18766	-0.0061	-0.6438	-0.8801	0.78836	-0.546	-0.1595	-0.2669	0.32729	0.00697	-0.37	-0.3949
NM_013105	LOC498198	0.01804	-0.0379	-0.1014	-0.0244	-0.1279	-0.088	-0.1943	0.12173	0.00015	-0.0321	-0.117	-0.0215	-0.0708	-0.0232
XM_573417.1	LOC498199	0.0017	0.00619	0.13958	0.03524	0.02438	-0.0207	0.04179	-0.0547	0.08328	0.05655	0.05289	-0.051	-0.0085	-0.0277
XM_573419.1	LOC498201	-0.0824	0.0777	-0.0109	0.17262	0.00308	0.23061	0.04797	-0.0478	0.1604	-0.0785	0.1658	0.29303	-0.1244	-0.0069
XM_573420.1	LOC498202	0.17429	0.11626	0.0958	0.06828	0.1665	0.18783	0.04201	0.03497	0.27714	0.33256	0.13249	0.2545	-0.0175	0.04467
XM_573421.1	LOC498203	-0.0311	-0.1039	-0.1199	0.04442	0.01501	-0.0944	0.02977	-0.0654	0.09427	-0.0795	-0.1137	-0.0617	0.00026	-0.0626
XM_573422.1	LOC498204	-0.0877	0.02747	-0.2544	0.0776	-0.1547	0.18337	-0.1288	-0.0901	-0.2092	-0.3488	-0.1147	-0.0409	-0.1427	-0.1022
XM_579796.1	LOC498208	0.0118	-0.4101	-0.3224	-0.609	-0.382	-0.7733	-0.556	-0.2969	-0.4212	-0.3295	-0.5614	-0.4909	-0.5008	-0.2901
XM_573429.1	LOC498212	-0.7835	-0.6206	-0.2423	-0.7396	-0.6049	-0.3908	-0.4022	-0.4795	-0.5594	-0.5074	-0.4792	0.40626	0.42767	0.3559
XM_573431.1	LOC498213	-0.0179	0.14238	0.15359	0.08013	-0.0793	-0.0325	0.01094	0.13085	-0.0438	-0.1184	-0.0648	0.02576	0.02134	-0.1181
XM_573433.1	LOC498215	0.03916	-0.076	-0.0408	0.05806	0.00665	0.08525	-0.0034	0.0236	-0.0489	0.05151	-0.0302	-0.0012	0.1124	-0.0109
XM_573434.1	LOC498216	-0.1687	-0.1243	-0.0489	-0.1667	-0.042	0.01748	-0.1081	-0.0371	-0.1341	-0.0572	-0.0786	0.03693	-0.1469	-0.1648
XM_573435.1	LOC498217	-0.2853	0.05771	0.02791	0.22612	-0.2054	-0.2761	0.11163	-0.1472	-0.2846	-0.061	-0.0125	-0.2859	-0.1669	-0.1376
XM_573438.1	LOC498219	-0.1288	-0.0461	-0.2095	-0.1014	-0.254	-0.2172	-0.0286	-0.1215	-0.1247	-0.0188	-0.2565	0.0109	-0.118	-0.1596
XM_573440.1	LOC498220	0.07067	0.15918	0.2321	0.06752	0.04576	0.02255	-0.1037	0.05996	0.20955	-0.1688	0.2602	-0.151	0.14795	0.17237
XM_573441.1	LOC498221	0.01566	-0.2993	-0.167	-0.1745	-0.1484	-0.0401	-0.0617	-0.289	-0.0945	-0.0641	-0.1873	-0.1481	0.17358	-0.1992
NM_001100788	LOC498222	0.01439	0.1836	0.08341	0.12618	0.08036	0.17909	0.03787	-0.0032	0.12485	0.1403	0.29401	0.12331	-0.1197	0.06444
NM_001109068	LOC498226	0.15209	0.21403	0.11756	0.21345	-0.1492	0.052	-0.1619	0.18174	0.08687	0.00641	0.06349	0.00771	0.098	0.21921
NM_001109069	LOC498228	-0.1798	-0.3712	-0.252	-0.32	-0.254	-0.1441	-0.4035	-0.2386	-0.2422	-0.2698	-0.3223	0.12554	-0.1893	-0.2189
NM_001047925	LOC498231	0.05279	0.10264	0.01488	-0.0387	-0.0763	0.23172	0.08572	0.11518	-0.169	-0.0056	0.02081	0.29249	0.00228	-0.0472
XM_579799.1	LOC498235	0.1399	-0.1004	0.085	-0.1122	-0.0908	-0.447	-0.3189	-0.5004	-0.3768	-0.3364	-0.1682	-0.4667	-0.1957	-0.1016
NM_001047926	LOC498236	-0.1064	-0.1258	-0.0786	-0.1021	-0.0793	-0.1629	-0.0783	0.03482	-0.1783	-0.1818	-0.0596	-0.0949	-0.0287	-0.1267
XM_579801.1	LOC498238	-0.0188	-0.0789	-0.0287	-0.0195	0.10731	0.07423	0.02429	0.23772	-0.021	0.11179	0.07343	-0.0117	-0.0813	-0.0749
XM_573462.1	LOC498239	0.12251	0.01237	0.05667	0.35496	0.09962	0.33177	0.28727	0.12493	-0.0008	0.24269	0.38572	0.27054	0.05248	0.1174
NM_001134792	LOC498241	0.1016	-0.0342	0.0787	0.17505	0.00899	0.0214	0.01438	0.19306	0.05384	-0.0547	0.22058	0.02592	0.05608	0.0742
XM_579802.1	LOC498243	0.06315	0.05207	0.03571	0.16708	0.05325	0.11404	0.23012	0.06967	-0.0367	0.14345	0.07397	0.04	0.03092	0.08033
XM_573468.1	LOC498245	0.27984	0.21729	0.51156	0.96523	0.11138	0.17501	1.107	0.37385	0.42248	0.0948	0.31442	0.2109	-0.0014	0.38156
XM_573469.1	LOC498246	0.18616	0.23776	0.03497	0.05196	0.19757	0.06181	0.17097	0.25542	0.08236	0.09361	0.17007	0.15592	0.05668	0.10458
NM_001134417	LOC498249	-0.2781	-0.3776	-0.2867	-0.3279	-0.3029	-0.3923	-0.3816	-0.2943	-0.2762	-0.2111	-0.3472	-0.2581	-0.3106	-0.2372
XM_579803.1	LOC498250	0.06732	0.2383	0.20531	0.90902	-0.3431	-0.2061	0.32453	-0.1755	-0.045	0.38085	0.11854	-0.0219	-0.3657	-0.3766
XM_573477.1	LOC498253	0.10621	0.05313	-0.0048	-0.072	0.05675	0.00293	0.15514	0.20767	-0.0481	0.12473	0.07326	0.02684	-0.0461	0.06245
XM_573478.1	LOC498254	-0.2508	-0.2402	-0.1097	-0.2624	-0.1912	-0.1705	-0.2507	0.10622	-0.2517	-0.2991	-0.2516	-0.1616	-0.2519	-0.2918
XM_573479.2	LOC498255	-0.287	-0.1603	-0.1461	-0.1169	-0.1618	0.00495	-0.2159	-0.2902	-0.3109	0.0682	-0.1669	-0.0989	-0.1192	-0.1311
NM_001025137	LOC498256	0.45455	0.33775	-0.3085	0.20366	0.16437	-0.5042	0.16713	-0.1519	0.33033	0.16753	0.07522	-0.3047	0.13251	0.15225
XM_573484.1	LOC498259	0.14903	0.23721	0.40083	0.03699	0.24764	0.18295	0.09749	0.09979	0.14474	0.03033	0.10671	0.46594	0.07209	0.09484
XM_573485.1	LOC498260	0.04401	0.12099	0.01412	0.16979	0.1498	0.12121	0.0724	0.09127	0.07575	0.11364	0.11936	0.24531	0.00375	-0.0036
XM_573486.1	LOC498261	-0.2807	0.207	-0.1629	0.14894	-0.1884	-0.1093	-0.0974	-0.0592	0.09971	0.12276	-0.1067	0.09737	-0.0293	0.00272
NM_001017493	LOC498265	-0.2667	-0.5228	0.47104	0.07844	-0.6439	-0.5323	-0.1695	-0.578	-0.2761	-0.4718	-0.3962	-0.1123	-0.1617	-0.1112
XM_579805.1	LOC498268	-0.137	0.00395	-0.015	0.19768	0.14334	-0.3373	-0.0069	-0.0183	-0.0441	-0.1754	-0.335	-0.0854	-0.1001	-0.1526
XM_573494.1	LOC498269	0.09913	0.08729	0.1337	0.06782	0.03052	0.0219	-0.08	0.21858	0.01939	-0.1088	0.11714	0.02604	0.05057	0.0944
XM_579806.1	LOC498271	0.21517	0.03622	0.09036	-0.1237	-0.017	-0.0921	-0.1162	0.15923	0.13079	0.01212	0.00019	0.09078	-0.147	0.08592
NM_001135992	LOC498276	-0.4667	1.3019	0.12107	0.00592	-1.3048	-0.8626	0.55937	-1.2755	1.0327	1.0958	0.8681	1.2613	-0.0285	-0.1047
XR_007542.1	LOC498277	-0.2584	1.0006	0.84564	0.21693	-0.905	-0.8842	0.79238	-1.0418	1.4178	1.2586	1.2211	1.3182	0.38755	0.47176
NM_001131001	LOC498279	-0.0441	0.23806	0.42387	0.05505	-0.1852	-0.3054	0.2742	-0.272	-0.1572	-0.0135	0.06755	0.40771	0.04689	-0.1498
XM_579807.1	LOC498280	0.09814	-0.0577	0.14108	0.05193	-0.0804	-0.0133	-0.0003	0.11572	0.01919	-0.092	-0.055	-0.1724	0.12687	-0.0306
NM_001034946	LOC498284	0.05193	0.15938	0.21379	0.09245	0.00875	0.16044	0.02293	0.03615	0.16352	0.11042	0.15482	0.05799	0.13214	0.05798

XM_573515.1	LOC498288	0.1489	-0.0209	-0.0305	0.31003	-0.0714	-0.1056	0.26066	-0.1123	-0.073	-0.1428	-0.0093	-0.0364	-0.0102	0.0693
XR_007707.1	LOC498289	0.05756	0.08043	0.22713	0.03656	-0.0489	0.23314	0.35348	-0.0205	0.34212	0.3669	0.19577	0.39885	0.42704	0.34667
NM_001109079	LOC498294	-0.0711	-0.0888	-0.2786	-0.2575	-0.1751	-0.1026	-0.1954	-0.1785	-0.0622	-0.0572	-0.1528	0.16182	-0.1476	-0.0714
NM_001025762	LOC498295	-0.1763	-0.0789	0.02388	-0.0835	-0.0301	-0.135	-0.0311	0.16201	-0.2074	0.03475	-0.1055	-0.2527	0.02862	0.02861
XM_573524.1	LOC498296	0.12661	0.19006	0.27357	0.20173	0.10584	0.42948	0.11643	0.07404	0.45442	0.08876	0.09587	0.02772	0.15538	0.21357
XM_573525.1	LOC498297	0.08515	-0.0832	0.04087	0.083	-0.0484	0.02447	0.0439	-0.0149	-0.1168	0.04251	0.04708	0.05505	0.03856	0.25648
XM_579809.1	LOC498298	-0.0923	0.02651	0.0073	-0.0845	-0.0903	-0.1678	-0.1671	-0.1472	-0.1389	-0.1379	-0.2768	-0.0379	-0.1536	-0.1759
XM_579810.1	LOC498307	-0.1593	-0.1018	0.05751	-0.002	-0.1127	-0.0352	-0.0661	-0.0736	-0.1281	0.08593	0.06868	-0.0814	-0.141	-0.1249
NM_001047927	LOC498308	-0.0899	0.0068	-0.1321	-0.2077	0.19106	-0.0732	-0.1106	-0.0909	-0.1349	-0.1711	-0.1332	-0.3296	-0.3287	-0.0612
XM_341173.2	LOC498312	-0.0685	-0.0964	0.14963	-0.14	-0.0893	-0.0992	0.02696	-0.0775	-0.0548	0.14179	-0.0008	-0.0792	0.06796	0.05244
XM_579811.1	LOC498313	0.27019	0.08812	0.29275	0.2945	0.14672	0.22811	0.0913	0.29079	0.29621	0.32634	0.19938	0.41655	0.25099	0.05335
XM_573541.1	LOC498314	-0.0958	-0.1322	-0.158	-0.0585	-0.0294	-0.1767	0.00027	-0.0254	0.11104	0.04289	0.00891	-0.008	-0.0692	-0.1059
XM_579812.1	LOC498315	0.16554	0.12165	0.13251	0.07217	0.03785	0.07693	0.07197	0.01926	0.09274	0.10027	0.05835	0.11385	0.06027	0.07075
XM_579814.1	LOC498318	-0.0594	0.04879	-0.0593	0.03036	-0.0147	-0.0422	-0.002	0.03032	0.01432	-0.0149	0.11714	-0.014	0.01945	0.07165
XM_573546.1	LOC498320	-0.1586	0.14274	-0.1244	-0.1472	-0.1479	-0.2076	-0.0127	-0.2086	-0.1334	0.0949	-0.1936	-0.1198	-0.0026	-0.0719
XM_579816.1	LOC498322	-0.1324	0.03671	-0.0404	0.07551	0.07332	0.07324	-0.1177	-0.0649	-0.0261	-0.0643	-0.0336	0.12017	0.07952	0.00079
NM_183402	LOC498325	0.06896	0.31187	0.16847	0.11473	0.16275	0.52154	0.06353	0.31695	0.27506	0.22741	0.09068	0.68966	0.17725	-0.0354
XM_573550.1	LOC498326	0.05176	0.34706	-0.6575	-0.2699	0.4159	0.31135	0.00099	0.40091	0.18607	0.24624	0.00548	-0.0256	0.34699	0.22259
NM_001014133	LOC498327	-0.0151	0.07368	-0.0321	0.11962	-0.0064	-0.012	-0.1065	-0.1119	0.02072	-0.0876	0.0839	0.22385	0.06099	0.04555
XM_579817.1	LOC498329	-0.1112	0.099	0.01977	0.49017	-0.3318	-0.1773	-0.255	-0.3007	0.4823	-0.0839	0.01968	0.79637	-0.1709	0.1019
NM_001017495	LOC498330	-0.1409	-0.1445	-0.0715	-0.0564	0.0031	0.01705	-0.094	0.03665	0.10437	-0.1408	0.01642	-0.0585	0.18295	0.11907
NM_001100789	LOC498331	0.09833	0.06978	0.0201	0.25398	0.28705	0.02316	0.21781	0.19438	0.34582	0.07761	0.18313	0.13252	0.11695	-0.049
NM_001100789	LOC498331	-0.0568	0.32966	0.07771	0.03186	-0.0623	-0.0317	0.2389	-0.0336	0.12409	0.23629	0.2986	-0.0011	-0.2066	-0.2184
XM_573562.1	LOC498336	0.01675	0.04275	0.27119	-0.0122	0.04093	-0.0138	0.22454	0.01367	0.04539	-0.0145	0.33706	0.18746	0.01645	0.00089
XM_573567.1	LOC498338	0.01234	0.17814	0.16162	0.00847	0.0308	0.11497	0.08632	0.03355	-0.0307	-0.0993	0.02677	0.10354	0.04406	0.05495
NM_001047928	LOC498339	0.00454	0.14784	-0.0507	0.08655	0.36886	-0.002	0.10224	0.0175	0.08344	0.10824	0.06391	-0.0032	-0.1351	0.23158
XM_573574.1	LOC498342	-0.0223	-0.133	-0.1869	-0.1379	-0.1108	0.14515	-0.2698	-0.2314	-0.0635	-0.2179	-0.2127	0.00473	-0.1956	0.03949
XM_573577.1	LOC498344	0.13932	0.29666	0.1215	0.09094	0.0277	0.1061	0.12486	0.12859	0.24654	0.09252	0.0293	0.18889	0.06493	0.10302
XM_573579.1	LOC498346	0.23192	-0.0258	0.57714	0.42476	0.31461	-0.3112	0.61476	0.22497	0.2381	-0.1351	0.04795	-0.0856	0.00058	-0.1246
XM_573580.1	LOC498347	0.00426	0.06997	0.09329	-0.0778	0.00317	-0.1109	0.05046	0.10084	-0.0174	0.02309	-0.0418	0.00224	-0.0403	0.01966
NM_001017498	LOC498350	-0.0653	0.10248	0.04886	0.14009	0.19174	0.22724	0.09471	0.00861	0.11915	0.17911	0.05634	0.17058	0.33102	0.22141
NM_001170601	LOC498351	-0.599	-0.6106	0.008	0.8275	-0.5878	-0.6662	0.40412	-0.7227	-0.5212	-0.6643	-0.357	-0.7751	-0.7407	-0.3888
NM_001017499	LOC498353	0.18115	0.56185	0.33429	-0.0427	0.37468	0.348	0.18437	0.11127	0.4001	0.55366	0.26842	0.59639	0.3356	0.32443
XM_573588.1	LOC498354	0.07184	-0.1328	-0.1505	-0.085	-0.1734	-0.0497	-0.2679	0.14288	0.24692	0.06043	-0.1385	0.05191	0.06387	-0.2046
XM_573590.1	LOC498355	-0.0081	0.04223	0.05268	-0.0624	0.09702	0.04058	0.15663	0.13356	-0.0533	-0.0626	0.14653	-0.0069	-0.0437	-0.0843
XM_579818.1	LOC498359	0.27174	0.19646	0.38254	0.0654	0.12031	0.36586	0.09393	0.17526	0.2161	0.08717	0.50186	0.05298	0.31972	0.02988
XM_579819.1	LOC498362	-0.8851	-0.5052	0.13707	-0.6884	-1.1173	-0.8623	-0.1612	-0.8177	-0.2419	-0.2046	-0.274	0.39255	-0.2781	-0.3117
XM_347184.2	LOC498363	-0.0476	-0.6369	-0.9124	-0.5627	-0.6008	0.2228	-0.9891	-0.1811	0.08621	-0.599	-0.1752	-0.1675	-0.3902	-0.5684
XM_573597.1	LOC498364	-0.0741	-0.0195	0.05522	0.12722	-0.0488	0.08285	0.04029	-0.0171	-0.0245	-0.1706	-0.0986	0.01888	-0.1227	0.01335
NM_001025138	LOC498365	-0.1202	-0.2634	-0.2719	-0.1571	-0.1513	-0.1769	-0.4253	-0.369	-0.3991	-0.1959	0.15081	-0.0328	-0.1803	-0.302
XM_573600.1	LOC498366	-0.1148	-0.2031	-0.2061	0.03356	-0.1746	-0.0937	-0.2085	-0.0491	0.32044	-0.2724	-0.0339	0.08113	-0.0722	-0.0165
NM_001017500	LOC498368	-1.4984	-1.5131	-1.5499	-1.4198	-1.6236	-1.593	-1.5852	-1.4947	-1.5675	-1.5404	-1.2862	-1.4728	-1.5329	-1.4699
XM_573605.1	LOC498370	-0.1556	-0.1928	-0.0941	-0.1788	-0.1082	-0.1281	-0.0673	-0.0609	-0.1116	0.00822	-0.1471	-0.1246	-0.1005	-0.1989
XM_573606.1	LOC498371	0.42299	0.25314	0.33441	0.69858	0.29728	0.05342	0.65114	0.38962	0.1509	0.40785	0.52862	0.08781	0.10181	0.23126
XM_573608.1	LOC498373	-0.0198	0.00472	0.06776	0.09627	0.10173	0.03418	0.17262	0.10692	-0.0547	0.03912	0.05932	-0.0761	0.27777	0.02923
XM_573609.2	LOC498374	0.09989	0.20819	-0.067	0.10389	0.1005	0.05967	0.23927	0.07135	-0.0516	0.02405	0.26583	0.16978	0.05538	-0.0754
XM_573611.1	LOC498376	-0.2698	-0.1299	-0.1616	-0.1307	-0.4469	-0.4604	0.1374	-0.2444	-0.396	-0.1394	-0.2059	0.08028	-0.4314	-0.4717
XM_573613.1	LOC498378	-0.2202	-0.1598	0.24469	0.51213	-0.8554	-0.7286	0.57721	-0.2757	-0.19	-0.5494	-0.1925	-0.4665	-0.4875	-0.4924
XM_573615.1	LOC498380	-0.2121	0.08841	-0.2672	-0.2089	-0.3776	-0.5212	-0.1664	-0.0941	0.15491	-0.1452	-0.1118	-0.1376	-0.3881	-0.2707

XM_573616.1	LOC498381	-0.0131	-0.0246	-0.0635	0.20541	-0.0279	0.09933	-0.0067	0.02436	0.11655	0.03884	0.19999	-0.0928	0.00861	-0.1166
XM_579820.1	LOC498382	-0.2303	-0.0014	-0.1323	-0.1728	-0.0617	0.04519	-0.1228	-0.1488	-0.1901	-0.0833	-0.0713	0.05732	-0.1817	-0.1311
XM_573636.2	LOC498396	0.0293	0.13583	-0.0054	0.05879	0.00964	0.07478	-0.048	0.07561	-0.0109	-0.0095	-0.0148	0.10957	0.19921	0.16817
XM_573636.2	LOC498396	-0.2448	-0.2828	-0.1679	-0.0104	-0.2822	-0.1983	-0.2467	-0.0313	0.01615	-0.1185	-0.1111	0.02103	-0.3351	-0.2282
NM_001017501	LOC498400	0.10245	0.18466	0.06613	0.31612	0.0545	0.09081	0.14942	0.04979	0.0419	0.13868	0.23629	-0.0225	0.09292	0.11407
XM_573643.1	LOC498401	-0.1241	-0.2522	-0.3545	-0.1831	-0.3984	-0.2917	-0.2559	-0.345	-0.3612	-0.2422	-0.377	-0.2022	-0.4531	-0.4417
NM_001017503	LOC498407	0.2689	-0.4178	-0.0102	-0.1202	0.18737	0.01381	0.07497	0.06013	-0.2082	-0.3593	-0.035	0.00679	-0.107	-0.0504
XM_573654.1	LOC498408	0.12894	0.03216	0.13609	0.17663	-0.0076	0.15497	0.13743	0.08367	0.27578	0.07632	0.15045	0.0945	0.16709	0.0496
NM_001109092	LOC498411	0.04284	-0.3084	-0.1127	-0.1632	0.06608	0.10233	-0.1134	-0.1759	-0.1322	-0.1333	-0.2363	0.20102	-0.1195	0.2604
XM_573659.2	LOC498412	0.11404	0.07886	0.06241	0.07877	0.08656	0.03361	-0.0103	-0.0222	-0.1188	-0.0566	0.1	0.00684	0.0812	0.24009
XM_573661.1	LOC498414	-0.125	-0.1099	-0.2283	-0.1566	-0.1286	-0.0098	0.03704	-0.1145	0.1555	-0.0797	-0.0384	-0.1284	-0.0041	-0.037
NM_001127559	LOC498418	-0.0466	0.42732	0.19603	-0.1817	0.00292	-0.0089	0.28424	0.03769	0.30143	0.60786	0.41103	-0.0852	0.50422	0.17505
XM_573671.1	LOC498422	0.01759	0.10573	0.01676	0.1688	0.17532	0.13559	-0.0342	0.04126	0.20125	-0.0428	0.10939	0.02014	0.01896	0.24869
NM_001024903	LOC498423	-0.3427	-0.3709	-0.2194	-0.2536	-0.3474	-0.0028	-0.4158	-0.3321	-0.2376	-0.1585	-0.1	-0.3664	-0.2588	-0.1603
XM_573673.1	LOC498424	-0.3012	-0.0469	-0.0255	0.26382	-0.2427	-0.4225	0.0898	-0.2689	-0.1994	-0.1094	-0.1635	-0.4288	-0.328	-0.2421
XM_573676.1	LOC498427	0.05817	0.12934	0.14738	0.28203	-0.1056	0.05377	0.16173	-0.1127	0.21141	0.09284	0.108	0.03892	0.03531	-0.0425
XM_573679.1	LOC498428	0.16462	-0.0733	-0.0385	-0.0161	-0.0929	-0.0041	-0.0257	0.02596	-0.0198	-0.004	-0.0334	-0.0225	-0.039	0.02376
NM_001025140	LOC498433	-0.4135	-0.255	-0.0132	0.24478	-0.0099	-0.1343	-0.246	0.14268	-0.0728	-0.3529	-0.2724	0.01847	-0.3868	-0.4783
NM_001109096	LOC498434	0.14124	0.16794	0.19861	0.24051	0.26784	0.52783	0.19138	0.39794	0.14761	0.09677	0.14838	0.38428	0.31419	0.13678
XM_573689.1	LOC498437	-0.1286	-0.1744	-0.2586	-0.1303	-0.0768	-0.1067	-0.2049	0.04282	-0.1098	-0.1788	-0.1618	-0.0527	-0.1019	0.10148
XM_344284.2	LOC498438	0.0328	-0.0263	0.04706	-0.045	0.10501	0.06376	0.05201	0.10879	-0.0031	0.0572	0.2084	0.18283	0.08567	0.04284
XM_579824.1	LOC498439	-0.0758	0.04144	0.32993	0.17078	0.05556	0.00373	-0.0326	0.15918	0.01994	0.03245	0.21472	0.14357	0.07842	0.09978
XM_573696.1	LOC498443	-0.0845	-0.1341	-0.1862	0.0129	-0.1612	-0.1503	-0.1634	-0.1839	-0.1898	-0.1642	-0.1615	-0.1785	0.05496	-0.169
NM_001011701	LOC498444	0.06929	0.18995	0.05928	0.08673	0.07842	0.16838	-0.0955	0.16058	0.30084	0.21881	0.15488	0.27361	0.0195	-0.1107
XM_573700.1	LOC498445	-0.0619	0.00126	-0.0043	0.04443	0.0017	0.06732	0.05745	-0.1072	-0.0186	-0.101	0.0498	-0.1095	0.10961	-0.1552
XM_001079520.1	LOC498446	0.02899	0.22237	0.19481	0.0146	0.08558	0.03997	0.01727	0.23653	0.10768	-0.0256	0.2747	0.03895	0.06661	0.11895
XM_573706.1	LOC498447	-0.0022	-0.1227	-0.1154	-0.0754	0.00078	-0.049	-0.131	-0.0818	-0.1562	-0.1228	0.00977	-0.0227	0.03056	7E-05
XM_573707.1	LOC498448	-0.0521	0.1591	0.00336	0.20944	0.00041	0.00079	0.10243	0.29607	0.08801	0.04811	0.05001	-0.0057	0.18045	-0.0551
XM_573708.1	LOC498449	0.10831	-0.0834	-0.2755	-0.0731	0.0345	0.01165	0.08343	0.26473	0.16176	0.0621	0.33118	0.00714	0.16201	-0.0812
XM_573709.1	LOC498450	-0.1241	-0.4129	-0.4024	-0.362	-0.3441	-0.1461	-0.3255	-0.2827	-0.2517	-0.107	-0.2412	-0.3779	-0.2545	-0.1754
XM_573710.1	LOC498451	0.00741	0.13294	0.12519	0.08454	0.34622	0.13395	0.19073	-0.0308	0.10484	0.2367	0.00376	0.00894	0.00386	0.18959
XM_573711.1	LOC498452	-0.1064	0.33471	-0.9203	-0.4992	-0.4014	-0.4877	-0.1018	-0.0216	0.20547	0.41912	0.14579	-0.166	-0.4138	-0.4612
NM_001025735	LOC498453	0.21546	0.37219	-0.6935	-0.0578	0.19969	-0.1948	0.48757	0.39018	0.34735	0.47398	0.08593	-0.3692	0.443	0.34608
NM_001025735	LOC498453	-0.4292	-0.1415	-0.4508	-0.4186	-0.6409	-0.4542	-0.1357	-0.2944	-0.1231	-0.3686	-0.3153	-0.227	-0.0782	-0.017
XM_573714.1	LOC498455	0.09509	-0.0172	-0.0112	0.00316	0.03086	-0.027	0.09472	-0.0014	-0.0164	0.01153	-0.0398	-0.0066	0.01106	0.21272
XM_573716.1	LOC498457	-0.059	-0.1644	-0.1332	-0.0053	-0.0038	-0.1628	0.03426	0.00797	-0.0496	-0.2405	0.01926	0.06975	0.27319	0.26996
XM_579825.1	LOC498458	-0.1879	-0.1456	-0.0495	-0.1692	0.02174	0.14755	-0.0369	-0.0188	-0.1334	-0.1829	-0.0773	-0.0297	-0.0954	0.00413
NM_001047931	LOC498460	0.74481	0.31962	0.06871	0.64086	1.115	1.0502	0.70416	1.1798	-0.0401	0.3996	0.83544	0.5808	0.53168	0.37519
XM_579826.1	LOC498462	0.07426	0.08927	0.01274	0.13925	0.05855	0.03191	0.1176	0.11272	0.05912	0.09154	0.1159	0.0386	0.19203	0.24557
XM_573720.2	LOC498463	0.00577	0.07051	0.13666	0.051	-0.0015	-0.0478	0.05187	0.01644	-0.0164	-0.1068	0.01607	0.15704	-0.042	0.25324
XM_573720.1	LOC498463	0.16467	0.22444	0.16815	-0.0459	0.05249	0.30798	0.15337	0.17123	-0.0228	0.37878	0.19406	-0.0185	0.10725	0.09644
XM_573720.2	LOC498463	-0.0616	-0.1075	-0.0496	-0.0531	-0.0332	-0.0256	-0.1439	-0.0811	-0.02	-0.1507	-0.1623	0.3226	-0.0171	-0.0363
XM_573720.2	LOC498463	-0.1002	-0.2338	-0.2581	-0.3671	-0.1802	-0.318	-0.3899	-0.1759	-0.4352	-0.3939	-0.1561	-0.0001	-0.2398	-0.0935
XM_573721.1	LOC498464	0.35656	0.13303	0.15768	0.11495	-0.0401	0.06909	0.07343	0.06631	-0.0186	0.22466	0.00849	0.18679	0.22239	0.12631
XM_573721.1	LOC498464	0.04238	-0.0795	-0.1013	-0.0417	0.0294	-0.0981	-0.1087	0.05829	-0.0725	0.08691	0.05667	0.16534	-0.1011	-0.04
XM_573721.1	LOC498464	-0.0119	0.01433	0.06271	0.10041	-0.0868	0.03258	0.0363	-0.0646	-0.0588	0.07877	-0.0431	-0.0207	0.127	-0.0173
XM_573723.1	LOC498466	-0.0508	0.04668	-0.0615	0.0227	-0.0578	0.07558	-0.0728	-0.0211	-0.0824	-0.0747	-0.1489	0.01835	-0.1554	-0.0512
XM_573724.1	LOC498467	-0.0138	-0.1189	-0.0576	-0.1884	0.01823	-0.0671	-0.1101	-0.0534	-0.2273	-0.0843	-0.1955	-0.1797	0.03421	-0.2149
XM_573724.1	LOC498467	0.19383	0.04892	0.06491	0.12399	0.10772	0.08552	0.03754	-0.0185	0.05232	0.0209	0.11279	-0.0639	-0.0041	0.04399

XM_573724.1	LOC498467	0.12199	0.15888	0.04974	0.10201	0.28114	0.01382	0.34263	0.22791	0.05927	0.04936	0.09368	-0.0107	0.0202	-0.0044
XM_573724.1	LOC498467	0.09485	-0.0278	0.03572	0.1223	0.00688	0.28358	-0.035	-0.0129	-0.0517	-0.0517	-0.0268	0.06094	-0.0188	-0.0878
XM_573725.1	LOC498468	0.19707	0.18675	0.21724	0.03606	0.08532	0.04342	0.09162	0.05661	0.00029	0.01553	0.05525	0.03863	0.06428	0.51493
XM_579827.1	LOC498469	-0.3643	0.08035	0.6497	0.23212	-0.3738	0.05088	0.24045	-0.3156	-0.0264	0.28028	0.17496	0.30775	0.31969	0.42052
XM_001062043.1	LOC498470	-0.0544	-0.053	-0.12	-0.149	-0.0346	-0.0968	-0.0128	-0.1056	0.05348	-0.0355	-0.0541	-0.051	0.04137	-0.0038
XM_001062043.1	LOC498470	0.3682	0.20486	0.2367	0.04219	0.2404	0.05026	0.19232	-0.0037	0.06662	0.19218	0.07202	-0.0162	-0.0047	0.19923
XM_001062043.1	LOC498470	-0.1647	-0.0223	-0.0644	-0.014	0.04323	0.16339	-0.0599	0.12129	-0.2088	0.03008	-0.096	0.08953	-0.1914	0.01491
XM_573730.1	LOC498473	0.06509	0.09729	0.08072	0.09525	0.11061	0.09727	0.09233	0.02686	0.18471	0.0318	0.15221	0.28029	0.23835	0.10059
XM_573731.2	LOC498474	0.22744	0.01047	0.06113	0.01362	0.04535	0.11709	-0.0173	0.02163	0.07754	0.19357	0.04859	0.30478	0.11609	0.10605
XM_573733.1	LOC498476	0.23952	-0.0455	-0.0697	-0.0253	0.10259	0.1747	-0.0599	0.30256	0.30144	0.05503	-0.0582	-0.0391	0.22149	0.10116
XM_573735.1	LOC498477	-0.0724	0.20316	0.03362	-0.083	0.13394	0.11762	0.10959	0.04716	0.0603	0.05108	0.30154	0.03589	0.04657	-0.0776
XM_573740.1	LOC498482	0.04885	0.05502	0.02242	0.09316	-0.0144	-0.0098	0.00045	0.13371	0.06206	0.28514	-0.0447	-0.0214	0.02795	0.0441
XM_573741.1	LOC498483	-0.0302	0.10786	0.31248	-0.0504	0.02744	-0.0211	-0.0742	0.12557	0.04577	-0.0875	0.07138	-0.0503	-0.0189	0.03959
XM_573755.1	LOC498494	-0.251	-0.2881	-0.3552	-0.2018	0.01968	-0.2668	-0.1425	-0.2102	-0.2424	-0.2496	-0.1802	-0.1863	-0.3918	-0.152
XM_573758.1	LOC498496	0.08829	0.23139	0.08983	0.15286	0.58195	0.38183	0.28417	0.84905	0.5413	0.35844	0.21988	0.14658	0.10669	0.35126
XM_579829.1	LOC498498	-0.0034	-0.0007	0.08806	0.00885	-0.1139	0.09512	-0.0462	-0.1126	0.09578	-0.1203	0.0902	0.16358	0.20398	-0.0709
XM_573764.1	LOC498500	0.07684	0.1417	0.05797	0.10218	0.05175	0.06655	0.0425	0.00898	-0.0812	0.18298	0.19016	0.08377	-0.0928	0.21952
XM_573765.1	LOC498501	-0.0475	-0.0336	0.04925	-0.1956	-0.0874	-0.0958	-0.0695	-0.1899	-0.0447	-0.1401	-0.0822	0.13227	-0.1097	-0.0132
XM_573766.1	LOC498502	0.12327	0.15178	0.16659	0.0076	-0.0439	0.20606	0.31461	0.06009	0.00287	0.01383	0.16212	0.06661	0.2732	0.07703
XM_573767.1	LOC498503	0.08458	-0.1328	-0.0732	0.15169	0.1299	0.08214	0.03167	0.06061	-0.0446	-0.0506	-0.1009	-0.0883	0.19828	0.00377
XM_573768.1	LOC498504	-0.0708	-0.071	-0.0407	0.04581	-0.0748	-0.0189	-0.0691	0.05785	0.03984	0.12193	-0.0508	0.26378	0.01639	-0.032
XM_224065.2	LOC498505	-0.0135	-0.0028	0.14266	0.04066	0.01633	0.01939	-0.0206	0.14293	0.46943	0.03345	0.09001	-0.0836	0.23465	0.0093
XM_573769.1	LOC498506	-0.0093	-0.0043	-0.2194	-0.0684	-0.1019	0.28933	0.19997	0.02054	0.16879	-0.0924	0.12475	0.22781	-0.0467	0.06999
XM_344363.2	LOC498507	0.15815	0.26498	0.19034	0.02735	0.03897	0.15214	0.21934	0.10011	0.25787	0.16514	0.13917	0.14584	0.05257	0.23232
XM_344363.2	LOC498507	-0.0112	-0.0635	0.08093	0.09798	0.09223	0.02027	0.0866	0.08325	0.03641	0.32935	-0.0325	0.01464	0.02093	0.00695
XM_224098.2	LOC498508	-0.1255	-0.0596	-0.1432	-0.1638	-0.1301	-0.0348	-0.0792	-0.2287	-0.191	-0.2069	-0.174	-0.0814	-0.2504	0.0152
XM_573770.1	LOC498509	0.13081	0.12192	0.25172	0.29332	0.18538	0.02088	0.06042	0.06338	-0.0536	-0.0734	0.22539	-0.0455	0.05752	-0.0231
XM_344397.2	LOC498510	0.07358	-0.07	-0.1493	0.07996	0.0221	-0.1204	0.0038	-0.0918	0.01213	0.06167	-0.0087	0.08048	0.03509	-0.1791
XM_573772.1	LOC498512	-0.3781	-0.2409	-0.0766	-0.2135	-0.2762	-0.1772	-0.1226	-0.3414	-0.4128	-0.3322	-0.2347	-0.1874	-0.2259	-0.2208
XM_579830.1	LOC498513	0.03007	0.18719	0.10792	0.2398	0.23677	0.2369	0.10719	0.03423	0.03411	0.00529	0.03153	0.07672	0.2805	0.08454
XM_579831.1	LOC498516	-0.1446	-0.1649	-0.2064	-0.1695	-0.3634	-0.1614	-0.0524	-0.4388	-0.2594	-0.3483	-0.2291	-0.2068	-0.1534	0.04826
XM_573790.1	LOC498521	-0.0591	0.21088	0.04591	-0.0202	0.08769	0.16408	0.02545	0.0569	0.02132	-0.0457	0.10941	-0.0903	-0.0565	0.08975
XM_573793.1	LOC498524	-0.0646	-0.0299	-0.1679	-0.0801	-0.0344	-0.0565	0.00251	0.05211	-0.1041	-0.0972	-0.0773	0.04368	0.00633	0.07363
XM_573794.1	LOC498525	0.07279	0.36948	0.18129	-0.2289	0.24133	0.6706	0.14499	0.27032	0.47886	0.57517	0.32386	0.7283	0.04362	0.03673
XM_573795.1	LOC498526	0.06554	0.10753	-0.0764	0.17523	-0.0687	0.11487	-0.0682	0.00245	-0.012	0.44607	0.10955	0.04351	-0.0609	0.3221
XM_579832.1	LOC498527	0.05425	-0.0456	-0.061	-0.0045	-0.2261	-0.1035	0.01239	-0.0766	-0.0034	-0.1151	-0.1003	0.10834	-0.107	-0.0276
XM_573802.1	LOC498532	0.09015	0.01372	-0.1494	0.09079	-0.0652	0.22203	0.10158	0.01139	-0.1044	0.05707	0.00606	0.03359	0.23623	-0.0228
NM_001109102	LOC498533	-0.1933	0.07431	-0.0963	-0.0241	-0.1382	-0.004	-0.0033	0.07479	-0.1443	-0.0972	-0.1275	-0.0803	-0.1068	0.10308
XM_579834.1	LOC498535	-0.1904	-0.1845	-0.1083	-0.1818	-0.2824	-0.0833	-0.2199	-0.2442	-0.3631	-0.2897	-0.1871	-0.2087	-0.2834	-0.3183
XM_573807.1	LOC498536	-0.3131	-0.3391	-0.2521	-0.3253	-0.3	-0.3187	-0.2756	-0.1947	-0.3198	-0.3342	-0.2435	-0.2853	-0.1877	-0.0408
XM_573813.1	LOC498542	0.11338	0.00509	0.0137	0.0399	0.11094	-0.072	0.09466	-0.0656	0.00808	-0.0344	0.16645	0.03287	0.06657	0.09732
XM_573818.1	LOC498544	0.39279	0.12312	0.58347	1.205	0.63735	0.69511	0.84462	0.75953	0.45357	0.26058	0.37447	0.48532	0.21218	0.59387
NM_001109912	LOC498545	-0.1984	0.0437	-0.063	-0.1356	0.03359	-0.0372	-0.0772	-0.0053	-0.0967	-0.066	-0.136	-0.0799	0.00019	-0.0776
NM_001109912	LOC498545	0.04922	-0.1346	-0.0338	-0.1342	-0.0758	-0.1455	-0.1456	-0.0545	-0.0882	0.13505	-0.0888	-0.0643	-0.1067	-0.1199
NM_001044257	LOC498547	0.16411	0.17775	0.08902	0.03347	0.12036	0.14037	-0.0497	0.08173	0.1458	0.0472	0.06555	0.04045	0.1375	0.08159
XM_573822.1	LOC498548	-0.1892	0.10449	-0.0593	-0.0903	-0.1285	-0.0615	0.11382	-0.0191	-0.0921	0.00604	-0.0989	-0.1444	-0.16	-0.1256
NM_012773	LOC498549	-0.0674	-0.0149	-0.0242	-0.0117	0.27714	-0.0928	0.0864	-0.098	0.03435	0.17475	0.07632	-0.1786	-0.0853	0.01097
XM_573826.1	LOC498551	0.17013	0.08482	0.10019	0.05825	0.32476	0.1595	0.22525	0.0795	0.19519	0.16673	0.17066	0.17855	0.21355	0.11089
XM_573828.1	LOC498552	-0.289	-0.1544	0.03964	0.57355	-0.4388	0.31744	0.69044	-0.0354	-0.2368	-0.1357	-0.165	0.22785	-0.2623	-0.1622

XM_573829.1	LOC498553	-0.173	-0.1993	-0.1236	-0.1747	0.04009	0.03851	0.11668	-0.2168	-0.0863	-0.0961	0.03432	0.16766	-0.1256	0.08821
XM_573830.1	LOC498554	-0.0285	0.21935	0.1422	0.0223	-0.0301	-0.0329	-0.0573	-0.0729	0.08515	0.06278	-0.1251	0.13145	-0.0409	-0.1002
NM_001030021	LOC498555	0.04345	-0.2859	-0.1306	-0.3606	-0.1779	-0.0942	-0.3483	-0.2295	-0.1368	0.11065	-0.0756	-0.1773	-0.3649	-0.202
XM_573833.1	LOC498557	0.34158	0.48205	-0.2784	0.36837	0.29931	-0.0989	0.02253	0.56048	0.43999	0.4366	0.18677	-0.4136	0.20441	0.30215
XM_579835.1	LOC498558	0.19894	-0.1079	0.02852	-0.0944	-0.165	-0.0916	-0.0123	-0.0983	-0.0954	-0.0961	-0.0392	0.09383	0.09234	-0.2195
NM_001109105	LOC498559	0.05838	-0.0386	0.10503	0.02578	-0.3141	-0.2249	-0.0691	-0.0633	0.02504	0.08284	-0.0045	-0.0691	-0.0874	-0.0389
XM_573836.1	LOC498561	0.06522	-0.03	-0.0676	-0.0806	-0.0942	0.04103	-0.1902	-0.1235	0.03628	-0.223	-0.1454	0.07531	-0.1331	0.1534
XM_573837.1	LOC498562	-0.0023	-0.0876	-0.0678	0.00879	0.20978	-0.2442	-0.1194	0.09838	-0.0242	-0.1808	0.10556	-0.1292	-0.0937	0.03598
XM_573838.1	LOC498563	0.04241	0.01025	-0.0007	-0.0126	-0.0467	-0.0124	0.04663	0.08094	0.12117	0.18524	-0.0169	-0.0328	0.04875	0.0089
NM_001011701	LOC498565	0.0793	-0.0453	-0.0146	0.1473	0.05337	0.06116	0.10363	0.06628	-0.0094	0.22933	0.07737	0.1078	-0.0224	0.05855
XM_229664.3	LOC498566	-0.3161	-0.1561	-0.3108	-0.1989	-0.2014	-0.0585	-0.2472	-0.2577	-0.2152	-0.2113	-0.1984	-0.1847	-0.2894	-0.1879
XM_573842.1	LOC498567	0.06583	-0.0337	0.05365	0.05172	0.07281	0.02424	-0.0262	-0.0114	-0.0839	0.16	0.15243	0.20921	-0.0113	0.03616
XM_573843.1	LOC498568	0.12852	0.03757	-0.0808	-0.0265	-0.038	0.12489	0.1409	0.12044	0.049	0.02878	0.00113	0.00961	0.17851	0.04891
XM_573844.1	LOC498570	0.15394	0.04337	-0.005	-0.0117	0.00594	0.21699	0.18118	-0.0052	0.05989	-0.0275	-0.025	0.20178	0.12404	-0.0454
XM_573847.2	LOC498573	0.06365	-0.1132	0.29454	0.18152	0.04114	-0.0932	0.00787	-0.0766	0.20137	-0.1495	-0.0392	0.32591	0.25256	0.18995
XM_573848.1	LOC498574	0.01476	-0.0091	0.29054	0.01528	0.04012	0.10161	0.12143	0.07864	0.14397	0.01737	0.07006	0.05579	-9E-05	0.31724
XM_573854.1	LOC498578	-0.0688	0.10966	0.42098	0.17674	0.24781	0.03384	0.05236	0.16509	0.1845	0.17699	0.12359	0.15627	0.23068	-0.0204
XM_573857.1	LOC498581	0.17771	0.27305	-0.0406	0.23899	0.04665	0.05129	0.09947	0.25721	0.06395	0.12903	0.24399	0.04857	0.05709	0.08003
XM_573859.1	LOC498583	-0.0917	-0.0487	0.07395	-0.0991	-0.1259	0.10522	-0.0141	-0.0082	-0.0159	-0.1929	-0.2667	0.05464	-0.0776	-0.0913
XM_573860.1	LOC498585	-0.022	0.04761	-0.1566	-0.1866	0.00958	0.10811	-0.2147	-0.1482	-0.089	-0.309	-0.0891	-0.0393	-0.2706	-0.2164
NM_001166307	LOC498592	0.16016	-0.0853	0.03806	-0.1407	0.04773	-0.0557	-0.1518	0.07458	-0.0879	-0.2117	-0.1213	-0.1927	-0.1122	-0.0242
XM_573870.1	LOC498593	0.23932	0.24383	0.22547	0.0977	0.10161	0.08407	0.24709	-0.0307	0.3848	0.14544	0.05421	0.20588	0.08913	0.07001
XM_579837.1	LOC498603	0.08469	0.10419	0.06467	0.0329	0.0613	-0.0525	0.01387	-0.0257	0.18432	0.01335	-0.0024	0.01404	0.0825	-0.0359
NM_001100908	LOC498604	0.19989	0.06	0.06077	0.14777	0.18775	-0.0033	0.07523	0.01135	0.10796	0.20571	-0.0022	0.01642	0.04744	-0.003
NM_001025143	LOC498606	0.09894	0.0225	0.16398	-0.2592	-0.222	-0.1796	-0.2264	-0.2138	-0.0702	0.03081	0.05315	0.0707	-0.2473	-0.0682
XM_573889.1	LOC498611	-0.1051	-0.0102	0.14813	0.27297	-0.0343	0.0267	0.60416	-0.257	-0.0747	-0.2365	0.19859	-0.4294	-0.3312	-0.0253
XM_579838.1	LOC498615	0.01219	-0.1009	-0.0508	-0.0507	-0.0855	0.08682	-0.169	-0.0265	0.03688	-0.056	-0.0699	-0.1335	-0.2051	-0.1149
XM_573895.1	LOC498617	0.1582	0.06027	0.08912	-0.037	-0.0778	0.03481	0.07846	-0.0208	0.23612	0.11545	-0.0156	0.01733	0.09389	0.07793
XM_573898.1	LOC498620	-0.0258	-0.0996	0.10047	-0.0486	-0.0623	-0.047	-0.0309	-0.0331	0.02322	0.1616	-0.0239	0.10452	-0.0698	-0.0973
XM_001071367.1	LOC498621	-0.3324	-0.1576	-0.4389	-0.2374	-0.2191	-0.1511	-0.3594	-0.35	-0.3417	-0.3715	-0.4799	-0.1669	-0.2644	-0.1876
XM_573902.1	LOC498623	-0.6711	-0.1594	-0.2448	0.12994	-0.7175	-0.1638	0.72631	-0.488	-0.1788	-0.0599	-0.3052	-0.0127	-0.0576	-0.1245
XM_573905.1	LOC498625	0.22182	0.04593	-0.0141	0.22883	0.29118	0.2268	0.12652	0.11355	0.41192	0.08243	-0.0595	0.24158	0.12206	0.16787
XM_573910.1	LOC498629	-0.0393	-0.0451	0.0657	-0.012	-0.0686	-0.0292	0.13599	0.25079	-0.0149	0.007	0.07739	0.05266	0.00355	0.05148
XM_579840.1	LOC498631	-0.4027	-0.3312	-0.2373	-0.4395	-0.4619	-0.2115	0.08382	-0.5007	-0.2727	-0.3299	-0.2716	-0.2197	-0.435	-0.2859
XM_579841.1	LOC498632	0.15317	0.13806	-0.0995	-0.0353	-0.0171	-0.196	-0.1589	-0.0416	0.01895	-0.1034	0.03841	-0.0755	-0.1374	-0.0995
XM_573917.1	LOC498636	-0.0143	0.09404	0.05912	-0.0328	0.07081	0.04752	-0.0077	0.09709	0.07912	0.13362	-0.0275	0.12213	0.03286	-0.0736
XM_573918.1	LOC498637	-0.0403	-0.1085	-0.0396	-0.2101	-0.1979	-0.2728	0.08698	-0.3166	0.05494	-0.3692	-0.3788	-0.146	-0.3077	-0.0539
XM_579842.1	LOC498641	0.19633	0.07716	0.01719	0.13329	0.11331	-0.0081	-0.0742	0.17369	0.51012	0.07483	0.03531	0.19535	0.16754	-0.0032
XM_573926.1	LOC498644	-0.4898	0.14318	-1.3922	-0.8187	-1.0462	-1.2907	-0.0938	-0.7685	-0.3284	0.32567	-0.3459	-0.6834	-1.1516	-1.0058
XM_573927.1	LOC498645	0.06694	0.15393	0.30217	0.16882	-0.1678	-0.1487	0.10803	0.25063	0.11517	0.19627	-0.0159	-0.1404	0.10717	0.13055
XM_573928.1	LOC498646	-0.0154	0.20322	0.30494	0.15818	0.02651	0.02568	0.19378	-0.0233	0.36014	0.09285	0.18609	0.09671	-0.0214	-0.0074
XM_573930.1	LOC498648	-0.0894	-0.1145	-0.0647	-0.1279	-0.0312	0.0234	-0.0256	-0.0532	0.40878	0.02363	-0.0785	0.28752	-0.072	-0.0432
XM_573931.1	LOC498649	-0.0867	0.0547	-0.1881	0.11185	0.05727	0.07856	0.04763	-0.0144	0.07628	-0.0131	0.02454	0.11781	0.17318	0.06119
XM_579843.1	LOC498652	-0.239	-0.1358	-0.2128	-0.1776	-0.0321	-0.1885	-0.134	-0.2431	-0.0322	-0.1276	-0.2565	-0.1563	-0.1851	-0.2035
NM_020303	LOC498654	-0.0555	-0.0388	-0.1808	-0.135	-0.2155	-0.1579	-0.0606	0.09868	-0.2286	-0.0814	-0.2205	-0.0338	-0.1694	-0.0341
XM_573937.1	LOC498655	-0.2057	-0.1563	0.0519	-0.1549	-0.0428	-0.0759	-0.1023	0.01734	-0.0455	0.02134	0.1611	-0.0564	-0.0513	-0.1315
XM_579844.1	LOC498656	-0.1226	0.26458	-0.1122	-0.1049	-0.1042	0.01702	-0.1125	-0.0759	-0.0853	-0.2058	-0.0532	-0.0647	-0.0041	-0.1399
NM_001025763	LOC498658	0.1226	-0.0279	0.11071	0.03127	0.20213	-0.0324	-0.0584	0.07539	-0.0006	0.14392	0.04596	0.05054	0.02593	0.00573
NM_001100791	LOC498662	0.58138	0.86583	0.50586	0.7836	0.04616	-0.1112	0.10524	0.25412	0.84461	0.89076	0.81304	0.21716	0.22822	0.43486

XM_573944.1	LOC498663	-0.0397	-0.0009	-0.0073	0.10999	-0.1165	-0.0538	-0.0033	0.00124	-0.0502	0.07092	-0.0238	0.02298	-0.0621	-0.0458
NM_001017508	LOC498664	-0.2609	-0.0097	-0.0777	-0.2033	0.08339	-0.0068	-0.0926	-0.1727	-0.0789	-0.1025	-0.1782	-0.2325	-0.2049	0.00184
XM_579846.1	LOC498665	0.1372	0.20953	0.06885	0.07683	0.05794	0.25625	-0.0036	0.15631	0.08404	-0.0353	0.02888	0.03936	0.10518	0.02305
XM_573952.1	LOC498669	-0.1966	-0.1653	0.39374	0.39365	-0.642	-0.2838	0.72821	-0.2353	-0.0985	-0.3826	0.22163	-0.1827	-0.6329	-0.1902
XM_573953.1	LOC498670	0.07965	-0.1128	0.02548	0.28774	-0.1034	0.10547	0.03035	-0.043	-0.1732	-0.1144	-0.1379	-0.222	-0.0077	-0.2533
XM_573954.1	LOC498671	0.05572	-0.0865	0.0972	-0.038	0.01253	0.0967	0.03584	0.12061	-0.0298	-0.0937	0.21641	0.19365	0.14817	-0.0421
XM_573955.2	LOC498672	0.06271	0.1057	0.27446	0.05451	0.13958	0.24083	0.19269	0.15013	0.08492	0.11662	0.01835	0.20328	0.14739	0.1711
XM_573956.1	LOC498673	0.09516	0.47641	0.17084	0.06536	0.22837	0.14185	0.13861	0.10549	-0.0339	0.02192	4E-05	0.08429	0.10204	0.08588
XM_579847.1	LOC498674	-0.2055	-0.1763	-0.4493	0.32746	-0.2583	-0.0502	-0.0621	-0.2308	-0.1582	0.01084	-0.072	0.0866	-0.4614	-0.5018
NM_001109113	LOC498675	-0.2423	-0.2997	0.21058	-0.2464	-0.1214	-0.2453	-0.0497	-0.2935	-0.1915	-0.0977	-0.1608	0.05283	-0.2688	-0.0551
XM_573958.1	LOC498676	0.01138	0.13408	0.07248	0.0279	0.09459	0.05916	0.05581	-0.0268	-0.0803	-0.0574	0.03557	0.02083	0.01616	-0.0184
XM_579848.1	LOC498679	0.23393	-0.0262	-0.0244	-0.0241	-0.0476	0.03986	-0.1426	-0.0361	-0.0583	-0.0349	-0.0307	0.0521	-0.0519	0.12396
NM_001109063	LOC498683	0.07378	0.28233	0.16272	-0.023	0.00966	0.39393	0.12691	0.10182	-0.0479	-0.0995	-0.1664	-0.0179	0.22572	0.16168
XM_573972.1	LOC498687	0.6921	0.29863	-0.1019	1.9101	1.3385	1.4295	0.3208	1.3804	0.56017	0.51393	0.38852	0.22795	-0.024	-0.3594
XM_573976.1	LOC498691	-0.049	-0.0347	0.12863	-0.0617	-0.0628	-0.0123	0.02617	0.14789	0.13182	0.02441	0.04617	0.04394	0.08134	-0.0877
NM_001134606	LOC498693	-0.0601	-0.0105	-0.11	-0.2008	-0.1032	-0.1224	-0.1395	-0.1528	-0.116	-0.1912	-0.1796	-0.1731	-0.0195	-0.1892
XM_579851.1	LOC498694	-0.1485	-0.1442	-0.012	-0.1489	0.04478	-0.1913	-0.0405	-0.0411	-0.1795	-0.2209	-0.0855	-0.1239	0.06521	0.01214
NM_001037362	LOC498695	0.17447	0.06113	-0.0526	-0.1936	0.16731	0.37075	0.06275	-0.039	0.10534	-0.1267	0.1273	-0.1744	0.26825	0.06019
XM_573981.1	LOC498696	-0.324	-0.2297	-0.1827	-0.9562	-0.6169	-0.4598	-0.6401	-0.5719	-0.0058	-0.2842	-0.3537	0.32882	0.18217	0.16979
NM_001113788	LOC498699	0.03195	-0.1271	0.03293	-0.1022	-0.0462	0.04825	-0.141	-0.0369	-0.2006	-0.0307	-0.0284	0.39717	-0.0811	0.10465
XM_579854.1	LOC498700	0.08935	0.08009	0.26706	0.13936	-0.1991	0.00821	-0.0715	0.23231	0.22842	0.00811	-0.1401	0.20045	-0.0671	0.03593
NM_001047934	LOC498705	0.0689	-0.0067	0.24518	0.21943	0.33016	0.22283	0.15174	0.30656	0.21062	0.12915	0.1711	0.37422	0.12271	0.06486
XM_573991.1	LOC498708	0.2155	-0.049	0.0428	0.01095	0.0776	0.00868	0.14278	0.18308	0.05162	-0.1192	0.05265	-0.0177	0.36893	0.2549
XM_579857.1	LOC498710	-0.027	-0.1255	-0.0516	-0.1029	-0.168	-0.1287	-0.2557	-0.0969	0.26814	-0.1089	-0.0777	0.05706	-0.2017	-0.1788
XM_579859.1	LOC498714	-0.1823	-0.3652	0.29136	0.29013	-0.2712	-0.1362	0.12934	-0.2366	-0.0194	-0.1524	0.01926	-0.1219	-0.2575	-0.2834
XM_579860.1	LOC498715	0.00378	0.09031	-0.1603	-0.1933	-0.176	-0.2904	-0.0501	-0.1887	-0.0703	0.04298	-0.0435	-0.0608	-0.2279	-0.1432
XM_579861.1	LOC498717	-0.0115	-0.0194	-0.0299	0.09128	-0.1189	0.09163	0.09068	0.00666	-0.0747	-0.0334	0.00584	0.27784	-0.0983	0.03556
XM_579863.1	LOC498720	0.21382	0.15846	0.29897	0.13319	0.22611	0.19769	0.08113	0.18256	0.18971	0.19882	0.1266	0.11602	-0.0268	0.23183
XM_579864.1	LOC498721	-0.0105	0.19742	0.14224	-0.0028	0.0355	0.04489	-0.0225	0.08686	0.09163	0.07571	0.0662	0.1962	0.24946	0.05212
XM_579865.1	LOC498722	0.05776	0.13524	0.00958	0.13767	-0.003	0.1909	0.47404	0.32543	0.02612	0.19136	0.04632	0.17023	0.10989	0.15312
XM_579867.1	LOC498724	-0.0538	-0.0252	0.23695	0.17704	0.01697	-0.0517	0.0849	0.00812	-0.05	0.32736	-0.0304	-0.0246	0.2362	0.15727
XM_579868.1	LOC498725	0.12692	0.06991	-0.0432	0.07778	0.22094	0.05758	0.08704	0.02687	-0.0071	0.13289	-0.0293	0.05461	0.1345	0.15398
XM_574005.1	LOC498730	0.00155	-0.0051	-0.0808	-0.1415	-0.0644	0.00092	-0.0275	-0.1041	-0.0475	0.18238	-0.072	-0.1013	0.03538	-0.0278
XM_574009.1	LOC498733	0.2427	-0.2084	-0.7367	-1.0783	0.16558	-0.1244	-1.0177	0.11972	0.10587	-0.0056	-0.2542	-0.0628	0.1939	0.34689
XM_574010.1	LOC498734	0.09704	0.0431	-0.1711	0.14867	0.0069	0.23769	-0.0281	-0.0041	-0.0448	0.0664	-0.1283	0.02102	0.03751	0.14256
XM_574011.2	LOC498735	0.30082	0.18254	0.30462	0.19866	0.15035	0.33031	0.27109	0.25983	0.25331	0.17134	0.19496	0.28519	0.05527	0.25138
NM_001109119	LOC498736	-0.3152	-0.8751	0.20143	-0.6578	-0.7943	-0.568	-1.4141	-0.7269	-0.3296	-0.6748	-0.9008	0.43922	0.11471	0.20677
XM_574014.1	LOC498737	0.38971	0.06719	-0.0409	-0.0136	0.0815	-0.0554	-0.1531	0.09956	-0.0317	0.24366	0.07321	0.19204	-0.0329	-0.0132
XM_574015.1	LOC498738	0.1114	-0.0006	0.13137	-0.0506	0.03451	0.0891	0.30408	0.02902	0.06705	0.02334	-0.0406	0.24689	-0.0892	0.06115
XM_574017.1	LOC498739	-0.0068	0.05767	0.05958	0.25014	0.26045	0.01631	-0.0407	-0.0181	0.13397	0.028	0.09539	0.25352	-0.0689	-0.0678
XM_574018.1	LOC498740	0.2143	0.19603	0.1069	0.21286	0.2152	0.16637	0.27289	0.17055	0.32371	0.14357	0.05297	0.4231	0.42054	0.09026
XM_574020.1	LOC498742	-0.3211	-0.3144	-0.205	-0.2618	-0.2719	-0.2611	-0.4264	-0.4115	-0.4685	-0.3713	-0.2583	-0.2713	-0.2977	-0.306
XM_574023.1	LOC498745	-0.0606	-0.0535	0.52357	0.81944	-0.6516	0.13024	1.1453	-0.1785	0.01471	-0.2322	0.10459	0.11605	-0.2621	-0.1961
NM_001017510	LOC498750	-0.4644	-0.0133	-0.2206	-0.931	-0.82	-1.3788	-0.0082	-0.8415	-0.3988	-0.3073	-0.1687	-0.8318	-0.6215	-0.5607
NM_001024282	LOC498753	-0.0855	-0.1406	0.19063	-0.0311	-0.1322	-0.0378	-0.0051	-0.0854	-0.1697	-0.003	-0.1901	0.00164	-0.1993	0.16291
XR_009616.1	LOC498759	-1.8245	-1.3748	0.33405	-0.3262	-1.3989	-1.9432	0.67461	-1.2403	-1.6839	-1.1839	-1.4448	-1.7908	-1.0948	-1.094
XM_574045.1	LOC498760	-0.1262	-0.1657	-0.1969	-0.1713	-0.2226	0.14639	-0.1902	-0.0879	-0.1227	0.00228	-0.1003	-0.0543	-0.1444	-0.0681
XM_574046.1	LOC498761	-0.0796	0.08274	-0.0087	0.11585	0.10856	-0.0263	-0.1524	0.10035	-0.0788	-0.031	0.21372	-0.0589	0.04194	-0.02
XM_574048.1	LOC498762	-0.0348	0.22722	0.26517	0.08179	0.02248	0.1503	0.06188	-0.0087	-0.0515	0.1501	0.18247	0.14745	0.04877	-0.0219

XM_574049.1	LOC498763	-0.2268	-0.1529	-0.2706	-0.1689	-0.1091	-0.2662	-0.1517	-0.1134	-0.3018	-0.2669	-0.1488	-0.2842	-0.1102	-0.2173
XM_574051.1	LOC498765	0.31105	-0.0838	0.22438	0.22632	0.05159	-0.4184	-0.0625	-0.0729	-0.1236	0.16031	0.28006	0.45217	0.87391	0.62028
XM_574052.1	LOC498766	0.07527	0.08275	0.28814	0.30613	0.15855	-0.0877	0.06728	0.38704	0.05143	0.08202	0.12626	0.22609	0.03481	0.07834
NM_001170421	LOC498767	-0.1154	-0.0059	-0.153	-0.1236	0.03706	0.03805	-0.0487	-0.0659	-0.0461	-0.0069	-0.1326	-0.1538	0.06207	-0.0368
XM_574054.1	LOC498768	-0.0837	0.02209	-0.1079	-0.1055	-0.0153	-0.0219	0.04976	0.13877	0.22521	-0.0223	-0.102	0.1826	-0.1001	0.07306
XM_574057.1	LOC498771	-0.0396	0.2006	0.12698	0.01472	0.19761	-0.0069	-0.0178	0.31454	-0.048	-0.1043	-0.0668	-0.0201	-0.0491	0.08861
XM_574058.1	LOC498772	0.10503	-0.0383	0.12759	0.10091	0.00899	0.01518	-0.0378	-0.0113	0.18353	-0.0014	0.07452	0.0439	0.25104	0.11731
XM_574059.1	LOC498773	0.06225	0.0807	-0.0904	-0.0314	0.02592	-0.1017	0.09126	-0.0683	0.18148	-0.0577	0.13434	0.10786	0.08513	-0.1065
XM_574061.1	LOC498775	-0.1029	-0.1144	-0.0792	-0.0288	-0.1541	-0.1153	0.04683	-0.1531	-0.0031	-0.1114	0.0577	-0.0845	-0.0146	-0.124
XM_574063.1	LOC498777	-0.0891	0.29096	-0.0902	0.04467	-0.1043	-0.1021	0.18751	-0.1168	-0.1024	-0.1176	-0.0098	0.10446	0.08383	0.05338
XM_574064.1	LOC498778	-0.0703	0.23123	0.14401	-0.0541	0.08579	-0.0085	-0.1491	-0.1919	-0.0577	-0.075	0.18987	0.00719	-0.0464	-0.0947
XM_574066.1	LOC498780	0.13431	-0.1597	-0.1555	-0.0355	0.02186	-0.1034	-0.0616	-0.0639	0.04808	0.14267	-0.0189	-0.0938	0.06512	0.06774
XM_001063340.1	LOC498781	0.12998	-0.0616	0.03799	-0.0468	-0.0163	-0.0248	-0.1139	-0.0384	-0.0983	-0.0564	0.02349	0.17313	0.15269	-0.0092
XM_001063340.1	LOC498781	-0.0426	0.00035	0.13705	-0.031	-0.0297	0.16464	0.03249	0.03493	0.1012	-0.031	0.03455	0.1249	-0.1583	-0.013
XM_574070.1	LOC498785	-0.0808	-0.1012	0.19646	-0.1351	-0.0981	-0.1272	-0.2012	0.08172	-0.0508	3E-05	-0.1608	-0.0803	-0.012	-0.2344
XM_574071.1	LOC498786	-0.4778	-0.2295	-0.0902	-0.3357	-0.0983	-0.3194	-0.0146	-0.0129	-0.1131	0.01997	-0.3067	-0.4214	0.1894	0.05389
XM_574072.1	LOC498787	0.05106	0.09641	-0.0118	-0.1323	0.21218	0.07188	-0.0299	-0.0041	0.10087	0.03302	0.10946	0.20841	0.01489	0.25408
XM_574073.1	LOC498788	-0.1534	-0.1254	-0.1125	-0.0017	-0.1166	0.09316	-0.0295	-0.1157	-0.1172	-0.0841	0.08126	-0.1356	-0.1004	-0.0804
XR_009617.1	LOC498793	-0.1541	0.03633	-0.0325	-0.1322	-0.0945	-0.024	-0.1057	-0.141	0.17165	-0.0398	-0.0504	-0.0916	-0.0114	-0.0311
XM_574081.1	LOC498795	-0.1269	-0.0927	-0.0086	-0.0771	-0.0511	0.16413	-0.0886	-0.0798	0.06071	-0.1312	0.00501	-0.081	-0.0271	-0.0817
XM_574084.1	LOC498799	-0.3146	-0.1464	0.27808	0.34304	-0.3682	-0.6368	0.67861	-0.4472	0.05496	-0.1549	0.13623	-0.3178	-0.3047	-0.2398
XM_579870.1	LOC498801	-0.3198	-0.2132	-0.3534	-0.1547	-0.2203	-0.358	-0.3829	-0.3166	-0.4213	-0.3659	-0.4449	-0.3729	-0.3874	-0.3167
XM_579871.1	LOC498802	-0.1569	-0.1549	-0.1276	-0.1585	0.14555	0.13589	0.09927	-0.0918	-0.0642	-0.1391	0.09643	0.00751	-0.1408	-0.1364
XM_574096.1	LOC498812	0.03974	-0.0824	0.00659	-0.0698	0.09752	0.09071	0.07019	0.01373	0.07361	0.03227	0.02997	0.06099	-0.105	0.14847
XM_579872.1	LOC498813	0.00697	-0.0279	-0.055	-0.1282	0.35644	0.61951	0.05316	-0.0909	0.11476	0.03482	-0.1	0.41686	0.13865	0.00324
XM_574098.1	LOC498814	0.1831	0.17473	0.59538	0.56794	0.28376	0.2048	0.2702	0.18614	0.00871	0.17803	0.19622	0.09195	0.0414	0.13667
XM_579873.1	LOC498815	0.04972	0.06607	0.33791	0.6026	0.56931	0.72024	0.11337	0.71555	0.1746	-0.0987	0.15266	0.12998	-0.0515	0.06129
XM_574099.1	LOC498817	-0.0634	-0.0634	0.14457	-0.0625	0.01619	-0.0796	0.05817	0.01433	-0.1333	-0.0098	-0.1636	0.09327	-0.0878	-0.0968
XM_574102.1	LOC498821	-0.0255	-0.1056	-0.152	-0.1557	-0.0144	-0.1282	-0.1425	-0.3138	0.02282	0.02346	-0.0306	0.01805	-0.0547	-0.208
XM_574104.1	LOC498823	0.1207	0.24134	-0.0185	-0.1179	-0.0914	0.21567	0.17346	0.10729	0.03615	-0.0082	0.2276	0.16967	-0.0291	0.0493
XM_574105.1	LOC498824	-0.7781	-0.4455	-0.1418	-0.4162	-0.6507	-0.625	-0.0647	-0.5466	-0.2262	-0.4251	-0.116	-0.1216	-0.1062	-0.3591
XM_574107.1	LOC498826	-0.1454	-0.0348	0.13762	0.16546	-0.0137	-0.1243	-0.0261	-0.1779	-0.0145	-0.1554	0.1755	-0.0883	0.00062	-0.0964
XR_005439.1	LOC498829	-0.5019	-0.756	0.64032	1.8608	-0.6228	-1.0618	-0.1954	-0.438	-0.9984	-1.0508	-0.7311	-1.1064	-0.3034	-0.3887
XM_574114.1	LOC498833	0.2115	0.16388	-0.0296	0.036	0.09294	-0.0638	0.01445	-0.0358	0.09377	-0.1905	0.21958	-0.0251	-0.0025	0.03572
NM_001047935	LOC498836	-0.1683	-0.1804	-0.0753	0.01206	-0.1801	-0.1759	-0.1633	-0.0929	0.07762	0.0446	0.15864	-0.0892	0.0912	-0.0669
XM_574122.1	LOC498838	0.09131	-0.1064	0.0502	-0.1375	-0.0719	-0.1512	0.08312	-0.03	-0.1957	0.1029	-0.123	0.01495	-0.1652	-0.0625
XM_574127.1	LOC498842	0.05332	0.02771	-0.0829	-0.0887	-0.0793	-0.1822	0.04569	0.17104	-0.0978	-0.0969	0.17304	-0.1512	-0.002	0.1142
XM_574130.1	LOC498845	0.27441	0.56552	0.1089	0.21587	0.05917	0.05346	0.54137	0.09531	0.23341	0.20696	0.23724	0.12136	0.08231	0.0454
NM_001037139	LOC498846	-0.177	0.11511	-0.2605	-0.4543	-0.4002	-0.3735	-0.1677	-0.0289	-0.0627	0.25414	0.20893	-0.0231	-0.2749	-0.2894
NM_001037154	LOC498847	-0.0123	0.3304	0.0512	0.04391	0.0788	0.11612	-0.0496	-0.0336	0.1904	0.07987	0.0767	-0.0002	-0.0732	0.01046
XM_574132.1	LOC498848	0.12987	0.36366	0.56739	0.10369	0.24007	0.23427	0.48949	0.31226	0.4536	0.29303	0.36731	0.06303	0.07741	0.24261
XM_579874.1	LOC498851	0.04104	-0.0536	-0.0257	-0.059	-0.0249	0.03955	0.01786	-0.0132	-0.004	-0.0338	-0.0068	0.04095	0.00144	0.04423
XM_579875.1	LOC498852	0.01418	0.17155	-0.0551	0.09993	0.13243	0.07237	0.06567	0.01251	0.03454	0.02223	0.26705	-0.0006	-0.0077	-0.1451
XM_574138.1	LOC498853	-0.1543	-0.2866	-0.2446	-0.1022	-0.1266	-0.207	-0.2254	-0.2365	-0.189	-0.1073	-0.0976	-0.3371	-0.1995	-0.2224
XM_574140.1	LOC498855	0.06194	0.17318	0.20448	0.06723	0.22296	0.08234	0.05918	-0.0416	-0.0518	0.03177	0.2625	0.11	0.10875	0.06466
XM_574145.1	LOC498859	-0.0826	-0.0023	0.08553	0.20387	0.06007	0.12007	0.10351	0.15144	0.25376	-0.0003	0.0455	0.15144	0.13801	0.09462
XM_574146.1	LOC498860	-0.0539	-0.2071	-0.2185	-0.1961	-0.2291	0.12332	-0.2646	-0.0394	-0.1313	-0.1268	0.06454	-0.1291	-0.3117	-0.252
XM_574147.1	LOC498861	0.0078	0.19089	0.00694	-0.0212	-0.0393	-0.0819	-0.0458	0.12297	0.06798	-0.1167	-0.1483	-0.0111	-0.1638	0.0038
XM_579876.1	LOC498862	-0.2358	-0.2565	-0.4227	-0.2504	-0.3557	0.18782	-0.3892	-0.1826	-0.3796	-0.1625	-0.138	-0.3017	-0.3437	-0.4959

XM_574148.1	LOC498863	0.03732	0.10961	-0.135	0.26997	0.23002	0.08251	0.04347	0.10923	-0.1398	0.00577	-0.0262	0.19434	0.26085	0.25431
XM_574152.1	LOC498867	-0.0302	0.06932	-0.0602	-0.0053	-0.0163	-0.0254	-0.0394	0.01092	0.06468	0.30305	0.1285	0.07762	0.01454	-0.0227
XM_579877.1	LOC498868	0.08385	0.02947	0.13319	-0.105	0.07706	0.38494	0.08427	0.02528	0.01422	-0.0629	-0.1118	0.17468	-0.0886	-0.093
XM_574154.1	LOC498869	-0.0387	-0.0122	-0.0221	-0.1004	-0.056	0.00713	-0.0627	0.02695	-0.0088	-0.0125	-0.0121	-0.0214	-0.0296	-0.0881
XM_574158.1	LOC498872	-0.1597	-0.0884	-0.0654	0.03689	-0.0434	-0.1114	-0.071	0.19975	0.06903	-0.0044	-0.0804	-0.2428	-0.1862	-0.0897
XM_579878.1	LOC498876	-0.1815	-0.183	-0.1621	-0.1586	-0.067	-0.2911	-0.3008	-0.1191	-0.2306	-0.2579	-0.3141	-0.2336	0.01508	-0.3073
XM_579879.1	LOC498877	-0.1592	-0.2636	-0.4018	-0.2795	-0.1454	-0.0349	-0.3325	-0.2449	-0.434	-0.1651	-0.2406	-0.2272	-0.4732	-0.5115
XM_574163.1	LOC498878	-0.2065	-0.1371	-0.1338	-0.0448	0.02799	-0.1273	-0.0263	0.00076	0.03101	0.09401	0.07229	-0.0739	-0.0569	-0.1547
XM_574169.1	LOC498882	0.05375	0.09277	0.00214	0.04091	0.10901	0.05284	0.0579	-0.1589	0.17124	-0.0105	-0.0112	0.03295	0.09533	0.00089
XM_574170.1	LOC498883	0.03598	0.07129	-0.1213	-0.0128	-0.0314	-0.0084	-0.1619	-0.1275	-0.0161	0.05295	0.07854	-0.0026	0.03749	-0.1336
XM_574172.1	LOC498884	0.32613	0.35305	0.00542	-0.0726	-0.0368	0.07289	-0.05	0.10929	-0.1517	0.32444	0.26426	-0.0278	0.21448	0.15976
XM_574175.1	LOC498887	0.14116	-0.0012	-0.0568	-0.1437	0.08613	0.18509	-0.0771	0.05102	-0.0042	0.02066	0.12486	-0.0469	-0.0344	0.25152
XM_574177.1	LOC498889	-0.5219	-0.3928	-0.593	-0.4119	-0.6131	-0.5407	-0.3822	-0.5205	-0.2604	-0.3388	-0.4541	-0.5041	-0.1829	-0.3991
XM_574180.1	LOC498893	0.11223	-0.0356	0.23646	0.11529	0.17981	0.04594	-0.0223	-0.0152	0.25152	0.02024	-0.0823	-0.0382	-0.0077	0.01377
XM_574181.1	LOC498894	0.01503	-0.155	-0.0669	0.73184	0.01283	0.05215	0.19896	-0.0946	0.13333	-0.0473	0.06002	-0.1098	-0.1435	0.25428
XM_574183.1	LOC498896	-0.5935	-0.1086	-0.918	-0.6585	-0.8108	-0.5501	-0.1799	-0.4485	-0.3285	-0.33	-0.4149	-0.2419	-0.8781	-1.0034
XM_574186.1	LOC498899	0.03896	-0.2532	-0.0041	0.15832	0.02342	-0.2305	-0.2101	0.08487	0.00586	-0.3494	0.01028	-0.0307	-0.3527	-0.1701
XM_574187.1	LOC498900	0.00459	-0.0433	-0.0613	0.03659	0.08028	0.11795	-0.09	0.15063	0.06144	0.14916	0.06477	0.14397	-0.0052	-0.0947
XM_574188.1	LOC498901	0.09393	0.04104	0.33054	0.38738	0.0979	-0.2568	0.53547	-0.0203	-0.0986	-0.3139	0.05999	-0.0749	-0.0765	0.24492
NM_001172151	LOC498902	-0.2619	0.27055	-0.4061	-0.1813	-0.2552	-0.2564	-0.2108	-0.2179	-0.187	0.2217	-0.1996	-0.1354	-0.1421	-0.1412
XM_574194.1	LOC498905	0.10987	-0.0656	0.11367	-0.1461	-0.1253	0.01663	-0.1411	-0.0314	0.05616	0.02478	-0.1251	-0.0423	-0.1265	-0.0094
XM_574196.1	LOC498907	-0.4565	-0.2407	0.15425	0.71724	-0.4611	0.18535	0.41559	0.04036	-0.2564	-0.0399	-0.0316	0.194	-0.1946	-0.3568
XM_574197.1	LOC498908	-0.0203	0.0155	-0.0097	0.16917	0.1067	-0.1204	-0.0738	0.02776	0.2248	0.01437	0.29081	-0.0511	-0.0099	0.0904
NM_001035257	LOC498909	0.23745	-0.0247	0.36941	0.18858	0.45431	0.53331	0.18748	0.55842	0.12377	0.15577	0.56583	0.60055	0.30909	0.13408
NM_001035257	LOC498909	0.41135	0.5088	0.50312	-0.1208	0.36207	0.42761	0.49867	0.17478	0.4312	0.42065	0.65021	0.56965	0.12141	0.19185
NM_001024906	LOC498910	0.11736	0.24334	0.09669	-0.0034	0.14881	-0.1886	-0.0066	-0.0984	-0.1826	-0.0468	-0.0069	0.29736	-0.0173	0.0934
XM_574204.1	LOC498914	1.0268	0.9602	-0.0239	0.65606	0.85975	0.97797	0.38163	0.76889	0.86999	0.79868	0.85982	0.32659	0.47775	0.52597
XM_574206.1	LOC498916	0.27984	-0.1101	-0.0535	-0.0366	-0.068	-0.1818	-0.129	0.36648	0.08717	-0.1099	-0.0672	0.05926	0.22424	-0.0311
NM_001030044	LOC498918	-0.0457	0.14992	0.48087	0.13695	0.10327	-0.0891	0.33756	0.13176	-0.0654	0.25477	-0.1239	0.44853	0.22698	0.34904
XM_579883.1	LOC498919	-0.0689	-0.0957	0.01142	0.08002	-0.0698	-0.1971	0.02715	-0.1182	-0.0899	0.01561	-0.0537	0.04426	-0.0948	-0.0159
XM_579884.1	LOC498920	0.11703	-0.0391	-0.2206	0.05053	-0.1895	0.14191	-0.2245	-0.2202	-0.1446	-0.2307	-0.2168	-0.1333	0.10254	0.08537
XM_579885.1	LOC498921	0.06484	-0.0356	-0.0584	-0.0974	0.00634	0.08609	0.12035	0.10762	0.13545	0.1665	0.07864	0.05847	-0.4054	-0.0054
XM_574212.1	LOC498924	-0.1716	-0.1597	0.10897	-0.0531	-0.1353	-0.0724	-0.068	-0.0416	0.16465	0.00122	-0.1647	-0.0654	0.06834	-0.087
XM_574213.1	LOC498925	-0.0857	0.13412	0.07675	0.05952	-0.0803	0.07658	-0.0362	-0.0173	0.03485	0.12671	0.04134	0.06091	0.0331	-0.0231
XM_579886.1	LOC498926	-0.0188	0.03267	-0.0132	0.1024	-0.0007	0.12243	0.11243	0.0118	0.0023	0.01522	0.03412	0.01466	0.09345	0.00951
XM_574214.1	LOC498927	0.10499	0.05766	0.01688	0.1353	-0.0196	0.09817	-0.0412	-0.0622	0.05392	0.21132	-0.0882	0.03586	0.1412	-0.019
XM_579887.1	LOC498928	-0.0008	0.00241	-0.076	-0.0505	-0.0685	0.02329	0.0272	0.077	-0.1311	-0.0404	-0.0621	-0.1291	-0.0514	-0.0795
XM_579888.1	LOC498929	-0.09	0.05095	-0.0363	-0.0027	-0.1817	-0.0881	-0.146	-0.0811	0.0313	-0.156	-0.0852	-0.024	0.15799	-0.1301
XM_574216.1	LOC498930	-0.041	0.22395	-0.3632	-0.2793	0.16796	0.43014	-0.6872	-0.1213	0.07374	0.10246	-0.0447	0.20155	0.05582	0.25672
XM_574218.1	LOC498932	-0.0775	-0.0374	-0.0638	0.36258	0.00434	-0.0563	-0.141	-0.0106	0.15184	-0.1735	-0.0784	-0.2001	0.12203	0.11124
NM_001025764	LOC498933	-0.1524	-0.0247	-0.171	-0.0312	-0.0116	-0.081	-0.1509	-0.1475	-0.0622	-0.1395	-0.0675	-0.2887	-0.2485	-0.0225
XM_574221.1	LOC498935	0.00274	0.0288	0.08689	-0.0729	-0.1493	-0.1829	0.01226	0.17955	0.00863	-0.1222	-0.1789	0.03324	-0.0472	-0.1814
XM_574223.1	LOC498938	0.0019	0.02597	-0.02	-0.0259	0.01503	0.04025	0.19362	-0.0083	0.0963	-0.0443	0.24604	-0.0489	0.10595	-0.1271
NM_053985	LOC498939	-0.02	0.0017	0.2206	0.48906	-0.0146	0.19564	0.02877	-0.0682	-0.0067	0.17091	0.10078	0.39038	0.23961	0.12123
NM_001044258	LOC498940	0.10784	0.00597	-0.0313	0.03906	0.13775	0.19803	0.03851	-0.0207	0.11813	-0.0331	0.17319	0.14173	-0.0292	0.02197
XM_574237.1	LOC498948	0.19137	-0.1047	-0.1186	0.01624	0.01281	-0.1485	-0.1357	-0.0596	-0.2079	-0.0412	-0.0961	-0.091	-0.1435	0.09121
NM_001025766	LOC498951	-0.1105	-0.0061	-0.128	0.03258	0.1125	-0.0796	-0.0985	0.17666	0.01322	0.15753	-0.0113	0.14565	-0.0385	-0.0462
XM_574243.1	LOC498954	-0.1125	-0.0391	-0.1179	-0.0779	0.19574	0.50164	-0.2653	0.15258	0.48122	0.33443	0.20955	0.45013	0.21085	-0.1075
XM_579892.1	LOC498955	-0.2128	-0.0455	-0.1369	-0.1556	-0.0452	-0.061	-0.0156	-0.1514	0.02415	-0.2424	-0.1346	-0.0815	0.02086	0.01606

XM_574245.1	LOC498956	0.08154	0.15576	0.08814	-0.0131	-0.0574	-0.1332	0.11996	0.12802	-0.0436	0.19261	-0.0171	0.11914	-0.0052	0.16133
NM_001017511	LOC498957	0.72096	0.27746	0.00744	0.2367	1.9111	2.0076	0.17136	1.9107	0.21397	0.44732	0.33174	-0.0974	0.28741	0.34848
XM_579893.1	LOC498958	0.10126	-0.0103	0.02907	-0.0292	-0.0153	0.01378	0.08523	-0.0321	-0.0612	0.06902	0.15692	0.11144	0.03439	0.35536
XM_579894.1	LOC498960	-0.0211	-0.1162	-0.0679	0.07296	-0.1108	-0.0485	-0.1467	-0.0915	-0.0835	-0.0292	-0.0129	-0.0201	-0.1278	-0.0018
XM_579895.1	LOC498961	-0.0256	0.03585	-0.0152	-0.0012	0.03393	-0.034	0.00261	-0.04	0.04614	0.027	0.10192	0.14157	-0.0307	-0.0923
XM_574249.1	LOC498962	0.19116	0.1926	0.10361	0.22736	0.02699	0.23711	-0.0326	0.17725	0.11194	0.20331	0.06869	0.25596	-0.0895	0.10753
XM_574257.1	LOC498970	0.0927	-0.1553	-0.002	0.09015	0.03403	-0.0086	-0.0077	-0.0985	-0.1615	-0.097	0.0085	-0.0763	-0.0224	-0.0736
XR_009548.1	LOC498972	0.00892	0.16708	0.06286	0.04121	0.03355	-0.3085	0.27497	-0.0044	0.37083	0.06453	-0.0374	0.13117	0.17823	0.35452
XR_009548.1	LOC498972	0.32322	0.00109	0.603	-0.28	-0.0787	-0.3147	0.40227	-0.0316	0.42378	0.13528	0.35004	0.06171	0.30356	0.54224
XM_574260.1	LOC498973	-0.131	0.07632	0.30905	0.62919	-0.282	-0.1653	0.62597	0.11733	0.02136	-0.0277	0.32921	0.06943	-0.0888	-0.0334
XM_574268.1	LOC498979	-0.4969	-0.3368	-0.3659	-0.0589	-0.7129	-0.3231	0.2733	-0.6753	-0.2849	-0.1809	-0.468	-0.5395	-0.3432	-0.1
XM_574270.1	LOC498981	0.15676	-0.0045	-0.1171	-0.053	0.13593	-0.076	0.06686	0.13275	0.08405	-0.0074	0.13421	0.24289	0.05367	0.08759
XM_574273.1	LOC498983	-0.1607	-0.2462	-0.2616	-0.3309	-0.3218	-0.2031	-0.3614	-0.1257	-0.2587	-0.2165	-0.3685	-0.2684	-0.1961	-0.3137
XM_574274.1	LOC498984	-0.2832	-0.1634	0.0185	0.1239	-0.1683	-0.2609	-0.0339	-0.2448	-0.2571	-0.0962	-0.2286	0.05226	-0.1808	-0.1123
XM_574275.1	LOC498985	0.0044	0.02204	0.12505	0.06275	0.07129	0.25259	0.20982	-0.0265	-0.0158	0.20335	0.07486	0.00999	0.02907	0.07292
NM_001109135	LOC498987	0.12657	0.10769	-0.0132	0.12794	0.30701	0.10911	0.08386	-0.0204	0.35131	0.27864	0.11001	0.11717	0.14217	0.15831
XM_574280.1	LOC498989	-0.0461	-1.2549	0.49013	-0.4605	-0.3268	-0.9819	-0.0592	-0.5294	-0.8281	-1.13	-0.5443	-0.0476	-0.2083	-0.1005
XM_574283.1	LOC498993	-0.5796	-0.616	-0.4844	-0.5165	-0.3611	-0.3436	-0.5438	-0.5067	-0.4739	-0.3953	-0.4428	-0.4062	-0.5716	-0.4911
XM_574284.1	LOC498994	0.28452	-0.0493	0.10006	-0.004	0.03184	0.13688	0.19548	-0.0199	0.0922	0.09295	0.20107	0.0469	0.15441	0.02828
NM_001039342	LOC499000	0.14516	-0.0218	-0.0822	0.23668	-0.1077	0.0064	-0.097	-0.0889	0.06213	0.01944	-0.1398	0.12218	-0.0171	-0.0154
XM_574295.1	LOC499002	-0.2011	-0.0538	0.06637	0.10478	-0.0261	0.33688	-0.0116	-0.0785	-0.2227	-0.0107	-0.0486	0.12991	0.06206	-0.0256
XM_574296.1	LOC499003	-0.0219	-0.0121	0.03311	0.17777	0.20474	0.19087	0.07586	0.13655	0.19989	0.17968	0.27233	0.00736	0.07035	0.03481
XM_574297.1	LOC499004	0.59052	0.0192	-0.2799	0.17609	0.42507	0.25408	0.32714	0.64938	0.04318	0.15874	0.20487	-0.1885	-0.2985	-0.0039
XM_574298.1	LOC499005	-0.0671	-0.0272	0.041	0.00643	0.09	-0.0412	-0.0583	0.34918	-0.027	0.10679	0.15975	-0.0177	-0.0387	-0.0086
XM_574301.1	LOC499008	-0.0144	0.029	-0.0914	-0.0357	0.01169	-0.1439	-0.0302	0.05839	0.06455	-0.0988	-0.0308	0.06115	-0.0841	-0.0343
XM_574302.1	LOC499009	0.00171	0.00034	-0.2522	-0.0934	-0.0942	0.39054	-0.3309	0.20344	-0.0118	-0.2479	0.17252	-0.197	-0.2378	-0.1838
XM_574312.1	LOC499019	-0.0454	-0.046	-0.0726	-0.0301	-0.0107	-0.0701	0.11954	0.01951	-0.0571	-0.0435	-0.037	0.04115	-0.0527	-0.0296
NM_001077677	LOC499021	0.17315	-0.0116	-0.01	-0.0985	-0.0703	-0.0292	0.0725	0.27974	0.18771	0.01293	0.38828	0.13126	0.07239	-0.0876
XM_579901.1	LOC499024	0.17977	0.21935	0.13267	0.26902	0.08254	0.24923	0.09914	0.25895	0.00266	0.01989	0.07208	0.14835	-0.0693	-0.0126
XM_579902.1	LOC499026	0.00851	0.10372	0.20776	0.20536	0.02806	0.03531	0.00131	-0.0603	0.31246	0.0801	0.01026	0.0715	0.07488	0.20262
XM_574319.1	LOC499027	0.11934	0.20865	0.17349	0.18012	0.18054	0.19272	0.13269	0.25135	0.03193	0.04826	0.18803	0.33194	0.11693	-0.0002
XM_579903.1	LOC499029	0.05794	0.12539	0.08699	0.54237	-0.1069	0.45939	0.77834	0.19998	0.30207	0.07944	-0.0588	-0.0719	0.17286	-0.0427
XM_579904.1	LOC499030	0.01892	0.04722	0.05471	0.02623	-0.1212	0.05659	-0.022	0.01365	0.04088	-0.0172	0.03928	0.10561	-0.0076	0.08272
XM_574321.1	LOC499031	-0.1095	0.08377	0.16666	0.4847	0.26317	0.07823	-0.0501	-0.001	-0.0695	0.05579	0.21276	0.18531	-0.0726	0.14424
XM_579907.1	LOC499035	0.01369	0.16957	0.01172	0.23497	0.0287	0.0437	0.14071	0.00063	0.00139	0.04802	-0.0663	-0.2026	-0.0117	-0.0212
XM_574323.1	LOC499036	0.05943	0.00414	0.08327	0.15914	0.02513	-0.0379	0.00367	0.03533	-0.0114	-0.0434	0.03608	-0.0048	0.05129	0.05408
XM_574324.1	LOC499039	0.1405	-0.0096	0.05135	0.02657	0.2964	0.04081	0.00598	-0.0122	-0.0071	-0.0117	-0.0072	0.01282	0.19577	0.28953
XM_579910.1	LOC499040	0.35881	-0.0189	-0.2884	-0.0517	-0.1749	-0.0924	-0.2896	0.1638	-0.1729	-0.1837	-0.2165	-0.1045	-0.1351	-0.1214
XM_574326.1	LOC499044	0.00954	-0.0163	0.22628	-0.0609	-0.0075	0.14162	-0.0118	0.05515	-0.0087	0.09924	0.16313	0.06626	0.0601	0.27309
XM_579913.1	LOC499045	0.07884	0.13878	0.26052	0.22769	0.29934	0.22848	0.13923	0.38712	0.25991	0.36861	0.31729	0.0143	0.1122	0.12806
XM_574329.1	LOC499048	0.01826	-0.0805	-0.0177	-0.051	-0.0582	-0.0084	-0.0318	-0.1009	0.0139	0.04251	-0.0313	-0.0309	0.00121	-0.0554
XM_579914.1	LOC499049	0.01856	0.15657	0.11769	0.08915	0.05818	0.12956	0.24574	0.02613	0.24795	0.04351	0.13238	0.26684	0.1454	0.01333
XM_574332.1	LOC499052	0.03606	0.03797	0.05459	0.16373	0.037	0.06107	0.03684	0.05069	0.1097	0.07822	0.14788	0.06589	0.03991	0.10139
XM_579915.1	LOC499053	0.00777	-0.043	-0.0577	-0.0131	-0.0457	0.03527	-0.0219	-0.0729	-0.0429	-0.0398	0.18615	0.26931	0.18597	-0.1164
XM_579916.1	LOC499054	0.05121	0.10019	0.04054	0.06204	0.08016	0.0238	0.13511	0.13644	0.05585	-0.0112	0.09625	0.14786	0.30891	0.15413
NM_001107467	LOC499056	0.00616	-0.1483	-0.0049	-0.1455	-0.4488	0.33892	-0.2284	0.06836	-0.0695	0.06914	-0.129	-0.1904	0.24687	0.24439
XM_574337.1	LOC499058	0.07725	-0.1247	-0.1716	-0.0368	-0.1636	-0.148	0.1063	-0.0281	-0.1112	-0.2891	-0.0411	-0.2136	-0.2358	-0.2197
XM_574339.1	LOC499059	0.13059	0.09709	0.06112	0.07643	0.14867	0.00598	0.04857	-0.0414	0.11227	0.00182	0.03796	0.19921	0.15102	0.09512
XM_574340.1	LOC499060	-0.0439	-0.0695	-0.0754	0.13415	-0.0833	-0.1892	-0.0414	0.22794	-0.1699	-0.1648	0.00411	0.00216	-0.0156	-0.003

XM_574346.1	LOC499065	0.03792	0.04406	-0.066	-0.0046	-0.093	0.05495	-0.0185	-0.0617	0.06384	-0.1779	0.02284	-0.2139	-0.0317	-0.2142
XM_574347.1	LOC499066	-0.0433	0.07851	0.0403	-0.0431	-0.0451	0.20374	0.15446	0.20676	0.03129	0.11024	0.13358	0.5658	0.17141	0.05584
NM_001134609	LOC499068	-0.0229	0.07164	0.02203	-0.0434	-0.0958	0.02888	-0.2252	0.17078	0.34904	0.18338	0.17028	0.08309	0.0823	0.01512
XM_001071078.1	LOC499076	-0.2014	-0.1134	0.97961	0.37636	0.11791	0.26869	0.15635	-0.0172	-0.2265	-0.0642	-0.1208	-0.4299	-0.1663	-0.0422
XM_574361.1	LOC499077	-0.0348	0.15894	-0.0564	0.03443	-0.1433	-0.1035	-0.1625	0.19876	-0.0845	-0.0321	0.00218	-0.0396	-0.0811	-0.1788
NM_001013894	LOC499078	-0.1758	-0.2383	0.06967	-0.0614	-0.1127	-0.216	-0.222	-0.2204	-0.1937	-0.2996	-0.167	-0.2895	-0.2276	-0.205
XM_574363.1	LOC499079	-0.1505	0.03661	0.1431	0.26042	0.08542	-0.0732	0.46884	-0.2576	0.09429	-0.1773	-0.1286	0.12324	-0.0366	-0.2297
XM_574364.1	LOC499080	0.06288	0.23368	-0.0803	0.21637	0.09065	-0.0132	0.24683	-0.0659	0.11691	0.20468	-0.0992	0.4447	0.27285	0.10412
XM_574376.1	LOC499089	0.01877	0.24244	-0.0967	0.1853	-0.0921	0.10418	-0.0612	-0.1457	0.17172	-0.0664	-0.0578	-0.1551	-0.0687	-0.1542
XM_574382.1	LOC499091	-0.0438	0.15576	0.14211	-0.1967	-0.0528	0.4822	0.17483	0.23344	-0.1028	-0.0797	0.02026	-0.2717	-0.0299	-0.2032
XM_574384.1	LOC499093	0.10115	-0.0849	0.08137	0.1296	-0.0548	-0.2346	-0.1444	-0.0882	-0.0355	-0.1324	-0.1982	-0.0043	-0.173	-0.1491
XM_574387.1	LOC499096	0.04598	0.12941	0.0911	0.13197	-0.0016	0.24005	0.03072	0.11621	0.06608	0.04691	0.01444	0.11163	0.13811	0.09081
XM_574388.1	LOC499097	-0.1837	-0.2255	-0.0751	-0.1011	-0.0144	-0.1867	-0.2186	-0.0421	0.0325	-0.1971	-0.1913	-0.0267	-0.1743	-0.0334
NM_001109140	LOC499100	0.17339	0.34178	0.07575	0.02498	0.11662	0.16755	0.12648	0.2356	0.15432	0.25607	0.08826	-0.0728	0.37883	-0.0094
XM_574393.1	LOC499102	-0.1906	-0.1328	0.03847	-0.2223	-0.0535	-0.1627	-0.2668	-0.1831	0.03314	-0.2023	-0.2597	-0.0593	-0.1269	-0.0619
XM_574394.1	LOC499103	-0.7404	-0.4789	-0.3555	-0.566	-0.9171	-0.8832	-0.1879	-0.9293	-0.7447	-0.5325	-0.6393	-0.6807	-0.9139	-0.5679
XM_574403.1	LOC499109	0.16366	0.13657	0.37768	0.27007	0.14571	0.02446	-0.0022	0.12556	-0.0176	0.0871	0.05352	0.20421	0.09079	0.05644
XM_001077990.1	LOC499110	0.00165	0.14942	0.12231	0.23321	0.1926	0.28295	0.05947	0.0965	0.02011	0.00405	0.09749	0.10351	0.10375	0.04476
XM_574406.1	LOC499111	-0.0506	0.09052	-0.0537	0.07122	-0.0327	-0.0453	-0.0953	0.07573	-0.0025	-0.0207	0.05011	-0.1125	-0.024	-0.0525
XM_574407.1	LOC499112	-0.3545	-0.225	-0.2574	-0.521	-0.2922	-0.4857	-0.2308	-0.2818	-0.4371	-0.0934	-0.3647	-0.0451	-0.6392	-0.3255
NM_001110491	LOC499114	-0.0699	0.1009	-0.0415	0.12536	-0.0429	-0.0091	0.18476	-0.0552	0.20069	0.05058	-0.0882	0.0002	-0.0978	-0.1651
XM_574413.1	LOC499119	-0.0889	0.24652	0.23981	0.04284	0.22945	0.13578	0.17149	0.44274	0.16739	-0.0365	0.18279	0.21924	0.11404	0.2773
XM_001078762.1	LOC499120	0.04603	0.19368	0.30132	0.01948	-0.0161	0.11769	0.05267	-0.0104	0.1059	0.11773	0.15832	-0.0632	-0.0399	0.02542
XM_001078762.1	LOC499120	-0.2234	0.40253	-0.1781	0.53127	0.0539	-0.5473	0.27738	0.04962	0.13596	0.0348	0.16508	-0.0009	0.11416	-0.0533
XM_574416.1	LOC499122	-0.3085	-0.236	-0.2704	-0.4195	-0.2561	0.05263	-0.427	-0.3703	-0.3034	-0.4127	-0.267	-0.0484	-0.2548	-0.0025
XM_574417.1	LOC499123	-0.2225	-0.5247	0.06936	-0.1529	-0.3183	-0.4472	-0.0679	-0.4201	-0.3313	-0.3061	-0.347	-0.231	-0.3026	-0.3644
NM_001100991	LOC499124	0.17987	0.31232	0.20736	0.36515	0.043	0.18354	0.16597	0.10264	0.29716	0.30365	0.18548	-0.1824	0.25792	-0.0821
NM_001100991	LOC499124	-0.1485	-0.186	-0.1684	-0.2092	-0.3308	-0.3569	0.08556	-0.0888	-0.059	-0.144	-0.0864	-0.1591	0.22831	0.21191
XM_574420.1	LOC499127	-0.0578	-0.072	-0.0961	-0.1746	-0.2171	-0.0836	0.08765	-0.1023	-0.1815	-0.1346	-0.0887	-0.0328	-0.1163	-0.055
XM_574421.1	LOC499128	0.01996	-0.0007	0.00551	-0.1345	-0.101	-0.0045	-0.0568	0.08985	0.01971	0.19847	-0.0861	0.19785	-0.0436	0.0933
NM_001109141	LOC499129	0.02857	-0.2147	-0.1359	-0.1361	-0.1194	-0.2158	-0.2251	-0.2789	-0.2417	-0.3314	-0.2573	-0.2082	-0.3466	-0.2444
NM_001037338	LOC499131	0.43295	0.10739	-0.0952	0.4018	0.60764	0.82805	-0.0784	0.6078	0.35835	0.373	0.35661	0.54563	0.2775	0.1469
NM_001047937	LOC499136	-0.128	0.19217	0.27003	0.48308	0.42156	0.69173	-0.1067	0.30601	0.70294	0.32708	0.32279	0.47424	0.36533	0.38965
XM_574432.1	LOC499138	-0.0493	-0.0639	0.00254	-0.0916	0.00933	0.13896	-0.0886	-0.1244	0.05668	-0.0433	-0.101	-0.0586	-0.0088	-0.0188
XM_574435.1	LOC499141	0.05372	-0.0203	0.0813	0.15714	0.09582	0.08902	0.03385	0.07026	-0.0225	0.05398	0.04827	0.09394	0.04895	0.01861
XM_574438.1	LOC499144	-0.2466	-0.1376	-0.1226	-0.1105	-0.0189	-0.0802	-0.0431	-0.1221	-0.0343	0.10779	0.08399	-0.0406	-0.1197	-0.142
XM_574440.1	LOC499145	0.02239	-0.0049	0.19814	-0.0014	-0.0164	-0.0636	-0.0182	0.21847	-0.0861	-0.0326	0.15629	0.11668	-0.0681	0.06594
XM_574443.1	LOC499148	-0.1144	0.46328	-0.1537	-0.3512	-0.2593	0.13164	-0.0996	-0.103	0.54558	0.53991	0.49385	0.16746	-0.2042	-0.4532
XM_579919.1	LOC499153	-0.0383	-0.0093	0.02027	0.19229	0.10444	-0.045	0.01198	-0.0831	-0.0716	-0.0556	0.20459	0.02606	0.03113	0.06108
XM_574458.1	LOC499160	0.16345	-0.0067	0.02093	0.00797	-0.038	0.10921	-0.0193	0.03754	-0.0671	0.01771	-0.0401	0.01539	-0.0273	0.0381
XM_579922.1	LOC499162	0.02168	-0.0657	-0.0602	-0.0928	-0.1031	-0.0906	-0.0483	-0.0535	-0.1032	-0.0397	-0.0818	-0.0305	0.11259	-0.0512
XM_574461.1	LOC499164	-0.1018	-0.1344	-0.1725	-0.0942	-0.0857	-0.0556	-0.144	-0.0345	0.00519	-0.1097	-0.1062	-0.0909	0.0604	-0.0996
XM_574462.1	LOC499165	-0.1689	-0.1901	0.02103	-0.2569	-0.0689	-0.0193	-0.1528	-0.1621	-0.2218	-0.0897	0.15778	-0.2623	-0.1769	-0.3117
XM_579923.1	LOC499168	-0.066	0.11798	-0.0807	-0.0819	0.05903	0.03005	-0.1533	0.02873	-0.1757	0.00508	-0.0063	-0.1382	-0.0588	-0.0376
XM_574466.1	LOC499170	0.1668	-0.0569	-0.0489	0.13986	0.03264	-0.0582	-0.0787	0.17866	-0.0289	-0.0911	-0.0254	0.19565	0.17328	0.01767
XM_579924.1	LOC499173	0.02984	0.15063	-0.0267	0.15773	0.07979	-0.0137	-0.098	0.12047	-0.1093	0.06718	0.01052	-0.0573	0.19193	0.01311
NM_001127561	LOC499174	-0.0843	-0.0583	-0.098	-0.1034	-0.0614	0.03627	0.0089	-0.0163	-0.0283	-0.0336	-0.0666	-0.0719	0.05932	0.06162
XM_579925.1	LOC499177	0.00033	0.14513	-0.053	0.04449	0.13593	0.11543	-0.0594	0.00036	0.03126	0.03777	-0.0598	-0.0104	-0.0527	0.14032
XM_579926.1	LOC499178	-0.2573	-0.4754	-0.2294	-0.0419	-0.4261	-0.5356	0.03407	-0.4716	-0.2272	-0.3831	-0.3719	-0.3147	-0.5689	-0.423

XM_574473.1	LOC499180	-0.1187	-0.752	0.22527	-0.2846	-0.4632	-0.5515	-0.6923	-0.7422	-0.0477	-0.5459	-0.2572	0.64632	0.33048	0.2388
XM_574475.1	LOC499182	0.01859	0.18691	-0.0893	0.09976	-0.0781	0.30963	-0.1201	-0.0212	0.19588	0.06114	-0.1263	0.10685	-0.1513	-0.1624
XM_574477.1	LOC499184	-0.1527	-0.1699	-0.3072	-0.0922	-0.4948	-0.496	0.0517	-0.5278	-0.3895	-0.2487	-0.3597	-0.2749	-0.3957	-0.4358
XM_579927.1	LOC499186	0.14206	0.04913	0.02061	0.29306	0.35421	0.36932	0.14644	0.18634	0.15925	0.34133	0.16559	0.24515	0.37218	0.28488
XM_579928.1	LOC499187	-0.0035	0.14221	0.08121	0.21471	0.03589	-0.071	-0.0865	-0.0393	-0.022	-0.0735	-0.0838	-0.0122	-0.1109	0.04996
XM_579930.1	LOC499190	0.19677	-0.1327	-0.1516	-0.3723	-0.2953	-0.1512	-0.2286	-0.0993	-0.168	-0.1458	-0.2277	0.15089	-0.1249	0.15949
XM_579931.1	LOC499192	0.00999	-0.1266	-0.0127	0.06361	0.01434	0.03277	-0.0764	-0.2513	0.2344	-0.1667	-0.2314	-0.0019	-0.1058	-0.0842
XM_574480.1	LOC499193	0.04716	0.11165	0.12625	0.20787	-0.0491	0.25863	0.07094	0.09262	0.10194	0.07814	0.13767	0.25397	0.05395	0.13851
XM_579933.1	LOC499196	-0.113	0.39777	-0.2364	-0.3851	0.23402	0.09662	-0.0913	-0.0754	-0.1396	0.22259	0.1482	-0.1206	-0.509	-0.4316
XM_574484.1	LOC499197	0.03171	-0.023	0.33281	0.53009	-0.0606	0.03325	0.50426	-0.0428	0.15125	0.25547	0.14957	0.19414	-0.1884	0.18333
XM_574485.1	LOC499198	0.04551	0.06405	0.05768	0.17134	0.17516	0.04095	0.02984	-0.0102	-0.0723	0.28846	0.14606	0.01148	-0.077	0.1027
XM_574486.1	LOC499199	0.14675	0.06635	0.24859	0.20263	0.20008	0.20645	0.25451	0.18483	0.25882	0.08387	0.0795	0.17457	0.13584	0.13769
XM_579934.1	LOC499201	2.5363	1.5341	0.81731	1.1087	1.9874	2.2878	0.05558	1.98	0.96992	1.253	1.2533	0.87656	1.6272	1.5736
XM_574488.1	LOC499202	0.17571	0.0573	0.03853	0.22151	0.2051	0.15221	0.1358	0.22776	0.21705	-0.0246	0.32413	0.12347	0.14288	0.10704
XM_574492.1	LOC499206	-0.067	0.20326	0.36735	0.74522	0.07464	0.17874	0.96675	0.25663	0.34309	0.01705	0.52095	0.08922	0.20477	-0.0172
XM_574494.1	LOC499208	0.12729	-0.099	0.17705	0.3289	0.02239	-0.1452	0.11419	0.05848	-0.0066	-0.0935	0.04463	-0.019	-0.1471	0.0244
XM_574501.1	LOC499213	-0.3898	-0.2143	-0.0438	0.09487	-0.1924	-0.2405	-0.3366	-0.0908	-0.1866	-0.1693	-0.1709	-0.2677	-0.0533	-0.019
NM_001047938	LOC499219	0.18696	-0.0469	0.02378	0.10477	-0.1897	0.10731	-0.1329	-0.1914	-0.1112	-0.0054	0.00132	0.09226	0.10155	-0.1777
XM_574514.1	LOC499221	-0.0771	0.28934	0.20095	-0.512	-0.052	0.2297	0.03976	-0.0713	0.16156	0.29462	0.14991	0.34061	0.19175	0.27102
XM_574527.1	LOC499234	0.03145	0.09451	0.22832	0.05683	-0.0465	-0.0711	0.00933	0.02757	0.12016	0.13103	0.18479	0.07937	-0.0108	0.09789
XM_574528.2	LOC499235	-0.0285	0.2208	-0.4521	-0.3117	0.00509	-0.2292	-0.1447	0.25905	0.40084	0.15934	-0.0691	0.02407	0.06486	0.35288
NM_001024287	LOC499240	-0.007	0.18044	-0.0086	0.03184	0.04082	0.02555	0.01132	0.13748	0.00691	0.02016	0.20035	0.01333	-0.0128	0.1201
NM_001024287	LOC499240	0.02919	0.03767	0.1167	0.08783	0.12551	0.11172	-0.0004	0.05077	0.08198	0.14154	0.13743	0.0938	0.21531	-0.0346
XM_574535.1	LOC499242	-0.1755	-0.2431	-0.1663	-0.1135	0.01361	0.2452	0.031	-0.0722	0.06254	-0.1318	-0.1669	-0.15	-0.0612	-0.1496
XM_579935.1	LOC499243	-0.1714	0.08666	-0.2052	-0.2363	-0.1399	0.00384	-0.3521	-0.024	-0.1073	-0.1203	-0.0123	0.07093	-0.1359	-0.0367
XM_574540.1	LOC499247	-0.0002	0.10503	0.26844	0.84376	0.42182	0.30669	0.18097	0.78451	0.30356	0.2349	0.16621	0.45469	0.75222	0.59785
XM_574545.1	LOC499252	-0.03	-0.0916	0.11144	-0.0557	0.09491	-0.0523	-0.0378	0.38664	-0.0798	-0.0957	0.16532	-0.0395	-0.0112	0.01435
NM_001134611	LOC499255	-0.0611	-0.0609	0.131	0.6964	0.1171	0.01655	-0.1201	0.23379	-0.0843	0.13116	0.22143	0.04266	0.04462	0.19141
XM_574558.1	LOC499263	-0.1443	-0.2533	-0.1919	-0.1778	-0.1065	-0.1458	-0.2739	-0.2199	-0.2035	-0.1388	-0.2393	-0.1624	-0.1886	-0.2705
XM_574566.1	LOC499269	0.04431	0.28003	0.01872	0.02112	0.03434	0.06189	0.16948	0.18169	0.05668	-0.0116	0.01724	0.0329	0.1403	0.03516
XM_579937.1	LOC499272	0.30118	-0.0326	-0.0026	-0.1009	0.04851	0.09182	-0.0366	0.02254	-0.024	-0.0285	0.05322	0.01992	0.11005	0.22413
XM_001053146.1	LOC499276	-0.1298	-0.0232	0.22588	0.01137	-0.087	-0.12	-0.1073	-0.1602	-0.0039	-0.1296	0.03409	-0.0597	-0.1902	0.19259
NM_001109158	LOC499278	0.10992	0.13404	-0.0943	-0.0374	0.10307	0.14062	0.20418	-0.0195	0.03132	-0.0216	-0.0307	0.04291	0.07503	0.03834
NM_001024288	LOC499279	0.03062	0.124	-0.0902	-0.0562	-0.1257	-0.0644	-0.0871	0.11937	0.00151	-0.0807	0.0854	0.02789	0.00031	0.01363
NM_001024288	LOC499279	0.00089	-0.023	-0.0487	-0.0099	0.13046	0.1599	0.06355	-0.003	0.08286	-0.0142	-0.0271	0.03454	0.01563	-0.0232
NM_001143756	LOC499281	-0.0301	0.09812	0.01278	0.10543	0.10905	-0.0755	-0.0839	0.01403	-0.0604	-0.0674	-0.0179	0.20436	0.01796	0.14649
XM_574584.1	LOC499285	-0.0259	-0.0581	0.11397	0.10746	-0.0337	0.03104	0.12806	-0.0712	-0.0694	0.03195	-0.0659	-0.0937	0.0099	0.07618
XM_579941.1	LOC499291	0.00325	0.05383	-0.0546	-0.0363	0.036	0.03448	0.1819	0.04683	0.04475	-0.1293	0.15981	-0.0814	0.01296	-0.0097
XM_574588.1	LOC499292	-0.04	0.16217	-0.03	-0.1143	-0.1723	0.20469	0.00802	0.14561	-0.2624	-0.1304	-0.142	0.01841	0.12292	-0.0861
XM_579942.1	LOC499293	0.16736	0.00187	0.15643	0.05009	-0.0477	0.08783	-0.1501	-0.0919	-0.155	-0.0059	-0.1712	-0.0234	0.01613	0.11863
XM_579943.1	LOC499294	-0.0297	-0.0242	-0.0137	0.09985	-0.0731	-0.0436	0.01885	-0.0162	-0.0073	-0.0801	-0.1167	0.07837	-0.1018	-0.0735
XM_579944.1	LOC499295	-0.2403	-0.1692	0.14239	-0.2585	0.08625	-0.2031	0.11815	-0.4568	0.07643	-0.2088	-0.0381	0.27475	-0.0455	0.23524
XM_574589.1	LOC499296	-0.3191	-0.251	-0.2081	-0.1484	-0.2425	-0.1532	-0.0011	-0.2095	-0.3597	-0.1435	-0.3221	-0.3036	-0.3547	-0.0529
XM_574596.1	LOC499299	-0.0391	-0.1022	-0.0224	0.14248	-0.0672	0.13031	0.00345	-0.0772	0.03805	0.15185	-0.0426	0.07836	-0.1544	0.17614
XM_579946.1	LOC499304	0.02388	-0.0581	0.06088	-0.1332	-0.0962	0.20508	-0.1804	-0.2131	-0.1756	0.00514	-0.233	0.07901	-0.1432	-0.0387
XM_574605.1	LOC499305	-0.0419	0.03881	0.09801	0.0663	-0.0306	0.16936	0.08067	-0.087	0.23774	0.11894	0.23325	0.15671	0.0126	0.00553
NM_001024291	LOC499306	0.19825	0.03089	-0.1005	-0.0054	0.19081	0.1285	0.03447	-0.0154	-0.0877	0.02962	0.30575	0.04196	0.09779	0.16372
NM_001009636	LOC499307	0.00788	-0.2056	-0.2369	-0.6523	0.08037	0.17249	-0.4715	-0.2862	-0.2199	-0.5474	-0.6548	-0.5001	0.10702	0.03751
XM_579947.1	LOC499311	-0.098	-0.0672	-0.1635	-0.0892	-0.0988	-0.115	-0.0941	0.1382	-0.0658	0.04904	0.01592	0.12601	-0.156	-0.185

XR_005457.1	LOC499315	-0.4039	-0.249	-0.4393	-0.2369	-0.3361	-0.3626	-0.3188	-0.2233	-0.1187	-0.2172	-0.2738	-0.3708	-0.1509	-0.2902
XM_574620.1	LOC499316	-0.056	-0.105	-0.0839	-0.0086	0.11003	-0.0086	-0.1347	-0.0093	-0.0152	-0.0247	0.09102	0.22695	0.06357	0.0137
XM_574624.1	LOC499319	0.19263	0.17828	0.10148	0.14137	-0.0444	0.00676	0.15455	0.17917	0.0476	0.05304	0.02512	0.2004	0.12534	0.12145
NM_001106290	LOC499321	-0.4446	-0.3436	-0.0083	0.13645	-0.5456	-0.4937	0.00738	-0.5617	-0.4699	-0.5019	-0.3283	-0.4991	-0.3549	-0.2974
XM_574633.1	LOC499327	0.268	0.05493	0.01634	-0.0535	0.0114	0.09253	-0.0742	0.03223	0.03382	0.02058	-0.005	-0.0947	0.02115	0.0098
NM_001024292	LOC499330	0.16989	-0.216	-0.395	-0.3131	-0.4689	-0.2039	-0.2071	-0.386	0.19108	-0.4158	-0.0429	-0.0182	-0.3999	-0.18
NM_001024293	LOC499331	0.34117	0.15384	0.19293	0.38964	0.54925	0.30528	0.14979	0.70321	0.24903	0.31465	0.06977	0.12561	0.32982	0.47636
XM_579949.1	LOC499333	0.03303	-0.0706	-0.1335	-0.1515	-0.2102	-0.1658	-0.1308	-0.112	-0.1575	-0.2073	-0.0904	-0.175	-0.2962	-0.2138
NM_001037793	LOC499337	-0.1419	0.24815	0.32353	0.19554	-0.2071	-0.311	0.19878	-0.1535	0.32128	0.17487	0.10567	0.07689	0.23883	0.22553
NM_001025036	LOC499339	-0.2395	-0.1135	-0.137	-0.2116	-0.2535	-0.0592	0.21766	-0.0348	-0.2089	-0.0889	-0.1943	0.0012	0.07788	0.03693
XM_574651.1	LOC499341	0.11337	0.15545	0.22961	0.0043	0.10264	0.09355	0.20042	0.15003	0.05664	0.16399	0.03234	0.14134	-0.0018	0.1579
XM_574652.1	LOC499342	0.07598	0.01206	0.07711	0.01572	0.02546	0.01956	0.01752	0.04539	0.30172	0.15538	0.06759	0.17156	0.08661	0.11681
XM_574654.1	LOC499344	-0.1677	-0.1268	-0.0239	0.16681	-0.1776	-0.0418	-0.1057	0.01304	0.04543	-0.0969	0.00133	-0.0976	-0.0835	-0.137
XM_574658.1	LOC499348	-0.0245	-0.137	0.05911	0.17115	0.06719	0.04218	0.16766	-0.0485	-0.0248	0.01768	0.11826	0.05476	0.1161	0.04509
XM_574663.1	LOC499351	0.1243	-0.0361	-0.0905	0.15752	-0.0398	-0.0727	0.09814	-0.0563	-0.0334	-0.0112	-0.1464	-0.0615	0.31522	-0.0948
XM_574667.1	LOC499355	0.04943	0.05425	0.01436	0.23695	0.05945	0.08994	0.06117	0.21123	-0.048	0.07487	0.2529	0.03096	-0.028	0.06315
XM_579951.1	LOC499357	-0.4094	-0.1568	-0.1227	-0.193	-0.454	-0.426	0.02195	-0.5484	-0.5022	-0.4394	-0.2571	-0.093	-0.4299	-0.3223
XM_574671.1	LOC499358	0.1206	-0.0772	0.06138	-0.0297	0.06666	-0.1191	-0.0638	-0.0523	-0.0767	0.05866	-0.0731	-0.1319	0.10062	-0.045
XM_574672.1	LOC499359	0.25077	0.19722	0.075	0.1298	0.09181	0.26652	0.29082	0.07514	0.1234	0.3352	0.12156	0.18635	0.09525	0.01611
NM_001134612	LOC499360	0.25333	0.25622	0.02018	0.29096	0.15569	0.18932	-0.0229	0.37253	0.24585	0.09233	0.18879	0.07949	0.10298	0.00526
XM_579952.1	LOC499365	-0.1924	-0.1655	0.14212	0.35311	-0.063	-0.1195	0.00823	-0.0855	-0.2794	-0.1573	-0.2554	-0.2694	-0.2941	-0.2432
XM_574679.1	LOC499366	0.11134	0.09873	0.2563	0.11058	0.01922	0.38495	0.011	0.06023	-0.066	-0.0363	0.00911	0.13332	0.33621	0.10323
XM_579953.1	LOC499369	-0.0235	0.22768	-0.3896	-0.52	-0.2024	-0.0728	-0.1929	-0.0167	0.1709	0.28002	0.26664	-0.1592	-0.2368	-0.2553
XM_574686.1	LOC499371	-0.262	0.02926	0.12367	-0.0736	0.09519	0.12677	0.1305	0.0649	0.0428	-0.0447	0.026	0.34176	0.19607	0.00352
XM_574687.1	LOC499372	0.17075	-0.2453	0.41294	0.5867	0.00281	-0.0003	0.85021	0.17752	-0.0723	-0.1685	0.05352	-0.1686	-0.1648	-0.0739
XM_579954.1	LOC499375	-0.0781	-0.0278	0.14636	-0.0131	-0.0002	0.40161	-0.1226	0.12963	0.12154	0.11742	0.15613	-0.0275	0.02734	0.07672
XM_579955.2	LOC499376	-0.0084	-0.1223	-0.2966	-0.0163	0.03945	-0.1238	-0.1774	-0.2415	0.14277	-0.0676	-0.1002	-0.0189	-0.1332	-0.2798
XM_574695.1	LOC499378	0.18727	-0.0975	0.00906	0.40907	0.3657	0.26327	0.12016	0.33838	-0.1076	0.09059	0.25246	-0.0296	-0.1186	0.10166
XM_574699.1	LOC499381	-0.0575	-0.1643	0.01858	-0.2183	-0.0734	-0.1525	0.07332	-0.1122	-0.0639	-0.2136	-0.1054	-0.005	-0.1935	-0.1986
XM_345054.2	LOC499383	0.00717	0.02526	0.00378	0.03145	-0.0104	-0.0037	-0.0044	0.07359	-0.0637	0.15861	-0.0339	-0.0883	-0.091	0.18861
XM_579956.1	LOC499384	0.02113	-0.0408	-0.1136	-0.035	-0.1062	0.09913	-0.0549	-0.0427	-0.1493	-0.1416	-0.0958	0.08669	-0.0255	-0.0917
XM_579957.1	LOC499386	0.10345	0.27283	0.10052	0.0317	0.25923	0.13252	0.0607	0.11796	0.1045	0.11867	-0.0209	0.07433	0.014	0.12462
XM_579958.1	LOC499387	-0.3394	-0.2678	-0.1369	-0.3156	-0.0973	0.08935	-0.2323	-0.292	0.03489	-0.2042	-0.1912	-0.3175	-0.278	-0.1922
XM_574702.1	LOC499388	-0.0016	0.17754	0.234	0.03628	0.01476	0.09016	0.00178	0.13629	0.0123	-0.0914	0.27901	0.26615	0.0555	0.23807
NM_001135780	LOC499392	-0.0079	0.02367	0.0903	0.00041	0.01012	-0.0612	0.04818	0.21932	0.07998	0.26299	-0.0046	0.15593	0.16361	0.19735
XM_574709.1	LOC499393	0.0034	0.08843	-0.0022	0.03636	0.08711	-0.0211	-0.0144	-0.0157	0.17855	-0.0484	-0.0332	0.11149	0.02951	0.17115
XM_574710.1	LOC499394	0.13885	-0.1306	-0.1657	-0.0246	-0.0461	-0.152	-0.1273	0.00012	-0.0407	0.13286	0.18606	-0.1274	0.04491	-0.0065
NM_001168353	LOC499400	0.09778	0.20435	0.09062	0.07309	0.10426	0.16872	-0.0773	-0.0735	0.1062	-0.035	-0.0883	0.08272	0.01258	0.0324
XM_579960.1	LOC499404	0.16834	0.14856	0.80337	0.18172	0.03179	0.10722	0.26112	-0.1891	0.26394	0.31175	0.61678	0.3121	0.29594	0.20379
NM_001047939	LOC499407	-0.0036	-0.1285	-0.0048	0.12091	0.05258	-0.0508	0.00261	0.04043	-0.0834	-0.0642	0.0451	0.06426	0.13543	0.04277
XM_579962.1	LOC499408	-0.2939	-0.2551	-0.104	-0.2715	-0.3403	-0.2414	-0.1091	0.03425	-0.2927	-0.2423	-0.3633	-0.093	-0.1027	-0.1902
XM_579963.1	LOC499409	-0.2068	0.21287	0.04738	0.02501	-0.1912	-0.0718	-0.055	-0.0863	0.0704	-0.1268	-0.1914	0.25394	-0.1675	0.09388
XM_574728.1	LOC499412	0.09202	-0.0907	-0.0902	-0.1401	-0.0616	-0.0476	0.09915	-0.2279	-0.1929	0.11379	0.14536	-0.078	-0.0126	-0.1159
XM_579966.1	LOC499414	-0.1536	-0.3041	-0.2787	-0.3867	0.05595	0.01387	-0.4068	-0.1229	-0.1824	-0.3452	-0.2652	-0.212	-0.249	-0.2175
XM_579966	LOC499414	-0.0248	0.03345	-0.0451	-0.2888	0.02768	0.23634	-0.1205	0.33052	-0.0971	-0.1638	-0.0828	0.25022	0.14729	-0.1606
XM_574731.1	LOC499415	0.09167	-0.1193	0.11556	0.06901	-0.156	-0.0326	0.11657	-0.0773	-0.1418	0.11158	-0.0444	0.32445	-0.0451	-0.0018
XM_574731	LOC499415	-0.1039	-0.0813	-0.132	-0.021	-0.0798	-0.1087	-0.0756	-0.0961	-0.0945	-0.1021	-0.1898	-0.1215	0.06272	-0.2464
XM_574732.1	LOC499416	-0.0143	0.07248	0.09338	-0.0143	0.33745	0.08587	0.09513	0.17435	0.24167	-0.0027	-0.0027	-0.057	0.0521	0.03015
NM_001024296	LOC499418	0.27148	0.23858	0.02681	0.04443	0.20734	-0.1273	0.05748	0.24587	0.37428	0.16321	0.14089	0.04515	0.1865	0.26463

XM_574739.1	LOC499421	0.19208	0.33663	0.15235	0.22795	0.25355	0.39678	0.11969	0.30723	0.40477	0.10665	0.29571	0.31479	0.20872	0.25371
NM_001137643	LOC499422	0.11936	0.03359	0.09062	-0.0072	0.1	0.1517	0.09125	0.09445	-0.086	-0.0155	0.11333	-0.0921	-0.0994	0.1137
XM_574752.1	LOC499427	-0.2248	0.04655	-0.2349	-0.4598	-0.2457	0.10717	-0.3859	-0.3543	0.05051	0.12797	0.04438	0.40784	0.07621	0.02407
XM_579967.1	LOC499428	-0.2823	-0.1296	-0.0169	0.58437	-0.1626	0.0062	-0.0914	-0.1179	0.12106	-0.1268	-0.1213	-0.3239	-0.0821	-0.2648
XM_574753.1	LOC499429	-0.0534	-0.0485	-0.0738	0.10289	-0.142	-0.0587	-0.1962	0.03019	-0.0008	-0.038	-0.2301	0.2066	-0.1202	-0.0371
NM_001109172	LOC499431	0.15624	0.07773	0.20015	0.0268	0.75737	0.87016	0.19708	0.38173	0.28703	-0.0596	-0.0803	0.10194	0.38534	0.15982
XM_574757.1	LOC499434	-0.195	-0.0973	-0.0456	0.12388	0.13373	-0.2197	0.0272	-0.0043	0.00913	-0.0337	-0.1379	0.20483	-0.2054	-0.0674
XM_574760.1	LOC499437	0.08151	0.17719	0.10293	-0.1015	-0.1859	-0.2296	0.20642	-0.0113	0.19849	0.14018	0.01275	0.32037	0.16592	0.20974
XM_574761.1	LOC499438	0.05775	0.26424	0.21293	0.10887	0.09759	0.02821	-0.0058	0.02031	-0.0448	-0.0251	-0.0077	0.29542	0.0465	0.12951
XM_574768.1	LOC499445	0.02236	0.08944	-0.0491	0.13237	-0.0576	0.06195	-0.1003	-0.0609	0.09301	-0.0258	0.03187	0.02785	0.12303	-0.0026
XM_574769.1	LOC499446	0.02674	-0.0444	-0.0808	-0.1069	0.04204	0.13417	0.02844	-0.0748	0.11353	-0.0216	-0.047	0.1137	-0.004	-0.0329
XM_574770.1	LOC499447	0.02037	-0.1162	0.09941	-0.106	0.07275	-0.1723	-0.1206	0.13859	-0.0262	-0.1932	-0.0754	-0.1108	-0.1355	-0.0759
XM_574771.1	LOC499448	-0.0822	-0.0357	-0.0079	-0.0297	-0.059	0.06284	-0.0634	-0.1068	-0.1081	0.0936	0.03078	-0.0684	-0.0093	-0.076
XM_574773.1	LOC499450	0.03663	0.08104	0.13436	-0.0075	0.09379	0.0645	0.18037	0.12204	0.16977	0.0488	0.09858	0.00192	0.03352	0.04987
XM_574774.1	LOC499451	0.02929	-0.0185	0.03071	-0.0402	-0.0576	0.01736	-0.0194	0.24354	-0.1185	-0.011	0.10895	-0.015	0.09187	-0.0001
XM_574782.1	LOC499458	0.04191	-0.0803	0.01116	-0.0983	-0.096	0.0532	-0.0708	-0.0326	-0.109	0.42104	0.04028	-0.049	0.13572	-0.0602
XM_574783.1	LOC499459	-0.0535	0.07709	0.02327	0.13592	-0.0252	0.19588	-0.022	-0.0773	-0.0875	-0.0776	-0.0668	0.16622	0.10868	0.02701
XM_574784.1	LOC499460	0.23111	0.04034	0.02185	0.00794	-0.0639	0.01326	0.07484	0.15474	0.02576	-0.0192	-0.1206	-0.0623	0.04667	-0.1163
XM_574786.1	LOC499462	-0.0266	0.24408	-0.1489	-0.0294	0.18781	0.11684	-0.0732	-0.0169	0.11574	-0.238	0.04324	0.33361	0.18043	0.15692
XM_574788.1	LOC499464	-0.3512	-0.0075	-0.0768	0.34671	-0.1591	-0.2241	0.33807	-0.1529	-0.0721	-0.0546	0.17828	-0.0669	-0.3239	-0.2922
NM_001025038	LOC499465	-0.0367	0.10123	0.04839	0.10293	0.30695	0.0837	-0.2646	0.3515	-0.1974	0.08973	-0.1317	-0.3558	-0.1334	0.02418
NM_001047940	LOC499469	0.06901	-0.0524	0.10761	0.30948	0.32188	-0.0164	0.06148	0.29304	0.06685	-0.0027	0.03118	0.08057	-0.0381	0.00302
XM_579969.1	LOC499472	0.11366	0.16648	0.1367	0.0698	0.12653	0.16867	0.29321	0.03816	0.0601	0.03782	0.05228	0.10395	0.31924	0.00716
XM_574799.1	LOC499476	0.12771	0.28395	0.08719	0.04803	0.18903	-0.0307	0.0283	0.21495	0.32165	0.15368	0.25018	-0.2047	-0.0065	0.02429
XM_574800.1	LOC499477	-0.296	-0.2663	-0.0527	-0.2441	-0.3431	-0.1544	-0.1921	-0.2477	-0.2701	-0.1579	-0.1586	-0.2509	0.04423	0.00309
XM_574801.1	LOC499478	0.03462	-0.1144	0.05057	0.04006	0.02123	-0.0614	0.12339	0.13741	0.01525	0.22914	0.06053	-0.1781	0.05516	0.04867
XM_574803.1	LOC499480	-0.4296	-0.0268	-0.0149	-0.2544	-0.3112	-0.363	0.09669	-0.4132	-0.305	-0.0632	-0.2883	-0.111	-0.544	-0.4468
XM_574804.1	LOC499481	-0.0838	-0.014	0.31966	0.58795	-0.1629	-0.1437	0.86937	0.1707	0.01523	-0.2078	0.20797	-0.0943	-0.2451	-0.13
XM_574817.1	LOC499492	0.17704	0.1067	-0.1876	0.08535	0.04961	0.12186	0.08442	0.05658	-0.0544	-0.2436	-0.0364	0.06013	0.14554	-0.0685
XM_574820.1	LOC499495	0.63474	0.35237	-0.388	0.31268	0.19617	-0.5031	0.07389	0.24736	0.19097	0.25452	-0.0672	-0.4707	0.25416	0.46753
XM_579970.1	LOC499496	-0.1958	-0.3448	-0.3234	-0.3016	0.11771	-0.2203	0.02477	-0.0737	-0.4717	-0.19	-0.2906	-0.36	-0.2564	-0.3096
XM_574825.1	LOC499501	-0.1855	-1.1106	0.42911	-0.5747	-0.8667	-0.9831	-0.9332	-0.8868	-0.2403	-0.8741	-0.6292	0.52634	0.35143	0.24499
XM_574826.1	LOC499502	-0.11	-0.0508	-0.1268	-0.0232	-0.008	-0.0698	-0.1099	0.02631	-0.0976	-0.0052	0.16956	-0.0786	-0.0608	-0.1125
XM_574827.1	LOC499503	0.22133	0.21133	0.07582	0.36375	0.01257	0.15743	0.37401	0.06676	0.08777	-0.0487	0.04434	0.24401	0.04746	0.10828
XM_574828.1	LOC499504	0.11994	-0.1015	0.0269	-0.043	-0.048	-0.0077	-0.004	-0.0264	-0.0389	0.04458	0.00773	0.00918	-0.0082	-0.0374
NM_001109174	LOC499506	-0.0561	-0.0604	0.05863	0.11566	0.09103	0.26553	0.01704	-0.0667	0.05462	-0.2033	-0.1865	-0.0344	-0.0095	-0.0622
XM_574836.1	LOC499511	-0.156	-0.0642	0.09379	-0.2432	0.18054	-0.2157	-0.0232	-0.1313	-0.2881	0.44156	-0.2386	-0.019	-0.177	-0.1265
XM_579971.1	LOC499513	-0.0869	0.1038	-0.0166	0.01436	0.13283	0.14186	-0.0115	0.07468	-0.1331	0.02904	0.15916	0.09995	-0.0413	0.01038
XM_574838.1	LOC499514	0.06344	-0.037	0.13958	0.13862	-0.0499	-0.0197	0.12913	0.10156	0.17441	-0.0529	0.04953	-0.0452	0.08774	0.08448
XM_579972.1	LOC499515	-0.0256	0.06901	-0.118	0.07974	-0.0275	0.04787	0.04988	-0.0955	0.01631	-0.1214	0.09766	-0.0794	0.01602	0.05872
XM_579973.1	LOC499519	0.10644	0.2804	0.1265	0.00135	0.00214	0.06897	0.15585	0.01647	0.32301	0.19205	0.43585	0.06927	0.50924	0.06533
XM_574845.1	LOC499521	-0.0435	0.03356	0.09847	-0.1367	0.0508	-0.1197	0.01335	0.15581	0.48582	0.03432	0.13409	-0.0048	-0.0479	-0.0637
NM_001108283	LOC499523	0.14896	0.1019	-0.2544	0.17012	0.12164	0.22717	-0.2896	0.0942	0.07266	0.20627	0.01525	-0.0186	0.01953	-0.0039
XM_574849.1	LOC499524	-0.178	0.01605	-0.0371	-0.1161	-0.2093	0.23755	0.11005	-0.0308	-0.1228	-0.2549	0.06653	-0.0752	-0.013	-0.0048
XM_579975.1	LOC499527	-0.0528	-0.0089	-0.0326	-0.0348	-0.0859	0.04218	-0.0643	0.06161	-0.1195	-0.0996	-0.0828	0.0913	-0.0604	0.07389
NM_001025146	LOC499529	-0.0455	-0.0074	-0.0177	0.10457	-0.0159	0.09862	-0.0539	0.13771	0.07338	0.05851	0.02407	-0.0258	-0.0238	0.14361
NM_001047941	LOC499530	-0.097	0.15465	0.11654	0.13805	0.20739	0.32505	-0.0754	0.10978	-0.0939	-0.0571	0.36677	0.24697	0.33364	0.17125
XM_574855.1	LOC499531	-0.4122	-0.1174	0.41997	0.67272	-0.5536	-0.543	0.83998	-0.2895	-0.0082	-0.1321	0.23623	-0.4205	-0.5412	-0.2692
XM_579976.1	LOC499533	0.04448	0.14665	0.04112	-0.0201	-0.0054	-0.0246	-0.0105	0.16176	0.27666	0.08654	0.24014	0.08232	0.21502	0.23512

XM_579977.1	LOC499534	-0.033	0.04999	0.02678	0.03639	-0.1616	0.02687	0.02307	-0.1794	-0.1942	-0.0662	0.11251	0.08828	0.11455	-0.0765
XM_579978.1	LOC499535	0.00026	0.00916	0.26915	0.08757	0.08736	0.08831	0.06131	0.15025	0.02423	0.35469	0.01438	0.06224	0.08381	0.20473
NM_001109176	LOC499537	0.07472	0.05776	0.01548	0.05932	0.198	-0.0101	0.13921	0.05074	0.18705	0.04839	0.22492	0.19561	-0.0323	0.31266
XM_574863.1	LOC499540	-0.1686	0.02268	0.0464	0.08216	-0.1529	-0.1573	0.08838	0.05074	-0.1154	0.15429	-0.0282	0.16472	0.00732	0.05953
NM_001047942	LOC499541	-0.0057	-0.1229	0.09656	-0.1083	-0.0952	0.07151	-0.0575	-0.1568	-0.2046	0.03463	-0.1002	-0.0606	-0.0754	0.01173
NM_001047943	LOC499542	-0.1739	-0.27	-0.1365	-0.2532	-0.2775	-0.1952	-0.1433	-0.1398	-0.2055	-0.2203	-0.2828	-0.0207	-0.1694	-0.1809
NM_001047944	LOC499544	0.04366	-0.0204	-0.0842	-0.0566	-0.0142	-0.0173	0.03571	0.00895	0.08607	-0.0604	0.22356	0.00631	-0.0152	-0.068
XM_579979.1	LOC499545	0.0265	0.02591	0.22805	0.14169	0.01241	0.12575	0.14825	-0.0306	0.08319	0.06622	0.02345	0.13991	0.0414	-0.0334
XM_574872.1	LOC499547	0.19966	0.12519	0.00189	0.32763	-0.0686	0.10232	0.13167	0.19059	0.00948	0.00327	0.11837	-0.0238	0.21952	0.23234
XM_574873.1	LOC499548	-0.1425	-0.0362	-0.1534	-0.0802	-0.0728	-0.1469	-0.0297	0.03677	-0.1171	0.07285	-0.1778	-0.0828	-0.1211	-0.0364
XM_574875.1	LOC499550	-0.0454	0.13077	-0.1198	-0.0435	-0.0546	-0.0679	0.1061	-0.0474	-0.0038	-0.007	-0.0974	-0.0793	-0.0394	0.03131
XM_574876.1	LOC499551	-0.0341	-0.0091	0.00646	0.12623	-0.0777	0.04593	0.0695	-0.044	-0.0183	0.4459	0.01563	-0.0117	-0.0627	-0.0443
XM_574878.1	LOC499553	-0.0212	0.03824	0.13828	0.16314	0.13198	0.22012	-0.0225	0.02114	-0.0316	0.31776	0.04614	0.07875	0.36275	0.0491
XM_574879.1	LOC499554	-0.5736	-0.2703	-0.7261	-0.3861	-1.302	-1.2634	0.40326	-0.8425	-0.3428	-0.3197	-0.3392	-0.31	-1.1606	-1.3321
XM_574880.1	LOC499555	0.30681	0.1998	0.21528	0.24507	0.15993	-0.2761	0.54021	0.37081	-0.1281	-0.1833	0.03453	-0.0709	-0.0703	-0.0625
XM_579980.1	LOC499559	0.10005	-0.0349	-0.1091	-0.0772	0.01539	0.02997	-0.152	0.13912	-0.1557	0.02805	-0.079	-0.0223	-0.1821	0.02333
XM_574884.1	LOC499560	-0.6024	-0.2256	-1.435	-1.0885	-1.0324	-1.5286	-0.3455	-0.7759	-0.3049	0.01592	-0.1924	-0.598	-0.8225	-1.2396
XM_574886.1	LOC499562	0.0835	0.03783	0.05231	0.00468	-0.007	0.13643	0.10684	0.04369	0.07072	0.09078	0.08988	0.23587	0.30193	0.11648
XM_574888.1	LOC499564	-0.5204	-0.3619	0.06137	0.3209	-0.9196	-0.7664	0.36711	-0.4599	-0.3576	-0.5178	-0.0203	-0.4743	-0.6547	-0.5292
NM_001047946	LOC499565	0.02006	-0.1103	-0.0864	0.21884	-0.1571	-0.105	0.04184	0.08156	-0.1455	0.15211	0.05999	0.03306	-0.0374	0.0784
NM_001134993	LOC499566	0.12781	0.03958	-0.3306	-0.1745	-0.0902	-0.1543	-0.2653	0.01262	-0.2456	0.31564	-0.1019	-0.2757	0.09634	-0.1229
XM_574893.1	LOC499568	-0.0885	-0.1378	-0.0121	0.15226	-0.2178	-0.0254	-0.0588	-0.0898	-0.0273	0.00588	-0.214	-0.0475	0.04813	0.01431
XM_579981.1	LOC499569	0.01218	0.21741	-0.1321	0.07689	0.18561	-0.1842	0.11416	0.16933	0.29261	0.28407	-0.025	-0.0243	0.08891	0.08953
XM_574896.1	LOC499572	0.23214	0.04689	-0.0103	-0.0155	0.00593	0.21754	-0.0155	-0.038	-0.0474	0.0636	0.07028	0.19659	-0.015	0.24335
NM_001047947	LOC499573	0.00672	0.11412	0.0696	-0.0738	0.05842	0.17349	-0.0423	0.00095	0.1132	-0.112	0.16558	0.00582	0.19757	0.20871
XM_574900.1	LOC499575	0.04141	-0.0279	0.16579	0.37785	-0.0942	0.0571	0.67791	0.08885	0.04323	-0.1217	-0.053	0.11823	-0.119	-0.0325
XM_574902.1	LOC499577	0.07011	-0.0008	-0.013	0.06195	0.042	0.00734	-0.0011	0.00974	-0.0627	0.00253	0.07032	0.03592	0.0086	0.14391
XM_574903.1	LOC499578	0.09441	0.12947	0.10019	0.06471	0.12871	0.28015	0.14134	0.1019	0.05318	0.17842	0.02262	0.06288	0.11919	-0.0263
XM_574906.1	LOC499581	0.01105	0.00211	0.01188	0.53819	-0.2066	0.06344	0.10518	-0.0206	-0.1059	-0.1163	0.06919	0.07492	-0.0731	-0.0589
XM_574907.1	LOC499582	-0.1955	-0.1342	0.16702	0.6459	-0.1774	-0.0428	0.97537	0.03151	-0.0021	-0.1718	0.25468	-0.4058	-0.0128	0.0141
NM_001047948	LOC499584	-0.0293	-0.0346	0.07	0.33797	0.04464	0.05776	-0.0213	0.10648	0.27922	0.2082	0.22694	0.25672	-0.0162	0.10146
XM_574910.1	LOC499585	0.01662	-0.1197	-0.0302	0.12265	-0.051	0.09585	-0.0588	-0.0381	0.08872	-0.1738	0.09661	0.11289	0.04418	-0.1441
NM_001164439	LOC499586	-0.1332	0.02172	-0.0839	-0.0859	-0.1895	-0.2075	-0.2668	-0.2245	0.02736	-0.2644	-0.3345	-0.2128	-0.1925	0.01393
NM_001134615	LOC499587	-0.3428	-0.0915	-0.1858	-0.2648	-0.0337	-0.3076	0.01548	-0.2701	-0.1023	-0.2706	-0.3471	-0.2191	-0.225	0.02197
XM_574915.1	LOC499590	0.31185	0.57497	0.22197	0.41573	0.60302	0.1235	0.82016	0.72544	0.48002	0.21795	0.4986	0.06296	0.28732	0.0347
XM_574917.1	LOC499591	0.05106	-0.0301	0.01642	-0.018	-0.0682	-0.1326	-0.0181	-0.0677	-0.1067	0.11472	0.31737	-0.0721	-0.1603	-0.0327
XM_579983.1	LOC499594	-0.089	-0.1181	-0.148	0.00377	-0.1593	-0.03	-0.2155	-0.111	-0.0362	-0.1909	-0.0756	-0.0748	-0.1148	-0.0123
XM_579984.1	LOC499595	-0.1035	-0.1051	-0.227	-0.1836	-0.0034	-0.235	-0.1398	-0.2218	0.13362	-0.2001	-0.203	0.14571	-0.0841	-0.0438
XM_574920.1	LOC499596	-0.0201	0.18896	0.03862	0.05437	0.18765	-0.0906	0.03841	-0.0529	-0.0424	0.0312	0.12482	0.05186	-0.0338	0.05168
XM_574922.1	LOC499597	-0.6567	-0.7138	-0.0395	0.22707	-1.0421	-0.8384	-0.1952	-0.7322	-0.3379	-0.4883	-0.5871	-0.0351	-0.3339	-0.1885
NM_001025039	LOC499602	0.09816	0.18147	-0.0681	0.16202	0.24536	-0.2449	0.11224	-0.0645	-0.1821	0.2026	0.19348	-0.1168	-0.0741	-0.0451
XM_574931.1	LOC499605	0.03256	0.04612	0.04374	0.2063	-0.2475	0.09103	0.04368	-0.1919	-0.2236	-0.0199	-0.0703	-0.2208	-0.0662	-0.0235
XM_574933.1	LOC499607	-0.0326	-0.0612	-0.0293	-0.0996	-0.1607	-0.0868	-0.0509	-0.0557	-0.0638	-0.089	-0.0505	-0.0646	0.03727	0.02871
XM_574934.1	LOC499608	-0.061	0.35102	0.13403	0.2938	-0.1307	-0.1523	0.46274	-0.0913	0.23737	0.28628	0.33691	0.09559	-0.2076	0.00393
XM_574935.1	LOC499609	0.13117	0.81842	0.83698	1.0084	0.20445	-0.0173	0.97979	0.24357	0.66162	0.88897	0.70343	0.80645	0.28534	0.32953
XM_574937.1	LOC499611	-0.2835	-0.0936	-0.3003	-0.1546	-0.1616	0.12873	-0.1354	-0.2654	-0.0452	-0.1037	-0.3004	0.10992	-0.0305	0.05879
XM_574939.1	LOC499612	0.15328	-0.1106	0.42382	0.57642	0.22669	0.08609	0.18208	0.17443	-0.2506	0.03201	0.27978	0.27232	0.09127	-0.0417
XM_579985.1	LOC499613	-0.4687	-0.3741	-0.3622	0.22013	-0.3148	-0.226	-0.1946	-0.4765	-0.1661	-0.0628	-0.1795	-0.2902	-0.5303	-0.4767
XM_579986.1	LOC499614	-0.507	-0.4198	-0.4743	-0.6507	-0.6622	-0.6069	-0.0285	-0.5182	-0.3831	-0.1438	-0.3568	-0.3118	-0.8154	-0.6628

XM_579987.1	LOC499616	0.19429	-0.0659	0.01286	-0.0113	0.19543	0.33094	-0.3358	0.29087	0.05351	-0.0087	0.24815	0.18071	-0.1319	0.03993
NM_001044263	LOC499617	-0.1388	-0.1532	-0.0089	-0.0699	-0.085	-0.1205	-0.0759	-0.2341	0.05478	0.00924	-0.122	-0.3349	-0.2342	-0.1441
NM_001109184	LOC499618	-0.0807	-0.0397	-0.1754	-0.1588	-0.2342	0.00691	-0.2212	-0.1175	-0.1592	-0.2244	-0.2075	0.01493	-0.1248	-0.1611
XM_579988.1	LOC499620	-0.6014	0.06949	0.20615	0.73812	-0.9966	-0.4708	0.34053	-0.6255	-0.2242	-0.2885	-0.0623	0.29845	-0.8914	-1.042
XM_579989.1	LOC499623	0.01932	-0.0539	0.01976	-0.079	-0.0672	-0.2124	-0.0042	-0.0685	0.0719	-0.0026	0.25666	-0.0985	0.09862	-0.1969
XM_574948.1	LOC499624	0.1908	0.02441	0.4605	0.12713	-0.0277	-0.0101	0.27306	-0.0289	-0.0445	0.03887	-0.224	-0.1416	0.05876	0.22918
XM_574949.1	LOC499625	0.17568	0.43548	-0.4553	-0.6094	0.06472	-0.4454	0.01685	0.18599	0.48578	0.13749	0.1931	0.44539	0.61265	0.51103
XM_579991.1	LOC499632	0.09015	0.13327	0.18664	0.10942	0.09953	0.09108	0.21905	0.21847	0.11265	0.38831	0.12212	0.10231	0.0922	0.32473
XM_574956.1	LOC499633	-0.0589	-0.058	-0.1004	-0.0964	0.07619	-0.0211	-0.14	0.03316	-0.1414	-0.0479	0.03358	-0.1279	-0.148	-0.0857
XM_574957.1	LOC499634	-0.0685	-0.0936	-0.1639	-0.1082	0.06219	0.00852	-0.1192	0.00896	-0.0171	-0.0275	0.18135	-0.0345	-0.1042	-0.1643
XM_574958.1	LOC499635	0.04765	0.04934	0.0441	0.06575	-0.0289	0.07127	-0.0194	-0.0341	0.00281	-0.1593	-0.0108	0.29237	-0.1114	0.0195
NM_001007678	LOC499636	-0.2094	-0.2873	0.10118	-0.5184	-0.386	-0.2999	-0.3925	-0.4792	-0.0701	-0.1162	-0.3634	0.57684	0.21123	0.39713
XM_579992.1	LOC499637	0.09439	-0.0278	0.05736	0.02693	-0.0331	-0.041	0.031	-0.0065	-0.0753	0.06638	0.09643	-0.0038	-0.0084	-0.0409
XM_574960.1	LOC499638	-0.0308	-0.071	0.42475	0.83462	-0.0962	0.10214	1.1083	0.08464	-0.0149	-0.1702	0.08439	-0.0291	-0.0829	0.00022
XM_579993.1	LOC499639	0.06306	0.03058	0.02989	0.1076	0.02329	-0.033	-0.037	0.20935	0.17647	0.07529	-0.053	0.07676	-0.018	0.05627
XM_574961.1	LOC499640	-0.1518	-0.1289	-0.0116	0.05823	-0.0937	-0.1193	-0.1183	-0.1102	-0.1127	-0.1151	0.02094	-0.1112	0.31191	-0.1385
XM_579994.1	LOC499642	-0.2375	-0.2563	-0.0934	-0.1294	-0.1659	-0.235	0.04071	-0.09	-0.2785	-0.2343	-0.2991	-0.123	-0.1127	0.09493
NM_001024301	LOC499643	-0.0799	-0.2468	-0.2121	-0.0876	-0.1846	-0.1426	0.03386	-0.2252	-0.0607	-0.125	-0.0728	-0.1522	-0.032	-0.2367
XM_579995.1	LOC499646	0.10259	0.0347	0.13205	0.02164	0.19967	0.14028	0.06885	-0.0227	0.201	0.03656	0.12124	0.14026	0.05554	0.03138
XM_579996.1	LOC499647	-0.0924	-0.085	-0.036	-0.0435	0.0565	0.11946	0.14451	-0.0489	0.14117	0.03187	-0.017	0.06298	-0.0684	-0.0032
NM_001024797	LOC499653	0.26429	0.22424	0.26104	0.07243	0.14678	0.01707	0.08339	0.05368	0.04764	-0.0259	-0.0294	0.0639	0.19314	0.10743
XM_574978.1	LOC499654	0.15239	0.02668	-0.2701	-0.1804	-0.1877	-0.2253	-0.138	-0.0124	-0.1302	-0.2241	-0.0283	-0.5181	-0.2243	-0.1031
XM_001057607.1	LOC499658	0.15264	-0.0031	-0.0238	-0.0835	-0.0515	0.00902	-0.1049	0.19889	0.10667	-0.0738	0.07554	-0.0699	-0.0057	-0.0064
XM_574983.1	LOC499659	0.00364	0.00556	-0.063	-0.0253	0.01106	0.14445	0.23714	0.02915	0.36528	-0.0069	0.07937	0.26502	0.30891	0.03559
XM_001056859.1	LOC499660	2.7494	0.8839	1.8474	1.9705	2.5088	2.6877	1.5884	2.5466	0.76285	0.84388	0.91897	0.1543	1.3792	1.3938
XM_579998.1	LOC499661	0.17295	0.20986	0.14389	0.02591	0.08974	0.08986	-0.029	-0.0162	-0.0134	0.01183	0.07074	-0.0624	0.29043	0.04548
NM_001109493	LOC499664	-0.1759	-0.1662	0.19761	0.6115	0.06268	0.3752	-0.0293	0.16422	-0.2022	0.00118	-0.0019	0.24586	0.03926	0.27879
XM_580001.1	LOC499665	0.44149	0.08768	0.17353	0.27931	0.23693	0.21049	0.13235	0.12775	0.132	0.37141	0.35128	0.07331	0.14295	0.069
XM_580002.1	LOC499666	-0.1583	-0.0808	-0.0427	-0.0815	-0.1006	0.26759	0.06905	0.10843	-0.0898	-0.1562	0.08342	-0.2439	-0.1064	-0.3076
NM_001109190	LOC499669	0.15093	0.08123	-0.0944	0.01739	0.10503	0.24295	0.32265	-0.0813	0.00435	-0.0941	-0.1321	-0.084	0.00196	-0.1193
XM_574997.1	LOC499675	-0.1045	-0.365	0.10789	0.00303	0.25839	1.02	-0.0463	0.05563	0.18222	-0.421	0.05945	1.1739	0.14048	0.1666
XM_574998.1	LOC499676	-0.4148	-0.1782	-0.061	-0.1162	-0.3637	-0.1745	-0.0212	-0.3628	-0.3293	-0.4422	-0.309	-0.1004	-0.1376	-0.2849
NM_001024303	LOC499677	-0.1098	0.11162	-0.2336	0.01454	-0.567	-0.4425	0.2698	-0.5195	0.03543	-0.0814	0.05475	0.21286	-0.3169	-0.4581
XM_575004.1	LOC499679	-0.0335	0.0744	0.03191	0.06858	0.22576	-0.0409	0.0917	0.01166	-0.1037	-0.0775	-0.0773	0.31319	0.00727	0.08956
XM_575005.1	LOC499680	-0.3362	-0.3305	-0.1642	-0.1658	-0.2672	-0.3217	-0.2333	-0.2482	-0.1694	-0.2333	-0.2111	-0.1016	-0.247	-0.2999
XM_580004.1	LOC499683	0.18309	0.25739	0.13612	0.01723	0.03963	0.11986	0.25893	0.06339	0.22943	0.52291	0.17015	0.39179	0.19121	0.01079
XM_580005.1	LOC499684	0.21066	0.05856	0.25942	0.07634	0.1413	0.04089	0.27263	0.32795	0.22402	0.08269	0.07109	0.50504	0.42945	0.52008
XM_575009.1	LOC499686	0.6572	0.11682	0.07411	-0.0152	0.04302	-0.1227	-0.0484	-0.0022	0.23838	0.26686	0.02839	0.15084	0.07305	0.01718
XM_575011.1	LOC499687	0.06346	0.02092	0.07397	-0.0107	0.01717	0.16146	-0.0064	0.16205	0.06223	0.1248	-0.0144	0.04733	0.10346	-0.04
NM_001024305	LOC499691	-0.0032	-0.0293	-0.1108	0.36682	0.17112	-0.1184	0.38638	0.12378	0.07081	0.08234	-0.0146	-0.1731	0.03525	-0.1797
XM_575017.1	LOC499692	-0.1447	-0.2246	-0.1687	-0.2033	-0.0901	-0.1557	-0.2203	-0.1451	-0.1422	-0.2003	-0.0921	-0.1373	-0.2333	-0.2221
NM_031502	LOC499694	-0.0588	-0.0709	0.06329	0.0452	-0.1455	-0.0022	0.04896	0.03742	0.1358	-0.2148	-0.2373	0.01	0.01722	0.05545
XM_575022.1	LOC499696	-0.1647	0.1218	-0.0165	-0.0129	-0.03	-0.0772	-0.0814	0.01667	0.11849	0.22413	-0.1351	-0.0006	-0.11	0.06379
XM_575026.1	LOC499698	-0.0839	-0.2272	-0.0673	-0.0654	-0.18	-0.2645	-0.2465	-0.1532	-0.0042	-0.2878	-0.3054	-0.3027	-0.4206	-0.258
XM_575028.1	LOC499699	0.3817	0.28121	-0.4283	-0.3862	0.26421	-0.1815	-0.0373	0.38714	0.27945	0.40348	0.00323	0.05975	0.30294	0.37843
XM_575029.1	LOC499700	0.00749	0.19193	0.02283	0.04837	0.00606	0.09516	-0.0034	-0.045	0.09906	0.04186	0.09891	-0.0461	0.10011	0.05467
XM_580006.1	LOC499701	-0.6552	-0.5571	-0.1076	-0.4983	-0.6593	-0.6066	-0.2652	-0.5549	-0.6113	-0.3542	-0.5584	-0.4918	-0.7795	-0.697
XM_575030.1	LOC499702	0.06718	0.14211	-0.0107	0.02221	-0.1683	-0.0304	-0.0787	-0.0915	-0.1185	0.06377	0.05852	-0.0791	-0.0798	-0.0211
XM_575031.1	LOC499703	-0.0222	-0.0228	-0.0594	-0.0434	0.0865	-0.1301	-0.0433	0.08497	0.00303	0.00927	-0.0026	-0.0803	0.05289	-0.0916

XM_575032.1	LOC499704	0.15155	0.18284	0.17493	0.19945	0.097	0.1034	0.27538	0.08632	0.13955	0.09392	0.10506	0.24849	0.12431	0.10406
XM_575035.1	LOC499707	-0.1893	-0.1633	-0.032	0.20152	-0.1156	-0.0146	0.02051	-0.1894	-0.202	-0.182	0.02121	-0.0276	-0.1106	-0.0595
XM_575036.1	LOC499708	0.27121	-0.1036	0.60951	0.75578	0.29791	0.38325	-0.2783	0.52	0.22259	0.01762	0.09326	0.07211	0.38085	0.2342
XM_575038.1	LOC499710	-0.3234	-0.2329	-0.2684	-0.2872	-0.0811	-0.2317	-0.2441	-0.2189	-0.1765	-0.2618	-0.2346	-0.2986	-0.1044	-0.1384
NM_001047950	LOC499715	-0.1582	0.00676	-0.1435	-0.1786	-0.1225	-0.1887	-0.1198	-0.1105	-0.1139	-0.1466	0.04921	-0.0451	-0.0226	0.09003
XM_001078145.1	LOC499716	0.08275	-0.3078	-0.0271	-0.7901	-0.5824	-0.6161	0.05986	-0.3293	0.19872	-0.4351	-0.0798	0.13893	0.21921	0.22443
XM_001078222.1	LOC499718	0.12914	0.12839	0.12554	0.10787	0.05756	0.02819	0.1045	0.13202	0.17399	0.07127	0.0076	0.05316	0.04222	0.33848
XM_575052.1	LOC499720	0.01948	0.02158	-0.0205	0.14096	-0.0494	-0.0993	-0.1448	-0.1587	-0.0278	-0.0782	-0.0303	-0.0532	0.02515	-0.2014
NM_001134616	LOC499724	0.01434	0.13266	-0.0867	0.25103	-0.0494	-0.1608	0.21976	-0.1038	0.2601	0.03474	-0.0077	0.14825	0.03688	0.10671
XM_580007.1	LOC499726	0.15208	0.13342	0.19802	0.11361	-0.0336	-0.0775	0.5435	-0.0094	0.27826	0.27589	0.24721	0.44873	0.00199	0.11032
XM_580008.1	LOC499727	0.07204	0.10387	0.00737	0.18787	0.12058	0.1061	0.01434	0.05416	0.02248	0.1834	-0.0206	0.13897	-0.015	0.16987
NM_001024307	LOC499732	0.15528	0.19702	0.11419	0.14296	-0.0155	0.0933	0.06398	0.16411	0.19356	0.10459	0.18241	0.03112	-0.0393	0.02091
XM_575068.1	LOC499733	-0.7037	-0.2343	-0.8628	-0.5326	-0.8801	-0.7505	-0.3101	-0.5458	-0.3946	-0.2729	-0.3282	-0.343	-0.7873	-0.8004
XM_580009.1	LOC499734	-0.1034	-0.0253	-0.054	0.08794	-0.033	-0.1366	0.09463	-0.0362	-0.0163	0.0057	-0.0933	-0.0075	-0.1567	0.00424
XM_575071.1	LOC499737	0.31498	0.19025	0.06428	0.30033	0.14723	0.11191	0.45141	-0.0855	0.1035	0.00371	-0.0102	0.05171	0.06495	0.0471
XM_575072.1	LOC499738	-0.0389	-0.0007	0.09863	-0.0609	-0.0991	-0.0108	0.00523	0.13864	-0.0531	-0.0356	0.04675	0.00286	-0.0158	0.16914
XM_575076.1	LOC499741	0.02898	0.04219	-0.05	0.25924	-0.0732	-0.0247	-0.0349	-0.0612	0.05564	0.15655	0.02806	0.25763	0.06385	-0.024
NM_001024308	LOC499742	0.32264	0.3159	0.21637	0.22145	0.26964	0.20776	0.23756	0.09089	0.17287	0.11089	0.1932	0.1655	0.07883	0.11221
NM_001037190	LOC499743	0.38353	0.13434	0.37597	0.32425	-0.0033	0.62399	0.15469	0.32502	0.43484	0.23912	0.18094	0.25253	0.08991	0.37843
XM_575081.1	LOC499746	0.0383	0.24755	0.01501	0.20501	0.11448	-0.0839	0.1111	0.17922	-0.1503	0.04071	-0.0894	-0.1618	0.05534	0.11153
NM_001024309	LOC499749	-0.1497	-0.3221	0.39034	0.27997	0.2551	0.37766	0.33274	0.11605	-0.1124	0.12961	0.04794	0.16785	0.09168	-0.0356
XM_575085.1	LOC499750	0.05398	0.10018	0.07803	0.08581	0.13054	0.06165	-0.0665	-0.0342	0.11612	0.10117	0.10899	0.02308	0.01264	-0.0664
NM_001025040	LOC499754	0.38784	0.33821	0.09845	0.15445	0.20266	0.22187	0.32437	0.30889	0.10228	-0.0571	0.05443	0.0562	0.14682	0.21938
NM_001128137	LOC499756	-0.0679	-0.0529	-0.1531	-0.0166	0.02086	-0.1083	-0.0114	-0.1441	-0.108	-0.1567	0.05645	-0.1296	-0.1166	-0.0438
XM_575093.1	LOC499757	0.03673	0.09814	0.24705	0.09873	0.063	0.18476	-0.0043	0.18773	0.08141	0.24369	0.16078	0.08447	0.15728	0.13854
NM_001134617	LOC499759	-0.0648	-0.1034	-0.11	-0.04	-0.0549	0.05845	-0.0529	0.37395	-0.0258	0.02476	0.02332	0.15462	0.07188	-0.038
XM_575096.1	LOC499760	0.02141	-0.1199	0.41049	-0.063	-0.3582	-0.3031	0.00544	-0.3066	-0.1029	-0.0843	-0.2853	-0.2529	-0.0576	-0.1559
XM_231041.3	LOC499761	0.01206	0.09904	-0.0077	0.21342	-0.047	-0.0353	-0.1671	-0.1198	0.05582	0.15941	0.00691	-0.167	-0.1019	0.0678
NM_001127563	LOC499765	-0.0663	0.08881	0.0167	-0.0155	0.19065	-0.0667	0.10498	0.23818	-0.0373	0.01672	-0.0094	-0.0862	0.02982	0.04918
XM_575105.1	LOC499767	-1.181	-0.9525	0.00156	-1.3138	-1.3534	-1.3605	-0.5707	-1.5712	-0.4672	-0.7298	-0.713	-0.0945	-0.055	-0.0527
XM_575106.2	LOC499768	-0.0366	0.03501	0.07482	-0.115	0.18628	-0.0732	-0.0898	-0.1231	0.08347	0.0235	0.12556	0.1026	-0.0358	0.00769
XM_580011.1	LOC499769	0.23865	-0.0254	-0.0028	0.11519	-0.0486	0.06218	0.14596	0.00556	-0.0656	0.13715	0.0996	-0.0104	-0.0606	-0.0058
NM_001047110	LOC499770	-0.3545	-0.4881	0.27649	-0.6689	-0.5652	-0.5258	-0.0734	-0.6517	-0.2611	-0.4632	-0.0483	-0.1478	-0.2136	-0.1818
XM_580012.1	LOC499773	-0.2784	-0.1094	-0.2705	-0.2935	-0.2641	-0.2888	-0.0434	-0.3596	-0.2003	-0.304	-0.1672	-0.1498	-0.1897	-0.316
XM_580013.1	LOC499774	0.00707	-0.0601	0.37012	0.30477	-0.0182	-0.0392	0.09569	-0.0477	0.12216	-0.0734	-0.0147	0.01115	0.02593	-0.0591
XM_580014.1	LOC499775	-0.3771	0.03413	0.02914	0.36786	-0.6115	-0.4435	-0.5264	-0.5673	-0.1445	-0.1336	-0.2511	0.01216	-0.383	-0.4056
XM_575112.2	LOC499779	0.00712	-0.0908	0.09274	-0.1542	0.03566	0.18244	0.17182	-0.1288	0.10249	0.03534	0.13788	0.32583	0.07552	0.10431
XM_575114.2	LOC499781	-0.0034	-0.2206	-0.1268	-0.1479	0.01909	0.16975	0.18609	0.08716	-0.0322	0.10214	0.07473	-0.0017	0.20372	-0.1056
NM_001109198	LOC499782	0.03885	0.1039	0.09268	0.14879	0.00543	-0.1929	0.00627	-0.0607	0.02498	0.02078	0.01202	-0.0469	0.05114	-0.0021
XM_575119.1	LOC499784	0.01132	0.029	0.19938	0.37222	0.34087	-0.0462	0.11202	-0.0241	-0.0368	-0.0948	0.15508	0.44819	-0.1372	0.07321
XM_580016.1	LOC499785	-0.2511	-0.2807	0.95533	0.35007	-0.2475	-0.3514	0.0802	-0.3849	-0.0219	-0.2703	0.04265	0.3523	0.66502	0.69281
XM_575123.1	LOC499788	0.01605	0.03839	-0.0047	0.05704	0.05801	0.06448	0.06187	0.02724	0.0115	0.17655	-0.007	0.06641	0.06516	0.0496
XM_580017.1	LOC499790	-0.2925	-0.2022	-0.2001	0.02646	-0.1991	-0.3139	-0.2034	-0.3502	-0.1208	-0.1165	-0.2358	0.14066	0.00388	-0.2725
XM_575126.1	LOC499791	0.10454	-0.1138	0.06988	0.05862	0.19285	0.09756	0.11142	0.21841	-0.0826	-0.1008	0.0909	-0.1137	-0.0111	0.01128
XM_575127.1	LOC499792	-0.2162	-0.0756	-0.0262	-0.137	-0.2308	-0.1744	-0.03	-0.1824	-0.0052	0.09444	-0.183	-0.0983	0.26891	-0.0406
XM_575129.1	LOC499793	-0.2295	-0.1232	-0.6245	-0.5242	-0.4632	-0.1145	-1.1584	-0.7479	-0.3174	-0.0868	-0.2469	-0.1903	-0.6358	-0.5802
NM_031100	LOC499794	-1.2884	-1.1132	0.42539	-0.5379	-1.5221	-1.2122	-0.3616	-1.4706	-1.0052	-1.0568	-0.8289	-0.5624	-1.2255	-1.1537
NM_001013917	LOC499795	0.03186	0.1311	0.09058	-0.0612	-0.0051	-0.0983	-0.1195	-0.1026	-0.016	-0.1078	-0.108	-0.0928	-0.0099	0.00385
NM_001047951	LOC499796	-0.067	0.11647	0.01303	-0.0256	-0.1204	-0.0127	-0.0266	0.0117	-0.0613	0.00047	0.07036	-0.0858	-0.0675	0.03713

XM_575136.1	LOC499799	0.00689	0.00376	-0.091	-0.2366	0.27701	0.17342	-0.0414	-0.0624	0.09521	-0.1223	0.06741	-0.0553	-0.1319	0.16496
XM_575142.1	LOC499802	0.10641	0.07032	0.16855	-0.0158	0.08806	0.06419	0.17972	0.04263	-0.0864	-0.0314	0.12053	-0.0585	0.04943	0.05756
XM_575144.1	LOC499804	-0.0083	0.0798	0.02148	0.07659	-0.0779	0.07703	-0.0982	0.0444	-0.0799	-0.0737	0.03074	-0.0974	0.02287	-0.1395
XM_575145.1	LOC499805	0.07605	0.14288	-0.0209	0.17253	-0.036	-0.031	0.14697	-0.0079	0.0078	0.01798	0.09676	-0.0246	0.14684	0.09902
NM_001024311	LOC499806	0.10492	0.05632	-0.0366	-0.0532	0.05011	0.12768	0.08311	0.19488	-0.067	0.21076	0.0383	0.03794	0.17952	0.14843
XM_580018.1	LOC499808	-0.0266	0.10525	0.23354	-0.0262	0.05993	-0.1357	-0.0073	-0.2112	-0.0384	-0.0676	0.11695	-0.1239	0.1742	0.04414
XM_580019.1	LOC499811	0.21764	0.09026	0.09479	0.24934	0.14483	0.21687	0.18322	0.35975	0.23961	0.35798	0.10144	0.13242	0.30669	0.13548
XM_575152.1	LOC499812	0.50934	0.59596	0.68917	-0.3061	0.65974	1.0962	0.51355	0.52768	0.93062	0.68591	0.85476	1.3487	0.592	0.41626
XM_580020.1	LOC499813	-0.1594	-0.1899	-0.1429	-0.226	-0.0735	-0.1654	-0.0906	-0.1852	0.1572	-0.3139	-0.1828	-0.3082	-0.1108	-0.2085
XM_580021.1	LOC499817	-0.1015	-0.0735	-0.1358	-0.0037	-0.1132	-0.2301	-0.1811	-0.0783	-0.2688	-0.216	-0.0183	-0.2684	-0.271	-0.1277
XM_575158.1	LOC499819	-0.094	-0.0787	-0.1069	-0.2304	-0.2967	-0.1389	-0.1584	-0.2132	-0.3377	-0.2408	-0.1517	-0.0304	-0.0702	-0.2634
XM_575161.1	LOC499822	0.0539	-0.0509	0.07829	0.01141	0.01162	0.25082	0.0186	0.04829	0.29829	0.09975	0.18763	0.17808	0.04351	-0.0495
XM_575162.1	LOC499823	-0.1795	0.23731	0.03705	-0.1561	-0.3774	0.19952	-0.2032	-0.054	-0.1675	0.07932	-0.1618	1.3154	-0.2154	-0.2276
NM_001108750	LOC499828	-0.0359	0.14325	-0.0283	-0.0152	0.04547	-0.0394	0.66071	0.04992	0.23707	0.3779	0.09582	0.05729	0.04681	0.17171
XM_575171.1	LOC499830	0.01277	-0.1634	-0.2093	-0.588	0.27538	-0.0044	-0.1332	0.08992	0.119	-0.1386	0.1607	0.19639	0.02667	0.14391
XM_580022.1	LOC499834	-0.1231	-0.0473	-0.1429	-0.1211	-0.0324	0.18856	-0.1971	-0.1112	0.21622	-0.0309	0.03307	-0.171	0.10456	-0.2086
XM_575175.1	LOC499835	0.14455	-0.0032	-0.0619	0.10919	0.26627	0.02652	0.25913	-0.0732	0.0546	-0.0499	-0.0568	0.02367	0.00521	0.02827
XM_580023.1	LOC499837	-0.7348	-0.3012	-0.2024	-0.1202	-0.3869	-0.1227	0.01918	-0.3385	-0.3202	-0.1128	-0.2368	-0.0188	-0.4847	-0.3191
XM_580024.1	LOC499838	-0.0298	0.26998	-0.0073	-0.0012	0.10491	-0.188	-0.163	-0.028	0.22539	0.19433	0.17184	0.45865	-0.0586	-0.085
XM_580025.1	LOC499840	-0.0864	-0.1896	-0.1607	0.36399	-0.2931	-0.0331	-0.3556	-0.0871	-0.0887	-0.229	0.33123	-0.0592	-0.1279	0.01405
NM_001047953	LOC499843	0.05853	0.00465	0.11702	-0.0467	0.11951	-0.0464	0.1633	0.12154	0.09997	0.07723	-0.0033	-0.0198	-0.135	-0.0312
XM_580026.1	LOC499844	-0.0254	-0.0413	-0.0477	0.01657	0.07091	0.11325	0.12977	0.07362	0.01088	-0.02	-0.0537	-0.0467	-0.0681	0.14143
NM_001109202	LOC499846	-0.1254	-0.1919	-0.1584	-0.0975	-0.0883	0.11032	-0.1185	-0.0081	-0.2543	-0.0991	-0.2393	-0.1651	-0.1803	-0.1818
XM_580027.1	LOC499849	-0.1613	0.08284	-0.0765	-0.0885	-0.0997	-0.0594	0.0965	-0.0141	-0.1051	0.07874	0.01492	-0.0738	-0.0872	-0.0929
XM_575189.1	LOC499850	0.01901	0.05646	0.12785	0.06011	-0.1303	-0.1361	0.27617	-0.1435	0.15081	-0.1031	-0.0334	0.10781	-0.1263	0.08163
XM_575194.1	LOC499853	-0.0558	-0.0647	-0.027	0.09509	0.14764	-0.0876	-0.1966	0.12555	0.22688	0.31081	0.14523	0.05202	0.15004	0.19572
XM_575195.1	LOC499854	0.06449	-0.0265	0.1575	0.47971	-0.0806	-0.2137	0.82909	0.19266	0.20075	0.06599	0.30441	-0.124	-0.1978	-0.0099
XM_575196.1	LOC499855	0.24648	0.1346	0.15002	0.33316	-0.0247	0.13903	0.04482	0.12042	0.02044	0.16367	0.02105	0.19572	1.0066	0.12118
NM_001025042	LOC499856	-0.7046	2.0173	-0.2076	0.90654	-1.4269	-1.4196	0.67012	-1.5309	1.8227	1.9784	2.3375	1.0171	-0.434	-0.5835
XM_575203.1	LOC499862	-0.0586	0.03977	0.07363	0.08474	-0.0772	0.02394	-0.065	-0.0373	0.08302	0.11089	-0.0191	0.04036	-0.0579	-0.0851
XM_575206.1	LOC499864	0.04634	0.0887	0.12558	0.04677	0.10917	0.15068	0.06883	0.18943	0.10797	0.05198	0.07664	0.01897	0.07026	0.16465
XM_575214.1	LOC499873	-0.0385	0.0073	-0.0094	-0.1853	-0.1538	0.02618	-0.0567	-0.117	-0.2972	0.11369	-0.0895	-0.0474	-0.089	-0.0511
XM_575223.1	LOC499880	0.26736	-0.0392	1.0901	-0.0468	-0.12	5E-05	0.17855	0.26535	0.23924	0.10153	-0.0535	-0.0191	-0.0528	0.1741
NM_001135015	LOC499883	0.15102	0.22736	0.10048	-0.0183	-0.0331	0.13781	-0.1216	0.10409	0.05697	0.1227	0.16626	0.06263	0.01438	0.15449
XM_575229.1	LOC499884	-0.0611	0.04199	-0.0376	0.03294	-0.065	0.03918	0.09358	0.09044	-0.0755	0.06748	0.02809	0.11509	0.0431	0.00583
NM_001025043	LOC499885	-0.0683	0.08622	-0.1513	-0.1144	0.00034	0.01999	-0.0996	0.16085	0.08603	-0.0961	-0.2032	-0.0626	0.01345	-0.0322
NM_001024312	LOC499886	-0.1523	0.07853	0.02507	0.00668	-0.0919	-0.094	-0.1464	-0.0927	0.17463	0.28142	0.02777	-0.0876	-0.2018	-0.1135
XM_580029.1	LOC499887	-0.0422	0.0294	0.06052	0.0079	0.15357	0.10073	-0.0027	0.02926	0.06075	0.27831	-0.0326	-0.0593	-0.0591	-0.0528
XM_575234.1	LOC499888	0.10109	0.20614	0.06357	0.01777	0.16052	-0.0023	0.02399	0.08448	0.05531	0.11223	0.0453	-0.0086	0.13899	-0.0243
XM_575236.1	LOC499890	-0.7142	-0.7347	-0.0921	-0.4207	-0.8253	-0.9998	-0.286	-0.7511	-0.3383	-0.5535	-0.3892	-0.6016	-0.4672	-0.4212
XM_575238.1	LOC499892	-0.2427	-0.2135	-0.1541	-0.1933	-0.2942	-0.3185	-0.1881	-0.2881	-0.1825	-0.3246	-0.3629	-0.2234	-0.2996	-0.2373
NM_001037190	LOC499897	0.43288	0.49968	-0.113	-0.0629	0.38052	0.29102	-0.0874	0.77695	0.01725	0.49666	0.3827	-0.1191	0.71283	0.43181
XM_575244.1	LOC499898	0.30672	-0.5149	-0.3986	-0.1693	0.39939	0.46941	-0.527	0.16263	-0.2658	-0.0125	-0.5907	0.05875	-0.3565	-0.2675
NM_001024313	LOC499900	-0.197	-0.1143	-0.3429	0.0796	-0.1279	0.12304	-0.1111	0.07536	-0.2491	0.153	-0.118	-0.015	-0.2263	-0.2849
XM_575248.1	LOC499902	-0.0826	-0.0466	-0.2251	-0.0301	-0.2548	0.13616	-0.0257	-0.1725	-0.1174	-0.1029	-0.187	-0.0754	0.04267	-0.1617
XM_575249.1	LOC499903	-0.0726	-0.0679	-0.1115	-0.1048	-0.1166	0.08084	-0.132	0.02345	0.09824	-0.0645	0.13933	-0.053	-0.0423	0.0786
XM_575250.1	LOC499904	0.01287	-0.0078	-0.0246	0.16235	0.00118	-0.0205	0.14102	0.07743	0.25709	0.01558	0.19117	0.00533	-0.0162	-0.0073
XM_575251.1	LOC499905	0.04682	0.02303	0.12037	-0.1485	0.00994	0.0528	-0.0095	-0.1104	0.01446	-0.1122	0.09643	0.028	-0.0028	0.02769
XM_580030.1	LOC499907	0.07962	-0.0669	0.0222	0.01671	0.1893	-0.131	-0.0549	-0.0317	0.1688	0.2101	0.00859	0.04007	0.03242	-0.1009

XM_575254.1	LOC499909	-0.0239	-0.0605	-0.0236	-0.082	-0.1675	0.66133	-0.0935	-0.1854	0.02176	0.14195	0.00372	-0.0864	-0.1701	0.02919
XM_580031.1	LOC499910	0.14318	0.04622	0.30944	0.11434	-0.0389	0.03911	0.12812	0.07556	0.05779	-0.0268	0.07778	0.1796	0.16395	-0.0841
XM_575255.1	LOC499911	-0.0776	-0.2126	-0.0521	-0.1146	-0.0055	-0.0855	-0.1519	-0.094	-0.0464	0.11221	-0.1818	-0.1391	-0.025	-0.1568
XM_580032.1	LOC499915	-0.2106	-0.0669	-0.0361	-0.1187	-0.0117	-0.0741	-0.0217	-0.1704	0.07092	-0.1907	-0.0885	0.06422	-0.084	-0.0004
NM_001109208	LOC499924	-0.0021	0.07949	0.0397	0.05957	0.13657	0.11977	0.25208	0.18076	0.1626	0.33955	0.0328	-0.0097	-0.0142	-0.0363
XM_575269.1	LOC499925	-0.2003	-0.2582	-0.1004	-0.1173	-0.1478	-0.2249	0.01583	-0.201	-0.1562	-0.257	-0.1758	-0.1343	0.18239	-0.1024
XM_575272.1	LOC499928	-0.242	-0.0399	-0.249	0.00511	-0.3012	-0.3216	-0.0258	-0.0814	-0.1188	-0.1394	-0.3233	-0.0751	-0.2531	-0.1189
NM_001172118	LOC499931	-0.1902	-0.1133	-0.2005	-0.1774	0.00116	-0.1393	-0.1032	0.04032	-0.1757	-0.1818	-0.0178	-0.0821	-0.1778	-0.1101
XM_575277.1	LOC499932	-0.0958	-0.185	-0.0822	0.21762	0.06623	0.24939	-0.1443	-0.0022	-0.127	-0.025	-0.1125	-0.2141	-0.1426	0.00359
NM_001025768	LOC499933	-0.4946	-0.3499	0.72902	0.0406	-0.6625	-0.3939	-0.1282	-0.5851	-0.3533	-0.3869	-0.0296	-0.1253	-0.1376	-0.1055
XM_575282.1	LOC499938	0.02306	0.02047	0.14997	-0.0035	-0.0262	0.04288	-0.039	0.09856	-0.0188	0.05032	0.09591	0.03047	0.03835	-0.0234
XM_580035.1	LOC499939	-0.0244	0.06389	-0.102	-0.0353	0.04785	-0.0354	-0.0792	-0.0328	0.04353	-0.045	0.01846	-0.104	-0.0575	-0.004
NM_001047111	LOC499941	-0.0182	-0.0525	0.07308	-0.5185	-0.0188	0.25815	0.48526	0.03519	-0.1099	-0.0171	0.07914	-0.1397	0.28726	0.41755
XM_580036.1	LOC499944	0.57595	0.43446	0.40374	0.06504	0.26573	0.43343	0.16994	0.14079	0.59948	0.37934	0.44818	0.61603	0.16891	0.29745
XM_580037.1	LOC499946	0.07127	0.15862	0.00113	0.16153	0.03384	0.13606	0.02619	-0.0733	0.01276	0.05633	0.02733	0.03582	-0.0063	0.12275
XM_580038.1	LOC499948	-0.0839	-0.1513	-0.0343	-0.0853	-0.1227	-0.2075	-0.0138	-0.119	0.01058	-0.3384	0.16131	-0.0877	-0.2737	-0.049
NM_001024315	LOC499949	-0.085	-0.0513	-0.1865	0.03125	-0.0901	-0.021	-0.0235	0.17297	0.12557	-0.1804	0.04919	-0.0714	-0.1605	0.08164
NM_001106224	LOC499959	-0.0393	-0.2412	0.0694	-0.0563	-0.1037	0.28143	-0.3664	-0.0582	-0.1084	-0.2466	-0.3762	0.48324	0.17523	0.25733
XM_575309.1	LOC499960	0.23832	0.26579	0.05557	0.0805	0.16607	0.1592	0.04269	0.08753	-0.0054	0.11854	0.21775	0.24938	0.07816	0.35933
NM_130400	LOC499963	-0.2365	-0.147	-0.2791	-0.1042	-0.217	0.09744	-0.1668	-0.0944	-0.1059	0.14449	-0.1563	-0.0126	-0.0052	-0.0724
XM_575316.1	LOC499965	0.10203	-0.081	0.16363	-0.0282	0.08086	0.04803	-0.0065	0.01802	0.07164	0.1336	-0.0556	-0.0717	-0.0276	0.10385
XM_575323.1	LOC499970	-0.0005	0.02157	0.11731	0.04016	-0.0034	0.19228	0.10006	0.07772	-0.0298	0.04194	0.11352	-0.0425	0.09894	-0.0309
NM_001024317	LOC499971	-0.2726	-0.3935	-0.2403	-0.2591	-0.3302	-0.3463	-0.2217	0.19334	-0.3049	-0.0086	-0.4607	-0.1908	-0.2423	-0.4182
XM_575331.1	LOC499977	0.11104	0.11534	-0.0074	0.03665	0.12649	0.01548	0.0465	0.07128	0.07666	0.04899	0.13483	0.07139	0.14539	0.11864
XM_575332.1	LOC499978	0.33641	0.27159	-0.1529	0.16511	0.47547	0.62398	0.38942	0.69279	0.41533	0.31444	0.33839	0.11466	-0.3681	-0.3519
NM_001025044	LOC499980	-0.2117	-0.2838	-0.107	0.16365	-0.0773	-0.1077	0.0151	-0.0672	0.01811	-0.1264	-0.1139	0.004	-0.0958	0.01361
XM_575335.1	LOC499981	-0.1722	-0.1629	-0.0148	-0.1761	-0.2233	-0.3499	-0.1566	-0.1661	-0.2763	-0.2793	-0.3274	-0.0746	-0.0757	-0.0592
XM_575336.1	LOC499982	0.05988	0.10286	0.22263	0.04755	0.02679	0.13443	0.01733	0.04748	0.06261	-0.0374	0.05717	0.0065	0.25136	0.12951
XM_575337.1	LOC499983	-0.1769	-0.1799	-0.0579	0.13325	-0.1774	-0.0733	-0.174	-0.0759	-0.2225	-0.0381	-0.2071	0.00153	-0.124	-0.1867
NM_001109218	LOC499985	-0.0425	-0.1674	-0.0061	-0.0866	-0.0644	-0.1491	-0.1659	-0.0357	0.06725	-0.1576	-0.1696	0.18862	-0.0223	-0.0724
XM_575341.1	LOC499987	0.07278	0.04238	0.0888	0.09097	0.09565	0.01176	0.0219	0.02336	0.03465	0.15431	0.07114	0.08395	-0.0236	0.17838
XM_575342.1	LOC499988	-0.0177	0.0606	0.03127	0.00679	0.09033	0.09481	0.27132	0.00695	0.04594	0.08222	-0.0073	0.20013	0.03913	0.04237
NM_001044265	LOC499991	0.12197	0.00012	0.02786	0.3748	-0.009	0.58355	0.11788	-0.0138	0.00563	-0.1215	0.04281	-0.0514	-0.0566	0.24366
XM_575347.1	LOC499992	-0.2779	0.02831	-0.0591	0.02911	-0.4263	-0.468	0.07059	0.0331	-0.2118	-0.1467	-0.2602	-0.0997	-0.7044	-0.5106
XM_580042.1	LOC500001	0.20038	0.44502	0.00413	-0.4784	-0.3498	-0.5763	0.32489	-0.3555	-0.0704	0.35501	0.13491	-0.07	-0.2707	-0.2157
XM_575359.2	LOC500005	-0.0359	-0.5113	-0.8654	-0.7031	-0.4284	-0.5426	-0.487	-0.4004	-0.5208	-0.4326	-0.6089	-0.7083	-0.533	-0.4458
XM_575361.1	LOC500007	0.02277	-0.0182	-0.0315	0.10844	-0.0455	0.03627	-0.0436	-0.0715	0.00251	-0.0059	0.20358	0.06662	0.05156	0.0112
XM_575369.2	LOC500015	1.2835	1.3849	1.2486	0.27959	1.0238	0.57222	2.0668	0.94721	1.057	1.0314	1.4396	0.30769	1.3368	1.2103
NM_001047954	LOC500028	0.20559	0.07695	0.18131	0.07416	0.2983	0.25634	0.38667	0.4295	0.18043	0.04598	-0.1521	-0.0285	0.40956	0.34617
NM_001024319	LOC500033	0.62262	0.30786	0.19365	0.63527	0.29361	0.41211	0.25938	-0.0192	0.11511	0.30928	0.19376	0.23248	0.34437	0.37392
NM_001047955	LOC500035	0.02191	0.13323	0.22338	0.00986	0.37729	0.0039	0.22211	-0.016	0.03011	0.17857	0.1873	-0.0547	0.01836	0.27122
NM_001039344	LOC500040	-0.333	-0.9882	-0.8258	-1.3411	-0.9366	-0.6975	-1.8856	-0.8797	-0.5054	-0.8863	-0.9439	-0.1208	-1.3312	-1.2289
XM_001061323.1	LOC500046	-0.0651	-0.0902	-0.1205	-0.0668	-0.3781	-0.4146	-0.0743	-0.4193	0.035	-0.229	0.07826	0.04154	-0.2423	-0.2544
XM_575406.1	LOC500050	-0.1061	0.00708	0.0668	-0.0072	0.21205	0.62621	0.18179	0.04266	0.30917	0.28347	0.02773	0.23729	-0.0715	0.14177
NM_001024320	LOC500051	-0.0438	0.18131	0.05065	0.0213	0.33644	0.2589	0.12349	-0.0398	-0.0406	0.10336	-0.0671	0.15192	0.04162	0.09076
NM_001024322	LOC500054	0.12272	-0.0112	0.05136	0.1817	-0.1241	-0.429	0.04378	-0.1071	0.18972	0.18453	0.06761	-0.1896	0.03061	-0.1576
XM_575411.1	LOC500056	-0.217	-0.0621	-0.1951	-0.1697	-0.0446	-0.0678	-0.1588	-0.077	-0.2092	-0.1883	-0.0602	-0.1467	-0.1103	-0.216
XM_575413.1	LOC500058	-0.2435	-0.4198	-0.4222	-0.3407	-0.3078	-0.3954	-0.212	-0.3808	-0.1984	-0.2367	-0.3987	-0.218	-0.2038	-0.2718
XM_580044.1	LOC500062	0.11483	0.24571	0.34034	0.05987	0.09022	0.11649	0.06256	0.10886	0.10701	0.08965	0.03631	0.05337	0.54409	0.05625

XM_575417.1	LOC500063	-0.1602	-0.2913	-0.4321	0.12308	-0.1625	0.10239	-0.4653	-0.0963	-0.366	-0.1201	-0.2796	-0.7116	-0.8508	-0.6614
XM_575418.1	LOC500064	-0.039	0.15348	0.01082	0.18251	0.02461	0.02798	0.19	0.01532	0.0543	-0.0173	0.05091	-0.0694	0.05329	0.03261
XM_580045.1	LOC500067	-0.0067	-0.3726	-0.1975	-0.3746	-0.2715	-0.333	-0.2718	-0.3409	-0.3091	-0.3071	-0.428	-0.2445	-0.3019	-0.4721
XM_575423.1	LOC500072	0.11293	-0.1226	-0.193	0.08232	-0.2818	-0.3708	-0.028	-0.243	-0.1828	-0.2715	-0.2439	0.00962	-0.2489	-0.3041
NM_001024325	LOC500077	0.31513	-0.0965	0.05554	0.59364	0.12491	0.16626	-0.1012	-0.161	-0.0699	0.10951	0.17976	0.46154	-0.1452	0.14134
XM_575430.1	LOC500080	0.07176	0.08292	0.04462	0.03567	0.07029	0.06236	0.00207	0.20589	-0.0532	0.01054	0.04352	0.00889	-0.0125	0.03268
NM_198782	LOC500082	-0.1812	-0.1061	0.00179	-0.0108	-0.1994	-0.1848	-0.1506	-0.045	-0.0537	-0.2728	-0.1475	0.07963	-0.0317	-0.094
NM_001044266	LOC500084	-0.0612	-0.2153	0.11214	-0.2608	-0.0598	-0.1797	-0.2142	0.33305	-0.0327	-0.3915	-6E-05	-0.4048	-0.1416	-0.172
NM_001044266	LOC500084	-0.0932	-0.1495	-0.1108	-0.244	0.06818	0.00672	-0.1304	0.24703	0.02334	0.11921	-0.0478	-0.1537	-0.1392	-0.1711
XM_575436.1	LOC500085	-0.0427	0.31878	0.04576	0.07758	0.17183	0.15962	0.06718	-0.0538	-0.1729	0.14595	0.1586	0.00866	0.10701	-0.1568
XM_575444.1	LOC500093	0.00946	0.14845	0.18853	0.01523	0.10032	0.14688	0.10548	0.09674	0.03264	0.0568	-0.0478	0.08557	0.00379	0.01113
XM_575445.1	LOC500094	-0.1177	0.02294	0.07199	-0.1389	0.07037	-0.115	0.05492	-0.0762	-0.1631	-0.1177	-0.109	-0.0458	-0.0363	-0.0662
XM_575446.1	LOC500095	-0.1688	-0.1083	-0.1876	-0.0962	-0.1133	-0.0793	-0.1176	-0.0569	-0.1147	0.06222	-0.1247	-0.2754	-0.0655	-0.1622
XM_575447.1	LOC500096	-0.0396	-0.0052	-0.0408	0.19868	-0.003	0.07508	-0.0401	-0.1449	0.15957	-0.248	-0.1297	-0.1916	0.04651	-0.1255
XM_575448.1	LOC500097	0.16852	0.18635	0.11725	0.13927	0.13464	0.2156	0.03543	0.07078	0.04186	0.11481	0.17071	0.10867	0.0401	0.04172
XM_575449.1	LOC500098	-0.2409	-0.0641	-0.2596	-0.0243	-0.1436	-0.0943	-0.2782	-0.1225	-0.2964	-0.1911	-0.2587	-0.2287	-0.0769	0.02619
XM_575450.1	LOC500099	-0.0666	-0.0333	-0.1126	-0.0833	-0.0903	-0.104	-0.0675	-0.1062	0.09299	0.06313	-0.1224	0.01024	-0.0917	-0.0666
XM_575451.1	LOC500100	-0.0659	-0.1429	-0.0403	-0.015	0.03363	-0.0662	-0.0135	-0.0934	0.05916	-0.0055	-0.0666	0.09341	-0.0239	-0.0324
XM_575455.1	LOC500104	0.53947	-0.0074	0.32871	-0.3506	0.4574	0.40545	-0.5446	0.586	0.22934	0.03284	0.28599	-0.0448	0.4384	0.37229
XM_575457.1	LOC500106	0.19537	-0.0159	-0.084	0.02712	-0.0448	-0.0877	0.01704	-0.0079	-0.1429	0.21156	-0.0945	0.28204	-0.1234	-0.1693
XM_575458.1	LOC500107	-0.1756	-0.2646	-0.315	-0.3075	-0.1386	-0.33	-0.2013	-0.1007	0.00335	-0.2299	-0.2724	-0.2488	-0.0466	-0.2813
NM_001109230	LOC500108	0.11465	0.06013	0.16247	0.11697	0.16689	0.18843	0.19707	0.1902	0.28654	0.05968	0.14319	0.39719	0.34127	0.29711
NM_024146	LOC500109	-0.0299	0.09082	0.07858	-0.067	-0.1547	0.12787	-0.1613	-0.0179	-0.1652	-0.2441	0.0074	0.08661	-0.1989	-0.2387
NM_001025771	LOC500118	0.07023	-0.4992	-0.6041	-0.1089	-0.4303	-0.6335	-0.5349	-0.2818	-0.2725	-0.4187	-0.6374	-0.4504	-0.4224	-0.4725
XM_575473.1	LOC500121	-0.0815	-0.1752	0.02949	-0.0251	-0.0081	0.21622	-0.0927	0.09666	-0.1697	-0.0505	-0.1016	0.2031	-0.0549	0.02415
XM_575475.1	LOC500123	0.28232	0.056	0.08867	0.01937	0.21982	0.51785	0.21346	0.07386	0.3411	0.46309	0.25723	0.17106	0.2111	0.2858
NM_001024329	LOC500124	0.20679	-0.0087	-0.2584	-0.1767	-0.0167	-0.1949	-0.0524	0.09053	-0.2869	-0.041	-0.0663	-0.2355	-0.1345	-0.1661
XM_575481.1	LOC500129	-0.5889	-0.3129	-0.4291	-0.3101	-0.4798	-0.5137	-0.4482	-0.461	-0.2553	-0.3396	-0.0935	-0.3744	-0.4525	-0.3541
XM_575482.1	LOC500130	0.06996	0.23284	0.28853	0.05642	0.39941	0.11485	0.08501	0.49879	0.20667	0.21011	0.09587	0.16931	0.15306	0.1243
XM_575487.1	LOC500135	-0.0727	0.03299	-0.0571	-0.0278	0.06588	-0.0698	-0.0727	0.03797	0.21034	0.08265	0.1467	-0.0725	-0.037	-0.0211
XM_575490.1	LOC500138	-0.4994	-0.5022	-0.54	-0.426	-0.3178	-0.1271	-0.443	-0.6338	-0.4454	-0.3533	-0.5188	-0.4031	-0.4805	-0.3353
XM_575492.1	LOC500140	-0.0484	0.05262	-0.1304	0.0244	0.02225	0.11696	0.105	0.01563	-0.0587	0.05344	0.19791	-0.0405	-0.0756	0.04407
XM_575493.1	LOC500141	-0.0517	0.15532	-0.1345	-0.0322	0.00945	0.04203	0.04912	-0.1324	-0.1085	0.09517	-0.0117	0.26151	-0.2016	0.06891
XM_575494.1	LOC500142	0.17351	0.18217	0.1642	0.22105	0.04828	0.00254	0.01889	0.16372	0.15358	0.23161	0.17042	0.27418	0.15481	0.25836
NM_001035253	LOC500148	-0.0437	-0.07	-0.0762	-0.2092	-0.049	0.24541	-0.2416	-0.097	-0.1464	-0.0681	-0.0563	-0.0811	-0.2253	-0.0789
XM_575502.1	LOC500150	-0.0785	-0.1537	-0.3763	-0.6433	-0.4871	-0.5086	-0.2411	-0.4096	-0.2226	-0.2957	-0.1899	-0.0592	-0.277	-0.3315
XM_575503.1	LOC500151	0.18098	0.13854	0.05298	0.48316	0.0238	-0.013	0.52855	0.11467	-0.0007	0.26367	0.14321	0.08788	-0.0254	0.01787
NM_001134622	LOC500153	0.03162	-0.0009	0.00496	0.03844	0.05607	0.04493	0.04427	0.18505	0.02784	0.09883	0.09642	0.1428	0.31536	0.03268
XM_575507.1	LOC500155	-0.4473	-0.2894	0.71025	-0.7531	-0.1508	-0.1361	-0.1453	-0.2474	-0.0519	-0.2321	-0.0775	0.35207	0.3173	0.36546
XM_575510.1	LOC500158	0.03001	-0.1353	-0.1112	-0.0771	0.04273	0.39494	-0.0978	-0.1298	0.04669	0.00673	-0.1213	0.17749	-0.0433	0.01285
XM_575511.1	LOC500159	-0.1675	-0.0839	-0.1138	-0.1208	-0.129	0.1735	-0.2445	-0.1137	-0.056	-0.2308	0.22397	0.05602	-0.1935	-0.2293
XM_575512.1	LOC500160	-0.1159	-0.1824	-0.1275	-0.13	0.07404	-0.0339	-0.1871	-0.1717	-0.1384	-0.1836	-0.1555	-0.1209	-0.0125	-0.1286
XM_575513.1	LOC500161	0.02187	-0.0267	0.00239	0.1182	-0.0328	-0.0105	0.08801	0.10613	0.02437	-0.023	0.0947	0.01042	0.08055	0.10197
XM_575514.1	LOC500162	-0.0903	-0.1268	-0.2777	-0.0902	-0.1596	-0.2728	-0.0903	-0.3243	-0.1541	-0.1632	-0.3102	-0.0052	-0.2929	-0.0395
XM_575515.1	LOC500163	0.07615	0.01892	-0.1245	-0.0124	-0.1177	0.12591	-0.0064	-0.1861	-0.0752	-0.0768	0.09657	0.00766	-0.1836	-0.0726
XM_575516.1	LOC500164	0.06156	0.11728	-0.0023	0.09233	0.05823	0.03358	0.01634	0.05437	0.35947	0.09119	0.12384	0.06461	-0.0111	0.05507
XM_575517.1	LOC500165	0.01985	0.02472	-0.0364	-0.0624	-0.0294	0.06111	0.13609	0.26092	-0.2217	-0.0008	-0.0284	0.02619	-0.1127	0.06258
XM_575518.1	LOC500166	0.00469	0.08222	0.01196	0.04343	0.12875	0.11966	0.08493	0.0366	0.19459	0.01018	0.05571	0.17863	0.12411	0.17227
XM_575519.1	LOC500167	-0.0072	0.03243	-0.0206	-0.0117	0.03901	-0.0023	0.0429	0.00717	0.09483	-0.0507	0.10005	-0.0381	0.129	0.04919

XM_575520.1	LOC500168	-0.0072	-0.0045	0.02385	0.13968	-0.0083	0.06598	0.18027	0.04728	0.20107	0.32968	-0.1389	0.15165	0.0261	0.15626
XM_575521.1	LOC500169	-0.0717	0.0196	-0.0891	-0.0152	-0.0776	0.01911	-0.019	-0.0382	0.07202	-0.0303	0.00054	-0.0632	-0.0444	0.19223
XM_575522.1	LOC500170	0.10381	-0.1851	0.10024	-0.0988	0.06236	0.09698	-0.0809	0.02829	-0.0395	0.07306	-0.1041	0.07212	-0.1138	0.00469
XM_575523.1	LOC500171	0.15735	0.17382	0.09498	-0.0217	0.11781	0.13245	0.13698	0.23105	0.05939	0.22421	0.08061	0.06149	0.32219	0.09789
XM_575524.1	LOC500172	0.16209	0.14373	0.10818	0.05941	0.16219	0.09715	0.20235	0.03906	0.34047	0.01795	0.27349	0.05858	0.08122	0.13297
XM_575525.1	LOC500173	-0.0759	-0.0487	-0.067	-0.0604	-0.1369	-0.0555	0.00464	0.15417	0.06936	-0.1197	-0.111	0.02167	-0.1295	-0.1044
XM_575526.1	LOC500174	-0.1632	-0.256	-0.2412	-0.0813	-0.1713	-0.2828	-0.2191	-0.1906	-0.1382	-0.1986	-0.0731	-0.0362	0.00628	-0.1951
XM_575527.1	LOC500175	-0.0898	-0.0418	-0.1086	-0.0166	-0.0863	-0.0328	-0.1015	-0.2113	-0.1174	-0.1665	-0.0506	-0.1752	-0.0326	-0.1449
XM_575528.1	LOC500176	0.01371	0.02785	0.08123	0.00563	0.24657	0.05854	0.06782	-0.0121	0.05345	0.04265	0.00704	-0.0198	0.06549	0.01043
XM_575529.1	LOC500177	-0.2302	-0.1663	-0.3503	-0.2585	-0.052	-0.174	-0.1978	0.02407	-0.022	-0.3192	-0.1464	-0.0148	-0.1236	-0.1727
XM_575530.1	LOC500178	-0.0081	-0.0117	0.07163	0.03085	-0.0223	0.11344	0.05171	0.05526	0.03031	0.15813	0.03689	0.3196	0.02726	0.14627
XM_575531.1	LOC500179	0.20935	0.30364	0.22063	0.51444	0.06102	0.15255	0.14409	0.21723	0.33812	0.27899	-0.0376	0.05656	0.01564	0.14828
XM_575532.1	LOC500180	-0.1507	0.06584	-0.0624	0.00375	-0.1047	-0.0631	0.0145	-0.1356	-0.0353	-0.0077	0.16554	-0.0265	0.01171	0.00593
XM_575533.1	LOC500181	-0.0336	-0.0662	-0.0063	-0.2069	-0.0543	-0.0258	-0.1309	-0.037	0.17099	-0.2445	0.13867	0.23106	-0.1309	0.01494
XM_575534.1	LOC500182	-0.0749	-0.1751	-0.1454	-0.1803	-0.1581	-0.0488	0.09902	-0.1748	-0.0319	-0.1908	0.0725	-0.1448	-0.1349	-0.1075
XM_575535.1	LOC500183	-0.055	-0.2156	-0.1809	0.05803	-0.16	0.00149	-0.0707	-0.235	-0.059	-0.2424	0.1421	-0.0846	-0.0195	-0.0127
XM_575537.1	LOC500185	-0.0894	-0.092	-0.1192	0.08159	-0.0756	-0.1757	-0.0769	-0.1793	-0.0649	-0.0043	-0.0827	-0.105	0.02103	-0.03
XM_575538.1	LOC500186	-0.1631	-0.0645	0.05222	0.01669	-0.2436	-0.0663	0.08536	-0.1719	-0.0102	0.03836	-0.1074	-0.1354	0.05831	0.00197
XM_575540.1	LOC500188	0.01909	-0.1772	-0.1047	-0.1068	0.09493	-0.0322	-0.178	-0.089	0.03809	-0.084	0.06385	-0.0803	-0.1035	-0.0996
XM_575541.1	LOC500189	0.23514	0.17186	0.15557	0.08026	0.19838	0.29699	0.35007	0.06807	0.0137	0.27761	0.35679	0.12067	0.03975	0.30052
XM_575542.1	LOC500190	0.0515	0.08682	0.03983	0.04706	0.08971	0.04651	0.01615	0.02834	0.06941	0.09891	0.02812	-0.0439	0.10593	0.02604
XM_575543.1	LOC500191	-0.0905	-0.2571	-0.2396	-0.061	-0.2638	-0.1896	-0.1623	-0.2069	-0.1706	-0.2127	-0.3241	-0.3318	-0.0487	-0.0081
XM_575544.1	LOC500192	0.00037	0.03232	0.36872	-0.0612	-0.0122	-0.0446	0.03927	-0.1028	-0.0606	-0.0224	-0.0597	-0.012	-0.0856	-0.0807
XM_575545.1	LOC500193	-0.0705	-0.1516	0.06096	-0.0791	-0.0972	-0.0174	-0.1031	-0.0399	-0.0234	0.04533	0.02751	0.05105	-0.0139	-0.1116
XM_575546.1	LOC500194	-0.2162	0.11323	0.00163	-0.0924	0.0463	-0.1014	-0.0378	0.1242	0.0551	-0.0921	-0.1224	0.14732	-0.0329	-0.0322
NM_001134718	LOC500199	-0.693	-0.949	-0.2615	-0.4621	-0.8003	-0.7064	-0.2154	-0.7018	-0.6046	-0.8007	-0.5529	-0.6261	-0.2255	-0.2889
XM_575553.1	LOC500201	0.20652	0.23066	0.1196	0.25274	0.04362	0.03493	-0.0485	0.01747	-0.0152	0.10235	0.05322	0.23669	0.01837	0.02108
XM_575559.1	LOC500207	0.01386	0.12724	0.00319	-0.0034	-0.104	0.01144	0.07159	-0.0114	0.01106	-0.0416	-0.001	0.08987	-0.0334	0.04858
XM_575561.1	LOC500209	0.03365	0.10358	-0.125	0.07133	-0.0621	-0.1232	-0.0207	-0.0507	0.07736	0.00092	0.04619	-0.0043	-0.0619	-0.0186
XM_575563.1	LOC500211	-0.0371	0.13079	-0.1326	0.18175	-0.1405	0.23418	0.27995	-0.007	-0.1422	-0.0254	0.02124	-0.1734	0.04755	-0.1952
XM_575564.2	LOC500213	-0.2167	-0.1272	-0.0753	-0.1458	-0.2021	0.02375	0.13175	-0.1029	-0.2033	-0.0574	-0.1217	-0.1578	-0.1764	-0.1567
XM_575567.1	LOC500216	0.0917	-0.0387	0.24438	0.2553	-0.0983	0.01445	0.12063	-0.0307	0.14346	-0.1723	0.0587	0.03458	0.03249	0.20464
XM_575569.1	LOC500218	0.10056	0.0999	0.13465	-0.0511	-0.0156	0.0734	0.01215	-0.0133	0.01406	0.02725	0.03957	-0.0254	0.03806	0.07437
XM_575574.1	LOC500223	0.16197	-0.0665	0.10314	0.20858	-0.0556	-0.0806	-0.0527	0.06537	0.02888	0.28155	0.21971	0.0627	0.01444	0.11798
NM_001109244	LOC500224	0.01584	0.02423	-0.0774	-0.1467	-0.1913	-0.0342	-0.2552	0.00462	0.01322	-0.2465	-0.0791	-0.0049	0.03588	0.00455
NM_001024330	LOC500227	-0.1824	0.01591	-0.0249	-0.1539	-0.1402	-0.0132	-0.0811	0.02799	0.09515	0.15715	-0.1625	-0.1995	0.06414	-0.1023
XM_575582.1	LOC500231	-0.1851	0.13729	-0.0545	0.13599	-0.0997	0.07536	-0.0411	-0.0524	-0.064	-1E-06	-0.1293	0.0356	0.04564	0.14118
XM_580049.1	LOC500232	0.03129	-0.043	-0.0491	-0.1095	-0.0202	-0.0139	-0.0963	-0.0543	0.02067	-0.1327	-0.0673	0.13571	-0.1182	-0.0667
NM_001109246	LOC500233	-0.0281	0.0627	0.07101	-0.0103	-0.0059	-0.0244	0.23722	-0.1601	0.17018	0.06447	-0.0474	-0.0023	-0.1715	-0.0061
XM_580050.1	LOC500234	-0.0023	-0.012	0.25509	0.01401	0.04094	-0.1583	0.0238	-0.0273	0.01144	0.05576	-0.0712	-0.0215	0.18561	-0.3174
XM_575587.1	LOC500237	-0.2336	-0.259	-0.2861	-0.1026	-0.4865	-0.3566	-0.3094	0.02983	-0.2699	-0.1797	0.03043	-0.2694	-0.2222	-0.3617
XM_575587	LOC500237	-0.1258	-0.1063	-0.1691	-0.1636	-0.1979	0.14195	-0.1463	-0.1906	-0.0794	-0.2387	-0.095	-0.1791	-0.0756	-0.2453
XM_575590.1	LOC500240	0.08928	-0.0528	0.17204	0.1133	-0.0028	-0.0382	0.03652	0.27717	-0.0003	-0.0007	-0.0095	-0.0609	-0.2008	-0.0497
XM_580051.1	LOC500241	0.20878	-0.0442	-0.0097	-0.0557	0.08168	0.21403	0.06098	0.14868	-0.1387	-0.0304	0.17792	0.11371	0.02581	-0.0272
XM_575594.1	LOC500244	0.13336	0.03566	-0.0223	0.30007	0.12282	0.06551	0.05637	0.09858	0.06118	-0.0658	0.16357	0.14202	0.01545	0.04493
NM_001031824	LOC500245	0.09058	0.1392	-0.1164	0.69733	0.01838	0.09307	-0.0178	0.24401	-0.1392	-0.0301	0.20079	-0.2175	-0.1701	0.11955
XM_575597.1	LOC500247	-0.407	-0.1636	0.46488	0.67442	-0.7891	-0.6827	0.52716	-0.7648	-0.2205	-0.0353	-0.1172	-0.2081	-0.5456	-0.5457
XM_580052.1	LOC500248	-0.0887	-0.1583	-0.0243	-0.156	-0.1923	0.03265	0.01927	0.04798	-0.0294	-0.1086	-0.0695	-0.1046	-0.0254	-0.1004
XM_580053.1	LOC500250	-0.0199	0.00158	-0.0194	-0.0687	0.02244	0.07214	-0.0396	-0.0687	0.23896	-0.0024	-0.1404	0.25611	-0.1162	0.13129

NM_001025047	LOC500251	0.21787	0.41632	-0.1204	0.0546	0.1975	0.19317	0.04494	-0.0012	0.20525	0.44641	0.37219	0.00925	0.12017	-0.0628
NM_001134623	LOC500258	-0.0259	-0.011	0.29974	0.07204	0.07074	0.08032	0.00336	-0.0217	0.00057	-0.0318	0.10395	0.16102	-0.0477	0.0595
XR_007267.1	LOC500265	0.05444	-0.0216	0.0325	-0.1384	0.04081	0.08303	0.08371	0.1207	0.0049	0.13302	-0.0296	-0.0113	-0.0347	-0.0306
XM_575616.1	LOC500266	-0.0374	0.25606	0.04508	-0.0667	-0.0313	0.0195	-0.2443	0.17882	0.08175	-0.0619	0.22889	-0.103	0.07691	-0.033
NM_001047956	LOC500270	0.31231	0.3048	0.09293	0.15682	0.10727	0.0892	0.0267	-0.0096	0.10472	0.05527	0.18621	-0.0803	0.13102	0.18256
XM_575624.1	LOC500274	-0.0345	-0.1607	-0.1101	-0.0221	-0.0198	0.05011	-0.1103	-0.1354	0.20524	-0.1385	-0.0116	-0.131	0.00744	-0.199
XM_575626.1	LOC500276	-0.0211	0.04853	0.06979	0.17852	0.00764	0.2034	0.06493	0.0054	-0.0303	0.0083	0.04352	0.09151	0.12609	0.05784
NM_133565	LOC500277	-0.2547	-0.5299	0.07988	-0.2952	-0.1398	-0.5188	-0.485	-0.2109	-0.4166	-0.4551	-0.4337	-0.5405	0.0159	-0.009
XM_575634.1	LOC500284	-0.1845	-0.28	0.06029	-0.217	-0.2559	0.21374	-0.1457	-0.1318	-0.4743	-0.0819	-0.1607	-0.6036	-0.3826	-0.3614
XM_575635.1	LOC500285	-0.0518	0.25577	0.55341	0.91948	-0.4484	-0.0424	1.2585	0.04708	0.12721	0.15778	0.1653	-0.0395	-0.0848	0.11211
XM_580054.1	LOC500289	-0.2833	-0.4306	-0.3114	-0.148	-0.3078	-0.3605	-0.2433	-0.2936	-0.446	-0.3034	-0.2808	-0.4229	-0.4268	-0.388
XM_575639.1	LOC500290	0.15442	0.00481	0.16343	0.00268	0.09399	-0.0087	0.18638	0.04881	0.09178	0.09343	0.04457	0.0936	0.01507	-0.017
XM_580055.1	LOC500291	0.02755	-0.0386	0.19007	0.01737	0.17121	0.09415	-0.0885	0.05774	0.2147	0.01973	0.05049	0.08151	0.07569	0.15603
NM_001109252	LOC500295	-0.2243	-0.2705	-0.186	-0.0546	-0.3253	-0.311	0.05743	-0.2439	-0.244	0.03517	-0.1887	-0.0602	-0.2749	-0.1864
XM_580056.1	LOC500296	-0.0375	0.11267	0.16367	-0.0869	-0.0526	0.06599	-0.0257	-0.0748	-0.167	0.02745	-0.0306	0.10629	0.10846	-0.0102
XM_575645.1	LOC500297	-0.0175	-0.3756	0.22158	0.05636	-0.4019	-0.2883	0.39651	-0.2651	-0.3831	-0.1882	-0.3909	0.42513	-0.2593	0.11577
NM_001024334	LOC500300	1.5058	-0.1732	-0.5142	-0.3546	0.425	0.31703	-0.7833	0.32075	-0.3129	-0.1317	-0.2228	-0.839	-0.7952	-1.0005
XM_575651.1	LOC500302	0.15105	0.19054	0.03381	0.05053	0.02337	0.07569	0.07869	-0.0133	-0.013	-0.0006	-0.0215	0.2563	0.1474	-0.0477
XM_575656.1	LOC500307	-0.1493	0.01825	-0.0757	0.20466	-0.2664	-0.1626	0.30499	-0.1377	-0.1542	-0.1099	-0.0864	-0.1684	0.07597	-0.1864
NM_001109671	LOC500311	0.13319	-0.033	0.05613	-0.0455	0.01063	-0.0277	-0.0189	-0.1224	-0.1385	0.10243	-0.0619	-0.148	-0.1331	-0.0584
NM_001099507	LOC500312	-0.0243	-0.0682	-0.1346	-0.091	-0.0766	-0.0241	-0.1305	-0.0701	-0.083	-0.1278	-0.0175	-0.1426	-0.1189	0.05833
XM_580057.1	LOC500314	-0.3101	-0.2005	-0.1098	-0.2109	0.0198	-0.2525	-0.2676	-0.2174	-0.1982	-0.1425	-0.3108	-0.2858	-0.1637	-0.0476
NM_001025049	LOC500316	0.16246	0.08745	0.09358	0.00942	-0.1475	0.08741	-0.1255	-0.0143	-0.0292	0.09862	0.22381	0.11218	-0.1324	0.28623
NM_022298	LOC500319	0.00729	-0.0054	-0.0259	0.11151	0.06795	0.12417	0.05511	0.20347	0.14117	0.0176	0.04704	0.04103	0.02389	-0.0198
XM_580058.1	LOC500320	0.16516	0.29542	-0.0773	-0.1567	-0.0388	-0.0272	-0.0848	0.11782	0.04148	0.13791	0.23709	0.00434	0.05133	0.02567
XM_575676.1	LOC500325	0.08402	0.12883	0.14942	0.13031	0.1876	-0.1128	-0.0733	0.06536	0.09523	-0.0364	0.07734	0.17905	-0.0257	0.24342
XM_575678.1	LOC500326	0.15027	0.03889	0.20621	0.27306	0.32696	0.37978	0.13438	0.06687	-0.0386	0.23983	-0.0122	-0.0151	-0.0371	0.19318
NM_001024337	LOC500331	0.1041	0.06083	0.16258	0.02302	0.02723	0.20991	0.10391	-0.025	0.02683	0.12677	0.00991	0.01292	0.00368	0.02865
XM_575684.1	LOC500332	-0.2265	-0.2303	-0.1904	-0.1734	0.06184	-0.2062	-0.1655	-0.1393	-0.1844	-0.1718	-0.1979	-0.2403	0.14944	-0.1075
XM_580059.1	LOC500333	-0.0214	0.43126	-0.0315	0.19411	-0.0023	-0.1751	-0.0961	-0.1309	-0.1667	-0.0763	0.11201	0.17517	0.05362	-0.0753
XM_575685.1	LOC500334	-0.1944	-0.0039	-0.0773	0.36752	0.34741	-0.0618	0.07475	-0.1268	-0.1388	0.14493	-0.2752	-0.0316	-0.0763	-0.1934
XM_575690.1	LOC500339	-0.0413	-0.1995	-0.2192	0.03324	-0.1587	-0.0855	-0.0339	-0.129	-0.0939	-0.2404	-0.1791	-0.0891	-0.2028	-0.039
XM_575693.1	LOC500341	-0.0042	0.32294	0.17205	-0.0138	0.06542	0.10051	-0.0204	0.01709	0.01587	-0.0778	0.00555	0.09823	0.07534	0.11344
XM_575695.1	LOC500343	-0.4184	-0.4945	-0.1595	0.05225	-0.6005	-0.3315	0.25853	-0.5151	-0.2738	-0.4591	-0.427	-0.1986	-0.3296	-0.2718
XM_575696.1	LOC500344	-0.2483	-0.7054	-1.3179	-1.1598	-0.837	-1.1173	-0.8818	-0.7617	-0.4117	-0.5875	-0.4167	-0.6314	-0.3616	-0.6836
XM_575698.1	LOC500346	0.0345	-0.1156	0.11849	0.22762	-0.0944	-0.1382	0.16954	0.02079	-0.0865	-0.0163	0.22099	-0.1148	0.05836	0.42142
NM_001047957	LOC500350	0.06768	0.20633	0.02462	-0.0594	0.02073	-0.0217	0.08886	0.04735	0.01979	0.22729	-0.072	0.28897	0.17135	0.10302
NM_001123469	LOC500351	-0.0379	-0.0635	0.10852	-0.071	0.21971	0.12683	0.03919	-0.0517	0.05984	-0.2211	0.01849	-0.1541	-0.0451	0.0782
XM_575706.1	LOC500353	0.02354	0.15645	-0.0409	0.09143	-0.0822	-0.0526	0.10345	0.05855	0.02322	0.07242	-0.0282	0.11242	0.05452	0.00081
NM_001037797	LOC500354	-0.1261	-0.134	0.01326	-0.0024	-0.0714	0.00725	0.05119	0.09107	0.00074	-0.1295	0.01759	-0.0655	-0.2869	0.02987
XM_575709.1	LOC500355	0.20342	-0.0446	0.57968	0.1485	0.14378	0.31237	0.15939	-0.0722	-0.1631	0.11519	0.09991	0.10602	-0.0371	0.06956
XM_575712.1	LOC500357	-0.0293	-0.04	-0.1682	-0.022	-0.1232	0.25019	-0.2373	-0.1266	-0.1726	-0.3	-0.0743	0.13799	-0.3284	-0.1008
XM_575713.1	LOC500358	-0.0774	-0.0677	-0.0687	-0.1167	0.06301	-0.0056	-0.0626	-0.0505	-0.0231	-0.1113	-0.0119	-0.021	-0.0696	0.00172
XM_575719.1	LOC500362	-0.1952	-0.2549	-0.2561	-0.1781	0.00844	-0.1433	-0.1413	-0.375	-0.2942	-0.29	-0.1865	-0.1133	-0.0713	-0.0353
XM_575723.1	LOC500365	-0.2326	-0.1146	-0.2929	-0.1768	-0.228	-0.0837	-0.2702	-0.3226	-0.1279	-0.2292	-0.378	-0.0827	-0.2335	-0.2154
XM_575726.1	LOC500368	0.07248	-0.0526	-0.0638	0.01469	0.03941	0.1011	0.04853	-0.0575	0.01884	0.08921	-0.0138	0.0044	0.14708	-0.0481
XM_575732.1	LOC500374	-0.0921	0.05721	-0.0476	-0.2173	-0.0923	-0.1339	-0.1416	-0.0962	-0.0318	-0.0737	0.12493	-0.0988	-0.1115	-0.0185
XM_575733.1	LOC500375	-0.0364	-0.0665	0.0643	-0.0793	-0.1591	-0.0688	-0.0103	-0.1233	-0.1501	-0.2804	0.11657	-0.1513	-0.1141	0.03566
NM_001014175	LOC500378	0.22237	0.15182	-0.1536	0.08026	0.14858	0.31193	0.05499	0.42099	-0.059	0.32311	-0.0506	0.11797	0.00692	-0.2453

XM_575737.1	LOC500379	-0.2048	-0.128	-0.2408	-0.2452	-0.1476	-0.2369	-0.1499	-0.1378	-0.1731	-0.1728	-0.1548	-0.2439	-0.2605	-0.0205
XM_575738.1	LOC500380	-0.343	-0.264	-0.3456	0.17551	-0.5144	-0.1454	0.57976	-0.4899	-0.3666	-0.2618	-0.197	0.38108	-0.274	-0.149
XM_575739.1	LOC500381	-0.0536	0.01466	0.07711	-0.0228	0.00281	-0.2056	-0.1933	0.02162	0.27431	0.06086	0.06438	0.12793	-0.0395	-0.0477
XM_575744.1	LOC500386	-0.147	0.22643	0.05499	0.09016	-0.2044	-0.0345	-0.249	-0.1504	-0.075	-0.0536	-0.2327	-0.0909	-0.2197	0.01326
XM_575748.1	LOC500389	0.25753	0.21436	0.47064	0.52356	0.08285	0.4205	0.69403	0.31073	0.17204	0.07858	0.43056	0.17408	0.06809	0.1076
XM_575750.1	LOC500391	-0.0432	0.23538	0.21954	0.2851	0.28295	0.16852	0.62643	0.01307	0.11857	-0.0148	0.03844	-0.0585	-0.0951	0.09108
NM_001024340	LOC500392	-0.342	-0.0477	0.01995	-0.1975	-0.591	-0.3697	0.21323	-0.4242	0.01722	-0.0008	-0.1737	-0.0646	-0.4629	-0.3968
XM_575756.1	LOC500397	-0.373	0.09623	0.00555	0.30014	-0.4904	-0.0795	0.60529	-0.2355	0.04332	-0.1589	0.20114	0.27379	-0.0158	0.08734
XM_575757.1	LOC500398	-0.3973	0.09719	-1.7439	-1.2078	-0.9732	-1.4252	-0.4001	-0.7241	-0.0411	0.14656	-0.1894	-0.6593	-0.8361	-1.2015
XM_575761.1	LOC500399	-0.2398	-0.0102	-0.8914	-0.3069	-0.6669	-0.876	0.21347	-0.5924	-0.2148	-0.1904	-0.2024	-0.1532	-0.8046	-0.3579
XM_575765.1	LOC500402	-0.0067	-0.0253	0.0893	0.1902	0.00343	0.06982	-0.0424	-0.0674	-0.07	-0.0363	0.02425	-0.0264	-0.0609	0.00373
XM_580060.1	LOC500405	-0.0172	-0.0481	0.12232	-0.1568	0.13408	-0.0385	-0.0503	0.02153	0.06878	-0.139	-0.0081	0.05057	0.12961	-0.1857
XM_575769.1	LOC500406	0.07617	-0.0464	-0.0329	-0.0346	-0.0069	-0.0319	-0.0363	0.01057	0.10689	0.14021	-0.0838	0.06414	0.02529	-0.0142
XM_575770.1	LOC500407	0.19312	-0.0597	0.14192	0.1643	-0.1179	0.03143	0.0608	-0.0939	-0.007	0.10039	0.02747	0.19383	-0.1491	0.05718
XM_580061.1	LOC500408	0.03579	0.0887	0.14259	-0.0457	0.19207	0.01935	0.11366	0.16743	0.03913	-0.0171	-0.0568	0.18112	0.17869	0.07933
NM_001127564	LOC500409	0.02293	0.12427	0.15705	0.16644	0.1256	0.25046	0.20946	0.17995	0.10548	0.20299	0.29335	0.21608	0.09381	0.00273
XM_575772.1	LOC500410	-0.0593	0.07228	-0.0035	0.07621	-0.0024	-0.0184	0.12634	0.02161	0.0114	-0.1253	-0.0399	0.2081	0.07535	0.07024
XM_575774.1	LOC500412	0.17731	0.00571	-0.0294	0.06389	-0.0939	-0.0059	0.06019	0.01364	0.02467	0.06355	-0.0501	0.1059	0.03441	0.18943
XM_575776.1	LOC500414	-0.0116	0.25173	0.08537	-0.1315	0.22037	-0.0279	-0.0034	-0.053	0.13834	0.27314	-0.1081	-0.1025	0.30044	-0.0601
XM_575783.2	LOC500420	0.94773	0.82603	0.08602	0.13813	0.82081	0.59832	0.46855	0.72974	0.63895	0.70153	0.82004	0.65489	0.59205	0.68052
XM_575785.1	LOC500421	0.04333	0.10077	0.09085	0.06935	0.31332	-0.0472	0.24282	0.03157	0.10429	-0.0425	0.17699	0.22501	0.13454	0.17358
XM_580064.1	LOC500422	-0.2566	-0.3995	-0.2086	-0.2643	-0.1592	-0.3112	-0.1696	-0.1419	-0.0749	-0.1284	-0.2439	-0.3583	-0.2411	-0.183
XM_580065.1	LOC500423	0.01423	0.37296	0.07713	0.14264	0.23084	0.12578	0.10857	0.04372	0.08217	0.12732	0.30707	0.061	0.00197	0.19691
XM_575787.1	LOC500425	-0.0662	-0.1093	0.01659	-0.1144	-0.1513	0.16311	0.01024	0.0915	-0.077	0.00214	-0.0836	-0.0224	0.03869	-0.1064
XM_575788.1	LOC500426	0.17471	0.05041	0.0364	0.04825	0.04549	0.12465	0.04006	0.13503	0.20703	0.07648	0.11263	0.07874	0.09574	0.06643
XM_575789.1	LOC500427	0.11735	0.0205	0.10541	0.07289	0.06535	-0.0078	-0.0192	0.01123	-0.0025	-0.0222	0.11635	-0.0051	0.05041	0.01376
XM_575790.1	LOC500428	-0.1295	0.0928	0.06938	0.6617	-0.1406	0.19657	0.60709	-0.0251	-0.0876	-0.0939	0.18373	-0.0011	0.00808	-0.1372
XM_575791.1	LOC500429	0.12994	0.01055	-0.0254	-0.3168	-0.1344	0.003	-0.019	-0.1193	-0.1918	-0.1209	-0.1984	0.26825	0.0166	-0.0308
NM_001134969	LOC500430	-0.0258	0.09248	-0.106	-0.0769	-0.049	0.32661	0.00193	-0.1302	-0.1802	-0.1324	-0.0995	-0.1573	0.01855	-0.1496
XM_575793.1	LOC500431	-0.038	0.01022	0.22933	-0.0022	0.02343	-0.034	-0.1138	-0.0417	-0.0735	-0.1205	0.02397	0.1327	-0.0348	0.16461
XM_575798.1	LOC500436	-0.2066	-0.0946	0.22547	0.0995	-0.2253	-1.1616	0.13435	-0.1289	-0.0916	-0.1928	-0.173	-0.8738	0.17294	0.16018
XM_575799.1	LOC500437	-1.5887	-0.4398	0.55198	-0.2792	-1.3996	-1.3949	1.2311	-1.3486	-0.7712	-0.2145	-0.7924	-1.0473	-0.5594	-0.3906
XM_575800.1	LOC500438	-0.3286	-0.1485	-1.2925	-0.8117	-0.7663	-0.9618	-0.3074	-0.52	-0.304	-0.2155	-0.1103	-0.6175	-0.8989	-0.955
XM_580066.1	LOC500439	0.34409	-0.0793	-0.0783	-0.0135	-0.0962	0.0676	-0.0053	0.04525	-0.1302	0.01076	0.04639	-0.0449	-0.0095	0.00831
XM_575806.1	LOC500444	-0.2058	-0.0979	-0.0589	-0.1906	-0.1127	0.01644	0.04978	-0.1458	-0.1514	-0.064	0.02553	-0.0133	-0.0178	-0.0958
NM_001024343	LOC500445	0.01775	-0.0654	0.07078	0.02061	0.12775	0.05462	-0.0328	-0.1017	-0.2127	-0.1371	-0.0184	0.14849	0.16308	-0.111
XM_575809.1	LOC500447	0.10208	0.31972	0.0704	0.13534	0.38433	0.02538	-0.008	0.12471	-0.0353	0.24189	0.21329	0.103	0.14102	0.02319
XM_575820.1	LOC500456	0.03154	0.31985	0.02321	0.02758	0.01361	0.03532	0.07336	0.04418	0.2228	0.15744	0.25976	0.05185	0.0792	0.06899
XM_575821.1	LOC500457	-0.055	-0.1397	-0.0258	-0.0861	-0.1591	-0.2118	-0.0129	-0.1487	-0.0062	-0.1603	0.12935	0.01316	-0.0696	-0.1689
XM_575822.1	LOC500458	-0.1079	0.06111	0.02432	-0.0549	0.11842	0.23724	0.25554	0.01072	0.03869	0.10852	0.36952	0.10574	-0.0225	0.12578
XM_580069.1	LOC500459	0.0237	0.03044	-0.0128	-0.0296	0.21848	0.14716	0.05631	0.02092	0.04803	-0.045	0.10511	0.01437	-0.0021	0.00609
NM_001000405	LOC500460	-0.0284	0.02644	0.05178	-0.1014	0.0134	0.10406	0.11603	0.08653	0.06217	0.05442	0.01466	-0.0562	0.06145	-0.1211
XM_575824.1	LOC500461	0.08486	0.11529	0.1237	-0.0047	-0.0191	0.20872	0.02588	0.10464	0.05113	0.10025	0.08207	0.05962	0.4437	0.30832
XM_580070.1	LOC500466	-0.237	-0.2723	-0.2422	-0.2534	-0.1432	-0.1304	-0.2369	-0.1645	-0.1735	-0.2077	-0.1763	-0.1874	-0.0829	-0.2623
XM_575831.1	LOC500468	-0.0823	-0.0338	0.06376	-0.1017	-0.1529	-0.0305	-0.2445	0.01242	-0.1527	0.04532	-0.1516	-0.1853	-0.1117	-0.2665
XM_575833.1	LOC500469	-0.3198	0.30086	-1.3257	-0.5226	-0.8241	-1.1087	-0.0188	-0.5672	-0.1991	-0.0468	-0.1968	-0.4591	-1.2057	-0.8607
XM_575834.1	LOC500470	-0.0521	-0.0352	-0.0148	-0.0107	-0.0582	0.00214	-0.0242	0.01772	-0.0993	-0.0779	-0.0347	-0.0353	0.00595	-0.1551
XM_575835.1	LOC500471	0.02121	-0.0458	0.05029	0.11644	-0.0543	0.17607	0.06016	0.09682	-0.0079	-0.067	0.01167	0.00844	-0.0864	0.09952
XM_575836.1	LOC500472	-0.0071	-0.1457	-0.0749	-0.08	0.10167	-0.0642	-0.1335	-0.0469	-0.0663	-0.0388	-0.0063	-0.1487	0.08781	-0.0172

NM_203512	LOC500473	0.16733	0.02665	-0.0277	-0.0472	0.00663	0.02893	0.23924	0.02942	0.00239	0.19902	-0.0366	0.10928	-0.0312	-0.0874
NM_001024346	LOC500475	0.25056	0.04997	0.11927	-0.1197	0.04698	-0.1084	-0.0822	-0.0358	-0.0887	-0.0371	0.03448	-0.0614	-0.076	0.12222
XM_575843.1	LOC500477	-0.1317	0.00464	-0.0345	-0.001	0.06389	0.03556	-0.0857	-0.0868	-0.0612	0.03339	0.00126	0.04372	-0.0283	-0.1133
XM_575847.1	LOC500480	0.19882	0.00245	-0.0359	0.06187	-0.1155	0.05214	0.04813	-0.1144	0.04503	-0.2014	-0.2448	-0.1305	-0.0134	0.07207
XM_580071.1	LOC500483	-0.3036	-0.3369	0.00864	-0.3283	-0.2201	-0.0686	-0.1208	-0.259	0.19756	-0.4268	-0.2682	0.1291	-0.1921	-0.19
XM_580072.1	LOC500488	-0.3713	-0.2272	-0.682	-0.3812	-0.516	-0.5586	-0.7058	-0.3897	-0.374	-0.6524	-0.5285	-0.4211	-0.5824	-0.4696
XM_580072	LOC500488	0.03484	0.00417	0.04836	0.08913	0.04566	0.01639	0.00828	0.08051	0.00412	0.07837	0.0312	0.05388	-0.0643	-0.0411
XM_575855.1	LOC500490	-0.4288	0.11499	-1.2353	-0.6114	-1.0182	-0.8491	-0.001	-0.5722	-0.2217	0.07212	-0.2502	-0.032	-0.7798	-0.7523
XM_580073.1	LOC500493	-0.0463	0.20449	0.07736	0.00383	0.13421	0.16458	0.32153	0.02277	0.37525	0.06233	0.1782	0.46784	-0.0118	0.1554
XM_575859.1	LOC500495	-0.4061	-0.2968	0.21203	0.30502	-0.6329	-0.405	0.48815	-0.2946	-0.0259	-0.3494	0.03586	-0.2929	-0.5069	-0.4543
XM_575863.1	LOC500499	0.05893	0.02398	-0.0313	0.04739	-0.0309	-0.0411	0.11079	0.10069	0.18921	-0.0868	0.06271	-0.0321	0.02717	-0.0585
XM_580075.1	LOC500500	0.19097	-0.2162	0.09252	-0.0511	-0.1636	0.25451	0.10647	-0.0288	-0.1279	0.03147	0.23664	0.31203	0.1643	-0.0035
NM_001039345	LOC500502	0.05609	-0.0256	-0.1214	0.04883	-0.2322	-0.053	0.26003	0.04338	-0.108	0.27028	-0.0809	-0.1714	0.05121	-0.0988
XM_580076.1	LOC500503	-0.0126	0.00142	0.15151	0.06319	0.06034	-0.009	0.06626	-0.0357	0.28317	0.0639	-0.0314	0.02328	-0.1118	-0.0029
XM_580077.1	LOC500504	-1.1275	-0.595	0.14777	0.11191	-1.2806	-1.1522	0.3691	-0.9643	-0.5541	-0.3888	-0.5675	-0.4907	-1.6801	-1.5603
NM_001134625	LOC500505	-0.1999	-0.1236	-0.0838	-0.1246	-0.1721	-0.2017	-0.176	-0.0518	-0.2339	-0.2519	-0.2099	-0.1716	-0.1971	0.13778
XM_575868.1	LOC500506	0.43096	0.47072	-0.2193	-0.8028	0.32518	-0.6309	-0.0895	0.42266	0.39083	0.4203	0.16964	-0.2707	0.5014	0.38641
XM_575869.1	LOC500507	-0.1441	-0.0871	0.0289	0.40008	-0.3961	-0.2669	0.36401	-0.3276	0.00552	-0.5084	0.10806	0.06685	-0.3566	-0.1418
XM_580078.1	LOC500508	-0.0588	0.02862	-0.0122	-0.0431	0.11196	0.102	-0.038	0.0412	0.21816	0.02585	0.09622	0.31695	0.09249	0.04817
XM_575871.1	LOC500509	-0.0203	0.08025	-0.0235	0.10547	0.0396	0.00751	-0.0095	-0.0585	-0.069	0.02539	0.1461	0.0228	-0.056	0.0313
XM_575874.1	LOC500511	-0.2433	-0.2136	-0.153	-0.1868	-0.0615	-0.1562	-0.0889	-0.1158	-0.0593	-0.0689	-0.1353	-0.0757	-0.0204	-0.0242
XM_580079.1	LOC500512	-0.1343	-0.1088	-0.1966	-0.2304	-0.087	0.03317	-0.0234	-0.2165	-0.114	-0.1431	-0.1459	-0.2	-0.1364	-0.1086
XM_575875.1	LOC500513	-0.2432	-0.262	-0.0279	-0.2141	-0.2863	-0.3056	-0.0732	0.24865	-0.2741	-0.1867	-0.3078	-0.3012	-0.152	-0.2194
XM_580080.1	LOC500515	0.00271	-0.0938	-0.0154	0.01229	0.01366	0.10282	-0.0694	-0.0456	0.04696	-0.1421	0.12126	-0.0486	-0.0533	-0.1355
XM_575878.1	LOC500517	-0.0129	-0.0974	-0.1126	-0.0098	-0.1603	-0.1232	-0.1943	-0.1417	-0.0946	-0.1773	0.03544	0.16205	-0.2042	-0.2647
XM_575881.1	LOC500520	-0.3377	-0.326	-0.3073	-0.3664	-0.1838	-0.1327	-0.335	-0.1996	-0.1724	-0.2441	0.21464	-0.3149	-0.3007	-0.0799
XM_575882.1	LOC500521	-0.0089	0.0941	-0.0263	0.08977	0.09796	-0.0525	0.00021	-0.0071	0.22698	-0.0123	0.03761	-0.0871	0.21508	0.1851
XM_575883.1	LOC500523	0.09319	0.14027	0.20742	0.22203	0.10953	0.10526	0.0728	0.03729	0.11547	0.13696	0.16557	0.11677	0.13292	0.00929
NM_001014067	LOC500528	-0.216	-0.1436	-0.1301	-0.2077	-0.2734	-0.1204	-0.1888	-0.175	-0.0751	0.05878	0.01275	-0.0242	-0.1027	-0.1287
XM_575890.1	LOC500529	-0.0557	-0.2439	-0.2039	-0.2415	-0.1698	-0.4292	-0.2857	-0.1463	-0.0774	-0.1738	-0.0955	-0.2768	-0.2295	-0.3715
XM_575894.1	LOC500533	0.11986	-0.0918	0.20658	-0.1245	0.21795	0.16575	0.05322	0.07268	0.22266	-0.1266	0.132	0.25572	0.04876	0.05374
XM_575896.1	LOC500535	-0.0373	-0.1437	-0.1381	-0.1072	-0.1746	-0.0376	-0.124	-0.1131	0.16503	-0.1606	-0.1966	0.00454	-0.0847	0.03393
XM_575897.1	LOC500536	0.55438	0.16955	-0.1895	-0.2299	0.57509	0.95749	-0.3722	0.37086	0.08146	-0.1519	0.26892	0.38059	0.28697	0.13197
NM_031563	LOC500538	0.20781	-0.2796	-0.2829	-0.2803	0.15107	-0.0647	-0.064	-0.0294	-0.1061	-0.0652	-0.2175	-0.0734	-0.0105	0.19894
XM_575902.1	LOC500541	-0.0927	-0.099	0.14229	0.0234	-0.3369	-0.602	-0.3339	-0.362	-0.1468	0.04143	0.15417	0.03721	-0.2397	-0.1779
XM_575903.1	LOC500542	0.06184	-0.1545	-0.1927	-0.097	0.08763	-0.2373	0.2199	-0.0093	-0.2025	-0.1327	-0.2259	-0.167	-0.2309	-0.2437
XM_575905.1	LOC500543	0.00392	-0.0328	0.12486	0.04249	-0.0098	0.06902	-0.1111	0.10852	0.03601	0.17316	0.13331	0.09703	-0.0141	0.29731
XM_580082.1	LOC500544	-0.0455	-0.043	-0.0068	-0.0263	0.03483	0.07099	0.18145	0.06246	-0.0174	-0.1008	-0.005	0.45309	0.10341	-0.0208
NM_001109264	LOC500546	-0.0821	-0.0273	-0.1947	0.0548	-0.0745	0.02641	-0.0994	-0.1028	-0.0466	-0.0766	-0.0035	-0.215	0.00851	-0.0108
XM_575910.1	LOC500548	0.53333	0.13274	-0.0542	0.03187	0.05442	0.06002	0.29891	-0.0982	-0.1094	-0.0101	-0.2431	-0.0616	-0.0061	0.38902
XM_575911.1	LOC500549	-0.354	-0.2859	0.11402	0.25968	-0.2359	-0.0528	-0.0645	-0.0618	0.00363	-0.1633	-0.097	-0.3825	-0.1968	-0.029
XM_575912.1	LOC500550	0.07298	0.00084	0.03605	0.20571	0.08386	0.21898	-0.0662	0.27984	0.00698	0.19875	-0.1211	0.39958	-0.1322	0.04989
NM_053602	LOC500560	-0.0395	0.03414	-0.0436	0.02444	-0.0381	0.03848	0.02802	0.13035	0.1243	0.43604	0.18296	0.14098	0.22553	0.00234
NM_001109269	LOC500562	0.21725	0.06763	0.19835	0.12193	-0.2156	-0.0302	-0.0211	-0.0801	-0.0493	0.03053	-0.156	-0.098	0.08846	-0.0465
NM_001024348	LOC500567	0.19729	0.34554	0.50786	0.42235	0.24399	0.30207	0.03881	0.46184	0.23196	0.27651	0.23255	0.21161	0.13621	0.12376
XM_580085.1	LOC500570	-0.0873	-0.0496	0.16314	0.2025	0.16132	0.03973	0.04606	0.1529	-0.0113	0.10157	0.0421	-0.0869	0.05136	0.06059
XM_580086.1	LOC500582	0.14053	0.04672	0.16714	0.03062	0.17304	0.0266	0.0816	0.17625	0.03495	0.29169	0.04952	0.17153	0.09117	0.15557
XM_575952.1	LOC500583	-0.0067	0.04031	-0.161	-0.0817	-0.1316	0.00451	-0.0729	-0.0415	-0.0952	0.07094	-0.0167	-0.0361	-0.0835	0.03531
XM_575954.1	LOC500585	0.21246	0.27806	0.20252	0.08354	0.1001	0.65692	0.29819	0.5135	0.18699	0.40137	-0.1638	0.08513	0.09706	0.09205

XM_575955.1	LOC500586	0.03664	0.07972	0.3885	0.62491	-0.1464	0.18998	0.75694	0.38758	0.15387	0.21663	0.2589	0.09503	0.114	0.22972
NM_001025773	LOC500590	-0.0392	0.07943	0.10261	-0.0375	0.02775	0.07037	-0.1133	-0.0861	-0.0169	0.09914	0.16457	0.08818	0.07124	0.04581
XM_001076183.1	LOC500591	-0.2546	-0.1864	-0.1049	0.0716	0.03253	-0.1888	-0.0651	-0.1746	-0.1373	-0.0406	-0.1287	-0.116	-0.2103	-0.1525
NM_001137644	LOC500592	-0.3624	-0.3388	-0.0174	-0.2374	-0.1982	-0.413	-0.1899	-0.2475	-0.3994	-0.2946	-0.2621	-0.1025	-0.3579	-0.2977
NM_001047958	LOC500594	0.11791	0.21953	0.36503	0.15472	0.41234	0.4342	0.34169	0.1224	0.22764	0.19677	0.06374	0.10544	0.15946	0.35671
NM_001163723	LOC500595	0.03104	0.11361	0.18223	0.1069	0.10095	0.16958	-0.0393	0.22982	0.00854	0.09722	0.2424	0.07499	0.04318	0.10533
NM_001024349	LOC500598	0.3676	0.00876	-0.0193	0.09892	-0.0321	0.25691	-0.005	-0.1118	0.07276	0.02112	0.04168	-0.0912	0.06682	-0.1021
XM_575973.1	LOC500599	-0.1837	-0.232	0.0286	-0.0233	-0.1565	0.16963	-0.2408	-0.0416	-0.1864	-0.2624	-0.1725	-0.0905	0.14608	-0.1667
XM_580089.1	LOC500600	0.04559	-0.036	0.08163	0.08557	0.19493	0.06443	0.11378	0.09549	0.06758	-0.0063	0.19076	0.05543	0.13989	0.11943
XM_575976.1	LOC500603	0.07213	-0.0166	-0.0475	0.10838	0.12268	-0.0696	-0.0656	-0.1474	0.00409	0.11935	0.2068	-0.0325	0.03823	0.04541
XM_580090.1	LOC500608	-0.055	0.06471	0.01577	0.03118	0.02464	0.24648	0.04264	0.14775	0.07392	0.00865	-0.014	0.00189	0.04494	0.00607
XM_580091.1	LOC500609	-0.1902	0.0623	-0.0612	-0.1264	-0.0932	-0.0105	-0.1595	0.13853	0.12054	-0.1411	-0.019	-0.1319	-0.1814	-0.1278
XM_580092.1	LOC500611	-0.0264	-0.0499	-0.0171	-0.0455	-0.1345	-0.1135	-0.1235	-0.0931	-0.1236	-0.0297	-0.143	-0.0634	0.0897	-0.1474
XM_575985.1	LOC500614	-0.1209	0.11008	-0.2819	0.28719	-0.2283	-0.5893	-0.0111	-0.2599	0.10597	-0.0386	-0.1927	-0.2931	0.09321	0.02774
XM_580094.1	LOC500615	-0.0878	-0.0712	0.0715	0.06591	-0.1792	-0.0766	0.31869	-0.1204	-0.0962	0.12016	0.04934	-0.0378	-0.1743	0.25202
XM_575991.1	LOC500617	0.0305	-0.0206	0.43041	0.40869	-0.1148	0.22213	0.4535	0.27798	-0.0348	0.12153	0.24434	-0.0187	0.1462	0.03935
XM_580095.1	LOC500618	0.05362	-0.123	0.09966	0.06687	0.0413	-0.0474	-0.1419	0.09727	-0.0767	0.11264	-0.1159	-0.0898	-0.1282	-0.1483
XM_575993.1	LOC500619	-0.155	-0.3224	0.20397	0.14047	-0.3094	-0.4433	-0.0009	-0.2629	-0.4023	-0.3439	0.10342	-0.3292	-0.3811	-0.147
NM_130826	LOC500621	0.50837	1.0487	-0.6052	-0.4412	0.62312	0.00842	-0.0532	0.52094	0.8725	0.88936	0.38501	-0.0662	0.10202	0.17144
XM_580096.1	LOC500622	0.00012	-0.0294	0.00826	0.10076	0.12274	-0.0768	-0.1536	0.13024	0.05746	-0.1807	0.03294	-0.0518	0.01104	0.0104
NM_001025151	LOC500625	-0.0817	-0.2553	-0.2276	-0.2756	-0.0141	-0.3034	-0.0524	-0.2786	-0.1261	-0.1639	-0.1141	-0.1246	-0.1181	-0.1482
XM_580097.1	LOC500626	-0.1349	-0.5326	0.47244	0.45755	-0.2097	-0.3923	0.58981	-0.4024	-0.303	-0.1496	-0.046	-0.0119	-0.3618	-0.0576
XM_580098.1	LOC500628	0.15877	-0.0657	-0.0573	0.01958	0.00831	0.02517	0.0425	-0.0195	-0.0858	-0.1517	-0.0148	-0.1088	-0.0108	0.04717
XM_576008.1	LOC500634	0.04515	-0.0003	-0.1677	-0.2648	0.12748	-0.1573	-0.0338	-0.2585	-0.1139	0.06262	-0.0181	-0.1946	-0.0179	0.01165
XM_576014.1	LOC500639	-0.0814	-0.1326	-0.1389	-0.0933	-0.0852	-0.0791	-0.1137	-0.0953	-0.0637	0.12658	-0.0138	-0.0321	0.03847	-0.0893
XM_576017.1	LOC500642	-0.1388	-0.0053	-0.0447	-0.1492	0.21654	0.05202	-0.1051	-0.1851	-0.1342	0.03214	-0.1187	-0.1499	0.2898	-0.0523
XM_576018.1	LOC500643	-1.7338	-1.9371	-1.8072	-1.5331	-0.8193	-0.8013	-1.6991	-0.7194	-1.9035	-1.8159	-1.8511	-1.8956	-1.7055	-1.8297
XM_580101.1	LOC500646	-0.1152	-0.2458	-0.2851	-0.1605	-0.0413	-0.1219	-0.125	-0.1732	-0.061	-0.261	-0.2357	-0.0032	-0.1337	-0.115
XM_576024.1	LOC500647	0.10472	0.1161	0.20057	0.14083	0.18507	0.09582	0.36357	0.32218	0.2459	0.0215	0.08956	0.08018	-0.0078	0.15622
XM_576026.1	LOC500649	-0.1217	0.04585	-0.1857	-0.2135	0.08295	0.01231	-0.0536	-0.1255	0.02711	-0.0651	-0.0733	-0.1311	-0.0862	-0.0784
XM_576030.1	LOC500652	0.39868	0.05152	-0.3858	0.32597	0.83285	1.0422	-0.309	0.80127	-0.0557	-0.1233	0.02141	-0.0559	-0.0806	0.11468
XM_576034.1	LOC500656	-0.2251	-0.2846	0.02033	-0.0007	-0.1319	-0.1906	0.03568	-0.1832	-0.0056	-0.0284	-0.0793	-0.2647	-0.2655	-0.2306
XM_576035.1	LOC500657	-0.0311	0.05693	0.25731	-0.0599	0.16715	0.01526	0.09846	0.05808	0.0211	0.06241	-0.0485	-0.0212	0.06639	0.28465
XM_576036.1	LOC500658	-0.2179	-0.1462	-0.1468	-0.1034	-0.0329	-0.1726	-0.0778	-0.1232	-0.0978	-0.1169	-0.102	-0.1528	-0.0959	-0.1779
XM_576037.1	LOC500659	0.11409	0.17375	0.05894	0.07382	0.16162	0.11436	0.5104	0.10024	0.23748	0.1126	0.08231	0.12978	0.06405	0.16565
XM_576041.1	LOC500663	-0.1668	0.11197	0.17817	0.2225	-0.2333	-0.381	0.22255	-0.159	-0.2654	-0.0516	-0.0559	-0.2224	-0.1926	-0.1227
XM_576042.1	LOC500664	0.16634	0.21269	-0.0195	0.12602	0.25148	-0.0092	0.10177	0.06286	0.44118	0.27301	0.25585	0.22841	-0.006	0.64715
XM_580103.1	LOC500667	0.00719	-0.1242	-0.1777	0.02653	-0.1132	-0.0059	-0.117	0.09228	0.0711	-0.1468	-0.154	-0.0675	0.13243	0.0582
NM_001164400	LOC500668	0.35767	0.21474	0.0675	0.52888	0.07556	0.13958	0.46494	0.25919	0.23103	0.19117	0.22067	0.05512	0.19193	0.13486
NM_001164400	LOC500668	-0.0994	-0.0404	0.26364	-0.1317	-0.098	0.03621	-0.1488	-0.1328	-0.2564	0.15599	-0.0598	0.02226	-0.0008	-0.0659
XM_576046.1	LOC500669	-0.0216	0.08566	-0.2115	0.0109	0.00559	-0.0115	-0.2559	-0.1567	0.07417	0.07259	0.00796	-0.1424	0.04801	0.02491
XM_576053.1	LOC500675	-0.0729	0.07126	0.1978	0.52293	0.04558	0.00204	0.15149	-0.0166	-0.3456	0.08759	-0.4104	-0.181	-0.2733	0.10013
XM_580104.1	LOC500676	-0.0029	0.09943	0.13858	0.05643	0.41435	0.25229	0.10321	0.39221	0.00231	0.27995	0.33094	0.1106	0.18398	0.24889
XM_576054.1	LOC500677	0.00442	-0.0176	-0.0339	-0.1505	0.01524	-0.2457	0.09028	-0.0942	0.14826	0.02578	0.1924	0.12974	-0.0045	0.05217
XM_576063.1	LOC500683	-0.009	0.24166	0.30359	0.38565	0.32252	0.29241	0.27125	0.31125	0.20675	0.01671	0.07305	0.26673	0.08692	0.0726
NM_001047959	LOC500684	-0.1054	-0.0282	0.11136	0.06371	-0.1541	0.01911	0.08185	-0.091	-0.0793	-0.1354	-0.1227	0.02281	-0.1685	0.29054
NM_001127566	LOC500685	-0.0705	0.09789	0.01385	0.03446	-0.0688	-0.015	-0.0181	0.1133	0.04456	0.06467	-0.0101	-0.057	-0.0938	0.05761
XM_580105.1	LOC500687	-0.0394	-0.5714	-0.0318	0.40412	-0.0509	0.38616	0.05623	0.01687	-0.3839	-0.648	-0.3711	-0.2993	-0.4138	-0.2892
NM_001109277	LOC500688	-0.138	-0.312	-0.0979	0.02075	-0.2727	-0.277	0.00745	-0.3712	-0.2148	-0.1495	-0.1429	-0.2922	-0.1233	-0.1974

XM_576078.1	LOC500696	-0.1541	-0.1284	-0.1557	-0.2152	-0.0963	-0.1811	-0.2599	-0.2047	-0.2011	-0.2476	-0.1723	-0.1563	-0.1613	-0.0887
XM_576081.1	LOC500699	0.15157	0.11856	0.07614	0.15537	0.06488	0.20775	0.12799	0.09754	0.20361	0.07182	0.05807	0.02259	0.0191	-0.0253
XR_006574.1	LOC500700	0.08169	0.12274	0.10683	0.03058	0.12862	0.07934	0.13437	0.05015	0.25187	0.03872	0.02798	0.34808	0.34236	0.36077
NM_001047960	LOC500702	-0.1443	0.04542	-0.0465	0.28149	-0.0094	0.21986	-0.0613	0.09144	0.08567	0.09917	0.01961	0.05349	-0.055	0.05019
XM_576086.1	LOC500704	-0.139	-0.1685	-0.2272	-0.0025	-0.157	-0.1557	-0.1678	-0.0221	-0.2522	-0.0884	-0.0924	0.09755	-0.1925	-0.1235
NM_001024350	LOC500705	-0.0857	0.09662	0.31893	0.13789	-0.1926	0.10397	-0.0203	-0.2652	0.10527	-0.1447	-0.117	0.53366	0.14923	0.52407
XM_580107.1	LOC500706	0.10169	-0.0736	-0.0175	-0.204	0.07997	0.03209	0.03478	-0.1136	-0.0284	-0.1402	-0.1776	-0.1138	-0.1142	0.02984
XM_580108.1	LOC500708	-0.1249	-0.0827	-0.1945	-0.2409	0.0472	-0.1165	-0.2619	-0.059	0.03088	-0.1575	0.03193	-0.0142	0.12282	-0.1179
XM_576090.1	LOC500709	0.09181	0.05968	0.07363	0.29665	0.37489	0.08379	0.1997	0.0869	0.22836	0.10824	0.27413	0.05188	0.30974	0.12138
XM_576095.1	LOC500712	-0.1147	0.18595	-0.0586	-0.2468	-0.1887	-0.2607	-0.2219	0.06529	-0.0352	0.09983	-0.0067	0.2776	-0.1455	-0.1542
XM_580109.1	LOC500713	0.02862	-0.1203	-0.2453	-0.0502	-0.1048	-0.6201	-0.2629	0.09444	-0.2192	-0.1789	0.04888	-0.647	-0.2156	-0.311
NM_001109672	LOC500715	0.04271	-0.145	-0.2038	-0.1058	-0.1741	-0.0805	-0.1446	-0.1534	-0.1305	-0.1002	-0.1451	-0.0484	-0.089	-0.1074
XM_580111.1	LOC500718	-0.077	-0.0855	-0.0583	-0.0285	-0.0904	0.04586	-0.0208	0.0033	-0.0045	0.01555	-0.0377	-0.0107	-0.0466	-0.0317
XM_580113.1	LOC500720	-0.0419	-0.1254	-0.1234	-0.1203	-0.1071	-0.0815	-0.0421	0.08355	-0.0404	-0.1485	0.01343	0.04359	-0.0849	-0.0545
XM_580114.1	LOC500721	-0.2171	-0.0649	-0.092	-0.0302	-0.1689	-0.1643	-0.1185	-0.0873	-0.1719	-0.1131	-0.203	-0.0713	-0.198	-0.1539
XM_580115.1	LOC500725	0.1163	0.01411	-0.0966	-0.0172	-0.0486	0.01852	-0.0403	0.11395	0.06708	-0.0108	0.25497	0.00509	0.14034	0.01919
XM_001072852.1	LOC500726	-0.1687	-0.2783	-0.1638	0.04774	0.16717	-0.1296	-0.2914	0.18084	0.02013	-0.1695	-0.069	-0.2516	0.04909	-0.2371
NM_001012146	LOC500729	0.01252	-0.0524	0.12279	0.03306	-0.0898	0.10957	-0.0099	0.01894	0.23874	0.02726	0.2422	0.01291	0.05646	-0.0048
XM_576110.1	LOC500731	-0.0528	0.22674	0.07328	0.06918	-0.0131	0.12315	0.02153	0.02821	-0.0066	0.14067	0.15122	0.04503	0.03576	0.11557
XM_576111.1	LOC500732	-0.0175	-0.0634	-0.0393	-0.0459	0.05327	0.20784	0.10093	-0.0927	0.0803	0.06173	-0.0456	0.01573	-0.0084	0.01139
XM_576112.1	LOC500733	0.25827	0.0933	0.06147	0.45539	0.16251	0.15302	0.10918	0.23053	0.03763	0.03297	0.18339	0.05183	-0.0779	0.15621
XM_576113.1	LOC500734	-0.0106	-0.138	0.05001	0.07122	-0.0352	0.03258	0.16114	-0.1102	0.02391	0.02843	-0.0414	-0.0074	-0.1241	-0.1184
XM_576114.1	LOC500735	0.06493	0.01844	-0.1951	0.03131	-0.3577	0.11326	0.13622	0.08502	0.08208	-0.1968	0.01799	0.28325	0.12839	0.18585
XM_576115.1	LOC500736	0.2039	-0.0446	-0.1448	0.032	-0.1542	-0.0783	-0.0954	0.29059	-0.0906	0.07703	0.19941	-0.2504	-0.0708	-0.0234
XM_576116.1	LOC500737	0.02085	0.0691	0.07084	-0.037	-0.141	0.11437	-0.0012	0.23239	-0.0524	0.00098	-0.1077	-0.023	-0.1958	-0.1202
XM_576118.1	LOC500738	0.23656	0.09033	0.14377	0.22135	0.09751	0.06795	0.07066	0.2814	0.1483	0.0387	0.07004	0.02901	0.03723	0.1152
XM_576119.1	LOC500739	-0.1379	-0.0673	-0.1089	-0.1268	-0.1424	-0.1375	-0.0888	-0.0699	-0.1095	0.01357	0.02358	-0.106	-0.0487	-0.1107
XM_576120.1	LOC500740	-0.0664	0.0146	-0.0505	-0.1744	-0.1753	-0.0086	-0.0989	0.0555	-0.0876	-0.125	0.06745	-0.1188	-0.0659	-0.0322
XM_234646.2	LOC500741	0.04552	0.22212	0.20058	0.17457	0.09891	0.04642	0.23284	0.01679	0.05619	0.11684	0.1016	0.09485	0.08197	0.14418
XM_576121.1	LOC500742	-0.1417	-0.1366	-0.3653	-0.166	-0.1839	-0.093	-0.0958	0.03457	-0.0527	-0.2313	-0.223	-0.2342	0.07185	0.11201
XM_576122.1	LOC500743	-0.1692	-0.1696	0.01046	-0.1443	-0.1446	-0.1463	0.07766	-0.0782	-0.1211	-0.0745	-0.2232	-0.1145	-0.0812	-0.0657
XM_576123.1	LOC500744	-0.1339	0.01321	-0.0648	0.09888	-0.0178	0.00846	0.00432	0.05948	-0.1097	-0.0287	-0.1047	-0.1365	0.0873	0.13321
XM_576124.1	LOC500745	-0.166	-0.0009	0.08042	-0.0348	-0.0547	-0.0747	-0.0349	-0.129	0.06743	-0.065	-0.0873	0.00621	-0.1035	-0.084
XM_576125.1	LOC500746	0.06386	0.10394	0.02317	0.14617	0.04732	-0.1541	-0.0425	-0.1569	0.08725	-0.0136	-0.0236	-0.133	0.05569	-0.158
XM_576126.1	LOC500747	-0.1317	-0.4444	-0.2223	-0.2128	-0.2878	-0.2207	-0.1874	-0.2881	-0.1167	-0.1999	0.13267	-0.0443	0.14469	-0.1217
XM_576127.1	LOC500748	-0.1743	-0.0202	-0.0585	-0.0975	0.13977	-0.0337	0.02142	-0.0187	-0.1225	-0.1513	-0.0712	-0.0623	-0.0793	-0.0207
XM_576129.1	LOC500751	-0.2069	-0.1157	-0.1239	-0.079	-0.0581	-0.1596	-0.1033	-0.1804	-0.2466	0.06868	-0.2262	0.11586	0.00487	-0.1135
XM_576130.1	LOC500752	0.13978	-0.0746	0.0835	0.26635	0.02493	0.14532	-0.0836	0.16707	0.28873	0.01361	-0.0422	0.07544	0.04051	0.13509
XM_580118.1	LOC500753	-0.0609	-0.1518	-0.188	-0.1392	0.10599	-0.1776	-0.1147	-0.2443	-0.1779	-0.0854	-0.0712	-0.0329	-0.1626	-0.0297
XM_580119.1	LOC500754	-0.1944	-0.0494	0.03001	0.10144	0.04139	-0.3694	-0.1956	0.2254	-0.1079	-0.1576	-0.0345	-0.2463	-0.0406	-0.1174
XM_576133.1	LOC500755	-0.0812	0.32539	-0.2895	-0.2071	-0.1482	-0.1303	0.07893	-0.0839	0.15613	0.01321	-0.1639	-0.0886	-0.1229	-0.2351
XM_576134.1	LOC500756	-0.1737	-0.1351	-0.1528	-0.0614	-0.136	-0.0934	-0.0932	-0.0081	-0.0828	-0.0325	-0.1736	-0.1003	-0.119	-0.1173
XM_576136.1	LOC500758	-0.0031	0.07956	-0.0136	0.1356	0.13189	-0.0237	0.03912	0.05071	-0.0004	0.08409	-0.0294	0.04191	0.0438	0.31885
XM_576174.1	LOC500788	-0.153	-0.0927	0.33745	0.26509	-0.1077	0.13707	0.76998	0.26085	-0.1068	-0.4478	0.28435	-0.1725	-0.4382	-0.1335
XM_580121.1	LOC500792	-0.1163	0.20226	-0.1708	-0.0309	-0.1697	0.01168	-0.2612	-0.1739	-0.0477	0.00107	-0.123	0.11656	-0.111	-0.0131
NM_001109284	LOC500797	0.46529	0.50685	0.3015	0.22198	0.31192	0.19164	-0.0082	0.42911	0.26886	0.23902	0.2793	0.31177	0.3839	0.3962
XM_576188.1	LOC500799	0.00444	-0.0592	0.09036	0.00442	-0.0948	0.00204	-0.0858	0.09745	-0.027	0.0605	-0.153	-0.023	0.03826	-0.0555
NM_001025774	LOC500800	0.16813	0.36969	0.0153	0.08097	0.29632	0.21372	0.30048	0.13216	0.09439	0.00833	0.19079	0.08648	0.144	0.18309
XM_576190.1	LOC500801	0.09128	0.33182	0.22872	0.26261	0.19952	0.33955	0.30996	-0.0031	0.29242	0.0705	0.20777	0.21493	0.15544	0.18751

XM_580122.1	LOC500803	0.19627	0.11675	0.30759	0.13198	0.14254	0.09291	0.0266	0.00773	0.12388	0.25781	0.10813	0.16355	0.22287	0.17007
XM_576196.1	LOC500807	-0.1783	0.03277	-0.0473	-0.1651	-0.2873	-0.0355	-0.1096	-0.2269	-0.2375	-0.2435	-0.1134	0.0469	-0.0653	-0.0056
XM_580123.1	LOC500808	-0.0712	0.07665	0.18245	-0.0272	0.0157	-0.0282	0.03856	0.08074	0.21889	0.33918	0.02727	-0.0378	0.02903	0.08664
XM_580124.1	LOC500811	0.08197	0.17836	0.10526	0.04027	0.06917	0.11145	0.11761	0.06839	0.18626	0.08594	0.10733	0.05379	0.08961	0.0627
XM_576199.1	LOC500812	-0.1514	-0.2221	-0.3817	-0.3745	-0.2009	-0.1609	-0.2592	-0.3311	-0.1569	-0.0602	-0.1954	-0.5226	-0.3492	-0.2914
NM_001013952	LOC500815	-0.1113	-0.0778	0.01628	-0.0587	-0.0485	-0.1005	-0.0169	0.1344	0.03373	-0.0088	-0.0711	-0.0016	0.05436	-0.0123
XM_580125.1	LOC500816	-0.1089	0.26042	0.09722	-0.0436	0.04461	-0.0969	0.06557	0.1205	0.08933	0.31204	0.21942	-0.0454	0.06004	-0.0725
XM_576211.1	LOC500821	-0.096	0.02045	0.01783	0.07073	-0.0278	0.03	-0.0351	-0.0165	-0.0018	0.05304	0.06241	0.08519	0.12551	0.07192
NM_001137645	LOC500824	-0.3084	-0.2991	0.18933	0.19807	0.02185	-0.0693	-0.2775	0.0161	0.04056	0.01867	-0.015	0.3744	-0.1191	-0.0328
NM_001025052	LOC500825	-0.0757	-0.0591	0.0034	0.05573	0.01344	-0.1019	0.09521	-0.0441	-0.0442	-0.0723	-0.0944	-0.0791	-0.0597	-0.0353
XM_576218.1	LOC500828	-0.1671	-0.069	-0.0458	0.12196	0.09776	-0.2104	0.10021	-0.123	0.03247	-0.0012	-0.0561	-0.0552	-0.1124	0.06261
XM_576219.1	LOC500829	-0.524	0.25936	-1.5097	-0.7986	-0.8345	-1.1538	-0.0341	-0.4648	-0.1122	0.11233	-0.2537	-0.4942	-0.9196	-0.7986
XM_576220.1	LOC500830	0.12456	0.08719	0.24646	-0.0022	-0.1022	0.10296	-0.0004	0.22	0.01865	0.20757	-0.0966	-0.0808	-0.048	-0.0216
NM_001109286	LOC500832	0.05349	-0.0996	0.0391	-0.0632	0.01327	-0.0046	0.01132	-0.0446	0.16153	-0.0574	-0.0295	-0.0865	-0.1173	-0.0796
XM_576230.1	LOC500838	-0.1962	-0.1306	-0.2149	-0.0099	-0.2073	-0.1525	0.01502	-0.2773	-0.2447	-0.2372	0.19364	0.00269	-0.1503	-0.111
XM_580126.1	LOC500839	-0.1568	-0.0308	0.08552	-0.2402	-0.4807	-0.3192	-0.1838	-0.3066	-0.2743	-0.3216	-0.2746	-0.3432	-0.2683	-0.2709
XM_580127.1	LOC500840	-0.587	-0.6228	0.096	-0.48	-0.8017	-0.6462	-0.5053	-0.939	-0.4983	-0.5274	-0.4833	-0.043	-0.0081	-0.1957
NM_001127567	LOC500841	0.1913	0.03053	0.11467	0.00925	0.03328	0.02771	0.0997	0.01907	0.15484	0.02051	-0.0577	-0.0224	-0.0638	-0.0132
XM_576233.1	LOC500842	-0.077	-0.0669	-0.079	-0.0632	-0.1747	-0.0322	-0.065	0.09783	-0.119	0.0151	-0.0799	-0.0599	-0.1113	-0.1227
XR_009634.1	LOC500845	-0.0289	-0.1071	-0.0285	-0.0761	-0.0616	0.09511	-0.1818	-0.1317	-0.0678	-0.1868	-0.1916	-0.0829	-0.2313	-0.1826
NM_001047962	LOC500846	-0.1218	0.0185	0.02369	0.00693	-0.0372	-0.1389	-0.1787	0.05282	-0.091	-0.0827	0.10012	-0.0101	-0.11	-0.0901
XM_580128.1	LOC500848	-0.0858	-0.0685	-0.1137	0.17161	-0.1254	0.0866	0.03172	-0.133	0.01391	0.02753	0.04364	-0.0291	0.1388	0.03728
XM_576243.1	LOC500850	0.00946	-0.0364	-0.0704	-0.0798	0.07427	0.21598	-0.1147	0.04097	0.0598	-0.0748	-0.0701	-0.0612	0.26072	-0.0697
XM_576245.1	LOC500851	-0.0223	-0.071	-0.1563	0.09967	-0.1118	0.19621	-0.059	-0.0606	-0.1409	-0.105	-0.1135	-0.0533	-0.116	-0.1536
XM_576246.1	LOC500852	-0.1059	-0.2138	-0.2193	-0.3268	-0.2647	0.12033	-0.1636	-0.1627	-0.0648	-0.3343	-0.0941	-0.1543	-0.2275	-0.2247
XM_576248.1	LOC500853	-0.1292	-0.1114	-0.5306	-0.2526	-0.5389	-0.4091	-0.1583	-0.2294	-0.1802	-0.3212	-0.3916	-0.3531	-0.4332	0.03524
XM_580129.1	LOC500854	0.1674	0.03415	0.11425	0.07796	0.12643	0.39858	0.14619	0.16914	0.19622	0.10779	0.03751	0.34727	0.01674	0.0646
NM_001126087	LOC500855	-0.56	-0.2006	-0.3227	-1.0569	-0.6541	-0.724	-0.71	-0.6416	-0.1289	-0.3658	-0.3419	-0.2442	-0.0101	-0.029
XM_576257.1	LOC500860	0.18616	0.23912	0.07418	0.02842	0.3192	0.18292	0.02136	0.2362	0.17906	-0.0541	0.32613	0.07041	0.22124	0.07902
XM_576259.1	LOC500861	0.25329	0.06198	0.00824	-0.064	-0.035	0.02064	0.06482	0.22351	-0.0646	0.00885	-0.0434	-0.0222	0.01636	-0.0064
XM_576260.1	LOC500862	0.20308	0.02837	-0.0122	0.12472	0.09205	0.11738	0.15124	0.15192	0.16007	0.14296	0.03693	0.09248	0.23044	0.08648
XM_576265.1	LOC500867	-0.9124	-0.1432	-0.5754	-0.2492	-1.7204	-1.3191	0.53317	-1.3244	-1.0561	-0.5394	-0.4161	-0.6931	-1.5927	-1.3749
XM_576266.1	LOC500868	-0.0142	0.00403	-0.1228	-0.0595	0.10833	-0.1699	-0.1698	-0.0751	-0.0977	0.06974	0.02213	-0.048	-0.1401	-0.1084
XM_576267.1	LOC500869	0.15971	0.11269	-0.1606	0.04817	0.1435	-0.0088	0.07671	-0.0172	-0.1855	0.05004	-0.0617	0.21168	-0.118	-0.0684
XM_576271.1	LOC500872	-0.0755	-0.145	-0.0691	-0.1583	-0.0736	-0.109	-0.1168	-0.0642	-0.0759	-0.0971	-0.0749	0.0994	-0.1235	-0.1632
XM_576274.1	LOC500875	0.03268	-0.0183	-0.0002	0.06474	-0.0144	0.11735	0.04653	-0.0317	0.03637	0.07511	-0.1306	0.08872	0.00082	0.17435
NM_001145141	LOC500876	-0.0895	-0.2418	-0.0984	-0.1231	-0.2358	-0.0185	-0.1058	-0.1002	-0.2028	-0.1277	-0.1873	0.0724	-0.1671	-0.0841
NM_001047963	LOC500877	0.01421	-0.012	0.26837	0.04279	-0.0483	-0.0955	-0.0401	0.10179	0.04165	0.18668	0.0196	0.01947	0.14226	-0.0498
XM_576278.1	LOC500878	-0.0226	0.19375	0.14106	0.14677	0.22475	0.09482	0.04899	0.06075	0.07742	0.14026	0.13074	-0.0036	-0.0027	-0.08
XM_576279.1	LOC500879	0.13418	0.07559	-0.0492	-0.0056	0.18939	-0.0174	-0.0482	0.03379	0.09973	-0.0697	0.13937	-0.0627	0.04084	-0.0181
XM_576284.1	LOC500883	-0.6232	-0.3549	-0.3148	-0.2105	-0.2747	-0.2183	0.12112	-0.1627	0.01705	-0.3825	-0.1749	-0.3576	-0.2951	-0.1455
XM_580132.1	LOC500884	0.20219	0.02263	0.15726	0.07997	-0.0495	0.0775	0.35986	0.11478	0.04238	0.30395	0.13656	0.22978	0.05027	0.02107
XM_576286.1	LOC500886	-0.0028	-0.1614	-0.1118	-0.1976	-0.1273	-0.186	-0.1781	-0.2816	-0.1954	-0.2277	-0.2199	-0.0992	-0.13	-0.1817
XM_576288.1	LOC500887	-0.0641	0.78733	0.05721	0.15516	-0.0011	-0.0654	-0.0943	-0.022	0.91644	1.03	0.71396	0.21654	0.02627	-0.1378
XM_576292.1	LOC500891	-0.0982	-0.0617	0.01071	-0.1164	-0.1194	0.26157	-0.1445	-0.0592	0.16856	-0.0821	-0.1439	-0.2063	-0.0584	0.03551
NM_001029926	LOC500893	-0.0365	-0.0289	-0.0546	-0.1622	-0.1509	-0.3286	-0.0878	-0.244	-0.0497	0.24553	-0.155	-0.1109	0.0126	-0.0818
XM_576296.1	LOC500894	-0.4008	-0.3286	-0.3347	-0.8059	-0.5299	-0.4629	-0.3574	-0.4471	-0.2634	-0.3678	-0.1559	-0.1206	-0.2695	-0.4359
XM_576298.1	LOC500896	-0.3889	-0.2801	-0.1475	-0.1527	-0.2243	-0.0936	-0.3246	-0.4032	-0.2277	-0.2719	-0.3904	-0.2046	-0.3499	-0.3959
XM_576300.1	LOC500898	-0.2406	0.02257	-0.2782	-0.1657	0.16832	0.16175	-0.3342	0.01066	-0.1444	-0.2321	0.09786	0.1009	-0.2126	-0.0494

NM_173122	LOC500901	0.1836	-0.0424	-0.0749	0.08444	-0.0455	0.08513	-0.0155	0.07361	0.11807	0.05394	0.00529	0.28825	-0.0274	-0.0137
XM_576305.1	LOC500903	0.2282	0.25742	0.03304	0.01401	0.12361	0.18437	0.19846	0.24026	0.18849	0.30217	0.27044	0.27214	0.12878	0.02272
NM_001127304	LOC500904	0.03318	0.19324	0.22366	0.07039	-0.0166	-0.1218	0.15404	-0.1015	0.05182	0.15582	0.20135	-0.1198	-0.0486	-0.0012
XM_576314.1	LOC500908	0.03163	0.25377	0.47825	-0.0448	0.12238	0.32089	0.12911	-0.0637	0.10454	-0.0189	0.46552	0.49656	0.3331	0.03943
XM_576325.1	LOC500916	-0.268	-0.268	0.2945	0.54558	-0.5837	-0.4648	0.76225	-0.0576	-0.4893	-0.297	-0.1869	-0.2592	-0.5476	-0.5124
XM_580134.1	LOC500919	-0.1847	-0.2157	-0.2114	-0.2098	-0.2675	-0.1646	-0.2622	-0.2402	-0.2239	-0.1308	-0.2917	-0.2321	-0.1953	-0.225
XM_576331.1	LOC500922	-0.0723	-0.0411	0.03223	0.13954	-0.0558	0.04507	-0.079	-0.0474	0.05762	-0.0086	-0.0103	-0.0622	-0.0808	-0.033
NM_001024909	LOC500925	-0.0203	0.02549	-0.0475	0.03105	-0.0589	0.21214	-0.0978	-0.0993	-0.0573	0.03239	-0.0494	0.08517	-0.0106	-0.1782
XM_576335.1	LOC500926	-0.1566	0.02475	0.00615	-0.1562	-0.1545	-0.0646	-0.0955	-0.0604	-0.1025	0.13311	0.00663	-0.1848	-0.0537	-0.0581
XM_576344.1	LOC500932	-0.0544	-0.1224	0.05547	0.08813	-0.056	-0.0712	0.10094	0.19139	-0.0024	0.13126	-0.1181	0.17586	-0.061	0.0301
XM_580136.1	LOC500934	0.12907	-0.0127	-0.0799	0.01634	-0.0725	-0.0229	0.0075	0.02662	0.00322	0.15584	0.0724	-0.0518	-0.054	0.08939
XM_576346.1	LOC500935	-0.0658	0.10206	-0.2719	0.01497	-0.1406	0.12686	-0.2391	0.02998	-0.2384	-0.1748	0.02995	-0.1851	-0.0839	-0.0788
XM_580137.1	LOC500936	0.03425	0.19462	0.45939	0.28853	-0.0031	-0.0618	0.12948	-0.0405	-0.3339	-0.009	-0.0571	0.05639	-0.0717	-0.2438
XM_576350.1	LOC500940	0.03401	0.07827	0.00758	0.10101	-0.1611	0.13603	0.20379	0.00443	0.02819	0.09987	0.26695	-0.0989	0.18882	0.15393
XM_576352.1	LOC500942	-0.0371	-0.0371	0.06294	0.11381	-0.0483	0.14184	-0.0201	-0.0015	-0.0482	0.11306	-0.001	0.16142	0.22418	-0.0236
XM_576357.1	LOC500946	-0.0784	-0.0046	-0.0092	0.00995	-0.0501	-0.1002	-0.0998	-0.0005	0.09806	-0.0414	0.01942	-0.0675	0.01623	-0.0599
NM_001144870	LOC500947	-0.4394	-0.5345	-0.4081	-0.4187	-0.3743	-0.3265	-0.3758	-0.2524	-0.3013	-0.457	-0.5012	-0.4046	-0.4523	-0.4074
NM_001047964	LOC500948	0.1973	0.02087	0.01162	-0.061	-0.0479	0.04539	-0.03	0.04386	-0.0133	-0.0615	0.02823	-0.0049	0.18554	-0.0315
XM_576360.1	LOC500949	-0.0129	0.20797	0.28031	0.36369	0.06226	-0.0898	0.98931	0.23875	0.1262	-0.1145	0.47367	0.00236	-0.2868	-0.0377
NM_001134634	LOC500950	-0.3368	-0.0999	-0.0933	0.12714	-0.301	-0.6076	0.41355	-0.3206	-0.0864	0.00669	0.21493	-0.4983	0.01364	-0.3276
XM_576364.1	LOC500952	-0.0353	-0.048	-0.0425	-0.0862	0.0056	0.06297	0.00135	0.00853	-0.0244	-0.0258	-0.1392	-0.0859	-0.1022	0.0203
XM_576365.1	LOC500953	0.01972	0.07259	-0.0026	0.06138	-0.0101	0.09227	-0.059	0.39188	0.17943	0.23442	0.1077	0.20298	-0.0253	-0.1056
NM_001048243	LOC500954	-0.3453	-0.1205	-0.3569	-0.2659	-0.2271	-0.3282	-0.2849	-0.3879	-0.2176	-0.3532	-0.3273	-0.2666	-0.2969	-0.221
NM_001025054	LOC500956	0.03612	0.09751	0.00946	0.09261	0.15913	0.02017	0.04855	0.0241	0.00143	0.13147	0.00711	0.11031	0.05611	0.05803
NM_001025054	LOC500956	0.15486	0.11888	0.1162	-0.0423	0.04655	0.07311	-0.0007	0.00464	0.13045	0.00963	0.08746	-0.0092	0.0856	0.06917
XM_580140.1	LOC500957	-0.1575	-0.1836	0.22272	-0.0075	-0.0126	-0.0039	0.25143	-0.2583	0.01079	0.0567	0.15384	0.05971	-0.0897	-0.1851
NM_022922	LOC500959	0.7042	0.42041	0.1826	0.1025	0.37873	0.45016	-0.7689	0.53238	0.40423	0.23152	0.58833	-0.0217	0.44397	0.2263
NM_022922	LOC500959	1.1746	0.96044	0.10146	0.38226	0.76073	-0.1181	-0.5271	0.83073	0.48983	1.0465	0.73547	-0.4693	0.90879	0.92817
XM_576371.1	LOC500960	-0.5711	-0.1638	-0.8934	-0.7697	-1.2642	-1.3551	0.10263	-1.0763	-0.4372	-0.3486	-0.2052	-0.4953	-1.2225	-1.2504
XM_580143.1	LOC500966	-0.039	0.03117	-0.0378	-0.0802	-0.0688	-0.1075	0.00674	0.04134	-0.0667	-0.1223	-0.1095	-0.0131	-0.0718	-0.0311
XM_576375.1	LOC500967	0.08453	0.21555	0.05326	0.12776	0.0367	0.01633	0.02241	-0.0309	-0.0802	0.06422	0.15491	-0.0792	0.05778	0.10467
XM_576377.1	LOC500969	-0.2247	-0.1262	-0.1334	-0.0438	-0.1468	-0.1342	0.05801	-0.0028	-0.089	0.06596	-0.1697	-0.1595	-0.0932	-0.079
XM_576380.1	LOC500970	-0.2665	-0.1856	-0.2267	0.0278	-0.2755	-0.1861	0.02465	-0.2277	-0.065	-0.3711	0.04158	-0.0657	-0.214	-0.2106
XM_576381.1	LOC500971	0.09159	0.02266	0.00441	0.04851	0.02326	-0.0136	0.01306	0.04151	0.07761	0.02033	0.06531	0.05522	0.01294	0.06212
XR_009636.1	LOC500974	-0.2995	-0.305	0.54247	1.1654	-0.8153	-0.6711	0.11797	-0.5973	-0.5761	-0.4364	-0.3916	0.21051	-0.2786	-0.29
XM_576385.1	LOC500975	0.02547	0.06764	0.00786	0.09895	0.04801	0.21117	-0.0109	0.01724	0.06027	0.24742	-0.0148	0.09481	0.07348	0.0363
XM_576388.1	LOC500978	-0.3488	-0.1788	-0.2764	-0.2133	-0.1341	-0.1893	-0.188	-0.0847	-0.2935	-0.4371	-0.243	-0.0194	-0.0022	0.06991
XM_576389.1	LOC500979	-0.0628	-0.0443	-0.1538	-0.1982	-0.0015	0.00759	-0.0129	-0.1491	0.11176	-0.1438	0.01295	0.15895	-0.1728	0.06493
NM_001037364	LOC500990	-0.1197	-0.1211	-0.1358	-0.0639	-0.0376	-0.0713	-0.0086	0.13731	-0.0129	-0.1285	-0.0936	-0.125	0.06574	-0.0605
XM_576408.1	LOC500994	0.15936	0.06255	-0.0574	-0.3353	0.0049	-0.2905	-0.0241	0.2501	-0.0611	-0.1635	-0.0834	-0.3177	0.05573	-0.1595
NM_001134636	LOC500995	0.06902	-0.0888	-0.2093	-0.0426	-0.1167	-0.058	-0.0915	-0.1389	-0.079	-0.1576	-0.103	-0.0436	0.03606	-0.0199
XM_576411.1	LOC500997	-0.1517	0.00023	-0.1729	0.00268	-0.1109	-0.2332	-0.1901	-0.17	0.02003	0.05325	-0.0613	0.09212	-0.0667	0.22038
XM_580145.1	LOC500998	0.10348	0.02248	-0.0026	-0.015	0.18264	0.258	0.29112	0.00495	0.16308	0.13047	0.1352	-0.0132	0.23392	-0.0153
XM_580146.1	LOC500999	-0.1177	-0.0063	-0.0307	-0.0174	0.09668	0.01272	0.01656	0.02961	-0.1966	-0.0219	-0.1584	-0.0063	0.01638	-0.1022
XM_580147.1	LOC501000	-0.0046	-0.0701	-0.0468	-0.1322	0.04669	-0.1683	-0.088	-0.118	0.1609	0.15274	-0.1738	-0.0638	-0.1132	0.06551
XM_580148.1	LOC501001	0.07682	0.07019	-0.0034	-0.0337	-0.019	-0.026	0.03449	0.02117	0.23816	0.04055	0.06064	0.03089	0.07282	0.10453
XM_576413.1	LOC501003	-0.2199	-0.0889	-0.1463	0.09576	-0.2062	0.1555	-0.0472	-0.187	-0.1806	0.1116	-0.2191	-0.2039	-0.179	-0.0617
XM_576414.1	LOC501004	0.00721	-0.1784	-0.0608	-0.0771	-0.2049	-0.128	-0.2168	-0.2463	0.18752	-0.1418	-0.196	-0.1743	-0.1221	-0.0793
XM_580149.1	LOC501008	0.00207	0.02379	-0.012	-0.0639	-0.0094	-0.0166	0.05457	-0.0423	-0.0484	-0.1005	0.02077	0.00515	0.0684	0.02449

XM_576423.1	LOC501011	-0.096	-0.0828	0.12148	0.08184	-0.021	0.01181	0.02003	0.05228	-0.0306	0.00727	-0.0314	0.05494	0.16297	0.20041
XM_580150.1	LOC501014	0.02859	0.02478	0.03271	-0.0064	-0.0039	0.03579	0.07143	0.12535	0.09761	0.05031	0.03211	0.05922	0.08262	0.07775
XM_576426.2	LOC501015	-0.0343	-0.0755	-0.0831	-0.0309	-0.0451	0.14921	-0.1366	-0.041	0.03119	-0.0824	-0.027	-0.0771	-0.0525	0.06825
XM_576427.1	LOC501016	-0.0366	0.02923	-0.039	0.04106	0.0752	0.04242	0.05127	0.05242	-0.0412	-0.0148	0.12269	0.17583	0.00605	0.01096
XM_580151.1	LOC501017	0.25436	0.02685	0.15841	0.0004	0.24495	0.59364	0.17602	0.39429	0.02535	0.14981	0.0382	0.03392	0.109	0.05909
XM_580153.1	LOC501020	0.21814	0.14289	0.08086	0.06049	0.25496	0.21318	0.24402	0.21473	0.14567	0.15209	-0.0107	0.13956	-0.0117	0.09584
XM_576430.1	LOC501021	0.14053	0.09173	-0.0108	0.0672	0.12451	-0.0421	-0.1421	0.09424	-0.0399	-0.2225	0.08477	-0.1442	0.15043	0.03251
XM_576431.1	LOC501022	-0.196	0.0068	-0.256	-0.1242	-0.2147	-0.0958	-0.0288	-0.0831	0.07881	0.01579	-0.2307	0.03508	-0.2483	-0.0052
XM_576434.1	LOC501024	0.09314	0.07428	0.15462	0.09925	0.11421	0.13423	0.04079	0.1431	0.2162	0.08095	0.09322	0.29748	0.10014	0.30461
NM_001137647	LOC501026	0.09347	0.09194	0.21971	0.0388	0.09642	0.15232	0.08681	0.19273	-0.0053	0.26463	0.00165	0.22403	-0.0817	0.06988
XM_576443.1	LOC501032	-0.0981	-0.0949	0.20249	-0.067	-0.1551	-0.0401	-0.0897	0.13238	-0.0796	0.03407	0.16991	0.09652	0.0057	-0.0045
XM_580154.1	LOC501037	0.23433	0.16205	0.00816	-0.0365	-0.059	0.14352	0.01568	0.14994	0.03905	0.15451	0.15651	0.10973	0.00189	0.335
NM_001047965	LOC501038	-0.1109	0.1442	-0.1044	-0.0899	-0.0695	-0.019	-0.0174	-0.0025	0.09516	-0.1453	0.03854	-0.0985	0.02594	-0.0946
XM_576451.1	LOC501039	-0.1248	-0.2009	-0.0496	-0.2617	-0.2013	-0.0374	-0.1041	-0.1108	-0.2064	-0.0555	-0.0842	-0.2009	-0.1054	-0.0858
XM_576452.1	LOC501040	-0.0678	-0.2838	-0.1415	-0.2882	-0.2623	-0.2709	-0.1672	-0.285	-0.1771	-0.2136	-0.1441	-0.0061	-0.2349	-0.2346
XM_576453.1	LOC501041	0.16687	-0.1569	-0.087	-0.0511	-0.0375	-0.0256	-0.1178	-0.0516	-0.0114	0.22671	0.04118	-0.1401	-0.0577	-0.0747
XM_576455.1	LOC501042	0.26062	0.21375	0.38548	0.17113	0.05844	0.08463	0.14533	0.2876	0.0258	0.27574	0.24313	0.00929	0.12629	0.12451
XM_576460.1	LOC501045	-0.5579	0.22285	0.04067	-0.6923	-0.7852	-0.9914	-0.4277	-0.8515	0.64899	0.17728	0.20167	0.67113	0.3134	0.32307
XM_576462.1	LOC501047	-0.0544	0.12614	-0.1653	0.06652	-0.0864	0.37554	-0.1413	-0.1626	-0.003	0.02586	-0.0577	-0.0633	-0.0678	0.15183
NM_001109297	LOC501052	0.19972	0.11935	0.01737	0.17483	0.42683	0.50925	0.24161	0.27475	0.17401	0.27995	0.20358	-0.0075	-0.035	0.15967
XM_580156.1	LOC501053	-0.0679	-0.1779	-0.1339	-0.1056	-0.1779	-0.0897	0.03765	-0.1563	-0.0311	-0.2069	-0.1391	-0.1016	0.09153	-0.0949
XM_576470.1	LOC501055	0.12116	-0.0185	0.23831	0.23303	0.01896	-0.0324	0.17336	-0.1595	0.06284	0.15771	0.06205	-0.08	-0.006	-0.0823
XM_345984.2	LOC501057	0.04677	0.16992	-0.0022	0.15058	0.5203	-0.0276	0.29249	0.25354	0.23247	0.2463	0.06551	-0.0252	0.04155	0.13213
XM_576478.1	LOC501062	0.17494	0.19947	0.0174	0.06788	0.22	0.28815	-0.0548	0.11121	0.18962	0.48384	0.36818	0.07471	0.40721	0.15888
XM_580157.1	LOC501063	0.04126	-0.0028	0.20229	0.07067	0.02684	-0.0064	0.01427	0.0985	0.04692	0.0356	0.03507	0.15087	0.06717	0.05064
XM_576479.1	LOC501064	-0.2221	-0.0848	-0.054	-0.0082	-0.1411	-0.2252	0.02586	-0.0082	-0.1236	-0.1151	-0.085	-0.3232	-0.0688	0.03452
XM_580158.1	LOC501066	0.00737	0.00244	0.13615	0.13642	0.01796	0.12407	0.12404	0.15992	0.17879	0.01256	0.09737	0.05542	0.11212	0.01602
XM_576481.1	LOC501067	-0.1289	-0.1407	0.04942	-0.0903	-0.0964	0.02013	0.01006	-0.1259	-0.1334	-0.1273	0.0877	-0.1504	-0.0828	-0.1454
XM_576482.1	LOC501068	-0.0063	0.09381	0.14834	0.00348	0.16643	0.06338	0.02219	0.01785	0.01084	0.07474	0.05347	0.16984	0.30759	0.08936
XM_576483.1	LOC501070	0.33259	0.14596	0.07791	0.09371	0.08159	0.24173	0.00712	0.28026	0.46462	0.1668	0.15582	0.14111	0.21215	0.20722
XM_580159.1	LOC501073	0.21884	0.10786	0.05924	-0.0358	-0.1018	-0.0079	0.12159	0.04364	-0.0242	-0.0694	0.13758	0.09424	-0.1057	0.04774
XM_576486.1	LOC501074	0.09851	-0.0558	0.1233	-0.0411	0.0864	0.11992	0.19328	-0.0223	-0.0034	0.1109	0.10495	0.06115	0.20439	0.1167
XM_576487.1	LOC501075	-0.0647	-0.5066	-0.0359	0.27587	-0.3047	0.01697	-0.2181	-0.0915	-0.0498	-0.3097	-0.3551	0.03537	0.17414	0.39086
XM_576488.1	LOC501076	-0.1246	-0.1931	-0.2665	-0.2197	-0.2537	-0.2657	-0.2541	-0.1222	-0.1471	-0.2418	-0.2073	-0.0835	-0.1775	-0.2469
XM_576489.1	LOC501077	0.04911	0.07669	0.14055	0.07723	0.25276	0.07956	-0.0055	0.02567	0.02935	0.10904	0.15372	0.11651	0.18995	0.13846
XM_576491.1	LOC501079	-0.1314	-0.2262	-0.1264	0.09472	-0.0344	0.04617	-0.1644	-0.1326	-0.0323	-0.1986	0.0153	-0.1907	-0.0491	-0.2222
XM_576497.1	LOC501085	0.13387	-0.0482	0.10545	0.24789	0.09649	-0.1112	0.12675	-0.0249	0.02986	0.14535	0.00813	0.06707	-0.017	0.0479
XM_576498.1	LOC501086	-0.1356	-0.0504	0.13217	0.12046	0.23491	-0.0223	-0.1779	-0.1663	-0.1402	-0.1856	-0.0711	0.17941	-0.0794	0.05699
XM_576501.1	LOC501087	-1.0447	-0.2606	-0.6506	-0.4125	-1.6802	-1.3757	0.46436	-1.0579	-0.8296	-0.4289	-0.568	-0.6827	-1.6456	-1.3582
XM_576504.1	LOC501089	-0.0797	-0.1009	-0.0496	0.2638	-0.0913	0.01143	-0.0635	-0.0969	0.00186	-0.1059	-0.0476	0.00619	-0.1004	-0.0701
XM_576504.1	LOC501089	-0.0791	-0.0476	0.07851	0.07536	0.13052	-0.0926	0.11434	0.15648	-0.0516	0.15359	-0.0605	-0.1529	0.18876	0.08977
XM_576505.1	LOC501090	-0.1764	-0.1161	-0.2048	-0.0833	-0.1804	-0.2192	-0.118	-0.0427	-0.1774	-0.1315	-0.0922	-0.1728	-0.0771	-0.0038
XM_576507.1	LOC501092	0.014	-0.0564	0.24896	-0.0108	0.05688	-0.0413	-0.031	0.1767	-0.0449	0.11729	0.11812	0.0139	-0.0327	0.03221
XM_576508.1	LOC501093	-0.1503	-0.0571	-0.0167	0.00054	-0.1174	0.1528	-0.0856	0.06419	-0.0399	-0.0766	-0.021	0.00096	-0.1073	-0.0507
XM_576511.1	LOC501096	0.0274	0.14505	0.13915	-0.0255	0.0181	0.13144	0.12032	0.05816	0.04019	-0.0117	-0.0153	0.15078	0.09563	0.14171
NM_001013178	LOC501098	0.18865	0.18681	-0.2211	0.18644	0.12625	0.3961	0.19869	0.4423	0.31696	0.21069	0.1627	0.30373	0.35835	0.29887
NM_001109303	LOC501100	0.05618	0.02418	-0.063	0.18196	0.013	0.02582	-0.1031	0.06138	0.17282	0.04361	-0.0624	-0.0134	0.06825	-0.0175
XM_580161.1	LOC501102	-0.4182	-0.3176	-0.3723	-0.5527	-0.7298	-0.4747	-0.6487	-0.6067	-0.3683	-0.3174	-0.5838	-0.2363	-0.2539	-0.2676
XM_576519.1	LOC501104	0.16407	0.29491	0.27336	0.04284	0.19152	0.27937	0.12743	0.18753	0.07356	0.17856	0.21938	0.08295	0.19251	0.08227

XM_576521.1	LOC501106	-0.0787	-0.2353	-0.1442	-0.1445	-0.2483	-0.1597	-0.3513	-0.4733	-0.0477	0.01067	-0.1476	0.04721	-0.1259	-0.1552
XM_576526.1	LOC501108	-0.3436	-0.3511	-0.1465	0.00727	-0.2455	-0.2651	-0.2985	-0.2487	0.05049	-0.3004	-0.1079	-0.1755	0.01604	-0.1407
NM_001024361	LOC501110	-0.0839	-0.0409	0.00275	0.07294	-0.0005	0.03639	-0.0497	0.04218	0.22578	-0.0009	0.014	0.12661	0.05126	-0.0264
XM_576529.1	LOC501111	-0.2187	-0.2384	0.13962	-0.0958	-0.2088	0.27095	-0.1459	-0.2125	-0.1646	-0.034	-0.2441	-0.1637	-0.153	-0.1949
XM_576530.1	LOC501112	0.13852	0.02305	0.06038	0.03165	0.17006	-0.0437	0.04654	0.04015	-0.0263	0.05109	0.25211	-0.0278	-0.0856	0.02853
XM_576531.1	LOC501113	-0.0325	0.09947	-0.0133	-0.0843	0.14241	-0.2734	0.12393	0.06872	0.15763	0.23186	0.09384	0.02549	-0.1688	0.06241
XM_576532.1	LOC501114	0.027	-0.0444	-0.0432	0.31108	-0.1	0.10156	-0.0461	0.1554	0.1524	0.03967	-0.0065	0.02594	0.00924	0.00962
NM_001017374	LOC501116	0.01226	-0.0604	0.09702	-0.0315	0.02256	0.02798	0.02546	0.01272	0.01988	0.16655	0.00427	0.05722	0.142	-0.0598
NM_001017374	LOC501116	0.01716	0.489	-1.7452	-1.1702	0.12778	-0.9934	-0.6316	0.20751	-0.2405	0.01106	-0.4615	-0.6112	0.05845	0.02414
XM_576535.1	LOC501117	0.02787	-0.1347	-0.1215	-0.0766	0.04804	-0.1491	-0.0921	0.04634	-0.1073	0.02007	-0.0846	0.04324	-0.1046	0.03886
XM_580162.1	LOC501120	-0.7996	-0.2382	0.01723	0.45369	-1.0458	-0.6017	-0.0627	-0.7489	-0.6607	-0.7167	-0.3836	0.13409	-0.958	-1.0006
XM_576539.1	LOC501122	-0.0521	0.04526	0.08553	0.23534	0.11064	0.26228	0.11765	-0.0334	0.03647	0.1342	0.2653	0.01057	0.49425	0.01107
XM_576540.1	LOC501123	-0.2181	0.06957	-0.0323	-0.0719	0.11457	-0.1988	-0.0713	0.05129	-0.1224	-0.2349	0.12853	0.01342	-0.1009	-0.175
XM_580163.1	LOC501124	-0.2223	-0.1494	-0.0838	-0.07	-0.0466	-0.0519	0.09048	-0.0764	0.2173	-0.0895	0.23974	0.04232	0.02655	-0.0629
XM_001055017.1	LOC501126	-0.152	-0.1935	-0.0544	-0.1358	-0.2409	-0.0874	-0.1346	0.00865	-0.0694	-0.0548	0.13587	-0.0663	-0.0541	0.04399
XM_576550.1	LOC501130	0.02502	-0.1005	0.19982	-0.081	-0.1435	-0.1436	-0.1071	-0.0342	0.08675	-0.0878	0.00106	0.00816	-0.1226	-0.1607
XM_576551.1	LOC501131	-0.1845	-0.0262	-0.1663	-0.0092	0.03687	0.20044	-0.069	-0.02	-0.0707	-0.1811	-0.0607	0.07129	-0.0234	0.01418
NM_001037767	LOC501132	-0.1862	-0.0153	-0.0095	0.022	-0.2517	-0.0343	-0.0518	0.16932	-0.0893	-0.0968	0.25514	0.49546	0.11697	0.05922
XM_576553.1	LOC501133	-0.0584	0.0559	0.01879	-0.0339	-0.1122	-0.0287	-0.0506	0.01081	-0.0005	-0.1201	0.21374	0.08796	-0.1533	0.0568
XM_576556.1	LOC501136	0.05769	-0.0094	0.34833	-0.0661	0.02524	-0.0799	0.26889	0.19232	-0.0566	0.02545	0.11165	-0.0269	0.17853	-0.0606
XM_576559.1	LOC501139	-0.0928	-0.1957	-0.0135	0.20105	-0.0852	0.02982	0.11075	-0.2007	-0.0797	0.30179	0.08929	-0.112	0.16068	-0.1609
NM_001113370	LOC501143	-0.0242	0.07729	-0.1259	-0.1067	-0.037	-0.0674	-0.0644	-0.0705	-0.1003	-0.0182	-0.0905	0.06631	-0.1473	-0.0904
NM_001109306	LOC501145	-0.7278	-0.4689	-0.4265	-1.0634	-0.8349	-0.7199	-0.6857	-0.827	-0.2544	-0.3838	-0.5324	-0.6799	-0.6269	-0.8
XM_580164.1	LOC501148	-0.0317	-0.0006	0.03319	-0.0957	-0.0647	0.0151	0.06842	0.12814	0.10748	-0.1318	0.09054	0.21642	0.17327	0.04435
XM_576576.1	LOC501151	-0.3535	-0.2213	-0.2323	0.07992	-0.2762	-0.1799	-0.1567	0.00269	-0.3231	0.08851	-0.1714	-0.1484	-0.3087	-0.1564
XM_580165.1	LOC501154	0.00927	0.15765	0.01026	0.02263	0.26864	0.0558	0.22546	0.12051	0.30946	0.11002	0.04584	0.21861	0.17965	0.03632
XM_580166.1	LOC501155	-0.3835	-0.6005	-0.3137	-0.4245	-0.4023	-0.5688	-0.4825	-0.5719	-0.4451	-0.5504	-0.4364	-0.3129	-0.4732	-0.2976
XM_576580.1	LOC501156	-0.3215	-0.1253	-0.1744	0.05896	-0.6116	0.14543	0.69509	-0.3657	-0.0204	0.13236	0.03176	0.3278	-0.0804	-0.3112
XM_576583.1	LOC501159	-0.077	-0.0219	-0.2337	0.01372	0.29384	-0.0363	-0.0287	0.05829	-0.281	-0.3385	-0.1581	-0.2223	-0.1052	-0.1592
NM_001109309	LOC501164	0.25286	0.01612	0.17672	0.14074	0.0747	0.12052	0.08456	0.02687	0.04408	0.13036	0.28941	0.38699	0.0597	-0.0221
XM_576590.1	LOC501165	-0.0391	-0.0342	0.02755	-0.1006	-0.0915	-0.1713	0.11803	0.02438	-0.0273	0.05781	0.00178	-0.0571	-0.2042	-0.1137
XM_576593.1	LOC501168	-0.2153	-0.2169	-0.0895	-0.1342	-0.0068	-0.0461	-0.0641	-0.176	0.21928	-0.0774	-0.056	-0.008	-0.1784	-0.1333
XM_576594.1	LOC501169	-0.3126	-0.2887	-0.1576	0.36464	-0.381	-0.1084	-0.1893	-0.5402	-0.1722	-0.2091	0.07253	0.13644	-0.4345	-0.154
XM_576597.1	LOC501170	0.6505	0.31904	-0.5785	-0.0721	0.6856	0.29607	-0.1725	0.87968	0.08276	0.19932	-0.1245	-0.2	0.43628	0.47108
XM_576599.1	LOC501172	-0.586	-0.5628	-0.2827	-0.0577	-0.0592	-0.1083	-0.3857	0.15319	-0.5604	-0.5889	-0.3522	-0.8253	-0.5951	-0.374
XM_576600.1	LOC501173	-0.1233	-0.2776	-0.0583	-0.1581	-0.2056	-0.077	-0.013	-0.2102	-0.1392	-0.1143	-0.3057	-0.2355	-0.254	-0.2925
XM_576603.1	LOC501175	0.08517	-0.128	-0.0288	0.02738	-0.0326	-0.1229	-0.0212	-0.0325	-0.0423	-0.1235	-0.0461	0.09301	-0.0573	-0.0745
XM_580168.1	LOC501179	-0.0848	-0.0175	-0.067	0.14971	0.03488	0.27894	-0.0451	-0.0315	0.0228	0.14997	-0.0325	0.04715	-0.0185	-0.0804
NM_001024364	LOC501180	0.06648	-0.1021	-0.0823	0.255	0.01975	0.09922	0.09356	-0.0651	-0.1105	-0.0576	-0.0564	0.03914	-0.0779	0.17504
XM_576613.1	LOC501184	-0.2429	-0.2705	0.17226	-0.0718	-0.2129	-0.2981	0.06835	-0.2783	0.01551	-0.187	-0.1712	-0.2286	-0.1875	-0.2076
XM_580170.1	LOC501186	0.03282	-0.0557	0.07918	-0.1101	-0.183	-0.1577	0.10847	0.0245	0.03046	0.07111	-0.0681	-0.1727	0.16842	0.05644
XM_576615.1	LOC501187	-0.0057	-0.2148	0.38966	0.42074	-0.036	-0.0499	0.66267	0.33143	-0.1283	-0.0411	0.10179	-0.2652	0.09277	0.234
XM_576616.1	LOC501188	0.66162	0.71675	-0.4961	0.23386	0.99014	0.21395	0.55869	0.94776	0.74127	0.47556	0.64445	0.15229	0.28647	0.52485
XM_576618.1	LOC501190	-0.0296	0.03784	0.02834	0.08708	0.19675	-0.0368	0.14394	-0.0977	0.01914	0.14381	-0.0552	-0.2406	-0.0198	0.18135
XM_576619.1	LOC501191	-0.3229	-0.2021	-0.5918	-0.3426	-0.4273	-0.6217	-0.2892	-0.4782	-0.1871	-0.1118	-0.039	-0.6485	-0.4615	-0.4436
XM_576620.1	LOC501192	0.10822	0.05043	-0.0387	0.01469	0.03266	0.09352	0.09695	0.03416	0.1227	0.02513	-0.0787	-0.1084	0.02681	0.12055
NM_001025775	LOC501194	-0.2951	0.1047	0.57754	1.1413	-0.7146	-0.7437	0.64839	-0.6385	-0.1533	-0.0359	0.18806	-0.4814	-0.2577	-0.4218
XM_576624.1	LOC501196	-0.1426	-0.1041	0.15358	0.03444	-0.1255	0.06634	-0.0587	-0.0478	0.02438	0.04944	-0.1039	0.10873	0.02963	-0.0769
XM_576626.1	LOC501198	0.12381	-0.1029	-0.1476	0.01334	-0.0817	0.10436	-0.1362	-0.0854	0.10944	0.12148	0.01284	0.24136	-0.0883	-0.0103

XM_576629.1	LOC501202	-0.5669	-0.4962	-0.3984	-0.5638	-0.5995	-0.2221	-0.3592	-0.2134	-0.3479	-0.5267	-0.3588	-0.5965	-0.5773	-0.6193
NM_001135017	LOC501203	-0.0977	0.10364	0.07589	0.00432	0.03295	-0.0337	0.12515	0.06018	0.05308	0.07092	-0.0082	-0.1203	0.10459	0.06954
NM_001014102	LOC501207	-0.0441	-0.0486	-0.295	-0.0982	-0.1126	0.10536	-0.3594	-0.1103	0.07397	-0.2607	0.02707	-0.0045	-0.1022	0.18954
XM_576634.1	LOC501208	0.0498	-0.0383	-0.0945	0.02415	0.01228	0.00069	-0.0808	-0.1462	0.27875	0.00443	-0.0399	0.07593	0.07314	-0.1137
XM_580173.1	LOC501211	0.68851	-0.3506	0.32093	-1.4657	0.06347	0.07209	-0.0807	0.04134	-0.0886	-0.4089	-0.2735	0.02272	0.21303	0.34897
XM_580174.1	LOC501212	-0.4228	-0.2827	-0.1529	-0.2459	-0.3377	-0.4019	-0.1622	-0.2223	0.10797	-0.1885	-0.012	-0.3428	-0.2222	-0.1314
XM_576637.1	LOC501214	-0.1643	-0.2619	-0.0841	-0.1956	-0.1319	-0.0692	-0.0048	-0.1568	-0.2809	-0.0197	-0.0518	0.11042	-0.1391	-0.1067
XM_576638.1	LOC501215	-0.1697	-0.0185	-0.1057	0.12613	-0.2333	0.06658	0.02619	-0.1968	-0.0715	-0.1567	0.01067	0.19711	-0.065	0.02297
XM_576640.1	LOC501217	-0.0957	-0.0544	-0.4679	-0.1972	0.1488	0.46704	-0.1451	0.2115	0.1167	-0.3031	-0.2023	0.19677	0.02956	-0.0369
XM_576641.1	LOC501218	-0.025	0.20489	-0.0063	0.05563	0.00177	0.18525	0.18066	-0.0127	-0.0072	0.21106	0.01372	-0.0619	0.00955	0.06136
XM_576644.1	LOC501221	-0.0438	-0.0267	-0.0122	0.14057	0.16112	0.05149	0.01898	0.27704	0.19772	0.15667	-0.0677	-0.0578	0.0596	-0.0711
XM_576645.2	LOC501222	0.0303	-0.052	-0.0107	0.02198	0.03628	-0.0022	-0.0123	-0.0234	0.06165	-0.0039	0.1012	0.05043	0.0725	0.076
XM_576646.1	LOC501223	0.03393	0.18517	0.06794	0.13448	-0.0796	0.29139	0.16209	-0.0216	0.18625	0.06961	0.1564	0.17282	0.04778	0.14104
XM_576646.1	LOC501223	0.06903	0.17982	0.05087	-0.0967	0.03634	0.18904	-0.0976	-0.0309	-0.1195	-0.1135	0.04281	0.04318	0.03863	-0.0266
XM_576646.1	LOC501223	-0.0226	0.17207	0.27012	0.14171	0.12118	0.09099	0.24723	-0.0306	0.31712	0.04608	0.18322	0.14518	0.24387	0.34283
XM_576647.1	LOC501224	0.16589	0.09795	0.08663	0.0868	0.16617	0.16461	0.02238	-0.0267	0.09622	-0.0332	0.16811	0.04968	-0.0093	0.05485
XM_576648.1	LOC501225	0.13425	0.02561	0.07539	0.10792	-0.0054	0.05494	0.12504	0.08703	0.06418	0.06515	0.00645	0.01936	0.01322	0.18657
XM_576649.2	LOC501226	-0.1179	-0.0223	0.0095	0.01304	0.04589	0.00711	0.1097	0.03823	0.01543	0.06736	-0.03	-0.044	0.0561	0.03154
XM_580175.1	LOC501227	0.089	0.04998	0.02649	-0.0125	0.12215	-0.01	-0.0234	0.06725	0.10344	-0.0018	-0.039	-0.0093	-0.0395	0.12989
XM_580176.1	LOC501229	-0.0645	-0.029	-0.0566	-0.054	-0.1214	-0.1088	0.06101	-0.0791	-0.0485	0.02621	-0.0437	0.02348	-0.0873	0.05593
NM_001109312	LOC501231	-0.001	0.03411	0.00503	0.10334	0.05731	0.17218	-0.0933	0.093	0.11387	-0.0017	-0.0851	0.03305	-0.0874	-0.0477
NM_001103359	LOC501232	-0.1395	-0.1136	-0.1022	0.00395	-0.1424	-0.1234	0.01784	-0.0072	-0.0804	-0.1036	-0.0357	0.06815	-0.2004	-0.0802
NM_001047967	LOC501233	0.20797	0.13193	0.13435	0.22542	0.19064	0.11627	0.15779	0.223	0.12444	0.25895	0.00035	0.43594	0.26771	0.10382
XM_580177.1	LOC501236	0.14204	0.27834	0.24708	0.14155	0.35257	0.16544	0.08621	0.16714	0.05463	0.19202	0.27181	0.0972	0.06125	0.22166
NM_183402	LOC501237	0.43481	0.06922	-0.1239	-0.1988	0.37566	0.20426	-0.0334	0.39983	-0.0298	-0.1256	-0.122	0.24542	0.30929	0.29759
XM_576657.1	LOC501238	-0.0789	-0.1421	0.0245	0.16617	-0.1974	-0.2055	-0.0059	-0.2366	-0.1547	-0.0949	-0.0186	-0.1729	-0.1774	-0.1607
XM_576658.1	LOC501239	0.10545	-0.1735	0.00338	-0.1038	-0.004	0.05035	-0.0282	0.1129	0.12334	-0.0519	0.15756	0.18415	0.09404	0.16509
XM_580179.1	LOC501240	0.08545	-0.1235	-0.1125	-0.153	0.04593	-0.1263	-0.1412	-0.0023	0.07128	0.06854	-0.109	-0.045	-0.0964	0.04329
XM_576660.1	LOC501242	-0.2815	0.30427	-0.4351	-0.3254	-0.5575	-0.2914	0.23605	-0.2453	-0.1145	-0.2	0.25698	-0.0892	-0.359	-0.5777
XM_576663.1	LOC501244	-0.1092	-0.0644	0.03268	0.06235	0.09839	0.31234	0.18129	-0.0538	-0.0506	-0.0042	-0.015	0.17377	0.39398	0.36164
XM_576664.1	LOC501245	-0.1365	-0.0298	0.07071	0.06005	0.04885	-0.1514	0.21386	0.0704	-0.0316	-0.0109	-0.0174	-0.0282	-0.0375	-0.0748
XM_576665.1	LOC501246	-0.1851	-0.2585	-0.2774	-0.2622	-0.0298	-0.2911	0.01757	0.01161	-0.2116	0.10416	-0.1625	-0.2986	-0.1046	-0.1359
XM_576666.1	LOC501247	0.19793	0.08541	0.241	0.06507	0.04008	0.06626	0.1732	0.05413	0.02064	0.34317	0.06703	0.05177	0.05913	0.22224
XM_576667.2	LOC501248	-0.1419	-0.2232	-0.1209	-0.1199	-0.1557	-0.2702	-0.1099	-0.3164	-0.1054	-0.2655	-0.0284	-0.0125	-0.1313	-0.2654
XM_576668.1	LOC501249	-0.1024	-0.2209	0.02853	-0.1949	-0.0561	-0.1163	-0.14	-0.0742	0.06539	-0.0773	-0.0494	-0.1964	-0.1619	0.07782
XM_576669.1	LOC501250	0.05319	-0.0601	0.03616	0.03288	0.10109	-0.0269	0.01904	0.08077	0.00096	0.06869	0.08534	-0.0034	0.01259	0.0514
XM_576670.1	LOC501251	0.19356	-0.0241	-0.0291	0.17443	0.11877	0.02081	0.06097	0.15701	0.19485	0.25395	0.14026	0.14686	0.0714	-0.0163
XM_576673.1	LOC501254	0.03261	-0.0372	0.1184	-0.0887	-0.0127	0.10346	0.00485	0.0526	-0.0077	-0.0548	0.06954	-0.1052	-0.0161	-0.0114
XM_576674.1	LOC501255	-0.1374	-0.1182	-0.1895	-0.0467	-0.1254	-0.1056	-0.1794	-0.0648	0.02563	-0.1413	0.03442	-0.1946	-0.0776	-0.0997
XM_576677.1	LOC501258	-0.1556	-0.0978	-0.1211	-0.0406	-0.0772	-0.0596	0.01224	0.00471	0.001	0.02362	-0.1387	-0.123	0.00466	-0.1479
XM_576679.1	LOC501259	0.13975	0.11012	-0.1194	-0.0058	-0.1306	0.03629	0.01975	-0.0858	-0.0243	0.00165	0.02344	0.15419	-0.0625	-0.0631
XM_580180.1	LOC501260	-0.2206	-0.0395	-0.1161	-0.2267	-0.0097	-0.2346	-0.0014	0.07455	-0.0661	0.12343	-0.148	-0.0042	-0.0058	-0.3061
XM_576681.1	LOC501262	-0.0028	-0.1439	-0.254	-0.1154	-0.1982	-0.1125	-0.0879	0.04979	-0.3042	-0.088	-0.2076	-0.0576	-0.1998	-0.0986
XM_580181.1	LOC501264	0.20009	0.34193	0.10415	0.16925	0.21736	0.10433	0.03634	0.32399	0.23766	0.12936	0.09941	0.04265	0.45884	0.05448
XR_009551.1	LOC501266	0.0077	-0.1334	-0.1284	-0.0213	-0.1256	-0.1422	-0.2335	0.13624	-0.1197	-0.1516	-0.0634	-0.2366	-0.1429	-0.1951
XR_009551.1	LOC501266	-0.077	0.04089	-0.0092	0.03763	-0.0241	0.18424	0.13601	0.13516	-0.0845	-0.0494	-0.2224	-0.126	0.11971	0.06967
XM_580182.1	LOC501267	-0.127	-0.0914	0.04921	0.0582	0.03545	0.11005	0.03898	0.04054	0.03337	0.10151	-0.0269	0.11656	-0.108	-0.0184
XM_576684.1	LOC501268	-0.1398	-0.2296	0.02157	-0.116	-7E-05	-0.1083	0.10969	-0.1709	-0.1526	-0.1648	-0.1377	-0.0259	-0.159	-0.0093
XM_576685.1	LOC501269	-0.0121	0.02611	-0.0363	0.00404	0.00841	-0.0274	0.08353	-0.0162	0.15232	-0.0064	0.06715	0.02817	0.09598	-0.0254

XM_576687.1	LOC501271	-0.0338	0.04833	0.03015	0.08914	0.05677	0.05729	0.16574	-0.0486	-0.1111	-0.0614	0.11762	-0.0045	-0.1475	0.07956
XM_576687.1	LOC501271	0.00188	-0.0146	-0.092	0.09795	-0.1047	0.05725	0.00362	0.019	0.05432	-0.1548	-0.0809	0.11818	-0.0144	-0.1017
XM_576688.1	LOC501272	-0.0664	0.01639	0.03113	-0.0432	0.07195	-0.1943	-0.0283	0.08955	-0.086	0.04475	0.00229	-0.0913	-0.0311	0.0027
XM_580183.1	LOC501273	0.12843	0.16993	-0.0976	-0.0587	0.01378	0.04752	-0.1347	-0.0773	-0.0986	-0.0005	-0.06	-0.1414	-0.0303	-0.0512
NM_001014221	LOC501274	0.1143	0.12578	0.21532	-0.0318	0.02754	0.05253	0.17749	0.17509	0.08238	0.09322	0.14134	-0.0466	0.01308	0.01139
XM_576690.1	LOC501275	-0.0231	-0.2319	-0.4086	0.08241	-0.1138	0.02086	0.01957	-0.0129	-0.0983	-0.1821	-0.1886	-0.1736	-0.3034	0.09089
XM_576691.1	LOC501276	-0.0094	0.0078	0.12519	-0.0565	0.12971	-0.1628	-0.0313	0.29597	0.04388	0.10278	0.06065	-0.14	-0.0192	-0.1785
XM_576694.1	LOC501279	0.0302	-0.0487	0.25638	0.10289	-0.0024	-0.035	0.08466	-0.0079	-0.0237	0.02002	-0.0845	-0.0495	0.26825	0.01437
NM_017343	LOC501280	0.60045	0.30706	-0.4017	1.1539	0.75827	0.83249	0.03094	0.99468	0.68044	0.35757	0.37591	-0.0349	0.10206	0.17375
NM_017343	LOC501280	-0.056	0.15109	-0.2696	-0.2591	0.13511	-0.3287	0.08612	0.12573	-0.2787	0.00473	-0.0252	-0.272	0.20802	0.19678
XM_001056647.1	LOC501281	-0.2729	-0.2591	0.54928	-0.0946	-0.225	-0.3263	0.15809	-0.304	-0.1641	-0.1214	-0.0994	-0.1598	0.00155	0.03215
XR_007919.1	LOC501282	0.36354	0.4182	-0.6355	-0.366	0.30182	0.1641	-0.0852	0.22728	0.44535	0.14979	0.16384	0.2087	0.46609	0.66964
NM_001134637	LOC501283	0.02516	0.04295	0.00665	-0.0378	0.14816	0.021	0.01895	0.0685	-0.0057	0.13518	0.37356	0.10417	0.06826	-0.007
XM_576699.1	LOC501284	-0.1353	-0.0416	-0.0983	-0.0029	-0.0834	0.08571	-0.1103	0.05049	0.16188	-0.0462	-0.0614	-0.0318	0.09279	-0.1171
NM_001109673	LOC501285	0.03543	-0.0298	0.14135	-0.2443	0.03102	0.04924	-0.2638	0.04308	0.26092	-0.0219	0.10079	-0.3173	0.16357	-0.0186
XM_576701.1	LOC501286	-0.1165	-0.1054	-0.1758	-0.1394	-0.022	-0.2302	0.01796	-0.1474	-0.2242	-0.1656	-0.0524	-0.073	-0.1611	-0.0994
XM_576702.1	LOC501287	-0.0249	0.14756	-0.162	-0.0831	-0.1018	-0.0563	-0.1913	-0.0075	0.08649	-0.1035	-0.0443	-0.1401	-0.1648	-0.202
XM_580184.1	LOC501288	0.05365	-0.0409	0.00673	0.05892	0.13285	0.04506	0.06647	-0.003	0.01012	0.03655	0.12268	0.01921	-0.0183	-0.0198
NM_183402	LOC501289	-0.0843	0.29112	0.25889	0.14759	0.11438	0.27438	0.54439	0.19503	0.08225	0.19583	0.24087	0.05991	-0.0402	0.1562
XM_576704.1	LOC501290	-0.0104	-0.1422	-0.0861	-0.0035	-0.0421	-0.0679	0.04322	-0.0293	0.0124	-0.1126	-0.1212	-0.1475	0.26094	0.03749
XM_576706.1	LOC501293	0.14198	0.33687	0.17893	0.10013	0.24039	0.25041	0.33259	0.12495	0.23739	0.2061	0.35078	0.32028	0.21328	0.05221
XM_576708.1	LOC501295	0.04338	0.11602	0.095	0.21326	0.13183	0.16497	0.16046	0.19	0.17441	0.12591	0.21188	0.28364	0.12636	0.17861
NM_001014221	LOC501296	-0.0782	-0.1431	-0.038	-0.0544	0.18794	-0.1285	-0.1092	-0.1033	0.28398	0.03383	0.13347	-0.0537	-0.1267	-0.0631
NM_183402	LOC501297	0.32134	-0.0606	0.02731	0.18776	0.30174	0.03509	0.04575	0.01544	0.04202	0.07272	0.05328	0.09656	0.03117	0.01721
NM_183402	LOC501299	-0.2197	-0.1068	-0.169	-0.1927	0.1639	0.01787	0.08139	-0.1767	-0.1283	-0.0964	0.04067	-0.2074	0.19575	-0.0763
XM_576713.1	LOC501300	0.27275	0.31238	0.31668	0.54807	0.46875	0.06052	0.07663	0.00777	0.32544	0.33876	0.35569	0.39018	0.14067	0.21872
XM_576714.1	LOC501301	-0.0052	0.03432	-0.1187	0.06018	0.07926	0.06503	0.10995	0.01731	0.03421	-0.0013	0.1412	0.04707	0.1899	-0.0033
NM_001014221	LOC501302	0.04139	-0.0135	0.11948	0.08667	-0.0024	0.42456	0.34264	0.08989	-0.026	0.17472	0.12928	0.21359	0.2067	0.11992
NM_001014221	LOC501302	0.18966	1.2228	0.6719	0.42327	0.94149	1.2865	0.20619	0.97111	1.1777	1.2453	1.8499	0.34426	0.43734	0.19436
XM_576717.1	LOC501304	0.18342	0.09366	0.0655	-0.0005	0.04584	-0.0593	-0.0224	-0.0567	-0.0148	-0.0672	0.16542	0.16896	0.09344	-0.0736
XM_576718.2	LOC501305	0.09788	0.16018	0.03836	0.16044	0.20346	0.20198	0.10599	0.18794	0.08678	0.24912	0.24731	0.11696	0.0254	0.05931
NM_001014221	LOC501306	-0.1309	0.07736	-0.0268	-0.0527	-0.0794	-0.056	0.01676	-0.0584	0.03127	0.24428	-0.0017	-0.1567	-0.0362	0.03605
NM_001014221	LOC501306	0.67194	1.5662	1.1883	0.56735	0.94181	1.1674	0.81911	0.93117	1.53	1.605	2.1498	0.92741	0.42958	0.5702
XM_576721.1	LOC501308	-0.3636	-0.3262	-0.0032	-0.1023	-0.2533	-0.1582	-0.1257	-0.2253	-0.1666	0.04664	-0.1941	-0.2233	0.04605	-0.323
XM_576722.1	LOC501309	0.00733	-0.0291	0.11637	0.06109	-0.0025	0.08896	0.41027	0.24283	0.08836	0.06491	0.02742	0.17183	-0.0518	0.21337
XM_576724.1	LOC501311	0.09036	0.1762	-0.5184	-0.1554	0.20182	0.14091	-0.0162	0.12904	0.11274	-0.0127	-0.0538	-0.0581	0.10844	0.06635
XM_576727.1	LOC501314	0.27407	0.5829	0.34164	0.4982	0.17735	0.26939	0.26949	0.36657	0.61688	0.49553	0.71412	0.45544	0.18917	0.13483
NM_001014221	LOC501315	0.1354	-0.0087	-0.1089	0.06247	-0.1093	-0.0104	-0.1826	-0.2357	-0.0485	-0.2294	-0.1111	-0.0156	-0.2252	-0.0456
NM_001014221	LOC501315	0.17102	1.0933	0.89148	0.15644	0.81789	0.29858	0.34122	0.87675	0.88872	0.83165	1.1948	0.45925	0.41705	0.50608
XM_580187.1	LOC501316	0.21704	0.58115	0.50173	0.18664	0.36707	0.40341	0.27651	0.37651	0.36479	0.72866	0.81274	0.06344	0.5362	0.46909
XM_576730.1	LOC501318	-0.0462	-0.0295	-0.0406	-0.0366	-0.0758	-0.0106	0.18774	0.11855	0.17882	-0.0859	0.09862	0.15956	0.03266	0.01604
XM_576731.1	LOC501319	0.16948	0.37008	0.2549	0.17667	0.30218	0.40617	0.12979	0.22576	0.40242	0.62586	0.29923	0.07356	0.29414	0.40913
XM_576733.1	LOC501321	0.35677	0.06829	0.18471	0.07542	0.37561	0.31938	0.36967	0.23578	0.19567	0.324	0.21579	0.25776	0.19056	0.4286
XM_576734.1	LOC501322	0.10581	0.05135	-0.0331	-0.1773	0.10832	0.18013	-0.1502	0.01607	0.04758	0.23018	-0.1862	0.14779	-0.3126	0.08527
XM_576735.1	LOC501323	-0.2856	-0.3779	-0.2833	0.00383	-0.1529	-0.2628	-0.0805	-0.2645	-0.1227	-0.2705	-0.0694	-0.1017	-0.0386	-0.2032
XM_580188.1	LOC501324	0.04662	0.04187	0.22622	0.13417	0.13414	0.39747	0.18973	0.00326	0.17413	0.10761	0.16535	0.10693	0.29384	0.11723
NM_183402	LOC501326	-0.0325	0.15198	-0.4496	-0.2463	0.12126	0.08091	-0.0366	0.15244	-0.3291	-0.2316	-0.2273	-0.1391	0.16543	0.21819
XM_576738.1	LOC501327	-0.0081	0.03443	0.00645	0.0946	0.0685	0.17783	0.04535	0.14797	0.13606	0.12637	0.03099	-0.0347	0.05319	0.12676
XM_576739.1	LOC501328	0.05767	-0.1227	0.20146	-0.0954	0.00636	0.13499	-0.0866	-0.0358	0.09522	-0.0104	-0.023	0.01309	-0.1295	0.22041

XM_576744.2	LOC501333	-0.3969	0.37005	0.10991	0.09897	0.24198	0.01923	0.04956	-0.029	0.14367	-0.1776	0.17023	-0.0792	-0.0818	-0.0291
XM_576744.1	LOC501333	-0.0359	-0.0798	0.03036	-0.1313	-0.0846	-0.1495	-0.0937	-0.0474	-0.0087	-0.0268	-0.1697	0.06461	-0.1397	-0.1257
XM_576749.1	LOC501338	0.14457	0.40418	0.52846	0.26144	0.22275	0.14709	0.29752	0.35546	0.71838	0.48995	0.7773	0.34308	0.10688	0.18692
NM_183402	LOC501341	0.30248	0.1172	-0.2038	#######	0.18707	0.27037	0.11699	0.3375	0.34132	-0.0852	0.16424	0.0728	0.08713	0.17555
XM_576754.1	LOC501343	0.3932	0.38991	0.36329	0.38932	0.28501	0.375	0.35879	0.54011	0.06221	0.51392	0.67017	0.25287	0.09858	0.17348
NM_183402	LOC501346	0.03773	-0.0092	0.04603	0.03777	0.18987	0.11364	0.03563	0.02459	0.19492	0.34113	0.03011	0.1067	0.03866	0.06999
XM_576758.1	LOC501347	-0.0188	0.45578	-0.1235	-0.072	-0.0448	-0.1508	0.14217	0.01677	0.15157	0.46375	0.42912	-0.1792	0.01944	-0.1405
XM_576762.1	LOC501351	0.13214	0.03475	-0.1908	0.06265	0.4645	0.25291	-0.0013	0.21814	0.06092	-0.4745	-0.0402	-0.0883	-0.1501	0.08777
XM_576763.1	LOC501352	-0.0407	0.08229	-0.0686	-0.0518	-0.0596	-0.05	0.01269	-0.0477	-0.0658	0.02822	0.12403	-0.0599	-0.0425	-0.0562
XM_576766.2	LOC501355	0.01381	0.1892	0.0359	0.05255	0.05238	0.45304	0.15027	0.18052	0.30422	0.31738	-0.0508	0.27249	0.12023	0.15234
XM_576770.2	LOC501358	0.82899	0.95038	0.86329	1.1335	1.0882	1.4019	0.87333	1.0381	1.1529	1.1953	1.5357	0.74451	0.52521	0.36414
NM_001014221	LOC501361	0.1614	0.69912	0.3838	0.17996	0.49137	0.63454	0.13479	0.28522	0.72524	0.54417	0.95927	0.14355	0.45239	0.07624
NM_001014221	LOC501362	0.40675	0.31815	0.52616	0.29003	0.23302	0.19346	0.28485	0.34438	0.5184	0.43083	0.53068	0.67142	0.31273	0.28591
XM_576776.1	LOC501363	0.06813	0.10901	-0.012	-0.0287	0.13311	-0.0419	0.21635	-0.1085	-0.0183	-0.167	0.08486	-0.095	-0.1065	0.08142
XM_576778.1	LOC501365	0.00524	-0.0959	-0.2082	-0.1668	-0.1546	-0.0128	-0.2322	-0.2017	-0.2644	-0.0372	-0.1814	-0.1911	-0.233	-0.1873
XM_576779.1	LOC501366	-0.1188	0.08865	0.15283	0.30781	0.01485	0.25531	0.22365	0.10622	0.29097	0.06671	-0.0137	0.15652	0.16239	0.05827
XM_576781.1	LOC501368	-0.0649	-0.1567	-0.1693	-0.1556	-0.1039	-0.1338	-0.1169	-0.1098	-0.2654	-0.017	-0.1452	-0.2225	0.1229	-0.0811
XM_576782.2	LOC501369	0.51541	0.87532	0.6324	0.70744	0.78468	0.87641	0.68365	0.92758	1.0322	0.86026	1.1775	0.73383	0.62007	0.18709
XM_576784.1	LOC501371	0.41554	0.35358	-0.1	0.02953	0.37242	0.19976	-0.0655	0.40906	-0.1019	0.28985	0.21983	0.21697	0.0377	0.09221
NM_183402	LOC501373	-0.1146	-0.1158	-0.1454	-0.0406	-0.0251	-0.0534	-0.1031	-0.0823	0.10619	-0.0413	-0.1324	0.12361	-0.2033	-0.1599
NM_183402	LOC501373	0.08947	0.54117	0.20867	-0.0012	0.45695	0.07976	0.08286	-0.1326	0.48273	0.54204	0.38917	0.08094	0.08933	0.01944
XM_576788.2	LOC501375	0.04698	-0.0093	-0.1086	0.03373	0.23892	-0.0802	0.11593	-0.0918	0.17685	0.09266	-0.0342	0.01928	0.15386	0.03105
XM_576789.1	LOC501376	-0.169	-0.1019	-0.1542	-0.1827	0.15845	-0.0235	-0.0129	-0.101	0.04076	0.06901	0.08733	0.04723	0.05901	-0.1098
XM_576791.1	LOC501378	-0.16	-0.0984	0.07321	-0.0024	-0.1972	-0.1442	0.07758	0.00562	-0.0849	-0.0455	-0.1225	-0.1644	-0.2075	-0.0506
XM_576792.1	LOC501379	0.60065	1.0083	1.0339	0.80443	0.83758	1.0164	1.0809	1.1633	1.2988	1.3446	1.5972	0.89225	0.28502	0.50535
NM_001014221	LOC501383	0.1264	0.10327	0.219	0.26645	-0.0112	0.01728	-0.0212	0.12654	0.0674	0.06041	0.23775	-0.0265	0.0666	0.21427
NM_001014221	LOC501384	0.0775	-0.0477	0.06488	-0.03	0.20506	0.23706	0.02693	0.05289	0.19106	-0.0135	-0.0031	0.01794	0.00611	0.03736
XM_576798.1	LOC501385	0.11381	-0.062	0.06619	0.06805	-0.031	0.01502	0.06841	-0.0738	0.0671	0.0376	0.01613	0.00367	0.03819	0.05243
NM_001014221	LOC501386	0.46214	0.39928	-0.0769	0.01852	0.46545	0.29254	0.23316	0.43418	0.40471	0.23833	0.15177	0.30751	0.3423	0.42866
NM_001014221	LOC501386	0.18658	-0.0313	0.05386	-0.1221	0.2013	-0.1904	-0.2094	-0.1882	-0.0415	-0.3085	-0.0564	-0.1775	0.08736	-0.2567
XM_576800.1	LOC501387	-0.3565	-0.1288	0.00847	0.21171	-0.1849	-0.3218	0.06574	0.03171	-0.3212	-0.2417	-0.1521	0.12864	-0.3141	-0.2042
XM_576801.1	LOC501388	0.04171	-0.2181	-0.1161	-0.0761	-0.2431	0.00796	0.01153	0.13458	0.07497	-0.0861	-0.2068	-0.0953	-0.1558	-0.2048
XM_580189.2	LOC501391	0.01328	0.05792	0.06171	0.02522	0.08826	-0.0026	0.27768	0.09881	0.09213	0.36014	0.1313	0.1783	-0.0126	0.0024
XM_576804.1	LOC501392	-0.2688	0.01477	-0.1709	-0.1278	-0.2233	-0.018	-0.1216	-0.1243	-0.1856	-0.2122	-0.2297	-0.2486	-0.1323	-0.0505
XM_576807.1	LOC501395	-0.0395	-0.2652	-0.2287	0.03785	-0.1268	-0.1784	-0.0615	-0.1296	0.07744	-0.1105	-0.0362	-0.0494	-0.1583	-0.2879
XM_576811.1	LOC501398	-0.4024	-0.3426	-0.4018	-0.29	-0.3744	-0.3615	-0.3433	-0.2567	-0.3476	-0.317	-0.0523	-0.2546	-0.4275	-0.4467
XM_576812.2	LOC501399	-0.0884	-0.0176	0.08855	0.0652	-0.039	0.05867	0.09972	-0.0171	-0.0511	0.0142	0.07031	-0.0679	-0.0554	0.1272
XM_576813.2	LOC501400	0.14599	-0.0947	0.07313	0.52872	0.08154	0.06828	0.0797	0.19127	-0.107	-0.0649	-0.1439	-0.054	0.05108	-0.2058
XM_576813.2	LOC501400	0.01308	-0.0882	-0.0302	-0.0825	-0.056	0.0183	-0.1107	-0.2087	0.16493	-0.1325	-0.1085	-0.0077	-0.0569	-0.1694
XM_576813.1	LOC501400	-0.1721	-0.0913	-0.1455	-0.0796	-0.1524	-0.0807	-0.1316	-0.1634	-0.0216	-0.0979	-0.1712	-0.1053	-0.0705	-0.1139
XM_576814.1	LOC501401	-0.0637	-0.072	-0.0096	-0.0212	0.0329	-0.0497	-0.0705	0.00555	0.23172	0.00014	-0.0042	-0.0512	-0.0279	0.00715
XM_580190.1	LOC501402	-0.2087	-0.1072	-0.0364	-0.0512	0.00115	-0.2451	-0.231	-0.209	-0.1072	-0.1746	0.00691	0.00871	-0.1055	-0.1408
XM_576815.2	LOC501403	0.17278	0.12565	0.24576	0.55226	0.22091	-0.0645	-0.0957	0.47679	0.04739	0.02376	0.01483	0.19693	0.05096	0.18753
XM_576817.1	LOC501405	-0.1199	-0.2406	-0.1397	0.05888	-0.1824	-0.0699	0.1648	-0.3046	-0.1599	-0.1962	-0.0954	-0.2144	0.00268	-0.019
XM_001063978.1	LOC501406	0.03374	-0.0155	-0.0495	0.05465	0.13507	-0.0035	0.07832	0.05301	-0.0044	-0.0044	0.0034	0.05028	-0.0107	0.05287
XM_576819.1	LOC501407	0.0656	-0.277	-0.0722	-0.2975	-0.0374	-0.156	-0.0089	-0.052	-0.303	-0.1368	0.13594	-0.2437	-0.2749	0.01079
XM_576820.1	LOC501408	-0.0376	0.05671	-0.0435	-0.0556	-0.092	0.0981	-0.0132	-0.0451	0.04422	-0.1498	-0.0846	-0.1061	-0.0576	-0.0556
XM_576821.1	LOC501409	-0.1046	-0.2538	-0.1746	-0.0342	-0.3192	-0.1541	-0.2431	-0.1383	-0.2234	-0.2792	-0.1638	-0.2546	-0.2932	-0.3644
XM_576825.1	LOC501413	0.27965	0.31757	0.00631	0.05109	0.44059	0.39075	0.02026	0.47761	0.3676	0.14372	0.25272	0.14214	0.34975	0.18643

XM_580191.1	LOC501414	-0.1708	-0.2008	-0.1251	-0.0422	-0.4407	-0.2556	-0.2249	-0.2852	-0.3428	-0.5042	-0.3814	-0.0697	-0.3954	-0.3042
XM_576827.1	LOC501416	0.34348	0.02813	-0.2641	-0.0845	0.36532	0.44703	0.17597	0.5987	0.46547	0.18924	0.28722	0.3287	0.30704	0.2673
XM_576829.1	LOC501418	-0.0453	0.11173	0.19176	0.13686	0.25627	0.26996	0.00502	0.32467	0.09555	0.10726	0.28534	0.07563	0.21682	-0.028
XM_576830.1	LOC501419	0.00621	-0.006	0.10535	0.10821	0.05303	0.15545	0.10743	0.07119	0.15421	0.13558	0.00919	-0.0252	0.0005	-0.0955
NM_183402	LOC501420	0.26148	0.18104	0.26304	0.18781	0.12769	0.23328	0.3203	0.19288	0.05115	0.39164	0.42151	0.06617	0.07655	0.11615
NM_001014221	LOC501421	#######	0.7788	0.39059	0.14203	0.21268	-0.0356	0.16877	0.16657	0.55412	0.64978	0.95886	-0.0173	0.05161	0.01946
NM_183402	LOC501422	0.01806	0.1671	0.19198	0.1035	0.10116	0.0943	0.31701	0.07958	0.19211	0.18386	0.09569	0.02225	0.07724	0.04012
XM_580193.1	LOC501423	-0.1727	-0.2947	-0.2399	-0.1869	-0.1202	-0.2431	-0.3058	-0.2126	-0.0592	-0.2965	0.05896	-0.161	-0.0156	-0.0666
XM_576834.1	LOC501424	0.22817	0.13541	0.32435	0.05089	0.15241	0.45926	0.2212	0.10828	0.03246	0.17051	0.02552	0.56837	0.16159	0.18145
XM_576835.1	LOC501425	-0.0518	0.01892	-0.0768	-0.0375	0.00577	-0.2063	-0.0095	0.16297	-0.0746	-0.0017	0.02365	0.20873	0.00394	0.16709
XM_576836.1	LOC501426	0.05904	0.1615	-0.0066	0.02552	0.12819	0.13199	0.0839	0.10501	0.01218	0.0374	0.0368	0.11941	0.06674	0.05837
XM_576840.1	LOC501430	0.27609	0.00037	0.18278	0.06725	0.1454	-0.0685	0.11151	0.13869	0.08317	-0.0505	-0.0023	-0.0379	0.06572	0.04283
XM_576841.1	LOC501431	-0.0165	0.09833	0.09608	-0.0372	0.12874	0.0604	0.03744	0.02719	0.12155	0.02416	-0.0416	-0.0157	0.31083	0.01493
XM_576843.1	LOC501433	0.20691	0.18177	0.25354	0.14683	0.04568	0.02685	0.29118	-0.0183	0.02977	0.27681	0.02929	0.0465	0.17054	0.25254
XM_576845.1	LOC501436	0.06382	0.02325	0.11259	0.07097	0.01611	-0.0532	0.21086	0.04346	-0.0469	-0.0849	-0.0096	-0.0125	-0.0666	-0.1083
XM_576846.1	LOC501437	0.11326	0.0144	0.09687	0.05149	0.08675	0.06318	0.07573	0.14971	0.00019	0.05279	0.06728	0.0846	0.17029	0.1145
XM_576847.1	LOC501438	0.01765	0.09722	0.07102	0.06032	-0.0014	0.11115	-0.0888	-0.029	0.10507	-0.0339	0.20058	-0.0705	0.13765	-0.024
XM_576848.1	LOC501439	-0.1235	-0.1631	-0.0958	0.03243	-0.1074	0.11939	0.03406	-0.0787	-0.031	-0.0486	-0.0903	0.01762	-0.0323	-0.1264
XM_576849.1	LOC501440	0.04313	0.05184	0.16429	0.01735	-0.0602	-0.074	-0.0147	0.14006	-0.1008	-0.064	0.10519	0.20628	-0.1119	0.05034
NM_022706	LOC501441	0.7559	0.55569	-0.1091	0.52576	0.71242	0.68962	0.61134	0.65936	0.30374	0.3145	0.64668	0.09146	0.19001	0.20719
XM_576852.1	LOC501442	-0.231	0.02325	-0.2376	0.02785	-0.197	-0.1464	-0.1929	-0.2682	-0.0701	-0.0849	-0.3083	-0.308	-0.3055	-0.1274
XM_576853.1	LOC501443	-0.0806	-0.0704	0.1374	0.05288	-0.0677	0.07261	0.02523	-0.0789	-0.0097	0.00736	0.22083	0.07776	-0.1169	-0.1378
XM_576855.1	LOC501444	-0.1631	-0.0187	-0.112	-0.2233	-0.2454	-0.2219	-0.1844	-0.2066	-0.0369	0.01988	-0.0348	-0.0521	-0.1402	0.16409
XM_576859.1	LOC501448	0.04629	-0.078	-0.0306	0.11797	0.16066	0.17575	0.09056	0.16198	0.00381	0.01035	0.12379	-0.2724	0.13261	0.12205
XM_576860.1	LOC501449	-0.3638	-0.0288	-0.0535	-0.127	-0.4787	0.02605	0.20871	-0.0881	-0.3619	0.02628	0.12039	-0.6017	-0.7393	-0.2737
XM_576861.1	LOC501450	0.20695	-0.0046	-0.0105	0.01026	0.01241	-0.0813	-0.0476	0.11086	-0.0294	0.06223	0.15548	0.0078	0.08097	0.07607
XM_576862.1	LOC501451	0.05905	0.01848	0.30217	0.15165	-0.1243	0.10999	0.14508	0.24902	0.22614	0.02082	0.13158	0.14323	0.19837	0.31219
XM_580194.1	LOC501452	-0.1833	-0.2762	-0.2809	0.48249	-0.1451	-0.0514	-0.1567	-0.2703	-0.0822	-0.0907	-0.1588	-0.1367	-0.209	-0.203
XM_576863.1	LOC501453	0.2579	-0.1032	-0.0181	0.07432	0.02777	0.13763	-0.0437	-0.0418	0.0155	0.04643	0.08537	0.17713	-0.0596	-0.066
NM_001007635	LOC501456	-0.1924	-0.1516	0.01879	-0.1363	-0.2273	-0.0941	-0.0571	-0.2428	-0.0982	-0.1883	-0.1205	0.03257	-0.1233	-0.1893
XM_576867.1	LOC501457	0.19668	0.06307	-0.029	0.23563	0.08567	0.0626	-0.0734	-0.0375	0.12589	0.09178	0.02718	0.18184	-0.0036	0.25155
XM_576869.1	LOC501459	-0.2118	-0.0847	-0.1351	-0.1086	-0.1897	-0.1341	-0.0286	-0.1648	-0.2119	-0.1584	-0.2364	-0.0611	-0.1708	-0.174
XM_580195.1	LOC501460	-0.0506	0.03945	-0.0273	0.25776	0.00164	0.14107	-0.0041	-0.0906	0.2002	0.09988	0.03201	0.09152	0.17496	0.13827
XM_576871.1	LOC501462	0.18212	0.14126	0.09583	0.03225	0.16304	0.06639	-0.0215	0.0973	0.03626	0.07165	-0.0562	0.29861	0.07347	0.20959
XM_576875.1	LOC501464	-0.1265	-0.1268	0.04795	0.05174	-0.1159	0.06128	0.35319	-0.0499	-0.0768	0.16588	0.15592	-0.0843	0.0915	-0.0047
NM_183402	LOC501467	-0.0194	0.06669	-0.0525	0.02028	0.06762	-0.0317	0.21018	0.07387	-0.0605	0.10985	0.01386	-0.0436	-0.0283	0.02672
NM_001014221	LOC501469	0.11272	0.66943	0.44049	0.32585	0.31388	0.44868	0.21546	0.33446	0.42497	0.35172	0.88155	0.1096	0.07148	0.17533
NM_001014221	LOC501469	-0.1013	-0.0483	0.19337	-0.0042	-0.2228	-0.1283	-0.0198	-0.101	-0.1113	0.08748	0.08876	-0.0867	0.23848	-0.1552
XM_576880.1	LOC501470	-0.0827	-0.1366	0.01153	-0.0544	-0.0864	-0.2631	-0.0198	-0.076	0.02275	-0.0896	-0.1901	-0.2204	0.06752	0.01639
XM_580196.1	LOC501471	0.10894	0.05091	0.17449	0.1794	0.03976	0.07573	0.04842	0.17641	0.05333	0.04943	-0.0604	0.03042	0.09452	-0.0162
XM_580197.1	LOC501472	-0.1561	-0.1593	-0.1576	-0.1362	0.07526	-0.1712	0.13953	-0.1337	0.03837	-0.0629	0.06658	-0.0097	-0.2054	-0.1262
XM_576884.1	LOC501476	0.15785	0.08291	-0.038	0.29065	0.1975	0.09263	-0.0226	0.10285	0.03715	0.13559	0.04584	0.03881	0.11044	0.18106
XM_576886.1	LOC501478	0.07323	0.27148	0.30275	-0.0634	-0.0022	-0.0065	0.13384	-0.0468	0.14212	0.04348	0.11054	0.06071	0.27529	0.03901
XM_580198.1	LOC501479	-0.1579	-0.1127	-0.193	-0.146	-0.086	-0.0539	-0.12	-0.1239	-0.0601	0.01805	-0.1082	-0.1008	-0.1909	0.00824
XM_001081012.1	LOC501479	-0.0041	0.03822	-0.1416	-0.0402	-0.1832	-0.0322	0.1722	0.15318	-0.0016	0.18936	0.16831	0.18058	0.01567	0.07955
NM_001014221	LOC501482	0.12074	-0.0173	0.29864	0.03522	0.07815	-0.1141	0.39387	0.03346	-0.1092	0.04708	0.0458	-0.0796	0.01607	0.11481
XM_576891.1	LOC501484	0.02313	-0.2356	0.11386	-0.0908	0.19748	0.28036	0.14806	-0.0393	0.21497	0.11641	0.22391	0.3614	0.1109	0.04394
XM_580199.1	LOC501485	-0.068	-0.0343	-0.051	-0.0082	-0.0392	-0.0588	0.12235	-0.0074	0.03107	0.14333	0.05177	-0.0404	-0.0331	-0.1079
XM_576892.1	LOC501486	0.2212	1.0785	0.91102	0.44013	0.47437	0.5155	0.45871	0.1891	0.94324	0.89122	1.1945	0.36861	0.38765	0.14728

NM_001014221	LOC501488	0.56542	1.7259	0.98044	0.87584	1.0678	1.1538	1.031	1.0895	1.1757	1.6305	2.0576	0.40817	0.52086	0.52437
NM_001014221	LOC501488	0.09524	0.1783	0.03021	-0.0234	0.08811	0.11704	0.1007	0.23322	0.15797	0.2104	0.53342	0.07032	-0.0487	-0.2368
XM_576896.1	LOC501490	-0.1628	0.02326	0.09026	-0.1381	-0.063	0.23992	-0.3081	-0.2393	-0.1023	-0.1041	-0.0808	-0.0368	-0.391	-0.2642
XM_576897.2	LOC501491	-0.2852	-0.0716	-0.1861	0.21031	-0.1116	-0.7627	0.07795	-0.2114	-0.1678	-0.0204	-0.1442	-0.4322	-0.2124	0.05924
XM_236855.3	LOC501493	-0.211	-0.0757	-0.1768	-0.1238	-0.0958	-0.1427	-0.0132	-0.0234	-0.0995	0.3398	0.0345	0.2109	-0.2345	0.07933
XM_580200.1	LOC501494	-0.0473	-0.0305	0.16424	0.1151	0.14189	-0.0582	-0.1699	-0.0132	0.02716	0.34504	-0.0079	0.09272	0.02939	0.13829
XM_580201.1	LOC501495	-0.0432	0.00292	-0.0511	-0.0057	-0.0097	0.36203	-0.028	0.12338	0.18473	0.11798	0.24216	0.11415	0.09926	-0.0023
XM_580202.1	LOC501497	-0.0424	0.231	0.34006	-0.1021	-0.0472	-0.0933	0.15558	-0.0207	-0.1113	-0.1084	-0.0235	0.03635	-0.0156	0.19023
XM_580203.1	LOC501498	-0.0463	-0.1019	-0.0418	0.02153	-0.144	0.02102	-0.1274	0.33963	-0.101	-0.1383	-0.1177	-0.0133	-0.0715	-0.1595
XM_576900.1	LOC501499	0.03363	-0.1687	-0.072	-0.0939	-0.0351	-0.1584	-0.291	0.07866	-0.2113	-0.0458	0.00847	-0.1588	0.04007	-0.2221
XM_576901.1	LOC501500	0.09851	0.4651	0.56292	0.20691	-0.1865	-0.1492	0.7856	-0.0582	0.43286	0.61239	0.52796	-0.0234	0.00877	0.15438
XM_576902.1	LOC501501	-0.086	-0.1312	-0.2295	-0.1386	0.08471	-0.126	-0.0272	-0.1568	0.07215	-0.2126	0.13683	-0.2396	-0.0257	-0.0426
XM_576904.1	LOC501503	-0.4305	0.02945	-0.3157	0.08768	-0.5991	-0.0948	0.60033	-0.3782	-0.1981	0.20189	-0.0552	0.00057	-0.2613	-0.3536
XM_576905.1	LOC501504	-0.0121	0.15974	0.0938	-0.0986	-0.0059	-0.1251	-0.1587	0.24906	-0.0299	0.14949	0.0299	-0.0728	0.2274	0.14281
XM_580204.1	LOC501505	0.01765	-0.0482	-0.0105	-0.0209	0.02801	0.0404	0.01116	0.05879	0.1911	0.14825	-0.0052	0.03276	0.08731	0.00751
NM_001109314	LOC501506	-0.0612	0.13906	-0.1646	-0.0753	0.17247	0.44982	0.17594	0.32482	0.03537	0.14216	0.12178	0.28504	0.26506	0.19544
XM_576908.1	LOC501509	-0.0205	0.06669	-0.0399	0.03653	-0.0162	-0.0801	-0.0221	0.0063	-0.069	0.00861	0.01919	-0.0871	0.10978	0.18835
XM_576909.1	LOC501510	0.04468	0.10123	-0.1826	0.02593	-0.024	0.03399	-0.1368	0.12654	-0.1724	-0.0424	-0.0238	-0.0013	0.16909	0.16263
XM_576913.1	LOC501512	0.0037	0.17195	-0.0051	-0.0051	0.05992	0.34765	0.06036	-0.0928	0.13205	-0.099	0.09384	0.27195	-0.0166	0.13154
XM_576918.1	LOC501517	-0.0492	0.07915	-0.315	0.14182	0.03193	-0.2221	-0.1441	-0.1688	-0.2481	0.09468	0.10678	-0.108	-0.2876	-0.1612
XM_576919.1	LOC501518	-0.0298	-0.0726	-0.2332	-0.1705	-0.1088	0.00284	-0.1853	-0.0382	-0.2321	-0.2567	-0.1412	-0.0413	-0.178	-0.2896
XM_576920.1	LOC501519	-0.0224	-0.0218	0.27214	0.08782	0.05633	0.06449	0.14245	0.1122	0.03573	0.04131	0.2042	0.21652	0.29141	0.09127
XM_576924.1	LOC501522	-0.1034	-0.0522	-0.1852	-0.0233	-0.089	0.09153	-0.0116	0.1248	-0.0643	-0.119	-0.0656	0.01492	0.01796	-0.0746
XM_576925.1	LOC501523	-0.084	-0.1892	-0.0642	-0.2096	-0.0405	-0.2333	-0.0813	-0.2575	-0.208	-0.2423	-0.168	-0.1067	-0.1335	-0.0695
XM_576928.1	LOC501527	-0.1662	-0.0463	-0.154	-0.1488	-0.1844	-0.1605	-0.1116	-0.2777	-0.1846	-0.1516	-0.2776	-0.1862	-0.1142	-0.2874
XM_580207.1	LOC501530	-0.1587	-0.1127	-0.1685	-0.0389	-0.1378	-0.1707	0.03749	-0.071	-0.0524	-0.0888	-0.2047	-0.0602	-0.1469	-0.1205
XM_576931.1	LOC501531	0.20257	0.1498	0.09275	0.14396	0.32473	0.06035	0.10652	0.24064	0.07017	0.02892	0.19386	0.20512	0.07418	0.07748
XM_580208.1	LOC501533	-0.0086	-0.1037	0.03069	0.03693	0.0852	0.01567	0.08682	0.01453	-0.0338	0.03305	-0.0521	-0.058	0.01071	-0.0576
XM_576934.1	LOC501534	-0.2918	-0.2776	0.00173	0.00691	-0.1719	0.0707	0.11081	-0.3894	-0.291	-0.2435	-0.0976	-0.0091	-0.1895	-0.1152
XM_576935.1	LOC501535	0.05366	0.20728	0.16386	0.05632	0.01011	0.00713	0.06809	0.01201	0.0518	0.12066	0.0213	0.13144	0.12798	0.10948
XM_576940.1	LOC501537	0.02979	0.3132	0.29577	0.2264	0.04023	0.15602	0.22532	0.05167	-0.0068	0.01546	0.0957	0.05167	0.3452	0.30162
XM_580209.1	LOC501538	-0.4386	-1.1534	-0.9864	-0.9343	-1.0136	-0.7888	-0.9934	-0.8072	-0.917	-0.8902	-0.6714	-0.7791	-0.7923	-0.5392
XM_580210.1	LOC501540	-0.0124	0.16651	0.06846	0.02882	0.07149	0.0301	0.03098	0.23945	-0.0092	0.08468	0.1635	0.25839	0.16572	0.04347
XM_576942.1	LOC501541	0.19441	0.21743	0.37434	0.13945	0.15624	0.30764	0.1534	0.09737	0.182	0.21426	0.08964	0.59169	0.1434	0.45465
XM_576943.1	LOC501542	0.04435	0.07678	0.07091	0.30313	-0.0539	0.14143	0.05673	0.11848	0.04777	0.05076	-0.0228	0.00959	0.00769	0.1087
XM_576949.1	LOC501547	-0.0103	0.00596	-0.0252	-0.0985	-0.0709	-0.0005	-0.0254	-0.2216	0.11453	0.52594	-0.1387	-0.1491	-0.2298	-0.1007
XM_576950.1	LOC501548	-0.6207	-0.3523	0.36702	0.61119	-1.1895	-1.3991	1.3334	-0.9275	-0.349	-0.2394	0.12007	-0.5827	-0.7972	-0.7087
XM_576951.1	LOC501549	0.22681	-0.08	0.1195	0.01616	-0.0088	-0.0425	0.09566	-0.089	-0.0546	-0.1182	-0.0083	0.22292	0.00906	-0.043
XM_576955.1	LOC501553	-0.2794	0.2256	-1.3953	-0.6961	-0.7277	-1.345	-0.1264	-0.7186	-0.0085	0.34896	0.08417	-0.5363	-0.7755	-0.8601
XM_576960.1	LOC501556	-0.1124	0.23178	-0.0838	-0.1323	-0.09	-0.0454	-0.0597	-0.0529	0.05312	-0.1525	-0.0632	0.11201	-0.0446	0.09991
NM_001127568	LOC501559	-0.0302	-0.1691	-0.1461	0.31577	-0.2717	-0.5722	0.08259	-0.1713	-0.3891	-0.0476	-0.4423	-0.2513	0.04577	-0.2424
XM_576965.1	LOC501560	0.2476	0.0173	0.00101	0.01722	0.03898	0.0749	-0.1153	-0.0474	-0.0588	-0.0516	-0.0321	0.10488	-0.0718	-0.0427
XM_576967.1	LOC501562	-0.2763	0.20234	-1.174	-0.696	-0.8465	-1.099	-0.023	-0.7149	-0.1458	-0.2187	-0.2341	-0.4327	-0.8137	-0.8618
XM_580211.1	LOC501564	0.0351	0.1679	0.05369	-0.048	0.06011	0.00686	-0.027	0.04618	0.12533	0.15467	0.12882	0.00862	-0.0421	-0.0647
XM_576969.1	LOC501565	-0.0468	0.03596	-0.1704	0.04807	-0.1654	-0.0456	-0.1138	-0.0914	0.02538	-0.0139	0.07878	-0.0924	0.00812	-0.031
XM_576971.1	LOC501567	0.03469	-0.0357	0.02292	0.08367	0.36914	-0.0019	0.04929	0.00718	0.04831	0.14501	0.11497	-0.0193	0.01427	0.14677
XM_576976.1	LOC501572	-0.0169	-0.037	0.01386	-0.0421	-0.1165	0.23093	-0.1323	0.09385	-0.0929	0.31215	0.10875	0.29669	0.07644	-0.0382
XM_576977.1	LOC501573	-0.1475	-0.2174	-0.2593	0.00606	-0.1659	-0.0997	-0.1579	-0.1897	0.12724	-0.1367	-0.0993	-0.1816	-0.3291	-0.2914
XM_580212.1	LOC501581	0.28433	0.03064	0.33051	0.13925	0.39104	0.48396	0.11262	0.08917	0.16859	0.07478	0.29811	0.03339	0.22147	0.16981

NM_001109319	LOC501582	-0.0371	0.07654	-0.0689	-0.0562	-0.066	0.05821	-0.0146	0.0021	0.03248	-0.0565	-0.0084	0.14633	0.01313	0.09175
XM_580213.1	LOC501583	-0.1739	-0.0309	-0.1591	-0.1776	0.01905	-0.0344	-0.0765	-0.1699	0.05292	-0.1033	-0.1895	-0.0289	-0.0971	-0.2338
XM_576987.1	LOC501585	-0.0505	-0.3222	-0.2542	-0.1548	-0.1876	0.04805	-0.2157	-0.1587	-0.0391	-0.2757	-0.2953	-0.1938	-0.2642	-0.1786
XM_576988.1	LOC501586	-0.0074	-0.0093	0.05416	0.04886	-0.0121	-0.037	0.01835	0.01044	0.08691	0.00177	-0.0202	0.08942	0.01628	0.08841
XM_576993.1	LOC501589	-0.2071	-0.2412	-0.0103	-0.1097	-0.1709	-0.149	-0.0075	-0.056	0.0329	-0.3047	-0.1903	0.09675	-0.0462	-0.0978
XM_580214.1	LOC501590	-0.2457	-0.0336	-0.0847	0.02725	-0.0159	-0.1633	-0.2131	-0.3472	-0.1686	-0.1329	-0.1938	-0.117	-0.0576	-0.0638
XM_576994.1	LOC501591	-0.0654	-0.0988	-0.1157	0.10784	-0.1389	-0.1443	-0.1059	-0.1508	-0.1319	-0.2051	-0.0892	-0.1426	0.01403	0.02828
XM_580216.1	LOC501593	-0.0782	-0.09	-0.1236	-0.1279	-0.0138	-0.1394	0.18924	-0.1642	-0.0942	-0.077	-0.1746	-0.0082	-0.1719	-0.1985
XM_580217.1	LOC501594	0.05072	0.0359	0.30635	0.23213	0.31668	-0.0083	0.24808	0.33554	0.19716	-0.0385	0.13992	0.05473	0.56743	0.53824
XM_576996.1	LOC501596	-0.0285	0.13927	0.17158	0.00751	0.09986	0.10058	-0.0645	0.0471	0.03455	0.01118	-0.0191	-0.0294	0.16711	0.01773
NM_001014106	LOC501601	1.1775	0.83585	-0.684	0.16652	1.6996	1.4503	0.54529	1.6143	0.80387	1.0722	0.6555	0.31454	0.01447	-0.1363
XM_577005.1	LOC501605	0.28175	-0.3069	-0.1164	-0.549	0.30994	0.7831	-0.5618	0.16704	0.3046	-0.0217	0.10688	0.97705	0.3894	0.37754
XM_577006.1	LOC501606	-0.1077	-0.1023	-0.0219	-0.0964	-0.0544	-0.0948	-0.1953	-0.2681	-0.1833	-0.1949	-0.0187	-0.3168	-0.0973	-0.2416
XM_577007.1	LOC501607	0.18241	0.1266	0.17616	0.10607	0.04575	0.22891	0.08396	0.00807	0.21781	0.0635	0.03693	0.09453	0.14329	0.03637
XM_580218.1	LOC501609	-0.2034	-0.1366	0.12166	0.3837	-0.1316	0.00953	0.10305	0.03754	-0.4878	-0.0456	-0.3412	-0.1978	-0.0398	-0.1177
XM_580218.1	LOC501609	-0.0054	-0.0664	-0.0963	0.02153	-0.0939	0.07101	0.07676	-0.1235	0.07464	0.14406	-0.0732	0.17585	-0.0562	0.05334
XM_577009.1	LOC501610	-0.0559	-0.1166	0.11389	0.01871	-0.0865	-0.2203	0.05106	0.02715	-0.1708	-0.0223	-0.07	-0.2007	-0.1266	-0.1284
XM_577010.1	LOC501611	0.04467	0.08551	0.14789	0.07937	0.02638	-0.0229	0.11266	0.09898	0.05814	0.22632	0.05049	0.0185	0.285	0.00089
XM_580219.1	LOC501614	0.00788	0.00142	0.18541	0.46784	-0.2262	-0.2105	0.0912	-0.2803	-0.1495	-0.0615	0.14756	0.06913	-0.3592	-0.2121
XM_577013.1	LOC501615	0.24042	0.07329	-0.0921	-0.0933	0.05947	0.01909	-0.1101	0.17365	0.04836	0.05353	-0.0158	0.07623	0.16683	0.15716
XM_577014.1	LOC501616	0.05108	0.23917	-0.0582	-0.033	-0.0678	0.07655	-0.05	-0.0833	-0.0211	-0.1161	0.28705	-0.0145	0.08568	0.07331
NM_001024366	LOC501617	0.07868	0.19727	0.1424	0.04106	0.16356	0.15646	0.1827	0.1622	0.2891	0.02545	0.03269	0.07389	0.10091	0.26516
NM_001047970	LOC501618	0.22697	-0.02	0.16558	0.2709	0.14526	0.16429	-0.0542	0.02217	0.15145	0.06183	0.02807	-0.0478	0.13678	0.02116
NM_001134854	LOC501620	-0.2549	-0.1832	0.00297	-0.1629	-0.3685	-0.1682	0.01877	-0.0497	-0.141	-0.2981	-0.157	-0.1367	-0.1537	-0.1943
NM_001037216	LOC501621	-0.207	-0.2217	-0.2229	-0.0848	-0.2525	-0.1003	-0.2001	-0.1278	-0.1685	-0.2765	-0.406	-0.0853	-0.1368	0.04999
XM_577022.1	LOC501622	-0.1106	-0.1882	-0.2261	-0.1636	-0.1673	-0.2038	-0.261	-0.2647	-0.2381	-0.0918	-0.1395	0.01947	-0.228	-0.2806
XM_580220.1	LOC501623	-0.1009	-0.1788	-0.1201	-0.1905	-0.0616	-0.0028	-0.0767	-0.1412	-0.1675	-0.2284	0.01106	-0.1077	-0.0915	0.02338
NM_001037554	LOC501624	-0.1209	-0.1072	0.02847	0.03527	0.0488	0.32173	0.06559	-0.0913	0.14984	0.23936	-0.0472	0.12714	-0.1329	0.0206
XM_577025.1	LOC501627	-0.0377	0.03303	0.02493	-0.0181	0.16428	0.0084	-0.0146	0.00778	-0.0019	0.04507	0.05509	0.21847	-0.0493	0.18904
XM_577027.1	LOC501629	0.0872	0.03247	0.04181	0.03263	0.1233	0.17645	#######	0.02847	0.19338	-0.0003	0.10389	0.01148	0.15072	0.00151
XM_577030.1	LOC501633	-0.5175	-0.6666	-0.8977	-1.3741	-0.4736	-0.6387	-0.98	-0.548	-0.7268	-0.9282	-0.7789	-0.7247	-0.6195	-0.7721
XM_577031.1	LOC501634	0.0162	0.24248	0.06526	0.12257	-0.0823	0.01111	0.25131	0.11087	-0.1124	0.1209	0.08923	0.33271	0.1247	0.20149
XM_577033.1	LOC501636	0.17629	0.13795	0.0245	0.24182	0.10052	0.17092	0.20139	0.00581	0.05421	0.09938	0.07198	0.17715	0.22438	0.00973
XM_577034.1	LOC501637	-0.6382	-0.1821	0.49727	0.63271	-1.1874	-1.1798	1.3978	-0.9182	-0.1175	-0.2276	-0.0115	-0.4328	-0.8955	-0.7207
XM_577038.1	LOC501641	-0.2715	-0.2013	-0.1924	-0.1746	-0.1637	-0.2387	-0.1945	-0.1926	-0.2696	-0.0674	-0.2355	-0.0143	-0.1796	-0.1681
XM_577039.1	LOC501642	-0.0323	0.1044	-0.0338	0.14151	-0.0586	0.01178	0.00042	-0.0287	0.14996	-0.023	0.01811	0.00938	0.10788	0.24384
XM_577040.1	LOC501643	0.09022	0.05723	0.15501	0.07044	0.11128	0.14244	0.10147	0.05223	0.13994	0.09648	0.01879	0.11698	0.30051	0.15315
XM_580223.1	LOC501646	-0.045	0.10206	0.00708	-0.0693	-0.0913	0.01139	0.17748	0.21282	0.0532	0.05623	0.04221	0.05791	-0.1013	-0.0378
XM_577044.1	LOC501648	0.01727	0.07329	-0.1257	0.09085	-0.1282	-0.0605	0.19486	0.24048	-0.1224	0.26676	0.09309	0.14073	0.10468	0.01468
XM_580224.1	LOC501649	0.1806	0.18586	0.17398	0.05213	0.0338	0.17799	0.19096	0.09687	0.05338	0.15992	0.24818	0.09043	-0.0739	0.10122
XM_577045.1	LOC501650	0.09549	0.06745	0.12172	0.12299	0.15352	0.21998	0.10137	0.13549	0.24473	0.24484	0.06543	0.18754	0.43862	0.09959
XM_577046.1	LOC501651	0.09771	0.13696	0.20883	0.18271	-0.0612	0.0781	0.34316	0.03154	0.18315	0.13967	0.11683	-0.0223	0.04533	-0.1063
XM_577047.1	LOC501652	-0.2621	-0.1163	0.13334	0.27088	-0.2108	-0.2737	0.4165	-0.1165	-0.0476	-0.1654	-0.1139	0.08545	-0.2865	-0.2362
XM_577049.1	LOC501654	-0.0402	-0.0074	-0.1314	-0.0503	-0.0864	0.03233	-0.123	-0.07	-0.1824	-0.0204	0.00016	0.1502	0.06665	-0.0608
XM_577051.1	LOC501656	0.08319	0.3197	0.0809	0.01919	0.10358	0.16821	0.23239	0.06798	0.29015	0.00934	0.00746	0.08196	-0.0031	0.20828
NM_001166576	LOC501659	-0.2485	-0.0759	-0.1925	-0.3142	0.03699	-0.3279	-0.0677	-0.1356	-0.2329	-0.0506	-0.3078	-0.1247	-0.195	-0.001
XM_580225.1	LOC501663	-0.039	0.11897	-0.0554	-0.0134	0.16799	-0.0329	0.18297	-0.0478	0.05124	0.25252	0.03059	0.18009	-0.1003	-0.0162
XM_577062.1	LOC501666	0.37688	0.33269	0.14079	0.11649	0.38771	0.2165	0.19857	0.43174	0.22533	0.24475	0.1687	0.12664	0.14478	0.17153
XM_577063.1	LOC501667	-0.1161	-0.1061	-0.2847	-0.1991	0.00957	0.24279	-0.0625	-0.0354	-0.2384	0.50815	-0.1147	-0.1806	0.15652	-0.0658

XM_577066.1	LOC501669	-0.0775	-0.1338	-0.0362	-0.0875	-0.0747	0.01621	0.0389	-0.0165	-0.0333	-0.0913	0.0028	-0.1587	0.04066	-0.0077
NM_001127377	LOC501670	0.02742	0.09587	0.05103	0.16469	0.14612	0.25816	0.07937	0.06067	0.04766	0.07355	0.03584	0.08606	0.24657	0.10545
XM_577068.1	LOC501671	-0.0571	-0.0067	-0.0359	-0.0218	0.09238	-0.0041	0.0121	0.07295	0.01364	0.05805	0.01337	-0.0666	-0.0228	0.05706
XM_577069.1	LOC501672	0.01992	0.2317	0.13232	0.0013	-0.0576	-0.0974	0.08234	0.09986	0.01321	-0.023	0.06489	0.03482	0.10867	-0.0605
XM_577070.1	LOC501673	-0.0642	-0.0462	-0.234	-0.2069	-0.2963	-0.3086	-0.327	-0.1963	-0.1765	-0.3344	-0.1247	-0.3062	-0.13	-0.2543
XM_577071.1	LOC501674	0.23354	0.07924	0.09206	0.0665	0.17307	0.03769	0.1341	0.13459	0.20261	0.09449	0.09275	0.05524	0.12603	0.30084
XM_577073.1	LOC501676	0.01205	-0.1451	0.09022	0.00809	0.03969	0.07838	-0.0012	-0.1092	0.07036	-0.0355	-0.1221	0.07272	0.06883	-0.021
XM_577075.1	LOC501678	-0.121	-0.1126	0.07799	-0.0975	-0.0546	0.05962	-0.0689	-0.1219	-0.1854	0.0052	0.06391	0.19147	0.0718	-0.1171
XM_220266.2	LOC501681	0.02044	0.02008	0.0657	0.11346	0.00889	-0.0049	0.03232	-0.0265	0.03015	0.01237	-0.0531	-0.1106	-0.1096	0.21267
XM_577081.1	LOC501684	0.05044	0.00373	-0.1479	-0.038	-0.0686	0.31199	-0.0105	0.10048	0.15856	0.15168	0.01343	0.3802	0.06229	0.01041
XM_577082.1	LOC501685	-0.174	-0.197	-0.2126	-0.2137	-0.1843	-0.1028	-0.006	-0.0646	0.14383	-0.2672	-0.0615	-0.21	0.0583	-0.088
XM_577092.1	LOC501691	-0.0959	-0.1314	-0.2873	0.08421	-0.3014	-0.3694	-0.0691	-0.3054	-0.2179	-0.4174	-0.3212	-0.3862	-0.2936	-0.356
XM_577093.1	LOC501692	0.14662	0.03026	0.03222	-0.0199	0.11955	0.00563	-0.0471	-0.058	0.17709	0.06456	0.12135	-0.0505	0.13863	0.08956
XM_577095.1	LOC501694	0.10248	0.0108	-0.0009	-0.0183	-0.028	0.02453	0.06462	0.06786	0.00887	0.00787	-0.0058	0.04419	-0.0642	0.05546
XM_577099.1	LOC501698	-0.0776	0.0487	-0.0679	0.03372	0.04449	-0.1436	-0.1013	-0.1397	0.12092	-0.0918	-0.137	0.10008	0.06065	0.00401
XM_577104.1	LOC501702	-0.0869	-0.1109	-0.0398	-0.1217	-0.0819	-0.0465	-0.053	-0.0093	-0.0049	0.01112	-0.0626	-0.0814	-0.1426	-0.1275
XM_577110.1	LOC501705	-0.3705	-0.3869	-0.1592	-0.2377	-0.4223	-0.4214	-0.3295	-0.3347	-0.3385	-0.4294	-0.3687	-0.3375	-0.3035	-0.3133
NM_001047972	LOC501706	-0.2072	-0.2413	-0.6963	-0.3419	-0.0168	-0.998	-0.3228	0.11477	0.2038	-0.1779	0.08534	-0.3488	0.31238	0.44519
XM_577112.1	LOC501707	0.05154	-0.0479	0.02655	0.04491	0.1801	0.11715	0.17983	0.1338	0.22311	0.36909	0.0823	0.06511	0.05451	0.09226
XM_577113.1	LOC501708	-0.2212	-0.1937	-0.1593	-0.0744	-0.0316	0.10058	-0.0722	0.18332	-0.1148	-0.0475	-0.0645	0.06064	0.05451	-0.1851
XM_577115.1	LOC501710	0.05397	0.07882	0.16962	0.09561	0.08287	-0.0025	0.18941	0.0662	0.28892	0.09702	-0.0501	0.07495	0.18362	0.01785
XM_577116.1	LOC501711	0.02784	-0.014	0.17543	-0.0368	0.04304	0.03024	0.08405	-0.0584	-0.0008	0.01585	-0.0218	0.14413	0.08059	-0.0233
XM_577117.1	LOC501712	-0.1228	-0.0515	-0.0438	0.04442	0.07715	-0.1305	0.00088	-0.0396	-0.1361	-0.0461	-0.1299	-0.0807	-0.0767	0.06686
XM_577118.1	LOC501713	-0.1419	-0.0918	-0.1884	-0.1363	-0.2334	-0.2319	-0.2425	-0.1717	-0.1486	-0.2127	0.0549	-0.193	-0.0661	-0.1781
XM_577121.1	LOC501716	-0.0659	-0.1783	-0.0742	-0.1789	-0.0555	-0.1171	-0.2565	0.08599	-0.2725	0.08385	-0.0919	0.01526	-0.2543	-0.2906
XM_580226.1	LOC501717	0.06345	0.07855	0.10583	0.09007	0.07532	-0.0047	0.04753	-0.0738	0.11941	0.23122	-0.051	0.04743	0.04615	0.07431
XM_580227.1	LOC501722	-0.2505	-0.295	-0.2363	-0.192	-0.3033	-0.2432	-0.2415	-0.2821	-0.1382	-0.2918	-0.2834	-0.0741	-0.0174	-0.2907
XM_580228.1	LOC501723	-0.0789	-0.4036	-0.3064	-0.3625	-0.1774	-0.4544	-0.3561	-0.421	0.01284	-0.1109	-0.4716	-0.3484	-0.1481	-0.3062
XM_580229.1	LOC501724	-0.0576	-0.0858	0.06873	-0.1806	-0.1515	-0.1683	-0.1304	-0.1023	-0.0038	-0.0338	-0.1275	-0.1784	-0.0667	-0.1182
XM_577130.1	LOC501731	0.0893	0.10342	0.10165	-0.0013	0.16872	0.21548	0.08066	0.08317	0.13937	0.06714	0.04204	-0.0082	0.08242	0.03143
XM_577131.1	LOC501732	0.47135	0.08881	0.14104	0.15066	0.10715	0.38829	0.09282	0.03934	0.11804	-0.0254	0.08358	0.0077	0.11247	0.19214
XM_577134.1	LOC501734	0.06821	0.09706	0.09055	-0.0103	0.00692	0.18679	0.01411	0.04258	0.08839	0.01783	0.2556	0.13883	0.23466	-0.013
XM_577137.1	LOC501736	0.03156	-0.1214	0.02803	0.05554	0.12965	-0.1269	-0.106	-0.0328	0.07764	-0.0671	-0.0045	0.00771	0.04345	-0.0391
XM_577138.1	LOC501737	0.22897	-0.0754	-0.1884	-0.1306	0.14043	-0.0581	0.11903	0.09314	0.05899	-0.0831	-0.1362	0.03576	-0.0645	-0.0906
NM_001167664	LOC501738	-0.111	-0.0045	0.02487	-0.1467	-0.1546	0.2044	0.14948	-0.022	-0.0493	0.04526	-0.0009	-0.1178	-0.0764	0.11399
XM_221091.3	LOC501740	-0.0112	-0.1364	-0.1178	0.05963	0.0058	0.17863	-0.0412	0.02303	0.14289	0.10932	-0.004	-0.0224	-0.095	-0.0641
XM_577144.1	LOC501743	0.15591	0.15376	0.0922	-0.1285	0.32085	0.04642	0.09965	0.03684	0.07239	0.18986	0.14873	0.08947	-0.0289	0.14603
XM_577145.1	LOC501744	0.05962	0.01259	0.01073	-0.0501	0.16637	0.12028	0.03982	-0.0368	-0.0735	-0.0615	-0.0523	-0.0459	-0.089	0.02898
XM_577146.1	LOC501745	0.01656	0.0717	0.21559	0.10018	0.04781	0.07348	0.04563	0.18438	0.10802	0.05344	0.16046	-0.021	0.18958	-0.006
XM_577147.1	LOC501746	-0.2161	0.3682	0.2443	-0.1971	-0.0385	-0.0054	0.07952	-0.2124	0.48176	0.1218	0.37542	0.44712	0.09412	0.13841
XM_577149.1	LOC501748	0.15064	0.12276	0.16514	0.12683	0.05299	0.41821	0.11198	0.19352	0.15734	0.23818	0.10425	0.13195	0.19567	0.1869
XM_577150.1	LOC501749	0.18883	-0.0004	0.42228	0.04065	0.19814	0.09265	0.00136	0.11616	0.11405	0.00888	0.06197	0.33402	0.40403	0.13412
XM_577151.1	LOC501751	-0.1541	-0.2358	-0.1322	-0.0499	-0.2448	-0.0883	-0.1159	-0.1566	-0.2398	-0.2006	-0.1529	-0.1819	-0.148	-0.1941
XM_577154.1	LOC501754	-0.066	0.02375	0.02499	-0.0258	-0.0048	-0.0239	0.37824	0.08861	0.01603	0.04638	0.06961	-0.0748	-0.044	-0.0087
XM_577155.1	LOC501755	0.02778	-0.0703	-0.115	-0.0144	-0.0849	-0.088	-0.031	-0.0811	0.00145	-0.011	0.00584	0.0535	-0.1094	-0.0945
XM_577156.1	LOC501756	-0.3268	-0.0818	-0.2838	-0.0346	-0.4338	-0.2943	-0.1723	-0.1986	-0.1332	-0.1724	-0.2886	-0.2579	-0.4014	-0.3275
XM_577158.1	LOC501758	0.04321	0.20497	0.1651	0.30838	0.36619	0.28146	0.19378	-0.0297	-0.0943	0.05418	0.19494	-0.0409	-0.1738	0.08223
XM_577160.1	LOC501760	-0.0011	-0.0187	0.03609	-0.0717	0.02122	-0.0512	0.06533	-0.0846	-0.0748	0.04665	-0.0336	-0.0308	0.04844	0.0362
XM_577161.1	LOC501761	-0.107	-0.0582	-0.1612	0.02756	0.08083	0.01791	-0.1276	-0.1218	0.02206	-0.0872	-0.1361	-0.0565	-0.158	-0.0419

XM_577162.1	LOC501762	0.17728	0.03223	0.01243	0.12957	-0.083	-0.0129	0.01327	-0.0219	0.07382	-0.0371	0.15734	-0.0702	-0.1084	-0.0873
XM_577163.1	LOC501763	-0.0035	0.02987	0.10229	-0.0217	0.03759	0.04829	0.01798	0.20444	0.10112	0.10675	0.10692	-0.0087	0.0253	0.08106
XM_577164.1	LOC501764	-0.0181	-0.0334	-0.0144	0.02201	-0.0326	0.05058	-0.0694	-0.0494	-0.0466	-0.0023	-0.0402	-0.0114	-0.0347	-0.0475
XM_580231.1	LOC501770	0.05471	-0.0215	0.08819	0.03883	0.10475	0.00957	0.05396	-0.0994	0.30969	0.19313	0.13143	-0.0183	0.07049	0.04162
XM_577170.1	LOC501771	0.14196	0.01141	-0.1368	0.06068	-0.1048	-0.1318	-0.0849	-0.077	-0.0977	-0.0605	-0.0401	-0.017	-0.1076	-0.1031
XM_577171.1	LOC501772	0.02614	0.1497	-0.0588	-0.0883	0.10723	-0.0094	0.0192	-0.0986	0.21839	-0.064	0.04494	0.10288	-0.1283	0.05451
XM_577172.1	LOC501773	0.08263	0.14576	-0.024	-0.0483	0.04364	0.0483	-0.072	0.20953	0.23123	-0.0029	0.05254	0.14817	-0.0393	-0.038
XM_577178.1	LOC501777	-0.0635	-0.268	0.03211	-0.2159	0.11264	-0.1779	-0.1302	-0.0331	0.0048	0.16275	0.17684	-0.0667	0.16312	0.06297
XM_577179.1	LOC501778	-0.0105	0.0533	-0.0636	-0.1043	0.02767	-0.0746	0.008	0.1048	0.1181	0.01364	-0.0225	0.0868	-0.0265	-0.0974
XM_577184.1	LOC501781	-0.0701	0.02523	-0.0858	0.13474	0.14436	-0.0745	0.21147	-0.0026	0.3084	0.00544	0.0344	0.15318	0.03775	0.25625
XM_577194.1	LOC501786	-0.0602	0.05092	0.08571	0.04381	0.01905	0.3869	-0.0488	-0.0778	0.21509	-0.0759	-0.0683	0.08724	-0.0176	0.14226
XM_577198.1	LOC501789	-0.1121	0.1295	-0.0061	0.11832	0.02851	0.02442	0.13593	0.05488	0.05744	0.15589	0.05418	0.16741	0.03887	-0.0472
XM_577199.1	LOC501790	-0.0349	0.12737	-0.0724	0.00674	0.00337	0.14117	0.02771	0.13505	-0.0549	-0.0456	0.10907	0.07652	0.03648	-0.0653
XM_577201.1	LOC501792	0.00089	-0.0495	-0.0048	-0.1379	0.07084	0.0604	-0.0883	0.07916	-0.0887	0.08342	0.08422	0.07644	0.09698	-0.0506
XM_577207.1	LOC501798	-0.0711	-0.1567	0.02573	-0.0808	-0.1231	-0.1258	0.02603	-0.1826	-0.1947	-0.0723	-0.1404	0.02489	-0.1076	-0.0605
XM_577211.1	LOC501803	0.15615	-0.0628	-0.084	-0.0382	0.00081	-0.1069	-0.0303	0.02775	0.03312	-0.1843	-0.0141	-0.1416	-0.0168	0.00586
XM_577212.1	LOC501804	-0.0363	-0.091	0.01242	-0.0658	-0.0287	0.0984	-0.017	0.03719	-0.0441	-0.0643	0.02025	0.03731	-0.0751	0.05167
XR_008976.1	LOC501810	0.07109	0.11893	0.01527	0.07019	0.01138	0.00702	0.13827	0.06555	0.14702	0.14801	0.0518	0.14215	0.1311	0.00472
XM_577230.1	LOC501818	-0.0443	0.00383	0.05117	-0.0605	0.01173	0.06501	-0.0289	0.13594	0.01701	-0.029	-0.0177	-0.0194	0.03225	0.11613
XM_577231.1	LOC501819	0.15217	-0.1948	0.01834	-0.0372	-0.1453	0.04589	-0.0791	-0.2045	-0.0625	-0.1622	-0.0119	-0.0529	-0.0953	-0.0842
XM_577233.1	LOC501821	-0.0618	-0.0401	-0.0356	-0.0312	0.05635	0.13497	-0.0685	-0.0167	0.15239	0.09112	-0.0916	0.0717	0.16014	-0.0077
XM_577235.1	LOC501823	0.05458	0.01233	0.00879	0.17827	-0.0114	0.04753	0.02014	0.05851	0.2415	0.04826	0.0475	0.25071	-0.0583	0.03487
XM_577236.1	LOC501824	-0.1745	-0.3325	-0.2264	-0.3614	-0.2122	-0.0723	-0.3279	-0.1087	0.16213	-0.1592	-0.2765	-0.1639	-0.0318	-0.3276
XM_577237.1	LOC501825	0.22307	0.05583	0.14036	0.46083	0.22999	-0.028	0.32452	0.03358	0.05077	0.28584	0.14973	0.33157	0.12565	0.0043
XM_577238.1	LOC501826	-0.0316	0.10999	0.0541	0.12809	0.15267	0.16307	0.06573	0.0016	0.08844	0.10412	-0.0092	0.07966	0.35372	0.0957
XM_577239.1	LOC501827	-0.1016	0.06461	-0.1238	-0.1583	-0.1459	0.0988	-0.0339	-0.2537	-0.3671	-0.215	0.24111	0.09186	0.09251	-0.1754
XM_577240.1	LOC501828	-0.4092	-0.3143	-0.1462	-0.3137	-0.2844	-0.1404	-0.2228	-0.3669	-0.0153	-0.3868	-0.2678	-0.2629	-0.3861	-0.3172
XM_577241.1	LOC501829	0.04682	0.06779	0.05033	0.09036	0.1125	0.04065	0.02702	0.45679	0.1222	0.07743	-0.0183	0.08961	0.05914	0.02663
XM_577242.1	LOC501830	-0.3998	-0.3657	-0.4246	-0.3733	-0.4139	-0.1924	-0.2164	-0.4994	-0.4522	-0.4015	-0.2671	-0.2641	-0.2214	-0.3177
XM_577244.1	LOC501832	0.33588	0.41351	-0.634	-0.4576	-0.1464	-0.3595	-0.0497	-0.214	0.08263	0.32841	-0.1822	0.01006	0.25285	0.26324
XM_577245.1	LOC501833	-0.0859	-0.1461	-0.1229	-0.049	-0.1957	-0.1123	-0.0438	-0.0141	0.12049	-0.2348	-0.0419	0.1604	-0.2014	-0.1166
XM_577250.1	LOC501837	-0.4059	-0.3912	-0.2523	-0.1898	-0.3113	-0.2405	-0.3573	-0.4303	-0.4185	-0.3009	-0.4382	-0.3497	-0.3469	-0.191
XM_577252.1	LOC501839	0.14195	0.11691	0.11219	-0.0121	0.06019	-0.0638	-0.0045	-0.0186	0.0703	0.04503	0.09872	-0.0573	-0.0871	-0.0529
XM_577256.1	LOC501843	0.00488	-0.1044	0.01411	-0.0589	0.0043	0.05068	-0.0736	0.07016	-0.1618	-0.1363	0.00285	-0.1081	-0.1601	-0.1659
XM_577257.1	LOC501844	0.08696	-0.018	0.14696	0.10552	0.29333	0.18721	0.02932	0.13967	0.08223	0.13095	0.12724	0.25364	0.14611	0.20362
XM_577258.1	LOC501845	-0.1111	-0.0183	-0.0963	-0.0359	0.02424	0.02471	-0.0329	-0.0748	-0.0444	-0.0537	0.12484	-0.0107	-0.0253	-0.0098
XM_577259.1	LOC501846	0.12801	0.10824	0.07484	0.11331	0.08831	0.10853	0.14611	0.04223	0.19692	0.08718	0.1371	0.17006	0.11526	0.1543
XM_577260.1	LOC501847	0.00805	0.13475	-0.0356	-0.0518	-0.0918	0.09019	0.06444	0.12092	0.11807	0.16151	-0.0359	0.05741	0.00265	-0.0243
XM_577263.1	LOC501850	0.07704	0.02514	0.01957	-0.0297	0.41403	0.02535	0.07025	0.24263	0.1982	0.01977	0.0627	0.14519	0.00161	0.08383
NM_001025732	LOC501852	0.07027	0.07258	-0.0198	0.13414	0.03269	0.13974	0.3546	-0.0682	0.05935	0.01667	-0.0651	-0.0671	0.11657	-0.0245
XM_577270.1	LOC501855	0.04938	-0.1249	0.02588	-0.0057	0.02725	-0.0162	-0.001	0.12897	-0.0742	-0.0795	-0.0562	0.1414	0.11809	0.03104
XM_577272.1	LOC501857	-0.0711	0.15997	0.19483	0.05946	-0.0989	0.14324	0.04297	-0.124	-0.1495	-0.2461	-0.0171	0.3031	-0.2138	0.1019
XM_580232.1	LOC501859	-0.0429	0.04137	0.12992	-0.0054	0.11287	0.00876	0.07872	0.20354	0.10776	0.16655	-0.0428	0.0253	0.05343	0.00075
XM_580233.1	LOC501860	0.10579	0.01476	-0.0709	0.25018	-0.0849	-0.0762	0.01624	-0.0051	-0.1309	-0.022	-0.0027	-0.1497	-0.1006	-0.0005
XM_577277.1	LOC501862	0.22265	0.1749	0.04001	0.12673	0.08228	0.23019	0.22459	0.12112	0.06521	-0.0699	-0.0008	-0.1051	0.18759	0.06884
XM_577279.1	LOC501864	-0.0016	0.02118	0.04891	0.01033	0.01157	-0.1174	0.01763	0.08876	-0.0721	0.15745	0.14306	0.04862	-0.0562	-0.0221
XM_577283.1	LOC501867	-0.1901	-0.3261	-0.2151	-0.3339	-0.2199	-0.3149	-0.3827	-0.3065	-0.3105	-0.3649	-0.0919	-0.1653	-0.3069	-0.2678
XM_577284.1	LOC501868	-0.0492	-0.1147	0.07017	0.04097	-0.0787	0.06921	-0.1152	0.0904	-0.0581	-0.0307	-0.1373	-0.0959	0.0345	-0.1036
XM_577287.1	LOC501870	0.17908	0.33782	0.33264	0.19282	0.35998	0.06256	0.07303	0.25042	0.17971	0.31077	0.22528	0.42427	0.26324	0.22651

XM_577288.1	LOC501871	0.00215	0.02881	0.01849	0.18802	0.06404	0.09719	-0.1052	-0.0289	0.02218	-0.1081	0.01374	-0.0015	-0.0624	-0.0162
XM_577292.1	LOC501873	0.18037	0.13738	-0.0275	0.15389	0.25075	0.26186	0.09185	0.31102	0.06526	0.39827	0.03299	0.24906	0.24216	0.06888
XM_577293.1	LOC501874	-0.0982	-0.1094	-0.1571	-0.1282	-0.1254	-0.0991	-0.118	0.07576	0.12766	-0.0743	-0.1034	-0.1728	-0.1089	-0.147
XM_577294.1	LOC501875	-0.0317	-0.0432	0.00849	-0.0714	0.15014	-0.0035	0.09162	0.09383	0.16581	-0.0341	-0.0838	-0.0985	0.06977	-0.1362
XM_577297.1	LOC501878	-0.2802	-0.2172	-0.02	-0.2069	-0.1989	-0.2364	-0.2866	-0.1677	-0.271	-0.2377	-0.2506	-0.2758	-0.2716	-0.2255
XM_577298.1	LOC501879	0.1037	0.04753	-0.0559	-0.0224	-0.0234	0.28011	-0.0023	-0.0639	-0.0065	0.05064	0.13753	0.16231	-0.0036	0.18644
XM_577303.1	LOC501884	-0.0168	-0.1219	0.04491	-0.0792	-0.134	-0.0628	-0.0671	0.0376	-0.1745	-0.0693	0.05995	-0.1065	-0.0125	0.07113
XM_577307.1	LOC501887	0.02646	-0.1253	0.26155	0.0593	0.33672	0.46203	0.02347	0.2497	0.17358	0.40122	0.0493	0.1137	0.26452	0.15482
XM_577308.1	LOC501888	0.09249	-0.0305	0.08682	0.01206	0.03137	0.05222	-0.0423	0.08784	0.01241	-0.1121	0.0675	0.22242	-0.067	-0.0288
XM_577313.1	LOC501890	-0.049	0.1282	0.0071	0.00227	0.07009	-0.1108	-0.0959	-0.1735	-0.0873	-0.1236	0.05697	-0.0102	0.0917	0.00901
XM_577314.1	LOC501891	0.16481	-0.0397	0.02932	-0.0361	-0.0319	0.3659	0.03548	0.09341	-0.0187	0.14701	-0.0581	0.09966	-0.0516	-0.0619
XM_577315.1	LOC501892	0.11093	0.07228	0.05937	0.08258	0.03944	0.16956	0.23787	-0.0205	0.15411	0.15549	0.7666	-0.1451	-0.054	0.27005
XM_577319.1	LOC501896	0.2	0.22755	-0.0447	0.13247	0.10511	0.18004	0.0874	0.28069	0.25945	-0.0044	0.46739	-0.0588	0.22778	0.15734
NM_001109329	LOC501899	0.01662	-0.0713	-0.0675	-0.0222	-0.1172	-0.1305	-0.1256	-0.0995	-0.041	-0.1499	-0.1222	-0.0958	-0.1002	-0.0315
XM_577326.1	LOC501902	0.32852	0.21955	0.27109	0.176	0.09068	0.1899	0.14519	0.16626	0.36055	0.30719	0.20873	0.1874	0.25086	0.27519
XM_577329.1	LOC501905	0.14178	0.07001	0.33891	0.10873	0.23973	0.1727	0.08941	0.09679	0.176	0.20795	0.17081	0.22327	0.13553	0.39333
XM_577330.1	LOC501906	-0.0942	-0.0977	-0.1921	0.32098	0.12939	0.09701	-0.0362	-0.0776	-0.2224	0.07515	-0.0316	0.04063	-0.107	0.08571
XM_577332.1	LOC501908	-0.1449	-0.1373	-0.1303	-0.0457	-0.1069	-0.0864	-0.1066	-0.2035	-0.1336	0.10636	-0.0342	-0.0631	-0.1465	-0.0748
XM_577335.1	LOC501911	-0.0458	-0.0191	-0.0902	-0.0752	0.09406	0.00375	-0.055	0.03171	-0.0217	-0.0532	0.18074	-0.0481	-0.0144	-0.0188
NM_001134639	LOC501923	0.09983	-0.05	0.01439	-0.0737	-0.0303	-0.0338	-0.079	-0.0616	-0.0618	-0.1303	0.13962	-0.0477	-0.1661	-0.0438
XM_001062303.1	LOC501925	-0.0156	0.049	0.03457	0.07934	-0.0499	-0.0245	0.14902	-0.0092	0.00395	-0.1162	-0.0271	-0.0878	0.08081	0.1211
XM_577352.1	LOC501927	0.13193	0.09707	0.00957	0.0777	-0.014	0.04801	0.00147	0.00842	0.13464	0.03371	0.19304	-0.0046	0.18078	0.05556
XM_577353.1	LOC501928	-0.1467	-0.0535	-0.2628	-0.0707	0.05629	-0.0084	-0.1801	-0.1884	-0.1225	0.07203	-0.1104	-0.0793	0.0069	-0.0987
XM_577355.1	LOC501929	-0.0448	0.25821	0.11185	0.02698	0.01885	0.13513	-0.0855	-0.0058	0.19703	0.07326	0.00806	0.0727	-0.0291	-0.0933
XM_577357.1	LOC501931	-0.2639	-0.2944	-0.2294	-0.3192	-0.1934	-0.3558	-0.2148	-0.0031	-0.3268	-0.2259	-0.2946	-0.1996	-0.299	-0.1397
XM_577358.1	LOC501932	0.03725	-0.1356	-0.0914	-0.1235	-0.0559	-0.1281	-0.0049	-0.1329	-0.0871	0.2117	-0.0521	-0.0253	-0.048	-0.0846
XM_577360.1	LOC501934	0.00843	0.20553	0.18826	-0.5104	0.10755	0.31077	0.05056	-0.1303	0.16229	0.21171	0.38173	0.37934	0.66101	0.5618
XM_577361.1	LOC501935	-0.026	0.14161	0.02827	-0.0683	-0.137	0.00625	0.02818	-0.0698	0.1221	0.03645	-0.0085	-0.0278	0.05621	-0.0131
XM_577362.1	LOC501936	-0.052	-0.0443	-0.0294	-0.0852	-0.1292	0.1573	0.12423	0.28629	-0.0711	0.17147	-0.136	0.14909	0.03909	0.18531
XM_577363.1	LOC501937	0.01176	0.01039	0.13303	0.07015	0.1495	0.19333	0.08816	-0.0205	0.11633	0.08284	0.12572	-0.0001	0.14061	0.06096
XM_577366.1	LOC501940	0.1707	0.09268	0.26247	0.24904	0.16057	0.15497	0.02146	0.09577	0.00791	0.12908	0.06505	0.31049	0.22191	0.24915
XM_577369.1	LOC501943	0.02512	-0.0528	-0.0434	-0.022	0.14877	0.01449	-0.0131	0.03172	0.03631	0.00724	-0.0357	0.01021	0.09629	0.26105
XM_577375.1	LOC501948	-0.0121	-0.0955	0.0932	0.1594	0.08274	-0.0244	0.16313	-0.0608	0.00662	-0.0166	-0.0587	0.08598	0.03853	0.04275
XM_577376.1	LOC501949	0.0745	0.11969	0.11297	-0.0381	0.19409	-0.0121	-0.0295	-0.1538	0.17934	0.14917	0.18576	0.20086	-0.1071	-0.1205
XM_577377.1	LOC501950	-0.2041	-0.3038	-0.1557	-0.1118	-0.253	0.21761	-0.1054	-0.076	-0.049	-0.1941	-0.1971	-0.1736	0.00515	-0.2972
XM_577380.1	LOC501953	0.07163	-0.0355	0.03574	0.10031	0.20894	0.03129	0.01456	0.00898	0.07826	0.06774	0.29079	0.0504	0.24866	0.04819
XM_577381.1	LOC501954	0.10777	-0.0112	0.01317	0.20253	0.02229	0.15775	0.22838	0.16141	0.03827	-0.0537	0.0202	0.20591	0.08804	0.06898
XM_577382.1	LOC501955	0.0756	0.04837	-0.1396	-0.1861	-0.0648	-0.1538	0.02441	-0.1678	-0.1791	-0.1775	0.16837	-0.1374	-0.1032	-0.2121
XM_577384.1	LOC501957	0.06326	0.02585	0.12257	-0.015	0.00214	0.11628	0.12497	-0.0088	0.25999	0.01851	-0.0481	0.20798	-0.02	0.06497
XM_577390.1	LOC501962	0.20524	0.04606	0.0819	0.02439	-0.0399	0.32461	0.05703	0.09561	-0.0309	0.11349	0.03269	0.23696	0.28419	0.01616
XM_577392.1	LOC501964	0.10583	0.18163	0.16004	0.03891	0.24343	0.10083	0.32558	0.01489	0.07849	0.21445	0.22479	0.14148	0.11834	0.24374
XM_577393.1	LOC501965	-0.0243	-0.0578	-0.1688	-0.2015	-0.0354	-0.2011	0.18092	0.04883	-0.132	-0.3024	-0.211	-0.057	0.2134	0.05087
XM_577396.1	LOC501968	0.07628	-0.0257	0.07157	0.08416	-0.0002	#######	0.01314	0.20513	0.03925	0.1739	0.12127	0.03317	0.09507	0.174
XM_577399.1	LOC501971	-0.0539	-0.042	0.27331	-0.0913	0.03543	0.22991	-0.0133	0.08887	-0.0705	0.06225	0.13867	-0.0733	-0.0202	-0.1488
XM_577400.1	LOC501972	0.18546	-0.0711	-0.2029	-0.1565	-0.0859	-0.296	0.01076	0.03104	-0.1142	-0.2053	-0.185	-0.2199	-0.094	-0.3171
XM_577401.1	LOC501973	-0.0291	-0.0586	0.07509	-0.0968	-0.1345	0.04077	-0.1554	-0.0498	-0.0141	0.1827	-0.0338	0.05078	0.01741	-0.0153
XM_577402.1	LOC501974	-0.1325	-0.1502	0.0505	-0.1791	-0.1981	-0.0341	-0.1264	-0.0575	-0.1666	0.04069	-0.1239	-0.2223	-0.1799	0.02173
XM_577404.1	LOC501976	0.01455	-0.0467	-0.0525	0.00808	0.0386	0.054	-0.0236	-0.0699	0.03252	-0.0436	0.08362	0.08262	-0.085	-0.0138
NM_053985	LOC501977	-0.1666	-0.1431	-0.0184	-0.037	-0.2069	-0.0034	-0.2223	0.01372	0.07101	0.05476	-0.0367	-0.0692	-0.0551	-0.0024

XM_577408.1	LOC501979	-0.1772	-0.2532	0.06793	-0.1439	-0.0767	0.18782	-0.0596	-0.1729	-0.0514	-0.2504	-0.1304	-0.0466	-0.3499	-0.23
XM_577410.1	LOC501981	0.18209	-0.0306	-0.1363	0.22235	-0.0088	0.13459	0.13033	-0.0168	0.00044	-0.0466	0.0098	0.26219	-0.1337	0.09968
XM_577411.1	LOC501982	-0.064	-0.0081	-0.107	0.07343	-0.1395	-0.1168	0.08738	-0.0384	0.023	-0.0763	-0.0496	0.01762	0.06013	0.03866
XM_577412.1	LOC501983	-0.0108	-0.005	-0.0527	-0.0843	0.0361	0.09476	0.06708	0.02133	-0.1122	-0.1192	-0.1177	-0.1366	0.06141	-0.0616
XM_577413.1	LOC501984	0.09961	0.01155	-0.0503	0.10032	0.04801	-0.0464	-0.012	0.19363	-0.0351	0.14336	0.06199	-0.0391	-0.0842	-0.0049
XM_577414.1	LOC501985	0.03946	0.0161	0.0603	-0.0456	0.00911	0.10335	-0.1007	0.00166	0.17307	0.05099	-0.002	-0.0621	0.00919	0.23909
XM_577415.1	LOC501986	-0.029	-0.0825	-0.1323	-0.0349	-0.1081	-0.2258	-0.1942	-0.2025	-0.2902	-0.3195	-0.3345	0.03749	-0.2188	-0.3129
XM_224104.3	LOC501987	-0.1445	-0.0537	0.11043	0.05063	0.091	0.0099	-0.0521	-0.1221	-0.1054	0.42415	-0.0923	-0.0177	0.04659	-0.0208
XM_224104.3	LOC501987	0.04223	-0.022	-0.0422	-0.0288	0.12449	-0.0501	0.04065	0.0693	0.03946	-0.0082	-0.0287	0.00465	0.00196	0.0363
XM_577416.1	LOC501988	-0.0773	-0.0825	-0.0467	0.01627	0.05665	-0.0863	0.07103	0.0958	0.23592	0.07663	0.25941	0.0458	-0.1362	-0.0267
XM_344365.2	LOC501989	0.17019	0.17096	-0.1219	0.03203	0.09283	0.01115	-0.0552	-0.1279	0.17512	0.15185	-0.0663	-0.0044	0.0163	0.21673
XM_577418.1	LOC501990	-0.1017	-0.1284	-0.0389	-0.0523	0.12339	-0.1542	0.04918	-0.2853	-0.0187	-0.0968	-0.3175	0.371	-0.2461	-0.2238
XM_577420.1	LOC501992	0.03043	0.01622	-0.0129	0.0744	-0.057	0.5618	-0.0069	0.04222	-0.0289	0.00613	0.20369	0.10261	0.08022	-0.046
XM_577420.1	LOC501992	0.10065	0.3175	0.20825	0.25038	0.24332	0.2625	0.35427	0.21203	0.28252	0.41093	0.50357	0.34252	0.20353	0.215
XM_344391.1	LOC501993	-0.2218	0.24365	0.04107	0.28473	-0.1493	-0.075	0.01433	-0.1875	-0.125	0.06476	0.0024	0.12118	-0.1337	-0.1823
XM_577421.1	LOC501994	-0.1853	-0.1293	-0.1857	-0.1291	-0.1567	-0.2645	-0.1811	0.01363	-0.1195	-0.0209	-0.1455	-0.1891	-0.1756	-0.303
XM_577422.1	LOC501995	-0.0757	-0.0717	-0.0892	0.1213	-0.1426	-0.1277	-0.0195	-0.0125	-0.1042	-0.0643	0.01235	-0.0812	0.17123	-0.0083
XM_577424.1	LOC501997	0.05232	0.15326	-0.0907	-0.0156	-0.0491	-0.0753	-0.0324	-0.002	-0.0444	-0.1145	-0.016	0.00416	-0.0082	0.16677
XM_577426.1	LOC501999	-0.1747	-0.0186	-0.0484	-0.1739	-0.2327	0.02662	-0.093	-0.0909	-0.1929	-0.3282	0.11375	0.27419	-0.072	-0.0792
XM_577427.1	LOC502000	0.34553	0.25515	0.32983	-0.0049	0.23112	0.20497	-0.0198	-0.0052	0.04794	0.08682	0.03586	-0.0559	0.14937	0.01161
XM_577428.1	LOC502001	-0.14	-0.0555	-0.1242	-0.131	-0.2023	0.1124	0.03129	-0.1672	-0.1034	0.25915	-0.3339	-0.0439	0.16051	-0.1455
XM_577431.1	LOC502004	0.29788	0.32047	0.15716	0.06377	0.16503	0.16544	0.17875	0.15934	0.01238	0.30177	0.17483	0.12804	0.08937	0.13412
XM_577433.1	LOC502005	-0.087	0.09705	0.16322	0.41428	0.0577	0.378	0.23368	-0.0332	0.04341	0.19271	0.18904	0.14286	0.25591	0.09763
XM_577434.1	LOC502006	0.59345	-0.0548	0.07236	-0.0653	0.39406	-0.055	0.0197	0.4722	0.19751	-0.2535	0.17729	0.07705	-0.1365	0.00024
XM_577437.1	LOC502009	0.00876	0.16713	0.15778	0.04835	-0.0828	-0.0706	-0.0098	-0.0587	0.08978	0.11153	-0.0166	-0.0832	-0.0007	-0.0757
XM_577438.1	LOC502010	0.05004	-0.0575	-0.1579	-0.0535	-0.0298	0.01336	-0.1022	-0.1699	-0.1677	0.03726	0.02734	-0.1812	-0.111	-0.0524
XM_577439.1	LOC502011	-0.0788	0.02744	-0.0461	0.11736	-0.1044	-0.0879	0.04354	-0.0629	0.07647	-0.1747	-0.1153	-0.0936	0.03577	-0.0817
XM_577440.1	LOC502012	0.22383	0.1917	0.15132	0.1444	0.16646	0.1138	0.12407	0.1631	0.13863	0.03585	0.14101	0.1861	0.07311	0.12687
XM_577441.1	LOC502013	-0.0768	0.15738	0.12824	0.09	0.00491	0.22765	0.10322	0.20779	0.39922	0.06972	0.16506	0.1535	0.3149	0.19149
XM_577442.1	LOC502014	0.02415	0.01326	0.12755	0.05343	0.02101	-0.0086	-0.0121	0.1457	-0.0514	0.03051	0.21128	-0.0001	0.02189	0.06953
XM_577443.1	LOC502015	-0.0606	0.09552	-0.0422	-0.0821	-0.0068	-0.095	-0.1378	0.13314	-0.1105	-0.1495	-0.0388	-0.0307	-0.161	0.09432
XM_577446.1	LOC502018	-0.1813	-0.1553	-0.1305	0.00036	-0.1166	-0.1993	-0.1334	-0.2195	-0.0397	-0.0076	-0.2224	-0.1498	-0.1127	-0.1947
XM_577447.1	LOC502019	0.05494	0.05671	0.04269	0.00584	0.14704	0.04497	0.07537	0.06444	-0.0366	-0.0469	0.06896	0.20536	0.07475	0.00091
NM_001134727	LOC502020	0.09314	0.07407	0.01422	0.1871	0.14925	0.09169	-0.0075	0.00169	0.33235	0.10666	0.12473	0.07778	0.09003	-0.0649
XM_577449.1	LOC502021	0.008	0.03121	-0.0052	0.18525	-0.0129	0.01551	-0.0008	0.13005	0.22147	0.47099	0.11617	-0.0051	0.17693	0.0269
XM_577459.1	LOC502028	0.08752	0.30535	0.00748	0.07743	-0.0348	-0.0395	0.00089	-0.0666	0.07455	0.04357	0.05023	0.23387	-0.0697	-0.0216
XM_577463.1	LOC502031	-0.0912	-0.0896	-0.2066	-0.1108	-0.1137	-0.1742	-0.1571	-0.1756	-0.1361	-0.1375	-0.0133	0.08036	-0.1922	-0.2569
XM_577464.1	LOC502032	-0.1154	-0.0998	0.04337	-0.1004	0.03205	0.17153	-0.2257	-0.0567	-0.2132	-0.1214	-0.0386	-0.0857	-0.0883	0.10563
XM_577465.1	LOC502033	-0.0012	-0.2894	-0.1396	-0.1112	-0.2281	-0.1824	-0.0762	-0.2435	-0.2002	0.03572	-0.0562	-0.2068	-0.1731	-0.2025
XM_580234.1	LOC502034	-0.1038	-0.1451	-0.1461	-0.0908	0.01507	-0.0882	-0.1515	-0.0133	-0.1056	0.04207	-0.1291	-0.059	-0.1075	-0.1312
XM_577466.1	LOC502035	0.10244	0.05735	-0.0607	0.00566	0.15614	-0.0527	-0.0565	-0.1133	0.0671	0.07368	0.26448	-0.0732	-0.0253	#######
XM_577468.1	LOC502036	-0.5179	-0.0294	-0.2005	-0.1989	-0.4463	-0.3891	-0.2233	-0.2488	-0.2429	-0.3743	-0.1418	-0.3776	-0.0292	-0.4611
XM_577470.1	LOC502038	-0.1271	0.09971	0.05692	0.0575	0.09335	-0.0188	-0.0178	-0.0071	0.08152	0.20089	-0.03	-0.1765	0.16673	0.13823
XM_577471.1	LOC502039	-0.0073	-0.0646	0.08151	-0.1091	-0.0649	-0.0921	0.02494	-0.0213	0.12099	-0.0555	0.16979	-0.0253	-0.034	0.03541
XM_577473.1	LOC502040	0.16399	0.09338	-0.0533	0.08969	0.05437	-0.036	0.2339	0.37312	0.18569	0.32744	0.24618	-0.0142	0.12998	0.08647
XM_577474.1	LOC502042	-0.0115	0.00224	0.08204	-0.0405	0.0752	0.2043	0.0563	0.22262	0.04784	-0.0274	-0.0899	-0.1574	-0.015	0.10673
XM_577475.1	LOC502043	-0.1303	0.14315	-0.1649	-0.0169	-0.1711	-0.023	-0.04	-0.1341	-0.0734	-0.1129	-0.0842	-0.1155	0.4192	-0.088
XM_577478.1	LOC502045	-0.3503	-0.435	-0.1664	-0.2137	-0.3643	-0.2687	-0.316	-0.0732	-0.3096	-0.5474	-0.4343	-0.2169	-0.2151	-0.4478
XM_577481.1	LOC502048	-0.0744	-0.0729	-0.0454	-0.085	-0.074	-0.0369	-0.024	-0.0243	-0.0652	-0.0308	0.09458	-0.0951	0.02878	0.00023

XM_577482.1	LOC502049	-0.2069	-0.1487	-0.1317	-0.0605	-0.1157	0.01013	-0.1572	-0.095	-0.0851	-0.0505	-0.1214	-0.0808	-0.0045	-0.176
XM_577483.1	LOC502050	-0.0814	-0.1483	-0.1862	0.07771	-0.1094	#######	-0.0785	-0.0394	0.12118	0.13734	-0.0273	-0.1182	-0.1039	-0.1483
XM_577484.1	LOC502051	-0.16	0.04357	-0.1587	-0.1421	-0.1277	0.04958	-0.0953	-0.0379	-0.2001	-0.0966	-0.1259	0.02011	-0.1377	-0.0598
XM_577485.1	LOC502052	0.23004	0.11093	0.01993	0.13915	0.07938	0.17611	0.14089	0.06906	0.15036	0.27751	0.11613	0.20309	0.35062	0.15995
XM_577486.1	LOC502053	0.07106	0.11706	-0.0408	0.02338	-0.0851	0.13949	-0.0336	0.23743	-0.0356	0.13726	-0.0415	0.01359	0.06047	-0.0523
XM_577490.1	LOC502057	-0.1414	0.0374	0.01068	-0.0897	-0.105	0.02716	-0.0993	0.08138	-0.0754	0.13743	-0.123	0.07844	-0.004	-0.0064
XM_577491.1	LOC502058	0.05721	-0.0382	0.0666	-0.0519	-0.0783	0.3515	-0.0296	-0.1225	0.0869	-0.0095	-0.0344	-0.0174	0.03428	-0.0089
XM_577492.1	LOC502059	0.03586	0.07475	-0.1208	-0.0765	-0.0463	-0.0119	-0.0678	0.02722	0.09164	-0.1203	0.27868	0.03727	-0.1173	-0.1256
XM_577493.1	LOC502060	0.03136	0.13891	0.08415	-0.053	-0.0126	0.11531	-0.0549	0.1577	-0.0723	0.01673	-0.0389	0.13645	-0.0349	0.03234
XM_577496.1	LOC502063	-0.274	-0.1107	-0.1949	-0.2455	-0.4711	-0.4694	-0.2329	-0.3171	0.061	-0.3552	-0.2677	-0.15	-0.2525	-0.328
XM_577499.1	LOC502066	0.05192	0.03622	0.08089	0.10133	0.08095	0.09213	0.26811	0.01503	-0.0703	0.07932	0.0528	0.12736	0.15495	0.30056
XM_580236.1	LOC502067	-0.0972	-0.1776	-0.0804	-0.0931	-0.0529	-0.0157	-0.1125	-0.2909	-0.0832	-0.1904	-0.2264	0.01479	0.0173	-0.1321
XM_580237.1	LOC502068	0.1999	0.06567	0.06383	0.19797	0.04583	0.06064	0.10548	0.01552	0.24381	0.05728	0.1958	0.09067	-0.0065	0.19364
XM_577501.1	LOC502069	-0.0223	0.01103	-0.019	-0.0153	0.00684	-0.0612	0.00514	-0.0824	0.00936	0.09187	0.04276	-0.0264	0.07541	-0.0788
XM_577503.1	LOC502071	-0.0253	-0.1637	-0.141	-0.0983	-0.2088	-0.0708	-0.1515	-0.0255	0.14096	-0.0128	-0.1071	-0.1564	-0.0538	0.08922
XM_577507.1	LOC502073	0.09727	0.00932	0.03597	-0.01	0.08908	0.05632	0.10358	0.07493	0.02673	0.0612	0.07491	0.09793	0.12706	0.05122
XM_577508.1	LOC502074	-0.1112	-0.0563	-0.0917	-0.0163	-0.0543	0.20839	-0.1618	-0.0258	-0.1528	-0.1055	-0.0429	-0.0385	-0.124	0.0638
XM_577509.1	LOC502075	0.01982	-0.0225	-0.0163	-0.0478	-0.0337	0.04033	0.12849	0.16528	-0.0041	-0.04	-0.0634	0.14401	-0.0126	0.33315
XM_577511.1	LOC502077	0.17416	0.29902	0.11632	-0.0801	0.00255	0.23225	-0.0197	0.08971	0.03963	0.18944	-0.117	0.02378	0.21487	0.50128
XM_577515.1	LOC502080	0.01335	-0.0744	0.01955	-0.0995	-0.0084	0.03837	-0.0171	-0.1409	-0.1536	0.01019	-0.0521	-0.0054	0.16393	-0.0665
XM_577516.1	LOC502081	0.01829	0.03117	-0.0763	0.06856	-0.0554	-0.0541	0.3291	-0.1133	0.28593	-0.0728	0.09636	0.05032	-0.1318	-0.0927
NM_001034994	LOC502084	0.14954	-0.1131	-0.0696	0.37505	0.12151	-0.2041	-0.1672	0.00305	0.02769	0.09527	0.10384	0.45395	-0.0033	0.24047
XM_577524.1	LOC502087	-0.0128	0.03455	0.01426	0.08165	-0.0478	-0.078	-0.1164	-0.1217	-0.1198	-0.1017	-0.0816	0.24616	-0.0421	0.19444
XM_577525.1	LOC502088	0.08938	0.16923	0.40794	0.11799	0.09704	0.266	0.19467	0.4438	0.41565	0.5327	0.15197	0.26939	0.20837	0.16231
XM_577528.1	LOC502090	-0.0308	0.04901	0.04191	-0.0336	-0.0605	-0.0239	0.01799	0.01469	0.08093	-0.0766	-0.0269	-0.0434	-0.0731	-0.0277
XM_577532.1	LOC502092	-0.0216	0.22016	0.08982	-0.0959	-0.0299	0.08236	-0.0111	-0.0373	0.05417	-0.1039	0.01799	0.26115	-0.0063	0.1759
XM_344535.1	LOC502094	0.32371	0.28931	0.24171	0.132	0.17795	0.06706	0.07978	0.08323	0.13523	0.22382	0.24	-0.0352	0.30636	0.08176
XM_577536.1	LOC502096	0.13268	0.20315	-0.1721	-0.0216	-0.0901	0.1733	0.076	0.24849	0.18683	0.18366	0.00346	0.30244	0.23585	0.01475
XM_577538.1	LOC502098	-0.1917	-0.1072	-0.0157	0.10948	0.06687	-0.0495	-0.0719	0.06672	-0.1332	0.08978	-0.1746	-0.1393	-0.0763	-0.1219
XM_577539.1	LOC502099	0.03573	0.0272	-0.0721	0.12291	0.08382	0.02119	0.00084	-0.0376	0.04057	0.15691	0.05739	0.13158	-0.0136	0.02761
XM_577540.1	LOC502100	-0.095	0.09779	0.0424	-0.0984	-0.0675	0.06256	0.06165	0.08364	-0.0633	-0.0818	0.04756	-0.084	-0.1091	0.1608
XM_577541.1	LOC502101	0.00296	-0.0121	-0.0024	-0.0153	0.24519	-0.1296	0.03239	0.16094	-0.0456	-0.0054	-0.0287	-0.0784	-0.0459	-0.0376
XM_577542.1	LOC502102	0.09963	0.00783	0.00169	0.06011	0.05866	0.12631	0.02325	0.13516	-0.0734	0.27228	0.18126	0.03431	0.14204	0.09744
XM_577544.1	LOC502103	-0.076	-0.074	-0.0693	-0.0939	0.09896	-0.0777	-0.0885	-0.0336	-0.0554	0.03778	-0.1064	-0.0405	-0.0911	-0.1048
XM_577548.1	LOC502105	-0.0105	-0.0354	0.16041	0.23888	-0.041	0.05678	0.1098	0.19919	0.27069	0.09846	0.03546	0.06712	0.33639	0.35429
XM_577549.1	LOC502106	-0.2424	-0.1153	-0.0013	-0.0165	0.03737	0.02473	-0.0233	-0.0865	0.06147	-0.039	-0.0092	-0.0246	0.33754	-0.0929
XM_577550.1	LOC502107	0.19838	-0.0291	0.19225	0.22123	0.03355	0.14413	0.23837	-0.0105	-0.0369	0.02003	0.17048	0.18215	0.13655	0.02037
XM_580238.1	LOC502108	-0.2126	-0.3375	-0.1155	-0.2791	-0.2073	-0.2376	-0.2745	-0.2251	-0.2449	-0.2375	-0.2243	-0.1851	-0.2387	-0.2445
XM_577551.1	LOC502109	-0.1477	-0.2042	-0.1765	-0.2247	-0.2813	-0.1984	-0.0459	-0.167	-0.3507	-0.1708	-0.3036	-0.3918	-0.2648	-0.375
XM_577553.1	LOC502112	-0.1424	-0.0866	-0.042	-0.1104	0.03194	0.15659	-0.0045	-0.0165	-0.1104	-0.0736	-0.1058	-0.017	-0.017	0.00354
XM_577556.1	LOC502114	0.11869	-0.1403	-0.1287	-0.0959	-0.0548	-0.0833	-0.21	-0.0291	-0.0127	-0.0949	-0.0614	0.04422	-0.0312	-0.0541
XM_577558.1	LOC502116	0.14882	0.3527	0.00607	0.24188	-0.0294	0.06084	0.15895	0.26911	0.15659	0.05595	0.24247	-0.1062	-0.11	0.12834
XM_577559.1	LOC502117	-0.0264	-0.2064	-0.208	-0.1211	-0.108	-0.2082	-0.058	-0.0126	-0.1921	-0.0314	-0.3024	-0.1319	-0.0704	-0.1582
XM_577564.1	LOC502119	0.18373	0.23136	0.09191	-0.058	-0.1222	0.30835	-0.0112	0.03801	0.11869	0.1298	0.17673	0.05444	0.05249	0.1312
XM_577569.1	LOC502121	-0.0596	0.05174	-0.102	-0.1392	-0.0741	-0.1353	-0.0139	-0.0154	-0.0779	0.17226	-0.0588	0.11062	-0.1612	0.04023
XM_577572.1	LOC502124	0.15191	0.03674	0.22477	0.14598	0.19439	0.17994	0.24962	0.13347	0.30908	0.14563	0.04497	0.06531	0.32386	0.12897
XM_577574.1	LOC502126	-0.0887	0.03453	-0.0186	0.01318	0.06	0.09665	-0.0135	-0.0862	-0.1973	-0.0674	-0.0455	-0.0351	0.01474	-0.0149
XM_577575.1	LOC502127	-0.0464	-0.0942	-0.0258	-0.1449	0.02703	-0.1941	0.07318	-0.0772	0.00645	-0.1292	0.12025	-0.0128	-0.1121	-0.0751
XM_577582.1	LOC502131	0.02514	-0.1454	-0.1705	-0.1813	-0.1276	-0.0827	-0.1607	-0.1683	-0.2015	-0.1375	-0.1241	-0.1347	-0.1225	-0.0143

XM_577583.1	LOC502132	-0.0072	0.08224	-0.0369	-0.0401	0.04491	0.14541	-0.0125	0.00799	0.0046	0.07615	0.02329	-0.0534	0.05883	0.06612
XM_577589.1	LOC502137	-0.0521	0.03734	0.06366	-0.0684	0.04678	-0.0597	-0.0626	-0.0275	0.0201	0.01644	-0.0797	0.1461	-0.0995	-0.1308
XM_577590.1	LOC502138	-0.1246	-0.1124	0.09071	-0.1012	-0.1302	-0.1705	-0.1078	-0.1612	-0.1648	-0.0317	-0.0514	-0.0221	-0.1293	-0.1576
XM_577591.1	LOC502139	-0.1273	-0.1658	-0.1561	-0.0872	-0.1576	-0.1755	-0.0683	-0.1827	-0.161	-0.0545	-0.0179	-0.0416	-0.0228	-0.1548
XM_577592.1	LOC502140	0.14175	0.07971	0.16193	-0.02	0.04309	-0.0089	0.22107	-0.0167	0.04305	0.12447	0.27379	-0.0701	0.22428	0.1484
XM_577598.1	LOC502145	-0.1382	0.00844	-0.3142	-0.1377	-0.258	0.43742	-0.3008	-0.26	-0.2264	0.2445	-0.0708	0.04953	0.01407	-0.3028
XM_577599.1	LOC502146	-0.0453	-0.0715	0.02157	0.09917	-0.0715	-0.017	0.01863	-0.1679	-0.1876	-0.0393	-0.2015	-0.1481	-0.2272	-0.1416
XM_577601.1	LOC502147	0.14467	0.22497	0.07704	0.12251	0.15053	0.05599	0.13233	0.28011	0.01333	0.19302	0.03217	0.12577	0.46327	0.26727
XM_577605.1	LOC502150	-0.0426	0.20718	-0.013	0.106	0.09765	0.01339	0.04898	-0.0521	0.20523	-0.0195	0.09324	0.03571	0.05029	0.00038
XM_577615.1	LOC502159	0.11606	0.17918	0.13876	0.28005	0.10791	0.21081	0.07276	0.16329	0.1076	0.15674	0.16415	0.12921	0.36425	0.11185
XM_577618.1	LOC502161	-0.1298	-0.0734	-0.0884	-0.0536	-0.1851	-0.005	-0.1509	-0.1568	-0.1329	-0.1181	-0.1527	-0.1904	-0.188	0.0818
XM_577619.1	LOC502162	0.01612	0.00529	0.23499	0.14244	-0.0396	0.0038	-0.0043	0.17943	-0.0354	0.04939	-0.0515	0.00799	-0.0756	0.05353
XM_577621.1	LOC502164	-0.0638	-0.0822	-0.0962	-0.089	0.08953	0.18132	-0.0074	-0.0354	0.07608	-0.096	0.14486	-0.0105	-0.0666	0.17088
XM_577625.1	LOC502168	-0.0421	0.15782	0.08018	-0.0788	-0.078	-0.0454	-0.0128	-0.0311	0.01083	0.13386	-0.0061	-0.028	-0.0741	-0.0246
NM_031826	LOC502170	-0.2604	-0.0421	0.03662	-0.126	-0.1298	-0.1111	-0.0226	-0.0915	-0.0417	-0.219	-0.2426	-0.292	-0.0652	-0.1235
XM_577630.1	LOC502173	-0.1738	-0.129	0.05882	0.13573	-0.1032	0.09738	-0.1375	0.11506	-0.0215	-0.0396	0.04472	0.03005	0.07638	0.00267
NM_001025057	LOC502176	0.05418	0.09685	-0.0232	0.02974	0.0832	-0.0002	-0.0339	-0.0326	0.12361	0.15607	0.17369	0.48404	0.05282	0.44015
XM_577636.1	LOC502178	0.0713	-0.0496	0.04478	-0.0235	-0.0116	0.05784	-0.0201	0.02384	-0.0041	0.03426	0.01922	-0.0326	-0.0397	0.05917
XM_580239.1	LOC502181	0.00394	0.02312	0.06699	0.10606	-0.0164	0.18311	-0.1312	-0.1957	-0.0114	-0.0899	0.00851	-0.1261	0.17373	0.07755
XM_577639.1	LOC502182	0.1483	0.0933	0.08533	0.24356	0.17591	-0.0157	0.16693	0.07927	-0.0816	0.09786	-0.1322	-0.1615	0.08299	-0.0204
XM_577640.1	LOC502183	0.25503	0.29239	0.09678	-0.0201	0.08364	0.01194	-0.103	0.27991	0.12598	-0.0525	-0.0028	-0.0194	0.25467	-0.0032
XM_577641.1	LOC502184	-0.1284	-0.0565	-0.1558	-0.1868	-0.016	-0.2784	-0.1616	-0.208	-0.1199	-0.0008	-0.1333	-0.1355	-0.2638	-0.1658
XM_580240.1	LOC502185	-0.1053	-0.1496	0.06848	0.16792	0.02082	0.03931	0.01778	0.10737	0.03672	0.04902	0.05803	-0.1507	-0.0997	0.10254
XM_577644.1	LOC502186	-0.0288	0.04608	0.02364	0.07271	0.00475	-0.0173	-0.0322	0.01847	0.08815	0.26578	0.16899	0.0328	-0.0401	-0.0361
XM_577649.1	LOC502190	-0.0881	0.17735	0.04668	-0.0067	0.03493	0.06972	0.10764	0.03223	-0.0326	0.08302	-0.0075	-0.0857	-0.1163	-0.066
XM_577654.1	LOC502193	-0.1653	-0.2492	-0.1523	-0.0517	-0.1661	-0.1525	-0.1642	-0.1298	-0.1038	-0.1237	-0.168	-0.1669	-0.0768	-0.0778
XM_577655.1	LOC502194	0.00024	0.15717	-0.0895	0.14301	0.07059	0.26453	0.01811	-0.0386	-0.0275	0.00267	-0.0207	-0.1357	0.175	0.11555
XM_577656.1	LOC502195	-0.3389	-0.1581	-0.3103	-0.2111	-0.2464	-0.2891	-0.4886	-0.288	-0.3751	0.00313	-0.3235	-0.2479	-0.2588	-0.2192
XM_577658.1	LOC502197	0.02504	-0.1081	-0.0309	0.03231	0.18061	0.06082	0.0792	0.00598	-0.0634	-0.0584	0.10475	-0.1233	-0.0622	-0.0007
XM_577661.1	LOC502200	0.23126	0.16889	-0.1369	0.09412	-0.0139	0.2787	-0.2246	0.09925	-0.1115	0.043	0.10097	0.20083	0.06403	0.26824
XM_577662.2	LOC502201	-0.0218	-0.2726	0.06547	-0.0309	-0.1359	-0.1182	-0.1179	-0.0327	-0.1308	0.04109	0.02158	-0.0621	-0.0516	-0.1059
XM_580241.1	LOC502202	0.12387	0.35425	0.0786	0.04652	0.26955	0.09442	0.1839	0.34682	0.04829	0.20044	0.17164	0.02548	0.00599	0.06064
XM_577663.1	LOC502203	-0.1752	-0.1684	-0.1774	-0.1247	0.0851	-0.1387	-0.1925	-0.1497	-0.0652	-0.0742	-0.1584	-0.1188	-0.1815	-0.1634
XM_577664.1	LOC502204	-0.0993	0.10883	0.12331	-0.1368	-0.0923	-0.1784	-0.0625	-0.146	-0.0955	-0.0723	-0.0038	0.02944	0.08357	-0.1672
XM_577665.1	LOC502205	-0.0233	-0.0676	-0.1153	-0.1038	-0.064	0.06138	-0.1013	-0.0602	-0.2401	-0.2283	-0.1483	-0.2697	-0.2144	0.1051
XM_577666.1	LOC502206	0.00446	-0.1911	0.01676	-0.2178	0.16563	-0.1384	-0.0647	-0.1161	-0.1475	-0.042	0.00334	-0.1028	-0.1076	-0.0676
XM_577667.1	LOC502207	0.49176	0.08173	0.05496	-0.0088	0.01717	0.07624	0.32028	-0.0111	-0.0567	-0.0439	-0.0622	-0.0279	0.01802	0.07162
XM_577668.1	LOC502208	-0.0999	-0.1086	0.0051	-0.0102	0.07507	0.10278	0.01762	0.07404	0.04742	-0.0134	-0.0084	0.08993	0.02242	0.11149
XM_577670.1	LOC502209	-0.0515	-0.0861	-0.0671	-0.0748	-0.0942	-0.0557	-0.0491	0.00217	-0.039	0.00572	0.04088	-0.0485	-0.0939	-0.0297
XR_007393.1	LOC502210	0.01523	-0.1323	-0.1139	-0.164	-0.1034	-0.129	-0.0298	-0.1621	0.01416	-0.1137	-0.0417	-0.0414	-0.1891	-0.1654
XM_577673.1	LOC502211	0.10535	0.03392	0.0349	0.05782	-0.0092	0.04518	0.0228	0.0073	0.00729	0.08989	-0.0027	0.25367	0.0208	0.16982
NM_001161691	LOC502216	-0.0138	-0.2199	-0.0812	-0.1966	-0.0528	-0.1303	-0.0986	-0.0427	0.01971	-0.2925	-0.1979	-0.1691	-0.0306	-0.0316
XM_577680.1	LOC502218	-0.1183	-0.0211	-0.0005	-0.0519	0.00947	-0.0671	-0.0351	0.103	-0.0344	-0.0677	0.028	-0.1201	0.1136	-0.0476
XM_580242.1	LOC502222	0.2094	-0.094	-0.0851	-0.1693	0.08213	0.11597	-0.0097	0.06117	-0.2531	0.19246	-0.2433	-0.1978	-0.2254	-0.1498
XM_577694.1	LOC502231	-0.1168	-0.2752	-0.2405	-0.5138	-0.1748	-0.1631	-0.2915	-0.1361	0.09022	-0.3893	-0.1516	0.02267	-0.0896	-0.0478
XM_577697.1	LOC502234	0.05674	0.13169	0.15032	-0.1247	-0.0356	-0.0728	0.13653	-0.0634	-0.0055	-0.0191	-0.0584	-0.0467	0.03113	0.02443
XM_217965.3	LOC502240	-0.0717	-0.0858	0.04103	-0.0594	-0.0153	0.16573	-0.0998	0.10192	-0.1113	-0.0439	-0.091	-0.0493	-0.0259	0.20661
XM_577701.1	LOC502241	0.0965	-0.1967	-0.1346	-0.0598	-0.129	-0.0586	-0.1391	-0.0189	0.03456	-0.1785	0.14699	-0.1627	-0.1288	-0.2343
XR_006315.1	LOC502243	0.06952	0.14946	0.24164	0.0357	0.11551	0.04067	0.07064	0.1117	0.05582	0.1561	0.07199	0.0137	0.37732	0.11173

XM_577704.1	LOC502244	0.05624	0.1737	-0.0162	-0.023	0.29684	0.20222	0.11255	0.31765	0.11976	0.06374	0.11473	-0.0075	0.20105	-0.0704
XM_344844.2	LOC502245	0.15214	-0.2494	-0.003	0.11	-0.2681	-0.2514	-0.1312	-0.2736	-0.3783	-0.2005	-0.3534	-0.143	-0.163	-0.2727
XM_577706.1	LOC502247	0.01434	-0.0993	-0.1593	-0.0074	-0.1479	-0.0703	-0.1167	-0.0595	-0.0325	-0.1106	0.02807	-0.0389	-0.0959	-0.171
XM_577707.1	LOC502248	0.10603	0.02506	-0.0555	-0.141	-0.0277	-0.0923	-0.0981	0.1449	-0.0874	0.01405	-0.1343	-0.1174	-0.1068	-0.0847
XM_577710.1	LOC502251	-0.0416	0.01427	0.04286	0.00151	-0.0767	0.08966	0.2571	-0.0061	-0.0457	-0.025	0.08919	-0.0098	0.02764	-0.0109
XM_577713.1	LOC502253	0.06135	0.01702	0.08931	0.05983	0.08762	-0.0051	0.06915	0.0666	0.11883	-0.0282	0.08713	0.10635	0.03452	0.08222
XM_577714.1	LOC502254	0.00823	0.22474	0.01243	0.2044	-0.0865	0.14517	0.17093	0.02557	-0.0967	0.02062	0.12111	-0.0106	0.01666	0.07389
XM_577715.1	LOC502255	-0.0299	0.03034	0.11407	0.01872	0.0216	0.16249	-0.005	0.10675	0.09309	0.1323	0.08142	0.18314	0.07242	-0.0034
XM_577716.1	LOC502256	-0.0915	-0.0712	-0.0418	-0.0515	-0.0234	0.07122	0.14307	0.01471	0.00201	0.01674	0.03839	-0.0727	-0.0107	0.0828
XM_577719.1	LOC502258	-0.054	-0.0625	-0.0599	0.09537	0.08875	-0.1023	-0.0948	-0.1255	-0.1202	-0.0065	-0.0819	0.1128	-0.0513	-0.1305
XM_577720.1	LOC502259	0.04803	-0.0993	0.08832	-0.0755	0.10416	0.01298	0.00527	-0.0439	0.06715	-0.1418	-0.1219	-0.0137	0.03697	-0.0505
XM_577721.1	LOC502260	0.0459	0.06658	0.05355	-0.0475	0.07158	0.27376	0.14975	-0.019	0.12406	0.15417	0.00328	-0.0191	0.23698	0.1358
XM_577721.1	LOC502260	0.09166	0.17757	0.05143	0.0658	0.06698	0.20524	0.09139	0.20968	0.18907	0.18342	0.18508	0.41681	0.04689	0.29423
XM_580243.1	LOC502261	-0.0305	0.11938	-0.0648	0.14263	0.18725	0.01902	-0.0279	-0.0172	0.1084	0.0612	-0.0307	-0.0099	-0.1003	0.07316
XM_577723.1	LOC502262	0.22816	-0.0043	-0.0595	0.09229	0.16933	0.08545	-0.1049	-0.0854	-0.0779	0.00733	0.16615	0.06639	-0.0181	-0.0741
XM_577724.1	LOC502263	0.06507	-0.0377	0.01396	0.10019	-0.0806	-0.047	-0.1135	-0.1561	0.07936	-0.0793	-0.0967	0.1152	-0.1134	-0.0122
XM_577726.1	LOC502265	-0.0475	-0.2299	-0.2574	0.09716	-0.119	-0.0885	0.03442	-0.0024	0.10971	0.05872	0.00184	0.05092	0.23981	0.11145
XM_577727.1	LOC502266	-0.0037	-0.0583	-0.0777	0.00052	-0.0295	-0.0995	-0.1123	-0.0142	-0.0217	0.17357	-0.0829	0.04092	0.1345	-0.0966
XM_577728.1	LOC502267	0.15756	0.08016	0.04752	0.05493	0.06101	-0.0009	0.16671	0.2228	0.2178	-0.0124	0.20009	-0.0048	0.07952	-0.0024
XM_577731.1	LOC502270	0.25117	0.03866	0.00305	0.07039	0.06525	-0.0429	-0.1078	0.09904	0.06646	-0.0465	0.00203	-0.161	0.12345	0.00723
XM_577736.1	LOC502275	0.20629	0.12625	0.10481	0.13838	-0.1092	0.1665	0.18265	-0.043	0.12046	0.00309	0.06106	0.13252	-0.021	0.28548
XM_577738.1	LOC502277	-0.0143	-0.3039	-0.0363	-0.0863	-0.1606	-0.2655	-0.2792	-0.1098	-0.3311	-0.1708	-0.3842	-0.1939	-0.2895	-0.1032
XM_577739.1	LOC502278	0.07139	0.12745	-0.0329	0.11398	0.0075	0.1332	0.02202	#######	0.14305	0.10824	0.02792	-0.0427	0.10861	0.08496
XM_577741.1	LOC502280	0.03311	0.04553	-0.0333	0.03971	-0.0085	-0.0215	0.08605	0.13797	-0.0223	0.14057	-0.0065	0.15431	-0.0359	0.07152
XM_577744.1	LOC502282	0.34294	0.65899	0.05829	0.39502	0.56194	0.35985	0.45514	0.9202	0.50464	0.62108	0.23219	0.08611	0.26822	0.5616
XM_577758.1	LOC502295	0.00215	-0.0694	0.03691	0.0907	-0.0521	0.10022	-0.019	-0.0249	0.00618	0.13477	-0.0343	0.02357	0.16664	0.03377
NM_001109334	LOC502300	-0.2289	-0.0087	-0.004	0.00289	-0.1036	-0.1574	-0.1228	-0.2151	-0.2143	-0.1097	-0.1879	-0.0431	-0.1108	0.18329
NM_001099483	LOC502301	0.09619	0.02554	-0.0256	0.02082	-0.0492	0.02978	-0.0113	0.12153	0.1132	0.1703	-0.0382	0.08675	0.08699	0.16607
XM_218303.1	LOC502302	0.06719	0.02323	-0.3232	0.01624	-0.0563	0.03733	-0.196	-0.1688	0.05526	-0.0468	-0.0198	-0.0543	-0.169	-0.1862
XM_580244.1	LOC502304	0.16698	-0.029	0.09762	0.14103	-0.1255	-0.0046	0.05389	0.05827	0.07872	0.02081	0.07864	-0.0331	-0.0274	0.32813
XM_577771.1	LOC502307	-0.1368	-0.1498	-0.1356	-0.0626	-0.0602	-0.0803	-0.0513	-0.124	-0.0324	-0.1808	-0.2258	-0.1091	-0.1807	-0.1678
XM_577776.1	LOC502312	0.11784	-0.0047	0.13438	-0.0683	0.14665	0.04996	0.06177	-0.0121	-0.0933	-0.1903	0.03755	-0.0224	0.07125	0.08938
XM_577777.1	LOC502313	-0.5023	-0.3015	-0.4961	-0.0695	-0.3792	-0.4095	-0.2652	-0.4473	-0.4341	-0.42	-0.265	-0.4026	-0.247	-0.4087
XM_577779.1	LOC502315	0.21634	0.02836	0.1812	0.01105	0.07794	0.01017	-0.0036	-0.0391	0.04992	-0.1232	-0.1171	-0.0341	0.1419	0.12544
NM_001134726	LOC502316	-0.0595	-0.4117	-0.1729	0.09419	-0.3927	-0.3038	-0.0172	-0.2317	-0.1931	-0.432	-0.3476	-0.4244	-0.3359	0.03224
XM_577784.1	LOC502319	0.0208	0.11856	0.0948	0.04627	0.06891	-0.0252	0.1445	0.12872	0.00336	0.0036	0.00521	0.0518	-0.0987	-0.0484
XM_577786.1	LOC502321	-0.0074	-0.1828	0.02911	0.02997	-0.0451	-0.2262	-0.0338	-0.0553	-0.1291	-0.2025	0.02188	-0.1993	-0.0418	0.11158
XM_577787.1	LOC502322	-0.0191	0.03869	0.08478	-0.0558	0.09486	-0.0105	0.09422	0.27338	0.01037	-0.0229	0.06392	0.27361	0.21675	0.12399
XM_577789.1	LOC502324	0.21566	0.17535	0.02344	0.27398	0.00873	0.08452	0.08879	0.26938	0.24802	0.16117	0.24609	0.18271	0.00238	0.01896
XM_577790.1	LOC502325	-0.0608	0.03778	-0.203	-0.0506	-0.1014	0.21431	-0.2101	-0.0632	-0.0857	0.08352	-0.1524	0.27148	0.00336	-0.2905
XM_577792.1	LOC502327	-0.0491	-0.0698	0.09132	-0.0353	0.06362	0.04507	-0.3295	0.06224	0.04371	-0.2785	-0.2385	-0.2943	-0.0658	-0.1915
XM_577793.1	LOC502328	-0.0292	-0.005	-0.2007	0.13462	0.04198	-0.1174	0.02445	0.04455	0.14743	0.15225	-0.0283	0.17142	-0.1608	-0.0501
XM_577794.1	LOC502329	-0.0266	-0.1149	-0.1138	0.08002	-0.1321	0.03925	-0.0347	-0.0685	0.12033	0.07537	-0.0656	-0.0307	0.04061	-0.0564
XM_577795.1	LOC502330	-0.1207	-0.0278	-0.0332	-0.117	0.1416	-0.0273	-0.1136	-0.0398	-0.1053	-0.0736	-0.1231	-0.118	0.00351	-0.0482
XM_577796.1	LOC502331	-0.055	-0.0293	-0.0245	-0.0439	-0.0209	-0.0418	0.04161	0.0134	-0.0746	-0.1298	0.10079	0.16738	0.04766	-0.0325
XM_577797.1	LOC502332	-0.0073	-0.3062	0.21975	0.01962	0.21116	0.39915	0.11075	-0.1126	0.08585	-0.2254	-0.077	0.64048	0.1993	0.1707
NM_001002285	LOC502338	-0.0504	-0.0608	-0.1739	-0.1205	0.31148	-0.1495	-0.1222	-0.1345	-0.1681	-0.1264	0.15922	0.40717	0.01195	0.16507
XM_577806.1	LOC502340	-0.1831	-0.1677	-0.1906	-0.0933	-0.079	-0.1482	-0.0096	-0.1631	-0.1275	-0.2585	-0.0175	0.00455	-0.156	-0.1333
XM_577807.1	LOC502341	-0.0097	-0.0522	-0.1	-0.122	0.06962	0.12446	-0.113	-0.0568	-0.0985	-0.1326	-0.1002	-0.0124	-0.0794	-0.0458

XM_577809.1	LOC502343	0.00308	0.16868	-0.0014	0.06414	0.05184	0.20579	0.02978	0.18525	-0.0482	0.06999	0.19547	-0.027	-0.0035	0.07613
XM_577811.1	LOC502344	-0.2623	-0.3806	-0.3483	-0.2137	-0.3589	-0.2255	-0.3613	-0.1728	-0.2501	-0.2798	-0.2366	-0.3824	0.05901	-0.0537
XM_577813.1	LOC502345	0.0138	0.03157	0.0016	0.11218	0.10931	0.13846	0.0532	0.11349	-0.0011	0.10892	0.08926	0.06309	0.09248	0.00701
XM_577814.1	LOC502346	0.00926	0.14065	-0.2114	-0.1051	-0.019	-0.0484	0.20939	-0.1332	-0.2084	-0.1281	-0.0939	-0.2358	-0.1141	-0.01
XM_577815.1	LOC502347	-0.0661	-0.1168	-0.3107	-0.2375	-0.2038	-0.0511	-0.1721	-0.1138	0.07447	-0.1271	-0.2062	0.01155	-0.358	-0.1036
XM_577821.1	LOC502351	0.13907	0.01832	0.01684	0.09328	0.04755	0.18919	-0.014	0.14545	0.08844	0.19814	0.09275	0.30474	0.14698	-0.0023
XM_577822.1	LOC502352	-0.0793	-0.0373	0.04114	-0.0301	-0.0764	-0.1719	-0.0312	-0.1453	-0.1042	-0.1279	-0.1399	0.11373	-0.1651	-0.0404
NM_001109336	LOC502353	-0.4376	-0.0636	-0.4018	-0.0748	-0.0063	-0.0758	-0.1453	-0.0738	-0.1853	-0.4546	-0.3207	-0.3814	-0.0519	-0.1764
XM_577833.1	LOC502361	0.05824	0.1245	0.22275	0.21685	0.07515	0.52005	0.05999	0.04301	0.11148	0.02582	0.01011	0.22767	0.04164	0.26935
XM_577835.1	LOC502363	-0.134	-0.1966	-0.1521	-0.2055	-0.184	-0.1216	-0.1879	-0.2644	-0.1713	-0.0935	-0.0844	-0.1582	-0.0073	-0.0539
XM_577836.1	LOC502364	-0.1044	0.05908	0.06642	-0.0332	0.02617	-0.0551	-0.0138	-0.0735	0.07981	0.15635	0.02197	-0.1179	-0.1137	0.18704
XM_577838.1	LOC502366	-0.1016	-0.2036	-0.0387	-0.1798	-0.1055	-0.1817	-0.1616	0.18014	-0.1568	0.00694	-0.154	-0.1281	-0.0693	-0.0753
XM_577839.1	LOC502367	-0.287	-0.3504	-0.351	-0.3159	-0.5193	-0.6922	-0.2654	-0.6063	-0.3938	-0.3297	-0.4993	-0.4569	-0.4482	-0.3669
NM_078617	LOC502370	-0.1135	-0.2401	-0.2465	-0.2715	-0.2237	-0.3052	-0.1113	-0.1831	-0.3704	-0.3627	-0.2445	-0.1733	-0.0339	-0.3764
NM_001025058	LOC502372	0.04011	0.02525	0.07761	0.03513	0.00159	-0.1113	0.06497	-0.0226	-0.0993	0.25493	0.04678	0.38114	0.16802	0.17376
XM_346329.2	LOC502373	-0.0134	-0.0669	-0.0487	-0.0092	-0.1527	-0.0519	-0.0161	-0.0571	-0.0777	-0.052	-0.0503	-0.0499	-0.09	-0.0541
NM_001143911	LOC502374	0.16991	0.03081	0.1545	0.12871	0.27547	0.18	0.18733	0.1779	0.45558	0.27682	0.08507	0.24299	0.05576	-0.0568
NM_022506	LOC502376	-0.0055	0.15752	0.40873	0.11339	0.1243	0.18897	-0.0137	0.10133	0.1255	0.1995	0.04763	0.10098	0.13182	0.03085
XM_577853.1	LOC502378	-0.1669	-0.1432	-0.2701	-0.2434	-0.0509	-0.2368	-0.3415	-0.2526	-0.1364	-0.301	-0.3066	-0.2046	-0.1635	-0.0079
XM_577855.1	LOC502380	-0.0074	0.00129	-0.0621	0.09025	0.06887	0.0246	0.02289	0.05097	-0.0717	0.23718	0.13156	0.03586	-0.0169	0.16286
XM_577859.1	LOC502383	0.30214	0.37044	0.31467	0.38806	0.649	0.58656	0.40937	0.41879	0.11216	0.203	0.3127	0.71044	0.529	0.41473
XM_577865.1	LOC502387	0.05577	-0.0808	0.07726	0.02747	-0.0067	0.1382	-0.0415	-0.0482	0.14263	0.00946	-0.0924	0.00588	0.02663	0.04029
XM_577866.1	LOC502388	0.32605	0.26884	0.04858	0.14732	0.12287	0.0896	0.05721	-0.0157	0.17143	0.0735	0.22297	0.21468	0.16128	0.0549
XM_577870.1	LOC502391	0.01286	-0.0276	-0.0331	0.06846	0.01034	0.0805	0.04706	0.03232	0.21802	0.0376	-0.0334	-0.0366	-0.0242	-0.0597
XM_577871.1	LOC502392	0.15631	0.08997	0.18118	0.78469	0.54994	0.04778	0.15575	0.12085	-0.0464	0.0189	0.17484	0.14279	0.21715	0.31596
XM_577872.1	LOC502393	0.16885	0.02033	0.02384	0.05812	0.10222	0.0618	0.1355	0.07829	-0.0779	0.13929	0.05045	0.00097	0.04803	0.04963
XM_577875.1	LOC502395	-0.0296	-0.1846	-0.1729	-0.2466	-0.3532	-0.1097	-0.4923	-0.0473	-0.0871	-0.3086	-0.1852	-0.1719	-0.1133	-0.187
XM_577876.1	LOC502396	0.08907	0.03775	-0.1881	-0.1162	-0.1336	-0.0593	-0.1842	-0.0154	-0.1172	-0.0074	-0.1782	-0.0947	-0.1564	-0.1705
XM_577878.1	LOC502398	-0.1857	-0.159	-0.1599	-0.2656	-0.0531	-0.2971	-0.2208	-0.1585	-0.2641	-0.1981	-0.1688	-0.2122	-0.1903	-0.171
XM_577882.1	LOC502402	0.08632	0.16728	0.25406	0.13929	0.16769	0.21717	0.13163	0.32066	0.46251	0.03284	0.13086	0.08215	0.11061	0.03448
XM_577892.1	LOC502411	0.15017	0.10593	0.13368	0.13168	-0.1092	0.14838	0.12685	0.09445	0.04652	-0.0618	0.12702	-0.0828	-0.1109	-0.0913
NM_001166267	LOC502412	0.18695	0.20018	0.14968	0.00342	-0.0066	0.08141	0.10552	0.07358	0.1388	0.1787	0.03278	0.012	0.20293	0.07844
XM_577894.1	LOC502413	-0.2102	0.01868	-0.2466	-0.1452	0.0161	-0.2223	-0.1108	0.02187	-0.2349	-0.1367	-0.2547	-0.2261	0.35846	-0.2313
XM_580246.1	LOC502416	-0.0692	0.03201	0.00983	-0.0774	0.11928	0.04576	-0.0246	0.01761	-0.1671	-0.0193	-0.0811	-0.0577	0.06152	-0.0021
XM_577902.1	LOC502422	-0.1225	0.11701	0.02634	-0.0645	-0.0134	0.04728	-0.0294	-0.1253	-0.0774	0.10584	0.00271	-0.1172	-0.0826	-0.1461
XM_577903.1	LOC502423	0.14297	0.03844	0.16981	0.25267	0.21906	0.22403	0.03377	0.03329	-0.029	0.06691	0.00456	0.20067	0.17011	0.03442
XM_577906.1	LOC502424	-0.163	0.18678	-0.1301	0.03454	-0.1676	0.09054	0.08006	0.01456	-0.1779	0.08773	0.24709	0.16595	-0.0453	-0.1068
XM_577907.1	LOC502425	-0.0685	-0.1042	0.03832	-0.0699	0.03141	-0.1585	-0.1028	-0.1606	-0.0042	-0.0894	-0.1947	-0.1486	-0.0936	-0.0834
XM_577909.1	LOC502427	-0.0335	0.02165	0.12686	-0.0522	-0.0817	-0.0046	-0.0113	0.03057	-0.079	-0.0113	0.06287	0.09932	0.06818	-0.0773
XM_577910.1	LOC502428	0.12772	0.11674	0.07544	0.15733	0.11623	0.27869	0.12762	0.10815	0.28971	-0.0311	0.24419	0.15309	0.23446	0.03957
XM_577911.1	LOC502429	0.01367	0.19507	0.05087	0.06617	-0.1324	-0.0037	0.14737	0.13197	0.22354	-0.0044	0.04021	0.16217	0.27986	-0.0057
XM_577912.1	LOC502430	-0.1577	-0.0697	-0.0993	-0.1045	-0.0442	-0.062	-0.0189	-0.0344	-0.0955	0.24364	0.07644	-0.0615	-0.1132	-0.05
XM_577913.1	LOC502431	0.34989	-0.0246	0.06786	0.23551	0.0792	0.05911	0.05281	0.26022	0.53129	0.33714	0.34436	0.16162	0.15312	0.05916
XM_577914.1	LOC502432	-0.0571	-0.0212	0.07981	0.00124	0.074	-0.0098	-0.0453	-0.0267	-0.0114	0.12896	0.00895	0.13117	0.05391	0.01809
XM_580247.1	LOC502433	0.0263	0.13683	0.03422	0.00115	0.04436	0.1954	0.08706	0.10477	-0.0499	0.10561	-0.0453	0.01091	0.06124	0.00418
XM_577915.1	LOC502434	-0.0602	-0.02	-0.0457	0.07703	-0.1098	-0.1109	0.00693	-0.0468	-0.0505	0.01433	0.30833	0.01861	-0.0214	-0.0906
XM_577916.1	LOC502435	0.04536	-0.052	0.103	0.07268	0.16322	0.03077	0.03935	0.10556	-0.0137	0.06212	0.14686	0.16272	0.00337	0.00111
XM_577917.1	LOC502436	-0.3161	-0.4378	-0.289	-0.2729	-0.2675	-0.3695	-0.1668	-0.3316	-0.3198	-0.379	-0.3432	-0.223	-0.2479	-0.3128
XM_577918.1	LOC502437	-0.0081	-0.0553	-0.0482	-0.0365	0.12668	-0.0331	0.06542	0.1114	0.06617	-0.0781	-0.1777	-0.0769	-0.0223	-0.0325

XM_577919.1	LOC502438	-0.1259	0.04576	-0.0647	0.09688	0.02462	-0.1153	-0.0454	0.00633	-0.1173	-0.165	-0.1377	0.01618	-0.1309	0.02127
XM_577920.1	LOC502439	0.08367	-0.0648	-0.0692	0.15365	0.03297	-0.1291	0.00969	0.03997	0.12561	0.05725	-0.055	0.07043	-0.0552	-0.0851
XM_577921.1	LOC502440	0.05127	-0.0613	0.29444	-0.0903	0.18002	0.00255	-0.0499	-0.0285	-0.1026	0.03953	-0.01	0.01776	-0.0753	-0.0987
XM_577922.1	LOC502441	-0.0837	-0.1331	0.03285	-0.0828	0.00339	0.20904	-0.0731	0.09033	0.12349	0.08087	-0.1327	0.09657	-0.0774	0.03678
XM_577923.1	LOC502442	0.14306	0.0975	0.01132	0.02613	0.13722	0.01447	0.19678	0.04473	0.17097	0.03212	0.0152	0.03586	0.05022	0.09907
XM_577924.1	LOC502443	0.08359	-0.0393	-0.0199	-0.0467	0.1451	0.04181	-0.1189	0.05103	-0.1642	-0.0933	0.06353	-0.0376	0.02946	0.16146
XM_577925.1	LOC502444	0.20718	0.19563	-0.0092	0.33988	0.13169	0.1398	0.03412	0.23942	0.11678	0.2723	0.10308	0.1836	0.20455	0.31829
XM_577926.1	LOC502445	-0.0048	0.19421	0.02916	0.08321	0.01725	0.65371	-0.0004	0.20239	0.02581	0.04319	0.03628	0.19236	0.16581	0.08482
XM_577927.1	LOC502446	-0.1034	-0.1076	-0.11	-0.1792	0.01622	-0.2508	-0.1663	-0.0533	-0.0167	0.00118	-0.0216	-0.2098	-0.0821	-0.2578
XM_577928.1	LOC502447	-0.1335	-0.2001	-0.034	-0.1548	-0.1866	-0.1966	0.00931	-0.1932	-0.1053	-0.2136	-0.1585	-0.1103	-0.1507	-0.0713
XM_577930.1	LOC502449	-0.0161	0.14877	-0.0356	0.01898	0.1473	-0.0024	-0.0577	0.14911	-0.0124	0.26121	-0.0507	0.02808	-0.0173	-0.0611
XM_577931.1	LOC502450	0.21618	-0.0343	0.00835	-0.0212	0.07806	0.02029	-0.0362	-0.0353	-0.0111	-0.0602	-0.0481	0.1752	0.00156	0.01918
XM_577932.1	LOC502451	0.05428	0.13088	0.05876	0.05239	0.00651	-0.0172	0.07978	0.08718	0.17764	-0.0556	0.00895	0.04951	0.03476	0.03313
XM_577933.1	LOC502452	-0.1385	0.02925	-0.0642	-0.011	0.11986	0.06289	-0.0621	-0.0955	-0.2409	0.07414	0.0521	0.13906	0.00359	0.06002
XM_577934.1	LOC502453	0.01048	-0.039	-0.0474	-0.0491	0.05816	-0.0534	0.09178	-0.0274	-0.0705	-0.0807	0.09299	-0.042	-0.1018	0.10086
XM_577936.1	LOC502455	0.10768	-0.009	0.07501	-0.0689	-0.0033	0.04642	-0.0393	-0.1427	0.12339	0.10659	-0.0063	-0.0297	0.0088	0.03926
XM_577937.1	LOC502456	0.21992	0.04712	0.04063	0.07596	0.04852	0.10027	0.13966	0.07648	0.13952	0.1566	0.31771	0.0648	-0.0583	0.19956
XM_577940.1	LOC502459	-0.1221	-0.1586	-0.0861	-0.1298	-0.2684	-0.044	-0.0799	-0.0539	-0.2313	-0.1602	-0.2158	-0.1743	-0.0439	-0.0739
XM_577944.1	LOC502463	0.15057	0.16706	0.15908	0.05599	0.1822	0.22607	0.06131	0.14907	0.2957	0.01671	0.33961	0.27685	0.17864	0.09322
XM_580248.1	LOC502464	0.4186	0.12384	0.03202	-0.0908	0.18757	0.10781	-0.1377	0.31452	-0.19	-0.1072	0.05352	-0.0766	-0.066	-0.0782
XM_577945.1	LOC502465	0.20574	0.07782	0.18093	0.22856	0.2154	0.28793	0.03248	0.23369	-0.1069	0.16012	0.02843	0.51031	0.26264	0.01225
XM_577946.1	LOC502466	-0.1079	0.00866	-0.097	-0.1106	-0.0977	-0.0194	-0.0199	-0.059	0.01947	-0.019	0.10316	0.00933	0.06893	0.17522
XM_577947.1	LOC502467	-0.082	0.06435	0.01991	-0.0379	0.03183	-0.0158	-0.0149	#######	-0.1277	-0.0731	0.18455	-0.0589	-0.1238	-0.001
XM_577948.1	LOC502468	-0.0073	0.12217	0.06741	0.03366	0.078	0.04148	0.07789	0.25659	0.00746	0.00079	0.04661	0.00761	0.08267	0.27654
XM_580249.1	LOC502471	-0.2283	-0.1282	-0.057	-0.1464	-0.142	-0.2095	-0.0996	-0.2166	-0.2311	-0.0439	-0.1125	-0.2313	-0.2704	-0.2505
XM_577951.1	LOC502472	0.03897	0.05608	0.01061	-0.0656	-0.0404	-0.0413	0.0413	-0.0717	0.17636	-0.0071	-0.028	-0.0416	-0.0003	-0.0077
XM_577953.1	LOC502474	0.01212	0.09368	0.05007	-0.0216	-0.1111	-0.1305	0.02896	0.01713	-0.0265	-0.1376	0.15453	-0.0134	0.2372	-0.0098
XM_577956.1	LOC502476	-0.0833	-0.228	0.10174	0.01253	0.07629	0.05476	0.02371	-0.187	0.01315	-0.037	-0.0254	0.2358	-0.0954	-0.1381
XM_577958.1	LOC502478	0.19248	0.13716	0.0624	0.48736	0.21478	0.17586	0.28256	0.33598	0.40421	0.286	0.27843	0.1947	0.11412	0.22057
XM_577960.1	LOC502480	0.05147	-0.2436	-0.0515	-0.1617	-0.1199	-0.1209	-0.1555	-0.0147	-0.1957	-0.2184	-0.0561	-0.1319	-0.2133	-0.181
XM_577961.1	LOC502481	0.15042	0.39837	0.40194	0.12852	0.05305	0.13941	0.35644	0.23603	0.08671	0.30027	0.06752	0.22122	0.09718	0.08065
XM_577965.1	LOC502484	0.09553	-0.0341	-0.1278	-0.0941	-0.1014	0.09948	-0.0156	-0.1043	0.04551	0.05492	-0.0412	0.0091	0.00955	0.05736
XM_577966.1	LOC502485	-0.1042	-0.0788	-0.0708	-0.2838	0.07192	-0.0027	-0.0666	-0.0626	0.07241	0.25409	-0.068	-0.1956	0.04423	-0.1592
XM_577967.1	LOC502486	-0.0824	0.00912	0.09377	0.13628	-0.0102	0.0351	0.01403	-0.1196	0.01148	0.04692	-0.0401	0.09424	-0.0232	0.03792
XM_577969.1	LOC502488	0.21648	0.20643	-0.0136	-0.0019	0.09181	0.21498	0.21	0.19864	0.09291	-0.0297	0.13128	0.17032	0.20685	0.10527
XM_577971.1	LOC502490	0.03936	-0.1162	0.06706	-0.3677	-0.0158	0.60704	-0.0187	0.51536	0.06406	0.08071	-0.1417	-0.2082	-0.1249	0.0126
XM_577975.1	LOC502493	-0.0976	-0.0783	-0.0076	0.0215	-0.087	0.05479	-0.0702	0.02344	-0.0649	0.14693	-0.1186	0.00761	-0.0973	0.12276
XM_577976.1	LOC502494	0.18338	0.18672	0.02472	-0.0029	0.07352	0.166	-0.119	-0.0084	-0.0789	0.31473	0.03789	0.00265	0.03231	0.22309
XM_577977.1	LOC502495	-0.1581	-0.2161	-0.1135	-0.1946	-0.2486	-0.2802	0.11063	-0.1537	-0.1206	-0.0886	0.08485	-0.4159	-0.1936	-0.0204
XM_580250.1	LOC502497	0.06831	0.04005	0.07431	0.028	-0.0395	-0.0462	0.0457	0.01281	0.02028	0.03938	0.1071	-0.0096	0.18184	-0.0508
XM_577979.1	LOC502498	0.04918	0.10986	0.04171	-0.0687	-0.0186	0.0708	-0.0305	-0.0568	-0.009	0.0527	-0.0604	0.01268	0.06923	0.00308
XM_577981.1	LOC502500	0.17525	-0.0119	-0.1103	-0.15	0.00214	-0.0519	0.03633	-0.0638	-0.1084	0.1917	-0.1921	-0.1653	-0.2243	-0.2903
XM_580251.1	LOC502502	-0.0391	0.00431	-0.0221	-0.0309	-0.0413	-0.0748	0.07445	-0.0139	0.09335	-0.1038	0.23461	-0.1226	-0.0543	-0.1535
XM_577987.1	LOC502506	0.15836	0.34918	0.03822	0.06985	0.16818	0.57145	0.22856	0.09488	0.16899	0.02726	0.16205	0.20909	0.31881	0.21996
XM_577988.1	LOC502507	0.21709	-0.0641	0.08084	0.12309	0.03832	0.07846	0.0064	0.01072	0.12233	0.00681	0.11491	-0.0315	-0.0045	0.03943
XM_577990.1	LOC502508	0.10714	0.15366	0.04201	0.24692	-0.0044	-0.0108	0.07939	0.02026	0.13079	0.0063	0.02898	0.10953	0.00181	0.0208
XM_577992.1	LOC502509	0.11507	0.06187	0.34983	0.37202	0.24049	0.20102	0.36503	0.3045	0.43049	0.24423	0.16764	0.07794	0.20277	0.25601
XM_577993.1	LOC502510	-0.0717	0.0889	-0.0517	0.07153	0.21138	0.06009	-0.0943	0.00401	0.2447	0.04071	-0.105	0.18081	-0.0028	-0.0324
XM_577995.1	LOC502511	0.07337	0.02904	0.12604	0.05333	0.09233	0.0534	0.09289	0.10159	0.15946	0.1674	0.10349	0.06934	0.16486	0.05214

XM_577999.1	LOC502515	-0.2849	-0.1311	-0.2243	-0.2448	-0.3965	-0.223	-0.3313	-0.3915	-0.4198	-0.3573	-0.1493	-0.3057	-0.4524	-0.3179
XM_578000.1	LOC502516	-0.0885	-0.0473	0.81066	0.37438	-0.2528	-0.0525	0.56583	-0.1898	0.13925	-0.0374	0.11847	-0.0209	0.17965	0.09093
XM_578001.1	LOC502517	0.03484	-0.0569	0.14505	-0.052	0.14046	0.05801	-0.1542	-0.1329	-0.1368	0.00548	-0.0082	0.32545	0.14209	0.07682
XM_578003.1	LOC502519	-0.1852	-0.1687	-0.1172	-0.1477	-0.0632	-0.1571	-0.2133	-0.2256	-0.1967	-0.1556	-0.3096	-0.0923	-0.175	0.02861
XM_578004.1	LOC502520	0.32496	-0.0249	0.23672	0.34616	0.0604	0.26495	-0.0384	0.07131	0.01018	0.18948	0.37119	0.12068	0.0691	0.01989
XM_578005.1	LOC502521	-0.0121	-0.0209	-0.0481	-0.0696	-0.2447	-0.1558	-0.3061	-0.2106	-0.2052	-0.2575	-0.0809	-0.2489	-0.0729	-0.2628
XM_578007.1	LOC502523	-0.1697	-0.0418	-0.018	-0.1533	-0.0684	-0.0051	-0.0855	-0.1161	-0.0688	0.05201	-0.0301	-0.1485	-0.1834	-0.2048
XM_578008.1	LOC502524	0.24564	0.06564	0.19005	0.06825	0.20333	0.13226	0.10648	0.09908	0.13034	0.10604	0.0149	0.23226	0.09463	0.09272
XM_578011.1	LOC502526	-0.1872	-0.0157	0.1923	-0.1343	-0.021	-0.0698	-0.1708	0.16889	-0.1736	-0.0462	0.05636	-0.1652	0.06763	0.28694
XM_580252.1	LOC502527	-0.0307	-0.0439	-0.1638	0.04842	0.16097	0.05857	0.08837	0.17282	-0.0611	0.00911	-0.0029	0.15268	-0.0556	-0.1404
XM_578015.1	LOC502529	-0.2469	-0.1765	-0.2087	-0.212	-0.2192	-0.0844	-0.1144	-0.268	-0.17	-0.2417	-0.1971	-0.1595	-0.1962	-0.2466
XM_578016.1	LOC502530	-0.3063	-0.2978	-0.2197	-0.331	-0.258	-0.3832	-0.3194	-0.1904	-0.2921	-0.2515	-0.3342	-0.3335	-0.1359	-0.3694
NM_001109339	LOC502531	-0.0505	0.12092	0.00898	0.09109	0.16259	0.07885	0.24041	0.05778	0.06304	0.04265	0.1899	-0.0819	0.34387	0.1319
XM_578018.1	LOC502532	0.10193	0.07647	0.04147	0.14771	0.15039	0.07761	0.18799	0.16381	0.23057	0.14939	0.0584	0.23971	0.01885	0.13985
XM_578020.1	LOC502534	-0.0917	-0.0063	0.06665	-0.0766	-0.0759	-0.0202	-0.0667	-0.0165	-0.0236	-0.061	-0.0332	-0.0279	-0.0242	0.123
XM_578021.1	LOC502535	0.05784	-0.0683	-0.0147	0.03464	-0.0379	0.02242	0.08264	0.15258	0.1544	-0.0234	0.1467	0.17019	0.25571	-0.0183
XM_578023.1	LOC502536	-0.1582	-0.1443	-0.0022	0.21409	-0.2172	0.05331	0.25667	0.11529	0.18446	0.00286	0.08501	0.02675	0.09818	-0.0712
XM_578025.1	LOC502537	0.03023	0.13908	0.10084	-0.0558	0.07466	-0.0313	0.13147	0.13702	-0.0253	-0.0649	-0.0028	-0.043	-0.017	-0.0244
XM_578027.1	LOC502539	0.04591	-0.0443	-0.0123	-0.0372	-0.0786	0.22974	0.00938	-0.0058	0.07895	0.20611	0.12937	-0.0533	-0.0506	-0.0071
XM_580253.1	LOC502541	0.38213	0.1377	0.27374	0.1048	0.02243	0.06032	-0.0014	0.15436	0.06334	0.02529	0.00682	0.08328	0.0648	0.1006
XM_580254.1	LOC502542	0.87627	0.5825	0.29707	0.51684	1.019	1.4635	0.97499	0.85654	0.71299	0.75351	0.79744	0.92002	0.39759	0.34997
XM_578032.1	LOC502546	0.15322	0.07642	0.12486	0.13807	0.13498	0.13713	0.1025	0.28724	0.05652	0.12551	0.35882	0.02084	0.01468	0.24625
XM_578034.1	LOC502548	-0.0292	0.04375	-0.0825	0.00805	0.1624	0.02583	-0.0147	-0.0501	-0.0319	0.13872	-0.1383	0.30953	-0.0359	-0.0135
XM_578037.1	LOC502551	0.05843	-0.0654	-0.0786	-0.1593	-0.1913	-0.0149	-0.081	0.01997	-0.0062	0.04881	-0.1469	-0.0484	-0.0331	0.00536
XR_006577.1	LOC502561	-0.0554	0.0125	-0.0825	-0.1371	0.05938	-0.0845	0.05196	-0.0313	0.13021	0.00236	-0.0608	0.01633	-0.1664	-0.1283
XM_578049.1	LOC502563	-0.0134	0.07835	0.07598	-0.1107	0.00765	-0.1088	0.01376	-0.1646	-0.0351	0.05778	-0.0342	0.07634	-0.1024	0.17019
XM_578050.1	LOC502564	0.06958	0.2379	0.23189	0.28966	0.30152	0.44935	0.16188	0.10506	0.19809	0.17679	0.32019	0.05394	0.38382	0.10391
NM_001100996	LOC502570	-0.0824	-0.1772	-0.0335	-0.0987	-0.1374	0.02521	0.0201	-0.0666	0.01172	-0.0874	-0.0978	-0.1534	-0.0575	0.04469
XM_578065.1	LOC502579	-0.1013	-0.1207	0.04671	-0.0654	-0.0155	0.19938	-0.0309	-0.1334	-0.141	-0.2182	0.21723	0.1292	0.02103	-0.0542
XM_578066.1	LOC502580	-0.1825	-0.187	0.33849	0.15883	-0.4318	-0.3872	-0.0597	-0.4681	-0.3541	-0.4379	-0.2459	0.0681	-0.5496	-0.4433
XM_578067.1	LOC502581	0.13846	0.09451	0.06266	0.06973	0.04508	0.03572	-0.043	-0.0398	0.02542	0.08238	0.06367	-0.0142	0.16662	-0.0332
XM_578068.1	LOC502582	0.117	0.06979	0.00646	-0.1867	-0.0004	0.27085	-0.0911	-0.1654	0.33597	-0.1585	0.08862	-0.1275	-0.0866	0.2487
XM_578072.1	LOC502586	0.06594	0.10214	-0.045	0.11156	0.12047	-0.0227	0.05475	-0.091	-0.0177	0.04368	-0.006	-0.0253	0.04479	-0.0874
XM_578076.1	LOC502590	-0.1919	-0.2255	-0.1531	-0.1632	0.04134	0.1682	0.13729	0.17038	-0.2381	-0.0657	-0.0524	-0.009	-0.1929	-0.124
XM_578079.1	LOC502593	-0.0388	-0.1101	0.04203	0.01213	-0.0923	-0.0567	0.01183	0.11952	-0.1376	0.04058	-0.002	0.31502	-0.0605	-0.0688
XM_578081.1	LOC502595	0.17678	0.17943	0.14513	0.19755	0.17527	0.33194	0.28573	0.08689	0.10093	0.20079	0.3301	0.14301	0.07973	0.2359
XM_578084.1	LOC502597	-0.1507	-0.0587	0.09848	0.02829	-0.0519	-0.0071	-0.0299	-0.1793	-0.0737	0.15673	0.11135	-0.0257	0.01476	0.06702
XM_578085.1	LOC502598	0.20941	0.10323	0.00933	0.04972	-0.0329	0.21241	0.04824	0.12351	0.01603	0.07854	-0.0735	-0.1045	-0.0455	-0.0902
XM_578086.1	LOC502599	-0.3214	-0.1156	-0.2654	0.08899	-0.152	0.57416	-0.5554	-0.2499	0.21226	0.0361	0.11063	0.7005	-0.0745	-0.3787
XM_578087.1	LOC502600	-0.0395	0.00439	0.27576	0.13732	0.14959	-0.0116	-0.0054	0.11836	0.27124	0.10518	0.16683	0.01252	0.3852	-0.0103
XM_578093.1	LOC502605	0.05091	0.13717	0.1187	0.04956	0.04941	0.08044	0.08803	0.01947	-0.0513	-0.0314	0.10133	0.07167	-0.0821	-0.0256
XM_578094.1	LOC502606	-0.0033	0.04146	0.00214	0.07462	-0.0029	0.07105	0.05828	0.0542	0.10836	-0.0292	-0.0158	0.0702	0.0077	0.22887
XM_578096.1	LOC502608	-0.2135	-0.1797	-0.1674	-0.1965	-0.2354	-0.0896	-0.1936	-0.168	-0.0021	-0.2818	-0.084	-0.0541	-0.1536	-0.145
XM_578097.1	LOC502609	-0.0383	-0.0174	0.02292	0.059	0.15463	0.09055	-0.0942	-0.1088	0.06863	0.16191	0.12699	-0.0019	0.15629	0.09479
XM_578098.1	LOC502610	0.1771	0.09275	0.12075	0.26675	0.14351	0.30338	0.18995	-0.0123	0.16399	0.2245	0.16203	0.17302	0.01546	0.05343
XM_578100.1	LOC502611	-0.0424	0.27984	0.22759	0.20771	0.15914	0.26351	0.01673	0.08754	0.1019	-0.0082	0.18474	0.08232	0.09373	-0.0441
NM_021842	LOC502613	0.00502	0.18665	-0.3496	-0.321	-0.0217	-0.3277	-0.1936	0.00652	-0.2649	-0.1075	-0.129	-0.4422	-0.1109	-0.1115
XM_578107.1	LOC502616	0.10754	0.26834	0.04965	0.05123	0.1151	-0.0635	0.03673	0.10502	-0.0294	0.15715	0.27008	0.0575	0.18296	0.21579
NM_001109341	LOC502617	0.04123	-0.0002	-0.0181	0.02614	0.12556	-0.0932	-0.1624	0.05555	-0.0521	-0.1057	-0.0715	0.2042	0.28452	0.02455

XM_578118.1	LOC502625	0.22944	-0.0168	0.02549	0.0624	0.23944	0.10762	0.06115	-0.0134	0.1604	0.00217	-0.0344	0.03511	0.08624	0.07695
XM_578119.1	LOC502626	-0.0385	0.11354	-0.0477	0.1116	0.14396	0.13548	0.07639	0.10112	0.03044	0.18007	-0.0743	0.08892	0.12953	0.11598
NM_001114181	LOC502632	0.02356	0.01259	-0.1103	-0.0801	-0.0468	0.00853	0.05397	0.02481	-0.0904	0.00103	-0.0284	0.24404	-0.0176	0.088
NM_138523	LOC502635	-0.0456	-0.2404	-0.6769	-0.7898	-0.6149	-0.8655	-0.4098	-0.2335	-0.2927	-0.086	-0.3045	-0.4263	-0.2905	-0.0692
XM_578131.1	LOC502636	0.16087	0.16425	-0.0823	0.29198	0.29407	0.49861	-0.3719	0.42287	0.18337	-0.2008	0.18875	0.28978	-0.1827	-0.1085
XM_578134.1	LOC502639	-0.0743	0.02308	-0.1003	-0.0862	-0.0176	0.05455	-0.0462	-0.1131	-0.0336	-0.0075	0.01703	-0.06	0.01485	0.04248
XM_578136.1	LOC502641	-0.1463	-0.022	-0.0949	0.18285	-0.0452	0.11187	-0.0348	-0.0486	-0.071	0.19404	-0.0052	-0.0445	0.03467	-0.0317
XM_578146.1	LOC502652	0.09438	0.20848	0.03826	0.31947	0.30281	0.03623	0.05855	-0.0398	0.02386	0.34421	0.10754	0.126	0.35786	0.05931
NM_001077592	LOC502654	0.30346	0.13074	0.00257	0.2209	0.28784	0.09644	0.08101	0.15759	0.30551	0.21057	-0.0415	0.20682	0.07797	-0.0445
XM_578149.1	LOC502655	-0.0428	0.12181	0.09813	0.01278	0.04613	-0.1643	-0.0156	0.08875	0.24129	0.22769	0.65705	0.08516	0.10677	0.11919
XM_578150.1	LOC502656	-0.1213	-0.2451	0.03776	-0.0415	-0.0349	-0.0589	-0.1193	-0.1168	0.01299	-0.1674	0.04377	0.02342	0.06121	-0.2318
XM_578151.1	LOC502657	-0.1027	-0.1868	-0.1507	-0.1988	-0.1374	0.09579	-0.1358	0.25515	-0.043	-0.1292	-0.0878	-0.0642	0.11139	-0.1361
NM_031706	LOC502659	-0.0497	-0.1875	-0.0052	-0.0514	-0.198	-0.0517	-0.1565	-0.0831	-0.1056	0.00285	-0.1696	-0.1042	0.00138	-0.1256
XM_578154.1	LOC502660	0.04714	-0.1641	0.02845	-0.0825	0.06348	-0.1499	-0.0795	0.00797	0.14051	0.01466	0.04253	-0.0485	0.04532	-0.1643
XM_578156.1	LOC502662	-0.1182	-0.1897	0.00202	-0.0388	-0.1027	0.37594	-0.0697	0.00931	-0.0649	-0.1496	-0.1117	0.12872	0.03779	-0.0994
NM_001098782	LOC502663	-0.3957	-0.4491	0.03374	-0.3488	-0.2496	-0.4016	-0.17	-0.2622	-0.4746	-0.1746	-0.1435	-0.3404	-0.3182	-0.3217
XM_578159.1	LOC502664	0.04376	-0.0357	-0.0954	-0.023	0.2036	-0.0664	-0.1174	0.41271	0.05761	0.13032	0.02408	-0.0035	0.16621	0.22723
XM_578162.1	LOC502666	0.13316	0.18265	0.23971	0.1894	0.15579	0.05288	0.09606	0.23101	0.24727	0.16365	0.09828	0.17524	0.14911	0.18182
XM_578163.1	LOC502667	0.15104	0.1528	0.15585	0.02546	0.11016	0.06394	0.15852	-0.0017	-0.0058	0.26811	0.17204	-0.0756	0.18034	0.10836
XM_578164.1	LOC502668	0.18194	0.05655	0.29039	0.54563	0.47808	0.11741	0.39962	0.28884	0.31803	-0.0501	0.20187	0.16719	0.31419	0.39571
XM_578166.1	LOC502669	0.09539	0.06177	0.01107	0.07528	-0.1495	0.21688	-0.0346	0.2408	-0.0369	0.14212	-0.0245	0.1691	-0.0558	0.25508
XM_578168.1	LOC502671	-0.0752	-0.16	-0.0387	-0.1884	-0.0398	0.13436	-0.0667	-0.1353	-0.1565	-0.1441	-0.1187	0.08441	-0.1238	-0.091
NM_001033666	LOC502674	0.12049	0.05845	0.18265	-0.0329	0.20754	0.33473	0.18795	0.1696	0.23042	0.11509	0.13711	-0.0243	0.12207	0.09129
XM_578172.1	LOC502675	-0.0214	-0.1832	-0.0325	-0.0514	-0.0149	0.04292	-0.1688	-0.0878	-0.1054	0.14939	0.05239	0.03551	-0.017	-0.1711
XM_578175.1	LOC502678	0.01969	-0.1231	-0.1787	-0.0998	-0.127	-0.1724	-0.0767	-0.1363	-0.2414	-0.0158	0.00183	-0.1995	-0.1961	-0.073
NM_001025060	LOC502684	-0.1111	-0.1876	-0.1423	-0.0787	0.01899	0.1318	-0.0899	0.03264	-0.0611	-0.0287	-0.0321	-0.1642	-0.0643	-0.1072
XM_578182.1	LOC502685	0.18921	0.11421	0.02126	-0.0321	0.03698	0.24004	0.16635	0.02073	0.01066	0.04129	0.02484	-0.016	-0.0675	0.0241
XM_578184.1	LOC502687	-0.0349	-0.0533	-0.0466	0.08655	0.25408	-0.0313	-0.0317	0.13354	0.26141	0.02994	0.00271	-0.0299	0.20536	0.00209
XM_578186.1	LOC502689	-0.0763	-0.1345	-0.0841	0.036	0.09404	0.24499	-0.005	-0.1191	0.06996	0.00521	0.10663	0.06525	-0.1235	-0.125
XM_578190.1	LOC502693	0.02825	-0.0017	-0.001	-0.0132	-0.0338	0.00936	0.08136	0.01652	-0.0792	-0.0317	-0.1026	0.04961	-0.0466	-0.1006
XM_578194.1	LOC502695	-0.0725	0.00085	-0.0835	-0.0233	-0.0827	-0.0356	0.05304	-0.0866	-0.0844	0.16003	-0.02	-0.0572	-0.0181	-0.0363
XM_578195.1	LOC502696	0.02386	0.0204	0.17669	0.27876	-0.0635	0.23089	0.06448	0.02682	0.21565	-0.0858	-0.0548	0.19525	0.16604	0.04787
XM_578196.1	LOC502697	0.08383	0.12402	0.19496	0.08269	0.08155	0.0446	-0.0341	0.10163	0.07843	0.02907	0.09979	0.3707	0.21017	0.10929
XM_578197.1	LOC502698	0.01718	-0.0194	0.10053	0.19198	-0.0261	-0.0189	0.24132	0.06731	0.02125	-0.0266	0.03905	0.30532	0.0895	0.02219
XM_578199.1	LOC502700	-0.0182	0.13486	0.20849	0.05406	0.13023	-0.0731	-0.0211	0.00694	0.0695	0.20377	0.07326	0.22483	0.03399	0.08335
XM_578205.1	LOC502705	-0.0105	-0.1898	-0.1729	-0.0952	-0.1286	0.07015	-0.1012	-0.1702	-0.0548	-0.1452	-0.1627	-0.1048	-0.1291	-0.1615
XM_578206.1	LOC502706	0.21219	0.04192	0.03695	0.0193	0.10241	-0.0565	0.03443	-0.0578	0.01938	0.05711	0.0032	0.01875	0.10401	0.03781
XM_578209.1	LOC502709	-0.1358	0.08415	-0.0811	-0.1711	-0.103	-0.1454	-0.0921	-0.1448	0.12832	0.06595	-0.079	-0.041	0.1255	-0.1081
XM_578211.1	LOC502712	0.09281	-0.0593	-0.0715	0.06844	-0.1382	-0.0353	0.02648	-0.054	0.13179	0.06519	-0.0287	0.24716	-0.0842	-0.0428
XM_578212.1	LOC502713	-0.0132	-0.1604	0.21733	0.29719	0.00347	-0.0228	-0.011	-0.1078	-0.0313	0.35523	-0.1159	-0.0208	-0.1412	-0.0283
XM_578216.1	LOC502717	0.05423	0.15649	0.09465	0.03822	0.38677	0.10143	-0.0893	0.03495	-0.0058	0.11136	0.04948	0.03143	0.22051	-0.0081
XM_578218.1	LOC502719	0.08902	0.06524	0.02496	0.24647	0.22698	0.26937	0.16008	0.27028	0.20742	0.07359	0.08258	-0.0206	0.28126	0.19775
XM_578225.1	LOC502726	0.09969	0.11592	0.10116	0.22754	0.20244	0.14697	0.07425	0.15515	0.31137	0.05682	0.33698	0.29837	0.0421	0.17565
XM_578227.1	LOC502728	0.29081	0.08012	0.07326	-0.0176	-0.0642	0.11112	0.00561	-0.0172	0.26621	-0.0376	-0.0225	0.15528	0.2178	0.24708
XM_578228.1	LOC502729	-0.2126	-0.1259	-0.2245	0.02648	-0.0183	-0.1843	-0.2643	-0.2395	-0.092	-0.2027	-0.2503	-0.0956	-0.0513	-0.1662
XM_578230.1	LOC502731	0.03933	0.03605	-0.0571	0.06508	0.0156	-0.0308	0.0443	-0.0169	-0.0976	0.19794	0.03926	-0.0993	0.14534	-0.0253
XM_578234.1	LOC502735	-0.1707	-0.1109	-0.0266	-0.1	-0.0859	0.00738	-0.1616	-0.0566	-0.1791	-0.1405	-0.1648	0.10661	-0.1308	-0.0503
XM_578241.1	LOC502741	-0.1255	-0.0044	-0.0538	-0.0207	-0.2206	-0.1913	0.14066	-0.0298	0.01845	-0.0086	0.07071	-0.0596	-0.0719	-0.1567
XM_580255.1	LOC502744	0.02255	-0.0153	0.01457	-0.0417	-0.0787	-0.0551	-0.0457	0.06639	-0.0212	0.13574	-0.0004	0.14172	-0.008	0.04942

XM_578244.1	LOC502745	0.3049	0.14832	0.20087	0.41969	0.16351	0.20952	0.05974	0.3315	0.06054	-0.0036	-0.0034	0.10893	0.17059	0.14778
NM_001077592	LOC502746	-0.0126	-0.0849	-0.0624	-0.0781	0.06436	0.16644	0.01531	0.09645	0.29054	-0.1292	-0.1155	0.03221	0.00321	-0.1363
XM_578249.1	LOC502750	-0.1802	-0.1654	-0.4224	-0.1979	-0.2386	-0.3337	-0.2184	0.13713	-0.4494	-0.3184	-0.2089	-0.3267	-0.7365	-0.5642
XM_580256.1	LOC502751	-0.1051	-0.0465	-0.1215	-0.0718	-0.1176	0.12258	-0.113	0.1508	-0.0121	-0.1099	-0.006	0.18684	-0.0624	-0.0726
XM_578250.1	LOC502752	0.05901	0.12459	-0.0468	0.16271	0.04301	0.57379	0.12899	0.06615	0.05246	0.0249	0.02878	0.38908	0.11474	0.05578
XM_578251.1	LOC502753	-0.1566	-0.1371	-0.0316	-0.1352	-0.0913	-0.0796	-0.0718	-0.1994	-0.107	0.24517	0.04994	0.07212	-0.0359	-0.0081
XM_578252.1	LOC502754	0.19024	0.03616	-0.0362	-0.048	-0.1786	0.05553	0.15949	0.12468	0.00351	-0.1112	-0.0618	0.0627	0.02688	0.14822
NM_001109347	LOC502758	0.06193	-0.0219	-0.0438	-0.0016	0.07587	0.12485	0.06362	-0.0099	0.05955	0.07687	0.0526	0.00405	0.15181	0.00706
XM_578260.1	LOC502762	-0.1366	-0.0801	0.06882	0.00224	-0.1081	-0.1467	-0.1046	-0.0843	-0.0084	-0.1331	0.03051	-0.2144	0.00735	-0.0384
XM_578261.1	LOC502763	0.03687	0.03221	-0.03	0.09877	-0.058	-0.0055	-0.0339	0.15145	-0.0519	-0.0314	-0.075	0.02523	-0.0169	-0.0644
NM_001109348	LOC502764	0.11434	0.03995	0.01312	0.12672	0.07395	0.17542	0.08891	-0.0097	0.05145	0.03282	0.15423	0.08668	0.08381	0.09047
XM_578264.1	LOC502766	0.1667	-0.1152	0.04448	-0.0543	-0.0149	-0.109	-0.0171	0.03184	-0.1368	-0.024	-0.0651	0.09187	-0.0158	0.01265
XM_578266.1	LOC502768	0.07628	-0.0101	0.08248	0.21249	0.14832	0.15945	0.07772	0.09249	0.01424	0.15981	0.1795	0.07638	0.03866	0.15277
XM_578267.1	LOC502769	0.08179	0.10401	-0.0734	0.23395	-0.0873	0.00573	0.15237	0.16582	0.10795	-0.0076	0.00127	0.06823	0.02074	0.06119
XM_578268.1	LOC502770	0.52348	0.12274	0.62314	-0.5192	0.33793	-0.0932	-0.3947	0.44743	-0.1581	-0.0733	-0.0139	-0.6012	0.34725	0.40045
NM_001114391	LOC502773	0.24607	0.40568	-0.1024	-0.0234	0.0983	0.22146	0.00461	0.34176	-0.0505	0.13516	-0.1111	0.23055	0.26437	-0.0389
NM_001029927	LOC502774	0.14136	-0.0101	0.11137	0.07019	-0.036	0.09398	-0.0206	-0.0401	0.0346	0.20897	0.20874	-0.0693	0.06057	-0.0205
XM_578273.1	LOC502775	-0.4638	-0.222	-0.3174	-0.2534	-0.0471	-0.255	-0.2755	-0.2998	-0.2057	-0.2201	-0.185	-0.2242	-0.4358	-0.0382
XM_578279.1	LOC502781	-0.066	0.01396	-0.0278	-0.0595	0.21971	0.05344	-0.0166	-0.0668	0.02329	0.1963	-0.0089	-0.0231	0.11732	0.02315
XM_578283.1	LOC502785	0.11161	0.19977	0.04287	0.07517	0.10954	0.1177	0.3274	0.28654	0.25241	0.17226	0.13818	0.11068	0.10001	0.20328
XM_578288.1	LOC502789	0.17713	-0.0872	0.08427	-0.0766	0.1859	0.2209	0.0617	-0.0999	0.03172	0.13965	0.1615	0.05943	0.3064	-0.022
XM_578289.1	LOC502790	0.12359	0.11308	0.17266	0.10994	-0.1875	0.13241	0.04595	0.53966	0.13598	0.1425	0.14977	0.20071	0.21868	0.5335
XM_578290.1	LOC502791	0.11186	0.15001	0.1591	0.17311	0.16953	0.35575	0.15843	0.14031	0.27217	0.43355	0.22249	0.13117	0.35267	0.24519
XM_578291.1	LOC502792	-0.0118	-0.0577	-0.0174	0.02235	0.14967	0.0263	0.03322	0.09337	0.08791	0.02217	0.08845	0.08204	0.11951	-0.0016
XM_578292.1	LOC502793	0.01047	-0.0138	0.13254	0.1139	-0.0187	0.16344	0.00905	0.22483	0.08947	0.07263	0.14593	-0.0107	0.16485	0.14886
XM_578293.1	LOC502794	-0.0396	-0.0627	0.06067	0.1732	-0.0129	0.06412	0.14855	-0.0088	0.12909	0.05803	0.05666	0.518	0.27335	-0.0031
XM_578294.1	LOC502795	-0.2794	-0.2773	-0.2196	-0.1739	-0.1396	-0.2348	-0.0755	-0.1494	-0.1471	-0.2063	-0.0299	-0.0278	-0.2227	-0.0435
XM_578295.1	LOC502796	-0.2317	-0.1445	-0.1376	-0.1463	-0.2363	-0.2509	-0.1518	-0.1157	-0.3043	-0.1851	-0.1562	-0.1288	-0.3259	-0.2295
XM_578296.1	LOC502797	0.02553	-0.0518	-0.0587	-0.0349	-0.1825	-0.1261	0.08011	-0.0443	0.04906	-0.1563	-0.1463	-0.0152	-0.1514	-0.164
XM_578298.1	LOC502799	0.0556	0.15998	0.39123	-0.0104	0.35238	0.21122	-0.1271	0.21722	-0.1808	0.04147	-0.0429	-0.0142	-0.1298	0.12604
XM_578299.1	LOC502800	-0.245	-0.0125	-0.1486	0.28468	0.0323	0.08447	-0.3293	0.01631	0.02956	-0.2621	-0.0203	0.09572	-0.045	0.2403
XM_578301.1	LOC502802	0.08469	-0.0346	0.00891	0.0441	0.12413	0.1373	-0.1086	0.10304	0.01867	0.19897	0.08047	0.05683	-0.063	-0.0371
XM_578302.1	LOC502803	0.11763	0.08279	-0.0697	0.10161	-0.0099	-0.0311	-0.1272	-0.0528	-0.0638	-0.0886	0.16677	0.03028	0.09724	-0.0431
XM_578303.1	LOC502804	0.14481	0.07938	0.18729	0.091	0.05112	0.08784	0.02138	0.14795	0.33901	0.37021	0.07126	0.04633	0.10233	0.06157
XM_578304.1	LOC502805	-0.0822	0.07109	-0.0657	0.07399	-0.0028	0.07857	0.1065	-0.0564	-0.1087	0.25895	-0.0697	0.14895	0.09119	0.2283
XM_578305.1	LOC502806	0.1488	-0.0212	0.10931	0.05286	0.03772	0.14634	-0.1468	0.20614	0.08912	0.04275	-0.0422	0.00356	-0.0673	0.2725
XM_578306.1	LOC502807	-0.024	0.11362	0.16069	-0.0025	0.08052	0.10155	0.12814	0.09988	-0.0883	-0.0397	0.1854	-0.0007	0.03454	-0.0492
XM_578307.1	LOC502808	-0.265	-0.1493	-0.0672	-0.332	-0.0614	-0.2227	-0.0911	-0.4181	-0.027	0.0299	-0.2439	-0.4194	-0.0045	-0.293
XM_578308.1	LOC502809	0.14149	0.05589	-0.0094	0.14899	0.09236	0.10738	0.15318	0.22619	0.03424	0.16959	0.34157	0.21437	0.23979	0.16474
XM_578309.1	LOC502810	0.16661	0.00481	0.15243	0.08702	0.04676	0.15242	0.2851	0.05662	0.14673	0.34475	0.15083	0.05005	0.14479	0.03489
XM_578310.1	LOC502811	-0.1348	-0.055	-0.0941	-0.1054	-0.3498	-0.205	-0.1407	0.02124	0.0168	-0.1896	-0.2528	-0.0633	0.08996	-0.0951
XM_578311.1	LOC502812	0.04311	-0.0062	-0.0233	0.03977	-0.0388	0.07704	-0.0237	0.01134	0.10817	0.06357	0.01364	-0.0363	0.16103	-0.0531
XM_578312.1	LOC502813	0.0184	0.1041	-0.0455	-0.0102	0.11707	0.10439	0.05882	0.30193	0.21817	0.0286	0.24963	0.04658	0.36102	-0.0854
XM_578313.1	LOC502814	0.05584	0.18669	0.1169	-0.1342	-0.1745	-0.0593	0.07905	-0.0802	0.16806	0.02743	0.0735	0.05026	0.06074	-0.0305
XM_578315.1	LOC502816	-0.1721	0.21374	-0.1218	-0.1541	-0.0787	-0.1109	-0.0486	0.23828	-0.0281	-0.0645	-0.0097	-0.042	-0.0717	0.06008
XM_578316.1	LOC502817	0.21352	0.0744	0.07347	0.06783	0.10189	0.15794	0.05209	0.01787	0.11502	0.09362	0.08284	0.11936	0.08404	0.17227
XR_000313.1	LOC502818	0.09433	0.08772	0.06218	0.02062	0.07465	0.00264	0.02963	0.11138	0.0727	0.07157	0.1142	0.13798	0.10514	0.00038
XM_578319.1	LOC502819	0.2262	0.20658	0.08933	0.19927	0.14532	0.24527	0.23652	0.26215	0.04295	0.39501	0.18733	0.23146	0.13895	0.17536
XM_578320.1	LOC502820	0.15461	0.04014	0.14917	0.12728	0.11794	0.22443	0.14368	-0.0151	0.07586	0.23361	0.13463	0.01852	0.03169	-0.0541

XM_578321.1	LOC502821	0.02148	0.06875	-0.0517	-0.0192	0.33735	0.24047	0.01114	0.03724	0.17918	0.03256	0.05898	-0.0381	0.04352	0.38925
XM_578324.1	LOC502824	0.35896	0.10063	0.19535	0.26273	0.196	0.14259	0.1738	0.31105	0.12357	0.45229	0.15643	0.15787	0.14447	0.0672
XM_578326.1	LOC502826	-0.0604	-0.0825	-0.0964	-0.1214	-0.0207	-0.1243	0.03614	-0.1085	-0.1288	0.04931	0.03193	-0.079	-0.0643	-0.1325
XM_578327.1	LOC502827	-0.1617	-0.1525	0.0213	0.02002	-0.2363	0.13345	-0.0444	-0.1324	-0.3445	-0.0002	-0.2403	-0.317	-0.0762	0.03954
XM_578328.1	LOC502828	-0.1327	-0.188	0.06007	-0.0145	-0.0689	-0.1392	0.03517	-0.0343	-0.0471	-0.006	-0.1546	-0.0002	-0.0463	0.09584
XM_578329.1	LOC502829	-0.2614	-0.2601	-0.1152	-0.2341	-0.2217	-0.2184	-0.2232	-0.1458	-0.0966	-0.0444	-0.0752	-0.0914	-0.2592	-0.235
XM_578331.1	LOC502831	-0.1655	-0.2664	-0.1397	-0.3165	-0.1946	-0.0379	-0.1828	-0.3885	-0.5453	-0.4154	-0.372	-0.5076	-0.1561	0.02293
XM_578332.1	LOC502832	0.09717	0.17798	0.06291	0.28753	0.06372	0.02004	0.10483	-0.0125	0.15182	0.07372	-0.0232	0.10029	0.4734	0.08959
XM_578334.1	LOC502834	0.0105	0.14621	0.04858	0.10517	0.12813	0.02926	-0.1375	0.157	-0.0139	0.01134	0.09472	0.16917	0.03488	-0.0029
XM_578335.1	LOC502835	-0.0038	0.17988	-0.0746	0.00255	-0.0223	-0.0311	0.07528	0.0419	-0.0689	0.12791	0.05767	0.11163	0.00423	0.02944
XM_578337.1	LOC502837	0.07116	0.16109	0.15952	0.05791	0.0522	0.027	0.28362	-0.0033	0.15028	-0.0048	0.13224	0.13626	0.14171	0.34201
XM_578338.1	LOC502838	-0.106	0.17351	0.00397	-0.1126	-0.0216	0.06884	-0.0736	-0.0104	0.13723	0.2179	-0.0903	0.0726	0.05241	0.15528
XM_578339.1	LOC502839	0.11092	-0.0309	-0.0548	-0.0196	-0.0008	0.07511	-0.0206	-0.0448	-0.0446	-0.0283	0.10499	0.02218	-0.0541	0.01584
XM_578340.1	LOC502840	-0.2316	-0.1633	0.15375	-0.073	-0.1367	0.17662	0.05223	-0.0067	-0.0958	0.11015	0.12524	0.24728	-0.0084	-0.2073
XM_578341.1	LOC502841	-0.1971	0.13888	0.05609	-0.0075	0.04079	0.37342	0.10866	-0.0015	-0.1442	-0.1967	-0.1245	0.27733	0.21616	-0.0206
XM_578342.1	LOC502842	0.20969	0.08321	0.30609	0.03799	-0.0005	0.16131	0.05329	0.05903	0.06002	-0.0269	-0.0155	0.03163	0.03793	0.08552
XM_578343.1	LOC502843	0.03262	0.10048	0.16602	0.01762	-0.0037	-0.0993	0.12273	0.11321	-0.0173	0.01043	-0.0393	0.22064	0.05179	0.02146
XM_578344.1	LOC502844	0.04251	0.0993	0.32674	0.3194	0.13986	0.10053	0.2317	0.33406	0.17022	0.05243	0.27597	-0.0018	0.0401	0.1174
XM_578345.1	LOC502845	0.31386	0.17086	0.15197	0.23853	0.11267	-0.0149	0.13386	-0.0967	0.22411	0.26586	0.04097	0.14002	0.03338	0.1267
XM_578347.1	LOC502847	-0.0156	-0.1021	-0.0526	-0.1649	-0.0016	0.03196	-0.1149	-0.0272	0.04109	-0.0897	-0.247	0.00411	-0.033	-0.1484
XM_578349.1	LOC502849	0.13387	0.22372	0.10521	0.12651	0.22535	0.14786	0.11657	0.0214	0.15637	-0.0331	0.04705	0.28602	0.22657	0.17764
XM_578359.1	LOC502859	0.02689	-0.0066	0.2958	0.13115	0.13367	0.12458	0.05155	0.23825	0.11933	0.07774	0.01454	0.03187	0.18249	0.06201
XM_578361.1	LOC502861	0.06827	0.12598	0.08986	0.29573	0.26909	0.06143	0.03897	0.04442	0.23259	0.00236	0.12766	0.03528	0.23531	0.07794
XM_578362.1	LOC502862	0.08238	0.09652	0.09426	0.03612	0.19296	0.06134	0.08621	-0.045	0.13155	0.09015	-0.0704	0.03707	-0.0003	0.1121
XM_578365.1	LOC502865	0.16213	0.08191	0.06547	0.12713	0.03215	0.01542	0.01262	0.16382	0.16594	0.20363	0.08364	0.21724	0.06243	-0.0083
NM_001114391	LOC502867	-0.0855	-0.0612	-0.0681	-0.0135	-0.1112	-0.0331	-0.0274	-0.0612	-0.0652	-0.0531	-0.1064	-0.0932	-0.0134	-0.0879
XM_578369.1	LOC502868	-0.167	-0.0755	0.05736	0.1546	-0.0216	0.02242	-0.2291	0.11771	-0.026	-0.1751	0.00611	-0.0105	-0.0228	0.14608
XM_578372.1	LOC502871	-0.0843	-0.1726	-0.2669	-0.1578	-0.1425	-0.1799	-0.2395	-0.1879	-0.0355	-0.0101	-0.1811	0.20192	-0.2175	-0.2282
NM_001044272	LOC502872	0.0094	-0.08	-0.0054	0.04568	0.28123	-0.0054	0.20763	0.14705	0.17243	0.03769	0.33046	0.15104	0.02353	0.18105
XM_578375.1	LOC502874	-0.1512	-0.1869	-0.1498	-0.0124	-0.1123	-0.2533	-0.0295	-0.1941	-0.2857	-0.3135	-0.1369	-0.2083	-0.2035	-0.2133
XM_578378.1	LOC502877	-0.1152	0.07821	-0.0206	-0.0156	-0.1194	-0.1165	0.2766	0.20223	-0.1623	-0.0862	0.29342	0.09763	0.02792	-0.1076
XM_578381.1	LOC502880	0.04424	0.26057	0.01475	0.12779	0.19592	-0.0323	-0.0075	0.08932	0.02268	0.06534	0.20797	0.22343	0.08665	0.09102
XM_578382.1	LOC502881	-0.014	0.01588	0.3808	0.41031	0.0457	0.02961	-0.0151	0.11517	0.22699	-0.0799	0.02401	0.05043	0.12766	0.04057
XM_578384.1	LOC502883	0.19302	0.0712	-0.0234	0.11366	0.2218	-0.064	-0.0123	0.02525	-0.0488	0.0807	0.16983	0.20148	-0.0759	0.0606
XM_578386.1	LOC502885	0.02283	0.09942	0.05491	0.08517	0.06036	-0.0314	0.21414	0.16964	0.059	0.11087	0.15825	0.10505	0.00791	0.03931
XM_578389.1	LOC502888	-0.3631	-0.2006	0.10532	0.15969	-0.1891	-0.4074	-0.0014	-0.3684	0.01343	-0.0805	-0.0563	-0.1294	-0.3576	-0.2626
XM_578395.1	LOC502893	0.09253	0.09435	0.1688	0.15944	-0.0897	-0.2806	-0.0684	-0.0644	0.04701	-0.001	-0.0027	0.20806	0.0858	0.18662
NM_001025064	LOC502894	0.18832	-0.0582	-0.0634	0.03329	-0.1314	-0.0605	-0.0176	0.09079	-0.162	0.02983	-0.0385	-0.0584	-0.0134	-0.1244
XM_578397.1	LOC502895	-0.1947	0.03938	0.14565	-0.0308	-0.0293	-0.0858	0.08168	-0.0472	0.04903	-0.1864	-0.0005	-0.0214	-0.0554	0.01102
XM_578398.1	LOC502896	-0.1751	-0.1123	-0.1237	-0.2196	-0.2071	-0.0789	-0.2163	-0.1858	-0.2008	-0.0918	-0.0461	-0.057	-0.0977	-0.094
XM_578400.1	LOC502898	0.28119	-0.0212	-0.0501	-0.1682	0.14879	0.01641	-0.1063	-0.0286	0.00473	0.05922	-0.1063	0.0626	-0.0384	-0.0034
NM_001109353	LOC502900	0.12709	0.05328	0.03931	-0.0196	0.07018	0.04759	0.00083	0.20416	0.18076	0.03897	0.04685	0.12168	0.11602	-0.0737
NM_001009497	LOC502907	-0.0068	-0.0739	0.17626	0.1457	0.17484	0.02943	0.11912	0.04829	0.04302	-0.0324	0.06332	0.07804	-0.0282	0.20183
XM_578410.1	LOC502908	0.06051	-0.1008	-0.136	-0.1089	-0.073	-0.0362	-0.0428	-0.0227	-0.0628	-0.0197	-0.0546	-0.0519	0.12459	-0.0016
XM_578411.1	LOC502909	0.00388	-0.2108	-0.1854	-0.1312	0.008	-0.2064	-0.2588	-0.296	0.01782	-0.1447	-0.0493	0.15562	-0.2682	-0.2983
XM_578412.1	LOC502910	-0.0473	0.03116	0.1688	0.09876	0.12889	0.30889	0.02335	-0.0388	-0.0374	0.01999	0.10237	-0.0902	0.0218	-0.0919
XM_578413.1	LOC502911	0.12903	0.08316	0.03167	0.04366	0.16733	0.04245	0.1176	0.18163	0.01443	-0.0583	0.02198	0.03053	0.06367	-0.0394
XM_578414.1	LOC502912	0.11029	0.07152	0.1505	0.07012	0.16034	0.1341	0.0155	-0.0009	0.0765	0.06301	0.13694	0.18978	0.17718	-0.0435
NM_001123469	LOC502913	-0.0441	0.04503	0.07718	0.0105	0.03304	-0.0122	0.18319	-0.0027	0.01545	0.01486	0.01767	0.14306	-0.0066	-0.084

XM_580257.1	LOC502915	0.0614	-0.3123	-0.2996	-0.3372	-0.267	-0.1889	-0.2105	-0.3657	-0.0474	-0.0929	-0.2933	-0.3131	-0.0756	-0.2881
XM_578421.1	LOC502920	0.08026	-0.0163	-0.0476	0.05838	0.15111	0.1164	0.05612	-0.0729	0.00589	0.08873	0.08846	0.02209	0.05847	-0.0634
XM_578422.1	LOC502921	0.42179	0.14161	0.1209	-0.0304	0.03572	0.20138	-0.0398	0.03575	-0.0822	0.16562	0.11295	0.06246	0.02604	0.22839
XM_578423.1	LOC502922	0.03997	0.22917	0.09011	0.07853	-0.0019	-0.0396	0.19007	0.16608	-0.0992	-0.0273	0.20016	0.03652	0.01442	-0.1744
XM_578425.1	LOC502924	0.0487	0.03111	0.36501	-0.0642	0.17739	0.12731	0.08893	-0.0207	-0.045	0.14744	0.1723	0.10952	0.16572	-0.0177
XM_578431.1	LOC502929	-0.0267	0.09154	0.02594	0.09065	0.38823	0.00369	0.10752	0.00745	0.19661	0.05691	0.07555	-0.0004	0.01287	0.26965
XM_578433.1	LOC502931	-0.0988	0.01252	-0.0247	0.15623	0.09656	-0.0089	-0.0254	-0.0985	-0.1369	0.02013	-0.1007	0.15112	0.10615	0.12731
XM_578437.1	LOC502935	-0.0537	-0.0524	-0.0889	0.18995	0.00421	0.00844	-0.0327	0.03018	-0.0545	-0.0111	0.05852	0.37804	-0.0099	0.05059
XM_578438.1	LOC502936	0.05378	-0.0049	0.00026	0.09775	0.01238	0.04421	0.13987	-0.0019	0.00844	0.20042	-0.0404	0.08162	0.22002	0.02157
XR_007268.1	LOC502940	-0.0333	-0.0371	0.05865	-0.0104	-0.0152	-0.0873	0.08505	0.04921	-0.077	-0.1685	-0.1553	-0.0144	-0.0557	0.00221
XM_578443.1	LOC502941	-0.2339	-0.2165	-0.1894	-0.0749	-0.3255	-0.0952	-0.2167	-0.327	-0.1824	-0.2475	-0.1884	-0.099	-0.2459	-0.2424
XM_578451.1	LOC502947	-0.0596	0.10692	0.11121	-0.2342	-0.0629	0.12039	-0.0311	-0.2326	-0.0766	-0.1353	-0.0044	-0.1662	-0.0561	-0.125
XM_578452.1	LOC502948	0.03121	0.19997	0.00504	0.26286	0.07475	0.17065	0.07512	0.08084	-0.0076	0.15499	0.01374	0.06982	0.04292	0.02766
XM_578455.1	LOC502950	0.0697	-0.0595	-0.1188	-0.0536	0.03154	-0.1437	-0.1876	-0.0861	-0.1481	-0.0659	-0.1021	-0.0782	0.11477	-0.0717
XM_578457.1	LOC502952	0.05267	0.26673	0.23919	0.18927	0.01447	0.13134	0.22511	0.15804	0.15239	0.24077	0.03866	0.17406	0.05351	-0.0207
XM_578458.1	LOC502953	-0.0662	-0.0128	-0.0509	-0.0058	0.0164	-0.0383	0.10543	-0.0689	-0.076	0.45519	-0.0576	0.03838	0.10539	0.03743
XM_578460.1	LOC502955	-0.126	-0.1561	0.03424	-0.1432	0.0713	0.14794	-0.1214	-0.2372	-0.1424	-0.0543	0.04867	-0.1412	0.20708	-0.0843
XR_007277.1	LOC502958	-0.2327	-0.3054	-0.1429	-0.1377	-0.3068	0.21665	-0.1092	-0.3027	-0.37	0.03657	-0.1685	-0.2523	-0.2688	-0.2376
XM_578469.1	LOC502964	-0.047	0.01708	-0.0176	0.10978	0.01851	0.02738	-0.0229	-0.0159	0.15325	-0.0149	0.03211	-0.0197	-0.039	0.04228
NM_001025065	LOC502970	-0.1157	0.06463	0.02498	0.08802	0.03786	-0.0088	-0.1942	-0.0423	-0.1762	-0.1503	-0.1665	-0.1759	0.07923	-0.1555
XM_578477.1	LOC502971	-0.0316	0.04899	-0.0593	-0.0276	0.01993	0.01869	-0.0475	-0.0029	0.04418	0.08426	-0.0251	-0.0531	0.07638	-0.0037
XM_578478.1	LOC502972	-0.1266	-0.176	-0.165	-0.0451	0.10806	-0.085	-0.2257	-0.156	0.13671	-0.2017	-0.1846	-0.1118	-0.2468	-0.1464
XM_578479.1	LOC502973	-0.0541	-0.1113	-0.0437	-0.1151	-0.008	0.15931	0.11088	0.01464	0.04928	-0.0547	0.06128	-0.1538	0.15715	-0.0502
XM_578480.1	LOC502974	-0.002	0.09968	-0.0381	-0.0557	-0.061	-0.0348	-0.0411	-0.0035	0.06138	-0.0031	-0.0693	-0.0754	0.00167	0.01398
XM_578481.1	LOC502975	0.15472	0.28644	0.08008	0.3038	0.38348	0.36404	0.33537	0.01882	0.20117	0.02035	0.09146	0.22729	-0.0341	0.21909
XM_578485.1	LOC502979	0.05858	0.11878	0.06376	0.01725	0.13636	0.25705	0.19197	0.25231	0.20508	0.04506	-0.0318	0.20014	0.02195	0.11415
XM_578488.1	LOC502982	-0.2989	-0.3513	-0.1628	-0.3136	-0.3616	-0.0799	-0.3683	-0.3221	-0.2748	-0.3421	-0.294	-0.3456	-0.3125	-0.242
XM_578489.1	LOC502983	0.12751	0.06654	0.24741	0.13979	0.07259	-0.0048	0.23265	0.06662	-0.0418	-0.0638	0.0215	0.01723	0.09082	0.0023
XM_578492.1	LOC502985	0.18145	0.1663	0.03818	0.04225	0.13652	0.12233	0.26676	0.05522	0.03797	0.17211	0.16114	0.24935	0.02962	0.18158
XM_578494.1	LOC502986	-0.0105	0.01321	-0.06	-0.1463	0.02127	-0.1366	-0.1057	-0.087	0.13219	-0.1022	-0.1739	-0.1117	-0.0989	-0.0995
XM_578495.1	LOC502987	0.06509	-0.2431	-0.1851	-0.2986	-0.2954	-0.2186	-0.2333	-0.0425	-0.3547	-0.2333	-0.3319	-0.2596	-0.2106	-0.3396
NM_001109358	LOC502988	-0.0489	-0.0871	0.10675	-0.0429	-0.054	0.03924	0.05693	-0.1178	0.05206	-0.0567	0.07479	-0.0177	0.19399	0.13728
XM_578497.1	LOC502989	0.06764	0.00289	0.0785	-0.0969	0.04647	0.09931	-0.0422	-0.0732	0.02385	0.01276	-0.0834	0.07042	0.03923	0.02109
XM_578498.1	LOC502990	-0.0414	-0.1364	-0.1386	-0.1775	0.1442	-0.163	0.0869	0.21856	-0.0636	-0.0692	-0.1481	-0.0187	-0.0595	-0.0415
XM_578499.1	LOC502991	0.01767	0.02189	0.12489	0.06278	0.07082	0.1521	-0.1058	-0.0199	-0.0725	-0.0754	-0.0702	0.09752	0.45079	-0.1016
XM_001078135.1	LOC503000	0.22765	-0.4874	-0.1718	-0.6069	0.1795	0.35203	-0.8313	0.07642	0.06613	0.0087	-0.1543	0.47577	-0.0444	0.1104
XM_578513.1	LOC503001	-0.1267	-0.0173	0.14393	-0.0806	0.02241	0.03708	-0.02	0.1123	0.04402	0.09639	-0.2075	-0.0014	-0.1139	-0.0266
XM_578515.1	LOC503003	0.1417	0.0545	0.3331	0.08533	-0.1002	0.01989	0.01564	0.13285	0.19418	-0.1533	0.03187	0.00788	-0.0626	0.1322
XM_578518.1	LOC503005	0.33541	0.09902	0.16271	0.30738	0.48853	0.51346	0.19669	0.6548	0.5605	0.49769	0.36241	0.09491	0.30747	0.28203
XM_578522.1	LOC503009	0.06548	0.03715	0.09407	0.12463	0.15426	0.10388	0.02771	0.06352	0.13267	0.27409	0.0632	0.11501	-0.0215	0.09253
XM_578524.1	LOC503011	0.217	0.07042	0.15855	0.14197	-0.0156	0.00795	0.06856	-0.0047	0.18485	0.01321	0.0692	0.00038	0.04273	0.10099
XM_578525.1	LOC503012	0.02933	0.07183	-0.1416	-0.131	-0.1078	-0.1182	-0.0837	-0.0646	0.07538	0.00801	-0.2475	-0.0161	0.01938	-0.0336
XM_578526.1	LOC503013	-0.2305	-0.139	-0.0917	-0.0251	-0.1453	-0.0866	-0.0678	-0.1582	-0.1941	-0.1299	-0.0764	-0.0715	0.03611	-0.3188
XM_578537.1	LOC503020	0.02155	-0.057	-0.0427	-0.0009	0.0933	0.10655	-0.095	0.00091	-0.1093	-0.0971	0.01815	0.0691	-0.064	0.08423
XM_578538.1	LOC503021	-0.0124	0.20401	0.28909	0.1658	-0.0906	0.08203	-0.012	-0.0346	-0.3029	-0.1204	0.0157	-0.013	-0.1074	-0.1379
XM_578540.1	LOC503022	0.16754	0.11442	0.10916	0.01268	0.07124	0.01478	0.15803	0.04171	0.01237	0.08034	0.17026	0.03089	0.13291	0.15657
XM_578541.1	LOC503023	-0.2229	-0.1786	-0.0629	0.03446	0.0112	-0.255	-0.1098	0.02409	-0.186	-0.2179	-0.1657	-0.2273	-0.2266	-0.1973
XM_578545.1	LOC503025	0.06613	-0.0343	0.01844	0.03159	-0.0228	-0.0439	0.00813	0.15096	0.01676	-0.1282	0.23413	-0.0016	0.02029	-0.0424
XM_578546.1	LOC503026	-0.0422	-0.108	-0.0954	-0.0639	0.0194	-0.0267	-0.1299	-0.095	0.1315	0.19215	-0.0093	0.02435	-0.0419	-0.0497

XM_578549.1	LOC503029	0.15047	-0.0204	-0.021	0.08907	-0.0606	-0.1206	0.10208	-0.1068	0.00833	0.04306	0.09714	0.02288	0.12897	0.25448
XM_578550.1	LOC503030	-0.1306	-0.1911	-0.2058	-0.1402	-0.2089	-0.2104	-0.2412	-0.2469	-0.2492	-0.2337	-0.2668	-0.1725	-0.1203	-0.2189
XM_578553.1	LOC503032	0.05438	-0.046	-0.0793	-0.0839	0.10432	0.02189	0.07356	-0.0754	-0.1107	-0.1087	0.00074	-0.0231	-0.2461	-0.0378
XM_578554.1	LOC503033	-0.1573	-0.0651	0.06838	-0.1756	-0.0853	-0.1946	0.02063	-0.0474	-0.1909	-0.1773	-0.2008	0.01011	-0.0323	-0.0949
XM_578558.1	LOC503037	-0.0446	0.04527	-0.0857	-0.0626	0.04741	0.06772	-0.1049	-0.0734	0.1066	0.06078	-0.1403	-0.0505	-0.0387	-0.016
XM_578559.1	LOC503038	0.03665	0.06272	0.05148	0.1244	0.04637	0.05959	-0.1267	0.16137	0.12273	-0.0011	0.02641	0.00686	0.05857	0.06934
XM_578561.1	LOC503040	-0.026	0.25773	-0.0178	-0.0256	0.06127	0.08053	0.08649	-0.0005	0.02936	-0.0539	-0.0452	0.11556	0.06971	0.01751
XM_578564.1	LOC503042	-0.0376	-0.0201	0.01591	-0.254	0.01227	0.15611	0.07318	0.05214	0.24678	-0.0913	0.00469	0.04266	0.09315	-0.256
XM_578570.1	LOC503047	0.01894	-0.105	-0.0912	-0.024	-0.0415	-0.0867	0.0082	0.2582	0.03854	0.01094	-0.09	0.00665	0.03947	0.09359
XM_578571.1	LOC503048	0.31059	0.2729	0.33384	0.36991	0.13637	0.21695	0.20027	0.17972	0.14552	0.03458	0.14782	0.41259	0.18483	0.20023
XM_578573.1	LOC503050	-0.0043	-0.0752	-0.0333	-0.0326	0.24008	-0.0495	0.02092	0.03522	0.03785	-0.0715	0.09752	-0.0167	0.03876	0.01693
XM_578579.1	LOC503056	0.05787	0.03287	-0.03	0.01248	-0.1149	-0.0572	-0.0049	0.23826	0.18153	-0.1262	-0.153	-0.0275	0.10865	0.21772
XM_578582.1	LOC503059	0.09459	0.31505	0.07831	0.05818	0.15023	0.12747	0.01187	-0.0108	0.19111	0.18955	-0.0033	0.07014	-0.0366	-0.0169
XM_580258.1	LOC503060	-0.1123	-0.0218	0.0431	-0.0168	0.10591	0.02535	-0.0873	-0.0775	-0.1268	0.20431	0.00214	-0.1948	-0.0058	0.00227
XM_580259.1	LOC503061	0.08374	0.11141	0.06576	0.30437	0.06278	0.04422	-0.0351	0.01963	0.00895	0.01723	-0.0738	0.044	-0.0816	0.07425
XM_578583.1	LOC503062	-0.0449	-0.0054	0.00431	-0.0106	-0.0514	-0.0861	-0.0353	-0.014	0.13762	-0.0408	-0.002	-0.1091	0.06323	-0.0152
XM_578584.1	LOC503063	-0.1614	-0.1283	-0.0386	-0.0117	0.00678	-0.0599	-0.1401	-0.1664	-0.0702	-0.0274	-0.0587	0.04957	0.10586	0.12983
XM_578585.1	LOC503065	0.07375	0.08739	-0.0467	0.07494	0.05519	0.16616	-0.0276	0.1112	-0.088	-0.0779	0.17473	-0.0326	0.00868	0.01143
XM_578586.1	LOC503066	0.10517	-0.1106	-0.1093	-0.0054	0.19896	0.28415	0.07139	-0.1901	-0.0221	-0.0575	0.21558	-0.1237	0.11788	-0.0584
XM_578588.1	LOC503068	0.04335	0.19407	0.07006	0.08387	-0.0386	0.12206	0.05219	-0.0045	-0.0158	0.12637	0.00538	0.22524	0.12673	0.11879
XM_578589.1	LOC503069	-0.3174	-0.3181	-0.1756	-0.1399	-0.1374	0.00977	-0.2036	-0.0891	-0.2043	-0.1551	-0.1539	-0.1379	-0.0081	-0.0182
XM_578590.1	LOC503070	-0.4538	-0.385	-0.3003	0.04321	0.06101	0.02759	-0.3693	-0.4707	-0.051	-0.287	-0.3106	-0.2734	-0.2317	-0.2138
XM_578593.1	LOC503073	0.1208	0.04305	0.15567	0.02283	-0.0094	0.09968	-0.036	0.18503	0.12969	0.27539	0.04776	-0.029	0.12904	0.07539
XM_578594.1	LOC503074	0.02599	0.06366	0.08839	0.13072	0.02501	-0.0183	0.21083	-0.0539	0.15497	0.44664	0.04912	0.08967	-0.0929	0.0673
XM_578595.1	LOC503075	-0.093	0.04757	-0.0313	0.08215	-0.053	-0.0002	-0.0836	0.01054	0.08032	-0.0822	0.02728	0.0768	-0.0422	0.04118
XM_578597.1	LOC503077	-0.2991	-0.1257	-0.2911	-0.2259	-0.1508	-0.1701	-0.3089	-0.2475	-0.2731	-0.127	-0.2139	-0.317	-0.1258	-0.1463
XM_578598.1	LOC503078	0.03776	0.22369	0.10856	-0.0639	0.2353	-0.0251	0.19152	0.17028	0.30152	0.35933	0.11225	0.17718	0.39885	0.5793
XM_578599.1	LOC503079	-0.0203	0.00936	-0.0826	0.24393	-0.036	-0.1185	-0.1543	-0.3142	0.0618	0.04044	-0.128	-0.0834	-0.0601	-0.0418
XM_578600.1	LOC503080	0.14924	0.06864	-0.0347	-0.1045	-0.0367	0.14915	-0.0517	-0.0012	-0.0065	0.14612	-0.0207	0.0188	0.03797	0.09523
XM_578601.1	LOC503081	-0.0529	0.04168	0.08885	0.06487	-0.0183	0.13995	0.20988	0.06327	0.07282	-0.0391	-0.0528	-0.0502	0.2225	-0.0267
XM_578602.1	LOC503082	-0.3227	-0.0904	-0.0541	-0.1229	0.04874	-0.0035	-0.1393	0.04796	-0.1641	0.05115	-0.0481	-0.0453	-0.057	-0.1587
XM_578605.1	LOC503085	0.25485	0.20375	0.07062	0.02456	0.19342	0.22595	-0.0228	-0.0485	0.17276	0.08529	0.16828	0.27991	0.13496	0.1704
XM_578606.1	LOC503086	-0.0009	0.12837	0.02435	0.16795	0.01891	0.08783	-0.0193	-0.0296	0.13504	0.04905	-0.0288	-0.0086	-0.0743	0.07435
XM_578607.1	LOC503087	0.05314	0.05197	0.11361	0.1581	0.26552	-0.0464	0.20521	0.12781	0.16608	-0.06	0.13611	0.0179	-0.043	-0.0064
XM_578609.1	LOC503089	-0.0299	-0.0923	-0.1963	-0.1145	0.16943	0.00431	-0.0193	-0.1323	-0.0704	-0.1427	-0.1716	0.01962	0.23163	-0.0144
XM_578610.1	LOC503090	-0.0155	0.1006	-0.0639	0.0814	-0.141	-0.0269	0.0017	-0.0814	-0.0473	-0.1054	0.04019	0.52569	-0.059	0.00802
XM_578611.1	LOC503091	0.37976	0.13384	0.20042	0.14989	0.19124	0.1549	0.25553	0.15563	0.39215	0.15396	0.17306	0.09751	0.13384	0.04753
XM_578613.1	LOC503093	0.0133	-0.0616	-0.103	-0.0214	-0.07	0.08249	0.01244	0.19858	0.26189	-0.0093	0.18926	0.10583	0.0394	-0.008
XM_578615.1	LOC503095	-0.0086	-0.088	0.12314	-0.1145	-0.146	-0.0416	0.0215	-0.1407	0.03474	0.01061	0.05044	0.05106	-0.0398	-0.06
XM_578616.1	LOC503096	0.06228	0.09768	-0.0903	-0.0701	-0.096	-0.1222	-0.0213	0.10461	-0.0403	0.0168	0.04799	-0.0682	0.10715	0.11235
XM_578617.1	LOC503097	-0.0769	0.23618	-0.0237	-0.0279	-0.0049	-0.1522	-0.1636	-0.0158	-0.0308	0.0382	-0.0764	0.05755	-0.0394	0.07714
XM_578618.1	LOC503098	0.15876	-0.0947	0.07006	0.05685	-0.1081	-0.1056	0.19425	0.12382	-0.093	-0.0066	-0.1193	-0.1015	-0.1174	0.04105
XM_578619.1	LOC503099	-0.169	-0.0184	0.09988	-0.0645	-0.0859	0.01886	-0.045	-0.0584	-0.0167	-0.0878	-0.0752	-0.0501	-0.0843	-0.1224
XM_578620.1	LOC503100	-0.0646	-0.1873	0.12588	-0.1427	0.07238	-0.1121	-0.0271	-0.0479	-0.0405	-0.197	-0.0884	-0.0419	-0.163	-0.01
XM_578621.1	LOC503101	-0.366	-0.3918	-0.2143	-0.3961	-0.0698	-0.2717	-0.2935	-0.063	-0.2332	-0.198	-0.3204	-0.2194	-0.3247	-0.2498
XM_578622.1	LOC503102	0.10683	-0.0431	-0.0823	-0.1162	0.05759	0.06839	-0.1441	-0.1179	-0.1707	-0.1333	-0.0538	-0.095	-0.164	-0.0364
XM_578625.1	LOC503105	0.10107	#######	-0.1297	-0.0936	-0.1053	-0.089	-0.0933	-0.179	-0.1325	0.01608	-0.1067	-0.0559	-0.0776	-0.1685
XM_578626.1	LOC503106	0.30641	0.03203	0.21749	0.07764	0.20146	0.12851	0.24517	0.14259	0.07458	0.12024	0.14397	0.16788	0.04589	0.1178
XM_578627.1	LOC503107	-0.0384	-0.019	0.00977	-0.0271	-0.0853	0.06366	-0.0434	-0.0909	0.25858	0.27072	0.10403	0.00196	-0.0102	0.08559

XM_578630.1	LOC503110	0.16737	0.1471	-0.1376	0.2345	0.0224	0.16529	-0.1602	0.06145	0.27624	0.043	0.08452	-0.0197	-0.2513	-0.0873
XM_578631.1	LOC503111	-0.3351	-0.3728	-0.2964	-0.3792	-0.2383	-0.406	-0.3885	-0.4287	-0.197	-0.2123	-0.2538	-0.3252	-0.0769	-0.1554
XM_578638.1	LOC503118	-0.1385	-0.0623	-0.1025	-0.1068	-0.2016	0.02164	0.09176	-0.1449	-0.016	-0.1341	-0.0798	-0.085	-0.0309	-0.042
XM_578639.1	LOC503119	0.22384	0.16811	0.05538	0.21385	0.08973	0.19592	0.05303	0.05249	0.07318	-0.06	0.08284	0.20757	0.24492	0.21225
XM_578645.1	LOC503125	0.0133	0.0133	0.09092	0.10477	0.01082	0.1019	0.04166	0.14034	0.03391	0.05292	0.02259	-0.0192	0.23098	0.13584
XM_578650.1	LOC503129	-0.0199	-0.116	0.09684	-0.0476	-0.1169	-0.1012	0.0201	-0.0542	0.14075	0.01028	-0.0694	0.02559	-0.0004	0.05339
XM_580260.1	LOC503130	0.1241	-0.0583	-0.1957	0.23761	0.06953	-0.1627	-0.0156	0.0267	-0.0523	0.39939	0.09478	0.10079	-0.2281	-0.1501
XM_578653.1	LOC503132	0.05685	-0.1633	-0.0031	-0.1974	-0.0438	-0.2049	-0.2043	-0.0682	0.10842	-0.2813	-0.08	-0.1634	-0.1354	0.01476
XM_578656.1	LOC503135	0.06085	-0.0459	-0.0578	-0.0149	0.10849	-0.01	-0.0346	-0.0914	-0.0453	0.19359	0.01093	-0.0409	0.05602	-0.0266
XM_578659.1	LOC503137	-0.2052	-0.0444	-0.1567	-0.1154	-0.2476	-0.2476	-0.1166	-0.1989	-0.1343	-0.0925	-0.1742	-0.0904	-0.1928	-0.0655
XM_580261.1	LOC503138	-0.1028	-0.0237	-0.1238	-0.0686	-0.177	0.01305	-0.02	-0.0673	-0.0856	-0.1392	-0.1631	-0.0343	-0.1948	0.04379
XM_578661.1	LOC503140	0.07035	-0.01	0.12777	0.0545	0.02338	0.12301	0.04603	0.21877	0.1901	0.06605	0.0846	0.14363	0.17092	0.16772
XM_578662.1	LOC503141	0.14454	0.14056	0.09603	0.12782	0.2263	0.1697	0.1128	0.19444	0.20768	0.14274	0.01317	0.15162	0.1659	0.2206
XM_578663.1	LOC503142	0.23482	0.1993	0.50891	0.42739	-0.0638	0.03578	0.51557	0.15847	-0.0131	0.1778	0.20505	0.13072	0.32198	0.4957
XM_578665.1	LOC503144	-0.0603	0.01252	0.11864	0.00488	0.01937	-0.065	-0.0297	0.01738	0.07824	0.13552	0.12108	0.12552	0.04413	0.13917
XM_578666.1	LOC503145	-0.0077	0.01094	0.06807	0.09459	0.02407	0.0131	0.09677	0.04834	0.13784	0.03679	0.11416	0.12221	0.05004	0.1582
XM_578668.1	LOC503147	-0.0021	0.18328	0.19211	0.17297	0.02034	0.0735	-0.0143	0.06669	0.02439	0.28174	0.10871	0.06728	0.01811	0.02568
XM_578671.1	LOC503150	0.07824	0.0553	0.02448	0.19797	-0.0042	0.08055	-0.0269	0.16334	0.11642	0.09538	-0.0213	0.00171	0.17145	-0.0014
XM_578680.1	LOC503156	0.0942	0.03075	0.05724	0.01494	0.06027	-0.0477	0.0894	-0.0918	-0.0423	-0.1138	0.42083	0.25609	-0.1087	0.19229
XM_578682.1	LOC503158	-0.1296	-0.0029	0.00363	-0.0148	-0.0352	-0.0522	-0.0208	0.11721	-0.0376	0.16116	-0.0451	-0.0525	-0.1024	0.0494
XM_578684.1	LOC503160	0.04861	-0.0842	-0.0445	-0.1771	-0.1714	-0.0693	-0.0552	-0.0876	-0.1454	-0.0411	-0.1154	0.00956	-0.2067	-0.0749
XM_578685.1	LOC503161	-0.0478	0.05278	-0.1036	0.12744	0.11917	0.23133	-0.0046	0.19785	-0.0226	-0.1184	-0.0115	-0.0495	-0.0597	0.05501
NM_001113792	LOC503162	-0.2595	-0.0688	-0.0626	-0.1585	-0.199	-0.1212	-0.0047	-0.1081	-0.0917	-0.1504	-0.113	-0.1733	0.01565	-0.1194
XM_578690.1	LOC503166	0.19679	0.01576	-0.0202	0.19366	-0.0063	0.09323	0.0098	0.16656	-0.0067	0.13568	0.27482	0.01508	0.0554	0.01515
NM_001025698	LOC503168	0.08754	0.09415	0.12019	-0.0599	0.13662	-0.0007	-0.0859	0.26034	0.14335	0.19362	0.07091	-0.062	-0.0005	-0.0419
NM_031100	LOC503169	-0.1668	-0.1739	-0.2522	-0.1995	-0.112	-0.1276	-0.2794	-0.081	0.00127	-0.164	0.0054	-0.0254	0.06706	-0.2418
XM_580262.1	LOC503170	0.21906	0.25642	0.32352	0.26852	0.15618	0.18901	0.1003	0.35682	0.43269	0.30497	0.389	0.45401	0.12459	0.16318
XM_578694.1	LOC503171	0.01136	0.03745	0.06926	0.08416	0.09842	0.0195	0.16639	-0.0123	0.1414	0.12958	0.1596	0.17345	0.12258	0.01072
XM_578695.1	LOC503172	0.08584	0.64044	-0.6222	-0.3356	0.08297	-0.4112	-0.218	0.09259	0.04796	0.2651	-0.028	-0.2412	0.22586	0.2264
XM_578697.1	LOC503173	0.09148	0.24365	0.32119	0.1487	0.12315	0.19548	0.32059	0.21531	0.08013	0.27285	0.52575	0.24389	0.27594	0.19573
XM_578698.1	LOC503174	0.04401	-0.1938	-0.063	0.10182	-0.2323	-0.1985	-0.1545	0.04312	-0.1167	-0.0942	-0.0811	-0.1193	-0.0594	0.0064
XM_578699.2	LOC503175	-0.0408	-0.105	-0.0271	-0.1503	-0.0967	-0.068	-0.0603	0.03401	-0.0211	-0.1648	0.01173	-0.0534	-0.0239	-0.0918
XM_578700.1	LOC503176	-0.7102	-0.4325	0.1923	0.49893	-0.7075	-0.8876	0.08764	-0.687	-0.8469	-0.4885	-0.3966	-0.5888	-0.8886	-0.7789
XM_578701.1	LOC503177	-0.2193	-0.0981	-0.1562	-0.0191	-0.1775	-0.2507	-0.0904	-0.2105	-0.1109	-0.1506	-0.2361	0.0483	0.02621	0.02456
XM_578704.1	LOC503181	0.14246	0.1685	-0.0008	-0.103	0.08607	0.23952	0.01795	0.25223	-0.1048	0.09643	0.0072	-0.1788	-0.1305	0.02721
XM_578705.1	LOC503182	0.0298	0.0483	0.0473	-0.0426	-0.0257	0.00047	0.07876	-0.0312	0.13714	0.08223	0.09168	-0.0729	0.30447	0.01146
XM_578708.1	LOC503185	0.05607	-0.1099	-0.1457	-0.0998	0.01309	-0.0902	0.02849	-0.2228	0.12814	0.07009	-0.1657	-0.0985	-0.0054	0.07917
XM_578709.1	LOC503186	0.15772	0.07416	0.06298	0.13691	0.07786	0.26885	0.28247	0.16072	0.06885	0.02587	0.17622	0.07979	0.04418	0.10513
XM_578714.1	LOC503191	-0.0141	0.0663	-0.0873	-0.074	-0.0557	-0.089	0.00175	-0.0535	0.07756	-0.0968	-0.007	-0.0257	0.05178	0.25907
XM_578715.1	LOC503192	-0.2811	0.00875	-0.6176	-0.4096	-0.2962	-0.8552	-0.2738	-0.3062	-0.3738	-0.3499	-0.2215	-0.5458	-0.3498	-0.7585
XM_578716.1	LOC503193	-0.3011	-0.1942	-0.2685	-0.253	-0.1537	-0.0524	-0.0758	-0.1936	-0.2751	-0.0337	-0.1669	-0.1023	-0.1641	-0.2619
XM_578719.1	LOC503195	-0.0991	0.04513	0.03512	0.04747	-0.0983	-0.2367	0.0598	-0.2005	0.09858	-0.0741	0.09683	0.22057	-0.0753	-0.01
XM_578721.1	LOC503197	0.15282	0.13359	0.13484	0.66859	0.14051	0.02784	-0.0854	0.0571	-0.0111	0.0181	0.11092	0.2973	0.03406	0.06656
XM_578723.1	LOC503199	-0.1983	-0.0331	0.09555	-0.0408	0.14832	0.14953	-0.0936	-0.123	-0.1721	-0.0058	-0.0101	-0.1124	-0.097	-0.0784
XM_578725.1	LOC503201	-0.0054	0.18689	-0.06	-0.0562	-0.0184	0.03123	-0.0753	-0.0428	-0.0924	0.11739	-0.0169	-0.0372	-0.0067	-0.106
NM_001000472	LOC503202	0.134	0.01763	0.21753	0.18615	0.08924	0.4203	-0.001	0.12758	0.18454	-0.0062	0.4455	0.04396	0.1439	0.09626
XM_578731.1	LOC503206	-0.0476	0.04015	-0.0361	-0.0817	-0.065	0.07833	-0.0954	0.0293	-0.0358	-0.0051	-0.0717	-0.0767	-0.1137	0.05238
XM_578732.1	LOC503207	0.1816	-0.0152	0.05308	0.00775	-0.0212	-0.0573	-0.0792	-0.0836	-0.0119	-0.0032	0.08623	0.0812	0.06718	-0.0487
XM_578733.1	LOC503208	-0.0007	-0.04	-0.0382	0.03972	0.10994	0.01982	-0.0291	0.00174	0.08996	0.12765	-0.0563	0.10718	0.04171	0.03416

XM_578735.1	LOC503210	-0.1943	-0.0908	-0.0288	-0.0446	-0.0831	-0.2199	-0.0437	-0.1326	-0.0427	-0.2221	-0.2224	0.04581	0.03653	0.06813
XM_578736.1	LOC503211	-0.1624	-0.2132	-0.1102	-0.2546	-0.4006	-0.0601	-0.2679	-0.2763	-0.3253	-0.3117	-0.0852	-0.1533	0.09358	-0.1762
XM_578737.1	LOC503212	-0.013	0.1008	-0.0354	0.13187	0.04299	0.07852	-0.0038	0.08238	-0.0069	0.07558	-0.044	-0.0678	0.04635	0.08837
XM_580263.1	LOC503213	0.05952	0.1897	0.03926	0.08199	0.08689	0.06165	-0.0008	0.21728	0.08929	0.14911	0.101	0.08931	-0.0439	0.14716
XM_578739.1	LOC503215	0.07722	0.13823	0.01162	-0.0187	0.01996	0.232	-0.0841	-0.0065	0.09665	-0.0572	-0.0066	-0.0498	-0.0274	-0.013
XM_578740.1	LOC503216	0.05346	-0.0323	0.19129	0.04565	-0.052	-0.1555	0.20603	0.01854	0.12103	0.04775	0.12119	-0.0509	-0.0571	-0.0304
XM_578741.1	LOC503217	-0.3053	-0.0301	0.33107	0.03209	-0.1949	-0.3688	-0.0673	-0.1483	-0.1016	-0.1482	0.0679	-0.3665	-0.3684	-0.4148
XM_578742.1	LOC503218	0.03056	-0.0759	0.06203	-0.0128	0.15052	0.01923	0.05119	-0.0667	-0.0715	-0.1838	-0.1027	0.03465	-0.1833	-0.1525
XM_580264.1	LOC503222	0.09285	0.2255	0.11822	0.13558	-0.0091	-0.0023	0.10576	0.10049	0.01685	0.29076	0.01855	0.08526	0.05588	0.0197
XM_578748.1	LOC503223	-0.0159	0.11654	0.36596	0.05248	0.05921	0.11307	-0.0411	0.10575	-0.0332	0.10469	0.13236	0.05246	0.32306	0.22682
XM_001067730.1	LOC503224	0.08189	0.07361	0.02436	0.11944	0.16859	0.12709	0.10131	0.24402	0.11824	0.0516	0.09616	0.20458	0.11857	0.19713
XM_578752.1	LOC503226	-0.0389	-0.0207	0.06914	-0.0602	0.0516	-0.0443	0.03516	-0.0394	-0.0355	-0.1025	0.03883	0.0013	-0.0478	-0.004
XM_578758.1	LOC503230	-0.0539	0.08624	-0.1183	-0.0858	0.0483	0.13667	-0.0609	-0.0019	-0.0198	0.2266	0.03395	0.07592	-0.0584	0.07268
XM_578759.1	LOC503231	0.06244	-0.0292	-0.0211	0.02048	-0.0545	-0.0205	0.2094	0.08976	0.01106	-0.007	0.22324	0.10539	0.07473	0.28346
XM_578763.1	LOC503233	-0.0542	-0.1053	-0.0345	-0.0704	-0.1168	-0.0262	0.04592	-0.1153	-0.0481	0.02817	-0.0545	-0.1206	-0.0813	-0.1051
XM_578765.1	LOC503235	-0.1252	-0.1825	-0.2589	-0.1386	-0.1988	-0.1681	-0.2352	-0.28	0.03208	-0.1621	-0.1592	-0.2254	-0.1845	-0.2573
XM_578766.1	LOC503236	0.08779	0.32186	0.12265	0.15885	0.01875	0.14027	0.23523	0.13642	-0.118	-0.1337	0.2383	0.14467	0.42328	0.34498
XM_578771.1	LOC503240	-0.1563	0.01561	-0.1476	-0.0493	-0.0481	-0.1243	-0.0792	-0.0541	-0.1229	0.11563	-0.1515	0.11485	-0.1347	0.00228
XM_578772.1	LOC503241	0.04251	-0.0647	0.10073	-0.0293	-0.0986	-0.1114	-0.1479	-0.019	0.0463	-0.097	-0.0726	-0.0192	0.10149	0.01292
XM_578773.1	LOC503242	0.06093	-0.1251	0.01512	-0.118	0.14402	-0.0972	-0.0986	0.32096	-0.0238	0.13636	-0.0865	0.1023	-0.0678	-0.0975
XM_578775.1	LOC503244	0.0843	0.03137	-0.0496	0.1002	0.02235	0.17191	0.02721	-0.0243	-0.0095	-0.0319	-0.0029	-0.0415	0.10513	0.07544
XM_578776.1	LOC503245	-0.0557	0.00656	-0.1866	-0.1689	0.07152	-0.0699	-0.1882	-0.2463	-0.0094	-0.0737	-0.0843	0.13927	-0.1776	0.09628
XM_578777.1	LOC503246	-0.1361	-0.1264	-0.1302	-0.0164	-0.1073	-0.1371	-0.2347	-0.2423	-0.172	-0.0246	-0.1615	-0.043	-0.0973	0.15247
XM_578780.1	LOC503248	0.04558	0.00962	-0.0139	-0.1017	0.02908	-0.0829	0.07132	0.02808	0.00052	-0.0575	-0.0612	0.04929	0.02604	0.098
XM_578781.1	LOC503249	-0.0981	-0.0693	-0.1207	0.04023	-0.1201	0.11328	0.12771	-0.092	-0.1453	-0.1053	-0.0248	-0.0361	0.00755	-0.0384
XM_578782.1	LOC503250	-0.1021	-0.1728	-0.0598	-0.1811	-0.0818	0.07738	-0.21	-0.3204	-0.1409	-0.0199	-0.25	-0.1234	-0.1342	-0.1007
XM_578784.1	LOC503251	0.20663	-0.0004	0.06902	-0.0315	0.16606	0.43999	-0.0124	0.18463	0.05958	0.1452	-0.0338	-0.0551	0.26813	-0.0091
XM_578789.1	LOC503255	-0.1818	-0.1834	-0.2626	-0.0874	-0.131	0.01329	-0.1814	-0.2797	-0.1981	-0.2582	-0.2851	-0.1773	-0.1186	-0.0413
XM_578790.1	LOC503256	0.05586	0.09839	0.09393	0.01675	0.11068	0.24578	-0.0325	-0.0313	0.2868	0.04453	-0.0209	-0.0716	0.04083	-0.0076
XM_578793.1	LOC503258	0.05064	-0.1857	-0.1173	-0.0702	-0.2294	-0.1843	-0.0443	-0.1941	0.03828	-0.2578	-0.0979	-0.1015	-0.1299	-0.0061
XM_578794.1	LOC503259	-0.0333	-0.0564	0.006	-0.2133	-0.0296	-0.2267	-0.0075	0.00634	-0.1805	-0.0938	-0.0866	-0.2851	-0.1924	-0.0386
XM_578795.1	LOC503260	-0.1387	-0.0679	-0.0714	-0.1242	0.08174	-0.0738	-0.0854	-0.0701	0.0618	-0.0143	-0.0489	-0.0467	-0.1069	-0.0398
XM_578796.1	LOC503261	0.01139	0.23671	-0.0891	0.08401	0.093	0.20234	0.13191	-0.0438	0.01341	0.33666	0.0172	0.0684	0.18317	0.16078
XM_578799.1	LOC503264	-0.0911	-0.019	-0.1991	0.04935	0.02279	-0.0397	-0.1312	-0.0882	-0.148	0.0858	0.02679	-0.1139	0.01447	0.18757
XM_578800.1	LOC503265	0.1429	-0.0673	0.0952	-0.0828	-0.0989	0.04902	-0.0663	-0.0523	-0.0907	0.10649	-0.0359	0.02934	-0.0018	0.0068
XM_578802.1	LOC503267	-0.0509	0.04453	0.0725	0.19039	-0.2098	0.01854	0.2529	-0.0365	-0.0505	0.05943	0.27938	-0.0935	-0.052	0.14289
XM_578803.1	LOC503268	-0.1355	-0.1097	-0.0242	-0.2133	-0.0809	-0.2105	-0.0852	-0.1332	-0.1303	0.1199	-0.0088	-0.1186	-0.1047	-0.2392
XM_578806.1	LOC503271	-0.1718	0.16206	0.08889	0.0599	-0.1047	0.09411	-0.2057	-0.1029	0.07972	0.00589	0.15288	0.10284	0.09076	-0.1297
XM_578808.1	LOC503273	0.0224	0.01302	-0.155	0.16796	0.23148	0.11614	-0.0824	-0.1803	-0.0945	0.10852	0.03198	0.04647	-0.0013	-0.118
XM_578810.1	LOC503275	0.24542	0.07742	0.16715	0.14507	0.09191	0.27943	0.14879	0.23679	0.07922	0.0874	0.20406	0.06754	0.17337	0.18577
XM_578814.1	LOC503279	-0.0053	0.14534	-0.0568	-0.0441	-0.096	-0.0159	0.09814	0.27318	-0.0299	0.00369	0.23188	-0.16	0.0112	0.00087
XM_578817.1	LOC503280	0.01621	0.04796	0.02664	0.03179	0.01277	-0.0338	0.05464	-0.1174	0.26016	0.11984	0.06662	-0.0259	0.05296	0.00798
XR_007520.1	LOC503280	0.00639	-0.0541	0.07265	0.08456	0.01309	0.01217	0.10605	-0.0168	0.0157	-0.1429	0.12525	0.14539	0.01058	0.06406
XM_578821.1	LOC503286	0.18608	0.24608	0.06295	0.06703	-0.0617	0.11691	0.09987	0.28558	0.07911	-0.0301	0.09253	0.18925	0.01874	0.07789
XM_229456.3	LOC503290	-0.0262	0.07184	-0.1045	-0.1581	-0.1013	0.04283	-0.1023	-0.2117	0.0186	-0.1536	-0.0979	-0.0276	-0.1	-0.0199
XM_578826.1	LOC503292	-0.16	-0.1487	-0.1198	0.0172	-0.2154	-0.1805	-0.1002	-0.1101	-0.1248	-0.0303	-0.1811	-0.0313	0.02374	-0.2107
XM_578827.1	LOC503293	-0.0443	-0.068	-0.0083	-0.0392	-0.1716	-0.0486	0.09483	-0.0832	-0.0357	0.00693	-0.0016	0.02026	0.03162	-0.121
XM_578830.1	LOC503294	0.11178	0.276	0.09323	0.3997	0.43128	0.62815	0.00489	0.10924	0.23763	0.39022	0.2162	0.20405	0.06083	0.1819
XM_578831.1	LOC503295	0.09091	-0.0818	0.04625	0.02067	0.06276	0.03683	-0.1196	-0.2542	0.04199	-0.0046	-0.015	-0.1775	0.02258	-0.1273

XM_578832.1	LOC503296	-0.1164	-0.0521	-0.1193	-0.1389	-0.0234	-0.1084	-0.0555	-0.1195	-0.0491	0.03124	-0.0983	-0.0487	-0.0711	-0.1214
XM_578834.1	LOC503298	0.04497	-0.0113	-0.0372	0.2318	0.00749	-0.0041	0.12228	0.19673	0.04563	0.05487	0.03122	-0.0007	-0.0402	0.05063
XM_578835.1	LOC503299	0.02169	0.08476	0.08937	0.33568	0.11357	-0.0946	0.04851	0.0411	-0.0635	-0.0934	0.06431	-0.0004	0.10774	0.18555
XM_578837.1	LOC503301	0.25246	0.17358	0.07137	0.24765	0.30637	0.25641	0.09187	-0.0666	-0.0061	0.03722	0.00847	0.27525	0.06486	0.05686
NM_001109364	LOC503306	-0.3673	-0.3003	-0.2494	-0.3161	-0.3393	-0.2283	-0.3863	-0.1995	-0.4115	-0.1691	-0.357	-0.3086	0.07041	-0.3239
XM_578845.1	LOC503310	-0.0341	0.12564	0.08545	0.19404	0.08634	0.08227	0.08202	-0.0236	0.29842	0.2654	-0.0941	0.12939	0.10012	0.04263
XM_578846.1	LOC503311	0.13695	0.55676	0.46246	0.44965	0.13813	0.29554	1.024	-0.0457	0.27281	0.46931	0.32807	0.26829	0.41503	0.31992
XM_578848.1	LOC503313	-0.2338	-0.0978	0.04444	-0.1152	-0.1106	0.12838	-0.2497	0.09168	0.02046	-0.006	-0.0214	0.01175	-0.0112	-0.1783
XM_578854.1	LOC503320	0.12358	0.0602	-0.1116	0.10643	-0.0098	0.18709	-0.0107	0.08426	-0.0129	0.10009	-0.0945	0.23482	-0.1633	-0.0038
XM_578859.1	LOC503325	-0.1544	1.3103	-1.1418	-1.3328	-0.891	0.72103	-1.0782	-0.0211	0.55674	1.0226	-0.349	2.0191	-1.1112	-1.2244
XM_578861.1	LOC503327	-0.0683	-0.0695	-0.0855	-0.1396	-0.1815	-0.1044	0.10941	-0.0456	-0.1034	-0.1736	-0.0027	-0.1767	-0.0793	0.04767
XM_578862.1	LOC503328	0.02852	-0.0335	-0.1183	-0.0508	-0.0083	0.02614	0.002	0.16356	0.16469	-0.0312	0.02949	-0.2118	0.12068	0.04162
XM_578864.1	LOC503329	-0.0983	-0.1048	0.01212	0.00119	-0.1049	-0.116	-0.1921	0.12713	-0.174	-0.0355	-0.1254	-0.0299	0.21284	-0.15
XM_578870.1	LOC503336	0.00731	0.11811	0.13666	0.01508	0.07563	0.08097	0.08293	0.093	0.3222	-0.019	0.09506	0.0639	0.07532	0.08343
XM_580265.1	LOC503339	0.04875	0.07342	-0.0085	0.07208	0.04581	0.17263	0.06112	0.06695	0.00643	0.04878	0.00537	0.04464	-0.0205	0.22428
XM_578878.1	LOC503344	0.22398	0.25033	0.08916	0.23453	-0.0076	0.11228	0.01778	0.053	0.06941	0.04359	0.04594	0.28007	0.05492	0.03792
XM_578881.1	LOC503345	-0.0672	-0.0814	0.35903	0.18455	-0.0489	0.21281	0.4537	0.0966	0.36328	0.1533	0.24919	-0.0013	0.07243	-0.0163
XM_578889.1	LOC503351	0.21321	0.4143	-0.149	-0.0094	0.4759	-0.0906	0.02172	0.57829	0.45039	0.35261	0.13862	-0.0114	0.66324	0.35197
XM_578892.1	LOC503354	-0.1706	-0.2445	-0.2153	-0.249	-0.1818	0.09917	-0.1889	-0.0937	0.06572	0.30505	-0.1151	0.12598	-0.0734	-0.0163
XM_578893.1	LOC503355	0.12997	0.69808	1.0799	1.0226	0.21084	-0.0015	1.2232	0.03377	0.59851	0.81118	0.47132	0.23714	0.54597	0.68345
XM_578899.1	LOC503360	0.11653	0.21015	0.17521	0.34783	0.37945	0.20107	0.106	0.10302	0.11536	0.10528	0.44407	0.20452	0.28056	0.31188
XM_578901.1	LOC503362	-0.1056	0.02675	-0.0021	0.17685	0.00582	0.14083	0.09595	0.10112	0.07356	0.01572	0.01593	0.17762	-0.0423	-0.137
XM_578903.1	LOC503364	0.21052	-0.0892	-0.087	-0.0566	-0.0236	-0.0759	-0.0252	-0.0656	-0.0076	-0.0976	-0.1252	-0.0818	0.00826	0.00644
XM_578904.1	LOC503365	-0.7159	-0.0886	0.06889	-0.8657	-0.7591	-0.2656	-0.3204	-0.821	-0.0822	-0.2553	0.07457	0.63554	-0.2891	-0.481
XM_578905.1	LOC503366	-0.1391	-0.0306	0.1482	-0.0617	-0.079	-0.0266	-0.109	-0.0737	0.11442	0.01457	-0.0198	0.05319	-0.0081	-0.0066
XM_578906.1	LOC503367	0.01054	0.1246	0.15149	0.18922	0.02687	-0.0409	0.11899	0.13036	0.23473	0.32657	0.0287	0.01616	-0.0206	0.02164
XM_578909.1	LOC503369	-0.0783	-0.0057	0.08282	0.2619	0.03098	0.10317	0.11712	-0.0035	0.01619	-0.0889	0.22129	0.11917	0.1262	0.21479
XM_580266.1	LOC503375	0.02751	0.01616	-0.0606	0.05965	0.02882	0.03234	0.01879	-0.03	0.0441	-0.0422	0.00602	0.04409	-0.0322	-0.0303
XM_578915.1	LOC503376	-0.0419	-0.1347	-0.0477	0.03	-0.1749	-0.0642	-0.0478	-0.0285	0.27248	-0.0252	-0.0263	0.008	0.02387	-0.0936
XM_580267.1	LOC503377	0.0199	0.11331	0.25773	-0.033	-0.0002	0.0537	0.06795	-0.1121	0.10418	0.09834	-0.0424	0.10944	0.01838	0.17353
XM_578916.1	LOC503378	0.18911	0.03132	-0.0297	0.13304	0.0895	0.27406	0.30084	-0.0529	0.15214	0.11021	-0.0069	0.28839	0.03249	0.1429
XM_578923.1	LOC503386	-0.0567	0.04191	-0.0186	0.0527	0.05227	0.01355	-0.0163	0.04509	0.12513	0.01163	0.23902	0.00635	0.00569	-0.0076
XM_578925.2	LOC503388	0.29446	0.2576	0.3016	0.39246	0.01776	0.18445	0.15028	0.17457	0.04849	0.08795	-0.0339	0.0122	0.05757	0.16917
XM_578927.1	LOC503389	-0.1036	-0.2015	-0.203	-0.1654	-0.1886	-0.1943	-0.133	-0.0512	-0.0061	0.21006	-0.0901	-0.1654	-0.1996	-0.1702
XM_578928.1	LOC503390	-0.0665	-0.2302	-0.2823	-0.1318	-0.1854	-0.1881	-0.0501	0.00299	0.07219	-0.1075	-0.0961	-0.1262	-0.186	-0.2411
XM_578930.1	LOC503392	0.05251	-0.1905	-0.092	-0.0314	0.03682	-0.0043	-0.1648	0.06373	-0.0436	-0.1899	-0.1645	0.00609	-0.1235	-0.0925
XM_578931.1	LOC503393	-0.1408	-0.0268	-0.1546	-0.0264	-0.1471	-0.0903	-0.1254	-0.1558	0.09111	-0.026	-0.1948	0.13683	0.15542	-0.134
XM_578932.1	LOC503394	0.01588	-0.0798	0.07256	-0.0413	0.13585	-0.0855	-0.0456	-0.0484	0.26987	-0.0081	0.10315	-0.0161	0.03694	-0.0526
XM_578934.1	LOC503396	-0.6681	-0.2832	-0.8804	-0.6541	-0.6942	-0.6758	-0.4867	-0.7713	-0.2747	-0.5755	-0.5833	-0.2818	-0.0481	-0.1855
XR_009201.1	LOC503397	0.05731	0.25059	0.23821	0.26528	-0.0007	-0.0335	0.30872	-0.074	0.01395	0.24621	0.07644	0.05991	-0.0091	0.14337
XM_578937.1	LOC503399	0.04684	-0.0695	-0.0011	-0.0867	-0.0094	-0.0681	0.11113	0.1057	-0.0775	-0.0153	0.02415	-0.0031	0.03175	-0.0302
XM_578938.1	LOC503400	-0.0027	-0.088	0.02676	-0.0331	-0.1199	0.12302	-0.0338	-0.0799	-0.0161	0.02952	-0.0184	-0.0438	-0.1168	0.23922
XM_578943.1	LOC503403	0.05204	0.14486	0.04112	-0.0008	-0.0326	0.20775	0.0236	0.01	0.02271	0.35456	0.1119	0.01309	0.07564	-0.0019
XM_578945.1	LOC503405	-0.0196	0.16514	-0.12	0.46722	-0.0502	0.05917	-0.0658	0.04809	-0.1133	-0.0539	-0.2219	-0.1058	-0.1971	-0.1021
XM_580268.1	LOC503406	0.09346	0.07253	-0.2046	-0.2018	-0.0327	-0.1055	-0.182	-0.2005	-0.1098	0.15837	-0.1732	-0.1975	-0.1401	-0.0181
XM_578947.1	LOC503408	0.15298	0.12338	-0.0448	0.03533	0.11875	-0.0253	-0.066	0.13215	-0.1082	0.09782	0.09324	0.02627	0.14997	-0.0971
XM_578948.1	LOC503409	0.70294	0.59996	-1.4397	-0.7119	0.17735	0.59424	0.33467	0.52472	0.65219	0.51079	0.63463	-0.0172	0.13848	0.12644
XM_578949.1	LOC503410	-0.2827	-0.3179	-0.2655	-0.1441	-0.2833	-0.1911	-0.3675	-0.2363	-0.3414	-0.145	-0.2284	0.20003	-0.2735	-0.2861
XM_578953.1	LOC503414	-0.1593	-0.2027	-0.2054	-0.0611	-0.0025	-0.1057	-0.0718	-0.061	-0.2518	-0.1553	-0.0872	-0.0397	-0.238	-0.2247

XR_007149.1	LOC503416	0.09905	-0.1121	0.10425	0.1939	0.05884	0.05427	0.04496	-0.0077	0.07409	-0.0596	-0.0311	0.1134	0.04398	0.15204
XM_578956.1	LOC503417	-0.1937	-0.0079	-0.2964	-0.345	-0.3006	-0.3305	-0.1809	-0.1617	-0.0722	-0.1957	0.0957	-0.3043	-0.1893	-0.2952
XM_580269.1	LOC503418	-0.0307	-0.5392	0.15187	-0.2351	-0.5525	-0.1647	-0.5385	-0.4885	-0.2301	-0.1615	-0.4209	0.2994	0.32506	0.18716
NM_001108630	LOC503419	-0.4148	-0.267	-0.2544	-0.2758	-0.4389	-0.2769	-0.4581	-0.6419	-0.3176	-0.4535	-0.5086	-0.4327	-0.4225	-0.4103
XM_578959.1	LOC503421	0.00156	0.00483	0.06351	0.11709	-0.0393	0.29116	-0.0427	-0.0899	-0.0645	-0.031	-0.0381	0.00692	0.10331	0.00345
XM_578960.1	LOC503422	-0.0153	-0.005	0.11767	-0.0741	0.13789	0.13126	0.10147	0.15767	0.17918	0.04115	-0.019	0.07037	0.13342	0.00313
XM_578967.1	LOC503427	-0.1578	-0.0652	-0.0212	0.0036	-0.044	-0.142	-0.0847	-0.199	0.06041	0.17745	-0.1444	0.20212	-0.0376	-0.0693
XM_578971.1	LOC503430	-0.0648	-0.0498	-0.1205	-0.1008	-0.0064	-0.1298	0.00253	-0.1581	-0.1319	0.04531	-0.1601	0.38328	-0.0379	-0.0498
XM_578972.1	LOC503431	-0.0103	0.08567	0.02832	0.41178	0.02054	0.04938	0.11667	0.08902	-0.0029	0.06095	-0.0003	0.02011	-0.0849	0.22537
XM_578973.1	LOC503432	0.01623	0.02639	-0.1197	-0.1436	0.01659	0.23487	0.03133	-0.0178	0.2677	-0.0463	0.16332	-0.0721	-0.0132	-0.0058
XM_578974.1	LOC503433	0.01652	0.05147	0.12073	0.02851	0.04934	0.08516	0.02525	0.0837	0.05313	0.14294	0.04337	0.01445	0.03764	0.01339
XM_578979.1	LOC503434	-0.1526	-0.0311	-0.0402	-0.1091	-0.2222	-0.1634	-0.2171	-0.2195	-0.1861	-0.1571	-0.1021	-0.1319	-0.1173	0.06283
XM_578982.1	LOC503436	0.12686	-0.0953	0.05319	-0.0826	-0.0809	0.11577	0.01755	-0.0514	-0.0638	-0.1094	0.06179	0.2099	0.16789	-0.1013
XM_578985.1	LOC503439	0.08185	0.13137	0.24087	-0.0553	0.29431	0.00452	-0.0574	-0.0864	0.08629	-0.012	-0.1128	0.04199	0.12026	0.10794
XM_578986.1	LOC503440	-0.1385	-0.0484	-0.015	0.04689	-0.1126	0.01368	-0.099	-0.0535	-0.1918	-0.0892	0.04971	0.05586	-0.0636	-0.0918
XM_578998.1	LOC503448	-0.205	-0.0722	-0.0248	0.18309	-0.1362	-0.0854	-0.0774	-0.229	-0.2504	-0.1017	-0.1047	0.04682	-0.1708	-0.2312
XM_578999.1	LOC503449	-0.1539	-0.2049	-0.1118	-0.2476	-0.1667	-0.1831	-0.2129	-0.0073	-0.2965	-0.2519	-0.192	-0.0073	-0.1241	-0.1113
XM_579000.1	LOC503450	-0.1384	0.13192	0.07961	-0.017	0.11066	0.10191	-0.0045	-0.0952	-0.0388	-0.0452	-0.023	-0.1375	-0.06	-0.0984
XM_579001.1	LOC503451	-0.0345	-0.1086	-0.0431	-0.0776	-0.0693	-0.0668	0.08185	0.01028	0.08829	-0.0998	0.09196	0.11797	-0.1037	-0.0148
XM_579003.1	LOC503453	-0.0084	-0.0593	-0.0212	0.02581	0.02993	-0.0139	0.10267	-0.0518	-0.0563	0.09906	0.02619	0.18028	0.02166	0.06875
XM_579013.1	LOC503460	-0.0444	0.00587	-0.0377	0.06615	0.16309	0.04014	-0.0744	0.02389	-0.0282	-0.0401	-0.0131	-0.0075	0.04606	0.0046
XM_579014.1	LOC503461	0.17721	-0.0438	-0.0976	-0.022	0.07326	-0.2068	-0.1139	-0.0146	-0.1003	-0.1563	0.10365	0.22012	0.32641	0.09497
NM_001109368	LOC503464	-0.0152	-0.0261	0.03154	-0.0707	-0.0014	-0.1058	-0.0935	-0.0049	-0.1682	0.08198	-0.0126	-0.1859	-0.0913	0.06906
XM_579021.1	LOC503467	-0.0508	-0.1126	-0.1131	-0.0593	-0.0249	0.08767	0.07373	-0.1343	0.12226	-0.137	-0.002	-0.0328	0.00253	0.03632
XM_579022.1	LOC503468	0.00759	0.21363	0.03085	0.23277	-0.0814	-0.0407	-0.1385	0.1217	0.07265	-0.0615	0.33642	-0.1012	0.15983	0.21592
XM_579023.1	LOC503469	0.06804	-0.0534	-0.0464	-0.0325	0.25468	0.12108	0.04247	-0.0702	-0.035	-0.0606	-0.1166	0.08327	0.02059	-0.0305
XM_579025.1	LOC503471	-0.0226	0.01382	0.23221	0.37426	-0.0322	0.12599	-0.0312	0.41765	0.16685	0.39323	-0.0338	0.0614	0.01207	0.06619
XM_580270.1	LOC503473	-0.3133	-0.1091	-0.0396	-0.1447	-0.1681	-0.3652	-0.2392	-0.2982	-0.0135	-0.008	-0.246	-0.1122	-0.3429	-0.3678
XM_579029.1	LOC503476	0.3304	-0.0607	0.02961	0.01622	-0.066	-0.0139	-0.0115	0.04133	0.01679	-0.0124	-0.0777	-0.0873	0.11447	0.095
XM_579036.1	LOC503479	0.50176	0.5288	0.31416	0.50564	0.26683	0.29149	0.13262	0.29473	0.29935	0.28033	0.32877	0.24251	0.28963	0.42591
XM_579038.1	LOC503481	-0.2473	-0.0688	-0.2606	0.02611	-0.1862	0.04724	-0.1925	-0.3499	-0.4261	-0.4058	-0.1793	-0.2757	-0.3444	-0.4853
XM_580271.1	LOC503482	-0.0875	-0.1507	-0.1914	-0.1688	-0.098	-0.1143	-0.1868	-0.1305	-0.0466	0.11551	-0.0189	0.50164	-0.1501	-0.1543
XM_580272.1	LOC503483	-0.169	-0.2291	-0.2051	-0.1449	-0.1611	-0.1665	-0.0019	-0.0529	-0.1933	-0.1263	-0.1094	-0.019	-0.0574	-0.1914
XM_229156.2	LOC503485	0.03317	0.00185	0.0339	0.03897	0.00444	0.00018	0.10151	0.0042	0.0828	0.09671	-0.0105	0.0702	-0.0007	0.10535
XM_579041.1	LOC503486	-0.1728	0.03548	0.04992	0.03246	0.24826	0.02799	-0.0085	0.13245	-0.1104	-0.2427	0.19223	-0.0186	-0.134	-0.088
XM_579044.1	LOC503488	0.08864	0.11884	0.10616	0.00228	0.18926	0.09517	0.24127	0.0662	0.16866	0.00928	0.08187	0.11241	0.0137	0.09544
NM_001167664	LOC503490	0.12406	0.22041	-0.0279	0.12975	0.17444	0.0824	0.01882	0.39587	0.28656	0.06259	0.03979	0.0349	0.15204	0.10998
NM_020089	LOC56764	-0.1079	-0.1115	-0.0666	-0.1047	0.06708	0.21528	-0.0103	0.04212	-0.0126	-0.0752	0.01383	-0.0823	0.11125	0.07986
NM_020091	LOC56825	0.0737	0.13752	0.08519	0.02917	0.00611	0.05651	0.05124	0.16413	0.0439	0.02134	-0.036	0.05771	0.07327	-0.0196
NM_001031627	LOC606294	-0.7038	-0.1766	-0.6821	-1.3028	-0.3697	-0.5497	-0.6247	-0.5509	-0.1231	-0.1289	-0.2294	-0.1331	-0.4017	-0.4082
NM_022271	LOC64038	-0.0025	0.07984	-0.0311	-0.1372	0.11962	0.66076	0.09499	-0.03	0.07099	0.05073	0.39899	0.44822	0.35541	0.10705
XM_001053214.1	LOC678741	-0.8814	-0.0756	-0.0271	-0.6793	-1.1101	-1.2686	-0.0587	-1.062	-0.0639	-0.3004	-0.0606	-0.0291	-0.0826	-0.0336
XM_001053168.1	LOC678785	-0.2955	-0.0293	-0.3949	-0.3527	-0.1794	-0.3001	-0.1706	-0.3452	-0.1455	0.23468	-0.1112	-0.0542	-0.258	-0.2627
XR_007278.1	LOC678786	-0.1109	-0.0577	-0.2944	-0.1872	-0.2224	-0.0475	-0.24	-0.39	-0.247	-0.0547	-0.3945	-0.2331	-0.3371	-0.2174
XM_001053354.1	LOC678825	-0.1277	-0.1379	-0.1687	-0.1069	-0.0735	0.09874	-0.1135	0.32326	-0.0663	0.00192	-0.2113	-0.1076	-0.1928	-0.1079
XM_001053398.1	LOC678833	0.07836	-0.0046	-0.1153	-0.0621	0.19563	0.06031	0.07019	-0.1149	0.03248	0.2958	-0.143	0.1205	0.08778	0.03234
XM_001053577.1	LOC678867	0.03189	-0.0971	0.19598	-0.0275	-0.025	0.14655	0.17894	-0.1322	-0.1227	-0.1614	0.03396	0.40029	0.02481	-0.0208
XM_001053582.1	LOC678868	0.17961	0.16134	0.10422	0.02801	-0.0683	0.09217	0.02775	-0.0024	-0.0225	0.16639	0.04917	0.04826	-0.0379	0.3435
XM_001053686.1	LOC678896	-0.2865	-0.5091	-0.3195	-0.5689	-0.785	-0.7364	-0.6368	-0.5752	-0.1954	-0.2461	-0.3816	0.07339	0.19105	0.26828

NM_001170326	LOC678897	0.183	0.02785	0.07112	-0.0016	0.06493	0.10526	0.13402	0.0079	-0.0123	-0.0335	0.11421	0.13387	0.02488	-0.0684
XM_001053866.1	LOC678928	-0.1518	-0.1448	0.06882	0.09718	0.01536	-0.0869	0.06272	-0.1135	0.20712	0.22378	-0.0061	0.12656	0.00689	-0.0273
XM_001054208.1	LOC679010	-0.1276	-0.2327	-0.0613	0.14688	0.01725	0.14219	0.00973	-0.0401	0.03263	-0.1848	-0.0683	-0.1626	0.1324	-0.0356
NM_001170429	LOC679020	0.10751	0.06477	0.24781	0.06809	0.13472	-0.0953	-0.0992	-0.1235	0.02679	0.03706	0.05208	-0.1168	0.1426	0.11704
NM_001106631	LOC679028	0.56843	0.39134	0.2516	0.41218	0.41211	0.26061	0.38168	0.18325	0.53564	0.24715	0.13065	0.22542	0.74125	0.49019
NM_001110165	LOC679036	0.31234	0.0262	0.23877	0.32946	-0.0164	0.20108	0.01026	0.30002	0.16792	0.03526	0.25822	0.17596	-0.0595	0.27924
NM_001110165	LOC679036	-0.303	-0.282	-0.0324	-0.1392	-0.0845	-0.1089	-0.2335	-0.244	-0.1194	-0.1644	-0.0436	-0.1868	-0.3103	-0.2904
XM_001054590.1	LOC679075	0.04759	0.07276	-0.0771	0.19899	0.26517	0.23168	0.20827	0.1114	-0.0141	0.11906	0.02975	0.08577	0.12884	0.1248
NM_001106351	LOC679081	-0.0685	0.00393	-0.1068	-0.0514	-0.1591	-0.1123	-0.1333	-0.2874	-0.1744	-0.0921	0.02866	-0.1388	-0.1396	-0.1211
XM_001054685.1	LOC679096	0.0977	0.11748	0.08626	-0.1032	-0.1553	-0.0596	0.03132	0.12693	0.12813	0.1904	0.06191	-0.047	0.15225	-0.0751
XM_001055210.1	LOC679107	0.13021	0.00525	0.12854	-0.0063	-0.0103	0.09158	0.02614	-0.072	0.02318	0.19367	0.07381	0.01918	0.2136	0.13489
XM_001054818.1	LOC679129	-0.0364	0.10378	-0.1448	0.13049	-0.0974	-0.0531	-0.1734	-0.378	-0.0526	-0.1182	0.24001	0.21696	0.22295	-0.2033
XM_001054250.1	LOC679161	0.65062	2.201	-0.1712	-0.3822	-0.6157	-0.7754	0.97484	-0.5746	1.2772	1.6226	1.7514	-0.1601	0.47606	0.34529
XM_001055133.1	LOC679188	-0.051	-0.0237	-0.0297	0.07395	0.0022	-0.0128	0.01373	0.16109	0.05737	0.22578	0.0983	0.05304	0.11347	0.14432
NM_001101000	LOC679221	0.02566	0.00103	0.04953	0.06321	0.02592	0.02241	0.01232	-0.0562	0.13234	0.13209	0.02451	0.065	0.07234	0.07039
NM_001106684	LOC679271	-0.1543	-0.0788	-0.0822	-0.0385	-0.1262	-0.0906	-0.2373	-0.0293	-0.0203	-0.1827	-0.1064	-0.0776	0.04318	0.16623
NM_001143858	LOC679295	-0.6881	-0.3437	-0.5327	-0.7331	-0.3517	-0.4852	-0.4749	-0.5007	-0.2444	-0.233	-0.3615	-0.2736	-0.6288	-0.449
NM_001134702	LOC679409	-0.1784	0.45063	0.59263	0.28366	-0.0533	0.16153	0.19969	-0.0891	0.56272	0.56066	0.5111	0.6525	0.40568	0.64502
NM_001127452	LOC679430	0.63233	0.47713	0.11011	0.25858	0.65069	1.0213	0.12794	0.78322	0.14421	0.2125	0.50964	0.00174	0.21851	-0.0455
XM_001053385.1	LOC679534	-0.0245	0.09178	0.1125	0.07231	0.19967	-0.0063	0.01163	0.12685	0.11795	0.15411	0.04905	0.10547	0.09891	0.09661
XM_001053629.1	LOC679595	0.04358	-0.0038	0.21923	0.05233	0.12091	0.10574	0.2157	0.03428	0.1981	0.24172	0.31577	0.05299	0.21684	0.0984
XM_001053265.1	LOC679610	0.03406	-0.0292	-0.0792	-0.0541	-0.0093	-0.0163	0.04295	0.05663	-0.084	0.01999	0.0703	-0.033	-0.0831	-0.0189
XM_001053861.1	LOC679649	0.00265	0.10527	-0.0253	-0.0587	0.06613	0.03669	0.18842	-0.0381	-0.0364	-0.072	-0.0126	0.17949	0.00721	0.04386
NM_022593	LOC679663	0.33665	-0.2653	0.1191	0.69894	0.1056	0.33986	0.02232	0.32781	0.29202	-0.0075	-0.0395	0.31644	0.24299	0.22579
XR_005538.1	LOC679664	-0.1027	-0.1307	-0.1783	-0.0134	-0.1396	-0.0971	-0.1437	-0.0452	0.02598	0.05783	-0.1397	-0.0654	-0.2129	-0.0815
NM_001014221	LOC679718	0.14991	0.0705	0.17846	0.19291	0.03743	0.02525	0.02561	0.06275	-0.0542	-0.0338	0.16139	0.44354	0.08724	0.03913
NM_001014221	LOC679726	0.4975	1.7885	1.4092	0.85622	1.6282	1.849	0.90842	1.5333	1.7064	2.0217	2.4759	1.0151	0.77342	0.49414
NM_001111295	LOC679731	-0.437	-0.4148	-0.0938	-0.8324	-0.3763	-0.1879	-0.2331	-0.1614	-0.4275	-0.6482	-0.2827	0.12367	0.09737	0.16921
NM_001014221	LOC679745	0.01198	0.4358	0.29201	0.07246	0.51156	0.34296	0.00058	0.34457	0.54301	0.44545	0.77134	-0.1384	-0.0938	-0.1268
XM_001054782.1	LOC679867	-0.1177	0.19854	-0.0061	-0.2233	-0.1867	0.24496	-0.0642	-0.062	0.02378	0.13513	0.172	0.21881	-0.0783	-0.1052
XM_001054844.1	LOC679869	-0.0266	-0.0294	-0.1608	-0.075	-0.068	-0.3637	0.13882	-0.1027	0.13588	0.05601	0.07791	-0.2076	#######	-0.1111
XM_001054800.1	LOC679873	0.16085	-0.029	-0.1717	0.11053	-0.0213	-0.0249	-0.1103	-0.0542	0.09175	-0.1369	-0.011	-0.1548	0.01526	-0.2125
XM_001054829.1	LOC679886	-0.0209	-0.1555	-0.7586	-0.7209	-0.1089	-0.0902	-0.3312	-0.2882	-0.0063	-0.0311	-0.0511	-0.3217	-0.4652	-0.4228
NM_001109381	LOC679890	0.43933	0.03334	-0.227	-0.0924	0.45455	-0.0591	0.12034	0.3592	-0.0064	0.16737	-0.1423	-0.2228	0.11834	0.22054
NM_001109381	LOC679890	0.14518	0.04521	0.17983	0.38122	0.42769	0.28467	0.50064	0.40394	-0.0465	-0.1919	0.17986	0.07019	0.0462	0.26245
NM_001135582	LOC679898	0.29245	0.56394	-0.0975	0.12703	0.34135	0.22028	0.1714	0.38575	0.61287	0.72983	0.4416	0.40265	0.36224	0.49692
XM_001055134.1	LOC679974	0.21697	0.08316	0.00272	0.04475	0.0092	0.07601	0.12979	0.07744	0.00884	0.0372	-0.0096	0.13046	0.2099	0.01218
NM_001161691	LOC679977	0.26032	0.2208	0.30439	-0.0125	0.07704	0.03603	-0.0553	0.06579	0.03038	0.2411	0.13777	-0.0796	0.06761	0.14552
XM_001055313.1	LOC680012	0.2662	0.18047	0.37844	0.33444	0.31246	0.15184	0.20631	0.0208	0.20758	0.23359	0.20815	0.50104	0.19852	0.10573
NM_001126299	LOC680014	-0.0492	-0.1264	-0.1834	-0.4284	-0.501	-0.2894	0.29776	-0.2045	-0.0294	0.01094	-0.1869	-0.2998	-0.4357	-0.4216
NM_001014779	LOC680020	-0.018	0.10362	-0.1219	0.00733	-0.0043	-0.161	0.09902	0.17238	-0.065	-0.0466	0.12995	0.10411	0.15686	0.04728
NM_001109389	LOC680045	0.05496	-0.0505	-0.1221	-0.0405	-0.0655	-0.1959	-0.1336	-0.0216	0.00403	-0.0661	-0.1543	-0.0063	0.01178	-0.1774
NM_001109390	LOC680047	0.0107	0.04836	0.06819	0.06302	0.02123	0.02783	0.10062	0.07503	0.05239	0.10495	0.03961	-0.0203	0.02444	0.07206
XM_001054790.1	LOC680069	-0.1136	-0.1499	-0.0298	-0.1271	0.0114	0.11644	0.00668	-0.0955	-0.0928	-0.1365	-0.0939	-0.209	-0.1102	-0.136
XM_001055834.1	LOC680128	-0.1038	-0.0989	-0.0587	-0.1217	-0.1133	-0.2262	0.02846	-0.1933	-0.0912	-0.1435	-0.2261	-0.1456	0.03106	-0.1969
XR_005658.1	LOC680162	-0.0352	-0.1486	-0.1966	0.01704	-0.1007	-0.0195	-0.0687	-0.161	0.07834	-0.1721	-0.1162	0.02902	-0.0328	-0.0339
NM_001014221	LOC680166	0.17934	0.69813	0.09174	0.07691	0.39742	0.43748	0.23656	0.30354	0.70162	0.52824	1.1872	0.08208	0.27843	0.31629
XM_001056100.1	LOC680199	0.27982	0.15579	0.11924	0.1246	-0.0332	-0.0187	0.02154	0.03964	0.01318	0.63044	0.19096	0.18238	-0.0254	0.0916
XM_001056172.1	LOC680222	-0.1299	-0.0534	-0.2048	-0.16	-0.1429	-0.2225	-0.0111	0.18335	-0.1293	-0.2339	0.10172	0.07496	-0.2802	-0.2788

XR_005694.1	LOC680293	0.10014	0.17961	0.08015	0.10214	0.22253	0.05249	0.01077	0.17147	0.1524	0.03344	0.04994	-0.0081	0.09676	0.21197
XR_005695.1	LOC680294	-0.2106	-0.3365	-0.4741	0.11943	-0.1652	0.24285	-0.3631	0.03126	-0.0277	-0.3099	-0.1046	-0.1012	-0.5051	-0.5615
XM_001056619.1	LOC680317	-0.1627	-0.1278	-0.0963	-0.0332	-0.1126	-0.2059	-0.1837	0.01507	-0.1704	0.0477	-0.0328	0.09421	0.00494	-0.2186
XR_005709.1	LOC680325	-0.1474	0.04172	-0.1104	-0.0513	0.07925	-0.135	-0.0447	-0.0995	0.12609	-0.027	-0.0918	-0.0713	-0.003	-0.0334
XM_001056707.1	LOC680328	-0.0834	0.09478	0.00037	-0.1404	-0.0166	-0.0809	-0.112	-0.0804	-0.0918	-0.0888	-0.1383	0.00132	0.13972	0.18476
XM_574133.2	LOC680361	0.10504	0.51426	0.21456	0.34114	0.08451	0.23635	0.17081	0.0263	0.28765	0.84205	0.28177	0.22197	0.11884	0.28329
NM_001109405	LOC680424	0.05273	0.07409	-0.1742	-0.2054	0.1747	-0.1568	-0.1286	0.06118	-0.1889	0.02539	-0.0431	-0.1894	0.11181	0.0754
XM_001057154.1	LOC680431	0.27187	0.25695	0.17384	0.33293	-0.0258	0.04886	-0.0442	0.07559	-0.003	-0.0395	0.00503	0.01381	0.12705	0.05981
XR_005755.1	LOC680448	-0.1274	-0.0217	-0.0805	0.02073	-0.1653	-0.0974	-0.0431	-0.027	0.1752	-0.2454	-0.1829	-0.093	-0.0665	0.00943
NM_001034913	LOC680460	-0.175	0.18602	-0.4812	-0.268	-0.0702	-0.4312	-0.0145	0.01726	-0.1115	0.11494	-0.101	-0.0298	-0.1062	-0.1027
NM_001034913	LOC680460	-0.154	-0.0537	-0.1655	0.02119	-0.2838	-0.2377	0.06801	-0.276	-0.2762	-0.1331	-0.3187	-0.2871	-0.4303	-0.2394
XM_001057332.1	LOC680470	-0.0297	0.1219	0.13377	0.06052	0.10131	0.1738	0.12943	0.05224	0.01908	0.11807	-0.0294	0.12724	0.01305	-0.0654
XM_001057350.1	LOC680475	-0.1062	-0.1594	-0.1738	0.00488	-0.1854	0.03613	-0.1678	-0.1153	-0.1458	-0.1445	-0.1491	-0.2775	0.05599	-0.2601
XM_001057350.1	LOC680475	-0.0574	-0.1356	0.29169	0.14924	-0.0551	-0.0681	0.02787	0.04988	-0.0203	-0.0216	0.03196	-0.0566	0.02519	0.09996
NM_001047959	LOC680519	-0.2715	-0.2043	-0.1594	-0.2046	-0.1188	0.01253	0.09579	-0.1874	0.11755	-0.2445	-0.0593	0.0512	-0.1866	-0.145
XM_001057551.1	LOC680523	-0.0683	0.14722	-0.1895	-0.1809	-0.1257	-0.2423	-0.2225	-0.1127	0.02586	-0.1966	-0.0581	-0.0352	-0.0667	-0.2073
XR_005795.1	LOC680561	0.16166	0.19903	0.24735	0.25829	0.20031	0.14413	0.01061	0.12969	0.33844	0.2965	0.1256	0.09057	0.15345	0.2194
XM_001057880.1	LOC680582	0.16023	0.20036	0.09825	0.07727	0.19045	0.01701	0.044	0.11369	0.02508	-0.0241	0.0359	0.46699	0.29999	0.04222
XM_001057963.1	LOC680606	0.07178	0.20394	0.00862	-0.0802	-0.0825	-0.0315	0.02601	-0.0055	-0.0463	-0.0052	-0.0297	0.16305	-0.0594	0.14964
NM_001081751	LOC680620	-0.1388	-0.0578	-0.1011	0.03174	0.11407	0.36735	0.09898	-0.2162	-0.0476	-0.1301	-0.1493	-0.1551	-0.0817	0.03289
XM_001058142.1	LOC680647	0.17297	0.03383	-0.0346	0.13798	0.02648	0.13263	-0.0613	0.0267	-0.1463	-0.017	-0.0035	0.0847	-0.0771	-0.1237
XM_001058249.1	LOC680665	0.07329	0.01156	0.02806	0.02063	-0.0664	0.05082	0.03034	0.04913	0.12457	-0.027	-0.086	-0.2234	0.1093	0.021
XM_001056700.1	LOC680682	0.352	0.839	0.97375	0.76401	0.41197	0.42055	0.78092	0.59481	0.54028	0.76962	0.84531	0.36796	0.47649	0.30491
XM_001056892.1	LOC680711	-0.1118	0.12113	0.0112	-0.0705	0.04008	0.00375	-0.0239	-0.1251	-0.1039	0.11072	0.1695	0.02412	-0.0546	-0.051
XR_005855.1	LOC680736	-0.1137	-0.3411	-0.1821	-0.077	-0.0385	0.02764	-0.1235	-0.0681	-0.3666	-0.1392	-0.3053	-0.2714	-0.2507	-0.0305
NM_183402	LOC680831	0.59626	0.61372	-0.6628	-0.141	0.61152	0.14498	0.04441	0.49882	0.60378	0.40601	0.13368	0.07813	0.30596	0.4231
XR_005889.1	LOC680843	-0.063	-0.1452	-0.2215	-0.2152	-0.1982	0.0277	-0.1878	0.04282	-0.2852	-0.173	-0.1507	-0.0021	-0.0952	-0.0473
XM_001059150.1	LOC680844	0.19736	0.22247	-0.098	0.09315	0.18252	0.21928	0.1173	0.02427	0.056	0.09432	0.07448	0.17478	0.17959	0.16025
XR_005890.1	LOC680847	-0.01	-0.4592	0.43609	-0.3968	-0.5645	-0.7595	-0.8211	-0.6685	-0.3007	-0.355	-0.4	-0.1931	0.4355	0.33984
XR_005890.1	LOC680847	-0.2959	-0.9047	0.16057	-0.8655	-0.7875	-0.8687	-1.1374	-0.9788	-0.6515	-0.5698	-0.4606	-0.1626	0.0313	0.03168
XM_001059198.1	LOC680856	-0.5469	-0.4249	-0.1999	-0.1822	-0.0875	0.22153	-0.1879	0.01335	-0.4929	-0.4283	-0.5937	0.11227	-0.1908	-0.3066
NM_022298	LOC680876	-0.15	0.03859	0.0133	-0.19	-0.2595	-0.7647	-0.3255	-0.3815	-0.0935	0.13597	-0.0939	0.16663	-0.1617	-0.2348
XR_005909.1	LOC680907	0.07035	0.13665	0.14854	0.08668	0.21134	0.08242	0.26761	-0.0432	-0.1309	0.23198	0.00413	0.04095	0.16003	0.14409
NM_001109433	LOC680945	0.19153	-0.5419	0.5341	-0.0746	0.28101	1.0675	-0.207	0.34843	-0.0195	-0.2635	-0.6122	0.6398	0.60358	0.4609
NM_031108	LOC681065	0.02929	-0.0715	-0.1002	-0.05	0.03785	-0.1159	-0.0807	0.01157	-0.0243	-0.044	-0.1121	0.15318	-0.0662	0.16502
NM_001101675	LOC681126	-0.1093	-0.1086	-0.0091	0.02869	-0.0779	-0.0303	-0.1136	0.04061	-0.1197	0.05921	-0.042	-0.1132	0.0413	0.12079
XM_001060454.1	LOC681139	0.09828	0.15768	0.03924	0.13099	0.19548	0.0492	-0.0061	0.03785	0.10065	0.06238	0.23123	0.13109	0.0921	0.13199
XM_001060542.1	LOC681153	0.15263	0.02635	0.05979	0.07478	0.03918	0.09915	0.00685	0.12167	0.01447	0.04145	0.04295	0.09789	0.06233	0.14771
XM_001060542.1	LOC681153	0.02178	0.03057	0.05963	0.19996	0.10316	-0.0414	0.01228	0.29768	0.0295	-0.0133	-0.0774	0.16123	-0.0234	0.00424
XM_001059937.1	LOC681180	-0.0705	-0.0363	0.0288	0.0069	0.13605	-0.0975	0.00289	-0.0901	-0.1692	-0.2411	-0.1173	-0.1556	-0.057	0.05203
XR_005993.1	LOC681189	-0.1205	0.08233	0.14383	0.02207	-0.1653	-0.0537	0.17326	0.49544	0.02373	0.25094	-0.0185	0.14479	0.08281	-0.041
XM_001060874.1	LOC681237	0.37406	0.02861	0.02814	0.14908	0.17994	0.21263	0.15952	0.34003	0.10929	0.31387	-0.0589	0.14673	0.07378	0.01089
XM_001060954.1	LOC681252	-0.4472	-0.1722	-0.3798	-0.2938	-0.2879	-0.379	-0.341	-0.2223	-0.2178	-0.2632	-0.2734	-0.1785	-0.31	-0.2277
XR_006018.1	LOC681273	-0.2316	0.00529	-0.0064	-0.1929	-0.0104	-0.1802	-0.0757	0.05827	-0.0953	-0.133	-0.2076	-0.116	-0.1145	-0.0925
NM_001161835	LOC681288	-0.1095	-0.2619	0.01373	-0.0549	-0.2413	-0.0088	0.0201	0.12032	-0.1072	0.05637	0.03271	-0.1537	0.27173	-0.0346
NM_017059	LOC681314	-0.0411	-0.5536	-0.4269	-0.1216	-0.2924	-0.253	-0.0768	-0.5219	-0.4309	-0.7557	-0.5053	-0.3709	-0.5264	-0.6655
XM_001061313.1	LOC681336	0.00982	0.2572	0.40533	0.33135	0.40801	0.74921	0.47934	0.58657	0.1696	0.3269	0.64101	0.12141	0.09028	0.10275
NM_001109553	LOC681467	0.40205	-0.1217	0.28845	0.2409	-0.0353	-0.1495	0.18297	0.1094	0.05711	-0.0216	-0.0987	0.10353	0.21932	-0.1423
XM_001057095.1	LOC681492	-0.0944	0.11939	0.06143	0.03759	0.04885	-0.0023	0.02265	-0.0329	0.00566	0.14971	0.10067	0.13326	0.02444	0.0273

NM_001109402	LOC681539	0.09692	0.15086	-0.0623	-0.0621	-0.0422	0.05292	0.05658	0.09417	0.03774	-0.0392	0.0445	0.06141	-0.1028	0.0732
NM_001109503	LOC681542	0.11312	-0.1693	-0.0264	-0.0259	0.17368	0.37708	0.02502	0.12117	0.34665	0.05644	0.0621	0.51616	0.3461	0.26383
NM_001047921	LOC681549	-0.1676	-0.0489	-0.0748	-0.1216	0.18684	0.48465	-0.0839	-0.2468	0.01844	-0.1749	-0.024	0.72406	-0.1581	0.1309
XM_001054845.1	LOC681591	-0.2807	-0.3522	-0.0447	-0.253	-0.5935	-0.6423	-0.167	-0.4498	-0.2615	-0.4176	-0.4345	-0.2223	-0.3102	-0.3706
NM_001170749	LOC681618	0.13869	0.07266	-0.0003	0.42259	0.02913	0.27196	-0.0908	-0.0233	0.28201	-0.0273	-0.0168	0.21661	-0.1377	-0.1256
XM_001058194.1	LOC681732	-0.1158	-0.1154	-0.242	-0.1961	-0.2059	0.02547	-0.1642	-0.1225	-0.1785	-0.1134	-0.0711	-0.1555	-0.1145	-0.155
XM_001058666.1	LOC681840	-0.0204	-0.1044	0.15221	-0.1107	-0.0593	-0.053	-0.1836	-0.142	-0.1536	-0.1652	#######	-0.0238	-0.1393	0.00862
XM_001058779.1	LOC681872	0.96801	0.01782	0.01844	0.20415	0.14872	0.10047	0.96208	0.07202	-0.06	0.01757	-0.0583	0.00578	0.03471	0.06724
NM_001109546	LOC681886	-0.1803	-0.1525	-0.2663	-0.1501	-0.2083	-0.0027	-0.2856	-0.3085	-0.1513	-0.1331	-0.1159	-0.1187	0.01061	-0.0409
XM_001058857.1	LOC681895	0.15523	0.03582	-0.0123	0.10152	-0.0199	0.07769	-0.0062	-0.0711	0.14301	0.00165	0.00866	0.11548	0.09599	-0.0449
XR_007710.1	LOC681929	0.00623	0.07699	0.15921	0.05286	0.05683	-0.0396	0.00245	0.008	0.15667	0.1742	0.11842	0.01617	-0.058	0.24935
NM_053483	LOC681932	-0.6447	-1.7183	0.43673	-1.0402	-1.2193	-1.4175	-1.477	-1.2181	-0.8376	-0.988	-1.1496	0.18307	0.35135	0.47757
NM_001108948	LOC681982	-0.0401	-0.0698	-0.304	-0.1892	-0.2764	-0.0695	-0.4151	0.04919	-0.027	-0.1311	-0.3262	-0.1034	-0.4339	-0.2552
XM_001057688.1	LOC681994	0.09828	0.34928	0.02717	-0.0185	0.00139	0.16237	0.13446	-0.027	0.01794	0.048	0.01861	0.06512	0.13686	0.05293
NM_001115034	LOC681996	0.07713	0.00558	0.10051	-0.0063	0.01634	-0.0275	0.05109	0.04508	0.00456	-0.0227	0.01266	0.08971	0.00856	-0.0313
XM_001059323.1	LOC682004	0.1357	0.05227	0.27612	0.11678	0.13913	0.2258	-0.0357	0.08336	0.15139	0.02196	0.12294	0.36595	-0.0373	0.04659
XM_001059436.1	LOC682021	-0.08	-0.0275	0.00642	0.05741	0.17083	0.1849	-0.0894	-0.06	0.1774	-0.0911	-0.0089	0.3033	-0.1591	0.11362
NM_001000107	LOC682056	-0.026	0.08979	0.09087	0.0768	0.00796	-0.0394	0.03718	0.32968	-0.005	0.071	0.05858	0.0348	0.26532	-0.0172
XM_001056160.1	LOC682072	-0.0595	-0.2813	-0.2466	-0.408	-0.0088	0.05406	-0.4354	0.08511	0.00092	-0.0059	-0.0935	-0.1006	-0.0826	-0.1555
NM_001127565	LOC682088	0.06812	-0.0016	0.15975	0.28404	0.27556	0.21085	0.30838	0.25869	0.05173	0.14111	0.38732	0.27641	0.05343	0.18124
XM_001059846.1	LOC682097	-0.0267	-0.04	-0.051	0.03067	-0.0163	0.04434	-0.1182	0.12113	-0.044	-0.1134	-0.0854	-0.1621	-0.1204	-0.1641
NM_001135918	LOC682159	-0.3415	-0.962	0.9895	0.05193	-0.7019	-0.4938	-0.639	-0.7349	-0.4358	-0.7138	-0.7771	0.407	0.2832	0.56581
NM_031851	LOC682174	0.27805	0.02405	0.30169	0.1705	0.54807	0.8991	0.04169	0.33095	0.51561	0.51267	0.32362	0.82642	0.77949	0.72196
XM_001060297.1	LOC682179	0.13908	0.10454	0.10704	0.13166	0.28887	0.18912	0.12549	0.17194	0.14184	0.20641	0.16297	0.084	0.16539	0.22923
NM_001134730	LOC682182	-0.283	-0.0747	-1.0005	-0.0701	-0.1583	-0.5484	-0.6259	-0.3688	-0.2203	0.0996	-0.2088	-0.9277	-0.6461	-0.5908
XM_001060443.1	LOC682210	0.11919	0.07365	0.12255	0.00475	-0.0643	0.12071	0.0768	0.17128	0.15013	-0.0287	-0.1273	0.09074	0.1484	0.00733
XR_007853.1	LOC682235	0.05192	0.16479	0.19864	-0.0649	0.02986	0.03882	-0.0136	-0.0644	-0.0264	0.18295	-0.0914	0.19718	0.04886	-0.0893
NM_001109475	LOC682248	-0.1469	-0.576	-0.6693	-0.7668	-0.2419	0.01391	-0.4552	-0.5626	-0.8332	-0.8305	-0.5397	-0.7936	-0.8745	-0.6402
XM_001060731.1	LOC682264	0.25196	0.00805	0.04172	0.07177	0.47519	0.19405	-0.0093	0.55453	0.01386	0.04861	0.19247	0.05466	0.09153	0.19027
NM_001106142	LOC682303	0.52444	0.14916	0.25121	0.49606	0.77164	0.48421	0.18119	0.6118	0.15863	-0.0425	-0.0581	0.15649	0.58983	0.31243
NM_001047907	LOC682323	-0.31	-0.2045	0.1215	-1.3588	-0.6083	-0.8162	-0.3262	-0.6003	-0.0377	-0.1211	-0.1635	0.26355	0.63673	0.51884
XM_001061225.1	LOC682368	0.03787	0.09522	0.07802	0.04385	0.05152	0.03866	0.30026	0.21679	0.05163	0.04342	0.03825	0.13956	0.08023	0.12613
XR_007914.1	LOC682393	-0.2313	-0.3464	-0.1366	-0.1628	-0.2899	-0.4218	-0.0561	-0.4883	-0.2954	-0.5744	-0.2857	-0.1109	-0.2152	-0.3407
XM_001061636.1	LOC682469	-0.2624	-0.2093	-0.1848	-0.2045	-0.1863	-0.2609	-0.2104	-0.3982	-0.1456	-0.2119	-0.1798	-0.2675	0.03475	-0.1699
XM_001059809.1	LOC682475	0.08386	0.20714	0.12175	0.04745	0.00793	-0.0647	0.02876	0.03689	0.11822	0.25546	0.02076	0.27383	-0.1308	-0.1324
XM_001061691.1	LOC682483	-0.0474	-0.0847	-0.0497	-0.0842	-0.054	-0.1308	0.02451	-0.016	-0.0099	0.03386	-0.0097	-0.1165	-0.0411	0.01646
XM_001061786.1	LOC682504	-0.1541	0.21426	-0.0288	-0.0275	0.15791	0.17148	0.26825	0.30948	-0.0288	0.02289	-0.0836	0.09499	0.02909	0.09969
XM_001061896.1	LOC682529	0.0496	0.04343	-0.0888	-0.0222	-0.1531	0.04447	-0.0171	0.22653	-0.1256	0.00262	0.13624	-0.0692	0.27134	-0.0057
XR_007969.1	LOC682546	0.08619	0.08197	0.22181	-0.0347	0.07349	-0.064	0.02837	-0.0203	-0.0847	0.21439	-0.0063	0.19845	0.12988	0.16776
NM_001024796	LOC682571	-0.214	0.16936	-0.3855	-0.3262	-0.0154	-0.0236	-0.3216	0.10394	0.01677	0.20488	-0.2754	-0.0606	0.19933	0.08999
XM_001062202.1	LOC682593	0.02387	0.38861	-0.4584	-0.2848	0.14491	-0.4162	-0.0508	0.25375	-0.0701	0.08097	-0.0648	-0.1801	-0.077	-0.1396
XM_001062202.1	LOC682593	-0.3058	-0.009	-0.4723	-0.1019	-0.1557	-0.3062	0.25298	-0.0915	0.03955	0.24872	-0.0792	-0.2106	-0.2774	-0.4443
NM_203512	LOC682605	0.16077	0.02016	0.08686	-0.0316	0.10486	0.06385	-0.0098	-0.0192	-0.0197	0.07822	0.18467	-0.0122	0.13872	0.16787
NM_001134542	LOC682714	-0.1367	-0.4895	0.10068	-1.0177	-0.6246	-0.5041	-0.8092	-0.6748	-0.0354	-0.1332	-0.3746	0.45868	-0.3402	-0.2202
XR_009552.1	LOC682762	-0.2572	-0.5068	-0.1991	0.07305	-0.1541	0.00911	-0.4055	-0.224	0.05934	-0.4039	-0.0419	-0.1105	-0.5315	-0.4094
NM_001135090	LOC682767	-0.045	-0.0481	-0.0368	-0.0536	0.12202	0.05102	0.01183	0.04799	0.18178	0.13861	0.10311	0.15644	-0.0511	0.0377
NM_133594	LOC682787	0.19884	0.1304	-0.1796	-0.0829	0.10747	0.13422	-0.1421	0.20064	0.02997	0.26943	0.21604	0.10254	0.35025	0.15402
NM_001099483	LOC682835	0.14244	-0.0307	0.17159	-0.0623	-0.1637	-0.1448	0.05194	0.15676	-0.1613	0.06564	-0.0731	-0.0001	-0.0607	0.10375
XM_001063392.1	LOC682851	-0.1987	-0.0928	0.02218	-0.0943	0.00857	0.01063	-0.029	-0.2234	-0.1092	-0.1321	-0.129	0.17772	-0.0325	-0.0379

NM_001134642	LOC682855	0.08657	0.19073	0.04207	0.05164	0.26342	-0.0356	0.09557	0.0311	0.05721	0.11544	0.08069	0.10882	0.34073	0.27338
XM_001061265.1	LOC682869	0.64062	-0.246	-0.3997	-0.2931	3.2101	3.0897	0.17599	3.0968	-0.5191	0.03488	-0.4254	-0.2542	-0.5929	-0.7311
NM_001126096	LOC682893	-0.0457	0.21098	0.23497	-0.768	-0.2847	-0.2365	0.36951	-0.2564	0.2805	0.38069	0.22702	0.44853	0.47037	0.58322
XM_001063823.1	LOC682948	0.13095	0.03382	0.34512	0.33762	0.1088	0.02134	0.20273	0.04128	0.14697	0.26976	0.11601	0.12051	0.29163	0.00208
NM_001109520	LOC682965	-0.056	-0.0593	-0.1256	0.00836	-0.1385	-0.1131	-0.1291	-0.2195	-0.1596	-0.1756	-0.0449	-0.1906	-0.0986	0.06254
XM_001063980.1	LOC682990	0.17499	0.11207	0.20742	0.32355	0.23581	0.20504	0.04456	0.17831	0.22289	0.20902	0.50167	0.23837	0.33602	0.31717
XM_001064709.1	LOC683156	0.05487	0.22412	0.00754	0.12315	0.02081	0.19182	0.14805	0.20237	0.02037	0.01314	0.17689	-0.034	0.2954	0.244
XM_001064716.1	LOC683159	0.00622	0.05807	0.13414	0.08058	0.10609	0.2815	-0.0299	-0.0589	0.17018	0.15238	0.12262	-0.0177	-0.0244	-0.0364
XR_008208.1	LOC683212	0.21812	-0.0459	-0.0075	-0.0632	-0.0813	0.08197	0.02158	-0.0575	-0.0573	0.12134	0.0757	-0.0458	0.01325	-0.1135
XM_001065095.1	LOC683249	0.07136	-0.1111	-0.179	0.1297	-0.2464	0.14389	-0.3159	0.12157	0.38214	-0.1488	-0.1959	0.22532	0.1646	-0.1141
XM_001065131.1	LOC683258	-0.0869	-0.287	-0.4383	-0.4498	-0.4234	-0.4228	-0.1569	-0.2814	-0.178	-0.3364	-0.3054	-0.3272	-0.2449	-0.2243
XM_001065191.1	LOC683268	0.0173	-0.0357	-0.068	0.10756	0.15386	-0.0341	0.02483	0.08745	-0.0778	-0.0003	-0.0558	-0.0813	0.15395	-0.0546
XM_001065309.1	LOC683302	0.27606	0.39687	0.11662	0.26269	0.01529	-0.1501	0.1153	0.00111	0.37833	0.14791	0.20788	0.12616	0.04233	-0.109
XM_001062351.1	LOC683334	-0.3017	-0.3446	0.18013	-0.029	-0.3746	-0.2193	0.26829	-0.354	-0.0919	-0.3535	0.12809	0.01992	-0.4429	-0.523
NM_181378	LOC683347	-0.0416	0.09126	-0.0509	-0.1274	-0.0489	-0.1278	0.06843	-0.1644	0.11211	-0.0639	-0.1786	0.06188	-0.0547	-0.0264
NM_181378	LOC683347	0.17155	-0.0088	-0.0963	-0.0867	-0.0651	-0.0561	-0.0295	-0.0932	-0.0898	-0.1151	-0.1433	0.0306	-0.1125	-0.1341
NM_001109584	LOC683383	0.3014	-0.3661	0.17407	0.88958	0.23008	0.79385	-0.1644	0.33581	-0.1903	-0.2131	-0.2089	0.40225	0.22981	0.31242
NM_022392	LOC683385	-1.7337	-1.6924	0.31292	0.72872	-1.4039	-1.8159	-0.6512	-1.1042	-1.3263	-1.7786	-1.8065	-0.9075	-1.3708	-1.4969
XM_001065985.1	LOC683447	1.0883	0.66455	0.55006	0.38467	0.93857	1.2503	1.1389	0.94026	0.95856	0.7841	0.80215	0.61096	0.60369	0.51695
XM_001066042.1	LOC683463	-0.0544	-0.0438	0.29877	0.0874	-0.0071	-0.109	-0.0717	0.37634	-0.112	-0.0828	-0.1155	-0.1741	0.07396	0.16761
XM_001061932.1	LOC683474	-0.1043	-0.1361	-0.1638	-0.051	0.08214	0.07733	-0.1312	0.1148	-0.0849	-0.0767	-0.1383	-0.0807	0.10932	-0.0874
XM_001066346.1	LOC683522	0.0628	0.15938	0.06101	1.0257	-0.0232	0.02108	0.02074	-0.0723	-0.2298	0.0041	-0.0231	0.06581	0.22062	0.08179
XM_001061448.1	LOC683529	0.3564	0.07603	0.28439	0.07937	0.18946	0.34206	0.18806	0.22865	0.25306	0.21112	0.33448	0.37357	0.31769	0.21634
XM_001061448.1	LOC683529	-0.098	0.11626	-0.1455	-0.1858	-0.037	-0.104	-0.1075	-0.1769	-0.1626	-0.4132	0.08713	-0.2398	-0.0049	-0.0306
NM_001109411	LOC683534	0.28846	-0.0573	0.33184	0.00155	0.22192	0.12158	-0.2219	0.3272	0.25999	0.16325	0.02175	0.4736	1.0243	0.88496
XM_001066436.1	LOC683538	0.24019	0.03258	0.25775	-0.035	0.26252	0.35927	0.1737	0.33802	0.11192	0.21538	0.24106	0.37903	0.23691	-0.0028
NM_001108721	LOC683544	0.15984	-0.0167	0.13198	0.05501	-0.0567	0.07418	0.0307	0.09856	0.01168	-0.0902	0.18	0.10595	0.11429	-0.0308
NM_022188	LOC683548	-0.3286	-0.3369	-0.0111	-0.2809	-0.2577	-0.3022	-0.1489	-0.0007	-0.0251	-0.2712	-0.2371	-0.1285	-0.21	-0.3398
XM_001066537.1	LOC683564	-0.0369	-0.0747	0.0537	0.21935	0.13573	0.02341	-0.0005	0.07549	-0.0172	0.06661	-0.044	0.14294	-0.0124	0.09718
XM_001066540.1	LOC683565	0.16255	0.08685	0.12201	0.13607	0.00031	0.13249	0.00706	-0.0203	0.028	0.00465	0.15529	0.02401	0.02603	0.14895
XM_001067751.1	LOC683578	-0.0116	0.07755	0.08125	-0.0786	0.02051	-0.0452	0.15696	0.0124	0.03411	-0.013	-0.0927	0.11568	-0.059	0.0059
XM_001066643.1	LOC683585	-0.1636	-0.1225	0.06611	0.08018	-0.0549	-0.2504	-0.1046	-0.0775	0.01137	-0.162	-0.1077	-0.2201	-0.1073	-0.1474
XM_001066690.1	LOC683598	-0.095	-0.2426	-0.1607	-0.2621	-0.3466	-0.2537	-0.1622	-0.3159	-0.2509	-0.2797	-0.1954	-0.1216	-0.2818	-0.167
XM_001063166.1	LOC683618	-0.0164	0.04015	0.0031	-0.7381	0.17416	-0.2344	0.12544	0.13411	-0.1247	-0.0302	0.0141	-0.2648	0.06752	-0.0425
XM_001066890.1	LOC683649	-0.0264	0.20296	0.25769	-0.0316	0.08496	0.27647	-0.0444	0.11556	-0.0851	0.34099	0.15064	0.11904	0.08916	0.10506
XM_001064018.1	LOC683674	0.1122	0.08521	0.11438	0.06868	0.12007	0.04644	0.08889	0.00175	0.02531	0.13098	0.27764	0.17251	0.49442	0.28313
NM_001106658	LOC683686	-0.227	0.00974	-0.6792	-0.2697	0.16525	-0.6113	-0.0492	0.01371	-0.2985	-0.1149	-0.5041	-0.2611	-0.0991	0.00354
NM_001113754	LOC683694	0.14863	0.15293	-0.0563	-0.2072	-0.0488	0.28808	-0.2003	-0.0271	0.22286	-0.1214	0.10606	0.05218	-0.1482	-0.0183
XM_001067187.1	LOC683719	-0.0591	0.04383	-0.184	-0.0867	-0.0551	0.10282	-0.0324	0.1102	0.23886	-0.0669	-0.1245	0.05742	0.11456	-0.085
XR_009563.1	LOC683720	0.20565	-0.0903	0.25807	-0.3607	-0.0888	-0.0836	-0.1657	-0.214	-0.3995	0.05069	-0.059	-0.0784	0.15456	0.17155
XM_001067312.1	LOC683748	0.20098	0.07649	0.15283	0.16647	0.11332	0.05691	-0.0169	0.01456	0.26769	0.01617	0.01972	-0.0003	0.12309	0.01957
XM_001067412.1	LOC683767	-0.0118	0.34237	-0.2215	-0.4892	-0.1264	-0.605	-0.0503	-0.0758	0.09228	0.24726	0.39039	-0.0923	0.15675	0.12692
NM_057114	LOC683813	0.07731	0.02668	0.27189	0.28944	-0.0253	0.01163	0.13608	-0.0428	0.03926	0.04899	0.02311	0.14188	0.0726	0.09914
XR_008476.1	LOC683893	-0.1368	0.06483	0.23862	0.1367	0.10735	0.37507	0.02515	0.35501	-0.0067	-0.0814	0.13053	0.24052	0.19523	0.16652
XM_001068011.1	LOC683907	-0.0902	-0.0592	0.35882	0.23643	0.10051	0.45808	0.02598	-0.0385	-0.2285	-0.0658	-0.1561	0.41791	0.04006	0.04765
XM_001068058.1	LOC683919	0.1018	0.16525	-0.1916	-0.5788	0.17648	0.34261	-0.3354	0.21048	-0.1819	-0.144	0.33093	0.27467	-0.0891	0.07745
NM_001108998	LOC683941	-0.0771	0.19405	0.09431	0.17821	-0.0679	0.08685	0.04776	-0.117	0.10211	0.02925	0.19974	-0.123	0.00943	0.21182
XM_001068421.1	LOC683999	-0.3547	-0.1528	-0.1401	-0.4168	-0.3234	-0.0614	-0.3843	-0.1255	-0.0674	-0.0381	-0.2146	-0.2274	-0.4991	-0.3384
XM_001068510.1	LOC684012	-0.2718	-0.3893	-0.049	-0.0467	-0.2723	-0.5302	-0.1648	-0.1895	-0.3496	0.0329	-0.6473	-0.4545	-0.2908	-0.0082

NM_001009497	LOC684059	-0.0682	-0.0701	-0.0531	0.02591	0.05463	0.11014	-0.0618	-0.0688	0.0238	0.03523	-0.0331	-0.0663	-0.1351	0.0162
XM_001068777.1	LOC684069	0.10018	-0.0112	0.16827	-0.0098	0.1239	0.27063	-0.028	-0.1179	0.16717	0.08938	0.03789	0.19754	-0.025	-0.0223
XM_001068928.1	LOC684101	0.08342	0.12027	0.06825	-0.0415	-0.0426	0.21697	0.09831	-0.0641	-0.0178	0.03967	0.13096	0.1683	0.12405	0.1001
NM_001134790	LOC684106	0.48976	0.25335	-0.364	-0.673	0.39957	0.18967	-0.0313	0.47636	0.39712	0.09931	0.2695	-0.0464	0.42583	0.45446
XM_001069100.1	LOC684139	0.12419	0.05597	0.13842	0.09103	0.09582	0.08988	0.09158	0.01847	0.08165	0.14166	0.37906	0.1095	0.19865	-0.0057
XM_001069294.1	LOC684180	-0.0275	-0.0055	0.09845	-0.0027	-0.0605	0.03454	0.12048	0.04323	-0.0649	0.07901	0.0318	0.04827	0.02886	0.06311
XM_001069391.1	LOC684204	-0.0206	-0.0285	0.01906	0.41339	0.04349	0.03276	0.19835	0.14461	0.05194	0.01213	0.0329	0.1213	-0.0342	0.05467
XM_001069399.1	LOC684205	0.02692	0.2476	0.30529	0.08455	0.06649	0.20324	0.2214	0.10966	0.19291	0.12423	0.49732	0.42008	0.12017	0.19677
XM_001069729.1	LOC684287	-0.046	0.20107	0.01931	-0.0595	0.00439	-0.0578	0.03645	-0.0908	0.00197	0.18949	0.16172	0.10894	0.27167	0.09927
NM_001025055	LOC684289	0.05514	0.06389	0.04899	0.05579	-0.0002	0.02532	-0.0292	0.10889	-0.0171	0.15518	-0.0213	0.00619	-0.0777	-0.0053
XM_001069325.1	LOC684302	0.48101	0.28804	-0.0208	0.27775	0.37515	0.48593	0.09277	0.23994	0.17629	0.15575	0.0722	0.0109	0.21761	0.47219
NM_001166576	LOC684318	-0.3181	-0.4253	-0.6457	-0.8873	-0.2465	-0.3655	-0.661	-0.3308	-0.3743	-0.9767	-0.4936	-0.4421	-0.3864	-0.3441
NM_001037352	LOC684355	-0.1961	-0.5482	0.07847	-0.8245	0.74908	0.61609	-0.1929	0.69495	-0.5396	-0.3263	-0.3919	-0.3655	-0.0899	0.01394
XM_001070057.1	LOC684363	-0.0891	-0.0614	-0.0874	-0.1322	-0.09	-0.156	-0.1091	0.09582	-0.1526	0.07186	-0.1471	-0.0669	0.38545	-0.0738
XM_001070069.1	LOC684368	0.03078	-0.0518	-0.0316	-0.026	0.00944	-0.0105	0.12836	0.03318	0.10052	0.0121	-0.0409	0.24415	-0.0892	0.01263
NM_001015029	LOC684447	-0.107	0.03141	-0.1885	-0.1636	0.49374	0.06487	-0.0617	0.19288	-0.1308	-0.1478	-0.1962	-0.2385	0.20821	0.40178
NM_001011920	LOC684448	-0.0738	0.11236	0.05509	0.03429	0.0015	-0.0797	-0.0705	-0.1652	0.08628	-0.03	-0.1066	-0.0097	-0.0911	0.06914
NM_001109398	LOC684490	0.32954	-1.3501	-0.5448	-1.1157	-0.6639	-0.7304	-1.4789	-0.6228	-0.7788	-1.1588	-1.2169	-0.431	-0.1819	-0.0536
NM_001109525	LOC684506	-0.0109	0.03951	-0.018	-0.0254	0.09273	-0.0824	0.28464	0.06926	0.04696	0.10313	0.14405	-0.0409	-0.0288	-0.0208
NM_001109525	LOC684506	0.38689	0.54902	-0.0554	0.72213	0.1851	0.19223	0.41785	0.36187	0.68873	0.55403	0.50158	0.39778	0.248	-0.009
NM_001004129	LOC684525	-0.0163	0.00486	0.10409	0.15651	0.06109	0.14443	0.08228	0.0516	0.02409	-0.0024	0.07225	0.01616	0.06429	-0.0432
NM_001109425	LOC684528	0.03756	0.11852	-0.193	-0.0168	0.17789	0.01035	-0.062	0.11848	0.22711	0.04805	0.0035	-0.1048	-0.2015	-0.0816
NM_001127639	LOC684536	0.25025	-0.2242	0.35024	0.28456	0.08995	0.20919	0.18513	0.22865	-0.0593	-0.2292	0.05315	0.04455	-0.013	0.00109
NM_001127590	LOC684597	0.02058	0.17554	0.00033	-0.0185	0.06596	0.00802	-0.0739	0.16523	0.11279	0.05617	-0.1087	-0.1108	0.18225	0.03951
XM_001071213.1	LOC684606	-0.0826	0.01384	-0.0498	-0.0314	0.14019	0.31258	0.08061	0.09423	-0.0073	0.16376	0.17475	0.10115	-0.0227	0.11547
NM_001126085	LOC684624	0.1165	-0.6987	0.86475	0.34289	-0.6398	-0.3319	-0.0529	-0.6946	-0.0328	-0.3547	-0.1539	0.58588	0.49234	0.39954
XM_001071515.1	LOC684666	0.08514	0.13476	0.20533	0.08917	0.10582	0.05926	0.1756	0.14964	0.08064	0.10189	0.1604	0.09345	0.15371	0.14324
XM_001071565.1	LOC684681	0.13331	0.96984	-0.4795	1.2354	0.59517	0.41489	0.51995	0.78588	0.73378	0.44091	0.49679	0.73952	-0.6078	-0.6467
NM_001123469	LOC684686	-0.5039	-0.1317	-0.1226	-0.0067	-0.261	-0.1303	0.1258	-0.2966	0.20396	0.21679	-0.3152	0.61205	-0.2693	-0.2429
XM_001071647.1	LOC684696	0.39121	0.1675	-0.1907	0.09957	0.225	0.5384	-0.4033	0.21616	0.0709	0.31557	0.22616	0.17273	0.06834	0.02484
XM_001071718.1	LOC684722	0.05263	0.24174	0.08165	0.11464	0.40604	0.08217	0.45649	0.07626	0.21161	0.05226	0.25859	0.29312	0.02294	0.34567
NM_001115042	LOC684771	-0.024	-0.017	0.05723	-0.2317	-0.2669	-0.1327	-0.1627	-0.3821	-0.1055	-0.2186	-0.2305	0.08865	0.20652	0.41891
XM_001071937.1	LOC684785	0.13799	-0.0702	-0.0468	-0.0265	0.12097	-0.0663	-0.1492	-0.1626	-0.0207	0.05493	-0.1288	-0.0155	-0.1265	0.00163
NM_001108567	LOC684829	-0.0223	-0.1036	-0.13	-0.1783	-0.1936	-0.1683	-0.1225	-0.2229	-0.057	-0.0354	-0.0402	0.00249	-0.2581	0.01505
XM_001072229.1	LOC684861	-0.1105	-0.0086	-0.052	-0.106	-0.0567	-0.0038	-0.1789	-0.1227	-0.078	-0.1806	-0.0606	-0.0558	-0.1471	0.00982
NM_021840	LOC684872	-0.7638	-0.8735	-0.1735	-0.5271	-0.4272	0.05064	-0.2946	-0.3319	-0.175	-0.278	-0.4444	0.71236	-0.4821	-0.3579
XM_001072334.1	LOC684886	0.43983	0.07331	0.03288	-0.1142	-0.1194	0.25376	0.27455	0.07445	-0.088	-0.122	-0.0708	-0.0747	0.18663	0.22316
NM_001109403	LOC684921	0.05045	0.20522	0.0255	0.20051	0.0792	0.30743	0.09834	0.00768	-0.0356	0.20504	-0.0352	0.14975	0.24114	0.17017
NM_001134849	LOC684972	0.94349	-3.5206	-2.0194	-2.5659	0.66606	1.1723	-1.953	0.38699	-3.2635	-2.8677	-2.6911	-2.8576	-2.1676	-2.299
XM_001061779.1	LOC684984	-0.0764	-0.0263	0.19471	0.16409	0.03607	-0.0067	-0.034	0.03222	-0.0593	0.13827	-0.0684	-0.0324	-0.0878	-0.06
NM_130432	LOC684988	-0.3172	-0.3836	-0.3938	-0.1537	-0.257	0.03349	-0.5939	-0.2435	-0.1936	-0.473	-0.2335	-0.1571	-0.3732	-0.468
XM_001061802.1	LOC684990	0.40794	0.82422	0.38026	0.69922	0.47645	0.44915	0.7007	0.7882	0.47985	0.7135	0.97304	0.39166	0.25887	0.49481
XM_001061864.1	LOC685004	-0.045	-0.0908	-0.0179	0.28706	0.24306	0.14831	0.17874	0.26116	0.04027	0.0319	0.02259	-0.1005	0.06603	0.00041
XM_001061892.1	LOC685010	0.01194	0.11056	0.06322	-0.0658	-0.079	-0.0359	-0.0339	0.21436	0.06304	0.02106	-0.1092	-0.0159	-0.029	-0.0148
XM_001061948.1	LOC685019	0.12594	0.0694	0.26422	0.04444	0.10087	-0.0577	0.15748	0.10541	0.05355	0.04084	0.0537	0.36126	0.18916	0.10298
XM_001061948.1	LOC685019	0.15152	-0.0349	0.21007	0.40204	0.04186	0.10569	0.14251	0.26945	-0.056	0.27403	0.16283	-0.0047	0.02145	0.0935
XM_001061977.1	LOC685024	-0.4395	-0.4697	-0.2139	-0.5372	-0.6529	-0.6447	-0.3899	-0.542	-0.4456	-0.4457	-0.3735	-0.5461	-0.0089	0.0085
NM_001014221	LOC685053	0.04313	0.01948	0.00528	0.09467	-0.0483	0.07864	-0.1008	0.06708	-0.027	0.0049	0.29182	0.09904	0.06827	-0.1039
NM_001109170	LOC685068	0.00893	-0.2593	0.33288	-0.276	-0.1182	0.14681	0.0424	-0.3128	0.01493	-0.1167	0.08083	0.68825	0.03089	0.29367

XM_001062249.1	LOC685088	-0.01	-0.093	-0.085	-0.0234	-0.0446	0.02847	-0.068	-0.0979	-0.1186	0.03983	-0.0485	0.00444	0.02898	-0.0487
NM_001024798	LOC685152	0.28529	-0.1053	-0.1249	-0.1002	0.05765	-0.2163	0.02459	-0.0345	0.00631	0.3491	-0.0165	-0.1768	-0.0233	-0.0082
XM_001062796.1	LOC685205	-0.0124	-0.2368	-0.4456	-0.1504	-0.1736	0.11457	-0.1965	-0.1615	-0.16	-0.1332	-0.0741	-0.2761	-0.2613	-0.4593
NM_001159493	LOC685245	-0.0345	-0.3671	-0.1325	-0.1816	-0.1535	0.30421	-0.2009	0.18397	-0.317	-0.1976	-0.1805	-0.1588	-0.0726	-0.1739
NM_001159493	LOC685245	0.2846	0.01197	0.1316	-0.0372	0.18726	-0.1156	0.04828	0.71846	-0.0088	0.08567	-0.1359	-0.1958	-0.0373	-0.0477
XM_001062997.1	LOC685246	0.09948	0.77088	-0.7955	-0.8379	0.12583	-0.605	-0.1939	0.58468	0.31772	0.30215	0.25214	-0.1855	0.27842	0.34354
NM_001014221	LOC685285	0.0967	-0.0118	-0.0002	0.10383	0.02117	-0.0474	-0.0002	0.04686	0.03455	0.12865	0.12415	0.06403	-0.0576	0.12118
NM_001014221	LOC685300	0.11264	0.15599	0.37274	0.12558	-0.053	0.05948	0.32929	0.23802	-0.0186	0.30516	0.06299	0.19729	0.02699	-0.0374
NM_001014221	LOC685300	0.29548	0.63414	0.27671	0.35896	0.42466	0.3274	0.40297	0.47943	0.5514	0.76087	0.85371	0.55103	0.11399	-0.026
NM_183402	LOC685331	0.04134	0.13843	0.05885	0.05868	-0.0004	-0.006	0.0214	0.14425	0.00588	0.03778	0.01996	0.02144	0.29959	0.06523
NM_183402	LOC685331	-0.0563	-0.3089	-0.0606	0.03788	0.06344	0.39489	-0.1336	0.01473	-0.2478	-0.1409	0.21539	-0.029	0.36601	-0.1565
XM_001063469.1	LOC685357	-0.0118	0.04689	0.00535	0.23853	0.03135	0.0603	0.04748	0.07769	0.11226	0.11227	-0.0278	0.12544	0.05582	0.09677
XM_001063496.1	LOC685363	0.09042	0.12414	-0.0446	0.35662	0.17704	0.49293	0.07247	0.26855	-0.0943	0.09483	0.18667	0.04659	-0.0237	-0.028
XM_001063509.1	LOC685368	0.06035	0.05051	0.07254	0.06827	0.04107	0.01066	0.07435	0.11207	0.02881	0.06837	-0.0664	0.05877	0.0656	0.03898
XM_001062165.1	LOC685374	0.52997	0.42027	0.09314	0.1512	0.31993	0.10687	0.18016	0.27921	0.13246	0.28183	0.14145	0.20868	0.10021	0.45097
XM_001063618.1	LOC685393	-0.0953	-0.0349	-0.0666	0.03923	-0.0454	0.08789	-0.048	-0.0826	0.04335	-0.0273	-0.2139	-0.0154	0.0305	-0.0763
NM_001130581	LOC685444	-0.1161	-0.1654	-0.1879	-0.1612	-0.2441	-0.0302	-0.0897	-0.2142	-0.2318	-0.1544	-0.1523	-0.0479	-0.0241	-0.1572
XR_006192.1	LOC685455	-0.3896	-0.4508	-0.2774	-0.2902	-0.3139	-0.0868	-0.1293	-0.3655	-0.4594	-0.1949	-0.4132	-0.1747	-0.1648	-0.288
NM_001109467	LOC685462	-0.1512	-0.0309	-0.1302	-0.1342	-0.1033	0.29525	-0.3694	-0.227	0.23467	-0.2831	-0.1425	-0.2497	-0.0852	-0.0644
NM_001107912	LOC685491	-0.0538	0.13607	-0.1308	0.15518	0.16997	-0.0289	-0.2587	0.38828	0.22835	0.26544	0.11663	0.26241	0.23657	0.11523
NM_001044280	LOC685529	0.01017	0.08151	0.15293	0.07725	0.24797	0.16676	-0.0444	-0.0206	0.17895	0.13733	0.0612	0.14104	-0.038	0.10881
NM_001013236	LOC685579	0.06605	0.2176	-0.4253	-0.6896	-0.0918	-0.5694	-0.189	0.25854	0.27426	0.16273	-0.2403	-0.1017	0.75959	0.77756
XM_001064447.1	LOC685593	-0.0414	-0.0664	-0.0332	0.0253	-0.1379	0.14867	-0.0822	-0.1131	-0.0538	-0.0865	0.08228	-0.0015	-0.0149	0.05958
XM_001064652.1	LOC685645	-0.0347	-0.1018	-0.008	-0.0815	-0.0113	-0.076	-0.0113	0.15597	-0.0395	-0.1164	-0.0723	-0.0318	0.40326	0.07835
NM_001109477	LOC685664	0.48209	-0.1198	-0.1554	-0.2635	0.17323	0.29693	-0.3293	0.06715	-0.032	-0.1629	-0.0694	-0.1964	-0.0604	-0.1749
NM_001109085	LOC685669	-0.2228	-0.2196	-0.1344	-0.2038	-0.1559	-0.2069	-0.1766	-0.2122	-0.2167	-0.488	-0.2895	-0.3683	-0.4329	-0.3555
NM_183402	LOC685745	0.20339	0.12224	-0.4214	-0.0356	0.38081	0.14535	0.14003	0.26281	-0.032	0.01205	-0.0122	-0.0663	0.19916	0.04723
NM_001109480	LOC685756	0.29643	0.09885	0.14761	0.109	0.25265	0.00839	0.08724	0.11126	0.24008	0.2927	0.23579	0.34678	0.03974	0.53933
NM_001109437	LOC685758	0.23368	0.07437	0.10204	0.07278	0.07842	0.21045	0.21271	0.18016	0.13428	0.08315	0.09326	0.29183	0.13682	0.17724
XM_001081538.1	LOC685766	-0.0015	-0.0545	0.00865	-0.0749	-0.0723	0.01105	-0.1266	-0.0347	-0.1504	0.03268	-0.0225	0.14321	-0.0743	-0.115
NM_001004072	LOC685778	0.22583	0.13625	0.00756	0.78553	0.4397	0.38231	0.41293	0.611	0.39712	0.02528	0.35031	0.12551	0.42214	0.39309
NM_001004072	LOC685778	0.19815	0.12152	-0.135	0.06714	0.35246	-0.0242	-0.0108	0.52206	-0.0411	-0.039	0.09652	-0.3411	0.31345	0.23641
XM_001065244.1	LOC685782	-0.009	-0.0688	-0.0399	-0.0094	0.00687	0.17532	-0.0915	-0.0451	0.0623	0.01219	-0.0842	-0.0215	0.08514	0.02619
XM_001065293.1	LOC685793	0.08384	0.05046	0.00688	0.11196	0.23986	0.04569	0.00966	0.2768	0.17174	0.12816	0.02567	0.17307	0.27524	0.02416
XM_001065502.1	LOC685850	-0.027	-0.0743	-0.032	-0.1087	0.01564	0.06426	-0.0913	-0.0534	-0.0228	0.04423	0.02699	0.15953	0.12204	0.04696
XM_001065605.1	LOC685878	-0.3619	-0.6751	-0.325	-0.9063	-0.4173	-0.2559	-0.4404	-0.2695	-0.3072	-0.5076	-0.2355	0.14725	0.26004	0.02729
XM_001062701.1	LOC685888	1.2128	0.79606	0.59664	1.256	0.97024	1.2167	0.3979	0.92233	0.85044	0.85891	0.96442	0.61661	0.65387	0.58005
NM_001106019	LOC685909	-0.4395	-0.7663	-0.1463	-0.4579	-0.5686	-0.3177	-0.4621	-0.7768	-0.4708	-0.8784	-0.4908	0.54104	-0.6394	-0.5102
XM_001065728.1	LOC685912	0.0105	0.12624	0.04459	0.05562	-0.0085	-0.0053	-0.0531	0.26631	0.0224	0.16356	0.1346	-0.0521	-0.0241	0.0002
XM_001066078.1	LOC685988	-0.0685	0.17487	0.2676	0.28813	0.24925	0.61123	0.13086	0.48818	0.21637	0.4198	0.43663	-0.2009	-0.0925	-0.052
NM_183402	LOC685989	0.00565	0.42235	-0.027	0.19212	0.06591	0.13093	0.25647	0.02212	0.19115	0.17543	0.30323	0.00027	0.04607	0.07526
XM_001066294.1	LOC686038	0.00296	0.12216	-0.011	0.05353	0.06778	-0.0527	0.14605	0.17149	-0.0383	0.09237	0.07035	0.05358	-0.0042	0.19381
NM_001014221	LOC686053	0.12057	0.70622	0.48457	0.31058	0.95224	1.2471	0.49635	1.1114	0.90638	0.69242	1.284	0.43323	-0.0268	0.0483
XR_006360.1	LOC686063	0.16424	-0.0024	0.36701	0.1539	0.09448	0.11435	0.39378	0.0873	0.18234	0.52064	-0.009	0.14889	0.15224	-0.0248
XM_001066470.1	LOC686082	-0.0601	0.07397	-0.0629	0.29575	-0.0975	0.02848	-0.0515	-0.003	0.02647	0.16448	-0.0951	-0.1041	-0.0654	-0.1167
NM_001014107	LOC686087	0.20994	0.19099	-0.4413	-0.082	0.18355	-0.3828	-0.0939	0.08005	-0.0858	-0.0322	-0.0502	-0.1042	0.33418	0.09196
XM_001067579.1	LOC686132	0.11808	0.08878	0.08855	-0.1121	-0.2611	0.19722	-0.1588	-0.0402	-0.055	-0.0372	-0.1497	-0.2429	0.13964	-0.239
XM_573722.1	LOC686141	0.08842	0.26081	-0.037	-0.0232	0.04329	-0.0018	0.12991	-0.0536	-0.0547	-0.0987	0.03165	0.10625	-0.0595	0.10552
XM_573722.1	LOC686141	0.07058	0.06714	0.2936	-0.0211	0.16314	0.31131	0.03146	0.28032	0.16975	-0.0178	0.06737	0.18683	0.23853	0.07607

XM_001072675.1	LOC686154	0.09162	0.1358	0.23321	0.15096	0.15073	-0.0134	0.13726	0.12821	0.28907	0.24436	0.29958	0.07675	0.22442	0.11576
NM_001109551	LOC686162	0.0075	0.15416	0.20146	0.04401	0.0636	0.08293	0.13016	0.1068	0.10222	0.13805	0.01366	0.29274	0.06513	0.13221
XM_001072775.1	LOC686176	-0.0543	0.15267	0.01438	-0.1471	-0.1511	-0.1562	-0.1174	0.17455	0.06102	-0.0741	-0.0634	-0.0363	-0.1278	0.01388
NM_001108606	LOC686212	0.15843	0.26101	0.23253	-0.0216	0.44645	0.77675	0.51435	0.39067	0.20245	0.13265	0.13999	0.25322	0.45272	0.45671
XM_001072911.1	LOC686213	0.54519	0.04098	-0.5611	-0.0138	0.59041	0.07408	-0.0751	0.80327	0.15416	-0.1139	-0.1678	-0.5547	0.25629	0.24646
NM_001107123	LOC686219	-0.2603	-0.0699	-0.1183	-0.1522	-0.1059	-0.127	0.20743	-0.143	0.31767	0.08042	-0.0031	-0.0228	-0.0861	-0.2323
XM_001072956.1	LOC686223	-0.0615	-0.0747	-0.1422	-0.0937	-0.1333	-0.2683	-0.0517	-0.1703	-0.0429	-0.1663	-0.0767	0.04861	-0.2508	-0.0734
NM_022514	LOC686255	0.02784	0.02178	0.05858	0.10377	0.26591	0.14476	0.17995	0.07439	0.05848	-0.0369	0.07031	0.07907	0.04144	0.00359
NM_001134994	LOC686257	-0.1143	0.08135	-0.1582	-0.0405	-0.0039	-0.1657	-0.1171	0.14332	-0.2266	-0.0041	-0.1273	-0.2104	-0.0008	0.1432
XM_001073234.1	LOC686274	-0.1571	-0.1185	0.09612	0.66645	-0.149	-0.1076	0.08369	-0.2018	-0.0654	0.17651	-0.2016	0.03508	-0.1506	0.13914
NM_001037190	LOC686275	0.51029	0.21892	0.47367	-0.5385	0.31377	0.12336	-0.3049	0.28773	-0.0044	-0.0273	0.10482	-0.1587	0.52516	0.46093
XM_001073483.1	LOC686312	-0.0092	0.00179	0.06985	0.19227	0.09112	0.0692	0.13706	0.12999	0.16969	0.15432	0.07546	0.09388	-0.0453	0.00607
XM_001073521.1	LOC686318	0.20735	0.09761	0.11517	0.05991	0.54207	0.26339	0.08321	0.09963	0.07831	0.1649	0.21519	0.10384	0.43685	0.12234
NM_001113752	LOC686344	0.39663	0.54628	-0.4788	-0.3521	0.53062	0.1083	0.08744	0.42744	0.1089	0.3183	0.13318	-0.2241	0.37182	0.4318
XM_001073737.1	LOC686361	0.02543	0.06389	0.138	-0.0208	0.53192	0.5601	0.08955	0.27018	0.13831	0.01695	-0.1001	0.14009	0.12926	0.04918
XM_001073880.1	LOC686384	0.23247	0.13529	0.25054	0.16376	0.01652	0.30249	0.157	-0.0141	0.2903	0.07134	-0.0574	0.28795	0.62233	0.56623
NM_053982	LOC686415	0.12422	0.14209	0.04185	0.1958	0.17067	0.2302	0.12273	0.05325	0.03825	0.16082	0.15318	-0.0494	0.07745	0.21351
XM_001069996.1	LOC686428	-0.0346	0.41396	0.16565	0.23003	0.08058	0.04805	0.05015	-0.0538	0.07268	0.13247	0.10089	0.02463	0.0637	0.09793
NM_001109629	LOC686457	0.21688	0.09153	0.00076	0.13997	0.31568	0.24595	0.06426	0.21249	0.17597	0.14416	-0.032	0.1018	0.05574	0.25812
XM_001074249.1	LOC686462	-0.1747	-0.3366	-0.14	-0.1787	-0.2901	-0.314	-0.2545	-0.2466	-0.2768	-0.1268	-0.1918	-0.2877	-0.3212	-0.2239
XR_008948.1	LOC686477	0.23151	0.0533	0.3678	0.06843	0.17606	0.23245	0.12574	0.3496	-0.0101	0.0716	0.03265	0.06656	0.05957	0.13215
XM_001074611.1	LOC686530	0.07757	-0.1391	0.11398	-0.0168	-0.1094	-0.1339	-0.0616	-0.0905	-0.0954	0.04265	-0.0714	-0.0986	-0.0374	-0.0566
XM_001074155.1	LOC686547	-0.0921	-0.1415	-0.2252	-0.147	0.15652	-0.1479	-0.2581	0.12687	-0.2566	-0.0952	0.02931	-0.2312	-0.047	-0.0557
NM_031051	LOC686548	0.65929	0.47416	-0.1585	-0.0643	0.38433	0.99353	-1.0632	0.20275	0.19749	0.23553	0.48914	0.32771	-0.0173	0.05743
NM_001108283	LOC686564	0.06928	-0.3415	-0.4531	0.32846	0.10549	0.41985	-0.5492	0.06208	0.10777	-0.6188	0.10039	-0.0597	-0.4906	-0.573
XM_001074769.1	LOC686566	-0.1572	-0.0517	0.2453	-0.2154	0.05192	-0.0201	0.11463	-0.0814	0.04319	-0.0789	-0.0918	-0.0273	-0.0679	-0.1202
XM_001074823.1	LOC686577	-0.0042	-0.0216	-0.0679	-0.1211	0.00527	-0.1796	-0.006	-0.0538	-0.0584	-0.0487	-0.0583	-0.098	0.20934	0.3275
NM_001047860	LOC686701	0.26421	0.60413	0.17883	0.08307	0.16777	-0.1853	0.33835	0.241	0.23426	0.22011	0.39212	-0.1323	-0.0477	0.3765
XM_001075418.1	LOC686725	0.05326	0.01322	0.02271	0.00816	0.09094	0.05879	0.13327	0.1922	0.22247	0.04844	0.08913	0.0231	0.04249	0.00638
XM_001075602.1	LOC686765	0.27705	0.20665	-0.0335	-0.0382	0.03915	0.29998	0.12561	-0.1744	0.50235	0.32417	0.57882	0.70094	0.07372	0.02692
NM_001109605	LOC686771	-0.5405	-0.5953	-0.4011	-0.8491	-0.4938	-0.5994	-0.7313	-0.6402	-0.6541	-0.6732	-0.5348	-0.6965	-0.6416	-0.6032
NM_001134801	LOC686810	-0.2411	-0.3021	-0.1075	-0.2164	-0.2292	-0.2149	-0.2349	-0.2778	-0.2534	-0.0076	-0.2481	0.03659	-0.212	-0.2126
XM_001076022.1	LOC686853	-0.1599	-0.1906	-0.0414	-0.1108	-0.1338	0.08419	0.07241	-0.2074	-0.0035	-0.165	-0.2189	-0.1991	-0.1981	-0.1837
NM_001109422	LOC686871	0.06728	0.22113	0.05876	0.14935	0.32361	0.27777	0.3212	0.31949	0.13753	0.09525	0.38285	0.1571	-0.0152	0.02942
NM_001109489	LOC686877	-0.0867	-0.1811	-0.1633	-0.2469	-0.241	-0.2056	-0.1502	-0.1848	0.03854	-0.1011	-0.1658	0.00654	-0.1695	-0.0887
NM_183402	LOC686886	0.0821	-0.0311	-0.1734	-0.0821	0.18426	-0.1019	-0.0928	-0.1808	-0.0466	-0.061	0.10334	0.10255	0.10232	0.01749
XM_001075403.1	LOC686935	-0.1803	0.19632	-0.0554	-0.1846	-0.2328	-0.0922	-0.0723	-0.0754	0.32125	0.29531	0.12724	0.31077	-0.0665	-0.2146
XM_001076515.1	LOC686963	-0.0297	-0.0692	-0.0833	-0.0597	-0.088	-0.1885	-0.1559	-0.1857	-0.0397	-0.0664	-0.0616	-0.0383	-0.183	-0.1496
NM_031764	LOC686988	0.10474	0.18087	0.15817	0.40603	-0.1388	0.06336	0.15133	0.03471	-0.0782	-0.1867	0.01521	0.14713	0.05577	0.23609
XM_001076743.1	LOC687016	-0.3229	-0.1395	-0.1349	-0.2336	-0.298	-0.1443	-0.2554	-0.3016	-0.2858	-0.1032	-0.2229	-0.1007	-0.3296	-0.2221
XM_001076778.1	LOC687026	0.12732	0.05076	0.14297	0.06113	-0.0324	-0.0136	0.03517	0.02078	0.07352	0.01859	0.01113	-0.0686	-0.0392	0.04232
NM_001107495	LOC687052	0.05137	-0.0028	0.15198	0.0983	0.02695	0.11393	0.02893	0.15717	0.00699	0.03912	0.08604	0.24393	0.16084	0.07752
NM_001109614	LOC687055	-0.1511	0.28523	-0.1045	-0.0046	0.18554	0.47655	0.23361	0.00834	0.25458	0.29453	0.23405	0.44075	-0.1136	0.08284
NM_001135913	LOC687058	-0.1941	-0.1173	-0.0605	-0.1438	-0.139	-0.2816	-0.2382	-0.0957	-0.2294	-0.2818	-0.0179	-0.3483	-0.1803	-0.0601
NM_001109488	LOC687177	0.00221	-0.0618	-0.1949	-0.0497	-0.0672	0.20632	-0.0259	-0.025	-0.1169	0.098	0.03463	0.3808	-0.0254	-0.0896
XM_001077062.1	LOC687191	-0.3372	-0.3569	-0.3341	-0.2837	-0.3041	-0.4298	-0.2727	-0.218	-0.1659	-0.3436	-0.324	-0.3517	-0.3637	-0.1734
XM_001077535.1	LOC687212	0.07198	0.01858	-0.0085	-0.1215	0.06853	0.07145	-0.0785	0.02884	-0.1858	-0.2077	0.10117	0.06535	0.36968	0.03729
XM_001077552.1	LOC687215	0.16914	-0.0216	0.03685	-0.0134	0.00207	0.03084	-0.0101	0.06153	-0.0659	-0.0221	-0.0444	-0.0245	0.17892	-0.0176
XM_001077570.1	LOC687222	-0.153	-0.0825	-0.0264	-0.0826	-0.1336	-0.118	-0.0748	-0.1263	-0.1878	-0.2995	-0.2395	-0.1761	-0.0039	-0.0883

XM_001077650.1	LOC687237	-0.1111	0.02437	0.62478	0.22427	0.12598	0.55075	0.06888	-0.0717	0.24359	0.22446	0.52202	0.71268	0.2981	0.16358
XR_009164.1	LOC687248	0.09641	0.06772	0.05657	-0.5793	-0.0255	0.06571	0.11098	0.07537	0.17858	-0.041	0.1802	0.25234	0.27105	0.56529
NM_001128138	LOC687251	0.14401	0.19936	0.06366	0.14286	-0.0605	0.03938	0.18153	0.24661	0.34043	0.21643	0.19669	0.02065	0.13136	0.00028
XM_001077865.1	LOC687292	-0.1584	-0.0679	-0.1598	0.03088	0.01341	-0.0476	0.14544	-0.0554	-0.013	-0.2307	-0.1152	-0.1117	-0.2377	0.01099
NM_001037346	LOC687298	0.31218	-0.1622	-0.1891	0.52757	0.15132	0.75385	-0.4715	0.02215	-0.2239	-0.2126	0.19584	0.44554	-0.3755	-0.3453
NM_001169139	LOC687334	0.19758	-1.1507	0.41947	0.88146	-0.6037	-0.6914	-0.9459	-0.5328	-0.601	-0.8768	-0.684	0.07765	0.76742	0.51729
NM_001011903	LOC687365	-0.1216	-0.2244	-0.0314	0.26651	-0.0158	0.02745	0.09485	-0.0359	-0.0838	-0.0077	-0.038	-0.0111	-0.0159	-0.2258
XM_001077717.1	LOC687381	-0.0239	-0.043	-0.2657	-0.152	-0.2178	-0.1049	-0.1369	0.03047	-0.0658	-0.2383	-0.1174	0.09451	0.2427	-0.1742
NM_001024261	LOC687406	0.45792	0.35193	-0.5443	0.27643	0.51858	0.17021	0.31782	0.90763	-0.0091	0.43013	-0.1544	-0.2828	0.55256	0.27672
NM_001024261	LOC687406	0.66507	0.41497	-0.322	0.3861	0.47319	0.09178	0.27905	0.68192	0.42129	0.29693	0.2247	-0.2008	0.45235	0.46074
XM_001078452.1	LOC687414	-0.1139	0.01255	0.02947	0.0094	0.02571	-0.0161	-0.0772	-0.0432	-0.0439	0.03565	0.07809	-0.0209	0.02218	0.10126
XM_001076559.1	LOC687424	-0.099	-0.0384	-0.1041	0.49548	-0.1206	-0.0501	-0.0152	-0.1102	-0.0293	-0.1344	-0.0456	0.16879	-0.1632	-0.0144
XM_001076559.1	LOC687424	0.31181	0.10332	0.01697	0.08694	0.02042	0.05095	0.05653	0.15158	0.09221	0.03958	0.00143	-0.0052	0.20144	0.01021
XM_001076559.1	LOC687424	0.06762	-0.0641	-0.0575	-0.1403	-0.0781	-0.0008	-0.0328	0.02761	-0.078	-0.0646	0.11308	0.04388	-0.0635	-0.0789
XR_009266.1	LOC687523	0.07555	0.31161	0.37269	0.30666	0.05491	0.0367	0.27446	0.04769	0.02063	0.17881	0.06036	0.18785	0.14477	-0.0244
XM_001078970.1	LOC687533	0.073	-0.1479	-0.0892	-0.1245	0.08939	0.15174	0.08699	0.08493	-0.138	0.22406	-0.1823	0.1891	0.18307	0.04507
NM_001105791	LOC687570	0.05518	-0.0873	0.27031	0.00319	-0.0378	-0.2784	-0.0903	-0.2733	-0.0007	-0.0091	0.07491	-0.1704	0.37492	0.27353
NM_001145273	LOC687571	-0.0355	-0.0655	-0.1342	-0.0505	-0.1415	-0.1095	-0.0667	0.02579	-0.0707	-0.0863	-0.0279	-0.0708	-0.0047	0.00901
XM_001079200.1	LOC687573	0.02393	-0.0233	0.32956	0.03323	0.03074	0.20059	0.04826	0.05252	0.10008	-0.0004	0.11302	0.10625	0.06534	0.02073
NM_001100136	LOC687579	0.81631	0.36424	0.16151	0.42079	0.81659	1.2835	0.25704	0.90277	0.33902	0.13482	0.31179	0.48511	0.38697	0.16284
XM_001079239.1	LOC687581	0.27896	0.0907	-0.0314	0.0266	0.15985	0.03881	0.14876	0.14061	0.06292	0.0289	0.11687	-0.096	0.04964	0.11598
NM_001134708	LOC687582	0.36376	0.00678	0.3622	-0.0434	-0.236	-0.3738	0.10707	0.04952	0.20008	-0.037	-0.1153	-0.1042	0.12744	0.26516
XM_001079268.1	LOC687588	0.02407	-0.2165	-0.1618	-0.1056	-0.1297	-0.0957	-0.1576	-0.0962	-0.1045	-0.062	-0.0065	-0.0896	-0.0468	-0.1365
NM_001109630	LOC687625	-0.0091	0.00853	-0.0697	0.05493	-0.1335	-0.1346	0.19282	0.06216	-0.0851	-0.1025	-0.1376	0.06566	-0.1274	-0.1367
NM_001109639	LOC687718	0.30465	-0.0588	0.01101	0.06393	-0.1071	-0.1161	0.10495	-0.0725	-0.0116	0.03178	0.09509	0.10936	-0.0318	0.07328
XM_001079922.1	LOC687721	0.22606	0.06611	-0.0292	0.10641	0.07345	0.00709	-0.0034	0.15296	0.26347	0.04641	-0.0211	0.04849	-0.0118	0.23549
NM_001134699	LOC687770	0.07412	0.16794	-0.2835	-0.4566	0.09071	0.40505	-0.2445	0.06179	0.16428	0.16444	0.13988	0.07242	0.0899	0.12548
NM_001012739	LOC687780	-0.0383	0.19286	-0.1835	0.15313	0.02104	0.07345	0.03732	-0.0653	0.1933	0.14294	0.21187	0.10861	-0.161	-0.0646
XM_001078497.1	LOC687796	-0.5563	-0.5441	0.15176	-0.2539	-0.6238	-0.7909	-0.3616	-0.5392	-0.5055	-0.461	-0.2452	-0.3719	-0.377	-0.3635
XM_001078482.1	LOC687796	-0.3876	-0.2077	0.11602	0.08543	-0.4096	-0.2178	0.07329	-0.6849	-0.372	-0.4083	-0.0257	0.0278	-0.1949	-0.469
XM_001080182.1	LOC687799	-0.3127	-0.6579	0.1192	-0.1902	-0.4249	0.24947	-0.34	-0.4244	0.28371	-0.4582	-0.2887	1.0679	-0.1385	-0.2888
NM_001106840	LOC687840	-0.0128	-0.0589	-0.5854	0.91248	-1.2807	-0.9975	0.68732	-1.1895	0.14771	0.12609	-0.1778	0.13173	0.33112	0.12677
XM_001080339.1	LOC687849	-0.5157	-0.5975	-0.4096	-0.9547	-0.2947	-0.0537	-0.7058	-0.3042	-0.0132	-0.4381	-0.2876	0.09368	-0.1885	-0.2466
XM_001080365.1	LOC687856	0.16086	-0.1608	-0.1202	0.15298	-0.0323	0.05096	0.00183	-0.1152	0.02636	-0.0816	0.01439	0.07126	-0.1154	-0.0529
XM_001080663.1	LOC687958	0.16059	0.01153	-0.1426	-0.0921	-0.092	-0.0937	0.07421	-0.047	-0.0888	0.04154	0.03145	-0.0874	-0.1068	0.14858
XM_001080764.1	LOC687984	0.08294	0.17219	0.09284	0.22724	-0.0111	0.05356	0.11031	0.08144	0.12158	-0.0515	0.23833	0.04894	-0.0201	0.11941
XR_009418.1	LOC688018	0.02346	-0.1503	-0.1343	-0.3462	0.121	-0.0969	0.04924	0.18337	-0.0493	-0.3947	-0.3748	-0.361	0.12426	0.08553
NM_001108548	LOC688103	-0.1894	-0.5378	0.26233	-0.8144	-0.1493	-0.2582	-0.3318	-0.4093	-0.3032	-0.2439	-0.4574	0.04887	-0.3846	-0.3297
NM_001107777	LOC688116	0.1149	0.1912	0.06958	0.06082	-0.0014	0.12837	-0.003	0.06531	0.02942	0.178	0.00998	0.12955	0.05457	0.15905
NM_001134699	LOC688144	0.03139	0.32715	-0.4227	-0.6472	0.22985	-0.2673	-0.0678	0.12196	0.15128	-0.1012	-0.0721	-0.235	0.19137	0.12005
NM_001106477	LOC688159	0.09194	0.14112	0.12864	0.23782	0.16257	0.18224	0.04551	0.10094	0.25098	0.43119	0.17807	0.2845	0.18335	0.17116
XR_009475.1	LOC688240	-0.3952	-0.4038	-0.4059	-0.3768	-0.4849	-0.2172	-0.135	-0.6349	-0.457	-0.5767	-0.4659	-0.3393	-0.5399	-0.3664
XM_001081619.1	LOC688243	0.00904	0.0567	-0.0128	0.1629	0.07987	0.21934	-0.0381	0.29968	-0.032	0.05763	0.09156	0.16998	-0.0187	-0.0163
NM_001109568	LOC688258	0.03578	0.04474	0.12899	-0.024	-0.1128	-0.0353	-0.0915	-0.1304	-0.0205	-0.1396	-0.0069	-0.0898	-0.1213	0.05414
XR_009493.1	LOC688264	0.27398	0.02056	-0.074	0.01384	-0.1102	-0.0228	-0.0905	-0.0205	-0.1197	-0.0437	0.03526	0.05055	-0.1152	-0.0301
XM_001081700.1	LOC688266	-0.432	-0.3078	-0.254	-0.2376	-0.0815	-0.284	-0.4802	-0.1877	-0.2834	-0.0549	-0.3428	0.14194	-0.0403	-0.0976
NM_001109585	LOC688269	-0.6759	-0.5514	0.6649	0.36852	-0.3824	-0.4177	0.67005	-0.4034	-0.4642	-0.6306	-0.4684	-0.1426	-0.2342	-0.1846
NM_001170348	LOC688273	0.08411	0.24103	-0.0968	0.07086	0.33958	0.29654	-0.0333	0.28705	0.26795	0.12496	-0.0757	-0.0433	0.2343	0.21193
XM_001081771.1	LOC688296	-0.1228	-0.2276	0.085	-0.3366	-0.2824	-0.3972	-0.0681	-0.2993	-0.3608	-0.1782	-0.3447	-0.19	-0.0671	-0.0971

XM_001081855.1	LOC688338	0.1028	0.45789	-0.0369	0.3053	0.26577	0.48213	-0.0441	0.41608	0.2186	0.42155	0.32033	-0.1089	0.35714	0.37698
XM_001067063.1	LOC688464	0.16189	0.08606	0.0054	0.15154	0.11554	0.07111	0.05998	0.29051	-0.0596	0.03983	0.06681	0.34066	0.30363	0.02116
XM_001067146.1	LOC688490	0.1341	0.91988	0.17398	0.16462	-0.0551	0.06271	0.34767	0.00325	0.50536	0.86733	0.78775	0.10615	0.11601	0.39655
XM_001067241.1	LOC688519	0.0782	0.10416	0.01313	0.05162	-0.0095	0.03385	0.00032	0.35155	-0.0465	-0.0461	-0.0301	0.04035	0.02713	0.01106
XR_006415.1	LOC688528	0.75321	0.33261	0.75369	-0.5176	0.36719	0.05167	-0.0857	0.25248	0.06723	0.02266	0.08421	-0.3307	0.71631	0.7204
NM_183402	LOC688538	-0.0612	1.0624	0.122	0.15354	-0.0796	0.03514	0.09885	0.0127	0.33791	0.8672	0.33695	-0.0736	0.02888	-0.0209
XM_001067487.1	LOC688578	-0.0878	-0.0748	0.04663	0.14392	0.38725	0.11932	-0.1041	-0.0481	-0.0116	0.02572	-0.0382	-0.1701	0.04656	-0.1116
NM_183402	LOC688587	0.08773	0.78872	-0.0077	-0.0963	0.13056	0.12315	0.08401	-0.0903	0.74654	0.98543	1.0548	-0.0673	-0.0057	-0.0336
XM_001067593.1	LOC688606	0.08776	0.1747	-0.1253	0.90057	0.89726	1.1344	0.0555	0.60275	0.33691	0.19394	0.35346	0.44715	-0.1322	0.0122
NM_001077647	LOC688622	-0.0084	0.12638	-0.059	0.15639	0.29907	0.07759	0.06188	-0.0198	0.20492	-0.0661	0.01749	0.07568	-0.0675	0.25603
XM_001067670.1	LOC688625	0.10746	-0.035	-0.0065	0.13358	0.00255	-0.123	-0.1644	-0.1039	-0.0458	-0.0587	-0.1089	-0.2003	-0.108	-0.1454
XM_001067693.1	LOC688631	0.00357	0.01132	0.18752	0.1754	-0.2528	-0.4471	-0.027	-0.3058	0.12644	0.09275	-0.0178	-0.1061	-0.0193	0.02431
XM_001067706.1	LOC688637	-0.2486	-0.2151	0.21176	0.40638	-0.1029	-0.0086	0.11528	0.10737	-0.2164	-0.0882	-0.1134	0.14359	0.2132	0.15404
NM_001014221	LOC688644	0.08351	-0.0381	-0.0211	0.04416	-0.0061	0.00851	0.13044	-0.0362	0.03244	0.05285	0.19939	-0.0134	0.02931	0.15614
NM_001014221	LOC688644	0.13813	-0.0304	0.06738	-0.0947	0.06439	0.27658	-0.1294	-0.0167	-0.0559	-0.0246	0.08795	-0.1301	-0.0706	-0.0776
XM_001067739.1	LOC688646	-0.1141	0.13177	0.20596	0.09961	-0.0798	0.10469	0.12248	-0.0014	0.17574	0.02557	0.09444	-0.0766	0.13404	0.15338
XM_001067795.1	LOC688663	0.08476	0.1492	0.15345	-0.0302	0.08119	-0.022	0.03126	0.16437	0.04286	0.03013	0.14861	0.03882	0.05886	0.04602
NM_001037097	LOC688673	0.16732	-0.2203	0.08009	-0.3177	-0.0687	-0.1734	0.03277	-0.1343	-0.1511	-0.0697	0.04065	-0.2529	-0.2986	-0.1318
XM_001058650.1	LOC688679	-0.2818	-0.2458	-0.1472	-0.2122	-0.3092	-0.0794	-0.1926	-0.1799	-0.2382	-0.1231	-0.2619	-0.0763	-0.0792	0.00017
XM_001067988.1	LOC688702	0.13028	0.23845	0.16243	0.06703	0.01791	0.27229	-0.1018	-0.0326	-0.1216	0.05493	0.03762	0.13265	-0.112	-0.0765
NM_001108548	LOC688712	-0.0555	-0.6219	0.04064	-0.9279	-0.3551	-0.1488	-0.536	-0.2204	-0.2644	-0.3381	-0.3447	-0.3912	-0.4472	-0.5632
XM_001068161.1	LOC688750	-0.0692	0.2214	0.15532	0.2592	0.13803	0.08221	0.01779	0.14598	-0.0285	0.06355	0.12064	0.20895	0.12766	0.11437
XM_001068185.1	LOC688758	0.02882	-0.1024	0.1215	-0.1361	0.04566	-0.1876	-0.2107	-0.0145	0.06519	-0.0642	0.07501	0.20872	-0.0844	0.2594
NM_001128083	LOC688785	-0.7181	-0.254	-0.2838	-1.7469	-0.9914	-0.9409	-0.3709	-1.0581	-0.04	-0.2232	-0.101	-0.0594	-0.37	-0.2706
NM_001108046	LOC688806	-0.1427	-0.0027	0.08358	-0.0986	0.05922	0.01455	0.0982	0.06714	0.01463	0.1254	-0.1271	-0.0023	0.00791	-0.1043
XM_001068612.1	LOC688862	0.0866	-0.0071	-0.0458	-0.1226	-0.0899	0.0537	-0.016	0.02578	0.19907	0.20873	0.22192	0.20985	0.0677	-0.0248
NM_001145273	LOC688869	0.43302	0.43894	0.59698	0.13945	0.77392	1.1287	0.55678	0.44787	0.52703	0.43567	0.49397	0.69894	0.36189	0.53201
XM_001068684.1	LOC688878	0.21312	0.22463	-0.0774	0.09295	-0.0571	0.14147	-0.1219	-0.2922	-0.2782	-0.1742	-0.0095	-0.1232	0.01778	-0.0088
XM_001068704.1	LOC688887	0.09953	0.22893	0.28128	0.1232	0.06699	0.17612	-0.0226	0.1309	0.024	-0.139	0.04872	0.34735	0.21775	0.01451
XM_001068751.1	LOC688900	0.04548	0.05508	-0.0219	0.04518	-0.1733	-0.1586	0.10785	-0.1551	-0.2629	-0.0496	-0.0637	-0.0961	0.06261	-0.013
NM_001014221	LOC688909	0.23548	0.25629	0.21525	0.25296	0.21244	0.28981	0.46148	0.33418	0.10336	0.09237	0.31366	0.27765	0.29313	-0.0404
XM_001069010.1	LOC688970	0.06946	0.15626	0.19816	0.24597	0.22221	0.27635	0.13659	0.09589	0.15936	0.34091	0.18855	0.15395	-0.0079	0.13746
NM_001014221	LOC689001	0.65629	1.7093	1.1618	0.73411	1.2972	1.6221	0.91993	1.3091	1.5995	1.3695	1.9459	0.30253	0.63807	0.29113
XM_001069149.1	LOC689004	-0.2477	-0.015	-0.1522	-0.1907	-0.2204	0.08746	-0.209	-0.2243	-0.0958	-0.1879	-0.0033	-0.1754	-0.0599	-0.1452
XM_001069267.1	LOC689035	0.11617	0.03461	0.15704	-0.0563	0.05857	0.10683	-0.0861	-0.0817	0.42618	0.03052	0.03983	0.03235	-0.0399	0.11181
XM_001069291.1	LOC689039	0.17356	0.12909	0.08438	0.07531	0.01775	0.02026	0.08009	0.02196	0.00421	0.05787	-0.0055	0.42839	0.01226	0.03396
NM_001014221	LOC689058	-0.0662	0.2606	0.08476	-0.0204	0.10109	0.14427	0.19659	0.04936	0.48963	0.30005	0.64229	0.00713	0.10509	-0.0442
NM_001100985	LOC689080	0.11003	-0.0094	0.23744	-0.0591	-0.0384	-0.2106	0.05924	-0.1996	-0.1031	-0.0114	-0.0901	-0.1232	0.2913	0.00157
XM_001069448.1	LOC689083	-0.1533	-0.0967	0.1297	-0.0755	0.0602	0.02582	-0.0704	-0.0425	-0.0186	-0.0256	-0.0041	0.04614	-0.0728	-0.0272
NM_001014221	LOC689084	-0.0326	0.02306	0.03696	0.08521	-0.0081	-0.0344	-0.0057	0.05521	-0.0035	0.04187	0.07338	-0.0112	0.01241	0.01948
XM_001069558.1	LOC689108	-0.0426	-0.0654	0.01681	-0.0923	-0.0314	0.3625	0.06479	-0.0995	0.0732	0.15581	-0.2353	-0.0668	0.42272	-0.0267
XM_237012.4	LOC689123	-0.7666	-0.4091	-0.1184	-0.454	-0.6799	-0.7745	-0.4286	-0.7959	0.09195	-0.2709	-0.4554	-0.1175	-0.2596	-0.2684
NM_001105712	LOC689135	0.08243	0.11742	#######	0.19986	0.20701	0.44292	0.04147	0.08439	-0.0901	0.13659	0.02424	0.03755	0.04342	0.23419
XM_001069739.1	LOC689147	-1.2021	-1.123	-0.9717	-1.1526	-1.0964	-1.161	-0.9599	-1.2922	-1.2363	-1.1624	-0.9502	-1.2349	-1.1006	-1.1695
XM_001070116.1	LOC689246	0.21771	0.12858	-0.2824	-0.4737	-0.08	-0.4193	-0.1369	0.04235	-0.0481	0.04752	-0.3316	-0.0667	0.28125	0.22418
XM_001070133.1	LOC689253	-0.4935	-0.2476	-0.0739	-0.0548	-0.5976	-1.1939	0.07722	-0.7686	-0.2671	-0.243	-0.1672	-0.4562	-0.3193	-0.3962
XM_001070183.1	LOC689265	0.03835	0.04242	0.12138	0.0662	0.21187	0.07506	0.03868	0.25666	0.03222	0.06157	-0.0119	-0.003	0.11381	0.06715
XM_001070282.1	LOC689290	0.87209	1.6851	1.0193	1.1297	0.8228	0.95972	1.3631	0.97041	1.4955	1.6912	2.0131	0.923	0.53265	0.58746
XM_001070282.1	LOC689290	0.05171	-0.0121	0.06521	0.17815	0.01297	0.27022	0.26246	0.02943	0.03045	0.18017	0.2717	0.01204	0.12973	-0.0641

XM_001070335.1	LOC689299	0.0821	-0.0949	0.11237	-0.0776	0.00501	-0.0421	0.03227	0.00206	-0.0034	-0.0907	0.09022	0.26298	-0.0403	0.0519
NM_001014221	LOC689326	0.52431	0.68099	0.82225	1.0398	1.0374	1.2788	0.75923	1.0338	1.1984	0.83374	1.3298	0.61063	0.44111	0.40977
NM_001044282	LOC689330	0.11372	0.06295	-0.0571	0.04242	0.01257	0.09949	0.09699	0.1142	-0.1135	0.09192	-0.0225	0.09488	0.03605	-0.0338
XM_001070518.1	LOC689362	0.29267	1.3579	0.99485	0.428	0.48451	0.88518	0.51213	0.91334	1.1668	1.3224	1.7713	0.61141	0.7594	0.58501
NM_001099460	LOC689403	0.34352	1.7839	1.2062	0.48315	-0.063	0.57978	1.1214	0.08222	1.2607	1.724	1.6446	0.28922	0.36472	0.28239
NM_001168674	LOC689421	-0.3983	-0.4204	-0.3642	-0.2544	-0.3278	-0.4461	-0.31	-0.3836	-0.2845	-0.3992	-0.2544	-0.2638	-0.3716	-0.2259
XM_001070809.1	LOC689443	-0.0047	0.05173	0.02133	-0.0012	0.13467	0.02709	0.06043	0.16752	-0.0216	0.05471	0.12543	0.20975	0.12614	0.14159
XM_001070964.1	LOC689484	0.14918	-0.1307	-0.2957	0.18092	0.26476	0.46737	-0.008	0.35349	0.46035	-0.0027	0.2416	0.0452	-0.0546	-0.1391
NM_001014221	LOC689497	0.26649	0.52536	0.28075	0.08761	0.28871	0.43666	0.17549	0.22271	0.16766	0.36691	0.66369	0.25768	0.43424	0.22854
XM_001071141.1	LOC689541	0.30926	0.76785	0.42954	0.26101	1.1089	1.2739	0.39413	1.437	0.641	0.932	1.0531	0.06048	0.30588	0.23892
XM_001071174.1	LOC689554	0.00516	-0.0644	0.13077	-0.0914	-0.0557	-0.1028	-0.0704	-0.1439	0.19378	-0.1153	0.20727	-0.073	0.00893	0.05111
NM_032078	LOC689560	-0.1091	-0.0232	-0.1897	-0.1982	-0.0954	0.06182	-0.0397	-0.2441	-0.1743	-0.147	-0.0196	-0.2953	-0.1325	-0.2676
NM_032078	LOC689560	-0.0999	-0.0509	0.0067	0.19933	0.05344	0.23819	0.0593	-0.0027	0.12239	0.11554	0.11832	-0.1176	-0.1126	0.20288
NM_032078	LOC689560	-0.0992	-0.2286	0.08499	-0.1549	-0.2028	-0.1193	0.05589	-0.1166	0.27628	-0.1072	-0.2287	0.13747	-0.1891	-0.0515
NM_032078	LOC689560	-0.0019	0.03515	0.10537	0.02834	0.21039	0.29091	0.12637	0.03606	-0.0027	0.07132	0.04368	-0.0756	0.12066	-0.0282
XM_001071243.1	LOC689577	0.08308	0.00128	0.0357	0.03999	-0.0117	-0.0688	-0.1783	-0.0129	0.07155	0.09563	0.24891	-0.0834	0.188	0.14775
NM_001110141	LOC689581	0.00136	0.20092	0.00556	0.15369	0.37501	0.35279	0.12678	0.40736	0.1216	0.06565	-0.0511	-0.1181	0.10199	0.29807
NM_183402	LOC689658	0.14138	0.02332	0.16788	-0.0303	-0.0255	-0.0705	0.14037	0.00717	0.08608	0.05663	0.10607	0.02241	0.031	-0.0875
NM_001014221	LOC689664	0.07488	-0.1005	-0.1919	-0.087	-0.1301	-0.0699	-0.0918	-0.1883	-0.2637	-0.1131	-0.0851	0.11586	-0.2392	0.11417
NM_183402	LOC689667	-0.0515	0.02143	0.00904	-0.0835	-0.0052	-0.0084	-0.0071	-0.0913	-0.0271	-0.0498	-0.0075	-0.0258	0.00466	0.03024
XM_001071625.1	LOC689679	0.31676	-0.0388	0.02099	0.10749	0.04808	0.13801	0.01145	0.02812	0.04378	0.06148	0.13038	0.06346	0.06294	0.04584
NM_183402	LOC689690	-0.1442	0.54346	0.09817	0.03056	-0.0348	0.10092	-0.0291	0.06385	0.12287	0.46296	0.5263	0.08127	0.07887	-0.1103
XM_001071683.1	LOC689695	-0.1997	-0.11	0.00856	0.14243	-0.0697	0.15847	-0.0426	0.03429	0.03383	-0.0264	0.10123	0.15484	-0.1193	-0.1187
XM_001071863.1	LOC689746	-0.2179	-0.4127	-0.089	-0.3216	-0.088	-0.0471	-0.0135	-0.2047	0.20704	-0.2311	-0.202	-0.1939	0.11558	-0.1044
NM_001107063	LOC689752	-0.4135	-0.2793	-0.1185	-0.2549	-0.0371	-0.2134	-0.1691	-0.3005	-0.409	-0.2836	-0.4431	-0.2816	-0.3003	-0.418
XM_001071886.1	LOC689753	-0.206	0.12129	0.69599	1.0921	0.11174	0.00714	0.97247	-0.0462	-0.0126	0.142	0.91447	0.05616	-0.1768	0.23407
NM_001142304	LOC689770	1.498	0.48734	0.73432	1.3118	0.39846	0.59755	0.49123	0.3862	0.73316	0.31513	0.27534	0.59816	0.23835	0.43109
XM_001072032.1	LOC689796	0.0643	0.41255	0.39786	0.29671	0.35685	0.79545	0.06214	0.49955	0.22898	0.32276	0.7173	-0.084	0.06201	-0.0448
NM_183402	LOC689811	-0.0574	0.12561	-0.5058	0.14945	0.23771	-0.1461	-0.1451	0.25671	-0.124	-0.0009	0.00695	-0.2726	0.18921	0.22148
NM_183402	LOC689811	-0.0162	0.15514	-0.065	0.0515	0.17947	0.11745	0.03071	-0.0295	0.14161	0.16947	0.02123	0.08712	0.07106	0.14326
XM_001072149.1	LOC689820	-0.611	-0.6962	0.29267	-0.4243	-0.8524	-0.8171	0.0248	-0.7041	-0.4439	-0.3002	-0.4666	-0.2812	-0.1034	0.11686
NM_001014221	LOC689827	0.92782	2.2009	1.5516	1.3097	1.5528	1.5456	1.2008	1.5258	1.8191	2.3162	2.5366	0.72645	0.68184	0.77658
NM_053689	LOC689842	-0.1114	-0.0237	0.30584	0.13295	-0.5221	-0.698	0.0353	-0.1238	-0.3045	-0.0118	-0.0766	0.03892	0.03157	0.32913
NM_001014221	LOC689878	0.09783	0.04283	0.01331	0.03972	0.26241	0.20372	0.11755	0.05262	-0.003	0.17049	0.15478	0.10752	-0.03	-0.0083
NM_001109555	LOC689927	-0.0008	0.01446	-0.02	0.22074	0.02088	0.11058	0.02735	0.02985	-0.003	0.24585	-0.0075	0.00376	0.11488	-0.0181
XR_005453.1	LOC689945	-0.2502	-0.121	-0.2487	-0.2533	-0.1892	-0.0443	-0.3019	-0.1969	-0.2143	-0.1534	-0.407	-0.1922	-0.1041	-0.1593
XM_001072807.1	LOC689985	0.18532	0.07661	0.34919	0.07305	0.13364	0.11661	0.1269	0.31266	0.22596	0.11143	0.18537	0.19156	0.22608	0.19211
XM_001072857.1	LOC689998	-0.0076	0.02052	0.1342	-0.0504	0.08318	0.06276	0.05498	0.0224	-0.025	0.01788	0.05022	0.27976	-0.0464	-0.0623
NM_175760	LOC690021	0.01544	0.13914	0.22782	-0.0348	0.17406	0.14328	0.05768	-0.029	0.10639	0.10572	-0.0205	0.07221	-0.0546	0.01114
NM_001128195	LOC690038	0.43801	0.60458	0.13511	0.28826	0.60969	-0.1638	0.29254	0.69338	0.71599	0.68089	0.3434	-0.0993	1.2009	0.96779
XM_001073060.1	LOC690054	0.19901	0.14224	0.04275	0.00828	-0.0337	-0.0473	0.28789	0.14648	0.1312	0.22524	0.09058	0.24067	-0.024	0.09443
NM_183402	LOC690081	0.08495	0.22734	0.07363	0.02685	0.03723	-0.046	0.10679	0.12671	0.22397	0.01273	0.14659	-0.0672	-0.0588	0.06154
XR_006791.1	LOC690093	-0.1108	-0.0102	-0.1915	-0.0438	0.10398	-0.2214	0.03789	-0.0528	-0.0006	-0.0322	-0.0898	-0.0823	0.08217	0.21564
XM_001073319.1	LOC690115	-0.0996	-0.0562	-0.1683	0.14975	-0.1121	-0.1946	0.00275	-0.2308	-0.0707	-0.0631	-0.038	-0.2115	-0.1317	-0.0317
XM_001073516.1	LOC690161	-0.1272	-0.0453	-0.2204	-0.1725	-0.0577	0.03873	0.05896	-0.0071	-0.1444	-0.0236	-0.0714	0.03034	-0.1293	0.22745
XM_001070526.1	LOC690211	-0.0524	0.07305	0.03324	0.1851	-0.0053	0.0532	0.05082	0.11623	0.16808	0.11038	0.24901	0.20615	0.01653	0.07959
NM_001014221	LOC690250	0.32119	0.52764	0.35331	0.22169	0.48221	0.74304	-0.0788	0.25518	0.5884	0.31582	0.76078	0.48379	-0.0597	0.04649
NM_001134871	LOC690262	0.35334	0.44233	-0.1781	0.59455	0.52466	0.07819	-0.0309	0.60965	0.48137	0.59717	0.2335	-0.0039	0.34315	0.40675
NM_001108999	LOC690279	-0.0146	-0.098	0.03849	-0.0889	-0.0131	-0.0489	-0.0392	-0.0143	-0.0342	0.05478	-0.1189	-0.0501	-0.0446	-0.1

XM_001073998.1	LOC690295	0.02976	-0.074	0.27388	-0.0331	-0.0128	0.2877	0.12275	0.09902	0.08027	0.13386	-0.0407	0.10769	0.00589	0.3443
NM_001100682	LOC690312	0.4352	0.52441	0.27508	0.7823	0.30129	0.36502	1.5726	0.38225	-0.0048	0.42897	0.31759	0.51351	0.08376	-0.0356
NM_001109577	LOC690315	-0.1847	-0.169	-0.1673	-0.0502	-0.1439	-0.1439	-0.0341	-0.0573	-0.1402	-0.2001	-0.1467	-0.0781	-0.118	-0.1516
XR_006857.1	LOC690317	-0.0093	0.12173	0.06255	0.07415	0.04389	-0.0304	0.09851	-0.0239	0.14385	0.05861	0.22438	0.02181	0.15713	-0.0233
XM_001074151.1	LOC690340	0.26714	0.17621	0.12301	-0.0375	0.15972	0.0901	0.24012	0.16227	0.07329	0.1701	0.15182	0.11168	0.37694	0.18256
NM_001126095	LOC690345	-0.1718	-0.1197	-0.0663	-0.1863	-0.1764	-0.2457	-0.234	-0.1437	-0.0887	-0.1731	-0.1637	-0.0905	-0.1156	-0.2355
NM_053330	LOC690364	-0.1271	-0.2652	-0.047	-0.1176	-0.2114	-0.0057	0.0311	-0.2581	-0.1276	-0.1919	-0.0961	-0.1253	-0.1181	-0.0165
NM_001109587	LOC690388	0.0906	0.0406	-0.0618	0.09034	-0.0258	-0.2556	-0.3287	0.06459	-0.0938	-0.0228	-0.0235	-0.286	0.18743	0.0505
NM_001014221	LOC690395	0.05574	0.59511	0.69993	0.10226	0.67058	0.68318	0.32942	0.82826	0.64973	0.68267	1.1551	-0.1345	-0.1068	0.32953
NM_001109589	LOC690407	0.12308	0.37475	0.10657	0.0094	0.07126	0.18367	0.02651	0.03958	0.06377	-0.0444	0.0948	0.09103	0.14353	0.10713
NM_183402	LOC690413	0.23557	0.17708	0.57661	0.29503	0.18595	0.0722	0.57072	0.14432	0.39505	0.40504	0.66286	0.33644	0.01449	0.09325
XM_001074429.1	LOC690428	0.30889	0.0334	0.00224	0.4137	0.45278	0.6968	0.03739	0.42611	0.25205	0.2032	0.21936	0.51688	0.31188	0.10951
XM_001074567.1	LOC690482	0.1455	0.22842	0.16312	-0.0248	0.22172	0.23495	0.15792	0.2672	0.17467	-0.0632	0.41799	0.07822	0.60148	0.30347
XM_215885.4	LOC690571	-0.0327	-0.1094	0.06062	0.66599	-0.0979	-0.0827	-0.0362	-0.1889	-0.0871	-0.0257	-0.0734	-0.2296	-0.0753	-0.2258
NM_001014221	LOC690577	0.40362	1.792	1.3225	0.95966	1.1547	1.3549	0.93871	1.1817	1.4395	1.4575	2.232	0.58032	0.48872	0.53247
XM_001075162.1	LOC690672	-0.2289	-0.1404	-0.2791	-0.1477	-0.3011	-0.1864	-0.2962	-0.27	-0.2528	-0.1396	-0.1213	-0.1336	-0.1243	-0.221
XM_001075438.1	LOC690739	-0.5257	-0.3606	-0.0513	-0.4232	-0.4873	-0.4512	-0.3538	-0.4524	-0.3853	-0.3407	-0.2266	-0.4124	0.13306	-0.4286
NM_001100811	LOC690745	0.01245	0.02584	-0.0682	0.16308	-0.1399	0.06645	0.18617	-0.0343	0.10674	0.16522	0.02143	0.04475	0.0423	0.00497
NM_001168586	LOC690777	-0.3371	0.00211	-0.017	-0.0451	0.02921	-0.3976	0.12637	-0.0788	-0.0075	0.01983	0.05833	-0.2428	-0.2011	-0.2587
NM_001108357	LOC690779	0.16143	0.06854	0.11348	0.18983	0.23586	0.16054	0.05588	0.01724	-0.0406	0.16962	-0.076	0.22372	0.21551	0.0754
NM_001108357	LOC690779	-0.0337	0.02159	-0.1734	-0.1276	0.17738	-0.0999	0.03474	0.29408	0.13393	0.1138	-0.1135	-0.1298	0.24089	0.21499
NM_001099493	LOC690816	0.25767	0.20834	0.24075	0.19381	-0.0706	0.29764	-0.056	-0.2042	-0.1463	0.10375	0.03465	0.22117	0.1385	0.0092
XM_001075967.1	LOC690874	0.04006	-0.0235	0.22585	0.02336	-0.041	0.02528	-0.0142	-0.0248	0.0576	-0.0219	0.07185	0.06839	0.11643	0.00672
XR_006981.1	LOC690911	-0.6361	-0.4221	-0.1136	0.06154	-0.436	-0.3066	-0.0807	-0.619	-0.3686	-0.1674	-0.2371	-0.3246	-0.3338	-0.297
NM_001014221	LOC690943	0.7297	1.0805	0.92942	0.84615	1.1396	1.3954	1.0083	1.1336	1.123	1.1962	1.2817	0.7827	0.46058	0.29785
XM_001076415.1	LOC690989	0.01116	0.02631	0.23716	-0.4271	-0.2207	-0.3897	0.07733	-0.3989	0.01899	-0.2267	-0.2422	0.13876	0.2234	0.22586
XR_007009.1	LOC691045	-0.0318	-0.066	-0.119	-0.0794	-0.0985	0.01962	-0.0652	0.0498	-0.0139	-0.0755	-0.0756	0.08542	0.00941	-0.0895
NM_001135894	LOC691052	0.16317	0.0688	0.19539	0.19898	0.25122	0.07616	0.08322	-0.045	0.05507	-0.0499	0.39052	0.29248	-0.0585	-0.0336
XM_001076998.1	LOC691146	0.19201	0.16711	0.03317	0.07192	0.02019	0.28709	0.00158	0.36454	0.30151	0.26043	0.33459	0.16571	-0.0336	0.29871
XM_001077034.1	LOC691157	0.00344	-0.1	-0.2208	0.0307	-0.0393	-0.1986	-0.1571	-0.0719	0.01715	-0.2193	-0.0957	-0.1785	0.0702	-0.035
NM_001109632	LOC691259	1.1911	-0.062	0.38842	0.60632	0.362	0.67889	0.98337	0.63829	-0.0137	-0.0152	0.2544	-0.0183	-0.0526	0.28662
NM_173124	LOC691312	0.2004	0.10354	0.10712	0.02983	0.00896	0.24115	-0.0562	0.11164	0.01117	0.0648	0.03458	0.01612	0.08299	0.03475
XM_001077680.1	LOC691318	-0.0675	0.23447	-0.1473	0.19099	0.15798	-0.2543	-0.2821	-0.1377	-0.0381	0.0771	-0.161	-0.2575	-0.0689	-0.1619
XM_001077719.1	LOC691325	-0.0099	-0.122	-0.1306	-0.1513	0.08302	-0.0748	-0.0077	-0.1224	0.0148	0.11519	-0.1492	-0.097	0.11237	0.16282
XR_007077.1	LOC691361	0.07325	0.03302	0.03633	0.06406	0.09537	0.13529	0.0836	0.02624	0.08899	0.20184	0.16995	0.08669	0.09821	0.07607
XM_001078077.1	LOC691401	-0.1339	0.33175	0.03433	0.0826	0.26291	0.39991	-0.0621	0.18177	0.31593	0.1403	0.46034	-0.0508	-0.1283	0.14743
NM_001014221	LOC691406	-0.0398	0.18007	0.12632	0.13274	-0.067	0.04036	-0.0085	0.15632	0.14942	0.15545	-0.0067	0.12204	-0.0206	0.03046
NM_001127539	LOC691411	0.12514	0.06058	-0.1104	-0.046	-0.0113	0.11449	-0.1474	0.26077	0.07621	0.10512	-0.2512	0.02726	0.01688	0.17489
XM_001078417.1	LOC691468	0.23744	0.08475	0.02191	0.32059	0.18403	0.42378	0.00188	0.06802	0.1912	0.03153	0.2561	0.34558	0.10091	0.15639
XM_001078495.1	LOC691487	-0.0314	-0.0254	-0.091	-0.0029	-0.0194	0.05257	-0.0149	-0.0784	0.02121	-0.068	-0.0863	-0.1623	-0.1207	0.12523
XM_001078524.1	LOC691491	-0.0169	0.00281	-0.0231	0.11006	0.1501	0.22709	0.06127	-0.0375	0.03226	-0.0107	0.08946	0.09418	-0.0053	-0.0257
NM_001109644	LOC691506	0.2816	0.38563	-0.0147	0.20379	0.63597	0.35997	0.01914	0.64289	0.07763	0.12459	0.20803	-0.1292	0.21236	0.45547
NM_001083940	LOC691552	-0.1701	-0.0366	-0.0875	-0.2178	-0.1596	-0.114	-0.0102	-0.0605	-0.1241	-0.0592	-0.1742	-0.1099	0.06114	-0.067
NM_001083940	LOC691552	0.10187	0.08741	0.03779	0.0571	-0.0897	0.10469	0.1325	-0.0386	-0.1193	-0.0742	0.09491	-0.1245	0.10796	-0.0536
XM_001078865.1	LOC691569	-0.0844	0.00013	-0.0343	-0.0591	-0.0666	-0.0291	-0.0556	-0.0767	-0.003	-0.0161	-0.0095	0.01883	-0.0005	0.08179
XM_001078898.1	LOC691575	0.41784	-0.0001	0.3103	0.63084	0.40754	0.31749	0.31877	0.46043	0.07249	0.05869	0.03405	0.32506	0.24378	0.02543
NM_001014221	LOC691586	0.02219	-0.0338	0.14421	0.21337	-0.058	0.1406	0.08127	0.06417	0.02368	0.01464	0.00199	-0.0507	0.18323	0.12466
NM_001135157	LOC691644	0.42187	0.02597	0.18307	-0.1042	-0.0989	0.15954	0.21959	0.15766	0.21254	0.30948	0.49999	0.06734	0.05917	0.07437
XM_001079209.1	LOC691669	-0.0863	-0.0981	-0.1618	-0.0611	-0.1338	-0.1102	-0.0977	-0.1841	-0.0769	-0.1624	-0.0398	-0.1112	-0.1279	-0.1362

XM_001079224.1	LOC691672	-0.1271	-0.0023	-0.0799	-0.0581	0.05109	0.15454	0.15979	-0.1075	-0.0069	-0.0262	-0.0738	-0.108	-0.013	-0.0601
XM_001079298.1	LOC691693	-0.0512	-0.0969	-0.0282	-0.0041	-0.1729	-0.0897	-0.0195	-0.1815	-0.065	-0.046	-0.1324	-0.1106	-0.0941	0.11353
XM_001079329.1	LOC691703	0.19071	-0.0362	0.03896	0.21424	0.11608	0.15961	0.07603	-0.0052	0.19213	0.29626	0.00375	0.28043	0.13798	-0.0231
NM_001127573	LOC691708	0.33601	-0.0166	0.28212	0.24705	0.44032	0.62994	0.3882	0.26197	0.05174	-0.0196	0.41806	0.44205	0.02504	0.14312
NM_183402	LOC691712	-0.1251	0.0992	-0.0982	-0.1087	-0.195	-0.1015	0.02791	-0.0493	0.01536	-0.0703	-0.0373	0.18511	-0.0375	-0.0827
NM_001014221	LOC691762	0.54317	0.95578	0.85592	0.87274	0.90977	1.0786	0.98983	0.82735	1.055	1.0059	1.2191	1.0645	0.56927	0.40135
XM_001080044.1	LOC691903	-0.0197	0.2845	0.09384	0.11935	0.02146	-0.0244	0.16365	-0.0473	0.00099	0.22463	0.07786	0.33126	0.11854	0.19454
XM_001080051.1	LOC691905	-0.3413	-0.0455	-0.0577	-0.2926	-0.2805	0.01306	-0.2788	-0.0934	-0.1103	-0.0752	-0.1483	-0.1407	0.02964	-0.2352
NM_001134694	LOC691909	0.14838	0.08841	0.06598	0.16417	0.37956	0.65969	0.33419	0.19624	0.08144	0.65807	0.32829	0.04468	-0.1449	-0.0189
XM_001080091.1	LOC691918	-1.0141	-1.3044	-1.6873	-1.4277	-1.2111	-0.9206	-1.2109	-1.03	-1.006	-1.142	-1.1648	-1.1014	-0.8044	-1.019
NM_001134887	LOC691922	0.1409	0.17721	-0.0447	0.03851	0.10796	-0.083	0.19158	-0.0114	0.16252	0.30068	0.19229	0.00608	0.21608	0.15912
XM_001080153.1	LOC691929	-1.5203	-1.9754	-1.8056	-2.1078	-2.4788	-2.2316	-1.9684	-2.4945	-1.5202	-1.3963	-1.6871	-0.8697	-1.4932	-1.47
NM_001077670	LOC691947	-0.133	-0.4038	0.07169	-0.6584	-0.2454	-0.365	0.00712	-0.264	-0.1973	-0.2546	-0.4129	-0.0338	0.31817	0.21949
NM_001047913	LOC691966	1.1966	0.87377	0.93415	0.55602	1.3509	0.95925	0.87186	1.4508	0.67424	0.82041	0.76915	-0.0558	0.81692	0.91677
NM_001134799	LOC691982	-0.3017	-0.1465	-0.3894	-0.1561	-0.4129	-0.1864	-0.4212	-0.0971	-0.2808	-0.3299	-0.2655	-0.4761	-0.3163	-0.1399
NM_001109331	LOC692000	0.44975	0.14937	0.28294	0.20061	0.24941	0.83239	0.14534	0.33502	0.43268	0.39271	0.27821	0.70598	0.11122	0.14872
XM_001080954.1	LOC692006	-0.0735	-0.0927	0.00967	-0.0001	0.05279	0.19481	-0.015	-0.0261	0.09138	-0.0148	0.20474	0.18001	0.10569	0.07433
NM_198755	Loh11cr2a	-0.1274	-0.0707	-0.0159	0.2847	-0.0223	-0.1847	-0.0209	-0.3466	-0.1141	-0.3515	-0.0949	0.06687	-0.1246	-0.0449
NM_001108650	Loh12cr1_predicted	0.29418	-0.0165	-0.2521	0.09769	0.09524	0.49817	-0.4063	0.47989	-0.3341	-0.1979	-0.2063	0.16382	-0.0274	-0.0685
NM_133404	Lonp1	0.63362	0.08194	0.55191	0.01682	0.28331	0.19429	0.11021	0.24029	-0.1538	-0.0465	0.11774	-0.452	0.19753	0.13667
XM_001066614.1	Lonrf1_predicted	0.03505	-0.1233	-0.2904	0.02902	-0.094	-0.1002	0.11542	0.05021	-0.1615	-0.1024	-0.1484	-0.1079	-0.2186	-0.2047
XM_001058206.1	Lonrf2_predicted	0.26438	0.38344	0.27759	-0.136	-0.1942	-0.1217	0.1193	0.1189	0.08514	0.05305	0.44649	0.28042	0.35576	0.57998
NM_017061	Lox	0.02215	0.10785	0.12213	-0.1215	0.22578	-0.0522	-0.0496	0.10518	0.04291	0.03714	-0.0102	0.24561	0.33285	0.25846
NM_001012125	Loxl1	-0.1999	-0.1809	0.10996	-0.7182	0.54788	0.46849	-0.2827	0.59882	0.14921	-0.0254	0.03264	0.30666	0.35144	0.49967
NM_001012125	Loxl1	-0.0287	-0.0164	0.26022	-0.5626	0.70074	0.42981	-0.0262	0.76206	0.25058	0.23283	0.06838	0.37015	0.549	0.62738
NM_001106047	Loxl2_predicted	-0.6281	-0.8074	-1.6086	-1.5021	0.21917	-0.6683	-1.9672	-0.0881	-0.7756	-0.6724	-0.8908	-1.7389	-1.6936	-1.5654
NM_001107866	Loxl3_predicted	1.4042	1.2353	0.81287	1.0875	1.0353	1.1548	0.67031	0.86437	1.3912	1.0635	1.1717	1.2944	0.62329	0.71795
NM_001107592	Loxl4_predicted	0.20162	0.15926	0.03376	0.11166	0.31376	0.31071	0.21886	0.1866	0.30745	0.09691	0.08582	0.14292	0.30937	0.03605
NM_022962	Lphn1	-0.2467	-0.3012	-0.6697	-0.4764	-0.1861	-0.1519	-0.4736	-0.016	-0.6025	-0.6359	-0.4361	-0.455	-0.4326	-0.5688
NM_134408	Lphn2	-0.0607	0.62642	-0.5043	0.02069	-1.1612	-1.1971	0.33786	-1.0636	0.40515	0.35159	0.75945	0.31412	-0.3208	-0.2577
NM_130822	Lphn3	-0.1094	-0.0472	-0.0794	-0.092	-0.0017	-0.043	-0.1634	-0.1055	-0.193	-0.1164	-0.1305	-0.1579	-0.1317	-0.0801
NM_001012111	Lpin1	-0.0993	0.11531	0.31931	0.3581	0.19145	0.13586	0.18069	0.32272	0.01011	0.12803	0.07235	-0.0609	-0.2995	-0.1326
NM_001012111	Lpin1_predicted	-0.4535	-0.5514	-0.0521	-0.1986	-0.5223	-0.6987	-0.2091	-0.6122	-0.4481	-0.5016	-0.3755	-0.4631	-0.7172	-0.6297
NM_001108236	Lpin2_predicted	0.11512	-0.2196	-0.1493	-0.0152	-0.1063	-0.0233	0.05062	0.11631	-0.1215	-0.1414	-0.0618	0.08677	0.1426	0.0169
NM_012598	Lpl	0.14857	1.5563	0.0612	0.0843	0.08741	0.51294	0.0935	0.12039	1.4994	1.4897	1.2777	0.05383	0.02822	0.06453
NM_001105829	Lpo_predicted	-0.0227	0.02308	0.06972	0.1978	0.16269	0.08469	0.00727	0.02878	-0.1043	-0.1168	-0.0782	-0.1714	-0.0139	0.21511
NM_001005881	Lppr2	-0.0806	0.06675	0.092	-0.1504	0.00096	-0.0986	0.0471	0.02647	-0.0843	0.09715	0.13634	0.03359	0.06417	0.11165
NM_001009649	Lpxn	-0.3236	-0.029	-0.255	-0.3248	-0.2077	-0.218	-0.2734	-0.1566	0.04716	-0.0621	-0.2161	-0.0786	0.0332	-0.0469
NM_001108555	Lrba_predicted	0.63788	0.10852	0.1066	0.58001	0.62232	0.40242	0.14397	0.85805	-0.0227	0.19076	0.15595	-0.1998	0.06264	0.19523
NM_001107088	Lrch3_predicted	0.02984	-0.0645	0.06499	-0.0478	0.09583	-0.0444	-0.0592	-0.0171	0.11711	0.13114	0.05769	0.14452	0.04841	0.11931
NM_001127551	Lrch4_predicted	-0.0701	-0.168	-0.1253	0.4984	0.41618	0.33039	-0.1129	0.09259	-0.2344	-0.0168	-0.0576	-0.1616	-0.1671	-0.1204
NM_001106318	Lrdd_predicted	0.06516	-0.2106	0.56065	0.4431	-0.1872	-0.22	0.12598	0.00074	-0.1992	-0.3311	-0.0858	-0.2019	0.06419	-0.0478
NM_001108910	Lrfn1_predicted	0.19501	0.01684	0.14348	0.02124	-0.0625	-0.037	0.04174	0.04572	-0.0541	0.19783	0.25881	0.06039	-0.0687	0.0259
NM_001039699	Lrfn2	0.21023	-0.0531	0.07668	-0.0367	0.05234	0.04435	0.04775	-0.0153	-0.1264	0.06573	0.13815	0.02024	-0.0021	-0.0293
NM_001107502	Lrfn3_predicted	0.06638	-0.16	-0.2284	-0.0502	-0.1098	-0.0785	-0.0998	0.01554	-0.2583	-0.0958	-0.1694	-0.2926	-0.1029	-0.0366
NM_001108024	Lrfn5_predicted	-0.0735	0.01453	0.34797	0.05078	0.07865	0.10711	0.06433	-0.1125	-0.0669	0.19076	0.12135	0.02166	-0.0782	-0.0434
NM_001009717	Lrg1	-0.2118	0.00251	0.01119	0.03599	-0.0137	-0.0836	-0.0772	-0.0302	0.04357	-0.086	-0.0058	0.03785	0.09893	-0.2261
XM_001076882.1	Lrig1_predicted	0.02977	-0.0043	0.22802	-0.0235	0.29633	0.00311	0.08833	0.2339	0.09484	-0.1134	0.33886	0.03341	0.26951	0.19158
NM_001107710	Lrig2_predicted	-0.114	-0.1778	0.0634	0.32489	0.20342	0.11336	0.18303	0.1978	-0.1103	0.14846	-0.0877	-0.1793	-0.0956	0.01753

NM_001130490	Lrp1	-0.464	0.80418	0.4611	0.84284	-0.6069	-0.5447	0.52739	-0.4242	0.47004	0.48668	0.78301	0.17529	-0.2609	-0.4202
NM_001037777	Lrp10	0.72915	0.59677	1.0061	1.3243	0.94351	0.57524	1.03	0.74257	0.26433	0.53282	0.4973	-0.0857	0.7866	0.8061
NM_001106217	Lrp11_predicted	-0.0826	-0.0657	-0.179	-0.0664	0.08374	0.06128	0.50315	0.19715	0.14139	0.09987	0.22618	0.37537	-0.2339	-0.1825
NM_001134883	Lrp12_predicted	0.11098	0.31844	0.20752	0.52616	0.18316	0.06509	0.02726	0.28727	0.23905	0.28376	0.28632	0.00073	0.52804	0.64124
NM_001107843	Lrp1b_predicted	-0.0077	-0.1249	0.07636	-0.1469	-0.0917	-0.0048	-0.1386	-0.0483	-0.1415	-0.1152	-0.1677	-0.0192	-0.234	-0.1102
NM_001017443	Lrp2bp	0.05923	0.04307	0.2328	0.04657	0.11941	-0.0425	0.06355	0.20825	0.20876	0.32674	0.13686	0.15229	0.25423	0.13626
NM_053541	Lrp3	-0.0537	-0.0755	0.17924	-0.0488	0.07211	-0.1033	-0.1	-0.0971	0.18947	0.02677	-0.0176	0.12847	0.05574	0.05496
NM_031322	Lrp4	-0.223	-0.1533	-0.3797	-0.172	0.01253	-0.0702	-0.1742	-0.0467	0.08609	-0.0606	-0.1806	0.09529	-0.2977	-0.1188
NM_001106321	Lrp5_predicted	-0.2839	0.27597	-0.2854	-0.1367	0.24196	0.11477	-0.2689	0.26222	0.03048	0.08756	0.05029	-0.3703	-0.3204	-0.4844
NM_001107892	Lrp6_predicted	-0.2943	-0.1578	-0.5991	-0.1406	-0.4186	-0.6734	-0.1654	-0.3761	-0.3609	-0.6038	-0.3171	-0.2919	-0.287	0.01156
XM_342877.2	Lrp8_predicted	-0.072	-0.2858	0.56057	0.68556	0.39892	0.20965	0.09541	0.23713	-0.1513	0.09674	-0.0149	-0.0591	0.55269	0.35108
NM_001169113	Lrpap1	-0.1115	-0.0689	-0.3091	-0.2624	-0.0611	-0.2908	-0.3912	-0.4346	-0.4998	-0.8544	-0.6469	-0.2902	-0.4232	-0.4118
NM_001008519	Lrpprc	-0.1517	-0.0111	0.31776	0.12996	-0.0585	-0.2937	0.12888	-0.3548	-0.1922	-0.1005	-0.0672	-0.5701	0.00785	0.13375
NM_001014268	Lrrc1	-0.3791	-0.5342	-0.2933	-0.0708	-0.1479	-0.339	-0.2155	-0.2128	-0.4262	-0.3777	-0.5371	-0.7164	-0.7516	-0.47
NM_001108096	Lrrc10_predicted	-0.0653	-0.0964	-0.1247	-0.051	-0.2486	-0.1799	-0.0552	-0.0297	-0.0624	-0.0282	-0.0301	-0.1464	-0.109	-0.1452
NM_001024354	Lrrc14	-0.1397	-0.2528	0.17573	-0.4291	-0.4965	-0.9213	-0.2321	-0.4372	-0.32	-0.0858	-0.1959	-0.1521	-0.3565	-0.5041
NM_145083	Lrrc15	-0.1223	-0.1746	-0.1298	-0.0003	-0.0833	-0.0637	-0.1301	-0.0152	0.03415	-0.1484	0.13406	-0.0094	0.14794	-0.0974
XM_225336.3	Lrrc16_predicted	-0.0375	0.10078	0.04776	-0.0818	-0.0679	0.00161	-0.0894	-0.0461	-0.0198	0.08646	0.13333	-0.0227	0.02241	-0.0997
XM_001070748.1	Lrrc16_predicted	-0.167	-0.0976	-0.1767	-0.3067	0.3037	0.02176	0.16869	0.57699	-0.2576	-0.3015	-0.0432	-0.2874	-0.5005	-0.4461
NM_001025155	Lrrc17	0.25617	0.18992	0.24773	-0.025	0.36539	0.18812	0.07744	0.22871	0.41593	0.22972	0.23646	0.44166	0.56553	0.23447
NM_139331	Lrrc21	-0.0467	-0.1255	-0.2294	-0.1152	-0.0574	-0.1731	0.07438	0.04068	-0.1089	-0.0146	-0.1557	-0.2154	-0.0093	-0.3532
NM_001013165	Lrrc23	-0.069	-0.0683	0.06303	-0.0483	-0.0443	-0.0392	-0.1563	-0.0506	0.27178	-0.0432	-0.1156	-0.0523	0.26453	-0.1256
NM_001014053	Lrrc26	0.26483	0.07172	0.09264	0.07304	0.03779	0.15543	0.1113	0.05866	0.10104	0.11819	-0.0301	0.15903	-0.0062	0.26237
NM_001108486	Lrrc28_predicted	0.54356	-0.3307	-0.2032	-0.2385	-0.0426	-0.2704	-0.4114	0.02405	0.12333	-0.3486	-0.2	0.38449	-0.1074	-0.1348
NM_145679	Lrrc3	0.09627	0.11587	0.09472	0.0329	0.09546	0.15472	0.02514	0.10575	0.05336	0.18962	0.15343	0.08459	-0.012	0.06762
XM_001054231.1	Lrrc30_predicted	0.01406	0.187	0.0349	0.19651	0.02116	0.21372	0.03065	-0.2189	-0.1261	-0.1163	0.2676	0.25952	0.00815	-0.0817
NM_001024995	Lrrc33	0.00551	0.08948	0.21951	0.10019	-0.0383	-0.062	0.18386	-0.0848	0.20585	-0.0725	-0.1358	0.10117	-0.0932	-0.0584
NM_001004088	Lrrc36	0.14543	0.01497	0.44305	0.77321	0.33978	0.13507	0.17105	0.00396	0.04948	0.28176	0.43791	0.26455	0.23772	0.13899
NM_001107991	Lrrc38_predicted	-0.0094	0.08344	0.01843	0.06582	0.02112	0.07846	0.02954	0.06078	0.07789	0.03454	0.11692	0.02367	0.00828	-0.0541
NM_001107991	Lrrc38_predicted	0.08872	0.12406	0.08429	0.01951	0.1687	0.10632	0.232	0.07457	0.10081	0.01955	0.1296	-0.0246	0.21554	0.22387
XR_008527.1	Lrrc3b_predicted	-0.0025	0.08856	0.07703	0.01228	-0.087	0.0349	-0.0314	-0.042	0.19533	-0.0977	0.05682	-0.0378	-0.0656	-0.0099
NM_001009710	Lrrc41	-0.1721	0.23886	-0.157	-0.0062	0.06176	-0.0573	0.1973	0.04752	-0.1278	-0.0029	0.04041	-0.1018	-0.0394	0.31392
NM_001025653	Lrrc42	0.26021	-0.0101	0.08659	0.65569	0.39256	0.46231	0.33042	0.20838	0.15209	0.14057	0.11186	0.0486	0.00621	-0.0525
NM_001170396	Lrrc43	0.13385	0.04459	0.28339	-0.0665	0.06905	-0.1068	0.13175	-0.1011	-0.1084	-0.1655	0.0483	-0.03	-0.008	-0.1175
NM_001004201	Lrrc46	-0.0366	-0.1316	-0.0873	0.02581	-0.012	0.177	-0.5419	-0.017	0.06996	-0.0031	-0.1422	-0.0788	0.02234	0.05199
NM_001135666	Lrrc47_predicted	0.11931	0.40071	0.14167	0.3509	0.30132	-0.0188	0.25368	0.25513	-0.1224	0.20901	0.04582	-0.0866	0.35292	0.25471
NM_001013857	Lrrc48	-0.0335	-0.192	-0.0721	-0.0152	0.08188	0.17394	-0.0178	0.13293	-0.0474	0.11907	0.02765	0.12458	-0.0068	-0.0298
NM_001134469	Lrrc49_predicted	-0.0338	0.01931	-0.023	-1.024	0.12147	-0.0049	-0.0303	-0.0925	-0.0101	0.04736	-0.0786	-0.0939	-0.4162	-0.3931
NM_001107753	Lrrc4c_predicted	-0.0056	0.20194	0.24592	0.21464	0.11908	0.12541	0.28177	0.08781	0.04805	0.12224	0.23049	0.32809	0.23522	0.12002
NM_001014154	Lrrc50	-0.0267	-0.1065	0.16419	-0.1362	-0.0686	-0.1117	0.07195	-0.1882	-0.0942	0.08978	-0.1483	-0.1429	-0.107	-0.1932
NM_001024902	Lrrc56	-0.3261	-0.3418	-0.1007	-0.3689	-0.3389	-0.285	-0.363	-0.4792	-0.193	-0.3319	-0.2524	-0.1677	-0.4234	-0.6005
NM_001008280	Lrrc59	-0.4245	-0.7592	0.28627	-1.082	-0.102	-0.3773	-0.6389	-0.1716	-0.3329	-0.4676	-0.4673	-0.1528	0.59746	0.69026
NM_001024782	Lrrc8a	-0.139	-0.1933	-0.1234	-0.3093	-0.2073	-0.1655	-0.0523	-0.0615	-0.2651	-0.0165	-0.0841	-0.1772	-0.1894	-0.1209
NM_001037179	Lrrc8c	-0.4315	-0.3744	-0.3398	-0.2522	-0.0763	-0.183	-0.4519	-0.2074	-0.293	-0.5262	-0.6453	0.11862	-0.1482	-0.3769
NM_001008338	Lrrc8d	-0.3624	-0.3374	-0.9736	-1.1658	-0.1831	-0.5027	-0.8769	-0.1897	-0.5388	-0.5026	-0.4615	-0.4815	-0.6263	-0.6336
NM_001034139	Lrrc8e	0.07072	-0.0863	-0.0062	0.05424	0.10991	0.06655	-0.041	0.05678	-0.067	-0.1167	-0.1117	0.08145	0.10953	0.22244
NM_001024761	Lrrfip2	0.38013	-0.0736	0.2337	0.67323	0.59106	0.80039	0.02944	0.48457	-0.3066	-0.1314	0.00918	-0.0028	0.08391	0.10538
NM_001107096	Lrriq2_predicted	0.0561	0.13618	0.3882	0.22743	-0.1969	-0.2458	-0.1401	-0.0603	-0.2609	0.22441	-0.0902	0.05616	0.19067	0.09518
XM_218760.4	Lrrk1_predicted	-0.931	-0.8743	-0.5496	-1.8745	-1.4676	-1.6496	-0.7983	-1.3454	-0.7752	-0.6956	-0.7772	-0.5899	-0.9978	-0.9498

XM_235581.4	Lrrk2	-0.042	0.23328	0.21163	-0.0056	-0.0127	-0.2666	0.14162	-0.1883	0.28923	0.61845	0.42387	0.07373	-0.1928	-0.1358
NM_001037363	Lrrn1	0.15957	0.14229	0.06895	0.21469	0.08342	0.10031	0.05325	-0.0202	0.01021	-0.0119	0.21138	0.23847	0.04719	0.13208
XM_001060363.1	Lrrn2_predicted	-0.0932	-0.0322	-0.0154	0.10492	-0.0739	-0.0188	-0.019	-0.1808	-0.0118	-0.0553	-0.0513	0.02449	0.24386	-0.154
NM_030856	Lrrn3	2.2437	4.4696	1.8455	1.3416	0.86784	0.53813	4.6719	0.66861	3.9102	4.1631	4.1518	0.51491	0.39452	0.41285
NM_001100722	Lrrn6a	0.06501	0.03567	0.04701	0.04259	0.15802	0.1933	0.17952	0.08935	0.05009	0.03781	0.08233	-0.0171	0.04378	0.03421
NM_001106387	Lrrtm3_predicted	-0.0136	-0.183	-0.2565	0.03254	0.24924	-0.428	-0.1232	-0.0547	-0.2434	-0.1175	-0.0925	-0.216	-0.393	0.03077
NM_017242	Lsamp	0.2591	-0.0418	-0.0088	-0.3497	-0.1247	-0.1933	0.08837	-0.1232	-0.093	-0.2492	0.18195	-0.1881	-0.138	0.02331
NM_001013421	Lsg1	-0.3138	-0.1864	0.59899	0.55009	-0.1754	-0.0832	0.38982	-0.141	-0.0955	-0.0708	-0.1687	0.15624	0.05791	0.27872
NM_001108876	Lsm1_predicted	0.08267	-0.0196	0.09295	0.10756	-0.0835	0.07718	0.10718	-0.0779	-0.1568	-0.0537	-0.1062	-0.1614	-0.1996	-0.0317
XM_001072079.1	Lsm16_predicted	0.34727	0.21228	0.08488	0.09049	0.45162	0.5146	0.06415	0.27265	0.00467	0.03744	0.1124	0.39913	0.1944	0.05021
XM_001072079.1	Lsm16_predicted	-0.0286	-0.0395	0.04283	-0.0707	0.13332	0.05134	-0.03	0.06575	0.21174	0.23767	-0.0904	0.06889	0.15204	0.16491
NM_001106611	Lsm3_predicted	-0.194	-0.7699	0.21583	-0.416	-0.233	0.40393	-0.7172	-0.4391	-0.2892	-0.6616	-0.6204	0.80716	0.06201	0.23248
NM_001106073	Lsm4_predicted	-0.0995	-0.9524	0.28313	-0.6804	-0.2468	0.36966	-0.7588	-0.5496	-0.838	-0.7054	-0.5596	0.6573	0.09758	0.22597
NM_001107289	Lsm5_predicted	0.02594	-0.9507	-0.0374	-0.1944	-0.5235	-0.2831	-0.5539	-0.5385	-0.3207	-0.7289	-0.5577	-0.1573	-0.2511	-0.3296
NM_001108732	Lsm7_predicted	-0.0647	-0.1228	0.38383	0.34477	-0.3452	0.37645	-0.0734	-0.3125	0.18157	0.02544	0.25715	0.96251	0.21797	0.20675
NM_001106585	Lsm8_predicted	-0.3936	-0.9624	-0.0503	-0.3918	-1.0289	-0.8212	-0.4995	-0.8811	-0.6872	-0.513	-0.7512	-0.2953	-0.202	-0.4958
NM_032616	Lsr	-0.4175	-0.3401	0.20376	-0.2459	0.24325	0.59239	-0.4207	0.2985	-0.4397	0.00891	-0.3292	0.15063	-0.0878	-0.0044
NM_022634	Lst1	-0.0016	-0.0086	-0.0994	-0.0795	-0.0955	0.17082	-0.066	0.09035	-0.009	0.12618	0.13334	0.09789	-0.1516	-0.0725
NM_080769	Lta	-0.0826	-0.0929	-0.1274	0.03621	-0.1031	-0.0967	0.06743	-0.2036	0.00345	0.05653	-0.1779	-0.0348	-0.1286	-0.1587
NM_001030031	Lta4h	0.21606	0.73778	0.47872	0.21375	0.10099	0.26991	-0.0019	0.26824	0.74986	0.74675	0.74077	0.55859	0.59751	0.56175
NM_212507	Ltb	-0.0603	0.09918	-0.035	0.00936	0.03223	0.13968	-0.0039	0.15454	0.05142	-0.0831	-0.0176	-0.1069	0.0036	0.02618
NM_138863	Ltb4dh	1.1721	1.0753	1.3225	1.6033	1.2418	1.3957	0.85205	1.2374	0.87214	1.0442	1.117	1.1378	2.0311	2.0501
NM_021656	Ltb4r1	-0.0026	0.04018	0.02165	-0.0104	0.17841	0.19662	0.15241	0.15881	0.1448	0.17695	0.27145	-0.0358	0.11053	0.53076
NM_053640	Ltb4r2	-0.0671	-0.2433	-0.1677	0.02088	0.1004	-0.0026	-0.1027	-0.1128	0.17704	-0.37	-0.0324	-0.0588	0.08519	-0.0135
NM_021587	Ltbp1	0.83082	0.81204	1.0417	0.04933	0.76	0.57966	0.43701	0.86106	0.56141	0.95089	0.7062	0.2407	0.8343	0.98958
NM_021586	Ltbp2	-0.8108	0.01582	0.00225	-0.7102	-1.1541	-1.621	0.33222	-1.4453	-0.2043	-0.2118	0.11784	-0.3926	-0.7302	-0.5364
XM_341997.2	Ltbp3	-0.6	-0.1707	-0.7584	-0.741	-0.9488	-1.0795	-0.448	-1.1263	-0.5258	-0.3811	-0.4289	-0.7017	-1.4327	-1.4086
NM_001170336	Ltbp4	-1.6561	-0.7886	-0.2349	-0.388	-0.6251	-0.8421	-0.0453	-0.554	-0.5963	-0.9485	-0.1442	-0.2748	-0.555	-0.5665
NM_001008315	Ltbr	0.11861	0.17761	-0.1886	-0.1768	-0.0282	-0.0813	-0.1328	-0.4334	0.12791	-0.0384	-0.0415	-0.0823	0.14922	-0.0287
NM_053639	Ltc4s	0.18743	0.23318	0.19149	0.06966	0.0919	0.39809	0.00639	0.11742	0.56164	0.27505	0.07658	0.33885	-0.0091	0.14932
NM_001106864	Ltf_predicted	0.04177	0.08051	0.16607	0.05939	0.20293	0.06369	0.12313	0.11226	0.02276	0.17402	0.03754	0.20773	0.10731	0.09931
NM_001107763	Ltk_predicted	0.04656	0.00375	-0.0492	-0.0712	0.00013	0.0578	0.03485	-0.0173	-0.0476	0.10257	-0.0424	0.20892	0.20794	0.09601
NM_001014157	Ltv1	0.05327	-0.1403	0.11377	0.53374	0.15757	0.12398	0.15899	-0.0902	-0.1351	-0.0377	0.01349	0.15149	0.1022	0.25934
NM_001024269	Luc7l_predicted	-0.4101	-0.2406	0.42066	1.8819	-0.0178	0.14706	0.53849	0.00036	-0.2145	-0.5508	-0.1883	0.3041	0.06272	0.00519
NM_001107853	Luc7l2_predicted	-0.4137	-0.3205	-0.6054	0.62079	-0.6319	-1.2439	0.11705	-0.5607	-0.4908	-0.4152	-0.4981	-0.9089	-0.4345	-0.4094
NM_031050	Lum	-3.4385	2.1863	-1.4131	-1.1156	-5.276	-5.0855	-1.0852	-5.2211	1.8638	2.2771	1.7604	1.093	-3.1836	-3.2046
NM_030830	Luzp1	-0.8578	-0.7374	-0.5181	-0.7027	-0.4456	-0.4181	-0.9126	-0.4398	-0.8696	-0.7143	-0.8015	-0.4781	-0.387	-0.4501
XM_001061369.1	Luzp5_predicted	-0.1561	-0.0961	-0.0061	-0.1172	-0.1412	-0.6947	-0.0578	-0.1379	-0.0258	0.00632	0.01822	-0.2746	0.5057	0.55101
NM_031655	Lxn	-0.3845	-0.4897	-0.5825	-1.1871	-0.2472	-0.0037	-0.6678	-0.165	-0.3317	-0.474	-0.3118	-0.2029	-0.3323	-0.4081
NM_001009499	Ly49i3	-0.0505	0.05577	-0.0662	0.05658	-0.0359	-0.0157	0.02997	0.18033	-0.0112	-0.0165	0.02964	-0.0094	-0.0585	0.02022
NM_001009495	Ly49i4	0.18777	-0.028	0.02298	0.16157	0.25037	-0.1352	0.2294	-0.071	-0.0205	0.07529	-0.0874	-0.1267	-0.0599	0.12234
NM_001009495	Ly49i4	0.13856	0.11821	-0.1047	0.02678	0.13071	-0.1086	0.04664	0.10784	0.02361	0.0869	0.22761	0.00229	0.26332	-0.0567
NM_001009501	Ly49i5	-0.1178	-0.0437	-0.007	-0.046	-0.0169	0.139	-0.0947	0.01849	-0.0176	0.11309	-0.0236	-0.13	0.01982	-0.0215
NM_001009718	Ly49i6	-0.0784	-0.2091	-0.2459	-0.1639	0.076	-0.1563	-0.221	-0.1341	-0.0419	-0.0071	-0.1719	-0.0676	-0.1597	0.10438
NM_001009500	Ly49i7	-0.1499	-0.1488	-0.0272	-0.158	-0.116	-0.0502	-0.0733	-0.0148	-0.042	-0.1183	-0.1185	-0.0758	0.12631	-0.078
NM_001009487	Ly49s4	0.23127	0.03287	0.13756	0.13432	0.14583	0.05105	0.16584	0.00632	0.15815	0.16384	0.02635	0.15196	0.09563	0.08295
NM_001009488	Ly49s6	-0.0052	-0.0292	0.09287	0.09546	-0.0413	0.24372	0.07227	0.02842	0.14118	0.00583	0.10229	0.03905	0.24568	0.16828
NM_001009494	Ly49s7	0.11427	0.23288	0.06244	0.09776	0.04637	0.15475	-0.0082	0.03184	0.05986	0.0001	0.01485	0.14683	0.12707	-0.018
NM_001009497	Ly49si1	-0.001	0.07244	-0.0671	0.03949	0.24663	-0.0093	0.17061	-0.0302	0.12381	0.32424	0.125	0.10864	-0.0116	0.1361

NM_001009498	Ly49si2	0.04891	-0.09	-0.0692	-0.0446	0.1252	-0.0454	-0.073	-0.082	-0.0452	-0.0286	0.05701	-0.0146	-0.0401	-0.0196
NM_139257	Ly6b	-0.1456	-0.1348	-0.0807	-0.1834	0.15977	0.30339	-0.0677	0.07762	-0.1841	-0.2706	-0.0381	-0.2018	-0.1741	-0.2333
NM_020103	Ly6c	0.35218	0.0913	-0.0113	0.0084	0.49634	0.78587	0.04598	0.5342	0.16188	0.12266	0.22087	-0.0232	-0.0019	-0.045
NM_001130552	Ly6d_predicted	0.08972	0.00341	-0.0189	0.16031	0.0137	0.2092	0.10401	0.04255	0.03133	0.05709	0.12048	0.05632	0.17043	0.08058
NM_001017467	Ly6e	-0.0142	-0.0014	0.14563	0.76535	0.16671	0.01266	1.2762	-0.0002	0.02606	0.07323	0.12829	-0.0255	0.03758	0.13329
NM_001001934	Ly6g5b	-0.1107	0.1145	0.05659	-0.0565	0.14927	0.10155	-0.0729	0.36954	0.00606	-0.1356	-0.0208	-0.0837	0.09004	-0.0994
NM_198739	Ly6g5c	0.30518	0.08985	0.11399	-0.0185	0.08182	-0.0441	0.00797	0.04888	0.06253	0.20258	0.20535	0.06127	-0.0333	0.10241
NM_001001969	Ly6g6c	-0.293	-0.1559	0.2052	0.17844	-0.2398	-0.0553	0.18156	-0.2324	0.11767	-0.1674	-0.3359	-0.0302	-0.2425	-0.2164
NM_001001970	Ly6g6d	-0.1652	-0.0855	-0.1826	-0.1126	-0.1906	-0.1883	-0.0422	-0.0328	-0.0532	-0.0858	-0.1743	-0.1717	-0.2175	-0.1723
NM_001001972	Ly6g6e	0.04665	0.029	-0.0995	0.15615	0.06344	-0.3093	0.16741	-0.2285	-0.0037	0.08402	-0.1042	-0.0968	0.09132	-0.1246
NM_001003691	Ly6g6f	0.01544	-0.0097	0.03087	-0.0204	0.04038	-0.1415	0.09541	0.0039	-0.2732	-0.064	0.04452	0.34839	0.20664	0.06706
NM_001134839	Ly6h_predicted	0.10078	-0.0858	0.21276	0.05132	-0.0565	0.10799	0.00872	0.10167	-0.1265	0.00094	-0.0751	0.18837	-0.0534	0.34272
NM_001106405	Ly78_predicted	-0.0193	-0.0755	0.20626	-0.0841	-0.0766	-0.0303	0.0227	-0.0586	0.05162	0.01226	-0.0353	0.09323	-0.0544	0.04105
NM_001106128	Ly86_predicted	0.18002	0.04948	-0.0197	0.10455	0.05317	0.1563	0.05462	0.33471	0.2137	0.23821	0.06583	-0.0814	-0.0219	0.14844
XM_222892.3	Ly9_predicted	0.05759	-0.0851	-0.0481	0.01406	-0.0457	0.07176	-0.0549	0.02661	-0.0642	-0.086	-0.0698	-0.192	0.05056	0.07887
NM_001011911	Lyar	-0.257	-1.1184	0.68394	1.2171	0.58972	0.40353	0.11134	0.56178	-0.2805	-0.6643	-0.5573	-0.0559	0.43039	0.57152
NM_182820	Lyk5	0.43394	0.01501	-0.0208	0.12753	-0.0478	0.18871	-0.1619	-0.3494	-0.297	0.14501	0.05908	-0.2385	0.11809	0.10418
NM_001007677	Lyl1	-0.1083	-0.0528	0.90171	0.46543	-0.3293	-0.3868	0.4492	-0.3367	-0.1451	-0.0707	0.06119	0.15103	-0.1253	0.17976
NM_030857	Lyn	0.05796	0.02494	0.08259	-0.2649	-0.0031	0.01378	-0.0551	-0.1841	0.14852	-0.3128	-0.2732	-0.1682	-0.0206	-0.1719
NM_001130546	Lynx1_predicted	0.09876	0.0684	0.04185	0.05767	-0.0186	0.01411	0.17073	-0.0975	0.07278	-0.0225	-0.0748	0.10257	0.20001	0.07047
NM_001007727	Lypd1	-0.5054	-0.3151	-0.4271	-0.5449	-0.4571	-0.5863	-0.4709	-0.4343	-0.4679	-0.5152	-0.5174	-0.3576	-0.5732	-0.491
NM_001130545	Lypd2_predicted	0.05587	0.13935	0.15908	0.08059	0.0466	0.09716	-0.0552	0.01806	-0.0486	0.04663	0.10851	0.01454	-0.0348	0.02562
NM_021759	Lypd3	0.00072	0.12675	0.11241	0.12807	0.12236	0.01677	0.08137	0.03603	0.23119	0.06321	0.10482	0.03121	0.18041	0.12297
NM_013006	Lypla1	-0.2859	-0.198	-0.1427	-0.8156	-0.4708	-0.5948	-0.0223	-0.4931	0.09327	-0.1049	-0.106	-0.0275	0.04498	-0.0647
NM_031342	Lypla2	0.2452	0.27041	0.24949	-0.1615	0.38093	-0.0842	-0.0811	0.13959	0.1326	0.34621	0.16681	-0.1094	-0.0439	-0.211
NM_001004277	Lypla3	1.1996	0.79154	0.02183	0.13113	0.52715	-0.4027	0.4131	0.64962	0.49523	0.47624	0.46877	-0.184	0.66367	0.80871
NM_001105986	Lyplal1_predicted	-0.6768	-0.8027	-1.4333	-0.7952	-0.7152	-0.6557	-1.1558	-0.7403	-0.9751	-0.6797	-0.8704	-1.2124	-1.4448	-1.6028
NM_001024302	Lysmd1	0.17554	0.15553	0.06167	0.33204	0.39286	0.495	-0.1681	0.3114	0.06189	-0.0184	-0.0907	0.00868	-0.0389	0.16932
NM_001106839	Lysmd2_predicted	-0.0579	-0.1124	-0.2721	-0.7708	-0.2244	0.00994	-0.1421	-0.1836	-0.0839	-0.1206	-0.0394	0.22773	0.44536	0.22407
NM_001009698	Lysmd3	0.36091	-0.0811	-0.2307	1.1365	0.52473	0.66879	0.24383	0.43428	0.05688	-0.1006	0.076	-0.0937	-0.206	-0.0672
NM_001108882	Lyzl1_predicted	0.04283	-0.0377	0.10127	0.11704	-0.0288	0.05653	0.06474	-0.0972	0.06895	-0.0736	0.07547	-0.1075	-0.1013	-0.0643
XM_343507.3	Lyzl4_predicted	0.11048	0.07611	0.03839	0.31168	0.72181	1.0509	0.12902	0.5339	0.13412	-0.1869	0.20137	-0.0961	0.02384	0.04277
NM_001135833	Lyzl6_predicted	-0.0469	0.00084	-0.1118	0.01661	-0.0908	-0.0662	-0.0333	-0.1396	-0.0176	0.09202	-0.1808	0.15868	-0.0432	-0.0334
NM_001013241	Lzic	-0.152	-0.1674	-0.0802	-0.0564	-0.1012	-0.2639	-0.2789	-0.1599	0.01806	-0.1216	-0.2787	0.26452	0.01911	-0.0439
NM_001024266	Lztfl1	0.65863	0.62519	-0.4427	0.66651	0.44591	0.36286	0.48395	0.29677	0.33205	0.54469	0.44753	-0.0471	-0.0064	-0.2462
NM_001108326	Lztr1_predicted	-0.1329	0.07812	0.00052	-0.384	-0.1989	-0.0913	-0.316	-0.2773	-0.1162	0.12887	-0.1722	0.00452	-0.4314	-0.3944
NM_153470	Lzts1	0.22826	0.14942	0.27993	0.04056	0.37296	0.35262	0.04778	0.39051	0.18114	0.22444	0.12688	0.43912	0.17608	0.40275
NM_001007700	M6pr	0.2296	-0.0863	0.10416	0.08672	0.04068	0.15655	-0.0815	-0.1841	-0.1011	-0.1001	0.07089	-0.0765	-0.0123	0.15852
NM_139337	Macrod1	0.00461	0.00807	-0.685	-0.516	-0.4275	-0.1808	-0.2821	-0.465	-0.1059	-0.3311	-0.1444	-0.4538	-0.6837	-0.581
NM_001106594	Mad2l1_predicted	-0.2823	-0.4192	0.22229	-0.0467	-0.2829	-0.3244	-0.1729	-0.3765	-0.4522	-0.2094	-0.3619	0.35309	-0.0576	0.07397
NM_001009699	Mad2l1bp	-0.0361	0.05702	0.1615	0.09732	0.16498	0.20733	-0.1094	0.34898	0.18327	0.09927	0.04719	0.08125	0.28693	0.40221
NM_001012106	Mad2l2	0.24677	-0.2772	0.57301	-0.1979	-0.1702	0.22542	-0.2521	-0.1783	-0.1595	-0.5219	-0.177	0.30654	0.28337	0.27849
NM_019317	Madcam1	0.00639	-0.0107	-0.0595	0.12842	0.06028	0.14974	-0.023	-0.1227	-0.0176	-0.1253	0.03819	0.27249	0.05475	-0.0413
NM_053585	Madd	-0.0494	0.11979	-0.1584	-0.1307	-0.1767	-0.2375	0.16781	0.00725	0.16904	0.0226	-0.0882	-0.124	-0.1303	-0.1572
NM_030858	Madh7	-0.6657	-0.7131	-0.8038	-1.3997	-1.3696	-1.2073	-0.5095	-1.2772	-0.4471	-0.5332	-0.7036	-0.0756	-0.7699	-0.7565
NM_001008319	Maea	-0.1656	-0.5081	-0.0743	0.50371	0.32628	0.47252	0.31099	0.26798	-0.6081	-0.6769	-0.4504	-0.1762	-0.1655	-0.1728
NM_001108857	Mael_predicted	0.1755	0.0628	-0.0146	0.11224	0.97826	1.081	0.07139	1.2302	-0.0143	0.04107	-0.0638	-0.0059	0.01972	-0.1062
NM_019318	Maf	-0.679	-0.594	-0.7854	-1.1729	-0.7591	-0.4108	-1.0116	-0.6557	-0.6854	-0.7957	-0.7792	-0.7269	-1.7047	-1.7466
NM_001007673	Maf	0.6557	1.9938	1.8018	0.40869	0.88242	0.47589	2.5867	1.057	1.5353	1.879	1.9426	0.42528	1.7986	1.7936

NM_001014085	Maf1	-0.4035	0.19284	-0.357	-0.187	-0.2322	-0.415	-0.1809	-0.2693	-0.0732	0.1858	0.07065	-0.12	-0.6229	-0.5965
NM_019316	Mafb	0.10999	-0.0968	-0.0566	-0.0798	-0.0293	-0.1444	0.05519	0.05603	-0.0146	0.10144	-0.0352	-0.048	-0.0666	-0.1098
NM_001130573	Maff_predicted	0.38905	0.55284	0.04698	0.13511	0.17565	0.40476	0.27912	0.355	0.37932	0.23244	0.11954	0.35954	0.33688	0.43601
NM_022386	Mafg	-0.9545	-1.0665	-0.549	-0.548	-1.2244	-1.4293	-0.3681	-1.2389	-0.7946	-0.8845	-1.0234	-0.9404	-0.9281	-0.8557
NM_145673	Mafk	0.11533	-0.1462	0.12316	0.38106	0.51034	0.54035	0.42428	0.40909	-0.134	-0.0432	-0.052	-0.3447	0.14311	0.10825
NM_017190	Mag	0.10213	-0.1846	0.14516	-0.142	-0.1162	-0.2401	-0.0433	-0.0483	-0.1901	-0.1227	0.0013	0.1899	-0.0807	0.05491
XR_007284.1	Magea10_predicted	0.1237	0.14713	0.17906	0.2066	0.22562	0.38106	-0.0168	0.15954	0.17694	0.24332	0.17323	0.0723	0.33655	0.15048
NM_001044246	Mageb18_predicted	0.04403	0.15611	0.1192	0.0297	0.10704	0.06186	-0.0439	0.1785	0.1495	-0.0149	0.05901	0.16694	-0.0454	0.06248
NM_001106946	Mageb5_predicted	-0.1265	-0.2244	-0.1238	-0.1557	-0.113	-0.162	-0.0456	-0.2189	-0.1495	0.07872	0.05164	0.04711	-0.1076	-0.1911
NM_053409	Maged1	0.11657	0.58411	-0.0032	0.93063	0.79044	0.53463	-0.2258	0.80373	0.864	0.51462	0.53712	0.81028	0.57404	0.50142
NM_080479	Maged2	0.07625	0.02861	-0.2389	0.36908	-0.145	0.03692	-0.3529	-0.0926	0.10238	-0.3697	-0.0384	0.40399	-0.2001	0.03023
NM_001079891	Magee1_predicted	0.87151	0.99804	0.63104	0.43289	0.73522	0.72678	0.52729	0.79316	0.94738	1.1902	0.99994	0.66607	0.57306	0.53434
NM_001106941	Magee2_predicted	-0.103	-0.1418	-0.0117	-0.0047	-0.0319	0.05701	-0.1031	-0.2075	-0.085	-0.0375	0.15573	0.04821	0.07546	-0.1247
NM_001030045	Magi1	0.06315	-0.1083	0.07709	0.05966	-0.1432	0.08009	-0.0896	-0.14	-0.12	-0.0618	0.08279	0.06729	-0.0627	0.04551
NM_053621	Magi2	-0.4561	-0.2016	-0.2923	-0.3068	-0.5595	-0.6834	-0.2271	-0.5267	-0.2723	-0.4241	-0.4169	-0.1716	-0.3082	-0.0753
NM_139084	Magi3	-0.1061	-0.8482	-0.9023	-0.8016	-0.4207	-0.6489	-1.3313	-0.1728	-0.764	-0.7101	-0.8961	-0.7255	-0.5549	-0.7268
NM_001014109	Magix	-0.0709	-0.0948	0.00316	-0.1407	0.00455	0.01223	0.04116	0.12776	0.02927	-0.0468	-0.0188	0.10205	0.18639	-0.0863
NM_001100536	Magoh_predicted	0.24019	-0.0734	0.46661	0.27297	-0.0048	0.24809	0.26877	0.08244	-0.0072	-0.2464	0.01634	0.37959	0.5331	0.46924
NM_013136	Mak	0.18103	0.0864	0.01022	-0.0256	0.13074	0.0412	0.04958	0.15493	-0.0543	0.01121	-0.0046	-0.003	0.11518	0.06005
NM_133324	Mak10	0.08624	0.0673	0.27284	0.59097	0.1883	0.31572	0.28999	0.28585	-0.0478	0.19985	0.14911	-0.0779	0.12092	-0.0133
NM_001105881	Mak3_predicted	0.31882	-0.1507	0.54591	0.47317	0.44368	0.73875	0.01574	0.56885	0.10099	0.2044	-0.1899	0.39496	0.6372	0.73653
NM_012798	Mal	1.3417	0.31992	0.23021	0.37675	1.8403	1.4952	0.20637	1.4655	0.48696	0.68925	0.4388	-0.03	0.11625	0.22294
NM_198786	Mal2	-0.013	0.05131	-0.0717	-0.0159	0.05416	-0.0125	0.04859	-0.0483	-0.049	0.12545	-0.0191	-0.0618	0.01065	0.04695
XM_225927.4	Malt1_predicted	-0.0052	-0.0039	0.098	0.31465	-0.019	-0.209	0.06332	0.0162	-0.0456	-0.06	-0.1849	0.04524	0.11853	-0.2513
XM_001078660.1	Mamdc2	-0.0463	0.43879	0.07688	-0.0212	0.00584	0.01166	0.16303	0.05916	0.41071	0.51262	0.55771	0.05714	-0.0855	-0.1466
NM_145768	Mamdc4	-0.1898	-0.0282	-0.0258	-0.1984	-0.2342	-0.0562	-0.1387	-0.082	-0.2592	-0.3159	-0.1719	-0.0487	-0.227	-0.0581
NM_001106997	Maml1_predicted	-0.1365	-0.1094	-0.2277	-0.2379	-0.0035	-0.3158	-0.1325	-0.0761	-0.1807	-0.1385	-0.1993	-0.3411	-0.251	-0.1934
NM_001033656	Man1a_predicted	-0.0459	-0.0748	0.01736	-0.0567	-0.1288	0.06355	-0.0808	-0.0538	-0.1561	-0.143	-0.0535	0.11033	0.1924	0.02073
NM_001106452	Man1a2_predicted	0.08535	-0.2595	-0.4843	-0.9818	-0.0906	-0.235	-0.3127	0.0666	-0.2142	-0.0599	-0.4391	-0.0934	0.23076	0.15869
NM_012979	Man2a1	-0.0894	-0.1203	0.07694	-0.1758	-0.0408	0.0158	0.02139	-0.2502	-0.0311	0.07144	-0.0467	0.05679	-0.1123	-0.0999
NM_001107527	Man2a2_predicted	0.28788	0.20427	-0.2906	0.1672	-0.0577	-0.3508	0.28163	-0.2383	0.06411	0.07323	-0.0916	-0.2421	0.04927	-0.3568
NM_199404	Man2b1	-0.2678	0.14851	0.40709	0.52425	-0.2114	0.00109	0.2963	-0.3848	-0.3097	0.00634	0.01329	0.1273	-0.2825	-0.2647
NM_139256	Man2c1	-0.0368	0.25848	0.1193	-0.443	0.24575	0.11823	-0.1779	0.16649	0.01304	0.12324	0.1515	-0.0971	-0.5273	-0.5574
NM_001031655	Manba	0.11359	0.07072	0.2326	0.2074	0.15429	0.31158	0.15874	0.13172	0.16421	0.09096	0.27032	0.10335	0.01733	-0.1216
NM_001031655	Manba	-0.091	-0.1252	-0.1486	-0.085	-0.1186	-0.0159	-0.029	-0.1676	-0.1173	-0.1318	0.00823	0.41082	-0.2009	0.01453
NM_033653	Maoa	1.2223	1.5636	0.23112	0.88928	2.2154	2.1479	0.63542	2.3228	1.1171	1.2633	1.2398	0.36125	1.129	1.1421
NM_013198	Maob	-0.2055	-0.1251	-0.1432	-0.0392	0.03542	0.04406	0.06515	0.09474	-0.0514	-0.0875	-0.1142	-0.089	-0.1992	-0.0807
NM_019217	Map1b	0.02394	0.04738	0.15805	0.37278	0.08185	0.17556	0.21334	0.04979	0.28961	0.05876	0.1214	0.18185	0.12156	-0.0493
NM_019217	Map1b	-0.6859	-0.3729	-0.9122	-0.8136	-0.1754	-1.1418	-0.838	-0.03	-0.3929	-0.4843	-0.6313	-0.9169	-0.4678	-0.3641
NM_199500	Map1lc3a	0.39377	1.072	-0.7433	-0.4611	0.32807	0.27265	0.38026	0.21728	0.64779	0.93313	1.0003	-0.1442	-0.3053	-0.2834
NM_022867	Map1lc3b	0.15175	0.27505	0.03767	0.11956	-0.0626	-0.4621	0.52886	0.11132	-0.0444	0.14899	0.1953	-0.3552	-0.2355	-0.2583
NM_031643	Map2k1	0.31133	0.32635	0.1669	-0.12	0.24821	-0.0132	0.02751	0.18348	0.1637	0.18749	0.30646	-0.3554	0.11766	0.22637
NM_001008375	Map2k1ip1	0.90632	0.48802	-0.2209	-0.6337	0.51953	0.54921	0.31543	0.56017	0.6168	0.47681	0.38299	0.48725	-0.0996	-0.1982
NM_133283	Map2k2	-1.234	-1.1605	-0.847	-1.5396	-1.1451	-1.3165	-1.0838	-1.2439	-1.2024	-1.1791	-1.1361	-0.7437	-0.9694	-1.0042
NM_001100674	Map2k3	-0.5251	-0.0401	-0.1217	-0.5705	-0.4667	-0.6565	-0.0935	-0.6334	0.00993	-0.0706	-0.0834	0.02884	-0.2036	-0.1749
NM_001030023	Map2k4_predicted	-0.307	-0.4686	-0.6076	-1.2475	-0.4151	-0.5847	-0.5121	-0.3224	-0.4216	-0.4598	-0.5132	-0.0878	-0.2445	-0.3065
NM_017246	Map2k5	-0.175	-0.3589	-0.1195	-0.3584	-0.3075	-0.3396	-0.3925	-0.1512	-0.333	-0.0312	-0.4229	0.02462	-0.2911	-0.3894
NM_053703	Map2k6	-0.2708	-0.1649	-0.2927	-0.1702	-0.002	0.14763	0.09611	0.24015	-0.0002	-0.1611	-0.2326	-0.2427	-0.0579	-0.3084
NM_053887	Map3k1	0.30826	0.05468	0.1056	-0.0698	0.08235	-0.001	0.0512	0.19297	0.12752	0.09381	0.00937	0.14646	0.02097	0.04556

XM_001073032.1	Map3k10	-0.291	-0.2614	-0.1409	-0.1351	-0.1386	-0.2323	-0.0608	0.08852	-0.2955	-0.2914	-0.3263	-0.233	-0.0926	-0.1761
NM_001013150	Map3k11	-0.0461	-0.0424	0.20865	-0.2503	-0.1866	-0.0448	-0.0393	-0.122	-0.2762	-0.2511	0.04421	0.0518	-0.104	-0.0387
NM_013055	Map3k12	-0.5819	0.40898	-0.5044	-0.9197	-0.6178	-0.5364	-0.2929	-0.6406	-0.1319	0.2407	0.09037	0.46493	-0.3528	-0.3711
NM_001108301	Map3k14_predicted	-0.0406	-0.1295	0.20471	0.10381	-0.0239	-0.2306	-0.1784	-0.1606	-0.1079	-0.3291	-0.0731	-0.1759	-0.067	0.18209
NM_001107058	Map3k3_predicted	0.09223	0.08716	0.38695	0.00349	0.13175	-0.0071	0.22671	0.03661	0.14393	0.1251	0.29507	-0.0541	0.32064	0.47053
NM_001107456	Map3k4_predicted	0.14527	0.13149	-0.2323	-0.1981	0.23703	0.32256	-0.3885	0.24412	0.07186	-0.0524	0.11719	0.21289	-0.2982	-0.0312
NM_001107909	Map3k6_predicted	-0.3035	-0.438	0.39294	-0.2024	-0.5244	-0.5686	0.0267	-0.6329	-0.5921	-0.1837	-0.307	-0.1954	-0.1412	-0.2517
NM_001107920	Map3k7_predicted	0.12002	-0.1471	-0.3853	-0.4406	-0.0996	-0.3529	-0.2721	-0.0742	-0.0569	-0.1084	-0.0527	-0.1901	-0.0059	0.12712
NM_001109976	Map3k7ip1_predicted	-0.17	-0.1806	0.01796	-0.1137	-0.2304	0.13738	-0.3543	-0.0795	-0.3635	-0.0891	-0.194	-0.0967	-0.0569	-0.0036
NM_001012062	Map3k7ip2	-0.0772	0.05589	-0.3417	0.50198	0.25291	0.11343	-0.007	0.08588	-0.1403	0.01383	-0.0602	-0.0268	-0.0869	0.0363
NM_053847	Map3k8	0.16639	-0.0032	-0.0736	0.14665	0.3402	0.32639	0.77437	0.51084	-0.0361	-0.0094	-0.0042	-0.1879	-0.5376	-0.6473
NM_001106243	Map4k1_predicted	1.4282	0.47097	0.06959	0.47653	1.2283	1.2145	0.22762	1.2593	0.17476	0.20057	0.77992	-0.0402	0.00048	0.0826
NM_001106329	Map4k2_predicted	-0.1338	-0.1404	-0.0597	-0.1027	-0.1946	-0.0623	-0.0817	-0.1487	-0.1691	0.09043	-0.079	-0.1583	0.04669	-0.1607
NM_133407	Map4k3	0.03703	0.68093	0.49197	0.63158	0.5384	0.47287	0.86648	0.53148	0.65552	0.47222	0.62504	0.46676	0.13565	0.04336
NM_001106904	Map4k4_predicted	-0.1597	-0.1576	0.23968	0.66411	0.17367	0.01943	-0.0832	-0.1557	-0.2001	-0.2073	-0.083	0.03829	-0.042	0.24274
NM_053842	Mapk1	0.18994	0.23652	-0.2236	-0.2544	0.15914	-0.0946	0.29034	0.21478	-0.0784	-0.0768	-0.1227	-0.2838	0.37411	0.46219
NM_012806	Mapk10	0.07063	0.21768	0.06283	0.10486	0.17055	-0.0102	0.09933	0.01044	0.04898	0.16158	0.12316	0.0312	0.01957	0.18957
NM_021746	Mapk12	-0.1663	0.07569	-0.0084	0.12465	-0.0428	0.26279	-0.0861	-0.1104	-0.0977	-0.0804	0.23024	0.28631	-0.0355	-0.1926
NM_019231	Mapk13	-0.0401	-0.0426	-0.0362	-0.0334	0.12025	-0.0036	-0.1236	-0.0113	-0.0166	-0.0561	0.03206	0.23099	0.10403	0.00266
NM_031020	Mapk14	-0.8088	-0.3029	-0.0392	-0.4299	-0.8131	-0.1774	-0.4035	-0.8329	-0.6597	-0.4719	-0.5211	-0.218	-0.7876	-0.6102
NM_173331	Mapk15	0.09692	0.16849	-0.0811	0.02145	-0.0956	0.04661	-0.0576	-0.0574	0.10688	-0.0262	0.02069	0.05461	-0.0102	0.02638
NM_017347	Mapk3	0.32825	0.17729	-0.8282	-0.4285	0.42432	0.07248	-0.0432	0.3563	-0.3462	-0.0636	-0.1349	-0.5585	-0.214	-0.1879
XM_225726.4	Mapk4	0.20609	-0.0208	0.16412	0.23965	0.03614	0.02972	0.05604	0.0702	0.21389	0.18371	0.13331	0.089	0.06574	0.29039
NM_031622	Mapk6	-0.8616	-0.9961	-0.2447	0.03314	-0.1251	-0.4419	-0.5036	-0.1282	-0.9253	-0.8841	-0.9065	-1.2794	-0.428	-0.4538
XM_340813.2	Mapk7	-0.3713	-0.3293	-0.3564	-0.571	-0.3465	-0.5881	-0.3917	-0.3407	-0.542	-0.3343	-0.5024	-0.5483	-0.3561	-0.2534
XM_341399.2	Mapk8	-0.0193	-0.0306	0.07984	-0.1741	0.02841	0.13343	0.12741	#######	0.04379	-0.1121	-0.2386	-0.2504	-0.0945	0.06985
NM_053777	Mapk8ip1	-1.017	-0.6222	-0.833	-1.1209	-0.9391	-0.9224	-0.8292	-0.9963	-0.6863	-0.8304	-0.8409	-0.2677	-0.8841	-1.0605
XM_235565.4	Mapk8ip2	0.07706	-0.0391	0.06507	-0.0913	0.06139	0.17631	0.05282	0.17776	-0.2294	-0.1806	-0.3015	-0.2629	0.03158	0.32454
NM_001100673	Mapk8ip3	-0.9315	-0.6414	0.71023	1.6565	-0.6935	-0.8376	0.12766	-0.7991	-0.9441	-0.9264	-0.6318	-0.587	-1.1259	-1.0688
NM_017322	Mapk9	0.09963	0.23516	-0.1376	0.03286	-0.0657	-0.2323	0.01705	0.29682	-0.2067	0.21736	0.16359	0.09025	0.08984	0.12335
NM_001011964	Mapkap1	0.10935	-0.0789	-0.0144	0.12523	0.14676	0.65523	-0.051	0.17004	0.09869	0.11781	0.18604	0.18749	-0.0227	0.11651
NM_178102	Mapkapk2	-0.1255	-0.1332	0.43661	0.04777	-0.019	-0.0218	-0.2992	-0.1362	0.06548	-0.1226	-0.0691	0.13725	-0.0581	0.05263
NM_001012127	Mapkapk3	-0.3163	-0.1888	-0.1462	-0.2106	-0.3187	-0.2956	-0.2507	-0.3378	-0.347	-0.3552	-0.1881	-0.2536	-0.2643	-0.0805
NM_001012127	Mapkapk3_predicted	-0.1595	-0.0399	0.0446	-0.0096	0.02801	-0.2694	0.23167	0.00116	-0.1808	-0.0513	-0.1065	-0.0134	-0.1196	-0.1264
NM_001025761	Mapkapk5	-0.4189	-0.7123	-0.3084	-0.2703	-0.5662	-0.3924	-0.371	-0.5247	-0.6134	-0.6945	-0.5128	-0.1797	-0.509	-0.5357
NM_001108589	Mapkbp1_predicted	-0.116	-0.1868	-0.0989	-0.0739	0.04772	-0.1066	-0.0852	0.01223	-0.164	0.18663	0.09395	-0.164	0.03713	-0.1203
NM_138509	Mapre1	0.81955	0.92483	-0.8839	-0.1221	1.2157	0.04808	0.25384	1.1401	0.81636	1.0706	0.44958	0.19121	1.1927	1.1755
NM_001007656	Mapre3	0.13111	-0.3163	-0.1982	-0.3021	0.11104	0.34533	0.01637	0.18214	-0.0541	-0.3611	-0.311	0.04326	0.03269	0.04569
NM_001135838	March1_predicted	0.13582	-0.1104	0.25641	0.1167	-0.0967	0.04734	0.18677	-0.0184	-0.1421	0.04494	0.00822	-0.0301	0.07802	0.088
NM_001012196	3/2/10	0.70535	1.3394	-0.3712	-0.0517	0.70688	0.60314	0.35373	0.51132	1.1233	1.1957	1.0474	0.2885	0.18094	0.21991
NM_001012196	3/2/10	0.36369	0.98178	-0.3298	-0.1259	0.6842	0.40616	0.5811	0.50237	1.0861	1.0018	1.1132	0.34023	0.17174	0.13095
NM_001007759	3/3/10	0.80869	1.5882	0.80365	0.9804	1.067	0.76723	1.4098	1.1729	1.4616	1.5855	1.3131	0.73045	1.0864	1.0744
NM_001106372	March5_predicted	0.30471	0.37036	-0.3235	-0.223	0.36585	0.1707	0.23621	0.31613	0.53561	0.39617	0.39619	0.21366	0.37762	0.21229
NM_001012087	3/7/10	0.66999	0.71842	-0.2497	0.16798	0.70802	0.14528	0.09719	0.90683	0.41036	0.66112	0.48002	0.17829	0.31897	0.12851
NM_001107882	March8_predicted	0.11891	0.02807	0.09925	-0.2177	-0.0531	-0.1145	-0.1387	-0.0339	-0.0338	0.02641	0.20104	0.17125	0.00253	0.16099
NM_030862	Marcksl1	-0.6027	0.26056	-0.2416	-0.3593	-0.246	-0.0759	-0.2196	-0.2731	0.18276	0.07533	0.01931	0.46894	0.00983	-0.2029
NM_001034936	Mare	-0.1623	-0.2973	0.08048	-0.2388	-0.3587	-0.1545	-0.2284	-0.4862	-0.1968	0.01054	-0.0204	-0.0448	-0.2081	-0.221
NM_053947	Mark1	-0.6338	-0.5612	-0.4254	-0.0712	-0.6918	-0.7828	-0.4089	-0.6509	-0.2836	-0.6479	-0.3956	-0.1885	-0.4138	-0.4196
NM_021699	Mark2	-0.7568	-0.6365	-0.2067	-0.7429	-0.5067	-0.961	-0.42	-0.5735	-0.6524	-0.6288	-0.7391	-0.6084	-0.6792	-0.5809

NM_130749	Mark3	-0.2962	-0.2747	0.19471	0.24001	-0.2452	0.03163	0.0308	-0.1143	-0.5099	-0.3204	-0.4333	-0.0985	-0.3865	-0.3562
NM_001127659	Mars_predicted	-0.4751	-0.7398	-0.0204	-0.0629	-0.4378	-0.5471	-0.6219	-0.5887	-0.6867	-0.6813	-0.7566	-0.4093	-0.093	-0.1647
XM_001066991.1	Mars2_predicted	-0.2093	-0.2079	-0.3905	-0.4997	-0.1321	-0.1533	-0.0626	-0.1444	-0.2732	-0.3055	-0.4321	-0.3919	-0.2002	-0.0837
NM_001108936	Marveld2_predicted	0.16133	0.31453	0.06075	0.29033	0.08929	0.01867	0.01685	0.2357	0.04873	0.29905	0.12698	0.238	0.05996	0.06266
NM_012757	Mas1	-0.1335	-0.2234	-0.3469	-0.2322	-0.2823	-0.1747	-0.2188	-0.3582	-0.2505	-0.1552	-0.2595	-0.2375	-0.3065	-0.154
NM_172043	Masp2	-0.2814	0.06542	0.06662	-0.1327	-0.1473	0.0277	-0.1172	-0.2335	0.09061	-0.1603	-0.2058	0.05075	-0.2077	-0.0336
NM_181089	MAST1	-0.0514	-0.2667	0.14163	0.10813	-0.2105	-0.3599	0.04071	-0.3726	-0.079	-0.1747	-0.0069	-0.3361	-0.1248	-0.179
NM_001108005	Mast2_predicted	-0.2368	-0.6372	0.49851	0.01214	-0.5983	-0.1791	-0.3168	-0.5685	-0.1843	-0.792	-0.5648	-0.0087	-0.1773	-0.1437
NM_001107369	Mastl_predicted	-0.0624	-0.0848	-0.1243	-0.1223	-0.2009	0.05932	-0.1183	0.13619	0.09846	-0.0794	-0.2228	-0.1661	0.4194	0.40614
NM_001107369	Mastl_predicted	-0.5485	-0.8446	0.83126	0.14752	-0.7968	-0.8677	-0.556	-0.6324	-0.1471	-0.5137	-0.5651	0.39967	0.92648	0.81521
NM_012860	Mat1a	-0.318	-0.2324	-0.296	-0.2602	0.173	-0.3954	-0.3552	-0.188	-0.2218	-0.2725	-0.2031	-0.0705	-0.346	-0.1809
NM_134351	Mat2a	-0.1854	-0.0006	-0.7107	-0.6892	-0.5002	-0.9233	-0.1945	-0.2327	-0.2008	-0.2264	-0.254	-0.7993	0.1142	0.0806
NM_021859	Matk	-0.3815	-0.209	0.00862	-0.1213	-0.2701	-0.0638	-0.2084	-0.3271	-0.2983	-0.3511	-0.4491	0.11657	-0.1645	0.06987
NM_001006979	Matn1	-0.0445	-0.0741	-0.1064	0.12335	1.4807	1.4814	-0.1526	1.7683	-0.1097	0.21555	0.03159	-0.162	-0.0252	-0.0225
XM_216941.4	Matn2_predicted	0.07241	-0.0227	0.10273	0.15823	-0.0279	-0.0129	0.09912	-0.0722	-0.0013	0.0499	0.02334	0.01663	-0.0225	-0.0503
NM_001108013	Matn3_predicted	-0.0137	-0.0092	0.2642	0.24656	-0.0219	0.04	-0.0276	0.18216	0.04539	-0.0213	0.02566	0.17812	0.26253	0.28186
NM_001106539	Matn4_predicted	-0.2965	-0.065	-0.1285	-0.1883	-0.0912	-0.0448	-0.1113	-0.2162	-0.0828	-0.0332	-0.0892	-0.0812	-0.099	-0.146
NM_001107653	Matp_predicted	0.2079	-0.054	0.09621	0.02926	-0.0184	-0.0012	0.0014	-0.082	-0.0526	0.04337	0.07489	0.01665	0.32587	0.05096
NM_022210	Max	-0.187	0.12951	-0.4971	-0.4193	-0.5209	-0.5321	-0.2274	-0.1978	0.03105	-0.0357	-0.2677	-0.4287	0.58409	0.41755
NM_021588	Mb	0.07915	0.23305	0.12454	0.17186	0.06823	0.10695	0.20608	0.00715	-0.0135	0.12374	0.17511	0.0893	0.16817	-0.0513
NM_017249	Mbc2	-0.4119	0.12857	0.33065	0.03205	-0.7219	-0.539	-0.0844	-0.7237	-0.1144	0.19292	-0.0365	-0.3109	-0.062	-0.1909
NM_001011924	Mbd1	-0.1413	-0.1457	-0.0225	0.32269	0.2704	0.13867	0.15238	0.30175	-0.3938	-0.2422	-0.1774	-0.5191	-0.4211	-0.5543
NM_001011924	Mbd1_predicted	-0.2742	0.03544	-0.4304	-0.2561	0.17028	-0.2539	-0.4495	0.27381	-0.32	-0.1731	-0.2174	-0.411	-0.4337	-0.2626
NM_001108735	Mbd3_predicted	-0.0411	-0.4503	0.29108	-0.6942	-0.4006	-0.536	-0.6114	-0.6535	-0.0171	-0.2378	-0.4541	0.33642	0.16491	0.45267
NM_001106803	Mbd3l1_predicted	0.05475	0.03174	-0.0301	0.05104	-0.0652	-0.1071	0.13092	0.21451	-0.0057	0.07478	0.01209	-0.0633	0.16163	0.01625
XM_001075118.1	Mbd3l2_predicted	-0.0667	-0.071	-0.0336	-0.1155	0.02807	0.31305	-0.0178	0.22339	0.00175	-0.006	0.08036	0.17323	0.11906	0.07575
NM_001170566	Mbd6_predicted	-0.6952	-0.3629	0.29698	-0.1944	-0.9508	-0.9803	-0.0687	-0.9253	-0.159	-0.4008	-0.3587	-0.0575	-0.4935	-0.5149
NM_001108712	Mbip_predicted	-0.8761	-0.7525	-1.1564	-0.6191	-0.6991	-0.8712	-0.719	-0.7354	-0.7967	-0.7441	-0.8016	-1.0082	-1.0673	-1.1456
NM_012599	Mbl1	-0.0897	-0.0122	-0.0002	0.0016	-0.1071	0.01362	-0.0394	-0.0171	-0.0905	-0.0675	-0.0945	0.0574	-0.1668	-0.0964
NM_022704	Mbl2	0.02377	0.21179	0.26688	-0.0313	0.29351	0.11375	0.05738	0.00603	0.02923	0.06503	-0.0996	-0.0578	-0.0066	0.25845
XM_001062557.1	Mbnl1	0.37712	0.50269	-0.2578	0.28468	0.52209	0.44768	0.21374	0.43817	0.11808	0.34504	0.20841	0.34458	0.34214	0.30495
XM_214253.3	Mbnl2_predicted	-0.1188	-0.0612	0.00932	-0.0651	-0.0591	-0.0605	-0.0927	-0.1719	-0.1573	-0.1772	-0.0361	0.08587	-0.1257	-0.0474
NM_001012189	Mboat5	0.60744	0.22736	0.33762	-0.6125	0.50439	0.25305	0.29719	0.38989	0.0569	0.1132	0.09439	-0.4325	-0.2704	-0.19
NM_001012189	Mboat5	0.29714	0.05516	0.12883	-0.4544	0.44611	0.40578	-0.0169	0.3795	-0.0428	-0.0242	0.08643	-0.2202	-0.3222	-0.2056
NM_001025289	Mbp	0.14671	0.13829	0.08626	0.22041	-0.0052	0.21372	0.06362	0.13669	0.26134	0.50574	-0.082	0.52449	0.14523	0.2252
NM_053569	Mbtps1	0.11786	0.15375	0.23174	0.2147	-0.3764	-0.0916	0.1721	-0.4379	-0.2154	0.16803	-0.0224	-0.0872	-0.3857	-0.2441
NM_001035007	Mbtps2	0.10175	-0.0449	0.09649	0.26665	0.30033	-0.0556	0.13544	0.11269	-0.2135	0.24655	-0.0219	-0.0446	0.19746	0.24463
XM_226554.2	Mc1r_predicted	0.0353	0.01337	0.1572	0.05216	-0.0181	0.03344	-0.0426	0.04932	0.0945	-0.0478	0.2698	0.06359	0.12889	-0.0127
NM_001100491	Mc2r	-0.0123	0.00402	0.03005	-0.0091	0.0249	0.16234	0.00725	0.03703	0.03242	0.09882	0.10282	-0.0645	-0.0802	-0.1259
NM_001025270	Mc3r	-0.313	-0.2284	-0.0713	-0.0711	0.03935	0.13457	-0.0799	0.11025	-0.055	0.08495	-0.1127	0.0423	0.10718	-0.1222
NM_013099	Mc4r	-0.0289	-0.2274	-0.2009	-0.1879	-0.1432	-0.2271	-0.2063	-0.1434	-0.1778	0.08389	-0.1636	-0.0532	-0.1849	-0.2095
NM_013182	Mc5r	0.08951	0.01774	0.06869	-0.0277	0.03956	0.00296	-0.0071	-0.0357	0.14192	-0.051	0.06236	0.02606	-0.054	-0.0384
NM_021585	Mca32	-0.0156	-0.0666	0.19469	-0.057	-0.1119	-0.0285	-0.0257	-0.0921	-0.0151	-0.0748	0.08322	-0.0306	-0.0535	-0.1169
NM_001034009	Mcam	0.28089	0.01449	0.01162	-0.0748	0.04223	0.00102	0.03126	0.13269	0.02825	0.12673	-0.0709	0.1133	-0.0221	0.1219
NM_001024785	Mcart1	-0.1534	0.01244	-0.8924	-0.9783	-0.1487	-0.796	-0.3125	-0.2209	0.10137	-0.0166	-0.2719	-0.6034	0.3891	0.29886
XM_223434.3	Mcart2_predicted	0.27335	0.07303	-0.2539	-0.3257	0.08159	-0.256	-0.0636	0.14394	-0.0943	-0.1086	-0.0781	-0.215	0.07155	0.10369
NM_001009653	Mccc1	-0.1436	0.01569	0.06259	0.03308	-0.0442	0.03671	-0.0559	-0.0112	0.12143	0.1297	0.00589	0.20517	0.01797	-0.0268
NM_001009653	Mccc1	-0.1443	-0.1193	0.11716	0.25286	-0.2268	-0.154	0.01555	-0.1981	-0.2114	-0.066	-0.1091	-0.1098	-0.4115	-0.4427
NM_001012177	Mccc2	0.02978	-0.0853	0.07636	-0.0938	-0.0385	-0.106	0.03268	0.11187	-0.1169	-0.1457	-0.0559	-0.1145	-0.1179	0.02673

NM_001012177	Mccc2_predicted	0.09423	0.29029	0.16184	0.05059	0.23286	0.00039	0.21182	0.00617	0.10347	0.12463	0.03276	0.0346	0.25008	-0.0613
NM_001106341	Mcee_predicted	0.60693	0.53531	-0.378	0.11956	0.44767	0.75466	0.13548	0.40443	0.52908	0.37452	0.54334	0.50076	0.06563	0.07264
XM_229172.4	Mcf2_predicted	0.05237	-0.0418	0.10711	0.24221	0.02416	-0.0465	-0.0602	0.28496	0.34842	0.17989	0.25915	0.07177	0.11429	0.22552
NM_053951	Mcf2l	0.24889	-0.0543	0.15379	0.15352	0.20798	0.1623	0.01259	0.01769	-0.0683	-0.0674	-0.0988	0.01298	0.0875	-0.0824
NM_139253	Mcfd2	0.63369	0.39723	0.3847	0.06653	1.1672	0.99034	0.58263	1.2999	0.50346	0.53746	0.62298	-0.0186	0.4183	0.52711
NM_031758	Mchr1	-0.0803	-0.0055	0.09028	-0.2042	-0.0131	-0.2331	-0.0326	-0.1789	-0.0279	0.16944	0.09825	0.03642	0.024	-0.1761
NM_021846	Mcl1	0.73667	0.41008	0.24686	-0.4156	0.38077	-0.0607	0.75557	0.41125	-0.0999	0.22623	0.23813	-0.3404	0.47515	0.3486
NM_001107366	Mcm10_predicted	-0.7265	-0.5387	0.26447	-0.7772	-0.8752	-1.0336	-0.5016	-0.8417	-0.1427	-0.4506	-0.4445	-0.0422	0.47298	0.45804
NM_001107873	Mcm2_predicted	-0.3742	-0.9086	0.3283	-0.929	-0.8714	-1.152	-0.9672	-1.0267	-0.7272	-0.5897	-0.9279	-0.3543	0.12033	0.23288
XM_236988.4	Mcm3_predicted	-0.304	-0.9077	0.57733	-0.7498	-0.9658	-0.8235	-0.6019	-0.8208	-0.5656	-0.5234	-0.7612	0.3183	0.42536	0.57437
NM_001106382	Mcm3ap_predicted	-0.575	-0.4551	0.34278	0.7546	-0.5299	-0.0638	0.14158	-0.4726	-0.3761	-0.3061	-0.282	0.00676	-0.5974	-0.4999
NM_033651	Mcm4	-0.169	-0.4484	0.29917	-0.4229	-0.3261	-0.8371	-0.3167	-0.4307	-0.1653	-0.3636	-0.2965	-0.1661	0.61714	0.62821
NM_001106170	Mcm5_predicted	-0.4094	-0.5585	-0.0019	-0.9861	-0.8721	-0.6482	-0.8183	-0.8686	-0.4048	-0.5951	-0.2914	0.21161	0.36236	0.54493
NM_001106170	Mcm5_predicted	-0.1517	-0.5263	0.15274	-0.602	-0.3043	-0.5306	-0.6171	-0.6198	-0.2748	-0.3599	-0.2472	-0.1334	0.69305	0.74043
NM_017287	Mcm6	-0.412	-0.6288	0.79667	-0.7332	-1.0001	-1.1654	-0.8204	-1.0398	-0.5984	-0.5409	-0.653	0.23777	0.6931	0.63086
NM_001004203	Mcm7	-1.2335	-1.628	0.64083	-0.9992	-1.8272	-1.6627	-0.9396	-1.9189	-1.0435	-1.2759	-1.07	-0.3213	0.28512	0.31991
NM_001106514	Mcm8_predicted	0.10818	-0.1906	0.41647	-0.1819	-0.3002	-0.1282	-0.1606	-0.408	-0.1469	-0.4455	-0.3156	0.07099	0.36145	0.05718
NM_001105903	Mcoln1_predicted	0.01871	-0.0636	-0.0507	0.08348	-0.1179	0.14085	0.10612	-0.0101	-0.0716	-0.2212	0.00214	-0.0228	-0.0515	-0.2502
NM_001039005	Mcoln2_predicted	-0.0419	-0.0289	0.15076	0.05784	0.13184	0.20637	-0.0238	0.09711	0.10276	0.07891	-0.0619	-0.0562	-0.0159	-0.0587
NM_001012059	Mcoln3	2.0901	0.34119	0.13732	0.65713	0.28497	0.18226	0.26751	0.24286	0.23813	-0.0718	0.09581	0.65014	0.2229	0.12334
NM_001012059	Mcoln3_predicted	2.091	0.09892	-0.0146	0.49131	-0.0399	-0.0127	0.12688	-0.0032	0.13959	0.08371	0.10787	0.13653	-0.0048	0.13065
NM_017145	Mcpt1	0.24355	0.15183	0.26257	0.08735	0.16025	0.00042	0.03146	0.05537	-0.0167	0.0772	0.21861	0.10676	-0.0179	0.20327
NM_017146	Mcpt10	-0.164	3.5125	0.38118	0.21981	-0.04	-0.0733	0.37546	-0.0273	3.1114	3.2272	3.1658	0.01222	0.41968	0.46726
XM_001058060.1	Mcpt1l2	-0.1533	1.3377	0.17818	0.02509	0.22142	-0.1029	-0.0724	-0.1343	1.1951	1.1895	1.2963	0.12246	-0.0121	0.33159
XM_573785.1	Mcpt1l3	-0.0623	-0.0637	0.04818	0.01022	0.00514	0.10852	0.01062	-0.1155	-0.067	-0.0412	0.10182	-0.0381	0.02309	0.27756
XM_573784.1	Mcpt1l4	-0.2742	-0.238	-0.087	-0.1139	-0.2166	-0.3523	-0.0031	0.27771	-0.1741	-0.3086	-0.2226	-0.2447	-0.1037	-0.1426
NM_172044	Mcpt2	0.0737	-0.0382	0.01147	0.12599	0.01907	-0.0438	0.0281	0.31307	-0.029	0.20061	0.19696	-0.07	0.09375	0.05141
NM_001170466	Mcpt3	-0.1975	-0.0859	0.00492	0.03224	0.01795	0.05655	-0.1814	0.02529	0.07732	-0.0511	0.07973	0.15499	-0.0867	-0.1439
NM_019321	Mcpt4	0.27053	-0.0976	0.06825	0.08735	0.39655	-0.1561	-0.0484	-0.0549	0.01966	0.00313	-0.0582	-0.1401	0.13624	0.02644
XM_001056983.1	Mcpt4l1	0.03505	0.2057	0.44343	0.24521	0.12564	0.12704	0.27785	0.01071	0.15358	0.11587	0.19104	0.02302	0.26978	-0.0777
NM_021598	Mcpt8	0.22754	1.4665	0.11542	-0.0665	-0.1156	-0.0663	0.12363	-0.0051	1.4039	1.4146	1.5589	0.1793	-0.0858	0.09271
NM_021598	Mcpt8l2	-0.1261	3.3708	0.67164	0.32742	-0.0075	0.30408	0.31409	-0.2099	2.9876	3.3952	3.2525	0.29651	0.54546	0.49128
NM_017146	Mcpt8l3	-0.4134	-0.3112	-0.1453	-0.2727	-0.2673	-0.2615	-0.1981	-0.246	-0.1195	-0.0131	-0.26	-0.2881	-0.2287	-0.1489
NM_019323	Mcpt9	0.04303	-0.1324	-0.1047	0.07911	0.05218	-0.1576	0.07963	-0.1578	-0.3128	-0.0519	-0.0853	-0.049	-0.236	-0.0501
NM_001013106	Mcrs1	-0.5897	-0.3494	0.5249	-0.417	-0.7681	-0.8107	-0.27	-0.8931	-0.133	-0.0369	-0.2506	0.45526	0.05709	-0.075
NM_031536	Mcsp	-0.131	-0.2245	-0.3713	-0.1037	-0.2928	-0.1456	-0.2681	-0.2314	-0.1616	-0.1342	-0.2162	0.04422	-0.2423	-0.2475
NM_001044237	Mcts1	0.44572	0.00502	0.26254	0.37504	0.42051	0.6578	0.07967	0.61955	0.31598	-0.043	0.13594	0.62389	0.41196	0.28375
NM_001166275	Mdc1	-0.3505	-0.4251	0.50452	-0.5048	-0.4675	-0.4789	-0.1467	-0.5503	-0.6423	-0.3808	-0.3123	-0.0106	0.12986	0.10818
NM_001105668	Mdfic_predicted	0.07399	0.06533	0.07371	-0.2957	-0.2359	-0.354	-0.3956	-0.2124	0.03521	0.0277	-0.1275	0.09333	0.18988	0.17747
NM_199269	Mdga2	2.603	3.4396	1.5135	1.3559	-0.3425	-0.3191	3.7061	-0.3986	3.0163	3.4713	3.8316	0.82439	3.1515	3.3011
NM_033235	Mdh1	0.46194	0.29838	0.1033	-0.0756	0.28984	0.24267	-0.0269	0.10028	0.41413	0.15716	0.32662	0.1057	0.09483	0.05766
NM_001108221	Mdh1b_predicted	-0.1788	-0.2954	0.00031	-0.3311	-0.2358	-0.2266	-0.0786	-0.113	-0.3869	-0.3487	-0.3347	-0.1827	-0.5351	-0.1376
NM_031151	Mdh2	0.24669	-0.2547	0.33965	-0.2865	0.22209	0.02924	0.21904	0.22838	-0.0387	0.33807	-0.1052	0.05697	0.18533	0.53414
NM_030859	Mdk	-0.1648	-0.0334	-0.017	-0.3558	-0.2597	-0.1109	-0.0029	-0.0786	0.11338	-0.1535	-0.1024	-0.1862	-0.1392	0.02962
NM_001017459	Mdm1	0.28448	0.05901	1.0729	0.83276	-0.2194	-0.4262	0.30946	-0.1293	-0.135	0.17281	0.15439	0.35084	0.78147	0.72675
NM_001108099	Mdm2_predicted	0.47438	0.83906	0.23466	1.6785	0.73142	0.31996	0.82288	0.99685	0.17003	0.60385	0.40846	-0.3707	0.46198	0.41319
NM_001012026	Mdm4	-0.7643	-0.3174	0.12635	-0.6823	-1.0557	-1.3379	0.08284	-0.9803	-0.1117	-0.2283	-0.3702	-0.3802	-0.4856	-0.433
NM_012600	Me1	0.4316	0.34692	0.02308	0.48667	0.42949	0.19123	-0.3351	0.39343	0.30472	0.2398	-0.1673	0.20615	0.54048	0.41037
NM_001107376	Me2_predicted	0.92852	0.6963	0.24956	-0.4197	0.62208	0.29295	0.06418	0.66998	0.75509	0.84463	0.57274	0.1155	0.66798	0.69438

NM_001108491	Me3_predicted	0.19519	0.04481	0.05931	0.07518	0.13288	0.00082	-0.0676	0.01746	-0.0443	-0.0461	0.06733	0.16329	0.11475	0.03251
NM_022673	Mecp2	-0.2061	-0.5858	-0.0825	0.9695	-0.0421	0.27326	-0.1648	0.31324	-0.6324	-0.5626	-0.5232	-0.2987	-0.3614	-0.335
NM_017209	Mecr	0.20361	0.08709	0.51977	0.28693	0.38435	0.32866	0.09373	0.22425	0.13189	0.31545	0.12158	0.36872	0.39113	0.48752
NM_001107741	Med19_predicted	0.76333	-0.0052	0.58679	0.36923	1.0183	1.1246	0.43896	0.85226	0.33141	0.27659	0.60356	0.20007	0.77454	0.84256
NM_001013178	Med20	0.10439	0.05739	-0.5315	0.02926	-0.0414	-0.6319	-0.1329	-0.047	-0.07	0.15734	-0.0374	-0.2694	0.05569	0.18521
NM_001170426	Med25_predicted	-0.2301	-0.0133	-0.5243	-0.7751	-0.3442	-0.6458	-0.6285	-0.3491	-0.1163	-0.1783	-0.2021	-0.2456	-0.7893	-0.7677
NM_001107217	Med28_predicted	0.76241	-0.053	-0.1637	0.3334	0.79289	0.95602	0.16384	0.46963	0.16524	-0.004	-0.1378	0.37537	0.16816	0.28049
NM_001135813	Med31_predicted	0.12159	-0.1321	-0.2567	0.24108	-0.0769	0.5079	-0.4822	0.16914	0.13352	-0.0125	0.04191	0.03677	-0.4763	-0.3731
NM_001024256	Med4	0.00149	-0.1428	-0.4034	-0.2604	0.035	-0.11	-0.2814	0.20877	0.02724	-0.0919	-0.0724	0.05002	-0.0173	0.00033
NM_001106742	Med6_predicted	0.25805	0.14437	0.00258	0.40067	0.48508	0.67741	0.33065	0.31153	0.19562	0.34526	0.36867	0.16063	0.20389	0.09116
NM_001106742	Med6_predicted	0.4328	0.25642	0.06028	0.49336	0.25022	0.58538	0.46326	0.40837	0.35441	0.40368	0.29832	0.14514	0.2786	0.04771
NM_001108673	Med8_predicted	-0.2675	-0.5288	-0.3005	-0.5336	-0.104	0.00515	-0.3495	-0.1557	-0.643	-0.4282	-0.7168	-0.1772	-0.1096	0.05623
NM_001017507	Mef2b	0.17383	-0.1873	-0.1172	0.21545	0.14253	0.28897	-0.1303	0.07108	-0.2605	-0.3097	0.09338	0.0653	-0.3287	0.20496
NM_030860	Mef2d	0.02162	-0.0277	0.23408	0.01348	-0.0711	0.27496	0.11062	0.14309	-0.161	0.1138	0.34706	0.02265	-0.1059	0.30527
NM_031634	Mefv	-0.2108	0.08498	-0.12	-0.0051	0.03558	-0.0686	-0.1265	-0.0088	-0.1829	-0.2526	-0.0395	-0.0685	-0.2681	-0.088
NM_001100657	Megf10	0.01369	0.18707	-0.3841	-0.405	0.50131	0.03897	-0.0087	0.6332	0.05252	0.19144	-0.2316	-0.1969	0.38516	0.5692
NM_022955	Megf6	-0.0924	0.00332	0.02483	-0.1379	-0.0218	-0.1979	-0.0059	-0.2357	-0.1536	-0.1293	-0.0359	-0.1007	-0.2396	-0.1672
NM_001130555	Mei1_predicted	0.20994	0.07329	0.12577	0.19465	0.03414	-0.0079	0.05381	0.09012	0.15067	0.12436	0.0452	0.2143	0.05272	0.00663
NM_001107758	Meis2_predicted	0.0662	0.18071	-0.2278	-0.1153	0.2099	0.00068	0.0638	0.09799	0.71627	0.09094	0.15064	0.49649	0.32654	0.30889
NM_001108472	Meis3_predicted	0.13081	0.19779	0.19025	-0.5841	-0.0422	-0.0094	0.1659	-0.142	0.26645	0.34346	0.26638	0.20671	-0.0894	-0.2321
NM_001108662	Melk_predicted	-0.2574	-0.4919	0.31984	-0.4119	-0.6284	-0.5982	-0.5153	-0.6449	-0.2148	-0.276	-0.1983	0.1063	0.22721	0.22527
NM_001107997	Mell1_predicted	0.31434	0.19168	-0.0148	0.09607	0.19715	0.17824	0.16314	0.22051	0.08454	0.26698	0.02299	0.33539	0.11822	0.03494
NM_001029917	Memo1	-0.4799	-0.3609	0.06183	-0.1863	-0.5802	-0.4751	-0.1836	-0.5793	-0.4374	-0.5505	-0.5203	-0.0781	0.06733	-0.0583
NM_019208	Men1	-0.4156	-0.3241	-0.1084	0.17161	-0.5207	-0.3229	-0.1904	-0.0898	-0.3116	-0.2206	-0.3366	-0.2256	-0.5589	-0.3482
NM_001108837	Meox1_predicted	-0.1919	-0.2179	-0.191	-0.2661	-0.2589	-0.1021	-0.3119	-0.2673	-0.2423	-0.0507	-0.2281	-0.1758	-0.2344	-0.2848
NM_017149	Meox2	0.00796	0.21035	-1.4266	-0.6884	-1.6135	-1.716	-0.3693	-1.6129	0.14272	0.26481	0.21959	-1.596	-1.4719	-1.5419
NM_013143	Mep1a	0.23481	0.09756	0.01274	0.03938	-0.0022	0.09301	0.00661	0.09903	0.02807	-0.0044	0.05241	0.09526	-0.0142	-0.0011
NM_022943	Mertk	0.0547	0.04929	0.07478	-0.0354	-0.0848	0.0863	-0.0327	0.28	-0.2359	-0.0173	0.26162	0.33551	-0.0377	0.03769
NM_001013149	Mesdc1	-0.7461	0.12222	-0.2241	-1.146	-0.7599	-0.6098	-0.7697	-0.8404	-0.1053	0.1763	-0.1109	-0.1424	-0.1836	-0.085
NM_001008345	Mesdc2	0.97249	1.1804	0.10196	0.22067	0.69311	0.64263	0.51389	0.67436	0.94145	1.0208	1.0902	0.42225	0.5205	0.51246
NM_001107531	Mesp1_predicted	-0.0398	0.06124	0.18865	0.02455	-0.056	-0.013	0.00492	0.19219	0.09898	0.04003	-0.0103	0.05677	0.02922	0.14927
NM_001106273	Mesp2_predicted	0.46978	0.56806	0.02375	0.15616	0.18317	0.28407	0.07702	0.31016	0.32156	0.49135	0.38906	0.07261	0.10017	0.14015
NM_001106476	Metap1_predicted	-0.0511	0.10612	-0.0926	0.42735	-0.1784	0.00456	0.01495	-0.2431	-0.0578	-0.064	-0.1372	-0.0118	-0.0785	-0.1166
NM_022539	Metap2	-0.4658	-0.3771	0.05816	-0.3831	-0.4036	-0.4909	-0.4525	-0.3013	-0.0795	-0.2889	-0.2859	-0.0206	0.26041	0.13906
NM_001107812	Metapl1_predicted	-0.0413	-0.0741	0.34398	-0.2389	0.05418	-0.088	0.31763	-0.0921	0.33448	-0.0416	0.03595	0.27479	0.23416	0.37943
NM_001009962	Metrn	0.49984	-0.0441	-0.1372	-0.388	-0.0049	0.29634	-0.4049	-0.0514	-0.0042	-0.0695	0.07816	0.41439	0.24168	-0.0183
NM_001014104	Metrnl	0.02546	-0.3945	-0.2314	0.32411	0.37147	0.45636	-0.6587	0.32003	0.08098	-0.4069	-0.2366	0.29707	0.25889	0.17842
NM_001108839	Mettl2_predicted	0.0621	-0.1238	0.08689	-0.1738	0.09545	0.05077	-0.2461	0.07215	-0.437	-0.1104	-0.1573	-0.2521	0.33762	0.25398
NM_001024794	Mettl3	-0.6025	-0.3541	-0.0172	-0.2097	-0.4858	-0.5962	0.25825	-0.4576	-0.18	-0.1432	-0.4087	-0.371	-0.1544	-0.223
NM_001007623	Mettl6	-0.0213	-0.0382	-0.0501	-0.0129	0.11417	0.02825	-0.035	-0.0906	0.06078	0.15785	0.0642	0.38584	0.04227	-0.1486
NM_001037355	Mettl7a	0.25012	0.8938	0.13701	0.4448	0.34478	0.55339	0.53551	0.44478	0.69818	0.63937	0.59495	0.54125	-0.1467	-0.2534
NM_001037355	Mettl7a	-0.1317	0.44615	-0.4127	-0.0439	0.01754	-0.5072	-0.1518	-0.1226	-0.0366	-0.0165	-0.0069	-0.6008	-0.3364	-0.382
NM_001024276	Mettl7b	-0.1832	-0.1501	-0.0237	-0.2175	-0.1545	-0.2042	0.00647	-0.067	-0.0517	-0.1576	-0.1946	-0.047	-0.1432	-0.1317
NM_001107989	Mfap2_predicted	0.07864	0.12332	0.0184	0.22746	0.0777	0.17244	0.26273	0.07847	-0.1009	-0.0132	0.23018	0.06412	0.39572	-0.1274
NM_001007609	Mfap3	0.13346	0.00484	0.07513	-0.0165	0.19449	0.0724	-0.0098	0.3105	-0.1505	-0.0011	-0.1235	-0.3197	-0.0083	0.11929
NM_001012049	Mfap3l	0.05193	0.4074	0.25342	0.30853	-0.0046	-0.0538	0.36965	0.09346	-0.0675	-0.0675	0.06632	-0.0835	0.06675	0.11027
NM_001034124	Mfap4	0.00168	0.06764	0.16887	0.2129	0.21548	0.17597	0.05812	-0.0374	0.00732	0.0053	-0.024	0.19524	0.38375	0.04454
NM_001108644	Mfap5_predicted	2.355	0.25236	0.49265	0.12146	-0.0585	-0.005	0.40585	0.11165	0.10086	0.18014	0.12715	0.0486	0.22922	-0.0012
NM_012811	Mfge8	-0.8331	-0.3552	-0.8114	-0.9744	-0.5084	-1.2872	-0.869	-0.5439	-0.7478	-0.6452	-0.5188	-0.8875	-0.4773	-0.5705

NM_001107316	Mfhas1_predicted	0.31132	0.02502	0.72637	0.48781	0.51425	0.20642	0.24796	0.23773	0.08176	0.56536	0.01529	0.25113	0.20587	0.43087
NM_001105872	Mfi2_predicted	0.26004	0.14119	0.04147	0.04927	-0.0371	0.06714	-0.085	-0.0388	0.10631	0.16024	0.04131	0.03334	0.09674	-0.0792
NM_138976	Mfn1	-0.0071	-0.1311	-0.3958	0.57843	0.02513	0.18617	0.18261	0.2488	-0.113	-0.0317	0.01413	0.06888	0.19179	0.19874
NM_130894	Mfn2	-0.0387	0.21083	0.49045	-0.3441	0.23427	0.12669	0.0169	0.25963	-0.0318	0.16975	0.16738	-0.0243	0.18926	0.18373
NM_199110	Mfng	-0.0323	-0.2208	0.0245	-0.2189	-0.0687	-0.0956	-0.1414	-0.2217	-0.1637	-0.1409	-0.1086	0.06973	-0.1451	-0.1641
NM_001108137	Mfrp_predicted	0.07957	0.07148	-0.0218	0.02839	0.16446	-0.027	-0.0243	-0.0324	0.00011	0.03437	-0.0607	0.15117	0.04623	-0.1109
XM_001061503.1	Mfsd1_predicted	0.54677	0.51368	0.08393	0.46348	0.586	0.30144	0.2919	0.52478	0.29007	0.13557	0.33831	-0.1191	0.7133	0.76088
NM_001024908	Mfsd3	-0.2351	-0.3483	-0.2463	-1.2543	-0.6334	-0.4967	-0.5964	-0.5714	-0.2125	-0.2905	-0.0088	-0.0855	-0.2686	-0.2654
NM_001108563	Mg29_predicted	-0.0489	-0.1238	0.01108	-0.2103	0.0827	-0.0684	-0.0417	-0.0074	-0.0304	0.03266	0.1279	-0.1058	0.08569	-0.0228
XM_231714.4	Mgam_predicted	0.21974	0.0776	-0.0382	-0.0309	-0.1438	0.04682	-0.0817	0.17177	-0.0049	0.07411	0.14081	0.05777	0.14346	0.11684
NM_030861	Mgat1	-0.0404	-0.0927	0.02099	0.48885	0.3526	0.6444	0.13574	0.50687	-0.3024	-0.0851	-0.0943	0.30398	-0.0061	-0.243
NM_053604	Mgat2	-0.0793	0.24331	-0.0299	-0.0042	0.14261	0.55638	0.1036	0.27451	0.33309	0.09687	0.09401	0.52795	0.0489	0.10467
NM_019239	Mgat3	-0.2227	-0.0724	-0.377	-0.1709	-0.1154	-0.1856	-0.2615	-0.3418	-0.1023	-0.0474	-0.2355	-0.142	-0.2359	-0.3722
NM_001012225	Mgat4a	0.10499	-0.0415	0.09645	0.04893	0.14467	0.12706	0.10811	0.08077	0.15792	0.09137	0.18446	-0.037	0.12773	0.17351
NM_001135814	Mgat4c_predicted	-0.1144	0.0128	-0.2419	0.13761	-0.1373	-0.1461	0.25262	-0.0422	-0.0416	-0.0211	0.15934	0.18522	-0.0654	0.08918
NM_023095	Mgat5	-0.0978	0.04183	0.13616	0.00912	-0.0263	-0.017	-0.0341	0.09753	0.09164	-0.0149	-0.122	-0.1362	0.25112	-0.0121
NM_001107068	Mgat5b_predicted	0.24799	0.09921	0.00874	-0.0563	0.05033	0.01484	-0.0349	0.02782	0.17285	0.0777	0.12044	-0.0609	0.05935	0.10982
NM_001024357	MGC105560	0.25289	0.45909	-0.8169	-0.0571	0.34595	-0.0538	0.30442	0.67218	0.45425	0.42751	0.46617	-0.1318	0.36489	0.15168
NM_001024284	MGC105567	-0.0337	-0.0269	-0.0423	0.06183	0.05474	-0.1658	-0.0571	-0.1014	0.07329	-0.0261	0.17007	0.09654	0.05812	-0.1552
NM_001007008	MGC105647	0.28379	0.054	-0.1188	-0.0374	0.26764	0.69292	0.09497	0.39064	-0.0731	-0.0168	0.22274	-0.0656	-0.1178	0.09721
NM_001008518	MGC105649	0.90189	0.54641	0.69731	0.13227	0.26614	0.71656	0.89629	0.25079	0.3257	0.77868	0.66847	-0.8611	0.32983	0.44351
NM_001008370	MGC105830	0.07552	0.04845	-0.1315	-0.1625	-0.0018	-0.1383	-0.1043	-0.0727	0.14909	-0.0449	0.08788	-0.1297	-0.2034	0.23565
NM_001009628	MGC108747	-0.9462	-0.7811	-0.9639	-0.8271	-0.3365	-0.5854	-0.8163	-0.5091	-0.771	-0.8749	-0.6227	-1.0609	-0.977	-1.0036
NM_001017466	MGC108776	-0.2408	0.43725	-0.3002	0.02691	-0.3325	-0.0558	0.47678	-0.4162	0.62679	0.39113	0.4816	0.86677	-0.4355	-0.2842
NM_031154	MGC108896	0.66151	0.52884	0.28044	-0.0249	0.36338	0.06782	0.12482	0.33603	0.3068	0.18402	0.3293	-0.156	0.03537	0.28384
NM_001017513	MGC108974	-0.171	0.16561	-0.0606	0.05857	0.19323	-0.2744	-0.1074	-0.0548	0.06442	-0.088	0.01876	0.09417	-0.0272	0.13874
XM_001067976.1	MGC109145	-0.2084	-0.0266	-0.0043	-0.1007	-0.2788	-0.0249	-0.0871	0.17199	-0.0819	-0.2913	-0.1089	-0.075	0.00709	-0.2656
NM_001017473	MGC112682	-0.0983	0.43206	-0.0765	-0.4375	0.27615	-0.2724	0.11496	0.01903	0.31216	0.09337	0.42142	0.14083	0.24548	0.15909
NM_001017473	MGC112682	0.03447	-0.0329	-0.0045	0.00029	0.04283	0.02083	0.13728	0.08162	0.01636	0.19757	-0.049	-0.0198	0.01758	0.03075
NM_001025759	MGC112692	0.31504	-0.1528	0.21793	0.05768	-0.067	-0.2751	-0.161	0.09737	0.02419	-0.1976	-0.2682	-0.2181	-0.2532	-0.0709
NM_001037787	MGC112727	-0.1046	-0.2489	-0.0429	-0.4389	-0.3658	-0.2712	-0.0624	-0.2864	-0.0108	-0.1682	-0.0148	-0.101	-0.1562	-0.169
NM_001044267	MGC112775	0.13089	0.65516	0.23134	0.47697	0.33257	0.03079	0.36869	0.00398	0.40978	0.22931	0.63027	0.19123	-0.0484	0.03331
NM_001044251	MGC112790	-0.8499	-0.7619	-0.2042	-0.746	-0.6033	-0.44	-0.618	-0.5722	-0.5312	-0.5149	-0.7311	-0.2238	0.53293	0.76061
NM_001025718	MGC112830	-0.7143	-1.0781	-0.2364	-0.987	-1.2164	-1.3545	-0.8512	-1.0558	-0.8348	-0.9457	-0.9864	-0.3381	-0.2969	-0.2742
NM_001044248	MGC112844	-0.1445	-0.0155	-0.0062	-0.1063	-0.0156	-0.1998	-0.0825	0.20397	0.00849	-0.1827	0.13683	-0.0789	-0.1649	-0.0945
NM_001047743	MGC112883	-0.2374	-0.2267	-0.0465	-0.1462	-0.1856	-0.1896	-0.8231	0.00015	-0.3434	-0.3134	-0.4732	-0.075	0.07255	-0.0031
NM_001037795	MGC112899	0.23541	-0.0122	0.10698	0.1207	0.28142	0.87671	0.09129	0.11224	0.16644	0.03529	0.27135	0.65344	0.0127	0.14201
NM_001017509	MGC114246	0.02494	-0.0781	0.05931	0.01709	0.06612	-0.008	0.03804	0.08187	0.08124	0.03392	0.04159	-0.0864	0.00396	0.18215
NM_001025287	MGC114381	-0.1188	-0.2568	-0.3083	0.19831	-0.1135	-0.2022	-0.1218	0.151	-0.0306	-0.262	-0.2946	-0.1048	-0.0504	0.04854
NM_001024877	MGC114417	-0.5177	-0.4867	0.34703	-0.0631	-0.8193	-0.6263	-0.2988	-0.809	-0.1008	-0.4684	-0.0973	0.44446	0.10847	-0.037
NM_001025694	MGC114439	-0.1606	-0.1607	-0.0686	-0.2381	-0.2258	-0.1348	-0.1889	-0.0768	-0.1242	0.12531	-0.2619	-0.0425	-0.2061	-0.4106
NM_001025772	MGC114440	0.31556	0.18091	0.16525	0.06655	0.37919	0.52992	0.13677	0.45589	0.03197	0.31805	0.31896	0.085	0.25867	0.18216
NM_001024880	MGC114483	0.22792	0.00879	0.12657	0.14086	-0.0032	-0.1705	0.07121	-0.0315	-0.1343	0.35135	0.05238	0.08296	0.13256	0.10507
NM_001024895	MGC114492	-0.2341	-0.2793	-0.2199	-0.2489	-0.2407	-0.2225	-0.2556	-0.2145	-0.0753	-0.0689	-0.1932	-0.1144	-0.1341	-0.2165
NM_001024907	MGC114499	-0.0929	0.30567	0.52513	-0.0793	0.0192	0.14797	0.01222	0.29192	-0.0598	0.05909	-0.0196	-0.0938	0.01108	0.01355
NM_001024907	MGC114499	-0.0688	-0.1337	-0.2439	0.0402	-0.2353	-0.1784	-0.2811	-0.1193	-0.2129	-0.1827	-0.2677	-0.2574	0.13672	-0.0279
NM_001024890	MGC114520	-0.1406	-0.1516	-0.0848	-0.1384	-0.0194	-0.0375	-0.1545	-0.0309	0.10613	-0.1043	-0.035	-0.149	-0.185	-0.2008
NM_001025713	MGC114529	-0.1492	-0.0923	-0.1187	-0.1983	-0.2478	0.01203	1.5971	-0.2248	-0.1612	-0.1172	-0.2277	-0.0002	-0.164	-0.119
NM_001024905	MGC116121	-0.0147	0.16273	0.34504	0.38491	0.29997	0.29523	0.25087	0.33895	0.23453	0.19555	0.06151	0.19165	0.29365	0.18094

NM_001025755	MGC116197	-0.3308	-0.2908	-0.3921	-0.3232	-0.3917	-0.1802	-0.1086	-0.4066	-0.3137	-0.4022	-0.3197	-0.2271	-0.4224	-0.3607
NM_001025755	MGC116197	-0.0347	0.01717	0.12513	0.08513	-0.0182	-0.1466	-0.1818	0.10012	0.00708	0.19985	-0.1738	0.09218	-0.0804	-0.045
NM_001025755	MGC116197	-0.1519	0.05406	0.0556	0.13554	-0.067	0.0948	0.22529	0.04488	0.13102	-0.1076	-0.0781	-0.0197	0.20877	0.00482
NM_001044255	MGC124824	0.07749	-0.058	-0.1125	-0.2098	0.01339	-0.0512	-0.109	0.03621	-0.0628	0.21824	0.06935	-0.1373	0.03508	0.09073
NM_001108357	MGC124888	0.03493	0.11172	0.07492	0.16854	0.1239	0.04889	-0.0121	0.18997	-0.0199	0.09005	-0.0441	0.00451	-0.0435	0.16264
NM_001108357	MGC124888	0.41734	0.31167	-0.5889	-0.6656	0.25703	-0.2322	-0.2131	0.06884	-0.0924	0.01373	-0.0874	-0.2235	0.32249	0.45661
NM_001033902	MGC124992	-0.1283	-0.3318	0.09758	1.2383	-0.2696	-0.1869	0.24262	-0.225	-0.3599	-0.3047	-0.312	-0.2971	-0.2486	-0.3682
NM_001034154	MGC125002	0.39713	0.37303	0.05965	0.62618	0.44469	0.30118	0.47458	0.58781	0.45525	0.54311	0.26143	0.23771	0.0183	0.0607
NM_001034153	MGC125213	0.1541	0.36872	0.38453	0.25673	0.10702	0.17659	0.20197	0.17065	0.32345	0.35493	0.04175	0.21143	0.15417	0.17751
NM_001134857	MGC125214	-0.0473	0.25035	-0.1306	-0.0738	0.25017	0.02922	0.32995	0.52555	-0.1464	0.03752	-0.1176	-0.4349	-0.0843	-0.1403
NM_001037794	MGC125233	0.24051	0.15559	0.10444	0.04097	0.14619	-0.0169	0.3004	0.32896	0.03961	0.13169	0.00032	0.3204	0.04697	0.00238
NM_199102	MGC72560	0.34374	0.23209	0.00925	-0.6729	0.39653	0.73222	-0.1237	0.16267	0.18165	0.26348	0.04667	0.57654	0.48473	0.47867
NM_001009533	MGC72567	-0.1091	0.33233	0.09682	-0.0036	-0.0697	-0.0324	0.08565	0.15597	0.57242	0.46061	0.36821	0.57014	0.26074	0.13036
NM_001009538	MGC72612	-0.1202	0.04755	0.32289	0.02906	-0.0269	-0.3111	0.10696	-0.1416	-0.1401	-0.064	0.01828	0.23869	0.2975	-0.2235
NM_199105	MGC72614	1.3987	1.1703	0.42548	1.0777	-0.0281	0.10668	0.38586	0.16944	1.3472	1.3535	1.3939	1.3462	1.6984	1.6027
NM_212516	MGC72942	0.36802	0.11309	0.30512	0.18036	0.5976	0.55751	0.34594	0.32727	0.08239	0.24307	0.28838	0.38008	0.16565	0.1804
NM_198776	MGC72973	0.00851	-0.0256	0.01721	-0.056	0.00325	-0.0293	0.16588	-0.1668	0.02512	0.10501	-0.0269	0.01551	-0.18	0.16781
NM_198772	MGC72974	0.66373	0.7666	0.42827	3.2641	-0.1267	-0.0484	1.9979	-0.0372	0.61436	0.94181	1.1803	-0.3317	0.57348	0.45952
NM_001080153	MGC72996	0.11587	-0.2534	-0.0078	-0.3952	-0.1761	-0.1914	-0.2089	-0.5162	-0.4521	-0.3761	-0.047	-0.1424	-0.2191	-0.2593
NM_001009537	MGC72997	-0.513	-0.3602	-0.4968	-0.376	-0.3179	-0.5419	-0.1419	-0.7156	-0.3509	-0.6188	-0.5327	-0.4563	-0.3187	-0.4347
NM_198777	MGC73003	-0.1612	-0.4103	0.15802	-0.1341	-0.0944	0.05502	-0.421	-0.44	-0.0841	-0.3924	-0.3755	0.20793	-0.1225	-0.1999
NM_001004221	MGC93975	-0.293	-0.0074	-0.1324	-0.5657	-0.271	-0.28	-0.0211	-0.2965	0.05435	-0.2293	0.0581	0.05011	0.04327	0.24777
NM_001004204	MGC94190	0.09055	0.26362	-0.0533	0.27241	0.73661	0.58427	0.57883	0.57714	0.10077	0.12183	0.20091	0.14147	0.25338	0.34202
NM_001004272	MGC94192	-0.3289	-0.0095	0.01316	-0.0285	-0.0794	-0.1497	0.08521	-0.0022	-0.0163	-0.1279	-0.0895	0.23504	0.0679	0.26649
NM_001007746	MGC94199	-0.2388	0.04881	-0.5581	0.18429	-0.3033	-0.3624	0.03874	-0.0668	0.01899	0.02095	0.01265	-0.1667	-1.0467	-1.0809
NM_001007751	MGC94207	-0.1445	0.08628	0.37733	-0.0078	-0.1811	0.06514	-0.0164	-0.2499	0.19124	0.02057	0.48706	0.58399	0.22246	0.29116
NM_001007649	MGC94282	0.22249	-0.5535	-0.2359	-0.93	-0.2189	-0.1534	-0.5532	-0.0991	-0.2293	-0.6034	-0.4357	-0.2153	-0.3111	-0.1889
NM_001005538	MGC94288	-0.119	-0.2195	0.14244	-0.3118	-0.2994	-0.2389	0.37611	-0.2708	-0.2056	-0.1132	-0.2904	-0.397	-0.3774	-0.4289
NM_001004251	MGC94335	0.05394	#######	-0.4737	-0.6311	-0.2288	-0.151	-0.0556	-0.1779	-0.0614	-0.1377	-0.2249	-0.2025	-0.2667	-0.1302
NM_001006964	MGC94542	0.0356	-0.177	0.03551	-0.5294	0.17375	0.21168	-0.0704	0.01483	-0.249	-0.2425	-0.1338	0.07787	-0.0977	0.00075
NM_001006989	MGC94600	0.20156	0.30326	-0.2185	-0.302	0.18808	0.24339	0.01969	0.04298	0.29829	0.47249	0.38692	0.40163	0.27448	0.09616
NM_001006974	MGC94720	-0.1405	-0.2596	0.31922	0.04393	-0.1627	0.05799	-0.1296	-0.0946	-0.1629	-0.1713	-0.2044	-0.0285	0.27619	0.4074
NM_001007009	MGC94915	0.07842	-0.0745	-0.0668	0.00498	0.11435	0.04969	0.10622	0.17253	-0.1796	-0.0078	0.20238	-0.0214	0.04576	0.02351
NM_001004233	MGC94941	0.26915	0.41689	-0.4601	-0.3074	0.49768	0.30565	0.35989	0.40999	0.00024	0.21256	0.12173	-0.1096	-0.1593	-0.1856
NM_001007645	MGC95152	-0.1311	-0.2885	-0.2814	0.08152	0.37643	0.49689	-0.3014	0.34751	-0.384	-0.5914	-0.5503	-0.0833	-0.2726	-0.3996
NM_001005546	MGC95155	0.12139	-0.1432	-0.1105	-0.1271	-0.0403	0.00999	0.07911	0.04196	-0.0819	-0.01	-0.1302	-0.0126	0.01774	0.02001
NM_001005546	MGC95155	0.0868	-0.0105	-0.117	-0.0839	-0.0718	-0.1062	-0.0161	-0.0865	-0.1726	-0.2545	-0.1412	-0.1226	-0.086	-0.0462
NM_001005552	MGC95208	0.05593	-0.1496	0.03134	-0.6395	-0.4984	-0.627	0.06203	-0.2452	0.11944	-0.1289	-0.1875	0.24281	0.04427	0.34894
NM_001005532	MGC95210	0.11684	0.25805	0.20279	0.31786	0.27059	0.01002	0.24891	0.13027	0.10961	0.18297	0.42313	-0.073	-0.0659	0.00777
NM_131904	Mgea5	0.13131	0.74631	-0.4633	0.92496	0.44611	-0.1585	-0.0541	0.50662	0.17503	0.49479	0.13594	-0.3936	0.02446	-0.0765
NM_001106734	Mgea6_predicted	-0.8018	-0.9367	-0.2569	-0.5962	-1.1722	-1.1446	-0.5078	-1.2409	-0.8856	-0.6985	-0.6081	-0.512	-0.4872	-0.5526
NM_001106734	Mgea6_predicted	0.01385	0.02714	0.05234	0.11502	0.01602	0.2088	0.00776	-0.0165	0.08069	0.05095	0.02422	0.20283	0.05671	0.0157
NM_022393	Mgl1	0.98281	0.21114	0.01667	0.23421	0.14256	0.09331	0.20363	0.28911	0.08082	0.26168	0.43936	0.40221	0.50164	0.29226
NM_138502	Mgll	-1.7173	-1.0265	-1.653	-1.4791	-1.1399	-1.0225	-1.2732	-1.384	-1.4341	-1.3595	-1.4918	-1.2914	-1.4451	-1.384
NM_012861	Mgmt	0.18793	-0.0531	0.06333	0.13246	-0.0129	-0.081	0.03606	0.06775	0.16334	-0.0074	0.23011	-0.095	0.15091	0.00392
NM_012862	Mgp	-1.1515	-0.0471	-1.3597	-1.6672	-1.5916	-1.3639	2.2462	-1.5817	-0.3272	-0.0493	-0.137	-1.2038	-1.6099	-1.6739
NM_001013964	Mgrn1	-0.2765	-0.318	-0.2585	-0.2963	-0.1507	-0.0815	-0.2073	-0.0249	-0.3982	-0.3754	0.05676	-0.6438	-0.0636	-0.0273
NM_134349	Mgst1	2.7371	2.5904	0.76133	0.95286	2.0769	2.1731	1.7421	2.1469	2.615	2.6326	2.591	1.1287	0.58518	0.46551
NM_001106430	Mgst2_predicted	-0.04	0.30354	-0.057	0.04173	-0.4338	-0.2369	0.42163	-0.4517	0.25277	0.35413	-0.0494	0.13832	0.18293	0.15422

XM_001076104.1	Mgst3_predicted	0.90176	0.63791	-1.0348	0.12032	0.38605	0.61591	0.12739	0.36779	0.50556	0.61524	0.61247	0.05766	-0.1782	-0.0921
NM_030852	Mia1	-0.1057	-0.0617	0.12395	0.30318	0.00863	0.17604	-0.0831	0.0273	0.04181	-0.0295	-0.0472	0.52818	0.28742	-0.0731
NM_001107405	Mib1_predicted	-0.3439	-0.2852	-0.0524	0.03756	-0.1553	-0.3738	-0.2311	-0.0182	-0.2713	-0.3031	-0.3415	-0.4261	0.23894	0.0151
NM_001106397	Mical1_predicted	-0.3124	-0.3691	0.34016	0.03068	0.07252	0.05376	-0.3082	-0.1751	-0.1666	-0.0216	-0.1141	0.01858	-0.2076	-0.1157
NM_182669	Micalcl	-0.2465	-0.3667	-0.3488	-0.2718	-0.195	-0.3416	-0.3412	-0.0755	-0.3102	-0.2677	-0.2972	-0.2445	-0.3198	-0.257
NM_022927	Mid1	-0.0506	-0.1071	-0.1511	-0.1187	-0.1746	-0.18	-0.1546	-0.1637	-0.0484	-0.1317	-0.096	-0.0587	-0.0149	-0.1952
NM_206950	Mid1ip1	-0.466	-0.7667	0.21288	-0.2392	-0.719	-0.768	-0.0526	-0.6197	-0.4444	-0.6017	-0.8617	-0.3776	-0.3009	-0.3174
XM_001053301.1	Mid2_predicted	0.00264	0.22741	-0.0522	-0.014	0.12762	-0.0632	0.05188	0.17574	0.03279	0.10912	-0.0983	-0.1253	-0.0195	0.13918
XM_001076784.1	Midn_predicted	-0.1595	0.02572	-0.0324	-0.0728	-0.0845	-0.0561	-0.0425	-0.079	0.14469	-0.0319	-0.0133	0.01229	0.07869	0.07438
XM_001076784.1	Midn_predicted	-0.1326	-0.1175	-0.2333	-0.2918	-0.125	-0.2251	0.16661	-0.0383	-0.0285	-0.2143	-0.2049	-0.0353	0.13432	-0.0088
NM_031051	Mif	0.65006	0.3363	0.52504	0.20413	0.3452	0.94804	-0.8725	0.32997	0.11008	0.45647	0.36435	0.26434	0.21532	0.13727
NM_001014122	Mif4gd	-0.3385	-0.5801	0.04022	-1.0263	-0.9126	-0.7681	-0.4774	-0.8877	-0.4041	-0.1062	-0.2613	-0.1033	-0.002	0.05909
NM_001011931	Mill1	0.01481	-0.0574	0.20857	-0.0702	0.01088	-0.0163	0.04889	0.24759	-0.0589	-0.0758	0.03515	0.04834	-0.0293	-0.0464
NM_001011931	Mill1	0.03846	0.0388	0.02055	0.08297	-0.0928	-0.1243	0.18285	-0.1527	0.00275	-0.0591	0.10566	0.05784	-0.0673	-0.0329
NM_001017468	Mill2	0.03956	-0.0906	0.01444	-0.131	-0.1736	-0.0437	-0.0765	0.11034	-0.1935	-0.0915	-0.1787	-0.1941	0.00211	-0.2248
NM_153309	Mina	-0.1294	-0.0924	0.39707	-0.09	-0.0642	-0.1879	-0.0096	0.00868	0.17539	-0.2072	-0.183	0.01502	0.0784	0.08334
NM_019263	Minpp1	-0.1751	-0.0634	-0.4347	-0.4218	-0.1662	-0.2539	-0.2206	-0.2211	-0.17	0.083	-0.19	-0.3014	0.06961	-0.1353
NM_145771	Miox	0.02969	-0.0563	-0.018	-0.0369	-0.0327	-0.012	-0.0447	-0.0218	-0.0972	-0.0122	0.00223	0.0906	-0.1105	-0.0364
NM_001105719	Mip	0.04037	-0.0767	-0.0387	0.08302	0.03391	0.03566	0.16173	-0.0072	0.1044	-0.1152	-0.0556	0.17591	-0.0193	-0.0029
NM_031052	Mipep	0.29539	0.41247	-0.1316	-0.4608	0.39096	0.3016	-0.0984	0.255	0.37879	0.327	0.27997	0.18951	-0.222	-0.1417
NM_012863	Mist1	0.01153	-0.0961	-0.0319	-0.0918	-0.0345	-0.0015	-0.0347	-0.0783	-0.1094	-0.0413	-0.0752	0.12025	0.05259	-0.123
NM_001009714	Mitd1	0.03386	0.25099	0.41343	-0.0172	0.03449	0.32925	0.42767	0.13008	0.08344	0.33288	0.16039	0.44463	0.34183	0.3269
NM_001105979	Mixl1_predicted	-0.0206	0.08006	0.03665	0.0826	-0.0084	0.14176	0.01286	0.09065	0.13863	0.07386	0.10747	0.22891	0.17995	0.16518
XR_008376.1	Mizf_predicted	-0.3322	-0.1175	0.16175	0.0845	-0.1855	-0.6411	0.20653	-0.0548	-0.4036	-0.536	0.0648	-0.4703	-0.2115	0.08799
NM_134399	Mk1	-0.3598	-0.5665	0.05251	-0.8342	-0.2306	-0.2275	-0.7014	-0.3795	-0.3454	-0.4121	-0.3121	-0.0425	0.37633	0.28631
XM_001056221.1	Mki67_predicted	-0.0402	-0.3657	-0.3267	-0.3964	-0.4779	-0.7681	-0.4939	-0.331	-0.104	-0.5517	-0.4137	-0.0155	0.82787	0.73299
NM_139186	Mki67ip	-0.2327	0.13236	-0.0645	-0.0675	-0.0734	0.09753	-0.0457	-0.2684	-0.1562	-0.1992	0.05468	0.44512	0.22143	0.02397
NM_001008353	Mkks	-0.5845	-0.4309	-0.1034	-0.5444	-0.6963	-0.7783	-0.2064	-0.7565	-0.2158	-0.2727	-0.1118	-0.1515	0.01426	-0.0246
XM_001077101.1	Mkl1_predicted	-0.0435	0.09353	0.34919	-0.1318	0.10072	-0.0423	-0.0532	0.06552	0.28257	0.35123	0.36923	0.31167	0.16341	0.15658
NM_031359	Mkln1	0.20878	0.12593	-0.0904	0.56488	0.90722	0.93871	0.2968	0.87242	-0.0503	-0.0426	0.11145	-0.2428	-0.0656	0.01239
NM_001011985	Mknk2	-0.4726	-0.0464	0.21627	-0.5178	-0.7153	-1.0882	-0.4212	-0.8505	0.00966	-0.1862	-0.2048	0.14541	-0.5338	-0.44
NM_001008314	Mkrn2	-0.0651	-0.1257	-0.211	-0.2702	-0.2156	-0.3845	0.04412	-0.1739	0.04993	-0.0142	0.05102	-0.39	-0.2646	-0.3915
XM_001057262.1	Mkrn3_predicted	-0.3171	-0.3339	-0.0264	-0.098	-0.2161	-0.2711	-0.1223	-0.0066	-0.3259	-0.5201	-0.2949	-0.1791	-0.2625	-0.1848
NM_001034917	Mks1	0.04779	0.02032	-0.094	-0.0438	0.12246	0.12638	-0.0751	-0.0549	-0.2291	-0.0412	0.33241	-0.0834	-0.0072	-0.0048
NM_001106348	Mlana_predicted	0.17189	-0.0357	-0.0223	0.29469	0.33742	0.44567	0.27911	0.28836	0.22118	0.05505	0.17479	0.13135	0.24263	0.17044
NM_001108105	Mlc1_predicted	-0.14	0.06578	-0.1077	-0.1668	-0.1047	-0.0364	-0.0243	-0.042	-0.0791	0.01876	-0.1089	-0.0155	-0.138	-0.0907
NM_020104	Mlc3	1.0921	0.14332	0.19194	0.27222	0.01277	0.18395	0.43785	0.44487	-0.0764	-0.0335	0.03939	0.1297	0.16655	0.06695
NM_001107680	Mlf1_predicted	-0.0801	-0.7092	-0.1417	-0.582	-0.3121	-0.3327	-0.5197	-0.4561	-0.5042	-0.7139	-0.5535	-0.3665	0.24266	-0.0143
NM_001107889	Mlf2_predicted	-0.0943	0.06517	-0.4504	-0.4571	0.12852	-0.6421	-0.2521	-0.4043	-0.4981	-0.3629	-0.1285	-0.5367	0.03092	0.15291
NM_001108043	Mlh3_predicted	0.24766	0.26134	0.39507	0.42857	0.09342	0.23067	0.04872	0.36877	0.31439	-0.0614	0.12127	0.02925	0.09779	0.34093
NM_001108139	Mll_mapped	-0.439	-0.3319	0.44625	0.06142	-0.5002	-0.5715	-0.0014	-0.4558	-0.505	-0.0289	-0.3767	-0.4497	-0.3905	-0.4622
XM_001062568.1	Mll2_predicted	-0.1009	-0.0938	0.09785	-0.1831	-0.173	-0.1577	0.065	-0.1857	-0.1892	-0.2263	0.04496	-0.1294	0.03597	0.01666
XM_231287.4	Mll5	-0.4833	0.18411	0.2123	0.11682	-0.9755	-0.8371	0.29549	-1.0712	-0.2493	-0.0599	-0.1442	-0.1807	-0.8445	-0.816
NM_001012162	Mllt10	0.03156	-0.1219	0.10907	0.02407	-0.1294	0.159	-0.0371	-0.1382	0.09819	0.03125	-0.2065	0.13121	0.02964	-0.106
NM_001012162	Mllt10_predicted	0.10921	0.13709	-0.0276	0.00211	0.0895	0.11854	0.06154	0.23867	0.34472	-0.0655	0.14132	-0.0391	-0.098	-0.0583
NM_001013912	Mllt11	0.20771	-0.2808	-0.1087	-0.2671	0.05447	0.05215	-0.1481	0.30414	-0.1209	-0.1696	-0.2439	-0.1212	0.16245	0.24779
NM_001107206	Mllt2_predicted	-0.0094	0.22437	-0.019	0.10434	0.11793	0.10634	0.08201	0.0539	-0.0308	0.10608	0.01296	0.15251	0.04288	0.24807
NM_053718	Mllt3	0.05112	-0.4744	-0.4385	-0.3854	-0.1774	-0.5259	-0.3749	-0.1797	0.08433	-0.5461	-0.4052	0.08453	-0.1941	-0.0247
NM_013217	Mllt4	-0.4362	0.16186	-0.3503	-0.2339	-0.1517	-0.3409	-0.3388	-0.0358	-0.3566	-0.2971	-0.579	-0.6925	-0.1508	-0.5336

XM_239329.3	Mllt6_predicted	-0.2217	-0.2006	0.01144	0.16642	-0.1749	-0.2169	-0.05	-0.3498	-0.2072	-0.1423	-0.2085	-0.2867	-0.1358	-0.1526
NM_001012135	Mlph	0.28444	0.22244	0.12145	0.07892	-0.0304	-0.0045	0.09986	0.22776	0.30079	0.27213	0.2035	0.07676	0.11301	-0.038
NM_001034112	Mlx	0.10745	-0.0761	0.15155	0.54521	-0.2325	0.28537	0.02562	-0.2112	-0.2423	-0.1734	0.0697	0.07874	0.09912	0.3105
NM_053477	Mlycd	0.38834	-0.0578	0.11053	-0.3124	0.72927	0.72126	-0.1128	0.68425	-0.1325	0.07538	-0.1106	-0.2118	-0.4644	-0.0782
NM_001134495	Mlze_predicted	0.06107	-0.1289	-0.0444	-0.2176	-0.0488	-0.1426	-0.1022	-0.244	-0.1436	-0.2022	-0.0283	-0.0536	-0.2779	-0.1054
NM_001106174	Mmaa_predicted	-0.224	-0.4659	0.84399	0.54801	-0.125	-0.0303	-0.2237	-0.1143	-0.1452	-0.0956	-0.2768	0.24801	-0.1879	-0.1064
NM_001107962	Mmachc_predicted	-0.0035	0.17827	-0.1925	-0.4965	-0.1303	-0.1914	-0.2851	0.15312	0.07709	-0.0838	-0.2286	-0.3353	-0.2188	0.03347
NM_001037217	Mmd2_predicted	-0.1501	-0.2429	-0.0774	0.05669	-0.1511	0.2109	-0.2145	0.21229	-0.2572	#######	0.00704	-0.2791	-0.291	-0.1434
NM_012608	Mme	-2.4573	1.7269	-1.0049	-1.3056	-2.4602	-2.5933	-0.8897	-2.4131	1.3301	1.3709	1.1373	1.0318	-0.5105	-0.4012
NM_133514	Mmp10	0.48072	0.08462	0.01085	0.00182	1.8604	2.1384	-0.0078	1.8156	0.23131	-0.0245	0.16056	0.00205	-0.0786	0.02411
NM_012980	Mmp11	-0.4502	0.00312	-0.4	-0.453	-0.0026	-0.3056	-0.3996	-0.2637	-0.1621	0.03829	0.0621	-0.2524	-0.6559	-0.6239
NM_053963	Mmp12	-0.2107	-0.2362	-0.1181	-0.2045	-0.0144	-0.1431	-0.2288	-0.2052	-0.0398	-0.1828	-0.2194	-0.1894	-0.1578	-0.1631
NM_133530	Mmp13	-0.2062	-0.2623	-0.2046	-0.0102	-0.0884	-0.2138	-0.0528	0.05409	-0.1156	0.01643	-0.0469	0.3446	0.00945	-0.1702
NM_031056	Mmp14	-0.3504	0.13811	0.21834	-0.3477	-0.3999	-1.0127	0.2088	-0.5129	0.01934	0.26169	0.12373	-0.6032	-0.0179	0.25127
NM_001106168	Mmp15_predicted	0.01781	-0.1275	-0.0921	-0.0493	-0.0995	-0.0533	0.00406	-0.2309	0.02043	-0.1011	-0.1646	0.00039	-0.1333	-0.1967
NM_080776	Mmp16	-0.3076	-0.1081	-0.1761	-0.1753	0.14921	0.19247	-0.0547	-0.0515	-0.0158	-0.3269	0.04112	0.09251	-0.1817	-0.2332
NM_001105925	Mmp17_predicted	0.01506	-0.075	0.10564	0.11038	-0.0737	0.13473	-0.0619	-0.0213	-0.1528	-0.0931	0.02534	-0.0418	-0.0027	0.01808
NM_001107159	Mmp19_predicted	0.5833	0.64333	0.40285	0.27917	0.54238	0.42725	0.1407	0.37805	0.80657	0.64043	0.7678	0.82872	0.15716	0.34668
NM_001134530	Mmp1a_predicted	0.12003	0.14736	-0.0143	0.00703	0.08358	0.07294	0.12381	0.25954	0.08728	0.03352	0.15144	0.03306	0.0791	0.02313
XM_001072313.1	Mmp1b_predicted	0.12097	0.05642	0.22723	0.04443	0.01887	-0.0479	0.42173	-0.1012	-0.0249	-0.1548	-0.0346	-0.2031	0.23857	0.07799
NM_031054	Mmp2	-0.1563	-0.069	-0.0031	-0.2883	-0.0463	0.16963	0.0418	-0.2381	0.06619	0.10328	-0.2402	0.16089	0.08818	0.02571
NM_001106800	Mmp20_predicted	-0.1516	0.11331	-0.0949	-0.1102	-0.1838	-0.1468	-0.0686	0.05881	-0.0241	-0.1851	-0.0562	-0.2168	-0.1733	-0.1746
NM_001106308	Mmp21_predicted	-0.1022	0.15606	-0.1627	-0.0132	0.07164	-0.0757	-0.0528	-0.0157	-0.1523	0.0001	0.20958	-0.1897	-0.1035	-0.1505
NM_053606	Mmp23	0.08741	-0.0405	0.13333	-0.0106	-0.0338	0.06159	0.04499	-0.0334	-0.0159	0.04861	-0.0258	0.041	0.0082	0.07019
XM_001055465.1	Mmp25_predicted	0.15781	0.06171	0.18127	0.11179	0.13975	0.24179	0.03969	0.16606	0.14055	0.21978	-0.031	0.0651	0.1927	0.17371
NM_001106799	Mmp27_predicted	-0.0489	-0.0596	0.01933	0.0287	0.30204	0.05906	0.16471	-0.0092	0.00193	0.02219	0.03518	-0.0407	-0.0132	0.05078
NM_001079888	Mmp28_predicted	0.02237	-0.0118	0.04224	0.0401	0.00537	0.10096	-0.044	-0.124	-0.071	-0.0834	-0.0069	-0.0234	0.02094	-0.0629
NM_133523	Mmp3	0.20609	-0.0528	-0.0827	0.10914	1.3333	1.0088	0.03671	1.2648	-0.1315	-0.0131	-0.0897	-0.0745	-0.0093	-0.0675
NM_012864	Mmp7	-0.0194	0.07248	-0.2216	-0.0637	-0.2916	-0.1161	-0.1718	-0.1315	-0.0795	-0.1754	-0.1674	0.03054	-0.1689	0.05464
NM_022221	Mmp8	-0.0362	0.01039	-0.0453	0.06274	-0.0223	0.03816	0.20885	-0.0198	0.0828	0.1267	-0.0399	-0.0257	-0.0977	-0.1338
NM_031055	Mmp9	-0.5321	-0.4244	-0.3609	-0.3889	-0.451	-0.2885	-0.2933	-0.3386	-0.4983	-0.4853	-0.4736	-0.2613	-0.3947	-0.4518
NM_001107298	Mmrn2_predicted	-0.1401	-0.2465	0.41237	-0.1251	0.18356	-0.1717	-0.0564	0.59531	-0.0102	0.1767	0.25555	0.6613	0.4275	0.2734
NM_001107839	Mnab_predicted	-0.067	0.01039	0.20558	0.26078	0.02435	-0.1321	0.19296	0.03594	0.11758	0.10662	0.0164	0.24195	0.20454	0.20594
NM_153472	Mnat1	-0.0398	0.04397	-0.2611	0.73308	0.24208	0.29527	0.46552	0.06892	0.27098	0.19393	0.20768	0.08271	-0.1637	-0.2527
NM_001012029	Mnda_predicted	-0.1292	-0.0154	-0.0601	-0.0365	-0.1397	-0.0798	-0.1361	0.04283	-0.0781	-0.2447	-0.0889	-0.1267	0.02302	-0.1574
NM_001007752	Mns1	0.03996	0.01358	0.34256	0.37171	0.36843	0.14538	-0.0121	0.22145	0.14612	-0.1286	-0.111	0.21961	0.08165	0.22705
NM_001105807	Mnt_predicted	-0.1449	-0.1844	0.28038	-0.0536	-0.4379	-0.377	0.11046	-0.3685	-0.1377	-0.2201	-0.0928	-0.2481	-0.4918	-0.2993
NM_001108357	Mobkl1a_predicted	-0.0389	-0.078	-0.1129	-0.0405	0.01442	-0.0998	-0.0152	-0.0639	-0.1573	-0.0202	-0.0754	-0.0213	-0.0583	-0.0908
NM_001108734	Mobkl2b_predicted	0.09049	0.21014	0.05836	-0.1575	0.06454	-0.0956	0.05122	0.18841	0.32917	0.1466	0.10688	0.18326	0.36495	0.60964
NM_001107960	Mobkl2c_predicted	-0.2443	-0.1899	-0.1708	-0.059	-0.1955	-0.1173	-0.0778	-0.2102	-0.1733	-0.0655	-0.0605	0.13981	-0.133	-0.1573
NM_133528	Mobkl3	0.37246	0.43989	-0.3325	0.12896	0.53361	0.43542	0.25044	0.174	0.4221	0.40683	0.51347	0.31186	0.24857	0.32238
NM_001108425	Mocos_predicted	0.30723	0.70963	0.23086	0.54604	0.68384	0.65683	0.48221	0.62698	0.42132	0.59772	0.63426	0.18598	0.01273	0.10803
NM_001106881	Mocs1_predicted	-0.0232	0.16748	0.07447	0.45644	0.06198	0.11879	0.00414	0.27041	0.03542	0.22228	0.03793	0.18533	0.11063	-0.0298
NM_001162413	Mocs2	0.43816	0.32438	-0.0518	0.88986	0.62921	0.7603	0.19728	0.37849	0.43684	0.6366	0.34604	0.51648	0.13517	0.23118
NM_001107804	Mocs3_predicted	-0.0378	0.20194	0.29582	-0.1905	-0.0449	-0.1557	0.17048	-0.0912	-0.1146	0.09552	-0.0749	0.16981	0.16302	0.10648
NM_001107804	Mocs3_predicted	-0.1951	-0.0845	0.39362	-0.1077	-0.0485	-0.0652	0.15244	0.03736	-0.0624	-0.064	0.08207	-0.0678	0.09692	0.06527
NM_022668	Mog	0.07819	-0.1185	-0.2212	-0.1554	-0.1234	-0.0659	-0.2577	-0.1242	-0.0713	-0.2847	-0.2854	-0.0236	-0.2115	-0.1576
NM_001108803	Mogat1_predicted	-0.079	-0.1053	0.0102	-0.2087	0.02786	-0.1311	0.0014	-0.0626	-0.1709	-0.006	-0.2374	-0.3328	0.15714	-0.1074
NM_001126284	Mon1a_predicted	0.27336	0.10637	0.38633	1.1093	0.32644	0.55763	0.25874	0.36421	0.01563	0.06776	0.45147	0.13896	0.01816	0.08966

XM_001053843.1	Mon2	-0.3888	-0.0263	-0.1683	0.42598	-0.1456	-0.4839	0.15951	0.05289	-0.1184	-0.058	-0.0912	-0.2153	-0.0942	-0.32
NM_001107109	Morc3_predicted	-0.166	-0.0297	-0.1216	0.10283	-0.4586	-0.9514	0.93568	0.03885	-0.5752	-0.2356	-0.4676	-0.7982	-0.4253	-0.5559
XM_001053814.1	Morc4_predicted	0.04179	-0.0275	0.28185	0.31782	0.27558	-0.0017	0.02045	0.02691	0.03611	0.04251	0.05148	-0.0878	0.11836	-0.0074
NM_001007714	Morf4l2	-0.0529	-0.2734	0.15449	0.53993	-0.1463	0.04576	0.15326	-0.1174	-0.2597	-0.3554	-0.3899	0.03055	-0.1696	-0.1049
NM_001005544	Morn1	-0.16	0.03232	-0.2448	-0.1245	-0.0745	-0.0619	-0.161	-0.0211	-0.2307	-0.3743	-0.211	-0.1188	-0.2945	0.0504
NM_134410	Mosc2	-0.1395	-0.1913	-0.5345	-0.2574	-0.4783	-0.6974	-0.4813	-0.5906	-0.0838	-0.5301	-0.2085	-0.2398	-0.3008	-0.0812
NM_001025629	Mospd3	0.21624	0.15375	-0.2389	-0.122	0.00773	-0.0651	-0.1002	-0.0546	0.34434	0.30733	0.37942	-0.0562	-0.2017	-0.0779
NM_001108241	Mospd4_predicted	0.18508	0.00694	0.19985	0.15567	0.04022	-0.0409	0.18851	0.00268	0.15495	0.0808	-0.0186	0.0017	-0.0018	0.27862
NM_001106787	Mov10l1_predicted	-0.0174	-0.1843	-0.0704	-0.1357	0.02827	-0.0793	-0.0415	-0.0947	-0.1209	-0.0414	-0.0695	-0.0162	-0.1797	-0.152
XM_220095.4	Moxd1	-0.0134	0.04097	-0.0259	-0.0281	0.07886	0.00981	0.1032	0.06706	0.04028	0.06101	0.17171	0.06603	0.03031	-0.01
NM_001107011	Mpdu1	-0.0516	-0.0528	-0.5021	-0.1279	-0.1977	0.17892	-0.2821	-0.3988	0.08511	-0.0675	-0.0086	0.13405	-0.516	-0.5888
NM_019196	Mpdz	-0.1794	-0.1594	-0.3778	0.08342	-0.5874	-0.3254	-0.0205	-0.3437	-0.018	-0.2169	-0.2685	-0.0187	-0.4078	0.00434
NM_022617	Mpeg1	0.24172	0.0811	-0.024	0.13107	0.18199	0.1992	0.29294	0.37225	0.16719	0.09865	0.05783	0.01558	0.42581	0.27105
NM_012601	Mpg	0.17431	0.22991	0.4936	0.13881	0.21474	0.19887	0.38578	-0.0623	0.03529	-0.0058	-0.018	0.40168	0.0979	0.226
NM_001107609	Mphosph1_predicted	0.33433	-0.6	0.55758	-0.0682	-0.3779	-0.4422	-0.4126	-0.2142	-0.2582	-0.2786	-0.4452	0.08009	0.74957	0.55408
NM_001106340	Mphosph10_predicted	0.08513	-0.0061	0.20524	0.47973	0.30239	0.29896	0.33008	0.34671	-0.2662	0.07258	-0.0199	-0.1148	0.28373	0.35
NM_001129881	Mphosph6_predicted	-0.1302	0.11823	0.16714	-0.0489	0.28894	0.13253	0.08398	0.09054	0.13162	0.07646	-0.0525	0.15354	-0.0191	0.10636
NM_001004081	Mpi	0.88016	0.53292	0.25003	0.16794	1.2327	1.0322	0.37822	1.1503	0.26035	0.48657	0.45763	0.1718	0.41853	0.19888
XM_345572.2	Mpl_predicted	-0.0211	0.01222	0.1351	0.1428	-0.0331	-0.0979	0.11226	0.01946	0.1311	0.17991	-0.0543	-0.0946	0.1407	0.01363
NM_001107036	Mpo_mapped	0.01548	0.16203	-0.0845	0.0948	0.0071	-0.0927	0.03585	0.10854	0.02566	0.04536	-0.1141	-0.0126	-0.0449	0.2428
NM_053513	Mpp2	-0.0128	-0.0943	-0.0539	-0.0891	-0.1067	0.06957	-0.0272	0.11332	0.00186	-0.0373	-0.0665	-0.0796	-0.0194	-0.1166
XM_340911.3	Mpp3	-0.1505	-0.0452	-0.044	-0.09	-0.0742	-0.0807	0.0513	0.39264	0.02455	0.00039	-0.0373	0.07867	-0.0495	-0.0502
NM_021265	Mpp4	0.15507	-0.0023	-0.0433	0.37474	-0.0585	0.05935	0.16061	-0.0804	-0.0631	-0.0412	0.05618	-0.002	0.24079	0.09949
NM_001108034	Mpp5_predicted	0.03186	-0.1565	-0.1669	0.50912	0.31299	0.23009	-0.1201	0.35441	-0.0497	-0.1738	-0.3376	-0.2201	0.03949	0.03274
NM_001134982	Mpp6_predicted	-1.1823	-1.7857	-1.6549	-1.4481	-1.4359	-1.8177	-1.6752	-1.471	-1.6967	-1.9631	-1.9228	-1.4273	-1.4186	-0.8673
XM_001061023.1	Mpp7	0.36013	0.03438	0.08611	0.08056	0.04749	-0.0089	-0.1039	0.08061	-0.1496	0.12921	-0.0107	-0.0673	-0.1109	0.36775
NM_001108435	Mppe1_predicted	-0.153	-0.069	-0.1029	-0.8281	-0.789	-0.7262	0.20064	-0.7427	-0.0306	-0.019	-0.1887	-0.0039	-0.5777	-0.4729
NM_001130569	Mpped1_predicted	0.08503	0.06874	0.12534	-0.0063	0.13195	0.32963	0.08515	0.26955	0.06459	0.6013	0.2199	0.08076	0.12208	0.04533
NM_138843	Mpst	-0.46	-0.2378	-0.5172	-0.2157	-0.3605	-0.3287	-0.9035	-0.3545	-0.3225	-0.3209	-0.2288	-0.2296	-0.773	-0.4182
NM_001098240	Mpv17l_predicted	-0.0923	-0.3077	-0.2056	-0.1231	-0.2632	-0.0796	-0.1259	-0.0962	-0.1636	-0.1122	-0.2655	-0.1325	-0.2328	-0.1829
NM_017027	Mpz	-0.1016	-0.0009	-0.0015	-0.1633	-0.0366	-0.1126	-0.0723	0.16557	-0.0151	-0.0808	-0.1522	-0.168	-0.0613	-0.0592
NM_001007728	Mpzl1	-0.3782	-0.2502	-0.6491	-0.1182	-0.7632	-0.4528	-0.434	-0.7479	-0.5325	-0.2836	-0.2675	-0.0157	-0.4441	-0.5238
NM_001135834	Mrap_predicted	0.18371	0.0612	0.28116	-0.0768	0.14509	-0.0858	0.15905	0.09671	0.20175	0.01048	0.12128	0.09395	-0.0472	0.26617
NM_012981	Mras	-0.1223	0.22713	0.26123	-0.2235	-0.312	-0.1709	0.03979	-0.2778	0.25373	0.11661	-0.007	0.12942	0.20127	0.11228
NM_001106123	Mrc1_predicted	-0.0344	-0.0599	-0.1762	0.05235	-0.0247	0.08519	0.13021	-0.0301	-0.0263	0.11297	-0.1525	-0.085	0.20061	-0.0288
NM_001100837	Mrc2_predicted	0.07711	0.01333	-0.066	-0.0238	-0.1118	-0.0758	0.32871	0.07075	0.0763	0.41108	-0.1093	0.00163	-0.203	-0.1592
NM_022279	Mre11a	-0.0537	-0.2958	-0.0501	-0.1609	0.1358	0.34009	-0.3197	-0.0417	-0.2016	-0.1561	-0.4653	0.00025	-0.0215	0.02788
NM_145787	Mrga10	-0.1637	-0.2092	-0.1555	-0.1605	-0.1265	-0.2326	-0.253	-0.1199	-0.0428	-0.1585	-0.1534	-0.1042	-0.1452	-0.2069
NM_001002283	Mrgprb13	-0.0384	0.1662	0.02751	-0.039	0.19394	0.01321	0.1738	0.1721	-0.0268	-0.0054	0.04865	0.02874	0.01169	0.0196
NM_001002285	Mrgprb2	0.04981	-0.0952	-0.0826	0.18102	-0.1294	-0.1666	-0.0328	-0.1369	-0.111	0.04484	0.05097	-0.0726	0.08086	0.08136
NM_001002287	Mrgprb4	0.15253	0.11023	0.09745	0.21287	-0.0416	-0.0376	0.18089	0.10535	0.22859	-0.0369	-0.0739	-0.0836	0.00925	-0.0063
NM_001002284	Mrgprb5	0.09183	-0.0494	0.09028	-0.0512	0.01579	0.03984	-0.0827	0.12749	-0.032	-0.0075	0.0202	0.13572	0.12843	0.27471
NM_001001506	Mrgprd	-0.0883	-0.1162	-0.1356	-0.1432	-0.1601	0.27629	-0.1645	-0.0662	-0.1	-0.1172	-0.1851	-0.1351	-0.1258	-0.1968
NM_001002288	Mrgpre	0.32034	0.08015	-0.034	-0.2417	0.05377	0.18142	-0.0726	-0.112	-0.0859	0.25397	-0.1004	-0.0233	0.13176	0.1789
NM_153722	Mrgprf	0.83258	1.0433	0.84824	0.93912	0.85897	0.93574	1.1442	0.54262	0.8611	0.92789	1.1788	0.11714	0.31238	0.44572
NM_203470	Mrgprg	-0.0477	-0.1187	-0.0104	-0.1913	-0.1392	0.03457	0.03503	0.09561	-0.1479	-0.0962	0.05324	-0.2318	-0.1553	-0.1532
NM_001002281	Mrgprh	0.06789	-0.0842	-0.213	-0.096	-0.1392	-0.2075	-0.2345	-0.2045	-0.1632	-0.2043	-0.1554	-0.0431	-0.1304	-0.2023
NM_001002286	Mrgprx1	-0.0141	0.02965	0.04763	0.02879	-0.0255	-0.0295	0.01977	-0.0411	0.01151	0.03411	-0.0017	0.05107	-0.0231	-0.0088
NM_001002280	Mrgprx2	-0.1264	-0.0457	-0.1049	-0.0708	-0.063	-0.1071	-0.0657	-0.0168	0.07689	-0.0899	0.07087	-0.1317	-0.0944	-0.0656

NM_001034022	M-rip	-0.4347	-0.0249	-0.3551	-0.9235	-0.7865	-0.8673	-0.4784	-0.777	-0.2534	-0.2551	-0.1556	-0.2129	-0.1032	0.02066
NM_017343	Mrlcb	0.30736	-0.1668	-0.6256	0.54639	0.36181	0.31874	-0.2019	0.7727	0.28608	-0.3338	-0.0662	-0.3453	-0.4278	-0.4127
NM_001105997	Mrpl1_predicted	0.21466	-0.5193	-0.3715	-0.166	0.14341	0.42642	-0.1226	0.21069	0.05088	-0.1984	-0.1873	0.0191	-0.1946	-0.1333
NM_001006973	mrpl11	0.08629	-0.3238	-0.0619	-0.4391	0.0167	0.29002	-0.247	-0.0513	0.07336	-0.2572	0.06211	0.1427	0.21634	0.14987
NM_001006985	Mrpl13	0.73185	0.66786	0.43456	1.0436	0.65686	0.72959	0.52621	0.62954	0.84152	0.66769	0.64622	0.76125	0.50958	0.47627
NM_001106890	Mrpl14_predicted	0.09551	0.07883	0.20795	0.06775	0.08277	0.41552	-0.0889	-0.1031	-0.043	0.10199	-0.0945	0.58876	0.17424	0.04102
NM_001106633	Mrpl15_predicted	0.09505	-0.3224	-0.1033	-0.3026	0.18959	0.55464	-0.0837	-0.0794	-0.094	-0.1638	-0.0967	0.25473	-0.0143	-0.0373
NM_001009647	Mrpl16	0.33072	0.01582	0.12104	0.10831	0.19812	0.60026	0.09878	-0.0367	0.1553	0.06586	0.25414	0.18812	0.0779	-0.0345
NM_133539	Mrpl17	-0.1285	-0.456	-0.2694	-0.5983	-0.1328	-0.0289	-0.3877	-0.2293	-0.1021	-0.3598	-0.3331	0.15746	-0.1191	-0.0943
NM_001106205	Mrpl18_predicted	0.07706	-0.3868	-0.24	0.08509	-0.1312	0.05994	-0.1061	0.37375	-0.0295	-0.1881	-0.2536	-0.075	0.2532	0.07694
NM_001029898	Mrpl19	0.34613	-0.129	-0.1458	-0.0701	0.35563	0.6752	-0.1901	0.65226	-0.1137	0.03331	-0.1025	0.05139	0.13835	0.18553
NM_001107567	Mrpl21_predicted	0.17852	-0.3178	0.02339	-0.1225	0.14344	0.51301	-0.2277	0.21694	-0.142	-0.1545	-0.2362	0.25848	0.38376	0.20337
NM_001105781	Mrpl22_predicted	0.24644	-0.0245	0.18186	0.63021	0.48979	0.77903	0.24244	0.414	0.29118	0.17047	0.15926	0.39038	0.15564	0.24661
NM_022529	Mrpl23	-0.0471	-0.2747	-0.1579	-1.0732	-0.282	0.22411	-0.3339	-0.4507	-0.1269	-0.1912	-0.273	0.49051	-0.0411	-0.2279
NM_001007637	mrpl24	0.59441	0.15239	0.4086	1.3258	0.63312	0.74436	0.57764	0.62317	0.22155	0.24143	0.49956	-0.21	-0.1013	-0.009
NM_001105831	Mrpl27_predicted	0.45816	0.10396	0.45854	0.13574	0.3986	0.74421	0.14658	0.31112	0.26863	0.24228	0.31014	0.87826	0.598	0.77952
NM_001106852	Mrpl3_predicted	0.38314	0.29638	0.11181	-0.0262	0.57088	0.36359	0.10163	0.29543	0.15064	0.1125	-0.021	0.23441	0.28094	0.37051
NM_001106903	Mrpl30_predicted	-0.1633	0.15489	-0.1677	-0.2102	0.07119	0.17646	-0.136	-0.0872	-0.172	0.0084	-0.0969	-0.1598	-0.1264	-0.0615
NM_001106116	Mrpl32_predicted	-0.1787	-0.0077	0.18503	0.28984	-0.017	0.28763	0.20906	0.13995	0.14364	-0.017	0.05988	0.3002	0.11364	0.10006
NM_001006965	Mrpl34	-0.141	0.12164	0.00321	-0.1858	-0.4822	0.16696	-0.2117	-0.3647	0.40019	0.22767	0.24711	0.67991	-0.087	-0.0689
NM_001106596	Mrpl35_predicted	0.79992	0.06798	0.19879	0.34701	0.86492	0.89138	0.22398	1.0328	0.15797	0.00272	0.20408	0.31331	0.51987	0.43526
NM_001108879	Mrpl36_predicted	-0.3744	-0.1251	-0.3694	-1.0769	-0.5086	-0.3129	-0.5388	-0.7505	0.33424	-0.0018	0.03911	0.39242	-0.009	0.15277
NM_001004235	Mrpl37	0.06278	-0.3822	-0.0462	-0.3862	0.28958	0.19427	-0.2099	0.09344	-0.2608	-0.234	-0.2404	-0.157	-0.0533	-0.0606
NM_001108754	Mrpl4_predicted	0.13108	0.12262	0.08568	-0.3426	0.02553	-0.0809	-0.0672	0.00961	0.02808	-0.2204	-0.2311	-0.0028	0.08454	0.05148
NM_001024865	Mrpl40	0.37591	0.25464	0.36239	-0.0149	0.48929	0.93394	0.19411	0.37653	0.51944	0.34853	0.28746	0.7156	0.40464	0.38506
NM_001013426	Mrpl41	0.31873	-0.0833	0.57949	0.17624	0.41274	0.74912	0.14616	0.48442	0.15397	0.11332	0.08675	0.38141	0.28013	0.35996
NM_001106782	Mrpl42_predicted	0.27777	0.39555	0.51863	0.16294	0.51079	0.49707	0.19397	0.5677	0.77265	0.36961	0.6032	0.67148	0.98414	0.94475
NM_001107598	Mrpl43_predicted	0.08886	-0.0652	-0.1221	0.05548	0.25303	0.47281	0.09802	0.15106	-0.1856	-0.1075	-0.0929	0.17491	-0.185	-0.148
NM_001031650	Mrpl44	0.22531	0.17668	0.05037	0.19042	0.34169	0.29108	0.38811	0.41726	0.01352	0.07831	0.15204	0.06911	0.26007	0.29039
NM_001105834	Mrpl45_predicted	0.94766	0.32592	0.64669	1.1337	1.015	1.0113	0.30062	0.95201	0.13633	0.39954	0.37604	0.3426	0.81911	0.88604
NM_001013068	Mrpl46	0.25311	0.01502	0.26288	0.41893	0.26916	0.57663	0.0311	0.12205	0.1095	-0.0833	0.04306	0.22328	0.03707	0.07347
NM_001037183	Mrpl47	0.46749	0.15145	0.11332	0.9055	0.91268	1.1407	0.58765	0.92435	0.12107	0.16348	0.31704	0.08344	0.46403	0.62046
NM_001106282	Mrpl48_predicted	-0.0964	-0.1978	-0.4487	-0.3668	-0.3153	-0.1887	-0.4481	-0.4493	0.25021	-0.1383	0.26864	0.16883	0.20255	0.02091
NM_001047883	Mrpl49	0.19654	0.11014	-0.0459	-0.5327	0.13301	0.23306	-0.0016	-0.0503	0.10104	0.29978	0.00294	0.12873	0.66488	0.54133
NM_001108665	Mrpl50_predicted	0.19467	0.16124	0.18113	0.00653	0.42278	0.68947	-0.0091	0.38884	0.42143	0.16771	0.20133	0.34874	0.50018	0.51209
NM_001106621	Mrpl51_predicted	0.37604	-0.2042	0.16596	0.02388	0.0612	0.3934	-0.2434	0.17601	-0.0932	-0.3515	-0.1172	0.0849	0.23124	0.32188
NM_001108375	Mrpl52_predicted	0.30235	0.12724	-0.1883	-0.1552	-0.0224	0.30044	-0.122	-0.0957	-0.2176	-0.116	-0.0679	0.12917	-0.2945	-0.2502
NM_001108635	Mrpl53_predicted	0.52848	0.04243	0.46508	0.76957	0.48036	0.84256	0.28525	0.23622	-0.007	0.1143	0.09635	0.10336	-0.1236	0.01008
NM_001106770	Mrpl54_predicted	-0.5922	-0.8065	-0.1215	-0.6769	-0.7943	-0.5337	-0.6585	-1.033	-0.5546	-0.6157	-0.5216	-0.0303	-0.2264	0.08129
NM_001105782	Mrpl55_predicted	0.31839	0.01243	0.13195	0.35003	0.30174	0.8326	-0.2197	0.44411	0.01726	0.04286	0.13993	0.18239	0.31612	0.2848
NM_001007696	mrpl9	0.54718	0.1671	-0.1334	0.37266	0.17681	0.06406	-0.1594	0.26161	0.03379	-0.0477	0.28911	-0.2307	-0.3036	-0.1908
NM_001008859	Mrps10	0.40363	-0.2492	-0.349	-0.6392	0.51864	0.25638	-0.1816	0.53223	-0.0685	-0.3165	-0.1788	-0.5301	-0.2638	-0.0536
NM_001106239	Mrps12_predicted	-0.1815	-0.4032	0.14038	-0.1413	-0.4971	-0.071	-0.4922	-0.6152	-0.0723	-0.2967	-0.1627	0.21538	0.21565	0.27214
NM_001105963	Mrps14_predicted	0.27974	0.42661	-0.129	-0.2936	-0.2117	-0.1683	0.25725	-0.1196	0.15221	0.38173	0.15636	0.25553	0.39462	0.28826
NM_001007653	Mrps15	-0.1049	0.19953	0.16557	0.07412	-0.1305	-0.0139	-0.0203	0.05162	-0.0156	-0.035	-0.1121	0.20925	-0.2466	0.20155
NM_001105923	Mrps17_predicted	-0.0372	0.04993	0.06972	-0.0148	-0.1408	0.21272	0.23564	-0.2127	0.26059	0.16571	0.13642	0.74244	0.21204	0.24047
NM_198756	Mrps18a	0.54721	0.02181	0.42778	0.34373	0.41603	0.88741	0.02893	0.22187	0.24284	0.07595	0.18837	0.67545	0.42594	0.42915
NM_212534	Mrps18b	-0.1704	-0.5229	-0.1467	0.22847	-0.1197	0.12661	-0.3865	-0.1052	-0.2194	-0.2453	-0.3434	-0.1379	-0.0331	-0.036
NM_001105996	Mrps18c_predicted	0.59493	-0.1396	0.59075	-0.0999	0.53965	0.62238	0.27041	0.30772	0.17879	0.09909	0.24	0.42156	0.34384	0.49167

NM_001108576	Mrps2_predicted	-0.5637	-0.295	-0.0083	-0.3554	-0.0897	-0.4305	0.07479	-0.4868	-0.4315	-0.3123	-0.096	-0.4108	-0.0186	-0.0116
XM_344706.2	Mrps21_predicted	-0.064	0.25195	-0.1099	-0.132	0.11654	-0.1194	-0.1002	0.07625	0.15898	-0.0319	0.08989	0.34309	-0.01	0.13946
NM_001108289	Mrps23_predicted	0.66955	-0.0385	0.14799	0.67026	0.78611	1.1403	0.25716	0.70605	0.38221	0.23795	0.43697	0.73683	0.42513	0.49833
NM_001025408	Mrps25	-0.7634	0.01418	0.17071	0.12089	0.15815	0.6451	-0.1609	-0.1795	0.0361	0.20926	0.28119	0.34227	0.13621	-0.064
NM_001013206	Mrps26_predicted	0.06086	-0.1865	-0.0806	-0.1613	0.13011	0.6068	-0.2617	0.00959	-0.1206	-0.1068	0.07779	0.21441	-0.1126	-0.1778
NM_001108543	Mrps27_predicted	0.37181	-0.1656	0.35363	0.30832	0.3272	0.31668	0.05711	0.13035	0.02232	-0.0427	0.00269	-0.1658	0.30859	0.42164
NM_001106412	Mrps30_predicted	-0.0995	-0.0149	0.21244	0.69374	0.29918	0.32873	0.38781	0.32725	-0.2046	0.07344	-0.0249	-0.2119	0.36471	0.10064
NM_001106091	Mrps31_predicted	0.18901	0.21577	0.07611	0.02612	0.02992	0.01383	0.23447	0.11389	-0.0164	-0.0043	0.22949	0.03947	0.07384	-0.0098
NM_001047863	Mrps33_predicted	0.40955	0.0112	-0.1678	-0.1968	0.1993	0.27083	-0.1824	0.0281	0.20569	0.22922	0.26812	0.26072	0.11935	-0.0865
NM_001105771	Mrps34_predicted	-0.0873	-0.3639	0.23904	0.14088	0.18753	0.56496	0.07015	0.04517	-0.2293	-0.3637	-0.2295	0.15123	0.29573	0.37452
NM_001106628	Mrps35_predicted	0.05731	-0.9799	-0.9102	0.09314	-0.4157	-0.0416	-0.7756	-0.4759	-0.9066	-0.9219	-0.8237	-0.7196	-0.919	-0.8795
XM_215468.3	Mrps36_predicted	0.52443	0.32269	0.09403	0.30685	0.43642	0.73561	0.23764	0.42687	0.60125	0.35106	0.57177	0.38573	0.08963	0.157
NM_001106505	Mrps5_predicted	0.20872	-0.0655	0.10534	0.08437	0.08107	0.49893	0.06636	-0.1941	-0.0824	-0.0105	-0.1117	0.3262	0.13778	0.13993
XM_001081692.1	Mrps7	-0.1811	-0.1778	0.14094	-0.6161	-0.0971	0.18762	-0.1781	-0.2177	0.12094	0.25251	0.08621	0.31889	0.17968	0.22151
NM_001100549	Mrps9	0.22138	0.10698	-0.4351	0.36172	0.15604	0.13639	-0.1709	-0.0067	-0.0207	0.02035	0.12699	-0.158	-0.2786	-0.2708
NM_001008354	Mrrf	0.01849	0.13	-0.3015	-0.4424	-0.121	-0.3532	0.00721	-0.3252	-0.1078	-0.0775	-0.0088	-0.1716	-0.2926	-0.2781
NM_024001	Mrs2	-0.2593	-0.5086	0.16933	0.57716	-0.0956	-0.0567	-0.207	-0.0137	-0.2837	-0.4352	-0.1885	-0.1915	0.1855	0.03579
NM_001105211	Mrvi1_predicted	0.28249	0.19643	0.22205	0.25904	0.28155	0.28593	0.0456	0.21881	0.18304	0.23688	-0.0763	0.28285	0.06677	0.08266
NM_001107590	Mrvldc1_predicted	-0.8413	-0.4911	-1.0288	-1.8944	-1.0147	-1.0641	-1.1364	-0.7816	-0.4312	-0.3827	-0.4761	-0.423	-0.5864	-0.7563
NM_001107590	Mrvldc1_predicted	-1.1611	-0.8099	-1.1553	-1.9925	-1.3212	-1.1813	-1.1928	-1.1785	-0.7274	-0.6938	-0.5816	-0.5487	-0.7481	-0.7514
NM_001107578	Ms4a1_predicted	0.19834	-0.0011	0.23103	0.02282	0.08595	0.16375	0.12388	0.01739	0.06039	0.09198	0.12846	0.04552	-0.053	0.25877
NM_001106336	Ms4a10_predicted	0.07296	0.03	-0.0211	-0.0434	-0.0337	-0.0304	-0.0125	0.03418	0.07345	0.0773	-0.0101	0.07196	0.16526	-0.0282
XM_001075502.1	Ms4a11_predicted	0.47771	0.43789	0.26278	0.09378	0.57702	0.66685	0.19937	0.74294	0.42093	0.31676	0.60044	0.0839	0.98803	0.79333
NM_001106337	Ms4a12_predicted	0.0854	0.10956	0.07212	-0.0898	0.03555	-0.1112	0.00929	0.04925	0.05212	-0.0182	-0.0938	-0.014	-0.0088	-0.0975
NM_012845	Ms4a2	0.09424	-0.0203	-0.0366	0.02051	-0.0765	-0.0313	-0.128	-0.0997	-0.1028	0.00983	-0.0199	-0.0841	0.11055	-0.0307
XM_001075551.1	Ms4a3_predicted	0.03605	0.13639	0.10156	0.05038	0.1952	0.02024	0.18097	0.08327	0.07918	-0.0017	-0.0202	0.00134	-0.0165	0.01854
XM_001075551.1	Ms4a3_predicted	-0.1433	-0.1866	-0.1866	-0.2366	-0.1703	-0.1978	-0.1892	-0.208	-0.0428	-0.241	-0.1333	-0.1708	-0.0511	-0.2515
XM_001075321.1	Ms4a4a_predicted	0.10651	0.09923	0.02919	0.03931	0.15316	0.28052	0.10044	0.11975	0.04398	0.13128	0.16287	0.39564	0.10181	0.01554
XM_001075462.1	Ms4a6a_predicted	0.2419	0.2177	0.07718	0.09119	0.00937	-0.0771	0.06517	-0.1024	0.3684	0.34067	0.19647	0.0115	0.16818	0.0791
XM_001075462.1	Ms4a6a_predicted	-0.1371	0.24249	-0.0964	0.11762	-0.0854	0.00758	0.27884	-0.3234	0.26147	0.13318	0.19928	0.28901	0.3402	0.21564
NM_001106338	Ms4a7_predicted	0.03731	-0.179	-0.0176	0.02112	-0.1013	-0.0793	-0.0016	-0.0104	0.0905	0.15826	-0.0184	-0.0751	-0.0106	0.04159
NM_001108519	Ms4a8b_predicted	-0.3329	-0.2422	-0.0557	0.17961	-0.0142	-0.0079	0.10528	-0.0971	0.02008	-0.1386	-0.1604	0.18684	-0.0374	-0.3819
XM_001063707.1	Msc_predicted	-0.0414	0.10588	0.00468	0.03244	0.0101	0.00484	0.00954	0.14408	0.13044	0.01292	0.24495	0.19263	0.16429	-0.0876
NM_031058	Msh2	-0.5339	-0.7275	0.18182	-0.6673	-0.7905	-0.6168	-0.6141	-0.8725	-0.3842	-0.5956	-0.4396	-0.1955	0.06735	-0.0697
NM_212536	Msh5	-0.12	-0.3813	0.02854	-0.0988	-0.3816	-0.4152	-0.2232	-0.284	0.00629	-0.0384	-0.3226	-0.2897	-0.3602	-0.3141
XM_345633.2	Msh6_predicted	-0.5165	-0.6629	-0.0146	-0.2598	-0.5356	-0.6983	-0.6696	-0.465	-0.5035	-0.5431	-0.5193	-0.4932	-0.1032	-0.2598
NM_148890	Msi1	-0.1046	-0.084	0.13132	-0.1618	-0.0377	0.05503	-0.1246	-0.0099	-0.0354	0.09948	0.09038	0.32153	-0.01	0.16228
XM_236567.4	Msl2l1_predicted	-0.7478	-0.3568	-0.2784	-0.4494	-0.7038	-1.0679	-0.1579	-0.5442	-0.327	-0.4941	-0.3483	-0.613	-0.5247	-0.3433
NM_031658	Msln	0.84961	1.1225	0.37876	1.2379	0.55941	0.40338	1.1568	0.48701	0.84692	1.194	1.0557	-0.2404	0.06752	0.05462
NM_019188	Msmb	-0.0383	0.05108	-0.0196	0.14727	0.16924	0.12901	0.0303	-0.0725	0.09811	0.13481	0.0995	0.07523	0.01449	0.02935
NM_030863	Msn	0.6279	1.1187	-1.4288	-0.7816	0.69911	-0.6111	-0.1372	0.89205	0.58261	1.0791	0.20055	-0.6643	0.92822	0.76981
NM_001107702	Msr2_predicted	0.034	0.13338	-0.0496	0.24892	-0.0014	0.20246	-0.0052	0.25808	0.18204	0.11637	0.08097	0.18799	0.26237	0.0388
NM_053307	Msra	-0.1761	0.0734	-0.0645	0.16111	0.06958	0.47663	-0.0648	-0.1831	0.03931	-0.021	0.1261	0.58393	0.19735	0.14581
NM_001031660	Msrb2	0.03304	-0.2297	0.16252	0.02829	-0.0964	-0.0718	0.16005	-0.0158	0.17536	0.06826	-0.0836	0.47843	0.26117	0.12124
NM_001007678	Mss4	-0.0365	-0.127	-0.0312	-0.4684	-0.6164	-0.3652	-0.0979	-0.7076	0.12079	0.08392	-0.2022	0.60311	0.45815	0.48877
NM_024352	Mst1	0.18702	0.15713	0.35748	0.21868	0.2059	0.08633	0.35925	0.08892	0.15595	0.12604	0.01895	0.11088	0.28153	0.25322
NM_001106855	Mst1r_predicted	-0.1422	0.07737	-0.0847	-0.0635	-0.0657	-0.0486	0.04456	-0.0561	-0.042	-0.1222	-0.039	-0.0663	0.0563	-0.1109
NM_019151	Mstn	-0.1713	0.01518	-0.2374	-0.211	-0.1447	-0.2229	-0.2455	-0.1653	-0.2081	-0.0822	-0.2051	-0.2819	0.16127	-0.0334
NM_001106443	Msto1_predicted	-0.5113	-0.1028	0.40946	0.07686	-0.5114	-0.4757	0.23883	-0.6946	-0.2633	-0.0947	-0.1184	-0.1348	-0.482	-0.3625

NM_001106443	Msto1_predicted	-0.344	-0.2703	0.23598	-0.2317	-0.4292	-0.3828	-0.3472	-0.5653	-0.3224	-0.1702	-0.5687	-0.0887	-0.2444	-0.1755
NM_031059	Msx1	-0.0186	-0.0123	0.00427	-0.1212	0.21849	0.00588	-0.1504	0.0456	-0.1398	-0.2015	-0.1325	-0.0256	0.06539	0.12379
NM_012982	Msx2	-0.3426	-0.1889	-0.2753	-0.1084	-0.2671	-0.1107	-0.1832	-0.1032	-0.3554	-0.3167	-0.4253	-0.1018	-0.2212	-0.3224
NM_053712	Msx3	-0.1273	-0.0915	-0.0577	-0.1613	-0.1092	-0.0187	-0.0721	0.1352	-0.0067	-0.1251	-0.0462	-0.1522	-0.1655	-0.088
NM_053712	Msx3	0.09446	0.10048	0.03764	0.11688	0.24876	0.05059	0.17007	0.08787	0.04683	0.11126	0.19641	0.09616	0.19786	0.12131
NM_138826	Mt1a	1.2044	0.68554	1.1789	0.16339	-0.4089	0.44433	0.2082	-0.4019	0.87614	0.7931	1.089	1.6051	1.1259	1.2324
NM_053968	Mt3	-0.1541	-0.1304	-0.1227	-0.1726	-0.0344	-0.0638	-0.1909	-0.2078	-0.2329	0.0129	-0.212	0.02561	-0.1856	-0.0914
NM_022588	Mta1	-0.5331	-0.3734	-0.2747	-0.6648	-0.5813	-0.834	-0.675	-0.317	-0.3194	-0.3368	-0.4748	-0.6918	-0.3198	-0.2833
NM_001100740	Mta2	-0.1345	-0.0283	0.14499	0.40195	-0.0444	0.12075	0.1345	-0.1627	0.11012	-0.2244	0.01147	0.30877	0.04185	-0.1106
NM_001106705	Mta3_predicted	0.38886	-0.1727	-0.2215	0.07838	1.0096	0.7966	-0.2671	0.68016	0.00011	0.0151	-0.2572	0.15888	0.40228	0.50015
NM_001047867	Mtap_predicted	-0.066	-0.0253	0.04973	-0.0899	-0.2194	0.0119	0.04306	-0.0605	-0.0647	0.15576	-0.0685	0.20833	-0.1782	-0.0023
NM_030995	Mtap1a	0.43423	0.46677	0.44888	0.33174	0.50988	0.39369	-0.047	0.47857	0.20968	0.45996	0.39286	0.4811	0.30207	0.4756
NM_013066	Mtap2	0.28259	0.21953	0.43829	0.35496	0.26704	0.24015	0.30959	0.00892	0.45366	0.61975	0.32258	0.14647	0.3577	0.39417
NM_001024278	Mtap4	0.09669	0.02896	0.00907	0.19605	0.05777	-0.0002	0.03127	0.11845	-0.0463	0.16027	0.01226	0.0497	0.08209	0.03986
NM_017204	Mtap6	0.4051	0.3435	0.19002	-0.7561	0.21073	0.07158	0.15151	0.34794	0.18805	0.4125	0.34369	-0.512	0.22618	-0.0076
NM_001106270	Mtap7_predicted	0.00746	0.12851	0.18143	0.05181	0.06536	-0.0527	-0.0838	-0.178	-0.0768	0.16052	0.39972	0.15225	0.00856	0.02051
NM_001100833	Mtch1	0.00281	0.07703	-0.1364	-0.2431	0.24994	0.46481	0.15249	0.08019	0.15844	-0.0441	0.13099	0.15503	-0.3822	-0.2215
NM_001106488	Mtch2_predicted	-0.8452	-0.2573	-0.0146	-1.4532	-0.7609	-0.9171	-0.1723	-0.5827	-0.0292	0.0205	-0.2739	0.30799	0.38973	0.25406
NM_133398	Mtdh	-0.3571	-0.1957	-0.1848	-0.1057	-0.1912	-0.5195	-0.0485	-0.2902	0.02987	-0.2529	-0.1399	-0.1363	0.20005	0.39201
NM_053499	Mterf	0.26702	0.1168	-0.2684	0.07364	-0.2033	-0.2213	0.25102	-0.0529	0.00915	-0.2906	-0.0989	-0.2744	-0.3646	-0.5073
NM_199387	Mterfd1	-0.5893	-0.4893	-0.3358	-0.1723	-0.4885	-0.689	-0.1623	-0.4068	-0.1277	-0.3707	-0.4755	-0.1328	0.03419	0.20307
NM_001037209	Mterfd2	0.10225	0.48885	-0.0974	0.42672	0.25594	0.0954	0.32205	0.21633	0.2005	0.37933	0.50634	-0.0787	0.19137	0.12929
NM_001014265	Mterfd3	0.26612	-0.0168	0.25689	0.09175	0.13509	0.13673	0.03758	0.1443	0.08446	0.10764	0.18092	0.08905	0.08959	0.28023
NM_001108677	Mtf1_predicted	-0.1167	0.25374	0.37647	0.53924	-0.0554	0.56015	0.22517	-0.1602	-0.0025	-0.0853	-0.1388	-0.357	0.03453	0.27035
NM_001100898	Mtf2	-0.19	-0.1921	0.12026	0.39222	-0.1473	-0.2845	-0.0074	-0.0937	-0.3572	-0.417	-0.1718	-0.2808	0.07685	0.16871
NM_001009697	Mtfmt	-0.071	-0.2994	-0.3342	0.51811	0.17763	0.13544	-0.5385	0.33751	-0.2915	-0.2526	-0.3528	-0.5134	-0.7414	-0.6265
NM_001100977	Mtfr1_predicted	0.33367	0.28848	-0.059	-0.1676	0.69159	0.14531	0.35598	0.75453	0.12781	0.47097	-0.069	-0.0686	0.48136	0.62774
NM_022508	Mthfd1	-0.4589	-0.7686	0.47881	-0.8743	-0.4652	-0.5361	-0.6961	-0.3947	-0.4972	-0.5158	-0.7083	-0.0701	0.23953	0.35283
NM_001108462	Mthfd1l_predicted	0.30053	-0.2097	0.50902	0.11538	0.09053	0.2022	-0.3391	0.06722	-0.2802	-0.0664	-0.1313	-0.3412	0.25842	0.42618
XM_001074061.1	Mthfr_predicted	0.11343	0.06586	0.14765	0.25454	0.0758	0.08303	0.07294	0.19759	0.21408	0.0781	0.10617	-0.0443	0.03676	0.10519
NM_001009349	Mthfs	0.3157	0.02848	0.06323	-0.4805	-0.3694	-0.2327	0.14634	-0.1334	0.15207	0.30411	0.16192	-0.1885	-0.0674	0.065
NM_001004254	Mtif2	-0.1127	-0.1706	0.26684	0.50131	-0.3374	-0.4729	0.66751	-0.2393	-0.1345	-0.0991	0.06988	-0.2561	-0.2961	-0.33
NM_001012069	Mtl5	-0.0361	0.08511	0.00218	-0.0103	0.02798	0.06858	0.05502	0.10055	0.04334	-0.0808	0.08944	0.25897	-0.0481	-0.0832
XM_228644.4	Mtmr1_predicted	0.06494	0.05596	-0.0828	0.15849	0.00645	-0.112	0.05328	-0.032	0.18572	0.28388	-0.0319	-0.0411	-0.0135	-0.0732
XM_228644.4	Mtmr1_predicted	0.30889	0.16647	0.0863	0.14632	0.0488	-0.0321	0.19988	0.04942	0.34999	0.0157	0.28128	0.52015	0.11639	0.01853
NM_001012077	Mtmr12	0.06602	-0.0455	0.07683	-0.0336	0.12814	0.0091	-0.0239	0.06448	-0.152	0.16623	0.01191	-0.0057	0.14881	0.01781
NM_001108123	Mtmr2_predicted	0.0852	0.41117	-0.0278	-0.24	0.32873	-0.2966	0.02064	0.27127	0.03965	0.31861	0.19704	-0.1734	0.36592	0.55232
NM_001012038	Mtmr3	0.22558	-0.185	-0.5218	-0.0558	0.05664	0.24435	0.10038	0.37093	-0.1664	-0.1355	-0.0817	-0.1297	-0.163	-0.1402
NM_001105827	Mtmr4_predicted	-0.0614	-0.0425	-0.0997	-0.2662	-0.235	-0.4609	-0.0159	-0.2151	-0.1177	0.18058	-0.0712	-0.057	-0.0177	0.03142
NM_001107268	Mtmr6_predicted	0.11795	0.40139	-0.9164	-0.6069	-0.0758	-0.421	-0.068	0.25032	0.1917	0.28597	-0.0525	-0.3485	0.07335	-0.0185
NM_001107312	Mtmr7_predicted	-0.1104	-0.1012	0.68872	1.1634	0.22908	-0.2259	-0.0326	-0.0192	-0.0086	-0.1161	-0.0023	-0.2378	-0.0371	0.01538
NM_001005761	Mtmr9	-0.0268	-0.0303	0.13466	0.1763	-0.2525	-0.5149	-0.0021	-0.0958	-0.2735	-0.1285	-0.2028	-0.1598	-0.244	-0.1207
NM_053676	Mtnr1a	-0.0259	-0.0838	-0.1218	0.02451	-0.0364	-0.0172	-0.084	0.03553	-0.0354	-0.1168	0.13048	-0.1151	0.04941	-0.0202
NM_001100641	Mtnr1b	0.17268	0.20906	0.27779	0.02772	0.03889	0.05019	0.14034	0.06723	0.10376	0.05061	0.06709	0.1674	0.05786	-0.0314
NM_001106841	Mto1_predicted	-0.0746	-0.1462	0.37463	0.52303	0.49523	0.22595	0.4092	0.22745	-0.1756	0.05253	-0.0379	-0.0898	-0.0812	0.18421
NM_024374	Mtpn	0.88247	0.25437	-0.7658	-0.0267	0.59308	0.41552	-0.2707	0.87454	0.20677	0.27934	-0.0938	-0.2807	0.28865	0.311
NM_030864	Mtr	-0.1512	-0.2614	0.2621	0.08947	-0.0614	-0.1194	-0.1656	-0.0773	-0.0207	-0.1806	-0.1456	-0.0201	0.08638	-0.0842
NM_001025723	Mtrf1l	0.02331	-0.0045	-0.0289	0.27641	0.31804	0.08622	0.41091	-0.0359	0.14582	-0.1746	0.16862	0.08824	0.27988	0.12767
NM_001025723	Mtrf1l	0.02472	0.30129	0.14042	0.39653	0.40995	0.53262	0.2291	0.35276	0.25085	0.21624	0.16878	0.35658	-0.0822	0.12861

NM_001039003	Mtrr	-0.002	0.14139	0.04186	0.11717	0.24264	-0.0492	0.14625	0.12203	0.09853	-0.053	-0.059	-0.2683	0.16071	-0.1805
NM_001130563	Mtss1_predicted	-0.017	0.33968	0.06683	0.19473	0.22924	0.20639	-0.1267	0.26065	0.11682	0.0029	-0.1065	-0.0501	-0.1163	0.05738
NM_001107727	Mttp	0.24994	0.14476	0.31241	0.17045	0.02106	0.36502	0.1421	0.27527	0.0259	0.24116	0.08009	0.29917	0.26747	0.09371
NM_178093	Mtus1	-0.3458	-0.5572	-0.4097	-0.1204	-0.3366	-0.3288	-0.5529	-0.304	-0.5779	-0.7839	-0.3975	-0.6153	-0.4046	-0.5274
NM_001015013	Mtvr2	-0.3812	-0.3378	0.20978	-0.0575	-0.2666	-0.2728	-0.3258	-0.4179	-0.1423	-0.2696	0.15336	0.15009	0.34902	0.26691
NM_001008286	Mtx2	0.45381	-0.0552	-0.1628	-0.0845	0.35031	0.14933	0.11184	0.19255	0.05811	-0.0457	-0.1662	0.04448	0.09425	-0.0641
NM_012602	Muc1	-0.1027	0.19572	0.15497	0.46035	0.19856	0.29432	0.13397	0.19652	0.43763	0.18895	0.23346	-0.0346	-0.0506	0.14181
NM_133513	Muc10	0.44555	0.65206	0.54649	0.32509	0.26953	0.16137	0.33382	0.34062	0.30507	0.05008	0.42247	0.15619	0.32546	0.17232
XM_235593.4	Muc19_predicted	0.16713	0.14608	0.1538	0.02379	-0.0031	-0.0285	0.13069	0.23446	0.07222	-0.017	0.06923	0.05247	0.07088	0.08614
NM_001107089	Muc20_predicted	0.11991	0.13335	0.11208	0.18503	0.13396	-0.0528	0.04577	-0.0769	0.2292	-0.0646	0.04371	0.08238	0.16511	0.02747
XM_001072593.1	Muc4	0.04949	-0.0762	0.06111	0.00273	0.05095	0.15038	0.02162	0.08871	0.09529	0.04438	-0.0574	0.16514	0.06769	-0.0606
XM_001063115.1	Muc6	-0.0032	-0.0134	0.037	-0.0514	0.06503	0.1189	0.10613	0.14451	0.09054	0.00625	0.00249	0.2722	-0.0204	0.07956
NM_138525	Mucdhl	0.36908	-0.0214	0.8906	0.29536	-0.3131	-0.3309	0.90633	-0.4253	-0.1686	0.17854	0.06263	-0.0681	0.77479	0.82068
NM_001037975	Mug2	-0.0736	-0.2022	-0.0942	-0.225	-0.232	-0.1559	-0.0981	-0.3378	-0.1883	-0.2513	-0.0821	-0.2477	-0.1206	-0.0655
NM_001108736	Mum1_predicted	-0.2084	-0.0366	0.20249	-0.1341	-0.2414	-0.1999	-0.0375	-0.2307	-0.2121	-0.092	-0.0083	0.03357	-0.0154	-0.0137
NM_203512	Mup4	-0.0064	0.10246	0.0592	0.02057	0.03825	0.01545	-0.0247	-0.0629	0.00583	-0.0411	0.06857	0.07853	-0.0372	-0.0754
NM_203512	Mup5	-0.184	-0.1856	-0.2064	-0.1729	-0.0574	-0.0296	-0.0785	-0.2316	-0.054	-0.1639	-0.0342	-0.1368	-0.2109	-0.2004
NM_001025645	Mus81	0.05298	0.02154	0.13226	-0.2792	0.03689	0.27532	0.06533	0.07342	-0.1283	-0.0341	-0.1397	-0.1188	-0.0919	-0.0718
NM_031061	Musk	-0.1148	0.09071	-0.0376	0.00082	-0.025	0.03754	0.06919	0.01901	0.00201	-0.0629	0.05768	0.01563	0.20404	0.03014
NM_181368	Mustn1	0.9231	0.40651	0.56545	0.00605	0.62204	0.9995	0.28045	0.81181	0.33223	0.50758	1.1004	1.0335	0.78747	0.6809
NM_001107347	Muted_predicted	0.07118	-0.0919	-0.1153	-0.1188	0.06892	-0.1408	-0.063	-0.0531	0.00126	0.02782	-0.0891	-0.0631	-0.0851	-0.1251
NM_001107347	Muted_predicted	-0.1083	-0.0547	-0.3926	-0.0697	0.00709	-0.1414	-0.1352	0.11246	-0.2365	0.01567	-0.2066	0.00702	-0.0748	0.06666
NM_133316	Mutyh	0.03979	0.18512	0.4972	0.46204	-0.2209	-0.1184	0.41546	-0.1155	0.13881	-0.1065	0.18601	-0.0018	-0.086	-0.2933
NM_031062	Mvd	-0.2262	-0.6907	1.8863	0.87929	-0.3856	-0.2707	0.11789	-0.3013	-0.1743	-0.5142	-0.5404	0.42714	0.22938	0.43346
NM_022715	Mvp	0.26872	0.42242	-0.4715	-0.1062	0.76152	0.84505	0.12367	0.81832	0.10487	0.20012	0.15673	-0.7356	-0.453	-0.5191
NM_173096	Mx1	0.41657	0.41104	0.08164	0.71952	1.8872	1.8343	0.45872	1.7857	-0.0293	0.54022	0.51125	0.09431	0.35346	0.54613
NM_134350	Mx2	0.3108	0.18582	0.34265	0.41559	1.3538	1.2366	0.46124	1.3716	0.0895	0.15754	0.14111	0.20411	0.17992	0.21122
NM_001100749	Mxd1	-0.1417	-0.1404	-0.2309	-0.1417	-0.2971	-0.1662	-0.2254	-0.2703	-0.2698	-0.2601	-0.2957	-0.2665	-0.0459	-0.2468
NM_145773	Mxd3	-0.0722	-1.09	0.46992	-0.5495	-1.0981	-1.0869	-0.6937	-1.1693	-0.5693	-1.1289	-0.7651	0.4924	0.25237	0.33231
NM_001108364	Mxd4_predicted	-0.7248	-0.5906	-0.8492	-0.7095	-0.2004	0.0126	-0.6765	-0.3176	-0.1199	-0.7003	-0.6558	0.28746	-0.7308	-0.6516
NM_001007002	Mxra8	-0.7388	-0.3746	-0.0155	-0.4479	-1.4965	-1.5506	-0.173	-1.4924	-0.3687	-0.4053	-0.4079	-0.3276	-0.7399	-0.728
NM_183332	Myadm	-1.0248	-0.6261	-0.0845	-1.0673	-0.8438	-0.8599	-0.4606	-0.8462	-0.8034	-0.6032	-0.5593	-0.7961	-0.5406	-0.8119
NM_031668	Mybbp1a	-0.6377	-0.7183	0.23577	-0.1026	-0.6099	-0.4868	-0.4576	-0.6189	-0.8643	-0.9919	-0.8463	-0.8144	-0.3561	-0.1797
NM_001106632	Mybl1_predicted	0.63104	-0.5385	0.93898	1.6115	0.49482	0.57489	0.28168	0.69683	-0.3231	0.06973	-0.0769	0.0855	0.67079	0.56355
NM_001106536	Mybl2_predicted	0.02656	-0.6885	1.0465	-0.3938	-0.2132	-0.2979	-0.2894	-0.2268	0.10912	0.13324	-0.3023	0.43446	0.83764	0.79029
NM_001100758	Mybpc1	0.12589	-0.0244	0.28661	0.06601	0.05103	0.35695	0.28216	0.17881	-0.0059	0.33043	0.12555	-0.008	0.09671	0.07147
NM_001106257	Mybpc2_predicted	-0.137	-0.1175	-0.0516	0.09082	-0.2148	-0.2767	0.07626	-0.0246	0.06162	-0.1113	-0.1032	-0.3144	-0.1291	-0.1184
NM_001106490	Mybpc3_predicted	0.03553	-0.0779	0.10897	-0.0098	-0.1162	0.23785	-0.0399	-0.059	-0.007	0.00548	0.11109	0.06716	-0.0602	-0.0161
NM_031813	Mybph	-0.0747	-0.0337	0.10012	0.37133	0.10399	0.00521	-0.0809	-0.0148	-0.1985	-0.175	-0.1569	0.181	-0.0857	-0.0778
NM_001014042	Mybphl	-0.4148	-0.0922	-0.297	-0.2955	-0.2866	-0.2145	-0.301	-0.3566	-0.0198	-0.2833	-0.3223	-0.2448	-0.4004	-0.0811
NM_012603	Myc	-1.4743	-2.6344	-1.2293	-1.21	-2.2716	-2.2863	-1.9718	-2.0206	-2.6331	-2.4089	-2.519	-2.3072	-1.5082	-1.3911
XM_216518.3	Mycbp_predicted	-0.0554	-0.063	-0.0016	0.00355	0.02157	-0.0216	0.01073	0.01901	0.0488	0.08918	0.07795	0.0764	-0.0647	-0.0293
XM_001054123.1	Mycl1_mapped	-0.1141	-0.1735	-0.0306	-0.0547	-0.1731	-0.0586	0.03263	0.06181	0.08319	-0.2063	-0.0392	-0.1832	-0.1188	-0.1851
NM_001013096	Mycn	-0.1209	0.03273	-0.6118	-0.1242	0.69042	0.38587	0.22169	0.67593	-0.0784	0.11215	-0.0882	-0.1726	-0.4268	-0.358
NM_021837	Mycs	0.05473	-0.0129	-0.01	0.01008	-0.0622	0.28134	0.04185	0.03838	-0.0046	0.13236	0.06032	0.04754	-0.0185	0.00952
NM_001106207	Myct1_predicted	0.182	-0.1441	-0.0172	-0.0006	-0.0805	0.20637	-0.2069	-0.076	-0.3099	0.1695	0.02185	0.34971	-0.0835	0.19187
NM_133546	Myd116	0.58089	0.00734	0.12808	0.60737	0.67432	0.89581	0.2658	0.7948	-0.2148	0.0658	0.00352	-0.1015	-0.0348	0.11827
NM_198130	Myd88	0.01662	0.22874	-0.0131	-0.2961	0.0046	0.11286	0.2421	-0.0597	0.0687	0.1628	0.15796	-0.3921	-0.2144	-0.034
NM_001013205	Myef2	0.35941	0.07259	0.20372	0.25802	0.46975	0.61431	0.01894	0.46308	0.15831	0.47866	0.3209	0.27049	0.46883	0.34308

NM_001013205	Myef2	-0.2155	-0.1726	-0.3009	0.072	-0.5335	-0.9289	0.15096	-0.3833	-0.39	-0.1482	-0.5057	-0.5419	-0.1974	-0.4614
NM_001106783	Myf5_predicted	0.11803	0.16729	0.13938	0.13766	0.08404	0.06633	0.03633	0.07131	-0.0258	-0.008	0.07109	0.17558	-0.0313	0.11936
[truncated: 1,831,425 more chars]
